# Longitudinal *APOE4*- and amyloid-dependent changes in the blood transcriptome in cognitively intact older adults

**DOI:** 10.1186/s13195-023-01242-5

**Published:** 2023-07-12

**Authors:** Emma S. Luckett, Magdalena Zielonka, Amine Kordjani, Jolien Schaeverbeke, Katarzyna Adamczuk, Steffi De Meyer, Koen Van Laere, Patrick Dupont, Isabelle Cleynen, Rik Vandenberghe

**Affiliations:** 1 Laboratory for Cognitive Neurology, Leuven Brain Institute, KU Leuven, Leuven, 3000 Belgium; 2 Alzheimer Research Centre KU Leuven, Leuven Brain Institute, Leuven, 3000 Belgium; 3 Laboratory for Complex Genetics, KU Leuven, Leuven, 3000 Belgium; 4 Laboratory for the Research of Neurodegenerative Diseases, VIB-KU Leuven, KU Leuven, Leuven, 3000 Belgium; 5 Laboratory of Neuropathology, Leuven Brain Institute, KU Leuven, Leuven, 3000 Belgium; 6 BioClinica, Newark, CA USA; 7 Laboratory of Molecular Neurobiomarker Research, KU Leuven, Leuven, 3000 Belgium; 8 Division of Nuclear Medicine, UZ Leuven, Leuven, 3000 Belgium; 9 Nuclear Medicine and Molecular Imaging, Department of Imaging and Pathology, KU Leuven, Leuven, 3000 Belgium; 10 Neurology Department, University Hospitals Leuven, Herestraat 49, Leuven, 3000 Belgium

**Keywords:** Alzheimer’s disease, RNA sequencing, Blood, Transcriptome, Longitudinal study, Amyloid accumulation, APOE4

## Abstract

**Background:**

Gene expression is dysregulated in Alzheimer’s disease (AD) patients, both in peripheral blood and post mortem brain. We investigated peripheral whole-blood gene (co)expression to determine molecular changes prior to symptom onset.

**Methods:**

RNA was extracted and sequenced for 65 cognitively healthy F-PACK participants (65 (56–80) years, 34 *APOE4* non-carriers, 31 *APOE4* carriers), at baseline and follow-up (interval: 5.0 (3.4–8.6) years). Participants received amyloid PET at both time points and amyloid rate of change derived. Accumulators were defined with rate of change ≥ 2.19 Centiloids. We performed differential gene expression and weighted gene co-expression network analysis to identify differentially expressed genes and networks of co-expressed genes, respectively, with respect to traits of interest (*APOE4* status, amyloid accumulation (binary/continuous)), and amyloid positivity status, followed by Gene Ontology annotation.

**Results:**

There were 166 significant differentially expressed genes at follow-up compared to baseline in *APOE4* carriers only, whereas 12 significant differentially expressed genes were found only in *APOE4* non-carriers, over time. Among the significant genes in *APOE4* carriers, several had strong evidence for a pathogenic role in AD based on direct association scores generated from the DISQOVER platform: *NGRN*, *IGF2*, *GMPR*, *CLDN5*, *SMIM24*. Top enrichment terms showed upregulated mitochondrial and metabolic pathways, and an exacerbated upregulation of ribosomal pathways in *APOE4* carriers compared to non-carriers. Similarly, there were 33 unique significant differentially expressed genes at follow-up compared to baseline in individuals classified as amyloid negative at baseline and positive at follow-up or amyloid positive at both time points and 32 unique significant differentially expressed genes over time in individuals amyloid negative at both time points. Among the significant genes in the first group, the top five with the highest direct association scores were as follows: *RPL17-C18orf32*, *HSP90AA1*, *MBP*, *SIRPB1*, and *GRINA*. Top enrichment terms included upregulated metabolism and focal adhesion pathways. Baseline and follow-up gene co-expression networks were separately built. Seventeen baseline co-expression modules were derived, with one significantly negatively associated with amyloid accumulator status (*r*^2^ =  − 0.25, *p* = 0.046). This was enriched for proteasomal protein catabolic process and myeloid cell development. Thirty-two follow-up modules were derived, with two significantly associated with *APOE4* status: one downregulated (*r*^2^ =  − 0.27, *p* = 0.035) and one upregulated (*r*^2^ = 0.26, *p* = 0.039) module. Top enrichment processes for the downregulated module included proteasomal protein catabolic process and myeloid cell homeostasis. Top enrichment processes for the upregulated module included cytoplasmic translation and rRNA processing.

**Conclusions:**

We show that there are longitudinal gene expression changes that implicate a disrupted immune system, protein removal, and metabolism in cognitively intact individuals who carry *APOE4* or who accumulate in cortical amyloid. This provides insight into the pathophysiology of AD, whilst providing novel targets for drug and therapeutic development.

**Supplementary Information:**

The online version contains supplementary material available at 10.1186/s13195-023-01242-5.

## Background

The major hallmarks of Alzheimer’s disease (AD) are, by biological definition, the presence of amyloid-β plaques and tau tangles [1]. However, AD-related (brain) changes begin more than a decade prior to symptom onset in the preclinical or asymptomatic phase [2].

Recent genome-wide association studies (GWAS) have enhanced our knowledge of genetic risk factors, highlighting variants associated with AD risk beyond the apolipoprotein E ε4 (*APOE4*) gene [3–6]. Furthermore, it is possible to investigate changes in gene expression to provide further insight into AD-related changes at the functional level. These studies may enable recruitment stratification for clinical trials and early disease diagnosis or provide targets for further research for drug development and treatments.

Post-mortem brain transcriptomic studies in AD cases versus controls have implicated several dysregulated pathways in AD, for example, DNA repair, mitochondrial pathways, inflammation, and calcium signalling [7–10]. Moreover, AD case–control studies have also investigated transcriptome changes in peripheral blood due to the less invasive nature of sampling (compared to cerebrospinal fluid collection) and the ability to perform longitudinal analyses, which is inherently impossible in post mortem studies. Such studies have observed similar transcriptomic changes as those in post mortem brain studies, in addition to providing evidence for dysfunctions in pathways such as protein synthesis and apoptosis [11–13]. Importantly, these blood-based transcriptomic analyses have also shown associations with changes in peripheral gene expression and AD-related brain changes, such as changes in hippocampal volume and amyloid deposition [14].

Transcriptomic analyses exclusively in the earliest asymptomatic disease stages in relation to AD genotypes and phenotypes in a longitudinal study design are distinctly lacking. Thus, we aimed to investigate longitudinal changes in peripheral blood RNA expression from individuals participating in the Flemish Prevent AD Cohort KU Leuven (F-PACK) to better understand the molecular changes occurring in asymptomatic AD. We hypothesise that expression profiles differ depending on genetic carrier status of *APOE4* and with respect to amyloid accumulation.

## Methods

### Study participants

The Laboratory for Cognitive Neurology follows a cohort of 180 deeply phenotyped elderly individuals, who were cognitively intact at recruitment, known as F-PACK [15–17]. Individuals were recruited between 2009 and 2015, in three waves of 60 participants, based on an inclusion age of 50–80 years, with a mini-mental state examination (MMSE) score ≥ 27 and Clinical Dementia Rating (CDR) score of 0. Furthermore, participants had to score within published norms on an extensive neuropsychological test battery [15, 18]. Exclusion criteria included a history of neurological or psychiatric illness, contraindication for magnetic resonance imaging (MRI), focal brain lesions on MRI, history of cancer, or exposure to radiation one year prior to the baseline positron emission tomography (PET) scan. Recruitment was stratified for two genetic factors: *APOE4* (present or absent) and brain-derived neurotrophic factor (BDNF) *66 met* (present or absent). This was carried out such that per 5-year age bin each factorial cell contained the same number of individuals matched for age, sex, and education. Participants are invited for 2-yearly neuropsychological evaluations over a 10-year period.

Sixty-five of these 180 F-PACK participants have received a baseline and follow-up ^18^F-Flutemetamol amyloid PET scan, structural MRI scan, and PAXgene RNA blood tube sampling (PreAnalytiX GmbH-BD Biosciences, Mississauga, ON, Canada, time interval: 5.1 (3.4–8.6) years).

The protocol was approved by the Ethics Committee University Hospitals Leuven. All participants provided written informed consent in accordance with the declaration of Helsinki.

### Imaging

#### Structural MRI

At baseline and follow-up, participants received a high-resolution T1-weighted structural MRI scan. A 3T Philips Achieva dstream 32-channel headcoil MRI scanner was used (Philips, Best, The Netherlands). Sixty-five baseline and 57 follow-up scans were acquired using a 3D turbo field echo sequence: repetition time = 9.6 ms; echo time = 4.6 ms; flip angle = 8°; field of view = 250 × 250 mm; 182 slices; voxel size 0.98 × 0.98 × 1.2 mm^3^. Five follow-up scans were acquired using a three-dimensional magnetisation-prepared rapid gradient-echo sequence, due to being acquired as part of The Amyloid imaging to prevent Alzheimer’s disease (AMYPAD) study: repetition time = 6.6 ms; echo time = 3.1 ms; flip angle = 9°; field of view = 270 × 252 mm; 170 slices; voxel size 1.05 × 1.05 × 1.2 mm^3^. Three individuals refused a follow-up MRI scan.

#### ^18^F-Flutemetamol PET

^18^F-Flutemetamol PET scans were acquired on a 16-slice Biograph PET/CT scanner (Siemens, Erlangen, Germany) at baseline and follow-up, with a net injected intravenous activity of 149 MBq (127–162 MBq) and 149 MBq (77–194 MBq), respectively, and an acquisition window of 90–120 min post-injection, as previously described [18–22]. Scans were reconstructed as frames of 5 min using ordered subsets expectation maximisation. All 65 baseline scans and 64 follow-up scans were reconstructed with 5 iterations and 8 subsets. One follow-up scan was reconstructed with 4 iterations and 21 subsets, due to being reconstructed as part of AMYPAD prior to a protocol amendment. The spatial resolution of the scanner is 4.6 mm full width at half maximum 1 cm off-centre measured with the NEMA protocol. All scans were smoothed with a 5 mm full width at half maximum isotropic Gaussian filter.

Statistical Parametric Mapping version 12 (Wellcome Trust Centre for Neuroimaging, London, UK, http://www.fil.ion.ucl.ac.uk/spm) running on MATLAB R2018b (Mathworks, Natick, MA, USA) was used to process the images, as described previously [18–22].

We used the Automated Anatomic Labelling Atlas (AAL) to calculate mean standardised uptake value ratios (SUVRs) in the spatially normalised images (voxel size: 2 × 2 × 2 mm^3^) in a composite cortical volume of interest (SUVR_*comp*_) within the acquisition window 90–110 min post injection to allow for conversion of SUVR_*comp*_ values to Centiloids (CL, below) [23]. This composite volume of interest included the following bilateral regions: frontal (AAL areas 3–10, 13–16, 23–28), parietal (AAL 57–70), anterior cingulate (AAL 31–32), posterior cingulate (AAL 35–36), and lateral temporal (AAL 81–82, 85–90) and was masked with the participant-specific grey matter segmentation map (intensity threshold = 0.3) [18, 24]. Cerebellar grey matter was used as the reference region to calculate SUVR_*comp*_, defined as AAL areas 91–108, and was masked by the participant-specific grey matter map (intensity threshold = 0.3) [18]. SUVR_*comp*_ were then converted to CL using the formula CL = 127.6 × SUVR_*comp*_ – 149 [23, 25]. We used an amyloid positivity threshold of CL = 23.5, a pathologically confirmed threshold for amyloid positivity [26]. Furthermore, to model amyloid change over the longitudinal time period, we calculated rate of change as follows: (follow-up CL – baseline CL)/time interval (years). Amyloid accumulators were defined as having a rate of change at least 1.5 standard deviations above the median rate of change of the subgroup of individuals who remained amyloid negative at both time points (CL rate of change ≥ 2.19).

### RNA

#### Extraction and sequencing

RNA was extracted from PAXgene RNA blood tubes using the QIAGEN PAXgene Blood RNA Kit according to the manufacturer protocol. RNA concentration and quality were assessed prior to sequencing using a NanoDrop Spectrophotometer (NanoDrop Technologies), and RNA Integrity Number was assessed with an Agilent 2100 Bioanalyzer (Agilent Technologies).

RNA was sequenced in collaboration with the UZ/KU Leuven Genomics Core Facility. Sequencing libraries were prepared using the Lexogen QuantSeq 3′ mRNA-Seq library prep kit as per the manufacturer protocol, with indexing to allow for multiplexing. Library quality and size were assessed using a Bioanalyzer RNA 6000 nano or pico kit (depending on RNA concentration). Libraries were sequenced on an Illumina HiSeq4000 instrument. Raw files were demultiplexed, by Genomics Core, into FastQ files for further analyses.

#### Quality control and processing

Processing was performed on the Vlaams Supercomputer Centrum (www.vscentrum.be). TrimGalore (Version 0.11.5) was used to remove adaptor sequences and low-quality end bases from raw sequencing reads (https://www.bioinformatics.babraham.ac.uk/projects/trimgalore/) using Cutadapt [27] and FastQC (https://www.bioinformatics.babraham.ac.uk/projects/fastqc/). Reads were trimmed to remove the first 12 base pairs from the 3′ end as per Lexogen’s recommendation (https://www.lexogen.com/quantseq-3mrna-sequencing/). Reads less than 12 base pairs were removed. If necessary, FastQ files from the same sample were merged.

Processed FastQ files underwent selective alignment for quantification using Salmon (Version 1.0.0) and a decoy-aware transcriptome, created using the GENCODE *Homo sapiens* GRCH38.p13 genome assembly, reference genome hg38, and kmer size of 23 [28–30]. Transcript abundances for each sample were then imported to R and summarised at the gene level in a *txi* object using *tximport*, with the *countsFromAbundance* = ”no” argument used given the QuantSeq library prep was 3′ tagged [31].

### Statistical analyses

Statistical analyses were performed in R version 4.2.1 (2022–06-23; The R Foundation for Statistical Computing; https://cran.r-project.org/).

F-PACK characteristics were stratified for *APOE4* status and assessed using Wilcoxon rank sum tests with continuity correction or Welch two-sample *t*-tests for continuous data, depending on normality (assessed using Shapiro–Wilk tests), and *χ*^2^ tests for categorical data. Only Trail Making Test part B divided by part A had a normal distribution after log transformation.

#### Filtering of the DESeqDataset

*DESeq2* [32] was downloaded from *Bioconductor* and used to create a DESeqDataset object, using the *DESeqDataSetFromTximport* function and the imported *txi* object for expression analyses. Subsequently, our primary analyses investigated changes in single gene expression and our secondary analyses investigated changes in gene co-expression networks, as detailed below.

The DESeqDataset was subjected to filtering prior to any analyses. The following were removed: the top globin genes (ENSG00000206172.8, ENSG00000188536.13, ENSG00000244734.4, ENSG00000229988.2, ENSG00000223609.11, ENSG00000213931.7, ENSE00001494261.2, ENSG00000086506.3, ENSG00000213934.9, ENSG00000196565.15, ENSG00000206177.7, ENSG00000130656.6, ENSG00000206178.2) [33], genes that had zero counts across all samples, non-protein coding genes, and low count samples (samples with less than 700,000 reads). Lowly expressed genes were also removed prior to individual analyses. In the case of a two-way differential expression analysis, genes with < 5 counts in > 50% of samples in both subgroups were removed; in the case of a differential expression analysis with a continuous outcome variable, genes with < 5 counts in > 30% of samples were removed; in the case of a weighted gene co-expression network analysis, genes with < 10 counts in > 90% of samples were removed.

#### Primary analyses: differential gene expression

In order to examine changes in single genes over time or between groups of interest, *DESeq2* was used to perform differential gene expression analyses between traits of interest, with baseline age and sex included as covariates.

We first performed differential expression analyses of follow-up expression data versus baseline expression data in *APOE4* carriers or non-carriers separately. To test whether there was a significant difference in the change in expression over time between the groups, we performed a time-series paired analysis, including an interaction between *APOE4* status and time point (binary). To further examine any significant differences, we also performed cross-sectional differential expression analyses between *APOE4* carriers and non-carriers at either baseline or follow-up.

We then performed differential expression analyses as above with individuals classified by amyloid accumulators (*N* = 12) and non-accumulators (*N* = 53). Furthermore, to determine whether there were differences in gene expression at baseline in response to future amyloid rate of change, we performed a differential expression analysis using baseline expression data with amyloid rate of change as a continuous outcome variable.

Finally, we grouped individuals based on amyloid positivity status: amyloid negative at both time points (*N* = 56); amyloid negative at baseline, positive at follow-up or amyloid positive at both time points (*N* = 9) [16]. Then, we performed differential expression analyses as above, based on this amyloid positivity status.

In order to determine whether results from the differential expression analyses were driven by cell-type contributions, we performed a cell deconvolution using CIBERSORT [34]. Normalised gene expression data were uploaded into the online tool as the “mixture file” and the LM22 dataset provided by CIBERSORT was used as the “signature matrix”. The algorithm was run in absolute mode, with the batch correction in “B-mode” and 100 permutations. Cell composition was then compared between groups of interest as per each contrast from the above differential gene expression using paired or unpaired Wilcoxon rank sum tests or linear regression as appropriate. If a cell-type was significantly associated with a contrast, then differential expression analyses were repeated with the cell-type contribution included as an additional covariate. However, some cell estimates were essentially zero for all samples; therefore, these were removed (median contribution < 0.2).

The Benjamini–Hochberg procedure was used to adjust *p*-values for multiple comparisons per analysis [35]. Genes were considered significant if the false discovery rate (FDR)-adjusted *p*-value was less than *α* = 0.05 and log2FoldChange was ± 1. Results were visualised using the *EnhancedVolcano* package [36].

##### Differentially expressed genes with highest direct association score

For the significant differentially expressed genes in the above analyses, we determined the direct association score using “gene name” and “Alzheimer disease” as search terms using the DISQOVER platform (https://www.disqover.com/). Genes with the highest direct association score are those where there is strongest evidence of the gene being a direct target of AD from previously published literature.

##### Functional enrichment of differentially expressed genes

If there were significant differentially expressed genes (DEGs) in the differential expression analyses, functional enrichment was performed by means of gene set enrichment analysis using Gene Ontology (GO, biological processes (BP), molecular function (MF), cellular component (CC)) using *clusterProfiler* and *enrichGO* [37–40]. We chose this method to rank the genes as this is a threshold-free method that ranks all the genes from the differential gene expression based on a gene significance score that combines the *p*-value and ± logFoldChange. This then determines whether groups of genes of an established gene set are randomly distributed or clustered at either end of the list. This allows the genes from the differential expression analysis to then be assigned to a group of upregulated or downregulated terms accordingly. All *p*-values of enriched terms were FDR-corrected and considered significant when *p*_*FDR*_ < 0.05.

#### Secondary analyses: weighted gene co-expression network analysis

In order to examine whether networks of co-expressed genes are significantly associated with traits of interest or change over time, the *WGCNA* package was used to perform weighted gene co-expression network analysis (WGCNA) [41] of baseline and follow-up expression data separately. The *variance stabilising transformation* function was used on the filtered DESeqDataset, as above, and sample clustering was performed to check for outliers. A soft power threshold value was then derived, using the *pickSoftThreshold* function with default parameters, which was selected based on the scale independence and mean connectivity outputs. The soft threshold was used to create a signed hybrid adjacency matrix using the *adjacency* function. This adjacency matrix was used to obtain a topological overlap matrix using the *TOMsimilarity* function. A dissimilarity measure was calculated as 1-(topological overlap matrix) and used to obtain modules of genes using the dynamic tree cutting method (*cutreeDynamic* function with default parameters). Dynamic modules were assigned a unique colour (unrelated to the properties of the module), and module eigengenes were obtained using the *moduleEigengenes* function. The dissimilarity between module eigengenes was calculated as 1-cor(module eigengenes), which was used for clustering. Clusters per time point were merged using the *mergeCloseModules* function with *cutHeight* = 0.25, resulting in the final set of merged modules for each time point. Merged modules were then correlated with traits of interest (*APOE4* status, amyloid rate of change as a continuous variable, amyloid accumulator status as a binary variable, amyloid positivity status as a binary variable as above) using a Pearson’s correlation analysis and visualised in a matrix using the *labeledHeatmap* function. Modules were considered significant at uncorrected *p* < 0.05.

Note that *DESeq2* performs an internal normalisation to avoid removing samples, which is not performed within the *WGCNA* package.

##### Functional analysis, hub gene detection and module visualisation, and transcriptional regulators

Functional analysis was performed on WGCNA modules that had significant associations with traits of interest using GO over-representation analysis. Over-representation analysis was performed given this is a threshold-based method to detect over-represented enrichment terms among the genes within a specific module (*p*_*FDR*_-value < 0.05 and log2FoldChange ± 1).

Modules with significant associations with traits of interest were subjected to hub gene detection and network visualisation. We considered hub genes to be those that were most highly interconnected with other genes within a given module; thus, the module eigen-based connectivity measures were calculated, and genes were considered as highly interconnected if the eigen-based connectivity was > 0.7 [42–45]. These genes were extracted and uploaded into STRING [46] to create a protein–protein interaction (PPI) network. This PPI network was then exported to Cytoscape (version 3.7.1) for visualisation [47]. Using the CytoHubba plugin [48] in Cytoscape, the Maximal Clique Centrality (MCC) was calculated for each node and the top four intramodular hub genes were selected as those that had the highest MCC values. Furthermore, we performed a cell-type specific enrichment analysis of these highly interconnected genes using WebCSEA, to assess which cell-types the genes may be linked to [49].

We also extracted all module genes for those modules with significant associations with traits of interest to determine potential upstream regulators using ChEA3 [50]. The top 10 regulators were visualised in the Bar Chart tab using the Mean Rank results (average integrated ranks across libraries: GTEx Coexpression, ReMAP CHiP-seq, Enrichr Queries, ENCODE CHiP-seq, ARCHS4 Coexpression, and literature CHiP-seq). Those regulators that remained when selecting only for ReMAP CHiP-seq, ENCODE CHiP-seq and literature CHiP-seq libraries were then considered as potential upstream regulators of the genes within a given module. Note that a lower score indicates a higher relevancy to the potential transcription factor.

##### Module preservation over time and consensus analyses

We used the *modulePreservation* function in order to determine whether modules of genes at baseline were preserved in follow-up expression data and vice versa. Baseline or follow-up co-expression modules were built as described in the above WGCNA and were used as the “reference set”. This reference set was subjected to 200 rounds of permutation testing in the “test set”, i.e. the other time point expression data, to assess whether the module node connectivity patterns are preserved. *Z*-scores were computed for each permutation during the preservation analysis (*Z* = observed – mean_*permuted*_/SD_*permuted*_), and these were combined into a composite *Zsummary* statistic to quantify the overall degree of preservation. A *Zsummary* < 2 suggests low preservation; > 2 *Zsummary* < 10 suggests moderate preservation; *Zsummary* > 10 suggests high preservation. To ensure module reliability, each module was also subjected to permutation testing on the reference set. Lowly preserved modules (*Zsummary* < 2) were subjected to functional enrichment analyses as described above.

Further to the preservation analyses, we wanted to determine whether the genes within modules at baseline or follow-up were significantly overlapping using a consensus analysis [41]. A soft threshold was chosen that was optimal for both expression datasets, which was used for consensus network construction using the *blockwiseConsensusModules* function, with *maxBlockSize* = 20,000 and default parameters. Following construction of the consensus modules, overlap of each pair of baseline-consensus modules or follow-up-consensus modules were calculated, and the Fisher’s exact test was used to assign a *p*-value to each of these pairwise overlaps. The results were then visualised using the *labeledHeatmap* function. The stronger the red colour, the more significant the overlap of the set-specific module with the consensus module.

## Results

### F-PACK characteristics

**Fig. 1:**
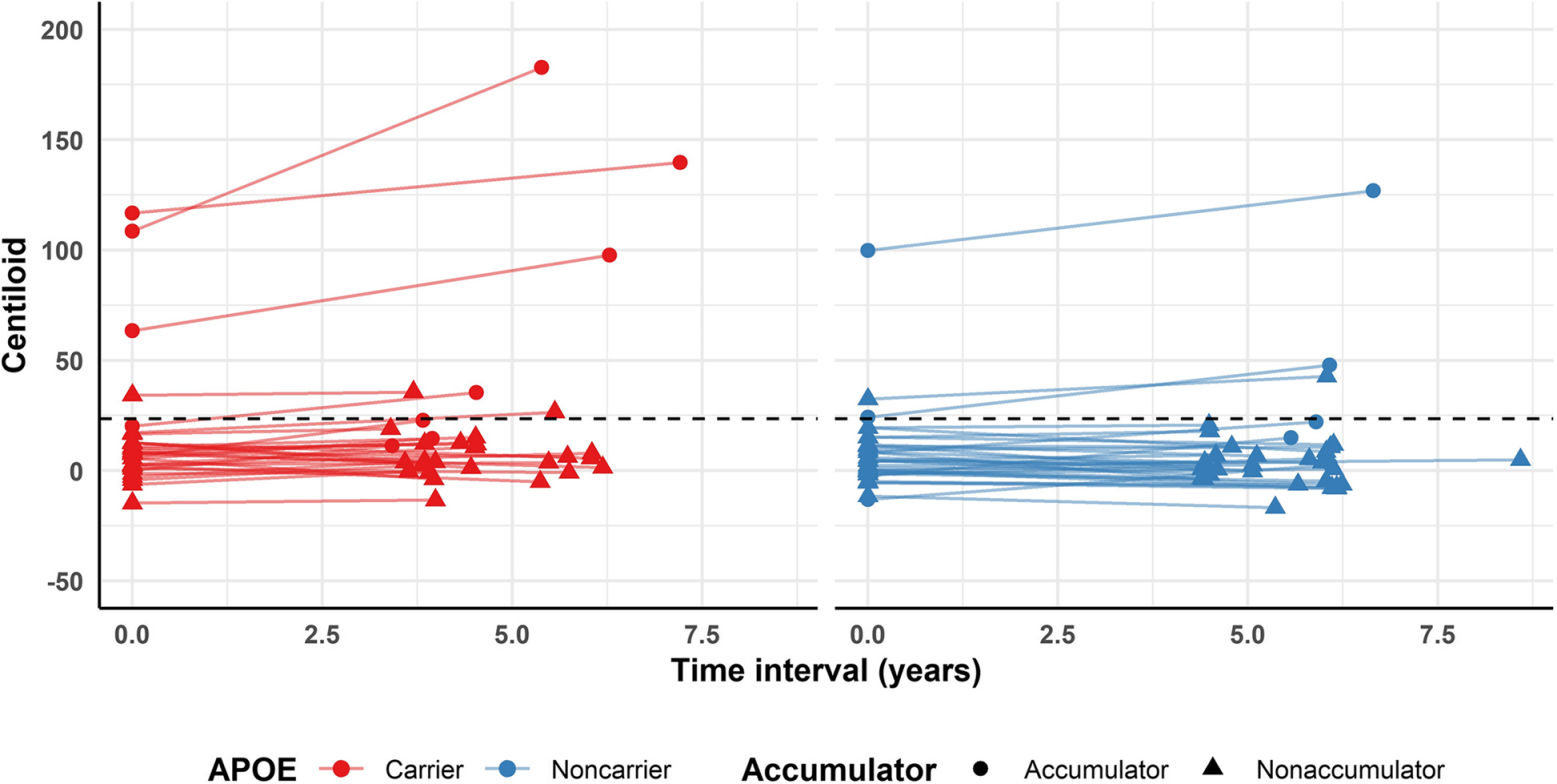
Amyloid change in F-PACK participants, stratified for *APOE4* status. The dotted line represents the threshold for amyloid positivity = 23.5. Red = *APOE4* carrier (*N* = 31), blue = *APOE4* non-carrier (*N* = 34)

Seven (10.8%) F-PACK individuals were amyloid positive at baseline, which increased to nine (13.8%) at follow-up (Fig. 1). Twelve participants were amyloid accumulators, of which seven were *APOE4* carriers. One individual had a CDR that had evolved to 0.5 at the time of follow-up amyloid PET, with corresponding MMSE score of 26. The full cohort characteristics can be found in Table 1, stratified for *APOE4* status.

**Table 1:** Characteristics of F-PACK participants stratified for *APOE4* status. Data are reported as median and range (minimum to maximum) for continuous variables, and numerical for categorical variables. *N* = 65: *ε*2*ε*3 *N* = 5; *ε*2*ε*4 *N* = 1; *ε*3*ε*3 *N* = 29; *ε*3*ε*4 *N* = 28; *ε*4*ε*4 *N* = 2

	***APOE4* non-carrier** **(*N* = 34)**	***APOE4* carrier** **(*N* = 31)**	**Statistics**
Sex (male/female)	17/17	18/13	*Χ*^*2*^ = 0.16, *p* = 0.69
BDNF *66 met* carriers (*N*)	18	16	*Χ*^*2*^ = 0, *p* = 1
Age (years)	68 (56–80)	69 (56–80)	*T* = − 0.42, *p* = 0.68
Education (years)	14 (8–20)	16 (9–23.5)	*T* = 2.17, *p* = 0.03
MMSE	29.5 (27–30)	30 (28–30)	*W* = 555.5, *p* = 0.68
CDR	0	0	*NA*
AVLT TL (/75)	46 (31–68)	46 (35–68)	*T* = 0.32, *p* = 0.75
AVLT %DR	82.6 (30–107.7)	84.6 (58.3–107.7)	*W* = 550, *p* = 0.77
BSRT TR (/12)	7.7 (5.6–10.8)	7.8 (4.9–10.3)	*T* = − 0.74, *p* = 0.46
BSRT DR (/12)	8 (2–12)	8 (3–12)	*W* = 481.5, *p* = 0.55
BNT (/60)	57 (46–60)	57 (41–60)	*W* = 530, *p* = 0.97
AVF (# words)	24 (16–33)	24 (18–38)	*T* = 0.78, *p* = 0.44
LVF (# words)	35 (14–65)	38 (9–61)	*T* = 0.72, *p* = 0.47
PALPA49 (/30)	28 (20–30)	27 (23–29)	*W* = 468, *p* = 0.43
RPM (/60)	45 (22–57)	45 (22–57)	*W* = 515.5, *p* = 0.88
TMT B/A	2.3 (1.3–4.8)	2.4 (1.0–4.8)	*T* = − 0.22, *p* = 0.83
Baseline Centiloid	4.6 (− 13.1–99.8)	7.7 (− 14.8–116.8)	*W* = 620, *p* = 0.23
Baseline amyloid positivity (*N*)	3 (8.8%)	4 (12.9%)	*Χ*^*2*^ = 0.02, *p* = 0.90
Follow-up Centiloid	3.1 (− 16.8–126.9)	7.9 (− 13.4–182.8)	*W* = 666, *p* = 0.07
Follow-up amyloid positivity (*N*)	3 (8.8%)	6 (19.4%)	*Χ*^*2*^ = 0.75, *p* = 0.39
Time interval (years)	5.7 (4.4–8.6)	4.5 (3.4–7.2)	*W* = 272, *p* = 0.0008
Amyloid rate of change	− 0.15 (− 2.8–4.1)	0.86 (− 2.6–13.8)	*W* = 672, *p* = 0.06
Amyloid accumulators (*N*)	5 (14.7%)	7 (22.6%)	*Χ*^*2*^ = 0.25, *p* = 0.62

*Abbreviations*: *AVF *Animal Verbal Fluency Test, *AVLT TL/DR *Rey Auditory Verbal Learning Test total learning/delayed recall, *BDNF *brain-derived neurotrophic factor, *BNT *Boston Naming Test, *BSRT TR/DR *Buschke Selective Reminding Test total retention/delayed recall, *CDR *Clinical Dementia Rating scale, *LVF *Letter Verbal Fluency Test, *MMSE *Mini Mental State Examination, *PALPA49 *Psycholinguistic Assessment of Language Processing in Aphasia (PALPA) subtest 49, *RPM *Raven’s Progressive Matrices, *TMT B/A *Trail Making Test part B divided by part A

### Sequencing mapping and quality control

Importing of sequence data into R and summarising at the gene level resulted in 8,788,419 ± 4,243,830 reads per sample. After filtering and removal of globin genes, there were 4,201,718 ± 1,054,566 reads remaining per sample. There were 19,955 genes remaining after the removal of globin genes and non-protein coding genes for expression analyses.

### Primary analysis: differential gene expression

#### *APOE4* status

**Fig. 2:**
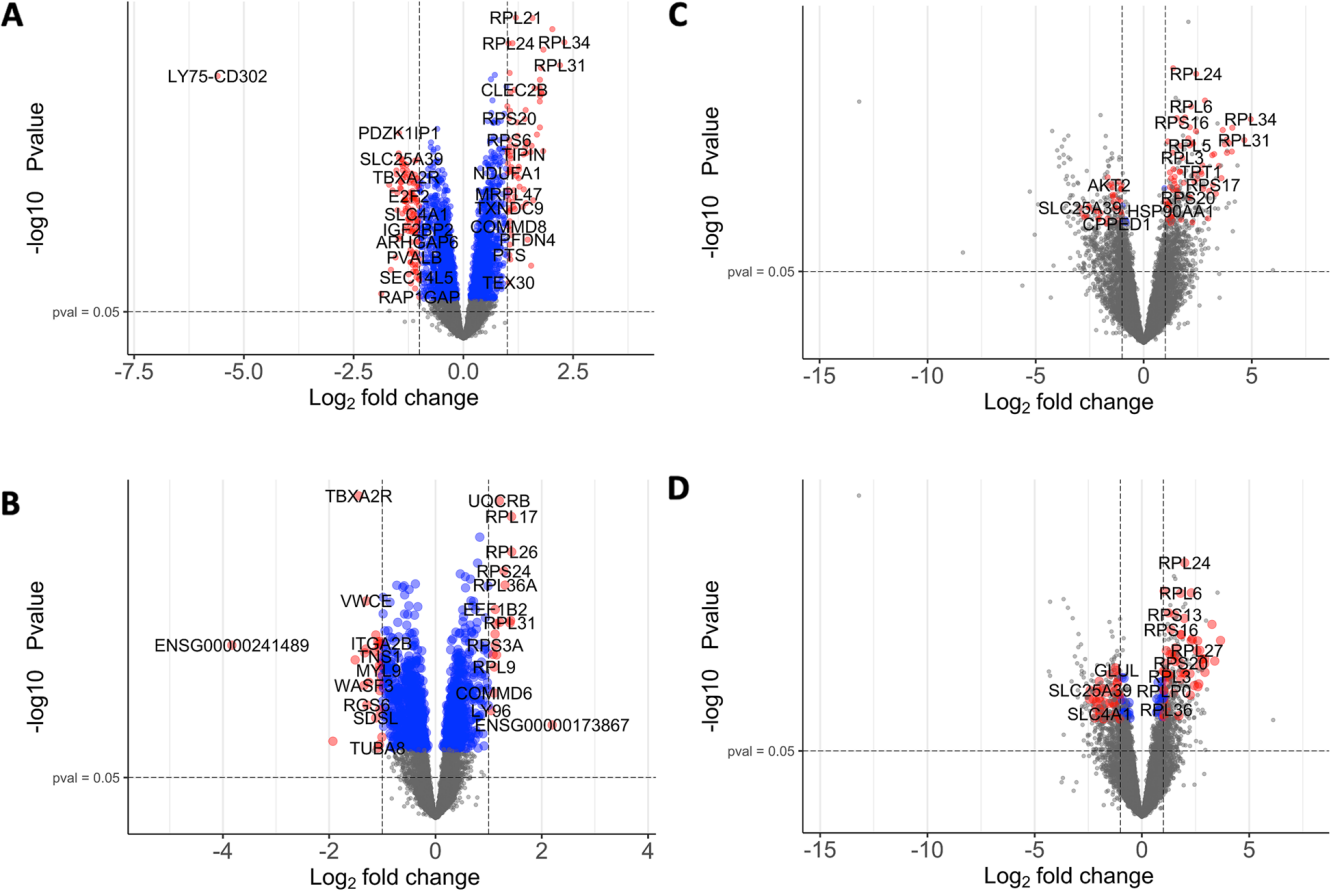
Differentially expressed genes at follow-up compared to baseline stratified for *APOE4* or amyloid positivity status. **A** Volcano plot showing the differentially expressed genes at follow-up compared to baseline in *APOE4* carriers. **B** Volcano plot showing the differentially expressed genes at follow-up compared to baseline in *APOE4* non-carriers. **C** Volcano plot showing the differentially expressed genes at follow-up compared to baseline in individuals amyloid positive at both time points or amyloid negative at baseline, positive at follow-up. **D** Volcano plot showing the differentially expressed genes at follow-up compared to baseline in individuals amyloid negative at both time points. Data points are coloured based on significance: grey = non-significant, blue = non-significant but with *p*_*FDR*_-value < 0.05, red = significant with *p*_*FDR*_-value < 0.05 and log2FoldChange ± 1. *APOE4* carriers *N* = 31, *APOE4* non-carriers *N* = 34. Amyloid positive-positive or amyloid negative–positive *N* = 9, amyloid negative-negative *N* = 56

From the 11,727 genes that passed quality control, there were 201 significant differentially expressed genes at follow-up compared to baseline found in *APOE4* carriers, 166 of which were “unique” to *APOE4* carriers as they were not found to be significantly differentially expressed in *APOE4* non-carriers (Fig. 2A). In contrast, there were 47 significant differentially expressed genes in *APOE4* non-carriers at follow-up compared to baseline, 12 of which were unique to non-carriers (Fig. 2B). Thirty-five genes were significantly differentially expressed at follow-up compared to baseline in both the *APOE4* carrier and non-carrier analyses. The top unique five upregulated and downregulated genes are listed in Table 2 (see Supplementary Tables 1 and 2 for full lists of genes). In the time-series paired analysis, there was only one significant differentially expressed gene that had a significantly different response to time when comparing *APOE4* carriers and non-carriers: *LY75-CD302* (*p*_*FDR*_-value = 0.04, log2FoldChange =  − 4.78). In this analysis, the *p*_*FDR*_-value denotes the significance of the difference between *APOE4* and non-*APOE4* expression profiles at follow-up compared to baseline, and the log2FoldChange is the relative difference between the log2FoldChange in *APOE4* carriers versus non-carriers. In the cross-sectional analyses, from the 12,013 genes that passed quality control, there were no significant differentially expressed genes at baseline nor at follow-up when comparing *APOE4* carriers versus non-carriers (all *p*_*FDR*_-values > 0.05).

**Table 2:** Top unique differentially expressed genes stratified for *APOE4* or amyloid positivity status at follow-up compared to baseline

Direction	*APOE4* carriers			*APOE4* non-carriers		
	**Gene**	**Log2FoldChange**	***P***_***FDR***_**-value**	**Gene**	**Log2FoldChange**	***P***_***FDR***_**-value**
Up	*COX6C*	1.68	3.6 × 10^−8^	*ENSG00000173867*	2.12	1.5 × 10^−2^
	*RPS27*	1.55	9.7 × 10^−8^	*ZNF208*	1.15	6.0 × 10^−4^
	*TOMM5*	1.54	2.6 × 10^−3^	-	-	-
	*COX7B*	1.53	1.5 × 10^−7^	-	-	-
	*RPS17*	1.47	7.0 × 10^−8^	-	-	-
Down	*LY75-CD302*	− 5.60	1.8 × 10^−10^	*ENSG00000241489*	− 3.83	4.0 × 10^−4^
	*ENSG00000268614*	− 1.65	3.8 × 10^−3^	*SHISA7*	− 1.51	7.6 × 10^−4^
	*YPEL4*	− 1.62	3.4 × 10^−6^	*WASF3*	− 1.34	2.3 × 10^−3^
	*SHISA4*	− 1.53	6.4 × 10^−7^	*RNF208*	− 1.33	5.6 × 10^−4^
	*SMIM24*	− 1.52	9.1 × 10^−7^	*PDGFA*	− 1.14	5.3 × 10^−4^
	**Amyloid negative–positive or positive-positive**			**Amyloid negative-negative**		
Up	*RPL17-C18orf32*	3.85	0.01	*FAU*	1.14	0.04
	*EEF1A1*	2.17	0.03	*RPS9*	1.00	0.05
	*RPL14*	1.43	0.02	-	-	-
	*RPS11*	1.34	0.01	-	-	-
	*B2M*	1.30	0.04	-	-	-
Down	*SELENBP1*	− 2.88	0.04	*NRGN*	− 2.44	0.03
	*PDZK1IP1*	− 2.75	0.04	*BCL2L1*	− 2.28	0.03
	*GRINA*	− 2.07	0.05	*ALAS2*	− 2.20	0.05
	*NCF1*	− 1.72	0.05	*DMTN*	− 2.14	0.04
	*AKT2*	− 1.60	0.02	*PSMF1*	− 2.07	0.03

The top five unique upregulated or downregulated genes are shown for each genotype group listed by log2FoldChange values. *APOE4* carriers *N* = 31, *APOE4* non-carriers *N* = 34, amyloid negative–positive or amyloid positive-positive *N* = 9, amyloid negative-negative *N* = 56

After performing the cell deconvolution (Supplementary Table 3), only CD8 T cells were significantly different between *APOE4* carriers and non-carriers at baseline. After including the cell-type contribution as a covariate in the repeated differential gene expression, the results did not change.

#### Amyloid rate of change

From the 11,727 genes that passed quality control, sixty-two genes were found to be significantly differentially expressed at follow-up compared to baseline in amyloid non-accumulators (Supplementary Fig. 1). The top upregulated and downregulated genes can be found in Supplementary Table 4. There were no significant differentially expressed genes in amyloid accumulators at follow-up compared to baseline. In the time-series paired analysis, there were no significant differentially expressed genes in amyloid accumulators versus non-accumulators in response to time. Similarly, no genes were significantly differentially expressed between amyloid accumulators and non-accumulators at baseline nor at follow-up from the 12,013 genes that passed quality control. Likewise, there were no differentially expressed genes in baseline expression data in response to amyloid rate of change as a continuous variable, from the 16,421 that passed quality control. All *p*_*FDR*_-values > 0.05.

Only CD4 memory resting T cells were significantly different at follow-up compared to baseline in amyloid non-accumulators, and results did not change when including this contribution as an additional covariate. Similarly, only monocytes (at baseline) were significantly associated with amyloid rate of change, and including this contribution as an additional covariate did not change the results.

#### Amyloid positivity status

From the 11,727 genes that passed quality control, 107 genes were found to be significantly differentially expressed at follow-up compared to baseline in individuals who were amyloid negative at baseline and positive at follow-up or amyloid positive at both time points (Fig. 2C). Thirty-three of these were unique to these individuals, where the top five unique upregulated and downregulated genes can be found in Table 2. There were 106 significant differentially expressed genes at follow-up compared to baseline in individuals classified as amyloid negative at both time points. Thirty-two of these genes were unique to amyloid negative-negative individuals, where the top five unique upregulated and downregulated genes can be found in Table 2 (Fig. 2D). See Supplementary Tables 5 and 6 for full lists of genes. In the time-series paired analysis, there were no significant differentially expressed genes found. Similarly, no genes were significantly differentially expressed in the cross-sectional analyses at either time point from the 12,013 genes that passed quality control.

After performing the cell deconvolution (Supplementary Table 3), only monocytes were significantly different at follow-up between individuals classified as amyloid negative at both time points versus individuals amyloid negative at baseline-positive at follow-up or positive at both time points. After including the cell-type contribution as a covariate in the repeated analysis, the results did not change.

#### Differentially expressed genes with highest direct association score

From the 166 unique significant differentially expressed genes obtained from *APOE4* carriers over time, we determined the top five genes that had the highest direct association score, based on “gene name” and “Alzheimer disease” as search terms (Supplementary Table 7). These top five genes were *NRGN* (direct association score = 0.11), *IGF2* (direct association score = 0.09), *GMPR* (direct association score = 0.07), *CLDN5* (direct association score = 0.05), and *SMIM24* (direct association score = 0.04). All genes were downregulated in *APOE4* carriers at follow-up compared to baseline: *NRGN* log2FoldChange =  − 1.16 (*p*_*FDR*_ = 1.2 × 10^−4^); *IGF2* log2FoldChange =  − 1.24 (*p*_*FDR*_ = 5.7 × 10^−3^); *GMPR* log2FoldChange =  − 1.41 (*p*_*FDR*_ = 2.73 × 10^−7^); *CDLN5* log2FoldChange =  − 1.21 (*p*_*FDR*_ = 5.57 × 10^−5^); *SMIM24* log2FoldChange =  − 1.52, (*p*_*FDR*_ = 9.07 × 10^−7^).

From the 33 unique significant differentially expressed genes obtained from amyloid negative–positive and amyloid positive-positive individuals over time, the top five genes with the highest direct association score were *RPL17-C18orf32* (direct association score = 0.05, log2FoldChange = 3.85, *p*_*FDR*_ = 0.01), *HSP90AA1* (direct association score = 0.02, log2FoldChange = 1.26, *p*_*FDR*_ = 0.04), *MBP* (direct association score = 0.02, log2FoldChange =  − 1.18, *p*_*FDR*_ = 0.02), *SIRPB1* (direct association score = 0.01, log2FoldChange =  − 1.18, *p*_*FDR*_ = 0.05), and *GRINA* (direct association score = 0.01, log2FoldChange = -2.07, *p*_*FDR*_ = 0.05, Supplementary Table 8).

#### Functional enrichment gene set enrichment analysis

Between follow-up and baseline, there were 118 significantly upregulated GO terms shared by both *APOE4* carriers and non-carriers, which mainly included ribosomal terms (Supplementary Tables 9 and 10). *APOE4* carriers had 40 significantly upregulated GO enrichment terms that were not significantly upregulated in non-carriers between time points. *APOE4* non-carriers had six significantly upregulated GO terms not significantly upregulated in *APOE4* carriers between time points. There were 906 significantly downregulated GO enrichment terms shared by both *APOE4* carriers and non-carriers. *APOE4* carriers had 220 significantly downregulated GO terms not significantly downregulated in non-carriers, whilst *APOE4* non-carriers had 187 that reached significance, not significantly downregulated in carriers. The top five upregulated and downregulated terms can be seen in Table 3 (Supplementary Tables 11 and 12 for full lists of terms).

**Table 3:** Top unique significant Gene Ontology terms stratified for *APOE4* or amyloid positivity status at follow-up compared to baseline

Direction	*APOE4* carriers				*APOE4* non-carriers			
	**GO identifier (Ontology)**	**Description**	***p***_***FDR***_**-value**	**Normalised enrichment score**	**GO identifier (Ontology)**	**Description**	***p***_***FDR***_**-value**	**Normalised enrichment score**
Up	GO:0,005,759 (CC)	Mitochondrial matrix	7.3 × 10^−7^	1.73	-	-	-	-
	GO:0,006,091 (BP)	Generation of precursor metabolites and energy	8.5 × 10^−4^	1.52	-	-	-	-
	GO:0,032,543 (BP)	Mitochondrial translation	1.0 × 10^−3^	1.88	-	-	-	-
	GO:0,005,689 (CC)	U12-type spliceosomal complex	1.4 × 10^−3^	1.97	-	-	-	-
	GO:0,006,457 (CC)	Protein folding	2.0 × 10^−3^	1.64	-	-	-	-
Down	GO:0,099,537 (BP)	Trans-synaptic signalling	1.1 × 10^−5^	− 1.68	GO:0,110,020 (BP)	Regulation of actomyosin structure organisation	8.1 × 10^−5^	− 1.99
	GO:0,099,177 (BP)	Regulation of trans-synaptic signalling	2.3 × 10^−4^	− 1.65	GO:0,032,231 (BP)	Regulation of actin filament bundle assembly	1.2 × 10^−4^	− 1.95
	GO:0,016,323 (CC)	Basolateral plasma membrane	2.4 × 10^−4^	− 1.78	GO:0,051,492 (BP)	Regulation of stress fibre assembly	1.3 × 10^−4^	− 1.97
	GO:0,099,003 (BP)	Vesicle-mediated transport in synapse	7.1 × 10^−4^	− 1.76	GO:0,046,939 (BP)	Nucleotide phosphorylation	2.8 × 10^−4^	− 1.86
	GO:0,042,581 (CC)	Specific granule	8.0 × 10^−4^	− 1.71	GO:0,006,165 (BP)	Nucleoside diphosphate phosphorylation	4.7 × 10^−4^	− 1.88
	**Amyloid negative–positive and amyloid-positive**				**Amyloid negative-negative**			
Up	GO:0,046,034 (BP)	ATP metabolic process	6.5 × 10^−3^	1.50	GO:0,071,013 (CC)	Catalytic step 2 spliceosome	7.8 × 10^−3^	1.66
	GO:0,005,925 (CC)	Focal adhesion	6.5 × 10^−3^	1.40	-	-	-	-
	GO:0,030,055 (CC)	Cell-substrate junction	9.91 × 10^−3^	1.40	-	-	-	-
	-	-	-	-	-	-	-	-
	-	-	-	-	-	-	-	-
Down	-	-	-	-	GO:0,014,855 (BP)	Striated muscle cell proliferation	6.81 × 10^−3^	− 1.80
	-	-	-	-	-	-	-	-
	-	-	-	-	-	-	-	-
	-	-	-	-	-	-	-	-
	-	-	-	-	-	-	-	-

The top five significant upregulated or downregulated gene set enrichment analysis GO are shown for each genotype group listed by *p*_*FDR*_-values, if the term had a *p*_*FDR*_-value < 0.01. *APOE4* carriers N, 31, *APOE4* non-carriers N, 34, amyloid negative–positive or amyloid positive-positive N, 9, amyloid negative-negative N, 56. *Abbreviations*: *CC *cellular compartment, *BP *biological processes, *MF *molecular function. The normalised enrichment score accounts for size differences in gene sets and correlations between gene sets and the expression dataset to allow direct comparisons across gene set results. A negative score means the enrichment is downregulated

Amyloid non-accumulators had 182 significantly upregulated GO enrichment terms, and 1071 significantly downregulated GO enrichment terms at follow-up compared to baseline (Supplementary Tables 13 and 14).

Between follow-up and baseline, there were 192 significantly upregulated GO terms shared by both amyloid negative-negative and amyloid negative–positive/amyloid positive-positive participants, which mainly included ribosomal and metabolism terms. Amyloid negative–positive/amyloid positive-positive individuals had 20 significantly upregulated GO enrichment terms that were not significantly upregulated in amyloid negative-negative participants between time points. Amyloid negative-negative participants had 14 unique significantly upregulated GO terms. See Supplementary Tables 15 and 16 for full list of terms. There were 1301 significantly downregulated GO enrichment terms shared by both amyloid negative-negative and amyloid negative–positive/amyloid positive-positive participants. Amyloid negative–positive/amyloid positive-positive individuals had 105 unique significantly downregulated GO terms, whilst amyloid negative-negative had 212 that reached significance, at follow-up compared to baseline (Supplementary Tables 17 and 18). The top five unique upregulated and downregulated terms can be seen in Table 3.

### Secondary analyses: weighted gene co-expression network analysis

#### Baseline expression data

In order to determine whether networks of co-expressed genes were significantly associated with our traits of interest (*APOE4* status, amyloid rate of change as a continuous variable, amyloid accumulator status as a binary variable, and amyloid positivity status), a baseline gene co-expression network was built using 9200 genes that passed quality control. Sample clustering highlighted two outliers, leaving 63 samples for network construction. A soft power threshold of 11 was selected based on the scale independence and mean connectivity (Supplementary Fig. 2A, B). Using the dynamic tree cutting method, 28 co-expression modules were derived ranging in size from 53 to 4538 genes. Similar modules were merged resulting in the final 17 co-expression modules (Supplementary Fig. 2C, D).

**Fig. 3:**
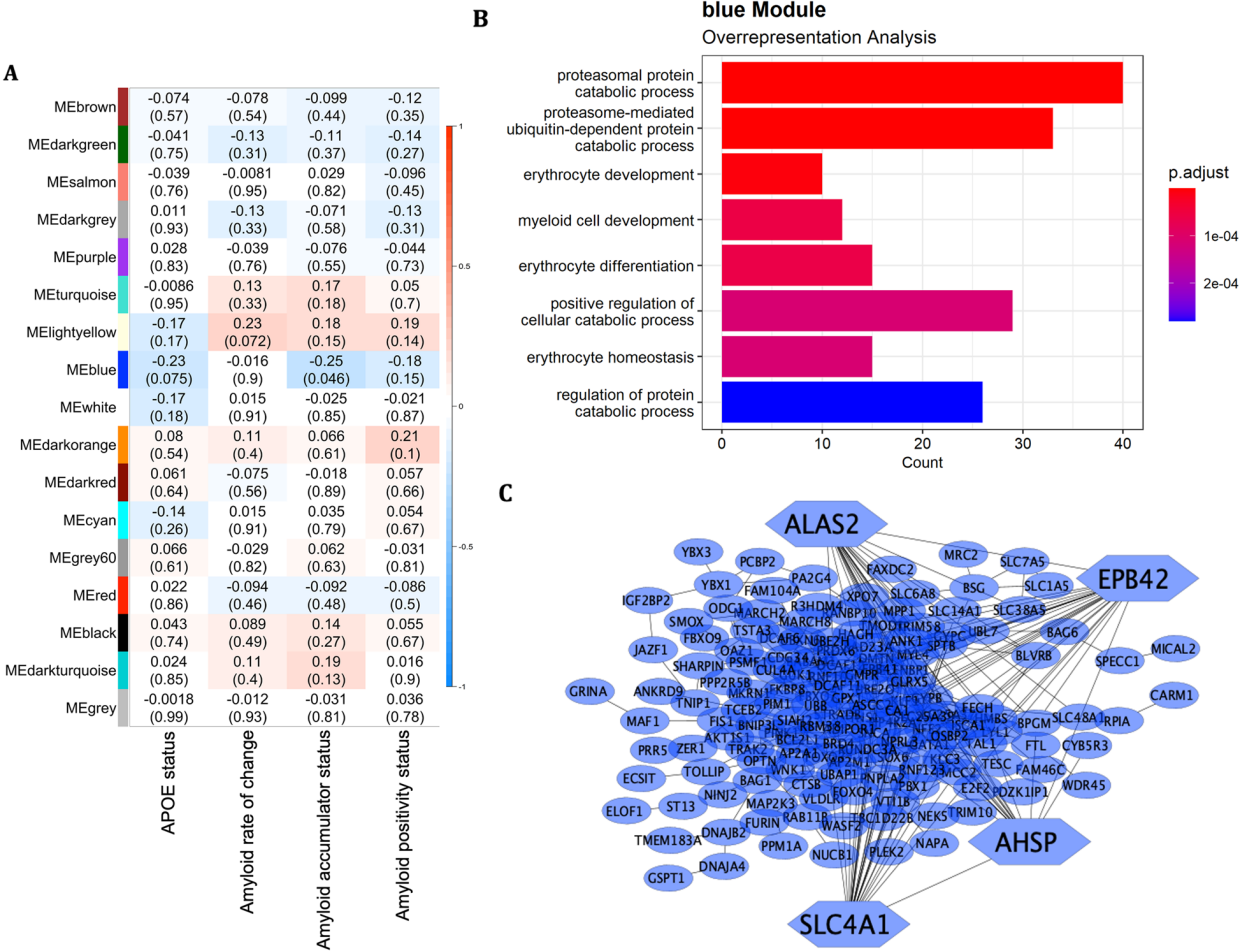
WGCNA of baseline expression data. **A** Correlation heatmap of traits of interest with WGCNA modules depicting the Pearson’s correlation coefficient with the *p*-value in brackets. **B** Gene ontology enrichment terms derived from over-representation analysis. **C** Protein–protein interaction network of highly interconnected genes with hub genes shaped as hexagonal

One module was significantly associated with amyloid accumulator status (blue module, suppressed network: *r*^2^ =  − 0.25, *p* = 0.046, Fig. 3A), meaning amyloid accumulators had a suppression of this module compared to non-accumulators. Top GO enrichment processes included proteasomal protein catabolic process (GO:0,010,498, *p*_*FDR*_-value = 3.4 × 10^−9^), proteasome-mediated ubiquitin-dependent protein catabolic process (GO:0,043,161, *p*_*FDR*_-value = 2.5 × 10^−7^), and myeloid cell development (GO:0,061,515, *p*_*FDR*_-value = 5.3 × 10^−5^, Fig. 3B, Supplementary Table 19). Highly interconnected genes were extracted and used to create the PPI network in Fig. 3C. From these genes, the top four hub genes were selected with the highest MCC: *SLC4A1* (MCC = 3224), *ALAS2* (MCC = 2389), *EPB42* (MCC = 2377), and *AHSP* (MCC = 2235). Furthermore, cell-type-specific enrichment analysis of these highly interconnected genes showed that they were mainly related to erythrocytes (Supplementary Fig. 3). Upstream regulators of this module included *KLF1* (score = 1.4), *GATA1* (score = 3.0), *TAL1* (score = 3.3), *GFI1B* (score = 12.4), and *MXI1* (score = 15.8). Furthermore, this module contained *SHISA4* and *SMIM24*, significantly downregulated genes in the primary analysis in response to *APOE4* carriership, and *SELENBP1*, *PDZKIP1*, and *GRINA*, significantly downregulated genes in the primary analyses in response to a positive amyloid status (Table 2). No modules were significantly correlated with *APOE4* status, amyloid rate of change as a continuous variable, or amyloid positivity status.

#### Follow-up expression data

To determine whether co-expression networks at follow-up were associated with our traits of interest, a gene co-expression network was built using 9200 genes that passed quality control from the follow-up expression data. Sample clustering highlighted two outliers, leaving 63 samples for network construction. A soft power threshold of five was selected based on the scale independence and mean connectivity (Supplementary Fig. 4A, B). Using the dynamic tree cutting method, 46 co-expression modules were derived ranging in size from 32 to 1668 genes. Similar modules were merged resulting in the final 35 co-expression modules (Supplementary Fig. 4C, D).

**Fig. 4:**
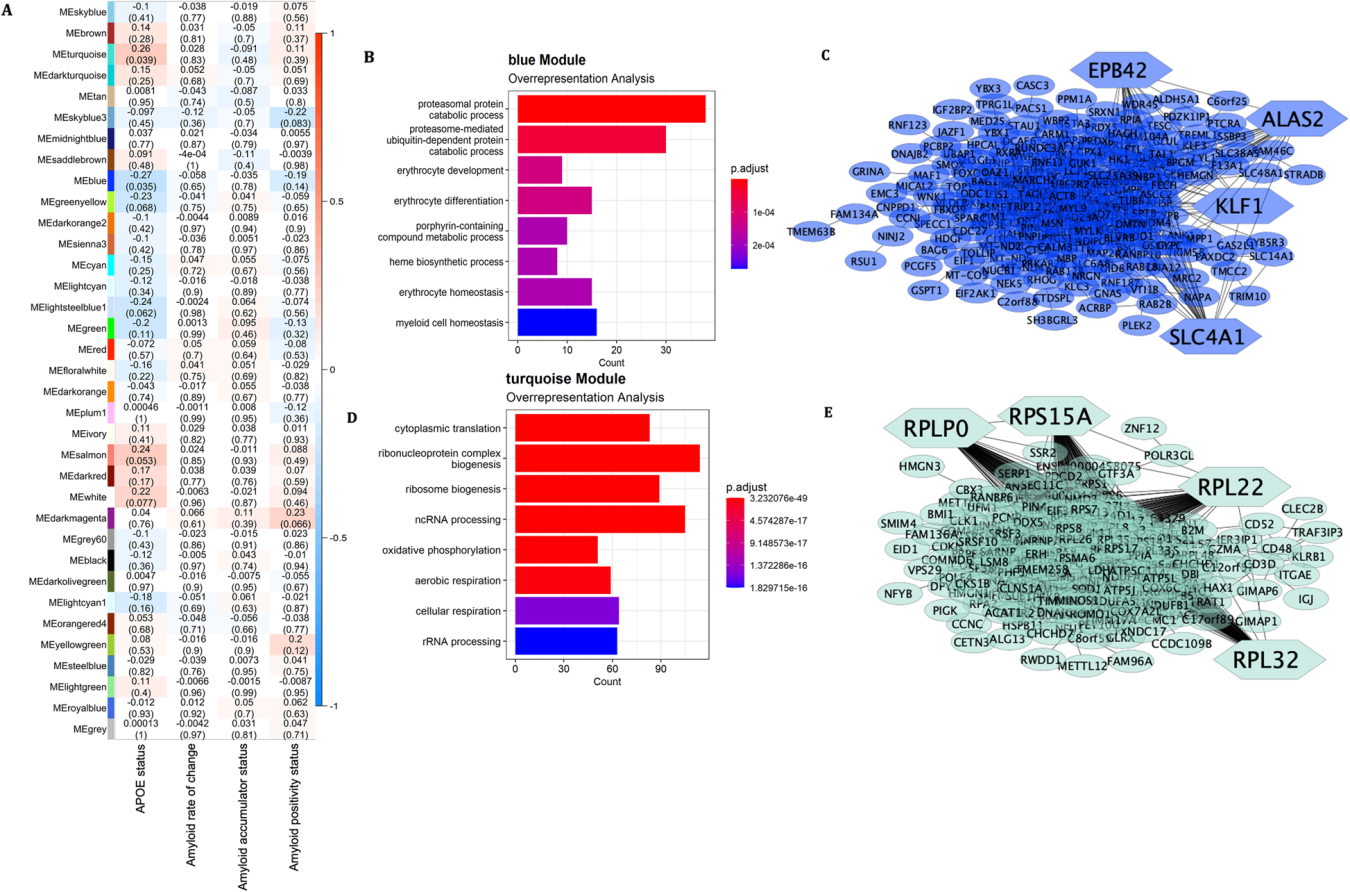
WGCNA of follow-up expression data. **A** Correlation heatmap of traits of interest with WGCNA modules depicting the Pearson’s correlation coefficient and the *p*-value in brackets. **B** Gene ontology enrichment terms derived from over-representation analysis for the blue module. **C** Protein–protein interaction network of highly interconnected genes with hub genes shaped as hexagonal for the blue module. **D** Gene ontology enrichment terms derived from over-representation analysis for the turquoise module. **E** Protein–protein interaction network of highly interconnected genes with hub genes shaped as hexagonal for the turquoise module. Note the differing sliding colour scale for the enrichment plots

Two modules were significantly associated with *APOE4* status: blue (downregulated network, *r*^2^ =  − 0.27, *p* = 0.035) and turquoise (upregulated network: *r*^2^ = 0.26, *p* = 0.039, Fig. 4A). Top GO enrichment processes for the blue module included proteasomal protein catabolic process (GO:0,010,498, *p*_*FDR*_-value = 1.9 × 10^−7^) and proteasome-mediated ubiquitin-dependent protein catabolic process (GO:0,043,161, *p*_*FDR*_-value = 9.0 × 10^−6^, Fig. 4B, Supplementary Table 20). Highly interconnected genes were extracted and used to create the PPI network in Fig. 4C. From these genes, the top four hub genes were selected with the highest MCC: *SLC4A1* (MCC = 2830), *EPB42* (MCC = 1969), *ALAS2* (MCC = 1929), and *KLF1* (MCC = 1844). Furthermore, cell-type-specific enrichment analysis of these highly interconnected genes showed that genes were mainly related to erythrocytes, as well as further cell-types such as megakaryocyte, haematopoietic stem cell, and epithelial cell (Supplementary Fig. 5). Upstream regulators of this module included *KLF1* (score = 2.2), *GATA1* (score = 2.5), *TAL1* (score = 2.7), *GFI1B* (score = 16.2), *MXI1* (score = 18.8), and *MAZ* (score = 39.6). Moreover, this module contained *SHISA4* and *SMIM24*, significantly downregulated genes in the primary analysis in response to *APOE4* carriership, and *SELENBP1*, *PDZKIP1*, and *GRINA*, significantly downregulated genes in the primary analyses in response to a positive amyloid status (Table 2). These genes were also found to be suppressed in the baseline WGCNA in response to amyloid rate of change.

Top GO enrichment processes for the turquoise module included cytoplasmic translation (GO:0,002,181, *p*_*FDR*_-value = 3.1 × 10^−49^) and ribonucleoprotein complex biogenesis (GO:0,022,613, *p*_*FDR*_-value = 1.2 × 10^−26^, Fig. 4D, Supplementary Table 21). Highly interconnected genes were extracted and used to create the PPI network in Fig. 4E. From these genes, the top four hub genes were selected with the highest MCC: *RPL22* (MCC = 9.2 × 10^13^), *RPS15A* (MCC = 9.2 × 10^13^), *RPL32* (MCC = 9.2 × 10^13^), and *RPLP0* (MCC = 9.2 × 10^13^). Furthermore, cell-type-specific enrichment analysis of these highly interconnected genes showed that they were related to T cells, followed by epithelial cells, monocytes, erythroid progenitor cell, and B cells. There were also other cell-types enriched, although to a lesser extent, such as natural killer cell, neutrophil and dendritic cell (Supplementary Fig. 6). ChEA3 identified *CEBPZ* as an upstream regulator (score = 48.6). Lastly, this module contained *COX6C*, *RPS27*, *COX7b*, and *RPS17*, which were significantly upregulated genes in the primary analysis in response to *APOE4* carriership, as well as *RPL17-C18orf32*, *EEF1A1*, *RPL14*, *RPS11*, and *B2M*, which were significantly upregulated genes in the primary analysis in response to a positive amyloid status (Table 2).

No modules were significantly correlated with amyloid rate of change as a continuous variable, amyloid accumulator status, or amyloid positivity status.

### Module preservation over time and consensus analyses

**Fig. 5:**
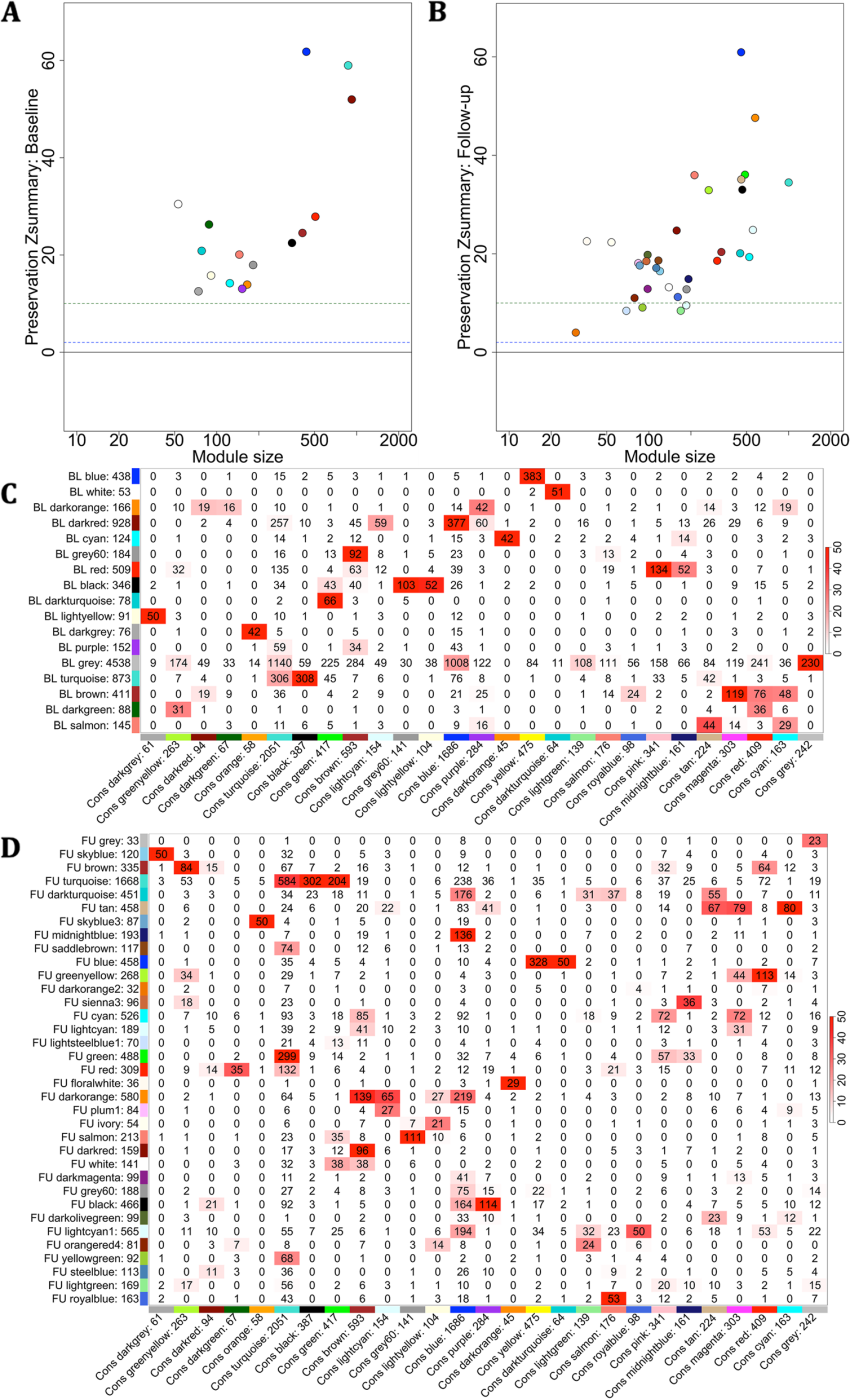
Module preservation and consensus of baseline or follow-up co-expression modules. *Zsummary* scores of **A** baseline WGCNA modules in follow-up expression data and **B** follow-up co-expression modules in baseline expression data. A *Zsummary* < 2 suggests low preservation (below blue dotted line); > 2 *Zsummary* < 10 suggests moderate preservation; *Zsummary* > 10 suggests high preservation (above green dotted line). Correspondence of **C** baseline set-specific modules or **D** follow-up set-specific modules with consensus modules. Each row is the set-specific module and each column is a consensus module. The more red the cell, the more significant the Fisher’s exact *p*-value (encoded by -log(*p*) signifying the gene overlap. For module labels in plots A and B for the preservation analyses please see y-axes module

All baseline WGCNA co-expression modules were well preserved in follow-up expression data (*Zsummary* > 10, Fig. 5A). Five follow-up co-expression modules were moderately preserved in baseline expression data (> 2 *Zsummary* < 10). All other modules were well preserved (Fig. 5B). No modules were lowly preserved in either analysis.

A soft threshold of 5 was chosen for consensus network construction based on the mean independence and scale connectivity (Supplementary Fig. 7A), which resulted in 27 consensus modules (Supplementary Fig. 7B). Pairwise Fisher’s exact tests showed that the genes within the blue WGCNA module at baseline and the blue WGCNA module at follow-up were significantly overlapping, as shown by the consensus yellow module -log(*p*) values of 383 and 328, respectively, in Fig. 5B, C.

## Discussion

Our study highlighted that peripheral whole-blood RNA sequencing detects longitudinal differences in single gene expression, as well gene co-expression networks, enriched for upregulated mitochondrial and ribosomal pathways and downregulated immune and proteasomal pathways, in those individuals that are at risk of developing AD (*APOE4* carriers or amyloid accumulators). Several of the genes and pathways highlighted from this study have already been implicated in AD pathogenesis from both post mortem brain and peripheral blood case–control studies.

Studies investigating gene expression allow for studying the functional changes at the RNA level occurring as a result of AD. Many transcriptional studies have been performed in case–control datasets using post mortem brain tissue or cell lines, highlighting dysfunctions in calcium signalling, (neuro)inflammation, metabolism, ribosomal function, and DNA repair in cases compared to controls [7–9, 51–54]. More recently, blood has been proposed as a surrogate tissue for transcriptomic studies since gene expression in the blood has a large overlap with gene expression in the brain [55, 56]. Moreover, blood is more easily obtainable than brain tissue and enables serial studies. Similarly, AD case–control peripheral blood studies have shown that AD cases have dysregulation in mitochondrial and ribosomal pathways, as well as apoptosis [11, 57].

A recent study investigating gene expression differences using lymphoblastoid cells and brain tissue found that there was a higher number of differentially expressed genes in Alzheimer’s cases (sporadic early-onset or autosomal dominant AD) compared to controls [58]. Enrichment for these differentially expressed genes showed downregulation of mitochondrial pathways and synaptic signalling, as well as upregulation of immune-related pathways. Our study can be considered an extension of this study, but in blood and in the asymptomatic phase. Despite the cell-types that were significantly associated with our differential gene expression contrasts, when we accounted for these in the analyses, our results were not influenced. Therefore, our study complements these previous results by also highlighting a higher number of significant differentially expressed genes in those individuals at a higher risk of developing AD, which are enriched for similar pathways. Specifically, we found 201 significant differentially expressed genes in *APOE4* carriers at follow-up compared to baseline (47 in non-carriers), 121 of which were downregulated. Unique downregulated terms in *APOE4* carriers were enriched for trans-synaptic signalling, similar to the previous study. Furthermore, unique upregulated terms in *APOE4* carriers were enriched for mitochondrial pathways and metabolism, which were also upregulated pathways observed in individuals classified as amyloid negative at baseline and positive at follow-up or positive at both time points. These results, therefore, suggest that the synaptic and metabolic changes present in those with AD are already occurring in those at a higher risk of developing the disease (given they carry *APOE4* and accumulate in amyloid) prior to developing symptoms. These results observed in our study are corroborated by altered glucose metabolism in AD case–control studies, as measured with fluro-2-deoxyglucose PET, for example, perhaps due to impaired mitochondrial machinery, or increased oxidative stress (as reviewed in [59]). Similarly, a recent neuropathological study analysing brain homogenate from the AD spectrum (individuals without neurodegeneration, preclinical AD, and symptomatic AD) found that vesicle endocytosis and the secretory pathway are key pathways that are altered in early AD pathogenesis [60]. We confirm the disruption of such pathways that are significantly downregulated in *APOE4* carriers (vesicle-mediated transport in synapse) that were not significantly downregulated in non-carriers. It is important to note, however, that these changes may also be present in non-carriers, but below the threshold for detection.

In addition to the similar enrichment pathways found in case–control studies and our differential gene expression results, some of the specific top unique significant differentially expressed genes within these enrichments found in *APOE4* carriers have already been implicated in AD. *COX6C* and *COX7b* are part of Complex IV in the electron transport chain of oxidative phosphorylation and have been found to be downregulated in blood of AD cases compared to controls [61]. We found an upregulation of these genes, potentially due to the different stages of AD being analysed: we are examining changes in an asymptomatic population, which may be potentially highlighting an upregulation prior to symptom onset, followed by a downregulation once the disease has progressed. However, with our data, we cannot exclude that with AD cases results may be different. *RPS27* and *RPS17* are part of the 40S ribosomal subunit and were previously found to be differentially expressed in AD cases compared to controls, where they were also identified as potential hub genes in a blood-based transcriptomic analysis [57]. *YPEL4*, which was downregulated in *APOE4* carriers, is predicted to enable metal ion binding activity and has recently been found to be downregulated in AD brain compared to controls [62]. Finally, *SHISA4*, a component of membranes, was another gene downregulated in *APOE4* carriers that has recently been found to be downregulated in mildly cognitively impaired and AD individuals in blood expression analyses using Alzheimer’s Disease Neuroimaging Initiative data [63].

Similarly, some of the genes that were significantly upregulated or downregulated in individuals classified as amyloid negative at baseline and positive at follow-up or amyloid positive at both time points have also been implicated in the AD literature. *EEF1A* is a eukaryotic elongation factor that has previously been shown to have reduced expression in AD brain regions such as CA1 [64]. *RPL14* was identified recently as a potential hub gene in AD patients compared with controls using temporal cortex Gene Expression Omnibus expression data [65]. *RPS11* was shown to be a methylation site in AD [66], which has more recently also been found to also be a methylation site in a Chinese population [67], thereby potentially affecting subsequent gene expression. Cognition has been shown to be negatively regulated by *B2M* in both healthy ageing and in stages of cognitive decline, including dementia [68]. Finally, *NCF1* was significantly overexpressed in blood of AD patients, which may be involved in an increased production of reactive oxygen species in AD [69].

Those significant differentially expressed genes described above are selected based on statistical results from the paired expression analysis, i.e. having the largest log2FoldChange. However, an alternative method for gene selection can be by using direct association scores, which are generated by relying on known associations between a given gene and disease from a priori literature evidence, e.g. using Open Targets. The top significant differentially expressed genes in *APOE4* carriers with the highest direct association scores generated from the DISQOVER platform were *NRGN*, *IGF2*, *GMPR*, *CLDN5*, and *SMIM24*. *NRGN* has been thoroughly studied as a potential AD biomarker in blood and cerebrospinal fluid [70, 71]. These studies show that *NRGN* levels are increased in AD individuals compared with controls, whilst in the current gene expression study levels were decreased. *IGF-2* has been implicated in AD for several decades based on an AD mouse model study [72], where it was shown to have a critical role in memory consolidation. This study also showed that *IGF-2* expression was decreased in the hippocampus of those individuals with AD, similar to the decreased expression we observe in *APOE4* carriers. *GMPR* is involved in nucleotide metabolism and was found to increase as AD progresses in a post mortem brain case–control differential expression analysis study [73]. *CLDN5* is an important component of endothelial cell tight junctions constituting the blood brain barrier. In a recent study, *CLDN5* methylation has been shown to be associated with cognitive decline in the Religious Order Study-Rush Memory and Aging Project study [74]. Hypermethylation of *CDLN5* occurs in dorsolateral prefrontal cortex in association with episodic memory and working memory decline; however, this association was only partly mediated by AD neuropathological changes. Furthermore, this also occurred in cases with low or no Alzheimer’s neuropathology, and there was no relationship between the hypermethylation and gene expression. Here, we show decreased expression levels of *CLDN5* over time in *APOE4* carriers, some of whom have brain amyloid pathology. Lastly, *SMIM24* is predicted to be a membrane component, where it was identified as a genome-wide significant single nucleotide polymorphism in an Alzheimer’s genome-wide association study [75].

Of the significant differentially expressed genes in the amyloid status-based primary analysis, the top five genes with highest direct association score were *RPL17-C18orf32*, *HSP90AA1*, *MBP*, *SIRPB1*, and *GRINA*. In a recent AD case–control study characterising hippocampal subfields, stereological data showed that the chaperone *HSP90AA1* protein was downregulated in AD and associated with astrocytes, where the authors hypothesised that this is related to chaperone-mediated autophagy of amyloid and tau within AD [76]. Disruption of cerebral white matter in AD is not a new concept, where myelin breakdown is often observed. An AD post mortem brain study has previously shown that the protein encoded by *MBP* (myelin basic protein) was located at the margins of amyloid plaques, suggesting an interaction between the two. Furthermore, the authors provide evidence of myelin injury in the AD brain by means of increased levels of LC3B and MBP [76]. The *SIRBP1* protein has been shown to be upregulated in AD brain, where knockdown in primary microglial cells resulted in impaired amyloid phagocytosis, highlighting a potential role for this protein in amyloid removal in AD [77]. Finally, *GRINA* encodes an NMDA receptor subunit, dysregulation of which has been linked to AD via calcium signalling alterations, as well as activation of NMDA receptors resulting in amyloid-induced mitochondrial toxicity and neuronal dysfunction when this activation is mediated by amyloid (reviewed in [78]).

Aside from those top genes discussed above, there were also other significant differentially expressed genes associated with AD found in *APOE4* carriers that had direct association scores not present in the results. These did not meet the requirements for being top genes, but nonetheless they have previously been implicated in AD. Based on gene expression data from brain tissue in cases versus controls, Brooks and Mias (2019) derived the top 25 upregulated or downregulated genes [79]. Among the latter list, three genes were also present in the current analysis: *LSM3* (ranked 14th), *COX7B* (ranked 8th) and *MRPS18C* (ranked 13th) [79]. Whilst they are downregulated in Alzheimer’s brain tissue, all three were significantly upregulated in blood from baseline to follow-up in *APOE4* carriers. Furthermore, based on open gene expression datasets, Tao et al. (2020) found several major pathogenic genes in AD and mild cognitively impaired individuals that were also bridge genes [80]. Four of these were significantly upregulated over time in *APOE4* carriers in the current study: *LSM3*, *RPS3A*, *S100A8*, and *SNRPG*. All four genes are closely related to spliceosomal and ribosomal function. Despite the expression of these four genes being decreased in the previous study, the expression of these genes in the current study was increased in the asymptomatic *APOE4* carriers over time.

It must be noted, however, that there was only one gene significantly differentially expressed in the time-series paired analysis involving *APOE4* carriers and non-carriers: *LY75-CD302*, a readthrough transcript of the two neighbouring parent genes *LY75* and *CD302*. Although both parent genes have known roles (*LY75* in the immune response as an endocytic receptor [81], and *CD302* in cell migration and endo- and phagocytosis as a lectin receptor [82]), the readthrough transcript is less well annotated. Similarly, the readthrough gene *RPL17-C18orf32* was obtained as a top differentially expressed gene in the amyloid-related analysis. Although *RPL17* is known to encode a protein that is part of the 60S ribosomal subunit [83], and *C18orf32* is thought to activate the NF-kappa-B signalling pathway [84], the readthrough transcript is also less well annotated. Readthrough genes are a relatively new phenomenon [85], and their functions are not yet well known or documented. Furthermore, with the short-read 3′ QuantSeq library prep and sequencing we performed, it is difficult to attribute readthrough genes when mapping. Therefore, these results should be interpreted with care.

It remains to be said that several studies based on gene expression analyses of brain samples of AD cases and controls, e.g. based on the Gene Expression Omnibus data, did not show any obvious overlap in terms of gene expression with our study [86]. This is also true for some of the studies based on AD case–control brain studies from academic cohorts [87]. This may be due to the difference in study population used here compared to those published (cognitively intact versus patient cohorts), the difference between brain and blood samples, and the challenges of replicability in complex datasets.

Those genes and pathways that are shared with *APOE4* carriers and non-carriers have more significant *p*_*FDR*_-values and larger log2FoldChange values in *APOE4* carriers. This dysregulation from our differential gene expression analyses was substantiated by the follow-up WGCNA analyses. The follow-up WGCNA highlights a significant activation of the turquoise module in *APOE4* carriers compared to non-carriers at follow-up. This module was enriched for similar pathways as with the differential gene expression analysis (oxidative phosphorylation, cytoplasmic translation), where hub genes were centred around ribosomal genes, highlighting ribosomal activity dysregulation in an early disease stage. Furthermore, *CEBPZ* was determined to be a potential upstream regulator of this module. Other members of the *CEBP* family have been implicated in AD, where it is suggested they play a role in altered transcription regulation (also in response to amyloid) [88–90]. Our results overall suggest that carriership of the *APOE4* AD risk allele has an exacerbating effect on single gene and network expression changes over time in a stage where individuals are still cognitively intact.

We have shown that with amyloid accumulation as a binary trait we see a significant downregulation of the blue module at baseline in those that are categorised as (future) accumulators. This module was enriched for proteasomal protein catabolic processes and myeloid cell development. This suggests that those individuals who will accumulate amyloid already have downregulated proteasomal processing and a reduced immune system function prior to this future increase. In particular, proteasomal and ubiquitin processes have already been suggested to be dysregulated in AD [10, 91, 92]. Hub gene analysis of this module highlighted several genes to be centred around erythrocytes and haemoglobin. To accompany this, the cell-type enrichment also showed that the genes within this module were related to erythrocytes, suggesting an association with amyloid accumulation. This association was also observed with *APOE4* carriership, given the profile of the blue module at follow-up was largely similar. Altered metabolism of erythrocytes has been suggested to be a potential AD risk factor, given there are cerebral blood flow alterations, vascular pathology, and altered glucose metabolism associated with AD (reviewed in [93, 94]). Our results do support altered erythrocyte function in our cohort of cognitively intact older adults, some of whom are already presenting with abnormal brain amyloid levels. Furthermore, the identified transcription factors are also involved in erythroid development, differentiation, or maturation: *GATA1* and *TAL1* were identified as potential upstream regulators, where both have been previously implicated as AD risk genes in a genome-wide meta-analysis [95]. Furthermore, *GFI1B* has been shown to be downregulated in an AD in silico study in response to epigenetic modifications [96]. This subsequently resulted in interrupted gene expression, further highlighting the importance of upstream regulators in differential gene expression [96]. Lastly, *MXI1* has been recently labelled an age-related gene associated with AD that had a high performance at discriminating between AD cases from controls and correlated with pathological progression of AD by means of tau load [97]. Altogether, these results have shown the dysregulation of known AD-associated genes and pathways, as well as regulatory transcription factors in asymptomatic individuals.

The differential gene expression and WGCNA results are not independent: many of the genes present in the primary analyses in response to *APOE4* carriership or amyloid positivity were also present in the modules in the secondary network analyses. This shared profile of transcription dysregulation is further evident when comparing the genes and enrichment profiles of the blue module at baseline and the blue module at follow-up. At baseline, the blue module was significantly correlated with amyloid accumulator status, whereas at follow-up, the blue module was significantly correlated with *APOE4* status. The baseline blue module was also the most well-preserved module in follow-up expression data and vice versa, as observed in the preservation analyses. This was further substantiated by the consensus analyses, in which there is a large overlap of genes in both blue modules, shown by the significant pairwise overlap. This highlights the similar transcriptional profiles associated with these two traits over time that appear to be dysregulated in association with known AD risk factors.

### Limitations

Our study analysed bulk RNA sequencing data from whole-blood that did not take into consideration all species of RNA. Many of these other RNA species (e.g. long non-coding RNA or microRNA) are often regulatory, but we were not able to determine whether these are having an effect in our study population due to the type of sequencing performed. Furthermore, the modules and genes found to have altered expression may be driven by healthy ageing since we are not investigating a clinical AD population. However, many of the genes and pathways we found have also been previously implicated in AD. Additionally, in the differential gene expression, we included age and sex as covariates, and in the WGCNA analyses, we correlated the modules with age and sex, and these did not have a significant association with those that did have a significant association with *APOE4* and amyloid accumulation. Therefore, it is less likely these gene co-expression networks are being driven by those other factors. It is, however, important to highlight that correction for multiple comparisons was not performed for the WGCNA module-trait associations, and the significant associations we found would not survive this. They should therefore be considered as hypothesis-generating, so require further confirmation. Finally, some expression analyses did not yield any significant results potentially due to the sample size; hence, increasing this may result in a higher statistical power and more significance. However, the lack of significance for some analyses may be due to the investigation of gene expression in peripheral blood; thus, some differences may not be observed in the periphery due to biological reasons.

## Conclusion

*APOE4* status has already been shown to modify transcription in older adults as well as those with AD [98, 99], and our study adds to this by highlighting transcriptional differences in blood in both upregulated and downregulated genes, co-expression networks, and enrichment terms in *APOE4* carriers who are still cognitively intact. We show that carriage of *APOE4* exacerbates differences in peripheral gene expression, where carriers have a significant downregulation in the proteasome and myeloid cell development and a significant upregulation in oxidative phosphorylation and ribosomal pathways, compared to non-carriers. These downregulated pathways seen in *APOE4* carriers are also shown to be downregulated in those individuals who accumulate in amyloid, compared to those who do not. Consequently, the results implicate a disrupted immune system, protein removal, and metabolism in the asymptomatic phase of AD, particularly in those individuals who are at higher risk of developing the disease. This provides insight into the pathophysiology of AD in blood, whilst providing potential targets for drug and therapeutic development, and potential blood biomarkers.

## Supplementary Information

**Additional file 1: Supplementary Figure 1.** Differentially expressed genes at follow-up compared to baseline in amyloid non-accumulators. Data points are based coloured on significance: grey = non-significant, blue = non-significant but with FDR *p*-value < 0.05, red = significant with FDR *p*-value < 0.05 and log2FoldChange ± 1. *N* = 53. Pval on *y*-axis represents the uncorrected *p*-value < 0.05 threshold for visualisation. **Supplementary Figure 2.** Detection of modules using baseline expression data. (A) Scale independence and (B) mean connectivity used to derive the soft power threshold. (C) Clustering of module eigengenes, where similar clusters were merged using a cutHeight =0.25 (red line). (D) Cluster dendrogram of co-expression modules shown, both with the 28 dynamic modules (top row) and final merged 17 modules (bottom row). **Supplementary Figure 3.** Top 20 general cell types derived from cell-specific enrichment of the highly interconnected genes from the baseline blue WGCNA module. The genes are displayed from left to right ranked by the most significant human tissue-cell-type. The red dotted line is the Bonferronicorrected significance (*p* = 3.69 × 10 × 10^−5^) by 1355 tissue-cell types. The grey line is the nominal significance (*p* = 1 × 10 × 10^−3^). The *Y*-axis indicates the tissue-cell-type specificity (–log10 (combined *p* value)) for each tissue-cell-type from the cell-specific enrichment. **Supplementary Figure 4.** Detection of modules using follow-up expression data. (A) Scale independence and (B) mean connectivity used to derive the soft power threshold. (C) Clustering of module eigengenes, where similar clusters were merged using a cutHeight =0.25 (red line). (D) Cluster dendrogram of co-expression modules shown, both with the 32 dynamic modules (top row) and final merged 35 modules (bottom row). **Supplementary Figure 5.** Top 20 general cell types derived from cell-specific enrichment of the highly interconnected genes from the follow-up blue WGCNA module. The genes are displayed from left to right ranked by the most significant human tissue-cell-type. The red dotted line is the Bonferronicorrected significance (*p* = 3.69 × 10 × 10^−5^) by 1355 tissue-cell types. The grey line is the nominal significance (*p* = 1 × 10 × 10^−3^). The *Y*-axis indicates the tissue-cell-type specificity (–log10 (combined *p* value)) for each tissue-cell-type from the cell-specific enrichment. **Supplementary Figure 6.** Top 20 general cell types derived from cell-specific enrichment of the highly interconnected genes from the follow-up turquoise WCGNA module. The genes are displayed from left to right ranked by the most significant human tissue-cell-type. The red dotted line is the Bonferroni-corrected significance (*p* = 3.69 × 10 × 10^−5^) by 1355 tissue-cell types. The grey line is the nominal significance (*p* = 1 × 10 × 10^−3^). The *Y*-axis indicates the tissue-cell-type specificity (–log10 (combined *p*-value)) for each tissue-cell-type from the cell-specific enrichment. **Supplementary Figure 7.** Detection of modules using follow-up expression data. (A) Scale independence and mean connectivity used to derive the soft power threshold. (B) Cluster dendrogram of consensus modules.

**Additional file 2: Supplementary Table 1.** APOE4 follow-up versus baseline significant differentially expressed genes. **Supplementary Table 2.** APOE4 non-carriers follow-up versus baseline significant differentially expressed genes. **Supplementary Table 3.** Cell deconvolution contributions generated using CIBERSORT. **Supplementary Table 4.** Amyloid non-accumulators follow-up versus baseline significant differentially expressed genes. **Supplementary Table 5.** Follow-up versus baseline significant differentially expressed genes in amyloid negative-positive and amyloid positive-positive individuals. **Supplementary Table 6.** Follow-up versus baseline significant differentially expressed genes in amyloid negative individuals. **Supplementary Table 7.** Direct association scores of significant differentially expressed genes at follow-up versus baseline in APOE4 carriers. **Supplementary Table 8.** Direct association scores of significant differentially expressed genes at follow-up versus baseline in amyloid negativepositive and amyloid positive-positive individuals. **Supplementary Table 9.** APOE4 carriers gene set enrichment analysis gene ontology upregulated. **Supplementary Table 10.** APOE4 non-carriers gene set enrichment analysis gene ontology upregulated. **Supplementary Table 11.** APOE4 carriers gene set enrichment analysis gene ontology downregulated. **Supplementary Table 12.** APOE4 non-carriers gene set enrichment analysis gene ontology downregulated. **Supplementary Table 13.** Amyloid non-accumulators gene set enrichment analysis gene ontology upregulated. **Supplementary Table 14**. Amyloid non-accumulators gene set enrichment analysis gene ontology downregulated. **Supplementary Table 15.** Amyloid negative-positive/positive-positive gene set enrichment analysis gene ontology upregulated. **Supplementary Table 16.** Amyloid negative-negative gene set enrichment analysis gene ontology upregulated. **Supplementary Table 17.** Amyloid negative-positive/positive-positive gene set enrichment analysis gene ontology downregulated. **Supplementary Table 18.** Amyloid negative-negative gene set enrichment analysis gene ontology downregulated. **Supplementary Table 19.** Baseline Blue module enrichment. **Supplementary Table 20.** Follow-up Blue module enrichment. **Supplementary Table 21.** Follow-up Turquoise module enrichment.

## Data Availability

Data will be deposited to a dedicated repository, to be decided upon publication.

## Abbreviations

AAL: Automated Anatomic Labelling Atlas
AD: Alzheimer’s disease
AMYPAD: Amyloid imaging to Prevent Alzheimer’s Disease study
*APOE4*: Apolipoprotein E ε4
AVF: Animal Verbal Fluency
AVLT: Rey’s Auditory Verbal Learning Test
BDNF: Brain-derived neurotrophic factor
BNT: Boston Naming Test
BSRT: Buschke Selective Reminding Test
BP: Biological processes
CC: Cellular component
CDR: Clinical Dementia Rating
CL: Centiloid
DR: Delayed recall
FDR: False discovery rate
F-PACK: Flemish Prevent Alzheimer’s disease Cohort KU Leuven
GO: Gene Ontology
GWAS: Genome-Wide Association Study
KEGG: Kyoto Encyclopaedia of Genes and Genomes
LVF: Letter Verbal Fluency
MCC: Maximal Clique Centrality
MF: Molecular function
MMSE: Mini-Mental State Examination
MRI: Magnetic resonance imaging
PALPA49: Psycholinguistic Assessment of Language Processing in Aphasia item 9
PET: Positron emission tomography
PPI: Protein-protein interaction
RPM: Raven’s Progressive Matrices
SUVR: Standardised uptake value ratio
SUVR_*comp*_: Standardised uptake value ratio in the composite cortical volume of interest
TL: Total learning
TMT B/A: Trail Making Test part B divided by part A
TR: Total retention
WGCNA: Weighted gene co-expression network analysis
