# Supplementary material for: Longitudinal APOE4- and amyloid-dependent changes in the blood transcriptome in cognitively intact older adults
Source: Alzheimers Res Ther. 2023 Jul 12;15:121. doi: 10.1186/s13195-023-01242-5 (PMC10337180; doi:10.1186/s13195-023-01242-5)
Supplement: Supplementary file 1 — Additional file 1: Supplementary Figure 1. Differentially expressed genes at follow-up compared to baseline in amyloid non-accumulators. Data points are based coloured on significance: grey = non-significant, blue = non-significant but with FDR p-value < 0.05, red = significant with FDR p-value < 0.05 and log2FoldChange ± 1. N = 53. Pval on y-axis represents the uncorrected p-value < 0.05 threshold for visualisation. Supplementary Figure 2. Detection of modules using baseline expression data. (A) Scale independence and (B) mean connectivity used to derive the soft power threshold. (C) Clustering of module eigengenes, where similar clusters were merged using a cutHeight =0.25 (red line). (D) Cluster dendrogram of co-expression modules shown, both with the 28 dynamic modules (top row) and final merged 17 modules (bottom row). Supplementary Figure 3. Top 20 general cell types derived from cell-specific enrichment of the highly interconnected genes from the baseline blue WGCNA module. The genes are displayed from left to right ranked by the most significant human tissue-cell-type. The red dotted line is the Bonferronicorrected significance (p = 3.69 × 10 × 10−5) by 1355 tissue-cell types. The grey line is the nominal significance (p = 1 × 10 × 10−3). The Y-axis indicates the tissue-cell-type specificity (–log10 (combined p value)) for each tissue-cell-type from the cell-specific enrichment. Supplementary Figure 4. Detection of modules using follow-up expression data. (A) Scale independence and (B) mean connectivity used to derive the soft power threshold. (C) Clustering of module eigengenes, where similar clusters were merged using a cutHeight =0.25 (red line). (D) Cluster dendrogram of co-expression modules shown, both with the 32 dynamic modules (top row) and final merged 35 modules (bottom row). Supplementary Figure 5. Top 20 general cell types derived from cell-specific enrichment of the highly interconnected genes from the follow-up blue WGCNA module. The genes are [file 13195_2023_1242_MOESM1_ESM.pdf]

Supplementary Figures

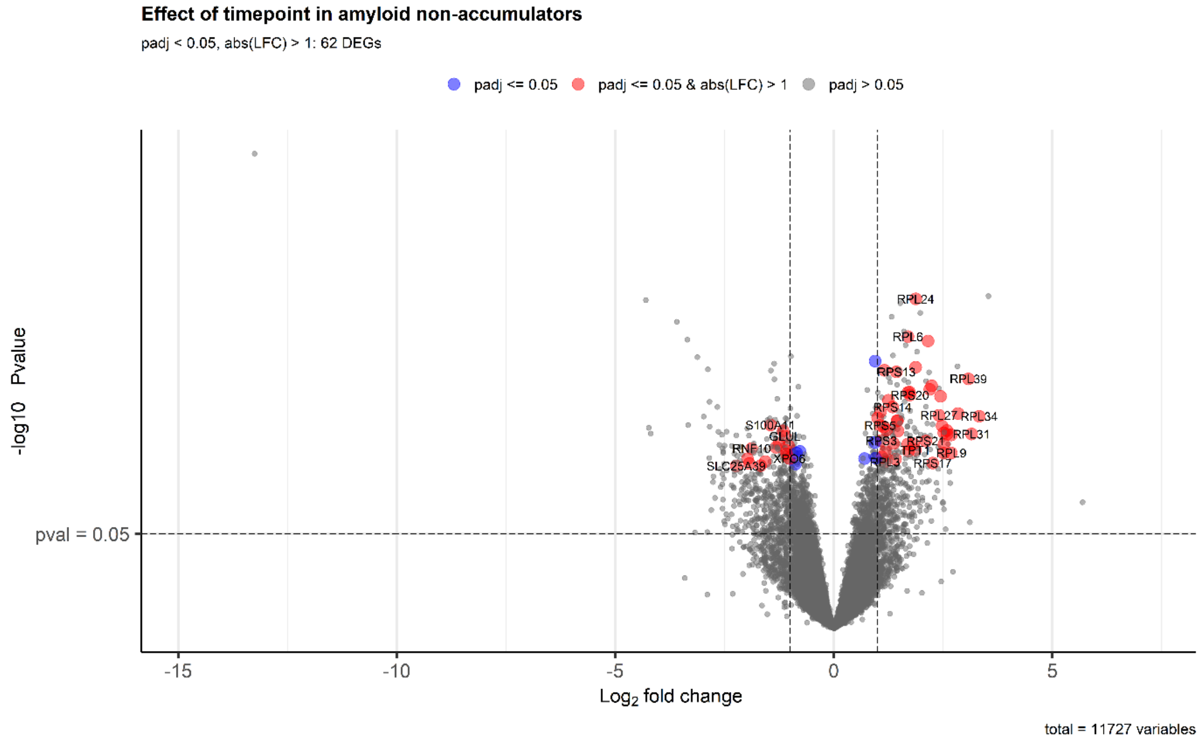

Supplementary Figure 1: Differentially expressed genes at follow-up compared to baseline in amyloid non-accumulators. Data points are based coloured on significance: grey = non-significant, blue = non-significant but with FDR *p*-value < 0.05, red = significant with FDR *p*-value < 0.05 and log2FoldChange ± 1. N = 53. Pval on y-axis represents the uncorrected *p*-value < 0.05 threshold for visualisation.

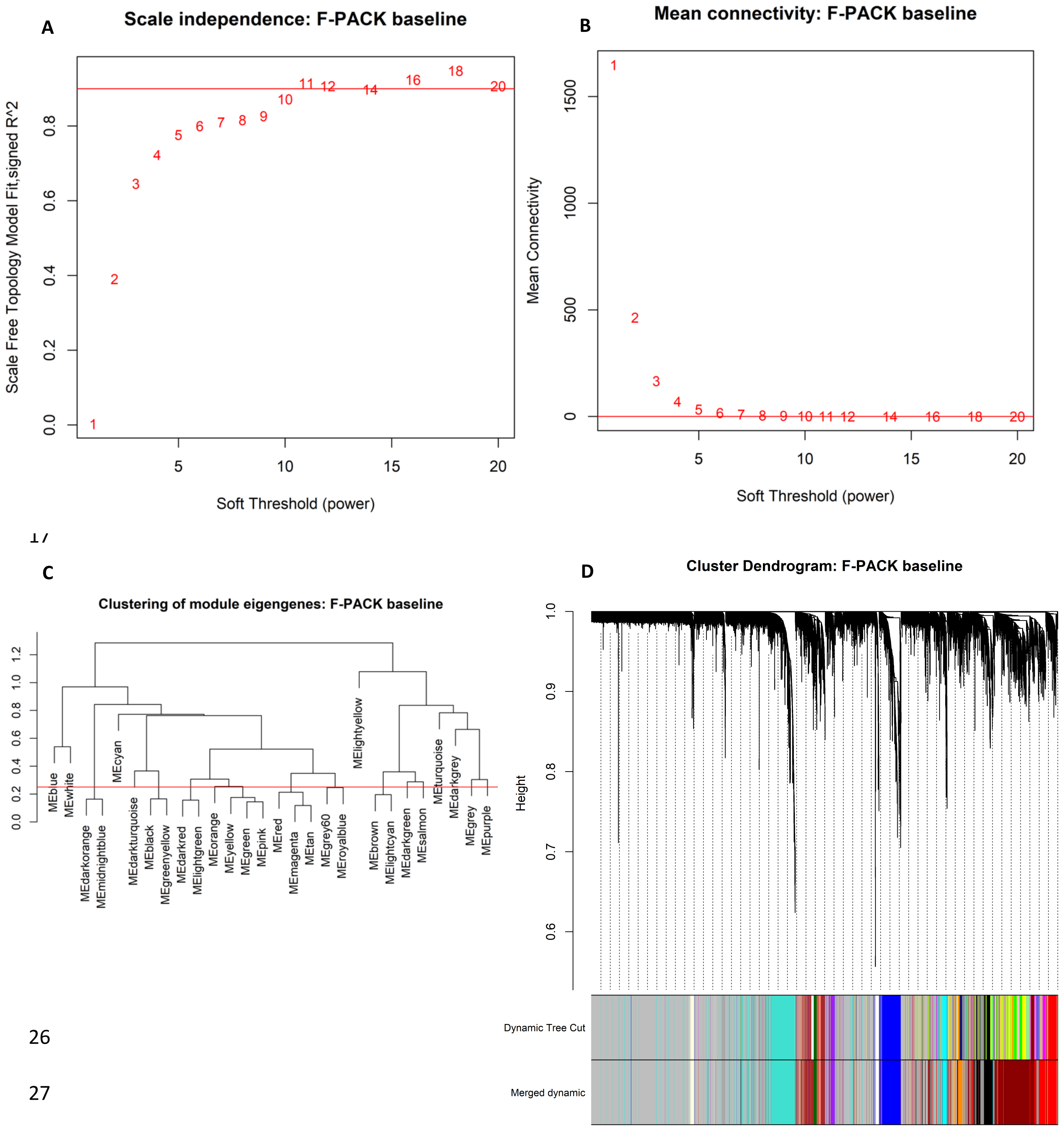

Supplementary Figure 2: Detection of modules using baseline expression data. (A) Scale independence and **(B)** mean connectivity used to derive the soft power threshold. **(C)** Clustering of module eigengenes, where similar clusters were merged using a cutHeight =0.25 (red line). **(D)** Cluster dendrogram of co-expression modules shown, both with the 28 dynamic modules (top row) and final merged 17 modules (bottom row).

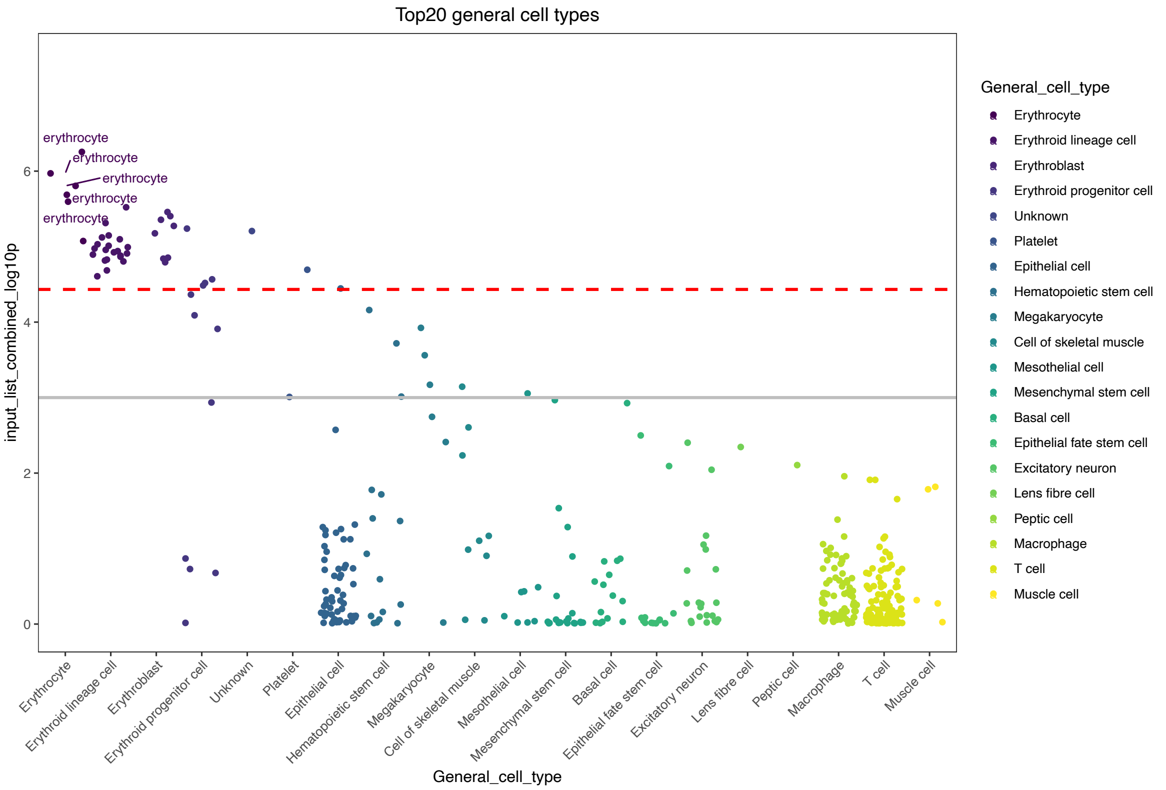

Supplementary Figure 3: Top 20 general cell types derived from cell-specific enrichment of the highly interconnected genes from the baseline blue WGCNA module. The genes are displayed from left to right ranked by the most significant human tissue-cell-type. The red dotted line is the Bonferroni-corrected significance (*p* = 3.69 x 10x10^{-5}) by 1,355 tissue-cell types. The grey line is the nominal significance (*p* = 1 x 10x10^{-3}). The Y-axis indicates the tissue-cell-type specificity (–log10 (combined *p*-value)) for each tissue-cell-type from the cell-specific enrichment.

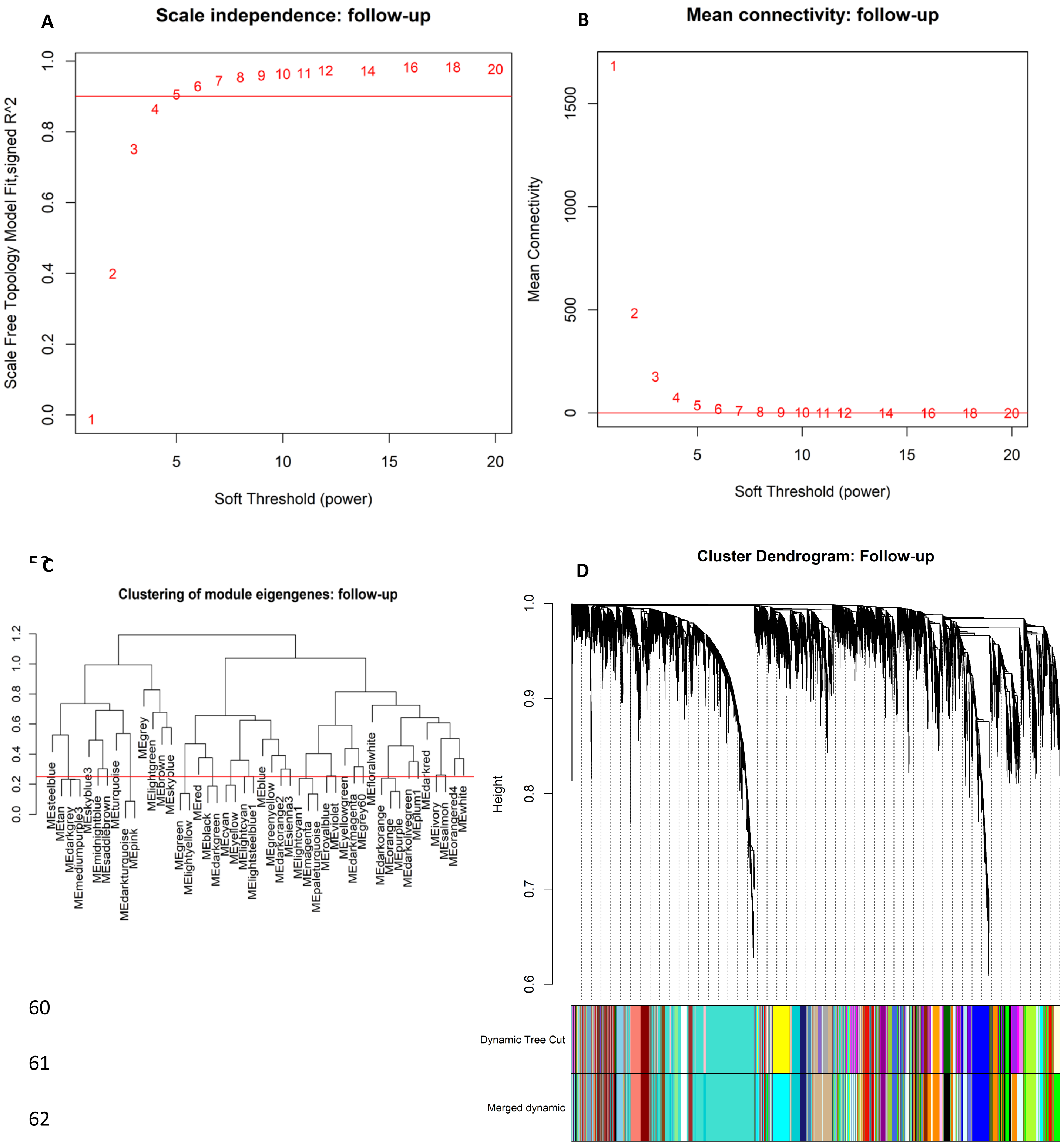

Supplementary Figure 4: Detection of modules using follow-up expression data. (A) Scale independence and **(B)** mean connectivity used to derive the soft power threshold. **(C)** Clustering of module eigengenes, where similar clusters were merged using a cutHeight =0.25 (red line). **(D)** Cluster dendrogram of co-expression modules shown, both with the 32 dynamic modules (top row) and final merged 35 modules (bottom row).

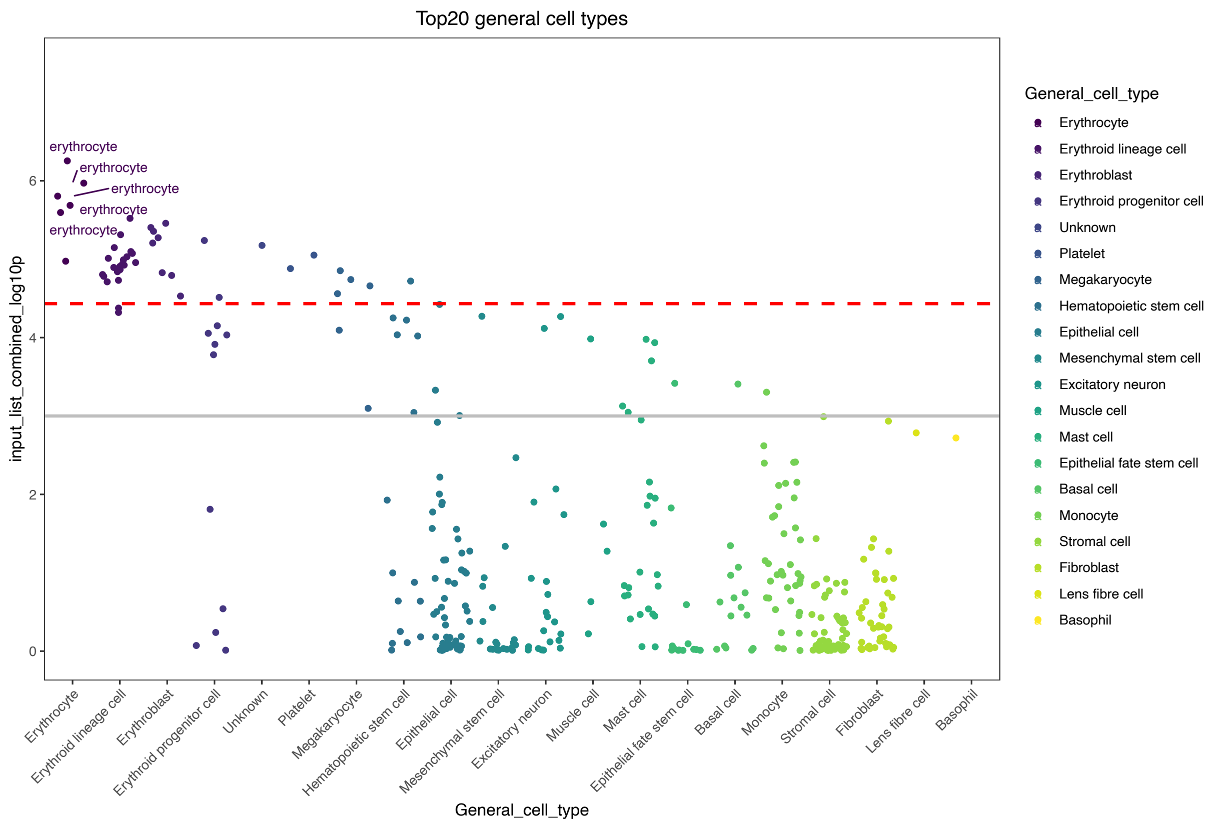

Supplementary Figure 5: Top 20 general cell types derived from cell-specific enrichment of the highly interconnected genes from the follow-up blue WGCNA module. The genes are displayed from left to right ranked by the most significant human tissue-cell-type. The red dotted line is the Bonferroni-corrected significance (*p* = 3.69 x 10x10^{-5}) by 1,355 tissue-cell types. The grey line is the nominal significance (*p* = 1 x 10x10^{-3}). The Y-axis indicates the tissue-cell-type specificity (–log10 (combined *p*-value)) for each tissue-cell-type from the cell-specific enrichment.

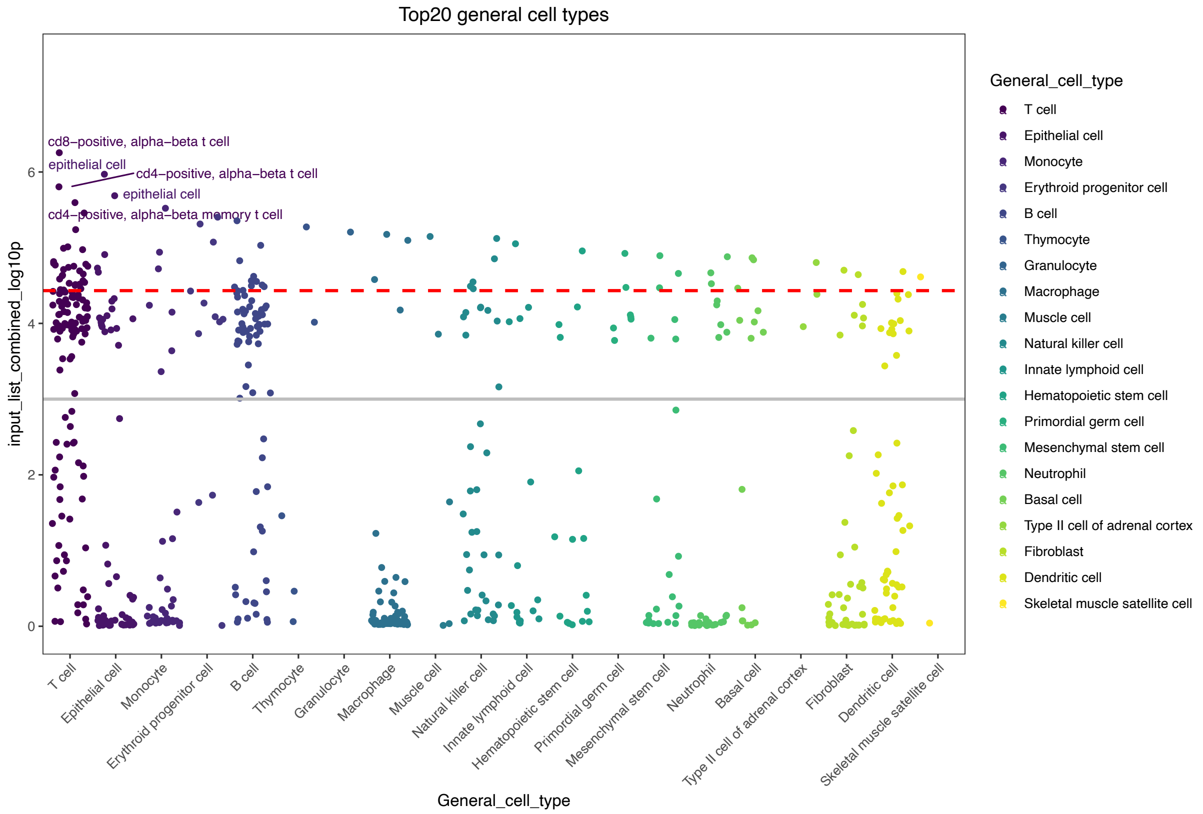

Supplementary Figure 6: Top 20 general cell types derived from cell-specific enrichment of the highly interconnected genes from the follow-up turquoise WCGNA module. The genes are displayed from left to right ranked by the most significant human tissue-cell-type. The red dotted line is the Bonferroni-corrected significance (p = 3.69 x 10x10^{-5}) by 1,355 tissue-cell types. The grey line is the nominal significance (p = 1 x 10x10^{-3}). The Y-axis indicates the tissue-cell-type specificity (–log10 (combined p-value)) for each tissue-cell-type from the cell-specific enrichment.

A

B

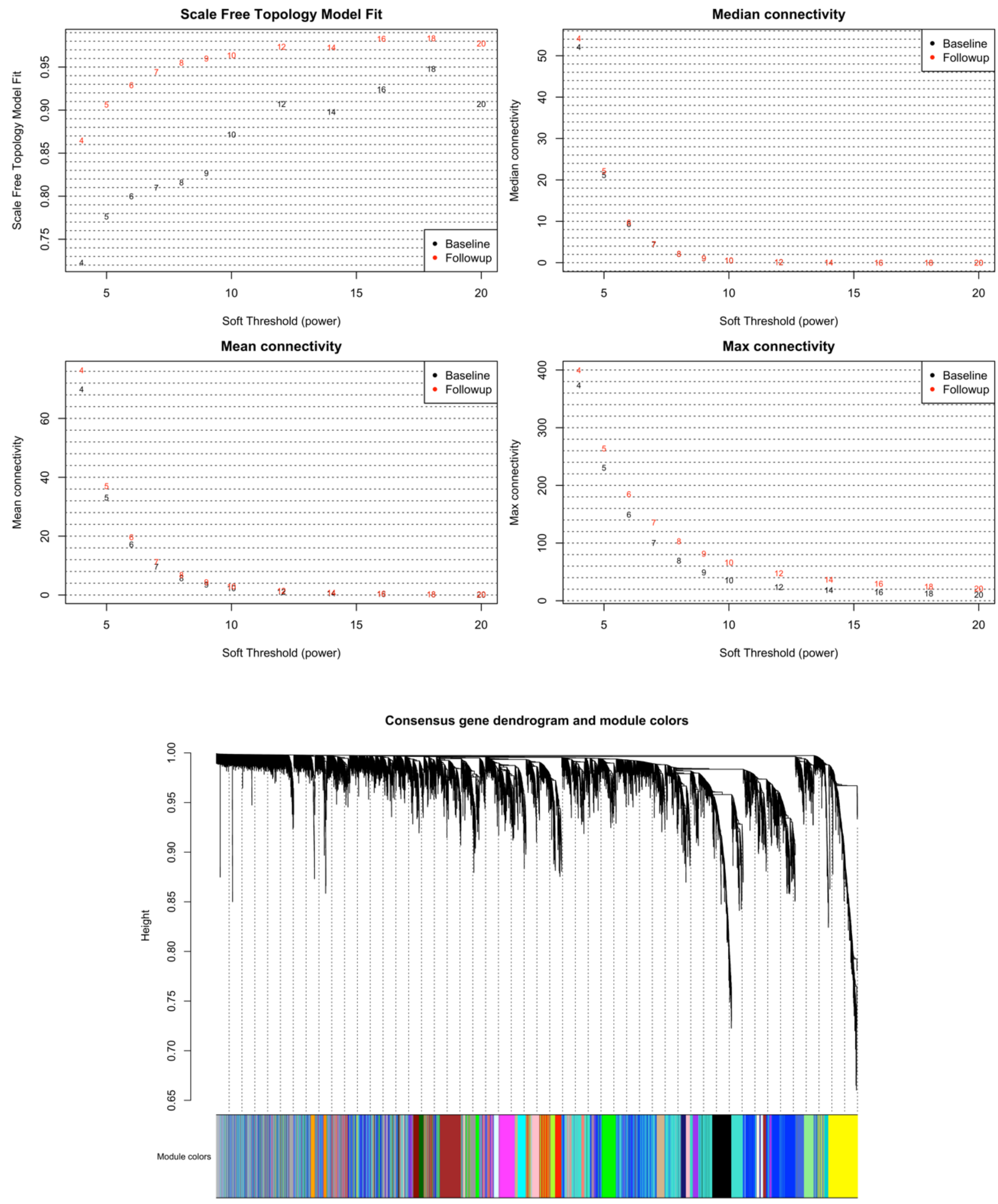

Supplementary Figure 7: Detection of modules using follow-up expression data. (A) Scale independence and mean connectivity used to derive the soft power threshold. **(B)** Cluster dendrogram of consensus modules.
