# Supplementary material for: Longitudinal APOE4- and amyloid-dependent changes in the blood transcriptome in cognitively intact older adults
Source: Alzheimers Res Ther. 2023 Jul 12;15:121. doi: 10.1186/s13195-023-01242-5 (PMC10337180; doi:10.1186/s13195-023-01242-5)
Supplement: Supplementary file 2 — Additional file 2: Supplementary Table 1. APOE4 follow-up versus baseline significant differentially expressed genes. Supplementary Table 2. APOE4 non-carriers follow-up versus baseline significant differentially expressed genes. Supplementary Table 3. Cell deconvolution contributions generated using CIBERSORT. Supplementary Table 4. Amyloid non-accumulators follow-up versus baseline significant differentially expressed genes. Supplementary Table 5. Follow-up versus baseline significant differentially expressed genes in amyloid negative-positive and amyloid positive-positive individuals. Supplementary Table 6. Follow-up versus baseline significant differentially expressed genes in amyloid negative individuals. Supplementary Table 7. Direct association scores of significant differentially expressed genes at follow-up versus baseline in APOE4 carriers. Supplementary Table 8. Direct association scores of significant differentially expressed genes at follow-up versus baseline in amyloid negativepositive and amyloid positive-positive individuals. Supplementary Table 9. APOE4 carriers gene set enrichment analysis gene ontology upregulated. Supplementary Table 10. APOE4 non-carriers gene set enrichment analysis gene ontology upregulated. Supplementary Table 11. APOE4 carriers gene set enrichment analysis gene ontology downregulated. Supplementary Table 12. APOE4 non-carriers gene set enrichment analysis gene ontology downregulated. Supplementary Table 13. Amyloid non-accumulators gene set enrichment analysis gene ontology upregulated. Supplementary Table 14. Amyloid non-accumulators gene set enrichment analysis gene ontology downregulated. Supplementary Table 15. Amyloid negative-positive/positive-positive gene set enrichment analysis gene ontology upregulated. Supplementary Table 16. Amyloid negative-negative gene set enrichment analysis gene ontology upregulated. Supplementary Table 17. Amyloid negative-positive/positive-positive gene set enrichment analysis gene ontolog [file 13195_2023_1242_MOESM2_ESM.pdf]

Supplementary Table 1 APOE4 follow-up versus baseline significant differentially expressed genes

Gene	baseMean	log2FoldChange	lfcSE	stat	pvalue	padj
LY75-CD30	79.20273	-5.598681237	0.760791	-7.35902	1.85E-13	1.81E-10
ATP6V0C	3413.391	-1.87314109	0.690113	-2.71425	0.006643	0.031334
KEL	21.66106	-1.69013473	0.306324	-5.51748	3.44E-08	2.11E-06
ENSG0000(	6.743152	-1.649011269	0.470789	-3.50265	0.000461	0.003802
YPEL4	9.01876	-1.615089466	0.298765	-5.40589	6.45E-08	3.42E-06
ODAD4	10.93416	-1.545522069	0.401164	-3.8526	0.000117	0.001247
SHISA4	67.93525	-1.525675833	0.263465	-5.7908	7.01E-09	6.42E-07
SMIM24	74.83143	-1.517357817	0.265405	-5.71715	1.08E-08	9.07E-07
RAB3IL1	17.3886	-1.503444256	0.300944	-4.99576	5.86E-07	1.90E-05
GCAT	11.14519	-1.503005857	0.280781	-5.35295	8.65E-08	4.31E-06
RNF182	10.55344	-1.47536839	0.34029	-4.33562	1.45E-05	0.000239
SMOX	109.6234	-1.474250876	0.241558	-6.10309	1.04E-09	1.63E-07
PDZK1IP1	725.3783	-1.467541528	0.22726	-6.45755	1.06E-10	3.04E-08
ANK1	252.0236	-1.46438479	0.24347	-6.01464	1.80E-09	2.45E-07
IFITM10	5.214603	-1.441245575	0.327673	-4.39843	1.09E-05	0.000191
ACKR1	29.99039	-1.440712052	0.264133	-5.45449	4.91E-08	2.81E-06
SLC6A8	328.0936	-1.427462434	0.256259	-5.57039	2.54E-08	1.72E-06
GNAZ	37.38343	-1.420140226	0.261199	-5.43701	5.42E-08	3.06E-06
PLVAP	32.84342	-1.417441627	0.255037	-5.55779	2.73E-08	1.76E-06
OSBP2	198.9142	-1.407141602	0.237965	-5.91324	3.35E-09	3.75E-07
SLC25A39	8333.375	-1.406168445	0.234113	-6.00637	1.90E-09	2.53E-07
GMPR	495.4253	-1.405688031	0.234626	-5.99119	2.08E-09	2.73E-07
GP1BA	31.71893	-1.388878415	0.282149	-4.9225	8.54E-07	2.56E-05
FAXDC2	148.0613	-1.380342309	0.238166	-5.79571	6.80E-09	6.33E-07
SELENBP1	737.2073	-1.378868167	0.234795	-5.87265	4.29E-09	4.49E-07
TNS1	314.2138	-1.37625038	0.244247	-5.63466	1.75E-08	1.28E-06
MYL9	110.6409	-1.356192954	0.261336	-5.18946	2.11E-07	8.65E-06
DMTN	1141.372	-1.349382834	0.227822	-5.92297	3.16E-09	3.60E-07
PEAK3	40.74859	-1.34709094	0.236025	-5.70742	1.15E-08	9.47E-07
VWCE	83.03884	-1.343011873	0.257793	-5.20966	1.89E-07	8.13E-06
TMPRSS9	14.94427	-1.338559051	0.294144	-4.55069	5.35E-06	0.000111
ALAS2	4004.627	-1.330915379	0.248695	-5.3516	8.72E-08	4.31E-06

KLC3	48.67827	-1.329874557	0.25644	-5.18591	2.15E-07	8.75E-06
RUNDC3A	121.2742	-1.327833302	0.253599	-5.23596	1.64E-07	7.26E-06
XK	28.42498	-1.319734349	0.229613	-5.74765	9.05E-09	8.02E-07
TSPAN9	14.60901	-1.318683965	0.264947	-4.97716	6.45E-07	2.04E-05
TMEM158	91.54618	-1.317784167	0.253469	-5.19899	2.00E-07	8.45E-06
GYPB	120.5002	-1.31279605	0.261136	-5.02726	4.98E-07	1.68E-05
SPTB	63.10904	-1.304717665	0.280127	-4.6576	3.20E-06	7.27E-05
TBXA2R	25.31024	-1.301224809	0.22977	-5.66315	1.49E-08	1.13E-06
TMCC2	77.57464	-1.292755956	0.219242	-5.89648	3.71E-09	4.03E-07
KLF1	94.99688	-1.290077248	0.249897	-5.16243	2.44E-07	9.69E-06
SMIM1	86.02493	-1.287398649	0.222188	-5.79418	6.87E-09	6.34E-07
EPB42	328.5195	-1.265656867	0.250358	-5.05539	4.30E-07	1.49E-05
ABLIM3	14.89804	-1.259188415	0.261849	-4.80884	1.52E-06	4.02E-05
PLEK2	43.87496	-1.252986765	0.261705	-4.78778	1.69E-06	4.39E-05
ESPN	36.69572	-1.250043248	0.276175	-4.52627	6.00E-06	0.000121
IGF2	7.737475	-1.244861138	0.369816	-3.36616	0.000762	0.0057
E2F2	161.0436	-1.240989392	0.234725	-5.28699	1.24E-07	5.76E-06
SLC38A5	203.8791	-1.229345638	0.221198	-5.55767	2.73E-08	1.76E-06
GJC2	5.467039	-1.228011501	0.310061	-3.96055	7.48E-05	0.000873
MAP2K3	2629.62	-1.224348032	0.215594	-5.67895	1.36E-08	1.06E-06
CAPN5	27.37665	-1.217563794	0.252475	-4.82252	1.42E-06	3.80E-05
CLDN5	17.61434	-1.213652722	0.256764	-4.72673	2.28E-06	5.57E-05
MRC2	71.26921	-1.211620954	0.225866	-5.36433	8.12E-08	4.09E-06
GPR146	94.36382	-1.211102093	0.26362	-4.59412	4.35E-06	9.42E-05
AQP10	13.9567	-1.207181101	0.243879	-4.94992	7.42E-07	2.28E-05
SLC6A9	14.29352	-1.206546697	0.324401	-3.71931	0.0002	0.001915
FZD5	7.799811	-1.202288529	0.322845	-3.72405	0.000196	0.001892
DGKK	6.621084	-1.201435896	0.296066	-4.05801	4.95E-05	0.000624
MYZAP	6.585932	-1.19617048	0.389251	-3.07301	0.002119	0.012991
NPRL3	275.032	-1.190586195	0.212098	-5.61337	1.98E-08	1.39E-06
MPL	10.22133	-1.188887389	0.279351	-4.25589	2.08E-05	0.000313
RHOBTB1	10.84179	-1.185058056	0.262877	-4.50804	6.54E-06	0.00013
MPIG6B	135.5966	-1.184572849	0.228175	-5.19151	2.09E-07	8.61E-06
KRT1	337.7379	-1.183367033	0.2214	-5.34494	9.04E-08	4.44E-06

STRADB	816.8492	-1.180530873	0.205773	-5.73705	9.63E-09	8.25E-07
ITGB5	42.47679	-1.168513486	0.255433	-4.57463	4.77E-06	0.000102
TREML1	95.59164	-1.16728602	0.233202	-5.00548	5.57E-07	1.83E-05
ITGB3	189.5839	-1.165635581	0.22222	-5.24541	1.56E-07	6.95E-06
NRGN	1589.382	-1.160342093	0.256369	-4.52607	6.01E-06	0.000121
AQP1	26.60189	-1.159297056	0.290265	-3.99393	6.50E-05	0.00078
TRIM58	583.9372	-1.158673538	0.214803	-5.39412	6.89E-08	3.60E-06
ATP1B2	7.776182	-1.15618273	0.31702	-3.64703	0.000265	0.00241
LTBP1	24.40168	-1.152541159	0.240891	-4.78449	1.71E-06	4.43E-05
DIPK1B	6.050744	-1.148939826	0.333371	-3.44643	0.000568	0.004504
NFIX	446.601	-1.14876181	0.234414	-4.90057	9.56E-07	2.77E-05
YBX3	3361.782	-1.139725903	0.216798	-5.25709	1.46E-07	6.55E-06
FBXO7	1992.629	-1.137042547	0.20507	-5.54465	2.95E-08	1.87E-06
FAM124A	181.9596	-1.135085135	0.288224	-3.93821	8.21E-05	0.000941
SMIM5	58.52839	-1.12687958	0.229358	-4.91318	8.96E-07	2.67E-05
MARCHF8	995.2548	-1.11896885	0.201002	-5.56696	2.59E-08	1.73E-06
GYPC	4560.939	-1.11663119	0.211993	-5.26731	1.38E-07	6.29E-06
DCAF12	1903.247	-1.116203674	0.210929	-5.29185	1.21E-07	5.68E-06
PVALB	8.411634	-1.11500065	0.289084	-3.85702	0.000115	0.001226
TSPAN5	536.9703	-1.103750769	0.193233	-5.71202	1.12E-08	9.29E-07
ACRBP	117.111	-1.101154739	0.184384	-5.97209	2.34E-09	2.99E-07
RGS6	10.93488	-1.100121634	0.379235	-2.90089	0.003721	0.020097
ASAP2	8.629296	-1.099075318	0.300723	-3.65477	0.000257	0.002344
TMOD1	91.03976	-1.097330853	0.245611	-4.46775	7.90E-06	0.000151
IFIT1B	42.59664	-1.094743429	0.244747	-4.47296	7.71E-06	0.000148
FAAH2	7.581559	-1.092319947	0.307252	-3.55513	0.000378	0.003215
TAL1	147.97	-1.091147079	0.226527	-4.81685	1.46E-06	3.89E-05
SFRP2	20.83041	-1.081119262	0.31377	-3.44558	0.00057	0.004515
BCL2L1	976.6138	-1.080757138	0.231587	-4.66675	3.06E-06	7.02E-05
ASCC2	885.9662	-1.074529467	0.237063	-4.53267	5.82E-06	0.000119
ZNF385D	11.99077	-1.073721697	0.269175	-3.98894	6.64E-05	0.000793
TGFB1I1	7.884104	-1.072914703	0.312039	-3.4384	0.000585	0.004618
TENT5C	383.6532	-1.069814258	0.207444	-5.15713	2.51E-07	9.84E-06
SEC14L5	5.742155	-1.068255024	0.326632	-3.27052	0.001074	0.00748

SLC4A1	1119.873	-1.067556733	0.217546	-4.90727	9.24E-07	2.72E-05
UBXN6	1638.285	-1.061121236	0.202825	-5.23171	1.68E-07	7.35E-06
GUCD1	903.8292	-1.051377726	0.175507	-5.99052	2.09E-09	2.73E-07
SH3BGRL2	56.30614	-1.050903827	0.19194	-5.47518	4.37E-08	2.54E-06
CDC34	924.4921	-1.048107725	0.186035	-5.63391	1.76E-08	1.28E-06
ARHGAP6	19.3074	-1.047116494	0.245955	-4.25735	2.07E-05	0.000313
NINJ2	273.1317	-1.046829265	0.185285	-5.64982	1.61E-08	1.21E-06
PTCRA	43.2275	-1.045831937	0.201618	-5.1872	2.13E-07	8.72E-06
CTTN	53.26811	-1.045602217	0.222295	-4.70368	2.56E-06	6.09E-05
R3HDM4	4250.072	-1.036590668	0.218511	-4.74387	2.10E-06	5.21E-05
ENSG0000(	458.2362	-1.031643921	0.21572	-4.78233	1.73E-06	4.45E-05
IGF2BP2	228.8169	-1.029344192	0.225851	-4.55763	5.17E-06	0.000108
DAW1	153.992	-1.028863411	0.282075	-3.64748	0.000265	0.002407
TMEM63B	176.9955	-1.023952971	0.239789	-4.27022	1.95E-05	0.000299
SLC7A5	41.06439	-1.01823277	0.184963	-5.50506	3.69E-08	2.24E-06
RNF10	1761.75	-1.017917424	0.186277	-5.46453	4.64E-08	2.67E-06
RAB6B	9.428403	-1.017571304	0.314682	-3.23365	0.001222	0.008323
SLC48A1	203.1913	-1.013105375	0.199061	-5.08942	3.59E-07	1.30E-05
DNM3	22.74739	-1.00878653	0.264653	-3.81173	0.000138	0.001433
CCDC190	9.964104	-1.006231596	0.315412	-3.19022	0.001422	0.009424
RAP1GAP	19.28541	-1.003590721	0.388219	-2.58511	0.009735	0.041797
RPS10	2187.607	1.001581496	0.145293	6.893549	5.44E-12	2.55E-09
NDUFA1	206.6057	1.006100778	0.175213	5.742166	9.35E-09	8.18E-07
CARD16	77.36409	1.006629436	0.163182	6.168735	6.88E-10	1.24E-07
RPL11	1786.138	1.006900342	0.164862	6.107533	1.01E-09	1.63E-07
ZNF181	27.05407	1.01343451	0.171228	5.918623	3.25E-09	3.66E-07
RPS21	1807.141	1.014568315	0.204696	4.956459	7.18E-07	2.23E-05
NDUFB1	192.489	1.021432927	0.188335	5.423496	5.84E-08	3.22E-06
COA6	21.77764	1.028524857	0.197169	5.216474	1.82E-07	7.95E-06
COMMD8	26.45054	1.028597087	0.222224	4.62864	3.68E-06	8.18E-05
RPL24	897.4281	1.02903072	0.13141	7.83067	4.85E-15	9.48E-12
TEX30	7.094487	1.029139209	0.331597	3.103586	0.001912	0.011964
MRPL47	26.15426	1.034014997	0.193993	5.330158	9.81E-08	4.76E-06
ATP5ME	184.1819	1.040487428	0.18049	5.764788	8.18E-09	7.32E-07

RPS6	3513.645	1.040981991	0.164306	6.335642	2.36E-10	5.71E-08
TXNDC9	21.11096	1.043699682	0.207135	5.038734	4.69E-07	1.59E-05
RPS20	3066.996	1.04556436	0.156077	6.699036	2.10E-11	8.20E-09
PTS	7.112631	1.047753654	0.267058	3.923322	8.73E-05	0.000981
RPL35	980.3456	1.047894624	0.168281	6.227047	4.75E-10	9.95E-08
MRPS18C	68.99507	1.05233625	0.14213	7.404062	1.32E-13	1.55E-10
NDUFB3	53.42891	1.056301454	0.251954	4.192434	2.76E-05	0.000393
BEX2	24.03755	1.056349075	0.181715	5.813216	6.13E-09	5.89E-07
RBIS	61.83855	1.058611198	0.222801	4.751378	2.02E-06	5.04E-05
EEF1E1	13.91573	1.062806693	0.233056	4.560302	5.11E-06	0.000108
SUB1	239.746	1.063144052	0.155862	6.821078	9.04E-12	3.92E-09
RPL35A	1659.169	1.065140265	0.175785	6.059342	1.37E-09	2.05E-07
HSPE1	70.65427	1.066821335	0.21369	4.992367	5.96E-07	1.93E-05
YAE1	6.616441	1.070693314	0.281885	3.798329	0.000146	0.001497
DBI	219.7462	1.073609836	0.150827	7.118168	1.09E-12	6.75E-10
TIMM8B	57.51034	1.081334385	0.179818	6.013509	1.82E-09	2.45E-07
SNRPE	58.4161	1.08531128	0.176837	6.137354	8.39E-10	1.46E-07
RPL41	9291.688	1.092718294	0.186732	5.851797	4.86E-09	4.92E-07
METTL18	10.94884	1.097759755	0.247845	4.42921	9.46E-06	0.000172
SLIRP	55.77165	1.116484057	0.215912	5.171011	2.33E-07	9.36E-06
RPS25	1659.969	1.118218546	0.142748	7.833509	4.74E-15	9.48E-12
RPS29	7686.161	1.14353348	0.225742	5.065659	4.07E-07	1.43E-05
BCL2A1	173.2939	1.144987923	0.199241	5.746738	9.10E-09	8.02E-07
S100A8	4477.439	1.147829673	0.212314	5.40628	6.43E-08	3.42E-06
CLEC2B	146.9915	1.164465961	0.162844	7.150804	8.63E-13	5.62E-10
ZNF681	9.711806	1.172433639	0.233547	5.020114	5.16E-07	1.73E-05
BDH2	13.79558	1.174185535	0.222468	5.277993	1.31E-07	5.96E-06
ATP5MJ	220.2191	1.187443812	0.190652	6.228333	4.71E-10	9.95E-08
RPL21	2154.788	1.195164969	0.146006	8.185752	2.71E-16	1.67E-12
SMIM30	16.66794	1.209181521	0.212571	5.688365	1.28E-08	1.02E-06
RPS18	1956.204	1.225211168	0.184702	6.633453	3.28E-11	1.07E-08
LSM3	56.25364	1.231099354	0.192108	6.408379	1.47E-10	4.01E-08
HAUS1	16.81395	1.259595704	0.223135	5.644998	1.65E-08	1.23E-06
TOMM7	548.4334	1.263944823	0.217474	5.811929	6.18E-09	5.89E-07

RPL27	1221.987	1.264095058	0.215735	5.859478	4.64E-09	4.73E-07
RSL24D1	100.7617	1.27907221	0.233422	5.47966	4.26E-08	2.50E-06
MRPS28	10.90051	1.352930314	0.267017	5.06683	4.04E-07	1.42E-05
TIPIN	13.77302	1.368920811	0.225014	6.083704	1.17E-09	1.81E-07
RPL22L1	32.61671	1.376063427	0.253586	5.426421	5.75E-08	3.18E-06
RPL7	1087.23	1.398283233	0.208984	6.690877	2.22E-11	8.39E-09
SNRPG	96.73275	1.409930487	0.226933	6.212968	5.20E-10	1.06E-07
TMA7	457.6265	1.418535563	0.207584	6.833554	8.28E-12	3.74E-09
RPS15A	1408.382	1.420468569	0.225588	6.29674	3.04E-10	6.95E-08
COX7C	397.2887	1.426557754	0.233717	6.103775	1.04E-09	1.63E-07
POLR2K	29.42571	1.427953294	0.277609	5.143749	2.69E-07	1.04E-05
PFDN4	21.72947	1.463936905	0.339243	4.315299	1.59E-05	0.000256
RPS17	1622.303	1.465340678	0.232943	6.290565	3.16E-10	7.00E-08
COX7B	75.15248	1.532945004	0.250285	6.124786	9.08E-10	1.52E-07
TOMM5	30.42526	1.543334216	0.425873	3.623927	0.00029	0.002586
RPS27	4006.362	1.554191305	0.249195	6.236846	4.46E-10	9.70E-08
UQCRB	395.0948	1.571195044	0.192084	8.179728	2.84E-16	1.67E-12
LY96	45.50433	1.591938288	0.305995	5.2025	1.97E-07	8.35E-06
EEF1B2	640.2826	1.622874862	0.226168	7.175518	7.20E-13	4.97E-10
COX6C	161.8269	1.676170896	0.260684	6.429902	1.28E-10	3.57E-08
RPL23	1217.637	1.736652591	0.241902	7.179147	7.01E-13	4.97E-10
RPL39	2100.614	1.740854757	0.249773	6.969753	3.17E-12	1.55E-09
RPL9	1324.591	1.743234491	0.266133	6.550233	5.74E-11	1.73E-08
RPS7	909.9927	1.744033502	0.238827	7.302498	2.82E-13	2.37E-10
RPL36A	1449.693	1.747659184	0.247798	7.052743	1.75E-12	9.35E-10
RPS24	1318.311	1.767815936	0.236339	7.479986	7.43E-14	9.69E-11
RPS3A	1526.443	1.782147418	0.250816	7.105393	1.20E-12	7.04E-10
RPL17-C18	777.3826	1.787874802	0.252164	7.090132	1.34E-12	7.48E-10
COMMD6	178.1257	1.817384555	0.295798	6.144002	8.05E-10	1.43E-07
RPL17	235.9116	1.823526602	0.235525	7.742384	9.76E-15	1.63E-11
RPL26	1309.917	2.027382442	0.252507	8.02903	9.82E-16	3.84E-12
RPL31	1217.307	2.20205104	0.29288	7.518601	5.54E-14	8.12E-11
RPL34	1191.319	2.301580918	0.293337	7.846193	4.29E-15	9.48E-12

Supplementary Table 2 APOE4 non-carriers follow-up versus baseline significant differentially expressed genes

Gene	baseMean	log2FoldChange	lfcSE	stat	pvalue	padj
ENSG0000(	223.1322	-3.83121248	0.817441	-4.68684	2.77E-06	0.000398
ATP6V0C	3413.391	-1.929960091	0.658949	-2.92885	0.003402	0.035506
SHISA7	6.517932	-1.510746292	0.338817	-4.45889	8.24E-06	0.000755
TBXA2R	25.31024	-1.442767625	0.218887	-6.59137	4.36E-11	3.67E-07
WASF3	6.048373	-1.344246568	0.333272	-4.03348	5.50E-05	0.002298
RNF208	7.195146	-1.329536533	0.290988	-4.56904	4.90E-06	0.000555
KEL	21.66106	-1.306921047	0.282368	-4.62844	3.68E-06	0.00048
VWCE	83.03884	-1.298387876	0.244094	-5.3192	1.04E-07	5.53E-05
RGS6	10.93488	-1.292107642	0.35102	-3.68101	0.000232	0.005727
SEC14L5	5.742155	-1.252041634	0.306329	-4.08724	4.37E-05	0.001969
PDGFA	31.2621	-1.143236323	0.249142	-4.58869	4.46E-06	0.000534
SDSL	5.589816	-1.124652382	0.328087	-3.42791	0.000608	0.011059
ANK1	252.0236	-1.121314122	0.231679	-4.83995	1.30E-06	0.00025
TUBA8	24.42435	-1.088140866	0.393226	-2.76722	0.005654	0.048739
TMEM63B	176.9955	-1.084141325	0.22861	-4.74231	2.11E-06	0.000339
MYL9	110.6409	-1.069670575	0.249474	-4.28771	1.81E-05	0.001108
OSBP2	198.9142	-1.06545292	0.226423	-4.70559	2.53E-06	0.000381
LTBP1	24.40168	-1.05983859	0.228071	-4.64697	3.37E-06	0.000449
TMEM158	91.54618	-1.052490719	0.240902	-4.36896	1.25E-05	0.000923
ODAD4	10.93416	-1.051495592	0.369218	-2.8479	0.004401	0.04213
TNS1	314.2138	-1.049790676	0.232681	-4.51172	6.43E-06	0.000656
ESPN	36.69572	-1.048823491	0.258547	-4.05661	4.98E-05	0.002131
GJC2	5.467039	-1.046755187	0.291512	-3.59078	0.00033	0.007309
ELOVL7	11.02203	-1.045256808	0.265014	-3.94416	8.01E-05	0.002908
DMTN	1141.372	-1.031158377	0.21736	-4.74402	2.10E-06	0.000339
SLC6A8	328.0936	-1.017724635	0.244051	-4.17013	3.04E-05	0.001545
ITGA2B	110.1208	-1.014887688	0.215339	-4.71299	2.44E-06	0.000372
SMOX	109.6234	-1.011621223	0.229094	-4.41574	1.01E-05	0.000814
NBPF26	23.34265	-1.007075175	0.333162	-3.02278	0.002505	0.028572
LY96	45.50433	1.045971179	0.292814	3.572136	0.000354	0.007727
RPL39	2100.614	1.084013653	0.238477	4.545561	5.48E-06	0.000584
COMMD6	178.1257	1.101418138	0.282388	3.900371	9.60E-05	0.003209

RPL9	1324.591	1.104228457	0.254114	4.345399	1.39E-05	0.000974
RPL23	1217.637	1.120930298	0.231	4.852511	1.22E-06	0.000238
RPS3A	1526.443	1.122261806	0.239475	4.686346	2.78E-06	0.000398
EEF1B2	640.2826	1.123951176	0.216009	5.203251	1.96E-07	7.94E-05
RPS7	909.9927	1.141252182	0.228064	5.004083	5.61E-07	0.000153
ZNF208	16.46769	1.148770473	0.253182	4.537328	5.70E-06	0.000602
UQCRB	395.0948	1.199571438	0.183492	6.537457	6.26E-11	3.67E-07
RPL17-C18	777.3826	1.209308515	0.240773	5.0226	5.10E-07	0.000146
RPS24	1318.311	1.287847058	0.225667	5.706859	1.15E-08	1.93E-05
RPL36A	1449.693	1.308349866	0.236642	5.52881	3.22E-08	2.68E-05
RPL31	1217.307	1.402089135	0.27958	5.01499	5.30E-07	0.000148
RPL34	1191.319	1.413800882	0.280069	5.048048	4.46E-07	0.000141
RPL17	235.9116	1.430240316	0.224883	6.359924	2.02E-10	7.89E-07
RPL26	1309.917	1.433589576	0.241093	5.946208	2.74E-09	6.44E-06
ENSG0000(	9.689362	2.196864714	0.668721	3.285172	0.001019	0.015462

Supplementary Table 3 Cell deconvolution contributions generated using CIBERSORT

Timepoint	B cells naive	B cells memory	Plasma cells	T cells CD8	T cells CD4 naive	T cells CD4 memory resting	T cells CD4 memory activated	T cells follicular helper	T cells regulatory (Tregs)	T cells gamma delta	NK cells resting	NK cells activated	Monocytes
Baseline	0.000	0.061	0.000	0.223	0.388	0.479	0.011	0.000	0.000	0.000	0.194	0.021	0.365
Baseline	0.000	0.103	0.000	0.199	0.563	0.335	0.048	0.000	0.007	0.000	0.190	0.008	0.352
Baseline	0.000	0.091	0.000	0.022	0.426	0.270	0.035	0.000	0.000	0.000	0.125	0.105	0.364
Baseline	0.000	0.109	0.000	0.038	0.260	0.306	0.000	0.000	0.037	0.000	0.213	0.051	0.439
Baseline	0.023	0.133	0.007	0.192	0.562	0.425	0.047	0.000	0.007	0.000	0.029	0.039	0.239
Baseline	0.005	0.073	0.000	0.326	0.319	0.456	0.006	0.000	0.000	0.000	0.166	0.060	0.370
Baseline	0.022	0.046	0.000	0.344	0.170	0.213	0.010	0.000	0.000	0.000	0.227	0.016	0.406
Baseline	0.000	0.103	0.000	0.224	0.505	0.294	0.028	0.000	0.020	0.000	0.047	0.083	0.469
Baseline	0.082	0.099	0.000	0.064	0.513	0.271	0.000	0.000	0.032	0.000	0.154	0.053	0.303
Baseline	0.002	0.035	0.000	0.077	0.244	0.416	0.000	0.000	0.000	0.000	0.202	0.019	0.406
Baseline	0.014	0.152	0.000	0.242	0.394	0.369	0.000	0.000	0.006	0.000	0.100	0.090	0.386
Baseline	0.005	0.067	0.002	0.346	0.540	0.241	0.034	0.000	0.000	0.000	0.147	0.078	0.310
Baseline	0.081	0.025	0.000	0.129	0.461	0.278	0.072	0.000	0.000	0.000	0.264	0.013	0.374
Baseline	0.031	0.072	0.000	0.233	0.407	0.289	0.015	0.000	0.000	0.000	0.080	0.064	0.380
Baseline	0.000	0.078	0.000	0.092	0.296	0.229	0.000	0.000	0.050	0.000	0.354	0.059	0.519
Baseline	0.003	0.062	0.000	0.220	0.336	0.309	0.000	0.000	0.000	0.000	0.396	0.000	0.302
Baseline	0.055	0.129	0.000	0.171	0.359	0.403	0.000	0.000	0.012	0.000	0.013	0.093	0.321
Baseline	0.037	0.081	0.000	0.078	0.584	0.162	0.035	0.000	0.022	0.000	0.174	0.007	0.409
Baseline	0.000	0.117	0.000	0.206	0.418	0.378	0.000	0.000	0.000	0.000	0.229	0.064	0.252
Baseline	0.000	0.271	0.000	0.135	0.149	0.330	0.017	0.000	0.000	0.000	0.089	0.020	0.351
Baseline	0.000	0.111	0.000	0.230	0.379	0.395	0.000	0.000	0.000	0.000	0.062	0.112	0.320
Baseline	0.000	0.077	0.000	0.387	0.034	0.275	0.000	0.000	0.072	0.000	0.130	0.088	0.519
Baseline	0.007	0.153	0.000	0.325	0.281	0.271	0.003	0.000	0.003	0.000	0.175	0.035	0.536
Baseline	0.000	0.173	0.000	0.226	0.380	0.515	0.029	0.000	0.000	0.000	0.145	0.001	0.354
Baseline	0.029	0.091	0.000	0.258	0.623	0.336	0.039	0.000	0.000	0.000	0.053	0.056	0.349
Baseline	0.027	0.193	0.000	0.224	0.671	0.343	0.008	0.000	0.038	0.000	0.065	0.046	0.372
Baseline	0.026	0.104	0.000	0.174	0.371	0.253	0.000	0.000	0.043	0.000	0.122	0.144	0.437
Baseline	0.006	0.093	0.000	0.177	0.462	0.363	0.020	0.000	0.043	0.000	0.060	0.047	0.451

Baseline	0.008	0.085	0.022	0.163	0.363	0.293	0.000	0.000	0.042	0.000	0.155	0.094	0.395
Baseline	0.045	0.100	0.000	0.119	0.331	0.562	0.000	0.000	0.000	0.000	0.131	0.082	0.558
Baseline	0.000	0.091	0.000	0.194	0.414	0.401	0.000	0.000	0.000	0.000	0.186	0.072	0.422
Baseline	0.016	0.133	0.000	0.233	0.388	0.477	0.000	0.000	0.000	0.000	0.122	0.071	0.269
Baseline	0.000	0.088	0.003	0.451	0.185	0.380	0.000	0.000	0.017	0.000	0.288	0.103	0.349
Baseline	0.015	0.081	0.000	0.170	0.444	0.290	0.000	0.000	0.063	0.000	0.164	0.079	0.532
Baseline	0.004	0.087	0.000	0.159	0.287	0.467	0.000	0.000	0.000	0.000	0.087	0.082	0.484
Baseline	0.000	0.136	0.000	0.202	0.369	0.473	0.011	0.000	0.000	0.000	0.103	0.059	0.331
Baseline	0.018	0.096	0.004	0.115	0.511	0.423	0.022	0.000	0.013	0.000	0.145	0.071	0.337
Baseline	0.023	0.063	0.000	0.076	0.719	0.193	0.023	0.000	0.028	0.000	0.091	0.004	0.437
Baseline	0.006	0.067	0.000	0.094	0.551	0.351	0.000	0.000	0.032	0.000	0.173	0.062	0.570
Baseline	0.000	0.053	0.000	0.291	0.422	0.173	0.037	0.000	0.031	0.000	0.122	0.052	0.516
Baseline	0.013	0.111	0.000	0.330	0.678	0.197	0.000	0.000	0.050	0.000	0.115	0.090	0.348
Baseline	0.000	0.167	0.000	0.076	0.324	0.295	0.000	0.000	0.028	0.000	0.076	0.027	0.677
Baseline	0.006	0.130	0.000	0.133	0.342	0.287	0.000	0.000	0.037	0.000	0.120	0.079	0.479
Baseline	0.002	0.134	0.000	0.220	0.380	0.406	0.015	0.000	0.032	0.000	0.103	0.036	0.422
Baseline	0.033	0.097	0.000	0.200	0.379	0.366	0.000	0.000	0.000	0.000	0.148	0.087	0.387
Baseline	0.166	0.279	0.000	0.122	0.499	0.179	0.002	0.000	0.054	0.000	0.027	0.075	0.295
Baseline	0.040	0.099	0.000	0.244	0.328	0.460	0.000	0.000	0.000	0.000	0.072	0.101	0.402
Baseline	0.000	0.099	0.000	0.251	0.337	0.390	0.000	0.000	0.039	0.000	0.086	0.097	0.472
Baseline	0.009	0.081	0.000	0.428	0.269	0.463	0.000	0.000	0.012	0.000	0.142	0.103	0.415
Baseline	0.000	0.123	0.019	0.156	0.310	0.395	0.000	0.000	0.048	0.000	0.080	0.070	0.421
Baseline	0.001	0.129	0.000	0.293	0.240	0.467	0.010	0.000	0.025	0.000	0.144	0.032	0.385
Baseline	0.029	0.033	0.000	0.157	0.431	0.299	0.030	0.000	0.000	0.000	0.111	0.045	0.434
Baseline	0.011	0.094	0.000	0.354	0.321	0.352	0.003	0.000	0.012	0.000	0.115	0.089	0.317
Baseline	0.053	0.138	0.000	0.036	0.416	0.284	0.000	0.000	0.000	0.000	0.168	0.095	0.409
Baseline	0.000	0.144	0.000	0.366	0.393	0.376	0.000	0.000	0.005	0.000	0.060	0.084	0.293
Baseline	0.000	0.111	0.000	0.219	0.500	0.296	0.000	0.000	0.009	0.000	0.231	0.064	0.607
Baseline	0.004	0.087	0.000	0.121	0.518	0.327	0.034	0.000	0.001	0.000	0.070	0.058	0.467
Baseline	0.044	0.145	0.003	0.157	0.790	0.155	0.007	0.000	0.043	0.000	0.064	0.044	0.350
Baseline	0.021	0.135	0.000	0.313	0.338	0.151	0.056	0.000	0.052	0.000	0.234	0.011	0.351
Baseline	0.008	0.094	0.001	0.149	0.391	0.315	0.000	0.000	0.000	0.000	0.020	0.126	0.365

Timepoint	B cells naive	B cells memory	Plasma cells	T cells CD8	T cells CD4 naive	T cells CD4 memory resting	T cells CD4 memory activated	T cells follicular helper	T cells regulatory (Tregs)	T cells gamma delta	NK cells resting	NK cells activated	Monocytes
Baseline	0.052	0.062	0.002	0.254	0.367	0.453	0.000	0.000	0.000	0.000	0.040	0.088	0.363
Baseline	0.042	0.031	0.000	0.132	0.454	0.384	0.023	0.000	0.000	0.000	0.049	0.074	0.457
Baseline	0.030	0.089	0.000	0.269	0.512	0.075	0.009	0.000	0.067	0.000	0.065	0.071	0.373
Baseline	0.000	0.088	0.003	0.191	0.322	0.471	0.032	0.000	0.000	0.000	0.205	0.032	0.456
Baseline	0.048	0.075	0.000	0.219	0.311	0.396	0.072	0.000	0.000	0.000	0.117	0.083	0.443
Followup	0.017	0.133	0.000	0.165	0.333	0.519	0.000	0.000	0.000	0.000	0.104	0.086	0.379
Followup	0.066	0.049	0.000	0.711	0.207	0.229	0.000	0.000	0.094	0.000	0.000	0.191	0.524
Followup	0.212	0.000	0.000	0.121	0.489	0.469	0.000	0.000	0.031	0.000	0.000	0.176	0.598
Followup	0.044	0.047	0.000	0.201	0.416	0.281	0.068	0.000	0.000	0.000	0.100	0.103	0.428
Followup	0.089	0.096	0.000	0.272	0.448	0.355	0.037	0.000	0.000	0.000	0.205	0.000	0.320
Followup	0.046	0.090	0.002	0.268	0.136	0.352	0.039	0.000	0.000	0.000	0.262	0.016	0.642
Followup	0.005	0.084	0.000	0.445	0.236	0.489	0.001	0.000	0.000	0.000	0.057	0.116	0.239
Followup	0.000	0.126	0.000	0.496	0.381	0.330	0.000	0.000	0.000	0.000	0.115	0.181	0.486
Followup	0.020	0.173	0.000	0.349	0.338	0.351	0.000	0.000	0.029	0.000	0.108	0.075	0.372
Followup	0.000	0.084	0.000	0.408	0.242	0.447	0.031	0.000	0.000	0.000	0.137	0.060	0.384
Followup	0.081	0.032	0.000	0.222	0.325	0.358	0.000	0.000	0.011	0.000	0.100	0.105	0.254
Followup	0.027	0.047	0.000	0.079	0.521	0.302	0.053	0.000	0.024	0.000	0.019	0.043	0.301
Followup	0.080	0.045	0.000	0.178	0.484	0.344	0.011	0.000	0.000	0.000	0.125	0.027	0.280
Followup	0.011	0.075	0.000	0.281	0.355	0.386	0.000	0.000	0.000	0.000	0.177	0.066	0.194
Followup	0.023	0.036	0.000	0.436	0.173	0.474	0.016	0.000	0.000	0.000	0.220	0.057	0.486
Followup	0.111	0.072	0.001	0.123	0.400	0.346	0.001	0.000	0.000	0.000	0.268	0.000	0.284
Followup	0.020	0.099	0.000	0.167	0.629	0.402	0.034	0.000	0.006	0.000	0.016	0.052	0.335
Followup	0.069	0.058	0.000	0.097	0.431	0.510	0.012	0.000	0.000	0.000	0.074	0.021	0.287
Followup	0.000	0.029	0.002	0.037	0.375	0.191	0.041	0.000	0.000	0.000	0.115	0.000	0.910
Followup	0.004	0.151	0.000	0.219	0.500	0.406	0.025	0.000	0.006	0.000	0.057	0.041	0.344
Followup	0.000	0.052	0.000	0.190	0.281	0.255	0.009	0.000	0.000	0.000	0.110	0.054	0.646
Followup	0.010	0.082	0.000	0.020	0.331	0.309	0.021	0.000	0.000	0.000	0.092	0.079	0.579
Followup	0.005	0.092	0.000	0.251	0.407	0.464	0.000	0.000	0.000	0.000	0.002	0.068	0.421
Followup	0.000	0.135	0.000	0.062	0.361	0.251	0.000	0.000	0.046	0.000	0.123	0.138	0.477

Followup	0.012	0.186	0.000	0.222	0.469	0.388	0.000	0.000	0.000	0.000	0.092	0.055	0.359
Followup	0.000	0.058	0.000	0.204	0.578	0.355	0.048	0.000	0.013	0.000	0.105	0.017	0.499
Followup	0.007	0.138	0.004	0.100	0.496	0.375	0.035	0.000	0.000	0.000	0.152	0.051	0.339
Followup	0.000	0.061	0.000	0.204	0.404	0.279	0.105	0.000	0.000	0.000	0.141	0.092	0.465
Followup	0.000	0.093	0.000	0.139	0.416	0.611	0.000	0.000	0.000	0.000	0.110	0.134	0.364
Followup	0.020	0.108	0.000	0.390	0.558	0.125	0.000	0.000	0.088	0.000	0.090	0.089	0.319
Followup	0.058	0.142	0.000	0.166	0.292	0.528	0.019	0.000	0.000	0.000	0.089	0.068	0.538
Followup	0.031	0.112	0.000	0.272	0.297	0.480	0.000	0.000	0.042	0.000	0.111	0.130	0.383
Followup	0.084	0.060	0.000	0.017	0.426	0.354	0.000	0.000	0.081	0.000	0.118	0.102	0.296
Followup	0.000	0.124	0.000	0.160	0.494	0.289	0.000	0.000	0.000	0.000	0.196	0.033	0.372
Followup	0.042	0.094	0.004	0.112	0.607	0.422	0.000	0.000	0.000	0.000	0.076	0.090	0.299
Followup	0.052	0.155	0.000	0.220	0.564	0.388	0.000	0.000	0.000	0.000	0.018	0.062	0.320
Followup	0.007	0.094	0.000	0.092	0.263	0.401	0.044	0.000	0.000	0.000	0.082	0.058	0.471
Followup	0.005	0.082	0.025	0.093	0.443	0.394	0.000	0.000	0.000	0.000	0.157	0.019	0.274
Followup	0.011	0.085	0.000	0.166	0.393	0.514	0.000	0.000	0.006	0.000	0.014	0.080	0.393
Followup	0.028	0.101	0.010	0.163	0.380	0.544	0.000	0.000	0.000	0.000	0.107	0.021	0.220
Followup	0.000	0.094	0.002	0.173	0.276	0.416	0.003	0.000	0.023	0.000	0.023	0.086	0.415
Followup	0.078	0.128	0.000	0.047	0.561	0.299	0.000	0.000	0.033	0.000	0.147	0.079	0.364
Followup	0.000	0.128	0.000	0.175	0.250	0.298	0.000	0.000	0.003	0.000	0.088	0.082	0.555
Followup	0.071	0.104	0.000	0.263	0.376	0.484	0.000	0.000	0.024	0.000	0.079	0.046	0.331
Followup	0.001	0.054	0.000	0.353	0.179	0.162	0.041	0.000	0.037	0.000	0.110	0.072	0.588
Followup	0.000	0.084	0.000	0.293	0.424	0.404	0.021	0.000	0.000	0.000	0.075	0.078	0.347
Followup	0.000	0.132	0.000	0.085	0.194	0.530	0.027	0.000	0.000	0.000	0.103	0.088	0.382
Followup	0.286	0.568	0.003	0.092	0.207	0.105	0.007	0.000	0.049	0.000	0.000	0.045	0.095
Followup	0.038	0.162	0.000	0.138	0.498	0.244	0.000	0.000	0.025	0.000	0.031	0.176	0.383
Followup	0.049	0.125	0.000	0.353	0.295	0.378	0.000	0.000	0.041	0.000	0.160	0.057	0.346
Followup	0.000	0.200	0.000	0.402	0.173	0.603	0.018	0.000	0.000	0.021	0.015	0.071	0.315
Followup	0.000	0.100	0.000	0.325	0.358	0.410	0.036	0.000	0.000	0.000	0.132	0.076	0.296
Followup	0.000	0.102	0.000	0.173	0.402	0.496	0.029	0.000	0.000	0.000	0.000	0.118	0.491
Followup	0.000	0.123	0.000	0.249	0.388	0.278	0.028	0.000	0.000	0.000	0.061	0.109	0.512
Followup	0.022	0.121	0.000	0.124	0.524	0.302	0.012	0.000	0.042	0.000	0.045	0.069	0.446
Followup	0.000	0.075	0.000	0.262	0.400	0.473	0.013	0.000	0.000	0.000	0.130	0.055	0.248

Followup	0.000	0.141	0.000	0.286	0.257	0.489	0.008	0.000	0.020	0.000	0.018	0.069	0.407
Followup	0.000	0.136	0.000	0.263	0.586	0.379	0.021	0.000	0.039	0.000	0.000	0.100	0.290
Followup	0.000	0.149	0.000	0.291	0.414	0.360	0.028	0.000	0.027	0.000	0.000	0.088	0.427
Followup	0.009	0.086	0.000	0.302	0.209	0.277	0.030	0.000	0.034	0.000	0.200	0.066	0.416
Followup	0.008	0.081	0.000	0.144	0.321	0.316	0.000	0.000	0.102	0.000	0.189	0.033	0.456
Followup	0.066	0.038	0.000	0.304	0.383	0.211	0.000	0.000	0.096	0.000	0.070	0.185	0.551
Followup	0.000	0.073	0.000	0.071	0.417	0.442	0.052	0.000	0.000	0.000	0.093	0.063	0.581
Followup	0.030	0.076	0.000	0.041	0.361	0.140	0.061	0.000	0.026	0.000	0.184	0.049	0.551
Followup	0.010	0.081	0.000	0.272	0.400	0.300	0.117	0.000	0.010	0.000	0.147	0.037	0.366

Timepoint	Macrophages M0	Macrophages M1	Macrophages M2	Dendritic cells resting	Dendritic cells activated	Mast cells resting	Mast cells activated	Eosinophils	Neutrophils	P-value
Baseline	0.004	0.000	0.000	0.000	0.052	0.059	0.000	0.000	0.675	0.000
Baseline	0.000	0.000	0.000	0.000	0.057	0.028	0.000	0.000	0.748	0.000
Baseline	0.000	0.000	0.000	0.000	0.061	0.142	0.000	0.000	1.705	0.000
Baseline	0.142	0.000	0.000	0.000	0.000	0.091	0.000	0.000	1.583	0.000
Baseline	0.000	0.000	0.020	0.006	0.016	0.040	0.000	0.000	0.758	0.000
Baseline	0.000	0.000	0.004	0.000	0.085	0.056	0.000	0.000	0.822	0.000
Baseline	0.023	0.000	0.000	0.000	0.066	0.058	0.000	0.000	1.597	0.000
Baseline	0.002	0.000	0.000	0.000	0.032	0.090	0.000	0.000	0.771	0.000
Baseline	0.000	0.000	0.000	0.000	0.036	0.060	0.000	0.000	1.066	0.000
Baseline	0.000	0.000	0.010	0.000	0.001	0.070	0.000	0.000	1.452	0.000
Baseline	0.000	0.000	0.000	0.000	0.022	0.079	0.000	0.000	0.591	0.000
Baseline	0.018	0.000	0.000	0.000	0.031	0.071	0.000	0.000	0.609	0.000
Baseline	0.049	0.000	0.000	0.000	0.023	0.024	0.000	0.000	0.979	0.000
Baseline	0.016	0.000	0.000	0.000	0.019	0.065	0.000	0.000	1.304	0.000
Baseline	0.032	0.000	0.022	0.000	0.007	0.078	0.000	0.000	1.151	0.000
Baseline	0.042	0.000	0.000	0.000	0.005	0.031	0.000	0.000	0.861	0.000
Baseline	0.000	0.000	0.000	0.000	0.029	0.086	0.000	0.000	1.153	0.000
Baseline	0.005	0.000	0.000	0.000	0.023	0.051	0.000	0.000	0.898	0.000
Baseline	0.000	0.000	0.000	0.000	0.001	0.038	0.000	0.000	0.970	0.000
Baseline	0.038	0.000	0.000	0.000	0.011	0.074	0.000	0.000	1.573	0.000
Baseline	0.005	0.000	0.000	0.000	0.000	0.089	0.000	0.000	1.127	0.000
Baseline	0.017	0.000	0.000	0.000	0.004	0.080	0.000	0.000	1.271	0.000
Baseline	0.077	0.000	0.000	0.000	0.018	0.070	0.000	0.000	0.918	0.000
Baseline	0.003	0.000	0.000	0.000	0.000	0.043	0.000	0.000	0.897	0.000
Baseline	0.000	0.000	0.000	0.000	0.018	0.086	0.000	0.000	0.940	0.000
Baseline	0.005	0.000	0.000	0.000	0.027	0.057	0.000	0.009	0.514	0.000
Baseline	0.007	0.000	0.000	0.000	0.015	0.140	0.000	0.000	0.841	0.000
Baseline	0.000	0.000	0.000	0.000	0.030	0.064	0.000	0.000	0.809	0.000

Baseline	0.015	0.000	0.000	0.000	0.017	0.087	0.000	0.000	0.987	0.000
Baseline	0.024	0.000	0.000	0.000	0.000	0.064	0.000	0.000	0.564	0.000
Baseline	0.009	0.000	0.000	0.000	0.002	0.062	0.000	0.000	0.732	0.000
Baseline	0.040	0.000	0.000	0.000	0.035	0.057	0.000	0.000	0.965	0.000
Baseline	0.002	0.000	0.000	0.000	0.026	0.086	0.000	0.010	0.630	0.000
Baseline	0.051	0.000	0.000	0.000	0.002	0.053	0.000	0.000	0.777	0.000
Baseline	0.000	0.000	0.000	0.000	0.000	0.080	0.000	0.000	0.810	0.000
Baseline	0.009	0.000	0.000	0.000	0.012	0.064	0.000	0.000	0.872	0.000
Baseline	0.022	0.000	0.000	0.000	0.028	0.090	0.000	0.000	1.197	0.000
Baseline	0.000	0.000	0.000	0.000	0.048	0.084	0.000	0.000	1.191	0.000
Baseline	0.016	0.000	0.000	0.000	0.003	0.074	0.000	0.000	0.390	0.000
Baseline	0.107	0.000	0.000	0.000	0.000	0.110	0.000	0.000	1.302	0.000
Baseline	0.016	0.000	0.000	0.000	0.017	0.066	0.000	0.000	0.583	0.000
Baseline	0.000	0.057	0.000	0.000	0.038	0.081	0.000	0.000	1.184	0.000
Baseline	0.000	0.000	0.000	0.000	0.025	0.102	0.000	0.000	1.093	0.000
Baseline	0.000	0.000	0.000	0.000	0.026	0.060	0.000	0.000	0.758	0.000
Baseline	0.000	0.000	0.000	0.011	0.000	0.031	0.000	0.056	0.676	0.000
Baseline	0.000	0.000	0.000	0.000	0.056	0.058	0.000	0.000	0.626	0.000
Baseline	0.039	0.000	0.000	0.000	0.004	0.066	0.000	0.000	0.683	0.000
Baseline	0.023	0.000	0.000	0.000	0.000	0.092	0.000	0.000	0.952	0.000
Baseline	0.000	0.000	0.000	0.005	0.017	0.070	0.000	0.000	0.537	0.000
Baseline	0.019	0.000	0.000	0.000	0.026	0.085	0.000	0.000	0.983	0.000
Baseline	0.004	0.000	0.000	0.000	0.020	0.053	0.000	0.000	1.024	0.000
Baseline	0.005	0.000	0.000	0.000	0.023	0.090	0.000	0.000	1.003	0.000
Baseline	0.014	0.000	0.000	0.000	0.020	0.083	0.000	0.000	0.846	0.000
Baseline	0.000	0.000	0.000	0.000	0.014	0.097	0.000	0.000	1.090	0.000
Baseline	0.000	0.000	0.000	0.000	0.009	0.080	0.000	0.000	1.016	0.000
Baseline	0.043	0.000	0.000	0.000	0.008	0.030	0.000	0.000	0.480	0.000
Baseline	0.033	0.000	0.000	0.000	0.014	0.100	0.000	0.000	1.049	0.000
Baseline	0.000	0.000	0.000	0.000	0.029	0.055	0.000	0.000	0.506	0.000
Baseline	0.004	0.000	0.000	0.000	0.061	0.085	0.000	0.000	0.720	0.000
Baseline	0.000	0.000	0.000	0.000	0.016	0.124	0.000	0.000	0.703	0.000

Timepoint	Macrophages M0	Macrophages M1	Macrophages M2	Dendritic cells resting	Dendritic cells activated	Mast cells resting	Mast cells activated	Eosinophils	Neutrophils	P-value
Baseline	0.000	0.000	0.000	0.000	0.016	0.064	0.000	0.000	0.947	0.000
Baseline	0.062	0.000	0.000	0.000	0.033	0.131	0.000	0.000	1.245	0.000
Baseline	0.099	0.000	0.000	0.001	0.062	0.075	0.000	0.000	0.728	0.000
Baseline	0.042	0.000	0.000	0.000	0.023	0.041	0.000	0.000	0.721	0.000
Baseline	0.000	0.000	0.000	0.003	0.007	0.125	0.000	0.000	0.901	0.000
Followup	0.000	0.000	0.005	0.000	0.015	0.087	0.000	0.008	0.821	0.000
Followup	0.000	0.001	0.000	0.000	0.004	0.104	0.000	0.000	0.785	0.000
Followup	0.000	0.000	0.007	0.000	0.019	0.121	0.000	0.000	0.888	0.000
Followup	0.000	0.000	0.000	0.000	0.033	0.119	0.000	0.038	0.668	0.000
Followup	0.009	0.000	0.000	0.000	0.034	0.054	0.000	0.000	0.872	0.000
Followup	0.000	0.000	0.000	0.000	0.031	0.045	0.000	0.000	0.776	0.000
Followup	0.000	0.000	0.000	0.030	0.012	0.083	0.000	0.000	1.168	0.000
Followup	0.000	0.000	0.021	0.000	0.058	0.033	0.000	0.000	0.622	0.000
Followup	0.000	0.000	0.005	0.000	0.030	0.046	0.000	0.000	0.835	0.000
Followup	0.000	0.000	0.000	0.000	0.011	0.060	0.000	0.006	0.998	0.000
Followup	0.000	0.000	0.030	0.000	0.035	0.083	0.000	0.000	1.153	0.000
Followup	0.000	0.000	0.000	0.000	0.092	0.093	0.000	0.036	1.125	0.000
Followup	0.000	0.000	0.000	0.000	0.078	0.053	0.000	0.000	0.747	0.000
Followup	0.000	0.000	0.000	0.019	0.024	0.049	0.000	0.000	1.010	0.000
Followup	0.000	0.000	0.000	0.010	0.019	0.043	0.000	0.000	0.718	0.000
Followup	0.070	0.000	0.000	0.000	0.030	0.058	0.000	0.000	0.975	0.000
Followup	0.000	0.000	0.000	0.000	0.051	0.076	0.000	0.000	0.675	0.000
Followup	0.011	0.000	0.000	0.000	0.047	0.071	0.000	0.000	0.660	0.000
Followup	0.054	0.000	0.000	0.000	0.075	0.104	0.000	0.000	1.211	0.000
Followup	0.012	0.000	0.000	0.000	0.022	0.057	0.000	0.021	0.807	0.000
Followup	0.011	0.000	0.000	0.000	0.061	0.125	0.000	0.000	1.458	0.000
Followup	0.019	0.000	0.000	0.000	0.023	0.128	0.000	0.000	1.266	0.000
Followup	0.000	0.000	0.000	0.000	0.058	0.073	0.000	0.000	0.851	0.000
Followup	0.069	0.000	0.000	0.000	0.042	0.101	0.000	0.000	2.121	0.000

Followup	0.000	0.000	0.000	0.000	0.056	0.068	0.000	0.000	0.738	0.000
Followup	0.020	0.000	0.000	0.000	0.007	0.066	0.000	0.000	0.547	0.000
Followup	0.010	0.000	0.000	0.000	0.055	0.046	0.000	0.000	0.777	0.000
Followup	0.000	0.000	0.000	0.000	0.024	0.140	0.000	0.000	0.913	0.000
Followup	0.000	0.009	0.004	0.000	0.024	0.090	0.000	0.001	1.133	0.000
Followup	0.000	0.000	0.000	0.000	0.022	0.078	0.000	0.000	0.725	0.000
Followup	0.013	0.000	0.000	0.000	0.023	0.059	0.000	0.000	0.432	0.000
Followup	0.025	0.000	0.000	0.000	0.020	0.117	0.000	0.000	0.739	0.000
Followup	0.069	0.000	0.000	0.000	0.006	0.029	0.000	0.000	1.477	0.000
Followup	0.000	0.000	0.000	0.000	0.098	0.024	0.000	0.000	0.475	0.000
Followup	0.000	0.000	0.000	0.000	0.032	0.061	0.000	0.000	0.484	0.000
Followup	0.019	0.000	0.000	0.000	0.045	0.033	0.000	0.000	0.407	0.000
Followup	0.059	0.000	0.000	0.000	0.020	0.115	0.000	0.000	1.030	0.000
Followup	0.000	0.000	0.000	0.014	0.051	0.040	0.000	0.000	1.168	0.000
Followup	0.003	0.000	0.000	0.000	0.064	0.087	0.000	0.058	0.831	0.000
Followup	0.001	0.000	0.000	0.000	0.015	0.030	0.000	0.000	0.815	0.000
Followup	0.000	0.000	0.000	0.000	0.016	0.137	0.000	0.087	0.825	0.000
Followup	0.000	0.000	0.000	0.000	0.003	0.085	0.000	0.000	0.794	0.000
Followup	0.022	0.000	0.000	0.000	0.034	0.083	0.000	0.000	1.254	0.000
Followup	0.000	0.000	0.000	0.000	0.036	0.027	0.000	0.000	0.913	0.000
Followup	0.004	0.000	0.000	0.000	0.045	0.096	0.000	0.000	1.234	0.000
Followup	0.000	0.000	0.000	0.000	0.045	0.102	0.000	0.000	0.902	0.000
Followup	0.002	0.000	0.000	0.000	0.029	0.114	0.000	0.000	1.100	0.000
Followup	0.000	0.000	0.000	0.000	0.049	0.043	0.000	0.000	0.289	0.000
Followup	0.000	0.000	0.000	0.000	0.019	0.161	0.000	0.000	0.706	0.000
Followup	0.004	0.000	0.000	0.000	0.042	0.065	0.000	0.000	0.736	0.000
Followup	0.000	0.000	0.000	0.000	0.037	0.048	0.000	0.000	0.704	0.000
Followup	0.000	0.000	0.000	0.000	0.061	0.090	0.000	0.026	0.649	0.000
Followup	0.000	0.000	0.000	0.000	0.005	0.126	0.000	0.000	0.714	0.000
Followup	0.000	0.000	0.000	0.000	0.007	0.149	0.000	0.000	1.198	0.000
Followup	0.000	0.000	0.000	0.000	0.039	0.115	0.000	0.000	1.024	0.000
Followup	0.000	0.000	0.000	0.000	0.035	0.079	0.000	0.000	1.298	0.000

Followup	0.022	0.000	0.000	0.000	0.019	0.084	0.000	0.000	1.224	0.000
Followup	0.000	0.000	0.002	0.000	0.047	0.095	0.000	0.000	0.675	0.000
Followup	0.000	0.000	0.000	0.000	0.043	0.092	0.000	0.000	0.979	0.000
Followup	0.000	0.000	0.000	0.000	0.021	0.092	0.000	0.000	0.720	0.000
Followup	0.001	0.000	0.000	0.000	0.031	0.050	0.000	0.000	0.779	0.000
Followup	0.000	0.000	0.000	0.000	0.043	0.054	0.000	0.000	0.636	0.000
Followup	0.000	0.000	0.000	0.000	0.037	0.088	0.000	0.000	0.759	0.000
Followup	0.025	0.000	0.000	0.000	0.044	0.112	0.000	0.000	1.124	0.000
Followup	0.000	0.000	0.000	0.000	0.054	0.082	0.000	0.000	0.926	0.000

Correlation	RMSE	Absolute score (sig.score)
0.900	0.525	2.534
0.915	0.503	2.639
0.942	0.370	3.346
0.946	0.366	3.269
0.925	0.485	2.542
0.919	0.492	2.751
0.942	0.373	3.199
0.911	0.503	2.667
0.905	0.480	2.732
0.932	0.393	2.936
0.909	0.527	2.444
0.887	0.545	2.500
0.922	0.466	2.772
0.932	0.412	2.976
0.895	0.486	2.966
0.888	0.514	2.568
0.926	0.438	2.825
0.913	0.476	2.564
0.907	0.483	2.674
0.954	0.341	3.056
0.915	0.459	2.829
0.928	0.419	2.953
0.911	0.491	2.873
0.925	0.474	2.766
0.910	0.492	2.878
0.856	0.591	2.597
0.914	0.494	2.679
0.922	0.479	2.628

0.898	0.496	2.729
0.893	0.546	2.580
0.898	0.519	2.583
0.924	0.471	2.807
0.894	0.535	2.618
0.923	0.485	2.723
0.929	0.463	2.548
0.920	0.479	2.639
0.923	0.453	3.094
0.911	0.457	2.979
0.900	0.549	2.389
0.899	0.475	3.214
0.850	0.588	2.614
0.902	0.469	3.031
0.928	0.439	2.834
0.911	0.503	2.594
0.919	0.499	2.470
0.883	0.562	2.437
0.911	0.512	2.539
0.898	0.501	2.838
0.903	0.533	2.552
0.920	0.465	2.736
0.914	0.473	2.825
0.923	0.451	2.691
0.930	0.469	2.632
0.930	0.438	2.800
0.928	0.456	2.828
0.904	0.538	2.598
0.920	0.459	2.882
0.905	0.535	2.391
0.896	0.535	2.532
0.841	0.582	2.312

Correlation	RMSE	Absolute score (sig.score)
0.900	0.495	2.708
0.889	0.494	3.115
0.907	0.516	2.527
0.875	0.550	2.628
0.883	0.531	2.800
0.926	0.481	2.673
0.917	0.500	2.964
0.858	0.567	3.131
0.918	0.512	2.547
0.935	0.472	2.791
0.950	0.443	2.705
0.926	0.446	2.966
0.892	0.543	2.850
0.902	0.512	2.731
0.951	0.421	2.867
0.940	0.416	2.789
0.946	0.403	2.760
0.933	0.475	2.452
0.938	0.433	2.647
0.906	0.511	2.711
0.861	0.551	2.739
0.922	0.499	2.562
0.918	0.503	2.349
0.938	0.393	3.144
0.920	0.489	2.672
0.923	0.418	3.253
0.937	0.397	2.958
0.932	0.463	2.693
0.924	0.402	3.924

0.919	0.504	2.645
0.918	0.514	2.517
0.924	0.490	2.587
0.923	0.478	2.828
0.886	0.512	3.129
0.908	0.513	2.612
0.912	0.543	2.428
0.893	0.538	2.758
0.931	0.405	3.118
0.907	0.539	2.265
0.922	0.517	2.324
0.920	0.528	2.283
0.941	0.422	2.735
0.943	0.403	2.764
0.913	0.496	2.705
0.930	0.462	2.436
0.918	0.485	2.576
0.911	0.504	2.618
0.936	0.405	2.973
0.920	0.478	2.754
0.936	0.407	2.976
0.923	0.478	2.774
0.942	0.417	2.783
0.837	0.622	1.838
0.910	0.521	2.581
0.906	0.520	2.651
0.915	0.510	2.605
0.885	0.552	2.559
0.886	0.539	2.656
0.896	0.488	3.102
0.901	0.493	2.884
0.881	0.504	3.068

0.889	0.494	3.043
0.889	0.543	2.633
0.913	0.485	2.898
0.882	0.539	2.461
0.927	0.474	2.511
0.897	0.531	2.636
0.919	0.491	2.676
0.933	0.421	2.824
0.911	0.495	2.802

Supplementary Table 4 amyloid non-accumulators follow-up versus baseline significant differentially expressed genes

Gene	baseMean	log2FoldChange	lfcSE	stat	pvalue	padj
SLC25A39	8333.375	-2.234728579	0.809815	-2.75955	0.005788	0.04924
PSMF1	925.0686	-1.975263667	0.697422	-2.83224	0.004622	0.044185
PCGF5	1029.707	-1.934524542	0.694774	-2.78439	0.005363	0.047666
RNF10	1761.75	-1.881366817	0.640761	-2.93614	0.003323	0.04122
MT-ND5	3362.684	-1.67829982	0.607491	-2.76268	0.005733	0.04924
MT-ND4	12652.17	-1.568866308	0.559194	-2.80559	0.005023	0.046797
S100A11	3071.025	-1.449940647	0.458835	-3.16005	0.001577	0.036119
ZYX	1372.57	-1.322655163	0.448706	-2.94771	0.003201	0.040852
LSP1	1048.375	-1.258196274	0.421193	-2.98722	0.002815	0.039118
LITAF	4363.529	-1.161890175	0.374158	-3.10534	0.001901	0.036119
GLUL	918.5887	-1.117698514	0.365898	-3.05467	0.002253	0.036737
LILRB3	1554.375	-1.085547428	0.376511	-2.88318	0.003937	0.041267
FLOT2	930.0029	-1.083111818	0.371769	-2.9134	0.003575	0.04122
IQGAP1	1986.602	-1.038086585	0.350594	-2.96093	0.003067	0.040008
XPO6	1178.564	-1.013079835	0.357238	-2.83587	0.00457	0.044185
NSA2	981.6404	1.000984031	0.310348	3.225357	0.001258	0.035168
RPS5	1586.337	1.060068137	0.335851	3.156363	0.001597	0.036119
EEF1G	1812.806	1.070948193	0.326316	3.281936	0.001031	0.035168
RPS3	4933.792	1.081389063	0.35892	3.0129	0.002588	0.039118
RPL13A	6646.236	1.104024901	0.387375	2.850013	0.004372	0.044185
RPL22	1487.431	1.143213923	0.363794	3.142473	0.001675	0.036119
RPL29	1102.106	1.156630145	0.318306	3.6337	0.000279	0.024603
RPL3	2841.264	1.168174737	0.417163	2.800282	0.005106	0.04683
RPSAP58	1654.001	1.172054385	0.40388	2.901986	0.003708	0.04122
RPL15	2298.715	1.202227597	0.386374	3.111565	0.001861	0.036119
RPS16	3965.088	1.24942949	0.369489	3.381506	0.000721	0.028211
RPS14	5357.569	1.342739516	0.404122	3.32261	0.000892	0.032718
RPL30	5438.029	1.3657595	0.457528	2.985086	0.002835	0.039118
RPS27A	5146.043	1.410837092	0.498676	2.829168	0.004667	0.044185
RPS13	2538.653	1.4325593	0.395618	3.621068	0.000293	0.024603
RPS8	4161.184	1.44341019	0.451351	3.197975	0.001384	0.035364
RPL37	3333.447	1.455487254	0.455178	3.197624	0.001386	0.035364

RPL5	2050.788	1.467757318	0.472819	3.104268	0.001908	0.036119
RPL11	1786.138	1.691039246	0.567554	2.979524	0.002887	0.039118
RPL6	2936.009	1.696303237	0.434777	3.901548	9.56E-05	0.021521
RPS25	1659.969	1.698593403	0.492363	3.44988	0.000561	0.026665
RPL35	980.3456	1.704255983	0.585878	2.908894	0.003627	0.04122
RPS10	2187.607	1.7301305	0.501223	3.451819	0.000557	0.026665
RPS20	3066.996	1.73806949	0.507251	3.426448	0.000612	0.026665
RPL35A	1659.169	1.823105344	0.611233	2.98267	0.002857	0.039118
TPT1	5981.477	1.865048378	0.638654	2.92028	0.003497	0.04122
RPL21	2154.788	1.871622858	0.511487	3.659179	0.000253	0.024603
RPL24	897.4281	1.87302792	0.44789	4.181889	2.89E-05	0.01697
RPS21	1807.141	2.102757941	0.698903	3.008655	0.002624	0.039118
RPS6	3513.645	2.155839402	0.557435	3.867429	0.00011	0.021521
RPS18	1956.204	2.193051765	0.631107	3.474927	0.000511	0.026665
RPL41	9291.688	2.239078058	0.638694	3.505712	0.000455	0.026665
RPS17	1622.303	2.261011856	0.810555	2.789461	0.00528	0.047666
RPL27	1221.987	2.40579096	0.740645	3.24824	0.001161	0.035168
RPL7	1087.23	2.439812683	0.714273	3.415796	0.000636	0.026665
RPS15A	1408.382	2.475924581	0.785895	3.150453	0.00163	0.036119
RPS27	4006.362	2.513109426	0.869827	2.889206	0.003862	0.04122
RPS24	1318.311	2.516487984	0.815159	3.087114	0.002021	0.036237
RPL36A	1449.693	2.516855434	0.84607	2.974761	0.002932	0.039118
RPL23	1217.637	2.585665802	0.841426	3.072955	0.00212	0.036237
RPS7	909.9927	2.586138018	0.83215	3.107779	0.001885	0.036119
RPL9	1324.591	2.685221858	0.928545	2.89186	0.00383	0.04122
RPS3A	1526.443	2.68729581	0.876136	3.067214	0.002161	0.036237
RPL26	1309.917	2.841185066	0.870462	3.263998	0.001099	0.035168
RPL39	2100.614	3.080865172	0.864578	3.563432	0.000366	0.026665
RPL31	1217.307	3.149761003	1.024186	3.075379	0.002102	0.036237
RPL34	1191.319	3.324657597	1.026454	3.238974	0.0012	0.035168

Supplementary Table 5 follow-up versus baseline significant differentially expressed genes in amyloid negative-positive and amyloid positive-positive individuals

Gene	baseMean	log2FoldChange	lfcSE	stat	pvalue	padj
SLC25A39	8333.375	-2.946763142	1.007914	-2.92363	0.00346	0.034665
SELENBP1	737.207	-2.877332923	1.010452	-2.84757	0.00441	0.039845
PDZK1IP1	725.378	-2.748864693	0.982759	-2.79709	0.00516	0.044183
R3HDM4	4250.072	-2.652620208	0.941028	-2.81885	0.00482	0.042642
MARCHF8	995.255	-2.537753177	0.861486	-2.94579	0.00322	0.033192
PCGF5	1029.707	-2.525243103	0.863323	-2.92503	0.00344	0.034665
RNF10	1761.750	-2.309136105	0.798265	-2.89269	0.00382	0.036578
MT-ND5	3362.684	-2.153309858	0.748204	-2.87797	0.00400	0.037448
GUCD1	903.829	-2.116041806	0.760194	-2.78355	0.00538	0.045029
GRINA	741.962	-2.070110142	0.746931	-2.77149	0.00558	0.045454
MT-ND4	12652.174	-1.992221794	0.697858	-2.85477	0.00431	0.039391
NCF1	784.551	-1.716766872	0.632402	-2.71468	0.00663	0.048219
IFITM2	16158.488	-1.674027362	0.601977	-2.78088	0.00542	0.045029
TLN1	1757.221	-1.670012553	0.505630	-3.30283	0.00096	0.019738
AKT2	1038.989	-1.600192639	0.498970	-3.20699	0.00134	0.021673
UBN1	2231.752	-1.501052245	0.465400	-3.22529	0.00126	0.021341
LITAF	4363.529	-1.437983707	0.465436	-3.08954	0.00200	0.024976
GLUL	918.589	-1.436762221	0.451659	-3.18108	0.00147	0.021811
LILRB3	1554.375	-1.426664911	0.464794	-3.06945	0.00214	0.026054
PIK3CD	1422.517	-1.381001593	0.460438	-2.99932	0.00271	0.029764
FLOT2	930.003	-1.344277152	0.461067	-2.91558	0.00355	0.034818
IQGAP1	1986.602	-1.339147868	0.433868	-3.08653	0.00203	0.024976
MARF1	727.002	-1.236694566	0.423419	-2.92074	0.00349	0.034665
CPPED1	665.886	-1.233213565	0.455753	-2.70588	0.00681	0.048219
SIRPB1	1059.771	-1.183711987	0.428741	-2.76090	0.00576	0.045547
MBP	2227.451	-1.17528944	0.361437	-3.25172	0.00115	0.020842
IQSEC1	698.991	-1.1678449	0.386015	-3.02539	0.00248	0.028469
KLF3	886.972	-1.146319159	0.423475	-2.70694	0.00679	0.048219
MAP4K4	1079.011	-1.074792982	0.356680	-3.01333	0.00258	0.028813
FMNL1	721.086	-1.058709293	0.368745	-2.87112	0.00409	0.037835
RIPOR2	4744.470	-1.042524287	0.329182	-3.16702	0.00154	0.021811
HCLS1	2234.326	-1.031835265	0.326866	-3.15676	0.00160	0.021811
STAT6	853.372	-1.030160294	0.321578	-3.20345	0.00136	0.021673
WIPF1	2335.337	-1.013951643	0.358185	-2.83080	0.00464	0.041533
RPLP1	7966.526	1.03587072	0.343681	3.01404	0.00258	0.028813
HNRNPA1	951.029	1.059724988	0.356440	2.97308	0.00295	0.031999
NSA2	981.640	1.109252827	0.401065	2.76577	0.00568	0.045454
RPL10	5254.909	1.112118143	0.375302	2.96326	0.00304	0.032603
RACK1	2362.251	1.124521526	0.303689	3.70287	0.00021	0.009486
RPL38	3145.480	1.185994566	0.401606	2.95313	0.00315	0.032884
RPS26	1256.480	1.211727712	0.360788	3.35856	0.00078	0.018222
RPS2	4303.426	1.218759592	0.382617	3.18533	0.00145	0.021811
RPL27A	7617.693	1.22340569	0.448917	2.72524	0.00643	0.047985
NPM1	678.777	1.252238793	0.445772	2.80915	0.00497	0.043477
HSP90AA1	1744.463	1.255939079	0.435894	2.88129	0.00396	0.037448
RPL22	1487.431	1.265043554	0.467028	2.70871	0.00675	0.048219
RPL12	1381.251	1.267261015	0.385218	3.28973	0.00100	0.019738
RPL13	5277.402	1.296043124	0.447514	2.89609	0.00378	0.036578
B2M	25067.347	1.298204006	0.463564	2.80049	0.00510	0.044183

RPS5	1586.337	1.322193542	0.416616	3.17365	0.00151	0.021811
RPS11	4565.613	1.341247856	0.374573	3.58074	0.00034	0.011245
RPL23A	5701.424	1.352202375	0.490320	2.75780	0.00582	0.045547
RPLP0	2162.997	1.356425527	0.430287	3.15237	0.00162	0.021811
RPL32	4642.894	1.363997365	0.308690	4.41866	0.00001	0.005118
EEF1G	1812.806	1.374683097	0.404886	3.39524	0.00069	0.018005
RPL10A	2322.747	1.402859084	0.430234	3.26069	0.00111	0.020842
RPS3	4933.792	1.426882978	0.442363	3.22559	0.00126	0.021341
RPL14	1654.390	1.433637451	0.433586	3.30646	0.00094	0.019738
RPL19	2517.236	1.519550667	0.482445	3.14968	0.00163	0.021811
RPL13A	6646.236	1.53490099	0.481890	3.18517	0.00145	0.021811
RPL29	1102.106	1.537392295	0.389953	3.94251	0.00008	0.009402
RPS14	5357.569	1.578415159	0.505402	3.12309	0.00179	0.023123
RPL36	1768.697	1.600524164	0.524896	3.04922	0.00229	0.026680
RPSAP58	1654.001	1.663639633	0.494851	3.36190	0.00077	0.018222
RPL4	1599.619	1.678967825	0.498070	3.37095	0.00075	0.018222
RPL15	2298.715	1.728336772	0.472764	3.65581	0.00026	0.009486
RPS27A	5146.043	1.74000756	0.629342	2.76480	0.00570	0.045454
RPS16	3965.088	1.758510878	0.451007	3.89908	0.00010	0.009402
RPL3	2841.264	1.792039151	0.508600	3.52348	0.00043	0.012382
RPL37	3333.447	1.852325786	0.572120	3.23765	0.00121	0.021326
RPS13	2538.653	1.915080068	0.484823	3.95006	0.00008	0.009402
RPS20	3066.996	2.055929798	0.673796	3.05127	0.00228	0.026680
RPS8	4161.184	2.067755107	0.553635	3.73487	0.00019	0.009486
RPL5	2050.788	2.128484055	0.581677	3.65922	0.00025	0.009486
RPL30	5438.029	2.132675534	0.553953	3.84992	0.00012	0.009402
EEF1A1	10481.048	2.16638163	0.709062	3.05528	0.00225	0.026680
RPL6	2936.009	2.195195607	0.540885	4.05853	0.00005	0.009402
DEFA1	1018.224	2.252366061	0.825914	2.72712	0.00639	0.047985
RPS25	1659.969	2.256529706	0.611438	3.69053	0.00022	0.009486
RPS10	2187.607	2.283092157	0.620887	3.67715	0.00024	0.009486
RPL35	980.346	2.346463527	0.721886	3.25046	0.00115	0.020842
RPL11	1786.138	2.357654068	0.705094	3.34374	0.00083	0.018364
RPL21	2154.788	2.418215929	0.634594	3.81065	0.00014	0.009402
RPL24	897.428	2.425579674	0.555387	4.36737	0.00001	0.005118
RPL35A	1659.169	2.482241756	0.755541	3.28538	0.00102	0.019738
TPT1	5981.477	2.646284113	0.786479	3.36472	0.00077	0.018222
RPL41	9291.688	2.676765937	0.801183	3.34102	0.00083	0.018364
RPS18	1956.204	2.714713768	0.789273	3.43951	0.00058	0.016174
RPS21	1807.141	2.750789713	0.870032	3.16171	0.00157	0.021811
RPS6	3513.645	2.836991078	0.689204	4.11633	0.00004	0.009402
RPL36A	1449.693	2.981767637	1.071874	2.78183	0.00541	0.045029
RPS29	7686.161	3.029367876	0.965795	3.13666	0.00171	0.022436
RPL7	1087.230	3.171897864	0.892724	3.55305	0.00038	0.011480
RPS17	1622.303	3.208942332	0.999766	3.20969	0.00133	0.021673
RPL27	1221.987	3.258066019	0.913840	3.56525	0.00036	0.011381
RPS27	4006.362	3.340972252	1.076237	3.10431	0.00191	0.024258
RPS24	1318.311	3.479622261	1.013467	3.43339	0.00060	0.016174
RPL26	1309.917	3.594855946	1.091932	3.29220	0.00099	0.019738
RPS15A	1408.382	3.658887427	0.957362	3.82184	0.00013	0.009402
RPS7	909.993	3.748260754	1.020909	3.67150	0.00024	0.009486
RPL23	1217.637	3.842812436	1.032856	3.72057	0.00020	0.009486

RPL17-C18orf32	777.383	3.852412831	1.076497	3.57866	0.00035	0.011245
RPS3A	1526.443	3.951585317	1.072349	3.68498	0.00023	0.009486
RPL9	1324.591	4.074933697	1.134145	3.59296	0.00033	0.011245
RPL39	2100.614	4.097424271	1.065690	3.84486	0.00012	0.009402
RPL31	1217.307	4.658624091	1.253730	3.71581	0.00020	0.009486
RPL34	1191.319	4.951725458	1.259251	3.93228	0.00008	0.009402

Supplementary Table 6 follow-up versus baseline significant differentially expressed genes in amyloid negative individuals

Gene	baseMean	log2FoldChan	lfcSE	stat	pvalue	padj
NRGN	1589.382	-2.441992	0.86491681	-2.82338	0.00475	0.033607
SLC25A39	8333.375	-2.41133	0.81731993	-2.95029	0.00317	0.028886
BCL2L1	976.614	-2.2754155	0.80559594	-2.82451	0.00474	0.033607
ALAS2	4004.627	-2.2032909	0.86908573	-2.53518	0.01124	0.048605
DMTN	1141.372	-2.1374255	0.79805465	-2.67829	0.00740	0.043007
R3HDM4	4250.072	-2.1276689	0.76310067	-2.78819	0.00530	0.035356
PCGF5	1029.707	-2.0753817	0.70008472	-2.96447	0.00303	0.028251
PSMF1	925.069	-2.074104	0.7079265	-2.92983	0.00339	0.029276
RNF10	1761.750	-1.9893221	0.64737438	-3.07291	0.00212	0.023749
MT-ATP8	4305.259	-1.9879935	0.70086453	-2.83649	0.00456	0.033607
SLC4A1	1119.873	-1.9785284	0.76118603	-2.59927	0.00934	0.045718
DCAF12	1903.247	-1.9766061	0.73761818	-2.67971	0.00737	0.043007
YBX3	3361.782	-1.9499155	0.7581601	-2.57190	0.01011	0.046052
MARCHF8	995.255	-1.9494233	0.69864556	-2.79029	0.00527	0.035356
GYPC	4560.939	-1.9424887	0.73958703	-2.62645	0.00863	0.045718
FAM210B	1695.587	-1.8191057	0.6820774	-2.66701	0.00765	0.044025
FBXO7	1992.629	-1.8129626	0.71978641	-2.51875	0.01178	0.048658
MT-ND5	3362.684	-1.8013913	0.60677527	-2.96879	0.00299	0.028251
UBXN6	1638.285	-1.7789849	0.70558365	-2.52130	0.01169	0.048658
GUCD1	903.829	-1.7057606	0.61649461	-2.76687	0.00566	0.035983
MT-ND4	12652.174	-1.6535725	0.56591293	-2.92196	0.00348	0.029592
PPDPF	1593.152	-1.5180533	0.54981684	-2.76102	0.00576	0.035983
S100A11	3071.025	-1.4773348	0.46634559	-3.16790	0.00154	0.020961
MKRN1	1650.054	-1.4725129	0.58271921	-2.52697	0.01151	0.048658
ZYX	1372.570	-1.3588389	0.45486586	-2.98734	0.00281	0.027520
IFITM2	16158.488	-1.3483568	0.48815544	-2.76215	0.00574	0.035983
BASP1	2819.756	-1.2880475	0.46925686	-2.74487	0.00605	0.036960
LSP1	1048.375	-1.2657243	0.43064347	-2.93915	0.00329	0.028886
YPEL3	2096.562	-1.2442074	0.47870186	-2.59913	0.00935	0.045718
LITAF	4363.529	-1.227065	0.37744095	-3.25101	0.00115	0.020455
WAS	2026.496	-1.1901008	0.46465878	-2.56124	0.01043	0.046736
TLN1	1757.221	-1.1841325	0.41012081	-2.88728	0.00389	0.031121
GLUL	918.589	-1.1795875	0.36638837	-3.21950	0.00128	0.020935
NIBAN1	3048.448	-1.1676599	0.44799734	-2.60640	0.00915	0.045718
LILRB3	1554.375	-1.1543767	0.37695597	-3.06236	0.00220	0.023749
FLOT2	930.003	-1.139429	0.37395057	-3.04700	0.00231	0.024228
VASP	1971.670	-1.1370007	0.43710428	-2.60121	0.00929	0.045718
IQGAP1	1986.602	-1.1037953	0.35188284	-3.13683	0.00171	0.021771
UBN1	2231.752	-1.0953055	0.37748393	-2.90160	0.00371	0.030695
RHOG	1462.773	-1.0619715	0.41726711	-2.54506	0.01093	0.047861
FTL	9186.091	-1.0559664	0.41090545	-2.56985	0.01017	0.046052
CNN2	1134.577	-1.0449807	0.36787134	-2.84061	0.00450	0.033607
XPO6	1178.564	-1.041784	0.36094961	-2.88623	0.00390	0.031121
MT-CO1	4168.057	-1.0382135	0.41195026	-2.52024	0.01173	0.048658
MSN	2674.536	-1.0114291	0.3594955	-2.81347	0.00490	0.033651
RALBP1	901.437	-1.0014777	0.38328709	-2.61287	0.00898	0.045718
RPL12	1381.251	1.00305756	0.31251884	3.20959	0.00133	0.020935
RPS9	2458.962	1.00465956	0.38777357	2.59084	0.00957	0.045805
RPL27A	7617.693	1.02500343	0.36406219	2.81546	0.00487	0.033651

RPLP0	2162.997	1.02559199	0.34900713	2.93860	0.00330	0.028886
RPL10A	2322.747	1.02825631	0.34893367	2.94685	0.00321	0.028886
RPL23A	5701.424	1.03131422	0.39762447	2.59369	0.00950	0.045805
RPL4	1599.619	1.03227089	0.40394658	2.55546	0.01060	0.046804
RPL32	4642.894	1.03604421	0.25037866	4.13791	0.00004	0.005672
NSA2	981.640	1.03928806	0.32544105	3.19348	0.00141	0.020935
RPL19	2517.236	1.0849283	0.39126373	2.77288	0.00556	0.035877
RPS5	1586.337	1.11826273	0.33796705	3.30879	0.00094	0.017989
RPL36	1768.697	1.13405177	0.4257173	2.66386	0.00772	0.044025
EEF1G	1812.806	1.13888783	0.32844899	3.46747	0.00053	0.016231
FAU	4141.028	1.14279174	0.42106381	2.71406	0.00665	0.039811
RPS3	4933.792	1.14573406	0.35876062	3.19359	0.00141	0.020935
RPL22	1487.431	1.18568032	0.37881605	3.12996	0.00175	0.021771
RPL13A	6646.236	1.19519822	0.39079182	3.05840	0.00223	0.023749
RPL29	1102.106	1.23524067	0.31635733	3.90457	0.00009	0.010091
RPSAP58	1654.001	1.26721494	0.40137767	3.15716	0.00159	0.021254
RPL3	2841.264	1.29402518	0.41246376	3.13731	0.00171	0.021771
RPL15	2298.715	1.31099805	0.38343122	3.41912	0.00063	0.017134
RPS16	3965.088	1.35820333	0.36576445	3.71333	0.00020	0.013194
RPS14	5357.569	1.40274378	0.40987722	3.42235	0.00062	0.017134
RPS27A	5146.043	1.48330491	0.51037171	2.90632	0.00366	0.030667
RPS13	2538.653	1.5268544	0.39320822	3.88307	0.00010	0.010091
RPL30	5438.029	1.53068576	0.44922234	3.40741	0.00066	0.017134
RPL37	3333.447	1.546425	0.46397974	3.33296	0.00086	0.017989
RPS8	4161.184	1.57228429	0.44897505	3.50194	0.00046	0.016231
RPL5	2050.788	1.60436204	0.47173012	3.40102	0.00067	0.017134
DEFA1	1018.224	1.72172094	0.67018841	2.56901	0.01020	0.046052
RPL6	2936.009	1.80524747	0.43865578	4.11541	0.00004	0.005672
RPS20	3066.996	1.80923809	0.54642883	3.31102	0.00093	0.017989
RPL35	980.346	1.81443474	0.58548903	3.09901	0.00194	0.022796
RPS25	1659.969	1.81507953	0.49587466	3.66036	0.00025	0.013194
RPL11	1786.138	1.82104137	0.57182033	3.18464	0.00145	0.020935
RPS10	2187.607	1.84052856	0.50355073	3.65510	0.00026	0.013194
RPL35A	1659.169	1.95211732	0.61273829	3.18589	0.00144	0.020935
RPL21	2154.788	1.97791124	0.5146449	3.84325	0.00012	0.010181
RPL24	897.428	1.99320631	0.4505187	4.42425	0.00001	0.005672
TPT1	5981.477	2.02779898	0.63778235	3.17945	0.00148	0.020935
RPS21	1807.141	2.2402697	0.70557576	3.17509	0.00150	0.020935
RPS29	7686.161	2.25559333	0.783181	2.88004	0.00398	0.031121
RPS18	1956.204	2.29658645	0.64007865	3.58798	0.00033	0.013194
RPS6	3513.645	2.30229889	0.5589135	4.11924	0.00004	0.005672
RPL41	9291.688	2.34482691	0.64970234	3.60908	0.00031	0.013194
RPS17	1622.303	2.44380436	0.81075445	3.01423	0.00258	0.026074
RPL27	1221.987	2.56981108	0.74111584	3.46749	0.00053	0.016231
RPL7	1087.230	2.59552544	0.72400781	3.58494	0.00034	0.013194
RPL36A	1449.693	2.60675447	0.86925722	2.99883	0.00271	0.026964
RPS27	4006.362	2.65072661	0.87274847	3.03722	0.00239	0.024589
RPS24	1318.311	2.68935232	0.82186303	3.27226	0.00107	0.019571
RPS15A	1408.382	2.69503198	0.77637351	3.47131	0.00052	0.016231
RPS7	909.993	2.78107236	0.82790389	3.35917	0.00078	0.017650
RPL23	1217.637	2.81994362	0.83757657	3.36679	0.00076	0.017650
RPS3A	1526.443	2.92538724	0.86963639	3.36392	0.00077	0.017650

RPL26	1309.917	2.92653835	0.88551003	3.30492	0.00095	0.017989
RPL9	1324.591	2.9368741	0.9197076	3.19327	0.00141	0.020935
RPL39	2100.614	3.26336331	0.86421543	3.77610	0.00016	0.011689
RPL31	1217.307	3.3935645	1.01667182	3.33792	0.00084	0.017989
RPL34	1191.319	3.66540359	1.02118056	3.58938	0.00033	0.013194

Supplementary Table 7 direct association scores of significant differentially expressed genes at follow-up versus baseline in APOE4 carriers

preferredLabel	Target	Direct Association Score
NRGN is associated with Alzheimer disease	Gene: NRGN [Bos taurus] | Protein: ENSP00000284292	0.112894
IGF2 is associated with Alzheimer disease	Gene: IGF2 [Bos taurus] | Protein: Insulin-like growth factor II [Bos taurus]	0.094575
ANK1 is associated with Alzheimer disease	Gene: ANK1 [Homo sapiens] | Protein: Ankyrin-1 [Homo sapiens]	0.071808
GMPR is associated with Alzheimer disease	Gene: GMPR [Homo sapiens] | Protein: GMP reductase 1 [Bos taurus]	0.071247
CLDN5 is associated with Alzheimer disease	Gene: CLDN5 [Bos taurus] | Protein: Claudin-5 [Bos taurus]	0.047344
SMIM24 is associated with Alzheimer disease	Gene: SMIM24 [Homo sapiens] | Protein: Small integral membrane protein 24 [Homo sapiens]	0.042477
NINJ2 is associated with Alzheimer disease	Gene: NINJ2 [Homo sapiens] | Protein: Ninjurin-2 [Bos taurus]	0.041980
RPS6 is associated with Alzheimer disease	Gene: RPS6 [Bos taurus] | Protein: 40S ribosomal protein S6 [Bos taurus]	0.034258
BCL2L1 is associated with Alzheimer disease	Gene: BCL2L1 [Homo sapiens] | Protein: Bcl-2-like protein 1	0.028414
PVALB is associated with Alzheimer disease	Gene: PVALB [Bos taurus] | Protein: ENSP00000216200	0.026059
S100A8 is associated with Alzheimer disease	Gene: S100A8 [Bos taurus] | Protein: Protein S100-A8 [Bos taurus]	0.025831
DNM3 is associated with Alzheimer disease	Gene: DNM3 [Homo sapiens] | Protein: Dynamin-3 [Bos taurus]	0.025255
RNF182 is associated with Alzheimer disease	Gene: RNF182 [Homo sapiens] | Protein: E3 ubiquitin-protein ligase RNF182 [Bos taurus]	0.025078
TBXA2R is associated with Alzheimer disease	Gene: TBXA2R [Bos taurus] | Protein: Thromboxane A2 receptor	0.024708
COX6C is associated with Alzheimer disease	Gene: COX6C [Bos taurus] | Protein: Cytochrome c oxidase subunit 6C [Bos taurus]	0.024452
RPL17-C18orf32 is associated with Alzheimer disease	Gene: RPL17-C18orf32 [Homo sapiens] | Protein: RPL17-C18orf32	0.024139
RPL17 is associated with Alzheimer disease	Gene: RPL17 [Homo sapiens] | Protein: 60S ribosomal protein L17 [Bos taurus]	0.022498
TSPAN5 is associated with Alzheimer disease	Gene: TSPAN5 [Bos taurus] | Protein: Tetraspanin-5 [Bos taurus]	0.020754
GJC2 is associated with Alzheimer disease	Gene: Cx47 | Protein: ENSP00000355675	0.019957
TREML1 is associated with Alzheimer disease	Gene: TREML1 [Homo sapiens] | Protein: Trem-like transcript 1 protein [Homo sapiens]	0.018291
TNS1 is associated with Alzheimer disease	Gene: TNS1 [Homo sapiens] | Protein: Tensin-1 [Bos taurus]	0.017900
SLC7A5 is associated with Alzheimer disease	Gene: L-type amino acid transporter 1 | Protein: Large neutral amino acids transporter small subunit 1	0.017089
SELENBP1 is associated with Alzheimer disease	Gene: SELENBP1 [Bos taurus] | Protein: ENSP00000357861	0.015198

ITGB5 is associated with Alzheimer disease	Gene: ITGB5 [Homo sapiens] | Protein: ENSP00000296181	0.014139
MAP2K3 is associated with Alzheimer disease	Gene: MAP2K3 [Homo sapiens] | Protein: Dual specificity mitogen-activated protein kinase kinase 3	0.013931
DAW1 is associated with Alzheimer disease	Gene: DAW1 [Bos taurus] | Protein: Dynein assembly factor with WDR repeat domains 1 [Bos taurus]	0.012566
HSPE1 is associated with Alzheimer disease	Gene: HSPE1 [Bos taurus] | Protein: 10 kDa heat shock protein, mitochondrial [Bos taurus]	0.011087
YBX3 is associated with Alzheimer disease	Gene: YBX3 [Homo sapiens] | Protein: ENSP00000228251	0.010837
SLIRP is associated with Alzheimer disease	Gene: SLIRP [Homo sapiens] | Protein: SRA stem-loop-interacting RNA-binding protein, mitochondrial [Bos taurus]	0.010025
AQP1 is associated with Alzheimer disease	Gene: AQP1 | Protein: Aquaporin-1	0.009996
KRT1 is associated with Alzheimer disease	Gene: KRT1 [Homo sapiens] | Protein: Keratin, type II cytoskeletal 1 [Homo sapiens]	0.008911
BCL2A1 is associated with Alzheimer disease	Gene: BCL2A1 [Bos taurus] | Protein: Bcl-2-related protein A1 [Bos taurus]	0.008131
FBXO7 is associated with Alzheimer disease	Gene: FBXO7 [Homo sapiens] | Protein: F-box only protein 7 [Bos taurus]	0.007914
SLC6A9 is associated with Alzheimer disease	Gene: GlyT1 | Protein: Sodium- and chloride-dependent glycine transporter 1	0.007761
POLR2K is associated with Alzheimer disease	Gene: POLR2K [Bos taurus] | Protein: DNA-directed RNA polymerases I, II, and III subunit RPABC4 [Bos taurus]	0.007392
RPL7 is associated with Alzheimer disease	Gene: RPL7 [Bos taurus] | Protein: 60S ribosomal protein L7 [Bos taurus]	0.007392
SMOX is associated with Alzheimer disease	Gene: SMOX [Homo sapiens] | Protein: Spermine oxidase [Bos taurus]	0.007368
RAB6B is associated with Alzheimer disease	Gene: RAB6B [Homo sapiens] | Protein: Ras-related protein Rab-6B [Bos taurus]	0.007170
MRPL47 is associated with Alzheimer disease	Gene: MRPL47 [Homo sapiens] | Protein: 39S ribosomal protein L47, mitochondrial [Bos taurus]	0.007155
SH3BGRL2 is associated with Alzheimer disease	Gene: SH3BGRL2 [Bos taurus] | Protein: SH3 domain-binding glutamic acid-rich-like protein 2 [Bos taurus]	0.007136
PTS is associated with Alzheimer disease	Gene: PTS [Homo sapiens] | Protein: 6-pyruvoyl tetrahydrobiopterin synthase [Bos taurus]	0.006884
NRGN is associated with familial Alzheimer disease	Gene: NRGN [Bos taurus] | Protein: ENSP00000284292	0.006652

ATP5MJ is associated with Alzheimer disease	Gene: ATP5MJ [Bos taurus] | Protein: ATP synthase subunit ATP5MJ, mitochondrial [Bos taurus]	0.006310
XK is associated with Alzheimer disease	Gene: XK [Homo sapiens] | Protein: Endoplasmic reticulum membrane adapter protein XK [Homo sapiens]	0.006172
EEF1E1 is associated with Alzheimer disease	Gene: EEF1E1 [Homo sapiens] | Protein: Eukaryotic translation elongation factor 1 epsilon-1 [Bos taurus]	0.005974
DIPK1B is associated with Alzheimer disease	Gene: DIPK1B [Homo sapiens] | Protein: Divergent protein kinase domain 1B [Homo sapiens]	0.005255
ACKR1 is associated with Alzheimer disease	Gene: ACKR1 | Protein: Atypical chemokine receptor 1 [Bos taurus]	0.005174
CDC34 is associated with Alzheimer disease	Gene: CDC34 [Homo sapiens] | Protein: ENSP00000215574	0.005106
MARCHF8 is associated with Alzheimer disease	Gene: MARCHF8 [Bos taurus] | Protein: E3 ubiquitin-protein ligase MARCHF8 [Bos taurus]	0.004990
CTTN is associated with Alzheimer disease	Gene: CTTN [Homo sapiens] | Protein: ENSP00000317189	0.004936
LTBP1 is associated with Alzheimer disease	Gene: LTBP1 [Homo sapiens] | Protein: Latent-transforming growth factor beta-binding protein 1 [Homo sapiens]	0.004782
ATP5ME is associated with Alzheimer disease	Gene: ATP5ME [Bos taurus] | Protein: ATP synthase subunit e, mitochondrial [Bos taurus]	0.004620
UQCRB is associated with Alzheimer disease	Gene: UQCRB [Bos taurus] | Protein: Cytochrome b-c1 complex subunit 7 [Bos taurus]	0.004620
COX7C is associated with Alzheimer disease	Gene: COX7C [Homo sapiens] | Protein: Cytochrome c oxidase subunit 7C, mitochondrial [Homo sapiens]	0.004435
FZD5 is associated with Alzheimer disease	Gene: FZD5 | Protein: Frizzled-5 [Homo sapiens]	0.004435
TIPIN is associated with Alzheimer disease	Gene: TIPIN [Homo sapiens] | Protein: TIMELESS-interacting protein [Homo sapiens]	0.004435
TMOD1 is associated with Alzheimer disease	Gene: TMOD1 [Bos taurus] | Protein: Tropomodulin-1 [Bos taurus]	0.003954
SFRP2 is associated with Alzheimer disease	Gene: SFRP2 [Homo sapiens] | Protein: ENSP00000274063	0.003699
COA6 is associated with Alzheimer disease	Gene: COA6 [Bos taurus] | Protein: Cytochrome c oxidase assembly factor 6 homolog [Bos taurus]	0.003696
SEC14L5 is associated with Alzheimer disease	Gene: SEC14L5 [Homo sapiens] | Protein: SEC14-like protein 5 [Bos taurus]	0.003696
COMMD8 is associated with Alzheimer disease	Gene: COMMD8 [Homo sapiens] | Protein: COMM domain-containing protein 8 [Homo sapiens]	0.003696

RPS27 is associated with Alzheimer disease	Gene: RPS27 [Bos taurus] | Protein: 40S ribosomal protein S27 [Bos taurus]	0.003696
ESPN is associated with Alzheimer disease	Gene: ESPN [Homo sapiens] | Protein: Espin [Homo sapiens]	0.003696
TMCC2 is associated with Alzheimer disease	Gene: TMCC2 [Homo sapiens] | Protein: Transmembrane and coiled-coil domains protein 2 [Bos taurus]	0.003696
TAL1 is associated with Alzheimer disease	Gene: TAL1 [Homo sapiens] | Protein: T-cell acute lymphocytic leukemia protein 1 [Bos taurus]	0.003696
SLC6A8 is associated with Alzheimer disease	Gene: CT1 | Protein: ENSP00000253122	0.003696
RPL11 is associated with Alzheimer disease	Gene: RPL11 [Homo sapiens] | Protein: 60S ribosomal protein L11 [Bos taurus]	0.003696
TXNDC9 is associated with Alzheimer disease	Gene: TXNDC9 [Homo sapiens] | Protein: Thioredoxin domain-containing protein 9 [Bos taurus]	0.003468
ATP6V0C is associated with Alzheimer disease	Gene: ATP6V0C [Homo sapiens] | Protein: V-type proton ATPase 16 kDa proteolipid subunit c [Bos taurus]	0.003326
TGFB1I1 is associated with Alzheimer disease	Gene: TGFB1I1 [Bos taurus] | Protein: ENSP00000355117	0.003313
COX7B is associated with Alzheimer disease	Gene: COX7B [Homo sapiens] | Protein: Cytochrome c oxidase subunit 7B, mitochondrial [Bos taurus]	0.002957
RPS17 is associated with Alzheimer disease	Gene: RPS17 [Homo sapiens] | Protein: 40S ribosomal protein S17 [Bos taurus]	0.002957
RPL31 is associated with Alzheimer disease	Gene: RPL31 [Homo sapiens] | Protein: 60S ribosomal protein L31 [Bos taurus]	0.002957
RBIS is associated with Alzheimer disease	Gene: RBIS [Homo sapiens] | Protein: ENSP00000481745	0.002826
RPS10 is associated with Alzheimer disease	Gene: RPS10 [Bos taurus] | Protein: 40S ribosomal protein S10 [Bos taurus]	0.001848
ZNF385D is associated with Alzheimer disease	Gene: ZNF385D [Homo sapiens] | Protein: Zinc finger protein 385D [Homo sapiens]	0.001820
LSM3 is associated with Alzheimer disease	Gene: LSM3 [Homo sapiens] | Protein: U6 snRNA-associated Sm-like protein LSm3 [Bos taurus]	0.001757
IGF2BP2 is associated with Alzheimer disease	Gene: IGF2BP2 [Homo sapiens] | Protein: Insulin-like growth factor 2 mRNA-binding protein 2 [Bos taurus]	0.001685
RPL41 is associated with Alzheimer disease	Gene: RPL41 [Homo sapiens] | Protein: 60S ribosomal protein L41 [Homo sapiens]	0.001478

RUNDC3A is associated with Alzheimer disease	Gene: RUNDC3A [Bos taurus] | Protein: RUN domain-containing protein 3A [Bos taurus]	0.001478
GYPC is associated with Alzheimer disease	Gene: GYPC [Homo sapiens] | Protein: Glycophorin-C [Bos taurus]	0.001478
PLVAP is associated with Alzheimer disease	Gene: PLVAP [Homo sapiens] | Protein: Plasmalemma vesicle-associated protein [Bos taurus]	0.001478
RGS6 is associated with Alzheimer disease	Gene: RGS6 [Homo sapiens] | Protein: ENSP00000450936	0.001478
RPL26 is associated with Alzheimer disease	Gene: RPL26 [Bos taurus] | Protein: 60S ribosomal protein L26 [Bos taurus]	0.001478
RPS3A is associated with Alzheimer disease	Gene: RPS3A [Bos taurus] | Protein: 40S ribosomal protein S3a [Bos taurus]	0.001478
RPL35 is associated with Alzheimer disease	Gene: RPL35 [Bos taurus] | Protein: 60S ribosomal protein L35 [Bos taurus]	0.001478
RPL34 is associated with Alzheimer disease	Gene: RPL34 [Homo sapiens] | Protein: 60S ribosomal protein L34 [Bos taurus]	0.001478
RPL9 is associated with Alzheimer disease	Gene: RPL9 [Bos taurus] | Protein: 60S ribosomal protein L9 [Bos taurus]	0.001478
RPL21 is associated with Alzheimer disease	Gene: RPL21 [Homo sapiens] | Protein: 60S ribosomal protein L21 [Bos taurus]	0.001478
RPL23 is associated with Alzheimer disease	Gene: RPL23 [Bos taurus] | Protein: 60S ribosomal protein L23 [Bos taurus]	0.001478
BEX2 is associated with Alzheimer disease	Gene: BEX2 [Bos taurus] | Protein: Protein BEX2 [Bos taurus]	0.001405
YPEL4 is associated with Alzheimer disease	Gene: YPEL4 [Homo sapiens] | Protein: ENSP00000300022	0.001390

Supplementary Table 8 direct association scores of significant differentially expressed genes at follow-up versus baseline in amyloid negative-positive and amyloid positive-positive individuals

preferredLabel	Target	Direct Association Score
HNRNPA1 is associated with Alzheimer disease	Gene: HNRNPA1 [Homo sapiens] | Protein: ENSP00000341826	0.080129
TPT1 is associated with Alzheimer disease	Gene: TPT1 [Homo sapiens] | Protein: ENSP00000368344	0.073238
RPL17-C18orf32 is associated with Alzheimer disease	Gene: RPL17-C18orf32 [Homo sapiens] | Protein: RPL17-C18orf32	0.050010
GLUL is associated with Alzheimer disease	Gene: GLUL [Homo sapiens] | Protein: ENSP00000307900	0.044200
RPL15 is associated with Alzheimer disease	Gene: RPL15 [Homo sapiens] | Protein: 60S ribosomal protein L15 [Homo sapiens]	0.029566
HSP90AA1 is associated with Alzheimer disease	Gene: HSP90AA1 [Homo sapiens] | Protein: ENSP00000216281	0.020612
MBP is associated with Alzheimer disease	Gene: MBP [Homo sapiens] | Protein: ENSP00000348273	0.019378
GLUL is associated with familial Alzheimer disease	Gene: GLUL [Homo sapiens] | Protein: ENSP00000307900	0.017740
RPLP0 is associated with Alzheimer disease	Gene: RPLP0 [Homo sapiens] | Protein: 60S acidic ribosomal protein P0 [Homo sapiens]	0.014783
DEFA1 is associated with Alzheimer disease	Gene: DEFA1 [Homo sapiens] | Protein: DEFA1	0.012894
SIRPB1 is associated with Alzheimer disease	Gene: SIRPB1 [Homo sapiens] | Protein: SIRPB1	0.010841
GRINA is associated with Alzheimer disease	Gene: GRINA [Homo sapiens] | Protein: Protein lifeguard 1 [Homo sapiens]	0.009609
KLF3 is associated with Alzheimer disease	Gene: KLF3 [Homo sapiens] | Protein: Krueppel-like factor 3 [Homo sapiens]	0.009052
RACK1 is associated with Alzheimer disease	Gene: RACK1 [Homo sapiens] | Protein: ENSP00000426909	0.008487
B2M is associated with Alzheimer disease	Gene: B2M [Homo sapiens] | Protein: Beta-2-microglobulin [Homo sapiens]	0.007862
IQGAP1 is associated with Alzheimer disease	Gene: IQGAP1 [Homo sapiens] | Protein: ENSP00000268182	0.007609
RPL30 is associated with Alzheimer disease	Gene: RPL30 [Homo sapiens] | Protein: 60S ribosomal protein L30 [Homo sapiens]	0.007392
AKT2 is associated with Alzheimer disease	Gene: AKT serine/threonine kinase 2 | Protein: RAC-beta serine/threonine-protein kinase	0.007392
NPM1 is associated with Alzheimer disease	Gene: NPM1 [Homo sapiens] | Protein: ENSP00000296930	0.006652
MAP4K4 is associated with Alzheimer disease	Gene: MAP4K4 [Homo sapiens] | Protein: Mitogen-activated protein kinase kinase kinase kinase 4	0.006562

UBN1 is associated with Alzheimer disease	Gene: UBN1 [Homo sapiens] | Protein: Ubinuclein-1 [Homo sapiens]	0.005930
MARF1 is associated with Alzheimer disease	Gene: MARF1 [Homo sapiens] | Protein: Meiosis regulator and mRNA stability factor 1 [Homo sapiens]	0.005163
MARCHF8 is associated with Alzheimer disease	Gene: MARCHF8 [Homo sapiens] | Protein: E3 ubiquitin-protein ligase MARCHF8 [Homo sapiens]	0.004990
PCGF5 is associated with Alzheimer disease	Gene: PCGF5 [Homo sapiens] | Protein: Polycomb group RING finger protein 5 [Homo sapiens]	0.004104
IQSEC1 is associated with Alzheimer disease	Gene: IQSEC1 [Homo sapiens] | Protein: IQ motif and SEC7 domain-containing protein 1 [Homo sapiens]	0.003838
IFITM2 is associated with Alzheimer disease	Gene: IFITM2 [Homo sapiens] | Protein: Interferon-induced transmembrane protein 2 [Homo sapiens]	0.003074
RPS3A is associated with Alzheimer disease	Gene: RPS3A [Homo sapiens] | Protein: 40S ribosomal protein S3a [Homo sapiens]	0.002957
RPS6 is associated with Alzheimer disease	Gene: RPS6 [Homo sapiens] | Protein: 40S ribosomal protein S6 [Homo sapiens]	0.002957
RPL11 is associated with Alzheimer disease	Gene: RPL11 [Homo sapiens] | Protein: 60S ribosomal protein L11 [Homo sapiens]	0.002217
RPL5 is associated with Alzheimer disease	Gene: RPL5 [Homo sapiens] | Protein: 60S ribosomal protein L5 [Homo sapiens]	0.002217
RPL22 is associated with Alzheimer disease	Gene: RPL22 [Homo sapiens] | Protein: 60S ribosomal protein L22 [Homo sapiens]	0.002217
SELENBP1 is associated with Alzheimer disease	Gene: SELENBP1 [Homo sapiens] | Protein: ENSP00000357861	0.001658
FLOT2 is associated with Alzheimer disease	Gene: FLOT2 [Homo sapiens] | Protein: ENSP00000378368	0.001478
EEF1A1 is associated with Alzheimer disease	Gene: EEF1A1 [Homo sapiens] | Protein: ENSP00000330054	0.001478

Supplementary table 9 APOE4 carriers gene set enrichment analysis gene ontology upreulated

ONTOLOGY	ID	Description	setSize	enrichment Score	NES	pvalue	p.adjust	qvalue	rank	leading_edge	core_enrichment
BP	GO:0034660	ncRNA metabolic process	500	0.47	1.87	1.00E-10	3.79E-09	2.32E-09	1808	tags=25%, list=15%, signal=22%	RPL26/RPS25/RPS24/LARP7/RPS7/RPL5/RPL7/RPS6/ESF1/RPS17/RPS27/RPL35/RPS8/RPL11/RPL35A/SSB/RPL27/RPS14/TRMT10C/NOP58/NSA2/NIFK/RTRAF/RPS19/RPS21/ZNHIT3/TPRKB/NGDN/KRR1/DDX5/EXOSC9/NARS1/MPHOSPH10/METTL18/FBL/RPL14/GTF3A/THUMPD1/POP5/MPHOSPH6/TRMO/RIOK2/RPF1/METTL5/RPS16/BRIX1/ZBTB8OS/FRG1/MRPL1/GTF2H5/PIN4/DTWD1/SBDS/EBNA1BP2/POLR1E/DALRD3/RPF2/DDX1/C1D/TSNAX/RPL10A/MTO1/EMG1/LYAR/TRMT12/LSM6/EXOSC1/TRMT13/DCAF13/DIS3/RSL1D1/NHP2/MTREX/ZC3H8/THUMPD2/UTP3/SRFBP1/MRPS11/CDK5RAP1/INTS10/GTF3C3/SNAPC1/PRKRA/TRMT6/WDR43/GATB/MTERF4/SNAPC5/METTL3/NOP53/DTD2/TSEN15/HENMT1/TYW3/RPP30/NOL8/IPPK/ELAC1/TRMT1/EXOSC8/OSGEP/RARS1/INTS6L/UTP23/TYW1/CARS2/TARS1/FCF1/YBEY/UTP15/CLP1/T
CC	GO:0031966	mitochondrial membrane	613	0.51	2.05	1.00E-10	3.79E-09	2.32E-09	1472	tags=27%, list=13%, signal=25%	UQCRB/MRPS18C/ATP5PO/COX6C/ATP5MJ/RPS27A/COX7B/COX7C/NDUFA5/TIMM8B/DNAJC19/UQCRH/NDUFS5/TOMM7/ATP5ME/BCL2A1/NDUFA1/NDUFA6/MRPL48/COX16/PET100/MRPS21/MRPS22/NDUFB1/TOMM20/UQCRQ/MRPL47/DNAJC15/SMDT1/MRPL46/MRPS28/ATP5PF/NDUFB2/TIMM17A/COX14/MRPS33/MRPL40/GRPEL2/COQ5/MRPL27/CCDC90B/ATP5MG/MICU2/TMEM14C/ATP5PD/COX20/ATP5MK/NDUFA4/MRPL17/RPS3/TIMM9/ATAD1/COX7A2/MRPL3/NDUFS3/ATP5MF/TMEM126A/LDHB/MRPS15/NDUFB3/TIMMDC1/MCUB/MRPS35/ATP5F1C/NDUFAF2/MRPL32/COX11/TMEM126B/MRPL13/ATP5F1E/SMIM20/COX4I1/MRPL51/MRPL57/MPC1/HIGD1A/MRPL35/PNPLA8/MRPL18/CISD1/MRPL22/COX6A1/SFXN4/IFI27L2/DHFR2/ATP5PB/UQCR10/PPP1CC/NDUFAF5/NDUFS4/TIMM10/MRPL50/MRPL30/NDUFV2/MICOS10/MRPL1/C12orf73/TOMM5/NDUFAF4/CYCS/BNIP1/TRAF3IP3/MRPS31/MRPL45/UQCRFS1/BNIP3/MRPL36/UQCRC2/NIPSNAP2/MRPL15/MRPL53/NDUFA12/IMMP1L/DMAC1/SLC25A32/MRPS36/MRPS17/ROMO1/MOAP1/MRPS14/CHCHD1/VDAC2/FAM162A/PMAIP1/NDUFB4/COX5A/MRPL14/UQCC2/TMEM70/NDUFAB1/HTRA2/MRPS5/FLVCR1/ABCB10/MRPS11/MRPS30/MRPL33/MRPS25/GNPAT/MFF/MRPL11/MICU3/COX7A2L/DAP3/NDU

CC	GO:0005740	mitochondrial envelope	658	0.51	2.06	1.00E-10	3.79E-09	2.32E-09	1472	tags=27%, list=13%, signal=25%	UQCRB/MRPS18C/ATP5PO/COX6C/ATP5MJ/RPS27A/COX7B/COX7C/NDUFA5/TIMM8B/DNAJC19/UQCRH/SOD1/NDUFS5/TOMM7/ATP5ME/BCL2A1/NDUFA1/NDUFA6/MRPL48/COX16/PET100/MRPS21/MRPS22/NDUFB1/TOMM20/UQCRQ/MRPL47/DNAJC15/COA6/SMDT1/MRPL46/MRPS28/ATP5PF/NDUFB2/TIMM17A/COX14/MRPS33/MRPL40/GRPEL2/COQ5/MRPL27/CCDC90B/ATP5MG/MICU2/TMEM14C/ATP5PD/COX20/ATP5MK/NDUFA4/MRPL17/RPS3/TIMM9/ATAD1/COX7A2/MRPL3/NDUFS3/ATP5MF/TMEM126A/LDHB/MRPS15/NDUFB3/TIMMDC1/MCUB/MRPS35/ATP5F1C/NDUFAF2/MRPL32/COX11/CHCHD7/TMEM126B/MRPL13/ATP5F1E/SMIM20/COX4I1/MRPL51/MRPL57/MPC1/HIGD1A/MRPL35/PNPLA8/MRPL18/CISD1/MRPL22/COX6A1/SFXN4/IFI27L2/DHFR2/SDHAF3/ATP5PB/UQCR10/PPP1CC/NDUFAF5/NDUFS4/TIMM10/MRPL50/MRPL30/PRELID3B/NDUFV2/MICOS10/MRPL1/C12orf73/TRIAP1/TOMM5/NDUFAF4/CYCS/BNIP1/TRAF3IP3/MRPS31/MRPL45/UQCRFS1/BNIP3/MRPL36/UQCRC2/NIPSNAP2/MRPL15/MRPL53/NDUFA12/IMMP1L/DMAC1/SLC25A32/MRPS36/PARK7/MRPS17/ROMO1/MOAP1/MRPS14/CHCHD1/VDAC2/FAM162A/PMAIP1/NDUFB4/COX5A/MRPL14/UQCC2/TMEM70/COX17/NDUFAB1/HTRA2/MRPS5/FLVCR1/ABCB10/MRPS11/MRPS30/MRPL33/MRPS25/GNPAT/MFF/MRPL11/MICU3/COX7A2L/DAP3/NDUFB5/SLC25A25/SFXN1/CHCHD4/MRPS6/COA4/MRPL58/CAT/NDUFA13/PMPCB/MIX23/OGT/VDAC3/MRPS10/GPAM/DEGS1/MRPL20/MRPL39/SLC25A46/CEBPZOS/HAX1/TIMM8A/NDUFC1/CMC4/TMEM242/ACADM/CARD19
BP	GO:0006518	peptide metabolic process	697	0.53	2.15	1.00E-10	3.79E-09	2.32E-09	1054	tags=23%, list=9%, signal=22%	RPL21/RPL26/RPL34/RPS25/RPL24/RPL17/RPL31/RPS24/MRPS18C/RPS7/RPL23/EEF1B2/RPS3A/RPL36A/RPL39/RPS10/EIF3E/RPL5/RPS20/RPL7/RPL22/RPL6/RPS18/NPM1/RPL9/RPS6/RPS15A/RPS17/RPS27/RPL35/RPS27A/RWDD1/RPS8/RPL11/RPL35A/RPS13/RPL3/RPS11/SOD1/RPL37/RPL13A/RPL27/RPL41/AIMP1/RPS14/RPL27A/TRMT10C/RPL19/RPS27L/MRPS21/RSL24D1/RPL22L1/RPL32/RPS23/EIF4A2/MRPL47/RPS26/RPL29/BDH2/RPL4/RPL12/SARNP/MRPS28/RPS29/RPS19/RPL30/MRPS33/USP16/SEC11C/RPS21/EIF2S2/RPL15/NGDN/RPLP0/MRPL27/RPL38/RPL36/RPS12/EXOSC9/EEF1E1/NARS1/EIF2A/MCTS1/UPF3A/EIF4E/MTRES1/METTL18/SRP9/ZC3H15/RBM3/RPS3/EIF3H/RPS9/EEF1D/RPL14/MRPL3/RPL10/LSM1/COPS5/MRPS15/DPH5/RPLP1/MRPL32/MRPL13/EIF3M/CNBP/MRPL51/MRPL57/MRPL35/RPL37A/MRPL18/EEF1G/METTL5/RPS2/RPS16/RPL8/EEF1A1/MAGOH/MTIF3/UPF3B/SELENOT/RPSA/SERP1/LARP1B/ZNF540/RPL26L1/RPL36AL/PAIP1/DALRD3/MRPL36/DDX1/RPL13/ATF4/RPL10A/SEC11A/PUM3/PARK7/MRPS17/DNAJC1/MRPS14/DENR/EIF3J/AASDH/ABCE1/UQCC2/GSTO1/TIA1/DIS3/MTIF2/MRPS5/RPS5/FXR1/EIF3K/TMED2/PRNP/MRPS11/CDK5RAP1

BP	GO:0043604	amide biosynthetic process	676	0.54	2.20	1.00E-10	3.79E-09	2.32E-09	1054	tags=23%, list=9%, signal=22%	RPL21/RPL26/RPL34/RPS25/RPL24/RPL17/RPL31/RPS24/MRPS18C/RPS7/RPL23/EEF1B2/RPS3A/RPL36A/RPL39/RPS10/EIF3E/RPL5/RPS20/RPL7/RPL22/RPL6/RPS18/NPM1/RPL9/RPS6/RPS15A/RPS17/RPS27/RPL35/RPS27A/RWDD1/RPS8/RPL11/RPL35A/RPS13/RPL3/RPS11/RPL37/RPL13A/RPL27/RPL41/AIMP1/RPS14/RPL27A/TRMT10C/RPL19/RPS27L/MRPS21/RSL24D1/RPL22L1/RPL32/RPS23/EIF4A2/MRPL47/RPS26/RPL29/BDH2/RPL4/RPL12/SARNP/MRPS28/RPS29/RPS19/RPL30/MRPS33/USP16/RPS21/EIF2S2/RPL15/NGDN/RPLP0/MRPL27/RPL38/RPL36/RPS12/EXOSC9/EEF1E1/NARS1/EIF2A/MCTS1/UPF3A/EIF4E/PPCS/MTRES1/METTL18/SRP9/ZC3H15/RBM3/RPS3/EIF3H/RPS9/EEF1D/RPL14/MRPL3/RPL10/LSM1/COPS5/MRPS15/DPH5/RPLP1/MRPL32/MRPL13/EIF3M/CNBP/MRPL51/DLD/MRPL57/MRPL35/RPL37A/MRPL18/EEF1G/METTL5/RPS2/RPS16/RPL8/EEF1A1/MAGOH/MTIF3/UPF3B/SELENOT/ORMDL1/RPSA/SERP1/LARP1B/ZNF540/RPL26L1/RPL36AL/PAIP1/DALRD3/MRPL36/DDX1/RPL13/ATF4/RPL10A/PUM3/MRPS17/DNAJC1/MRPS14/DENR/EIF3J/AASDH/ABCE1/UQCC2/TIA1/DIS3/MTIF2/MRPS5/RPS5/FXR1/EIF3K/TMED2/ACAT1/MRPS11/CDK5RAP1
BP	GO:0034470	ncRNA processing	388	0.54	2.12	1.00E-10	3.79E-09	2.32E-09	1711	tags=27%, list=15%, signal=24%	RPL26/RPS25/RPS24/LARP7/RPS7/RPL5/RPL7/RPS6/ESF1/RPS17/RPS27/RPL35/RPS8/RPL11/RPL35A/SSB/RPL27/RPS14/TRMT10C/NOP58/NSA2/NIFK/RTRAF/RPS19/RPS21/ZNHIT3/TPRKB/NGDN/KRR1/DDX5/EXOSC9/MPHOSPH10/METTL18/FBL/RPL14/THUMPD1/POP5/MPHOSPH6/TRMO/RIOK2/RPF1/METTL5/RPS16/BRIX1/ZBTB8OS/FRG1/MRPL1/GTF2H5/PIN4/DTWD1/SBDS/EBNA1BP2/DALRD3/RPF2/DDX1/C1D/TSNAX/RPL10A/MTO1/EMG1/LYAR/TRMT12/LSM6/EXOSC1/TRMT13/DCAF13/DIS3/RSL1D1/NHP2/MTREX/THUMPD2/UTP3/SRFBP1/MRPS11/CDK5RAP1/INTS10/PRKRA/TRMT6/WDR43/MTERF4/METTL3/NOP53/TSEN15/HENMT1/TYW3/RPP30/NOL8/ELAC1/TRMT1/EXOSC8/OSGEP/INTS6L/UTP23/TYW1/FCF1/YBEY/UTP15/CLP1/TRMT10A/DPH3/TRUB1/REXO4/WDR75/TFB2M/HELQ

CC	GO:0019866	organelle inner membrane	462	0.56	2.23	1.00E-10	3.79E-09	2.32E-09	1284	tags=28%, list=11%, signal=26%	UQCRB/MRPS18C/ATP5PO/COX6C/ATP5MJ/COX7B/COX7C/NDUFA5/TIMM8B/DNAJC19/UQCRH/NDUFS5/ATP5ME/NDUFA1/NDUFA6/MRPL48/COX16/PET100/MRPS21/MRPS22/CBX3/NDUFB1/UQCRQ/MRPL47/DNAJC15/SMDT1/MRPL46/MRPS28/ATP5PF/NDUFB2/TIMM17A/TRA2B/MRPS33/MRPL40/GRPEL2/COQ5/MRPL27/ATP5MG/MICU2/ATP5PD/COX20/ATP5MK/NDUFA4/MRPL17/RPS3/TIMM9/COX7A2/MRPL3/NDUFS3/ATP5MF/TMEM126A/LDHB/MRPS15/NDUFB3/TIMMDC1/MCUB/MRPS35/ATP5F1C/NDUFAF2/MRPL32/COX11/TMEM126B/MRPL13/ATP5F1E/SMIM20/COX4I1/MRPL51/MRPL57/MPC1/HIGD1A/MRPL35/MRPL18/MRPL22/COX6A1/SFXN4/DHFR2/ATP5PB/UQCR10/NDUFAF5/NDUFS4/TIMM10/MRPL50/MRPL30/NDUFV2/MICOS10/MRPL1/C12orf73/NDUFAF4/CYCS/MRPS31/MRPL45/UQCRFS1/MRPL36/UQCRC2/MRPL15/MRPL53/NDUFA12/IMMP1L/DMAC1/SLC25A32/MRPS36/MATR3/MRPS17/ROMO1/MRPS14/CHCHD1/NDUFB4/COX5A/MRPL14/UQCC2/TMEM70/NDUFAB1/MRPS5/FLVCR1/ABCB10/MRPS11/MRPS30/MRPL33/MRPS25/MRPL11/MICU3/COX7A2L/DAP3/NDUFB5/SLC25A25/SFXN1/MRPS6/MRPL58/NDUFA13/PMPCB
BP	GO:0043043	peptide biosynthetic process	584	0.57	2.31	1.00E-10	3.79E-09	2.32E-09	1054	tags=26%, list=9%, signal=25%	RPL21/RPL26/RPL34/RPS25/RPL24/RPL17/RPL31/RPS24/MRPS18C/RPS7/RPL23/EEF1B2/RPS3A/RPL36A/RPL39/RPS10/EIF3E/RPL5/RPS20/RPL7/RPL22/RPL6/RPS18/NPM1/RPL9/RPS6/RPS15A/RPS17/RPS27/RPL35/RPS27A/RWDD1/RPS8/RPL11/RPL35A/RPS13/RPL3/RPS11/RPL37/RPL13A/RPL27/RPL41/AIMP1/RPS14/RPL27A/TRMT10C/RPL19/RPS27L/MRPS21/RSL24D1/RPL22L1/RPL32/RPS23/EIF4A2/MRPL47/RPS26/RPL29/BDH2/RPL4/RPL12/SARNP/MRPS28/RPS29/RPS19/RPL30/MRPS33/USP16/RPS21/EIF2S2/RPL15/NGDN/RPLP0/MRPL27/RPL38/RPL36/RPS12/EXOSC9/EEF1E1/NARS1/EIF2A/MCTS1/UPF3A/EIF4E/MTRES1/METTL18/SRP9/ZC3H15/RBM3/RPS3/EIF3H/RPS9/EEF1D/RPL14/MRPL3/RPL10/LSM1/COPS5/MRPS15/DPH5/RPLP1/MRPL32/MRPL13/EIF3M/CNBP/MRPL51/MRPL57/MRPL35/RPL37A/MRPL18/EEF1G/METTL5/RPS2/RPS16/RPL8/EEF1A1/MAGOH/MTIF3/UPF3B/SELENOT/RPSA/SERP1/LARP1B/ZNF540/RPL26L1/RPL36AL/PAIP1/DALRD3/MRPL36/DDX1/RPL13/ATF4/RPL10A/PUM3/MRPS17/DNAJC1/MRPS14/DENR/EIF3J/AASDH/ABCE1/UQCC2/TIA1/DIS3/MTIF2/MRPS5/RPS5/FXR1/EIF3K/TMED2/MRPS11/CDK5RAP1

BP	GO:0006412	translation	564	0.58	2.35	1.00E-10	3.79E-09	2.32E-09	1054	tags=26%, list=9%, signal=25%	RPL21/RPL26/RPL34/RPS25/RPL24/RPL17/RPL31/RPS24/MRPS18C/RPS7/RPL23/EEF1B2/RPS3A/RPL36A/RPL39/RPS10/EIF3E/RPL5/RPS20/RPL7/RPL22/RPL6/RPS18/NPM1/RPL9/RPS6/RPS15A/RPS17/RPS27/RPL35/RPS27A/RWDD1/RPS8/RPL11/RPL35A/RPS13/RPL3/RPS11/RPL37/RPL13A/RPL27/RPL41/AIMP1/RPS14/RPL27A/TRMT10C/RPL19/RPS27L/MRPS21/RSL24D1/RPL22L1/RPL32/RPS23/EIF4A2/MRPL47/RPS26/RPL29/RPL4/RPL12/SARNP/MRPS28/RPS29/RPS19/RPL30/MRPS33/USP16/RPS21/EIF2S2/RPL15/NGDN/RPLP0/MRPL27/RPL38/RPL36/RPS12/EXOSC9/EEF1E1/NARS1/EIF2A/MCTS1/UPF3A/EIF4E/MTRES1/METTL18/SRP9/ZC3H15/RBM3/RPS3/EIF3H/RPS9/EEF1D/RPL14/MRPL3/RPL10/LSM1/COPS5/MRPS15/DPH5/RPLP1/MRPL32/MRPL13/EIF3M/CNBP/MRPL51/MRPL57/MRPL35/RPL37A/MRPL18/EEF1G/METTL5/RPS2/RPS16/RPL8/EEF1A1/MAGOH/MTIF3/UPF3B/SELENOT/RPSA/SERP1/LARP1B/ZNF540/RPL26L1/RPL36AL/PAIP1/DALRD3/MRPL36/DDX1/RPL13/ATF4/RPL10A/PUM3/MRPS17/DNAJC1/MRPS14/DENR/EIF3J/ABCE1/UQCC2/TIA1/DIS3/MTIF2/MRPS5/RPS5/FXR1/EIF3K/TMED2/MRPS11/CDK5RAP1
BP	GO:0016072	rRNA metabolic process	253	0.59	2.21	1.00E-10	3.79E-09	2.32E-09	1796	tags=31%, list=15%, signal=27%	RPL26/RPS25/RPS24/RPS7/RPL5/RPL7/RPS6/ESF1/RPS17/RPS27/RPL35/RPS8/RPL11/RPL35A/RPL27/RPS14/NOP58/NSA2/NIFK/RPS19/RPS21/ZNHIT3/NGDN/KRR1/EXOSC9/MPHOSPH10/METTL18/FBL/RPL14/GTF3A/POP5/MPHOSPH6/RIOK2/RPF1/METTL5/RPS16/BRIX1/FRG1/MRPL1/GTF2H5/PIN4/SBDS/EBNA1BP2/POLR1E/RPF2/C1D/RPL10A/EMG1/LYAR/LSM6/EXOSC1/DCAF13/DIS3/RSL1D1/NHP2/MTREX/UTP3/SRFBP1/MRPS11/GTF3C3/WDR43/MTERF4/NOP53/RPP30/NOL8/IPPK/EXOSC8/UTP23/FCF1/YBEY/UTP15/REXO4/WDR75/TFB2M/HELQ/EXOSC3/TAF1B/DROSHA

CC	GO:0005743	mitochondrial inner membrane	416	0.59	2.32	1.00E-10	3.79E-09	2.32E-09	1284	tags=31%, list=11%, signal=28%	UQCRB/MRPS18C/ATP5PO/COX6C/ATP5MJ/COX7B/COX7C/NDUFA5/TIMM8B/DNAJC19/UQCRH/NDUFS5/ATP5ME/NDUFA1/NDUFA6/MRPL48/COX16/PET100/MRPS21/MRPS22/NDUFB1/UQCRQ/MRPL47/DNAJC15/SMDT1/MRPL46/MRPS28/ATP5PF/NDUFB2/TIMM17A/MRPS33/MRPL40/GRPEL2/COQ5/MRPL27/ATP5MG/MICU2/ATP5PD/COX20/ATP5MK/NDUFA4/MRPL17/RPS3/TIMM9/COX7A2/MRPL3/NDUFS3/ATP5MF/TMEM126A/LDHB/MRPS15/NDUFB3/TIMMDC1/MCUB/MRPS35/ATP5F1C/NDUFAF2/MRPL32/COX11/TMEM126B/MRPL13/ATP5F1E/SMIM20/COX4I1/MRPL51/MRPL57/MPC1/HIGD1A/MRPL35/MRPL18/MRPL22/COX6A1/SFXN4/DHFR2/ATP5PB/UQCR10/NDUFAF5/NDUFS4/TIMM10/MRPL50/MRPL30/NDUFV2/MICOS10/MRPL1/C12orf73/NDUFAF4/CYCS/MRPS31/MRPL45/UQCRFS1/MRPL36/UQCRC2/MRPL15/MRPL53/NDUFA12/IMMP1L/DMAC1/SLC25A32/MRPS36/MRPS17/ROMO1/MRPS14/CHCHD1/NDUFB4/COX5A/MRPL14/UQCC2/TMEM70/NDUFAB1/MRPS5/FLVCR1/ABCB10/MRPS11/MRPS30/MRPL33/MRPS25/MRPL11/MICU3/COX7A2L/DAP3/NDUFB5/SLC25A25/SFXN1/MRPS6/MRPL58/NDUFA13/PMPCB
BP	GO:0045333	cellular respiration	183	0.59	2.15	1.00E-10	3.79E-09	2.32E-09	1265	tags=30%, list=11%, signal=27%	UQCRB/ATP5PO/COX7C/NDUFA5/UQCRH/NDUFS5/ATP5ME/NDUFA1/NDUFA6/NDUFB1/UQCRQ/DNAJC15/COA6/ATP5PF/NDUFB2/ATP5MG/ATP5PD/NDUFA4/COX7A2/NDUFS3/ATP5MF/NDUFB3/ATP5F1C/ATP5F1E/COX4I1/DLD/CISD1/COX6A1/ATP5PB/UQCR10/NDUFS4/NDUFV2/CYCS/UQCRFS1/BNIP3/UQCRC2/NIPSNAP2/NDUFA12/MRPS36/PARK7/NDUFB4/COX5A/UQCC2/ADSL/NDUFAB1/C2orf69/ETFRF1/COX7A2L/MSH2/NDUFB5/SLC25A25/SUCLG1/NOP53/CAT
BP	GO:0022613	ribonucleoprotein complex biogenesis	392	0.60	2.36	1.00E-10	3.79E-09	2.32E-09	1257	tags=29%, list=11%, signal=27%	RPL26/RPS25/RPL24/RPS24/RPS7/RPL5/RPL7/RPL6/HSP90AA1/NPM1/SNRPD2/RPS6/ESF1/RPS17/RPS27/RPL35/SNRPG/RPS8/SNRPE/RPL11/RPL35A/RPL3/RPL13A/RPL27/RPS14/RPS27L/RSL24D1/NOP58/NSA2/NIFK/LTV1/SNRPD1/RPS19/RPS21/EIF2S2/ZNHIT3/NGDN/KRR1/RPLP0/RBIS/RPL38/EXOSC9/EIF2A/MCTS1/LUC7L3/MPHOSPH10/METTL18/FBL/RPL14/GTF3A/RPL10/ZRSR2/CLNS1A/POP5/MPHOSPH6/RIOK2/SLU7/RPF1/MRPL22/METTL5/SRSF10/COIL/RPS16/BRIX1/NMD3/FRG1/RPSA/MRPL1/GTF2H5/PIN4/PRPF39/NUP88/SBDS/EBNA1BP2/RPL26L1/PSIP1/RPF2/MRPL36/SF3B1/DDX1/C1D/RPL10A/EMG1/SNRPC/LYAR/LSM6/EXOSC1/DENR/TAF9/ABCE1/SNRPF/DCAF13/NUDT21/DIS3/NOP16/RSL1D1/NHP2/MTREX/RAN/SNRPD3/RPS5/UTP3/SRFBP1/PTGES3/MRPS11/GEMIN6/ZNF622/RPL23A/GPATCH4/WDR43/GNL2/MTERF4/NCBP1/NOP53

MF	GO:0005198	structural molecule activity	376	0.61	2.38	1.00E-10	3.79E-09	2.32E-09	722	tags=27%, list=6%, signal=26%	RPL21/RPL26/RPL34/RPS25/RPL24/RPL17/RPL31/RPS24/MRPS18C/RPS7/RPL23/RPS3A/RPL36A/RPL39/RPS10/RPL5/RPS20/RPL7/RPL22/RPL6/RPS18/RPL9/RPS6/RPS15A/RPS17/RPS27/RPL35/RPS27A/RPS8/RPL11/RPL35A/RPS13/RPL3/RPS11/RPL37/RPL13A/RPL27/RPL41/RPS14/RPL27A/RPL19/RPS27L/MRPS21/MRPS22/RSL24D1/RPL22L1/RPL32/RPS23/MRPL47/RPS26/RPL29/RPL4/RPL12/MRPL46/RPS29/RPS19/RPL30/MRPS33/RPS21/RPL15/RPLP0/MRPL27/RPL38/RPL36/RPS12/UPF3A/MRPL17/RPS3/RPS9/RPL14/MRPL3/RPL10/MRPS15/MRPS35/RPLP1/MRPL32/MRPL13/MRPL51/MRPL57/MRPL35/RPL37A/MRPL18/MRPL22/RPS2/RPS16/RPL8/MYL6B/PNN/UPF3B/MRPL30/RPSA/MRPL1/NUP88/RPL26L1/RPL36AL/MRPS31/MRPL36/RPL13/YEATS4/MRPL15/RPL10A
BP	GO:0009060	aerobic respiration	152	0.62	2.21	1.00E-10	3.79E-09	2.32E-09	1265	tags=34%, list=11%, signal=30%	UQCRB/ATP5PO/COX7C/NDUFA5/UQCRH/NDUFS5/ATP5ME/NDUFA1/NDUFA6/NDUFB1/UQCRQ/DNAJC15/COA6/ATP5PF/NDUFB2/ATP5MG/ATP5PD/NDUFA4/COX7A2/NDUFS3/ATP5MF/NDUFB3/ATP5F1C/ATP5F1E/COX4I1/DLD/COX6A1/ATP5PB/UQCR10/NDUFS4/NDUFV2/CYCS/UQCRFS1/BNIP3/UQCRC2/NIPSNAP2/NDUFA12/MRPS36/PARK7/NDUFB4/COX5A/UQCC2/ADSL/NDUFAB1/C2orf69/COX7A2L/MSH2/NDUFB5/SUCLG1/NOP53/CAT
BP	GO:0006364	rRNA processing	215	0.63	2.32	1.00E-10	3.79E-09	2.32E-09	1051	tags=27%, list=9%, signal=25%	RPL26/RPS25/RPS24/RPS7/RPL5/RPL7/RPS6/ESF1/RPS17/RPS27/RPL35/RPS8/RPL11/RPL35A/RPL27/RPS14/NOP58/NSA2/NIFK/RPS19/RPS21/ZNHIT3/NGDN/KRR1/EXOSC9/MPHOSPH10/METTL18/FBL/RPL14/POP5/MPHOSPH6/RIOK2/RPF1/METTL5/RPS16/BRIX1/FRG1/MRPL1/GTF2H5/PIN4/SBDS/EBNA1BP2/RPF2/C1D/RPL10A/EMG1/LYAR/LSM6/EXOSC1/DCAF13/DIS3/RSL1D1/NHP2/MTREX/UTP3/SRFBP1/MRPS11
BP	GO:0042254	ribosome biogenesis	289	0.63	2.41	1.00E-10	3.79E-09	2.32E-09	1711	tags=36%, list=15%, signal=31%	RPL26/RPS25/RPL24/RPS24/RPS7/RPL5/RPL7/RPL6/NPM1/RPS6/ESF1/RPS17/RPS27/RPL35/RPS8/RPL11/RPL35A/RPL3/RPL27/RPS14/RPS27L/RSL24D1/NOP58/NSA2/NIFK/LTV1/RPS19/RPS21/ZNHIT3/NGDN/KRR1/RPLP0/RBIS/RPL38/EXOSC9/EIF2A/MPHOSPH10/METTL18/FBL/RPL14/GTF3A/RPL10/POP5/MPHOSPH6/RIOK2/RPF1/MRPL22/METTL5/RPS16/BRIX1/NMD3/FRG1/RPSA/MRPL1/GTF2H5/PIN4/NUP88/SBDS/EBNA1BP2/RPL26L1/RPF2/MRPL36/C1D/RPL10A/EMG1/LYAR/LSM6/EXOSC1/ABCE1/DCAF13/DIS3/NOP16/RSL1D1/NHP2/MTREX/RAN/RPS5/UTP3/SRFBP1/MRPS11/ZNF622/RPL23A/GPATCH4/WDR43/GNL2/MTERF4/NOP53/RPP30/NOL8/METTL17/MRPL20/EXOSC8/UTP23/FCF1/NIP7/YBEY/UTP15/MTERF3/NGRN/REXO4/WDR75/TFB2M/HELQ

CC	GO:1990904	ribonucleoprotein complex	625	0.65	2.61	1.00E-10	3.79E-09	2.32E-09	1241	tags=35%, list=11%, signal=33%	RPL21/RPL26/RPL34/RPS25/RPL24/RPL17/RPL31/RPS24/MRPS18C/LARP7/RPS7/RPL23/RPS3A/RPL36A/SRP14/RPL39/RPS10/EIF3E/RPL5/RPS20/RPL7/RPL22/RPL6/RPS18/NPM1/RPL9/LSM3/SNRPD2/RPS6/RPS15A/RPS17/RPS27/RPL35/SNRPG/RPS27A/RWDD1/SF3B6/RPS8/SNRPE/RPL11/RPL35A/RPS13/RPL3/RPS11/SSB/RPL37/RPL13A/PSMA6/RPL27/RPL41/MRPL48/RPS14/RPL27A/TRMT10C/VBP1/RPL19/RPS27L/MRPS21/MRPS22/NOP58/NSA2/RPL32/RPS23/LSM5/MRPL47/RPS26/RPL29/RPL4/RPL12/ZCRB1/SLIRP/LTV1/HNRNPA1/SNRPD1/MRPL46/MRPS28/RPS29/SYF2/RPS19/TRA2B/RPL30/MRPS33/ZNF830/RPS21/MRPL40/ZNHIT3/RPL15/NGDN/KRR1/RPLP0/MRPL27/CWC15/DDX5/ZCCHC17/RPL38/AKAP17A/RPL36/RPS12/SNRPB2/EIF2A/EIF4E/LSM8/LUC7L3/MPHOSPH10/MRPL17/SRP9/FBL/RBM3/RPS3/EIF3H/RPS9/RPL14/MRPL3/RPL10/MRPS15/ZRSR2/MRPS35/CLNS1A/RPLP1/MRPL32/POP5/MRPL13/EIF3M/RIOK2/MRPL51/MRPL57/MRPL35/RPL37A/SLU7/BCAS2/MRPL18/LSM7/RPF1/MRPL22/PHF5A/RPS2/RPS16/RPL8/PRPF38B/MAGOH/PNN/WBP4/MRPL50/MRPL30/SRP19/FRG1/RPSA/RHEB/MRPL1/CCDC12/PRPF39/BTF3/EBNA1BP2/RPL26L1/RPL36AL/RNPC3/MRPL45/MRPL36/HSPA8/SF3B1/DDX1/RPL13/MRPL15/MRPL53/RPL10A/CWC22/PPWD1/MAGOHB/EMG1/SNRPC/PRPF4B/PRPF40A/SRP72/DHX15/SNRNP48/HNRNPH3/MRPS17/LSM6/MRPS14/SRSF2/RBM45/TAF9/EIF3J/MRPL14/SNRPF/PRPF38A/DCAF13/PABPC1L/PPIL3/RSL1D1/SNRPA1/NHP2/MTREX/SNRPD3/MRPS5/RPS5/FXR1/UTP3/SRFBP1/PDCD7/EIF3K/PTGES3/MRPS11/MRPS30/MRPL33/MRPL11/CWF19L2/ZNF622/RBMX2/RPL23A/DAP3/PLRG1/PSMA1/MTERF4/MRPS6/CDC40/MRPL58/NCBP1
BP	GO:0006119	oxidative phosphorylation	110	0.69	2.36	1.00E-10	3.79E-09	2.32E-09	1188	tags=41%, list=10%, signal=37%	UQCRB/ATP5PO/COX7C/NDUFA5/UQCRH/NDUFS5/ATP5ME/NDUFA1/NDUFA6/NDUFB1/UQCRQ/DNAJC15/COA6/ATP5PF/NDUFB2/ATP5MG/ATP5PD/NDUFA4/COX7A2/NDUFS3/ATP5MF/NDUFB3/ATP5F1C/ATP5F1E/COX4I1/DLD/COX6A1/ATP5PB/UQCR10/NDUFS4/NDUFV2/CYCS/UQCRFS1/UQCRC2/NIPSNAP2/NDUFA12/PARK7/NDUFB4/COX5A/UQCC2/NDUFAB1/C2orf69/COX7A2L/MSH2/NDUFB5
BP	GO:0033108	mitochondrial respiratory chain complex assembly	89	0.71	2.36	1.00E-10	3.79E-09	2.32E-09	1829	tags=47%, list=16%, signal=40%	NDUFA5/NDUFS5/NDUFA1/NDUFA6/COX16/PET100/NDUFB1/COA5/NDUFB2/COX14/COX20/NDUFAF8/NDUFS3/TMEM126A/NDUFB3/NDUFAF2/TMEM126B/SMIM20/SDHAF3/NDUFAF5/NDUFS4/C12orf73/NDUFAF4/UQCRFS1/NDUFA12/DMAC1/TFAM/NDUFB4/UQCC2/COX17/NDUFAB1/NDUFB5/CHCHD4/COA4/NDUFA13/NDUFC1/PET117/NDUFB6/SLC25A33/SCO1/NDUFC2/SDHAF1

CC	GO:0098798	mitochondrial protein-containing complex	254	0.74	2.77	1.00E-10	3.79E-09	2.32E-09	1455	tags=42%, list=12%, signal=37%	UQCRB/MRPS18C/ATP5PO/COX6C/ATP5MJ/COX7B/COX7C/NDUFA5/TIMM8B/DNAJC19/UQCRH/NDUFS5/TOMM7/ATP5ME/NDUFA1/NDUFA6/MRPL48/TRMT10C/MRPS21/MRPS22/NDUFB1/TOMM20/UQCRQ/MRPL47/DNAJC15/SMDT1/MRPL46/MRPS28/ATP5PF/NDUFB2/TIMM17A/MRPS33/MRPL40/GRPEL2/MRPL27/ATP5MG/MICU2/ATP5PD/ATP5MK/NDUFA4/MRPL17/TIMM9/COX7A2/MRPL3/NDUFS3/ATP5MF/MRPS15/NDUFB3/MCUB/MRPS35/ATP5F1C/MRPL32/MRPL13/ATP5F1E/COX4I1/MRPL51/DLD/MRPL57/MRPL35/MRPL18/MRPL22/COX6A1/ATP5PB/UQCR10/NDUFS4/TIMM10/MRPL50/MRPL30/NDUFV2/MICOS10/TOMM5/MRPL45/UQCRFS1/MRPL36/UQCRC2/MRPL15/MRPL53/NDUFA12/IMMP1L/MRPS36/MRPS17/ROMO1/MRPS14/NDUFB4/COX5A/MRPL14/NDUFAB1/MRPS5/MRPS11/MRPS30/MRPL33/MRPL11/MICU3/COX7A2L/DAP3/NDUFB5/MTERF4/MRPS6/MRPL58/SUCLG1/NDUFA13/MRPS10/MRPL20/MRPL39/TIMM8A/NDUFC1
CC	GO:0070469	respirasome	78	0.74	2.43	1.00E-10	3.79E-09	2.32E-09	1283	tags=44%, list=11%, signal=39%	UQCRB/COX6C/COX7B/COX7C/NDUFA5/UQCRH/NDUFS5/NDUFA1/NDUFA6/NDUFB1/UQCRQ/COA6/NDUFB2/NDUFA4/COX7A2/NDUFS3/NDUFB3/COX4I1/HIGD1A/COX6A1/UQCR10/NDUFS4/NDUFV2/CYCS/UQCRFS1/UQCRC2/NDUFA12/NDUFB4/COX5A/NDUFAB1/C2orf69/COX7A2L/NDUFB5/NDUFA13
CC	GO:0098800	inner mitochondrial membrane protein complex	131	0.75	2.62	1.00E-10	3.79E-09	2.32E-09	869	tags=37%, list=7%, signal=35%	UQCRB/ATP5PO/COX6C/ATP5MJ/COX7B/COX7C/NDUFA5/DNAJC19/UQCRH/NDUFS5/ATP5ME/NDUFA1/NDUFA6/NDUFB1/UQCRQ/DNAJC15/SMDT1/ATP5PF/NDUFB2/TIMM17A/GRPEL2/ATP5MG/MICU2/ATP5PD/ATP5MK/NDUFA4/TIMM9/COX7A2/NDUFS3/ATP5MF/NDUFB3/MCUB/ATP5F1C/ATP5F1E/COX4I1/COX6A1/ATP5PB/UQCR10/NDUFS4/TIMM10/NDUFV2/MICOS10/UQCRFS1/UQCRC2/NDUFA12/IMMP1L/ROMO1/NDUFB4/COX5A
BP	GO:0042273	ribosomal large subunit biogenesis	69	0.76	2.44	1.00E-10	3.79E-09	2.32E-09	1143	tags=45%, list=10%, signal=41%	RPL26/RPL24/RPL5/RPL7/RPL6/NPM1/RPL35/RPL11/RPL35A/RPL3/RSL24D1/NSA2/NIFK/ZNHIT3/RPLP0/RPL38/RPL14/GTF3A/RPL10/RPF1/BRIX1/MRPL1/EBNA1BP2/RPL26L1/RPF2/RPL10A/NOP16/RSL1D1/NHP2/ZNF622/RPL23A
CC	GO:0098803	respiratory chain complex	68	0.77	2.46	1.00E-10	3.79E-09	2.32E-09	1283	tags=46%, list=11%, signal=41%	UQCRB/COX6C/COX7B/COX7C/NDUFA5/UQCRH/NDUFS5/NDUFA1/NDUFA6/NDUFB1/UQCRQ/COA6/NDUFB2/NDUFA4/COX7A2/NDUFS3/NDUFB3/COX4I1/COX6A1/UQCR10/NDUFS4/NDUFV2/UQCRFS1/UQCRC2/NDUFA12/NDUFB4/COX5A/NDUFAB1/COX7A2L/NDUFB5/NDUFA13
MF	GO:0019843	rRNA binding	64	0.77	2.45	1.00E-10	3.79E-09	2.32E-09	1506	tags=53%, list=13%, signal=47%	MRPS18C/RPL23/RPL5/RPS18/RPL9/RPL11/RPS13/RPL3/RPS11/RPL37/RPS14/RPL12/RPLP0/RPS3/RPS9/GTF3A/MRPL18/RPF1/RPL8/BRIX1/SBDS/RPF2/EMG1/MRPS17/RPS5/MRPS11/MRPL11/RPL23A/MTERF4/MRPS6/NOP53/RPS4X/MRPL20/UTP23

CC	GO:0000313	organellar ribosome	80	0.78	2.58	1.00E-10	3.79E-09	2.32E-09	1416	tags=52%, list=12%, signal=46%	MRPS18C/MRPL48/MRPS21/MRPS22/MRPL47/MRPL46/MRPS28/MRPS33/MRPL40/MRPL27/MRPL17/MRPL3/MRPS15/MRPS35/MRPL32/MRPL13/MRPL51/MRPL57/MRPL35/MRPL18/MRPL22/MRPL50/MRPL30/MRPL45/MRPL36/MRPL15/MRPL53/MRPS17/MRPS14/MRPL14/MRPS5/MRPS11/MRPS30/MRPL33/MRPL11/DAP3/MTERF4/MRPS6/MRPL58/MRPS10/MRPL20/MRPL39
CC	GO:0005761	mitochondrial ribosome	80	0.78	2.58	1.00E-10	3.79E-09	2.32E-09	1416	tags=52%, list=12%, signal=46%	MRPS18C/MRPL48/MRPS21/MRPS22/MRPL47/MRPL46/MRPS28/MRPS33/MRPL40/MRPL27/MRPL17/MRPL3/MRPS15/MRPS35/MRPL32/MRPL13/MRPL51/MRPL57/MRPL35/MRPL18/MRPL22/MRPL50/MRPL30/MRPL45/MRPL36/MRPL15/MRPL53/MRPS17/MRPS14/MRPL14/MRPS5/MRPS11/MRPS30/MRPL33/MRPL11/DAP3/MTERF4/MRPS6/MRPL58/MRPS10/MRPL20/MRPL39
CC	GO:0005840	ribosome	195	0.83	3.02	1.00E-10	3.79E-09	2.32E-09	1058	tags=59%, list=9%, signal=55%	RPL21/RPL26/RPL34/RPS25/RPL24/RPL17/RPL31/RPS24/MRPS18C/RPS7/RPL23/RPS3A/RPL36A/RPL39/RPS10/RPL5/RPS20/RPL7/RPL22/RPL6/RPS18/RPL9/RPS6/RPS15A/RPS17/RPS27/RPL35/RPS27A/RPS8/RPL11/RPL35A/RPS13/RPL3/RPS11/RPL37/RPL13A/RPL27/RPL41/MRPL48/RPS14/RPL27A/RPL19/RPS27L/MRPS21/MRPS22/RSL24D1/RPL22L1/RPL32/RPS23/MRPL47/RPS26/RPL29/RPL4/RPL12/MRPL46/MRPS28/RPS29/RPS19/RPL30/MRPS33/RPS21/MRPL40/RPL15/RPLP0/MRPL27/ZCCHC17/RPL38/RPL36/RPS12/EIF2A/MRPL17/RPS3/EIF3H/RPS9/RPL14/MRPL3/RPL10/MRPS15/MRPS35/RPLP1/MRPL32/MRPL13/MRPL51/MRPL57/MRPL35/RPL37A/MRPL18/MRPL22/RPS2/RPS16/RPL8/MRPL50/MRPL30/RPSA/MRPL1/SERP1/BTF3/RPL26L1/RPL36AL/MRPL45/MRPL36/RPL13/MRPL15/MRPL53/RPL10A/MRPS36/MRPS17/MRPS14/DNAJC21/MRPL14/HSPA14/MRPS5/RPS5/MRPS11/MRPS30/MRPL33
BP	GO:0002181	cytoplasmic translation	139	0.85	2.97	1.00E-10	3.79E-09	2.32E-09	722	tags=56%, list=6%, signal=53%	RPL21/RPL26/RPL34/RPS25/RPL24/RPL17/RPL31/RPS24/RPS7/RPL23/RPS3A/RPL36A/RPL39/RPS10/RPL5/RPS20/RPL7/RPL22/RPL6/RPS18/RPL9/RPS6/RPS15A/RPS17/RPS27/RPL35/RPS27A/RWDD1/RPS8/RPL11/RPL35A/RPS13/RPL3/RPS11/RPL37/RPL13A/RPL27/RPL41/RPS14/RPL27A/RPL19/RPL22L1/RPL32/RPS23/EIF4A2/RPS26/RPL29/RPL4/RPL12/RPS29/RPS19/RPL30/RPS21/EIF2S2/RPL15/RPLP0/RPL38/RPL36/RPS12/MCTS1/ZC3H15/RPS3/RPS9/RPL14/RPL10/DPH5/RPLP1/EIF3M/CNBP/RPL37A/RPS2/RPS16/RPL8/RPSA/RPL26L1/PAIP1/RPL13/RPL10A
CC	GO:0015935	small ribosomal subunit	64	0.85	2.71	1.00E-10	3.79E-09	2.32E-09	1051	tags=64%, list=9%, signal=59%	RPS25/RPS24/MRPS18C/RPS7/RPS3A/RPS10/RPS20/RPS18/RPS6/RPS15A/RPS17/RPS27/RPS27A/RPS8/RPS13/RPS11/RPS14/RPS27L/MRPS21/MRPS22/RPS23/RPS26/MRPS28/RPS29/RPS19/MRPS33/RPS21/RPS12/EIF2A/RPS3/RPS9/MRPS15/MRPS35/RPS2/RPS16/RPSA/MRPS17/MRPS14/MRPS5/RPS5/MRPS11

CC	GO:0015934	large ribosomal subunit	108	0.86	2.91	1.00E-10	3.79E-09	2.32E-09	722	tags=59%, list=6%, signal=56%	RPL21/RPL26/RPL34/RPL24/RPL17/RPL31/RPL23/RPL36A/RPL39/RPL5/RPL7/RPL22/RPL6/RPL9/RPL35/RPL11/RPL35A/RPL3/RPL37/RPL13A/RPL27/RPL41/MRPL48/RPL27A/RPL19/RPL32/MRPL47/RPL29/RPL4/RPL12/MRPL46/RPL30/MRPL40/RPL15/RPLP0/MRPL27/ZCCHC17/RPL38/RPL36/MRPL17/RPL14/MRPL3/RPL10/RPLP1/MRPL32/MRPL13/MRPL51/MRPL57/MRPL35/RPL37A/MRPL18/MRPL22/RPL8/MRPL50/MRPL30/MRPL1/RPL26L1/RPL36AL/MRPL45/MRPL36/RPL13/MRPL15/MRPL53/RPL10A
CC	GO:0044391	ribosomal subunit	169	0.86	3.09	1.00E-10	3.79E-09	2.32E-09	722	tags=59%, list=6%, signal=56%	RPL21/RPL26/RPL34/RPS25/RPL24/RPL17/RPL31/RPS24/MRPS18C/RPS7/RPL23/RPS3A/RPL36A/RPL39/RPS10/RPL5/RPS20/RPL7/RPL22/RPL6/RPS18/RPL9/RPS6/RPS15A/RPS17/RPS27/RPL35/RPS27A/RPS8/RPL11/RPL35A/RPS13/RPL3/RPS11/RPL37/RPL13A/RPL27/RPL41/MRPL48/RPS14/RPL27A/RPL19/RPS27L/MRPS21/MRPS22/RPL32/RPS23/MRPL47/RPS26/RPL29/RPL4/RPL12/MRPL46/MRPS28/RPS29/RPS19/RPL30/MRPS33/RPS21/MRPL40/RPL15/RPLP0/MRPL27/ZCCHC17/RPL38/RPL36/RPS12/EIF2A/MRPL17/RPS3/RPS9/RPL14/MRPL3/RPL10/MRPS15/MRPS35/RPLP1/MRPL32/MRPL13/MRPL51/MRPL57/MRPL35/RPL37A/MRPL18/MRPL22/RPS2/RPS16/RPL8/MRPL50/MRPL30/RPSA/MRPL1/RPL26L1/RPL36AL/MRPL45/MRPL36/RPL13/MRPL15/MRPL53/RPL10A
MF	GO:0003735	structural constituent of ribosome	154	0.87	3.08	1.00E-10	3.79E-09	2.32E-09	722	tags=62%, list=6%, signal=59%	RPL21/RPL26/RPL34/RPS25/RPL24/RPL17/RPL31/RPS24/MRPS18C/RPS7/RPL23/RPS3A/RPL36A/RPL39/RPS10/RPL5/RPS20/RPL7/RPL22/RPL6/RPS18/RPL9/RPS6/RPS15A/RPS17/RPS27/RPL35/RPS27A/RPS8/RPL11/RPL35A/RPS13/RPL3/RPS11/RPL37/RPL13A/RPL27/RPL41/RPS14/RPL27A/RPL19/RPS27L/MRPS21/MRPS22/RSL24D1/RPL22L1/RPL32/RPS23/MRPL47/RPS26/RPL29/RPL4/RPL12/MRPL46/RPS29/RPS19/RPL30/MRPS33/RPS21/RPL15/RPLP0/MRPL27/RPL38/RPL36/RPS12/MRPL17/RPS3/RPS9/RPL14/MRPL3/RPL10/MRPS15/MRPS35/RPLP1/MRPL32/MRPL13/MRPL51/MRPL57/MRPL35/RPL37A/MRPL18/MRPL22/RPS2/RPS16/RPL8/MRPL30/RPSA/MRPL1/RPL26L1/RPL36AL/MRPS31/MRPL36/RPL13/MRPL15/RPL10A
CC	GO:0042788	polysomal ribosome	29	0.89	2.43	1.00E-10	3.79E-09	2.32E-09	722	tags=69%, list=6%, signal=65%	RPL24/RPL31/RPL36A/RPL39/RPL6/RPL11/RPL41/RPL19/RPL32/RPS23/RPS26/RPS29/RPL30/RPS21/RPL38/RPL36/EIF3H/RPL8/BTF3/RPL10A
CC	GO:0022627	cytosolic small ribosomal subunit	39	0.91	2.63	1.00E-10	3.79E-09	2.32E-09	590	tags=74%, list=5%, signal=71%	RPS25/RPS24/RPS7/RPS3A/RPS10/RPS20/RPS18/RPS6/RPS15A/RPS17/RPS27/RPS27A/RPS8/RPS13/RPS11/RPS14/RPS27L/RPS23/RPS26/RPS29/RPS19/RPS21/RPS12/EIF2A/RPS3/RPS9/RPS2/RPS16/RPSA

CC	GO:0022626	cytosolic ribosome	93	0.91	3.06	1.00E-10	3.79E-09	2.32E-09	722	tags=78%, list=6%, signal=74%	RPL21/RPL26/RPL34/RPS25/RPL24/RPL17/RPL31/RPS24/RPS7/RPL23/RPS3A/RPL36A/RPL39/RPS10/RPL5/RPS20/RPL7/RPL22/RPL6/RPS18/RPL9/RPS6/RPS15A/RPS17/RPS27/RPL35/RPS27A/RPS8/RPL11/RPL35A/RPS13/RPL3/RPS11/RPL37/RPL13A/RPL27/RPL41/RPS14/RPL27A/RPL19/RPS27L/RPL32/RPS23/RPS26/RPL29/RPL4/RPL12/RPS29/RPS19/RPL30/RPS21/RPL15/RPLP0/ZCCHC17/RPL38/RPL36/RPS12/EIF2A/RPS3/RPS9/RPL14/RPL10/RPLP1/RPL37A/RPS2/RPS16/RPL8/RPSA/MRPL1/RPL26L1/RPL36AL/RPL13/RPL10A
CC	GO:0022625	cytosolic large ribosomal subunit	54	0.91	2.81	1.00E-10	3.79E-09	2.32E-09	722	tags=81%, list=6%, signal=77%	RPL21/RPL26/RPL34/RPL24/RPL17/RPL31/RPL23/RPL36A/RPL39/RPL5/RPL7/RPL22/RPL6/RPL9/RPL35/RPL11/RPL35A/RPL3/RPL37/RPL13A/RPL27/RPL41/RPL27A/RPL19/RPL32/RPL29/RPL4/RPL12/RPL30/RPL15/RPLP0/ZCCHC17/RPL38/RPL36/RPL14/RPL10/RPLP1/RPL37A/RPL8/MRPL1/RPL26L1/RPL36AL/RPL13/RPL10A
CC	GO:0005746	mitochondrial respirasome	72	0.73	2.38	1.41E-10	5.21E-09	3.20E-09	1283	tags=42%, list=11%, signal=37%	UQCRB/COX6C/COX7B/COX7C/NDUFA5/UQCRH/NDUFS5/NDUFA1/NDUFA6/NDUFB1/UQCRQ/NDUFB2/NDUFA4/COX7A2/NDUFS3/NDUFB3/COX4I1/COX6A1/UQCR10/NDUFS4/NDUFV2/UQCRFS1/UQCRC2/NDUFA12/NDUFB4/COX5A/NDUFAB1/COX7A2L/NDUFB5/NDUFA13
BP	GO:0042274	ribosomal small subunit biogenesis	71	0.73	2.36	3.64E-10	1.21E-08	7.46E-09	1159	tags=38%, list=10%, signal=34%	RPS25/RPS24/RPS7/NPM1/RPS6/RPS17/RPS27/RPS8/RPS14/RPS27L/LTV1/RPS19/RPS21/NGDN/RPL38/RIOK2/RPS16/RPSA/GTF2H5/EMG1/LSM6/DCAF13/RPS5/UTP3/SRFBP1/MRPS11/WDR43
CC	GO:0000315	organellar large ribosomal subunit	54	0.77	2.37	4.30E-10	1.38E-08	8.46E-09	1416	tags=52%, list=12%, signal=46%	MRPL48/MRPL47/MRPL46/MRPL40/MRPL27/MRPL17/MRPL3/MRPL32/MRPL13/MRPL51/MRPL57/MRPL35/MRPL18/MRPL22/MRPL50/MRPL30/MRPL45/MRPL36/MRPL15/MRPL53/MRPL14/MRPS30/MRPL33/MRPL11/MTERF4/MRPL58/MRPL20/MRPL39
CC	GO:0005762	mitochondrial large ribosomal subunit	54	0.77	2.37	4.30E-10	1.38E-08	8.46E-09	1416	tags=52%, list=12%, signal=46%	MRPL48/MRPL47/MRPL46/MRPL40/MRPL27/MRPL17/MRPL3/MRPL32/MRPL13/MRPL51/MRPL57/MRPL35/MRPL18/MRPL22/MRPL50/MRPL30/MRPL45/MRPL36/MRPL15/MRPL53/MRPL14/MRPS30/MRPL33/MRPL11/MTERF4/MRPL58/MRPL20/MRPL39
BP	GO:0042773	ATP synthesis coupled electron transport	70	0.72	2.33	5.36E-10	1.68E-08	1.03E-08	1188	tags=43%, list=10%, signal=39%	UQCRB/COX7C/NDUFA5/UQCRH/NDUFS5/NDUFA1/NDUFA6/NDUFB1/UQCRQ/DNAJC15/COA6/NDUFB2/NDUFA4/NDUFS3/NDUFB3/COX4I1/DLD/COX6A1/UQCR10/NDUFS4/NDUFV2/CYCS/UQCRFS1/NDUFA12/PARK7/NDUFB4/COX5A/NDUFAB1/COX7A2L/NDUFB5
BP	GO:0042775	mitochondrial ATP synthesis coupled electron transport	70	0.72	2.33	5.36E-10	1.68E-08	1.03E-08	1188	tags=43%, list=10%, signal=39%	UQCRB/COX7C/NDUFA5/UQCRH/NDUFS5/NDUFA1/NDUFA6/NDUFB1/UQCRQ/DNAJC15/COA6/NDUFB2/NDUFA4/NDUFS3/NDUFB3/COX4I1/DLD/COX6A1/UQCR10/NDUFS4/NDUFV2/CYCS/UQCRFS1/NDUFA12/PARK7/NDUFB4/COX5A/NDUFAB1/COX7A2L/NDUFB5

BP	GO:0022904	respiratory electron transport chain	87	0.69	2.29	1.23E-09	3.52E-08	2.16E-08	1188	tags=36%, list=10%, signal=32%	UQCRB/COX7C/NDUFA5/UQCRH/NDUFS5/NDUFA1/NDUFA6/NDUFB1/UQCRQ/DNAJC15/COA6/NDUFB2/NDUFA4/NDUFS3/NDUFB3/COX4I1/DLD/COX6A1/UQCR10/NDUFS4/NDUFV2/CYCS/UQCRFS1/NDUFA12/PARK7/NDUFB4/COX5A/NDUFAB1/ETFRF1/COX7A2L/NDUFB5
BP	GO:0022618	ribonucleoprotein complex assembly	153	0.59	2.10	1.32E-09	3.66E-08	2.25E-08	1257	tags=31%, list=11%, signal=28%	RPL24/RPL5/RPL6/HSP90AA1/SNRPD2/RPS27/SNRPG/SNRPE/RPL11/RPL3/RPL13A/RPS14/RPS27L/SNRPD1/RPS19/EIF2S2/ZNHIT3/RPLP0/RPL38/MCTS1/LUC7L3/RPL10/ZRSR2/CLNS1A/SLU7/SRSF10/COIL/BRIX1/RPSA/PRPF39/PSIP1/RPF2/SF3B1/DDX1/SNRPC/DENR/TAF9/SNRPF/NUDT21/SNRPD3/RPS5/PTGES3/MRPS11/GEMIN6/RPL23A/NCBP1/NOP53
BP	GO:0022900	electron transport chain	125	0.61	2.13	1.95E-09	5.20E-08	3.19E-08	1188	tags=27%, list=10%, signal=25%	UQCRB/COX7C/NDUFA5/UQCRH/NDUFS5/NDUFA1/NDUFA6/NDUFB1/UQCRQ/DNAJC15/COA6/NDUFB2/NDUFA4/NDUFS3/NDUFB3/COX11/COX4I1/DLD/COX6A1/UQCR10/NDUFS4/NDUFV2/CYCS/UQCRFS1/NDUFA12/PARK7/ADH5/NDUFB4/COX5A/NDUFAB1/FDX1/ETFRF1/COX7A2L/NDUFB5
BP	GO:0071826	ribonucleoprotein complex subunit organization	161	0.57	2.03	3.42E-09	8.56E-08	5.26E-08	1257	tags=30%, list=11%, signal=27%	RPL24/RPL5/RPL6/HSP90AA1/SNRPD2/RPS27/SNRPG/SNRPE/RPL11/RPL3/RPL13A/RPS14/RPS27L/SNRPD1/RPS19/EIF2S2/ZNHIT3/RPLP0/RPL38/MCTS1/LUC7L3/RPL10/ZRSR2/CLNS1A/SLU7/SRSF10/COIL/BRIX1/RPSA/ZFAND1/PRPF39/PSIP1/RPF2/SF3B1/DDX1/SNRPC/DENR/TAF9/SNRPF/NUDT21/SNRPD3/RPS5/PTGES3/MRPS11/GEMIN6/RPL23A/NCBP1/NOP53
BP	GO:0008380	RNA splicing	362	0.46	1.78	5.27E-09	1.25E-07	7.66E-08	1242	tags=25%, list=11%, signal=23%	LARP7/LSM3/SNRPD2/PPIG/SNRPG/SF3B6/SNRPE/RPS13/RBM39/LSM5/RPS26/RTRAF/ZCRB1/HNRNPA1/SNRPD1/SYF2/TRA2B/ZNF830/SREK1IP1/SRSF7/CWC15/DDX5/AKAP17A/SNRPB2/LSM8/LUC7L3/MPHOSPH10/RBM3/CLK1/LSM1/SRSF3/ZRSR2/CLNS1A/SLU7/BCAS2/ZRANB2/LSM7/PHF5A/RP9/SRSF10/COIL/PRPF38B/MAGOH/PNN/WBP4/ZBTB8OS/FRG1/SFPQ/PRPF39/RNPC3/PSIP1/HSPA8/SF3B1/DDX1/CWC22/PPWD1/MAGOHB/SNRPC/PRPF4B/PRPF40A/SRSF11/DHX15/SNRNP48/HNRNPH3/LSM6/THOC7/ARL6IP4/SRSF2/UBL5/SNRPF/PRPF38A/CCNL2/PPIL3/TIA1/CLK4/SNRPA1/SRSF8/MTREX/SNRPD3/FXR1/PDCD7/GEMIN6/ZNF326/CWF19L2/RBMX2/PLRG1/CDC40/NCBP1/METTL3
BP	GO:0019646	aerobic electron transport chain	64	0.72	2.30	6.62E-09	1.54E-07	9.44E-08	1188	tags=44%, list=10%, signal=40%	UQCRB/COX7C/NDUFA5/UQCRH/NDUFS5/NDUFA1/NDUFA6/NDUFB1/UQCRQ/DNAJC15/NDUFB2/NDUFA4/NDUFS3/NDUFB3/COX4I1/DLD/COX6A1/UQCR10/NDUFS4/NDUFV2/CYCS/UQCRFS1/PARK7/NDUFB4/COX5A/NDUFAB1/COX7A2L/NDUFB5
CC	GO:0005844	polysome	61	0.72	2.27	1.11E-08	2.46E-07	1.51E-07	722	tags=41%, list=6%, signal=39%	RPL24/RPL31/RPL36A/RPL39/RPL6/RPS6/RWDD1/RPL11/PSMA6/RPL41/VBP1/RPL19/RPL32/RPS23/RPS26/RPS29/RPL30/RPS21/RPL38/RPL36/RPS3/EIF3H/RPL8/BTF3/RPL10A

BP	GO:0015980	energy derivation by oxidation of organic compounds	235	0.50	1.89	1.27E-08	2.77E-07	1.70E-07	1265	tags=26%, list=11%, signal=24%	UQCRB/ATP5PO/COX7C/NDUFA5/UQCRH/NDUFS5/ATP5ME/NDUFA1/NDUFA6/NDUFB1/UQCRQ/DNAJC15/COA6/ATP5PF/NDUFB2/ATP5MG/ATP5PD/NDUFA4/COX7A2/NDUFS3/ATP5MF/NDUFB3/ATP5F1C/ATP5F1E/COX4I1/DLD/CISD1/COX6A1/ATP5PB/UQCR10/PPP1CC/NDUFS4/NDUFV2/CYCS/UQCRFS1/BNIP3/UQCRC2/NIPSNAP2/NDUFA12/UGP2/MRPS36/PARK7/SELENOS/NDUFB4/COX5A/UQCC2/ADSL/NDUFAB1/EPM2AIP1/C2orf69/PTGES3/ETFRF1/RB1CC1/PASK/COX7A2L/MSH2/NDUFB5/SLC25A25/SUCLG1/NOP53/CAT
MF	GO:0015453	oxidoreduction-driven active transmembrane transporter activity	55	0.73	2.25	1.79E-08	3.79E-07	2.33E-07	1283	tags=42%, list=11%, signal=37%	COX7B/NDUFA5/UQCRH/NDUFS5/NDUFA1/NDUFA6/NDUFB1/NDUFB2/NDUFA4/NDUFS3/NDUFB3/COX4I1/COX6A1/UQCR10/NDUFS4/NDUFV2/UQCRFS1/NDUFA12/NDUFB4/COX5A/COX7A2L/NDUFB5/NDUFA13
BP	GO:0042255	ribosome assembly	56	0.72	2.24	2.69E-08	5.53E-07	3.40E-07	1617	tags=50%, list=14%, signal=43%	RPL24/RPL5/RPL6/NPM1/RPS27/RPL11/RPL3/RPS14/RPS27L/RPS19/RPLP0/RPL38/EIF2A/RPL10/MRPL22/BRIX1/RPSA/SBDS/RPF2/RPS5/MRPS11/RPL23A/MTERF4/NOP53/MRPL20/NIP7/MTERF3/NGRN
CC	GO:0005681	spliceosomal complex	185	0.54	1.96	3.13E-08	6.32E-07	3.88E-07	1233	tags=31%, list=11%, signal=29%	LSM3/SNRPD2/SNRPG/SF3B6/SNRPE/LSM5/ZCRB1/HNRNPA1/SNRPD1/SYF2/TRA2B/ZNF830/CWC15/DDX5/AKAP17A/SNRPB2/LSM8/LUC7L3/RBM3/ZRSR2/CLNS1A/SLU7/BCAS2/LSM7/PHF5A/PRPF38B/MAGOH/PNN/WBP4/FRG1/RHEB/CCDC12/PRPF39/RNPC3/HSPA8/SF3B1/CWC22/PPWD1/MAGOHB/SNRPC/PRPF4B/PRPF40A/DHX15/SNRNP48/HNRNPH3/LSM6/SRSF2/SNRPF/PRPF38A/PPIL3/SNRPA1/MTREX/SNRPD3/PDCD7/CWF19L2/RBMX2/PLRG1/CDC40
CC	GO:0005759	mitochondrial matrix	413	0.44	1.73	3.69E-08	7.34E-07	4.51E-07	1967	tags=26%, list=17%, signal=23%	MRPS18C/DNAJC19/SOD1/MRPL48/TRMT10C/MRPS21/MRPS22/MRPL47/DNAJC15/SMDT1/MRPL46/MRPS28/HSPE1/MRPS33/MRPL40/GRPEL2/COQ5/MRPL27/PPA2/MTRES1/MRPL17/RPS3/MRPL3/NDUFS3/MRPS15/MRPS35/ATP5F1C/MRPL32/MRPL13/ATP5F1E/MRPL51/DLD/MRPL57/MRPL35/MRPL18/MRPL22/DHFR2/SDHAF3/ATP5PB/LIPT1/MRPL50/MRPL30/PIN4/ACAA2/MRPL45/MRPL36/MRPL15/ACOT13/MRPL53/MTERF2/MRPS36/MTHFD2/PARK7/MRPS17/MRPS14/SSBP1/VDAC2/TFAM/MRPL14/ABCE1/UQCC2/ACOT2/NDUFAB1/C2orf69/MRPS5/FDX1/PRDX3/ACAT1/MRPS11/MRPS30/MRPL33/MRPL11/ISCA2/DAP3/MTERF4/MRPS6/MRPL58/GCSH/SUCLG1/MRPS10/METTL17/MRPL20/MRPL39/NADK2/FASTKD1/ACADM/CBR4/CARS2/MTERF1/DNA2/HADH/GPT2/DNAJA3/SUCLA2/MRPL24/BCKDHB/TFB2M/MRPS16/PRIMPOL/PYROXD2/SDHAF1/MAIP1/HSPA9/MTRF1L/ATP5F1A/GLS/PAM16/PUS1

MF	GO:0009055	electron transfer activity	93	0.64	2.15	3.92E-08	7.73E-07	4.74E-07	1455	tags=32%, list=12%, signal=28%	COX7B/NDUFA5/UQCRH/NDUFS5/NDUFA1/NDUFA6/NDUFB1/NDUFB2/NDUFA4/COX7A2/NDUFS3/NDUFB3/COX11/COX4I1/COX6A1/UQCR10/NDUFS4/NDUFV2/CYCS/UQCRFS1/NDUFA12/ADH5/NDUFB4/COX5A/FDX1/COX7A2L/NDUFB5/NDUFA13/DEGS1/NDUFC1
BP	GO:0000377	RNA splicing, via transesterification reactions with bulged adenosine as nucleophile	259	0.47	1.80	1.51E-07	2.59E-06	1.59E-06	1242	tags=25%, list=11%, signal=23%	LARP7/LSM3/SNRPD2/SNRPG/SF3B6/SNRPE/RBM39/LSM5/ZCRB1/HNRNPA1/SNRPD1/SYF2/TRA2B/SRSF7/CWC15/DDX5/SNRPB2/LSM8/LUC7L3/RBM3/SRSF3/ZRSR2/CLNS1A/SLU7/BCAS2/LSM7/PHF5A/SRSF10/COIL/MAGOH/PNN/WBP4/FRG1/SFPQ/PRPF39/RNPC3/PSIP1/HSPA8/SF3B1/DDX1/CWC22/PPWD1/MAGOHB/SNRPC/PRPF4B/PRPF40A/HNRNPH3/LSM6/SRSF2/UBL5/SNRPF/PRPF38A/PPIL3/TIA1/SNRPA1/SRSF8/MTREX/SNRPD3/FXR1/GEMIN6/CWF19L2/RBMX2/PLRG1/CDC40/NCBP1/METTL3
BP	GO:0000398	mRNA splicing, via spliceosome	259	0.47	1.80	1.51E-07	2.59E-06	1.59E-06	1242	tags=25%, list=11%, signal=23%	LARP7/LSM3/SNRPD2/SNRPG/SF3B6/SNRPE/RBM39/LSM5/ZCRB1/HNRNPA1/SNRPD1/SYF2/TRA2B/SRSF7/CWC15/DDX5/SNRPB2/LSM8/LUC7L3/RBM3/SRSF3/ZRSR2/CLNS1A/SLU7/BCAS2/LSM7/PHF5A/SRSF10/COIL/MAGOH/PNN/WBP4/FRG1/SFPQ/PRPF39/RNPC3/PSIP1/HSPA8/SF3B1/DDX1/CWC22/PPWD1/MAGOHB/SNRPC/PRPF4B/PRPF40A/HNRNPH3/LSM6/SRSF2/UBL5/SNRPF/PRPF38A/PPIL3/TIA1/SNRPA1/SRSF8/MTREX/SNRPD3/FXR1/GEMIN6/CWF19L2/RBMX2/PLRG1/CDC40/NCBP1/METTL3
BP	GO:0000375	RNA splicing, via transesterification reactions	263	0.47	1.80	1.56E-07	2.66E-06	1.64E-06	1242	tags=26%, list=11%, signal=24%	LARP7/LSM3/SNRPD2/SNRPG/SF3B6/SNRPE/RBM39/LSM5/ZCRB1/HNRNPA1/SNRPD1/SYF2/TRA2B/SRSF7/CWC15/DDX5/SNRPB2/LSM8/LUC7L3/MPHOSPH10/RBM3/LSM1/SRSF3/ZRSR2/CLNS1A/SLU7/BCAS2/LSM7/PHF5A/SRSF10/COIL/MAGOH/PNN/WBP4/FRG1/SFPQ/PRPF39/RNPC3/PSIP1/HSPA8/SF3B1/DDX1/CWC22/PPWD1/MAGOHB/SNRPC/PRPF4B/PRPF40A/HNRNPH3/LSM6/SRSF2/UBL5/SNRPF/PRPF38A/PPIL3/TIA1/SNRPA1/SRSF8/MTREX/SNRPD3/FXR1/GEMIN6/CWF19L2/RBMX2/PLRG1/CDC40/NCBP1/METTL3
BP	GO:0010257	NADH dehydrogenase complex assembly	53	0.71	2.18	3.12E-07	5.06E-06	3.11E-06	1283	tags=40%, list=11%, signal=35%	NDUFA5/NDUFS5/NDUFA1/NDUFA6/NDUFB1/NDUFB2/NDUFAF8/NDUFS3/TMEM126A/NDUFB3/NDUFAF2/TMEM126B/NDUFAF5/NDUFS4/NDUFAF4/NDUFA12/DMAC1/NDUFB4/NDUFAB1/NDUFB5/NDUFA13
BP	GO:0032981	mitochondrial respiratory chain complex I assembly	53	0.71	2.18	3.12E-07	5.06E-06	3.11E-06	1283	tags=40%, list=11%, signal=35%	NDUFA5/NDUFS5/NDUFA1/NDUFA6/NDUFB1/NDUFB2/NDUFAF8/NDUFS3/TMEM126A/NDUFB3/NDUFAF2/TMEM126B/NDUFAF5/NDUFS4/NDUFAF4/NDUFA12/DMAC1/NDUFB4/NDUFAB1/NDUFB5/NDUFA13

MF	GO:0016655	oxidoreductase activity, acting on NAD(P)H, quinone or similar compound as acceptor	45	0.74	2.21	4.16E-07	6.49E-06	3.98E-06	1824	tags=49%, list=16%, signal=41%	NDUFA5/NDUFS5/NDUFA1/NDUFA6/NDUFB1/NDUFB2/NDUFA4/NDUFS3/NDUFB3/NDUFS4/NDUFV2/CRYZ/NDUFA12/NDUFB4/AKR1C3/NDUFB5/NDUFA13/NDUFC1/CBR4/NDUFS6/NDUFB6/NDUFC2
CC	GO:0070069	cytochrome complex	26	0.82	2.20	4.78E-07	7.43E-06	4.56E-06	869	tags=58%, list=7%, signal=54%	UQCRB/COX6C/COX7B/COX7C/UQCRH/UQCRQ/COA6/NDUFA4/COX7A2/COX4I1/COX6A1/UQCR10/UQCRFS1/UQCRC2/COX5A
BP	GO:0008033	tRNA processing	121	0.57	1.99	8.42E-07	1.23E-05	7.55E-06	2931	tags=47%, list=25%, signal=36%	SSB/TRMT10C/RTRAF/TPRKB/THUMPD1/POP5/TRMO/ZBTB8OS/DTWD1/DALRD3/DDX1/MTO1/TRMT12/LSM6/TRMT13/THUMPD2/CDK5RAP1/TRMT6/TSEN15/TYW3/RPP30/ELAC1/TRMT1/OSGEP/TYW1/CLP1/TRMT10A/DPH3/TRUB1/MOCS3/LCMT2/PUS1/DUS1L/GRSF1/METTL8/TSEN54/TRMT10B/NSUN6/THUMPD3/OSGEPL1/ANKRD16/WDR6/TYW5/POLR3K/TRIT1/PUS3/RTCB/RPP14/ADAT2/RPP38/CTU2/POP4/TARBP1/TRNT1/GTPBP3/TRMT61B/TP53RK
MF	GO:0008137	NADH dehydrogenase (ubiquinone) activity	35	0.75	2.13	1.88E-06	2.56E-05	1.57E-05	1824	tags=54%, list=16%, signal=46%	NDUFA5/NDUFS5/NDUFA1/NDUFA6/NDUFB1/NDUFB2/NDUFA4/NDUFS3/NDUFB3/NDUFS4/NDUFV2/NDUFA12/NDUFB4/NDUFB5/NDUFA13/NDUFC1/NDUFS6/NDUFB6/NDUFC2
MF	GO:0050136	NADH dehydrogenase (quinone) activity	35	0.75	2.13	1.88E-06	2.56E-05	1.57E-05	1824	tags=54%, list=16%, signal=46%	NDUFA5/NDUFS5/NDUFA1/NDUFA6/NDUFB1/NDUFB2/NDUFA4/NDUFS3/NDUFB3/NDUFS4/NDUFV2/NDUFA12/NDUFB4/NDUFB5/NDUFA13/NDUFC1/NDUFS6/NDUFB6/NDUFC2
CC	GO:0000314	organellar small ribosomal subunit	25	0.82	2.18	2.09E-06	2.77E-05	1.70E-05	1376	tags=56%, list=12%, signal=50%	MRPS18C/MRPS21/MRPS22/MRPS28/MRPS33/MRPS15/MRPS35/MRPS17/MRPS14/MRPS5/MRPS11/DAP3/MRPS6/MRPS10
CC	GO:0005763	mitochondrial small ribosomal subunit	25	0.82	2.18	2.09E-06	2.77E-05	1.70E-05	1376	tags=56%, list=12%, signal=50%	MRPS18C/MRPS21/MRPS22/MRPS28/MRPS33/MRPS15/MRPS35/MRPS17/MRPS14/MRPS5/MRPS11/DAP3/MRPS6/MRPS10
CC	GO:0005747	mitochondrial respiratory chain complex I	41	0.73	2.15	2.87E-06	3.61E-05	2.21E-05	1824	tags=49%, list=16%, signal=41%	NDUFA5/NDUFS5/NDUFA1/NDUFA6/NDUFB1/NDUFB2/NDUFA4/NDUFS3/NDUFB3/NDUFS4/NDUFV2/NDUFA12/NDUFB4/NDUFAB1/NDUFB5/NDUFA13/NDUFC1/NDUFS6/NDUFB6/NDUFC2
CC	GO:0030964	NADH dehydrogenase complex	41	0.73	2.15	2.87E-06	3.61E-05	2.21E-05	1824	tags=49%, list=16%, signal=41%	NDUFA5/NDUFS5/NDUFA1/NDUFA6/NDUFB1/NDUFB2/NDUFA4/NDUFS3/NDUFB3/NDUFS4/NDUFV2/NDUFA12/NDUFB4/NDUFAB1/NDUFB5/NDUFA13/NDUFC1/NDUFS6/NDUFB6/NDUFC2
CC	GO:0045271	respiratory chain complex I	41	0.73	2.15	2.87E-06	3.61E-05	2.21E-05	1824	tags=49%, list=16%, signal=41%	NDUFA5/NDUFS5/NDUFA1/NDUFA6/NDUFB1/NDUFB2/NDUFA4/NDUFS3/NDUFB3/NDUFS4/NDUFV2/NDUFA12/NDUFB4/NDUFAB1/NDUFB5/NDUFA13/NDUFC1/NDUFS6/NDUFB6/NDUFC2

MF	GO:0003955	NAD(P)H dehydrogenase (quinone) activity	37	0.75	2.15	4.08E-06	4.99E-05	3.07E-05	1824	tags=54%, list=16%, signal=46%	NDUFA5/NDUFS5/NDUFA1/NDUFA6/NDUFB1/NDUFB2/NDUFA4/NDUFS3/NDUFB3/NDUFS4/NDUFV2/NDUFA12/NDUFB4/NDUFB5/NDUFA13/NDUFC1/CBR4/NDUFS6/NDUFB6/NDUFC2
BP	GO:0006397	mRNA processing	416	0.40	1.59	4.44E-06	5.35E-05	3.28E-05	2386	tags=33%, list=20%, signal=27%	LARP7/LSM3/SNRPD2/SNRPG/SF3B6/SNRPE/RBM39/LSM5/ZCRB1/HNRNPA1/SNRPD1/SYF2/TRA2B/ZNF830/SREK1IP1/SRSF7/CWC15/DDX5/AKAP17A/SNRPB2/LSM8/LUC7L3/CMTR2/RBM3/LSM1/SRSF3/ZRSR2/CLNS1A/SLU7/BCAS2/ZRANB2/LSM7/PHF5A/SRSF10/COIL/PRPF38B/RPRD1A/KIN/MAGOH/PNN/WBP4/PCF11/FRG1/SFPQ/PRPF39/RNPC3/PSIP1/HSPA8/SF3B1/DDX1/CWC22/PPWD1/MAGOHB/SNRPC/PRPF4B/PRPF40A/SRSF11/DHX15/SNRNP48/HNRNPH3/LSM6/THOC7/ARL6IP4/SRSF2/UBL5/CSTF3/SNRPF/PRPF38A/PPIL3/TIA1/NUDT21/SNRPA1/SRSF8/MTREX/SNRPD3/FXR1/SLTM/GEMIN6/ZNF326/CWF19L2/RBMX2/PLRG1/CDC40/NCBP1/METTL3/TSEN15/NSRP1/PPP1R8/RNMT/TRA2A/PPP4R2/LSM11/CLP1/NCBP2/HTATSF1/RBM41/DDX39A/METTL14/SAP18/RBMX/SF3A3/SF3B5/GEMIN2/RBM17/THOC1/LUC7L/CLASRP/NONO/SRSF5/RBM25/SNRNP70/MFAP1/GRSF1/STRAP/NRDE2/TBRG4/TSEN54/SREK1/CWC25/PAXBP1/CDC5L/WTAP/CIRBP/HNRNPLL/POLR2D/GCFC2/FIP1L1/HNRNPA2B1/SLBP/PABPN1/HNRNPA0/YTHDC1/CSTF2T/SUGP2/RBM8A/ZPR1/ZC3H14
CC	GO:0120114	Sm-like protein family complex	76	0.60	1.97	8.05E-06	8.92E-05	5.48E-05	1142	tags=34%, list=10%, signal=31%	LARP7/LSM3/SNRPD2/SNRPG/SF3B6/SNRPE/LSM5/SNRPD1/SNRPB2/LSM8/LUC7L3/LSM1/CLNS1A/SLU7/LSM7/PHF5A/PRPF39/SF3B1/SNRPC/PRPF40A/LSM6/SNRPF/SNRPA1/SNRPD3/GEMIN6/RBMX2
MF	GO:0003954	NADH dehydrogenase activity	36	0.74	2.11	8.94E-06	9.79E-05	6.01E-05	1824	tags=53%, list=16%, signal=45%	NDUFA5/NDUFS5/NDUFA1/NDUFA6/NDUFB1/NDUFB2/NDUFA4/NDUFS3/NDUFB3/NDUFS4/NDUFV2/NDUFA12/NDUFB4/NDUFB5/NDUFA13/NDUFC1/NDUFS6/NDUFB6/NDUFC2
BP	GO:0046034	ATP metabolic process	211	0.47	1.72	1.39E-05	0.00014444	8.87E-05	1204	tags=24%, list=10%, signal=22%	UQCRB/ATP5PO/COX7C/NDUFA5/UQCRH/NDUFS5/ATP5ME/NDUFA1/NDUFA6/NDUFB1/UQCRQ/DNAJC15/COA6/ATP5PF/NDUFB2/ATP5IF1/ATP5MG/ATP5PD/NDUFA4/COX7A2/NDUFS3/ATP5MF/OLA1/NDUFB3/ATP5F1C/ATP5F1E/COX4I1/DLD/COX6A1/ATP5PB/UQCR10/NDUFS4/NDUFV2/CYCS/UQCRFS1/HSPA8/UQCRC2/NIPSNAP2/NDUFA12/PARK7/NDUFB4/COX5A/PRKAG1/UQCC2/NDUFAB1/C2orf69/COX7A2L/MSH2/NDUFB5/SLC25A25
CC	GO:1990204	oxidoreductase complex	97	0.57	1.92	1.52E-05	0.00015501	9.52E-05	1728	tags=33%, list=15%, signal=28%	UQCRB/NDUFA5/UQCRH/NDUFS5/NDUFA1/NDUFA6/NDUFB1/UQCRQ/NDUFB2/NDUFA4/NDUFS3/LDHB/NDUFB3/DLD/UQCR10/NDUFS4/NDUFV2/UQCRFS1/UQCRC2/NDUFA12/MRPS36/NDUFB4/NDUFAB1/NDUFB5/GCSH/CAT/NDUFA13/NDUFC1/CBR4/BCKDHB/NDUFS6/NDUFB6
CC	GO:0005684	U2-type spliceosomal complex	89	0.57	1.90	2.08E-05	0.00020337	0.00012486	1233	tags=35%, list=11%, signal=31%	LSM3/SNRPD2/SNRPG/SF3B6/SNRPE/LSM5/SNRPD1/SYF2/CWC15/SNRPB2/LSM8/LUC7L3/BCAS2/LSM7/PHF5A/WBP4/CCDC12/PRPF39/SF3B1/CWC22/MAGOHB/SNRPC/PRPF40A/LSM6/SNRPF/PRPF38A/SNRPA1/SNRPD3/RBMX2/PLRG1/CDC40

BP	GO:0006400	tRNA modification	87	0.58	1.93	2.08E-05	0.00020337	0.00012486	2902	tags=45%, list=25%, signal=34%	SSB/TRMT10C/TPRKB/THUMPD1/TRMO/DTWD1/DALRD3/MTO1/TRMT12/TRMT13/THUMPD2/CDK5RAP1/TRMT6/TYW3/TRMT1/OSGEP/TYW1/TRMT10A/DPH3/TRUB1/MOCS3/LCMT2/PUS1/DUS1L/METTL8/TRMT10B/NSUN6/THUMPD3/OSGEPL1/ANKRD16/WDR6/TYW5/TRIT1/PUS3/ADAT2/CTU2/TARBP1/GTPBP3/TRMT61B
BP	GO:0006399	tRNA metabolic process	178	0.47	1.71	2.36E-05	0.0002257	0.00013857	2937	tags=40%, list=25%, signal=31%	SSB/TRMT10C/RTRAF/TPRKB/EXOSC9/NARS1/THUMPD1/POP5/TRMO/ZBTB8OS/DTWD1/DALRD3/DDX1/MTO1/TRMT12/LSM6/TRMT13/THUMPD2/CDK5RAP1/GTF3C3/TRMT6/GATB/DTD2/TSEN15/TYW3/RPP30/ELAC1/TRMT1/EXOSC8/OSGEP/RARS1/TYW1/CARS2/TARS1/CLP1/TRMT10A/DPH3/TRUB1/EXOSC3/MOCS3/LCMT2/GTF3C6/PUS1/DUS1L/MARS1/GRSF1/METTL8/TSEN54/TRMT10B/NSUN6/THUMPD3/OSGEPL1/MARS2/ANKRD16/WDR6/TYW5/POLR3K/TRIT1/PUS3/RTCB/RPP14/ADAT2/RPP38/CTU2/POP4/TARBP1/GATC/TRNT1/GTPBP3/TRMT61B/TP53RK/DARS1
BP	GO:0044743	protein transmembrane import into intracellular organelle	35	0.72	2.03	2.38E-05	0.00022713	0.00013944	813	tags=26%, list=7%, signal=24%	DNAJC19/TOMM7/TOMM20/DNAJC15/TIMM17A/GRPEL2/PEX2/PEX13/ROMO1
CC	GO:0030532	small nuclear ribonucleoprotein complex	66	0.60	1.93	4.75E-05	0.00042474	0.00026077	1142	tags=35%, list=10%, signal=32%	LARP7/LSM3/SNRPD2/SNRPG/SF3B6/SNRPE/LSM5/SNRPD1/SNRPB2/LSM8/LUC7L3/SLU7/LSM7/PHF5A/PRPF39/SF3B1/SNRPC/PRPF40A/LSM6/SNRPF/SNRPA1/SNRPD3/RBMX2
BP	GO:0140053	mitochondrial gene expression	103	0.53	1.82	5.40E-05	0.00047484	0.00029152	2983	tags=45%, list=25%, signal=34%	TRMT10C/MRPS21/MRPL47/MRPS28/MTRES1/MRPS15/MRPL51/MRPL57/MTIF3/MTO1/MTERF2/MRPS17/MRPS14/TFAM/UQCC2/MTIF2/MRPS11/CDK5RAP1/GATB/MTERF4/MRPS6/MRPL58/FASTKD1/MTERF1/NGRN/TFB2M/MRPS16/SLC25A33/MTRF1L/NOA1/PUS1/TBRG4/PTCD3/RPUSD3/TRIT1/MTRF1/RMND1/FASTKD2/GATC/FASTKD3/TRNT1/POLRMT/PRKAA1/TRMT61B/MRPL23/RCC1L
BP	GO:0001510	RNA methylation	78	0.57	1.87	7.57E-05	0.00063127	0.00038757	2312	tags=35%, list=20%, signal=28%	LARP7/TRMT10C/CMTR2/FBL/TRMO/METTL5/DALRD3/MTO1/EMG1/TRMT12/TRMT13/THUMPD2/TRMT6/METTL3/HENMT1/TYW3/TRMT1/RNMT/TRMT10A/METTL14/TFB2M/LCMT2/METTL8/WTAP/TRMT10B/NSUN6/THUMPD3
CC	GO:0071011	precatalytic spliceosome	52	0.64	1.97	7.65E-05	0.00063642	0.00039073	1142	tags=42%, list=10%, signal=38%	LSM3/SNRPD2/SNRPG/SF3B6/SNRPE/LSM5/SNRPD1/SNRPB2/LSM8/LSM7/PHF5A/PRPF38B/WBP4/SF3B1/CWC22/MAGOHB/LSM6/SNRPF/PRPF38A/SNRPA1/SNRPD3/RBMX2
CC	GO:0031304	intrinsic component of mitochondrial inner membrane	54	0.63	1.93	8.48E-05	0.00069835	0.00042875	2744	tags=41%, list=23%, signal=31%	COX16/PET100/SMDT1/TIMM17A/MCUB/COX11/MPC1/SFXN4/MICOS10/SFXN1/MPC2/MICOS13/SCO1/PPOX/HSPA9/CCDC51/MTX3/GHITM/APOO/UQCC3/COQ2/COX18
CC	GO:0097525	spliceosomal snRNP complex	57	0.62	1.91	0.000101377	0.00081429	0.00049993	1142	tags=37%, list=10%, signal=33%	LSM3/SNRPD2/SNRPG/SF3B6/SNRPE/LSM5/SNRPD1/SNRPB2/LSM8/LUC7L3/LSM7/PHF5A/PRPF39/SF3B1/SNRPC/PRPF40A/LSM6/SNRPF/SNRPA1/SNRPD3/RBMX2

BP	GO:0006091	generation of precursor metabolites and energy	361	0.39	1.52	0.000106493	0.00084853	0.00052095	1283	tags=20%, list=11%, signal=18%	UQCRB/ATP5PO/COX6C/COX7C/NDUFA5/UQCRH/NDUFS5/ATP5ME/NDUFA1/NDUFA6/NDUFB1/UQCRQ/DNAJC15/COA6/ATP5PF/NDUFB2/ATP5IF1/ATP5MG/ATP5PD/NDUFA4/COX7A2/NDUFS3/ATP5MF/NDUFB3/ATP5F1C/COX11/ATP5F1E/COX4I1/DLD/CISD1/COX6A1/ATP5PB/UQCR10/PPP1CC/NDUFS4/NDUFV2/CYCS/UQCRFS1/BNIP3/UQCRC2/NIPSNAP2/NDUFA12/UGP2/MRPS36/PARK7/ADH5/SELENOS/NDUFB4/COX5A/PRKAG1/UQCC2/ADSL/COX17/NDUFAB1/EPM2AIP1/C2orf69/FDX1/PTGES3/ACAT1/ETFRF1/RB1CC1/PASK/COX7A2L/MSH2/NDUFB5/SLC25A25/SUCLG1/NOP53/CAT/AK9/NDUFA13
BP	GO:0017004	cytochrome complex assembly	35	0.69	1.96	0.000106985	0.00084905	0.00052127	1795	tags=43%, list=15%, signal=36%	COX16/PET100/COA6/COA5/COX14/COX20/SMIM20/C12orf73/UQCRFS1/UQCC2/COX17/COA4/PET117/SLC25A33/SCO1
BP	GO:0006120	mitochondrial electron transport, NADH to ubiquinone	42	0.67	1.96	0.000107644	0.00085257	0.00052344	956	tags=38%, list=8%, signal=35%	NDUFA5/NDUFS5/NDUFA1/NDUFA6/NDUFB1/DNAJC15/NDUFB2/NDUFA4/NDUFS3/NDUFB3/DLD/NDUFS4/NDUFV2/PARK7/NDUFB4/NDUFAB1
BP	GO:0032543	mitochondrial translation	71	0.58	1.88	0.000130118	0.00100262	0.00061555	1237	tags=24%, list=11%, signal=22%	TRMT10C/MRPS21/MRPL47/MRPS28/MRPS15/MRPL51/MRPL57/MTIF3/MRPS17/MRPS14/UQCC2/MTIF2/MRPS11/CDK5RAP1/GATB/MRPS6/MRPL58
CC	GO:0031305	integral component of mitochondrial inner membrane	53	0.62	1.91	0.00015109	0.00114425	0.00070251	2744	tags=40%, list=23%, signal=30%	COX16/PET100/SMDT1/TIMM17A/MCUB/COX11/MPC1/SFXN4/MICOS10/SFXN1/MPC2/MICOS13/SCO1/HSPA9/CCDC51/MTX3/GHITM/APOO/UQCC3/COQ2/COX18
CC	GO:1990351	transporter complex	179	0.45	1.64	0.00016353	0.0012176	0.00074754	2084	tags=27%, list=18%, signal=23%	UQCRB/NDUFA5/TIMM8B/UQCRH/NDUFS5/NDUFA1/NDUFA6/NDUFB1/UQCRQ/SMDT1/NDUFB2/MICU2/NDUFA4/TIMM9/NDUFS3/NDUFB3/MCUB/UQCR10/NDUFS4/TIMM10/NDUFV2/UQCRFS1/UQCRC2/PEX13/NDUFA12/NDUFB4/DLG3/NDUFAB1/CALM2/MICU3/NDUFB5/NDUFA13/TIMM8A/NDUFC1/FXYD1/KCNA3/ABCB8/NDUFS6/NDUFB6/ATP1B3/NDUFC2/PEX12/TMEM30A/CACNA1D/CCDC51/PACC1/ABCD4/OSTM1/TIMM10B
CC	GO:0005689	U12-type spliceosomal complex	26	0.74	1.97	0.000191641	0.00137788	0.00084594	1027	tags=62%, list=9%, signal=56%	SNRPD2/SNRPG/SF3B6/SNRPE/ZCRB1/SNRPD1/ZRSR2/LSM7/PHF5A/RNPC3/SF3B1/DHX15/SNRNP48/SNRPF/SNRPD3/PDCD7
CC	GO:0071013	catalytic step 2 spliceosome	86	0.54	1.81	0.000236315	0.00166594	0.0010228	998	tags=31%, list=9%, signal=29%	LSM3/SNRPD2/SNRPG/SF3B6/SNRPE/HNRNPA1/SNRPD1/SYF2/CWC15/DDX5/SNRPB2/SLU7/BCAS2/LSM7/MAGOH/PNN/FRG1/SF3B1/CWC22/PPWD1/MAGOHB/PRPF4B/SNRPF/PPIL3/SNRPA1/MTREX/SNRPD3
MF	GO:0015399	primary active transmembrane transporter activity	113	0.50	1.74	0.000283694	0.0019515	0.00119812	1824	tags=30%, list=16%, signal=26%	COX7B/NDUFA5/UQCRH/NDUFS5/NDUFA1/NDUFA6/NDUFB1/TOMM20/NDUFB2/NDUFA4/NDUFS3/ATP6V1G1/NDUFB3/COX4I1/COX6A1/UQCR10/NDUFS4/NDUFV2/UQCRFS1/NDUFA12/NDUFB4/COX5A/ATP6V1E2/ABCB10/COX7A2L/NDUFB5/NDUFA13/NDUFC1/ABCB8/NDUFS6/NDUFB6/ABCD3/ABCB1/NDUFC2

BP	GO:0006457	protein folding	167	0.46	1.64	0.000284891	0.00195297	0.00119902	1532	tags=27%, list=13%, signal=24%	TBCA/DNAJA1/HSP90AA1/B2M/PPIG/CCT2/DNAJC19/DNAJC2/VBP1/TXNDC9/HSPE1/GRPEL2/MKKS/TBCC/PDCD5/PFDN4/SACS/NUDCD2/PFDN2/DNAJC10/HSPA8/CCT8/HSPA13/PFDN6/PRDX4/DNAJC1/QSOX2/DNAJC21/PPIA/PPIL3/CDC37L1/HSPA14/PTGES3/PFDN1/ERP29/CCT5/CHCHD4/SELENOF/DNAJC25/DNAJB11/CHORDC1/DNAJC7/TBCE/PFDN5/DNAJC24
BP	GO:0030490	maturation of SSU-rRNA	49	0.63	1.89	0.000286606	0.00196135	0.00120417	1159	tags=29%, list=10%, signal=26%	RPS8/RPS14/RPS19/RPS21/NGDN/RIOK2/RPS16/GTF2H5/LSM6/DCAF13/UTP3/SRFBP1/MRPS11/WDR43
BP	GO:0009451	RNA modification	153	0.47	1.66	0.000306246	0.00206751	0.00126934	2902	tags=39%, list=25%, signal=29%	LARP7/SSB/TRMT10C/TPRKB/CMTR2/FBL/THUMPD1/TRMO/METTL5/DTWD1/DALRD3/MTO1/EMG1/TRMT12/TRMT13/NHP2/THUMPD2/CDK5RAP1/TRMT6/METTL3/DNAJB11/APOBEC3A/HENMT1/TYW3/TRMT1/RNMT/OSGEP/TYW1/TRMT10A/DPH3/TRUB1/METTL14/TFB2M/MOCS3/LCMT2/PUS1/DUS1L/METTL8/WTAP/TRMT10B/PUS7L/NSUN6/THUMPD3/OSGEPL1/APOBEC3G/HNRNPAB/RPUSD3/ANKRD16/WDR6/TYW5/TRIT1/PUS3/MRM3/ADAT2/CTU2/METTL15/TARBP1/GTPBP3/TRMT61B
MF	GO:0140318	protein transporter activity	27	0.71	1.93	0.00031137	0.00209477	0.00128608	337	tags=19%, list=3%, signal=18%	TIMM8B/TOMM7/TOMM20/TIMM17A/ATAD1
BP	GO:0000462	maturation of SSU-rRNA from tricistronic rRNA transcript (SSU-rRNA, 5.8S rRNA, LSU-rRNA)	36	0.67	1.92	0.000317069	0.00212591	0.0013052	1159	tags=31%, list=10%, signal=28%	RPS8/RPS14/RPS19/RPS21/NGDN/RPS16/GTF2H5/DCAF13/UTP3/MRPS11/WDR43
MF	GO:0051082	unfolded protein binding	94	0.52	1.75	0.000391051	0.00256995	0.00157781	1057	tags=28%, list=9%, signal=25%	DNAJA1/HSP90AA1/NPM1/CCT2/PET100/VBP1/TOMM20/HSPE1/GRPEL2/MKKS/PFDN4/NACA/SRSF10/ERLEC1/NUDCD2/PFDN2/HSPA8/CCT8/HSPA13/PFDN6/PPIA/CDC37L1/HSPA14/HTRA2/PTGES3/PFDN1

BP	GO:0016071	mRNA metabolic process	612	0.34	1.35	0.000394999	0.00259161	0.00159111	2074	tags=25%, list=18%, signal=22%	LARP7/EIF3E/NPM1/LSM3/SNRPD2/SNRPG/SF3B6/SNRPE/SSB/RBM39/TRMT10C/LSM5/TAF7/ZCRB1/HNRNPA1/SNRPD1/SYF2/TRA2B/ZNF830/SREK1IP1/SRSF7/CWC15/PNRC2/DDX5/AKAP17A/ZBTB1/SNRPB2/EXOSC9/UPF3A/LSM8/LUC7L3/CMTR2/RBM3/TAF11/LSM1/SRSF3/ZRSR2/CLNS1A/SLU7/BCAS2/ZRANB2/LSM7/PHF5A/SRSF10/COIL/PRPF38B/RPRD1A/KIN/MAGOH/PNN/WBP4/UPF3B/PCF11/FRG1/SFPQ/PRPF39/RNPC3/PAIP1/PSIP1/HSPA8/SF3B1/DDX1/ATF4/CWC22/PPWD1/MAGOHB/SNRPC/PRPF4B/PRPF40A/SRSF11/DHX15/SNRNP48/HNRNPH3/LSM6/THOC7/ARL6IP4/SRSF2/TAF9/UBL5/PCID2/CSTF3/SNRPF/PRPF38A/ANGEL2/PPIL3/TIA1/NUDT21/DIS3/SNRPA1/SRSF8/MTREX/SNRPD3/FXR1/SLTM/GEMIN6/ZNF326/CWF19L2/RBMX2/TRMT6/TAF13/PLRG1/CDC40/NCBP1/METTL3/DNAJB11/TSEN15/NSRP1/PPP1R8/UPF2/RNMT/EXOSC8/FASTKD1/CNOT8/TRA2A/PPP4R2/LSM11/PYM1/CLP1/NCBP2/HTATSF1/SKIV2L/RBM41/DDX39A/TRUB1/METTL14/SAP18/RBMX/SF3A3/SF3B5/GEMIN2/EXOSC3/RBM17/THOC1/LUC7L/CLASRP/APEX1/NONO/ATF2/SRSF5/RBM25/SNRNP70/PUS1/MFAP1/GRSF1/STRAP/CEBPG/NRDE2/MRTO4/TBRG4/METTL8/TNRC6A/TSEN54/YTHDF2/SREK1/CWC25
CC	GO:0071005	U2-type precatalytic spliceosome	50	0.62	1.88	0.000402808	0.00262983	0.00161457	1142	tags=40%, list=10%, signal=36%	LSM3/SNRPD2/SNRPG/SNRPE/LSM5/SNRPD1/SNRPB2/LSM8/LSM7/PHF5A/WBP4/SF3B1/CWC22/MAGOHB/LSM6/SNRPF/PRPF38A/SNRPA1/SNRPD3/RBMX2
MF	GO:0000049	tRNA binding	70	0.56	1.81	0.000404947	0.00263946	0.00162049	2498	tags=31%, list=21%, signal=25%	RPL35A/SSB/AIMP1/TRMT10C/EIF2A/MTRES1/EEF1A1/DALRD3/IFIT5/DTD2/TRMT1/RARS1/NEMF/TARS1/TRMT10A/PUS1/MARS1/TRMT10B/MTRFR/NSUN6/TRNAU1AP/TYW5
CC	GO:0098796	membrane protein complex	756	0.32	1.33	0.000415846	0.0026841	0.00164789	1374	tags=16%, list=12%, signal=15%	UQCRB/ATP5PO/COX6C/B2M/ATP5MJ/COX7B/COX7C/NDUFA5/DNAJC19/UQCRH/NDUFS5/TOMM7/ATP5ME/NDUFA1/NDUFA6/CD3D/NDUFB1/TOMM20/UQCRQ/DNAJC15/COA6/LY96/SMDT1/SNX2/ATP5PF/NDUFB2/TIMM17A/SEC11C/GRPEL2/VPS29/ATP5MG/MICU2/ATP5PD/HMGB1/ATP5MK/NDUFA4/SRP9/TIMM9/COX7A2/UFD1/PIGH/NDUFS3/ATP5MF/OSTC/ATP6V1G1/VAMP8/TRAT1/ITGAE/NDUFB3/MCUB/ATP5F1C/UBXN1/ATP5F1E/SNX4/COX4I1/AP5M1/AP1S2/COX6A1/RP9/ATP5PB/UQCR10/SAR1B/CHMP1B/NDUFS4/TIMM10/NDUFV2/MICOS10/ORMDL1/LAMTOR5/STX16/TOMM5/SNX5/BNIP1/UQCRFS1/UQCRC2/NDUFA12/SEC11A/IMMP1L/BIRC2/VAMP7/ROMO1/PIGK/SELENOS/VDAC2/VAMP4/NDUFB4/COX5A/DLG3/NDUFAB1/ATP6V1E2/AP3S1/HTRA2/HLA-DMB/EMC2/PDCD6/LAMTOR3/BET1/AUP1/SEC23A/NECAP1/TMED7/USE1/CALM2/NECAP2/MICU3/STX8/COX7A2L/PIGP/NDUFB5/STX7/STX2/TGFBR1/NDUFA13/CASP3/SOCS6/SNX6/PIGU/SOCS5/SRPRB/SPCS1/VDAC3
CC	GO:0046540	U4/U6 x U5 tri-snRNP complex	31	0.70	1.95	0.000465851	0.00294004	0.00180503	998	tags=35%, list=9%, signal=33%	LSM3/SNRPD2/SNRPG/SNRPE/LSM5/SNRPD1/LSM8/LSM7/LSM6/SNRPF/SNRPD3

CC	GO:0097526	spliceosomal tri-snRNP complex	31	0.70	1.95	0.000465851	0.00294004	0.00180503	998	tags=35%, list=9%, signal=33%	LSM3/SNRPD2/SNRPG/SNRPE/LSM5/SNRPD1/LSM8/LSM7/LSM6/SNRPF/SNRPD3
BP	GO:0007005	mitochondrion organization	456	0.36	1.41	0.000560764	0.0034675	0.00212886	1973	tags=25%, list=17%, signal=21%	HSP90AA1/NDUFA5/TIMM8B/DNAJC19/RALA/NDUFS5/TOMM7/NDUFA1/NDUFA6/COX16/PET100/NDUFB1/TOMM20/DNAJC15/ATG3/SLIRP/COA5/BLOC1S2/NDUFB2/TIMM17A/COX14/GRPEL2/ATP5IF1/PDCD5/COX20/TIMM9/COX7A2/NDUFAF8/NDUFS3/TMEM126A/NDUFB3/NDUFAF2/TMEM126B/SMIM20/HIGD1A/SDHAF3/GGCT/PSMD10/NDUFAF5/NDUFS4/TIMM10/MICOS10/C12orf73/TRIAP1/TOMM5/NDUFAF4/ACAA2/UQCRFS1/BNIP3/ATG5/NIPSNAP2/NDUFA12/IMMP1L/DMAC1/HEBP2/PARK7/ROMO1/MOAP1/SSBP1/VDAC2/FAM162A/PMAIP1/TFAM/NDUFB4/UBL5/UQCC2/TMEM70/COX17/NDUFAB1/HTRA2/PRDX3/ATG12/MFF/RB1CC1/COX7A2L/NDUFB5/MTERF4/CHCHD4/COA4/NDUFA13/PMPCB/PRMT6/SLC25A46/HAX1/TIMM8A/ZDHHC6/NDUFC1/TMEM242/SLC25A36/PET117/DNA2/ARMC1/FMC1/DNAJA3/MICOS13/GABPB1/NDUFB6/SLC25A33/PARL/PRIMPOL/SCO1/PYROXD2/NDUFC2/SDHAF1/MYO19/MAIP1/HSPA9/ATF2/ATPAF1/PAM16/TIGAR/MTFR1L/IFIT2
BP	GO:0000470	maturation of LSU-rRNA	28	0.70	1.88	0.000725476	0.00433107	0.00265905	983	tags=39%, list=8%, signal=36%	RPL7/RPL35/NSA2/NIFK/ZNHIT3/RPF1/MRPL1/RPF2/RPL10A/RSL1D1/NHP2
MF	GO:0003729	mRNA binding	248	0.40	1.51	0.000769692	0.00453377	0.00278349	1351	tags=21%, list=12%, signal=19%	RPL26/RPL24/RPS7/RPS3A/RPL5/RPL7/RPL35/SF3B6/RPS13/SSB/RPL13A/RPL41/RPS14/RPS26/TRA2B/EIF2S2/DDX5/EXOSC9/EIF2A/LUC7L3/RPS3/GNL3/MRPL13/CNBP/DHFR2/RPS2/UPF3B/PCF11/CRYZ/SF3B1/PUM3/SNRPC/PARK7/DENR/CSTF3/ANGEL2/PABPC1L/TIA1/NUDT21/RSL1D1/NHP2/RPS5/FXR1/MRPS11/CHTOP/CCT5/CDC40/NCBP1/METTL3/DHFR/NSRP1/PPP1R8
BP	GO:1901796	regulation of signal transduction by p53 class mediator	85	0.52	1.72	0.001068035	0.0059081	0.00362726	858	tags=19%, list=7%, signal=18%	RPL26/RPS7/RPL23/RPL5/RPS20/RPL11/RPL37/DDX5/EEF1E1/ING4/PSMD10/TRIAP1/RPF2/SMYD2/TAF9/PMAIP1
CC	GO:0071007	U2-type catalytic step 2 spliceosome	29	0.68	1.87	0.001233075	0.00666129	0.00408968	1233	tags=48%, list=11%, signal=43%	SNRPD2/SNRPG/SNRPE/SNRPD1/SYF2/CWC15/SNRPB2/BCAS2/CWC22/SNRPF/SNRPA1/SNRPD3/PLRG1/CDC40
MF	GO:0015078	proton transmembrane transporter activity	79	0.51	1.69	0.001343268	0.00710217	0.00436036	1171	tags=25%, list=10%, signal=23%	ATP5PO/COX7B/UQCRH/ATP5ME/ATP5PF/ATP5MG/ATP5PD/ATP5MF/ATP6V1G1/ATP5F1C/ATP5F1E/COX4I1/COX6A1/ATP5PB/UQCR10/UQCRFS1/COX5A/ATP6V1E2/SLC15A2/COX7A2L
MF	GO:0048027	mRNA 5'-UTR binding	25	0.71	1.89	0.001362055	0.00717289	0.00440377	408	tags=32%, list=3%, signal=31%	RPL26/RPS7/RPS3A/RPL5/RPS13/RPL41/RPS14/GNL3
BP	GO:1990542	mitochondrial transmembrane transport	77	0.51	1.66	0.001763804	0.00873336	0.00536182	2026	tags=35%, list=17%, signal=29%	DNAJC19/TOMM7/TOMM20/DNAJC15/SMDT1/TIMM17A/GRPEL2/MICU2/MCUB/MPC1/MRPL18/SFXN4/SLC25A32/ROMO1/MICU3/SFXN1/CHCHD4/PMPCB/SLC25A36/MPC2/ABCB8/SLC25A33/MAIP1/MRS2/PAM16/CCDC51/SLC39A8

CC	GO:0032592	integral component of mitochondrial membrane	81	0.50	1.65	0.001771732	0.00873928	0.00536545	2634	tags=35%, list=22%, signal=27%	COX16/PET100/TOMM20/SMDT1/TIMM17A/MCUB/COX11/MPC1/SFXN4/MICOS10/BNIP3/TMEM70/ABCB10/MFF/SFXN1/MPC2/MICOS13/SCO1/HSPA9/CCDC51/MTX3/GHITM/APOO/SLC25A4/RHOT2/UQCC3/SYNJ2BP/COQ2
CC	GO:0016469	proton-transporting two-sector ATPase complex	41	0.62	1.82	0.001794549	0.00882535	0.0054183	514	tags=29%, list=4%, signal=28%	ATP5PO/ATP5MJ/ATP5ME/ATP5PF/ATP5MG/ATP5PD/ATP5MK/ATP5MF/ATP6V1G1/ATP5F1C/ATP5F1E/ATP5PB
CC	GO:0030684	preribosome	74	0.51	1.66	0.00185369	0.00905439	0.00555891	1558	tags=27%, list=13%, signal=24%	RPS7/NOP58/NSA2/LTV1/NGDN/KRR1/MPHOSPH10/FBL/RIOK2/RPF1/EBNA1BP2/EMG1/DCAF13/RSL1D1/UTP3/SRFBP1/ZNF622/UTP23/FCF1/NIP7
CC	GO:1902495	transmembrane transporter complex	160	0.43	1.53	0.001874391	0.00911073	0.00559351	2044	tags=26%, list=17%, signal=21%	UQCRB/NDUFA5/UQCRH/NDUFS5/NDUFA1/NDUFA6/NDUFB1/UQCRQ/SMDT1/NDUFB2/MICU2/NDUFA4/NDUFS3/NDUFB3/MCUB/UQCR10/NDUFS4/NDUFV2/UQCRFS1/UQCRC2/NDUFA12/NDUFB4/DLG3/NDUFAB1/CALM2/MICU3/NDUFB5/NDUFA13/NDUFC1/FXYD1/KCNA3/ABCB8/NDUFS6/NDUFB6/ATP1B3/NDUFC2/CACNA1D/CCDC51/PACC1/ABCD4/OSTM1
MF	GO:0016651	oxidoreductase activity, acting on NAD(P)H	65	0.54	1.72	0.001944628	0.0093833	0.00576085	1824	tags=34%, list=16%, signal=29%	NDUFA5/NDUFS5/NDUFA1/NDUFA6/NDUFB1/NDUFB2/NDUFA4/NDUFS3/NDUFB3/NDUFS4/NDUFV2/CRYZ/NDUFA12/NDUFB4/AKR1C3/NDUFB5/NDUFA13/NDUFC1/CBR4/NDUFS6/NDUFB6/NDUFC2
BP	GO:0000387	spliceosomal snRNP assembly	35	0.62	1.75	0.002136717	0.0101138	0.00620933	1093	tags=31%, list=9%, signal=29%	SNRPD2/SNRPG/SNRPE/SNRPD1/CLNS1A/SRSF10/COIL/SNRPC/SNRPF/SNRPD3/GEMIN6
BP	GO:0051438	regulation of ubiquitin-protein transferase activity	50	0.57	1.75	0.00214916	0.0101606	0.00623807	1542	tags=30%, list=13%, signal=26%	RPS7/RPL23/RPL5/RPS20/RPL11/RPL37/TOPORS/RPS2/PARK7/DCUN1D5/BMI1/UBE2N/DCUN1D4/FEM1B/BUB3
BP	GO:0008535	respiratory chain complex IV assembly	25	0.69	1.85	0.002586527	0.01185935	0.00728101	1795	tags=44%, list=15%, signal=37%	COX16/PET100/COA6/COA5/COX14/COX20/SMIM20/COX17/COA4/PET117/SCO1
MF	GO:0008173	RNA methyltransferase activity	62	0.55	1.74	0.002847749	0.01282293	0.0078726	2312	tags=35%, list=20%, signal=29%	TRMT10C/CMTR2/FBL/TRMO/METTL5/EMG1/TRMT12/TRMT13/THUMPD2/METTL3/HENMT1/TYW3/TRMT1/RNMT/TRMT10A/METTL14/TFB2M/LCMT2/METTL8/TRMT10B/NSUN6/THUMPD3
BP	GO:0006413	translational initiation	112	0.46	1.58	0.002863333	0.01286397	0.00789779	1441	tags=26%, list=12%, signal=23%	RPS3A/EIF3E/NPM1/RPS17/RPL13A/EIF4A2/EIF2S2/EIF2A/MCTS1/RPS3/EIF3H/COPS5/EIF3M/MTIF3/PAIP1/DDX1/ATF4/DENR/EIF3J/ABCE1/MTIF2/RPS5/EIF3K/TMED2/NCBP1/METTL3/EIF3F/EIF2S1/EIF3I
CC	GO:0005758	mitochondrial intermembrane space	76	0.49	1.59	0.003935959	0.01688174	0.01036449	1461	tags=30%, list=12%, signal=27%	TIMM8B/SOD1/NDUFS5/COA6/MICU2/TIMM9/CHCHD7/SDHAF3/TIMM10/PRELID3B/TRIAP1/CYCS/PARK7/UQCC2/COX17/HTRA2/CHCHD4/COA4/CAT/MIX23/HAX1/TIMM8A/CMC4

MF	GO:0016741	transferase activity, transferring one-carbon groups	177	0.40	1.44	0.003965458	0.01696462	0.01041538	2358	tags=28%, list=20%, signal=22%	CSKMT/TRMT10C/HNMT/COQ5/METTL18/CMTR2/FBL/DPH5/TRMO/METTL5/NDUFAF5/SMYD2/ARMT1/EMG1/VCPKMT/TRMT12/TRMT13/FAM98B/PCMT1/THUMPD2/GCSH/METTL3/PRMT6/HENMT1/TYW3/METTL17/TRMT1/RNMT/SETD6/N6AMT1/TRMT10A/CARNMT1/METTL14/PCMTD2/TFB2M/METTL23/ICMT/METTL21A/METTL13/LCMT2/PRMT3/METTL8/WDR5/FAM98A/TRMT10B/NSUN6/THUMPD3/SETDB2/SHMT1
MF	GO:0090079	translation regulator activity, nucleic acid binding	96	0.47	1.59	0.004084798	0.01738882	0.01067581	2005	tags=35%, list=17%, signal=30%	EEF1B2/EIF3E/RPS27L/EIF4A2/EIF2S2/EIF2A/MCTS1/EIF4E/EIF3H/EEF1D/COPS5/EIF3M/EEF1G/EEF1A1/MTIF3/ZNF540/PAIP1/DENR/EIF3J/MTIF2/EIF3K/MRPL58/EIF3F/HBS1L/DHFR/EIF2S1/EIF3I/EIF4E2/EIF4A1/EIF5/MTRF1L/DHX29/EIF3C/EIF4B
MF	GO:0003743	translation initiation factor activity	50	0.56	1.69	0.004469787	0.0184931	0.01135378	2005	tags=46%, list=17%, signal=38%	EIF3E/EIF4A2/EIF2S2/EIF2A/MCTS1/EIF4E/EIF3H/COPS5/EIF3M/MTIF3/DENR/EIF3J/MTIF2/EIF3K/EIF3F/EIF2S1/EIF3I/EIF4E2/EIF4A1/EIF5/DHX29/EIF3C/EIF4B
BP	GO:0051204	protein insertion into mitochondrial membrane	26	0.65	1.74	0.004629419	0.01899543	0.01166218	2208	tags=46%, list=19%, signal=38%	HSP90AA1/TIMM8B/PDCD5/TIMM9/TIMM10/ROMO1/MOAP1/NDUFA13/TIMM8A/MAIP1/TIMM10B/TRMT10B
BP	GO:0030488	tRNA methylation	41	0.59	1.74	0.005070754	0.02043746	0.01254752	3492	tags=63%, list=30%, signal=45%	TRMT10C/TRMO/DALRD3/MTO1/TRMT12/TRMT13/THUMPD2/TRMT6/TYW3/TRMT1/TRMT10A/LCMT2/METTL8/TRMT10B/NSUN6/THUMPD3/WDR6/TARBP1/GTPBP3/TRMT61B/METTL2A/ALKBH8/BCDIN3D/TRMT11/WDR4/METTL6
MF	GO:0008135	translation factor activity, RNA binding	80	0.49	1.60	0.005296195	0.02114224	0.01298021	2005	tags=38%, list=17%, signal=31%	EEF1B2/EIF3E/EIF4A2/EIF2S2/EIF2A/MCTS1/EIF4E/EIF3H/EEF1D/COPS5/EIF3M/EEF1G/EEF1A1/MTIF3/DENR/EIF3J/MTIF2/EIF3K/MRPL58/EIF3F/HBS1L/EIF2S1/EIF3I/EIF4E2/EIF4A1/EIF5/MTRF1L/DHX29/EIF3C/EIF4B
MF	GO:0008168	methyltransferase activity	171	0.40	1.45	0.005676648	0.02230272	0.01369269	2349	tags=28%, list=20%, signal=23%	CSKMT/TRMT10C/HNMT/COQ5/METTL18/CMTR2/FBL/DPH5/TRMO/METTL5/NDUFAF5/SMYD2/ARMT1/EMG1/VCPKMT/TRMT12/TRMT13/FAM98B/PCMT1/THUMPD2/GCSH/METTL3/PRMT6/HENMT1/TYW3/METTL17/TRMT1/RNMT/SETD6/N6AMT1/TRMT10A/CARNMT1/METTL14/PCMTD2/TFB2M/METTL23/ICMT/METTL21A/METTL13/LCMT2/PRMT3/METTL8/WDR5/FAM98A/TRMT10B/NSUN6/THUMPD3/SETDB2
CC	GO:0000793	condensed chromosome	189	0.39	1.43	0.0062121	0.02397991	0.01472239	1740	tags=23%, list=15%, signal=20%	CBX3/NIFK/HMGB2/HMGB1/NUP37/CHMP5/MIS12/ANAPC16/CHMP1B/PPP1CC/CENPQ/SGO2/NUDCD2/BOD1/CFDP1/DYNLT3/FBXO28/SMC6/GPATCH11/SMC3/SYCP2/NDC80/SEPTIN7/SKA2/ITGB3BP/DYNLL1/ORC2/NSMCE4A/MLH3/DCTN6/SS18L1/SEPTIN2/NUP107/EID3/SMC2/LIG4/CCNB1IP1/ZWILCH/BUB3/SUGT1/NSMCE2/MEAF6/RAD50
BP	GO:0016556	mRNA modification	25	0.66	1.76	0.006261069	0.0241221	0.01480969	2566	tags=48%, list=22%, signal=38%	TRMT10C/CMTR2/TRMT6/METTL3/DNAJB11/TRUB1/METTL14/PUS1/METTL8/WTAP/HNRNPAB/PUS3
BP	GO:0065002	intracellular protein transmembrane transport	50	0.55	1.67	0.006573175	0.02512975	0.01542833	813	tags=20%, list=7%, signal=19%	DNAJC19/TOMM7/TOMM20/DNAJC15/TIMM17A/GRPEL2/SEC62/PEX2/PEX13/ROMO1

BP	GO:0071806	protein transmembrane transport	58	0.52	1.62	0.006832085	0.02584621	0.0158682	813	tags=17%, list=7%, signal=16%	DNAJC19/TOMM7/TOMM20/DNAJC15/TIMM17A/GRPEL2/SEC62/PEX2/PEX13/ROMO1
CC	GO:0098573	intrinsic component of mitochondrial membrane	83	0.47	1.55	0.007975369	0.02906514	0.01784445	2634	tags=35%, list=22%, signal=27%	COX16/PET100/TOMM20/SMDT1/TIMM17A/MCUB/COX11/MPC1/SFXN4/MICOS10/BNIP3/TMEM70/ABCB10/MFF/SFXN1/MPC2/MICOS13/SCO1/PPOX/HSPA9/CCDC51/MTX3/GHITM/APOO/SLC25A4/RHOT2/UQCC3/SYNJ2BP/COQ2
BP	GO:0043516	regulation of DNA damage response, signal transduction by p53 class mediator	30	0.63	1.73	0.007986451	0.02907887	0.01785288	858	tags=23%, list=7%, signal=22%	RPL26/DDX5/EEF1E1/ING4/PSMD10/SMYD2/PMAIP1
BP	GO:0043628	ncRNA 3'-end processing	38	0.59	1.67	0.00835028	0.03015505	0.0185136	326	tags=16%, list=3%, signal=15%	LARP7/SSB/TRMT10C/RPS21/EXOSC9/FBL
BP	GO:0006414	translational elongation	50	0.54	1.64	0.009134702	0.0324862	0.0199448	1636	tags=32%, list=14%, signal=28%	EEF1B2/USP16/MTRES1/SRP9/EEF1D/DPH5/RPLP1/EEF1G/EEF1A1/SELENOT/HBS1L/NEMF/RPLP2/DNAJC24/RACK1/DPH3
MF	GO:0045182	translation regulator activity	124	0.42	1.46	0.010690324	0.03654749	0.02243822	1441	tags=27%, list=12%, signal=24%	EEF1B2/EIF3E/RPL22/RPS14/RPS27L/EIF4A2/EIF2S2/EIF2A/MCTS1/EIF4E/EIF3H/RPS9/EEF1D/RPL10/COPS5/EIF3M/CNBP/EEF1G/EEF1A1/MTIF3/ZNF540/PAIP1/DENR/EIF3J/MTIF2/FXR1/EIF3K/MRPL58/EIF3F/HBS1L/DHFR/EIF2S1/EIF3I
BP	GO:0072331	signal transduction by p53 class mediator	137	0.40	1.40	0.010711948	0.03658996	0.02246429	890	tags=15%, list=8%, signal=14%	RPL26/RPS7/RPL23/RPL5/RPS20/RPL11/RPL37/RPS27L/DDX5/HINT1/EEF1E1/ING4/TOPORS/PSMD10/TRIAP1/RPF2/SMYD2/CDKN1B/TAF9/PMAIP1/CASP6
BP	GO:0002183	cytoplasmic translational initiation	26	0.63	1.67	0.01172521	0.03917599	0.02405198	2072	tags=50%, list=18%, signal=41%	RPL13A/EIF4A2/EIF2S2/MCTS1/EIF3M/DENR/METTL3/EIF3I/EIF4A1/EIF5/DHX29/EIF4B/YTHDF2
MF	GO:0140101	catalytic activity, acting on a tRNA	120	0.42	1.45	0.01222204	0.04030925	0.02474774	3128	tags=39%, list=27%, signal=29%	TRMT10C/PTRHD1/NARS1/POP5/TRMO/DTWD1/DALRD3/PTRH2/TRMT12/TRMT13/THUMPD2/GATB/MRPL58/DTD2/TYW3/RPP30/ELAC1/TRMT1/RARS1/TYW1/CARS2/TARS1/TRMT10A/LCMT2/PUS1/DUS1L/MARS1/METTL8/TRMT10B/NSUN6/THUMPD3/MARS2/TYW5/TRIT1/PUS3/RPP14/RPP38/POP4/TARBP1/GATC/TRNT1/TRMT61B/DARS1/METTL2A/ALKBH8/TARS3/YARS2
CC	GO:0031970	organelle envelope lumen	84	0.45	1.49	0.012226598	0.04030925	0.02474774	1461	tags=30%, list=12%, signal=26%	TIMM8B/SOD1/NDUFS5/COA6/MICU2/CACYBP/TIMM9/CHCHD7/SDHAF3/TIMM10/PRELID3B/TRIAP1/CYCS/PARK7/CCAR1/UQCC2/COX17/HTRA2/CHCHD4/COA4/CAT/MIX23/HAX1/TIMM8A/CMC4
CC	GO:0032040	small-subunit processome	36	0.57	1.64	0.012275711	0.04040416	0.02480601	1544	tags=31%, list=13%, signal=27%	RPS7/NOP58/NGDN/KRR1/MPHOSPH10/FBL/EMG1/DCAF13/UTP3/UTP23/FCF1
MF	GO:0051087	chaperone binding	80	0.46	1.52	0.012329441	0.04054744	0.02489398	1164	tags=26%, list=10%, signal=24%	TBCA/DNAJA1/SOD1/HSPE1/GRPEL2/TBCC/TIMM9/PFDN4/SACS/TIMM10/DNAJC10/HSPA8/CDKN1B/PFDN6/BIRC2/DNAJC1/CDC37L1/PTGES3/PRNP/ERP29/DNAJB9

BP	GO:0072655	establishment of protein localization to mitochondrion	112	0.42	1.44	0.012668306	0.04128622	0.02534754	1284	tags=23%, list=11%, signal=21%	DNAJA1/HSP90AA1/TIMM8B/DNAJC19/RALA/TOMM7/TOMM20/DNAJC15/TIMM17A/GRPEL2/ATP5IF1/DDIT3/PDCD5/TIMM9/TIMM10/TOMM5/IMMP1L/ROMO1/MOAP1/UBL5/HTRA2/MFF/MTERF4/CHCHD4/NDUFA13/PMPCB
BP	GO:0051123	RNA polymerase II preinitiation complex assembly	25	0.64	1.70	0.012889036	0.04186831	0.02570491	1185	tags=36%, list=10%, signal=32%	PSMC6/TAF7/GTF2B/HMGB1/TAF11/CAND1/TAF9/GTF2A2/TAF13
MF	GO:0008175	tRNA methyltransferase activity	33	0.58	1.62	0.013370346	0.04325508	0.02655632	3492	tags=64%, list=30%, signal=45%	TRMT10C/TRMO/TRMT12/TRMT13/THUMPD2/TYW3/TRMT1/TRMT10A/LCMT2/METTL8/TRMT10B/NSUN6/THUMPD3/TARBP1/TRMT61B/METTL2A/ALKBH8/BCDIN3D/TRMT11/WDR4/METTL6
BP	GO:0007007	inner mitochondrial membrane organization	39	0.56	1.61	0.014034293	0.04481956	0.02751683	2545	tags=49%, list=22%, signal=38%	TIMM8B/TIMM9/TIMM10/MICOS10/ROMO1/NDUFA13/TIMM8A/MICOS13/MAIP1/HSPA9/MYC/TIMM10B/OPA1/MTX3/OMA1/GHITM/TRMT10B/APOO/UQCC3
MF	GO:0015036	disulfide oxidoreductase activity	35	0.57	1.61	0.014491268	0.0459468	0.02820889	1410	tags=34%, list=12%, signal=30%	TXNDC17/TMX3/TMX1/GLRX/SELENOT/DNAJC10/TXN/QSOX2/GSTO1/CHCHD4/TXNL1/TXNDC12
BP	GO:0031397	negative regulation of protein ubiquitination	70	0.47	1.51	0.014632072	0.04607382	0.02828688	899	tags=20%, list=8%, signal=19%	RPS7/RPL23/RPL5/RPS20/DNAJA1/RPL11/RPL37/RPS3/UBXN1/UFL1/ATG5/CAMLG/PARK7/PPIA
BP	GO:0045005	DNA-templated DNA replication maintenance of fidelity	40	0.54	1.57	0.015148643	0.04727708	0.02902562	1994	tags=28%, list=17%, signal=23%	TIPIN/ZNF830/PCNA/GEN1/ETAA1/DNA2/PRIMPOL/THOC1/MUS81/TIMELESS/NUCKS1
CC	GO:0032391	photoreceptor connecting cilium	25	0.63	1.68	0.015177005	0.04732845	0.02905715	1507	tags=36%, list=13%, signal=31%	CETN3/TBCC/IFT57/IFT20/TOPORS/KIAA1549/SEPTIN2/IQCB1/CEP290

Supplementary table 10 APOE4 non-carriers gene set enrichment analysis gene ontology upreulated

ONTOLOGY	ID	Description	setSize	enrichment Score	NES	pvalue	p.adjust	qvalue	rank	leading_edge	core_enrichment
BP	GO:0006518	peptide metabolic process	697	0.43	1.88	1.00E-10	4.69E-09	2.91E-09	1117	tags=21%, list=10%, signal=20%	RPL17/MRPS18C/RPL26/RPS25/RPS24/NPM1/RPL36A/RPL24/EEF1B2/RPL22/RPL34/RPL31/RPS7/RPL5/CNBP/RPL21/NGDN/RPL23/RPL3/RPS23/RPS18/RPS3A/RPL7/RPS20/RPS27A/RPS10/RPL39/METTL5/RPL13A/EIF3E/RPL9/RPL37/RPL6/RPS8/RPL27/RPS17/MRPS33/RPS6/MCTS1/MRPL32/EEF1D/RPL22L1/RPL35/RPL11/RPS13/USP16/MTIF3/RPS27L/MRPS21/RPS15A/RWDD1/EIF2A/RSL24D1/RPS27/UPF3B/RPL27A/SARNP/AIMP1/RPL35A/NEMF/RPL12/ZC3H15/EIF4A2/RPL38/RPS21/DHX29/RPS12/MRPS11/TRMT10C/RPL29/RPL32/MRPL3/RPS14/MRPL47/SEC11C/PUM3/SELENOT/MRPL51/BDH2/RPL41/RPSA/SOD1/RPL19/MAGOH/RPS29/RPLP0/ATF4/EIF4E/MRPL18/METTL18/RPL36AL/SEC11A/RPL36/RPL15/EIF5/EIF2S2/RPL14/RPL4/RPS11/RPS4X/RGS2/TARBP2/EXOSC8/HBS1L/RPS2/MTIF2/NGRN/EEF1E1/MRPS15/RPL37A/EIF3M/SRP9/MTRES1/RBM3/RPS3/RPL10A/HARS2/PAIP1/CPEB3/EEF1G/RPS19/MRPS28/RPS9/MRPL24/MRPL57/RPS26/MAPKAPK5/RPL10/RPL8/YARS2/DPH5/RPLP1/MARS1/UPF3A/NARS1/MRPL13/CD28/HABP4/COPS5/RPL30/RNF139/PATL2/DENR
BP	GO:0043604	amide biosynthetic process	676	0.44	1.92	1.00E-10	4.69E-09	2.91E-09	1117	tags=21%, list=10%, signal=21%	RPL17/MRPS18C/RPL26/RPS25/RPS24/NPM1/RPL36A/RPL24/EEF1B2/RPL22/RPL34/RPL31/RPS7/RPL5/CNBP/RPL21/NGDN/RPL23/RPL3/RPS23/RPS18/RPS3A/RPL7/RPS20/RPS27A/RPS10/RPL39/METTL5/RPL13A/EIF3E/RPL9/RPL37/RPL6/RPS8/RPL27/RPS17/MRPS33/RPS6/MCTS1/MRPL32/EEF1D/RPL22L1/RPL35/RPL11/DLD/RPS13/USP16/MTIF3/RPS27L/MRPS21/RPS15A/RWDD1/EIF2A/RSL24D1/RPS27/ORMDL1/UPF3B/RPL27A/SARNP/AIMP1/RPL35A/NEMF/ASAH1/RPL12/ZC3H15/EIF4A2/RPL38/RPS21/DHX29/RPS12/MRPS11/TRMT10C/RPL29/RPL32/MRPL3/RPS14/MRPL47/PUM3/SELENOT/MRPL51/BDH2/RPL41/RPSA/RPL19/MAGOH/RPS29/RPLP0/ATF4/EIF4E/MRPL18/B4GALT4/METTL18/RPL36AL/RPL36/RPL15/EIF5/EIF2S2/RPL14/RPL4/RPS11/RPS4X/RGS2/TARBP2/EXOSC8/HBS1L/RPS2/MTIF2/NGRN/EEF1E1/MRPS15/RPL37A/EIF3M/SRP9/MTRES1/RBM3/RPS3/RPL10A/HARS2/PAIP1/CPEB3/EEF1G/RPS19/MRPS28/RPS9/MRPL24/HSD17B12/MRPL57/RPS26/MAPKAPK5/RPL10/RPL8/YARS2/DPH5/RPLP1/MARS1/UPF3A/NARS1/MRPL13/CD28/HABP4/COPS5/RPL30/RNF139/PATL2/DENR

BP	GO:0034470	ncRNA processing	388	0.45	1.87	1.00E-10	4.69E-09	2.91E-09	2303	tags=31%, list=20%, signal=26%	RPL26/RPS25/RPS24/LARP7/RPS7/RPL5/NGDN/RPL7/NOP58/ESF1/METTL5/RPS8/RPL27/RPS17/SSB/RPS6/RPL35/RPL11/KRR1/RPS27/TRNT1/POP5/RPL35A/DDX5/NSA2/RPS21/NIFK/MRPS11/TRMT10C/RPS14/RTRAF/TRMT12/ZBTB8OS/THUMPD1/C1D/TSNAX/INTS10/METTL18/DTWD1/LYAR/RPL14/TARBP2/EXOSC8/BRIX1/UTP6/RPF2/PRORP/TRMT10A/TRMT1/PIN4/FBL/RPL10A/MPHOSPH10/RIOK2/FRG1/MTO1/ZNHIT3/RPS19/EXOSC1/TRMO/MPHOSPH6/PUS3/RPS16/GTPBP3/ELP4/EBNA1BP2/RSL1D1/THUMPD3/WDR12/MTERF4/MTREX/TRMT13/TOE1/TPRKB/DCAF13/MRPL1/SRFBP1/GTF2H5/PPAN/DDX52/DDX18/OSGEP/DALRD3/SBDS/TARBP1/DDX1/DDX17/DIS3/NHP2/UTP23/EXOSC9/ERI2/ELAC1/NOL8/TRIT1/ERCC2/ELP1/THUMPD2/TYW1/METTL3/TRMT6/LCMT2/RNF113A/RPL7A/LSM6/INTS6L/ZMPSTE24/AGO3/PTCD1/INTS3/WDR75/TSN/RPF1/REXO4/INTS11/TRMT2B/ADAT2/ERI3/QTRT1/POP4/METTL2B/DIMT1
CC	GO:0019866	organelle inner membrane	462	0.46	1.95	1.00E-10	4.69E-09	2.91E-09	1992	tags=30%, list=17%, signal=26%	UQCRB/MRPS18C/ATP5PO/TRA2B/NDUFS5/SMIM20/COX16/NDUFA1/ATP5ME/MRPS33/COX7B/PET100/NDUFA5/MRPL32/NDUFA6/DPY19L4/COX7C/MRPS22/COX6C/NDUFAB1/UQCRQ/TIMM8B/ATP5MJ/NDUFS4/MRPS21/CBX3/UQCRC2/DMAC1/UQCRH/COX20/NDUFB1/NDUFAF2/ATP5MF/MRPL50/MICU2/CRLS1/IMMP1L/MRPS11/ATP5PF/MRPL3/MRPL47/SMDT1/MPC1/NDUFB2/MRPL51/MRPL17/TIMM17A/ATP5PD/MRPS23/UQCR10/CCDC51/COX6A1/ATP5MG/MRPL18/MRPS31/RNF13/ATP5PB/CPOX/TMEM70/NDUFV2/ADCK1/DNAJC15/MRPS15/MCUB/UCP3/DPY19L3/COX4I1/DNAJC19/MRPS35/TIMM10B/RPS3/DHFR2/MRPL15/ATP5F1E/MRPS28/MRPL24/BDH1/MRPL57/LDHB/TMEM126B/MATR3/NDUFA4/MRPL40/TIMMDC1/ATP5MK/MRPL13/FLVCR1/MRPS26/ATP5F1C/NDUFS3/NDUFAF4/TTC19/COQ8B/NOA1/TMEM126A/PARL/NDUFA13/MRPL30/MRPL33/SLC25A32/MICOS10/UNC50/GHITM/ROMO1/CYCS/MRPL53/PGS1/HIGD1A/NDUFA12/COQ2/MRPL1/PTPMT1/TIMM23/SLC25A4/NDUFB3/TAFAZZIN/UQCRHL/LRPPRC/MRPL35/BCS1L/MRPL11/COX11/MRPS36/SLC9B2/MRPL48/COA1/PHB2/COQ10A/YME1L1/NDUFAF5/MRPS6/MRPS14/MRPL52/SFXN4/ATP23/NDUFC1/MRPL58/ZMPSTE24/MRPL39

CC	GO:0005743	mitochondrial inner membrane	416	0.47	1.99	1.00E-10	4.69E-09	2.91E-09	1893	tags=30%, list=16%, signal=26%	UQCRB/MRPS18C/ATP5PO/NDUFS5/SMIM20/COX16/NDUFA1/ATP5ME/MRPS33/COX7B/PET100/NDUFA5/MRPL32/NDUFA6/COX7C/MRPS22/COX6C/NDUFAB1/UQCRQ/TIMM8B/ATP5MJ/NDUFS4/MRPS21/UQCRC2/DMAC1/UQCRH/COX20/NDUFB1/NDUFAF2/ATP5MF/MRPL50/MICU2/CRLS1/IMMP1L/MRPS11/ATP5PF/MRPL3/MRPL47/SMDT1/MPC1/NDUFB2/MRPL51/MRPL17/TIMM17A/ATP5PD/MRPS23/UQCR10/CCDC51/COX6A1/ATP5MG/MRPL18/MRPS31/ATP5PB/CPOX/TMEM70/NDUFV2/ADCK1/DNAJC15/MRPS15/MCUB/UCP3/COX4I1/DNAJC19/MRPS35/TIMM10B/RPS3/DHFR2/MRPL15/ATP5F1E/MRPS28/MRPL24/BDH1/MRPL57/LDHB/TMEM126B/NDUFA4/MRPL40/TIMMDC1/ATP5MK/MRPL13/FLVCR1/MRPS26/ATP5F1C/NDUFS3/NDUFAF4/TTC19/COQ8B/NOA1/TMEM126A/PARL/NDUFA13/MRPL30/MRPL33/SLC25A32/MICOS10/GHITM/ROMO1/CYCS/MRPL53/PGS1/HIGD1A/NDUFA12/COQ2/MRPL1/PTPMT1/TIMM23/SLC25A4/NDUFB3/TAFAZZIN/UQCRHL/MRPL35/BCS1L/MRPL11/COX11/MRPS36/SLC9B2/MRPL48/COA1/PHB2/COQ10A/YME1L1/NDUFAF5/MRPS6/MRPS14/MRPL52/SFXN4
BP	GO:0043043	peptide biosynthetic process	584	0.47	2.05	1.00E-10	4.69E-09	2.91E-09	1117	tags=24%, list=10%, signal=23%	RPL17/MRPS18C/RPL26/RPS25/RPS24/NPM1/RPL36A/RPL24/EEF1B2/RPL22/RPL34/RPL31/RPS7/RPL5/CNBP/RPL21/NGDN/RPL23/RPL3/RPS23/RPS18/RPS3A/RPL7/RPS20/RPS27A/RPS10/RPL39/METTL5/RPL13A/EIF3E/RPL9/RPL37/RPL6/RPS8/RPL27/RPS17/MRPS33/RPS6/MCTS1/MRPL32/EEF1D/RPL22L1/RPL35/RPL11/RPS13/USP16/MTIF3/RPS27L/MRPS21/RPS15A/RWDD1/EIF2A/RSL24D1/RPS27/UPF3B/RPL27A/SARNP/AIMP1/RPL35A/NEMF/RPL12/ZC3H15/EIF4A2/RPL38/RPS21/DHX29/RPS12/MRPS11/TRMT10C/RPL29/RPL32/MRPL3/RPS14/MRPL47/PUM3/SELENOT/MRPL51/BDH2/RPL41/RPSA/RPL19/MAGOH/RPS29/RPLP0/ATF4/EIF4E/MRPL18/METTL18/RPL36AL/RPL36/RPL15/EIF5/EIF2S2/RPL14/RPL4/RPS11/RPS4X/RGS2/TARBP2/EXOSC8/HBS1L/RPS2/MTIF2/NGRN/EEF1E1/MRPS15/RPL37A/EIF3M/SRP9/MTRES1/RBM3/RPS3/RPL10A/HARS2/PAIP1/CPEB3/EEF1G/RPS19/MRPS28/RPS9/MRPL24/MRPL57/RPS26/MAPKAPK5/RPL10/RPL8/YARS2/DPH5/RPLP1/MARS1/UPF3A/NARS1/MRPL13/CD28/HABP4/COPS5/RPL30/RNF139/PATL2/DENR

BP	GO:0006412	translation	564	0.48	2.09	1.00E-10	4.69E-09	2.91E-09	1117	tags=25%, list=10%, signal=23%	RPL17/MRPS18C/RPL26/RPS25/RPS24/NPM1/RPL36A/RPL24/EEF1B2/RPL22/RPL34/RPL31/RPS7/RPL5/CNBP/RPL21/NGDN/RPL23/RPL3/RPS23/RPS18/RPS3A/RPL7/RPS20/RPS27A/RPS10/RPL39/METTL5/RPL13A/EIF3E/RPL9/RPL37/RPL6/RPS8/RPL27/RPS17/MRPS33/RPS6/MCTS1/MRPL32/EEF1D/RPL22L1/RPL35/RPL11/RPS13/USP16/MTIF3/RPS27L/MRPS21/RPS15A/RWDD1/EIF2A/RSL24D1/RPS27/UPF3B/RPL27A/SARNP/AIMP1/RPL35A/NEMF/RPL12/ZC3H15/EIF4A2/RPL38/RPS21/DHX29/RPS12/MRPS11/TRMT10C/RPL29/RPL32/MRPL3/RPS14/MRPL47/PUM3/SELENOT/MRPL51/RPL41/RPSA/RPL19/MAGOH/RPS29/RPLP0/ATF4/EIF4E/MRPL18/METTL18/RPL36AL/RPL36/RPL15/EIF5/EIF2S2/RPL14/RPL4/RPS11/RPS4X/RGS2/TARBP2/EXOSC8/HBS1L/RPS2/MTIF2/NGRN/EEF1E1/MRPS15/RPL37A/EIF3M/SRP9/MTRES1/RBM3/RPS3/RPL10A/HARS2/PAIP1/CPEB3/EEF1G/RPS19/MRPS28/RPS9/MRPL24/MRPL57/RPS26/MAPKAPK5/RPL10/RPL8/YARS2/DPH5/RPLP1/MARS1/UPF3A/NARS1/MRPL13/CD28/HABP4/COPS5/RPL30/RNF139/PATL2/DENR
MF	GO:0005198	structural molecule activity	376	0.51	2.12	1.00E-10	4.69E-09	2.91E-09	1085	tags=27%, list=9%, signal=25%	RPL17/MRPS18C/RPL26/RPS25/RPS24/RPL36A/RPL24/RPL22/RPL34/RPL31/RPS7/RPL5/RPL21/RPL23/RPL3/RPS23/RPS18/RPS3A/RPL7/RPS20/RPS27A/RPS10/RPL39/RPL13A/RPL9/RPL37/RPL6/TUBE1/RPS8/RPL27/RPS17/MRPS33/RPS6/MRPL32/RPL22L1/RPL35/MRPS22/RPL11/RPS13/RPS27L/MRPS21/RPS15A/RSL24D1/RPS27/UPF3B/RPL27A/DCTN3/RPL35A/RPL12/RPL38/RPS21/PNN/RPS12/MRPS11/RPL29/RPL32/MRPL3/RPS14/MRPL47/MRPL51/SEPTIN7/MRPL17/RPL41/RPSA/RPL19/MRPS23/ASPH/RPS29/RPLP0/ARPC3/MRPL18/MRPS31/RPL36AL/RPL36/RPL15/RPL14/RPL4/CPOX/RPS11/RPS4X/RPS2/MRPS15/RPL37A/MPZ/MRPS35/RPS3/RPL10A/MRPL15/RPS19/RPS9/MRPL24/MRPL57/RPS26/RPL10/RPL8/MATR3/RPLP1/UPF3A/MRPL13/RPL30

BP	GO:0022613	ribonucleoprotein complex biogenesis	392	0.51	2.13	1.00E-10	4.69E-09	2.91E-09	1803	tags=32%, list=15%, signal=28%	RPL26/RPS25/RPS24/NPM1/RPL24/RPS7/RPL5/SNRPD1/NGDN/RPL3/RPL7/SNRPG/NOP58/ESF1/METTL5/RPL13A/SNRPE/HSP90AA1/RPL6/RPS8/RPL27/RPS17/RPS6/MCTS1/RPL35/RPL11/LUC7L3/RPS27L/SLU7/KRR1/EIF2A/RSL24D1/RPS27/PSIP1/POP5/ZRSR2/SNRPD2/RPL35A/RBIS/RPL38/NSA2/SF3B1/RPS21/DHX29/NIFK/MRPS11/RPS14/RPSA/GTF3A/C1D/RPLP0/METTL18/LYAR/EIF5/EIF2S2/RPL14/COIL/GNL2/EXOSC8/BRIX1/NGRN/UTP6/RPF2/RAN/PIN4/FBL/SNRPC/RPL10A/MPHOSPH10/RIOK2/FRG1/NMD3/SRSF10/ZNHIT3/RPS19/RPL10/PTBP2/LTV1/SRSF5/EXOSC1/PTGES3/DENR/CLNS1A/GTPBP10/MPHOSPH6/GEMIN6/RPS16/XPO1/EBNA1BP2/RSL1D1/WDR12/MTERF4/POLR2D/MTREX/CPSF7/DCAF13/MRPL1/SRFBP1/ZNF622/SRSF6/GTF2H5/RPL26L1/PPAN/DDX52/DDX18/SNRPF/PRPF39/SDAD1/SBDS/DDX1/DDX17/DIS3/NHP2/UTP23/GEMIN7/EXOSC9/SNRPD3/XRCC5/ERI2/NOL8/RIOX2/LTO1/ERCC2/HEATR3
BP	GO:0042254	ribosome biogenesis	289	0.52	2.12	1.00E-10	4.69E-09	2.91E-09	1803	tags=32%, list=15%, signal=28%	RPL26/RPS25/RPS24/NPM1/RPL24/RPS7/RPL5/NGDN/RPL3/RPL7/NOP58/ESF1/METTL5/RPL6/RPS8/RPL27/RPS17/RPS6/RPL35/RPL11/RPS27L/KRR1/EIF2A/RSL24D1/RPS27/POP5/RPL35A/RBIS/RPL38/NSA2/RPS21/DHX29/NIFK/MRPS11/RPS14/RPSA/GTF3A/C1D/RPLP0/METTL18/LYAR/RPL14/GNL2/EXOSC8/BRIX1/NGRN/UTP6/RPF2/RAN/PIN4/FBL/RPL10A/MPHOSPH10/RIOK2/FRG1/NMD3/ZNHIT3/RPS19/RPL10/LTV1/EXOSC1/GTPBP10/MPHOSPH6/RPS16/XPO1/EBNA1BP2/RSL1D1/WDR12/MTERF4/MTREX/DCAF13/MRPL1/SRFBP1/ZNF622/GTF2H5/RPL26L1/PPAN/DDX52/DDX18/SDAD1/SBDS/DDX17/DIS3/NHP2/UTP23/EXOSC9/XRCC5/ERI2/NOL8/RIOX2/LTO1/ERCC2/HEATR3

CC	GO:1990904	ribonucleoprotein complex	625	0.55	2.39	1.00E-10	4.69E-09	2.91E-09	1710	tags=35%, list=15%, signal=31%	RPL17/MRPS18C/RPL26/RPS25/RPS24/LARP7/NPM1/RPL36A/RPL24/RPL22/LSM3/RPL34/RPL31/RPS7/RPL5/SNRPD1/RPL21/NGDN/RPL23/RPL3/TRA2B/RPS23/RPS18/RPS3A/RPL7/RPS20/RPS27A/ZCCHC17/SNRPG/RPS10/RPL39/NOP58/RPL13A/EIF3E/SNRPE/RPL9/AKAP17A/RPL37/RPL6/RPS8/RPL27/LSM8/RPS17/MRPS33/SSB/RPS6/MRPL32/SRP14/SLIRP/RPL35/MRPS22/RPL11/LUC7L3/SYF2/RPS13/SF3B6/PRPF38B/RPS27L/SLU7/MRPS21/RPS15A/KRR1/RWDD1/EIF2A/RPS27/POP5/ZRSR2/RPL27A/SNRPD2/RPL35A/RBM45/DDX5/RPL12/HNRNPA1/VBP1/CWF19L2/TRA2A/RPL38/NSA2/SF3B1/RPS21/MRPL50/PRPF4B/ZNF830/DHX29/PLRG1/PNN/RPS12/SNRNP48/MRPS11/TRMT10C/PRPF40A/RPL29/RPL32/CWC15/MRPL3/RPS14/MRPL47/LSM5/RNPC3/MRPL51/SRP19/MRPL17/RPL41/RPSA/RPL19/MAGOH/SREK1/IVNS1ABP/RPS29/RPLP0/SNRPB2/EIF4E/PSMA6/MRPL18/UPF2/RPL36AL/RPL36/RPL15/RPL14/RPL4/RPS11/RPS4X/TARBP2/CWC25/RPS2/MRPS15/UTP6/RPL37A/PRORP/EIF3M/SRP9/NSRP1/RBM3/ZCRB1/PSMA1/FBL/PPWD1/MRPS35/RPS3/SNRPC/RPL10A/SRP72/MRPL15/MPHOSPH10/MAGOHB/HTATSF1/RIOK2/FRG1/WBP4/CPEB3/ZNHIT3/RPS19/MRPS28/RPS9/MRPL24/PPIL3/PHF5A/MRPL57/RPS26/RPL10/RPL8/CWC22/PTBP2/LTV1/MRPL40/RPLP1/MRPL13/DHX16/RPL30/PTGES3/MRPS26/CLNS1A/RPS16/XPO1/EBNA1BP2/MRPL30/MRPL33/RSL1D1/PHAX/LSM7/WDR12/PPIL1/MTERF4/EIF3I/MRPL53/MTREX/RBMX2/DCAF13/MRPL1/SRFBP1/ZNF622/R[illegible]
CC	GO:0098798	mitochondrial protein-containing complex	254	0.61	2.47	1.00E-10	4.69E-09	2.91E-09	1764	tags=35%, list=15%, signal=30%	UQCRB/MRPS18C/ATP5PO/NDUFS5/NDUFA1/TOMM7/ATP5ME/MRPS33/COX7B/NDUFA5/MRPL32/NDUFA6/COX7C/MRPS22/COX6C/DLD/NDUFAB1/UQCRQ/TIMM8B/ATP5MJ/NDUFS4/MRPS21/UQCRC2/UQCRH/NDUFB1/ATP5MF/MRPL50/MICU2/IMMP1L/MRPS11/TRMT10C/ATP5PF/MRPL3/MRPL47/SMDT1/NDUFB2/MRPL51/MRPL17/TIMM17A/ATP5PD/UQCR10/COX6A1/ATP5MG/MRPL18/ATP5PB/TOMM20/NDUFV2/DNAJC15/MRPS15/PRORP/MCUB/COX4I1/DNAJC19/MRPS35/TIMM10B/MRPL15/ATP5F1E/MRPS28/MRPL24/MRPL57/NDUFA4/MRPL40/ATP5MK/MRPL13/MRPS26/ATP5F1C/NDUFS3/TOMM5/NDUFA13/MRPL30/MRPL33/MICOS10/ROMO1/MTERF4/TOMM40L/MRPL53/NDUFA12/TIMM23/SLC25A4/NDUFB3/UQCRHL/MCCC1/MRPL35/BCS1L/MRPL11/MRPS36/MRPL48/PDHA1/PHB2

CC	GO:0098800	inner mitochondrial membrane protein complex	131	0.66	2.49	1.00E-10	4.69E-09	2.91E-09	1594	tags=37%, list=14%, signal=32%	UQCRB/ATP5PO/NDUFS5/NDUFA1/ATP5ME/COX7B/NDUFA5/NDUFA6/COX7C/COX6C/NDUFAB1/UQCRQ/ATP5MJ/NDUFS4/UQCRC2/UQCRH/NDUFB1/ATP5MF/MICU2/IMMP1L/ATP5PF/SMDT1/NDUFB2/TIMM17A/ATP5PD/UQCR10/COX6A1/ATP5MG/ATP5PB/NDUFV2/DNAJC15/MCUB/COX4I1/DNAJC19/TIMM10B/ATP5F1E/NDUFA4/ATP5MK/ATP5F1C/NDUFS3/NDUFA13/MICOS10/ROMO1/NDUFA12/TIMM23/NDUFB3/UQCRHL/BCS1L
CC	GO:0005840	ribosome	195	0.75	2.95	1.00E-10	4.69E-09	2.91E-09	1106	tags=49%, list=9%, signal=45%	RPL17/MRPS18C/RPL26/RPS25/RPS24/RPL36A/RPL24/RPL22/RPL34/RPL31/RPS7/RPL5/RPL21/RPL23/RPL3/RPS23/RPS18/RPS3A/RPL7/RPS20/RPS27A/ZCCHC17/RPS10/RPL39/RPL13A/RPL9/RPL37/RPL6/RPS8/RPL27/RPS17/MRPS33/RPS6/MRPL32/RPL22L1/RPL35/MRPS22/RPL11/RPS13/RPS27L/MRPS21/RPS15A/EIF2A/RSL24D1/RPS27/RPL27A/RPL35A/RPL12/RPL38/RPS21/MRPL50/DHX29/RPS12/MRPS11/RPL29/RPL32/MRPL3/RPS14/MRPL47/MRPL51/MRPL17/RPL41/RPSA/RPL19/HSPA14/RPS29/RPLP0/MRPL18/RPL36AL/RPL36/RPL15/RPL14/RPL4/RPS11/RPS4X/RPS2/MRPS15/RPL37A/MRPS35/RPS3/RPL10A/MRPL15/DNAJC21/RPS19/MRPS28/RPS9/MRPL24/MRPL57/RPS26/RPL10/RPL8/MRPL40/RPLP1/MRPL13/RPL30/MRPS26
BP	GO:0002181	cytoplasmic translation	139	0.77	2.95	1.00E-10	4.69E-09	2.91E-09	1014	tags=57%, list=9%, signal=53%	RPL17/RPL26/RPS25/RPS24/RPL36A/RPL24/RPL22/RPL34/RPL31/RPS7/RPL5/CNBP/RPL21/RPL23/RPL3/RPS23/RPS18/RPS3A/RPL7/RPS20/RPS27A/RPS10/RPL39/RPL13A/RPL9/RPL37/RPL6/RPS8/RPL27/RPS17/RPS6/MCTS1/RPL22L1/RPL35/RPL11/RPS13/RPS15A/RWDD1/RPS27/RPL27A/RPL35A/NEMF/RPL12/ZC3H15/EIF4A2/RPL38/RPS21/DHX29/RPS12/RPL29/RPL32/RPS14/RPL41/RPSA/RPL19/RPS29/RPLP0/RPL36/RPL15/EIF5/EIF2S2/RPL14/RPL4/RPS11/RPS4X/RPS2/RPL37A/EIF3M/RPS3/RPL10A/PAIP1/CPEB3/RPS19/RPS9/RPS26/RPL10/RPL8/DPH5/RPLP1
CC	GO:0015934	large ribosomal subunit	108	0.77	2.83	1.00E-10	4.69E-09	2.91E-09	1457	tags=56%, list=12%, signal=49%	RPL17/RPL26/RPL36A/RPL24/RPL22/RPL34/RPL31/RPL5/RPL21/RPL23/RPL3/RPL7/ZCCHC17/RPL39/RPL13A/RPL9/RPL37/RPL6/RPL27/MRPL32/RPL35/RPL11/RPL27A/RPL35A/RPL12/RPL38/MRPL50/RPL29/RPL32/MRPL3/MRPL47/MRPL51/MRPL17/RPL41/RPL19/RPLP0/MRPL18/RPL36AL/RPL36/RPL15/RPL14/RPL4/RPL37A/RPL10A/MRPL15/MRPL24/MRPL57/RPL10/RPL8/MRPL40/RPLP1/MRPL13/RPL30/MRPL30/MRPL33/MTERF4/MRPL53/MRPL1/RPL13/RPL26L1

CC	GO:0044391	ribosomal subunit	169	0.79	3.04	1.00E-10	4.69E-09	2.91E-09	1106	tags=54%, list=9%, signal=50%	RPL17/MRPS18C/RPL26/RPS25/RPS24/RPL36A/RPL24/RPL22/RPL34/RPL31/RPS7/RPL5/RPL21/RPL23/RPL3/RPS23/RPS18/RPS3A/RPL7/RPS20/RPS27A/ZCCHC17/RPS10/RPL39/RPL13A/RPL9/RPL37/RPL6/RPS8/RPL27/RPS17/MRPS33/RPS6/MRPL32/RPL35/MRPS22/RPL11/RPS13/RPS27L/MRPS21/RPS15A/EIF2A/RPS27/RPL27A/RPL35A/RPL12/RPL38/RPS21/MRPL50/DHX29/RPS12/MRPS11/RPL29/RPL32/MRPL3/RPS14/MRPL47/MRPL51/MRPL17/RPL41/RPSA/RPL19/RPS29/RPLP0/MRPL18/RPL36AL/RPL36/RPL15/RPL14/RPL4/RPS11/RPS4X/RPS2/MRPS15/RPL37A/MRPS35/RPS3/RPL10A/MRPL15/RPS19/MRPS28/RPS9/MRPL24/MRPL57/RPS26/RPL10/RPL8/MRPL40/RPLP1/MRPL13/RPL30/MRPS26
MF	GO:0003735	structural constituent of ribosome	154	0.81	3.08	1.00E-10	4.69E-09	2.91E-09	1085	tags=58%, list=9%, signal=53%	RPL17/MRPS18C/RPL26/RPS25/RPS24/RPL36A/RPL24/RPL22/RPL34/RPL31/RPS7/RPL5/RPL21/RPL23/RPL3/RPS23/RPS18/RPS3A/RPL7/RPS20/RPS27A/RPS10/RPL39/RPL13A/RPL9/RPL37/RPL6/RPS8/RPL27/RPS17/MRPS33/RPS6/MRPL32/RPL22L1/RPL35/MRPS22/RPL11/RPS13/RPS27L/MRPS21/RPS15A/RSL24D1/RPS27/RPL27A/RPL35A/RPL12/RPL38/RPS21/RPS12/MRPS11/RPL29/RPL32/MRPL3/RPS14/MRPL47/MRPL51/MRPL17/RPL41/RPSA/RPL19/MRPS23/RPS29/RPLP0/MRPL18/MRPS31/RPL36AL/RPL36/RPL15/RPL14/RPL4/RPS11/RPS4X/RPS2/MRPS15/RPL37A/MRPS35/RPS3/RPL10A/MRPL15/RPS19/RPS9/MRPL24/MRPL57/RPS26/RPL10/RPL8/RPLP1/MRPL13/RPL30
CC	GO:0015935	small ribosomal subunit	64	0.81	2.73	1.00E-10	4.69E-09	2.91E-09	1173	tags=62%, list=10%, signal=57%	MRPS18C/RPS25/RPS24/RPS7/RPS23/RPS18/RPS3A/RPS20/RPS27A/RPS10/RPS8/RPS17/MRPS33/RPS6/MRPS22/RPS13/RPS27L/MRPS21/RPS15A/EIF2A/RPS27/RPS21/DHX29/RPS12/MRPS11/RPS14/RPSA/RPS29/RPS11/RPS4X/RPS2/MRPS15/MRPS35/RPS3/RPS19/MRPS28/RPS9/RPS26/MRPS26/RPS16
CC	GO:0022625	cytosolic large ribosomal subunit	54	0.85	2.78	1.00E-10	4.69E-09	2.91E-09	1085	tags=76%, list=9%, signal=69%	RPL17/RPL26/RPL36A/RPL24/RPL22/RPL34/RPL31/RPL5/RPL21/RPL23/RPL3/RPL7/ZCCHC17/RPL39/RPL13A/RPL9/RPL37/RPL6/RPL27/RPL35/RPL11/RPL27A/RPL35A/RPL12/RPL38/RPL29/RPL32/RPL41/RPL19/RPLP0/RPL36AL/RPL36/RPL15/RPL14/RPL4/RPL37A/RPL10A/RPL10/RPL8/RPLP1/RPL30
CC	GO:0022626	cytosolic ribosome	93	0.87	3.11	1.00E-10	4.69E-09	2.91E-09	1014	tags=76%, list=9%, signal=70%	RPL17/RPL26/RPS25/RPS24/RPL36A/RPL24/RPL22/RPL34/RPL31/RPS7/RPL5/RPL21/RPL23/RPL3/RPS23/RPS18/RPS3A/RPL7/RPS20/RPS27A/ZCCHC17/RPS10/RPL39/RPL13A/RPL9/RPL37/RPL6/RPS8/RPL27/RPS17/RPS6/RPL35/RPL11/RPS13/RPS27L/RPS15A/EIF2A/RPS27/RPL27A/RPL35A/RPL12/RPL38/RPS21/DHX29/RPS12/MRPS11/RPL29/RPL32/RPS14/RPL41/RPSA/RPL19/RPS29/RPLP0/RPL36AL/RPL36/RPL15/RPL14/RPL4/RPS11/RPS4X/RPS2/RPL37A/RPS3/RPL10A/RPS19/RPS9/RPS26/RPL10/RPL8/RPLP1

CC	GO:0022627	cytosolic small ribosomal subunit	39	0.88	2.65	1.00E-10	4.69E-09	2.91E-09	955	tags=79%, list=8%, signal=73%	RPS25/RPS24/RPS7/RPS23/RPS18/RPS3A/RPS20/RPS27A/RPS10/RPS8/RPS17/RPS6/RPS13/RPS27L/RPS15A/EIF2A/RPS27/RPS21/DHX29/RPS12/MRPS11/RPS14/RPSA/RPS29/RPS11/RPS4X/RPS2/RPS3/RPS19/RPS9/RPS26
CC	GO:0031966	mitochondrial membrane	613	0.39	1.70	1.77E-10	7.65E-09	4.74E-09	1992	tags=27%, list=17%, signal=24%	UQCRB/MRPS18C/ATP5PO/RPS27A/NDUFS5/SMIM20/COX16/NDUFA1/TOMM7/ATP5ME/MRPS33/COX7B/PET100/NDUFA5/MRPL32/NDUFA6/COX7C/BCL2A1/MRPS22/COX6C/NDUFAB1/UQCRQ/TIMM8B/ATP5MJ/NDUFS4/MRPS21/CCDC90B/COX14/UQCRC2/DMAC1/UQCRH/COX20/NDUFB1/NDUFAF2/PNPLA8/ATP5MF/MRPL50/MICU2/PMAIP1/CRLS1/IMMP1L/MRPS11/ATP5PF/MRPL3/MRPL47/SMDT1/MPC1/MIGA1/NDUFB2/MRPL51/MRPL17/TIMM17A/ATP5PD/MRPS23/UQCR10/CCDC51/COX6A1/ATP5MG/MRPL18/MTARC1/MRPS31/ATP5PB/CPOX/TOMM20/TMEM70/NDUFV2/ADCK1/DNAJC15/NGRN/MRPS15/MCUB/UCP3/PPP1CC/COX4I1/DNAJC19/MRPS35/TIMM10B/RPS3/DHFR2/MRPL15/ATAD1/TMEM14C/TRAF3IP3/ATP5F1E/CISD1/MRPS28/MRPL24/BDH1/MRPL57/PIGBOS1/LDHB/TMEM126B/NDUFA4/MOAP1/SYNJ2BP/MRPL40/TIMMDC1/HAX1/ATP5MK/MRPL13/FLVCR1/MRPS26/ATP5F1C/NDUFS3/NDUFAF4/TTC19/TMEM14A/BNIP1/COQ8B/RAF1/IFI27L2/NOA1/TOMM5/TMEM126A/PARL/NDUFA13/MRPL30/MRPL33/VRK2/SLC25A32/MICOS10/GHITM/ROMO1/CYCS/TOMM40L/MRPL53/PGS1/HIGD1A/NDUFA12/COQ2/MRPL1/PTPMT1/TIMM23/SLC25A4/NDUFB3/TAFAZZIN/UQCRHL/MRPL35/ARMC1/BCS1L/MRPL11/COX11/MRPS36/SLC9B2/MRPL48/GNPAT/BNIP3/COA1/PHB2/COQ10A/MFF/AIFM2/YME1L1/NDUFAF5/MRPS6/NIPSNAP2/SLC25A46/MRPS14/MRPL52/SFXN4/ATP23/NDUFC1/ARG1/VAMP1/MRPL58/MRPL39

CC	GO:0005740	mitochondrial envelope	658	0.38	1.68	1.83E-10	7.84E-09	4.86E-09	1893	tags=26%, list=16%, signal=23%	UQCRB/MRPS18C/ATP5PO/RPS27A/NDUFS5/SMIM20/COX16/NDUFA1/TOMM7/ATP5ME/MRPS33/COX7B/PET100/NDUFA5/MRPL32/NDUFA6/COX7C/BCL2A1/MRPS22/COX6C/NDUFAB1/UQCRQ/TIMM8B/ATP5MJ/NDUFS4/MRPS21/CCDC90B/COX14/UQCRC2/DMAC1/UQCRH/COX20/NDUFB1/NDUFAF2/PNPLA8/ATP5MF/MRPL50/MICU2/PMAIP1/CRLS1/IMMP1L/MRPS11/ATP5PF/MRPL3/MRPL47/SMDT1/MPC1/MIGA1/NDUFB2/MRPL51/MRPL17/TIMM17A/ATP5PD/SOD1/CHCHD7/MRPS23/UQCR10/CCDC51/COX6A1/ATP5MG/MRPL18/MTARC1/MRPS31/ATP5PB/CPOX/TOMM20/TMEM70/NDUFV2/ADCK1/DNAJC15/NGRN/MRPS15/PRELID3B/MCUB/UCP3/PPP1CC/COX4I1/DNAJC19/MRPS35/TIMM10B/RPS3/DHFR2/MRPL15/ATAD1/TMEM14C/TRAF3IP3/ATP5F1E/CISD1/MRPS28/MRPL24/BDH1/MRPL57/PIGBOS1/LDHB/TMEM126B/NDUFA4/MOAP1/SYNJ2BP/MRPL40/TIMMDC1/HAX1/ATP5MK/MRPL13/FLVCR1/MRPS26/ATP5F1C/NDUFS3/NDUFAF4/TTC19/TMEM14A/BNIP1/COQ8B/RAF1/IFI27L2/NOA1/TOMM5/TMEM126A/PARL/NDUFA13/MRPL30/MRPL33/VRK2/SLC25A32/MICOS10/GHITM/ROMO1/COX17/CYCS/TOMM40L/MRPL53/PGS1/CAPN2/HIGD1A/NDUFA12/COQ2/MRPL1/PTPMT1/TIMM23/SLC25A4/NDUFB3/TAFAZZIN/TRIAP1/PARK7/UQCRHL/MRPL35/ARMC1/BCS1L/MRPL11/BLOC1S1/COX11/MRPS36/SLC9B2/MRPL48/GNPAT/BNIP3/COA1/PHB2/COQ10A/MFF/AIFM2/YME1L1/NDUFAF5/MRPS6/NIPSNAP2/SLC25A46/MRPS14/MRPL52/SFXN4
BP	GO:0033108	mitochondrial respiratory chain complex assembly	89	0.64	2.27	1.58E-09	5.43E-08	3.36E-08	2653	tags=49%, list=23%, signal=39%	NDUFS5/SMIM20/COX16/NDUFA1/PET100/NDUFA5/NDUFA6/NDUFAB1/NDUFS4/COX14/DMAC1/COX20/NDUFB1/NDUFAF2/NDUFB2/TMEM126B/NDUFS3/NDUFAF4/TTC19/NDUFAF8/TMEM126A/NDUFA13/COX17/NDUFA12/COA5/NDUFB3/TAFAZZIN/BCS1L/COA1/NDUFAF5/NUBPL/NDUFC1/C12orf73/COA4/NDUFB4/COA8/NDUFC2/NDUFAF1/TFAM/SCO1/UQCC1/LYRM7/UQCRFS1/CHCHD4
BP	GO:0006364	rRNA processing	215	0.50	1.99	1.81E-09	6.16E-08	3.82E-08	1794	tags=30%, list=15%, signal=26%	RPL26/RPS25/RPS24/RPS7/RPL5/NGDN/RPL7/NOP58/ESF1/METTL5/RPS8/RPL27/RPS17/RPS6/RPL35/RPL11/KRR1/RPS27/POP5/RPL35A/NSA2/RPS21/NIFK/MRPS11/RPS14/C1D/METTL18/LYAR/RPL14/EXOSC8/BRIX1/UTP6/RPF2/PIN4/FBL/RPL10A/MPHOSPH10/RIOK2/FRG1/ZNHIT3/RPS19/EXOSC1/MPHOSPH6/RPS16/EBNA1BP2/RSL1D1/WDR12/MTERF4/MTREX/DCAF13/MRPL1/SRFBP1/GTF2H5/PPAN/DDX52/DDX18/SBDS/DDX17/DIS3/NHP2/UTP23/EXOSC9/ERI2/NOL8/ERCC2

BP	GO:0034660	ncRNA metabolic process	500	0.39	1.69	3.15E-09	9.85E-08	6.11E-08	2303	tags=28%, list=20%, signal=24%	RPL26/RPS25/RPS24/LARP7/RPS7/RPL5/NGDN/RPL7/NOP58/ESF1/METTL5/RPS8/RPL27/RPS17/SSB/RPS6/RPL35/RPL11/KRR1/RPS27/TRNT1/POP5/RPL35A/DDX5/NSA2/RPS21/NIFK/MRPS11/TRMT10C/RPS14/RTRAF/TRMT12/ZBTB8OS/THUMPD1/GTF3A/SNAPC1/C1D/RPAP2/TSNAX/INTS10/METTL18/DTWD1/LYAR/RPL14/TARBP2/EXOSC8/BRIX1/UTP6/METTL4/RPF2/PRORP/TRMT10A/TRMT1/PIN4/FBL/RPL10A/HARS2/MPHOSPH10/RIOK2/FRG1/MTO1/ZNHIT3/RPS19/YARS2/MARS1/EXOSC1/NARS1/TRMO/MPHOSPH6/PUS3/RPS16/GTPBP3/ELP4/EBNA1BP2/RSL1D1/SNAPC5/THUMPD3/WDR12/MTERF4/MTREX/TRMT13/TOE1/TPRKB/DCAF13/MRPL1/SRFBP1/IPPK/GTF2H5/PPAN/TENT2/DDX52/DDX18/OSGEP/DALRD3/SBDS/TARBP1/ZC3H8/DDX1/DDX17/DIS3/MACROH2A1/NHP2/UTP23/EXOSC9/ERI2/ELAC1/NOL8/TRIT1/SEPSECS/ERCC2/ELP1/THUMPD2/TYW1/METTL3/TRMT6/LCMT2/RNF113A/RPL7A/LSM6/INTS6L/ZMPSTE24/AGO3/PTCD1/TARS3/INTS3/WDR75/TSN/NARS2/RARS2/RPF1/REXO4/INTS11/TRMT2B/ADAT2/DARS1/ERI3/QTRT1/USPL1/POP4/METTL2B/DIMT1
BP	GO:0042273	ribosomal large subunit biogenesis	69	0.67	2.28	3.35E-09	1.04E-07	6.46E-08	1803	tags=49%, list=15%, signal=42%	RPL26/NPM1/RPL24/RPL5/RPL3/RPL7/RPL6/RPL35/RPL11/RSL24D1/RPL35A/RPL38/NSA2/NIFK/GTF3A/RPLP0/RPL14/BRIX1/RPF2/RPL10A/ZNHIT3/RPL10/EBNA1BP2/RSL1D1/WDR12/MRPL1/ZNF622/RPL26L1/PPAN/DDX18/SDAD1/NHP2/LTO1/HEATR3
BP	GO:0008380	RNA splicing	362	0.44	1.82	3.46E-09	1.07E-07	6.62E-08	2032	tags=31%, list=17%, signal=27%	LARP7/PPIG/LSM3/SNRPD1/TRA2B/RBM39/SNRPG/SNRPE/AKAP17A/LSM8/LUC7L3/SYF2/RPS13/SF3B6/PRPF38B/SLU7/PSIP1/ZRANB2/ZRSR2/SNRPD2/DDX5/HNRNPA1/CWF19L2/RP9/TRA2A/SF3B1/PRPF4B/ZNF830/PLRG1/PNN/SNRNP48/SRSF7/PRPF40A/CWC15/LSM5/RNPC3/RTRAF/CLK4/ZBTB8OS/MAGOH/SREK1/IVNS1ABP/SNRPB2/CLK1/SRSF11/COIL/CIR1/CWC25/METTL4/NSRP1/RBM3/ZCRB1/PPWD1/SNRPC/HNRNPLL/MPHOSPH10/MAGOHB/HTATSF1/SRSF3/SREK1IP1/FRG1/WBP4/SRSF10/THOC7/PPIL3/PHF5A/RPS26/CWC22/ZNF326/RBM25/PTBP2/SRSF5/DHX16/HABP4/CLASRP/CLNS1A/GEMIN6/TIA1/CCNL2/LSM7/PPIL1/MTREX/ZNF638/FAM172A/RBMX2/SRSF6/SNRPF/SRSF2/PRPF39/HNRNPH3/BCAS2/HSPA8/DDX1/DDX17/MFAP1/GEMIN7/SUGP2/SNRPD3/PPP2CA/UBL5/WTAP/LUC7L/METTL3/YTHDC1/SNRPA1/LSM1/RNF113A/SNRNP70/LSM6/DHX15/MBNL2/HNRNPH1/CDC40
MF	GO:0019843	rRNA binding	64	0.69	2.31	3.85E-09	1.16E-07	7.19E-08	967	tags=36%, list=8%, signal=33%	MRPS18C/RPL5/RPL23/RPL3/RPS18/RPL9/RPL37/RPL11/RPS13/RPL12/MRPS11/RPS14/GTF3A/RPLP0/MRPL18/RPS11/RPS4X/BRIX1/NGRN/RPF2/RPS3/RPS9/RPL8

BP	GO:0006119	oxidative phosphorylation	110	0.59	2.16	4.08E-09	1.22E-07	7.57E-08	1530	tags=35%, list=13%, signal=31%	UQCRB/ATP5PO/NDUFS5/NDUFA1/ATP5ME/NDUFA5/NDUFA6/COX7C/DLD/NDUFAB1/UQCRQ/NDUFS4/UQCRC2/UQCRH/NDUFB1/ATP5MF/ATP5PF/NDUFB2/ATP5PD/UQCR10/COX6A1/ATP5MG/ATP5PB/NDUFV2/DNAJC15/COX4I1/ATP5F1E/NDUFA4/ATP5F1C/NDUFS3/GHITM/CYCS/C2orf69/MSH2/NDUFA12/NDUFB3/TAFAZZIN/PARK7/UQCRHL
BP	GO:0016072	rRNA metabolic process	253	0.47	1.89	9.11E-09	2.41E-07	1.50E-07	1794	tags=27%, list=15%, signal=24%	RPL26/RPS25/RPS24/RPS7/RPL5/NGDN/RPL7/NOP58/ESF1/METTL5/RPS8/RPL27/RPS17/RPS6/RPL35/RPL11/KRR1/RPS27/POP5/RPL35A/NSA2/RPS21/NIFK/MRPS11/RPS14/GTF3A/C1D/METTL18/LYAR/RPL14/EXOSC8/BRIX1/UTP6/RPF2/PIN4/FBL/RPL10A/MPHOSPH10/RIOK2/FRG1/ZNHIT3/RPS19/MARS1/EXOSC1/MPHOSPH6/RPS16/EBNA1BP2/RSL1D1/WDR12/MTERF4/MTREX/DCAF13/MRPL1/SRFBP1/IPPK/GTF2H5/PPAN/DDX52/DDX18/SBDS/DDX17/DIS3/MACROH2A1/NHP2/UTP23/EXOSC9/ERI2/NOL8/ERCC2
CC	GO:0098803	respiratory chain complex	68	0.68	2.29	1.00E-08	2.62E-07	1.63E-07	1594	tags=38%, list=14%, signal=33%	UQCRB/NDUFS5/NDUFA1/COX7B/NDUFA5/NDUFA6/COX7C/COX6C/NDUFAB1/UQCRQ/NDUFS4/UQCRC2/UQCRH/NDUFB1/NDUFB2/UQCR10/COX6A1/NDUFV2/COX4I1/NDUFA4/NDUFS3/NDUFA13/NDUFA12/NDUFB3/UQCRHL/BCS1L
BP	GO:0042773	ATP synthesis coupled electron transport	70	0.65	2.22	3.30E-08	7.90E-07	4.90E-07	1530	tags=39%, list=13%, signal=34%	UQCRB/NDUFS5/NDUFA1/NDUFA5/NDUFA6/COX7C/DLD/NDUFAB1/UQCRQ/NDUFS4/UQCRH/NDUFB1/NDUFB2/UQCR10/COX6A1/NDUFV2/DNAJC15/COX4I1/NDUFA4/NDUFS3/GHITM/CYCS/NDUFA12/NDUFB3/TAFAZZIN/PARK7/UQCRHL
BP	GO:0042775	mitochondrial ATP synthesis coupled electron transport	70	0.65	2.22	3.30E-08	7.90E-07	4.90E-07	1530	tags=39%, list=13%, signal=34%	UQCRB/NDUFS5/NDUFA1/NDUFA5/NDUFA6/COX7C/DLD/NDUFAB1/UQCRQ/NDUFS4/UQCRH/NDUFB1/NDUFB2/UQCR10/COX6A1/NDUFV2/DNAJC15/COX4I1/NDUFA4/NDUFS3/GHITM/CYCS/NDUFA12/NDUFB3/TAFAZZIN/PARK7/UQCRHL
BP	GO:0071826	ribonucleoprotein complex subunit organization	161	0.51	1.96	5.06E-08	1.15E-06	7.13E-07	1726	tags=33%, list=15%, signal=28%	RPL24/RPL5/SNRPD1/RPL3/SNRPG/RPL13A/SNRPE/HSP90AA1/RPL6/MCTS1/RPL11/LUC7L3/RPS27L/SLU7/RPS27/ZFAND1/PSIP1/ZRSR2/SNRPD2/RPL38/SF3B1/DHX29/MRPS11/RPS14/RPSA/RPLP0/EIF5/EIF2S2/COIL/BRIX1/RPF2/SNRPC/SRSF10/ZNHIT3/RPS19/RPL10/KIF5B/PTBP2/SRSF5/PTGES3/DENR/CLNS1A/GEMIN6/POLR2D/CPSF7/SRSF6/PPAN/SNRPF/PRPF39/DDX1/GEMIN7/SNRPD3/XRCC5
BP	GO:0022618	ribonucleoprotein complex assembly	153	0.52	1.99	6.59E-08	1.46E-06	9.04E-07	1726	tags=33%, list=15%, signal=29%	RPL24/RPL5/SNRPD1/RPL3/SNRPG/RPL13A/SNRPE/HSP90AA1/RPL6/MCTS1/RPL11/LUC7L3/RPS27L/SLU7/RPS27/PSIP1/ZRSR2/SNRPD2/RPL38/SF3B1/DHX29/MRPS11/RPS14/RPSA/RPLP0/EIF5/EIF2S2/COIL/BRIX1/RPF2/SNRPC/SRSF10/ZNHIT3/RPS19/RPL10/PTBP2/SRSF5/PTGES3/DENR/CLNS1A/GEMIN6/POLR2D/CPSF7/SRSF6/PPAN/SNRPF/PRPF39/DDX1/GEMIN7/SNRPD3/XRCC5

CC	GO:0070469	respirasome	78	0.63	2.19	1.01E-07	2.17E-06	1.34E-06	1594	tags=38%, list=14%, signal=33%	UQCRB/NDUFS5/NDUFA1/COX7B/NDUFA5/NDUFA6/COX7C/COX6C/NDUFAB1/UQCRQ/NDUFS4/UQCRC2/UQCRH/NDUFB1/NDUFB2/UQCR10/COX6A1/NDUFV2/COX4I1/NDUFA4/NDUFS3/TTC19/NDUFA13/CYCS/C2orf69/HIGD1A/NDUFA12/NDUFB3/UQCRHL/BCS1L
BP	GO:0000377	RNA splicing, via transesterification reactions with bulged adenosine as nucleophile	259	0.44	1.78	1.19E-07	2.49E-06	1.54E-06	2032	tags=32%, list=17%, signal=27%	LARP7/LSM3/SNRPD1/TRA2B/RBM39/SNRPG/SNRPE/LSM8/LUC7L3/SYF2/SF3B6/SLU7/PSIP1/ZRSR2/SNRPD2/DDX5/HNRNPA1/CWF19L2/TRA2A/SF3B1/PRPF4B/PLRG1/PNN/SRSF7/PRPF40A/CWC15/LSM5/RNPC3/MAGOH/SNRPB2/COIL/CWC25/NSRP1/RBM3/ZCRB1/PPWD1/SNRPC/MAGOHB/HTATSF1/SRSF3/FRG1/WBP4/SRSF10/PPIL3/PHF5A/CWC22/RBM25/PTBP2/SRSF5/DHX16/CLNS1A/GEMIN6/TIA1/LSM7/PPIL1/MTREX/FAM172A/RBMX2/SRSF6/SNRPF/SRSF2/PRPF39/HNRNPH3/BCAS2/HSPA8/DDX1/DDX17/MFAP1/GEMIN7/SNRPD3/UBL5/WTAP/LUC7L/METTL3/YTHDC1/SNRPA1/RNF113A/SNRNP70/LSM6/MBNL2/HNRNPH1/CDC40
BP	GO:0000398	mRNA splicing, via spliceosome	259	0.44	1.78	1.19E-07	2.49E-06	1.54E-06	2032	tags=32%, list=17%, signal=27%	LARP7/LSM3/SNRPD1/TRA2B/RBM39/SNRPG/SNRPE/LSM8/LUC7L3/SYF2/SF3B6/SLU7/PSIP1/ZRSR2/SNRPD2/DDX5/HNRNPA1/CWF19L2/TRA2A/SF3B1/PRPF4B/PLRG1/PNN/SRSF7/PRPF40A/CWC15/LSM5/RNPC3/MAGOH/SNRPB2/COIL/CWC25/NSRP1/RBM3/ZCRB1/PPWD1/SNRPC/MAGOHB/HTATSF1/SRSF3/FRG1/WBP4/SRSF10/PPIL3/PHF5A/CWC22/RBM25/PTBP2/SRSF5/DHX16/CLNS1A/GEMIN6/TIA1/LSM7/PPIL1/MTREX/FAM172A/RBMX2/SRSF6/SNRPF/SRSF2/PRPF39/HNRNPH3/BCAS2/HSPA8/DDX1/DDX17/MFAP1/GEMIN7/SNRPD3/UBL5/WTAP/LUC7L/METTL3/YTHDC1/SNRPA1/RNF113A/SNRNP70/LSM6/MBNL2/HNRNPH1/CDC40
BP	GO:0022904	respiratory electron transport chain	87	0.59	2.09	1.50E-07	2.98E-06	1.85E-06	1530	tags=32%, list=13%, signal=28%	UQCRB/NDUFS5/NDUFA1/NDUFA5/NDUFA6/COX7C/DLD/NDUFAB1/UQCRQ/NDUFS4/UQCRH/NDUFB1/ETFRF1/NDUFB2/UQCR10/COX6A1/NDUFV2/DNAJC15/COX4I1/NDUFA4/NDUFS3/GHITM/CYCS/NDUFA12/NDUFB3/TAFAZZIN/PARK7/UQCRHL
CC	GO:0005746	mitochondrial respirasome	72	0.63	2.13	1.63E-07	3.22E-06	1.99E-06	1594	tags=36%, list=14%, signal=31%	UQCRB/NDUFS5/NDUFA1/COX7B/NDUFA5/NDUFA6/COX7C/COX6C/NDUFAB1/UQCRQ/NDUFS4/UQCRC2/UQCRH/NDUFB1/NDUFB2/UQCR10/COX6A1/NDUFV2/COX4I1/NDUFA4/NDUFS3/NDUFA13/NDUFA12/NDUFB3/UQCRHL/BCS1L

CC	GO:0005681	spliceosomal complex	185	0.48	1.89	2.30E-07	4.43E-06	2.75E-06	2032	tags=37%, list=17%, signal=31%	LSM3/SNRPD1/TRA2B/SNRPG/SNRPE/AKAP17A/LSM8/LUC7L3/SYF2/SF3B6/PRPF38B/SLU7/ZRSR2/SNRPD2/DDX5/HNRNPA1/CWF19L2/TRA2A/SF3B1/PRPF4B/ZNF830/PLRG1/PNN/SNRNP48/PRPF40A/CWC15/LSM5/RNPC3/MAGOH/SREK1/IVNS1ABP/SNRPB2/CWC25/RBM3/ZCRB1/PPWD1/SNRPC/MAGOHB/HTATSF1/FRG1/WBP4/PPIL3/PHF5A/CWC22/PTBP2/DHX16/CLNS1A/LSM7/PPIL1/MTREX/RBMX2/SNRPF/SRSF2/PRPF39/HNRNPH3/BCAS2/HSPA8/MFAP1/CCDC12/SNRPD3/LUC7L/SNRPA1/RNF113A/SNRNP70/LSM6/DHX15/HNRNPH1/CDC40
CC	GO:0000313	organellar ribosome	80	0.62	2.14	2.65E-07	4.97E-06	3.08E-06	1992	tags=41%, list=17%, signal=34%	MRPS18C/MRPS33/MRPL32/MRPS22/MRPS21/MRPL50/MRPS11/MRPL3/MRPL47/MRPL51/MRPL17/MRPL18/MRPS15/MRPS35/MRPL15/MRPS28/MRPL24/MRPL57/MRPL40/MRPL13/MRPS26/MRPL30/MRPL33/MTERF4/MRPL53/MRPL35/MRPL11/MRPL48/MRPS6/MRPS14/MRPL52/MRPL58/MRPL39
CC	GO:0005761	mitochondrial ribosome	80	0.62	2.14	2.65E-07	4.97E-06	3.08E-06	1992	tags=41%, list=17%, signal=34%	MRPS18C/MRPS33/MRPL32/MRPS22/MRPS21/MRPL50/MRPS11/MRPL3/MRPL47/MRPL51/MRPL17/MRPL18/MRPS15/MRPS35/MRPL15/MRPS28/MRPL24/MRPL57/MRPL40/MRPL13/MRPS26/MRPL30/MRPL33/MTERF4/MRPL53/MRPL35/MRPL11/MRPL48/MRPS6/MRPS14/MRPL52/MRPL58/MRPL39
BP	GO:0019646	aerobic electron transport chain	64	0.64	2.15	4.45E-07	7.86E-06	4.87E-06	1530	tags=38%, list=13%, signal=33%	UQCRB/NDUFS5/NDUFA1/NDUFA5/NDUFA6/COX7C/DLD/NDUFAB1/UQCRQ/NDUFS4/UQCRH/NDUFB1/NDUFB2/UQCR10/COX6A1/NDUFV2/DNAJC15/COX4I1/NDUFA4/NDUFS3/CYCS/NDUFB3/PARK7/UQCRHL
BP	GO:0000375	RNA splicing, via transesterification reactions	263	0.43	1.76	7.05E-07	1.19E-05	7.36E-06	2032	tags=32%, list=17%, signal=27%	LARP7/LSM3/SNRPD1/TRA2B/RBM39/SNRPG/SNRPE/LSM8/LUC7L3/SYF2/SF3B6/SLU7/PSIP1/ZRSR2/SNRPD2/DDX5/HNRNPA1/CWF19L2/TRA2A/SF3B1/PRPF4B/PLRG1/PNN/SRSF7/PRPF40A/CWC15/LSM5/RNPC3/MAGOH/SNRPB2/COIL/CWC25/NSRP1/RBM3/ZCRB1/PPWD1/SNRPC/MPHOSPH10/MAGOHB/HTATSF1/SRSF3/FRG1/WBP4/SRSF10/PPIL3/PHF5A/CWC22/RBM25/PTBP2/SRSF5/DHX16/CLNS1A/GEMIN6/TIA1/LSM7/PPIL1/MTREX/FAM172A/RBMX2/SRSF6/SNRPF/SRSF2/PRPF39/HNRNPH3/BCAS2/HSPA8/DDX1/DDX17/MFAP1/GEMIN7/SNRPD3/UBL5/WTAP/LUC7L/METTL3/YTHDC1/SNRPA1/LSM1/RNF113A/SNRNP70/LSM6/MBNL2/HNRNPH1/CDC40
MF	GO:0016655	oxidoreductase activity, acting on NAD(P)H, quinone or similar compound as acceptor	45	0.69	2.13	9.79E-07	1.57E-05	9.73E-06	2116	tags=49%, list=18%, signal=40%	NDUFS5/NDUFA1/NDUFA5/NDUFA6/NDUFS4/NDUFB1/NDUFB2/CBR4/NDUFV2/CRYZL1/NDUFA4/NDUFS3/NQO2/AKR1C3/NDUFA13/NDUFA12/NDUFB3/CBR1/CRYZ/AIFM2/NDUFC1/NDUFB4

BP	GO:0045333	cellular respiration	183	0.47	1.82	1.11E-06	1.74E-05	1.08E-05	1797	tags=27%, list=15%, signal=23%	UQCRB/ATP5PO/NDUFS5/NDUFA1/ATP5ME/NDUFA5/NDUFA6/COX7C/DLD/NDUFAB1/UQCRQ/NDUFS4/UQCRC2/UQCRH/NDUFB1/ATP5MF/ATP5PF/ETFRF1/NDUFB2/ATP5PD/UQCR10/COX6A1/ATP5MG/ATP5PB/NDUFV2/DNAJC15/COX4I1/ATP5F1E/CISD1/NDUFA4/MDH1/ATP5F1C/NDUFS3/NOA1/GHITM/CYCS/C2orf69/MSH2/NDUFA12/NDUFB3/TAFAZZIN/PARK7/UQCRHL/BLOC1S1/MRPS36/BNIP3/PDHA1/COQ10A/AIFM2
CC	GO:0042788	polysomal ribosome	29	0.76	2.14	1.58E-06	2.40E-05	1.49E-05	1085	tags=62%, list=9%, signal=56%	RPL36A/RPL24/RPL31/RPS23/RPL39/RPL6/RPL11/RPL38/RPS21/RPL32/RPL41/RPL19/RPS29/RPL36/RPL10A/RPS26/RPL8/RPL30
BP	GO:0009060	aerobic respiration	152	0.49	1.89	1.64E-06	2.48E-05	1.54E-05	1747	tags=29%, list=15%, signal=25%	UQCRB/ATP5PO/NDUFS5/NDUFA1/ATP5ME/NDUFA5/NDUFA6/COX7C/DLD/NDUFAB1/UQCRQ/NDUFS4/UQCRC2/UQCRH/NDUFB1/ATP5MF/ATP5PF/NDUFB2/ATP5PD/UQCR10/COX6A1/ATP5MG/ATP5PB/NDUFV2/DNAJC15/COX4I1/ATP5F1E/NDUFA4/MDH1/ATP5F1C/NDUFS3/GHITM/CYCS/C2orf69/MSH2/NDUFA12/NDUFB3/TAFAZZIN/PARK7/UQCRHL/BLOC1S1/MRPS36/BNIP3/PDHA1
BP	GO:0042274	ribosomal small subunit biogenesis	71	0.61	2.05	2.28E-06	3.32E-05	2.06E-05	1794	tags=38%, list=15%, signal=32%	RPS25/RPS24/NPM1/RPS7/NGDN/RPS8/RPS17/RPS6/RPS27L/RPS27/RPL38/RPS21/MRPS11/RPS14/RPSA/UTP6/RIOK2/RPS19/LTV1/RPS16/DCAF13/SRFBP1/GTF2H5/DDX52/UTP23/XRCC5/ERCC2
BP	GO:0010257	NADH dehydrogenase complex assembly	53	0.65	2.12	2.55E-06	3.65E-05	2.26E-05	2361	tags=51%, list=20%, signal=41%	NDUFS5/NDUFA1/NDUFA5/NDUFA6/NDUFAB1/NDUFS4/DMAC1/NDUFB1/NDUFAF2/NDUFB2/TMEM126B/NDUFS3/NDUFAF4/NDUFAF8/TMEM126A/NDUFA13/NDUFA12/NDUFB3/TAFAZZIN/BCS1L/COA1/NDUFAF5/NUBPL/NDUFC1/NDUFB4/NDUFC2/NDUFAF1
BP	GO:0032981	mitochondrial respiratory chain complex I assembly	53	0.65	2.12	2.55E-06	3.65E-05	2.26E-05	2361	tags=51%, list=20%, signal=41%	NDUFS5/NDUFA1/NDUFA5/NDUFA6/NDUFAB1/NDUFS4/DMAC1/NDUFB1/NDUFAF2/NDUFB2/TMEM126B/NDUFS3/NDUFAF4/NDUFAF8/TMEM126A/NDUFA13/NDUFA12/NDUFB3/TAFAZZIN/BCS1L/COA1/NDUFAF5/NUBPL/NDUFC1/NDUFB4/NDUFC2/NDUFAF1
BP	GO:0042255	ribosome assembly	56	0.65	2.11	3.17E-06	4.35E-05	2.70E-05	965	tags=36%, list=8%, signal=33%	NPM1/RPL24/RPL5/RPL3/RPL6/RPL11/RPS27L/EIF2A/RPS27/RPL38/DHX29/MRPS11/RPS14/RPSA/RPLP0/BRIX1/NGRN/RPF2/RPS19/RPL10
CC	GO:0005844	polysome	61	0.61	2.03	8.01E-06	9.71E-05	6.02E-05	1085	tags=43%, list=9%, signal=39%	RPL36A/RPL24/RPL31/RPS23/RPL39/RPL6/RPS6/RPL11/RWDD1/VBP1/RPL38/RPS21/RPL32/RPL41/RPL19/RPS29/PSMA6/UPF2/RPL36/RPS4X/PSMA1/RPS3/RPL10A/RPS26/RPL8/RPL30
CC	GO:0000315	organellar large ribosomal subunit	54	0.63	2.06	8.33E-06	0.00010007	6.20E-05	3023	tags=57%, list=26%, signal=43%	MRPL32/MRPL50/MRPL3/MRPL47/MRPL51/MRPL17/MRPL18/MRPL15/MRPL24/MRPL57/MRPL40/MRPL13/MRPL30/MRPL33/MTERF4/MRPL53/MRPL35/MRPL11/MRPL48/MRPL52/MRPL58/MRPL39/MRPL45/MRPL42/MRPL4/MRPL9/MRPL46/MRPL2/MRPL20/MRPL22/MRPL27

CC	GO:0005762	mitochondrial large ribosomal subunit	54	0.63	2.06	8.33E-06	0.00010007	6.20E-05	3023	tags=57%, list=26%, signal=43%	MRPL32/MRPL50/MRPL3/MRPL47/MRPL51/MRPL17/MRPL18/MRPL15/MRPL24/MRPL57/MRPL40/MRPL13/MRPL30/MRPL33/MTERF4/MRPL53/MRPL35/MRPL11/MRPL48/MRPL52/MRPL58/MRPL39/MRPL45/MRPL42/MRPL4/MRPL9/MRPL46/MRPL2/MRPL20/MRPL22/MRPL27
CC	GO:1990204	oxidoreductase complex	97	0.53	1.92	8.71E-06	0.00010335	6.41E-05	1747	tags=30%, list=15%, signal=26%	UQCRB/NDUFS5/NDUFA1/NDUFA5/NDUFA6/DLD/NDUFAB1/UQCRQ/NDUFS4/UQCRC2/UQCRH/NDUFB1/NDUFB2/CBR4/UQCR10/MTARC1/NDUFV2/LDHB/NDUFA4/NDUFS3/NDUFA13/GMPR2/NDUFA12/NDUFB3/UQCRHL/BCS1L/MRPS36/LDHA/PDHA1
BP	GO:0006397	mRNA processing	416	0.36	1.53	1.34E-05	0.00014748	9.14E-05	2032	tags=27%, list=17%, signal=23%	LARP7/LSM3/SNRPD1/TRA2B/RBM39/SNRPG/SNRPE/AKAP17A/LSM8/LUC7L3/SYF2/SF3B6/PRPF38B/SLU7/PSIP1/ZRANB2/ZRSR2/SNRPD2/DDX5/HNRNPA1/CWF19L2/TRA2A/SF3B1/PRPF4B/ZNF830/PLRG1/PNN/SNRNP48/SRSF7/PRPF40A/CWC15/LSM5/RNPC3/MAGOH/SREK1/KIN/SNRPB2/SRSF11/CSTF2T/COIL/CIR1/CWC25/CMTR2/NSRP1/RBM3/ZCRB1/PPWD1/SNRPC/PCF11/HNRNPL/MAGOHB/HTATSF1/SRSF3/SREK1IP1/FRG1/WBP4/CPEB3/SRSF10/THOC7/TBRG4/PPIL3/PHF5A/CWC22/ZNF326/RBM25/PTBP2/SRSF5/DHX16/HABP4/CLASRP/CLNS1A/GEMIN6/TIA1/LSM7/PPIL1/POLR2D/MTREX/CPSF7/FAM172A/RBMX2/SRSF6/TENT2/SNRPF/SRSF2/PRPF39/HNRNPH3/RPRD1A/BCAS2/HSPA8/DDX1/DDX17/MFAP1/GEMIN7/SUGP2/SNRPD3/UBL5/WTAP/LUC7L/METTL3/YTHDC1/SNRPA1/LSM1/RNF113A/RBBP6/HSF1/SNRNP70/LSM6/DHX15/MBNL2/HNRNPH1/CDC40
MF	GO:0003955	NAD(P)H dehydrogenase (quinone) activity	37	0.69	2.10	1.72E-05	0.00018374	0.00011393	1469	tags=41%, list=13%, signal=36%	NDUFS5/NDUFA1/NDUFA5/NDUFA6/NDUFS4/NDUFB1/NDUFB2/CBR4/NDUFV2/NDUFA4/NDUFS3/NQO2/NDUFA13/NDUFA12/NDUFB3
BP	GO:0022900	electron transport chain	125	0.49	1.82	2.87E-05	0.00028281	0.00017535	1530	tags=26%, list=13%, signal=22%	UQCRB/NDUFS5/NDUFA1/NDUFA5/NDUFA6/COX7C/DLD/NDUFAB1/UQCRQ/NDUFS4/UQCRH/NDUFB1/ETFRF1/NDUFB2/UQCR10/ASPH/COX6A1/NDUFV2/DNAJC15/COX4I1/AOC2/NDUFA4/NDUFS3/NQO2/NDUFA13/GHITM/CYCS/NDUFA12/NDUFB3/TAFAZZIN/PARK7/UQCRHL
MF	GO:0008137	NADH dehydrogenase (ubiquinone) activity	35	0.68	2.02	3.91E-05	0.00036959	0.00022916	1469	tags=37%, list=13%, signal=33%	NDUFS5/NDUFA1/NDUFA5/NDUFA6/NDUFS4/NDUFB1/NDUFB2/NDUFV2/NDUFA4/NDUFS3/NDUFA13/NDUFA12/NDUFB3
MF	GO:0050136	NADH dehydrogenase (quinone) activity	35	0.68	2.02	3.91E-05	0.00036959	0.00022916	1469	tags=37%, list=13%, signal=33%	NDUFS5/NDUFA1/NDUFA5/NDUFA6/NDUFS4/NDUFB1/NDUFB2/NDUFV2/NDUFA4/NDUFS3/NDUFA13/NDUFA12/NDUFB3
MF	GO:0003954	NADH dehydrogenase activity	36	0.68	2.03	4.00E-05	0.00037575	0.00023298	1469	tags=36%, list=13%, signal=32%	NDUFS5/NDUFA1/NDUFA5/NDUFA6/NDUFS4/NDUFB1/NDUFB2/NDUFV2/NDUFA4/NDUFS3/NDUFA13/NDUFA12/NDUFB3

MF	GO:0015453	oxidoreduction-driven active transmembrane transporter activity	55	0.61	1.99	5.43E-05	0.00049135	0.00030465	1469	tags=33%, list=13%, signal=29%	NDUFS5/NDUFA1/COX7B/NDUFA5/NDUFA6/NDUFS4/UQCRH/NDUFB1/NDUFB2/UQCR10/COX6A1/NDUFV2/COX4I1/NDUFA4/NDUFS3/NDUFA13/NDUFA12/NDUFB3
CC	GO:0005747	mitochondrial respiratory chain complex I	41	0.65	1.97	6.26E-05	0.00054607	0.00033858	1469	tags=34%, list=13%, signal=30%	NDUFS5/NDUFA1/NDUFA5/NDUFA6/NDUFAB1/NDUFS4/NDUFB1/NDUFB2/NDUFV2/NDUFA4/NDUFS3/NDUFA13/NDUFA12/NDUFB3
CC	GO:0030964	NADH dehydrogenase complex	41	0.65	1.97	6.26E-05	0.00054607	0.00033858	1469	tags=34%, list=13%, signal=30%	NDUFS5/NDUFA1/NDUFA5/NDUFA6/NDUFAB1/NDUFS4/NDUFB1/NDUFB2/NDUFV2/NDUFA4/NDUFS3/NDUFA13/NDUFA12/NDUFB3
CC	GO:0045271	respiratory chain complex I	41	0.65	1.97	6.26E-05	0.00054607	0.00033858	1469	tags=34%, list=13%, signal=30%	NDUFS5/NDUFA1/NDUFA5/NDUFA6/NDUFAB1/NDUFS4/NDUFB1/NDUFB2/NDUFV2/NDUFA4/NDUFS3/NDUFA13/NDUFA12/NDUFB3
BP	GO:0015980	energy derivation by oxidation of organic compounds	235	0.40	1.60	7.08E-05	0.00060239	0.0003735	1797	tags=24%, list=15%, signal=21%	UQCRB/ATP5PO/NDUFS5/NDUFA1/ATP5ME/NDUFA5/NDUFA6/COX7C/DLD/NDUFAB1/UQCRQ/NDUFS4/UQCRC2/UQCRH/NDUFB1/EPM2AIP1/ATP5MF/ATP5PF/ETFRF1/NDUFB2/ATP5PD/UQCR10/COX6A1/ATP5MG/ATP5PB/NDUFV2/DNAJC15/PPP1CC/COX4I1/RB1CC1/ATP5F1E/CISD1/NDUFA4/PTGES3/MDH1/ATP5F1C/NDUFS3/NOA1/GHITM/SELENOS/CYCS/C2orf69/MSH2/UGP2/NDUFA12/NDUFB3/TAFAZZIN/PARK7/UQCRHL/PPP1CB/BLOC1S1/MRPS36/BNIP3/PDHA1/COQ10A/AIFM2
CC	GO:0005684	U2-type spliceosomal complex	89	0.51	1.82	8.16E-05	0.0006777	0.0004202	2032	tags=43%, list=17%, signal=36%	LSM3/SNRPD1/SNRPG/SNRPE/LSM8/LUC7L3/SYF2/SF3B6/SNRPD2/SF3B1/PLRG1/PRPF40A/CWC15/LSM5/SNRPB2/CWC25/SNRPC/MAGOHB/HTATSF1/WBP4/PHF5A/CWC22/DHX16/LSM7/PPIL1/RBMX2/SNRPF/PRPF39/BCAS2/MFAP1/CCDC12/SNRPD3/LUC7L/SNRPA1/RNF113A/SNRNP70/LSM6/CDC40
CC	GO:0120114	Sm-like protein family complex	76	0.54	1.88	8.68E-05	0.00071421	0.00044283	1971	tags=39%, list=17%, signal=33%	LARP7/LSM3/SNRPD1/SNRPG/SNRPE/LSM8/LUC7L3/SF3B6/SLU7/SNRPD2/SF3B1/PRPF40A/LSM5/SNRPB2/SNRPC/HTATSF1/PHF5A/CLNS1A/GEMIN6/LSM7/RBMX2/SNRPF/PRPF39/GEMIN7/SNRPD3/LUC7L/SNRPA1/LSM1/SNRNP70/LSM6
CC	GO:0070069	cytochrome complex	26	0.72	2.00	8.74E-05	0.00071702	0.00044458	988	tags=42%, list=8%, signal=39%	UQCRB/COX7B/COX7C/COX6C/UQCRQ/UQCRC2/UQCRH/UQCR10/COX6A1/COX4I1/NDUFA4
BP	GO:0017004	cytochrome complex assembly	35	0.66	1.95	9.64E-05	0.00077743	0.00048204	2638	tags=51%, list=22%, signal=40%	SMIM20/COX16/PET100/COX14/COX20/TTC19/COX17/COA5/BCS1L/COA1/C12orf73/COA4/COA8/COA6/SCO1/UQCC1/LYRM7/UQCRFS1
CC	GO:0030532	small nuclear ribonucleoprotein complex	66	0.55	1.85	9.77E-05	0.0007846	0.00048648	1971	tags=39%, list=17%, signal=33%	LARP7/LSM3/SNRPD1/SNRPG/SNRPE/LSM8/LUC7L3/SF3B6/SLU7/SNRPD2/SF3B1/PRPF40A/LSM5/SNRPB2/SNRPC/HTATSF1/PHF5A/LSM7/RBMX2/SNRPF/PRPF39/SNRPD3/LUC7L/SNRPA1/SNRNP70/LSM6

MF	GO:0016651	oxidoreductase activity, acting on NAD(P)H	65	0.55	1.84	0.00017052	0.00125318	0.00077702	2116	tags=40%, list=18%, signal=33%	NDUFS5/NDUFA1/NDUFA5/NDUFA6/NDUFS4/NDUFB1/NDUFB2/CBR4/NDUFV2/CRYZL1/NDUFA4/NDUFS3/NQO2/AKR1C3/MTRR/NDUFA13/GMPR2/NDUFA12/NDUFB3/CYB5R4/CBR1/CRYZ/AIFM2/NDUFC1/CYB5RL/NDUFB4
BP	GO:0006120	mitochondrial electron transport, NADH to ubiquinone	42	0.61	1.88	0.00026657	0.00184974	0.00114691	1524	tags=36%, list=13%, signal=31%	NDUFS5/NDUFA1/NDUFA5/NDUFA6/DLD/NDUFAB1/NDUFS4/NDUFB1/NDUFB2/NDUFV2/DNAJC15/NDUFA4/NDUFS3/NDUFB3/PARK7
BP	GO:0008033	tRNA processing	121	0.46	1.73	0.00032532	0.00219232	0.00135932	2868	tags=40%, list=24%, signal=31%	SSB/TRNT1/POP5/TRMT10C/RTRAF/TRMT12/ZBTB8OS/THUMPD1/DTWD1/PRORP/TRMT10A/TRMT1/MTO1/TRMO/PUS3/GTPBP3/ELP4/THUMPD3/TRMT13/TPRKB/OSGEP/DALRD3/TARBP1/DDX1/ELAC1/TRIT1/ELP1/THUMPD2/TYW1/TRMT6/LCMT2/LSM6/PTCD1/TRMT2B/ADAT2/QTRT1/POP4/METTL2B/CDK5RAP1/TYW5/TSEN15/AARS2/TSEN2/TP53RK/PUS1/TRMT61B/OSGEPL1/RPUSD4/GRSF1
CC	GO:0097525	spliceosomal snRNP complex	57	0.53	1.76	0.00048283	0.00303273	0.0018804	1971	tags=42%, list=17%, signal=35%	LSM3/SNRPD1/SNRPG/SNRPE/LSM8/LUC7L3/SF3B6/SNRPD2/SF3B1/PRPF40A/LSM5/SNRPB2/SNRPC/HTATSF1/PHF5A/LSM7/RBMX2/SNRPF/PRPF39/SNRPD3/LUC7L/SNRPA1/SNRNP70/LSM6
BP	GO:0044743	protein transmembrane import into intracellular organelle	35	0.61	1.83	0.00075385	0.00440657	0.00273223	1430	tags=26%, list=12%, signal=23%	TOMM7/PEX2/TIMM17A/TOMM20/DNAJC15/DNAJC19/ROMO1/TOMM40L/TIMM23
CC	GO:0031304	intrinsic component of mitochondrial inner membrane	54	0.55	1.79	0.00080642	0.00467396	0.00289803	1893	tags=26%, list=16%, signal=22%	COX16/PET100/SMDT1/MPC1/TIMM17A/CCDC51/MCUB/MICOS10/GHITM/COQ2/TIMM23/COX11/COA1/SFXN4
CC	GO:0031305	integral component of mitochondrial inner membrane	53	0.55	1.80	0.00083418	0.00479987	0.00297609	1893	tags=26%, list=16%, signal=22%	COX16/PET100/SMDT1/MPC1/TIMM17A/CCDC51/MCUB/MICOS10/GHITM/COQ2/TIMM23/COX11/COA1/SFXN4
BP	GO:0008535	respiratory chain complex IV assembly	25	0.67	1.83	0.00111283	0.00623933	0.00386861	2466	tags=52%, list=21%, signal=41%	SMIM20/COX16/PET100/COX14/COX20/COX17/COA5/BCS1L/COA1/COA4/COA8/COA6/SCO1
BP	GO:1901796	regulation of signal transduction by p53 class mediator	85	0.46	1.62	0.00141444	0.00766187	0.00475064	974	tags=20%, list=8%, signal=18%	RPL26/RPS7/RPL5/RPL23/RPS20/RPL37/ARMC10/RPL11/DDX5/PMAIP1/ING4/EEF1E1/RPF2/PSMD10/PTTG1IP/MAPKAPK5/PIP4P1
CC	GO:0032592	integral component of mitochondrial membrane	81	0.48	1.68	0.00144069	0.00773869	0.00479827	1893	tags=27%, list=16%, signal=23%	COX16/PET100/SMDT1/MPC1/TIMM17A/CCDC51/TOMM20/TMEM70/ADCK1/MCUB/PIGBOS1/SYNJ2BP/MICOS10/GHITM/COQ2/TIMM23/SLC25A4/COX11/BNIP3/COA1/MFF/SFXN4

CC	GO:0000314	organellar small ribosomal subunit	25	0.66	1.79	0.00162329	0.00851479	0.00527948	1106	tags=36%, list=9%, signal=33%	MRPS18C/MRPS33/MRPS22/MRPS21/MRPS11/MRPS15/MRPS35/MRPS28/MRPS26
CC	GO:0005763	mitochondrial small ribosomal subunit	25	0.66	1.79	0.00162329	0.00851479	0.00527948	1106	tags=36%, list=9%, signal=33%	MRPS18C/MRPS33/MRPS22/MRPS21/MRPS11/MRPS15/MRPS35/MRPS28/MRPS26
CC	GO:0071013	catalytic step 2 spliceosome	86	0.47	1.63	0.0017331	0.00897551	0.00556514	969	tags=26%, list=8%, signal=24%	LSM3/SNRPD1/SNRPG/SNRPE/SYF2/SF3B6/SLU7/SNRPD2/DDX5/HNRNPA1/SF3B1/PRPF4B/PLRG1/PNN/CWC15/MAGOH/SNRPB2/PPWD1/MAGOHB/FRG1/PPIL3/CWC22
MF	GO:0009055	electron transfer activity	93	0.47	1.68	0.00175921	0.00908394	0.00563237	2116	tags=31%, list=18%, signal=26%	NDUFS5/NDUFA1/COX7B/NDUFA5/NDUFA6/NDUFS4/UQCRH/NDUFB1/NDUFB2/UQCR10/ASPH/COX6A1/NDUFV2/COX4I1/AOC2/NDUFA4/NDUFS3/NQO2/NDUFA13/CYCS/NDUFA12/NDUFB3/COX11/AIFM2/NDUFC1/ADH5/DEGS1/COX7A2/NDUFB4
BP	GO:0051438	regulation of ubiquitin-protein transferase activity	50	0.52	1.67	0.00203177	0.01025169	0.00635642	888	tags=26%, list=8%, signal=24%	RPS7/RPL5/TOPORS/RPL23/RPS20/RPL37/RPL11/MAD2L1/BMI1/DCUN1D4/RPS2/UBE2N/MASTL
CC	GO:0071011	precatalytic spliceosome	52	0.53	1.69	0.00221985	0.01099144	0.00681509	1971	tags=46%, list=17%, signal=39%	LSM3/SNRPD1/SNRPG/SNRPE/LSM8/SF3B6/PRPF38B/SNRPD2/SF3B1/LSM5/SNRPB2/MAGOHB/WBP4/PHF5A/CWC22/DHX16/LSM7/RBMX2/SNRPF/MFAP1/SNRPD3/SNRPA1/RNF113A/LSM6
BP	GO:0140053	mitochondrial gene expression	103	0.43	1.58	0.00262353	0.01255252	0.00778302	1869	tags=27%, list=16%, signal=23%	MTIF3/MRPS21/TRNT1/MTERF2/MRPS11/TRMT10C/MRPL47/MRPL51/MTERF1/MTIF2/NGRN/MRPS15/METTL4/PRORP/MTRES1/FASTKD1/MTO1/MRPS28/TBRG4/MRPL57/YARS2/NOA1/MTERF4/LRPPRC/TRIT1/MRPS6/MRPS14/RMND1
BP	GO:0001510	RNA methylation	78	0.47	1.63	0.00301049	0.0139507	0.00864994	3330	tags=46%, list=28%, signal=33%	LARP7/METTL5/TRMT10C/TRMT12/METTL4/TRMT10A/CMTR2/TRMT1/FBL/MTO1/TRMO/GTPBP3/THUMPD3/TRMT13/DALRD3/TARBP1/THUMPD2/WTAP/METTL3/TRMT6/LCMT2/TRMT2B/METTL2B/DIMT1/FDXACB1/TRMT2A/CBLL1/TRMT61B/METTL15/TRMT11/RNMT/METTL6/EMG1/NSUN6/ALKBH8/FTSJ1
BP	GO:0051204	protein insertion into mitochondrial membrane	26	0.63	1.75	0.00343719	0.01554751	0.00964002	1293	tags=31%, list=11%, signal=27%	HSP90AA1/TIMM8B/PDCD5/TIMM10B/MOAP1/NDUFA13/MAPK8/ROMO1

BP	GO:0016071	mRNA metabolic process	612	0.29	1.26	0.00380607	0.01687775	0.01046481	2032	tags=23%, list=17%, signal=20%	LARP7/NPM1/LSM3/SNRPD1/TRA2B/RBM39/SNRPG/TAF7/EIF3E/SNRPE/AKAP17A/LSM8/SSB/LUC7L3/SYF2/SF3B6/PRPF38B/SLU7/PSIP1/ZRANB2/ZRSR2/UPF3B/SNRPD2/DDX5/HNRNPA1/CWF19L2/TRA2A/TAF13/SF3B1/PRPF4B/ZNF830/PLRG1/PNN/SNRNP48/SRSF7/TRMT10C/PRPF40A/CWC15/LSM5/RNPC3/MAGOH/SREK1/KIN/SNRPB2/ATF4/SLC11A1/UPF2/SRSF11/PNRC2/CSTF2T/COIL/CIR1/EXOSC8/CWC25/CMTR2/NSRP1/RBM3/ZCRB1/ZBTB1/PPWD1/SNRPC/PCF11/HNRNPLL/FASTKD1/MAGOHB/HTATSF1/SRSF3/SREK1IP1/PAIP1/FRG1/WBP4/CPEB3/SRSF10/THOC7/TBRG4/PPIL3/PHF5A/CWC22/ZNF326/RBM25/PTBP2/SRSF5/UPF3A/DHX16/HABP4/CLASRP/PATL2/CLNS1A/PUS3/TAF11/GEMIN6/TIA1/TRAF5/LSM7/PPIL1/POLR2D/ANGEL2/MTREX/CPSF7/FAM172A/RBMX2/SRSF6/TENT2/SNRPF/SRSF2/PRPF39/HNRNPH3/RPRD1A/DCP2/BCAS2/HSPA8/DNAJB11/DDX1/DDX17/DIS3/MFAP1/GEMIN7/SUGP2/EXOSC9/SNRPD3/UBL5/WTAP/LUC7L/METTL3/YTHDC1/TRMT6/SNRPA1/LSM1/RNF113A/CNOT8/RBBP6/ROCK1/HSF1/SNRNP70/LSM6/AGO3/DHX15/MBNL2/HNRNPH1/CDC40
BP	GO:0006400	tRNA modification	87	0.45	1.58	0.00382014	0.01689531	0.0104757	3343	tags=47%, list=29%, signal=34%	SSB/TRMT10C/TRMT12/THUMPD1/DTWD1/TRMT10A/TRMT1/MTO1/TRMO/PUS3/GTPBP3/ELP4/THUMPD3/TRMT13/TPRKB/OSGEP/DALRD3/TARBP1/TRIT1/ELP1/THUMPD2/TYW1/TRMT6/LCMT2/TRMT2B/ADAT2/QTRT1/METTL2B/CDK5RAP1/TYW5/AARS2/PUS1/TRMT61B/OSGEPL1/RPUSD4/TRMT11/METTL6/NSUN6/ALKBH8/FTSJ1/CTU1
BP	GO:0006413	translational initiation	112	0.41	1.51	0.00426035	0.01831261	0.01135448	2161	tags=33%, list=18%, signal=27%	NPM1/RPS3A/RPL13A/EIF3E/RPS17/MCTS1/MTIF3/EIF2A/EIF4A2/DHX29/ATF4/EIF5/EIF2S2/MTIF2/EIF3M/RPS3/PAIP1/HABP4/COPS5/DENR/EIF3I/POLR2D/PPP1R15B/EIF3F/EIF3J/DDX1/EIF3H/EIF4E2/LTO1/EIF1AX/EIF3K/METTL3/PAIP2B/NCBP1/RPS5/EIF3D/ABCE1
MF	GO:0051082	unfolded protein binding	94	0.42	1.51	0.00425743	0.01831261	0.01135448	1829	tags=29%, list=16%, signal=24%	NPM1/NACA/HSP90AA1/PET100/VBP1/HSPA14/HSPA13/PFDN2/PFDN4/TOMM20/DNAJA1/CCT2/HSPE1/TIMM10B/ERLEC1/SRSF10/PTGES3/CDC37L1/NUDCD2/PFDN6/PPIA/MKKS/HSPA8/DNAJB11/AIPL1/HEATR3/TTC1
CC	GO:0071007	U2-type catalytic step 2 spliceosome	29	0.60	1.69	0.00433566	0.01857608	0.01151784	2032	tags=52%, list=17%, signal=43%	SNRPD1/SNRPG/SNRPE/SYF2/SNRPD2/PLRG1/CWC15/SNRPB2/CWC22/PPIL1/SNRPF/BCAS2/SNRPD3/SNRPA1/CDC40
BP	GO:0043628	ncRNA 3'-end processing	38	0.54	1.65	0.00459019	0.01943621	0.01205115	2016	tags=34%, list=17%, signal=28%	LARP7/SSB/TRNT1/RPS21/TRMT10C/EXOSC8/FBL/TOE1/EXOSC9/ERI2/ELAC1/INTS6L/PTCD1
MF	GO:0003743	translation initiation factor activity	50	0.51	1.61	0.00464964	0.01956277	0.01212962	1852	tags=40%, list=16%, signal=34%	EIF3E/MCTS1/MTIF3/EIF2A/EIF4A2/DHX29/EIF4E/EIF5/EIF2S2/MTIF2/EIF3M/COPS5/DENR/EIF3I/EIF3F/EIF3J/EIF3H/EIF4E2/EIF1AX/EIF3K

BP	GO:0046034	ATP metabolic process	211	0.36	1.42	0.00514381	0.02117297	0.01312801	1530	tags=20%, list=13%, signal=18%	UQCRB/ATP5PO/NDUFS5/NDUFA1/ATP5ME/NDUFA5/NDUFA6/COX7C/DLD/NDUFAB1/UQCRQ/ATP5IF1/NDUFS4/UQCRC2/UQCRH/NDUFB1/ATP5MF/ATP5PF/NDUFB2/ATP5PD/UQCR10/COX6A1/ATP5MG/ATP5PB/NDUFV2/DNAJC15/OLA1/COX4I1/AK9/ATP5F1E/NDUFA4/ATP5F1C/NDUFS3/GHITM/CYCS/C2orf69/FIGNL1/MSH2/NDUFA12/NDUFB3/TAFAZZIN/PARK7/UQCRHL
CC	GO:0098573	intrinsic component of mitochondrial membrane	83	0.45	1.57	0.00516676	0.02120025	0.01314492	1893	tags=27%, list=16%, signal=22%	COX16/PET100/SMDT1/MPC1/TIMM17A/CCDC51/TOMM20/TMEM70/ADCK1/MCUB/PIGBOS1/SYNJ2BP/MICOS10/GHITM/COQ2/TIMM23/SLC25A4/COX11/BNIP3/COA1/MFF/SFXN4
BP	GO:0000387	spliceosomal snRNP assembly	35	0.55	1.65	0.00518494	0.02125291	0.01317757	1710	tags=34%, list=15%, signal=29%	SNRPD1/SNRPG/SNRPE/SNRPD2/COIL/SNRPC/SRSF10/CLNS1A/GEMIN6/SNRPF/GEMIN7/SNRPD3
CC	GO:0071005	U2-type precatalytic spliceosome	50	0.50	1.59	0.00650946	0.02544897	0.01577928	1971	tags=44%, list=17%, signal=37%	LSM3/SNRPD1/SNRPG/SNRPE/LSM8/SNRPD2/SF3B1/LSM5/SNRPB2/MAGOHB/WBP4/PHF5A/CWC22/DHX16/LSM7/RBMX2/SNRPF/MFAP1/SNRPD3/SNRPA1/RNF113A/LSM6
CC	GO:0032391	photoreceptor connecting cilium	25	0.61	1.67	0.00663994	0.02583209	0.01601682	1133	tags=32%, list=10%, signal=29%	TBCC/TOPORS/IFT20/CEP290/RPGRIP1/SEPTIN2/IFT57/CETN3
MF	GO:0048027	mRNA 5'-UTR binding	25	0.61	1.66	0.00705235	0.02706578	0.01678176	398	tags=32%, list=3%, signal=31%	RPL26/RPS7/RPL5/RPS3A/RPS13/MRPS11/RPS14/RPL41
CC	GO:0016469	proton-transporting two-sector ATPase complex	41	0.54	1.64	0.00741504	0.0282823	0.01753604	1395	tags=34%, list=12%, signal=30%	ATP5PO/ATP5ME/ATP5MJ/ATP5MF/ATP5PF/ATP5PD/ATP5MG/ATP5PB/ATP6V1C1/ATP6V1G1/ATP5F1E/ATP5MK/ATP5F1C/ATP6V1E1
BP	GO:0009451	RNA modification	153	0.37	1.42	0.00761179	0.02890591	0.0179227	2892	tags=35%, list=25%, signal=26%	LARP7/METTL5/SSB/PUS7L/TRMT10C/TRMT12/THUMPD1/DTWD1/APOBEC3A/METTL4/TRMT10A/CMTR2/TRMT1/FBL/MTO1/TRMO/PUS3/GTPBP3/ELP4/THUMPD3/TRMT13/TPRKB/OSGEP/DALRD3/DNAJB11/TARBP1/NHP2/TRIT1/ELP1/THUMPD2/WTAP/TYW1/METTL3/TRMT6/LCMT2/TRMT2B/ADAT2/QTRT1/METTL2B/DIMT1/CDK5RAP1/TYW5/APOBEC3G/AARS2/FDXACB1/TRMT2A/PUS1/CBLL1/TRMT61B/METTL15/OSGEPL1/RPUSD4/NUDT16
BP	GO:0006399	tRNA metabolic process	178	0.36	1.39	0.00939406	0.03426679	0.02124664	2628	tags=32%, list=22%, signal=25%	SSB/TRNT1/POP5/TRMT10C/RTRAF/TRMT12/ZBTB8OS/THUMPD1/DTWD1/EXOSC8/PRORP/TRMT10A/TRMT1/HARS2/MTO1/YARS2/MARS1/NARS1/TRMO/PUS3/GTPBP3/ELP4/THUMPD3/TRMT13/TPRKB/OSGEP/DALRD3/TARBP1/DDX1/EXOSC9/ELAC1/TRIT1/SEPSECS/ELP1/THUMPD2/TYW1/TRMT6/LCMT2/LSM6/PTCD1/TARS3/NARS2/RARS2/TRMT2B/ADAT2/DARS1/QTRT1/POP4/METTL2B/CDK5RAP1/TYW5/TSEN15/AARS2/TSEN2/QARS1/TP53RK/PUS1

CC	GO:1990351	transporter complex	179	0.35	1.38	0.00988334	0.03552997	0.02202986	1617	tags=18%, list=14%, signal=16%	UQCRB/NDUFS5/NDUFA1/NDUFA5/NDUFA6/NDUFAB1/UQCRQ/TIMM8B/NDUFS4/UQCRC2/UQCRH/NDUFB1/MICU2/SMDT1/NDUFB2/UQCR10/CCDC51/NDUFV2/MCUB/TIMM10B/NDUFA4/DLG3/NDUFS3/BEST1/NDUFA13/ABCG8/NDUFA12/NDUFB3/CLIC1/UQCRHL/BCS1L/OSTM1
MF	GO:0015078	proton transmembrane transporter activity	79	0.43	1.48	0.01066867	0.03770545	0.02337874	871	tags=20%, list=7%, signal=19%	ATP5PO/ATP5ME/COX7B/UQCRH/ATP5MF/ATP5PF/ATP5PD/UQCR10/SLC11A1/COX6A1/ATP5MG/ATP5PB/COX4I1/ATP6V1C1/ATP6V1G1/ATP5F1E
CC	GO:0046540	U4/U6 x U5 tri-snRNP complex	31	0.56	1.62	0.01080026	0.03800161	0.02356237	365	tags=23%, list=3%, signal=22%	LSM3/SNRPD1/SNRPG/SNRPE/LSM8/SNRPD2/LSM5
CC	GO:0097526	spliceosomal tri-snRNP complex	31	0.56	1.62	0.01080026	0.03800161	0.02356237	365	tags=23%, list=3%, signal=22%	LSM3/SNRPD1/SNRPG/SNRPE/LSM8/SNRPD2/LSM5
CC	GO:1902495	transmembrane transporter complex	160	0.36	1.36	0.01204384	0.04156797	0.02577364	1617	tags=19%, list=14%, signal=16%	UQCRB/NDUFS5/NDUFA1/NDUFA5/NDUFA6/NDUFAB1/UQCRQ/NDUFS4/UQCRC2/UQCRH/NDUFB1/MICU2/SMDT1/NDUFB2/UQCR10/CCDC51/NDUFV2/MCUB/NDUFA4/DLG3/NDUFS3/BEST1/NDUFA13/ABCG8/NDUFA12/NDUFB3/CLIC1/UQCRHL/BCS1L/OSTM1
MF	GO:0015399	primary active transmembrane transporter activity	113	0.39	1.43	0.01237759	0.04246185	0.02632788	1469	tags=22%, list=13%, signal=20%	NDUFS5/NDUFA1/COX7B/NDUFA5/NDUFA6/NDUFS4/UQCRH/NDUFB1/ABCD3/NDUFB2/UQCR10/COX6A1/TOMM20/NDUFV2/COX4I1/ATP6V1C1/ATP6V1G1/NDUFA4/NDUFS3/NDUFA13/ABCG8/ATP6V1E1/NDUFA12/ABCC2/NDUFB3
BP	GO:0000470	maturation of LSU-rRNA	28	0.57	1.59	0.01340825	0.04510254	0.0279652	2162	tags=50%, list=18%, signal=41%	RPL7/RPL35/NSA2/NIFK/RPF2/RPL10A/ZNHIT3/RSL1D1/WDR12/MRPL1/DDX18/NHP2/RPL7A/RPF1
MF	GO:0008135	translation factor activity, RNA binding	80	0.43	1.49	0.01405834	0.04673575	0.02897785	2129	tags=36%, list=18%, signal=30%	EEF1B2/EIF3E/MCTS1/EEF1D/MTIF3/EIF2A/EIF4A2/DHX29/EIF4E/EIF5/EIF2S2/HBS1L/MTIF2/EIF3M/CPEB3/EEF1G/COPS5/DENR/EIF3I/EIF3F/EIF3J/EIF3H/EIF4E2/EEF1A1/EIF1AX/EIF3K/MRPL58/EIF5A2/EIF3D
BP	GO:0043484	regulation of RNA splicing	127	0.41	1.52	0.00240998	0.01182971	0.00733485	2115	tags=31%, list=18%, signal=26%	LARP7/TRA2B/RBM39/AKAP17A/RPS13/DDX5/HNRNPA1/TRA2A/SRSF7/CLK4/MAGOH/CLK1/METTL4/NSRP1/RBM3/HNRNPLL/SRSF3/SRSF10/RPS26/CWC22/ZNF326/RBM25/PTBP2/SRSF5/HABP4/TIA1/CCNL2/FAM172A/SRSF6/SRSF2/HNRNPH3/HSPA8/DDX17/WTAP/YTHDC1/SNRNP70/MBNL2/HNRNPH1/RBM12/NCBP1
BP	GO:0000245	spliceosomal complex assembly	51	0.53	1.68	0.00401351	0.01747833	0.01083719	1891	tags=37%, list=16%, signal=31%	SNRPD1/SNRPG/SNRPE/LUC7L3/SLU7/PSIP1/ZRSR2/SNRPD2/SF3B1/SNRPC/SRSF10/PTBP2/SRSF5/GEMIN6/SRSF6/PRPF39/DDX1/LUC7L/YTHDC1
MF	GO:0036002	pre-mRNA binding	30	0.58	1.64	0.00970562	0.03498599	0.02169258	780	tags=27%, list=7%, signal=25%	TRA2B/SLU7/ZRSR2/DDX5/HNRNPA1/RNPC3/TARBP2/SNRPC
BP	GO:0007062	sister chromatid cohesion	43	0.50	1.54	0.01110869	0.03881207	0.02406488	3016	tags=47%, list=26%, signal=35%	ESCO1/NSMCE2/CTNNB1/BOD1/SLF1/HDAC8/NAA10/SMC3/SGO2/SMC5/MAU2/MACROH2A1/PHB2/NAA50/FBXW7/RAD51C/SFPQ/TENT4A/STAG3/STAG2

CC	GO:0035869	ciliary transition zone	48	0.51	1.62	0.01306591	0.04440177	0.02753069	2504	tags=33%, list=21%, signal=26%	TBCC/TOPORS/CFAP36/IFT20/CEP290/RPGRIP1/SEPTIN2/IFT57/CETN3/TCTN1/TMEM80/FAM161A/DYNC2LI1/IQCB1/KIFAP3/ARL3
BP	GO:2001244	positive regulation of intrinsic apoptotic signaling pathway	51	0.49	1.56	0.01477539	0.04871223	0.03020334	775	tags=18%, list=7%, signal=17%	RPL26/DDIT3/RPS7/S100A8/PTPN2/PMAIP1/SOD1/S100A9/RPS3

Supplementary table 11 APOE4 carriers gene set enrichment analysis gene ontology downregulated

ONTOLOGY	ID	Description	setSize	enrichment Score	NES	pvalue	p.adjust	qvalue	rank	leading_edge	core_enrichment
BP	GO:0099537	trans-synaptic signaling	327	-0.44	-1.68	7.46E-07	1.12E-05	6.90E-06	2755	tags=32%, list=23%, signal=26%	KIT/APBA1/SYNGAP1/CNP/ZDHHC2/SLC12A7/SNPH/SV2A/NCSTN/CACNB4/P2RY1/MCTP1/STAU2/YWHAG/BTBD9/SCRIB/CD38/SLC1A4/PRKAR1B/ZDHHC3/EPHB2/VPS18/ATXN3/SNAP25/DISC1/SRF/FYN/PTEN/LRRC4/SRC/EXOC4/YWHAH/ABAT/ADNP/PRKCE/GRIK2/EXT1/DGKZ/OR10H1/PNKD/DLGAP4/SLC12A6/SDCBP/CTBP2/SYAP1/RPH3A/LRRK2/PLCB1/SYNJ1/NPTXR/SYN3/DLG4/DBN1/MME/EPHB1/CACNB1/STX4/CA2/S1PR2/SIPA1L1/FLOT1/CLSTN1/CHRNE/CLN3/EPHA4/SHISA7/ARF1/GSK3A/PIP5K1C/HRH2/PRKACA/RARA/PTK2B/NEURL1/BCR/RAPGEF2/MAP1A/PPP3CA/APBA3/FBXL20/CALM3/PVALB/DNAJC5/GABARAP/KIF1B/GSK3B/TPRG1L/GNAI2/TSHZ3/PRKCB/STAU1/ARRB2/NF1/PPP1R9B/PINK1/NRGN/RPS6KA1/SNCA/GUCY1A1/PRKAR2B/SQSTM1/NAPA/EIF4EBP2/MBP/P2RX1/GLUL
BP	GO:0099177	regulation of trans-synaptic signaling	220	-0.45	-1.65	2.34E-05	0.00022516	0.00013824	2069	tags=32%, list=18%, signal=27%	NCSTN/P2RY1/MCTP1/STAU2/YWHAG/BTBD9/CD38/PRKAR1B/ZDHHC3/EPHB2/VPS18/SNAP25/DISC1/SRF/FYN/PTEN/LRRC4/SRC/YWHAH/ADNP/PRKCE/GRIK2/DGKZ/PNKD/DLGAP4/SYAP1/LRRK2/PLCB1/NPTXR/SYN3/DLG4/DBN1/MME/EPHB1/STX4/CA2/S1PR2/SIPA1L1/FLOT1/CLSTN1/CLN3/EPHA4/SHISA7/ARF1/PRKACA/RARA/PTK2B/NEURL1/BCR/RAPGEF2/MAP1A/PPP3CA/FBXL20/CALM3/GSK3B/TPRG1L/GNAI2/TSHZ3/PRKCB/STAU1/ARRB2/NF1/PPP1R9B/PINK1/NRGN/SNCA/PRKAR2B/SQSTM1/EIF4EBP2/P2RX1/GLUL
CC	GO:0016323	basolateral plasma membrane	134	-0.51	-1.78	2.50E-05	0.00023679	0.00014538	1869	tags=24%, list=16%, signal=20%	ERBB2/ATP7A/SLC12A6/NUMB/EZR/C5AR1/STX4/FLOT1/SLC16A3/AQP9/LEPR/SLC2A1/SLC3A2/SLC9A1/SLC6A9/B4GALT1/SLC14A1/ABCC4/AQP1/LRP1/FLOT2/ABCC3/ATP2B4/ARRB2/MSN/EPB41/SLC4A1/AQP10/BSG/SLC7A5/WASF2/ANK1
BP	GO:0099003	vesicle-mediated transport in synapse	106	-0.52	-1.76	8.61E-05	0.00070559	0.00043319	2615	tags=39%, list=22%, signal=30%	SNPH/SV2A/P2RY1/BTBD9/KIAA1109/SCRIB/PRKAR1B/VPS18/SNAP25/PTEN/ITSN1/RAB27B/SYNJ2/CTBP2/ACTG1/NUMB/LRRK2/SYNJ1/SYN3/STX4/CLSTN1/VPS35/PIP5K1C/CANX/SH3GL1/FBXL20/CALM3/DNM3/ACTB/DNAJC5/GSK3B/DNM2/GRIPAP1/PRKCB/SNCA/NAPA/AP2M1/GAK/STON2/ITGB3/P2RX1
CC	GO:0042581	specific granule	143	-0.49	-1.71	9.92E-05	0.00079985	0.00049106	2234	tags=35%, list=19%, signal=29%	HMOX2/ADAM8/CHI3L1/ORMDL3/SNAP25/CD93/CD177/CMTM6/ALDH3B1/SLC2A3/PDXK/ANO6/CKAP4/TSPAN14/ITGAM/SPTAN1/TMBIM1/STX4/STK10/PTPRJ/PTPN6/ADGRG3/HVCN1/AP1M1/KPNB1/QSOX1/ITGB2/NFKB1/OSCAR/TIMP2/PGLYRP1/VCL/HGSNAT/SLC44A2/LAMTOR1/LCN2/UBR4/TOM1/DOCK2/LTF/DNAJC5/TNFRSF1B/CNN2/ITGAL/LRG1/CANT1/ANXA11/CTSD/P2RX1/TOLLIP

BP	GO:0001816	cytokine production	538	-0.35	-1.41	0.00015831	0.00119211	0.00073189	2419	tags=28%, list=21%, signal=23%	SPON2/SPN/SCRIB/ALOX5/BAP1/BTN3A2/ADAM17/CYBB/NCKAP1L/DDX58/NRROS/HDAC7/MUL1/IFI16/EPHB2/RNF135/MAVS/ADAM8/TRIM38/RPS6KA5/PLD4/FCER1G/IRAK3/TLR2/ATP2B1/CHI3L1/RFTN1/CR1/TLR5/HEG1/LGALS9C/FGR/TIRAP/NLRP3/SRC/PER1/KPNA6/MAP2K5/TGFB1/SIRPA/DICER1/TNFSF4/G3BP1/LILRA5/BCL3/POU2F2/CPTP/ROCK2/GSTP1/ARRB1/LRRK2/HGF/PLCB1/STAT5B/NFAT5/EZR/EGR1/ARID5A/CMKLR1/ZBTB20/RIPK1/NFATC1/C5AR1/MAPK14/EIF2AK2/YY1/PTPRJ/FLOT1/PTPN6/PYCARD/RELB/TREM1/ITCH/MAST2/PML/PANX2/CSF1R/ARHGEF2/AKIRIN2/KAT5/FOXP1/ARNT/ERMAP/NLRC4/CHID1/NDRG2/SPTBN1/VSIR/NFKB1/NFAM1/TSPO/NFATC2/SORL1/IRF1/PGLYRP1/RARA/RPS6KA4/TRAF6/FRMD8/OTUD5/PRKD2/TBX21/IL6R/CSK/PTPRC/SYK/THBS1/PTAFR/ILRUN/NPLOC4/TLR6/IL17RA/NLRX1/FZD5/LTF/PIK3CG/JAK3/HSPA1A/INPP5D/PF4/AGPAT1/CD14/RAC1/LYN/RIOK3/LGALS9/LAPTM5/HSPB1/STMP1/TNFRSF1B/NFATC3/CLU/ARRB2/HK1/IGF2BP2/STAT3/LTBP1/MAPKAPK2/N4BP1/PIK3CD/LITAF/MBP/ACKR1/SLC7A5/IQGAP1/MAP2K3/FURIN/RELA
CC	GO:0005766	primary lysosome	138	-0.48	-1.68	0.00018003	0.0013134	0.00080636	2137	tags=34%, list=18%, signal=28%	GLB1/PA2G4/PADI2/GUSB/UNC13D/CPNE3/CD68/CMTM6/LAMP2/SDCBP/CKAP4/ACLY/CPNE1/PYCARD/CD63/TRAPPC1/ATP6V0C/CAP1/PSMD1/CPPED1/RNASET2/NFAM1/DYNC1H1/ARL8A/IMPDH1/LAMTOR1/PYGB/GAA/IST1/TOM1/B4GALT1/VCP/TUBB4B/DNAJC5/CYB5R3/PSAP/GNS/FTL/ARHGAP45/TUBB/ADA2/GRN/ANXA11/LAMP1/PRDX6/RAB3D/TOLLIP
CC	GO:0042582	azurophil granule	138	-0.48	-1.68	0.00018003	0.0013134	0.00080636	2137	tags=34%, list=18%, signal=28%	GLB1/PA2G4/PADI2/GUSB/UNC13D/CPNE3/CD68/CMTM6/LAMP2/SDCBP/CKAP4/ACLY/CPNE1/PYCARD/CD63/TRAPPC1/ATP6V0C/CAP1/PSMD1/CPPED1/RNASET2/NFAM1/DYNC1H1/ARL8A/IMPDH1/LAMTOR1/PYGB/GAA/IST1/TOM1/B4GALT1/VCP/TUBB4B/DNAJC5/CYB5R3/PSAP/GNS/FTL/ARHGAP45/TUBB/ADA2/GRN/ANXA11/LAMP1/PRDX6/RAB3D/TOLLIP
BP	GO:0045446	endothelial cell differentiation	67	-0.57	-1.79	0.00025242	0.00176074	0.001081	2072	tags=37%, list=18%, signal=31%	HEG1/EDF1/ATOH8/VEZF1/PDE2A/ROCK2/BTG1/PLCB1/EZR/S1PR2/TJP2/RBPJ/RAPGEF2/VCL/PECAM1/BMP6/FOXJ2/MYADM/GPX1/FASN/MSN/KDM6B/ADD1/RAPGEF1/CLDN5
CC	GO:0016528	sarcoplasm	42	-0.63	-1.84	0.00027155	0.00188099	0.00115483	1204	tags=29%, list=10%, signal=26%	ITPR2/CHERP/ATP2A3/TMEM38A/GSN/THBS1/PLEC/FKBP1A/RTN2/STIM1/ANK1/IRAG1
BP	GO:0002275	myeloid cell activation involved in immune response	76	-0.54	-1.72	0.00028816	0.0019686	0.00120862	2149	tags=39%, list=18%, signal=32%	FCER1G/FGR/UNC13D/CD177/PRKCE/ADGRE2/ITGAM/STX4/PTPN6/PYCARD/SBNO2/SPI1/PDPK1/ITGB2/FES/GAB2/BCR/SYK/PTAFR/DOCK2/PIK3CG/PRAM1/LYN/LGALS9/RAC2/DYSF/GATA1/GRN/PIK3CD/CBL
CC	GO:0030426	growth cone	116	-0.48	-1.65	0.00033	0.00220147	0.00135158	2285	tags=36%, list=19%, signal=29%	MYO5A/MYO9A/GDPD5/ENO2/NIN/KATNB1/SNAP25/DISC1/FEZ1/EXOC4/EPS8/CPEB4/NECTIN1/EXOC7/SYAP1/LRRK2/NDEL1/DBN1/CDKL5/SETX/EXOC3/CTNND1/EPHA4/ARPC5/ADGRL1/PALLD/NDRG2/FXR2/PTK2B/COPA/USP9X/CALM3/BASP1/FLNA/DNM2/TSHZ3/PPP1R9B/PINK1/SNCA/CTTN/CBL/IQGAP1
BP	GO:0043297	apical junction assembly	42	-0.62	-1.82	0.00041284	0.00267339	0.00164132	2999	tags=52%, list=26%, signal=39%	IKBKB/TNF/AFDN/F11R/TBCD/FBF1/STRN/SRF/ROCK2/PARD3/GPBAR1/WDR1/PRKACA/OCEL1/VCL/PECAM1/CTNNA1/ESAM/FZD5/RHOA/CLDN5/PAK2

BP	GO:1901615	organic hydroxy compound metabolic process	305	-0.38	-1.47	0.00041442	0.00267923	0.0016449	1966	tags=25%, list=17%, signal=21%	RETSAT/ACSS1/PTEN/SULT1A3/ABAT/GALK1/STARD3/ALDH3B1/ACSS2/ATP7A/OSBPL1A/ITPKB/SYNJ2/PDXK/PNKD/SCAP/ABCB11/ACLY/ITGAM/FAH/PLCB1/SYNJ1/SNX17/LIPE/SPTLC2/CPQ/CEBPA/OSBPL2/PER2/OSBPL5/VPS35/DHCR24/ITGB2/ACADVL/TKTL1/SULT1B1/LEPR/DGAT2/ALPL/RDH11/DHRS13/NFKB1/VLDLR/NUDT4/PTK2B/APOBR/INPP5K/RAPGEF2/LDLRAP1/CYP27A1/PIAS4/LPCAT3/BMP6/POR/PTAFR/MED1/G6PD/GDE1/ALOX12/PLEK/RXRA/FDFT1/CYB5R3/PNPLA2/ITPK1/SNCA/MTPN/IP6K1/ACO2/HDLBP/DPM2/CTSB/HAGH/DAB2/FAXDC2
BP	GO:0002090	regulation of receptor internalization	37	-0.65	-1.88	0.00043138	0.00277087	0.00170117	1719	tags=46%, list=15%, signal=39%	SDCBP/ANKRD13A/NUMB/ARRB1/DLG4/FLOT1/ATXN2/CD63/ARF1/INSR/GREM1/SYK/ARRB2/RIN3/TBC1D5/AP2M1/ITGB3
MF	GO:0038023	signaling receptor activity	532	-0.34	-1.38	0.00045525	0.0028824	0.00176964	2278	tags=22%, list=19%, signal=19%	SCARB1/TRIM5/KIR2DL1/SLC20A1/EPHB2/IL2RG/PTK7/INTS6/FCER1G/TLR2/PTGIR/CR1/THRA/TLR5/CD93/IFNAR2/LILRA1/EPHB4/RXRB/IFNAR1/JMJD6/IL17RD/CD300C/PAQR8/SDC4/GRIK2/THBD/ERBB2/VDR/AGTRAP/ADGRE2/OR10H1/LILRA5/NOTCH2/NECTIN1/OPRL1/CMKLR1/C5AR1/NR6A1/CD300LF/EPHB1/OR52K2/S1PR2/OR52K1/SORT1/PTPN6/CHRNE/ADGRG3/SEMA4D/NR1H2/TREM1/EPHA4/GPBAR1/ITGB2/CCR1/CSF1R/KLRD1/LEPR/ADIPOR2/HRH2/ARNT/ANPEP/INSR/AMOT/GUCY1B1/TNFRSF10C/ADGRL1/NFAM1/CD300LB/OSCAR/TSPO/VLDLR/SORL1/PGLYRP1/RARA/PTK2B/CXCR2/INPP5K/IL6R/PECAM1/IL13RA1/RRBP1/TAS2R30/PLXNC1/GP6/PTPRC/LILRB3/PTAFR/ADIPOR1/NR3C1/TLR6/IL17RA/FZD5/ABHD2/SLC1A5/RXRA/CD14/MMD/ADGRE5/IGF2R/LRP1/TNFRSF1B/MPL/TMEM63B/ITGAL/CXCR1/ITGB5/GPR146/GUCY1A1/STAT3/LTBP1/GP1BA/TREML1/ITGB3/KRT1/MRC2/ACKR1/P2RX1/TBXA2R
MF	GO:0060089	molecular transducer activity	532	-0.34	-1.38	0.00045525	0.0028824	0.00176964	2278	tags=22%, list=19%, signal=19%	SCARB1/TRIM5/KIR2DL1/SLC20A1/EPHB2/IL2RG/PTK7/INTS6/FCER1G/TLR2/PTGIR/CR1/THRA/TLR5/CD93/IFNAR2/LILRA1/EPHB4/RXRB/IFNAR1/JMJD6/IL17RD/CD300C/PAQR8/SDC4/GRIK2/THBD/ERBB2/VDR/AGTRAP/ADGRE2/OR10H1/LILRA5/NOTCH2/NECTIN1/OPRL1/CMKLR1/C5AR1/NR6A1/CD300LF/EPHB1/OR52K2/S1PR2/OR52K1/SORT1/PTPN6/CHRNE/ADGRG3/SEMA4D/NR1H2/TREM1/EPHA4/GPBAR1/ITGB2/CCR1/CSF1R/KLRD1/LEPR/ADIPOR2/HRH2/ARNT/ANPEP/INSR/AMOT/GUCY1B1/TNFRSF10C/ADGRL1/NFAM1/CD300LB/OSCAR/TSPO/VLDLR/SORL1/PGLYRP1/RARA/PTK2B/CXCR2/INPP5K/IL6R/PECAM1/IL13RA1/RRBP1/TAS2R30/PLXNC1/GP6/PTPRC/LILRB3/PTAFR/ADIPOR1/NR3C1/TLR6/IL17RA/FZD5/ABHD2/SLC1A5/RXRA/CD14/MMD/ADGRE5/IGF2R/LRP1/TNFRSF1B/MPL/TMEM63B/ITGAL/CXCR1/ITGB5/GPR146/GUCY1A1/STAT3/LTBP1/GP1BA/TREML1/ITGB3/KRT1/MRC2/ACKR1/P2RX1/TBXA2R

BP	GO:0001817	regulation of cytokine production	528	-0.34	-1.37	0.00057133	0.00351643	0.0021589	2419	tags=27%, list=21%, signal=22%	SPON2/SPN/SCRIB/ALOX5/BAP1/BTN3A2/ADAM17/CYBB/NCKAP1L/DDX58/HDAC7/MUL1/IFI16/EPHB2/RNF135/MAVS/ADAM8/TRIM38/RPS6KA5/PLD4/FCER1G/IRAK3/TLR2/ATP2B1/CHI3L1/RFTN1/CR1/TLR5/HEG1/LGALS9C/FGR/TIRAP/NLRP3/SRC/PER1/KPNA6/MAP2K5/TGFB1/SIRPA/DICER1/TNFSF4/G3BP1/LILRA5/BCL3/POU2F2/CPTP/ROCK2/GSTP1/ARRB1/LRRK2/HGF/PLCB1/STAT5B/EZR/EGR1/ARID5A/CMKLR1/ZBTB20/RIPK1/C5AR1/MAPK14/EIF2AK2/YY1/PTPRJ/FLOT1/PTPN6/PYCARD/RELB/ITCH/MAST2/PML/PANX2/CSF1R/ARHGEF2/AKIRIN2/KAT5/FOXP1/ARNT/ERMAP/NLRC4/CHID1/NDRG2/SPTBN1/VSIR/NFKB1/NFAM1/TSPO/SORL1/IRF1/PGLYRP1/RARA/RPS6KA4/TRAF6/FRMD8/OTUD5/PRKD2/TBX21/IL6R/CSK/PTPRC/SYK/THBS1/PTAFR/ILRUN/NPLOC4/TLR6/IL17RA/NLRX1/FZD5/LTF/PIK3CG/JAK3/HSPA1A/INPP5D/PF4/AGPAT1/CD14/RAC1/LYN/RIOK3/LGALS9/LAPTM5/HSPB1/STMP1/TNFRSF1B/CLU/ARRB2/HK1/IGF2BP2/STAT3/LTBP1/MAPKAPK2/N4BP1/PIK3CD/LITAF/MBP/ACKR1/SLC7A5/IQGAP1/MAP2K3/FURIN/RELA
MF	GO:0038024	cargo receptor activity	43	-0.62	-1.82	0.0005713	0.00351643	0.0021589	1654	tags=26%, list=14%, signal=22%	ITGAM/TEX261/ITGB2/CXCL16/INSR/LRP10/VLDLR/SORL1/APOBR/LRP1/DAB2
CC	GO:0045211	postsynaptic membrane	106	-0.49	-1.66	0.00057876	0.00355666	0.0021836	2412	tags=30%, list=21%, signal=24%	SCRIB/UTRN/EPHB2/STRN/LRRC4/SSPN/DBNL/GRIK2/LRFN3/SYAP1/RPH3A/DLG4/DBN1/FXYD6/CLSTN1/SLC16A3/CHRNE/EPHA4/SHISA7/CANX/CNIH3/SLC8A1/SLC6A9/DNM3/HIP1/DNM2/LRFN1/ARRB2/NRGN/ITGB3/RNF10/P2RX1
CC	GO:0044304	main axon	30	-0.69	-1.90	0.00058985	0.0036192	0.002222	1727	tags=33%, list=15%, signal=28%	NAV1/DLG4/PARD3/SPTBN1/MAP1A/SCN1B/GJC2/TNFRSF1B/CLDN5/MBP
BP	GO:0099504	synaptic vesicle cycle	100	-0.50	-1.68	0.00074871	0.00442327	0.00271565	2615	tags=37%, list=22%, signal=29%	SNPH/SV2A/P2RY1/BTBD9/KIAA1109/SCRIB/PRKAR1B/VPS18/SNAP25/PTEN/ITSN1/RAB27B/SYNJ2/CTBP2/ACTG1/LRRK2/SYNJ1/SYN3/STX4/PIP5K1C/CANX/SH3GL1/FBXL20/CALM3/DNM3/ACTB/DNAJC5/GSK3B/DNM2/GRIPAP1/PRKCB/SNCA/NAPA/AP2M1/GAK/STON2/P2RX1
BP	GO:0090090	negative regulation of canonical Wnt signaling pathway	77	-0.52	-1.67	0.00075583	0.00445871	0.00273741	2402	tags=36%, list=20%, signal=29%	AMFR/SLC9A3R1/CTNNBIP1/AXIN2/FOXO1/STK4/G3BP1/EGR1/MAPK14/TLE4/MAD2L2/FUZ/CTNND1/INVS/GSK3A/TMEM131L/GREM1/FRMD8/OTUD5/SFRP2/STK11/CHD8/GSK3B/TCF7L2/SIAH2/TLE3/DAB2/FOXO3
BP	GO:0045123	cellular extravasation	53	-0.57	-1.76	0.00079036	0.00463489	0.00284558	2180	tags=43%, list=19%, signal=35%	FADD/TNF/F11R/ADD2/SPN/ADAM8/CD99L2/CD177/EXT1/SIRPA/PLCB1/SELPLG/SELP/BCR/PECAM1/JAM3/PTAFR/PIK3CG/SELL/ITGAL/RIPOR2/PIK3CD/PLVAP
CC	GO:0099240	intrinsic component of synaptic membrane	50	-0.59	-1.80	0.00084688	0.00491559	0.00301791	2599	tags=38%, list=22%, signal=30%	APH1A/NCSTN/P2RY1/EPHB2/ATP2B1/LRRC4/LRFN3/NECTIN1/DLG4/FXYD6/SLC16A3/EPHA4/SHISA7/CANX/SLC8A1/SLC6A9/ATP2B4/LRFN1/P2RX1
BP	GO:0032640	tumor necrosis factor production	125	-0.45	-1.57	0.00085313	0.00493032	0.00302695	2419	tags=34%, list=21%, signal=27%	SPON2/SPN/CYBB/DDX58/EPHB2/MAVS/IRAK3/TLR2/TIRAP/SIRPA/DICER1/LILRA5/BCL3/GSTP1/LRRK2/ARID5A/ZBTB20/RIPK1/PTPRJ/PTPN6/PYCARD/ARHGEF2/FOXP1/VSIR/TSPO/RARA/FRMD8/PTPRC/SYK/THBS1/PTAFR/ILRUN/FZD5/LTF/PF4/CD14/LGALS9/HSPB1/CLU/ARRB2/STAT3/MAPKAPK2

BP	GO:0032680	regulation of tumor necrosis factor production	125	-0.45	-1.57	0.00085313	0.00493032	0.00302695	2419	tags=34%, list=21%, signal=27%	SPON2/SPN/CYBB/DDX58/EPHB2/MAVS/IRAK3/TLR2/TIRAP/SIRPA/DICER1/LILRA5/BCL3/GSTP1/LRRK2/ARID5A/ZBTB20/RIPK1/PTPRJ/PTPN6/PYCARD/ARHGEF2/FOXP1/VSIR/TSPO/RARA/FRMD8/PTPRC/SYK/THBS1/PTAFR/ILRUN/FZD5/LTF/PF4/CD14/LGALS9/HSPB1/CLU/ARRB2/STAT3/MAPKAPK2
MF	GO:0032451	demethylase activity	35	-0.64	-1.81	0.00088751	0.00512156	0.00314437	2712	tags=46%, list=23%, signal=35%	KDM5A/KDM6A/KDM7A/KDM4A/ALKBH5/PHF2/KDM5B/PHF8/FTO/JMJD6/KDM3B/KDM8/KDM2A/KDM6B/KDM4B/JARID2
CC	GO:0042383	sarcolemma	71	-0.53	-1.69	0.00090232	0.00519613	0.00319015	976	tags=23%, list=8%, signal=21%	CORO1C/SLC2A1/SLC8A1/VCL/PPP3CA/SLC9A1/SCN1B/PLEC/ALOX12/AQP1/DYSF/ATP2B4/RTN2/PPP3R1/LAMP1/BSG
BP	GO:0048660	regulation of smooth muscle cell proliferation	86	-0.51	-1.67	0.00091326	0.00523219	0.00321229	2098	tags=31%, list=18%, signal=26%	CTNNBIP1/PTGIR/SOD2/PTEN/XRCC6/TGFB1/ERN1/TRIB1/S1PR2/FOXP1/MAP3K5/TPM1/NDRG2/LDLRAP1/IL6R/MMP9/THBS1/PTAFR/SF1/TCF7L2/GNAI2/RHOA/FOXJ2/GNA12/CDKN1A/MFN2/PRKDC
CC	GO:0099699	integral component of synaptic membrane	45	-0.59	-1.76	0.00095538	0.00539995	0.00331528	2599	tags=40%, list=22%, signal=31%	APH1A/NCSTN/P2RY1/EPHB2/ATP2B1/LRRC4/LRFN3/NECTIN1/FXYD6/SLC16A3/EPHA4/SHISA7/CANX/SLC8A1/SLC6A9/ATP2B4/LRFN1/P2RX1
BP	GO:0016577	histone demethylation	28	-0.67	-1.82	0.00097664	0.00547689	0.00336252	2712	tags=57%, list=23%, signal=44%	KDM5C/KDM5A/KDM6A/KDM7A/KDM4A/UBE2B/PHF2/KDM5B/PHF8/JMJD6/KDM3B/KDM8/KDM2A/KDM6B/KDM4B/JARID2
CC	GO:0005902	microvillus	49	-0.58	-1.75	0.00116603	0.00633354	0.00388846	2503	tags=43%, list=21%, signal=34%	FSCN1/SPN/SLC9A3R1/MYO1G/MYO1E/EXOC4/ATP6V1B2/ATP7A/TGFB1/AOC3/LRRK2/EZR/PDGFA/JAM3/IQGAP2/MYO1F/ATP6V1A/MSN/ESPN/ITGB3/SLC7A5
BP	GO:0042982	amyloid precursor protein metabolic process	60	-0.56	-1.74	0.00122659	0.00663524	0.00407368	1342	tags=27%, list=11%, signal=24%	CLN3/EPHA4/DHCR24/RTN3/GSK3A/AGO2/SORL1/LDLRAP1/LYN/FLOT2/FKBP1A/RTN2/CLU/SP1/TMCC2/RELA
CC	GO:0101003	ficolin-1-rich granule membrane	53	-0.56	-1.73	0.00124359	0.00670896	0.00411894	2651	tags=51%, list=23%, signal=40%	FPR1/LGALS3/SIGLEC14/NCKAP1L/MGAM/ADAM8/FCER1G/CR1/CD93/SIRPA/SLC2A3/AP2A2/LAMP2/NBEAL2/ATP6V0C/SIGLEC5/ITGB2/ATP6V0A1/ARL8A/LAMTOR1/UBR4/GAA/DOK3/RAC1/RHOA/LAMP1/DIAPH1
BP	GO:0030178	negative regulation of Wnt signaling pathway	93	-0.49	-1.63	0.0012909	0.00688943	0.00422974	2402	tags=34%, list=20%, signal=28%	AMFR/SLC9A3R1/CTNNBIP1/AXIN2/FOXO1/STK4/G3BP1/TSC2/RNF213/EGR1/NFATC1/MAPK14/TLE4/MAD2L2/FUZ/CTNND1/INVS/GSK3A/TMEM131L/GREM1/FRMD8/OTUD5/SFRP2/STK11/CHD8/GSK3B/LRP1/TCF7L2/SIAH2/TLE3/DAB2/FOXO3
BP	GO:0006482	protein demethylation	30	-0.67	-1.84	0.00143141	0.00746886	0.00458548	2712	tags=53%, list=23%, signal=41%	KDM5C/KDM5A/KDM6A/KDM7A/KDM4A/UBE2B/PHF2/KDM5B/PHF8/JMJD6/KDM3B/KDM8/KDM2A/KDM6B/KDM4B/JARID2
BP	GO:0008214	protein dealkylation	30	-0.67	-1.84	0.00143141	0.00746886	0.00458548	2712	tags=53%, list=23%, signal=41%	KDM5C/KDM5A/KDM6A/KDM7A/KDM4A/UBE2B/PHF2/KDM5B/PHF8/JMJD6/KDM3B/KDM8/KDM2A/KDM6B/KDM4B/JARID2
CC	GO:0035578	azurophil granule lumen	78	-0.51	-1.65	0.00143028	0.00746886	0.00458548	2137	tags=36%, list=18%, signal=30%	GLB1/PA2G4/PADI2/GUSB/UNC13D/SDCBP/ACLY/PYCARD/TRAPPC1/CAP1/PSMD1/CPPED1/RNASET2/DYNC1H1/IMPDH1/PYGB/IST1/VCP/TUBB4B/CYB5R3/GNS/FTL/ARHGAP45/TUBB/ADA2/GRN/PRDX6/TOLLIP

BP	GO:0048015	phosphatidylinositol-mediated signaling	112	-0.46	-1.58	0.00149912	0.00769096	0.00472184	2910	tags=36%, list=25%, signal=27%	TNF/PLEKHA1/PTPN13/PI4KB/NKX3-1/PIK3CB/PLCE1/KIT/FBXL2/PLCB4/SLC9A3R1/PLCD1/FGR/PTEN/SRC/ERBB2/TSC2/HGF/PLCB1/EZR/ZFP36L1/PTPN6/SEMA4D/NCF1/CSF1R/PIP5K1C/ENTPD5/PDGFA/INSR/PITPNM2/GAB2/SELP/PLCB3/PTAFR/PIK3CG/NF1/HCLS1/PIK3CD/PRR5/CBL
BP	GO:0036465	synaptic vesicle recycling	46	-0.59	-1.75	0.00150507	0.00770161	0.00472838	2447	tags=46%, list=21%, signal=36%	BTBD9/KIAA1109/SCRIB/ITSN1/RAB27B/SYNJ2/ACTG1/LRRK2/SYNJ1/PIP5K1C/CANX/SH3GL1/CALM3/DNM3/ACTB/DNM2/GRIPAP1/SNCA/AP2M1/GAK/STON2
CC	GO:0044291	cell-cell contact zone	45	-0.58	-1.73	0.00162149	0.00823379	0.00505511	1979	tags=38%, list=17%, signal=32%	YWHAH/TIAM1/NECTIN1/FLOT1/PKP4/TJP2/SLC2A1/SLC8A1/PAK1/VCL/CTNNA1/SLC9A1/JAM3/SCN1B/FLOT2/FLCN/ACTN1
BP	GO:0007519	skeletal muscle tissue development	87	-0.48	-1.59	0.0016359	0.00828576	0.00508701	1576	tags=26%, list=13%, signal=23%	EGR1/EPHB1/MAPK14/FLOT1/HIVEP3/NEURL1/SVIL/KLF5/IGF2/PPP3CA/SELENON/PLEC/SKI/NIBAN2/RHOA/GPX1/NF1/BCL9/RIPOR2/YBX3/KEL/XK/HLX
CC	GO:0016529	sarcoplasmic reticulum	37	-0.62	-1.78	0.0016396	0.00828981	0.0050895	1204	tags=27%, list=10%, signal=24%	ITPR2/CHERP/ATP2A3/TMEM38A/THBS1/FKBP1A/RTN2/STIM1/ANK1/IRAG1
BP	GO:0071706	tumor necrosis factor superfamily cytokine production	129	-0.45	-1.57	0.00165367	0.00833332	0.00511621	2419	tags=33%, list=21%, signal=27%	SPON2/SPN/CYBB/DDX58/EPHB2/MAVS/ADAM8/IRAK3/TLR2/TIRAP/SIRPA/DICER1/LILRA5/BCL3/GSTP1/LRRK2/ARID5A/ZBTB20/RIPK1/PTPRJ/PTPN6/PYCARD/ARHGEF2/FOXP1/VSIR/TSPO/RARA/FRMD8/PTPRC/SYK/THBS1/PTAFR/ILRUN/FZD5/LTF/PF4/CD14/LGALS9/HSPB1/CLU/ARRB2/STAT3/MAPKAPK2
BP	GO:1903555	regulation of tumor necrosis factor superfamily cytokine production	129	-0.45	-1.57	0.00165367	0.00833332	0.00511621	2419	tags=33%, list=21%, signal=27%	SPON2/SPN/CYBB/DDX58/EPHB2/MAVS/ADAM8/IRAK3/TLR2/TIRAP/SIRPA/DICER1/LILRA5/BCL3/GSTP1/LRRK2/ARID5A/ZBTB20/RIPK1/PTPRJ/PTPN6/PYCARD/ARHGEF2/FOXP1/VSIR/TSPO/RARA/FRMD8/PTPRC/SYK/THBS1/PTAFR/ILRUN/FZD5/LTF/PF4/CD14/LGALS9/HSPB1/CLU/ARRB2/STAT3/MAPKAPK2
MF	GO:0005085	guanyl-nucleotide exchange factor activity	179	-0.41	-1.49	0.00170709	0.00849484	0.00521538	2110	tags=27%, list=18%, signal=22%	ARHGEF11/PTGIR/ARHGEF3/RGP1/ITSN1/TRIO/RHOF/ARHGEF12/TIAM1/MCF2L2/DOCK5/DENND1C/MYCBP2/SOS2/PREX1/HERC2/ARHGEF2/PCP2/SH3BP5L/PLEKHG3/DOCK8/FGD4/BCR/RAPGEF2/HERC1/RIN1/SBF2/RASGRP4/SH2D3C/SBF1/FGD3/ELMO1/VAV1/LAMTOR1/DOCK2/MADD/GAPVD1/AKAP13/IQSEC1/RIN3/NUCB1/RAPGEF1/FLCN/CYTH4/HPS1/DENND1A/RAB3IL1/TBXA2R
CC	GO:0043204	perikaryon	77	-0.50	-1.61	0.00176786	0.00873787	0.00536459	2673	tags=39%, list=23%, signal=30%	KCNE3/HDAC6/AMIGO1/SEPTIN4/ENDOG/PI4K2A/DIP2B/GIGYF2/ENO2/DMWD/TOP1/DBNL/GRIK2/ATP7A/SLC2A3/G3BP1/CPNE5/SYAP1/LRRK2/EPHA4/PPP1CA/MCRS1/NEURL1/SCN1B/FZD5/FLNA/GJC2/ROGDI/WDFY3/GLUL
BP	GO:0071621	granulocyte chemotaxis	76	-0.50	-1.61	0.0017957	0.00882535	0.0054183	1573	tags=36%, list=13%, signal=31%	CMKLR1/PIKFYVE/C5AR1/TREM1/PREX1/ITGB2/CXCL5/CSF1R/PIP5K1C/PF4V1/CXCR2/VAV1/SYK/JAM3/THBS1/IL17RA/PIK3CG/CAMK1D/PF4/RAC1/RAC2/CXCR1/MAPK3/RIPOR2/PIK3CD/BSG/MPP1

CC	GO:0098552	side of membrane	385	-0.35	-1.37	0.00184268	0.00903389	0.00554633	1671	tags=25%, list=14%, signal=23%	TF/TAPBP/CLPTM1/IL2RG/TNK2/MICB/FCER1G/ATP2B1/GNA15/HEG1/SNAP25/FGR/FYN/PTEN/SRC/SPPL3/THBD/HCK/TIAM1/NCAM1/SYAP1/ITGAM/LRRK2/SAMD12/EZR/LY6G5C/RASAL3/DLG4/TGM3/CHMP4B/OSBPL2/MAP2K2/FLOT1/FCGRT/GNAQ/PKP4/ITGB2/CCR1/CDIP1/KLRD1/LEPR/ABCG2/CANX/ANPEP/CA4/ERMAP/INSR/AMOT/MAP3K5/FES/MYZAP/BCAP31/PTK2B/SPTA1/SELP/CXCR2/TRAF6/LDLRAP1/IL6R/IL13RA1/PPP3CA/ITGA5/PTPRC/SYK/THBS1/PTPN1/GNB1/ATP1B2/B4GALT1/G6PD/ABCC4/CD14/RAC1/SLA/LYN/HIP1/ITGA2B/GNAI2/FKBP1A/MPL/RHOA/ITGAL/CXCR1/PPP1R9B/GNA12/ECE1/SPTB/LAMP1/SLC4A1/GP1BA/LITAF/CTSB/GNAZ/SLC7A5/P2RX1/MYH9/IQGAP1/ANK1
BP	GO:0007033	vacuole organization	164	-0.42	-1.49	0.0020029	0.00958284	0.00588335	2564	tags=31%, list=22%, signal=25%	AMBRA1/VPS4A/FIG4/ANXA2/RALB/CORO1A/MAP1LC3B2/RAB43/ACP3/ATG2B/VPS18/FEZ1/SH3GLB1/HOOK2/EXT1/ACP2/LYST/LRRK2/TFEB/PIKFYVE/PIP4K2C/CHMP4B/PHF23/HOOK3/CLN3/VPS35/MAP1LC3B/MTMR3/VPS11/TPP1/NSFL1C/RAB7A/RAB14/LAMTOR1/WDR45/ATG4D/GAA/ATG7/WIPI2/ULK1/GABARAP/LAPTM5/ELAPOR1/ATG2A/GRN/ATG13/PINK1/PIP4K2A/HPS1/MFN2/RAB1B
MF	GO:0032452	histone demethylase activity	27	-0.67	-1.79	0.00200526	0.00958284	0.00588335	2712	tags=56%, list=23%, signal=43%	KDM5C/KDM5A/KDM6A/KDM7A/KDM4A/PHF2/KDM5B/PHF8/JMJD6/KDM3B/KDM8/KDM2A/KDM6B/KDM4B/JARID2
MF	GO:0140457	protein demethylase activity	27	-0.67	-1.79	0.00200526	0.00958284	0.00588335	2712	tags=56%, list=23%, signal=43%	KDM5C/KDM5A/KDM6A/KDM7A/KDM4A/PHF2/KDM5B/PHF8/JMJD6/KDM3B/KDM8/KDM2A/KDM6B/KDM4B/JARID2
BP	GO:0048488	synaptic vesicle endocytosis	39	-0.60	-1.75	0.00210036	0.00997734	0.00612556	2447	tags=51%, list=21%, signal=41%	BTBD9/KIAA1109/SCRIB/ITSN1/RAB27B/SYNJ2/ACTG1/LRRK2/SYNJ1/PIP5K1C/CANX/SH3GL1/CALM3/DNM3/ACTB/DNM2/SNCA/AP2M1/GAK/STON2
BP	GO:0140238	presynaptic endocytosis	39	-0.60	-1.75	0.00210036	0.00997734	0.00612556	2447	tags=51%, list=21%, signal=41%	BTBD9/KIAA1109/SCRIB/ITSN1/RAB27B/SYNJ2/ACTG1/LRRK2/SYNJ1/PIP5K1C/CANX/SH3GL1/CALM3/DNM3/ACTB/DNM2/SNCA/AP2M1/GAK/STON2
BP	GO:0016051	carbohydrate biosynthetic process	130	-0.44	-1.52	0.00216073	0.01017897	0.00624935	1972	tags=29%, list=17%, signal=25%	IRS2/CHST15/EXT1/TGFB1/PGD/B4GALT5/ST3GAL2/ST3GAL3/PER2/B3GNT7/LEPR/GSK3A/PPP1CA/DGAT2/PDK2/INSR/SESN2/FBP1/NFKB1/AP2A1/B3GNT8/PTK2B/INPP5K/CHST11/IGF2/STK11/PTAFR/CLTC/B4GALT1/G6PD/NDST1/GPI/PLEK/GSK3B/DDB1/SNCA/AKT2/USP7
BP	GO:0043299	leukocyte degranulation	65	-0.53	-1.67	0.00220475	0.01034957	0.00635408	2051	tags=40%, list=17%, signal=33%	FGR/UNC13D/NKG7/CD177/AP1G1/HCK/ADGRE2/ITGAM/STX4/SPI1/PDPK1/ITGB2/FES/GAB2/BCR/SYK/PTAFR/PIK3CG/PRAM1/LYN/LGALS9/RAC2/GATA1/LAMP1/PIK3CD/CBL
BP	GO:0007272	ensheathment of neurons	92	-0.48	-1.59	0.00229022	0.01070025	0.00656939	2596	tags=37%, list=22%, signal=29%	ABCA2/TSPAN2/ARHGEF10/TNF/ERCC2/EPB41L3/NCSTN/AMIGO1/FIG4/SERINC5/TLR2/ORMDL3/PTEN/ERBB2/DICER1/HGF/B4GALT5/PIKFYVE/WASF3/PARD3/RARA/SBF2/JAM3/PLEC/SKI/TNFRSF1B/CLU/NF1/CLDN5/AKT2/MBP/RNF10/KEL/XK
BP	GO:0008366	axon ensheathment	92	-0.48	-1.59	0.00229022	0.01070025	0.00656939	2596	tags=37%, list=22%, signal=29%	ABCA2/TSPAN2/ARHGEF10/TNF/ERCC2/EPB41L3/NCSTN/AMIGO1/FIG4/SERINC5/TLR2/ORMDL3/PTEN/ERBB2/DICER1/HGF/B4GALT5/PIKFYVE/WASF3/PARD3/RARA/SBF2/JAM3/PLEC/SKI/TNFRSF1B/CLU/NF1/CLDN5/AKT2/MBP/RNF10/KEL/XK
BP	GO:0106027	neuron projection organization	60	-0.55	-1.70	0.00228534	0.01070025	0.00656939	2573	tags=40%, list=22%, signal=31%	STAU2/HDAC6/ACTR2/EPHB2/FYN/PTEN/DBNL/LRRK2/DLG4/DBN1/EPHB1/SIPA1L1/ABI3/VPS35/EPHA4/ARF1/INSR/MAP1A/PDLIM5/DCTN1/DNM3/GSK3B/CFL1/CTTN
BP	GO:0001885	endothelial cell development	46	-0.58	-1.72	0.00229901	0.01072871	0.00658686	1854	tags=39%, list=16%, signal=33%	HEG1/VEZF1/PDE2A/ROCK2/PLCB1/EZR/S1PR2/TJP2/RAPGEF2/VCL/PECAM1/MYADM/GPX1/FASN/MSN/ADD1/RAPGEF1/CLDN5

BP	GO:0120192	tight junction assembly	38	-0.61	-1.76	0.00239822	0.01112641	0.00683103	2999	tags=50%, list=26%, signal=37%	IKBKB/TNF/AFDN/F11R/TBCD/EPHB2/STRN/SRF/ACTG1/ROCK2/PARD3/GPBAR1/PRKACA/OCEL1/PECAM1/ESAM/FZD5/CLDN5/PAK2
BP	GO:1903557	positive regulation of tumor necrosis factor superfamily cytokine production	81	-0.48	-1.56	0.00242456	0.01122241	0.00688996	2419	tags=37%, list=21%, signal=30%	SPON2/SPN/CYBB/DDX58/EPHB2/MAVS/ADAM8/TLR2/TIRAP/LILRA5/LRRK2/ARID5A/ZBTB20/RIPK1/PTPRJ/PYCARD/ARHGEF2/FRMD8/PTPRC/SYK/THBS1/PTAFR/FZD5/PF4/CD14/LGALS9/HSPB1/CLU/STAT3/MAPKAPK2
BP	GO:0018958	phenol-containing compound metabolic process	44	-0.58	-1.72	0.00249154	0.0114923	0.00705566	1966	tags=34%, list=17%, signal=28%	SULT1A3/ABAT/ATP7A/PNKD/ITGAM/FAH/CPQ/VPS35/ITGB2/SULT1B1/RAPGEF2/MED1/SNCA/MTPN/CTSB
BP	GO:0042552	myelination	91	-0.48	-1.59	0.00248865	0.0114923	0.00705566	2596	tags=37%, list=22%, signal=29%	ABCA2/TSPAN2/ARHGEF10/TNF/ERCC2/EPB41L3/NCSTN/AMIGO1/FIG4/SERINC5/TLR2/ORMDL3/PTEN/ERBB2/DICER1/HGF/B4GALT5/PIKFYVE/WASF3/PARD3/RARA/SBF2/JAM3/PLEC/SKI/TNFRSF1B/CLU/NF1/CLDN5/AKT2/MBP/RNF10/KEL/XK
BP	GO:0032526	response to retinoic acid	55	-0.54	-1.64	0.00266653	0.01215839	0.0074646	2541	tags=40%, list=22%, signal=31%	TWF2/CREB1/CD38/SLC10A3/PTK7/ASXL1/CDKN2D/MICB/RXRB/ATM/SYNJ1/SETX/PDGFA/RARA/PTK2B/BMP6/RXRA/AQP1/LYN/GSK3B/IGF2R/TESC
BP	GO:0044706	multi-multicellular organism process	106	-0.46	-1.54	0.00285751	0.01285232	0.00789064	1967	tags=26%, list=17%, signal=22%	KPNA6/H3-3B/THBD/VDR/H3-3A/ATP7A/ASH1L/STAT5B/ARHGDIB/TRIM28/AGO2/YTHDF3/RARA/SLC2A1/PITHD1/MMP9/ITGA5/PTAFR/MED1/B4GALT1/UCP2/LGALS9/EPN1/BSG/CTSB/KLF1/P2RX1/TIMP1
BP	GO:0051147	regulation of muscle cell differentiation	81	-0.48	-1.55	0.00292742	0.01310747	0.00804729	2903	tags=40%, list=25%, signal=30%	MECP2/MDM2/NOTCH1/KIT/SMAD1/ATP11A/CCN3/MAML1/SOD2/SRF/ANKRD17/HDAC4/TGFB1/CTDP1/NFATC1/MAPK14/YY1/MSX1/PTBP1/GSK3A/GREM1/NFATC2/YBX1/SOX6/IGF2/FOXO4/SUPT6H/G6PD/NIBAN2/NFATC3/ARRB2/RBM38
CC	GO:0035580	specific granule lumen	54	-0.54	-1.66	0.00295486	0.01321545	0.00811358	1755	tags=33%, list=15%, signal=28%	PDXK/SPTAN1/PTPN6/KPNB1/QSOX1/NFKB1/OSCAR/TIMP2/PGLYRP1/VCL/LCN2/DOCK2/LTF/CNN2/LRG1/CANT1/CTSD/TOLLIP
BP	GO:0030593	neutrophil chemotaxis	64	-0.53	-1.66	0.00297494	0.01329029	0.00815953	1537	tags=34%, list=13%, signal=30%	PIKFYVE/C5AR1/TREM1/PREX1/ITGB2/CXCL5/PIP5K1C/PF4V1/CXCR2/VAV1/SYK/JAM3/PIK3CG/CAMK1D/PF4/RAC1/RAC2/CXCR1/RIPOR2/PIK3CD/BSG/MPP1
BP	GO:0120193	tight junction organization	41	-0.59	-1.73	0.00298663	0.01332754	0.0081824	2999	tags=51%, list=26%, signal=38%	IKBKB/TNF/AFDN/F11R/TBCD/EPHB2/STRN/SRF/EXT1/ACTG1/ROCK2/PARD3/GPBAR1/PRKACA/OCEL1/PECAM1/PLEC/ESAM/FZD5/CLDN5/PAK2
CC	GO:0098889	intrinsic component of presynaptic membrane	25	-0.68	-1.78	0.00299342	0.01334289	0.00819182	2599	tags=52%, list=22%, signal=41%	APH1A/NCSTN/P2RY1/EPHB2/ATP2B1/LRFN3/NECTIN1/FXYD6/EPHA4/CANX/SLC6A9/ATP2B4/P2RX1
BP	GO:0034113	heterotypic cell-cell adhesion	39	-0.59	-1.72	0.00305345	0.01356481	0.00832807	1942	tags=33%, list=17%, signal=28%	NINJ1/MAP2K5/SIRPA/FLOT1/ITGB2/ITGA5/PTPRC/JAM3/IL1RN/MYADM/ITGB3/MBP/WNK1

BP	GO:0060538	skeletal muscle organ development	93	-0.47	-1.57	0.00322995	0.01430096	0.00878003	1576	tags=26%, list=13%, signal=23%	EGR1/EPHB1/MAPK14/FLOT1/HIVEP3/NEURL1/SVIL/KLF5/IGF2/PPP3CA/SELENON/PLEC/BASP1/SKI/NIBAN2/RHOA/GPX1/NF1/BCL9/RIPOR2/YBX3/KEL/XK/HLX
BP	GO:0010507	negative regulation of autophagy	68	-0.51	-1.63	0.00325489	0.01439536	0.00883799	2169	tags=34%, list=18%, signal=28%	KLHL22/FEZ1/USP36/TSC2/CPTP/LRRK2/HGF/CHMP4B/PHF23/DAP/QSOX1/CLEC16A/LEPR/TSPO/HERC1/EIF4G2/ATG7/EIF4G1/RUBCN/GOLGA2/PINK1/SNCA/STAT3
BP	GO:0032760	positive regulation of tumor necrosis factor production	78	-0.49	-1.58	0.00337463	0.01485885	0.00912254	2419	tags=37%, list=21%, signal=30%	SPON2/SPN/CYBB/DDX58/EPHB2/MAVS/TLR2/TIRAP/LILRA5/LRRK2/ARID5A/ZBTB20/RIPK1/PTPRJ/PYCARD/ARHGEF2/FRMD8/PTPRC/SYK/THBS1/PTAFR/FZD5/PF4/CD14/LGALS9/HSPB1/CLU/STAT3/MAPKAPK2
CC	GO:0098936	intrinsic component of postsynaptic membrane	36	-0.61	-1.75	0.0036261	0.01577394	0.00968436	2232	tags=36%, list=19%, signal=29%	EPHB2/LRRC4/LRFN3/DLG4/FXYD6/SLC16A3/EPHA4/SHISA7/CANX/SLC8A1/SLC6A9/LRFN1/P2RX1
BP	GO:0060996	dendritic spine development	64	-0.52	-1.65	0.00366007	0.01588694	0.00975373	2573	tags=34%, list=22%, signal=27%	STAU2/HDAC6/ACTR2/EPHB2/DISC1/PTEN/DBNL/LRRK2/DLG4/DBN1/EPHB1/SIPA1L1/ABI3/EPHA4/NEURL1/EIF4G2/PDLIM5/ASAP1/DNM3/IQSEC1/CFL1/PAK2
CC	GO:0044306	neuron projection terminus	55	-0.53	-1.61	0.00369635	0.01597535	0.00980802	1973	tags=29%, list=17%, signal=24%	ELK1/GRIK2/ACTG1/LRRK2/SYNJ1/DLG4/MME/EPHA4/SLC8A1/ACTB/TPRG1L/PRKCB/SNCA/ATP6V0D1/RNF40/GLUL
BP	GO:1902991	regulation of amyloid precursor protein catabolic process	33	-0.62	-1.74	0.00369651	0.01597535	0.00980802	1293	tags=36%, list=11%, signal=32%	ROCK2/EPHA4/RTN3/GSK3A/SORL1/LYN/FLOT2/FKBP1A/RTN2/CLU/SP1/RELA
CC	GO:0030118	clathrin coat	32	-0.62	-1.72	0.00379578	0.01638657	0.01006048	1861	tags=47%, list=16%, signal=40%	CLTB/AP1G1/AP2A2/AFTPH/AP2B1/SYNJ1/AP2S1/SYNRG/KCNQ5/AP2A1/CLTC/IGF2R/EPN1/EPS15L1/AP2M1
CC	GO:0070820	tertiary granule	142	-0.42	-1.48	0.00382279	0.0164674	0.01011011	2387	tags=36%, list=20%, signal=29%	SIGLEC14/CYBB/NCKAP1L/MGAM/ADAM8/FCER1G/CR1/SNAP25/CYSTM1/CD93/CD177/DBNL/SIRPA/SLC2A3/AP2A2/LAMP2/ANO6/TSPAN14/ITGAM/NBEAL2/SPTAN1/PTPN6/ATP6V0C/QSOX1/SIGLEC5/ITGB2/ATP6V0A1/OSCAR/TIMP2/PGLYRP1/RAB14/HGSNAT/ARL8A/MMP9/ALDOA/LAMTOR1/UBR4/PTAFR/GAA/LTF/DOK3/RAC1/CDA/CNN2/RHOA/LRG1/CANT1/FTH1/LAMP1/CTSD/DIAPH1
BP	GO:0032006	regulation of TOR signaling	87	-0.47	-1.52	0.00392265	0.01687927	0.01036297	2439	tags=36%, list=21%, signal=28%	GPR137/RFFL/ENDOG/NCKAP1L/DYRK3/HDAC3/KLHL22/RRAGD/ATM/AKT1S1/LARS1/SZT2/TSC2/WAC/CLEC16A/GSK3A/SESN3/SESN2/RRAGC/SIK3/OTUD5/LAMTOR1/STK11/UBR2/FBXO9/CASTOR2/GNA12/FLCN/ARAF/TMEM127/NPRL3
BP	GO:0030316	osteoclast differentiation	68	-0.51	-1.61	0.00396808	0.01696462	0.01041538	3269	tags=53%, list=28%, signal=38%	SH3PXD2A/PRXL2A/UBASH3B/JUNB/CEBPB/TLR4/FARP2/TNF/RASSF2/FOS/CREB1/ANXA2/GPR137/IREB2/TF/SRC/PPARGC1B/NOTCH2/MAPK14/SBNO2/LRRK1/CCR1/CSF1R/ATP6AP1/FOXP1/OSCAR/GAB2/SNX10/TRAF6/TOB2/TFE3/LILRB3/MAFB/LTF/INPP5D/NF1

BP	GO:0035249	synaptic transmission, glutamatergic	40	-0.57	-1.65	0.00396808	0.01696462	0.01041538	2065	tags=32%, list=18%, signal=27%	DISC1/GRIK2/EXT1/DGKZ/LRRK2/CLN3/PTK2B/TPRG1L/TSHZ3/NF1/NAPA/P2RX1/GLUL
BP	GO:1903305	regulation of regulated secretory pathway	90	-0.48	-1.58	0.00404087	0.01722024	0.01057231	2141	tags=33%, list=18%, signal=27%	VPS18/FGR/UNC13D/CD177/AP1G1/ADGRE2/ITGAM/LRRK2/STX4/SPI1/PDPK1/ITGB2/FES/GAB2/BCR/SYK/PTAFR/FBXL20/CALM3/PRAM1/LYN/LGALS9/GSK3B/RAC2/GNAI2/PRKCB/GATA1/LAMP1/RAB3D/P2RX1
BP	GO:0007584	response to nutrient	88	-0.48	-1.58	0.0041268	0.01753009	0.01076254	2156	tags=32%, list=18%, signal=26%	CDKN2D/ATP2B1/SOD2/SRF/APAF1/FKBP1B/MTHFR/VDR/MGMT/ALAD/F5/PDK2/ALPL/FES/KANK2/TSPO/RARA/SLC8A1/USF2/POR/SFRP2/MED1/RXRA/GNAI2/GPX1/PIM1/NCOA1/TBXA2R
BP	GO:0061028	establishment of endothelial barrier	37	-0.59	-1.71	0.00426531	0.01800304	0.01105291	1768	tags=41%, list=15%, signal=35%	PDE2A/ROCK2/PLCB1/EZR/S1PR2/TJP2/RAPGEF2/VCL/PECAM1/MYADM/FASN/MSN/ADD1/RAPGEF1/CLDN5
CC	GO:0042734	presynaptic membrane	62	-0.52	-1.63	0.00427332	0.01801771	0.01106192	2735	tags=44%, list=23%, signal=34%	APBA1/APH1A/NCSTN/P2RY1/SCRIB/PI4K2A/EPHB2/CLTB/ATP2B1/SNAP25/ITSN1/GRIK2/LRFN3/PDE2A/NECTIN1/SYAP1/STX4/FXYD6/EXOC3/EPHA4/CANX/ADGRL1/SLC6A9/HIP1/ATP2B4/DENND1A/P2RX1
BP	GO:0016311	dephosphorylation	305	-0.35	-1.35	0.00428421	0.01804449	0.01107836	2038	tags=24%, list=17%, signal=20%	PTEN/PPM1F/SRC/PPP4R1/FKBP1B/PNKP/PPP1R15A/SPPL3/SMG6/ACP2/TGFB1/PPARGC1B/SYNJ2/CTDP1/ROCK2/LRRK2/SYNJ1/PHLPP1/EYA3/PPM1M/MGAT5/CTDSP2/PTPRJ/PTPN6/SEMA4D/PPM1L/LRRK1/PTPN18/PTPN12/PTBP1/SSH1/CHP1/PPP1CA/CPPED1/MTMR3/ALPL/PPP2R2D/FBP1/YWHAB/PFKFB3/HTT/MFHAS1/VRK3/INPP5K/SBF1/STK11/PPP3CA/PTPRC/PTPN1/PFKFB4/PHOSPHO1/CTDSPL/CALM3/PPP2R5B/LPIN2/INPP5D/PLEK/NT5M/GSK3B/GNAI2/CTDSP1/FKBP1A/PPP1R9B/SSH2/GNA12/SMG7/PINK1/PTPN23/PPP3R1/PTP4A3/PPM1A/CTDNEP1/IQGAP1
BP	GO:0031347	regulation of defense response	441	-0.33	-1.31	0.00433781	0.01821237	0.01118143	2258	tags=26%, list=19%, signal=22%	NFE2L2/USP27X/TRIM5/MUL1/IFI16/EPHB2/RNF135/MAVS/ADAM8/TRIM38/MICB/IRAK3/TLR2/SERPING1/CR1/FGR/FYN/TIRAP/NLRP3/SRC/NKG7/ATM/NINJ1/XIAP/XRCC6/AP1G1/HCK/SIRPA/AREL1/PDE2A/TNFSF4/LILRA5/LSM14A/GSTP1/LRRK2/IKBKE/HGF/GPS2/ASH1L/METRNL/STAT5B/RIPK1/MAPK14/CEBPA/PTPN6/PYCARD/NR1H2/SBNO2/SPATA2/VPS35/SPI1/ITCH/PTGES/NCF1/AKIRIN2/CCDC92/KLRD1/ALOX5AP/FOXP1/OPTN/NLRC4/YTHDF3/NFKB1/IRF1/PGLYRP1/MFHAS1/BCR/TRAF6/LPCAT3/PAK1/MMP9/VAV1/AKNA/PTPRC/SYK/ADAR/PTPN1/ILRUN/NPLOC4/MED1/MGLL/IL17RA/NLRX1/RBM14/PIK3CG/ZYX/LYN/RIOK3/LGALS9/SIN3A/CREBBP/APOBEC3F/CDK19/TNFRSF1B/TNIP1/GPX1/ARRB2/GRN/IFIT1B/ZNFX1/SEC14L1/MAPK3/SNCA/DTX3L/PCBP2/LAMP1/N4BP1/PRKDC/BRD4/PAK2/SHARPIN/KRT1/FURIN/RELA
BP	GO:0072666	establishment of protein localization to vacuole	42	-0.56	-1.64	0.00435922	0.01824449	0.01120115	1745	tags=36%, list=15%, signal=31%	LAMP2/VPS13D/HGS/SORT1/VPS41/MON1B/VPS37B/VPS37C/NCOA4/SORL1/RAB7A/VPS28/LAPTM5/CLU/PTPN23
MF	GO:0015291	secondary active transmembrane transporter activity	127	-0.43	-1.49	0.00443187	0.01836542	0.01127539	3273	tags=35%, list=28%, signal=25%	MFSD12/CLCN7/SLC4A7/SLC26A8/SLC9A6/SLC25A1/SLC22A18/MFSD3/SLC35C1/SLC5A6/SLC35E1/SLC12A7/SLC37A2/SLC13A4/SLCO3A1/SLC1A4/SLC19A1/SLC10A3/TMEM241/SLC20A1/LETM1/SLC28A2/SLC24A4/SLC12A6/MFSD4B/CLCN5/SLC35E2B/SLC16A3/SLC36A1/SLC45A4/SLC25A11/SLC8A1/ANKH/SLC6A20/SLC24A3/SLC3A2/SLC9A1/SLC6A9/SLC1A5/SLC15A3/SLC4A1/MFSD2B/SLC7A5/SLC6A8

BP	GO:0051591	response to cAMP	48	-0.55	-1.66	0.00442583	0.01836542	0.01127539	1993	tags=44%, list=17%, signal=36%	PER1/THBD/PPARGC1B/PDE2A/HSPA5/EZR/ZFP36L1/AQP9/ITPR2/PTK2B/SLC8A1/INPP5K/RAPGEF2/PTAFR/PIK3CG/AQP1/GATA1/RAPGEF1/BSG/CARM1/DMTN
BP	GO:0070830	bicellular tight junction assembly	34	-0.62	-1.73	0.00440897	0.01836542	0.01127539	2999	tags=50%, list=26%, signal=37%	IKBKB/TNF/AFDN/F11R/TBCD/STRN/SRF/ROCK2/PARD3/GPBAR1/PRKACA/OCEL1/PECAM1/ESAM/FZD5/CLDN5/PAK2
BP	GO:0043507	positive regulation of JUN kinase activity	33	-0.62	-1.73	0.0045414	0.01871152	0.01148788	2329	tags=55%, list=20%, signal=44%	MAP4K2/ARHGEF5/MAP3K4/ERN2/ERN1/EPHA4/SASH1/MAP3K5/TAOK3/PTK2B/TRAF6/PAK1/MAP2K7/SYK/DVL3/PTPN1/TLR6/FZD5
BP	GO:0006470	protein dephosphorylation	217	-0.37	-1.37	0.00461181	0.01894273	0.01162983	2391	tags=28%, list=20%, signal=22%	CALM1/DUSP16/NCKAP1L/CRY2/PPP2R5D/DUSP1/ACP3/PPP6R1/PTEN/PPM1F/PPP4R1/FKBP1B/PPP1R15A/SPPL3/TGFB1/CTDP1/ROCK2/LRRK2/PHLPP1/EYA3/PPM1M/MGAT5/CTDSP2/PTPRJ/PTPN6/PPM1L/LRRK1/PTPN18/PTPN12/PTBP1/SSH1/PPP1CA/CPPED1/MTMR3/PPP2R2D/YWHAB/HTT/MFHAS1/VRK3/SBF1/STK11/PPP3CA/PTPRC/PTPN1/CTDSPL/CALM3/PPP2R5B/GSK3B/GNAI2/CTDSP1/FKBP1A/PPP1R9B/SSH2/GNA12/PINK1/PTPN23/PPP3R1/PTP4A3/PPM1A/CTDNEP1
BP	GO:0045601	regulation of endothelial cell differentiation	27	-0.65	-1.73	0.00468388	0.01915957	0.01176296	3110	tags=59%, list=27%, signal=44%	TNFRSF1A/IKBKB/TNF/NOTCH1/F11R/ATOH8/VEZF1/ROCK2/BTG1/PLCB1/S1PR2/VCL/BMP6/FOXJ2/ADD1/CLDN5
BP	GO:1901222	regulation of NIK/NF-kappaB signaling	80	-0.49	-1.58	0.00470119	0.0192106	0.01179429	3307	tags=51%, list=28%, signal=37%	EP300/NOL3/IRAK1/NFKBIA/TLR4/SPHK1/NLRP12/TLR7/TNF/TNFSF14/AKIP1/RASSF2/CCN3/RC3H1/CALR/TERF2IP/HDAC7/LIMS1/TLR2/NLRP3/RC3H2/BCL3/NFAT5/CPNE1/EIF2AK2/ADGRG3/SPI1/SASH1/GREM1/TRAF6/ADIPOR1/CD14/LGALS9/RBCK1/LAPTM5/RHOA/ACTN4/LITAF/PTP4A3/PPM1A/RELA
BP	GO:0006816	calcium ion transport	231	-0.37	-1.39	0.0048507	0.01970009	0.01209481	1421	tags=19%, list=12%, signal=17%	PTPN6/TMBIM6/PDPK1/PML/MICU1/CCR1/ITPR2/PRKACA/CHERP/ATP2A3/ERO1A/TSPO/TMEM38A/HTT/PTK2B/SLC8A1/INPP5K/SLC24A3/SLC3A2/PPP3CA/PTPRC/SLC9A1/SELENON/EHD3/CALM3/PIK3CG/G6PD/JAK3/ANXA6/GJC2/LYN/GP9/ATP2B4/GNAI2/FKBP1A/RHOA/PRKCB/ARRB2/MYLK/SNCA/EPB41/GP1BA/STIM1/P2RX1/DIAPH1
BP	GO:0046942	carboxylic acid transport	138	-0.42	-1.49	0.00513602	0.02060626	0.01265115	1423	tags=20%, list=12%, signal=17%	PER2/SLC16A3/SLC36A1/AQP9/SLC25A11/CLN3/PTGES/TSPO2/ABCG2/SLC27A1/LRRC8A/SLC6A20/SLC3A2/SLC25A38/SYK/SLC6A9/SLC1A5/ABCC4/SLC43A2/PSAP/SLC43A1/ABCC3/NF1/SNCA/SLC7A5/SLC38A5/SLC6A8
BP	GO:0051148	negative regulation of muscle cell differentiation	39	-0.58	-1.68	0.00521473	0.02087993	0.01281917	1963	tags=49%, list=17%, signal=41%	MECP2/NOTCH1/SMAD1/CCN3/ANKRD17/HDAC4/CTDP1/NFATC1/YY1/MSX1/PTBP1/GSK3A/NFATC2/YBX1/SOX6/IGF2/FOXO4/G6PD/NFATC3
BP	GO:0045834	positive regulation of lipid metabolic process	87	-0.46	-1.50	0.0052655	0.02106202	0.01293096	2051	tags=31%, list=17%, signal=26%	AMBRA1/CREB1/GPLD1/SCARB1/FGR/SRC/IRS2/PRKCE/TGFB1/DGKZ/SCAP/ZBTB20/NR1H2/ABHD5/CHP1/DGAT2/SLC27A1/PTK2B/LDLRAP1/BMP6/POR/CPT1A/LYN/PNPLA2/AKT2/DAB2/CTDNEP1

BP	GO:0120162	positive regulation of cold-induced thermogenesis	63	-0.51	-1.60	0.00532699	0.02122254	0.01302951	2490	tags=33%, list=21%, signal=26%	EHMT1/SIRT6/HDAC3/THRA/ZNF516/PPARGC1B/CMKLR1/PER2/ALMS1/LEPR/ADIPOR2/ALPL/STAT6/BSCL2/DYNC1H1/LCN2/SYK/ADIPOR1/UCP2/KDM6B/MFN2
BP	GO:0060349	bone morphogenesis	47	-0.56	-1.67	0.0053863	0.02141593	0.01314824	1868	tags=30%, list=16%, signal=25%	EXT1/CHSY1/MSX1/ALPL/FOXN3/RARA/BMP6/POR/SFRP2/GLG1/PHOSPHO1/LTF/SKI/ANXA6
BP	GO:0030500	regulation of bone mineralization	40	-0.56	-1.62	0.00539799	0.02141957	0.01315048	2404	tags=42%, list=20%, signal=34%	ALOX5/TENT5A/ATP2B1/TGFB1/ANO6/NBR1/CCR1/GREM1/PTK2B/SLC8A1/ANKH/BMP6/BMP2K/PHOSPHO1/LTF/RFLNB/GATA1
BP	GO:0032615	interleukin-12 production	50	-0.55	-1.66	0.00549344	0.02175488	0.01335634	2148	tags=40%, list=18%, signal=33%	IRAK3/TLR2/TIRAP/TNFSF4/LILRA5/PLCB1/CMKLR1/MAPK14/MAST2/FOXP1/NFKB1/IRF1/TRAF6/SYK/THBS1/JAK3/LGALS9/LAPTM5/ARRB2/RELA
BP	GO:0032655	regulation of interleukin-12 production	50	-0.55	-1.66	0.00549344	0.02175488	0.01335634	2148	tags=40%, list=18%, signal=33%	IRAK3/TLR2/TIRAP/TNFSF4/LILRA5/PLCB1/CMKLR1/MAPK14/MAST2/FOXP1/NFKB1/IRF1/TRAF6/SYK/THBS1/JAK3/LGALS9/LAPTM5/ARRB2/RELA
CC	GO:0099055	integral component of postsynaptic membrane	33	-0.61	-1.71	0.00552712	0.02184474	0.01341151	2232	tags=36%, list=19%, signal=30%	EPHB2/LRRC4/LRFN3/FXYD6/SLC16A3/EPHA4/SHISA7/CANX/SLC8A1/SLC6A9/LRFN1/P2RX1
BP	GO:0006066	alcohol metabolic process	221	-0.37	-1.39	0.00554821	0.02190634	0.01344933	2092	tags=24%, list=18%, signal=20%	RETSAT/ACSS1/PTEN/SULT1A3/GALK1/STARD3/ALDH3B1/ACSS2/OSBPL1A/ITPKB/SYNJ2/SCAP/ACLY/PLCB1/SYNJ1/SNX17/LIPE/SPTLC2/CEBPA/OSBPL5/DHCR24/ACADVL/TKTL1/SULT1B1/LEPR/DGAT2/RDH11/DHRS13/NFKB1/VLDLR/NUDT4/PTK2B/APOBR/INPP5K/LDLRAP1/CYP27A1/LPCAT3/BMP6/POR/PTAFR/G6PD/GDE1/PLEK/RXRA/FDFT1/CYB5R3/PNPLA2/ITPK1/SNCA/IP6K1/ACO2/HDLBP/DPM2/DAB2
BP	GO:0042987	amyloid precursor protein catabolic process	46	-0.56	-1.67	0.00559349	0.02201954	0.01351882	1342	tags=28%, list=11%, signal=25%	CLN3/EPHA4/DHCR24/RTN3/GSK3A/SORL1/LYN/FLOT2/FKBP1A/RTN2/CLU/SP1/RELA
BP	GO:0034219	carbohydrate transmembrane transport	77	-0.48	-1.53	0.00563423	0.02215796	0.01360381	1972	tags=26%, list=17%, signal=22%	IRS2/ZDHHC7/SLC2A3/ABCB11/MFSD4B/MAPK14/SORT1/AQP9/TRIB3/GSK3A/INSR/SESN2/SLC27A1/SLC2A1/INPP5K/AQP1/PRKCB/RTN2/AKT2/AQP10
CC	GO:0005776	autophagosome	98	-0.45	-1.51	0.00570038	0.02232975	0.01370928	1795	tags=28%, list=15%, signal=24%	WDR81/LAMP2/LRRK2/NBR1/NRBF2/TBC1D17/PIP4K2C/CHMP4B/CLN3/MAP1LC3B/OPTN/ZFYVE1/VPS11/NCOA4/HTT/RAB7A/ULK1/CHMP1A/GABARAP/TBC1D25/FTL/WDFY3/FTH1/PIP4K2A/LAMP1/SQSTM1/TBC1D5
BP	GO:0097061	dendritic spine organization	53	-0.52	-1.60	0.00590363	0.02308046	0.01417017	2573	tags=40%, list=22%, signal=31%	STAU2/HDAC6/ACTR2/EPHB2/FYN/PTEN/DBNL/LRRK2/DLG4/DBN1/EPHB1/SIPA1L1/ABI3/VPS35/EPHA4/ARF1/INSR/PDLIM5/DNM3/CFL1/CTTN

BP	GO:0002274	myeloid leukocyte activation	179	-0.39	-1.42	0.00598583	0.02333301	0.01432523	2149	tags=30%, list=18%, signal=25%	FCER1G/TLR2/FGR/CD93/UNC13D/CD177/ATM/JMJD6/MYO18A/PRKCE/ADGRE2/NOTCH2/ITGAM/LRRK2/C5AR1/CD300LF/STX4/CEBPA/PTPN6/PYCARD/SBNO2/RELB/SPI1/PREX1/PDPK1/ITGB2/FOXP1/FES/RBPJ/MFHAS1/GAB2/BCR/CXCR2/TRAF6/PTPRC/SYK/THBS1/PTAFR/TLR6/DOCK2/PIK3CG/PRAM1/JUND/LYN/LGALS9/RAC2/DYSF/GATA1/CLU/GRN/SNCA/PIK3CD/CBL
MF	GO:0042562	hormone binding	31	-0.62	-1.72	0.00600855	0.02337573	0.01435145	1573	tags=39%, list=13%, signal=34%	THRA/CMKLR1/SHC1/NRDC/LEPR/ADIPOR2/INSR/TSPO/ADIPOR1/NR3C1/ABHD2/ECE1
CC	GO:0030175	filopodium	68	-0.50	-1.58	0.00604145	0.02341209	0.01437378	2541	tags=37%, list=22%, signal=29%	TWF2/FSCN1/UTRN/SLC9A3R1/MYO5A/ACP3/MYO1G/SYNE2/EZR/EPHB1/NF2/ACTA2/EPHA4/LCP1/FXR2/AP2A1/FGD4/DYNC1H1/IQGAP2/B4GALT1/MSN/PPP1R9B/RIPOR2/ITGB3/VASP
BP	GO:0002252	immune effector process	441	-0.33	-1.30	0.00623328	0.02403833	0.01475826	2419	tags=26%, list=21%, signal=21%	SPON2/SPN/BTN3A2/ADAM17/CORO1A/NCKAP1L/DDX58/CR1L/C1QA/EPHB2/PKN1/MAVS/MYO1G/FCER1G/IRAK3/SERPING1/RFTN1/SLA2/CR1/FGR/FYN/UNC13D/PRDX2/NLRP3/SRC/NKG7/CD177/ANKRD17/MYO18A/PRKCE/TNFSF13/AP1G1/LYST/ATP7A/HCK/TGFB1/ADGRE2/TNFSF4/RC3H2/BCL3/NOTCH2/ITGAM/STAT5B/ARID5A/STX4/PTPRJ/PTPN6/PYCARD/MAD2L2/TMBIM6/SBNO2/RELB/TREM1/SPI1/PDPK1/NCF1/ITGB2/WDR1/KLRD1/LCP1/FOXP1/FES/PLEKHM2/PGLYRP1/RARA/PTK2B/GAB2/SEMA4A/BCR/STAT6/TRAF6/TBX21/IL6R/PAK1/VAV1/PTPRC/SYK/PTAFR/SUPT6H/ST3GAL1/PLEC/DOCK2/FZD5/PIK3CG/PRAM1/JAK3/TUBB4B/INPP5D/GPI/LYN/LGALS9/LAPTM5/RAC2/LRP1/TNFRSF1B/DYSF/PRF1/ITGAL/GATA1/TUBB/CLU/ARRB2/GRN/WAS/HK1/APBB1IP/STAT3/LAMP1/PIK3CD/KRT1/SLC7A5/CBL/HLX
BP	GO:0048588	developmental cell growth	127	-0.42	-1.47	0.00628704	0.02419871	0.01485672	2665	tags=34%, list=23%, signal=26%	ARHGAP4/PLXNA4/HDAC6/TWF2/KIAA0319/DIP2B/MUL1/BCL11A/NIN/DISC1/MACF1/SRF/ADNP/DBNL/EXT1/CPNE5/CTDP1/NDEL1/KMT2D/DBN1/CPNE1/CDKL5/YY1/SEMA4D/ARIH2/GSK3A/DPYSL2/SEMA4A/EIF4G2/VCL/STK11/USP9X/PDLIM5/IST1/G6PD/ULK1/SIN3A/CYFIP2/GSK3B/DNM2/AKAP13/CTTN/IQGAP1
MF	GO:0032182	ubiquitin-like protein binding	109	-0.44	-1.48	0.0063938	0.02458583	0.01509439	1271	tags=25%, list=11%, signal=22%	HGS/UBXN7/UBR5/NBR1/HERC2/PML/OTUB1/PELP1/IKBKG/MARK4/NSFL1C/RAD23A/ILRUN/NPLOC4/TOM1/UBAP1/VPS28/RBCK1/HSPB1/ASCC2/N4BP1/SQSTM1/DNAJB2/SHARPIN/JARID2/FBXO7/TOLLIP
BP	GO:0006023	aminoglycan biosynthetic process	55	-0.51	-1.58	0.00646821	0.02484794	0.01525531	1884	tags=31%, list=16%, signal=26%	CHST15/EXT1/TGFB1/CHSY1/B4GALT7/XYLT1/B4GALT5/ST3GAL2/ST3GAL3/B3GNT7/NFKB1/AP2A1/B3GNT8/CHST11/ST3GAL1/CLTC/NDST1
BP	GO:1902414	protein localization to cell junction	61	-0.52	-1.62	0.00660568	0.02520144	0.01547234	2072	tags=31%, list=18%, signal=26%	HEG1/SNAP25/RAB27B/ZDHHC7/NECTIN1/ACTG1/DLG4/CLSTN1/VPS35/TJP2/MAP1A/VCL/PECAM1/JAM3/ACTB/HSPB1/GRIPAP1/JAK1/PAK2
BP	GO:2000377	regulation of reactive oxygen species metabolic process	105	-0.44	-1.48	0.00690074	0.02605634	0.01599721	2107	tags=30%, list=18%, signal=24%	FOXO1/SOD2/FYN/PRDX2/CD177/TGFB1/GSTP1/ITGAM/RIPK1/PIKFYVE/MAPK14/HVCN1/ITGB2/GRB2/EIF6/TSPO/PTK2B/BCR/SYK/THBS1/PDK3/TLR6/G6PD/RAC1/RAC2/GNAI2/RHOA/PINK1/SNCA/CDKN1A/FOXO3
BP	GO:0051928	positive regulation of calcium ion transport	69	-0.49	-1.54	0.00696231	0.0261646	0.01606367	1286	tags=33%, list=11%, signal=30%	LGALS3/CAPN3/BAK1/TRPC3/CALM1/STIMATE/ATP2B1/LILRA5/PDPK1/CCR1/TSPO/HTT/SLC9A1/EHD3/CALM3/G6PD/JAK3/GJC2/ARRB2/MYLK/SNCA/STIM1/P2RX1

MF	GO:0004888	transmembrane signaling receptor activity	389	-0.34	-1.32	0.00702514	0.02627654	0.0161324	2150	tags=20%, list=18%, signal=17%	INTS6/FCER1G/TLR2/PTGIR/CR1/TLR5/IFNAR2/LILRA1/EPHB4/IFNAR1/IL17RD/CD300C/GRIK2/THBD/ERBB2/AGTRAP/ADGRE2/OR10H1/LILRA5/OPRL1/CMKLR1/C5AR1/CD300LF/EPHB1/OR52K2/S1PR2/OR52K1/SORT1/PTPN6/CHRNE/ADGRG3/SEMA4D/EPHA4/GPBAR1/ITGB2/CCR1/CSF1R/KLRD1/LEPR/HRH2/INSR/TNFRSF10C/ADGRL1/NFAM1/CD300LB/OSCAR/VLDLR/SORL1/PTK2B/CXCR2/INPP5K/IL6R/PECAM1/IL13RA1/TAS2R30/PLXNC1/GP6/PTPRC/LILRB3/PTAFR/TLR6/IL17RA/FZD5/CD14/ADGRE5/IGF2R/TNFRSF1B/MPL/TMEM63B/ITGAL/CXCR1/GPR146/LTBP1/GP1BA/TREML1/ACKR1/P2RX1/TBXA2R
BP	GO:0008643	carbohydrate transport	98	-0.44	-1.48	0.00701318	0.02627654	0.0161324	1811	tags=24%, list=15%, signal=21%	IRS2/ZDHHC7/SLC2A3/ABCB11/EZR/MFSD4B/MAPK14/SORT1/SLC35A4/SLC45A4/AQP9/TRIB3/GSK3A/INSR/SESN2/SLC27A1/SLC2A1/INPP5K/AQP1/PRKCB/RTN2/AKT2/AQP10/MFSD2B
BP	GO:0038034	signal transduction in absence of ligand	42	-0.55	-1.60	0.00702514	0.02627654	0.0161324	2089	tags=43%, list=18%, signal=35%	BCL2L11/PRDX2/MAP2K5/EYA3/RIPK1/GSK3A/PPP1CA/MKNK2/CTNNA1/CASP2/HSPA1A/PF4/GSK3B/GATA1/NF1/BCL2L1/FOXO3/STRADB
BP	GO:0097192	extrinsic apoptotic signaling pathway in absence of ligand	42	-0.55	-1.60	0.00702514	0.02627654	0.0161324	2089	tags=43%, list=18%, signal=35%	BCL2L11/PRDX2/MAP2K5/EYA3/RIPK1/GSK3A/PPP1CA/MKNK2/CTNNA1/CASP2/HSPA1A/PF4/GSK3B/GATA1/NF1/BCL2L1/FOXO3/STRADB
BP	GO:0034198	cellular response to amino acid starvation	44	-0.55	-1.62	0.00745816	0.02755916	0.01691986	2010	tags=39%, list=17%, signal=32%	RRAGD/SH3GLB1/GCN1/LARS1/SZT2/TFEB/EIF2AK2/DAP/SESN3/SESN2/MAP3K5/RRAGC/UCP2/MAPK3/CDKN1A/FLCN/NPRL3
BP	GO:0051963	regulation of synapse assembly	38	-0.57	-1.65	0.00745761	0.02755916	0.01691986	2573	tags=45%, list=22%, signal=35%	STAU2/AMIGO1/EPHB2/TLR2/ADNP/LRFN3/NECTIN1/EPHB1/CLSTN1/SEMA4D/ABI3/VPS35/SEMA4A/SETD5/PDLIM5/EIF4G1/SNCA
BP	GO:2000177	regulation of neural precursor cell proliferation	40	-0.55	-1.59	0.00768796	0.0283031	0.0173766	2107	tags=32%, list=18%, signal=27%	FOXO1/DISC1/LRRK2/NF2/SMARCD3/ZNF335/CTNNA1/GJC2/LYN/GNAI2/RHOA/NF1/FOXO3
BP	GO:0071320	cellular response to cAMP	33	-0.60	-1.67	0.00771255	0.02833409	0.01739563	1768	tags=45%, list=15%, signal=39%	PDE2A/HSPA5/EZR/ZFP36L1/AQP9/ITPR2/SLC8A1/INPP5K/RAPGEF2/PTAFR/PIK3CG/AQP1/GATA1/RAPGEF1/DMTN
BP	GO:0071260	cellular response to mechanical stimulus	55	-0.51	-1.57	0.00780026	0.02858418	0.01754917	3275	tags=53%, list=28%, signal=38%	MYD88/MAP3K1/TNFRSF1A/TLR8/TLR4/ENG/CRADD/FADD/TLR7/CASP5/TNFSF14/F11R/KCNJ2/BAK1/MAP2K4/TLR5/PDE2A/MAP3K2/NFKB1/IRF1/SLC2A1/BMP6/SLC9A1/PLEC/CASP2/AQP1/CNN2/MAPK3/MTPN
CC	GO:0016235	aggresome	27	-0.63	-1.69	0.00780856	0.02858824	0.01755166	2908	tags=44%, list=25%, signal=34%	HSPA1B/UBQLN1/URB2/HDAC6/FGR/EEF2/RANGAP1/TRIM66/HSPA1A/SQSTM1/STRADB/CABIN1
BP	GO:0051058	negative regulation of small GTPase mediated signal transduction	40	-0.55	-1.59	0.00783166	0.02864644	0.01758739	2232	tags=30%, list=19%, signal=24%	EPHB2/ARHGAP24/SPRY3/HEG1/RASA2/RASAL3/FBP1/NF1/FLCN/RIPOR2/SH3BP1/MFN2

BP	GO:0000041	transition metal ion transport	65	-0.49	-1.55	0.00818224	0.02965594	0.01820717	782	tags=18%, list=7%, signal=17%	LCN2/CLTC/SLC6A9/LTF/DNM2/RAB11B/FTL/FTH1/MMGT1/SLC48A1/SLC25A37/ARHGAP1
BP	GO:0006820	anion transport	267	-0.36	-1.35	0.00824683	0.02983566	0.01831751	2509	tags=26%, list=21%, signal=21%	SLC25A23/SLC37A2/SLC13A4/SERINC5/SLCO3A1/SLC1A4/SLC19A1/SLC10A3/CRY2/LRRC8B/SLC9A3R1/SLC20A1/ANO10/SLC25A15/CR1/ABAT/SLC24A4/SLC25A41/SLC66A1/SLC2A3/ANO6/SLC12A6/CPTP/GET3/ABCB11/RIPK1/TTYH3/CLCN5/CA2/PER2/SLC16A3/SLC36A1/AQP9/SLC25A11/CLN3/PTGES/CLCC1/TSPO2/ABCG2/MFSD5/CA4/SLC27A1/TSPO/LRRC8A/SLC2A1/ANKH/SLC6A20/SLC24A3/SLC3A2/CLIC3/SLC25A38/SYK/SLC9A1/PTAFR/SLC6A9/SLC1A5/ABCC4/SLC43A2/PSAP/SLC43A1/RAB11B/ABCC3/NF1/SNCA/AHCYL1/SLC4A1/SLC7A5/SLC38A5/SLC6A8
BP	GO:0048469	cell maturation	92	-0.45	-1.49	0.00826288	0.02986656	0.01833648	1775	tags=20%, list=15%, signal=17%	ZBTB7A/PTBP3/LRRK2/B4GALT5/ROPN1L/CEBPA/CDKN1C/PRKACA/SLFN14/PTK2B/FZD5/ABHD2/G6PD/TAL1/FAM210B/EPB42/TRIM58/FOXO3
CC	GO:0035579	specific granule membrane	82	-0.46	-1.48	0.00837411	0.03021368	0.01854959	2467	tags=38%, list=21%, signal=30%	PLAUR/CYBB/HMOX2/ADAM8/ORMDL3/SNAP25/CD93/CD177/CMTM6/ALDH3B1/SLC2A3/ANO6/CKAP4/TSPAN14/ITGAM/TMBIM1/STK10/PTPRJ/ADGRG3/HVCN1/AP1M1/ITGB2/HGSNAT/SLC44A2/LAMTOR1/UBR4/TOM1/DNAJC5/TNFRSF1B/ITGAL/P2RX1
MF	GO:0071889	14-3-3 protein binding	27	-0.63	-1.68	0.00839236	0.03022466	0.01855634	2263	tags=48%, list=19%, signal=39%	KIF13B/HDAC7/KLHL22/IRS2/PRKCE/TMCC3/ZFP36L1/TBC1D22A/TBC1D22B/RIPOR1/SRPK2/ARRB2/RIPOR2
CC	GO:0043679	axon terminus	44	-0.54	-1.61	0.00861668	0.03097642	0.01901787	1973	tags=32%, list=17%, signal=27%	ELK1/GRIK2/ACTG1/LRRK2/SYNJ1/EPHA4/SLC8A1/ACTB/TPRG1L/PRKCB/SNCA/ATP6V0D1/RNF40/GLUL
BP	GO:0044703	multi-organism reproductive process	102	-0.45	-1.50	0.00879448	0.031445	0.01930556	1967	tags=25%, list=17%, signal=21%	KPNA6/H3-3B/THBD/VDR/H3-3A/ATP7A/ASH1L/STAT5B/ARHGDIB/TRIM28/AGO2/YTHDF3/RARA/SLC2A1/MMP9/ITGA5/PTAFR/MED1/UCP2/LGALS9/EPN1/BSG/CTSB/KLF1/P2RX1/TIMP1
BP	GO:0048705	skeletal system morphogenesis	104	-0.44	-1.48	0.00887839	0.03171652	0.01947226	2229	tags=28%, list=19%, signal=23%	MTHFD1/AXIN2/THRA/FGR/EXT1/NIPBL/CHSY1/PPARGC1B/MAPK14/MSX1/FUZ/ALPL/FOXN3/SGPL1/GREM1/RARA/SOX6/CHST11/BMP6/POR/SFRP2/GLG1/PHOSPHO1/LTF/SKI/NDST1/ANXA6/RFLNB/CSRNP1
MF	GO:0008022	protein C-terminus binding	137	-0.40	-1.41	0.00912766	0.0324862	0.0199448	2002	tags=28%, list=17%, signal=23%	SRC/DBNL/NCF2/XRCC6/ERBB2/NIPBL/SDCBP/OPRL1/PRRC2C/SYNJ1/SNX17/EZR/DLG4/CTBP1/EML2/ATXN2/NUMA1/SASH1/OPTN/AGO2/FOXN3/MKI67/PTK2B/PIAS4/CSK/ZBTB16/MAP2K7/NPAT/SIPA1/TBL1X/ATXN1/SP1/PPP1R9B/TERF2/EPB41/USP7/DAB2/POLR2A
BP	GO:0002768	immune response-regulating cell surface receptor signaling pathway	221	-0.37	-1.36	0.0091625	0.03253443	0.01997441	2425	tags=26%, list=21%, signal=21%	FPR3/GPLD1/CD38/BTN3A2/NCKAP1L/MAP2K4/KIR2DL1/MYO1G/MICB/FCER1G/RFTN1/SLA2/CR1/FGR/FYN/SRC/PRKCE/SPPL3/HCK/DGKZ/RC3H2/FYB1/GPS2/EZR/CMKLR1/SH2B2/C5AR1/PTPRJ/PTPN6/KLRD1/FOXP1/ERMAP/SPG21/NFAM1/NFATC2/IKBKG/TRAF6/PRKD2/PAK1/CSK/VAV1/STK11/MAP2K7/PTPRC/SYK/PRAM1/INPP5D/LYN/RBCK1/LAPTM5/CREBBP/PRKCB/PIK3CD/PAK2/WNK1/BAG6/RELA
BP	GO:0046580	negative regulation of Ras protein signal transduction	35	-0.57	-1.62	0.00916463	0.03253443	0.01997441	2232	tags=31%, list=19%, signal=26%	EPHB2/ARHGAP24/SPRY3/HEG1/RASA2/RASAL3/FBP1/NF1/FLCN/RIPOR2/MFN2

BP	GO:0006623	protein targeting to vacuole	34	-0.58	-1.64	0.00928682	0.03288015	0.02018667	1745	tags=38%, list=15%, signal=33%	LAMP2/VPS13D/HGS/SORT1/VPS41/MON1B/VPS37B/VPS37C/NCOA4/SORL1/RAB7A/LAPTM5/CLU
MF	GO:0001228	DNA-binding transcription activator activity, RNA polymerase II-specific	250	-0.36	-1.35	0.00930455	0.03291359	0.0202072	1973	tags=26%, list=17%, signal=22%	FOXO1/KLF6/SRF/ELK1/RXRB/ZNF48/ATF6/VEZF1/ETV6/GTF2I/TFDP2/ZFAT/STAT5B/NFAT5/NFIA/EGR1/NFATC1/NR6A1/CTCFL/CEBPA/YY1/MSX1/ZNF175/NR1H2/IRF2/NFKB2/CREBRF/USF3/SPI1/RFX2/MTF1/KLF13/E2F3/NFKB1/RBPJ/NFATC2/IRF1/STAT6/MEIS1/NFIC/TBX21/KLF5/ZBTB16/TFE3/FOXO4/FOSL2/NR3C1/MAFB/JUND/MLXIP/GFI1B/FOXJ2/NFATC3/TFDP1/GATA1/SP1/PBX1/STAT3/CSRNP1/LITAF/E2F2/FOXO3/ELF4/RELA
BP	GO:1900271	regulation of long-term synaptic potentiation	28	-0.61	-1.65	0.00935812	0.03307369	0.02030549	1518	tags=39%, list=13%, signal=34%	ZDHHC2/NCSTN/EPHB2/MME/EPHA4/SHISA7/GSK3B/STAU1/NF1/NRGN/SQSTM1
BP	GO:0032007	negative regulation of TOR signaling	36	-0.59	-1.67	0.00941068	0.0331595	0.02035817	1948	tags=36%, list=17%, signal=30%	ATM/AKT1S1/SZT2/TSC2/GSK3A/SESN3/SESN2/STK11/UBR2/CASTOR2/FLCN/TMEM127/NPRL3
BP	GO:0035303	regulation of dephosphorylation	101	-0.44	-1.49	0.00941068	0.0331595	0.02035817	2564	tags=37%, list=22%, signal=29%	AMBRA1/LGALS3/GPLD1/CALM1/NCKAP1L/CRY2/PPP2R5D/PPP6R1/SRC/FKBP1B/PPP1R15A/SPPL3/SMG6/TGFB1/PPARGC1B/ROCK2/MGAT5/SEMA4D/PTBP1/CHP1/MTMR3/YWHAB/HTT/MFHAS1/VRK3/INPP5K/PTPRC/CALM3/PLEK/GSK3B/GNAI2/FKBP1A/PPP1R9B/GNA12/SMG7/PINK1/IQGAP1
BP	GO:0038061	NIK/NF-kappaB signaling	103	-0.45	-1.50	0.00946466	0.03325211	0.02041503	3052	tags=44%, list=26%, signal=33%	TLR4/SPHK1/NLRP12/ZFP91/TLR7/TNF/TNFSF14/AKIP1/RASSF2/CCN3/RC3H1/CALR/TERF2IP/HDAC7/LIMS1/TLR2/CHI3L1/TIRAP/NLRP3/DICER1/RC3H2/BCL3/IKBKE/NFAT5/CPNE1/EIF2AK2/ADGRG3/NFKB2/RELB/SPI1/SASH1/GREM1/TRAF6/ADIPOR1/TLR6/CD14/LGALS9/RBCK1/LAPTM5/RHOA/ACTN4/LITAF/PTP4A3/PPM1A/RELA
BP	GO:0043300	regulation of leukocyte degranulation	43	-0.54	-1.59	0.00946715	0.03325211	0.02041503	2051	tags=49%, list=17%, signal=40%	FGR/UNC13D/CD177/AP1G1/ADGRE2/ITGAM/STX4/SPI1/PDPK1/ITGB2/FES/GAB2/BCR/SYK/PTAFR/PRAM1/LYN/LGALS9/RAC2/GATA1/LAMP1
BP	GO:1990845	adaptive thermogenesis	109	-0.43	-1.45	0.00962054	0.03365923	0.02066498	2379	tags=29%, list=20%, signal=24%	ADAM17/SIRT6/LNPEP/HDAC3/THRA/PCTP/ZNF516/PPARGC1B/CMKLR1/NRDC/PER2/NR1H2/ALMS1/LEPR/ADIPOR2/ALPL/RBPJ/SORL1/STAT6/BSCL2/DYNC1H1/STK11/LCN2/TFE3/SYK/ADIPOR1/UCP2/KDM6B/FLCN/IP6K1/TLE3/MFN2
BP	GO:0071356	cellular response to tumor necrosis factor	146	-0.40	-1.40	0.00962539	0.03365923	0.02066498	1657	tags=23%, list=14%, signal=20%	GSTP1/GPS2/SMPD4/RIPK1/CPNE1/MAPK14/CEBPA/ZFP36L1/EIF5A/PYCARD/SPATA2/ARHGEF2/CDIP1/MAP3K5/NFKB1/HIPK1/PTK2B/INPP5K/TRAF6/PIAS4/SYK/THBS1/HSPA1A/LAPTM5/TNFRSF1B/PRPF8/ACTN4/MAPK3/H2BC11/YBX3/SHARPIN/PLVAP/FOXO3/RELA
BP	GO:0007193	adenylate cyclase-inhibiting G protein-coupled receptor signaling pathway	28	-0.61	-1.65	0.00964386	0.0336942	0.02068645	2886	tags=39%, list=25%, signal=30%	GNAI3/PTGDR2/MARCO/P2RY1/INSL3/PDE2A/OPRL1/FLNA/PSAP/GNAI2/GNAZ

BP	GO:0006611	protein export from nucleus	53	-0.51	-1.55	0.00965462	0.03370216	0.02069134	1881	tags=32%, list=16%, signal=27%	NUP214/XPO5/TGFB1/RANGAP1/NUTF2/CHP1/PRKACA/RBM22/FRAT1/STRADA/XPO7/GSK3B/TCF7L2/XPO6/AHCYL1/PPM1A/STRADB
BP	GO:0048013	ephrin receptor signaling pathway	31	-0.60	-1.68	0.00981655	0.0342049	0.02099999	2232	tags=42%, list=19%, signal=34%	EPHB2/EFNB1/FYN/SRC/EPHB4/TIAM1/EPHB1/SIPA1L1/EPHA4/RBPJ/PAK1/MMP9/LYN
BP	GO:0051640	organelle localization	409	-0.33	-1.29	0.00982445	0.0342049	0.02099999	2463	tags=28%, list=21%, signal=23%	ACTR2/TRAK1/RALB/UBE2B/SCRIB/ARCN1/CALM1/ASPM/ATP9A/MAP4K2/F8A1/SLC9A3R1/ARFGAP2/HPS6/MYO5A/SCFD2/MUL1/STK25/MYO1G/VPS18/VPS4B/KATNB1/MYO1E/SNAP25/FGR/LIMK2/PTEN/FEZ1/UNC13D/RAB27B/MAP1S/PDZD8/BICD2/MOSPD3/ATM/STARD3/BLOC1S3/SDC4/STX6/SUN2/ADGRE2/BORCS6/SDCBP/HGS/SYNE2/CTBP2/LRRK2/NDEL1/SYNJ1/EZR/TFEB/SYN3/STX4/PEF1/CHMP4B/AGTPBP1/PARD3/HOOK3/CLN3/MAP4/KPNB1/PDPK1/NUMA1/YWHAZ/KIF3C/EIF6/STX5/VPS11/FES/KIF1C/PLEKHM2/ARHGAP21/IKBKG/HTT/GAB2/KIF13A/DYNC1H1/NSFL1C/RAB7A/NDE1/LAMTOR1/STK11/SYK/CLUH/LMNA/DCTN1/PIK3CG/MYO1F/EML3/RIPOR1/PLEK/CHMP1A/MARK2/LYN/LGALS9/RAC2/DNM2/RAB11B/SYTL4/TRAK2/GATA1/ACTN4/CFL1/PINK1/SNCA/FLCN/LAMP1/UBB/AHCYL1/PIK3CD/MFN2/RAB3D/TRIM58/MYH9/CBL/SEC16A
MF	GO:0005547	phosphatidylinositol-3,4,5-trisphosphate binding	32	-0.59	-1.63	0.00991058	0.03441438	0.0211286	1699	tags=34%, list=14%, signal=29%	ARHGAP9/WASHC2C/PARD3/ARAP1/ZFYVE1/ADAP2/GAB2/ASAP1/IQGAP2/NRGN/IQGAP1
BP	GO:0006509	membrane protein ectodomain proteolysis	32	-0.59	-1.63	0.00991058	0.03441438	0.0211286	2910	tags=44%, list=25%, signal=33%	ADAM9/TNF/SPPL2B/APH1A/NCSTN/GPLD1/ADAM17/ADAM8/NRDC/TIMP2/TNFRSF1B/MYH9/TIMP1/FURIN
BP	GO:0045638	negative regulation of myeloid cell differentiation	58	-0.48	-1.49	0.00992975	0.03445085	0.02115099	1786	tags=31%, list=15%, signal=26%	ITPKB/STAT5B/TRIB1/ZFP36L1/KLF13/RARA/PTK2B/MEIS1/TOB2/ZBTB16/LILRB3/MAFB/LTF/INPP5D/PF4/LYN/CUL4A/NF1
BP	GO:0006836	neurotransmitter transport	103	-0.45	-1.50	0.01003395	0.03466116	0.02128011	1753	tags=29%, list=15%, signal=25%	SNPH/SV2A/P2RY1/MCTP1/SCRIB/SLC28A2/VPS18/SNAP25/PNKD/CTBP2/RPH3A/LRRK2/SYNJ1/SYN3/STX4/FLOT1/PER2/PIP5K1C/FBXL20/CALM3/SLC6A9/DNAJC5/GSK3B/PRKCB/NF1/SNCA/NAPA/ITGB3/P2RX1/SLC6A8
BP	GO:0014065	phosphatidylinositol 3-kinase signaling	91	-0.45	-1.50	0.01019577	0.03512858	0.02156708	2910	tags=32%, list=25%, signal=24%	TNF/PLEKHA1/PTPN13/NKX3-1/PIK3CB/KIT/FBXL2/SLC9A3R1/FGR/PTEN/SRC/ERBB2/TSC2/HGF/ZFP36L1/PTPN6/SEMA4D/NCF1/PIP5K1C/ENTPD5/PDGFA/INSR/SELP/PIK3CG/NF1/HCLS1/PIK3CD/PRR5/CBL
BP	GO:0001738	morphogenesis of a polarized epithelium	53	-0.50	-1.54	0.0103916	0.03571046	0.02192432	2554	tags=38%, list=22%, signal=30%	MED12/SCRIB/SLC9A3R1/MYO9A/PTK7/VANGL1/MKS1/ZDHHC7/TIAM1/ACTG1/FUZ/WDR1/SFRP2/DVL3/ACTB/RAC1/RHOA/MSN/SH3BP1/DAB2
CC	GO:0016234	inclusion body	61	-0.50	-1.58	0.01051974	0.03608843	0.02215638	2080	tags=34%, list=18%, signal=28%	ATXN3/FGR/ARIH1/EEF2/LRRK2/RANGAP1/KEAP1/TRIM66/NUP98/HTT/HSPA1A/WDFY3/GPX1/CLU/ATXN1/PINK1/SNCA/SQSTM1/FBXO7/STRADB/CABIN1

BP	GO:1902115	regulation of organelle assembly	169	-0.39	-1.41	0.01058301	0.03624294	0.02225124	2043	tags=29%, list=17%, signal=24%	LIMK2/FEZ1/SRC/KCTD17/SH3GLB1/SDC4/HCK/G3BP1/SDCBP/SYNE2/TCHP/LRRK2/EZR/PIKFYVE/PIP4K2C/CDKL5/CHMP4B/PHF23/FUZ/ABI3/MAP4/NUMA1/ALMS1/EVI5L/LCP1/MTMR3/CNOT6/VPS11/HTT/DYNC1H1/MARK4/GSN/WDR45/ASAP1/RBM14/ULK1/EML3/HSPA1A/CHMP1A/GSK3B/ELAPOR1/DNM2/RHOA/ATG2A/MSN/PINK1/PIP4K2A/CNOT1/RAB1B
BP	GO:0001704	formation of primary germ layer	54	-0.51	-1.56	0.01075025	0.03665781	0.02250595	2473	tags=39%, list=21%, signal=31%	SETD2/ARMC5/WLS/TBX19/DUSP1/PAF1/SRF/EXOC4/ATOH8/NF2/ITGB2/PRKACA/MMP9/SFRP2/ITGA5/GPI/ETS2/KDM6B/ITGB5/TAL1/ITGB3
BP	GO:0060560	developmental growth involved in morphogenesis	128	-0.40	-1.39	0.01090156	0.03711012	0.02278364	2665	tags=32%, list=23%, signal=25%	ARHGAP4/PLXNA4/HDAC6/MED12/TWF2/KIAA0319/DIP2B/MUL1/BCL11A/PTK7/NIN/DISC1/MACF1/SRF/KDM5B/ADNP/DBNL/EXT1/CPNE5/NDEL1/DBN1/CPNE1/CDKL5/SEMA4D/TRIM28/DPYSL2/SEMA4A/EIF4G2/VCL/SFRP2/STK11/USP9X/MED1/IST1/ULK1/SIN3A/CYFIP2/GSK3B/DNM2/CTTN/IQGAP1
BP	GO:0048844	artery morphogenesis	33	-0.59	-1.65	0.01096731	0.03723827	0.02286232	999	tags=21%, list=9%, signal=19%	RBPJ/ARID2/ZMIZ1/LRP1/NF1/MYLK/NPRL3
BP	GO:0048016	inositol phosphate-mediated signaling	40	-0.53	-1.55	0.01099299	0.03725632	0.0228734	2206	tags=40%, list=19%, signal=33%	STIMATE/FHL2/SPPL3/NFAT5/NFATC1/PTBP1/ITPR2/CHP1/CHERP/NFATC2/PPP3CA/SLC9A1/GSK3B/ATP2B4/NFATC3/PPP3R1
BP	GO:0055067	monovalent inorganic cation homeostasis	88	-0.46	-1.50	0.0109882	0.03725632	0.0228734	1868	tags=24%, list=16%, signal=20%	EXT1/SLC12A6/LRRK2/CA2/KCNH2/HVCN1/CLN3/ATP6AP1/CHP1/PDK2/ATP6V0A1/SLC8A1/RAB7A/SLC9A1/ATP1B2/GNAI2/GRN/MLLT6/MAPK3/SLC4A1/ATP6V0D1
BP	GO:0045927	positive regulation of growth	144	-0.39	-1.39	0.01105135	0.03730064	0.02290061	2541	tags=35%, list=22%, signal=28%	TWF2/CAPN3/CREB1/DDX49/CD38/ADAM17/MUL1/KLHL22/RFTN1/DISC1/MACF1/SRF/PPM1F/HDGFL2/ADNP/DBNL/MAP2K5/H3-3B/ERBB2/NIPBL/H3-3A/CPNE5/SDCBP/PLCB1/NDEL1/STAT5B/EZR/DBN1/MAPK14/CDKL5/SEMA4D/CXCL16/INSR/CSNK2A1/RBPJ/PTK2B/EIF4G2/IGF2/SFRP2/EXTL3/EXOSC4/SLC9A1/IST1/BASP1/EIF4G1/RHOA/PIM1/RPS6KA1/MTPN/YBX3/HLX
BP	GO:0072657	protein localization to membrane	417	-0.33	-1.30	0.01107854	0.0373607	0.02293748	2097	tags=23%, list=18%, signal=19%	RFTN1/TTC7A/FRMPD1/SNAP25/MACF1/FYN/LRRC4/EXOC4/SH3GLB1/PRKCE/GRIK2/ERBB2/TGFB1/ZDHHC7/ATP13A1/SDCBP/HGS/ZDHHC19/HSPA5/GET3/NUMB/EXOC7/TSPAN14/ROCK2/ITGAM/FYB1/AP2B1/EZR/TMBIM1/DLG4/PIKFYVE/STX4/PLEKHF1/CHMP4B/FLOT1/CLSTN1/PARD3/CLN3/ABI3/VPS35/SHISA7/PDPK1/NCF1/ITGB2/ATP6AP1/CHP1/OPTN/VPS37B/VPS37C/EMC3/SPTBN1/KIF13A/INPP5K/RAPGEF2/LDLRAP1/RAB7A/CSK/GSN/TSPAN33/FIS1/RAB31/EHD3/CLTC/CALM3/PRAM1/FLNA/ACTB/RAB11B/LRP1/FLOT2/ATP2B4/GRIPAP1/ITGAL/PACS1/MYADM/BBC3/PPP1R9B/TESC/BCL2L1/EHD1/SQSTM1/AKT2/SLC4A1/AP2M1/GAK/MMGT1/BSG/RAB3D/DAB2/SEC16A/TSPAN5/RHOG/DMTN/BAG6/ANK1
CC	GO:0030666	endocytic vesicle membrane	134	-0.41	-1.44	0.01114439	0.03755094	0.02305428	2642	tags=37%, list=23%, signal=29%	RAB22A/MARCO/CALR/CD4/VAMP3/KIAA0319/CYBB/CORO1A/WLS/SCARB1/RAB43/TF/TAPBP/UBC/TLR2/WNT1/ATP6V0B/UBA52/MCOLN1/ATP7A/AP2A2/LAMP2/AP2B1/AP2S1/DLG4/PIKFYVE/STX4/HVCN1/ATP6V0C/ATP6V0A1/AP2A1/LDLRAP1/TGOLN2/RAB7A/RAB31/TLR6/CLTC/FZD5/RAB11FIP1/LYN/RAC2/DNM2/IGF2R/RAB11B/LRP1/RILP/LAMP1/UBB/ATP6V0D1/AP2M1

BP	GO:0097242	amyloid-beta clearance	27	-0.62	-1.66	0.01116543	0.03758996	0.02307824	2049	tags=37%, list=17%, signal=31%	SRF/ITGAM/C5AR1/MME/ITGB2/INSR/CLTC/RAB11B/LRP1/CLU
BP	GO:0010921	regulation of phosphatase activity	62	-0.49	-1.53	0.0112315	0.03778039	0.02319515	2521	tags=37%, list=21%, signal=29%	LGALS3/GPLD1/CALM1/NCKAP1L/CRY2/PPP6R1/FKBP1B/PPP1R15A/PPARGC1B/ROCK2/MGAT5/SEMA4D/CHP1/HTT/VRK3/PTPRC/CALM3/PLEK/GSK3B/GNAI2/FKBP1A/PPP1R9B/GNA12
BP	GO:0006885	regulation of pH	65	-0.48	-1.53	0.01136817	0.03817554	0.02343775	1650	tags=22%, list=14%, signal=19%	LRRK2/CA2/HVCN1/CLN3/ATP6AP1/CHP1/PDK2/ATP6V0A1/RAB7A/SLC9A1/GRN/MAPK3/SLC4A1/ATP6V0D1
MF	GO:0051019	mitogen-activated protein kinase binding	26	-0.64	-1.69	0.01145733	0.03834541	0.02354204	2573	tags=50%, list=22%, signal=39%	STAU2/DUSP16/DUSP1/NBR1/NFATC1/MAPK14/PTPRJ/ATF7/PTAFR/MAPKAPK3/TNIP1/ARRB2/MAPKAPK2
BP	GO:0006949	syncytium formation	31	-0.60	-1.67	0.01151147	0.0384942	0.02363339	3269	tags=61%, list=28%, signal=44%	SH3PXD2A/ADAM9/IL4R/NOS1/NPHS1/TNFSF14/PLEKHO1/ADAMTS5/CFLAR/MYOF/WNT1/DYRK1B/MAPK14/FLOT1/SBNO2/NFATC2/EHD1/RIPOR2/MYH9
MF	GO:0031490	chromatin DNA binding	82	-0.44	-1.44	0.01195272	0.03980306	0.02443696	2107	tags=30%, list=18%, signal=25%	FOXO1/THRA/TOX2/SRF/PER1/H3-3B/H3-3A/SMARCC1/SMARCA4/KDM3B/CTCFL/SBNO2/RARA/SMARCC2/GATAD2B/ACTB/CREBBP/CHD4/GATA1/KDM6B/ACTN4/WBP2/STAT3/FOXO3/RELA
BP	GO:1901224	positive regulation of NIK/NF-kappaB signaling	56	-0.50	-1.53	0.01212977	0.04029071	0.02473636	3052	tags=45%, list=26%, signal=33%	TLR4/SPHK1/NLRP12/TLR7/TNF/TNFSF14/RC3H1/CALR/TERF2IP/LIMS1/TLR2/RC3H2/NFAT5/EIF2AK2/SASH1/GREM1/TRAF6/CD14/LGALS9/RBCK1/LAPTM5/RHOA/ACTN4/PTP4A3/RELA
BP	GO:0106106	cold-induced thermogenesis	102	-0.44	-1.46	0.01217149	0.04029581	0.02473948	2379	tags=30%, list=20%, signal=24%	ADAM17/SIRT6/LNPEP/HDAC3/THRA/PCTP/ZNF516/PPARGC1B/CMKLR1/NRDC/PER2/NR1H2/ALMS1/LEPR/ADIPOR2/ALPL/RBPJ/STAT6/BSCL2/DYNC1H1/STK11/LCN2/TFE3/SYK/ADIPOR1/UCP2/KDM6B/FLCN/IP6K1/TLE3/MFN2
BP	GO:0120161	regulation of cold-induced thermogenesis	102	-0.44	-1.46	0.01217149	0.04029581	0.02473948	2379	tags=30%, list=20%, signal=24%	ADAM17/SIRT6/LNPEP/HDAC3/THRA/PCTP/ZNF516/PPARGC1B/CMKLR1/NRDC/PER2/NR1H2/ALMS1/LEPR/ADIPOR2/ALPL/RBPJ/STAT6/BSCL2/DYNC1H1/STK11/LCN2/TFE3/SYK/ADIPOR1/UCP2/KDM6B/FLCN/IP6K1/TLE3/MFN2
MF	GO:0008083	growth factor activity	43	-0.53	-1.57	0.0122133	0.04030925	0.02474774	1092	tags=23%, list=9%, signal=21%	TGFB1/HGF/PDGFA/IGF2/BMP6/GPI/ADA2/GRN/HDGF/TIMP1
BP	GO:0002931	response to ischemia	36	-0.58	-1.65	0.01224091	0.04032301	0.02475618	1772	tags=47%, list=15%, signal=40%	NOL3/PANX1/TP53/PIK3CB/PPIF/AIFM1/CPEB4/ROCK2/EGR1/PER2/PANX2/CSF1R/MAP3K5/HYOU1/PINK1/SQSTM1/MAP2K3
BP	GO:0002683	negative regulation of immune system process	299	-0.34	-1.30	0.01240601	0.04074509	0.02501532	2284	tags=25%, list=19%, signal=21%	CR1L/DUSP1/MUL1/IFI16/PKN1/TMEM176B/IRAK3/SERPING1/SLA2/CR1/LGALS9C/FGR/FYN/PADI2/PRDX2/SRC/ATM/SDC4/ERBB2/CD68/HCK/TGFB1/DGKZ/TNFSF4/RC3H2/GPS2/PLCB1/EZR/PAG1/CD300LF/MAPK14/TRIB1/PTPRJ/PTPN6/NR1H2/TMBIM6/SPI1/ITCH/KLRD1/YTHDF3/RUNX3/TMEM131L/VSIR/GREM1/IRF1/PGLYRP1/RARA/BCR/STAT6/TBX21/TOB2/PTPRC/LILRB3/SYK/ADAR/THBS1/MAFB/NLRX1/LTF/TSC22D3/JAK3/INPP5D/LYN/LGALS9/LAPTM5/PDE5A/CUL4A/GPX1/ARRB2/NF1/GRN/RIN3/RIPOR2/FBXO7/HLX/FURIN

BP	GO:0046683	response to organophosphorus	70	-0.48	-1.52	0.01243053	0.04074509	0.02501532	1993	tags=33%, list=17%, signal=27%	PER1/THBD/PPARGC1B/PDE2A/HSPA5/EZR/ZFP36L1/AQP9/ITPR2/SSH1/PTK2B/SLC8A1/INPP5K/RAPGEF2/PTAFR/PIK3CG/AQP1/GATA1/RAPGEF1/BSG/CARM1/P2RX1/DMTN
BP	GO:0002366	leukocyte activation involved in immune response	229	-0.37	-1.36	0.01245345	0.04078659	0.0250408	2083	tags=26%, list=18%, signal=22%	CR1/FGR/UNC13D/NLRP3/NKG7/CD177/PRKCE/TNFSF13/AP1G1/ATP7A/TGFB1/ADGRE2/TNFSF4/RC3H2/BCL3/NOTCH2/ITGAM/STX4/PTPN6/PYCARD/MAD2L2/SBNO2/RELB/SPI1/PDPK1/ITGB2/LCP1/FOXP1/FES/PGLYRP1/RARA/PTK2B/GAB2/SEMA4A/BCR/STAT6/TBX21/IL6R/PTPRC/SYK/PTAFR/SUPT6H/ST3GAL1/DOCK2/PIK3CG/PRAM1/JAK3/LYN/LGALS9/RAC2/DYSF/ITGAL/GATA1/GRN/APBB1IP/STAT3/LAMP1/PIK3CD/CBL/HLX
MF	GO:0001216	DNA-binding transcription activator activity	253	-0.36	-1.34	0.0124765	0.04079486	0.02504588	1973	tags=25%, list=17%, signal=22%	FOXO1/KLF6/SRF/ELK1/RXRB/ZNF48/ATF6/VEZF1/ETV6/GTF2I/TFDP2/ZFAT/STAT5B/NFAT5/NFIA/EGR1/NFATC1/NR6A1/CTCFL/CEBPA/YY1/MSX1/ZNF175/NR1H2/IRF2/NFKB2/CREBRF/USF3/SPI1/RFX2/MTF1/KLF13/E2F3/NFKB1/RBPJ/NFATC2/IRF1/STAT6/MEIS1/NFIC/TBX21/KLF5/ZBTB16/TFE3/FOXO4/FOSL2/NR3C1/MAFB/JUND/MLXIP/GFI1B/FOXJ2/NFATC3/TFDP1/GATA1/SP1/PBX1/STAT3/CSRNP1/LITAF/E2F2/FOXO3/ELF4/RELA
BP	GO:0030198	extracellular matrix organization	122	-0.40	-1.39	0.0125226	0.0408492	0.02507923	3135	tags=35%, list=27%, signal=26%	ADAMTS4/TNFRSF1A/DNAJB6/ENG/NID1/NPHS1/TNF/CTSV/NOTCH1/ERCC2/SMAD3/ADAMTS5/SPOCK2/CLASP1/CST3/COLGALT2/FSCN1/CFLAR/MPZL3/ADAM8/TNXB/MYO1E/CRTAP/EXT1/ATP7A/TGFB1/BCL3/CRISPLD2/FLOT1/NFKB2/QSOX1/LCP1/GREM1/ERO1A/COLGALT1/GFOD2/MMP9/SFRP2/B4GALT1/LRP1/TNFRSF1B/NF1/FURIN
BP	GO:0043062	extracellular structure organization	122	-0.40	-1.39	0.0125226	0.0408492	0.02507923	3135	tags=35%, list=27%, signal=26%	ADAMTS4/TNFRSF1A/DNAJB6/ENG/NID1/NPHS1/TNF/CTSV/NOTCH1/ERCC2/SMAD3/ADAMTS5/SPOCK2/CLASP1/CST3/COLGALT2/FSCN1/CFLAR/MPZL3/ADAM8/TNXB/MYO1E/CRTAP/EXT1/ATP7A/TGFB1/BCL3/CRISPLD2/FLOT1/NFKB2/QSOX1/LCP1/GREM1/ERO1A/COLGALT1/GFOD2/MMP9/SFRP2/B4GALT1/LRP1/TNFRSF1B/NF1/FURIN
BP	GO:0061756	leukocyte adhesion to vascular endothelial cell	33	-0.58	-1.63	0.01252394	0.0408492	0.02507923	3178	tags=48%, list=27%, signal=35%	IRAK1/ITGB7/TNF/ADD2/SPN/ALOX5/EXT1/NFAT5/ITGB2/SELPLG/SELP/TRAF6/PTAFR/SELL/RHOA/RELA
BP	GO:0070098	chemokine-mediated signaling pathway	44	-0.53	-1.57	0.01281632	0.04166615	0.0255808	1573	tags=25%, list=13%, signal=22%	CMKLR1/CXCL5/CCR1/PF4V1/PTK2B/CXCR2/PF4/MPL/CXCR1/ACKR1/WNK1
BP	GO:0006022	aminoglycan metabolic process	80	-0.46	-1.48	0.0129109	0.0419051	0.0257275	2137	tags=32%, list=18%, signal=27%	CTBS/HYAL3/SPOCK2/CHST14/GLB1/CHI3L1/GUSB/CHST15/EXT1/TGFB1/CHSY1/B4GALT7/XYLT1/B4GALT5/ST3GAL2/ST3GAL3/B3GNT7/NFKB1/AP2A1/B3GNT8/PGLYRP1/CHST11/ST3GAL1/CLTC/NDST1/GNS
BP	GO:0051346	negative regulation of hydrolase activity	199	-0.37	-1.36	0.01307473	0.04233315	0.0259903	2181	tags=30%, list=19%, signal=25%	SH3RF1/SERPINA1/CDKN2D/SERPING1/CR1/KNG1/SRC/FKBP1B/MAP2K5/PPP1R15A/XIAP/ROCK2/LRRK2/HGF/PI3/MGAT5/CAST/UBE2Z/SORT1/SEMA4D/TMBIM6/DHCR24/BIRC6/LEPR/CHP1/PCSK1N/AMOT/CSNK2A1/SORL1/PRDX5/TIMP2/BCAS3/GMIP/MMP9/POR/SFRP2/APLP2/THBS1/LTF/GPI/AQP1/GSK3B/LRP1/GNAI2/FKBP1A/GPX1/ARRB2/PPP1R9B/TERF2/UBE2O/RPS6KA1/SNCA/SIAH2/PSMF1/CTSB/PAK2/WNK1/TIMP1/FURIN
BP	GO:0007411	axon guidance	100	-0.44	-1.46	0.01363012	0.04389478	0.02694906	2305	tags=32%, list=20%, signal=26%	BCL11B/EPHB2/RPS6KA5/EFNB1/FYN/FEZ1/EPHB4/TRIO/ERBB2/EXT1/RNF165/NCAM1/NOTCH2/NECTIN1/EPHB1/SEMA4D/MYCBP2/EPHA4/LYPLA2/CSF1R/DPYSL2/PALLD/SEMA4A/PLXNC1/SCN1B/NIBAN2/RAC1/RAC2/ECE1/BSG/VASP/RHOG

BP	GO:0097485	neuron projection guidance	100	-0.44	-1.46	0.01363012	0.04389478	0.02694906	2305	tags=32%, list=20%, signal=26%	BCL11B/EPHB2/RPS6KA5/EFNB1/FYN/FEZ1/EPHB4/TRIO/ERBB2/EXT1/RNF165/NCAM1/NOTCH2/NECTIN1/EPHB1/SEMA4D/MYCBP2/EPHA4/LYPLA2/CSF1R/DPYSL2/PALLD/SEMA4A/PLXNC1/SCN1B/NIBAN2/RAC1/RAC2/ECE1/BSG/VASP/RHOG
BP	GO:0099024	plasma membrane invagination	53	-0.49	-1.51	0.01363432	0.04389478	0.02694906	1257	tags=28%, list=11%, signal=25%	ANO6/ITGAM/ITGB2/ARF1/ELMO1/GSN/THBS1/RAB31/RAC1/RAC2/RHOA/RHOBTB1/SH3BP1/MYH9/RHOG
BP	GO:0002263	cell activation involved in immune response	232	-0.37	-1.37	0.01376068	0.04413861	0.02709876	2083	tags=26%, list=18%, signal=22%	CR1/FGR/UNC13D/NLRP3/NKG7/CD177/PRKCE/TNFSF13/AP1G1/ATP7A/TGFB1/ADGRE2/TNFSF4/RC3H2/BCL3/NOTCH2/ITGAM/STX4/PTPN6/PYCARD/MAD2L2/SBNO2/RELB/SPI1/PDPK1/ITGB2/LCP1/FOXP1/FES/PGLYRP1/RARA/PTK2B/GAB2/SEMA4A/BCR/STAT6/TBX21/IL6R/PTPRC/SYK/PTAFR/SUPT6H/ST3GAL1/DOCK2/PIK3CG/PRAM1/JAK3/LYN/LGALS9/RAC2/LRP1/DYSF/ITGAL/GATA1/GRN/APBB1IP/STAT3/LAMP1/PIK3CD/CBL/HLX
BP	GO:0043647	inositol phosphate metabolic process	39	-0.55	-1.60	0.01376068	0.04413861	0.02709876	2038	tags=33%, list=17%, signal=28%	PTEN/ITPKB/SYNJ2/PLCB1/SYNJ1/NUDT4/PTK2B/INPP5K/PTAFR/PLEK/ITPK1/SNCA/IP6K1
BP	GO:0001938	positive regulation of endothelial cell proliferation	52	-0.52	-1.57	0.01379826	0.0442078	0.02714124	1286	tags=23%, list=11%, signal=21%	PDPK1/ARNT/PRKD2/IGF2/BMP6/STAT5A/NF1/SP1/LRG1/STAT3/PIK3CD/ITGB3
BP	GO:0035305	negative regulation of dephosphorylation	37	-0.56	-1.60	0.01400902	0.04477997	0.02749252	2521	tags=46%, list=21%, signal=36%	LGALS3/NCKAP1L/CRY2/FKBP1B/PPP1R15A/ROCK2/MGAT5/SEMA4D/CHP1/YWHAB/MFHAS1/INPP5K/GSK3B/GNAI2/FKBP1A/PPP1R9B/IQGAP1
BP	GO:0015844	monoamine transport	26	-0.62	-1.65	0.01437636	0.04576494	0.02809724	742	tags=50%, list=6%, signal=47%	SYT11/DTNBP1/SYT12/NOS1/P2RY1/ABAT/GRK2/SYK/ACTB/PINK1/SNCA/ITGB3/P2RX1
BP	GO:1990849	vacuolar localization	67	-0.48	-1.51	0.01443296	0.04587166	0.02816276	2051	tags=33%, list=17%, signal=27%	FGR/UNC13D/ADGRE2/BORCS6/NDEL1/TFEB/PDPK1/FES/PLEKHM2/GAB2/LAMTOR1/SYK/PIK3CG/LYN/LGALS9/RAC2/SYTL4/GATA1/FLCN/PIK3CD/MYH9/CBL
BP	GO:0032418	lysosome localization	67	-0.48	-1.51	0.01443296	0.04587166	0.02816276	2051	tags=33%, list=17%, signal=27%	FGR/UNC13D/ADGRE2/BORCS6/NDEL1/TFEB/PDPK1/FES/PLEKHM2/GAB2/LAMTOR1/SYK/PIK3CG/LYN/LGALS9/RAC2/SYTL4/GATA1/FLCN/PIK3CD/MYH9/CBL
BP	GO:0042180	cellular ketone metabolic process	125	-0.40	-1.37	0.01448281	0.0459468	0.02820889	1972	tags=22%, list=17%, signal=18%	IRS2/STARD3/PRKCE/PNKD/SCAP/ABCB11/STAT5B/EGR1/NR1H2/CLN3/ACADVL/TRIB3/DGAT2/PDK2/EIF6/GOT2/BCKDK/BMP6/CPT1A/PDK3/NCOR2/ATP2B4/WDTC1/SNCA/AKT2/HAGH/DAB2
BP	GO:0060191	regulation of lipase activity	52	-0.51	-1.56	0.0145441	0.04607257	0.02828611	1528	tags=29%, list=13%, signal=25%	C5AR1/SORT1/NR1H2/ABHD5/PDPK1/GNAQ/PCSK6/RASGRP4/POR/PTAFR/LRP1/ARHGAP6/RHOA/SNCA/FURIN
MF	GO:0004879	nuclear receptor activity	29	-0.59	-1.59	0.01467039	0.04607382	0.02828688	2077	tags=34%, list=18%, signal=28%	THRA/RXRB/VDR/NR6A1/NR1H2/ARNT/RARA/NR3C1/RXRA/STAT3
BP	GO:0032411	positive regulation of transporter activity	66	-0.47	-1.49	0.01463956	0.04607382	0.02828688	1161	tags=24%, list=10%, signal=22%	CHP1/KMT2A/HTT/SLC9A1/PTAFR/ATP1B2/EHD3/CALM3/SLC6A9/RXRA/DNM2/ACTN4/PINK1/TESC/STIM1/WNK1

BP	GO:0048736	appendage development	90	-0.44	-1.47	0.01462022	0.04607382	0.02828688	2488	tags=27%, list=21%, signal=21%	PBX2/BAK1/IFT122/KAT2B/BCL2L11/EXT1/NIPBL/RNF165/MKS1/RC3H2/NOTCH2/MSX1/GREM1/RARA/CHST11/PIAS4/ZBTB16/SFRP2/MED1/SKI/CREBBP/GNA12/ECE1/PBX1
BP	GO:0060173	limb development	90	-0.44	-1.47	0.01462022	0.04607382	0.02828688	2488	tags=27%, list=21%, signal=21%	PBX2/BAK1/IFT122/KAT2B/BCL2L11/EXT1/NIPBL/RNF165/MKS1/RC3H2/NOTCH2/MSX1/GREM1/RARA/CHST11/PIAS4/ZBTB16/SFRP2/MED1/SKI/CREBBP/GNA12/ECE1/PBX1
MF	GO:0098531	ligand-activated transcription factor activity	29	-0.59	-1.59	0.01467039	0.04607382	0.02828688	2077	tags=34%, list=18%, signal=28%	THRA/RXRB/VDR/NR6A1/NR1H2/ARNT/RARA/NR3C1/RXRA/STAT3
BP	GO:1901186	positive regulation of ERBB signaling pathway	25	-0.63	-1.64	0.01469893	0.04609065	0.02829721	1267	tags=28%, list=11%, signal=25%	NCF1/RBPJ/NEURL1/MMP9/HIP1/SHKBP1/CBL
BP	GO:0031098	stress-activated protein kinase signaling cascade	187	-0.38	-1.41	0.01484775	0.0465206	0.02856117	2267	tags=32%, list=19%, signal=26%	MAP2K4/DUSP1/GPS1/HDAC3/SH3RF1/STK25/MAP4K4/FOXO1/MAP3K4/TIRAP/PER1/XIAP/GRIK2/SIRPA/SDCBP/SH3RF3/GSTP1/LRRK2/HGF/NBR1/GPS2/PLCB1/EZR/PHLPP1/RIPK1/KAT7/EPHB1/MAPK14/TRIB1/MAP2K2/EIF2AK2/ZFP36L1/PYCARD/SPI1/ITCH/NCF1/SASH1/MAP3K5/NFKB1/TAOK3/PTK2B/MFHAS1/TRAF6/SH2D3C/TAOK1/MAP4K1/SFRP2/MAP2K7/MTURN/HIPK2/LYN/LGALS9/MAP3K3/RHOA/PINK1/MAPK3/MAPKAPK2/MAP2K3/STRADB
BP	GO:0061387	regulation of extent of cell growth	62	-0.48	-1.50	0.01490187	0.04665342	0.02864272	2665	tags=37%, list=23%, signal=29%	ARHGAP4/PLXNA4/TWF2/KIAA0319/DIP2B/DISC1/MACF1/SRF/ADNP/DBNL/NDEL1/DBN1/CDKL5/SEMA4D/DPYSL2/SEMA4A/EIF4G2/IST1/ULK1/SIN3A/GSK3B/DNM2/CTTN
BP	GO:0030031	cell projection assembly	392	-0.32	-1.26	0.01522182	0.04739385	0.0290973	3013	tags=32%, list=26%, signal=25%	DTNBP1/PFN1/AUTS2/SEPTIN9/ODAD3/TTC26/CNTROB/ATMIN/RABL2B/INPPL1/CEP164/MPHOSPH9/NOTCH1/CEP131/TRAPPC14/SEPTIN6/STK36/PLCE1/KCNQ1/KIT/ARL13B/RO60/GBF1/CARMIL1/MAK/EMP3/FGD6/STAU2/HDAC6/CFAP410/EHD4/TWF2/ABLIM1/CDC42EP1/FSCN1/ACTR2/FBF1/UBE2B/NCKAP1L/SCIN/SLC9A3R1/IFT122/EPHB2/ARHGAP24/DISC1/SRF/LIMK2/WNT1/SRC/KCTD17/EXOC4/DBNL/ATP7A/EPS8/MKS1/BBOF1/UNC119B/ANO6/ARMC2/SDCBP/SYNE2/TCHP/TMEM250/NDEL1/EZR/ALPK1/TMEM67/WASF3/CDKL5/S1PR2/RAP1GAP/PARVG/CDC42EP4/ARAP1/FUZ/CLN3/ABI3/MAP4/RFX2/EVI5L/PDGFA/FXR2/CEP350/CORO1C/HTT/FGD4/NEURL1/SNX10/INPP5K/RAPGEF2/BCAS3/KLF5/MARK4/VCL/FGD3/GSN/DYNLL2/ASAP1/DAW1/EHD3/DCTN1/FLNA/DNM3/ABCC4/ODAD4/RAC1/GSK3B/RAC2/DNM2/CAPZB/RILP/PPP1R9B/WAS/ESPN/MYLK/PARVB/PTPN23/EHD1/CTTN/RIPOR2/PLEK2/ABLIM3/ATP6V0D1/KLC3/WASF2/RHOG/DMTN
BP	GO:0033619	membrane protein proteolysis	42	-0.52	-1.52	0.01528105	0.04754103	0.02918767	2910	tags=36%, list=25%, signal=27%	TNF/SPPL2B/APH1A/NCSTN/GPLD1/ADAM17/ADAM8/SPPL3/TGFB1/NRDC/TIMP2/TNFRSF1B/MYH9/TIMP1/FURIN
BP	GO:0015837	amine transport	32	-0.57	-1.58	0.01562005	0.04844409	0.02974209	3183	tags=44%, list=27%, signal=32%	SYT11/DTNBP1/SYT12/TNF/SV2A/P2RY1/ABAT/GRK2/PER2/AQP9/SLC43A2/SLC43A1/PINK1/SNCA
BP	GO:0046503	glycerolipid catabolic process	37	-0.55	-1.59	0.01610951	0.04992316	0.03065017	1624	tags=32%, list=14%, signal=28%	PLCB1/LIPE/SMPD4/ABHD5/LYPLA2/SORL1/MGLL/PIK3CG/ABHD2/GDE1/PNPLA2/PRDX6

BP	GO:0000902	cell morphogenesis	591	-0.48	-1.96	1.00E-10	3.79E-09	2.32E-09	2769	tags=39%, list=24%, signal=31%	DNMBP/AKIP1/ALCAM/KIT/EPHA1/F11R/SYNGAP1/ARL13B/BCL9L/PLEKHO1/LAMC3/NEO1/CNP/ST6GAL1/ARHGAP4/CARMIL1/PLXNA4/CALR/EPB41L3/FGD6/P2RY1/STAU2/HDAC6/AMIGO1/CFAP410/TWF2/CDC42EP1/VAMP3/CREB1/ACTR2/TBCD/SCRIB/KIAA0319/CORO1A/NCKAP1L/DIP2B/SLC9A3R1/BCL11B/MYO9A/KIF13B/MUL1/EPHB2/BCL11A/LIMS1/ADAM8/STK25/MAP4K4/RPS6KA5/EFNB1/ASXL1/NIN/SPRY3/PARD6B/ZNF385A/SHROOM1/HEG1/DISC1/MACF1/FGR/SRF/FYN/PTEN/FEZ1/UNC13D/SRC/TRIOBP/MAP1S/EPHB4/PDZD8/YWHAH/TRIO/RHOF/KIDINS220/ADNP/DBNL/STK4/ERBB2/EXT1/VDR/RNF165/ATP7A/HCK/CASS4/EPS8/TIAM1/MEGF9/DICER1/NCAM1/CUL7/CPNE5/SZT2/NOTCH2/NECTIN1/NUMB/LRRK2/HGF/B4GALT5/NBEAL2/SIDT2/NDEL1/EZR/DLG4/DBN1/DOCK5/WASF3/EPHB1/CPNE1/MAPK14/CDKL5/SIPA1L1/FLOT1/MSX1/PARVG/CAMSAP1/PTPN6/WASHC2C/CDC42EP4/PARD3/FRY/SEMA4D/MYCBP2/ARAP1/ABI3/DAPK3/AGO4/SPI1/PREX1/EPHA4/FERMT3/LYPLA2/ITGB2/CSF1R/ARHGEF2/WDR1/CAP1/SSH1/DPYSL2/ZRANB1/ZNF335/NEDD9/BRWD3/SH3KBP1/PALLD/TPM1/FES/TAOK3/GREM1/VLDLR/CORO1C/PTK2B/FGD4/SPTA1/SEMA4A/RAPGEF2/EIF4G2/ZMYM3/PAK1/MAP1A/VCL/RERE/FGD3/TAOK1/ALDOA/STK11/PLXNC1/PPP3CA/APLP2/USP9X/PDLIM5/DVL3/SCN1B/PLEC/MED1/IST1/FLNA/ULK1/NIBAN2/DNM3/FRYL/ACTB/FMNL1/RAC1/MARK2/SIN3A/CYFIP2/GSK3B/RAC2/DNM2/PXN/MPL/RHOA/CAPZB/GATA1/MYADM/CLU/MSN/ACTN4/GNA12/ECE1/CFL1/RHOBTB1/EPB41/TBC1D20/PARVB/SPARC/CTTN/GAS7/TAL1/UBB/ACTN1/GP1BA/EPB42/BSG/PRKDC/MPIG6B/PAK2/ITGB3/VASP/DAB2/MBP/CUX1/KEL/MYH9/IQGAP1/PEAK3/WASF2/STRADB/XK/RHOG/DMTN/DIAPH1
BP	GO:0001525	angiogenesis	296	-0.52	-1.98	1.00E-10	3.79E-09	2.32E-09	1942	tags=32%, list=17%, signal=27%	SRF/PTEN/EPHB4/NINJ1/JMJD6/MAP2K5/STK4/PROK2/ERBB2/VEZF1/RRAS/NOTCH2/SLC12A6/GTF2I/HGS/RNF213/MTDH/ACTG1/ROCK2/BTG1/HGF/GAB1/C5AR1/SHC1/EPHB1/MAPK14/PDPK1/PML/LEPR/ADIPOR2/SASH1/YWHAZ/AGO2/ANPEP/PDGFA/PKM/AMOT/GREM1/HIPK1/RBPJ/PTK2B/SEMA4A/MEIS1/WARS1/BCAS3/PRKD2/KLF5/CALD1/SFRP2/ITGA5/SYK/SH2D2A/FOXO4/JAM3/THBS1/AGO1/MED1/FZD5/PIK3CG/HIPK2/B4GALT1/FLNA/NIBAN2/PF4/AQP1/MAP3K3/HSPB1/ITGA2B/SRPK2/ATP2B4/EPN1/RHOA/FOXJ2/PRKCB/GPX1/NF1/SP1/LRG1/GRN/JAK1/STAT3/SPARC/CLDN5/TAL1/PIK3CD/BSG/STIM1/ITGB3/E2F2/KRT1/MYH9/GLUL/TBXA2R/WASF2
BP	GO:0001568	blood vessel development	391	-0.51	-2.00	1.00E-10	3.79E-09	2.32E-09	2483	tags=36%, list=21%, signal=29%	BAK1/SETD2/ATP5F1B/ANXA2/SOCS3/CFLAR/ALOX5/GPLD1/CYBB/SIRT6/NFE2L2/HDAC7/EPHB2/ARHGAP24/PTK7/ADAM8/CHI3L1/FOXO1/MYO1E/HEG1/SRF/PTEN/EPHB4/ANKRD17/NINJ1/JMJD6/MAP2K5/STK4/PROK2/ERBB2/VEZF1/ATP7A/TGFB1/PDE2A/RRAS/CUL7/NOTCH2/SLC12A6/GTF2I/HGS/RNF213/MTDH/ACTG1/ROCK2/BTG1/HGF/AP2B1/SNX17/GAB1/EGR1/C5AR1/SHC1/EPHB1/MAPK14/ACTA2/ZFP36L1/SPI1/PDPK1/PML/HIF1AN/LEPR/ADIPOR2/SASH1/YWHAZ/AGO2/ANPEP/PDGFA/PKM/AMOT/SGPL1/SUFU/GREM1/HIPK1/RBPJ/PTK2B/SEMA4A/MEIS1/WARS1/ARID2/RAPGEF2/BCAS3/PRKD2/KLF5/IL6R/CALD1/PECAM1/ZMIZ1/SFRP2/ITGA5/SYK/SH2D2A/FOXO4/JAM3/THBS1/AGO1/MED1/FZD5/PIK3CG/HIPK2/B4GALT1/FLNA/NIBAN2/NDST1/PF4/AQP1/MAP3K3/LRP1/HSPB1/ITGA2B/SRPK2/TCF7L2/ATP2B4/EPN1/RHOA/FOXJ2/PRKCB/GPX1/NF1/SP1/LRG1/GRN/JAK1/MYLK/STAT3/SPARC/CLDN5/TAL1/PIK3CD/BSG/STIM1/ITGB3/E2F2/KRT1/LYL1/MYH9/GLUL/NPRL3/TBXA2R/WASF2

BP	GO:0001667	ameboidal-type cell migration	254	-0.53	-2.01	1.00E-10	3.79E-09	2.32E-09	2665	tags=37%, list=23%, signal=30%	ARHGAP4/SVBP/RIN2/CCN3/CALR/ZEB2/CLASP1/HDAC6/INSL3/ATP5F1B/MAPRE2/RFFL/GPLD1/ADAM17/NFE2L2/SCARB1/HDAC7/PKN1/MAP4K4/EFNB1/GLIPR2/MACF1/SRF/PTEN/PPM1F/SRC/EPHB4/MAP2K5/PRKCE/ATOH8/SDC4/TGFB1/PHACTR4/RRAS/ROCK2/DOCK5/ACTA2/ARHGDIB/SEMA4D/CLN3/PDPK1/PML/CAP1/FOXP1/SASH1/AGO2/AMOT/YTHDF3/SGPL1/GREM1/KANK2/CORO1C/PTK2B/SLC8A1/SEMA4A/BCAS3/PRKD2/MMP9/THBS1/PLEC/ADIPOR1/PIK3CG/ANXA6/GFUS/GPI/RAC1/MAP3K3/STAT5A/HSPB1/ATP2B4/PXN/IQSEC1/RHOA/GPX1/PDLIM1/NF1/SP1/GRN/GNA12/PTPN23/SPARC/SH3BP1/PIK3CD/PTP4A3/DNAJA4/ITGB3/MYH9/GLUL/TIMP1/TNS1/IQGAP1/TBXA2R/MAP2K3/WASF2/DMTN
BP	GO:0001932	regulation of protein phosphorylation	706	-0.41	-1.68	1.00E-10	3.79E-09	2.32E-09	2211	tags=29%, list=19%, signal=25%	SH2B3/HDAC3/PKN1/AXIN2/MAVS/TNK2/ADAM8/RPS6KA5/TADA3/CDKN2D/IRAK3/TNXB/CHI3L1/SPRY3/HEG1/FGR/FYN/LIMK2/WNT1/PTEN/MAP3K4/TIRAP/PPM1F/SRC/FNIP2/ATM/AKT1S1/MLLT1/ADNP/CDK12/MAP2K5/STK4/PRKCE/SDC4/XRCC6/ERBB2/CCNY/CASS4/WDFY2/MAGED1/TGFB1/SIRPA/LDLRAD4/ITPKB/ERN2/LILRA5/ERN1/SDCBP/HGS/TSC2/SYAP1/ROCK2/GSTP1/ARRB1/LRRK2/HGF/PIP5KL1/STK38/CACUL1/EGR1/RIPK1/DLG4/ROPN1L/C5AR1/SMG8/SHC1/TRIB1/CEBPA/S1PR2/CTDSP2/NF2/MAP2K2/PTPRJ/FLOT1/PTPN6/PYCARD/PARD3/SEMA4D/MAD2L2/CDKN1C/DAZAP2/CLN3/LRRK1/EPHA4/PDPK1/GNAQ/NCF1/CCNF/PML/RALBP1/ITGB2/CSF1R/ARHGEF2/NSD1/TRIB3/LEPR/CHP1/SASH1/PDGFA/NEDD9/INSR/SESN2/MAP3K5/SLC27A1/TAOK3/FXR2/GREM1/VLDLR/SH3BP5L/SORL1/CORO1C/HTT/PTK2B/NEURL1/SLC8A1/WARS1/INPP5K/RAPGEF2/RPS6KA4/CCND3/TRAF6/PRKD2/IL6R/SH2D3C/PAK1/MOB3A/IGF2/PECAM1/CSK/BMP6/MMP9/TAOK1/SFRP2/STK11/ITGA5/MAP2K7/PELI2/STRADA/PTPRC/SYK/ADAR/DVL3/THBS1/PTPN1/PLEC/TLR6/CTDSPL/CALM3/HEXIM2/FZD5/LTF/PIK3CG/HIPK2/PPP2R5B/EIF4G1/ACTB/MADD/GJC2/RAC1/MARK2/LYN/MMD/PDE5A/LRP1/HSPB1/AKAP13/ATP2B4/CTDSP1/RHOA/GATA1/MYADM/CLU/ARRB2/NF1/CCNI/FKBP8/PPP1R9B/HCLS1/PINK1/MAPK3/SNCA/CDKN1A/FLCN/NIBAN1/PRKAR2A/TAL1/PRKAR2B/SQSTM1/AKT2/ARAF/CCNJL/PRKDC/BRD4/PAK2/ITGB3/PRR5/DAB2/WNK1/FBXO7/CBL/IQGAP1/MAP2K3/EIF2AK1/STRADB/FAXDC2/CNPPD1/DMTN
BP	GO:0001944	vasculature development	413	-0.51	-2.00	1.00E-10	3.79E-09	2.32E-09	2483	tags=35%, list=21%, signal=29%	BAK1/SETD2/ATP5F1B/ANXA2/SOCS3/CFLAR/ALOX5/GPLD1/CYBB/SIRT6/NFE2L2/HDAC7/EPHB2/ARHGAP24/PTK7/ADAM8/CHI3L1/FOXO1/SOD2/MYO1E/HEG1/SRF/PTEN/UNC13D/EPHB4/ANKRD17/NINJ1/JMJD6/MAP2K5/STK4/PROK2/ERBB2/VEZF1/ATP7A/TGFB1/PDE2A/RRAS/CUL7/NOTCH2/SLC12A6/GTF2I/HGS/RNF213/MTDH/ACTG1/ROCK2/BTG1/HGF/AP2B1/SNX17/GAB1/EGR1/NFATC1/C5AR1/SHC1/EPHB1/MAPK14/ACTA2/ZFP36L1/SPI1/PDPK1/PML/HIF1AN/LEPR/ADIPOR2/SASH1/YWHAZ/AGO2/ANPEP/PDGFA/PKM/AMOT/SGPL1/SUFU/GREM1/HIPK1/RBPJ/NFATC2/PTK2B/SEMA4A/MEIS1/WARS1/ARID2/RAPGEF2/BCAS3/PRKD2/KLF5/IL6R/CALD1/PECAM1/ZMIZ1/SFRP2/STK11/ITGA5/SYK/SH2D2A/FOXO4/JAM3/THBS1/AGO1/MED1/FZD5/PIK3CG/HIPK2/B4GALT1/FLNA/NIBAN2/NDST1/PF4/AQP1/MAP3K3/LRP1/HSPB1/ITGA2B/SRPK2/TCF7L2/ATP2B4/EPN1/RHOA/FOXJ2/NFATC3/PRKCB/GPX1/NF1/SP1/LRG1/GRN/JAK1/MYLK/STAT3/SPARC/CLDN5/TAL1/PIK3CD/BSG/STIM1/ITGB3/E2F2/KRT1/LYL1/MYH9/GLUL/NPRL3/TBXA2R/WASF2

MF	GO:0003779	actin binding	291	-0.57	-2.17	1.00E-10	3.79E-09	2.32E-09	2158	tags=37%, list=18%, signal=31%	MYO1G/VPS18/FLII/SHROOM1/MYO1E/MACF1/TRIOBP/MAP1S/YWHAH/PHACTR2/MSRB2/DIAPH2/ACTR3C/DBNL/MYO18A/PRKCE/MARCKS/LSP1/EPS8/TPM3/PHACTR4/SYNE2/LRRK2/ENC1/EZR/SPTAN1/ARPC1B/DBN1/INF2/WASF3/NF2/PARVG/MYOM1/FHL3/CTNNAL1/ABI3/ARPC5/WDR1/CAP1/LCP1/SSH1/TPM4/WIPF2/PALLD/TPM1/S100A4/SPTBN1/CORO1C/PLEKHG3/FGD4/ZNF185/SPTA1/MSRB1/SVIL/CALD1/MAP1A/VCL/PDLIM7/CTNNA1/ALDOA/GSN/PDLIM5/MYO9B/PLEC/IQGAP2/MYO1F/FLNA/ANXA6/FMNL1/HIP1/MYL4/PPP1R18/CNN2/LASP1/WIPF1/COTL1/CAPZB/GAS2L1/PDLIM1/MSN/ACTN4/ADD1/PPP1R9B/SSH2/HCLS1/TMOD1/ARPC4/CFL1/WAS/ESPN/MYLK/SNCA/EPB41/PARVB/SPTB/CTTN/GAS7/ABLIM3/ACTN1/VASP/MYH9/TNS1/IQGAP1/MICAL2/WASF2/DMTN/DIAPH1/TLN1
MF	GO:0004672	protein kinase activity	407	-0.56	-2.19	1.00E-10	3.79E-09	2.32E-09	2329	tags=35%, list=20%, signal=29%	MAP4K2/AATK/MAST4/MAP2K4/PIM3/AAK1/EPHB2/DYRK3/PKN1/PTK7/TNK2/STK25/MAP4K4/SRPK1/RPS6KA5/IRAK3/CDC42BPA/GRK3/FGR/FYN/LIMK2/MAP3K4/SRC/TOP1/EPHB4/TRIO/ATM/CDC42BPB/CPNE3/CDK12/MAP2K5/STK4/PRKCE/ERBB2/HCK/ERN2/ERN1/ROCK2/LRRK2/IKBKE/STK38/DYRK1B/ALPK1/RIPK1/PIKFYVE/CLK3/EPHB1/MAPK14/PHKA2/STK10/CDKL5/GRK2/MAP2K2/EIF2AK2/MAP3K2/RSKR/DAPK3/CSNK1G2/MARK3/LRRK1/EPHA4/PDPK1/MAST2/CDK13/CSF1R/TRIM28/GSK3A/GRK6/PDK2/PRKACA/BCKDK/INSR/STK24/CSNK2A1/MAP3K5/PSKH1/FES/TAOK3/HIPK1/MKNK2/PIM2/PTK2B/VRK3/BCR/RPS6KA4/SIK3/CCND3/PRKD2/MARK4/NEK5/PAK1/CSK/TAOK1/MAP4K1/STK11/MAP2K7/SYK/BMP2K/STK40/PDK3/MAPKAPK3/PIK3CG/HIPK2/CAMK1D/ULK1/JAK3/MARK2/LYN/RIOK3/MAP3K3/MMD/GSK3B/IGF2R/CDK19/AKAP13/SRPK2/PRKCB/CSNK1G1/SCYL1/PPP1R9B/JAK1/PIM1/PINK1/RPS6KA1/MYLK/MAPK3/CDKN1A/NUAK2/LTBP1/MAPKAPK2/SQSTM1/AKT2/ARAF/GAK/PRKDC/BRD4/PAK2/WNK1/MAST3/MAP2K3/EIF2AK1/PEAK3
MF	GO:0004674	protein serine/threonine kinase activity	327	-0.55	-2.13	1.00E-10	3.79E-09	2.32E-09	2329	tags=36%, list=20%, signal=29%	MAP4K2/AATK/MAST4/MAP2K4/PIM3/AAK1/DYRK3/PKN1/TNK2/STK25/MAP4K4/SRPK1/RPS6KA5/IRAK3/CDC42BPA/GRK3/LIMK2/MAP3K4/TOP1/TRIO/ATM/CDC42BPB/CPNE3/CDK12/MAP2K5/STK4/PRKCE/ERN2/ERN1/ROCK2/LRRK2/IKBKE/STK38/DYRK1B/ALPK1/RIPK1/PIKFYVE/CLK3/MAPK14/PHKA2/STK10/CDKL5/GRK2/MAP2K2/EIF2AK2/MAP3K2/RSKR/DAPK3/CSNK1G2/MARK3/LRRK1/PDPK1/MAST2/CDK13/GSK3A/GRK6/PRKACA/BCKDK/STK24/CSNK2A1/MAP3K5/PSKH1/TAOK3/HIPK1/MKNK2/PIM2/PTK2B/VRK3/BCR/RPS6KA4/SIK3/CCND3/PRKD2/MARK4/NEK5/PAK1/TAOK1/MAP4K1/STK11/MAP2K7/SYK/BMP2K/STK40/PDK3/MAPKAPK3/PIK3CG/HIPK2/CAMK1D/ULK1/MARK2/RIOK3/MAP3K3/GSK3B/CDK19/AKAP13/SRPK2/PRKCB/CSNK1G1/PIM1/PINK1/RPS6KA1/MYLK/MAPK3/NUAK2/LTBP1/MAPKAPK2/SQSTM1/AKT2/ARAF/GAK/PRKDC/BRD4/PAK2/WNK1/MAST3/MAP2K3/EIF2AK1

MF	GO:0004712	protein serine/threonine/tyrosine kinase activity	325	-0.56	-2.17	1.00E-10	3.79E-09	2.32E-09	2329	tags=36%, list=20%, signal=29%	MAP4K2/AATK/MAST4/MAP2K4/PIM3/AAK1/EPHB2/DYRK3/PKN1/TNK2/STK25/MAP4K4/SRPK1/RPS6KA5/CDC42BPA/FGR/FYN/LIMK2/MAP3K4/SRC/EPHB4/TRIO/ATM/CDC42BPB/CDK12/MAP2K5/STK4/PRKCE/ERBB2/HCK/ERN2/ERN1/ROCK2/LRRK2/STK38/DYRK1B/ALPK1/RIPK1/PIKFYVE/CLK3/EPHB1/MAPK14/STK10/CDKL5/MAP2K2/EIF2AK2/MAP3K2/RSKR/DAPK3/CSNK1G2/MARK3/LRRK1/EPHA4/PDPK1/MAST2/CDK13/CSF1R/GSK3A/PRKACA/INSR/STK24/CSNK2A1/MAP3K5/PSKH1/FES/TAOK3/HIPK1/MKNK2/PIM2/PTK2B/BCR/RPS6KA4/SIK3/PRKD2/MARK4/NEK5/PAK1/CSK/TAOK1/MAP4K1/STK11/MAP2K7/SYK/BMP2K/STK40/MAPKAPK3/PIK3CG/HIPK2/CAMK1D/ULK1/JAK3/MARK2/LYN/RIOK3/MAP3K3/GSK3B/CDK19/SRPK2/PRKCB/CSNK1G1/JAK1/PIM1/PINK1/RPS6KA1/MAPK3/NUAK2/MAPKAPK2/AKT2/ARAF/GAK/PRKDC/PAK2/WNK1/MAST3/MAP2K3/EIF2AK1
MF	GO:0005096	GTPase activator activity	207	-0.55	-2.04	1.00E-10	3.79E-09	2.32E-09	2665	tags=40%, list=23%, signal=32%	ARHGAP4/RUNDC1/RIN2/RGS3/AGAP1/RGS7/TBCD/ARHGAP32/NCKAP1L/RGS16/ARFGAP2/TBC1D8/SGSM1/MYO9A/RANBP3/ARHGAP24/ARHGEF11/ARHGEF12/RASA2/ARHGAP19/LARS1/ACAP2/TSC2/ARHGAP9/ARRB1/LRRK2/RANGAP1/RALGAPA1/PLCB1/TBC1D17/RASAL3/TBC1D3I/DOCK5/GIT2/SIPA1L1/RAP1GAP/ARHGDIB/ARAP1/PREX1/TBC1D22A/GNAQ/RALBP1/RALGAPA2/EVI5L/TBC1D10B/RABGAP1/RGS6/ARHGAP31/ARHGAP21/ADAP2/BCR/RAPGEF2/TBC1D22B/RIN1/GMIP/ADAP1/SMAP2/SIPA1L2/MYO9B/ASAP1/ASAP2/ARHGAP8/DOCK2/IQGAP2/TBC1D13/SIPA1/GAPVD1/ARHGAP6/TBC1D25/ARHGAP45/NF1/ARHGDIA/RIN3/ARHGAP26/TBC1D20/FLCN/TBC1D5/SH3BP1/TBC1D1/ARHGAP1/TBC1D2/NPRL3/IQGAP1
CC	GO:0005768	endosome	749	-0.42	-1.71	1.00E-10	3.79E-09	2.32E-09	2700	tags=34%, list=23%, signal=28%	SNX18/VTI1B/HYAL3/ATP11A/ZDHHC2/VPS26B/CCDC22/CARMIL1/ABCA3/RAB22A/APH1A/NCSTN/CD4/MCTP1/HDAC6/VPS4A/GGA3/EHD4/STOML1/UBE2D3/PMEPA1/VAMP3/FIG4/ANXA2/TRAK1/DNAJC13/RFFL/KIAA0319/ARHGAP32/ZFYVE21/MGRN1/CORO1A/MIB2/TOM1L2/WLS/PRKAR1B/ATP9A/F8A1/PI4K2A/LNPEP/ANTXR2/GIGYF2/HPS6/MYO5A/TF/IL2RG/PKN1/TNK2/VPS53/UBC/PLD4/VPS18/AVL9/VPS4B/RFTN1/SLA2/RAB11FIP5/FYN/TPCN1/ATP6V0B/ITSN1/UBA52/UNC13D/SLC29A3/SRC/RAB27B/ZNRF1/KIDINS220/IFNAR1/DIAPH2/MCOLN1/DBNL/STARD3/MARCHF3/ERBB2/CD68/AP1G1/CMTM6/NCF4/ATP7A/WDFY2/STX6/LDLRAD4/SNX3/WDR81/OSBPL1A/SUN2/AOC3/AP2A2/ACAP2/LAMP2/HGS/ANKRD13A/CPTP/NUMB/RAB11FIP4/ABCB11/LRRK2/NBR1/AP2B1/SNX17/EZR/AP2S1/TMBIM1/RIPK1/TBC1D17/PIKFYVE/RNF11/VPS51/MME/EPHB1/CLCN5/STX4/PLEKHF1/CHMP4B/NF2/MAP2K2/RAP1GAP/FLOT1/CLSTN1/SORT1/WASHC2C/CD63/GRAP2/IFITM3/VPS41/CLN3/VPS35/ATP6V0C/ITCH/FCGRT/EPHA4/MON1B/ARPC5/PML/CLEC16A/ATP6AP1/CDIP1/BIRC6/TMEM184A/PIP5K1C/GRB2/SNX8/OPTN/VPS37B/VPS37C/CHID1/INSR/ATP6V0A1/SPG21/USP10/VPS11/AP2A1/TPP1/PLEKHM2/SORL1/CORO1C/SH3GL1/HTT/KIF13A/SNX10/RAPGEF2/TRAF6/SBF2/LDLRAP1/TGOLN2/RAB7A/ANKFY1/RAB14/ARL8A/LAMTOR1/SNX11/RAB5B/SLC66A2/PTPN1/SCAMP2/RAB31/EHD3/TOM1/PLIN3/UBAP1/CLTC/SLC6A9/FZD5/LTF/RAB11FIP1/ULK1/JAK3/RUBCN/ANXA6/YIPF2/VPS28/TGFBRAP1/CD14/CHMP1A/RAC1/SLA/SNX27/MMD/ELAPOR1/GAPVD1/UBA1/DNM2/IGF2R/PSAP/RAB11B/LRP1/FLOT2/RUFY1/IFITM2/SYTL4/DYSF/TRAK2/EPN1/GRIPAP1/PRF1/RHOA/RILP/ARRB2/GRN/JAK1/RIN3/ECE1/RHOBTB1/MARCHF2/MAPK3/SIAH2/DTX3L/RAPGEF1/PTPN23/EHD1/LAMP1/SLC15A3/UBB/SQSTM1/TBC1D5/AKT2/TMEM127/LITAF/ATP6V0D1/AP2M1/PTP4A3/MMGT1/BSG/CTSB/SLC48A1/RAB3D/CTSD/UBXN6/ARHGAP1/ACKR1/MARCHF8/WASF2/FURIN

CC	GO:0005911	cell-cell junction	287	-0.54	-2.06	1.00E-10	3.79E-09	2.32E-09	2202	tags=36%, list=19%, signal=30%	LIMS1/ARHGAP24/PTK7/TNK2/STRN/PARD6B/SHROOM1/MYO1E/HEG1/CDC42BPA/CD99L2/YWHAH/CDC42BPB/TIAM1/SDCBP/MTDH/NECTIN1/TCHP/ACTG1/ABCB11/ASH1L/EZR/GAB1/DBN1/PIKFYVE/NF2/MAP2K2/PTPRJ/FLOT1/PTPN6/CDC42EP4/PARD3/CTNND1/FRMD4B/EPHA4/PKP4/PANX2/ARHGEF2/WDR1/PCDH1/TJP2/PIP5K1C/GRB2/PPP1CA/OCEL1/AMOT/S100A11/SH3KBP1/NHS/SLC2A1/SLC8A1/RAPGEF2/EIF4G2/PAK1/FRMD4A/VCL/PECAM1/PDLIM7/CTNNA1/CSK/VAV1/PPP3CA/ITGA5/PDLIM5/TSPAN33/SLC9A1/JAM3/SCN1B/ESAM/FZD5/B4GALT1/FLNA/NIBAN2/ACTB/ZYX/GJC2/LYN/UBA1/FLOT2/CNN2/PXN/RHOA/MYADM/PDLIM1/MSN/UBN1/ADD1/PPP1R9B/WAS/FLCN/CLDN5/ACTN1/SH3BP1/MPP1/PAK2/ITGB3/YBX3/VASP/MYH9/IQGAP1/WASF2/TLN1
CC	GO:0005938	cell cortex	212	-0.62	-2.30	1.00E-10	3.79E-09	2.32E-09	2206	tags=49%, list=19%, signal=41%	FSCN1/SEPTIN4/ACTR2/TRAK1/FNBP1/ARHGAP32/CORO1A/UTRN/SCIN/STIMATE/ENO2/CLTB/SEPTIN10/FRMPD1/PARD6B/SHROOM1/MYO1E/EXOC4/RHOF/DBNL/MARCKS/DCTN4/EPS8/AGTRAP/CTBP2/TCHP/EXOC7/TMEM250/EZR/SPTAN1/RASAL3/DLG4/DBN1/CTBP1/NF2/FLOT1/PARD3/FRY/EXOC3/CTNND1/CSNK1G2/ITCH/NUMA1/WDR1/EXOC6B/ITPR2/CAP1/TPM4/SEPTIN1/NEDD9/GUCY1B1/SPTBN1/MYZAP/CORO1C/PTK2B/SLC2A1/SPTA1/TRAF6/DYNC1H1/CALD1/GSN/NRBP1/MYO9B/DCTN1/FLNA/PVALB/FRYL/ACTB/FMNL1/RAC1/RAC2/FLOT2/AKAP13/LASP1/PXN/COTL1/RHOA/CAPZB/ACTR1A/PRKCB/MYADM/ACTN4/PPP1R9B/HCLS1/TMOD1/RHOBTB1/SNCA/EPB41/SPTB/CTTN/CLDN5/ACTN1/AKT2/SLC4A1/ENO1/EPB42/STIM1/MPP1/MYL9/GYPC/MYH9/IQGAP1/RHOG/DMTN
BP	GO:0006897	endocytosis	389	-0.51	-2.01	1.00E-10	3.79E-09	2.32E-09	2074	tags=33%, list=18%, signal=28%	MYO1E/SNAP25/GRK3/TPCN1/ITSN1/SRC/RAB27B/CD177/HOOK2/DBNL/JMJD6/MYO18A/MARCHF3/AP1G1/SYNJ2/AP2A2/ANO6/SDCBP/TSC2/ANKRD13A/ACTG1/NUMB/ITGAM/ARRB1/LRRK2/AP2B1/SYNJ1/SNX17/EZR/AP2S1/DLG4/PIKFYVE/CLCN5/GRK2/DENND1C/FLOT1/TEX261/SORT1/PYCARD/ATXN2/NR1H2/CD63/CLN3/SYNRG/CSNK1G2/RALBP1/ITGB2/CAP1/ARF1/DPYSL2/PIP5K1C/CXCL16/GRB2/CANX/INSR/LRP10/SH3KBP1/GREM1/AP2A1/VLDLR/SORL1/SH3GL1/APOBR/SNX10/CXCR2/RIN1/LDLRAP1/PDLIM7/RAB7A/ANKFY1/ELMO1/GSN/PPP3CA/RAB5B/SYK/THBS1/BMP2K/PTPN1/RAB31/EHD3/MAPKAPK3/TOM1/CD151/CLTC/CALM3/DOCK2/PIK3CG/DNM3/RUBCN/ACTB/VPS28/CD14/RAC1/HIP1/HNRNPK/GAPVD1/RAC2/DNM2/IGF2R/LRP1/REPS2/RUFY1/EPN1/EPS15L1/CSNK1G1/CLU/ARRB2/CXCR1/RIN3/RHOBTB1/MARCHF2/MAPK3/SNCA/BCL2L1/EHD1/CTTN/MAPKAPK2/TBC1D5/SH3BP1/DENND1A/AP2M1/GAK/STON2/ITGB3/DAB2/MRC2/MYH9/CBL/WASF2/RHOG
BP	GO:0007015	actin filament organization	308	-0.54	-2.07	1.00E-10	3.79E-09	2.32E-09	2079	tags=37%, list=18%, signal=32%	CORO1A/PPFIA1/NCKAP1L/SCIN/ARHGEF5/MYO5A/CORO7/TF/MYO1G/FLII/SHROOM1/MYO1E/SRF/PPM1F/SRC/TRIOBP/MSRB2/RHOF/DIAPH2/DBNL/PRKCE/MARCKS/SDC4/HCK/CASS4/EPS8/TPM3/PPARGC1B/CYRIA/ACTG1/ROCK2/ARRB1/EZR/SPTAN1/ARPC1B/DBN1/WASF3/NF2/WASHC2C/PYCARD/CDC42EP4/MAD2L2/ARAP1/PREX1/ARPC5/ARHGEF2/WDR1/ALMS1/LCP1/ARF1/SSH1/GRB2/TPM4/NEDD9/SH3KBP1/TPM1/SPTBN1/CORO1C/PTK2B/SPTA1/MSRB1/INPP5K/SVIL/CALD1/PAK1/CTNNA1/ALDOA/ELMO1/GSN/SLC9A1/PLEC/ESAM/IQGAP2/MYO1F/FLNA/PLEK/ZYX/RAC1/RFLNB/HIP1/CYFIP2/RAC2/PXN/ARHGAP6/WIPF1/COTL1/RHOA/CAPZB/GAS2L1/MYADM/PDLIM1/ADD1/PPP1R9B/SSH2/HCLS1/TMOD1/ARPC4/CFL1/RHOBTB1/WAS/ESPN/ITGB5/SPTB/MTPN/CTTN/GAS7/ACTN1/SH3BP1/PAK2/VASP/MICAL2/WASF2/RHOG/DMTN/DIAPH1

BP	GO:0007167	enzyme linked receptor protein signaling pathway	538	-0.52	-2.10	1.00E-10	3.79E-09	2.32E-09	2311	tags=35%, list=20%, signal=30%	LNPEP/SLC9A3R1/GIGYF2/MVP/NRROS/TF/VIM/EPHB2/SH2B3/TNK2/RPS6KA5/EFNB1/FOXO1/SPRY3/PTGIR/MYO1E/FGR/FYN/WNT1/SRC/EPHB4/IRS2/TRIO/AKT1S1/CPNE3/IL17RD/ATOH8/XIAP/ERBB2/EXT1/RNF165/HCK/CASS4/TGFB1/LDLRAD4/TIAM1/ZBTB7A/BTBD11/NOTCH2/SDCBP/HGS/TSC2/HSPA5/SMARCC1/SAMD12/HGF/ZC3H3/PLCB1/NDEL1/STAT5B/GAB1/EGR1/NOMO3/SH2B2/PIP4K2C/SHC1/EPHB1/MAPK14/PTPRJ/SIPA1L1/MSX1/SETX/SORT1/DOK1/CD63/FUZ/CDKN1C/PPM1L/FAM89B/SPI1/PTPN18/EPHA4/PDPK1/RNF115/PTPN12/NCF1/PML/NUMA1/CSF1R/GSK3A/GRB2/PDK2/ARNT/FIBP/PDGFA/NEDD9/INSR/SESN3/SGPL1/SPTBN1/FES/GREM1/RBPJ/SORL1/RNF126/PCSK6/PTK2B/NEURL1/GAB2/STAT6/INPP5K/RAPGEF2/CCND3/CHST11/PRKD2/RASGRP4/PAK1/IGF2/RAB7A/ZMIZ1/IL13RA1/RAB14/ZNF106/BMP6/MMP9/VAV1/TGFB1I1/SFRP2/STK11/GP6/USP9X/ITGA5/SYK/LTBP2/FOXO4/THBS1/PTPN1/NUP93/ADIPOR1/GLG1/MAPKAPK3/RBM14/HIPK2/PPP2R5B/SKI/NIBAN2/JAK3/DOK3/NDST1/HSPA1A/TGFBRAP1/ZYX/RAC1/SLA/LYN/HIP1/CYFIP2/GSK3B/STAT5A/DNM2/CREBBP/IGF2R/LRP1/HSPB1/REPS2/TXNIP/GNAI2/PXN/FKBP1A/PRKCB/SHKBP1/ARRB2/FKBP8/LRG1/JAK1/UBE2O/MAPK3/SNCA/ITGB5/RAPGEF1/FLCN/STAT3/CSRNP1/PIP4K2A/CLDN5/LTBP1/ZNF703/MAPKAPK2/TAL1/AKT2/PIK3CD/PTP4A3/PPM1A/EIF4EBP2/PAK2/ITGB3/DAB2/CBL/IQGAP1/FURIN
BP	GO:0007169	transmembrane receptor protein tyrosine kinase signaling pathway	362	-0.52	-2.03	1.00E-10	3.79E-09	2.32E-09	2309	tags=36%, list=20%, signal=30%	SLC9A3R1/GIGYF2/MVP/EPHB2/SH2B3/TNK2/RPS6KA5/EFNB1/FOXO1/SPRY3/PTGIR/MYO1E/FGR/FYN/WNT1/SRC/EPHB4/IRS2/AKT1S1/CPNE3/ERBB2/EXT1/HCK/CASS4/TGFB1/TIAM1/HGS/TSC2/SMARCC1/SAMD12/HGF/PLCB1/NDEL1/STAT5B/GAB1/SH2B2/PIP4K2C/SHC1/EPHB1/MAPK14/PTPRJ/SIPA1L1/SETX/SORT1/DOK1/CD63/FUZ/PTPN18/EPHA4/PDPK1/RNF115/PTPN12/NCF1/CSF1R/GSK3A/GRB2/PDK2/ARNT/FIBP/PDGFA/NEDD9/INSR/SESN3/SGPL1/FES/GREM1/RBPJ/SORL1/RNF126/PTK2B/NEURL1/GAB2/STAT6/INPP5K/RAPGEF2/CCND3/PRKD2/RASGRP4/PAK1/IGF2/RAB7A/RAB14/ZNF106/MMP9/VAV1/ITGA5/SYK/FOXO4/THBS1/PTPN1/ADIPOR1/MAPKAPK3/PPP2R5B/NIBAN2/JAK3/DOK3/NDST1/RAC1/SLA/LYN/HIP1/CYFIP2/GSK3B/STAT5A/IGF2R/LRP1/HSPB1/REPS2/TXNIP/GNAI2/PXN/PRKCB/SHKBP1/JAK1/SNCA/RAPGEF1/STAT3/CSRNP1/PIP4K2A/MAPKAPK2/TAL1/AKT2/PIK3CD/PTP4A3/EIF4EBP2/PAK2/ITGB3/CBL/IQGAP1
BP	GO:0007596	blood coagulation	148	-0.63	-2.22	1.00E-10	3.79E-09	2.32E-09	2303	tags=43%, list=20%, signal=35%	NFE2L2/HPS6/EPHB2/SH2B3/SERPINA1/FCER1G/SERPING1/CSRP1/KNG1/DGKA/SRF/PRDX2/SRC/ABAT/BLOC1S3/THBD/DGKG/DGKZ/ANO6/ACTG1/MAPK14/PTPN6/PAPSS2/FERMT3/GNAQ/F5/FIBP/PDGFA/PF4V1/SELP/F13A1/VCL/VAV1/GP6/SYK/THBS1/PIK3CG/FLNA/ACTB/PF4/ALOX12/PLEK/LYN/DGKK/GP9/HSPB1/MPL/GATA1/ITPK1/GNA12/WAS/SLC4A1/GP1BA/TREML1/MYL9/MPIG6B/ITGB3/KRT1/P2RX1/MYH9/TBXA2R/DMTN/TLN1
BP	GO:0007599	hemostasis	152	-0.63	-2.25	1.00E-10	3.79E-09	2.32E-09	2303	tags=43%, list=20%, signal=35%	NFE2L2/HPS6/EPHB2/SH2B3/SERPINA1/FCER1G/SERPING1/CSRP1/ZNF385A/KNG1/DGKA/SRF/PRDX2/SRC/ABAT/BLOC1S3/THBD/DGKG/DGKZ/ANO6/ACTG1/MAPK14/PTPN6/PAPSS2/FERMT3/GNAQ/F5/FIBP/PDGFA/PF4V1/SELP/F13A1/VCL/VAV1/GP6/SYK/THBS1/PIK3CG/FLNA/ACTB/PF4/ALOX12/GPI/PLEK/LYN/DGKK/GP9/HSPB1/MPL/GATA1/ITPK1/GNA12/WAS/SLC4A1/GP1BA/TREML1/NFE2/MYL9/MPIG6B/ITGB3/KRT1/P2RX1/MYH9/TBXA2R/DMTN/TLN1

BP	GO:0009611	response to wounding	342	-0.50	-1.91	1.00E-10	3.79E-09	2.32E-09	2467	tags=36%, list=21%, signal=29%	PLAUR/CFLAR/MYOF/SCRIB/KIAA0319/ALOX5/ADAM17/SIGLEC10/NFE2L2/HPS6/SCARB1/MAP2K4/EPHB2/SH2B3/ARHGAP24/PTK7/SERPINA1/FCER1G/SERPING1/CSRP1/SOD2/KNG1/DGKA/MACF1/SRF/WNT1/PTEN/PRDX2/SRC/FKBP1B/ABAT/BLOC1S3/PRKCE/SDC4/C6orf89/THBD/ERBB2/EXT1/DGKG/ATP7A/TGFB1/DGKZ/ANO6/NOTCH2/ACTG1/RANGAP1/NDEL1/MAPK14/CHMP4B/ACTA2/ZFP36L1/PTPN6/PAPSS2/EPHA4/FERMT3/GNAQ/CCR1/ADIPOR2/F5/FIBP/PDGFA/PF4V1/STK24/MAP3K5/TPM1/TSPO/SELP/F13A1/MAX/PAK1/VCL/CTNNA1/VAV1/GP6/PPP3CA/ITGA5/SYK/THBS1/PLEC/CD151/PIK3CG/ABHD2/B4GALT1/FLNA/RIPOR1/ACTB/ANXA6/PF4/ALOX12/PLEK/CHMP1A/RAC1/LYN/DGKK/GP9/HSPB1/SYTL4/CNN2/DYSF/MPL/RHOA/GATA1/GPX1/ITPK1/NF1/GRN/GNA12/WAS/MYLK/CDKN1A/SLC4A1/GP1BA/TREML1/MYL9/MPIG6B/ITGB3/KRT1/P2RX1/MYH9/TIMP1/TBXA2R/DMTN/TLN1
BP	GO:0009968	negative regulation of signal transduction	796	-0.42	-1.72	1.00E-10	3.79E-09	2.32E-09	2415	tags=31%, list=21%, signal=26%	LGALS3/UBE2D3/PMEPA1/PPIF/RGS7/PLAUR/SOCS7/SOCS3/CFLAR/UBE2B/RFFL/AMFR/DUSP16/ADAM17/MGRN1/ENDOG/CRY2/RGS16/SLC9A3R1/NFE2L2/MVP/IFT122/NRROS/DUSP1/HDAC7/MUL1/EPHB2/CTNNBIP1/DYRK3/SH2B3/HDAC3/ARHGAP24/AXIN2/FHL2/SH3RF1/ASXL1/CDKN2D/IRAK3/VPS18/FOXO1/SPRY3/PTGIR/SOD2/SLA2/ZNF385A/HEG1/WWC3/GRK3/WNT1/PADI2/PTEN/PRDX2/NLRP3/SRC/PER1/ATM/AKT1S1/SLC24A4/RASA2/IL17RD/MAP2K5/PPP1R15A/STK4/XIAP/DGKG/DGKZ/SIRPA/LDLRAD4/ZBTB7A/PDE2A/PHACTR4/G3BP1/BCL3/SZT2/HGS/TSC2/RNF213/HSPA5/GSTP1/ARRB1/LRRK2/OPRL1/HGF/SMARCA4/GPS2/ASH1L/STK38/EZR/PHLPP1/EYA3/TMBIM1/EGR1/RIPK1/RASAL3/NOMO3/NFATC1/PIP4K2C/CD300LF/CPNE1/MAPK14/TRIB1/TLE4/S1PR2/GRK2/NF2/PTPRJ/PTPN6/PYCARD/ADGRG3/MAD2L2/NR1H2/TMBIM6/FUZ/CTNND1/FAM89B/MARK3/SPI1/ITCH/PTPN18/EPHA4/INVS/PDPK1/RNF115/PTPN12/HIF1AN/ARHGEF2/BIRC6/FOXP1/GSK3A/CHP1/RGS6/OPTN/PRKACA/YTHDF3/SESN3/SESN2/USP10/CSNK2A1/FBP1/TMEM131L/NDRG2/YWHAB/SUFU/VPS11/TAOK3/GREM1/KANK2/SORL1/RNF126/HTT/NEURL1/MFHAS1/VRK3/INPP5K/FRMD8/CHST11/OTUD5/PIAS4/RAB7A/CTNNA1/CSK/MMP9/TGFB1I1/SFRP2/STK11/THOC5/PTPRC/ADAR/THBS1/PSME3/PTPN1/NPLOC4/LMNA/NCOR2/ADIPOR1/TLR6/IL1RN/GLG1/NLRX1/LTF/HIPK2/CHD8/SKI/NIBAN2/VCP/HSPA1A/UBR2/INPP5D/PF4/PLEK/LYN/RIOK3/ITPRIP/LAPTM5/GSK3B/DNM2/HYOU1/LRP1/HSPB1/WWP2/TCF7L2/PPP1R10/ATP2B4/GNAI2/FKBP1A/RHOA/TNIP1/PRKCB/CASTOR2/GATA1/MYADM/GPX1/CLU/ARRB2/NF1/PINK1/SEC14L1/SNCA/SIAH2/H2BC11/BCL2L1/FLCN/CTTN/PIP4K2A/RIPOR2/LTBP1/TLE3/GRINA/SH3BP1/TMEM127/LITAF/ENO1/MFN2/PPM1A/MPIG6B/YBX3/DAB2/CNOT1/NPRL3/FOXO3/CBL/CDC34/MAP2K3/STRADB/RELA

BP	GO:0010243	response to organonitrogen compound	655	-0.43	-1.75	1.00E-10	3.79E-09	2.32E-09	2309	tags=30%, list=20%, signal=26%	SLC9A3R1/NFE2L2/FOXRED2/MYO5A/AIFM1/KAT2B/VIM/EPHB2/USP19/BCL11A/KLHL22/FCER1G/IRAK3/TLR2/ATP2B1/FOXO1/GNA15/BCL2L11/ATXN3/FYN/WNT1/RRAGD/SRC/PER1/IRS2/SULT1A3/MTHFR/ABAT/SLC24A4/PRKCE/THBD/EXT1/CD68/LARS1/ATP7A/ZDHHC7/AGTRAP/PPARGC1B/CPEB4/PDE2A/OR10H1/MGMT/TSC2/SCAP/HSPA5/SYAP1/ROCK2/UBE2J2/SMARCC1/LRRK2/BTG1/RANGAP1/PLCB1/NDEL1/STAT5B/KLF16/EZR/GAB1/EGR1/MAN1A1/KAT7/MARCHF6/SH2B2/TMEM67/C5AR1/PIP4K2C/SHC1/MAPK14/CACNB1/CA2/YOD1/ZFP36L1/SORT1/TMBIM6/AQP9/ALAD/EFTUD2/VPS35/EPHA4/PDPK1/TRDMT1/ITGB2/ARHGEF2/ITPR2/TRIB3/SSH1/GSK3A/GRB2/CANX/HRH2/PDK2/PRKACA/EIF6/PKM/INSR/UBE4B/SESN3/SESN2/SLC27A1/FBP1/NFKB1/TSPO/SORL1/TMEM38A/BCAP31/PTK2B/SLC2A1/NEURL1/SLC8A1/RRAGC/STAT6/INPP5K/RAPGEF2/CCND3/RCN3/MAX/IGF2/CTNNA1/CSK/ZNF106/MMP9/POR/LAMTOR1/STK11/CPT1A/PPP3CA/SYK/SLC9A1/FOXO4/PTAFR/PTPN1/NPLOC4/UQCRC1/GNB1/NCOR2/RAB31/ADIPOR1/TLR6/CALM3/PIK3CG/FLNA/LPIN2/JUND/JAK3/VCP/UBR2/ACTB/GPI/UCP2/AQP1/LYN/SIN3A/GSK3B/STAT5A/DNM2/LRP1/CDA/ATP2B4/GNAI2/PXN/RHOA/PRKCB/CASTOR2/GATA1/GPX1/ARRB2/SP1/PPP1R9B/JAK1/WDTC1/LARP1/MAPK3/SNCA/TGM2/RAPGEF1/BCL2L1/STAT3/PIP4K2A/PRKAR2B/AHCYL1/AKT2/DNAJB2/BSG/PRKDC/KLF1/RNF40/EIF4EBP2/UBXN6/GNAZ/CARM1/P2RX1/TIMP1/FOXO3/DMTN/BAG6/KLF3/DIAPH1/RELA
CC	GO:0015629	actin cytoskeleton	333	-0.55	-2.14	1.00E-10	3.79E-09	2.32E-09	2400	tags=42%, list=20%, signal=34%	ARHGAP32/ADAM17/CORO1A/UTRN/DDX58/SCIN/ARHGEF5/SLC9A3R1/MYO5A/MYO9A/KAT2B/ADAM8/MYO1G/INTS6/VPS18/FLII/SHROOM1/MYO1E/CDC42BPA/MACF1/FGR/FYN/SRC/TRIOBP/CDC42BPB/DIAPH2/DBNL/MYO18A/MARCKS/LSP1/HCK/DCTN4/TPM3/ACTG1/FYB1/EZR/SPTAN1/ARPC1B/DBN1/KEAP1/NF2/ACTA2/SIPA1L1/FLOT1/PARVG/CDC42EP4/FHL3/FERMT3/ARPC5/PTPN12/WDR1/CAP1/LCP1/TPM4/WIPF2/AMOT/PALLD/TPM1/SPTBN1/MYZAP/ARHGAP21/CORO1C/SH3GL1/RARA/ZNF185/SLC2A1/SPTA1/MSRB1/SVIL/CALD1/PAK1/VCL/PDLIM7/CTNNA1/ALDOA/GSN/DYNLL2/PDLIM5/JAM3/MYO9B/ASAP1/SPECC1/IQGAP2/MYO1F/FLNA/PVALB/ACTB/MSRA/ZYX/RAC1/RFLNB/GABARAP/MARK2/HNRNPK/RAC2/MYL4/FLOT2/AKAP13/CNN2/LPP/LASP1/PXN/ARHGAP6/WIPF1/COTL1/TMEM63B/CAPZB/ACTR1A/PRKCB/GAS2L1/MYADM/PDLIM1/ACTN4/ADD1/PPP1R9B/HCLS1/TMOD1/ARPC4/CFL1/WAS/ESPN/MYLK/SNCA/PARVB/SPTB/MTPN/CTTN/CLDN5/GAS7/ABLIM3/ACTN1/MYL9/VASP/MYH9/IQGAP1/PEAK3/WASF2/DMTN/DIAPH1

MF	GO:0016301	kinase activity	536	-0.52	-2.09	1.00E-10	3.79E-09	2.32E-09	2329	tags=32%, list=20%, signal=27%	MAP4K2/PI4K2A/AATK/MAST4/MAP2K4/PIM3/AAK1/EPHB2/FAM20B/DYRK3/AK4/PKN1/PTK7/TNK2/STK25/MAP4K4/SRPK1/RPS6KA5/IRAK3/CDC42BPA/DGKA/GRK3/FGR/FYN/LIMK2/MAP3K4/SRC/TOP1/EPHB4/TRIO/ATM/DCAKD/GALK1/CDC42BPB/CPNE3/PNKP/CDK12/MAP2K5/STK4/PRKCE/ERBB2/DGKG/HCK/DGKZ/ITPKB/ERN2/PDXK/ERN1/ROCK2/LRRK2/IKBKE/PIP5KL1/STK38/NME4/DYRK1B/ALPK1/RIPK1/PIKFYVE/PIP4K2C/CLK3/EPHB1/MAPK14/PHKA2/STK10/CDKL5/GRK2/MAP2K2/EIF2AK2/MAP3K2/RSKR/CKB/DAPK3/CSNK1G2/MARK3/LRRK1/PAPSS2/EPHA4/PDPK1/MAST2/CDK13/CSF1R/NOL9/TRIM28/TJP2/GSK3A/PIP5K1C/GRK6/PDK2/PRKACA/BCKDK/PKM/INSR/STK24/CSNK2A1/MAP3K5/PSKH1/FES/TAOK3/HIPK1/MKNK2/PIM2/PFKFB3/PTK2B/VRK3/BCR/RPS6KA4/SIK3/CCND3/PRKD2/MARK4/NEK5/PAK1/CSK/TAOK1/MAP4K1/STK11/MAP2K7/SYK/BMP2K/STK40/PDK3/PFKFB4/MAPKAPK3/PIK3CG/HIPK2/CAMK1D/ULK1/JAK3/MARK2/LYN/RIOK3/MAP3K3/MMD/GSK3B/DGKK/IGF2R/CDK19/AKAP13/AK1/SRPK2/PRKCB/CSNK1G1/SCYL1/ITPK1/PPP1R9B/JAK1/PIM1/PINK1/HK1/RPS6KA1/MYLK/MAPK3/CDKN1A/NUAK2/GUK1/PIP4K2A/IP6K1/LTBP1/MAPKAPK2/SQSTM1/AKT2/ARAF/PIK3CD/GAK/PRKDC/BRD4/MPP1/PAK2/WNK1/MAST3/MAP2K3/EIF2AK1/PEAK3
MF	GO:0016772	transferase activity, transferring phosphorus-containing groups	668	-0.45	-1.84	1.00E-10	3.79E-09	2.32E-09	2329	tags=28%, list=20%, signal=24%	MAP4K2/PI4K2A/AATK/TENT5A/MAST4/MAP2K4/PIM3/AAK1/EPHB2/FAM20B/DYRK3/AK4/PKN1/PTK7/TNK2/STK25/MAP4K4/SRPK1/RPS6KA5/IRAK3/CDC42BPA/DGKA/GRK3/FGR/FYN/LIMK2/MAP3K4/SRC/TOP1/EPHB4/TRIO/ATM/DCAKD/GALK1/CDC42BPB/CDIPT/CPNE3/PNKP/CDK12/MAP2K5/POLG/STK4/PRKCE/ERBB2/DGKG/HCK/DGKZ/ITPKB/ERN2/PDXK/ERN1/ROCK2/LRRK2/IKBKE/PIP5KL1/STK38/NME4/DYRK1B/ALPK1/RIPK1/PIKFYVE/PIP4K2C/CLK3/EPHB1/MAPK14/PHKA2/STK10/CDKL5/GRK2/POLR1A/MAP2K2/EIF2AK2/MAP3K2/RSKR/CKB/DAPK3/CSNK1G2/MARK3/LRRK1/PAPSS2/EPHA4/PDPK1/MAST2/CDK13/POLR2E/CHRAC1/CSF1R/NOL9/TRIM28/POLR2J/TJP2/GSK3A/PIP5K1C/GRK6/PDK2/SELENOO/PRKACA/BCKDK/PKM/INSR/TENT4B/STK24/CSNK2A1/MAP3K5/PSKH1/FES/TAOK3/HIPK1/MKNK2/PIM2/PFKFB3/PTK2B/VRK3/BCR/RPS6KA4/SIK3/CCND3/PRKD2/MARK4/NEK5/PAK1/CSK/TAOK1/MAP4K1/STK11/MAP2K7/SYK/BMP2K/STK40/PDK3/PFKFB4/MAPKAPK3/PIK3CG/HIPK2/CAMK1D/ULK1/JAK3/POLL/MARK2/LYN/RIOK3/MAP3K3/MMD/GSK3B/DGKK/IGF2R/CDK19/AKAP13/AK1/SRPK2/PRKCB/CSNK1G1/SCYL1/ITPK1/PPP1R9B/TERF2/JAK1/PIM1/PINK1/HK1/RPS6KA1/MYLK/MAPK3/CDKN1A/NUAK2/GUK1/PIP4K2A/IP6K1/LTBP1/MAPKAPK2/SQSTM1/AKT2/ARAF/PIK3CD/GAK/PRKDC/TENT5C/BRD4/MPP1/PAK2/WNK1/MAST3/MAP2K3/EIF2AK1/PEAK3/POLR2A

MF	GO:0016773	phosphotransferase activity, alcohol group as acceptor	494	-0.54	-2.17	1.00E-10	3.79E-09	2.32E-09	2329	tags=34%, list=20%, signal=28%	MAP4K2/PI4K2A/AATK/MAST4/MAP2K4/PIM3/AAK1/EPHB2/FAM20B/DYRK3/PKN1/PTK7/TNK2/STK25/MAP4K4/SRPK1/RPS6KA5/IRAK3/CDC42BPA/DGKA/GRK3/FGR/FYN/LIMK2/MAP3K4/SRC/TOP1/EPHB4/TRIO/ATM/DCAKD/GALK1/CDC42BPB/CPNE3/PNKP/CDK12/MAP2K5/STK4/PRKCE/ERBB2/DGKG/HCK/DGKZ/ITPKB/ERN2/PDXK/ERN1/ROCK2/LRRK2/IKBKE/PIP5KL1/STK38/DYRK1B/ALPK1/RIPK1/PIKFYVE/PIP4K2C/CLK3/EPHB1/MAPK14/PHKA2/STK10/CDKL5/GRK2/MAP2K2/EIF2AK2/MAP3K2/RSKR/DAPK3/CSNK1G2/MARK3/LRRK1/PAPSS2/EPHA4/PDPK1/MAST2/CDK13/CSF1R/NOL9/TRIM28/GSK3A/PIP5K1C/GRK6/PDK2/PRKACA/BCKDK/PKM/INSR/STK24/CSNK2A1/MAP3K5/PSKH1/FES/TAOK3/HIPK1/MKNK2/PIM2/PFKFB3/PTK2B/VRK3/BCR/RPS6KA4/SIK3/CCND3/PRKD2/MARK4/NEK5/PAK1/CSK/TAOK1/MAP4K1/STK11/MAP2K7/SYK/BMP2K/STK40/PDK3/PFKFB4/MAPKAPK3/PIK3CG/HIPK2/CAMK1D/ULK1/JAK3/MARK2/LYN/RIOK3/MAP3K3/MMD/GSK3B/DGKK/IGF2R/CDK19/AKAP13/SRPK2/PRKCB/CSNK1G1/SCYL1/ITPK1/PPP1R9B/JAK1/PIM1/PINK1/HK1/RPS6KA1/MYLK/MAPK3/CDKN1A/NUAK2/PIP4K2A/IP6K1/LTBP1/MAPKAPK2/SQSTM1/AKT2/ARAF/PIK3CD/GAK/PRKDC/BRD4/PAK2/WNK1/MAST3/MAP2K3/EIF2AK1/PEAK3
BP	GO:0022603	regulation of anatomical structure morphogenesis	546	-0.52	-2.09	1.00E-10	3.79E-09	2.32E-09	2755	tags=39%, list=23%, signal=31%	DNMBP/KIT/EPHA1/F11R/SYNGAP1/BCL9L/PLEKHO1/SMAD1/ST6GAL1/JMJD8/ARHGAP4/CARMIL1/RC3H1/PLXNA4/CALR/EPB41L3/FGD6/P2RY1/STAU2/CLASP1/AMIGO1/TGIF2/CFAP410/MED12/CST3/TWF2/CDC42EP1/ACTR2/KIAA0319/ALOX5/CYBB/CORO1A/SIRT6/DIP2B/SLC9A3R1/NFE2L2/MYO9A/KIF13B/MUL1/EPHB2/BCL11A/MTCH2/LIMS1/PKN1/PTK7/STK25/NIN/CHI3L1/SPRY3/DISC1/MACF1/FGR/SRF/FYN/PTEN/VANGL1/UNC13D/SRC/TRIOBP/PDZD8/YWHAH/RHOF/NINJ1/ADNP/DBNL/ERBB2/HCK/CASS4/EPS8/MAGED1/MKS1/TGFB1/TIAM1/RRAS/CUL7/CPNE5/GTF2I/HGS/MTDH/ROCK2/LRRK2/BTG1/HGF/NDEL1/EZR/TMBIM1/GAB1/DLG4/DBN1/INF2/DOCK5/C5AR1/WASF3/CDKL5/SIPA1L1/MSX1/CAMSAP1/WASHC2C/CDC42EP4/SEMA4D/ARAP1/FUZ/ABI3/DAPK3/AGO4/VPS35/PREX1/EPHA4/PDPK1/PML/RALBP1/ITGB2/CSF1R/WDR1/FOXP1/ZRANB1/SASH1/AGO2/PDGFA/NEDD9/PKM/BRWD3/AMOT/SH3KBP1/TPM1/FES/GREM1/HIPK1/CORO1C/PTK2B/FGD4/SPTA1/SEMA4A/BCR/WARS1/RAPGEF2/EIF4G2/PRKD2/ZMYM3/PAK1/FGD3/ALDOA/SFRP2/STK11/PLXNC1/PPP3CA/ITGA5/PDLIM5/FOXO4/DVL3/THBS1/FIS1/AGO1/IST1/HIPK2/BTBD7/FLNA/ULK1/NIBAN2/DNM3/PF4/FMNL1/RAC1/MARK2/AQP1/GSK3B/RAC2/DNM2/HSPB1/HUWE1/ATP2B4/EPN1/MPL/RHOA/FOXJ2/CAPZB/PRKCB/MYADM/NF1/MSN/SP1/ACTN4/LRG1/GRN/JAK1/GNA12/CFL1/RHOBTB1/PINK1/EPB41/PARVB/STAT3/SPARC/CLDN5/PIK3CD/EPB42/STIM1/PRKDC/PAK2/E2F2/DAB2/MBP/KRT1/CUX1/KEL/MYH9/GLUL/TBXA2R/PEAK3/XK/RHOG/DMTN/DIAPH1

BP	GO:0030029	actin filament-based process	525	-0.53	-2.11	1.00E-10	3.79E-09	2.32E-09	2115	tags=34%, list=18%, signal=29%	FLII/ARHGEF11/CSRP1/ATXN3/SHROOM1/MYO1E/CDC42BPA/SRF/LIMK2/PPM1F/SRC/TRIOBP/PHACTR2/MSRB2/RHOF/CDC42BPB/DIAPH2/DBNL/MYO18A/PRKCE/MARCKS/SDC4/HCK/CASS4/EPS8/TPM3/PPARGC1B/SUN2/ACAP2/PHACTR4/NOTCH2/SDCBP/SYNE2/CYRIA/ACTG1/ROCK2/ARRB1/EZR/SPTAN1/GAB1/ARPC1B/DBN1/INF2/WASF3/SHC1/S1PR2/NF2/KCNH2/ACTA2/SIPA1L1/ARHGDIB/PARVG/WASHC2C/PYCARD/CDC42EP4/MAD2L2/ARAP1/FHL3/CLN3/ABI3/DAPK3/PREX1/PDPK1/ARPC5/CSF1R/ARHGEF2/WDR1/ALMS1/CAP1/LCP1/ARF1/SSH1/PIP5K1C/GRB2/TPM4/WIPF2/PDGFA/NEDD9/AMOT/SH3KBP1/PALLD/TPM1/SPTBN1/FES/CORO1C/PTK2B/FGD4/SPTA1/BCR/MSRB1/INPP5K/BCAS3/SVIL/CALD1/PAK1/PDLIM7/CTNNA1/FGD3/TAOK1/ALDOA/ELMO1/GSN/PDLIM5/SLC9A1/JAM3/PTPN1/MYO9B/SCN1B/PLEC/SPECC1/ESAM/DOCK2/IQGAP2/MYO1F/FLNA/ACTB/PLEK/ZYX/FMNL1/RAC1/RFLNB/AQP1/HIP1/CYFIP2/RAC2/LRP1/AKAP13/CNN2/PXN/ARHGAP6/WIPF1/COTL1/IQSEC1/RHOA/CAPZB/GAS2L1/MYADM/PDLIM1/NF1/ACTN4/ADD1/PPP1R9B/SSH2/HCLS1/ARHGDIA/TMOD1/ARPC4/CFL1/RHOBTB1/WAS/ESPN/ARHGAP26/ITGB5/EPB41/PARVB/SPTB/NUAK2/MTPN/CTTN/GAS7/PLEK2/ABLIM3/ACTN1/SH3BP1/MYL9/PAK2/VASP/MYH9/IQGAP1/MICAL2/PEAK3/WASF2/RHOG/DMTN/DIAPH1/TLN1
BP	GO:0030036	actin cytoskeleton organization	484	-0.54	-2.17	1.00E-10	3.79E-09	2.32E-09	2115	tags=35%, list=18%, signal=30%	FLII/ARHGEF11/CSRP1/ATXN3/SHROOM1/MYO1E/CDC42BPA/SRF/LIMK2/PPM1F/SRC/TRIOBP/PHACTR2/MSRB2/RHOF/CDC42BPB/DIAPH2/DBNL/MYO18A/PRKCE/MARCKS/SDC4/HCK/CASS4/EPS8/TPM3/PPARGC1B/PHACTR4/NOTCH2/SDCBP/CYRIA/ACTG1/ROCK2/ARRB1/EZR/SPTAN1/GAB1/ARPC1B/DBN1/INF2/WASF3/SHC1/S1PR2/NF2/SIPA1L1/ARHGDIB/PARVG/WASHC2C/PYCARD/CDC42EP4/MAD2L2/ARAP1/FHL3/CLN3/ABI3/DAPK3/PREX1/PDPK1/ARPC5/CSF1R/ARHGEF2/WDR1/ALMS1/CAP1/LCP1/ARF1/SSH1/PIP5K1C/GRB2/TPM4/PDGFA/NEDD9/AMOT/SH3KBP1/PALLD/TPM1/SPTBN1/FES/CORO1C/PTK2B/FGD4/SPTA1/BCR/MSRB1/INPP5K/BCAS3/SVIL/CALD1/PAK1/PDLIM7/CTNNA1/FGD3/TAOK1/ALDOA/ELMO1/GSN/PDLIM5/SLC9A1/JAM3/PTPN1/PLEC/SPECC1/ESAM/DOCK2/IQGAP2/MYO1F/FLNA/ACTB/PLEK/ZYX/FMNL1/RAC1/RFLNB/AQP1/HIP1/CYFIP2/RAC2/LRP1/AKAP13/CNN2/PXN/ARHGAP6/WIPF1/COTL1/IQSEC1/RHOA/CAPZB/GAS2L1/MYADM/PDLIM1/NF1/ACTN4/ADD1/PPP1R9B/SSH2/HCLS1/ARHGDIA/TMOD1/ARPC4/CFL1/RHOBTB1/WAS/ESPN/ARHGAP26/ITGB5/EPB41/PARVB/SPTB/NUAK2/MTPN/CTTN/GAS7/PLEK2/ABLIM3/ACTN1/SH3BP1/MYL9/PAK2/VASP/MYH9/IQGAP1/MICAL2/PEAK3/WASF2/RHOG/DMTN/DIAPH1/TLN1

CC	GO:0030141	secretory granule	613	-0.44	-1.79	1.00E-10	3.79E-09	2.32E-09	2149	tags=34%, list=18%, signal=29%	FCER1G/TLR2/GLB1/PA2G4/CHI3L1/SERPING1/ORMDL3/RAB11FIP5/CR1/KNG1/SNAP25/FGR/CYSTM1/CD93/PADI2/GUSB/UNC13D/RAB27B/APAF1/PHACTR2/CD177/RHOF/DBNL/CPNE3/NCF2/XRCC6/CD68/CMTM6/ALDH3B1/ATP7A/EEF2/TGFB1/SIRPA/SLC2A3/AP2A2/PDXK/LAMP2/ANO6/SDCBP/ARHGAP9/CKAP4/TSPAN14/ACLY/GSTP1/ITGAM/RPH3A/HGF/NBEAL2/SPTAN1/TMBIM1/PSMD2/PSMD3/C5AR1/MME/CLK3/CPNE1/MAPK14/STX4/STK10/CRISPLD2/PTPRJ/PTPN6/PYCARD/ADGRG3/HVCN1/EXOC3/CD63/TRAPPC1/ALAD/AP1M1/ATP6V0C/KPNB1/QSOX1/FERMT3/SIGLEC5/ARPC5/ITGB2/CDK13/PGAM1/ITPR2/CAP1/TMEM184A/F5/PSMD1/CPPED1/ANPEP/PRKACA/CA4/PCSK1N/PDGFA/PKM/S100A11/RNASET2/ATP6V0A1/NFKB1/NFAM1/OSCAR/TIMP2/PGLYRP1/PAFAH1B2/SELP/SNX10/CXCR2/PYGL/DYNC1H1/F13A1/PSMD11/IGF2/VCL/PECAM1/RAB7A/CTNNA1/RAB14/HGSNAT/ARL8A/IMPDH1/MMP9/SLC44A2/ALDOA/LAMTOR1/LCN2/GSN/UBR4/APLP2/PYGB/RAB5B/PTPRC/LILRB3/THBS1/PTAFR/GAA/RAB31/ATG7/IST1/TOM1/SLC6A9/DOCK2/IQGAP2/LTF/ABHD2/B4GALT1/VCP/DOK3/ABCC4/HSPA1A/TUBB4B/CAPN1/SELL/PF4/GPI/DNAJC5/CD14/RAC1/CYB5R3/ADGRE5/SIRPB1/IGF2R/PSAP/FLOT2/ITGA2B/TNFRSF1B/SYTL4/CDA/CNN2/HUWE1/COTL1/RHOA/ITGAL/GNS/FTL/ARHGAP45/TUBB/CLU/CXCR1/ATP6V1A/ADA2/ACTN4/LRG1/CANT1/FTH1/GRN/ECE1/PLEKHO2/SNCA/ANXA11/SPARC/LAMP1/PRDX6/ACTN1/TREML1/BSG/CTSB/RAB3D/PAK2/CTSD/ITGB3/KRT1/P2RX1/MYH9/TOLLIP/TIMP1/IQGAP1/TBXA2R/RHOG/ACRBP/DIAPH1
BP	GO:0030334	regulation of cell migration	568	-0.49	-1.99	1.00E-10	3.79E-09	2.32E-09	2053	tags=29%, list=18%, signal=25%	CD99L2/MACF1/FGR/SRF/PADI2/PTEN/TIRAP/PPM1F/SRC/IRS2/ATM/CPNE3/MAP2K5/STK4/PRKCE/ATOH8/SDC4/NIPBL/ATP7A/CASS4/TGFB1/LDLRAD4/TIAM1/SUN2/RRAS/ANO6/SDCBP/SYNE2/HSPA5/ACTG1/NUMB/ROCK2/HGF/PIP5KL1/PLCB1/GAB1/CMKLR1/ELP3/DOCK5/C5AR1/MGAT5/TRIB1/STX4/STK10/S1PR2/NF2/ACTA2/PTPRJ/ARHGDIB/PYCARD/ADGRG3/SEMA4D/FUZ/CLN3/ABI3/FAM89B/DAPK3/SPI1/EPHA4/FERMT3/PDPK1/CCR1/CSF1R/ARHGEF2/FOXP1/CXCL16/SASH1/AGO2/PDGFA/NEDD9/INSR/AMOT/YTHDF3/S100A11/STK24/TPM1/VSIR/GREM1/SORL1/CORO1C/PLEKHG3/DOCK8/PTK2B/SLC8A1/SELP/SEMA4A/BCR/ARID2/RAPGEF2/BCAS3/PRKD2/IL6R/PAK1/VCL/PECAM1/MMP9/SFRP2/PLXNC1/PPP3CA/ITGA5/PTPRC/FOXO4/JAM3/THBS1/PTAFR/ZNF609/LMNA/ATP1B2/ADIPOR1/CD151/PIK3CG/ABHD2/CAMK1D/FLNA/RIPOR1/GFUS/GPI/RAC1/LYN/LGALS9/MAP3K3/STAT5A/RAC2/LRP1/HSPB1/CNN2/ATP2B4/GNAI2/IQSEC1/RHOA/MYADM/NF1/MSN/SP1/ACTN4/GRN/RIN3/GNA12/MYLK/MAPK3/FLCN/STAT3/PTPN23/SPARC/CLDN5/RIPOR2/ZNF703/AKT2/SH3BP1/PIK3CD/MPP1/DNAJA4/ITGB3/DAB2/WNK1/PLVAP/GLUL/TIMP1/FOXO3/TBXA2R/MAP2K3/RHOG/DMTN/DIAPH1

BP	GO:0030335	positive regulation of cell migration	336	-0.50	-1.93	1.00E-10	3.79E-09	2.32E-09	2416	tags=37%, list=21%, signal=30%	MAPRE2/SPN/GPLD1/ADAM17/CORO1A/NCKAP1L/NFE2L2/SCARB1/HDAC7/EPHB2/ADAM8/MAP4K4/GLIPR2/SOD2/CD99L2/FGR/TIRAP/PPM1F/SRC/IRS2/ATM/CPNE3/STK4/PRKCE/ATOH8/NIPBL/ATP7A/CASS4/TGFB1/TIAM1/SUN2/RRAS/ANO6/SDCBP/SYNE2/HSPA5/ACTG1/NUMB/ROCK2/HGF/GAB1/CMKLR1/ELP3/DOCK5/C5AR1/MGAT5/STX4/ACTA2/PYCARD/SEMA4D/FAM89B/DAPK3/SPI1/EPHA4/FERMT3/PDPK1/CCR1/CSF1R/ARHGEF2/FOXP1/CXCL16/SASH1/AGO2/PDGFA/NEDD9/INSR/S100A11/VSIR/DOCK8/PTK2B/SLC8A1/SELP/SEMA4A/RAPGEF2/BCAS3/PRKD2/IL6R/PAK1/PECAM1/MMP9/PPP3CA/ITGA5/PTPRC/FOXO4/JAM3/THBS1/PTAFR/ZNF609/CD151/PIK3CG/CAMK1D/RIPOR1/GFUS/GPI/RAC1/LYN/LGALS9/MAP3K3/STAT5A/RAC2/HSPB1/GNAI2/IQSEC1/RHOA/MYADM/SP1/ACTN4/GRN/MYLK/MAPK3/STAT3/SPARC/RIPOR2/ZNF703/AKT2/PIK3CD/ITGB3/DAB2/WNK1/PLVAP/MAP2K3/DMTN/DIAPH1
CC	GO:0030863	cortical cytoskeleton	70	-0.73	-2.34	1.00E-10	3.79E-09	2.32E-09	1597	tags=59%, list=14%, signal=51%	EZR/SPTAN1/DLG4/DBN1/NF2/FLOT1/NUMA1/WDR1/CAP1/TPM4/SPTBN1/MYZAP/SLC2A1/SPTA1/CALD1/GSN/PVALB/ACTB/FLOT2/AKAP13/LASP1/COTL1/CAPZB/PRKCB/MYADM/ACTN4/PPP1R9B/HCLS1/TMOD1/EPB41/SPTB/CTTN/CLDN5/ACTN1/SLC4A1/EPB42/MPP1/GYPC/MYH9/IQGAP1/DMTN
BP	GO:0034330	cell junction organization	354	-0.50	-1.95	1.00E-10	3.79E-09	2.32E-09	3022	tags=44%, list=26%, signal=34%	ANK3/DTNBP1/PFN1/IKBKB/DUSP22/PLXNB2/PLXND1/TNF/MECP2/TANC2/NUMBL/POLDIP2/TESK2/KIFC3/AFDN/SMAD3/F11R/SYNGAP1/ZDHHC2/SPOCK2/ADD2/EPB41L3/PPFIBP2/CACNB4/STAU2/CLASP1/HDAC6/AMIGO1/FSCN1/ACTR2/TBCD/FBF1/MAPRE2/PPFIA1/MYO9A/HDAC7/C1QA/EPHB2/LIMS1/MAP4K4/TLR2/STRN/PARD6B/HEG1/DISC1/MACF1/SRF/FYN/PTEN/LRRC4/PPM1F/SRC/CD177/ADNP/DBNL/SDC4/ERBB2/EXT1/TGFB1/LRFN3/SDCBP/CTBP2/NECTIN1/ACTG1/NUMB/ROCK2/ITGAM/LRRK2/NFIA/DLG4/DBN1/C5AR1/WASF3/EPHB1/MAPK14/CACNB1/CDKL5/NF2/CAST/PTPRJ/SIPA1L1/FLOT1/CLSTN1/PARD3/SEMA4D/CTNND1/CLN3/ABI3/DAPK3/VPS35/EPHA4/SHISA7/GPBAR1/PKP4/CSF1R/WDR1/ARF1/TJP2/PIP5K1C/YWHAZ/PRKACA/OCEL1/INSR/ADGRL1/GREM1/HIPK1/CORO1C/PTK2B/NEURL1/SEMA4A/BCR/RAPGEF2/BCAS3/VCL/PECAM1/CTNNA1/CSK/BMP6/SETD5/ITGA5/PDLIM5/SLC9A1/JAM3/THBS1/PLEC/ESAM/DCTN1/FZD5/FLNA/EIF4G1/DNM3/VCP/ACTB/RAC1/GRIPAP1/ARHGAP6/IQSEC1/RHOA/TUBB/STAU1/ADD1/CFL1/SNCA/RAPGEF1/FLCN/PTPN23/CTTN/CLDN5/ZNF703/ACTN1/SH3BP1/PAK2/WASF2/DMTN/TLN1
BP	GO:0035239	tube morphogenesis	471	-0.46	-1.85	1.00E-10	3.79E-09	2.32E-09	2232	tags=31%, list=19%, signal=26%	EPHB2/MTHFD1/CTNNBIP1/ARHGAP24/PTK7/ADAM8/CHI3L1/BCL2L11/MYO1E/HEG1/SRF/WNT1/PTEN/KDM5B/SRC/APAF1/EPHB4/MTHFR/NINJ1/JMJD6/MAP2K5/STK4/ATOH8/SDC4/PROK2/ERBB2/EXT1/NIPBL/VDR/VEZF1/MAGED1/MKS1/TGFB1/PHACTR4/RRAS/CUL7/NOTCH2/SLC12A6/GTF2I/HGS/TSC2/RNF213/MTDH/ACTG1/ROCK2/BTG1/HGF/GAB1/C5AR1/SHC1/EPHB1/MAPK14/ZFP36L1/FUZ/SPI1/EPHA4/PDPK1/PML/HIF1AN/CSF1R/FOXP1/LEPR/ADIPOR2/SASH1/YWHAZ/PPP1CA/AGO2/ANPEP/PRKACA/PDGFA/PKM/AMOT/SGPL1/SUFU/GREM1/HIPK1/RBPJ/RARA/PTK2B/SEMA4A/MEIS1/WARS1/ARID2/RAPGEF2/BCAS3/TRAF6/PRKD2/KLF5/CALD1/PAK1/ZMIZ1/SFRP2/ITGA5/SYK/SH2D2A/FOXO4/DVL3/JAM3/THBS1/AGO1/MED1/FZD5/PIK3CG/HIPK2/B4GALT1/SKI/FLNA/NIBAN2/PF4/AQP1/MAP3K3/LRP1/HSPB1/ITGA2B/SRPK2/ATP2B4/EPN1/RHOA/FOXJ2/PRKCB/GPX1/NF1/SP1/LRG1/GRN/JAK1/MYLK/TGM2/PBX1/STAT3/SPARC/CLDN5/TAL1/PIK3CD/BSG/STIM1/ITGB3/VASP/E2F2/KRT1/MYH9/GLUL/NPRL3/TBXA2R/MICAL2/WASF2/HLX

BP	GO:0035295	tube development	572	-0.45	-1.83	1.00E-10	3.79E-09	2.32E-09	2232	tags=30%, list=19%, signal=25%	EPHB2/MTHFD1/CTNNBIP1/ARHGAP24/PTK7/ADAM8/ASXL1/CHI3L1/BCL2L11/THRA/MYO1E/HEG1/SRF/WNT1/PTEN/KDM5B/SRC/APAF1/EPHB4/ELK1/MTHFR/NINJ1/JMJD6/MAP2K5/STK4/ATOH8/SDC4/PROK2/ERBB2/EXT1/NIPBL/VDR/VEZF1/ATP7A/MAGED1/MKS1/TGFB1/PHACTR4/RC3H2/RRAS/CUL7/NOTCH2/SLC12A6/GTF2I/HGS/TSC2/RNF213/MTDH/ACTG1/ROCK2/BTG1/HGF/NFIA/GAB1/C5AR1/MME/SHC1/EPHB1/MAPK14/CEBPA/CRISPLD2/ZFP36L1/PTPN6/FUZ/SPATA2/SPI1/EPHA4/PDPK1/PML/HIF1AN/CSF1R/FOXP1/LEPR/ADIPOR2/SASH1/YWHAZ/PPP1CA/AGO2/ANPEP/PRKACA/PDGFA/PKM/AMOT/SGPL1/SUFU/GREM1/HIPK1/RBPJ/RARA/PTK2B/SEMA4A/MEIS1/WARS1/ARID2/RAPGEF2/BCAS3/TRAF6/RCN3/PRKD2/KLF5/CALD1/PAK1/ZMIZ1/SFRP2/ITGA5/SYK/SH2D2A/FOXO4/DVL3/JAM3/THBS1/STK40/AGO1/MED1/FZD5/PIK3CG/HIPK2/B4GALT1/BASP1/CHD8/SKI/FLNA/NIBAN2/ODAD4/PF4/AQP1/MAP3K3/LRP1/HSPB1/ITGA2B/SRPK2/ATP2B4/EPN1/RHOA/FOXJ2/PRKCB/GPX1/ITPK1/NF1/FKBP8/SP1/LRG1/GRN/JAK1/ECE1/MYLK/MAPK3/TGM2/TBC1D20/PBX1/STAT3/SPARC/CLDN5/TAL1/UBB/PIK3CD/BSG/STIM1/ITGB3/VASP/E2F2/KRT1/MYH9/GLUL/NPRL3/TBXA2R/MICAL2/WASF2/HLX/BAG6
BP	GO:0040012	regulation of locomotion	614	-0.48	-1.93	1.00E-10	3.79E-09	2.32E-09	2053	tags=28%, list=18%, signal=24%	CD99L2/MACF1/FGR/SRF/PADI2/PTEN/TIRAP/PPM1F/SRC/IRS2/ATM/CPNE3/MAP2K5/STK4/PRKCE/ATOH8/SDC4/NIPBL/ATP7A/CASS4/TGFB1/LDLRAD4/TIAM1/SUN2/RRAS/ANO6/SDCBP/SYNE2/HSPA5/ACTG1/NUMB/ROCK2/LRRK2/HGF/PIP5KL1/PLCB1/GAB1/CMKLR1/ELP3/DOCK5/C5AR1/MGAT5/TRIB1/STX4/STK10/S1PR2/NF2/ACTA2/PTPRJ/ARHGDIB/PYCARD/ADGRG3/SEMA4D/MYCBP2/FUZ/CLN3/ABI3/FAM89B/DAPK3/VPS35/SPI1/EPHA4/FERMT3/PDPK1/CCR1/CSF1R/ARHGEF2/FOXP1/CXCL16/SASH1/AGO2/PDGFA/NEDD9/INSR/AMOT/YTHDF3/S100A11/STK24/TPM1/VSIR/FES/GREM1/SORL1/CORO1C/PLEKHG3/DOCK8/PTK2B/SLC8A1/SELP/SEMA4A/BCR/ARID2/RAPGEF2/BCAS3/PRKD2/IL6R/PAK1/VCL/PECAM1/CTNNA1/MMP9/SFRP2/PLXNC1/PPP3CA/ITGA5/PTPRC/FOXO4/JAM3/THBS1/PTAFR/ZNF609/LMNA/ATP1B2/ADIPOR1/CD151/PIK3CG/ABHD2/CAMK1D/FLNA/RIPOR1/GFUS/GPI/RAC1/LYN/LGALS9/MAP3K3/STAT5A/RAC2/LRP1/HSPB1/CNN2/ATP2B4/GNAI2/IQSEC1/RHOA/MYADM/NF1/MSN/SP1/ACTN4/GRN/RIN3/GNA12/WAS/MYLK/MAPK3/SNCA/FLCN/STAT3/PTPN23/SPARC/CLDN5/RIPOR2/ZNF703/AKT2/SH3BP1/PIK3CD/MPP1/DNAJA4/ITGB3/DAB2/WNK1/FBXO7/PLVAP/GLUL/TIMP1/FOXO3/TBXA2R/MAP2K3/RHOG/DMTN/DIAPH1
BP	GO:0040017	positive regulation of locomotion	348	-0.49	-1.91	1.00E-10	3.79E-09	2.32E-09	2416	tags=36%, list=21%, signal=29%	MAPRE2/SPN/GPLD1/ADAM17/CORO1A/NCKAP1L/NFE2L2/SCARB1/HDAC7/TF/EPHB2/ADAM8/MAP4K4/GLIPR2/SOD2/CD99L2/FGR/TIRAP/PPM1F/SRC/IRS2/ATM/CPNE3/STK4/PRKCE/ATOH8/NIPBL/ATP7A/CASS4/TGFB1/TIAM1/SUN2/RRAS/ANO6/SDCBP/SYNE2/HSPA5/ACTG1/NUMB/ROCK2/HGF/GAB1/CMKLR1/ELP3/DOCK5/C5AR1/MGAT5/STX4/ACTA2/PYCARD/SEMA4D/FAM89B/DAPK3/VPS35/SPI1/EPHA4/FERMT3/PDPK1/CCR1/CSF1R/ARHGEF2/FOXP1/CXCL16/SASH1/AGO2/PDGFA/NEDD9/INSR/S100A11/VSIR/DOCK8/PTK2B/SLC8A1/SELP/SEMA4A/RAPGEF2/BCAS3/PRKD2/IL6R/PAK1/PECAM1/MMP9/PPP3CA/ITGA5/PTPRC/FOXO4/JAM3/THBS1/PTAFR/ZNF609/CD151/PIK3CG/CAMK1D/RIPOR1/GFUS/GPI/RAC1/LYN/LGALS9/MAP3K3/STAT5A/RAC2/HSPB1/GNAI2/IQSEC1/RHOA/MYADM/SP1/ACTN4/GRN/MYLK/MAPK3/STAT3/SPARC/RIPOR2/ZNF703/AKT2/PIK3CD/ITGB3/DAB2/WNK1/PLVAP/MAP2K3/DMTN/DIAPH1

BP	GO:0042060	wound healing	273	-0.53	-2.01	1.00E-10	3.79E-09	2.32E-09	2467	tags=38%, list=21%, signal=31%	PLAUR/CFLAR/MYOF/SCRIB/ALOX5/ADAM17/NFE2L2/HPS6/SCARB1/EPHB2/SH2B3/ARHGAP24/PTK7/SERPINA1/FCER1G/SERPING1/CSRP1/KNG1/DGKA/MACF1/SRF/PTEN/PRDX2/SRC/ABAT/BLOC1S3/PRKCE/SDC4/C6orf89/THBD/ERBB2/EXT1/DGKG/TGFB1/DGKZ/ANO6/NOTCH2/ACTG1/MAPK14/CHMP4B/ACTA2/PTPN6/PAPSS2/FERMT3/GNAQ/ADIPOR2/F5/FIBP/PDGFA/PF4V1/MAP3K5/TPM1/SELP/F13A1/PAK1/VCL/VAV1/GP6/PPP3CA/ITGA5/SYK/THBS1/PLEC/CD151/PIK3CG/B4GALT1/FLNA/ACTB/ANXA6/PF4/ALOX12/PLEK/CHMP1A/LYN/DGKK/GP9/HSPB1/SYTL4/CNN2/DYSF/MPL/RHOA/GATA1/GPX1/ITPK1/NF1/GNA12/WAS/MYLK/CDKN1A/SLC4A1/GP1BA/TREML1/MYL9/MPIG6B/ITGB3/KRT1/P2RX1/MYH9/TIMP1/TBXA2R/DMTN/TLN1
CC	GO:0043005	neuron projection	736	-0.43	-1.75	1.00E-10	3.79E-09	2.32E-09	2673	tags=33%, list=23%, signal=27%	KCNE3/ARHGAP4/MAK/NDUFS7/KCNJ2/CCN3/CETN2/PTGDR2/PRR12/SNPH/SV2A/EPB41L3/HNRNPU/P2RY1/STAU2/HDAC6/AMIGO1/CFAP410/TWF2/FOS/FSCN1/VAMP3/SEPTIN4/TRAK1/AMFR/ARHGAP32/SLC1A4/CYBB/CORO1A/PPFIA1/PI4K2A/DIP2B/SLC9A3R1/BCL11B/GIGYF2/IFT122/MYO5A/MYO9A/KIF13B/MUL1/AAK1/VIM/EPHB2/GDPD5/ENO2/NIN/ATP2B1/STRN/KATNB1/ZNF385A/SNAP25/DISC1/FYN/PTEN/DMWD/FEZ1/LRRC4/ITSN1/SRC/DNAJB1/RAB27B/TRIOBP/MAP1S/EXOC4/EPHB4/ELK1/ABAT/SLC24A4/ADNP/DBNL/BLOC1S3/GRIK2/ATP7A/EPS8/LRFN3/CPEB4/OR10H1/NCAM1/CPNE5/NAV1/NECTIN1/ACTG1/EXOC7/SYAP1/RPH3A/LRRK2/OPRL1/RANGAP1/MPST/NDEL1/SYNJ1/DLG4/DBN1/MME/EPHB1/STX4/CDKL5/NF2/SIPA1L1/AGTPBP1/RAP1GAP/CLSTN1/SETX/CHRNE/PARD3/CKB/MYCBP2/EXOC3/CTNND1/CLN3/KPNA1/ABI3/MAP4/VPS35/MARK3/EPHA4/SHISA7/ARPC5/GNAQ/NCF1/NUMA1/ARF1/GSK3A/TXNRD2/PPP1CA/CANX/HRH2/AGO2/PRKACA/FAM168B/INSR/SH3KBP1/ADGRL1/PALLD/NDRG2/S100A4/SPTBN1/CNIH3/FXR2/MCRS1/KIF1C/ERO1A/HTT/RARA/PTK2B/NEURL1/SLC8A1/SPTA1/BCR/INPP5K/RAPGEF2/EIF4G2/SBF2/LDLRAP1/COPA/DYNC1H1/MARK4/MAX/SH2D3C/PAK1/MAP1A/PTGS1/ARL8A/VCAN/PPP3CA/USP9X/APBA3/GNB1/SCN1B/ASAP1/PLEC/DCTN1/CALM3/FZD5/BASP1/GLRX5/FLNA/ULK1/DNM3/PVALB/ACTB/MADD/GPI/GJC2/RAC1/KIF1B/SLA/MARK2/CYFIP2/GSK3B/DNM2/TPRG1L/ROGDI/FLOT2/HSPB1/TNFRSF1B/ATP2B4/GNAI2/TRAK2/GRIPAP1/MPL/TSHZ3/RHOA/PRKCB/WDFY3/STAU1/CLU/ARRB2/NF1/ACTN4/PPP1R9B/TERF2/RIN3/GNA12/CASC3/PINK1/NRGN/ESPN/SNCA/SIAH2/MTPN/CTTN/PIP4K2A/CLDN5/RIPOR2/PRKAR2B/UBB/ACTN1/ATP6V0D1/STON2/BSG/MPP1/KLC3/RNF40/SHARPIN/MBP/GNAZ/P2RX1/FBXO7/GLUL/CBL/IQGAP1/OSBP2/ANK1

BP	GO:0045597	positive regulation of cell differentiation	505	-0.46	-1.84	1.00E-10	3.79E-09	2.32E-09	2816	tags=37%, list=24%, signal=30%	ACVR1B/SMAD3/NFKBID/SOX4/TNFSF14/KIT/F11R/BCL9L/SMAD1/ATP11A/CARMIL1/RIN2/SART1/SPEN/PLXNA4/CALR/ZEB2/HNRNPU/CD4/STAU2/AMIGO1/AMBRA1/TGIF2/TWF2/FOS/KDM4A/MAML1/CREB1/ACTR2/SOCS3/ASPM/RCOR1/NCKAP1L/SCIN/TENT5A/EPHB2/CTNNBIP1/MTCH2/GDPD5/LIMS1/AXIN2/ADAM8/STK25/NIN/TLR2/PA2G4/GLIPR2/SOD2/CR1/ZNF385A/DISC1/MACF1/SRF/FEZ1/UNC13D/NLRP3/TRIOBP/ADNP/DBNL/STK4/ATOH8/XRCC6/VDR/VEZF1/CASS4/WDFY2/TGFB1/TIAM1/PPARGC1B/ITPKB/DICER1/TNFSF4/RRAS/CUL7/NOTCH2/SDCBP/NUMB/SYAP1/BTG1/HGF/PLCB1/METRNL/NDEL1/STAT5B/SYNJ1/CMKLR1/RIPK1/DBN1/KAT7/DOCK5/MME/CPNE1/MAPK14/TRIB1/CEBPA/CDKL5/S1PR2/NF2/ZFP36L1/SMARCD3/SEMA4D/NCOA3/PREX1/EPHA4/CCR1/NUMA1/HIF1AN/ARHGEF2/ARNT/ZNF335/NEDD9/RUNX3/MAP3K5/VSIR/NFKB1/FES/GREM1/TSPO/LRRC8A/RARA/NEURL1/RAPGEF2/SOX6/EIF4G2/TRAF6/PITHD1/KLF5/IL6R/PDLIM7/ZMIZ1/BMP6/ZBTB16/ASXL2/TGFB1I1/POR/SFRP2/STK11/PLXNC1/TFE3/PTPRC/SYK/MED1/IST1/MTURN/LTF/FLNA/JUND/EIF4G1/NIBAN2/HSPA1A/INPP5D/PF4/GJC2/RAC1/LYN/LGALS9/SIN3A/MMD/GSK3B/PDE5A/DNM2/TNFRSF1B/TCF7L2/MPL/RHOA/GATA1/MYADM/ARRB2/ADD1/HCLS1/PIM1/RPS6KA1/TESC/TGM2/STAT3/CLDN5/RIPOR2/ZNF703/TAL1/NCOA1/FAM210B/PRKDC/DAB2/CUX1/CARM1/FOXO3/FAXDC2/HLX/DMTN
BP	GO:0045765	regulation of angiogenesis	161	-0.60	-2.13	1.00E-10	3.79E-09	2.32E-09	1752	tags=30%, list=15%, signal=26%	RRAS/GTF2I/HGS/MTDH/ROCK2/BTG1/HGF/GAB1/C5AR1/PDPK1/PML/SASH1/AGO2/PKM/AMOT/GREM1/HIPK1/PTK2B/SEMA4A/WARS1/PRKD2/SFRP2/ITGA5/FOXO4/THBS1/AGO1/HIPK2/NIBAN2/PF4/AQP1/HSPB1/ATP2B4/EPN1/FOXJ2/PRKCB/NF1/SP1/LRG1/GRN/JAK1/STAT3/SPARC/CLDN5/PIK3CD/STIM1/E2F2/KRT1/GLUL/TBXA2R
BP	GO:0048514	blood vessel morphogenesis	342	-0.52	-2.01	1.00E-10	3.79E-09	2.32E-09	2074	tags=32%, list=18%, signal=27%	MYO1E/HEG1/SRF/PTEN/EPHB4/NINJ1/JMJD6/MAP2K5/STK4/PROK2/ERBB2/VEZF1/TGFB1/RRAS/CUL7/NOTCH2/SLC12A6/GTF2I/HGS/RNF213/MTDH/ACTG1/ROCK2/BTG1/HGF/GAB1/C5AR1/SHC1/EPHB1/MAPK14/ZFP36L1/SPI1/PDPK1/PML/HIF1AN/LEPR/ADIPOR2/SASH1/YWHAZ/AGO2/ANPEP/PDGFA/PKM/AMOT/SGPL1/GREM1/HIPK1/RBPJ/PTK2B/SEMA4A/MEIS1/WARS1/ARID2/RAPGEF2/BCAS3/PRKD2/KLF5/CALD1/ZMIZ1/SFRP2/ITGA5/SYK/SH2D2A/FOXO4/JAM3/THBS1/AGO1/MED1/FZD5/PIK3CG/HIPK2/B4GALT1/FLNA/NIBAN2/PF4/AQP1/MAP3K3/LRP1/HSPB1/ITGA2B/SRPK2/ATP2B4/EPN1/RHOA/FOXJ2/PRKCB/GPX1/NF1/SP1/LRG1/GRN/JAK1/MYLK/STAT3/SPARC/CLDN5/TAL1/PIK3CD/BSG/STIM1/ITGB3/E2F2/KRT1/MYH9/GLUL/NPRL3/TBXA2R/WASF2

BP	GO:0048646	anatomical structure formation involved in morphogenesis	616	-0.46	-1.89	1.00E-10	3.79E-09	2.32E-09	2473	tags=33%, list=21%, signal=28%	SETD2/FIG4/ATP5F1B/ANXA2/CFLAR/MYOF/SCRIB/ALOX5/GPLD1/ARMC5/ADAM17/CYBB/SIRT6/WLS/NFE2L2/TBX19/IFT122/DUSP1/HDAC7/EPHB2/MTHFD1/ARHGAP24/PKN1/AXIN2/PTK7/FHL2/ADAM8/TLR2/CHI3L1/FLII/CSRP1/BCL2L11/ZNF385A/PAF1/SRF/WNT1/PTEN/UNC13D/APAF1/EXOC4/EPHB4/MTHFR/ATM/NINJ1/SLC24A4/JMJD6/MAP2K5/STK4/ATOH8/SDC4/PROK2/ERBB2/EXT1/VEZF1/ATP7A/MKS1/TGFB1/PPARGC1B/DICER1/PHACTR4/WDR74/RRAS/BCL3/NOTCH2/SLC12A6/GTF2I/HGS/TSC2/RNF213/MTDH/NECTIN1/ACTG1/ROCK2/BTG1/HGF/NBEAL2/DYRK1B/GAB1/PIKFYVE/C5AR1/SHC1/EPHB1/MAPK14/PEF1/NF2/AGTPBP1/FLOT1/MSX1/PTPN6/SMARCD3/SBNO2/FUZ/NFKB2/PTPN18/PDPK1/PML/ITGB2/WDR1/RFX2/LEPR/ADIPOR2/GRB2/SASH1/YWHAZ/AGO2/ANPEP/PRKACA/PDGFA/PKM/MBOAT7/AMOT/TPM1/SUFU/GREM1/HIPK1/RBPJ/NFATC2/RARA/PTK2B/SEMA4A/MEIS1/WARS1/HERC1/BCAS3/TRAF6/PRKD2/KLF5/CALD1/MMP9/SFRP2/ITGA5/SYK/SH2D2A/FOXO4/DVL3/JAM3/THBS1/AGO1/SUPT6H/PLEC/MED1/MAFB/FZD5/PIK3CG/HIPK2/B4GALT1/SKI/FLNA/NIBAN2/PF4/GPI/AQP1/MAP3K3/ETS2/HSPB1/AKAP13/ITGA2B/SRPK2/ATP2B4/EPN1/FKBP1A/MPL/RHOA/FOXJ2/PRKCB/GATA1/GPX1/NF1/KDM6B/SP1/LRG1/GRN/JAK1/TMOD1/PIM1/MAPK3/ITGB5/TGM2/TBC1D20/STAT3/MTPN/EHD1/SPARC/CLDN5/RIPOR2/TAL1/ACTN1/PIK3CD/MFN2/BSG/STIM1/PRKDC/MYL9/MPIG6B/ITGB3/VASP/E2F2/KRT1/CNOT1/MYH9/GLUL/TBXA2R/WASF2/FURIN/ACRBP
BP	GO:0050817	coagulation	149	-0.63	-2.22	1.00E-10	3.79E-09	2.32E-09	2303	tags=42%, list=20%, signal=34%	NFE2L2/HPS6/EPHB2/SH2B3/SERPINA1/FCER1G/SERPING1/CSRP1/KNG1/DGKA/SRF/PRDX2/SRC/ABAT/BLOC1S3/THBD/DGKG/DGKZ/ANO6/ACTG1/MAPK14/PTPN6/PAPSS2/FERMT3/GNAQ/F5/FIBP/PDGFA/PF4V1/SELP/F13A1/VCL/VAV1/GP6/SYK/THBS1/PIK3CG/FLNA/ACTB/PF4/ALOX12/PLEK/LYN/DGKK/GP9/HSPB1/MPL/GATA1/ITPK1/GNA12/WAS/SLC4A1/GP1BA/TREML1/MYL9/MPIG6B/ITGB3/KRT1/P2RX1/MYH9/TBXA2R/DMTN/TLN1
BP	GO:0050878	regulation of body fluid levels	223	-0.59	-2.19	1.00E-10	3.79E-09	2.32E-09	2303	tags=39%, list=20%, signal=32%	NFE2L2/HPS6/EPHB2/SH2B3/SERPINA1/FCER1G/SERPING1/CSRP1/ZNF385A/KNG1/HEG1/DGKA/SRF/PRDX2/SRC/ABAT/BLOC1S3/PRKCE/THBD/EXT1/DGKG/VDR/ATP7A/DGKZ/ANO6/ACTG1/OPRL1/STAT5B/MAPK14/PTPN6/NR1H2/PAPSS2/FERMT3/GNAQ/F5/PRKACA/FIBP/PDGFA/PF4V1/NEURL1/SELP/INPP5K/COPA/F13A1/USF2/VCL/VAV1/GP6/SYK/THBS1/NCOR2/PLEC/MED1/PIK3CG/FLNA/ACTB/PF4/ALOX12/GPI/PLEK/AQP1/LYN/DGKK/STAT5A/GP9/HSPB1/GNAI2/MPL/GATA1/ITPK1/GNA12/WAS/MLLT6/SLC4A1/GP1BA/TREML1/NFE2/MYL9/MPIG6B/ITGB3/KRT1/WNK1/P2RX1/MYH9/TBXA2R/DMTN/TLN1

BP	GO:0051093	negative regulation of developmental process	469	-0.46	-1.84	1.00E-10	3.79E-09	2.32E-09	2260	tags=32%, list=19%, signal=27%	HDAC7/MUL1/EPHB2/USP19/BCL11A/AXIN2/TMEM176B/ASXL1/SFMBT1/TLR2/FOXO1/SPRY3/SOD2/CR1/PAF1/WWC3/WNT1/PTEN/PRDX2/YWHAH/ANKRD17/HDAC4/TRIO/CDK12/IL17RD/STK4/ERBB2/TGFB1/LDLRAD4/ITPKB/DICER1/TNFSF4/RC3H2/GTF2I/HGS/CTDP1/ROCK2/NBR1/GPS2/STAT5B/JDP2/NFATC1/EPHB1/TRIB1/SRA1/YY1/ZFP36L1/RAP1GAP/MSX1/SORT1/HOOK3/SEMA4D/MAD2L2/DPF2/NR1H2/DDX6/FUZ/USF3/SPI1/EPHA4/CMTM5/PML/CDK13/PTBP1/CCR1/TRIB3/KLF13/GSK3A/AMOT/RUNX3/TMEM131L/SUFU/GREM1/RBPJ/TSPO/NFATC2/SORL1/CORO1C/YBX1/IRF1/PGLYRP1/RARA/PTK2B/SEMA4A/BCR/MEIS1/RAPGEF2/SOX6/TBX21/TOB2/IGF2/ZHX2/CTNNA1/ZBTB16/MMP9/TGFB1I1/SFRP2/STK11/PPP3CA/LILRB3/FOXO4/THBS1/AGO1/ASAP1/ADIPOR1/MED1/MAFB/LTF/G6PD/SKI/ULK1/NIBAN2/DNM3/JAK3/INPP5D/PF4/RFLNB/LYN/GSK3B/HUWE1/ATP2B4/CTDSP1/TRIM11/EPN1/RHOA/FOXJ2/CUL4A/NFATC3/GATA1/NF1/ACTN4/PINK1/CDKN1A/PBX1/FLCN/STAT3/SPARC/CLDN5/TENT5C/ITGB3/YBX3/E2F2/MBP/JARID2/RNF10/CARM1/MBNL3/FBXO7/FOXO3/HLX/DMTN
BP	GO:0051094	positive regulation of developmental process	736	-0.45	-1.85	1.00E-10	3.79E-09	2.32E-09	2246	tags=30%, list=19%, signal=26%	MUL1/EPHB2/CTNNBIP1/MTCH2/GDPD5/LIMS1/AXIN2/ADAM8/STK25/NIN/TLR2/ATP2B1/PA2G4/GLIPR2/CHI3L1/SOD2/CR1/ZNF385A/DISC1/MACF1/SRF/WNT1/KDM5B/FEZ1/UNC13D/NLRP3/SRC/TRIOBP/FKBP1B/NINJ1/ADNP/DBNL/STK4/ATOH8/TNFSF13/XRCC6/NIPBL/VDR/VEZF1/CASS4/WDFY2/MAGED1/TGFB1/TIAM1/PPARGC1B/ITPKB/DICER1/TNFSF4/RRAS/CUL7/CPNE5/ANO6/NOTCH2/SDCBP/MTDH/NUMB/SYAP1/BTG1/HGF/B4GALT5/PLCB1/METRNL/NDEL1/STAT5B/SYNJ1/EZR/GAB1/CMKLR1/RIPK1/DLG4/DBN1/KAT7/DOCK5/C5AR1/MME/EPHB1/CPNE1/MAPK14/TRIB1/CEBPA/CDKL5/S1PR2/NF2/ZFP36L1/FLOT1/CLSTN1/SMARCD3/SEMA4D/MAD2L2/VPS35/NCOA3/PREX1/EPHA4/PDPK1/RALBP1/CCR1/NUMA1/HIF1AN/ARHGEF2/SASH1/AGO2/ARNT/ZNF335/NEDD9/PKM/INSR/AMOT/STK24/ADGRL1/RUNX3/MAP3K5/VSIR/NFKB1/FES/GREM1/HIPK1/RBPJ/TSPO/VLDLR/NFATC2/SORL1/CORO1C/LRRC8A/RARA/PTK2B/NEURL1/SLC8A1/SEMA4A/STAT6/RAPGEF2/SOX6/EIF4G2/TRAF6/PITHD1/PRKD2/TBX21/KLF5/IL6R/IGF2/PDLIM7/ZMIZ1/BMP6/ZBTB16/ASXL2/TGFB1I1/POR/SFRP2/STK11/PLXNC1/TFE3/ITGA5/PTPRC/SYK/DVL3/THBS1/FIS1/MED1/IST1/MTURN/LTF/HIPK2/BASP1/FLNA/JUND/EIF4G1/NIBAN2/HSPA1A/INPP5D/PF4/GJC2/RAC1/AQP1/LYN/LGALS9/SIN3A/MMD/GSK3B/PDE5A/DNM2/HSPB1/TNFRSF1B/TCF7L2/MPL/RHOA/PRKCB/GATA1/MYADM/ARRB2/SP1/LRG1/ADD1/GRN/JAK1/HCLS1/PIM1/CFL1/PINK1/RPS6KA1/TESC/TGM2/STAT3/EHD1/CLDN5/RIPOR2/ZNF703/TAL1/NCOA1/FAM210B/PIK3CD/STIM1/PRKDC/YBX3/DAB2/CUX1/CARM1/FOXO3/TBXA2R/FAXDC2/HLX/DMTN

BP	GO:0051241	negative regulation of multicellular organismal process	575	-0.43	-1.75	1.00E-10	3.79E-09	2.32E-09	2260	tags=30%, list=19%, signal=25%	HDAC7/MUL1/EPHB2/BCL11A/SH2B3/AXIN2/RPS6KA5/TMEM176B/SFMBT1/IRAK3/TLR2/ATP2B1/SERPING1/FOXO1/RAB11FIP5/CR1/KNG1/PCTP/WWC3/LGALS9C/SRF/PTEN/PRDX2/NLRP3/YWHAH/FKBP1B/ABAT/IL17RD/MAP2K5/STK4/THBD/ERBB2/TGFB1/SIRPA/LDLRAD4/CHSY1/DICER1/TNFSF4/RC3H2/LILRA5/BCL3/GTF2I/HGS/CTDP1/CPTP/ROCK2/GSTP1/ARRB1/HGF/EZR/CMKLR1/DOCK5/TRIB1/GRK2/NRDC/YY1/RAP1GAP/ARHGDIB/PTPN6/PYCARD/HOOK3/SEMA4D/MAD2L2/NR1H2/FUZ/RELB/USF3/SPI1/ITCH/EPHA4/PML/CCR1/KAT5/GSK3A/PDGFA/CHID1/AMOT/RUNX3/TMEM131L/NDRG2/VSIR/NFKB1/GREM1/RBPJ/TSPO/SORL1/CORO1C/IRF1/PGLYRP1/RARA/PTK2B/SEMA4A/BCR/RAPGEF2/SOX6/RPS6KA4/OTUD5/TBX21/TOB2/CTNNA1/CSK/SFRP2/STK11/PPP3CA/TFE3/PTPRC/LILRB3/FOXO4/THBS1/ILRUN/NPLOC4/AGO1/LMNA/ADIPOR1/MAFB/TLR6/NLRX1/LTF/PIK3CG/RAB11FIP1/G6PD/SKI/ULK1/NIBAN2/JAK3/INPP5D/PF4/ALOX12/RAC1/RFLNB/LYN/LGALS9/LAPTM5/GSK3B/PDE5A/TNFRSF1B/ATP2B4/GNAI2/CTDSP1/TRIM11/EPN1/RHOA/FOXJ2/CUL4A/GATA1/ARRB2/NF1/GRN/GUCY1A1/FLCN/PTPN23/SPARC/KHSRP/CLDN5/IP6K1/N4BP1/TLE3/GP1BA/DNAJA4/E2F2/MBP/KRT1/JARID2/RNF10/WNK1/FBXO7/TIMP1/TBXA2R/HLX/FURIN
BP	GO:0051270	regulation of cellular component movement	635	-0.46	-1.87	1.00E-10	3.79E-09	2.32E-09	2053	tags=27%, list=18%, signal=24%	CD99L2/MACF1/FGR/SRF/PADI2/PTEN/FEZ1/TIRAP/PPM1F/SRC/IRS2/ATM/CPNE3/MAP2K5/STK4/PRKCE/ATOH8/SDC4/NIPBL/ATP7A/CASS4/TGFB1/LDLRAD4/TIAM1/SUN2/RRAS/ANO6/SDCBP/SYNE2/HSPA5/ACTG1/NUMB/ROCK2/HGF/PIP5KL1/PLCB1/GAB1/CMKLR1/ELP3/DOCK5/C5AR1/MGAT5/TRIB1/STX4/STK10/S1PR2/NF2/ACTA2/PTPRJ/ARHGDIB/PYCARD/ADGRG3/SEMA4D/MYCBP2/ARAP1/FUZ/CLN3/ABI3/FAM89B/DAPK3/SPI1/EPHA4/FERMT3/PDPK1/CCR1/CSF1R/ARHGEF2/FOXP1/CXCL16/SASH1/AGO2/PDGFA/NEDD9/INSR/AMOT/YTHDF3/S100A11/STK24/TPM1/VSIR/FES/GREM1/SORL1/CORO1C/PLEKHG3/DOCK8/PTK2B/SLC8A1/SELP/SEMA4A/BCR/ARID2/RAPGEF2/BCAS3/PRKD2/IL6R/PAK1/VCL/PECAM1/CTNNA1/MMP9/SFRP2/STK11/PLXNC1/PPP3CA/ITGA5/PTPRC/FOXO4/JAM3/THBS1/PTAFR/ZNF609/LMNA/ATP1B2/ADIPOR1/CD151/PIK3CG/ABHD2/CAMK1D/FLNA/RIPOR1/GFUS/GPI/RAC1/LYN/LGALS9/MAP3K3/STAT5A/RAC2/LRP1/HSPB1/CNN2/ATP2B4/GNAI2/IQSEC1/RHOA/MYADM/NF1/MSN/SP1/ACTN4/GRN/RIN3/GNA12/WAS/MYLK/MAPK3/FLCN/STAT3/PTPN23/SPARC/CLDN5/RIPOR2/LAMP1/ZNF703/ACTN1/AKT2/SH3BP1/PIK3CD/MPP1/DNAJA4/ITGB3/DAB2/TRIM58/WNK1/PLVAP/GLUL/TIMP1/FOXO3/TBXA2R/MAP2K3/RHOG/DMTN/DIAPH1
BP	GO:0051272	positive regulation of cellular component movement	349	-0.49	-1.88	1.00E-10	3.79E-09	2.32E-09	2416	tags=36%, list=21%, signal=30%	MAPRE2/SPN/GPLD1/ADAM17/CORO1A/NCKAP1L/NFE2L2/SCARB1/HDAC7/TF/EPHB2/ADAM8/MAP4K4/GLIPR2/SOD2/CD99L2/FGR/FEZ1/TIRAP/PPM1F/SRC/IRS2/ATM/CPNE3/STK4/PRKCE/ATOH8/NIPBL/ATP7A/CASS4/TGFB1/TIAM1/SUN2/RRAS/ANO6/SDCBP/SYNE2/HSPA5/ACTG1/NUMB/ROCK2/HGF/GAB1/CMKLR1/ELP3/DOCK5/C5AR1/MGAT5/STX4/ACTA2/PYCARD/SEMA4D/FAM89B/DAPK3/SPI1/EPHA4/FERMT3/PDPK1/CCR1/CSF1R/ARHGEF2/FOXP1/CXCL16/SASH1/AGO2/PDGFA/NEDD9/INSR/S100A11/VSIR/DOCK8/PTK2B/SLC8A1/SELP/SEMA4A/RAPGEF2/BCAS3/PRKD2/IL6R/PAK1/PECAM1/MMP9/STK11/PPP3CA/ITGA5/PTPRC/FOXO4/JAM3/THBS1/PTAFR/ZNF609/CD151/PIK3CG/CAMK1D/RIPOR1/GFUS/GPI/RAC1/LYN/LGALS9/MAP3K3/STAT5A/RAC2/HSPB1/GNAI2/IQSEC1/RHOA/MYADM/SP1/ACTN4/GRN/MYLK/MAPK3/STAT3/SPARC/RIPOR2/ZNF703/AKT2/PIK3CD/ITGB3/DAB2/WNK1/PLVAP/MAP2K3/DMTN/DIAPH1

BP	GO:0070848	response to growth factor	390	-0.52	-2.03	1.00E-10	3.79E-09	2.32E-09	2045	tags=32%, list=17%, signal=27%	FYN/WNT1/KDM5B/FEZ1/SRC/APAF1/ELK1/AKT1S1/KIDINS220/CPNE3/IL17RD/MAP2K5/XIAP/ERBB2/EXT1/RNF165/ATP7A/TGFB1/LDLRAD4/ZBTB7A/PDE2A/ACAP2/ERN1/NOTCH2/SDCBP/HGS/HSPA5/SYAP1/ROCK2/HGF/STAT5B/GAB1/EGR1/RIPK1/SHC1/MAPK14/ZFP36L1/RAP1GAP/MSX1/SETX/SORT1/CD63/FUZ/CDKN1C/FAM89B/SPI1/PDPK1/PTPN12/PML/NUMA1/ZFHX3/ARNT/FIBP/PDGFA/INSR/RUNX3/GREM1/RBPJ/SORL1/PCSK6/IER2/INPP5K/RAPGEF2/SOX6/CHST11/PRKD2/ZMIZ1/RAB14/BMP6/TGFB1I1/SFRP2/STK11/USP9X/ITGA5/LTBP2/THBS1/PTPN1/MED1/NR3C1/GLG1/HIPK2/PPP2R5B/SKI/NIBAN2/NDST1/HSPA1A/TGFBRAP1/ZYX/CYFIP2/DNM2/CREBBP/HSPB1/TNFRSF1B/ATP2B4/PXN/FKBP1A/PRKCB/ARRB2/FKBP8/LRG1/PPP1R9B/UBE2O/MAPK3/ITGB5/RAPGEF1/FLCN/STAT3/EHD1/CLDN5/LTBP1/ZNF703/MAPKAPK2/PIK3CD/PTP4A3/PPM1A/PAK2/ITGB3/DAB2/FOXO3/CBL/CDC34/IQGAP1/MAP2K3/FURIN/RELA
BP	GO:0071363	cellular response to growth factor stimulus	373	-0.52	-2.02	1.00E-10	3.79E-09	2.32E-09	2045	tags=32%, list=17%, signal=27%	FYN/WNT1/KDM5B/FEZ1/SRC/APAF1/AKT1S1/KIDINS220/CPNE3/IL17RD/MAP2K5/XIAP/ERBB2/EXT1/RNF165/ATP7A/TGFB1/LDLRAD4/ZBTB7A/PDE2A/ACAP2/ERN1/NOTCH2/SDCBP/HGS/HSPA5/SYAP1/HGF/STAT5B/GAB1/EGR1/RIPK1/SHC1/MAPK14/ZFP36L1/RAP1GAP/MSX1/SETX/SORT1/CD63/FUZ/CDKN1C/FAM89B/SPI1/PDPK1/PTPN12/PML/NUMA1/ARNT/FIBP/PDGFA/INSR/GREM1/RBPJ/SORL1/PCSK6/INPP5K/RAPGEF2/SOX6/CHST11/PRKD2/ZMIZ1/RAB14/BMP6/TGFB1I1/SFRP2/STK11/USP9X/ITGA5/LTBP2/THBS1/PTPN1/MED1/NR3C1/GLG1/HIPK2/PPP2R5B/SKI/NIBAN2/NDST1/HSPA1A/TGFBRAP1/ZYX/CYFIP2/DNM2/CREBBP/HSPB1/TNFRSF1B/ATP2B4/PXN/FKBP1A/PRKCB/ARRB2/FKBP8/LRG1/PPP1R9B/UBE2O/MAPK3/ITGB5/RAPGEF1/FLCN/STAT3/EHD1/CLDN5/LTBP1/ZNF703/MAPKAPK2/PIK3CD/PTP4A3/PPM1A/PAK2/ITGB3/DAB2/FOXO3/CBL/IQGAP1/MAP2K3/FURIN/RELA
BP	GO:0071417	cellular response to organonitrogen compound	396	-0.50	-1.95	1.00E-10	3.79E-09	2.32E-09	1843	tags=29%, list=16%, signal=25%	LARS1/ATP7A/ZDHHC7/AGTRAP/CPEB4/PDE2A/OR10H1/TSC2/HSPA5/SYAP1/ROCK2/SMARCC1/LRRK2/RANGAP1/PLCB1/NDEL1/STAT5B/KLF16/EZR/GAB1/EGR1/SH2B2/PIP4K2C/SHC1/CACNB1/CA2/ZFP36L1/AQP9/VPS35/EPHA4/PDPK1/ARHGEF2/ITPR2/TRIB3/SSH1/GSK3A/GRB2/HRH2/PDK2/PRKACA/PKM/INSR/SESN3/SESN2/SLC27A1/FBP1/NFKB1/SORL1/TMEM38A/NEURL1/SLC8A1/RRAGC/STAT6/INPP5K/RAPGEF2/CCND3/MAX/IGF2/CTNNA1/CSK/ZNF106/POR/LAMTOR1/SLC9A1/FOXO4/PTAFR/PTPN1/GNB1/RAB31/ADIPOR1/TLR6/PIK3CG/FLNA/LPIN2/JAK3/UBR2/ACTB/UCP2/AQP1/LYN/SIN3A/GSK3B/STAT5A/DNM2/LRP1/ATP2B4/GNAI2/PXN/PRKCB/CASTOR2/GATA1/ARRB2/SP1/PPP1R9B/JAK1/WDTC1/LARP1/MAPK3/SNCA/TGM2/RAPGEF1/BCL2L1/STAT3/PIP4K2A/AHCYL1/AKT2/PRKDC/KLF1/EIF4EBP2/GNAZ/FOXO3/DMTN/KLF3/DIAPH1/RELA

BP	GO:0071495	cellular response to endogenous stimulus	794	-0.45	-1.85	1.00E-10	3.79E-09	2.32E-09	2253	tags=31%, list=19%, signal=27%	AIFM1/KAT2B/VIM/EPHB2/BCL11A/LIMS1/PKN1/SAFB/KLHL22/TADA3/VPS18/ATP2B1/FOXO1/SPRY3/GNA15/BCL2L11/THRA/FYN/WNT1/PADI2/KDM5B/RRAGD/SRC/PER1/APAF1/YWHAH/IRS2/SULT1A3/RXRB/DDX54/KIDINS220/IL17RD/PRKCE/PAQR8/XIAP/ERBB2/EXT1/RNF165/LARS1/ATP7A/TGFB1/ZDHHC7/LDLRAD4/AGTRAP/PPARGC1B/ZBTB7A/CPEB4/PDE2A/OR10H1/ACAP2/TNFSF4/NOTCH2/SDCBP/TSC2/UBR5/HSPA5/SYAP1/ROCK2/SMARCC1/LRRK2/RANGAP1/SMARCA4/PLCB1/NDEL1/STAT5B/KLF16/EZR/GAB1/EGR1/ARID5A/KMT2D/SH2B2/PIP4K2C/SHC1/CACNB1/SRA1/CA2/ZFP36L1/RAP1GAP/MSX1/SETX/SORT1/SMC1A/FUZ/AQP9/CDKN1C/FAM89B/VPS35/SPI1/NCOA3/EPHA4/PDPK1/PTPN12/PML/NUMA1/ARHGEF2/ITPR2/TRIB3/FOXP1/LEPR/SSH1/GSK3A/UBE2L3/ADIPOR2/GRB2/HRH2/PDK2/PRKACA/FIBP/PDGFA/PKM/INSR/SESN3/SESN2/SLC27A1/FBP1/NFKB1/VPS11/GREM1/KANK2/RBPJ/NCOA4/NCOA2/SORL1/TMEM38A/PELP1/PCSK6/RARA/NEURL1/SLC8A1/RRAGC/STAT6/INPP5K/RAPGEF2/SOX6/BCAS3/CCND3/CHST11/PRKD2/MAX/PAK1/IGF2/ZMIZ1/CTNNA1/CSK/RAB14/ZNF106/BMP6/TGFB1I1/POR/LAMTOR1/SFRP2/STK11/USP9X/LTBP2/SLC9A1/FOXO4/THBS1/PTAFR/PTPN1/GNB1/NCOR2/RAB31/ADIPOR1/MED1/NR3C1/TLR6/GLG1/PIK3CG/ABHD2/HIPK2/SKI/FLNA/LPIN2/JAK3/NDST1/HSPA1A/UBR2/ACTB/TGFBRAP1/UCP2/RXRA/ZYX/AQP1/LYN/SIN3A/GSK3B/STAT5A/DNM2/CREBBP/LRP1/ATP2B4/GNAI2/PXN/FKBP1A/RHOA/PRKCB/GAS2L1/CASTOR2/GATA1/ARRB2/FKBP8/SP1/LRG1/PPP1R9B/JAK1/WDTC1/LARP1/ARID1A/UBE2O/WBP2/MAPK3/SNCA/ITGB5/TGM2/RAPGEF1/BCL2L1/FLCN/STAT3/EHD1/PIP4K2A/CLDN5/LTBP1/ZNF703/AHCYL1/AKT2/NCOA1/PPM1A/CTSB/PRKDC/KLF1/EIF4EBP2/PAK2/DAB2/CNOT1/GNAZ/CARM1/FOXO3/CBL/IQGAP1/FURIN/DMTN/KLF3/DIAPH1/RELA
BP	GO:0072359	circulatory system development	623	-0.47	-1.90	1.00E-10	3.79E-09	2.32E-09	2303	tags=30%, list=20%, signal=26%	NFE2L2/TBX19/IFT122/HDAC7/KAT2B/EPHB2/MTHFD1/ARHGAP24/AXIN2/PTK7/FHL2/ADAM8/ASXL1/CHI3L1/FOXO1/SOD2/MYO1E/HEG1/SRF/PTEN/UNC13D/EPHB4/ANKRD17/MOSPD3/ATM/NINJ1/JMJD6/MAP2K5/STK4/PROK2/ERBB2/EXT1/NIPBL/VEZF1/ATP7A/MKS1/TGFB1/PDE2A/RRAS/CUL7/NOTCH2/SLC12A6/GTF2I/HGS/CTDP1/TSC2/RNF213/MTDH/ACTG1/TFDP2/ROCK2/BTG1/HGF/AP2B1/SNX17/GAB1/EGR1/NFATC1/C5AR1/SHC1/EPHB1/MAPK14/GRK2/ACTA2/YY1/ZFP36L1/MSX1/SMARCD3/SPI1/PDPK1/PML/HIF1AN/LEPR/GSK3A/ADIPOR2/SASH1/YWHAZ/AGO2/ANPEP/PDGFA/PKM/INSR/AMOT/TPM1/SGPL1/PSKH1/SUFU/GREM1/HIPK1/RBPJ/NFATC2/ADAP2/RARA/PTK2B/SLC8A1/SEMA4A/MEIS1/WARS1/ARID2/RAPGEF2/SOX6/EIF4G2/BCAS3/PRKD2/KLF5/IL6R/CALD1/PECAM1/PDLIM7/ZMIZ1/SFRP2/STK11/ITGA5/PDLIM5/SYK/SH2D2A/SLC9A1/FOXO4/JAM3/THBS1/AGO1/LMNA/GAA/PLEC/DAW1/MED1/FZD5/PIK3CG/HIPK2/B4GALT1/G6PD/BASP1/FLNA/NIBAN2/NDST1/ODAD4/PF4/AQP1/MAP3K3/LRP1/HSPB1/AKAP13/ITGA2B/TNFRSF1B/SRPK2/TCF7L2/ATP2B4/EPN1/FKBP1A/RHOA/FOXJ2/NFATC3/PRKCB/GPX1/PDLIM1/ARRB2/NF1/KDM6B/SP1/NCOA6/LRG1/GRN/JAK1/PIM1/ECE1/MYLK/MAPK3/CDKN1A/AP1B1/STAT3/SPARC/CLDN5/TAL1/PIK3CD/BSG/STIM1/PRKDC/ITGB3/E2F2/KRT1/JARID2/LYL1/MYH9/GLUL/NPRL3/TBXA2R/MAP2K3/MICAL2/WASF2

BP	GO:0097435	supramolecular fiber organization	504	-0.48	-1.91	1.00E-10	3.79E-09	2.32E-09	2567	tags=36%, list=22%, signal=29%	CLASP1/HDAC6/CST3/COLGALT2/TWF2/CDC42EP1/CAPN3/FSCN1/ACTR2/CFLAR/TBCD/NAA80/KRT15/TUBGCP6/TPPP3/CORO1A/PPFIA1/NCKAP1L/SCIN/ARHGEF5/MYO5A/CORO7/TF/PRUNE1/VIM/MYO1G/NIN/TNXB/FLII/CSRP1/KATNB1/SHROOM1/MYO1E/SRF/LIMK2/PPM1F/SRC/TRIOBP/MAP1S/CRTAP/ATXN7/MSRB2/RHOF/HOOK2/DIAPH2/DBNL/PRKCE/MARCKS/SDC4/EXT1/ATP7A/HCK/CASS4/EPS8/TPM3/PPARGC1B/B4GALT7/CYRIA/ACTG1/ROCK2/ARRB1/CKAP5/NDEL1/EZR/SPTAN1/RIPK1/ARPC1B/DBN1/WASF3/NF2/CAMSAP1/EML2/WASHC2C/PYCARD/CDC42EP4/HOOK3/MAD2L2/SLAIN2/ARAP1/CLIP4/DAPK3/KPNB1/PREX1/ARPC5/NUMA1/NCKAP5L/ARHGEF2/WDR1/ALMS1/LCP1/ARF1/SSH1/CHP1/GRB2/TPM4/NEDD9/SH3KBP1/TPM1/SPTBN1/FES/GREM1/CORO1C/PTK2B/SPTA1/MSRB1/INPP5K/COLGALT1/SVIL/DYNC1H1/CALD1/PAK1/MAP1A/CTNNA1/TAOK1/ALDOA/ELMO1/NDE1/SFRP2/GSN/USP9X/LTBP2/SLC9A1/PLEC/ESAM/DCTN1/IQGAP2/MYO1F/FLNA/HSPA1A/PLEK/ZYX/RAC1/RFLNB/GOLGA2/HIP1/CYFIP2/RAC2/AKAP13/PXN/FKBP1A/ARHGAP6/WIPF1/COTL1/RHOA/CAPZB/GAS2L1/MYADM/GPX1/PDLIM1/CLU/NF1/ADD1/PPP1R9B/SSH2/HCLS1/TMOD1/ARPC4/CFL1/RHOBTB1/WAS/ESPN/SNCA/ITGB5/SIAH2/SPTB/MTPN/CTTN/GAS7/ACTN1/SH3BP1/MYL9/PAK2/VASP/KRT1/MICAL2/WASF2/RHOG/FURIN/DMTN/DIAPH1
CC	GO:0098590	plasma membrane region	652	-0.47	-1.92	1.00E-10	3.79E-09	2.32E-09	2395	tags=30%, list=20%, signal=25%	CD38/ASPM/ADAM17/UTRN/SLC19A1/DDX58/MAP4K2/PI4K2A/SLC9A3R1/SCARB1/MGAM/TF/AAK1/EPHB2/ENO2/PKN1/TNK2/SLC28A2/CLTB/REPS1/ATP2B1/STRN/PARD6B/CR1/SHROOM1/SNAP25/MACF1/FGR/PTEN/TIRAP/LRRC4/ITSN1/SRC/RAB27B/CD177/NINJ1/SSPN/SLC24A4/ATP6V1B2/DBNL/GRIK2/THBD/ERBB2/ATP7A/HCK/EPS8/LRFN3/ADGRE2/AP2A2/PDE2A/PDE4A/SLC12A6/SYNE2/MTDH/NECTIN1/NUMB/SYAP1/RAB11FIP4/ABCB11/ITGAM/ARRB1/RPH3A/LRRK2/AP2B1/SYNJ1/LIPE/EZR/AP2S1/DLG4/MFSD4B/DBN1/TMEM67/C5AR1/STX4/CDKL5/NF2/PTPRJ/FXYD6/FLOT1/CLSTN1/SORT1/SLC16A3/CHRNE/PARD3/HVCN1/EXOC3/AQP9/CLN3/EPHA4/SHISA7/ITGB2/ARHGEF2/LCP1/LEPR/SSH1/PIP5K1C/ABCG2/CANX/PRKACA/CA4/OCEL1/INSR/LRP10/ADGRL1/NHS/TPM1/SLC27A1/SPTBN1/CNIH3/AP2A1/VLDLR/CORO1C/DOCK8/SLC2A1/SLC8A1/SELPLG/CAVIN2/SLC6A20/INPP5K/RAPGEF2/SVIL/LDLRAP1/SLC3A2/IL6R/SH2D3C/PAK1/CTNNA1/NDE1/ITGA5/SLC9A1/ASAP1/ATP1B2/EHD3/CLTC/SLC6A9/B4GALT1/SLC1A5/DNM3/SLC14A1/ABCC4/GPI/PLEK/RAC1/AQP1/HIP1/DNM2/LRP1/FLOT2/ABCC3/ATP2B4/EPN1/RHOA/EPS15L1/ARHGAP45/ITPK1/LRFN1/ARRB2/ATP6V1A/MSN/PPP1R9B/GNA12/CFL1/NRGN/TESC/MYLK/MAPK3/EPB41/EHD1/CTTN/CLDN5/RIPOR2/PLEK2/PRKAR2A/AHCYL1/AKT2/SLC4A1/DENND1A/AQP10/ATP6V0D1/AP2M1/BSG/CTSB/STIM1/ITGB3/VASP/DAB2/RNF10/SLC7A5/P2RX1/MYH9/PLVAP/CBL/WASF2/DMTN/ANK1/DIAPH1/TLN1

CC	GO:0099503	secretory vesicle	705	-0.44	-1.81	1.00E-10	3.79E-09	2.32E-09	2180	tags=33%, list=19%, signal=28%	ADAM8/SERPINA1/CLTB/FCER1G/TLR2/GLB1/ATP2B1/PA2G4/CHI3L1/SERPING1/ORMDL3/RAB11FIP5/CR1/KNG1/SNAP25/DISC1/FGR/CYSTM1/CD93/PADI2/GUSB/UNC13D/RAB27B/APAF1/PHACTR2/CD177/ZNRF1/RHOF/DBNL/CPNE3/NCF2/XRCC6/CD68/CMTM6/ALDH3B1/ATP7A/EEF2/STX6/TGFB1/SIRPA/SLC2A3/AP2A2/PDXK/LAMP2/ANO6/SDCBP/ARHGAP9/CKAP4/TSPAN14/ACLY/GSTP1/ITGAM/RPH3A/LRRK2/HGF/NBEAL2/NDEL1/SPTAN1/TMBIM1/SYTL3/SYN3/DLG4/PSMD2/PSMD3/C5AR1/MME/CLK3/CPNE1/CLCN5/MAPK14/STX4/STK10/CRISPLD2/PTPRJ/PTPN6/PYCARD/ADGRG3/HVCN1/EXOC3/CD63/TRAPPC1/ALAD/AP1M1/CLN3/ATP6V0C/KPNB1/QSOX1/FERMT3/SIGLEC5/ARPC5/ITGB2/CDK13/PGAM1/ITPR2/CAP1/TMEM184A/F5/PSMD1/CPPED1/ANPEP/PRKACA/CA4/SEPTIN1/PCSK1N/PDGFA/PKM/S100A11/RNASET2/ATP6V0A1/NFKB1/NFAM1/OSCAR/TIMP2/PGLYRP1/PAFAH1B2/SELP/SNX10/CXCR2/PYGL/DYNC1H1/F13A1/PSMD11/IGF2/VCL/PECAM1/RAB7A/CTNNA1/RAB14/HGSNAT/ARL8A/IMPDH1/MMP9/SLC44A2/ALDOA/LAMTOR1/LCN2/GSN/UBR4/APLP2/PYGB/RAB5B/PTPRC/LILRB3/THBS1/PTAFR/GAA/RAB31/ATG7/IST1/TOM1/CALM3/SLC6A9/DOCK2/IQGAP2/LTF/ABHD2/B4GALT1/VCP/DOK3/ABCC4/HSPA1A/TUBB4B/CAPN1/SELL/PF4/GPI/DNAJC5/CD14/RAC1/KIF1B/CYB5R3/ADGRE5/SIRPB1/IGF2R/PSAP/TPRG1L/RAB11B/ROGDI/FLOT2/ITGA2B/TNFRSF1B/SYTL4/CDA/CNN2/HUWE1/COTL1/IQSEC1/RHOA/ITGAL/GNS/FTL/ARHGAP45/TUBB/CLU/CXCR1/ATP6V1A/ADA2/ACTN4/LRG1/CANT1/FTH1/GRN/ECE1/PLEKHO2/SNCA/ANXA11/BCL2L1/SPARC/LAMP1/PRDX6/ACTN1/ATP6V0D1/RAB3IL1/TREML1/STON2/BSG/CTSB/RAB3D/PAK2/CTSD/ITGB3/KRT1/P2RX1/MYH9/TOLLIP/TIMP1/IQGAP1/TBXA2R/RHOG/ACRBP/DIAPH1
MF	GO:0106310	protein serine kinase activity	275	-0.56	-2.11	1.00E-10	3.79E-09	2.32E-09	2329	tags=36%, list=20%, signal=29%	MAP4K2/AATK/MAST4/MAP2K4/PIM3/AAK1/DYRK3/PKN1/TNK2/STK25/MAP4K4/SRPK1/RPS6KA5/CDC42BPA/LIMK2/MAP3K4/TRIO/ATM/CDC42BPB/CDK12/MAP2K5/STK4/PRKCE/ERN2/ERN1/ROCK2/LRRK2/STK38/DYRK1B/ALPK1/RIPK1/PIKFYVE/CLK3/MAPK14/STK10/CDKL5/MAP2K2/EIF2AK2/MAP3K2/RSKR/DAPK3/CSNK1G2/MARK3/LRRK1/PDPK1/MAST2/CDK13/GSK3A/PRKACA/STK24/CSNK2A1/MAP3K5/PSKH1/TAOK3/HIPK1/MKNK2/PIM2/BCR/RPS6KA4/SIK3/PRKD2/MARK4/NEK5/PAK1/TAOK1/MAP4K1/STK11/MAP2K7/BMP2K/STK40/MAPKAPK3/PIK3CG/HIPK2/CAMK1D/ULK1/MARK2/RIOK3/MAP3K3/GSK3B/CDK19/SRPK2/PRKCB/CSNK1G1/PIM1/PINK1/RPS6KA1/MAPK3/NUAK2/MAPKAPK2/AKT2/ARAF/GAK/PRKDC/PAK2/WNK1/MAST3/MAP2K3/EIF2AK1

BP	GO:1901698	response to nitrogen compound	706	-0.42	-1.71	1.00E-10	3.79E-09	2.32E-09	2369	tags=31%, list=20%, signal=26%	CYBB/EPG5/RHBDD2/DDX58/CRY2/SLC9A3R1/NFE2L2/FOXRED2/MYO5A/AIFM1/KAT2B/MUL1/VIM/EPHB2/USP19/BCL11A/MAVS/KLHL22/FCER1G/IRAK3/TLR2/ATP2B1/FOXO1/GNA15/RFTN1/BCL2L11/ATXN3/FYN/WNT1/RRAGD/SRC/PER1/IRS2/SULT1A3/MTHFR/ABAT/SLC24A4/PRKCE/RTF2/THBD/EXT1/CD68/LARS1/ATP7A/ZDHHC7/AGTRAP/PPARGC1B/CPEB4/PDE2A/OR10H1/TNFSF4/MGMT/TSC2/SCAP/HSPA5/SYAP1/ROCK2/UBE2J2/SMARCC1/LRRK2/BTG1/RANGAP1/PLCB1/NDEL1/STAT5B/KLF16/EZR/GAB1/EGR1/MAN1A1/KAT7/MARCHF6/SH2B2/TMEM67/C5AR1/PIP4K2C/SHC1/MAPK14/CACNB1/CA2/GRK2/YOD1/ZFP36L1/FLOT1/SORT1/TMBIM6/AQP9/ALAD/EFTUD2/VPS35/EPHA4/PDPK1/TRDMT1/GNAQ/ITGB2/ARHGEF2/ITPR2/TRIB3/SSH1/GSK3A/GRB2/CANX/HRH2/PDK2/PRKACA/EIF6/PKM/INSR/UBE4B/GUCY1B1/SESN3/SESN2/MAP3K5/SLC27A1/FBP1/NFKB1/TSPO/SORL1/TMEM38A/BCAP31/PTK2B/SLC2A1/NEURL1/SLC8A1/RRAGC/STAT6/INPP5K/RAPGEF2/CCND3/RCN3/SLC3A2/MAX/IGF2/CTNNA1/CSK/ZNF106/MMP9/POR/LAMTOR1/STK11/CPT1A/PPP3CA/SYK/SLC9A1/FOXO4/PTAFR/PTPN1/NPLOC4/UQCRC1/GNB1/NCOR2/RAB31/ADIPOR1/TLR6/CALM3/PIK3CG/FLNA/LPIN2/JUND/JAK3/VCP/UBR2/ACTB/GPI/UCP2/AQP1/LYN/RIOK3/SIN3A/GSK3B/STAT5A/DNM2/LRP1/CDA/ATP2B4/GNAI2/PXN/RHOA/PRKCB/CASTOR2/GATA1/GPX1/ARRB2/SP1/PPP1R9B/JAK1/WDTC1/LARP1/IFIT1B/MAPK3/SNCA/TGM2/RAPGEF1/BCL2L1/STAT3/PIP4K2A/PRKAR2B/AHCYL1/AKT2/DNAJB2/BSG/PRKDC/KLF1/RNF40/EIF4EBP2/UBXN6/GNAZ/CARM1/P2RX1/TIMP1/FOXO3/DMTN/BAG6/KLF3/DIAPH1/RELA
BP	GO:1901699	cellular response to nitrogen compound	436	-0.48	-1.90	1.00E-10	3.79E-09	2.32E-09	2463	tags=35%, list=21%, signal=29%	ACTR2/SOCS3/CFLAR/RALB/GPLD1/CYBB/EPG5/DDX58/SLC9A3R1/NFE2L2/MYO5A/AIFM1/KAT2B/MUL1/VIM/EPHB2/BCL11A/MAVS/KLHL22/ATP2B1/FOXO1/GNA15/BCL2L11/FYN/WNT1/RRAGD/SRC/IRS2/SULT1A3/RTF2/LARS1/ATP7A/ZDHHC7/AGTRAP/CPEB4/PDE2A/OR10H1/TSC2/HSPA5/SYAP1/ROCK2/SMARCC1/LRRK2/RANGAP1/PLCB1/NDEL1/STAT5B/KLF16/EZR/GAB1/EGR1/SH2B2/PIP4K2C/SHC1/CACNB1/CA2/GRK2/ZFP36L1/FLOT1/AQP9/VPS35/EPHA4/PDPK1/GNAQ/ARHGEF2/ITPR2/TRIB3/SSH1/GSK3A/GRB2/HRH2/PDK2/PRKACA/PKM/INSR/GUCY1B1/SESN3/SESN2/MAP3K5/SLC27A1/FBP1/NFKB1/SORL1/TMEM38A/NEURL1/SLC8A1/RRAGC/STAT6/INPP5K/RAPGEF2/CCND3/MAX/IGF2/CTNNA1/CSK/ZNF106/POR/LAMTOR1/SLC9A1/FOXO4/PTAFR/PTPN1/GNB1/RAB31/ADIPOR1/TLR6/PIK3CG/FLNA/LPIN2/JAK3/UBR2/ACTB/UCP2/AQP1/LYN/RIOK3/SIN3A/GSK3B/STAT5A/DNM2/LRP1/ATP2B4/GNAI2/PXN/PRKCB/CASTOR2/GATA1/ARRB2/SP1/PPP1R9B/JAK1/WDTC1/LARP1/IFIT1B/MAPK3/SNCA/TGM2/RAPGEF1/BCL2L1/STAT3/PIP4K2A/AHCYL1/AKT2/PRKDC/KLF1/EIF4EBP2/GNAZ/FOXO3/DMTN/KLF3/DIAPH1/RELA

BP	GO:1901701	cellular response to oxygen-containing compound	751	-0.42	-1.71	1.00E-10	3.79E-09	2.32E-09	2309	tags=31%, list=20%, signal=27%	SLC9A3R1/NFE2L2/MYO5A/SCARB1/PIM3/TRIM5/AIFM1/KAT2B/VIM/EPHB2/BCL11A/FANCC/PTK7/STK25/KLHL22/TLR2/ATP2B1/FOXO1/GNA15/SOD2/BCL2L11/RAB11FIP5/PAF1/SRF/FYN/WNT1/TIRAP/RRAGD/PRDX2/NLRP3/SRC/ELK1/IRS2/SULT1A3/FKBP1B/ATM/PRKCE/CD68/LCOR/VDR/LARS1/ATP7A/HCK/TGFB1/ZDHHC7/SIRPA/AGTRAP/PPARGC1B/CPEB4/PDE2A/OR10H1/TNFSF4/ERN1/TSC2/MTDH/HSPA5/SYAP1/ROCK2/GSTP1/SMARCC1/LRRK2/RANGAP1/HGF/SMARCA4/PLCB1/SIDT2/NDEL1/STAT5B/KLF16/EZR/GAB1/EGR1/ARID5A/ZBTB20/RIPK1/SH2B2/PIP4K2C/SHC1/MAPK14/CACNB1/TRIB1/STX4/CA2/GRK2/ZFP36L1/SETX/PYCARD/SBNO2/AQP9/VPS35/SPI1/NCOA3/EPHA4/PDPK1/GPBAR1/GNAQ/NCF1/CXCL5/ARHGEF2/KAT5/ITPR2/TRIB3/SSH1/GSK3A/GRB2/SASH1/HRH2/DGAT2/PDK2/PRKACA/PKM/INSR/RDH11/PF4V1/GUCY1B1/SESN3/SESN2/MAP3K5/TPM1/SLC27A1/FBP1/NFKB1/FES/KANK2/TSPO/UPF1/NCOA4/SORL1/PRDX5/RARA/PTK2B/NEURL1/SLC8A1/RRAGC/BCR/STAT6/INPP5K/RAPGEF2/CCND3/TRAF6/PLSCR3/MAX/IGF2/CTNNA1/CSK/ZNF106/BMP6/MMP9/MAP4K1/POR/LAMTOR1/CPT1A/PPP3CA/MAP2K7/SLC9A1/FOXO4/PTAFR/PTPN1/PDK3/GNB1/RAB31/PLEC/ADIPOR1/MED1/NR3C1/TLR6/LTF/PIK3CG/FLNA/LPIN2/JAK3/VCP/UBR2/ACTB/PF4/SIPA1/UCP2/RXRA/CD14/AQP1/LYN/SIN3A/GSK3B/STAT5A/DNM2/CDK19/RAB11B/LRP1/TNFRSF1B/ATP2B4/GNAI2/PXN/RHOA/TNIP1/PRPF8/PRKCB/CASTOR2/GATA1/GPX1/CLU/ARRB2/MSN/KDM6B/SP1/PPP1R9B/JAK1/WDTC1/LARP1/PIM1/PINK1/TESC/MAPK3/SNCA/TGM2/RAPGEF1/BCL2L1/STAT3/PIP4K2A/ZNF703/AHCYL1/AKT2/FAM210B/LITAF/PRKDC/KLF1/EIF4EBP2/GNAZ/FOXO3/TBXA2R/MAP2K3/DMTN/KLF3/RELA
BP	GO:1905114	cell surface receptor signaling pathway involved in cell-cell signaling	303	-0.51	-1.96	1.00E-10	3.79E-09	2.32E-09	2415	tags=34%, list=21%, signal=28%	UBE2B/AMFR/ASPM/WLS/SLC9A3R1/PORCN/CTNNBIP1/AXIN2/PTK7/TLR2/STRN/FOXO1/PAF1/DISC1/MACF1/WNT1/PTEN/VANGL1/SRC/ABAT/STK4/XIAP/GRIK2/EXT1/CCNY/MKS1/TGFB1/SNX3/TIAM1/G3BP1/TSC2/UBR5/RNF213/ITGAM/LRRK2/SMARCA4/EGR1/DLG4/NFATC1/MAPK14/TLE4/S1PR2/CHRNE/MAD2L2/FUZ/CTNND1/KPNA1/DAPK3/VPS35/CSNK1G2/ATP6V0C/LRRK1/INVS/GNAQ/GSK3A/ZRANB1/PPP1CA/GRK6/CSNK2A1/TMEM131L/NDRG2/NFKB1/GREM1/RBPJ/FRAT1/PTK2B/FRMD8/OTUD5/PIAS4/TBL1XR1/TGFB1I1/SFRP2/STK11/PPP3CA/DVL3/FZD5/USP34/CHD8/SKI/VCP/RAC1/MARK2/GSK3B/LRP1/DDB1/TCF7L2/RHOA/TBL1X/CSNK1G1/GID8/ARRB2/BCL9/SNCA/SIAH2/ZNF703/TLE3/PPM1A/DAB2/WNK1/FAM53B/P2RX1/CTDNEP1/FOXO3

BP	GO:2000026	regulation of multicellular organismal development	775	-0.44	-1.79	1.00E-10	3.79E-09	2.32E-09	2240	tags=28%, list=19%, signal=25%	PRUNE1/EPHB2/CTNNBIP1/CLPTM1/AXIN2/SH3RF1/ADAM8/STK25/TMEM176B/NIN/TLR2/ATP2B1/CHI3L1/CR1/PAF1/DISC1/MACF1/SRF/WNT1/PTEN/PRDX2/NLRP3/YWHAH/NINJ1/ADNP/DBNL/ATOH8/MYCL/XRCC6/ERBB2/NIPBL/VDR/VEZF1/TGFB1/TIAM1/PPARGC1B/ITPKB/DICER1/TNFSF4/RC3H2/RRAS/CUL7/ANO6/NOTCH2/GTF2I/HGS/CTDP1/MTDH/NUMB/ROCK2/BTG1/HGF/NBR1/B4GALT5/PLCB1/NDEL1/STAT5B/SYNJ1/GAB1/RIPK1/DBN1/KAT7/KEAP1/C5AR1/MME/WASF3/EPHB1/MAPK14/TRIB1/CDKL5/S1PR2/NF2/YY1/ZFP36L1/CLSTN1/PER2/PTPN6/SMARCD3/PARD3/HOOK3/SEMA4D/CDKN1C/SOS2/SPI1/NCOA3/EPHA4/PDPK1/PML/CCR1/NUMA1/FOXP1/KLF13/GSK3A/SASH1/AGO2/TRPS1/ARNT/EIF6/ZNF335/CNOT4/PKM/INSR/AMOT/RUNX3/TMEM131L/VSIR/NFAM1/FES/GREM1/HIPK1/RBPJ/TSPO/SORL1/IRF1/PGLYRP1/RARA/PTK2B/NEURL1/SLC8A1/SEMA4A/ANKH/MEIS1/WARS1/RAPGEF2/SOX6/EIF4G2/TRAF6/PITHD1/PRKD2/TBX21/TOB2/VCL/ZMIZ1/CTNNA1/RAB14/BMP6/ZBTB16/MMP9/POR/SFRP2/STK11/PLXNC1/PPP3CA/TFE3/ITGA5/PTPRC/LILRB3/SYK/FOXO4/THBS1/BMP2K/AGO1/MED1/MAFB/GLG1/IST1/MTURN/PHOSPHO1/LTF/HIPK2/TRIP12/G6PD/BASP1/SKI/ULK1/NIBAN2/JAK3/HSPA1A/INPP5D/PF4/GJC2/RFLNB/AQP1/LYN/LGALS9/GFI1B/HSPB1/TNFRSF1B/ATP2B4/CTDSP1/TRIM11/EPN1/MPL/RHOA/FOXJ2/CUL4A/PRKCB/GATA1/NF1/SP1/LRG1/ADD1/GRN/JAK1/HCLS1/PIM1/CFL1/TESC/TGM2/STAT3/SPARC/CLDN5/TAL1/FAM210B/PIK3CD/STIM1/PRKDC/E2F2/MBP/KRT1/JARID2/CUX1/RNF10/FBXO7/GLUL/FOXO3/TBXA2R/FAXDC2/HLX/BAG6
BP	GO:2000145	regulation of cell motility	596	-0.48	-1.93	1.00E-10	3.79E-09	2.32E-09	2416	tags=32%, list=21%, signal=27%	MAPRE2/SPN/RFFL/GPLD1/ADAM17/CORO1A/NCKAP1L/MIIP/DDX58/SLC9A3R1/NFE2L2/SCARB1/DUSP1/HDAC7/TF/EPHB2/PKN1/ADAM8/MAP4K4/GLIPR2/SOD2/PARD6B/CD99L2/MACF1/FGR/SRF/PADI2/PTEN/TIRAP/PPM1F/SRC/IRS2/ATM/CPNE3/MAP2K5/STK4/PRKCE/ATOH8/SDC4/NIPBL/ATP7A/CASS4/TGFB1/LDLRAD4/TIAM1/SUN2/RRAS/ANO6/SDCBP/SYNE2/HSPA5/ACTG1/NUMB/ROCK2/HGF/PIP5KL1/PLCB1/GAB1/CMKLR1/ELP3/DOCK5/C5AR1/MGAT5/TRIB1/STX4/STK10/S1PR2/NF2/ACTA2/PTPRJ/ARHGDIB/PYCARD/ADGRG3/SEMA4D/FUZ/CLN3/ABI3/FAM89B/DAPK3/SPI1/EPHA4/FERMT3/PDPK1/CCR1/CSF1R/ARHGEF2/FOXP1/CXCL16/SASH1/AGO2/PDGFA/NEDD9/INSR/AMOT/YTHDF3/S100A11/STK24/TPM1/VSIR/FES/GREM1/SORL1/CORO1C/PLEKHG3/DOCK8/PTK2B/SLC8A1/SELP/SEMA4A/BCR/ARID2/RAPGEF2/BCAS3/PRKD2/IL6R/PAK1/VCL/PECAM1/CTNNA1/MMP9/SFRP2/PLXNC1/PPP3CA/ITGA5/PTPRC/FOXO4/JAM3/THBS1/PTAFR/ZNF609/LMNA/ATP1B2/ADIPOR1/CD151/PIK3CG/ABHD2/CAMK1D/FLNA/RIPOR1/GFUS/GPI/RAC1/LYN/LGALS9/MAP3K3/STAT5A/RAC2/LRP1/HSPB1/CNN2/ATP2B4/GNAI2/IQSEC1/RHOA/MYADM/NF1/MSN/SP1/ACTN4/GRN/RIN3/GNA12/WAS/MYLK/MAPK3/FLCN/STAT3/PTPN23/SPARC/CLDN5/RIPOR2/ZNF703/AKT2/SH3BP1/PIK3CD/MPP1/DNAJA4/ITGB3/DAB2/WNK1/PLVAP/GLUL/TIMP1/FOXO3/TBXA2R/MAP2K3/RHOG/DMTN/DIAPH1

CC	GO:0031252	cell leading edge	300	-0.51	-1.93	1.02E-10	3.82E-09	2.35E-09	2713	tags=40%, list=23%, signal=32%	PLEKHO1/CDK6/GBF1/CARMIL1/RAB22A/EPB41L3/FGD6/HDAC6/TWF2/ABLIM1/FSCN1/DPP9/ACTR2/SCRIB/ADAM17/CORO1A/DDX58/SLC9A3R1/MYO5A/AAK1/VIM/SH3RF1/MYO1G/ATP2B1/CDC42BPA/MACF1/FGR/TIRAP/ITSN1/SRC/CD177/ATP6V1B2/CDC42BPB/DBNL/ATP7A/EPS8/DGKZ/ADGRE2/ACAP2/PHACTR4/PDE4A/SYNE2/NDEL1/EZR/DBN1/WASF3/SRA1/STX4/CDKL5/NF2/ACTA2/PTPRJ/FLOT1/CTNND1/FRMD4B/ABI3/FAM89B/SHISA7/ARPC5/ARHGEF2/LCP1/ARF1/SSH1/PIP5K1C/ARHGAP31/NEDD9/INSR/AMOT/S100A11/PALLD/NHS/TPM1/SPTBN1/CORO1C/DOCK8/PTK2B/FGD4/INPP5K/BCAS3/SH2D3C/PAK1/PDLIM7/CTNNA1/FGD3/GSN/NRBP1/ITGA5/SLC9A1/IQGAP2/ACTB/PLEK/RAC1/RAC2/DNM2/FLOT2/PXN/WIPF1/RHOA/CAPZB/MYADM/ARHGAP45/PPP1R9B/CFL1/TESC/MYLK/APBB1IP/PARVB/CTTN/RIPOR2/PLEK2/ABLIM3/ACTN1/AKT2/SH3BP1/ITGB3/VASP/MYH9/IQGAP1/WASF2/DIAPH1/TLN1
BP	GO:1901342	regulation of vasculature development	162	-0.59	-2.12	1.21E-10	4.50E-09	2.76E-09	1752	tags=30%, list=15%, signal=26%	RRAS/GTF2I/HGS/MTDH/ROCK2/BTG1/HGF/GAB1/C5AR1/PDPK1/PML/SASH1/AGO2/PKM/AMOT/GREM1/HIPK1/PTK2B/SEMA4A/WARS1/PRKD2/SFRP2/ITGA5/FOXO4/THBS1/AGO1/HIPK2/NIBAN2/PF4/AQP1/HSPB1/ATP2B4/EPN1/FOXJ2/PRKCB/NF1/SP1/LRG1/GRN/JAK1/STAT3/SPARC/CLDN5/PIK3CD/STIM1/E2F2/KRT1/GLUL/TBXA2R
BP	GO:2000147	positive regulation of cell motility	342	-0.49	-1.90	1.44E-10	5.27E-09	3.24E-09	2416	tags=36%, list=21%, signal=30%	MAPRE2/SPN/GPLD1/ADAM17/CORO1A/NCKAP1L/NFE2L2/SCARB1/HDAC7/TF/EPHB2/ADAM8/MAP4K4/GLIPR2/SOD2/CD99L2/FGR/TIRAP/PPM1F/SRC/IRS2/ATM/CPNE3/STK4/PRKCE/ATOH8/NIPBL/ATP7A/CASS4/TGFB1/TIAM1/SUN2/RRAS/ANO6/SDCBP/SYNE2/HSPA5/ACTG1/NUMB/ROCK2/HGF/GAB1/CMKLR1/ELP3/DOCK5/C5AR1/MGAT5/STX4/ACTA2/PYCARD/SEMA4D/FAM89B/DAPK3/SPI1/EPHA4/FERMT3/PDPK1/CCR1/CSF1R/ARHGEF2/FOXP1/CXCL16/SASH1/AGO2/PDGFA/NEDD9/INSR/S100A11/VSIR/DOCK8/PTK2B/SLC8A1/SELP/SEMA4A/RAPGEF2/BCAS3/PRKD2/IL6R/PAK1/PECAM1/MMP9/PPP3CA/ITGA5/PTPRC/FOXO4/JAM3/THBS1/PTAFR/ZNF609/CD151/PIK3CG/CAMK1D/RIPOR1/GFUS/GPI/RAC1/LYN/LGALS9/MAP3K3/STAT5A/RAC2/HSPB1/GNAI2/IQSEC1/RHOA/MYADM/SP1/ACTN4/GRN/MYLK/MAPK3/STAT3/SPARC/RIPOR2/ZNF703/AKT2/PIK3CD/ITGB3/DAB2/WNK1/PLVAP/MAP2K3/DMTN/DIAPH1

BP	GO:0009894	regulation of catabolic process	785	-0.40	-1.64	1.57E-10	5.67E-09	3.48E-09	2109	tags=29%, list=18%, signal=26%	ORMDL3/FOXO1/BCL2L11/ATXN3/THRA/ALKBH5/DISC1/RGP1/CSDE1/FYN/WNT1/PTEN/TPCN1/FEZ1/TIRAP/ATP6V0B/FTO/RRAGD/ARIH1/APAF1/EXOC4/IRS2/SH3GLB1/HDAC4/ATM/ATP6V1B2/COP1/PRKCE/ATF6/USP36/DET1/ZDHHC7/SNX3/AREL1/RNF144B/PUM1/ZBTB7A/RC3H2/VPS13D/ERN1/SDCBP/HGS/TSC2/MTDH/CPTP/EXOC7/SH3RF3/ROCK2/ABCB11/SMARCC1/LRRK2/IKBKE/HGF/EZR/SPTLC2/ZBTB20/TFEB/PSMD2/KEAP1/PSMD3/PIP4K2C/PIAS1/MAPK14/TRIB1/CEBPA/PLEKHF1/WAC/CHMP4B/NRDC/ZFP36L1/AGTPBP1/PHF23/PYCARD/CBFA2T3/MAD2L2/MYCBP2/DAP/ARIH2/SF3B3/ALAD/CLN3/DAPK3/VPS35/ATP6V0C/QSOX1/ITCH/ABHD5/EPHA4/TRDMT1/PML/ZC3HAV1/CLEC16A/PGAM1/KAT5/TRIB3/LEPR/GSK3A/PPP1CA/OPTN/PSMD1/AGO2/ENTPD5/ARNT/MTMR3/PRKACA/EIF6/INSR/YTHDF3/SESN3/TENT4B/SESN2/STX5/ATP6V0A1/USP10/CNOT6/CSNK2A1/FBP1/VSIR/SUFU/VPS11/FXR2/TSPO/UPF1/ODC1/SORL1/PIM2/BCAP31/IKBKG/YBX1/TIMP2/HTT/PTK2B/PAFAH1B2/RRAGC/HERC1/BSCL2/EIF4G2/PITHD1/MAP1A/RAB7A/RAD23A/STK11/CPT1A/FZR1/WDR45/PSME3/PTPN1/GTPBP1/FBXL20/ATG7/FZD5/PIK3CG/HIPK2/FLNA/EIF4G1/ULK1/VCP/RUBCN/HSPA1A/PSMC4/VPS28/CAPN1/CAPNS1/GOLGA2/GABARAP/LAPTM5/GSK3B/ELAPOR1/ZC3H18/PSAP/LRP1/HSPB1/WWP2/BNIP3L/DDB1/TNFRSF1B/SUPT5H/HUWE1/ATP2B4/PNPLA2/TBC1D25/TRIM14/RILP/GPX1/CLU/ATP6V1A/ATG2A/MSN/LARP1/GNA12/OAZ1/CASC3/PINK1/MARCHF2/MAPK3/SNCA/DTX3L/FLCN/STAT3/CTTN/KHSRP/PIP4K2A/PSMF1/MAPKAPK2/N4BP1/UBB/AKT2/DNAJB2/SLC4A1/USP7/ARAF/ATP6V0D1/SAMD4B/TENT5C/RNF40/YBX3/DCAF12/DAB2/CNOT1/FBXO7/TIMP1/NPRL3/FOXO3/ZER1/FURIN/BAG6/RYBP/RELA
BP	GO:0000904	cell morphogenesis involved in differentiation	403	-0.47	-1.83	1.65E-10	5.89E-09	3.62E-09	2768	tags=36%, list=24%, signal=29%	AKIP1/ALCAM/EPHA1/SYNGAP1/ARL13B/LAMC3/NEO1/CNP/ST6GAL1/ARHGAP4/CARMIL1/PLXNA4/CALR/STAU2/HDAC6/AMIGO1/TWF2/VAMP3/CREB1/ACTR2/TBCD/SCRIB/KIAA0319/DIP2B/SLC9A3R1/BCL11B/KIF13B/EPHB2/LIMS1/STK25/RPS6KA5/EFNB1/NIN/PARD6B/ZNF385A/HEG1/DISC1/MACF1/SRF/FYN/PTEN/FEZ1/UNC13D/SRC/TRIOBP/MAP1S/EPHB4/YWHAH/TRIO/KIDINS220/ADNP/DBNL/ERBB2/EXT1/RNF165/CASS4/TIAM1/MEGF9/NCAM1/CUL7/SZT2/NOTCH2/NECTIN1/NUMB/LRRK2/B4GALT5/NBEAL2/NDEL1/DLG4/DBN1/DOCK5/EPHB1/CDKL5/SIPA1L1/FLOT1/PARVG/PTPN6/WASHC2C/PARD3/SEMA4D/MYCBP2/ABI3/SPI1/PREX1/EPHA4/FERMT3/LYPLA2/CSF1R/WDR1/DPYSL2/NEDD9/PALLD/VLDLR/CORO1C/SEMA4A/RAPGEF2/EIF4G2/PAK1/MAP1A/VCL/RERE/STK11/PLXNC1/PPP3CA/APLP2/USP9X/PDLIM5/SCN1B/IST1/FLNA/ULK1/NIBAN2/DNM3/ACTB/RAC1/MARK2/SIN3A/GSK3B/RAC2/DNM2/PXN/MPL/RHOA/GATA1/MYADM/ACTN4/ECE1/CFL1/TBC1D20/PARVB/CTTN/TAL1/ACTN1/BSG/PRKDC/MPIG6B/PAK2/ITGB3/VASP/DAB2/MBP/CUX1/KEL/MYH9/XK/RHOG/DMTN
MF	GO:0051015	actin filament binding	145	-0.60	-2.10	2.14E-10	7.59E-09	4.66E-09	2541	tags=48%, list=22%, signal=38%	TWF2/ABLIM1/FSCN1/ACTR2/CORO1A/UTRN/SCIN/MYO5A/CORO7/MYO1G/FLII/SHROOM1/MYO1E/MACF1/TRIOBP/MAP1S/ACTR3C/DBNL/MYO18A/MARCKS/TPM3/SYNE2/EZR/SPTAN1/ARPC1B/DBN1/MYOM1/CTNNAL1/ABI3/ARPC5/WDR1/LCP1/TPM4/TPM1/SPTBN1/CORO1C/ZNF185/SPTA1/SVIL/CTNNA1/GSN/IQGAP2/MYO1F/FLNA/ANXA6/FMNL1/HIP1/MYL4/LASP1/COTL1/CAPZB/GAS2L1/ACTN4/ADD1/PPP1R9B/HCLS1/TMOD1/ARPC4/CFL1/ESPN/SPTB/CTTN/GAS7/ABLIM3/ACTN1/MYH9/IQGAP1/DMTN/TLN1

MF	GO:0008092	cytoskeletal protein binding	686	-0.41	-1.67	2.71E-10	9.53E-09	5.85E-09	2099	tags=28%, list=18%, signal=24%	CSRP1/BCL2L11/RAB11FIP5/KATNB1/SHROOM1/MYO1E/DISC1/MACF1/FYN/FEZ1/RAB27B/TRIOBP/MAP1S/YWHAH/PHACTR2/BICD2/MSRB2/HOOK2/ADNP/DIAPH2/ACTR3C/DBNL/MYO18A/PRKCE/MARCKS/AP1G1/LSP1/EPS8/TPM3/TTLL4/SUN2/PHACTR4/TTLL5/SYNE2/GPAA1/ROCK2/LRRK2/ENC1/CKAP5/NDEL1/EZR/SPTAN1/ARPC1B/DLG4/DBN1/INF2/TMEM67/WASF3/NF2/AGTPBP1/CLSTN1/PARVG/CAMSAP1/EML2/PYCARD/HOOK3/CLIP4/MYOM1/FHL3/VPS41/CTNNAL1/ABI3/MAP4/MARK3/NEIL2/MAST2/ARPC5/MAP1LC3B/NUMA1/ARHGEF2/WDR1/ALMS1/CAP1/LCP1/SSH1/RABGAP1/GSK3A/DPYSL2/CHP1/KIF3C/TPM4/WIPF2/PALLD/TPM1/S100A4/SPTBN1/NFKB1/FES/KIF1C/CEP350/PLEKHM2/CORO1C/PLEKHG3/HTT/RARA/FGD4/ZNF185/SLC8A1/SPTA1/KIF13A/MSRB1/RAB6B/BCAS3/SVIL/MARK4/CALD1/PAK1/MAP1A/VCL/PDLIM7/CTNNA1/RAB14/ARL8A/TAOK1/ALDOA/NDE1/GSN/PDLIM5/PTPRC/MYO9B/PLEC/DCTN1/CALM3/IQGAP2/MYO1F/B4GALT1/FLNA/EML3/DNM3/ACTB/ANXA6/FMNL1/RFLNB/GOLGA2/GABARAP/KIF1B/MARK2/LYN/HIP1/GSK3B/DNM2/MYL4/PPP1R18/RAB11B/CNN2/TRAK2/LASP1/PXN/WIPF1/COTL1/RHOA/CAPZB/GAS2L1/PDLIM1/CLU/MSN/ACTN4/ADD1/PPP1R9B/SSH2/HCLS1/TMOD1/ARPC4/CFL1/WAS/ESPN/MYLK/SNCA/EPB41/PARVB/SPTB/CTTN/GAS7/ABLIM3/ACTN1/SLC4A1/RAB3D/STIM1/KLC3/MYL9/VASP/MYH9/TNS1/IQGAP1/MICAL2/WASF2/DMTN/ANK1/DIAPH1/TLN1/RELA
BP	GO:0032970	regulation of actin filament-based process	262	-0.51	-1.94	2.86E-10	9.98E-09	6.13E-09	2007	tags=31%, list=17%, signal=27%	PPM1F/TRIOBP/RHOF/DBNL/PRKCE/SDC4/HCK/EPS8/NOTCH2/CYRIA/ACTG1/ROCK2/SPTAN1/ARPC1B/DBN1/WASF3/NF2/ACTA2/ARHGDIB/WASHC2C/PYCARD/CDC42EP4/ARAP1/ABI3/DAPK3/ARPC5/CSF1R/WDR1/ALMS1/ARF1/SSH1/GRB2/PDGFA/TPM1/SPTBN1/FES/PTK2B/SPTA1/INPP5K/BCAS3/SVIL/PAK1/TAOK1/GSN/SLC9A1/JAM3/ESAM/IQGAP2/FLNA/PLEK/RAC1/CYFIP2/RAC2/LRP1/AKAP13/CNN2/PXN/ARHGAP6/COTL1/RHOA/CAPZB/MYADM/ADD1/SSH2/HCLS1/ARHGDIA/TMOD1/ARPC4/RHOBTB1/WAS/SPTB/MTPN/CTTN/SH3BP1/PAK2/VASP/MYH9/IQGAP1/PEAK3/WASF2/RHOG/DMTN
BP	GO:0071375	cellular response to peptide hormone stimulus	194	-0.56	-2.05	3.09E-10	1.07E-08	6.55E-09	1811	tags=31%, list=15%, signal=27%	ZDHHC7/AGTRAP/TSC2/SYAP1/ROCK2/SMARCC1/RANGAP1/PLCB1/NDEL1/STAT5B/GAB1/SH2B2/PIP4K2C/SHC1/CA2/ZFP36L1/PDPK1/TRIB3/GSK3A/GRB2/PDK2/PKM/INSR/SESN3/SLC27A1/FBP1/NFKB1/SORL1/STAT6/INPP5K/CCND3/MAX/IGF2/CSK/ZNF106/POR/SLC9A1/FOXO4/PTPN1/RAB31/ADIPOR1/LPIN2/JAK3/UCP2/LYN/GSK3B/STAT5A/GNAI2/PXN/PRKCB/SP1/JAK1/WDTC1/STAT3/PIP4K2A/AHCYL1/AKT2/PRKDC/EIF4EBP2/RELA
BP	GO:0022604	regulation of cell morphogenesis	219	-0.54	-2.00	3.18E-10	1.09E-08	6.69E-09	2769	tags=45%, list=24%, signal=35%	DNMBP/KIT/F11R/BCL9L/PLEKHO1/ST6GAL1/CARMIL1/PLXNA4/CALR/EPB41L3/FGD6/P2RY1/STAU2/CFAP410/CDC42EP1/ACTR2/CORO1A/SLC9A3R1/MYO9A/MUL1/EPHB2/BCL11A/LIMS1/SPRY3/MACF1/FGR/FYN/UNC13D/SRC/TRIOBP/PDZD8/RHOF/DBNL/HCK/CASS4/EPS8/CUL7/CPNE5/EZR/DLG4/DBN1/DOCK5/WASF3/CDKL5/CAMSAP1/WASHC2C/CDC42EP4/SEMA4D/ARAP1/DAPK3/AGO4/PREX1/EPHA4/ITGB2/CSF1R/WDR1/ZRANB1/NEDD9/BRWD3/SH3KBP1/TPM1/FES/CORO1C/PTK2B/FGD4/SPTA1/SEMA4A/ZMYM3/FGD3/ALDOA/PLXNC1/DVL3/FLNA/FMNL1/RAC1/MARK2/RAC2/DNM2/MPL/RHOA/CAPZB/MYADM/MSN/ACTN4/GNA12/CFL1/RHOBTB1/EPB41/PARVB/SPARC/EPB42/PRKDC/DAB2/CUX1/MYH9/PEAK3/RHOG/DMTN/DIAPH1

BP	GO:0031589	cell-substrate adhesion	208	-0.55	-2.03	3.22E-10	1.09E-08	6.72E-09	2864	tags=44%, list=24%, signal=34%	NOTCH1/POLDIP2/TESK2/PIK3CB/SMAD3/AKIP1/EPHA1/LAMC3/CDK6/ST6GAL1/SPOCK2/CARMIL1/RIN2/CALR/CLASP1/VAMP3/TBCD/CORO1A/UTRN/PPFIA1/LIMS1/PLEKHA2/MAP4K4/MYO1G/TNXB/BCL2L11/ATXN3/DISC1/MACF1/SRF/WNT1/PTEN/UNC13D/PPM1F/SRC/TRIOBP/STK4/PRKCE/SDC4/CASS4/TIAM1/MEGF9/RRAS/ACTG1/ROCK2/ITGAM/DOCK5/EPHB1/NF2/PTPRJ/PARVG/WASHC2C/CD63/DAPK3/PREX1/FERMT3/ITGB2/NEDD9/GREM1/CORO1C/PTK2B/BCR/BCAS3/VCL/ITGA5/SLC9A1/JAM3/THBS1/ATP1B2/FLNA/GFUS/ZYX/RAC1/GSK3B/RAC2/DNM2/ITGA2B/PXN/ARHGAP6/RHOA/ITGAL/MYADM/NF1/ACTN4/RSU1/ITGB5/PARVB/CTTN/ACTN1/ITGB3/DAB2/DMTN
CC	GO:0005773	vacuole	658	-0.41	-1.67	3.55E-10	1.20E-08	7.35E-09	2039	tags=27%, list=17%, signal=23%	PADI2/TPCN1/GUSB/ATP6V0B/UBA52/UNC13D/RRAGD/SLC29A3/SRC/NKG7/FNIP2/SH3GLB1/ZNRF1/ATP6V1B2/IFNAR1/MCOLN1/CPNE3/STARD3/NCF2/SDC4/MARCHF3/THBD/CD68/AP1G1/CMTM6/LARS1/ACP2/NCF4/HCK/SLC66A1/WDR81/AP2A2/LAMP2/SZT2/USP4/SDCBP/HGS/TSC2/CKAP4/ACLY/ARRB1/LRRK2/NBR1/SIDT2/AP2B1/AP2S1/TMBIM1/NRBF2/TFEB/TBC1D17/CPQ/PIP4K2C/CPNE1/CLCN5/STX4/PLEKHF1/CHMP4B/PGAP6/FLOT1/SORT1/PYCARD/SLC36A1/CD63/IFITM3/TRAPPC1/AP1M1/VPS41/CLN3/VPS35/ATP6V0C/GNAQ/NCF1/CLEC16A/MAP1LC3B/CDIP1/CAP1/OPTN/PSMD1/CPPED1/ANPEP/CHID1/INSR/RNASET2/ATP6V0A1/ZFYVE1/YWHAB/NFAM1/VPS11/AP2A1/TPP1/VLDLR/PLEKHM2/NCOA4/HTT/LRRC8A/RRAGC/CXCR2/SBF2/SLC3A2/DYNC1H1/RAB7A/ANKFY1/RAB14/HGSNAT/ARL8A/IMPDH1/SLC44A2/LAMTOR1/VCAN/PYGB/GNB1/GAA/IST1/TOM1/CLTC/B4GALT1/ULK1/VCP/RUBCN/ANXA6/TUBB4B/CAPN1/DNAJC5/CHMP1A/GABARAP/CYB5R3/LYN/MMD/LAPTM5/ELAPOR1/UBA1/PSAP/AP5B1/LRP1/IFITM2/TBC1D25/PRF1/GNS/FTL/RILP/WDFY3/ARHGAP45/SHKBP1/TUBB/BBC3/ATP6V1A/ADA2/FTH1/GRN/ECE1/MARCHF2/SNCA/ANXA11/AP1B1/DTX3L/FLCN/PIP4K2A/LAMP1/PRDX6/HPS1/SLC15A3/SQSTM1/TBC1D5/LITAF/ATP6V0D1/AP2M1/CTSB/SLC48A1/RAB3D/CTSD/UBXN6/DAB2/SLC7A5/MARCHF8/TOLLIP/NPRL3
BP	GO:0032956	regulation of actin cytoskeleton organization	244	-0.53	-1.97	3.69E-10	1.22E-08	7.51E-09	2007	tags=32%, list=17%, signal=27%	PPM1F/TRIOBP/RHOF/DBNL/PRKCE/SDC4/HCK/EPS8/NOTCH2/CYRIA/ACTG1/ROCK2/SPTAN1/ARPC1B/DBN1/WASF3/NF2/ARHGDIB/WASHC2C/PYCARD/CDC42EP4/ARAP1/ABI3/DAPK3/ARPC5/CSF1R/WDR1/ALMS1/ARF1/SSH1/GRB2/PDGFA/TPM1/SPTBN1/FES/PTK2B/SPTA1/INPP5K/BCAS3/SVIL/PAK1/TAOK1/GSN/SLC9A1/JAM3/ESAM/IQGAP2/FLNA/PLEK/RAC1/CYFIP2/RAC2/LRP1/AKAP13/PXN/ARHGAP6/COTL1/RHOA/CAPZB/MYADM/ADD1/SSH2/HCLS1/ARHGDIA/TMOD1/ARPC4/RHOBTB1/WAS/SPTB/MTPN/CTTN/SH3BP1/PAK2/VASP/IQGAP1/PEAK3/WASF2/RHOG/DMTN
BP	GO:0034329	cell junction assembly	200	-0.55	-2.04	4.22E-10	1.38E-08	8.46E-09	2999	tags=48%, list=26%, signal=36%	IKBKB/DUSP22/PLXNB2/PLXND1/TNF/MECP2/POLDIP2/TESK2/AFDN/SMAD3/F11R/SPOCK2/ADD2/EPB41L3/STAU2/CLASP1/AMIGO1/FSCN1/TBCD/FBF1/MYO9A/HDAC7/EPHB2/LIMS1/MAP4K4/TLR2/STRN/PARD6B/HEG1/MACF1/SRF/PTEN/LRRC4/PPM1F/SRC/ADNP/DBNL/SDC4/LRFN3/SDCBP/NECTIN1/ACTG1/ROCK2/EPHB1/PTPRJ/FLOT1/CLSTN1/PARD3/SEMA4D/CTNND1/ABI3/DAPK3/VPS35/GPBAR1/PKP4/WDR1/PIP5K1C/PRKACA/OCEL1/GREM1/HIPK1/CORO1C/PTK2B/SEMA4A/BCR/RAPGEF2/BCAS3/VCL/PECAM1/CTNNA1/SETD5/ITGA5/PDLIM5/SLC9A1/JAM3/THBS1/PLEC/ESAM/FZD5/EIF4G1/DNM3/ACTB/RAC1/ARHGAP6/RHOA/SNCA/RAPGEF1/FLCN/CTTN/CLDN5/ZNF703/ACTN1/SH3BP1/PAK2/DMTN/TLN1

BP	GO:0198738	cell-cell signaling by wnt	265	-0.51	-1.92	4.27E-10	1.38E-08	8.46E-09	2415	tags=35%, list=21%, signal=29%	UBE2B/AMFR/ASPM/WLS/SLC9A3R1/PORCN/CTNNBIP1/AXIN2/PTK7/TLR2/STRN/FOXO1/PAF1/DISC1/MACF1/WNT1/PTEN/VANGL1/SRC/STK4/XIAP/EXT1/CCNY/MKS1/TGFB1/SNX3/TIAM1/G3BP1/TSC2/UBR5/RNF213/LRRK2/SMARCA4/EGR1/NFATC1/MAPK14/TLE4/MAD2L2/FUZ/CTNND1/KPNA1/DAPK3/VPS35/CSNK1G2/ATP6V0C/LRRK1/INVS/GNAQ/GSK3A/ZRANB1/PPP1CA/GRK6/CSNK2A1/TMEM131L/NDRG2/NFKB1/GREM1/RBPJ/FRAT1/FRMD8/OTUD5/PIAS4/TBL1XR1/TGFB1I1/SFRP2/STK11/DVL3/FZD5/USP34/CHD8/SKI/VCP/RAC1/MARK2/GSK3B/LRP1/DDB1/TCF7L2/RHOA/TBL1X/CSNK1G1/GID8/BCL9/SIAH2/PTPN23/ZNF703/TLE3/PPM1A/DAB2/WNK1/FAM53B/CTDNEP1/FOXO3
BP	GO:0090066	regulation of anatomical structure size	295	-0.50	-1.90	4.38E-10	1.39E-08	8.55E-09	2671	tags=38%, list=23%, signal=30%	SLC12A7/ARHGAP4/CARMIL1/RAB22A/ADD2/PLXNA4/P2RY1/TWF2/CDC42EP1/CREB1/NAA80/KIAA0319/CD38/CORO1A/NCKAP1L/SCIN/DIP2B/SLC9A3R1/ASXL1/ATP2B1/FLII/SOD2/KNG1/DISC1/MACF1/SRF/PTEN/TRIOBP/AKT1S1/ADNP/DBNL/PPP1R15A/PRKCE/EXT1/LARS1/ATP7A/HCK/EPS8/ANO6/SLC12A6/CYRIA/ROCK2/LRRK2/NDEL1/EZR/SPTAN1/ARPC1B/DBN1/DOCK5/CDKL5/ACTA2/PER2/WASHC2C/PYCARD/CDC42EP4/SEMA4D/CLN3/ARPC5/WDR1/SSH1/DPYSL2/GRB2/PPP1CA/HRH2/SPTBN1/LRRC8A/PTK2B/SLC8A1/SPTA1/SEMA4A/EIF4G2/SVIL/VAV1/GSN/IST1/ESAM/ULK1/PLEK/RAC1/AQP1/SIN3A/CYFIP2/GSK3B/DNM2/COTL1/RHOA/CAPZB/RILP/MYADM/GPX1/MSN/ADD1/SSH2/WDTC1/HCLS1/TMOD1/RIN3/ARPC4/ECE1/WAS/GUCY1A1/SPTB/MTPN/CTTN/SH3BP1/AP2M1/VASP/KEL/P2RX1/TBXA2R/XK/DMTN
BP	GO:0031401	positive regulation of protein modification process	671	-0.40	-1.64	5.89E-10	1.83E-08	1.12E-08	2045	tags=26%, list=17%, signal=23%	FYN/LIMK2/WNT1/PTEN/MAP3K4/TIRAP/SRC/FNIP2/HDAC4/ATM/ADNP/MAP2K5/PPP1R15A/STK4/FANCI/SPPL3/SDC4/XIAP/C6orf89/XRCC6/ERBB2/NIPBL/CCNY/CASS4/WDFY2/MAGED1/TGFB1/ERN2/LILRA5/ERN1/SDCBP/HSPA5/SYAP1/ROCK2/ARRB1/LRRK2/HGF/CACUL1/JDP2/EGR1/ARID5A/RIPK1/DLG4/KAT7/PHF20/PIAS1/CTBP1/PEF1/S1PR2/MAP2K2/PTPRJ/FLOT1/PHF23/SEMA4D/MAD2L2/MYCBP2/DAZAP2/CLN3/LRRK1/EPHA4/PDPK1/NCF1/PML/RALBP1/PTBP1/CSF1R/ARHGEF2/KAT5/TRIB3/LEPR/GSK3A/UBE2L3/CHP1/BRMS1/SASH1/KMT2A/ARNT/PDGFA/NEDD9/INSR/MAP3K5/SLC27A1/TAOK3/FXR2/MCRS1/GREM1/VLDLR/PTK2B/NEURL1/VRK3/RAPGEF2/RPS6KA4/CCND3/TRAF6/PRKD2/IL6R/SH2D3C/RASSF5/PIAS4/PAK1/MOB3A/IGF2/PECAM1/CSK/BMP6/MMP9/TAOK1/SFRP2/STK11/ITGA5/MAP2K7/PELI2/FZR1/STRADA/PTPRC/SYK/DVL3/THBS1/PTPN1/LMNA/ATG7/TLR6/CALM3/FZD5/LTF/PIK3CG/HIPK2/EIF4G1/VCP/MADD/RAC1/GOLGA2/GABARAP/MARK2/LYN/MMD/LAPTM5/PDE5A/AKAP13/HUWE1/ATP2B4/FKBP1A/RHOA/TNIP1/GATA1/CLU/ARRB2/HCLS1/GNA12/PINK1/WBP2/MAPK3/SNCA/CDKN1A/FLCN/NIBAN1/TAL1/UBB/SQSTM1/AKT2/ARAF/BRD4/RNF40/PAK2/ITGB3/PRR5/DAB2/JARID2/WNK1/TOLLIP/IQGAP1/MAP2K3/STRADB/FAXDC2/HCFC1

BP	GO:0009725	response to hormone	524	-0.44	-1.75	6.21E-10	1.91E-08	1.17E-08	2193	tags=31%, list=19%, signal=26%	PKN1/FHL2/MMS19/SAFB/TADA3/TLR2/VPS18/GLB1/ATP2B1/FOXO1/BCL2L11/THRA/SRF/WNT1/PADI2/SRC/PER1/YWHAH/IRS2/RXRB/DDX54/SLC24A4/PRKCE/PAQR8/TGFB1/ZDHHC7/AGTRAP/PPARGC1B/ZBTB7A/TNFSF4/TSC2/UBR5/SCAP/SYAP1/ROCK2/ABCB11/SMARCC1/BTG1/RANGAP1/SMARCA4/PLCB1/NDEL1/STAT5B/GAB1/EGR1/ARID5A/KMT2D/ARPC1B/SH2B2/PIP4K2C/SHC1/MAPK14/SRA1/CA2/YY1/ZFP36L1/SORT1/ALAD/NCOA3/PDPK1/DHCR24/TRIB3/FOXP1/LEPR/GSK3A/UBE2L3/ADIPOR2/GRB2/PDK2/ALPL/PRKACA/EIF6/PKM/INSR/SESN3/SESN2/SLC27A1/FBP1/NFKB1/VPS11/KANK2/TSPO/NCOA4/NCOA2/SORL1/PELP1/TIMP2/RARA/PTK2B/SLC2A1/STAT6/INPP5K/BCAS3/CCND3/MAX/PAK1/IGF2/ZMIZ1/CTNNA1/CSK/ZNF106/BMP6/POR/STK11/SLC9A1/FOXO4/THBS1/PTAFR/PTPN1/GNB1/NCOR2/RAB31/ADIPOR1/MED1/NR3C1/IL1RN/RBM14/CALM3/ABHD2/LPIN2/JUND/JAK3/HSPA1A/GPI/UCP2/RXRA/AQP1/LYN/GSK3B/STAT5A/TXNIP/GNAI2/PXN/RHOA/PRKCB/GAS2L1/GATA1/GPX1/SP1/NCOA6/PPP1R9B/JAK1/WDTC1/HCLS1/ARID1A/WBP2/STAT3/PIP4K2A/AHCYL1/AKT2/NCOA1/BSG/CTSB/PRKDC/RNF40/EIF4EBP2/DAB2/MBP/CNOT1/CARM1/TIMP1/FOXO3/RELA
BP	GO:0030182	neuron differentiatio n	745	-0.39	-1.61	6.49E-10	1.99E-08	1.22E-08	2312	tags=26%, list=20%, signal=22%	DIP2B/SLC9A3R1/BCL11B/NFE2L2/GIGYF2/MYO9A/KIF13B/C1QA/MUL1/VIM/EPHB2/BCL11A/GDPD5/PTK7/STK25/MAP4K4/RPS6KA5/EFNB1/NIN/STRN/SPRY3/SOD2/PARD6B/KATNB1/SNAP25/DISC1/MACF1/SRF/FYN/WNT1/PTEN/FEZ1/TRIOBP/MAP1S/EPHB4/YWHAH/FKBP1B/TRIO/KIDINS220/ADNP/DBNL/PIGT/BLOC1S3/ATOH8/SDC4/MYCL/ERBB2/EXT1/DGKG/RNF165/ATP7A/MKS1/SNX3/TIAM1/DICER1/NCAM1/CUL7/CPNE5/SZT2/NOTCH2/HSPA5/NECTIN1/RETREG3/NUMB/LRRK2/HGF/B4GALT5/ENC1/NDEL1/DLG4/DBN1/EPHB1/CPNE1/CDKL5/SIPA1L1/AGTPBP1/RAP1GAP/FLOT1/SETX/CAMSAP1/PARD3/FRY/SEMA4D/MYCBP2/DDX6/CDKN1C/ABI3/MAP4/EPHA4/LYPLA2/CSF1R/ARHGEF2/ZFHX3/GSK3A/DPYSL2/YWHAZ/ZNF335/STK24/PALLD/RUNX3/SUFU/SF3A2/FES/TAOK3/HIPK1/RBPJ/TSPO/VLDLR/RARA/PTK2B/NEURL1/SEMA4A/IER2/MEIS1/RAPGEF2/HERC1/EIF4G2/PAK1/MAP1A/VCL/ZHX2/ZMIZ1/CTNNA1/BMP6/RERE/TAOK1/SFRP2/STK11/PLXNC1/PPP3CA/APLP2/USP9X/PDLIM5/DVL3/SCN1B/ASAP1/ATP1B2/MED1/IST1/FZD5/HIPK2/CAMK1D/PPP2R5B/FLNA/EIF4G1/ULK1/NIBAN2/DNM3/FRYL/ACTB/RAC1/MARK2/LYN/SIN3A/MMD/CYFIP2/GSK3B/RAC2/DNM2/CTDSP1/IQSEC1/RHOA/SCYL1/TRAPPC9/GRN/PPP1R9B/ECE1/CFL1/PBX1/RAPGEF1/STAT3/MTPN/EHD1/CTTN/GAS7/TAL1/UBB/NAPA/NCOA1/GAK/BSG/PAK2/VASP/DAB2/MBP/CUX1/CARM1/WNK1/KEL/FBXO7/FOXO3/IQGAP1/XK/RHOG
BP	GO:0043434	response to peptide hormone	259	-0.51	-1.91	6.65E-10	2.02E-08	1.24E-08	1811	tags=34%, list=15%, signal=30%	CREB1/SOCS3/CFLAR/GPLD1/CYBB/CRY2/NFE2L2/MYO5A/KAT2B/TLR2/ATP2B1/FOXO1/WNT1/SRC/IRS2/SLC24A4/ZDHHC7/AGTRAP/TSC2/SCAP/SYAP1/ROCK2/SMARCC1/BTG1/RANGAP1/PLCB1/NDEL1/STAT5B/GAB1/EGR1/SH2B2/PIP4K2C/SHC1/MAPK14/CA2/ZFP36L1/SORT1/PDPK1/TRIB3/GSK3A/GRB2/PDK2/EIF6/PKM/INSR/SESN3/SESN2/SLC27A1/FBP1/NFKB1/SORL1/SLC2A1/STAT6/INPP5K/CCND3/MAX/IGF2/CSK/ZNF106/POR/STK11/SLC9A1/FOXO4/PTPN1/RAB31/ADIPOR1/LPIN2/JUND/JAK3/UCP2/LYN/GSK3B/STAT5A/GNAI2/PXN/PRKCB/SP1/JAK1/WDTC1/STAT3/PIP4K2A/AHCYL1/AKT2/BSG/PRKDC/RNF40/EIF4EBP2/TIMP1/RELA

BP	GO:0048534	hematopoietic or lymphoid organ development	674	-0.41	-1.66	6.94E-10	2.09E-08	1.28E-08	2254	tags=30%, list=19%, signal=26%	IREB2/TF/GON4L/IFI16/CTNNBIP1/CLPTM1/DYRK3/SH2B3/PKN1/SH3RF1/ADAM8/TMEM176B/ASXL1/PLD4/FCER1G/TLR2/SOD2/TTC7A/BCL2L11/CMTM7/CR1/ZNF385A/THRA/MYO1E/PAF1/KLF6/SRF/WNT1/TIRAP/PRDX2/NLRP3/SRC/HDAC4/ATM/JMJD6/STK4/XRCC6/ERBB2/EXT1/ATP7A/EEF2/TGFB1/PPARGC1B/ITPKB/ZBTB7A/ETV6/TNFSF4/RC3H2/RRAS/BCL3/NOTCH2/PTBP3/ZFAT/GPS2/PRRC2C/NBEAL2/STAT5B/EGR1/RIPK1/KAT7/MAPK14/TRIB1/CEBPA/EIF2AK2/YY1/PTPRJ/ZFP36L1/PTPN6/ADGRG3/CBFA2T3/DPF2/ARIH2/SBNO2/NFKB2/CDKN1C/RELB/SOS2/SPI1/ITCH/LRRK1/PREX1/PML/CDK13/CCR1/CSF1R/WDR1/TSPO2/ATP6AP1/FOXP1/LEPR/KLF13/KMT2A/ARNT/EIF6/CNOT4/RUNX3/SGPL1/TMEM131L/VSIR/NFAM1/FES/HIPK1/OSCAR/RBPJ/MKNK2/IRF1/PGLYRP1/LRRC8A/RARA/PTK2B/MFHAS1/SELPLG/GAB2/SPTA1/SEMA4A/BCR/SNX10/STAT6/MEIS1/TRAF6/PITHD1/RASGRP4/TBX21/IL6R/TOB2/ZMIZ1/ZBTB16/MMP9/VAV1/SFRP2/STK11/SLC25A38/TFE3/THOC5/PTPRC/LILRB3/SYK/ADAR/KDELR1/JAM3/ST3GAL1/MED1/MAFB/MTURN/DOCK2/FZD5/LTF/HIPK2/G6PD/GLRX5/JAK3/HSPA1A/INPP5D/PF4/PLEK/LYN/LGALS9/SIN3A/GFI1B/ROGDI/CNN2/BPGM/MPL/RHOA/CUL4A/GATA1/FASN/NF1/NCOA6/AHSP/JAK1/HCLS1/TRIM10/PIM1/TESC/MAPK3/PBX1/FLCN/STAT3/PIP4K2A/TAL1/ACTN1/SLC4A1/FAM210B/PIK3CD/EPB42/IKZF1/KAT6A/PRKDC/KLF1/MPIG6B/ALAS2/TRIM58/JARID2/LYL1/FBXO7/MYH9/FOXO3/EIF2AK1/WASF2/FAXDC2/HLX/DMTN
BP	GO:0048699	generation of neurons	782	-0.39	-1.62	8.85E-10	2.64E-08	1.62E-08	2312	tags=26%, list=20%, signal=23%	DIP2B/SLC9A3R1/BCL11B/NFE2L2/GIGYF2/MYO9A/KIF13B/C1QA/MUL1/VIM/EPHB2/BCL11A/GDPD5/PTK7/SH3RF1/STK25/MAP4K4/RPS6KA5/EFNB1/NIN/STRN/SPRY3/SOD2/PARD6B/KATNB1/SNAP25/DISC1/MACF1/SRF/FYN/WNT1/PTEN/FEZ1/TRIOBP/MAP1S/EPHB4/YWHAH/FKBP1B/TRIO/KIDINS220/ADNP/DBNL/PIGT/BLOC1S3/ATOH8/SDC4/MYCL/ERBB2/EXT1/DGKG/NIPBL/RNF165/ATP7A/MKS1/SNX3/TIAM1/DICER1/NCAM1/CUL7/CPNE5/SZT2/NOTCH2/NAV1/HSPA5/NECTIN1/RETREG3/NUMB/LRRK2/HGF/B4GALT5/ENC1/NDEL1/TUBB2A/DLG4/ELP3/DBN1/EPHB1/CPNE1/CDKL5/SIPA1L1/AGTPBP1/RAP1GAP/FLOT1/SETX/CAMSAP1/SMARCD3/PARD3/FRY/POMGNT2/SEMA4D/MYCBP2/DDX6/CDKN1C/ABI3/MAP4/EPHA4/LYPLA2/CSF1R/ARHGEF2/ZFHX3/GSK3A/DPYSL2/YWHAZ/ZNF335/STK24/PALLD/RUNX3/SUFU/SF3A2/FES/TAOK3/HIPK1/RBPJ/TSPO/VLDLR/RARA/PTK2B/NEURL1/SEMA4A/IER2/MEIS1/RAPGEF2/HERC1/EIF4G2/PAK1/MAP1A/VCL/ZHX2/ZMIZ1/CTNNA1/BMP6/RERE/TAOK1/NDE1/SFRP2/STK11/AKNA/PLXNC1/PPP3CA/APLP2/USP9X/PDLIM5/DVL3/ZNF609/SCN1B/ASAP1/ATP1B2/MED1/IST1/FZD5/HIPK2/CAMK1D/PPP2R5B/FLNA/EIF4G1/ULK1/NIBAN2/DNM3/FRYL/ACTB/RAC1/MARK2/LYN/SIN3A/MMD/CYFIP2/GSK3B/RAC2/DNM2/CTDSP1/IQSEC1/RHOA/SCYL1/NF1/TRAPPC9/GRN/PPP1R9B/ECE1/CFL1/PBX1/RAPGEF1/STAT3/MTPN/EHD1/CTTN/GAS7/TAL1/UBB/NAPA/NCOA1/GAK/BSG/PAK2/VASP/DAB2/MBP/CUX1/CARM1/WNK1/KEL/FBXO7/FOXO3/IQGAP1/XK/RHOG

BP	GO:0016055	Wnt signaling pathway	263	-0.51	-1.91	9.08E-10	2.69E-08	1.65E-08	2415	tags=35%, list=21%, signal=28%	UBE2B/AMFR/ASPM/WLS/SLC9A3R1/PORCN/CTNNBIP1/AXIN2/PTK7/TLR2/STRN/FOXO1/PAF1/DISC1/MACF1/WNT1/PTEN/VANGL1/SRC/STK4/XIAP/EXT1/CCNY/MKS1/TGFB1/SNX3/TIAM1/G3BP1/TSC2/UBR5/RNF213/LRRK2/SMARCA4/EGR1/NFATC1/MAPK14/TLE4/MAD2L2/FUZ/CTNND1/KPNA1/DAPK3/VPS35/CSNK1G2/ATP6V0C/LRRK1/INVS/GNAQ/GSK3A/ZRANB1/PPP1CA/GRK6/CSNK2A1/TMEM131L/NDRG2/NFKB1/GREM1/RBPJ/FRAT1/FRMD8/OTUD5/PIAS4/TBL1XR1/TGFB1I1/SFRP2/STK11/DVL3/FZD5/USP34/CHD8/SKI/VCP/RAC1/MARK2/GSK3B/LRP1/DDB1/TCF7L2/RHOA/TBL1X/CSNK1G1/GID8/BCL9/SIAH2/ZNF703/TLE3/PPM1A/DAB2/WNK1/FAM53B/CTDNEP1/FOXO3
CC	GO:0000323	lytic vacuole	589	-0.41	-1.68	1.02E-09	2.99E-08	1.84E-08	2039	tags=27%, list=17%, signal=24%	PADI2/TPCN1/GUSB/ATP6V0B/UBA52/UNC13D/RRAGD/SLC29A3/SRC/NKG7/FNIP2/ZNRF1/ATP6V1B2/IFNAR1/MCOLN1/CPNE3/STARD3/NCF2/SDC4/MARCHF3/CD68/AP1G1/CMTM6/LARS1/ACP2/NCF4/HCK/SLC66A1/WDR81/AP2A2/LAMP2/SZT2/USP4/SDCBP/HGS/TSC2/CKAP4/ACLY/ARRB1/LRRK2/NBR1/SIDT2/AP2B1/AP2S1/TMBIM1/TFEB/CPQ/CPNE1/CLCN5/PLEKHF1/CHMP4B/PGAP6/FLOT1/SORT1/PYCARD/SLC36A1/CD63/IFITM3/TRAPPC1/AP1M1/VPS41/CLN3/VPS35/ATP6V0C/GNAQ/NCF1/CLEC16A/CDIP1/CAP1/PSMD1/CPPED1/ANPEP/CHID1/INSR/RNASET2/ATP6V0A1/NFAM1/VPS11/AP2A1/TPP1/VLDLR/PLEKHM2/NCOA4/LRRC8A/RRAGC/CXCR2/SLC3A2/DYNC1H1/RAB7A/ANKFY1/RAB14/HGSNAT/ARL8A/IMPDH1/SLC44A2/LAMTOR1/VCAN/PYGB/GNB1/GAA/IST1/TOM1/CLTC/B4GALT1/VCP/RUBCN/ANXA6/TUBB4B/CAPN1/DNAJC5/CHMP1A/GABARAP/CYB5R3/LYN/MMD/LAPTM5/ELAPOR1/UBA1/PSAP/AP5B1/LRP1/IFITM2/PRF1/GNS/FTL/RILP/ARHGAP45/SHKBP1/TUBB/BBC3/ATP6V1A/ADA2/FTH1/GRN/ECE1/MARCHF2/SNCA/ANXA11/AP1B1/DTX3L/FLCN/PIP4K2A/LAMP1/PRDX6/HPS1/SLC15A3/SQSTM1/LITAF/ATP6V0D1/AP2M1/CTSB/SLC48A1/RAB3D/CTSD/UBXN6/DAB2/SLC7A5/MARCHF8/TOLLIP/NPRL3
CC	GO:0005764	lysosome	589	-0.41	-1.68	1.02E-09	2.99E-08	1.84E-08	2039	tags=27%, list=17%, signal=24%	PADI2/TPCN1/GUSB/ATP6V0B/UBA52/UNC13D/RRAGD/SLC29A3/SRC/NKG7/FNIP2/ZNRF1/ATP6V1B2/IFNAR1/MCOLN1/CPNE3/STARD3/NCF2/SDC4/MARCHF3/CD68/AP1G1/CMTM6/LARS1/ACP2/NCF4/HCK/SLC66A1/WDR81/AP2A2/LAMP2/SZT2/USP4/SDCBP/HGS/TSC2/CKAP4/ACLY/ARRB1/LRRK2/NBR1/SIDT2/AP2B1/AP2S1/TMBIM1/TFEB/CPQ/CPNE1/CLCN5/PLEKHF1/CHMP4B/PGAP6/FLOT1/SORT1/PYCARD/SLC36A1/CD63/IFITM3/TRAPPC1/AP1M1/VPS41/CLN3/VPS35/ATP6V0C/GNAQ/NCF1/CLEC16A/CDIP1/CAP1/PSMD1/CPPED1/ANPEP/CHID1/INSR/RNASET2/ATP6V0A1/NFAM1/VPS11/AP2A1/TPP1/VLDLR/PLEKHM2/NCOA4/LRRC8A/RRAGC/CXCR2/SLC3A2/DYNC1H1/RAB7A/ANKFY1/RAB14/HGSNAT/ARL8A/IMPDH1/SLC44A2/LAMTOR1/VCAN/PYGB/GNB1/GAA/IST1/TOM1/CLTC/B4GALT1/VCP/RUBCN/ANXA6/TUBB4B/CAPN1/DNAJC5/CHMP1A/GABARAP/CYB5R3/LYN/MMD/LAPTM5/ELAPOR1/UBA1/PSAP/AP5B1/LRP1/IFITM2/PRF1/GNS/FTL/RILP/ARHGAP45/SHKBP1/TUBB/BBC3/ATP6V1A/ADA2/FTH1/GRN/ECE1/MARCHF2/SNCA/ANXA11/AP1B1/DTX3L/FLCN/PIP4K2A/LAMP1/PRDX6/HPS1/SLC15A3/SQSTM1/LITAF/ATP6V0D1/AP2M1/CTSB/SLC48A1/RAB3D/CTSD/UBXN6/DAB2/SLC7A5/MARCHF8/TOLLIP/NPRL3

MF	GO:0030695	GTPase regulator activity	377	-0.47	-1.83	1.04E-09	3.01E-08	1.85E-08	2400	tags=32%, list=20%, signal=27%	ARHGAP32/MCF2L/NCKAP1L/RGS16/ARHGEF5/ARFGAP2/TBC1D8/SGSM1/MYO9A/GPS1/RANBP3/ARHGAP24/TNK2/ARHGEF11/PTGIR/ARHGEF3/RGP1/ITSN1/TRIO/RHOF/ARHGEF12/RASA2/ARHGAP19/LARS1/SIRPA/TIAM1/ACAP2/MCF2L2/TSC2/ARHGAP9/ARRB1/LRRK2/RANGAP1/RALGAPA1/GPS2/PLCB1/TBC1D17/RASAL3/TBC1D3I/DOCK5/GIT2/SIPA1L1/RAP1GAP/DENND1C/ARHGDIB/MYCBP2/ARAP1/SOS2/PREX1/TBC1D22A/HERC2/GNAQ/RALBP1/ARHGEF2/RALGAPA2/EVI5L/TBC1D10B/PCP2/RABGAP1/RGS6/ARHGAP31/SESN2/ARHGAP21/SH3BP5L/PLEKHG3/ADAP2/DOCK8/FGD4/BCR/RAPGEF2/HERC1/TBC1D22B/RIN1/SBF2/RASGRP4/GMIP/SH2D3C/SBF1/FGD3/ELMO1/ADAP1/VAV1/LAMTOR1/SMAP2/SIPA1L2/MYO9B/ASAP1/ASAP2/ARHGAP8/DOCK2/IQGAP2/TBC1D13/MADD/SIPA1/GAPVD1/AKAP13/ARHGAP6/TBC1D25/IQSEC1/ARHGAP45/NF1/ARHGDIA/RIN3/WAS/NUCB1/ARHGAP26/TBC1D20/RAPGEF1/FLCN/CYTH4/HPS1/TBC1D5/SH3BP1/DENND1A/TBC1D1/RAB3IL1/RUNDC3A/ARHGAP1/TBC1D2/NPRL3/IQGAP1/TBXA2R
MF	GO:0060589	nucleoside-triphosphatase regulator activity	377	-0.47	-1.83	1.04E-09	3.01E-08	1.85E-08	2400	tags=32%, list=20%, signal=27%	ARHGAP32/MCF2L/NCKAP1L/RGS16/ARHGEF5/ARFGAP2/TBC1D8/SGSM1/MYO9A/GPS1/RANBP3/ARHGAP24/TNK2/ARHGEF11/PTGIR/ARHGEF3/RGP1/ITSN1/TRIO/RHOF/ARHGEF12/RASA2/ARHGAP19/LARS1/SIRPA/TIAM1/ACAP2/MCF2L2/TSC2/ARHGAP9/ARRB1/LRRK2/RANGAP1/RALGAPA1/GPS2/PLCB1/TBC1D17/RASAL3/TBC1D3I/DOCK5/GIT2/SIPA1L1/RAP1GAP/DENND1C/ARHGDIB/MYCBP2/ARAP1/SOS2/PREX1/TBC1D22A/HERC2/GNAQ/RALBP1/ARHGEF2/RALGAPA2/EVI5L/TBC1D10B/PCP2/RABGAP1/RGS6/ARHGAP31/SESN2/ARHGAP21/SH3BP5L/PLEKHG3/ADAP2/DOCK8/FGD4/BCR/RAPGEF2/HERC1/TBC1D22B/RIN1/SBF2/RASGRP4/GMIP/SH2D3C/SBF1/FGD3/ELMO1/ADAP1/VAV1/LAMTOR1/SMAP2/SIPA1L2/MYO9B/ASAP1/ASAP2/ARHGAP8/DOCK2/IQGAP2/TBC1D13/MADD/SIPA1/GAPVD1/AKAP13/ARHGAP6/TBC1D25/IQSEC1/ARHGAP45/NF1/ARHGDIA/RIN3/WAS/NUCB1/ARHGAP26/TBC1D20/RAPGEF1/FLCN/CYTH4/HPS1/TBC1D5/SH3BP1/DENND1A/TBC1D1/RAB3IL1/RUNDC3A/ARHGAP1/TBC1D2/NPRL3/IQGAP1/TBXA2R

MF	GO:0005102	signaling receptor binding	724	-0.40	-1.64	1.24E-09	3.52E-08	2.16E-08	2250	tags=28%, list=19%, signal=24%	TF/TAPBP/AAK1/EPHB2/RNF135/ICAM4/SH2B3/TNK2/EFNB1/ASXL1/MICB/TLR2/TNXB/ARHGEF11/GNA15/CMTM7/KNG1/TLR5/GRK3/FGR/FYN/WNT1/GUSB/TIRAP/SRC/YWHAH/IRS2/CD177/FKBP1B/DDX54/ARHGEF12/DIAPH2/CPNE3/TNFSF13/PROK2/ERBB2/HCK/TGFB1/KIR3DL2/TNFSF4/ERN1/USP4/SDCBP/ICAM3/ITGAM/ARRB1/HGF/FYB1/METRNL/CNPY3/SNX17/NOTCH2NLA/TMBIM1/SYTL3/RIPK1/DLG4/SH2B2/CD300LF/SHC1/BABAM2/SECTM1/S1PR2/GRK2/NF2/PTPRJ/SIPA1L1/FLOT1/EML2/SMARCD3/PYCARD/SEMA4D/ATXN2/NR1H2/CTNND1/DAZAP2/VPS35/ITCH/EPHA4/SHISA7/FERMT3/CMTM5/GNAQ/ITGB2/CXCL5/HIF1AN/KLRD1/LCP1/GSK3A/CXCL16/GRB2/CANX/ARNT/ERMAP/PCSK1N/PDGFA/INSR/PF4V1/PITPNM2/SPG21/S100A4/FES/GREM1/AP2A1/TACC1/NCOA2/BCAP31/RNF126/IKBKG/RARA/PTK2B/SELPLG/GAB2/SEMA4A/RAPGEF2/TRAF6/LDLRAP1/COPA/IL6R/IGF2/IL13RA1/BMP6/ASXL2/TGFB1I1/SFRP2/PLXNC1/ITGA5/PTPRC/SYK/DVL3/JAM3/THBS1/PTPN1/MYO9B/NCOR2/MED1/TLR6/IL1RN/GLG1/IL17RA/CD151/CLTC/CALM3/DOCK2/PIK3CG/FLNA/DNM3/JAK3/VCP/HSPA1A/TUBB4B/TGFBRAP1/MADD/PF4/HMGA1/GPI/GABARAP/SLA/LYN/HIP1/DNM2/LRP1/ITGA2B/SYTL4/TAF6/GNAI2/TRAK2/PXN/FKBP1A/ITGAL/TUBB/CLU/ARRB2/MSN/ADA2/ACTN4/LRG1/GRN/PPP1R9B/JAK1/GNA12/SEC14L1/ITGB5/STAT3/SQSTM1/ACTN1/SH3BP1/NCOA1/AP2M1/HDGF/MPP1/ITGB3/DAB2/GNAZ/MYH9/MARCHF8/TOLLIP/TIMP1/CBL/DMTN/BAG6/DIAPH1/TLN1
CC	GO:0030667	secretory granule membrane	245	-0.51	-1.92	1.25E-09	3.52E-08	2.16E-08	2395	tags=41%, list=20%, signal=33%	CD38/SIGLEC14/CYBB/NCKAP1L/MGAM/HMOX2/ACP3/ADAM8/FCER1G/TLR2/ORMDL3/CR1/SNAP25/CYSTM1/CD93/RAB27B/PHACTR2/CD177/RHOF/CPNE3/CD68/CMTM6/ALDH3B1/SIRPA/SLC2A3/AP2A2/LAMP2/ANO6/CKAP4/TSPAN14/ITGAM/NBEAL2/TMBIM1/C5AR1/MME/CPNE1/STK10/PTPRJ/ADGRG3/HVCN1/EXOC3/CD63/AP1M1/ATP6V0C/SIGLEC5/ITGB2/ITPR2/TMEM184A/ANPEP/CA4/ATP6V0A1/NFAM1/SELP/CXCR2/PECAM1/RAB7A/RAB14/HGSNAT/ARL8A/SLC44A2/LAMTOR1/UBR4/APLP2/RAB5B/PTPRC/LILRB3/PTAFR/GAA/RAB31/TOM1/IQGAP2/B4GALT1/DOK3/ABCC4/SELL/DNAJC5/CD14/RAC1/ADGRE5/SIRPB1/IGF2R/PSAP/FLOT2/ITGA2B/TNFRSF1B/SYTL4/RHOA/ITGAL/CXCR1/SNCA/SPARC/LAMP1/BSG/RAB3D/ITGB3/P2RX1/IQGAP1/RHOG/ACRBP/DIAPH1

BP	GO:0044087	regulation of cellular component biogenesis	679	-0.40	-1.64	1.30E-09	3.64E-08	2.23E-08	2144	tags=29%, list=18%, signal=25%	NIN/TLR2/FLII/VPS4B/ORMDL3/BCL2L11/THRA/MACF1/SRF/PHF2/LIMK2/WNT1/PTEN/PHF8/FEZ1/TIRAP/PPM1F/SRC/DNAJB1/TRIOBP/KCTD17/FNIP2/SH3GLB1/ATM/ADNP/DBNL/H3-3B/PRKCE/SDC4/H3-3A/ATP7A/HCK/EPS8/TGFB1/LDLRAD4/LRFN3/G3BP1/SETDB1/SDCBP/HGS/SYNE2/CYRIA/HSPA5/CPTP/NECTIN1/TCHP/ACTG1/ROCK2/LRRK2/IKBKE/CKAP5/NDEL1/EZR/SPTAN1/TMC8/ARPC1B/DBN1/PIKFYVE/PIP4K2C/EPHB1/CDKL5/CHMP4B/NF2/EIF2AK2/PTPRJ/RAP1GAP/FLOT1/CLSTN1/CAMSAP1/PHF23/EML2/WASHC2C/PYCARD/CDC42EP4/SEMA4D/NR1H2/SLAIN2/ARAP1/FUZ/CLN3/ABI3/MAP4/DAPK3/VPS35/ARPC5/GPBAR1/NUMA1/SAMD1/ALMS1/EVI5L/LCP1/TRIM28/SSH1/GRB2/MTMR3/PRKACA/CNOT6/TPM1/SPTBN1/VPS11/FES/FXR2/GREM1/SORL1/CORO1C/HTT/PTK2B/NEURL1/SPTA1/SELP/SEMA4A/WARS1/INPP5K/RAPGEF2/BCAS3/SVIL/DYNC1H1/KLF5/MARK4/PAK1/VCL/RAB7A/SETD5/GSN/PDLIM5/WDR45/SYK/SLC9A1/JAM3/THBS1/ASAP1/TLR6/ESAM/DCTN1/RBM14/FZD5/PPP2R5B/FLNA/EIF4G1/ULK1/EML3/DNM3/VCP/HSPA1A/GNL3L/PLEK/CHMP1A/RAC1/RIOK3/SIN3A/CYFIP2/GSK3B/ELAPOR1/RAC2/DNM2/STMP1/PXN/ARHGAP6/COTL1/RHOA/CAPZB/MYADM/CLU/ATG2A/MSN/ADD1/SSH2/TERF2/HCLS1/TMOD1/ARPC4/WAS/PINK1/SNCA/RAPGEF1/SPTB/MTPN/CTTN/PIP4K2A/CLDN5/RIPOR2/TFIP11/PLEK2/TAL1/NAPA/DNAJB2/PPM1A/DNAJA4/PAK2/MED25/VASP/CNOT1/CDC34/RAB1B/WASF2/RHOG/DMTN/HCFC1
BP	GO:0043087	regulation of GTPase activity	255	-0.51	-1.92	1.35E-09	3.72E-08	2.28E-08	1714	tags=29%, list=15%, signal=25%	TSC2/ARHGAP9/LRRK2/RANGAP1/RALGAPA1/NDEL1/TBC1D17/RASAL3/TBC1D3I/CDKL5/SIPA1L1/RAP1GAP/PYCARD/SEMA4D/ARAP1/SOS2/PREX1/EPHA4/TBC1D22A/PKP4/RALBP1/RALGAPA2/EVI5L/TBC1D10B/RABGAP1/RGS6/NEDD9/AMOT/CORO1C/ADAP2/DOCK8/PTK2B/FGD4/BCR/RAPGEF2/BCAS3/TBC1D22B/SBF2/RASGRP4/GMIP/SBF1/FGD3/ADAP1/VAV1/PLXNC1/SIPA1L2/DVL3/ASAP1/ASAP2/IQGAP2/TBC1D13/SIPA1/GSK3B/GAPVD1/ARHGAP6/TBC1D25/IQSEC1/ARHGAP45/ARRB2/NF1/RSU1/TGM2/TBC1D20/RAPGEF1/FLCN/RIPOR2/TBC1D5/AKT2/SH3BP1/TBC1D1/WNK1/TBC1D2/IQGAP1/RHOG
BP	GO:0009887	animal organ morphogenesis	474	-0.44	-1.76	1.48E-09	4.05E-08	2.48E-08	2328	tags=28%, list=20%, signal=23%	OTX1/SLC9A3R1/BCL11B/TBX19/IFT122/EPHB2/MTHFD1/CTNNBIP1/FANCC/AXIN2/PTK7/FHL2/TMEM176B/ASXL1/BCL2L11/THRA/HEG1/FGR/RFNG/SRF/WNT1/PTEN/KDM5B/VANGL1/SRC/TRIOBP/EXOC4/EPHB4/SLC24A4/EXT1/NIPBL/VDR/MAGED1/MKS1/TGFB1/TIAM1/CHSY1/PPARGC1B/MEGF9/PHACTR4/NOTCH2/NECTIN1/SMARCC1/LRRK2/HGF/ASH1L/VPS51/EPHB1/MAPK14/NF2/ACTA2/YY1/AGTPBP1/MSX1/SMARCD3/FUZ/PML/DGCR2/CSF1R/GSK3A/ALPL/ZNF335/PDGFA/INSR/FOXN3/TPM1/SGPL1/SUFU/GREM1/HIPK1/RBPJ/RARA/BCR/SNX10/STAT6/MEIS1/ARID2/SOX6/TRAF6/CHST11/IGF2/ZHX2/ZMIZ1/CTNNA1/BMP6/ASXL2/POR/SFRP2/SYK/DVL3/STK40/GAA/MED1/MAFB/GLG1/PHOSPHO1/FZD5/LTF/HIPK2/BTBD7/BASP1/SKI/FLNA/NDST1/ANXA6/CAPN1/RAC1/RFLNB/AQP1/ROGDI/FKBP1A/RHOA/NF1/MSN/PIM1/RPS6KA1/MYLK/MAPK3/TGM2/TBC1D20/PBX1/STAT3/CSRNP1/CLDN5/TLE3/MFN2/BSG/STIM1/DAB2/FOXO3/MICAL2/HLX/RELA

BP	GO:0030097	hemopoiesis	656	-0.41	-1.66	1.54E-09	4.20E-08	2.58E-08	2254	tags=30%, list=19%, signal=26%	IREB2/TF/GON4L/IFI16/CTNNBIP1/CLPTM1/DYRK3/SH2B3/SH3RF1/ADAM8/TMEM176B/ASXL1/PLD4/FCER1G/TLR2/SOD2/TTC7A/CMTM7/CR1/ZNF385A/THRA/MYO1E/PAF1/KLF6/SRF/WNT1/TIRAP/PRDX2/NLRP3/SRC/HDAC4/ATM/JMJD6/STK4/XRCC6/ERBB2/EXT1/ATP7A/EEF2/TGFB1/PPARGC1B/ITPKB/ZBTB7A/ETV6/TNFSF4/RC3H2/RRAS/BCL3/NOTCH2/PTBP3/ZFAT/GPS2/PRRC2C/NBEAL2/STAT5B/EGR1/RIPK1/KAT7/MAPK14/TRIB1/CEBPA/EIF2AK2/YY1/PTPRJ/ZFP36L1/PTPN6/ADGRG3/CBFA2T3/DPF2/ARIH2/SBNO2/CDKN1C/RELB/SOS2/SPI1/ITCH/LRRK1/PREX1/PML/CDK13/CCR1/CSF1R/WDR1/TSPO2/ATP6AP1/FOXP1/LEPR/KLF13/KMT2A/ARNT/EIF6/CNOT4/RUNX3/SGPL1/TMEM131L/VSIR/NFAM1/FES/HIPK1/OSCAR/RBPJ/MKNK2/IRF1/PGLYRP1/LRRC8A/RARA/PTK2B/MFHAS1/SELPLG/GAB2/SPTA1/SEMA4A/BCR/SNX10/STAT6/MEIS1/TRAF6/PITHD1/RASGRP4/TBX21/IL6R/TOB2/ZMIZ1/ZBTB16/MMP9/VAV1/SFRP2/STK11/SLC25A38/TFE3/THOC5/PTPRC/LILRB3/SYK/ADAR/KDELR1/JAM3/ST3GAL1/MED1/MAFB/MTURN/DOCK2/FZD5/LTF/HIPK2/G6PD/GLRX5/JAK3/HSPA1A/INPP5D/PF4/PLEK/LYN/LGALS9/SIN3A/GFI1B/ROGDI/CNN2/BPGM/MPL/RHOA/CUL4A/GATA1/FASN/NF1/NCOA6/AHSP/JAK1/HCLS1/TRIM10/PIM1/TESC/PBX1/FLCN/STAT3/PIP4K2A/TAL1/ACTN1/SLC4A1/FAM210B/PIK3CD/EPB42/IKZF1/KAT6A/PRKDC/KLF1/MPIG6B/ALAS2/TRIM58/LYL1/FBXO7/MYH9/FOXO3/EIF2AK1/WASF2/FAXDC2/HLX/DMTN
BP	GO:0030168	platelet activation	94	-0.65	-2.17	1.70E-09	4.60E-08	2.82E-08	2211	tags=46%, list=19%, signal=37%	SH2B3/FCER1G/CSRP1/DGKA/SRF/SRC/ABAT/BLOC1S3/THBD/DGKG/DGKZ/ACTG1/MAPK14/PTPN6/FERMT3/FIBP/PDGFA/PF4V1/SELP/VCL/VAV1/GP6/SYK/PIK3CG/FLNA/ACTB/PF4/ALOX12/PLEK/LYN/DGKK/GP9/HSPB1/MPL/GATA1/GP1BA/TREML1/MYL9/ITGB3/P2RX1/MYH9/DMTN/TLN1
BP	GO:0001775	cell activation	773	-0.39	-1.60	1.95E-09	5.20E-08	3.19E-08	2248	tags=28%, list=19%, signal=24%	GON4L/C1QA/EPHB2/CLPTM1/SH2B3/PKN1/SH3RF1/ADAM8/EFNB1/MICB/FCER1G/TLR2/CSRP1/SLA2/CMTM7/CR1/KLF6/LGALS9C/DGKA/FGR/SRF/FYN/CD93/WNT1/TIRAP/UNC13D/PRDX2/NLRP3/SRC/NKG7/IRS2/CD177/FKBP1B/HDAC4/ATM/ABAT/JMJD6/MYO18A/BLOC1S3/PRKCE/TNFSF13/SDC4/THBD/XRCC6/ERBB2/DGKG/AP1G1/ATP7A/TGFB1/DGKZ/SIRPA/ITPKB/ADGRE2/ZBTB7A/TNFSF4/RC3H2/LILRA5/BCL3/NOTCH2/ACTG1/ITGAM/LRRK2/GPS2/PLCB1/STAT5B/EGR1/PAG1/RASAL3/KAT7/C5AR1/CD300LF/EPHB1/MAPK14/STX4/CEBPA/ACTA2/YY1/PTPRJ/ZFP36L1/PTPN6/PYCARD/ADGRG3/MAD2L2/SBNO2/NFKB2/RELB/SOS2/KPNA1/SPI1/ITCH/PREX1/FERMT3/PDPK1/ITGB2/LCP1/FOXP1/LEPR/FIBP/ZNF335/PDGFA/PF4V1/SH3KBP1/RUNX3/TMEM131L/VSIR/NFAM1/FES/RBPJ/NFATC2/IRF1/PGLYRP1/LRRC8A/DOCK8/RARA/PTK2B/MFHAS1/SELPLG/GAB2/SPTA1/SELP/SEMA4A/BCR/STAT6/CXCR2/CCND3/TRAF6/TBX21/KLF5/IL6R/IGF2/VCL/SKAP2/ZMIZ1/CSK/ZBTB16/VAV1/STK11/GSN/GP6/PPP3CA/PTPRC/SYK/KDELR1/THBS1/PTAFR/SUPT6H/ST3GAL1/MAFB/TLR6/IL17RA/CD151/DOCK2/FZD5/PIK3CG/PRAM1/FLNA/JUND/JAK3/ACTB/INPP5D/PF4/ALOX12/PLEK/LYN/SNX27/LGALS9/LAPTM5/PDE5A/DGKK/RAC2/SIRPB1/GP9/LRP1/FLOT2/HSPB1/TNFRSF1B/DYSF/FKBP1A/MPL/PRF1/RHOA/ITGAL/PRKCB/GATA1/CLU/MSN/GRN/WAS/RPS6KA1/SNCA/CDKN1A/APBB1IP/PBX1/STAT3/RIPOR2/LAMP1/GP1BA/PIK3CD/TREML1/IKZF1/PRKDC/MYL9/ITGB3/LYL1/P2RX1/FBXO7/MYH9/TOLLIP/TIMP1/FOXO3/CBL/ELF4/HLX/DMTN/BAG6/TLN1

BP	GO:0030099	myeloid cell differentiation	300	-0.49	-1.86	2.24E-09	5.94E-08	3.65E-08	1812	tags=32%, list=15%, signal=27%	TGFB1/PPARGC1B/ITPKB/ZBTB7A/NOTCH2/PTBP3/NBEAL2/STAT5B/RIPK1/KAT7/MAPK14/TRIB1/CEBPA/ZFP36L1/PTPN6/CBFA2T3/SBNO2/CDKN1C/RELB/SPI1/LRRK1/PML/CCR1/CSF1R/WDR1/TSPO2/ATP6AP1/FOXP1/KLF13/ARNT/EIF6/CNOT4/FES/OSCAR/RBPJ/RARA/PTK2B/MFHAS1/GAB2/SNX10/MEIS1/TRAF6/PITHD1/RASGRP4/TOB2/ZBTB16/MMP9/SLC25A38/TFE3/THOC5/LILRB3/ADAR/MED1/MAFB/MTURN/LTF/HIPK2/G6PD/JAK3/HSPA1A/INPP5D/PF4/LYN/BPGM/MPL/CUL4A/GATA1/FASN/NF1/NCOA6/AHSP/HCLS1/TRIM10/TESC/STAT3/PIP4K2A/TAL1/ACTN1/SLC4A1/FAM210B/PIK3CD/EPB42/IKZF1/KAT6A/PRKDC/KLF1/MPIG6B/ALAS2/TRIM58/MYH9/FOXO3/EIF2AK1/WASF2/FAXDC2/DMTN
BP	GO:0007186	G protein-coupled receptor signaling pathway	365	-0.46	-1.80	2.26E-09	5.96E-08	3.66E-08	2110	tags=25%, list=18%, signal=21%	ARHGEF11/GNA15/PTGIR/FRMPD1/DGKA/GRK3/SRC/SLC24A4/ARHGEF12/PROK2/DGKG/ZDHHC7/DGKZ/AGTRAP/ADGRE2/PDE2A/OR10H1/PDE4A/ROCK2/ARRB1/OPRL1/PLCB1/EZR/CMKLR1/C5AR1/OR52K2/CA2/GIT2/S1PR2/GRK2/OR52K1/SORT1/PTPN6/PARD3/ADGRG3/PREX1/GPBAR1/GNAQ/CCR1/GSK3A/RGS6/HRH2/GRK6/PCSK1N/INSR/ADGRL1/YWHAB/SORL1/PTK2B/CXCR2/INPP5K/RAPGEF2/RASGRP4/TAS2R30/VAV1/PLCB3/APLP2/PTAFR/GNB1/MGLL/CALM3/FZD5/IQGAP2/PIK3CG/FLNA/PF4/PLEK/RAC1/DGKK/RAC2/ADGRE5/DNM2/IGF2R/PSAP/AKAP13/ATP2B4/GNAI2/ARRB2/CXCR1/PPP1R9B/GNA12/ECE1/SNCA/TGM2/GPR146/AHCYL1/GP1BA/GNAZ/ACKR1/TBXA2R/WASF2/RELA
BP	GO:1901652	response to peptide	318	-0.47	-1.82	2.73E-09	7.15E-08	4.39E-08	1714	tags=26%, list=15%, signal=23%	TSC2/SCAP/SYAP1/ROCK2/SMARCC1/BTG1/RANGAP1/PLCB1/NDEL1/STAT5B/GAB1/EGR1/KAT7/SH2B2/PIP4K2C/SHC1/MAPK14/CACNB1/CA2/ZFP36L1/SORT1/EPHA4/PDPK1/ARHGEF2/TRIB3/GSK3A/GRB2/PDK2/EIF6/PKM/INSR/SESN3/SESN2/SLC27A1/FBP1/NFKB1/SORL1/SLC2A1/STAT6/INPP5K/CCND3/MAX/IGF2/CSK/ZNF106/MMP9/POR/STK11/SLC9A1/FOXO4/PTPN1/RAB31/ADIPOR1/TLR6/LPIN2/JUND/JAK3/UCP2/LYN/GSK3B/STAT5A/LRP1/GNAI2/PXN/PRKCB/SP1/PPP1R9B/JAK1/WDTC1/STAT3/PIP4K2A/AHCYL1/AKT2/BSG/PRKDC/KLF1/RNF40/EIF4EBP2/TIMP1/FOXO3/KLF3/RELA
BP	GO:0032535	regulation of cellular component size	241	-0.51	-1.91	2.80E-09	7.28E-08	4.47E-08	2065	tags=39%, list=18%, signal=32%	SLC12A7/ARHGAP4/CARMIL1/RAB22A/ADD2/PLXNA4/TWF2/CDC42EP1/CREB1/NAA80/KIAA0319/CORO1A/NCKAP1L/SCIN/DIP2B/SLC9A3R1/FLII/DISC1/MACF1/SRF/PTEN/TRIOBP/AKT1S1/ADNP/DBNL/PPP1R15A/PRKCE/LARS1/ATP7A/HCK/EPS8/ANO6/SLC12A6/CYRIA/NDEL1/EZR/SPTAN1/ARPC1B/DBN1/CDKL5/WASHC2C/PYCARD/CDC42EP4/SEMA4D/CLN3/ARPC5/WDR1/SSH1/DPYSL2/GRB2/PPP1CA/SPTBN1/LRRC8A/PTK2B/SPTA1/SEMA4A/EIF4G2/SVIL/VAV1/GSN/IST1/ESAM/ULK1/PLEK/RAC1/AQP1/SIN3A/CYFIP2/GSK3B/DNM2/COTL1/RHOA/CAPZB/RILP/MYADM/MSN/ADD1/SSH2/WDTC1/HCLS1/TMOD1/RIN3/ARPC4/WAS/SPTB/MTPN/CTTN/SH3BP1/AP2M1/VASP/KEL/XK/DMTN

BP	GO:0010562	positive regulation of phosphorus metabolic process	549	-0.42	-1.69	2.86E-09	7.33E-08	4.50E-08	2051	tags=26%, list=17%, signal=23%	FGR/FYN/LIMK2/WNT1/MAP3K4/TIRAP/SRC/EPHB4/FNIP2/ATM/GCN1/ADNP/MAP2K5/PPP1R15A/STK4/KCTD20/SPPL3/SDC4/XRCC6/ERBB2/CCNY/CASS4/WDFY2/MAGED1/TGFB1/DGKZ/PPARGC1B/ERN2/LILRA5/ERN1/SDCBP/SYAP1/ROCK2/ARRB1/LRRK2/HGF/CACUL1/EGR1/ZBTB20/RIPK1/DLG4/EPHB1/S1PR2/MAP2K2/PTPRJ/FLOT1/SEMA4D/MAD2L2/DAZAP2/CLN3/LRRK1/EPHA4/PDPK1/NCF1/RALBP1/PTBP1/CSF1R/ARHGEF2/LEPR/CHP1/SASH1/ENTPD5/ARNT/PDGFA/NEDD9/INSR/MAP3K5/SLC27A1/TAOK3/FXR2/GREM1/VLDLR/PTK2B/NEURL1/VRK3/RAPGEF2/RPS6KA4/CCND3/TRAF6/PRKD2/IL6R/SH2D3C/PAK1/MOB3A/IGF2/PECAM1/CSK/BMP6/MMP9/TAOK1/SFRP2/STK11/ITGA5/MAP2K7/PELI2/STRADA/PTPRC/SYK/DVL3/THBS1/PTAFR/PTPN1/TLR6/CALM3/FZD5/LTF/PIK3CG/HIPK2/EIF4G1/VCP/MADD/PLEK/RAC1/MARK2/LYN/MMD/PDE5A/AKAP13/ATP2B4/RHOA/GATA1/CLU/ARRB2/HCLS1/GNA12/PINK1/MAPK3/SNCA/CDKN1A/FLCN/NIBAN1/TAL1/SQSTM1/AKT2/ARAF/ENO1/PAK2/ITGB3/PRR5/DAB2/WNK1/IQGAP1/MAP2K3/STRADB/FAXDC2
BP	GO:0045937	positive regulation of phosphate metabolic process	549	-0.42	-1.69	2.86E-09	7.33E-08	4.50E-08	2051	tags=26%, list=17%, signal=23%	FGR/FYN/LIMK2/WNT1/MAP3K4/TIRAP/SRC/EPHB4/FNIP2/ATM/GCN1/ADNP/MAP2K5/PPP1R15A/STK4/KCTD20/SPPL3/SDC4/XRCC6/ERBB2/CCNY/CASS4/WDFY2/MAGED1/TGFB1/DGKZ/PPARGC1B/ERN2/LILRA5/ERN1/SDCBP/SYAP1/ROCK2/ARRB1/LRRK2/HGF/CACUL1/EGR1/ZBTB20/RIPK1/DLG4/EPHB1/S1PR2/MAP2K2/PTPRJ/FLOT1/SEMA4D/MAD2L2/DAZAP2/CLN3/LRRK1/EPHA4/PDPK1/NCF1/RALBP1/PTBP1/CSF1R/ARHGEF2/LEPR/CHP1/SASH1/ENTPD5/ARNT/PDGFA/NEDD9/INSR/MAP3K5/SLC27A1/TAOK3/FXR2/GREM1/VLDLR/PTK2B/NEURL1/VRK3/RAPGEF2/RPS6KA4/CCND3/TRAF6/PRKD2/IL6R/SH2D3C/PAK1/MOB3A/IGF2/PECAM1/CSK/BMP6/MMP9/TAOK1/SFRP2/STK11/ITGA5/MAP2K7/PELI2/STRADA/PTPRC/SYK/DVL3/THBS1/PTAFR/PTPN1/TLR6/CALM3/FZD5/LTF/PIK3CG/HIPK2/EIF4G1/VCP/MADD/PLEK/RAC1/MARK2/LYN/MMD/PDE5A/AKAP13/ATP2B4/RHOA/GATA1/CLU/ARRB2/HCLS1/GNA12/PINK1/MAPK3/SNCA/CDKN1A/FLCN/NIBAN1/TAL1/SQSTM1/AKT2/ARAF/ENO1/PAK2/ITGB3/PRR5/DAB2/WNK1/IQGAP1/MAP2K3/STRADB/FAXDC2
BP	GO:0001934	positive regulation of protein phosphorylation	453	-0.43	-1.72	2.93E-09	7.46E-08	4.58E-08	2203	tags=29%, list=19%, signal=25%	HDAC3/AXIN2/MAVS/TNK2/ADAM8/RPS6KA5/CHI3L1/FYN/LIMK2/WNT1/MAP3K4/TIRAP/SRC/FNIP2/ATM/ADNP/MAP2K5/STK4/SDC4/XRCC6/ERBB2/CCNY/CASS4/WDFY2/MAGED1/TGFB1/ERN2/LILRA5/ERN1/SDCBP/SYAP1/ROCK2/ARRB1/LRRK2/HGF/CACUL1/EGR1/RIPK1/DLG4/S1PR2/MAP2K2/PTPRJ/FLOT1/SEMA4D/MAD2L2/DAZAP2/CLN3/LRRK1/EPHA4/PDPK1/NCF1/RALBP1/CSF1R/ARHGEF2/LEPR/SASH1/PDGFA/NEDD9/INSR/MAP3K5/SLC27A1/TAOK3/FXR2/GREM1/VLDLR/PTK2B/NEURL1/RAPGEF2/RPS6KA4/CCND3/TRAF6/PRKD2/IL6R/SH2D3C/PAK1/MOB3A/IGF2/PECAM1/CSK/BMP6/MMP9/TAOK1/SFRP2/STK11/ITGA5/MAP2K7/PELI2/STRADA/PTPRC/SYK/DVL3/THBS1/PTPN1/TLR6/CALM3/FZD5/LTF/PIK3CG/HIPK2/EIF4G1/MADD/RAC1/MARK2/LYN/MMD/PDE5A/AKAP13/ATP2B4/RHOA/GATA1/CLU/ARRB2/HCLS1/PINK1/MAPK3/SNCA/CDKN1A/FLCN/NIBAN1/TAL1/SQSTM1/AKT2/ARAF/PAK2/ITGB3/PRR5/DAB2/WNK1/IQGAP1/MAP2K3/STRADB/FAXDC2

BP	GO:0045944	positive regulation of transcription by RNA polymerase II	787	-0.39	-1.59	3.39E-09	8.53E-08	5.24E-08	2005	tags=26%, list=17%, signal=23%	NLRP3/TAF8/PER1/MED13/ELK1/KPNA6/HDAC4/RXRB/ATM/GCN1/CDK12/JMJD6/MAP2K5/ZNF48/ATF6/XRCC6/NIPBL/VDR/VEZF1/POU2F1/MLLT10/TGFB1/PPARGC1B/ETV6/BCL3/ZNF148/GTF2I/POU2F2/CTBP2/HSPA5/TFDP2/SMARCC1/ARRB1/ZFAT/HGF/IRF2BPL/SMARCA4/GPS2/ASH1L/STAT5B/NFAT5/NFIA/EGR1/ARID5A/KMT2D/RIPK1/TFEB/KAT7/NFATC1/NR6A1/CTCFL/MAPK14/CEBPA/ELL/YY1/MSX1/SETX/ATF7/EIF5A/ZNF175/DPF2/NR1H2/IRF2/SBNO2/NFKB2/CREBRF/USF3/SPI1/NCOA3/MAML2/CDK13/ZNF296/ARHGEF2/RFX2/AKIRIN2/KAT5/ZFHX3/HELZ2/MTF1/SMARCA2/KLF13/KMT2A/AGO2/ARNT/MED16/ZNF335/E2F3/SESN2/NFKB1/MCRS1/GREM1/RBPJ/NCOA2/NFATC2/PELP1/IKBKG/YBX1/IRF1/RARA/IER2/STAT6/MEIS1/NFIC/RPS6KA4/BCAS3/TRAF6/PRKD2/TBX21/KLF5/MAX/USF2/IGF2/ZMIZ1/SSBP3/BMP6/TBL1XR1/ZBTB16/ASXL2/SFRP2/AKNA/PPP3CA/TFE3/SLC9A1/FOXO4/DVL3/NPAT/PTMS/FOSL2/ZNF609/AGO1/SUPT6H/BPTF/MED1/NR3C1/MAFB/DCAF6/RBM14/FZD5/TET3/HIPK2/PPP2R5B/CHD8/SKI/MAML3/LPIN2/JUND/PF4/HMGA1/RXRA/HNRNPK/MLXIP/SIN3A/ETS2/ARID3B/GFI1B/CREBBP/WWP2/TCF7L2/SUPT5H/TAF6/MEPCE/TNIP1/FOXJ2/NFATC3/TBL1X/TFDP1/KMT2C/GATA1/KDM6B/SP1/NCOA6/ARID3A/BCL9/HCLS1/WAS/RPS6KA1/WBP2/MAPK3/PBX1/FLCN/STAT3/CSRNP1/PPP3R1/PCBP2/ABLIM3/TAL1/SQSTM1/NFIX/NCOA1/PCGF5/LITAF/HDGF/KAT6A/PRKDC/BRD4/MED25/E2F2/DAB2/RNF10/FOXO3/ELF4/MICAL2/HCFC1/RELA
BP	GO:0090287	regulation of cellular response to growth factor stimulus	148	-0.59	-2.08	3.39E-09	8.53E-08	5.24E-08	1916	tags=35%, list=16%, signal=30%	IL17RD/XIAP/RNF165/TGFB1/LDLRAD4/ZBTB7A/NOTCH2/SDCBP/HGS/HSPA5/MSX1/CD63/FUZ/CDKN1C/FAM89B/PDPK1/NUMA1/ARNT/GREM1/RBPJ/SORL1/PCSK6/CHST11/PRKD2/TGFB1I1/SFRP2/STK11/ITGA5/THBS1/PTPN1/GLG1/HIPK2/PPP2R5B/SKI/NIBAN2/HSPA1A/CYFIP2/DNM2/CREBBP/ATP2B4/FKBP1A/PRKCB/FKBP8/LRG1/UBE2O/FLCN/LTBP1/ZNF703/PTP4A3/PPM1A/ITGB3/DAB2
BP	GO:0051493	regulation of cytoskeleton organization	381	-0.45	-1.78	3.48E-09	8.66E-08	5.31E-08	2007	tags=29%, list=17%, signal=25%	PPM1F/TRIOBP/MAP1S/ATXN7/BICD2/RHOF/DBNL/PRKCE/SDC4/HCK/EPS8/NOTCH2/CYRIA/ACTG1/ROCK2/CKAP5/SPTAN1/ARPC1B/DBN1/TMEM67/WASF3/CHMP4B/NF2/CDK2AP2/ARHGDIB/CAMSAP1/EML2/WASHC2C/PYCARD/CDC42EP4/MYCBP2/SLAIN2/ARAP1/CLN3/ABI3/DAPK3/ARPC5/CCNF/NUMA1/CSF1R/ARHGEF2/WDR1/ALMS1/ARF1/SSH1/GRB2/PDGFA/TPM1/SPTBN1/FES/PTK2B/SPTA1/INPP5K/BCAS3/SVIL/DYNC1H1/PAK1/MAP1A/TAOK1/GSN/SLC9A1/JAM3/ESAM/DCTN1/CLTC/RBM14/IQGAP2/TACC3/FLNA/EML3/HSPA1A/PLEK/CHMP1A/RAC1/MARK2/CYFIP2/GSK3B/RAC2/LRP1/AKAP13/PXN/ARHGAP6/COTL1/RHOA/CAPZB/GAS2L1/MYADM/ADD1/SSH2/HCLS1/ARHGDIA/TMOD1/ARPC4/RHOBTB1/WAS/MAPK3/SNCA/SPTB/MTPN/CTTN/SH3BP1/PAK2/VASP/IQGAP1/PEAK3/WASF2/RHOG/DMTN/DIAPH1
BP	GO:0003013	circulatory system process	297	-0.48	-1.84	3.86E-09	9.54E-08	5.86E-08	2395	tags=31%, list=20%, signal=25%	CD38/SLCO3A1/CALM1/SLC1A4/SLC19A1/LNPEP/SLC9A3R1/TBC1D8/CTNNBIP1/SLC28A2/ATP2B1/SERPING1/SOD2/THRA/KNG1/HEG1/FYN/SRC/FKBP1B/ABAT/EXT1/TGFB1/AGTRAP/SLC2A3/PDE2A/ROCK2/OPRL1/DOCK5/GRK2/KCNH2/ACTA2/PTPRJ/PER2/TJP2/LEPR/GSK3A/ABCG2/F5/HRH2/ANPEP/PRKACA/INSR/GUCY1B1/ATP2A3/TPM1/SLC27A1/TMEM38A/SLC2A1/SLC8A1/BCR/SLC6A20/SLC24A3/PTGS1/BMP6/GSN/PLCB3/SLC9A1/PTAFR/GAA/SCN1B/PLEC/ATP1B2/EHD3/CALM3/SLC6A9/PIK3CG/FLNA/SLC1A5/ABCC4/CYB5R3/PDE5A/MYL4/LRP1/AKAP13/ABCC3/ATP2B4/GNAI2/RHOA/GPX1/GNA12/ECE1/GUCY1A1/CLDN5/PTP4A3/WNK1/SLC7A5/KEL/P2RX1/SLC38A5/PLVAP/TBXA2R/MAP2K3

CC	GO:0098978	glutamatergic synapse	175	-0.55	-1.98	4.04E-09	9.91E-08	6.09E-08	2141	tags=39%, list=18%, signal=33%	VPS18/ATP2B1/SNAP25/LRRC4/SRC/DNAJB1/GRIK2/EPS8/LRFN3/DLGAP4/CTBP2/NUMB/LRRK2/PLCB1/AP2B1/NPTXR/SYN3/DLG4/DBN1/WASF3/EPHB1/MAPK14/STX4/CTBP1/CDKL5/FXYD6/FLOT1/CLSTN1/CTNND1/KPNA1/VPS35/EPHA4/SHISA7/YWHAZ/PPP1CA/CANX/GUCY1B1/SPTBN1/SH3GL1/PTK2B/BCR/PPP3CA/DYNLL2/FBXL20/DNM3/VCP/ACTB/RAC1/LYN/HIP1/GSK3B/DNM2/FLOT2/ATP2B4/GRIPAP1/RHOA/STAU1/NRGN/GUCY1A1/ABLIM3/PRKAR2B/ACTN1/NAPA/AP2M1/PAK2/ITGB3/RNF10/P2RX1/RELA
BP	GO:0007264	small GTPase mediated signal transduction	381	-0.45	-1.78	4.11E-09	1.00E-07	6.16E-08	2110	tags=30%, list=18%, signal=26%	MYO9A/NKIRAS2/EPHB2/ARHGAP24/TNK2/MAP4K4/ARHGEF11/SPRY3/ARHGEF3/HEG1/ITSN1/SRC/TRIO/RHOF/ARHGEF12/DBNL/RASA2/ARHGAP19/EPS8/TIAM1/ITPKB/PHACTR4/G3BP1/RRAS/SETDB1/NOTCH2/RIT1/SDCBP/TSC2/ARHGAP9/ROCK2/ARRB1/RALGAPA1/RASAL3/DOCK5/SIPA1L1/RAP1GAP/ARHGDIB/FLOT1/DOK1/CDC42EP4/ARAP1/GRAP2/CTNNAL1/SOS2/KPNB1/PREX1/DHCR24/RALBP1/ARHGEF2/RALGAPA2/TRIM28/CHP1/GRB2/ARHGAP31/AMOT/FBP1/KANK2/ARHGAP21/PLEKHG3/DOCK8/FGD4/RRAGC/BCR/RAPGEF2/LRRC59/RASGRP4/GMIP/SH2D3C/FGD3/ELMO1/VAV1/SIPA1L2/MYO9B/GNB1/ARHGAP8/DOCK2/PIK3CG/DOK3/RIPOR1/MADD/SIPA1/RAC1/GABARAP/RAC2/DNM2/AKAP13/RUFY1/ARHGAP6/IQSEC1/RHOA/ARHGAP45/NF1/ARHGDIA/GNA12/CFL1/RHOBTB1/WAS/NUCB1/ARHGAP26/SIAH2/CDKN1A/TGM2/RAPGEF1/FLCN/CYTH4/RIPOR2/SQSTM1/SH3BP1/DENND1A/MFN2/RUNDC3A/ARHGAP1/CBL/WASF2/RHOG
BP	GO:0002520	immune system development	729	-0.39	-1.61	4.45E-09	1.08E-07	6.62E-08	2254	tags=29%, list=19%, signal=25%	IREB2/TF/GON4L/IFI16/CTNNBIP1/CLPTM1/DYRK3/SH2B3/PKN1/SH3RF1/ADAM8/TMEM176B/ASXL1/PLD4/FCER1G/IRAK3/TLR2/SOD2/TTC7A/BCL2L11/CMTM7/CR1/ZNF385A/THRA/MYO1E/PAF1/KLF6/SRF/WNT1/TIRAP/PRDX2/NLRP3/SRC/HDAC4/ATM/JMJD6/STK4/MCM3AP/TNFSF13/XRCC6/ERBB2/EXT1/ATP7A/EEF2/TGFB1/PPARGC1B/ITPKB/ZBTB7A/ETV6/TNFSF4/RC3H2/RRAS/BCL3/NOTCH2/PTBP3/ZFAT/GPS2/PRRC2C/NBEAL2/STAT5B/PHLPP1/EGR1/RIPK1/KAT7/MAPK14/TRIB1/CEBPA/EIF2AK2/YY1/PTPRJ/ZFP36L1/PTPN6/ADGRG3/CBFA2T3/MAD2L2/DPF2/ARIH2/SBNO2/NFKB2/CDKN1C/RELB/SOS2/SPI1/ITCH/LRRK1/PREX1/PML/CDK13/CCR1/CSF1R/WDR1/TSPO2/ATP6AP1/FOXP1/LEPR/KLF13/KMT2A/ARNT/EIF6/CNOT4/RUNX3/SGPL1/TMEM131L/VSIR/NFAM1/FES/HIPK1/OSCAR/RBPJ/MKNK2/IRF1/PGLYRP1/LRRC8A/RARA/PTK2B/MFHAS1/SELPLG/GAB2/SPTA1/SEMA4A/BCR/SNX10/STAT6/MEIS1/TRAF6/PITHD1/RASGRP4/TBX21/IL6R/TOB2/ZMIZ1/ZBTB16/MMP9/VAV1/SFRP2/STK11/SLC25A38/TFE3/THOC5/PTPRC/LILRB3/SYK/ADAR/KDELR1/JAM3/SUPT6H/ST3GAL1/MED1/MAFB/MTURN/DOCK2/FZD5/LTF/HIPK2/G6PD/GLRX5/JAK3/HSPA1A/POLL/INPP5D/PF4/PLEK/LYN/LGALS9/SIN3A/GFI1B/ROGDI/CNN2/BPGM/MPL/RHOA/CUL4A/GATA1/FASN/NF1/NCOA6/AHSP/JAK1/HCLS1/TRIM10/PIM1/TESC/MAPK3/PBX1/FLCN/STAT3/PIP4K2A/TAL1/ACTN1/SLC4A1/FAM210B/PIK3CD/EPB42/IKZF1/KAT6A/PRKDC/KLF1/MPIG6B/ALAS2/TRIM58/JARID2/LYL1/FBXO7/MYH9/FOXO3/EIF2AK1/WASF2/FAXDC2/HLX/DMTN

CC	GO:0030424	axon	364	-0.46	-1.79	4.74E-09	1.13E-07	6.96E-08	2285	tags=32%, list=19%, signal=27%	MYO5A/MYO9A/KIF13B/MUL1/AAK1/VIM/EPHB2/GDPD5/ENO2/NIN/KATNB1/SNAP25/DISC1/FEZ1/RAB27B/EXOC4/ELK1/ADNP/BLOC1S3/GRIK2/ATP7A/EPS8/LRFN3/CPEB4/NAV1/NECTIN1/ACTG1/EXOC7/SYAP1/LRRK2/RANGAP1/NDEL1/SYNJ1/DLG4/DBN1/MME/EPHB1/CDKL5/AGTPBP1/RAP1GAP/SETX/PARD3/MYCBP2/EXOC3/CTNND1/MAP4/EPHA4/ARPC5/GSK3A/TXNRD2/CANX/FAM168B/INSR/ADGRL1/PALLD/NDRG2/SPTBN1/FXR2/KIF1C/HTT/PTK2B/SLC8A1/SPTA1/BCR/EIF4G2/SBF2/LDLRAP1/COPA/DYNC1H1/SH2D3C/PAK1/MAP1A/ARL8A/USP9X/SCN1B/PLEC/DCTN1/CALM3/FZD5/BASP1/FLNA/ULK1/DNM3/PVALB/ACTB/MADD/GJC2/KIF1B/GSK3B/DNM2/TPRG1L/ROGDI/HSPB1/TNFRSF1B/TRAK2/GRIPAP1/TSHZ3/RHOA/PRKCB/WDFY3/NF1/PPP1R9B/TERF2/RIN3/PINK1/NRGN/SNCA/MTPN/CTTN/CLDN5/ATP6V0D1/BSG/RNF40/MBP/GLUL/CBL/IQGAP1
BP	GO:0060627	regulation of vesicle-mediated transport	362	-0.45	-1.77	4.71E-09	1.13E-07	6.96E-08	2012	tags=38%, list=17%, signal=33%	P2RY1/MCTP1/CLASP1/VPS4A/EHD4/LGALS3/SEPTIN4/ANXA2/BTBD9/DNAJC13/SCRIB/CORO1A/NCKAP1L/PRKAR1B/ATP9A/SCARB1/TF/AAK1/IL2RG/TNK2/FCER1G/TLR2/VPS18/VPS4B/FGR/UNC13D/SRC/RAB27B/CD177/MYO18A/SDC4/AP1G1/HCK/SIRPA/SNX3/ADGRE2/ANO6/SDCBP/HGS/TSC2/ANKRD13A/NUMB/ITGAM/ARRB1/LRRK2/AP2B1/SYNJ1/SNX17/EZR/AP2S1/DLG4/CD300LF/STX4/MAP2K2/PTPRJ/FLOT1/PYCARD/ATXN2/NR1H2/CD63/CLN3/SPI1/PDPK1/ITGB2/ARF1/SEPTIN1/INSR/VPS11/FES/GREM1/SORL1/GAB2/BCR/LDLRAP1/RAB7A/ANKFY1/CSK/PPP3CA/RAB5B/PTPRC/SYK/PTAFR/BMP2K/PTPN1/FBXL20/RAB31/ARHGAP8/CD151/CALM3/DOCK2/CAMK1D/PRAM1/RUBCN/VPS28/DNAJC5/CD14/RAC1/LYN/HIP1/LGALS9/HNRNPK/GSK3B/RAC2/SIRPB1/DNM2/RAB11B/LRP1/RUFY1/SYTL4/CNN2/DYSF/GNAI2/GRIPAP1/PRKCB/GATA1/CLU/ARRB2/MSN/RIN3/MAPK3/SNCA/TGM2/TBC1D20/PTPN23/EHD1/LAMP1/TBC1D5/AKT2/USP7/AP2M1/STON2/RAB3D/ITGB3/DAB2/ARHGAP1/P2RX1/CBL/RAB2B
MF	GO:0031267	small GTPase binding	224	-0.52	-1.95	5.22E-09	1.24E-07	7.63E-08	2295	tags=37%, list=20%, signal=30%	HPS6/MYO5A/SGSM1/PKN1/PPP6R1/RAB11FIP5/TNPO1/RGP1/UNC13D/TRIOBP/EXOC4/BICD2/CDC42BPB/DIAPH2/NCF2/AP1G1/XPO5/ATP7A/TNPO2/EPS8/TSC2/TNPO3/CYRIA/ROCK2/RAB11FIP4/RPH3A/LRRK2/RANGAP1/IPO8/NUTF2/SYTL3/INF2/DOCK5/CDKL5/RAP1GAP/ARHGDIB/GCC1/CDC42EP4/MYCBP2/DAPK3/KPNB1/RALBP1/ARHGEF2/ATP6AP1/EVI5L/RABGAP1/OPTN/SORL1/CORO1C/FGD4/RIN1/RASSF5/PAK1/RAB7A/ANKFY1/FGD3/DVL3/MYO9B/DOCK2/IQGAP2/RAB11FIP1/FLNA/ULK1/TBC1D13/YIPF2/XPO7/FMNL1/CYFIP2/GAPVD1/AKAP13/SYTL4/RILP/RIN3/WAS/XPO6/TBC1D20/EHD1/TMEM127/PAK2/YBX3/ARHGAP1/IQGAP1/DIAPH1
BP	GO:0051056	regulation of small GTPase mediated signal transduction	228	-0.51	-1.92	5.83E-09	1.37E-07	8.42E-08	2416	tags=35%, list=21%, signal=28%	MAPRE2/ARHGAP32/MCF2L/ARHGEF5/MYO9A/EPHB2/ARHGAP24/MAP4K4/ARHGEF11/SPRY3/ARHGEF3/HEG1/ITSN1/SRC/TRIO/RHOF/ARHGEF12/RASA2/ARHGAP19/EPS8/TIAM1/ITPKB/NOTCH2/TSC2/ARHGAP9/ARRB1/RALGAPA1/RASAL3/SIPA1L1/RAP1GAP/ARHGDIB/FLOT1/ARAP1/SOS2/PREX1/RALBP1/ARHGEF2/RALGAPA2/ARHGAP31/AMOT/FBP1/KANK2/ARHGAP21/PLEKHG3/DOCK8/FGD4/BCR/LRRC59/RASGRP4/GMIP/FGD3/VAV1/SIPA1L2/MYO9B/ARHGAP8/DOCK2/PIK3CG/MADD/SIPA1/RAC1/GABARAP/DNM2/AKAP13/ARHGAP6/IQSEC1/ARHGAP45/NF1/ARHGDIA/ARHGAP26/TGM2/FLCN/CYTH4/RIPOR2/SQSTM1/SH3BP1/DENND1A/MFN2/ARHGAP1/CBL

BP	GO:1901653	cellular response to peptide	242	-0.50	-1.89	6.36E-09	1.49E-07	9.13E-08	2303	tags=33%, list=20%, signal=27%	NFE2L2/MYO5A/KAT2B/VIM/EPHB2/ATP2B1/FOXO1/BCL2L11/FYN/WNT1/SRC/IRS2/ZDHHC7/AGTRAP/TSC2/SYAP1/ROCK2/SMARCC1/RANGAP1/PLCB1/NDEL1/STAT5B/GAB1/SH2B2/PIP4K2C/SHC1/CACNB1/CA2/ZFP36L1/EPHA4/PDPK1/ARHGEF2/TRIB3/GSK3A/GRB2/PDK2/PKM/INSR/SESN3/SLC27A1/FBP1/NFKB1/SORL1/STAT6/INPP5K/CCND3/MAX/IGF2/CSK/ZNF106/POR/SLC9A1/FOXO4/PTPN1/RAB31/ADIPOR1/TLR6/LPIN2/JAK3/UCP2/LYN/GSK3B/STAT5A/LRP1/GNAI2/PXN/PRKCB/SP1/PPP1R9B/JAK1/WDTC1/STAT3/PIP4K2A/AHCYL1/AKT2/PRKDC/KLF1/EIF4EBP2/FOXO3/KLF3/RELA
BP	GO:0030155	regulation of cell adhesion	495	-0.42	-1.70	7.71E-09	1.78E-07	1.09E-07	3003	tags=38%, list=26%, signal=30%	NID1/CCDC88B/DUSP22/SERPINE1/PLXNB2/FADD/PPP2R1A/PLXND1/HLA-A/TNF/CARD11/NOTCH1/POLDIP2/AFDN/PIK3CB/SMAD3/NFKBID/SOX4/TNFSF14/EPHA1/F11R/CDK6/ZDHHC2/ST6GAL1/SPOCK2/CARMIL1/IFNL1/RIN2/SART1/RC3H1/PLXNA4/CALR/CD4/CLASP1/AMBRA1/LGALS3/PLAUR/ATP5F1B/TBCD/SPN/SCRIB/ALOX5/CORO1A/UTRN/NCKAP1L/DUSP1/EPHB2/SH2B3/LIMS1/ADAM8/PLEKHA2/MAP4K4/EFNB1/CR1/ATXN3/KNG1/DISC1/LGALS9C/MACF1/SRF/FYN/WNT1/PTEN/UNC13D/PRDX2/PPM1F/NLRP3/SRC/TRIOBP/ATM/ABAT/MAP2K5/STK4/PRKCE/SDC4/ERBB2/CASS4/TGFB1/SIRPA/LRFN3/ITPKB/TNFSF4/RC3H2/RRAS/ACTG1/ROCK2/STAT5B/NFAT5/PAG1/RASAL3/DOCK5/MAPK14/STX4/NF2/PTPRJ/ARHGDIB/FLOT1/PTPN6/WASHC2C/PYCARD/SEMA4D/MAD2L2/DAPK3/SPI1/ITCH/PREX1/EPHA4/FERMT3/PML/PKP4/ITGB2/ZFHX3/NEDD9/RUNX3/TPM1/TMEM131L/VSIR/FES/GREM1/CORO1C/IRF1/DOCK8/RARA/PTK2B/SPTA1/SELP/BCAS3/TRAF6/PRKD2/TBX21/IGF2/VCL/ZMIZ1/CSK/BMP6/ZBTB16/VAV1/SFRP2/AKNA/PLXNC1/GP6/PPP3CA/TFE3/ITGA5/PTPRC/SYK/SLC9A1/JAM3/THBS1/PTAFR/IL1RN/PIK3CG/FLNA/JAK3/GFUS/ALOX12/SIPA1/RAC1/LYN/LGALS9/LAPTM5/GSK3B/PDE5A/RAC2/SIRPB1/DNM2/FLOT2/ARHGAP6/RHOA/MYADM/NF1/ACTN4/JAK1/ARHGDIA/RSU1/TESC/APBB1IP/TGM2/FLCN/PTPN23/RIPOR2/ZNF703/DAB2/MBP/WNK1/FOXO3/HLX/DMTN/RELA
BP	GO:0044057	regulation of system process	263	-0.49	-1.86	7.74E-09	1.78E-07	1.09E-07	2136	tags=30%, list=18%, signal=25%	ATP2B1/ORMDL3/FOXO1/THRA/SRF/PTEN/FTO/SRC/FKBP1B/HDAC4/ABAT/PROK2/DICER1/DLGAP4/CTDP1/ROCK2/LRRK2/OPRL1/HGF/NPTXR/DLG4/DOCK5/WASF3/S1PR2/GRK2/KCNH2/YY1/PER2/PARD3/NR1H2/DAPK3/SHISA7/GSK3A/HRH2/PRKACA/ATP2A3/TPM1/TMEM38A/PTK2B/SLC8A1/INPP5K/LPCAT3/BMP6/PPP3CA/SLC9A1/PTAFR/LMNA/GAA/SCN1B/PPP1R12B/ATP1B2/MGLL/EHD3/CALM3/PIK3CG/G6PD/FLNA/ANXA6/AQP1/PDE5A/MYL4/TNFRSF1B/ATP2B4/GNAI2/TSHZ3/RHOA/ECE1/EPB41/GUCY1A1/MTPN/CTTN/ENO1/MYL9/DAB2/JARID2/RNF10/WNK1/P2RX1/FOXO3/TBXA2R
BP	GO:0034109	homotypic cell-cell adhesion	66	-0.69	-2.19	8.62E-09	1.97E-07	1.21E-07	1421	tags=36%, list=12%, signal=32%	PTPN6/FERMT3/TJP2/FIBP/VCL/GP6/SYK/PIK3CG/FLNA/ACTB/ALOX12/PLEK/LYN/HSPB1/MPL/GATA1/JAK1/ZNF703/GP1BA/MYL9/ITGB3/MYH9/DMTN/TLN1
BP	GO:0007178	transmembrane receptor protein serine/threonine kinase signaling pathway	196	-0.53	-1.94	8.75E-09	1.99E-07	1.22E-07	1916	tags=33%, list=16%, signal=28%	IL17RD/ATOH8/XIAP/EXT1/RNF165/TGFB1/LDLRAD4/ZBTB7A/BTBD11/NOTCH2/SDCBP/HSPA5/ZC3H3/EGR1/NOMO3/MAPK14/MSX1/CDKN1C/PPM1L/FAM89B/SPI1/PDPK1/PML/NUMA1/SPTBN1/GREM1/RBPJ/SORL1/PCSK6/CHST11/ZMIZ1/BMP6/TGFB1I1/SFRP2/STK11/USP9X/LTBP2/THBS1/NUP93/GLG1/RBM14/HIPK2/SKI/HSPA1A/TGFBRAP1/ZYX/DNM2/CREBBP/LRP1/PXN/FKBP1A/ARRB2/FKBP8/LRG1/UBE2O/MAPK3/ITGB5/FLCN/STAT3/CLDN5/LTBP1/ZNF703/PPM1A/DAB2/FURIN

BP	GO:0032989	cellular component morphogenesis	418	-0.44	-1.74	9.02E-09	2.04E-07	1.25E-07	2604	tags=33%, list=22%, signal=27%	PLXNA4/EPB41L3/STAU2/CLASP1/HDAC6/AMIGO1/TWF2/CAPN3/FIG4/CREB1/ACTR2/CFLAR/KIAA0319/NCKAP1L/DIP2B/BCL11B/MYO9A/KIF13B/MUL1/EPHB2/BCL11A/STK25/MAP4K4/RPS6KA5/EFNB1/NIN/TLR2/FLII/SPRY3/CSRP1/PARD6B/DISC1/MACF1/SRF/FYN/PTEN/FEZ1/SRC/MAP1S/EPHB4/YWHAH/TRIO/KIDINS220/ADNP/DBNL/ERBB2/EXT1/RNF165/ATP7A/TIAM1/DICER1/NCAM1/CUL7/CPNE5/SZT2/NOTCH2/NECTIN1/ACTG1/NUMB/LRRK2/B4GALT5/NDEL1/DLG4/DBN1/PIKFYVE/EPHB1/CPNE1/CDKL5/SIPA1L1/FLOT1/PARD3/SEMA4D/MYCBP2/ABI3/EPHA4/LYPLA2/CSF1R/WDR1/RFX2/DPYSL2/ZNF335/PALLD/TPM1/TAOK3/VLDLR/CORO1C/SEMA4A/RAPGEF2/EIF4G2/PAK1/MAP1A/VCL/RERE/TAOK1/STK11/PLXNC1/PPP3CA/APLP2/USP9X/PDLIM5/DVL3/FIS1/SCN1B/PLEC/IST1/ULK1/NIBAN2/DNM3/ACTB/RAC1/MARK2/SIN3A/CYFIP2/GSK3B/RAC2/DNM2/AKAP13/RHOA/TMOD1/ECE1/CFL1/TBC1D20/BCL2L1/CTTN/GAS7/UBB/BSG/MYL9/PAK2/VASP/MBP/CUX1/KEL/IQGAP1/WASF2/XK/RHOG/ACRBP
BP	GO:0045596	negative regulation of cell differentiation	354	-0.46	-1.78	9.22E-09	2.07E-07	1.27E-07	2232	tags=34%, list=19%, signal=28%	DIP2B/NFE2L2/HDAC7/EPHB2/BCL11A/AXIN2/TMEM176B/ASXL1/FOXO1/SOD2/CR1/PAF1/WNT1/PTEN/PRDX2/YWHAH/ANKRD17/HDAC4/TRIO/CDK12/IL17RD/ERBB2/TGFB1/LDLRAD4/ITPKB/DICER1/TNFSF4/RC3H2/CTDP1/NBR1/GPS2/STAT5B/JDP2/NFATC1/EPHB1/TRIB1/SRA1/YY1/ZFP36L1/RAP1GAP/MSX1/SORT1/HOOK3/SEMA4D/MAD2L2/DPF2/NR1H2/DDX6/FUZ/USF3/EPHA4/CMTM5/CDK13/PTBP1/TRIB3/KLF13/GSK3A/RUNX3/TMEM131L/SUFU/GREM1/RBPJ/TSPO/NFATC2/SORL1/CORO1C/YBX1/IRF1/PGLYRP1/RARA/PTK2B/SEMA4A/MEIS1/RAPGEF2/SOX6/TBX21/TOB2/IGF2/ZHX2/CTNNA1/ZBTB16/MMP9/TGFB1I1/SFRP2/PPP3CA/LILRB3/FOXO4/ADIPOR1/MED1/MAFB/LTF/G6PD/SKI/ULK1/JAK3/INPP5D/PF4/RFLNB/LYN/GSK3B/CTDSP1/TRIM11/RHOA/FOXJ2/CUL4A/NFATC3/NF1/ACTN4/PBX1/FLCN/STAT3/TENT5C/ITGB3/MBP/RNF10/MBNL3/FBXO7/FOXO3/HLX/DMTN

BP	GO:0043085	positive regulation of catalytic activity	794	-0.38	-1.57	9.92E-09	2.22E-07	1.36E-07	2296	tags=30%, list=20%, signal=26%	SERINC5/MAPRE2/SCRIB/GPLD1/CALM1/ADAM17/EGLN3/NCKAP1L/RGS16/MAP4K2/ARHGEF5/PCOLCE/TBC1D8/SGSM1/SCARB1/MYO9A/AIFM1/MUL1/IFI16/EPHB2/LIMS1/ARHGAP24/BCL2L13/AXIN2/ADAM8/MAP4K4/CHI3L1/GNA15/BCL2L11/CR1/RGP1/FGR/FYN/PTEN/MAP3K4/TIRAP/PPM1F/NLRP3/SRC/APAF1/EPHB4/GCN1/ADNP/PNKP/MAP2K5/PPP1R15A/STK4/SDC4/C6orf89/XRCC6/ERBB2/VDR/LARS1/CCNY/ATP7A/CASS4/MAGED1/TGFB1/DGKZ/TIAM1/PPARGC1B/ERN2/PHACTR4/LILRA5/ERN1/CHTF8/TSC2/SYAP1/ROCK2/ITGAM/LRRK2/RANGAP1/RALGAPA1/NDEL1/CACUL1/EGR1/TBC1D17/DLG4/TBC1D3I/C5AR1/EPHB1/MAPK14/STX4/CDKL5/MAP2K2/SIPA1L1/RAP1GAP/PYCARD/SEMA4D/NR1H2/DAP/ARAP1/DAZAP2/SOS2/ABHD5/PREX1/EPHA4/PDPK1/TBC1D22A/GNAQ/NCF1/PML/PKP4/RALBP1/ITGB2/PSME4/CSF1R/RALGAPA2/AKIRIN2/EVI5L/TBC1D10B/CAP1/TRIB3/RABGAP1/GSK3A/UBE2L3/SASH1/RGS6/NLRC4/PDGFA/NEDD9/INSR/ATP2A3/MAP3K5/SLC27A1/YWHAB/VSIR/TAOK3/GREM1/VLDLR/SORL1/CORO1C/BCAP31/ADAP2/DOCK8/PTK2B/NEURL1/VRK3/BCR/RAPGEF2/BCAS3/CCND3/TRAF6/RCN3/TBC1D22B/PRKD2/RASGRP4/GMIP/IL6R/PAK1/MOB3A/IGF2/CSK/TAOK1/ADAP1/VAV1/POR/SFRP2/STK11/GSN/MAP2K7/FZR1/STRADA/PTPRC/SIPA1L2/SYK/DVL3/THBS1/FIS1/PSME3/PTAFR/PTPN1/ASAP1/ASAP2/TLR6/CASP2/CALM3/FZD5/LTF/PIK3CG/VCP/TBC1D13/HSPA1A/MADD/SIPA1/PLEK/MARK2/LYN/HIP1/LGALS9/MMD/CYFIP2/LAPTM5/GSK3B/PDE5A/PSAP/AKAP13/ATP2B4/GNAI2/ARHGAP6/TBC1D25/IQSEC1/RHOA/ARHGAP45/BBC3/CLU/NF1/GRN/TERF2/RSU1/GNA12/PINK1/MAPK3/SNCA/CDKN1A/TGM2/TBC1D20/DTX3L/RAPGEF1/FLCN/STAT3/TAL1/TBC1D5/AKT2/SH3BP1/TBC1D1/STIM1/PAK2/CTSD/ITGB3/MBP/WNK1/TBC1D2/P2RX1/IQGAP1/MAP2K3/STRADB/RHOG
BP	GO:0051345	positive regulation of hydrolase activity	396	-0.44	-1.73	1.09E-08	2.43E-07	1.49E-07	2416	tags=35%, list=21%, signal=29%	MAPRE2/SCRIB/GPLD1/CALM1/EGLN3/RGS16/ARHGEF5/PCOLCE/TBC1D8/SGSM1/MYO9A/AIFM1/MUL1/IFI16/LIMS1/ARHGAP24/BCL2L13/MAP4K4/GNA15/BCL2L11/CR1/RGP1/FYN/PPM1F/NLRP3/APAF1/PPP1R15A/C6orf89/ERBB2/LARS1/TIAM1/PPARGC1B/TSC2/ROCK2/LRRK2/RANGAP1/RALGAPA1/NDEL1/TBC1D17/TBC1D3I/C5AR1/CDKL5/SIPA1L1/RAP1GAP/PYCARD/SEMA4D/NR1H2/DAP/ARAP1/SOS2/ABHD5/PREX1/EPHA4/PDPK1/TBC1D22A/GNAQ/PML/PKP4/RALBP1/PSME4/RALGAPA2/AKIRIN2/EVI5L/TBC1D10B/RABGAP1/RGS6/NLRC4/NEDD9/ATP2A3/MAP3K5/VSIR/CORO1C/BCAP31/ADAP2/DOCK8/PTK2B/VRK3/BCR/RAPGEF2/BCAS3/RCN3/TBC1D22B/PRKD2/RASGRP4/GMIP/ADAP1/VAV1/SFRP2/GSN/PTPRC/SIPA1L2/SYK/DVL3/FIS1/PSME3/PTAFR/ASAP1/ASAP2/CASP2/CALM3/VCP/TBC1D13/HSPA1A/SIPA1/PLEK/LYN/HIP1/LGALS9/CYFIP2/LAPTM5/GSK3B/PSAP/ARHGAP6/TBC1D25/IQSEC1/RHOA/ARHGAP45/BBC3/NF1/GRN/RSU1/GNA12/PINK1/SNCA/TGM2/TBC1D20/RAPGEF1/FLCN/STAT3/TBC1D5/AKT2/SH3BP1/TBC1D1/CTSD/MBP/TBC1D2/P2RX1/RHOG

MF	GO:0008047	enzyme activator activity	406	-0.44	-1.75	1.20E-08	2.63E-07	1.62E-07	1932	tags=28%, list=16%, signal=24%	ARHGEF12/DBNL/RASA2/PPP1R15A/STK4/PRKCE/NCF2/ARHGAP19/LARS1/NCF4/CCNY/ACAP2/PHACTR4/TSC2/ARHGAP9/ARRB1/LRRK2/RANGAP1/RALGAPA1/PLCB1/TBC1D17/RASAL3/TBC1D3I/DOCK5/STX4/GIT2/MAP2K2/SIPA1L1/RAP1GAP/ARHGDIB/PYCARD/ARAP1/DAZAP2/PREX1/PDPK1/TBC1D22A/GNAQ/NCF1/RALBP1/PSME4/RALGAPA2/EVI5L/TBC1D10B/ALOX5AP/RABGAP1/UBE2L3/RGS6/ARHGAP31/NLRC4/ATP2A3/VSIR/GREM1/ARHGAP21/ADAP2/BCR/RAPGEF2/BCAS3/TBC1D22B/RIN1/GMIP/MOB3A/IGF2/TAOK1/BRPF1/ADAP1/SFRP2/STK11/SMAP2/FZR1/STRADA/SIPA1L2/PSME3/MYO9B/ASAP1/PPP1R12B/ASAP2/ARHGAP8/CALM3/DOCK2/IQGAP2/LTF/PPP2R5B/VCP/TBC1D13/MADD/SIPA1/MARK2/GAPVD1/PSAP/ARHGAP6/TBC1D25/ARHGAP45/NF1/ARHGDIA/RIN3/PINK1/ARHGAP26/CDKN1A/TBC1D20/DTX3L/FLCN/TAL1/DPM2/TBC1D5/SH3BP1/TBC1D1/PAK2/ARHGAP1/WNK1/TBC1D2/NPRL3/IQGAP1
BP	GO:0051336	regulation of hydrolase activity	644	-0.40	-1.62	1.28E-08	2.78E-07	1.70E-07	2416	tags=32%, list=21%, signal=27%	MAPRE2/SCRIB/RFFL/GPLD1/CALM1/EGLN3/NCKAP1L/CRY2/BCL2L12/RGS16/ARHGEF5/PCOLCE/TBC1D8/SGSM1/MYO9A/AIFM1/MUL1/IFI16/LIMS1/ARHGAP24/BCL2L13/SH3RF1/SERPINA1/MAP4K4/CDKN2D/PPP6R1/SERPING1/GNA15/BCL2L11/CR1/KNG1/RGP1/FYN/PPM1F/NLRP3/SRC/APAF1/FKBP1B/RASA2/MAP2K5/PPP1R15A/XIAP/C6orf89/ERBB2/LARS1/TIAM1/PPARGC1B/MGMT/TSC2/ARHGAP9/ROCK2/LRRK2/RANGAP1/HGF/RALGAPA1/NDEL1/OASL/TBC1D17/RASAL3/PI3/TBC1D3I/C5AR1/MGAT5/CDKL5/NRDC/CAST/UBE2Z/SIPA1L1/RAP1GAP/SORT1/PYCARD/SEMA4D/NR1H2/DAP/TMBIM6/ARAP1/SOS2/ABHD5/PREX1/EPHA4/PDPK1/DHCR24/TBC1D22A/GNAQ/PML/PKP4/RALBP1/PSME4/RALGAPA2/AKIRIN2/EVI5L/TBC1D10B/BIRC6/LEPR/RABGAP1/CHP1/RGS6/PCSK1N/NLRC4/NEDD9/AMOT/ATP2A3/CSNK2A1/MAP3K5/VSIR/SORL1/CORO1C/BCAP31/PRDX5/ADAP2/TIMP2/HTT/PCSK6/DOCK8/PTK2B/FGD4/VRK3/BCR/RAPGEF2/BCAS3/RCN3/TBC1D22B/SBF2/PRKD2/RASGRP4/GMIP/SBF1/FGD3/MMP9/ADAP1/VAV1/POR/SFRP2/GSN/PLXNC1/APLP2/PTPRC/SIPA1L2/SYK/DVL3/THBS1/FIS1/PSME3/PTAFR/ASAP1/ASAP2/CASP2/CALM3/IQGAP2/LTF/VCP/TBC1D13/HSPA1A/GPI/SIPA1/PLEK/AQP1/LYN/HIP1/LGALS9/CYFIP2/LAPTM5/GSK3B/GAPVD1/PSAP/LRP1/GNAI2/FKBP1A/ARHGAP6/TBC1D25/IQSEC1/RHOA/GPX1/ARHGAP45/BBC3/ARRB2/NF1/GRN/PPP1R9B/TERF2/RSU1/GNA12/UBE2O/PINK1/RPS6KA1/SNCA/SIAH2/TGM2/TBC1D20/RAPGEF1/FLCN/STAT3/RIPOR2/PSMF1/TBC1D5/AKT2/SH3BP1/TBC1D1/CTSB/PAK2/CTSD/MBP/WNK1/TBC1D2/P2RX1/TIMP1/IQGAP1/RHOG/FURIN

BP	GO:0051130	positive regulation of cellular component organization	717	-0.39	-1.60	1.32E-08	2.86E-07	1.76E-07	2573	tags=32%, list=22%, signal=27%	STAU2/CLASP1/AMIGO1/AMBRA1/BMF/TWF2/CDC42EP1/LGALS3/FSCN1/CALCOCO2/PPIF/BAK1/FIG4/PLAUR/ACTR2/ANXA2/CFLAR/RALB/CCT7/MAPRE2/UBE2B/ENDOG/TPPP3/SIRT6/NCKAP1L/SCIN/ARHGEF5/NFE2L2/TF/MUL1/EPHB2/LIMS1/PTK7/STK25/MAP4K4/NIN/TNXB/VPS4B/BCL2L11/KATNB1/DISC1/MACF1/SRF/FYN/WNT1/FEZ1/MAP3K4/TIRAP/PPM1F/SRC/CDC27/KCTD17/FNIP2/SH3GLB1/FKBP1B/ATM/KIDINS220/ADNP/DBNL/PNKP/PPP1R15A/PRKCE/SDC4/ATP7A/HCK/EPS8/TGFB1/SNX3/TIAM1/G3BP1/CUL7/SETDB1/VPS13D/ANO6/SDCBP/HSPA5/RETREG3/ROCK2/ARRB1/LRRK2/HGF/ENC1/PLCB1/CKAP5/AP2B1/NDEL1/SYNJ1/DLG4/DBN1/RAD21/PIP4K2C/WASF3/EPHB1/MAPK14/CDKL5/NF2/PTPRJ/FLOT1/MSX1/CLSTN1/SETX/EIF5A/WASHC2C/PYCARD/CDC42EP4/SEMA4D/NR1H2/CD63/SLAIN2/ARAP1/FUZ/CLN3/VPS35/EPHA4/PML/RALBP1/NUMA1/WDR1/LCP1/TRIM28/GSK3A/GRB2/INSR/STK24/ADGRL1/CNOT6/TPM1/SF3A2/FES/GREM1/VLDLR/PLEKHM2/NFATC2/CORO1C/HTT/PTK2B/NEURL1/SEMA4A/WARS1/RAPGEF2/EIF4G2/BCAS3/COLGALT1/LDLRAP1/DYNC1H1/MARK4/PAK1/IGF2/ANKFY1/MMP9/STK11/GSN/PLXNC1/PPP3CA/MAP2K7/WDR45/SYK/DVL3/FIS1/SCN1B/ASAP1/RAB31/ATP1B2/TLR6/IST1/CD151/DCTN1/CAMK1D/PPP2R5B/FLNA/EIF4G1/ULK1/DNM3/VCP/HSPA1A/VPS28/GNL3L/PLEK/CD14/RAC1/MARK2/LYN/HIP1/HNRNPK/SIN3A/GSK3B/ELAPOR1/RAC2/DNM2/LRP1/STMP1/PPP1R10/PXN/IQSEC1/RHOA/GPX1/BBC3/CLU/ARRB2/ATG2A/MSN/ADD1/GRN/TERF2/HCLS1/WAS/PINK1/MAPK3/SNCA/RAPGEF1/PTPN23/EHD1/CTTN/PIP4K2A/CLDN5/RIPOR2/PLEK2/TAL1/TBC1D5/AKT2/AP2M1/MED25/VASP/DAB2/TRIM58/CNOT1/CUX1/FBXO7/CBL/CDC34/WASF2
BP	GO:0006898	receptor-mediated endocytosis	172	-0.54	-1.96	1.58E-08	3.39E-07	2.08E-07	2250	tags=40%, list=19%, signal=32%	TF/AAK1/TNK2/CLTB/FCER1G/REPS1/SNAP25/GRK3/ITSN1/DBNL/AP1G1/AP2A2/SDCBP/ANKRD13A/NUMB/ITGAM/ARRB1/AP2B1/SYNJ1/SNX17/EZR/AP2S1/DLG4/PIKFYVE/GRK2/FLOT1/ATXN2/CD63/CLN3/ITGB2/CAP1/ARF1/PIP5K1C/CXCL16/GRB2/CANX/INSR/GREM1/AP2A1/VLDLR/SORL1/CXCR2/LDLRAP1/PDLIM7/SYK/BMP2K/RAB31/CLTC/DNM3/CD14/RAC1/HIP1/HNRNPK/DNM2/IGF2R/LRP1/CLU/ARRB2/CXCR1/RIN3/SNCA/CTTN/TBC1D5/AP2M1/GAK/ITGB3/DAB2/CBL
BP	GO:0042327	positive regulation of phosphorylation	504	-0.42	-1.66	1.71E-08	3.65E-07	2.24E-07	2051	tags=27%, list=17%, signal=23%	FGR/FYN/LIMK2/WNT1/MAP3K4/TIRAP/SRC/EPHB4/FNIP2/ATM/GCN1/ADNP/MAP2K5/STK4/KCTD20/SDC4/XRCC6/ERBB2/CCNY/CASS4/WDFY2/MAGED1/TGFB1/DGKZ/PPARGC1B/ERN2/LILRA5/ERN1/SDCBP/SYAP1/ROCK2/ARRB1/LRRK2/HGF/CACUL1/EGR1/ZBTB20/RIPK1/DLG4/EPHB1/S1PR2/MAP2K2/PTPRJ/FLOT1/SEMA4D/MAD2L2/DAZAP2/CLN3/LRRK1/EPHA4/PDPK1/NCF1/RALBP1/CSF1R/ARHGEF2/LEPR/SASH1/ENTPD5/ARNT/PDGFA/NEDD9/INSR/MAP3K5/SLC27A1/TAOK3/FXR2/GREM1/VLDLR/PTK2B/NEURL1/RAPGEF2/RPS6KA4/CCND3/TRAF6/PRKD2/IL6R/SH2D3C/PAK1/MOB3A/IGF2/PECAM1/CSK/BMP6/MMP9/TAOK1/SFRP2/STK11/ITGA5/MAP2K7/PELI2/STRADA/PTPRC/SYK/DVL3/THBS1/PTPN1/TLR6/CALM3/FZD5/LTF/PIK3CG/HIPK2/EIF4G1/MADD/RAC1/MARK2/LYN/MMD/PDE5A/AKAP13/ATP2B4/RHOA/GATA1/CLU/ARRB2/HCLS1/PINK1/MAPK3/SNCA/CDKN1A/FLCN/NIBAN1/TAL1/SQSTM1/AKT2/ARAF/PAK2/ITGB3/PRR5/DAB2/WNK1/IQGAP1/MAP2K3/STRADB/FAXDC2

BP	GO:0042692	muscle cell differentiation	204	-0.52	-1.92	1.79E-08	3.79E-07	2.33E-07	3049	tags=44%, list=26%, signal=33%	SYNE1/SPAG9/ENG/NOS1/HIRA/NPHS1/MECP2/MDM2/NOTCH1/TNFSF14/TMOD3/KIT/PLEKHO1/MYEF2/SMAD1/ADAMTS5/ATP11A/MRTFA/CCN3/CALR/HNRNPU/CAPN3/MAML1/CFLAR/MYOF/FHL2/FLII/CSRP1/SOD2/SRF/WNT1/HDGFL2/ANKRD17/HDAC4/NINJ1/H3-3B/H3-3A/TGFB1/CTDP1/ACTG1/DYRK1B/NFATC1/MAPK14/YY1/FLOT1/MSX1/SORT1/SMARCD3/PTBP1/WDR1/GSK3A/TPM1/GREM1/RBPJ/NFATC2/YBX1/RARA/SLC8A1/SOX6/KLF5/IGF2/PPP3CA/PDLIM5/SLC9A1/FOXO4/SUPT6H/SELENON/LMNA/PLEC/G6PD/SKI/NIBAN2/AKAP13/DYSF/NFATC3/GPX1/ARRB2/KDM6B/ACTN4/BCL9/TMOD1/MTPN/EHD1/RIPOR2/RBM38/ACTN1/MYL9/KEL/MYH9/XK
BP	GO:0043547	positive regulation of GTPase activity	189	-0.54	-1.97	1.83E-08	3.85E-07	2.36E-07	2416	tags=38%, list=21%, signal=30%	MAPRE2/SCRIB/RGS16/ARHGEF5/TBC1D8/SGSM1/MYO9A/LIMS1/ARHGAP24/MAP4K4/RGP1/ERBB2/LARS1/TIAM1/TSC2/RANGAP1/RALGAPA1/NDEL1/TBC1D17/TBC1D3I/CDKL5/SIPA1L1/RAP1GAP/SEMA4D/ARAP1/SOS2/PREX1/TBC1D22A/PKP4/RALBP1/RALGAPA2/EVI5L/TBC1D10B/RABGAP1/RGS6/NEDD9/CORO1C/ADAP2/DOCK8/PTK2B/BCR/RAPGEF2/BCAS3/TBC1D22B/RASGRP4/GMIP/ADAP1/VAV1/SIPA1L2/DVL3/ASAP1/ASAP2/TBC1D13/SIPA1/GSK3B/ARHGAP6/TBC1D25/IQSEC1/ARHGAP45/NF1/RSU1/TGM2/TBC1D20/RAPGEF1/FLCN/TBC1D5/AKT2/SH3BP1/TBC1D1/TBC1D2/RHOG
MF	GO:0051020	GTPase binding	251	-0.49	-1.85	2.19E-08	4.58E-07	2.81E-07	2085	tags=34%, list=18%, signal=29%	RAB11FIP5/TNPO1/RGP1/UNC13D/RRAGD/TRIOBP/EXOC4/BICD2/CDC42BPB/DIAPH2/NCF2/AP1G1/XPO5/ATP7A/TNPO2/EPS8/TSC2/TNPO3/CYRIA/ROCK2/RAB11FIP4/RPH3A/LRRK2/RANGAP1/IPO8/NUTF2/SYTL3/INF2/DOCK5/CDKL5/RAP1GAP/ARHGDIB/GCC1/CDC42EP4/MYCBP2/DAPK3/KPNB1/RALBP1/ARHGEF2/ATP6AP1/EVI5L/LCP1/RABGAP1/OPTN/SPTBN1/SORL1/CORO1C/YBX1/FGD4/RRAGC/RIN1/RASSF5/PAK1/RAB7A/ANKFY1/FGD3/LAMTOR1/DVL3/MYO9B/GNB1/DOCK2/IQGAP2/RAB11FIP1/FLNA/ULK1/TBC1D13/YIPF2/XPO7/FMNL1/CYFIP2/GAPVD1/AKAP13/SYTL4/RILP/RIN3/WAS/XPO6/TBC1D20/EHD1/TMEM127/ENO1/PAK2/YBX3/ARHGAP1/IQGAP1/DIAPH1
BP	GO:0031175	neuron projection development	545	-0.41	-1.65	2.36E-08	4.91E-07	3.01E-07	2312	tags=28%, list=20%, signal=24%	DIP2B/SLC9A3R1/BCL11B/NFE2L2/MYO9A/KIF13B/MUL1/VIM/EPHB2/BCL11A/PTK7/STK25/MAP4K4/RPS6KA5/EFNB1/NIN/STRN/SPRY3/PARD6B/KATNB1/SNAP25/DISC1/MACF1/SRF/FYN/PTEN/FEZ1/TRIOBP/MAP1S/EPHB4/YWHAH/FKBP1B/TRIO/KIDINS220/ADNP/DBNL/BLOC1S3/SDC4/ERBB2/EXT1/DGKG/RNF165/ATP7A/MKS1/SNX3/TIAM1/DICER1/NCAM1/CUL7/CPNE5/SZT2/NOTCH2/HSPA5/NECTIN1/RETREG3/NUMB/LRRK2/HGF/B4GALT5/ENC1/NDEL1/DLG4/DBN1/EPHB1/CPNE1/CDKL5/SIPA1L1/FLOT1/SETX/CAMSAP1/PARD3/FRY/SEMA4D/MYCBP2/ABI3/MAP4/EPHA4/LYPLA2/CSF1R/GSK3A/DPYSL2/ZNF335/STK24/PALLD/SF3A2/FES/TAOK3/TSPO/VLDLR/PTK2B/NEURL1/SEMA4A/RAPGEF2/HERC1/EIF4G2/PAK1/MAP1A/VCL/CTNNA1/RERE/TAOK1/SFRP2/STK11/PLXNC1/PPP3CA/APLP2/USP9X/PDLIM5/DVL3/SCN1B/ASAP1/ATP1B2/IST1/CAMK1D/PPP2R5B/FLNA/ULK1/NIBAN2/DNM3/FRYL/ACTB/RAC1/MARK2/LYN/SIN3A/CYFIP2/GSK3B/RAC2/DNM2/IQSEC1/RHOA/GRN/PPP1R9B/ECE1/CFL1/RAPGEF1/EHD1/CTTN/GAS7/UBB/GAK/BSG/PAK2/VASP/DAB2/MBP/CUX1/CARM1/KEL/FBXO7/IQGAP1/XK/RHOG

BP	GO:0098609	cell-cell adhesion	521	-0.42	-1.67	2.56E-08	5.30E-07	3.25E-07	2215	tags=27%, list=19%, signal=23%	ICAM4/SH2B3/LIMS1/ADAM8/EFNB1/TNXB/CSRP1/CR1/LGALS9C/SRF/FYN/CD93/WNT1/LRRC4/PRDX2/PPM1F/NLRP3/SRC/CD177/ABAT/NINJ1/MAP2K5/SDC4/ERBB2/EXT1/TGFB1/SIRPA/LRFN3/ITPKB/TNFSF4/RC3H2/NECTIN1/ACTG1/ICAM3/ITGAM/STAT5B/NFAT5/EZR/PAG1/RASAL3/DLG4/SHC1/MAPK14/STK10/NF2/ZFP36L1/FLOT1/CLSTN1/PTPN6/PYCARD/SEMA4D/MAD2L2/CTNND1/SPI1/ITCH/FERMT3/PKP4/ITGB2/PCDH1/TJP2/PIP5K1C/PPP1CA/FIBP/S100A11/ADGRL1/PALLD/RUNX3/TMEM131L/VSIR/IRF1/DOCK8/RARA/SELPLG/SPTA1/SELP/TRAF6/TBX21/IGF2/VCL/PECAM1/ZMIZ1/CTNNA1/CSK/BMP6/ZBTB16/VAV1/AKNA/GP6/ITGA5/PDLIM5/PTPRC/SYK/JAM3/PTAFR/ATP1B2/IL1RN/ESAM/PIK3CG/FLNA/JAK3/ACTB/GFUS/SELL/ALOX12/PLEK/LYN/LGALS9/CYFIP2/LAPTM5/PDE5A/RAC2/SIRPB1/FLOT2/HSPB1/ITGA2B/LPP/MPL/RHOA/ITGAL/TNIP1/GATA1/MYADM/PDLIM1/MSN/JAK1/ITGB5/PTPN23/CLDN5/RIPOR2/ZNF703/GP1BA/BSG/MYL9/ITGB3/MBP/WNK1/MYH9/FOXO3/NINJ2/HLX/DMTN/TLN1/RELA
CC	GO:0099512	supramolecular fiber	538	-0.41	-1.64	2.78E-08	5.69E-07	3.49E-07	2437	tags=32%, list=21%, signal=26%	CCT7/MTA1/TBCD/FBF1/MAPRE2/KRT15/CALM1/ASPM/SLC1A4/TUBGCP6/TPPP3/CORO1A/VMAC/MYO5A/MAP1LC3B2/KIF13B/KAT2B/TUBA8/VIM/FHL2/KLHL22/NIN/VPS18/CSRP1/KATNB1/SHROOM1/DISC1/MACF1/FYN/FEZ1/SRC/MAP1S/FKBP1B/HOOK2/DIAPH2/DBNL/SDC4/XIAP/HCK/DCTN4/TPM3/TTLL4/TTLL5/DNAL4/NAV1/SYNE2/TCHP/ACTG1/NBR1/CKAP5/NDEL1/SYNJ1/EZR/TUBB2A/DBN1/KEAP1/CHMP4B/MAP2K2/CDK2AP2/ACTA2/CAMSAP1/EML2/HOOK3/SLAIN2/CLIP4/MYOM1/FHL3/MAP4/INVS/MAP1LC3B/NUMA1/NCKAP5L/ARHGEF2/LCP1/ARF1/DPYSL2/KIF3C/TPM4/EIF6/WIPF2/AMOT/PALLD/TPM1/SPTBN1/MYZAP/KIF1C/CORO1C/SLC2A1/SLC8A1/KIF13A/BCAS3/SVIL/LDLRAP1/DYNC1H1/CALD1/RASSF5/PAK1/TUBA4A/MAP1A/VCL/PDLIM7/ALDOA/NDE1/STK11/AKNA/PPP3CA/DYNLL2/PDLIM5/TPGS2/JAM3/MYO9B/LMNA/PPP1R12B/PLEC/SPECC1/DCTN1/CLTC/CALM3/FLNA/DYNLRB1/EML3/DNM3/ACTB/TUBB4B/CHMP1A/RAC1/GOLGA2/GABARAP/KIF1B/MARK2/RAC2/DNM2/MYL4/HSPB1/AK1/ATP2B4/FKBP1A/ARHGAP6/WIPF1/COTL1/CAPZB/GAS2L1/RTN2/PDLIM1/TUBB/ACTN4/HCLS1/TMOD1/WAS/ESPN/TUBB1/SNCA/PARVB/CTTN/GAS7/LTBP1/SQSTM1/ACTN1/SLC4A1/ENO1/RAB3D/STIM1/KLC3/MYL9/KRT1/MYH9/IQGAP1/DMTN/ANK1/DIAPH1

CC	GO:0099081	supramolecular polymer	539	-0.41	-1.64	2.84E-08	5.79E-07	3.55E-07	2437	tags=32%, list=21%, signal=26%	CCT7/MTA1/TBCD/FBF1/MAPRE2/KRT15/CALM1/ASPM/SLC1A4/TUBGCP6/TPPP3/CORO1A/VMAC/MYO5A/MAP1LC3B2/KIF13B/KAT2B/TUBA8/VIM/FHL2/KLHL22/NIN/VPS18/CSRP1/KATNB1/SHROOM1/DISC1/MACF1/FYN/FEZ1/SRC/MAP1S/FKBP1B/HOOK2/DIAPH2/DBNL/SDC4/XIAP/HCK/DCTN4/TPM3/TTLL4/TTLL5/DNAL4/NAV1/SYNE2/TCHP/ACTG1/NBR1/CKAP5/NDEL1/SYNJ1/EZR/TUBB2A/DBN1/KEAP1/CHMP4B/MAP2K2/CDK2AP2/ACTA2/CAMSAP1/EML2/HOOK3/SLAIN2/CLIP4/MYOM1/FHL3/MAP4/INVS/MAP1LC3B/NUMA1/NCKAP5L/ARHGEF2/LCP1/ARF1/DPYSL2/KIF3C/TPM4/EIF6/WIPF2/AMOT/PALLD/TPM1/SPTBN1/MYZAP/KIF1C/CORO1C/SLC2A1/SLC8A1/KIF13A/BCAS3/SVIL/LDLRAP1/DYNC1H1/CALD1/RASSF5/PAK1/TUBA4A/MAP1A/VCL/PDLIM7/ALDOA/NDE1/STK11/AKNA/PPP3CA/DYNLL2/PDLIM5/TPGS2/JAM3/MYO9B/LMNA/PPP1R12B/PLEC/SPECC1/DCTN1/CLTC/CALM3/FLNA/DYNLRB1/EML3/DNM3/ACTB/TUBB4B/CHMP1A/RAC1/GOLGA2/GABARAP/KIF1B/MARK2/RAC2/DNM2/MYL4/HSPB1/AK1/ATP2B4/FKBP1A/ARHGAP6/WIPF1/COTL1/CAPZB/GAS2L1/RTN2/PDLIM1/TUBB/ACTN4/HCLS1/TMOD1/WAS/ESPN/TUBB1/SNCA/PARVB/CTTN/GAS7/LTBP1/SQSTM1/ACTN1/SLC4A1/ENO1/RAB3D/STIM1/KLC3/MYL9/KRT1/MYH9/IQGAP1/DMTN/ANK1/DIAPH1
BP	GO:0010810	regulation of cell-substrate adhesion	135	-0.57	-2.00	3.05E-08	6.19E-07	3.80E-07	3003	tags=48%, list=26%, signal=36%	NID1/DUSP22/SERPINE1/NOTCH1/POLDIP2/PIK3CB/SMAD3/EPHA1/CDK6/ST6GAL1/SPOCK2/CARMIL1/RIN2/CALR/CLASP1/TBCD/UTRN/LIMS1/PLEKHA2/MAP4K4/ATXN3/DISC1/MACF1/WNT1/PTEN/UNC13D/PPM1F/SRC/TRIOBP/STK4/PRKCE/SDC4/CASS4/RRAS/ACTG1/ROCK2/DOCK5/NF2/PTPRJ/WASHC2C/DAPK3/PREX1/NEDD9/GREM1/CORO1C/PTK2B/BCAS3/VCL/ITGA5/SLC9A1/THBS1/FLNA/GFUS/RAC1/GSK3B/RAC2/DNM2/ARHGAP6/RHOA/MYADM/NF1/ACTN4/RSU1/DAB2/DMTN
MF	GO:0017124	SH3 domain binding	87	-0.64	-2.08	3.40E-08	6.82E-07	4.19E-07	1421	tags=36%, list=12%, signal=32%	SIRPA/SYNJ2/SYNJ1/PTPN6/ABI3/PTPN12/NCF1/GRB2/ARHGAP31/SH3KBP1/PLSCR3/ZNF106/ELMO1/SH2D2A/INPP5D/LYN/DNM2/RUFY1/ARHGAP6/WIPF1/GPX1/HCLS1/WAS/ESPN/RAPGEF1/SH3BP1/VASP/ARHGAP1/SH3BGRL2/CBL/WASF2
BP	GO:0030111	regulation of Wnt signaling pathway	196	-0.52	-1.91	3.63E-08	7.26E-07	4.46E-07	2415	tags=38%, list=21%, signal=30%	UBE2B/AMFR/ASPM/WLS/SLC9A3R1/CTNNBIP1/AXIN2/PTK7/TLR2/FOXO1/DISC1/MACF1/SRC/STK4/XIAP/CCNY/MKS1/TGFB1/SNX3/TIAM1/G3BP1/TSC2/UBR5/RNF213/LRRK2/SMARCA4/EGR1/NFATC1/MAPK14/TLE4/MAD2L2/FUZ/CTNND1/KPNA1/DAPK3/VPS35/CSNK1G2/ATP6V0C/LRRK1/INVS/GNAQ/GSK3A/ZRANB1/PPP1CA/CSNK2A1/TMEM131L/NFKB1/GREM1/RBPJ/FRAT1/FRMD8/OTUD5/TBL1XR1/SFRP2/STK11/USP34/CHD8/SKI/VCP/GSK3B/LRP1/TCF7L2/TBL1X/CSNK1G1/GID8/SIAH2/ZNF703/TLE3/PPM1A/DAB2/WNK1/FAM53B/CTDNEP1/FOXO3
BP	GO:0099536	synaptic signaling	345	-0.45	-1.75	3.83E-08	7.58E-07	4.65E-07	2102	tags=26%, list=18%, signal=22%	GNA15/ATXN3/SNAP25/DISC1/SRF/FYN/PTEN/LRRC4/SRC/EXOC4/YWHAH/ABAT/ADNP/PRKCE/GRIK2/EXT1/DGKZ/OR10H1/PNKD/DLGAP4/SLC12A6/SDCBP/CTBP2/SYAP1/RPH3A/LRRK2/PLCB1/SYNJ1/NPTXR/SYN3/DLG4/DBN1/MME/EPHB1/CACNB1/STX4/CA2/S1PR2/GRK2/SIPA1L1/FLOT1/CLSTN1/CHRNE/CLN3/KPNA1/EPHA4/SHISA7/GNAQ/ARF1/GSK3A/PIP5K1C/HRH2/PRKACA/RARA/PTK2B/NEURL1/BCR/RAPGEF2/MAP1A/PPP3CA/APBA3/FBXL20/GNB1/CALM3/PVALB/DNAJC5/GABARAP/KIF1B/GSK3B/TPRG1L/GNAI2/TSHZ3/PRKCB/STAU1/ARRB2/NF1/PPP1R9B/PINK1/NRGN/RPS6KA1/SNCA/GUCY1A1/PRKAR2B/SQSTM1/NAPA/EIF4EBP2/MBP/RNF10/P2RX1/GLUL/RELA

BP	GO:0071559	response to transforming growth factor beta	153	-0.55	-1.97	4.54E-08	8.89E-07	5.46E-07	2045	tags=34%, list=17%, signal=28%	FYN/WNT1/SRC/APAF1/IL17RD/TGFB1/LDLRAD4/ZBTB7A/PDE2A/SDCBP/HSPA5/ROCK2/ZFP36L1/CDKN1C/FAM89B/SPI1/PDPK1/PML/ZFHX3/PDGFA/RUNX3/SOX6/CHST11/ZMIZ1/TGFB1I1/STK11/USP9X/LTBP2/THBS1/NR3C1/GLG1/HIPK2/SKI/HSPA1A/TGFBRAP1/ZYX/DNM2/CREBBP/PXN/FKBP1A/ARRB2/LRG1/ITGB5/FLCN/STAT3/CLDN5/LTBP1/ZNF703/PPM1A/PAK2/DAB2/FURIN
BP	GO:0032870	cellular response to hormone stimulus	387	-0.43	-1.70	4.65E-08	9.06E-07	5.56E-07	2193	tags=31%, list=19%, signal=26%	PKN1/SAFB/TADA3/VPS18/ATP2B1/FOXO1/BCL2L11/THRA/WNT1/PADI2/SRC/PER1/YWHAH/IRS2/RXRB/DDX54/PRKCE/PAQR8/ZDHHC7/AGTRAP/PPARGC1B/ZBTB7A/TNFSF4/TSC2/UBR5/SYAP1/ROCK2/SMARCC1/RANGAP1/SMARCA4/PLCB1/NDEL1/STAT5B/GAB1/ARID5A/KMT2D/SH2B2/PIP4K2C/SHC1/SRA1/CA2/ZFP36L1/NCOA3/PDPK1/TRIB3/FOXP1/LEPR/GSK3A/UBE2L3/ADIPOR2/GRB2/PDK2/PRKACA/PKM/INSR/SESN3/SLC27A1/FBP1/NFKB1/VPS11/KANK2/NCOA4/NCOA2/SORL1/PELP1/RARA/STAT6/INPP5K/BCAS3/CCND3/MAX/PAK1/IGF2/ZMIZ1/CSK/ZNF106/POR/SLC9A1/FOXO4/PTPN1/GNB1/NCOR2/RAB31/ADIPOR1/MED1/NR3C1/ABHD2/LPIN2/JAK3/HSPA1A/UCP2/RXRA/AQP1/LYN/GSK3B/STAT5A/GNAI2/PXN/RHOA/PRKCB/GAS2L1/GATA1/SP1/JAK1/WDTC1/ARID1A/WBP2/STAT3/PIP4K2A/AHCYL1/AKT2/NCOA1/CTSB/PRKDC/EIF4EBP2/DAB2/CNOT1/CARM1/FOXO3/RELA
MF	GO:0019904	protein domain specific binding	507	-0.41	-1.65	5.06E-08	9.81E-07	6.02E-07	2233	tags=30%, list=19%, signal=26%	VIM/CTNNBIP1/PRRG4/MAVS/TNK2/PLEKHA2/TADA3/REPS1/ATP2B1/STRN/THRA/FYN/WNT1/PTEN/GUSB/SRC/RAB27B/TOP1/EXOC4/YWHAH/IRS2/MOSPD3/KIDINS220/DBNL/GRIK2/NIPBL/SIRPA/PPARGC1B/SYNJ2/AP2A2/ETV6/DICER1/PDE2A/LAMP2/HGS/HSPA5/TFDP2/SYNJ1/JDP2/RRM1/EZR/RIPK1/PAG1/DLG4/KEAP1/SH2B2/CTBP1/MAP2K2/KCNH2/PTPN6/PYCARD/DAP/DDX6/DAZAP2/ABI3/DAPK3/KPNB1/NCOA3/EPHA4/SCAF1/PTPN12/NCF1/NUMA1/HIF1AN/TRIM28/POLR2J/ARF1/TJP2/GRB2/YWHAZ/TRPS1/PRKACA/ARHGAP31/INSR/SH3KBP1/MAP3K5/YWHAB/VPS11/NCOA2/IKBKG/RARA/WARS1/RAPGEF2/ACOX1/PLSCR3/ZNF106/ZBTB16/ELMO1/NDE1/STK11/SYK/SH2D2A/MED1/IST1/CASP2/CLTC/CALM3/BASP1/CHD8/SKI/VCP/HSPA1A/PHRF1/INPP5D/RXRA/CHMP1A/LYN/HNRNPK/ETS2/DNM2/DDB1/RUFY1/TCF7L2/ATP2B4/TRIM11/ARHGAP6/WIPF1/RHOA/TBL1X/TFDP1/GATA1/GPX1/TUBB/ARRB2/FKBP8/SP1/HCLS1/WAS/PINK1/ESPN/RAPGEF1/BCL2L1/PPP3R1/LAMP1/PRKAR2A/PRKAR2B/SQSTM1/ACTN1/SH3BP1/LITAF/AP2M1/NFE2/IKZF1/PRKDC/VASP/ARHGAP1/CNOT1/SH3BGRL2/MYH9/CBL/IQGAP1/WASF2/TLN1/RELA
BP	GO:0022411	cellular component disassembly	357	-0.45	-1.73	5.54E-08	1.07E-06	6.56E-07	2274	tags=35%, list=19%, signal=29%	MAP1LC3B2/SCAF4/AIFM1/C1QA/MUL1/DYRK3/ATG2B/ADAM8/MAP4K4/SSRP1/IRAK3/FLII/VPS4B/DHX8/KATNB1/TRIOBP/APAF1/MAP1S/ATXN7/MCOLN1/USP36/EPS8/TGFB1/ERN2/DICER1/LAMP2/VPS13D/TSC2/RETREG3/SMARCC1/ITGAM/SMARCA4/CKAP5/NDEL1/SYNJ1/SPTAN1/CHMP4B/FLOT1/SETX/CAMSAP1/PHF23/SMARCD3/EIF5A/WASHC2C/CLN3/EPHA4/CLEC16A/MAP1LC3B/NCKAP5L/ARHGEF2/DEDD2/WDR1/LCP1/GLE1/GSK3A/PPP1CA/OPTN/INSR/STX5/SPTBN1/TSPO/UPF1/SH3GL1/HTT/FRAT1/SPTA1/ARID2/SVIL/SMARCC2/MAP1A/MMP9/TAOK1/GSN/WDR45/GRWD1/ATG4D/FIS1/ATG7/IST1/DCTN1/WIPI2/FZD5/ULK1/VCP/RUBCN/HMGA1/PLEK/CHMP1A/GOLGA2/GABARAP/MARK2/GSK3B/LRP1/BNIP3L/HUWE1/TBC1D25/IQSEC1/CAPZB/PRKCB/ATG2A/ADD1/PPP1R9B/TMOD1/ARID1A/ATG13/CFL1/PINK1/SPTB/MTPN/CTTN/TFIP11/SQSTM1/NAPA/SH3BP1/MFN2/GAK/NFE2/SHARPIN/GSPT1/TRIM58/CTDNEP1/FBXO7/MICAL2/FURIN/DMTN

BP	GO:0060070	canonical Wnt signaling pathway	182	-0.53	-1.93	5.62E-08	1.08E-06	6.63E-07	2415	tags=37%, list=21%, signal=30%	UBE2B/AMFR/ASPM/WLS/SLC9A3R1/PORCN/CTNNBIP1/AXIN2/PTK7/FOXO1/DISC1/WNT1/PTEN/SRC/STK4/XIAP/EXT1/CCNY/MKS1/TGFB1/G3BP1/UBR5/LRRK2/EGR1/MAPK14/TLE4/MAD2L2/FUZ/CTNND1/KPNA1/DAPK3/VPS35/CSNK1G2/LRRK1/INVS/GNAQ/GSK3A/PPP1CA/TMEM131L/NFKB1/GREM1/RBPJ/FRAT1/FRMD8/OTUD5/TBL1XR1/SFRP2/STK11/DVL3/FZD5/USP34/CHD8/VCP/GSK3B/TCF7L2/TBL1X/CSNK1G1/GID8/BCL9/SIAH2/ZNF703/TLE3/PPM1A/DAB2/WNK1/FAM53B/CTDNEP1/FOXO3
MF	GO:0008289	lipid binding	505	-0.41	-1.63	6.15E-08	1.18E-06	7.22E-07	2546	tags=33%, list=22%, signal=27%	AGAP1/GGA3/TWF2/APOL6/DBT/SEC14L4/ANXA2/MYOF/FNBP1/ZCCHC2/SPON2/GRAMD1A/ARHGAP32/TECPR1/MCF2L/PCYT1A/TOM1L2/SCIN/ARHGEF5/SCARB1/ZFYVE26/PLCD1/ATG2B/HS1BP3/PLEKHA2/MYO1G/TLR2/FLII/SAP30L/MYO1E/PCTP/DGKA/SNX29/PTEN/TPCN1/TIRAP/PDZD8/SH3GLB1/STARD7/NINJ1/CDIPT/MCOLN1/CPNE3/RASA2/STARD3/PAQR8/DGKG/VDR/NCF4/SNX3/TIAM1/OSBPL1A/UNC119B/AP2A2/CPNE5/SDCBP/HGS/SCAP/ARHGAP9/CPTP/FITM1/TTPAL/GPAA1/GSTP1/RPH3A/FYB1/PLCB1/SNX17/NME4/SYTL3/CD300LF/MME/SHC1/CPNE1/PLEKHF1/S1PR2/OSBPL2/DENND1C/WASHC2C/PARD3/ARAP1/CLN3/OSBPL5/PITPNC1/PREX1/NCF1/MAP1LC3B/NUMA1/TSPO2/ITPR2/ALOX5AP/GLE1/SNX8/PITPNM2/ZFYVE1/SPTBN1/FES/PITPNA/TPP1/TSPO/SH3GL1/ADAP2/RARA/GAB2/SELP/CAVIN2/SNX10/RAPGEF2/BSCL2/BCAS3/SVIL/SBF2/LDLRAP1/ACOX1/RASGRP4/NSFL1C/ANKFY1/ADAP1/SNX11/GSN/WDR45/SLC9A1/THBS1/PTAFR/ASAP1/NR3C1/TLR6/TOM1/WIPI2/FZD5/IQGAP2/LTF/PRAM1/VCP/RUBCN/ANXA6/SELL/RXRA/CD14/MARK2/LYN/SNX27/HIP1/IGF2R/PSAP/RUFY1/SYTL4/DYSF/EPN1/IQSEC1/WDFY3/NF1/ATG2A/NRGN/ARHGAP26/SNCA/EPB41/ANXA11/H2BC11/CYTH4/PLEK2/HDLBP/DENND1A/AP2M1/IQGAP1/OSBP2/TLN1
BP	GO:0048858	cell projection morphogenesis	351	-0.45	-1.73	6.73E-08	1.28E-06	7.86E-07	2312	tags=31%, list=20%, signal=25%	DIP2B/BCL11B/MYO9A/KIF13B/MUL1/EPHB2/BCL11A/STK25/MAP4K4/RPS6KA5/EFNB1/NIN/SPRY3/PARD6B/DISC1/MACF1/SRF/FYN/PTEN/FEZ1/SRC/MAP1S/EPHB4/YWHAH/TRIO/KIDINS220/ADNP/DBNL/ERBB2/EXT1/RNF165/ATP7A/TIAM1/DICER1/NCAM1/CUL7/CPNE5/SZT2/NOTCH2/NECTIN1/NUMB/LRRK2/B4GALT5/NDEL1/DLG4/DBN1/EPHB1/CPNE1/CDKL5/SIPA1L1/FLOT1/PARD3/SEMA4D/MYCBP2/ABI3/EPHA4/LYPLA2/CSF1R/DPYSL2/ZNF335/PALLD/TAOK3/VLDLR/CORO1C/SEMA4A/RAPGEF2/EIF4G2/PAK1/MAP1A/VCL/RERE/TAOK1/STK11/PLXNC1/PPP3CA/APLP2/USP9X/PDLIM5/DVL3/SCN1B/IST1/ULK1/NIBAN2/DNM3/ACTB/RAC1/MARK2/SIN3A/CYFIP2/GSK3B/RAC2/DNM2/RHOA/ECE1/CFL1/CTTN/GAS7/UBB/BSG/PAK2/VASP/MBP/CUX1/KEL/IQGAP1/WASF2/XK/RHOG
CC	GO:0030027	lamellipodium	156	-0.55	-1.95	6.90E-08	1.31E-06	8.02E-07	2062	tags=37%, list=18%, signal=31%	SH3RF1/MYO1G/CDC42BPA/ITSN1/CD177/CDC42BPB/DBNL/DGKZ/PHACTR4/SYNE2/DBN1/WASF3/STX4/NF2/ACTA2/FLOT1/CTNND1/ABI3/FAM89B/ARPC5/SSH1/ARHGAP31/NEDD9/AMOT/PALLD/NHS/CORO1C/DOCK8/PTK2B/FGD4/PAK1/CTNNA1/FGD3/GSN/NRBP1/SLC9A1/IQGAP2/ACTB/RAC1/RAC2/DNM2/FLOT2/PXN/RHOA/CAPZB/PPP1R9B/CFL1/TESC/MYLK/APBB1IP/PARVB/CTTN/PLEK2/ABLIM3/SH3BP1/ITGB3/VASP/WASF2

BP	GO:0048666	neuron development	621	-0.39	-1.59	7.16E-08	1.35E-06	8.28E-07	2312	tags=26%, list=20%, signal=22%	DIP2B/SLC9A3R1/BCL11B/NFE2L2/MYO9A/KIF13B/C1QA/MUL1/VIM/EPHB2/BCL11A/PTK7/STK25/MAP4K4/RPS6KA5/EFNB1/NIN/STRN/SPRY3/SOD2/PARD6B/KATNB1/SNAP25/DISC1/MACF1/SRF/FYN/PTEN/FEZ1/TRIOBP/MAP1S/EPHB4/YWHAH/FKBP1B/TRIO/KIDINS220/ADNP/DBNL/BLOC1S3/SDC4/ERBB2/EXT1/DGKG/RNF165/ATP7A/MKS1/SNX3/TIAM1/DICER1/NCAM1/CUL7/CPNE5/SZT2/NOTCH2/HSPA5/NECTIN1/RETREG3/NUMB/LRRK2/HGF/B4GALT5/ENC1/NDEL1/DLG4/DBN1/EPHB1/CPNE1/CDKL5/SIPA1L1/AGTPBP1/FLOT1/SETX/CAMSAP1/PARD3/FRY/SEMA4D/MYCBP2/CDKN1C/ABI3/MAP4/EPHA4/LYPLA2/CSF1R/GSK3A/DPYSL2/YWHAZ/ZNF335/STK24/PALLD/RUNX3/SF3A2/FES/TAOK3/TSPO/VLDLR/PTK2B/NEURL1/SEMA4A/RAPGEF2/HERC1/EIF4G2/PAK1/MAP1A/VCL/ZMIZ1/CTNNA1/RERE/TAOK1/SFRP2/STK11/PLXNC1/PPP3CA/APLP2/USP9X/PDLIM5/DVL3/SCN1B/ASAP1/ATP1B2/IST1/CAMK1D/PPP2R5B/FLNA/ULK1/NIBAN2/DNM3/FRYL/ACTB/RAC1/MARK2/LYN/SIN3A/CYFIP2/GSK3B/RAC2/DNM2/IQSEC1/RHOA/SCYL1/GRN/PPP1R9B/ECE1/CFL1/PBX1/RAPGEF1/EHD1/CTTN/GAS7/UBB/GAK/BSG/PAK2/VASP/DAB2/MBP/CUX1/CARM1/WNK1/KEL/FBXO7/IQGAP1/XK/RHOG
BP	GO:0009896	positive regulation of catabolic process	413	-0.43	-1.70	7.88E-08	1.48E-06	9.08E-07	2197	tags=32%, list=19%, signal=27%	USP5/SH3RF1/ADAM8/TRIM38/ORMDL3/FOXO1/BCL2L11/ATXN3/DISC1/CSDE1/PTEN/TPCN1/FTO/ARIH1/IRS2/SH3GLB1/ATM/COP1/PRKCE/ATF6/DET1/AREL1/RNF144B/PUM1/RC3H2/VPS13D/TSC2/MTDH/SH3RF3/ROCK2/LRRK2/EZR/SPTLC2/ZBTB20/TFEB/KEAP1/PIP4K2C/PIAS1/TRIB1/CEBPA/PLEKHF1/WAC/NRDC/ZFP36L1/AGTPBP1/CBFA2T3/ARIH2/VPS35/ITCH/ABHD5/ZC3HAV1/KAT5/TRIB3/GSK3A/OPTN/AGO2/ENTPD5/ARNT/INSR/YTHDF3/SESN3/TENT4B/SESN2/STX5/CNOT6/CSNK2A1/VSIR/VPS11/UPF1/SORL1/PIM2/BCAP31/IKBKG/YBX1/HTT/PTK2B/PAFAH1B2/RAB7A/RAD23A/STK11/CPT1A/FZR1/WDR45/PTPN1/GTPBP1/ATG7/ULK1/VCP/HSPA1A/PSMC4/VPS28/GABARAP/LAPTM5/GSK3B/ELAPOR1/ZC3H18/LRP1/WWP2/BNIP3L/DDB1/TNFRSF1B/SUPT5H/HUWE1/PNPLA2/TRIM14/RILP/CLU/ATG2A/MSN/LARP1/OAZ1/PINK1/MARCHF2/MAPK3/SNCA/DTX3L/FLCN/KHSRP/PIP4K2A/AKT2/DNAJB2/SAMD4B/RNF40/DAB2/CNOT1/FBXO7/NPRL3/FOXO3/ZER1/FURIN/BAG6
BP	GO:0014070	response to organic cyclic compound	523	-0.40	-1.62	7.94E-08	1.48E-06	9.10E-07	2204	tags=29%, list=19%, signal=25%	ENO2/PKN1/AXIN2/MAVS/SAFB/TADA3/CDKN2D/IRAK3/TLR2/VPS18/GLB1/ATP2B1/GNA15/RFTN1/SOD2/BCL2L11/PADI2/SRC/PER1/YWHAH/ELK1/SULT1A3/FKBP1B/MTHFR/RXRB/DDX54/ABAT/PRKCE/PAQR8/THBD/LCOR/VDR/TGFB1/PPARGC1B/ZBTB7A/PDE2A/OR10H1/MGMT/UBR5/HSPA5/ROCK2/ABCB11/LRRK2/OPRL1/SMARCA4/PLCB1/STAT5B/KLF16/EZR/EGR1/KMT2D/ARPC1B/CEBPA/ZFP36L1/FLOT1/AQP9/ALAD/EFTUD2/VPS35/NCOA3/GPBAR1/KAT5/ITPR2/FOXP1/SSH1/GSK3A/UBE2L3/HRH2/ALPL/PRKACA/PDGFA/NFKB1/VPS11/FES/KANK2/TSPO/NCOA4/NCOA2/TMEM38A/RARA/PTK2B/SLC8A1/INPP5K/RAPGEF2/SLC3A2/PAK1/ZMIZ1/CTNNA1/BMP6/POR/CPT1A/SLC9A1/THBS1/PTAFR/GNB1/NCOR2/MED1/NR3C1/IL1RN/CALM3/PIK3CG/ABHD2/G6PD/FLNA/JUND/HSPA1A/ACTB/GPI/RXRA/AQP1/LYN/RIOK3/SIN3A/GSK3B/DNM2/TXNIP/CDA/ATP2B4/RHOA/GATA1/GPX1/CLU/ARRB2/MSN/PPP1R9B/LARP1/IFIT1B/ARID1A/PIM1/WBP2/MAPK3/SNCA/TGM2/RAPGEF1/BCL2L1/STAT3/ZNF703/PRKAR2B/FAM210B/BSG/PAK2/DAB2/MBP/CNOT1/GNAZ/CARM1/P2RX1/FOXO3/CBL/TBXA2R/DMTN/DIAPH1

BP	GO:0045216	cell-cell junction organization	113	-0.60	-2.03	8.66E-08	1.61E-06	9.87E-07	2910	tags=50%, list=25%, signal=38%	TNF/NUMBL/KIFC3/AFDN/SMAD3/F11R/EPB41L3/FSCN1/TBCD/FBF1/HDAC7/EPHB2/LIMS1/STRN/PARD6B/HEG1/SRF/SRC/CD177/EXT1/TGFB1/NECTIN1/ACTG1/NUMB/ROCK2/NF2/PARD3/CTNND1/EPHA4/GPBAR1/PKP4/CSF1R/WDR1/TJP2/PIP5K1C/PRKACA/OCEL1/HIPK1/VCL/PECAM1/CTNNA1/CSK/BMP6/JAM3/PLEC/ESAM/FZD5/FLNA/ACTB/RHOA/ADD1/FLCN/PTPN23/CLDN5/ZNF703/PAK2/TLN1
BP	GO:0060348	bone development	130	-0.56	-1.97	8.87E-08	1.64E-06	1.01E-06	2273	tags=37%, list=19%, signal=30%	BBX/SH2B3/AXIN2/ASXL1/ZNF385A/SRF/WNT1/SRC/EXT1/CHSY1/NOTCH2/XYLT1/MAPK14/MSX1/PTPN6/BNC2/SEMA4D/LRRK1/PAPSS2/ATP6AP1/FOXP1/LEPR/ALPL/FOXN3/GREM1/RARA/SNX10/MEIS1/BMP6/ZBTB16/POR/SFRP2/PTPRC/MED1/GLG1/PHOSPHO1/LTF/SKI/ANXA6/RFLNB/AKAP13/RHOA/SLC38A10/TGM2/PIP4K2A/TAL1/MPIG6B/WASF2
BP	GO:0008154	actin polymerization or depolymerization	152	-0.55	-1.96	9.56E-08	1.76E-06	1.08E-06	1995	tags=35%, list=17%, signal=29%	TRIOBP/MSRB2/DIAPH2/DBNL/PRKCE/HCK/EPS8/CYRIA/SPTAN1/ARPC1B/DBN1/WASF3/WASHC2C/PYCARD/CDC42EP4/PREX1/ARPC5/WDR1/SSH1/GRB2/SPTBN1/PTK2B/SPTA1/MSRB1/SVIL/GSN/ESAM/PLEK/RAC1/CYFIP2/ARHGAP6/WIPF1/COTL1/RHOA/CAPZB/MYADM/ADD1/PPP1R9B/SSH2/HCLS1/TMOD1/ARPC4/CFL1/WAS/SPTB/MTPN/CTTN/GAS7/SH3BP1/VASP/MICAL2/DMTN/DIAPH1
CC	GO:0005887	integral component of plasma membrane	725	-0.37	-1.53	9.68E-08	1.77E-06	1.08E-06	2765	tags=28%, list=24%, signal=23%	ALCAM/KCNQ1/KIT/EPHA1/SLC5A6/DIABLO/TM7SF2/NEO1/ATP11A/ZDHHC2/SLC12A7/IFNL1/NCR1/KCNJ2/PTGDR2/MARCO/PLXNA4/APH1A/NCSTN/CD4/P2RY1/LTB4R/PLPPR2/PLAUR/TMC4/KIR2DL3/TRPC3/SLC13A4/SPN/SLCO3A1/CALM1/SLC1A4/ADAM17/CYBB/SLC19A1/NCKAP1L/SIGLEC7/LRRC8B/PI4K2A/LNPEP/PORCN/PCDHB1/SCARB1/KIR2DL1/SLC20A1/EPHB2/CLPTM1/ICAM4/IL2RG/PTK7/ADAM8/SLC28A2/EFNB1/FCER1G/TLR2/ATP2B1/PTGIR/CR1/TLR5/LRRC4/IFNAR2/NKG7/EPHB4/SSPN/SLC24A4/IFNAR1/MCOLN1/CD300C/NCF2/SDC4/GRIK2/THBD/ERBB2/GYPA/KCNJ15/TSPAN18/NCF4/EPS8/KIR3DL2/SIRPA/LRFN3/SLC2A3/ADGRE2/OR10H1/TNFSF4/NOTCH2/SLC12A6/SDCBP/NECTIN1/ICAM3/TSPAN14/ITGAM/OPRL1/TMC8/CMKLR1/DLG4/C5AR1/MME/EPHB1/CLCN5/KCNH2/PTPRJ/FXYD6/PGAP6/SLC16A3/CHRNE/ADGRG3/SEMA4D/HVCN1/TMBIM6/CD63/AQP9/SLC25A11/CD37/EPHA4/SHISA7/GPBAR1/NCF1/ITGB2/KCNQ5/CCR1/CSF1R/KLRD1/PCDH1/CANX/HRH2/INSR/ADGRL1/SLC27A1/CNIH3/SORL1/BCAP31/LRRC8A/PTK2B/SLC2A1/SLC8A1/SELPLG/SELP/SEMA4A/ANKH/SLC6A20/CXCR2/RAPGEF2/TRAF6/SLC24A3/IL6R/PECAM1/IL13RA1/PLXNC1/GP6/ITGA5/TSPAN33/PTPRC/LILRB3/SLC9A1/JAM3/PTAFR/SCN1B/ATP1B2/TLR6/IL17RA/CD151/CALM3/SLC6A9/FZD5/SLC1A5/SLC14A1/SELL/AQP1/LYN/MMD/LAPTM5/ELAPOR1/ADGRE5/SIRPB1/IGF2R/SLC43A1/GP9/LRP1/ITGA2B/ABCC3/ATP2B4/ITGAL/LRFN1/TMPRSS9/ITGB5/LAMP1/SLC4A1/GP1BA/AQP10/TSPAN9/MFSD2B/GYPB/BSG/STIM1/ITGB3/GYPC/P2RX1/SLC38A5/SLC6A8/NINJ2/TBXA2R/TSPAN5
BP	GO:0007179	transforming growth factor beta receptor signaling pathway	117	-0.58	-1.99	9.69E-08	1.77E-06	1.08E-06	2042	tags=35%, list=17%, signal=29%	WNT1/SRC/IL17RD/TGFB1/LDLRAD4/ZBTB7A/SDCBP/HSPA5/CDKN1C/FAM89B/SPI1/PDPK1/PML/CHST11/ZMIZ1/TGFB1I1/STK11/USP9X/LTBP2/THBS1/GLG1/HIPK2/SKI/HSPA1A/TGFBRAP1/ZYX/DNM2/CREBBP/PXN/FKBP1A/ARRB2/LRG1/ITGB5/FLCN/STAT3/CLDN5/LTBP1/ZNF703/PPM1A/DAB2/FURIN

CC	GO:0036477	somatodendritic compartment	456	-0.42	-1.66	1.00E-07	1.82E-06	1.12E-06	2402	tags=30%, list=20%, signal=25%	AMFR/ARHGAP32/SLC1A4/ENDOG/CYBB/PPFIA1/PI4K2A/DIP2B/GIGYF2/MUL1/EPHB2/ENO2/NIN/ATP2B1/STRN/KATNB1/ZNF385A/SNAP25/FYN/DMWD/FEZ1/LRRC4/DNAJB1/TOP1/MAP1S/ELK1/ADNP/DBNL/GRIK2/ATP7A/LRFN3/SLC2A3/CPEB4/OR10H1/G3BP1/CPNE5/NECTIN1/SYAP1/RPH3A/LRRK2/RANGAP1/ENC1/NDEL1/DLG4/DBN1/MME/EPHB1/STX4/CDKL5/SIPA1L1/RAP1GAP/PYCARD/CKB/CTNND1/KPNA1/ABI3/VPS35/MARK3/EPHA4/SHISA7/NCF1/NUMA1/GSK3A/TXNRD2/PPP1CA/CANX/HRH2/AGO2/PRKACA/PDE1A/INSR/CNIH3/FXR2/MCRS1/KIF1C/ERO1A/SORL1/HTT/RARA/PTK2B/NEURL1/SLC8A1/BCR/RAPGEF2/SLC3A2/TBX21/MARK4/MAX/PAK1/MAP1A/PPP3CA/APBA3/SCN1B/ASAP1/PLEC/DCTN1/FZD5/GLRX5/FLNA/DNM3/PVALB/GJC2/RAC1/KIF1B/SLA/MARK2/GSK3B/ROGDI/FLOT2/TNFRSF1B/GNAI2/TRAK2/GRIPAP1/MPL/RHOA/WDFY3/STAU1/CLU/ARRB2/NF1/PPP1R9B/RIN3/GNA12/CASC3/NRGN/SNCA/SIAH2/CTTN/PRKAR2B/UBB/ACTN1/SHARPIN/MBP/GNAZ/GLUL/OSBP2/HCFC1
BP	GO:0061061	muscle structure development	349	-0.44	-1.72	1.02E-07	1.84E-06	1.13E-06	2115	tags=28%, list=18%, signal=23%	FLII/CSRP1/SOD2/HEG1/SRF/WNT1/HDGFL2/ANKRD17/HDAC4/NINJ1/H3-3B/H3-3A/RNF165/TGFB1/CTDP1/ACTG1/BTG1/PLCB1/UNC45A/DYRK1B/EGR1/NFATC1/EPHB1/MAPK14/SRA1/YY1/ZFP36L1/FLOT1/MSX1/SORT1/SMARCD3/FHL3/CMTM5/PTBP1/HIF1AN/WDR1/ZFHX3/GSK3A/HIVEP3/MAP3K5/TPM1/GREM1/RBPJ/NFATC2/YBX1/LRRC8A/RARA/NEURL1/SLC8A1/SOX6/SVIL/KLF5/IGF2/PDLIM7/PPP3CA/PDLIM5/SLC9A1/FOXO4/ZNF609/SUPT6H/SELENON/LMNA/PLEC/MED1/G6PD/BASP1/SKI/NIBAN2/FLOT2/AKAP13/TCF7L2/DYSF/FKBP1A/RHOA/NFATC3/GPX1/PDLIM1/ARRB2/NF1/KDM6B/ACTN4/BCL9/TMOD1/MYLK/MTPN/EHD1/RIPOR2/RBM38/ACTN1/MYL9/YBX3/MBNL3/KEL/MYH9/XK/HLX
BP	GO:0010975	regulation of neuron projection development	260	-0.47	-1.78	1.12E-07	2.02E-06	1.24E-06	2604	tags=34%, list=22%, signal=27%	PLXNA4/STAU2/AMIGO1/TWF2/FIG4/ACTR2/CFLAR/KIAA0319/CD38/DIP2B/NFE2L2/KIF13B/VIM/EPHB2/BCL11A/PTK7/STK25/NIN/KATNB1/SNAP25/DISC1/MACF1/SRF/FYN/PTEN/FEZ1/YWHAH/FKBP1B/KIDINS220/ADNP/DBNL/DGKG/SNX3/TIAM1/CUL7/HSPA5/RETREG3/LRRK2/HGF/ENC1/NDEL1/DBN1/CDKL5/SIPA1L1/SETX/SEMA4D/ABI3/EPHA4/GSK3A/STK24/SF3A2/FES/VLDLR/PTK2B/SEMA4A/RAPGEF2/EIF4G2/PAK1/SFRP2/STK11/PLXNC1/PPP3CA/PDLIM5/SCN1B/ATP1B2/IST1/CAMK1D/PPP2R5B/FLNA/ULK1/DNM3/MARK2/LYN/GSK3B/RHOA/GRN/CFL1/RAPGEF1/EHD1/GAK/PAK2/DAB2/MBP/CUX1/CARM1/KEL/FBXO7/XK
BP	GO:0060429	epithelium development	565	-0.40	-1.60	1.15E-07	2.07E-06	1.27E-06	2077	tags=25%, list=18%, signal=22%	THRA/MYO1E/HEG1/SRF/WNT1/PTEN/KDM5B/VANGL1/EDF1/SRC/TRIOBP/APAF1/MTHFR/ATM/STK4/ATOH8/SDC4/MYCL/EXT1/VDR/VEZF1/ATP7A/MAGED1/MKS1/TGFB1/ZDHHC7/TIAM1/PDE2A/PHACTR4/NOTCH2/MGMT/TSC2/ACTG1/ROCK2/BTG1/HGF/PLCB1/SIDT2/STAT5B/EZR/TGM3/KEAP1/CA2/S1PR2/NF2/ACTA2/ZFP36L1/MSX1/FUZ/CDKN1C/NCOA3/EPHA4/PDPK1/PML/ACADVL/NUMA1/CSF1R/SULT1B1/WDR1/TRIM28/FOXP1/TJP2/GSK3A/GRB2/PPP1CA/TAGLN2/PRKACA/PDGFA/PALLD/SUFU/GREM1/TPP1/KANK2/RBPJ/RARA/BCR/STAT6/RAPGEF2/TRAF6/RCN3/PRKD2/KLF5/PIAS4/PAK1/VCL/PECAM1/BMP6/MMP9/TGFB1I1/SFRP2/CPT1A/ITGA5/FZR1/DVL3/FOSL2/PLEC/MED1/CD151/FZD5/BTBD7/B4GALT1/BASP1/SKI/FLNA/ACTB/RAC1/GSK3B/PSAP/TXNIP/RHOA/FOXJ2/GATA1/MYADM/GPX1/ITPK1/TUBB/FASN/NF1/MSN/KDM6B/FKBP8/ADD1/TMOD1/ECE1/TGM2/TBC1D20/PBX1/RAPGEF1/CLDN5/ZNF703/TST/AKT2/SH3BP1/PIK3CD/CTSB/PRKDC/SHARPIN/VASP/DAB2/KRT1/TOLLIP/IQGAP1/MICAL2/RELA

BP	GO:0031329	regulation of cellular catabolic process	686	-0.38	-1.55	1.23E-07	2.19E-06	1.35E-06	2109	tags=29%, list=18%, signal=25%	ORMDL3/FOXO1/BCL2L11/ATXN3/ALKBH5/DISC1/RGP1/CSDE1/WNT1/PTEN/TPCN1/FEZ1/TIRAP/ATP6V0B/FTO/RRAGD/ARIH1/APAF1/EXOC4/IRS2/SH3GLB1/ATM/ATP6V1B2/COP1/ATF6/USP36/DET1/RNF144B/PUM1/RC3H2/VPS13D/ERN1/SDCBP/TSC2/MTDH/CPTP/EXOC7/SH3RF3/ROCK2/ABCB11/SMARCC1/LRRK2/IKBKE/HGF/EZR/SPTLC2/ZBTB20/TFEB/KEAP1/PIP4K2C/PIAS1/MAPK14/TRIB1/CEBPA/PLEKHF1/WAC/CHMP4B/NRDC/ZFP36L1/AGTPBP1/PHF23/PYCARD/CBFA2T3/DAP/ARIH2/ALAD/CLN3/DAPK3/VPS35/ATP6V0C/QSOX1/ITCH/ABHD5/EPHA4/TRDMT1/PML/ZC3HAV1/CLEC16A/PGAM1/KAT5/TRIB3/LEPR/GSK3A/PPP1CA/OPTN/AGO2/ENTPD5/ARNT/MTMR3/PRKACA/INSR/YTHDF3/SESN3/TENT4B/SESN2/ATP6V0A1/USP10/CNOT6/CSNK2A1/SUFU/VPS11/FXR2/TSPO/UPF1/SORL1/PIM2/BCAP31/IKBKG/YBX1/TIMP2/HTT/PTK2B/PAFAH1B2/RRAGC/HERC1/EIF4G2/PITHD1/MAP1A/RAD23A/STK11/CPT1A/FZR1/WDR45/PSME3/PTPN1/GTPBP1/ATG7/FZD5/PIK3CG/HIPK2/EIF4G1/ULK1/VCP/RUBCN/HSPA1A/PSMC4/CAPN1/CAPNS1/GOLGA2/GABARAP/LAPTM5/GSK3B/ELAPOR1/ZC3H18/PSAP/LRP1/HSPB1/BNIP3L/TNFRSF1B/SUPT5H/ATP2B4/PNPLA2/TBC1D25/TRIM14/GPX1/CLU/ATP6V1A/ATG2A/MSN/LARP1/GNA12/CASC3/PINK1/MARCHF2/MAPK3/SNCA/DTX3L/FLCN/STAT3/CTTN/KHSRP/PIP4K2A/PSMF1/MAPKAPK2/N4BP1/UBB/AKT2/DNAJB2/USP7/ARAF/ATP6V0D1/SAMD4B/TENT5C/RNF40/YBX3/DCAF12/DAB2/CNOT1/FBXO7/TIMP1/NPRL3/FOXO3/ZER1/FURIN/BAG6/RYBP
BP	GO:0071560	cellular response to transforming growth factor beta stimulus	149	-0.55	-1.94	1.27E-07	2.25E-06	1.38E-06	2045	tags=33%, list=17%, signal=28%	FYN/WNT1/SRC/APAF1/IL17RD/TGFB1/LDLRAD4/ZBTB7A/PDE2A/SDCBP/HSPA5/ZFP36L1/CDKN1C/FAM89B/SPI1/PDPK1/PML/PDGFA/SOX6/CHST11/ZMIZ1/TGFB1I1/STK11/USP9X/LTBP2/THBS1/NR3C1/GLG1/HIPK2/SKI/HSPA1A/TGFBRAP1/ZYX/DNM2/CREBBP/PXN/FKBP1A/ARRB2/LRG1/ITGB5/FLCN/STAT3/CLDN5/LTBP1/ZNF703/PPM1A/PAK2/DAB2/FURIN
BP	GO:0048812	neuron projection morphogenesis	338	-0.45	-1.75	1.29E-07	2.28E-06	1.40E-06	2312	tags=31%, list=20%, signal=26%	DIP2B/BCL11B/MYO9A/KIF13B/MUL1/EPHB2/BCL11A/STK25/MAP4K4/RPS6KA5/EFNB1/NIN/SPRY3/PARD6B/DISC1/MACF1/SRF/FYN/PTEN/FEZ1/MAP1S/EPHB4/YWHAH/TRIO/KIDINS220/ADNP/DBNL/ERBB2/EXT1/RNF165/ATP7A/TIAM1/DICER1/NCAM1/CUL7/CPNE5/SZT2/NOTCH2/NECTIN1/NUMB/LRRK2/B4GALT5/NDEL1/DLG4/DBN1/EPHB1/CPNE1/CDKL5/SIPA1L1/FLOT1/PARD3/SEMA4D/MYCBP2/ABI3/EPHA4/LYPLA2/CSF1R/DPYSL2/ZNF335/PALLD/TAOK3/VLDLR/SEMA4A/RAPGEF2/EIF4G2/PAK1/MAP1A/VCL/RERE/TAOK1/STK11/PLXNC1/PPP3CA/APLP2/USP9X/PDLIM5/DVL3/SCN1B/IST1/ULK1/NIBAN2/DNM3/ACTB/RAC1/MARK2/SIN3A/CYFIP2/GSK3B/RAC2/DNM2/RHOA/ECE1/CFL1/CTTN/GAS7/UBB/BSG/PAK2/VASP/MBP/CUX1/KEL/IQGAP1/XK/RHOG
BP	GO:0090130	tissue migration	193	-0.51	-1.89	1.32E-07	2.33E-06	1.43E-06	2659	tags=37%, list=23%, signal=29%	SVBP/RIN2/CCN3/CALR/ZEB2/CLASP1/HDAC6/INSL3/ATP5F1B/MAPRE2/GPLD1/ADAM17/NFE2L2/SCARB1/HDAC7/PKN1/MAP4K4/GLIPR2/MACF1/SRF/PTEN/PPM1F/SRC/EPHB4/MAP2K5/PRKCE/ATOH8/TGFB1/RRAS/ROCK2/DOCK5/ACTA2/PDPK1/FOXP1/SASH1/AMOT/GREM1/KANK2/CORO1C/PTK2B/SEMA4A/BCAS3/PRKD2/MMP9/THBS1/ADIPOR1/PIK3CG/GFUS/GPI/RAC1/MAP3K3/STAT5A/HSPB1/ATP2B4/PXN/IQSEC1/RHOA/GPX1/NF1/SP1/GRN/PTPN23/SPARC/SH3BP1/PIK3CD/PTP4A3/DNAJA4/ITGB3/MYH9/GLUL/TBXA2R/MAP2K3
CC	GO:0030864	cortical actin cytoskeleton	52	-0.71	-2.17	1.40E-07	2.44E-06	1.50E-06	1592	tags=54%, list=14%, signal=47%	SPTAN1/DBN1/NF2/FLOT1/WDR1/CAP1/SPTBN1/MYZAP/SLC2A1/SPTA1/CALD1/GSN/PVALB/FLOT2/AKAP13/LASP1/COTL1/PRKCB/MYADM/ACTN4/PPP1R9B/HCLS1/SPTB/CTTN/CLDN5/ACTN1/MYH9/IQGAP1

BP	GO:0120039	plasma membrane bounded cell projection morphogenesis	350	-0.45	-1.74	1.39E-07	2.44E-06	1.50E-06	2312	tags=31%, list=20%, signal=26%	DIP2B/BCL11B/MYO9A/KIF13B/MUL1/EPHB2/BCL11A/STK25/MAP4K4/RPS6KA5/EFNB1/NIN/SPRY3/PARD6B/DISC1/MACF1/SRF/FYN/PTEN/FEZ1/SRC/MAP1S/EPHB4/YWHAH/TRIO/KIDINS220/ADNP/DBNL/ERBB2/EXT1/RNF165/ATP7A/TIAM1/DICER1/NCAM1/CUL7/CPNE5/SZT2/NOTCH2/NECTIN1/NUMB/LRRK2/B4GALT5/NDEL1/DLG4/DBN1/EPHB1/CPNE1/CDKL5/SIPA1L1/FLOT1/PARD3/SEMA4D/MYCBP2/ABI3/EPHA4/LYPLA2/CSF1R/DPYSL2/ZNF335/PALLD/TAOK3/VLDLR/CORO1C/SEMA4A/RAPGEF2/EIF4G2/PAK1/MAP1A/VCL/RERE/TAOK1/STK11/PLXNC1/PPP3CA/APLP2/USP9X/PDLIM5/DVL3/SCN1B/IST1/ULK1/NIBAN2/DNM3/ACTB/RAC1/MARK2/SIN3A/CYFIP2/GSK3B/RAC2/DNM2/RHOA/ECE1/CFL1/CTTN/GAS7/UBB/BSG/PAK2/VASP/MBP/CUX1/KEL/IQGAP1/WASF2/XK/RHOG
BP	GO:0046777	protein autophosphorylation	174	-0.52	-1.88	1.45E-07	2.52E-06	1.55E-06	2407	tags=39%, list=21%, signal=32%	MKNK1/CALM1/AATK/MVP/PIM3/AAK1/STK25/IRAK3/FGR/FYN/SRC/EPHB4/ATM/CDK12/STK4/ERBB2/HCK/ERN1/LRRK2/DYRK1B/RIPK1/PIKFYVE/CLK3/EPHB1/STK10/CDKL5/MAP2K2/EIF2AK2/DAPK3/MARK3/EPHA4/PDPK1/CSF1R/TRIM28/CHP1/PRKACA/PDGFA/INSR/STK24/FES/TAOK3/GREM1/MKNK2/PIM2/PTK2B/PRKD2/PAK1/CSK/TAOK1/MAP4K1/STK11/PTPRC/SYK/MAPKAPK3/CALM3/PPP2R5B/ULK1/MARK2/LYN/MAP3K3/GSK3B/PIM1/ATG13/PINK1/MAPK3/MAPKAPK2/PAK2/EIF2AK1
CC	GO:0009898	cytoplasmic side of plasma membrane	122	-0.57	-1.98	1.55E-07	2.65E-06	1.63E-06	2183	tags=38%, list=19%, signal=31%	TNK2/ATP2B1/GNA15/SNAP25/FGR/FYN/PTEN/SRC/HCK/TIAM1/SYAP1/SAMD12/EZR/DLG4/TGM3/CHMP4B/OSBPL2/MAP2K2/GNAQ/PKP4/FES/MYZAP/PTK2B/SPTA1/TRAF6/LDLRAP1/PPP3CA/PTPRC/SYK/GNB1/G6PD/RAC1/SLA/LYN/HIP1/GNAI2/RHOA/PPP1R9B/GNA12/SPTB/SLC4A1/LITAF/GNAZ/MYH9/IQGAP1/ANK1
BP	GO:0010631	epithelial cell migration	192	-0.51	-1.88	1.62E-07	2.73E-06	1.67E-06	2659	tags=37%, list=23%, signal=29%	SVBP/RIN2/CCN3/CALR/ZEB2/CLASP1/HDAC6/INSL3/ATP5F1B/MAPRE2/GPLD1/ADAM17/NFE2L2/SCARB1/HDAC7/PKN1/MAP4K4/GLIPR2/MACF1/SRF/PTEN/PPM1F/SRC/EPHB4/MAP2K5/PRKCE/ATOH8/TGFB1/RRAS/ROCK2/DOCK5/PDPK1/FOXP1/SASH1/AMOT/GREM1/KANK2/CORO1C/PTK2B/SEMA4A/BCAS3/PRKD2/MMP9/THBS1/ADIPOR1/PIK3CG/GFUS/GPI/RAC1/MAP3K3/STAT5A/HSPB1/ATP2B4/PXN/IQSEC1/RHOA/GPX1/NF1/SP1/GRN/PTPN23/SPARC/SH3BP1/PIK3CD/PTP4A3/DNAJA4/ITGB3/MYH9/GLUL/TBXA2R/MAP2K3
BP	GO:0090092	regulation of transmembrane receptor protein serine/threonine kinase signaling pathway	133	-0.56	-1.96	1.62E-07	2.73E-06	1.67E-06	1916	tags=44%, list=16%, signal=38%	PELO/TP53/NOTCH1/ACVR1B/SMAD3/FBXL15/BCL9L/NEO1/CCN3/ZEB2/UBE2D3/PMEPA1/KIAA0319/ADAM17/NRROS/WNT1/IL17RD/XIAP/RNF165/TGFB1/LDLRAD4/ZBTB7A/NOTCH2/SDCBP/HSPA5/ZC3H3/NOMO3/MSX1/CDKN1C/FAM89B/PDPK1/NUMA1/GREM1/RBPJ/SORL1/PCSK6/CHST11/BMP6/TGFB1I1/SFRP2/STK11/THBS1/NUP93/GLG1/HIPK2/SKI/HSPA1A/DNM2/CREBBP/LRP1/FKBP1A/FKBP8/LRG1/UBE2O/FLCN/LTBP1/ZNF703/PPM1A/DAB2
BP	GO:0090132	epithelium migration	192	-0.51	-1.88	1.62E-07	2.73E-06	1.67E-06	2659	tags=37%, list=23%, signal=29%	SVBP/RIN2/CCN3/CALR/ZEB2/CLASP1/HDAC6/INSL3/ATP5F1B/MAPRE2/GPLD1/ADAM17/NFE2L2/SCARB1/HDAC7/PKN1/MAP4K4/GLIPR2/MACF1/SRF/PTEN/PPM1F/SRC/EPHB4/MAP2K5/PRKCE/ATOH8/TGFB1/RRAS/ROCK2/DOCK5/PDPK1/FOXP1/SASH1/AMOT/GREM1/KANK2/CORO1C/PTK2B/SEMA4A/BCAS3/PRKD2/MMP9/THBS1/ADIPOR1/PIK3CG/GFUS/GPI/RAC1/MAP3K3/STAT5A/HSPB1/ATP2B4/PXN/IQSEC1/RHOA/GPX1/NF1/SP1/GRN/PTPN23/SPARC/SH3BP1/PIK3CD/PTP4A3/DNAJA4/ITGB3/MYH9/GLUL/TBXA2R/MAP2K3

BP	GO:0018105	peptidyl-serine phosphorylation	219	-0.50	-1.84	1.68E-07	2.82E-06	1.73E-06	2038	tags=30%, list=17%, signal=25%	PTEN/PPM1F/SRC/TOP1/FNIP2/ATM/STK4/PRKCE/TGFB1/ERN1/ROCK2/LRRK2/IKBKE/HGF/STK38/DYRK1B/RIPK1/PIKFYVE/MAPK14/GRK2/MAP2K2/MAD2L2/CSNK1G2/MARK3/PDPK1/MAST2/NSD1/PRKACA/CSNK2A1/HIPK1/MKNK2/VRK3/INPP5K/RPS6KA4/PRKD2/SH2D3C/PAK1/MAP4K1/SFRP2/SYK/PDK3/MAPKAPK3/HIPK2/CAMK1D/EIF4G1/ULK1/MARK2/GSK3B/SRPK2/ATP2B4/PRKCB/CSNK1G1/ARRB2/HCLS1/PINK1/RPS6KA1/SNCA/MAPKAPK2/AKT2/ARAF/PRKDC/PAK2/WNK1/MAST3/DMTN
BP	GO:0031344	regulation of cell projection organization	412	-0.43	-1.68	1.78E-07	2.97E-06	1.83E-06	2312	tags=29%, list=20%, signal=24%	DIP2B/NFE2L2/MYO9A/KIF13B/VIM/EPHB2/BCL11A/ARHGAP24/PTK7/STK25/NIN/SPRY3/KATNB1/SNAP25/DISC1/MACF1/SRF/FYN/LIMK2/WNT1/PTEN/FEZ1/SRC/KCTD17/YWHAH/FKBP1B/KIDINS220/ADNP/DBNL/DGKG/ATP7A/EPS8/SNX3/TIAM1/CUL7/SYNE2/HSPA5/TCHP/RETREG3/LRRK2/HGF/ENC1/NDEL1/EZR/DLG4/DBN1/CDKL5/SIPA1L1/RAP1GAP/SETX/CDC42EP4/SEMA4D/ARAP1/FUZ/CLN3/ABI3/MAP4/VPS35/EPHA4/EVI5L/GSK3A/STK24/SF3A2/FES/FXR2/VLDLR/CORO1C/HTT/PTK2B/NEURL1/SEMA4A/RAPGEF2/EIF4G2/BCAS3/KLF5/MARK4/PAK1/SFRP2/STK11/PLXNC1/PPP3CA/PDLIM5/DVL3/SCN1B/ATP1B2/IST1/CAMK1D/PPP2R5B/FLNA/ULK1/DNM3/RAC1/MARK2/LYN/GSK3B/RAC2/DNM2/RHOA/CAPZB/GRN/CFL1/WAS/RAPGEF1/EHD1/RIPOR2/PLEK2/GAK/PAK2/DAB2/MBP/CUX1/CARM1/KEL/FBXO7/WASF2/XK/RHOG/DMTN
BP	GO:0072659	protein localization to plasma membrane	204	-0.50	-1.85	1.90E-07	3.15E-06	1.93E-06	1678	tags=25%, list=14%, signal=22%	NUMB/TSPAN14/ROCK2/FYB1/EZR/TMBIM1/STX4/PLEKHF1/FLOT1/CLN3/ABI3/VPS35/PDPK1/ATP6AP1/OPTN/SPTBN1/KIF13A/INPP5K/RAPGEF2/LDLRAP1/RAB7A/CSK/TSPAN33/RAB31/EHD3/CLTC/PRAM1/FLNA/ACTB/LRP1/FLOT2/ATP2B4/GRIPAP1/PACS1/MYADM/PPP1R9B/TESC/BCL2L1/EHD1/SQSTM1/AKT2/SLC4A1/AP2M1/GAK/BSG/RAB3D/DAB2/SEC16A/TSPAN5/RHOG/ANK1
BP	GO:0120035	regulation of plasma membrane bounded cell projection organization	403	-0.42	-1.66	1.89E-07	3.15E-06	1.93E-06	2233	tags=28%, list=19%, signal=23%	VIM/EPHB2/BCL11A/ARHGAP24/PTK7/STK25/NIN/KATNB1/SNAP25/DISC1/MACF1/SRF/FYN/LIMK2/WNT1/PTEN/FEZ1/SRC/KCTD17/YWHAH/FKBP1B/KIDINS220/ADNP/DBNL/DGKG/ATP7A/EPS8/SNX3/TIAM1/CUL7/SYNE2/HSPA5/TCHP/RETREG3/LRRK2/HGF/ENC1/NDEL1/EZR/DBN1/CDKL5/SIPA1L1/RAP1GAP/SETX/CDC42EP4/SEMA4D/ARAP1/FUZ/CLN3/ABI3/MAP4/VPS35/EPHA4/EVI5L/GSK3A/STK24/SF3A2/FES/FXR2/VLDLR/CORO1C/HTT/PTK2B/NEURL1/SEMA4A/RAPGEF2/EIF4G2/BCAS3/KLF5/MARK4/PAK1/SFRP2/STK11/PLXNC1/PPP3CA/PDLIM5/SCN1B/ATP1B2/IST1/CAMK1D/PPP2R5B/FLNA/ULK1/DNM3/RAC1/MARK2/LYN/GSK3B/RAC2/DNM2/RHOA/CAPZB/GRN/CFL1/WAS/RAPGEF1/EHD1/RIPOR2/PLEK2/GAK/PAK2/DAB2/MBP/CUX1/CARM1/KEL/FBXO7/WASF2/XK/RHOG/DMTN
BP	GO:0032990	cell part morphogenesis	369	-0.43	-1.69	2.03E-07	3.35E-06	2.06E-06	2312	tags=30%, list=20%, signal=25%	DIP2B/BCL11B/MYO9A/KIF13B/MUL1/EPHB2/BCL11A/STK25/MAP4K4/RPS6KA5/EFNB1/NIN/SPRY3/PARD6B/DISC1/MACF1/SRF/FYN/PTEN/FEZ1/SRC/MAP1S/EPHB4/YWHAH/TRIO/KIDINS220/ADNP/DBNL/ERBB2/EXT1/RNF165/ATP7A/TIAM1/DICER1/NCAM1/CUL7/CPNE5/SZT2/NOTCH2/NECTIN1/NUMB/LRRK2/B4GALT5/NDEL1/DLG4/DBN1/EPHB1/CPNE1/CDKL5/SIPA1L1/FLOT1/PARD3/SEMA4D/MYCBP2/ABI3/EPHA4/LYPLA2/CSF1R/DPYSL2/ZNF335/PALLD/TAOK3/VLDLR/CORO1C/SEMA4A/RAPGEF2/EIF4G2/PAK1/MAP1A/VCL/RERE/TAOK1/STK11/PLXNC1/PPP3CA/APLP2/USP9X/PDLIM5/DVL3/FIS1/SCN1B/PLEC/IST1/ULK1/NIBAN2/DNM3/ACTB/RAC1/MARK2/SIN3A/CYFIP2/GSK3B/RAC2/DNM2/RHOA/ECE1/CFL1/BCL2L1/CTTN/GAS7/UBB/BSG/PAK2/VASP/MBP/CUX1/KEL/IQGAP1/WASF2/XK/RHOG

BP	GO:0051146	striated muscle cell differentiatio n	147	-0.54	-1.91	2.17E-07	3.57E-06	2.19E-06	2770	tags=43%, list=24%, signal=33%	TNFSF14/TMOD3/PLEKHO1/MYEF2/ADAMTS5/ATP11A/CCN3/CALR/HNRNPU/CAPN3/MAML1/CFLAR/MYOF/FHL2/FLII/CSRP1/SRF/WNT1/HDAC4/TGFB1/CTDP1/ACTG1/DYRK1B/MAPK14/YY1/FLOT1/MSX1/SORT1/WDR1/GSK3A/TPM1/GREM1/RBPJ/NFATC2/YBX1/RARA/SLC8A1/SOX6/KLF5/IGF2/PPP3CA/PDLIM5/SLC9A1/SELENON/LMNA/PLEC/G6PD/SKI/NIBAN2/AKAP13/GPX1/ARRB2/KDM6B/BCL9/TMOD1/MTPN/EHD1/RIPOR2/RBM38/MYL9/KEL/MYH9/XK
BP	GO:0071900	regulation of protein serine/threon ine kinase activity	256	-0.47	-1.76	2.48E-07	4.05E-06	2.49E-06	2391	tags=37%, list=20%, signal=30%	CALM1/DUSP16/ADAM17/PRKAR1B/MAP4K2/ARHGEF5/DUSP1/KAT2B/SH2B3/ADAM8/CDKN2D/IRAK3/TNXB/SPRY3/HEG1/PTEN/MAP3K4/SRC/CDK12/MAP2K5/STK4/ERBB2/CCNY/MAGED1/TGFB1/ERN2/ERN1/HGS/SYAP1/GSTP1/LRRK2/STK38/TRIB1/CEBPA/MAP2K2/PTPRJ/PTPN6/PYCARD/CDKN1C/DAZAP2/EPHA4/CCNF/CSF1R/TRIB3/SASH1/PDGFA/INSR/SESN2/MAP3K5/SLC27A1/TAOK3/SORL1/HTT/PTK2B/SLC8A1/INPP5K/RAPGEF2/CCND3/TRAF6/PAK1/IGF2/CSK/TAOK1/SFRP2/MAP2K7/STRADA/PTPRC/SYK/DVL3/THBS1/PTPN1/TLR6/CALM3/HEXIM2/FZD5/LTF/PIK3CG/ACTB/LYN/PDE5A/AKAP13/ATP2B4/RHOA/NF1/CCNI/SNCA/CDKN1A/PRKAR2A/PRKAR2B/CCNJL/DAB2/FBXO7/IQGAP1/CNPPD1
CC	GO:0000785	chromatin	730	-0.37	-1.51	3.19E-07	5.15E-06	3.16E-06	2133	tags=25%, list=18%, signal=22%	H2BC21/NSD3/FOXO1/CBX5/ZNF385A/THRA/PAF1/KLF6/SRF/PADI2/GATAD2A/WDR82/ELK1/ANKRD17/RXRB/ADNP/VPS72/H3-3B/BAZ2A/ATOH8/SRCAP/ATF6/MYCL/NIPBL/STAG1/VDR/H3-3A/POU2F1/MAGED1/H2AC6/WAPL/ETV6/EP400/FANCE/POU2F2/TFDP2/SMARCC1/ARRB1/SMARCA4/ENC1/PLCB1/KDM3B/STAT5B/FOXP2/NFAT5/JDP2/KLF16/FANCG/NFIA/EGR1/TFEB/ASF1B/NFATC1/RAD21/NR6A1/CEBPA/ELL/MXD1/YY1/MSX1/ATF7/SMARCD3/BNC2/DPF2/NR1H2/IRF2/NFKB2/RELB/MAFK/SPI1/NCOA3/PML/ARID1B/CHRAC1/NSD1/RFX2/AKIRIN2/KAT5/ZFHX3/TRIM28/SMARCA2/FOXP1/KLF13/BRMS1/TRPS1/ARNT/SAP130/E2F3/TRRAP/RUNX3/CSNK2A1/FOXN3/NFKB1/MCRS1/RBPJ/UPF1/NCOA2/NFATC2/PELP1/IRF1/RARA/USP3/STAT6/MEIS1/NFIC/ARID2/SOX6/BCAS3/SMARCC2/KLF5/MAX/USF2/ZHX2/SETD5/MXD4/TFE3/EXOSC4/FOXO4/DVL3/FOSL2/SUPT6H/NCOR2/BICRAL/BPTF/NR3C1/MAFB/IST1/GATAD2B/CREB5/BASP1/CHD8/JUND/UBR2/ACTB/HMGA1/RXRA/HNRNPK/MLXIP/SIN3A/ETS2/UBA1/STAT5A/RFX1/CREBBP/SRPK2/TCF7L2/PPP1R10/CHD4/CBX6/TSHZ3/FOXJ2/BRD3/NFATC3/TFDP1/GATA1/H3C10/SP1/ARID1A/PINK1/WBP2/TGM2/PBX1/H2BC11/STAT3/CSRNP1/SP2/TAL1/NFIX/NCOA1/NFE2/IKZF1/KAT6A/KLF1/BRD4/RNF40/E2F2/MXI1/LYL1/CUX1/FOXO3/ELF4/HLX/KLF3/RELA
BP	GO:0071496	cellular response to external stimulus	235	-0.48	-1.78	3.33E-07	5.35E-06	3.29E-06	2303	tags=34%, list=20%, signal=28%	NFE2L2/MAP1LC3B2/MAP2K4/AIFM1/IFI16/ATP2B1/FOXO1/TLR5/SRF/RRAGD/SH3GLB1/GCN1/ADNP/CD68/VDR/LARS1/CPEB4/PDE2A/LAMP2/SZT2/HSPA5/LRRK2/TFEB/EIF2AK2/MAP3K2/DAP/VPS41/CLEC16A/MAP1LC3B/PDK2/MTMR3/SESN3/SESN2/STK24/ZFYVE1/MAP3K5/NFKB1/FES/KANK2/IRF1/SLC2A1/RRAGC/MAX/USF2/BMP6/SFRP2/PTPRC/WDR45/SLC9A1/ATG7/PLEC/MED1/CASP2/WIPI2/EIF4G1/ULK1/RIPOR1/SIPA1/UCP2/RXRA/GABARAP/AQP1/LYN/ELAPOR1/CDA/CNN2/GAS2L1/PPP1R9B/PIM1/MAPK3/CDKN1A/NUAK2/FLCN/MTPN/NCOA1/GLUL/NPRL3/FOXO3/CBL

CC	GO:0005774	vacuolar membrane	387	-0.42	-1.65	3.37E-07	5.40E-06	3.31E-06	2036	tags=27%, list=17%, signal=23%	TPCN1/ATP6V0B/UBA52/SLC29A3/NKG7/FNIP2/SH3GLB1/ATP6V1B2/MCOLN1/CPNE3/STARD3/THBD/CD68/AP1G1/CMTM6/ACP2/SLC66A1/WDR81/AP2A2/LAMP2/SZT2/CKAP4/ARRB1/SIDT2/AP2B1/AP2S1/TMBIM1/TFEB/CPNE1/CLCN5/PLEKHF1/CHMP4B/PGAP6/FLOT1/SORT1/SLC36A1/CD63/IFITM3/AP1M1/VPS41/CLN3/VPS35/ATP6V0C/GNAQ/CLEC16A/MAP1LC3B/CDIP1/ANPEP/ATP6V0A1/YWHAB/NFAM1/VPS11/AP2A1/VLDLR/PLEKHM2/LRRC8A/SBF2/SLC3A2/RAB7A/ANKFY1/RAB14/HGSNAT/ARL8A/SLC44A2/LAMTOR1/GNB1/GAA/TOM1/CLTC/B4GALT1/ULK1/ANXA6/DNAJC5/CHMP1A/GABARAP/LYN/MMD/LAPTM5/ELAPOR1/UBA1/PSAP/AP5B1/LRP1/IFITM2/RILP/WDFY3/ATP6V1A/GRN/ECE1/MARCHF2/AP1B1/DTX3L/FLCN/LAMP1/SLC15A3/LITAF/ATP6V0D1/AP2M1/SLC48A1/RAB3D/CTSD/UBXN6/DAB2/SLC7A5/MARCHF8/NPRL3
BP	GO:0060828	regulation of canonical Wnt signaling pathway	155	-0.54	-1.91	3.40E-07	5.42E-06	3.33E-06	2415	tags=39%, list=21%, signal=31%	UBE2B/AMFR/ASPM/WLS/SLC9A3R1/CTNNBIP1/AXIN2/PTK7/FOXO1/SRC/STK4/XIAP/CCNY/MKS1/TGFB1/G3BP1/UBR5/LRRK2/EGR1/MAPK14/TLE4/MAD2L2/FUZ/CTNND1/KPNA1/DAPK3/VPS35/CSNK1G2/LRRK1/INVS/GNAQ/GSK3A/PPP1CA/TMEM131L/NFKB1/GREM1/RBPJ/FRAT1/FRMD8/OTUD5/TBL1XR1/SFRP2/STK11/USP34/CHD8/VCP/GSK3B/TCF7L2/TBL1X/CSNK1G1/GID8/SIAH2/ZNF703/TLE3/PPM1A/DAB2/WNK1/FAM53B/CTDNEP1/FOXO3
BP	GO:0003018	vascular process in circulatory system	141	-0.55	-1.94	3.48E-07	5.54E-06	3.40E-06	2395	tags=38%, list=20%, signal=30%	CD38/SLCO3A1/SLC1A4/SLC19A1/SLC9A3R1/CTNNBIP1/SLC28A2/ATP2B1/SOD2/KNG1/SRC/EXT1/TGFB1/SLC2A3/PDE2A/ROCK2/DOCK5/ACTA2/PTPRJ/PER2/TJP2/LEPR/ABCG2/HRH2/INSR/ATP2A3/SLC27A1/SLC2A1/SLC8A1/BCR/SLC6A20/SLC24A3/BMP6/PLEC/ATP1B2/SLC6A9/SLC1A5/ABCC4/LRP1/ABCC3/ATP2B4/RHOA/GPX1/ECE1/GUCY1A1/CLDN5/PTP4A3/SLC7A5/KEL/P2RX1/SLC38A5/PLVAP/TBXA2R
BP	GO:0007268	chemical synaptic transmission	321	-0.44	-1.70	3.78E-07	5.97E-06	3.66E-06	2755	tags=33%, list=23%, signal=26%	KIT/APBA1/SYNGAP1/CNP/ZDHHC2/SLC12A7/SNPH/SV2A/NCSTN/CACNB4/P2RY1/MCTP1/STAU2/YWHAG/BTBD9/SCRIB/CD38/SLC1A4/PRKAR1B/ZDHHC3/EPHB2/VPS18/ATXN3/SNAP25/DISC1/SRF/FYN/PTEN/LRRC4/SRC/EXOC4/YWHAH/ABAT/ADNP/PRKCE/GRIK2/EXT1/DGKZ/OR10H1/PNKD/DLGAP4/SLC12A6/SDCBP/CTBP2/SYAP1/RPH3A/LRRK2/PLCB1/SYNJ1/NPTXR/SYN3/DLG4/DBN1/MME/EPHB1/CACNB1/STX4/CA2/S1PR2/SIPA1L1/FLOT1/CLSTN1/CHRNE/CLN3/EPHA4/SHISA7/ARF1/GSK3A/PIP5K1C/HRH2/PRKACA/RARA/PTK2B/NEURL1/BCR/RAPGEF2/MAP1A/PPP3CA/APBA3/FBXL20/CALM3/PVALB/DNAJC5/GABARAP/KIF1B/GSK3B/TPRG1L/GNAI2/TSHZ3/PRKCB/STAU1/ARRB2/NF1/PPP1R9B/PINK1/NRGN/RPS6KA1/SNCA/GUCY1A1/PRKAR2B/SQSTM1/NAPA/EIF4EBP2/MBP/P2RX1/GLUL
BP	GO:0098916	anterograde trans-synaptic signaling	321	-0.44	-1.70	3.78E-07	5.97E-06	3.66E-06	2755	tags=33%, list=23%, signal=26%	KIT/APBA1/SYNGAP1/CNP/ZDHHC2/SLC12A7/SNPH/SV2A/NCSTN/CACNB4/P2RY1/MCTP1/STAU2/YWHAG/BTBD9/SCRIB/CD38/SLC1A4/PRKAR1B/ZDHHC3/EPHB2/VPS18/ATXN3/SNAP25/DISC1/SRF/FYN/PTEN/LRRC4/SRC/EXOC4/YWHAH/ABAT/ADNP/PRKCE/GRIK2/EXT1/DGKZ/OR10H1/PNKD/DLGAP4/SLC12A6/SDCBP/CTBP2/SYAP1/RPH3A/LRRK2/PLCB1/SYNJ1/NPTXR/SYN3/DLG4/DBN1/MME/EPHB1/CACNB1/STX4/CA2/S1PR2/SIPA1L1/FLOT1/CLSTN1/CHRNE/CLN3/EPHA4/SHISA7/ARF1/GSK3A/PIP5K1C/HRH2/PRKACA/RARA/PTK2B/NEURL1/BCR/RAPGEF2/MAP1A/PPP3CA/APBA3/FBXL20/CALM3/PVALB/DNAJC5/GABARAP/KIF1B/GSK3B/TPRG1L/GNAI2/TSHZ3/PRKCB/STAU1/ARRB2/NF1/PPP1R9B/PINK1/NRGN/RPS6KA1/SNCA/GUCY1A1/PRKAR2B/SQSTM1/NAPA/EIF4EBP2/MBP/P2RX1/GLUL

BP	GO:0060341	regulation of cellular localization	584	-0.38	-1.54	3.81E-07	5.98E-06	3.67E-06	2112	tags=28%, list=18%, signal=24%	VPS4B/ORMDL3/PARD6B/RAB11FIP5/HSPA1L/FGR/FYN/LIMK2/PTEN/FEZ1/UNC13D/SRC/SH3GLB1/FKBP1B/PRKCE/SDC4/NUP214/ERBB2/USP36/SUMO3/MAGED1/TGFB1/ZDHHC7/WAPL/SNX3/ADGRE2/NPEPPS/SDCBP/HGS/UBR5/ANKRD13A/NUMB/ROCK2/UBE2J2/ITGAM/LRRK2/RANGAP1/SIDT2/AP2B1/NDEL1/EZR/TMBIM1/DLG4/KAT7/MAPK14/STX4/POLR1A/NF2/MAP2K2/YOD1/ZFP36L1/PTPN6/TMBIM6/ARIH2/CLN3/ABI3/VPS35/SPI1/SHISA7/PDPK1/PML/ITGB2/NUMA1/LCP1/TRIM28/GSK3A/UBE2L3/CHP1/PRKACA/SESN2/SPTBN1/SUFU/VPS11/FES/MCRS1/RBM22/SORL1/TMEM38A/BCAP31/HTT/FRAT1/PTK2B/MFHAS1/SLC8A1/GAB2/INPP5K/BCAS3/LDLRAP1/DYNC1H1/MARK4/RASSF5/NSFL1C/MAP1A/RAB7A/CTNNA1/CSK/STK11/GSN/SYK/FIS1/PTPN1/ILRUN/SUPT6H/SELENON/LMNA/ARHGAP8/CLTC/CALM3/SLC6A9/FZD5/FLNA/VCP/RIPOR1/ACTB/VPS28/GNL3L/UCP2/LYN/LGALS9/SIN3A/GSK3B/RAC2/RAB11B/LRP1/BNIP3L/TCF7L2/HUWE1/ATP2B4/FKBP1A/GRIPAP1/MEPCE/GATA1/BBC3/NF1/MSN/PPP1R9B/HCLS1/OAZ1/ATG13/PINK1/MAPK3/SNCA/EPB41/TBC1D20/DTX3L/BCL2L1/FLCN/PTPN23/EHD1/RIPOR2/LAMP1/ABLIM3/SQSTM1/AKT2/USP7/AP2M1/PPM1A/DAB2/ARHGAP1/TRIM58/P2RX1/GLUL/RHOG/DMTN/DIAPH1
CC	GO:0031253	cell projection membrane	197	-0.50	-1.85	4.14E-07	6.48E-06	3.98E-06	2379	tags=32%, list=20%, signal=26%	ADAM17/UTRN/SLC19A1/DDX58/SLC9A3R1/SLC28A2/CLTB/ATP2B1/MACF1/FGR/TIRAP/SRC/ATP7A/EPS8/ADGRE2/PDE4A/SYNE2/EZR/TMEM67/STX4/CDKL5/NF2/PTPRJ/SHISA7/ARHGEF2/LCP1/PIP5K1C/ABCG2/CA4/INSR/TPM1/SPTBN1/CORO1C/DOCK8/INPP5K/SH2D3C/PAK1/ITGA5/ASAP1/ATP1B2/EHD3/B4GALT1/GPI/PLEK/RAC1/AQP1/DNM2/RHOA/ARHGAP45/MSN/PPP1R9B/GNA12/CFL1/TESC/EHD1/RIPOR2/PLEK2/AKT2/ITGB3/VASP/SLC7A5/DMTN/DIAPH1/TLN1
CC	GO:0031226	intrinsic component of plasma membrane	761	-0.36	-1.50	4.93E-07	7.63E-06	4.69E-06	2765	tags=28%, list=24%, signal=23%	ALCAM/KCNQ1/KIT/EPHA1/SLC5A6/DIABLO/TM7SF2/NEO1/ATP11A/ZDHHC2/SLC12A7/IFNL1/NCR1/KCNJ2/PTGDR2/MARCO/PLXNA4/APH1A/NCSTN/CD4/P2RY1/LTB4R/PLPPR2/PLAUR/TMC4/KIR2DL3/TRPC3/SLC13A4/SPN/SLCO3A1/CALM1/SLC1A4/ADAM17/CYBB/SLC19A1/NCKAP1L/SIGLEC7/LRRC8B/PI4K2A/LNPEP/PORCN/PCDHB1/SCARB1/KIR2DL1/SLC20A1/EPHB2/CLPTM1/ICAM4/IL2RG/PTK7/ADAM8/SLC28A2/EFNB1/FCER1G/TLR2/ATP2B1/PTGIR/CR1/TLR5/LRRC4/IFNAR2/NKG7/EPHB4/CD177/SSPN/SLC24A4/IFNAR1/MCOLN1/CD300C/NCF2/SDC4/GRIK2/THBD/ERBB2/GYPA/KCNJ15/TSPAN18/NCF4/EPS8/KIR3DL2/SIRPA/LRFN3/SLC2A3/ADGRE2/OR10H1/TNFSF4/NOTCH2/SLC12A6/SDCBP/NECTIN1/ICAM3/TSPAN14/ITGAM/OPRL1/TMC8/CMKLR1/DLG4/C5AR1/MME/EPHB1/CLCN5/KCNH2/PTPRJ/FXYD6/PGAP6/SLC16A3/CHRNE/ADGRG3/SEMA4D/HVCN1/TMBIM6/CD63/AQP9/SLC25A11/CD37/EPHA4/SHISA7/GPBAR1/NCF1/ITGB2/KCNQ5/CCR1/CSF1R/KLRD1/PCDH1/ADIPOR2/CANX/HRH2/CA4/INSR/ADGRL1/SLC27A1/CNIH3/SORL1/BCAP31/LRRC8A/PTK2B/SLC2A1/SLC8A1/SELPLG/SPTA1/SELP/SEMA4A/ANKH/SLC6A20/CXCR2/RAPGEF2/TRAF6/SLC24A3/IL6R/PECAM1/IL13RA1/PLXNC1/GP6/ITGA5/TSPAN33/PTPRC/LILRB3/SLC9A1/JAM3/PTAFR/SCN1B/ATP1B2/ADIPOR1/TLR6/IL17RA/CD151/CALM3/SLC6A9/FZD5/SLC1A5/SLC14A1/SELL/CD14/AQP1/LYN/MMD/LAPTM5/ELAPOR1/ADGRE5/SIRPB1/IGF2R/SLC43A1/GP9/LRP1/ITGA2B/ABCC3/ATP2B4/ITGAL/LRFN1/TMPRSS9/ITGB5/TGM2/SPTB/LAMP1/SLC4A1/GP1BA/AQP10/TSPAN9/MFSD2B/GYPB/BSG/STIM1/ITGB3/GYPC/P2RX1/SLC38A5/SLC6A8/NINJ2/TBXA2R/TSPAN5

BP	GO:0110053	regulation of actin filament organization	192	-0.50	-1.84	5.03E-07	7.76E-06	4.76E-06	2007	tags=31%, list=17%, signal=26%	PPM1F/TRIOBP/DBNL/PRKCE/SDC4/HCK/EPS8/CYRIA/ACTG1/ROCK2/SPTAN1/ARPC1B/DBN1/WASF3/NF2/WASHC2C/PYCARD/CDC42EP4/ARAP1/ARPC5/WDR1/ALMS1/ARF1/SSH1/GRB2/TPM1/SPTBN1/PTK2B/SPTA1/INPP5K/SVIL/PAK1/GSN/SLC9A1/ESAM/FLNA/PLEK/RAC1/CYFIP2/PXN/ARHGAP6/COTL1/RHOA/CAPZB/MYADM/ADD1/SSH2/HCLS1/TMOD1/ARPC4/WAS/SPTB/MTPN/CTTN/SH3BP1/PAK2/VASP/WASF2/DMTN
BP	GO:0070527	platelet aggregation	53	-0.69	-2.12	6.09E-07	9.35E-06	5.74E-06	1421	tags=40%, list=12%, signal=35%	PTPN6/FERMT3/FIBP/VCL/GP6/SYK/PIK3CG/FLNA/ACTB/ALOX12/PLEK/LYN/HSPB1/MPL/GATA1/GP1BA/MYL9/ITGB3/MYH9/DMTN/TLN1
BP	GO:0045087	innate immune response	541	-0.39	-1.57	6.16E-07	9.42E-06	5.78E-06	2499	tags=31%, list=21%, signal=25%	CALCOCO2/VAMP3/SETD2/ACTR2/TRIM35/NUB1/SERINC5/SPON2/CYBB/CORO1A/DDX58/SIGLEC10/MAP4K2/NFE2L2/PADI4/USP27X/TRIM5/RAB43/C1QA/MUL1/IFI16/VIM/RNF135/MAVS/TNK2/ADAM8/TRIM38/SRPK1/PLD4/FCER1G/IRAK3/APOBEC3D/TLR2/H2BC21/SERPING1/CR1/TLR5/FGR/FYN/TIRAP/UNC13D/NLRP3/IFNAR2/SRC/NKG7/CD177/ANKRD17/IFNAR1/NCF2/XIAP/XRCC6/AP1G1/LYST/HCK/SIRPA/G3BP1/LILRA5/ACTG1/LSM14A/ITGAM/IKBKE/STAT5B/CNPY3/OASL/ARID5A/ALPK1/PI3/STX4/EIF2AK2/PTPN6/PYCARD/CDC42EP4/NR1H2/IFITM3/RELB/TREM1/DAPK3/SPI1/ITCH/NCF1/PML/ZC3HAV1/CSF1R/ARHGEF2/AKIRIN2/CCDC92/KLRD1/TRIM28/CXCL16/OPTN/NLRC4/CHID1/YTHDF3/RNASET2/MAP3K5/FES/PLEKHM2/IKBKG/IRF1/PGLYRP1/PTK2B/MFHAS1/MSRB1/TRAF6/PAK1/VAV1/LCN2/GSN/SYK/ADAR/PTPN1/ILRUN/ECSIT/MED1/IL17RA/NLRX1/RBM14/LTF/PIK3CG/JAK3/TUBB4B/ZYX/CD14/SLA/LYN/RIOK3/LGALS9/SIN3A/CREBBP/APOBEC3F/FBXO9/STMP1/IFITM2/SRPK2/TRIM11/TRIM14/TUBB/CLU/ARRB2/APOBEC3C/GRN/JAK1/IFIT1B/TRIM10/RNF187/WAS/MARCHF2/ZNFX1/HK1/SEC14L1/SNCA/TRIM26/H2BC11/DTX3L/PCBP2/LAMP1/SLC15A3/N4BP1/PIK3CD/TREML1/PRKDC/PAK2/KRT1/TRIM58/TOLLIP/ELF4/RELA
CC	GO:0005884	actin filament	77	-0.63	-2.02	6.20E-07	9.45E-06	5.80E-06	2045	tags=53%, list=17%, signal=44%	FYN/SRC/DIAPH2/DBNL/HCK/TPM3/ACTG1/EZR/DBN1/KEAP1/LCP1/TPM4/WIPF2/AMOT/PALLD/TPM1/PAK1/PDLIM7/PDLIM5/JAM3/MYO9B/SPECC1/ACTB/RAC1/MARK2/RAC2/ARHGAP6/WIPF1/COTL1/GAS2L1/PDLIM1/HCLS1/TMOD1/WAS/ESPN/CTTN/GAS7/ACTN1/IQGAP1/DMTN/DIAPH1

BP	GO:0034097	response to cytokine	619	-0.38	-1.54	6.72E-07	1.02E-05	6.26E-06	2499	tags=31%, list=21%, signal=26%	CALCOCO2/VAMP3/SETD2/CREB1/ATP5F1B/ACTR2/SOCS3/NUB1/RFFL/STIP1/MYBL2/CD38/ADAM17/RAD23B/ENDOG/CORO1A/DDX58/NFE2L2/USP27X/NKIRAS2/DUSP1/RAB43/MUL1/IFI16/VIM/SH2B3/IL2RG/LIMS1/MAVS/RPS6KA5/FCER1G/IRAK3/TLR2/CHI3L1/SRF/PADI2/KDM5B/IFNAR2/SRC/LILRA1/MTHFR/HDAC4/IFNAR1/IL17RD/MAP2K5/XIAP/EXT1/LSP1/HCK/EPS8/SIRPA/PDE2A/LILRA5/CTBP2/HSPA5/ACTG1/LSM14A/GSTP1/IKBKE/GPS2/PLCB1/STAT5B/NFAT5/SYNJ1/SMPD4/OASL/EGR1/CMKLR1/RIPK1/KEAP1/CD300LF/MME/CPNE1/MAPK14/STX4/CEBPA/MT2A/TLE4/EIF2AK2/YY1/PTPRJ/ZFP36L1/PTPN6/EIF5A/DOK1/PYCARD/CDC42EP4/NR1H2/SBNO2/IFITM3/ALAD/RELB/SPATA2/DAPK3/SPI1/PML/CXCL5/CCR1/CSF1R/ARHGEF2/RFX2/CDIP1/LEPR/GSK3A/ADIPOR2/CXCL16/PRKACA/YTHDF3/PF4V1/USP10/MAP3K5/SLC27A1/NFKB1/HIPK1/UPF1/YBX1/IRF1/TIMP2/DOCK8/RARA/PTK2B/SELPLG/SELP/SNX10/STAT6/CXCR2/INPP5K/RPS6KA4/TRAF6/LDLRAP1/KLF5/IL6R/PIAS4/IL13RA1/GSN/MAP2K7/PTPRC/LILRB3/SYK/ADAR/THBS1/PTPN1/ADIPOR1/MED1/IL1RN/IL17RA/MAPKAPK3/JAK3/HSPA1A/PF4/AGPAT1/ZYX/CD14/LGALS9/LAPTM5/GSK3B/STAT5A/TNFRSF1B/IFITM2/MPL/RHOA/PRPF8/FASN/CXCR1/ACTN4/JAK1/HCLS1/IFIT1B/WAS/MAPK3/SNCA/H2BC11/BCL2L1/STAT3/KHSRP/RIPOR2/MAPKAPK2/PTP4A3/HDGF/YBX3/SHARPIN/MBP/JARID2/ACKR1/WNK1/PLVAP/TOLLIP/TIMP1/FOXO3/CBL/CDC34/RELA
BP	GO:0030100	regulation of endocytosis	145	-0.53	-1.87	6.92E-07	1.05E-05	6.42E-06	1738	tags=34%, list=15%, signal=29%	ANO6/SDCBP/TSC2/ANKRD13A/NUMB/ARRB1/LRRK2/AP2B1/SYNJ1/SNX17/AP2S1/DLG4/FLOT1/ATXN2/NR1H2/CD63/CLN3/ARF1/INSR/GREM1/LDLRAP1/ANKFY1/PPP3CA/RAB5B/SYK/BMP2K/PTPN1/RAB31/CD151/CALM3/RUBCN/VPS28/CD14/RAC1/HIP1/HNRNPK/DNM2/LRP1/RUFY1/CLU/ARRB2/RIN3/SNCA/TBC1D5/AP2M1/STON2/ITGB3/DAB2/CBL
CC	GO:0005769	early endosome	289	-0.46	-1.73	7.62E-07	1.14E-05	7.00E-06	2699	tags=38%, list=23%, signal=30%	VTI1B/HYAL3/ATP11A/VPS26B/RAB22A/APH1A/NCSTN/CD4/VPS4A/GGA3/EHD4/PMEPA1/VAMP3/FIG4/TRAK1/DNAJC13/KIAA0319/MGRN1/CORO1A/MIB2/WLS/ATP9A/F8A1/LNPEP/HPS6/MYO5A/TF/PLD4/VPS18/RFTN1/RAB11FIP5/TPCN1/DIAPH2/DBNL/MARCHF3/ERBB2/CMTM6/ATP7A/WDFY2/STX6/LDLRAD4/SNX3/WDR81/AOC3/HGS/NUMB/SNX17/PIKFYVE/RNF11/MME/EPHB1/CLCN5/NF2/MAP2K2/RAP1GAP/FLOT1/SORT1/WASHC2C/IFITM3/VPS41/CLN3/VPS35/ITCH/EPHA4/PML/TMEM184A/SNX8/USP10/VPS11/SORL1/SH3GL1/HTT/LDLRAP1/ANKFY1/RAB14/RAB5B/PTPN1/RAB31/EHD3/TOM1/FZD5/LTF/RUBCN/VPS28/TGFBRAP1/CHMP1A/SNX27/IGF2R/LRP1/RUFY1/DYSF/TRAK2/GRIPAP1/RIN3/MAPK3/SIAH2/DTX3L/RAPGEF1/PTPN23/EHD1/AKT2/TMEM127/LITAF/ATP6V0D1/PTP4A3/MMGT1/UBXN6/ACKR1/MARCHF8/WASF2
BP	GO:0045055	regulated exocytosis	150	-0.53	-1.88	7.59E-07	1.14E-05	6.99E-06	2141	tags=33%, list=18%, signal=28%	VPS18/RAB11FIP5/SNAP25/FGR/UNC13D/NKG7/CD177/AP1G1/HCK/ADGRE2/CTBP2/ITGAM/LRRK2/SYNJ1/STX4/SPI1/PDPK1/ITGB2/PIP5K1C/FES/GAB2/BCR/SYK/PTAFR/FBXL20/RAB31/CALM3/PIK3CG/RAB11FIP1/PRAM1/ABCC4/PLEK/DNAJC5/LYN/LGALS9/GSK3B/RAC2/RAB11B/GNAI2/PRKCB/GATA1/SNCA/LAMP1/NAPA/PIK3CD/RAB3D/P2RX1/MYH9/CBL/DMTN
BP	GO:0007160	cell-matrix adhesion	139	-0.54	-1.89	7.74E-07	1.15E-05	7.07E-06	2202	tags=35%, list=19%, signal=29%	LIMS1/PLEKHA2/MAP4K4/TNXB/BCL2L11/DISC1/MACF1/SRF/PTEN/PPM1F/SRC/SDC4/TIAM1/RRAS/ACTG1/ROCK2/ITGAM/NF2/PTPRJ/PARVG/CD63/DAPK3/FERMT3/ITGB2/GREM1/CORO1C/PTK2B/BCR/BCAS3/VCL/ITGA5/SLC9A1/JAM3/THBS1/GFUS/ZYX/RAC1/GSK3B/ITGA2B/PXN/ARHGAP6/RHOA/ITGAL/NF1/ITGB5/CTTN/ACTN1/ITGB3/DMTN

BP	GO:0033993	response to lipid	537	-0.39	-1.56	7.77E-07	1.15E-05	7.07E-06	2419	tags=31%, list=21%, signal=26%	SPON2/GRAMD1A/GPLD1/CD38/ADAM17/ENDOG/CYBB/SLC10A3/CRY2/SCARB1/TRIM5/AIFM1/VIM/EPHB2/ENO2/PKN1/PTK7/SAFB/TADA3/ASXL1/CDKN2D/MICB/IRAK3/TLR2/VPS18/GLB1/ATP2B1/VPS4B/FOXO1/PTGIR/SOD2/BCL2L11/PAF1/PADI2/TIRAP/PRDX2/NLRP3/SRC/PER1/YWHAH/ELK1/RXRB/DDX54/ATM/IFNAR1/PRKCE/PAQR8/THBD/CD68/LCOR/VDR/HCK/TGFB1/SIRPA/PPARGC1B/ZBTB7A/TNFSF4/UBR5/MTDH/ROCK2/ABCB11/GSTP1/OPRL1/SMARCA4/PLCB1/STAT5B/SYNJ1/ARID5A/KMT2D/ARPC1B/MAPK14/TRIB1/ZFP36L1/SETX/PYCARD/SBNO2/ALAD/SPI1/NCOA3/GPBAR1/CXCL5/AKIRIN2/KAT5/FOXP1/UBE2L3/SASH1/DGAT2/ALPL/PDGFA/PF4V1/NFKB1/VPS11/FES/KANK2/TSPO/UPF1/NCOA4/NCOA2/RARA/PTK2B/SELP/BCR/TRAF6/OTUD5/PLSCR3/PAK1/ZMIZ1/BMP6/MAP4K1/POR/STK11/CPT1A/SYK/THBS1/PTAFR/PDK3/NCOR2/MED1/NR3C1/IL1RN/MAPKAPK3/CALM3/LTF/ABHD2/JUND/HSPA1A/PF4/GPI/UCP2/RXRA/CD14/AQP1/LYN/LGALS9/GSK3B/IGF2R/CDK19/TNFRSF1B/TXNIP/RHOA/TNIP1/PRPF8/GATA1/GPX1/CLU/MSN/PPP1R9B/ARID1A/PIM1/TESC/WBP2/MAPK3/SNCA/STAT3/ZNF703/MAPKAPK2/FAM210B/LITAF/DAB2/MBP/CNOT1/CARM1/FOXO3/CBL/TBXA2R/MAP2K3/RELA
BP	GO:1902532	negative regulation of intracellular signal transduction	374	-0.42	-1.66	8.14E-07	1.20E-05	7.39E-06	2415	tags=32%, list=21%, signal=26%	UBE2B/RFFL/DUSP16/MGRN1/ENDOG/SLC9A3R1/NFE2L2/DUSP1/HDAC7/MUL1/EPHB2/DYRK3/SH2B3/HDAC3/ARHGAP24/FHL2/CDKN2D/IRAK3/FOXO1/SPRY3/SOD2/SLA2/ZNF385A/HEG1/WWC3/PTEN/NLRP3/SRC/PER1/ATM/AKT1S1/SLC24A4/RASA2/DGKG/SIRPA/PDE2A/SZT2/TSC2/GSTP1/LRRK2/OPRL1/GPS2/ASH1L/STK38/EZR/PHLPP1/RIPK1/RASAL3/CPNE1/MAPK14/NF2/PTPRJ/PTPN6/PYCARD/ADGRG3/TMBIM6/MARK3/SPI1/ITCH/EPHA4/ARHGEF2/GSK3A/CHP1/OPTN/SESN3/SESN2/USP10/FBP1/NDRG2/TAOK3/SORL1/VRK3/INPP5K/CSK/MMP9/SFRP2/STK11/THOC5/PTPRC/THBS1/PTPN1/NPLOC4/ADIPOR1/NLRX1/HSPA1A/UBR2/PLEK/LYN/RIOK3/GSK3B/HYOU1/HSPB1/PPP1R10/ATP2B4/RHOA/TNIP1/CASTOR2/MYADM/GPX1/CLU/ARRB2/NF1/PINK1/SEC14L1/BCL2L1/FLCN/RIPOR2/GRINA/SH3BP1/TMEM127/LITAF/ENO1/MFN2/PPM1A/YBX3/DAB2/NPRL3/CDC34/MAP2K3/RELA
BP	GO:0019221	cytokine-mediated signaling pathway	314	-0.44	-1.68	8.25E-07	1.21E-05	7.45E-06	2282	tags=30%, list=19%, signal=25%	USP27X/NKIRAS2/MUL1/SH2B3/IL2RG/LIMS1/MAVS/RPS6KA5/FCER1G/IRAK3/PADI2/IFNAR2/SRC/LILRA1/IFNAR1/IL17RD/XIAP/EXT1/HCK/LILRA5/LSM14A/GSTP1/IKBKE/GPS2/PLCB1/STAT5B/OASL/EGR1/CMKLR1/RIPK1/CD300LF/CPNE1/CEBPA/PTPRJ/PTPN6/EIF5A/DOK1/PYCARD/NR1H2/IFITM3/SPATA2/SPI1/CXCL5/CCR1/CSF1R/CDIP1/LEPR/ADIPOR2/PRKACA/YTHDF3/PF4V1/SLC27A1/HIPK1/IRF1/PTK2B/STAT6/CXCR2/RPS6KA4/TRAF6/IL6R/PIAS4/IL13RA1/PTPRC/LILRB3/SYK/ADAR/PTPN1/ADIPOR1/MED1/IL1RN/IL17RA/JAK3/HSPA1A/PF4/AGPAT1/LAPTM5/STAT5A/TNFRSF1B/IFITM2/MPL/CXCR1/ACTN4/JAK1/MAPK3/H2BC11/STAT3/SHARPIN/ACKR1/WNK1/PLVAP/TOLLIP/FOXO3/CBL/RELA
CC	GO:0030017	sarcomere	110	-0.57	-1.94	8.33E-07	1.22E-05	7.51E-06	1360	tags=34%, list=12%, signal=30%	MYOM1/FHL3/ARF1/TPM4/PALLD/TPM1/SPTBN1/MYZAP/CORO1C/SLC2A1/SLC8A1/PAK1/PDLIM7/ALDOA/STK11/PPP3CA/PDLIM5/PPP1R12B/PLEC/CALM3/FLNA/MYL4/HSPB1/ATP2B4/FKBP1A/CAPZB/RTN2/PDLIM1/ACTN4/TMOD1/PARVB/SQSTM1/ACTN1/SLC4A1/ENO1/MYL9/ANK1

BP	GO:0018209	peptidyl-serine modification	230	-0.48	-1.79	8.64E-07	1.26E-05	7.72E-06	2038	tags=28%, list=17%, signal=24%	PTEN/PPM1F/SRC/TOP1/FNIP2/ATM/STK4/PRKCE/TGFB1/ERN1/ROCK2/LRRK2/IKBKE/HGF/STK38/DYRK1B/RIPK1/PIKFYVE/MAPK14/GRK2/MAP2K2/MAD2L2/CSNK1G2/MARK3/PDPK1/MAST2/NSD1/PRKACA/CSNK2A1/HIPK1/MKNK2/VRK3/INPP5K/RPS6KA4/PRKD2/SH2D3C/PAK1/MAP4K1/SFRP2/SYK/PDK3/MAPKAPK3/HIPK2/CAMK1D/EIF4G1/ULK1/MARK2/GSK3B/SRPK2/ATP2B4/PRKCB/CSNK1G1/ARRB2/HCLS1/PINK1/RPS6KA1/SNCA/MAPKAPK2/AKT2/ARAF/PRKDC/PAK2/WNK1/MAST3/DMTN
BP	GO:0098542	defense response to other organism	690	-0.37	-1.53	8.72E-07	1.27E-05	7.77E-06	2436	tags=29%, list=21%, signal=24%	TRIM35/NUB1/SERINC5/SPON2/SPN/ARMC5/ADAM17/CYBB/CORO1A/DDX58/SIGLEC10/MAP4K2/NFE2L2/PADI4/USP27X/TRIM5/RAB43/TF/C1QA/MUL1/IFI16/VIM/RNF135/MAVS/TNK2/ADAM8/TRIM38/SRPK1/MICB/PLD4/FCER1G/IRAK3/APOBEC3D/TLR2/H2BC21/SERPING1/CR1/TLR5/FGR/FYN/TIRAP/UNC13D/NLRP3/IFNAR2/SRC/NKG7/CD177/ANKRD17/IFNAR1/NCF2/XIAP/XRCC6/AP1G1/LYST/HCK/SIRPA/PUM1/TNFSF4/G3BP1/LILRA5/BCL3/NOTCH2/RNF213/ACTG1/LSM14A/ITGAM/IKBKE/STAT5B/CNPY3/OASL/ARID5A/ALPK1/PI3/C5AR1/SHC1/DDX60L/STX4/EIF2AK2/PTPN6/PYCARD/CDC42EP4/ZNF175/NR1H2/IRF2/DCD/IFITM3/RELB/TREM1/DAPK3/SPI1/ITCH/NCF1/PML/ZC3HAV1/CSF1R/ARHGEF2/AKIRIN2/CCDC92/KLRD1/TRIM28/FOXP1/CXCL16/OPTN/NLRC4/CHID1/YTHDF3/RNASET2/MAP3K5/FES/RBPJ/PLEKHM2/IKBKG/IRF1/PGLYRP1/PTK2B/MFHAS1/SELP/MSRB1/TRAF6/IL6R/PAK1/RAB14/VAV1/LCN2/GSN/EXOSC4/PTPRC/SYK/ADAR/PTPN1/ILRUN/NPLOC4/ECSIT/ATG7/MED1/TLR6/IL17RA/NLRX1/RBM14/WIPI2/LTF/PIK3CG/JAK3/TUBB4B/PF4/ZYX/CD14/SLA/AQP1/LYN/RIOK3/LGALS9/RBCK1/SIN3A/CREBBP/APOBEC3F/FBXO9/STMP1/BNIP3L/IFITM2/SRPK2/TRIM11/COTL1/PRF1/TRIM14/TUBB/CLU/ARRB2/APOBEC3C/GRN/JAK1/IFIT1B/TRIM10/RNF187/WAS/MARCHF2/ZNFX1/HK1/SEC14L1/MAPK3/SNCA/TRIM26/H2BC11/DTX3L/BCL2L1/PCBP2/LAMP1/SLC15A3/N4BP1/PIK3CD/TREML1/PRKDC/PAK2/SHARPIN/KRT1/TRIM58/TOLLIP/ELF4/RELA
BP	GO:0019932	second-messenger-mediated signaling	175	-0.50	-1.81	9.95E-07	1.44E-05	8.83E-06	1966	tags=32%, list=17%, signal=27%	SULT1A3/FKBP1B/SLC24A4/ADNP/MCOLN1/SPPL3/PDE2A/LRRK2/OPRL1/NFAT5/CMKLR1/NFATC1/PTPRJ/PDPK1/PTBP1/CCR1/ITPR2/CAP1/CHP1/PDK2/PRKACA/CHERP/GUCY1B1/ADGRL1/NFATC2/TMEM38A/BCAP31/NUDT4/HTT/PTK2B/SLC8A1/SELP/CXCR2/RAPGEF2/PPP3CA/PTPRC/SYK/SLC9A1/THBS1/FIS1/CALM3/PLEK/AQP1/GSK3B/PDE5A/ATP2B4/NFATC3/CXCR1/PPP1R9B/GUCY1A1/PPP3R1/RUNDC3A/CDC34/TBXA2R/DMTN/IRAG1

BP	GO:0140352	export from cell	512	-0.39	-1.56	1.07E-06	1.54E-05	9.44E-06	2763	tags=31%, list=24%, signal=25%	KCNQ1/KIT/PFKL/APBA1/VTI1B/HYAL3/KCNE3/SVBP/CCN3/SNPH/SV2A/RHBDF2/P2RY1/MCTP1/CLASP1/VPS4A/VAMP3/SEPTIN4/CREB1/RALB/SCRIB/ALOX5/GPLD1/CD38/CALM1/CORO1A/NCKAP1L/CRY2/WLS/SCIN/ATP9A/PORCN/HPS6/SCFD2/PIM3/ADAM8/MYO1G/TLR2/VPS18/ATP2B1/VPS4B/RAB11FIP5/SNAP25/FGR/KDM5B/ITSN1/UNC13D/RAB27B/NKG7/EXOC4/IRS2/CD177/FKBP1B/ABAT/SLC24A4/MYO18A/BLOC1S3/PRKCE/SDC4/AP1G1/HCK/TGFB1/ADGRE2/PNKD/ANO6/SDCBP/HGS/MIDN/CTBP2/EXOC7/ITGAM/RPH3A/LRRK2/SIDT2/SYNJ1/EZR/SYTL3/SYN3/STX4/GRK2/KCNH2/PER2/NR1H2/EXOC3/MYOM1/VPS35/SPI1/PTGES/PDPK1/RALBP1/ITGB2/CCR1/EXOC6B/PIP5K1C/ABCG2/CANX/SEPTIN1/SLC27A1/VPS11/FES/LRRC8A/PCSK6/SLC8A1/GAB2/BCR/RCN3/PAK1/FRMD4A/RAB7A/BMP6/CPT1A/PPP3CA/SYK/LTBP2/SLC9A1/PTAFR/FBXL20/RAB31/ATP1B2/IL1RN/CALM3/PIK3CG/RAB11FIP1/PRAM1/SLC1A5/NIBAN2/ABCC4/UCP2/PLEK/DNAJC5/LYN/LGALS9/GSK3B/RAC2/RAB11B/TNFRSF1B/SYTL4/TCF7L2/GNAI2/PRKCB/GATA1/NF1/PINK1/SNCA/PTPN23/LAMP1/NAPA/PIK3CD/RAB3IL1/TANGO2/RAB3D/DAB2/P2RX1/MYH9/GLUL/CBL/RAB2B/FURIN/DMTN/ANK1
BP	GO:0010632	regulation of epithelial cell migration	146	-0.52	-1.85	1.09E-06	1.56E-05	9.55E-06	2659	tags=40%, list=23%, signal=32%	SVBP/RIN2/CALR/CLASP1/HDAC6/INSL3/ATP5F1B/MAPRE2/GPLD1/ADAM17/NFE2L2/SCARB1/HDAC7/MAP4K4/GLIPR2/MACF1/PTEN/PPM1F/SRC/MAP2K5/PRKCE/ATOH8/TGFB1/RRAS/ROCK2/DOCK5/PDPK1/FOXP1/SASH1/CORO1C/PTK2B/SEMA4A/BCAS3/PRKD2/MMP9/THBS1/ADIPOR1/PIK3CG/GFUS/GPI/RAC1/MAP3K3/STAT5A/HSPB1/ATP2B4/IQSEC1/RHOA/NF1/SP1/GRN/PTPN23/SPARC/SH3BP1/PIK3CD/DNAJA4/ITGB3/GLUL/TBXA2R/MAP2K3
BP	GO:0034762	regulation of transmembrane transport	293	-0.44	-1.67	1.09E-06	1.56E-05	9.55E-06	1564	tags=22%, list=13%, signal=19%	RIPK1/DLG4/MAPK14/CACNB1/CA2/KCNH2/FXYD6/PER2/PTPN6/HVCN1/CD63/SHISA7/PDPK1/KCNQ5/TRIB3/GSK3A/CHP1/PRKACA/INSR/CNIH3/TMEM38A/HTT/PTK2B/SLC8A1/INPP5K/CLIC3/MMP9/SLC9A1/THBS1/PTAFR/SELENON/SCN1B/ATP1B2/EHD3/CALM3/SLC6A9/PIK3CG/G6PD/FLNA/ACTB/CAPN1/SLC43A2/GJC2/LYN/DNM2/SLC43A1/WWP2/ATP2B4/FKBP1A/PRKCB/RTN2/ACTN4/PIM1/OAZ1/PINK1/TESC/SNCA/AHCYL1/AKT2/STIM1/WNK1/KEL/P2RX1/DIAPH1
CC	GO:0045121	membrane raft	220	-0.47	-1.75	1.13E-06	1.61E-05	9.87E-06	2579	tags=35%, list=22%, signal=28%	CD4/HDAC6/STOML1/RGS7/ANXA2/CFLAR/MYOF/ADAM17/PI4K2A/SLC9A3R1/SCARB1/ENO2/EFNB1/TLR2/ATP2B1/RFTN1/CR1/FYN/SRC/CD177/SDC4/ATP7A/HCK/SYNJ2/SDCBP/ITGAM/LRRK2/LIPE/EZR/RIPK1/PAG1/PIKFYVE/MME/EPHB1/EFHD2/FLOT1/CLN3/ITGB2/ABCG2/PRKACA/INSR/NFAM1/TPP1/CORO1C/PTK2B/SLC2A1/SELPLG/CAVIN2/STAT6/PECAM1/CTNNA1/CSK/LAMTOR1/GP6/PTPRC/SLC9A1/TLR6/INPP5D/CD14/LYN/FLOT2/TNFRSF1B/ATP2B4/GNAI2/MYADM/TUBB/ARID3A/HK1/MAPK3/PRKAR2A/PRKAR2B/BSG/STIM1/CTSD/P2RX1/PLVAP/CBL/FURIN
BP	GO:1990778	protein localization to cell periphery	238	-0.47	-1.76	1.17E-06	1.67E-05	1.02E-05	1678	tags=24%, list=14%, signal=21%	NUMB/EXOC7/TSPAN14/ROCK2/FYB1/EZR/TMBIM1/DLG4/STX4/PLEKHF1/FLOT1/CLSTN1/CLN3/ABI3/VPS35/PDPK1/NUMA1/ATP6AP1/OPTN/SPTBN1/KIF13A/INPP5K/RAPGEF2/LDLRAP1/RAB7A/CSK/TSPAN33/RAB31/EHD3/CLTC/PRAM1/FLNA/ACTB/LRP1/FLOT2/ATP2B4/GRIPAP1/PACS1/MYADM/PPP1R9B/TESC/EPB41/BCL2L1/EHD1/SQSTM1/AKT2/SLC4A1/AP2M1/GAK/BSG/RAB3D/DAB2/SEC16A/TSPAN5/RHOG/ANK1

CC	GO:0005765	lysosomal membrane	352	-0.42	-1.64	1.32E-06	1.86E-05	1.14E-05	2036	tags=28%, list=17%, signal=24%	TPCN1/ATP6V0B/UBA52/SLC29A3/NKG7/FNIP2/ATP6V1B2/MCOLN1/CPNE3/STARD3/CD68/AP1G1/CMTM6/ACP2/SLC66A1/WDR81/AP2A2/LAMP2/SZT2/CKAP4/ARRB1/SIDT2/AP2B1/AP2S1/TMBIM1/TFEB/CPNE1/CLCN5/PLEKHF1/CHMP4B/PGAP6/FLOT1/SORT1/SLC36A1/CD63/IFITM3/AP1M1/VPS41/CLN3/VPS35/ATP6V0C/GNAQ/CLEC16A/CDIP1/ANPEP/ATP6V0A1/NFAM1/VPS11/AP2A1/VLDLR/PLEKHM2/LRRC8A/SLC3A2/RAB7A/ANKFY1/RAB14/HGSNAT/ARL8A/SLC44A2/LAMTOR1/GNB1/GAA/TOM1/CLTC/B4GALT1/ANXA6/DNAJC5/CHMP1A/LYN/MMD/LAPTM5/ELAPOR1/UBA1/PSAP/AP5B1/LRP1/IFITM2/RILP/ATP6V1A/GRN/ECE1/MARCHF2/AP1B1/DTX3L/FLCN/LAMP1/SLC15A3/LITAF/ATP6V0D1/AP2M1/SLC48A1/RAB3D/CTSD/UBXN6/DAB2/SLC7A5/MARCHF8/NPRL3
CC	GO:0098852	lytic vacuole membrane	352	-0.42	-1.64	1.32E-06	1.86E-05	1.14E-05	2036	tags=28%, list=17%, signal=24%	TPCN1/ATP6V0B/UBA52/SLC29A3/NKG7/FNIP2/ATP6V1B2/MCOLN1/CPNE3/STARD3/CD68/AP1G1/CMTM6/ACP2/SLC66A1/WDR81/AP2A2/LAMP2/SZT2/CKAP4/ARRB1/SIDT2/AP2B1/AP2S1/TMBIM1/TFEB/CPNE1/CLCN5/PLEKHF1/CHMP4B/PGAP6/FLOT1/SORT1/SLC36A1/CD63/IFITM3/AP1M1/VPS41/CLN3/VPS35/ATP6V0C/GNAQ/CLEC16A/CDIP1/ANPEP/ATP6V0A1/NFAM1/VPS11/AP2A1/VLDLR/PLEKHM2/LRRC8A/SLC3A2/RAB7A/ANKFY1/RAB14/HGSNAT/ARL8A/SLC44A2/LAMTOR1/GNB1/GAA/TOM1/CLTC/B4GALT1/ANXA6/DNAJC5/CHMP1A/LYN/MMD/LAPTM5/ELAPOR1/UBA1/PSAP/AP5B1/LRP1/IFITM2/RILP/ATP6V1A/GRN/ECE1/MARCHF2/AP1B1/DTX3L/FLCN/LAMP1/SLC15A3/LITAF/ATP6V0D1/AP2M1/SLC48A1/RAB3D/CTSD/UBXN6/DAB2/SLC7A5/MARCHF8/NPRL3
CC	GO:0019898	extrinsic component of membrane	213	-0.48	-1.77	1.37E-06	1.93E-05	1.18E-05	2467	tags=36%, list=21%, signal=29%	PLAUR/SOCS7/SOCS3/GRAMD1A/SCRIB/MCF2L/TF/AAK1/ATG2B/TNK2/UBC/GOLGA3/GNA15/SLA2/BCL2L11/SNAP25/FGR/FYN/SRC/TRIO/HCK/TIAM1/WDR81/VPS13D/NUMB/SYAP1/RPH3A/EZR/SYTL3/SYN3/DLG4/TGM3/OSBPL2/CTNND1/GNAQ/NCF1/PML/ACADVL/NUMA1/RGS6/MTMR3/ZFYVE1/FES/MYZAP/PTK2B/SNX10/RAB7A/CTNNA1/PPP3CA/WDR45/SYK/GNB1/MGLL/WIPI2/PIK3CG/ULK1/RAC1/SLA/LYN/HIP1/SYTL4/GNAI2/FKBP1A/GRIPAP1/RHOA/WDFY3/ATG2A/GNA12/ATG13/UBB/PIK3CD/RNF40/UBXN6/GNAZ/RNF10/IQGAP1
BP	GO:0007229	integrin-mediated signaling pathway	73	-0.63	-2.01	1.38E-06	1.94E-05	1.19E-05	2051	tags=40%, list=17%, signal=33%	FGR/SRC/CD177/HCK/PHACTR4/ITGAM/FYB1/CD63/FERMT3/ITGB2/NEDD9/PTK2B/CTNNA1/VAV1/ITGA5/SYK/PRAM1/FLNA/PLEK/ZYX/ITGA2B/ITGAL/ITGB5/MPIG6B/ITGB3/MYH9/TIMP1/DMTN/TLN1
CC	GO:0098857	membrane microdomain	221	-0.47	-1.76	1.44E-06	2.01E-05	1.23E-05	2579	tags=36%, list=22%, signal=28%	CD4/HDAC6/STOML1/RGS7/ANXA2/CFLAR/MYOF/ADAM17/PI4K2A/SLC9A3R1/SCARB1/ENO2/EFNB1/TLR2/ATP2B1/RFTN1/CR1/FYN/SRC/CD177/SDC4/ATP7A/HCK/SYNJ2/LAMP2/SDCBP/ITGAM/LRRK2/LIPE/EZR/RIPK1/PAG1/PIKFYVE/MME/EPHB1/EFHD2/FLOT1/CLN3/ITGB2/ABCG2/PRKACA/INSR/NFAM1/TPP1/CORO1C/PTK2B/SLC2A1/SELPLG/CAVIN2/STAT6/PECAM1/CTNNA1/CSK/LAMTOR1/GP6/PTPRC/SLC9A1/TLR6/INPP5D/CD14/LYN/FLOT2/TNFRSF1B/ATP2B4/GNAI2/MYADM/TUBB/ARID3A/HK1/MAPK3/PRKAR2A/PRKAR2B/BSG/STIM1/CTSD/P2RX1/PLVAP/CBL/FURIN

BP	GO:0000165	MAPK cascade	484	-0.39	-1.57	1.62E-06	2.25E-05	1.38E-05	2329	tags=30%, list=20%, signal=25%	MAP4K2/ARHGEF5/SLC9A3R1/MAP2K4/DUSP1/TRIM5/GPS1/TF/EPHB2/SH2B3/HDAC3/SH3RF1/ADAM8/STK25/MAP4K4/TRAF7/IRAK3/GLIPR2/CHI3L1/FOXO1/SPRY3/PTEN/MAP3K4/TIRAP/PRDX2/SRC/PER1/SULT1A3/MAP2K5/STK4/PRKCE/XIAP/GRIK2/ERBB2/MAGED1/TGFB1/SIRPA/ITPKB/ERN2/RRAS/LILRA5/ERN1/NOTCH2/SDCBP/SH3RF3/ROCK2/GSTP1/ARRB1/LRRK2/HGF/NBR1/GPS2/ASH1L/PLCB1/STK38/EZR/PHLPP1/RIPK1/C5AR1/SHC1/EPHB1/MAPK14/TRIB1/NF2/MAP2K2/EIF2AK2/ACTA2/PTPRJ/ZFP36L1/MAP3K2/SETX/PTPN6/PYCARD/PPM1L/SPI1/ITCH/EPHA4/GPBAR1/NCF1/CCR1/CSF1R/GRB2/SASH1/YWHAZ/PDGFA/INSR/MAP3K5/NDRG2/NFKB1/TAOK3/SORL1/PTK2B/MFHAS1/VRK3/INPP5K/RAPGEF2/TRAF6/PRKD2/SH2D3C/PAK1/IGF2/CSK/TAOK1/MAP4K1/LAMTOR1/SFRP2/MAP2K7/PELI2/PTPRC/SYK/DVL3/THBS1/STK40/PTPN1/TLR6/MTURN/MAPKAPK3/ARHGAP8/FZD5/PIK3CG/HIPK2/NDST1/MADD/LYN/LGALS9/MAP3K3/LAPTM5/PDE5A/AKAP13/GNAI2/TNIP1/ARRB2/NF1/PINK1/MAPK3/FLCN/MAPKAPK2/ARAF/PAK2/DAB2/MBP/PLVAP/IQGAP1/MAP2K3/STRADB
BP	GO:0040007	growth	565	-0.38	-1.54	1.62E-06	2.25E-05	1.38E-05	2110	tags=26%, list=18%, signal=22%	ARHGEF11/RFTN1/BCL2L11/HEG1/DISC1/WWC3/MACF1/SRF/PTEN/KDM5B/FTO/PPM1F/SRC/TAF8/HDGFL2/ZMAT3/ATM/NINJ1/ADNP/DBNL/MAP2K5/STK4/H3-3B/ERBB2/EXT1/NIPBL/H3-3A/TGFB1/RC3H2/CPNE5/NOTCH2/SDCBP/CTDP1/TCHP/BTG1/SMARCA4/PLCB1/NDEL1/STAT5B/EZR/TMC8/KMT2D/DBN1/KAT7/CPQ/SHC1/CPNE1/MAPK14/MT2A/CDKL5/YY1/PTPRJ/ZFP36L1/MSX1/CLSTN1/BNC2/SEMA4D/MAD2L2/ARIH2/CDKN1C/KPNA1/NAIF1/ITCH/PTPN12/PML/KAT5/TRIM28/SMARCA2/LEPR/GSK3A/DPYSL2/CXCL16/NEDD9/INSR/SESN2/CSNK2A1/SGPL1/FBP1/GREM1/RBPJ/RARA/PTK2B/SEMA4A/ARID2/EIF4G2/NDRG3/CHST11/KLF5/IGF2/VCL/ZMIZ1/TBL1XR1/POR/LAMTOR1/SFRP2/EXTL3/STK11/PPP3CA/EXOSC4/USP9X/PDLIM5/SLC9A1/STK40/PLEC/ADIPOR1/MED1/IST1/G6PD/BASP1/EIF4G1/ULK1/HSPA1A/ANXA6/SIPA1/SIN3A/CYFIP2/GSK3B/STAT5A/DNM2/PSAP/AKAP13/CDA/RHOA/GPX1/FKBP8/BCL9/PPP1R9B/WDTC1/PIM1/RPS6KA1/CDKN1A/BCL2L1/FLCN/STAT3/MTPN/CTTN/SP2/DNAJB2/ENO1/PAK2/YBX3/DAB2/JARID2/FOXO3/IQGAP1/NINJ2/HLX
BP	GO:0031331	positive regulation of cellular catabolic process	369	-0.42	-1.63	1.70E-06	2.35E-05	1.44E-05	2197	tags=32%, list=19%, signal=27%	USP5/SH3RF1/ADAM8/TRIM38/ORMDL3/FOXO1/BCL2L11/ATXN3/DISC1/CSDE1/PTEN/TPCN1/FTO/ARIH1/IRS2/SH3GLB1/ATM/COP1/ATF6/DET1/RNF144B/PUM1/RC3H2/VPS13D/TSC2/MTDH/SH3RF3/ROCK2/LRRK2/EZR/SPTLC2/ZBTB20/TFEB/KEAP1/PIP4K2C/PIAS1/TRIB1/CEBPA/PLEKHF1/WAC/NRDC/ZFP36L1/AGTPBP1/CBFA2T3/ARIH2/VPS35/ITCH/ABHD5/ZC3HAV1/KAT5/TRIB3/GSK3A/OPTN/AGO2/ENTPD5/ARNT/INSR/YTHDF3/SESN3/TENT4B/SESN2/CNOT6/VPS11/UPF1/PIM2/BCAP31/IKBKG/YBX1/HTT/PTK2B/PAFAH1B2/RAD23A/STK11/CPT1A/FZR1/WDR45/PTPN1/GTPBP1/ULK1/VCP/HSPA1A/PSMC4/GABARAP/LAPTM5/GSK3B/ELAPOR1/ZC3H18/LRP1/BNIP3L/TNFRSF1B/SUPT5H/PNPLA2/TRIM14/CLU/ATG2A/MSN/LARP1/PINK1/MARCHF2/MAPK3/SNCA/DTX3L/FLCN/KHSRP/PIP4K2A/AKT2/DNAJB2/SAMD4B/RNF40/DAB2/CNOT1/FBXO7/NPRL3/FOXO3/ZER1/FURIN/BAG6
CC	GO:0001726	ruffle	141	-0.53	-1.88	1.74E-06	2.40E-05	1.47E-05	2052	tags=35%, list=17%, signal=29%	MACF1/FGR/TIRAP/SRC/ATP6V1B2/DBNL/EPS8/ADGRE2/ACAP2/PDE4A/EZR/CDKL5/NF2/PTPRJ/FRMD4B/ARHGEF2/LCP1/PIP5K1C/AMOT/S100A11/PALLD/TPM1/CORO1C/FGD4/INPP5K/SH2D3C/PAK1/PDLIM7/FGD3/ITGA5/PLEK/RAC1/DNM2/WIPF1/RHOA/MYADM/ARHGAP45/PPP1R9B/CFL1/TESC/CTTN/ACTN1/AKT2/ITGB3/MYH9/IQGAP1/WASF2/DIAPH1/TLN1

BP	GO:0030041	actin filament polymerization	130	-0.54	-1.87	1.94E-06	2.65E-05	1.63E-05	1995	tags=35%, list=17%, signal=30%	TRIOBP/MSRB2/DIAPH2/DBNL/PRKCE/HCK/EPS8/CYRIA/SPTAN1/ARPC1B/DBN1/WASF3/WASHC2C/PYCARD/CDC42EP4/PREX1/ARPC5/SSH1/GRB2/SPTBN1/PTK2B/SPTA1/MSRB1/SVIL/GSN/ESAM/RAC1/CYFIP2/ARHGAP6/COTL1/RHOA/CAPZB/MYADM/ADD1/SSH2/HCLS1/TMOD1/ARPC4/WAS/SPTB/MTPN/CTTN/GAS7/VASP/DMTN/DIAPH1
BP	GO:0032940	secretion by cell	481	-0.39	-1.55	1.98E-06	2.68E-05	1.65E-05	2633	tags=29%, list=22%, signal=24%	CCN3/SNPH/SV2A/RHBDF2/P2RY1/MCTP1/CLASP1/VPS4A/VAMP3/SEPTIN4/CREB1/RALB/SCRIB/ALOX5/GPLD1/CD38/CORO1A/NCKAP1L/CRY2/WLS/SCIN/ATP9A/PORCN/HPS6/SCFD2/PIM3/ADAM8/MYO1G/TLR2/VPS18/VPS4B/RAB11FIP5/SNAP25/FGR/KDM5B/ITSN1/UNC13D/RAB27B/NKG7/EXOC4/IRS2/CD177/FKBP1B/ABAT/MYO18A/BLOC1S3/PRKCE/SDC4/AP1G1/HCK/TGFB1/ADGRE2/PNKD/SDCBP/HGS/MIDN/CTBP2/EXOC7/ITGAM/RPH3A/LRRK2/SIDT2/SYNJ1/EZR/SYTL3/SYN3/STX4/GRK2/PER2/NR1H2/EXOC3/MYOM1/VPS35/SPI1/PTGES/PDPK1/ITGB2/CCR1/EXOC6B/PIP5K1C/CANX/SEPTIN1/VPS11/FES/LRRC8A/PCSK6/GAB2/BCR/RCN3/PAK1/FRMD4A/RAB7A/BMP6/CPT1A/PPP3CA/SYK/LTBP2/PTAFR/FBXL20/RAB31/IL1RN/CALM3/PIK3CG/RAB11FIP1/PRAM1/SLC1A5/NIBAN2/ABCC4/UCP2/PLEK/DNAJC5/LYN/LGALS9/GSK3B/RAC2/RAB11B/TNFRSF1B/SYTL4/TCF7L2/GNAI2/PRKCB/GATA1/NF1/PINK1/SNCA/PTPN23/LAMP1/NAPA/PIK3CD/RAB3IL1/TANGO2/RAB3D/DAB2/P2RX1/MYH9/GLUL/CBL/RAB2B/FURIN/DMTN/ANK1
BP	GO:0090630	activation of GTPase activity	80	-0.61	-1.99	1.98E-06	2.68E-05	1.65E-05	1641	tags=36%, list=14%, signal=31%	RANGAP1/RALGAPA1/NDEL1/TBC1D17/TBC1D3I/SIPA1L1/RAP1GAP/TBC1D22A/RALGAPA2/EVI5L/TBC1D10B/RABGAP1/NEDD9/CORO1C/PTK2B/BCR/BCAS3/TBC1D22B/GMIP/SIPA1L2/TBC1D13/SIPA1/TBC1D25/ARHGAP45/TBC1D5/AKT2/TBC1D1/TBC1D2/RHOG
BP	GO:0008360	regulation of cell shape	111	-0.56	-1.88	2.02E-06	2.71E-05	1.67E-05	1597	tags=49%, list=14%, signal=42%	ARHGAP18/DNMBP/KIT/F11R/PLEKHO1/PLXNA4/EPB41L3/FGD6/P2RY1/CFAP410/CDC42EP1/CORO1A/SLC9A3R1/FGR/FYN/RHOF/HCK/EPS8/EZR/WASF3/CDC42EP4/SEMA4D/ARAP1/DAPK3/ITGB2/CSF1R/WDR1/BRWD3/SH3KBP1/TPM1/FES/PTK2B/FGD4/SPTA1/SEMA4A/FGD3/ALDOA/PLXNC1/FMNL1/RAC1/MARK2/RAC2/RHOA/MSN/GNA12/RHOBTB1/EPB41/PARVB/EPB42/MYH9/PEAK3/RHOG/DMTN/DIAPH1
CC	GO:0070161	anchoring junction	573	-0.38	-1.54	2.01E-06	2.71E-05	1.66E-05	1719	tags=29%, list=15%, signal=26%	SDCBP/SYNE2/MTDH/HSPA5/NECTIN1/TCHP/ACTG1/NUMB/ABCB11/ASH1L/EZR/GAB1/ARPC1B/DBN1/PIKFYVE/MME/GIT2/NF2/MAP2K2/PTPRJ/FLOT1/PARVG/PTPN6/CDC42EP4/PARD3/IRF2/CTNND1/FHL3/FRMD4B/ATP6V0C/EPHA4/FERMT3/PDPK1/ARPC5/PTPN12/PKP4/ITGB2/PANX2/ARHGEF2/WDR1/CAP1/PCDH1/LCP1/ARF1/TJP2/PIP5K1C/CHP1/GRB2/YWHAZ/PPP1CA/TPM4/ARHGAP31/OCEL1/NEDD9/AMOT/S100A11/SH3KBP1/PALLD/NHS/YWHAB/FES/CORO1C/PTK2B/ZNF185/SLC2A1/SLC8A1/RAPGEF2/EIF4G2/SVIL/PAK1/FRMD4A/VCL/PECAM1/PDLIM7/CTNNA1/CSK/VAV1/TGFB1I1/GSN/PPP3CA/ITGA5/PDLIM5/TSPAN33/PTPRC/SLC9A1/JAM3/SCN1B/PLEC/DCAF6/EHD3/CD151/ESAM/CLTC/FZD5/B4GALT1/FLNA/NIBAN2/HSPA1A/ACTB/ANXA6/CAPN1/HMGA1/ZYX/GJC2/RAC1/SLA/LYN/HNRNPK/UBA1/RAC2/ADGRE5/DNM2/FOCAD/IGF2R/HYOU1/LRP1/FLOT2/HSPB1/ITGA2B/CNN2/LPP/LASP1/PXN/RHOA/MYADM/PDLIM1/MSN/UBN1/ACTN4/ADD1/PPP1R9B/JAK1/RSU1/GNA12/CFL1/WAS/ARHGAP26/MAPK3/ITGB5/APBB1IP/TGM2/PARVB/FLCN/CTTN/CLDN5/PCBP2/PRKAR2A/CAPN5/ACTN1/SH3BP1/TSPAN9/GAK/BSG/MPP1/PAK2/ITGB3/YBX3/VASP/DAB2/MRC2/MYH9/CBL/TNS1/IQGAP1/PEAK3/WASF2/RHOG/TLN1

MF	GO:0005543	phospholipid binding	321	-0.43	-1.65	2.03E-06	2.72E-05	1.67E-05	2546	tags=34%, list=22%, signal=27%	AGAP1/GGA3/TWF2/ANXA2/MYOF/ZCCHC2/ARHGAP32/TECPR1/MCF2L/PCYT1A/TOM1L2/SCIN/SCARB1/ZFYVE26/PLCD1/ATG2B/HS1BP3/MYO1G/FLII/SAP30L/MYO1E/PCTP/DGKA/SNX29/TPCN1/TIRAP/CPNE3/RASA2/NCF4/SNX3/OSBPL1A/CPNE5/SDCBP/HGS/ARHGAP9/CPTP/TTPAL/GPAA1/RPH3A/PLCB1/SNX17/NME4/SYTL3/CD300LF/MME/SHC1/CPNE1/PLEKHF1/OSBPL2/DENND1C/WASHC2C/PARD3/ARAP1/OSBPL5/PITPNC1/PREX1/NCF1/NUMA1/ITPR2/GLE1/SNX8/PITPNM2/ZFYVE1/SPTBN1/FES/PITPNA/TPP1/ADAP2/GAB2/CAVIN2/SNX10/RAPGEF2/BSCL2/BCAS3/SVIL/SBF2/LDLRAP1/ANKFY1/ADAP1/SNX11/GSN/WDR45/SLC9A1/THBS1/PTAFR/ASAP1/TOM1/WIPI2/IQGAP2/RUBCN/ANXA6/SNX27/HIP1/PSAP/SYTL4/DYSF/EPN1/WDFY3/NF1/ATG2A/NRGN/ARHGAP26/SNCA/EPB41/ANXA11/PLEK2/DENND1A/IQGAP1/TLN1
CC	GO:0030016	myofibril	124	-0.55	-1.89	2.04E-06	2.73E-05	1.67E-05	1360	tags=32%, list=12%, signal=29%	MYOM1/FHL3/ARF1/TPM4/PALLD/TPM1/SPTBN1/MYZAP/CORO1C/SLC2A1/SLC8A1/SVIL/CALD1/PAK1/VCL/PDLIM7/ALDOA/STK11/PPP3CA/PDLIM5/PPP1R12B/PLEC/CALM3/FLNA/MYL4/HSPB1/ATP2B4/FKBP1A/CAPZB/RTN2/PDLIM1/ACTN4/TMOD1/PARVB/SQSTM1/ACTN1/SLC4A1/ENO1/MYL9/ANK1
BP	GO:0043542	endothelial cell migration	140	-0.54	-1.90	2.15E-06	2.83E-05	1.74E-05	1180	tags=24%, list=10%, signal=22%	PDPK1/FOXP1/SASH1/AMOT/GREM1/PTK2B/SEMA4A/BCAS3/PRKD2/THBS1/PIK3CG/GFUS/GPI/RAC1/MAP3K3/STAT5A/HSPB1/ATP2B4/PXN/RHOA/GPX1/NF1/SP1/GRN/SPARC/SH3BP1/PIK3CD/PTP4A3/DNAJA4/ITGB3/MYH9/GLUL/TBXA2R/MAP2K3
BP	GO:0007043	cell-cell junction assembly	76	-0.61	-1.95	2.17E-06	2.84E-05	1.75E-05	2596	tags=45%, list=22%, signal=35%	EPB41L3/FSCN1/TBCD/FBF1/HDAC7/EPHB2/STRN/PARD6B/HEG1/SRF/ACTG1/ROCK2/PARD3/CTNND1/GPBAR1/PKP4/WDR1/PIP5K1C/PRKACA/OCEL1/HIPK1/VCL/PECAM1/CTNNA1/JAM3/ESAM/FZD5/ACTB/RHOA/FLCN/CLDN5/ZNF703/PAK2/TLN1
BP	GO:0031668	cellular response to extracellular stimulus	185	-0.49	-1.80	2.17E-06	2.84E-05	1.75E-05	2303	tags=36%, list=20%, signal=29%	NFE2L2/MAP1LC3B2/AIFM1/IFI16/ATP2B1/FOXO1/SRF/RRAGD/SH3GLB1/GCN1/ADNP/CD68/VDR/LARS1/CPEB4/LAMP2/SZT2/HSPA5/LRRK2/TFEB/EIF2AK2/DAP/VPS41/CLEC16A/MAP1LC3B/PDK2/MTMR3/SESN3/SESN2/STK24/ZFYVE1/MAP3K5/FES/KANK2/SLC2A1/RRAGC/MAX/USF2/SFRP2/PTPRC/WDR45/ATG7/PLEC/MED1/WIPI2/EIF4G1/ULK1/RIPOR1/SIPA1/UCP2/RXRA/GABARAP/LYN/ELAPOR1/GAS2L1/PPP1R9B/PIM1/MAPK3/CDKN1A/NUAK2/FLCN/NCOA1/GLUL/NPRL3/FOXO3/CBL
BP	GO:0051050	positive regulation of transport	583	-0.38	-1.52	2.15E-06	2.84E-05	1.74E-05	2207	tags=28%, list=19%, signal=24%	IL2RG/STIMATE/HDAC3/MAVS/ADAM8/FCER1G/TLR2/ATP2B1/VPS4B/HSPA1L/FGR/FYN/FEZ1/UNC13D/SRC/RAB27B/IRS2/CD177/SH3GLB1/ABAT/MYO18A/PRKCE/SDC4/ERBB2/AP1G1/USP36/TGFB1/SIRPA/LILRA5/ANO6/NPEPPS/SDCBP/HGS/UBR5/UBE2J2/ABCB11/ITGAM/ARRB1/LRRK2/OPRL1/GPS2/AP2B1/SYNJ1/EZR/CD300LF/MAPK14/STX4/CA2/GRK2/KCNH2/PTPRJ/FLOT1/PYCARD/NR1H2/CD63/ARIH2/MYOM1/CLN3/VPS35/PTGES/PDPK1/ITGB2/CCR1/NUMA1/TRIM28/GSK3A/UBE2L3/CHP1/KMT2A/PRKACA/INSR/VPS11/GREM1/TSPO/RBM22/SORL1/BCAP31/HTT/LRRC8A/GAB2/BCR/INPP5K/BCAS3/LDLRAP1/DYNC1H1/LPCAT3/FRMD4A/RAB7A/ANKFY1/BMP6/STK11/PPP3CA/PTPRC/SYK/SLC9A1/FIS1/PTAFR/SCN1B/RAB31/ATP1B2/EHD3/CD151/CALM3/SLC6A9/DOCK2/FZD5/G6PD/CAMK1D/FLNA/DNM3/JAK3/RIPOR1/ACTB/VPS28/RXRA/CD14/GJC2/AQP1/HIP1/HNRNPK/GSK3B/RAC2/SIRPB1/DNM2/LRP1/SYTL4/TCF7L2/HUWE1/GNAI2/GATA1/BBC3/CLU/ARRB2/MSN/ACTN4/HCLS1/OAZ1/ATG13/PINK1/MLLT6/TESC/MYLK/SNCA/TBC1D20/PTPN23/EHD1/LAMP1/ABLIM3/TBC1D5/AHCYL1/AKT2/AP2M1/PPM1A/RAB3D/STIM1/DAB2/WNK1/P2RX1/GLUL/CBL/RAB2B

BP	GO:0046903	secretion	549	-0.38	-1.55	2.20E-06	2.87E-05	1.76E-05	2412	tags=27%, list=21%, signal=22%	SCRIB/ALOX5/GPLD1/CD38/CORO1A/NCKAP1L/CRY2/WLS/SCIN/ATP9A/SLC9A3R1/PORCN/HPS6/SCFD2/PIM3/ADAM8/MYO1G/TLR2/VPS18/VPS4B/RAB11FIP5/SNAP25/FGR/KDM5B/ITSN1/UNC13D/RAB27B/NKG7/EXOC4/IRS2/CD177/FKBP1B/ABAT/MYO18A/BLOC1S3/PRKCE/SDC4/AP1G1/VDR/ATP7A/HCK/TGFB1/ADGRE2/PNKD/SDCBP/HGS/MIDN/CTBP2/EXOC7/ABCB11/ITGAM/RPH3A/LRRK2/OPRL1/SIDT2/STAT5B/SYNJ1/EZR/SYTL3/SYN3/STX4/CA2/GRK2/PER2/NR1H2/EXOC3/AQP9/MYOM1/VPS35/SPI1/PTGES/PDPK1/ITGB2/CCR1/EXOC6B/PIP5K1C/CANX/HRH2/SEPTIN1/VPS11/FES/LRRC8A/PCSK6/NEURL1/GAB2/BCR/SNX10/RCN3/COPA/USF2/PAK1/FRMD4A/RAB7A/BMP6/CPT1A/PPP3CA/SYK/LTBP2/PTAFR/FBXL20/NCOR2/RAB31/MED1/IL1RN/CALM3/PIK3CG/RAB11FIP1/PRAM1/SLC1A5/NIBAN2/ABCC4/UCP2/PLEK/DNAJC5/AQP1/LYN/LGALS9/GSK3B/STAT5A/RAC2/RAB11B/TNFRSF1B/SYTL4/TCF7L2/GNAI2/PRKCB/GATA1/NF1/PINK1/SNCA/PTPN23/LAMP1/NAPA/PIK3CD/RAB3IL1/TANGO2/RAB3D/DAB2/WNK1/P2RX1/MYH9/GLUL/CBL/RAB2B/FURIN/DMTN/ANK1
MF	GO:0005509	calcium ion binding	309	-0.44	-1.67	2.35E-06	3.05E-05	1.88E-05	2632	tags=34%, list=22%, signal=27%	CETN2/AOAH/CALR/PLCB4/MCTP1/EHD4/CAPN3/SLC25A23/ANXA2/CALM1/TKT/SCIN/USP32/PADI4/PCDHB1/PLCD1/LETM1/PRRG4/ADAM8/REPS1/NIN/EFCAB14/HEG1/DGKA/MACF1/CD93/PADI2/ITSN1/THBD/DGKG/ADGRE2/AOC3/NOTCH2/MGMT/HSPA5/RAB11FIP4/RPH3A/PLCB1/NOTCH2NLA/SPTAN1/MAN1A1/TGM3/CPNE1/PEF1/EFHD2/CLSTN1/CABP5/MICU1/ITPR2/PCDH1/LCP1/CHP1/CANX/TPM4/ALPL/S100A11/PITPNM2/S100A4/VLDLR/SLC8A1/SPTA1/SELP/RAPGEF2/RCN3/RASGRP4/PLSCR3/NHSL2/VCAN/GSN/PLCB3/PPP3CA/LTBP2/THBS1/SELENON/EHD3/CALM3/PVALB/ANXA6/CAPN1/SELL/CAPNS1/ADGRE5/MYL4/LRP1/REPS2/DYSF/PRF1/EPS15L1/ACTN4/CANT1/NUCB1/TESC/SNCA/TGM2/ANXA11/EHD1/SPARC/PPP3R1/LTBP1/ACTN1/STIM1/MYL9/VWCE/HPCAL1/CBL/IQGAP1
BP	GO:0045444	fat cell differentiation	142	-0.52	-1.85	2.42E-06	3.13E-05	1.92E-05	2159	tags=36%, list=18%, signal=30%	ASXL1/FOXO1/SOD2/ZNF385A/WNT1/ZNF516/FTO/TAF8/TRIO/STK4/NIPBL/WDFY2/TGFB1/ZBTB7A/FAM120B/CTBP2/SYAP1/GPS2/PLCB1/METRNL/ARL4A/JDP2/CMKLR1/PIAS1/MAPK14/CTBP1/CEBPA/ZFP36L1/SORT1/PER2/TRIB3/ERO1A/BSCL2/KLF5/TBL1XR1/ZBTB16/ASXL2/TGFB1I1/SFRP2/TFE3/MED1/MAFB/TMEM120A/FBXO9/TCF7L2/GPX1/LRG1/PIM1/FLCN/AKT2/CARM1
BP	GO:0045859	regulation of protein kinase activity	452	-0.39	-1.56	2.44E-06	3.14E-05	1.93E-05	2391	tags=33%, list=20%, signal=27%	CALM1/DUSP16/ADAM17/NCKAP1L/PRKAR1B/MAP4K2/ARHGEF5/SLC9A3R1/MVP/DUSP1/KAT2B/EPHB2/SH2B3/PKN1/AXIN2/ADAM8/CDKN2D/IRAK3/TNXB/CHI3L1/SPRY3/HEG1/FGR/PTEN/MAP3K4/TIRAP/PPM1F/SRC/AKT1S1/MLLT1/ADNP/CDK12/MAP2K5/STK4/SDC4/XRCC6/ERBB2/CCNY/CASS4/MAGED1/TGFB1/ERN2/LILRA5/ERN1/HGS/TSC2/SYAP1/GSTP1/LRRK2/HGF/STK38/CACUL1/EGR1/DLG4/C5AR1/SMG8/SHC1/TRIB1/CEBPA/NF2/MAP2K2/PTPRJ/PTPN6/PYCARD/CDKN1C/DAZAP2/EPHA4/PDPK1/GNAQ/NCF1/CCNF/CSF1R/TRIB3/CHP1/SASH1/PDGFA/NEDD9/INSR/SESN2/MAP3K5/SLC27A1/TAOK3/GREM1/VLDLR/SH3BP5L/SORL1/CORO1C/HTT/PTK2B/NEURL1/SLC8A1/WARS1/INPP5K/RAPGEF2/CCND3/TRAF6/IL6R/PAK1/MOB3A/IGF2/CSK/TAOK1/SFRP2/STK11/MAP2K7/STRADA/PTPRC/SYK/ADAR/DVL3/THBS1/PTPN1/PLEC/TLR6/CALM3/HEXIM2/FZD5/LTF/PIK3CG/ACTB/MADD/MARK2/LYN/MMD/PDE5A/HSPB1/AKAP13/ATP2B4/RHOA/CLU/NF1/CCNI/PINK1/SNCA/CDKN1A/PRKAR2A/TAL1/PRKAR2B/CCNJL/PAK2/ITGB3/DAB2/WNK1/FBXO7/CBL/IQGAP1/MAP2K3/STRADB/CNPPD1

BP	GO:0048878	chemical homeostasis	684	-0.37	-1.51	2.78E-06	3.58E-05	2.20E-05	2404	tags=28%, list=20%, signal=24%	ALOX5/GPLD1/CD38/CALM1/ENDOG/CORO1A/SIRT6/CRY2/SLC9A3R1/HPS6/SCARB1/NKIRAS2/PIM3/IREB2/TF/ACO1/HMOX2/LETM1/TLCD4/MTCH2/ATP2B1/CNNM3/ORMDL3/FOXO1/GNA15/PTGIR/SOD2/TTC7A/RAB11FIP5/KNG1/DISC1/COX19/SRF/FYN/TPCN1/KCTD17/PDZD8/MED13/IRS2/FKBP1B/SLC24A4/ADNP/MCOLN1/PRKCE/SPPL3/XIAP/GRIK2/PROK2/EXT1/SLC39A7/VDR/ATP7A/SLC66A1/ATP13A1/ERN1/SLC12A6/ABCB11/GSTP1/LRRK2/OPRL1/SMARCA4/SIDT2/TMC8/CMKLR1/ZBTB20/DLG4/C5AR1/STX4/CA2/CEBPA/MT2A/KCNH2/PTPN6/CKB/HVCN1/NR1H2/TMBIM6/AQP9/CLN3/ABHD5/PDPK1/PML/ABHD4/MICU1/CCR1/ATP6AP1/ITPR2/ARF1/LEPR/ADIPOR2/CHP1/DGAT2/PDK2/PRKACA/CHERP/INSR/SESN3/TENT4B/SESN2/ATP6V0A1/ATP2A3/ERO1A/NCOA4/TMEM38A/BCAP31/HTT/LRRC8A/PTK2B/SLC8A1/SNX10/CXCR2/INPP5K/SLC24A3/RCN3/LDLRAP1/ACOX1/PYGL/PLSCR3/USF2/RAB7A/BMP6/LAMTOR1/STK11/LCN2/HECTD4/PPP3CA/PTPRC/SLC9A1/FIS1/SELENON/PDK3/PLEC/ATP1B2/ADIPOR1/CALM3/SLC6A9/LTF/PIK3CG/JAK3/ANKRD9/ANXA6/ALOX12/GPI/UCP2/AQP1/LYN/SIN3A/RALY/GP9/RAB11B/DDB1/TCF7L2/ATP2B4/GNAI2/FKBP1A/PNPLA2/FTL/PRKCB/GATA1/GPX1/STAU1/CXCR1/ATP6V1A/FTH1/GRN/WDTC1/MLLT6/HK1/MAPK3/SNCA/TGM2/TBC1D20/STAT3/EHD1/GRINA/SLC4A1/GP1BA/ATP6V0D1/EPB42/SLC25A37/STIM1/KRT1/ALAS2/WNK1/KEL/P2RX1/FOXO3/TBXA2R/EIF2AK1/XK/DIAPH1
BP	GO:0006914	autophagy	458	-0.40	-1.57	2.87E-06	3.61E-05	2.21E-05	2274	tags=32%, list=19%, signal=27%	MAP1LC3B2/TRIM5/RAB43/MUL1/IFI16/ATG2B/TRIM38/KLHL22/VPS18/VPS4B/ORMDL3/FOXO1/BCL2L11/TPCN1/FEZ1/ATP6V0B/RRAGD/SRC/MAP1S/EXOC4/SH3GLB1/ATM/ATP6V1B2/MCOLN1/ATF6/USP36/WDR81/LAMP2/VPS13D/ERN1/HGS/TSC2/RNF213/MTDH/CPTP/RETREG3/EXOC7/LRRK2/HGF/NBR1/SPTLC2/NRBF2/TFEB/TBC1D17/KEAP1/PIKFYVE/PIP4K2C/VPS51/PLEKHF1/WAC/CHMP4B/YOD1/PHF23/PYCARD/DAP/TMBIM6/VPS41/CLN3/DAPK3/VPS35/ATP6V0C/QSOX1/CLEC16A/MAP1LC3B/KAT5/TRIB3/LEPR/GSK3A/OPTN/VPS37B/MTMR3/PRKACA/VPS37C/SESN3/SESN2/ATP6V0A1/USP10/ZFYVE1/VPS11/TSPO/PIM2/IKBKG/HTT/PAFAH1B2/RRAGC/HERC1/EIF4G2/BCAS3/SBF2/TECPR2/NSFL1C/RAB7A/STK11/WDR45/ATG4D/FIS1/ILRUN/ATG7/CLTC/WIPI2/FZD5/EIF4G1/ULK1/VCP/RUBCN/VPS28/CAPN1/TGFBRAP1/CAPNS1/CHMP1A/GOLGA2/GABARAP/MARK2/GSK3B/ELAPOR1/PSAP/HSPB1/BNIP3L/SUPT5H/HUWE1/TBC1D25/TRIM14/WDFY3/CLU/ATP6V1A/ATG2A/LARP1/ATG13/PINK1/MAPK3/SNCA/FLCN/STAT3/CTTN/PIP4K2A/SQSTM1/TBC1D5/ATP6V0D1/MFN2/UBXN6/DCAF12/FBXO7/TOLLIP/NPRL3/FOXO3/SEC16A/RAB1B
BP	GO:0008064	regulation of actin polymerization or depolymerization	119	-0.54	-1.84	2.81E-06	3.61E-05	2.21E-05	1995	tags=34%, list=17%, signal=28%	TRIOBP/DBNL/PRKCE/HCK/EPS8/CYRIA/SPTAN1/ARPC1B/DBN1/WASHC2C/PYCARD/CDC42EP4/ARPC5/WDR1/SSH1/GRB2/SPTBN1/PTK2B/SPTA1/SVIL/GSN/ESAM/PLEK/CYFIP2/COTL1/RHOA/CAPZB/MYADM/ADD1/SSH2/HCLS1/TMOD1/ARPC4/WAS/SPTB/MTPN/CTTN/SH3BP1/VASP/DMTN
BP	GO:0045639	positive regulation of myeloid cell differentiation	73	-0.62	-1.98	2.85E-06	3.61E-05	2.21E-05	1812	tags=40%, list=15%, signal=34%	TGFB1/PPARGC1B/NOTCH2/STAT5B/RIPK1/KAT7/MAPK14/TRIB1/ZFP36L1/CCR1/ARNT/FES/TRAF6/PITHD1/MED1/MTURN/HSPA1A/INPP5D/PF4/MPL/GATA1/HCLS1/TESC/STAT3/TAL1/FAM210B/PRKDC/FOXO3/FAXDC2

BP	GO:0061919	process utilizing autophagic mechanism	458	-0.40	-1.57	2.87E-06	3.61E-05	2.21E-05	2274	tags=32%, list=19%, signal=27%	MAP1LC3B2/TRIM5/RAB43/MUL1/IFI16/ATG2B/TRIM38/KLHL22/VPS18/VPS4B/ORMDL3/FOXO1/BCL2L11/TPCN1/FEZ1/ATP6V0B/RRAGD/SRC/MAP1S/EXOC4/SH3GLB1/ATM/ATP6V1B2/MCOLN1/ATF6/USP36/WDR81/LAMP2/VPS13D/ERN1/HGS/TSC2/RNF213/MTDH/CPTP/RETREG3/EXOC7/LRRK2/HGF/NBR1/SPTLC2/NRBF2/TFEB/TBC1D17/KEAP1/PIKFYVE/PIP4K2C/VPS51/PLEKHF1/WAC/CHMP4B/YOD1/PHF23/PYCARD/DAP/TMBIM6/VPS41/CLN3/DAPK3/VPS35/ATP6V0C/QSOX1/CLEC16A/MAP1LC3B/KAT5/TRIB3/LEPR/GSK3A/OPTN/VPS37B/MTMR3/PRKACA/VPS37C/SESN3/SESN2/ATP6V0A1/USP10/ZFYVE1/VPS11/TSPO/PIM2/IKBKG/HTT/PAFAH1B2/RRAGC/HERC1/EIF4G2/BCAS3/SBF2/TECPR2/NSFL1C/RAB7A/STK11/WDR45/ATG4D/FIS1/ILRUN/ATG7/CLTC/WIPI2/FZD5/EIF4G1/ULK1/VCP/RUBCN/VPS28/CAPN1/TGFBRAP1/CAPNS1/CHMP1A/GOLGA2/GABARAP/MARK2/GSK3B/ELAPOR1/PSAP/HSPB1/BNIP3L/SUPT5H/HUWE1/TBC1D25/TRIM14/WDFY3/CLU/ATP6V1A/ATG2A/LARP1/ATG13/PINK1/MAPK3/SNCA/FLCN/STAT3/CTTN/PIP4K2A/SQSTM1/TBC1D5/ATP6V0D1/MFN2/UBXN6/DCAF12/FBXO7/TOLLIP/NPRL3/FOXO3/SEC16A/RAB1B
BP	GO:0006954	inflammatory response	506	-0.39	-1.55	2.94E-06	3.69E-05	2.27E-05	2280	tags=28%, list=19%, signal=23%	NRROS/NKIRAS2/C1QA/IFI16/EPHB2/CTNNBIP1/ADAM8/SERPINA1/RPS6KA5/PLD4/TLR2/CHI3L1/PTGIR/KNG1/TLR5/FGR/FYN/TIRAP/UNC13D/PRDX2/NLRP3/SRC/NKG7/PER1/KPNA6/HDAC4/ATM/NINJ1/XIAP/PROK2/EXT1/CD68/HCK/TGFB1/SIRPA/AREL1/ADGRE2/AOC3/PDE2A/TNFSF4/LILRA5/ANO6/NOTCH2/GSTP1/ITGAM/LRRK2/HGF/GPS2/ASH1L/METRNL/STAT5B/CMKLR1/RIPK1/C5AR1/MAPK14/CEBPA/PYCARD/SBNO2/RELB/TREM1/SPATA2/VPS35/ITCH/PTGES/NCF1/ITGB2/CXCL5/CCR1/CSF1R/ALOX5AP/FOXP1/NLRC4/CHID1/PF4V1/NFKB1/NFAM1/RBPJ/PRDX5/IKBKG/PGLYRP1/MFHAS1/SELP/BCR/CXCR2/RPS6KA4/IL6R/LPCAT3/PTGS1/BMP6/MMP9/AKNA/PTPRC/SYK/JAM3/THBS1/PTAFR/TLR6/IL1RN/MGLL/IL17RA/NLRX1/PIK3CG/B4GALT1/CAMK1D/NDST1/PF4/ZYX/CD14/RAC1/LYN/LGALS9/ADGRE5/CDK19/LRP1/TNFRSF1B/ITGAL/TNIP1/NFATC3/SCYL1/GPX1/FASN/CLU/KDM6B/GRN/HK1/SNCA/STAT3/MAPKAPK2/PIK3CD/BRD4/SHARPIN/KRT1/ACKR1/P2RX1/TOLLIP/TIMP1/TBXA2R/MAP2K3/EIF2AK1/FURIN/RELA
CC	GO:0010008	endosome membrane	422	-0.40	-1.58	3.09E-06	3.85E-05	2.36E-05	2551	tags=33%, list=22%, signal=27%	VPS4A/GGA3/EHD4/STOML1/UBE2D3/PMEPA1/VAMP3/FIG4/ANXA2/DNAJC13/RFFL/KIAA0319/ARHGAP32/WLS/ATP9A/ANTXR2/HPS6/TF/VPS53/UBC/VPS18/VPS4B/SLA2/RAB11FIP5/TPCN1/ATP6V0B/UBA52/SLC29A3/RAB27B/MCOLN1/STARD3/MARCHF3/ERBB2/CD68/CMTM6/NCF4/ATP7A/LDLRAD4/SNX3/WDR81/SUN2/AP2A2/ACAP2/LAMP2/HGS/CPTP/NUMB/RAB11FIP4/ABCB11/AP2B1/SNX17/AP2S1/TMBIM1/RIPK1/PIKFYVE/EPHB1/CLCN5/PLEKHF1/CHMP4B/SORT1/CD63/IFITM3/VPS41/CLN3/VPS35/ATP6V0C/ITCH/FCGRT/EPHA4/PML/CLEC16A/ATP6AP1/CDIP1/TMEM184A/PIP5K1C/SNX8/OPTN/VPS37B/VPS37C/INSR/ATP6V0A1/SPG21/VPS11/AP2A1/PLEKHM2/SORL1/CORO1C/SH3GL1/KIF13A/SNX10/TRAF6/SBF2/RAB7A/ANKFY1/RAB14/ARL8A/LAMTOR1/RAB5B/SCAMP2/RAB31/EHD3/PLIN3/UBAP1/CLTC/FZD5/ANXA6/YIPF2/VPS28/CD14/CHMP1A/RAC1/SNX27/MMD/ELAPOR1/UBA1/IGF2R/RAB11B/RUFY1/IFITM2/GRIPAP1/RILP/ECE1/RHOBTB1/MARCHF2/DTX3L/EHD1/LAMP1/SLC15A3/UBB/TBC1D5/LITAF/ATP6V0D1/AP2M1/MMGT1/SLC48A1/CTSD/UBXN6/ARHGAP1/MARCHF8/FURIN

BP	GO:0030855	epithelial cell differentiation	312	-0.43	-1.64	3.08E-06	3.85E-05	2.36E-05	2077	tags=28%, list=18%, signal=23%	THRA/MYO1E/HEG1/WNT1/KDM5B/EDF1/SRC/TRIOBP/STK4/ATOH8/MYCL/EXT1/VDR/VEZF1/ZDHHC7/PDE2A/NOTCH2/MGMT/ROCK2/BTG1/PLCB1/SIDT2/STAT5B/EZR/TGM3/KEAP1/S1PR2/NF2/ACTA2/ZFP36L1/CDKN1C/NCOA3/PDPK1/ACADVL/NUMA1/SULT1B1/TJP2/GSK3A/TAGLN2/PALLD/GREM1/TPP1/RBPJ/RARA/BCR/RAPGEF2/KLF5/VCL/PECAM1/BMP6/MMP9/TGFB1I1/CPT1A/FZR1/FOSL2/PLEC/MED1/B4GALT1/BASP1/GSK3B/PSAP/TXNIP/RHOA/FOXJ2/GATA1/MYADM/GPX1/TUBB/FASN/MSN/KDM6B/ADD1/TMOD1/TBC1D20/RAPGEF1/CLDN5/ZNF703/TST/AKT2/SH3BP1/CTSB/SHARPIN/DAB2/KRT1/TOLLIP/IQGAP1
BP	GO:0071407	cellular response to organic cyclic compound	363	-0.41	-1.60	3.26E-06	4.05E-05	2.49E-05	2193	tags=29%, list=19%, signal=25%	PKN1/AXIN2/MAVS/SAFB/TADA3/VPS18/ATP2B1/GNA15/BCL2L11/PADI2/SRC/PER1/YWHAH/ELK1/SULT1A3/RXRB/DDX54/PAQR8/LCOR/VDR/TGFB1/PPARGC1B/ZBTB7A/PDE2A/OR10H1/MGMT/UBR5/HSPA5/ROCK2/LRRK2/SMARCA4/PLCB1/KLF16/EZR/EGR1/KMT2D/CEBPA/ZFP36L1/FLOT1/AQP9/VPS35/NCOA3/GPBAR1/KAT5/ITPR2/FOXP1/SSH1/GSK3A/UBE2L3/HRH2/ALPL/PRKACA/NFKB1/VPS11/FES/KANK2/NCOA4/TMEM38A/SLC8A1/INPP5K/RAPGEF2/PAK1/ZMIZ1/CTNNA1/SLC9A1/PTAFR/GNB1/NCOR2/MED1/NR3C1/PIK3CG/ABHD2/FLNA/HSPA1A/ACTB/RXRA/AQP1/RIOK3/SIN3A/GSK3B/DNM2/ATP2B4/RHOA/GATA1/CLU/ARRB2/MSN/PPP1R9B/LARP1/IFIT1B/ARID1A/PIM1/WBP2/MAPK3/SNCA/TGM2/RAPGEF1/ZNF703/FAM210B/PAK2/DAB2/CNOT1/GNAZ/CARM1/FOXO3/DMTN/DIAPH1
BP	GO:0031623	receptor internalization	73	-0.62	-1.97	3.46E-06	4.29E-05	2.63E-05	1719	tags=41%, list=15%, signal=35%	SDCBP/ANKRD13A/NUMB/ARRB1/EZR/DLG4/GRK2/FLOT1/ATXN2/CD63/ITGB2/ARF1/GRB2/INSR/GREM1/CXCR2/LDLRAP1/SYK/RAB31/CLTC/DNM3/DNM2/LRP1/ARRB2/CXCR1/RIN3/SNCA/TBC1D5/AP2M1/ITGB3
BP	GO:0007163	establishment or maintenance of cell polarity	160	-0.51	-1.81	3.61E-06	4.45E-05	2.73E-05	2503	tags=40%, list=21%, signal=32%	FSCN1/ACTR2/FBF1/SPN/SCRIB/NCKAP1L/SLC9A3R1/MYO9A/PTK7/STK25/ARHGEF11/PARD6B/FEZ1/RHOF/CDC42BPB/MYO18A/ROCK2/CKAP5/NDEL1/EPHB1/PARVG/PARD3/FRMD4B/MAP4/FAM89B/ARPC5/NUMA1/WDR1/CAP1/AMOT/PLEKHG3/HTT/DOCK8/PTK2B/BCAS3/NSFL1C/FRMD4A/CTNNA1/NDE1/STK11/GSN/SLC9A1/JAM3/LMNA/DCTN1/DOCK2/ACTB/RAC1/MARK2/AQP1/SNX27/GSK3B/RAC2/FLOT2/RHOA/PDLIM1/MSN/RHOBTB1/PARVB/RIPOR2/SH3BP1/MYH9/RHOG/ANK1
BP	GO:0098657	import into cell	125	-0.53	-1.85	3.66E-06	4.50E-05	2.76E-05	2069	tags=28%, list=18%, signal=23%	SNAP25/FYN/IRS2/SLC24A4/KCNJ15/SLC2A3/SLC12A6/KCNH2/FLOT1/PER2/SLC36A1/TSPO2/SLC27A1/LRRC8A/SLC2A1/SLC8A1/SLC6A20/SLC3A2/SLC9A1/THBS1/ATP1B2/SLC6A9/SLC1A5/ACTB/VPS28/SLC43A2/SLC43A1/ATP2B4/SNCA/SLC15A3/AKT2/ITGB3/WNK1/SLC7A5/P2RX1
BP	GO:0006887	exocytosis	242	-0.45	-1.68	3.93E-06	4.83E-05	2.96E-05	2615	tags=35%, list=22%, signal=28%	SNPH/SV2A/P2RY1/CLASP1/VPS4A/VAMP3/SEPTIN4/RALB/SCRIB/CORO1A/NCKAP1L/SCIN/ATP9A/SCFD2/MYO1G/VPS18/VPS4B/RAB11FIP5/SNAP25/FGR/ITSN1/UNC13D/RAB27B/NKG7/EXOC4/CD177/SDC4/AP1G1/HCK/ADGRE2/SDCBP/HGS/CTBP2/EXOC7/ITGAM/LRRK2/SYNJ1/SYTL3/STX4/EXOC3/SPI1/PDPK1/ITGB2/CCR1/EXOC6B/PIP5K1C/SEPTIN1/VPS11/FES/GAB2/BCR/PAK1/RAB7A/SYK/PTAFR/FBXL20/RAB31/CALM3/PIK3CG/RAB11FIP1/PRAM1/ABCC4/PLEK/DNAJC5/LYN/LGALS9/GSK3B/RAC2/RAB11B/SYTL4/GNAI2/PRKCB/GATA1/SNCA/LAMP1/NAPA/PIK3CD/RAB3IL1/RAB3D/P2RX1/MYH9/CBL/RAB2B/DMTN/ANK1

MF	GO:0019900	kinase binding	583	-0.37	-1.50	4.12E-06	5.03E-05	3.09E-05	2416	tags=30%, list=21%, signal=25%	MAPRE2/RFFL/CALM1/DUSP16/UTRN/TOM1L2/CRY2/PRKAR1B/MAP4K2/TTC28/MVP/DUSP1/KIF13B/HDAC7/TRIM5/KAT2B/BCL11A/SH2B3/LIMS1/PKN1/AXIN2/MAVS/TRIM38/CDKN2D/IRAK3/NIN/INKA1/BCL2L14/BCL2L11/PARD6B/WWC3/FGR/PTEN/FEZ1/TIRAP/IFNAR2/SRC/PER1/IRS2/RHOF/GCN1/CPNE3/CDK12/PPP1R15A/MARCKS/SDC4/XIAP/CCNY/LYST/CASS4/EEF2/SIRPA/TIAM1/AP2A2/MIDN/CTBP2/GSTP1/BTG1/NBR1/NBEAL2/STK38/CACUL1/EZR/DLG4/NFATC1/SH2B2/SHC1/MAPK14/TRIB1/CEBPA/ACTA2/PTPRJ/MAP3K2/ATF7/PTPN6/MAD2L2/MYOM1/CTNND1/RELB/DAZAP2/EPHA4/ITGB2/CDK13/PGAM1/TRIB3/TJP2/GSK3A/CHP1/GRB2/SASH1/YWHAZ/PRKACA/NEDD9/PITPNM2/MAP3K5/SUFU/AP2A1/HTT/RARA/PTK2B/SLC2A1/GAB2/CAVIN2/WARS1/CCND3/TRAF6/PRKD2/TUBA4A/CSK/RAD23A/GSN/GP6/PDLIM5/MAP2K7/STRADA/PTPRC/WDR45/SYK/PTAFR/PTPN1/PPP1R12B/ADIPOR1/NR3C1/MAPKAPK3/DCTN1/CLTC/CALM3/FZD5/PRAM1/SKI/FLNA/ACTB/PLEK/RAC1/GOLGA2/GSK3B/RAC2/DNM2/HSPB1/TCF7L2/ATP2B4/RHOA/TNIP1/PRKCB/TRIM14/ARRB2/MSN/PPP1R9B/HCLS1/ATG13/RHOBTB1/WAS/PINK1/CDKN1A/AP1B1/BCL2L1/STAT3/PTPN23/MAPKAPK2/PRKAR2A/PRKAR2B/SQSTM1/PAK2/WNK1/FBXO7/TOLLIP/FOXO3/CBL/IQGAP1/MAP2K3/RHOG/CNPPD1/POLR2A/RELA
BP	GO:0001501	skeletal system development	260	-0.44	-1.65	4.20E-06	5.10E-05	3.13E-05	2229	tags=30%, list=19%, signal=25%	MTHFD1/SH2B3/AXIN2/ASXL1/CHI3L1/ZNF385A/THRA/FGR/SRF/WNT1/SRC/EXT1/NIPBL/VDR/ATP7A/MKS1/TGFB1/CHSY1/PPARGC1B/NOTCH2/XYLT1/ASH1L/ARID5A/CMKLR1/MAPK14/MSX1/PTPN6/BNC2/SEMA4D/FUZ/CDKN1C/LRRK1/PAPSS2/ATP6AP1/FOXP1/LEPR/TRPS1/ALPL/RUNX3/FOXN3/SGPL1/SUFU/GREM1/RARA/SNX10/ANKH/MEIS1/SOX6/CHST11/BMP6/ZBTB16/MMP9/POR/SFRP2/VCAN/PTPRC/MED1/GLG1/PHOSPHO1/LTF/SKI/NDST1/ANXA6/RFLNB/ETS2/AKAP13/RHOA/SLC38A10/MAPK3/TGM2/PBX1/CSRNP1/PIP4K2A/TAL1/MPIG6B/TIMP1/WASF2
BP	GO:0048871	multicellular organismal homeostasis	292	-0.43	-1.64	4.21E-06	5.11E-05	3.13E-05	2447	tags=32%, list=21%, signal=26%	BTBD9/GPR137/CD38/ADAM17/CORO1A/SIRT6/LNPEP/GIGYF2/NKIRAS2/TF/HDAC3/ADAM8/SLC28A2/FOXO1/THRA/PCTP/SRF/ZNF516/FTO/SRC/ABAT/EXT1/MKS1/PPARGC1B/ACTG1/METRNL/EGR1/CMKLR1/CHMP4B/NRDC/PER2/NR1H2/LRRK1/PTGES/ACADVL/CSF1R/ALMS1/MTF1/TJP2/LEPR/ADIPOR2/ALPL/PRKACA/SLC27A1/TPP1/RBPJ/PTK2B/AMPD2/SLC2A1/SNX10/STAT6/INPP5K/BSCL2/TRAF6/RCN3/DYNC1H1/VCL/PECAM1/RAB7A/CSK/BMP6/STK11/LCN2/TFE3/SYK/JAM3/PLEC/ATP1B2/ADIPOR1/ESAM/LTF/EIF4G1/ACTB/INPP5D/ALOX12/UCP2/AQP1/HSPB1/GATA1/GPX1/NF1/KDM6B/FLCN/STAT3/CLDN5/IP6K1/TLE3/UBB/SQSTM1/MFN2/RAB3D/ITGB3/MBP/KRT1
MF	GO:0050839	cell adhesion molecule binding	372	-0.41	-1.59	4.26E-06	5.15E-05	3.16E-05	1692	tags=30%, list=14%, signal=26%	SDCBP/HSPA5/NECTIN1/ICAM3/NUMB/ITGAM/RANGAP1/CKAP5/STK38/EZR/SPTAN1/UNC45A/DBN1/S1PR2/CHMP4B/NF2/CAST/PTPRJ/EFHD2/PTPN6/EXOC3/DDX6/CTNND1/CTNNAL1/FERMT3/LYPLA2/ZC3HAV1/PKP4/ITGB2/LCP1/TJP2/YWHAZ/PPP1CA/TAGLN2/PKM/S100A11/STX5/STK24/ADGRL1/PALLD/SPTBN1/YWHAB/PUF60/SH3GL1/LRRC59/EIF4G2/SLC3A2/CALD1/IGF2/VCL/CTNNA1/ALDOA/SFRP2/PLCB3/ITGA5/PDLIM5/SYK/JAM3/THBS1/PTPN1/ASAP1/PLEC/IST1/PLIN3/CD151/ESAM/FLNA/NIBAN2/HSPA1A/GOLGA2/MARK2/LYN/HNRNPK/GAPVD1/RAB11B/ITGA2B/CNN2/LASP1/ITGAL/CAPZB/EPS15L1/SCYL1/PDLIM1/FASN/MSN/H3C10/ACTN4/ADD1/LARP1/ITGB5/EHD1/CTTN/PRDX6/SH3GLB2/HDLBP/ACTN1/ENO1/BSG/PAK2/ITGB3/VASP/ARHGAP1/TBC1D2/MYH9/CBL/IQGAP1/WASF2/SND1/TLN1/HCFC1

BP	GO:0030832	regulation of actin filament length	120	-0.54	-1.84	4.51E-06	5.42E-05	3.33E-05	1995	tags=33%, list=17%, signal=28%	TRIOBP/DBNL/PRKCE/HCK/EPS8/CYRIA/SPTAN1/ARPC1B/DBN1/WASHC2C/PYCARD/CDC42EP4/ARPC5/WDR1/SSH1/GRB2/SPTBN1/PTK2B/SPTA1/SVIL/GSN/ESAM/PLEK/CYFIP2/COTL1/RHOA/CAPZB/MYADM/ADD1/SSH2/HCLS1/TMOD1/ARPC4/WAS/SPTB/MTPN/CTTN/SH3BP1/VASP/DMTN
BP	GO:1902903	regulation of supramolecular fiber organization	268	-0.44	-1.68	4.53E-06	5.42E-05	3.33E-05	2144	tags=31%, list=18%, signal=26%	NIN/TNXB/FLII/KATNB1/PPM1F/TRIOBP/MAP1S/ATXN7/DBNL/PRKCE/SDC4/HCK/EPS8/CYRIA/ACTG1/ROCK2/CKAP5/SPTAN1/ARPC1B/DBN1/WASF3/NF2/CAMSAP1/EML2/WASHC2C/PYCARD/CDC42EP4/SLAIN2/ARAP1/DAPK3/ARPC5/NUMA1/ARHGEF2/WDR1/ALMS1/ARF1/SSH1/GRB2/TPM1/SPTBN1/FES/PTK2B/SPTA1/INPP5K/COLGALT1/SVIL/PAK1/MAP1A/TAOK1/GSN/SLC9A1/ESAM/DCTN1/FLNA/HSPA1A/PLEK/RAC1/CYFIP2/AKAP13/PXN/ARHGAP6/COTL1/RHOA/CAPZB/MYADM/GPX1/CLU/ADD1/SSH2/HCLS1/TMOD1/ARPC4/WAS/SNCA/SPTB/MTPN/CTTN/SH3BP1/PAK2/VASP/WASF2/DMTN
BP	GO:0003012	muscle system process	217	-0.46	-1.70	4.55E-06	5.43E-05	3.33E-05	2136	tags=29%, list=18%, signal=25%	ATP2B1/ARHGEF11/ORMDL3/FOXO1/SRF/FKBP1B/HDAC4/ABAT/SSPN/PROK2/TPM3/CTDP1/ROCK2/DOCK5/GRK2/KCNH2/ACTA2/YY1/CHRNE/MYOM1/DAPK3/GSK3A/TPM4/PRKACA/TPM1/TMEM38A/SLC8A1/CALD1/ALDOA/GSN/PPP3CA/PDLIM5/SLC9A1/PTAFR/LMNA/GAA/SCN1B/PPP1R12B/EHD3/CALM3/PIK3CG/G6PD/FLNA/ANXA6/PDE5A/MYL4/AKAP13/TNFRSF1B/ATP2B4/RHOA/TMOD1/MYLK/GUCY1A1/MTPN/CTTN/ENO1/MYL9/PAK2/JARID2/P2RX1/SLC6A8/FOXO3/TBXA2R/MAP2K3
BP	GO:0032868	response to insulin	185	-0.49	-1.78	4.69E-06	5.58E-05	3.43E-05	2506	tags=34%, list=21%, signal=27%	YWHAG/SOCS3/CFLAR/GPLD1/CRY2/MYO5A/KAT2B/TLR2/ATP2B1/FOXO1/SRC/IRS2/ZDHHC7/TSC2/SCAP/SYAP1/SMARCC1/NDEL1/GAB1/EGR1/SH2B2/PIP4K2C/SHC1/MAPK14/ZFP36L1/SORT1/PDPK1/TRIB3/GSK3A/GRB2/PDK2/EIF6/PKM/INSR/SESN3/SESN2/SLC27A1/FBP1/SORL1/SLC2A1/INPP5K/CCND3/MAX/IGF2/ZNF106/SLC9A1/FOXO4/PTPN1/RAB31/ADIPOR1/LPIN2/UCP2/LYN/GSK3B/GNAI2/PRKCB/SP1/WDTC1/PIP4K2A/AKT2/PRKDC/EIF4EBP2
BP	GO:0017015	regulation of transforming growth factor beta receptor signaling pathway	76	-0.60	-1.92	4.85E-06	5.75E-05	3.53E-05	2042	tags=36%, list=17%, signal=30%	WNT1/IL17RD/TGFB1/LDLRAD4/ZBTB7A/SDCBP/HSPA5/CDKN1C/FAM89B/PDPK1/CHST11/TGFB1I1/STK11/THBS1/GLG1/HIPK2/SKI/HSPA1A/DNM2/CREBBP/FKBP1A/LRG1/FLCN/LTBP1/ZNF703/PPM1A/DAB2
CC	GO:0042641	actomyosin	55	-0.65	-1.98	5.05E-06	5.94E-05	3.65E-05	2062	tags=47%, list=18%, signal=39%	CDC42BPA/CDC42BPB/MYO18A/DCTN4/TPM3/DBN1/ACTA2/FHL3/LCP1/TPM4/PALLD/TPM1/PDLIM7/PDLIM5/ZYX/CNN2/LPP/PXN/GAS2L1/PDLIM1/ACTN4/MYLK/ABLIM3/ACTN1/MYL9/MYH9
BP	GO:0045766	positive regulation of angiogenesis	97	-0.57	-1.91	5.04E-06	5.94E-05	3.65E-05	1752	tags=30%, list=15%, signal=26%	RRAS/MTDH/BTG1/HGF/GAB1/C5AR1/PDPK1/SASH1/AGO2/PKM/GREM1/HIPK1/PTK2B/PRKD2/SFRP2/ITGA5/THBS1/HIPK2/AQP1/HSPB1/PRKCB/SP1/LRG1/GRN/JAK1/STAT3/PIK3CD/STIM1/TBXA2R
BP	GO:1904018	positive regulation of vasculature development	97	-0.57	-1.91	5.04E-06	5.94E-05	3.65E-05	1752	tags=30%, list=15%, signal=26%	RRAS/MTDH/BTG1/HGF/GAB1/C5AR1/PDPK1/SASH1/AGO2/PKM/GREM1/HIPK1/PTK2B/PRKD2/SFRP2/ITGA5/THBS1/HIPK2/AQP1/HSPB1/PRKCB/SP1/LRG1/GRN/JAK1/STAT3/PIK3CD/STIM1/TBXA2R
CC	GO:0032432	actin filament bundle	52	-0.68	-2.06	5.07E-06	5.95E-05	3.65E-05	1895	tags=48%, list=16%, signal=40%	MARCKS/DCTN4/TPM3/ACTA2/FHL3/LCP1/TPM4/PALLD/TPM1/PDLIM7/PDLIM5/FLNA/ZYX/RFLNB/CNN2/LPP/PXN/GAS2L1/PDLIM1/ACTN4/MYLK/ABLIM3/ACTN1/MYL9/MYH9

BP	GO:0048589	developmental growth	355	-0.41	-1.62	5.09E-06	5.95E-05	3.66E-05	2089	tags=27%, list=18%, signal=23%	BCL2L11/HEG1/DISC1/WWC3/MACF1/SRF/PTEN/KDM5B/FTO/SRC/TAF8/HDGFL2/ATM/NINJ1/ADNP/DBNL/STK4/H3-3B/EXT1/NIPBL/H3-3A/RC3H2/CPNE5/NOTCH2/CTDP1/PLCB1/NDEL1/STAT5B/EZR/KMT2D/DBN1/CPQ/CPNE1/MAPK14/CDKL5/YY1/ZFP36L1/BNC2/SEMA4D/ARIH2/CDKN1C/KPNA1/PTPN12/TRIM28/LEPR/GSK3A/DPYSL2/INSR/SGPL1/RBPJ/RARA/SEMA4A/ARID2/EIF4G2/CHST11/KLF5/IGF2/VCL/ZMIZ1/TBL1XR1/POR/SFRP2/STK11/PPP3CA/USP9X/PDLIM5/STK40/PLEC/MED1/IST1/G6PD/BASP1/ULK1/ANXA6/SIN3A/CYFIP2/GSK3B/STAT5A/DNM2/PSAP/AKAP13/GPX1/FKBP8/BCL9/WDTC1/PIM1/CDKN1A/STAT3/MTPN/CTTN/SP2/YBX3/JARID2/FOXO3/IQGAP1/NINJ2/HLX
BP	GO:0045637	regulation of myeloid cell differentiation	146	-0.51	-1.80	5.24E-06	6.11E-05	3.75E-05	1812	tags=32%, list=15%, signal=28%	TGFB1/PPARGC1B/ITPKB/NOTCH2/STAT5B/RIPK1/KAT7/MAPK14/TRIB1/ZFP36L1/SPI1/CCR1/FOXP1/KLF13/ARNT/EIF6/CNOT4/FES/RARA/PTK2B/MEIS1/TRAF6/PITHD1/TOB2/ZBTB16/TFE3/LILRB3/MED1/MAFB/MTURN/LTF/HSPA1A/INPP5D/PF4/LYN/MPL/CUL4A/GATA1/NF1/HCLS1/TESC/STAT3/TAL1/FAM210B/PRKDC/FOXO3/FAXDC2
BP	GO:0061564	axon development	255	-0.45	-1.69	5.26E-06	6.11E-05	3.75E-05	2312	tags=36%, list=20%, signal=30%	ALCAM/EPHA1/SYNGAP1/NEO1/CNP/ARHGAP4/SVBP/PLXNA4/HDAC6/AMIGO1/TWF2/CREB1/KIAA0319/DIP2B/BCL11B/KIF13B/EPHB2/STK25/RPS6KA5/EFNB1/NIN/SPRY3/PARD6B/DISC1/MACF1/SRF/FYN/PTEN/FEZ1/MAP1S/EPHB4/FKBP1B/TRIO/ADNP/DBNL/ERBB2/EXT1/RNF165/TIAM1/NCAM1/SZT2/NOTCH2/NECTIN1/NUMB/B4GALT5/NDEL1/DBN1/EPHB1/CDKL5/SIPA1L1/FLOT1/PARD3/SEMA4D/MYCBP2/EPHA4/LYPLA2/CSF1R/DPYSL2/STK24/PALLD/TSPO/SEMA4A/EIF4G2/PAK1/MAP1A/VCL/CTNNA1/STK11/PLXNC1/APLP2/USP9X/SCN1B/IST1/FLNA/ULK1/NIBAN2/ACTB/RAC1/MARK2/SIN3A/GSK3B/RAC2/DNM2/GRN/ECE1/CTTN/BSG/PAK2/VASP/MBP/KEL/XK/RHOG
CC	GO:0005912	adherens junction	108	-0.56	-1.91	5.54E-06	6.42E-05	3.94E-05	1719	tags=41%, list=15%, signal=35%	CDC42EP1/ANXA2/TBCD/SCRIB/ARHGAP24/TNK2/SHROOM1/MYO1E/CD99L2/SDCBP/NECTIN1/EZR/NF2/FLOT1/CDC42EP4/PARD3/CTNND1/FRMD4B/EPHA4/PKP4/TJP2/PIP5K1C/PPP1CA/S100A11/EIF4G2/FRMD4A/VCL/PDLIM7/CTNNA1/PDLIM5/TSPAN33/ESAM/NIBAN2/ACTB/ZYX/LYN/FLOT2/PDLIM1/MSN/ADD1/PPP1R9B/SH3BP1/MYH9/TLN1
BP	GO:0008015	blood circulation	238	-0.46	-1.70	5.61E-06	6.48E-05	3.98E-05	2136	tags=26%, list=18%, signal=22%	ATP2B1/SERPING1/SOD2/THRA/KNG1/SRC/FKBP1B/ABAT/EXT1/TGFB1/AGTRAP/PDE2A/ROCK2/OPRL1/DOCK5/GRK2/KCNH2/ACTA2/PTPRJ/PER2/TJP2/GSK3A/F5/HRH2/ANPEP/PRKACA/GUCY1B1/ATP2A3/TPM1/TMEM38A/SLC8A1/BCR/PTGS1/BMP6/GSN/PLCB3/SLC9A1/PTAFR/GAA/SCN1B/PLEC/ATP1B2/EHD3/CALM3/PIK3CG/FLNA/CYB5R3/PDE5A/MYL4/ATP2B4/RHOA/GPX1/GNA12/ECE1/GUCY1A1/CLDN5/PTP4A3/WNK1/KEL/P2RX1/PLVAP/TBXA2R/MAP2K3
BP	GO:0048667	cell morphogenesis involved in neuron differentiation	305	-0.42	-1.62	5.96E-06	6.87E-05	4.22E-05	2765	tags=34%, list=24%, signal=27%	ALCAM/EPHA1/SYNGAP1/NEO1/CNP/ARHGAP4/PLXNA4/STAU2/HDAC6/AMIGO1/TWF2/CREB1/ACTR2/TBCD/SCRIB/KIAA0319/DIP2B/SLC9A3R1/BCL11B/KIF13B/EPHB2/STK25/RPS6KA5/EFNB1/NIN/PARD6B/DISC1/MACF1/SRF/FYN/PTEN/FEZ1/TRIOBP/MAP1S/EPHB4/YWHAH/TRIO/KIDINS220/ADNP/DBNL/ERBB2/EXT1/RNF165/TIAM1/NCAM1/CUL7/SZT2/NOTCH2/NECTIN1/NUMB/LRRK2/B4GALT5/NDEL1/DLG4/DBN1/EPHB1/CDKL5/SIPA1L1/FLOT1/PARD3/SEMA4D/MYCBP2/ABI3/EPHA4/LYPLA2/CSF1R/DPYSL2/PALLD/VLDLR/SEMA4A/RAPGEF2/EIF4G2/PAK1/MAP1A/VCL/RERE/STK11/PLXNC1/PPP3CA/APLP2/USP9X/PDLIM5/SCN1B/IST1/ULK1/NIBAN2/DNM3/ACTB/RAC1/MARK2/SIN3A/GSK3B/RAC2/DNM2/ECE1/CFL1/CTTN/BSG/PAK2/VASP/MBP/CUX1/KEL/XK/RHOG

CC	GO:0009925	basal plasma membrane	146	-0.51	-1.79	6.17E-06	7.09E-05	4.35E-05	1869	tags=24%, list=16%, signal=20%	ERBB2/ATP7A/SLC12A6/NUMB/EZR/C5AR1/STX4/FLOT1/SLC16A3/AQP9/LEPR/SLC27A1/SLC2A1/LDLRAP1/SLC3A2/SLC9A1/SLC6A9/B4GALT1/SLC1A5/SLC14A1/ABCC4/AQP1/LRP1/FLOT2/ABCC3/ATP2B4/ARRB2/MSN/EPB41/SLC4A1/AQP10/BSG/SLC7A5/WASF2/ANK1
BP	GO:0007044	cell-substrate junction assembly	61	-0.64	-2.00	6.28E-06	7.19E-05	4.41E-05	2202	tags=46%, list=19%, signal=37%	LIMS1/MAP4K4/MACF1/PTEN/PPM1F/SRC/SDC4/ACTG1/ROCK2/PTPRJ/DAPK3/GREM1/CORO1C/PTK2B/BCR/BCAS3/VCL/ITGA5/SLC9A1/THBS1/PLEC/RAC1/ARHGAP6/RHOA/CTTN/ACTN1/DMTN/TLN1
BP	GO:0060485	mesenchyme development	142	-0.51	-1.80	6.29E-06	7.19E-05	4.41E-05	2192	tags=33%, list=19%, signal=27%	AXIN2/PTK7/EFNB1/GLIPR2/PTEN/EXOC4/IL17RD/EXT1/TGFB1/LDLRAD4/TIAM1/PHACTR4/CUL7/SDCBP/ROCK2/HGF/PEF1/ACTA2/ZFP36L1/MSX1/MCRIP1/BNC2/SEMA4D/MAD2L2/FUZ/USF3/EPHA4/TRIM28/S100A4/GREM1/RBPJ/CORO1C/SEMA4A/OTUD5/TGFB1I1/SFRP2/AKNA/ADIPOR1/BASP1/FLNA/ANXA6/RFLNB/GSK3B/TCF7L2/MAPK3/ZNF703/DAB2
BP	GO:1901888	regulation of cell junction assembly	101	-0.57	-1.89	6.32E-06	7.20E-05	4.42E-05	2232	tags=43%, list=19%, signal=35%	EPHB2/LIMS1/MAP4K4/TLR2/MACF1/PTEN/PPM1F/SRC/ADNP/SDC4/LRFN3/NECTIN1/ACTG1/ROCK2/EPHB1/PTPRJ/FLOT1/CLSTN1/SEMA4D/ABI3/DAPK3/VPS35/GPBAR1/PRKACA/GREM1/CORO1C/SEMA4A/RAPGEF2/BCAS3/VCL/SETD5/PDLIM5/SLC9A1/THBS1/FZD5/EIF4G1/RAC1/ARHGAP6/RHOA/SNCA/RAPGEF1/CLDN5/DMTN
BP	GO:0060284	regulation of cell development	296	-0.43	-1.62	6.47E-06	7.35E-05	4.51E-05	2240	tags=30%, list=19%, signal=25%	PRUNE1/EPHB2/BCL11A/LIMS1/AXIN2/STK25/NIN/TLR2/DISC1/MACF1/SRF/PTEN/UNC13D/TRIOBP/YWHAH/ADNP/DBNL/CASS4/TGFB1/TIAM1/DICER1/CUL7/NOTCH2/NUMB/ROCK2/LRRK2/PLCB1/NDEL1/SYNJ1/DBN1/DOCK5/MME/TRIB1/CDKL5/S1PR2/NF2/PER2/SMARCD3/HOOK3/SEMA4D/PREX1/EPHA4/GSK3A/ZNF335/NEDD9/TSPO/SORL1/CORO1C/NEURL1/SEMA4A/RAPGEF2/EIF4G2/VCL/CTNNA1/STK11/PLXNC1/PPP3CA/IST1/LTF/SKI/FLNA/ULK1/NIBAN2/GJC2/RAC1/RFLNB/LYN/GSK3B/PDE5A/DNM2/TNFRSF1B/CTDSP1/TRIM11/MPL/MYADM/NF1/ACTN4/ADD1/GRN/TGM2/CLDN5/PRKDC/DAB2/MBP/TRIM58/CUX1/RNF10/DMTN
MF	GO:0005516	calmodulin binding	114	-0.54	-1.85	6.75E-06	7.65E-05	4.70E-05	2635	tags=43%, list=22%, signal=34%	FBXL2/SNTB2/ADD2/SNTB1/MKNK1/ASPM/PCYT1A/RGS16/MYO5A/MYO1G/ATP2B1/STRN/MYO1E/EDF1/SLC24A4/MARCKS/ITPKB/RIT1/PLCB1/SPTAN1/PHKA2/CAMSAP1/INVS/KCNQ5/PDE1A/SPTBN1/MKNK2/SLC8A1/CALD1/PLCB3/UBR4/PPP3CA/SLC9A1/MYO9B/MAPKAPK3/IQGAP2/MYO1F/CAMK1D/CNN2/ATP2B4/ADD1/NRGN/MYLK/EPB41/PPP3R1/MAPKAPK2/MBP/MYH9/IQGAP1
MF	GO:0019901	protein kinase binding	520	-0.38	-1.51	6.78E-06	7.66E-05	4.70E-05	2416	tags=30%, list=21%, signal=25%	MAPRE2/RFFL/CALM1/DUSP16/UTRN/TOM1L2/CRY2/PRKAR1B/MAP4K2/MVP/DUSP1/KIF13B/HDAC7/TRIM5/KAT2B/BCL11A/SH2B3/LIMS1/PKN1/AXIN2/MAVS/TRIM38/CDKN2D/IRAK3/INKA1/BCL2L14/BCL2L11/PARD6B/FGR/PTEN/FEZ1/TIRAP/IFNAR2/SRC/IRS2/RHOF/GCN1/CPNE3/CDK12/PPP1R15A/MARCKS/SDC4/XIAP/CCNY/LYST/CASS4/EEF2/SIRPA/AP2A2/CTBP2/GSTP1/NBR1/NBEAL2/STK38/CACUL1/EZR/DLG4/NFATC1/SH2B2/SHC1/MAPK14/TRIB1/ACTA2/PTPRJ/MAP3K2/ATF7/PTPN6/MAD2L2/CTNND1/RELB/DAZAP2/EPHA4/ITGB2/CDK13/PGAM1/TRIB3/TJP2/GSK3A/GRB2/SASH1/YWHAZ/PRKACA/NEDD9/PITPNM2/MAP3K5/SUFU/AP2A1/RARA/PTK2B/GAB2/CAVIN2/WARS1/CCND3/TRAF6/PRKD2/TUBA4A/CSK/GP6/PDLIM5/MAP2K7/PTPRC/WDR45/SYK/PTAFR/PTPN1/PPP1R12B/ADIPOR1/NR3C1/MAPKAPK3/DCTN1/CLTC/CALM3/FZD5/PRAM1/SKI/FLNA/ACTB/PLEK/RAC1/GOLGA2/GSK3B/RAC2/DNM2/HSPB1/TCF7L2/ATP2B4/RHOA/TNIP1/PRKCB/TRIM14/ARRB2/MSN/HCLS1/ATG13/RHOBTB1/WAS/PINK1/CDKN1A/AP1B1/BCL2L1/STAT3/PTPN23/MAPKAPK2/PRKAR2A/PRKAR2B/SQSTM1/PAK2/WNK1/FBXO7/FOXO3/CBL/IQGAP1/MAP2K3/RHOG/CNPPD1/RELA

BP	GO:0031346	positive regulation of cell projection organization	232	-0.46	-1.72	6.83E-06	7.69E-05	4.72E-05	2604	tags=35%, list=22%, signal=28%	PLXNA4/STAU2/AMIGO1/TWF2/CDC42EP1/FSCN1/FIG4/ACTR2/CFLAR/NFE2L2/EPHB2/PTK7/STK25/NIN/KATNB1/DISC1/MACF1/SRF/FYN/WNT1/FEZ1/SRC/KCTD17/FKBP1B/KIDINS220/ADNP/DBNL/ATP7A/EPS8/SNX3/TIAM1/CUL7/HSPA5/RETREG3/HGF/ENC1/NDEL1/DLG4/DBN1/CDKL5/SETX/CDC42EP4/SEMA4D/ARAP1/FUZ/EPHA4/STK24/SF3A2/FES/VLDLR/CORO1C/HTT/PTK2B/NEURL1/RAPGEF2/EIF4G2/BCAS3/MARK4/STK11/PLXNC1/DVL3/SCN1B/ATP1B2/IST1/CAMK1D/PPP2R5B/FLNA/DNM3/RAC1/MARK2/LYN/GSK3B/RAC2/DNM2/GRN/RAPGEF1/EHD1/RIPOR2/PLEK2/CUX1/WASF2
BP	GO:0002573	myeloid leukocyte differentiation	151	-0.50	-1.78	7.39E-06	8.30E-05	5.10E-05	1812	tags=34%, list=15%, signal=29%	NRROS/IREB2/TF/IFI16/CTNNBIP1/TLR2/SRC/TGFB1/PPARGC1B/NOTCH2/RIPK1/MAPK14/TRIB1/CEBPA/ZFP36L1/CBFA2T3/SBNO2/RELB/SPI1/LRRK1/CCR1/CSF1R/ATP6AP1/FOXP1/FES/OSCAR/RBPJ/RARA/GAB2/SNX10/TRAF6/TOB2/MMP9/TFE3/THOC5/LILRB3/MED1/MAFB/LTF/INPP5D/PF4/LYN/CUL4A/GATA1/FASN/NF1/HCLS1/TESC/TAL1/PIK3CD/MYH9/EIF2AK1
BP	GO:1903844	regulation of cellular response to transforming growth factor beta stimulus	77	-0.59	-1.90	7.71E-06	8.64E-05	5.30E-05	2042	tags=42%, list=17%, signal=35%	ZEB2/PMEPA1/CFLAR/ADAM17/NRROS/WNT1/IL17RD/TGFB1/LDLRAD4/ZBTB7A/SDCBP/HSPA5/CDKN1C/FAM89B/PDPK1/CHST11/TGFB1I1/STK11/THBS1/GLG1/HIPK2/SKI/HSPA1A/DNM2/CREBBP/FKBP1A/LRG1/FLCN/LTBP1/ZNF703/PPM1A/DAB2
BP	GO:0007417	central nervous system development	581	-0.37	-1.49	7.96E-06	8.85E-05	5.43E-05	2514	tags=30%, list=21%, signal=25%	KDM7A/KDM4A/PBX2/RGS7/BAK1/SETD2/CREB1/SCRIB/ARCN1/CALM1/ASPM/WLS/SCIN/OTX1/AATK/BCL11B/GIGYF2/TBX19/NRROS/C1QA/VIM/EPHB2/FANCC/NIN/TLR2/ATP2B1/BCL2L11/DISC1/SRF/FYN/WNT1/PADI2/PTEN/PHF8/FEZ1/SRC/DNAJB1/APAF1/MAP1S/YWHAH/IRS2/TRIO/ATM/ABAT/SNRPB/STK4/MARCKS/ERBB2/EXT1/NIPBL/ATP7A/TGFB1/SYNJ2/SZT2/ZNF148/SYNE2/HSPA5/NUMB/GSTP1/ITGAM/LRRK2/B4GALT5/PLCB1/MPST/NDEL1/FOXP2/SYNJ1/NOTCH2NLA/ELP3/C5AR1/VPS51/WASF3/EPHB1/NF2/AGTPBP1/MSX1/HOOK3/CKB/MYCBP2/CTNND1/FPGS/EPHA4/ARPC5/CSF1R/WDR1/ZFHX3/MTF1/UBE2L3/DPYSL2/ZNF335/MBOAT7/NDRG2/SUFU/TPP1/TACC1/RBPJ/VLDLR/SH3GL1/PFKFB3/RARA/SLC2A1/NEURL1/SLC8A1/PAFAH1B2/BCR/MEIS1/RAPGEF2/HERC1/SOX6/ALDH5A1/PIAS4/ZMIZ1/CSK/RERE/ZBTB16/NDE1/SFRP2/VCAN/GSN/AKNA/PPP3CA/APLP2/SCN1B/ATP1B2/BPTF/MED1/MAFB/CASP2/DCTN1/CALM3/G6PD/TACC3/BASP1/CHD8/SKI/NDST1/ODAD4/ACTB/GJC2/AQP1/LYN/SIN3A/GSK3B/ROGDI/LRP1/TNFRSF1B/ATP2B4/RHOA/SCYL1/CLU/ATXN1/ARRB2/NF1/NCOA6/TRAPPC9/GRN/PPP1R9B/NRGN/MAPK3/PBX1/STAT3/MTPN/TAL1/UBB/NAPA/ATP6V0D1/PRKDC/SHARPIN/MBP/JARID2/FOXO3/SEC16A/BAG6
CC	GO:0030133	transport vesicle	264	-0.43	-1.64	7.94E-06	8.85E-05	5.43E-05	2085	tags=28%, list=18%, signal=23%	RAB11FIP5/SNAP25/DISC1/UNC13D/RAB27B/ZNRF1/SSPN/BLOC1S3/AP1G1/ATP7A/HCK/STX6/SCAP/AFTPH/COPE/RPH3A/LRRK2/NDEL1/SYTL3/SYN3/DLG4/MME/CLCN5/STX4/PEF1/CRISPLD2/SORT1/CLN3/SYNRG/COPB2/TMEM184A/CHP1/CA4/SEPTIN1/STX5/SPG21/CNIH3/AP2A1/SORL1/COPA/SEC31A/TGOLN2/RAB7A/RAB14/RAB5B/KDELR1/PLIN3/CLTC/CALM3/SLC6A9/YIPF2/SIPA1/DNAJC5/KIF1B/LAPTM5/IGF2R/TPRG1L/RAB11B/ROGDI/SYTL4/IQSEC1/ATP6V1A/NRGN/SNCA/BCL2L1/LAMP1/ATP6V0D1/RAB3IL1/STON2/RAB3D/SEC16A/RAB1B/FURIN

BP	GO:0071345	cellular response to cytokine stimulus	552	-0.37	-1.50	7.97E-06	8.85E-05	5.43E-05	2282	tags=28%, list=19%, signal=24%	USP27X/NKIRAS2/DUSP1/RAB43/MUL1/IFI16/VIM/SH2B3/IL2RG/LIMS1/MAVS/RPS6KA5/FCER1G/IRAK3/TLR2/CHI3L1/PADI2/KDM5B/IFNAR2/SRC/LILRA1/IFNAR1/IL17RD/XIAP/EXT1/LSP1/HCK/EPS8/SIRPA/PDE2A/LILRA5/CTBP2/HSPA5/ACTG1/LSM14A/GSTP1/IKBKE/GPS2/PLCB1/STAT5B/NFAT5/SMPD4/OASL/EGR1/CMKLR1/RIPK1/KEAP1/CD300LF/MME/CPNE1/MAPK14/STX4/CEBPA/MT2A/TLE4/YY1/PTPRJ/ZFP36L1/PTPN6/EIF5A/DOK1/PYCARD/CDC42EP4/NR1H2/SBNO2/IFITM3/ALAD/SPATA2/DAPK3/SPI1/PML/CXCL5/CCR1/CSF1R/ARHGEF2/RFX2/CDIP1/LEPR/GSK3A/ADIPOR2/PRKACA/YTHDF3/PF4V1/USP10/MAP3K5/SLC27A1/NFKB1/HIPK1/UPF1/YBX1/IRF1/DOCK8/PTK2B/SELPLG/SNX10/STAT6/CXCR2/INPP5K/RPS6KA4/TRAF6/LDLRAP1/KLF5/IL6R/PIAS4/IL13RA1/GSN/PTPRC/LILRB3/SYK/ADAR/THBS1/PTPN1/ADIPOR1/MED1/IL1RN/IL17RA/JAK3/HSPA1A/PF4/AGPAT1/ZYX/LGALS9/LAPTM5/GSK3B/STAT5A/TNFRSF1B/IFITM2/MPL/RHOA/PRPF8/FASN/CXCR1/ACTN4/JAK1/HCLS1/IFIT1B/WAS/MAPK3/H2BC11/STAT3/KHSRP/RIPOR2/PTP4A3/HDGF/YBX3/SHARPIN/JARID2/ACKR1/WNK1/PLVAP/TOLLIP/FOXO3/CBL/CDC34/RELA
CC	GO:0001725	stress fiber	49	-0.67	-2.02	8.40E-06	9.25E-05	5.68E-05	1458	tags=45%, list=12%, signal=39%	DCTN4/TPM3/ACTA2/FHL3/LCP1/TPM4/PALLD/TPM1/PDLIM7/PDLIM5/ZYX/CNN2/LPP/PXN/GAS2L1/PDLIM1/ACTN4/MYLK/ABLIM3/ACTN1/MYL9/MYH9
CC	GO:0097517	contractile actin filament bundle	49	-0.67	-2.02	8.40E-06	9.25E-05	5.68E-05	1458	tags=45%, list=12%, signal=39%	DCTN4/TPM3/ACTA2/FHL3/LCP1/TPM4/PALLD/TPM1/PDLIM7/PDLIM5/ZYX/CNN2/LPP/PXN/GAS2L1/PDLIM1/ACTN4/MYLK/ABLIM3/ACTN1/MYL9/MYH9
CC	GO:0045202	synapse	715	-0.35	-1.42	8.92E-06	9.79E-05	6.01E-05	2080	tags=26%, list=18%, signal=23%	ATXN3/SNAP25/DISC1/FYN/PTEN/LRRC4/ITSN1/UBA52/SRC/DNAJB1/RAB27B/MAP1S/EXOC4/ELK1/ZNRF1/MTHFR/NINJ1/SSPN/DBNL/GRIK2/EXT1/ATP7A/EPS8/EEF2/STX6/LRFN3/TIAM1/SYNJ2/CPEB4/PDE2A/OR10H1/DLGAP4/SLC12A6/SDCBP/TSC2/CTBP2/NECTIN1/ACTG1/NUMB/SYAP1/RPH3A/LRRK2/PLCB1/MPST/AP2B1/NDEL1/SYNJ1/NPTXR/SYN3/DLG4/DBN1/MME/WASF3/EPHB1/CLCN5/MAPK14/CACNB1/STX4/CTBP1/CDKL5/GRK2/SIPA1L1/FXYD6/FLOT1/CLSTN1/SLC16A3/CHRNE/EXOC3/CTNND1/CLN3/KPNA1/ABI3/VPS35/EPHA4/SHISA7/PDPK1/GNAQ/PKP4/RTN3/ARF1/GSK3A/UBE2L3/PIP5K1C/YWHAZ/PPP1CA/CANX/HRH2/PRKACA/SEPTIN1/GUCY1B1/SH3KBP1/ADGRL1/PALLD/SPTBN1/CNIH3/FXR2/CORO1C/SH3GL1/HTT/PTK2B/SLC2A1/NEURL1/SLC8A1/BCR/RAB6B/RAPGEF2/SLC3A2/MAP1A/RAB7A/ARL8A/NDE1/PLCB3/PPP3CA/DYNLL2/ITGA5/PDLIM5/APBA3/RAB5B/FBXL20/GNB1/ASAP1/ATP1B2/CALM3/SLC6A9/FZD5/DNM3/VCP/PVALB/ACTB/DNAJC5/RAC1/GABARAP/KIF1B/SLA/LYN/HIP1/CYFIP2/GSK3B/DNM2/TPRG1L/RAB11B/ROGDI/FLOT2/ATP2B4/GNAI2/GRIPAP1/IQSEC1/RHOA/EIF3B/CAPZB/PRKCB/STAU1/LRFN1/CLU/ARRB2/NF1/ADD1/PPP1R9B/NRGN/RPS6KA1/SNCA/GUCY1A1/BCL2L1/CTTN/PCBP2/LAMP1/ABLIM3/CAPN5/PRKAR2B/ACTN1/NAPA/DENND1A/ATP6V0D1/AP2M1/GAK/STON2/RAB3D/RNF40/PAK2/ITGB3/SHARPIN/MBP/RNF10/P2RX1/MYH9/GLUL/WASF2/DMTN/RELA

BP	GO:0043269	regulation of ion transport	336	-0.41	-1.58	9.05E-06	9.89E-05	6.07E-05	2579	tags=30%, list=22%, signal=24%	CD4/CACNB4/P2RY1/AMIGO1/LGALS3/CAPN3/TMEM109/PPIF/RGS7/BAK1/TRPC3/CALM1/CYBB/CORO1A/UTRN/CRY2/SLC9A3R1/TF/EPHB2/STIMATE/ATP2B1/FYN/TPCN1/PER1/YWHAH/FKBP1B/ABAT/PRKCE/KCNJ15/ATP7A/LILRA5/ANO6/ABCB11/OPRL1/RIPK1/DLG4/CACNB1/CA2/KCNH2/FXYD6/PER2/PTPN6/HVCN1/TMBIM6/CD63/PTGES/SHISA7/PDPK1/PML/KCNQ5/CCR1/CHP1/PRKACA/CNIH3/TSPO/TMEM38A/HTT/PTK2B/SLC8A1/INPP5K/CLIC3/MMP9/SYK/SLC9A1/PTAFR/SELENON/SCN1B/ATP1B2/EHD3/CALM3/SLC6A9/PIK3CG/G6PD/FLNA/JAK3/CAPN1/SLC43A2/GJC2/LYN/DNM2/SLC43A1/RAB11B/WWP2/ATP2B4/GNAI2/FKBP1A/RHOA/ARRB2/ACTN4/PINK1/MLLT6/TESC/MYLK/SNCA/EPB41/AHCYL1/STIM1/ITGB3/WNK1/KEL/P2RX1/DIAPH1
BP	GO:0009991	response to extracellular stimulus	309	-0.42	-1.62	9.10E-06	9.91E-05	6.08E-05	2156	tags=30%, list=18%, signal=25%	CDKN2D/ATP2B1/FOXO1/SOD2/SRF/RRAGD/SRC/APAF1/SH3GLB1/FKBP1B/MTHFR/GCN1/ADNP/CD68/VDR/LARS1/CHSY1/CPEB4/LAMP2/SZT2/MGMT/HSPA5/LRRK2/TFEB/MAPK14/EIF2AK2/NR1H2/DAP/ALAD/VPS41/CLEC16A/MAP1LC3B/F5/PDK2/MTMR3/ALPL/PCSK1N/SESN3/SESN2/STK24/ZFYVE1/MAP3K5/FES/KANK2/TSPO/SORL1/RARA/SLC2A1/SLC8A1/RRAGC/BCAS3/RASGRP4/MAX/USF2/TBL1XR1/POR/SFRP2/CPT1A/PTPRC/WDR45/FOXO4/ATG7/PLEC/MED1/WIPI2/G6PD/EIF4G1/ULK1/RIPOR1/SIPA1/UCP2/RXRA/GABARAP/LYN/ELAPOR1/GNAI2/GAS2L1/GPX1/PPP1R9B/LARP1/PIM1/MAPK3/CDKN1A/NUAK2/FLCN/TBC1D5/NCOA1/GLUL/NPRL3/FOXO3/CBL/TBXA2R/EIF2AK1
BP	GO:0021700	developmental maturation	161	-0.49	-1.76	9.34E-06	0.00010141	6.23E-05	2065	tags=29%, list=18%, signal=24%	DISC1/WNT1/PTEN/ANKRD17/SLC24A4/BLOC1S3/EXT1/AP1G1/ZBTB7A/PTBP3/XYLT1/LRRK2/B4GALT5/NFIA/DLG4/ROPN1L/CEBPA/CLSTN1/SEMA4D/CD63/CDKN1C/AP1M1/YWHAZ/PRKACA/ADGRL1/GREM1/RBPJ/SLFN14/PTK2B/NEURL1/SNX10/RERE/ZBTB16/PHOSPHO1/FZD5/LTF/ABHD2/G6PD/RFLNB/RHOA/NF1/TAL1/FAM210B/EPB42/TRIM58/LYL1/FOXO3
BP	GO:0032869	cellular response to insulin stimulus	150	-0.50	-1.78	9.37E-06	0.00010152	6.23E-05	2506	tags=34%, list=21%, signal=27%	YWHAG/SOCS3/CFLAR/GPLD1/MYO5A/KAT2B/ATP2B1/FOXO1/SRC/IRS2/ZDHHC7/TSC2/SYAP1/SMARCC1/NDEL1/GAB1/SH2B2/PIP4K2C/SHC1/ZFP36L1/PDPK1/TRIB3/GSK3A/GRB2/PDK2/PKM/INSR/SESN3/SLC27A1/FBP1/SORL1/INPP5K/CCND3/IGF2/ZNF106/SLC9A1/FOXO4/PTPN1/RAB31/ADIPOR1/LPIN2/UCP2/GSK3B/GNAI2/PRKCB/SP1/WDTC1/PIP4K2A/AKT2/PRKDC/EIF4EBP2
BP	GO:0031032	actomyosin structure organization	122	-0.52	-1.81	9.56E-06	0.00010329	6.34E-05	2803	tags=42%, list=24%, signal=32%	SMAD3/TMOD3/EPHA1/F11R/FRMD3/CARMIL1/EPB41L3/CLASP1/VPS4A/CAPN3/CFLAR/PPFIA1/ARHGEF5/FLII/CSRP1/CDC42BPA/SRF/PPM1F/SRC/CDC42BPB/MYO18A/SDC4/ACTG1/ROCK2/ARRB1/NF2/ARAP1/WDR1/ALMS1/TPM1/PTK2B/INPP5K/PAK1/SLC9A1/PLEC/ZYX/RAC1/AKAP13/CNN2/PXN/ARHGAP6/RHOA/PDLIM1/TMOD1/WAS/ITGB5/EPB41/MYL9/PAK2/MYH9/WASF2
BP	GO:0051261	protein depolymerization	81	-0.59	-1.89	9.64E-06	0.00010392	6.38E-05	2660	tags=51%, list=23%, signal=39%	CARMIL1/ADD2/CLASP1/HDAC6/VPS4A/TWF2/SCIN/FLII/VPS4B/KATNB1/TRIOBP/MAP1S/ATXN7/EPS8/CKAP5/SYNJ1/SPTAN1/CAMSAP1/WASHC2C/NCKAP5L/ARHGEF2/WDR1/SPTBN1/SH3GL1/SPTA1/SVIL/MAP1A/TAOK1/GSN/PLEK/CAPZB/ADD1/PPP1R9B/TMOD1/CFL1/SPTB/MTPN/SH3BP1/GAK/MICAL2/DMTN

BP	GO:0007409	axonogenesis	229	-0.45	-1.69	9.99E-06	0.0001073	6.59E-05	2765	tags=37%, list=24%, signal=29%	ALCAM/EPHA1/SYNGAP1/NEO1/CNP/ARHGAP4/PLXNA4/HDAC6/AMIGO1/TWF2/CREB1/KIAA0319/DIP2B/BCL11B/KIF13B/EPHB2/STK25/RPS6KA5/EFNB1/NIN/PARD6B/DISC1/MACF1/SRF/FYN/PTEN/FEZ1/MAP1S/EPHB4/TRIO/ADNP/DBNL/ERBB2/EXT1/RNF165/TIAM1/NCAM1/SZT2/NOTCH2/NECTIN1/NUMB/B4GALT5/NDEL1/DBN1/EPHB1/CDKL5/SIPA1L1/FLOT1/PARD3/SEMA4D/MYCBP2/EPHA4/LYPLA2/CSF1R/DPYSL2/PALLD/SEMA4A/EIF4G2/PAK1/MAP1A/VCL/STK11/PLXNC1/APLP2/USP9X/SCN1B/IST1/ULK1/NIBAN2/ACTB/RAC1/MARK2/SIN3A/GSK3B/RAC2/DNM2/ECE1/CTTN/BSG/PAK2/VASP/MBP/KEL/XK/RHOG
BP	GO:0150115	cell-substrate junction organization	67	-0.62	-1.95	1.00E-05	0.0001073	6.59E-05	2202	tags=43%, list=19%, signal=35%	LIMS1/MAP4K4/MACF1/PTEN/PPM1F/SRC/SDC4/ACTG1/ROCK2/PTPRJ/DAPK3/GREM1/CORO1C/PTK2B/BCR/BCAS3/VCL/ITGA5/SLC9A1/THBS1/PLEC/RAC1/ARHGAP6/IQSEC1/RHOA/CTTN/ACTN1/DMTN/TLN1
BP	GO:0031667	response to nutrient levels	286	-0.43	-1.63	1.01E-05	0.00010759	6.61E-05	2156	tags=30%, list=18%, signal=25%	CDKN2D/ATP2B1/FOXO1/SOD2/SRF/RRAGD/SRC/APAF1/SH3GLB1/FKBP1B/MTHFR/GCN1/CD68/VDR/LARS1/CHSY1/CPEB4/LAMP2/SZT2/MGMT/HSPA5/LRRK2/TFEB/MAPK14/EIF2AK2/NR1H2/DAP/ALAD/VPS41/CLEC16A/MAP1LC3B/F5/PDK2/MTMR3/ALPL/PCSK1N/SESN3/SESN2/STK24/ZFYVE1/MAP3K5/FES/KANK2/TSPO/SORL1/RARA/SLC2A1/SLC8A1/RRAGC/BCAS3/MAX/USF2/TBL1XR1/POR/SFRP2/CPT1A/WDR45/FOXO4/ATG7/PLEC/MED1/WIPI2/G6PD/EIF4G1/ULK1/RIPOR1/UCP2/RXRA/GABARAP/ELAPOR1/GNAI2/GAS2L1/GPX1/LARP1/PIM1/MAPK3/CDKN1A/NUAK2/FLCN/TBC1D5/NCOA1/GLUL/NPRL3/FOXO3/CBL/TBXA2R/EIF2AK1
BP	GO:0060537	muscle tissue development	198	-0.47	-1.74	1.02E-05	0.000109	6.69E-05	1508	tags=24%, list=13%, signal=21%	EGR1/EPHB1/MAPK14/YY1/FLOT1/MSX1/GSK3A/HIVEP3/TPM1/GREM1/RBPJ/RARA/NEURL1/SLC8A1/ARID2/SOX6/SVIL/KLF5/IGF2/PPP3CA/PDLIM5/SLC9A1/SELENON/LMNA/PLEC/MED1/G6PD/SKI/NIBAN2/AKAP13/FKBP1A/RHOA/GPX1/ARRB2/NF1/KDM6B/BCL9/PIM1/MYLK/FLCN/MTPN/RIPOR2/YBX3/JARID2/KEL/NPRL3/XK/HLX
BP	GO:0007265	Ras protein signal transduction	261	-0.44	-1.66	1.06E-05	0.00011216	6.89E-05	1932	tags=26%, list=16%, signal=22%	ARHGEF12/DBNL/RASA2/EPS8/TIAM1/ITPKB/PHACTR4/G3BP1/RRAS/SETDB1/NOTCH2/RIT1/SDCBP/ROCK2/ARRB1/RASAL3/ARHGDIB/FLOT1/DOK1/CDC42EP4/GRAP2/CTNNAL1/SOS2/KPNB1/DHCR24/RALBP1/ARHGEF2/TRIM28/GRB2/FBP1/KANK2/BCR/RAPGEF2/LRRC59/RASGRP4/ELMO1/MYO9B/GNB1/PIK3CG/DOK3/RIPOR1/MADD/RAC1/GABARAP/RAC2/DNM2/AKAP13/ARHGAP6/IQSEC1/RHOA/NF1/ARHGDIA/GNA12/CFL1/WAS/CDKN1A/RAPGEF1/FLCN/CYTH4/RIPOR2/SQSTM1/SH3BP1/DENND1A/MFN2/ARHGAP1/CBL/WASF2/RHOG
BP	GO:2001236	regulation of extrinsic apoptotic signaling pathway	109	-0.55	-1.86	1.10E-05	0.00011697	7.18E-05	2181	tags=39%, list=19%, signal=32%	SH3RF1/BCL2L14/PTEN/PRDX2/SRC/MAP2K5/STK4/GSTP1/HGF/EYA3/TMBIM1/TMC8/RIPK1/STX4/PYCARD/PML/ARHGEF2/DEDD2/BIRC6/PPP1CA/HTT/CTNNA1/SFRP2/PTPRC/THBS1/PSME3/LMNA/HSPA1A/MADD/PF4/ITPRIP/RBCK1/GSK3B/TCF7L2/GATA1/GPX1/NF1/SIAH2/BCL2L1/CTTN/PAK2/STRADB/RELA
CC	GO:0030018	Z disc	68	-0.61	-1.93	1.13E-05	0.00011937	7.33E-05	1049	tags=32%, list=9%, signal=30%	PALLD/MYZAP/SLC2A1/SLC8A1/PAK1/PDLIM7/STK11/PPP3CA/PDLIM5/PPP1R12B/PLEC/FLNA/HSPB1/ATP2B4/FKBP1A/RTN2/PDLIM1/ACTN4/PARVB/ACTN1/SLC4A1/MYL9
BP	GO:0010594	regulation of endothelial cell migration	107	-0.55	-1.86	1.20E-05	0.00012707	7.80E-05	1286	tags=26%, list=11%, signal=24%	PDPK1/FOXP1/SASH1/PTK2B/SEMA4A/BCAS3/PRKD2/THBS1/PIK3CG/GFUS/GPI/RAC1/MAP3K3/STAT5A/HSPB1/ATP2B4/RHOA/NF1/SP1/GRN/SPARC/SH3BP1/PIK3CD/DNAJA4/ITGB3/GLUL/TBXA2R/MAP2K3

CC	GO:0030312	external encapsulating structure	183	-0.47	-1.72	1.22E-05	0.000128	7.86E-05	1123	tags=18%, list=10%, signal=17%	ALPL/PKM/S100A4/GREM1/TIMP2/PCSK6/F13A1/GFOD2/MMP9/TGFB1I1/SFRP2/VCAN/LTBP2/THBS1/GLG1/PHOSPHO1/CD151/ANXA6/PF4/LGALS9/PSAP/CLU/TGM2/ANXA11/SPARC/TFIP11/LTBP1/GP1BA/CTSB/HDGF/CTSD/KRT1/TIMP1
BP	GO:0031669	cellular response to nutrient levels	162	-0.50	-1.78	1.26E-05	0.00013205	8.11E-05	2303	tags=36%, list=20%, signal=30%	NFE2L2/MAP1LC3B2/AIFM1/IFI16/ATP2B1/FOXO1/SRF/RRAGD/SH3GLB1/GCN1/CD68/VDR/LARS1/CPEB4/LAMP2/SZT2/HSPA5/LRRK2/TFEB/EIF2AK2/DAP/VPS41/CLEC16A/MAP1LC3B/PDK2/MTMR3/SESN3/SESN2/STK24/ZFYVE1/MAP3K5/FES/KANK2/SLC2A1/RRAGC/MAX/USF2/WDR45/ATG7/MED1/WIPI2/EIF4G1/ULK1/RIPOR1/UCP2/RXRA/GABARAP/ELAPOR1/GAS2L1/PIM1/MAPK3/CDKN1A/NUAK2/FLCN/NCOA1/GLUL/NPRL3/FOXO3/CBL
MF	GO:0008013	beta-catenin binding	55	-0.63	-1.94	1.32E-05	0.00013801	8.47E-05	2803	tags=47%, list=24%, signal=36%	SMAD3/BCL9L/HDAC6/MED12/SLC9A3R1/CTNNBIP1/AXIN2/FOXO1/MED12L/NUMB/PTPRJ/SUFU/VCL/CTNNA1/TBL1XR1/FOXO4/DVL3/CHD8/GSK3B/TCF7L2/PXN/TBL1X/KDM6B/BCL9/CARM1/FOXO3
CC	GO:0043025	neuronal cell body	266	-0.43	-1.62	1.37E-05	0.00014347	8.81E-05	2699	tags=33%, list=23%, signal=26%	VTI1B/KCNE3/NDUFS7/KCNJ2/CCN3/SV2A/STAU2/HDAC6/AMIGO1/SEPTIN4/AMFR/SLC1A4/ENDOG/CYBB/PI4K2A/DIP2B/GIGYF2/MUL1/EPHB2/ENO2/ATP2B1/STRN/KATNB1/ZNF385A/DMWD/FEZ1/DNAJB1/TOP1/MAP1S/ELK1/ADNP/DBNL/GRIK2/ATP7A/SLC2A3/G3BP1/CPNE5/SYAP1/LRRK2/ENC1/NDEL1/MME/RAP1GAP/PYCARD/CKB/VPS35/EPHA4/NCF1/NUMA1/GSK3A/TXNRD2/PPP1CA/CANX/PDE1A/INSR/FXR2/MCRS1/SORL1/PTK2B/NEURL1/SLC8A1/RAPGEF2/SLC3A2/TBX21/MAP1A/SCN1B/DCTN1/FZD5/GLRX5/FLNA/PVALB/GJC2/ROGDI/TNFRSF1B/MPL/WDFY3/STAU1/PPP1R9B/RIN3/GNA12/NRGN/SNCA/SIAH2/PRKAR2B/UBB/MBP/GLUL/HCFC1
CC	GO:0099513	polymeric cytoskeletal fiber	422	-0.39	-1.53	1.38E-05	0.00014364	8.82E-05	2437	tags=31%, list=21%, signal=26%	CCT7/MTA1/TBCD/FBF1/MAPRE2/KRT15/CALM1/ASPM/SLC1A4/TUBGCP6/TPPP3/CORO1A/VMAC/MYO5A/MAP1LC3B2/KIF13B/TUBA8/VIM/KLHL22/NIN/VPS18/KATNB1/SHROOM1/DISC1/MACF1/FYN/FEZ1/SRC/MAP1S/HOOK2/DIAPH2/DBNL/XIAP/HCK/TPM3/TTLL4/TTLL5/DNAL4/NAV1/TCHP/ACTG1/CKAP5/NDEL1/SYNJ1/EZR/TUBB2A/DBN1/KEAP1/CHMP4B/MAP2K2/CDK2AP2/CAMSAP1/EML2/HOOK3/SLAIN2/CLIP4/MAP4/INVS/MAP1LC3B/NUMA1/NCKAP5L/ARHGEF2/LCP1/DPYSL2/KIF3C/TPM4/EIF6/WIPF2/AMOT/PALLD/TPM1/KIF1C/SLC8A1/KIF13A/BCAS3/SVIL/LDLRAP1/DYNC1H1/RASSF5/PAK1/TUBA4A/MAP1A/PDLIM7/NDE1/AKNA/DYNLL2/PDLIM5/TPGS2/JAM3/MYO9B/LMNA/PLEC/SPECC1/DCTN1/CLTC/CALM3/DYNLRB1/EML3/DNM3/ACTB/TUBB4B/CHMP1A/RAC1/GOLGA2/GABARAP/KIF1B/MARK2/RAC2/DNM2/AK1/ARHGAP6/WIPF1/COTL1/GAS2L1/RTN2/PDLIM1/TUBB/HCLS1/TMOD1/WAS/ESPN/TUBB1/CTTN/GAS7/ACTN1/RAB3D/STIM1/KLC3/KRT1/IQGAP1/DMTN/DIAPH1
BP	GO:0032984	protein-containing complex disassembly	195	-0.47	-1.72	1.44E-05	0.00014883	9.14E-05	2567	tags=42%, list=22%, signal=33%	CLASP1/HDAC6/VPS4A/TWF2/CALCOCO2/TECPR1/CALM1/EPG5/SCIN/MAP1LC3B2/SCAF4/DYRK3/SSRP1/IRAK3/FLII/VPS4B/DHX8/KATNB1/TRIOBP/MAP1S/ATXN7/MCOLN1/EPS8/LAMP2/SMARCC1/SMARCA4/CKAP5/SYNJ1/SPTAN1/CHMP4B/SETX/CAMSAP1/PHF23/SMARCD3/EIF5A/WASHC2C/CLN3/CLEC16A/MAP1LC3B/NCKAP5L/ARHGEF2/WDR1/GLE1/PPP1CA/INSR/SPTBN1/UPF1/SH3GL1/FRAT1/SPTA1/ARID2/SVIL/SMARCC2/MAP1A/TAOK1/GSN/GRWD1/IST1/VCP/RUBCN/HMGA1/PLEK/CHMP1A/GSK3B/TBC1D25/CAPZB/ADD1/PPP1R9B/TMOD1/ARID1A/CFL1/SPTB/MTPN/TFIP11/NAPA/SH3BP1/GAK/NFE2/GSPT1/MICAL2/DMTN

BP	GO:0055001	muscle cell development	93	-0.56	-1.86	1.45E-05	0.00014996	9.21E-05	1222	tags=38%, list=10%, signal=34%	HNRNPU/CAPN3/MAML1/CFLAR/MYOF/FHL2/FLII/CSRP1/SRF/CTDP1/ACTG1/YY1/WDR1/GSK3A/TPM1/NFATC2/SLC8A1/PPP3CA/PDLIM5/SELENON/LMNA/PLEC/G6PD/SKI/NIBAN2/AKAP13/DYSF/GPX1/ACTN4/TMOD1/RIPOR2/ACTN1/MYL9/KEL/XK
BP	GO:0010232	vascular transport	59	-0.64	-1.97	1.48E-05	0.00015051	9.24E-05	1174	tags=29%, list=10%, signal=26%	LEPR/ABCG2/INSR/ATP2A3/SLC27A1/SLC2A1/SLC6A20/SLC24A3/ATP1B2/SLC6A9/SLC1A5/ABCC4/LRP1/ABCC3/ATP2B4/SLC7A5/SLC38A5
BP	GO:0016525	negative regulation of angiogenesis	51	-0.65	-1.98	1.47E-05	0.00015051	9.24E-05	1717	tags=35%, list=15%, signal=30%	GTF2I/HGS/ROCK2/PML/AMOT/SEMA4A/FOXO4/THBS1/AGO1/NIBAN2/PF4/ATP2B4/EPN1/FOXJ2/NF1/SPARC/CLDN5/E2F2
BP	GO:0150104	transport across blood-brain barrier	59	-0.64	-1.97	1.48E-05	0.00015051	9.24E-05	1174	tags=29%, list=10%, signal=26%	LEPR/ABCG2/INSR/ATP2A3/SLC27A1/SLC2A1/SLC6A20/SLC24A3/ATP1B2/SLC6A9/SLC1A5/ABCC4/LRP1/ABCC3/ATP2B4/SLC7A5/SLC38A5
BP	GO:1901343	negative regulation of vasculature development	51	-0.65	-1.98	1.47E-05	0.00015051	9.24E-05	1717	tags=35%, list=15%, signal=30%	GTF2I/HGS/ROCK2/PML/AMOT/SEMA4A/FOXO4/THBS1/AGO1/NIBAN2/PF4/ATP2B4/EPN1/FOXJ2/NF1/SPARC/CLDN5/E2F2
BP	GO:2000181	negative regulation of blood vessel morphogenesis	51	-0.65	-1.98	1.47E-05	0.00015051	9.24E-05	1717	tags=35%, list=15%, signal=30%	GTF2I/HGS/ROCK2/PML/AMOT/SEMA4A/FOXO4/THBS1/AGO1/NIBAN2/PF4/ATP2B4/EPN1/FOXJ2/NF1/SPARC/CLDN5/E2F2
CC	GO:0098793	presynapse	274	-0.43	-1.64	1.54E-05	0.00015599	9.58E-05	2615	tags=35%, list=22%, signal=28%	SNPH/SV2A/APH1A/NCSTN/PPFIBP2/P2RY1/MCTP1/YWHAG/SEPTIN4/KIAA1109/SCRIB/PPFIA1/PI4K2A/AAK1/EPHB2/CLTB/VPS18/ATP2B1/SNAP25/DISC1/ITSN1/RAB27B/ELK1/ZNRF1/GRIK2/STX6/LRFN3/SYNJ2/PDE2A/CTBP2/NECTIN1/ACTG1/SYAP1/RPH3A/LRRK2/NDEL1/SYNJ1/SYN3/DLG4/MME/CLCN5/STX4/CTBP1/FXYD6/FLOT1/EXOC3/CTNND1/CLN3/VPS35/EPHA4/UBE2L3/PIP5K1C/PPP1CA/CANX/SEPTIN1/GUCY1B1/ADGRL1/FXR2/SH3GL1/HTT/SLC2A1/SLC8A1/RAB6B/RAB7A/PDLIM5/RAB5B/FBXL20/CALM3/SLC6A9/DNM3/ACTB/DNAJC5/KIF1B/HIP1/DNM2/TPRG1L/RAB11B/ROGDI/ATP2B4/IQSEC1/PRKCB/NF1/SNCA/BCL2L1/LAMP1/NAPA/DENND1A/ATP6V0D1/AP2M1/GAK/STON2/RAB3D/RNF40/P2RX1/GLUL
BP	GO:0015850	organic hydroxy compound transport	116	-0.53	-1.80	1.55E-05	0.00015708	9.64E-05	1475	tags=25%, list=13%, signal=22%	GRK2/OSBPL2/SLC16A3/NR1H2/AQP9/OSBPL5/RALBP1/TSPO2/NFKB1/TSPO/LDLRAP1/LPCAT3/BMP6/SYK/ABCC4/ACTB/AQP1/LRP1/ABCC3/CLU/PINK1/SNCA/PIP4K2A/AQP10/ITGB3/DAB2/P2RX1/FURIN/OSBP2
BP	GO:0097191	extrinsic apoptotic signaling pathway	164	-0.48	-1.71	1.64E-05	0.00016582	0.0001018	2123	tags=34%, list=18%, signal=28%	BCL2L14/BCL2L11/PTEN/PRDX2/SRC/MAP2K5/STK4/TGFB1/GSTP1/HGF/EYA3/TMBIM1/TMC8/RIPK1/STX4/SORT1/PYCARD/SPI1/PDPK1/PML/ARHGEF2/DEDD2/BIRC6/GSK3A/PPP1CA/TNFRSF10C/HIPK1/MKNK2/HTT/IL6R/CTNNA1/SFRP2/PTPRC/THBS1/PSME3/LMNA/CASP2/HSPA1A/MADD/PF4/GABARAP/ITPRIP/RBCK1/GSK3B/TNFRSF1B/TCF7L2/GATA1/GPX1/NF1/SIAH2/BCL2L1/CTTN/PAK2/FOXO3/STRADB/RELA
CC	GO:0043292	contractile fiber	132	-0.52	-1.80	1.70E-05	0.00017108	0.00010503	1883	tags=36%, list=16%, signal=30%	SDC4/DCTN4/TPM3/SYNE2/ACTG1/NBR1/ACTA2/MYOM1/FHL3/ARF1/TPM4/PALLD/TPM1/SPTBN1/MYZAP/CORO1C/SLC2A1/SLC8A1/SVIL/CALD1/PAK1/VCL/PDLIM7/ALDOA/STK11/PPP3CA/PDLIM5/PPP1R12B/PLEC/CALM3/FLNA/MYL4/HSPB1/ATP2B4/FKBP1A/CAPZB/RTN2/PDLIM1/ACTN4/TMOD1/PARVB/SQSTM1/ACTN1/SLC4A1/ENO1/MYL9/ANK1

BP	GO:0002684	positive regulation of immune system process	622	-0.36	-1.45	1.72E-05	0.00017285	0.00010612	2425	tags=27%, list=21%, signal=22%	FPR3/SPON2/SPN/GPLD1/CD38/BTN3A2/ADAM17/CORO1A/NCKAP1L/DDX58/CR1L/USP27X/TRIM5/C1QA/IFI16/EPHB2/CTNNBIP1/MAVS/ADAM8/EFNB1/MYO1G/MICB/FCER1G/IRAK3/SERPING1/RFTN1/SLA2/CR1/CD99L2/FGR/FYN/TIRAP/UNC13D/NLRP3/SRC/IRS2/CD177/ANKRD17/MYO18A/BLOC1S3/PRKCE/TNFSF13/SPPL3/XRCC6/AP1G1/HCK/TGFB1/DGKZ/SIRPA/PPARGC1B/ITPKB/ADGRE2/TNFSF4/RC3H2/ANO6/NOTCH2/LSM14A/ITGAM/LRRK2/IKBKE/FYB1/GPS2/STAT5B/EZR/ARID5A/CMKLR1/RIPK1/RASAL3/SH2B2/C5AR1/TRIB1/STX4/CEBPA/PTPRJ/ZFP36L1/PTPN6/PYCARD/MAD2L2/SPI1/ITCH/PDPK1/ITGB2/CCR1/CSF1R/AKIRIN2/KLRD1/FOXP1/ZNF335/ERMAP/NLRC4/SPG21/SH3KBP1/RUNX3/VSIR/NFAM1/FES/NFATC2/IKBKG/DOCK8/RARA/PTK2B/GAB2/SPTA1/SELP/STAT6/TRAF6/PRKD2/TBX21/IL6R/PAK1/IGF2/ZMIZ1/CSK/ZBTB16/VAV1/STK11/PTPRC/SYK/JAM3/THBS1/PTAFR/MED1/TLR6/RBM14/FZD5/CAMK1D/PRAM1/JUND/JAK3/INPP5D/PF4/GPI/RAC1/LYN/RIOK3/LGALS9/RBCK1/SIN3A/LAPTM5/RAC2/SIRPB1/CREBBP/FLOT2/MPL/RHOA/PRKCB/GATA1/CLU/HCLS1/ZNFX1/HK1/TESC/MAPK3/CDKN1A/RIPOR2/LAMP1/PIK3CD/PRKDC/PAK2/KRT1/WNK1/SLC7A5/PLVAP/FOXO3/HLX/BAG6/RELA
CC	GO:0031674	I band	71	-0.61	-1.93	1.76E-05	0.00017584	0.00010796	1049	tags=32%, list=9%, signal=30%	PALLD/MYZAP/SLC2A1/SLC8A1/PAK1/PDLIM7/ALDOA/STK11/PPP3CA/PDLIM5/PPP1R12B/PLEC/FLNA/HSPB1/ATP2B4/FKBP1A/RTN2/PDLIM1/ACTN4/PARVB/ACTN1/SLC4A1/MYL9
BP	GO:0051017	actin filament bundle assembly	106	-0.54	-1.83	1.76E-05	0.00017629	0.00010823	2079	tags=36%, list=18%, signal=30%	SHROOM1/SRF/PPM1F/SRC/MARCKS/SDC4/EPS8/ACTG1/ROCK2/ARRB1/EZR/NF2/ARAP1/ALMS1/LCP1/NEDD9/TPM1/PTK2B/INPP5K/CALD1/PAK1/SLC9A1/FLNA/PLEK/ZYX/RAC1/PXN/ARHGAP6/RHOA/PDLIM1/ADD1/WAS/ESPN/ITGB5/ACTN1/PAK2/WASF2/DMTN
BP	GO:0019058	viral life cycle	253	-0.44	-1.63	1.86E-05	0.00018505	0.00011361	2278	tags=30%, list=19%, signal=25%	SCARB1/TRIM5/RAB43/IFI16/MAVS/TRIM38/SRPK1/APOBEC3D/VPS18/VPS4B/CR1/TPCN1/KPNA6/GYPA/SNX3/NCAM1/SETDB1/CTBP2/NECTIN1/ROCK2/NFIA/OASL/PIKFYVE/CTBP1/GRK2/CHMP4B/EIF2AK2/DDX6/IFITM3/ITCH/ZC3HAV1/TRIM28/VPS37B/ANPEP/VPS37C/INSR/SELPLG/INPP5K/SLC3A2/RAB7A/RAD23A/GSN/ITGA5/ADAR/IST1/LTF/SLC1A5/VCP/HSPA1A/VPS28/CHMP1A/LGALS9/APOBEC3F/WWP2/DDB1/IFITM2/SRPK2/TRIM11/TNIP1/TRIM14/STAU1/APOBEC3C/LARP1/TRIM10/ZNFX1/ITGB5/TRIM26/TBC1D20/PCBP2/LAMP1/N4BP1/BSG/CTSB/ITGB3/RAB1B/FURIN
CC	GO:0031234	extrinsic component of cytoplasmic side of plasma membrane	69	-0.60	-1.91	1.88E-05	0.00018641	0.00011445	2183	tags=39%, list=19%, signal=32%	TNK2/GNA15/SNAP25/FGR/FYN/SRC/HCK/TIAM1/SYAP1/DLG4/TGM3/OSBPL2/GNAQ/FES/MYZAP/PTK2B/SYK/GNB1/RAC1/SLA/LYN/HIP1/GNAI2/RHOA/GNA12/GNAZ/IQGAP1
BP	GO:0050804	modulation of chemical synaptic transmission	219	-0.45	-1.66	1.88E-05	0.0001866	0.00011456	2069	tags=32%, list=18%, signal=27%	NCSTN/P2RY1/MCTP1/STAU2/YWHAG/BTBD9/CD38/PRKAR1B/ZDHHC3/EPHB2/VPS18/SNAP25/DISC1/SRF/FYN/PTEN/LRRC4/SRC/YWHAH/ADNP/PRKCE/GRIK2/DGKZ/PNKD/DLGAP4/SYAP1/LRRK2/PLCB1/NPTXR/SYN3/DLG4/DBN1/MME/EPHB1/STX4/CA2/S1PR2/SIPA1L1/FLOT1/CLSTN1/CLN3/EPHA4/SHISA7/ARF1/PRKACA/RARA/PTK2B/NEURL1/BCR/RAPGEF2/MAP1A/PPP3CA/FBXL20/CALM3/GSK3B/TPRG1L/GNAI2/TSHZ3/PRKCB/STAU1/ARRB2/NF1/PPP1R9B/PINK1/NRGN/SNCA/PRKAR2B/SQSTM1/EIF4EBP2/P2RX1/GLUL

BP	GO:0006909	phagocytosis	176	-0.47	-1.71	1.93E-05	0.00019035	0.00011686	2158	tags=40%, list=18%, signal=33%	KIAA1109/SPON2/CORO1A/NCKAP1L/SCARB1/IL2RG/MYO1G/PLD4/FCER1G/TLR2/FGR/FYN/CD93/UNC13D/SRC/JMJD6/MYO18A/PRKCE/NCF2/NCF4/LYST/HCK/SIRPA/SNX3/ANO6/ICAM3/ITGAM/PIKFYVE/CD300LF/PTPRJ/PYCARD/CLN3/ITGB2/LEPR/PIP5K1C/CORO1C/RARA/BCR/PAK1/PECAM1/RAB7A/CSK/RAB14/ELMO1/VAV1/GSN/PTPRC/SYK/THBS1/RAB31/DOCK2/CAMK1D/RUBCN/CD14/RAC1/LYN/RAC2/SIRPB1/DNM2/LRP1/CNN2/DYSF/ITGAL/RHOBTB1/TGM2/ANXA11/SH3BP1/ITGB3/MYH9/EIF2AK1/RHOG
BP	GO:0043549	regulation of kinase activity	526	-0.37	-1.49	1.93E-05	0.00019035	0.00011686	2391	tags=31%, list=20%, signal=26%	CALM1/DUSP16/ADAM17/NCKAP1L/PRKAR1B/MAP4K2/ARHGEF5/SLC9A3R1/MVP/DUSP1/KAT2B/EPHB2/SH2B3/PKN1/AXIN2/ADAM8/CDKN2D/IRAK3/TNXB/CHI3L1/SPRY3/SLA2/HEG1/FGR/PTEN/MAP3K4/TIRAP/PPM1F/SRC/EPHB4/IRS2/AKT1S1/GCN1/MLLT1/ADNP/CDK12/MAP2K5/STK4/SDC4/XRCC6/ERBB2/CCNY/CASS4/MAGED1/TGFB1/DGKZ/WDR81/ERN2/LILRA5/ERN1/HGS/TSC2/MIDN/SYAP1/GSTP1/LRRK2/HGF/STK38/CACUL1/EGR1/NRBF2/DLG4/C5AR1/PIP4K2C/SMG8/SHC1/EPHB1/TRIB1/CEBPA/NF2/MAP2K2/PTPRJ/PTPN6/PYCARD/CDKN1C/DAZAP2/EPHA4/PDPK1/GNAQ/NCF1/CCNF/CSF1R/TRIB3/CHP1/SASH1/PDGFA/NEDD9/INSR/SESN2/MAP3K5/SLC27A1/TAOK3/GREM1/VLDLR/SH3BP5L/SORL1/CORO1C/HTT/PTK2B/NEURL1/SLC8A1/WARS1/INPP5K/RAPGEF2/CCND3/TRAF6/IL6R/PAK1/MOB3A/IGF2/CSK/TAOK1/SFRP2/STK11/MAP2K7/STRADA/PTPRC/SYK/ADAR/DVL3/THBS1/PTPN1/PLEC/TLR6/CALM3/HEXIM2/FZD5/LTF/PIK3CG/RUBCN/ACTB/MADD/MARK2/LYN/MMD/PDE5A/HSPB1/AKAP13/ATP2B4/RHOA/CLU/NF1/CCNI/PINK1/SNCA/CDKN1A/PIP4K2A/PRKAR2A/TAL1/PRKAR2B/CCNJL/PAK2/ITGB3/DAB2/WNK1/FBXO7/CBL/IQGAP1/MAP2K3/STRADB/CNPPD1
BP	GO:0071396	cellular response to lipid	376	-0.39	-1.53	1.96E-05	0.00019265	0.00011828	2278	tags=31%, list=19%, signal=25%	SCARB1/TRIM5/AIFM1/VIM/EPHB2/PKN1/PTK7/SAFB/TADA3/TLR2/VPS18/ATP2B1/BCL2L11/PAF1/PADI2/PRDX2/NLRP3/SRC/PER1/YWHAH/ELK1/RXRB/DDX54/ATM/PRKCE/PAQR8/CD68/LCOR/VDR/HCK/TGFB1/SIRPA/PPARGC1B/ZBTB7A/TNFSF4/UBR5/MTDH/ROCK2/GSTP1/SMARCA4/PLCB1/ARID5A/KMT2D/MAPK14/TRIB1/ZFP36L1/SETX/PYCARD/SBNO2/SPI1/NCOA3/GPBAR1/CXCL5/KAT5/FOXP1/UBE2L3/SASH1/DGAT2/PF4V1/NFKB1/VPS11/FES/KANK2/TSPO/UPF1/NCOA4/RARA/PTK2B/BCR/TRAF6/PLSCR3/PAK1/ZMIZ1/BMP6/MAP4K1/CPT1A/SYK/PTAFR/PDK3/NCOR2/MED1/NR3C1/LTF/ABHD2/HSPA1A/PF4/RXRA/CD14/AQP1/LYN/GSK3B/CDK19/TNFRSF1B/RHOA/TNIP1/PRPF8/GATA1/CLU/MSN/PPP1R9B/ARID1A/PIM1/TESC/WBP2/MAPK3/ZNF703/FAM210B/LITAF/DAB2/CNOT1/CARM1/FOXO3/TBXA2R/MAP2K3/RELA
BP	GO:0018108	peptidyl-tyrosine phosphorylation	229	-0.45	-1.66	2.01E-05	0.00019711	0.00012101	2320	tags=32%, list=20%, signal=26%	AATK/MVP/MAP2K4/EPHB2/DYRK3/SH2B3/TNK2/FGR/FYN/SRC/EPHB4/ADNP/MAP2K5/PRKCE/ERBB2/HCK/CASS4/TGFB1/LILRA5/HGF/DYRK1B/DLG4/CLK3/SHC1/EPHB1/NF2/MAP2K2/EIF2AK2/PTPRJ/PTPN6/SEMA4D/LRRK1/EPHA4/NCF1/ITGB2/CSF1R/ARHGEF2/PDGFA/NEDD9/INSR/FES/GREM1/HIPK1/SH3BP5L/PTK2B/NEURL1/BCR/IL6R/IGF2/PECAM1/CSK/BMP6/SFRP2/ITGA5/MAP2K7/PTPRC/SYK/PTPN1/HIPK2/PPP2R5B/JAK3/LYN/GATA1/SCYL1/ARRB2/JAK1/HCLS1/MAPK3/PAK2/ITGB3/CBL/MAP2K3/DMTN
BP	GO:0030177	positive regulation of Wnt signaling pathway	89	-0.57	-1.87	2.21E-05	0.00021568	0.00013242	2415	tags=40%, list=21%, signal=32%	UBE2B/ASPM/WLS/PTK7/TLR2/DISC1/MACF1/SRC/XIAP/TGFB1/UBR5/LRRK2/SMARCA4/DAPK3/VPS35/CSNK1G2/ATP6V0C/LRRK1/ZRANB1/CSNK2A1/NFKB1/RBPJ/FRAT1/TBL1XR1/SFRP2/USP34/SKI/VCP/TBL1X/CSNK1G1/GID8/PPM1A/DAB2/WNK1/FAM53B/CTDNEP1

BP	GO:0043408	regulation of MAPK cascade	406	-0.39	-1.55	2.27E-05	0.00022001	0.00013508	2329	tags=31%, list=20%, signal=26%	MAP4K2/ARHGEF5/SLC9A3R1/DUSP1/TRIM5/EPHB2/SH2B3/HDAC3/SH3RF1/ADAM8/STK25/MAP4K4/TRAF7/IRAK3/GLIPR2/CHI3L1/FOXO1/SPRY3/PTEN/MAP3K4/TIRAP/PRDX2/SRC/PER1/MAP2K5/STK4/PRKCE/XIAP/GRIK2/ERBB2/MAGED1/TGFB1/SIRPA/ERN2/RRAS/LILRA5/ERN1/NOTCH2/SDCBP/SH3RF3/ROCK2/GSTP1/ARRB1/LRRK2/HGF/NBR1/GPS2/ASH1L/PLCB1/STK38/EZR/PHLPP1/RIPK1/C5AR1/SHC1/EPHB1/NF2/MAP2K2/EIF2AK2/ACTA2/PTPRJ/PTPN6/PYCARD/SPI1/ITCH/EPHA4/GPBAR1/NCF1/CCR1/CSF1R/GRB2/SASH1/YWHAZ/PDGFA/INSR/MAP3K5/NDRG2/TAOK3/SORL1/PTK2B/MFHAS1/VRK3/INPP5K/RAPGEF2/TRAF6/PRKD2/PAK1/IGF2/CSK/TAOK1/MAP4K1/LAMTOR1/SFRP2/MAP2K7/PELI2/PTPRC/SYK/DVL3/THBS1/STK40/PTPN1/TLR6/MTURN/ARHGAP8/FZD5/PIK3CG/HIPK2/NDST1/MADD/LYN/LGALS9/MAP3K3/LAPTM5/PDE5A/AKAP13/GNAI2/TNIP1/ARRB2/NF1/PINK1/MAPK3/FLCN/PAK2/DAB2/IQGAP1/MAP2K3
CC	GO:0150034	distal axon	155	-0.49	-1.75	2.27E-05	0.00022001	0.00013508	2285	tags=35%, list=19%, signal=28%	MYO5A/MYO9A/AAK1/GDPD5/ENO2/NIN/KATNB1/SNAP25/DISC1/FEZ1/EXOC4/ELK1/GRIK2/EPS8/CPEB4/NECTIN1/ACTG1/EXOC7/SYAP1/LRRK2/NDEL1/SYNJ1/DBN1/CDKL5/SETX/EXOC3/CTNND1/EPHA4/ARPC5/ADGRL1/PALLD/NDRG2/FXR2/PTK2B/SLC8A1/COPA/USP9X/CALM3/BASP1/FLNA/ACTB/DNM2/TPRG1L/TSHZ3/PRKCB/PPP1R9B/PINK1/SNCA/CTTN/ATP6V0D1/RNF40/GLUL/CBL/IQGAP1
BP	GO:0061572	actin filament bundle organization	108	-0.55	-1.85	2.29E-05	0.00022132	0.00013588	2079	tags=36%, list=18%, signal=30%	SHROOM1/SRF/PPM1F/SRC/MARCKS/SDC4/EPS8/ACTG1/ROCK2/ARRB1/EZR/NF2/ARAP1/ALMS1/LCP1/NEDD9/TPM1/PTK2B/INPP5K/CALD1/PAK1/SLC9A1/FLNA/PLEK/ZYX/RAC1/RFLNB/PXN/ARHGAP6/RHOA/PDLIM1/ADD1/WAS/ESPN/ITGB5/ACTN1/PAK2/WASF2/DMTN
CC	GO:0031256	leading edge membrane	108	-0.55	-1.85	2.31E-05	0.00022328	0.00013708	2158	tags=40%, list=18%, signal=33%	MYO1G/ATP2B1/MACF1/FGR/TIRAP/SRC/EPS8/ADGRE2/PDE4A/SYNE2/EZR/STX4/CDKL5/NF2/PTPRJ/SHISA7/ARHGEF2/LCP1/PIP5K1C/INSR/TPM1/SPTBN1/CORO1C/DOCK8/INPP5K/SH2D3C/PAK1/ITGA5/PLEK/RAC1/DNM2/RHOA/ARHGAP45/PPP1R9B/CFL1/TESC/RIPOR2/PLEK2/AKT2/ITGB3/VASP/DIAPH1/TLN1
CC	GO:0043197	dendritic spine	99	-0.56	-1.87	2.33E-05	0.00022439	0.00013777	2232	tags=35%, list=19%, signal=29%	EPHB2/ATP2B1/STRN/LRRC4/DNAJB1/CPEB4/RPH3A/DLG4/STX4/SIPA1L1/CTNND1/ABI3/EPHA4/SHISA7/PPP1CA/CANX/PRKACA/FXR2/PTK2B/NEURL1/SLC8A1/BCR/PPP3CA/APBA3/ASAP1/DNM3/RAC1/SLA/RHOA/ARRB2/PPP1R9B/NRGN/CTTN/PRKAR2B/ACTN1
CC	GO:0045178	basal part of cell	157	-0.49	-1.74	2.36E-05	0.0002257	0.00013857	1869	tags=22%, list=16%, signal=19%	ERBB2/ATP7A/SLC12A6/NUMB/EZR/C5AR1/STX4/FLOT1/SLC16A3/AQP9/LEPR/SLC27A1/SLC2A1/LDLRAP1/SLC3A2/SLC9A1/SLC6A9/B4GALT1/SLC1A5/SLC14A1/ABCC4/AQP1/LRP1/FLOT2/ABCC3/ATP2B4/ARRB2/MSN/EPB41/SLC4A1/AQP10/BSG/SLC7A5/WASF2/ANK1
BP	GO:0051258	protein polymerization	213	-0.45	-1.65	2.45E-05	0.00023308	0.0001431	2618	tags=37%, list=22%, signal=29%	ADD2/UBE2S/CLASP1/AMBRA1/TWF2/CDC42EP1/TBCD/TUBGCP6/TPPP3/CORO1A/NCKAP1L/SCIN/CORO7/PRUNE1/RNF135/NIN/FLII/TRIOBP/MSRB2/DIAPH2/DBNL/PRKCE/HCK/EPS8/CYRIA/CKAP5/NDEL1/SPTAN1/ARPC1B/DBN1/WASF3/CHMP4B/CAMSAP1/EML2/WASHC2C/PYCARD/CDC42EP4/SLAIN2/PREX1/ARPC5/NUMA1/SSH1/GRB2/SPTBN1/FES/PTK2B/SPTA1/MSRB1/SVIL/PAK1/NDE1/GSN/ESAM/DCTN1/HSPA1A/RAC1/GOLGA2/CYFIP2/ARHGAP6/COTL1/RHOA/CAPZB/MYADM/ADD1/SSH2/HCLS1/TMOD1/ARPC4/WAS/PINK1/SNCA/SPTB/MTPN/CTTN/GAS7/VASP/DMTN/DIAPH1

BP	GO:0008285	negative regulation of cell population proliferation	439	-0.38	-1.51	2.46E-05	0.0002339	0.0001436	2413	tags=30%, list=21%, signal=25%	SPN/SCRIB/ALOX5/GPLD1/BAP1/SIRT6/SCIN/SLC9A3R1/BCL11B/DUSP1/KAT2B/CTNNBIP1/SH2B3/PKN1/AXIN2/CDKN2D/TLR2/STRN/PTGIR/SOD2/CR1/LGALS9C/SRF/PTEN/ATM/STK4/ATOH8/SDC4/ERBB2/VDR/MAGED1/H2AC6/TGFB1/DICER1/B4GALT7/HGS/TSC2/CTBP2/GSTP1/BTG1/EPHB1/TRIB1/CTBP1/CEBPA/S1PR2/NF2/EIF2AK2/PTPRJ/AGTPBP1/RAP1GAP/MSX1/PER2/PTPN6/CBFA2T3/FUZ/CDKN1C/ITCH/PTGES/DHCR24/PML/CSF1R/SMARCA2/KLF13/CHERP/E2F3/S100A11/RUNX3/TPM1/TMEM131L/NDRG2/VSIR/GREM1/KANK2/RBPJ/TSPO/PIM2/IRF1/RARA/PTK2B/NEURL1/WARS1/ARID2/RAPGEF2/WDR13/RASSF5/PAK1/TOB2/SKAP2/CTNNA1/CSK/ZBTB16/TGFB1I1/SFRP2/STK11/FOXO4/THBS1/LMNA/MED1/FZD5/TP53I11/B4GALT1/SKI/ULK1/NIBAN2/HSPA1A/INPP5D/HMGA1/LYN/LGALS9/PDE5A/SF1/RHOA/TFDP1/GATA1/NF1/FTH1/GNA12/ATG13/TESC/CDKN1A/FLCN/STAT3/SPARC/DNAJB2/TMEM127/MFN2/MED25/DAB2/JARID2/RNF10/EIF2AK1
BP	GO:0010506	regulation of autophagy	281	-0.42	-1.59	2.52E-05	0.00023824	0.00014627	2175	tags=32%, list=19%, signal=27%	TRIM38/KLHL22/ORMDL3/FOXO1/BCL2L11/TPCN1/FEZ1/ATP6V0B/RRAGD/EXOC4/SH3GLB1/ATM/ATP6V1B2/ATF6/USP36/VPS13D/ERN1/TSC2/MTDH/CPTP/EXOC7/LRRK2/HGF/SPTLC2/TFEB/KEAP1/PIP4K2C/PLEKHF1/WAC/CHMP4B/PHF23/PYCARD/DAP/CLN3/DAPK3/VPS35/ATP6V0C/QSOX1/CLEC16A/KAT5/TRIB3/LEPR/GSK3A/OPTN/MTMR3/PRKACA/SESN3/SESN2/ATP6V0A1/USP10/TSPO/PIM2/IKBKG/HTT/PAFAH1B2/RRAGC/HERC1/EIF4G2/STK11/WDR45/ATG7/FZD5/EIF4G1/ULK1/RUBCN/CAPN1/CAPNS1/GOLGA2/GSK3B/ELAPOR1/PSAP/HSPB1/BNIP3L/SUPT5H/TBC1D25/TRIM14/ATP6V1A/ATG2A/LARP1/PINK1/MAPK3/SNCA/FLCN/STAT3/CTTN/PIP4K2A/ATP6V0D1/DCAF12/FBXO7/NPRL3/FOXO3
BP	GO:0048762	mesenchymal cell differentiation	125	-0.51	-1.77	2.58E-05	0.00024262	0.00014895	2192	tags=33%, list=19%, signal=27%	AXIN2/EFNB1/GLIPR2/PTEN/IL17RD/EXT1/TGFB1/LDLRAD4/TIAM1/PHACTR4/CUL7/SDCBP/ROCK2/HGF/PEF1/MSX1/MCRIP1/SEMA4D/MAD2L2/FUZ/USF3/EPHA4/TRIM28/S100A4/GREM1/RBPJ/CORO1C/SEMA4A/OTUD5/TGFB1I1/SFRP2/AKNA/ADIPOR1/FLNA/ANXA6/RFLNB/GSK3B/TCF7L2/MAPK3/ZNF703/DAB2
BP	GO:0010959	regulation of metal ion transport	220	-0.44	-1.65	2.64E-05	0.00024776	0.00015211	1502	tags=33%, list=13%, signal=29%	CD4/CACNB4/AMIGO1/LGALS3/CAPN3/RGS7/BAK1/TRPC3/CALM1/CORO1A/UTRN/SLC9A3R1/TF/STIMATE/ATP2B1/FYN/PER1/YWHAH/FKBP1B/PRKCE/ATP7A/LILRA5/ANO6/OPRL1/CACNB1/KCNH2/FXYD6/PTPN6/TMBIM6/CD63/PDPK1/PML/CCR1/CHP1/PRKACA/TSPO/TMEM38A/HTT/PTK2B/SLC8A1/INPP5K/SLC9A1/SELENON/SCN1B/ATP1B2/EHD3/CALM3/SLC6A9/PIK3CG/G6PD/FLNA/JAK3/GJC2/LYN/DNM2/WWP2/ATP2B4/GNAI2/FKBP1A/RHOA/ARRB2/ACTN4/MLLT6/TESC/MYLK/SNCA/EPB41/AHCYL1/STIM1/WNK1/KEL/P2RX1/DIAPH1
CC	GO:0044309	neuron spine	100	-0.56	-1.86	2.84E-05	0.00026611	0.00016338	2232	tags=35%, list=19%, signal=29%	EPHB2/ATP2B1/STRN/LRRC4/DNAJB1/CPEB4/RPH3A/DLG4/STX4/SIPA1L1/CTNND1/ABI3/EPHA4/SHISA7/PPP1CA/CANX/PRKACA/FXR2/PTK2B/NEURL1/SLC8A1/BCR/PPP3CA/APBA3/ASAP1/DNM3/RAC1/SLA/RHOA/ARRB2/PPP1R9B/NRGN/CTTN/PRKAR2B/ACTN1
BP	GO:0018212	peptidyl-tyrosine modification	232	-0.45	-1.66	2.86E-05	0.00026802	0.00016455	2320	tags=32%, list=20%, signal=26%	AATK/TPST2/MVP/MAP2K4/EPHB2/DYRK3/SH2B3/TNK2/FGR/FYN/SRC/EPHB4/ADNP/MAP2K5/PRKCE/ERBB2/HCK/CASS4/TGFB1/LILRA5/HGF/DYRK1B/DLG4/CLK3/SHC1/EPHB1/NF2/MAP2K2/EIF2AK2/PTPRJ/PTPN6/SEMA4D/LRRK1/EPHA4/NCF1/ITGB2/CSF1R/ARHGEF2/PDGFA/NEDD9/INSR/FES/GREM1/HIPK1/SH3BP5L/PTK2B/NEURL1/BCR/IL6R/IGF2/PECAM1/CSK/BMP6/SFRP2/ITGA5/MAP2K7/PTPRC/SYK/PTPN1/HIPK2/PPP2R5B/JAK3/LYN/GATA1/SCYL1/ARRB2/JAK1/HCLS1/MAPK3/PAK2/ITGB3/CBL/MAP2K3/DMTN

CC	GO:0048471	perinuclear region of cytoplasm	520	-0.37	-1.47	2.91E-05	0.00027123	0.00016652	2431	tags=30%, list=21%, signal=25%	CTIF/SERINC5/ALOX5/AMFR/CYBB/EPG5/TPPP3/RHBDD2/ATP9A/AATK/LNPEP/SLC9A3R1/MVP/AIFM1/TF/GDPD5/LIMS1/TNK2/SH3RF1/VPS53/GLB1/CHI3L1/SNAP25/DISC1/FYN/LIMK2/MAP3K4/PPM1F/SRC/TAF8/MAP1S/RASA2/PRKCE/SRCAP/ERBB2/AP1G1/ATP7A/STX6/SYNJ2/CPEB4/DICER1/PDE2A/CUL7/PDE4A/BCL3/TSC2/UBR5/ZDHHC19/ANKRD13A/MTDH/AFTPH/CKAP4/SYAP1/RANGAP1/SYNJ1/EZR/UNC45A/INF2/PIKFYVE/STX4/PLEKHF1/NF2/MAP2K2/KCNH2/EIF2AK2/SORT1/PER2/DOK1/ATXN2/EXOC3/IFITM3/SYNRG/VPS35/PTGES/CCNF/PKP4/HIF1AN/KAT5/LCP1/ARF1/TMEM184A/OPTN/DGAT2/PRKACA/CA4/CHERP/FAM168B/ATP6V0A1/ZFYVE1/NDRG2/S100A4/YWHAB/BCAP31/PRDX5/HTT/RARA/PTK2B/NEURL1/INPP5K/RAPGEF2/TRAF6/SBF2/SEC31A/RAB14/SBF1/TAOK1/APBA3/SLC9A1/MYO9B/PLEC/EHD3/ARF3/FZD5/DNM3/VCP/HSPA1A/ANXA6/SIPA1/LYN/CYFIP2/LAPTM5/DNM2/IGF2R/CDK19/FLOT2/AKAP13/TNFRSF1B/CLU/MSN/ACTN4/ECE1/CASC3/PINK1/SNCA/TGM2/MTPN/EHD1/LAMP1/PRDX6/PSMF1/PRKAR2B/GAK/CTSB/PAK2/YBX3/DAB2/ARHGAP1/PLVAP/TOLLIP/CBL/SEC16A/RAB1B/OSBP2/DMTN/IRAG1
BP	GO:0010563	negative regulation of phosphorus metabolic process	314	-0.41	-1.57	2.92E-05	0.00027176	0.00016684	2521	tags=36%, list=21%, signal=29%	LGALS3/PMEPA1/YWHAG/BAK1/TERF2IP/SOCS3/UBE2B/CALM1/DUSP16/SIRT6/NCKAP1L/CRY2/PRKAR1B/MVP/DUSP1/KAT2B/EPHB2/SH2B3/PKN1/CDKN2D/IRAK3/SPRY3/HEG1/PTEN/PPM1F/IRS2/FKBP1B/HDAC4/AKT1S1/MLLT1/PPP1R15A/TGFB1/SIRPA/LDLRAD4/TSC2/MIDN/ROCK2/GSTP1/LRRK2/HGF/PIP5KL1/STK38/PIP4K2C/MGAT5/TRIB1/CEBPA/CTDSP2/NF2/PTPRJ/PTPN6/PYCARD/PARD3/CBFA2T3/SEMA4D/CDKN1C/LRRK1/PDPK1/GNAQ/TRIB3/GSK3A/CHP1/PDGFA/SLC27A1/FBP1/YWHAB/GREM1/SH3BP5L/SORL1/CORO1C/MFHAS1/SLC8A1/WARS1/INPP5K/CSK/SFRP2/PTPRC/ADAR/PTPN1/PLEC/CTDSPL/CALM3/HEXIM2/EIF4G1/RUBCN/PLEK/LYN/GSK3B/LRP1/HSPB1/CDA/GNAI2/CTDSP1/FKBP1A/MYADM/ARRB2/NF1/FKBP8/PPP1R9B/SNCA/CDKN1A/FLCN/STAT3/PIP4K2A/NIBAN1/PRKAR2A/PRKAR2B/SLC4A1/PRKDC/PAK2/FBXO7/CBL/IQGAP1/DMTN
BP	GO:0044089	positive regulation of cellular component biogenesis	349	-0.40	-1.56	2.95E-05	0.00027427	0.00016839	3052	tags=40%, list=26%, signal=31%	TLR4/WIPI1/CLIP1/PFN1/AUTS2/SEPTIN9/TPR/ARHGEF10/NPHS1/SORBS3/SLX4/ATF7IP/TNF/ATMIN/HSPA1B/TP53/MECP2/POLDIP2/PSRC1/SMAD3/PLCE1/KIT/EPHA1/SNX18/CARMIL1/ABCA3/STAU2/CLASP1/AMIGO1/AMBRA1/VPS4A/BMF/TWF2/CDC42EP1/LGALS3/FSCN1/BAK1/ACTR2/RALB/TPPP3/NCKAP1L/ARHGEF5/EPHB2/LIMS1/MAP4K4/NIN/VPS4B/BCL2L11/SRF/WNT1/TIRAP/PPM1F/SRC/KCTD17/FNIP2/SH3GLB1/ATM/ADNP/PRKCE/SDC4/ATP7A/HCK/EPS8/TGFB1/G3BP1/SETDB1/SDCBP/HGS/ROCK2/CKAP5/NDEL1/PIP4K2C/EPHB1/NF2/PTPRJ/FLOT1/CLSTN1/WASHC2C/PYCARD/CDC42EP4/SEMA4D/NR1H2/SLAIN2/ARAP1/FUZ/NUMA1/LCP1/TRIM28/GRB2/CNOT6/TPM1/FES/HTT/PTK2B/NEURL1/SEMA4A/WARS1/BCAS3/DYNC1H1/MARK4/PAK1/RAB7A/WDR45/SYK/ASAP1/TLR6/DCTN1/PPP2R5B/EIF4G1/ULK1/DNM3/VCP/HSPA1A/GNL3L/PLEK/RAC1/SIN3A/GSK3B/ELAPOR1/RAC2/DNM2/STMP1/PXN/RHOA/CLU/ATG2A/MSN/PINK1/SNCA/CTTN/PIP4K2A/CLDN5/RIPOR2/PLEK2/TAL1/MED25/VASP/CNOT1/CDC34/WASF2

BP	GO:0045936	negative regulation of phosphate metabolic process	313	-0.41	-1.57	3.01E-05	0.00027934	0.0001715	2521	tags=36%, list=21%, signal=29%	LGALS3/PMEPA1/YWHAG/BAK1/TERF2IP/SOCS3/UBE2B/CALM1/DUSP16/SIRT6/NCKAP1L/CRY2/PRKAR1B/MVP/DUSP1/KAT2B/EPHB2/SH2B3/PKN1/CDKN2D/IRAK3/SPRY3/HEG1/PTEN/PPM1F/IRS2/FKBP1B/HDAC4/AKT1S1/MLLT1/PPP1R15A/TGFB1/SIRPA/LDLRAD4/TSC2/MIDN/ROCK2/GSTP1/LRRK2/HGF/PIP5KL1/STK38/PIP4K2C/MGAT5/TRIB1/CEBPA/CTDSP2/NF2/PTPRJ/PTPN6/PYCARD/PARD3/CBFA2T3/SEMA4D/CDKN1C/LRRK1/PDPK1/GNAQ/TRIB3/CHP1/PDGFA/SLC27A1/FBP1/YWHAB/GREM1/SH3BP5L/SORL1/CORO1C/MFHAS1/SLC8A1/WARS1/INPP5K/CSK/SFRP2/PTPRC/ADAR/PTPN1/PLEC/CTDSPL/CALM3/HEXIM2/EIF4G1/RUBCN/PLEK/LYN/GSK3B/LRP1/HSPB1/CDA/GNAI2/CTDSP1/FKBP1A/MYADM/ARRB2/NF1/FKBP8/PPP1R9B/SNCA/CDKN1A/FLCN/STAT3/PIP4K2A/NIBAN1/PRKAR2A/PRKAR2B/SLC4A1/PRKDC/PAK2/FBXO7/CBL/IQGAP1/DMTN
BP	GO:0007507	heart development	317	-0.41	-1.57	3.19E-05	0.00029505	0.00018114	2293	tags=27%, list=20%, signal=22%	TBX19/IFT122/KAT2B/MTHFD1/AXIN2/PTK7/FHL2/ASXL1/SOD2/HEG1/SRF/PTEN/EPHB4/MOSPD3/ATM/JMJD6/MAP2K5/STK4/ERBB2/EXT1/NIPBL/MKS1/TGFB1/PDE2A/NOTCH2/CTDP1/TSC2/TFDP2/ROCK2/AP2B1/SNX17/NFATC1/SHC1/MAPK14/GRK2/YY1/ZFP36L1/MSX1/SMARCD3/GSK3A/INSR/TPM1/PSKH1/SUFU/GREM1/RBPJ/ADAP2/RARA/SLC8A1/ARID2/SOX6/EIF4G2/PDLIM7/ZMIZ1/SFRP2/PDLIM5/SLC9A1/LMNA/GAA/PLEC/DAW1/MED1/G6PD/BASP1/FLNA/NDST1/ODAD4/AKAP13/TNFRSF1B/FKBP1A/PDLIM1/ARRB2/NF1/KDM6B/NCOA6/PIM1/ECE1/MAPK3/CDKN1A/AP1B1/CLDN5/PRKDC/JARID2/NPRL3/MAP2K3/MICAL2
CC	GO:0030139	endocytic vesicle	256	-0.43	-1.60	3.23E-05	0.00029773	0.00018279	2369	tags=34%, list=20%, signal=27%	CYBB/CORO1A/WLS/SCARB1/RAB43/TF/TAPBP/VIM/ADAM8/CLTB/UBC/PLD4/TLR2/RAB11FIP5/MYO1E/WNT1/TIRAP/ATP6V0B/UBA52/MCOLN1/NCF2/NCF4/ATP7A/STX6/SNX3/AP2A2/LAMP2/HGS/ACTG1/RAB11FIP4/AP2B1/AP2S1/DLG4/PIKFYVE/STX4/CDC42EP4/HVCN1/ATP6V0C/NCF1/AMOT/ATP6V0A1/SH3KBP1/VPS11/AP2A1/PGLYRP1/RAPGEF2/RIN1/LDLRAP1/TGOLN2/RAB7A/RAB14/GSN/RAB5B/SYK/RAB31/TLR6/EHD3/CLTC/FZD5/LTF/RAB11FIP1/ZYX/FMNL1/LYN/GAPVD1/RAC2/DNM2/IGF2R/HYOU1/RAB11B/LRP1/FLOT2/DYSF/TRIM14/RILP/ARRB2/RIN3/WAS/ITGB5/ANXA11/EHD1/SPARC/LAMP1/UBB/ATP6V0D1/AP2M1
CC	GO:0062023	collagen-containing extracellular matrix	145	-0.49	-1.72	3.26E-05	0.00030048	0.00018448	1086	tags=19%, list=9%, signal=17%	PKM/S100A4/GREM1/TIMP2/PCSK6/F13A1/MMP9/TGFB1I1/SFRP2/VCAN/LTBP2/THBS1/CD151/ANXA6/PF4/LGALS9/PSAP/CLU/TGM2/ANXA11/SPARC/LTBP1/CTSB/HDGF/CTSD/KRT1/TIMP1
BP	GO:0034765	regulation of ion transmembrane transport	233	-0.43	-1.61	3.37E-05	0.0003091	0.00018977	1564	tags=31%, list=13%, signal=28%	CACNB4/AMIGO1/CAPN3/TMEM109/PPIF/RGS7/CALM1/CYBB/CORO1A/UTRN/SLC9A3R1/EPHB2/STIMATE/FYN/TPCN1/YWHAH/FKBP1B/PRKCE/KCNJ15/ATP7A/ANO6/OPRL1/RIPK1/DLG4/CACNB1/KCNH2/FXYD6/PER2/PTPN6/HVCN1/CD63/SHISA7/PDPK1/KCNQ5/CHP1/PRKACA/CNIH3/TMEM38A/HTT/PTK2B/SLC8A1/CLIC3/MMP9/SLC9A1/PTAFR/SELENON/SCN1B/ATP1B2/EHD3/CALM3/SLC6A9/PIK3CG/G6PD/FLNA/CAPN1/SLC43A2/GJC2/LYN/DNM2/SLC43A1/WWP2/ATP2B4/FKBP1A/ACTN4/PINK1/TESC/SNCA/AHCYL1/STIM1/WNK1/KEL/P2RX1/DIAPH1

BP	GO:0032880	regulation of protein localization	605	-0.35	-1.44	3.41E-05	0.00031249	0.00019185	2085	tags=25%, list=18%, signal=22%	RAB11FIP5/SNAP25/HSPA1L/FYN/LIMK2/PPM1F/SRC/IRS2/SH3GLB1/FKBP1B/ABAT/MYO18A/PRKCE/ERBB2/USP36/SUMO3/STX6/MAGED1/TGFB1/ZDHHC7/WAPL/SNX3/NPEPPS/UBR5/MIDN/ANKRD13A/NUMB/ROCK2/UBE2J2/ITGAM/LRRK2/RANGAP1/SIDT2/AP2B1/NDEL1/EZR/TMBIM1/DLG4/KAT7/MAPK14/STX4/POLR1A/NF2/YOD1/PER2/MYCBP2/NR1H2/ARIH2/MYOM1/CLN3/ABI3/VPS35/SPI1/SHISA7/PDPK1/ITGB2/NUMA1/LCP1/TRIM28/GSK3A/UBE2L3/CHP1/PRKACA/SESN2/SPTBN1/SUFU/VPS11/MCRS1/PLEKHM2/RBM22/SORL1/BCAP31/FRAT1/LRRC8A/MFHAS1/INPP5K/BCAS3/LDLRAP1/MARK4/RASSF5/NSFL1C/MAP1A/FRMD4A/VCL/PECAM1/CTNNA1/CSK/RAB14/BMP6/STK11/GSN/CPT1A/PPP3CA/DVL3/FIS1/PTPN1/ILRUN/LMNA/CLTC/FZD5/RAB11FIP1/FLNA/VCP/RIPOR1/ACTB/VPS28/GNL3L/UCP2/SIN3A/MMD/GSK3B/GAPVD1/RAC2/RAB11B/LRP1/WWP2/BNIP3L/SYTL4/TCF7L2/HUWE1/ATP2B4/FKBP1A/GRIPAP1/MEPCE/TRIM14/BBC3/NF1/MSN/PPP1R9B/HCLS1/ARHGDIA/OAZ1/ATG13/PINK1/EPB41/DTX3L/BCL2L1/FLCN/PTPN23/RIPOR2/ABLIM3/SQSTM1/AKT2/TBC1D1/AP2M1/PTP4A3/PPM1A/CTSD/ITGB3/DAB2/GLUL/RHOG/DMTN
BP	GO:0070482	response to oxygen levels	209	-0.44	-1.65	3.57E-05	0.00032652	0.00020047	2395	tags=35%, list=20%, signal=28%	CD38/ADAM17/EGLN3/ENDOG/CYBB/NFE2L2/AIFM1/HMOX2/USP19/AK4/ADAM8/TLR2/FOXO1/SOD2/ALKBH5/SRF/PTEN/SRC/APAF1/MTHFR/ATM/ABAT/PRKCE/ATP7A/AGTRAP/CPEB4/PLOD1/NPEPPS/SCAP/ROCK2/EGR1/ZFP36L1/CBFA2T3/TMBIM6/ALAD/EPHA4/PML/HIF1AN/ATP6AP1/ITPR2/TXNRD2/ARNT/PDGFA/GUCY1B1/RBPJ/ERO1A/PTK2B/SLC2A1/SLC8A1/SLC9A1/THBS1/PDK3/LMNA/ATG7/HIPK2/UCP2/AQP1/CREBBP/HYOU1/BNIP3L/MPL/RHOA/PDLIM1/NF1/ATP6V1A/ACTN4/PINK1/GUCY1A1/ATP6V0D1/ENO1/ALAS2/FOXO3/CBL
BP	GO:0071695	anatomical structure maturation	127	-0.50	-1.75	3.71E-05	0.00033815	0.00020761	2042	tags=28%, list=17%, signal=23%	WNT1/ANKRD17/SLC24A4/EXT1/ZBTB7A/PTBP3/XYLT1/LRRK2/B4GALT5/ROPN1L/CEBPA/SEMA4D/CDKN1C/PRKACA/GREM1/RBPJ/SLFN14/PTK2B/SNX10/RERE/ZBTB16/PHOSPHO1/FZD5/LTF/ABHD2/G6PD/RFLNB/RHOA/NF1/TAL1/FAM210B/EPB42/TRIM58/LYL1/FOXO3
BP	GO:0034446	substrate adhesion-dependent cell spreading	66	-0.60	-1.90	3.86E-05	0.00035088	0.00021542	2768	tags=47%, list=24%, signal=36%	AKIP1/EPHA1/LAMC3/ST6GAL1/CARMIL1/CALR/VAMP3/LIMS1/UNC13D/SRC/TRIOBP/CASS4/MEGF9/DOCK5/PARVG/WASHC2C/PREX1/FERMT3/NEDD9/CORO1C/FLNA/RAC1/DNM2/PXN/RHOA/MYADM/ACTN4/PARVB/ITGB3/DAB2/DMTN
CC	GO:0031012	extracellular matrix	182	-0.47	-1.72	3.91E-05	0.00035525	0.0002181	1123	tags=18%, list=10%, signal=17%	ALPL/PKM/S100A4/GREM1/TIMP2/PCSK6/F13A1/GFOD2/MMP9/TGFB1I1/SFRP2/VCAN/LTBP2/THBS1/GLG1/PHOSPHO1/CD151/ANXA6/PF4/LGALS9/PSAP/CLU/TGM2/ANXA11/SPARC/TFIP11/LTBP1/GP1BA/CTSB/HDGF/CTSD/KRT1/TIMP1
BP	GO:0010256	endomembrane system organization	406	-0.39	-1.53	4.02E-05	0.00036412	0.00022355	2174	tags=24%, list=19%, signal=21%	STK25/UBL4A/VPS18/VPS4B/CR1/ZNF385A/SNAP25/PTEN/UNC13D/SH3GLB1/MOSPD3/HOOK2/MYO18A/BLOC1S3/LYST/STX6/SNX3/SUN2/CUL7/ANO6/HGS/GET3/LRRK2/NDEL1/SYNJ1/SMPD4/VPS51/STX4/PLEKHF1/CHMP4B/OSBPL2/MAP2K2/FLOT1/HOOK3/COG1/TOR1AIP2/CLN3/RTN3/RFX2/YWHAZ/OPTN/VPS37B/VPS37C/EMC3/STX5/SPTBN1/VPS11/PLEKHM2/ARHGAP21/TMEM38A/CORO1C/HTT/SPTA1/CAVIN2/SNX10/BCAS3/PLSCR3/NSFL1C/SEC31A/LPCAT3/RAB7A/LAMTOR1/GSN/RAB5B/PTPRC/NPLOC4/LMNA/ASAP1/NUP93/PLEC/IST1/EHD3/DCTN1/ANXA6/VPS28/CHMP1A/GOLGA2/DNM2/SYTL4/DYSF/HUWE1/PRKCB/RILP/CLU/MAPK3/TBC1D20/SQSTM1/AKT2/SLC4A1/TMEM127/TMCC1/GAK/MMGT1/TANGO2/CTDNEP1/MYH9/SEC16A/BAG6/ACRBP

BP	GO:0014902	myotube differentiation	64	-0.61	-1.92	4.13E-05	0.00037321	0.00022913	3000	tags=52%, list=26%, signal=39%	NOS1/NPHS1/NOTCH1/TNFSF14/PLEKHO1/MYEF2/ADAMTS5/ATP11A/CCN3/MAML1/CFLAR/MYOF/WNT1/HDAC4/DYRK1B/MAPK14/FLOT1/SORT1/NFATC2/KLF5/PPP3CA/SELENON/PLEC/SKI/NIBAN2/GPX1/BCL9/EHD1/RIPOR2/RBM38/KEL/MYH9/XK
MF	GO:0035091	phosphatidylinositol binding	210	-0.45	-1.65	4.26E-05	0.00038392	0.00023571	2763	tags=39%, list=24%, signal=30%	KCNQ1/PHLDA3/SNX18/GBF1/KCNJ2/PHF12/GGA3/TWF2/ANXA2/ZCCHC2/ARHGAP32/TECPR1/MCF2L/TOM1L2/SCIN/SCARB1/ZFYVE26/PLCD1/ATG2B/HS1BP3/MYO1G/FLII/SAP30L/MYO1E/SNX29/TPCN1/TIRAP/NCF4/SNX3/SDCBP/HGS/ARHGAP9/TTPAL/GPAA1/RPH3A/PLCB1/SNX17/PLEKHF1/OSBPL2/DENND1C/WASHC2C/PARD3/ARAP1/OSBPL5/PITPNC1/NCF1/NUMA1/ITPR2/SNX8/PITPNM2/ZFYVE1/FES/PITPNA/ADAP2/GAB2/SNX10/BCAS3/SVIL/SBF2/LDLRAP1/ANKFY1/ADAP1/SNX11/GSN/WDR45/SLC9A1/ASAP1/TOM1/WIPI2/IQGAP2/RUBCN/SNX27/HIP1/WDFY3/ATG2A/NRGN/EPB41/PLEK2/DENND1A/IQGAP1/TLN1
CC	GO:0043296	apical junction complex	87	-0.55	-1.80	4.29E-05	0.00038542	0.00023663	2428	tags=36%, list=21%, signal=28%	TBCD/FBF1/DDX58/STRN/PARD6B/SHROOM1/MTDH/NECTIN1/ACTG1/ASH1L/PARD3/CTNND1/FRMD4B/ARHGEF2/TJP2/OCEL1/AMOT/NHS/RAPGEF2/FRMD4A/CTNNA1/JAM3/ESAM/FZD5/ACTB/RHOA/UBN1/CLDN5/SH3BP1/YBX3/VASP
CC	GO:0098797	plasma membrane protein complex	283	-0.41	-1.56	4.29E-05	0.00038542	0.00023663	2999	tags=36%, list=26%, signal=27%	IKBKB/ABCA2/APH1B/FADD/HLA-A/GNAS/GNAI3/CACNA1E/FCGR3A/KCNAB3/GNAL/ACVR1B/KCNQ1/DIABLO/NEO1/IFNL1/KCNJ2/SNTB2/APH1A/NCSTN/CD4/CACNB4/SNTB1/ANXA2/CFLAR/CALM1/CYBB/UTRN/SLC9A3R1/PORCN/TF/TNK2/ADAM8/CLTB/FCER1G/TLR2/GNA15/SSPN/NCF2/GRIK2/ERBB2/NCF4/EPS8/AP2A2/SDCBP/ITGAM/AP2B1/SYNJ1/AP2S1/RIPK1/DLG4/SHC1/CACNB1/KCNH2/FLOT1/PTPN6/CHRNE/CTNND1/SHISA7/GNAQ/NCF1/ITGB2/KCNQ5/GRB2/INSR/CNIH3/AP2A1/CORO1C/PTK2B/TRAF6/SLC3A2/IL6R/CTNNA1/IL13RA1/ITGA5/SYK/GNB1/SCN1B/ATP1B2/TLR6/CLTC/CALM3/GJC2/LYN/GP9/LRP1/FLOT2/ITGA2B/GNAI2/FKBP1A/ITGAL/EPS15L1/GNA12/ITGB5/APBB1IP/GP1BA/ATP6V0D1/AP2M1/ITGB3/GNAZ/SLC7A5/CBL
BP	GO:0050806	positive regulation of synaptic transmission	75	-0.58	-1.84	4.32E-05	0.00038645	0.00023726	2270	tags=33%, list=19%, signal=27%	ZDHHC3/EPHB2/SNAP25/PTEN/PRKCE/GRIK2/DLG4/MME/STX4/CA2/FLOT1/CLSTN1/EPHA4/SHISA7/PTK2B/GSK3B/TSHZ3/STAU1/ARRB2/NF1/PINK1/NRGN/SNCA/SQSTM1/GLUL
BP	GO:0060322	head development	468	-0.37	-1.47	4.89E-05	0.00043621	0.00026781	2065	tags=35%, list=18%, signal=30%	TSPAN2/POMK/EPOR/PLXNB2/PAFAH1B3/PFAS/TP53/MECP2/NUMBL/GNAI3/PLEKHA1/NOTCH1/NOTCH2NLC/CENPF/TACC2/CCDC141/CASP5/STK36/PDSS2/ATIC/ARL13B/CDK6/SMAD1/CNP/CSNK2A2/OGDH/PLXNA4/NCSTN/AMIGO1/KDM7A/PBX2/RGS7/BAK1/SETD2/CREB1/SCRIB/ARCN1/CALM1/ASPM/WLS/OTX1/AATK/BCL11B/TBX19/EPHB2/FANCC/NIN/ATP2B1/BCL2L11/DISC1/SRF/FYN/LIMK2/WNT1/PADI2/PTEN/PHF8/FEZ1/SRC/DNAJB1/APAF1/MAP1S/YWHAH/IRS2/ATM/ABAT/SNRPB/EXT1/NIPBL/ATP7A/MKS1/SYNJ2/RRAS/SZT2/ZNF148/SYNE2/HSPA5/NUMB/ITGAM/LRRK2/PLCB1/NDEL1/FOXP2/SYNJ1/NOTCH2NLA/VPS51/EPHB1/NF2/CRISPLD2/AGTPBP1/MSX1/HOOK3/CKB/CTNND1/FPGS/ARPC5/CSF1R/ZFHX3/UBE2L3/DPYSL2/ZNF335/MBOAT7/SGPL1/NDRG2/TACC1/RBPJ/PFKFB3/RARA/SLC2A1/NEURL1/SLC8A1/PAFAH1B2/BCR/MEIS1/RAPGEF2/HERC1/SOX6/ZMIZ1/CSK/RERE/NDE1/SFRP2/AKNA/PPP3CA/ATP1B2/BPTF/MED1/MAFB/CASP2/CALM3/G6PD/TACC3/BASP1/CHD8/SKI/NDST1/ODAD4/ACTB/GJC2/AQP1/SIN3A/GSK3B/ROGDI/ATP2B4/RHOA/ATXN1/ARRB2/NF1/NCOA6/TRAPPC9/PPP1R9B/NRGN/MAPK3/PBX1/MTPN/CSRNP1/CLDN5/UBB/NAPA/ATP6V0D1/PRKDC/SHARPIN/MBP/FOXO3/SEC16A/BAG6

BP	GO:0043410	positive regulation of MAPK cascade	281	-0.42	-1.57	4.91E-05	0.00043667	0.00026809	2181	tags=30%, list=19%, signal=25%	SH3RF1/ADAM8/STK25/TRAF7/GLIPR2/CHI3L1/PTEN/MAP3K4/TIRAP/PRDX2/SRC/MAP2K5/PRKCE/XIAP/ERBB2/MAGED1/TGFB1/ERN2/LILRA5/ERN1/NOTCH2/SDCBP/SH3RF3/ROCK2/ARRB1/LRRK2/HGF/PLCB1/RIPK1/C5AR1/SHC1/MAP2K2/EIF2AK2/ACTA2/PTPRJ/PYCARD/SPI1/EPHA4/GPBAR1/NCF1/CCR1/CSF1R/SASH1/PDGFA/INSR/MAP3K5/TAOK3/PTK2B/MFHAS1/RAPGEF2/TRAF6/PRKD2/PAK1/IGF2/CSK/TAOK1/MAP4K1/LAMTOR1/MAP2K7/PELI2/PTPRC/SYK/DVL3/THBS1/PTPN1/TLR6/MTURN/ARHGAP8/FZD5/PIK3CG/HIPK2/NDST1/MADD/LGALS9/MAP3K3/LAPTM5/PDE5A/AKAP13/GNAI2/ARRB2/MAPK3/IQGAP1/MAP2K3
BP	GO:0030833	regulation of actin filament polymerization	106	-0.53	-1.78	4.97E-05	0.00044067	0.00027055	1995	tags=34%, list=17%, signal=28%	TRIOBP/DBNL/PRKCE/HCK/EPS8/CYRIA/SPTAN1/ARPC1B/DBN1/WASHC2C/PYCARD/CDC42EP4/ARPC5/SSH1/GRB2/SPTBN1/PTK2B/SPTA1/SVIL/GSN/ESAM/CYFIP2/COTL1/RHOA/CAPZB/MYADM/ADD1/SSH2/HCLS1/TMOD1/ARPC4/SPTB/MTPN/CTTN/VASP/DMTN
BP	GO:0045321	leukocyte activation	687	-0.34	-1.39	5.27E-05	0.00046681	0.0002866	2248	tags=25%, list=19%, signal=22%	GON4L/C1QA/EPHB2/CLPTM1/PKN1/SH3RF1/ADAM8/EFNB1/MICB/FCER1G/TLR2/SLA2/CMTM7/CR1/KLF6/LGALS9C/FGR/SRF/FYN/CD93/WNT1/TIRAP/UNC13D/PRDX2/NLRP3/SRC/NKG7/IRS2/CD177/FKBP1B/HDAC4/ATM/JMJD6/MYO18A/BLOC1S3/PRKCE/TNFSF13/SDC4/XRCC6/ERBB2/AP1G1/ATP7A/TGFB1/SIRPA/ITPKB/ADGRE2/ZBTB7A/TNFSF4/RC3H2/BCL3/NOTCH2/ITGAM/LRRK2/GPS2/STAT5B/EGR1/PAG1/RASAL3/KAT7/C5AR1/CD300LF/EPHB1/STX4/CEBPA/YY1/PTPRJ/ZFP36L1/PTPN6/PYCARD/ADGRG3/MAD2L2/SBNO2/RELB/SOS2/SPI1/ITCH/PREX1/PDPK1/ITGB2/LCP1/FOXP1/LEPR/ZNF335/SH3KBP1/RUNX3/TMEM131L/VSIR/NFAM1/FES/RBPJ/NFATC2/IRF1/PGLYRP1/LRRC8A/DOCK8/RARA/PTK2B/MFHAS1/SELPLG/GAB2/SPTA1/SEMA4A/BCR/STAT6/CXCR2/CCND3/TRAF6/TBX21/IL6R/IGF2/SKAP2/ZMIZ1/CSK/ZBTB16/VAV1/STK11/GSN/PPP3CA/PTPRC/SYK/KDELR1/THBS1/PTAFR/SUPT6H/ST3GAL1/MAFB/TLR6/CD151/DOCK2/FZD5/PIK3CG/PRAM1/JUND/JAK3/INPP5D/LYN/SNX27/LGALS9/LAPTM5/PDE5A/RAC2/SIRPB1/FLOT2/TNFRSF1B/DYSF/FKBP1A/MPL/PRF1/RHOA/ITGAL/PRKCB/GATA1/CLU/MSN/GRN/WAS/SNCA/CDKN1A/APBB1IP/PBX1/STAT3/RIPOR2/LAMP1/PIK3CD/IKZF1/PRKDC/LYL1/FBXO7/MYH9/TOLLIP/FOXO3/CBL/ELF4/HLX/BAG6
BP	GO:0007034	vacuolar transport	138	-0.49	-1.73	5.35E-05	0.00047299	0.00029039	1861	tags=33%, list=16%, signal=28%	AP1G1/LYST/LAMP2/VPS13D/HGS/LRRK2/VPS51/CHMP4B/SORT1/HOOK3/VPS41/CLN3/VPS35/MON1B/CLEC16A/VPS37B/VPS37C/VPS11/NCOA4/SORL1/KIF13A/RAB7A/ANKFY1/HGSNAT/EHD3/VCP/HSPA1A/VPS28/TGFBRAP1/CHMP1A/SNX27/LAPTM5/IGF2R/PSAP/LRP1/RILP/CLU/GRN/PINK1/DTX3L/PTPN23/LAMP1/ATP6V0D1/GAK/UBXN6
BP	GO:0010720	positive regulation of cell development	186	-0.45	-1.66	5.37E-05	0.00047355	0.00029073	2611	tags=38%, list=22%, signal=30%	PLXNB2/PLXND1/TNF/NUMBL/NOTCH1/KIT/F11R/CARMIL1/SPEN/PLXNA4/CALR/STAU2/AMIGO1/TWF2/ACTR2/ASPM/EPHB2/LIMS1/STK25/NIN/TLR2/DISC1/MACF1/SRF/UNC13D/TRIOBP/ADNP/DBNL/CASS4/TGFB1/TIAM1/DICER1/CUL7/NUMB/NDEL1/SYNJ1/DBN1/DOCK5/MME/TRIB1/CDKL5/S1PR2/SMARCD3/SEMA4D/PREX1/EPHA4/ZNF335/NEDD9/TSPO/NEURL1/EIF4G2/STK11/PLXNC1/IST1/FLNA/NIBAN2/GJC2/RAC1/LYN/PDE5A/DNM2/TNFRSF1B/MPL/MYADM/ADD1/TGM2/CLDN5/PRKDC/DAB2/CUX1/DMTN
BP	GO:0001952	regulation of cell-matrix adhesion	84	-0.56	-1.83	5.59E-05	0.00049026	0.000301	2982	tags=46%, list=25%, signal=35%	DUSP22/SERPINE1/POLDIP2/PIK3CB/SMAD3/EPHA1/CDK6/RIN2/CLASP1/UTRN/LIMS1/PLEKHA2/MAP4K4/DISC1/MACF1/PTEN/PPM1F/SRC/SDC4/RRAS/ACTG1/ROCK2/NF2/PTPRJ/DAPK3/GREM1/CORO1C/PTK2B/BCAS3/VCL/SLC9A1/THBS1/GFUS/RAC1/GSK3B/ARHGAP6/RHOA/NF1/DMTN

BP	GO:0048041	focal adhesion assembly	55	-0.61	-1.87	5.61E-05	0.00049107	0.00030149	2202	tags=45%, list=19%, signal=37%	LIMS1/MAP4K4/MACF1/PTEN/PPM1F/SRC/SDC4/ACTG1/ROCK2/PTPRJ/DAPK3/GREM1/CORO1C/PTK2B/BCR/BCAS3/VCL/SLC9A1/THBS1/RAC1/ARHGAP6/RHOA/CTTN/ACTN1/DMTN
BP	GO:0090257	regulation of muscle system process	122	-0.50	-1.73	5.76E-05	0.00050295	0.00030878	2136	tags=34%, list=18%, signal=28%	ATP2B1/ORMDL3/FOXO1/SRF/FKBP1B/HDAC4/ABAT/PROK2/CTDP1/ROCK2/DOCK5/GRK2/YY1/DAPK3/GSK3A/PRKACA/TPM1/TMEM38A/SLC8A1/PPP3CA/SLC9A1/PTAFR/LMNA/PPP1R12B/EHD3/CALM3/PIK3CG/G6PD/ANXA6/PDE5A/TNFRSF1B/ATP2B4/RHOA/GUCY1A1/MTPN/CTTN/ENO1/MYL9/JARID2/P2RX1/FOXO3/TBXA2R
BP	GO:0006812	cation transport	593	-0.35	-1.42	5.81E-05	0.00050516	0.00031014	1505	tags=18%, list=13%, signal=16%	CLCN5/CACNB1/KCNH2/FXYD6/PTPN6/CHRNE/SLC36A1/HVCN1/TMBIM6/CD63/CLN3/SHISA7/PDPK1/PML/RALBP1/KCNQ5/PANX2/MICU1/CCR1/TSPO2/ITPR2/CHP1/PRKACA/CHERP/ATP2A3/CNIH3/ERO1A/TSPO/TMEM38A/HTT/PTK2B/SLC8A1/SLC6A20/INPP5K/SLC24A3/SLC3A2/MMP9/SLC44A2/SLC25A38/LCN2/PPP3CA/PTPRC/SYK/SLC9A1/UQCRC1/SELENON/SCN1B/ATP1B2/EHD3/CLTC/CALM3/SLC6A9/LTF/PIK3CG/G6PD/FLNA/SLC1A5/JAK3/ANXA6/CAPN1/UCP2/SLC43A2/GJC2/AQP1/LYN/DNM2/SLC43A1/GP9/RAB11B/WWP2/ATP2B4/GNAI2/FKBP1A/TMEM63B/RHOA/FTL/PRKCB/ARRB2/ATP6V1A/SLC38A10/ACTN4/FTH1/PINK1/MLLT6/TESC/SEC14L1/MYLK/SNCA/EPB41/SLC15A3/AHCYL1/GP1BA/ATP6V0D1/MMGT1/SLC48A1/SLC25A37/STIM1/ITGB3/ARHGAP1/WNK1/SLC7A5/KEL/P2RX1/SLC38A5/SLC6A8/DIAPH1
BP	GO:0032101	regulation of response to external stimulus	625	-0.35	-1.43	5.80E-05	0.00050516	0.00031014	2266	tags=27%, list=19%, signal=23%	DUSP1/TRIM5/MUL1/IFI16/EPHB2/RNF135/SH2B3/MAVS/ADAM8/TRIM38/MICB/IRAK3/TLR2/SERPING1/CR1/KNG1/FGR/FYN/PADI2/PTEN/TIRAP/PRDX2/PPM1F/NLRP3/SRC/NKG7/FKBP1B/ANKRD17/ATM/ABAT/NINJ1/XIAP/THBD/XRCC6/AP1G1/VDR/HCK/TGFB1/SIRPA/AREL1/PUM1/PDE2A/TNFSF4/LILRA5/ANO6/LSM14A/GSTP1/LRRK2/IKBKE/HGF/GPS2/ASH1L/METRNL/NDEL1/STAT5B/OASL/CMKLR1/RIPK1/C5AR1/MAPK14/TRIB1/STX4/CEBPA/PTPN6/PYCARD/SEMA4D/MYCBP2/NR1H2/SBNO2/SPATA2/VPS35/SPI1/ITCH/PTGES/EPHA4/NCF1/CCR1/CSF1R/AKIRIN2/CCDC92/KLRD1/ALOX5AP/FOXP1/SASH1/OPTN/NLRC4/PDGFA/YTHDF3/STK24/NFKB1/GREM1/KANK2/IRF1/PGLYRP1/PTK2B/MFHAS1/SEMA4A/BCR/TRAF6/PRKD2/IL6R/LPCAT3/PAK1/BMP6/MMP9/VAV1/AKNA/PTPRC/SYK/ADAR/JAM3/THBS1/PTPN1/ILRUN/NPLOC4/MED1/MGLL/IL17RA/NLRX1/RBM14/LTF/PIK3CG/CAMK1D/CHD8/FLNA/ALOX12/RXRA/ZYX/RAC1/LYN/RIOK3/LGALS9/SIN3A/RAC2/CREBBP/APOBEC3F/CDK19/HSPB1/TNFRSF1B/TNIP1/GPX1/ARRB2/GRN/RIN3/IFIT1B/ZNFX1/SEC14L1/MAPK3/SNCA/DTX3L/RIPOR2/PCBP2/LAMP1/N4BP1/GP1BA/PRKDC/BRD4/MPP1/PAK2/SHARPIN/KRT1/WNK1/TBXA2R/FURIN/DMTN/RELA

CC	GO:0097447	dendritic tree	336	-0.39	-1.52	5.83E-05	0.00050569	0.00031047	2687	tags=36%, list=23%, signal=28%	LYNX1/ALCAM/APBA1/SYNGAP1/ZDHHC5/KCNE3/KCNJ2/CCN3/SV2A/HNRNPU/P2RY1/STAU2/HDAC6/AMIGO1/SEPTIN4/TRAK1/AMFR/ARHGAP32/SLC1A4/CYBB/PPFIA1/PI4K2A/DIP2B/GIGYF2/EPHB2/NIN/ATP2B1/STRN/ZNF385A/FYN/DMWD/FEZ1/LRRC4/DNAJB1/MAP1S/ELK1/ADNP/DBNL/GRIK2/ATP7A/LRFN3/CPEB4/OR10H1/NECTIN1/SYAP1/RPH3A/LRRK2/RANGAP1/DLG4/DBN1/MME/EPHB1/STX4/CDKL5/SIPA1L1/RAP1GAP/CKB/CTNND1/KPNA1/ABI3/MARK3/EPHA4/SHISA7/NCF1/NUMA1/GSK3A/TXNRD2/PPP1CA/CANX/HRH2/AGO2/PRKACA/INSR/CNIH3/FXR2/MCRS1/KIF1C/ERO1A/HTT/RARA/PTK2B/NEURL1/SLC8A1/BCR/MARK4/MAX/PAK1/MAP1A/PPP3CA/APBA3/ASAP1/PLEC/FZD5/GLRX5/DNM3/RAC1/KIF1B/SLA/MARK2/GSK3B/ROGDI/FLOT2/GNAI2/TRAK2/GRIPAP1/RHOA/STAU1/CLU/ARRB2/NF1/PPP1R9B/RIN3/CASC3/NRGN/CTTN/PRKAR2B/ACTN1/SHARPIN/GNAZ/OSBP2
BP	GO:0048771	tissue remodeling	86	-0.56	-1.82	6.03E-05	0.00052244	0.00032075	2545	tags=42%, list=22%, signal=33%	CST3/BAK1/GPR137/CD38/TF/ADAM8/SRC/EXT1/VDR/ATP7A/TGFB1/PPARGC1B/NOTCH2/ROCK2/TMBIM1/LRRK1/CSF1R/LEPR/GREM1/TPP1/RBPJ/PTK2B/BCR/SNX10/TRAF6/RAB7A/CSK/PPP3CA/SYK/FLNA/CAPN1/INPP5D/NF1/RAB3D/ITGB3/TIMP1
BP	GO:0098742	cell-cell adhesion via plasma-membrane adhesion molecules	87	-0.55	-1.79	6.06E-05	0.00052361	0.00032147	2022	tags=29%, list=17%, signal=24%	LRRC4/CD177/MAP2K5/LRFN3/NECTIN1/ITGAM/MAPK14/CLSTN1/ITGB2/PCDH1/ADGRL1/PALLD/SELP/PECAM1/ITGA5/IL1RN/ESAM/SELL/ITGAL/MYADM/PTPN23/CLDN5/BSG/MBP/WNK1
CC	GO:0044853	plasma membrane raft	71	-0.59	-1.86	6.09E-05	0.00052502	0.00032233	2083	tags=35%, list=18%, signal=29%	CR1/SRC/CD177/HCK/ITGAM/LRRK2/LIPE/EZR/FLOT1/CLN3/ITGB2/PRKACA/INSR/CORO1C/SLC2A1/SELPLG/CAVIN2/CTNNA1/FLOT2/ATP2B4/MAPK3/PRKAR2A/STIM1/PLVAP/CBL
BP	GO:0003158	endothelium development	74	-0.57	-1.83	6.12E-05	0.00052703	0.00032357	2072	tags=38%, list=18%, signal=31%	HEG1/EDF1/ATOH8/VEZF1/PDE2A/ROCK2/BTG1/PLCB1/EZR/S1PR2/FOXP1/TJP2/RBPJ/RAPGEF2/PRKD2/VCL/PECAM1/BMP6/RHOA/FOXJ2/MYADM/GPX1/FASN/MSN/KDM6B/ADD1/RAPGEF1/CLDN5
BP	GO:0000003	reproduction	738	-0.34	-1.38	6.20E-05	0.00053241	0.00032687	2089	tags=22%, list=18%, signal=20%	BCL2L11/THRA/ALKBH5/HSPA1L/CSDE1/LIMK2/PTEN/KDM5B/MAP3K4/TOP3A/SRC/CRTAP/KPNA6/ATM/ABAT/ADNP/DIAPH2/STK4/H3-3B/PAQR8/THBD/PROK2/NIPBL/VDR/H3-3A/ATP7A/SUN2/PUM1/ETV6/CUL7/NOTCH2/ARMC2/ZNF148/HSPA5/LRRK2/OPRL1/BTG1/IRF2BPL/C3orf62/ASH1L/PLCB1/STAT5B/FANCG/EGR1/KMT2D/TFEB/ASF1B/PI3/RAD21/ROPN1L/NR6A1/MME/CTCFL/MAPK14/CEBPA/YY1/ZFP36L1/ARHGDIB/MSX1/SETX/PTPN6/SMC1A/CDKN1C/SPATA2/GMCL1/AGO4/MAST2/DHCR24/HERC2/CCNF/UBAP2L/PSME4/ZNF296/NUMA1/RFX2/BIRC6/TRIM28/SMARCA2/LEPR/GRB2/AGO2/ARNT/ALPL/PRKACA/PDGFA/INSR/YTHDF3/GNPDA1/SGPL1/RBPJ/NCOA4/BCAP31/LRRC8A/RARA/PTK2B/SLC2A1/NEURL1/RPA1/PAFAH1B2/NDRG3/PITHD1/ACOX1/TOB2/IGF2/ZMIZ1/CTNNA1/BMP6/SBF1/ZBTB16/MMP9/ALDOA/SFRP2/STK11/USP9X/ITGA5/FZR1/JAM3/PTAFR/NCOR2/BPTF/MED1/CASP2/FZD5/ABHD2/B4GALT1/BASP1/UBR2/SPOCD1/UCP2/GOLGA2/LGALS9/PDE5A/DNM2/IGF2R/PSAP/SF1/ATP2B4/EPN1/FOXJ2/GATA1/ARRB2/CCNI/PPP1R9B/TBC1D20/PBX1/BCL2L1/STAT3/UBB/NCOA1/BSG/CTSB/PRKDC/KLF1/KLC3/YBX3/P2RX1/CTDNEP1/MARF1/MYH9/TIMP1/FOXO3/CBL/OSBP2/BAG6/ACRBP

BP	GO:0030001	metal ion transport	442	-0.37	-1.46	6.30E-05	0.00053845	0.00033058	2069	tags=22%, list=18%, signal=19%	SNAP25/FYN/TPCN1/PER1/YWHAH/FKBP1B/SLC24A4/MCOLN1/PRKCE/KCNJ15/SLC39A7/VDR/ATP7A/ATP13A1/LILRA5/ANO6/SLC12A6/NECTIN1/OPRL1/MFSD4B/CACNB1/KCNH2/FXYD6/PTPN6/TMBIM6/CD63/PDPK1/PML/KCNQ5/MICU1/CCR1/ITPR2/CHP1/PRKACA/CHERP/ATP2A3/ERO1A/TSPO/TMEM38A/HTT/PTK2B/SLC8A1/SLC6A20/INPP5K/SLC24A3/SLC3A2/LCN2/PPP3CA/PTPRC/SLC9A1/SELENON/SCN1B/ATP1B2/EHD3/CLTC/CALM3/SLC6A9/LTF/PIK3CG/G6PD/FLNA/JAK3/ANXA6/GJC2/AQP1/LYN/DNM2/GP9/RAB11B/WWP2/ATP2B4/GNAI2/FKBP1A/RHOA/FTL/PRKCB/ARRB2/SLC38A10/ACTN4/FTH1/MLLT6/TESC/MYLK/SNCA/EPB41/AHCYL1/GP1BA/MMGT1/SLC48A1/SLC25A37/STIM1/ARHGAP1/WNK1/KEL/P2RX1/SLC6A8/DIAPH1
CC	GO:0044297	cell body	306	-0.40	-1.54	6.28E-05	0.00053845	0.00033058	2699	tags=33%, list=23%, signal=26%	VTI1B/KCNE3/NDUFS7/KCNJ2/CCN3/SV2A/P2RY1/STAU2/HDAC6/AMIGO1/SEPTIN4/CCT7/AMFR/SLC1A4/ENDOG/CYBB/PI4K2A/DIP2B/GIGYF2/MUL1/EPHB2/ENO2/TLR2/ATP2B1/STRN/KATNB1/ZNF385A/HSPA1L/DISC1/FYN/DMWD/FEZ1/DNAJB1/TOP1/MAP1S/ELK1/ADNP/DBNL/GRIK2/ATP7A/SLC2A3/G3BP1/CPNE5/SYAP1/LRRK2/ENC1/NDEL1/MME/NF2/ACTA2/RAP1GAP/PYCARD/CKB/VPS35/EPHA4/NCF1/NUMA1/GSK3A/TXNRD2/PPP1CA/CANX/PDE1A/INSR/FXR2/MCRS1/SORL1/PTK2B/NEURL1/SLC8A1/RAPGEF2/SLC3A2/TBX21/MAP1A/SCN1B/ATP1B2/DCTN1/FZD5/GLRX5/FLNA/PVALB/GJC2/ROGDI/TNFRSF1B/GNAI2/MPL/WDFY3/TUBB/STAU1/PPP1R9B/RIN3/GNA12/PINK1/NRGN/SNCA/SIAH2/PRKAR2B/UBB/MBP/GNAZ/GLUL/HCFC1
BP	GO:0003333	amino acid transmembrane transport	55	-0.61	-1.86	6.45E-05	0.00055016	0.00033777	1423	tags=27%, list=12%, signal=24%	PER2/SLC36A1/CLN3/TSPO2/LRRC8A/SLC6A20/SLC3A2/SLC25A38/SLC6A9/SLC1A5/SLC43A2/SLC43A1/SLC38A10/SLC7A5/SLC38A5
BP	GO:0010811	positive regulation of cell-substrate adhesion	79	-0.56	-1.82	6.50E-05	0.00055371	0.00033995	2860	tags=46%, list=24%, signal=35%	POLDIP2/SMAD3/EPHA1/CDK6/SPOCK2/CARMIL1/RIN2/CALR/UTRN/LIMS1/PLEKHA2/MAP4K4/DISC1/UNC13D/PPM1F/TRIOBP/STK4/PRKCE/SDC4/CASS4/RRAS/DOCK5/PTPRJ/PREX1/NEDD9/PTK2B/ITGA5/FLNA/GFUS/RAC1/GSK3B/DNM2/MYADM/RSU1/DAB2/DMTN
BP	GO:0006511	ubiquitin-dependent protein catabolic process	552	-0.36	-1.44	6.58E-05	0.00055906	0.00034323	1818	tags=24%, list=16%, signal=21%	DET1/AREL1/WDR81/RNF144B/GID4/RC3H2/CUL7/USP4/SDCBP/UBXN7/RNF213/HSPA5/SH3RF3/UBE2J2/SMARCC1/ARRB1/LRRK2/CACUL1/MAN1A1/PSMD2/KEAP1/MARCHF6/PSMD3/RNF11/TMEM67/PIAS1/TRIB1/CEBPA/WAC/CHMP4B/YOD1/UBE2Z/AGTPBP1/CBFA2T3/ARIH2/SPOP/ITCH/KCTD2/RNF115/HERC2/CCNF/PML/KAT5/TRIB3/GSK3A/UBE2L3/ZRANB1/CANX/PSMD1/VPS37B/VPS37C/FBXL18/CNOT4/UBE4B/USP10/CSNK2A1/SUFU/BCAP31/RNF126/PTK2B/USP3/TBX21/PSMD11/NSFL1C/MAP1A/TBL1XR1/RAD23A/TGFB1I1/UBR4/USP9X/FZR1/NPLOC4/FBXL20/UBAP1/HIPK2/USP34/TRIP12/VCP/HSPA1A/ANKRD9/UBR2/PSMC4/VPS28/CHMP1A/GABARAP/KLHL8/RBCK1/LAPTM5/GSK3B/UBA1/UBE2R2/FBXO9/WWP2/DDB1/HUWE1/CUL4A/RMND5A/TBL1X/GID8/UBL7/CLU/ARRB2/USP22/GNA12/RNF187/PINK1/ASCC2/SIAH2/DTX3L/PTPN23/PCBP2/PSMF1/N4BP1/SQSTM1/DNAJB2/USP7/ARAF/RNF40/SHARPIN/DCAF12/DAB2/TRIM58/FBXO7/TOLLIP/UBE2H/CBL/CDC34/ZER1/BAG6/RYBP/DCAF11
BP	GO:0043244	regulation of protein-containing complex disassembly	98	-0.54	-1.79	6.76E-05	0.00057315	0.00035188	2660	tags=47%, list=23%, signal=37%	CARMIL1/ADD2/CLASP1/HDAC6/TWF2/CALCOCO2/TECPR1/SCIN/SCAF4/IRAK3/FLII/KATNB1/TRIOBP/MAP1S/ATXN7/EPS8/SPTAN1/SETX/CAMSAP1/PHF23/EIF5A/WASHC2C/CLN3/CLEC16A/ARHGEF2/WDR1/GLE1/INSR/SPTBN1/UPF1/SPTA1/SVIL/MAP1A/TAOK1/GSN/RUBCN/PLEK/TBC1D25/CAPZB/ADD1/TMOD1/SPTB/MTPN/SH3BP1/GSPT1/DMTN

BP	GO:2000146	negative regulation of cell motility	184	-0.45	-1.65	6.94E-05	0.00058727	0.00036055	2049	tags=26%, list=17%, signal=21%	SRF/PADI2/PTEN/MAP2K5/TGFB1/LDLRAD4/RRAS/PIP5KL1/PLCB1/TRIB1/S1PR2/NF2/PTPRJ/ARHGDIB/FUZ/EPHA4/STK24/TPM1/GREM1/CORO1C/BCR/ARID2/VCL/CTNNA1/SFRP2/THBS1/ATP1B2/ADIPOR1/ABHD2/RAC1/LRP1/CNN2/ATP2B4/RHOA/NF1/RIN3/GNA12/WAS/FLCN/STAT3/PTPN23/CLDN5/RIPOR2/DNAJA4/TIMP1/FOXO3/TBXA2R
CC	GO:0098858	actin-based cell projection	126	-0.50	-1.73	6.98E-05	0.00058951	0.00036193	2541	tags=34%, list=22%, signal=27%	TWF2/FSCN1/SPN/UTRN/SLC9A3R1/MYO5A/ACP3/MYO1G/MYO1E/TRIOBP/EXOC4/ATP6V1B2/ATP7A/EPS8/TGFB1/AOC3/SYNE2/LRRK2/EZR/EPHB1/NF2/ACTA2/EPHA4/LCP1/PDGFA/FXR2/AP2A1/FGD4/DYNC1H1/JAM3/IQGAP2/MYO1F/B4GALT1/PVALB/ATP6V1A/MSN/PPP1R9B/ESPN/RIPOR2/MPP1/ITGB3/VASP/SLC7A5
MF	GO:0044325	transmembrane transporter binding	83	-0.56	-1.82	7.19E-05	0.00060545	0.00037171	1204	tags=33%, list=10%, signal=29%	FYN/SRC/YWHAH/FKBP1B/LRRK2/PYCARD/ITPR2/YWHAZ/CHERP/USP10/ATP2A3/TSPO/HTT/SLC8A1/SCN1B/CALM3/FLNA/LYN/FKBP1A/PACS1/ACTN4/PPP1R9B/ACTN1/GRINA/AP2M1/ANK1/DIAPH1
BP	GO:0098739	import across plasma membrane	96	-0.54	-1.79	7.21E-05	0.00060604	0.00037208	2045	tags=30%, list=17%, signal=25%	FYN/IRS2/SLC24A4/KCNJ15/SLC2A3/SLC12A6/KCNH2/PER2/SLC36A1/TSPO2/SLC27A1/LRRC8A/SLC2A1/SLC8A1/SLC6A20/SLC3A2/SLC9A1/THBS1/ATP1B2/SLC6A9/SLC1A5/SLC43A2/SLC43A1/ATP2B4/SLC15A3/AKT2/WNK1/SLC7A5/P2RX1
BP	GO:0051271	negative regulation of cellular component movement	190	-0.45	-1.65	7.30E-05	0.00061256	0.00037608	2049	tags=25%, list=17%, signal=21%	SRF/PADI2/PTEN/MAP2K5/TGFB1/LDLRAD4/RRAS/PIP5KL1/PLCB1/TRIB1/S1PR2/NF2/PTPRJ/ARHGDIB/FUZ/EPHA4/STK24/TPM1/GREM1/CORO1C/BCR/ARID2/VCL/CTNNA1/SFRP2/THBS1/ATP1B2/ADIPOR1/ABHD2/RAC1/LRP1/CNN2/ATP2B4/RHOA/NF1/RIN3/GNA12/WAS/FLCN/STAT3/PTPN23/CLDN5/RIPOR2/ACTN1/DNAJA4/TIMP1/FOXO3/TBXA2R
BP	GO:0045860	positive regulation of protein kinase activity	275	-0.42	-1.59	7.52E-05	0.00062975	0.00038663	2028	tags=28%, list=17%, signal=24%	MAP3K4/TIRAP/SRC/ADNP/MAP2K5/STK4/SDC4/XRCC6/ERBB2/CCNY/CASS4/MAGED1/TGFB1/ERN2/LILRA5/ERN1/SYAP1/LRRK2/CACUL1/EGR1/DLG4/MAP2K2/DAZAP2/EPHA4/PDPK1/NCF1/CSF1R/SASH1/PDGFA/NEDD9/INSR/MAP3K5/SLC27A1/TAOK3/GREM1/VLDLR/PTK2B/NEURL1/RAPGEF2/CCND3/TRAF6/IL6R/PAK1/MOB3A/IGF2/CSK/TAOK1/STK11/MAP2K7/STRADA/PTPRC/SYK/DVL3/THBS1/PTPN1/TLR6/CALM3/FZD5/LTF/PIK3CG/MADD/MARK2/MMD/PDE5A/AKAP13/ATP2B4/RHOA/CLU/PINK1/SNCA/CDKN1A/TAL1/PAK2/ITGB3/WNK1/IQGAP1/MAP2K3/STRADB
BP	GO:1904062	regulation of cation transmembrane transport	186	-0.45	-1.64	7.57E-05	0.00063127	0.00038757	1555	tags=24%, list=13%, signal=21%	DLG4/CACNB1/KCNH2/FXYD6/PTPN6/CD63/SHISA7/PDPK1/CHP1/PRKACA/CNIH3/TMEM38A/HTT/PTK2B/SLC8A1/MMP9/SLC9A1/SELENON/SCN1B/ATP1B2/EHD3/CALM3/SLC6A9/PIK3CG/G6PD/FLNA/CAPN1/SLC43A2/GJC2/LYN/DNM2/SLC43A1/WWP2/ATP2B4/FKBP1A/ACTN4/PINK1/TESC/SNCA/STIM1/WNK1/KEL/P2RX1/DIAPH1
BP	GO:0016032	viral process	344	-0.40	-1.54	7.92E-05	0.00065767	0.00040378	1759	tags=24%, list=15%, signal=21%	NCAM1/SETDB1/CTDP1/CTBP2/NECTIN1/ROCK2/SMARCA4/TMEM250/NFIA/OASL/PIKFYVE/ST3GAL2/MGAT5/CTBP1/GRK2/CHMP4B/EIF2AK2/ST3GAL3/DDX6/IFITM3/ITCH/MON1B/ZC3HAV1/PTBP1/CSF1R/TRIM28/GSK3A/CANX/VPS37B/ANPEP/VPS37C/INSR/MGAT4B/SELPLG/INPP5K/SLC3A2/USF2/RAB7A/RAD23A/GSN/ITGA5/ADAR/ST3GAL1/ATG7/IST1/LTF/SLC1A5/VCP/HSPA1A/VPS28/CHMP1A/LGALS9/GSK3B/IGF2R/APOBEC3F/ST6GALNAC4/WWP2/DDB1/IFITM2/SRPK2/TRIM11/TNIP1/EIF3B/TRIM14/STAU1/SP1/APOBEC3C/LARP1/TRIM10/CFL1/ZNFX1/ITGB5/TRIM26/TBC1D20/BCL2L1/PCBP2/LAMP1/N4BP1/BSG/CTSB/ITGB3/RAB1B/FURIN/HCFC1

BP	GO:0010876	lipid localization	262	-0.42	-1.57	8.20E-05	0.00067922	0.00041701	2557	tags=31%, list=22%, signal=25%	MEST/VPS4A/STOML1/APOL6/PRELID2/ANXA2/GRAMD1A/SLCO3A1/SLC10A3/CRY2/ATP9A/SCARB1/ATG2B/VPS4B/PCTP/KDM5B/FTO/PDZD8/IRS2/STARD3/OSBPL1A/ANO6/RNF213/CPTP/FITM1/ABCB11/IKBKE/STAT5B/NME4/VPS51/SLC27A3/OSBPL2/NR1H2/AQP9/CLN3/OSBPL5/PITPNC1/PTGES/ABHD5/TSPO2/ABCG2/DGAT2/GOT2/LRP10/PITPNM2/ZFYVE1/SLC27A1/NFKB1/PITPNA/TSPO/VLDLR/SLC2A1/APOBR/BSCL2/LDLRAP1/PLSCR3/LPCAT3/BMP6/CPT1A/SYK/THBS1/FIS1/SLC66A2/PLIN3/ABCC4/PSAP/LRP1/ABCC3/PNPLA2/CLU/ATG2A/EHD1/PIP4K2A/HDLBP/AKT2/SLC4A1/MFSD2B/ITGB3/DAB2/FURIN/OSBP2
MF	GO:0004713	protein tyrosine kinase activity	89	-0.54	-1.80	8.31E-05	0.00068513	0.00042063	2320	tags=38%, list=20%, signal=31%	AATK/MAP2K4/EPHB2/DYRK3/TNK2/FGR/FYN/SRC/EPHB4/MAP2K5/ERBB2/HCK/DYRK1B/CLK3/EPHB1/MAP2K2/EIF2AK2/EPHA4/CSF1R/INSR/FES/HIPK1/PTK2B/BCR/CSK/MAP2K7/SYK/HIPK2/JAK3/LYN/IGF2R/SCYL1/JAK1/MAP2K3
BP	GO:0045807	positive regulation of endocytosis	68	-0.57	-1.83	8.30E-05	0.00068513	0.00042063	1738	tags=43%, list=15%, signal=37%	ANO6/ARRB1/LRRK2/AP2B1/SYNJ1/FLOT1/CD63/CLN3/INSR/GREM1/LDLRAP1/ANKFY1/PPP3CA/SYK/RAB31/CD151/VPS28/CD14/HIP1/HNRNPK/DNM2/LRP1/CLU/ARRB2/SNCA/TBC1D5/AP2M1/DAB2/CBL
BP	GO:0044403	biological process involved in symbiotic interaction	211	-0.44	-1.61	8.61E-05	0.00070559	0.00043319	1802	tags=25%, list=15%, signal=21%	SNX3/NCAM1/CTDP1/NECTIN1/EXOC7/SMARCA4/PIKFYVE/GRK2/CHMP4B/DCD/IFITM3/TREM1/ITCH/CSF1R/VPS37B/ANPEP/INSR/PGLYRP1/SELPLG/INPP5K/SLC3A2/RAB7A/GSN/THOC5/ITGA5/ATG7/IST1/LTF/SLC1A5/HSPA1A/PF4/CHMP1A/LGALS9/IGF2R/WWP2/DDB1/IFITM2/TRIM11/TRIM14/GPX1/SP1/TRIM10/CFL1/ITGB5/TRIM26/TBC1D20/BCL2L1/LAMP1/BSG/CTSB/ITGB3/CBL
MF	GO:1901981	phosphatidylinositol phosphate binding	131	-0.50	-1.74	8.81E-05	0.00072057	0.00044239	2763	tags=41%, list=24%, signal=32%	KCNQ1/PHLDA3/SNX18/GBF1/KCNJ2/TWF2/ANXA2/ARHGAP32/TECPR1/SCIN/ZFYVE26/PLCD1/ATG2B/MYO1G/FLII/SAP30L/TPCN1/TIRAP/NCF4/SNX3/SDCBP/ARHGAP9/TTPAL/RPH3A/PLCB1/PLEKHF1/OSBPL2/DENND1C/WASHC2C/PARD3/ARAP1/OSBPL5/ZFYVE1/ADAP2/GAB2/SVIL/LDLRAP1/ANKFY1/ADAP1/SNX11/GSN/WDR45/SLC9A1/ASAP1/WIPI2/IQGAP2/RUBCN/SNX27/HIP1/ATG2A/NRGN/PLEK2/DENND1A/IQGAP1
MF	GO:0019902	phosphatase binding	163	-0.46	-1.66	8.92E-05	0.00072814	0.00044704	2348	tags=32%, list=20%, signal=26%	CRY2/SIGLEC10/SLC9A3R1/MVP/PPP6R1/STRN/FOXO1/CDC27/IRS2/PPP1R15A/ERBB2/SIRPA/SNX3/PHACTR4/TSC2/IKBKE/DLG4/MAPK14/ELL/MAST2/CSF1R/GRB2/PPP1CA/MTMR3/MAP3K5/NFATC2/MFHAS1/VRK3/STAT6/SBF2/CSK/MAP2K7/SYK/SLC9A1/PTPN1/JAK3/VCP/PPP1R18/PPP1R10/ATP2B4/PXN/STAU1/PPP1R9B/JAK1/GNA12/SMG7/MAPK3/STAT3/PPP3R1/WNK1/IQGAP1/ANK1

BP	GO:0006325	chromatin organization	454	-0.37	-1.47	8.95E-05	0.00072904	0.00044759	2566	tags=32%, list=22%, signal=26%	HDAC6/BANF1/KDM6A/KDM7A/KDM4A/TET2/CDAN1/IPO4/EHMT1/SETD2/KAT6B/MTA1/NFRKB/L3MBTL4/BAP1/RCOR1/SIRT6/PADI4/HDAC7/KAT2B/IFI16/RSBN1/HDAC3/CBX4/PKN1/SAFB/SRPK1/SSRP1/ASXL1/SFMBT1/H2BC21/NSD3/CBX5/PHF2/PADI2/KDM5B/GATAD2A/PHF8/TOP1/MTHFR/HDAC4/ATXN7L3/MLLT1/VPS72/JMJD6/H3-3B/MCM3AP/BAZ2A/SRCAP/H3-3A/RFWD3/ZBTB7A/SETDB1/EP400/EIF1/SMCHD1/SMARCC1/SMARCA4/ASH1L/KDM3B/KDM8/EYA3/KMT2D/ASF1B/KAT7/L3MBTL2/PHF20/PHF13/CTCFL/KMT2B/BABAM2/CTBP1/WAC/YY1/SMARCD3/CDKN1C/DAPK3/SPI1/KDM2A/ARID1B/PSME4/CHRAC1/NSD1/SAMD1/KAT5/UBN2/TRIM28/SMARCA2/SETD1B/KMT2A/BCORL1/ZNF335/TRRAP/MCRS1/UPF1/MKI67/USP3/ARID2/SMARCC2/PAK1/IGF2/SETD5/RERE/TBL1XR1/BRPF1/GRWD1/SUPT6H/BPTF/NR3C1/GATAD2B/TET3/CHD8/UBR2/ACTB/HMGA1/PHF21A/SIN3A/GFI1B/CHD4/BABAM1/CBX6/BRD3/PRKCB/KMT2C/GPX1/KDM6B/H3C10/KDM4B/UBN1/USP22/ARID1A/ZNFX1/WBP2/H2BC11/DTX3L/PCGF5/NFE2/IKZF1/KAT6A/BRD4/RNF40/JARID2/CARM1/CABIN1/BAG6/HCFC1/RELA
MF	GO:0003712	transcription coregulator activity	426	-0.37	-1.47	9.02E-05	0.00073321	0.00045015	2260	tags=31%, list=19%, signal=26%	HDAC7/TRIM5/KAT2B/GON4L/HDAC3/CBX4/PKN1/FHL2/MMS19/TRIM38/TADA3/ASXL1/SFMBT1/PA2G4/SAP30L/MED12L/TOX2/PHF2/KDM5B/PHF8/EDF1/DNAJB1/HDGFL2/CCDC124/MED13/ELK1/ATXN7L3/DDX54/SRCAP/LCOR/MAGED1/PPARGC1B/BCL3/MTDH/CTBP2/SMARCC1/ARRB1/MED27/BTG1/IRF2BPL/SMARCA4/GPS2/MIDEAS/KDM3B/XPC/DYRK1B/ARID5A/KMT2D/KAT7/PIAS1/SRA1/CTBP1/TLE4/PER2/SMARCD3/CBFA2T3/DPF2/FHL3/NCOA3/KDM2A/MAML2/ARID1B/NSD1/AKIRIN2/KAT5/TRIB3/HELZ2/TRIM28/SMARCA2/UBE2L3/BCORL1/MED16/TRRAP/CNOT6/SUFU/TACC1/NCOA4/NCOA2/NUP98/MED13L/SMARCC2/PIAS4/TOB2/ZMIZ1/RERE/TBL1XR1/TGFB1I1/NPAT/NCOR2/MED1/DCAF6/RBM14/HIPK2/BASP1/MAML3/LPIN2/NIBAN2/YY1AP1/HSPA1A/HMGA1/SIN3A/RALY/CREBBP/MED15/SF1/TAF6/PXN/PRKCB/TRIM14/TBL1X/KMT2C/PDLIM1/NCOA6/ARID3A/ACTN4/BCL9/USP22/JAZF1/ARID1A/WBP2/SIAH2/TLE3/ACTN1/NCOA1/ENO1/HDGF/KAT6A/BRD4/CARM1/SND1/RYBP/HCFC1
CC	GO:0030425	dendrite	335	-0.40	-1.53	9.42E-05	0.00076416	0.00046916	2687	tags=36%, list=23%, signal=28%	LYNX1/ALCAM/APBA1/SYNGAP1/ZDHHC5/KCNE3/KCNJ2/CCN3/SV2A/HNRNPU/P2RY1/STAU2/HDAC6/AMIGO1/SEPTIN4/TRAK1/AMFR/ARHGAP32/SLC1A4/CYBB/PPFIA1/PI4K2A/DIP2B/GIGYF2/EPHB2/NIN/ATP2B1/STRN/ZNF385A/FYN/DMWD/FEZ1/LRRC4/DNAJB1/MAP1S/ELK1/ADNP/DBNL/GRIK2/ATP7A/LRFN3/CPEB4/OR10H1/NECTIN1/SYAP1/RPH3A/LRRK2/RANGAP1/DLG4/DBN1/MME/EPHB1/STX4/CDKL5/SIPA1L1/RAP1GAP/CKB/CTNND1/KPNA1/ABI3/MARK3/EPHA4/SHISA7/NCF1/NUMA1/GSK3A/TXNRD2/PPP1CA/CANX/HRH2/AGO2/PRKACA/INSR/CNIH3/FXR2/MCRS1/KIF1C/ERO1A/HTT/RARA/PTK2B/NEURL1/SLC8A1/BCR/MARK4/MAX/PAK1/MAP1A/PPP3CA/APBA3/ASAP1/PLEC/FZD5/GLRX5/DNM3/RAC1/KIF1B/SLA/MARK2/GSK3B/ROGDI/FLOT2/GNAI2/TRAK2/GRIPAP1/RHOA/STAU1/CLU/ARRB2/NF1/PPP1R9B/RIN3/CASC3/NRGN/CTTN/PRKAR2B/ACTN1/SHARPIN/GNAZ/OSBP2
BP	GO:0007041	lysosomal transport	108	-0.52	-1.77	9.45E-05	0.0007653	0.00046985	2177	tags=39%, list=19%, signal=32%	VPS53/VPS18/VPS4B/HOOK2/AP1G1/LYST/LAMP2/HGS/LRRK2/VPS51/CHMP4B/SORT1/HOOK3/VPS41/CLN3/VPS35/CLEC16A/VPS11/NCOA4/SORL1/KIF13A/RAB7A/ANKFY1/HGSNAT/EHD3/VCP/HSPA1A/TGFBRAP1/CHMP1A/SNX27/LAPTM5/IGF2R/PSAP/LRP1/RILP/CLU/GRN/PINK1/DTX3L/LAMP1/GAK/UBXN6

BP	GO:0051235	maintenance of location	228	-0.42	-1.58	9.70E-05	0.00078384	0.00048123	2603	tags=35%, list=22%, signal=28%	CALR/HNRNPU/CD4/MEST/TWF2/CAPN3/SLC25A23/CALM1/ASPM/CORO1A/CRY2/SCIN/NRROS/SCARB1/LETM1/TPCN1/FTO/TAF8/FKBP1B/MCOLN1/PRKCE/SUN2/VPS13D/RNF213/HSPA5/FITM1/GPAA1/IKBKE/RANGAP1/STAT5B/DBN1/KEAP1/PTPN6/NR1H2/CIZ1/ABHD5/PDPK1/PML/ITPR2/DGAT2/PRKACA/CHERP/ZFYVE1/YWHAB/NFKB1/SUFU/ERO1A/TSPO/ARHGAP21/SORL1/TMEM38A/HTT/PTK2B/SLC8A1/BSCL2/LCN2/GSN/CPT1A/PTPRC/KDELR1/SELENON/GAA/PLIN3/CALM3/FLNA/ANXA6/LYN/GP9/FKBP1A/PNPLA2/FTL/FTH1/PINK1/HK1/SNCA/EHD1/LTBP1/GP1BA/ITGB3/DIAPH1
BP	GO:0007188	adenylate cyclase-modulating G protein-coupled receptor signaling pathway	89	-0.54	-1.79	0.00010104	0.00081325	0.00049929	2890	tags=38%, list=25%, signal=29%	GNAS/GNAI3/GNAL/ADGRE1/FPR1/PTGDR2/MARCO/P2RY1/INSL3/SLC9A3R1/GNA15/PTGIR/ADGRE2/PDE2A/PDE4A/OPRL1/S1PR2/ADGRG3/GNAQ/GSK3A/ADGRL1/RAPGEF2/GNB1/FLNA/PF4/ADGRE5/PSAP/AKAP13/ATP2B4/GNAI2/GNA12/GNAZ/TBXA2R/WASF2
BP	GO:0006936	muscle contraction	163	-0.46	-1.65	0.00010414	0.0008348	0.00051252	2136	tags=30%, list=18%, signal=25%	ATP2B1/ARHGEF11/ORMDL3/SRF/FKBP1B/ABAT/SSPN/PROK2/TPM3/ROCK2/DOCK5/GRK2/KCNH2/ACTA2/CHRNE/MYOM1/DAPK3/TPM4/PRKACA/TPM1/TMEM38A/SLC8A1/CALD1/ALDOA/GSN/SLC9A1/PTAFR/GAA/SCN1B/PPP1R12B/EHD3/CALM3/PIK3CG/FLNA/ANXA6/PDE5A/MYL4/ATP2B4/RHOA/TMOD1/MYLK/GUCY1A1/CTTN/ENO1/MYL9/P2RX1/SLC6A8/TBXA2R/MAP2K3
BP	GO:0030336	negative regulation of cell migration	176	-0.45	-1.65	0.00010441	0.00083531	0.00051284	2049	tags=26%, list=17%, signal=21%	SRF/PADI2/PTEN/MAP2K5/TGFB1/LDLRAD4/RRAS/PIP5KL1/PLCB1/TRIB1/S1PR2/NF2/PTPRJ/ARHGDIB/FUZ/EPHA4/STK24/TPM1/GREM1/CORO1C/BCR/ARID2/VCL/SFRP2/THBS1/ATP1B2/ADIPOR1/ABHD2/RAC1/LRP1/CNN2/ATP2B4/RHOA/NF1/RIN3/GNA12/FLCN/STAT3/PTPN23/CLDN5/RIPOR2/DNAJA4/TIMP1/FOXO3/TBXA2R
CC	GO:0019897	extrinsic component of plasma membrane	107	-0.52	-1.75	0.00010619	0.00084783	0.00052052	2417	tags=36%, list=21%, signal=28%	GRAMD1A/SCRIB/TF/AAK1/TNK2/GNA15/SNAP25/FGR/FYN/SRC/HCK/TIAM1/NUMB/SYAP1/SYTL3/DLG4/TGM3/OSBPL2/CTNND1/GNAQ/NUMA1/FES/MYZAP/PTK2B/CTNNA1/PPP3CA/SYK/GNB1/RAC1/SLA/LYN/HIP1/GNAI2/RHOA/GNA12/GNAZ/RNF10/IQGAP1
BP	GO:0048732	gland development	225	-0.43	-1.59	0.00010694	0.00084905	0.00052127	2470	tags=32%, list=21%, signal=26%	CREB1/CFLAR/SCRIB/ARMC5/WLS/SLC9A3R1/BCL11B/TBX19/ASXL1/SOD2/BCL2L11/THRA/SRF/PHF2/WNT1/PTEN/KDM5B/PRDX2/SRC/ELK1/IRS2/ATM/EXT1/VDR/ATP7A/TGFB1/NOTCH2/MGMT/HGF/ASH1L/MPST/STAT5B/CEBPA/RAP1GAP/MSX1/CDKN1C/FPGS/PML/CSF1R/PDGFA/INSR/RBPJ/TSPO/RARA/NEURL1/STAT6/USF2/IGF2/STK11/CPT1A/NCOR2/MED1/MAFB/BTBD7/CAPN1/UCP2/STAT5A/IGF2R/PSAP/GPX1/FASN/NF1/MSN/RPS6KA1/MAPK3/TGM2/PBX1/ACO2/ZNF703/AKT2/JARID2/HLX/RELA
BP	GO:0048010	vascular endothelial growth factor receptor signaling pathway	31	-0.71	-1.96	0.00010821	0.00085533	0.00052512	2045	tags=55%, list=17%, signal=45%	FYN/SRC/HGS/MAPK14/ARNT/PTK2B/PRKD2/VAV1/ITGA5/PTPN1/MAPKAPK3/NIBAN2/HSPB1/PRKCB/MAPKAPK2/PAK2/ITGB3
BP	GO:0006865	amino acid transport	81	-0.55	-1.77	0.00010897	0.00085965	0.00052778	1423	tags=22%, list=12%, signal=20%	PER2/SLC36A1/CLN3/TSPO2/LRRC8A/SLC6A20/SLC3A2/SLC25A38/SLC6A9/SLC1A5/SLC43A2/SLC43A1/NF1/SLC38A10/SNCA/SLC7A5/SLC38A5/XK

BP	GO:0019941	modification-dependent protein catabolic process	562	-0.35	-1.42	0.00010934	0.00086083	0.00052851	1798	tags=23%, list=15%, signal=21%	AREL1/WDR81/RNF144B/GID4/RC3H2/CUL7/USP4/SDCBP/UBXN7/RNF213/HSPA5/SH3RF3/UBE2J2/SMARCC1/ARRB1/LRRK2/CACUL1/MAN1A1/PSMD2/KEAP1/MARCHF6/PSMD3/RNF11/TMEM67/PIAS1/TRIB1/CEBPA/WAC/CHMP4B/YOD1/UBE2Z/AGTPBP1/CBFA2T3/ARIH2/SPOP/ITCH/KCTD2/RNF115/HERC2/CCNF/PML/KAT5/TRIB3/GSK3A/UBE2L3/ZRANB1/CANX/PSMD1/VPS37B/VPS37C/FBXL18/CNOT4/UBE4B/USP10/CSNK2A1/SUFU/BCAP31/RNF126/PTK2B/USP3/TBX21/PSMD11/NSFL1C/MAP1A/TBL1XR1/RAD23A/TGFB1I1/UBR4/USP9X/FZR1/NPLOC4/FBXL20/UBAP1/HIPK2/USP34/TRIP12/VCP/HSPA1A/ANKRD9/UBR2/PSMC4/VPS28/CHMP1A/GABARAP/KLHL8/RBCK1/LAPTM5/GSK3B/UBA1/UBE2R2/FBXO9/WWP2/DDB1/HUWE1/CUL4A/RMND5A/TBL1X/GID8/UBL7/CLU/ARRB2/USP22/GNA12/RNF187/PINK1/ASCC2/SIAH2/DTX3L/PTPN23/PCBP2/PSMF1/N4BP1/UBB/SQSTM1/DNAJB2/USP7/ARAF/RNF40/SHARPIN/DCAF12/DAB2/TRIM58/FBXO7/TOLLIP/UBE2H/CBL/CDC34/ZER1/BAG6/RYBP/DCAF11
BP	GO:2001237	negative regulation of extrinsic apoptotic signaling pathway	67	-0.58	-1.83	0.00011004	0.00086468	0.00053086	2008	tags=42%, list=17%, signal=35%	PRDX2/SRC/MAP2K5/GSTP1/HGF/EYA3/TMBIM1/RIPK1/ARHGEF2/BIRC6/HTT/CTNNA1/SFRP2/THBS1/PSME3/LMNA/HSPA1A/PF4/ITPRIP/GSK3B/TCF7L2/GATA1/GPX1/SIAH2/BCL2L1/CTTN/STRADB/RELA
BP	GO:0022414	reproductive process	734	-0.33	-1.37	0.0001105	0.00086653	0.000532	2089	tags=22%, list=18%, signal=19%	BCL2L11/THRA/ALKBH5/HSPA1L/CSDE1/LIMK2/PTEN/KDM5B/MAP3K4/TOP3A/SRC/CRTAP/KPNA6/ATM/ABAT/ADNP/DIAPH2/STK4/H3-3B/PAQR8/THBD/PROK2/NIPBL/VDR/H3-3A/ATP7A/SUN2/PUM1/ETV6/CUL7/NOTCH2/ARMC2/ZNF148/HSPA5/LRRK2/OPRL1/BTG1/IRF2BPL/C3orf62/ASH1L/PLCB1/STAT5B/FANCG/EGR1/KMT2D/TFEB/ASF1B/PI3/RAD21/ROPN1L/NR6A1/MME/CTCFL/MAPK14/CEBPA/YY1/ZFP36L1/ARHGDIB/MSX1/SETX/PTPN6/SMC1A/CDKN1C/SPATA2/GMCL1/AGO4/MAST2/DHCR24/HERC2/CCNF/UBAP2L/PSME4/ZNF296/NUMA1/RFX2/BIRC6/TRIM28/SMARCA2/GRB2/AGO2/ARNT/ALPL/PRKACA/PDGFA/INSR/YTHDF3/GNPDA1/SGPL1/RBPJ/NCOA4/BCAP31/LRRC8A/RARA/PTK2B/SLC2A1/NEURL1/RPA1/PAFAH1B2/NDRG3/PITHD1/ACOX1/TOB2/IGF2/ZMIZ1/CTNNA1/BMP6/SBF1/ZBTB16/MMP9/ALDOA/SFRP2/STK11/USP9X/ITGA5/FZR1/JAM3/PTAFR/NCOR2/BPTF/MED1/CASP2/FZD5/ABHD2/B4GALT1/BASP1/UBR2/SPOCD1/UCP2/GOLGA2/LGALS9/PDE5A/DNM2/IGF2R/PSAP/SF1/ATP2B4/EPN1/FOXJ2/GATA1/ARRB2/CCNI/PPP1R9B/TBC1D20/PBX1/BCL2L1/UBB/NCOA1/BSG/CTSB/PRKDC/KLF1/KLC3/YBX3/P2RX1/CTDNEP1/MARF1/MYH9/TIMP1/FOXO3/CBL/OSBP2/BAG6/ACRBP
BP	GO:0046849	bone remodeling	49	-0.63	-1.89	0.00011073	0.00086664	0.00053207	2439	tags=49%, list=21%, signal=39%	GPR137/CD38/TF/ADAM8/SRC/EXT1/TGFB1/PPARGC1B/NOTCH2/LRRK1/CSF1R/LEPR/GREM1/TPP1/PTK2B/SNX10/TRAF6/RAB7A/CSK/SYK/INPP5D/NF1/RAB3D/ITGB3
BP	GO:0002064	epithelial cell development	117	-0.49	-1.69	0.00011164	0.0008721	0.00053542	2305	tags=34%, list=20%, signal=28%	BCL11B/VIM/ASXL1/MYO1E/HEG1/SRC/VEZF1/PDE2A/NOTCH2/ROCK2/PLCB1/SIDT2/EZR/S1PR2/ACTA2/PDPK1/TJP2/GSK3A/PALLD/RARA/RAPGEF2/KLF5/VCL/PECAM1/BMP6/FOSL2/PLEC/B4GALT1/GSK3B/GATA1/MYADM/GPX1/FASN/MSN/ADD1/TMOD1/TBC1D20/RAPGEF1/CLDN5/IQGAP1
BP	GO:0048167	regulation of synaptic plasticity	102	-0.53	-1.77	0.0001142	0.00089032	0.00054661	2755	tags=36%, list=23%, signal=28%	KIT/SYNGAP1/ZDHHC2/NCSTN/MCTP1/STAU2/YWHAG/CD38/EPHB2/SNAP25/SRF/PTEN/YWHAH/GRIK2/SYAP1/DLG4/DBN1/MME/STX4/SIPA1L1/CLN3/EPHA4/SHISA7/ARF1/RARA/PTK2B/NEURL1/RAPGEF2/MAP1A/GSK3B/TSHZ3/STAU1/NF1/NRGN/SNCA/SQSTM1/EIF4EBP2

BP	GO:0043254	regulation of protein-containing complex assembly	304	-0.40	-1.52	0.00011634	0.00090519	0.00055574	2567	tags=37%, list=22%, signal=30%	CLASP1/HDAC6/AMBRA1/BMF/TWF2/CDC42EP1/LGALS3/PMEPA1/FSCN1/BAK1/RALB/TBCD/TPPP3/CORO1A/NCKAP1L/SCIN/ARHGEF5/PRUNE1/CTNNBIP1/NIN/FLII/BCL2L11/THRA/TIRAP/SRC/TRIOBP/FNIP2/SH3GLB1/ATM/DBNL/H3-3B/PRKCE/H3-3A/HCK/EPS8/TGFB1/LDLRAD4/CYRIA/HSPA5/CPTP/IKBKE/CKAP5/SPTAN1/TMC8/ARPC1B/DBN1/EIF2AK2/CAMSAP1/EML2/WASHC2C/PYCARD/CDC42EP4/NR1H2/SLAIN2/VPS35/ARPC5/NUMA1/LCP1/SSH1/GRB2/SPTBN1/FES/SORL1/PTK2B/SPTA1/SELP/WARS1/SVIL/PAK1/GSN/SYK/JAM3/ASAP1/TLR6/ESAM/DCTN1/PPP2R5B/EIF4G1/ULK1/VCP/HSPA1A/GNL3L/PLEK/RAC1/RIOK3/CYFIP2/GSK3B/STMP1/COTL1/RHOA/CAPZB/MYADM/CLU/MSN/ADD1/SSH2/HCLS1/TMOD1/ARPC4/PINK1/SNCA/SPTB/MTPN/CTTN/TFIP11/TAL1/NAPA/PPM1A/MED25/VASP/DMTN/HCFC1
CC	GO:0032587	ruffle membrane	74	-0.56	-1.78	0.00011857	0.00092074	0.00056529	2052	tags=43%, list=17%, signal=36%	MACF1/FGR/TIRAP/SRC/EPS8/ADGRE2/PDE4A/EZR/CDKL5/NF2/PTPRJ/ARHGEF2/LCP1/PIP5K1C/TPM1/CORO1C/INPP5K/SH2D3C/PAK1/ITGA5/PLEK/RAC1/DNM2/RHOA/ARHGAP45/PPP1R9B/CFL1/TESC/AKT2/ITGB3/DIAPH1/TLN1
BP	GO:0071867	response to monoamine	48	-0.63	-1.90	0.00012199	0.00094363	0.00057934	3013	tags=60%, list=26%, signal=45%	DTNBP1/FCSK/GNAS/GNAI3/GNAL/KCNQ1/NCSTN/SLC9A3R1/GNA15/SULT1A3/OR10H1/LRRK2/KLF16/VPS35/GSK3A/HRH2/PRKACA/SLC9A1/GNB1/FLNA/SIN3A/GSK3B/DNM2/ATP2B4/ARRB2/MAPK3/SNCA/TGM2/GNAZ
BP	GO:0071869	response to catecholamine	48	-0.63	-1.90	0.00012199	0.00094363	0.00057934	3013	tags=60%, list=26%, signal=45%	DTNBP1/FCSK/GNAS/GNAI3/GNAL/KCNQ1/NCSTN/SLC9A3R1/GNA15/SULT1A3/OR10H1/LRRK2/KLF16/VPS35/GSK3A/HRH2/PRKACA/SLC9A1/GNB1/FLNA/SIN3A/GSK3B/DNM2/ATP2B4/ARRB2/MAPK3/SNCA/TGM2/GNAZ
CC	GO:0097060	synaptic membrane	156	-0.47	-1.66	0.00012793	0.0009877	0.00060639	2648	tags=33%, list=23%, signal=26%	NDUFS7/APH1A/NCSTN/P2RY1/SCRIB/UTRN/PI4K2A/EPHB2/ENO2/CLTB/ATP2B1/STRN/SNAP25/LRRC4/ITSN1/NINJ1/SSPN/DBNL/GRIK2/LRFN3/PDE2A/NECTIN1/SYAP1/RPH3A/SYNJ1/DLG4/DBN1/STX4/FXYD6/CLSTN1/SLC16A3/CHRNE/EXOC3/EPHA4/SHISA7/CANX/ADGRL1/CNIH3/SLC8A1/SLC6A9/DNM3/HIP1/DNM2/ATP2B4/LRFN1/ARRB2/PPP1R9B/NRGN/DENND1A/ITGB3/RNF10/P2RX1
BP	GO:0010761	fibroblast migration	43	-0.64	-1.89	0.00013046	0.00100331	0.00061598	1899	tags=40%, list=16%, signal=33%	PRKCE/SDC4/TGFB1/ACTA2/CLN3/PML/SGPL1/CORO1C/SLC8A1/THBS1/PLEC/RAC1/PDLIM1/GNA12/TNS1/IQGAP1/DMTN
BP	GO:0071902	positive regulation of protein serine/threonine kinase activity	148	-0.48	-1.70	0.00013253	0.00101726	0.00062454	2028	tags=32%, list=17%, signal=27%	MAP3K4/SRC/MAP2K5/STK4/ERBB2/CCNY/MAGED1/TGFB1/ERN2/ERN1/SYAP1/LRRK2/MAP2K2/DAZAP2/EPHA4/CSF1R/SASH1/PDGFA/INSR/MAP3K5/SLC27A1/TAOK3/PTK2B/RAPGEF2/CCND3/TRAF6/PAK1/IGF2/CSK/TAOK1/MAP2K7/STRADA/PTPRC/SYK/DVL3/THBS1/PTPN1/TLR6/CALM3/FZD5/LTF/PIK3CG/PDE5A/AKAP13/ATP2B4/RHOA/SNCA/IQGAP1
BP	GO:0050900	leukocyte migration	246	-0.42	-1.56	0.00013598	0.00104175	0.00063958	2413	tags=34%, list=21%, signal=27%	SPN/ALOX5/ADAM17/CORO1A/NCKAP1L/ARHGEF5/DUSP1/ADAM8/MYO1G/FCER1G/CD99L2/FYN/PADI2/TIRAP/SRC/CD177/NINJ1/EXT1/LYST/HCK/EPS8/SIRPA/ADGRE2/ANO6/PLCB1/STAT5B/CMKLR1/PIKFYVE/C5AR1/STK10/PYCARD/TREM1/SPI1/PREX1/ITGB2/CXCL5/CCR1/CSF1R/WDR1/PIP5K1C/CXCL16/PF4V1/GREM1/DOCK8/PTK2B/SELPLG/SELP/BCR/CXCR2/TBX21/IL6R/PECAM1/MMP9/VAV1/SYK/JAM3/THBS1/PTAFR/PLEC/IL17RA/PIK3CG/B4GALT1/CAMK1D/SELL/PF4/RAC1/LYN/LGALS9/RAC2/RHOA/ITGAL/CXCR1/MSN/RIN3/MAPK3/RIPOR2/PIK3CD/BSG/MPP1/WNK1/MYH9/PLVAP/RHOG

BP	GO:0019935	cyclic-nucleotide-mediated signaling	37	-0.67	-1.93	0.00013851	0.00105904	0.00065019	2520	tags=46%, list=21%, signal=36%	INSL3/UBE2B/MGRN1/ADNP/PDE2A/OPRL1/CAP1/GUCY1B1/NUDT4/PTK2B/RAPGEF2/THBS1/AQP1/GUCY1A1/RUNDC3A/CDC34/IRAG1
BP	GO:0016358	dendrite development	144	-0.48	-1.68	0.00014385	0.00109781	0.000674	2232	tags=28%, list=19%, signal=23%	EPHB2/BCL11A/STRN/DISC1/FYN/PTEN/MAP1S/YWHAH/KIDINS220/DBNL/DGKG/CUL7/LRRK2/DLG4/DBN1/EPHB1/CDKL5/SIPA1L1/SEMA4D/ABI3/EPHA4/VLDLR/NEURL1/RAPGEF2/EIF4G2/MAP1A/RERE/STK11/PPP3CA/PDLIM5/ASAP1/CAMK1D/DNM3/GSK3B/IQSEC1/RHOA/PPP1R9B/CFL1/PAK2/CUX1/CARM1
BP	GO:0048259	regulation of receptor-mediated endocytosis	69	-0.57	-1.80	0.0001456	0.00110898	0.00068085	1719	tags=39%, list=15%, signal=34%	SDCBP/ANKRD13A/NUMB/ARRB1/SYNJ1/DLG4/FLOT1/ATXN2/CD63/ARF1/INSR/GREM1/LDLRAP1/SYK/BMP2K/RAC1/HIP1/HNRNPK/DNM2/CLU/ARRB2/RIN3/TBC1D5/AP2M1/ITGB3/DAB2/CBL
BP	GO:0051592	response to calcium ion	81	-0.54	-1.75	0.00014638	0.0011128	0.0006832	2588	tags=38%, list=22%, signal=30%	NCSTN/FOS/CAPN3/SLC25A23/PPIF/TRPC3/GPLD1/CALM1/ENDOG/MCOLN1/CPNE3/ITPKB/CPNE5/HSPA5/ACTG1/CPNE1/PEF1/ALOX5AP/PTK2B/SEC31A/PPP3CA/THBS1/CALM3/JUND/TXNIP/ADD1/ANXA11/AHCYL1/STIM1/IQGAP1/DMTN
BP	GO:0042176	regulation of protein catabolic process	316	-0.39	-1.51	0.00014864	0.00112781	0.00069241	1818	tags=28%, list=16%, signal=24%	DET1/SNX3/AREL1/RNF144B/SDCBP/HGS/SH3RF3/SMARCC1/LRRK2/EZR/PSMD2/KEAP1/PSMD3/PIAS1/TRIB1/CEBPA/WAC/NRDC/AGTPBP1/CBFA2T3/MAD2L2/MYCBP2/ARIH2/SF3B3/ALAD/VPS35/ITCH/EPHA4/PML/TRIB3/GSK3A/PSMD1/PRKACA/STX5/CSNK2A1/SUFU/VPS11/ODC1/SORL1/BCAP31/TIMP2/PTK2B/PITHD1/MAP1A/RAB7A/RAD23A/FZR1/PSME3/FBXL20/ATG7/HIPK2/FLNA/VCP/HSPA1A/PSMC4/VPS28/GABARAP/LAPTM5/GSK3B/LRP1/WWP2/DDB1/TNFRSF1B/HUWE1/RILP/GPX1/CLU/MSN/GNA12/OAZ1/PINK1/MARCHF2/SNCA/PSMF1/N4BP1/UBB/DNAJB2/USP7/ARAF/RNF40/DAB2/TIMP1/ZER1/FURIN/BAG6/RYBP/RELA
BP	GO:0007162	negative regulation of cell adhesion	193	-0.44	-1.62	0.00015274	0.00115452	0.00070882	2466	tags=34%, list=21%, signal=27%	ATP5F1B/TBCD/SPN/SCRIB/NCKAP1L/DUSP1/EPHB2/SH2B3/MAP4K4/CR1/KNG1/LGALS9C/WNT1/PTEN/PRDX2/PPM1F/SRC/ABAT/MAP2K5/SDC4/ERBB2/TGFB1/TNFSF4/RC3H2/PAG1/NF2/ARHGDIB/PTPN6/SEMA4D/MAD2L2/SPI1/ITCH/EPHA4/RUNX3/TMEM131L/VSIR/CORO1C/IRF1/BCAS3/TBX21/BMP6/AKNA/PLXNC1/PTPRC/JAM3/THBS1/IL1RN/JAK3/ALOX12/SIPA1/LGALS9/LAPTM5/PDE5A/ARHGAP6/RHOA/MYADM/NF1/ACTN4/ARHGDIA/RIPOR2/ZNF703/MBP/WNK1/HLX/DMTN
BP	GO:0045785	positive regulation of cell adhesion	307	-0.40	-1.53	0.00015749	0.00118818	0.00072948	2202	tags=27%, list=19%, signal=23%	LIMS1/ADAM8/PLEKHA2/MAP4K4/EFNB1/CR1/DISC1/FYN/UNC13D/PPM1F/NLRP3/SRC/TRIOBP/ATM/STK4/PRKCE/SDC4/ERBB2/CASS4/SIRPA/ITPKB/TNFSF4/RRAS/STAT5B/NFAT5/RASAL3/DOCK5/STX4/PTPRJ/FLOT1/PTPN6/PYCARD/PREX1/EPHA4/ITGB2/ZFHX3/NEDD9/RUNX3/TPM1/VSIR/DOCK8/RARA/PTK2B/SPTA1/SELP/TRAF6/PRKD2/IGF2/ZMIZ1/CSK/ZBTB16/VAV1/SFRP2/GP6/PPP3CA/TFE3/ITGA5/PTPRC/SYK/PTAFR/FLNA/JAK3/GFUS/RAC1/LYN/LGALS9/GSK3B/SIRPB1/DNM2/FLOT2/RHOA/MYADM/JAK1/RSU1/APBB1IP/TGM2/FLCN/PTPN23/DAB2/FOXO3/HLX/DMTN/RELA
BP	GO:1903522	regulation of blood circulation	111	-0.50	-1.69	0.00015873	0.001193	0.00073244	1539	tags=23%, list=13%, signal=21%	DOCK5/GRK2/KCNH2/PER2/GSK3A/HRH2/PRKACA/ATP2A3/TPM1/TMEM38A/SLC8A1/SLC9A1/GAA/SCN1B/ATP1B2/EHD3/CALM3/PIK3CG/FLNA/PDE5A/MYL4/ATP2B4/RHOA/ECE1/P2RX1/TBXA2R

BP	GO:0008284	positive regulation of cell population proliferation	525	-0.36	-1.43	0.00016115	0.00120672	0.00074086	1617	tags=22%, list=14%, signal=20%	ATP7A/HCK/TGFB1/WAPL/TIAM1/TNFSF4/ERN1/NOTCH2/SDCBP/TMEM250/STAT5B/CACUL1/EGR1/KMT2D/RASAL3/KAT7/C5AR1/SHC1/MAPK14/STX4/S1PR2/PTPN6/SMARCD3/PYCARD/EPHA4/PDPK1/GPBAR1/PML/CDK13/CXCL5/CSF1R/AKIRIN2/BIRC6/FOXP1/ARNT/ZNF335/E2F3/PDGFA/INSR/CNOT6/CSNK2A1/MAP3K5/GREM1/HIPK1/RBPJ/TSPO/ODC1/NFATC2/RARA/PTK2B/GAB2/SPTA1/MEIS1/CXCR2/TRAF6/LDLRAP1/PRKD2/IL6R/PAK1/IGF2/ZMIZ1/IL13RA1/BMP6/MMP9/SFRP2/FZR1/PTPRC/SYK/THBS1/PTAFR/FOSL2/MED1/LTF/HIPK2/B4GALT1/JAK3/CAPN1/CAPNS1/GJC2/AQP1/LYN/LGALS9/MAP3K3/STAT5A/RAC2/ROGDI/SRPK2/TCF7L2/GNAI2/MPL/FOXJ2/CUL4A/GATA1/GID8/NF1/SP1/LRG1/GRN/HCLS1/PIM1/RNF187/MAPK3/CDKN1A/PBX1/BCL2L1/STAT3/CLDN5/ZNF703/AKT2/PIK3CD/MFN2/ITGB3/CARM1/GLUL/TIMP1/RHOG/HLX/RELA
BP	GO:0051701	biological process involved in interaction with host	146	-0.46	-1.63	0.00016116	0.00120672	0.00074086	2278	tags=33%, list=19%, signal=27%	SCARB1/TRIM5/TRIM38/VPS18/VPS4B/CR1/TPCN1/SRC/GYPA/SNX3/NCAM1/NECTIN1/EXOC7/PIKFYVE/GRK2/CHMP4B/IFITM3/ITCH/VPS37B/ANPEP/INSR/PGLYRP1/SELPLG/SLC3A2/RAB7A/GSN/THOC5/ITGA5/ATG7/IST1/SLC1A5/HSPA1A/CHMP1A/LGALS9/WWP2/DDB1/IFITM2/TRIM11/TRIM14/TRIM10/ITGB5/TRIM26/BCL2L1/LAMP1/BSG/CTSB/ITGB3/CBL
BP	GO:0061515	myeloid cell development	65	-0.58	-1.82	0.00016147	0.00120678	0.0007409	2356	tags=48%, list=20%, signal=38%	NCKAP1L/NRROS/SH2B3/TLR2/ZNF385A/SRF/SRC/JMJD6/ZBTB7A/NOTCH2/PTBP3/PTPN6/LRRK1/ATP6AP1/FOXP1/MEIS1/MED1/LTF/G6PD/BPGM/GATA1/PIP4K2A/TAL1/SLC4A1/FAM210B/EPB42/MPIG6B/ALAS2/TRIM58/WASF2/DMTN
BP	GO:0045165	cell fate commitment	97	-0.52	-1.74	0.00016301	0.001216	0.00074656	1263	tags=21%, list=11%, signal=19%	PML/ARHGEF2/SUFU/RBPJ/RARA/STAT6/SOX6/TBX21/IL6R/TGFB1I1/SFRP2/ETS2/TCF7L2/RHOA/GATA1/KDM6B/FKBP8/STAT3/TAL1/PRKDC
BP	GO:0071868	cellular response to monoamine stimulus	47	-0.64	-1.90	0.00016637	0.00123414	0.0007577	2102	tags=57%, list=18%, signal=47%	GNAS/GNAI3/GNAL/KCNQ1/NCSTN/SLC9A3R1/GNA15/SULT1A3/OR10H1/LRRK2/KLF16/VPS35/GSK3A/HRH2/PRKACA/SLC9A1/GNB1/FLNA/SIN3A/GSK3B/DNM2/ATP2B4/ARRB2/MAPK3/SNCA/TGM2/GNAZ
BP	GO:0071870	cellular response to catecholamine stimulus	47	-0.64	-1.90	0.00016637	0.00123414	0.0007577	2102	tags=57%, list=18%, signal=47%	GNAS/GNAI3/GNAL/KCNQ1/NCSTN/SLC9A3R1/GNA15/SULT1A3/OR10H1/LRRK2/KLF16/VPS35/GSK3A/HRH2/PRKACA/SLC9A1/GNB1/FLNA/SIN3A/GSK3B/DNM2/ATP2B4/ARRB2/MAPK3/SNCA/TGM2/GNAZ
BP	GO:0008286	insulin receptor signaling pathway	86	-0.54	-1.76	0.00016709	0.00123712	0.00075953	2107	tags=34%, list=18%, signal=28%	FOXO1/SRC/IRS2/TSC2/SMARCC1/NDEL1/GAB1/SH2B2/PIP4K2C/SHC1/GSK3A/GRB2/PDK2/INSR/SESN3/SORL1/INPP5K/CCND3/IGF2/ZNF106/FOXO4/PTPN1/ADIPOR1/GSK3B/GNAI2/PRKCB/PIP4K2A/AKT2/EIF4EBP2

BP	GO:0009628	response to abiotic stimulus	707	-0.34	-1.38	0.00016773	0.00123961	0.00076106	2267	tags=26%, list=19%, signal=22%	MAP2K4/AIFM1/IFI16/HMOX2/LETM1/USP19/AK4/PKN1/ADAM8/CDKN2D/MICB/TLR2/ATP2B1/CHI3L1/FOXO1/SOD2/RAB11FIP5/ATXN3/THRA/TLR5/ALKBH5/DISC1/SRF/FYN/PTEN/ZNF516/MAP3K4/SRC/DNAJB1/PER1/APAF1/ELK1/MTHFR/ATM/ABAT/SLC24A4/MCOLN1/PNKP/COP1/PRKCE/GRIK2/THBD/XRCC6/EXT1/NIPBL/RFWD3/ATP7A/AGTRAP/CPEB4/PDE2A/BCL3/PLOD1/SLC12A6/NPEPPS/MGMT/SCAP/HSPA5/ACTG1/ROCK2/METRNL/NFAT5/FANCG/XPC/EYA3/UVSSA/EGR1/MME/EPHB1/MAPK14/BABAM2/YY1/ZFP36L1/MAP3K2/PER2/CBFA2T3/HVCN1/SMC1A/TMBIM6/AQP9/ALAD/RELB/CLN3/EPHA4/PDPK1/GNAQ/PML/ACADVL/MICU1/HIF1AN/ARHGEF2/ATP6AP1/KAT5/ITPR2/CHP1/GRB2/TXNRD2/PPP1CA/ARNT/PRKACA/PCSK1N/PDGFA/UBE4B/GUCY1B1/SLC27A1/NFKB1/RBPJ/ERO1A/TSPO/IRF1/HTT/LRRC8A/PTK2B/SLC2A1/SLC8A1/BMP6/MMP9/SFRP2/STK11/GSN/MAP2K7/PTPRC/SLC9A1/THBS1/PTAFR/PDK3/LMNA/ATG7/PLEC/CASP2/HIPK2/TSC22D3/JUND/DYNLRB1/VCP/HSPA1A/GPI/SIPA1/UCP2/CD14/FECH/AQP1/LYN/GSK3B/TMEM120A/DNM2/CREBBP/HYOU1/RAB11B/HSPB1/BNIP3L/DDB1/TXNIP/CNN2/ATP2B4/MPL/BABAM1/RHOA/GPX1/PDLIM1/ARRB2/NF1/ATP6V1A/ACTN4/PINK1/MYLK/MAPK3/CDKN1A/GUCY1A1/BCL2L1/MTPN/MAPKAPK2/N4BP1/AKT2/AQP10/ATP6V0D1/ENO1/PRKDC/DNAJA4/YBX3/ALAS2/TIMP1/FOXO3/CBL/GMPR/RELA
CC	GO:0030427	site of polarized growth	121	-0.50	-1.74	0.00017119	0.00126278	0.00077528	2325	tags=41%, list=20%, signal=33%	TWF2/FSCN1/AMFR/PI4K2A/MYO5A/MYO9A/GDPD5/ENO2/NIN/KATNB1/SNAP25/DISC1/FEZ1/EXOC4/DBNL/EPS8/CPEB4/NECTIN1/EXOC7/SYAP1/LRRK2/NDEL1/DBN1/CDKL5/SETX/FRY/EXOC3/CTNND1/EPHA4/ARPC5/ADGRL1/PALLD/NDRG2/FXR2/PTK2B/COPA/USP9X/CALM3/BASP1/FLNA/FRYL/DNM2/TSHZ3/PPP1R9B/HCLS1/PINK1/SNCA/CTTN/CBL/IQGAP1
BP	GO:0033002	muscle cell proliferation	111	-0.50	-1.68	0.00017151	0.00126282	0.0007753	2098	tags=30%, list=18%, signal=25%	PTGIR/SOD2/PTEN/XRCC6/TGFB1/ERN1/EPHB1/MAPK14/TRIB1/S1PR2/KPNA1/FOXP1/MAP3K5/TPM1/NDRG2/RBPJ/ARID2/LDLRAP1/IL6R/MMP9/THBS1/PTAFR/SF1/TCF7L2/GNAI2/RHOA/FOXJ2/PIM1/GNA12/CDKN1A/MFN2/PRKDC/JARID2
BP	GO:0033674	positive regulation of kinase activity	319	-0.39	-1.52	0.00017299	0.00127134	0.00078054	2051	tags=26%, list=17%, signal=22%	FGR/MAP3K4/TIRAP/SRC/EPHB4/GCN1/ADNP/MAP2K5/STK4/SDC4/XRCC6/ERBB2/CCNY/CASS4/MAGED1/TGFB1/DGKZ/ERN2/LILRA5/ERN1/SYAP1/LRRK2/CACUL1/EGR1/DLG4/EPHB1/MAP2K2/DAZAP2/EPHA4/PDPK1/NCF1/CSF1R/SASH1/PDGFA/NEDD9/INSR/MAP3K5/SLC27A1/TAOK3/GREM1/VLDLR/PTK2B/NEURL1/RAPGEF2/CCND3/TRAF6/IL6R/PAK1/MOB3A/IGF2/CSK/TAOK1/STK11/MAP2K7/STRADA/PTPRC/SYK/DVL3/THBS1/PTPN1/TLR6/CALM3/FZD5/LTF/PIK3CG/MADD/MARK2/LYN/MMD/PDE5A/AKAP13/ATP2B4/RHOA/CLU/PINK1/SNCA/CDKN1A/TAL1/PAK2/ITGB3/WNK1/IQGAP1/MAP2K3/STRADB
BP	GO:0034332	adherens junction organization	29	-0.71	-1.92	0.00017431	0.00127872	0.00078507	1293	tags=52%, list=11%, signal=46%	SRC/NUMB/EPHA4/PIP5K1C/HIPK1/VCL/CSK/BMP6/JAM3/PLEC/ACTB/ADD1/PTPN23/ZNF703/PAK2
BP	GO:0018107	peptidyl-threonine phosphorylation	85	-0.53	-1.73	0.00017582	0.00128742	0.00079041	2220	tags=35%, list=19%, signal=29%	DYRK3/PKN1/CHI3L1/CDC42BPA/CDC42BPB/TGFB1/ROCK2/LRRK2/DYRK1B/S1PR2/GRK2/PARD3/PDPK1/GSK3A/PRKACA/CSNK2A1/HIPK1/INPP5K/PRKD2/STK11/CALM3/HIPK2/EIF4G1/ULK1/MARK2/GSK3B/PRKCB/PRKDC/WNK1/DMTN

BP	GO:0001558	regulation of cell growth	266	-0.40	-1.51	0.00018097	0.00131786	0.0008091	2932	tags=37%, list=25%, signal=29%	PPP2R1A/VGLL4/HSPA1B/TP53/PSRC1/CHPT1/ACVR1B/SMAD3/EI24/FHL1/PLCE1/OGFR/ARHGAP4/CCN3/PLXNA4/EPB41L3/TWF2/KIAA1109/DDX49/KIAA0319/BAP1/CD38/ADAM17/DIP2B/MUL1/BCL11A/KLHL22/CDKN2D/ARHGEF11/DISC1/MACF1/SRF/HDGFL2/ADNP/DBNL/MAP2K5/H3-3B/ERBB2/H3-3A/TGFB1/CPNE5/SDCBP/CTDP1/TCHP/BTG1/SMARCA4/NDEL1/TMC8/DBN1/KAT7/CDKL5/YY1/PTPRJ/MSX1/CLSTN1/SEMA4D/MAD2L2/NAIF1/ITCH/PML/SMARCA2/GSK3A/DPYSL2/CXCL16/SESN2/CSNK2A1/FBP1/GREM1/PTK2B/SEMA4A/EIF4G2/NDRG3/LAMTOR1/SFRP2/EXTL3/STK11/EXOSC4/SLC9A1/ADIPOR1/IST1/G6PD/EIF4G1/ULK1/HSPA1A/SIPA1/SIN3A/GSK3B/DNM2/CDA/RHOA/PPP1R9B/RPS6KA1/CDKN1A/FLCN/MTPN/CTTN/DNAJB2/ENO1/DAB2
BP	GO:0009790	embryo development	604	-0.34	-1.39	0.00018484	0.00134354	0.00082486	2089	tags=23%, list=18%, signal=20%	BCL2L11/MYO1E/HEG1/PAF1/SRF/WNT1/TAF8/TRIOBP/APAF1/TOP1/EXOC4/MTHFR/ATM/KIDINS220/MAP2K5/STK4/ATOH8/SDC4/EXT1/NIPBL/ATP7A/MKS1/TGFB1/TTLL4/PHACTR4/WDR74/NOTCH2/TSC2/ROCK2/XYLT1/B4GALT5/PLCB1/NDEL1/TFEB/ASF1B/DBN1/KEAP1/CEBPA/PEF1/ELL/NF2/ZFP36L1/SLC35E2B/MSX1/FUZ/CDKN1C/PTPN18/ITGB2/AKIRIN2/BIRC6/TRIM28/GRB2/KMT2A/ARNT/PRKACA/ZNF335/PDGFA/INSR/AMOT/SUFU/GREM1/HIPK1/RBPJ/YBX1/PCSK6/RARA/BCR/MEIS1/INPP5K/ARID2/TRAF6/CHST11/IGF2/ZMIZ1/RAB14/TBL1XR1/ZBTB16/MMP9/TGFB1I1/SFRP2/THOC5/ITGA5/APBA3/DVL3/NPAT/SUPT6H/BPTF/MED1/MAFB/IL1RN/FZD5/TET3/HIPK2/TRIP12/BASP1/CHD8/SKI/NIBAN2/NDST1/PSMC4/GPI/SIN3A/ETS2/CREBBP/EPN1/CUL4A/CMIP/GATA1/MYADM/ITPK1/NF1/KDM6B/FKBP8/TERF2/WDTC1/USP22/GNA12/ECE1/CFL1/MAPK3/ITGB5/PBX1/BCL2L1/FLCN/TAL1/NCOA1/MFN2/PRKDC/TENT5C/ITGB3/YBX3/VASP/MBP/CNOT1/WNK1/MYH9/MICAL2/HLX/FURIN/BAG6
BP	GO:0006811	ion transport	774	-0.33	-1.36	0.00018534	0.00134474	0.0008256	1946	tags=20%, list=17%, signal=18%	ABAT/SLC24A4/ATP6V1B2/MCOLN1/PRKCE/SLC25A41/GRIK2/KCNJ15/SLC39A7/VDR/COX15/ATP7A/SLC66A1/SLC2A3/ATP13A1/LILRA5/ANO6/SLC12A6/CPTP/NECTIN1/GET3/ABCB11/OPRL1/TMC8/RIPK1/DLG4/MFSD4B/TTYH3/CLCN5/CACNB1/CA2/KCNH2/FXYD6/PER2/PTPN6/SLC16A3/CHRNE/SLC36A1/HVCN1/TMBIM6/CD63/AQP9/SLC25A11/CLN3/PTGES/SHISA7/PDPK1/PML/RALBP1/CLCC1/KCNQ5/PANX2/MICU1/CCR1/TSPO2/ITPR2/ABCG2/CHP1/MFSD5/PRKACA/CA4/CHERP/ATP2A3/SLC27A1/CNIH3/ERO1A/TSPO/SLC22A23/TMEM38A/HTT/LRRC8A/PTK2B/SLC2A1/SLC8A1/ANKH/SLC6A20/INPP5K/SLC24A3/SLC3A2/CLIC3/MMP9/SLC44A2/SLC25A38/LCN2/PPP3CA/PTPRC/SYK/SLC9A1/PTAFR/UQCRC1/SELENON/SCN1B/ATP1B2/EHD3/CLTC/CALM3/SLC6A9/LTF/PIK3CG/G6PD/FLNA/SLC1A5/JAK3/ABCC4/ANXA6/CAPN1/UCP2/SLC43A2/GJC2/AQP1/LYN/TMEM120A/DNM2/PSAP/SLC43A1/GP9/RAB11B/WWP2/ABCC3/ATP2B4/GNAI2/LASP1/FKBP1A/TMEM63B/RHOA/FTL/PRKCB/ARRB2/NF1/ATP6V1A/SLC38A10/ACTN4/FTH1/PINK1/MLLT6/TESC/SEC14L1/MYLK/SNCA/EPB41/SLC15A3/AHCYL1/SLC4A1/GP1BA/ATP6V0D1/MMGT1/SLC48A1/SLC25A37/STIM1/ITGB3/ARHGAP1/WNK1/SLC7A5/KEL/P2RX1/SLC38A5/SLC6A8/DIAPH1

BP	GO:0007420	brain development	439	-0.36	-1.43	0.00018671	0.00135057	0.00082918	2972	tags=36%, list=25%, signal=28%	TSPAN2/POMK/EPOR/PLXNB2/PAFAH1B3/TP53/MECP2/NUMBL/GNAI3/NOTCH1/NOTCH2NLC/CENPF/TACC2/CCDC141/CASP5/STK36/PDSS2/ATIC/ARL13B/CDK6/SMAD1/CNP/CSNK2A2/OGDH/PLXNA4/NCSTN/AMIGO1/KDM7A/PBX2/RGS7/BAK1/SETD2/CREB1/SCRIB/ARCN1/CALM1/ASPM/WLS/OTX1/AATK/BCL11B/TBX19/EPHB2/FANCC/NIN/ATP2B1/BCL2L11/DISC1/SRF/FYN/WNT1/PADI2/PTEN/PHF8/FEZ1/SRC/DNAJB1/APAF1/MAP1S/YWHAH/IRS2/ATM/ABAT/SNRPB/EXT1/NIPBL/ATP7A/SYNJ2/SZT2/ZNF148/SYNE2/HSPA5/NUMB/ITGAM/LRRK2/PLCB1/NDEL1/FOXP2/SYNJ1/NOTCH2NLA/VPS51/EPHB1/NF2/AGTPBP1/MSX1/HOOK3/CKB/CTNND1/FPGS/ARPC5/CSF1R/ZFHX3/UBE2L3/DPYSL2/ZNF335/MBOAT7/NDRG2/TACC1/RBPJ/PFKFB3/RARA/SLC2A1/NEURL1/SLC8A1/PAFAH1B2/BCR/MEIS1/RAPGEF2/HERC1/SOX6/ZMIZ1/CSK/RERE/NDE1/SFRP2/AKNA/PPP3CA/ATP1B2/BPTF/MED1/MAFB/CASP2/CALM3/G6PD/TACC3/BASP1/CHD8/SKI/NDST1/ODAD4/ACTB/GJC2/AQP1/SIN3A/GSK3B/ROGDI/ATP2B4/RHOA/ATXN1/ARRB2/NF1/NCOA6/TRAPPC9/PPP1R9B/NRGN/PBX1/MTPN/UBB/NAPA/ATP6V0D1/PRKDC/SHARPIN/MBP/FOXO3/SEC16A/BAG6
BP	GO:0050808	synapse organization	201	-0.43	-1.60	0.00018716	0.00135057	0.00082918	3022	tags=39%, list=26%, signal=29%	ANK3/DTNBP1/PFN1/PLXNB2/PLXND1/TNF/MECP2/TANC2/SYNGAP1/ZDHHC2/SPOCK2/ADD2/PPFIBP2/CACNB4/STAU2/HDAC6/AMIGO1/ACTR2/PPFIA1/C1QA/EPHB2/TLR2/DISC1/FYN/PTEN/LRRC4/ADNP/DBNL/ERBB2/LRFN3/SDCBP/CTBP2/NECTIN1/ACTG1/ITGAM/LRRK2/NFIA/DLG4/DBN1/C5AR1/WASF3/EPHB1/MAPK14/CACNB1/CDKL5/CAST/SIPA1L1/CLSTN1/SEMA4D/CLN3/ABI3/VPS35/EPHA4/SHISA7/ARF1/YWHAZ/INSR/ADGRL1/NEURL1/SEMA4A/SETD5/PDLIM5/DCTN1/FZD5/FLNA/EIF4G1/DNM3/VCP/ACTB/GRIPAP1/RHOA/TUBB/STAU1/CFL1/SNCA/CTTN/ACTN1/WASF2
CC	GO:0098562	cytoplasmic side of membrane	142	-0.46	-1.64	0.00018713	0.00135057	0.00082918	2183	tags=37%, list=19%, signal=30%	TNK2/ATP2B1/GNA15/SNAP25/FGR/FYN/PTEN/SRC/SPPL3/HCK/TIAM1/SYAP1/LRRK2/SAMD12/EZR/RASAL3/DLG4/TGM3/CHMP4B/OSBPL2/MAP2K2/GNAQ/PKP4/CDIP1/FES/MYZAP/PTK2B/SPTA1/TRAF6/LDLRAP1/PPP3CA/PTPRC/SYK/PTPN1/GNB1/G6PD/RAC1/SLA/LYN/HIP1/GNAI2/FKBP1A/RHOA/PPP1R9B/GNA12/SPTB/SLC4A1/LITAF/GNAZ/MYH9/IQGAP1/ANK1
BP	GO:0008361	regulation of cell size	113	-0.51	-1.72	0.00018926	0.00136319	0.00083693	2671	tags=39%, list=23%, signal=30%	SLC12A7/ARHGAP4/PLXNA4/TWF2/CREB1/KIAA0319/DIP2B/SLC9A3R1/DISC1/MACF1/SRF/PTEN/AKT1S1/ADNP/DBNL/LARS1/ATP7A/ANO6/SLC12A6/NDEL1/DBN1/CDKL5/SEMA4D/CLN3/DPYSL2/LRRC8A/SEMA4A/EIF4G2/VAV1/IST1/ULK1/PLEK/RAC1/AQP1/SIN3A/GSK3B/DNM2/RHOA/MSN/WDTC1/MTPN/CTTN/KEL/XK
MF	GO:0030276	clathrin binding	45	-0.63	-1.88	0.00019324	0.00138684	0.00085144	1861	tags=42%, list=16%, signal=36%	AP1G1/AP2A2/AFTPH/LRRK2/AP2B1/AP2S1/AP1M1/AP2A1/LDLRAP1/TOM1/CLTC/HIP1/LRP1/EPN1/AP1B1/AP2M1/GAK/STON2/DAB2

BP	GO:0051248	negative regulation of protein metabolic process	749	-0.33	-1.35	0.00019908	0.00142618	0.0008756	1812	tags=23%, list=15%, signal=21%	TGFB1/SIRPA/LDLRAD4/SNX3/PUM1/CPEB4/RC3H2/USP4/SDCBP/TSC2/UBR5/LSM14A/ROCK2/GSTP1/SMARCC1/ARRB1/LRRK2/HGF/PIP5KL1/GPS2/ENC1/STK38/PI3/MGAT5/TRIB1/CTBP1/CEBPA/CTDSP2/WAC/NF2/CAST/EIF2AK2/UBE2Z/PTPRJ/ZFP36L1/PER2/PTPN6/PYCARD/PARD3/FRY/SEMA4D/MAD2L2/MYCBP2/NR1H2/DDX6/SF3B3/CDKN1C/ALAD/CLN3/DAPK3/AGO4/VPS35/SPI1/LRRK1/EPHA4/PDPK1/DHCR24/GNAQ/PML/RTN3/TRIB3/BIRC6/CHP1/AGO2/EIF6/PCSK1N/YTHDF3/TENT4B/SESN2/CNOT6/CSNK2A1/OTUB1/YWHAB/NFKB1/SUFU/FXR2/GREM1/TSPO/UPF1/SH3BP5L/SORL1/CORO1C/PRDX5/YBX1/TIMP2/RARA/MFHAS1/SLC8A1/WARS1/INPP5K/MAP1A/MMP9/POR/SFRP2/APLP2/PTPRC/ADAR/THBS1/PTPN1/AGO1/SUPT6H/PLEC/GLG1/CTDSPL/CALM3/HEXIM2/LTF/HIPK2/TRIP12/G6PD/SKI/FLNA/EIF4G1/JAK3/HSPA1A/VPS28/GNL3L/GPI/AQP1/LYN/SIN3A/GSK3B/LRP1/FLOT2/HSPB1/ATP2B4/GNAI2/CTDSP1/FKBP1A/RILP/MYADM/RTN2/GPX1/CLU/ARRB2/NF1/FKBP8/PPP1R9B/LARP1/UBE2O/MLLT6/RPS6KA1/IGF2BP2/SNCA/SIAH2/CDKN1A/DTX3L/FLCN/KHSRP/NIBAN1/PSMF1/PRKAR2A/PRKAR2B/N4BP1/SQSTM1/USP7/SAMD4B/CTSB/PRKDC/EIF4EBP2/PAK2/ITGB3/CNOT1/JARID2/FBXO7/TIMP1/CBL/EIF2AK1/FURIN/DMTN/BAG6/RYBP/RELA
BP	GO:0008277	regulation of G protein-coupled receptor signaling pathway	87	-0.52	-1.72	0.0002058	0.00147167	0.00090353	2630	tags=36%, list=22%, signal=28%	PTGDR2/RGS3/RGS7/MGRN1/RGS16/ZDHHC3/ACP3/FRMPD1/GRK3/SLC24A4/ZDHHC7/PDE4A/ROCK2/ARRB1/OPRL1/PLCB1/GIT2/GRK2/GSK3A/RGS6/GRK6/YWHAB/RASGRP4/MGLL/PLEK/DNM2/ATP2B4/GNAI2/ARRB2/PPP1R9B/SNCA
BP	GO:1901879	regulation of protein depolymerization	63	-0.58	-1.82	0.00020661	0.00147485	0.00090548	2660	tags=51%, list=23%, signal=39%	TMOD3/CARMIL1/ADD2/CLASP1/HDAC6/TWF2/SCIN/FLII/KATNB1/TRIOBP/MAP1S/ATXN7/EPS8/SPTAN1/CAMSAP1/WASHC2C/ARHGEF2/WDR1/SPTBN1/SPTA1/SVIL/MAP1A/TAOK1/GSN/PLEK/CAPZB/ADD1/TMOD1/SPTB/MTPN/SH3BP1/DMTN
BP	GO:0010976	positive regulation of neuron projection development	99	-0.52	-1.76	0.00021225	0.00151235	0.0009285	2573	tags=41%, list=22%, signal=33%	STAU2/AMIGO1/TWF2/FIG4/ACTR2/CFLAR/NFE2L2/EPHB2/PTK7/KATNB1/DISC1/FYN/FEZ1/FKBP1B/KIDINS220/ADNP/DBNL/SNX3/HSPA5/RETREG3/HGF/ENC1/NDEL1/DBN1/SETX/STK24/SF3A2/FES/VLDLR/PTK2B/RAPGEF2/SCN1B/ATP1B2/CAMK1D/PPP2R5B/FLNA/MARK2/LYN/GRN/RAPGEF1/EHD1

BP	GO:0044257	cellular protein catabolic process	694	-0.33	-1.37	0.00021607	0.00153681	0.00094352	1798	tags=22%, list=15%, signal=20%	AREL1/WDR81/RNF144B/GID4/RC3H2/CUL7/LAMP2/USP4/SDCBP/UBXN7/RNF213/HSPA5/SH3RF3/UBE2J2/SMARCC1/ARRB1/LRRK2/ENC1/CACUL1/EZR/MAN1A1/RIPK1/PSMD2/KEAP1/MARCHF6/PSMD3/RNF11/TMEM67/PIAS1/TRIB1/CEBPA/WAC/CHMP4B/YOD1/UBE2Z/AGTPBP1/CBFA2T3/ARIH2/ALAD/CLN3/SPOP/VPS35/ITCH/EPHA4/KCTD2/RNF115/HERC2/CCNF/PML/PSME4/KAT5/TRIB3/GSK3A/UBE2L3/ZRANB1/CANX/PSMD1/VPS37B/PRKACA/VPS37C/FBXL18/CNOT4/UBE4B/USP10/CSNK2A1/SUFU/VPS11/TPP1/BCAP31/RNF126/PTK2B/USP3/RCN3/PITHD1/TBX21/PSMD11/NSFL1C/MAP1A/RAB7A/TBL1XR1/RAD23A/TGFB1I1/UBR4/USP9X/FZR1/PSME3/NPLOC4/FBXL20/UBAP1/HIPK2/USP34/TRIP12/VCP/HSPA1A/ANKRD9/UBR2/PSMC4/VPS28/CHMP1A/GABARAP/KLHL8/RBCK1/LAPTM5/GSK3B/UBA1/UBE2R2/FBXO9/LRP1/WWP2/BNIP3L/DDB1/HUWE1/CUL4A/RMND5A/TBL1X/GID8/GPX1/UBL7/CLU/ARRB2/MSN/USP22/GNA12/RNF187/PINK1/ASCC2/MARCHF2/SIAH2/DTX3L/PTPN23/PCBP2/PSMF1/N4BP1/UBB/SQSTM1/DNAJB2/USP7/ARAF/CTSB/RNF40/UBXN6/SHARPIN/DCAF12/DAB2/TRIM58/FBXO7/RNF123/TOLLIP/UBE2H/CBL/CDC34/ZER1/FURIN/BAG6/RYBP/DCAF11
BP	GO:0007266	Rho protein signal transduction	94	-0.52	-1.73	0.00022524	0.0015992	0.00098182	2110	tags=32%, list=18%, signal=26%	ARHGEF11/ARHGEF3/HEG1/ARHGEF12/EPS8/PHACTR4/ROCK2/ARRB1/ARHGDIB/FLOT1/CDC42EP4/CTNNAL1/RALBP1/ARHGEF2/KANK2/BCR/MYO9B/RIPOR1/RAC1/AKAP13/ARHGAP6/RHOA/ARHGDIA/GNA12/CFL1/WAS/FLCN/RIPOR2/ARHGAP1/RHOG
BP	GO:0062012	regulation of small molecule metabolic process	212	-0.42	-1.55	0.00022609	0.00160241	0.00098379	1711	tags=25%, list=15%, signal=22%	MIDN/SCAP/ABCB11/EGR1/ZBTB20/CBFA2T3/NR1H2/CLN3/ACADVL/PGAM1/TRIB3/LEPR/GSK3A/PPP1CA/DGAT2/PDK2/ENTPD5/ARNT/EIF6/BCKDK/INSR/SESN2/FBP1/NFKB1/NCOA2/PTK2B/INPP5K/LDLRAP1/LPCAT3/IGF2/BMP6/POR/STK11/CPT1A/PTAFR/PDK3/NCOR2/ADIPOR1/VCP/PLEK/GSK3B/DDB1/CDA/ATP2B4/WDTC1/PINK1/SNCA/FLCN/STAT3/AKT2/SLC4A1/USP7/ENO1/DAB2
BP	GO:0001659	temperature homeostasis	118	-0.49	-1.68	0.0002312	0.00163566	0.00100421	2379	tags=35%, list=20%, signal=28%	ADAM17/SIRT6/LNPEP/HDAC3/FOXO1/THRA/PCTP/ZNF516/FTO/ABAT/PPARGC1B/EGR1/CMKLR1/NRDC/PER2/NR1H2/PTGES/ACADVL/ALMS1/LEPR/ADIPOR2/ALPL/SLC27A1/RBPJ/STAT6/BSCL2/DYNC1H1/STK11/LCN2/TFE3/SYK/ADIPOR1/UCP2/GPX1/KDM6B/FLCN/STAT3/IP6K1/TLE3/SQSTM1/MFN2
BP	GO:0009612	response to mechanical stimulus	120	-0.49	-1.67	0.00023338	0.00164816	0.00101188	3275	tags=47%, list=28%, signal=34%	MYD88/TTN/DMD/MAP3K1/NFKBIA/TNFRSF1A/TLR8/TLR4/ENG/CRADD/FADD/TLR7/TNF/CASP5/TNFSF14/KCNQ1/KIT/F11R/KCNJ2/P2RY1/FOS/BAK1/KIAA0319/ENDOG/MAP2K4/CHI3L1/TLR5/FYN/SRC/PDE2A/ACTG1/XPC/MAPK14/MAP3K2/NFKB1/IRF1/HTT/PTK2B/SLC2A1/SLC8A1/BMP6/GSN/SLC9A1/THBS1/PLEC/CASP2/JUND/GPI/AQP1/TMEM120A/TXNIP/CNN2/RHOA/MAPK3/MTPN/RELA
BP	GO:0090263	positive regulation of canonical Wnt signaling pathway	68	-0.56	-1.79	0.00023879	0.00168037	0.00103166	2415	tags=40%, list=21%, signal=32%	UBE2B/ASPM/WLS/PTK7/SRC/XIAP/TGFB1/UBR5/LRRK2/DAPK3/VPS35/CSNK1G2/LRRK1/NFKB1/RBPJ/FRAT1/TBL1XR1/SFRP2/USP34/VCP/TBL1X/CSNK1G1/GID8/PPM1A/WNK1/FAM53B/CTDNEP1
MF	GO:0005200	structural constituent of cytoskeleton	60	-0.60	-1.86	0.00024158	0.00169706	0.0010419	1682	tags=37%, list=14%, signal=32%	ACTG1/SPTAN1/TUBB2A/ARPC1B/ARPC5/TPM1/SPTBN1/SPTA1/TUBA4A/PLEC/ACTB/TUBB4B/HIP1/TUBB/MSN/ARPC4/TUBB1/EPB41/SPTB/EPB42/ANK1/TLN1

BP	GO:0032271	regulation of protein polymerization	152	-0.46	-1.65	0.00024332	0.00170624	0.00104754	3081	tags=48%, list=26%, signal=36%	FCHSD1/DYRK1A/CLIP1/PFN1/NPHS1/HSPA1B/SSH3/MECP2/PSRC1/ARHGAP18/TMOD3/CARMIL1/ADD2/CLASP1/AMBRA1/TWF2/CDC42EP1/TBCD/TPPP3/CORO1A/NCKAP1L/SCIN/PRUNE1/NIN/FLII/TRIOBP/DBNL/PRKCE/HCK/EPS8/CYRIA/CKAP5/SPTAN1/ARPC1B/DBN1/CAMSAP1/EML2/WASHC2C/PYCARD/CDC42EP4/SLAIN2/ARPC5/NUMA1/SSH1/GRB2/SPTBN1/FES/PTK2B/SPTA1/SVIL/PAK1/GSN/ESAM/DCTN1/HSPA1A/RAC1/CYFIP2/COTL1/RHOA/CAPZB/MYADM/ADD1/SSH2/HCLS1/TMOD1/ARPC4/PINK1/SNCA/SPTB/MTPN/CTTN/VASP/DMTN
BP	GO:0090100	positive regulation of transmembrane receptor protein serine/threonine kinase signaling pathway	64	-0.58	-1.82	0.0002465	0.00172548	0.00105935	1845	tags=47%, list=16%, signal=40%	ENG/PELO/NOTCH1/ACVR1B/FBXL15/NEO1/ZEB2/KIAA0319/ADAM17/RNF165/TGFB1/NOTCH2/SDCBP/ZC3H3/MSX1/CDKN1C/NUMA1/RBPJ/BMP6/TGFB1I1/STK11/THBS1/NUP93/HIPK2/CREBBP/FKBP8/LRG1/UBE2O/FLCN/DAB2
BP	GO:0010634	positive regulation of epithelial cell migration	100	-0.52	-1.74	0.00025193	0.00176043	0.00108081	2656	tags=44%, list=23%, signal=34%	RIN2/CALR/CLASP1/HDAC6/INSL3/ATP5F1B/MAPRE2/GPLD1/ADAM17/NFE2L2/SCARB1/HDAC7/MAP4K4/GLIPR2/PPM1F/SRC/PRKCE/ATOH8/TGFB1/RRAS/ROCK2/DOCK5/PDPK1/FOXP1/SASH1/PTK2B/BCAS3/PRKD2/MMP9/THBS1/PIK3CG/GFUS/GPI/RAC1/MAP3K3/STAT5A/HSPB1/IQSEC1/SP1/GRN/SPARC/PIK3CD/ITGB3/MAP2K3
BP	GO:0089718	amino acid import across plasma membrane	25	-0.74	-1.93	0.00026272	0.00182938	0.00112314	1423	tags=40%, list=12%, signal=35%	PER2/SLC36A1/TSPO2/SLC6A20/SLC3A2/SLC6A9/SLC1A5/SLC43A2/SLC43A1/SLC7A5
BP	GO:0050770	regulation of axonogenesis	90	-0.53	-1.75	0.00026485	0.001841	0.00113028	2729	tags=41%, list=23%, signal=32%	SYNGAP1/ARHGAP4/PLXNA4/AMIGO1/TWF2/KIAA0319/DIP2B/KIF13B/EPHB2/STK25/NIN/DISC1/MACF1/SRF/PTEN/ADNP/DBNL/TIAM1/NDEL1/DBN1/CDKL5/SIPA1L1/SEMA4D/EPHA4/SEMA4A/EIF4G2/PAK1/STK11/PLXNC1/IST1/ULK1/MARK2/GSK3B/PAK2/MBP/KEL/XK
BP	GO:0032269	negative regulation of cellular protein metabolic process	699	-0.33	-1.35	0.00027102	0.00188061	0.0011546	2083	tags=26%, list=18%, signal=23%	CR1/KNG1/HEG1/RGP1/CSDE1/FYN/WNT1/PTEN/FTO/PPM1F/SRC/CRTAP/FKBP1B/AKT1S1/MLLT1/PNKP/MAP2K5/PPP1R15A/PRKCE/XIAP/TGFB1/SIRPA/LDLRAD4/PUM1/CPEB4/RC3H2/USP4/SDCBP/TSC2/UBR5/LSM14A/ROCK2/GSTP1/SMARCC1/ARRB1/LRRK2/HGF/PIP5KL1/GPS2/ENC1/STK38/PI3/MGAT5/TRIB1/CTBP1/CEBPA/CTDSP2/WAC/NF2/CAST/EIF2AK2/UBE2Z/PTPRJ/ZFP36L1/PER2/PTPN6/PYCARD/PARD3/FRY/SEMA4D/MAD2L2/NR1H2/DDX6/CDKN1C/ALAD/CLN3/DAPK3/AGO4/VPS35/SPI1/LRRK1/EPHA4/PDPK1/DHCR24/GNAQ/PML/TRIB3/BIRC6/CHP1/AGO2/EIF6/PCSK1N/YTHDF3/TENT4B/SESN2/CNOT6/CSNK2A1/OTUB1/YWHAB/NFKB1/SUFU/FXR2/GREM1/TSPO/UPF1/SH3BP5L/SORL1/CORO1C/PRDX5/YBX1/TIMP2/RARA/MFHAS1/SLC8A1/WARS1/INPP5K/MAP1A/MMP9/POR/SFRP2/APLP2/PTPRC/ADAR/THBS1/PTPN1/AGO1/SUPT6H/PLEC/GLG1/CTDSPL/CALM3/HEXIM2/LTF/HIPK2/TRIP12/G6PD/SKI/EIF4G1/HSPA1A/VPS28/GNL3L/GPI/AQP1/LYN/SIN3A/GSK3B/LRP1/HSPB1/ATP2B4/GNAI2/CTDSP1/FKBP1A/MYADM/GPX1/ARRB2/NF1/FKBP8/PPP1R9B/LARP1/UBE2O/MLLT6/RPS6KA1/IGF2BP2/SNCA/SIAH2/CDKN1A/DTX3L/FLCN/KHSRP/NIBAN1/PSMF1/PRKAR2A/PRKAR2B/N4BP1/SQSTM1/USP7/SAMD4B/CTSB/PRKDC/EIF4EBP2/PAK2/CNOT1/JARID2/FBXO7/TIMP1/CBL/EIF2AK1/FURIN/DMTN/BAG6/RYBP/RELA

BP	GO:0040013	negative regulation of locomotion	201	-0.43	-1.58	0.00028058	0.00194012	0.00119113	1812	tags=24%, list=15%, signal=21%	SRF/PADI2/PTEN/MAP2K5/TGFB1/LDLRAD4/RRAS/PIP5KL1/PLCB1/TRIB1/S1PR2/NF2/PTPRJ/ARHGDIB/SEMA4D/FUZ/EPHA4/STK24/TPM1/GREM1/CORO1C/SEMA4A/BCR/ARID2/VCL/CTNNA1/SFRP2/THBS1/ATP1B2/ADIPOR1/ABHD2/RAC1/LRP1/CNN2/ATP2B4/RHOA/NF1/RIN3/GNA12/WAS/FLCN/STAT3/PTPN23/CLDN5/RIPOR2/DNAJA4/TIMP1/FOXO3/TBXA2R
BP	GO:0019725	cellular homeostasis	548	-0.35	-1.41	0.00028369	0.0019515	0.00119812	2109	tags=24%, list=18%, signal=20%	ORMDL3/FOXO1/GNA15/PTGIR/TTC7A/RAB11FIP5/KNG1/DISC1/COX19/SRF/FYN/TPCN1/PRDX2/KCTD17/PDZD8/IRS2/FKBP1B/SLC24A4/MCOLN1/PRKCE/SPPL3/GRIK2/PROK2/SLC39A7/VDR/ATP7A/ATP13A1/LAMP2/ANO6/ERN1/SLC12A6/LRRK2/OPRL1/SMARCA4/SIDT2/CMKLR1/ZBTB20/DLG4/C5AR1/STX4/CA2/MT2A/PTPN6/CKB/HVCN1/TMBIM6/CLN3/PDPK1/PML/MICU1/CCR1/DEDD2/ATP6AP1/ITPR2/ARF1/CHP1/TXNRD2/DGAT2/PRKACA/CHERP/ATP6V0A1/ATP2A3/ERO1A/NCOA4/TMEM38A/BCAP31/PRDX5/HTT/LRRC8A/PTK2B/SLC8A1/CXCR2/SLC24A3/MAP1A/RAB7A/BMP6/TAOK1/ALDOA/LCN2/PPP3CA/PTPRC/SLC9A1/FIS1/SELENON/PDK3/GAA/ATP1B2/DCTN1/CALM3/SLC6A9/PIK3CG/JAK3/ANKRD9/ANXA6/UCP2/AQP1/LYN/SIN3A/GP9/RAB11B/ATP2B4/FKBP1A/FTL/PRKCB/GATA1/GPX1/CXCR1/ATP6V1A/FTH1/GRN/WDTC1/PINK1/HK1/MAPK3/SNCA/TGM2/PRDX6/GRINA/DNAJB2/SLC4A1/GP1BA/ATP6V0D1/STIM1/ALAS2/KEL/P2RX1/FOXO3/TBXA2R/XK/DIAPH1
CC	GO:0045335	phagocytic vesicle	124	-0.48	-1.68	0.00028369	0.0019515	0.00119812	2687	tags=42%, list=23%, signal=33%	ZDHHC5/VPS26B/RAB22A/CALR/VAMP3/CYBB/CORO1A/RAB43/TAPBP/VIM/ADAM8/PLD4/TLR2/RAB11FIP5/ATP6V0B/MCOLN1/NCF2/NCF4/ATP7A/STX6/SNX3/LAMP2/HGS/ACTG1/PIKFYVE/STX4/CDC42EP4/HVCN1/ATP6V0C/NCF1/ATP6V0A1/PGLYRP1/RAB7A/RAB14/GSN/SYK/RAB31/TLR6/LTF/RAB11FIP1/ZYX/FMNL1/RAC2/DNM2/RAB11B/TRIM14/RILP/WAS/ITGB5/ANXA11/LAMP1/ATP6V0D1
BP	GO:0043632	modification-dependent macromolecule catabolic process	573	-0.35	-1.40	0.00028426	0.00195203	0.00119844	1798	tags=23%, list=15%, signal=21%	AREL1/WDR81/RNF144B/GID4/RC3H2/CUL7/USP4/SDCBP/UBXN7/RNF213/HSPA5/SH3RF3/UBE2J2/SMARCC1/ARRB1/LRRK2/CACUL1/MAN1A1/PSMD2/KEAP1/MARCHF6/PSMD3/RNF11/TMEM67/PIAS1/TRIB1/CEBPA/WAC/CHMP4B/YOD1/UBE2Z/AGTPBP1/CBFA2T3/ARIH2/SPOP/ITCH/KCTD2/RNF115/HERC2/CCNF/PML/KAT5/TRIB3/GSK3A/UBE2L3/ZRANB1/CANX/PSMD1/VPS37B/VPS37C/FBXL18/CNOT4/UBE4B/TENT4B/USP10/CSNK2A1/SUFU/BCAP31/RNF126/PTK2B/USP3/TBX21/PSMD11/NSFL1C/MAP1A/TBL1XR1/RAD23A/TGFB1I1/UBR4/USP9X/FZR1/NPLOC4/FBXL20/UBAP1/HIPK2/USP34/TRIP12/VCP/HSPA1A/ANKRD9/UBR2/PSMC4/VPS28/CHMP1A/GABARAP/KLHL8/RBCK1/LAPTM5/GSK3B/UBA1/UBE2R2/FBXO9/WWP2/DDB1/HUWE1/CUL4A/RMND5A/TBL1X/GID8/UBL7/CLU/ARRB2/USP22/GNA12/RNF187/PINK1/ASCC2/SIAH2/DTX3L/PTPN23/PCBP2/PSMF1/N4BP1/UBB/SQSTM1/DNAJB2/USP7/ARAF/RNF40/SHARPIN/DCAF12/DAB2/TRIM58/FBXO7/TOLLIP/UBE2H/CBL/CDC34/ZER1/BAG6/RYBP/DCAF11

BP	GO:0040008	regulation of growth	397	-0.37	-1.44	0.00028968	0.00197561	0.00121292	2470	tags=29%, list=21%, signal=24%	CREB1/SOCS7/SOCS3/KIAA1109/DDX49/KIAA0319/BAP1/CD38/ADAM17/TKT/DIP2B/MUL1/BCL11A/KLHL22/CDKN2D/ARHGEF11/RFTN1/BCL2L11/DISC1/WWC3/MACF1/SRF/PTEN/FTO/PPM1F/HDGFL2/ZMAT3/ADNP/DBNL/MAP2K5/STK4/H3-3B/ERBB2/NIPBL/H3-3A/TGFB1/CPNE5/SDCBP/CTDP1/TCHP/BTG1/SMARCA4/PLCB1/NDEL1/STAT5B/EZR/TMC8/DBN1/KAT7/SHC1/MAPK14/MT2A/CDKL5/YY1/PTPRJ/MSX1/CLSTN1/SEMA4D/MAD2L2/NAIF1/ITCH/PML/KAT5/SMARCA2/GSK3A/DPYSL2/CXCL16/NEDD9/INSR/SESN2/CSNK2A1/SGPL1/FBP1/GREM1/RBPJ/PTK2B/SEMA4A/EIF4G2/NDRG3/IGF2/LAMTOR1/SFRP2/EXTL3/STK11/EXOSC4/SLC9A1/ADIPOR1/IST1/G6PD/BASP1/EIF4G1/ULK1/HSPA1A/SIPA1/SIN3A/GSK3B/STAT5A/DNM2/CDA/RHOA/PPP1R9B/PIM1/RPS6KA1/CDKN1A/BCL2L1/FLCN/STAT3/MTPN/CTTN/DNAJB2/ENO1/PAK2/YBX3/DAB2/JARID2/HLX
BP	GO:0016049	cell growth	307	-0.39	-1.50	0.00029069	0.00197909	0.00121506	2831	tags=35%, list=24%, signal=27%	PSRC1/CHPT1/ACVR1B/SMAD3/EI24/FHL1/PLCE1/ALCAM/OGFR/ARHGAP4/CCN3/PLXNA4/EPB41L3/HDAC6/TWF2/KIAA1109/DDX49/KIAA0319/BAP1/CD38/ADAM17/DIP2B/MUL1/BCL11A/KLHL22/CDKN2D/NIN/ARHGEF11/DISC1/MACF1/SRF/HDGFL2/ADNP/DBNL/MAP2K5/H3-3B/ERBB2/EXT1/H3-3A/TGFB1/CPNE5/SDCBP/CTDP1/TCHP/BTG1/SMARCA4/NDEL1/TMC8/KMT2D/DBN1/KAT7/CPNE1/CDKL5/YY1/PTPRJ/MSX1/CLSTN1/SEMA4D/MAD2L2/ARIH2/NAIF1/ITCH/PML/SMARCA2/GSK3A/DPYSL2/CXCL16/SESN2/CSNK2A1/FBP1/GREM1/PTK2B/SEMA4A/EIF4G2/NDRG3/VCL/LAMTOR1/SFRP2/EXTL3/STK11/EXOSC4/USP9X/PDLIM5/SLC9A1/ADIPOR1/IST1/G6PD/EIF4G1/ULK1/HSPA1A/SIPA1/SIN3A/CYFIP2/GSK3B/DNM2/AKAP13/CDA/RHOA/PPP1R9B/RPS6KA1/CDKN1A/FLCN/MTPN/CTTN/DNAJB2/ENO1/DAB2/IQGAP1
BP	GO:0050818	regulation of coagulation	40	-0.63	-1.84	0.00029136	0.00198025	0.00121577	2303	tags=40%, list=20%, signal=32%	NFE2L2/EPHB2/SH2B3/SERPING1/KNG1/PRDX2/ABAT/THBD/ANO6/PDGFA/THBS1/ALOX12/GP1BA/KRT1/TBXA2R/DMTN
BP	GO:0030851	granulocyte differentiation	29	-0.69	-1.89	0.00029746	0.00201819	0.00123906	1501	tags=41%, list=13%, signal=36%	TRIB1/CEBPA/CBFA2T3/SPI1/RARA/INPP5D/CUL4A/GATA1/FASN/HCLS1/TESC/TAL1
BP	GO:1903900	regulation of viral life cycle	110	-0.50	-1.69	0.00029796	0.00201819	0.00123906	2258	tags=32%, list=19%, signal=26%	TRIM5/IFI16/MAVS/TRIM38/SRPK1/APOBEC3D/KPNA6/SNX3/SETDB1/OASL/EIF2AK2/IFITM3/ZC3HAV1/TRIM28/VPS37B/INPP5K/RAD23A/GSN/ADAR/LTF/LGALS9/APOBEC3F/DDB1/IFITM2/SRPK2/TRIM11/TNIP1/TRIM14/STAU1/APOBEC3C/LARP1/TRIM10/ZNFX1/TRIM26/N4BP1

BP	GO:0002521	leukocyte differentiation	420	-0.37	-1.46	0.00030628	0.00206751	0.00126934	2254	tags=32%, list=19%, signal=27%	MFNG/FOS/TET2/BAK1/CREB1/ANXA2/GPR137/SPN/ARMC5/ADAM17/NCKAP1L/BCL11B/NRROS/IREB2/TF/GON4L/IFI16/CTNNBIP1/CLPTM1/SH3RF1/ADAM8/TMEM176B/FCER1G/TLR2/CMTM7/CR1/KLF6/SRF/WNT1/PRDX2/NLRP3/SRC/HDAC4/ATM/JMJD6/XRCC6/ERBB2/ATP7A/TGFB1/PPARGC1B/ITPKB/ZBTB7A/TNFSF4/RC3H2/RRAS/BCL3/NOTCH2/GPS2/STAT5B/EGR1/RIPK1/KAT7/MAPK14/TRIB1/CEBPA/YY1/PTPRJ/ZFP36L1/PTPN6/ADGRG3/CBFA2T3/SBNO2/RELB/SOS2/SPI1/LRRK1/PREX1/CCR1/CSF1R/ATP6AP1/FOXP1/LEPR/RUNX3/TMEM131L/VSIR/NFAM1/FES/OSCAR/RBPJ/IRF1/PGLYRP1/LRRC8A/RARA/PTK2B/GAB2/SEMA4A/SNX10/STAT6/TRAF6/TBX21/IL6R/TOB2/ZMIZ1/ZBTB16/MMP9/VAV1/STK11/TFE3/THOC5/PTPRC/LILRB3/SYK/KDELR1/ST3GAL1/MED1/MAFB/DOCK2/FZD5/LTF/JAK3/INPP5D/PF4/LYN/LGALS9/RHOA/CUL4A/GATA1/FASN/NF1/HCLS1/TESC/PBX1/STAT3/TAL1/PIK3CD/IKZF1/PRKDC/LYL1/FBXO7/MYH9/FOXO3/EIF2AK1/HLX
BP	GO:0044409	entry into host	111	-0.49	-1.65	0.00031046	0.00209216	0.00128447	2278	tags=33%, list=19%, signal=27%	SCARB1/TRIM5/TRIM38/VPS18/CR1/TPCN1/SRC/GYPA/SNX3/NCAM1/NECTIN1/EXOC7/PIKFYVE/GRK2/IFITM3/ITCH/ANPEP/INSR/SELPLG/SLC3A2/GSN/ITGA5/SLC1A5/HSPA1A/LGALS9/WWP2/IFITM2/TRIM11/TRIM14/TRIM10/ITGB5/TRIM26/LAMP1/BSG/CTSB/ITGB3/CBL
BP	GO:0070988	demethylation	57	-0.59	-1.83	0.00031621	0.00212374	0.00130386	2533	tags=44%, list=22%, signal=35%	KDM6A/KDM7A/KDM4A/TET2/UBE2B/APOBEC3D/ALKBH5/PHF2/KDM5B/PHF8/FTO/JMJD6/KDM3B/KDM8/KDM2A/TRIM28/POR/USP9X/TET3/APOBEC3F/KDM6B/KDM4B/APOBEC3C/USP7/JARID2
BP	GO:0031532	actin cytoskeleton reorganization	77	-0.53	-1.72	0.00031888	0.00213445	0.00131044	1828	tags=48%, list=16%, signal=41%	CDC42BPB/HCK/CASS4/EPS8/NOTCH2/EZR/GAB1/SHC1/S1PR2/SIPA1L1/ARHGDIB/PARVG/ARAP1/ABI3/DAPK3/CSF1R/GRB2/NEDD9/FES/PTK2B/BCAS3/PAK1/GSN/PTPN1/PLEC/ESAM/IQGAP2/FLNA/PLEK/RHOA/HCLS1/ARHGDIA/PARVB/CTTN/PLEK2/MYH9/DMTN
BP	GO:0006869	lipid transport	222	-0.42	-1.55	0.00032763	0.00218934	0.00134414	2551	tags=31%, list=22%, signal=24%	VPS4A/STOML1/APOL6/PRELID2/ANXA2/GRAMD1A/SLCO3A1/SLC10A3/CRY2/ATP9A/SCARB1/ATG2B/VPS4B/PCTP/KDM5B/PDZD8/IRS2/STARD3/OSBPL1A/ANO6/CPTP/ABCB11/NME4/VPS51/SLC27A3/OSBPL2/NR1H2/AQP9/CLN3/OSBPL5/PITPNC1/PTGES/TSPO2/ABCG2/GOT2/LRP10/PITPNM2/SLC27A1/NFKB1/PITPNA/TSPO/VLDLR/SLC2A1/APOBR/LDLRAP1/PLSCR3/LPCAT3/BMP6/CPT1A/SYK/THBS1/FIS1/SLC66A2/ABCC4/PSAP/LRP1/ABCC3/CLU/ATG2A/PIP4K2A/HDLBP/AKT2/SLC4A1/MFSD2B/ITGB3/DAB2/FURIN/OSBP2
CC	GO:0070160	tight junction	76	-0.54	-1.72	0.00033284	0.00221668	0.00136092	1698	tags=26%, list=14%, signal=23%	MTDH/ASH1L/PARD3/FRMD4B/ARHGEF2/TJP2/OCEL1/AMOT/NHS/RAPGEF2/FRMD4A/JAM3/ESAM/FZD5/ACTB/UBN1/CLDN5/SH3BP1/YBX3/VASP
CC	GO:0009986	cell surface	468	-0.35	-1.41	0.00033582	0.00223282	0.00137083	1812	tags=26%, list=15%, signal=23%	PLAUR/ATP5F1B/ANXA2/SPN/CD38/BTN3A2/SLC1A4/ADAM17/ANTXR2/NRROS/SCARB1/TF/EPHB2/CLPTM1/IL2RG/ENO2/ADAM8/MICB/FCER1G/TLR2/CR1/HEG1/CD99L2/CD93/WNT1/MYO18A/SDC4/THBD/TGFB1/SIRPA/LRFN3/AOC3/TNFSF4/NCAM1/RC3H2/LILRA5/ANO6/NOTCH2/HSPA5/TSPAN14/ABCB11/ITGAM/LY6G5C/MME/STX4/NRDC/KCNH2/PTPRJ/FLOT1/CLSTN1/SORT1/CD63/FCGRT/EPHA4/ITGB2/CCR1/CSF1R/FAM234A/KLRD1/LEPR/ABCG2/ANPEP/CA4/ERMAP/PDGFA/INSR/AMOT/TNFRSF10C/MAP3K5/GREM1/SORL1/LRRC8A/PCSK6/RARA/SELP/CXCR2/SLC3A2/IL6R/IL13RA1/GP6/ITGA5/TSPAN33/PTPRC/SLC9A1/THBS1/ATP1B2/CD151/FZD5/IQGAP2/LTF/B4GALT1/ABCC4/CD14/SIRPB1/IGF2R/ITGA2B/MPL/ITGAL/RTN2/LRFN1/CLU/CXCR1/MSN/ECE1/ITGB5/SPARC/LAMP1/CAPN5/GP1BA/ENO1/TREML1/CTSB/ITGB3/MBP/SLC7A5/P2RX1/PLVAP/TNS1/SMIM1/FURIN/TLN1

BP	GO:0052126	movement in host environment	133	-0.46	-1.63	0.00034509	0.0022906	0.00140631	2278	tags=33%, list=19%, signal=27%	SCARB1/TRIM5/TRIM38/VPS18/VPS4B/CR1/TPCN1/SRC/GYPA/SNX3/NCAM1/NECTIN1/EXOC7/PIKFYVE/GRK2/CHMP4B/IFITM3/ITCH/VPS37B/ANPEP/INSR/SELPLG/SLC3A2/RAB7A/GSN/ITGA5/IST1/SLC1A5/HSPA1A/CHMP1A/LGALS9/WWP2/DDB1/IFITM2/TRIM11/TRIM14/TRIM10/ITGB5/TRIM26/LAMP1/BSG/CTSB/ITGB3/CBL
MF	GO:0004842	ubiquitin-protein transferase activity	323	-0.38	-1.48	0.00036116	0.00239328	0.00146935	1910	tags=22%, list=16%, signal=19%	COP1/XIAP/MARCHF3/RFWD3/RNF165/AREL1/RNF144B/GID4/RC3H2/UBR5/RNF213/SH3RF3/UBE2J2/MED27/IRF2BPL/FBXW2/MARCHF6/RNF11/UBE2Z/MYCBP2/ARIH2/DTX2/UBE2M/ITCH/RNF115/HERC2/PML/BIRC6/TRIM28/RNF208/UBE2L3/CNOT4/UBE4B/VPS11/RNF126/NEURL1/HERC1/TRAF6/HECTD4/UBR4/PELI2/TRIP12/UBR2/RBCK1/UBE2R2/FBXO9/WWP2/HUWE1/TRIM11/RMND5A/TRIM14/RNF182/RNF24/TRIM10/RNF187/UBE2O/MARCHF2/SIAH2/TRIM26/DTX3L/MKRN1/RNF40/SHARPIN/TRIM58/RNF10/FBXO7/MARCHF8/RNF123/UBE2H/CBL/CDC34
BP	GO:0042330	taxis	335	-0.38	-1.47	0.00036739	0.0024305	0.0014922	2811	tags=37%, list=24%, signal=29%	GPSM3/PIK3CB/SMAD3/TNFSF14/ALCAM/KIT/EPHA1/NEO1/GBF1/ST6GAL1/FPR1/CCN3/PTGDR2/PLXNA4/CALR/LGALS3/PLAUR/FPR3/SPN/SCRIB/ALOX5/ADAM17/CORO1A/NCKAP1L/ARHGEF5/BCL11B/DUSP1/EPHB2/ADAM8/RPS6KA5/EFNB1/FCER1G/CMTM7/FYN/PADI2/FEZ1/TIRAP/PPM1F/EPHB4/TRIO/NINJ1/PROK2/ERBB2/EXT1/LSP1/RNF165/LYST/TGFB1/ADGRE2/NCAM1/ANO6/NOTCH2/TSC2/NECTIN1/HGF/GAB1/CMKLR1/PIKFYVE/C5AR1/EPHB1/MAPK14/STX4/PTPRJ/SEMA4D/MYCBP2/TREM1/SPI1/PREX1/EPHA4/LYPLA2/CMTM5/RALBP1/ITGB2/CXCL5/CCR1/CSF1R/DPYSL2/PIP5K1C/CXCL16/PDGFA/AMOT/PF4V1/PALLD/FES/GREM1/PTK2B/SEMA4A/CXCR2/PRKD2/IL6R/VAV1/PLXNC1/SYK/JAM3/THBS1/PTAFR/SCN1B/PLEC/IL17RA/DOCK2/PIK3CG/CAMK1D/NIBAN2/PF4/RAC1/LYN/LGALS9/RAC2/HSPB1/RHOA/ARRB2/CXCR1/RIN3/ECE1/MAPK3/RIPOR2/PIK3CD/BSG/MPP1/ITGB3/VASP/WNK1/RHOG
CC	GO:0005775	vacuolar lumen	128	-0.46	-1.60	0.00037896	0.00249898	0.00153424	2137	tags=30%, list=18%, signal=25%	GLB1/PA2G4/PADI2/GUSB/UNC13D/SDC4/ACP2/LAMP2/SDCBP/ACLY/PYCARD/TRAPPC1/CAP1/PSMD1/CPPED1/CHID1/RNASET2/TPP1/DYNC1H1/IMPDH1/VCAN/PYGB/GAA/IST1/VCP/TUBB4B/CYB5R3/PSAP/GNS/FTL/ARHGAP45/TUBB/ADA2/GRN/PRDX6/CTSB/CTSD/TOLLIP
BP	GO:0030278	regulation of ossification	60	-0.59	-1.83	0.000379	0.00249898	0.00153424	1865	tags=42%, list=16%, signal=35%	ALOX5/TENT5A/ATP2B1/NIPBL/TGFB1/CHSY1/ANO6/NBR1/MAPK14/CCR1/GREM1/RBPJ/PTK2B/SLC8A1/ANKH/TOB2/BMP6/ZBTB16/BMP2K/PHOSPHO1/LTF/RFLNB/GATA1/MAPK3/PBX1
BP	GO:0051603	proteolysis involved in cellular protein catabolic process	647	-0.33	-1.35	0.0003856	0.00253832	0.0015584	1798	tags=22%, list=15%, signal=20%	AREL1/WDR81/RNF144B/GID4/RC3H2/CUL7/USP4/SDCBP/UBXN7/RNF213/HSPA5/SH3RF3/UBE2J2/SMARCC1/ARRB1/LRRK2/ENC1/CACUL1/MAN1A1/PSMD2/KEAP1/MARCHF6/PSMD3/RNF11/TMEM67/PIAS1/TRIB1/CEBPA/WAC/CHMP4B/YOD1/UBE2Z/AGTPBP1/CBFA2T3/ARIH2/ALAD/SPOP/ITCH/EPHA4/KCTD2/RNF115/HERC2/CCNF/PML/PSME4/KAT5/TRIB3/GSK3A/UBE2L3/ZRANB1/CANX/PSMD1/VPS37B/PRKACA/VPS37C/FBXL18/CNOT4/UBE4B/USP10/CSNK2A1/SUFU/BCAP31/RNF126/PTK2B/USP3/RCN3/PITHD1/TBX21/PSMD11/NSFL1C/MAP1A/TBL1XR1/RAD23A/TGFB1I1/UBR4/USP9X/FZR1/PSME3/NPLOC4/FBXL20/UBAP1/HIPK2/USP34/TRIP12/VCP/HSPA1A/ANKRD9/UBR2/PSMC4/VPS28/CHMP1A/GABARAP/KLHL8/RBCK1/LAPTM5/GSK3B/UBA1/UBE2R2/FBXO9/WWP2/DDB1/HUWE1/CUL4A/RMND5A/TBL1X/GID8/GPX1/UBL7/CLU/ARRB2/USP22/GNA12/RNF187/PINK1/ASCC2/SIAH2/DTX3L/PTPN23/PCBP2/PSMF1/N4BP1/UBB/SQSTM1/DNAJB2/USP7/ARAF/CTSB/RNF40/UBXN6/SHARPIN/DCAF12/DAB2/TRIM58/FBXO7/RNF123/TOLLIP/UBE2H/CBL/CDC34/ZER1/BAG6/RYBP/DCAF11

MF	GO:0045296	cadherin binding	263	-0.40	-1.51	0.0003976	0.00260437	0.00159894	1692	tags=31%, list=14%, signal=27%	HSPA5/NUMB/RANGAP1/CKAP5/STK38/EZR/SPTAN1/UNC45A/DBN1/CHMP4B/CAST/PTPRJ/EFHD2/EXOC3/DDX6/CTNND1/CTNNAL1/LYPLA2/ZC3HAV1/PKP4/TJP2/YWHAZ/PPP1CA/TAGLN2/PKM/S100A11/STX5/STK24/SPTBN1/YWHAB/PUF60/SH3GL1/LRRC59/EIF4G2/SLC3A2/CALD1/VCL/CTNNA1/ALDOA/PLCB3/PDLIM5/PTPN1/ASAP1/PLEC/IST1/PLIN3/FLNA/NIBAN2/HSPA1A/GOLGA2/MARK2/HNRNPK/GAPVD1/RAB11B/CNN2/LASP1/CAPZB/EPS15L1/SCYL1/PDLIM1/FASN/H3C10/ADD1/LARP1/EHD1/CTTN/PRDX6/SH3GLB2/HDLBP/ENO1/BSG/PAK2/VASP/ARHGAP1/TBC1D2/MYH9/CBL/IQGAP1/WASF2/SND1/TLN1/HCFC1
BP	GO:0045453	bone resorption	41	-0.64	-1.86	0.00039906	0.00260967	0.0016022	2439	tags=49%, list=21%, signal=39%	GPR137/CD38/TF/ADAM8/SRC/EXT1/PPARGC1B/LRRK1/CSF1R/TPP1/PTK2B/SNX10/TRAF6/RAB7A/CSK/SYK/INPP5D/NF1/RAB3D/ITGB3
BP	GO:0010543	regulation of platelet activation	34	-0.67	-1.87	0.00040852	0.00265836	0.00163209	2211	tags=41%, list=19%, signal=34%	SH2B3/FCER1G/ABAT/THBD/PDGFA/SELP/GP6/SYK/ALOX12/PLEK/LYN/GP9/GP1BA/DMTN
BP	GO:0001503	ossification	222	-0.42	-1.54	0.00041008	0.00265982	0.00163299	2466	tags=33%, list=21%, signal=26%	ATP5F1B/ALYREF/DNAJC13/ALOX5/GPLD1/TUFT1/TENT5A/HDAC7/CTNNBIP1/AXIN2/FHL2/ATP2B1/THRA/FGR/H3-3B/EXT1/NIPBL/H3-3A/TGFB1/CHSY1/PPARGC1B/ANO6/XYLT1/HGF/NBR1/MAPK14/CEBPA/HEMGN/SORT1/SEMA4D/SBNO2/CCR1/TPM4/ALPL/PRKACA/RUNX3/SUFU/GREM1/RBPJ/PTK2B/SLC8A1/SNX10/ANKH/TRAF6/SLC24A3/IL6R/TOB2/IGF2/PDLIM7/RRBP1/BMP6/ZBTB16/MMP9/SFRP2/VCAN/ADAR/BMP2K/PHOSPHO1/CLTC/LTF/SKI/JUND/RFLNB/GSK3B/RHOA/GATA1/FASN/NF1/SNRNP200/MAPK3/PBX1/MRC2/SND1
BP	GO:0043242	negative regulation of protein-containing complex disassembly	62	-0.57	-1.80	0.00040959	0.00265982	0.00163299	2764	tags=52%, list=24%, signal=40%	TMOD3/CARMIL1/ADD2/CLASP1/HDAC6/TWF2/SCIN/SCAF4/IRAK3/FLII/KATNB1/TRIOBP/ATXN7/EPS8/SPTAN1/CAMSAP1/PHF23/WASHC2C/CLEC16A/ARHGEF2/SPTBN1/SPTA1/SVIL/TAOK1/GSN/RUBCN/CAPZB/ADD1/TMOD1/SPTB/MTPN/DMTN
BP	GO:0006638	neutral lipid metabolic process	78	-0.54	-1.74	0.00042444	0.00273511	0.00167921	2401	tags=38%, list=20%, signal=31%	SEL1L/PLCE1/PLB1/DGKD/GPLD1/SCARB1/TNXB/DGKA/DGKG/DGKZ/LIPE/NR1H2/ABHD5/LYPLA2/DGAT2/MBOAT7/SLC27A1/SORL1/APOBR/TBL1XR1/CPT1A/MGLL/PIK3CG/ABHD2/LPIN2/DGKK/PNPLA2/GPX1/SNCA/CTDNEP1
BP	GO:0051924	regulation of calcium ion transport	143	-0.46	-1.63	0.00042662	0.00274473	0.00168512	2579	tags=33%, list=22%, signal=26%	CD4/CACNB4/LGALS3/CAPN3/BAK1/TRPC3/CALM1/CORO1A/STIMATE/ATP2B1/FYN/FKBP1B/PRKCE/LILRA5/OPRL1/CACNB1/PTPN6/TMBIM6/PDPK1/PML/CCR1/PRKACA/TSPO/TMEM38A/HTT/PTK2B/SLC8A1/INPP5K/SLC9A1/SELENON/EHD3/CALM3/PIK3CG/G6PD/JAK3/GJC2/LYN/GNAI2/FKBP1A/RHOA/ARRB2/MYLK/SNCA/EPB41/STIM1/P2RX1/DIAPH1
CC	GO:0031983	vesicle lumen	264	-0.39	-1.48	0.0004328	0.00277106	0.00170129	2137	tags=33%, list=18%, signal=28%	GLB1/PA2G4/CHI3L1/SERPING1/KNG1/FGR/PADI2/GUSB/UNC13D/APAF1/DBNL/XRCC6/EEF2/TGFB1/PDXK/SDCBP/ARHGAP9/ACLY/GSTP1/HGF/SPTAN1/PSMD2/PSMD3/MAPK14/CRISPLD2/PTPN6/PYCARD/TRAPPC1/ALAD/KPNB1/QSOX1/FERMT3/ARPC5/PGAM1/CAP1/F5/PSMD1/CPPED1/PDGFA/PKM/S100A11/RNASET2/NFKB1/OSCAR/TIMP2/PGLYRP1/PAFAH1B2/PYGL/DYNC1H1/F13A1/PSMD11/IGF2/VCL/IMPDH1/ALDOA/LCN2/GSN/PYGB/THBS1/ATG7/IST1/DOCK2/LTF/VCP/TUBB4B/PF4/GPI/CYB5R3/CDA/CNN2/HUWE1/COTL1/GNS/FTL/ARHGAP45/TUBB/CLU/ADA2/ACTN4/LRG1/CANT1/GRN/SPARC/PRDX6/ACTN1/CTSD/TOLLIP/TIMP1

CC	GO:0060205	cytoplasmic vesicle lumen	264	-0.39	-1.48	0.0004328	0.00277106	0.00170129	2137	tags=33%, list=18%, signal=28%	GLB1/PA2G4/CHI3L1/SERPING1/KNG1/FGR/PADI2/GUSB/UNC13D/APAF1/DBNL/XRCC6/EEF2/TGFB1/PDXK/SDCBP/ARHGAP9/ACLY/GSTP1/HGF/SPTAN1/PSMD2/PSMD3/MAPK14/CRISPLD2/PTPN6/PYCARD/TRAPPC1/ALAD/KPNB1/QSOX1/FERMT3/ARPC5/PGAM1/CAP1/F5/PSMD1/CPPED1/PDGFA/PKM/S100A11/RNASET2/NFKB1/OSCAR/TIMP2/PGLYRP1/PAFAH1B2/PYGL/DYNC1H1/F13A1/PSMD11/IGF2/VCL/IMPDH1/ALDOA/LCN2/GSN/PYGB/THBS1/ATG7/IST1/DOCK2/LTF/VCP/TUBB4B/PF4/GPI/CYB5R3/CDA/CNN2/HUWE1/COTL1/GNS/FTL/ARHGAP45/TUBB/CLU/ADA2/ACTN4/LRG1/CANT1/GRN/SPARC/PRDX6/ACTN1/CTSD/TOLLIP/TIMP1
BP	GO:0036293	response to decreased oxygen levels	191	-0.44	-1.60	0.00043546	0.00278357	0.00170897	2395	tags=41%, list=20%, signal=33%	MDM4/TNF/TP53/MECP2/EGLN1/MDM2/NOTCH1/NKX3-1/PIK3CB/SMAD3/LONP1/UBQLN1/CFLAR/CD38/ADAM17/EGLN3/ENDOG/CYBB/NFE2L2/AIFM1/HMOX2/USP19/AK4/ADAM8/TLR2/SOD2/ALKBH5/SRF/PTEN/SRC/APAF1/MTHFR/ATM/ABAT/PRKCE/ATP7A/AGTRAP/CPEB4/PLOD1/NPEPPS/SCAP/ROCK2/EGR1/ZFP36L1/CBFA2T3/TMBIM6/ALAD/EPHA4/PML/HIF1AN/ITPR2/ARNT/PDGFA/RBPJ/ERO1A/PTK2B/SLC2A1/SLC8A1/SLC9A1/THBS1/PDK3/LMNA/HIPK2/UCP2/AQP1/CREBBP/HYOU1/BNIP3L/MPL/RHOA/PDLIM1/NF1/ACTN4/PINK1/ENO1/ALAS2/FOXO3/CBL
BP	GO:0030042	actin filament depolymerization	46	-0.61	-1.84	0.00044075	0.00281287	0.00172695	1592	tags=46%, list=14%, signal=40%	FLII/TRIOBP/EPS8/SPTAN1/WASHC2C/WDR1/SPTBN1/SPTA1/SVIL/GSN/PLEK/CAPZB/ADD1/PPP1R9B/TMOD1/CFL1/SPTB/MTPN/SH3BP1/MICAL2/DMTN
BP	GO:1903649	regulation of cytoplasmic transport	27	-0.71	-1.89	0.00044278	0.00282131	0.00173214	2438	tags=52%, list=21%, signal=41%	DNAJC13/ATP9A/SRC/SNX3/LRRK2/EZR/MAP2K2/VPS11/SORL1/MSN/MAPK3/PTPN23/USP7/DAB2
BP	GO:0006639	acylglycerol metabolic process	77	-0.53	-1.70	0.00045527	0.0028824	0.00176964	2840	tags=38%, list=24%, signal=29%	SEL1L/PLCE1/PLB1/DGKD/GPLD1/SCARB1/TNXB/DGKA/DGKG/DGKZ/LIPE/NR1H2/ABHD5/LYPLA2/DGAT2/MBOAT7/SLC27A1/SORL1/APOBR/TBL1XR1/CPT1A/MGLL/PIK3CG/ABHD2/LPIN2/DGKK/PNPLA2/GPX1/CTDNEP1
BP	GO:0009895	negative regulation of catabolic process	247	-0.40	-1.48	0.00045351	0.0028824	0.00176964	2057	tags=29%, list=18%, signal=25%	RGP1/CSDE1/FYN/WNT1/FEZ1/TIRAP/HDAC4/USP36/ZDHHC7/SNX3/SDCBP/TSC2/CPTP/SMARCC1/LRRK2/IKBKE/HGF/MAPK14/WAC/CHMP4B/PHF23/CBFA2T3/MAD2L2/MYCBP2/DAP/SF3B3/ALAD/VPS35/QSOX1/EPHA4/TRDMT1/PML/CLEC16A/LEPR/GSK3A/TENT4B/CSNK2A1/FBP1/SUFU/TSPO/SORL1/YBX1/TIMP2/HERC1/BSCL2/EIF4G2/MAP1A/ATG7/PIK3CG/HIPK2/FLNA/EIF4G1/RUBCN/GOLGA2/ATP2B4/RILP/LARP1/PINK1/SNCA/STAT3/PSMF1/MAPKAPK2/N4BP1/SLC4A1/USP7/TENT5C/YBX3/TIMP1/FURIN/BAG6/RYBP/RELA
BP	GO:0048729	tissue morphogenesis	305	-0.38	-1.47	0.00046662	0.00294022	0.00180514	2473	tags=30%, list=21%, signal=24%	SETD2/SOCS3/SCRIB/ARMC5/ADAM17/SIRT6/WLS/SLC9A3R1/TBX19/IFT122/MYO9A/MTHFD1/CTNNBIP1/ARHGAP24/PTK7/HEG1/SRF/WNT1/PTEN/KDM5B/VANGL1/SRC/APAF1/EXOC4/MTHFR/STK4/SDC4/EXT1/VDR/ATP7A/MAGED1/MKS1/TGFB1/ZDHHC7/TIAM1/PHACTR4/NOTCH2/TSC2/ACTG1/HGF/TGM3/CA2/NF2/ACTA2/MSX1/FUZ/EPHA4/PML/CSF1R/WDR1/TRIM28/FOXP1/GRB2/PPP1CA/PRKACA/PDGFA/TPM1/SUFU/GREM1/RBPJ/RARA/TRAF6/PRKD2/PAK1/VCL/TGFB1I1/SFRP2/ITGA5/DVL3/MED1/CD151/FZD5/BTBD7/SKI/FLNA/ACTB/GPI/RAC1/FKBP1A/RHOA/MSN/KDM6B/MYLK/TGM2/PBX1/TAL1/SH3BP1/PIK3CD/ITGB3/VASP/DAB2/MICAL2
BP	GO:0031644	regulation of nervous system process	62	-0.57	-1.79	0.00047235	0.0029716	0.00182441	2223	tags=35%, list=19%, signal=29%	ACP3/PTEN/SRC/ABAT/DICER1/DLGAP4/ROCK2/LRRK2/OPRL1/HGF/NPTXR/DLG4/WASF3/S1PR2/PARD3/SHISA7/PTK2B/SLC9A1/PTAFR/MGLL/TNFRSF1B/RNF10

BP	GO:0005975	carbohydrate metabolic process	414	-0.36	-1.41	0.00047625	0.00299143	0.00183658	2137	tags=23%, list=18%, signal=20%	GLB1/CHI3L1/MOGS/FOXO1/PTEN/GUSB/SRC/IRS2/HDAC4/GALK1/PRKCE/NPL/TPD52L2/CHST15/EXT1/TGFB1/SLC2A3/SYNJ2/ZBTB7A/PGD/B4GALT7/MIDN/B4GALT5/SYNJ1/MAN1A1/ZBTB20/ST3GAL2/MAPK14/PHKA2/ST3GAL3/PER2/CBFA2T3/B3GNT7/TKTL1/PGAM1/LEPR/GSK3A/PPP1CA/DGAT2/PDK2/ENTPD5/ARNT/EIF6/CHID1/BCKDK/PKM/INSR/SESN2/GNPDA1/FBP1/RPIA/NFKB1/AP2A1/B3GNT8/NCOA2/PFKFB3/PTK2B/SLC2A1/INPP5K/CHST11/B4GALT3/SLC3A2/PYGL/IGF2/ALDOA/STK11/HECTD4/CPT1A/PYGB/GANAB/PTAFR/STK40/PDK3/GAA/ADIPOR1/PFKFB4/CLTC/B4GALT1/G6PD/NDST1/GPI/PLEK/GSK3B/ST6GALNAC4/DDB1/BPGM/ITPK1/WDTC1/TALDO1/HK1/SNCA/STAT3/AKT2/SLC4A1/USP7/ENO1/GBGT1
BP	GO:0000122	negative regulation of transcription by RNA polymerase II	625	-0.34	-1.38	0.00047993	0.00300977	0.00184784	1775	tags=21%, list=15%, signal=19%	ZBTB7A/ETV6/DICER1/PDE2A/NOTCH2/SDCBP/ZNF148/MTDH/CTBP2/IRF2BPL/SMARCA4/GPS2/FOXP2/JDP2/KLF16/EZR/EGR1/ARID5A/ZNF608/ZBTB20/NR6A1/PIAS1/MYPOP/CTBP1/TLE4/MXD1/YY1/MSX1/PER2/ZNF175/SEMA4D/MAD2L2/DPF2/NR1H2/IRF2/TMBIM6/CDKN1C/SNAI3/MAFK/SPI1/ZNF296/HIF1AN/ZNF282/NSD1/SAMD1/AKIRIN2/KAT5/ZFHX3/TRIB3/TRIM28/SMARCA2/FOXP1/BRMS1/YWHAZ/TRPS1/BCORL1/SAP130/RUNX3/FBP1/NFKB1/SUFU/KANK2/RBPJ/NCOA2/NFATC2/YBX1/RARA/STAT6/NFIC/CCND3/TRAF6/TBX21/KLF5/MAX/PIAS4/IGF2/ZHX2/BMP6/TBL1XR1/ZBTB16/MXD4/USP9X/NCOR2/BPTF/MED1/NR3C1/GATAD2B/HEXIM2/HIPK2/CHD8/SKI/JUND/HSPA1A/RXRA/PHF21A/SIN3A/ETS2/GFI1B/CREBBP/WWP2/TXNIP/TCF7L2/SUPT5H/CHD4/MEPCE/CBX6/TBL1X/GATA1/ATXN1/WDTC1/HCLS1/JAZF1/SNCA/FLCN/STAT3/SP2/TAL1/SQSTM1/NFIX/ENO1/PPM1A/HDGF/MED25/YBX3/DAB2/MXI1/CNOT1/JARID2/CUX1/FOXO3/RYBP/KLF3/HCFC1/RELA
BP	GO:0006935	chemotaxis	334	-0.38	-1.47	0.00051219	0.00320701	0.00196893	2811	tags=37%, list=24%, signal=29%	GPSM3/PIK3CB/SMAD3/TNFSF14/ALCAM/KIT/EPHA1/NEO1/GBF1/ST6GAL1/FPR1/CCN3/PTGDR2/PLXNA4/CALR/LGALS3/PLAUR/FPR3/SPN/SCRIB/ALOX5/ADAM17/CORO1A/NCKAP1L/ARHGEF5/BCL11B/DUSP1/EPHB2/ADAM8/RPS6KA5/EFNB1/FCER1G/CMTM7/FYN/PADI2/FEZ1/TIRAP/PPM1F/EPHB4/TRIO/NINJ1/PROK2/ERBB2/EXT1/LSP1/RNF165/LYST/TGFB1/ADGRE2/NCAM1/ANO6/NOTCH2/TSC2/NECTIN1/HGF/GAB1/CMKLR1/PIKFYVE/C5AR1/EPHB1/MAPK14/STX4/PTPRJ/SEMA4D/MYCBP2/TREM1/SPI1/PREX1/EPHA4/LYPLA2/CMTM5/RALBP1/ITGB2/CXCL5/CCR1/CSF1R/DPYSL2/PIP5K1C/CXCL16/PDGFA/AMOT/PF4V1/PALLD/FES/GREM1/PTK2B/SEMA4A/CXCR2/PRKD2/IL6R/VAV1/PLXNC1/SYK/JAM3/THBS1/PTAFR/SCN1B/PLEC/IL17RA/DOCK2/PIK3CG/CAMK1D/NIBAN2/PF4/RAC1/LYN/LGALS9/RAC2/HSPB1/RHOA/ARRB2/CXCR1/RIN3/ECE1/MAPK3/RIPOR2/PIK3CD/BSG/MPP1/ITGB3/VASP/WNK1/RHOG
CC	GO:0070382	exocytic vesicle	129	-0.47	-1.64	0.00051583	0.00322478	0.00197984	3013	tags=40%, list=26%, signal=30%	DTNBP1/SYT12/RAB35/VTI1A/SEPTIN6/TRAPPC4/RABAC1/APBA1/VTI1B/SV2A/APH1A/NCSTN/MCTP1/SEPTIN4/CLTB/ATP2B1/SNAP25/DISC1/UNC13D/RAB27B/ZNRF1/STX6/RPH3A/LRRK2/NDEL1/SYTL3/SYN3/DLG4/MME/CLCN5/STX4/CLN3/SEPTIN1/RAB7A/RAB5B/CALM3/SLC6A9/DNAJC5/KIF1B/TPRG1L/RAB11B/ROGDI/SYTL4/IQSEC1/SNCA/BCL2L1/LAMP1/ATP6V0D1/RAB3IL1/STON2/RAB3D

BP	GO:0003006	developmental process involved in reproduction	504	-0.34	-1.37	0.00052946	0.00330477	0.00202895	2089	tags=23%, list=18%, signal=20%	BCL2L11/ALKBH5/CSDE1/LIMK2/PTEN/KDM5B/MAP3K4/SRC/CRTAP/ATM/DIAPH2/STK4/H3-3B/PAQR8/PROK2/NIPBL/VDR/H3-3A/PUM1/CUL7/NOTCH2/ARMC2/HSPA5/LRRK2/BTG1/IRF2BPL/C3orf62/ASH1L/STAT5B/FANCG/KMT2D/TFEB/ASF1B/ROPN1L/MME/MAPK14/CEBPA/YY1/ZFP36L1/SETX/PTPN6/CDKN1C/SPATA2/GMCL1/AGO4/MAST2/DHCR24/HERC2/CCNF/PSME4/ZNF296/RFX2/BIRC6/TRIM28/SMARCA2/GRB2/ARNT/ALPL/PRKACA/PDGFA/INSR/SGPL1/RBPJ/NCOA4/BCAP31/LRRC8A/RARA/PTK2B/NEURL1/PAFAH1B2/NDRG3/PITHD1/ACOX1/IGF2/CTNNA1/BMP6/SBF1/ZBTB16/SFRP2/STK11/JAM3/BPTF/MED1/CASP2/FZD5/ABHD2/B4GALT1/BASP1/UBR2/SPOCD1/PDE5A/DNM2/IGF2R/PSAP/SF1/ATP2B4/FOXJ2/GATA1/ARRB2/CCNI/PPP1R9B/TBC1D20/PBX1/BCL2L1/UBB/NCOA1/BSG/CTSB/PRKDC/KLC3/YBX3/MARF1/FOXO3/CBL/OSBP2/BAG6/ACRBP
BP	GO:0030193	regulation of blood coagulation	37	-0.65	-1.87	0.00054243	0.0033751	0.00207213	2303	tags=43%, list=20%, signal=35%	NFE2L2/EPHB2/SH2B3/SERPING1/KNG1/PRDX2/ABAT/THBD/ANO6/PDGFA/THBS1/ALOX12/GP1BA/KRT1/TBXA2R/DMTN
BP	GO:1900046	regulation of hemostasis	37	-0.65	-1.87	0.00054243	0.0033751	0.00207213	2303	tags=43%, list=20%, signal=35%	NFE2L2/EPHB2/SH2B3/SERPING1/KNG1/PRDX2/ABAT/THBD/ANO6/PDGFA/THBS1/ALOX12/GP1BA/KRT1/TBXA2R/DMTN
BP	GO:0051129	negative regulation of cellular component organization	475	-0.35	-1.41	0.00054963	0.00340926	0.0020931	2574	tags=32%, list=22%, signal=26%	MCTP1/CLASP1/HDAC6/CST3/TWF2/LGALS3/PMEPA1/PPIF/ANXA2/TERF2IP/CFLAR/TBCD/KIAA0319/CD38/CORO1A/PPFIA1/TOM1L2/SCIN/DIP2B/DUSP1/SCAF4/KAT2B/MUL1/VIM/EPHB2/CTNNBIP1/BCL11A/ARHGAP24/KLHL22/IRAK3/TLR2/FLII/SPRY3/KATNB1/THRA/PHF2/FYN/LIMK2/PTEN/PHF8/FEZ1/SRC/DNAJB1/TRIOBP/YWHAH/ATXN7/ATM/H3-3B/SMG6/H3-3A/EPS8/TGFB1/WAPL/LDLRAD4/SDCBP/TSC2/ANKRD13A/HSPA5/CPTP/TCHP/ROCK2/LRRK2/HGF/SPTAN1/TMC8/DLG4/RAD21/TMEM67/CHMP4B/RAP1GAP/CAMSAP1/PHF23/EML2/WASHC2C/SEMA4D/MAD2L2/ATXN2/NR1H2/ARAP1/ABI3/MAP4/VPS35/EPHA4/CCNF/CLEC16A/ARHGEF2/EVI5L/SSH1/TENT4B/SPTBN1/TSPO/SORL1/CORO1C/SPTA1/SEMA4A/INPP5K/RAPGEF2/BCAS3/SVIL/RAB7A/BMP6/TAOK1/GSN/PPP3CA/JAM3/THBS1/LMNA/RBM14/FLNA/ULK1/DNM3/RUBCN/HSPA1A/GNL3L/RAC1/RIOK3/GSK3B/DNM2/TNFRSF1B/HUWE1/ARHGAP6/RHOA/CAPZB/MYADM/GPX1/CLU/ARRB2/ADD1/SSH2/TERF2/TMOD1/RIN3/WAS/PINK1/SNCA/SPTB/BCL2L1/FLCN/MTPN/TFIP11/DNAJB2/PPM1A/GAK/DNAJA4/PAK2/ITGB3/DAB2/MBP/CARM1/WASF2/DMTN
BP	GO:1903706	regulation of hemopoiesis	272	-0.40	-1.50	0.00054963	0.00340926	0.0020931	1871	tags=25%, list=16%, signal=22%	XRCC6/ERBB2/TGFB1/PPARGC1B/ITPKB/TNFSF4/RC3H2/NOTCH2/STAT5B/RIPK1/KAT7/MAPK14/TRIB1/ZFP36L1/PTPN6/SOS2/SPI1/CCR1/FOXP1/KLF13/ARNT/EIF6/CNOT4/RUNX3/TMEM131L/VSIR/NFAM1/FES/IRF1/PGLYRP1/RARA/PTK2B/MEIS1/TRAF6/PITHD1/TBX21/TOB2/ZMIZ1/ZBTB16/TFE3/PTPRC/LILRB3/SYK/MED1/MAFB/MTURN/LTF/JAK3/HSPA1A/INPP5D/PF4/LYN/LGALS9/GFI1B/MPL/RHOA/CUL4A/GATA1/NF1/HCLS1/TESC/STAT3/TAL1/FAM210B/PRKDC/FBXO7/FOXO3/FAXDC2/HLX

BP	GO:0055082	cellular chemical homeostasis	484	-0.35	-1.41	0.00055574	0.00344177	0.00211307	2109	tags=24%, list=18%, signal=20%	ORMDL3/FOXO1/GNA15/PTGIR/TTC7A/RAB11FIP5/KNG1/DISC1/COX19/SRF/FYN/TPCN1/KCTD17/PDZD8/IRS2/FKBP1B/SLC24A4/MCOLN1/PRKCE/SPPL3/GRIK2/PROK2/SLC39A7/VDR/ATP7A/ATP13A1/ERN1/LRRK2/OPRL1/SMARCA4/SIDT2/CMKLR1/ZBTB20/DLG4/C5AR1/STX4/CA2/MT2A/PTPN6/CKB/HVCN1/TMBIM6/CLN3/PDPK1/PML/MICU1/CCR1/ATP6AP1/ITPR2/ARF1/CHP1/DGAT2/PRKACA/CHERP/ATP6V0A1/ATP2A3/ERO1A/NCOA4/TMEM38A/BCAP31/HTT/LRRC8A/PTK2B/SLC8A1/CXCR2/SLC24A3/RAB7A/BMP6/LCN2/PPP3CA/PTPRC/SLC9A1/FIS1/SELENON/PDK3/ATP1B2/CALM3/SLC6A9/PIK3CG/JAK3/ANKRD9/ANXA6/UCP2/AQP1/LYN/SIN3A/GP9/RAB11B/ATP2B4/FKBP1A/FTL/PRKCB/GATA1/GPX1/CXCR1/ATP6V1A/FTH1/GRN/WDTC1/HK1/MAPK3/SNCA/TGM2/GRINA/SLC4A1/GP1BA/ATP6V0D1/STIM1/ALAS2/KEL/P2RX1/FOXO3/TBXA2R/XK/DIAPH1
BP	GO:0150116	regulation of cell-substrate junction organization	49	-0.59	-1.79	0.00056244	0.00347244	0.0021319	2202	tags=55%, list=19%, signal=45%	DUSP22/POLDIP2/SMAD3/CLASP1/MAPRE2/LIMS1/MAP4K4/MACF1/PTEN/PPM1F/SRC/SDC4/ACTG1/ROCK2/PTPRJ/DAPK3/GREM1/CORO1C/BCAS3/VCL/SLC9A1/THBS1/RAC1/ARHGAP6/IQSEC1/RHOA/DMTN
BP	GO:0050801	ion homeostasis	447	-0.35	-1.39	0.00059478	0.00364385	0.00223713	2136	tags=25%, list=18%, signal=21%	ATP2B1/CNNM3/GNA15/PTGIR/SOD2/TTC7A/KNG1/DISC1/COX19/FYN/TPCN1/KCTD17/PDZD8/FKBP1B/SLC24A4/MCOLN1/PRKCE/SPPL3/XIAP/GRIK2/PROK2/EXT1/SLC39A7/VDR/ATP7A/ATP13A1/SLC12A6/LRRK2/OPRL1/TMC8/CMKLR1/DLG4/C5AR1/CA2/MT2A/KCNH2/PTPN6/CKB/HVCN1/TMBIM6/CLN3/PDPK1/PML/MICU1/CCR1/ATP6AP1/ITPR2/ARF1/CHP1/PDK2/PRKACA/CHERP/ATP6V0A1/ATP2A3/ERO1A/NCOA4/TMEM38A/BCAP31/HTT/PTK2B/SLC8A1/SNX10/CXCR2/SLC24A3/RAB7A/BMP6/LCN2/PTPRC/SLC9A1/FIS1/SELENON/ATP1B2/CALM3/SLC6A9/LTF/PIK3CG/JAK3/ANKRD9/ANXA6/LYN/GP9/ATP2B4/GNAI2/FKBP1A/FTL/PRKCB/GATA1/CXCR1/ATP6V1A/FTH1/GRN/MLLT6/MAPK3/SNCA/TGM2/GRINA/SLC4A1/GP1BA/ATP6V0D1/EPB42/SLC25A37/STIM1/ALAS2/WNK1/KEL/P2RX1/TBXA2R/EIF2AK1/XK/DIAPH1
BP	GO:0001666	response to hypoxia	182	-0.44	-1.59	0.00061254	0.00374113	0.00229685	2920	tags=41%, list=25%, signal=31%	MDM4/TNF/TP53/MECP2/EGLN1/MDM2/NOTCH1/NKX3-1/PIK3CB/SMAD3/LONP1/UBQLN1/CFLAR/CD38/ADAM17/EGLN3/ENDOG/CYBB/NFE2L2/HMOX2/USP19/AK4/ADAM8/TLR2/SOD2/ALKBH5/SRF/PTEN/SRC/APAF1/MTHFR/ATM/ABAT/PRKCE/ATP7A/AGTRAP/PLOD1/NPEPPS/SCAP/ROCK2/EGR1/ZFP36L1/CBFA2T3/TMBIM6/ALAD/EPHA4/PML/HIF1AN/ITPR2/ARNT/PDGFA/RBPJ/ERO1A/PTK2B/SLC2A1/SLC8A1/SLC9A1/THBS1/PDK3/LMNA/HIPK2/UCP2/AQP1/CREBBP/HYOU1/BNIP3L/MPL/RHOA/PDLIM1/NF1/ACTN4/PINK1/ENO1/ALAS2/FOXO3
BP	GO:1902905	positive regulation of supramolecular fiber organization	130	-0.46	-1.60	0.00061191	0.00374113	0.00229685	3133	tags=42%, list=27%, signal=31%	CDK5RAP2/FCHSD1/CLIP1/PFN1/ARHGEF10/NPHS1/SORBS3/HSPA1B/MECP2/PSRC1/SMAD3/EPHA1/CARMIL1/CLASP1/CDC42EP1/NCKAP1L/SCIN/ARHGEF5/NIN/TNXB/KATNB1/PPM1F/PRKCE/SDC4/HCK/ROCK2/CKAP5/WASF3/NF2/WASHC2C/PYCARD/CDC42EP4/SLAIN2/NUMA1/WDR1/GRB2/TPM1/FES/PTK2B/COLGALT1/PAK1/GSN/DCTN1/HSPA1A/PLEK/RAC1/PXN/RHOA/GPX1/CLU/WAS/CTTN/VASP/WASF2

BP	GO:0055080	cation homeostasis	435	-0.36	-1.41	0.00061388	0.00374351	0.00229832	2136	tags=25%, list=18%, signal=21%	ATP2B1/CNNM3/GNA15/PTGIR/SOD2/TTC7A/KNG1/DISC1/COX19/FYN/TPCN1/KCTD17/PDZD8/FKBP1B/SLC24A4/MCOLN1/PRKCE/SPPL3/XIAP/GRIK2/PROK2/EXT1/SLC39A7/VDR/ATP7A/ATP13A1/SLC12A6/LRRK2/OPRL1/TMC8/CMKLR1/DLG4/C5AR1/CA2/MT2A/KCNH2/PTPN6/HVCN1/TMBIM6/CLN3/PDPK1/PML/MICU1/CCR1/ATP6AP1/ITPR2/ARF1/CHP1/PDK2/PRKACA/CHERP/ATP6V0A1/ATP2A3/ERO1A/NCOA4/TMEM38A/BCAP31/HTT/PTK2B/SLC8A1/SNX10/CXCR2/SLC24A3/RAB7A/BMP6/LCN2/PTPRC/SLC9A1/FIS1/SELENON/ATP1B2/CALM3/SLC6A9/LTF/PIK3CG/JAK3/ANKRD9/ANXA6/LYN/GP9/ATP2B4/GNAI2/FKBP1A/FTL/PRKCB/GATA1/CXCR1/ATP6V1A/FTH1/GRN/MLLT6/MAPK3/SNCA/TGM2/GRINA/SLC4A1/GP1BA/ATP6V0D1/EPB42/SLC25A37/STIM1/ALAS2/WNK1/KEL/P2RX1/TBXA2R/EIF2AK1/XK/DIAPH1
BP	GO:0045598	regulation of fat cell differentiation	80	-0.53	-1.71	0.00063207	0.00384855	0.0023628	2591	tags=42%, list=22%, signal=33%	HNRNPU/HDAC6/CREB1/ALOX5/ASXL1/FOXO1/SOD2/ZNF385A/WNT1/FTO/TAF8/TRIO/STK4/WDFY2/TGFB1/SYAP1/GPS2/METRNL/JDP2/CMKLR1/MAPK14/CEBPA/ZFP36L1/SORT1/TRIB3/KLF5/ZBTB16/ASXL2/TGFB1I1/SFRP2/TFE3/PIM1/FLCN/CARM1
BP	GO:0016197	endosomal transport	198	-0.42	-1.56	0.00064014	0.00389171	0.0023893	1849	tags=29%, list=16%, signal=25%	CMTM6/LYST/STX6/SNX3/ACAP2/HGS/RAB11FIP4/LRRK2/SNX17/TBC1D17/PIKFYVE/VPS51/CHMP4B/DENND1C/SORT1/WASHC2C/VPS35/CLEC16A/ATP6AP1/ALMS1/TBC1D10B/SNX8/STX5/SORL1/CORO1C/RNF126/RAB6B/RAB7A/ANKFY1/RAB14/LAMTOR1/SLC66A2/EHD3/ARHGAP8/TOM1/DCTN1/CLTC/VCP/VPS28/CHMP1A/SNX27/AP5B1/RAB11B/REPS2/GRIPAP1/RILP/EPS15L1/UBE2O/WAS/PTPN23/EHD1/SQSTM1/TBC1D5/USP7/DENND1A/TMCC1/UBXN6/ARHGAP1
BP	GO:0070997	neuron death	238	-0.41	-1.52	0.00064599	0.00392132	0.00240748	2398	tags=30%, list=20%, signal=24%	MYBL2/EGLN3/ENDOG/CORO1A/MAP2K4/AIFM1/C1QA/ENO2/ADAM8/SOD2/BCL2L11/FYN/WNT1/APAF1/ELK1/SULT1A3/ATM/AKT1S1/ADNP/PIGT/XIAP/GRIK2/KIR3DL2/CPEB4/HSPA5/ITGAM/LRRK2/AP2B1/EGR1/EPHB1/CHMP4B/UBE2M/CLN3/VPS35/DHCR24/ITGB2/CHP1/MAP3K5/SORL1/IKBKG/PTK2B/MAX/MAP2K7/SLC9A1/TLR6/CASP2/HIPK2/G6PD/EIF4G1/GPI/DNAJC5/GSK3B/HYOU1/TNFRSF1B/SRPK2/RHOA/GPX1/CLU/ARRB2/NF1/GRN/PINK1/SNCA/BCL2L1/UBB/AKT2/HDGF/FBXO7/FOXO3/CBL/CDC34
CC	GO:0034774	secretory granule lumen	261	-0.39	-1.48	0.00065226	0.00395332	0.00242713	2137	tags=34%, list=18%, signal=28%	GLB1/PA2G4/CHI3L1/SERPING1/KNG1/FGR/PADI2/GUSB/UNC13D/APAF1/DBNL/XRCC6/EEF2/TGFB1/PDXK/SDCBP/ARHGAP9/ACLY/GSTP1/HGF/SPTAN1/PSMD2/PSMD3/MAPK14/CRISPLD2/PTPN6/PYCARD/TRAPPC1/ALAD/KPNB1/QSOX1/FERMT3/ARPC5/PGAM1/CAP1/F5/PSMD1/CPPED1/PDGFA/PKM/S100A11/RNASET2/NFKB1/OSCAR/TIMP2/PGLYRP1/PAFAH1B2/PYGL/DYNC1H1/F13A1/PSMD11/IGF2/VCL/IMPDH1/ALDOA/LCN2/GSN/PYGB/THBS1/ATG7/IST1/DOCK2/LTF/VCP/TUBB4B/PF4/GPI/CYB5R3/CDA/CNN2/HUWE1/COTL1/GNS/FTL/ARHGAP45/TUBB/CLU/ADA2/ACTN4/LRG1/CANT1/GRN/SPARC/PRDX6/ACTN1/CTSD/TOLLIP/TIMP1
BP	GO:0002761	regulation of myeloid leukocyte differentiation	79	-0.52	-1.69	0.0006789	0.00410228	0.00251858	1812	tags=30%, list=15%, signal=26%	TGFB1/PPARGC1B/NOTCH2/RIPK1/TRIB1/ZFP36L1/CCR1/FOXP1/FES/RARA/TRAF6/TOB2/TFE3/LILRB3/MAFB/LTF/INPP5D/PF4/LYN/CUL4A/NF1/HCLS1/TESC/TAL1

CC	GO:0005667	transcription regulator complex	353	-0.37	-1.43	0.0006789	0.00410228	0.00251858	1696	tags=24%, list=14%, signal=21%	CTBP2/TFDP2/MED27/GPS2/MIDEAS/NFAT5/EYA3/ARID5A/NFATC1/NR6A1/SRA1/CTBP1/CEBPA/TLE4/MXD1/YY1/ATF7/NR1H2/SNAI3/RELB/SPI1/AKIRIN2/KAT5/ZFHX3/TRIM28/ARNT/MED16/E2F3/TRRAP/RUNX3/CSNK2A1/RBPJ/NCOA2/NFATC2/RARA/STAT6/MEIS1/KLF5/MAX/TBL1XR1/ZBTB16/FOSL2/NCOR2/MED1/MAFB/GATAD2B/RBM14/HIPK2/SKI/JUND/GTF3C1/HMGA1/RXRA/SIN3A/GFI1B/CREBBP/MED15/TCF7L2/TAF6/CHD4/NFATC3/TBL1X/TFDP1/GATA1/PDLIM1/SP1/NCOA6/BCL9/HCLS1/JAZF1/ASCC2/PBX1/STAT3/TAL1/TLE3/NCOA1/NFE2/HDGF/PRKDC/MED25/E2F2/MXI1/FOXO3/RELA
BP	GO:0010770	positive regulation of cell morphogenesis involved in differentiation	59	-0.57	-1.77	0.00068332	0.00412272	0.00253113	2660	tags=44%, list=23%, signal=34%	CARMIL1/CALR/STAU2/ACTR2/EPHB2/LIMS1/UNC13D/TRIOBP/DBNL/CASS4/CUL7/DBN1/DOCK5/CDKL5/PREX1/EPHA4/NEDD9/FLNA/RAC1/DNM2/MPL/MYADM/PRKDC/DAB2/CUX1/DMTN
BP	GO:0030308	negative regulation of cell growth	113	-0.48	-1.64	0.00069618	0.00419397	0.00257487	2932	tags=42%, list=25%, signal=32%	PPP2R1A/VGLL4/HSPA1B/TP53/PSRC1/ACVR1B/SMAD3/EI24/FHL1/ARHGAP4/CCN3/KIAA0319/DIP2B/MUL1/BCL11A/CDKN2D/TGFB1/CTDP1/TCHP/BTG1/SMARCA4/YY1/PTPRJ/MSX1/SEMA4D/NAIF1/PML/SMARCA2/GSK3A/SESN2/FBP1/GREM1/SEMA4A/NDRG3/SFRP2/STK11/ADIPOR1/G6PD/ULK1/HSPA1A/SIPA1/CDA/PPP1R9B/CDKN1A/FLCN/DNAJB2/ENO1/DAB2
BP	GO:0045600	positive regulation of fat cell differentiation	37	-0.64	-1.85	0.00070472	0.00423901	0.00260252	2082	tags=49%, list=18%, signal=40%	HNRNPU/CREB1/ZNF385A/STK4/WDFY2/SYAP1/METRNL/CMKLR1/MAPK14/CEBPA/ZFP36L1/KLF5/ZBTB16/ASXL2/SFRP2/TFE3/PIM1/CARM1
BP	GO:0099565	chemical synaptic transmission, postsynaptic	45	-0.60	-1.80	0.00071082	0.00426924	0.00262109	2038	tags=31%, list=17%, signal=26%	PTEN/ABAT/GRIK2/LRRK2/DLG4/S1PR2/CHRNE/GSK3A/PTK2B/PPP3CA/GSK3B/ARRB2/SNCA/P2RX1
CC	GO:0000407	phagophore assembly site	30	-0.69	-1.88	0.00071219	0.00427097	0.00262215	1042	tags=40%, list=9%, signal=37%	ZFYVE1/BCAS3/RAB7A/WDR45/ILRUN/ATG7/WIPI2/ULK1/ATG2A/ATG13/SQSTM1/RAB1B
BP	GO:0050807	regulation of synapse organization	105	-0.49	-1.65	0.00072181	0.00432123	0.002653	2232	tags=34%, list=19%, signal=28%	EPHB2/TLR2/DISC1/FYN/PTEN/ADNP/DBNL/LRFN3/NECTIN1/LRRK2/DBN1/EPHB1/MAPK14/CDKL5/SIPA1L1/CLSTN1/SEMA4D/CLN3/ABI3/VPS35/EPHA4/YWHAZ/ADGRL1/NEURL1/SEMA4A/SETD5/PDLIM5/DCTN1/EIF4G1/DNM3/VCP/GRIPAP1/RHOA/TUBB/CFL1/SNCA
BP	GO:0098751	bone cell development	31	-0.67	-1.86	0.00072274	0.00432123	0.002653	2211	tags=52%, list=19%, signal=42%	SH2B3/ZNF385A/SRF/SRC/NOTCH2/PTPN6/LRRK1/ATP6AP1/FOXP1/MEIS1/MED1/LTF/PIP4K2A/TAL1/MPIG6B/WASF2
BP	GO:0015849	organic acid transport	164	-0.43	-1.55	0.00072863	0.00434338	0.0026666	1515	tags=21%, list=13%, signal=18%	SLC27A3/PER2/SLC16A3/SLC36A1/AQP9/SLC25A11/CLN3/PTGES/TSPO2/ABCG2/GOT2/SLC27A1/LRRC8A/SLC2A1/SLC6A20/SLC3A2/SLC25A38/CPT1A/SYK/THBS1/FIS1/SLC6A9/SLC1A5/ABCC4/SLC43A2/SLC43A1/ABCC3/NF1/SLC38A10/SNCA/AKT2/SLC7A5/SLC38A5/XK
BP	GO:0051893	regulation of focal adhesion assembly	44	-0.62	-1.82	0.00073215	0.00435128	0.00267146	2202	tags=48%, list=19%, signal=39%	LIMS1/MAP4K4/MACF1/PTEN/PPM1F/SRC/SDC4/ACTG1/ROCK2/PTPRJ/DAPK3/GREM1/CORO1C/BCAS3/VCL/SLC9A1/THBS1/RAC1/ARHGAP6/RHOA/DMTN

BP	GO:0090109	regulation of cell-substrate junction assembly	44	-0.62	-1.82	0.00073215	0.00435128	0.00267146	2202	tags=48%, list=19%, signal=39%	LIMS1/MAP4K4/MACF1/PTEN/PPM1F/SRC/SDC4/ACTG1/ROCK2/PTPRJ/DAPK3/GREM1/CORO1C/BCAS3/VCL/SLC9A1/THBS1/RAC1/ARHGAP6/RHOA/DMTN
BP	GO:1905475	regulation of protein localization to membrane	121	-0.48	-1.66	0.00074307	0.0044096	0.00270726	1899	tags=31%, list=16%, signal=26%	PRKCE/ERBB2/TGFB1/ZDHHC7/NUMB/ITGAM/AP2B1/EZR/TMBIM1/DLG4/STX4/CLN3/ABI3/SHISA7/PDPK1/ITGB2/CHP1/SPTBN1/INPP5K/LDLRAP1/CSK/GSN/FIS1/CLTC/ACTB/LRP1/ATP2B4/GRIPAP1/BBC3/PPP1R9B/BCL2L1/SQSTM1/AKT2/AP2M1/DAB2/RHOG/DMTN
CC	GO:0042470	melanosome	91	-0.50	-1.66	0.00074714	0.00442058	0.002714	1997	tags=33%, list=17%, signal=28%	RAB27B/ATP6V1B2/ATP7A/SDCBP/HSPA5/FLOT1/CD63/YWHAZ/CANX/ATP6V0A1/YWHAB/TPP1/SLC2A1/SLC3A2/RAB7A/GANAB/RAB5B/CLTC/SLC1A5/ANXA6/DNAJC5/RAC1/FASN/ANXA11/LAMP1/BSG/CTSB/CTSD/ITGB3/SND1
CC	GO:0048770	pigment granule	91	-0.50	-1.66	0.00074714	0.00442058	0.002714	1997	tags=33%, list=17%, signal=28%	RAB27B/ATP6V1B2/ATP7A/SDCBP/HSPA5/FLOT1/CD63/YWHAZ/CANX/ATP6V0A1/YWHAB/TPP1/SLC2A1/SLC3A2/RAB7A/GANAB/RAB5B/CLTC/SLC1A5/ANXA6/DNAJC5/RAC1/FASN/ANXA11/LAMP1/BSG/CTSB/CTSD/ITGB3/SND1
BP	GO:0050803	regulation of synapse structure or activity	107	-0.48	-1.63	0.00077724	0.00457143	0.00280662	2232	tags=34%, list=19%, signal=27%	EPHB2/TLR2/DISC1/FYN/PTEN/ADNP/DBNL/LRFN3/NECTIN1/LRRK2/DBN1/EPHB1/MAPK14/CDKL5/SIPA1L1/CLSTN1/SEMA4D/CLN3/ABI3/VPS35/EPHA4/YWHAZ/ADGRL1/NEURL1/SEMA4A/SETD5/PDLIM5/DCTN1/EIF4G1/DNM3/VCP/GRIPAP1/RHOA/TUBB/CFL1/SNCA
BP	GO:0001837	epithelial to mesenchymal transition	91	-0.50	-1.66	0.00078209	0.00459321	0.00281999	2192	tags=35%, list=19%, signal=29%	AXIN2/GLIPR2/PTEN/IL17RD/TGFB1/LDLRAD4/TIAM1/CUL7/SDCBP/ROCK2/HGF/PEF1/MSX1/MCRIP1/MAD2L2/FUZ/USF3/EPHA4/TRIM28/S100A4/GREM1/RBPJ/TGFB1I1/SFRP2/AKNA/ADIPOR1/FLNA/RFLNB/GSK3B/TCF7L2/ZNF703/DAB2
BP	GO:0032386	regulation of intracellular transport	262	-0.39	-1.47	0.00079461	0.00465296	0.00285667	1881	tags=27%, list=16%, signal=23%	NUP214/ERBB2/USP36/TGFB1/SNX3/NPEPPS/UBR5/UBE2J2/ITGAM/LRRK2/RANGAP1/NDEL1/EZR/MAPK14/MAP2K2/YOD1/ARIH2/VPS35/ITGB2/NUMA1/LCP1/TRIM28/GSK3A/UBE2L3/CHP1/PRKACA/SUFU/VPS11/RBM22/SORL1/BCAP31/FRAT1/INPP5K/BCAS3/LDLRAP1/DYNC1H1/STK11/FIS1/PTPN1/SUPT6H/ARHGAP8/FZD5/FLNA/RIPOR1/GSK3B/RAC2/RAB11B/BNIP3L/TCF7L2/HUWE1/GRIPAP1/BBC3/NF1/MSN/HCLS1/OAZ1/ATG13/PINK1/MAPK3/TBC1D20/PTPN23/EHD1/LAMP1/ABLIM3/AKT2/USP7/PPM1A/DAB2/ARHGAP1/TRIM58/DMTN
BP	GO:0030865	cortical cytoskeleton organization	47	-0.60	-1.80	0.00080216	0.00469029	0.00287959	1666	tags=47%, list=14%, signal=40%	IKBKB/RHOBTB2/TNF/CALR/EPB41L3/VPS4A/NCKAP1L/RHOF/ROCK2/EZR/WDR1/LCP1/PLEC/PLEK/FMNL1/RAC1/RAC2/RHOA/RHOBTB1/EPB41/RHOG/TLN1
BP	GO:0018210	peptidyl-threonine modification	90	-0.51	-1.69	0.00082661	0.00482612	0.00296298	2220	tags=33%, list=19%, signal=27%	DYRK3/PKN1/CHI3L1/CDC42BPA/CDC42BPB/TGFB1/ROCK2/LRRK2/DYRK1B/S1PR2/GRK2/PARD3/PDPK1/GSK3A/PRKACA/CSNK2A1/HIPK1/INPP5K/PRKD2/STK11/CALM3/HIPK2/EIF4G1/ULK1/MARK2/GSK3B/PRKCB/PRKDC/WNK1/DMTN
BP	GO:0006140	regulation of nucleotide metabolic process	57	-0.57	-1.77	0.00083387	0.00486137	0.00298463	1569	tags=35%, list=13%, signal=31%	HDAC4/ZBTB7A/ZBTB20/CBFA2T3/PGAM1/PDK2/ENTPD5/ARNT/EIF6/INSR/FBP1/PDK3/VCP/CDA/PINK1/SNCA/FLCN/STAT3/SLC4A1/ENO1
CC	GO:0030136	clathrin-coated vesicle	129	-0.46	-1.61	0.00083982	0.00488893	0.00300154	2250	tags=34%, list=19%, signal=28%	TF/AAK1/TNK2/CLTB/VPS18/MYO1E/UNC13D/RAB27B/DBNL/AP1G1/ATP7A/STX6/SNX3/AP2A2/AFTPH/NUMB/AP2B1/AP2S1/DENND1C/SORT1/AP1M1/VPS41/SYNRG/SPG21/VPS11/AP2A1/BCAP31/LDLRAP1/TGOLN2/RAB14/CLTC/FZD5/DNAJC5/HIP1/DNM2/IGF2R/EPN1/ECE1/NRGN/DENND1A/AP2M1/STON2/DAB2/FURIN

BP	GO:0010769	regulation of cell morphogenesis involved in differentiation	70	-0.54	-1.72	0.00084288	0.00489956	0.00300807	2676	tags=43%, list=23%, signal=33%	ST6GAL1/CARMIL1/CALR/STAU2/ACTR2/EPHB2/LIMS1/UNC13D/TRIOBP/DBNL/CASS4/CUL7/DBN1/DOCK5/CDKL5/WASHC2C/PREX1/EPHA4/NEDD9/CORO1C/FLNA/RAC1/DNM2/MPL/MYADM/ACTN4/PRKDC/DAB2/CUX1/DMTN
BP	GO:0046718	viral entry into host cell	105	-0.48	-1.64	0.00084994	0.00492617	0.00302441	2278	tags=32%, list=19%, signal=26%	SCARB1/TRIM5/TRIM38/VPS18/CR1/TPCN1/GYPA/SNX3/NCAM1/NECTIN1/PIKFYVE/GRK2/IFITM3/ITCH/ANPEP/INSR/SELPLG/SLC3A2/GSN/ITGA5/SLC1A5/HSPA1A/LGALS9/WWP2/IFITM2/TRIM11/TRIM14/TRIM10/ITGB5/TRIM26/LAMP1/BSG/CTSB/ITGB3
BP	GO:0060078	regulation of postsynaptic membrane potential	46	-0.60	-1.80	0.00090305	0.00519613	0.00319015	2038	tags=30%, list=17%, signal=25%	PTEN/ABAT/GRIK2/LRRK2/DLG4/S1PR2/CHRNE/GSK3A/PTK2B/PPP3CA/GSK3B/ARRB2/SNCA/P2RX1
BP	GO:0006641	triglyceride metabolic process	56	-0.57	-1.75	0.00090953	0.00522583	0.00320838	1598	tags=29%, list=14%, signal=25%	LIPE/NR1H2/ABHD5/DGAT2/MBOAT7/SLC27A1/SORL1/APOBR/TBL1XR1/CPT1A/MGLL/PIK3CG/LPIN2/PNPLA2/GPX1/CTDNEP1
BP	GO:0045732	positive regulation of protein catabolic process	188	-0.42	-1.55	0.0009117	0.00523075	0.0032114	2197	tags=32%, list=19%, signal=26%	USP5/SH3RF1/ADAM8/FOXO1/ATXN3/DISC1/PTEN/ARIH1/COP1/DET1/AREL1/RNF144B/SH3RF3/LRRK2/EZR/KEAP1/PIAS1/TRIB1/CEBPA/NRDC/AGTPBP1/CBFA2T3/ARIH2/VPS35/ITCH/TRIB3/GSK3A/STX5/CSNK2A1/VPS11/SORL1/BCAP31/PTK2B/RAB7A/RAD23A/FZR1/ATG7/VCP/HSPA1A/PSMC4/VPS28/GABARAP/LAPTM5/GSK3B/LRP1/WWP2/DDB1/TNFRSF1B/HUWE1/RILP/CLU/MSN/OAZ1/MARCHF2/DNAJB2/RNF40/DAB2/ZER1/FURIN/BAG6
BP	GO:1902533	positive regulation of intracellular signal transduction	661	-0.32	-1.32	0.00091503	0.00523479	0.00321388	2206	tags=26%, list=19%, signal=23%	STIMATE/HDAC3/LIMS1/MAVS/SH3RF1/ADAM8/TRIM38/STK25/MAP4K4/TRAF7/KLHL22/TLR2/GLIPR2/CHI3L1/BCL2L11/ZNF385A/ARHGEF3/FGR/FYN/PTEN/MAP3K4/TIRAP/RRAGD/PRDX2/SRC/ANKRD17/ATM/MAP2K5/PRKCE/SPPL3/XIAP/ERBB2/LARS1/CASS4/MAGED1/TGFB1/ITPKB/ERN2/PUM1/RC3H2/LILRA5/ERN1/NOTCH2/SDCBP/MTDH/SH3RF3/ROCK2/ARRB1/LRRK2/IKBKE/HGF/PLCB1/NFAT5/OASL/ALPK1/RIPK1/C5AR1/SHC1/CPNE1/SECTM1/PLEKHF1/WAC/MAP2K2/EIF2AK2/ACTA2/PTPRJ/MSX1/PTPN6/EIF5A/PYCARD/SEMA4D/SOS2/SPI1/LRRK1/EPHA4/GPBAR1/NCF1/PML/CLEC16A/PTBP1/CCR1/CSF1R/SASH1/CHERP/PDGFA/INSR/MAP3K5/S100A4/TAOK3/GREM1/PIM2/BCAP31/IKBKG/PTK2B/MFHAS1/RRAGC/SELP/RAPGEF2/LRRC59/SIK3/TRAF6/OTUD5/PRKD2/RASGRP4/PIAS4/PAK1/IGF2/CSK/SLC44A2/TAOK1/MAP4K1/LAMTOR1/MAP2K7/PELI2/PTPRC/SYK/SLC9A1/DVL3/THBS1/FIS1/PTPN1/TLR6/MTURN/ARHGAP8/FZD5/LTF/PIK3CG/HIPK2/FLNA/NDST1/HSPA1A/MADD/CD14/RAC1/LYN/HIP1/LGALS9/MAP3K3/RBCK1/LAPTM5/PDE5A/AKAP13/TCF7L2/GNAI2/FKBP1A/RHOA/PRKCB/TRIM14/GPX1/BBC3/ARRB2/ACTN4/CANT1/HCLS1/PINK1/MAPK3/TGM2/GUCY1A1/FLCN/SHISA5/SLC15A3/UBB/PIK3CD/LITAF/PTP4A3/PPM1A/BRD4/RUNDC3A/SHARPIN/PRR5/CBL/IQGAP1/MAP2K3/RELA
BP	GO:0034103	regulation of tissue remodeling	42	-0.60	-1.77	0.0009186	0.0052476	0.00322175	2545	tags=48%, list=22%, signal=37%	CST3/GPR137/CD38/TF/ADAM8/SRC/TGFB1/PPARGC1B/ROCK2/TMBIM1/CSF1R/LEPR/GREM1/BCR/CSK/PPP3CA/SYK/INPP5D/NF1/ITGB3

MF	GO:0061659	ubiquitin-like protein ligase activity	242	-0.39	-1.47	0.00092385	0.00527005	0.00323553	2617	tags=30%, list=22%, signal=24%	MED24/RC3H1/MED12/ZBED1/UBE2D3/UBE3B/TRIM35/RFFL/AMFR/MGRN1/MIB2/TRIM5/MUL1/RNF135/CBX4/SH3RF1/TRIM38/VPS18/ARIH1/ZNRF1/COP1/XIAP/RFWD3/RNF165/AREL1/RNF144B/GID4/RC3H2/UBR5/RNF213/SH3RF3/MED27/IRF2BPL/MARCHF6/RNF11/PIAS1/MYCBP2/ARIH2/DTX2/ITCH/RNF115/HERC2/RNF208/UBE4B/VPS11/RNF126/NEURL1/TRAF6/PIAS4/UBR4/PELI2/TRIP12/UBR2/WWP2/HUWE1/TRIM11/RMND5A/TRIM14/RNF24/TRIM10/RNF187/UBE2O/MARCHF2/SIAH2/TRIM26/DTX3L/MKRN1/RNF40/TRIM58/RNF10/MARCHF8/CBL
BP	GO:0042445	hormone metabolic process	99	-0.49	-1.65	0.0009253	0.00527076	0.00323596	1612	tags=26%, list=14%, signal=23%	STAT5B/LIPE/EGR1/CPQ/PAPSS2/SULT1B1/DGAT2/ARNT/PCSK1N/RDH11/DHRS13/SGPL1/NFKB1/ERO1A/TSPO/PCSK6/BMP6/POR/MED1/ALDH9A1/TCF7L2/PNPLA2/ECE1/CTSB/DAB2/FURIN
BP	GO:1902475	L-alpha-amino acid transmembrane transport	38	-0.63	-1.82	0.0009357	0.00532235	0.00326764	1423	tags=34%, list=12%, signal=30%	SLC25A15/SLC66A1/PER2/CLN3/SLC6A20/SLC3A2/SLC25A38/SLC6A9/SLC1A5/SLC43A2/SLC43A1/SLC7A5/SLC38A5
BP	GO:0097530	granulocyte migration	94	-0.49	-1.62	0.00094454	0.00536498	0.00329381	1573	tags=38%, list=13%, signal=33%	ADAM8/FCER1G/CD99L2/TIRAP/CD177/ADGRE2/CMKLR1/PIKFYVE/C5AR1/TREM1/PREX1/ITGB2/CXCL5/CSF1R/WDR1/PIP5K1C/PF4V1/CXCR2/PECAM1/VAV1/SYK/JAM3/THBS1/IL17RA/PIK3CG/CAMK1D/PF4/RAC1/RAC2/CXCR1/MAPK3/RIPOR2/PIK3CD/BSG/MPP1/RHOG
BP	GO:1901214	regulation of neuron death	208	-0.41	-1.50	0.00095045	0.00539087	0.00330971	2267	tags=30%, list=19%, signal=24%	MYBL2/EGLN3/CORO1A/MAP2K4/AIFM1/C1QA/ENO2/ADAM8/SOD2/BCL2L11/FYN/WNT1/ELK1/SULT1A3/ATM/AKT1S1/ADNP/GRIK2/KIR3DL2/CPEB4/ITGAM/LRRK2/AP2B1/EGR1/EPHB1/CHMP4B/UBE2M/CLN3/VPS35/DHCR24/ITGB2/CHP1/MAP3K5/SORL1/IKBKG/PTK2B/MAP2K7/TLR6/CASP2/HIPK2/G6PD/EIF4G1/GPI/DNAJC5/GSK3B/HYOU1/TNFRSF1B/SRPK2/RHOA/CLU/ARRB2/NF1/GRN/PINK1/SNCA/BCL2L1/UBB/HDGF/FBXO7/FOXO3/CBL/CDC34
BP	GO:0044092	negative regulation of molecular function	707	-0.32	-1.33	0.00095241	0.0053943	0.00331181	2252	tags=27%, list=19%, signal=23%	GPS1/KAT2B/IFI16/EPHB2/CTNNBIP1/SH2B3/PKN1/TNK2/SH3RF1/SERPINA1/CDKN2D/IRAK3/INKA1/SERPING1/ORMDL3/SPRY3/CR1/KNG1/HEG1/DISC1/PTEN/PRDX2/PPM1F/NLRP3/SRC/PHACTR2/MED13/IRS2/FKBP1B/HDAC4/AKT1S1/MLLT1/ADNP/MAP2K5/PPP1R15A/PRKCE/XIAP/SUMO3/WAPL/AOC3/TNFSF4/BCL3/TSC2/MIDN/ROCK2/GSTP1/ARRB1/LRRK2/OPRL1/HGF/GPS2/STK38/TMBIM1/TMC8/CMKLR1/PI3/KEAP1/PIP4K2C/MGAT5/CPNE1/TRIB1/CEBPA/NF2/CAST/UBE2Z/PTPRJ/RAP1GAP/MSX1/SORT1/PER2/PTPN6/PYCARD/FRY/ADGRG3/SEMA4D/MAD2L2/DAP/TMBIM6/CDKN1C/CLN3/SPI1/ITCH/PDPK1/DHCR24/GNAQ/TRIB3/BIRC6/LEPR/GSK3A/CHP1/BRMS1/PPP1CA/PCSK1N/AMOT/ATP2A3/CSNK2A1/YWHAB/NFKB1/SUFU/TAOK3/SH3BP5L/SORL1/CORO1C/PRDX5/TIMP2/SLC8A1/WARS1/INPP5K/BCAS3/GMIP/PIAS4/CSK/MMP9/POR/SFRP2/PPP3CA/APLP2/APBA3/PTPRC/ADAR/THBS1/PTPN1/ILRUN/PPP1R12B/PLEC/MTURN/CALM3/SLC6A9/HEXIM2/IQGAP2/LTF/FLNA/RUBCN/ACTB/GNL3L/GPI/GOLGA2/AQP1/LYN/ITPRIP/RBCK1/SIN3A/GSK3B/LRP1/HSPB1/WWP2/TXNIP/TCF7L2/PPP1R10/ATP2B4/GNAI2/FKBP1A/MEPCE/TFDP1/GATA1/GPX1/ARRB2/NF1/PPP1R9B/TERF2/WDTC1/PIM1/OAZ1/UBE2O/RPS6KA1/TESC/MAPK3/SNCA/SIAH2/CDKN1A/DTX3L/PIP4K2A/RIPOR2/TFIP11/PSMF1/PRKAR2A/PRKAR2B/DNAJB2/SLC4A1/USP7/CTSB/PAK2/ITGB3/DAB2/WNK1/FBXO7/TIMP1/CBL/IQGAP1/CABIN1/FURIN
BP	GO:0006937	regulation of muscle contraction	81	-0.51	-1.64	0.00095613	0.00539995	0.00331528	2136	tags=35%, list=18%, signal=28%	ATP2B1/ORMDL3/SRF/FKBP1B/ABAT/PROK2/DOCK5/GRK2/DAPK3/PRKACA/TPM1/TMEM38A/SLC8A1/SLC9A1/PTAFR/PPP1R12B/EHD3/CALM3/PIK3CG/ANXA6/PDE5A/RHOA/GUCY1A1/CTTN/ENO1/MYL9/P2RX1/TBXA2R

CC	GO:0031091	platelet alpha granule	64	-0.56	-1.75	0.00095987	0.00541339	0.00332354	1309	tags=34%, list=11%, signal=31%	QSOX1/FERMT3/F5/PDGFA/SELP/F13A1/IGF2/PECAM1/ALDOA/APLP2/THBS1/PF4/ITGA2B/SYTL4/CLU/ACTN4/SNCA/SPARC/ACTN1/TREML1/ITGB3/TIMP1
BP	GO:0009135	purine nucleoside diphosphate metabolic process	80	-0.52	-1.69	0.00096834	0.00544569	0.00334336	2204	tags=31%, list=19%, signal=26%	ENO2/AK4/HDAC4/GALK1/ZBTB7A/ZBTB20/CBFA2T3/PGAM1/TJP2/ENTPD5/ARNT/EIF6/PKM/INSR/FBP1/ALDOA/GPI/AK1/BPGM/HK1/STAT3/GUK1/SLC4A1/ENO1/MPP1
BP	GO:0009179	purine ribonucleoside diphosphate metabolic process	80	-0.52	-1.69	0.00096834	0.00544569	0.00334336	2204	tags=31%, list=19%, signal=26%	ENO2/AK4/HDAC4/GALK1/ZBTB7A/ZBTB20/CBFA2T3/PGAM1/TJP2/ENTPD5/ARNT/EIF6/PKM/INSR/FBP1/ALDOA/GPI/AK1/BPGM/HK1/STAT3/GUK1/SLC4A1/ENO1/MPP1
BP	GO:1903350	response to dopamine	39	-0.62	-1.80	0.00097477	0.00547412	0.00336082	3013	tags=62%, list=26%, signal=46%	DTNBP1/FCSK/GNAS/GNAI3/GNAL/NCSTN/SLC9A3R1/GNA15/SULT1A3/OR10H1/LRRK2/KLF16/VPS35/GSK3A/HRH2/GNB1/FLNA/SIN3A/GSK3B/DNM2/ARRB2/MAPK3/TGM2/GNAZ
BP	GO:0098876	vesicle-mediated transport to the plasma membrane	115	-0.48	-1.64	0.00098169	0.00549744	0.00337513	2544	tags=42%, list=22%, signal=33%	ZDHHC2/CCDC22/GGA3/EHD4/VAMP3/KIAA1109/SCRIB/EPG5/ATP9A/SYS1/VPS53/SNAP25/MACF1/EXOC4/CMTM6/STX6/SNX3/ACAP2/RAB11FIP4/SNX17/VPS51/DENND1C/CLSTN1/WASHC2C/CLN3/OSBPL5/VPS35/ATP6AP1/EXOC6B/OPTN/SPTBN1/SORL1/KIF13A/RAB7A/CSK/RAB14/RAB31/EHD3/ARHGAP8/SNX27/DNM2/RAB11B/GRIPAP1/PTPN23/EHD1/DENND1A/ARHGAP1/SEC16A
MF	GO:0005178	integrin binding	81	-0.50	-1.63	0.00099187	0.00554666	0.00340535	1679	tags=36%, list=14%, signal=31%	ADAM17/UTRN/ICAM4/TNXB/SRC/CD177/ICAM3/ITGAM/S1PR2/NF2/FERMT3/ITGB2/LCP1/IGF2/SFRP2/ITGA5/SYK/JAM3/THBS1/CD151/LYN/ITGA2B/ITGAL/ACTN4/ITGB5/ACTN1/ITGB3/MYH9/TLN1
BP	GO:0030038	contractile actin filament bundle assembly	74	-0.51	-1.63	0.00099932	0.00557266	0.00342132	2049	tags=47%, list=17%, signal=39%	PFN1/ARHGEF10/SORBS3/SMAD3/EPHA1/F11R/CARMIL1/CLASP1/PPFIA1/ARHGEF5/SRF/PPM1F/SRC/SDC4/ACTG1/ROCK2/ARRB1/NF2/ARAP1/ALMS1/TPM1/PTK2B/INPP5K/PAK1/SLC9A1/ZYX/RAC1/PXN/ARHGAP6/RHOA/PDLIM1/WAS/ITGB5/PAK2/WASF2
BP	GO:0043149	stress fiber assembly	74	-0.51	-1.63	0.00099932	0.00557266	0.00342132	2049	tags=47%, list=17%, signal=39%	PFN1/ARHGEF10/SORBS3/SMAD3/EPHA1/F11R/CARMIL1/CLASP1/PPFIA1/ARHGEF5/SRF/PPM1F/SRC/SDC4/ACTG1/ROCK2/ARRB1/NF2/ARAP1/ALMS1/TPM1/PTK2B/INPP5K/PAK1/SLC9A1/ZYX/RAC1/PXN/ARHGAP6/RHOA/PDLIM1/WAS/ITGB5/PAK2/WASF2
BP	GO:0016236	macroautophagy	273	-0.39	-1.47	0.00100221	0.00558096	0.00342641	2030	tags=29%, list=17%, signal=24%	FEZ1/ATP6V0B/SRC/EXOC4/SH3GLB1/ATP6V1B2/MCOLN1/USP36/WDR81/LAMP2/VPS13D/ERN1/HGS/TSC2/RNF213/RETREG3/EXOC7/LRRK2/NBR1/SPTLC2/PIKFYVE/PIP4K2C/WAC/CHMP4B/YOD1/PHF23/VPS41/CLN3/VPS35/ATP6V0C/QSOX1/CLEC16A/MAP1LC3B/OPTN/VPS37B/MTMR3/PRKACA/VPS37C/SESN3/SESN2/ATP6V0A1/ZFYVE1/IKBKG/HTT/PAFAH1B2/NSFL1C/RAB7A/WDR45/ATG4D/ILRUN/ATG7/WIPI2/ULK1/VCP/RUBCN/VPS28/CAPN1/CAPNS1/CHMP1A/GABARAP/ELAPOR1/BNIP3L/SUPT5H/HUWE1/TBC1D25/WDFY3/ATP6V1A/ATG2A/LARP1/ATG13/PINK1/MAPK3/PIP4K2A/SQSTM1/TBC1D5/ATP6V0D1/MFN2/UBXN6/RAB1B

BP	GO:0060548	negative regulation of cell death	634	-0.33	-1.33	0.00104958	0.00583652	0.00358331	2204	tags=26%, list=19%, signal=22%	ENO2/HDAC3/CBX4/FHL2/SH3RF1/ADAM8/MAP4K4/CDKN2D/PA2G4/VPS4B/ORMDL3/FOXO1/SOD2/ZNF385A/FYN/WNT1/PTEN/PRDX2/SRC/IRS2/SULT1A3/ADNP/MAP2K5/MYO18A/XIAP/GRIK2/PROK2/SON/HCK/KIR3DL2/AREL1/ITPKB/RNF144B/CPEB4/BCL3/NOTCH2/MGMT/MTDH/HSPA5/GSTP1/LRRK2/HGF/AP2B1/STAT5B/EYA3/TMBIM1/RIPK1/SHC1/BABAM2/CHMP4B/CAST/EIF2AK2/UBE2Z/MSX1/SETX/SEMA4D/TMBIM6/IER3/CLN3/AGO4/VPS35/ITCH/PDPK1/DHCR24/CSF1R/ARHGEF2/BIRC6/FOXP1/YWHAZ/CSNK2A1/NFKB1/GREM1/KANK2/SORL1/PIM2/PRDX5/IKBKG/HTT/DOCK8/PTK2B/CHST11/CTNNA1/MMP9/POR/SFRP2/ITGA5/SLC9A1/ADAR/THBS1/PSME3/STK40/PTMS/PTPN1/ST3GAL1/LMNA/MED1/CASP2/LTF/PIK3CG/HIPK2/CAMK1D/CHD8/TSC22D3/FLNA/EIF4G1/NIBAN2/JAK3/HSPA1A/PF4/ALOX12/GPI/UCP2/DNAJC5/CHMP1A/AQP1/HNRNPK/ITPRIP/RBCK1/SIN3A/LAPTM5/GSK3B/HYOU1/HSPB1/BNIP3L/DDB1/TNFRSF1B/TCF7L2/PPP1R10/GNAI2/RHOA/GATA1/GPX1/CLU/ARRB2/FKBP8/GRN/HCLS1/ARHGDIA/PIM1/CFL1/UBE2O/PINK1/RPS6KA1/SNCA/SIAH2/NUAK2/BCL2L1/CTTN/BAG1/GRINA/AKT2/ARAF/ENO1/HDGF/PRKDC/PTCRA/PAK2/YBX3/DAB2/FBXO7/TIMP1/CBL/STRADB/BAG6/RELA
BP	GO:0030522	intracellular receptor signaling pathway	190	-0.42	-1.52	0.0010525	0.00584462	0.00358828	2230	tags=34%, list=19%, signal=28%	RNF135/PKN1/SAFB/TADA3/ASXL1/VPS18/THRA/PADI2/SRC/PER1/YWHAH/ANKRD17/RXRB/DDX54/XIAP/VDR/PPARGC1B/PUM1/ZBTB7A/FAM120B/UBR5/CTBP2/LSM14A/SMARCA4/GPS2/OASL/ALPK1/KMT2D/NR6A1/NR1H2/ITCH/PML/FOXP1/ARNT/VPS11/KANK2/NCOA4/RARA/TRAF6/PAK1/ZMIZ1/ASXL2/NPLOC4/PDK3/NCOR2/MED1/NR3C1/NLRX1/HSPA1A/RXRA/RIOK3/RHOA/ACTN4/ARID1A/PIM1/SEC14L1/WBP2/STAT3/SLC15A3/NCOA1/DAB2/CNOT1/CARM1/RELA
BP	GO:0046578	regulation of Ras protein signal transduction	140	-0.46	-1.63	0.00106184	0.00588824	0.00361507	2232	tags=28%, list=19%, signal=23%	EPHB2/ARHGAP24/MAP4K4/SPRY3/ARHGEF3/HEG1/RASA2/EPS8/ITPKB/NOTCH2/ARRB1/RASAL3/ARHGDIB/FLOT1/RALBP1/ARHGEF2/FBP1/KANK2/BCR/LRRC59/RASGRP4/MYO9B/PIK3CG/MADD/RAC1/GABARAP/DNM2/AKAP13/IQSEC1/NF1/ARHGDIA/FLCN/CYTH4/RIPOR2/SQSTM1/SH3BP1/DENND1A/MFN2/CBL
CC	GO:0005923	bicellular tight junction	72	-0.53	-1.70	0.00106839	0.0059081	0.00362726	1698	tags=26%, list=14%, signal=23%	MTDH/ASH1L/PARD3/FRMD4B/ARHGEF2/TJP2/OCEL1/AMOT/NHS/RAPGEF2/FRMD4A/JAM3/ESAM/FZD5/UBN1/CLDN5/SH3BP1/YBX3/VASP
BP	GO:0030879	mammary gland development	70	-0.54	-1.71	0.00107806	0.00595331	0.00365502	2089	tags=31%, list=18%, signal=26%	BCL2L11/KDM5B/SRC/IRS2/VDR/ATP7A/MGMT/STAT5B/MSX1/PML/CSF1R/NEURL1/STAT6/USF2/NCOR2/MED1/CAPN1/STAT5A/GPX1/FASN/ZNF703/AKT2
MF	GO:0015175	neutral amino acid transmembrane transporter activity	28	-0.67	-1.82	0.00108222	0.00596501	0.0036622	911	tags=36%, list=8%, signal=33%	SLC36A1/SLC6A20/SLC3A2/SLC25A38/SLC6A9/SLC1A5/SLC43A2/SLC43A1/SLC7A5/SLC38A5

BP	GO:0042326	negative regulation of phosphorylation	270	-0.38	-1.44	0.00108318	0.00596501	0.0036622	2511	tags=34%, list=21%, signal=28%	PMEPA1/YWHAG/BAK1/TERF2IP/SOCS3/UBE2B/CALM1/DUSP16/SIRT6/PRKAR1B/MVP/DUSP1/KAT2B/EPHB2/SH2B3/PKN1/CDKN2D/IRAK3/SPRY3/HEG1/PTEN/PPM1F/IRS2/HDAC4/AKT1S1/MLLT1/TGFB1/SIRPA/LDLRAD4/TSC2/MIDN/GSTP1/LRRK2/HGF/PIP5KL1/STK38/PIP4K2C/TRIB1/CEBPA/CTDSP2/NF2/PTPRJ/PTPN6/PYCARD/PARD3/CBFA2T3/SEMA4D/CDKN1C/LRRK1/PDPK1/GNAQ/TRIB3/CHP1/FBP1/GREM1/SH3BP5L/SORL1/CORO1C/SLC8A1/WARS1/INPP5K/CSK/SFRP2/PTPRC/ADAR/PTPN1/PLEC/CTDSPL/CALM3/HEXIM2/EIF4G1/RUBCN/LYN/LRP1/HSPB1/CTDSP1/MYADM/ARRB2/NF1/FKBP8/SNCA/CDKN1A/STAT3/PIP4K2A/NIBAN1/PRKAR2A/PRKAR2B/SLC4A1/PRKDC/PAK2/FBXO7/CBL/DMTN
MF	GO:0140297	DNA-binding transcription factor binding	370	-0.36	-1.40	0.00109187	0.00600451	0.00368645	2203	tags=32%, list=19%, signal=27%	HDAC3/PKN1/FHL2/MMS19/TADA3/ASXL1/STRN/CBX5/SRF/PADI2/ZNF516/NLRP3/SRC/PER1/YWHAH/MED13/ELK1/HDAC4/DDX54/STK4/BAZ2A/ATOH8/LCOR/VDR/PPARGC1B/ZBTB7A/BCL3/NOTCH2/GTF2I/CTDP1/UBXN7/MTDH/CTBP2/SMARCA4/STAT5B/NFIA/OASL/ARID5A/KEAP1/NFATC1/PIAS1/CPNE1/MAPK14/TRIB1/CTBP1/CEBPA/TLE4/YY1/SMARCD3/MAD2L2/NR1H2/DAPK3/SPI1/NCOA3/HIF1AN/NSD1/KAT5/FOXP1/BRMS1/YWHAZ/ARNT/MED16/FBP1/TACC1/RBPJ/NCOA2/BCAS3/KLF5/MAX/USF2/TOB2/TGFB1I1/FOXO4/NCOR2/MED1/HIPK2/TRIP12/SKI/FLNA/JUND/ACTB/HMGA1/RXRA/SIN3A/ETS2/GSK3B/GFI1B/CREBBP/HSPB1/WWP2/TCF7L2/TAF6/CHD4/PRKCB/GAS2L1/TBL1X/TFDP1/GATA1/SP1/NCOA6/ACTN4/ADD1/HCLS1/ARID1A/WBP2/PBX1/DTX3L/STAT3/ZNF703/TAL1/NCOA1/KAT6A/PRKDC/MED25/CNOT1/HCFC1/RELA
BP	GO:0043090	amino acid import	32	-0.65	-1.81	0.00109771	0.00601999	0.00369595	1423	tags=34%, list=12%, signal=30%	PER2/SLC36A1/TSPO2/SLC6A20/SLC3A2/SLC25A38/SLC6A9/SLC1A5/SLC43A2/SLC43A1/SLC7A5
BP	GO:0048659	smooth muscle cell proliferation	89	-0.50	-1.66	0.00109637	0.00601999	0.00369595	2098	tags=29%, list=18%, signal=24%	PTGIR/SOD2/PTEN/XRCC6/TGFB1/ERN1/TRIB1/S1PR2/FOXP1/MAP3K5/TPM1/NDRG2/LDLRAP1/IL6R/MMP9/THBS1/PTAFR/SF1/TCF7L2/GNAI2/RHOA/FOXJ2/GNA12/CDKN1A/MFN2/PRKDC
BP	GO:0048511	rhythmic process	170	-0.43	-1.53	0.0011026	0.00603017	0.0037022	2038	tags=28%, list=17%, signal=24%	PTEN/KDM5B/SRC/PER1/TOP1/ADNP/PROK2/MAGED1/HSPA5/ROCK2/OPRL1/STAT5B/KDM8/PHLPP1/EGR1/SETX/PER2/MYCBP2/NFKB2/RELB/KDM2A/GNAQ/PML/ZFHX3/PPP1CA/KMT2A/CSNK2A1/SGPL1/NCOA2/USP9X/HEBP1/NCOR2/ATG7/CASP2/JUND/SIN3A/GSK3B/CREBBP/DDB1/HUWE1/CUL4A/ARRB2/SP1/SIAH2/USP7/SRRD/PRKDC/FOXO3
BP	GO:0051668	localization within membrane	482	-0.34	-1.37	0.00110193	0.00603017	0.0037022	1874	tags=24%, list=16%, signal=21%	RFTN1/TTC7A/FRMPD1/SNAP25/MACF1/FYN/LRRC4/EXOC4/SH3GLB1/PRKCE/GRIK2/ERBB2/CMTM6/STX6/TGFB1/ZDHHC7/SNX3/ATP13A1/ACAP2/SDCBP/HGS/ZDHHC19/HSPA5/GET3/NUMB/EXOC7/TSPAN14/ROCK2/RAB11FIP4/ITGAM/FYB1/AP2B1/SNX17/EZR/TMBIM1/DLG4/PIKFYVE/VPS51/STX4/PLEKHF1/CHMP4B/DENND1C/FLOT1/CLSTN1/WASHC2C/PARD3/CLN3/OSBPL5/ABI3/VPS35/SHISA7/PDPK1/NCF1/ITGB2/ATP6AP1/EXOC6B/CHP1/OPTN/VPS37B/VPS37C/EMC3/SPTBN1/SORL1/KIF13A/INPP5K/RAPGEF2/LDLRAP1/RAB7A/CSK/RAB14/GSN/TSPAN33/PTPRC/FIS1/RAB31/EHD3/ARHGAP8/CLTC/CALM3/DOCK2/PRAM1/FLNA/ACTB/RAC1/SNX27/DNM2/PSAP/RAB11B/LRP1/FLOT2/ATP2B4/GRIPAP1/ITGAL/PACS1/MYADM/BBC3/PPP1R9B/TESC/BCL2L1/PTPN23/EHD1/SQSTM1/AKT2/SLC4A1/DENND1A/AP2M1/GAK/MMGT1/BSG/RAB3D/DAB2/ARHGAP1/SEC16A/TSPAN5/RHOG/DMTN/BAG6/ANK1

BP	GO:0007568	aging	96	-0.49	-1.64	0.00110679	0.00604478	0.00371117	2575	tags=36%, list=22%, signal=29%	P2RY1/FOS/BAK1/AMFR/ENDOG/NFE2L2/C1QA/ATP2B1/SERPING1/SOD2/APAF1/ABAT/CD68/RNF165/SCAP/MME/CDKN1C/GRB2/CANX/TSPO/CTNNA1/PPP3CA/FOXO4/CASP2/JUND/UCP2/SIN3A/TNFRSF1B/GPX1/PPP1R9B/GNA12/MBP/CARM1/TIMP1/FOXO3
BP	GO:0016570	histone modification	414	-0.35	-1.39	0.00113284	0.00617853	0.00379329	2167	tags=30%, list=18%, signal=26%	HDAC6/NFYC/KDM6A/KDM7A/KDM4A/TET2/EHMT1/SETD2/KAT6B/MTA1/UBE2B/BAP1/RCOR1/SIRT6/PADI4/HDAC7/KAT2B/PKN1/RPS6KA5/TADA3/ASXL1/NSD3/PAF1/PHF2/PADI2/KDM5B/GATAD2A/PHF8/WDR82/PPM1F/PER1/ATXN7/MTHFR/HDAC4/ATXN7L3/ATM/MLLT1/VPS72/JMJD6/MCM3AP/BAZ2A/SRCAP/C6orf89/NIPBL/USP36/SETDB1/EP400/UBR5/ARRB1/LRRK2/ASH1L/MIDEAS/KDM3B/KDM8/JDP2/EYA3/ARID5A/KMT2D/KAT7/PHF20/CTCFL/KMT2B/CTBP1/WAC/PER2/SF3B3/SPI1/NCOA3/KDM2A/NSD1/KAT5/SETD1B/BRMS1/KMT2A/ZNF335/TRRAP/OTUB1/MCRS1/USP3/VRK3/RPS6KA4/PRKD2/CXXC1/IGF2/SETD5/TBL1XR1/BRPF1/SUPT6H/LMNA/GATAD2B/RBM14/TET3/TRIP12/SKI/UBR2/ACTB/SIN3A/CREBBP/DDB1/TAF6/HUWE1/CHD4/PRKCB/TBL1X/KMT2C/KDM6B/KDM4B/USP22/PINK1/MLLT6/WBP2/MAPK3/SNCA/DTX3L/FLCN/NCOA1/USP7/PCGF5/KAT6A/BRD4/RNF40/JARID2/CARM1/RYBP/HCFC1
BP	GO:0016052	carbohydrate catabolic process	110	-0.48	-1.61	0.00113534	0.00618369	0.00379646	2751	tags=45%, list=23%, signal=34%	FOXK2/GK5/FOXK1/MANBA/EP300/NCOR1/PFKP/PRKAG2/PYGM/PGM1/TP53/CTBS/PFKL/PHKG2/JMJD8/OGDH/AOAH/SIRT6/NAGA/MGAM/ENO2/GLB1/GUSB/HDAC4/GALK1/ZBTB7A/PGD/ZBTB20/CBFA2T3/TKTL1/PGAM1/GSK3A/PPP1CA/ENTPD5/ARNT/EIF6/PKM/INSR/FBP1/PYGL/ALDOA/PYGB/GAA/GPI/BPGM/HK1/STAT3/SLC4A1/ENO1
BP	GO:0030003	cellular cation homeostasis	387	-0.35	-1.37	0.00114379	0.00622122	0.0038195	2102	tags=24%, list=18%, signal=20%	GNA15/PTGIR/TTC7A/KNG1/DISC1/COX19/FYN/TPCN1/KCTD17/PDZD8/FKBP1B/SLC24A4/MCOLN1/PRKCE/SPPL3/GRIK2/PROK2/SLC39A7/VDR/ATP7A/ATP13A1/LRRK2/OPRL1/CMKLR1/DLG4/C5AR1/CA2/MT2A/PTPN6/HVCN1/TMBIM6/CLN3/PDPK1/PML/MICU1/CCR1/ATP6AP1/ITPR2/ARF1/CHP1/PRKACA/CHERP/ATP6V0A1/ATP2A3/ERO1A/NCOA4/TMEM38A/BCAP31/HTT/PTK2B/SLC8A1/CXCR2/SLC24A3/RAB7A/BMP6/LCN2/PTPRC/SLC9A1/FIS1/SELENON/ATP1B2/CALM3/SLC6A9/PIK3CG/JAK3/ANKRD9/ANXA6/LYN/GP9/ATP2B4/FKBP1A/FTL/PRKCB/GATA1/CXCR1/ATP6V1A/FTH1/GRN/MAPK3/SNCA/TGM2/GRINA/SLC4A1/GP1BA/ATP6V0D1/STIM1/ALAS2/KEL/P2RX1/TBXA2R/XK/DIAPH1
BP	GO:0098771	inorganic ion homeostasis	440	-0.35	-1.38	0.00117097	0.00635167	0.00389959	2136	tags=25%, list=18%, signal=21%	ATP2B1/CNNM3/GNA15/PTGIR/SOD2/TTC7A/KNG1/DISC1/COX19/FYN/TPCN1/KCTD17/PDZD8/FKBP1B/SLC24A4/MCOLN1/PRKCE/SPPL3/XIAP/GRIK2/PROK2/EXT1/SLC39A7/VDR/ATP7A/ATP13A1/SLC12A6/LRRK2/OPRL1/TMC8/CMKLR1/DLG4/C5AR1/CA2/MT2A/KCNH2/PTPN6/CKB/HVCN1/TMBIM6/CLN3/PDPK1/PML/MICU1/CCR1/ATP6AP1/ITPR2/ARF1/CHP1/PDK2/PRKACA/CHERP/ATP6V0A1/ATP2A3/ERO1A/NCOA4/TMEM38A/BCAP31/HTT/PTK2B/SLC8A1/SNX10/CXCR2/SLC24A3/RAB7A/BMP6/LCN2/PTPRC/SLC9A1/FIS1/SELENON/ATP1B2/CALM3/SLC6A9/LTF/PIK3CG/JAK3/ANKRD9/ANXA6/LYN/GP9/ATP2B4/GNAI2/FKBP1A/FTL/PRKCB/GATA1/CXCR1/ATP6V1A/FTH1/GRN/MLLT6/MAPK3/SNCA/TGM2/GRINA/SLC4A1/GP1BA/ATP6V0D1/EPB42/SLC25A37/STIM1/ALAS2/KEL/P2RX1/TBXA2R/EIF2AK1/XK/DIAPH1

BP	GO:0090101	negative regulation of transmembrane receptor protein serine/threonine kinase signaling pathway	65	-0.54	-1.71	0.00122181	0.00661843	0.00406336	2907	tags=46%, list=25%, signal=35%	TP53/NOTCH1/BCL9L/CCN3/UBE2D3/PMEPA1/ADAM17/NRROS/WNT1/IL17RD/LDLRAD4/ZBTB7A/HSPA5/NOMO3/FAM89B/PDPK1/GREM1/SORL1/CHST11/TGFB1I1/SFRP2/GLG1/HIPK2/SKI/HSPA1A/DNM2/LRP1/FKBP1A/LTBP1/PPM1A
BP	GO:0007623	circadian rhythm	125	-0.45	-1.55	0.0012494	0.0067221	0.00412701	3210	tags=40%, list=27%, signal=29%	NCOR1/RBM4/AHR/NFIL3/GFPT1/DYRK1A/TNF/TP53/IMPDH2/THRAP3/KDM5A/HNRNPU/CREB1/BTBD9/MTA1/CRY2/HDAC3/PTEN/PER1/TOP1/PROK2/MAGED1/ROCK2/OPRL1/KDM8/PHLPP1/EGR1/SETX/PER2/MYCBP2/RELB/KDM2A/GNAQ/PML/ZFHX3/PPP1CA/KMT2A/NCOA2/USP9X/HEBP1/ATG7/JUND/SIN3A/GSK3B/DDB1/HUWE1/SIAH2/USP7/SRRD/PRKDC
CC	GO:0030140	trans-Golgi network transport vesicle	26	-0.70	-1.86	0.00124937	0.0067221	0.00412701	2171	tags=58%, list=19%, signal=47%	CLTB/RAB27B/AP1G1/ATP7A/AFTPH/SORT1/SYNRG/SPG21/AP2A1/TGOLN2/RAB14/CLTC/IGF2R/NRGN/FURIN
BP	GO:0061448	connective tissue development	129	-0.45	-1.58	0.00127667	0.00685026	0.00420569	2449	tags=32%, list=21%, signal=25%	CFLAR/GPLD1/SCIN/AXIN2/CHI3L1/THRA/SRF/ZNF516/FTO/EXT1/ATP7A/TGFB1/CHSY1/DYRK1B/SPTLC2/EGR1/ARID5A/MAPK14/ACTA2/MSX1/SPI1/DGAT2/TRPS1/RUNX3/GREM1/SORL1/RARA/SOX6/CHST11/IL6R/BMP6/TBL1XR1/ZBTB16/POR/SFRP2/GLG1/ANXA6/RFLNB/MAPK3/UBB/TIMP1
MF	GO:0061630	ubiquitin protein ligase activity	230	-0.40	-1.49	0.00127623	0.00685026	0.00420569	2459	tags=30%, list=21%, signal=24%	MED24/RC3H1/MED12/UBE2D3/UBE3B/TRIM35/RFFL/AMFR/MGRN1/MIB2/TRIM5/MUL1/RNF135/SH3RF1/TRIM38/VPS18/ARIH1/ZNRF1/COP1/XIAP/RFWD3/RNF165/AREL1/RNF144B/GID4/RC3H2/UBR5/RNF213/SH3RF3/MED27/IRF2BPL/MARCHF6/RNF11/MYCBP2/ARIH2/DTX2/ITCH/RNF115/HERC2/RNF208/UBE4B/VPS11/RNF126/NEURL1/TRAF6/UBR4/PELI2/TRIP12/UBR2/WWP2/HUWE1/TRIM11/RMND5A/TRIM14/RNF24/TRIM10/RNF187/UBE2O/MARCHF2/SIAH2/TRIM26/DTX3L/MKRN1/RNF40/TRIM58/RNF10/MARCHF8/CBL
MF	GO:0015179	L-amino acid transmembrane transporter activity	35	-0.63	-1.77	0.00128387	0.0068796	0.00422371	911	tags=40%, list=8%, signal=37%	SERINC5/SLC1A4/SLC25A15/SLC66A1/SLC36A1/SLC6A20/SLC3A2/SLC25A38/SLC6A9/SLC1A5/SLC43A2/SLC43A1/SLC7A5/SLC38A5
BP	GO:0060249	anatomical structure homeostasis	174	-0.42	-1.52	0.00128629	0.00688328	0.00422597	1302	tags=34%, list=11%, signal=31%	MAK/ABCA3/FGGY/P2RY1/CAPN3/GPR137/CD38/CORO1A/LARGE2/GIGYF2/NKIRAS2/TF/ADAM8/SLC28A2/ATP2B1/SRF/SRC/EXT1/MKS1/PPARGC1B/LAMP2/ACTG1/CHMP4B/LRRK1/CSF1R/MTF1/TJP2/TPP1/PTK2B/SLC2A1/SNX10/TRAF6/RCN3/MAP1A/VCL/PECAM1/RAB7A/CSK/TAOK1/ALDOA/STK11/SYK/JAM3/GAA/ATP1B2/ESAM/DCTN1/LTF/ACTB/INPP5D/HSPB1/GATA1/NF1/CLDN5/DNAJB2/RAB3D/ITGB3/MBP/KRT1/P2RX1
BP	GO:0032388	positive regulation of intracellular transport	160	-0.43	-1.54	0.00128871	0.006887	0.00422825	2067	tags=31%, list=18%, signal=26%	HSPA1L/FYN/FEZ1/SH3GLB1/ERBB2/USP36/TGFB1/NPEPPS/UBR5/UBE2J2/ITGAM/EZR/MAPK14/ARIH2/ITGB2/NUMA1/TRIM28/GSK3A/UBE2L3/CHP1/PRKACA/VPS11/RBM22/SORL1/BCAP31/BCAS3/LDLRAP1/DYNC1H1/STK11/FIS1/FZD5/FLNA/RIPOR1/GSK3B/RAC2/TCF7L2/HUWE1/BBC3/MSN/HCLS1/OAZ1/ATG13/PINK1/TBC1D20/PTPN23/EHD1/ABLIM3/AKT2/PPM1A/DAB2

MF	GO:0043130	ubiquitin binding	90	-0.50	-1.66	0.00129605	0.00690764	0.00424092	1039	tags=22%, list=9%, signal=20%	OTUB1/IKBKG/MARK4/NSFL1C/RAD23A/ILRUN/NPLOC4/TOM1/UBAP1/VPS28/RBCK1/HSPB1/ASCC2/N4BP1/SQSTM1/DNAJB2/SHARPIN/JARID2/FBXO7/TOLLIP
BP	GO:0043069	negative regulation of programmed cell death	571	-0.33	-1.34	0.00129789	0.00690816	0.00424124	2203	tags=26%, list=19%, signal=23%	HDAC3/CBX4/FHL2/SH3RF1/ADAM8/MAP4K4/CDKN2D/PA2G4/ORMDL3/FOXO1/SOD2/ZNF385A/WNT1/PTEN/PRDX2/SRC/IRS2/ADNP/MAP2K5/MYO18A/XIAP/GRIK2/PROK2/SON/HCK/AREL1/ITPKB/RNF144B/CPEB4/BCL3/NOTCH2/MGMT/MTDH/HSPA5/GSTP1/LRRK2/HGF/STAT5B/EYA3/TMBIM1/RIPK1/SHC1/BABAM2/CAST/EIF2AK2/UBE2Z/MSX1/SETX/SEMA4D/TMBIM6/IER3/CLN3/AGO4/ITCH/PDPK1/DHCR24/CSF1R/ARHGEF2/BIRC6/FOXP1/YWHAZ/CSNK2A1/NFKB1/GREM1/KANK2/PIM2/PRDX5/HTT/DOCK8/PTK2B/CHST11/CTNNA1/MMP9/POR/SFRP2/ITGA5/SLC9A1/ADAR/THBS1/PSME3/STK40/PTMS/PTPN1/ST3GAL1/LMNA/MED1/CASP2/LTF/PIK3CG/HIPK2/CAMK1D/CHD8/TSC22D3/FLNA/NIBAN2/JAK3/HSPA1A/PF4/ALOX12/GPI/UCP2/DNAJC5/AQP1/HNRNPK/ITPRIP/RBCK1/SIN3A/LAPTM5/GSK3B/HYOU1/HSPB1/BNIP3L/DDB1/TCF7L2/PPP1R10/GNAI2/RHOA/GATA1/GPX1/CLU/ARRB2/FKBP8/GRN/HCLS1/ARHGDIA/PIM1/CFL1/UBE2O/PINK1/RPS6KA1/SNCA/SIAH2/NUAK2/BCL2L1/CTTN/BAG1/GRINA/AKT2/ARAF/ENO1/HDGF/PRKDC/PTCRA/PAK2/YBX3/DAB2/TIMP1/CBL/STRADB/BAG6/RELA
BP	GO:1900542	regulation of purine nucleotide metabolic process	56	-0.56	-1.72	0.0013084	0.00695478	0.00426987	1960	tags=34%, list=17%, signal=28%	HDAC4/ZBTB7A/ZBTB20/CBFA2T3/PGAM1/PDK2/ENTPD5/ARNT/EIF6/INSR/FBP1/PDK3/VCP/PINK1/SNCA/FLCN/STAT3/SLC4A1/ENO1
BP	GO:0006970	response to osmotic stress	53	-0.56	-1.73	0.0013162	0.00697763	0.0042839	2970	tags=51%, list=25%, signal=38%	OXSR1/TNF/TP53/MLC1/CAPN3/SLC25A23/TSC22D4/LETM1/PKN1/SLC12A6/NFAT5/ZFP36L1/AQP9/RELB/CLN3/PDPK1/MICU1/ARHGEF2/TSPO/LRRC8A/PTK2B/SLC2A1/MAP2K7/TSC22D3/AQP1/MYLK/YBX3
BP	GO:0032496	response to lipopolysaccharide	223	-0.40	-1.47	0.00131603	0.00697763	0.0042839	2278	tags=30%, list=19%, signal=25%	SCARB1/TRIM5/VIM/EPHB2/IRAK3/TLR2/PTGIR/SOD2/PAF1/TIRAP/PRDX2/NLRP3/SRC/IFNAR1/PRKCE/THBD/CD68/HCK/TGFB1/SIRPA/TNFSF4/MTDH/GSTP1/ARID5A/MAPK14/TRIB1/PYCARD/SBNO2/ALAD/SPI1/CXCL5/AKIRIN2/FOXP1/SASH1/ALPL/PF4V1/NFKB1/TSPO/UPF1/RARA/SELP/BCR/TRAF6/OTUD5/PLSCR3/BMP6/PTAFR/MAPKAPK3/LTF/JUND/PF4/CD14/LYN/LGALS9/CDK19/TNFRSF1B/RHOA/TNIP1/PRPF8/GATA1/MAPK3/SNCA/MAPKAPK2/LITAF/TBXA2R/MAP2K3/RELA
BP	GO:0044262	cellular carbohydrate metabolic process	197	-0.41	-1.49	0.00132067	0.00699201	0.00429272	2137	tags=28%, list=18%, signal=24%	GLB1/FOXO1/PTEN/GUSB/SRC/IRS2/HDAC4/GALK1/PRKCE/EXT1/SYNJ2/PGD/MIDN/B4GALT5/SYNJ1/PHKA2/PER2/CBFA2T3/B3GNT7/PGAM1/LEPR/GSK3A/PPP1CA/DGAT2/PDK2/BCKDK/INSR/SESN2/FBP1/B3GNT8/NCOA2/PFKFB3/PTK2B/INPP5K/PYGL/IGF2/STK11/PYGB/PTAFR/STK40/PDK3/GAA/ADIPOR1/PFKFB4/B4GALT1/NDST1/PLEK/GSK3B/DDB1/ITPK1/HK1/SNCA/STAT3/AKT2/SLC4A1/USP7
CC	GO:0008021	synaptic vesicle	119	-0.45	-1.56	0.00135026	0.00712968	0.00437724	3013	tags=39%, list=26%, signal=30%	DTNBP1/SYT12/RAB35/VTI1A/SEPTIN6/TRAPPC4/RABAC1/APBA1/VTI1B/SV2A/APH1A/NCSTN/MCTP1/SEPTIN4/CLTB/ATP2B1/SNAP25/DISC1/RAB27B/ZNRF1/STX6/RPH3A/LRRK2/NDEL1/SYN3/DLG4/MME/CLCN5/STX4/CLN3/SEPTIN1/RAB7A/RAB5B/CALM3/SLC6A9/DNAJC5/KIF1B/TPRG1L/RAB11B/ROGDI/IQSEC1/SNCA/BCL2L1/LAMP1/ATP6V0D1/STON2/RAB3D
BP	GO:0006826	iron ion transport	40	-0.60	-1.74	0.00135397	0.00713975	0.00438343	782	tags=30%, list=7%, signal=28%	LCN2/CLTC/SLC6A9/LTF/DNM2/RAB11B/FTL/FTH1/MMGT1/SLC48A1/SLC25A37/ARHGAP1

BP	GO:0034110	regulation of homotypic cell-cell adhesion	27	-0.68	-1.82	0.00137521	0.00723256	0.00444041	770	tags=52%, list=7%, signal=49%	UBASH3B/EMILIN2/ANK3/F11R/PLAUR/SH2B3/ABAT/GP6/SYK/ALOX12/LYN/JAK1/ZNF703/DMTN
BP	GO:0043405	regulation of MAP kinase activity	124	-0.46	-1.58	0.00138727	0.00728636	0.00447344	2381	tags=42%, list=20%, signal=34%	DUSP16/MAP4K2/ARHGEF5/DUSP1/SH2B3/ADAM8/IRAK3/TNXB/SPRY3/MAP3K4/SRC/CDK12/MAP2K5/ERBB2/MAGED1/TGFB1/ERN2/ERN1/HGS/GSTP1/LRRK2/STK38/TRIB1/PTPRJ/PTPN6/EPHA4/TRIB3/SASH1/PDGFA/INSR/MAP3K5/TAOK3/SORL1/PTK2B/INPP5K/TRAF6/PAK1/CSK/SFRP2/MAP2K7/PTPRC/SYK/DVL3/THBS1/PTPN1/TLR6/FZD5/PIK3CG/LYN/PDE5A/AKAP13/NF1
MF	GO:0019787	ubiquitin-like protein transferase activity	345	-0.36	-1.40	0.00140565	0.00737315	0.00452672	2617	tags=29%, list=22%, signal=23%	MED24/RC3H1/UBE2S/KLHL21/MED12/ZBED1/UBE2D3/UBE3B/TRIM35/UBE2B/RFFL/AMFR/MGRN1/MIB2/UBA7/HDAC7/TRIM5/MUL1/RNF135/CBX4/SH3RF1/TRIM38/TRAF7/UBL4A/VPS18/ARIH1/ZNRF1/HDAC4/COP1/XIAP/MARCHF3/RFWD3/RNF165/AREL1/RNF144B/GID4/RC3H2/UBR5/RNF213/SH3RF3/UBE2J2/MED27/IRF2BPL/FBXW2/MARCHF6/RNF11/PIAS1/UBE2Z/MYCBP2/ARIH2/DTX2/UBE2M/ITCH/RNF115/HERC2/PML/BIRC6/TRIM28/RNF208/UBE2L3/CNOT4/UBE4B/VPS11/RNF126/NEURL1/HERC1/TRAF6/PIAS4/HECTD4/UBR4/PELI2/TRIP12/UBR2/RBCK1/UBE2R2/FBXO9/WWP2/HUWE1/TRIM11/RMND5A/TRIM14/RNF182/RNF24/TRIM10/RNF187/UBE2O/MARCHF2/SIAH2/TRIM26/DTX3L/MKRN1/RNF40/SHARPIN/TRIM58/RNF10/FBXO7/MARCHF8/RNF123/UBE2H/CBL/CDC34
BP	GO:0045862	positive regulation of proteolysis	281	-0.38	-1.42	0.00141656	0.00742059	0.00455585	2483	tags=34%, list=21%, signal=28%	BAK1/ANXA2/CFLAR/NUB1/GPLD1/EGLN3/SIRT6/NFE2L2/PCOLCE/AIFM1/MUL1/IFI16/USP5/BCL2L13/SH3RF1/ADAM8/BCL2L11/CR1/ATXN3/DISC1/FYN/PTEN/PPM1F/NLRP3/SRC/ARIH1/APAF1/COP1/DET1/RNF144B/SH3RF3/ROCK2/LRRK2/KEAP1/PIAS1/TRIB1/CEBPA/NRDC/AGTPBP1/PYCARD/CBFA2T3/DAP/ARIH2/EPHA4/PML/PSME4/AKIRIN2/TRIB3/GSK3A/NLRC4/ATP2A3/MAP3K5/VSIR/BCAP31/PTK2B/RCN3/LPCAT3/RAD23A/SFRP2/GSN/FZR1/SYK/FIS1/PSME3/IST1/CASP2/VCP/HSPA1A/PSMC4/GABARAP/LYN/HIP1/LGALS9/CYFIP2/LAPTM5/GSK3B/TNFRSF1B/RHOA/TNIP1/BBC3/CLU/GRN/PINK1/SNCA/STAT3/DNAJB2/ENO1/RNF40/CTSD/DAB2/MBP/P2RX1/MYH9/ZER1/FURIN/BAG6
BP	GO:1903351	cellular response to dopamine	38	-0.62	-1.79	0.00143358	0.00747038	0.00458642	3013	tags=61%, list=26%, signal=45%	DTNBP1/GNAS/GNAI3/GNAL/NCSTN/SLC9A3R1/GNA15/SULT1A3/OR10H1/LRRK2/KLF16/VPS35/GSK3A/HRH2/GNB1/FLNA/SIN3A/GSK3B/DNM2/ARRB2/MAPK3/TGM2/GNAZ
MF	GO:0019887	protein kinase regulator activity	154	-0.44	-1.55	0.00143795	0.00748337	0.00459439	1554	tags=25%, list=13%, signal=22%	ELP3/TRIB1/MAP2K2/CDKN1C/DAZAP2/CCNF/TRIB3/CHP1/YWHAB/TAOK3/GREM1/SH3BP5L/CCND3/MOB3A/IGF2/TAOK1/STK11/STRADA/CALM3/HEXIM2/LTF/MADD/MARK2/ITPRIP/RAC2/HSPB1/CCNI/TESC/SNCA/CDKN1A/PRKAR2A/TAL1/PRKAR2B/CCNJL/PAK2/WNK1/IQGAP1/CNPPD1
BP	GO:0019216	regulation of lipid metabolic process	206	-0.39	-1.46	0.00144625	0.0075167	0.00461486	2109	tags=27%, list=18%, signal=22%	ORMDL3/THRA/FGR/EDF1/SRC/IRS2/PRKCE/TGFB1/DGKZ/SCAP/RNF213/ABCB11/GPS2/STAT5B/EGR1/ZBTB20/PIP4K2C/NR1H2/ABHD5/ACADVL/TRIB3/CHP1/DGAT2/PDK2/MTMR3/EIF6/PDGFA/MBOAT7/SLC27A1/NFKB1/TSPO/SORL1/PTK2B/BSCL2/LDLRAP1/LPCAT3/BMP6/TBL1XR1/POR/LAMTOR1/CPT1A/PDK3/ADIPOR1/PIK3CG/RUBCN/LYN/PSAP/SF1/PNPLA2/WDTC1/SNCA/PIP4K2A/AKT2/DAB2/CTDNEP1
BP	GO:1903825	organic acid transmembrane transport	95	-0.49	-1.62	0.00145262	0.00753013	0.0046231	1423	tags=24%, list=12%, signal=21%	PER2/SLC16A3/SLC36A1/AQP9/SLC25A11/CLN3/TSPO2/SLC27A1/LRRC8A/SLC2A1/SLC6A20/SLC3A2/SLC25A38/CPT1A/THBS1/SLC6A9/SLC1A5/SLC43A2/SLC43A1/SLC38A10/AKT2/SLC7A5/SLC38A5

BP	GO:1905039	carboxylic acid transmembrane transport	95	-0.49	-1.62	0.00145262	0.00753013	0.0046231	1423	tags=24%, list=12%, signal=21%	PER2/SLC16A3/SLC36A1/AQP9/SLC25A11/CLN3/TSPO2/SLC27A1/LRRC8A/SLC2A1/SLC6A20/SLC3A2/SLC25A38/CPT1A/THBS1/SLC6A9/SLC1A5/SLC43A2/SLC43A1/SLC38A10/AKT2/SLC7A5/SLC38A5
BP	GO:0003300	cardiac muscle hypertrophy	49	-0.57	-1.73	0.00146866	0.00759351	0.00466201	1715	tags=37%, list=15%, signal=31%	FOXO1/HDAC4/CTDP1/ROCK2/YY1/GSK3A/PPP3CA/PDLIM5/SLC9A1/LMNA/G6PD/PDE5A/AKAP13/TNFRSF1B/ATP2B4/MTPN/PAK2/JARID2
BP	GO:0014897	striated muscle hypertrophy	49	-0.57	-1.73	0.00146866	0.00759351	0.00466201	1715	tags=37%, list=15%, signal=31%	FOXO1/HDAC4/CTDP1/ROCK2/YY1/GSK3A/PPP3CA/PDLIM5/SLC9A1/LMNA/G6PD/PDE5A/AKAP13/TNFRSF1B/ATP2B4/MTPN/PAK2/JARID2
CC	GO:0005905	clathrin-coated pit	57	-0.56	-1.73	0.00147667	0.00761819	0.00467716	2250	tags=44%, list=19%, signal=36%	TF/AAK1/TNK2/CLTB/REPS1/ITSN1/AP2A2/NUMB/ARRB1/AP2B1/SYNJ1/AP2S1/SORT1/LRP10/AP2A1/VLDLR/CLTC/DNM2/LRP1/EPN1/EPS15L1/ARRB2/CTTN/AP2M1/DAB2
BP	GO:0030162	regulation of proteolysis	491	-0.34	-1.36	0.00147727	0.00761819	0.00467716	1553	tags=22%, list=13%, signal=20%	PI3/KEAP1/PIAS1/TRIB1/CEBPA/WAC/NRDC/CAST/UBE2Z/AGTPBP1/PYCARD/CBFA2T3/NR1H2/DAP/ARIH2/ALAD/CLN3/SPOP/ITCH/EPHA4/DHCR24/PML/PSME4/AKIRIN2/TRIB3/BIRC6/GSK3A/PRKACA/PCSK1N/NLRC4/ATP2A3/CSNK2A1/MAP3K5/VSIR/SUFU/SORL1/BCAP31/PRDX5/TIMP2/PTK2B/RCN3/PITHD1/LPCAT3/MAP1A/MMP9/RAD23A/POR/SFRP2/GSN/APLP2/FZR1/SYK/THBS1/FIS1/PSME3/GLG1/IST1/CASP2/LTF/HIPK2/VCP/HSPA1A/PSMC4/GPI/GABARAP/AQP1/LYN/HIP1/LGALS9/CYFIP2/LAPTM5/GSK3B/LRP1/TNFRSF1B/RHOA/TNIP1/GPX1/BBC3/CLU/ARRB2/GRN/GNA12/UBE2O/PINK1/RPS6KA1/SNCA/SIAH2/STAT3/PSMF1/N4BP1/UBB/DNAJB2/USP7/ARAF/ENO1/CTSB/RNF40/PAK2/CTSD/DAB2/MBP/P2RX1/MYH9/TIMP1/ZER1/FURIN/BAG6/RYBP
BP	GO:1904375	regulation of protein localization to cell periphery	85	-0.49	-1.60	0.00148072	0.00762607	0.004682	1899	tags=31%, list=16%, signal=26%	PRKCE/TGFB1/ZDHHC7/NUMB/EZR/TMBIM1/STX4/CLN3/ABI3/PDPK1/NUMA1/SPTBN1/LDLRAP1/CSK/CLTC/ACTB/LRP1/ATP2B4/GRIPAP1/PPP1R9B/EPB41/BCL2L1/SQSTM1/AP2M1/DAB2/RHOG
MF	GO:0051213	dioxygenase activity	69	-0.52	-1.66	0.0014889	0.00765831	0.00470179	2533	tags=38%, list=22%, signal=30%	KDM6A/KDM7A/KDM4A/TET2/ALOX5/EGLN3/RSBN1/ALKBH5/PHF2/KDM5B/PHF8/FTO/JMJD6/PLOD1/KDM3B/KDM8/KDM2A/HIF1AN/ALOX5AP/PTGS1/POR/HGD/TET3/ALOX12/KDM6B/KDM4B
MF	GO:0060090	molecular adaptor activity	301	-0.37	-1.41	0.00149912	0.00769096	0.00472184	2211	tags=27%, list=19%, signal=23%	SH2B3/MAVS/MMS19/SH3RF1/VPS18/SEPTIN10/CBX5/SLA2/SNAP25/DISC1/WWC3/GATAD2A/TIRAP/ITSN1/BICD2/AP1G1/COX15/EPS8/STX6/DET1/SUN2/AP2A2/BCL3/DLGAP4/SDCBP/SYNE2/LRRK2/TMEM250/AP2B1/AP2S1/PAG1/SH2B2/SHC1/STX4/PEF1/MAP2K2/ST13/AP1M1/SPATA2/DEDD2/TJP2/GRB2/SASH1/OPTN/SEPTIN1/STX5/VPS11/AP2A1/ADAP2/GAB2/LDLRAP1/MAP1A/FRMD4A/LAMTOR1/SLC9A1/GNB1/ATP1B2/DAW1/EIF4G1/ANKRD9/HIP1/ZC3H18/AKAP13/DDB1/CUL4A/EIF3B/GAS2L1/DCAF7/ARPC4/STAT3/NAPA/SLC4A1/AP2M1/STON2/DCAF12/DAB2/CNOT1/FBXO7/MYH9/IQGAP1/ANK1/HCFC1
BP	GO:1904064	positive regulation of cation transmembrane transport	78	-0.51	-1.65	0.00150135	0.0076925	0.00472278	1286	tags=26%, list=11%, signal=23%	KCNH2/PDPK1/CHP1/HTT/SLC9A1/ATP1B2/EHD3/CALM3/SLC6A9/G6PD/FLNA/GJC2/DNM2/ACTN4/PINK1/TESC/SNCA/STIM1/WNK1/P2RX1
BP	GO:0060291	long-term synaptic potentiation	45	-0.58	-1.74	0.00151632	0.00773925	0.00475149	2232	tags=33%, list=19%, signal=27%	EPHB2/SNAP25/PTEN/MME/STX4/EPHA4/SHISA7/PTK2B/GSK3B/TSHZ3/STAU1/NF1/NRGN/SNCA/SQSTM1

BP	GO:1901880	negative regulation of protein depolymerization	53	-0.56	-1.72	0.0015159	0.00773925	0.00475149	2764	tags=51%, list=24%, signal=39%	TMOD3/CARMIL1/ADD2/CLASP1/HDAC6/TWF2/SCIN/FLII/KATNB1/TRIOBP/ATXN7/EPS8/SPTAN1/CAMSAP1/WASHC2C/ARHGEF2/SPTBN1/SPTA1/SVIL/TAOK1/GSN/CAPZB/ADD1/TMOD1/SPTB/MTPN/DMTN
BP	GO:0010595	positive regulation of endothelial cell migration	72	-0.53	-1.67	0.00156149	0.00795957	0.00488675	2466	tags=44%, list=21%, signal=35%	RIN2/CALR/ATP5F1B/GPLD1/ADAM17/NFE2L2/SCARB1/HDAC7/ATOH8/TGFB1/RRAS/ROCK2/PDPK1/FOXP1/SASH1/PTK2B/BCAS3/PRKD2/THBS1/PIK3CG/GFUS/GPI/RAC1/MAP3K3/STAT5A/HSPB1/SP1/GRN/SPARC/PIK3CD/ITGB3/MAP2K3
BP	GO:0038066	p38MAPK cascade	42	-0.59	-1.73	0.00159638	0.00811661	0.00498317	2266	tags=40%, list=19%, signal=33%	DUSP1/MAP3K4/PER1/HGF/EZR/PHLPP1/MAPK14/ZFP36L1/SPI1/NCF1/SASH1/MAP3K5/MFHAS1/LGALS9/MAP3K3/MAPKAPK2/MAP2K3
BP	GO:0060079	excitatory postsynaptic potential	44	-0.60	-1.76	0.00159638	0.00811661	0.00498317	2038	tags=30%, list=17%, signal=25%	PTEN/GRIK2/LRRK2/DLG4/S1PR2/CHRNE/GSK3A/PTK2B/PPP3CA/GSK3B/ARRB2/SNCA/P2RX1
BP	GO:0043406	positive regulation of MAP kinase activity	80	-0.51	-1.65	0.00162572	0.00824474	0.00506183	2329	tags=41%, list=20%, signal=33%	MAP4K2/ARHGEF5/ADAM8/MAP3K4/SRC/MAP2K5/ERBB2/MAGED1/TGFB1/ERN2/ERN1/LRRK2/EPHA4/SASH1/PDGFA/INSR/MAP3K5/TAOK3/PTK2B/TRAF6/PAK1/CSK/MAP2K7/PTPRC/SYK/DVL3/THBS1/PTPN1/TLR6/FZD5/PIK3CG/PDE5A/AKAP13
BP	GO:0045926	negative regulation of growth	142	-0.43	-1.53	0.00164087	0.00828981	0.0050895	2932	tags=37%, list=25%, signal=28%	PPP2R1A/VGLL4/HSPA1B/TP53/PSRC1/ACVR1B/SMAD3/EI24/FHL1/ARHGAP4/CCN3/KIAA0319/DIP2B/MUL1/BCL11A/CDKN2D/WWC3/PTEN/STK4/TGFB1/CTDP1/TCHP/BTG1/SMARCA4/MT2A/YY1/PTPRJ/MSX1/SEMA4D/NAIF1/PML/SMARCA2/GSK3A/SESN2/FBP1/GREM1/SEMA4A/NDRG3/SFRP2/STK11/ADIPOR1/G6PD/ULK1/HSPA1A/SIPA1/CDA/PPP1R9B/CDKN1A/FLCN/DNAJB2/ENO1/DAB2/JARID2
BP	GO:0043255	regulation of carbohydrate biosynthetic process	66	-0.53	-1.67	0.00165957	0.00835248	0.00512798	1174	tags=33%, list=10%, signal=30%	LEPR/GSK3A/PPP1CA/DGAT2/PDK2/INSR/SESN2/FBP1/NFKB1/AP2A1/PTK2B/INPP5K/IGF2/STK11/PTAFR/CLTC/PLEK/GSK3B/DDB1/SNCA/AKT2/USP7
BP	GO:0030218	erythrocyte differentiation	110	-0.47	-1.59	0.00166458	0.00835654	0.00513047	1612	tags=34%, list=14%, signal=29%	STAT5B/KAT7/MAPK14/ZFP36L1/SPI1/TSPO2/KLF13/ARNT/MFHAS1/SLC25A38/ADAR/MED1/MAFB/HIPK2/G6PD/JAK3/HSPA1A/INPP5D/LYN/BPGM/GATA1/AHSP/HCLS1/TRIM10/STAT3/TAL1/SLC4A1/FAM210B/EPB42/IKZF1/PRKDC/KLF1/MPIG6B/ALAS2/TRIM58/FOXO3/DMTN
BP	GO:0055065	metal ion homeostasis	382	-0.35	-1.36	0.00166283	0.00835654	0.00513047	2254	tags=27%, list=19%, signal=23%	IREB2/TF/ACO1/HMOX2/LETM1/ATP2B1/CNNM3/GNA15/PTGIR/SOD2/TTC7A/KNG1/DISC1/COX19/FYN/TPCN1/KCTD17/PDZD8/FKBP1B/SLC24A4/MCOLN1/PRKCE/SPPL3/XIAP/GRIK2/PROK2/EXT1/SLC39A7/VDR/ATP7A/ATP13A1/SLC12A6/OPRL1/TMC8/CMKLR1/DLG4/C5AR1/MT2A/KCNH2/PTPN6/TMBIM6/CLN3/PDPK1/PML/MICU1/CCR1/ATP6AP1/ITPR2/ARF1/PRKACA/CHERP/ATP2A3/ERO1A/NCOA4/TMEM38A/BCAP31/HTT/PTK2B/SLC8A1/SNX10/CXCR2/SLC24A3/BMP6/LCN2/PTPRC/SLC9A1/FIS1/SELENON/ATP1B2/CALM3/SLC6A9/LTF/PIK3CG/JAK3/ANKRD9/ANXA6/LYN/GP9/ATP2B4/GNAI2/FKBP1A/FTL/PRKCB/GATA1/CXCR1/ATP6V1A/FTH1/MLLT6/SNCA/TGM2/GRINA/GP1BA/ATP6V0D1/EPB42/SLC25A37/STIM1/ALAS2/KEL/P2RX1/TBXA2R/EIF2AK1/XK/DIAPH1
BP	GO:1902893	regulation of miRNA transcription	40	-0.59	-1.72	0.00167254	0.00838591	0.0051485	2910	tags=52%, list=25%, signal=40%	TNF/TP53/SMAD3/SMAD1/MRTFA/FOS/SRF/ATOH8/TGFB1/SMARCA4/EGR1/YY1/NR1H2/SPI1/RARA/KLF5/NR3C1/NFATC3/STAT3/FOXO3/RELA

BP	GO:1990266	neutrophil migration	80	-0.51	-1.65	0.00167903	0.00840781	0.00516195	1537	tags=31%, list=13%, signal=27%	PIKFYVE/C5AR1/TREM1/PREX1/ITGB2/CXCL5/WDR1/PIP5K1C/PF4V1/CXCR2/PECAM1/VAV1/SYK/JAM3/PIK3CG/CAMK1D/PF4/RAC1/RAC2/CXCR1/RIPOR2/PIK3CD/BSG/MPP1/RHOG
BP	GO:0006109	regulation of carbohydrate metabolic process	126	-0.45	-1.55	0.00168794	0.00842065	0.00516983	2002	tags=33%, list=17%, signal=27%	SRC/IRS2/HDAC4/PRKCE/TGFB1/ZBTB7A/MIDN/ZBTB20/CBFA2T3/PGAM1/LEPR/GSK3A/PPP1CA/DGAT2/PDK2/ENTPD5/ARNT/EIF6/BCKDK/INSR/SESN2/FBP1/NFKB1/AP2A1/NCOA2/PTK2B/INPP5K/IGF2/STK11/PTAFR/PDK3/ADIPOR1/CLTC/PLEK/GSK3B/DDB1/SNCA/STAT3/AKT2/SLC4A1/USP7
BP	GO:0043161	proteasome-mediated ubiquitin-dependent protein catabolic process	370	-0.36	-1.38	0.00168742	0.00842065	0.00516983	1818	tags=23%, list=16%, signal=20%	DET1/AREL1/RNF144B/GID4/SDCBP/UBXN7/HSPA5/SH3RF3/UBE2J2/SMARCC1/ARRB1/LRRK2/MAN1A1/PSMD2/KEAP1/MARCHF6/TMEM67/PIAS1/TRIB1/CEBPA/WAC/YOD1/CBFA2T3/ARIH2/SPOP/ITCH/KCTD2/HERC2/CCNF/PML/KAT5/TRIB3/GSK3A/CANX/PSMD1/FBXL18/UBE4B/BCAP31/RNF126/TBX21/NSFL1C/MAP1A/TBL1XR1/RAD23A/FZR1/NPLOC4/FBXL20/TRIP12/VCP/HSPA1A/ANKRD9/UBR2/PSMC4/GABARAP/RBCK1/GSK3B/FBXO9/WWP2/DDB1/HUWE1/CUL4A/RMND5A/TBL1X/GID8/CLU/ARRB2/GNA12/RNF187/ASCC2/SIAH2/PCBP2/PSMF1/N4BP1/DNAJB2/USP7/ARAF/SHARPIN/DCAF12/DAB2/UBE2H/CDC34/ZER1/BAG6/RYBP/DCAF11
BP	GO:0050776	regulation of immune response	627	-0.33	-1.33	0.00168698	0.00842065	0.00516983	2425	tags=26%, list=21%, signal=22%	FPR3/SPN/GPLD1/CD38/BTN3A2/EPG5/NCKAP1L/DDX58/NFE2L2/CR1L/USP27X/MAP2K4/TRIM5/C1QA/MUL1/TAPBP/IFI16/KIR2DL1/EPHB2/RNF135/PKN1/MAVS/ADAM8/MYO1G/MICB/FCER1G/IRAK3/TLR2/SERPING1/RFTN1/SLA2/CR1/TLR5/FGR/FYN/TIRAP/UNC13D/NLRP3/SRC/CD177/ANKRD17/NINJ1/PRKCE/TNFSF13/SPPL3/XIAP/XRCC6/AP1G1/HCK/TGFB1/DGKZ/ADGRE2/PUM1/TNFSF4/RC3H2/LSM14A/ITGAM/IKBKE/FYB1/GPS2/STAT5B/EZR/OASL/ARID5A/CMKLR1/ALPK1/SH2B2/C5AR1/CD300LF/MAPK14/STX4/PTPRJ/FLOT1/PTPN6/PYCARD/MAD2L2/NR1H2/SPI1/ITCH/PDPK1/NCF1/ITGB2/AKIRIN2/KLRD1/FOXP1/ERMAP/NLRC4/YTHDF3/SPG21/VSIR/NFAM1/FES/NFATC2/IKBKG/IRF1/PGLYRP1/RARA/MFHAS1/GAB2/BCR/STAT6/TRAF6/PRKD2/TBX21/PAK1/CSK/VAV1/STK11/MAP2K7/PTPRC/SYK/ADAR/PTAFR/PTPN1/NPLOC4/SUPT6H/MED1/TLR6/MAPKAPK3/NLRX1/RBM14/FZD5/LTF/PRAM1/JAK3/HSPA1A/INPP5D/CD14/LYN/RIOK3/LGALS9/RBCK1/SIN3A/LAPTM5/RAC2/CREBBP/TNFRSF1B/FKBP1A/TNIP1/PRKCB/GATA1/GPX1/CLU/ARRB2/GRN/WAS/ZNFX1/SEC14L1/LAMP1/SLC15A3/MAPKAPK2/N4BP1/PIK3CD/PRKDC/PAK2/KRT1/WNK1/HLX/FURIN/BAG6/RELA
BP	GO:0045648	positive regulation of erythrocyte differentiation	29	-0.65	-1.78	0.00170009	0.00847061	0.0052005	1612	tags=45%, list=14%, signal=39%	STAT5B/KAT7/MAPK14/ARNT/MED1/HSPA1A/INPP5D/GATA1/STAT3/TAL1/FAM210B/PRKDC/FOXO3
BP	GO:0046850	regulation of bone remodeling	28	-0.66	-1.79	0.0017333	0.00861449	0.00528884	2439	tags=50%, list=21%, signal=40%	GPR137/CD38/TF/ADAM8/SRC/PPARGC1B/CSF1R/LEPR/GREM1/CSK/SYK/INPP5D/NF1/ITGB3
BP	GO:1990868	response to chemokine	51	-0.57	-1.71	0.00174673	0.00865959	0.00531653	1244	tags=25%, list=11%, signal=23%	CXCL5/CCR1/PF4V1/DOCK8/PTK2B/CXCR2/PF4/MPL/RHOA/CXCR1/RIPOR2/ACKR1/WNK1
BP	GO:1990869	cellular response to chemokine	51	-0.57	-1.71	0.00174673	0.00865959	0.00531653	1244	tags=25%, list=11%, signal=23%	CXCL5/CCR1/PF4V1/DOCK8/PTK2B/CXCR2/PF4/MPL/RHOA/CXCR1/RIPOR2/ACKR1/WNK1

MF	GO:0015171	amino acid transmembrane transporter activity	47	-0.59	-1.76	0.00176911	0.00873787	0.00536459	911	tags=34%, list=8%, signal=32%	SERINC5/SLC1A4/SLC9A3R1/SLC25A15/SLC66A1/SLC36A1/SLC6A20/SLC3A2/SLC25A38/SLC6A9/SLC1A5/SLC43A2/SLC43A1/SLC38A10/SLC7A5/SLC38A5
BP	GO:0009267	cellular response to starvation	126	-0.45	-1.55	0.00177379	0.00873928	0.00536545	2010	tags=34%, list=17%, signal=29%	NFE2L2/MAP1LC3B2/IFI16/FOXO1/RRAGD/SH3GLB1/GCN1/LARS1/CPEB4/LAMP2/SZT2/HSPA5/LRRK2/TFEB/EIF2AK2/DAP/VPS41/CLEC16A/MAP1LC3B/MTMR3/SESN3/SESN2/STK24/ZFYVE1/MAP3K5/SLC2A1/RRAGC/MAX/WDR45/ATG7/WIPI2/RIPOR1/UCP2/GABARAP/ELAPOR1/GAS2L1/MAPK3/CDKN1A/NUAK2/FLCN/GLUL/NPRL3/FOXO3
BP	GO:1904705	regulation of vascular associated smooth muscle cell proliferation	33	-0.64	-1.78	0.00180414	0.00885589	0.00543704	2096	tags=42%, list=18%, signal=35%	SOD2/PTEN/ERN1/S1PR2/MAP3K5/TPM1/LDLRAP1/MMP9/GNAI2/RHOA/FOXJ2/GNA12/CDKN1A/MFN2
BP	GO:0002764	immune response-regulating signaling pathway	345	-0.36	-1.39	0.00185347	0.00905439	0.00555891	2425	tags=26%, list=21%, signal=21%	FPR3/GPLD1/CD38/BTN3A2/EPG5/NCKAP1L/DDX58/MAP2K4/KIR2DL1/RNF135/MYO1G/MICB/FCER1G/IRAK3/TLR2/RFTN1/SLA2/CR1/TLR5/FGR/FYN/TIRAP/SRC/ANKRD17/NINJ1/PRKCE/SPPL3/XIAP/HCK/DGKZ/PUM1/RC3H2/LSM14A/FYB1/GPS2/EZR/OASL/CMKLR1/ALPK1/SH2B2/C5AR1/CD300LF/PTPRJ/FLOT1/PTPN6/ITCH/PDPK1/KLRD1/FOXP1/ERMAP/SPG21/NFAM1/NFATC2/IKBKG/IRF1/MFHAS1/TRAF6/PRKD2/PAK1/CSK/VAV1/STK11/MAP2K7/PTPRC/SYK/NPLOC4/TLR6/MAPKAPK3/NLRX1/LTF/PRAM1/HSPA1A/INPP5D/CD14/LYN/RIOK3/RBCK1/LAPTM5/CREBBP/TNIP1/PRKCB/ARRB2/SEC14L1/SLC15A3/MAPKAPK2/PIK3CD/PAK2/WNK1/BAG6/RELA
BP	GO:0031214	biomineral tissue development	81	-0.49	-1.58	0.00184986	0.00905439	0.00555891	2404	tags=37%, list=20%, signal=30%	ALOX5/TUFT1/TENT5A/SLC20A1/AXIN2/ATP2B1/FGR/SLC24A4/TGFB1/ANO6/NECTIN1/ROCK2/NBR1/SBNO2/CCR1/ALPL/GREM1/PTK2B/SLC8A1/SNX10/ANKH/SLC24A3/BMP6/BMP2K/PHOSPHO1/LTF/RFLNB/GATA1/TFIP11/STIM1
BP	GO:0030511	positive regulation of transforming growth factor beta receptor signaling pathway	26	-0.69	-1.84	0.00186074	0.00906656	0.00556639	1719	tags=38%, list=15%, signal=33%	SDCBP/CDKN1C/TGFB1I1/STK11/THBS1/HIPK2/CREBBP/LRG1/FLCN/DAB2
BP	GO:1903846	positive regulation of cellular response to transforming growth factor beta stimulus	26	-0.69	-1.84	0.00186074	0.00906656	0.00556639	1719	tags=38%, list=15%, signal=33%	SDCBP/CDKN1C/TGFB1I1/STK11/THBS1/HIPK2/CREBBP/LRG1/FLCN/DAB2
BP	GO:1902117	positive regulation of organelle assembly	69	-0.52	-1.65	0.00186807	0.00909111	0.00558146	2112	tags=41%, list=18%, signal=33%	FSCN1/RALB/ARHGEF5/VPS4B/SRC/KCTD17/SH3GLB1/SDC4/G3BP1/SDCBP/PIP4K2C/FUZ/NUMA1/LCP1/CNOT6/HTT/DYNC1H1/MARK4/WDR45/ASAP1/ULK1/GSK3B/ELAPOR1/RHOA/ATG2A/MSN/PIP4K2A/CNOT1

BP	GO:0009617	response to bacterium	372	-0.35	-1.38	0.00187917	0.00912279	0.00560091	2278	tags=28%, list=19%, signal=23%	SCARB1/TRIM5/TF/VIM/EPHB2/MAVS/FCER1G/IRAK3/TLR2/H2BC21/PTGIR/SOD2/TLR5/PAF1/FGR/TIRAP/PRDX2/NLRP3/SRC/ANKRD17/IFNAR1/PRKCE/THBD/CD68/LYST/HCK/TGFB1/SIRPA/SNX3/TNFSF4/BCL3/NOTCH2/RNF213/MTDH/GSTP1/ARID5A/PI3/C5AR1/SHC1/MAPK14/TRIB1/PYCARD/ST13/SBNO2/DCD/ALAD/TREM1/SPI1/CXCL5/AKIRIN2/FOXP1/SASH1/OPTN/ALPL/NLRC4/PF4V1/NFKB1/RBPJ/TSPO/UPF1/PGLYRP1/RARA/SELP/BCR/TRAF6/OTUD5/PYGL/PLSCR3/IL6R/RAB14/BMP6/LCN2/SYK/PTAFR/TLR6/MAPKAPK3/FZD5/LTF/JUND/PF4/CD14/AQP1/LYN/LGALS9/RBCK1/CDK19/TNFRSF1B/RHOA/TNIP1/PRPF8/GATA1/GPX1/LRG1/GRN/MARCHF2/ZNFX1/MAPK3/SNCA/H2BC11/MAPKAPK2/LITAF/SHARPIN/TBXA2R/MAP2K3/RELA
BP	GO:0072583	clathrin-dependent endocytosis	40	-0.59	-1.71	0.00190315	0.00922795	0.00566547	2239	tags=42%, list=19%, signal=35%	AAK1/TNK2/CLTB/ITSN1/AP2A2/AP2B1/AP2S1/PIP5K1C/CANX/AP2A1/BMP2K/CLTC/HIP1/DNM2/AP2M1/GAK/DAB2
BP	GO:0050679	positive regulation of epithelial cell proliferation	91	-0.49	-1.61	0.00192386	0.00931703	0.00572016	1915	tags=27%, list=16%, signal=23%	KDM5B/MAP2K5/ERBB2/ATP7A/NOTCH2/C5AR1/PDPK1/GPBAR1/ARNT/PRKD2/IGF2/BMP6/MED1/B4GALT1/STAT5A/TCF7L2/NF1/SP1/LRG1/GRN/STAT3/ZNF703/PIK3CD/ITGB3/GLUL
BP	GO:0014896	muscle hypertrophy	50	-0.57	-1.74	0.0019368	0.00936827	0.00575162	1715	tags=36%, list=15%, signal=31%	FOXO1/HDAC4/CTDP1/ROCK2/YY1/GSK3A/PPP3CA/PDLIM5/SLC9A1/LMNA/G6PD/PDE5A/AKAP13/TNFRSF1B/ATP2B4/MTPN/PAK2/JARID2
BP	GO:0010639	negative regulation of organelle organization	264	-0.38	-1.42	0.00194058	0.00937513	0.00575583	2660	tags=32%, list=23%, signal=25%	CARMIL1/MAK/ADD2/HNRNPU/CLASP1/HDAC6/TWF2/PPIF/TERF2IP/TBCD/CORO1A/PPFIA1/TOM1L2/SCIN/DUSP1/KAT2B/MUL1/KLHL22/FLII/KATNB1/LIMK2/FEZ1/SRC/TRIOBP/ATXN7/ATM/H3-3B/SMG6/H3-3A/EPS8/WAPL/TSC2/TCHP/LRRK2/HGF/SPTAN1/RAD21/TMEM67/CHMP4B/CAMSAP1/PHF23/EML2/WASHC2C/MAD2L2/ARAP1/MAP4/CCNF/ARHGEF2/EVI5L/SSH1/TENT4B/SPTBN1/TSPO/SPTA1/INPP5K/SVIL/RAB7A/TAOK1/GSN/LMNA/RBM14/HSPA1A/GNL3L/DNM2/HUWE1/ARHGAP6/CAPZB/MYADM/GPX1/CLU/ARRB2/ADD1/SSH2/TERF2/TMOD1/WAS/PINK1/SNCA/SPTB/BCL2L1/FLCN/MTPN/PAK2/WASF2/DMTN
BP	GO:1903829	positive regulation of protein localization	332	-0.36	-1.40	0.00195001	0.00939789	0.0057698	2232	tags=27%, list=19%, signal=22%	EPHB2/HDAC3/MAVS/ADAM8/TLR2/ORMDL3/HSPA1L/FYN/LIMK2/SRC/IRS2/SH3GLB1/ABAT/MYO18A/PRKCE/ERBB2/USP36/TGFB1/NPEPPS/UBR5/ROCK2/UBE2J2/ITGAM/AP2B1/EZR/DLG4/KAT7/MAPK14/STX4/NR1H2/ARIH2/MYOM1/CLN3/VPS35/PDPK1/ITGB2/NUMA1/TRIM28/GSK3A/UBE2L3/CHP1/PRKACA/SESN2/SPTBN1/VPS11/MCRS1/RBM22/SORL1/BCAP31/LRRC8A/BCAS3/MARK4/MAP1A/FRMD4A/PECAM1/BMP6/STK11/FIS1/FZD5/FLNA/RIPOR1/VPS28/GNL3L/GSK3B/RAC2/LRP1/SYTL4/TCF7L2/HUWE1/ATP2B4/MEPCE/BBC3/MSN/PPP1R9B/HCLS1/OAZ1/ATG13/PINK1/EPB41/DTX3L/PTPN23/ABLIM3/SQSTM1/AKT2/PTP4A3/PPM1A/GLUL/RHOG
BP	GO:0048017	inositol lipid-mediated signaling	115	-0.47	-1.60	0.00197307	0.00949747	0.00583094	2910	tags=36%, list=25%, signal=27%	TNF/PLEKHA1/PTPN13/PI4KB/NKX3-1/PIK3CB/PLCE1/KIT/FBXL2/PLCB4/SLC9A3R1/PLCD1/FGR/PTEN/SRC/ERBB2/TSC2/HGF/PLCB1/EZR/ZFP36L1/PTPN6/SEMA4D/NCF1/CSF1R/PIP5K1C/ENTPD5/PDGFA/INSR/PITPNM2/ADAP2/GAB2/SELP/PLCB3/PTAFR/PIK3CG/NF1/HCLS1/PIK3CD/PRR5/CBL
BP	GO:0009185	ribonucleoside diphosphate metabolic process	89	-0.49	-1.62	0.00199536	0.00958158	0.00588258	1960	tags=29%, list=17%, signal=25%	ENO2/AK4/HDAC4/GALK1/ZBTB7A/RRM1/ZBTB20/CBFA2T3/PGAM1/TJP2/ENTPD5/ARNT/EIF6/PKM/INSR/FBP1/ALDOA/GPI/AK1/BPGM/HK1/STAT3/GUK1/SLC4A1/ENO1/MPP1

BP	GO:1903034	regulation of response to wounding	91	-0.48	-1.60	0.00199446	0.00958158	0.00588258	2567	tags=36%, list=22%, signal=29%	CLASP1/INSL3/KIAA0319/ALOX5/SIGLEC10/NFE2L2/EPHB2/SH2B3/SERPING1/KNG1/PTEN/PRDX2/FKBP1B/ABAT/PRKCE/THBD/ATP7A/ANO6/ACTG1/NDEL1/ACTA2/EPHA4/PDGFA/STK24/THBS1/FLNA/ALOX12/GRN/MYLK/GP1BA/KRT1/TBXA2R/DMTN
BP	GO:0051494	negative regulation of cytoskeleton organization	109	-0.46	-1.57	0.00199912	0.00958284	0.00588335	2764	tags=42%, list=24%, signal=33%	TMOD3/F11R/CARMIL1/ADD2/CLASP1/HDAC6/TWF2/TBCD/CORO1A/PPFIA1/SCIN/KAT2B/FLII/KATNB1/TRIOBP/ATXN7/EPS8/SPTAN1/TMEM67/CAMSAP1/EML2/WASHC2C/ARAP1/CCNF/ARHGEF2/SSH1/SPTBN1/SPTA1/INPP5K/SVIL/TAOK1/GSN/RBM14/ARHGAP6/CAPZB/MYADM/ADD1/SSH2/TMOD1/WAS/SNCA/SPTB/MTPN/PAK2/WASF2/DMTN
BP	GO:0007189	adenylate cyclase-activating G protein-coupled receptor signaling pathway	56	-0.55	-1.67	0.00207021	0.00988134	0.00606662	1782	tags=29%, list=15%, signal=24%	ADGRE2/PDE4A/OPRL1/S1PR2/ADGRG3/GNAQ/GSK3A/ADGRL1/RAPGEF2/GNB1/PF4/ADGRE5/AKAP13/ATP2B4/GNAI2/TBXA2R
BP	GO:0006873	cellular ion homeostasis	395	-0.35	-1.36	0.002073	0.00988281	0.00606752	2102	tags=24%, list=18%, signal=20%	GNA15/PTGIR/TTC7A/KNG1/DISC1/COX19/FYN/TPCN1/KCTD17/PDZD8/FKBP1B/SLC24A4/MCOLN1/PRKCE/SPPL3/GRIK2/PROK2/SLC39A7/VDR/ATP7A/ATP13A1/LRRK2/OPRL1/CMKLR1/DLG4/C5AR1/CA2/MT2A/PTPN6/CKB/HVCN1/TMBIM6/CLN3/PDPK1/PML/MICU1/CCR1/ATP6AP1/ITPR2/ARF1/CHP1/PRKACA/CHERP/ATP6V0A1/ATP2A3/ERO1A/NCOA4/TMEM38A/BCAP31/HTT/PTK2B/SLC8A1/CXCR2/SLC24A3/RAB7A/BMP6/LCN2/PTPRC/SLC9A1/FIS1/SELENON/ATP1B2/CALM3/SLC6A9/PIK3CG/JAK3/ANKRD9/ANXA6/LYN/GP9/ATP2B4/FKBP1A/FTL/PRKCB/GATA1/CXCR1/ATP6V1A/FTH1/GRN/MAPK3/SNCA/TGM2/GRINA/SLC4A1/GP1BA/ATP6V0D1/STIM1/ALAS2/KEL/P2RX1/TBXA2R/XK/DIAPH1
BP	GO:0042594	response to starvation	148	-0.44	-1.56	0.00209234	0.00996307	0.00611679	2010	tags=30%, list=17%, signal=26%	RRAGD/SH3GLB1/GCN1/LARS1/CPEB4/LAMP2/SZT2/HSPA5/LRRK2/TFEB/EIF2AK2/DAP/VPS41/CLEC16A/MAP1LC3B/MTMR3/SESN3/SESN2/STK24/ZFYVE1/MAP3K5/SLC2A1/RRAGC/BCAS3/MAX/WDR45/ATG7/WIPI2/ULK1/RIPOR1/UCP2/GABARAP/ELAPOR1/GAS2L1/LARP1/MAPK3/CDKN1A/NUAK2/FLCN/TBC1D5/GLUL/NPRL3/FOXO3/CBL/EIF2AK1
BP	GO:0015807	L-amino acid transport	40	-0.58	-1.70	0.00211658	0.01003042	0.00615814	2424	tags=35%, list=21%, signal=28%	SERINC5/SLC1A4/SLC25A15/SLC66A1/PER2/SLC36A1/CLN3/SLC6A20/SLC3A2/SLC1A5/SLC43A2/SLC43A1/SLC7A5/SLC38A5
BP	GO:1900024	regulation of substrate adhesion-dependent cell spreading	40	-0.58	-1.69	0.00211658	0.01003042	0.00615814	2676	tags=48%, list=23%, signal=37%	ST6GAL1/CARMIL1/CALR/LIMS1/UNC13D/TRIOBP/CASS4/DOCK5/WASHC2C/PREX1/NEDD9/CORO1C/FLNA/RAC1/DNM2/MYADM/ACTN4/DAB2/DMTN
CC	GO:0005901	caveola	50	-0.57	-1.73	0.00215401	0.01017141	0.0062447	2002	tags=38%, list=17%, signal=32%	HDAC6/MYOF/SCARB1/SRC/HCK/LRRK2/LIPE/FLOT1/CLN3/INSR/CORO1C/SLC2A1/CAVIN2/CTNNA1/FLOT2/ATP2B4/MAPK3/PLVAP/CBL
BP	GO:0010508	positive regulation of autophagy	116	-0.45	-1.55	0.00216073	0.01017897	0.00624935	2175	tags=36%, list=19%, signal=30%	TRIM38/ORMDL3/FOXO1/BCL2L11/TPCN1/SH3GLB1/ATF6/VPS13D/TSC2/MTDH/LRRK2/SPTLC2/TFEB/PIP4K2C/PLEKHF1/WAC/KAT5/GSK3A/OPTN/SESN3/SESN2/PIM2/IKBKG/HTT/PAFAH1B2/STK11/WDR45/ULK1/GSK3B/ELAPOR1/BNIP3L/SUPT5H/TRIM14/ATG2A/LARP1/PINK1/MAPK3/FLCN/PIP4K2A/FBXO7/NPRL3/FOXO3

BP	GO:0035265	organ growth	82	-0.50	-1.61	0.00217931	0.01024407	0.00628931	2089	tags=33%, list=18%, signal=27%	BCL2L11/HEG1/WWC3/PTEN/STK4/EXT1/CTDP1/MAPK14/YY1/BNC2/LEPR/GSK3A/RBPJ/RARA/ARID2/IGF2/POR/PDLIM5/G6PD/BASP1/ANXA6/PSAP/AKAP13/PIM1/YBX3/JARID2/HLX
BP	GO:1903076	regulation of protein localization to plasma membrane	71	-0.51	-1.62	0.0021797	0.01024407	0.00628931	1899	tags=34%, list=16%, signal=29%	PRKCE/TGFB1/ZDHHC7/NUMB/EZR/TMBIM1/STX4/CLN3/ABI3/PDPK1/SPTBN1/LDLRAP1/CSK/CLTC/ACTB/LRP1/ATP2B4/GRIPAP1/PPP1R9B/BCL2L1/SQSTM1/AP2M1/DAB2/RHOG
BP	GO:0043270	positive regulation of ion transport	151	-0.43	-1.51	0.0022119	0.01037088	0.00636717	1301	tags=19%, list=11%, signal=17%	PTGES/PDPK1/CCR1/CHP1/TSPO/HTT/SLC9A1/PTAFR/SCN1B/ATP1B2/EHD3/CALM3/SLC6A9/G6PD/FLNA/JAK3/GJC2/DNM2/ARRB2/ACTN4/PINK1/MLLT6/TESC/MYLK/SNCA/AHCYL1/STIM1/WNK1/P2RX1
MF	GO:0030674	protein-macromolecule adaptor activity	246	-0.38	-1.44	0.00232552	0.01083972	0.00665501	2211	tags=28%, list=19%, signal=23%	SH2B3/MAVS/MMS19/SH3RF1/VPS18/CBX5/SLA2/SNAP25/GATAD2A/TIRAP/BICD2/AP1G1/EPS8/STX6/DET1/SUN2/AP2A2/BCL3/SDCBP/SYNE2/LRRK2/AP2B1/AP2S1/PAG1/SH2B2/SHC1/STX4/PEF1/MAP2K2/ST13/AP1M1/SPATA2/DEDD2/TJP2/GRB2/OPTN/STX5/VPS11/AP2A1/ADAP2/GAB2/LDLRAP1/MAP1A/FRMD4A/SLC9A1/GNB1/ATP1B2/DAW1/ANKRD9/HIP1/ZC3H18/AKAP13/DDB1/CUL4A/GAS2L1/DCAF7/ARPC4/STAT3/NAPA/SLC4A1/AP2M1/STON2/DCAF12/DAB2/FBXO7/MYH9/IQGAP1/ANK1/HCFC1
BP	GO:0032504	multicellular organism reproduction	444	-0.33	-1.32	0.00233904	0.01088997	0.00668586	2204	tags=23%, list=19%, signal=19%	ENO2/FANCC/SRPK1/SFMBT1/GOLGA3/BCL2L11/THRA/ALKBH5/LIMK2/PTEN/SRC/CRTAP/KPNA6/ATM/ABAT/ADNP/DIAPH2/H3-3B/PAQR8/PROK2/VDR/H3-3A/PUM1/ETV6/ARMC2/ZNF148/HSPA5/LRRK2/OPRL1/BTG1/C3orf62/ASH1L/STAT5B/FANCG/EGR1/KMT2D/ASF1B/PI3/ROPN1L/NR6A1/CTCFL/YY1/SETX/SPATA2/GMCL1/AGO4/MAST2/HERC2/PSME4/ZNF296/RFX2/SMARCA2/PRKACA/SGPL1/BCAP31/LRRC8A/RARA/PTK2B/NEURL1/PAFAH1B2/NDRG3/PITHD1/ACOX1/TOB2/ZMIZ1/SBF1/ZBTB16/STK11/USP9X/JAM3/PTAFR/NCOR2/MED1/CASP2/ABHD2/B4GALT1/UBR2/SPOCD1/LGALS9/PDE5A/DNM2/IGF2R/ATP2B4/FOXJ2/ARRB2/CCNI/PPP1R9B/TBC1D20/BCL2L1/UBB/BSG/CTSB/KLF1/KLC3/YBX3/P2RX1/CTDNEP1/MARF1/FOXO3/OSBP2/BAG6/ACRBP
BP	GO:0006886	intracellular protein transport	706	-0.31	-1.27	0.00234359	0.01089835	0.00669101	1881	tags=24%, list=16%, signal=21%	NUP214/ERBB2/AP1G1/USP36/XPO5/TNPO2/STX6/TGFB1/ZDHHC7/AP2A2/LAMP2/BCL3/VPS13D/NPEPPS/SDCBP/HGS/TSC2/UBR5/TNPO3/ZDHHC19/PEX10/HSPA5/GET3/UBE2J2/NUP188/ITGAM/RPH3A/LRRK2/RANGAP1/AP2B1/NDEL1/IPO8/ARL4A/SNX17/NUTF2/AP2S1/MAN1A1/SYTL3/PIKFYVE/MAPK14/STX4/CHMP4B/YOD1/SORT1/PARD3/ARIH2/AP1M1/VPS41/KPNA1/SYNRG/VPS35/KPNB1/COPB2/MON1B/HERC2/NCF1/PML/ITGB2/KPNA4/ARHGEF2/ATP6AP1/EXOC6B/FAM53C/LCP1/TRIM28/ARF1/GSK3A/UBE2L3/CHP1/YWHAZ/SNX8/VPS37B/PRKACA/VPS37C/E2F3/STX5/YWHAB/SUFU/VPS11/AP2A1/TSPO/NCOA4/RBM22/SORL1/NUP98/BCAP31/FRAT1/KIF13A/BCR/RAB6B/SNX10/INPP5K/BCAS3/COPA/TECPR2/SEC31A/MAP1A/RAB7A/RAB14/SNX11/PPP3CA/RAB5B/STRADA/SYK/POM121/LTBP2/FIS1/PTPN1/NPLOC4/LMNA/RAB31/NUP93/MED1/ARF3/CLTC/FZD5/FLNA/VCP/TBC1D13/RIPOR1/XPO7/VPS28/TGFBRAP1/GABARAP/SNX27/LAPTM5/GSK3B/POM121C/RAC2/HSPB1/BNIP3L/TXNIP/SYTL4/TCF7L2/PPP1R10/HUWE1/TRAK2/GRIPAP1/RTN2/BBC3/CLU/NF1/HCLS1/OAZ1/ATG13/PINK1/XPO6/CDKN1A/AP1B1/STAT3/PTPN23/EHD1/CTTN/ABLIM3/SQSTM1/AHCYL1/AKT2/NAPA/MFN2/AP2M1/PPM1A/FAM53B/FBXO7/SEC16A/RAB1B/STRADB/DMTN/BAG6

BP	GO:0043066	negative regulation of apoptotic process	557	-0.33	-1.33	0.00239473	0.01112321	0.00682906	2203	tags=27%, list=19%, signal=23%	HDAC3/CBX4/FHL2/SH3RF1/ADAM8/MAP4K4/CDKN2D/PA2G4/ORMDL3/FOXO1/SOD2/ZNF385A/WNT1/PTEN/PRDX2/SRC/IRS2/ADNP/MAP2K5/MYO18A/XIAP/GRIK2/PROK2/SON/HCK/AREL1/ITPKB/RNF144B/CPEB4/BCL3/NOTCH2/MGMT/MTDH/HSPA5/GSTP1/LRRK2/HGF/STAT5B/EYA3/TMBIM1/RIPK1/SHC1/BABAM2/CAST/EIF2AK2/UBE2Z/MSX1/SETX/SEMA4D/TMBIM6/IER3/CLN3/AGO4/ITCH/PDPK1/DHCR24/CSF1R/ARHGEF2/BIRC6/FOXP1/YWHAZ/CSNK2A1/NFKB1/GREM1/PIM2/PRDX5/HTT/DOCK8/PTK2B/CHST11/CTNNA1/MMP9/POR/SFRP2/ITGA5/SLC9A1/ADAR/THBS1/PSME3/STK40/PTMS/PTPN1/ST3GAL1/LMNA/MED1/CASP2/LTF/PIK3CG/HIPK2/CAMK1D/CHD8/TSC22D3/FLNA/NIBAN2/JAK3/HSPA1A/PF4/ALOX12/GPI/UCP2/DNAJC5/AQP1/HNRNPK/ITPRIP/SIN3A/GSK3B/HYOU1/HSPB1/BNIP3L/DDB1/TCF7L2/PPP1R10/GNAI2/RHOA/GATA1/GPX1/CLU/ARRB2/FKBP8/GRN/HCLS1/ARHGDIA/PIM1/CFL1/UBE2O/PINK1/RPS6KA1/SNCA/SIAH2/NUAK2/BCL2L1/CTTN/BAG1/GRINA/AKT2/ARAF/ENO1/HDGF/PRKDC/PTCRA/PAK2/YBX3/DAB2/TIMP1/CBL/STRADB/BAG6/RELA
BP	GO:0030219	megakaryocyte differentiation	43	-0.59	-1.72	0.00241514	0.01119185	0.0068712	1119	tags=33%, list=10%, signal=30%	EIF6/CNOT4/MEIS1/PITHD1/MED1/MTURN/PF4/GATA1/TESC/PIP4K2A/TAL1/MPIG6B/WASF2/FAXDC2
BP	GO:0043534	blood vessel endothelial cell migration	77	-0.49	-1.59	0.00248188	0.01147438	0.00704466	1078	tags=36%, list=9%, signal=33%	ATP5F1B/GPLD1/ADAM17/NFE2L2/SCARB1/HDAC7/SRF/EPHB4/MAP2K5/TGFB1/PDPK1/AMOT/GREM1/PTK2B/PRKD2/THBS1/MAP3K3/STAT5A/HSPB1/ATP2B4/RHOA/GPX1/NF1/SP1/SH3BP1/MYH9/TBXA2R/MAP2K3
BP	GO:0071219	cellular response to molecule of bacterial origin	158	-0.42	-1.51	0.00252208	0.01161968	0.00713387	2278	tags=34%, list=19%, signal=27%	SCARB1/TRIM5/VIM/EPHB2/TLR2/PAF1/TIRAP/PRDX2/NLRP3/SRC/PRKCE/CD68/HCK/TGFB1/SIRPA/TNFSF4/MTDH/GSTP1/ARID5A/MAPK14/TRIB1/PYCARD/SBNO2/SPI1/CXCL5/SASH1/PF4V1/NFKB1/TSPO/UPF1/RARA/BCR/TRAF6/PLSCR3/BMP6/PTAFR/TLR6/FZD5/LTF/PF4/CD14/LYN/CDK19/TNFRSF1B/RHOA/TNIP1/PRPF8/GATA1/MAPK3/LITAF/TBXA2R/MAP2K3/RELA
MF	GO:0061629	RNA polymerase II-specific DNA-binding transcription factor binding	273	-0.38	-1.43	0.00253979	0.01168774	0.00717565	2203	tags=34%, list=19%, signal=28%	HDAC3/PKN1/MMS19/TADA3/ASXL1/STRN/SRF/PADI2/SRC/YWHAH/MED13/ELK1/HDAC4/DDX54/STK4/BAZ2A/LCOR/VDR/PPARGC1B/ZBTB7A/NOTCH2/GTF2I/CTDP1/UBXN7/MTDH/CTBP2/SMARCA4/STAT5B/OASL/ARID5A/KEAP1/NFATC1/CPNE1/MAPK14/TRIB1/CTBP1/CEBPA/SMARCD3/MAD2L2/NR1H2/SPI1/NCOA3/HIF1AN/NSD1/FOXP1/BRMS1/ARNT/MED16/FBP1/TACC1/RBPJ/NCOA2/BCAS3/TOB2/TGFB1I1/NCOR2/MED1/HIPK2/TRIP12/JUND/ACTB/HMGA1/RXRA/SIN3A/ETS2/GSK3B/GFI1B/CREBBP/HSPB1/WWP2/TCF7L2/TAF6/CHD4/PRKCB/GAS2L1/GATA1/SP1/NCOA6/ACTN4/ADD1/HCLS1/ARID1A/WBP2/DTX3L/STAT3/TAL1/NCOA1/KAT6A/PRKDC/MED25/CNOT1/RELA
BP	GO:0030512	negative regulation of transforming growth factor beta receptor signaling pathway	40	-0.58	-1.68	0.00257902	0.01185453	0.00727805	2511	tags=48%, list=21%, signal=37%	PMEPA1/ADAM17/NRROS/WNT1/IL17RD/LDLRAD4/ZBTB7A/HSPA5/FAM89B/PDPK1/CHST11/TGFB1I1/GLG1/SKI/HSPA1A/DNM2/FKBP1A/LTBP1/PPM1A

BP	GO:0016482	cytosolic transport	153	-0.43	-1.52	0.00258465	0.01185935	0.00728101	2489	tags=36%, list=21%, signal=29%	VAMP3/ACTR2/DNAJC13/CORO1A/ATP9A/SYS1/CORO7/VPS53/RGP1/SRC/HOOK2/AP1G1/STX6/SNX3/WDR81/LRRK2/EZR/TBC1D17/PIKFYVE/VPS51/MAP2K2/SORT1/WASHC2C/HOOK3/CLN3/VPS35/TBC1D10B/SNX8/STX5/VPS11/AP2A1/KIF1C/SORL1/RNF126/RAB6B/RAB7A/ANKFY1/RAB14/SLC66A2/EHD3/DCTN1/CLTC/FLNA/LAPTM5/RILP/MSN/UBE2O/MAPK3/PTPN23/PIP4K2A/LAMP1/TBC1D5/USP7/GAK/DAB2
BP	GO:0045069	regulation of viral genome replication	77	-0.49	-1.59	0.00258901	0.01185935	0.00728101	2241	tags=32%, list=19%, signal=26%	IFI16/MAVS/TRIM38/SRPK1/APOBEC3D/SETDB1/OASL/EIF2AK2/IFITM3/ZC3HAV1/TRIM28/INPP5K/RAD23A/ADAR/LTF/APOBEC3F/DDB1/IFITM2/SRPK2/TNIP1/STAU1/APOBEC3C/LARP1/ZNFX1/N4BP1
BP	GO:0016241	regulation of macroautophagy	138	-0.44	-1.53	0.00262492	0.01201001	0.00737351	2030	tags=33%, list=17%, signal=27%	FEZ1/ATP6V0B/EXOC4/SH3GLB1/ATP6V1B2/USP36/VPS13D/ERN1/TSC2/EXOC7/LRRK2/SPTLC2/PIP4K2C/WAC/CHMP4B/PHF23/CLN3/VPS35/ATP6V0C/QSOX1/CLEC16A/OPTN/PRKACA/SESN3/SESN2/ATP6V0A1/IKBKG/HTT/PAFAH1B2/WDR45/ULK1/RUBCN/CAPN1/CAPNS1/ELAPOR1/BNIP3L/SUPT5H/TBC1D25/ATP6V1A/ATG2A/LARP1/PINK1/MAPK3/PIP4K2A/ATP6V0D1
BP	GO:0046649	lymphocyte activation	574	-0.32	-1.29	0.00262975	0.01201825	0.00737857	2263	tags=25%, list=19%, signal=21%	KIF13B/GON4L/EPHB2/CLPTM1/PKN1/SH3RF1/ADAM8/EFNB1/MICB/FCER1G/SLA2/CMTM7/CR1/KLF6/LGALS9C/FGR/SRF/FYN/WNT1/TIRAP/UNC13D/PRDX2/NLRP3/SRC/NKG7/IRS2/FKBP1B/HDAC4/ATM/JMJD6/BLOC1S3/TNFSF13/SDC4/XRCC6/ERBB2/AP1G1/ATP7A/TGFB1/SIRPA/ITPKB/ZBTB7A/TNFSF4/RC3H2/BCL3/NOTCH2/GPS2/STAT5B/EGR1/PAG1/RASAL3/KAT7/EPHB1/YY1/PTPRJ/ZFP36L1/PTPN6/PYCARD/ADGRG3/MAD2L2/RELB/SOS2/SPI1/ITCH/PREX1/LCP1/FOXP1/LEPR/ZNF335/SH3KBP1/RUNX3/TMEM131L/VSIR/NFAM1/RBPJ/NFATC2/IRF1/PGLYRP1/LRRC8A/DOCK8/RARA/PTK2B/SPTA1/SEMA4A/STAT6/CCND3/TRAF6/TBX21/IL6R/IGF2/SKAP2/ZMIZ1/CSK/ZBTB16/VAV1/STK11/GSN/PPP3CA/PTPRC/SYK/KDELR1/SUPT6H/ST3GAL1/MAFB/CD151/DOCK2/FZD5/PIK3CG/JAK3/INPP5D/LYN/SNX27/LGALS9/LAPTM5/PDE5A/RAC2/SIRPB1/FLOT2/TNFRSF1B/FKBP1A/MPL/PRF1/RHOA/ITGAL/PRKCB/MSN/WAS/CDKN1A/APBB1IP/PBX1/STAT3/RIPOR2/LAMP1/PIK3CD/IKZF1/PRKDC/LYL1/FBXO7/MYH9/FOXO3/ELF4/HLX/BAG6
BP	GO:0061180	mammary gland epithelium development	35	-0.61	-1.73	0.00264969	0.01209549	0.00742599	2037	tags=34%, list=17%, signal=28%	KDM5B/SRC/VDR/MGMT/MSX1/PML/CSF1R/STAT6/MED1/GPX1/ZNF703/AKT2
BP	GO:0009792	embryo development ending in birth or egg hatching	390	-0.34	-1.34	0.00267132	0.01216626	0.00746944	2089	tags=24%, list=18%, signal=20%	BCL2L11/MYO1E/HEG1/SRF/WNT1/TAF8/APAF1/MTHFR/ATM/KIDINS220/STK4/SDC4/EXT1/NIPBL/ATP7A/MKS1/TGFB1/TTLL4/PHACTR4/WDR74/NOTCH2/TSC2/XYLT1/NDEL1/TFEB/ASF1B/DBN1/KEAP1/CEBPA/PEF1/ELL/ZFP36L1/SLC35E2B/MSX1/FUZ/CDKN1C/PTPN18/AKIRIN2/BIRC6/TRIM28/GRB2/ARNT/PRKACA/ZNF335/AMOT/SUFU/RBPJ/YBX1/RARA/INPP5K/TRAF6/CHST11/IGF2/ZMIZ1/TBL1XR1/SFRP2/APBA3/DVL3/NPAT/SUPT6H/BPTF/MED1/FZD5/CHD8/SKI/NDST1/PSMC4/GPI/SIN3A/EPN1/CUL4A/CMIP/GATA1/ITPK1/NF1/FKBP8/TERF2/WDTC1/USP22/GNA12/ECE1/PBX1/BCL2L1/FLCN/NCOA1/MFN2/PRKDC/TENT5C/YBX3/VASP/CNOT1/MYH9/FURIN
BP	GO:0000768	syncytium formation by plasma membrane fusion	29	-0.64	-1.73	0.00270138	0.01227506	0.00753623	3269	tags=66%, list=28%, signal=47%	SH3PXD2A/ADAM9/IL4R/NOS1/NPHS1/TNFSF14/PLEKHO1/ADAMTS5/CFLAR/MYOF/WNT1/DYRK1B/MAPK14/FLOT1/SBNO2/NFATC2/EHD1/RIPOR2/MYH9

BP	GO:0140253	cell-cell fusion	29	-0.64	-1.73	0.00270138	0.01227506	0.00753623	3269	tags=66%, list=28%, signal=47%	SH3PXD2A/ADAM9/IL4R/NOS1/NPHS1/TNFSF14/PLEKHO1/ADAMTS5/CFLAR/MYOF/WNT1/DYRK1B/MAPK14/FLOT1/SBNO2/NFATC2/EHD1/RIPOR2/MYH9
BP	GO:0050877	nervous system process	467	-0.34	-1.34	0.00272012	0.01234611	0.00757985	2096	tags=22%, list=18%, signal=19%	SOD2/ZNF385A/THRA/SNAP25/SRF/FYN/PTEN/SRC/TRIOBP/ATXN7/FKBP1B/ABAT/SLC24A4/ADNP/ATF6/GRIK2/PROK2/NIPBL/GPR155/DICER1/OR10H1/PNKD/PDE4A/DLGAP4/ROCK2/LRRK2/OPRL1/HGF/PLCB1/SYNJ1/EYA3/NPTXR/DLG4/C5AR1/MME/WASF3/EPHB1/OR52K2/S1PR2/AGTPBP1/OR52K1/EML2/CHRNE/PARD3/MYCBP2/CLN3/VPS35/PTGES/SHISA7/DGCR2/WDR1/GSK3A/INSR/PITPNA/TPP1/VLDLR/HTT/PTK2B/BCR/HERC1/MAP1A/SETD5/TAS2R30/PPP3CA/ITGA5/SLC9A1/JAM3/PTAFR/GNB1/GAA/SCN1B/PLEC/DAW1/MGLL/DCTN1/HIPK2/CHD8/ODAD4/GPI/GSK3B/TMEM120A/TNFRSF1B/TSHZ3/TBL1X/GPX1/ATXN1/ARRB2/NF1/PPP1R9B/NRGN/ESPN/MAPK3/SNCA/CLDN5/RIPOR2/HPS1/PRKAR2B/EIF4EBP2/MBP/RNF10/P2RX1/DIAPH1
BP	GO:1990874	vascular associated smooth muscle cell proliferation	34	-0.62	-1.76	0.00279031	0.01265027	0.00776659	2096	tags=41%, list=18%, signal=34%	SOD2/PTEN/ERN1/S1PR2/MAP3K5/TPM1/LDLRAP1/MMP9/GNAI2/RHOA/FOXJ2/GNA12/CDKN1A/MFN2
MF	GO:0044389	ubiquitin-like protein ligase binding	278	-0.37	-1.39	0.00279876	0.0126741	0.00778122	2216	tags=32%, list=19%, signal=27%	USP19/AXIN2/TNK2/UBC/PA2G4/FOXO1/ATXN3/HSPA1L/UBA52/ARIH1/TRIOBP/PER1/ATF6/GRIK2/SUMO3/DET1/CUL7/HGS/UBXN7/HSPA5/ACTG1/UBE2J2/ARRB1/IKBKE/RANGAP1/CACUL1/RIPK1/PIAS1/TRIB1/KCNH2/YOD1/CKB/TMBIM6/SPOP/ATP6V0C/ITCH/HERC2/PML/MAP1LC3B/TRIB3/TRIM28/UBE2L3/YWHAZ/PSMD1/PRKACA/SH3KBP1/OTUB1/IKBKG/PTK2B/MFHAS1/TRAF6/PIAS4/VCL/NPLOC4/UQCRC1/FZD5/SKI/VCP/HSPA1A/GPI/GABARAP/LYN/LAPTM5/GSK3B/TNFRSF1B/TXNIP/CUL4A/TUBB/CLU/ARRB2/JAK1/CASC3/PINK1/CDKN1A/DTX3L/BAG1/PCBP2/PRDX6/PRKAR2A/PRKAR2B/UBB/SQSTM1/USP7/MFN2/RNF40/FBXO7/TOLLIP/BAG6/POLR2A/RELA
MF	GO:0003682	chromatin binding	435	-0.34	-1.35	0.00280562	0.0126796	0.0077846	2203	tags=27%, list=19%, signal=23%	HDAC3/CBX4/PKN1/SAFB/SSRP1/ASXL1/SFMBT1/FOXO1/CBX5/SAP30L/THRA/PAF1/TOX2/SRF/PHF8/WDR82/PER1/TOP1/ELK1/ANKRD17/SCML4/ADNP/POLG/H3-3B/MCM3AP/NIPBL/STAG1/H3-3A/MLLT10/SETDB1/EP400/CTBP2/SMARCC1/MBD6/SMARCA4/ASH1L/KDM3B/STAT5B/KDM8/JDP2/NFIA/EGR1/ARID5A/UBTF/L3MBTL2/RAD21/PHF13/CTCFL/CTBP1/CEBPA/TLE4/WAC/POLR1A/YY1/SMC1A/SBNO2/PHC2/SPI1/TNRC18/ARID1B/NSD1/SAMD1/TRIM28/SMARCA2/DNTTIP1/NFKB1/RBPJ/UPF1/NCOA2/NFATC2/NUP98/PELP1/YBX1/RARA/MEIS1/BCAS3/SMARCC2/WDR13/RERE/ASXL2/GRWD1/FOXO4/FOSL2/ZNF609/SUPT6H/NCOR2/MED1/GATAD2B/CHD8/ACTB/HMGA1/PHF21A/SIN3A/CREBBP/SUPT5H/CHD4/TSHZ3/BRD3/PRKCB/GATA1/KDM6B/NCOA6/ARID3A/ACTN4/ARID1A/MLLT6/WBP2/STAT3/TAL1/NCOA1/BRD4/JARID2/FOXO3/CABIN1/POLR2A/HCFC1/RELA
BP	GO:0015804	neutral amino acid transport	30	-0.65	-1.79	0.00280635	0.0126796	0.0077846	911	tags=33%, list=8%, signal=31%	SLC36A1/SLC6A20/SLC3A2/SLC25A38/SLC6A9/SLC1A5/SLC43A2/SLC43A1/SLC7A5/SLC38A5
BP	GO:0032890	regulation of organic acid transport	38	-0.61	-1.75	0.0028123	0.01269205	0.00779225	1972	tags=29%, list=17%, signal=24%	IRS2/ABAT/PER2/PTGES/SYK/THBS1/FIS1/SLC43A2/SLC43A1/SNCA/AKT2

BP	GO:0043009	chordate embryonic development	380	-0.34	-1.33	0.00281927	0.0127091	0.00780271	2089	tags=24%, list=18%, signal=20%	BCL2L11/MYO1E/HEG1/SRF/WNT1/TAF8/APAF1/MTHFR/ATM/KIDINS220/STK4/SDC4/EXT1/NIPBL/ATP7A/MKS1/TGFB1/TTLL4/PHACTR4/WDR74/NOTCH2/TSC2/XYLT1/NDEL1/TFEB/ASF1B/DBN1/KEAP1/CEBPA/PEF1/ELL/ZFP36L1/SLC35E2B/MSX1/FUZ/CDKN1C/PTPN18/BIRC6/TRIM28/GRB2/ARNT/PRKACA/ZNF335/AMOT/SUFU/RBPJ/YBX1/RARA/INPP5K/TRAF6/CHST11/IGF2/ZMIZ1/TBL1XR1/SFRP2/APBA3/DVL3/NPAT/SUPT6H/BPTF/MED1/FZD5/CHD8/SKI/NDST1/PSMC4/GPI/SIN3A/EPN1/CUL4A/CMIP/GATA1/ITPK1/NF1/FKBP8/TERF2/WDTC1/GNA12/ECE1/PBX1/BCL2L1/FLCN/NCOA1/MFN2/PRKDC/TENT5C/YBX3/VASP/CNOT1/MYH9/FURIN
BP	GO:0019722	calcium-mediated signaling	131	-0.45	-1.57	0.00288877	0.01294898	0.00794999	1966	tags=34%, list=17%, signal=28%	STIMATE/FHL2/SLA2/SULT1A3/FKBP1B/SLC24A4/MCOLN1/SPPL3/LRRK2/NFAT5/CMKLR1/NFATC1/PTPRJ/PDPK1/PTBP1/CCR1/CHP1/PDK2/PRKACA/CHERP/ADGRL1/NFATC2/TMEM38A/BCAP31/NUDT4/HTT/PTK2B/SLC8A1/SELP/CXCR2/PPP3CA/PTPRC/SYK/SLC9A1/FIS1/CALM3/PLEK/GSK3B/ATP2B4/NFATC3/CXCR1/PPP1R9B/PPP3R1/DMTN
BP	GO:0110148	biomineralization	82	-0.49	-1.58	0.00288801	0.01294898	0.00794999	2404	tags=37%, list=20%, signal=29%	ALOX5/TUFT1/TENT5A/SLC20A1/AXIN2/ATP2B1/FGR/SLC24A4/TGFB1/ANO6/NECTIN1/ROCK2/NBR1/SBNO2/CCR1/ALPL/GREM1/PTK2B/SLC8A1/SNX10/ANKH/SLC24A3/BMP6/BMP2K/PHOSPHO1/LTF/RFLNB/GATA1/TFIP11/STIM1
BP	GO:0030032	lamellipodium assembly	56	-0.54	-1.65	0.00300272	0.01336931	0.00820805	835	tags=38%, list=7%, signal=35%	PLCE1/KIT/CARMIL1/TWF2/ABLIM1/FSCN1/ACTR2/WNT1/ATP7A/WASF3/ABI3/VCL/RAC1/RAC2/DNM2/CAPZB/WAS/PARVB/ABLIM3/WASF2/DMTN
BP	GO:0050731	positive regulation of peptidyl-tyrosine phosphorylation	109	-0.45	-1.53	0.00300736	0.013375	0.00821154	2045	tags=30%, list=17%, signal=25%	FYN/SRC/ADNP/CASS4/TGFB1/LILRA5/HGF/DLG4/PTPRJ/SEMA4D/LRRK1/EPHA4/NCF1/CSF1R/ARHGEF2/NEDD9/GREM1/PTK2B/NEURL1/IL6R/IGF2/PECAM1/BMP6/ITGA5/PTPRC/SYK/PTPN1/LYN/GATA1/ARRB2/HCLS1/PAK2/ITGB3
BP	GO:0042752	regulation of circadian rhythm	74	-0.49	-1.56	0.0031878	0.01413009	0.00867512	1817	tags=31%, list=15%, signal=26%	MTA1/CRY2/HDAC3/PER1/MAGED1/ROCK2/OPRL1/PHLPP1/PER2/KDM2A/GNAQ/PML/ZFHX3/PPP1CA/USP9X/ATG7/SIN3A/GSK3B/DDB1/SIAH2/USP7/SRRD/PRKDC
BP	GO:0071222	cellular response to lipopolysaccharide	151	-0.42	-1.49	0.00318721	0.01413009	0.00867512	2278	tags=33%, list=19%, signal=27%	SCARB1/TRIM5/VIM/EPHB2/TLR2/PAF1/PRDX2/NLRP3/SRC/PRKCE/CD68/HCK/TGFB1/SIRPA/TNFSF4/MTDH/GSTP1/ARID5A/MAPK14/TRIB1/PYCARD/SBNO2/SPI1/CXCL5/SASH1/PF4V1/NFKB1/TSPO/UPF1/RARA/BCR/TRAF6/PLSCR3/BMP6/PTAFR/LTF/PF4/CD14/LYN/CDK19/TNFRSF1B/RHOA/TNIP1/PRPF8/GATA1/MAPK3/LITAF/TBXA2R/MAP2K3/RELA
BP	GO:0060402	calcium ion transport into cytosol	91	-0.47	-1.57	0.00326524	0.01442512	0.00885626	2517	tags=38%, list=21%, signal=30%	CAPN3/BAK1/TRPC3/CALM1/CORO1A/LETM1/FYN/TPCN1/FKBP1B/SLC24A4/MCOLN1/PRKCE/PTPN6/TMBIM6/PDPK1/PML/ITPR2/PRKACA/CHERP/ERO1A/TMEM38A/HTT/PTK2B/SLC8A1/PTPRC/SELENON/CALM3/LYN/GP9/ATP2B4/FKBP1A/SNCA/GP1BA/P2RX1/DIAPH1
MF	GO:0019903	protein phosphatase binding	123	-0.45	-1.54	0.00328168	0.01448166	0.00889097	2152	tags=31%, list=18%, signal=25%	PPP6R1/STRN/FOXO1/CDC27/IRS2/PPP1R15A/ERBB2/SIRPA/SNX3/PHACTR4/IKBKE/DLG4/MAPK14/CSF1R/GRB2/PPP1CA/MTMR3/MAP3K5/MFHAS1/VRK3/STAT6/CSK/MAP2K7/SLC9A1/PTPN1/JAK3/VCP/PPP1R10/ATP2B4/PXN/STAU1/PPP1R9B/JAK1/GNA12/SMG7/STAT3/IQGAP1/ANK1
BP	GO:0010717	regulation of epithelial to mesenchymal transition	55	-0.53	-1.63	0.00329177	0.01451008	0.00890842	2192	tags=44%, list=19%, signal=36%	NOTCH1/SMAD3/BCL9L/CLASP1/AXIN2/GLIPR2/PTEN/IL17RD/TGFB1/LDLRAD4/TIAM1/SDCBP/MCRIP1/MAD2L2/FUZ/USF3/EPHA4/GREM1/TGFB1I1/SFRP2/ADIPOR1/TCF7L2/ZNF703/DAB2

MF	GO:0003713	transcription coactivator activity	236	-0.38	-1.41	0.00338776	0.01490016	0.00914791	2258	tags=33%, list=19%, signal=27%	TRIM5/KAT2B/PKN1/MMS19/TRIM38/TADA3/ASXL1/MED12L/TOX2/PHF2/EDF1/CCDC124/MED13/ELK1/ATXN7L3/SRCAP/MAGED1/PPARGC1B/BCL3/MTDH/CTBP2/SMARCC1/ARRB1/MED27/SMARCA4/GPS2/XPC/DYRK1B/KMT2D/SRA1/PER2/SMARCD3/NCOA3/MAML2/ARID1B/KAT5/HELZ2/TRIM28/SMARCA2/UBE2L3/MED16/CNOT6/TACC1/NCOA4/NCOA2/NUP98/SMARCC2/ZMIZ1/RERE/TGFB1I1/NPAT/MED1/DCAF6/RBM14/HIPK2/MAML3/LPIN2/NIBAN2/HMGA1/SIN3A/CREBBP/TAF6/PRKCB/TRIM14/KMT2C/PDLIM1/NCOA6/ACTN4/BCL9/USP22/ARID1A/WBP2/ACTN1/NCOA1/KAT6A/BRD4/CARM1/HCFC1
BP	GO:0006814	sodium ion transport	113	-0.45	-1.54	0.00343341	0.01508425	0.00926093	2036	tags=27%, list=17%, signal=22%	SLC13A4/UTRN/SLC9A3R1/SLC20A1/TPCN1/PER1/YWHAH/SLC24A4/PRKCE/ANO6/MFSD4B/FXYD6/CHP1/SLC8A1/SLC6A20/SLC24A3/SLC3A2/SLC9A1/SCN1B/ATP1B2/SLC6A9/DNM2/ATP2B4/SLC38A10/ACTN4/MLLT6/TESC/AHCYL1/WNK1/SLC6A8
BP	GO:0010522	regulation of calcium ion transport into cytosol	55	-0.53	-1.62	0.00343889	0.01509164	0.00926547	2517	tags=44%, list=21%, signal=34%	CAPN3/BAK1/TRPC3/CALM1/CORO1A/FYN/FKBP1B/PRKCE/PTPN6/TMBIM6/PDPK1/PML/PRKACA/TMEM38A/HTT/PTK2B/SLC8A1/SELENON/CALM3/LYN/FKBP1A/SNCA/P2RX1/DIAPH1
BP	GO:0034764	positive regulation of transmembrane transport	120	-0.44	-1.52	0.00345933	0.01516458	0.00931025	1504	tags=21%, list=13%, signal=18%	MAPK14/CA2/KCNH2/PDPK1/CHP1/INSR/HTT/SLC9A1/PTAFR/ATP1B2/EHD3/CALM3/SLC6A9/G6PD/FLNA/GJC2/DNM2/ACTN4/PINK1/TESC/SNCA/AKT2/STIM1/WNK1/P2RX1
BP	GO:0050730	regulation of peptidyl-tyrosine phosphorylation	159	-0.42	-1.49	0.00346351	0.01516621	0.00931124	2045	tags=28%, list=17%, signal=23%	FYN/SRC/ADNP/PRKCE/CASS4/TGFB1/LILRA5/HGF/DLG4/SHC1/NF2/PTPRJ/PTPN6/SEMA4D/LRRK1/EPHA4/NCF1/ITGB2/CSF1R/ARHGEF2/PDGFA/NEDD9/GREM1/SH3BP5L/PTK2B/NEURL1/IL6R/IGF2/PECAM1/BMP6/SFRP2/ITGA5/PTPRC/SYK/PTPN1/PPP2R5B/LYN/GATA1/ARRB2/HCLS1/PAK2/ITGB3/CBL/DMTN
BP	GO:1900026	positive regulation of substrate adhesion-dependent cell spreading	30	-0.65	-1.77	0.00347704	0.01520871	0.00933734	2202	tags=50%, list=19%, signal=41%	CARMIL1/CALR/LIMS1/UNC13D/TRIOBP/CASS4/DOCK5/PREX1/NEDD9/FLNA/RAC1/DNM2/MYADM/DAB2/DMTN
BP	GO:0007517	muscle organ development	173	-0.41	-1.49	0.00354895	0.0155017	0.00951722	1589	tags=21%, list=14%, signal=19%	UNC45A/EGR1/EPHB1/MAPK14/FLOT1/MSX1/FHL3/ZFHX3/HIVEP3/TPM1/RBPJ/NEURL1/SOX6/SVIL/KLF5/IGF2/PPP3CA/FOXO4/ZNF609/SELENON/PLEC/MED1/BASP1/SKI/NIBAN2/FKBP1A/RHOA/GPX1/NF1/BCL9/MYLK/MTPN/RIPOR2/YBX3/KEL/XK/HLX
BP	GO:0014706	striated muscle tissue development	110	-0.45	-1.54	0.00355182	0.0155017	0.00951722	2072	tags=28%, list=18%, signal=23%	HEG1/SRF/PTEN/TGFB1/NOTCH2/CTDP1/MAPK14/YY1/MSX1/GSK3A/TPM1/GREM1/RBPJ/RARA/SLC8A1/ARID2/SOX6/PDLIM5/SLC9A1/LMNA/PLEC/MED1/G6PD/AKAP13/FKBP1A/ARRB2/KDM6B/PIM1/MTPN/JARID2/NPRL3
BP	GO:0032963	collagen metabolic process	44	-0.57	-1.69	0.00356701	0.01555091	0.00954743	1027	tags=23%, list=9%, signal=21%	VIM/TNXB/TGFB1/VSIR/RCN3/MMP9/ARRB2/CTSB/MRC2/FURIN

BP	GO:0050865	regulation of cell activation	457	-0.34	-1.34	0.00357609	0.01557342	0.00956125	2232	tags=26%, list=19%, signal=22%	EPHB2/CLPTM1/SH2B3/PKN1/SH3RF1/ADAM8/EFNB1/FCER1G/CR1/LGALS9C/FGR/FYN/TIRAP/UNC13D/PRDX2/NLRP3/SRC/IRS2/CD177/ATM/ABAT/MYO18A/BLOC1S3/TNFSF13/SDC4/THBD/XRCC6/ERBB2/AP1G1/TGFB1/SIRPA/ITPKB/ADGRE2/TNFSF4/RC3H2/LILRA5/ITGAM/LRRK2/STAT5B/PAG1/RASAL3/CD300LF/STX4/CEBPA/ACTA2/ZFP36L1/PTPN6/PYCARD/MAD2L2/SOS2/SPI1/ITCH/PDPK1/ITGB2/ZNF335/PDGFA/SH3KBP1/RUNX3/TMEM131L/VSIR/NFAM1/FES/NFATC2/IRF1/PGLYRP1/DOCK8/RARA/MFHAS1/GAB2/SPTA1/SELP/BCR/STAT6/TRAF6/TBX21/IGF2/ZMIZ1/CSK/ZBTB16/VAV1/GP6/PTPRC/SYK/THBS1/PTAFR/SUPT6H/TLR6/PRAM1/JUND/JAK3/INPP5D/ALOX12/PLEK/LYN/LGALS9/LAPTM5/PDE5A/RAC2/SIRPB1/GP9/FLOT2/TNFRSF1B/MPL/RHOA/GATA1/GRN/RPS6KA1/SNCA/CDKN1A/RIPOR2/LAMP1/GP1BA/PRKDC/FBXO7/FOXO3/HLX/DMTN
BP	GO:0010591	regulation of lamellipodium assembly	32	-0.62	-1.72	0.00365389	0.01587744	0.00974791	535	tags=41%, list=5%, signal=39%	TWF2/FSCN1/ACTR2/WNT1/ATP7A/ABI3/RAC1/RAC2/DNM2/CAPZB/WAS/WASF2/DMTN
BP	GO:0048609	multicellular organismal reproductive process	440	-0.34	-1.33	0.00366707	0.01589995	0.00976173	2204	tags=23%, list=19%, signal=20%	ENO2/FANCC/SRPK1/SFMBT1/GOLGA3/BCL2L11/THRA/ALKBH5/LIMK2/PTEN/SRC/CRTAP/KPNA6/ATM/ABAT/ADNP/DIAPH2/H3-3B/PAQR8/PROK2/VDR/H3-3A/PUM1/ETV6/ARMC2/ZNF148/HSPA5/LRRK2/OPRL1/BTG1/C3orf62/ASH1L/STAT5B/FANCG/EGR1/KMT2D/ASF1B/PI3/ROPN1L/NR6A1/CTCFL/YY1/SETX/SPATA2/GMCL1/AGO4/MAST2/HERC2/PSME4/ZNF296/RFX2/SMARCA2/PRKACA/SGPL1/BCAP31/LRRC8A/RARA/PTK2B/NEURL1/PAFAH1B2/NDRG3/PITHD1/ACOX1/TOB2/ZMIZ1/SBF1/ZBTB16/STK11/USP9X/JAM3/PTAFR/NCOR2/MED1/CASP2/ABHD2/B4GALT1/UBR2/SPOCD1/LGALS9/PDE5A/DNM2/IGF2R/ATP2B4/FOXJ2/ARRB2/CCNI/PPP1R9B/TBC1D20/BCL2L1/UBB/BSG/CTSB/KLF1/KLC3/YBX3/P2RX1/CTDNEP1/MARF1/FOXO3/OSBP2/BAG6/ACRBP
BP	GO:0002253	activation of immune response	247	-0.37	-1.38	0.0036821	0.01594776	0.00979108	2425	tags=28%, list=21%, signal=23%	FPR3/GPLD1/CD38/BTN3A2/NCKAP1L/CR1L/TRIM5/C1QA/IFI16/MAVS/MYO1G/MICB/FCER1G/SERPING1/RFTN1/SLA2/CR1/FGR/FYN/SRC/PRKCE/SPPL3/XRCC6/HCK/DGKZ/RC3H2/FYB1/GPS2/EZR/CMKLR1/SH2B2/C5AR1/PTPRJ/PTPN6/PYCARD/KLRD1/FOXP1/ERMAP/NLRC4/SPG21/NFAM1/NFATC2/IKBKG/TRAF6/PRKD2/PAK1/CSK/VAV1/STK11/PTPRC/SYK/RBM14/PRAM1/INPP5D/LYN/RBCK1/SIN3A/LAPTM5/CREBBP/PRKCB/CLU/ZNFX1/PIK3CD/PRKDC/PAK2/KRT1/WNK1/BAG6/RELA
CC	GO:0005798	Golgi-associated vesicle	64	-0.52	-1.64	0.00380502	0.01640862	0.01007402	1997	tags=36%, list=17%, signal=30%	CLTB/RAB27B/SPPL3/AP1G1/ATP7A/AFTPH/COPE/LRRK2/SORT1/SYNRG/COPB2/SPG21/AP2A1/COPA/TGOLN2/RAB14/KDELR1/CLTC/IGF2R/PACS1/SCYL1/NRGN/FURIN
BP	GO:0045124	regulation of bone resorption	25	-0.67	-1.75	0.00392691	0.01687935	0.01036303	2439	tags=48%, list=21%, signal=38%	GPR137/CD38/TF/ADAM8/SRC/PPARGC1B/CSF1R/CSK/SYK/INPP5D/NF1/ITGB3
BP	GO:0045921	positive regulation of exocytosis	56	-0.53	-1.63	0.00393596	0.01688174	0.01036449	2112	tags=39%, list=18%, signal=32%	VPS4B/FGR/UNC13D/RAB27B/CD177/SDC4/AP1G1/SDCBP/HGS/ITGAM/STX4/ITGB2/GAB2/RAB7A/SYK/PTAFR/SYTL4/GATA1/SNCA/LAMP1/RAB3D/RAB2B
BP	GO:0045071	negative regulation of viral genome replication	49	-0.55	-1.66	0.0039912	0.01704512	0.0104648	2241	tags=39%, list=19%, signal=31%	IFI16/MAVS/SRPK1/APOBEC3D/SETDB1/OASL/EIF2AK2/IFITM3/ZC3HAV1/TRIM28/INPP5K/LTF/APOBEC3F/IFITM2/SRPK2/TNIP1/APOBEC3C/ZNFX1/N4BP1

BP	GO:0097553	calcium ion transmembrane import into cytosol	82	-0.48	-1.55	0.00402404	0.01716694	0.01053959	2045	tags=33%, list=17%, signal=27%	FYN/TPCN1/FKBP1B/SLC24A4/MCOLN1/PRKCE/PTPN6/PDPK1/ITPR2/PRKACA/CHERP/ERO1A/TMEM38A/HTT/PTK2B/SLC8A1/PTPRC/SELENON/CALM3/LYN/GP9/ATP2B4/FKBP1A/SNCA/GP1BA/P2RX1/DIAPH1
MF	GO:0031625	ubiquitin protein ligase binding	263	-0.36	-1.37	0.00410335	0.01744913	0.01071284	2216	tags=33%, list=19%, signal=27%	USP19/AXIN2/TNK2/UBC/PA2G4/FOXO1/ATXN3/HSPA1L/UBA52/ARIH1/TRIOBP/PER1/ATF6/GRIK2/DET1/CUL7/UBXN7/HSPA5/ACTG1/UBE2J2/ARRB1/IKBKE/RANGAP1/CACUL1/RIPK1/PIAS1/TRIB1/KCNH2/YOD1/CKB/TMBIM6/SPOP/ATP6V0C/HERC2/PML/MAP1LC3B/TRIB3/TRIM28/UBE2L3/YWHAZ/PSMD1/PRKACA/SH3KBP1/OTUB1/IKBKG/PTK2B/MFHAS1/TRAF6/PIAS4/VCL/NPLOC4/UQCRC1/FZD5/SKI/VCP/HSPA1A/GPI/GABARAP/LYN/LAPTM5/GSK3B/TNFRSF1B/TXNIP/CUL4A/TUBB/CLU/ARRB2/JAK1/CASC3/PINK1/CDKN1A/BAG1/PCBP2/PRDX6/PRKAR2A/PRKAR2B/UBB/SQSTM1/USP7/MFN2/RNF40/FBXO7/TOLLIP/BAG6/POLR2A/RELA
BP	GO:0051222	positive regulation of protein transport	221	-0.38	-1.41	0.00413468	0.0175448	0.01077157	2401	tags=30%, list=20%, signal=24%	GPLD1/BAP1/CD38/WLS/ARHGEF5/HDAC3/MAVS/ADAM8/TLR2/HSPA1L/FYN/SRC/IRS2/SH3GLB1/ABAT/MYO18A/PRKCE/ERBB2/USP36/TGFB1/NPEPPS/UBR5/UBE2J2/ITGAM/EZR/MAPK14/STX4/NR1H2/ARIH2/MYOM1/CLN3/VPS35/ITGB2/TRIM28/GSK3A/UBE2L3/CHP1/PRKACA/VPS11/RBM22/SORL1/BCAP31/LRRC8A/BCAS3/FRMD4A/BMP6/FIS1/FZD5/FLNA/RIPOR1/VPS28/GSK3B/RAC2/SYTL4/TCF7L2/HUWE1/BBC3/HCLS1/OAZ1/ATG13/PINK1/PTPN23/ABLIM3/AKT2/PPM1A/GLUL
BP	GO:0001933	negative regulation of protein phosphorylation	238	-0.38	-1.42	0.00417451	0.01767609	0.01085218	2511	tags=34%, list=21%, signal=27%	PMEPA1/YWHAG/BAK1/TERF2IP/SOCS3/UBE2B/CALM1/DUSP16/PRKAR1B/MVP/DUSP1/KAT2B/EPHB2/SH2B3/PKN1/CDKN2D/IRAK3/SPRY3/HEG1/PTEN/PPM1F/AKT1S1/MLLT1/TGFB1/SIRPA/LDLRAD4/TSC2/GSTP1/LRRK2/HGF/PIP5KL1/STK38/TRIB1/CEBPA/CTDSP2/NF2/PTPRJ/PTPN6/PYCARD/PARD3/SEMA4D/CDKN1C/LRRK1/PDPK1/GNAQ/TRIB3/CHP1/GREM1/SH3BP5L/SORL1/CORO1C/SLC8A1/WARS1/INPP5K/SFRP2/PTPRC/ADAR/PTPN1/PLEC/CTDSPL/CALM3/HEXIM2/EIF4G1/LYN/LRP1/HSPB1/CTDSP1/MYADM/ARRB2/NF1/FKBP8/SNCA/CDKN1A/NIBAN1/PRKAR2A/PRKAR2B/PRKDC/PAK2/FBXO7/CBL/DMTN
BP	GO:0022407	regulation of cell-cell adhesion	316	-0.35	-1.36	0.00417349	0.01767609	0.01085218	2083	tags=23%, list=18%, signal=20%	CR1/LGALS9C/FYN/WNT1/PRDX2/PPM1F/NLRP3/SRC/ABAT/MAP2K5/SDC4/ERBB2/TGFB1/SIRPA/LRFN3/ITPKB/TNFSF4/RC3H2/STAT5B/NFAT5/PAG1/RASAL3/MAPK14/NF2/FLOT1/PTPN6/PYCARD/MAD2L2/SPI1/ITCH/FERMT3/ITGB2/RUNX3/TMEM131L/VSIR/IRF1/DOCK8/RARA/SPTA1/SELP/TRAF6/TBX21/IGF2/ZMIZ1/CSK/BMP6/ZBTB16/VAV1/AKNA/GP6/PTPRC/SYK/PTAFR/IL1RN/JAK3/ALOX12/LYN/LGALS9/LAPTM5/PDE5A/SIRPB1/FLOT2/RHOA/MYADM/JAK1/PTPN23/RIPOR2/ZNF703/MBP/WNK1/FOXO3/HLX/DMTN/RELA
BP	GO:0032456	endocytic recycling	64	-0.52	-1.63	0.00423986	0.01792149	0.01100284	2681	tags=50%, list=23%, signal=39%	ZDHHC2/CCDC22/GGA3/EHD4/KIAA1109/SCRIB/EPG5/ATP9A/VPS53/CMTM6/STX6/SNX3/ACAP2/RAB11FIP4/SNX17/VPS51/DENND1C/WASHC2C/VPS35/ATP6AP1/SORL1/RAB7A/RAB14/EHD3/ARHGAP8/SNX27/RAB11B/GRIPAP1/PTPN23/EHD1/DENND1A/ARHGAP1
BP	GO:1901654	response to ketone	110	-0.45	-1.52	0.00424148	0.01792149	0.01100284	996	tags=23%, list=8%, signal=21%	TSPO/NCOA4/NCOA2/MAP4K1/POR/THBS1/PTAFR/GNB1/NR3C1/CALM3/ABHD2/GPI/AQP1/SIN3A/TXNIP/CDA/MSN/PPP1R9B/LARP1/WBP2/BCL2L1/MBP/FOXO3/CBL/TBXA2R

BP	GO:0002429	immune response-activating cell surface receptor signaling pathway	197	-0.39	-1.43	0.00430243	0.01808295	0.01110197	2158	tags=27%, list=18%, signal=23%	FPR3/GPLD1/CD38/BTN3A2/NCKAP1L/MYO1G/MICB/FCER1G/RFTN1/SLA2/CR1/FGR/FYN/SRC/PRKCE/SPPL3/HCK/DGKZ/RC3H2/FYB1/GPS2/EZR/CMKLR1/SH2B2/C5AR1/PTPRJ/PTPN6/KLRD1/FOXP1/ERMAP/SPG21/NFAM1/NFATC2/IKBKG/TRAF6/PRKD2/PAK1/CSK/VAV1/STK11/PTPRC/SYK/PRAM1/INPP5D/LYN/RBCK1/LAPTM5/CREBBP/PRKCB/PIK3CD/PAK2/WNK1/BAG6/RELA
BP	GO:0002757	immune response-activating signal transduction	197	-0.39	-1.43	0.00430243	0.01808295	0.01110197	2158	tags=27%, list=18%, signal=23%	FPR3/GPLD1/CD38/BTN3A2/NCKAP1L/MYO1G/MICB/FCER1G/RFTN1/SLA2/CR1/FGR/FYN/SRC/PRKCE/SPPL3/HCK/DGKZ/RC3H2/FYB1/GPS2/EZR/CMKLR1/SH2B2/C5AR1/PTPRJ/PTPN6/KLRD1/FOXP1/ERMAP/SPG21/NFAM1/NFATC2/IKBKG/TRAF6/PRKD2/PAK1/CSK/VAV1/STK11/PTPRC/SYK/PRAM1/INPP5D/LYN/RBCK1/LAPTM5/CREBBP/PRKCB/PIK3CD/PAK2/WNK1/BAG6/RELA
CC	GO:0030055	cell-substrate junction	353	-0.35	-1.35	0.00435922	0.01824449	0.01120115	1315	tags=27%, list=11%, signal=25%	ATP6V0C/FERMT3/PDPK1/ARPC5/PTPN12/ITGB2/ARHGEF2/CAP1/LCP1/ARF1/PIP5K1C/CHP1/YWHAZ/TPM4/ARHGAP31/NEDD9/SH3KBP1/PALLD/NHS/YWHAB/FES/CORO1C/PTK2B/ZNF185/SVIL/PAK1/VCL/PDLIM7/CTNNA1/TGFB1I1/GSN/ITGA5/PTPRC/SLC9A1/PLEC/DCAF6/EHD3/CD151/CLTC/FLNA/HSPA1A/ACTB/ANXA6/CAPN1/HMGA1/ZYX/RAC1/SLA/HNRNPK/RAC2/ADGRE5/DNM2/FOCAD/IGF2R/HYOU1/LRP1/FLOT2/HSPB1/ITGA2B/CNN2/LPP/LASP1/PXN/RHOA/PDLIM1/MSN/ACTN4/ADD1/JAK1/RSU1/GNA12/CFL1/ARHGAP26/MAPK3/ITGB5/APBB1IP/TGM2/PARVB/CTTN/PCBP2/PRKAR2A/CAPN5/ACTN1/TSPAN9/GAK/BSG/ITGB3/VASP/DAB2/MRC2/MYH9/CBL/TNS1/IQGAP1/PEAK3/RHOG/TLN1
BP	GO:0051347	positive regulation of transferase activity	389	-0.34	-1.34	0.00435922	0.01824449	0.01120115	2051	tags=24%, list=17%, signal=21%	FGR/PTEN/MAP3K4/TIRAP/SRC/EPHB4/GCN1/ADNP/PNKP/MAP2K5/STK4/SDC4/XRCC6/ERBB2/CCNY/CASS4/MAGED1/TGFB1/DGKZ/ERN2/LILRA5/ERN1/CHTF8/SYAP1/LRRK2/CACUL1/EGR1/DLG4/EPHB1/MAP2K2/DAZAP2/EPHA4/PDPK1/NCF1/CSF1R/TRIB3/GSK3A/UBE2L3/SASH1/PDGFA/NEDD9/INSR/MAP3K5/SLC27A1/TAOK3/GREM1/VLDLR/SORL1/PTK2B/NEURL1/RAPGEF2/CCND3/TRAF6/IL6R/PAK1/MOB3A/IGF2/CSK/TAOK1/STK11/MAP2K7/FZR1/STRADA/PTPRC/SYK/DVL3/THBS1/PTPN1/TLR6/CALM3/FZD5/LTF/PIK3CG/MADD/MARK2/LYN/MMD/PDE5A/AKAP13/ATP2B4/RHOA/CLU/PINK1/MAPK3/SNCA/CDKN1A/DTX3L/TAL1/PAK2/ITGB3/WNK1/IQGAP1/MAP2K3/STRADB
BP	GO:0031929	TOR signaling	109	-0.44	-1.50	0.00436738	0.01825842	0.0112097	2439	tags=34%, list=21%, signal=27%	GPR137/RFFL/ENDOG/NCKAP1L/DYRK3/HDAC3/KLHL22/DISC1/RRAGD/ATM/AKT1S1/LARS1/SZT2/TSC2/SYAP1/WAC/CLEC16A/GSK3A/SESN3/SESN2/RRAGC/SIK3/OTUD5/LAMTOR1/STK11/UBR2/FBXO9/CASTOR2/LARP1/GNA12/PINK1/FLCN/ARAF/TMEM127/EIF4EBP2/PRR5/NPRL3
BP	GO:0033144	negative regulation of intracellular steroid hormone receptor signaling pathway	28	-0.64	-1.73	0.00437174	0.01825842	0.0112097	2603	tags=46%, list=22%, signal=36%	CALR/CRY2/VPS18/PER1/ZBTB7A/SMARCA4/FOXP1/VPS11/KANK2/NCOR2/RHOA/DAB2/CNOT1

BP	GO:0070371	ERK1 and ERK2 cascade	177	-0.40	-1.45	0.00439578	0.01833959	0.01125953	2309	tags=31%, list=20%, signal=25%	SLC9A3R1/TF/EPHB2/TRAF7/GLIPR2/CHI3L1/SPRY3/PTEN/TIRAP/SRC/SULT1A3/ERBB2/TGFB1/SIRPA/RRAS/NOTCH2/GSTP1/ARRB1/EZR/C5AR1/SHC1/EPHB1/MAP2K2/ACTA2/ZFP36L1/PTPN6/PYCARD/EPHA4/GPBAR1/CCR1/CSF1R/YWHAZ/PDGFA/NDRG2/PTK2B/MFHAS1/VRK3/RAPGEF2/PRKD2/CSK/MAP2K7/PTPRC/SYK/PTPN1/MTURN/ARHGAP8/LYN/LGALS9/GNAI2/TNIP1/ARRB2/MAPK3/FLCN/DAB2
CC	GO:0005925	focal adhesion	349	-0.35	-1.35	0.0044141	0.01836542	0.01127539	1286	tags=27%, list=11%, signal=25%	PDPK1/ARPC5/PTPN12/ITGB2/ARHGEF2/CAP1/LCP1/ARF1/PIP5K1C/CHP1/YWHAZ/TPM4/ARHGAP31/NEDD9/SH3KBP1/PALLD/NHS/YWHAB/FES/CORO1C/PTK2B/ZNF185/SVIL/PAK1/VCL/PDLIM7/CTNNA1/TGFB1I1/GSN/ITGA5/PTPRC/SLC9A1/PLEC/DCAF6/EHD3/CD151/CLTC/FLNA/HSPA1A/ACTB/ANXA6/CAPN1/HMGA1/ZYX/RAC1/SLA/HNRNPK/RAC2/ADGRE5/DNM2/FOCAD/IGF2R/HYOU1/LRP1/FLOT2/HSPB1/ITGA2B/CNN2/LPP/LASP1/PXN/RHOA/PDLIM1/MSN/ACTN4/ADD1/JAK1/RSU1/GNA12/CFL1/ARHGAP26/MAPK3/ITGB5/APBB1IP/TGM2/PARVB/CTTN/PCBP2/PRKAR2A/CAPN5/ACTN1/TSPAN9/GAK/BSG/ITGB3/VASP/DAB2/MRC2/MYH9/CBL/TNS1/IQGAP1/PEAK3/RHOG/TLN1
BP	GO:0030866	cortical actin cytoskeleton organization	32	-0.61	-1.70	0.00443431	0.01836542	0.01127539	2999	tags=44%, list=26%, signal=33%	IKBKB/TNF/CALR/EPB41L3/VPS4A/NCKAP1L/ROCK2/EZR/LCP1/PLEC/PLEK/FMNL1/EPB41/TLN1
BP	GO:0033273	response to vitamin	43	-0.56	-1.66	0.00442267	0.01836542	0.01127539	2156	tags=42%, list=18%, signal=34%	CDKN2D/ATP2B1/SOD2/FKBP1B/MTHFR/VDR/MGMT/ALAD/F5/ALPL/FES/KANK2/TSPO/RARA/MED1/RXRA/GPX1/PIM1
BP	GO:0048821	erythrocyte development	32	-0.61	-1.70	0.00443431	0.01836542	0.01127539	679	tags=34%, list=6%, signal=32%	MED1/G6PD/BPGM/GATA1/TAL1/SLC4A1/FAM210B/EPB42/ALAS2/TRIM58/DMTN
BP	GO:0050773	regulation of dendrite development	67	-0.50	-1.60	0.00448361	0.01853101	0.01137706	2573	tags=34%, list=22%, signal=27%	STAU2/ACTR2/KIAA0319/EPHB2/BCL11A/YWHAH/DBNL/DGKG/CUL7/DBN1/CDKL5/SIPA1L1/SEMA4D/EPHA4/VLDLR/RAPGEF2/STK11/PPP3CA/CAMK1D/GSK3B/RHOA/CUX1/CARM1
BP	GO:0030010	establishment of cell polarity	103	-0.46	-1.56	0.00449664	0.01856557	0.01139827	1666	tags=30%, list=14%, signal=26%	ROCK2/NDEL1/EPHB1/PARD3/FRMD4B/MAP4/FAM89B/NUMA1/AMOT/PLEKHG3/HTT/DOCK8/PTK2B/BCAS3/NSFL1C/FRMD4A/NDE1/STK11/GSN/JAM3/DCTN1/DOCK2/MARK2/SNX27/GSK3B/FLOT2/RHOA/MSN/RIPOR2/SH3BP1/MYH9
BP	GO:0098927	vesicle-mediated transport between endosomal compartments	44	-0.56	-1.67	0.00450332	0.01857388	0.01140337	2002	tags=41%, list=17%, signal=34%	DNAJC13/CORO1A/SRC/HOOK2/SNX3/WDR81/EZR/MAP2K2/HOOK3/VPS11/SORL1/RAB7A/FLNA/RILP/MSN/MAPK3/PTPN23/DAB2
BP	GO:0007009	plasma membrane organization	94	-0.45	-1.50	0.00458566	0.0188743	0.01158782	1499	tags=24%, list=13%, signal=22%	STX4/CHMP4B/OSBPL2/FLOT1/CLN3/SPTBN1/PLEKHM2/SPTA1/CAVIN2/PLSCR3/GSN/PTPRC/ASAP1/PLEC/ANXA6/CHMP1A/DNM2/SYTL4/DYSF/CLU/AKT2/SLC4A1/MYH9
CC	GO:0101002	ficolin-1-rich granule	173	-0.41	-1.47	0.00460306	0.01892633	0.01161976	2180	tags=38%, list=19%, signal=32%	ADAM8/SERPINA1/FCER1G/GLB1/CR1/CD93/GUSB/APAF1/DBNL/XRCC6/EEF2/SIRPA/SLC2A3/AP2A2/LAMP2/ACLY/GSTP1/NBEAL2/PSMD2/PSMD3/MAPK14/CRISPLD2/ALAD/ATP6V0C/KPNB1/SIGLEC5/ARPC5/ITGB2/CDK13/PGAM1/PKM/ATP6V0A1/TIMP2/PAFAH1B2/PYGL/PSMD11/VCL/ARL8A/IMPDH1/MMP9/ALDOA/LAMTOR1/GSN/UBR4/GAA/ATG7/VCP/DOK3/HSPA1A/CAPN1/GPI/RAC1/CDA/HUWE1/COTL1/RHOA/GNS/LRG1/CANT1/FTH1/PLEKHO2/LAMP1/CTSB/CTSD/KRT1/DIAPH1

BP	GO:0010611	regulation of cardiac muscle hypertrophy	37	-0.59	-1.69	0.00468143	0.01915957	0.01176296	1715	tags=35%, list=15%, signal=30%	CTDP1/ROCK2/YY1/GSK3A/PPP3CA/SLC9A1/LMNA/G6PD/PDE5A/TNFRSF1B/ATP2B4/MTPN/JARID2
BP	GO:0014743	regulation of muscle hypertrophy	37	-0.59	-1.69	0.00468143	0.01915957	0.01176296	1715	tags=35%, list=15%, signal=30%	CTDP1/ROCK2/YY1/GSK3A/PPP3CA/SLC9A1/LMNA/G6PD/PDE5A/TNFRSF1B/ATP2B4/MTPN/JARID2
BP	GO:0051480	regulation of cytosolic calcium ion concentration	186	-0.39	-1.42	0.00470908	0.01922309	0.01180195	2517	tags=31%, list=21%, signal=24%	CAPN3/BAK1/TRPC3/FPR3/CD38/CALM1/CORO1A/LETM1/ATP2B1/GNA15/PTGIR/KNG1/FYN/TPCN1/FKBP1B/SLC24A4/MCOLN1/PRKCE/SPPL3/PROK2/OPRL1/CMKLR1/DLG4/C5AR1/PTPN6/TMBIM6/CLN3/PDPK1/PML/CCR1/ITPR2/PRKACA/CHERP/ERO1A/TMEM38A/BCAP31/HTT/PTK2B/SLC8A1/CXCR2/PTPRC/FIS1/SELENON/CALM3/PIK3CG/JAK3/LYN/GP9/ATP2B4/FKBP1A/GATA1/CXCR1/SNCA/GP1BA/P2RX1/TBXA2R/DIAPH1
BP	GO:0051651	maintenance of location in cell	149	-0.41	-1.46	0.00475263	0.01938097	0.01189888	2603	tags=37%, list=22%, signal=29%	CALR/HNRNPU/CD4/TWF2/CAPN3/SLC25A23/CALM1/ASPM/CORO1A/SCIN/LETM1/TPCN1/TAF8/FKBP1B/MCOLN1/PRKCE/SUN2/VPS13D/HSPA5/GPAA1/RANGAP1/DBN1/PTPN6/CIZ1/PDPK1/PML/ITPR2/PRKACA/CHERP/ERO1A/TSPO/ARHGAP21/SORL1/TMEM38A/HTT/PTK2B/SLC8A1/LCN2/GSN/PTPRC/KDELR1/SELENON/GAA/CALM3/ANXA6/LYN/GP9/FKBP1A/FTL/FTH1/PINK1/HK1/SNCA/GP1BA/DIAPH1
MF	GO:0030295	protein kinase activator activity	73	-0.49	-1.56	0.00475852	0.01938512	0.01190144	1009	tags=23%, list=9%, signal=21%	MAP2K2/DAZAP2/GREM1/MOB3A/IGF2/TAOK1/STK11/STRADA/CALM3/LTF/MADD/MARK2/CDKN1A/TAL1/PAK2/WNK1/IQGAP1
BP	GO:0030856	regulation of epithelial cell differentiation	67	-0.50	-1.59	0.00477522	0.01943324	0.01193098	1898	tags=46%, list=16%, signal=39%	MAFG/CEBPB/TNFRSF1A/IKBKB/SERPINE1/TNF/NOTCH1/MAFF/F11R/KIAA1109/ATOH8/MYCL/VDR/VEZF1/ROCK2/BTG1/PLCB1/STAT5B/KEAP1/S1PR2/ZFP36L1/CDKN1C/NCOA3/NUMA1/VCL/BMP6/MMP9/MED1/FOXJ2/ADD1/CLDN5
BP	GO:0048738	cardiac muscle tissue development	102	-0.46	-1.55	0.00479568	0.01949656	0.01196985	2072	tags=29%, list=18%, signal=24%	HEG1/SRF/PTEN/TGFB1/NOTCH2/CTDP1/MAPK14/YY1/MSX1/GSK3A/TPM1/GREM1/RBPJ/RARA/SLC8A1/ARID2/SOX6/PDLIM5/SLC9A1/LMNA/PLEC/MED1/G6PD/AKAP13/FKBP1A/ARRB2/KDM6B/PIM1/JARID2/NPRL3
BP	GO:0051282	regulation of sequestering of calcium ion	74	-0.48	-1.52	0.00492569	0.01998424	0.01226926	2517	tags=39%, list=21%, signal=31%	CAPN3/SLC25A23/CALM1/CORO1A/LETM1/TPCN1/FKBP1B/MCOLN1/PRKCE/PTPN6/PDPK1/ITPR2/PRKACA/CHERP/ERO1A/TMEM38A/HTT/PTK2B/SLC8A1/PTPRC/SELENON/CALM3/ANXA6/LYN/GP9/FKBP1A/SNCA/GP1BA/DIAPH1
BP	GO:0071216	cellular response to biotic stimulus	182	-0.40	-1.46	0.00497331	0.02015684	0.01237523	2278	tags=34%, list=19%, signal=28%	SCARB1/TRIM5/VIM/EPHB2/TLR2/BCL2L11/PAF1/TIRAP/PRDX2/NLRP3/SRC/APAF1/PRKCE/CD68/HCK/TGFB1/SIRPA/TNFSF4/NOTCH2/MTDH/HSPA5/GSTP1/ARID5A/MAPK14/TRIB1/PYCARD/SMC1A/SBNO2/SPI1/CXCL5/SASH1/PF4V1/NFKB1/TSPO/UPF1/RARA/BCR/TRAF6/PLSCR3/BMP6/SYK/PTAFR/TLR6/FZD5/LTF/PF4/CD14/LYN/GSK3B/CDK19/TNFRSF1B/TXNIP/CDA/RHOA/TNIP1/PRPF8/GATA1/MAPK3/LITAF/TBXA2R/MAP2K3/RELA

BP	GO:0010498	proteasomal protein catabolic process	435	-0.33	-1.32	0.00498412	0.02017639	0.01238723	1818	tags=22%, list=16%, signal=20%	DET1/AREL1/RNF144B/GID4/SDCBP/UBXN7/HSPA5/SH3RF3/UBE2J2/SMARCC1/ARRB1/LRRK2/ENC1/MAN1A1/PSMD2/KEAP1/MARCHF6/TMEM67/PIAS1/TRIB1/CEBPA/WAC/YOD1/CBFA2T3/ARIH2/ALAD/SPOP/ITCH/KCTD2/HERC2/CCNF/PML/PSME4/KAT5/TRIB3/GSK3A/CANX/PSMD1/PRKACA/FBXL18/UBE4B/BCAP31/RNF126/RCN3/PITHD1/TBX21/NSFL1C/MAP1A/TBL1XR1/RAD23A/FZR1/PSME3/NPLOC4/FBXL20/TRIP12/VCP/HSPA1A/ANKRD9/UBR2/PSMC4/GABARAP/RBCK1/GSK3B/FBXO9/WWP2/DDB1/HUWE1/CUL4A/RMND5A/TBL1X/GID8/GPX1/CLU/ARRB2/GNA12/RNF187/PINK1/ASCC2/SIAH2/PCBP2/PSMF1/N4BP1/UBB/DNAJB2/USP7/ARAF/RNF40/UBXN6/SHARPIN/DCAF12/DAB2/UBE2H/CDC34/ZER1/BAG6/RYBP/DCAF11
BP	GO:0010565	regulation of cellular ketone metabolic process	77	-0.48	-1.54	0.00498828	0.02017639	0.01238723	1972	tags=29%, list=17%, signal=24%	IRS2/PRKCE/SCAP/ABCB11/EGR1/NR1H2/CLN3/ACADVL/TRIB3/DGAT2/PDK2/EIF6/BCKDK/BMP6/CPT1A/PDK3/NCOR2/ATP2B4/WDTC1/SNCA/AKT2/DAB2
BP	GO:0048638	regulation of developmental growth	173	-0.40	-1.46	0.00502471	0.02030308	0.01246501	2089	tags=26%, list=18%, signal=22%	BCL2L11/DISC1/WWC3/MACF1/SRF/PTEN/FTO/ADNP/DBNL/STK4/NIPBL/CPNE5/CTDP1/PLCB1/NDEL1/STAT5B/EZR/DBN1/MAPK14/CDKL5/YY1/SEMA4D/GSK3A/DPYSL2/INSR/SGPL1/RBPJ/SEMA4A/EIF4G2/IGF2/IST1/G6PD/BASP1/ULK1/SIN3A/GSK3B/STAT5A/DNM2/PIM1/CDKN1A/STAT3/CTTN/YBX3/JARID2/HLX
MF	GO:0005518	collagen binding	36	-0.60	-1.71	0.00503426	0.02032103	0.01247603	1092	tags=31%, list=9%, signal=28%	PCOLCE/TNXB/CRTAP/PDGFA/PAK1/MMP9/GP6/THBS1/SPARC/CTSB/MRC2
BP	GO:0009132	nucleoside diphosphate metabolic process	98	-0.45	-1.52	0.00507853	0.02043746	0.01254752	1960	tags=26%, list=17%, signal=21%	HDAC4/GALK1/ZBTB7A/RRM1/NME4/ZBTB20/CBFA2T3/PGAM1/TJP2/ENTPD5/ARNT/EIF6/PKM/INSR/FBP1/ALDOA/GPI/AK1/BPGM/HK1/STAT3/GUK1/SLC4A1/ENO1/MPP1
BP	GO:0071453	cellular response to oxygen levels	112	-0.43	-1.46	0.00507572	0.02043746	0.01254752	2449	tags=44%, list=21%, signal=35%	MDM4/TP53/EGLN1/MDM2/NOTCH1/NKX3-1/PIK3CB/UBQLN1/CFLAR/EGLN3/ENDOG/CYBB/NFE2L2/AIFM1/USP19/AK4/ADAM8/FOXO1/PTEN/SRC/PRKCE/ATP7A/CPEB4/NPEPPS/ROCK2/EGR1/ZFP36L1/TMBIM6/EPHA4/HIF1AN/ATP6AP1/RBPJ/ERO1A/SLC8A1/SLC9A1/PDK3/LMNA/ATG7/HIPK2/AQP1/HYOU1/BNIP3L/MPL/ATP6V1A/PINK1/ATP6V0D1/ENO1/FOXO3/CBL
BP	GO:0002237	response to molecule of bacterial origin	234	-0.38	-1.41	0.00509235	0.02047236	0.01256894	2278	tags=30%, list=19%, signal=25%	SCARB1/TRIM5/VIM/EPHB2/IRAK3/TLR2/PTGIR/SOD2/PAF1/TIRAP/PRDX2/NLRP3/SRC/IFNAR1/PRKCE/THBD/CD68/HCK/TGFB1/SIRPA/TNFSF4/MTDH/GSTP1/ARID5A/C5AR1/MAPK14/TRIB1/PYCARD/SBNO2/ALAD/SPI1/CXCL5/AKIRIN2/FOXP1/SASH1/ALPL/PF4V1/NFKB1/TSPO/UPF1/RARA/SELP/BCR/TRAF6/OTUD5/PLSCR3/BMP6/PTAFR/TLR6/MAPKAPK3/FZD5/LTF/JUND/PF4/CD14/LYN/LGALS9/CDK19/TNFRSF1B/RHOA/TNIP1/PRPF8/GATA1/MAPK3/SNCA/MAPKAPK2/LITAF/TBXA2R/MAP2K3/RELA
CC	GO:0030135	coated vesicle	214	-0.38	-1.40	0.00510467	0.02050117	0.01258663	2250	tags=29%, list=19%, signal=24%	TF/AAK1/TNK2/SERPINA1/SLC28A2/CLTB/VPS18/MYO1E/UNC13D/RAB27B/DBNL/AP1G1/ATP7A/STX6/SNX3/AP2A2/SCAP/AFTPH/NUMB/COPE/AP2B1/AP2S1/PEF1/DENND1C/ERGIC1/TEX261/SORT1/AP1M1/VPS41/SYNRG/COPB2/F5/STX5/SPG21/CNIH3/VPS11/AP2A1/BCAP31/LDLRAP1/COPA/SEC31A/TGOLN2/RAB14/KDELR1/CLTC/FZD5/DNAJC5/GOLGA2/HIP1/DNM2/IGF2R/EPN1/PACS1/SCYL1/ECE1/NRGN/DENND1A/AP2M1/STON2/DAB2/SEC16A/FURIN

BP	GO:0052547	regulation of peptidase activity	263	-0.36	-1.36	0.00515547	0.02066347	0.01268627	1467	tags=23%, list=13%, signal=21%	NRDC/CAST/UBE2Z/PYCARD/DAP/EPHA4/DHCR24/PML/PSME4/AKIRIN2/BIRC6/PCSK1N/NLRC4/ATP2A3/CSNK2A1/MAP3K5/VSIR/SORL1/BCAP31/PRDX5/TIMP2/RCN3/MMP9/POR/SFRP2/GSN/APLP2/SYK/THBS1/FIS1/PSME3/CASP2/LTF/VCP/GPI/AQP1/LYN/HIP1/LGALS9/CYFIP2/LAPTM5/LRP1/RHOA/GPX1/BBC3/ARRB2/GRN/UBE2O/PINK1/RPS6KA1/SNCA/SIAH2/STAT3/PSMF1/CTSB/PAK2/CTSD/MBP/P2RX1/TIMP1/FURIN
BP	GO:0045646	regulation of erythrocyte differentiation	41	-0.58	-1.69	0.00527516	0.02107942	0.01294164	1612	tags=44%, list=14%, signal=38%	STAT5B/KAT7/MAPK14/ZFP36L1/SPI1/KLF13/ARNT/MED1/MAFB/HSPA1A/INPP5D/LYN/GATA1/STAT3/TAL1/FAM210B/PRKDC/FOXO3
BP	GO:0098655	cation transmembrane transport	450	-0.33	-1.30	0.00532699	0.02122254	0.01302951	1505	tags=17%, list=13%, signal=15%	CLCN5/CACNB1/KCNH2/FXYD6/PTPN6/CHRNE/SLC36A1/HVCN1/CD63/CLN3/SHISA7/PDPK1/KCNQ5/MICU1/TSPO2/ITPR2/CHP1/PRKACA/CHERP/ATP2A3/CNIH3/ERO1A/TMEM38A/HTT/PTK2B/SLC8A1/SLC6A20/SLC24A3/SLC3A2/MMP9/SLC25A38/PTPRC/SLC9A1/UQCRC1/SELENON/SCN1B/ATP1B2/EHD3/CALM3/SLC6A9/PIK3CG/G6PD/FLNA/SLC1A5/ANXA6/CAPN1/UCP2/SLC43A2/GJC2/AQP1/LYN/DNM2/SLC43A1/GP9/WWP2/ATP2B4/FKBP1A/TMEM63B/ATP6V1A/ACTN4/PINK1/TESC/SNCA/SLC15A3/GP1BA/ATP6V0D1/MMGT1/SLC25A37/STIM1/WNK1/SLC7A5/KEL/P2RX1/SLC38A5/SLC6A8/DIAPH1
BP	GO:0071526	semaphorin-plexin signaling pathway	25	-0.66	-1.73	0.00536795	0.02136432	0.01311655	1759	tags=36%, list=15%, signal=31%	NCAM1/SEMA4D/SEMA4A/PLXNC1/FLNA/RAC1/ARHGDIA/ECE1/SH3BP1
BP	GO:0060419	heart growth	40	-0.56	-1.62	0.00539799	0.02141957	0.01315048	2072	tags=35%, list=18%, signal=29%	HEG1/PTEN/CTDP1/MAPK14/YY1/GSK3A/RBPJ/ARID2/PDLIM5/G6PD/BASP1/AKAP13/PIM1/JARID2
BP	GO:0048598	embryonic morphogenesis	293	-0.35	-1.33	0.00550908	0.02179511	0.01338104	2567	tags=30%, list=22%, signal=24%	CLASP1/TGIF2/MED12/PBX2/SETD2/SOCS3/SCRIB/ARMC5/WLS/OTX1/SLC9A3R1/TBX19/IFT122/DUSP1/EPHB2/MTHFD1/PTK7/BCL2L11/PAF1/SRF/WNT1/TRIOBP/APAF1/EXOC4/MTHFR/MAP2K5/STK4/ATOH8/SDC4/EXT1/NIPBL/MKS1/TGFB1/PHACTR4/NOTCH2/TSC2/ROCK2/NF2/ZFP36L1/MSX1/FUZ/CDKN1C/ITGB2/TRIM28/GRB2/PRKACA/AMOT/SUFU/GREM1/HIPK1/YBX1/RARA/BCR/TRAF6/CHST11/IGF2/ZBTB16/MMP9/TGFB1I1/SFRP2/ITGA5/DVL3/MED1/MAFB/IL1RN/FZD5/HIPK2/SKI/NDST1/GPI/ETS2/CREBBP/MYADM/KDM6B/GNA12/ECE1/MAPK3/ITGB5/PBX1/TAL1/NCOA1/ITGB3/VASP/MBP/WNK1/MICAL2/HLX
BP	GO:0000422	autophagy of mitochondrion	82	-0.47	-1.52	0.00559089	0.02201954	0.01351882	1240	tags=28%, list=11%, signal=25%	MAP1LC3B/GSK3A/OPTN/TSPO/HTT/WDR45/ATG4D/FIS1/ATG7/WIPI2/FZD5/ULK1/GABARAP/MARK2/BNIP3L/HUWE1/ATG2A/ATG13/PINK1/CTTN/SQSTM1/MFN2/FBXO7
BP	GO:0061726	mitochondrion disassembly	82	-0.47	-1.52	0.00559089	0.02201954	0.01351882	1240	tags=28%, list=11%, signal=25%	MAP1LC3B/GSK3A/OPTN/TSPO/HTT/WDR45/ATG4D/FIS1/ATG7/WIPI2/FZD5/ULK1/GABARAP/MARK2/BNIP3L/HUWE1/ATG2A/ATG13/PINK1/CTTN/SQSTM1/MFN2/FBXO7
BP	GO:0055074	calcium ion homeostasis	256	-0.37	-1.37	0.00568957	0.02230938	0.01369677	2136	tags=26%, list=18%, signal=22%	ATP2B1/GNA15/PTGIR/KNG1/DISC1/FYN/TPCN1/KCTD17/PDZD8/FKBP1B/SLC24A4/MCOLN1/PRKCE/SPPL3/GRIK2/PROK2/VDR/ATP13A1/OPRL1/CMKLR1/DLG4/C5AR1/PTPN6/TMBIM6/CLN3/PDPK1/PML/MICU1/CCR1/ITPR2/PRKACA/CHERP/ATP2A3/ERO1A/TMEM38A/BCAP31/HTT/PTK2B/SLC8A1/SNX10/CXCR2/SLC24A3/PTPRC/FIS1/SELENON/CALM3/PIK3CG/JAK3/ANXA6/LYN/GP9/ATP2B4/FKBP1A/PRKCB/GATA1/CXCR1/SNCA/TGM2/GRINA/GP1BA/STIM1/KEL/P2RX1/TBXA2R/XK/DIAPH1

BP	GO:0055085	transmembrane transport	799	-0.30	-1.24	0.00568397	0.02230938	0.01369677	1979	tags=19%, list=17%, signal=17%	YWHAH/IRS2/FKBP1B/SLC24A4/ATP6V1B2/MCOLN1/BLOC1S3/PRKCE/SLC25A41/GRIK2/KCNJ15/SLC39A7/COX15/ATP7A/GPR155/ZDHHC7/SLC66A1/SLC2A3/ATP13A1/ANO6/SLC12A6/PEX10/HSPA5/ABCB11/OPRL1/TMC8/RIPK1/DLG4/MFSD4B/TTYH3/CLCN5/MAPK14/CACNB1/CA2/KCNH2/SLC35E2B/FXYD6/SORT1/PER2/PTPN6/SLC16A3/CHRNE/SLC36A1/HVCN1/SLC35A4/CD63/AQP9/SLC25A11/CLN3/SHISA7/PDPK1/RALBP1/KCNQ5/PANX2/MICU1/TSPO2/ITPR2/TRIB3/GSK3A/ABCG2/CHP1/PRKACA/CHERP/INSR/SESN2/ATP2A3/SLC27A1/CNIH3/ERO1A/SLC22A23/TMEM38A/HTT/LRRC8A/PTK2B/SLC2A1/SLC8A1/BCR/ANKH/SLC6A20/INPP5K/SLC24A3/SLC3A2/CLIC3/MMP9/SLC44A2/SLC25A38/CPT1A/PTPRC/SLC9A1/THBS1/PTAFR/UQCRC1/SELENON/SCN1B/ATP1B2/EHD3/CALM3/SLC6A9/PIK3CG/G6PD/FLNA/SLC1A5/SLC14A1/ABCC4/ACTB/ANXA6/CAPN1/UCP2/SLC43A2/GJC2/AQP1/LYN/TMEM120A/DNM2/SLC43A1/GP9/WWP2/ABCC3/ATP2B4/LASP1/FKBP1A/TMEM63B/PRF1/PRKCB/RTN2/ATP6V1A/SLC38A10/ACTN4/PIM1/OAZ1/PINK1/TESC/SNCA/SLC15A3/TST/AHCYL1/AKT2/SLC4A1/GP1BA/AQP10/MFSD2B/ATP6V0D1/MMGT1/SLC25A37/STIM1/WNK1/SLC7A5/KEL/P2RX1/SLC38A5/SLC6A8/DIAPH1
BP	GO:0030834	regulation of actin filament depolymerization	41	-0.58	-1.68	0.00578447	0.02263688	0.01389784	2764	tags=56%, list=24%, signal=43%	TMOD3/CARMIL1/ADD2/TWF2/SCIN/FLII/TRIOBP/EPS8/SPTAN1/WASHC2C/WDR1/SPTBN1/SPTA1/SVIL/GSN/PLEK/CAPZB/ADD1/TMOD1/SPTB/MTPN/SH3BP1/DMTN
BP	GO:0002685	regulation of leukocyte migration	144	-0.41	-1.44	0.00592421	0.02313816	0.0142056	1624	tags=22%, list=14%, signal=19%	PLCB1/CMKLR1/C5AR1/STK10/PYCARD/SPI1/CCR1/CSF1R/GREM1/DOCK8/PTK2B/SELP/BCR/IL6R/JAM3/THBS1/PTAFR/CAMK1D/RAC1/LYN/LGALS9/RAC2/RHOA/MSN/RIN3/MAPK3/RIPOR2/MPP1/WNK1/PLVAP/RHOG
BP	GO:0034220	ion transmembrane transport	560	-0.32	-1.27	0.00597461	0.02331213	0.01431241	2517	tags=25%, list=21%, signal=21%	CAPN3/SLC25A23/TMEM109/PPIF/RGS7/BAK1/SLC37A2/ATP5F1B/TMC4/ANXA2/TRPC3/SLC13A4/CALM1/SLC1A4/CYBB/CORO1A/UTRN/SLC19A1/LRRC8B/SLC9A3R1/SLC20A1/EPHB2/LETM1/STIMATE/ATP2B1/ANO10/SLC25A15/SNAP25/FYN/TPCN1/YWHAH/FKBP1B/SLC24A4/ATP6V1B2/MCOLN1/PRKCE/SLC25A41/GRIK2/KCNJ15/SLC39A7/COX15/ATP7A/SLC66A1/ATP13A1/ANO6/SLC12A6/OPRL1/TMC8/RIPK1/DLG4/TTYH3/CLCN5/CACNB1/KCNH2/FXYD6/PER2/PTPN6/SLC16A3/CHRNE/SLC36A1/HVCN1/CD63/SLC25A11/CLN3/SHISA7/PDPK1/KCNQ5/MICU1/TSPO2/ITPR2/CHP1/PRKACA/CHERP/ATP2A3/SLC27A1/CNIH3/ERO1A/TMEM38A/HTT/LRRC8A/PTK2B/SLC8A1/ANKH/SLC6A20/SLC24A3/SLC3A2/CLIC3/MMP9/SLC25A38/PTPRC/SLC9A1/PTAFR/UQCRC1/SELENON/SCN1B/ATP1B2/EHD3/CALM3/SLC6A9/PIK3CG/G6PD/FLNA/SLC1A5/ABCC4/ANXA6/CAPN1/UCP2/SLC43A2/GJC2/AQP1/LYN/TMEM120A/DNM2/SLC43A1/GP9/WWP2/ATP2B4/LASP1/FKBP1A/TMEM63B/ATP6V1A/ACTN4/PINK1/TESC/SNCA/SLC15A3/AHCYL1/SLC4A1/GP1BA/ATP6V0D1/MMGT1/SLC25A37/STIM1/WNK1/SLC7A5/KEL/P2RX1/SLC38A5/SLC6A8/DIAPH1
BP	GO:0060048	cardiac muscle contraction	71	-0.49	-1.55	0.00600073	0.02336817	0.01434681	1475	tags=24%, list=13%, signal=21%	GRK2/KCNH2/PRKACA/TPM1/TMEM38A/SLC8A1/GSN/SLC9A1/GAA/SCN1B/EHD3/CALM3/PIK3CG/FLNA/PDE5A/MYL4/MAP2K3
BP	GO:0014074	response to purine-containing compound	76	-0.47	-1.52	0.00601457	0.02337628	0.01435179	1993	tags=32%, list=17%, signal=26%	PER1/THBD/PPARGC1B/PDE2A/HSPA5/EZR/ZFP36L1/AQP9/ITPR2/SSH1/TMEM38A/PTK2B/SLC8A1/INPP5K/RAPGEF2/PTAFR/PIK3CG/AQP1/GATA1/RAPGEF1/BSG/CARM1/P2RX1/DMTN

BP	GO:0030326	embryonic limb morphogenesis	58	-0.50	-1.54	0.00603834	0.02341209	0.01437378	2089	tags=26%, list=18%, signal=21%	BCL2L11/NIPBL/MKS1/NOTCH2/MSX1/GREM1/CHST11/ZBTB16/SFRP2/MED1/SKI/CREBBP/GNA12/ECE1/PBX1
BP	GO:0035113	embryonic appendage morphogenesis	58	-0.50	-1.54	0.00603834	0.02341209	0.01437378	2089	tags=26%, list=18%, signal=21%	BCL2L11/NIPBL/MKS1/NOTCH2/MSX1/GREM1/CHST11/ZBTB16/SFRP2/MED1/SKI/CREBBP/GNA12/ECE1/PBX1
BP	GO:0046031	ADP metabolic process	70	-0.49	-1.57	0.00611153	0.02366062	0.01452636	2204	tags=31%, list=19%, signal=26%	ENO2/AK4/HDAC4/GALK1/ZBTB7A/ZBTB20/CBFA2T3/PGAM1/ENTPD5/ARNT/EIF6/PKM/INSR/FBP1/ALDOA/GPI/AK1/BPGM/HK1/STAT3/SLC4A1/ENO1
BP	GO:0034767	positive regulation of ion transmembrane transport	90	-0.47	-1.55	0.0061491	0.0237829	0.01460143	1286	tags=22%, list=11%, signal=20%	PDPK1/CHP1/HTT/SLC9A1/PTAFR/ATP1B2/EHD3/CALM3/SLC6A9/G6PD/FLNA/GJC2/DNM2/ACTN4/PINK1/TESC/SNCA/STIM1/WNK1/P2RX1
BP	GO:0061024	membrane organization	571	-0.32	-1.28	0.00620971	0.02397991	0.01472239	1819	tags=19%, list=16%, signal=16%	STX6/SNX3/SUN2/SYNJ2/ANO6/HGS/SCAP/CPTP/GET3/ITGAM/NDEL1/SYNJ1/DLG4/PIKFYVE/STX4/PEF1/CHMP4B/OSBPL2/FLOT1/TOR1AIP2/VPS41/CLN3/OSBPL5/PITPNC1/NAIF1/SHISA7/DHCR24/ITGB2/ARF1/GSK3A/PIP5K1C/CHP1/EMC3/PITPNM2/STX5/SPTBN1/VPS11/PITPNA/TSPO/PLEKHM2/CORO1C/SPTA1/CAVIN2/PLSCR3/NSFL1C/SEC31A/LPCAT3/RAB7A/ANKFY1/ELMO1/GSN/PTPRC/SLC9A1/THBS1/SLC66A2/LMNA/ASAP1/RAB31/NUP93/PLEC/DCTN1/CALM3/DOCK2/HSPA1A/ANXA6/TGFBRAP1/CHMP1A/RAC1/LAPTM5/GSK3B/RAC2/DNM2/FLOT2/BNIP3L/SYTL4/DYSF/HUWE1/RHOA/PRKCB/RILP/MYADM/BBC3/CLU/ATG2A/RHOBTB1/PINK1/SNCA/TBC1D20/BCL2L1/PIP4K2A/SH3GLB2/AKT2/NAPA/SH3BP1/SLC4A1/TMCC1/MFN2/AP2M1/GAK/MMGT1/MBP/CTDNEP1/MYH9/SEC16A/RHOG/BAG6
BP	GO:1990089	response to nerve growth factor	30	-0.63	-1.72	0.00649179	0.02489041	0.01528139	1937	tags=30%, list=17%, signal=25%	KIDINS220/ACAP2/HSPA5/SORT1/RAPGEF2/RAPGEF1/EHD1/FOXO3/CBL
BP	GO:1990090	cellular response to nerve growth factor stimulus	30	-0.63	-1.72	0.00649179	0.02489041	0.01528139	1937	tags=30%, list=17%, signal=25%	KIDINS220/ACAP2/HSPA5/SORT1/RAPGEF2/RAPGEF1/EHD1/FOXO3/CBL
BP	GO:1902743	regulation of lamellipodium organization	41	-0.57	-1.67	0.00651207	0.02494412	0.01531436	2771	tags=41%, list=24%, signal=32%	PLCE1/CARMIL1/TWF2/FSCN1/ACTR2/WNT1/SRC/ATP7A/ABI3/CORO1C/RAC1/RAC2/DNM2/CAPZB/WAS/WASF2/DMTN
MF	GO:0019207	kinase regulator activity	176	-0.39	-1.43	0.00653355	0.02500232	0.0153501	1554	tags=26%, list=13%, signal=23%	GCN1/KIDINS220/STK4/CCNY/WDR81/GSTP1/ELP3/TRIB1/MAP2K2/CDKN1C/DAZAP2/CCNF/TRIB3/CHP1/YWHAB/TAOK3/GREM1/SH3BP5L/WARS1/CCND3/MOB3A/IGF2/TAOK1/STK11/STRADA/CALM3/HEXIM2/LTF/MADD/MARK2/ITPRIP/RAC2/HSPB1/CCNI/TESC/SNCA/CDKN1A/PRKAR2A/TAL1/PRKAR2B/CCNJL/PAK2/WNK1/IQGAP1/CNPPD1
BP	GO:0070372	regulation of ERK1 and ERK2 cascade	162	-0.40	-1.45	0.00659861	0.02520144	0.01547234	2232	tags=30%, list=19%, signal=25%	EPHB2/TRAF7/GLIPR2/CHI3L1/SPRY3/PTEN/TIRAP/SRC/ERBB2/TGFB1/SIRPA/RRAS/NOTCH2/GSTP1/ARRB1/EZR/C5AR1/SHC1/EPHB1/ACTA2/PTPN6/PYCARD/EPHA4/GPBAR1/CCR1/CSF1R/YWHAZ/PDGFA/NDRG2/PTK2B/MFHAS1/VRK3/RAPGEF2/PRKD2/CSK/MAP2K7/PTPRC/SYK/PTPN1/MTURN/ARHGAP8/LYN/LGALS9/GNAI2/TNIP1/ARRB2/MAPK3/FLCN/DAB2

BP	GO:1902105	regulation of leukocyte differentiation	210	-0.38	-1.40	0.00661094	0.02520144	0.01547234	2226	tags=28%, list=19%, signal=23%	CTNNBIP1/CLPTM1/SH3RF1/ADAM8/TMEM176B/CR1/PRDX2/NLRP3/XRCC6/ERBB2/TGFB1/PPARGC1B/ITPKB/TNFSF4/RC3H2/NOTCH2/STAT5B/RIPK1/TRIB1/ZFP36L1/PTPN6/SOS2/CCR1/FOXP1/RUNX3/TMEM131L/VSIR/NFAM1/FES/IRF1/PGLYRP1/RARA/TRAF6/TBX21/TOB2/ZMIZ1/ZBTB16/TFE3/PTPRC/LILRB3/SYK/MAFB/LTF/JAK3/INPP5D/PF4/LYN/LGALS9/RHOA/CUL4A/NF1/HCLS1/TESC/TAL1/PRKDC/FBXO7/FOXO3/HLX
BP	GO:0031330	negative regulation of cellular catabolic process	203	-0.38	-1.40	0.00663773	0.02527931	0.01552015	1719	tags=26%, list=15%, signal=22%	SDCBP/TSC2/CPTP/SMARCC1/LRRK2/IKBKE/HGF/MAPK14/WAC/CHMP4B/PHF23/DAP/ALAD/VPS35/QSOX1/EPHA4/TRDMT1/PML/CLEC16A/LEPR/GSK3A/TENT4B/CSNK2A1/SUFU/TSPO/SORL1/YBX1/TIMP2/HERC1/EIF4G2/MAP1A/ATG7/PIK3CG/HIPK2/EIF4G1/RUBCN/GOLGA2/ATP2B4/LARP1/PINK1/SNCA/STAT3/PSMF1/MAPKAPK2/N4BP1/USP7/TENT5C/YBX3/TIMP1/FURIN/BAG6/RYBP
BP	GO:0035725	sodium ion transmembrane transport	80	-0.48	-1.55	0.00671082	0.02551236	0.01566323	2365	tags=30%, list=20%, signal=24%	UTRN/SLC9A3R1/SLC20A1/TPCN1/YWHAH/SLC24A4/PRKCE/ANO6/FXYD6/CHP1/SLC8A1/SLC6A20/SLC24A3/SLC3A2/SLC9A1/SCN1B/ATP1B2/SLC6A9/DNM2/ATP2B4/ACTN4/TESC/WNK1/SLC6A8
BP	GO:0060326	cell chemotaxis	188	-0.39	-1.42	0.00671175	0.02551236	0.01566323	1640	tags=26%, list=14%, signal=22%	LYST/ADGRE2/ANO6/HGF/GAB1/CMKLR1/PIKFYVE/C5AR1/EPHB1/TREM1/SPI1/PREX1/ITGB2/CXCL5/CCR1/CSF1R/PIP5K1C/CXCL16/PF4V1/GREM1/PTK2B/CXCR2/PRKD2/IL6R/VAV1/SYK/JAM3/THBS1/PLEC/IL17RA/PIK3CG/CAMK1D/PF4/RAC1/LYN/LGALS9/RAC2/HSPB1/ARRB2/CXCR1/RIN3/MAPK3/RIPOR2/PIK3CD/BSG/MPP1/WNK1/RHOG
BP	GO:0045022	early endosome to late endosome transport	41	-0.57	-1.67	0.00673035	0.02555862	0.01569164	2002	tags=37%, list=17%, signal=30%	SRC/HOOK2/SNX3/WDR81/EZR/MAP2K2/HOOK3/VPS11/RAB7A/FLNA/RILP/MSN/MAPK3/PTPN23/DAB2
BP	GO:0007610	behavior	292	-0.35	-1.33	0.00681475	0.02584621	0.0158682	2472	tags=26%, list=21%, signal=21%	FIG4/CREB1/ACTR2/BTBD9/MTA1/ARCN1/AMFR/PRKAR1B/GIGYF2/EPHB2/TLR2/ORMDL3/STRN/SOD2/ZNF385A/THRA/SNAP25/SRF/FYN/PTEN/ABAT/SLC24A4/ADNP/PRKCE/GRIK2/EXT1/ATP7A/EPS8/PUM1/LRRK2/OPRL1/PLCB1/SYNJ1/EGR1/DLG4/PI3/MME/AGTPBP1/CLN3/VPS35/EPHA4/SHISA7/ZFHX3/LEPR/INSR/TSPO/VLDLR/NCOA2/HTT/ANKH/MEIS1/MAP1A/CPT1A/ITGA5/THBS1/FBXL20/GAA/ATP1B2/DCTN1/HIPK2/CHD8/EIF4G1/DYNLRB1/GPI/ATXN1/ARRB2/NF1/PPP1R9B/TMOD1/NRGN/SNCA/STAT3/CLDN5/TAL1/PRKAR2B/EIF4EBP2/P2RX1
CC	GO:0030672	synaptic vesicle membrane	51	-0.54	-1.62	0.00683209	0.02584621	0.0158682	2171	tags=45%, list=19%, signal=37%	VAMP2/DTNBP1/SYT12/RAB35/SV2A/MCTP1/CLTB/ATP2B1/RAB27B/ZNRF1/STX6/RPH3A/LRRK2/SYN3/RAB7A/RAB5B/CALM3/SLC6A9/DNAJC5/TPRG1L/RAB11B/SNCA/BCL2L1
CC	GO:0099501	exocytic vesicle membrane	51	-0.54	-1.62	0.00683209	0.02584621	0.0158682	2171	tags=45%, list=19%, signal=37%	VAMP2/DTNBP1/SYT12/RAB35/SV2A/MCTP1/CLTB/ATP2B1/RAB27B/ZNRF1/STX6/RPH3A/LRRK2/SYN3/RAB7A/RAB5B/CALM3/SLC6A9/DNAJC5/TPRG1L/RAB11B/SNCA/BCL2L1
BP	GO:0048260	positive regulation of receptor-mediated endocytosis	36	-0.59	-1.69	0.00686487	0.02594556	0.0159292	1653	tags=44%, list=14%, signal=38%	ARRB1/SYNJ1/CD63/INSR/GREM1/LDLRAP1/SYK/HIP1/HNRNPK/DNM2/CLU/ARRB2/TBC1D5/AP2M1/DAB2/CBL

BP	GO:0001701	in utero embryonic development	262	-0.36	-1.35	0.00693972	0.02615387	0.01605709	2089	tags=24%, list=18%, signal=20%	BCL2L11/MYO1E/HEG1/SRF/TAF8/KIDINS220/STK4/ATP7A/TTLL4/WDR74/NOTCH2/NDEL1/TFEB/ASF1B/DBN1/KEAP1/CEBPA/ELL/ZFP36L1/SLC35E2B/MSX1/CDKN1C/PTPN18/BIRC6/TRIM28/GRB2/ARNT/ZNF335/AMOT/RBPJ/YBX1/INPP5K/TRAF6/CHST11/IGF2/ZMIZ1/TBL1XR1/APBA3/NPAT/SUPT6H/BPTF/MED1/FZD5/CHD8/PSMC4/GPI/SIN3A/EPN1/CUL4A/CMIP/GATA1/TERF2/WDTC1/GNA12/BCL2L1/FLCN/NCOA1/MFN2/TENT5C/YBX3/CNOT1/MYH9/FURIN
BP	GO:0010035	response to inorganic substance	332	-0.35	-1.34	0.00693959	0.02615387	0.01605709	2603	tags=31%, list=22%, signal=25%	CALR/NCSTN/HDAC6/FOS/CAPN3/SLC25A23/PPIF/BAK1/CREB1/TRPC3/CFLAR/GPLD1/BAP1/CALM1/ENDOG/CYBB/NFE2L2/AIFM1/TF/ACO1/FANCC/STK25/FOXO1/SOD2/FYN/PRDX2/SRC/FKBP1B/ABAT/ADNP/MCOLN1/CPNE3/RTF2/ATP7A/SIRPA/ITPKB/TNFSF4/CPNE5/ERN1/HSPA5/ACTG1/LRRK2/HGF/PLCB1/RIPK1/DLG4/CPNE1/CEBPA/PEF1/MT2A/SETX/HVCN1/AQP9/ALAD/NCF1/MTF1/ALOX5AP/TXNRD2/PPP1CA/PDGFA/GUCY1B1/STK24/MAP3K5/FBP1/TSPO/SLFN14/PTK2B/SLC8A1/STAT6/SEC31A/BMP6/MMP9/PPP3CA/THBS1/PLEC/CALM3/G6PD/JUND/VCP/GPI/SIPA1/UCP2/CD14/AQP1/DNM2/TXNIP/ATP2B4/GPX1/KDM6B/ADD1/PINK1/MAPK3/SNCA/ANXA11/AHCYL1/BSG/STIM1/MBP/FOXO3/IQGAP1/DMTN/RELA
BP	GO:0032409	regulation of transporter activity	153	-0.41	-1.45	0.00696175	0.0261646	0.01606367	1649	tags=23%, list=14%, signal=20%	OPRL1/RIPK1/DLG4/CACNB1/FXYD6/SHISA7/CHP1/KMT2A/PRKACA/CNIH3/HTT/PTK2B/MMP9/SLC9A1/PTAFR/SELENON/SCN1B/ATP1B2/EHD3/CALM3/SLC6A9/ACTB/CAPN1/RXRA/DNM2/WWP2/FKBP1A/ACTN4/PIM1/PINK1/TESC/SNCA/AHCYL1/STIM1/WNK1
BP	GO:0097581	lamellipodium organization	69	-0.49	-1.54	0.00696231	0.0261646	0.01606367	2771	tags=38%, list=24%, signal=29%	PLCE1/KIT/PLEKHO1/CARMIL1/TWF2/ABLIM1/FSCN1/ACTR2/WNT1/SRC/ATP7A/WASF3/ABI3/ARPC5/CORO1C/VCL/RAC1/RAC2/DNM2/CAPZB/WAS/PARVB/CTTN/ABLIM3/WASF2/DMTN
BP	GO:0006875	cellular metal ion homeostasis	338	-0.35	-1.34	0.00698438	0.02622274	0.01609937	2102	tags=25%, list=18%, signal=21%	GNA15/PTGIR/TTC7A/KNG1/DISC1/COX19/FYN/TPCN1/KCTD17/PDZD8/FKBP1B/SLC24A4/MCOLN1/PRKCE/SPPL3/GRIK2/PROK2/SLC39A7/VDR/ATP7A/ATP13A1/OPRL1/CMKLR1/DLG4/C5AR1/MT2A/PTPN6/TMBIM6/CLN3/PDPK1/PML/MICU1/CCR1/ATP6AP1/ITPR2/ARF1/PRKACA/CHERP/ATP2A3/ERO1A/NCOA4/TMEM38A/BCAP31/HTT/PTK2B/SLC8A1/CXCR2/SLC24A3/BMP6/LCN2/PTPRC/SLC9A1/FIS1/SELENON/ATP1B2/CALM3/SLC6A9/PIK3CG/JAK3/ANKRD9/ANXA6/LYN/GP9/ATP2B4/FKBP1A/FTL/PRKCB/GATA1/CXCR1/ATP6V1A/FTH1/SNCA/TGM2/GRINA/GP1BA/ATP6V0D1/STIM1/ALAS2/KEL/P2RX1/TBXA2R/XK/DIAPH1
BP	GO:0002028	regulation of sodium ion transport	53	-0.52	-1.59	0.00707701	0.02644565	0.01623623	1993	tags=30%, list=17%, signal=25%	PER1/YWHAH/PRKCE/FXYD6/CHP1/SLC8A1/SLC9A1/SCN1B/ATP1B2/DNM2/ATP2B4/ACTN4/MLLT6/TESC/AHCYL1/WNK1

BP	GO:0043086	negative regulation of catalytic activity	478	-0.33	-1.32	0.00713295	0.02662966	0.0163492	2266	tags=29%, list=19%, signal=25%	DUSP1/GPS1/KAT2B/IFI16/EPHB2/SH2B3/PKN1/TNK2/SH3RF1/SERPINA1/CDKN2D/IRAK3/INKA1/SERPING1/ORMDL3/SPRY3/CR1/KNG1/HEG1/PTEN/PPM1F/SRC/PHACTR2/IRS2/FKBP1B/AKT1S1/MLLT1/MAP2K5/PPP1R15A/XIAP/AOC3/TSC2/MIDN/ROCK2/GSTP1/ARRB1/LRRK2/HGF/GPS2/STK38/TMBIM1/PI3/PIP4K2C/MGAT5/TRIB1/CEBPA/NF2/CAST/UBE2Z/PTPRJ/SORT1/PTPN6/PYCARD/FRY/SEMA4D/MAD2L2/TMBIM6/CDKN1C/CLN3/PDPK1/DHCR24/GNAQ/TRIB3/BIRC6/LEPR/GSK3A/CHP1/PCSK1N/AMOT/CSNK2A1/YWHAB/NFKB1/TAOK3/SH3BP5L/SORL1/CORO1C/PRDX5/TIMP2/SLC8A1/WARS1/INPP5K/BCAS3/GMIP/CSK/MMP9/POR/SFRP2/APLP2/APBA3/PTPRC/ADAR/THBS1/PTPN1/PPP1R12B/PLEC/HEXIM2/IQGAP2/LTF/RUBCN/GPI/AQP1/LYN/ITPRIP/GSK3B/LRP1/HSPB1/TXNIP/PPP1R10/ATP2B4/GNAI2/FKBP1A/GPX1/ARRB2/NF1/PPP1R9B/TERF2/WDTC1/OAZ1/UBE2O/RPS6KA1/TESC/SNCA/SIAH2/CDKN1A/DTX3L/PIP4K2A/TFIP11/PSMF1/PRKAR2A/PRKAR2B/SLC4A1/CTSB/PAK2/WNK1/FBXO7/TIMP1/CBL/IQGAP1/CABIN1/FURIN
BP	GO:0050792	regulation of viral process	129	-0.42	-1.45	0.00716541	0.02672578	0.01640821	2258	tags=29%, list=19%, signal=24%	TRIM5/IFI16/MAVS/TRIM38/SRPK1/APOBEC3D/KPNA6/SNX3/SETDB1/TMEM250/OASL/EIF2AK2/IFITM3/ZC3HAV1/TRIM28/VPS37B/INPP5K/RAB7A/RAD23A/GSN/ADAR/LTF/LGALS9/APOBEC3F/DDB1/IFITM2/SRPK2/TRIM11/TNIP1/TRIM14/STAU1/APOBEC3C/LARP1/TRIM10/ZNFX1/TRIM26/TBC1D20/N4BP1
BP	GO:1903169	regulation of calcium ion transmembrane transport	84	-0.47	-1.53	0.00718538	0.02675007	0.01642312	1649	tags=30%, list=14%, signal=26%	FYN/FKBP1B/PRKCE/OPRL1/CACNB1/PTPN6/PDPK1/PRKACA/TMEM38A/HTT/PTK2B/SLC8A1/SLC9A1/SELENON/EHD3/CALM3/PIK3CG/G6PD/GJC2/LYN/FKBP1A/SNCA/STIM1/P2RX1/DIAPH1
BP	GO:1903530	regulation of secretion by cell	320	-0.34	-1.32	0.00717883	0.02675007	0.01642312	2598	tags=28%, list=22%, signal=23%	RHBDF2/P2RY1/MCTP1/CLASP1/VPS4A/VAMP3/SEPTIN4/CREB1/ALOX5/GPLD1/CD38/NCKAP1L/CRY2/WLS/ATP9A/PIM3/ADAM8/TLR2/VPS18/VPS4B/RAB11FIP5/SNAP25/FGR/KDM5B/UNC13D/RAB27B/IRS2/CD177/FKBP1B/ABAT/MYO18A/PRKCE/SDC4/AP1G1/TGFB1/ADGRE2/PNKD/SDCBP/HGS/MIDN/ITGAM/LRRK2/SIDT2/EZR/STX4/GRK2/PER2/NR1H2/MYOM1/VPS35/SPI1/PTGES/PDPK1/ITGB2/SEPTIN1/FES/LRRC8A/GAB2/BCR/FRMD4A/RAB7A/BMP6/CPT1A/PPP3CA/SYK/PTAFR/FBXL20/CALM3/RAB11FIP1/PRAM1/UCP2/LYN/LGALS9/GSK3B/RAC2/TNFRSF1B/SYTL4/TCF7L2/GNAI2/PRKCB/GATA1/NF1/PINK1/SNCA/PTPN23/LAMP1/RAB3D/DAB2/P2RX1/GLUL/RAB2B
BP	GO:0051208	sequestering of calcium ion	78	-0.47	-1.52	0.007285	0.02709558	0.01663525	2603	tags=38%, list=22%, signal=30%	CALR/CAPN3/SLC25A23/CALM1/CORO1A/LETM1/TPCN1/FKBP1B/MCOLN1/PRKCE/PTPN6/PDPK1/ITPR2/PRKACA/CHERP/ERO1A/TMEM38A/HTT/PTK2B/SLC8A1/PTPRC/SELENON/CALM3/ANXA6/LYN/GP9/FKBP1A/SNCA/GP1BA/DIAPH1
BP	GO:0002763	positive regulation of myeloid leukocyte differentiation	37	-0.58	-1.66	0.00731822	0.02719369	0.01669548	2946	tags=49%, list=25%, signal=37%	FADD/TNF/CD4/FOS/CREB1/CTNNBIP1/TGFB1/PPARGC1B/NOTCH2/RIPK1/TRIB1/ZFP36L1/CCR1/FES/TRAF6/PF4/HCLS1/TESC
BP	GO:1903035	negative regulation of response to wounding	46	-0.55	-1.65	0.00733195	0.02721928	0.01671119	3264	tags=52%, list=28%, signal=38%	GIT1/CLASP2/KREMEN1/UBASH3B/RTN4R/SERPINE1/TNF/SMAD3/CLASP1/KIAA0319/ALOX5/SIGLEC10/SH2B3/SERPING1/KNG1/PTEN/ABAT/THBD/EPHA4/PDGFA/THBS1/ALOX12/GP1BA/KRT1

BP	GO:0072001	renal system development	159	-0.40	-1.43	0.00737475	0.02735262	0.01679306	3013	tags=36%, list=26%, signal=27%	DTNBP1/NID1/NPHS1/FADD/PLXND1/KLHL3/NOTCH1/CENPF/NKX3-1/SMAD3/SOX4/PLCE1/SMAD1/APH1A/CFLAR/CTNNBIP1/PTK7/ASXL1/BCL2L11/MYO1E/WNT1/APAF1/JMJD6/SDC4/EXT1/NIPBL/MAGED1/NOTCH2/LRRK2/MPST/AP2B1/SNX17/NFIA/EGR1/MME/ACTA2/CDKN1C/EPHA4/PDGFA/SGPL1/GREM1/KANK2/ODC1/RARA/IL6R/PECAM1/BMP6/ZBTB16/MMP9/BASP1/RHOA/NF1/PBX1/AP1B1/FLCN/IQGAP1/BAG6
MF	GO:0008134	transcription factor binding	473	-0.33	-1.30	0.00741543	0.02747786	0.01686995	2203	tags=29%, list=19%, signal=24%	HDAC3/PKN1/FHL2/MMS19/TADA3/ASXL1/STRN/CBX5/THRA/SRF/PADI2/ZNF516/EDF1/NLRP3/SRC/PER1/YWHAH/MED13/ELK1/HDAC4/DDX54/STK4/BAZ2A/ATOH8/LCOR/VDR/PPARGC1B/ZBTB7A/BCL3/NOTCH2/GTF2I/CTDP1/UBXN7/MTDH/CTBP2/TFDP2/ARRB1/SMARCA4/WIZ/STAT5B/NFAT5/NFIA/OASL/ARID5A/KEAP1/NFATC1/PIAS1/CPNE1/MAPK14/TRIB1/CTBP1/CEBPA/TLE4/YY1/PER2/ATF7/SMARCD3/MAD2L2/NR1H2/FAM89B/DAPK3/SPI1/NCOA3/HIF1AN/NSD1/KAT5/FOXP1/BRMS1/YWHAZ/ARNT/MED16/RUNX3/FBP1/SUFU/TACC1/RBPJ/NCOA2/NFATC2/RARA/BCAS3/KLF5/MAX/USF2/TOB2/ZBTB16/TGFB1I1/FOXO4/NCOR2/MED1/HIPK2/TRIP12/SKI/FLNA/JUND/ACTB/HMGA1/RXRA/SIN3A/ETS2/GSK3B/GFI1B/CREBBP/HSPB1/WWP2/TCF7L2/TAF6/CHD4/NFATC3/PRKCB/GAS2L1/TBL1X/TFDP1/GATA1/SP1/NCOA6/ACTN4/ADD1/HCLS1/ARID1A/PIM1/WBP2/PBX1/DTX3L/STAT3/ZNF703/TAL1/NCOA1/ENO1/HDGF/KAT6A/PRKDC/MED25/CNOT1/FOXO3/HCFC1/RELA
BP	GO:0009743	response to carbohydrate	133	-0.41	-1.44	0.00742979	0.02750543	0.01688687	2096	tags=27%, list=18%, signal=22%	SOD2/RAB11FIP5/SRF/IRS2/FKBP1B/ADNP/PRKCE/ERN1/SMARCA4/PLCB1/SIDT2/EGR1/ZBTB20/KAT7/STX4/ZFP36L1/PRKACA/SESN2/PTK2B/SLC8A1/PPP3CA/MAP2K7/THBS1/PDK3/UCP2/LYN/SIN3A/RAB11B/TXNIP/TCF7L2/RHOA/PRKCB/GPX1/GLUL/FOXO3/MAP2K3
BP	GO:0031099	regeneration	99	-0.45	-1.51	0.00751259	0.02773451	0.01702752	2042	tags=31%, list=17%, signal=26%	WNT1/PTEN/HDGFL2/FKBP1B/NINJ1/HGF/NDEL1/CPQ/KPNA1/FPGS/EPHA4/PTPN12/LCP1/STK24/TSPO/KLF5/CTNNA1/CPT1A/PPP3CA/MED1/FLNA/ULK1/UCP2/IGF2R/GATA1/GPX1/BCL9/GRN/CDKN1A/MTPN/NINJ2
BP	GO:0032103	positive regulation of response to external stimulus	295	-0.34	-1.31	0.00754427	0.02782561	0.01708345	2045	tags=25%, list=17%, signal=21%	FYN/TIRAP/PPM1F/SRC/NKG7/FKBP1B/ANKRD17/NINJ1/THBD/XRCC6/AP1G1/HCK/TGFB1/PUM1/PDE2A/TNFSF4/LILRA5/ANO6/LSM14A/LRRK2/IKBKE/NDEL1/STAT5B/OASL/CMKLR1/RIPK1/C5AR1/STX4/CEBPA/PYCARD/SPI1/CCR1/CSF1R/AKIRIN2/KLRD1/ALOX5AP/SASH1/OPTN/NLRC4/STK24/PTK2B/TRAF6/PRKD2/IL6R/PAK1/BMP6/VAV1/SYK/THBS1/MED1/IL17RA/RBM14/PIK3CG/CAMK1D/FLNA/RAC1/LYN/RIOK3/LGALS9/SIN3A/RAC2/CREBBP/CDK19/HSPB1/TNIP1/GRN/ZNFX1/MAPK3/SNCA/RIPOR2/LAMP1/PRKDC/PAK2/WNK1
BP	GO:0006096	glycolytic process	61	-0.52	-1.61	0.0076178	0.02807078	0.01723397	1960	tags=39%, list=17%, signal=33%	PFKL/JMJD8/OGDH/SIRT6/ENO2/HDAC4/GALK1/ZBTB7A/ZBTB20/CBFA2T3/PGAM1/ENTPD5/ARNT/EIF6/PKM/INSR/FBP1/ALDOA/GPI/BPGM/HK1/STAT3/SLC4A1/ENO1
MF	GO:0019209	kinase activator activity	77	-0.47	-1.51	0.00771776	0.02833409	0.01739563	1009	tags=22%, list=9%, signal=20%	MAP2K2/DAZAP2/GREM1/MOB3A/IGF2/TAOK1/STK11/STRADA/CALM3/LTF/MADD/MARK2/CDKN1A/TAL1/PAK2/WNK1/IQGAP1
BP	GO:0030510	regulation of BMP signaling pathway	47	-0.55	-1.64	0.00771255	0.02833409	0.01739563	3020	tags=47%, list=26%, signal=35%	ENG/PELO/NOTCH1/FBXL15/NEO1/UBE2D3/WNT1/XIAP/RNF165/NOTCH2/MSX1/NUMA1/GREM1/RBPJ/SORL1/PCSK6/SFRP2/HIPK2/SKI/FKBP8/UBE2O/PPM1A

BP	GO:0045744	negative regulation of G protein-coupled receptor signaling pathway	34	-0.60	-1.68	0.00772724	0.02834271	0.01740092	1653	tags=35%, list=14%, signal=30%	MGRN1/GRK3/ARRB1/OPRL1/GRK2/YWHAB/PLEK/DNM2/ATP2B4/GNAI2/ARRB2/SNCA
BP	GO:0010721	negative regulation of cell development	90	-0.46	-1.52	0.00783916	0.0286475	0.01758804	1402	tags=24%, list=12%, signal=22%	HOOK3/SEMA4D/GSK3A/TSPO/SORL1/CORO1C/SEMA4A/RAPGEF2/CTNNA1/PPP3CA/LTF/SKI/ULK1/RFLNB/GSK3B/CTDSP1/TRIM11/NF1/ACTN4/MBP/RNF10/DMTN
MF	GO:0032266	phosphatidylinositol-3-phosphate binding	37	-0.57	-1.65	0.00787579	0.02875493	0.017654	2393	tags=43%, list=20%, signal=35%	TECPR1/ZFYVE26/ATG2B/NCF4/SNX3/PLEKHF1/WASHC2C/PARD3/ZFYVE1/WDR45/WIPI2/SNX27/HIP1/ATG2A/PLEK2/DENND1A
BP	GO:0010942	positive regulation of cell death	422	-0.33	-1.30	0.00795999	0.02903571	0.01782638	2282	tags=26%, list=19%, signal=22%	USP27X/MAP2K4/AIFM1/C1QA/MTCH2/AXIN2/ADAM8/TRAF7/FOXO1/SOD2/BCL2L11/KATNB1/KNG1/FYN/PTEN/SRC/APAF1/ELK1/ATM/STK4/ATF6/GRIK2/VDR/MAGED1/ANO6/NOTCH2/ITGAM/LRRK2/BTG1/PIP5KL1/EGR1/RIPK1/PLEKHF1/UBE2Z/TEX261/MSX1/EIF5A/PYCARD/UBE2M/DAPK3/PML/ITGB2/DEDD2/BRMS1/PPP1CA/E2F3/NLRC4/TNFRSF10C/MAP3K5/TSPO/BCAP31/HTT/NEURL1/RAPGEF2/MARK4/PIAS4/CTNNA1/ZBTB16/MMP9/SFRP2/GSN/MAP2K7/PTPRC/SYK/SLC9A1/THBS1/FIS1/ATG7/TLR6/CASP2/B4GALT1/CAMK1D/EIF4G1/INPP5D/UCP2/LYN/LGALS9/RBCK1/LAPTM5/GSK3B/DNM2/IGF2R/CDK19/LRP1/BNIP3L/TNFRSF1B/TXNIP/SRPK2/PRF1/RHOA/BBC3/CLU/ARRB2/NF1/GRN/SNCA/CDKN1A/TGM2/FLCN/UBB/SQSTM1/NCOA1/PIK3CD/MFN2/PRKDC/PAK2/CTSD/FOXO3/CDC34/RYBP
BP	GO:0022898	regulation of transmembrane transporter activity	143	-0.42	-1.46	0.00802609	0.02919645	0.01792507	1649	tags=26%, list=14%, signal=23%	YWHAH/FKBP1B/PRKCE/ATP7A/OPRL1/RIPK1/DLG4/CACNB1/FXYD6/SHISA7/CHP1/PRKACA/CNIH3/HTT/PTK2B/MMP9/SLC9A1/PTAFR/SELENON/SCN1B/ATP1B2/EHD3/CALM3/SLC6A9/ACTB/CAPN1/DNM2/WWP2/FKBP1A/ACTN4/PIM1/PINK1/TESC/SNCA/AHCYL1/STIM1/WNK1
BP	GO:0051216	cartilage development	89	-0.46	-1.52	0.00806046	0.02929469	0.01798538	2803	tags=36%, list=24%, signal=28%	SMAD3/SMAD1/HYAL3/CCN3/GPLD1/SCIN/AXIN2/CHI3L1/THRA/SRF/EXT1/ATP7A/TGFB1/CHSY1/ARID5A/MAPK14/MSX1/TRPS1/RUNX3/GREM1/RARA/SOX6/CHST11/BMP6/ZBTB16/POR/SFRP2/GLG1/ANXA6/RFLNB/MAPK3/TIMP1
BP	GO:0007204	positive regulation of cytosolic calcium ion concentration	177	-0.39	-1.41	0.00813933	0.0295543	0.01814477	2517	tags=31%, list=21%, signal=25%	CAPN3/BAK1/TRPC3/FPR3/CD38/CALM1/CORO1A/LETM1/GNA15/PTGIR/KNG1/FYN/TPCN1/FKBP1B/SLC24A4/MCOLN1/PRKCE/SPPL3/PROK2/OPRL1/CMKLR1/DLG4/C5AR1/PTPN6/TMBIM6/PDPK1/PML/CCR1/ITPR2/PRKACA/CHERP/ERO1A/TMEM38A/BCAP31/HTT/PTK2B/SLC8A1/CXCR2/PTPRC/FIS1/SELENON/CALM3/PIK3CG/JAK3/LYN/GP9/ATP2B4/FKBP1A/GATA1/CXCR1/SNCA/GP1BA/P2RX1/TBXA2R/DIAPH1
BP	GO:0006757	ATP generation from ADP	62	-0.50	-1.56	0.00814839	0.02956022	0.01814841	1960	tags=31%, list=17%, signal=26%	HDAC4/GALK1/ZBTB7A/ZBTB20/CBFA2T3/PGAM1/ENTPD5/ARNT/EIF6/PKM/INSR/FBP1/ALDOA/GPI/BPGM/HK1/STAT3/SLC4A1/ENO1

BP	GO:0010927	cellular component assembly involved in morphogenesis	60	-0.52	-1.60	0.0082142	0.02974467	0.01826165	2841	tags=38%, list=24%, signal=29%	ERCC2/TMOD3/EPB41L3/CLASP1/CAPN3/FIG4/CFLAR/TLR2/FLII/CSRP1/SRF/DICER1/ACTG1/PIKFYVE/WDR1/RFX2/TPM1/PLEC/AKAP13/TMOD1/TBC1D20/MYL9/ACRBP
BP	GO:0006874	cellular calcium ion homeostasis	253	-0.36	-1.36	0.00838821	0.03022466	0.01855634	2136	tags=26%, list=18%, signal=21%	ATP2B1/GNA15/PTGIR/KNG1/DISC1/FYN/TPCN1/KCTD17/PDZD8/FKBP1B/SLC24A4/MCOLN1/PRKCE/SPPL3/GRIK2/PROK2/VDR/ATP13A1/OPRL1/CMKLR1/DLG4/C5AR1/PTPN6/TMBIM6/CLN3/PDPK1/PML/MICU1/CCR1/ITPR2/PRKACA/CHERP/ATP2A3/ERO1A/TMEM38A/BCAP31/HTT/PTK2B/SLC8A1/CXCR2/SLC24A3/PTPRC/FIS1/SELENON/CALM3/PIK3CG/JAK3/ANXA6/LYN/GP9/ATP2B4/FKBP1A/PRKCB/GATA1/CXCR1/SNCA/TGM2/GRINA/GP1BA/STIM1/KEL/P2RX1/TBXA2R/XK/DIAPH1
BP	GO:0033627	cell adhesion mediated by integrin	50	-0.54	-1.63	0.00855509	0.03078283	0.01889902	1421	tags=34%, list=12%, signal=30%	EXT1/ITGAM/PTPN6/FERMT3/ITGB2/SFRP2/ITGA5/SYK/JAM3/PIK3CG/LYN/ITGA2B/ITGAL/TESC/ITGB5/ITGB3/WNK1
CC	GO:0014704	intercalated disc	28	-0.62	-1.67	0.00864378	0.03104576	0.01906045	939	tags=29%, list=8%, signal=26%	SLC2A1/SLC8A1/PAK1/VCL/CTNNA1/SLC9A1/SCN1B/ACTN1
BP	GO:1904019	epithelial cell apoptotic process	64	-0.50	-1.57	0.00866727	0.03110203	0.019095	1464	tags=25%, list=12%, signal=22%	STK4/ANO6/CAST/ZFP36L1/PDPK1/MAP3K5/HIPK1/PIAS4/GSN/THBS1/PIK3CG/TCF7L2/ARRB2/BCL2L1/E2F2/FOXO3
BP	GO:0031058	positive regulation of histone modification	86	-0.46	-1.51	0.00871065	0.03122954	0.01917328	2167	tags=29%, list=18%, signal=24%	RPS6KA5/NSD3/PAF1/ATM/C6orf89/NIPBL/ARRB1/LRRK2/JDP2/ARID5A/KAT7/PHF20/CTBP1/KMT2A/MCRS1/RPS6KA4/PRKD2/LMNA/PINK1/WBP2/MAPK3/BRD4/RNF40/JARID2/HCFC1
BP	GO:0034101	erythrocyte homeostasis	116	-0.43	-1.47	0.00872848	0.03126525	0.0191952	783	tags=25%, list=7%, signal=24%	SLC25A38/ADAR/MED1/MAFB/HIPK2/G6PD/JAK3/HSPA1A/INPP5D/GPI/LYN/BPGM/GATA1/AHSP/HCLS1/TRIM10/STAT3/TAL1/SLC4A1/FAM210B/EPB42/IKZF1/PRKDC/KLF1/MPIG6B/ALAS2/TRIM58/FOXO3/DMTN
MF	GO:1902936	phosphatidylinositol bisphosphate binding	70	-0.48	-1.54	0.00875291	0.03132455	0.01923161	3270	tags=54%, list=28%, signal=39%	SNX21/SH3PXD2A/AMER1/WIPI1/PFN1/RAB35/KCNQ1/PHLDA3/SNX18/GBF1/KCNJ2/TWF2/ANXA2/SCIN/PLCD1/MYO1G/FLII/TPCN1/TIRAP/SNX3/SDCBP/TTPAL/RPH3A/PLCB1/OSBPL2/WASHC2C/PARD3/ADAP2/SVIL/LDLRAP1/ADAP1/GSN/WDR45/SLC9A1/ASAP1/WIPI2/HIP1/PLEK2
BP	GO:0050673	epithelial cell proliferation	200	-0.37	-1.38	0.00894069	0.03191038	0.01959128	1915	tags=24%, list=16%, signal=20%	MAP2K5/ATOH8/C6orf89/PROK2/ERBB2/VDR/ATP7A/MAGED1/TGFB1/ERN1/NOTCH2/HGF/SIDT2/C5AR1/ZFP36L1/RAP1GAP/CDKN1C/PDPK1/GPBAR1/ARNT/RUNX3/STAT6/PRKD2/WDR13/IGF2/BMP6/SFRP2/STK11/THBS1/MED1/B4GALT1/STAT5A/TCF7L2/GPX1/NF1/SP1/LRG1/GRN/RPS6KA1/STAT3/SPARC/ZNF703/PIK3CD/PRKDC/ITGB3/DAB2/GLUL
MF	GO:0016705	oxidoreductase activity, acting on paired donors, with incorporation or reduction of molecular oxygen	96	-0.44	-1.46	0.00898615	0.03204387	0.01967323	3169	tags=36%, list=27%, signal=27%	HMOX1/TBXAS1/NOS1/KDM5C/EGLN1/FADS2/CYP4F3/KDM5A/AKR1D1/DOHH/KDM6A/KDM7A/KDM4A/TET2/EGLN3/HMOX2/ALKBH5/PHF2/KDM5B/PHF8/FTO/JMJD6/PLOD1/KDM3B/KDM8/KDM2A/HIF1AN/CYP27A1/PTGS1/POR/TET3/KDM6B/KDM4B/MICAL2/FAXDC2

BP	GO:0016239	positive regulation of macroautophagy	62	-0.50	-1.55	0.00902928	0.03216882	0.01974995	1739	tags=34%, list=15%, signal=29%	VPS13D/TSC2/SPTLC2/PIP4K2C/WAC/OPTN/SESN3/SESN2/IKBKG/HTT/PAFAH1B2/WDR45/ULK1/ELAPOR1/BNIP3L/SUPT5H/ATG2A/LARP1/PINK1/MAPK3/PIP4K2A
BP	GO:1903532	positive regulation of secretion by cell	167	-0.39	-1.40	0.00923406	0.03275166	0.02010778	2575	tags=30%, list=22%, signal=24%	P2RY1/CLASP1/VPS4A/CREB1/GPLD1/CD38/WLS/ADAM8/TLR2/VPS4B/FGR/UNC13D/RAB27B/IRS2/CD177/ABAT/MYO18A/PRKCE/SDC4/AP1G1/TGFB1/SDCBP/HGS/ITGAM/EZR/STX4/GRK2/NR1H2/MYOM1/VPS35/PTGES/ITGB2/LRRC8A/GAB2/FRMD4A/RAB7A/BMP6/SYK/PTAFR/SYTL4/TCF7L2/GATA1/PINK1/SNCA/PTPN23/LAMP1/RAB3D/DAB2/GLUL/RAB2B
BP	GO:0030048	actin filament-based movement	66	-0.49	-1.55	0.00924993	0.03277871	0.02012438	1788	tags=30%, list=15%, signal=26%	MYO5A/MYO1G/MYO1E/SUN2/SYNE2/KCNH2/ACTA2/WIPF2/TPM1/GSN/SLC9A1/MYO9B/SCN1B/MYO1F/FLNA/WIPF1/ACTN4/WAS/MYH9/WASF2
BP	GO:0090316	positive regulation of intracellular protein transport	128	-0.40	-1.40	0.00937398	0.0331003	0.02032183	2203	tags=32%, list=19%, signal=26%	HDAC3/MAVS/HSPA1L/FYN/SH3GLB1/ERBB2/USP36/TGFB1/NPEPPS/UBR5/UBE2J2/ITGAM/MAPK14/ARIH2/ITGB2/TRIM28/GSK3A/UBE2L3/CHP1/PRKACA/VPS11/RBM22/SORL1/BCAP31/BCAS3/FIS1/FZD5/FLNA/RIPOR1/GSK3B/RAC2/TCF7L2/HUWE1/BBC3/HCLS1/OAZ1/ATG13/PINK1/ABLIM3/AKT2/PPM1A
BP	GO:0051283	negative regulation of sequestering of calcium ion	72	-0.48	-1.54	0.00941576	0.0331595	0.02035817	1421	tags=38%, list=12%, signal=33%	CALM1/CORO1A/LETM1/TPCN1/FKBP1B/MCOLN1/PRKCE/PTPN6/PDPK1/ITPR2/PRKACA/CHERP/ERO1A/TMEM38A/HTT/PTK2B/SLC8A1/PTPRC/SELENON/CALM3/ANXA6/LYN/GP9/FKBP1A/SNCA/GP1BA/DIAPH1
BP	GO:0048525	negative regulation of viral process	74	-0.46	-1.48	0.00945343	0.03325211	0.02041503	2258	tags=35%, list=19%, signal=29%	TRIM5/IFI16/MAVS/SRPK1/APOBEC3D/SNX3/SETDB1/OASL/EIF2AK2/IFITM3/ZC3HAV1/TRIM28/INPP5K/GSN/LTF/APOBEC3F/IFITM2/SRPK2/TRIM11/TNIP1/TRIM14/APOBEC3C/TRIM10/ZNFX1/TRIM26/N4BP1
BP	GO:0061458	reproductive system development	228	-0.36	-1.35	0.0094801	0.0332682	0.02042491	2089	tags=28%, list=18%, signal=23%	BCL2L11/CSDE1/PTEN/KDM5B/MAP3K4/SRC/ATM/STK4/H3-3B/NIPBL/VDR/H3-3A/CUL7/NOTCH2/HSPA5/ASH1L/STAT5B/FANCG/TFEB/MME/MAPK14/CEBPA/ZFP36L1/PTPN6/CDKN1C/SPATA2/AGO4/DHCR24/CCNF/BIRC6/TRIM28/GRB2/ARNT/PDGFA/INSR/SGPL1/RBPJ/NCOA4/RARA/IGF2/CTNNA1/BMP6/SFRP2/STK11/BPTF/MED1/CASP2/FZD5/BASP1/PSAP/SF1/GATA1/ARRB2/PPP1R9B/TBC1D20/BCL2L1/UBB/NCOA1/BSG/CTSB/YBX3/FOXO3/CBL
BP	GO:0071470	cellular response to osmotic stress	28	-0.61	-1.65	0.00950099	0.03331213	0.02045187	2517	tags=50%, list=21%, signal=39%	CAPN3/SLC25A23/LETM1/SLC12A6/ZFP36L1/RELB/CLN3/MICU1/ARHGEF2/TSPO/SLC2A1/AQP1/MYLK/YBX3
BP	GO:0070849	response to epidermal growth factor	33	-0.59	-1.66	0.00955219	0.03346213	0.02054397	1869	tags=36%, list=16%, signal=31%	ERBB2/SYAP1/STAT5B/ZFP36L1/PDPK1/PTPN12/INPP5K/MED1/PPP1R9B/MAPK3/DAB2/IQGAP1
BP	GO:0002009	morphogenesis of an epithelium	253	-0.36	-1.35	0.00979796	0.03417253	0.02098012	2473	tags=30%, list=21%, signal=25%	SETD2/SOCS3/SCRIB/ADAM17/SIRT6/SLC9A3R1/IFT122/MYO9A/MTHFD1/CTNNBIP1/ARHGAP24/PTK7/SRF/WNT1/PTEN/KDM5B/VANGL1/SRC/APAF1/MTHFR/STK4/SDC4/EXT1/VDR/ATP7A/MAGED1/MKS1/TGFB1/ZDHHC7/TIAM1/PHACTR4/NOTCH2/TSC2/ACTG1/HGF/TGM3/CA2/FUZ/EPHA4/PML/CSF1R/WDR1/TRIM28/FOXP1/GRB2/PPP1CA/PRKACA/PDGFA/SUFU/GREM1/RBPJ/RARA/TRAF6/PRKD2/PAK1/VCL/TGFB1I1/SFRP2/ITGA5/DVL3/MED1/CD151/FZD5/BTBD7/SKI/FLNA/ACTB/RAC1/RHOA/MSN/TGM2/PBX1/SH3BP1/PIK3CD/VASP/DAB2/MICAL2

BP	GO:1902895	positive regulation of miRNA transcription	32	-0.59	-1.63	0.00991058	0.03441438	0.0211286	2910	tags=56%, list=25%, signal=42%	TNF/TP53/SMAD3/SMAD1/MRTFA/FOS/SRF/ATOH8/TGFB1/SMARCA4/EGR1/NR1H2/SPI1/KLF5/NR3C1/STAT3/FOXO3/RELA
BP	GO:2001239	regulation of extrinsic apoptotic signaling pathway in absence of ligand	26	-0.64	-1.70	0.00999782	0.03465678	0.02127742	2008	tags=46%, list=17%, signal=38%	PRDX2/MAP2K5/EYA3/RIPK1/PPP1CA/CTNNA1/HSPA1A/PF4/GATA1/NF1/BCL2L1/STRADB
BP	GO:0030509	BMP signaling pathway	77	-0.46	-1.48	0.01002582	0.03466116	0.02128011	1879	tags=29%, list=16%, signal=24%	WNT1/XIAP/EXT1/RNF165/TGFB1/NOTCH2/EGR1/MSX1/NUMA1/GREM1/RBPJ/SORL1/PCSK6/BMP6/SFRP2/USP9X/HIPK2/SKI/FKBP8/UBE2O/MAPK3/PPM1A
CC	GO:0031093	platelet alpha granule lumen	45	-0.53	-1.58	0.01003395	0.03466116	0.02128011	2178	tags=42%, list=19%, signal=35%	SERPINA1/SERPING1/KNG1/TGFB1/HGF/QSOX1/FERMT3/F5/PDGFA/F13A1/IGF2/ALDOA/THBS1/PF4/CLU/ACTN4/SPARC/ACTN1/TIMP1
BP	GO:0060840	artery development	48	-0.53	-1.60	0.01003395	0.03466116	0.02128011	1768	tags=27%, list=15%, signal=23%	SRF/PDE2A/AP2B1/SNX17/SUFU/RBPJ/ARID2/ZMIZ1/NDST1/LRP1/NF1/MYLK/NPRL3
BP	GO:0043068	positive regulation of programmed cell death	384	-0.33	-1.28	0.01004529	0.03467019	0.02128565	1477	tags=19%, list=13%, signal=17%	PLEKHF1/UBE2Z/TEX261/MSX1/EIF5A/PYCARD/UBE2M/DAPK3/PML/DEDD2/BRMS1/PPP1CA/E2F3/NLRC4/TNFRSF10C/MAP3K5/TSPO/BCAP31/HTT/NEURL1/RAPGEF2/MARK4/PIAS4/CTNNA1/ZBTB16/MMP9/SFRP2/GSN/MAP2K7/PTPRC/SYK/SLC9A1/THBS1/FIS1/ATG7/CASP2/B4GALT1/CAMK1D/INPP5D/LYN/LGALS9/RBCK1/LAPTM5/DNM2/IGF2R/CDK19/BNIP3L/TNFRSF1B/TXNIP/SRPK2/PRF1/RHOA/BBC3/CLU/ARRB2/NF1/GRN/SNCA/CDKN1A/TGM2/FLCN/UBB/SQSTM1/NCOA1/PIK3CD/MFN2/PRKDC/PAK2/CTSD/FOXO3/CDC34/RYBP
BP	GO:0010675	regulation of cellular carbohydrate metabolic process	102	-0.44	-1.49	0.01006086	0.03469381	0.02130015	1218	tags=25%, list=10%, signal=23%	PGAM1/LEPR/GSK3A/PPP1CA/DGAT2/PDK2/BCKDK/INSR/SESN2/FBP1/NCOA2/PTK2B/INPP5K/IGF2/STK11/PTAFR/PDK3/ADIPOR1/PLEK/GSK3B/DDB1/SNCA/STAT3/AKT2/SLC4A1/USP7
BP	GO:0002687	positive regulation of leukocyte migration	89	-0.45	-1.50	0.01030332	0.0354684	0.02177571	2970	tags=42%, list=25%, signal=31%	OXSR1/SERPINE1/FADD/TNF/GPSM3/TNFSF14/CALR/LGALS3/SPN/ADAM17/NCKAP1L/ADAM8/CD99L2/TIRAP/ANO6/CMKLR1/C5AR1/PYCARD/SPI1/CCR1/CSF1R/DOCK8/PTK2B/SELP/IL6R/JAM3/THBS1/PTAFR/CAMK1D/RAC1/LGALS9/RAC2/RHOA/MAPK3/RIPOR2/WNK1/PLVAP
BP	GO:1901216	positive regulation of neuron death	67	-0.48	-1.53	0.01033023	0.03553028	0.0218137	2253	tags=36%, list=19%, signal=29%	AIFM1/C1QA/BCL2L11/FYN/ELK1/ATM/GRIK2/ITGAM/EGR1/UBE2M/ITGB2/MAP3K5/MAP2K7/TLR6/CASP2/GSK3B/SRPK2/RHOA/CLU/NF1/GRN/SNCA/FOXO3/CDC34

BP	GO:0031400	negative regulation of protein modification process	387	-0.32	-1.27	0.01043835	0.03584014	0.02200394	2521	tags=33%, list=21%, signal=27%	LGALS3/CAPN3/PMEPA1/KDM4A/YWHAG/BAK1/TERF2IP/SOCS3/UBE2B/CALM1/DUSP16/NCKAP1L/CRY2/PRKAR1B/MVP/DUSP1/KAT2B/EPHB2/SH2B3/PKN1/CDKN2D/IRAK3/SPRY3/HEG1/FYN/PTEN/PPM1F/CRTAP/FKBP1B/AKT1S1/MLLT1/PNKP/PPP1R15A/PRKCE/TGFB1/SIRPA/LDLRAD4/USP4/TSC2/UBR5/ROCK2/GSTP1/ARRB1/LRRK2/HGF/PIP5KL1/GPS2/STK38/MGAT5/TRIB1/CTBP1/CEBPA/CTDSP2/NF2/PTPRJ/PER2/PTPN6/PYCARD/PARD3/FRY/SEMA4D/MAD2L2/CDKN1C/SPI1/LRRK1/PDPK1/GNAQ/TRIB3/CHP1/OTUB1/YWHAB/GREM1/TSPO/SH3BP5L/SORL1/CORO1C/MFHAS1/SLC8A1/WARS1/INPP5K/SFRP2/PTPRC/ADAR/PTPN1/SUPT6H/PLEC/CTDSPL/CALM3/HEXIM2/TRIP12/G6PD/SKI/EIF4G1/HSPA1A/VPS28/GNL3L/LYN/SIN3A/GSK3B/LRP1/HSPB1/ATP2B4/GNAI2/CTDSP1/FKBP1A/MYADM/ARRB2/NF1/FKBP8/PPP1R9B/MLLT6/SNCA/CDKN1A/DTX3L/FLCN/NIBAN1/PRKAR2A/PRKAR2B/N4BP1/SQSTM1/PRKDC/PAK2/JARID2/FBXO7/CBL/DMTN/RELA
BP	GO:1902904	negative regulation of supramolecular fiber organization	106	-0.42	-1.43	0.01056378	0.03620828	0.02222996	1424	tags=25%, list=12%, signal=22%	CAMSAP1/EML2/WASHC2C/ARAP1/ARHGEF2/SSH1/SPTBN1/SPTA1/INPP5K/SVIL/TAOK1/GSN/ARHGAP6/CAPZB/MYADM/CLU/ADD1/SSH2/TMOD1/WAS/SNCA/SPTB/MTPN/PAK2/WASF2/DMTN
BP	GO:0002262	myeloid cell homeostasis	139	-0.40	-1.41	0.01066361	0.03648753	0.02240141	783	tags=23%, list=7%, signal=22%	SLC25A38/ADAR/JAM3/MED1/MAFB/HIPK2/G6PD/JAK3/HSPA1A/INPP5D/GPI/LYN/BPGM/MPL/GATA1/AHSP/HCLS1/TRIM10/STAT3/TAL1/SLC4A1/FAM210B/PIK3CD/EPB42/IKZF1/PRKDC/KLF1/MPIG6B/ALAS2/TRIM58/FOXO3/DMTN
BP	GO:0006941	striated muscle contraction	87	-0.44	-1.44	0.01072506	0.0366033	0.02247248	1475	tags=21%, list=13%, signal=18%	GRK2/KCNH2/PRKACA/TPM1/TMEM38A/SLC8A1/ALDOA/GSN/SLC9A1/GAA/SCN1B/EHD3/CALM3/PIK3CG/FLNA/PDE5A/MYL4/MAP2K3
BP	GO:0030835	negative regulation of actin filament depolymerization	35	-0.57	-1.60	0.01084362	0.03694448	0.02268195	2764	tags=57%, list=24%, signal=44%	TMOD3/CARMIL1/ADD2/TWF2/SCIN/FLII/TRIOBP/EPS8/SPTAN1/WASHC2C/SPTBN1/SPTA1/SVIL/GSN/CAPZB/ADD1/TMOD1/SPTB/MTPN/DMTN
BP	GO:1904377	positive regulation of protein localization to cell periphery	44	-0.54	-1.58	0.01093372	0.03718775	0.0228313	1597	tags=39%, list=14%, signal=34%	ZDHHC5/ZDHHC2/LGALS3/EPHB2/PRKCE/EZR/STX4/CLN3/PDPK1/NUMA1/SPTBN1/LRP1/ATP2B4/PPP1R9B/EPB41/SQSTM1/RHOG
BP	GO:0001892	embryonic placenta development	52	-0.52	-1.59	0.01096405	0.03723827	0.02286232	1562	tags=29%, list=13%, signal=25%	STK4/TFEB/CEBPA/ZFP36L1/CDKN1C/BIRC6/TRIM28/GRB2/ARNT/RBPJ/IGF2/BPTF/MED1/FZD5/NCOA1
MF	GO:0005342	organic acid transmembrane transporter activity	86	-0.46	-1.49	0.01101425	0.03725632	0.0228734	2424	tags=30%, list=21%, signal=24%	SERINC5/SLC1A4/SLC19A1/SLC10A3/SLC9A3R1/SLC25A15/SLC66A1/ABCB11/SLC16A3/SLC36A1/AQP9/SLC25A11/ABCG2/SLC27A1/SLC6A20/SLC3A2/SLC25A38/SLC6A9/SLC1A5/ABCC4/SLC43A2/SLC43A1/ABCC3/SLC38A10/SLC7A5/SLC38A5
BP	GO:0031670	cellular response to nutrient	27	-0.62	-1.66	0.01101948	0.03725632	0.0228734	2136	tags=41%, list=18%, signal=33%	ATP2B1/SRF/VDR/PDK2/FES/KANK2/USF2/MED1/RXRA/PIM1/NCOA1

MF	GO:0046943	carboxylic acid transmembrane transporter activity	86	-0.46	-1.49	0.01101425	0.03725632	0.0228734	2424	tags=30%, list=21%, signal=24%	SERINC5/SLC1A4/SLC19A1/SLC10A3/SLC9A3R1/SLC25A15/SLC66A1/ABCB11/SLC16A3/SLC36A1/AQP9/SLC25A11/ABCG2/SLC27A1/SLC6A20/SLC3A2/SLC25A38/SLC6A9/SLC1A5/ABCC4/SLC43A2/SLC43A1/ABCC3/SLC38A10/SLC7A5/SLC38A5
MF	GO:0001221	transcription coregulator binding	91	-0.45	-1.49	0.01105135	0.03730064	0.02290061	1859	tags=31%, list=16%, signal=26%	LCOR/ZBTB7A/CTBP2/SMARCA4/WIZ/NFATC1/CTBP1/PER2/ATF7/SMARCD3/FAM89B/RUNX3/RARA/ZBTB16/MED1/JUND/HMGA1/RXRA/CREBBP/CHD4/GATA1/SP1/PBX1/ENO1/HDGF/MED25/FOXO3/RELA
BP	GO:0022612	gland morphogenesis	55	-0.50	-1.53	0.011366	0.03817554	0.02343775	2926	tags=42%, list=25%, signal=32%	PLXND1/TNF/NOTCH1/NKX3-1/CFLAR/SCRIB/SLC9A3R1/KDM5B/SRC/VDR/TGFB1/NOTCH2/HGF/PML/CSF1R/PDGFA/STAT6/MED1/BTBD7/CAPN1/MSN/RPS6KA1/TGM2
BP	GO:1905477	positive regulation of protein localization to membrane	74	-0.46	-1.46	0.01138874	0.03819052	0.02344695	2045	tags=28%, list=17%, signal=24%	FYN/PRKCE/ERBB2/ITGAM/AP2B1/EZR/DLG4/STX4/CLN3/PDPK1/ITGB2/CHP1/SPTBN1/FIS1/LRP1/ATP2B4/BBC3/PPP1R9B/SQSTM1/AKT2/RHOG
BP	GO:0034612	response to tumor necrosis factor	163	-0.39	-1.40	0.01139184	0.03819052	0.02344695	1657	tags=23%, list=14%, signal=20%	GSTP1/GPS2/SMPD4/RIPK1/CPNE1/MAPK14/CEBPA/ZFP36L1/EIF5A/PYCARD/SPATA2/ARHGEF2/CDIP1/CXCL16/MAP3K5/NFKB1/HIPK1/PTK2B/INPP5K/TRAF6/PIAS4/MAP2K7/SYK/THBS1/HSPA1A/CD14/LAPTM5/TNFRSF1B/PRPF8/ACTN4/MAPK3/H2BC11/YBX3/SHARPIN/MBP/PLVAP/FOXO3/RELA
BP	GO:2001234	negative regulation of apoptotic signaling pathway	166	-0.40	-1.44	0.01143532	0.03830399	0.02351662	1657	tags=23%, list=14%, signal=20%	GSTP1/LRRK2/HGF/EYA3/TMBIM1/RIPK1/TMBIM6/ARHGEF2/BIRC6/CSNK2A1/HTT/CTNNA1/MMP9/SFRP2/THBS1/PSME3/PTPN1/LMNA/HSPA1A/PF4/ITPRIP/GSK3B/HYOU1/HSPB1/TCF7L2/GNAI2/GATA1/GPX1/CLU/ARRB2/PINK1/SIAH2/BCL2L1/CTTN/GRINA/ENO1/YBX3/STRADB/RELA
BP	GO:0000209	protein polyubiquitination	212	-0.36	-1.35	0.01179396	0.0393726	0.02417268	2643	tags=30%, list=23%, signal=23%	ANAPC1/RC3H1/UBE2S/HDAC6/AMBRA1/UBE2D3/UBE3B/UBE2B/RFFL/AMFR/MGRN1/LNPEP/TRIM5/MUL1/RNF135/THOP1/TRIM38/CDC27/ARIH1/ZNRF1/XIAP/RNF165/AREL1/RNF144B/RC3H2/NPEPPS/UBR5/RNF213/UBE2J2/GPS2/MARCHF6/ARIH2/SPOP/ITCH/RNF115/UBE2L3/SASH1/UBE4B/OTUB1/RNF126/TRAF6/PELI2/FZR1/DAW1/TRIP12/GABARAP/RBCK1/UBE2R2/WWP2/HUWE1/RMND5A/TRIM14/RNF187/UBE2O/DTX3L/MKRN1/RNF40/SHARPIN/TRIM58/MARCHF8/UBE2H/CBL/CDC34
BP	GO:0017157	regulation of exocytosis	131	-0.42	-1.45	0.01194568	0.03980306	0.02443696	2141	tags=31%, list=18%, signal=25%	VPS18/VPS4B/FGR/UNC13D/RAB27B/CD177/SDC4/AP1G1/ADGRE2/SDCBP/HGS/ITGAM/LRRK2/STX4/SPI1/PDPK1/ITGB2/SEPTIN1/FES/GAB2/BCR/RAB7A/SYK/PTAFR/FBXL20/CALM3/PRAM1/LYN/LGALS9/GSK3B/RAC2/SYTL4/GNAI2/PRKCB/GATA1/SNCA/LAMP1/RAB3D/P2RX1/RAB2B
CC	GO:0030658	transport vesicle membrane	125	-0.40	-1.38	0.01196294	0.03980306	0.02443696	2171	tags=27%, list=19%, signal=22%	CLTB/ATP2B1/RAB11FIP5/RAB27B/ZNRF1/AP1G1/STX6/SCAP/AFTPH/RPH3A/LRRK2/SYN3/PEF1/SYNRG/TMEM184A/CA4/STX5/CNIH3/AP2A1/SORL1/SEC31A/RAB7A/RAB5B/CLTC/CALM3/SLC6A9/DNAJC5/TPRG1L/RAB11B/SYTL4/NRGN/SNCA/BCL2L1/SEC16A
BP	GO:0061035	regulation of cartilage development	33	-0.58	-1.63	0.01195789	0.03980306	0.02443696	1134	tags=39%, list=10%, signal=36%	SMAD3/SMAD1/SCIN/AXIN2/TRPS1/GREM1/RARA/SOX6/BMP6/ZBTB16/POR/GLG1/RFLNB
BP	GO:0042310	vasoconstriction	30	-0.61	-1.68	0.01204952	0.0400576	0.02459324	1539	tags=33%, list=13%, signal=29%	DOCK5/ACTA2/PER2/HRH2/SLC8A1/RHOA/ECE1/KEL/P2RX1/TBXA2R

BP	GO:0001894	tissue homeostasis	146	-0.39	-1.38	0.01219212	0.04029581	0.02473948	1302	tags=31%, list=11%, signal=28%	GPR137/CD38/CORO1A/GIGYF2/NKIRAS2/TF/ADAM8/SLC28A2/SRF/SRC/EXT1/MKS1/PPARGC1B/ACTG1/CHMP4B/LRRK1/CSF1R/MTF1/TJP2/TPP1/PTK2B/SLC2A1/SNX10/TRAF6/RCN3/VCL/PECAM1/RAB7A/CSK/STK11/SYK/JAM3/ATP1B2/ESAM/LTF/ACTB/INPP5D/HSPB1/GATA1/NF1/CLDN5/RAB3D/ITGB3/MBP/KRT1
BP	GO:0008610	lipid biosynthetic process	474	-0.32	-1.28	0.01217149	0.04029581	0.02473948	2116	tags=21%, list=18%, signal=18%	CERS5/ORMDL3/TTC7A/SLA2/ACSS1/DGKA/PTEN/ATM/CDIPT/PIGT/STARD3/DGKG/ALDH3B1/ACSS2/DGKZ/OSBPL1A/ITPKB/SYNJ2/SCAP/FITM1/GPAA1/ABCB11/ACLY/GSTP1/PIP5KL1/B4GALT5/SYNJ1/SMPD4/SPTLC2/EGR1/ZBTB20/PIKFYVE/PIP4K2C/ST3GAL2/OSBPL2/ST3GAL3/PRXL2B/NR1H2/MCAT/CLN3/PPM1L/CSNK1G2/PTGES/ABHD5/DHCR24/ACADVL/ABHD4/ELOVL7/AGPAT3/TRIB3/PIP5K1C/CHP1/DGAT2/MTMR3/EIF6/PDGFA/MBOAT7/PITPNM2/SLC27A1/SGPL1/NFKB1/TSPO/INPP5K/B4GALT3/PRKD2/CYP27A1/PLSCR3/LPCAT3/PTGS1/BMP6/SLC44A2/POR/ST3GAL1/MED1/MGLL/PIK3CG/ABHD2/G6PD/LPIN2/INPP5D/ALOX12/AGPAT1/FDFT1/CYB5R3/SF1/ST6GALNAC4/PNPLA2/FASN/WDTC1/PBX1/PIP4K2A/IP6K1/DPM2/PIK3CD/DAB2/P2RX1/CTDNEP1/GBGT1/FAXDC2
BP	GO:0010762	regulation of fibroblast migration	30	-0.61	-1.68	0.01219212	0.04029581	0.02473948	1899	tags=37%, list=16%, signal=31%	PRKCE/SDC4/TGFB1/ACTA2/CLN3/CORO1C/SLC8A1/THBS1/RAC1/GNA12/DMTN
BP	GO:0050886	endocrine process	39	-0.56	-1.61	0.01219212	0.04029581	0.02473948	814	tags=15%, list=7%, signal=14%	BMP6/RAB11FIP1/NIBAN2/RHOA/ECE1/DAB2
BP	GO:0002062	chondrocyte differentiation	57	-0.49	-1.52	0.01243053	0.04074509	0.02501532	2401	tags=35%, list=20%, signal=28%	GPLD1/SCIN/AXIN2/EXT1/TGFB1/CHSY1/ARID5A/MAPK14/TRPS1/RUNX3/GREM1/SOX6/CHST11/BMP6/ZBTB16/POR/SFRP2/GLG1/ANXA6/RFLNB
BP	GO:0007498	mesoderm development	60	-0.50	-1.56	0.01243024	0.04074509	0.02501532	2803	tags=38%, list=24%, signal=29%	SMAD3/SMAD1/MEST/SETD2/ARMC5/WLS/TBX19/SRF/EXOC4/EXT1/HCK/SECTM1/NF2/ZFP36L1/PRKACA/SFRP2/GPI/ETS2/KDM6B/TAL1/IKZF1/ITGB3/CTDNEP1
BP	GO:0031348	negative regulation of defense response	167	-0.38	-1.37	0.0124765	0.04079486	0.02504588	2246	tags=28%, list=19%, signal=23%	MUL1/IFI16/TRIM38/MICB/IRAK3/SERPING1/CR1/FGR/FYN/NLRP3/SRC/HCK/SIRPA/GSTP1/HGF/GPS2/ASH1L/METRNL/MAPK14/NR1H2/VPS35/ITCH/KLRD1/YTHDF3/NFKB1/PGLYRP1/MFHAS1/BCR/LPCAT3/PTPRC/SYK/ADAR/ILRUN/NPLOC4/NLRX1/LYN/RIOK3/LGALS9/TNFRSF1B/GPX1/ARRB2/GRN/SEC14L1/PCBP2/SHARPIN/KRT1/FURIN
BP	GO:0001822	kidney development	157	-0.40	-1.41	0.01277842	0.04161096	0.02554692	3013	tags=36%, list=26%, signal=27%	DTNBP1/NID1/NPHS1/FADD/PLXND1/KLHL3/NOTCH1/CENPF/NKX3-1/SMAD3/SOX4/PLCE1/SMAD1/APH1A/CFLAR/CTNNBIP1/PTK7/ASXL1/BCL2L11/MYO1E/WNT1/APAF1/JMJD6/SDC4/EXT1/NIPBL/MAGED1/NOTCH2/LRRK2/MPST/AP2B1/SNX17/EGR1/MME/ACTA2/CDKN1C/EPHA4/PDGFA/SGPL1/GREM1/KANK2/ODC1/RARA/IL6R/PECAM1/BMP6/ZBTB16/MMP9/BASP1/RHOA/NF1/PBX1/AP1B1/FLCN/IQGAP1/BAG6
BP	GO:0038127	ERBB signaling pathway	82	-0.44	-1.43	0.01281159	0.04166615	0.0255808	2771	tags=38%, list=24%, signal=29%	PLCE1/RASSF2/DGKD/RHBDF2/PLAUR/ADAM17/MVP/RPS6KA5/SRC/CPNE3/TGFB1/GAB1/SHC1/PTPRJ/PTPN18/PDPK1/RNF115/PTPN12/NCF1/GRB2/RBPJ/RNF126/PTK2B/NEURL1/RAB7A/MMP9/HIP1/REPS2/SHKBP1/CBL/IQGAP1
MF	GO:0030374	nuclear receptor coactivator activity	51	-0.52	-1.57	0.01298129	0.04209919	0.02584667	2193	tags=47%, list=19%, signal=38%	PKN1/TADA3/MED13/ELK1/ATXN7L3/PPARGC1B/SRA1/NCOA3/HELZ2/CNOT6/TACC1/NCOA2/ZMIZ1/MED1/DCAF6/RBM14/HMGA1/PRKCB/NCOA6/ACTN4/USP22/ACTN1/NCOA1/CARM1

BP	GO:0051693	actin filament capping	32	-0.58	-1.60	0.01304769	0.04228006	0.02595771	2764	tags=62%, list=24%, signal=48%	TMOD3/CARMIL1/ADD2/TWF2/SCIN/FLII/TRIOBP/EPS8/SPTAN1/WASHC2C/SPTBN1/SPTA1/SVIL/GSN/CAPZB/ADD1/TMOD1/SPTB/MTPN/DMTN
BP	GO:0051209	release of sequestered calcium ion into cytosol	71	-0.47	-1.49	0.01339257	0.04329174	0.02657883	1421	tags=37%, list=12%, signal=32%	CALM1/CORO1A/LETM1/TPCN1/FKBP1B/MCOLN1/PRKCE/PTPN6/PDPK1/ITPR2/PRKACA/CHERP/ERO1A/TMEM38A/HTT/PTK2B/SLC8A1/PTPRC/SELENON/CALM3/LYN/GP9/FKBP1A/SNCA/GP1BA/DIAPH1
BP	GO:0061045	negative regulation of wound healing	36	-0.58	-1.64	0.01351452	0.04365047	0.02679907	2966	tags=47%, list=25%, signal=35%	UBASH3B/SERPINE1/TNF/SMAD3/CLASP1/ALOX5/SH2B3/SERPING1/KNG1/PTEN/ABAT/THBD/PDGFA/THBS1/ALOX12/GP1BA/KRT1
BP	GO:0045664	regulation of neuron differentiation	95	-0.44	-1.45	0.01363012	0.04389478	0.02694906	2603	tags=36%, list=22%, signal=28%	CALR/TGIF2/KDM4A/YWHAG/ASPM/BCL11B/BCL11A/GDPD5/FEZ1/YWHAH/MYCL/TIAM1/LRRK2/CPNE1/RAP1GAP/DDX6/ARHGEF2/ZFHX3/DPYSL2/RARA/MEIS1/ZHX2/BMP6/SFRP2/MED1/EIF4G1/SIN3A/MMD/GSK3B/CTDSP1/RHOA/PBX1/NCOA1/FOXO3
BP	GO:0090288	negative regulation of cellular response to growth factor stimulus	43	-0.53	-1.56	0.01365865	0.04393752	0.0269753	2515	tags=42%, list=21%, signal=33%	NOTCH1/PIK3CB/UBE2D3/CFLAR/SPRY3/WNT1/HGS/FUZ/GREM1/SORL1/SFRP2/THBS1/PTPN1/HIPK2/SKI/NIBAN2/ATP2B4/PPM1A
BP	GO:0061136	regulation of proteasomal protein catabolic process	164	-0.38	-1.38	0.0137193	0.04409695	0.02707318	1818	tags=26%, list=16%, signal=22%	DET1/RNF144B/SDCBP/SH3RF3/SMARCC1/LRRK2/KEAP1/PIAS1/TRIB1/CEBPA/WAC/CBFA2T3/ARIH2/ALAD/TRIB3/GSK3A/PRKACA/BCAP31/PITHD1/MAP1A/RAD23A/FZR1/PSME3/VCP/HSPA1A/PSMC4/GABARAP/GSK3B/GPX1/CLU/GNA12/PINK1/PSMF1/N4BP1/UBB/DNAJB2/USP7/ARAF/RNF40/DAB2/ZER1/BAG6/RYBP
BP	GO:0030282	bone mineralization	63	-0.48	-1.51	0.01376556	0.04413861	0.02709876	2404	tags=44%, list=20%, signal=36%	PHEX/ERCC2/SMAD3/FBXL15/ALOX5/TUFT1/TENT5A/AXIN2/ATP2B1/FGR/TGFB1/ANO6/NBR1/SBNO2/CCR1/ALPL/GREM1/PTK2B/SLC8A1/SNX10/ANKH/SLC24A3/BMP6/BMP2K/PHOSPHO1/LTF/RFLNB/GATA1
BP	GO:0048608	reproductive structure development	225	-0.36	-1.32	0.01388626	0.04445391	0.02729234	2089	tags=28%, list=18%, signal=23%	BCL2L11/CSDE1/PTEN/KDM5B/MAP3K4/SRC/ATM/STK4/H3-3B/NIPBL/VDR/H3-3A/CUL7/NOTCH2/HSPA5/ASH1L/STAT5B/FANCG/TFEB/MME/MAPK14/CEBPA/ZFP36L1/PTPN6/CDKN1C/SPATA2/AGO4/DHCR24/CCNF/BIRC6/TRIM28/GRB2/ARNT/PDGFA/INSR/SGPL1/RBPJ/NCOA4/RARA/IGF2/CTNNA1/BMP6/SFRP2/STK11/BPTF/MED1/CASP2/FZD5/BASP1/PSAP/SF1/GATA1/ARRB2/TBC1D20/BCL2L1/UBB/NCOA1/BSG/CTSB/YBX3/FOXO3/CBL
BP	GO:1903828	negative regulation of protein localization	145	-0.39	-1.36	0.01401063	0.04477997	0.02749252	1817	tags=28%, list=15%, signal=24%	MAGED1/TGFB1/SNX3/MIDN/ANKRD13A/NUMB/ROCK2/LRRK2/RANGAP1/TMBIM1/POLR1A/YOD1/ABI3/VPS35/SPI1/CHP1/SUFU/MFHAS1/INPP5K/NSFL1C/MAP1A/FRMD4A/CTNNA1/CSK/PPP3CA/ILRUN/CLTC/RAB11FIP1/UCP2/SIN3A/GSK3B/WWP2/SYTL4/NF1/BCL2L1/FLCN/RIPOR2/AP2M1/DAB2/DMTN
BP	GO:0009755	hormone-mediated signaling pathway	122	-0.40	-1.38	0.0142016	0.04528418	0.02780208	2193	tags=32%, list=19%, signal=26%	PKN1/SAFB/TADA3/VPS18/THRA/PADI2/SRC/PER1/YWHAH/RXRB/DDX54/PAQR8/PPARGC1B/ZBTB7A/UBR5/SMARCA4/KMT2D/FOXP1/ADIPOR2/SLC27A1/VPS11/KANK2/NCOA4/RARA/PAK1/ZMIZ1/NCOR2/ADIPOR1/MED1/NR3C1/ABHD2/RXRA/RHOA/ARID1A/WBP2/NCOA1/DAB2/CNOT1/CARM1

BP	GO:0046486	glycerolipid metabolic process	284	-0.34	-1.30	0.01420256	0.04528418	0.02780208	2903	tags=30%, list=25%, signal=23%	MECP2/INPPL1/PI4KB/SEL1L/CHPT1/PIK3CB/PGAP2/PLCE1/PLB1/PCYT2/DGKD/ABCA3/PIGQ/IMPA2/FIG4/SOCS7/SOCS3/SERINC5/GPLD1/PCYT1A/PI4K2A/SCARB1/PLCD1/TNXB/TTC7A/SLA2/DGKA/PTEN/ATM/CDIPT/PIGT/DGKG/DGKZ/ITPKB/SYNJ2/GPAA1/PIP5KL1/PLCB1/SYNJ1/LIPE/SMPD4/PIKFYVE/PIP4K2C/NR1H2/CLN3/OSBPL5/ABHD5/LYPLA2/ABHD4/CSF1R/AGPAT3/SERINC2/PIP5K1C/DGAT2/MTMR3/PDGFA/MBOAT7/PITPNM2/SLC27A1/SORL1/APOBR/INPP5K/PLSCR3/LPCAT3/TBL1XR1/SLC44A2/PLCB3/CPT1A/MGLL/PIK3CG/ABHD2/LPIN2/GDE1/INPP5D/PLEK/AGPAT1/DGKK/TMEM86B/PNPLA2/GPX1/PIP4K2A/PRDX6/IP6K1/DPM2/PIK3CD/CTDNEP1
BP	GO:0099601	regulation of neurotransmitter receptor activity	29	-0.59	-1.59	0.01423678	0.04535693	0.02784674	2232	tags=38%, list=19%, signal=31%	EPHB2/SRC/DLGAP4/NPTXR/DLG4/SHISA7/CNIH3/PTK2B/SLC6A9/CAPN1/PINK1
BP	GO:0038202	TORC1 signaling	45	-0.52	-1.55	0.01444598	0.04587638	0.02816566	2220	tags=40%, list=19%, signal=33%	DYRK3/KLHL22/RRAGD/ATM/LARS1/SZT2/WAC/CLEC16A/SESN3/SESN2/RRAGC/SIK3/OTUD5/STK11/CASTOR2/LARP1/FLCN/NPRL3
CC	GO:0072562	blood microparticle	44	-0.53	-1.56	0.01455411	0.04607257	0.02828611	2250	tags=43%, list=19%, signal=35%	TF/SERPING1/KNG1/HSPA1L/TGFB1/SDCBP/ACTG1/YWHAZ/SLC2A1/F13A1/GSN/HSPA1A/ACTB/ITGA2B/GRIPAP1/CLU/MSN/SLC4A1/KRT1
BP	GO:0035904	aorta development	31	-0.59	-1.65	0.01462022	0.04607382	0.02828688	3341	tags=55%, list=28%, signal=39%	CHD7/PRDM1/ENG/PLXND1/NOTCH1/NKX3-1/SOX4/SRF/PDE2A/AP2B1/SNX17/SUFU/RBPJ/NDST1/LRP1/MYLK/NPRL3
BP	GO:0050819	negative regulation of coagulation	26	-0.62	-1.65	0.01466826	0.04607382	0.02828688	2211	tags=38%, list=19%, signal=31%	SH2B3/SERPING1/KNG1/ABAT/THBD/PDGFA/THBS1/ALOX12/GP1BA/KRT1
BP	GO:0071772	response to BMP	80	-0.45	-1.47	0.01464196	0.04607382	0.02828688	1879	tags=28%, list=16%, signal=23%	WNT1/XIAP/EXT1/RNF165/TGFB1/NOTCH2/EGR1/MSX1/NUMA1/GREM1/RBPJ/SORL1/PCSK6/BMP6/SFRP2/USP9X/HIPK2/SKI/FKBP8/UBE2O/MAPK3/PPM1A
BP	GO:0071773	cellular response to BMP stimulus	80	-0.45	-1.47	0.01464196	0.04607382	0.02828688	1879	tags=28%, list=16%, signal=23%	WNT1/XIAP/EXT1/RNF165/TGFB1/NOTCH2/EGR1/MSX1/NUMA1/GREM1/RBPJ/SORL1/PCSK6/BMP6/SFRP2/USP9X/HIPK2/SKI/FKBP8/UBE2O/MAPK3/PPM1A
BP	GO:0051279	regulation of release of sequestered calcium ion into cytosol	44	-0.53	-1.56	0.01469893	0.04609065	0.02829721	1421	tags=41%, list=12%, signal=36%	CAPN3/CALM1/CORO1A/FKBP1B/PRKCE/PTPN6/PDPK1/PRKACA/TMEM38A/HTT/PTK2B/SLC8A1/SELENON/CALM3/LYN/FKBP1A/SNCA/DIAPH1
BP	GO:0006939	smooth muscle contraction	47	-0.53	-1.58	0.01493243	0.0467123	0.02867887	2136	tags=40%, list=18%, signal=33%	ATP2B1/ORMDL3/SRF/FKBP1B/ABAT/PROK2/ROCK2/DOCK5/ACTA2/DAPK3/SLC8A1/PTAFR/ATP2B4/RHOA/MYLK/GUCY1A1/CTTN/P2RX1/TBXA2R
CC	GO:0005903	brush border	59	-0.49	-1.52	0.01499057	0.04685733	0.02876791	2358	tags=36%, list=20%, signal=29%	SLC19A1/SCIN/SLC9A3R1/SLC28A2/FLII/MYO1E/ATP7A/EPS8/EZR/MME/ABCG2/CA4/PLEC/B4GALT1/FLNA/AQP1/CAPZB/GNA12/ESPN/ACTN1/MYH9
BP	GO:0019079	viral genome replication	117	-0.40	-1.38	0.01507356	0.04707972	0.02890445	2241	tags=38%, list=19%, signal=31%	TNF/NOTCH1/OAS2/RNASEL/GBP7/ATG16L2/FBXL2/BANF1/ZBED1/PCBP1/IFI16/MAVS/TRIM38/SRPK1/APOBEC3D/KPNA6/SETDB1/CTBP2/ROCK2/NFIA/OASL/CTBP1/GRK2/EIF2AK2/IFITM3/ZC3HAV1/TRIM28/INPP5K/RAD23A/ADAR/LTF/VCP/APOBEC3F/DDB1/IFITM2/SRPK2/TNIP1/STAU1/APOBEC3C/LARP1/ZNFX1/TBC1D20/PCBP2/N4BP1

BP	GO:0043535	regulation of blood vessel endothelial cell migration	62	-0.48	-1.50	0.01519695	0.0473535	0.02907253	878	tags=19%, list=7%, signal=18%	PRKD2/THBS1/MAP3K3/STAT5A/HSPB1/ATP2B4/RHOA/NF1/SP1/SH3BP1/TBXA2R/MAP2K3
MF	GO:0008509	anion transmembrane transporter activity	165	-0.39	-1.40	0.01544255	0.04800592	0.02947309	2509	tags=27%, list=21%, signal=21%	SLC25A23/SLC37A2/SLC13A4/SERINC5/SLCO3A1/SLC1A4/SLC19A1/LRRC8B/SLC9A3R1/ANO10/SLC25A15/SLC24A4/SLC25A41/SLC66A1/SLC2A3/ANO6/SLC12A6/GET3/TTYH3/CLCN5/SLC16A3/SLC36A1/SLC25A11/CLCC1/ABCG2/MFSD5/SLC27A1/LRRC8A/SLC2A1/ANKH/SLC6A20/SLC24A3/SLC3A2/CLIC3/SLC25A38/SLC9A1/SLC6A9/SLC1A5/ABCC4/ABCC3/SLC4A1/SLC7A5/SLC38A5/SLC6A8
BP	GO:1904951	positive regulation of establishment of protein localization	235	-0.35	-1.32	0.01548174	0.04809017	0.02952481	2437	tags=29%, list=21%, signal=23%	CCT7/GPLD1/BAP1/CD38/WLS/ARHGEF5/HDAC3/MAVS/ADAM8/TLR2/HSPA1L/FYN/SRC/IRS2/SH3GLB1/ABAT/MYO18A/PRKCE/ERBB2/USP36/TGFB1/NPEPPS/UBR5/UBE2J2/ITGAM/EZR/MAPK14/STX4/NR1H2/ARIH2/MYOM1/CLN3/VPS35/ITGB2/TRIM28/GSK3A/UBE2L3/CHP1/PRKACA/VPS11/RBM22/SORL1/BCAP31/LRRC8A/BCAS3/FRMD4A/BMP6/FIS1/FZD5/FLNA/RIPOR1/VPS28/GSK3B/RAC2/SYTL4/TCF7L2/HUWE1/BBC3/HCLS1/OAZ1/ATG13/PINK1/PTPN23/ABLIM3/AKT2/PTP4A3/PPM1A/GLUL
BP	GO:0097190	apoptotic signaling pathway	457	-0.32	-1.25	0.01559018	0.0483892	0.0297084	1657	tags=21%, list=14%, signal=19%	GSTP1/ITGAM/LRRK2/IKBKE/HGF/EYA3/TMBIM1/TMC8/RIPK1/STX4/PLEKHF1/MSX1/SORT1/EIF5A/PYCARD/DPF2/DAP/TMBIM6/DAPK3/SPI1/PDPK1/PML/ARHGEF2/DEDD2/CDIP1/TRIB3/BIRC6/GSK3A/PPP1CA/PDK2/UBE4B/TNFRSF10C/STK24/ATP2A3/CSNK2A1/MAP3K5/SGPL1/HIPK1/MKNK2/ERO1A/BCAP31/HTT/PLSCR3/IL6R/PIAS4/CTNNA1/MMP9/SFRP2/STK11/PTPRC/THBS1/FIS1/PSME3/PTPN1/LMNA/CASP2/HIPK2/HSPA1A/ANXA6/MADD/PF4/GABARAP/HIP1/HNRNPK/ITPRIP/RBCK1/GSK3B/HYOU1/HSPB1/BNIP3L/TNFRSF1B/TCF7L2/GNAI2/CUL4A/GATA1/GPX1/BBC3/CLU/ARRB2/NF1/PINK1/SIAH2/CDKN1A/BCL2L1/FLCN/CTTN/SHISA5/UBB/GRINA/ENO1/PRKDC/PAK2/YBX3/E2F2/FOXO3/STRADB/BAG6/RELA
BP	GO:0043502	regulation of muscle adaptation	49	-0.51	-1.53	0.01612286	0.0499256	0.03065167	2107	tags=33%, list=18%, signal=27%	FOXO1/HDAC4/CTDP1/ROCK2/YY1/GSK3A/PPP3CA/SLC9A1/LMNA/G6PD/PDE5A/TNFRSF1B/ATP2B4/MTPN/JARID2/FOXO3

Supplementary table 12 APOE4 non-carriers gene set enrichment analysis gene ontology downregulated

ONTOLOGY	ID	Description	setSize	enrichment Score	NES	pvalue	p.adjust	qvalue	rank	leading_edge	core_enrichment
BP	GO:0110020	regulation of actomyosin structure organization	66	-0.61	-1.99	6.59E-06	8.11E-05	5.03E-05	2222	tags=38%, list=19%, signal=31%	PFN1/ARAP1/ROCK2/SMAD3/EDN1/SORBS3/ACTG1/TPM1/RAC1/SLC9A1/NRP1/PXN/ABL1/PAK1/CLASP1/ARHGEF18/RHOA/ARHGAP6/WAS/INPP5K/NF2/SDC4/AKAP13/PAK2/WASF2
BP	GO:0032231	regulation of actin filament bundle assembly	70	-0.60	-1.95	1.08E-05	0.00012313	7.63E-05	1476	tags=30%, list=13%, signal=26%	SORBS3/ACTG1/PLEK/TPM1/RAC1/SLC9A1/NRP1/PXN/ABL1/PAK1/CLASP1/ARHGEF18/RHOA/ARHGAP6/WAS/FLNA/INPP5K/NF2/SDC4/PAK2/WASF2
BP	GO:0051492	regulation of stress fiber assembly	61	-0.62	-1.97	1.12E-05	0.00012772	7.92E-05	1476	tags=38%, list=13%, signal=33%	PFN1/ARAP1/ROCK2/SMAD3/SORBS3/ACTG1/TPM1/RAC1/SLC9A1/NRP1/PXN/ABL1/PAK1/CLASP1/ARHGEF18/RHOA/ARHGAP6/WAS/INPP5K/NF2/SDC4/PAK2/WASF2
BP	GO:0046939	nucleotide phosphorylation	74	-0.56	-1.86	2.84E-05	0.00028033	0.00017382	2017	tags=35%, list=17%, signal=29%	ENTPD5/PGAM1/ZBTB7A/EIF6/FOXK1/ENO2/GALK1/FOXK2/GIT1/CBFA2T3/JMJD8/ALDOA/NME4/ALDOC/PFKL/ARNT/AK1/PPARA/PKM/BPGM/STAT3/FBP1/HK1/SLC4A1/GPI/ENO1
BP	GO:0006165	nucleoside diphosphate phosphorylation	73	-0.57	-1.88	5.17E-05	0.00046913	0.00029088	2017	tags=36%, list=17%, signal=30%	ENTPD5/PGAM1/ZBTB7A/EIF6/FOXK1/ENO2/GALK1/FOXK2/GIT1/CBFA2T3/JMJD8/ALDOA/NME4/ALDOC/PFKL/ARNT/AK1/PPARA/PKM/BPGM/STAT3/FBP1/HK1/SLC4A1/GPI/ENO1
BP	GO:0061138	morphogenesis of a branching epithelium	76	-0.56	-1.86	7.66E-05	0.000644	0.0003993	1756	tags=34%, list=15%, signal=29%	EDN1/MAGED1/ENG/FZD5/GRB2/CTNNBIP1/ST14/SPINT2/ILK/PLXND1/DCHS1/SRC/PML/GREM1/SFRP2/PLXNA1/PPP1CA/BTBD7/NRP1/BTRC/ABL1/PAK1/MED1/PDGFA/TGM2/PBX1
BP	GO:0001763	morphogenesis of a branching structure	85	-0.53	-1.80	8.81E-05	0.00071927	0.00044597	1756	tags=33%, list=15%, signal=28%	EDN1/RERE/MAGED1/ENG/FZD5/GRB2/CTNNBIP1/ST14/SPINT2/BCL11A/ILK/PLXND1/DCHS1/SRC/PML/GREM1/SFRP2/PLXNA1/PPP1CA/BTBD7/NRP1/BTRC/ABL1/PAK1/MED1/PDGFA/TGM2/PBX1
BP	GO:0043393	regulation of protein binding	148	-0.46	-1.67	0.00016005	0.00118946	0.00073751	2665	tags=44%, list=23%, signal=34%	TRIM21/ADD2/PKD1/MAPRE1/ATP2A3/RALB/SORL1/AKT1/CBLB/ZFPM1/DISC1/ADNP/ATP2A2/HDAC4/HDAC5/CDK5/GTF2F1/SMARCD3/TMC8/PIN1/TFIP11/DERL1/TIAM1/TAF1/POLDIP2/MMP9/MARK3/DTNBP1/GSK3B/PRKACA/HSP90AB1/CTNNBIP1/MEN1/HIP1R/SPPL3/PLXND1/DZIP1/MARK2/SRC/PPARA/AMFR/ACTB/PPP1CA/NRP1/RFNG/ABL1/FKBP1A/DTX3L/ARRB2/GOLGA2/LDLRAP1/HIPK2/GNL3L/CLDN5/LRP1/EPB41/SPTA1/TCF7L2/UBASH3B/CAMK1/MAPK3/CDKN1A/DNAJB2/ADD1/DAB2
BP	GO:0032272	negative regulation of protein polymerization	57	-0.57	-1.79	0.00018541	0.0013452	0.00083407	2653	tags=47%, list=23%, signal=37%	ADD2/MAPRE1/EPS8/TMOD3/SPTAN1/TRIOBP/PFN1/SSH1/TBCD/MYADM/SVIL/SSH2/EML2/HIP1R/FLII/SSH3/GSN/SPTBN1/MTPN/SPTA1/SPTB/SNCA/TMOD1/SCIN/CAPZB/DMTN/ADD1
BP	GO:0051495	positive regulation of cytoskeleton organization	142	-0.46	-1.69	0.00024065	0.0016905	0.00104817	2222	tags=33%, list=19%, signal=27%	PFN1/MECP2/CDK5/CDK5RAP2/VPS4B/ROCK2/FES/NUMA1/SMAD3/FMN1/EDN1/GRB2/SLAIN2/BAIAP2/SORBS3/PYCARD/CYFIP1/GIT1/CDC42EP4/PLEK/WDR1/TPM1/KATNB1/ALOX15/BCAS3/RAC1/PTK2B/HSPA1A/HCLS1/DYNC1H1/NRP1/PXN/ABL1/PAK1/CLASP1/RHOA/CKAP5/WAS/NF2/SDC4/DCTN1/GSN/VASP/WASF3/SCIN/CTTN/WASF2
BP	GO:0048008	platelet-derived growth factor receptor signaling pathway	39	-0.63	-1.88	0.00035802	0.00236851	0.00146856	1323	tags=44%, list=11%, signal=39%	MYO1E/HIP1/HIP1R/PTPN12/SRC/NRP1/PDGFC/ABL1/PTPRJ/TXNIP/LRP1/PTPN1/IQGAP1/SNCA/CBL/SMPD3/PDGFA

BP	GO:0031334	positive regulation of protein-containing complex assembly	152	-0.44	-1.63	0.00039393	0.00255928	0.00158685	2002	tags=31%, list=17%, signal=26%	TLR6/LCP1/FES/LGALS3/NR1H2/NUMA1/FMN1/GRB2/SLAIN2/GSK3B/BAIAP2/PYCARD/TGFB1/GIT1/ASAP1/CDC42EP4/PLEK/ALOX15/HIP1R/PINK1/RAC1/PTK2B/HSPA1A/BCL2L11/SRC/PIH1D1/SYK/VCP/PAK1/FNIP2/CLASP1/WARS1/RHOA/CKAP5/GNL3L/DHX33/DCTN1/MED25/PPP2R5B/MSN/SNCA/EIF4G1/VASP/TAL1/CTTN/STMP1/CLU
MF	GO:0005506	iron ion binding	71	-0.54	-1.78	0.00045859	0.00291735	0.00180886	2016	tags=30%, list=17%, signal=25%	OGFOD1/PHF8/HAAO/HIF1AN/FTO/DOHH/PLOD1/CYP27A1/ALOX15/CALR/PHF2/EGLN3/LCN2/LTF/FTH1/ALOX12/SNCA/FTL/FAXDC2/FECH/ACO2
BP	GO:0007595	lactation	27	-0.69	-1.89	0.00049258	0.00307455	0.00190633	921	tags=33%, list=8%, signal=31%	OAS2/DDR1/SLC29A2/USF2/STAT5A/NEURL1/SLC29A1/MED1/NCOR2
CC	GO:0030662	coated vesicle membrane	125	-0.46	-1.65	0.00054592	0.00334968	0.00207692	1753	tags=30%, list=15%, signal=25%	KIAA0319/FZD5/CD74/HLA-C/SEC23IP/AP2B1/HLA-DQA1/SREBF1/SYNRG/COPA/CLTC/DNAJC5/DENND1A/HIP1/AP2S1/HIP1R/VTI1B/FZD2/CD9/KDELR1/HLA-DRB5/VTI1A/TGOLN2/COPB2/SEC31A/CNIH3/STX5/SCYL1/CLTB/EPN1/LDLRAP1/ARCN1/IGF2R/NRGN/AP2M1/SEC16A/DAB2
BP	GO:0045912	negative regulation of carbohydrate metabolic process	35	-0.63	-1.80	0.00058425	0.00355741	0.00220572	1521	tags=40%, list=13%, signal=35%	GSK3B/FAM3A/GSK3A/TGFB1/GIT1/CLTC/CBFA2T3/PLEK/PPARA/STAT3/FBP1/INPP5K/SLC4A1/SMPD3
MF	GO:0016922	nuclear receptor binding	118	-0.45	-1.63	0.000699	0.00413576	0.00256432	1998	tags=36%, list=17%, signal=30%	TACC1/SMARCD3/PPARGC1B/ZBTB7A/NR1H2/SMAD3/TAF1/TGFB1I1/TRERF1/JUND/FOXP1/NCOA1/TOB2/CRY2/BAZ2A/TRIP6/SMARCA4/NCOA2/NCOA6/PARP1/BCAS3/TADA3/RXRA/MED16/SRC/DCAF1/NCOA3/CALR/WBP2/DAXX/YWHAH/MED25/PRKCB/TCF7L2/TRIP12/HMGA1/ACTN4/MED1/ARID1A/GAS2L1/NCOR2/CNOT1
MF	GO:0042887	amide transmembrane transporter activity	26	-0.68	-1.85	0.00070209	0.00414169	0.00256799	2513	tags=58%, list=21%, signal=45%	SLC46A1/ABCB9/ABCD1/SLC33A1/TAPBP/SLC19A1/ABCC1/SLC27A1/AQP10/SLC5A6/SLC14A1/ABCG2/ABCC4/SLC15A3/SLC38A5
BP	GO:0006090	pyruvate metabolic process	75	-0.51	-1.71	0.00070337	0.0041431	0.00256887	2017	tags=36%, list=17%, signal=30%	ENTPD5/PGAM1/ZBTB7A/EIF6/FOXK1/ENO2/GALK1/PDK3/FOXK2/GIT1/CBFA2T3/JMJD8/ALDOA/ALDOC/PFKL/ARNT/PPARA/PKM/BPGM/STAT3/PDK2/VDAC1/FBP1/HK1/SLC4A1/GPI/ENO1
BP	GO:0032355	response to estradiol	62	-0.55	-1.75	0.00072175	0.00424511	0.00263212	1100	tags=24%, list=9%, signal=22%	RARA/BAD/BCL2L11/ARPC1B/NCOA3/CALR/STAT3/TXNIP/PPP1R9B/FAM210B/GPI/ZNF703/GPX1/PDGFA/NCOR2
BP	GO:0010638	positive regulation of organelle organization	380	-0.35	-1.41	0.00078264	0.00455606	0.00282492	1842	tags=22%, list=16%, signal=19%	NUMA1/ARHGAP35/SMAD3/FMN1/SYNJ1/EDN1/CHCHD10/STOML2/MMP9/BBC3/CCT3/GRB2/SLAIN2/GSK3B/BAIAP2/SORBS3/GSK3A/PYCARD/G3BP2/CYFIP1/GIT1/ASAP1/SPHK1/ATG2A/CDC42EP4/PLEK/WDR1/VPS13D/TPM1/KATNB1/ALOX15/HIP1R/BCAS3/CDC27/PINK1/RAC1/PTK2B/IGF2/HSPA1A/MAP2K7/BAD/DZIP1/HCLS1/BCL2L11/DYNC1H1/SRC/PML/SLX4/FIS1/CDK2/TRIM28/NRP1/VDAC1/PXN/ABL1/PAK1/CLASP1/NCAPH/SETDB1/RHOA/CKAP5/WAS/NF2/SDC4/DCTN1/GSN/TERF2/AKT2/MSN/GRN/SNCA/PPP1R10/VASP/PIP4K2A/MAPK3/ELAPOR1/FBXO7/WASF3/SMPD3/SCIN/WDR45/CTTN/HTT/WASF2/CNOT1

BP	GO:0043401	steroid hormone mediated signaling pathway	99	-0.48	-1.66	0.00112547	0.00628492	0.00389688	2428	tags=37%, list=21%, signal=30%	NR1D1/DDX54/UBR5/CNOT9/PKN1/ZNF366/PAQR8/NCOA4/PPARGC1B/ZBTB7A/NR3C1/TRERF1/FOXP1/CRY2/SMARCA4/PARP1/LBH/TADA3/RXRA/SRC/CALR/PPARA/WBP2/DAXX/ZMIZ1/YWHAH/PAK1/RHOA/KANK2/ABHD2/SAFB/MED1/ARID1A/NCOR2/CARM1/CNOT1/DAB2
BP	GO:0043277	apoptotic cell clearance	34	-0.61	-1.76	0.00117385	0.00653675	0.00405302	1799	tags=41%, list=15%, signal=35%	SCARB1/RHOBTB1/CD300LF/NR1H3/ALOX15/RAC1/RARA/RAB14/RAC2/LRP1/THBS1/RHOG/ITGB3/TGM2
BP	GO:0007589	body fluid secretion	46	-0.59	-1.80	0.00124557	0.00687829	0.00426479	2287	tags=33%, list=20%, signal=26%	OAS2/DDR1/NR1H2/SLC29A2/EDN1/NR1H3/COPA/AQP1/USF2/STAT5A/NEURL1/SLC29A1/MED1/NCOR2/WNK1
BP	GO:1902305	regulation of sodium ion transmembrane transport	40	-0.59	-1.76	0.00124787	0.00688147	0.00426676	2267	tags=42%, list=19%, signal=34%	CHP1/NETO2/FXYD6/ATP1B1/SNTA1/STOM/RANGRF/NEDD4L/SLC9A1/ATP1B2/YWHAH/DNM2/ATP2B4/ACTN4/TESC/SCN1B/WNK1
CC	GO:0098794	postsynapse	342	-0.36	-1.46	0.00134824	0.00734327	0.00455309	2253	tags=31%, list=19%, signal=26%	EIF4A3/EPHA4/MAGI2/NETO2/MECP2/CDK5/FXR2/ABI3/FYN/ABHD17C/FXYD6/CRKL/P2RX4/DOCK10/CTNND1/DNAJB1/SNTA1/SRGAP2/PIN1/MIB1/PKP4/SYNE1/PDE4B/LRFN1/DROSHA/CANX/EIF3A/AP2B1/PPP1R9A/KCNJ2/SEPTIN11/DTNBP1/GSK3B/BAIAP2/PRKACA/GSK3A/CYFIP1/ACTG1/GIT1/ASAP1/PRR7/TRAPPC4/STX4/KIF3B/BCL11A/HIP1/HIP1R/LYN/PRR12/RAC1/NPFF/PTK2B/ARF1/SIPA1L1/GRIPAP1/SRC/DLGAP4/IQSEC1/SLA/ARHGAP32/PPP3CA/ADGRL1/ACTB/PPP1CA/CNIH3/NRP1/BCR/APBA3/ABL1/ELAVL1/SYNGAP1/C1QA/ARRB2/PLCB3/CLTB/RHOA/DNM2/SLC6A9/DNM3/CLSTN1/DYNLL2/PPP1R9B/SPTBN1/SHARPIN/SNCA/LRFN3/CAMK1/NEURL1/P2RX1/RNF10/PCBP2/SLC29A1/NRGN/WASF3/PAK2/CAPZB/ACTN1/PRKAR2B/CTTN/AP2M1/HTT/SHISA7/ITGB3/DMTN/ADD1
BP	GO:0033143	regulation of intracellular steroid hormone receptor signaling pathway	56	-0.56	-1.75	0.00143565	0.00773462	0.00479574	1550	tags=29%, list=13%, signal=25%	FOXP1/CRY2/SMARCA4/PARP1/LBH/SRC/CALR/WBP2/PAK1/RHOA/KANK2/MED1/NCOR2/CARM1/CNOT1/DAB2
BP	GO:0060562	epithelial tube morphogenesis	163	-0.42	-1.54	0.00144225	0.00773869	0.00479827	2241	tags=33%, list=19%, signal=27%	EPHA4/HES1/PFN1/PRKD2/DDR1/DEAF1/CSF1R/MIB1/LZTS2/ARHGAP35/SMAD3/FMN1/EDN1/PHACTR4/MAGED1/ENG/RBPJ/SOX4/FOXP1/PRKACA/TGFB1/CTNNBIP1/ST14/SPINT2/SKI/TRAF6/MTHFR/PLXNB2/ILK/FZD2/RARA/PLXND1/DCHS1/SRC/PML/GREM1/BBS5/PIK3CD/SFRP2/DVL3/IFT52/PPP1CA/NRP1/BTRC/ABL1/PAK1/RHOA/NOTCH2/SUFU/SDC4/VASP/MICAL2/MED1/PBX1
BP	GO:0043500	muscle adaptation	59	-0.53	-1.69	0.00146923	0.00783066	0.00485529	2444	tags=49%, list=21%, signal=39%	NOTCH1/TRPC3/ATP2A2/HDAC4/EZH2/FOXO1/G6PD/IL1B/P2RX4/ROCK2/CDK9/SMAD3/EDN1/KDM4A/GSK3A/TNFRSF1B/PARP1/YY1/SLC9A1/PPARA/PPP3CA/JARID2/PDE5A/PDE9A/LMNA/GSN/MTPN/ATP2B4/FOXO3
MF	GO:0051219	phosphoprotein binding	71	-0.51	-1.69	0.0015416	0.00811839	0.00503369	2642	tags=41%, list=23%, signal=32%	FGR/MID2/CBLB/PTPN11/PFN1/CRKL/PTPN6/VAV2/PIN1/SHC1/PIK3R3/GRB2/MEN1/LYN/SRC/SH2D3C/YWHAB/VAV1/PIH1D1/BTRC/SYK/ABL1/LDLRAP1/IGF2R/EPB41/UBASH3B/SNCA/MAPK3/CBL

BP	GO:0016567	protein ubiquitination	658	-0.31	-1.33	0.00154019	0.00811839	0.00503369	1472	tags=18%, list=13%, signal=17%	GSK3A/HSP90AB1/SVBP/HDAC3/KLHL22/VHL/DET1/PRR7/ENC1/SPHK1/TSPAN17/CNOT4/BCOR/WDTC1/TRAF6/TRIM35/UBR2/UBE2G1/UBA52/DAW1/LNPEP/NEDD4L/TRIM26/DDA1/CDC27/SASH1/PINK1/HSPA1A/ANKIB1/IFI27/TRIM32/MARCHF6/FBXO31/DTX2/UBE2Z/PML/UBE2R2/DCAF1/RNF115/RBCK1/RMND5A/SEPTIN4/MYCBP2/HERC2/LAPTM5/DAXX/TRIM10/TRIM28/AMFR/UBA1/BTRC/USP9X/GABARAP/FBXO6/HERC1/DDB1/VCP/KCTD9/XIAP/FBH1/ABL1/ARIH2/FKBP1A/KLHL8/N4BP1/SAE1/RNF213/DTX3L/ARRB2/RNF11/WWP2/UBE2M/MAD2L2/UBE2S/ZNRF1/USP7/DCAF7/TRIM11/RNF24/GNL3L/VPS28/SIAH2/UBB/DCAF6/RASSF5/UBR4/PCGF5/USP22/FZR1/HUWE1/SQSTM1/TRIM14/UBE2L3/MKRN1/RNF187/PER2/MARCHF2/SHARPIN/PAF1/ANKRD9/NEURL1/RNF123/RNF10/MARCHF8/FBXO7/DCAF12/CBL/TRIP12/UBE2O/RYBP/RNF40/DNAJB2/CDC34/UBE2H/DCAF11/CUL4A/TRIM58/RNF208
BP	GO:0002040	sprouting angiogenesis	65	-0.52	-1.70	0.00162711	0.00852359	0.00528493	1835	tags=31%, list=16%, signal=26%	MAP3K3/PIK3CB/PIK3R3/KLF4/JMJD8/PTK2B/KLF2/GREM1/CEACAM1/PKM/NRP1/JAK1/ABL1/RNF213/EPN1/RHOA/GLUL/THBS1/E2F2/TBXA2R
BP	GO:0030837	negative regulation of actin filament polymerization	48	-0.57	-1.76	0.00166299	0.00868906	0.00538753	889	tags=48%, list=8%, signal=44%	ADD2/EPS8/TMOD3/SPTAN1/TRIOBP/PFN1/SSH1/MYADM/SVIL/SSH2/HIP1R/FLII/SSH3/GSN/SPTBN1/MTPN/SPTA1/SPTB/TMOD1/SCIN/CAPZB/DMTN/ADD1
BP	GO:0032446	protein modification by small protein conjugation	707	-0.31	-1.30	0.00168147	0.00876216	0.00543285	1472	tags=18%, list=13%, signal=17%	GSK3A/HSP90AB1/SVBP/HDAC3/KLHL22/VHL/DET1/PRR7/ENC1/SPHK1/TSPAN17/CNOT4/BCOR/WDTC1/TRAF6/TRIM35/BCL11A/UBR2/UBE2G1/UBA52/DAW1/LNPEP/NEDD4L/TRIM26/DDA1/EPAS1/CDC27/SASH1/PINK1/HSPA1A/ANKIB1/PIAS4/IFI27/TRIM32/MARCHF6/FBXO31/DTX2/UBE2Z/PML/UBE2R2/DCAF1/ARNT/RNF115/RBCK1/RMND5A/SEPTIN4/MYCBP2/HERC2/LAPTM5/DAXX/ZMIZ1/TRIM10/TRIM28/AMFR/UBA1/RANGAP1/BTRC/USP9X/GABARAP/FBXO6/HERC1/DDB1/VCP/KCTD9/XIAP/FBH1/ABL1/ARIH2/FKBP1A/KLHL8/N4BP1/RELA/SAE1/RNF213/DTX3L/ARRB2/RNF11/WWP2/UBE2M/MAD2L2/UBE2S/ZNRF1/USP7/ATG7/DCAF7/TRIM11/RNF24/GNL3L/VPS28/SIAH2/UBB/DCAF6/RASSF5/UBR4/PCGF5/USP22/FZR1/HUWE1/SQSTM1/TRIM14/UBE2L3/MKRN1/RNF187/PER2/MARCHF2/SHARPIN/PAF1/ANKRD9/NEURL1/RNF123/RNF10/MARCHF8/FBXO7/DCAF12/CBL/TRIP12/UBE2O/RYBP/RNF40/DNAJB2/CDC34/TOLLIP/UBE2H/DCAF11/CUL4A/TRIM58/RNF208
BP	GO:0042063	gliogenesis	172	-0.41	-1.53	0.00169067	0.00879856	0.00545542	2551	tags=36%, list=22%, signal=29%	AKT1/SH3TC2/DISC1/PTPN11/NOTCH1/NR1D1/SYNE2/EZH2/TSPO/EPHA4/HES1/CDK5/CRKL/IL1B/P2RX4/CSF1R/CERS2/NDRG1/SRGAP2/MBOAT7/NRROS/SOX13/SYNJ1/B4GALT5/SOX4/VIM/KDM4A/TGFB1/ASCL2/CDK6/TNFRSF1B/SKI/PRX/WDR1/LYN/ILK/PTK2B/CD9/NCSTN/SOX6/OLIG2/ATP1B2/ZMIZ1/STAT3/ABL1/PARD3/E2F1/C1QA/OLIG1/RHOA/NF2/PLEC/LRP1/CSK/AKT2/GJC2/GRN/MAPK3/RNF10/WASF3/TAL1/CLU
MF	GO:0001222	transcription corepressor binding	42	-0.58	-1.75	0.00169557	0.00881253	0.00546409	2428	tags=45%, list=21%, signal=36%	NR1D1/HDAC5/EZH2/HES1/ZBTB16/WIZ/ZBTB7A/EHMT1/CTBP1/EHMT2/HDAC3/ETS1/NEK6/RUNX3/FAM89B/PER2/ENO1/HDGF/PBX1
CC	GO:0098862	cluster of actin-based cell projections	85	-0.47	-1.62	0.0017337	0.00897551	0.00556514	1727	tags=40%, list=15%, signal=34%	KPTN/MYO1D/B4GALT1/SLC27A4/DOCK4/ATP7A/EPS8/SLC46A1/PEX19/CYBRD1/TRIOBP/MYO15A/RIPOR2/LIMA1/SLC19A1/HSP90AB1/EZR/CLIC5/MYO1E/SLC5A6/AQP1/FLNB/FLII/ABCG2/PVALB/FLNA/MPP1/GNA12/PLEC/ESPN/SCIN/CAPZB/ACTN1/MYH9
BP	GO:0002065	columnar/cuboidal epithelial cell differentiation	44	-0.58	-1.77	0.00178488	0.00918533	0.00569524	2703	tags=45%, list=23%, signal=35%	PDPK1/EXT1/AKT1/NOTCH1/PRDM1/HES1/SOX4/GSK3B/GSK3A/CDK6/MEN1/RARA/BAD/SRC/FAM20C/ABL1/BMP6/SIDT2/FASN/TUBB

BP	GO:0048545	response to steroid hormone	202	-0.40	-1.50	0.00178578	0.00918533	0.00569524	2444	tags=34%, list=21%, signal=27%	NOTCH1/CYBB/NR1D1/ATP1A1/CASP9/DDX54/ZFP36/UBR5/CNOT9/TSPO/PKN1/ZNF366/PAQR8/NCOA4/PPARGC1B/ZBTB7A/ZFP36L2/NR3C1/ALPL/EDN1/ENG/TRERF1/FOXP1/SREBF1/CRY2/NR1H3/TGFB1/SMARCA4/IL1RN/NCOA2/ALAD/PARP1/GLB1/AQP1/LBH/MBP/TADA3/HSPA1A/RXRA/BAD/BCL2L11/SRC/CALR/PPARA/WBP2/DAXX/ZMIZ1/YWHAH/PAK1/FOS/BMP6/RHOA/CALM3/KANK2/ABHD2/SAFB/TXNIP/PPP1R9B/THBS1/UBE2L3/GPI/FOXO3/MED1/ARID1A/NCOR2/CARM1/CNOT1/DAB2
BP	GO:0019082	viral protein processing	30	-0.64	-1.77	0.0019694	0.01006468	0.00624047	1931	tags=40%, list=16%, signal=33%	MGAT5/ST6GAL1/MAN2A1/CANX/GSK3B/GSK3A/ST3GAL3/EDEM2/ST6GALNAC4/MGAT4B/ST3GAL1/FURIN
MF	GO:0001664	G protein-coupled receptor binding	120	-0.43	-1.53	0.00208512	0.01047716	0.00649622	2956	tags=38%, list=25%, signal=28%	SAV1/PTCH1/DVL1/DNAJC14/CCRL2/SDCBP/USP20/BBS1/GNAQ/GNAO1/POMC/BICD1/DLG4/ARHGEF11/INSL3/MAGI2/FYN/YARS1/PPBP/GNA11/CXCL16/ARRB1/ADA2/EDN1/GNAZ/GRK2/RPGRIP1L/GNAS/NPFF/GNA15/HSPA1A/GNAI2/DVL3/JAK1/ARRB2/ARHGEF12/CALM3/DNM2/FLNA/DNM3/GNA12/PF4V1/PPP1R9B/CXCL5/PF4
BP	GO:0042476	odontogenesis	52	-0.54	-1.69	0.00212616	0.01064684	0.00660143	2340	tags=42%, list=20%, signal=34%	SLC24A4/PHEX/TUFT1/ALPL/EDN1/ACVR2B/SNX10/BCL11B/BCOR/TRAF6/AQP1/BCL2L11/SRC/PPARA/FAM20C/CTNNA1/STIM1/RHOA/BSG/NF2/ROGDI/SMPD3
BP	GO:2000649	regulation of sodium ion transmembrane transporter activity	33	-0.63	-1.79	0.00214223	0.01071386	0.00664298	2267	tags=42%, list=19%, signal=34%	CHP1/NETO2/FXYD6/ATP1B1/STOM/RANGRF/NEDD4L/SLC9A1/YWHAH/DNM2/ACTN4/TESC/SCN1B/WNK1
BP	GO:1905476	negative regulation of protein localization to membrane	25	-0.65	-1.78	0.00218254	0.01086081	0.0067341	485	tags=32%, list=4%, signal=31%	TGFB1/CLTC/INPP5K/BCL2L1/CSK/AP2M1/DMTN/DAB2
BP	GO:0051098	regulation of binding	275	-0.37	-1.43	0.00218854	0.01087707	0.00674417	2609	tags=35%, list=22%, signal=28%	PKD1/MAPRE1/ATP2A3/RALB/SORL1/AKT1/CBLB/ZFPM1/JUN/PEX19/DISC1/ADNP/EDF1/ATP2A2/HDAC4/HDAC5/EIF4A3/EPHA4/HES1/CDK5/SUMO3/CPNE1/GTF2F1/SMARCD3/ZBTB7A/CDK9/TMC8/PIN1/TFIP11/SMAD3/DERL1/GPSM1/TIAM1/RIPOR2/TAF1/POLDIP2/RAP1GAP/MMP9/KLF4/MARK3/DTNBP1/GSK3B/PRKACA/HSP90AB1/TGFB1/CTNNBIP1/MEPCE/MEN1/SKI/PARP1/TRAF6/HIP1R/SPPL3/RARA/PLXND1/DZIP1/MARK2/ILRUN/SRC/PPARA/PPP3CA/TRIM28/AMFR/ACTB/PPP1CA/NRP1/RFNG/FBH1/ABL1/FKBP1A/E2F1/DTX3L/ARRB2/GOLGA2/CALM3/LDLRAP1/MAD2L2/HIPK2/NIBAN2/GNL3L/MED25/CLDN5/LRP1/EPB41/SPTA1/TCF7L2/PER2/UBASH3B/CAMK1/EIF4G1/MAPK3/GATA1/CDKN1A/DNAJB2/CARM1/ADD1/DAB2
CC	GO:0030665	clathrin-coated vesicle membrane	69	-0.50	-1.64	0.00221586	0.0109903	0.00681438	1753	tags=52%, list=15%, signal=45%	EPN2/FCGR1A/CLTCL1/CD3G/AFTPH/VAMP3/HBEGF/AP2A1/AP2A2/SYT11/M6PR/HLA-DRB1/HLA-DQA2/KIAA0319/FZD5/CD74/AP2B1/HLA-DQA1/SYNRG/CLTC/DNAJC5/DENND1A/HIP1/AP2S1/HIP1R/FZD2/CD9/HLA-DRB5/TGOLN2/CLTB/EPN1/LDLRAP1/IGF2R/NRGN/AP2M1/DAB2
BP	GO:0010817	regulation of hormone levels	245	-0.37	-1.43	0.00223123	0.01103404	0.0068415	2638	tags=27%, list=22%, signal=22%	SLC16A1/RAB11FIP5/CYP2S1/STARD3/PIM3/POMC/CPQ/TIPARP/PTPN11/TRPM2/NR1D1/ATP1A1/EGR1/SGPL1/TSPO/DHRS13/HLA-DRB1/PCSK1N/IL1B/HSD17B4/DHCR7/LIPE/EDN1/UBE2Q1/CPT1A/SOX4/SREBF1/ACVR2B/CRY2/PCSK6/IL1RN/CCN3/STX4/UCP2/IL4I1/BACE2/LYN/RAB11FIP1/PAPSS2/BAD/PFKL/ARNT/LRRC8A/SLC7A8/PPP3CA/DHRS3/RDH11/PNPLA2/NFKB1/BMP6/SLC7A5/SIDT2/GALR1/RAB11B/FKBP1B/ALDH9A1/ECE1/NIBAN2/SYTL4/GLUL/TCF7L2/PER2/SMPD3/CTSB/FURIN/MED1/DAB2

BP	GO:0032102	negative regulation of response to external stimulus	251	-0.37	-1.45	0.00243171	0.01189233	0.00737367	2695	tags=34%, list=23%, signal=27%	SH2B3/TRIM21/CR1/FGR/CORO1B/MAPK14/RHBDF2/MACIR/FOXP3/MUL1/SYT11/SERPINF2/MAPK7/KNG1/NOTCH1/NR1D1/YTHDF3/ZFP36/BANF1/OAS3/EPHA4/HLA-DRB1/FYN/CNR2/PROS1/HLA-A/CERS2/PGLYRP1/GSTP1/ST6GAL1/TRIB1/NR1H2/SMAD3/PTPRC/EDN1/KIAA0319/EPPK1/KLF4/SEMA4D/SIGIRR/NLRX1/NR1H3/FAM3A/CACTIN/MICB/GIT1/TNFRSF1B/CCN3/LYN/SERPING1/ADAR/CD9/TTLL12/ILRUN/SRC/GREM1/CEACAM1/PPARA/RIN3/SEMA4A/NRP1/BCR/SYK/CLASP1/LTF/NFKB1/ARRB2/SIGLEC10/CHD8/MFHAS1/LGALS9/KRT1/RIOK3/GP1BA/ALOX12/THBS1/SEC14L1/GRN/UBASH3B/SHARPIN/PCBP2/GPX1/FURIN/PDGFA/NPLOC4
BP	GO:0051496	positive regulation of stress fiber assembly	38	-0.58	-1.71	0.00245109	0.0119724	0.00742332	2019	tags=32%, list=17%, signal=26%	ROCK2/SMAD3/SORBS3/TPM1/RAC1/NRP1/PXN/ABL1/PAK1/RHOA/NF2/SDC4
BP	GO:0032092	positive regulation of protein binding	67	-0.50	-1.63	0.00246247	0.01201321	0.00744862	1890	tags=39%, list=16%, signal=33%	PIN1/DERL1/TIAM1/TAF1/POLDIP2/MMP9/MARK3/GSK3B/HSP90AB1/MEN1/HIP1R/SPPL3/PLXND1/AMFR/NRP1/RFNG/ABL1/FKBP1A/DTX3L/HIPK2/CLDN5/LRP1/EPB41/SPTA1/TCF7L2/ADD1
BP	GO:0019320	hexose catabolic process	26	-0.64	-1.75	0.00250052	0.01209932	0.00750201	1845	tags=38%, list=16%, signal=32%	FOXK1/ENO2/GALK1/FOXK2/GLB1/BAD/PFKL/GALE/HK1/ENO1
BP	GO:0010633	negative regulation of epithelial cell migration	39	-0.59	-1.74	0.00253281	0.01220059	0.00756481	2606	tags=46%, list=22%, signal=36%	JUP/NOTCH1/HDAC5/MECP2/SPRED1/VASH1/EPPK1/KLF4/DNAJA4/TGFB1/SVBP/CORO1C/RHOA/ADIPOR1/THBS1/ATP2B4/PTPN23/TBXA2R
BP	GO:1903579	negative regulation of ATP metabolic process	25	-0.65	-1.76	0.00278231	0.01310721	0.00812694	2367	tags=56%, list=20%, signal=45%	HDAC4/FLCN/TSPO/PDE12/GIT1/CBFA2T3/PARP1/PPARA/FIS1/STAT3/FBP1/RHOA/SLC4A1/SNCA
MF	GO:0140030	modification-dependent protein binding	147	-0.41	-1.50	0.00284599	0.01334393	0.00827372	1702	tags=27%, list=15%, signal=24%	TAF1/OTUD7B/WDR81/WDR5/IKBKG/KDM4A/BAZ2A/SMARCA4/CXXC1/MINDY1/BABAM2/HDGFL2/ZRANB1/LYN/BPTF/TTLL12/PML/VAV1/LAPTM5/DAXX/TP53BP1/CDYL2/PHF2/VCP/OPTN/KMT2A/RAD23A/JARID2/CHD8/PSME4/PRPF8/SQSTM1/SHARPIN/BRD3/BRD4/UBL7/DNAJB2/NPLOC4/CARM1/BAG6
BP	GO:0031333	negative regulation of protein-containing complex assembly	105	-0.45	-1.57	0.00285367	0.01334848	0.00827654	2653	tags=40%, list=23%, signal=31%	ADD2/MAPRE1/ULK1/EPS8/SORL1/AIDA/TMOD3/SPTAN1/TRIOBP/PFN1/SSH1/THRA/TBCD/HSPA5/MYADM/SVIL/TMC8/SSH2/TFIP11/GSK3B/CTNNBIP1/EML2/HIP1R/JAM3/SRC/FLII/CPTP/SSH3/RIOK3/GSN/SPTBN1/MTPN/SPTA1/SPTB/SNCA/TMOD1/SCIN/CAPZB/PPM1A/CLU/DMTN/ADD1
BP	GO:0061614	miRNA transcription	41	-0.57	-1.71	0.00288342	0.01344018	0.0083334	2479	tags=51%, list=21%, signal=41%	JUN/EGR1/SPI1/SREBF2/NR3C1/NR1H2/SMAD3/KLF4/SREBF1/NFATC3/TGFB1/SMARCA4/ETS1/YY1/RARA/MRTFA/PPARA/STAT3/FOS/RELA/FOXO3
BP	GO:0006006	glucose metabolic process	138	-0.42	-1.54	0.00290523	0.01352601	0.00838661	2042	tags=29%, list=17%, signal=24%	SLC25A1/HECTD4/NLN/FOXK1/PHKG2/ENO2/CPT1A/PDK3/WDR5/GSK3B/SORD/FOXK2/FAM3A/GSK3A/BCKDK/NCOA2/INPPL1/WDTC1/SESN2/GDPGP1/IGF2/BAD/PFKL/SRC/PPARA/PPP1CA/PDK2/KAT2B/DDB1/FBP1/GAA/HK1/INPP5K/USP7/STK11/ADIPOR1/AKT2/PER2/GPI/ENO1
BP	GO:0060395	SMAD protein signal transduction	41	-0.57	-1.70	0.00305403	0.01410318	0.00874448	2008	tags=54%, list=17%, signal=45%	SMAD5/JUN/MAGI2/SLC33A1/SMAD3/KIAA0319/VIM/TGFB1/RBM14/CCN3/SKI/PARP1/LNPEP/FAM89B/ZMIZ1/FOS/BMP6/NUP93/HIPK2/LRP1/SPTBN1/DAB2

BP	GO:0032412	regulation of ion transmembrane transporter activity	138	-0.42	-1.53	0.00306574	0.01414083	0.00876782	1792	tags=24%, list=15%, signal=21%	PDE4B/EDN1/MMP9/EHD3/STOM/PRKACA/RANGRF/DAPK1/SELENON/OXSR1/FHL1/NEDD4L/PINK1/PTK2B/SLC9A1/ATP1B2/CNIH3/YWHAH/FKBP1A/STIM1/WWP2/CALM3/DNM2/FKBP1B/SLC6A9/AHCYL1/CAPN1/ACTN4/TESC/HTT/SHISA7/SCN1B/WNK1
BP	GO:0031109	microtubule polymerization or depolymerization	92	-0.46	-1.59	0.00312172	0.01434907	0.00889694	2601	tags=42%, list=22%, signal=33%	MAPRE1/ARL2/TUBGCP2/ATXN7/TUBGCP4/TUBGCP6/MECP2/NDE1/NCKAP5L/CDK5RAP2/TBCD/MAPRE2/TUBG1/TUBG2/FES/NUMA1/MAP1S/NDEL1/TPX2/SLAIN2/GIT1/TAOK1/EML2/RANGRF/KATNB1/PRUNE1/RAC1/HSPA1A/CAMSAP1/MAP1A/ARHGEF2/ABL1/PAK1/CLASP1/GOLGA2/CKAP5/DCTN1/SNCA/GAS2L1
MF	GO:0001784	phosphotyrosine residue binding	35	-0.59	-1.68	0.00316434	0.01451526	0.00899998	2642	tags=49%, list=23%, signal=38%	FGR/CBLB/PTPN11/PFN1/CRKL/PTPN6/VAV2/SHC1/PIK3R3/GRB2/SH2D3C/VAV1/SYK/ABL1/LDLRAP1/MAPK3/CBL
BP	GO:0030518	intracellular steroid hormone receptor signaling pathway	88	-0.47	-1.62	0.00319371	0.01462927	0.00907068	2394	tags=36%, list=20%, signal=29%	DDX54/UBR5/CNOT9/PKN1/ZNF366/NCOA4/PPARGC1B/ZBTB7A/NR3C1/TRERF1/FOXP1/CRY2/SMARCA4/PARP1/LBH/TADA3/SRC/CALR/WBP2/DAXX/ZMIZ1/YWHAH/PAK1/RHOA/KANK2/SAFB/MED1/ARID1A/NCOR2/CARM1/CNOT1/DAB2
BP	GO:0050678	regulation of epithelial cell proliferation	171	-0.39	-1.47	0.00339803	0.01545834	0.00958473	1454	tags=19%, list=12%, signal=17%	TGFB1/CDK6/MEN1/BCL11B/IGF2/BAD/CDKN1C/ARNT/CEACAM1/PIK3CD/SFRP2/RUNX3/IFT52/STAT3/NRP1/LRG1/BMP6/NOTCH2/STAT5A/STK11/GLUL/WDR13/THBS1/TCF7L2/SP1/GRN/PRKDC/ZNF703/GPX1/MED1/ITGB3/SPARC/DAB2
BP	GO:1905952	regulation of lipid localization	91	-0.46	-1.57	0.00342545	0.01553623	0.00963302	2551	tags=36%, list=22%, signal=29%	AKT1/ABCA3/POMC/PTPN11/ACACB/TSPO/IKBKE/IL1B/PLIN3/P2RX4/SREBF2/NR1H2/SCARB1/EDN1/CRY2/PLIN2/NR1H3/FITM1/PPARA/FIS1/SYK/PNPLA2/NFKB1/BMP6/GALR1/LDLRAP1/LRP1/THBS1/AKT2/EHD1/FURIN/ITGB3/DAB2
BP	GO:0051223	regulation of protein transport	358	-0.34	-1.36	0.00348323	0.01573788	0.00975805	2638	tags=30%, list=22%, signal=24%	SLC16A1/RAB11FIP5/JUP/MAPK14/SORL1/PKIG/RAB8A/RHBDF2/SELENOK/PIM3/SH3TC2/PTPN11/TRPM2/NR1D1/CNST/CLN3/UBR5/TSG101/CHP1/HLA-DRB1/PCNT/CDK5/HYAL2/CD81/FYN/IL1B/PARD6A/SREBF2/LCP1/RIPOR1/NR1H2/ITGAM/UBE2J2/SMAD3/FZD5/CPT1A/NDEL1/BBC3/SNX12/BCAP31/SOX4/TM9SF4/SREBF1/GSK3B/PRR5L/STOM/PRKACA/NR1H3/GSK3A/HSP90AB1/TGFB1/FRMD4A/HDAC3/CCN3/EDEM2/EZR/STX4/UCP2/BCAS3/MAVS/RAB11FIP1/LEPROT/PINK1/IFI27/BAD/PFKL/HCLS1/GRIPAP1/SRC/SNX3/LRRC8A/FIS1/PPP3CA/TRIM28/RANGAP1/NOLC1/USP36/ARIH2/ABLIM3/BMP6/SAE1/MYO18A/WWP2/SIDT2/SUFU/FLNA/FKBP1B/RAC2/INPP5K/VPS28/SYTL4/GLUL/HUWE1/CSK/YOD1/AKT2/UBE2L3/PTPN1/TCF7L2/PER2/CAMK1/ATG13/BNIP3L/GAPVD1/OAZ1/PTPN23/PPM1A/DMTN
BP	GO:0032233	positive regulation of actin filament bundle assembly	45	-0.54	-1.64	0.00356764	0.01604631	0.00994929	2520	tags=36%, list=21%, signal=28%	SWAP70/SERPINF2/PFN1/ROCK2/SMAD3/SORBS3/PLEK/TPM1/RAC1/NRP1/PXN/ABL1/PAK1/RHOA/NF2/SDC4
BP	GO:0007173	epidermal growth factor receptor signaling pathway	73	-0.49	-1.63	0.00367656	0.01646172	0.01020686	2703	tags=48%, list=23%, signal=37%	PDPK1/KIF16B/RHBDF2/AKT1/CBLB/PIK3CA/PTPN11/NCF1/PDE6G/MVB12B/TSG101/CNOT9/RNF126/SHC1/NEU3/CHMP6/MMP9/DGKD/GRB2/TGFB1/MVP/HIP1/HIP1R/PTPN12/RAB7A/PTK2B/SRC/CEACAM1/RNF115/ABL1/REPS2/PTPRJ/IQGAP1/NEURL1/CBL

BP	GO:0045666	positive regulation of neuron differentiation	39	-0.57	-1.70	0.00372405	0.01665557	0.01032706	2111	tags=36%, list=18%, signal=30%	CPNE1/FEZ1/CYB5D2/HMG20B/KDM4A/NCOA1/TGIF2/RARA/TRIM32/ARHGEF2/BMP6/RHOA/MMD/EIF4G1
BP	GO:2000736	regulation of stem cell differentiation	38	-0.57	-1.68	0.00373849	0.01666393	0.01033224	2728	tags=42%, list=23%, signal=32%	EIF2AK2/CDK12/NOTCH1/HES1/NELFB/ZFP36L2/GSK3B/CDK6/LBH/SOX6/PUS7/LTBP3/STAT3/ABL1/SETD1A/PRKDC
BP	GO:0030224	monocyte differentiation	25	-0.64	-1.73	0.00378391	0.0168475	0.01044606	2479	tags=60%, list=21%, signal=47%	THOC5/ZFP36L1/JUN/HLA-DRB1/CSF1R/FES/INPP5D/CD74/FOXP1/CTNNBIP1/CDK6/ACIN1/FASN/MED1/MYH9
MF	GO:0046983	protein dimerization activity	696	-0.31	-1.29	0.00383028	0.01691199	0.01048604	2350	tags=25%, list=20%, signal=21%	TSG101/CNOT9/BANF1/CBS/PTPA/H2AC8/ZNF318/PAPSS1/ABCB9/MAP3K13/G6PD/IL6R/HES1/GTF2A1/ABCD1/USF3/FXR2/ZBTB38/ACOX1/MAFG/ZBTB4/MLX/MITF/DRAP1/ATP1B1/GBP4/XPNPEP1/VPS4B/RIPK1/CSF1R/SREBF2/TLR6/ZBTB16/DARS2/GSTM4/ZDHHC3/MTPAP/ST6GAL1/PDE2A/HSD17B4/SRGAP2/MAN2A1/TFE3/SYNE1/SMAD3/ADA2/MICU1/RRAGD/HIF1AN/TFEB/DROSHA/ENG/TAF1/TAF6/RAP1GAP/CENPF/PNPO/FOXP2/CHMP4B/CDA/MAP3K11/DGKD/NR6A1/IKBKG/TOP2A/CHRAC1/SREBF1/NCOA1/STOM/PYCARD/HSP90AB1/TBC1D22A/JDP2/ASCL2/NCOA2/DAPK3/SMC1A/ATIC/PLEK/CANT1/MAP3K5/GLB1/NAGA/TPM1/KATNB1/HIP1/KARS1/ANO6/HIP1R/SPPL3/EPAS1/JAM3/STK10/ST13/CGAS/CHMP1A/SOX6/PML/GREM1/ARNT/CEACAM1/TAF4/NCOA3/STK25/USF2/OLIG2/PPP3CA/ATP1B2/TAF8/PSAP/GALE/MAX/RAB11FIP4/ACOT7/STAT3/PDK2/YWHAH/BTRC/NOLC1/PDGFC/ABCG2/NRBP1/ELAVL1/DNTTIP1/RRAGC/KMT2A/RELA/E2F1/BMP6/WARS1/SAE1/OLIG1/SHMT2/TPST2/GID8/ODC1/FLNA/S100Z/ECE1/ATG7/HSPB1/BCL2L1/MLXIP/KRT1/SLC4A1/PSMF1/TERF2/RILP/TRIM14/MXD4/SP1/H2BC11/ENO1/PIP4K2A/E2F2/BNIP3L/FBXO7/LYL1/ACTN4/RNF40/ACTN1/HPS1/SMIM1/TESC/MXI1/FECH/EIF2AK1/TAL1/H3C10/MYH9/MGLL/PDGFA/ADD1/SUPT5H
BP	GO:1903578	regulation of ATP metabolic process	67	-0.49	-1.58	0.00387188	0.01706719	0.01058228	2674	tags=43%, list=23%, signal=34%	ATP7A/ZBTB20/ARL2/HDAC4/FLCN/TSPO/ABCD1/SLC25A23/ENTPD5/PGAM1/ZBTB7A/EIF6/PDE12/GIT1/CBFA2T3/JMJD8/PARP1/PINK1/ARNT/PPARA/FIS1/STAT3/VCP/FBP1/SHMT2/RHOA/SLC4A1/SNCA/ENO1
BP	GO:0070201	regulation of establishment of protein localization	380	-0.33	-1.34	0.00388419	0.01710249	0.01060416	1706	tags=29%, list=15%, signal=26%	RAB11FIP5/JUP/MAPK14/SORL1/PKIG/RAB8A/RHBDF2/SELENOK/PIM3/SH3TC2/PTPN11/TRPM2/NR1D1/CNST/CLN3/UBR5/TSG101/CHP1/HLA-DRB1/PCNT/CDK5/HYAL2/CD81/FYN/IL1B/PARD6A/SREBF2/LCP1/RIPOR1/NR1H2/ITGAM/UBE2J2/SMAD3/FZD5/CPT1A/NDEL1/BBC3/CCT3/SNX12/BCAP31/SOX4/TM9SF4/SREBF1/GSK3B/PRR5L/STOM/PRKACA/NR1H3/GSK3A/HSP90AB1/TGFB1/FRMD4A/HDAC3/CCN3/EDEM2/EZR/STX4/UCP2/BCAS3/MAVS/RAB11FIP1/LEPROT/PINK1/IFI27/BAD/PFKL/HCLS1/GRIPAP1/SRC/SNX3/LRRC8A/FIS1/PTP4A3/PPP3CA/TRIM28/RANGAP1/NOLC1/USP36/ARIH2/ABLIM3/BMP6/SAE1/MYO18A/WWP2/SIDT2/SUFU/FLNA/FKBP1B/RAC2/INPP5K/VPS28/SYTL4/GLUL/HUWE1/CSK/YOD1/AKT2/UBE2L3/PTPN1/TCF7L2/PER2/CAMK1/ATG13/BNIP3L/CTSD/GAPVD1/OAZ1/PTPN23/PPM1A/DMTN
BP	GO:0007030	Golgi organization	128	-0.41	-1.49	0.0038933	0.01710469	0.01060552	1822	tags=23%, list=16%, signal=19%	SYNE1/CUL7/STX6/SEC23IP/NSFL1C/EHD3/TJAP1/BCAS3/VTI1B/PLEKHM2/STK25/VTI1A/MAP2K2/KIFC3/OPTN/CLASP1/STX5/MYO18A/GOLGA2/DNM2/TBC1D20/ARHGAP21/GAK/HUWE1/TANGO2/MAPK3/HTT/SEC16A/NPLOC4

BP	GO:0051726	regulation of cell cycle	763	-0.29	-1.25	0.00389119	0.01710469	0.01060552	2240	tags=24%, list=19%, signal=21%	HES1/BTG1/MECP2/ANAPC1/IRF1/CDK5/EME2/ASPM/XRCC3/CDK5RAP2/CASP2/CSNK2A1/DACH1/IL1B/FOXN3/VPS4B/PTPN6/MADD/ROCK2/DCUN1D3/CCNY/EP400/WIZ/ZFP36L2/SVIL/CHFR/CCAR2/CDK9/PIN1/BOP1/PKP4/NUMA1/RCC2/CDC37/TMEM8B/CUL7/BIRC6/WEE1/RFWD3/PTTG1/CDK2AP2/PTPRC/EDN1/PHACTR4/CHMP6/TAF1/TAF6/POLDIP2/VPS72/CTBP1/TPX2/CENPF/NSFL1C/KLF4/JUND/CHMP4B/WDR5/CTDSP1/PSME3/TRRAP/CABLES2/ZFHX3/PPM1G/ANKRD17/CETN2/PRKACA/MIIP/HSP90AB1/TGFB1/GIT1/MEPCE/MKI67/KLHL22/CDK6/CLTC/TAOK1/EML3/RBM14/CTDSP2/RPRD1B/SPHK1/DAPK3/MEN1/KIF3B/NEK6/BABAM2/SERTAD1/TP53BP2/TRIM35/PRCC/FHL1/YY1/CDC27/PUM1/CDKL5/RARA/TADA3/IGF2/HSPA1A/TTLL12/TACC3/DHCR24/TRIM32/BCL2L11/DYNC1H1/FBXO31/SRC/CHMP1A/PML/CCND3/KAT7/PRR11/CDKN1C/SRPK2/EIF4G2/CALR/RUVBL1/RUNX3/CDK2/TAOK2/TP53BP1/TFDP1/ACTB/RAB11FIP4/TTC28/STAT3/USP19/BCR/KAT2B/BTRC/FBXO6/MCRS1/DDB1/XIAP/ABL1/BABAM1/GADD45G/FOXO4/E2F1/PIM2/RAD23A/RHOA/CALM3/MAD2L2/KANK2/HIPK2/NF2/CDK19/BCL2L1/STK11/HEXIM2/SND1/DCTN1/USP22/DNA2/FZR1/TERF2/PPP1R9B/PPP2R5B/AKT2/CCNI/GJC2/IQGAP1/PER2/CTDSPL/ATP2B4/PAF1/PPP1R10/EIF4G1/PRKDC/ZNF703/E2F2/FBXO7/BRD4/CDKN1A/SMPD3/MED1/TAL1/PPM1A/CUL4A/GFI1B/YY1AP1/PBX1/HCFC1/CNPPD1
BP	GO:0031110	regulation of microtubule polymerization or depolymerization	67	-0.49	-1.58	0.00393922	0.01721136	0.01067166	2217	tags=45%, list=19%, signal=37%	MAPRE1/ARL2/ATXN7/MECP2/CDK5RAP2/TBCD/MAPRE2/FES/NUMA1/MAP1S/TPX2/SLAIN2/GIT1/TAOK1/EML2/RANGRF/KATNB1/PRUNE1/RAC1/HSPA1A/CAMSAP1/MAP1A/ARHGEF2/ABL1/PAK1/CLASP1/CKAP5/DCTN1/SNCA/GAS2L1
BP	GO:0043470	regulation of carbohydrate catabolic process	43	-0.54	-1.64	0.0040582	0.01762676	0.01092922	2649	tags=42%, list=23%, signal=33%	ZBTB20/ARL2/HDAC4/ENTPD5/PGAM1/ZBTB7A/EIF6/PHKG2/GSK3A/GIT1/CBFA2T3/JMJD8/ARNT/PPARA/PPP1CA/STAT3/FBP1/SLC4A1
BP	GO:0071241	cellular response to inorganic substance	134	-0.42	-1.52	0.00406089	0.01762676	0.01092922	2825	tags=41%, list=24%, signal=32%	NEK7/CEBPA/SLC40A1/FOSB/ATP13A2/CPNE2/ATP7A/TRAF2/AKT1/SYT11/JUN/TRPM2/CYBB/NCF1/DLG4/FOXO1/CPNE3/TSPO/EIF4A3/SLC25A23/CPNE1/P2RX4/HSPA5/EDN1/SLC41A1/MMP9/JUND/SMPD1/HVCN1/ALAD/PARP1/ALOX15/AQP1/MTF1/MT2A/BAD/NCSTN/CPNE5/CALR/CDK2/DAXX/SLFN14/DDI2/FOS/FBP1/BMP6/DNM2/MCOLN1/IQGAP1/SNCA/MAPK3/SMPD3/GUCY1B1/DMTN/ADD1
BP	GO:0002067	glandular epithelial cell differentiation	25	-0.63	-1.72	0.00418573	0.01808309	0.01121217	2703	tags=56%, list=23%, signal=43%	PDPK1/EXT1/AKT1/NOTCH1/HES1/GSK3B/GSK3A/CDK6/MEN1/RARA/BAD/BMP6/SIDT2/FASN
BP	GO:0031345	negative regulation of cell projection organization	107	-0.44	-1.55	0.00419206	0.01808309	0.01121217	2869	tags=36%, list=24%, signal=28%	KREMEN1/TRIM46/ARPIN/TESK1/MYLIP/RAB3IP/ULK1/DIP2B/EPHA4/HES1/MAP4/CDK5/ABI3/FYN/CERS2/PRAG1/ITM2C/PMP22/KIAA0319/RAP1GAP/SEMA4D/DTNBP1/VIM/BCL11A/MBP/EVI5L/PPP3CA/SEMA4A/NRP1/YWHAH/SYNGAP1/RHOA/DNM2/FLNA/DNM3/GAK/CAPZB/CARM1/DAB2
BP	GO:0070507	regulation of microtubule cytoskeleton organization	117	-0.44	-1.56	0.00428589	0.01840247	0.01141019	2609	tags=39%, list=22%, signal=31%	PKD1/MAPRE1/ARL2/BICD2/BICD1/ATXN7/MECP2/CDK5RAP2/VPS4B/TBCD/MAPRE2/FES/NUMA1/CDK2AP2/DIXDC1/CHMP6/MAP1S/TPX2/CHMP4B/SLAIN2/GSK3B/GIT1/CLTC/TAOK1/EML2/EML3/RANGRF/KATNB1/PRUNE1/RAC1/HSPA1A/TACC3/MARK2/DYNC1H1/CHMP1A/CAMSAP1/MAP1A/ARHGEF2/ABL1/PAK1/CLASP1/RHOA/CKAP5/DCTN1/SNCA/GAS2L1
BP	GO:0001707	mesoderm formation	32	-0.61	-1.72	0.00437127	0.01870847	0.01159993	2123	tags=34%, list=18%, signal=28%	EXOC4/SMAD3/KLF4/PRKACA/KDM6B/TWSG1/SFRP2/NF2/GPI/TAL1/ITGB3

BP	GO:0061041	regulation of wound healing	71	-0.49	-1.60	0.00438229	0.01873547	0.01161666	1816	tags=27%, list=15%, signal=23%	SMAD3/EDN1/EPPK1/ACTG1/ANO6/SERPING1/CD9/CEACAM1/CLASP1/ACTA2/MYLK/KRT1/GP1BA/ALOX12/THBS1/UBASH3B/PDGFA/DMTN/TBXA2R
BP	GO:0010629	negative regulation of gene expression	738	-0.30	-1.27	0.00443418	0.01893696	0.0117416	2650	tags=30%, list=23%, signal=25%	CR1/ZBTB20/TRAF2/ILF3/EXOSC10/CAPRIN1/SUPV3L1/BPI/MAPK14/MACIR/AKT1/FOXP3/MUL1/AGO4/POMC/SYT11/PIK3CA/ZFPM1/TIPARP/SERPINF2/EXOSC4/ADNP/NOTCH1/ACACB/BAK1/CD22/PCBP4/CD3E/YTHDF3/ZFP36/HDAC5/EZH2/FLCN/CNOT9/BANF1/DCP1A/TSPO/CCR1/OAS3/U2AF2/EIF4A3/IKBKE/PRDM1/HES1/HLA-DRB1/SMG9/ABCD1/MECP2/MOV10/FXR2/UNK/FYN/CSDE1/LGALS9C/CRKL/CPNE1/SPI1/RIPK1/PTPN6/PABPC1/ROCK2/LARP4B/TLR6/MYADM/SRSF4/SYNCRIP/PGLYRP1/MTPAP/PHF8/GSTP1/NDRG2/DCPS/ZFP36L2/EIF6/HELZ2/NR1H2/LSM4/AGO1/ARRB1/SMAD3/PATL1/CNOT11/SERBP1/ACVR1B/PTPRC/EDN1/CAST/PYM1/DROSHA/ENG/INPP5D/TAF1/FTO/CTBP1/OTUD7B/SNX12/PDE12/KLF4/SIGIRR/IGF2BP3/VIM/FOXP1/TNRC6C/KDM4A/SFSWAP/GSK3B/PRR5L/METTL16/NLRX1/PYCARD/CASC3/CACTIN/HSP90AB1/TGFB1/BAZ2A/GIT1/NBAS/SCMH1/LILRA5/TBX21/TTC5/VHL/ENC1/EZR/DAPK3/INPPL1/DAPK1/TWSG1/CHID1/SLC35C2/UBR2/SESN2/YY1/GTPBP1/PUM1/ADAR/PINK1/RAC1/RARA/HSPA1A/KLF2/ZC3HAV1/ILRUN/CHMP1A/PML/CDKN1C/PUS7/CEACAM1/CALR/SFRP2/PPARA/CDK2/PPP3CA/LAPTM5/PTBP1/DAXX/TRIM28/SLFN14/MAX/SPOCD1/STAT3/MAP2K2/KAT2B/UPF1/ALKBH5/PHF2/JAK3/CPTP/XIAP/ELAVL1/TNFSF4/N4BP1/OTUD5/E2F1/LTF/NFKB1/ARRB2/DCP1B/SETDB1/WWP2/HNRNPK/SHMT2/SLC7A5/NOTCH2/IGF2BP2/LGALS9/HNRNPM/MAPKAPK2/SAMD1/SLC24A3/TENT4B/SND1/PABPC4/CLDN5/LRP1/TERF2/CSK/THBS1/GLG1/YBX1/RPS6KA4/ZNFX1/ATP2B4/CHD4/EIF4G1/EIF4EBP2/CDKN1A/SMG7/AKIRIN2/KHSRP/YBX3/FURIN/EIF2AK1/GSPT1/NPLOC4/TENT5C/NCOR2/LARP1/CNOT1/MARF1
BP	GO:0001954	positive regulation of cell-matrix adhesion	42	-0.55	-1.67	0.00450894	0.01918137	0.01189314	1816	tags=43%, list=15%, signal=36%	JUP/DISC1/CD3E/SMAD3/FMN1/LIMS1/POLDIP2/GSK3B/CDK6/RAC1/PTK2B/RRAS/NRP1/ABL1/PLEKHA2/PTPRJ/SDC4/GFUS
CC	GO:0030660	Golgi-associated vesicle membrane	39	-0.57	-1.68	0.00461233	0.01950708	0.01209509	3081	tags=46%, list=26%, signal=34%	CLTCL1/AFTPH/COPG1/SPPL2B/AP2A1/COPZ1/PKD1/KDELR2/SYNRG/COPA/CLTC/SPPL3/KDELR1/COPB2/SCYL1/CLTB/ARCN1/NRGN
BP	GO:1903312	negative regulation of mRNA metabolic process	73	-0.48	-1.61	0.00462098	0.01950708	0.01209509	2416	tags=34%, list=21%, signal=27%	PCBP4/ZFP36/U2AF2/IKBKE/CSDE1/PABPC1/LARP4B/SRSF4/SYNCRIP/CDK9/SFSWAP/METTL16/NBAS/PTBP1/ELAVL1/E2F1/HNRNPK/MAPKAPK2/TENT4B/YBX1/RNF40/YBX3/TENT5C/LARP1/SUPT5H
BP	GO:0001655	urogenital system development	177	-0.39	-1.47	0.00476969	0.0200045	0.01240351	2666	tags=32%, list=23%, signal=25%	PECAM1/EXT1/PKD1/SMAD5/TIPARP/NOTCH1/CASP9/EGR1/FLCN/SGPL1/PRDM1/IL6R/EPHA4/HES1/MAGI2/HYAL2/CRKL/SNX17/ZBTB16/MPST/BASP1/LZTS2/SMAD3/FMN1/MAGED1/MMP9/CENPF/AP2B1/SOX4/DTNBP1/ACVR2B/CTNNBIP1/NFIA/RPGRIP1L/MYO1E/ILK/RARA/PLXND1/DCHS1/BCL2L11/GREM1/CDKN1C/PSAP/NRP1/BMP6/RHOA/NOTCH2/ODC1/ACTA2/AP1B1/KANK2/STK11/SDC4/IQGAP1/PDGFA/PBX1/BAG6
BP	GO:0048332	mesoderm morphogenesis	33	-0.61	-1.72	0.00486569	0.02037254	0.01263171	2123	tags=33%, list=18%, signal=27%	EXOC4/SMAD3/KLF4/PRKACA/KDM6B/TWSG1/SFRP2/NF2/GPI/TAL1/ITGB3

BP	GO:0008217	regulation of blood pressure	78	-0.48	-1.59	0.00491768	0.02056015	0.01274804	2701	tags=32%, list=23%, signal=25%	RPS6KA2/EXT1/PTAFR/POMC/SERPINF2/ATP1A1/MECP2/P2RX4/GNA11/SMAD3/EDN1/GSK3A/TPM1/LNPEP/PPARA/PLCB3/RHOA/ACTA2/GUCY1A1/ECE1/GNA12/P2RX1/PTGS1/WNK1/TBXA2R
BP	GO:0031060	regulation of histone methylation	68	-0.48	-1.58	0.00501659	0.02086397	0.01293641	1600	tags=25%, list=14%, signal=22%	WDR5/EHMT2/KDM4A/NELFA/BCOR/MTHFR/SUPT6H/KANSL1/PIH1D1/MCRS1/KMT2A/JARID2/LMNA/PAF1/BRD4/MLLT6/HCFC1
MF	GO:0019001	guanyl nucleotide binding	289	-0.35	-1.40	0.00503654	0.02090322	0.01296075	2380	tags=29%, list=20%, signal=23%	PDE6G/AGAP2/AGAP1/AGAP3/SEPTIN6/ARL4C/RAB30/RABL6/GTPBP2/TUBB2A/RNGTT/MTG1/ARL4A/GBP4/EEF2/AK3/TUBA1C/GNA11/TUBG1/TUBG2/PDE2A/ARHGAP35/GBP6/RAB20/RRAGD/GNAZ/SLC19A1/RHOBTB1/BMS1/RHOF/SEPTIN11/EFTUD2/EHD3/HSP90AB1/TUBA4A/URGCP/GNAS/DAPK1/EHD4/RAB31/GTPBP1/RAB7A/RAC1/GNA15/EEFSEC/ARF1/CGAS/RRAS/SEPTIN1/GNAI2/SEPTIN4/LRRK1/RAB6B/NOLC1/TUBA8/RRAGC/RAB14/RAB27B/ANXA6/TUBB4B/PDE5A/RHOA/MFHAS1/DNM2/RAB5B/RAB11B/GUCY1A1/ARL8A/RAB3D/RAC2/GNL3L/DNM3/GNA12/RAB1B/MFN2/RAB2B/RHOG/EHD1/TUBB/TUBB1/GSPT1/GUCY1B1/TGM2
MF	GO:0032561	guanyl ribonucleotide binding	289	-0.35	-1.40	0.00503654	0.02090322	0.01296075	2380	tags=29%, list=20%, signal=23%	PDE6G/AGAP2/AGAP1/AGAP3/SEPTIN6/ARL4C/RAB30/RABL6/GTPBP2/TUBB2A/RNGTT/MTG1/ARL4A/GBP4/EEF2/AK3/TUBA1C/GNA11/TUBG1/TUBG2/PDE2A/ARHGAP35/GBP6/RAB20/RRAGD/GNAZ/SLC19A1/RHOBTB1/BMS1/RHOF/SEPTIN11/EFTUD2/EHD3/HSP90AB1/TUBA4A/URGCP/GNAS/DAPK1/EHD4/RAB31/GTPBP1/RAB7A/RAC1/GNA15/EEFSEC/ARF1/CGAS/RRAS/SEPTIN1/GNAI2/SEPTIN4/LRRK1/RAB6B/NOLC1/TUBA8/RRAGC/RAB14/RAB27B/ANXA6/TUBB4B/PDE5A/RHOA/MFHAS1/DNM2/RAB5B/RAB11B/GUCY1A1/ARL8A/RAB3D/RAC2/GNL3L/DNM3/GNA12/RAB1B/MFN2/RAB2B/RHOG/EHD1/TUBB/TUBB1/GSPT1/GUCY1B1/TGM2
BP	GO:0006997	nucleus organization	114	-0.42	-1.51	0.00504434	0.02091375	0.01296728	1943	tags=25%, list=17%, signal=21%	PHF8/NUMA1/SYNE1/SERBP1/CHMP6/TBPL1/NDEL1/NSFL1C/CHMP4B/BAZ2A/ETS1/NEK6/SUN2/NUP98/CHMP1A/PML/SRPK2/NOLC1/PHF2/USP36/SF1/NUP93/LMNA/PSME4/HIPK2/DCTN1/PLEC/PRKCB/CTDNEP1
BP	GO:0010799	regulation of peptidyl-threonine phosphorylation	25	-0.63	-1.71	0.00505634	0.02094169	0.0129846	1454	tags=28%, list=12%, signal=25%	TGFB1/SPHK1/PARD3/CALM3/INPP5K/EIF4G1/DMTN
BP	GO:0061337	cardiac conduction	43	-0.54	-1.63	0.00514946	0.02117297	0.01312801	2606	tags=40%, list=22%, signal=31%	JUP/ATP2A3/KCNH2/ATP1A1/ATP2A2/ATP1B1/KCNJ2/EHD3/PRKACA/RANGRF/SLC9A1/ATP1B2/CALM3/FLNA/FKBP1B/ATP2B4/SCN1B
MF	GO:0045309	protein phosphorylated amino acid binding	46	-0.55	-1.68	0.00523696	0.02139994	0.01326873	2642	tags=43%, list=23%, signal=34%	FGR/CBLB/PTPN11/PFN1/CRKL/PTPN6/VAV2/PIN1/SHC1/PIK3R3/GRB2/SH2D3C/YWHAB/VAV1/BTRC/SYK/ABL1/LDLRAP1/MAPK3/CBL
BP	GO:0035150	regulation of tube size	60	-0.50	-1.61	0.00533387	0.02170673	0.01345895	716	tags=18%, list=6%, signal=17%	KLF2/ABL1/RHOA/ACTA2/GUCY1A1/ECE1/PER2/P2RX1/GPX1/KEL/TBXA2R
BP	GO:0035296	regulation of tube diameter	60	-0.50	-1.61	0.00533387	0.02170673	0.01345895	716	tags=18%, list=6%, signal=17%	KLF2/ABL1/RHOA/ACTA2/GUCY1A1/ECE1/PER2/P2RX1/GPX1/KEL/TBXA2R
BP	GO:0097746	blood vessel diameter maintenance	60	-0.50	-1.61	0.00533387	0.02170673	0.01345895	716	tags=18%, list=6%, signal=17%	KLF2/ABL1/RHOA/ACTA2/GUCY1A1/ECE1/PER2/P2RX1/GPX1/KEL/TBXA2R

BP	GO:0010038	response to metal ion	208	-0.38	-1.44	0.00545748	0.0221192	0.0137147	2825	tags=36%, list=24%, signal=27%	NEK7/CEBPA/FGF23/SLC40A1/FOSB/TTN/ATP13A2/CPNE2/ATP7A/AKT1/SYT11/JUN/TRPM2/CYBB/NCF1/CASP9/CYBRD1/TRPC3/DLG4/CPNE3/TSPO/G6PD/SLC25A23/CPNE1/P2RX4/HSPA5/D2HGDH/NDRG1/HAAO/EDN1/SLC41A1/CD14/MMP9/JUND/SMPD1/SORD/HVCN1/ACTG1/ALAD/PARP1/ALOX15/AQP1/NEDD4L/MTF1/MBP/PTK2B/MT2A/BAD/NCSTN/CPNE5/CALR/PPP3CA/DAXX/SLFN14/PPP1CA/SEC31A/FOS/FBP1/STIM1/BMP6/ANXA11/CALM3/BSG/MCOLN1/TXNIP/THBS1/IQGAP1/GPI/SNCA/AHCYL1/MAPK3/SMPD3/DMTN/ADD1
BP	GO:0010001	glial cell differentiation	122	-0.42	-1.52	0.00553209	0.02230789	0.01383169	2551	tags=34%, list=22%, signal=27%	AKT1/SH3TC2/PTPN11/NOTCH1/NR1D1/EPHA4/HES1/CDK5/IL1B/NDRG1/NRROS/SOX13/B4GALT5/SOX4/VIM/KDM4A/TGFB1/CDK6/TNFRSF1B/SKI/PRX/WDR1/LYN/ILK/NCSTN/SOX6/OLIG2/STAT3/ABL1/PARD3/C1QA/OLIG1/RHOA/PLEC/LRP1/CSK/AKT2/GRN/MAPK3/WASF3/TAL1/CLU
BP	GO:0003015	heart process	118	-0.42	-1.50	0.0059347	0.02357446	0.01461701	2735	tags=37%, list=23%, signal=29%	TTN/RPS6KA2/EXT1/JUP/ATP2A3/SMAD5/KCNH2/PIK3CA/ATP1A1/ATP2A2/FYN/THRA/ATP1B1/P2RX4/SNTA1/PDE4B/EDN1/GRK2/KCNJ2/EHD3/SREBF1/PRKACA/GSK3A/RANGRF/TPM1/NEDD4L/EPAS1/SLC9A1/SRC/GNAI2/ATP1B2/GAA/PDE5A/CALM3/FLNA/FKBP1B/MYL4/GSN/AKAP13/PIK3CG/ATP2B4/GPX1/MAP2K3/SCN1B
BP	GO:0048754	branching morphogenesis of an epithelial tube	60	-0.50	-1.59	0.00620607	0.02445522	0.01516312	1756	tags=30%, list=15%, signal=26%	EDN1/MAGED1/ENG/CTNNBIP1/ILK/PLXND1/DCHS1/SRC/PML/GREM1/SFRP2/PPP1CA/NRP1/BTRC/ABL1/PAK1/MED1/PBX1
BP	GO:0048813	dendrite morphogenesis	86	-0.46	-1.56	0.00623925	0.02453735	0.01521404	3363	tags=44%, list=29%, signal=32%	DBNL/CDC42/EEF2K/STAU2/ACTR2/HDAC6/PDLIM5/KIDINS220/DVL1/ABI2/EPHB1/CAPRIN1/MINK1/DLG4/EPHA4/CDK5/ABI3/FYN/DOCK10/CUL7/RERE/SEMA4D/DTNBP1/GSK3B/BAIAP2/NEDD4L/CDKL5/SIPA1L1/FBXO31/PPP3CA/TAOK2/NRP1/VLDLR/YWHAH/STK11/DNM3/CFL1/CUX1
MF	GO:0070851	growth factor receptor binding	72	-0.47	-1.57	0.00629333	0.02472559	0.01533075	2915	tags=40%, list=25%, signal=30%	HBEGF/ATXN2/FGF23/FRS2/SDCBP/CNOT9/IL6R/FYN/IL1B/RNF126/VAV2/TNK2/SHC1/CD300LF/GRB2/PYCARD/TRIP6/IL1RN/HIP1/LYN/NCSTN/SRC/GREM1/PSPN/PDGFC/PTPRJ/TOLLIP/ITGB3/PDGFA
MF	GO:0048029	monosaccharide binding	50	-0.52	-1.61	0.00636541	0.02495944	0.01547575	1711	tags=30%, list=15%, signal=26%	ENG/GALK1/PLOD1/ALDOA/PFKL/TALDO1/EGLN3/FBP1/LGALS9/HK1/BSG/IGF2R/GPI/RPIA/SELP
BP	GO:0007020	microtubule nucleation	35	-0.57	-1.63	0.00657075	0.02561303	0.015881	2517	tags=54%, list=21%, signal=43%	TUBGCP2/TUBGCP4/TUBGCP6/MECP2/NDE1/TUBG1/TUBG2/NDEL1/TPX2/SLAIN2/GIT1/EML2/RANGRF/HSPA1A/PAK1/CLASP1/GOLGA2/CKAP5/DCTN1
BP	GO:0071383	cellular response to steroid hormone stimulus	146	-0.41	-1.49	0.00701869	0.02698868	0.01673395	2428	tags=32%, list=21%, signal=26%	NR1D1/ATP1A1/CASP9/DDX54/ZFP36/UBR5/CNOT9/PKN1/ZNF366/PAQR8/NCOA4/PPARGC1B/ZBTB7A/ZFP36L2/NR3C1/EDN1/TRERF1/FOXP1/CRY2/SMARCA4/PARP1/AQP1/LBH/TADA3/HSPA1A/RXRA/BCL2L11/SRC/CALR/PPARA/WBP2/DAXX/ZMIZ1/YWHAH/PAK1/RHOA/KANK2/ABHD2/SAFB/UBE2L3/FOXO3/MED1/ARID1A/NCOR2/CARM1/CNOT1/DAB2
BP	GO:0007423	sensory organ development	244	-0.35	-1.38	0.0071269	0.02732553	0.01694281	3232	tags=40%, list=28%, signal=29%	SRF/PPP1R13L/ZHX2/STAU2/PRKCI/RARG/TTC39C/NF1/SCRIB/PBX2/RAB3GAP1/CITED2/PBX3/KIT/ANP32B/MYCL/LEF1/IFT122/DVL1/NECTIN1/ABI2/FLT1/CEBPA/MSX1/EPHB1/FRS2/MCM2/SDK2/BNC2/JUN/PTPN11/NOTCH1/BAK1/TRIOBP/PRDM1/EPHA4/HES1/SMG9/SPRED1/DDR1/MYO15A/CASP2/MITF/SMARCD3/MAN2A1/ARHGAP35/SMAD3/EDN1/PHACTR4/FZD5/RBPJ/KLF4/DTNBP1/VIM/ACVR2B/SMARCA4/ASCL2/RPGRIP1L/SIPA1L3/BCL11B/SKI/TGIF2/TWSG1/MFSD2A/YY1/FZD2/RARA/DCHS1/GNB1/BCL2L11/CDKN1C/MAX/STAT3/NRP1/BCR/MAFB/BLOC1S3/BMP6/NOTCH2/HIPK1/PVALB/TBC1D20/MAPKAPK2/ECE1/HIPK2/NF2/SDC4/FZR1/MFN2/MEIS1/FKBP8/ATP2B4/TMOD1/MAPK3/MED1/PDGFA/PBX1

BP	GO:0051047	positive regulation of secretion	181	-0.38	-1.43	0.00728885	0.02789267	0.01729446	2776	tags=30%, list=24%, signal=23%	GAB2/TTN/SDCBP/ATP13A2/GABBR1/FGR/PTAFR/ABCB11/PTPN11/TRPM2/TSG101/HLA-DRB1/CDK5/IL1B/P2RX4/VPS4B/PARD6A/NR1H2/ITGAM/HGS/EDN1/GRK2/SOX4/DTNBP1/HYAL3/TGFB1/FRMD4A/EZR/STX4/AQP1/RAB7A/PINK1/BAD/GNAI2/LRRC8A/SYK/CLASP1/RAB27B/BMP6/MYO18A/GALR1/RAB3D/SDC4/SYTL4/GLUL/RAB2B/TCF7L2/SNCA/LAMP1/GATA1/SMPD3/SCIN/PTPN23/DAB2
BP	GO:0051338	regulation of transferase activity	625	-0.30	-1.27	0.00769441	0.02916396	0.0180827	2581	tags=28%, list=22%, signal=23%	GNAQ/RALB/SORL1/PKIG/AKT1/AIDA/TAOK3/CBLB/PIK3CA/CCNJL/MLLT1/ADNP/MOB3B/NCF1/EGR1/GADD45B/AGAP2/ZFP36/CDKN2D/EZH2/DLG4/TSG101/PNKP/CHP1/MAP3K13/LMTK2/SERINC5/IL6R/EPHA4/PKN1/HLA-DRB1/SH3BP5L/PIP4K2C/CDK5/SPRED1/HYAL2/CD81/DDR1/CDK5RAP2/IL1B/ORMDL3/CSF1R/PTPN6/MADD/DCUN1D3/VAV2/TLR6/CCNY/GSTP1/MOB1B/TRIB1/PIN1/SOCS7/PRRC1/XRCC6/CDC37/SHC1/GCN1/HGS/PTPRC/EDN1/PIK3CB/MAGED1/CHMP6/FZD5/DGKZ/CD74/TPX2/PIK3R3/WDR81/KLF4/MAP3K11/SMPD1/DTNBP1/ACVR2B/GSK3B/NEDD9/GSK3A/PYCARD/HSP90AB1/TGFB1/MOB3A/LILRA5/TAOK1/YWHAG/JMJD8/MEN1/SLC27A1/MVP/SERTAD1/CORO1C/TRAF6/MAP3K5/SESN2/LYN/SASH1/ADAR/PINK1/PTK2B/IGF2/MAP2K7/BAD/MARK2/SRC/CCND3/GREM1/CDKN1C/CEACAM1/RUBCN/SFRP2/DVL3/TAOK2/DAXX/ACTB/PIH1D1/MAP2K2/VLDLR/KAT2B/BTRC/PDGFC/SYK/ABL1/PAK1/LTF/WARS1/DTX3L/PRKAR2A/PDE5A/RHOA/CALM3/MAD2L2/PTPRJ/UBE2S/INPP5K/HSPB1/NF2/STK11/SDC4/HEXIM2/PLEC/FZR1/CSK/THBS1/UBE2L3/CCNI/PTPN1/AKAP13/PIK3CG/IQGAP1/UBASH3B/ATP2B4/SNCA/MMD/CAMK1/NEURL1/PIP4K2A/MAPK3/FBXO7/CDKN1A/CBL/PAK2/STRADB/PRKAR2B/TAL1/HTT/ITGB3/PDGFA/CLU/MAP2K3/WNK1/DAB2/CNPPD1
BP	GO:0051960	regulation of nervous system development	244	-0.35	-1.37	0.00797968	0.0301017	0.01866414	2605	tags=30%, list=22%, signal=24%	CAPRIN1/ULK1/SORL1/AKT1/DIP2B/PLXNC1/DISC1/PLXNA4/ADNP/NOTCH1/NR1D1/EZH2/HOXB3/TSPO/MAP3K13/EPHA4/HES1/CDK5/ASPM/IL1B/SLITRK5/CERS2/MACF1/SMARCD3/MAN2A1/CUL7/SYNJ1/KIAA0319/TIAM1/DIXDC1/NDEL1/SEMA4D/CTDSP1/KDM4A/BAIAP2/CYFIP1/TGFB1/ASCL2/TNFRSF1B/SKI/PRUNE1/IST1/LYN/PLXNB2/CDKL5/MBP/RARA/PLXND1/TRIM32/FBXO31/HOOK3/EIF4G2/STK25/OLIG2/PLXNA1/PPP3CA/CTNNA1/SEMA4A/NRP1/YWHAH/PARD3/SYNGAP1/E2F1/NF2/TRIM11/STK11/CLSTN1/GJC2/PER2/CUX1/NEURL1/RNF10/WASF3/TGM2
BP	GO:0032368	regulation of lipid transport	65	-0.49	-1.57	0.00824837	0.03088089	0.01914726	2551	tags=37%, list=22%, signal=29%	AKT1/ABCA3/POMC/PTPN11/TSPO/IL1B/P2RX4/NR1H2/EDN1/CRY2/NR1H3/PPARA/FIS1/SYK/NFKB1/BMP6/GALR1/LDLRAP1/LRP1/THBS1/AKT2/FURIN/ITGB3/DAB2
BP	GO:0046365	monosaccharide catabolic process	28	-0.60	-1.64	0.00827832	0.03096389	0.01919873	1845	tags=36%, list=16%, signal=30%	FOXK1/ENO2/GALK1/FOXK2/GLB1/BAD/PFKL/GALE/HK1/ENO1
MF	GO:0019838	growth factor binding	72	-0.47	-1.55	0.00841052	0.03134042	0.01943219	1865	tags=29%, list=16%, signal=25%	NRROS/SHC1/ACVR1B/ENG/IL2RG/ACVR2B/IL2RB/PCSK6/IL1RN/CCN3/TWSG1/LTBP3/NRP1/SORT1/IGF2R/THBS1/GLG1/FURIN/ITGB3/PDGFA/LTBP1
BP	GO:1901890	positive regulation of cell junction assembly	43	-0.52	-1.58	0.00843065	0.03138602	0.01946046	2084	tags=33%, list=18%, signal=27%	SLITRK5/SMAD3/FMN1/LIMS1/POLDIP2/SEMA4D/RAC1/SEMA4A/NRP1/ABL1/PTPRJ/SDC4/CLSTN1/CLDN5
BP	GO:0019318	hexose metabolic process	172	-0.38	-1.44	0.00845867	0.03146089	0.01950688	2042	tags=27%, list=17%, signal=23%	SLC25A1/PFKFB4/HECTD4/NLN/MAN2A1/FOXK1/PHKG2/ENO2/GALK1/CPT1A/PDK3/WDR5/GSK3B/SORD/FOXK2/FAM3A/GSK3A/BCKDK/NCOA2/INPPL1/WDTC1/ALDOA/GLB1/SESN2/GDPGP1/IGF2/ALDOC/BAD/PFKL/SRC/PPARA/GALE/PPP1CA/PDK2/KAT2B/DDB1/FBP1/GAA/HK1/INPP5K/USP7/STK11/ADIPOR1/AKT2/PER2/GPI/ENO1

BP	GO:0010677	negative regulation of cellular carbohydrate metabolic process	29	-0.59	-1.65	0.00867287	0.03216728	0.01994487	1521	tags=38%, list=13%, signal=33%	GSK3B/FAM3A/GSK3A/GIT1/CBFA2T3/PLEK/PPARA/STAT3/FBP1/INPP5K/SLC4A1
BP	GO:0050764	regulation of phagocytosis	68	-0.47	-1.52	0.00872936	0.03234663	0.02005607	1925	tags=37%, list=16%, signal=31%	SIRPG/SCARB1/PTPRC/CD300LF/IL2RG/SIRPB1/IL2RB/PYCARD/SPHK1/ALOX15/RAB31/ANO6/DYSF/CALR/SNX3/CNN2/BCR/SYK/MYO18A/DNM2/PTPRJ/DOCK2/CAMK1D/CSK/TGM2
CC	GO:0035097	histone methyltransferase complex	59	-0.49	-1.56	0.00879331	0.03255326	0.02018419	1702	tags=37%, list=15%, signal=32%	TAF1/TAF6/WDR5/ASH2L/CXXC1/MEN1/KDM6B/NCOA6/KMT2B/SETD1B/KANSL1/TAF4/RUVBL1/PELP1/MAX/MCRS1/KMT2A/JARID2/KMT2C/CHD8/SETD1A/HCFC1
BP	GO:0005996	monosaccharide metabolic process	185	-0.37	-1.40	0.00880637	0.03257128	0.02019536	2042	tags=26%, list=17%, signal=22%	SLC25A1/PFKFB4/HECTD4/NLN/MAN2A1/FOXK1/PHKG2/ENO2/GALK1/CPT1A/PDK3/WDR5/GSK3B/SORD/FOXK2/FAM3A/GSK3A/BCKDK/NCOA2/INPPL1/WDTC1/ALDOA/GLB1/SESN2/GDPGP1/IGF2/ALDOC/BAD/PFKL/SRC/PPARA/GALE/PPP1CA/PDK2/KAT2B/DDB1/FBP1/SLC2A1/GAA/HK1/INPP5K/USP7/STK11/ADIPOR1/AKT2/PER2/GPI/RPIA/ENO1
BP	GO:0002066	columnar/cuboidal epithelial cell development	26	-0.61	-1.66	0.00888084	0.03278573	0.02032833	1521	tags=46%, list=13%, signal=40%	PDPK1/AKT1/PRDM1/GSK3B/GSK3A/CDK6/RARA/BAD/SRC/BMP6/SIDT2/FASN
BP	GO:0062014	negative regulation of small molecule metabolic process	57	-0.48	-1.52	0.00904561	0.03327043	0.02062887	1596	tags=32%, list=14%, signal=27%	CDA/FAM3A/GSK3A/GIT1/BCKDK/CBFA2T3/PLEK/WDTC1/PARP1/MFSD2A/CEACAM1/PPARA/STAT3/FBP1/NFKB1/ACADVL/SLC4A1/ATP2B4
MF	GO:0051018	protein kinase A binding	39	-0.54	-1.62	0.00913287	0.03349844	0.02077024	1521	tags=31%, list=13%, signal=27%	GSK3B/PRKACA/GSK3A/EZR/RARA/C2orf88/PRKAR2A/CSK/AKAP13/WASF3/PRKAR2B/WASF2
BP	GO:0031647	regulation of protein stability	250	-0.35	-1.37	0.00915099	0.0335068	0.02077542	1912	tags=25%, list=16%, signal=21%	CHFR/CCAR2/CTSA/PIN1/CDC37/SMAD3/DERL1/LSS/CRTAP/TAF1/B4GALT5/CCT3/WDR81/TMEM183A/SOX4/SREBF1/PYCARD/HSP90AB1/HDAC3/VHL/HIP1/HIP1R/NEDD4L/GLMP/PINK1/TADA3/HSPA1A/CREBBP/SRC/CALR/DVL3/ATP1B2/USP19/TBL1X/DDI2/BTRC/USP9X/USP36/AHSP/PIM2/RAD23A/FLNA/LMNA/USP7/NF2/GNL3L/GSN/GRN/SNCA/PRKDC/LAMP1/DPM2/BAG1/FBXO7/TESC/SERF2/HTT/SEC16A/CLU/PIM1/HCFC1/BAG6
BP	GO:0006110	regulation of glycolytic process	34	-0.56	-1.59	0.00925237	0.03381197	0.02096464	2649	tags=44%, list=23%, signal=34%	ZBTB20/ARL2/HDAC4/ENTPD5/PGAM1/ZBTB7A/EIF6/GIT1/CBFA2T3/JMJD8/ARNT/PPARA/STAT3/FBP1/SLC4A1
BP	GO:0036294	cellular response to decreased oxygen levels	101	-0.43	-1.51	0.00938897	0.03426679	0.02124664	3103	tags=40%, list=26%, signal=29%	IRAK1/ENDOG/CPEB4/CITED2/TMBIM6/ZFP36L1/NPEPPS/SLC8A1/ATP7A/AKT1/NOTCH1/CYBB/EGR1/HYOU1/EPHA4/ROCK2/NDRG1/EDN1/PIK3CB/HIF1AN/RBPJ/PDK3/VHL/AQP1/EPAS1/PINK1/SLC9A1/BAD/SRC/USP19/EGLN3/E2F1/MPL/LMNA/HIPK2/ENO1/BNIP3L/SLC29A1/CBL/FOXO3

BP	GO:0043065	positive regulation of apoptotic process	374	-0.33	-1.33	0.00944158	0.03437702	0.02131499	1779	tags=21%, list=15%, signal=19%	TRAF7/PTPRC/PIK3CB/MAGED1/INPP5D/MMP9/BBC3/MAP3K11/BCAP31/SOX4/SMPD1/TOP2A/NCOA1/PYCARD/TEX261/TNFRSF1B/PRR7/DAPK3/DAPK1/TP53BP2/TRIM35/MAP3K5/ANO6/HIP1R/LYN/PIAS4/MAP2K7/SLC9A1/BAD/BRMS1/PDCD1/BCL2L11/SRC/UBE2Z/PML/EIF5A/SRPK2/RBCK1/PIK3CD/SFRP2/SEPTIN4/FIS1/PLEKHF1/PPP1CA/CTNNA1/FBH1/ABL1/GADD45G/E2F1/ARRB2/RHOA/UBE2M/NOTCH2/DNM2/LGALS9/ATG7/CDK19/CAMK1D/UBB/GSN/TXNIP/IGF2R/SQSTM1/THBS1/MFN2/GRN/SNCA/NEURL1/PRKDC/BNIP3L/CTSD/SCIN/PAK2/RYBP/FOXO3/CDC34/HTT/CLU/TGM2
MF	GO:0003725	double-stranded RNA binding	69	-0.47	-1.52	0.00948962	0.03448877	0.02138428	2948	tags=42%, list=25%, signal=32%	RC3H1/TUBA1B/EIF4A1/LSM14A/EIF2AK2/ILF3/RFTN1/SUPV3L1/AGO4/OAS2/OAS3/ZFR/AGO1/DROSHA/VIM/DDX60/HSP90AB1/CLTC/ADAR/MTDH/DHX30/ELAVL1/STAU1/TUBB4B/SIDT2/SLC3A2/DHX33/MSN/ACTN1
BP	GO:0006940	regulation of smooth muscle contraction	27	-0.59	-1.62	0.00952406	0.03453103	0.02141048	1343	tags=33%, list=11%, signal=30%	ORMDL3/EDN1/SPHK1/DAPK3/RHOA/GUCY1A1/P2RX1/CTTN/TBXA2R
BP	GO:0031440	regulation of mRNA 3'-end processing	27	-0.59	-1.62	0.00952406	0.03453103	0.02141048	244	tags=15%, list=2%, signal=15%	PAF1/AHCYL1/RNF40/SUPT5H
BP	GO:0071277	cellular response to calcium ion	47	-0.52	-1.60	0.0095273	0.03453103	0.02141048	3487	tags=57%, list=30%, signal=41%	NEUROD2/ALOX5AP/BRAF/ITPKB/EEF2K/RASGRP2/ENDOG/FOSB/CPNE2/SYT11/JUN/TRPM2/CPNE3/SLC25A23/CPNE1/HSPA5/EDN1/JUND/SMPD1/ALOX15/NCSTN/CPNE5/FOS/MCOLN1/IQGAP1/DMTN/ADD1
BP	GO:0035924	cellular response to vascular endothelial growth factor stimulus	30	-0.58	-1.63	0.00961584	0.03473911	0.0215395	2578	tags=53%, list=22%, signal=42%	MAPK14/AKT1/PIK3CA/NOTCH1/PRKD2/PIK3CB/CD63/SPHK1/PIK3CD/PTP4A3/NRP1/RELA/MAPKAPK2/HSPB1/ATP2B4/MAP2K3
BP	GO:0035270	endocrine system development	56	-0.51	-1.60	0.00961128	0.03473911	0.0215395	2703	tags=45%, list=23%, signal=35%	PDPK1/AKT1/TBX19/BAK1/HOXB3/TSPO/IL6R/HES1/CRKL/THRA/SMAD3/EDN1/RBPJ/RAP1GAP/SOX4/GSK3B/GSK3A/CDK6/MEN1/BAD/CDKN1C/BMP6/SIDT2/MAPK3/PBX1
BP	GO:0033146	regulation of intracellular estrogen receptor signaling pathway	26	-0.60	-1.65	0.00982992	0.03536993	0.02193063	1270	tags=35%, list=11%, signal=31%	PARP1/LBH/SRC/WBP2/PAK1/KANK2/MED1/CARM1/CNOT1
BP	GO:0032434	regulation of proteasomal ubiquitin-dependent protein catabolic process	123	-0.41	-1.48	0.00993817	0.03563044	0.02209215	1917	tags=26%, list=16%, signal=22%	TRIB1/CHFR/CCAR2/KEAP1/SMARCC1/TAF1/BCAP31/GSK3B/GSK3A/HSP90AB1/CBFA2T3/DET1/DDA1/HSPA1A/ANKIB1/CDK2/MAP1A/GABARAP/VCP/ARIH2/N4BP1/RAD23A/USP7/GNA12/FZR1/ARAF/RYBP/ZER1/DNAJB2/CLU/DAB2/BAG6

MF	GO:0005525	GTP binding	281	-0.35	-1.36	0.00992071	0.03563044	0.02209215	2377	tags=28%, list=20%, signal=23%	AGAP2/AGAP1/AGAP3/SEPTIN6/ARL4C/RAB30/RABL6/GTPBP2/TUBB2A/RNGTT/MTG1/ARL4A/GBP4/EEF2/AK3/TUBA1C/GNA11/TUBG1/TUBG2/ARHGAP35/GBP6/RAB20/RRAGD/GNAZ/RHOBTB1/BMS1/RHOF/SEPTIN11/EFTUD2/EHD3/HSP90AB1/TUBA4A/URGCP/GNAS/DAPK1/EHD4/RAB31/GTPBP1/RAB7A/RAC1/GNA15/EEFSEC/ARF1/CGAS/RRAS/SEPTIN1/GNAI2/SEPTIN4/LRRK1/RAB6B/NOLC1/TUBA8/RRAGC/RAB14/RAB27B/ANXA6/TUBB4B/RHOA/MFHAS1/DNM2/RAB5B/RAB11B/GUCY1A1/ARL8A/RAB3D/RAC2/GNL3L/DNM3/GNA12/RAB1B/MFN2/RAB2B/RHOG/EHD1/TUBB/TUBB1/GSPT1/GUCY1B1/TGM2
BP	GO:0006338	chromatin remodeling	181	-0.38	-1.41	0.01012693	0.03614423	0.02241072	2675	tags=32%, list=23%, signal=25%	H3-3A/SSRP1/PBRM1/SMARCA2/FOXP3/MLLT1/CHD3/YEATS2/HDAC4/HDAC5/NAP1L4/ARID2/MTA2/TOP1/MTA1/GRWD1/H1-4/SMARCD3/ZBTB7A/MCM3AP/RERE/SMARCC1/VPS72/KDM4A/CHRAC1/BAZ2A/SMARCA4/SCMH1/KDM6B/YY1/SUPT6H/BPTF/SUPT16H/ASF1B/GATAD2B/RUVBL1/DAXX/ACTB/PIH1D1/KAT2B/MCRS1/PAK1/KDM4B/JARID2/KDM5C/CHD8/PSME4/KAT6A/NFE2/CHD4/BRD3/H2BC11/BRD4/HMGA1/SMARCC2/H3C10/ARID1A/HCFC1
CC	GO:0044665	MLL1/2 complex	31	-0.58	-1.62	0.01017628	0.03625526	0.02247957	1702	tags=48%, list=15%, signal=41%	TAF1/TAF6/WDR5/ASH2L/MEN1/KMT2B/KANSL1/TAF4/RUVBL1/PELP1/MAX/MCRS1/KMT2A/CHD8/HCFC1
BP	GO:1903362	regulation of cellular protein catabolic process	218	-0.36	-1.38	0.01035578	0.03686176	0.02285562	1776	tags=25%, list=15%, signal=21%	KEAP1/F8A1/SMARCC1/TAF1/PSME3/BCAP31/GSK3B/PRKACA/GSK3A/HSP90AB1/CBFA2T3/DET1/VGLL4/EZR/ALAD/DDA1/PINK1/PTK2B/HSPA1A/ANKIB1/PML/CDK2/LAPTM5/USP19/MAP1A/BTRC/GABARAP/RGP1/VCP/ARIH2/N4BP1/PITHD1/PSMC4/RAD23A/SUFU/USP7/HIPK2/UBB/GNA12/FZR1/PSMF1/LRP1/MSN/MARCHF2/ARAF/RYBP/ZER1/GPX1/RNF40/DNAJB2/FURIN/CLU/DAB2/BAG6
MF	GO:0022836	gated channel activity	109	-0.42	-1.47	0.01037875	0.03691049	0.02288583	2550	tags=24%, list=22%, signal=19%	TMEM109/KCNH2/TRPM2/CYBB/CDK5/P2RX4/ITPR2/TMC8/KCNJ2/KCNQ5/HVCN1/CLIC5/ANO6/AQP1/PTK2B/RASA3/CACNG6/VDAC1/ANXA6/CLCN5/FKBP1B/MCOLN1/P2RX1/TTYH3/TMEM63B/SCN1B
MF	GO:0002039	p53 binding	56	-0.51	-1.59	0.01042925	0.03705691	0.02297662	2260	tags=38%, list=19%, signal=30%	ZNF385A/CDK5/KMT5A/USP10/EHMT1/RFWD3/TAF1/PSME3/EHMT2/GSK3B/SMARCA4/TP53BP2/CREBBP/DAXX/TP53BP1/CHD8/USP7/STK11/GATA1/BRD4/HTT
BP	GO:0010883	regulation of lipid storage	39	-0.54	-1.60	0.01050621	0.03726955	0.02310846	2434	tags=46%, list=21%, signal=37%	ACACB/IKBKE/PLIN3/SREBF2/NR1H2/SCARB1/FTO/CPT1A/SREBF1/PLIN2/NR1H3/FITM1/PPARA/MEST/PNPLA2/NFKB1/EHD1/ITGB3
BP	GO:0048872	homeostasis of number of cells	228	-0.36	-1.38	0.01053576	0.03733528	0.02314922	1483	tags=23%, list=13%, signal=20%	NAPEPLD/TSPO2/CDK6/ACIN1/ETS1/CCN3/SLC39A3/SIT1/LYN/KLF13/EPAS1/ADAR/JAM3/HSPA1A/KLF2/UBAP2L/HCLS1/NCSTN/BCL2L11/KAT7/SLC25A38/ARNT/PIK3CD/SEPTIN4/TRIM10/BPGM/STAT3/BCR/JAK3/ABL1/MAFB/AHSP/MPL/MFHAS1/LGALS9/HIPK2/KLF1/SLC4A1/EPB42/SPTA1/FAM210B/GPI/PRKDC/IKZF1/GATA1/ALAS2/FOXO3/MPIG6B/MED1/TAL1/TRIM58/DMTN
BP	GO:0033157	regulation of intracellular protein transport	185	-0.37	-1.38	0.01060618	0.03755136	0.02328319	2267	tags=32%, list=19%, signal=26%	CHP1/PCNT/CDK5/HYAL2/CD81/FYN/IL1B/SREBF2/LCP1/RIPOR1/ITGAM/UBE2J2/SMAD3/FZD5/NDEL1/BBC3/BCAP31/TM9SF4/SREBF1/GSK3B/PRR5L/STOM/PRKACA/GSK3A/HSP90AB1/TGFB1/HDAC3/EDEM2/BCAS3/MAVS/LEPROT/PINK1/IFI27/HCLS1/GRIPAP1/FIS1/TRIM28/RANGAP1/NOLC1/USP36/ARIH2/ABLIM3/SAE1/SUFU/FLNA/RAC2/INPP5K/HUWE1/YOD1/AKT2/UBE2L3/PTPN1/TCF7L2/CAMK1/ATG13/BNIP3L/OAZ1/PPM1A/DMTN
BP	GO:0052372	modulation by symbiont of entry into host	35	-0.55	-1.57	0.01066401	0.03770545	0.02337874	2730	tags=43%, list=23%, signal=33%	FUCA2/TRIM21/MID2/EXOC7/HLA-DRB1/CD74/TRIM26/IFITM3/SNX3/TRIM10/IFITM2/LGALS9/TRIM11/GSN/TRIM14

BP	GO:0010812	negative regulation of cell-substrate adhesion	36	-0.56	-1.62	0.01074409	0.03787101	0.02348139	1312	tags=28%, list=11%, signal=25%	MEN1/CORO1C/BCAS3/SRC/RHOA/ARHGAP6/NF2/THBS1/ACTN4/DMTN
BP	GO:0060711	labyrinthine layer development	27	-0.59	-1.61	0.01087497	0.03823066	0.02370439	2928	tags=48%, list=25%, signal=36%	LEF1/ZFP36L1/AKT1/NSDHL/HES1/VASH1/BIRC6/FZD5/RBPJ/GRB2/NCOA1/ST14/SPINT2
CC	GO:0000139	Golgi membrane	499	-0.31	-1.27	0.01102087	0.03857304	0.02391667	2277	tags=23%, list=19%, signal=20%	CORO7/RAB30/CHP1/SMPD4/HLA-DRB1/GCC1/HYAL2/ACER3/HLA-DQA2/CHST14/GBP4/YKT6/PROS1/HLA-A/SREBF2/SLC9A7/SLC33A1/DOP1A/ZDHHC3/XYLT1/MGAT5/ST6GAL1/ERGIC1/MAN2A1/NUMA1/ARRB1/B3GNT5/GALNT10/GPSM1/PI4KB/TAPBP/CREB3/FZD5/LPCAT2/CD74/B4GALT5/RAP1GAP/HLA-C/STX6/ZDHHC19/BCAP31/HLA-DQA1/SREBF1/NAPEPLD/PYCARD/COPA/ST3GAL3/TEX261/TRAPPC3/CBFA2T3/MAN1C1/TRAPPC4/NDST1/YIF1A/B3GNT8/AP1M1/GBGT1/UNC93B1/ABO/LEPROT/CHST11/PLD3/ARF1/KDELR1/STK24/DHCR24/GALNT2/SLC35A4/PGAP2/HLA-DRB5/ARHGAP32/VTI1A/COPB2/RAB6B/LITAF/ARFGAP2/PDGFC/GABARAP/RFNG/RGP1/RAB14/OPTN/CYTH4/AGPAT3/GOLGA3/STX5/MYO18A/SLC2A1/CLCN5/GOLGA2/TPST2/NOTCH2/DNM2/AP1B1/MAN1A1/TBC1D20/ARHGAP21/BSG/RNF24/ST6GALNAC4/MMGT1/CLSTN1/RAB1B/HUWE1/ARCN1/IGF2R/GLG1/RAB2B/MARCHF2/MGAT4B/B4GALT3/SMPD3/ST3GAL1/FURIN/SEC16A/PDGFA
BP	GO:0008016	regulation of heart contraction	90	-0.43	-1.48	0.01120617	0.0391098	0.02424948	2606	tags=34%, list=22%, signal=27%	JUP/ATP2A3/KCNH2/ATP1A1/ATP2A2/THRA/ATP1B1/P2RX4/SNTA1/PDE4B/EDN1/GRK2/KCNJ2/EHD3/SREBF1/PRKACA/GSK3A/RANGRF/TPM1/EPAS1/SLC9A1/ATP1B2/GAA/PDE5A/CALM3/FLNA/FKBP1B/MYL4/PIK3CG/ATP2B4/SCN1B
BP	GO:0009746	response to hexose	113	-0.41	-1.47	0.01137583	0.03960622	0.02455728	1609	tags=23%, list=14%, signal=20%	PDK3/SOX4/SREBF1/ACVR2B/PRKACA/SMARCA4/MEN1/STX4/UCP2/SESN2/PTK2B/BAD/PFKL/PPP3CA/PIH1D1/SIDT2/RHOA/RAB11B/FKBP1B/GLUL/TXNIP/THBS1/TCF7L2/SLC29A1/GPX1/FOXO3
BP	GO:0034284	response to monosaccharide	116	-0.41	-1.46	0.01141617	0.03971189	0.0246228	1609	tags=22%, list=14%, signal=20%	PDK3/SOX4/SREBF1/ACVR2B/PRKACA/SMARCA4/MEN1/STX4/UCP2/SESN2/PTK2B/BAD/PFKL/PPP3CA/PIH1D1/SIDT2/RHOA/RAB11B/FKBP1B/GLUL/TXNIP/THBS1/TCF7L2/SLC29A1/GPX1/FOXO3
BP	GO:0010469	regulation of signaling receptor activity	82	-0.45	-1.53	0.0115295	0.04007106	0.0248455	2554	tags=37%, list=22%, signal=29%	MINK1/CBLB/NCF1/BICD1/DLG4/TSG101/SLC24A4/NETO2/LYNX1/SHC1/EDN1/CHMP6/NPTXR/TAF1/DAPK1/PINK1/PTK2B/SRC/GREM1/DLGAP4/NCOA3/PPARA/CNIH3/NRP1/SLC6A9/PPP2R5B/NEURL1/CAPN1/CBL/SHISA7
BP	GO:0010906	regulation of glucose metabolic process	82	-0.45	-1.52	0.01206889	0.0416183	0.02580484	1961	tags=32%, list=17%, signal=27%	NLN/FOXK1/PHKG2/PDK3/WDR5/GSK3B/FOXK2/FAM3A/GSK3A/BCKDK/NCOA2/SESN2/IGF2/BAD/SRC/PPARA/PPP1CA/PDK2/KAT2B/DDB1/FBP1/INPP5K/USP7/STK11/ADIPOR1/AKT2
BP	GO:0035637	multicellular organismal signaling	63	-0.47	-1.51	0.01215968	0.04189504	0.02597643	2606	tags=33%, list=22%, signal=26%	JUP/ATP2A3/KCNH2/ATP1A1/ATP2A2/ATP1B1/P2RX4/KCNJ2/EHD3/PRKACA/RANGRF/JAM3/SLC9A1/ATP1B2/CALM3/FLNA/FKBP1B/PLEC/ATP2B4/P2RX1/SCN1B
CC	GO:0030120	vesicle coat	46	-0.52	-1.58	0.01217669	0.04191734	0.02599026	1599	tags=46%, list=14%, signal=40%	SEC24D/PEF1/EPN2/CLTCL1/AFTPH/COPG1/AP2A1/COPZ1/AP2A2/AP2B1/SYNRG/COPA/CLTC/AP2S1/COPB2/SEC31A/SCYL1/CLTB/EPN1/ARCN1/AP2M1

BP	GO:0034504	protein localization to nucleus	236	-0.35	-1.34	0.01219957	0.04195978	0.02601657	2606	tags=35%, list=22%, signal=28%	JUP/MAPK14/PKIG/AKT1/ARL2/E2F3/CBLB/NOTCH1/KPNA3/BANP/UBR5/FLCN/KPNA6/CHP1/CDK5/HYAL2/SUMO3/FYN/ORMDL3/ZBTB16/IPO4/ZBTB7A/PIN1/FAM53C/LZTS2/SYNE1/SMAD3/CHCHD10/MAGED1/IPO8/CCT3/OTUD7B/GSK3B/CRY2/NUP188/HSP90AB1/TGFB1/HDAC3/MEPCE/RANGRF/PARP1/SESN2/SUN2/NUP98/POM121C/MAVS/HCLS1/ILRUN/SRC/PML/KAT7/CALR/PPP3CA/TAF8/TRIM28/STAT3/CTNNA1/RANGAP1/NOLC1/MCRS1/SYK/ELAVL1/CIZ1/DTX3L/MFHAS1/SUFU/POM121/FLNA/NUP93/LMNA/NF2/STK11/RASSF5/GLUL/TXNIP/SQSTM1/PAF1/PPP1R10/CDKN1A/FAM53B/CTDNEP1/MED1/HDGF
BP	GO:0046785	microtubule polymerization	67	-0.45	-1.48	0.01251878	0.04279853	0.02653663	2601	tags=45%, list=22%, signal=35%	MAPRE1/ARL2/TUBGCP2/TUBGCP4/TUBGCP6/MECP2/NDE1/CDK5RAP2/TBCD/TUBG1/TUBG2/FES/NUMA1/NDEL1/TPX2/SLAIN2/GIT1/EML2/RANGRF/PRUNE1/RAC1/HSPA1A/CAMSAP1/ABL1/PAK1/CLASP1/GOLGA2/CKAP5/DCTN1/SNCA
BP	GO:0098656	anion transmembrane transport	142	-0.39	-1.42	0.01251294	0.04279853	0.02653663	2571	tags=30%, list=22%, signal=24%	SLC16A1/PTAFR/SLC46A1/PCYOX1/CLN3/SLC24A4/SLC20A1/SLC25A11/RIPK1/SLC25A1/MFSD12/SLC9A7/SLC43A2/SLC7A1/SLC25A42/SLC37A2/SLC19A1/ABCC1/TSPO2/SLC27A1/ARL6IP1/SLC35C2/SLC5A6/ANO6/SLC9A1/SLC25A38/LRRC8A/SLC7A8/SLC1A5/SLC1A4/SLC6A20/VDAC1/ABCC4/SLC7A5/SLC3A2/SLC6A9/SLC24A3/SLC4A1/ANKH/SLC43A1/PER2/SLC6A8/SLC38A5
BP	GO:0051781	positive regulation of cell division	47	-0.51	-1.58	0.01268017	0.04327584	0.02683258	2108	tags=30%, list=18%, signal=25%	IL1B/PPBP/SVIL/PKP4/POLDIP2/TGFB1/KIF3B/LBH/IGF2/PDGFC/RHOA/YBX1/TAL1/PDGFA
BP	GO:0050728	negative regulation of inflammatory response	96	-0.42	-1.45	0.01278946	0.04357402	0.02701746	2642	tags=36%, list=23%, signal=28%	FGR/MAPK14/RHBDF2/MACIR/FOXP3/SYT11/MAPK7/NR1D1/ZFP36/HLA-DRB1/FYN/CNR2/PGLYRP1/GSTP1/SMAD3/PTPRC/KLF4/NLRX1/NR1H3/GIT1/TNFRSF1B/CCN3/LYN/SRC/PPARA/BCR/SYK/NFKB1/SIGLEC10/MFHAS1/KRT1/GRN/SHARPIN/GPX1/FURIN
BP	GO:0071248	cellular response to metal ion	112	-0.41	-1.45	0.01289078	0.04388162	0.02720818	2825	tags=42%, list=24%, signal=32%	NEK7/CEBPA/SLC40A1/FOSB/ATP13A2/CPNE2/ATP7A/AKT1/SYT11/JUN/TRPM2/CYBB/NCF1/DLG4/CPNE3/TSPO/SLC25A23/CPNE1/P2RX4/HSPA5/EDN1/SLC41A1/MMP9/JUND/SMPD1/HVCN1/ALAD/PARP1/ALOX15/AQP1/MTF1/MT2A/NCSTN/CPNE5/CALR/DAXX/SLFN14/FOS/FBP1/BMP6/MCOLN1/IQGAP1/SNCA/MAPK3/SMPD3/DMTN/ADD1
BP	GO:0042475	odontogenesis of dentin-containing tooth	36	-0.56	-1.61	0.01313164	0.04451098	0.02759841	1522	tags=33%, list=13%, signal=29%	ACVR2B/BCL11B/TRAF6/BCL2L11/PPARA/FAM20C/CTNNA1/STIM1/BSG/NF2/RODGI/SMPD3
BP	GO:0043903	regulation of biological process involved in symbiotic interaction	38	-0.53	-1.57	0.01313164	0.04451098	0.02759841	3186	tags=50%, list=27%, signal=37%	TRIM5/TRIM62/F2RL1/FUCA2/TRIM21/MID2/EXOC7/HLA-DRB1/CD74/TRIM26/IFITM3/SNX3/TRIM10/IFITM2/LTF/LGALS9/TRIM11/GSN/TRIM14
BP	GO:1903078	positive regulation of protein localization to plasma membrane	40	-0.53	-1.57	0.01316414	0.04452292	0.02760582	1363	tags=25%, list=12%, signal=22%	RANGRF/EZR/STX4/ZDHHC5/SQSTM1/LRP1/PPP1R9B/SPTBN1/ATP2B4/RHOG

BP	GO:0043124	negative regulation of I-kappaB kinase/NF-kappaB signaling	40	-0.53	-1.57	0.01316414	0.04452292	0.02760582	2067	tags=35%, list=18%, signal=29%	RIPK1/USP10/GSTP1/OTUD7B/NLRX1/PYCARD/CACTIN/ABL1/OPTN/RHOA/TLE1/RIOK3/TNIP1/PPM1A
BP	GO:0033500	carbohydrate homeostasis	142	-0.39	-1.42	0.01328423	0.0448371	0.02780061	1609	tags=23%, list=14%, signal=20%	PDK3/SOX4/FOXK2/CRY2/PRKACA/SMARCA4/MEN1/STX4/UCP2/SESN2/BACE2/BAD/ADIPOR2/LRRC8A/PPP3CA/PIH1D1/STAT3/PDK2/SESN3/SIDT2/HK1/RAB11B/FKBP1B/INPP5K/TENT4B/STK11/PLSCR3/ADIPOR1/TCF7L2/GPI/SLC29A1/GPX1/FOXO3
BP	GO:0009749	response to glucose	111	-0.41	-1.47	0.01377975	0.04611809	0.02859487	1609	tags=23%, list=14%, signal=20%	PDK3/SOX4/SREBF1/ACVR2B/PRKACA/SMARCA4/MEN1/STX4/UCP2/SESN2/PTK2B/BAD/PFKL/PPP3CA/PIH1D1/SIDT2/RHOA/RAB11B/FKBP1B/GLUL/TXNIP/THBS1/TCF7L2/SLC29A1/GPX1/FOXO3
BP	GO:0010821	regulation of mitochondrion organization	127	-0.39	-1.40	0.01377975	0.04611809	0.02859487	1741	tags=25%, list=15%, signal=22%	CHCHD10/SLC35F6/FZD5/STOML2/MMP9/BBC3/SREBF1/GSK3B/GSK3A/PYCARD/VPS13D/HIP1R/PINK1/HSPA1A/BAD/BCL2L11/INF2/FIS1/VDAC1/USP36/ARRB2/LMNA/BCL2L1/PLSCR3/HUWE1/SQSTM1/BNIP3L/FBXO7/GPX1/CTTN/HTT/CLU
BP	GO:0051046	regulation of secretion	351	-0.33	-1.31	0.01380815	0.04614885	0.02861395	2735	tags=28%, list=23%, signal=22%	TTN/SDCBP/ATP13A2/PDPK1/GABBR1/FGR/SLC16A1/RAB11FIP5/PTAFR/RAB8A/RHBDF2/PIM3/FBXL20/POMC/SYT11/NSF/ABCB11/PTPN11/NOTCH1/TRPM2/NR1D1/ATP2A2/TSG101/OAS2/HLA-DRB1/CDK5/IL1B/SPI1/P2RX4/VPS4B/PARD6A/FES/NR1H2/ITGAM/HGS/EDN1/CD74/CPT1A/GRK2/SOX4/DTNBP1/HYAL3/SREBF1/GSK3B/CRY2/NR1H3/TGFB1/ATP9A/FRMD4A/GIT1/TNFRSF1B/CCN3/EZR/STX4/UCP2/AQP1/HIP1R/LYN/RAB7A/RAB11FIP1/PINK1/BAD/PFKL/SEPTIN1/CEACAM1/GNAI2/LRRC8A/SEPTIN4/PPP3CA/BCR/SYK/CLASP1/RAB27B/BMP6/MYO18A/SIDT2/GALR1/CALM3/LGALS9/FKBP1B/RAB3D/RAC2/SDC4/SYTL4/GLUL/PRKCB/RAB2B/TCF7L2/PER2/SNCA/LAMP1/P2RX1/GATA1/SMPD3/SCIN/PTPN23/WNK1/DAB2
MF	GO:0005080	protein kinase C binding	43	-0.50	-1.53	0.0139139	0.0464108	0.02877637	2364	tags=40%, list=20%, signal=32%	HDAC5/PKN1/PRKD2/FEZ1/PARD6A/PARD6B/TOP2A/YWHAG/PLEK/SRC/ABL1/FLNA/CAVIN2/HSPB1/SDC4/SQSTM1/PRKCB
BP	GO:0033673	negative regulation of kinase activity	169	-0.38	-1.41	0.01390833	0.0464108	0.02877637	2856	tags=38%, list=24%, signal=29%	SLC8A1/PRKAR1B/TESK1/CEBPA/TSC2/PDPK1/SH2B3/GNAQ/SORL1/PKIG/AKT1/AIDA/CBLB/MLLT1/GADD45B/CDKN2D/TSG101/CHP1/PKN1/SH3BP5L/PIP4K2C/SPRED1/HYAL2/CDK5RAP2/IL1B/PTPN6/GSTP1/TRIB1/PTPRC/PIK3CB/CHMP6/SMPD1/DTNBP1/PYCARD/YWHAG/MEN1/MVP/CORO1C/LYN/ADAR/CDKN1C/CEACAM1/RUBCN/SFRP2/KAT2B/ABL1/WARS1/PRKAR2A/PTPRJ/INPP5K/HSPB1/NF2/HEXIM2/PLEC/CSK/PTPN1/UBASH3B/SNCA/PIP4K2A/FBXO7/CDKN1A/CBL/PAK2/PRKAR2B
BP	GO:0010977	negative regulation of neuron projection development	73	-0.46	-1.51	0.01403434	0.04673412	0.02897684	2241	tags=30%, list=19%, signal=25%	EPHA4/HES1/CDK5/CERS2/PRAG1/ITM2C/PMP22/KIAA0319/SEMA4D/DTNBP1/VIM/BCL11A/MBP/SEMA4A/NRP1/SYNGAP1/RHOA/FLNA/DNM3/GAK/CARM1/DAB2
MF	GO:0008514	organic anion transmembrane transporter activity	106	-0.41	-1.45	0.01405091	0.04673575	0.02897785	2245	tags=27%, list=19%, signal=22%	SERINC5/SLC25A11/SLC25A23/SLC25A1/MFSD12/SLC33A1/SLC7A1/SLC25A42/SLC19A1/ABCC1/SLC27A1/MFSD2A/SLC5A6/SLC25A38/LRRC8A/SLC7A8/SLC1A5/SLC1A4/SLC6A20/ABCG2/ABCC4/SLC2A1/SLC7A5/SLC3A2/SLC6A9/SLC4A1/SLC29A1/SLC6A8/SLC38A5
BP	GO:0001508	action potential	62	-0.47	-1.49	0.01447389	0.04803689	0.0297846	2606	tags=35%, list=22%, signal=28%	JUP/GNAQ/KCNH2/ATP1A1/CLN3/ATP2A2/ATP1B1/P2RX4/CNR2/GNA11/SNTA1/KCNJ2/RANGRF/NEDD4L/GNA15/SLC9A1/YWHAH/CALM3/FLNA/FKBP1B/P2RX1/SCN1B

BP	GO:0072507	divalent inorganic cation homeostasis	284	-0.33	-1.31	0.01466506	0.04850937	0.03007756	2609	tags=28%, list=22%, signal=22%	PKD1/ATP2A3/CORO1A/SELENOK/PTGIR/SWAP70/DISC1/KNG1/TRPM2/BAK1/CLN3/TRPC3/ATP2A2/DLG4/SLC24A4/CCR1/CDK5/FYN/SLC25A23/ATP1B1/P2RX4/PTPN6/ATP13A3/ITPR2/TMC8/LPAR4/SMAD3/MICU1/PTPRC/EDN1/PIK3CB/STOML2/SLC41A1/CXCR2/BCAP31/PRKACA/CXCR1/SNX10/CNNM3/SELENON/FPR3/CHERP/LYN/SPPL3/PTK2B/GNA15/MT2A/SLC39A4/PML/RASA3/CALR/ATP13A1/FIS1/JAK3/ABL1/FKBP1A/STIM1/ANXA6/XK/GALR1/CALM3/FKBP1B/MCOLN1/SLC24A3/GP1BA/PRKCB/GP9/HRH4/PIK3CG/UBASH3B/ATP2B4/SNCA/P2RX1/GATA1/GRINA/HTT/KEL/TGM2/DIAPH1/TBXA2R
BP	GO:0046854	phosphatidylinositol phosphate biosynthetic process	60	-0.47	-1.51	0.01477113	0.04871223	0.03020334	2923	tags=35%, list=25%, signal=26%	INPP1/IMPA2/ATM/PIP5KL1/PIK3CA/PIP4K2C/PI4K2A/SOCS7/SYNJ1/PI4KB/PIK3CB/PIK3R3/ITPKC/EFR3A/PIK3CD/TTC7B/PIP5K1B/IP6K1/TTC7A/PIK3CG/PIP4K2A
BP	GO:0051702	biological process involved in interaction with symbiont	69	-0.45	-1.49	0.01474442	0.04871223	0.03020334	1501	tags=20%, list=13%, signal=18%	STOM/NAPEPLD/DCD/SMARCA4/IFI27/DDB1/LTF/TBC1D20/INPP5K/CFL1/IGF2R/SP1/PF4/GPX1
BP	GO:0072073	kidney epithelium development	54	-0.49	-1.54	0.01479494	0.04873627	0.03021824	2666	tags=44%, list=23%, signal=34%	PECAM1/EXT1/PKD1/SMAD5/NOTCH1/EPHA4/HES1/MAGI2/BASP1/LZTS2/SMAD3/FMN1/MAGED1/CTNNBIP1/MYO1E/ILK/RARA/DCHS1/GREM1/NOTCH2/KANK2/SDC4/IQGAP1/PBX1
CC	GO:0080008	Cul4-RING E3 ubiquitin ligase complex	30	-0.57	-1.59	0.01487744	0.04888652	0.03031141	1361	tags=33%, list=12%, signal=30%	DET1/WDTC1/DDA1/DCAF1/DDB1/DCAF7/DCAF6/DCAF12/DCAF11/CUL4A
CC	GO:0071339	MLL1 complex	30	-0.57	-1.59	0.01487744	0.04888652	0.03031141	1702	tags=47%, list=15%, signal=40%	TAF1/TAF6/WDR5/ASH2L/MEN1/KANSL1/TAF4/RUVBL1/PELP1/MAX/MCRS1/KMT2A/CHD8/HCFC1
BP	GO:0071364	cellular response to epidermal growth factor stimulus	30	-0.57	-1.59	0.01487744	0.04888652	0.03031141	2938	tags=47%, list=25%, signal=35%	SYAP1/ZFP36L1/PDPK1/AKT1/PTPN11/ZFP36/ZFP36L2/BAIAP2/PTPN12/INPP5K/PPP1R9B/IQGAP1/MED1/DAB2
BP	GO:0045806	negative regulation of endocytosis	35	-0.54	-1.54	0.01490409	0.04893367	0.03034064	3027	tags=46%, list=26%, signal=34%	LRSAM1/ANKRD13A/ATXN2/SDCBP/SYT11/DLG4/SNX33/LGALS3/NR1H2/PACSIN1/NEU3/NR1H3/RAC1/RUBCN/RIN3/ITGB3
BP	GO:0050767	regulation of neurogenesis	208	-0.36	-1.37	0.01516472	0.04970727	0.03082029	2605	tags=33%, list=22%, signal=26%	CAPRIN1/ULK1/SORL1/DIP2B/PLXNC1/DISC1/PLXNA4/ADNP/NOTCH1/NR1D1/EZH2/HOXB3/TSPO/MAP3K13/EPHA4/HES1/CDK5/ASPM/IL1B/CERS2/MACF1/SMARCD3/MAN2A1/CUL7/SYNJ1/KIAA0319/TIAM1/DIXDC1/NDEL1/SEMA4D/CTDSP1/KDM4A/BAIAP2/CYFIP1/TGFB1/ASCL2/TNFRSF1B/SKI/PRUNE1/IST1/LYN/PLXNB2/CDKL5/MBP/PLXND1/TRIM32/FBXO31/HOOK3/EIF4G2/STK25/OLIG2/PLXNA1/PPP3CA/CTNNA1/SEMA4A/NRP1/YWHAH/SYNGAP1/E2F1/NF2/TRIM11/STK11/GJC2/PER2/CUX1/NEURL1/RNF10/TGM2
BP	GO:0048880	sensory system development	171	-0.36	-1.36	0.01523787	0.04986482	0.03091799	2247	tags=27%, list=19%, signal=23%	PRDM1/SMG9/SPRED1/CASP2/MITF/SMARCD3/MAN2A1/ITGAM/ARHGAP35/SMAD3/PHACTR4/FZD5/KLF4/DTNBP1/VIM/ACVR2B/SMARCA4/RPGRIP1L/SIPA1L3/BCL11B/SKI/TGIF2/TWSG1/MFSD2A/YY1/RARA/GNB1/CDKN1C/MAX/STAT3/NRP1/BLOC1S3/C1QA/BMP6/NOTCH2/HIPK1/TBC1D20/HIPK2/NF2/FZR1/MFN2/MEIS1/FKBP8/ATP2B4/TMOD1/MED1/PBX1

BP	GO:0150063	visual system development	171	-0.36	-1.36	0.01523787	0.04986482	0.03091799	2247	tags=27%, list=19%, signal=23%	PRDM1/SMG9/SPRED1/CASP2/MITF/SMARCD3/MAN2A1/ITGAM/ARHGAP35/SMAD3/PHACTR4/FZD5/KLF4/DTNBP1/VIM/ACVR2B/SMARCA4/RPGRIP1L/SIPA1L3/BCL11B/SKI/TGIF2/TWSG1/MFSD2A/YY1/RARA/GNB1/CDKN1C/MAX/STAT3/NRP1/BLOC1S3/C1QA/BMP6/NOTCH2/HIPK1/TBC1D20/HIPK2/NF2/FZR1/MFN2/MEIS1/FKBP8/ATP2B4/TMOD1/MED1/PBX1
BP	GO:0007044	cell-substrate junction assembly	61	-0.74	-2.36	1.00E-10	4.69E-09	2.91E-09	2019	tags=56%, list=17%, signal=46%	ROCK2/MACF1/TESK2/RCC2/SMAD3/FMN1/LIMS1/POLDIP2/ACTG1/TRIP6/DAPK3/CORO1C/BCAS3/RAC1/PTK2B/SLC9A1/SRC/GREM1/TAOK2/NRP1/BCR/ABL1/CLASP1/RHOA/VCL/ARHGAP6/PTPRJ/SDC4/PLEC/THBS1/ACTN1/CTTN/DMTN/TLN1
BP	GO:0150115	cell-substrate junction organization	67	-0.71	-2.31	1.00E-10	4.69E-09	2.91E-09	2048	tags=54%, list=17%, signal=45%	MAPRE2/ROCK2/MACF1/TESK2/RCC2/SMAD3/FMN1/LIMS1/POLDIP2/ACTG1/TRIP6/DAPK3/CORO1C/BCAS3/RAC1/PTK2B/SLC9A1/SRC/GREM1/IQSEC1/TAOK2/NRP1/BCR/ABL1/CLASP1/RHOA/VCL/ARHGAP6/PTPRJ/SDC4/PLEC/THBS1/ACTN1/CTTN/DMTN/TLN1
BP	GO:0030168	platelet activation	94	-0.68	-2.36	1.00E-10	4.69E-09	2.91E-09	1743	tags=43%, list=15%, signal=37%	PIK3CB/DGKZ/DGKD/ACTG1/TEC/GNAS/PLEK/LYN/ILK/CD9/SRC/CEACAM1/VAV1/ACTB/CSRP1/SYK/BLOC1S3/MPL/GP6/VCL/FLNA/FERMT3/HSPB1/GP1BA/ALOX12/PF4V1/GP9/PIK3CG/UBASH3B/SELP/P2RX1/GATA1/PF4/TREML1/MYL9/MYH9/ITGB3/PDGFA/DMTN/TLN1
BP	GO:0007599	hemostasis	152	-0.65	-2.40	1.00E-10	4.69E-09	2.91E-09	1756	tags=38%, list=15%, signal=32%	EDN1/PIK3CB/DGKZ/DGKD/DTNBP1/ACTG1/TEC/GNAS/PLEK/ANO6/LYN/ILK/F13A1/SERPING1/CD9/PAPSS2/SRC/CEACAM1/VAV1/ACTB/CSRP1/SYK/BLOC1S3/MPL/GP6/VCL/WAS/FLNA/FERMT3/ITPK1/HSPB1/KRT1/GNA12/PABPC4/SLC4A1/GP1BA/ALOX12/PF4V1/THBS1/GP9/PIK3CG/GPI/NFE2/UBASH3B/SELP/P2RX1/GATA1/PF4/TREML1/MYL9/MPIG6B/MYH9/ITGB3/PDGFA/DMTN/TLN1/TBXA2R
BP	GO:0007596	blood coagulation	148	-0.65	-2.39	1.00E-10	4.69E-09	2.91E-09	1756	tags=37%, list=15%, signal=32%	EDN1/PIK3CB/DGKZ/DGKD/DTNBP1/ACTG1/TEC/GNAS/PLEK/ANO6/LYN/ILK/F13A1/SERPING1/CD9/PAPSS2/SRC/CEACAM1/VAV1/ACTB/CSRP1/SYK/BLOC1S3/MPL/GP6/VCL/WAS/FLNA/FERMT3/ITPK1/HSPB1/KRT1/GNA12/PABPC4/SLC4A1/GP1BA/ALOX12/PF4V1/THBS1/GP9/PIK3CG/UBASH3B/SELP/P2RX1/GATA1/PF4/TREML1/MYL9/MPIG6B/MYH9/ITGB3/PDGFA/DMTN/TLN1/TBXA2R
BP	GO:0050817	coagulation	149	-0.65	-2.38	1.00E-10	4.69E-09	2.91E-09	1756	tags=37%, list=15%, signal=32%	EDN1/PIK3CB/DGKZ/DGKD/DTNBP1/ACTG1/TEC/GNAS/PLEK/ANO6/LYN/ILK/F13A1/SERPING1/CD9/PAPSS2/SRC/CEACAM1/VAV1/ACTB/CSRP1/SYK/BLOC1S3/MPL/GP6/VCL/WAS/FLNA/FERMT3/ITPK1/HSPB1/KRT1/GNA12/PABPC4/SLC4A1/GP1BA/ALOX12/PF4V1/THBS1/GP9/PIK3CG/UBASH3B/SELP/P2RX1/GATA1/PF4/TREML1/MYL9/MPIG6B/MYH9/ITGB3/PDGFA/DMTN/TLN1/TBXA2R
BP	GO:0050878	regulation of body fluid levels	223	-0.63	-2.40	1.00E-10	4.69E-09	2.91E-09	1796	tags=33%, list=15%, signal=29%	LSR/SLC29A2/EDN1/PIK3CB/DGKZ/DGKD/DTNBP1/PRKACA/NR1H3/COPA/ACTG1/TEC/GNAS/PLEK/ANO6/AQP1/LYN/ILK/F13A1/SERPING1/CD9/PAPSS2/SRC/CEACAM1/USF2/GNAI2/VAV1/ACTB/CSRP1/SYK/BLOC1S3/MPL/GP6/VCL/WAS/FLNA/FERMT3/INPP5K/ITPK1/HSPB1/STAT5A/KRT1/GNA12/PLEC/PABPC4/SLC4A1/GP1BA/ALOX12/PF4V1/THBS1/GP9/PIK3CG/GPI/NFE2/UBASH3B/SELP/NEURL1/P2RX1/GATA1/SLC29A1/PF4/TREML1/MYL9/MPIG6B/MED1/MLLT6/MYH9/ITGB3/PDGFA/DMTN/NCOR2/TLN1/WNK1/TBXA2R
CC	GO:0005938	cell cortex	212	-0.57	-2.19	1.00E-10	4.69E-09	2.91E-09	2151	tags=42%, list=18%, signal=35%	FMNL1/SPIRE2/EXOC4/PARD6A/ITPR2/CTNND1/MYADM/EPB41L2/PARD6B/NUMA1/FRY/GPSM1/SHROOM1/ENO2/TRAK1/RHOBTB1/CTBP1/RHOF/PPP1R9A/SEPTIN11/NEDD9/EZR/CLIC5/MYO1E/MYZAP/CORO1C/TRAF6/WDR1/HIP1R/DCTN4/RAC1/PTK2B/FLNB/EXOC3/HCLS1/MYO9B/DYNC1H1/SEPTIN1/SEPTIN4/ARHGAP32/ACTB/ACTR1A/CAP1/PXN/NRBP1/PARD3/CLASP1/STIM1/CALD1/SLC2A1/CLTB/RHOA/PVALB/FLNA/RAC2/LASP1/NF2/MPP1/DCTN1/GSN/SLC4A1/EPB42/CLDN5/PPP1R9B/PRKCB/SPTBN1/AKT2/EPB41/AKAP13/TRIP10/IQGAP1/SPTA1/COTL1/SPTB/SNCA/ENO1/GYPC/RHOG/TMOD1/SCIN/ACTN4/CAPZB/ACTN1/MYL9/CTTN/MYH9/GUCY1B1/DMTN

BP	GO:0001667	ameboidal-type cell migration	254	-0.55	-2.15	1.00E-10	4.69E-09	2.91E-09	2617	tags=41%, list=22%, signal=33%	ZEB2/CORO1B/JUP/TMEM201/MACIR/AKT1/PIK3CA/JUN/PTPN11/NOTCH1/CLN3/EFNB1/YTHDF3/HDAC5/SGPL1/INSL3/PKN1/PFN1/MECP2/PRKD2/SPRED1/HYAL2/MTA2/P2RX4/MAPRE2/VASH1/ROCK2/MACF1/ITGB7/TIMP1/SRGAP2/RCC2/MAP3K3/SCARB1/ACVR1B/EDN1/PIK3CB/LGALS12/PHACTR4/MMP9/EPPK1/PIK3R3/KLF4/SEMA4D/DNAJA4/FOXP1/PRR5L/TGFB1/SVBP/ETS1/AMOT/CCN3/CORO1C/BCAS3/ILK/SASH1/RAC1/PTK2B/PLXND1/RRAS/SRC/PML/GREM1/CEACAM1/IQSEC1/CALR/PIK3CD/PTP4A3/SEMA4A/NRP1/CAP1/PXN/ABL1/CLASP1/ANXA6/RHOA/ACTA2/KANK2/HSPB1/STAT5A/SDC4/GNA12/PLEC/ADIPOR1/GFUS/GLUL/THBS1/PIK3CG/IQGAP1/SP1/GPI/GRN/ATP2B4/GPX1/PDLIM1/PTPN23/MYH9/ITGB3/TNS1/SPARC/WASF2/SH3BP1/MAP2K3/DMTN/TBXA2R
CC	GO:0015629	actin cytoskeleton	333	-0.55	-2.19	1.00E-10	4.69E-09	2.91E-09	2013	tags=38%, list=17%, signal=33%	MACF1/SH3PXD2A/LCP1/MYADM/DIAPH2/SVIL/EPB41L2/PARVG/PDLIM7/RCSD1/ARHGAP35/FMN1/KEAP1/SHROOM1/ARPC5/STOML2/LIMA1/PPP1R9A/SEPTIN11/TPM3/BAIAP2/ACTG1/TRIP6/ZNF185/PSTPIP2/SPECC1L/ASAP1/AMOT/EZR/CLIC5/MYO1E/SIPA1L3/DAPK1/CDC42EP4/MYZAP/CORO1C/WDR1/ALDOA/TLNRD1/TPM1/CDC42BPB/ZYX/DCTN4/PTPN12/ILK/RAC1/JAM3/RARA/FLNB/SH3GL1/SIPA1L1/MARK2/HCLS1/MYO9B/SRC/GAS7/ARPC1B/ARHGAP32/FLII/TAOK2/ACTB/FHL3/CTNNA1/CNN2/KAT2B/GABARAP/RFLNB/ACTR1A/CAP1/PXN/ABL1/PAK1/ABLIM3/CALD1/MYO18A/SLC2A1/HNRNPK/ACTL10/VCL/ARPC4/ARHGAP6/ACTA2/PVALB/WAS/FLNA/ARHGAP21/FERMT3/RAC2/MYLK/LASP1/NF2/MYL4/SPECC1/AFAP1/GSN/CFL1/WIPF1/CLDN5/DYNLL2/PPP1R9B/PRKCB/SPTBN1/AKAP13/MTPN/IQGAP1/SPTA1/COTL1/SPTB/SNCA/PEAK3/VASP/TMOD1/PARVB/ESPN/SCIN/ACTN4/CAPZB/ACTN1/PDLIM1/MYL9/CTTN/MYH9/GAS2L1/WASF2/TMEM63B/DMTN/ADD1/DIAPH1
BP	GO:0042060	wound healing	273	-0.54	-2.13	1.00E-10	4.69E-09	2.91E-09	1817	tags=32%, list=15%, signal=28%	ARHGAP35/SMAD3/SCARB1/EDN1/PIK3CB/CHMP6/DGKZ/EPPK1/CHMP4B/SMPD1/DGKD/DTNBP1/TGFB1/ACTG1/TEC/GNAS/PLEK/MAP3K5/TPM1/ALOX15/ANO6/LYN/ILK/F13A1/SERPING1/CD9/DYSF/PAPSS2/SRC/CHMP1A/ADIPOR2/CEACAM1/MRTFA/PPARA/VAV1/PPP3CA/ACTB/MYOF/CNN2/CSRP1/SYK/PAK1/CLASP1/BLOC1S3/ANXA6/MPL/GP6/RHOA/VCL/NOTCH2/ACTA2/WAS/FLNA/FERMT3/MYLK/ITPK1/HSPB1/SDC4/KRT1/GNA12/PLEC/SYTL4/PABPC4/SLC4A1/GP1BA/ALOX12/PF4V1/THBS1/GP9/PIK3CG/CD151/UBASH3B/SELP/P2RX1/GATA1/CDKN1A/PF4/TREML1/GPX1/MYL9/MPIG6B/MYH9/ITGB3/PDGFA/DMTN/TLN1/TBXA2R
MF	GO:0003779	actin binding	291	-0.54	-2.15	1.00E-10	4.69E-09	2.91E-09	2173	tags=38%, list=19%, signal=32%	MYO1F/ABI3/MYO15A/FMNL1/SSH1/SPIRE2/VASH1/MSRB2/MACF1/LCP1/SNTA1/DIAPH2/SVIL/FMNL3/SSH2/EPB41L2/PARVG/PDLIM7/RCSD1/SYNE1/FMN1/SNTB1/SHROOM1/DIXDC1/PHACTR4/ARPC5/MAP1S/LIMA1/PPP1R9A/TPM3/CYFIP1/ZNF185/PSTPIP2/ACTR3B/ENC1/EZR/MYO1E/INPPL1/CORO1C/WDR1/ALDOA/TLNRD1/TPM1/HIP1/HIP1R/FLNB/S100A4/HCLS1/MYO9B/CEACAM1/MRTFA/GAS7/ARPC1B/INF2/FLII/FHL3/CTNNA1/CTNNAL1/CNN2/MAP1A/YWHAH/CAP1/ABL1/FGD4/ABLIM3/ANXA6/SSH3/CALD1/MYO18A/VCL/ARPC4/WAS/FLNA/PPP1R18/MYLK/LASP1/NF2/MYL4/AFAP1/GSN/PLEC/CFL1/WIPF1/PPP1R9B/SPTBN1/EPB41/IQGAP1/SPTA1/MSN/COTL1/SPTB/SNCA/VASP/TMOD1/PARVB/MICAL2/WASF3/ESPN/SCIN/ACTN4/CAPZB/ACTN1/PDLIM1/CTTN/MYH9/TNS1/GAS2L1/WASF2/DMTN/TLN1/ADD1/DIAPH1

BP	GO:0032956	regulation of actin cytoskeleton organization	244	-0.54	-2.12	1.00E-10	4.69E-09	2.91E-09	2488	tags=41%, list=21%, signal=33%	CORO1B/EPS8/CORO1A/SWAP70/PIK3CA/TMOD3/SERPINF2/TRPM2/DSTN/SPTAN1/TRIOBP/WASHC5/ARHGAP18/PFN1/CDK5/ABI3/SSH1/ARAP1/CSF1R/ROCK2/MYADM/SVIL/FES/SSH2/ARHGAP35/SMAD3/FMN1/EDN1/DIXDC1/ARPC5/LIMA1/RHOBTB1/RHOF/GRB2/BAIAP2/SORBS3/PYCARD/CYFIP1/ACTG1/TAOK1/ARHGAP17/DAPK3/CDC42EP4/PLEK/WDR1/TPM1/ALOX15/HIP1R/BCAS3/RAC1/JAM3/PTK2B/ARF1/SLC9A1/HCLS1/ARPC1B/FLII/TAOK2/NRP1/PXN/ABL1/PAK1/CLASP1/ARHGEF18/SSH3/RHOA/NOTCH2/ARPC4/ARHGAP6/ARHGDIA/WAS/FLNA/RAC2/INPP5K/NF2/SDC4/CYFIP2/GSN/LRP1/SPTBN1/AKAP13/MTPN/IQGAP1/SPTA1/COTL1/SPTB/PEAK3/VASP/RHOG/TMOD1/WASF3/SCIN/PAK2/CAPZB/CTTN/ESAM/PDGFA/WASF2/SH3BP1/DMTN/ADD1
BP	GO:0032970	regulation of actin filament-based process	262	-0.53	-2.09	1.00E-10	4.69E-09	2.91E-09	2610	tags=42%, list=22%, signal=33%	CORO1B/JUP/EPS8/CORO1A/SWAP70/PIK3CA/TMOD3/SERPINF2/TRPM2/DSTN/SPTAN1/ATP2A2/TRIOBP/WASHC5/ARHGAP18/PFN1/CDK5/ABI3/SSH1/ARAP1/CSF1R/ROCK2/MYADM/SVIL/FES/SSH2/ARHGAP35/SMAD3/FMN1/PDE4B/EDN1/DIXDC1/ARPC5/LIMA1/RHOBTB1/RHOF/KCNJ2/GRB2/BAIAP2/SORBS3/PYCARD/CYFIP1/ACTG1/TAOK1/RANGRF/ARHGAP17/DAPK3/CDC42EP4/PLEK/WDR1/TPM1/ALOX15/HIP1R/BCAS3/RAC1/JAM3/PTK2B/ARF1/SLC9A1/HCLS1/ARPC1B/FLII/TAOK2/NRP1/CNN2/PXN/ABL1/PAK1/CLASP1/ARHGEF18/SSH3/RHOA/NOTCH2/ARPC4/ARHGAP6/ACTA2/ARHGDIA/WAS/FLNA/RAC2/INPP5K/NF2/SDC4/CYFIP2/GSN/LRP1/SPTBN1/AKAP13/MTPN/IQGAP1/SPTA1/COTL1/SPTB/PEAK3/VASP/RHOG/TMOD1/WASF3/SCIN/PAK2/CAPZB/CTTN/MYH9/ESAM/PDGFA/WASF2/SH3BP1/DMTN/ADD1
BP	GO:0030036	actin cytoskeleton organization	484	-0.53	-2.21	1.00E-10	4.69E-09	2.91E-09	2488	tags=39%, list=21%, signal=32%	PIK3CA/TMOD3/SERPINF2/TPM4/TRPM2/SH3D21/DSTN/CLN3/SPTAN1/SPIRE1/AGAP2/TRIOBP/MSRB1/ARHGEF11/WASHC5/ARHGAP18/CORO7/PFN1/CDK5/MYO1F/FGD6/ABI3/MYO15A/FMNL1/SSH1/ARAP1/SPIRE2/SH3KBP1/CSF1R/NUAK2/MSRB2/ROCK2/LCP1/MYADM/ARHGAP26/DIAPH2/TESK2/PPARGC1B/SVIL/FES/FMNL3/SSH2/EPB41L2/PARVG/PDLIM7/PACSIN1/SHC1/ARRB1/ARHGAP35/SMAD3/FMN1/EDN1/SHROOM1/DIXDC1/PHACTR4/ARPC5/LIMA1/RHOBTB1/RHOF/PPP1R9A/DTNBP1/GRB2/TPM3/BAIAP2/NEDD9/SORBS3/PYCARD/CYFIP1/ACTG1/PSTPIP2/SPECC1L/TAOK1/PREX1/AMOT/EZR/ARHGAP17/MYO1E/DAPK3/INPPL1/CDC42EP4/PLEK/CORO1C/WDR1/ALDOA/TPM1/ALOX15/CDC42BPB/ZYX/HIP1/AQP1/HIP1R/BCAS3/RAC1/JAM3/PTK2B/ARF1/FLNB/SLC9A1/TRIM32/SIPA1L1/HCLS1/SRC/MRTFA/GAS7/ARPC1B/IQSEC1/INF2/CALR/FLII/TAOK2/ACTB/FHL3/CTNNA1/NRP1/CNN2/ARHGEF2/BCR/RFLNB/CAP1/CSRP1/PXN/ABL1/PAK1/FGD4/CLASP1/ARHGEF18/FRMD3/ABLIM3/SSH3/CALD1/PLEK2/MYO18A/RHOA/NOTCH2/ARPC4/MAD2L2/ARHGAP6/ARHGDIA/WAS/FLNA/RAC2/INPP5K/NF2/DOCK2/SDC4/SPECC1/CYFIP2/ITGB5/GSN/PLEC/CFL1/WIPF1/LRP1/PPP1R9B/SPTBN1/PTPN1/EPB41/AKAP13/TRIP10/MTPN/IQGAP1/SPTA1/COTL1/SPTB/PEAK3/VASP/RHOG/TMOD1/PARVB/MICAL2/WASF3/ESPN/SCIN/ACTN4/PAK2/CAPZB/ACTN1/PDLIM1/MYL9/CTTN/MYH9/ESAM/GAS2L1/PDGFA/WASF2/SH3BP1/DMTN/TLN1/ADD1/DIAPH1

BP	GO:0007015	actin filament organization	308	-0.53	-2.11	1.00E-10	4.69E-09	2.91E-09	2488	tags=40%, list=21%, signal=33%	PIK3CA/TMOD3/SERPINF2/TPM4/SH3D21/DSTN/SPTAN1/SPIRE1/TRIOBP/MSRB1/WASHC5/ARHGAP18/CORO7/PFN1/MYO1F/MYO15A/SSH1/ARAP1/SPIRE2/SH3KBP1/MSRB2/ROCK2/LCP1/MYADM/DIAPH2/PPARGC1B/SVIL/SSH2/PACSIN1/ARRB1/ARHGAP35/SMAD3/FMN1/SHROOM1/ARPC5/LIMA1/RHOBTB1/RHOF/PPP1R9A/GRB2/TPM3/BAIAP2/NEDD9/SORBS3/PYCARD/CYFIP1/ACTG1/PSTPIP2/PREX1/EZR/ARHGAP17/MYO1E/INPPL1/CDC42EP4/PLEK/CORO1C/WDR1/ALDOA/TPM1/ALOX15/ZYX/HIP1/HIP1R/RAC1/PTK2B/ARF1/SLC9A1/HCLS1/SRC/GAS7/ARPC1B/FLII/CTNNA1/NRP1/ARHGEF2/RFLNB/PXN/ABL1/PAK1/CLASP1/ARHGEF18/SSH3/CALD1/RHOA/ARPC4/MAD2L2/ARHGAP6/WAS/FLNA/RAC2/INPP5K/NF2/SDC4/CYFIP2/ITGB5/GSN/PLEC/CFL1/WIPF1/PPP1R9B/SPTBN1/MTPN/SPTA1/COTL1/SPTB/VASP/RHOG/TMOD1/MICAL2/WASF3/ESPN/SCIN/PAK2/CAPZB/ACTN1/PDLIM1/CTTN/ESAM/GAS2L1/WASF2/SH3BP1/DMTN/ADD1/DIAPH1
BP	GO:0030029	actin filament-based process	525	-0.53	-2.19	1.00E-10	4.69E-09	2.91E-09	2488	tags=39%, list=21%, signal=32%	PIK3CA/TMOD3/SERPINF2/TPM4/TRPM2/SH3D21/DSTN/SYNE2/ATP1A1/CLN3/SPTAN1/ATP2A2/SPIRE1/AGAP2/TRIOBP/MSRB1/ARHGEF11/WASHC5/ARHGAP18/CORO7/PFN1/CDK5/MYO1F/FGD6/ABI3/MYO15A/FMNL1/SSH1/ARAP1/SPIRE2/SH3KBP1/CSF1R/NUAK2/MSRB2/ROCK2/LCP1/MYADM/SNTA1/ARHGAP26/DIAPH2/TESK2/PPARGC1B/SVIL/FES/FMNL3/SRGAP2/SSH2/EPB41L2/PARVG/PDLIM7/PACSIN1/SHC1/ARRB1/ARHGAP35/SMAD3/FMN1/PDE4B/EDN1/SHROOM1/DIXDC1/PHACTR4/ARPC5/LIMA1/RHOBTB1/RHOF/PPP1R9A/KCNJ2/DTNBP1/GRB2/TPM3/BAIAP2/NEDD9/SORBS3/PYCARD/CYFIP1/ACTG1/PSTPIP2/SPECC1L/TAOK1/PREX1/RANGRF/AMOT/EZR/ARHGAP17/MYO1E/DAPK3/INPPL1/CDC42EP4/PLEK/CORO1C/WDR1/ALDOA/TPM1/SUN2/ALOX15/CDC42BPB/ZYX/HIP1/AQP1/HIP1R/BCAS3/NEDD4L/RAC1/JAM3/PTK2B/ARF1/FLNB/SLC9A1/TRIM32/SIPA1L1/HCLS1/MYO9B/SRC/MRTFA/GAS7/ARPC1B/IQSEC1/INF2/CALR/FLII/TAOK2/ACTB/FHL3/CTNNA1/NRP1/CNN2/ARHGEF2/BCR/RFLNB/CAP1/CSRP1/PXN/ABL1/PAK1/FGD4/CLASP1/ARHGEF18/FRMD3/ABLIM3/SSH3/CALD1/PLEK2/MYO18A/RHOA/NOTCH2/ARPC4/MAD2L2/ARHGAP6/ACTA2/ARHGDIA/WAS/FLNA/RAC2/INPP5K/NF2/DOCK2/SDC4/SPECC1/CYFIP2/ITGB5/GSN/PLEC/CFL1/WIPF1/LRP1/PPP1R9B/SPTBN1/PTPN1/EPB41/AKAP13/TRIP10/MTPN/IQGAP1/SPTA1/COTL1/SPTB/PEAK3/VASP/RHOG/TMOD1/PARVB/MICAL2/WASF3/ESPN/SCIN/ACTN4/PAK2/CAPZB/ACTN1/PDLIM1/MYL9/CTTN/MYH9/ESAM/GAS2L1/PDGFA/WASF2/SH3BP1/DMTN/TLN1/SCN1B/ADD1/DIAPH1
CC	GO:0005911	cell-cell junction	287	-0.51	-2.03	1.00E-10	4.69E-09	2.91E-09	2110	tags=34%, list=18%, signal=28%	ATP1B1/SH3KBP1/TBCD/FBF1/PARD6A/PTPN6/CTNND1/AJM1/MYADM/TNK2/NDRG1/PDLIM7/PKP4/PARD6B/FMN1/LSR/PMP22/FRMD4B/TIAM1/SHROOM1/FZD5/LIMS1/VSIG10/POLDIP2/EPPK1/KCNJ2/GRB2/UBN1/BAIAP2/ACTG1/FRMD4A/SPECC1L/RPGRIP1L/RANGRF/AMOT/CCN3/EZR/TJAP1/ARHGAP17/MYO1E/SIPA1L3/CDC42EP4/WDR1/CDC42BPB/ZYX/LYN/JAM3/DCHS1/SLC9A1/MTDH/CEACAM1/EIF4G2/VAV1/PPP3CA/ACTB/UBA1/PPP1CA/CTNNA1/MAP2K2/CNN2/ARHGEF2/YWHAH/TJP2/PXN/PAK1/KIFC3/PARD3/SLC2A1/PCDH1/RHOA/VCL/PTPRJ/WAS/FLNA/NIBAN2/NF2/MPP1/CLDN5/PPP1R9B/CSK/GJC2/IQGAP1/TSPAN33/MSN/VASP/PAK2/ACTN1/YBX3/PDLIM1/MYH9/ITGB3/ESAM/WASF2/SH3BP1/TLN1/SCN1B/ADD1

BP	GO:0009611	response to wounding	342	-0.51	-2.04	1.00E-10	4.69E-09	2.91E-09	2097	tags=32%, list=18%, signal=27%	P2RX4/PROS1/AK3/PTPN6/VASH1/CERS2/MACF1/VAV2/TIMP1/ZFP36L2/RIPOR1/ARHGAP35/SMAD3/SCARB1/EDN1/KIAA0319/PIK3CB/CHMP6/DGKZ/EPPK1/NDEL1/KLF4/CHMP4B/SMPD1/DGKD/DTNBP1/TGFB1/ACTG1/GIT1/TEC/GNAS/PLEK/MAP3K5/TPM1/ALOX15/ANO6/LYN/ILK/F13A1/SERPING1/RAC1/CD9/DYSF/PAPSS2/STK24/SRC/CHMP1A/ADIPOR2/CEACAM1/MRTFA/PPARA/VAV1/PPP3CA/MAX/ACTB/CTNNA1/MYOF/RANGAP1/CNN2/CSRP1/SYK/PAK1/CLASP1/BLOC1S3/ANXA6/SIGLEC10/MPL/GP6/RHOA/VCL/NOTCH2/ACTA2/WAS/FLNA/FKBP1B/FERMT3/MYLK/ITPK1/HSPB1/SDC4/KRT1/ABHD2/GNA12/PLEC/SYTL4/PABPC4/SLC4A1/GP1BA/ALOX12/PF4V1/THBS1/GP9/PIK3CG/CD151/GRN/UBASH3B/SELP/P2RX1/GATA1/CDKN1A/PF4/TREML1/GPX1/MYL9/MPIG6B/MYH9/ITGB3/PDGFA/DMTN/TLN1/TBXA2R
BP	GO:0001525	angiogenesis	296	-0.50	-1.99	1.00E-10	4.69E-09	2.91E-09	1848	tags=27%, list=16%, signal=23%	AGO1/MAP3K3/SHC1/RUNX1/HGS/EDN1/PIK3CB/ENG/FZD5/RBPJ/PIK3R3/KLF4/ACTG1/ETS1/AMOT/SPHK1/CCN3/JMJD8/AQP1/BCAS3/EPAS1/SASH1/JAM3/PTK2B/PLXND1/KLF2/MTDH/RRAS/PML/GREM1/ADIPOR2/SRPK2/CEACAM1/PIK3CD/SFRP2/PKM/STAT3/SEMA4A/NRP1/JAK1/SYK/LRG1/ABL1/FOXO4/STIM1/CALD1/WARS1/RNF213/EPN1/RHOA/NOTCH2/HIPK1/FLNA/BSG/HSPB1/FOXJ2/HIPK2/NIBAN2/KRT1/GLUL/CLDN5/PRKCB/THBS1/PIK3CG/MEIS1/SP1/GRN/ATP2B4/E2F2/PF4/GPX1/MED1/TAL1/MYH9/ITGB3/SPARC/PDGFA/WASF2/ITGA2B/TBXA2R
BP	GO:0034330	cell junction organization	354	-0.50	-2.02	1.00E-10	4.69E-09	2.91E-09	2241	tags=33%, list=19%, signal=28%	EPHA4/PFN1/MECP2/CDK5/ABI3/FYN/ABHD17C/CRKL/IL1B/SLITRK5/TBCD/DOCK10/FBF1/PARD6A/CSF1R/MAPRE2/ROCK2/CTNND1/MACF1/AJM1/SNTA1/TESK2/PIN1/ITGAM/PKP4/PARD6B/RCC2/SMAD3/FMN1/LSR/CAST/CHCHD10/FZD5/LIMS1/POLDIP2/SEMA4D/DTNBP1/BAIAP2/PRKACA/CYFIP1/TGFB1/ACTG1/TRIP6/NFIA/MESD/DAPK3/CORO1C/WDR1/BCAS3/PLXNB2/CDKL5/RAC1/JAM3/PTK2B/PLXND1/CD9/DCHS1/ARF1/SLC9A1/SIPA1L1/GRIPAP1/SRC/GREM1/IQSEC1/ADGRL1/TAOK2/ACTB/CTNNA1/SEMA4A/NRP1/BCR/VCP/TJP2/ABL1/KIFC3/PARD3/SYNGAP1/CLASP1/RAPGEF1/C1QA/BMP6/STAU1/RHOA/VCL/ARHGAP6/HIPK1/PTPRJ/FLNA/NF2/SDC4/DNM3/DCTN1/PLEC/CFL1/CLSTN1/CLDN5/CSK/THBS1/SNCA/LRFN3/CAMK1/EIF4G1/NEURL1/ZNF703/WASF3/TUBB/PAK2/ACTN1/PTPN23/CTTN/SHISA7/ESAM/WASF2/SH3BP1/DMTN/TLN1/ADD1
BP	GO:0048514	blood vessel morphogenesis	342	-0.50	-2.01	1.00E-10	4.69E-09	2.91E-09	2488	tags=33%, list=21%, signal=27%	PIK3CA/JUN/TIPARP/SERPINF2/MAPK7/NOTCH1/CYBB/BAK1/YWHAZ/HDAC5/SGPL1/HOXB3/PRDM1/HES1/BTG1/MECP2/PRKD2/SPRED1/ARID2/IL1B/SPI1/VASH1/ROCK2/VAV2/FMNL3/AGO1/MAP3K3/SHC1/CUL7/RUNX1/HGS/EDN1/PIK3CB/HIF1AN/ENG/FZD5/RBPJ/PIK3R3/KLF4/SOX4/TGFB1/ACTG1/ETS1/AMOT/SPHK1/CCN3/JMJD8/MYO1E/AQP1/BCAS3/EPAS1/SASH1/JAM3/PTK2B/PLXND1/KLF2/MTDH/RRAS/PML/GREM1/ADIPOR2/SRPK2/CEACAM1/PIK3CD/SFRP2/ZMIZ1/PKM/STAT3/SEMA4A/NRP1/JAK1/SYK/LRG1/ABL1/FOXO4/STIM1/CALD1/WARS1/RNF213/EPN1/RHOA/NOTCH2/HIPK1/FLNA/BSG/MYLK/HSPB1/FOXJ2/HIPK2/NIBAN2/KRT1/GLUL/CLDN5/LRP1/PRKCB/THBS1/PIK3CG/MEIS1/SP1/GRN/ATP2B4/E2F2/PF4/GPX1/NPRL3/MED1/TAL1/MYH9/ITGB3/SPARC/PDGFA/WASF2/ITGA2B/TBXA2R

BP	GO:0003013	circulatory system process	297	-0.50	-1.97	1.00E-10	4.69E-09	2.91E-09	2159	tags=31%, list=18%, signal=26%	FYN/THRA/ATP1B1/HRH2/P2RX4/GNA11/ROCK2/SNTA1/PDE2A/SLC7A1/ARHGAP35/SMAD3/PDE4B/SLC29A2/EDN1/SLC19A1/GRK2/ABCC1/KCNJ2/EHD3/SREBF1/PRKACA/GSK3A/TGFB1/CTNNBIP1/RANGRF/SLC27A1/MFSD2A/SLC5A6/TPM1/LNPEP/NEDD4L/EPAS1/SERPING1/KLF2/SLC9A1/SRC/CEACAM1/ABCC3/GNAI2/SLC7A8/PPARA/SLC1A5/PTP4A3/ATP1B2/MYOF/SLC1A4/BCR/SLC6A20/ABCG2/TJP2/ABCC4/ABL1/BMP6/SLC2A1/GAA/PLCB3/PDE5A/SLC7A5/RHOA/CALM3/ACTA2/PTPRJ/FLNA/GUCY1A1/FKBP1B/ECE1/MYL4/SLC6A9/SLC24A3/CYB5R3/GNA12/GSN/PLEC/CLDN5/LRP1/PLVAP/AKAP13/PIK3CG/PER2/ATP2B4/P2RX1/SLC29A1/PTGS1/GPX1/SLC38A5/GUCY1B1/KEL/MAP2K3/SCN1B/WNK1/TBXA2R
BP	GO:0071363	cellular response to growth factor stimulus	373	-0.49	-2.00	1.00E-10	4.69E-09	2.91E-09	2674	tags=39%, list=23%, signal=31%	ATP7A/FIBP/KIF16B/EXT1/ZEB2/CORO1B/MAPK14/SMAD5/SORL1/CORO1A/AKT1/PIK3CA/JUN/MAPK7/PTPN11/NOTCH1/EGR1/ZFP36/FLCN/CPNE3/EIF4A3/HES1/MAGI2/PRKD2/SPRED1/HYAL2/FYN/CRKL/IL1B/SPI1/FEZ1/RIPK1/HSPA5/SLC33A1/PDE2A/ZBTB7A/ZFP36L2/NR3C1/PIN1/NRROS/NUMA1/SHC1/SMAD3/ACVR1B/HGS/EDN1/PIK3CB/ENG/LIMS1/RBPJ/TGFB1I1/RAP1GAP/KLF4/BCL9L/ACVR2B/BAIAP2/CYFIP1/HSP90AB1/TGFB1/CD63/TNFRSF1B/PCSK6/SPHK1/SPINT2/MEN1/NDST1/SKI/EHD4/TWSG1/PARP1/ZYX/PTPN12/ILK/SHCBP1/C20orf27/HSPA1A/CHST11/CREBBP/SRC/SOX6/PML/GREM1/CDKN1C/ARNT/PIK3CD/SFRP2/PPARA/LTBP3/PTP4A3/FAM20C/FAM89B/ZMIZ1/STAT3/NRP1/USP9X/PXN/XIAP/LRG1/ABL1/FOS/RAB14/FKBP1A/RELA/E2F1/RAPGEF1/BMP6/ARRB2/NOTCH2/DNM2/MAPKAPK2/INPP5K/HSPB1/HIPK2/NIBAN2/STK11/CYFIP2/ITGB5/SORT1/CLDN5/PPP1R9B/PRKCB/PPP2R5B/THBS1/GLG1/PTPN1/IQGAP1/FKBP8/ATP2B4/ZNF703/MAPK3/TGFBRAP1/CBL/EHD1/SMPD3/PAK2/UBE2O/FOXO3/FURIN/MED1/PPM1A/ITGB3/PDGFA/LTBP1/MAP2K3/DAB2
BP	GO:0070848	response to growth factor	390	-0.49	-2.01	1.00E-10	4.69E-09	2.91E-09	2674	tags=39%, list=23%, signal=31%	ATP7A/FIBP/KIF16B/EXT1/ZEB2/CORO1B/MAPK14/SMAD5/SORL1/CORO1A/AKT1/PIK3CA/JUN/MAPK7/PTPN11/NOTCH1/EGR1/ZFP36/FLCN/CPNE3/EIF4A3/HES1/MAGI2/PRKD2/SPRED1/HYAL2/FYN/CRKL/IL1B/SPI1/FEZ1/RIPK1/HSPA5/ROCK2/SLC33A1/PDE2A/ZBTB7A/ZFP36L2/NR3C1/PIN1/NRROS/NUMA1/SHC1/SMAD3/ACVR1B/HGS/EDN1/PIK3CB/ENG/LIMS1/RBPJ/TGFB1I1/RAP1GAP/KLF4/BCL9L/ACVR2B/ZFHX3/BAIAP2/IER2/CYFIP1/HSP90AB1/TGFB1/CD63/TNFRSF1B/PCSK6/SPHK1/SPINT2/MEN1/NDST1/SKI/EHD4/TWSG1/PARP1/ZYX/PTPN12/ILK/SHCBP1/C20orf27/HSPA1A/CHST11/CREBBP/SRC/SOX6/PML/GREM1/CDKN1C/ARNT/PIK3CD/SFRP2/PPARA/LTBP3/PTP4A3/RUNX3/FAM20C/FAM89B/ZMIZ1/STAT3/NRP1/USP9X/PXN/XIAP/LRG1/ABL1/FOS/RAB14/FKBP1A/RELA/E2F1/RAPGEF1/BMP6/ARRB2/NOTCH2/DNM2/MAPKAPK2/INPP5K/HSPB1/HIPK2/NIBAN2/ELK1/STK11/CYFIP2/ITGB5/SORT1/CLDN5/PPP1R9B/PRKCB/PPP2R5B/THBS1/GLG1/PTPN1/IQGAP1/FKBP8/ATP2B4/ZNF703/MAPK3/TGFBRAP1/CBL/EHD1/SMPD3/PAK2/UBE2O/FOXO3/CDC34/FURIN/MED1/PPM1A/ITGB3/PDGFA/LTBP1/MAP2K3/DAB2

BP	GO:0007167	enzyme linked receptor protein signaling pathway	538	-0.49	-2.05	1.00E-10	4.69E-09	2.91E-09	2788	tags=41%, list=24%, signal=33%	FGF23/GAB2/MSX1/DOK1/SLC27A4/EPHB1/FRS2/SDCBP/TSC2/PDPK1/SH2B3/FIBP/FGR/KIF16B/EXT1/ZEB2/MAPK14/SMAD5/SORL1/RHBDF2/AKT1/SH3TC2/PTGIR/CBLB/PIK3CA/JUN/TIPARP/PTPN11/NOTCH1/NCF1/CD3E/EFNB1/EGR1/PDE6G/FLCN/MVB12B/SGPL1/FOXO1/TSG101/CPNE3/CNOT9/LMTK2/EPHA4/HES1/MAGI2/PIP4K2C/MAPKAPK3/PRKD2/SPRED1/DDR1/FYN/CRKL/IL1B/SPI1/CSF1R/RNF126/HSPA5/SLC33A1/VAV2/PTPN18/TNK2/ZBTB7A/FES/PIN1/NRROS/NUMA1/SHC1/SMAD3/ACVR1B/HGS/SH2B2/KIAA0319/NEU3/TIAM1/PIK3CB/CHMP6/ENG/SMARCC1/RBPJ/TGFB1I1/MMP9/ANKS1A/NDEL1/PIK3R3/SAMD10/DGKD/BCL9L/VIM/GRB2/SREBF1/ACVR2B/GSK3B/BAIAP2/NEDD9/GSK3A/CYFIP1/DOK3/HSP90AB1/TGFB1/GIT1/CD63/PCSK6/RBM14/ZNF106/CCN3/MYO1E/MEN1/NDST1/SKI/MVP/TGIF2/TWSG1/PARP1/ZYX/HIP1/LNPEP/HIP1R/LYN/PTPN12/RAB7A/ILK/LEPROT/NOMO3/RAC1/PPM1L/SHCBP1/PTK2B/IGF2/C20orf27/HSPA1A/CHST11/TRIO/CREBBP/SIPA1L1/SRC/PML/CCND3/GREM1/CDKN1C/ARNT/CEACAM1/RNF115/PIK3CD/SFRP2/GNAI2/SLA/PPARA/LTBP3/VAV1/PTP4A3/FAM20C/FAM89B/ZMIZ1/STAT3/NRP1/PDK2/USP9X/PDGFC/JAK1/SYK/PXN/JAK3/XIAP/LRG1/ABL1/PAK1/FOS/RAB14/FKBP1A/FOXO4/RAPGEF1/BMP6/ARRB2/REPS2/GP6/SESN3/NOTCH2/DNM2/PTPRJ/MAPKAPK2/NUP93/INPP5K/HSPB1/STAT5A/HIPK2/NIBAN2/STK11/CYFIP2/ITGB5/SORT1/ADIPOR1/TXNIP/CLDN5/IGF2R/LRP1/PRKCB/PPP2R5B/THBS1/GLG1/SPTBN1/AKT2/PTPN1/IQGAP1/FKBP8/UBASH3B/SNCA/NEURL1/ZNF703/PIP4K2A/MAPK3/EIF4EBP2/TGFBRAP1/CBL/SMPD3/PAK2/UBE2O/FURIN/TAL1/PPM1A/ITGB3/PDGFA/LTBP1/DAB2
BP	GO:0022603	regulation of anatomical structure morphogenesis	546	-0.49	-2.03	1.00E-10	4.69E-09	2.91E-09	2621	tags=37%, list=22%, signal=30%	DAAM1/CORO1B/JUP/CAPRIN1/ULK1/EPS8/PAK4/CORO1A/DIP2B/MUL1/PLXNC1/AGO4/DISC1/PLXNA4/MAPK7/ADNP/NOTCH1/CYBB/TMBIM1/SPIRE1/DLG4/TRIOBP/ARHGAP18/MAP3K13/EPHA4/HES1/PKN1/BTG1/MAGI2/MECP2/CDK5/PRKD2/SPRED1/MOV10/FGD6/ABI3/FYN/FMNL1/ARAP1/CRKL/IL1B/PLEKHO1/SH3KBP1/CSF1R/VASH1/ROCK2/MACF1/MYADM/PRAG1/RALBP1/ST6GAL1/FES/FMNL3/AGO1/RCC2/CUL7/ARHGAP35/RUNX1/HGS/EDN1/KIAA0319/TIAM1/PIK3CB/ZMYM4/DIXDC1/MAGED1/ENG/ZMYM3/LIMS1/RHOBTB1/NDEL1/RHOF/KLF4/SEMA4D/DTNBP1/BCL9L/FOXP1/GSK3B/BAIAP2/NEDD9/CYFIP1/TGFB1/PREX1/ETS1/AMOT/SPHK1/EZR/JMJD8/DAPK3/CDC42EP4/TGIF2/CORO1C/WDR1/ALDOA/MFSD2A/BCL11A/TPM1/ZRANB1/IST1/AQP1/NEDD4L/PLXNB2/ILK/SASH1/CDKL5/PINK1/FZD2/RAC1/MBP/PTK2B/PLXND1/KLF2/MTDH/KIF13B/SIPA1L1/MARK2/RRAS/FBXO31/SRC/PML/GREM1/CEACAM1/EIF4G2/INF2/CPNE5/STK25/CALR/PIK3CD/SFRP2/DVL3/FIS1/PLXNA1/PPP3CA/CAMSAP1/TAOK2/PKM/STAT3/SEMA4A/BTBD7/NRP1/BCR/YWHAH/JAK1/LRG1/ABL1/PAK1/SYNGAP1/FGD4/CLASP1/ARHGEF18/FOXO4/STIM1/WARS1/MPL/XK/EPN1/RHOA/DNM2/HIPK1/FLNA/RAC2/HSPB1/FOXJ2/HIPK2/NIBAN2/STK11/KRT1/DNM3/GNA12/CFL1/GLUL/EPB42/HUWE1/CLDN5/PRKCB/THBS1/EPB41/SPTA1/MSN/SP1/GRN/ATP2B4/CUX1/PRKDC/PEAK3/RHOG/E2F2/PARVB/PF4/WASF3/ACTN4/PAK2/CAPZB/MYH9/SPARC/PDGFA/KEL/DMTN/DAB2/DIAPH1/TBXA2R

BP	GO:0007169	transmembrane receptor protein tyrosine kinase signaling pathway	362	-0.49	-1.97	1.00E-10	4.69E-09	2.91E-09	2788	tags=40%, list=24%, signal=32%	FGF23/GAB2/DOK1/SLC27A4/EPHB1/FRS2/TSC2/PDPK1/SH2B3/FIBP/FGR/KIF16B/EXT1/MAPK14/SORL1/RHBDF2/AKT1/SH3TC2/PTGIR/CBLB/PIK3CA/TIPARP/PTPN11/NCF1/CD3E/EFNB1/PDE6G/MVB12B/SGPL1/FOXO1/TSG101/CPNE3/CNOT9/LMTK2/EPHA4/PIP4K2C/MAPKAPK3/PRKD2/DDR1/FYN/CRKL/IL1B/CSF1R/RNF126/VAV2/PTPN18/TNK2/FES/SHC1/HGS/SH2B2/NEU3/TIAM1/PIK3CB/CHMP6/SMARCC1/RBPJ/MMP9/ANKS1A/NDEL1/PIK3R3/SAMD10/DGKD/GRB2/SREBF1/GSK3B/BAIAP2/NEDD9/GSK3A/CYFIP1/DOK3/TGFB1/GIT1/CD63/ZNF106/MYO1E/NDST1/MVP/HIP1/HIP1R/LYN/PTPN12/RAB7A/LEPROT/RAC1/SHCBP1/PTK2B/IGF2/SIPA1L1/SRC/CCND3/GREM1/ARNT/CEACAM1/RNF115/PIK3CD/GNAI2/SLA/VAV1/PTP4A3/FAM20C/STAT3/NRP1/PDK2/PDGFC/JAK1/SYK/PXN/JAK3/ABL1/PAK1/RAB14/FOXO4/RAPGEF1/REPS2/SESN3/PTPRJ/MAPKAPK2/INPP5K/HSPB1/STAT5A/NIBAN2/CYFIP2/SORT1/ADIPOR1/TXNIP/IGF2R/LRP1/PRKCB/PPP2R5B/THBS1/AKT2/PTPN1/IQGAP1/UBASH3B/SNCA/NEURL1/PIP4K2A/EIF4EBP2/CBL/SMPD3/PAK2/TAL1/ITGB3/PDGFA
BP	GO:0006897	endocytosis	389	-0.48	-1.97	1.00E-10	4.69E-09	2.91E-09	1903	tags=32%, list=16%, signal=27%	TNK2/RALBP1/LGALS3/MIB1/NR1H2/ITGAM/PDLIM7/PACSIN1/CXCL16/ARRB1/TOM1/SCARB1/SYNJ1/PI4KB/NEU3/PIK3CB/CSNK1G1/CD14/ANKFY1/GRK2/RHOBTB1/CANX/SNX12/AP2B1/CXCR2/APOBR/SMPD1/DGKD/DTNBP1/GRB2/EHD3/NR1H3/SYNRG/PYCARD/ACTG1/REPS1/ATP9A/CXCR1/CD63/TEX261/CLTC/DENND1C/SPHK1/EZR/MYO1E/SNX10/DENND1A/INPPL1/EHD4/ALOX15/RAB31/HIP1/ANO6/AP2S1/ITSN1/HIP1R/NEDD4L/RAB7A/RAC1/DPYSL2/CD9/MICALL1/ARF1/SH3GL1/SRC/GREM1/CEACAM1/RUBCN/PPP3CA/RIN3/ACTB/VLDLR/EPS15L1/CAP1/SYK/ABL1/RAB27B/ARRB2/MYO18A/REPS2/CLCN5/HNRNPK/CLTB/EPN1/CALM3/DNM2/LDLRAP1/RAB5B/MAPKAPK2/RAC2/DOCK2/BCL2L1/VPS28/SORT1/DNM3/STON2/GSN/GAK/IGF2R/LRP1/THBS1/PTPN1/TRIP10/PIK3CG/CD151/MARCHF2/SNCA/MRC2/RHOG/TBC1D5/MAPK3/CBL/EHD1/GAPVD1/RUFY1/CTTN/AP2M1/MYH9/ITGB3/WASF2/CLU/SH3BP1/DAB2
BP	GO:0001568	blood vessel development	391	-0.48	-1.94	1.00E-10	4.69E-09	2.91E-09	2609	tags=35%, list=22%, signal=28%	PKD1/JUP/MAPK14/PAK4/AKT1/NSDHL/PIK3CA/JUN/TIPARP/SERPINF2/MAPK7/NOTCH1/CYBB/BAK1/YWHAZ/EGR1/HDAC5/SGPL1/FOXO1/HOXB3/PRDM1/IL6R/HES1/BTG1/MECP2/PRKD2/SPRED1/ARID2/CRKL/IL1B/SPI1/SNX17/VASH1/ROCK2/VAV2/PDE2A/FMNL3/MIB1/AGO1/MAP3K3/SHC1/CUL7/RUNX1/HGS/EDN1/PIK3CB/HIF1AN/ENG/FZD5/RBPJ/PIK3R3/KLF4/AP2B1/SOX4/ACVR2B/ANKRD17/TGFB1/ACTG1/ETS1/AMOT/SPHK1/CCN3/JMJD8/MYO1E/NDST1/AQP1/BCAS3/EPAS1/SASH1/JAM3/PTK2B/PLXND1/KLF2/MTDH/RRAS/PML/GREM1/ADIPOR2/SRPK2/CEACAM1/PIK3CD/SFRP2/ZMIZ1/PKM/STAT3/SEMA4A/NRP1/JAK1/SYK/LRG1/ABL1/FOXO4/STIM1/CALD1/WARS1/RNF213/EPN1/RHOA/NOTCH2/ACTA2/HIPK1/SUFU/FLNA/BSG/MYLK/HSPB1/FOXJ2/HIPK2/NIBAN2/KRT1/GLUL/CLDN5/LRP1/PRKCB/THBS1/PIK3CG/MEIS1/TCF7L2/SP1/GRN/ATP2B4/E2F2/LYL1/PF4/GPX1/NPRL3/MED1/TAL1/MYH9/ITGB3/SPARC/PDGFA/WASF2/ITGA2B/TBXA2R

BP	GO:0035239	tube morphogenesis	471	-0.48	-1.97	1.00E-10	4.69E-09	2.91E-09	2247	tags=30%, list=19%, signal=26%	PRDM1/EPHA4/HES1/BTG1/PFN1/MECP2/PRKD2/SPRED1/DDR1/ARID2/DEAF1/IL1B/SPI1/CSF1R/VASH1/ROCK2/VAV2/FMNL3/MIB1/LZTS2/AGO1/MAP3K3/SHC1/CUL7/ARHGAP35/SMAD3/FMN1/RUNX1/HGS/EDN1/PIK3CB/HIF1AN/PHACTR4/MAGED1/ENG/FZD5/RBPJ/PIK3R3/KLF4/SOX4/FOXP1/ACVR2B/PRKACA/TGFB1/ACTG1/CTNNBIP1/ETS1/ST14/AMOT/SPHK1/CCN3/JMJD8/MYO1E/SPINT2/SKI/TRAF6/MTHFR/AQP1/BCAS3/PLXNB2/EPAS1/ILK/SASH1/FZD2/JAM3/PTK2B/RARA/PLXND1/KLF2/DCHS1/MTDH/BCL2L11/RRAS/SRC/PML/GREM1/ADIPOR2/SRPK2/CEACAM1/BBS5/PIK3CD/SFRP2/DVL3/IFT52/ZMIZ1/PKM/PPP1CA/STAT3/SEMA4A/NRP1/BTRC/JAK1/SYK/LRG1/ABL1/PAK1/FOXO4/STIM1/CALD1/WARS1/RNF213/EPN1/RHOA/NOTCH2/HIPK1/SUFU/FLNA/BSG/MYLK/HSPB1/FOXJ2/HIPK2/NIBAN2/SDC4/KRT1/GLUL/CLDN5/LRP1/PRKCB/THBS1/PIK3CG/MEIS1/SP1/GRN/ATP2B4/VASP/E2F2/MICAL2/HLX/PF4/GPX1/NPRL3/MED1/TAL1/MYH9/ITGB3/SPARC/PDGFA/WASF2/ITGA2B/TGM2/PBX1/TBXA2R
BP	GO:0097435	supramolecular fiber organization	504	-0.47	-1.96	1.00E-10	4.69E-09	2.91E-09	2520	tags=36%, list=21%, signal=29%	SWAP70/TUBGCP2/PIK3CA/TMOD3/SERPINF2/TPM4/SH3D21/DSTN/SPTAN1/SPIRE1/ATXN7/KRT73/TRIOBP/MSRB1/WASHC5/ARHGAP18/CORO7/TUBGCP4/CHP1/TUBGCP6/PFN1/MECP2/NDE1/MYO1F/MYO15A/NCKAP5L/CDK5RAP2/SSH1/ARAP1/SPIRE2/SH3KBP1/TBCD/RIPK1/MSRB2/ROCK2/LCP1/MYADM/TUBG1/TUBG2/DIAPH2/PPARGC1B/SVIL/FES/SSH2/PACSIN1/NUMA1/ARRB1/ARHGAP35/SMAD3/FMN1/EDN1/SHROOM1/ARPC5/CRTAP/MAP1S/LIMA1/RHOBTB1/EPPK1/NDEL1/TPX2/RHOF/PPP1R9A/VIM/GRB2/SLAIN2/TPM3/BAIAP2/NEDD9/SORBS3/COLGALT1/PYCARD/CYFIP1/HSP90AB1/ACTG1/GIT1/PSTPIP2/TAOK1/PREX1/EML2/RANGRF/EZR/ARHGAP17/MYO1E/DAPK3/INPPL1/CDC42EP4/PLEK/CORO1C/WDR1/ALDOA/TPM1/KATNB1/PRUNE1/ALOX15/ZYX/HIP1/HIP1R/RAC1/PTK2B/HSPA1A/ARF1/SLC9A1/HCLS1/DYNC1H1/SRC/GREM1/HOOK3/GAS7/ARPC1B/SFRP2/LTBP3/FLII/CAMSAP1/CTNNA1/NRP1/MAP1A/ARHGEF2/USP9X/RFLNB/CSRP1/PXN/ABL1/PAK1/FKBP1A/CLASP1/ARHGEF18/SSH3/CALD1/GOLGA2/RHOA/ARPC4/MAD2L2/ARHGAP6/CKAP5/WAS/FLNA/RAC2/INPP5K/NF2/SDC4/SIAH2/CYFIP2/KRT1/ITGB5/DCTN1/GSN/PLEC/CFL1/WIPF1/PPP1R9B/SPTBN1/AKAP13/MTPN/SPTA1/COTL1/SPTB/SNCA/VASP/RHOG/TMOD1/MICAL2/WASF3/ESPN/SCIN/PAK2/CAPZB/GPX1/ACTN1/PDLIM1/MYL9/FURIN/CTTN/ESAM/GAS2L1/WASF2/CLU/SH3BP1/DMTN/ADD1/DIAPH1

BP	GO:0030334	regulation of cell migration	568	-0.47	-1.98	1.00E-10	4.69E-09	2.91E-09	2571	tags=33%, list=22%, signal=27%	PTAFR/SORL1/MACIR/CORO1A/MINK1/AKT1/PIP5KL1/SELENOK/SWAP70/PLXNC1/JUN/PLXNA4/NOTCH1/SYNE2/CLN3/DOCK8/YTHDF3/HDAC5/FLCN/CPNE3/INSL3/CCR1/IL6R/EPHA4/MAGI2/PFN1/MECP2/CDK5/PRKD2/SPRED1/HYAL2/CD81/ABI3/ARID2/CD99L2/ICAM1/CRKL/MTA2/MITF/DACH1/IL1B/SPI1/P2RX4/DOCK10/CSF1R/MAPRE2/VASH1/HSPA5/CERS2/ROCK2/MACF1/MYADM/TIMP1/MGAT5/TRIB1/RIPOR1/LGALS3/SRGAP2/PARD6B/RCC2/CXCL16/MAP3K3/SMAD3/SCARB1/ACVR1B/PTPRC/EDN1/TIAM1/PIK3CB/RIPOR2/CREB3/ENG/CD74/MMP9/EPPK1/PIK3R3/KLF4/SEMA4D/DNAJA4/FOXP1/PRR5L/NEDD9/MIIP/PYCARD/TGFB1/ACTG1/TRIP6/SVBP/ETS1/AMOT/SPHK1/CCN3/DAPK3/STX4/CORO1C/OXSR1/TPM1/SUN2/ANO6/LYN/BCAS3/PLXNB2/SASH1/RAC1/JAM3/PTK2B/STK10/PLXND1/CD9/SPN/STK24/TRIM32/RRAS/FBXO31/SRC/GREM1/CEACAM1/IQSEC1/CALR/PIK3CD/SFRP2/GNAI2/PLXNA1/PPP3CA/ATP1B2/RIN3/FAM89B/ZNF609/STAT3/SEMA4A/NRP1/CNN2/ARHGEF2/BCR/PDGFC/ABL1/PAK1/CLASP1/FOXO4/RHOA/VCL/ACTA2/PTPRJ/LGALS9/FLNA/FERMT3/RAC2/LMNA/MYLK/HSPB1/STAT5A/NF2/CAMK1D/SDC4/MPP1/ABHD2/GNA12/ADIPOR1/GFUS/GLUL/CLDN5/LRP1/PLVAP/THBS1/AKT2/PIK3CG/CD151/MSN/SP1/GPI/GRN/ATP2B4/SELP/ZNF703/RHOG/MAPK3/SMPD3/ACTN4/FOXO3/PTPN23/ITGB3/SPARC/PDGFA/SH3BP1/MAP2K3/DMTN/WNK1/DAB2/DIAPH1/TBXA2R
BP	GO:0051493	regulation of cytoskeleton organization	381	-0.47	-1.91	1.00E-10	4.69E-09	2.91E-09	2610	tags=38%, list=22%, signal=31%	CORO1B/PKD1/MAPRE1/EPS8/CORO1A/ARL2/SWAP70/BICD2/PIK3CA/TMOD3/SERPINF2/TRPM2/DSTN/CLN3/SPTAN1/BICD1/ATXN7/TRIOBP/WASHC5/ARHGAP18/PFN1/MECP2/CDK5/ABI3/CDK5RAP2/SSH1/ARAP1/VPS4B/TBCD/CSF1R/MAPRE2/ROCK2/MYADM/SVIL/FES/SSH2/NUMA1/ARHGAP35/SMAD3/FMN1/CDK2AP2/EDN1/DIXDC1/ARPC5/CHMP6/MAP1S/LIMA1/RHOBTB1/TPX2/RHOF/CHMP4B/GRB2/SLAIN2/GSK3B/BAIAP2/SORBS3/PYCARD/CYFIP1/ACTG1/GIT1/CLTC/TAOK1/EML2/EML3/RBM14/RANGRF/ARHGAP17/DAPK3/CDC42EP4/PLEK/WDR1/TPM1/KATNB1/PRUNE1/ALOX15/HIP1R/BCAS3/RAC1/JAM3/PTK2B/HSPA1A/ARF1/TACC3/SLC9A1/MARK2/HCLS1/DYNC1H1/CHMP1A/ARPC1B/MYCBP2/FLII/CAMSAP1/TAOK2/NRP1/MAP1A/ARHGEF2/KAT2B/PXN/ABL1/PAK1/CLASP1/ARHGEF18/SSH3/RHOA/NOTCH2/ARPC4/ARHGAP6/CKAP5/ARHGDIA/WAS/FLNA/RAC2/INPP5K/NF2/SDC4/CYFIP2/DCTN1/AFAP1/GSN/LRP1/SPTBN1/AKAP13/MTPN/IQGAP1/SPTA1/COTL1/SPTB/SNCA/PEAK3/VASP/RHOG/TMOD1/MAPK3/WASF3/SCIN/PAK2/CAPZB/CTTN/ESAM/GAS2L1/PDGFA/WASF2/SH3BP1/DMTN/ADD1/DIAPH1
BP	GO:0001944	vasculature development	413	-0.47	-1.92	1.00E-10	4.69E-09	2.91E-09	2609	tags=34%, list=22%, signal=27%	PKD1/JUP/MAPK14/PAK4/AKT1/NSDHL/PIK3CA/JUN/TIPARP/SERPINF2/MAPK7/NOTCH1/CYBB/BAK1/YWHAZ/EGR1/HDAC5/SGPL1/FOXO1/HOXB3/PRDM1/IL6R/HES1/BTG1/MECP2/PRKD2/SPRED1/ARID2/CRKL/IL1B/SPI1/SNX17/VASH1/ROCK2/VAV2/PDE2A/FMNL3/MIB1/AGO1/MAP3K3/SHC1/CUL7/RUNX1/HGS/EDN1/PIK3CB/LGALS12/HIF1AN/ENG/FZD5/RBPJ/PIK3R3/KLF4/AP2B1/SOX4/NFATC1/ACVR2B/NFATC3/ANKRD17/TGFB1/ACTG1/ETS1/AMOT/SPHK1/CCN3/JMJD8/MYO1E/NDST1/AQP1/BCAS3/EPAS1/SASH1/JAM3/PTK2B/PLXND1/KLF2/MTDH/RRAS/PML/GREM1/ADIPOR2/SRPK2/CEACAM1/PIK3CD/SFRP2/ZMIZ1/PKM/STAT3/SEMA4A/NRP1/JAK1/SYK/LRG1/ABL1/FOXO4/STIM1/CALD1/WARS1/RNF213/EPN1/RHOA/NOTCH2/ACTA2/HIPK1/SUFU/FLNA/BSG/MYLK/HSPB1/FOXJ2/HIPK2/NIBAN2/STK11/KRT1/GLUL/CLDN5/LRP1/PRKCB/THBS1/PIK3CG/MEIS1/TCF7L2/SP1/NFATC2/GRN/ATP2B4/E2F2/LYL1/PF4/GPX1/NPRL3/MED1/TAL1/MYH9/ITGB3/SPARC/PDGFA/WASF2/ITGA2B/TBXA2R

BP	GO:0048646	anatomical structure formation involved in morphogenesis	616	-0.47	-1.98	1.00E-10	4.69E-09	2.91E-09	2240	tags=29%, list=19%, signal=25%	HES1/PKN1/BTG1/PFN1/MECP2/CDK5/PRKD2/SPRED1/CD81/DEAF1/EXOC4/IL1B/PLEKHO1/TTLL1/PTPN6/VASH1/ROCK2/VAV2/SH3PXD2A/PTPN18/SMARCD3/PPARGC1B/FMNL3/MBOAT7/MIB1/AGO1/MAP3K3/SHC1/ARHGAP35/SMAD3/FMN1/RUNX1/HGS/PMP22/EDN1/PIK3CB/RIPOR2/PHACTR4/KLHL6/ENG/FZD5/RBPJ/TBPL1/MMP9/PIK3R3/KLF4/SOX4/GRB2/ACVR2B/PRKACA/TGFB1/ACTG1/DYRK1B/ETS1/ST14/AMOT/SPHK1/CCN3/JMJD8/SPINT2/KDM6B/SKI/TWSG1/TRAF6/WDR1/MTHFR/TPM1/AQP1/BCAS3/PLXNB2/EPAS1/SASH1/SUPT6H/FZD2/JAM3/PTK2B/RARA/PLXND1/CD9/KLF2/DCHS1/MTDH/BCL2L11/RRAS/PML/GREM1/ADIPOR2/SRPK2/CEACAM1/PIK3CD/SFRP2/PPARA/DVL3/FLII/IFT52/FAM20C/PKM/STAT3/SEMA4A/MYOF/NRP1/ETS2/NOLC1/JAK1/CSRP1/HERC1/SYK/LRG1/ABL1/MAFB/FKBP1A/CLASP1/FOXO4/STIM1/CALD1/WARS1/RNF213/MPL/EPN1/RHOA/NOTCH2/HIPK1/SUFU/FLNA/TBC1D20/BSG/HSPB1/FOXJ2/HIPK2/NIBAN2/NF2/SDC4/KRT1/ITGB5/PLEC/GLUL/CLDN5/PRKCB/THBS1/AKAP13/MTPN/PIK3CG/MFN2/MEIS1/SP1/GPI/NFATC2/GRN/ATP2B4/PAF1/CAMK1/PRKDC/VASP/E2F2/TMOD1/MAPK3/GATA1/PF4/EHD1/SMPD3/GPX1/ACTN1/MYL9/MPIG6B/FURIN/MED1/TAL1/MYH9/ITGB3/SPARC/PDGFA/WASF2/ITGA2B/ACRBP/TGM2/PIM1/CNOT1/TBXA2R
BP	GO:0035295	tube development	572	-0.47	-1.95	1.00E-10	4.69E-09	2.91E-09	2247	tags=29%, list=19%, signal=25%	PRDM1/EPHA4/HES1/BTG1/PFN1/MECP2/PRKD2/SPRED1/DDR1/ARID2/STK40/CRKL/DEAF1/THRA/IL1B/SPI1/CSF1R/PTPN6/VASH1/ROCK2/VAV2/BASP1/FMNL3/MAN2A1/MIB1/LZTS2/SPATA2/AGO1/MAP3K3/SHC1/CUL7/ARHGAP35/SMAD3/FMN1/RUNX1/HGS/EDN1/PIK3CB/HIF1AN/PHACTR4/MAGED1/ENG/FZD5/RBPJ/PIK3R3/KLF4/CRISPLD2/SOX4/FOXP1/SREBF1/ACVR2B/PRKACA/TGFB1/ACTG1/CTNNBIP1/NFIA/RPGRIP1L/ETS1/ST14/AMOT/SPHK1/CCN3/JMJD8/MYO1E/SPINT2/SKI/TRAF6/MTHFR/AQP1/BCAS3/PLXNB2/EPAS1/ILK/SASH1/FZD2/JAM3/PTK2B/RARA/PLXND1/KLF2/DCHS1/MTDH/BCL2L11/RRAS/SRC/PML/GREM1/ADIPOR2/SRPK2/CEACAM1/BBS5/PIK3CD/SFRP2/DVL3/LTBP3/IFT52/ZMIZ1/PKM/PPP1CA/STAT3/SEMA4A/NRP1/BTRC/PDGFC/JAK1/SYK/LRG1/ABL1/PAK1/FOXO4/ODAD4/STIM1/CALD1/WARS1/RNF213/EPN1/RHOA/CHD8/NOTCH2/HIPK1/SUFU/FLNA/TBC1D20/BSG/MYLK/ITPK1/ECE1/HSPB1/FOXJ2/HIPK2/NIBAN2/ELK1/SDC4/UBB/KRT1/GLUL/CLDN5/LRP1/PRKCB/THBS1/PIK3CG/MEIS1/SP1/FKBP8/GRN/ATP2B4/VASP/E2F2/MAPK3/MICAL2/HLX/PF4/SMPD3/GPX1/NPRL3/MED1/TAL1/MYH9/ITGB3/SPARC/PDGFA/WASF2/ITGA2B/TGM2/PBX1/BAG6/TBXA2R

BP	GO:2000145	regulation of cell motility	596	-0.46	-1.94	1.00E-10	4.69E-09	2.91E-09	2571	tags=33%, list=22%, signal=27%	PTAFR/SORL1/MACIR/CORO1A/MINK1/AKT1/PIP5KL1/SELENOK/SWAP70/PLXNC1/JUN/PLXNA4/NOTCH1/SYNE2/CLN3/DOCK8/YTHDF3/HDAC5/FLCN/CPNE3/INSL3/ARHGAP18/CCR1/IL6R/EPHA4/PKN1/MAGI2/PFN1/MECP2/CDK5/PRKD2/SPRED1/HYAL2/CD81/ABI3/ARID2/CD99L2/ICAM1/CRKL/MTA2/MITF/DACH1/IL1B/SPI1/P2RX4/DOCK10/CSF1R/MAPRE2/VASH1/HSPA5/CERS2/ROCK2/MACF1/MYADM/PRAG1/TIMP1/MGAT5/TRIB1/RIPOR1/FES/LGALS3/SRGAP2/PIN1/PARD6B/RCC2/CXCL16/MAP3K3/SMAD3/SCARB1/ACVR1B/PTPRC/EDN1/TIAM1/PIK3CB/RIPOR2/CREB3/ENG/CD74/MMP9/EPPK1/PIK3R3/KLF4/SEMA4D/DNAJA4/FOXP1/PRR5L/NEDD9/MIIP/PYCARD/TGFB1/ACTG1/TRIP6/SVBP/CDK6/ETS1/AMOT/SPHK1/CCN3/SPINT2/DAPK3/STX4/CORO1C/OXSR1/TPM1/SUN2/ANO6/LYN/BCAS3/PLXNB2/SASH1/RAC1/JAM3/PTK2B/STK10/PLXND1/CD9/SPN/STK24/TRIM32/RRAS/FBXO31/SRC/GREM1/CEACAM1/IQSEC1/CALR/PIK3CD/SFRP2/GNAI2/PLXNA1/PPP3CA/ATP1B2/RIN3/FAM89B/ZNF609/STAT3/CTNNA1/SEMA4A/NRP1/CNN2/ARHGEF2/BCR/PDGFC/ABL1/PAK1/CLASP1/FOXO4/RHOA/VCL/ACTA2/PTPRJ/LGALS9/WAS/FLNA/FERMT3/RAC2/LMNA/MYLK/HSPB1/STAT5A/NF2/CAMK1D/SDC4/MPP1/ABHD2/GNA12/ADIPOR1/GFUS/GLUL/CLDN5/LRP1/PLVAP/THBS1/AKT2/PIK3CG/CD151/MSN/SP1/GPI/GRN/ATP2B4/SELP/ZNF703/RHOG/MAPK3/SMPD3/ACTN4/FOXO3/PTPN23/ITGB3/SPARC/PDGFA/SH3BP1/MAP2K3/DMTN/WNK1/DAB2/DIAPH1/TBXA2R
BP	GO:0040012	regulation of locomotion	614	-0.46	-1.94	1.00E-10	4.69E-09	2.91E-09	2571	tags=33%, list=22%, signal=27%	PTAFR/SORL1/MACIR/CORO1A/MINK1/AKT1/PIP5KL1/SELENOK/SWAP70/PLXNC1/JUN/PLXNA4/NOTCH1/SYNE2/CLN3/DOCK8/YTHDF3/HDAC5/FLCN/CPNE3/INSL3/ARHGAP18/CCR1/IL6R/EPHA4/PKN1/MAGI2/PFN1/MECP2/CDK5/PRKD2/SPRED1/HYAL2/CD81/ABI3/ARID2/CD99L2/ICAM1/CRKL/MTA2/MITF/DACH1/IL1B/SPI1/P2RX4/DOCK10/CSF1R/MAPRE2/VASH1/HSPA5/CERS2/ROCK2/MACF1/MYADM/PRAG1/TIMP1/MGAT5/ST6GAL1/TRIB1/RIPOR1/FES/LGALS3/SRGAP2/PIN1/PARD6B/RCC2/CXCL16/MAP3K3/SMAD3/SCARB1/ACVR1B/PTPRC/EDN1/TIAM1/PIK3CB/RIPOR2/CREB3/ENG/CD74/MMP9/EPPK1/PIK3R3/KLF4/SEMA4D/DNAJA4/FOXP1/PRR5L/NEDD9/MIIP/PYCARD/TGFB1/ACTG1/TRIP6/SVBP/CDK6/ETS1/AMOT/SPHK1/CCN3/SPINT2/DAPK3/STX4/CORO1C/OXSR1/TPM1/SUN2/ANO6/LYN/BCAS3/PLXNB2/SASH1/RAC1/JAM3/PTK2B/STK10/PLXND1/CD9/SPN/STK24/TRIM32/RRAS/FBXO31/SRC/GREM1/CEACAM1/IQSEC1/CALR/PIK3CD/SFRP2/GNAI2/MYCBP2/PLXNA1/PPP3CA/ATP1B2/RIN3/FAM89B/ZNF609/STAT3/CTNNA1/SEMA4A/NRP1/CNN2/ARHGEF2/BCR/PDGFC/ABL1/PAK1/CLASP1/FOXO4/RHOA/VCL/ACTA2/PTPRJ/LGALS9/WAS/FLNA/FERMT3/RAC2/LMNA/MYLK/HSPB1/STAT5A/NF2/CAMK1D/SDC4/MPP1/ABHD2/GNA12/ADIPOR1/GFUS/GLUL/CLDN5/LRP1/PLVAP/THBS1/AKT2/PIK3CG/CD151/MSN/SP1/GPI/GRN/ATP2B4/SELP/SNCA/ZNF703/RHOG/MAPK3/FBXO7/SMPD3/ACTN4/FOXO3/PTPN23/ITGB3/SPARC/PDGFA/SH3BP1/MAP2K3/DMTN/WNK1/DAB2/DIAPH1/TBXA2R

BP	GO:0045597	positive regulation of cell differentiation	505	-0.46	-1.90	1.00E-10	4.69E-09	2.91E-09	2261	tags=31%, list=19%, signal=26%	MAP3K13/ZNF385A/IL6R/EPHA4/HES1/HLA-DRB1/BTG1/ASPM/CRKL/CPNE1/IL1B/FEZ1/RIPK1/CYB5D2/MACF1/ZBTB16/MYADM/SMARCD3/HMG20B/PPARGC1B/TRIB1/FES/MAN2A1/PDLIM7/XRCC6/NUMA1/SOX13/TFE3/CUL7/SMAD3/SYNJ1/ACVR1B/RUNX1/PTPRC/EDN1/TIAM1/HIF1AN/RIPOR2/DIXDC1/ENG/INPP5D/LIMS1/CD74/TGFB1I1/NDEL1/JUND/SEMA4D/SOX4/BCL9L/KDM4A/NCOA1/ACVR2B/GSK3B/BAIAP2/NAPEPLD/NEDD9/RPS6KA1/CYFIP1/HSP90AB1/TGFB1/CTNNBIP1/ACIN1/TNFRSF1B/PREX1/VHL/ETS1/TGIF2/IL4I1/PARP1/TRAF6/MAP3K5/IST1/LYN/PLXNB2/ILK/CDKL5/RAC1/RARA/HSPA1A/PLXND1/BAD/TRIM32/HCLS1/RRAS/FBXO31/SOX6/GREM1/KAT7/ARNT/CEACAM1/EIF4G2/NCOA3/STK25/CALR/SFRP2/LRRC8A/OLIG2/LTBP3/RUNX3/PLXNA1/FAM20C/ZMIZ1/STAT3/NRP1/ARHGEF2/SYK/ABL1/FOS/TNFSF4/E2F1/PITHD1/LTF/NFKB1/BMP6/ARRB2/MPL/PDE5A/RHOA/NOTCH2/DNM2/LGALS9/FLNA/MTURN/PA2G4/NIBAN2/NF2/STK11/ASXL2/CLDN5/GJC2/TCF7L2/FAM210B/CUX1/MMD/CAMK1/EIF4G1/NEURL1/PRKDC/ZNF703/SOX12/GATA1/HLX/PF4/SCIN/FOXO3/TESC/FAXDC2/MED1/TAL1/DMTN/CARM1/TGM2/ADD1/PIM1/DAB2
BP	GO:1901699	cellular response to nitrogen compound	436	-0.45	-1.86	1.00E-10	4.69E-09	2.91E-09	1834	tags=28%, list=16%, signal=25%	SHC1/RGS10/PDE4B/OR10H1/SLC29A2/RRAGD/SH2B2/EDN1/SMARCC1/TAF1/GNAZ/GRK2/NDEL1/PIK3R3/PDE12/ABCC1/DTNBP1/EHMT2/VIM/GRB2/SREBF1/GSK3B/BAIAP2/PRKACA/GSK3A/CYFIP1/KLHL22/YWHAG/ZNF106/EZR/GNAS/MEN1/UCP2/SLC27A1/WDTC1/PARP1/MAP3K5/BCL11A/UBR2/SESN2/RAB31/AQP1/LYN/MAVS/EPG5/LEPROT/GNA15/IGF2/KLF2/CGAS/SLC9A1/GNB1/NCSTN/BCL2L11/SRC/CCND3/CEACAM1/CA2/GNAI2/CDK2/DAXX/PKM/MAX/ACTB/STAT3/CTNNA1/RANGAP1/PDK2/ARHGEF2/KAT2B/DDI2/PDGFC/JAK1/PXN/JAK3/ABL1/RRAGC/FBP1/RELA/FOXO4/NFKB1/RAPGEF1/LPIN2/ARRB2/LAMTOR1/SESN3/DNM2/FLNA/IFIT1B/INPP5K/STAT5A/KLF1/BCL2L1/RIOK3/ADIPOR1/LRP1/PPP1R9B/PRKCB/CSK/AKT2/HRH4/PTPN1/PIK3CG/KLF16/SP1/ATP2B4/SNCA/NEURL1/PRKDC/AHCYL1/CASTOR2/PIP4K2A/MAPK3/GATA1/EIF4EBP2/SMPD3/FOXO3/GUCY1B1/KLF3/DMTN/LARP1/TGM2/DIAPH1
BP	GO:0000902	cell morphogenesis	591	-0.45	-1.90	1.00E-10	4.69E-09	2.91E-09	2622	tags=36%, list=22%, signal=29%	EXT1/CORO1B/CAPRIN1/ULK1/EPS8/MAPK14/RAB8A/CORO1A/MINK1/DIP2B/TAOK3/MUL1/PLXNC1/AGO4/ZFPM1/DISC1/PLXNA4/ADNP/PTPN11/NOTCH1/NBEAL2/CASP9/EFNB1/DLG4/NCAM1/TRIOBP/LYPLA2/ARHGAP18/MAP3K13/ZNF385A/LMTK2/EPHA4/HES1/CDK5/MOV10/UNK/DDR1/FGD6/ABI3/FYN/FMNL1/SSH1/ARAP1/CRKL/CPNE1/SPI1/FEZ1/PLEKHO1/SLITRK5/SH3KBP1/TBCD/DOCK10/CSF1R/PTPN6/MACF1/MYADM/PRAG1/ITGB7/ST6GAL1/FES/FMNL3/SRGAP2/PARVG/PACSIN1/PARD6B/RCC2/CUL7/ARHGAP35/WEE1/FRY/APLP2/EDN1/KIAA0319/TIAM1/RERE/SHROOM1/ZMYM4/DIXDC1/ZMYM3/LIMS1/MAP1S/B4GALT5/RHOBTB1/NDEL1/RHOF/SEMA4D/PSMB10/DTNBP1/BCL9L/GSK3B/BAIAP2/NEDD9/CYFIP1/HSP90AB1/TAOK1/PREX1/VHL/ST14/EZR/SIPA1L3/SPINT2/DAPK3/BCL11B/CDC42EP4/CORO1C/WDR1/ALDOA/MFSD2A/BCL11A/SLC39A3/TPM1/ZRANB1/IST1/NEDD4L/PLXNB2/ILK/CDKL5/RAC1/MBP/PTK2B/DPYSL2/PLXND1/KLF2/DCHS1/FLNB/TRIO/KIF13B/SIPA1L1/MARK2/FBXO31/SRC/GREM1/EIF4G2/GAS7/CPNE5/STK25/CALR/DVL3/MYCBP2/PLXNA1/PPP3CA/CAMSAP1/TAOK2/ACTB/SEMA4A/NRP1/MAP1A/ARHGEF2/VLDLR/YWHAH/USP9X/CAP1/PXN/ABL1/PAK1/PARD3/SYNGAP1/FGD4/ARHGEF18/MPL/XK/SIDT2/RHOA/VCL/NOTCH2/DNM2/FLNA/TBC1D20/FERMT3/RAC2/BSG/ECE1/NIBAN2/STK11/UBB/CYFIP2/DNM3/GNA12/PLEC/CFL1/GP1BA/EPB42/EPB41/IQGAP1/SPTA1/MSN/CUX1/PRKDC/PEAK3/VASP/RHOG/GATA1/PARVB/WASF3/ACTN4/PAK2/STRADB/CAPZB/ACTN1/MPIG6B/MED1/TAL1/CTTN/MYH9/ITGB3/SPARC/WASF2/KEL/CLU/DMTN/SCN1B/DAB2/DIAPH1

BP	GO:0051270	regulation of cellular component movement	635	-0.45	-1.90	1.00E-10	4.69E-09	2.91E-09	2571	tags=33%, list=22%, signal=27%	PTAFR/SORL1/MACIR/CORO1A/MINK1/AKT1/PIP5KL1/SELENOK/SWAP70/PLXNC1/JUN/PLXNA4/NOTCH1/SYNE2/CLN3/DOCK8/ATP2A2/YTHDF3/HDAC5/FLCN/CPNE3/INSL3/ARHGAP18/CCR1/IL6R/EPHA4/PKN1/MAGI2/PFN1/MECP2/CDK5/PRKD2/SPRED1/HYAL2/CD81/ABI3/ARID2/CD99L2/ICAM1/ARAP1/CRKL/MTA2/MITF/DACH1/IL1B/SPI1/FEZ1/P2RX4/DOCK10/CSF1R/MAPRE2/VASH1/HSPA5/CERS2/ROCK2/MACF1/MYADM/PRAG1/TIMP1/MGAT5/TRIB1/RIPOR1/FES/LGALS3/SRGAP2/PIN1/PARD6B/RCC2/CXCL16/MAP3K3/SMAD3/SCARB1/PDE4B/ACVR1B/PTPRC/EDN1/TIAM1/PIK3CB/RIPOR2/CREB3/ENG/CD74/MMP9/EPPK1/PIK3R3/KLF4/SEMA4D/DNAJA4/KCNJ2/FOXP1/PRR5L/NEDD9/MIIP/PYCARD/TGFB1/ACTG1/TRIP6/SVBP/CDK6/ETS1/RANGRF/AMOT/SPHK1/CCN3/SPINT2/DAPK3/STX4/CORO1C/OXSR1/TPM1/SUN2/ANO6/LYN/BCAS3/PLXNB2/SASH1/RAC1/JAM3/PTK2B/STK10/PLXND1/CD9/SPN/STK24/TRIM32/RRAS/FBXO31/SRC/GREM1/CEACAM1/IQSEC1/CALR/PIK3CD/SFRP2/GNAI2/MYCBP2/PLXNA1/PPP3CA/ATP1B2/RIN3/FAM89B/ZNF609/STAT3/CTNNA1/SEMA4A/NRP1/CNN2/ARHGEF2/BCR/PDGFC/ABL1/PAK1/CLASP1/FOXO4/RHOA/VCL/ACTA2/PTPRJ/LGALS9/WAS/FLNA/FERMT3/RAC2/LMNA/MYLK/HSPB1/STAT5A/NF2/CAMK1D/STK11/SDC4/MPP1/ABHD2/GNA12/ADIPOR1/GFUS/GLUL/CLDN5/LRP1/PLVAP/THBS1/AKT2/PIK3CG/CD151/MSN/SP1/GPI/GRN/ATP2B4/SELP/LAMP1/ZNF703/RHOG/MAPK3/SMPD3/ACTN4/ACTN1/FOXO3/PTPN23/TRIM58/ITGB3/SPARC/PDGFA/SH3BP1/MAP2K3/DMTN/WNK1/DAB2/DIAPH1/TBXA2R
MF	GO:0016773	phosphotransferase activity, alcohol group as acceptor	494	-0.45	-1.84	1.00E-10	4.69E-09	2.91E-09	2846	tags=35%, list=24%, signal=28%	MAP3K4/FLT1/PDXK/NEK7/TESK1/RNASEL/OBSCN/EPHB1/TTN/PRKAB1/EIF2AK2/FAM20B/PDPK1/RPS6KA2/CDK12/CAD/FGR/ULK1/MAPK14/PAK4/MARK4/MINK1/AKT1/PIP5KL1/BMP2K/PIM3/TAOK3/PIK3CA/MAPK7/NADK/POMK/CPNE3/PNKP/PAPSS1/MAP4K5/MAP3K13/LMTK2/IKBKE/EPHA4/PKN1/PIP4K2C/MAPKAPK3/PI4K2A/CDK5/PRKD2/DDR1/FYN/STK40/CSNK2A1/TOP1/RIPK1/CSF1R/NUAK2/PFKFB4/ROCK2/MKNK2/PRAG1/TNK2/TESK2/FES/CDK9/MAP3K3/WEE1/ACVR1B/PI4KB/PIK3CB/PHKG2/CSNK1G1/ENG/TAF1/DGKZ/GALK1/GRK2/PIK3R3/PDK3/MAP3K11/DGKD/MARK3/ACVR2B/GSK3B/PRKACA/GSK3A/RPS6KA1/CDK6/TEC/TAOK1/BCKDK/DYRK1B/ITPKC/SPHK1/DAPK3/DAPK1/NEK6/OXSR1/MAP3K5/CDC42BPB/MAP4K1/VRK3/LYN/ILK/CDKL5/PINK1/PTK2B/STK10/PAPSS2/MAP2K7/TRIO/STK24/PFKL/MARK2/SRC/CCND3/DCAF1/SRPK2/STK25/PIK3CD/NEK5/PSKH1/MAST3/CDK2/LRRK1/FAM20C/TAOK2/NOL9/PKM/TRIM28/NRP1/MAP2K2/PDK2/BCR/JAK1/SYK/JAK3/ABL1/PIP5K1B/PAK1/PIM2/SCYL1/HIPK1/HK1/MAPKAPK2/MYLK/ITPK1/HIPK2/CDK19/CAMK1D/STK11/SIK3/IP6K1/RIOK3/GAK/IGF2R/SQSTM1/PPP1R9B/PRKCB/CSK/AKT2/RPS6KA4/AKAP13/PIK3CG/ARAF/MMD/CAMK1/PRKDC/PEAK3/PIP4K2A/MAPK3/BRD4/CDKN1A/PAK2/EIF2AK1/LTBP1/MAP2K3/WNK1/PIM1

BP	GO:0071495	cellular response to endogenous stimulus	794	-0.44	-1.90	1.00E-10	4.69E-09	2.91E-09	2253	tags=33%, list=19%, signal=28%	PTPN11/NOTCH1/TRPM2/CYBB/NR1D1/ATP1A1/CASP9/EGR1/DDX54/ZFP36/UBR5/HDAC5/EZH2/FLCN/FOXO1/CNOT9/PHEX/EIF4A3/EPHA4/HES1/PKN1/MAGI2/PIP4K2C/CDK5/PRKD2/SPRED1/HYAL2/FYN/SSH1/ICAM1/CRKL/THRA/IL1B/SPI1/ZNF366/HRH2/P2RX4/ITPR2/HSPA5/GNA11/ROCK2/SLC33A1/TLR6/PAQR8/NCOA4/PPARGC1B/PDE2A/ZBTB7A/ZFP36L2/NR3C1/PIN1/NRROS/NUMA1/SHC1/SMAD3/PDE4B/OR10H1/SLC29A2/RRAGD/SH2B2/EDN1/ENG/SMARCC1/TAF1/LIMS1/RBPJ/GNAZ/TGFB1I1/RAP1GAP/NDEL1/TRERF1/PIK3R3/ABCC1/DTNBP1/EHMT2/BCL9L/VIM/FOXP1/GRB2/SREBF1/NCOA1/ACVR2B/GSK3B/BAIAP2/CRY2/PRKACA/NR1H3/GSK3A/CYFIP1/HSP90AB1/TGFB1/SMARCA4/KLHL22/YWHAG/PCSK6/ZNF106/EZR/NCOA2/GNAS/SPINT2/MEN1/UCP2/NDST1/SLC27A1/SKI/SMC1A/WDTC1/TWSG1/PARP1/BCL11A/UBR2/SESN2/RAB31/ZYX/AQP1/LYN/BCAS3/PTPN12/ILK/LEPROT/LBH/SHCBP1/RARA/GNA15/TADA3/IGF2/C20orf27/HSPA1A/RXRA/CHST11/KLF2/CREBBP/SLC9A1/GNB1/NCSTN/BCL2L11/SRC/SOX6/PML/CCND3/GREM1/CDKN1C/ADIPOR2/CEACAM1/CA2/NCOA3/CALR/SFRP2/GNAI2/PPARA/WBP2/SLX4/LTBP3/PELP1/FAM20C/DAXX/FAM89B/ZMIZ1/PKM/MAX/ACTB/STAT3/CTNNA1/RANGAP1/PDK2/ARHGEF2/KAT2B/YWHAH/USP9X/PDGFC/JAK1/PXN/JAK3/XIAP/LRG1/FBH1/ABL1/PAK1/RRAGC/FOS/TNFSF4/RAB14/FKBP1A/FBP1/RELA/FOXO4/E2F1/NFKB1/RAPGEF1/LPIN2/BMP6/ARRB2/LAMTOR1/SESN3/RHOA/NOTCH2/DNM2/FLNA/INPP5K/KANK2/STAT5A/HIPK2/KLF1/BCL2L1/STK11/ITGB5/SORT1/ABHD2/ADIPOR1/SAFB/CLDN5/LRP1/PPP1R9B/PRKCB/CSK/THBS1/GLG1/AKT2/UBE2L3/HRH4/PTPN1/PIK3CG/KLF16/IQGAP1/SP1/FKBP8/ATP2B4/SNCA/NEURL1/PRKDC/AHCYL1/ZNF703/CASTOR2/PIP4K2A/MAPK3/GATA1/EIF4EBP2/TGFBRAP1/CBL/EHD1/SMPD3/PAK2/UBE2O/CTSB/FOXO3/FURIN/MED1/ARID1A/PPM1A/GAS2L1/PDGFA/LTBP1/KLF3/DMTN/NCOR2/CARM1/LARP1/TGM2/CNOT1/DAB2/DIAPH1
BP	GO:0051093	negative regulation of developmental process	469	-0.44	-1.83	1.00E-10	4.69E-09	2.91E-09	2375	tags=34%, list=20%, signal=28%	RAI1/ZFP36/HDAC4/HDAC5/EZH2/FLCN/FOXO1/TSPO/CCR1/G6PD/EPHA4/HES1/MECP2/IRF1/CDK5/USF3/DPF2/SPRED1/NELFB/ASPM/CDK5RAP2/IL1B/SPI1/VASH1/CERS2/ROCK2/ZBTB16/PGLYRP1/ZFP36L2/TRIB1/MIB1/NR1H2/AGO1/RCC2/SMAD3/RUNX1/HGS/KIAA0319/PIK3CB/DIXDC1/INPP5D/RBPJ/CMTM5/CD74/TGFB1I1/RAP1GAP/MMP9/KLF4/SEMA4D/CTDSP1/DTNBP1/NFATC1/KDM4A/HYAL3/TOB2/GSK3B/NFATC3/ANKRD17/NR1H3/SFMBT1/GSK3A/TGFB1/JDP2/ASCL2/CDK6/TBX21/TMEM131L/ASAP1/AMOT/CCN3/VGLL4/MEN1/BCOR/SKI/TWSG1/CORO1C/BCL11A/LYN/YY1/KLF13/PINK1/LBH/MBP/PTK2B/RARA/IGF2/KLF2/TRIO/PDCD1/SOX6/PML/GREM1/HOOK3/CEACAM1/CALR/SFRP2/OLIG2/PPARA/LTBP3/RUNX3/PPP3CA/PTBP1/STAT3/USP19/CTNNA1/SEMA4A/NRP1/BCR/YWHAH/RFLNB/JAK3/MAFB/SYNGAP1/TNFSF4/FOXO4/E2F1/LTF/JARID2/EPN1/RHOA/MAD2L2/SUFU/GPR137/FOXJ2/NIBAN2/TRIM11/STK11/SORT1/DNM3/ADIPOR1/HUWE1/CLDN5/THBS1/YBX1/MEIS1/NFATC2/UBASH3B/ATP2B4/PAF1/E2F2/GATA1/RNF10/FBXO7/HLX/CDKN1A/PF4/ACTN4/YBX3/FOXO3/MED1/CUL4A/ITGB3/SPARC/TENT5C/DMTN/CARM1/MBNL3/PBX1

BP	GO:0072359	circulatory system development	623	-0.44	-1.87	1.00E-10	4.69E-09	2.91E-09	2773	tags=34%, list=24%, signal=27%	MEF2D/MSX1/EPHB1/FRS2/TTN/TSC2/PDPK1/RPS6KA2/DCTN5/ATP7A/PECAM1/CASP7/EXT1/PKD1/JUP/MAPK14/MOSPD3/SMAD5/PAK4/AKT1/NSDHL/PIK3CA/ZFPM1/JUN/TBX19/TIPARP/SERPINF2/PLXNA4/MAPK7/PTPN11/NOTCH1/ACACB/CYBB/BAK1/YWHAZ/EGR1/HDAC5/SGPL1/FOXO1/HOXB3/G6PD/PRDM1/IL6R/HES1/BTG1/SMG9/MECP2/PRKD2/SPRED1/ARID2/CRKL/IL1B/SPI1/SNX17/VASH1/GNA11/ROCK2/VAV2/SMARCD3/BASP1/PDE2A/FMNL3/MIB1/PDLIM7/AGO1/MAP3K3/SHC1/CUL7/SMAD3/RUNX1/HGS/EDN1/PIK3CB/LGALS12/HIF1AN/ADAP2/ENG/FZD5/RBPJ/GRK2/PIK3R3/KLF4/AP2B1/SOX4/NFATC1/ACVR2B/NFATC3/ANKRD17/GSK3A/TGFB1/ACTG1/TNFRSF1B/RPGRIP1L/ETS1/AMOT/SPHK1/CCN3/VGLL4/JMJD8/MYO1E/BCOR/KDM6B/NDST1/NCOA6/TPM1/DAW1/AQP1/YY1/BCAS3/EPAS1/ILK/SASH1/FZD2/JAM3/PTK2B/RARA/PLXND1/KLF2/DCHS1/SLC9A1/MTDH/DZIP1/RRAS/SOX6/PML/GREM1/ADIPOR2/SRPK2/CEACAM1/EIF4G2/BBS5/CALR/PIK3CD/SFRP2/PSKH1/PPARA/IFT52/ZMIZ1/PKM/DHRS3/STAT3/SEMA4A/NRP1/KAT2B/JAK1/SYK/LRG1/ABL1/FKBP1A/FOXO4/ODAD4/STIM1/CALD1/WARS1/RNF213/ARRB2/JARID2/GAA/EPN1/RHOA/NOTCH2/ACTA2/HIPK1/AP1B1/SUFU/FLNA/BSG/LMNA/MYLK/ECE1/HSPB1/FOXJ2/HIPK2/NIBAN2/STK11/KRT1/PLEC/GLUL/CLDN5/LRP1/PRKCB/THBS1/AKAP13/PIK3CG/MEIS1/TCF7L2/SP1/NFATC2/GRN/ATP2B4/PRKDC/E2F2/MAPK3/MICAL2/LYL1/CDKN1A/PF4/GPX1/PDLIM1/NPRL3/MED1/TAL1/MYH9/ITGB3/SPARC/PDGFA/WASF2/MAP2K3/ITGA2B/PIM1/TBXA2R
BP	GO:0051094	positive regulation of developmental process	736	-0.44	-1.87	1.00E-10	4.69E-09	2.91E-09	2111	tags=27%, list=18%, signal=24%	CPNE1/IL1B/FEZ1/SLITRK5/DHX37/RIPK1/CYB5D2/MACF1/ZBTB16/MYADM/SMARCD3/HMG20B/RALBP1/BASP1/PPARGC1B/TRIB1/FES/MAN2A1/PDLIM7/XRCC6/PACSIN1/NUMA1/SOX13/TFE3/CUL7/SMAD3/SYNJ1/ACVR1B/RUNX1/PTPRC/EDN1/TIAM1/HIF1AN/RIPOR2/DIXDC1/MAGED1/ENG/INPP5D/LIMS1/RBPJ/CD74/TGFB1I1/B4GALT5/NDEL1/KLF4/JUND/SEMA4D/SOX4/BCL9L/KDM4A/NCOA1/ACVR2B/GSK3B/BAIAP2/NAPEPLD/NEDD9/RPS6KA1/CYFIP1/HSP90AB1/TGFB1/CTNNBIP1/TBX21/ACIN1/TNFRSF1B/PREX1/VHL/ETS1/AMOT/SPHK1/EZR/JMJD8/TGIF2/IL4I1/CORO1C/PARP1/TRAF6/MAP3K5/IST1/ANO6/AQP1/LYN/NEDD4L/PLXNB2/ILK/SASH1/CDKL5/PINK1/LBH/RAC1/PTK2B/RARA/IGF2/HSPA1A/PLXND1/MTDH/BAD/STK24/TRIM32/HCLS1/RRAS/FBXO31/SRC/SOX6/GREM1/KAT7/ARNT/CEACAM1/EIF4G2/NCOA3/CPNE5/STK25/CALR/PIK3CD/SFRP2/LRRC8A/OLIG2/DVL3/LTBP3/FIS1/RUNX3/PLXNA1/FAM20C/ADGRL1/ZMIZ1/PKM/TP53BP1/HMCES/STAT3/SEMA4A/NRP1/ARHGEF2/VLDLR/JAK1/SYK/LRG1/ABL1/FOS/TNFSF4/CLASP1/E2F1/STIM1/PITHD1/LTF/NFKB1/BMP6/ARRB2/MPL/PDE5A/RHOA/NOTCH2/DNM2/MAD2L2/HIPK1/LGALS9/FLNA/FKBP1B/MTURN/PA2G4/HSPB1/HIPK2/NIBAN2/NF2/STK11/CFL1/CLSTN1/ASXL2/CLDN5/PRKCB/THBS1/GJC2/TCF7L2/FAM210B/SP1/NFATC2/GRN/CUX1/MMD/CAMK1/EIF4G1/NEURL1/PRKDC/ZNF703/SOX12/GATA1/HLX/PF4/EHD1/SCIN/YBX3/FOXO3/TESC/FAXDC2/MED1/TAL1/DMTN/CARM1/TGM2/ADD1/PIM1/DAB2/TBXA2R

MF	GO:0016301	kinase activity	536	-0.44	-1.82	1.00E-10	4.69E-09	2.91E-09	2846	tags=34%, list=24%, signal=27%	MAP3K4/FLT1/PDXK/NEK7/TESK1/RNASEL/OBSCN/EPHB1/TTN/PRKAB1/EIF2AK2/FAM20B/PDPK1/RPS6KA2/CDK12/CAD/FGR/ULK1/MAPK14/PAK4/MARK4/MINK1/AKT1/PIP5KL1/BMP2K/PIM3/TAOK3/PIK3CA/MAPK7/NADK/POMK/CPNE3/PNKP/PAPSS1/MAP4K5/MAP3K13/LMTK2/IKBKE/EPHA4/PKN1/PIP4K2C/MAPKAPK3/PI4K2A/CDK5/PRKD2/DDR1/FYN/STK40/CSNK2A1/TOP1/RIPK1/CSF1R/AK3/NUAK2/PFKFB4/ROCK2/MKNK2/PRAG1/TNK2/TESK2/FES/CDK9/MAP3K3/WEE1/ACVR1B/PI4KB/PIK3CB/PHKG2/CSNK1G1/ENG/TAF1/DGKZ/GALK1/GRK2/PIK3R3/PDK3/MAP3K11/DGKD/MARK3/ACVR2B/GSK3B/PRKACA/GSK3A/RPS6KA1/CDK6/TEC/TAOK1/BCKDK/DYRK1B/ITPKC/SPHK1/DAPK3/DAPK1/NEK6/OXSR1/MAP3K5/CDC42BPB/MAP4K1/VRK3/LYN/ILK/NME4/CDKL5/PINK1/PTK2B/STK10/PAPSS2/MAP2K7/TRIO/STK24/PFKL/MARK2/SRC/CCND3/DCAF1/SRPK2/STK25/PIK3CD/NEK5/AK1/PSKH1/MAST3/CDK2/LRRK1/FAM20C/TAOK2/NOL9/PKM/TRIM28/NRP1/MAP2K2/PDK2/BCR/JAK1/SYK/TJP2/JAK3/ABL1/PIP5K1B/PAK1/PIM2/CKB/SCYL1/HIPK1/HK1/MAPKAPK2/ALDH18A1/MYLK/ITPK1/HIPK2/CDK19/CAMK1D/STK11/SIK3/IP6K1/MPP1/RIOK3/GAK/IGF2R/SQSTM1/PPP1R9B/PRKCB/CSK/AKT2/RPS6KA4/AKAP13/PIK3CG/ARAF/MMD/CAMK1/PRKDC/PEAK3/PIP4K2A/MAPK3/GUK1/BRD4/CDKN1A/PAK2/EIF2AK1/LTBP1/MAP2K3/WNK1/PIM1
BP	GO:2000026	regulation of multicellular organismal development	775	-0.44	-1.85	1.00E-10	4.69E-09	2.91E-09	2261	tags=29%, list=19%, signal=25%	MAP3K13/G6PD/PRDM1/EPHA4/HES1/HLA-DRB1/BTG1/MECP2/IRF1/CDK5/PRKD2/SPRED1/ASPM/MAFG/MITF/IL1B/SPI1/SLITRK5/RIPK1/PTPN6/VASH1/CERS2/ROCK2/MACF1/ZBTB16/PGLYRP1/SMARCD3/BASP1/PPARGC1B/ZFP36L2/EIF6/TRIB1/FES/EHMT1/MAN2A1/XRCC6/AGO1/NUMA1/SOX13/TFE3/CUL7/SMAD3/MAFF/SYNJ1/ACVR1B/RUNX1/KEAP1/HGS/PTPRC/EDN1/KIAA0319/TIAM1/PIK3CB/DIXDC1/DROSHA/ENG/INPP5D/RBPJ/CD74/B4GALT5/MMP9/NDEL1/KLF4/SEMA4D/CTDSP1/SOX4/FOXP1/KDM4A/TOB2/ACVR2B/BAIAP2/GSK3A/CYFIP1/TGFB1/CTNNBIP1/ASCL2/CDK6/TBX21/TMEM131L/ACIN1/TNFRSF1B/ETS1/AMOT/SPHK1/VGLL4/JMJD8/CNOT4/BCOR/SKI/TGIF2/IL4I1/TRAF6/TRPS1/PRUNE1/IST1/ANO6/AQP1/LYN/YY1/PLXNB2/KLF13/SASH1/CDKL5/MBP/PTK2B/RARA/HSPA1A/PLXND1/KLF2/MTDH/BAD/TRIM32/HCLS1/RRAS/FBXO31/SOX6/PML/GREM1/KAT7/CDKN1C/ARNT/HOOK3/CEACAM1/EIF4G2/NCOA3/STK25/PIK3CD/SFRP2/OLIG2/PPARA/LTBP3/RUNX3/PLXNA1/PPP3CA/FAM20C/ZMIZ1/PKM/PHOSPHO1/STAT3/CTNNA1/SEMA4A/NRP1/YWHAH/RFLNB/JAK1/SYK/JAK3/LRG1/ABL1/MAFB/PARD3/FOS/SYNGAP1/TNFSF4/RAB14/CLASP1/FOXO4/E2F1/STIM1/PITHD1/LTF/BMP6/WARS1/JARID2/MPL/EPN1/RHOA/VCL/NOTCH2/HIPK1/LGALS9/GPR137/MTURN/HSPB1/FOXJ2/HIPK2/NIBAN2/NF2/TRIM11/STK11/KRT1/CFL1/CLSTN1/GLUL/CLDN5/PRKCB/THBS1/GLG1/ANKH/GJC2/MEIS1/FAM210B/PER2/SP1/GRN/UBASH3B/ATP2B4/CUX1/PAF1/NEURL1/PRKDC/E2F2/SOX12/GATA1/RNF10/FBXO7/HLX/PF4/WASF3/SMPD3/TRIP12/SCIN/FOXO3/TESC/FAXDC2/MED1/TAL1/CUL4A/SPARC/GFI1B/TGM2/ADD1/PIM1/BAG6/TBXA2R

BP	GO:0014070	response to organic cyclic compound	523	-0.43	-1.79	1.00E-10	4.69E-09	2.91E-09	2638	tags=35%, list=22%, signal=29%	SLC16A1/NRIP1/CASP7/JUP/RFTN1/RALB/SMAD5/PTAFR/PAK4/ABCA3/FOXP3/GRAMD1B/MUL1/GNAO1/PIK3CA/TIPARP/ABCB11/NOTCH1/TRPM2/ACACB/CYBB/NR1D1/BAK1/ATP1A1/CASP9/TRPC3/EGR1/DDX54/ZFP36/CDKN2D/UBR5/EZH2/CNOT9/TSPO/PHEX/EIF4A3/G6PD/HES1/PKN1/MLC1/CDK5/SSH1/IL1B/ZNF366/HRH2/P2RX4/OSBPL7/ITPR2/HSPA5/GNA11/ROCK2/PAQR8/NCOA4/PPARGC1B/PDE2A/ZBTB7A/ZFP36L2/NR3C1/FES/ALPL/PDE4B/OR10H1/EDN1/ENG/ENO2/TAF1/GNAZ/CPT1A/TRERF1/PDE12/JUND/CDA/SMPD1/DTNBP1/EHMT2/FOXP1/EFTUD2/SREBF1/GSK3B/CRY2/PRKACA/PLIN2/NR1H3/GSK3A/HSP90AB1/TGFB1/SMARCA4/IL1RN/EZR/NCOA2/GNAS/ALAD/PARP1/GLB1/MTHFR/AQP1/LYN/MAVS/EPG5/LBH/MBP/PTK2B/RARA/GNA15/TADA3/HSPA1A/RXRA/KLF2/MGMT/CGAS/SLC9A1/BAD/GNB1/NCSTN/BCL2L11/SRC/ARPC1B/NCOA3/CALR/PPARA/WBP2/DAXX/ZMIZ1/ACTB/STAT3/CTNNA1/YWHAH/BTRC/ABL1/PAK1/FOS/NFKB1/RAPGEF1/BMP6/ARRB2/RHOA/CALM3/DNM2/FLNA/SLC3A2/FKBP1B/IFIT1B/BSG/INPP5K/KANK2/ELK1/BCL2L1/ABHD2/RIOK3/SAFB/TXNIP/PPP1R9B/THBS1/UBE2L3/HRH4/PIK3CG/KLF16/FAM210B/MSN/GPI/ATP2B4/SNCA/ZNF703/P2RX1/MAPK3/GATA1/CBL/PAK2/GPX1/PRKAR2B/FOXO3/MED1/ARID1A/PDGFA/CLU/DMTN/NCOR2/CARM1/LARP1/TGM2/PIM1/CNOT1/DAB2/DIAPH1/TBXA2R
CC	GO:0098590	plasma membrane region	652	-0.42	-1.78	1.00E-10	4.69E-09	2.91E-09	2451	tags=31%, list=21%, signal=26%	NAALADL1/AP2A2/SLC46A1/SYT11/PEX19/ABCB11/NOTCH1/PSD4/SYNE2/ATP1A1/CLN3/CYBRD1/DOCK8/SPIRE1/SLC7A7/PDE6G/DLG4/SLC24A4/IQCE/SEPTIN6/RPH3A/IL6R/EPHA4/PKN1/PI4K2A/MLC1/SPRED1/NDE1/HYAL2/ASPM/CD81/ABHD17C/FXYD6/SSH1/CRKL/SPIRE2/MAP7/ATP1B1/PLEKHO1/PARD6A/SELPLG/MACF1/SCIMP/AJM1/LCP1/SNTA1/TNK2/PDE2A/SVIL/SRGAP2/SLC7A1/ITGAM/LIPE/PACSIN1/PARD6B/ARRB1/SYNE1/SCARB1/SYNJ1/SLC29A2/NEU3/LRFN1/SHROOM1/RIPOR2/ENO2/SLC41A1/LIMA1/SLC19A1/EPPK1/CANX/AP2B1/ABCC1/DGKD/DTNBP1/EHD3/BAIAP2/PRKACA/HVCN1/NAPEPLD/HSP90AB1/REPS1/CLTC/ASAP1/ST14/RANGRF/PRR7/SPHK1/EZR/TRAPPC4/CLIC5/DENND1A/GNAS/SIPA1L3/MEN1/STX4/SLC27A1/PLEK/CORO1C/AQP10/SLC5A6/TPM1/HIP1/AP2S1/AQP1/ITSN1/HIP1R/CDKL5/RAC1/PLXND1/SLC9A1/MTDH/EXOC3/NCSTN/SLC39A4/SRC/SH2D3C/CEACAM1/ABCC3/BBS5/ATP6V1A/SLC7A8/SLC1A5/SLC14A1/ATP1B2/ADGRL1/DHRS3/ARHGAP45/RAB11FIP4/CTNNA1/CNIH3/MYOF/NRP1/ARHGEF2/VLDLR/SLC6A20/EPS15L1/ABCG2/ABCC4/ATP6V0D1/PAK1/PARD3/ARHGEF18/STIM1/RAB27B/PLEK2/ARRB2/SLC2A1/CLTB/PRKAR2A/SLC7A5/EPN1/RHOA/PDE9A/DNM2/LDLRAP1/PTPRJ/SLC3A2/BSG/CAVIN2/MYLK/INPP5K/ITPK1/NF2/SLC6A9/SORT1/DNM3/GNA12/CFL1/CLSTN1/SLC4A1/CLDN5/LRP1/PPP1R9B/PLVAP/SPTBN1/AKT2/PDE4A/EPB41/MSN/GPI/ATP2B4/LRFN3/AHCYL1/VASP/P2RX1/MAPK3/RNF10/SLC29A1/CBL/NRGN/EHD1/CTSB/TESC/CTTN/AP2M1/MYH9/SHISA7/ITGB3/WASF2/DMTN/TLN1/ANK1/DAB2/DIAPH1

CC	GO:0070161	anchoring junction	573	-0.42	-1.76	1.00E-10	4.69E-09	2.91E-09	2076	tags=34%, list=18%, signal=30%	SH3KBP1/TBCD/FBF1/PARD6A/MAPRE2/PTPN6/HSPA5/PABPC1/CTNND1/AJM1/LCP1/MYADM/ARHGAP26/PRAG1/TNK2/ITGB7/SVIL/FES/NDRG1/EPB41L2/PARVG/PDLIM7/PKP4/PARD6B/FMN1/LSR/PMP22/SNTB1/PTPRC/FRMD4B/TIAM1/SHROOM1/DIXDC1/ARPC5/ENG/FZD5/LIMS1/TGFB1I1/VSIG10/LIMA1/POLDIP2/EPPK1/KCNJ2/ADGRE5/VIM/GRB2/EHD3/UBN1/BAIAP2/NEDD9/SORBS3/CYFIP1/ACTG1/TRIP6/FRMD4A/ZNF185/GIT1/TNS3/CLTC/SPECC1L/RPGRIP1L/YWHAG/RANGRF/AMOT/CCN3/EZR/TJAP1/ARHGAP17/MYO1E/SIPA1L3/CDC42EP4/CORO1C/WDR1/IRF2/PRUNE1/FHL1/CDC42BPB/ZYX/LYN/DCTN4/PTPN12/ILK/FZD2/RAC1/JAM3/PTK2B/HSPA1A/CD9/DCHS1/ARF1/FLNB/SLC9A1/MTDH/TSPAN9/NCSTN/RRAS/SRC/CEACAM1/EIF4G2/ARPC1B/CALR/YWHAB/SLA/VAV1/FLII/PPP3CA/ACTB/UBA1/PPP1CA/FHL3/CTNNA1/NRP1/MAP2K2/CNN2/ARHGEF2/YWHAH/JAK1/CAP1/CSRP1/TJP2/PXN/PAK1/KIFC3/PARD3/CLASP1/ANXA6/ATP6V0C/SLC2A1/APBB1IP/HNRNPK/PRKAR2A/PCDH1/RHOA/VCL/DNM2/PTPRJ/WAS/FLNA/ARHGAP31/FERMT3/RAC2/BSG/RSU1/HSPB1/LASP1/NIBAN2/NF2/SDC4/DCAF6/ITGB5/MPP1/GNA12/AFAP1/GSN/PLEC/CFL1/GAK/CLDN5/IGF2R/LRP1/PPP1R9B/CSK/GJC2/CD151/IQGAP1/TSPAN33/MSN/MRC2/PEAK3/CAPN1/VASP/RHOG/MAPK3/PARVB/PCBP2/CBL/CAPN5/HMGA1/ACTN4/PAK2/ACTN1/YBX3/PDLIM1/CTTN/MYH9/ITGB3/ESAM/TNS1/WASF2/SH3BP1/ITGA2B/TLN1/TGM2/SCN1B/ADD1/DAB2
BP	GO:0009968	negative regulation of signal transduction	796	-0.42	-1.78	1.00E-10	4.69E-09	2.91E-09	2535	tags=31%, list=22%, signal=26%	AIDA/PTGIR/TAOK3/MUL1/CBLB/LZTR1/FRMD8/MAPK7/DUSP8/NOTCH1/NR1D1/CD22/TMBIM1/CD3E/BICD1/EGR1/PPP1R15A/KCTD10/YTHDF3/RGS3/CDKN2D/EZH2/FLCN/FOXO1/TNFAIP8L1/TSG101/CNOT9/SLC24A4/HYOU1/CHP1/OAS3/ZNF385A/LRRC14/EIF4A3/EPHA4/MAGI2/PIP4K2C/SPRED1/HYAL2/ICAM1/CSNK2A1/CPNE1/IL1B/SPI1/ZNF366/RIPK1/OTUD4/PTPN6/USP10/RNF126/HSPA5/CTNND1/TLR6/PTPN18/MYADM/GSTP1/NDRG2/PDE2A/ZBTB7A/TRIB1/CCAR2/LGALS3/PIN1/NR1H2/SOCS7/NRROS/LZTS2/SOX13/ARRB1/RGS10/BIRC6/ARHGAP35/PDE4B/HGS/PTPRC/PIK3CB/HIF1AN/RIPOR2/PHACTR4/CREB3/SLC35F6/CHMP6/INPP5D/DGKZ/CD74/TGFB1I1/GRK2/MMP9/CD300LF/OTUD7B/RGS16/EIF3A/ITPRIP/SIGIRR/PSME3/SMPD1/DGKD/MARK3/BCL9L/FOXP1/NFATC1/GSK3B/PRR5L/RITA1/CRY2/PRKACA/NLRX1/NR1H3/GSK3A/PYCARD/CACTIN/CTNNBIP1/HDAC3/SMARCA4/TMEM131L/RPGRIP1L/VHL/IL1RN/CCN3/VGLL4/EZR/MEN1/SKI/MVP/PLEK/HIC1/TWSG1/UBR2/SESN2/VRK3/LYN/PTPN12/RAB7A/LEPROT/NOMO3/ADAR/PINK1/LBH/HSPA1A/CHST11/PIAS4/TTLL12/TRIM32/SRC/GREM1/CEACAM1/RASA3/RNF115/CALR/YWHAB/SFRP2/GNAI2/PPARA/LAPTM5/FAM89B/DHRS3/AMFR/TLE3/LITAF/PIH1D1/CTNNA1/NRP1/ARHGEF2/BTRC/PHLPP1/VCP/XIAP/ABL1/SYNGAP1/OPTN/FKBP1A/FBP1/RELA/OTUD5/LTF/RNF213/ARRB2/WWP2/TLE4/SESN3/RHOA/CHD8/MFHAS1/DNM2/MAD2L2/PTPRJ/SUFU/LMNA/INPP5K/KANK2/TLE1/HSPB1/HIPK2/NIBAN2/NF2/TMEM127/BCL2L1/STK11/SIAH2/RIOK3/ADIPOR1/LRP1/PRKCB/CSK/THBS1/GLG1/PTPN1/MFN2/SEC14L1/TCF7L2/RGS6/UBASH3B/ATP2B4/SNCA/PPP1R10/H2BC11/ENO1/NEURL1/CASTOR2/PIP4K2A/GATA1/GRINA/PF4/CBL/TNIP1/STRADB/GPX1/YBX3/NPRL3/FOXO3/MPIG6B/CDC34/CTTN/HTT/PPM1A/NPLOC4/LTBP1/CLU/SH3BP1/MAP2K3/NCOR2/CNOT1/DAB2

MF	GO:0008092	cytoskeletal protein binding	686	-0.41	-1.75	1.00E-10	4.69E-09	2.91E-09	2654	tags=37%, list=23%, signal=30%	MYOM2/ADD2/KIF16B/DAAM1/RAB11FIP5/CORO1B/MAPRE1/EPS8/TMEM201/MID2/RAB8A/MARK4/CORO1A/DIP2B/NCKIPSD/TUBGCP2/BICD2/SYT11/TMOD3/DISC1/ADNP/TPM4/DSTN/SYNE2/SPTAN1/BICD1/TTLL4/SPIRE1/PDE6G/RUSC1/DLG4/TRIOBP/MSRB1/DCP1A/ARL4C/CORO7/SYNM/TUBGCP4/CHP1/TUBGCP6/LMTK2/MAP4/PFN1/CDK5/NDE1/MYO1F/ABI3/FYN/MYO15A/FMNL1/CDK5RAP2/BICDL1/SSH1/MACO1/SPIRE2/FEZ1/TTLL1/TBCD/MAPRE2/VASH1/MSRB2/ROCK2/MACF1/LCP1/SNTA1/DIAPH2/SVIL/FES/NDRG1/FMNL3/PIN1/SSH2/EPB41L2/PARVG/PDLIM7/PACSIN1/RCSD1/NUMA1/RCC2/MAP1LC3B/SYNE1/FMN1/PDE4B/SNTB1/PTPRC/SHROOM1/DIXDC1/PHACTR4/ARPC5/TRAK1/MAP1S/LIMA1/NDEL1/VPS41/TPX2/CENPF/AGBL5/PPP1R9A/MARK3/CEP350/GSK3B/TPM3/KIF1C/RITA1/CETN2/SORBS3/GSK3A/PYCARD/CYFIP1/HSP90AB1/ZNF185/SVBP/GIT1/PSTPIP2/TAOK1/EML2/EML3/ACTR3B/ENC1/EZR/MYO1E/INPPL1/KIF3B/CCDC170/NEK6/CORO1C/WDR1/ALDOA/TLNRD1/TPM1/KATNB1/PRUNE1/SUN2/HIP1/HIP1R/LYN/BCAS3/GPAA1/RABGAP1/PLEKHM2/RARA/DPYSL2/ALDOC/FLNB/TTLL12/S100A4/KIF13B/TRIM32/MARK2/HCLS1/BCL2L11/MYO9B/HOOK3/CEACAM1/MRTFA/GAS7/ARPC1B/INF2/KIF1B/FLII/CAMSAP1/TAOK2/ACTB/RAB6B/FHL3/CTNNA1/CTNNAL1/CNN2/MAP1A/ARHGEF2/YWHAH/GABARAP/RFLNB/CAP1/CSRP1/TRAK2/PXN/ABL1/PAK1/KIFC3/RAB14/FGD4/CLASP1/FRMD3/ABLIM3/RELA/STIM1/RAB27B/NFKB1/ANXA6/SSH3/CALD1/MYO18A/GOLGA2/KLC3/RHOA/VCL/CALM3/ARPC4/DNM2/CKAP5/WAS/FLNA/RAB11B/ARL8A/RAB3D/PPP1R18/MYLK/LASP1/NF2/MYL4/DNM3/DCTN1/AFAP1/GSN/PLEC/CFL1/CLSTN1/WIPF1/SLC4A1/PPP1R9B/SPTBN1/EPB41/IQGAP1/SPTA1/MSN/COTL1/SPTB/SNCA/VASP/TMOD1/PARVB/MICAL2/WASF3/ESPN/SCIN/ACTN4/CAPZB/ACTN1/PDLIM1/MYL9/CTTN/HTT/MYH9/TNS1/GAS2L1/WASF2/CLU/DMTN/TLN1/ANK1/ADD1/DIAPH1
BP	GO:0010243	response to organonitrogen compound	655	-0.41	-1.73	1.00E-10	4.69E-09	2.91E-09	1837	tags=25%, list=16%, signal=22%	UBE2J2/SHC1/DERL1/PDE4B/RHBDD1/OR10H1/SLC29A2/RRAGD/SH2B2/EDN1/SMARCC1/TAF1/GNAZ/CPT1A/MMP9/NDEL1/CANX/PIK3R3/JUND/CDA/ABCC1/BCAP31/SMPD1/DTNBP1/EHMT2/VIM/GRB2/EFTUD2/SREBF1/GSK3B/BAIAP2/CRY2/PRKACA/GSK3A/CYFIP1/HSP90AB1/KLHL22/YWHAG/ZNF106/EDEM2/EZR/GNAS/INPPL1/MEN1/UCP2/SLC27A1/ALAD/WDTC1/PARP1/BCL11A/UBR2/SESN2/MTHFR/RAB31/AQP1/LYN/LEPROT/PTK2B/GNA15/IGF2/CD9/KLF2/MGMT/SLC9A1/BAD/GNB1/MARCHF6/NCSTN/BCL2L11/SRC/CCND3/KAT7/CEACAM1/CA2/CALR/GNAI2/PPARA/PPP3CA/PKM/AMFR/MAX/ACTB/STAT3/USP19/CTNNA1/RANGAP1/PDK2/ARHGEF2/KAT2B/PDGFC/FBXO6/JAK1/SYK/VCP/PXN/JAK3/ABL1/RRAGC/FOS/FBP1/RELA/FOXO4/NFKB1/RAPGEF1/LPIN2/ARRB2/LAMTOR1/SLC2A1/SESN3/RHOA/CALM3/DNM2/MAN1A1/FLNA/BSG/INPP5K/STAT5A/KLF1/BCL2L1/STK11/SORT1/ADIPOR1/LRP1/PPP1R9B/PRKCB/CSK/YOD1/AKT2/HRH4/PTPN1/PIK3CG/KLF16/SP1/GPI/ATP2B4/SNCA/NEURL1/PRKDC/AHCYL1/CASTOR2/PIP4K2A/P2RX1/MAPK3/GATA1/EIF4EBP2/SMPD3/GPX1/RNF40/DNAJB2/PRKAR2B/FOXO3/UBXN6/NPLOC4/KLF3/DMTN/NCOR2/CARM1/LARP1/TGM2/DIAPH1/BAG6

BP	GO:0051130	positive regulation of cellular component organization	717	-0.41	-1.73	1.00E-10	4.69E-09	2.91E-09	1910	tags=26%, list=16%, signal=23%	FES/LGALS3/MIB1/NR1H2/PACSIN1/NUMA1/ARRB1/CUL7/ARHGAP35/SMAD3/FMN1/SYNJ1/RUNX1/EDN1/TIAM1/CHCHD10/RIPOR2/DIXDC1/STOML2/LIMS1/CD14/ANKFY1/POLDIP2/MMP9/NDEL1/BBC3/CCT3/AP2B1/SEMA4D/SMPD1/DGKD/DTNBP1/GRB2/SLAIN2/GSK3B/BAIAP2/SORBS3/GSK3A/COLGALT1/PYCARD/G3BP2/CYFIP1/TGFB1/GIT1/CD63/EIF5AL1/ASAP1/ENC1/SPHK1/ATG2A/CDC42EP4/PLEK/CORO1C/WDR1/VPS13D/TPM1/KATNB1/ALOX15/IST1/RAB31/HIP1/ANO6/HIP1R/LYN/BCAS3/NEDD4L/PLXNB2/CDC27/CDKL5/PINK1/RAC1/PLEKHM2/PTK2B/IGF2/HSPA1A/PLXND1/MAP2K7/BAD/STK24/DZIP1/MARK2/HCLS1/BCL2L11/DYNC1H1/FBXO31/SRC/PML/GREM1/EIF5A/EIF4G2/IQSEC1/STK25/SNX3/DVL3/SLX4/FIS1/PLXNA1/CDK2/PPP3CA/ATP1B2/ADGRL1/TRIM28/PIH1D1/SEMA4A/NRP1/VLDLR/VDAC1/SYK/VCP/PXN/ABL1/PAK1/FNIP2/CLASP1/NCAPH/RAPGEF1/PLEK2/WARS1/ARRB2/SETDB1/HNRNPK/RHOA/DNM2/LDLRAP1/CKAP5/PTPRJ/WAS/FLNA/FKBP1B/RAC2/NF2/GNL3L/CAMK1D/STK11/VPS28/SDC4/DNM3/DHX33/DCTN1/GSN/CLSTN1/MED25/CLDN5/LRP1/TERF2/PPP2R5B/AKT2/CD151/MSN/NFATC2/GRN/CUX1/SNCA/PPP1R10/CAMK1/EIF4G1/NEURL1/VASP/PIP4K2A/TBC1D5/MAPK3/ELAPOR1/FBXO7/CBL/EHD1/WASF3/SMPD3/SCIN/GPX1/WDR45/CDC34/PTPN23/TAL1/CTTN/AP2M1/HTT/TRIM58/STMP1/WASF2/CLU/SCN1B/ADD1/CNOT1/DAB2
BP	GO:1901698	response to nitrogen compound	706	-0.41	-1.72	1.00E-10	4.69E-09	2.91E-09	1837	tags=25%, list=16%, signal=22%	UBE2J2/SHC1/RGS10/DERL1/PDE4B/RHBDD1/OR10H1/SLC29A2/RRAGD/SH2B2/EDN1/SMARCC1/TAF1/GNAZ/CPT1A/GRK2/MMP9/NDEL1/CANX/PIK3R3/PDE12/JUND/CDA/ABCC1/BCAP31/SMPD1/DTNBP1/EHMT2/VIM/GRB2/EFTUD2/SREBF1/GSK3B/BAIAP2/CRY2/PRKACA/GSK3A/CYFIP1/HSP90AB1/KLHL22/YWHAG/ZNF106/EDEM2/EZR/GNAS/INPPL1/MEN1/UCP2/SLC27A1/ALAD/WDTC1/PARP1/MAP3K5/BCL11A/UBR2/SESN2/MTHFR/RAB31/AQP1/LYN/MAVS/EPG5/LEPROT/PTK2B/GNA15/IGF2/CD9/KLF2/MGMT/CGAS/SLC9A1/BAD/GNB1/MARCHF6/NCSTN/BCL2L11/SRC/CCND3/KAT7/CEACAM1/CA2/CALR/GNAI2/PPARA/CDK2/PPP3CA/DAXX/PKM/AMFR/MAX/ACTB/STAT3/USP19/CTNNA1/RANGAP1/PDK2/ARHGEF2/KAT2B/DDI2/PDGFC/FBXO6/JAK1/SYK/VCP/PXN/JAK3/ABL1/RRAGC/FOS/TNFSF4/FBP1/RELA/FOXO4/NFKB1/RAPGEF1/LPIN2/ARRB2/LAMTOR1/SLC2A1/SESN3/RHOA/CALM3/DNM2/MAN1A1/FLNA/SLC3A2/IFIT1B/BSG/INPP5K/STAT5A/KLF1/BCL2L1/STK11/SORT1/RIOK3/ADIPOR1/LRP1/PPP1R9B/PRKCB/CSK/YOD1/AKT2/HRH4/PTPN1/PIK3CG/KLF16/SP1/GPI/ATP2B4/SNCA/NEURL1/PRKDC/AHCYL1/CASTOR2/PIP4K2A/P2RX1/MAPK3/GATA1/EIF4EBP2/SMPD3/GPX1/RNF40/DNAJB2/PRKAR2B/FOXO3/UBXN6/GUCY1B1/NPLOC4/KLF3/DMTN/NCOR2/CARM1/LARP1/TGM2/DIAPH1/BAG6

BP	GO:0001932	regulation of protein phosphorylation	706	-0.40	-1.71	1.00E-10	4.69E-09	2.91E-09	2581	tags=31%, list=22%, signal=26%	GNAQ/RALB/SORL1/PKIG/AKT1/PIP5KL1/AIDA/TAOK3/CBLB/PIK3CA/JUN/CCNJL/MLLT1/ADNP/PTPN11/MOB3B/BAK1/NCF1/CLN3/CD3E/EGR1/GADD45B/AGAP2/CDKN2D/EZH2/FLCN/DLG4/TSG101/CNOT9/CHP1/MAP3K13/IL6R/EPHA4/HES1/PKN1/HLA-DRB1/SH3BP5L/CDK5/PRKD2/SPRED1/FXR2/HYAL2/PELI2/CD81/FYN/CDK5RAP2/CRKL/IL1B/RIPK1/PARD6A/CSF1R/PTPN6/MADD/ROCK2/TLR6/CCNY/MYADM/TNK2/RALBP1/GSTP1/MOB1B/TRIB1/CDK9/PIN1/PRRC1/XRCC6/CDC37/SHC1/ARRB1/HGS/PTPRC/EDN1/PIK3CB/MAGED1/CHMP6/ENG/FZD5/CD74/MMP9/TPX2/PIK3R3/SEMA4D/MAP3K11/CTDSP1/SMPD1/DTNBP1/ACVR2B/PRR5L/NEDD9/PYCARD/CACTIN/HSP90AB1/TGFB1/MOB3A/HDAC3/LILRA5/TEC/TAOK1/YWHAG/CTDSP2/SPHK1/MEN1/SLC27A1/MVP/EHD4/TWSG1/SERTAD1/CORO1C/TRAF6/MAP3K5/SESN2/LYN/PLXNB2/MAVS/ILK/SASH1/ADAR/PINK1/RAC1/PTK2B/TADA3/IGF2/MAP2K7/MARK2/HCLS1/SRC/PML/CCND3/GREM1/CDKN1C/SH2D3C/CEACAM1/SFRP2/DVL3/LRRK1/TAOK2/DAXX/ACTB/PIH1D1/NRP1/MAP2K2/ARHGEF2/VLDLR/KAT2B/PDGFC/SYK/FBH1/ABL1/PAK1/PARD3/FNIP2/LTF/BMP6/WARS1/ARRB2/NIBAN1/PRKAR2A/PDE5A/RHOA/CALM3/MAD2L2/PPP4C/PTPRJ/INPP5K/HSPB1/HIPK2/NF2/STK11/SDC4/HEXIM2/PLEC/SQSTM1/LRP1/PPP1R9B/CSK/PPP2R5B/THBS1/AKT2/CCNI/PTPN1/RPS6KA4/AKAP13/GJC2/PIK3CG/IQGAP1/PRR5/FKBP8/CTDSPL/ARAF/UBASH3B/ATP2B4/SNCA/MMD/CAMK1/EIF4G1/NEURL1/PRKDC/MAPK3/GATA1/FBXO7/BRD4/CDKN1A/CBL/SMPD3/PAK2/STRADB/PRKAR2B/FAXDC2/EIF2AK1/TAL1/HTT/ITGB3/PDGFA/CLU/MAP2K3/DMTN/WNK1/DAB2/CNPPD1
MF	GO:0005102	signaling receptor binding	724	-0.40	-1.70	1.00E-10	4.69E-09	2.91E-09	2511	tags=31%, list=21%, signal=26%	CBLB/PLXNC1/GNAO1/POMC/NSF/ERMAP/KNG1/PTPN11/NOTCH1/CD22/TMBIM1/CD3E/EFNB1/BICD1/DDX54/KCTD10/DLG4/CPNE3/CNOT9/ARHGEF11/PTPA/INSL3/ABCB9/IL6R/EPHA4/HLA-DRB1/MAGI2/NETO2/CDK5/SPRED1/LYNX1/HYAL2/CD81/PCSK1N/FYN/ICAM1/YARS1/CRKL/MAP7/IL1B/P2RX4/SNX17/HLA-A/RIPK1/SELPLG/PPBP/MADD/RNF126/GNA11/CTNND1/VAV2/TLR6/TACC1/LCP1/CNPY3/DIAPH2/TNK2/ITGB7/SMARCD3/TIMP1/SECTM1/FES/LGALS3/NR1H2/ITGAM/KIR3DL2/CXCL16/SHC1/ARRB1/SMAD3/DERL1/KIR2DS4/ADA2/PTPRC/SH2B2/EDN1/TAPBP/LGALS12/HIF1AN/ENG/STOML2/TAF6/CMTM5/GNAZ/TRAK1/CD74/TGFB1I1/GRK2/ANKS1A/CD300LF/CANX/EIF3A/SEMA4D/BCAP31/IKBKG/GRB2/NCOA1/GSK3A/PYCARD/COPA/HSP90AB1/TGFB1/MICB/TRIP6/CLTC/EML2/RPGRIP1L/YWHAG/MESD/IL1RN/BEX3/CCN3/NCOA2/GNAS/BABAM2/TRAF6/VSTM1/BTN3A2/HIP1/UNC93B1/LYN/LEPROT/PIK3AP1/IL17RA/JAM3/NPFF/PTK2B/RARA/GNA15/IGF2/HSPA1A/CD9/S100A4/PITPNM2/SIPA1L1/NCSTN/MYO9B/SRC/GREM1/ARNT/PSPN/TAF4/CALR/SFRP2/GNAI2/SLA/DVL3/TAOK2/STAT3/SEMA4A/YWHAH/PDGFC/GABARAP/JAK1/SYK/VCP/TRAK2/PXN/JAK3/LRG1/ABL1/TNFSF4/FKBP1A/BMP6/ARRB2/TUBB4B/ITGAL/ARHGEF12/CALM3/DNM2/LDLRAP1/PTPRJ/FLNA/FKBP1B/FERMT3/NF2/DOCK2/ITGB5/SPG21/DNM3/MPP1/GNA12/SYTL4/ASXL2/PF4V1/SQSTM1/LRP1/PPP1R9B/THBS1/GLG1/PTPN1/PIK3CG/CD151/SEC14L1/MSN/GPI/GRN/CXCL5/MARCHF8/TGFBRAP1/PF4/CBL/HMGA1/TUBB/ACTN4/ACTN1/TOLLIP/MED1/AP2M1/HDGF/MYH9/SHISA7/ITGB3/PDGFA/CLU/SH3BP1/ITGA2B/DMTN/NCOR2/TLN1/DAB2/DIAPH1/BAG6

BP	GO:1901701	cellular response to oxygen-containing compound	751	-0.40	-1.70	1.00E-10	4.69E-09	2.91E-09	2143	tags=28%, list=18%, signal=25%	SSH1/ICAM1/CRKL/IL1B/SPI1/HRH2/P2RX4/OSBPL7/RIPK1/ADNP2/PPBP/ITPR2/HSPA5/GNA11/ROCK2/SCIMP/TLR6/NCOA4/GSTP1/PPARGC1B/PDE2A/DCPS/TRIB1/NR3C1/FES/SHC1/RGS10/SCARB1/PDE4B/OR10H1/SLC29A2/RRAGD/SH2B2/EDN1/CCS/SMARCC1/TAF1/GNAZ/CD14/CPT1A/GRK2/MMP9/NDEL1/TRERF1/PIK3R3/PDK3/ABCC1/SIGIRR/SOX4/DTNBP1/EHMT2/VIM/GRB2/SREBF1/GSK3B/BAIAP2/PRKACA/NR1H3/GSK3A/PYCARD/CYFIP1/CACTIN/TGFB1/SMARCA4/GIT1/KLHL22/TNFRSF1B/YWHAG/ZNF106/SPHK1/EZR/CD180/GNAS/DAPK1/MEN1/STX4/KDM6B/UCP2/SLC27A1/WDTC1/PARP1/TRAF6/MAP3K5/BCL11A/UBR2/SESN2/TPM1/RAB31/MAP4K1/AQP1/LYN/LEPROT/SASH1/PINK1/PTK2B/RARA/GNA15/IGF2/RXRA/KLF2/MAP2K7/SLC9A1/MTDH/BAD/GNB1/NCSTN/BCL2L11/SRC/CCND3/CEACAM1/CA2/NCOA3/STK25/GNAI2/CDK2/PPP3CA/PKM/MAX/ACTB/LITAF/PIH1D1/STAT3/CTNNA1/RANGAP1/PDK2/ARHGEF2/BCR/KAT2B/UPF1/PDGFC/JAK1/VCP/PXN/JAK3/ABL1/RRAGC/FOS/TNFSF4/FBP1/RDH11/RELA/FOXO4/E2F1/LTF/NFKB1/RAPGEF1/LPIN2/BMP6/ARRB2/LAMTOR1/SESN3/SIDT2/RHOA/DNM2/FLNA/RAB11B/FKBP1B/INPP5K/KANK2/STAT5A/CDK19/KLF1/ELK1/BCL2L1/PLSCR3/PRDX5/PRPF8/PLEC/ADIPOR1/PF4V1/LRP1/PPP1R9B/PRKCB/CSK/AKT2/HRH4/PTPN1/PIK3CG/KLF16/FAM210B/MSN/SP1/ATP2B4/SNCA/PAF1/CXCL5/NEURL1/PRKDC/AHCYL1/ZNF703/CASTOR2/PIP4K2A/MAPK3/GATA1/EIF4EBP2/SLC29A1/PF4/SMPD3/TNIP1/GPX1/FOXO3/TESC/MED1/GUCY1B1/CLU/MAP2K3/KLF3/DMTN/LARP1/TGM2/PIM1/TBXA2R
BP	GO:0009894	regulation of catabolic process	785	-0.40	-1.71	1.00E-10	4.69E-09	2.91E-09	2594	tags=34%, list=22%, signal=28%	SUPV3L1/ULK1/MAPK14/MID2/RALB/SORL1/RAB8A/AKT1/ARL2/UBQLN4/EXOC7/FBXL20/CBLB/MTMR3/PIK3CA/NSF/DDX49/PIAS1/TIPARP/DISC1/ABCB11/ACACB/CLN3/PCBP4/YTHDF3/BANP/RNF144B/AGAP2/ZFP36/HDAC4/FLCN/FOXO1/SNX33/WAC/DCP1A/TSPO/EIF4A3/IKBKE/EPHA4/PIP4K2C/ABCD1/CDK5/MOV10/FXR2/WDR24/CD81/NUB1/FYN/CSDE1/THRA/CSNK2A1/EXOC4/IL1B/FEZ1/OSBPL7/ORMDL3/SREBF2/USP10/PABPC1/ROCK2/LARP4B/ENTPD5/PGAM1/SYNCRIP/TIMP1/ZBTB7A/DCPS/ZFP36L2/EIF6/TRIB1/CHFR/CCAR2/CTSA/PIN1/FOXK1/SMAD3/PATL1/SCARB1/SERBP1/KEAP1/F8A1/HGS/GPSM1/RRAGD/PIK3CB/LGALS12/TFEB/PHKG2/CHMP6/FZD5/SMARCC1/TAF1/FTO/CPT1A/POLDIP2/PHF23/SNX12/PDE12/CHMP4B/PSME3/BCAP31/EHMT2/VIM/IKBKG/TNRC6C/KDM4A/SREBF1/TECPR1/GSK3B/PRR5L/METTL16/FOXK2/TIMP2/PRKACA/GSK3A/PYCARD/CASC3/DAP/HSP90AB1/GIT1/NBAS/KLHL22/TNFRSF1B/TTC5/CBFA2T3/DET1/VGLL4/EZR/JMJD8/ATG2A/DAPK3/DAPK1/ALAD/VPS13D/MFSD2A/SESN2/NEDD4L/GTPBP1/DDA1/RAB7A/BSCL2/PUM1/PINK1/ZC3H18/PTK2B/HSPA1A/ANKIB1/MTDH/BAD/TRIM32/ZC3HAV1/BCL2L11/PML/ARNT/EIF4G2/RUBCN/SNX3/ATP6V1A/PPARA/MYCBP2/PLEKHF1/CDK2/LAPTM5/PSAP/PPP1CA/STAT3/USP19/MAP1A/UPF1/BTRC/GABARAP/VDAC1/HERC1/ALKBH5/RGP1/DDB1/VCP/CPTP/USP36/ATP6V0D1/ABL1/ELAVL1/RRAGC/ARIH2/OPTN/FBP1/N4BP1/RELA/PNPLA2/E2F1/PITHD1/PSMC4/PIM2/STX5/RAD23A/DTX3L/ATP6V0C/DCP1B/CAPNS1/WWP2/GOLGA2/SESN3/ODC1/MAD2L2/SUFU/FLNA/HNRNPM/GPR137/MAPKAPK2/PSMD2/USP7/ATG7/SF3B3/HSPB1/HIPK2/ATP6V0A1/TENT4B/STK11/VPS28/UBB/GNA12/PABPC4/SLC4A1/FZR1/HUWE1/PSMF1/LRP1/RILP/TRIM14/AKT2/YBX1/PTPN1/PIK3CG/MSN/MARCHF2/ARAF/ATP2B4/SNCA/EIF4G1/CAPN1/PIP4K2A/MAPK3/ELAPOR1/BNIP3L/FBXO7/DCAF12/TBC1D25/RYBP/ZER1/GPX1/KHSRP/RNF40/DNAJB2/YBX3/WDR45/NPRL3/FOXO3/FURIN/OAZ1/CTTN/HTT/CLU/TENT5C/LARP1/CNOT1/DAB2/BAG6/SUPT5H

CC	GO:0043005	neuron projection	736	-0.40	-1.69	1.00E-10	4.69E-09	2.91E-09	2593	tags=33%, list=22%, signal=27%	ULK1/EPS8/GNAQ/RAB8A/SBF2/MARK4/CORO1A/MINK1/DIP2B/EXOC7/MUL1/SYT11/NSF/DISC1/ADNP/TRPM2/CYBB/NR1D1/NCF1/ATP1A1/CLN3/CD3E/PDE6G/DLG4/NCAM1/TRIOBP/SLC24A4/SEPTIN6/RPH3A/ZNF385A/EIF4A3/LMTK2/EPHA4/MAGI2/MAP4/DMWD/PI4K2A/CDK5/LYNX1/FXR2/ABI3/FYN/ABHD17C/MYO15A/MACO1/EXOC4/MAP7/FEZ1/HRH2/YKT6/P2RX4/CNR2/SH3KBP1/DOCK10/SMN1/MADD/GNA11/NAV1/CTNND1/DNAJB1/MPST/BASP1/NDRG2/SRGAP2/TMEM185A/PACSIN1/NUMA1/PDE4B/SYNJ1/OR10H1/RIPOR2/DIXDC1/ARPC5/ENO2/FZD5/GNAZ/TRAK1/MAP1S/RAP1GAP/ANKS1A/NDEL1/CANX/TPX2/PPP1R9A/KCNJ2/SMIM31/MARK3/SEPTIN11/DTNBP1/VIM/GSK3B/KIF1C/BAIAP2/CETN2/PRKACA/NAPEPLD/GSK3A/CASC3/CYFIP1/COPA/HSP90AB1/ACTG1/GIT1/TNFRSF1B/RPGRIP1L/TPRG1L/ASAP1/PRR7/CCN3/TRAPPC4/STX4/KIF3B/BCL11B/KATNB1/ITSN1/HIP1R/PRR12/CDKL5/PINK1/RAC1/MBP/NPFF/PTK2B/RARA/ARF1/S100A4/KIF13B/EXOC3/SIPA1L1/GNB1/MARK2/GRIPAP1/DYNC1H1/SRC/N4BP3/SH2D3C/EIF4G2/CPNE5/GNAI2/SLA/KIF1B/SEPTIN4/ARHGAP32/MYCBP2/VTI1A/IFT52/PPP3CA/RIN3/ADGRL1/TAOK2/DHRS3/AMFR/MAX/ACTB/PPP1CA/CNIH3/NRP1/RANGAP1/SLC1A4/MAP1A/BCR/USP9X/APBA3/MCRS1/TRAK2/ATP6V0D1/ABL1/PAK1/PARD3/FOS/SYNGAP1/BLOC1S3/RAB27B/FAM168B/ARRB2/STAU1/MPL/CKB/KLC3/RHOA/CALM3/DNM2/LDLRAP1/PVALB/FLNA/ARL8A/BSG/INPP5K/HSPB1/NF2/GLRX5/ELK1/ROGDI/SIAH2/ZDHHC5/UBB/CYFIP2/DNM3/MPP1/STON2/GNA12/DCTN1/PLEC/CLSTN1/GLUL/CLDN5/TERF2/PPP1R9B/PRKCB/SPTBN1/HRH4/GJC2/MTPN/IQGAP1/SPTA1/GPI/ATP2B4/SHARPIN/SNCA/LRFN3/NEURL1/PIP4K2A/P2RX1/FBXO7/CBL/NRGN/ESPN/ACTN4/PTGDR2/PTGS1/RNF40/ACTN1/PRKAR2B/CTTN/HTT/SHISA7/CLU/OSBP2/ANK1/SCN1B
BP	GO:0044087	regulation of cellular component biogenesis	679	-0.41	-1.71	1.06E-10	4.89E-09	3.04E-09	2248	tags=28%, list=19%, signal=24%	IKBKE/HES1/MAP4/PFN1/PIP4K2C/MECP2/FXR2/ABI3/CDK5RAP2/SSH1/ICAM1/ARAP1/THRA/IL1B/FEZ1/SLITRK5/ORMDL3/VPS4B/TBCD/HSPA5/ROCK2/MACF1/DNAJB1/TLR6/LCP1/MYADM/PHF8/SVIL/FES/LGALS3/TMC8/SSH2/NR1H2/TFIP11/NUMA1/RCC2/ARHGAP35/SMAD3/FMN1/HGS/RIPOR2/ARPC5/CHMP6/FZD5/LIMS1/POLDIP2/RAP1GAP/NDEL1/PHF23/CHMP4B/SEMA4D/DNAJA4/EHMT2/GRB2/SLAIN2/GSK3B/BAIAP2/PRKACA/SORBS3/PYCARD/G3BP2/CYFIP1/TGFB1/ACTG1/CTNNBIP1/GIT1/EML2/EML3/RBM14/ASAP1/RANGRF/SPHK1/EZR/ATG2A/DAPK3/CDC42EP4/PLEK/CORO1C/PARP1/TPM1/PRUNE1/ALOX15/HIP1R/BCAS3/RAB7A/CDKL5/PINK1/RAC1/JAM3/PTK2B/HSPA1A/EVI5L/SLC9A1/DZIP1/HCLS1/BCL2L11/DYNC1H1/SRC/CHMP1A/GREM1/ARPC1B/SLX4/FLII/CDK2/CAMSAP1/TRIM28/PIH1D1/SEMA4A/NRP1/KAT2B/SYK/PHF2/VCP/PXN/CPTP/ABL1/PAK1/FNIP2/CLASP1/ARHGEF18/RAPGEF1/SSH3/PLEK2/WARS1/SETDB1/RHOA/VCL/ARPC4/DNM2/ARHGAP6/CKAP5/PTPRJ/WAS/FLNA/RAC2/INPP5K/SAMD1/NF2/GNL3L/NAPA/SDC4/CYFIP2/DNM3/DHX33/RIOK3/DCTN1/GSN/CLSTN1/MED25/RAB1B/CLDN5/TERF2/PPP2R5B/THBS1/SPTBN1/MTPN/SPTA1/MSN/COTL1/SPTB/SELP/SNCA/LRFN3/EIF4G1/NEURL1/VASP/PIP4K2A/RHOG/TMOD1/ELAPOR1/SMPD3/SCIN/PAK2/CAPZB/DNAJB2/WDR45/CDC34/TAL1/CTTN/HTT/PPM1A/ESAM/STMP1/WASF2/CLU/DMTN/ADD1/CNOT1/HCFC1

BP	GO:0000904	cell morphogenesis involved in differentiation	403	-0.46	-1.87	1.11E-10	5.06E-09	3.14E-09	2622	tags=35%, list=22%, signal=28%	EXT1/CAPRIN1/ULK1/RAB8A/MINK1/DIP2B/PLXNC1/ZFPM1/DISC1/PLXNA4/ADNP/PTPN11/NOTCH1/NBEAL2/CASP9/EFNB1/DLG4/NCAM1/TRIOBP/LYPLA2/MAP3K13/ZNF385A/LMTK2/EPHA4/HES1/CDK5/UNK/ABI3/FYN/CRKL/SPI1/FEZ1/SLITRK5/TBCD/DOCK10/CSF1R/PTPN6/MACF1/MYADM/ITGB7/ST6GAL1/SRGAP2/PARVG/PARD6B/RCC2/CUL7/ARHGAP35/APLP2/EDN1/KIAA0319/TIAM1/RERE/DIXDC1/LIMS1/MAP1S/B4GALT5/NDEL1/SEMA4D/DTNBP1/GSK3B/BAIAP2/NEDD9/CYFIP1/HSP90AB1/PREX1/ST14/SIPA1L3/SPINT2/BCL11B/CORO1C/WDR1/MFSD2A/IST1/NEDD4L/PLXNB2/ILK/CDKL5/RAC1/MBP/DPYSL2/PLXND1/FLNB/TRIO/KIF13B/SIPA1L1/MARK2/FBXO31/SRC/EIF4G2/STK25/CALR/MYCBP2/PLXNA1/PPP3CA/TAOK2/ACTB/SEMA4A/NRP1/MAP1A/VLDLR/YWHAH/USP9X/PXN/ABL1/PAK1/PARD3/SYNGAP1/MPL/XK/RHOA/VCL/NOTCH2/DNM2/FLNA/TBC1D20/FERMT3/RAC2/BSG/ECE1/NIBAN2/STK11/DNM3/CFL1/CUX1/PRKDC/VASP/RHOG/GATA1/PARVB/ACTN4/PAK2/ACTN1/MPIG6B/TAL1/CTTN/MYH9/ITGB3/KEL/DMTN/SCN1B/DAB2
BP	GO:0090130	tissue migration	193	-0.55	-2.09	1.15E-10	5.19E-09	3.22E-09	2617	tags=40%, list=22%, signal=32%	ZEB2/CORO1B/JUP/AKT1/PIK3CA/JUN/PTPN11/NOTCH1/HDAC5/INSL3/PKN1/PFN1/MECP2/PRKD2/SPRED1/P2RX4/MAPRE2/VASH1/ROCK2/MACF1/MAP3K3/SCARB1/EDN1/PIK3CB/LGALS12/MMP9/EPPK1/PIK3R3/KLF4/DNAJA4/FOXP1/TGFB1/SVBP/ETS1/AMOT/CCN3/CORO1C/BCAS3/SASH1/RAC1/PTK2B/PLXND1/RRAS/SRC/GREM1/CEACAM1/IQSEC1/CALR/PIK3CD/PTP4A3/SEMA4A/NRP1/PXN/ABL1/CLASP1/RHOA/ACTA2/KANK2/HSPB1/STAT5A/ADIPOR1/GFUS/GLUL/THBS1/PIK3CG/SP1/GPI/GRN/ATP2B4/GPX1/PTPN23/MYH9/ITGB3/SPARC/SH3BP1/MAP2K3/TBXA2R
BP	GO:0030335	positive regulation of cell migration	336	-0.47	-1.90	1.64E-10	7.24E-09	4.49E-09	2733	tags=36%, list=23%, signal=29%	DOCK4/SDCBP/PDPK1/CMKLR1/ATP7A/PECAM1/FGR/PTAFR/CORO1A/AKT1/SELENOK/SWAP70/JUN/NOTCH1/SYNE2/DOCK8/CPNE3/INSL3/CCR1/IL6R/EPHA4/PFN1/PRKD2/CD99L2/ICAM1/CRKL/IL1B/SPI1/P2RX4/CSF1R/MAPRE2/HSPA5/ROCK2/MYADM/MGAT5/RIPOR1/LGALS3/CXCL16/MAP3K3/SMAD3/SCARB1/ACVR1B/PTPRC/EDN1/TIAM1/PIK3CB/RIPOR2/CREB3/CD74/MMP9/PIK3R3/SEMA4D/FOXP1/NEDD9/PYCARD/TGFB1/ACTG1/TRIP6/ETS1/SPHK1/DAPK3/STX4/OXSR1/SUN2/ANO6/LYN/BCAS3/SASH1/RAC1/JAM3/PTK2B/SPN/TRIM32/RRAS/FBXO31/SRC/IQSEC1/CALR/PIK3CD/GNAI2/PPP3CA/FAM89B/ZNF609/STAT3/SEMA4A/NRP1/ARHGEF2/PDGFC/ABL1/PAK1/CLASP1/FOXO4/RHOA/ACTA2/LGALS9/FERMT3/RAC2/MYLK/HSPB1/STAT5A/CAMK1D/GFUS/PLVAP/THBS1/AKT2/PIK3CG/CD151/SP1/GPI/GRN/SELP/ZNF703/MAPK3/ACTN4/ITGB3/SPARC/PDGFA/MAP2K3/DMTN/WNK1/DAB2/DIAPH1
MF	GO:0004672	protein kinase activity	407	-0.46	-1.85	1.63E-10	7.24E-09	4.49E-09	2846	tags=36%, list=24%, signal=29%	MAP3K4/FLT1/NEK7/TESK1/RNASEL/OBSCN/EPHB1/TTN/PRKAB1/EIF2AK2/PDPK1/RPS6KA2/CDK12/CAD/FGR/ULK1/MAPK14/PAK4/MARK4/MINK1/AKT1/BMP2K/PIM3/TAOK3/PIK3CA/MAPK7/POMK/CPNE3/MAP4K5/MAP3K13/LMTK2/IKBKE/EPHA4/PKN1/MAPKAPK3/CDK5/PRKD2/DDR1/FYN/STK40/CSNK2A1/TOP1/RIPK1/CSF1R/NUAK2/ROCK2/MKNK2/PRAG1/TNK2/TESK2/FES/CDK9/MAP3K3/WEE1/ACVR1B/PHKG2/CSNK1G1/ENG/TAF1/GRK2/PDK3/MAP3K11/MARK3/ACVR2B/GSK3B/PRKACA/GSK3A/RPS6KA1/CDK6/TEC/TAOK1/BCKDK/DYRK1B/DAPK3/DAPK1/NEK6/OXSR1/MAP3K5/CDC42BPB/MAP4K1/VRK3/LYN/ILK/CDKL5/PINK1/PTK2B/STK10/MAP2K7/TRIO/STK24/MARK2/SRC/CCND3/DCAF1/SRPK2/STK25/NEK5/PSKH1/MAST3/CDK2/LRRK1/FAM20C/TAOK2/TRIM28/NRP1/MAP2K2/PDK2/BCR/JAK1/SYK/JAK3/ABL1/PAK1/PIM2/SCYL1/HIPK1/MAPKAPK2/MYLK/HIPK2/CDK19/CAMK1D/STK11/SIK3/RIOK3/GAK/IGF2R/SQSTM1/PPP1R9B/PRKCB/CSK/AKT2/RPS6KA4/AKAP13/PIK3CG/ARAF/MMD/CAMK1/PRKDC/PEAK3/MAPK3/BRD4/CDKN1A/PAK2/EIF2AK1/LTBP1/MAP2K3/WNK1/PIM1

BP	GO:0060627	regulation of vesicle-mediated transport	362	-0.46	-1.87	1.67E-10	7.28E-09	4.51E-09	1925	tags=31%, list=16%, signal=27%	SIRPG/FES/LGALS3/MIB1/NR1H2/ITGAM/PACSIN1/ARRB1/SCARB1/SYNJ1/HGS/PTPRC/NEU3/PIK3CB/CD14/ANKFY1/CD300LF/IL2RG/SNX12/AP2B1/SIRPB1/SMPD1/DGKD/DTNBP1/HYAL3/GSK3B/IL2RB/NR1H3/PYCARD/ATP9A/GIT1/CD63/DNAJC5/SPHK1/EZR/STX4/EHD4/ALOX15/RAB31/HIP1/ANO6/AP2S1/HIP1R/LYN/NEDD4L/RAB7A/RAC1/ARF1/DYSF/GRIPAP1/SEPTIN1/SRC/GREM1/CEACAM1/CALR/RUBCN/GNAI2/SNX3/SEPTIN4/PPP3CA/RIN3/NRP1/MAP2K2/CNN2/BCR/SYK/ABL1/CLASP1/RAB27B/ARRB2/MYO18A/HNRNPK/CALM3/DNM2/LDLRAP1/PTPRJ/LGALS9/RAB5B/RAB11B/TBC1D20/RAB3D/RAC2/ARHGAP1/USP7/DOCK2/CAMK1D/VPS28/SDC4/STON2/SYTL4/LRP1/PRKCB/CSK/AKT2/PTPN1/CD151/RAB2B/MSN/SNCA/LAMP1/TBC1D5/P2RX1/MAPK3/GATA1/CBL/EHD1/SMPD3/RUFY1/PTPN23/AP2M1/ITGB3/CLU/TGM2/DAB2
CC	GO:0005768	endosome	749	-0.39	-1.67	1.92E-10	8.11E-09	5.03E-09	2597	tags=33%, list=22%, signal=27%	RFTN1/ULK1/SIGLEC1/GRAP2/STEAP3/SORL1/RAB8A/SBF2/CORO1A/STARD3/ABCA3/SH3TC2/AP2A2/SYT11/LZTR1/ABCB11/AP4E1/CD22/CLN3/LAMP2/TMBIM1/AGAP2/RUSC1/MVB12B/TSG101/M6PR/SNX27/WASHC5/CLEC18A/TUBGCP4/LMTK2/EPHA4/PKN1/HLA-DRB1/MAGI2/PI4K2A/MLC1/FYN/ABHD17C/HLA-DQA2/CMTM6/MARCHF3/VPS39/PLIN3/YKT6/SNX17/HLA-A/VPS4B/RIPK1/VPS51/ATP13A3/USP10/MFSD12/SLC9A7/TUBG1/ANKRD50/DIAPH2/DOP1A/TNK2/TCN2/NDRG1/TPP1/PACSIN1/RCC2/TOM1/BIRC6/DERL1/F8A1/HGS/PI4KB/KIAA0319/NEU3/WDFY4/ARPC5/CHMP6/FZD5/CD14/TRAK1/CD74/ANKFY1/RHOBTB1/RAP1GAP/HLA-C/VPS41/STX6/IL2RG/WDR81/SNX12/VPS37B/CHMP4B/AP2B1/SMPD1/WASHC2A/TM9SF4/DTNBP1/TRAPPC5/YIPF2/VPS53/GRB2/HLA-DQA1/EHD3/HYAL3/IL2RB/TBC1D17/NAPEPLD/ATP9A/CD63/CLTC/SPHK1/EZR/SNX10/STX4/ATP6AP1/EHD4/CORO1C/TRAF6/CHID1/PLD4/SUN2/BACE2/RAB31/UNC93B1/UBA52/AP2S1/ITSN1/LNPEP/NEDD4L/SNX15/VTI1B/RAB7A/RAB11FIP1/LEPROT/RAC1/PLEKHM2/VOPP1/IFITM3/PRF1/TMEM184A/MICALL1/PLD3/MON1B/DYSF/SH3GL1/NCSTN/GRIPAP1/SLC39A4/SRC/CHMP1A/PML/RUBCN/SLA/SNX3/HLA-DRB5/ARHGAP32/VTI1A/PTP4A3/TGOLN2/PLEKHF1/CDK2/RIN3/PSAP/RAB11FIP4/LITAF/IFITM2/UBA1/NRP1/MAP2K2/CTSE/DENND6B/JAK1/SNX11/TRAK2/JAK3/CPTP/ATP6V0D1/RAB14/OPTN/UBAP1/LTF/RAB27B/RAPGEF1/ANXA6/DTX3L/ARRB2/ATP6V0C/LAMTOR1/RNF11/CLCN5/EPN1/RHOA/DNM2/LDLRAP1/RAB5B/RAB11B/ARL8A/ZNRF1/RAB3D/BSG/ECE1/ARHGAP1/NF2/ATP6V0A1/TMEM127/MCOLN1/SLC6A9/CDIP1/VPS28/SIAH2/UBB/MMGT1/SPG21/SORT1/SYTL4/CLSTN1/SLC66A2/IGF2R/SQSTM1/LRP1/RILP/AKT2/PTPN1/MARCHF2/GRN/MMD/LAMP1/TBC1D5/MAPK3/ELAPOR1/MARCHF8/TGFBRAP1/ACKR1/CTSD/EHD1/GAPVD1/SLC48A1/CTSB/RUFY1/SLC15A3/FURIN/UBXN6/PTPN23/AP2M1/HTT/WASF2/SCAMP2

CC	GO:0099081	supramolecular polymer	539	-0.42	-1.74	2.50E-10	1.05E-08	6.50E-09	2276	tags=32%, list=19%, signal=27%	SYNM/TUBGCP4/TUBGCP6/HLA-DRB1/MAP4/PCNT/TUBB2A/CDK5/NDE1/ASPM/FYN/NCKAP5L/CDK5RAP2/MAP7/FEZ1/TTLL1/TBCD/FBF1/SMN1/MTA1/MAPRE2/TUBA1C/NAV1/MACF1/LCP1/TUBG1/TUBG2/DIAPH2/SVIL/EIF6/NDRG1/LZTS2/PDLIM7/RCSD1/NUMA1/RCC2/MAP1LC3B/SYNE1/FMN1/PDE4B/SYNJ1/CDK2AP2/KEAP1/SHROOM1/CHMP6/MAP1S/EPPK1/NDEL1/CCT3/TPX2/EIF3A/CHMP4B/MAP3K11/VIM/SLAIN2/TPM3/KIF1C/BAIAP2/AKNA/ACTG1/FLACC1/KLHL22/PSTPIP2/CLTC/SPECC1L/EML2/RPGRIP1L/EML3/TUBA4A/AMOT/EZR/KIF3B/NEK6/MYZAP/CORO1C/ALDOA/TPM1/KATNB1/BCAS3/DCTN4/ILK/RAC1/JAM3/DPYSL2/ARF1/FLNB/KIF13B/TRIM32/MARK2/HCLS1/MYO9B/DYNC1H1/SRC/CHMP1A/HOOK3/GAS7/AK1/KIF1B/PPP3CA/CAMSAP1/ACTB/FHL3/NRP1/MAP2K2/SLC1A4/MAP1A/ARHGEF2/KAT2B/GABARAP/CSRP1/TUBA8/XIAP/PAK1/KIFC3/FKBP1A/CLASP1/STIM1/CALD1/TUBB4B/SLC2A1/GOLGA2/TPGS2/KLC3/VCL/CALM3/DNM2/LDLRAP1/ARHGAP6/ACTA2/CKAP5/WAS/FLNA/FKBP1B/RAB3D/RAC2/LMNA/HSPB1/MYL4/STK11/SDC4/SPECC1/KRT1/DNM3/RASSF5/DCTN1/PLEC/WIPF1/SLC4A1/SQSTM1/DYNLL2/SPTBN1/IQGAP1/COTL1/ATP2B4/SNCA/ENO1/DYNLRB1/TMOD1/PARVB/ESPN/TUBB/ACTN4/CAPZB/ACTN1/PDLIM1/MYL9/TUBB1/CTTN/MYH9/GAS2L1/LTBP1/DMTN/ANK1/DIAPH1
BP	GO:0090287	regulation of cellular response to growth factor stimulus	148	-0.58	-2.14	2.58E-10	1.07E-08	6.61E-09	2240	tags=41%, list=19%, signal=33%	HES1/PRKD2/SPRED1/IL1B/HSPA5/ZBTB7A/PIN1/NRROS/NUMA1/SMAD3/HGS/PIK3CB/ENG/RBPJ/TGFB1I1/BCL9L/CYFIP1/HSP90AB1/TGFB1/CD63/PCSK6/MEN1/SKI/TWSG1/ILK/C20orf27/HSPA1A/CHST11/CREBBP/GREM1/CDKN1C/ARNT/SFRP2/PPARA/PTP4A3/FAM20C/FAM89B/XIAP/LRG1/ABL1/FKBP1A/NOTCH2/DNM2/HIPK2/NIBAN2/STK11/CYFIP2/PRKCB/PPP2R5B/THBS1/GLG1/PTPN1/FKBP8/ATP2B4/ZNF703/UBE2O/PPM1A/ITGB3/LTBP1/DAB2
BP	GO:0034109	homotypic cell-cell adhesion	66	-0.71	-2.32	2.95E-10	1.21E-08	7.49E-09	1452	tags=44%, list=12%, signal=39%	ACTG1/GNAS/PLEK/LYN/ILK/CD9/CEACAM1/ACTB/JAK1/CSRP1/SYK/TJP2/MPL/GP6/VCL/FLNA/FERMT3/HSPB1/GP1BA/ALOX12/PIK3CG/UBASH3B/ZNF703/GATA1/MYL9/MYH9/ITGB3/DMTN/TLN1
BP	GO:0031589	cell-substrate adhesion	208	-0.54	-2.04	3.06E-10	1.24E-08	7.69E-09	2609	tags=41%, list=22%, signal=33%	PKD1/JUP/SIGLEC1/CORO1A/MINK1/ARL2/DISC1/NOTCH1/CD3E/TRIOBP/CDK5/DDR1/CRKL/TBCD/ROCK2/MACF1/MYADM/ITGB7/TESK2/ST6GAL1/SRGAP2/ITGAM/PARVG/RCC2/TMEM8B/SMAD3/FMN1/TIAM1/PIK3CB/LIMS1/POLDIP2/GSK3B/NEDD9/SORBS3/ACTG1/TRIP6/CD63/CDK6/PREX1/DAPK3/MEN1/CORO1C/ALOX15/ZYX/BCAS3/ILK/RAC1/JAM3/PTK2B/SLC9A1/BCL2L11/RRAS/SRC/GREM1/CALR/ATP1B2/TAOK2/NRP1/BCR/PXN/ABL1/CLASP1/PLEKHA2/ITGAL/RHOA/VCL/DNM2/ARHGAP6/PTPRJ/FLNA/FERMT3/RAC2/RSU1/NF2/SDC4/ITGB5/GFUS/THBS1/PARVB/ACTN4/ACTN1/CTTN/ITGB3/ITGA2B/DMTN/DAB2
CC	GO:0030863	cortical cytoskeleton	70	-0.70	-2.29	3.09E-10	1.24E-08	7.70E-09	1336	tags=50%, list=11%, signal=45%	EZR/MYO1E/MYZAP/WDR1/HCLS1/ACTB/CAP1/CLASP1/CALD1/SLC2A1/PVALB/LASP1/NF2/MPP1/GSN/SLC4A1/EPB42/CLDN5/PPP1R9B/PRKCB/SPTBN1/EPB41/AKAP13/IQGAP1/SPTA1/COTL1/SPTB/GYPC/TMOD1/ACTN4/CAPZB/ACTN1/CTTN/MYH9/DMTN
BP	GO:0022604	regulation of cell morphogenesis	219	-0.52	-2.01	3.12E-10	1.24E-08	7.70E-09	2642	tags=45%, list=23%, signal=35%	FGR/CORO1B/CAPRIN1/EPS8/CORO1A/MUL1/PLXNC1/AGO4/PLXNA4/DLG4/TRIOBP/ARHGAP18/MAP3K13/EPHA4/MOV10/FGD6/FYN/FMNL1/ARAP1/CRKL/PLEKHO1/SH3KBP1/CSF1R/MACF1/MYADM/PRAG1/ST6GAL1/FES/FMNL3/RCC2/CUL7/ARHGAP35/ZMYM4/ZMYM3/LIMS1/RHOBTB1/RHOF/SEMA4D/BCL9L/BAIAP2/NEDD9/CYFIP1/PREX1/EZR/DAPK3/CDC42EP4/CORO1C/WDR1/ALDOA/MFSD2A/BCL11A/TPM1/ZRANB1/NEDD4L/PLXNB2/ILK/CDKL5/RAC1/PTK2B/PLXND1/MARK2/FBXO31/SRC/CPNE5/CALR/DVL3/PLXNA1/CAMSAP1/TAOK2/SEMA4A/NRP1/ABL1/FGD4/ARHGEF18/MPL/RHOA/DNM2/FLNA/RAC2/GNA12/CFL1/EPB42/EPB41/SPTA1/MSN/CUX1/PRKDC/PEAK3/RHOG/PARVB/WASF3/ACTN4/CAPZB/MYH9/SPARC/DMTN/DAB2/DIAPH1

BP	GO:0048534	hematopoietic or lymphoid organ development	674	-0.40	-1.68	3.43E-10	1.35E-08	8.38E-09	2485	tags=31%, list=21%, signal=26%	TMOD3/ZFPM1/JUN/TIPARP/PTPN11/NOTCH1/TRPM2/NBEAL2/BAK1/CD3E/CASP9/EGR1/ZFP36/HDAC4/HDAC5/EZH2/FLCN/SGPL1/HOXB3/CCR1/ZNF385A/G6PD/PRDM1/IL6R/HES1/PKN1/HLA-DRB1/IRF1/DPF2/HYAL2/BATF2/CD81/CRKL/THRA/MITF/IL1B/SPI1/EEF2/DOCK10/RIPK1/CSF1R/PTPN6/SELPLG/SH3PXD2A/ZBTB16/MKNK2/PGLYRP1/OSCAR/PPARGC1B/ZBTB7A/ZFP36L2/EIF6/TRIB1/FES/CIAPIN1/NRROS/XRCC6/SOX13/TFE3/ACVR1B/RUNX1/PTPRC/DROSHA/FZD5/INPP5D/RBPJ/CD74/MMP9/ARMC6/KLF4/SOX4/FOXP1/TOP2A/TOB2/TSPO2/TGFB1/CTNNBIP1/ASH2L/CDK6/TBX21/TMEM131L/ACIN1/PREX1/ETS1/CBFA2T3/PRR7/ETV6/MYO1E/SNX10/CNOT4/SIPA1L3/BCL11B/ATP6AP1/PLEK/IL4I1/NCOA6/TWSG1/PARP1/TRAF6/WDR1/PLD4/LYN/YY1/KLF13/EPAS1/ADAR/JAM3/PTK2B/RARA/HSPA1A/KLF2/KDELR1/SPN/BAD/HCLS1/BCL2L11/RRAS/SRC/PML/KAT7/CDKN1C/SLC25A38/DCAF1/PUS7/ARNT/CEACAM1/PIK3CD/SFRP2/LRRC8A/VAV1/RUNX3/LRRK1/FAM20C/ZMIZ1/TRIM10/BPGM/STAT3/SEMA4A/CNN2/BCR/JAK1/SYK/JAK3/ABL1/MAFB/ARIH2/FOS/TNFSF4/KMT2A/PITHD1/LTF/AHSP/JARID2/MPL/RHOA/MFHAS1/NOTCH2/HIPK1/PTPRJ/LGALS9/GPR137/MTURN/HIPK2/FASN/GLRX5/KLF1/DOCK2/STK11/ROGDI/TTC7A/PABPC4/KAT6A/SLC4A1/EPB42/SETD1A/SPTA1/MEIS1/FAM210B/UBASH3B/PAF1/PRKDC/PIP4K2A/SOX12/IKZF1/MAPK3/GATA1/FBXO7/HLX/LYL1/PF4/SMPD3/SCIN/ST3GAL1/ACTN1/ALAS2/FOXO3/TESC/MPIG6B/FAXDC2/MED1/EIF2AK1/TAL1/CUL4A/MYH9/TRIM58/GFI1B/WASF2/DMTN/PIM1/PBX1
BP	GO:0010631	epithelial cell migration	192	-0.55	-2.07	3.75E-10	1.45E-08	8.98E-09	2617	tags=40%, list=22%, signal=31%	ZEB2/CORO1B/JUP/AKT1/PIK3CA/JUN/PTPN11/NOTCH1/HDAC5/INSL3/PKN1/PFN1/MECP2/PRKD2/SPRED1/P2RX4/MAPRE2/VASH1/ROCK2/MACF1/MAP3K3/SCARB1/EDN1/PIK3CB/LGALS12/MMP9/EPPK1/PIK3R3/KLF4/DNAJA4/FOXP1/TGFB1/SVBP/ETS1/AMOT/CCN3/CORO1C/BCAS3/SASH1/RAC1/PTK2B/PLXND1/RRAS/SRC/GREM1/CEACAM1/IQSEC1/CALR/PIK3CD/PTP4A3/SEMA4A/NRP1/PXN/ABL1/CLASP1/RHOA/KANK2/HSPB1/STAT5A/ADIPOR1/GFUS/GLUL/THBS1/PIK3CG/SP1/GPI/GRN/ATP2B4/GPX1/PTPN23/MYH9/ITGB3/SPARC/SH3BP1/MAP2K3/TBXA2R
BP	GO:0090132	epithelium migration	192	-0.55	-2.07	3.75E-10	1.45E-08	8.98E-09	2617	tags=40%, list=22%, signal=31%	ZEB2/CORO1B/JUP/AKT1/PIK3CA/JUN/PTPN11/NOTCH1/HDAC5/INSL3/PKN1/PFN1/MECP2/PRKD2/SPRED1/P2RX4/MAPRE2/VASH1/ROCK2/MACF1/MAP3K3/SCARB1/EDN1/PIK3CB/LGALS12/MMP9/EPPK1/PIK3R3/KLF4/DNAJA4/FOXP1/TGFB1/SVBP/ETS1/AMOT/CCN3/CORO1C/BCAS3/SASH1/RAC1/PTK2B/PLXND1/RRAS/SRC/GREM1/CEACAM1/IQSEC1/CALR/PIK3CD/PTP4A3/SEMA4A/NRP1/PXN/ABL1/CLASP1/RHOA/KANK2/HSPB1/STAT5A/ADIPOR1/GFUS/GLUL/THBS1/PIK3CG/SP1/GPI/GRN/ATP2B4/GPX1/PTPN23/MYH9/ITGB3/SPARC/SH3BP1/MAP2K3/TBXA2R
BP	GO:0071417	cellular response to organonitrogen compound	396	-0.45	-1.84	5.02E-10	1.92E-08	1.19E-08	1792	tags=28%, list=15%, signal=24%	PDE4B/OR10H1/SLC29A2/RRAGD/SH2B2/EDN1/SMARCC1/TAF1/GNAZ/NDEL1/PIK3R3/ABCC1/DTNBP1/EHMT2/VIM/GRB2/SREBF1/GSK3B/BAIAP2/PRKACA/GSK3A/CYFIP1/KLHL22/YWHAG/ZNF106/EZR/GNAS/MEN1/UCP2/SLC27A1/WDTC1/PARP1/BCL11A/UBR2/SESN2/RAB31/AQP1/LYN/LEPROT/GNA15/IGF2/KLF2/SLC9A1/GNB1/NCSTN/BCL2L11/SRC/CCND3/CEACAM1/CA2/GNAI2/PKM/MAX/ACTB/STAT3/CTNNA1/RANGAP1/PDK2/ARHGEF2/KAT2B/PDGFC/JAK1/PXN/JAK3/ABL1/RRAGC/FBP1/RELA/FOXO4/NFKB1/RAPGEF1/LPIN2/ARRB2/LAMTOR1/SESN3/DNM2/FLNA/INPP5K/STAT5A/KLF1/BCL2L1/ADIPOR1/LRP1/PPP1R9B/PRKCB/CSK/AKT2/HRH4/PTPN1/PIK3CG/KLF16/SP1/ATP2B4/SNCA/NEURL1/PRKDC/AHCYL1/CASTOR2/PIP4K2A/MAPK3/GATA1/EIF4EBP2/SMPD3/FOXO3/KLF3/DMTN/LARP1/TGM2/DIAPH1

CC	GO:0099512	supramolecular fiber	538	-0.42	-1.74	5.43E-10	2.05E-08	1.27E-08	2276	tags=32%, list=19%, signal=27%	SYNM/TUBGCP4/TUBGCP6/HLA-DRB1/MAP4/PCNT/TUBB2A/CDK5/NDE1/ASPM/FYN/NCKAP5L/CDK5RAP2/MAP7/FEZ1/TTLL1/TBCD/FBF1/SMN1/MTA1/MAPRE2/TUBA1C/NAV1/MACF1/LCP1/TUBG1/TUBG2/DIAPH2/SVIL/EIF6/NDRG1/LZTS2/PDLIM7/RCSD1/NUMA1/RCC2/MAP1LC3B/SYNE1/FMN1/PDE4B/SYNJ1/CDK2AP2/KEAP1/SHROOM1/CHMP6/MAP1S/EPPK1/NDEL1/CCT3/TPX2/EIF3A/CHMP4B/MAP3K11/VIM/SLAIN2/TPM3/KIF1C/BAIAP2/AKNA/ACTG1/FLACC1/KLHL22/PSTPIP2/CLTC/SPECC1L/EML2/RPGRIP1L/EML3/TUBA4A/AMOT/EZR/KIF3B/NEK6/MYZAP/CORO1C/ALDOA/TPM1/KATNB1/BCAS3/DCTN4/ILK/RAC1/JAM3/DPYSL2/ARF1/FLNB/KIF13B/TRIM32/MARK2/HCLS1/MYO9B/DYNC1H1/SRC/CHMP1A/HOOK3/GAS7/AK1/KIF1B/PPP3CA/CAMSAP1/ACTB/FHL3/NRP1/MAP2K2/SLC1A4/MAP1A/ARHGEF2/KAT2B/GABARAP/CSRP1/TUBA8/XIAP/PAK1/KIFC3/FKBP1A/CLASP1/STIM1/CALD1/TUBB4B/SLC2A1/GOLGA2/TPGS2/KLC3/VCL/CALM3/DNM2/LDLRAP1/ARHGAP6/ACTA2/CKAP5/WAS/FLNA/FKBP1B/RAB3D/RAC2/LMNA/HSPB1/MYL4/STK11/SDC4/SPECC1/KRT1/DNM3/RASSF5/DCTN1/PLEC/WIPF1/SLC4A1/SQSTM1/DYNLL2/SPTBN1/IQGAP1/COTL1/ATP2B4/SNCA/ENO1/DYNLRB1/TMOD1/PARVB/ESPN/TUBB/ACTN4/CAPZB/ACTN1/PDLIM1/MYL9/TUBB1/CTTN/MYH9/GAS2L1/LTBP1/DMTN/ANK1/DIAPH1
BP	GO:0040017	positive regulation of locomotion	348	-0.46	-1.85	6.16E-10	2.31E-08	1.43E-08	2244	tags=35%, list=19%, signal=29%	DOCK4/SDCBP/PDPK1/CMKLR1/ATP7A/PECAM1/FGR/PTAFR/CORO1A/AKT1/SELENOK/SWAP70/JUN/NOTCH1/SYNE2/DOCK8/CPNE3/INSL3/CCR1/IL6R/EPHA4/PFN1/PRKD2/CD99L2/ICAM1/CRKL/IL1B/SPI1/P2RX4/CSF1R/MAPRE2/HSPA5/ROCK2/MYADM/MGAT5/RIPOR1/LGALS3/CXCL16/MAP3K3/SMAD3/SCARB1/ACVR1B/PTPRC/EDN1/TIAM1/PIK3CB/RIPOR2/CREB3/CD74/MMP9/PIK3R3/SEMA4D/FOXP1/NEDD9/PYCARD/TGFB1/ACTG1/TRIP6/ETS1/SPHK1/DAPK3/STX4/OXSR1/SUN2/ANO6/LYN/BCAS3/SASH1/RAC1/JAM3/PTK2B/SPN/TRIM32/RRAS/FBXO31/SRC/IQSEC1/CALR/PIK3CD/GNAI2/PPP3CA/FAM89B/ZNF609/STAT3/SEMA4A/NRP1/ARHGEF2/PDGFC/ABL1/PAK1/CLASP1/FOXO4/RHOA/ACTA2/LGALS9/FERMT3/RAC2/MYLK/HSPB1/STAT5A/CAMK1D/GFUS/PLVAP/THBS1/AKT2/PIK3CG/CD151/SP1/GPI/GRN/SELP/ZNF703/MAPK3/ACTN4/ITGB3/SPARC/PDGFA/MAP2K3/DMTN/WNK1/DAB2/DIAPH1

BP	GO:0002520	immune system development	729	-0.39	-1.65	6.44E-10	2.39E-08	1.48E-08	2544	tags=31%, list=22%, signal=26%	LY9/FOXP3/TNFSF13/SWAP70/CBLB/TMOD3/ZFPM1/JUN/TIPARP/PTPN11/NOTCH1/TRPM2/NBEAL2/BAK1/CD3E/CASP9/EGR1/ZFP36/HDAC4/HDAC5/EZH2/FLCN/SGPL1/HOXB3/CCR1/ZNF385A/G6PD/PRDM1/IL6R/HES1/PKN1/HLA-DRB1/IRF1/DPF2/HYAL2/BATF2/CD81/CRKL/THRA/MITF/IL1B/SPI1/EEF2/DOCK10/RIPK1/CSF1R/PTPN6/SELPLG/SH3PXD2A/ZBTB16/MKNK2/PGLYRP1/OSCAR/PPARGC1B/ZBTB7A/ZFP36L2/EIF6/TRIB1/FES/CIAPIN1/MCM3AP/NRROS/XRCC6/SOX13/TFE3/SMAD3/ACVR1B/RUNX1/PTPRC/DROSHA/FZD5/INPP5D/RBPJ/CD74/MMP9/ARMC6/KLF4/SOX4/FOXP1/TOP2A/TOB2/TSPO2/TGFB1/CTNNBIP1/ASH2L/CDK6/TBX21/TMEM131L/ACIN1/PREX1/ETS1/CBFA2T3/PRR7/ETV6/MYO1E/SNX10/CNOT4/SIPA1L3/BCL11B/ATP6AP1/PLEK/IL4I1/NCOA6/TWSG1/PARP1/TRAF6/WDR1/PLD4/LYN/YY1/KLF13/EPAS1/SUPT6H/ADAR/JAM3/PTK2B/RARA/HSPA1A/KLF2/KDELR1/SPN/BAD/PDCD1/HCLS1/BCL2L11/RRAS/SRC/PML/KAT7/CDKN1C/SLC25A38/DCAF1/PUS7/ARNT/CEACAM1/PIK3CD/SFRP2/LRRC8A/VAV1/RUNX3/LRRK1/FAM20C/ZMIZ1/TRIM10/TP53BP1/HMCES/BPGM/STAT3/SEMA4A/CNN2/BCR/PHLPP1/JAK1/SYK/JAK3/ABL1/MAFB/ARIH2/FOS/TNFSF4/KMT2A/PITHD1/LTF/AHSP/JARID2/MPL/RHOA/POLL/MFHAS1/NOTCH2/MAD2L2/HIPK1/PTPRJ/LGALS9/GPR137/MTURN/HIPK2/FASN/GLRX5/KLF1/DOCK2/STK11/ROGDI/TTC7A/PABPC4/KAT6A/SLC4A1/EPB42/SETD1A/SPTA1/MEIS1/FAM210B/UBASH3B/PAF1/PRKDC/PIP4K2A/SOX12/IKZF1/MAPK3/GATA1/FBXO7/HLX/LYL1/PF4/SMPD3/SCIN/ST3GAL1/ACTN1/ALAS2/FOXO3/TESC/MPIG6B/FAXDC2/MED1/EIF2AK1/TAL1/CUL4A/MYH9/TRIM58/GFI1B/WASF2/DMTN/PIM1/PBX1
BP	GO:0044057	regulation of system process	263	-0.50	-1.95	8.14E-10	3.00E-08	1.86E-08	2508	tags=35%, list=21%, signal=28%	KCNH2/POMC/PTPN11/NOTCH1/ATP1A1/TRPC3/ATP2A2/HDAC4/DLG4/FOXO1/EIF4A3/G6PD/MECP2/MTG1/THRA/ATP1B1/HRH2/P2RX4/ORMDL3/ROCK2/SNTA1/CDK9/NR1H2/SMAD3/PDE4B/EDN1/NPTXR/FTO/GRK2/KCNJ2/EHD3/SREBF1/BAIAP2/CRY2/PRKACA/NR1H3/GSK3A/TNFRSF1B/RANGRF/SPHK1/CCN3/DAPK3/PARP1/TPM1/AQP1/HIP1R/YY1/EPAS1/PTK2B/SLC9A1/SRC/DLGAP4/GNAI2/PPARA/PPP3CA/ATP1B2/ABL1/PARD3/ANXA6/BMP6/JARID2/GAA/PDE5A/GALR1/RHOA/PDE9A/CALM3/FLNA/GUCY1A1/FKBP1B/LMNA/INPP5K/ECE1/MYL4/EPB41/MTPN/PIK3CG/PER2/ATP2B4/ENO1/P2RX1/RNF10/WASF3/FOXO3/MYL9/CTTN/SHISA7/MGLL/SCN1B/WNK1/DAB2/TBXA2R

BP	GO:0051241	negative regulation of multicellular organismal process	575	-0.41	-1.72	8.69E-10	3.17E-08	1.97E-08	2615	tags=35%, list=22%, signal=28%	RAB11FIP5/JUP/ULK1/BPI/SORL1/MACIR/DIP2B/FOXP3/MUL1/POMC/SYT11/ZFPM1/SERPINF2/KNG1/PTPN11/NOTCH1/NR1D1/ATP1A1/ATP2A2/RAI1/ZFP36/HDAC5/EZH2/FLCN/FOXO1/BANF1/TSPO/CCR1/OAS3/G6PD/EPHA4/HES1/HLA-DRB1/ABCD1/MECP2/IRF1/CDK5/USF3/SPRED1/LGALS9C/IL1B/SPI1/P2RX4/PROS1/PTPN6/VASH1/CERS2/ROCK2/TLR6/PGLYRP1/PCTP/TIMP1/GSTP1/NDRG2/TRIB1/NR1H2/AGO1/ARRB1/TFE3/SMAD3/PDE4B/RUNX1/HGS/PTPRC/EDN1/KIAA0319/PIK3CB/INPP5D/RBPJ/CD74/GRK2/RAP1GAP/NOVA1/EPPK1/OTUD7B/KLF4/SEMA4D/DNAJA4/SIGIRR/CTDSP1/KDM4A/TOB2/ACVR2B/GSK3B/CRY2/NLRX1/NR1H3/SFMBT1/GSK3A/PYCARD/CACTIN/HSP90AB1/TGFB1/SVBP/GIT1/ASCL2/LILRA5/CDK6/TBX21/TMEM131L/TNFRSF1B/APOM/AMOT/CCN3/VGLL4/EZR/BCOR/SKI/TWSG1/CORO1C/CHID1/BCL11A/LNPEP/LYN/YY1/RAB11FIP1/SERPING1/RAC1/MBP/PTK2B/RARA/CD9/KLF2/PDCD1/ILRUN/SOX6/PML/GREM1/HOOK3/CEACAM1/CALR/SFRP2/GNAI2/PPARA/LTBP3/RUNX3/PPP3CA/LAPTM5/TLE3/CTNNA1/SEMA4A/NRP1/BCR/YWHAH/RFLNB/JAK3/CPTP/MAFB/SYNGAP1/TNFSF4/N4BP1/FOXO4/OTUD5/LTF/NFKB1/ARRB2/JARID2/PDE5A/EPN1/RHOA/MAD2L2/LGALS9/GUCY1A1/GPR137/FKBP1B/LMNA/FOXJ2/NIBAN2/TRIM11/STK11/IP6K1/KRT1/ADIPOR1/GP1BA/ALOX12/CLDN5/CSK/THBS1/RPS6KA4/PIK3CG/GRN/UBASH3B/ATP2B4/E2F2/GATA1/RNF10/FBXO7/HLX/PF4/PTGDR2/KHSRP/FURIN/PTPN23/CUL4A/SPARC/PDGFA/NPLOC4/WNK1/TBXA2R
BP	GO:0048041	focal adhesion assembly	55	-0.73	-2.29	1.28E-09	4.57E-08	2.84E-08	2019	tags=58%, list=17%, signal=48%	ROCK2/MACF1/TESK2/RCC2/SMAD3/FMN1/LIMS1/POLDIP2/ACTG1/TRIP6/DAPK3/CORO1C/BCAS3/RAC1/PTK2B/SLC9A1/SRC/GREM1/TAOK2/NRP1/BCR/ABL1/CLASP1/RHOA/VCL/ARHGAP6/PTPRJ/SDC4/THBS1/ACTN1/CTTN/DMTN
BP	GO:0060429	epithelium development	565	-0.41	-1.71	1.27E-09	4.57E-08	2.84E-08	2247	tags=28%, list=19%, signal=24%	PRDM1/EPHA4/HES1/BTG1/MAGI2/PFN1/PRKD2/SPRED1/DDR1/MAFG/ICAM1/DEAF1/THRA/IL1B/CSF1R/ROCK2/MYADM/BASP1/PDE2A/HSD17B4/LGALS3/MIB1/TPP1/LZTS2/NUMA1/ARHGAP35/SMAD3/FMN1/MAFF/ACVR1B/KEAP1/EDN1/TIAM1/PHACTR4/MAGED1/ENG/FZD5/RBPJ/TGFB1I1/CPT1A/MMP9/EPPK1/KLF4/SOX4/VIM/FOXP1/GRB2/ACVR2B/GSK3B/PRKACA/GSK3A/TGFB1/ACTG1/CTNNBIP1/CDK6/RPGRIP1L/ST14/EZR/MYO1E/SIPA1L3/SPINT2/MEN1/BCL11B/KDM6B/SKI/TRAF6/WDR1/MFSD2A/MTHFR/PLXNB2/ILK/FZD2/LBH/RAC1/RARA/ALDOC/PLXND1/PIAS4/KLF2/DCHS1/MGMT/FLNB/BAD/SRC/PML/GREM1/CDKN1C/CEACAM1/MRTFA/CA2/BBS5/NCOA3/PIK3CD/SFRP2/DVL3/PLXNA1/IFT52/FAM20C/PSAP/TRIM28/ACTB/PPP1CA/PIH1D1/BTBD7/NRP1/BCR/BTRC/VDAC1/TJP2/ABL1/PAK1/CLASP1/RELA/RAPGEF1/BMP6/ACADVL/SIDT2/RHOA/VCL/NOTCH2/ACTA2/SUFU/FLNA/TBC1D20/ITPK1/ECE1/KANK2/FOXJ2/NF2/FASN/SDC4/KRT1/PLEC/FZR1/TXNIP/CLDN5/AKT2/CD151/IQGAP1/TST/MSN/FKBP8/SHARPIN/PRKDC/VASP/ZNF703/TMOD1/GATA1/MICAL2/TUBB/CTSB/GPX1/TOLLIP/MED1/PDGFA/SH3BP1/TGM2/ADD1/PBX1/DAB2

BP	GO:0010562	positive regulation of phosphorus metabolic process	549	-0.41	-1.70	1.30E-09	4.57E-08	2.84E-08	2453	tags=30%, list=21%, signal=25%	ADNP/PTPN11/MOB3B/NCF1/CLN3/CD3E/EGR1/PPP1R15A/AGAP2/EZH2/FLCN/DLG4/CNOT9/PTPA/CHP1/MAP3K13/LMTK2/IL6R/EPHA4/HES1/HLA-DRB1/MAGI2/CDK5/PRKD2/FXR2/PELI2/CD81/DDR1/FYN/CRKL/IL1B/RIPK1/CSF1R/MADD/ROCK2/ENTPD5/VAV2/TLR6/CCNY/TNK2/RALBP1/PPARGC1B/MOB1B/CDK9/PIN1/PRRC1/XRCC6/ARRB1/GCN1/SMAD3/PTPRC/EDN1/MAGED1/ENG/FZD5/STOML2/DGKZ/CD74/MMP9/TPX2/PIK3R3/SEMA4D/MAP3K11/SMPD1/DTNBP1/ACVR2B/PRR5L/NEDD9/HSP90AB1/TGFB1/MOB3A/HDAC3/LILRA5/TEC/TAOK1/SPHK1/SLC27A1/PLEK/EHD4/TWSG1/TRAF6/MAP3K5/KCTD20/PPP1R7/VRK3/LYN/SPPL3/MAVS/ILK/SASH1/PINK1/RAC1/PTK2B/IGF2/MAP2K7/BAD/MARK2/HCLS1/SRC/CCND3/GREM1/ARNT/SH2D3C/SFRP2/PPARA/DVL3/LRRK1/TAOK2/PTBP1/DAXX/PIH1D1/NRP1/MAP2K2/ARHGEF2/VLDLR/PDGFC/SYK/VCP/FBH1/ABL1/PAK1/FNIP2/LTF/BMP6/ARRB2/NIBAN1/PDE5A/RHOA/CALM3/MAD2L2/PTPRJ/HIPK2/STK11/SDC4/GNA12/SQSTM1/CSK/THBS1/AKT2/PTPN1/RPS6KA4/AKAP13/PIK3CG/IQGAP1/PRR5/ARAF/ATP2B4/SNCA/MMD/CAMK1/EIF4G1/ENO1/NEURL1/MAPK3/GATA1/CDKN1A/PAK2/STRADB/FAXDC2/TAL1/ITGB3/PDGFA/CLU/MAP2K3/WNK1/DAB2
BP	GO:0045937	positive regulation of phosphate metabolic process	549	-0.41	-1.70	1.30E-09	4.57E-08	2.84E-08	2453	tags=30%, list=21%, signal=25%	ADNP/PTPN11/MOB3B/NCF1/CLN3/CD3E/EGR1/PPP1R15A/AGAP2/EZH2/FLCN/DLG4/CNOT9/PTPA/CHP1/MAP3K13/LMTK2/IL6R/EPHA4/HES1/HLA-DRB1/MAGI2/CDK5/PRKD2/FXR2/PELI2/CD81/DDR1/FYN/CRKL/IL1B/RIPK1/CSF1R/MADD/ROCK2/ENTPD5/VAV2/TLR6/CCNY/TNK2/RALBP1/PPARGC1B/MOB1B/CDK9/PIN1/PRRC1/XRCC6/ARRB1/GCN1/SMAD3/PTPRC/EDN1/MAGED1/ENG/FZD5/STOML2/DGKZ/CD74/MMP9/TPX2/PIK3R3/SEMA4D/MAP3K11/SMPD1/DTNBP1/ACVR2B/PRR5L/NEDD9/HSP90AB1/TGFB1/MOB3A/HDAC3/LILRA5/TEC/TAOK1/SPHK1/SLC27A1/PLEK/EHD4/TWSG1/TRAF6/MAP3K5/KCTD20/PPP1R7/VRK3/LYN/SPPL3/MAVS/ILK/SASH1/PINK1/RAC1/PTK2B/IGF2/MAP2K7/BAD/MARK2/HCLS1/SRC/CCND3/GREM1/ARNT/SH2D3C/SFRP2/PPARA/DVL3/LRRK1/TAOK2/PTBP1/DAXX/PIH1D1/NRP1/MAP2K2/ARHGEF2/VLDLR/PDGFC/SYK/VCP/FBH1/ABL1/PAK1/FNIP2/LTF/BMP6/ARRB2/NIBAN1/PDE5A/RHOA/CALM3/MAD2L2/PTPRJ/HIPK2/STK11/SDC4/GNA12/SQSTM1/CSK/THBS1/AKT2/PTPN1/RPS6KA4/AKAP13/PIK3CG/IQGAP1/PRR5/ARAF/ATP2B4/SNCA/MMD/CAMK1/EIF4G1/ENO1/NEURL1/MAPK3/GATA1/CDKN1A/PAK2/STRADB/FAXDC2/TAL1/ITGB3/PDGFA/CLU/MAP2K3/WNK1/DAB2

BP	GO:0030097	hemopoiesis	656	-0.40	-1.68	1.31E-09	4.57E-08	2.84E-08	2485	tags=31%, list=21%, signal=26%	TMOD3/ZFPM1/JUN/TIPARP/PTPN11/NOTCH1/TRPM2/NBEAL2/BAK1/CD3E/CASP9/EGR1/ZFP36/HDAC4/HDAC5/EZH2/FLCN/SGPL1/HOXB3/CCR1/ZNF385A/G6PD/PRDM1/IL6R/HES1/HLA-DRB1/IRF1/DPF2/HYAL2/BATF2/CD81/THRA/MITF/IL1B/SPI1/EEF2/DOCK10/RIPK1/CSF1R/PTPN6/SELPLG/SH3PXD2A/ZBTB16/MKNK2/PGLYRP1/OSCAR/PPARGC1B/ZBTB7A/ZFP36L2/EIF6/TRIB1/FES/CIAPIN1/NRROS/XRCC6/SOX13/TFE3/ACVR1B/RUNX1/PTPRC/DROSHA/FZD5/INPP5D/RBPJ/CD74/MMP9/ARMC6/KLF4/SOX4/FOXP1/TOP2A/TOB2/TSPO2/TGFB1/CTNNBIP1/ASH2L/CDK6/TBX21/TMEM131L/ACIN1/PREX1/ETS1/CBFA2T3/PRR7/ETV6/MYO1E/SNX10/CNOT4/SIPA1L3/BCL11B/ATP6AP1/PLEK/IL4I1/NCOA6/TWSG1/PARP1/TRAF6/WDR1/PLD4/LYN/YY1/KLF13/EPAS1/ADAR/JAM3/PTK2B/RARA/HSPA1A/KLF2/KDELR1/SPN/BAD/HCLS1/RRAS/SRC/PML/KAT7/CDKN1C/SLC25A38/DCAF1/PUS7/ARNT/CEACAM1/PIK3CD/SFRP2/LRRC8A/VAV1/RUNX3/LRRK1/FAM20C/ZMIZ1/TRIM10/BPGM/STAT3/SEMA4A/CNN2/BCR/JAK1/SYK/JAK3/ABL1/MAFB/ARIH2/FOS/TNFSF4/KMT2A/PITHD1/LTF/AHSP/MPL/RHOA/MFHAS1/NOTCH2/HIPK1/PTPRJ/LGALS9/GPR137/MTURN/HIPK2/FASN/GLRX5/KLF1/DOCK2/STK11/ROGDI/TTC7A/PABPC4/KAT6A/SLC4A1/EPB42/SETD1A/SPTA1/MEIS1/FAM210B/UBASH3B/PAF1/PRKDC/PIP4K2A/SOX12/IKZF1/GATA1/FBXO7/HLX/LYL1/PF4/SMPD3/SCIN/ST3GAL1/ACTN1/ALAS2/FOXO3/TESC/MPIG6B/FAXDC2/MED1/EIF2AK1/TAL1/CUL4A/MYH9/TRIM58/GFI1B/WASF2/DMTN/PIM1/PBX1
BP	GO:0051050	positive regulation of transport	583	-0.41	-1.72	1.46E-09	5.06E-08	3.14E-08	1925	tags=25%, list=16%, signal=22%	SIRPG/RIPOR1/LGALS3/MIB1/NR1H2/ITGAM/NUMA1/UBE2J2/ARRB1/SMAD3/SYNJ1/HGS/PTPRC/EDN1/CHCHD10/FZD5/CD14/ANKFY1/GRK2/BBC3/CD300LF/IL2RG/AP2B1/SIRPB1/KCNJ2/BCAP31/SOX4/SMPD1/DGKD/TM9SF4/DTNBP1/EHD3/HYAL3/GSK3B/PRR5L/IL2RB/STOM/PRKACA/NR1H3/GSK3A/PYCARD/HSP90AB1/TGFB1/FRMD4A/HDAC3/CD63/LILRA5/EDEM2/EZR/STX4/ARL6IP1/FHL1/RAB31/HIP1/ANO6/AQP1/HIP1R/BCAS3/NEDD4L/MAVS/RAB7A/LEPROT/PINK1/RXRA/SLC9A1/BAD/HCLS1/DYNC1H1/SRC/GREM1/CA2/CALR/GNAI2/LRRC8A/FIS1/PPP3CA/ATP1B2/TRIM28/ACTB/BCR/SYK/JAK3/USP36/ABL1/ARIH2/KMT2A/CLASP1/ABLIM3/STIM1/RAB27B/BMP6/SAE1/ARRB2/MYO18A/HNRNPK/GALR1/CALM3/DNM2/LDLRAP1/PTPRJ/FLNA/TBC1D20/RAB3D/RAC2/MYLK/INPP5K/SLC6A9/DOCK2/CAMK1D/STK11/VPS28/SDC4/DNM3/SYTL4/GLUL/HUWE1/LRP1/AKT2/UBE2L3/GJC2/CD151/RAB2B/TCF7L2/MSN/SNCA/CAMK1/ATG13/LAMP1/AHCYL1/TBC1D5/P2RX1/GATA1/CBL/EHD1/SMPD3/SCIN/ACTN4/TESC/OAZ1/PTPN23/AP2M1/HTT/PPM1A/MLLT6/CLU/SCN1B/WNK1/DAB2
BP	GO:0007178	transmembrane receptor protein serine/threonine kinase signaling pathway	196	-0.52	-1.98	1.86E-09	6.26E-08	3.88E-08	1926	tags=38%, list=16%, signal=32%	HSPA5/SLC33A1/ZBTB7A/PIN1/NRROS/NUMA1/SMAD3/ACVR1B/KIAA0319/ENG/RBPJ/TGFB1I1/BCL9L/VIM/ACVR2B/HSP90AB1/TGFB1/PCSK6/RBM14/CCN3/MEN1/SKI/TGIF2/TWSG1/PARP1/ZYX/LNPEP/ILK/NOMO3/PPM1L/C20orf27/HSPA1A/CHST11/CREBBP/SRC/PML/GREM1/CDKN1C/SFRP2/PPARA/LTBP3/FAM89B/ZMIZ1/STAT3/USP9X/PXN/XIAP/LRG1/ABL1/FOS/FKBP1A/BMP6/ARRB2/NOTCH2/DNM2/NUP93/HIPK2/STK11/ITGB5/CLDN5/LRP1/THBS1/GLG1/SPTBN1/FKBP8/ZNF703/MAPK3/TGFBRAP1/SMPD3/UBE2O/FURIN/PPM1A/LTBP1/DAB2

MF	GO:0051015	actin filament binding	145	-0.57	-2.09	2.01E-09	6.71E-08	4.16E-08	2953	tags=57%, list=25%, signal=43%	TLN2/KPTN/MYO1G/ABLIM1/MYO1D/FSCN1/MYH3/TTN/GAS2L3/MYOM2/ADD2/CORO1B/TMEM201/CORO1A/TPM4/DSTN/SYNE2/SPTAN1/TRIOBP/CORO7/MYO1F/ABI3/MYO15A/FMNL1/MACF1/LCP1/SVIL/FMNL3/RCSD1/SYNE1/SHROOM1/ARPC5/MAP1S/LIMA1/PPP1R9A/TPM3/CYFIP1/ZNF185/PSTPIP2/ACTR3B/EZR/MYO1E/CORO1C/WDR1/TPM1/HIP1/HIP1R/FLNB/HCLS1/GAS7/ARPC1B/FLII/CTNNA1/CTNNAL1/ABL1/ABLIM3/ANXA6/MYO18A/ARPC4/FLNA/LASP1/MYL4/GSN/CFL1/PPP1R9B/SPTBN1/IQGAP1/SPTA1/COTL1/SPTB/TMOD1/ESPN/SCIN/ACTN4/CAPZB/ACTN1/CTTN/MYH9/GAS2L1/DMTN/TLN1/ADD1
BP	GO:0051272	positive regulation of cellular component movement	349	-0.46	-1.84	2.15E-09	7.07E-08	4.39E-08	2244	tags=30%, list=19%, signal=25%	IL6R/EPHA4/PFN1/PRKD2/CD99L2/ICAM1/CRKL/IL1B/SPI1/FEZ1/P2RX4/CSF1R/MAPRE2/HSPA5/ROCK2/MYADM/MGAT5/RIPOR1/LGALS3/CXCL16/MAP3K3/SMAD3/SCARB1/ACVR1B/PTPRC/EDN1/TIAM1/PIK3CB/RIPOR2/CREB3/CD74/MMP9/PIK3R3/SEMA4D/FOXP1/NEDD9/PYCARD/TGFB1/ACTG1/TRIP6/ETS1/SPHK1/DAPK3/STX4/OXSR1/SUN2/ANO6/LYN/BCAS3/SASH1/RAC1/JAM3/PTK2B/SPN/TRIM32/RRAS/FBXO31/SRC/IQSEC1/CALR/PIK3CD/GNAI2/PPP3CA/FAM89B/ZNF609/STAT3/SEMA4A/NRP1/ARHGEF2/PDGFC/ABL1/PAK1/CLASP1/FOXO4/RHOA/ACTA2/LGALS9/FERMT3/RAC2/MYLK/HSPB1/STAT5A/CAMK1D/STK11/GFUS/PLVAP/THBS1/AKT2/PIK3CG/CD151/SP1/GPI/GRN/SELP/ZNF703/MAPK3/ACTN4/ITGB3/SPARC/PDGFA/MAP2K3/DMTN/WNK1/DAB2/DIAPH1
BP	GO:0009725	response to hormone	524	-0.42	-1.73	2.14E-09	7.07E-08	4.39E-08	2578	tags=34%, list=22%, signal=28%	MAPK14/PTAFR/SORL1/RAB8A/AKT1/ABCA3/POMC/PIK3CA/ABCB11/PTPN11/NOTCH1/CYBB/NR1D1/ATP1A1/CASP9/EGR1/DDX54/ZFP36/UBR5/HDAC5/FOXO1/CNOT9/SLC24A4/TSPO/PHEX/HES1/PKN1/BTG1/PIP4K2C/THRA/IL1B/ZNF366/ROCK2/PAQR8/SRSF4/NCOA4/TIMP1/PPARGC1B/ZBTB7A/ZFP36L2/EIF6/NR3C1/SHC1/ALPL/SLC29A2/SH2B2/EDN1/ENG/SMARCC1/NDEL1/TRERF1/PIK3R3/JUND/FOXP1/GRB2/SREBF1/NCOA1/GSK3B/SORD/BAIAP2/CRY2/TIMP2/PRKACA/NR1H3/GSK3A/CYFIP1/TGFB1/ASH2L/SMARCA4/YWHAG/RBM14/IL1RN/ZNF106/NCOA2/INPPL1/MEN1/UCP2/SLC27A1/ALAD/NCOA6/WDTC1/PARP1/GLB1/SESN2/RAB31/AQP1/LYN/YY1/BCAS3/LEPROT/LBH/MBP/PTK2B/RARA/TADA3/IGF2/HSPA1A/RXRA/SLC9A1/BAD/DHCR24/GNB1/HCLS1/BCL2L11/SRC/CCND3/ADIPOR2/CEACAM1/CA2/ARPC1B/NCOA3/CALR/GNAI2/PPARA/WBP2/PELP1/DAXX/ZMIZ1/PKM/MAX/STAT3/CTNNA1/RANGAP1/PDK2/KAT2B/YWHAH/JAK1/PXN/JAK3/PAK1/FOS/TNFSF4/FBP1/RELA/FOXO4/NFKB1/LPIN2/BMP6/SLC2A1/SESN3/RHOA/CALM3/BSG/INPP5K/KANK2/STAT5A/STK11/SORT1/ABHD2/ADIPOR1/SAFB/TXNIP/PPP1R9B/PRKCB/CSK/THBS1/AKT2/UBE2L3/PTPN1/SP1/GPI/PRKDC/AHCYL1/PIP4K2A/GATA1/EIF4EBP2/CTSB/GPX1/RNF40/FOXO3/MED1/ARID1A/GAS2L1/NCOR2/CARM1/CNOT1/DAB2
BP	GO:0043542	endothelial cell migration	140	-0.58	-2.11	2.26E-09	7.37E-08	4.57E-08	2217	tags=38%, list=19%, signal=31%	MECP2/PRKD2/SPRED1/P2RX4/VASH1/ROCK2/MAP3K3/SCARB1/EDN1/PIK3CB/LGALS12/PIK3R3/KLF4/DNAJA4/FOXP1/TGFB1/SVBP/ETS1/AMOT/CCN3/BCAS3/SASH1/RAC1/PTK2B/PLXND1/RRAS/GREM1/CEACAM1/CALR/PIK3CD/PTP4A3/SEMA4A/NRP1/PXN/ABL1/RHOA/HSPB1/STAT5A/GFUS/GLUL/THBS1/PIK3CG/SP1/GPI/GRN/ATP2B4/GPX1/MYH9/ITGB3/SPARC/SH3BP1/MAP2K3/TBXA2R
BP	GO:0045765	regulation of angiogenesis	161	-0.55	-2.05	2.29E-09	7.41E-08	4.59E-08	2232	tags=35%, list=19%, signal=29%	BTG1/MECP2/PRKD2/SPRED1/IL1B/VASH1/ROCK2/AGO1/RUNX1/HGS/PIK3CB/ENG/KLF4/ETS1/AMOT/SPHK1/JMJD8/AQP1/SASH1/PTK2B/PLXND1/KLF2/MTDH/RRAS/PML/GREM1/CEACAM1/PIK3CD/SFRP2/PKM/STAT3/SEMA4A/NRP1/JAK1/LRG1/ABL1/FOXO4/STIM1/WARS1/EPN1/HIPK1/HSPB1/FOXJ2/HIPK2/NIBAN2/KRT1/GLUL/CLDN5/PRKCB/THBS1/SP1/GRN/ATP2B4/E2F2/PF4/SPARC/TBXA2R

BP	GO:0031032	actomyosin structure organization	122	-0.59	-2.12	2.35E-09	7.54E-08	4.67E-08	1476	tags=34%, list=13%, signal=30%	EPB41L2/ARRB1/SMAD3/EDN1/SORBS3/ACTG1/WDR1/TPM1/CDC42BPB/ZYX/RAC1/PTK2B/SLC9A1/SRC/FLII/NRP1/CNN2/CSRP1/PXN/ABL1/PAK1/CLASP1/ARHGEF18/FRMD3/MYO18A/RHOA/ARHGAP6/WAS/INPP5K/NF2/SDC4/ITGB5/PLEC/EPB41/AKAP13/TMOD1/PAK2/PDLIM1/MYL9/MYH9/WASF2
BP	GO:1902903	regulation of supramolecular fiber organization	268	-0.49	-1.92	2.59E-09	8.24E-08	5.11E-08	2610	tags=40%, list=22%, signal=32%	CORO1B/MAPRE1/EPS8/CORO1A/ARL2/SWAP70/PIK3CA/TMOD3/SERPINF2/DSTN/SPTAN1/ATXN7/TRIOBP/WASHC5/ARHGAP18/PFN1/MECP2/CDK5RAP2/SSH1/ARAP1/TBCD/ROCK2/MYADM/SVIL/FES/SSH2/NUMA1/ARHGAP35/SMAD3/FMN1/EDN1/ARPC5/MAP1S/LIMA1/TPX2/GRB2/SLAIN2/BAIAP2/SORBS3/COLGALT1/PYCARD/CYFIP1/ACTG1/GIT1/TAOK1/EML2/RANGRF/DAPK3/CDC42EP4/PLEK/WDR1/TPM1/KATNB1/PRUNE1/ALOX15/HIP1R/RAC1/PTK2B/HSPA1A/ARF1/SLC9A1/HCLS1/ARPC1B/FLII/CAMSAP1/NRP1/MAP1A/ARHGEF2/PXN/ABL1/PAK1/CLASP1/ARHGEF18/SSH3/RHOA/ARPC4/ARHGAP6/CKAP5/WAS/FLNA/INPP5K/NF2/SDC4/CYFIP2/DCTN1/GSN/SPTBN1/AKAP13/MTPN/SPTA1/COTL1/SPTB/SNCA/VASP/TMOD1/WASF3/SCIN/PAK2/CAPZB/GPX1/CTTN/ESAM/WASF2/CLU/SH3BP1/DMTN/ADD1
BP	GO:0090066	regulation of anatomical structure size	295	-0.47	-1.88	3.10E-09	9.78E-08	6.07E-08	2593	tags=35%, list=22%, signal=28%	ULK1/EPS8/CORO1A/DIP2B/SWAP70/PIK3CA/TMOD3/DISC1/SERPINF2/PLXNA4/ADNP/KNG1/DSTN/CLN3/SPTAN1/PPP1R15A/TRIOBP/WASHC5/ARHGAP18/MAP3K13/PFN1/CDK5/SSH1/HRH2/ROCK2/MACF1/VAV2/MYADM/SNTA1/SVIL/SSH2/ARHGAP35/FMN1/EDN1/KIAA0319/ARPC5/LIMA1/NDEL1/SEMA4D/GRB2/GSK3B/BAIAP2/PYCARD/CYFIP1/HSP90AB1/EZR/CDC42EP4/PLEK/WDTC1/WDR1/ALOX15/IST1/ANO6/AQP1/HIP1R/CDKL5/RAC1/PTK2B/DPYSL2/KLF2/HCLS1/EIF4G2/ARPC1B/LRRC8A/VAV1/FLII/RIN3/PPP1CA/SEMA4A/NRP1/ABL1/SSH3/XK/RHOA/ARPC4/DNM2/ACTA2/WAS/GUCY1A1/ECE1/CYFIP2/GSN/RILP/SPTBN1/MTPN/SPTA1/PER2/MSN/COTL1/SPTB/VASP/TMOD1/P2RX1/SCIN/CAPZB/GPX1/CTTN/AP2M1/ESAM/KEL/SH3BP1/DMTN/ADD1/TBXA2R
BP	GO:0031401	positive regulation of protein modification process	671	-0.39	-1.65	3.58E-09	1.09E-07	6.78E-08	2538	tags=31%, list=22%, signal=26%	DIP2B/FOXP3/TAOK3/MUL1/CBLB/PIK3CA/PIAS1/ADNP/PTPN11/MOB3B/NCF1/CLN3/CD3E/EGR1/PPP1R15A/AGAP2/HDAC4/EZH2/FLCN/DLG4/CNOT9/PTPA/CHP1/MAP3K13/IL6R/EPHA4/HES1/HLA-DRB1/MAGI2/MECP2/CDK5/PRKD2/FXR2/GLYR1/PELI2/CD81/FYN/CRKL/IL1B/RIPK1/MTA1/CSF1R/MADD/HSPA5/ROCK2/DCUN1D3/TLR6/CCNY/TNK2/RALBP1/MOB1B/CHFR/CDK9/PIN1/PRRC1/XRCC6/ARRB1/DERL1/PTPRC/EDN1/MAGED1/ENG/FZD5/CD74/MMP9/CTBP1/TPX2/PIK3R3/PHF23/FANCI/WDR5/SEMA4D/MAP3K11/SOX4/SMPD1/DTNBP1/SREBF1/ACVR2B/PRR5L/NELFA/NEDD9/GSK3A/HSP90AB1/TGFB1/MOB3A/HDAC3/JDP2/LILRA5/TEC/TAOK1/SPHK1/SLC27A1/EHD4/TWSG1/TRAF6/MAP3K5/PPP1R7/VRK3/LYN/SPPL3/MAVS/ILK/SASH1/PINK1/RAC1/PTK2B/IGF2/PIAS4/MAP2K7/KANSL1/BRMS1/MARK2/HCLS1/SRC/PML/CCND3/GREM1/KAT7/ARNT/SH2D3C/SFRP2/DVL3/WBP2/SEPTIN4/MYCBP2/LRRK1/LAPTM5/TAOK2/PTBP1/DAXX/PIH1D1/NRP1/MAP2K2/ARHGEF2/VLDLR/BTRC/PDGFC/GABARAP/MCRS1/SYK/VCP/XIAP/FBH1/ABL1/PAK1/KMT2A/FNIP2/FKBP1A/LTF/BMP6/SAE1/ARRB2/JARID2/GOLGA2/NIBAN1/PDE5A/RHOA/CALM3/MAD2L2/PTPRJ/UBE2S/LMNA/ATG7/HIPK2/STK11/SDC4/UBB/RASSF5/GNA12/FZR1/HUWE1/SQSTM1/CSK/THBS1/AKT2/UBE2L3/PTPN1/RPS6KA4/AKAP13/PIK3CG/IQGAP1/PRR5/ARAF/ATP2B4/SNCA/PAF1/MMD/CAMK1/EIF4G1/NEURL1/MAPK3/GATA1/BRD4/CDKN1A/TNIP1/PAK2/STRADB/RNF40/FAXDC2/TOLLIP/TAL1/ITGB3/PDGFA/CLU/MAP2K3/WNK1/DAB2/HCFC1

BP	GO:0090092	regulation of transmembrane receptor protein serine/threonine kinase signaling pathway	133	-0.58	-2.10	3.71E-09	1.13E-07	6.99E-08	1926	tags=38%, list=16%, signal=32%	ZBTB7A/PIN1/NRROS/NUMA1/SMAD3/ACVR1B/KIAA0319/ENG/RBPJ/TGFB1I1/BCL9L/ACVR2B/HSP90AB1/TGFB1/PCSK6/CCN3/MEN1/SKI/TWSG1/PARP1/ILK/NOMO3/C20orf27/HSPA1A/CHST11/CREBBP/GREM1/CDKN1C/SFRP2/PPARA/FAM89B/XIAP/LRG1/ABL1/FKBP1A/BMP6/NOTCH2/DNM2/NUP93/HIPK2/STK11/LRP1/THBS1/GLG1/FKBP8/ZNF703/UBE2O/PPM1A/LTBP1/DAB2
BP	GO:0001934	positive regulation of protein phosphorylation	453	-0.42	-1.74	4.49E-09	1.33E-07	8.26E-08	2453	tags=31%, list=21%, signal=25%	ADNP/PTPN11/MOB3B/NCF1/CLN3/CD3E/EGR1/AGAP2/EZH2/FLCN/DLG4/CNOT9/MAP3K13/IL6R/EPHA4/HES1/HLA-DRB1/CDK5/PRKD2/FXR2/PELI2/CD81/FYN/CRKL/IL1B/RIPK1/CSF1R/MADD/ROCK2/TLR6/CCNY/TNK2/RALBP1/MOB1B/CDK9/PIN1/PRRC1/XRCC6/ARRB1/PTPRC/EDN1/MAGED1/ENG/FZD5/CD74/MMP9/TPX2/PIK3R3/SEMA4D/MAP3K11/DTNBP1/ACVR2B/PRR5L/NEDD9/HSP90AB1/TGFB1/MOB3A/HDAC3/LILRA5/TEC/TAOK1/SPHK1/SLC27A1/EHD4/TWSG1/TRAF6/MAP3K5/LYN/MAVS/ILK/SASH1/PINK1/RAC1/PTK2B/IGF2/MAP2K7/MARK2/HCLS1/SRC/CCND3/GREM1/SH2D3C/SFRP2/DVL3/LRRK1/TAOK2/DAXX/PIH1D1/NRP1/MAP2K2/ARHGEF2/VLDLR/PDGFC/SYK/FBH1/ABL1/PAK1/FNIP2/LTF/BMP6/ARRB2/NIBAN1/PDE5A/RHOA/CALM3/MAD2L2/PTPRJ/HIPK2/STK11/SDC4/SQSTM1/CSK/THBS1/AKT2/PTPN1/RPS6KA4/AKAP13/PIK3CG/IQGAP1/PRR5/ARAF/ATP2B4/SNCA/MMD/CAMK1/EIF4G1/NEURL1/MAPK3/GATA1/CDKN1A/PAK2/STRADB/FAXDC2/TAL1/ITGB3/PDGFA/CLU/MAP2K3/WNK1/DAB2
BP	GO:0034329	cell junction assembly	200	-0.52	-1.98	4.88E-09	1.44E-07	8.92E-08	2217	tags=36%, list=19%, signal=29%	MECP2/CDK5/ABI3/CRKL/IL1B/SLITRK5/TBCD/FBF1/ROCK2/CTNND1/MACF1/TESK2/PKP4/PARD6B/RCC2/SMAD3/FMN1/LSR/FZD5/LIMS1/POLDIP2/SEMA4D/PRKACA/ACTG1/TRIP6/DAPK3/CORO1C/WDR1/BCAS3/PLXNB2/RAC1/JAM3/PTK2B/PLXND1/CD9/DCHS1/SLC9A1/SRC/GREM1/TAOK2/ACTB/CTNNA1/SEMA4A/NRP1/BCR/ABL1/PARD3/CLASP1/RAPGEF1/RHOA/VCL/ARHGAP6/HIPK1/PTPRJ/SDC4/DNM3/PLEC/CLSTN1/CLDN5/THBS1/SNCA/LRFN3/EIF4G1/ZNF703/PAK2/ACTN1/CTTN/ESAM/SH3BP1/DMTN/TLN1

BP	GO:0030182	neuron differentiation	745	-0.38	-1.60	5.15E-09	1.51E-07	9.34E-08	2567	tags=30%, list=22%, signal=25%	RAB8A/PAK4/MINK1/AKT1/DIP2B/TAOK3/NCKIPSD/MUL1/PLXNC1/JUN/DISC1/PLXNA4/ADNP/PTPN11/NOTCH1/YWHAZ/CD3E/EFNB1/EZH2/DLG4/NCAM1/TRIOBP/LYPLA2/TSPO/MAP3K13/LMTK2/PRDM1/EPHA4/HES1/MAGI2/MAP4/MECP2/CDK5/MOV10/UNK/ASPM/DDR1/ABI3/FYN/CDK5RAP2/BICDL1/CRKL/CPNE1/FEZ1/SLITRK5/TTLL1/TBCD/DOCK10/CYB5D2/CSF1R/ADNP2/HSPA5/CERS2/MACF1/PRAG1/ITM2C/HMG20B/FES/SRGAP2/PIN1/MIB1/PACSIN1/PARD6B/CUL7/ARHGAP35/WEE1/FRY/RUNX1/APLP2/PMP22/EDN1/KIAA0319/TIAM1/RERE/DIXDC1/FZD5/RBPJ/MAP1S/B4GALT5/RAP1GAP/ANKS1A/NDEL1/KLF4/WDR5/SEMA4D/PPP1R9A/CTDSP1/SOX4/DTNBP1/EHMT2/VIM/KDM4A/NCOA1/GSK3B/ZFHX3/BAIAP2/GSK3A/IER2/CYFIP1/HSP90AB1/SVBP/GIT1/ASCL2/TAOK1/RPGRIP1L/YWHAG/ASAP1/ENC1/TRAPPC4/BCL11B/TGIF2/MFSD2A/BCL11A/KATNB1/IST1/LYN/NEDD4L/PLXNB2/CDKL5/FZD2/RAC1/MBP/PTK2B/RARA/DPYSL2/PLXND1/MICALL1/TRIO/KIF13B/STK24/TRIM32/SIPA1L1/MARK2/FBXO31/CDKN1C/EIF4G2/GAS7/IQSEC1/CPNE5/STK25/CALR/SFRP2/SNX3/OLIG2/DVL3/MYCBP2/RUNX3/PLXNA1/PPP3CA/ATP1B2/CAMSAP1/TAOK2/ZMIZ1/ACTB/STAT3/CTNNA1/SEMA4A/PIGT/TRAPPC9/NRP1/MAP1A/ARHGEF2/VLDLR/YWHAH/USP9X/HERC1/ABL1/PAK1/PARD3/SYNGAP1/BLOC1S3/RAPGEF1/C1QA/BMP6/OLIG1/XK/SCYL1/RHOA/VCL/NOTCH2/DNM2/HIPK1/SUFU/FLNA/FKBP1B/RAC2/BSG/ECE1/HIPK2/NIBAN2/CAMK1D/STK11/NAPA/SDC4/UBB/CYFIP2/DNM3/CFL1/GAK/PPP1R9B/PPP2R5B/MTPN/IQGAP1/MEIS1/GRN/CUX1/MMD/CAMK1/EIF4G1/NEURL1/VASP/RHOG/FBXO7/EHD1/PAK2/FOXO3/MED1/TAL1/CTTN/KEL/CARM1/SCN1B/WNK1/PBX1/DAB2
BP	GO:0110053	regulation of actin filament organization	192	-0.53	-1.99	5.24E-09	1.52E-07	9.43E-08	2610	tags=40%, list=22%, signal=32%	CORO1B/EPS8/CORO1A/SWAP70/PIK3CA/TMOD3/SERPINF2/DSTN/SPTAN1/TRIOBP/WASHC5/ARHGAP18/PFN1/SSH1/ARAP1/ROCK2/MYADM/SVIL/SSH2/ARHGAP35/SMAD3/FMN1/ARPC5/LIMA1/GRB2/BAIAP2/SORBS3/PYCARD/CYFIP1/ACTG1/CDC42EP4/PLEK/WDR1/TPM1/ALOX15/HIP1R/RAC1/PTK2B/ARF1/SLC9A1/HCLS1/ARPC1B/FLII/NRP1/PXN/ABL1/PAK1/CLASP1/ARHGEF18/SSH3/RHOA/ARPC4/ARHGAP6/WAS/FLNA/INPP5K/NF2/SDC4/CYFIP2/GSN/SPTBN1/MTPN/SPTA1/COTL1/SPTB/VASP/TMOD1/WASF3/SCIN/PAK2/CAPZB/CTTN/ESAM/WASF2/SH3BP1/DMTN/ADD1
BP	GO:0007160	cell-matrix adhesion	139	-0.57	-2.08	5.46E-09	1.56E-07	9.68E-08	2019	tags=40%, list=17%, signal=33%	ROCK2/MACF1/ITGB7/TESK2/ITGAM/PARVG/RCC2/TMEM8B/SMAD3/FMN1/TIAM1/PIK3CB/LIMS1/POLDIP2/GSK3B/ACTG1/TRIP6/CD63/CDK6/DAPK3/CORO1C/ZYX/BCAS3/ILK/RAC1/JAM3/PTK2B/SLC9A1/BCL2L11/RRAS/SRC/GREM1/TAOK2/NRP1/BCR/PXN/ABL1/CLASP1/PLEKHA2/ITGAL/RHOA/VCL/ARHGAP6/PTPRJ/FERMT3/NF2/SDC4/ITGB5/GFUS/THBS1/ACTN1/CTTN/ITGB3/ITGA2B/DMTN

BP	GO:0042327	positive regulation of phosphorylation	504	-0.41	-1.71	5.44E-09	1.56E-07	9.68E-08	2453	tags=30%, list=21%, signal=25%	ADNP/PTPN11/MOB3B/NCF1/CLN3/CD3E/EGR1/AGAP2/EZH2/FLCN/DLG4/CNOT9/MAP3K13/LMTK2/IL6R/EPHA4/HES1/HLA-DRB1/CDK5/PRKD2/FXR2/PELI2/CD81/DDR1/FYN/CRKL/IL1B/RIPK1/CSF1R/MADD/ROCK2/ENTPD5/VAV2/TLR6/CCNY/TNK2/RALBP1/PPARGC1B/MOB1B/CDK9/PIN1/PRRC1/XRCC6/ARRB1/GCN1/PTPRC/EDN1/MAGED1/ENG/FZD5/DGKZ/CD74/MMP9/TPX2/PIK3R3/SEMA4D/MAP3K11/DTNBP1/ACVR2B/PRR5L/NEDD9/HSP90AB1/TGFB1/MOB3A/HDAC3/LILRA5/TEC/TAOK1/SPHK1/SLC27A1/EHD4/TWSG1/TRAF6/MAP3K5/KCTD20/LYN/MAVS/ILK/SASH1/PINK1/RAC1/PTK2B/IGF2/MAP2K7/BAD/MARK2/HCLS1/SRC/CCND3/GREM1/ARNT/SH2D3C/SFRP2/DVL3/LRRK1/TAOK2/DAXX/PIH1D1/NRP1/MAP2K2/ARHGEF2/VLDLR/PDGFC/SYK/FBH1/ABL1/PAK1/FNIP2/LTF/BMP6/ARRB2/NIBAN1/PDE5A/RHOA/CALM3/MAD2L2/PTPRJ/HIPK2/STK11/SDC4/SQSTM1/CSK/THBS1/AKT2/PTPN1/RPS6KA4/AKAP13/PIK3CG/IQGAP1/PRR5/ARAF/ATP2B4/SNCA/MMD/CAMK1/EIF4G1/NEURL1/MAPK3/GATA1/CDKN1A/PAK2/STRADB/FAXDC2/TAL1/ITGB3/PDGFA/CLU/MAP2K3/WNK1/DAB2
BP	GO:2000147	positive regulation of cell motility	342	-0.46	-1.86	5.68E-09	1.61E-07	9.99E-08	2733	tags=36%, list=23%, signal=28%	DOCK4/SDCBP/PDPK1/CMKLR1/ATP7A/PECAM1/FGR/PTAFR/CORO1A/AKT1/SELENOK/SWAP70/JUN/NOTCH1/SYNE2/DOCK8/CPNE3/INSL3/CCR1/IL6R/EPHA4/PFN1/PRKD2/CD99L2/ICAM1/CRKL/IL1B/SPI1/P2RX4/CSF1R/MAPRE2/HSPA5/ROCK2/MYADM/MGAT5/RIPOR1/LGALS3/CXCL16/MAP3K3/SMAD3/SCARB1/ACVR1B/PTPRC/EDN1/TIAM1/PIK3CB/RIPOR2/CREB3/CD74/MMP9/PIK3R3/SEMA4D/FOXP1/NEDD9/PYCARD/TGFB1/ACTG1/TRIP6/ETS1/SPHK1/DAPK3/STX4/OXSR1/SUN2/ANO6/LYN/BCAS3/SASH1/RAC1/JAM3/PTK2B/SPN/TRIM32/RRAS/FBXO31/SRC/IQSEC1/CALR/PIK3CD/GNAI2/PPP3CA/FAM89B/ZNF609/STAT3/SEMA4A/NRP1/ARHGEF2/PDGFC/ABL1/PAK1/CLASP1/FOXO4/RHOA/ACTA2/LGALS9/FERMT3/RAC2/MYLK/HSPB1/STAT5A/CAMK1D/GFUS/PLVAP/THBS1/AKT2/PIK3CG/CD151/SP1/GPI/GRN/SELP/ZNF703/MAPK3/ACTN4/ITGB3/SPARC/PDGFA/MAP2K3/DMTN/WNK1/DAB2/DIAPH1

BP	GO:0048699	generation of neurons	782	-0.37	-1.59	5.80E-09	1.64E-07	1.01E-07	2567	tags=30%, list=22%, signal=25%	RAB8A/PAK4/MINK1/AKT1/DIP2B/TAOK3/NCKIPSD/TUBGCP2/MUL1/PLXNC1/JUN/DISC1/PLXNA4/ADNP/PTPN11/NOTCH1/POMK/YWHAZ/CD3E/EFNB1/EZH2/DLG4/NCAM1/TRIOBP/LYPLA2/TSPO/MAP3K13/LMTK2/PRDM1/EPHA4/HES1/MAGI2/MAP4/MECP2/TUBB2A/CDK5/NDE1/MOV10/UNK/ASPM/DDR1/ABI3/FYN/CDK5RAP2/BICDL1/CRKL/CPNE1/FEZ1/SLITRK5/TTLL1/TBCD/DOCK10/CYB5D2/CSF1R/ADNP2/HSPA5/NAV1/CERS2/MACF1/PRAG1/SMARCD3/ITM2C/HMG20B/POMGNT2/FES/SRGAP2/PIN1/MIB1/PACSIN1/PARD6B/CUL7/ARHGAP35/WEE1/FRY/RUNX1/APLP2/PMP22/EDN1/KIAA0319/TIAM1/RERE/DIXDC1/FZD5/RBPJ/MAP1S/B4GALT5/RAP1GAP/ANKS1A/NDEL1/KLF4/WDR5/SEMA4D/PPP1R9A/CTDSP1/SOX4/DTNBP1/EHMT2/VIM/KDM4A/NCOA1/GSK3B/ZFHX3/BAIAP2/GSK3A/IER2/CYFIP1/AKNA/HSP90AB1/SVBP/GIT1/ASCL2/CDK6/TAOK1/RPGRIP1L/YWHAG/ASAP1/ENC1/TRAPPC4/BCL11B/TGIF2/MFSD2A/BCL11A/KATNB1/IST1/LYN/NEDD4L/PLXNB2/CDKL5/FZD2/RAC1/MBP/PTK2B/RARA/DPYSL2/PLXND1/MICALL1/TRIO/KIF13B/STK24/TRIM32/SIPA1L1/MARK2/FBXO31/CDKN1C/EIF4G2/GAS7/IQSEC1/CPNE5/STK25/CALR/SFRP2/SNX3/OLIG2/DVL3/SEPTIN4/MYCBP2/RUNX3/PLXNA1/PPP3CA/ATP1B2/CAMSAP1/TAOK2/ZMIZ1/ACTB/ZNF609/STAT3/CTNNA1/SEMA4A/PIGT/TRAPPC9/NRP1/MAP1A/ARHGEF2/VLDLR/YWHAH/USP9X/HERC1/ABL1/PAK1/PARD3/SYNGAP1/BLOC1S3/RAPGEF1/C1QA/BMP6/OLIG1/XK/SCYL1/RHOA/VCL/NOTCH2/DNM2/HIPK1/SUFU/FLNA/FKBP1B/RAC2/BSG/ECE1/HIPK2/NIBAN2/CAMK1D/STK11/NAPA/SDC4/UBB/CYFIP2/DNM3/CFL1/GAK/PPP1R9B/PPP2R5B/MTPN/IQGAP1/MEIS1/GRN/CUX1/MMD/CAMK1/EIF4G1/NEURL1/VASP/RHOG/FBXO7/EHD1/PAK2/FOXO3/MED1/TAL1/CTTN/KEL/CARM1/SCN1B/WNK1/PBX1/DAB2
BP	GO:1901342	regulation of vasculature development	162	-0.55	-2.03	6.02E-09	1.69E-07	1.05E-07	2232	tags=35%, list=19%, signal=29%	BTG1/MECP2/PRKD2/SPRED1/IL1B/VASH1/ROCK2/AGO1/RUNX1/HGS/PIK3CB/ENG/KLF4/ETS1/AMOT/SPHK1/JMJD8/AQP1/SASH1/PTK2B/PLXND1/KLF2/MTDH/RRAS/PML/GREM1/CEACAM1/PIK3CD/SFRP2/PKM/STAT3/SEMA4A/NRP1/JAK1/LRG1/ABL1/FOXO4/STIM1/WARS1/EPN1/HIPK1/HSPB1/FOXJ2/HIPK2/NIBAN2/KRT1/GLUL/CLDN5/PRKCB/THBS1/SP1/GRN/ATP2B4/E2F2/PF4/SPARC/TBXA2R
BP	GO:0060341	regulation of cellular localization	584	-0.40	-1.70	6.61E-09	1.84E-07	1.14E-07	2578	tags=33%, list=22%, signal=27%	MAPK14/RALB/SORL1/PKIG/MARK4/CORO1A/AKT1/PIM3/SH3TC2/SYT11/PTPN11/NR1D1/BAK1/CNST/CLN3/TMBIM1/TRPC3/BICD1/ZFP36/UBR5/FLCN/DLG4/SNX33/TSG101/AAAS/CHP1/HLA-DRB1/ARHGAP8/PCNT/MLC1/CDK5/HYAL2/SUMO3/CD81/ABI3/FYN/ABHD17C/CRKL/IL1B/SPI1/FEZ1/P2RX4/ORMDL3/VPS4B/PARD6A/SREBF2/PTPN6/ROCK2/LCP1/RIPOR1/FES/LGALS3/PIN1/EPB41L2/ITGAM/LZTS2/PARD6B/NUMA1/UBE2J2/SMAD3/HGS/RIPOR2/MAGED1/FZD5/NDEL1/BBC3/CCT3/OTUD7B/SNX12/NSFL1C/AP2B1/BCAP31/TM9SF4/SREBF1/GSK3B/PRR5L/STOM/PRKACA/GSK3A/HSP90AB1/TGFB1/ATP9A/HDAC3/MEPCE/CLTC/MESD/RANGRF/EDEM2/EZR/STX4/UCP2/SELENON/PARP1/SESN2/LYN/BCAS3/NEDD4L/MAVS/VTI1B/RAB7A/LEPROT/SUPT6H/PINK1/PTK2B/IFI27/BAD/DZIP1/HCLS1/GRIPAP1/DYNC1H1/ILRUN/SRC/PML/KAT7/SNX3/FIS1/TRIM28/ACTB/CTNNA1/MAP2K2/RANGAP1/MAP1A/NOLC1/MCRS1/SYK/VCP/USP36/ABL1/ARIH2/FKBP1A/ABLIM3/SAE1/DTX3L/SIDT2/MFHAS1/CALM3/LDLRAP1/LGALS9/SUFU/FLNA/RAB11B/TBC1D20/FKBP1B/RAC2/LMNA/INPP5K/ARHGAP1/USP7/NF2/SLC6A9/GNL3L/BCL2L1/STK11/VPS28/SDC4/ZDHHC5/RASSF5/GSN/GLUL/HUWE1/SQSTM1/LRP1/PPP1R9B/CSK/YOD1/SPTBN1/AKT2/UBE2L3/PTPN1/EPB41/TCF7L2/MSN/UBASH3B/ATP2B4/SNCA/CAMK1/ATG13/LAMP1/RHOG/P2RX1/MAPK3/GATA1/BNIP3L/EHD1/SMPD3/OAZ1/PTPN23/AP2M1/HTT/PPM1A/SHISA7/TRIM58/DMTN/DAB2/DIAPH1

BP	GO:0070527	platelet aggregation	53	-0.72	-2.25	6.87E-09	1.90E-07	1.18E-07	1452	tags=49%, list=12%, signal=43%	ACTG1/GNAS/PLEK/LYN/ILK/CD9/CEACAM1/ACTB/CSRP1/SYK/MPL/GP6/VCL/FLNA/FERMT3/HSPB1/GP1BA/ALOX12/PIK3CG/UBASH3B/GATA1/MYL9/MYH9/ITGB3/DMTN/TLN1
BP	GO:0071407	cellular response to organic cyclic compound	363	-0.45	-1.80	7.26E-09	1.99E-07	1.23E-07	2638	tags=36%, list=22%, signal=29%	SLC16A1/NRIP1/CASP7/JUP/RALB/SMAD5/PTAFR/PAK4/GRAMD1B/MUL1/GNAO1/TIPARP/TRPM2/NR1D1/ATP1A1/CASP9/EGR1/DDX54/ZFP36/UBR5/EZH2/CNOT9/PHEX/PKN1/MLC1/SSH1/IL1B/ZNF366/HRH2/P2RX4/OSBPL7/ITPR2/HSPA5/GNA11/ROCK2/PAQR8/NCOA4/PPARGC1B/PDE2A/ZBTB7A/ZFP36L2/NR3C1/FES/ALPL/PDE4B/OR10H1/EDN1/TAF1/GNAZ/TRERF1/PDE12/DTNBP1/EHMT2/FOXP1/GSK3B/CRY2/PRKACA/GSK3A/HSP90AB1/TGFB1/SMARCA4/EZR/GNAS/PARP1/AQP1/MAVS/EPG5/LBH/GNA15/TADA3/HSPA1A/RXRA/KLF2/MGMT/CGAS/SLC9A1/GNB1/NCSTN/BCL2L11/SRC/NCOA3/CALR/PPARA/WBP2/DAXX/ZMIZ1/ACTB/CTNNA1/YWHAH/BTRC/ABL1/PAK1/NFKB1/RAPGEF1/ARRB2/RHOA/DNM2/FLNA/IFIT1B/INPP5K/KANK2/ELK1/ABHD2/RIOK3/SAFB/PPP1R9B/UBE2L3/HRH4/PIK3CG/KLF16/FAM210B/MSN/ATP2B4/SNCA/ZNF703/MAPK3/GATA1/PAK2/FOXO3/MED1/ARID1A/CLU/DMTN/NCOR2/CARM1/LARP1/TGM2/PIM1/CNOT1/DAB2/DIAPH1
BP	GO:0009896	positive regulation of catabolic process	413	-0.43	-1.77	7.62E-09	2.08E-07	1.29E-07	2594	tags=33%, list=22%, signal=26%	SUPV3L1/ULK1/MID2/RALB/SORL1/AKT1/CBLB/NSF/PIAS1/TIPARP/DISC1/YTHDF3/RNF144B/ZFP36/FLCN/FOXO1/SNX33/WAC/DCP1A/PIP4K2C/ABCD1/MOV10/CD81/NUB1/CSDE1/CSNK2A1/IL1B/OSBPL7/ORMDL3/PABPC1/ROCK2/ENTPD5/SYNCRIP/DCPS/ZFP36L2/TRIB1/CHFR/PATL1/KEAP1/GPSM1/PIK3CB/TFEB/PHKG2/TAF1/FTO/CPT1A/PDE12/BCAP31/IKBKG/TNRC6C/GSK3B/PRR5L/METTL16/GSK3A/TNFRSF1B/TTC5/CBFA2T3/DET1/VGLL4/EZR/ATG2A/DAPK1/VPS13D/SESN2/NEDD4L/GTPBP1/DDA1/RAB7A/PUM1/PINK1/ZC3H18/PTK2B/HSPA1A/ANKIB1/MTDH/BAD/TRIM32/ZC3HAV1/BCL2L11/ARNT/PPARA/PLEKHF1/LAPTM5/UPF1/GABARAP/VDAC1/DDB1/VCP/ARIH2/OPTN/PNPLA2/PSMC4/PIM2/STX5/RAD23A/DTX3L/DCP1B/WWP2/SESN3/ATG7/TENT4B/STK11/VPS28/FZR1/HUWE1/LRP1/RILP/TRIM14/AKT2/YBX1/PTPN1/MSN/MARCHF2/SNCA/PIP4K2A/MAPK3/ELAPOR1/BNIP3L/FBXO7/ZER1/KHSRP/RNF40/DNAJB2/WDR45/NPRL3/FOXO3/FURIN/OAZ1/HTT/CLU/LARP1/CNOT1/DAB2/BAG6/SUPT5H
BP	GO:0003018	vascular process in circulatory system	141	-0.57	-2.07	8.32E-09	2.25E-07	1.39E-07	2019	tags=36%, list=17%, signal=30%	ROCK2/SNTA1/PDE2A/SLC7A1/ARHGAP35/SLC29A2/EDN1/SLC19A1/ABCC1/TGFB1/CTNNBIP1/SLC27A1/MFSD2A/SLC5A6/KLF2/SRC/CEACAM1/ABCC3/SLC7A8/SLC1A5/PTP4A3/ATP1B2/SLC1A4/BCR/SLC6A20/ABCG2/TJP2/ABCC4/ABL1/BMP6/SLC2A1/SLC7A5/RHOA/ACTA2/PTPRJ/GUCY1A1/ECE1/SLC6A9/SLC24A3/PLEC/CLDN5/LRP1/PLVAP/PER2/ATP2B4/P2RX1/SLC29A1/GPX1/SLC38A5/KEL/TBXA2R
BP	GO:0032535	regulation of cellular component size	241	-0.49	-1.91	8.44E-09	2.27E-07	1.41E-07	2593	tags=38%, list=22%, signal=30%	ULK1/EPS8/CORO1A/DIP2B/SWAP70/PIK3CA/TMOD3/DISC1/PLXNA4/ADNP/DSTN/CLN3/SPTAN1/PPP1R15A/TRIOBP/WASHC5/ARHGAP18/MAP3K13/PFN1/CDK5/SSH1/MACF1/VAV2/MYADM/SVIL/SSH2/ARHGAP35/FMN1/EDN1/KIAA0319/ARPC5/LIMA1/NDEL1/SEMA4D/GRB2/GSK3B/BAIAP2/PYCARD/CYFIP1/HSP90AB1/EZR/CDC42EP4/PLEK/WDTC1/WDR1/ALOX15/IST1/ANO6/AQP1/HIP1R/CDKL5/RAC1/PTK2B/DPYSL2/HCLS1/EIF4G2/ARPC1B/LRRC8A/VAV1/FLII/RIN3/PPP1CA/SEMA4A/NRP1/ABL1/SSH3/XK/RHOA/ARPC4/DNM2/WAS/CYFIP2/GSN/RILP/SPTBN1/MTPN/SPTA1/MSN/COTL1/SPTB/VASP/TMOD1/SCIN/CAPZB/CTTN/AP2M1/ESAM/KEL/SH3BP1/DMTN/ADD1

BP	GO:1905114	cell surface receptor signaling pathway involved in cell-cell signaling	303	-0.47	-1.86	9.11E-09	2.41E-07	1.50E-07	2622	tags=37%, list=22%, signal=29%	EXT1/DAAM1/ZEB2/PKD1/JUP/GNAQ/MAPK14/AKT1/FRMD8/DISC1/NOTCH1/EGR1/UBR5/DLG4/FOXO1/EIF4A3/MAGI2/MECP2/CDK5/SPIN1/ASPM/CSNK2A1/MITF/P2RX4/CTNND1/MACF1/CCNY/NDRG2/CCAR2/PIN1/ITGAM/LZTS2/BCL9/SOX13/SMAD3/EDN1/TIAM1/CSNK1G1/DIXDC1/FZD5/RBPJ/TGFB1I1/KLF4/SOX4/BCL9L/NFATC1/GSK3B/BAIAP2/GSK3A/TGFB1/CTNNBIP1/SMARCA4/TMEM131L/MESD/VGLL4/DAPK3/SKI/HIC1/ZRANB1/ILK/FZD2/RAC1/NPFF/PTK2B/PIAS4/MARK2/TBL1XR1/SRC/GREM1/SFRP2/SNX3/DVL3/RUVBL1/PPP3CA/LRRK1/AMFR/TLE3/PPP1CA/TBL1X/BTRC/USP34/DDB1/VCP/XIAP/ABL1/OTUD5/FBXW4/NFKB1/RNF213/ARRB2/ATP6V0C/TLE4/RHOA/CHD8/GID8/MAD2L2/TLE1/STK11/SIAH2/LRP1/TCF7L2/SNCA/PAF1/ZNF703/P2RX1/SLC29A1/FAM53B/FOXO3/CTDNEP1/PPM1A/WNK1/DAB2
CC	GO:0031252	cell leading edge	300	-0.47	-1.85	9.29E-09	2.45E-07	1.52E-07	2165	tags=31%, list=18%, signal=26%	FGD6/ABI3/SSH1/PLEKHO1/PARD6A/PABPC1/CTNND1/MACF1/SCIMP/LCP1/MYADM/TUBG1/SRGAP2/PDLIM7/PACSIN1/FRMD4B/RIPOR2/PHACTR4/ARPC5/DGKZ/LIMA1/NDEL1/VIM/BAIAP2/NEDD9/CYFIP1/GIT1/CDK6/AMOT/EZR/INPPL1/STX4/PLEK/CORO1C/TPM1/CDC42BPB/ITSN1/HIP1R/BCAS3/ILK/CDKL5/RAC1/PTK2B/PLXND1/ARF1/SLC9A1/SRC/SH2D3C/FAM89B/ACTB/ARHGAP45/CTNNA1/ARHGEF2/PXN/NRBP1/ABL1/PAK1/FGD4/ABLIM3/PLEK2/APBB1IP/RHOA/PDE9A/DNM2/ACTA2/PTPRJ/ARHGAP31/RAC2/MYLK/INPP5K/NF2/GSN/CFL1/WIPF1/PPP1R9B/SPTBN1/AKT2/PDE4A/IQGAP1/VASP/PARVB/WASF3/CAPZB/ACTN1/TESC/CTTN/MYH9/SHISA7/ITGB3/WASF2/SH3BP1/TLN1/DIAPH1
BP	GO:0008154	actin polymerization or depolymerization	152	-0.55	-2.03	1.08E-08	2.81E-07	1.75E-07	2588	tags=46%, list=22%, signal=36%	EPS8/CORO1A/SWAP70/PIK3CA/TMOD3/DSTN/SPTAN1/SPIRE1/TRIOBP/MSRB1/WASHC5/ARHGAP18/CORO7/PFN1/SSH1/SPIRE2/MSRB2/MYADM/DIAPH2/SVIL/SSH2/ARHGAP35/FMN1/ARPC5/LIMA1/GRB2/BAIAP2/PYCARD/CYFIP1/PSTPIP2/PREX1/CDC42EP4/PLEK/WDR1/ALOX15/HIP1R/RAC1/PTK2B/HCLS1/GAS7/ARPC1B/FLII/ABL1/SSH3/RHOA/ARPC4/ARHGAP6/WAS/CYFIP2/GSN/CFL1/WIPF1/PPP1R9B/SPTBN1/MTPN/SPTA1/COTL1/SPTB/VASP/TMOD1/MICAL2/WASF3/SCIN/CAPZB/CTTN/ESAM/SH3BP1/DMTN/ADD1/DIAPH1
BP	GO:0010632	regulation of epithelial cell migration	146	-0.56	-2.07	1.11E-08	2.86E-07	1.77E-07	2222	tags=37%, list=19%, signal=30%	PFN1/MECP2/PRKD2/SPRED1/P2RX4/MAPRE2/VASH1/ROCK2/MACF1/MAP3K3/SCARB1/EDN1/PIK3CB/MMP9/EPPK1/KLF4/DNAJA4/FOXP1/TGFB1/SVBP/ETS1/CORO1C/BCAS3/SASH1/RAC1/PTK2B/RRAS/SRC/CEACAM1/IQSEC1/CALR/PIK3CD/SEMA4A/NRP1/ABL1/CLASP1/RHOA/HSPB1/STAT5A/ADIPOR1/GFUS/GLUL/THBS1/PIK3CG/SP1/GPI/GRN/ATP2B4/PTPN23/ITGB3/SPARC/SH3BP1/MAP2K3/TBXA2R
BP	GO:0009887	animal organ morphogenesis	474	-0.41	-1.71	1.16E-08	2.98E-07	1.85E-07	2358	tags=30%, list=20%, signal=25%	ZFPM1/JUN/TBX19/TIPARP/NOTCH1/BAK1/SGPL1/TRIOBP/SLC24A4/HOXB3/DGCR2/PHEX/EIF4A3/PRDM1/HES1/MAGI2/HYAL2/DDR1/ARID2/MYO15A/STK40/CRKL/THRA/EXOC4/FOXN3/CSF1R/VPS51/TUFT1/SMARCD3/BASP1/PPARGC1B/MAN2A1/MIB1/LZTS2/ALPL/SMAD3/FMN1/EDN1/TIAM1/PHACTR4/MAGED1/ENG/FZD5/SMARCC1/RBPJ/SOX4/ACVR2B/GSK3A/RPS6KA1/TGFB1/CTNNBIP1/RPGRIP1L/SNX10/INPPL1/BCL11B/BCOR/NDST1/SKI/TWSG1/TRAF6/MFSD2A/SLC39A3/TPM1/FHL1/AQP1/YY1/ILK/FZD2/RAC1/RARA/IGF2/PLXND1/CHST11/DCHS1/BCL2L11/SRC/SOX6/PML/GREM1/CEACAM1/BBS5/SFRP2/PPARA/DVL3/LTBP3/PLXNA1/IFT52/FAM20C/ZMIZ1/DHRS3/TLE3/PHOSPHO1/STAT3/CTNNA1/BTBD7/NRP1/BCR/BTRC/PDGFC/RFLNB/RFNG/SYK/ABL1/MAFB/FKBP1A/RELA/STIM1/LTF/ANXA6/BMP6/GAA/RHOA/NOTCH2/ACTA2/HIPK1/SUFU/FLNA/TBC1D20/BSG/MYLK/TLE1/HIPK2/NF2/ROGDI/ASXL2/CLDN5/GLG1/MFN2/MEIS1/MSN/CAPN1/MAPK3/MICAL2/HLX/SMPD3/FOXO3/MED1/PDGFA/TGM2/PIM1/PBX1/DAB2

BP	GO:0010975	regulation of neuron projection development	260	-0.48	-1.87	1.27E-08	3.23E-07	2.00E-07	2605	tags=36%, list=22%, signal=28%	CAPRIN1/ULK1/AKT1/DIP2B/NCKIPSD/PLXNC1/DISC1/PLXNA4/ADNP/EZH2/MAP3K13/EPHA4/HES1/MAGI2/CDK5/ABI3/FYN/CRKL/FEZ1/HSPA5/CERS2/MACF1/PRAG1/ITM2C/FES/PACSIN1/CUL7/ARHGAP35/PMP22/KIAA0319/TIAM1/DIXDC1/NDEL1/KLF4/SEMA4D/DTNBP1/VIM/GSK3B/BAIAP2/GSK3A/CYFIP1/ENC1/MFSD2A/BCL11A/KATNB1/IST1/LYN/NEDD4L/PLXNB2/CDKL5/MBP/PTK2B/PLXND1/KIF13B/STK24/SIPA1L1/MARK2/FBXO31/EIF4G2/STK25/SFRP2/SNX3/PLXNA1/PPP3CA/ATP1B2/SEMA4A/NRP1/VLDLR/YWHAH/ABL1/PAK1/SYNGAP1/RAPGEF1/XK/RHOA/FLNA/FKBP1B/CAMK1D/STK11/DNM3/CFL1/GAK/PPP2R5B/GRN/CUX1/CAMK1/FBXO7/EHD1/PAK2/KEL/CARM1/SCN1B/DAB2
BP	GO:0030099	myeloid cell differentiation	300	-0.47	-1.84	1.30E-08	3.28E-07	2.04E-07	2260	tags=36%, list=19%, signal=30%	ZNF385A/G6PD/HLA-DRB1/BATF2/CD81/THRA/MITF/SPI1/RIPK1/CSF1R/PTPN6/SH3PXD2A/ZBTB16/OSCAR/PPARGC1B/ZBTB7A/EIF6/TRIB1/FES/NRROS/TFE3/ACVR1B/RUNX1/INPP5D/RBPJ/CD74/MMP9/FOXP1/TOB2/TSPO2/TGFB1/CTNNBIP1/CDK6/ACIN1/ETS1/CBFA2T3/SNX10/CNOT4/ATP6AP1/NCOA6/PARP1/TRAF6/WDR1/LYN/KLF13/EPAS1/ADAR/PTK2B/RARA/HSPA1A/KLF2/HCLS1/SRC/PML/KAT7/CDKN1C/SLC25A38/ARNT/CEACAM1/PIK3CD/LRRK1/FAM20C/TRIM10/BPGM/STAT3/JAK3/MAFB/FOS/PITHD1/LTF/AHSP/MPL/MFHAS1/NOTCH2/GPR137/MTURN/HIPK2/FASN/KLF1/PABPC4/KAT6A/SLC4A1/EPB42/MEIS1/FAM210B/UBASH3B/PAF1/PRKDC/PIP4K2A/IKZF1/GATA1/PF4/SCIN/ACTN1/ALAS2/FOXO3/TESC/MPIG6B/FAXDC2/MED1/EIF2AK1/TAL1/CUL4A/MYH9/TRIM58/WASF2/DMTN
BP	GO:0071559	response to transforming growth factor beta	153	-0.55	-2.03	1.33E-08	3.36E-07	2.08E-07	2200	tags=41%, list=19%, signal=34%	SPRED1/HYAL2/FYN/CRKL/SPI1/HSPA5/ROCK2/PDE2A/ZBTB7A/ZFP36L2/NR3C1/PIN1/NRROS/SMAD3/EDN1/ENG/LIMS1/TGFB1I1/BCL9L/ZFHX3/HSP90AB1/TGFB1/MEN1/SKI/TWSG1/PARP1/ZYX/C20orf27/HSPA1A/CHST11/CREBBP/SRC/SOX6/PML/CDKN1C/PPARA/LTBP3/RUNX3/FAM89B/ZMIZ1/STAT3/USP9X/PXN/LRG1/ABL1/FOS/FKBP1A/ARRB2/DNM2/HIPK2/STK11/ITGB5/CLDN5/THBS1/GLG1/ZNF703/TGFBRAP1/PAK2/FURIN/PPM1A/PDGFA/LTBP1/DAB2
BP	GO:0030155	regulation of cell adhesion	495	-0.41	-1.71	1.43E-08	3.58E-07	2.22E-07	2609	tags=34%, list=22%, signal=28%	PKD1/JUP/RASAL3/MAPK14/PTAFR/CORO1A/MINK1/AKT1/ARL2/SELENOK/FOXP3/SWAP70/CBLB/PLXNC1/PIK3CA/DISC1/SERPINF2/PLXNA4/MAPK7/KNG1/PTPN11/NOTCH1/CD3E/EFNB1/DOCK8/FLCN/TRIOBP/EPHA4/HES1/HLA-DRB1/IRF1/PRKD2/CD81/DDR1/FYN/HLA-DQA2/LGALS9C/CRKL/IL1B/SPI1/HLA-A/TBCD/PTPN6/ROCK2/MACF1/ZBTB16/MYADM/ST6GAL1/SIRPG/FES/LGALS3/SLC7A1/PKP4/RCC2/SOX13/TFE3/SMAD3/FMN1/RUNX1/PTPRC/PIK3CB/RIPOR2/LIMS1/CD74/POLDIP2/KLF4/SEMA4D/SIRPB1/SOX4/HLA-DQA1/GSK3B/ZFHX3/NEDD9/PYCARD/AKNA/TGFB1/ACTG1/CDK6/TBX21/TMEM131L/PREX1/ETS1/IL1RN/SPINT2/DAPK3/MEN1/STX4/IL4I1/TWSG1/CORO1C/TRAF6/TPM1/ALOX15/LYN/BCAS3/PLXNB2/ILK/RAC1/MBP/JAM3/PTK2B/RARA/IGF2/PLXND1/CD9/SLC9A1/SPN/BAD/RRAS/SRC/PML/GREM1/CEACAM1/CALR/SFRP2/HLA-DRB5/PPARA/VAV1/RUNX3/PLXNA1/PPP3CA/LAPTM5/ZMIZ1/NRP1/JAK1/SYK/JAK3/ABL1/TNFSF4/CLASP1/PLEKHA2/RELA/BMP6/APBB1IP/GP6/PDE5A/RHOA/VCL/DNM2/MAD2L2/ARHGAP6/ARHGDIA/PTPRJ/LGALS9/FLNA/FERMT3/RAC2/RSU1/NF2/SDC4/GFUS/ALOX12/CSK/THBS1/PIK3CG/SPTA1/UBASH3B/SELP/LRFN3/ZNF703/SOX12/HLX/ACTN4/FOXO3/TESC/PTPN23/DMTN/TGM2/WNK1/DAB2

BP	GO:0007417	central nervous system development	581	-0.39	-1.65	1.77E-08	4.41E-07	2.73E-07	2250	tags=29%, list=19%, signal=24%	G6PD/EPHA4/HES1/SMG9/MECP2/CDK5/NDE1/ASPM/FYN/CDK5RAP2/MACO1/CASP2/CRKL/IL1B/FEZ1/TTLL1/DHX37/CSF1R/VPS51/HSPA5/CTNND1/DNAJB1/SPATA5/TACC1/ZBTB16/MPST/PHF8/BASP1/GSTP1/NDRG2/SRGAP2/MBOAT7/ITGAM/TPP1/NRROS/SOX13/ARHGAP35/B3GNT5/LSR/SYNJ1/APLP2/RERE/DIXDC1/ARPC5/TAF1/RBPJ/MAP1S/B4GALT5/NDEL1/CENPF/FOXP2/SOX4/DTNBP1/VIM/KDM4A/GSK3B/ZFHX3/BAIAP2/AKNA/HSP90AB1/TGFB1/GIT1/CDK6/TNFRSF1B/RPGRIP1L/THOC6/SPHK1/MEN1/CASP5/BCL11B/NDST1/SKI/ATIC/NCOA6/TWSG1/WDR1/SNRPB/MFSD2A/AQP1/LYN/PLXNB2/MTF1/MBP/BPTF/RARA/DPYSL2/ATXN1/PIAS4/TACC3/TRIO/SH3GL1/BAD/DHX30/NCSTN/BCL2L11/SRC/SOX6/PSPN/HOOK3/SFRP2/OLIG2/MYCBP2/PPP3CA/ATP1B2/ZMIZ1/ACTB/STAT3/TRAPPC9/NRP1/VLDLR/BCR/YWHAH/PDGFC/DPCD/HERC1/ATP6V0D1/ABL1/MAFB/ODAD4/E2F1/C1QA/ARRB2/OLIG1/JARID2/SLC2A1/CKB/SCYL1/ALDH5A1/RHOA/CHD8/CALM3/SUFU/AARS1/NF2/ROGDI/NAPA/UBB/DCTN1/GSN/ARCN1/LRP1/PPP1R9B/CSK/UBE2L3/GJC2/SETD1A/MTPN/MEIS1/GRN/ATP2B4/SHARPIN/NEURL1/PRKDC/SOX12/MAPK3/NRGN/WASF3/SCIN/FOXO3/MED1/TAL1/SEC16A/CLU/SCN1B/PBX1/BAG6
BP	GO:0071560	cellular response to transforming growth factor beta stimulus	149	-0.55	-2.01	1.86E-08	4.58E-07	2.84E-07	2200	tags=40%, list=19%, signal=33%	SPRED1/HYAL2/FYN/CRKL/SPI1/HSPA5/PDE2A/ZBTB7A/ZFP36L2/NR3C1/PIN1/NRROS/SMAD3/EDN1/ENG/LIMS1/TGFB1I1/BCL9L/HSP90AB1/TGFB1/MEN1/SKI/TWSG1/PARP1/ZYX/C20orf27/HSPA1A/CHST11/CREBBP/SRC/SOX6/PML/CDKN1C/PPARA/LTBP3/FAM89B/ZMIZ1/STAT3/USP9X/PXN/LRG1/ABL1/FOS/FKBP1A/ARRB2/DNM2/HIPK2/STK11/ITGB5/CLDN5/THBS1/GLG1/ZNF703/TGFBRAP1/PAK2/FURIN/PPM1A/PDGFA/LTBP1/DAB2
CC	GO:0099503	secretory vesicle	705	-0.38	-1.60	2.22E-08	5.45E-07	3.38E-07	2118	tags=27%, list=18%, signal=23%	CMTM6/CPNE1/IL1B/PROS1/EEF2/ORMDL3/PTPN6/PPBP/ITPR2/RNASE3/PGAM1/ALDH3B1/PGLYRP1/TIMP1/OSCAR/GSTP1/ENKUR/LGALS3/CTSA/ITGAM/APEH/XRCC6/TOM1/PDE4B/ADA2/APLP2/PTPRC/EDN1/ARPC5/CD14/MMP9/NDEL1/HLA-C/STX6/RHOF/CDA/SIRPB1/CXCR2/CRISPLD2/SMPD1/DTNBP1/ADGRE5/TRAPPC1/HYAL3/SERPINB6/LAIR1/STOM/BAIAP2/TIMP2/PRKACA/HVCN1/PYCARD/CYFIP1/DOK3/HSP90AB1/TGFB1/CXCR1/CD63/TNFRSF1B/TPRG1L/SYNGR1/SLC44A2/DNAJC5/TRAPPC4/SNX10/STX4/ALAD/MVP/IL4I1/ALDOA/CANT1/GLB1/AP1M1/IST1/RAB31/ANO6/VTI1B/RAB7A/F13A1/SERPING1/RAC1/IGF2/ALDOC/STK10/HSPA1A/TMEM184A/CD9/PFKL/EXOC3/NCSTN/DYNC1H1/SEPTIN1/CEACAM1/IQSEC1/CALR/SCFD2/KIF1B/ATP6V1A/SEPTIN4/VTI1A/PSAP/PKM/ARHGAP45/CTNNA1/CNN2/PSMD11/SELL/CAP1/VCP/ABCC4/ATP6V0D1/LRG1/LCN2/RAB14/PYGB/LTF/RAB27B/NFKB1/RAB3IL1/ATP6V0C/LAMTOR1/TUBB4B/CLCN5/ITGAL/GAA/ANXA11/CLTB/RHOA/VCL/CALM3/PTPRJ/RAB5B/RAB11B/ARL8A/FERMT3/ZNRF1/PSMD2/RAB3D/BSG/ECE1/PA2G4/ATG7/ATP6V0A1/SLC6A9/DOCK2/CYB5R3/BCL2L1/ROGDI/FTH1/KRT1/ABHD2/STON2/UBR4/GSN/SYTL4/PLEKHO2/HUWE1/IGF2R/THBS1/IQGAP1/GNS/GPI/COTL1/GRN/SELP/SNCA/PRDX6/LAMP1/CAPN1/RHOG/P2RX1/PF4/CTSD/TUBB/TREML1/FTL/ACTN4/PAK2/CTSB/ACTN1/TOLLIP/MYH9/ITGB3/SPARC/PDGFA/CLU/ITGA2B/ACRBP/DIAPH1/TBXA2R
BP	GO:0010810	regulation of cell-substrate adhesion	135	-0.56	-2.03	2.63E-08	6.42E-07	3.98E-07	2606	tags=42%, list=22%, signal=33%	JUP/MINK1/ARL2/DISC1/NOTCH1/CD3E/TRIOBP/DDR1/CRKL/TBCD/ROCK2/MACF1/MYADM/ST6GAL1/RCC2/SMAD3/FMN1/PIK3CB/LIMS1/POLDIP2/GSK3B/NEDD9/ACTG1/CDK6/PREX1/DAPK3/MEN1/CORO1C/ALOX15/BCAS3/ILK/RAC1/PTK2B/SLC9A1/RRAS/SRC/GREM1/CALR/NRP1/ABL1/CLASP1/PLEKHA2/RHOA/VCL/DNM2/ARHGAP6/PTPRJ/FLNA/RAC2/RSU1/NF2/SDC4/GFUS/THBS1/ACTN4/DMTN/DAB2

BP	GO:0031329	regulation of cellular catabolic process	686	-0.38	-1.62	3.17E-08	7.69E-07	4.77E-07	2253	tags=29%, list=19%, signal=25%	EIF4A3/IKBKE/EPHA4/PIP4K2C/ABCD1/CDK5/MOV10/FXR2/WDR24/CD81/NUB1/CSDE1/CSNK2A1/EXOC4/IL1B/FEZ1/OSBPL7/ORMDL3/SREBF2/USP10/PABPC1/ROCK2/LARP4B/ENTPD5/PGAM1/SYNCRIP/TIMP1/DCPS/ZFP36L2/TRIB1/CHFR/CCAR2/CTSA/FOXK1/PATL1/SCARB1/SERBP1/KEAP1/F8A1/GPSM1/RRAGD/PIK3CB/TFEB/PHKG2/FZD5/SMARCC1/TAF1/FTO/CPT1A/POLDIP2/PHF23/PDE12/CHMP4B/PSME3/BCAP31/EHMT2/VIM/IKBKG/TNRC6C/KDM4A/SREBF1/TECPR1/GSK3B/PRR5L/METTL16/FOXK2/TIMP2/PRKACA/GSK3A/PYCARD/CASC3/DAP/HSP90AB1/NBAS/KLHL22/TNFRSF1B/TTC5/CBFA2T3/DET1/VGLL4/EZR/ATG2A/DAPK3/DAPK1/ALAD/VPS13D/MFSD2A/SESN2/GTPBP1/DDA1/PUM1/PINK1/ZC3H18/PTK2B/HSPA1A/ANKIB1/MTDH/BAD/TRIM32/ZC3HAV1/BCL2L11/PML/ARNT/EIF4G2/RUBCN/ATP6V1A/PPARA/PLEKHF1/CDK2/LAPTM5/PSAP/PPP1CA/STAT3/USP19/MAP1A/UPF1/BTRC/GABARAP/VDAC1/HERC1/ALKBH5/RGP1/VCP/CPTP/USP36/ATP6V0D1/ABL1/ELAVL1/RRAGC/ARIH2/OPTN/N4BP1/PNPLA2/E2F1/PITHD1/PSMC4/PIM2/RAD23A/DTX3L/ATP6V0C/DCP1B/CAPNS1/GOLGA2/SESN3/SUFU/HNRNPM/GPR137/MAPKAPK2/USP7/ATG7/HSPB1/HIPK2/ATP6V0A1/TENT4B/STK11/UBB/GNA12/PABPC4/FZR1/PSMF1/LRP1/TRIM14/AKT2/YBX1/PTPN1/PIK3CG/MSN/MARCHF2/ARAF/ATP2B4/SNCA/EIF4G1/CAPN1/PIP4K2A/MAPK3/ELAPOR1/BNIP3L/FBXO7/DCAF12/TBC1D25/RYBP/ZER1/GPX1/KHSRP/RNF40/DNAJB2/YBX3/WDR45/NPRL3/FOXO3/FURIN/CTTN/HTT/CLU/TENT5C/LARP1/CNOT1/DAB2/BAG6/SUPT5H
BP	GO:0001775	cell activation	773	-0.37	-1.56	3.34E-08	7.95E-07	4.93E-07	2247	tags=26%, list=19%, signal=23%	PRDM1/IL6R/HES1/PKN1/HLA-DRB1/IRF1/HYAL2/BATF2/CD81/FYN/HLA-DQA2/ICAM1/LGALS9C/IL1B/SPI1/P2RX4/CNR2/HLA-A/SH3KBP1/DOCK10/TXLNA/PTPN6/SELPLG/VAV2/TLR6/ZBTB16/LCP1/PGLYRP1/TIMP1/ZBTB7A/SIRPG/ZFP36L2/FES/NDRG1/LGALS3/SLC7A1/ITGAM/XRCC6/SOX13/SMAD3/RUNX1/PTPRC/EDN1/PIK3CB/RIPOR2/WDFY4/DROSHA/FZD5/STOML2/INPP5D/DGKZ/RBPJ/CD74/CD300LF/NCR3/JUND/SIRPB1/CXCR2/SOX4/PSMB10/DGKD/FOXP1/HLA-DQA1/IL2RB/NR1H3/PYCARD/RPS6KA1/TGFB1/ACTG1/MICB/LILRA5/CDK6/TEC/TBX21/TMEM131L/TNFRSF1B/PREX1/PRR7/SPHK1/CD180/GNAS/STX4/BCL11B/PLEK/IL4I1/TWSG1/TRAF6/KARS1/SIT1/LYN/YY1/ILK/SUPT6H/IL17RA/PTK2B/PRF1/RARA/IGF2/CD9/DYSF/KDELR1/CGAS/SPN/BAD/KIF13B/NCSTN/SRC/CCND3/KAT7/DCAF1/CEACAM1/PIK3CD/LRRC8A/HLA-DRB5/VAV1/RUNX3/PPP3CA/LAPTM5/ZMIZ1/TP53BP1/ACTB/HMCES/STAT3/SEMA4A/BCR/CSRP1/SYK/JAK3/ABL1/MAFB/TNFSF4/FKBP1A/BLOC1S3/C1QA/MYO18A/ITGAL/APBB1IP/MPL/GP6/PDE5A/RHOA/MFHAS1/VCL/NOTCH2/MAD2L2/ACTA2/PTPRJ/LGALS9/WAS/FLNA/FKBP1B/FERMT3/RAC2/HSPB1/DOCK2/STK11/SDC4/GSN/GP1BA/ALOX12/PF4V1/LRP1/PRKCB/CSK/THBS1/GP9/PIK3CG/CD151/SPTA1/MSN/NFATC2/GRN/UBASH3B/ELF4/SELP/SNCA/PRKDC/LAMP1/SOX12/IKZF1/P2RX1/GATA1/FBXO7/HLX/LYL1/CDKN1A/PF4/CBL/TREML1/ST3GAL1/FOXO3/MYL9/TOLLIP/MYH9/ITGB3/PDGFA/CLU/DMTN/TLN1/PBX1/BAG6

BP	GO:0031331	positive regulation of cellular catabolic process	369	-0.43	-1.75	3.39E-08	8.02E-07	4.98E-07	2392	tags=31%, list=20%, signal=25%	YTHDF3/RNF144B/ZFP36/FLCN/FOXO1/SNX33/WAC/DCP1A/PIP4K2C/ABCD1/MOV10/CD81/NUB1/CSDE1/IL1B/OSBPL7/ORMDL3/PABPC1/ROCK2/ENTPD5/SYNCRIP/DCPS/ZFP36L2/TRIB1/CHFR/PATL1/KEAP1/GPSM1/PIK3CB/TFEB/PHKG2/TAF1/FTO/CPT1A/PDE12/BCAP31/IKBKG/TNRC6C/GSK3B/PRR5L/METTL16/GSK3A/TNFRSF1B/TTC5/CBFA2T3/DET1/VGLL4/EZR/ATG2A/DAPK1/VPS13D/SESN2/GTPBP1/DDA1/PUM1/PINK1/ZC3H18/PTK2B/HSPA1A/ANKIB1/MTDH/BAD/TRIM32/ZC3HAV1/BCL2L11/ARNT/PPARA/PLEKHF1/LAPTM5/UPF1/GABARAP/VDAC1/VCP/ARIH2/OPTN/PNPLA2/PSMC4/PIM2/RAD23A/DTX3L/DCP1B/SESN3/TENT4B/STK11/FZR1/LRP1/TRIM14/AKT2/YBX1/PTPN1/MSN/MARCHF2/SNCA/PIP4K2A/MAPK3/ELAPOR1/BNIP3L/FBXO7/ZER1/KHSRP/RNF40/DNAJB2/WDR45/NPRL3/FOXO3/FURIN/HTT/CLU/LARP1/CNOT1/DAB2/BAG6/SUPT5H
MF	GO:0019901	protein kinase binding	520	-0.40	-1.66	3.46E-08	8.14E-07	5.04E-07	2735	tags=36%, list=23%, signal=29%	TTN/DOCK4/PRKAB1/ATF7/CDK12/AP2A1/SH2B3/TRIM21/ADD2/FGR/TRAF2/PKD1/JUP/MAPRE1/MAPK14/PTAFR/RAB8A/AKT1/CEP68/AP2A2/CBLB/NSF/MAPK7/PTPN11/FBXW5/NBEAL2/YWHAZ/CD3E/CASP9/BICD1/PPP1R15A/AGAP2/ZFP36/CDKN2D/HDAC5/DLG4/CPNE3/INKA1/MAP3K13/EPHA4/HES1/PKN1/MAPKAPK3/CDK5/PRKD2/SPRED1/HYAL2/ZBTB4/CDK5RAP2/CRKL/ATP1B1/FEZ1/EEF2/PARD6A/MAPRE2/PTPN6/CTNND1/CCNY/PGAM1/GSTP1/TRIB1/NR3C1/CDK9/PIN1/PARD6B/RCC2/CDC37/SHC1/GCN1/SMAD3/LRBA/POLA1/PTPRC/SH2B2/FAM83F/DIXDC1/FZD5/LIMS1/RHOBTB1/TPX2/RHOF/EIF3A/USP37/MAP3K11/GRB2/TOP2A/NFATC1/SREBF1/GSK3B/CRY2/PRKACA/NEDD9/GSK3A/HSP90AB1/CLTC/YWHAG/TUBA4A/PRR7/EZR/NEK6/SKI/MVP/PLEK/PARP1/TRAF6/OXSR1/MAP3K5/BCL11A/MAVS/BCL2L14/ILK/SASH1/PINK1/RAC1/PTK2B/RARA/MAP2K7/BAD/KIF13B/PITPNM2/HCLS1/BCL2L11/SRC/CCND3/PSPN/CEACAM1/TAOK2/DAXX/ACTB/PIH1D1/STAT3/NRP1/KAT2B/VDAC1/SYK/TJP2/XIAP/ABL1/ELAVL1/RELA/E2F1/WARS1/ARRB2/GOLGA2/PRKAR2A/GP6/RHOA/CALM3/DNM2/MAD2L2/ACTA2/PTPRJ/AP1B1/SUFU/WAS/FLNA/MAPKAPK2/RAC2/CAVIN2/HSPB1/BCL2L1/SDC4/DCTN1/ADIPOR1/SQSTM1/PRKCB/TRIM14/CSK/PTPN1/IQGAP1/TCF7L2/MSN/ATP2B4/ATG13/RHOG/FBXO7/CDKN1A/CBL/TNIP1/PAK2/WDR45/PRKAR2B/FOXO3/PTPN23/MAP2K3/WNK1/CNPPD1
BP	GO:0022411	cellular component disassembly	357	-0.43	-1.75	3.55E-08	8.31E-07	5.16E-07	2539	tags=38%, list=22%, signal=30%	UBQLN4/SWAP70/MUL1/PIK3CA/NSF/TMOD3/DSTN/CLN3/LAMP2/SPTAN1/ATP2A2/ATXN7/ATG2B/TRIOBP/TSG101/TSPO/LIX1L/EPHA4/CDK5/DDR1/ARID2/NCKAP5L/VPS4B/GRWD1/SREBF2/MAPRE2/OGFOD1/LCP1/SMARCD3/SVIL/ITGAM/APEH/TFIP11/CDC37/MAP1LC3B/LRBA/SYNJ1/PYM1/CHMP6/FZD5/SMARCC1/MAP1S/LIMA1/MMP9/NDEL1/TPX2/PHF23/CHMP4B/TOP2A/SREBF1/TECPR1/GSK3B/GSK3A/TGFB1/SMARCA4/ACIN1/TAOK1/EIF5AL1/DHX8/ATG2A/NEK6/PLEK/WDR1/VPS13D/KATNB1/IST1/EPG5/PINK1/SUPT16H/SH3GL1/MARK2/CHMP1A/EIF5A/IQSEC1/RUBCN/FIS1/FLII/CAMSAP1/PPP1CA/MAP1A/ARHGEF2/UPF1/GABARAP/VDAC1/VCP/USP36/OPTN/CLASP1/ATG4D/C1QA/STX5/WIPI2/GOLGA2/CKAP5/ATG7/MCOLN1/NAPA/DCTN1/GSN/CFL1/GAK/HUWE1/SQSTM1/LRP1/PPP1R9B/PRKCB/SPTBN1/MTPN/SPTA1/MFN2/NFE2/SHARPIN/SPTB/ATG13/TMOD1/BNIP3L/MICAL2/FBXO7/TBC1D25/SCIN/HMGA1/CAPZB/WDR45/CTDNEP1/FURIN/SMARCC2/CTTN/HTT/ARID1A/GSPT1/TRIM58/SH3BP1/DMTN/ADD1

MF	GO:0019900	kinase binding	583	-0.39	-1.65	3.97E-08	9.23E-07	5.72E-07	2735	tags=36%, list=23%, signal=29%	TTN/DOCK4/PRKAB1/ATF7/CDK12/AP2A1/SH2B3/TRIM21/MYOM2/ADD2/FGR/TRAF2/PKD1/JUP/MAPRE1/MAPK14/PTAFR/RAB8A/AKT1/CEP68/AP2A2/CBLB/NSF/MAPK7/PTPN11/FBXW5/NBEAL2/YWHAZ/CD3E/CASP9/BICD1/PPP1R15A/AGAP2/ZFP36/CDKN2D/HDAC5/DLG4/CPNE3/CNOT9/INKA1/CHP1/MAP3K13/LRRC14/EPHA4/HES1/PKN1/BTG1/MAPKAPK3/CDK5/PRKD2/SPRED1/HYAL2/ZBTB4/CDK5RAP2/CRKL/ATP1B1/FEZ1/EEF2/PARD6A/MAPRE2/PTPN6/CTNND1/CCNY/PGAM1/GSTP1/MOB1B/TRIB1/NR3C1/CDK9/PIN1/PARD6B/RCC2/CDC37/SHC1/GCN1/SMAD3/LRBA/POLA1/PTPRC/SH2B2/TIAM1/FAM83F/DIXDC1/FZD5/LIMS1/RHOBTB1/TPX2/RHOF/EIF3A/USP37/MAP3K11/DGKD/GRB2/TOP2A/NFATC1/SREBF1/GSK3B/CRY2/PRKACA/NEDD9/GSK3A/HSP90AB1/TRIP6/RASGRP3/CLTC/YWHAG/TUBA4A/PRR7/EZR/NEK6/SKI/MVP/PLEK/PARP1/TRAF6/OXSR1/MAP3K5/BCL11A/SIT1/MAVS/BCL2L14/ILK/SASH1/PINK1/RAC1/PTK2B/RARA/MAP2K7/BAD/KIF13B/PFKL/PITPNM2/HCLS1/BCL2L11/SRC/CCND3/PSPN/CEACAM1/TAOK2/DAXX/ACTB/TTC28/PIH1D1/STAT3/NRP1/KAT2B/VDAC1/SYK/TJP2/XIAP/ABL1/ELAVL1/RELA/E2F1/WARS1/RAD23A/ARRB2/SLC2A1/GOLGA2/PRKAR2A/GP6/RHOA/CALM3/DNM2/MAD2L2/ACTA2/PTPRJ/AP1B1/SUFU/WAS/FLNA/MAPKAPK2/RAC2/CAVIN2/HSPB1/BCL2L1/SDC4/POLR2A/DCTN1/GSN/ADIPOR1/SQSTM1/PPP1R9B/PRKCB/TRIM14/CSK/PTPN1/IQGAP1/TCF7L2/MSN/ATP2B4/ATG13/RHOG/FBXO7/CDKN1A/CBL/TNIP1/PAK2/WDR45/PRKAR2B/FOXO3/TOLLIP/PTPN23/HTT/MAP2K3/WNK1/CNPPD1
BP	GO:0031344	regulation of cell projection organization	412	-0.42	-1.72	4.33E-08	1.00E-06	6.21E-07	2625	tags=32%, list=22%, signal=25%	RAB3IP/CORO1B/CAPRIN1/ULK1/EPS8/MARK4/AKT1/DIP2B/NCKIPSD/PLXNC1/PIK3CA/DISC1/PLXNA4/ADNP/TRPM2/SYNE2/CLN3/EZH2/DLG4/MAP3K13/EPHA4/HES1/MAGI2/MAP4/PFN1/CDK5/MOV10/FXR2/ABI3/FYN/ICAM1/ARAP1/CRKL/FEZ1/HSPA5/CERS2/MACF1/PRAG1/ITM2C/FES/PACSIN1/RCC2/CUL7/ARHGAP35/PMP22/KIAA0319/TIAM1/RIPOR2/DIXDC1/RAP1GAP/NDEL1/KLF4/SEMA4D/DTNBP1/VIM/GSK3B/BAIAP2/GSK3A/CYFIP1/ENC1/EZR/CDC42EP4/CORO1C/MFSD2A/BCL11A/KATNB1/IST1/LYN/BCAS3/NEDD4L/PLXNB2/CDKL5/RAC1/MBP/PTK2B/PLXND1/EVI5L/KIF13B/STK24/SIPA1L1/DZIP1/MARK2/FBXO31/SRC/EIF4G2/STK25/SFRP2/SNX3/DVL3/PLXNA1/PPP3CA/ATP1B2/SEMA4A/NRP1/VLDLR/YWHAH/ABL1/PAK1/SYNGAP1/RAPGEF1/PLEK2/XK/RHOA/DNM2/WAS/FLNA/FKBP1B/RAC2/CAMK1D/STK11/DNM3/CFL1/GAK/PPP2R5B/GRN/CUX1/CAMK1/NEURL1/RHOG/FBXO7/EHD1/PAK2/CAPZB/HTT/WASF2/KEL/DMTN/CARM1/SCN1B/DAB2
BP	GO:0048729	tissue morphogenesis	305	-0.45	-1.79	4.44E-08	1.02E-06	6.33E-07	1880	tags=27%, list=16%, signal=23%	MIB1/LZTS2/ARHGAP35/SMAD3/FMN1/EDN1/TIAM1/PHACTR4/MAGED1/ENG/FZD5/RBPJ/TGFB1I1/KLF4/SOX4/FOXP1/GRB2/PRKACA/TGFB1/ACTG1/CTNNBIP1/RPGRIP1L/ST14/SPINT2/KDM6B/SKI/TWSG1/TRAF6/WDR1/MTHFR/TPM1/PLXNB2/ILK/FZD2/RAC1/RARA/PLXND1/DCHS1/SRC/PML/GREM1/CEACAM1/MRTFA/CA2/BBS5/PIK3CD/SFRP2/DVL3/PLXNA1/IFT52/TRIM28/ACTB/PPP1CA/BTBD7/NRP1/BTRC/ABL1/PAK1/FKBP1A/CLASP1/RHOA/VCL/NOTCH2/ACTA2/SUFU/FLNA/MYLK/NF2/SDC4/CD151/MSN/GPI/VASP/MICAL2/MED1/TAL1/ITGB3/PDGFA/SH3BP1/TGM2/PBX1/DAB2
BP	GO:0051017	actin filament bundle assembly	106	-0.59	-2.06	4.91E-08	1.12E-06	6.95E-07	2134	tags=40%, list=18%, signal=33%	ARAP1/SPIRE2/ROCK2/LCP1/ARRB1/SMAD3/SHROOM1/LIMA1/BAIAP2/NEDD9/SORBS3/ACTG1/EZR/PLEK/TPM1/ZYX/RAC1/PTK2B/SLC9A1/SRC/NRP1/PXN/ABL1/PAK1/CLASP1/ARHGEF18/CALD1/RHOA/ARHGAP6/WAS/FLNA/INPP5K/NF2/SDC4/ITGB5/ESPN/PAK2/ACTN1/PDLIM1/WASF2/DMTN/ADD1
CC	GO:0030864	cortical actin cytoskeleton	52	-0.70	-2.16	6.22E-08	1.40E-06	8.68E-07	1323	tags=46%, list=11%, signal=41%	MYO1E/MYZAP/WDR1/HCLS1/CAP1/CALD1/SLC2A1/PVALB/LASP1/NF2/GSN/CLDN5/PPP1R9B/PRKCB/SPTBN1/AKAP13/IQGAP1/SPTA1/COTL1/SPTB/ACTN4/ACTN1/CTTN/MYH9

BP	GO:0045596	negative regulation of cell differentiation	354	-0.43	-1.75	6.24E-08	1.40E-06	8.68E-07	2445	tags=35%, list=21%, signal=29%	PTPN11/NOTCH1/NR1D1/ZFP36/HDAC4/HDAC5/EZH2/FLCN/FOXO1/TSPO/G6PD/EPHA4/HES1/MECP2/IRF1/CDK5/USF3/DPF2/SPRED1/NELFB/ASPM/CDK5RAP2/IL1B/CERS2/ZBTB16/PGLYRP1/ZFP36L2/TRIB1/MIB1/NR1H2/RCC2/SMAD3/RUNX1/KIAA0319/DIXDC1/INPP5D/RBPJ/CMTM5/CD74/TGFB1I1/RAP1GAP/MMP9/SEMA4D/CTDSP1/NFATC1/KDM4A/TOB2/GSK3B/NFATC3/ANKRD17/NR1H3/GSK3A/TGFB1/JDP2/ASCL2/CDK6/TBX21/TMEM131L/CCN3/MEN1/SKI/TWSG1/CORO1C/BCL11A/LYN/YY1/KLF13/LBH/MBP/PTK2B/RARA/IGF2/TRIO/SOX6/GREM1/HOOK3/CEACAM1/CALR/SFRP2/OLIG2/PPARA/LTBP3/RUNX3/PPP3CA/PTBP1/STAT3/CTNNA1/SEMA4A/NRP1/YWHAH/RFLNB/JAK3/MAFB/SYNGAP1/TNFSF4/FOXO4/E2F1/LTF/RHOA/MAD2L2/SUFU/GPR137/FOXJ2/TRIM11/SORT1/ADIPOR1/YBX1/MEIS1/NFATC2/UBASH3B/PAF1/RNF10/FBXO7/HLX/PF4/ACTN4/FOXO3/MED1/CUL4A/ITGB3/TENT5C/DMTN/MBNL3/PBX1
MF	GO:0004674	protein serine/threonine kinase activity	327	-0.44	-1.75	6.30E-08	1.41E-06	8.73E-07	2593	tags=33%, list=22%, signal=27%	ULK1/MAPK14/PAK4/MARK4/MINK1/AKT1/BMP2K/PIM3/TAOK3/PIK3CA/MAPK7/CPNE3/MAP4K5/MAP3K13/LMTK2/IKBKE/PKN1/MAPKAPK3/CDK5/PRKD2/STK40/CSNK2A1/TOP1/RIPK1/NUAK2/ROCK2/MKNK2/TNK2/TESK2/CDK9/MAP3K3/ACVR1B/PHKG2/CSNK1G1/ENG/TAF1/GRK2/PDK3/MAP3K11/MARK3/ACVR2B/GSK3B/PRKACA/GSK3A/RPS6KA1/CDK6/TAOK1/BCKDK/DYRK1B/DAPK3/DAPK1/NEK6/OXSR1/MAP3K5/CDC42BPB/MAP4K1/VRK3/ILK/CDKL5/PINK1/PTK2B/STK10/MAP2K7/TRIO/STK24/MARK2/CCND3/DCAF1/SRPK2/STK25/NEK5/PSKH1/MAST3/CDK2/LRRK1/FAM20C/TAOK2/MAP2K2/BCR/SYK/PAK1/PIM2/HIPK1/MAPKAPK2/MYLK/HIPK2/CDK19/CAMK1D/STK11/SIK3/RIOK3/GAK/SQSTM1/PRKCB/AKT2/RPS6KA4/AKAP13/PIK3CG/ARAF/CAMK1/PRKDC/MAPK3/BRD4/PAK2/EIF2AK1/LTBP1/MAP2K3/WNK1/PIM1
BP	GO:0120035	regulation of plasma membrane bounded cell projection organization	403	-0.42	-1.72	6.60E-08	1.46E-06	9.04E-07	2625	tags=32%, list=22%, signal=25%	RAB3IP/CORO1B/CAPRIN1/ULK1/EPS8/MARK4/AKT1/DIP2B/NCKIPSD/PLXNC1/PIK3CA/DISC1/PLXNA4/ADNP/TRPM2/SYNE2/CLN3/EZH2/MAP3K13/EPHA4/HES1/MAGI2/MAP4/PFN1/CDK5/FXR2/ABI3/FYN/ICAM1/ARAP1/CRKL/FEZ1/HSPA5/CERS2/MACF1/PRAG1/ITM2C/FES/PACSIN1/RCC2/CUL7/ARHGAP35/PMP22/KIAA0319/TIAM1/RIPOR2/DIXDC1/RAP1GAP/NDEL1/KLF4/SEMA4D/DTNBP1/VIM/GSK3B/BAIAP2/GSK3A/CYFIP1/ENC1/EZR/CDC42EP4/CORO1C/MFSD2A/BCL11A/KATNB1/IST1/LYN/BCAS3/NEDD4L/PLXNB2/CDKL5/RAC1/MBP/PTK2B/PLXND1/EVI5L/KIF13B/STK24/SIPA1L1/DZIP1/MARK2/FBXO31/SRC/EIF4G2/STK25/SFRP2/SNX3/PLXNA1/PPP3CA/ATP1B2/SEMA4A/NRP1/VLDLR/YWHAH/ABL1/PAK1/SYNGAP1/RAPGEF1/PLEK2/XK/RHOA/DNM2/WAS/FLNA/FKBP1B/RAC2/CAMK1D/STK11/DNM3/CFL1/GAK/PPP2R5B/GRN/CUX1/CAMK1/NEURL1/RHOG/FBXO7/EHD1/PAK2/CAPZB/HTT/WASF2/KEL/DMTN/CARM1/SCN1B/DAB2
BP	GO:0061572	actin filament bundle organization	108	-0.59	-2.07	6.85E-08	1.51E-06	9.34E-07	2134	tags=40%, list=18%, signal=33%	ARAP1/SPIRE2/ROCK2/LCP1/ARRB1/SMAD3/SHROOM1/LIMA1/BAIAP2/NEDD9/SORBS3/ACTG1/EZR/PLEK/TPM1/ZYX/RAC1/PTK2B/SLC9A1/SRC/NRP1/RFLNB/PXN/ABL1/PAK1/CLASP1/ARHGEF18/CALD1/RHOA/ARHGAP6/WAS/FLNA/INPP5K/NF2/SDC4/ITGB5/ESPN/PAK2/ACTN1/PDLIM1/WASF2/DMTN/ADD1

MF	GO:0004712	protein serine/threonine/tyrosine kinase activity	325	-0.44	-1.76	7.54E-08	1.65E-06	1.02E-06	3008	tags=38%, list=26%, signal=29%	KIT/UHMK1/ERN2/CLK3/ATM/BAZ1B/ALPK1/MAP3K4/FLT1/NEK7/TESK1/OBSCN/EPHB1/TTN/EIF2AK2/PDPK1/RPS6KA2/CDK12/FGR/ULK1/MAPK14/PAK4/MARK4/MINK1/AKT1/BMP2K/PIM3/TAOK3/PIK3CA/MAPK7/MAP4K5/MAP3K13/LMTK2/EPHA4/PKN1/MAPKAPK3/CDK5/PRKD2/DDR1/FYN/STK40/CSNK2A1/RIPK1/CSF1R/NUAK2/ROCK2/MKNK2/TNK2/TESK2/FES/CDK9/MAP3K3/WEE1/CSNK1G1/TAF1/MAP3K11/MARK3/ACVR2B/GSK3B/PRKACA/GSK3A/RPS6KA1/CDK6/TEC/TAOK1/DYRK1B/DAPK3/DAPK1/NEK6/OXSR1/MAP3K5/CDC42BPB/MAP4K1/LYN/ILK/CDKL5/PINK1/PTK2B/STK10/MAP2K7/TRIO/STK24/MARK2/SRC/DCAF1/SRPK2/STK25/NEK5/PSKH1/MAST3/CDK2/LRRK1/FAM20C/TAOK2/MAP2K2/BCR/JAK1/SYK/JAK3/ABL1/PAK1/PIM2/HIPK1/MAPKAPK2/HIPK2/CDK19/CAMK1D/STK11/SIK3/RIOK3/GAK/PRKCB/CSK/AKT2/RPS6KA4/PIK3CG/ARAF/CAMK1/PRKDC/MAPK3/PAK2/EIF2AK1/MAP2K3/WNK1/PIM1
MF	GO:0016772	transferase activity, transferring phosphorus-containing groups	668	-0.38	-1.60	8.85E-08	1.92E-06	1.19E-06	2290	tags=31%, list=20%, signal=27%	MAP3K4/FLT1/PDXK/NEK7/TESK1/RNASEL/OBSCN/POLR2J/EPHB1/CHPT1/TTN/PRKAB1/EIF2AK2/FAM20B/PRPSAP2/PDPK1/RPS6KA2/CDK12/POLR2J3/FHIT/CAD/FGR/ULK1/MAPK14/PAK4/MARK4/MINK1/AKT1/PIP5KL1/BMP2K/PIM3/TAOK3/PIK3CA/MAPK7/NADK/PTDSS1/POMK/CPNE3/PNKP/PAPSS1/OAS2/MAP4K5/OAS3/MAP3K13/LMTK2/IKBKE/EPHA4/PKN1/PIP4K2C/CDIPT/MAPKAPK3/PI4K2A/CDK5/PRKD2/RNGTT/POLD3/DDR1/FYN/STK40/CSNK2A1/TOP1/SELENOO/RIPK1/CSF1R/AK3/NUAK2/PFKFB4/ROCK2/POLD4/MKNK2/PRAG1/TNK2/MTPAP/TESK2/FES/CDK9/MAP3K3/WEE1/POLA1/ACVR1B/PI4KB/PIK3CB/PHKG2/CSNK1G1/ENG/TAF1/DGKZ/GALK1/GRK2/PIK3R3/PDK3/MAP3K11/DGKD/MARK3/CHRAC1/ACVR2B/GSK3B/PRKACA/GSK3A/RPS6KA1/CDK6/TEC/TAOK1/POLR2E/BCKDK/DYRK1B/ITPKC/SPHK1/DAPK3/DAPK1/NEK6/OXSR1/MAP3K5/CDC42BPB/MAP4K1/KARS1/VRK3/LYN/GDPGP1/ILK/NME4/CDKL5/PINK1/PTK2B/STK10/PAPSS2/MAP2K7/CGAS/TRIO/STK24/PFKL/MARK2/SRC/CCND3/DCAF1/SRPK2/STK25/PIK3CD/NEK5/AK1/PSKH1/MAST3/CDK2/LRRK1/FAM20C/TAOK2/NOL9/PKM/TRIM28/NRP1/MAP2K2/PDK2/BCR/JAK1/SYK/TJP2/JAK3/ABL1/PIP5K1B/PAK1/PIM2/CKB/SCYL1/POLL/HIPK1/HK1/MAPKAPK2/ALDH18A1/MYLK/ITPK1/HIPK2/CDK19/CAMK1D/TENT4B/STK11/SIK3/IP6K1/MPP1/RIOK3/POLR2A/GAK/IGF2R/SQSTM1/TERF2/PPP1R9B/PRKCB/CSK/AKT2/RPS6KA4/AKAP13/PIK3CG/ARAF/MMD/CAMK1/PRKDC/PEAK3/PIP4K2A/MAPK3/GUK1/BRD4/CDKN1A/PAK2/EIF2AK1/LTBP1/MAP2K3/TENT5C/WNK1/PIM1
CC	GO:0030141	secretory granule	613	-0.38	-1.60	9.26E-08	2.00E-06	1.24E-06	2118	tags=28%, list=18%, signal=24%	CMTM6/CPNE1/IL1B/PROS1/EEF2/ORMDL3/PTPN6/PPBP/ITPR2/RNASE3/PGAM1/ALDH3B1/PGLYRP1/TIMP1/OSCAR/GSTP1/ENKUR/LGALS3/CTSA/ITGAM/APEH/XRCC6/TOM1/ADA2/APLP2/PTPRC/EDN1/ARPC5/CD14/MMP9/HLA-C/RHOF/CDA/SIRPB1/CXCR2/CRISPLD2/SMPD1/ADGRE5/TRAPPC1/HYAL3/SERPINB6/LAIR1/STOM/BAIAP2/TIMP2/PRKACA/HVCN1/PYCARD/CYFIP1/DOK3/HSP90AB1/TGFB1/CXCR1/CD63/TNFRSF1B/SYNGR1/SLC44A2/DNAJC5/SNX10/STX4/ALAD/MVP/IL4I1/ALDOA/CANT1/GLB1/AP1M1/IST1/RAB31/ANO6/VTI1B/RAB7A/F13A1/SERPING1/RAC1/IGF2/ALDOC/STK10/HSPA1A/TMEM184A/CD9/PFKL/EXOC3/NCSTN/DYNC1H1/CEACAM1/CALR/SCFD2/ATP6V1A/PSAP/PKM/ARHGAP45/CTNNA1/CNN2/PSMD11/SELL/CAP1/VCP/ABCC4/LRG1/LCN2/RAB14/PYGB/LTF/RAB27B/NFKB1/ATP6V0C/LAMTOR1/TUBB4B/ITGAL/GAA/ANXA11/RHOA/VCL/PTPRJ/RAB5B/ARL8A/FERMT3/PSMD2/RAB3D/BSG/ECE1/PA2G4/ATG7/ATP6V0A1/SLC6A9/DOCK2/CYB5R3/FTH1/KRT1/ABHD2/UBR4/GSN/SYTL4/PLEKHO2/HUWE1/IGF2R/THBS1/IQGAP1/GNS/GPI/COTL1/GRN/SELP/SNCA/PRDX6/LAMP1/CAPN1/RHOG/P2RX1/PF4/CTSD/TUBB/TREML1/FTL/ACTN4/PAK2/CTSB/ACTN1/TOLLIP/MYH9/ITGB3/SPARC/PDGFA/CLU/ITGA2B/ACRBP/DIAPH1/TBXA2R

BP	GO:0198738	cell-cell signaling by wnt	265	-0.46	-1.82	9.39E-08	2.02E-06	1.25E-06	2622	tags=37%, list=22%, signal=29%	EXT1/DAAM1/ZEB2/PKD1/JUP/GNAQ/MAPK14/FRMD8/DISC1/NOTCH1/EGR1/UBR5/FOXO1/MAGI2/SPIN1/ASPM/CSNK2A1/MITF/CTNND1/MACF1/CCNY/NDRG2/CCAR2/PIN1/LZTS2/BCL9/SOX13/SMAD3/EDN1/TIAM1/CSNK1G1/DIXDC1/FZD5/RBPJ/TGFB1I1/KLF4/SOX4/BCL9L/NFATC1/GSK3B/GSK3A/TGFB1/CTNNBIP1/SMARCA4/TMEM131L/MESD/VGLL4/DAPK3/SKI/HIC1/ZRANB1/ILK/FZD2/RAC1/PIAS4/MARK2/TBL1XR1/SRC/GREM1/SFRP2/SNX3/DVL3/RUVBL1/LRRK1/AMFR/TLE3/PPP1CA/TBL1X/BTRC/USP34/DDB1/VCP/XIAP/ABL1/OTUD5/FBXW4/NFKB1/RNF213/ATP6V0C/TLE4/RHOA/CHD8/GID8/MAD2L2/TLE1/STK11/SIAH2/LRP1/TCF7L2/PAF1/ZNF703/FAM53B/FOXO3/CTDNEP1/PTPN23/PPM1A/WNK1/DAB2
BP	GO:0016055	Wnt signaling pathway	263	-0.46	-1.81	1.04E-07	2.22E-06	1.38E-06	2622	tags=37%, list=22%, signal=29%	EXT1/DAAM1/ZEB2/PKD1/JUP/GNAQ/MAPK14/FRMD8/DISC1/NOTCH1/EGR1/UBR5/FOXO1/MAGI2/SPIN1/ASPM/CSNK2A1/MITF/CTNND1/MACF1/CCNY/NDRG2/CCAR2/PIN1/LZTS2/BCL9/SOX13/SMAD3/EDN1/TIAM1/CSNK1G1/DIXDC1/FZD5/RBPJ/TGFB1I1/KLF4/SOX4/BCL9L/NFATC1/GSK3B/GSK3A/TGFB1/CTNNBIP1/SMARCA4/TMEM131L/MESD/VGLL4/DAPK3/SKI/HIC1/ZRANB1/ILK/FZD2/RAC1/PIAS4/MARK2/TBL1XR1/SRC/GREM1/SFRP2/SNX3/DVL3/RUVBL1/LRRK1/AMFR/TLE3/PPP1CA/TBL1X/BTRC/USP34/DDB1/VCP/XIAP/ABL1/OTUD5/FBXW4/NFKB1/RNF213/ATP6V0C/TLE4/RHOA/CHD8/GID8/MAD2L2/TLE1/STK11/SIAH2/LRP1/TCF7L2/PAF1/ZNF703/FAM53B/FOXO3/CTDNEP1/PPM1A/WNK1/DAB2
CC	GO:0000785	chromatin	730	-0.37	-1.56	1.13E-07	2.40E-06	1.49E-06	2810	tags=33%, list=24%, signal=27%	NFXL1/CEBPA/HELLS/KAT5/MEF2D/ZBED4/MSX1/FOSB/EPC2/KDM3B/MCM2/IRF9/BHLHE40/ATF7/ETV3/THAP11/H3-3A/SSRP1/BNC2/FANCG/NRIP1/PBRM1/ZFY/ZEB2/EXOSC10/SLF2/SMARCA2/SMAD5/CENPB/FOXP3/E2F3/RRP1B/ZFPM1/JUN/TBX19/CHD3/EXOSC4/ADNP/NR1D1/ELL/EGR1/GMEB2/RAD21/EZH2/FOXO1/PDS5A/HOXB3/H2AC8/NAP1L4/ZNF385A/ARID1B/TSHZ2/HES1/ELK4/MECP2/IRF1/DPF2/BATF2/WDR82/GLYR1/ARID2/MLX/DEAF1/THRA/CSNK2A1/MTA2/MITF/SPI1/FOXN3/MTA1/SREBF2/ADNP2/H1-4/EP400/IPO4/SMARCD3/BASP1/NR3C1/CCAR2/NR1H2/FOXK1/SOX13/ARRB1/TFE3/SMAD3/MAFF/POLA1/RUNX1/PRDM10/TFEB/MAGED1/CREB3/SMARCC1/TAF1/RBPJ/VPS72/CENPF/KLF4/JUND/FOXP2/BBX/SOX4/EHMT2/NFIC/NR6A1/FOXP1/NFATC1/KDM4A/TRRAP/CHRAC1/SREBF1/NCOA1/NFATC3/ZFHX3/FOXK2/ANKRD17/BICRAL/GMEB1/NR1H3/BAZ2A/ASH2L/SMARCA4/JDP2/ASCL2/NFIA/ETS1/ENC1/NCOA2/ETV6/MEN1/BCL11B/TGIF2/HIC1/IRF2/BCL11A/UBR2/TRPS1/IST1/YY1/BCAS3/KLF13/EPAS1/SUPT6H/PINK1/BPTF/RARA/SP2/SUPT16H/ASF1B/RXRA/KLF2/CREBBP/BRMS1/BCL7A/SOX6/PML/ARNT/SRPK2/TAF4/NCOA3/POU2F2/USF2/GATAD2B/OLIG2/PPARA/DVL3/WBP2/SLX4/RUVBL1/RUNX3/PELP1/BICRA/TRIM28/TFDP1/MAX/ACTB/UBA1/STAT3/ETS2/UPF1/MAFK/MCRS1/FBH1/MAFB/FOS/RELA/FOXO4/E2F1/NFKB1/DDX23/OLIG1/HNRNPK/CHD8/RFX1/PPP4C/FOXJ2/STAT5A/SAP130/KLF1/ELK1/DPF3/MLXIP/KAT6A/MXD4/SETD1A/KLF16/MEIS1/TCF7L2/SP1/NFE2/NFATC2/ELF4/CUX1/CHD4/BRD3/PAF1/PPP1R10/H2BC11/NFIX/E2F2/SOX12/IKZF1/GATA1/BRD4/HLX/LYL1/AKIRIN2/HMGA1/CBX6/RNF40/FOXO3/SMARCC2/MXI1/TAL1/H3C10/ARID1A/KLF3/NCOR2/TGM2/PBX1
BP	GO:0030038	contractile actin filament bundle assembly	74	-0.63	-2.09	1.22E-07	2.53E-06	1.57E-06	2222	tags=39%, list=19%, signal=32%	PFN1/ARAP1/ROCK2/ARRB1/SMAD3/SORBS3/ACTG1/TPM1/ZYX/RAC1/PTK2B/SLC9A1/SRC/NRP1/PXN/ABL1/PAK1/CLASP1/ARHGEF18/RHOA/ARHGAP6/WAS/INPP5K/NF2/SDC4/ITGB5/PAK2/PDLIM1/WASF2

BP	GO:0043149	stress fiber assembly	74	-0.63	-2.09	1.22E-07	2.53E-06	1.57E-06	2222	tags=39%, list=19%, signal=32%	PFN1/ARAP1/ROCK2/ARRB1/SMAD3/SORBS3/ACTG1/TPM1/ZYX/RAC1/PTK2B/SLC9A1/SRC/NRP1/PXN/ABL1/PAK1/CLASP1/ARHGEF18/RHOA/ARHGAP6/WAS/INPP5K/NF2/SDC4/ITGB5/PAK2/PDLIM1/WASF2
BP	GO:0051893	regulation of focal adhesion assembly	44	-0.73	-2.22	1.27E-07	2.59E-06	1.61E-06	2019	tags=57%, list=17%, signal=47%	ROCK2/MACF1/RCC2/SMAD3/FMN1/LIMS1/POLDIP2/ACTG1/DAPK3/CORO1C/BCAS3/RAC1/SLC9A1/SRC/GREM1/NRP1/ABL1/CLASP1/RHOA/VCL/ARHGAP6/PTPRJ/SDC4/THBS1/DMTN
BP	GO:0090109	regulation of cell-substrate junction assembly	44	-0.73	-2.22	1.27E-07	2.59E-06	1.61E-06	2019	tags=57%, list=17%, signal=47%	ROCK2/MACF1/RCC2/SMAD3/FMN1/LIMS1/POLDIP2/ACTG1/DAPK3/CORO1C/BCAS3/RAC1/SLC9A1/SRC/GREM1/NRP1/ABL1/CLASP1/RHOA/VCL/ARHGAP6/PTPRJ/SDC4/THBS1/DMTN
BP	GO:0051258	protein polymerization	213	-0.49	-1.87	1.28E-07	2.59E-06	1.61E-06	2601	tags=42%, list=22%, signal=33%	MAPRE1/EPS8/CORO1A/ARL2/TUBGCP2/TMOD3/SPTAN1/SPIRE1/TRIOBP/MSRB1/WASHC5/ARHGAP18/CORO7/TUBGCP4/TUBGCP6/PFN1/MECP2/NDE1/CDK5RAP2/SSH1/SPIRE2/TBCD/MSRB2/MYADM/TUBG1/TUBG2/DIAPH2/SVIL/FES/SSH2/NUMA1/FMN1/ARPC5/NDEL1/TPX2/CHMP4B/GRB2/SLAIN2/BAIAP2/PYCARD/CYFIP1/GIT1/PSTPIP2/PREX1/EML2/RANGRF/CDC42EP4/PRUNE1/ALOX15/HIP1R/PINK1/RAC1/PTK2B/HSPA1A/TRIM32/HCLS1/GAS7/ARPC1B/FLII/CAMSAP1/ABL1/PAK1/CLASP1/SSH3/GOLGA2/RHOA/ARPC4/ARHGAP6/CKAP5/UBE2S/WAS/CYFIP2/DCTN1/GSN/SPTBN1/MTPN/SPTA1/COTL1/SPTB/SNCA/VASP/TMOD1/WASF3/SCIN/CAPZB/CTTN/ESAM/DMTN/ADD1/DIAPH1
MF	GO:0019904	protein domain specific binding	507	-0.40	-1.65	1.28E-07	2.59E-06	1.61E-06	2540	tags=36%, list=22%, signal=29%	CEP68/AIDA/NCKIPSD/AP2A2/CBLB/KCNH2/NSF/CCDC6/BAK1/NCF1/LAMP2/YWHAZ/CD3E/CASP9/DLG4/M6PR/CNOT9/BANF1/U2AF2/EPHA4/HES1/MECP2/NDE1/ACOX1/ABI3/FYN/CASP2/THRA/EXOC4/TOP1/IL1B/SH3KBP1/RIPK1/PRRG4/GTF2F1/PTPN6/HSPA5/ZBTB16/SNTA1/TNK2/BASP1/PPARGC1B/PDE2A/ENKUR/EHMT1/SOCS7/EPB41L2/LSM4/NUMA1/RCC2/FMN1/PTTG1/SYNJ1/KEAP1/HGS/SNTB1/SH2B2/HIF1AN/DIXDC1/INPP5D/WBP11/CTBP1/IKZF4/EHMT2/VIM/IKBKG/GRB2/SCAF1/BAIAP2/PRKACA/PYCARD/DAP/HSP90AB1/CTNNBIP1/REPS1/JDP2/CLTC/YWHAG/ZNF106/EZR/NCOA2/ARHGAP17/ETV6/DAPK3/INPPL1/SKI/TP53BP2/MAP3K5/TRPS1/IST1/SIT1/HIP1R/LYN/PTPN12/MAVS/PINK1/FZD2/SHCBP1/RARA/TADA3/HSPA1A/RXRA/PLXND1/ARF1/HCLS1/SRC/CHMP1A/MRTFA/NCOA3/YWHAB/OLIG2/PPARA/CDK2/TRIM28/TFDP1/LITAF/MAP2K2/TBL1X/ETS2/YWHAH/PHRF1/NOLC1/PPP3R1/SYK/DDB1/VCP/TJP2/ABL1/FAM189B/SYNGAP1/PLEKHA2/RELA/RAB27B/RAPGEF1/WARS1/ARRB2/SIGLEC10/HNRNPK/PRKAR2A/RHOA/CHD8/CALM3/DNM2/ARHGAP6/WAS/HNRNPM/ARHGAP31/ARHGAP1/TRIM11/BCL2L1/STK11/PLSCR3/AFAP1/WIPF1/SQSTM1/IQGAP1/TCF7L2/SP1/NFE2/FKBP8/ATP2B4/PRKDC/LAMP1/VASP/IKZF1/GATA1/CBL/ESPN/TUBB/GPX1/RUFY1/ACTN1/PRKAR2B/MED1/AP2M1/MYH9/WASF2/SH3BP1/TLN1/CNOT1/SH3BGRL2
BP	GO:0030832	regulation of actin filament length	120	-0.55	-1.98	1.32E-07	2.64E-06	1.64E-06	2588	tags=42%, list=22%, signal=33%	EPS8/CORO1A/SWAP70/PIK3CA/TMOD3/DSTN/SPTAN1/TRIOBP/ARHGAP18/PFN1/SSH1/MYADM/SVIL/SSH2/ARHGAP35/FMN1/ARPC5/LIMA1/GRB2/BAIAP2/PYCARD/CYFIP1/CDC42EP4/PLEK/WDR1/ALOX15/HIP1R/PTK2B/HCLS1/ARPC1B/FLII/SSH3/RHOA/ARPC4/WAS/CYFIP2/GSN/SPTBN1/MTPN/SPTA1/COTL1/SPTB/VASP/TMOD1/SCIN/CAPZB/CTTN/ESAM/SH3BP1/DMTN/ADD1

MF	GO:0050839	cell adhesion molecule binding	372	-0.43	-1.73	1.31E-07	2.64E-06	1.64E-06	2142	tags=33%, list=18%, signal=28%	ICAM1/CRKL/IL1B/PLIN3/YKT6/P2RX4/EEF2/EFHD2/PTPN6/HSPA5/CTNND1/MACF1/DNAJB1/LCP1/ITGB7/NDRG1/ITGAM/PKP4/GCN1/AHSA1/SERBP1/CAST/LRRC59/LGALS12/CCS/VSIG10/LIMA1/CHMP4B/ABCF3/BAIAP2/HSP90AB1/ASAP1/UBAP2/CCN3/EZR/ATIC/EHD4/ALDOA/IST1/LYN/JAM3/IGF2/HSPA1A/CD9/DCHS1/MICALL1/FLNB/SH3GL1/STK24/EXOC3/MARK2/ZC3HAV1/SRC/EIF4G2/CALR/YWHAB/SFRP2/RUVBL1/ADGRL1/PKM/PPP1CA/CTNNA1/CTNNAL1/RANGAP1/CNN2/SH3GLB2/EPS15L1/UBFD1/SYK/TJP2/GOLGA3/CALD1/STX5/ITGAL/GOLGA2/HNRNPK/PLCB3/SCYL1/VCL/CKAP5/PTPRJ/FLNA/SLC3A2/RAB11B/FERMT3/BSG/ARHGAP1/LASP1/NIBAN2/NF2/FASN/ITGB5/SND1/PLEC/THBS1/SPTBN1/PTPN1/CD151/IQGAP1/MSN/PRDX6/ENO1/VASP/CBL/EHD1/GAPVD1/ACTN4/PAK2/CAPZB/ACTN1/PDLIM1/CTTN/H3C10/MYH9/ITGB3/ESAM/WASF2/HDLBP/ITGA2B/TLN1/LARP1/ADD1/HCFC1
BP	GO:0010594	regulation of endothelial cell migration	107	-0.59	-2.08	1.36E-07	2.71E-06	1.68E-06	2217	tags=40%, list=19%, signal=33%	MECP2/PRKD2/SPRED1/P2RX4/VASH1/ROCK2/MAP3K3/SCARB1/EDN1/PIK3CB/KLF4/DNAJA4/FOXP1/TGFB1/SVBP/ETS1/BCAS3/SASH1/RAC1/PTK2B/RRAS/CEACAM1/CALR/PIK3CD/SEMA4A/NRP1/ABL1/RHOA/HSPB1/STAT5A/GFUS/GLUL/THBS1/PIK3CG/SP1/GPI/GRN/ATP2B4/ITGB3/SPARC/SH3BP1/MAP2K3/TBXA2R
MF	GO:0017124	SH3 domain binding	87	-0.60	-2.07	1.61E-07	3.18E-06	1.97E-06	2518	tags=51%, list=21%, signal=40%	NCKIPSD/CBLB/CCDC6/NCF1/CD3E/CASP9/ABI3/SH3KBP1/PTPN6/ENKUR/SOCS7/FMN1/PTTG1/SYNJ1/INPP5D/GRB2/REPS1/ZNF106/ARHGAP17/INPPL1/TP53BP2/HIP1R/LYN/PTPN12/HCLS1/ABL1/SYNGAP1/RAPGEF1/DNM2/ARHGAP6/WAS/ARHGAP31/ARHGAP1/PLSCR3/AFAP1/WIPF1/VASP/CBL/ESPN/GPX1/RUFY1/WASF2/SH3BP1/SH3BGRL2
BP	GO:0045639	positive regulation of myeloid cell differentiation	73	-0.64	-2.12	1.67E-07	3.28E-06	2.03E-06	1934	tags=44%, list=16%, signal=37%	PPARGC1B/TRIB1/FES/ACVR1B/RUNX1/INPP5D/CD74/TGFB1/CTNNBIP1/ACIN1/ETS1/TRAF6/HSPA1A/HCLS1/KAT7/ARNT/STAT3/FOS/PITHD1/MPL/NOTCH2/MTURN/FAM210B/PRKDC/GATA1/PF4/SCIN/FOXO3/TESC/FAXDC2/MED1/TAL1
BP	GO:0061061	muscle structure development	349	-0.43	-1.72	1.94E-07	3.78E-06	2.35E-06	1961	tags=28%, list=17%, signal=24%	NLN/SMARCD3/HMG20B/BASP1/SVIL/CDK9/PDLIM7/FOXK1/BCL9/SYNE1/SMAD3/MAFF/EDN1/HIF1AN/RIPOR2/ENG/RBPJ/CMTM5/CENPF/NFATC1/NFATC3/ZFHX3/ANKRD17/SFMBT1/GSK3A/TGFB1/ACTG1/DYRK1B/CCN3/KDM6B/SKI/SELENON/WDR1/MAP3K5/HDGFL2/TPM1/FHL1/YY1/EPAS1/SUPT6H/FZD2/RARA/IGF2/CD9/PLD3/DYSF/FLNB/SLC9A1/TRIM32/SOX6/GREM1/MRTFA/CALR/LRRC8A/PPARA/FLII/PPP3CA/PTBP1/ZNF609/FHL3/USP19/MYOF/CSRP1/FOS/FKBP1A/FOXO4/ARRB2/XK/RHOA/LMNA/MYLK/RBM38/ZBTB42/NIBAN2/SORT1/PLEC/YBX1/AKAP13/MTPN/TCF7L2/NFATC2/CAMK1/NEURL1/TMOD1/HLX/EHD1/ACTN4/GPX1/ACTN1/YBX3/PDLIM1/MYL9/MED1/MYH9/KEL/HIVEP3/MBNL3

CC	GO:0036477	somatodendritic compartment	456	-0.41	-1.68	2.25E-07	4.36E-06	2.70E-06	2569	tags=34%, list=22%, signal=28%	SORL1/RAB8A/MARK4/MINK1/DIP2B/MUL1/SYT11/NSF/ADNP/TRPM2/CYBB/NR1D1/NCF1/CD22/CD3E/DLG4/RPH3A/ZNF385A/EIF4A3/LMTK2/EPHA4/MAGI2/DMWD/PI4K2A/CDK5/LYNX1/FXR2/ABI3/FYN/ABHD17C/TOP1/FEZ1/HRH2/YKT6/P2RX4/CNR2/DOCK10/SMN1/CTNND1/DNAJB1/SRGAP2/TMEM185A/NUMA1/PDE4B/OR10H1/DIXDC1/ENO2/FZD5/GNAZ/TRAK1/MAP1S/RAP1GAP/NDEL1/CANX/TPX2/PPP1R9A/KCNJ2/MARK3/SEPTIN11/DTNBP1/GSK3B/KIF1C/BAIAP2/PRKACA/GSK3A/PYCARD/CASC3/CYFIP1/HSP90AB1/TBX21/TNFRSF1B/PDE1A/ASAP1/PRR7/ENC1/CCN3/TRAPPC4/STX4/KIF3B/KATNB1/HIP1R/VTI1B/CDKL5/RAC1/MBP/NPFF/PTK2B/RARA/SIPA1L1/MARK2/GRIPAP1/FBXO31/N4BP3/CPNE5/GNAI2/SLA/KIF1B/SEPTIN4/ARHGAP32/VTI1A/IFT52/PPP3CA/RIN3/TAOK2/AMFR/MAX/PPP1CA/CNIH3/RANGAP1/SLC1A4/MAP1A/BCR/APBA3/MCRS1/TRAK2/ABL1/PAK1/SYNGAP1/ARRB2/STAU1/MPL/CKB/RHOA/PDE9A/PVALB/FLNA/SLC3A2/GLRX5/ELK1/ROGDI/SIAH2/ZDHHC5/UBB/DNM3/GNA12/DCTN1/PLEC/GLUL/PPP1R9B/HRH4/GJC2/SHARPIN/SNCA/LRFN3/NEURL1/NRGN/ACTN1/PRKAR2B/CTTN/HTT/SHISA7/CLU/OSBP2/SCN1B/HCFC1
MF	GO:0140297	DNA-binding transcription factor binding	370	-0.42	-1.68	2.39E-07	4.59E-06	2.85E-06	2141	tags=32%, list=18%, signal=27%	MLX/CRKL/MTA2/CPNE1/SPI1/ZNF366/MTA1/TACC1/SMARCD3/PPARGC1B/ZBTB7A/TRIB1/NR1H2/UBXN7/SMAD3/KEAP1/HIF1AN/TAF1/TAF6/RBPJ/TGFB1I1/CTBP1/TRERF1/IKZF4/CENPF/KLF4/JUND/FOXP1/NFATC1/NCOA1/TOB2/GSK3B/CRY2/BAZ2A/TRIP6/HDAC3/SMARCA4/NFIA/VHL/ETS1/PDCD11/NCOA2/DAPK3/BCOR/NEK6/SKI/NCOA6/PARP1/TP53BP2/YY1/BCAS3/EPAS1/TADA3/RXRA/IFI27/CREBBP/MTDH/MED16/TRIM32/BRMS1/HCLS1/SRC/DCAF1/ARNT/TAF4/BBS5/NCOA3/CALR/USF2/PPARA/WBP2/DAXX/TP53BP1/TFDP1/MAX/ACTB/STAT3/TBL1X/ETS2/KAT2B/YWHAH/FOS/FBP1/RELA/FOXO4/E2F1/DTX3L/WWP2/TLE4/NOTCH2/MAD2L2/FLNA/TLE1/HSPB1/HIPK2/ELK1/DHX33/KAT6A/MED25/PRKCB/SETD1A/TCF7L2/SP1/CHD4/PRKDC/ZNF703/GATA1/TRIP12/HMGA1/ACTN4/MED1/TAL1/ARID1A/GAS2L1/GFI1B/NCOR2/ADD1/CNOT1/PBX1/HCFC1
BP	GO:0033993	response to lipid	537	-0.39	-1.61	2.41E-07	4.61E-06	2.86E-06	2108	tags=26%, list=18%, signal=22%	IL1B/SPI1/ZNF366/OSBPL7/CNR2/VPS4B/ADNP2/PPBP/ROCK2/SCIMP/PAQR8/NCOA4/GSTP1/PPARGC1B/ZBTB7A/ZFP36L2/TRIB1/NR3C1/FES/ALPL/SCARB1/PDE4B/SYNJ1/EDN1/ENG/ENO2/CD14/CPT1A/TRERF1/KLF4/PDK3/JUND/SIGIRR/VIM/FOXP1/SREBF1/GSK3B/CRY2/NR1H3/PYCARD/CACTIN/TGFB1/MICB/SMARCA4/GIT1/TNFRSF1B/IL1RN/NCOA2/CD180/UCP2/ALAD/PARP1/TRAF6/GLB1/MAP4K1/AQP1/LYN/SASH1/LBH/MBP/PTK2B/RARA/TADA3/HSPA1A/RXRA/MTDH/BAD/BCL2L11/SRC/ARPC1B/NCOA3/CALR/PPARA/WBP2/DAXX/ZMIZ1/LITAF/STAT3/BCR/UPF1/YWHAH/SYK/ABL1/PAK1/FOS/TNFSF4/RELA/OTUD5/E2F1/LTF/NFKB1/BMP6/RHOA/CALM3/LGALS9/MAPKAPK2/KANK2/CDK19/ELK1/STK11/PLSCR3/PRPF8/ABHD2/SAFB/TXNIP/PF4V1/IGF2R/PPP1R9B/THBS1/UBE2L3/FAM210B/MSN/GPI/SELP/SNCA/PAF1/CXCL5/ZNF703/MAPK3/GATA1/PF4/AKIRIN2/CBL/TNIP1/GPX1/FOXO3/TESC/MED1/ARID1A/PDGFA/CLU/MAP2K3/NCOR2/CARM1/PIM1/CNOT1/DAB2/TBXA2R
BP	GO:0008015	blood circulation	238	-0.47	-1.83	2.52E-07	4.78E-06	2.97E-06	2128	tags=34%, list=18%, signal=29%	EXT1/JUP/ATP2A3/SMAD5/PTAFR/KCNH2/POMC/PIK3CA/SERPINF2/TBC1D8/KNG1/ATP1A1/ATP2A2/MECP2/THRA/ATP1B1/HRH2/P2RX4/GNA11/ROCK2/SNTA1/PDE2A/ARHGAP35/SMAD3/PDE4B/EDN1/GRK2/KCNJ2/EHD3/SREBF1/PRKACA/GSK3A/TGFB1/CTNNBIP1/RANGRF/TPM1/LNPEP/NEDD4L/EPAS1/SERPING1/KLF2/SLC9A1/SRC/CEACAM1/PPARA/PTP4A3/ATP1B2/MYOF/BCR/TJP2/ABL1/BMP6/GAA/PLCB3/PDE5A/RHOA/CALM3/ACTA2/PTPRJ/FLNA/GUCY1A1/FKBP1B/ECE1/MYL4/CYB5R3/GNA12/GSN/PLEC/CLDN5/PLVAP/PIK3CG/PER2/ATP2B4/P2RX1/PTGS1/GPX1/GUCY1B1/KEL/MAP2K3/SCN1B/WNK1/TBXA2R

BP	GO:0032870	cellular response to hormone stimulus	387	-0.42	-1.71	2.53E-07	4.78E-06	2.97E-06	1775	tags=27%, list=15%, signal=24%	NCOA4/PPARGC1B/ZBTB7A/ZFP36L2/NR3C1/SHC1/SLC29A2/SH2B2/EDN1/SMARCC1/NDEL1/TRERF1/PIK3R3/FOXP1/GRB2/SREBF1/NCOA1/GSK3B/BAIAP2/CRY2/PRKACA/NR1H3/GSK3A/CYFIP1/SMARCA4/YWHAG/ZNF106/NCOA2/MEN1/UCP2/SLC27A1/WDTC1/PARP1/RAB31/AQP1/LYN/BCAS3/LEPROT/LBH/RARA/TADA3/IGF2/HSPA1A/RXRA/SLC9A1/GNB1/BCL2L11/SRC/CCND3/ADIPOR2/CEACAM1/CA2/NCOA3/CALR/GNAI2/PPARA/WBP2/PELP1/DAXX/ZMIZ1/PKM/MAX/STAT3/RANGAP1/PDK2/KAT2B/YWHAH/JAK1/PXN/JAK3/PAK1/FOS/TNFSF4/FBP1/RELA/FOXO4/NFKB1/LPIN2/SESN3/RHOA/INPP5K/KANK2/STAT5A/ABHD2/ADIPOR1/SAFB/PRKCB/CSK/AKT2/UBE2L3/PTPN1/SP1/PRKDC/AHCYL1/PIP4K2A/GATA1/EIF4EBP2/CTSB/FOXO3/MED1/ARID1A/GAS2L1/NCOR2/CARM1/CNOT1/DAB2
BP	GO:0003012	muscle system process	217	-0.48	-1.82	2.70E-07	5.03E-06	3.12E-06	2606	tags=37%, list=22%, signal=30%	JUP/SMAD5/PTAFR/KCNH2/GNAO1/PIK3CA/TMOD3/NOTCH1/TPM4/ATP1A1/TRPC3/ATP2A2/HDAC4/EZH2/FOXO1/ARHGEF11/SYNM/G6PD/ATP1B1/IL1B/P2RX4/ORMDL3/ROCK2/SNTA1/CDK9/RCSD1/SMAD3/PDE4B/SNTB1/EDN1/GRK2/KCNJ2/EHD3/KDM4A/TPM3/PRKACA/GSK3A/TNFRSF1B/RANGRF/SPHK1/DAPK3/PARP1/ALDOA/TPM1/YY1/NEDD4L/SLC9A1/PPARA/PPP3CA/MYOF/ANXA6/CALD1/JARID2/GAA/PDE5A/RHOA/PDE9A/CALM3/ACTA2/FLNA/GUCY1A1/FKBP1B/LMNA/MYLK/MYL4/GSN/AKAP13/MTPN/PIK3CG/ATP2B4/ENO1/TMOD1/P2RX1/PAK2/SLC6A8/FOXO3/MYL9/CTTN/MAP2K3/SCN1B/TBXA2R
BP	GO:0071396	cellular response to lipid	376	-0.42	-1.70	2.77E-07	5.14E-06	3.19E-06	2108	tags=28%, list=18%, signal=24%	IL1B/SPI1/ZNF366/OSBPL7/ADNP2/PPBP/ROCK2/SCIMP/PAQR8/NCOA4/GSTP1/PPARGC1B/ZBTB7A/ZFP36L2/TRIB1/NR3C1/FES/SCARB1/PDE4B/EDN1/CD14/CPT1A/TRERF1/PDK3/SIGIRR/VIM/FOXP1/SREBF1/GSK3B/CRY2/NR1H3/PYCARD/CACTIN/TGFB1/SMARCA4/GIT1/TNFRSF1B/CD180/PARP1/TRAF6/MAP4K1/AQP1/LYN/SASH1/LBH/PTK2B/RARA/TADA3/HSPA1A/RXRA/MTDH/BAD/BCL2L11/SRC/NCOA3/CALR/PPARA/WBP2/DAXX/ZMIZ1/LITAF/BCR/UPF1/YWHAH/SYK/ABL1/PAK1/TNFSF4/RELA/E2F1/LTF/NFKB1/BMP6/RHOA/KANK2/CDK19/ELK1/PLSCR3/PRPF8/ABHD2/SAFB/PF4V1/PPP1R9B/UBE2L3/FAM210B/MSN/PAF1/CXCL5/ZNF703/MAPK3/GATA1/PF4/TNIP1/FOXO3/TESC/MED1/ARID1A/CLU/MAP2K3/NCOR2/CARM1/PIM1/CNOT1/DAB2/TBXA2R
BP	GO:1901888	regulation of cell junction assembly	101	-0.58	-2.02	3.16E-07	5.84E-06	3.62E-06	2161	tags=38%, list=18%, signal=31%	ABI3/IL1B/SLITRK5/ROCK2/MACF1/RCC2/SMAD3/FMN1/FZD5/LIMS1/POLDIP2/SEMA4D/PRKACA/ACTG1/DAPK3/CORO1C/BCAS3/RAC1/SLC9A1/SRC/GREM1/SEMA4A/NRP1/ABL1/CLASP1/RAPGEF1/RHOA/VCL/ARHGAP6/PTPRJ/SDC4/CLSTN1/CLDN5/THBS1/SNCA/LRFN3/EIF4G1/DMTN
BP	GO:0150116	regulation of cell-substrate junction organization	49	-0.70	-2.16	3.20E-07	5.89E-06	3.65E-06	2048	tags=55%, list=17%, signal=46%	MAPRE2/ROCK2/MACF1/RCC2/SMAD3/FMN1/LIMS1/POLDIP2/ACTG1/DAPK3/CORO1C/BCAS3/RAC1/SLC9A1/SRC/GREM1/IQSEC1/NRP1/ABL1/CLASP1/RHOA/VCL/ARHGAP6/PTPRJ/SDC4/THBS1/DMTN
BP	GO:0007179	transforming growth factor beta receptor signaling pathway	117	-0.57	-2.03	3.22E-07	5.91E-06	3.66E-06	1926	tags=38%, list=16%, signal=32%	ZBTB7A/PIN1/NRROS/SMAD3/ENG/TGFB1I1/BCL9L/HSP90AB1/TGFB1/MEN1/SKI/TWSG1/PARP1/ZYX/C20orf27/HSPA1A/CHST11/CREBBP/SRC/PML/CDKN1C/PPARA/LTBP3/FAM89B/ZMIZ1/STAT3/USP9X/PXN/LRG1/FOS/FKBP1A/ARRB2/DNM2/HIPK2/STK11/ITGB5/CLDN5/THBS1/GLG1/ZNF703/TGFBRAP1/FURIN/PPM1A/LTBP1/DAB2
BP	GO:0008064	regulation of actin polymerization or depolymerization	119	-0.55	-1.98	3.36E-07	6.13E-06	3.80E-06	2588	tags=43%, list=22%, signal=34%	EPS8/CORO1A/SWAP70/PIK3CA/TMOD3/DSTN/SPTAN1/TRIOBP/ARHGAP18/PFN1/SSH1/MYADM/SVIL/SSH2/ARHGAP35/FMN1/ARPC5/LIMA1/GRB2/BAIAP2/PYCARD/CYFIP1/CDC42EP4/PLEK/WDR1/ALOX15/HIP1R/PTK2B/HCLS1/ARPC1B/FLII/SSH3/RHOA/ARPC4/WAS/CYFIP2/GSN/SPTBN1/MTPN/SPTA1/COTL1/SPTB/VASP/TMOD1/SCIN/CAPZB/CTTN/ESAM/SH3BP1/DMTN/ADD1

CC	GO:0031253	cell projection membrane	197	-0.49	-1.86	3.47E-07	6.30E-06	3.90E-06	1727	tags=27%, list=15%, signal=24%	RIPOR2/LIMA1/SLC19A1/EHD3/NAPEPLD/HSP90AB1/ASAP1/EZR/STX4/PLEK/CORO1C/SLC5A6/TPM1/AQP1/HIP1R/CDKL5/RAC1/PLXND1/SRC/SH2D3C/CEACAM1/BBS5/SLC7A8/ATP1B2/DHRS3/ARHGAP45/ARHGEF2/ABCG2/PAK1/PLEK2/CLTB/SLC7A5/RHOA/PDE9A/DNM2/PTPRJ/INPP5K/NF2/GNA12/CFL1/PPP1R9B/SPTBN1/AKT2/PDE4A/MSN/GPI/VASP/EHD1/TESC/SHISA7/ITGB3/DMTN/TLN1/DIAPH1
BP	GO:0044089	positive regulation of cellular component biogenesis	349	-0.42	-1.70	3.70E-07	6.68E-06	4.14E-06	1910	tags=28%, list=16%, signal=24%	PFN1/PIP4K2C/MECP2/CDK5RAP2/ARAP1/SLITRK5/VPS4B/ROCK2/TLR6/LCP1/FES/LGALS3/NR1H2/NUMA1/ARHGAP35/SMAD3/FMN1/HGS/RIPOR2/LIMS1/POLDIP2/NDEL1/SEMA4D/GRB2/SLAIN2/GSK3B/BAIAP2/SORBS3/PYCARD/G3BP2/CYFIP1/TGFB1/GIT1/ASAP1/ATG2A/CDC42EP4/PLEK/TPM1/ALOX15/HIP1R/BCAS3/RAB7A/PINK1/RAC1/PTK2B/HSPA1A/DZIP1/BCL2L11/DYNC1H1/SRC/SLX4/CDK2/TRIM28/PIH1D1/SEMA4A/NRP1/SYK/VCP/PXN/ABL1/PAK1/FNIP2/CLASP1/PLEK2/WARS1/SETDB1/RHOA/DNM2/CKAP5/PTPRJ/RAC2/NF2/GNL3L/SDC4/DNM3/DHX33/DCTN1/CLSTN1/MED25/CLDN5/PPP2R5B/MSN/SNCA/EIF4G1/NEURL1/VASP/PIP4K2A/ELAPOR1/SMPD3/WDR45/CDC34/TAL1/CTTN/HTT/STMP1/WASF2/CLU/CNOT1
BP	GO:0045216	cell-cell junction organization	113	-0.57	-2.03	4.16E-07	7.48E-06	4.64E-06	2108	tags=37%, list=18%, signal=31%	IL1B/TBCD/FBF1/PARD6A/CSF1R/ROCK2/CTNND1/AJM1/PKP4/PARD6B/SMAD3/LSR/FZD5/LIMS1/PRKACA/TGFB1/ACTG1/WDR1/JAM3/CD9/DCHS1/SRC/ACTB/CTNNA1/TJP2/KIFC3/PARD3/BMP6/RHOA/VCL/HIPK1/FLNA/NF2/PLEC/CLDN5/CSK/ZNF703/PAK2/PTPN23/ESAM/TLN1/ADD1
BP	GO:0030111	regulation of Wnt signaling pathway	196	-0.48	-1.82	4.21E-07	7.54E-06	4.68E-06	2014	tags=38%, list=17%, signal=32%	ZEB2/JUP/GNAQ/MAPK14/FRMD8/DISC1/NOTCH1/EGR1/UBR5/FOXO1/SPIN1/ASPM/CSNK2A1/CTNND1/MACF1/CCNY/CCAR2/PIN1/LZTS2/SOX13/SMAD3/TIAM1/CSNK1G1/DIXDC1/RBPJ/SOX4/NFATC1/GSK3B/GSK3A/TGFB1/CTNNBIP1/SMARCA4/TMEM131L/VGLL4/DAPK3/SKI/HIC1/ZRANB1/ILK/TBL1XR1/SRC/GREM1/SFRP2/SNX3/RUVBL1/LRRK1/AMFR/TLE3/PPP1CA/TBL1X/BTRC/USP34/VCP/XIAP/ABL1/OTUD5/NFKB1/RNF213/ATP6V0C/TLE4/CHD8/GID8/MAD2L2/TLE1/STK11/SIAH2/LRP1/TCF7L2/ZNF703/FAM53B/FOXO3/CTDNEP1/PPM1A/WNK1/DAB2
BP	GO:0001501	skeletal system development	260	-0.45	-1.77	4.30E-07	7.66E-06	4.75E-06	2707	tags=37%, list=23%, signal=29%	PRPSAP2/CMKLR1/SH2B3/ATP7A/FGR/BNC2/EXT1/PKD1/TTC9/MAPK14/SMAD5/ZFPM1/TIPARP/PTPN11/RAI1/SGPL1/HOXB3/PHEX/PAPSS1/ZNF385A/EIF4A3/HYAL2/DEAF1/THRA/FOXN3/PTPN6/GNA11/ZBTB16/TIMP1/XYLT1/PPARGC1B/ALPL/SMAD3/FMN1/RUNX1/PTPRC/EDN1/ENG/MMP9/WDR5/SEMA4D/BBX/SOX4/FOXP1/HYAL3/ACVR2B/TGFB1/CCN3/SNX10/GNAS/INPPL1/NDST1/ATP6AP1/SKI/SLC39A3/TRPS1/RARA/CHST11/DCHS1/PAPSS2/SRC/SOX6/GREM1/CDKN1C/SFRP2/LTBP3/RUNX3/LRRK1/FAM20C/DHRS3/PHOSPHO1/ETS2/PDGFC/RFLNB/LTF/ANXA6/BMP6/RHOA/NOTCH2/SUFU/SLC38A10/GLG1/ANKH/AKAP13/MEIS1/PIP4K2A/MAPK3/SMPD3/SCIN/MPIG6B/MED1/TAL1/WASF2/TGM2/PBX1
CC	GO:0030017	sarcomere	110	-0.57	-2.01	4.38E-07	7.77E-06	4.82E-06	1288	tags=33%, list=11%, signal=29%	MYZAP/CORO1C/ALDOA/TPM1/DCTN4/ILK/ARF1/FLNB/TRIM32/PPP3CA/FHL3/KAT2B/CSRP1/PAK1/FKBP1A/SLC2A1/CALM3/FLNA/FKBP1B/HSPB1/MYL4/STK11/PLEC/SLC4A1/SQSTM1/SPTBN1/ATP2B4/ENO1/TMOD1/PARVB/ACTN4/CAPZB/ACTN1/PDLIM1/MYL9/ANK1

BP	GO:0045944	positive regulation of transcription by RNA polymerase II	787	-0.35	-1.51	5.40E-07	9.50E-06	5.89E-06	2240	tags=28%, list=19%, signal=24%	HES1/GTF2A1/ELK4/MECP2/IRF1/USF3/PRKD2/DPF2/ZBTB38/HYAL2/GLYR1/CD81/MAFG/MLX/THRA/MTA2/MITF/DRAP1/IL1B/SPI1/RIPK1/SREBF2/GTF2F1/HSPA5/ZBTB16/TCEA1/ZNF395/PPARGC1B/NR3C1/HELZ2/CDK9/PIN1/NR1H2/XRCC6/AGO1/BCL9/ARRB1/TFE3/GCN1/SMAD3/MAFF/RUNX1/MLLT10/EDN1/ZNF814/PRDM10/TFEB/CREB3/ENG/FZD5/SMARCC1/TAF1/TAF6/RBPJ/TRERF1/IKZF4/KLF4/JUND/SOX4/NFIC/BCL9L/NR6A1/IKBKG/TOP2A/NFATC1/SREBF1/NCOA1/NFATC3/ZFHX3/NELFA/FOXK2/GMEB1/NR1H3/RPS6KA1/IER2/AKNA/TGFB1/ASH2L/HDAC3/SMARCA4/MEPCE/ASCL2/ZNF668/NFIA/TBX21/TTC5/ETS1/RBM14/RPRD1B/NCOA2/ETV6/MEN1/MAML3/ARID3B/BCL11B/KDM6B/SKI/IL4I1/NCOA6/SERTAD1/PARP1/TRAF6/IRF2/SESN2/YY1/BCAS3/KLF13/MAVS/GLMP/EPAS1/SUPT6H/MTF1/BPTF/RARA/IGF2/SUPT16H/RXRA/KLF2/SSBP4/CREBBP/SLC9A1/MED16/HCLS1/TBL1XR1/GREM1/KAT7/ARNT/EIF5A/MRTFA/TAF4/NCOA3/POU2F2/SFRP2/USF2/ZNF296/PPARA/DVL3/WBP2/PELP1/PPP3CA/TAF8/ZMIZ1/TP53BP1/TFDP1/MAX/LITAF/ZNF609/STAT3/TBL1X/ETS2/ARHGEF2/KAT2B/MCRS1/PPP3R1/ABL1/MAFB/FOS/KMT2A/ABLIM3/RELA/FOXO4/E2F1/NFKB1/LPIN2/BMP6/TOX2/SSBP3/KMT2C/WWP2/HNRNPK/GALR1/CHD8/WAS/FOXJ2/HIPK2/ELK1/DPF3/MLXIP/DCAF6/PCGF5/ASXL2/KAT6A/MED25/SQSTM1/PPP2R5B/YBX1/RPS6KA4/MEIS1/TCF7L2/SP1/NFATC2/ELF4/CAMK1/NFIX/PRKDC/E2F2/SOX12/MAPK3/GATA1/RNF10/ARID3A/MICAL2/PCBP2/BRD4/PF4/AKIRIN2/HMGA1/TNIP1/TET3/FOXO3/PTMS/MED1/TAL1/HDGF/GFI1B/PBX1/DAB2/HCFC1/SUPT5H
BP	GO:0048878	chemical homeostasis	684	-0.37	-1.55	5.48E-07	9.60E-06	5.95E-06	2649	tags=30%, list=23%, signal=25%	ZBTB20/SLC16A1/MED13/EXT1/RAB11FIP5/PKD1/ATP2A3/STEAP3/CORO1A/AKT1/SELENOK/ABCA3/PIM3/PTGIR/SWAP70/GRAMD1B/SLC46A1/KCNH2/POMC/PIK3CA/DISC1/ADNP/ABCB11/KNG1/PTPN11/TRPM2/NR1D1/BAK1/ATP1A1/CLN3/CYBRD1/TRPC3/ATP2A2/DLG4/FOXO1/SLC24A4/CCR1/CHP1/HLA-DRB1/ABCD1/CDK5/HYAL2/ACOX1/MAFG/FYN/ICAM1/SLC25A23/ATP1B1/P2RX4/ORMDL3/SREBF2/PTPN6/ATP13A3/ITPR2/SLC9A7/HECTD4/NCOA4/GSTP1/TMC8/NR1H2/FOXK1/LPAR4/SMAD3/SCARB1/LSR/RAB20/MICU1/PTPRC/EDN1/PIK3CB/STOML2/SLC41A1/LIMA1/FRRS1/PDK3/KCNJ2/CXCR2/BCAP31/SOX4/TM9SF4/FOXK2/CRY2/PRKACA/HVCN1/NR1H3/SMARCA4/CXCR1/APOM/SNX10/MEN1/STX4/UCP2/CNNM3/ATP6AP1/SELENON/WDTC1/FPR3/SESN2/BACE2/TLCD4/CHERP/AQP1/LYN/NEDD4L/SPPL3/EPAS1/RAB7A/PTK2B/GNA15/MT2A/ARF1/PNPLA1/SLC9A1/BAD/SLC22A17/SLC39A4/PML/ADIPOR2/CA2/RASA3/CALR/USF2/GNAI2/ATP6V1A/LRRC8A/ATP13A1/SLC7A8/FIS1/PPP3CA/ATP1B2/PIH1D1/STAT3/PDK2/ABHD4/DDB1/JAK3/XIAP/ATP6V0D1/ABL1/LCN2/FKBP1A/PNPLA2/STIM1/LTF/ANXA6/BMP6/LAMTOR1/STAU1/XK/CKB/SESN3/SIDT2/GALR1/CALM3/LDLRAP1/HK1/RAB11B/TBC1D20/FKBP1B/INPP5K/ATP6V0A1/MCOLN1/SLC6A9/SLC24A3/TENT4B/STK11/PLSCR3/FTH1/KRT1/PLEC/TTC7A/ADIPOR1/SLC4A1/GP1BA/ALOX12/EPB42/PRKCB/GP9/HRH4/PIK3CG/TCF7L2/GPI/GRN/UBASH3B/ATP2B4/SNCA/ANKRD9/P2RX1/MAPK3/GATA1/GRINA/SLC29A1/EHD1/FTL/GPX1/ALAS2/SLC25A37/FOXO3/EIF2AK1/HTT/MLLT6/KEL/TGM2/WNK1/DIAPH1/TBXA2R
BP	GO:0030100	regulation of endocytosis	145	-0.53	-1.92	5.94E-07	1.04E-05	6.42E-06	1960	tags=38%, list=17%, signal=32%	TNK2/LGALS3/MIB1/NR1H2/PACSIN1/ARRB1/SYNJ1/NEU3/PIK3CB/CD14/ANKFY1/SNX12/AP2B1/SMPD1/DGKD/DTNBP1/NR1H3/CD63/SPHK1/EHD4/ALOX15/RAB31/HIP1/ANO6/AP2S1/HIP1R/NEDD4L/RAC1/ARF1/SRC/GREM1/RUBCN/PPP3CA/RIN3/SYK/ABL1/ARRB2/HNRNPK/CALM3/DNM2/LDLRAP1/RAB5B/VPS28/STON2/LRP1/PTPN1/CD151/SNCA/TBC1D5/CBL/RUFY1/AP2M1/ITGB3/CLU/DAB2

BP	GO:0034762	regulation of transmembrane transport	293	-0.44	-1.73	6.19E-07	1.08E-05	6.67E-06	1587	tags=21%, list=14%, signal=18%	KCNJ2/KCNQ5/EHD3/STOM/PRKACA/HVCN1/GSK3A/CD63/RANGRF/CLIC5/DAPK1/SELENON/ARL6IP1/OXSR1/FHL1/ANO6/LYN/NEDD4L/PINK1/PTK2B/SLC9A1/CA2/ATP1B2/CACNG6/ACTB/CNIH3/YWHAH/VDAC1/ABL1/FKBP1A/STIM1/WWP2/CALM3/DNM2/FLNA/FKBP1B/INPP5K/SLC6A9/PRKCB/THBS1/AKT2/GJC2/PIK3CG/SLC43A1/PER2/UBASH3B/ATP2B4/SNCA/AHCYL1/CAPN1/P2RX1/ACTN4/TESC/OAZ1/HTT/SHISA7/KEL/SCN1B/WNK1/PIM1/DIAPH1
BP	GO:0071900	regulation of protein serine/threonine kinase activity	256	-0.45	-1.74	6.26E-07	1.08E-05	6.71E-06	2856	tags=38%, list=24%, signal=29%	SLC8A1/MAP3K4/FLT1/PRKAR1B/SPATC1L/TESK1/CEBPA/CDK12/SH2B3/TRAF2/PKD1/RALB/SORL1/PKIG/AKT1/AIDA/TAOK3/CCNJL/CDKN2D/EZH2/TSG101/MAP3K13/EPHA4/HYAL2/CDK5RAP2/IL1B/CSF1R/PTPN6/TLR6/CCNY/GSTP1/TRIB1/CDC37/HGS/PTPRC/EDN1/PIK3CB/MAGED1/FZD5/MAP3K11/SMPD1/DTNBP1/PYCARD/HSP90AB1/TGFB1/TAOK1/MEN1/SLC27A1/SERTAD1/TRAF6/MAP3K5/SESN2/LYN/SASH1/PTK2B/IGF2/MAP2K7/SRC/CCND3/CDKN1C/SFRP2/DVL3/TAOK2/ACTB/PIH1D1/MAP2K2/KAT2B/PDGFC/SYK/ABL1/PAK1/LTF/PRKAR2A/PDE5A/RHOA/CALM3/PTPRJ/INPP5K/HEXIM2/CSK/THBS1/CCNI/PTPN1/AKAP13/PIK3CG/IQGAP1/ATP2B4/SNCA/CAMK1/FBXO7/CDKN1A/PRKAR2B/HTT/PDGFA/DAB2/CNPPD1
BP	GO:0002009	morphogenesis of an epithelium	253	-0.45	-1.76	6.50E-07	1.12E-05	6.94E-06	1880	tags=28%, list=16%, signal=24%	MIB1/LZTS2/ARHGAP35/SMAD3/FMN1/EDN1/TIAM1/PHACTR4/MAGED1/ENG/FZD5/RBPJ/TGFB1I1/KLF4/SOX4/FOXP1/GRB2/PRKACA/TGFB1/ACTG1/CTNNBIP1/RPGRIP1L/ST14/SPINT2/SKI/TRAF6/WDR1/MTHFR/PLXNB2/ILK/FZD2/RAC1/RARA/PLXND1/DCHS1/SRC/PML/GREM1/CEACAM1/MRTFA/CA2/BBS5/PIK3CD/SFRP2/DVL3/PLXNA1/IFT52/TRIM28/ACTB/PPP1CA/BTBD7/NRP1/BTRC/ABL1/PAK1/CLASP1/RHOA/VCL/NOTCH2/SUFU/FLNA/SDC4/CD151/MSN/VASP/MICAL2/MED1/PDGFA/SH3BP1/TGM2/PBX1/DAB2
BP	GO:0098609	cell-cell adhesion	521	-0.38	-1.60	6.54E-07	1.12E-05	6.95E-06	2666	tags=32%, list=23%, signal=26%	PECAM1/FIBP/SDK2/ADD2/CR1/EXT1/PKD1/JUP/SIGLEC1/RASAL3/MAPK14/PTAFR/PAK4/CORO1A/MINK1/AKT1/SELENOK/FOXP3/SWAP70/CBLB/PIK3CA/TMOD3/SERPINF2/MAPK7/PTPN11/NOTCH1/CD3E/EFNB1/DOCK8/DLG4/HES1/HLA-DRB1/IRF1/LPP/CD81/FYN/HLA-DQA2/ICAM1/LGALS9C/IL1B/SPI1/HLA-A/PTPN6/SELPLG/CTNND1/ZBTB16/MYADM/ITGB7/SIRPG/LGALS3/SLC7A1/ITGAM/PKP4/SHC1/SOX13/RUNX1/PTPRC/PIK3CB/RIPOR2/LIMS1/CD74/VSIG10/KLF4/SEMA4D/SIRPB1/SOX4/HLA-DQA1/BAIAP2/PYCARD/AKNA/TGFB1/ACTG1/TBX21/TMEM131L/ETS1/IL1RN/CCN3/EZR/GNAS/SPINT2/PLEK/IL4I1/TWSG1/TRAF6/LYN/PLXNB2/ILK/MBP/JAM3/RARA/IGF2/STK10/CD9/DCHS1/SPN/BAD/SRC/CEACAM1/HLA-DRB5/PPARA/VAV1/RUNX3/ATP1B2/ADGRL1/LAPTM5/ZMIZ1/ACTB/PPP1CA/CTNNA1/SELL/JAK1/CSRP1/SYK/TJP2/JAK3/ABL1/KIFC3/TNFSF4/RELA/BMP6/ITGAL/MPL/PCDH1/GP6/PDE5A/RHOA/VCL/MAD2L2/LGALS9/FLNA/FERMT3/RAC2/BSG/HSPB1/NF2/SDC4/CYFIP2/ITGB5/CLSTN1/GFUS/GP1BA/ALOX12/CLDN5/CSK/PIK3CG/SPTA1/MSN/UBASH3B/SELP/LRFN3/ZNF703/SOX12/GATA1/HLX/NINJ2/TNIP1/PDLIM1/FOXO3/MYL9/PTPN23/MYH9/ITGB3/ESAM/ITGA2B/DMTN/TLN1/WNK1
CC	GO:0001726	ruffle	141	-0.53	-1.92	6.57E-07	1.12E-05	6.95E-06	2096	tags=34%, list=18%, signal=28%	PLEKHO1/PARD6A/MACF1/SCIMP/LCP1/MYADM/PDLIM7/PACSIN1/FRMD4B/LIMA1/BAIAP2/CYFIP1/CDK6/AMOT/EZR/PLEK/CORO1C/TPM1/HIP1R/CDKL5/RAC1/SRC/SH2D3C/ARHGAP45/ARHGEF2/ABL1/PAK1/FGD4/RHOA/PDE9A/DNM2/PTPRJ/INPP5K/NF2/CFL1/WIPF1/PPP1R9B/AKT2/PDE4A/IQGAP1/ACTN1/TESC/CTTN/MYH9/ITGB3/WASF2/TLN1/DIAPH1

BP	GO:0030041	actin filament polymerization	130	-0.55	-1.98	6.69E-07	1.14E-05	7.04E-06	2402	tags=42%, list=20%, signal=34%	SPTAN1/SPIRE1/TRIOBP/MSRB1/WASHC5/ARHGAP18/CORO7/PFN1/SSH1/SPIRE2/MSRB2/MYADM/DIAPH2/SVIL/SSH2/FMN1/ARPC5/GRB2/BAIAP2/PYCARD/CYFIP1/PSTPIP2/PREX1/CDC42EP4/ALOX15/HIP1R/RAC1/PTK2B/HCLS1/GAS7/ARPC1B/FLII/ABL1/SSH3/RHOA/ARPC4/ARHGAP6/WAS/CYFIP2/GSN/SPTBN1/MTPN/SPTA1/COTL1/SPTB/VASP/TMOD1/WASF3/SCIN/CAPZB/CTTN/ESAM/DMTN/ADD1/DIAPH1
CC	GO:0030016	myofibril	124	-0.55	-1.99	7.00E-07	1.19E-05	7.35E-06	1288	tags=31%, list=11%, signal=28%	MYZAP/CORO1C/ALDOA/TPM1/DCTN4/ILK/ARF1/FLNB/TRIM32/PPP3CA/FHL3/KAT2B/CSRP1/PAK1/FKBP1A/CALD1/SLC2A1/VCL/CALM3/FLNA/FKBP1B/HSPB1/MYL4/STK11/SDC4/PLEC/SLC4A1/SQSTM1/SPTBN1/ATP2B4/ENO1/TMOD1/PARVB/ACTN4/CAPZB/ACTN1/PDLIM1/MYL9/ANK1
BP	GO:0043254	regulation of protein-containing complex assembly	304	-0.43	-1.72	7.18E-07	1.21E-05	7.47E-06	2604	tags=37%, list=22%, signal=29%	SLF2/MAPRE1/ULK1/EPS8/RALB/SORL1/CORO1A/ARL2/ABCA3/AIDA/TMOD3/PTPN11/BAK1/SPTAN1/TRIOBP/ARHGAP18/IKBKE/HES1/PFN1/MECP2/CDK5RAP2/SSH1/THRA/TBCD/HSPA5/TLR6/LCP1/MYADM/SVIL/FES/LGALS3/TMC8/SSH2/NR1H2/TFIP11/NUMA1/FMN1/ARPC5/GRB2/SLAIN2/GSK3B/BAIAP2/PYCARD/CYFIP1/TGFB1/CTNNBIP1/GIT1/EML2/ASAP1/RANGRF/CDC42EP4/PLEK/PARP1/PRUNE1/ALOX15/HIP1R/PINK1/RAC1/JAM3/PTK2B/HSPA1A/HCLS1/BCL2L11/SRC/ARPC1B/FLII/CAMSAP1/PIH1D1/SYK/VCP/CPTP/ABL1/PAK1/FNIP2/CLASP1/SSH3/WARS1/RHOA/ARPC4/CKAP5/GNL3L/NAPA/CYFIP2/DHX33/RIOK3/DCTN1/GSN/MED25/PPP2R5B/SPTBN1/MTPN/SPTA1/MSN/COTL1/SPTB/SELP/SNCA/EIF4G1/VASP/TMOD1/SCIN/CAPZB/TAL1/CTTN/PPM1A/ESAM/STMP1/CLU/DMTN/ADD1/HCFC1
BP	GO:0010563	negative regulation of phosphorus metabolic process	314	-0.43	-1.70	7.32E-07	1.22E-05	7.59E-06	2581	tags=36%, list=22%, signal=29%	GNAQ/SORL1/PKIG/AKT1/PIP5KL1/AIDA/SWAP70/CBLB/JUN/MLLT1/BAK1/PPP1R15A/GADD45B/CDKN2D/HDAC4/FLCN/TSG101/PTPA/CHP1/PKN1/SH3BP5L/PIP4K2C/SPRED1/HYAL2/CDK5RAP2/CRKL/IL1B/PARD6A/PTPN6/ROCK2/MYADM/GSTP1/MGAT5/TRIB1/LGALS3/PTPRC/PIK3CB/CHMP6/ENG/SEMA4D/CDA/CTDSP1/SMPD1/DTNBP1/GSK3B/PRR5L/CRY2/GSK3A/PYCARD/CACTIN/TGFB1/GIT1/YWHAG/CBFA2T3/CTDSP2/MEN1/SLC27A1/MVP/PLEK/CORO1C/PARP1/LYN/ADAR/GREM1/CDKN1C/CEACAM1/RUBCN/YWHAB/SFRP2/GNAI2/PPARA/LRRK1/STAT3/KAT2B/ABL1/PARD3/FKBP1A/FBP1/WARS1/ARRB2/NIBAN1/PRKAR2A/MFHAS1/CALM3/PPP4C/PTPRJ/FKBP1B/INPP5K/HSPB1/NF2/HEXIM2/PLEC/SLC4A1/LRP1/PPP1R9B/CSK/PTPN1/IQGAP1/FKBP8/CTDSPL/UBASH3B/SNCA/EIF4G1/PRKDC/PIP4K2A/FBXO7/CDKN1A/CBL/PAK2/PRKAR2B/PDGFA/DMTN
BP	GO:0072659	protein localization to plasma membrane	204	-0.47	-1.80	7.38E-07	1.23E-05	7.62E-06	1454	tags=22%, list=12%, signal=20%	TGFB1/CLTC/RANGRF/EZR/TSPAN17/STX4/ATP6AP1/EHD4/EFR3A/RAB31/VTI1B/RAB7A/DCHS1/GRIPAP1/PLEKHF1/PACS1/ACTB/TTC7B/OPTN/LDLRAP1/FLNA/RAB3D/BSG/INPP5K/BCL2L1/ZDHHC5/TTC7A/SLC4A1/GAK/SQSTM1/LRP1/PPP1R9B/CSK/SPTBN1/AKT2/TSPAN33/ATP2B4/RHOG/EHD1/TSPAN5/TESC/AP2M1/SEC16A/ANK1/DAB2
CC	GO:0042641	actomyosin	55	-0.67	-2.10	7.57E-07	1.25E-05	7.78E-06	1672	tags=51%, list=14%, signal=44%	LCP1/PDLIM7/LIMA1/SEPTIN11/TPM3/TRIP6/SIPA1L3/TLNRD1/TPM1/CDC42BPB/ZYX/DCTN4/ILK/FLNB/FHL3/CNN2/KAT2B/PXN/ABLIM3/MYO18A/ACTA2/MYLK/ACTN4/ACTN1/PDLIM1/MYL9/MYH9/GAS2L1
BP	GO:0048732	gland development	225	-0.46	-1.77	7.73E-07	1.28E-05	7.91E-06	2488	tags=32%, list=21%, signal=26%	PIK3CA/JUN/TBX19/NOTCH1/EZH2/HOXB3/TSPO/OAS2/HES1/DDR1/CRKL/DEAF1/THRA/CSF1R/MPST/MAN2A1/ARHGAP35/SMAD3/LSR/SLC29A2/EDN1/RBPJ/CPT1A/RAP1GAP/RPS6KA1/TGFB1/RPGRIP1L/BCL11B/UCP2/TWSG1/LBH/RARA/IGF2/PLXND1/MGMT/BCL2L11/SRC/PML/CDKN1C/CEACAM1/USF2/PLXNA1/PSAP/BTBD7/NRP1/BTRC/PHF2/ABL1/MAFB/RELA/JARID2/NOTCH2/STAT5A/FASN/ELK1/STK11/IGF2R/AKT2/MSN/NEURL1/CAPN1/ZNF703/MAPK3/HLX/SLC29A1/GPX1/MED1/ACO2/PDGFA/NCOR2/TGM2/PBX1

BP	GO:0060070	canonical Wnt signaling pathway	182	-0.49	-1.83	7.99E-07	1.31E-05	8.14E-06	2622	tags=38%, list=22%, signal=30%	EXT1/ZEB2/JUP/GNAQ/MAPK14/FRMD8/DISC1/NOTCH1/EGR1/UBR5/FOXO1/ASPM/MITF/CTNND1/CCNY/CCAR2/PIN1/LZTS2/BCL9/SOX13/SMAD3/EDN1/CSNK1G1/DIXDC1/FZD5/RBPJ/KLF4/SOX4/BCL9L/GSK3B/GSK3A/TGFB1/CTNNBIP1/TMEM131L/DAPK3/ILK/FZD2/TBL1XR1/SRC/GREM1/SFRP2/DVL3/RUVBL1/LRRK1/AMFR/TLE3/PPP1CA/TBL1X/BTRC/USP34/VCP/XIAP/OTUD5/NFKB1/TLE4/CHD8/GID8/MAD2L2/TLE1/STK11/SIAH2/TCF7L2/ZNF703/FAM53B/FOXO3/CTDNEP1/PPM1A/WNK1/DAB2
MF	GO:0008134	transcription factor binding	473	-0.40	-1.63	8.42E-07	1.38E-05	8.54E-06	2141	tags=31%, list=18%, signal=26%	MLX/CRKL/THRA/MTA2/DRAP1/CPNE1/SPI1/ZNF366/MTA1/PARD6A/GTF2F1/TACC1/ZBTB16/WIZ/SMARCD3/PPARGC1B/ZBTB7A/TRIB1/CDK9/EHMT1/NR1H2/UBXN7/ARRB1/SMAD3/KEAP1/HIF1AN/TAF1/TAF6/RBPJ/TGFB1I1/CTBP1/TRERF1/IKZF4/CENPF/KLF4/JUND/EHMT2/FOXP1/NFATC1/SREBF1/NCOA1/TOB2/GSK3B/NFATC3/BAIAP2/CRY2/BAZ2A/TRIP6/HDAC3/SMARCA4/NFIA/VHL/ETS1/PDCD11/VGLL4/NCOA2/DAPK3/BCOR/NEK6/SKI/NCOA6/PARP1/TP53BP2/YY1/BCAS3/EPAS1/RARA/TADA3/RXRA/IFI27/CREBBP/MTDH/MED16/TRIM32/BRMS1/HCLS1/SRC/DCAF1/ARNT/TAF4/BBS5/NCOA3/CALR/USF2/PPARA/WBP2/RUVBL1/RUNX3/DAXX/FAM89B/TP53BP1/TFDP1/MAX/ACTB/STAT3/TBL1X/ETS2/KAT2B/YWHAH/NOLC1/FOS/FBP1/RELA/FOXO4/E2F1/DTX3L/WWP2/TLE4/NOTCH2/MAD2L2/SUFU/FLNA/TLE1/HSPB1/HIPK2/ELK1/DHX33/KAT6A/MED25/PRKCB/SETD1A/TCF7L2/PER2/SP1/NFATC2/CHD4/ENO1/PRKDC/ZNF703/GATA1/TRIP12/HMGA1/ACTN4/FOXO3/MED1/TAL1/HDGF/ARID1A/GAS2L1/GFI1B/NCOR2/ADD1/PIM1/CNOT1/PBX1/HCFC1
CC	GO:0043292	contractile fiber	132	-0.54	-1.96	8.46E-07	1.38E-05	8.55E-06	1288	tags=30%, list=11%, signal=27%	MYZAP/CORO1C/ALDOA/TPM1/DCTN4/ILK/ARF1/FLNB/TRIM32/PPP3CA/FHL3/KAT2B/CSRP1/PAK1/FKBP1A/CALD1/SLC2A1/VCL/CALM3/ACTA2/FLNA/FKBP1B/HSPB1/MYL4/STK11/SDC4/PLEC/SLC4A1/SQSTM1/SPTBN1/ATP2B4/ENO1/TMOD1/PARVB/ACTN4/CAPZB/ACTN1/PDLIM1/MYL9/ANK1
CC	GO:0032432	actin filament bundle	52	-0.67	-2.08	8.55E-07	1.39E-05	8.61E-06	1672	tags=52%, list=14%, signal=45%	LCP1/PDLIM7/LIMA1/SEPTIN11/TPM3/TRIP6/SIPA1L3/TLNRD1/TPM1/ZYX/DCTN4/ILK/FLNB/FHL3/CNN2/RFLNB/PXN/ABLIM3/ACTA2/FLNA/MYLK/ACTN4/ACTN1/PDLIM1/MYL9/MYH9/GAS2L1
BP	GO:0007229	integrin-mediated signaling pathway	73	-0.62	-2.06	9.39E-07	1.52E-05	9.41E-06	1957	tags=41%, list=17%, signal=34%	ITGB7/TIMP1/ITGAM/RCC2/PHACTR4/LIMS1/NEDD9/CD63/TEC/PLEK/ZYX/ILK/PTK2B/SRC/CEACAM1/VAV1/CTNNA1/NRP1/SYK/ABL1/ITGAL/FLNA/FERMT3/ITGB5/MPIG6B/MYH9/ITGB3/ITGA2B/DMTN/TLN1
BP	GO:0042692	muscle cell differentiation	204	-0.47	-1.79	9.43E-07	1.52E-05	9.41E-06	1961	tags=42%, list=17%, signal=35%	ATP11A/SLC8A1/MYH3/OBSCN/MSX1/FRS2/TTN/H3-3A/MYOM2/MAPK14/AKT1/TMOD3/NOTCH1/ATP2A2/HDAC4/HDAC5/EZH2/G6PD/HES1/MECP2/CD81/PLEKHO1/NLN/SMARCD3/CDK9/BCL9/SYNE1/EDN1/RIPOR2/ENG/RBPJ/NFATC1/NFATC3/ANKRD17/GSK3A/TGFB1/ACTG1/DYRK1B/CCN3/KDM6B/SKI/SELENON/WDR1/HDGFL2/TPM1/YY1/SUPT6H/RARA/IGF2/CD9/PLD3/DYSF/SLC9A1/TRIM32/SOX6/GREM1/MRTFA/CALR/PPARA/FLII/PPP3CA/PTBP1/MYOF/CSRP1/FOXO4/ARRB2/XK/LMNA/RBM38/NIBAN2/SORT1/PLEC/YBX1/AKAP13/MTPN/NFATC2/CAMK1/TMOD1/EHD1/ACTN4/GPX1/ACTN1/MYL9/MYH9/KEL
BP	GO:0060348	bone development	130	-0.54	-1.96	9.87E-07	1.58E-05	9.78E-06	2773	tags=44%, list=24%, signal=34%	MEF2D/MSX1/PRPSAP2/SH2B3/BNC2/EXT1/TTC9/MAPK14/SMAD5/ZFPM1/PTPN11/PHEX/ZNF385A/FOXN3/PTPN6/ZBTB16/XYLT1/ALPL/PTPRC/ENG/SEMA4D/BBX/FOXP1/SNX10/GNAS/INPPL1/ATP6AP1/SKI/RARA/DCHS1/PAPSS2/SRC/GREM1/SFRP2/LTBP3/LRRK1/FAM20C/DHRS3/PHOSPHO1/PDGFC/RFLNB/LTF/ANXA6/BMP6/RHOA/NOTCH2/SLC38A10/GLG1/AKAP13/MEIS1/PIP4K2A/SMPD3/MPIG6B/MED1/TAL1/WASF2/TGM2

BP	GO:0031175	neuron projection development	545	-0.38	-1.58	1.02E-06	1.62E-05	1.00E-05	2567	tags=31%, list=22%, signal=25%	RAB8A/PAK4/MINK1/AKT1/DIP2B/TAOK3/NCKIPSD/MUL1/PLXNC1/JUN/DISC1/PLXNA4/ADNP/PTPN11/NOTCH1/CD3E/EFNB1/EZH2/DLG4/NCAM1/TRIOBP/LYPLA2/TSPO/MAP3K13/LMTK2/EPHA4/HES1/MAGI2/MAP4/MECP2/CDK5/MOV10/DDR1/ABI3/FYN/BICDL1/CRKL/CPNE1/FEZ1/SLITRK5/DOCK10/CSF1R/HSPA5/CERS2/MACF1/PRAG1/ITM2C/FES/SRGAP2/PACSIN1/PARD6B/CUL7/ARHGAP35/WEE1/FRY/APLP2/PMP22/EDN1/KIAA0319/TIAM1/RERE/DIXDC1/MAP1S/B4GALT5/NDEL1/KLF4/WDR5/SEMA4D/PPP1R9A/DTNBP1/VIM/GSK3B/BAIAP2/GSK3A/CYFIP1/HSP90AB1/SVBP/TAOK1/ASAP1/ENC1/TRAPPC4/BCL11B/MFSD2A/BCL11A/KATNB1/IST1/LYN/NEDD4L/PLXNB2/CDKL5/RAC1/MBP/PTK2B/DPYSL2/PLXND1/MICALL1/TRIO/KIF13B/STK24/TRIM32/SIPA1L1/MARK2/FBXO31/EIF4G2/GAS7/IQSEC1/CPNE5/STK25/SFRP2/SNX3/DVL3/MYCBP2/PLXNA1/PPP3CA/ATP1B2/CAMSAP1/TAOK2/ACTB/CTNNA1/SEMA4A/NRP1/MAP1A/VLDLR/YWHAH/USP9X/HERC1/ABL1/PAK1/PARD3/SYNGAP1/BLOC1S3/RAPGEF1/XK/RHOA/VCL/NOTCH2/DNM2/FLNA/FKBP1B/RAC2/BSG/ECE1/NIBAN2/CAMK1D/STK11/SDC4/UBB/CYFIP2/DNM3/CFL1/GAK/PPP1R9B/PPP2R5B/IQGAP1/GRN/CUX1/CAMK1/NEURL1/VASP/RHOG/FBXO7/EHD1/PAK2/CTTN/KEL/CARM1/SCN1B/DAB2
BP	GO:0048666	neuron development	621	-0.36	-1.53	1.04E-06	1.65E-05	1.02E-05	2567	tags=30%, list=22%, signal=24%	RAB8A/PAK4/MINK1/AKT1/DIP2B/TAOK3/NCKIPSD/MUL1/PLXNC1/JUN/DISC1/PLXNA4/ADNP/PTPN11/NOTCH1/YWHAZ/CD3E/EFNB1/EZH2/DLG4/NCAM1/TRIOBP/LYPLA2/TSPO/MAP3K13/LMTK2/PRDM1/EPHA4/HES1/MAGI2/MAP4/MECP2/CDK5/MOV10/UNK/DDR1/ABI3/FYN/BICDL1/CRKL/CPNE1/FEZ1/SLITRK5/TBCD/DOCK10/CSF1R/HSPA5/CERS2/MACF1/PRAG1/ITM2C/FES/SRGAP2/PACSIN1/PARD6B/CUL7/ARHGAP35/WEE1/FRY/RUNX1/APLP2/PMP22/EDN1/KIAA0319/TIAM1/RERE/DIXDC1/MAP1S/B4GALT5/ANKS1A/NDEL1/KLF4/WDR5/SEMA4D/PPP1R9A/DTNBP1/VIM/GSK3B/BAIAP2/GSK3A/CYFIP1/HSP90AB1/SVBP/GIT1/TAOK1/RPGRIP1L/ASAP1/ENC1/TRAPPC4/BCL11B/MFSD2A/BCL11A/KATNB1/IST1/LYN/NEDD4L/PLXNB2/CDKL5/FZD2/RAC1/MBP/PTK2B/DPYSL2/PLXND1/MICALL1/TRIO/KIF13B/STK24/TRIM32/SIPA1L1/MARK2/FBXO31/CDKN1C/EIF4G2/GAS7/IQSEC1/CPNE5/STK25/SFRP2/SNX3/DVL3/MYCBP2/RUNX3/PLXNA1/PPP3CA/ATP1B2/CAMSAP1/TAOK2/ZMIZ1/ACTB/CTNNA1/SEMA4A/NRP1/MAP1A/VLDLR/YWHAH/USP9X/HERC1/ABL1/PAK1/PARD3/SYNGAP1/BLOC1S3/RAPGEF1/C1QA/XK/SCYL1/RHOA/VCL/NOTCH2/DNM2/FLNA/FKBP1B/RAC2/BSG/ECE1/NIBAN2/CAMK1D/STK11/SDC4/UBB/CYFIP2/DNM3/CFL1/GAK/PPP1R9B/PPP2R5B/IQGAP1/GRN/CUX1/CAMK1/NEURL1/VASP/RHOG/FBXO7/EHD1/PAK2/CTTN/KEL/CARM1/SCN1B/WNK1/PBX1/DAB2
BP	GO:0032989	cellular component morphogenesis	418	-0.40	-1.65	1.08E-06	1.70E-05	1.05E-05	2622	tags=33%, list=22%, signal=26%	EXT1/CORO1B/CAPRIN1/SUPV3L1/ULK1/RAB8A/MINK1/DIP2B/TAOK3/MUL1/PLXNC1/TMOD3/DISC1/PLXNA4/ADNP/PTPN11/NOTCH1/EFNB1/DLG4/NCAM1/LYPLA2/MAP3K13/LMTK2/EPHA4/CDK5/MOV10/DDR1/ABI3/FYN/CPNE1/FEZ1/PLEKHO1/SLITRK5/DOCK10/CSF1R/MACF1/SRGAP2/PACSIN1/PARD6B/CUL7/ARHGAP35/WEE1/APLP2/PMP22/EDN1/KIAA0319/TIAM1/RERE/DIXDC1/MAP1S/B4GALT5/TBPL1/POLDIP2/NDEL1/SEMA4D/DTNBP1/GSK3B/BAIAP2/CYFIP1/HSP90AB1/ACTG1/TAOK1/BCL11B/CORO1C/WDR1/MFSD2A/BCL11A/TPM1/IST1/NEDD4L/PLXNB2/CDKL5/RAC1/MBP/DPYSL2/PLXND1/CD9/TRIO/KIF13B/SIPA1L1/MARK2/FBXO31/SRC/EIF4G2/GAS7/CPNE5/STK25/DVL3/MYCBP2/FIS1/FLII/PLXNA1/PPP3CA/TAOK2/ACTB/SEMA4A/NRP1/MAP1A/VLDLR/YWHAH/USP9X/CSRP1/ABL1/PAK1/PARD3/SYNGAP1/CLASP1/XK/RHOA/VCL/NOTCH2/DNM2/TBC1D20/RAC2/BSG/ECE1/NIBAN2/BCL2L1/STK11/UBB/CYFIP2/DNM3/PLEC/CFL1/AKAP13/IQGAP1/CUX1/VASP/RHOG/TMOD1/PAK2/MYL9/CTTN/WASF2/KEL/ACRBP/SCN1B

BP	GO:0006914	autophagy	458	-0.39	-1.62	1.15E-06	1.79E-05	1.11E-05	1845	tags=26%, list=16%, signal=23%	FOXK1/CDC37/MAP1LC3B/LRBA/KEAP1/HGS/GPSM1/RRAGD/PIK3CB/TFEB/WDFY4/CHMP6/FZD5/MAP1S/POLDIP2/VPS41/PHF23/WDR81/NSFL1C/VPS37B/CHMP4B/EHMT2/IKBKG/KDM4A/SREBF1/TECPR1/GSK3B/FOXK2/PRKACA/TBC1D17/GSK3A/PYCARD/DAP/KLHL22/CLTC/TECPR2/TRAPPC4/ATG2A/DAPK3/DAPK1/VPS13D/SESN2/BCAS3/VTI1B/RAB7A/EPG5/PINK1/MTDH/BAD/TRIM32/MARK2/BCL2L11/ILRUN/SRC/CHMP1A/EIF4G2/RUBCN/ATP6V1A/FIS1/VTI1A/PLEKHF1/PSAP/STAT3/GABARAP/VDAC1/HERC1/VCP/CPTP/USP36/ATP6V0D1/ZFYVE1/ABL1/RRAGC/OPTN/ATG4D/PIM2/RNF213/ATP6V0C/CAPNS1/WIPI2/GOLGA2/SESN3/GPR137/ATG7/HSPB1/ATP6V0A1/MCOLN1/STK11/VPS28/RAB1B/HUWE1/SQSTM1/TRIM14/YOD1/MFN2/SNCA/EIF4G1/ATG13/CAPN1/PIP4K2A/TBC1D5/MAPK3/ELAPOR1/BNIP3L/FBXO7/DCAF12/TGFBRAP1/TBC1D25/WDR45/NPRL3/FOXO3/UBXN6/TOLLIP/CTTN/HTT/SEC16A/CLU/LARP1/SUPT5H
BP	GO:0061919	process utilizing autophagic mechanism	458	-0.39	-1.62	1.15E-06	1.79E-05	1.11E-05	1845	tags=26%, list=16%, signal=23%	FOXK1/CDC37/MAP1LC3B/LRBA/KEAP1/HGS/GPSM1/RRAGD/PIK3CB/TFEB/WDFY4/CHMP6/FZD5/MAP1S/POLDIP2/VPS41/PHF23/WDR81/NSFL1C/VPS37B/CHMP4B/EHMT2/IKBKG/KDM4A/SREBF1/TECPR1/GSK3B/FOXK2/PRKACA/TBC1D17/GSK3A/PYCARD/DAP/KLHL22/CLTC/TECPR2/TRAPPC4/ATG2A/DAPK3/DAPK1/VPS13D/SESN2/BCAS3/VTI1B/RAB7A/EPG5/PINK1/MTDH/BAD/TRIM32/MARK2/BCL2L11/ILRUN/SRC/CHMP1A/EIF4G2/RUBCN/ATP6V1A/FIS1/VTI1A/PLEKHF1/PSAP/STAT3/GABARAP/VDAC1/HERC1/VCP/CPTP/USP36/ATP6V0D1/ZFYVE1/ABL1/RRAGC/OPTN/ATG4D/PIM2/RNF213/ATP6V0C/CAPNS1/WIPI2/GOLGA2/SESN3/GPR137/ATG7/HSPB1/ATP6V0A1/MCOLN1/STK11/VPS28/RAB1B/HUWE1/SQSTM1/TRIM14/YOD1/MFN2/SNCA/EIF4G1/ATG13/CAPN1/PIP4K2A/TBC1D5/MAPK3/ELAPOR1/BNIP3L/FBXO7/DCAF12/TGFBRAP1/TBC1D25/WDR45/NPRL3/FOXO3/UBXN6/TOLLIP/CTTN/HTT/SEC16A/CLU/LARP1/SUPT5H
BP	GO:1901652	response to peptide	318	-0.42	-1.69	1.19E-06	1.85E-05	1.15E-05	2578	tags=33%, list=22%, signal=27%	MAPK14/SORL1/RAB8A/AKT1/POMC/PIK3CA/PTPN11/NOTCH1/CYBB/EGR1/HDAC5/FOXO1/SLC24A4/PHEX/EPHA4/BTG1/PIP4K2C/CDK5/FYN/ICAM1/IL1B/ROCK2/TLR6/SRSF4/TIMP1/EIF6/SHC1/SLC29A2/SH2B2/EDN1/SMARCC1/MMP9/NDEL1/PIK3R3/JUND/ABCC1/VIM/GRB2/SREBF1/GSK3B/BAIAP2/CRY2/GSK3A/CYFIP1/YWHAG/ZNF106/INPPL1/MEN1/UCP2/SLC27A1/WDTC1/PARP1/SESN2/RAB31/LYN/LEPROT/IGF2/KLF2/SLC9A1/BCL2L11/SRC/CCND3/KAT7/CEACAM1/CA2/GNAI2/PPARA/PKM/MAX/STAT3/RANGAP1/PDK2/ARHGEF2/KAT2B/JAK1/PXN/JAK3/FBP1/RELA/FOXO4/NFKB1/LPIN2/SLC2A1/SESN3/BSG/INPP5K/STAT5A/KLF1/STK11/SORT1/ADIPOR1/LRP1/PPP1R9B/PRKCB/CSK/AKT2/PTPN1/SP1/PRKDC/AHCYL1/PIP4K2A/EIF4EBP2/SMPD3/RNF40/FOXO3/KLF3
BP	GO:0045936	negative regulation of phosphate metabolic process	313	-0.43	-1.70	1.37E-06	2.12E-05	1.31E-05	2581	tags=35%, list=22%, signal=28%	GNAQ/SORL1/PKIG/AKT1/PIP5KL1/AIDA/SWAP70/CBLB/JUN/MLLT1/BAK1/PPP1R15A/GADD45B/CDKN2D/HDAC4/FLCN/TSG101/PTPA/CHP1/PKN1/SH3BP5L/PIP4K2C/SPRED1/HYAL2/CDK5RAP2/CRKL/IL1B/PARD6A/PTPN6/ROCK2/MYADM/GSTP1/MGAT5/TRIB1/LGALS3/PTPRC/PIK3CB/CHMP6/ENG/SEMA4D/CDA/CTDSP1/SMPD1/DTNBP1/GSK3B/PRR5L/CRY2/PYCARD/CACTIN/TGFB1/GIT1/YWHAG/CBFA2T3/CTDSP2/MEN1/SLC27A1/MVP/PLEK/CORO1C/PARP1/LYN/ADAR/GREM1/CDKN1C/CEACAM1/RUBCN/YWHAB/SFRP2/GNAI2/PPARA/LRRK1/STAT3/KAT2B/ABL1/PARD3/FKBP1A/FBP1/WARS1/ARRB2/NIBAN1/PRKAR2A/MFHAS1/CALM3/PPP4C/PTPRJ/FKBP1B/INPP5K/HSPB1/NF2/HEXIM2/PLEC/SLC4A1/LRP1/PPP1R9B/CSK/PTPN1/IQGAP1/FKBP8/CTDSPL/UBASH3B/SNCA/EIF4G1/PRKDC/PIP4K2A/FBXO7/CDKN1A/CBL/PAK2/PRKAR2B/PDGFA/DMTN

CC	GO:0005884	actin filament	77	-0.61	-2.03	1.50E-06	2.32E-05	1.44E-05	1992	tags=48%, list=17%, signal=40%	LCP1/DIAPH2/PDLIM7/RCSD1/FMN1/KEAP1/TPM3/ACTG1/PSTPIP2/SPECC1L/AMOT/EZR/TPM1/RAC1/JAM3/MARK2/HCLS1/MYO9B/SRC/GAS7/ACTB/PAK1/ARHGAP6/WAS/RAC2/SPECC1/WIPF1/IQGAP1/COTL1/TMOD1/ESPN/ACTN1/PDLIM1/CTTN/GAS2L1/DMTN/DIAPH1
BP	GO:0032880	regulation of protein localization	605	-0.37	-1.55	1.55E-06	2.38E-05	1.48E-05	2578	tags=31%, list=22%, signal=25%	MAPK14/SORL1/PKIG/RAB8A/RHBDF2/MARK4/AKT1/SELENOK/PIM3/SH3TC2/SWAP70/SYT11/PTPN11/TRPM2/NR1D1/CNST/CLN3/TMBIM1/BICD1/UBR5/FLCN/DLG4/SNX33/TSG101/CHP1/HLA-DRB1/PCNT/CDK5/HYAL2/SUMO3/CD81/ABI3/FYN/ABHD17C/CRKL/IL1B/SPI1/ORMDL3/PARD6A/SREBF2/ROCK2/LCP1/RIPOR1/LGALS3/PIN1/NR1H2/EPB41L2/ITGAM/LZTS2/NUMA1/UBE2J2/SMAD3/RIPOR2/MAGED1/FZD5/CPT1A/NDEL1/BBC3/CCT3/OTUD7B/STX6/SNX12/NSFL1C/AP2B1/BCAP31/SOX4/TM9SF4/SREBF1/GSK3B/PRR5L/STOM/PRKACA/NR1H3/GSK3A/HSP90AB1/TGFB1/FRMD4A/HDAC3/MEPCE/CLTC/MESD/RANGRF/CCN3/EDEM2/EZR/STX4/UCP2/PARP1/SESN2/BCAS3/NEDD4L/MAVS/VTI1B/RAB11FIP1/LEPROT/PINK1/PLEKHM2/IFI27/BAD/PFKL/DZIP1/HCLS1/GRIPAP1/ILRUN/SRC/KAT7/SNX3/LRRC8A/DVL3/MYCBP2/FIS1/PTP4A3/PPP3CA/TRIM28/ACTB/CTNNA1/RANGAP1/MAP1A/NOLC1/MCRS1/VCP/USP36/ARIH2/RAB14/FKBP1A/ABLIM3/BMP6/SAE1/DTX3L/MYO18A/WWP2/SIDT2/MFHAS1/VCL/LDLRAP1/ARHGDIA/SUFU/FLNA/RAB11B/FKBP1B/RAC2/LMNA/INPP5K/NF2/GNL3L/BCL2L1/STK11/VPS28/ZDHHC5/RASSF5/GSN/SYTL4/GLUL/HUWE1/SQSTM1/LRP1/PPP1R9B/TRIM14/CSK/YOD1/SPTBN1/AKT2/UBE2L3/PTPN1/EPB41/TCF7L2/PER2/MSN/ATP2B4/MMD/CAMK1/ATG13/RHOG/BNIP3L/TBC1D1/CTSD/GAPVD1/OAZ1/PTPN23/AP2M1/PPM1A/SHISA7/ITGB3/DMTN/DAB2
BP	GO:0051248	negative regulation of protein metabolic process	749	-0.35	-1.50	1.57E-06	2.40E-05	1.49E-05	2398	tags=31%, list=20%, signal=26%	PPP1R15A/YTHDF3/BANP/GADD45B/AGAP2/ZFP36/CDKN2D/UBR5/FLCN/TSG101/CNOT9/WAC/DCP1A/PTPA/PNKP/TSPO/CDYL/CHP1/U2AF2/EIF4A3/G6PD/EPHA4/PKN1/SH3BP5L/CDK5/SPRED1/MOV10/FXR2/UNK/HYAL2/PCSK1N/FYN/CSDE1/CDK5RAP2/CRKL/CSNK2A1/IL1B/SPI1/PROS1/PARD6A/PTPN6/PABPC1/ROCK2/DCUN1D3/MYADM/SNTA1/SYNCRIP/ITM2C/HMG20B/TIMP1/GSTP1/MGAT5/DCPS/ZFP36L2/EIF6/TRIB1/CCAR2/LGALS3/CTSA/PIN1/NR1H2/AGO1/ARRB1/BIRC6/SMAD3/PATL1/CNOT11/PTTG1/FRY/APLP2/F8A1/PTPRC/EDN1/CAST/PIK3CB/CHMP6/ENG/CRTAP/SMARCC1/TAF1/FTO/MMP9/CTBP1/SNX12/PDE12/KLF4/WDR5/SEMA4D/CTDSP1/SOX4/IGF2BP3/SMPD1/DTNBP1/TNRC6C/KDM4A/SERPINB6/GSK3B/PRR5L/METTL16/CRY2/TIMP2/NR1H3/PYCARD/RPS6KA1/CACTIN/HSP90AB1/TGFB1/SVBP/YWHAG/CTDSP2/PRR7/ENC1/SPINT2/DAPK3/DAPK1/MEN1/BCOR/ALAD/SKI/MVP/CORO1C/ARL6IP1/SESN2/BACE2/AQP1/LYN/SUPT6H/SERPING1/PUM1/ADAR/RARA/HSPA1A/DHCR24/SRC/UBE2Z/PML/GREM1/CDKN1C/PUS7/CEACAM1/CALR/YWHAB/SFRP2/GNAI2/SNX3/MYCBP2/LRRK1/PIH1D1/USP19/MAP1A/KAT2B/UPF1/RGP1/JAK3/XIAP/ABL1/PARD3/FKBP1A/N4BP1/RELA/LTF/NFKB1/WARS1/DTX3L/ARRB2/DCP1B/JARID2/NIBAN1/SHMT2/PRKAR2A/MFHAS1/CALM3/MAD2L2/PPP4C/IGF2BP2/PTPRJ/SUFU/FLNA/FKBP1B/INPP5K/USP7/SF3B3/HSPB1/HIPK2/NF2/GNL3L/TENT4B/VPS28/SIAH2/PRDX5/HEXIM2/PLEC/PSMF1/SQSTM1/LRP1/RILP/PPP1R9B/THBS1/GLG1/YBX1/PTPN1/PER2/GPI/FKBP8/CTDSPL/UBASH3B/ATP2B4/SNCA/EIF4G1/PRKDC/EIF4EBP2/FBXO7/CDKN1A/CBL/TRIP12/PAK2/UBE2O/RYBP/CTSB/GPX1/KHSRP/PRKAR2B/FURIN/EIF2AK1/MLLT6/ITGB3/CLU/DMTN/LARP1/CNOT1/BAG6

BP	GO:0032271	regulation of protein polymerization	152	-0.51	-1.87	1.61E-06	2.45E-05	1.52E-05	2601	tags=42%, list=22%, signal=33%	MAPRE1/EPS8/CORO1A/ARL2/TMOD3/SPTAN1/TRIOBP/ARHGAP18/PFN1/MECP2/CDK5RAP2/SSH1/TBCD/MYADM/SVIL/FES/SSH2/NUMA1/FMN1/ARPC5/GRB2/SLAIN2/BAIAP2/PYCARD/CYFIP1/GIT1/EML2/RANGRF/CDC42EP4/PRUNE1/ALOX15/HIP1R/PINK1/RAC1/PTK2B/HSPA1A/HCLS1/ARPC1B/FLII/CAMSAP1/ABL1/PAK1/CLASP1/SSH3/RHOA/ARPC4/CKAP5/CYFIP2/DCTN1/GSN/SPTBN1/MTPN/SPTA1/COTL1/SPTB/SNCA/VASP/TMOD1/SCIN/CAPZB/CTTN/ESAM/DMTN/ADD1
BP	GO:0032269	negative regulation of cellular protein metabolic process	699	-0.35	-1.49	1.70E-06	2.56E-05	1.59E-05	2569	tags=33%, list=22%, signal=27%	SORL1/PKIG/AKT1/PIP5KL1/AIDA/FOXP3/SWAP70/CBLB/AGO4/JUN/SERPINF2/MLLT1/KNG1/BAK1/CLN3/PPP1R15A/YTHDF3/GADD45B/ZFP36/CDKN2D/UBR5/FLCN/TSG101/CNOT9/WAC/DCP1A/PTPA/PNKP/TSPO/CDYL/CHP1/U2AF2/EIF4A3/G6PD/EPHA4/PKN1/SH3BP5L/CDK5/SPRED1/MOV10/FXR2/UNK/HYAL2/PCSK1N/FYN/CSDE1/CDK5RAP2/CRKL/CSNK2A1/IL1B/SPI1/PROS1/PARD6A/PTPN6/PABPC1/ROCK2/DCUN1D3/MYADM/SNTA1/SYNCRIP/HMG20B/TIMP1/GSTP1/MGAT5/DCPS/ZFP36L2/EIF6/TRIB1/CCAR2/LGALS3/NR1H2/AGO1/ARRB1/BIRC6/PATL1/CNOT11/PTTG1/FRY/APLP2/F8A1/PTPRC/EDN1/CAST/PIK3CB/CHMP6/ENG/CRTAP/SMARCC1/TAF1/FTO/MMP9/CTBP1/SNX12/PDE12/KLF4/WDR5/SEMA4D/CTDSP1/SOX4/IGF2BP3/SMPD1/DTNBP1/TNRC6C/KDM4A/SERPINB6/GSK3B/PRR5L/METTL16/CRY2/TIMP2/NR1H3/PYCARD/RPS6KA1/CACTIN/HSP90AB1/TGFB1/SVBP/YWHAG/CTDSP2/PRR7/ENC1/SPINT2/DAPK3/DAPK1/MEN1/BCOR/ALAD/SKI/MVP/CORO1C/ARL6IP1/SESN2/AQP1/LYN/SUPT6H/SERPING1/PUM1/ADAR/RARA/HSPA1A/DHCR24/SRC/UBE2Z/PML/GREM1/CDKN1C/PUS7/CEACAM1/CALR/YWHAB/SFRP2/GNAI2/LRRK1/PIH1D1/USP19/MAP1A/KAT2B/UPF1/RGP1/XIAP/ABL1/PARD3/FKBP1A/N4BP1/RELA/LTF/NFKB1/WARS1/DTX3L/ARRB2/DCP1B/JARID2/NIBAN1/SHMT2/PRKAR2A/MFHAS1/CALM3/MAD2L2/PPP4C/IGF2BP2/PTPRJ/SUFU/FKBP1B/INPP5K/USP7/HSPB1/HIPK2/NF2/GNL3L/TENT4B/VPS28/SIAH2/PRDX5/HEXIM2/PLEC/PSMF1/SQSTM1/LRP1/PPP1R9B/THBS1/GLG1/YBX1/PTPN1/PER2/GPI/FKBP8/CTDSPL/UBASH3B/ATP2B4/SNCA/EIF4G1/PRKDC/EIF4EBP2/FBXO7/CDKN1A/CBL/TRIP12/PAK2/UBE2O/RYBP/CTSB/GPX1/KHSRP/PRKAR2B/FURIN/EIF2AK1/MLLT6/DMTN/LARP1/CNOT1/BAG6
BP	GO:0009790	embryo development	604	-0.36	-1.53	1.71E-06	2.57E-05	1.59E-05	2253	tags=25%, list=19%, signal=22%	EIF4A3/PRDM1/HES1/SMG9/PFN1/UNK/MAFG/ARID2/MYO15A/DEAF1/EXOC4/TOP1/TTLL1/VASH1/ROCK2/SH3PXD2A/PTPN18/ZBTB16/MYADM/XYLT1/BASP1/EHMT1/MAN2A1/MIB1/BIRC6/ARHGAP35/SMAD3/MAFF/ACVR1B/KEAP1/EDN1/PIK3CB/TFEB/PHACTR4/ENG/FZD5/RBPJ/TGFB1I1/B4GALT5/MMP9/NDEL1/OTUD7B/KLF4/SOX4/GRB2/TOP2A/NCOA1/ACVR2B/PRKACA/TGFB1/TRIP6/ASCL2/RPGRIP1L/PCSK6/ST14/IL1RN/AMOT/MYO1E/SPINT2/BCOR/KDM6B/NDST1/SKI/TGIF2/WDTC1/TWSG1/TRAF6/SLC39A3/MTHFR/PLXNB2/EPAS1/SUPT6H/FZD2/MBP/BPTF/RARA/IGF2/ASF1B/CHST11/KLF2/DCHS1/CREBBP/BCL2L11/TBL1XR1/GREM1/CDKN1C/ARNT/BBS5/SFRP2/DVL3/IFT52/TAF8/ZMIZ1/TRIM28/NRP1/ETS2/BCR/NOLC1/PDGFC/APBA3/ABL1/MAFB/RAB14/KMT2A/CLASP1/FBXW4/PSMC4/CMIP/EPN1/CHD8/NOTCH2/HIPK1/SUFU/INPP5K/ITPK1/ECE1/HIPK2/NIBAN2/NF2/BCL2L1/SDC4/ITGB5/GNA12/USP22/CFL1/TERF2/YBX1/MFN2/MEIS1/GPI/FKBP8/PAF1/PRKDC/VASP/MAPK3/GATA1/MICAL2/HLX/AKIRIN2/TRIP12/TET3/YBX3/FURIN/MED1/TAL1/CUL4A/MYH9/ITGB3/PDGFA/TENT5C/WNK1/CNOT1/PBX1/BAG6

BP	GO:0019932	second-messenger-mediated signaling	175	-0.49	-1.83	1.86E-06	2.78E-05	1.73E-05	2497	tags=35%, list=21%, signal=28%	POMC/MAPK7/ADNP/TRPM2/CD22/CD3E/ATP2A2/SLC24A4/INSL3/CCR1/CHP1/ATP1B1/P2RX4/ITPR2/PDE2A/PTPRC/EDN1/PPP1R9A/CXCR2/BCAP31/NFATC1/GSK3B/NFATC3/PRKACA/CXCR1/SPHK1/PLEK/CHERP/AQP1/SPPL3/PTK2B/CGAS/SLC9A1/NUDT4/FIS1/PPP3CA/ADGRL1/PTBP1/PDK2/IRAG1/CAP1/PPP3R1/SYK/PDE5A/PDE9A/CALM3/PTPRJ/GUCY1A1/FKBP1B/MCOLN1/PPP1R9B/THBS1/NFATC2/ATP2B4/SELP/RUNDC3A/PTGDR2/CDC34/HTT/GUCY1B1/DMTN/TBXA2R
BP	GO:0034097	response to cytokine	619	-0.37	-1.54	1.89E-06	2.81E-05	1.74E-05	2298	tags=28%, list=20%, signal=23%	USP27X/CCR1/OAS2/SMPD4/OAS3/IKBKE/IL6R/HES1/MAPKAPK3/IRF1/HYAL2/NUB1/ICAM1/CRKL/CPNE1/IL1B/SPI1/GBP4/RIPK1/CSF1R/OTUD4/PTPN6/SELPLG/PPBP/USP10/HSPA5/IL5RA/SYNCRIP/TIMP1/GSTP1/PDE2A/ZFP36L2/CDK9/NR1H2/SPATA2/CDC37/CXCL16/GBP6/RAB20/SYNJ1/KEAP1/PTPRC/EDN1/RIPOR2/LIMS1/CD14/CD74/CD300LF/IL2RG/PDE12/KLF4/CXCR2/SIGIRR/SMPD1/VIM/HYAL3/GSK3B/IL2RB/TIMP2/PRKACA/NR1H3/GSK3A/PYCARD/CACTIN/HSP90AB1/ACTG1/CXCR1/LILRA5/TNFRSF1B/SYNGR1/IL1RN/SPHK1/SNX10/DAPK3/DAPK1/STX4/SLC27A1/ALAD/ATIC/CDC42EP4/TRAF6/OXSR1/MAP3K5/MTHFR/ALOX15/ZYX/YY1/MAVS/ILK/IL17RA/ADAR/MBP/IFITM3/PTK2B/RARA/MT2A/HSPA1A/PIAS4/STIP1/KLF2/IFI27/MAP2K7/FLNB/TTLL12/BAD/TRIM32/HCLS1/SRC/PML/ADIPOR2/EIF5A/CEACAM1/PTP4A3/LAPTM5/IFITM2/STAT3/ARHGEF2/UPF1/JAK1/SYK/JAK3/XIAP/AGPAT1/FOS/RELA/NFKB1/JARID2/MPL/TLE4/SHMT2/RHOA/LDLRAP1/HIPK1/PTPRJ/LGALS9/WAS/MAPKAPK2/IFIT1B/INPP5K/STAT5A/FASN/CDIP1/BCL2L1/PRPF8/GSN/ADIPOR1/PF4V1/PLVAP/THBS1/YBX1/PTPN1/RPS6KA4/SHARPIN/SELP/SNCA/CXCL5/H2BC11/MAPK3/ACKR1/PF4/CBL/SMPD3/ACTN4/KHSRP/YBX3/FOXO3/CDC34/TOLLIP/MED1/HDGF/WNK1
CC	GO:0031256	leading edge membrane	108	-0.55	-1.95	1.91E-06	2.84E-05	1.76E-05	1336	tags=30%, list=11%, signal=26%	EZR/STX4/PLEK/CORO1C/TPM1/HIP1R/CDKL5/RAC1/PLXND1/SRC/SH2D3C/ARHGAP45/ARHGEF2/PAK1/PLEK2/RHOA/PDE9A/DNM2/PTPRJ/INPP5K/NF2/CFL1/PPP1R9B/SPTBN1/AKT2/PDE4A/VASP/TESC/SHISA7/ITGB3/TLN1/DIAPH1
BP	GO:0010543	regulation of platelet activation	34	-0.73	-2.08	2.13E-06	3.15E-05	1.95E-05	1409	tags=41%, list=12%, signal=36%	TEC/PLEK/LYN/CD9/CEACAM1/SYK/GP6/GP1BA/ALOX12/GP9/UBASH3B/SELP/PDGFA/DMTN
BP	GO:0071375	cellular response to peptide hormone stimulus	194	-0.48	-1.81	2.20E-06	3.25E-05	2.01E-05	1834	tags=29%, list=16%, signal=25%	SHC1/SLC29A2/SH2B2/EDN1/SMARCC1/NDEL1/PIK3R3/GRB2/SREBF1/GSK3B/BAIAP2/GSK3A/CYFIP1/YWHAG/ZNF106/MEN1/UCP2/SLC27A1/WDTC1/PARP1/RAB31/LYN/LEPROT/IGF2/SLC9A1/SRC/CCND3/CEACAM1/CA2/GNAI2/PKM/MAX/STAT3/RANGAP1/PDK2/KAT2B/JAK1/PXN/JAK3/FBP1/RELA/FOXO4/NFKB1/LPIN2/SESN3/INPP5K/STAT5A/ADIPOR1/PRKCB/CSK/AKT2/PTPN1/SP1/PRKDC/AHCYL1/PIP4K2A/EIF4EBP2
BP	GO:0030177	positive regulation of Wnt signaling pathway	89	-0.58	-1.99	2.22E-06	3.26E-05	2.02E-05	2189	tags=39%, list=19%, signal=32%	SPIN1/ASPM/CSNK2A1/MACF1/CCAR2/PIN1/SMAD3/CSNK1G1/DIXDC1/RBPJ/SOX4/TGFB1/SMARCA4/DAPK3/SKI/ZRANB1/ILK/TBL1XR1/SRC/SFRP2/RUVBL1/LRRK1/TBL1X/USP34/VCP/XIAP/ABL1/NFKB1/ATP6V0C/GID8/FAM53B/CTDNEP1/PPM1A/WNK1/DAB2
BP	GO:1901653	cellular response to peptide	242	-0.45	-1.76	2.24E-06	3.28E-05	2.03E-05	1775	tags=28%, list=15%, signal=24%	SLC29A2/SH2B2/EDN1/SMARCC1/NDEL1/PIK3R3/ABCC1/VIM/GRB2/SREBF1/GSK3B/BAIAP2/GSK3A/CYFIP1/YWHAG/ZNF106/MEN1/UCP2/SLC27A1/WDTC1/PARP1/RAB31/LYN/LEPROT/IGF2/KLF2/SLC9A1/BCL2L11/SRC/CCND3/CEACAM1/CA2/GNAI2/PKM/MAX/STAT3/RANGAP1/PDK2/ARHGEF2/KAT2B/JAK1/PXN/JAK3/FBP1/RELA/FOXO4/NFKB1/LPIN2/SESN3/INPP5K/STAT5A/KLF1/ADIPOR1/LRP1/PPP1R9B/PRKCB/CSK/AKT2/PTPN1/SP1/PRKDC/AHCYL1/PIP4K2A/EIF4EBP2/SMPD3/FOXO3/KLF3

BP	GO:0043549	regulation of kinase activity	526	-0.37	-1.55	2.33E-06	3.38E-05	2.09E-05	2856	tags=33%, list=24%, signal=26%	SLC8A1/MAP3K4/FLT1/PRKAR1B/LCP2/SPATC1L/TESK1/CEBPA/EPHB1/TNFRSF10A/TTN/TSC2/PDPK1/CDK12/SH2B3/FGR/TRAF2/PKD1/GNAQ/RALB/SORL1/PKIG/AKT1/AIDA/TAOK3/CBLB/PIK3CA/CCNJL/MLLT1/ADNP/MOB3B/NCF1/EGR1/GADD45B/AGAP2/CDKN2D/EZH2/DLG4/TSG101/CHP1/MAP3K13/LMTK2/IL6R/EPHA4/PKN1/HLA-DRB1/SH3BP5L/PIP4K2C/CDK5/SPRED1/HYAL2/CD81/DDR1/CDK5RAP2/IL1B/CSF1R/PTPN6/MADD/VAV2/TLR6/CCNY/GSTP1/MOB1B/TRIB1/SOCS7/PRRC1/XRCC6/CDC37/SHC1/GCN1/HGS/PTPRC/EDN1/PIK3CB/MAGED1/CHMP6/FZD5/DGKZ/CD74/TPX2/PIK3R3/WDR81/MAP3K11/SMPD1/DTNBP1/ACVR2B/NEDD9/PYCARD/HSP90AB1/TGFB1/MOB3A/LILRA5/TAOK1/YWHAG/MEN1/SLC27A1/MVP/SERTAD1/CORO1C/TRAF6/MAP3K5/SESN2/LYN/SASH1/ADAR/PINK1/PTK2B/IGF2/MAP2K7/BAD/MARK2/SRC/CCND3/GREM1/CDKN1C/CEACAM1/RUBCN/SFRP2/DVL3/TAOK2/DAXX/ACTB/PIH1D1/MAP2K2/VLDLR/KAT2B/PDGFC/SYK/ABL1/PAK1/LTF/WARS1/PRKAR2A/PDE5A/RHOA/CALM3/PTPRJ/INPP5K/HSPB1/NF2/STK11/SDC4/HEXIM2/PLEC/CSK/THBS1/CCNI/PTPN1/AKAP13/PIK3CG/IQGAP1/UBASH3B/ATP2B4/SNCA/MMD/CAMK1/NEURL1/PIP4K2A/FBXO7/CDKN1A/CBL/PAK2/STRADB/PRKAR2B/TAL1/HTT/ITGB3/PDGFA/CLU/MAP2K3/WNK1/DAB2/CNPPD1
BP	GO:0060284	regulation of cell development	296	-0.43	-1.70	2.45E-06	3.55E-05	2.20E-05	2605	tags=35%, list=22%, signal=28%	CAPRIN1/ULK1/SORL1/AKT1/DIP2B/PLXNC1/DISC1/PLXNA4/ADNP/NOTCH1/NR1D1/EZH2/TRIOBP/HOXB3/TSPO/MAP3K13/EPHA4/HES1/CDK5/ASPM/CRKL/IL1B/CERS2/ROCK2/MACF1/MYADM/SMARCD3/TRIB1/MAN2A1/RCC2/CUL7/SYNJ1/KIAA0319/TIAM1/DIXDC1/LIMS1/NDEL1/SEMA4D/CTDSP1/KDM4A/GSK3B/BAIAP2/NEDD9/GSK3A/CYFIP1/TGFB1/ASCL2/TNFRSF1B/PREX1/SKI/CORO1C/BCL11A/PRUNE1/IST1/LYN/PLXNB2/ILK/CDKL5/RAC1/MBP/PLXND1/BAD/TRIM32/FBXO31/HOOK3/EIF4G2/STK25/CALR/OLIG2/PLXNA1/PPP3CA/CTNNA1/SEMA4A/NRP1/YWHAH/RFLNB/ABL1/SYNGAP1/E2F1/LTF/MPL/PDE5A/VCL/NOTCH2/DNM2/FLNA/NIBAN2/NF2/TRIM11/STK11/CLDN5/GJC2/PER2/GRN/CUX1/NEURL1/PRKDC/RNF10/ACTN4/TRIM58/DMTN/TGM2/ADD1/DAB2
BP	GO:0007264	small GTPase mediated signal transduction	381	-0.40	-1.62	2.52E-06	3.63E-05	2.25E-05	2071	tags=25%, list=18%, signal=21%	DOCK10/MAPRE2/MADD/ROCK2/VAV2/ARHGAP26/PRAG1/TNK2/RALBP1/RIPOR1/SRGAP2/ARRB1/LPAR4/ARHGAP35/TIAM1/PIK3CB/LRRC59/RIPOR2/PHACTR4/RHOBTB1/RAP1GAP/RHOF/GRB2/G3BP2/CYFIP1/DOK3/RABIF/RASGRP3/PREX1/RANGRF/AMOT/ARHGAP17/DENND1A/SIPA1L3/CDC42EP4/ITSN1/RAC1/TRIO/DHCR24/SIPA1L1/GNB1/MYO9B/RRAS/SRC/SH2D3C/RASA3/IQSEC1/ARHGAP32/VAV1/CDK2/GMIP/TRIM28/ARHGAP45/CTNNAL1/NRP1/ARHGEF2/BCR/GABARAP/ABL1/RRAGC/SYNGAP1/FGD4/FBP1/ARHGEF18/CYTH4/RAPGEF1/SETDB1/RHOA/ARHGEF12/NOTCH2/DNM2/ARHGAP6/ARHGDIA/WAS/ARHGAP31/ARHGAP21/RAC2/KANK2/ARHGAP1/DOCK2/SIAH2/GNA12/CFL1/SQSTM1/AKAP13/PIK3CG/MFN2/RUNDC3A/RHOG/CDKN1A/CBL/NUCB1/RUFY1/WASF2/SH3BP1/TGM2
MF	GO:0061629	RNA polymerase II-specific DNA-binding transcription factor binding	273	-0.43	-1.70	2.65E-06	3.78E-05	2.35E-05	2141	tags=36%, list=18%, signal=30%	MLX/CRKL/MTA2/CPNE1/SPI1/ZNF366/MTA1/TACC1/SMARCD3/PPARGC1B/ZBTB7A/TRIB1/NR1H2/UBXN7/SMAD3/KEAP1/HIF1AN/TAF1/TAF6/RBPJ/TGFB1I1/CTBP1/TRERF1/KLF4/JUND/FOXP1/NFATC1/NCOA1/TOB2/GSK3B/CRY2/BAZ2A/TRIP6/HDAC3/SMARCA4/ETS1/PDCD11/NCOA2/NCOA6/PARP1/TP53BP2/BCAS3/EPAS1/TADA3/RXRA/IFI27/CREBBP/MTDH/MED16/TRIM32/BRMS1/HCLS1/SRC/DCAF1/ARNT/TAF4/BBS5/NCOA3/CALR/PPARA/WBP2/DAXX/TP53BP1/ACTB/STAT3/ETS2/YWHAH/FOS/FBP1/RELA/DTX3L/WWP2/NOTCH2/MAD2L2/HSPB1/HIPK2/ELK1/KAT6A/MED25/PRKCB/SETD1A/TCF7L2/SP1/CHD4/PRKDC/GATA1/TRIP12/HMGA1/ACTN4/MED1/TAL1/ARID1A/GAS2L1/GFI1B/NCOR2/ADD1/CNOT1

CC	GO:0030027	lamellipodium	156	-0.49	-1.82	2.70E-06	3.83E-05	2.38E-05	1722	tags=41%, list=15%, signal=35%	PTPN13/RAB3IP/CORO1B/CAPRIN1/CORO1A/AKT1/SWAP70/PIK3CA/SYNE2/DOCK8/CDK5/FGD6/ABI3/SSH1/PABPC1/CTNND1/SRGAP2/PHACTR4/ARPC5/DGKZ/BAIAP2/NEDD9/CYFIP1/GIT1/AMOT/INPPL1/STX4/CORO1C/CDC42BPB/ITSN1/ILK/RAC1/PTK2B/PLXND1/SLC9A1/FAM89B/ACTB/CTNNA1/PXN/NRBP1/PAK1/FGD4/ABLIM3/PLEK2/APBB1IP/RHOA/DNM2/ACTA2/ARHGAP31/RAC2/MYLK/NF2/GSN/CFL1/PPP1R9B/VASP/PARVB/WASF3/CAPZB/TESC/CTTN/ITGB3/WASF2/SH3BP1
MF	GO:0051020	GTPase binding	251	-0.44	-1.71	2.79E-06	3.95E-05	2.45E-05	2733	tags=38%, list=23%, signal=30%	DOCK4/TSC2/NCF2/ATP7A/RAB3IP/DAAM1/RAB11FIP5/ULK1/EPS8/SORL1/RAB8A/GNB2/BICD2/LZTR1/BICD1/TRIOBP/RPH3A/PPP6R1/PKN1/PFN1/GCC1/FGD6/FMNL1/BICDL1/EXOC4/PARD6A/ROCK2/LCP1/IPO4/DIAPH2/RALBP1/NDRG1/FMNL3/SRGAP2/PDE6D/RCC2/RRAGD/STOML2/ANKFY1/RAP1GAP/IPO8/MAP3K11/YIPF2/XPO6/CYFIP1/RASGRP3/RANGRF/DAPK3/KIF3B/ATP6AP1/CDC42EP4/CORO1C/CDC42BPB/RAB7A/RAB11FIP1/RABGAP1/CDKL5/MICALL1/EVI5L/GNB1/MYO9B/INF2/DVL3/MYCBP2/RIN3/RAB11FIP4/TBC1D13/RANGAP1/ARHGEF2/RGP1/PAK1/RRAGC/OPTN/FGD4/LAMTOR1/WAS/XPO7/FLNA/TBC1D20/ARHGAP1/TMEM127/DOCK2/CYFIP2/RASSF5/SYTL4/RILP/SPTBN1/YBX1/AKAP13/IQGAP1/ENO1/EHD1/GAPVD1/PAK2/YBX3/DIAPH1
CC	GO:0098978	glutamatergic synapse	175	-0.49	-1.82	2.82E-06	3.97E-05	2.46E-05	1709	tags=29%, list=15%, signal=25%	NPTXR/CANX/CTBP1/AP2B1/DTNBP1/GSK3B/BAIAP2/PRR7/STX4/HIP1/LYN/CDKL5/RAC1/PTK2B/SH3GL1/GRIPAP1/SRC/DLGAP4/PPP3CA/ACTB/PPP1CA/NRP1/BCR/VCP/ELAVL1/SYNGAP1/ABLIM3/RELA/STAU1/RHOA/DNM2/GUCY1A1/NAPA/DNM3/CLSTN1/DYNLL2/SPTBN1/ATP2B4/LRFN3/CAMK1/P2RX1/RNF10/NRGN/WASF3/PAK2/ACTN1/PRKAR2B/AP2M1/SHISA7/ITGB3/GUCY1B1
MF	GO:0106310	protein serine kinase activity	275	-0.43	-1.69	2.83E-06	3.98E-05	2.47E-05	2846	tags=36%, list=24%, signal=28%	MAP3K4/NEK7/TESK1/OBSCN/TTN/EIF2AK2/PDPK1/RPS6KA2/CDK12/ULK1/MAPK14/PAK4/MARK4/MINK1/AKT1/BMP2K/PIM3/TAOK3/PIK3CA/MAPK7/MAP4K5/MAP3K13/LMTK2/PKN1/MAPKAPK3/CDK5/PRKD2/STK40/CSNK2A1/RIPK1/NUAK2/ROCK2/MKNK2/TNK2/TESK2/CDK9/MAP3K3/CSNK1G1/TAF1/MAP3K11/MARK3/GSK3B/PRKACA/GSK3A/RPS6KA1/CDK6/TAOK1/DYRK1B/DAPK3/DAPK1/NEK6/OXSR1/MAP3K5/CDC42BPB/MAP4K1/ILK/CDKL5/PINK1/STK10/MAP2K7/TRIO/STK24/MARK2/DCAF1/SRPK2/STK25/NEK5/PSKH1/MAST3/CDK2/LRRK1/FAM20C/TAOK2/MAP2K2/BCR/PAK1/PIM2/HIPK1/MAPKAPK2/HIPK2/CDK19/CAMK1D/STK11/SIK3/RIOK3/GAK/PRKCB/AKT2/RPS6KA4/PIK3CG/ARAF/CAMK1/PRKDC/MAPK3/PAK2/EIF2AK1/MAP2K3/WNK1/PIM1
CC	GO:0048471	perinuclear region of cytoplasm	520	-0.37	-1.55	2.95E-06	4.14E-05	2.57E-05	2251	tags=27%, list=19%, signal=23%	LMTK2/SERINC5/MAGI2/ABCD1/MLC1/HYAL2/FYN/XRCC3/CDK5RAP2/MAP7/GBP4/P2RX4/OSBPL7/DCUN1D3/LCP1/TNK2/ITM2C/NDRG2/PDE2A/NDRG1/PKP4/PACSIN1/CUL7/SERBP1/PDE4B/SYNJ1/PI4KB/HIF1AN/FZD5/LIMS1/MAP1S/CENPF/STX6/ZDHHC19/BCAP31/VPS53/EHD3/STOM/PRKACA/SYNRG/CASC3/CYFIP1/HSP90AB1/ATP9A/RASGRP3/TAOK1/TNFRSF1B/RANGRF/PRR7/EZR/STX4/MVP/EHD4/TRAF6/TP53BP2/GLB1/CHERP/LNPEP/HIP1R/LYN/VTI1B/EPG5/PINK1/IFITM3/PTK2B/RARA/HSPA1A/TMEM184A/ARF1/SBF1/SLC9A1/MTDH/S100A4/EXOC3/MYO9B/SRC/GALNT2/INF2/CALR/YWHAB/VTI1A/PLEKHF1/LAPTM5/TAF8/AMFR/SEC31A/MAP2K2/RANGAP1/IRAG1/APBA3/VCP/ZFYVE1/ABL1/RAB14/OPTN/ANXA6/FAM168B/PDE9A/DNM2/INPP5K/ECE1/ARHGAP1/NF2/CDK19/ATP6V0A1/PRDX5/CYFIP2/SORT1/DNM3/PLEC/RAB1B/GAK/IGF2R/PSMF1/PLVAP/PDE4A/AKAP13/TRIP10/MTPN/PER2/MSN/SNCA/PRDX6/NEURL1/LAMP1/CBL/EHD1/ACTN4/PAK2/CTSB/YBX3/PRKAR2B/TOLLIP/HTT/SEC16A/CLU/OSBP2/DMTN/TGM2/DAB2

BP	GO:0060828	regulation of canonical Wnt signaling pathway	155	-0.50	-1.84	2.99E-06	4.17E-05	2.59E-05	2014	tags=31%, list=17%, signal=26%	CTNND1/CCNY/CCAR2/PIN1/LZTS2/SOX13/SMAD3/CSNK1G1/RBPJ/SOX4/GSK3B/GSK3A/TGFB1/CTNNBIP1/TMEM131L/DAPK3/ILK/TBL1XR1/SRC/GREM1/SFRP2/RUVBL1/LRRK1/AMFR/TLE3/PPP1CA/TBL1X/BTRC/USP34/VCP/XIAP/OTUD5/NFKB1/TLE4/CHD8/GID8/MAD2L2/TLE1/STK11/SIAH2/TCF7L2/ZNF703/FAM53B/FOXO3/CTDNEP1/PPM1A/WNK1/DAB2
BP	GO:0071868	cellular response to monoamine stimulus	47	-0.68	-2.08	3.04E-06	4.22E-05	2.61E-05	2099	tags=51%, list=18%, signal=42%	HRH2/GNA11/PDE4B/OR10H1/GNAZ/DTNBP1/GSK3B/PRKACA/GSK3A/GNAS/GNA15/SLC9A1/GNB1/NCSTN/ABL1/ARRB2/DNM2/FLNA/HRH4/KLF16/ATP2B4/SNCA/MAPK3/TGM2
BP	GO:0071870	cellular response to catecholamine stimulus	47	-0.68	-2.08	3.04E-06	4.22E-05	2.61E-05	2099	tags=51%, list=18%, signal=42%	HRH2/GNA11/PDE4B/OR10H1/GNAZ/DTNBP1/GSK3B/PRKACA/GSK3A/GNAS/GNA15/SLC9A1/GNB1/NCSTN/ABL1/ARRB2/DNM2/FLNA/HRH4/KLF16/ATP2B4/SNCA/MAPK3/TGM2
BP	GO:0098657	import into cell	125	-0.53	-1.92	3.14E-06	4.34E-05	2.69E-05	2551	tags=36%, list=22%, signal=28%	AKT1/SLC46A1/KCNH2/TRPM2/ATP1A1/CYBRD1/TSG101/SLC24A4/FYN/ATP1B1/SLC9A7/SLC43A2/SLC7A1/SLC29A2/SLC19A1/KCNJ2/TSPO2/SLC27A1/ARL6IP1/SLC5A6/SLC9A1/SLC39A4/LRRC8A/SLC7A8/SLC1A5/ATP1B2/ACTB/SLC1A4/SLC6A20/SLC2A1/SLC7A5/SLC3A2/SLC6A9/VPS28/THBS1/AKT2/SLC43A1/PER2/ATP2B4/SNCA/P2RX1/SLC29A1/SLC15A3/ITGB3/WNK1
BP	GO:0120039	plasma membrane bounded cell projection morphogenesis	350	-0.40	-1.62	3.17E-06	4.35E-05	2.70E-05	2622	tags=33%, list=22%, signal=27%	EXT1/CORO1B/CAPRIN1/ULK1/RAB8A/MINK1/DIP2B/TAOK3/MUL1/PLXNC1/DISC1/PLXNA4/ADNP/PTPN11/NOTCH1/EFNB1/DLG4/NCAM1/LYPLA2/MAP3K13/LMTK2/EPHA4/CDK5/MOV10/DDR1/ABI3/FYN/CPNE1/FEZ1/PLEKHO1/SLITRK5/DOCK10/CSF1R/MACF1/SRGAP2/PACSIN1/PARD6B/CUL7/ARHGAP35/WEE1/APLP2/EDN1/KIAA0319/TIAM1/RERE/DIXDC1/MAP1S/B4GALT5/NDEL1/SEMA4D/DTNBP1/GSK3B/BAIAP2/CYFIP1/HSP90AB1/TAOK1/BCL11B/CORO1C/MFSD2A/BCL11A/IST1/NEDD4L/PLXNB2/CDKL5/RAC1/MBP/DPYSL2/PLXND1/TRIO/KIF13B/SIPA1L1/MARK2/FBXO31/SRC/EIF4G2/GAS7/CPNE5/STK25/DVL3/MYCBP2/PLXNA1/PPP3CA/TAOK2/ACTB/SEMA4A/NRP1/MAP1A/VLDLR/YWHAH/USP9X/ABL1/PAK1/PARD3/SYNGAP1/XK/RHOA/VCL/NOTCH2/DNM2/RAC2/BSG/ECE1/NIBAN2/STK11/UBB/CYFIP2/DNM3/CFL1/IQGAP1/CUX1/VASP/RHOG/PAK2/CTTN/WASF2/KEL/SCN1B
BP	GO:0010256	endomembrane system organization	406	-0.39	-1.60	3.19E-06	4.36E-05	2.71E-05	1849	tags=23%, list=16%, signal=20%	PACSIN1/SYNE1/CUL7/SYNJ1/HGS/PTPRC/CHMP6/TBPL1/NDEL1/STX6/SEC23IP/NSFL1C/VPS37B/CHMP4B/SMPD1/DTNBP1/EHD3/BAIAP2/SYNGR1/ASAP1/TJAP1/SNX10/STX4/NEK6/PRX/CORO1C/ARL6IP1/ESYT1/SUN2/IST1/ANO6/BCAS3/VTI1B/RAB7A/PLEKHM2/CD9/MICALL1/DYSF/CHMP1A/HOOK3/STK25/TMCC1/SNX3/VTI1A/PLEKHF1/SEC31A/MYOF/MAP2K2/EMC3/KIFC3/OPTN/CLASP1/BLOC1S3/ANXA6/STX5/MYO18A/LAMTOR1/GOLGA2/DNM2/RAB5B/TBC1D20/ARHGAP21/NUP93/CAVIN2/LMNA/TMEM127/VPS28/PLSCR3/MMGT1/DCTN1/GSN/PLEC/SYTL4/SLC4A1/GAK/HUWE1/SQSTM1/RILP/PRKCB/SPTBN1/AKT2/TANGO2/SPTA1/MAPK3/CTDNEP1/HTT/MYH9/SEC16A/NPLOC4/CLU/ACRBP/BAG6

MF	GO:0008047	enzyme activator activity	406	-0.39	-1.60	3.25E-06	4.43E-05	2.74E-05	2488	tags=30%, list=21%, signal=24%	PIK3CA/JUN/TBC1D8/MOB3B/NCF1/CASP9/PPP1R15A/RGS3/AGAP2/FLCN/AGAP1/DCP1A/ARHGEF11/PTPA/AGAP3/ARHGAP18/MAP3K13/ARHGAP8/FYN/ARAP1/TBCD/GTF2F1/MADD/SH3PXD2A/CCNY/ARHGAP26/RALBP1/MOB1B/SRGAP2/CTSA/ARRB1/RGS10/ARHGAP35/SMAP2/ADAP2/PHACTR4/RAP1GAP/TPX2/RGS16/PSME3/TBC1D17/PYCARD/MOB3A/TBC1D22A/GIT1/RASGRP3/ASAP2/TAOK1/PREX1/ASAP1/BEX3/ARHGAP17/GNAS/SIPA1L3/STX4/BCAS3/RABGAP1/PINK1/BRPF1/IGF2/EVI5L/BAD/SIPA1L1/MARK2/MYO9B/SRC/GREM1/RASA3/SFRP2/SLX4/ARHGAP32/RIN3/TAOK2/DAXX/PSAP/GMIP/ARHGAP45/TBC1D13/ARFGAP2/NRP1/MAP2K2/RANGAP1/BCR/BTRC/VCP/SYNGAP1/LTF/SAE1/PPP2R5E/DTX3L/DCP1B/ARHGEF12/CALM3/ARHGAP6/TBC1D22B/ARHGDIA/TBC1D20/ARHGAP31/ARHGAP21/ARHGAP1/PSME4/DOCK2/STK11/BCL2L13/FZR1/PPP2R5B/UBE2L3/IQGAP1/RGS6/TBC1D5/DPM2/TBC1D25/CDKN1A/TBC1D1/GAPVD1/PAK2/NPRL3/TAL1/SH3BP1/WNK1
CC	GO:0032587	ruffle membrane	74	-0.59	-1.96	3.31E-06	4.49E-05	2.79E-05	1336	tags=35%, list=11%, signal=31%	EZR/PLEK/CORO1C/TPM1/HIP1R/CDKL5/RAC1/SRC/SH2D3C/ARHGAP45/ARHGEF2/PAK1/RHOA/PDE9A/DNM2/PTPRJ/INPP5K/NF2/CFL1/PPP1R9B/AKT2/PDE4A/TESC/ITGB3/TLN1/DIAPH1
BP	GO:0043434	response to peptide hormone	259	-0.43	-1.69	3.51E-06	4.74E-05	2.94E-05	2578	tags=34%, list=22%, signal=27%	MAPK14/SORL1/RAB8A/AKT1/POMC/PIK3CA/PTPN11/CYBB/EGR1/HDAC5/FOXO1/SLC24A4/PHEX/BTG1/PIP4K2C/IL1B/ROCK2/SRSF4/TIMP1/EIF6/SHC1/SLC29A2/SH2B2/EDN1/SMARCC1/NDEL1/PIK3R3/JUND/GRB2/SREBF1/GSK3B/BAIAP2/CRY2/GSK3A/CYFIP1/YWHAG/ZNF106/INPPL1/MEN1/UCP2/SLC27A1/WDTC1/PARP1/SESN2/RAB31/LYN/LEPROT/IGF2/SLC9A1/SRC/CCND3/CEACAM1/CA2/GNAI2/PPARA/PKM/MAX/STAT3/RANGAP1/PDK2/KAT2B/JAK1/PXN/JAK3/FBP1/RELA/FOXO4/NFKB1/LPIN2/SLC2A1/SESN3/BSG/INPP5K/STAT5A/STK11/SORT1/ADIPOR1/PRKCB/CSK/AKT2/PTPN1/SP1/PRKDC/AHCYL1/PIP4K2A/EIF4EBP2/RNF40
BP	GO:0007163	establishment or maintenance of cell polarity	160	-0.49	-1.82	3.64E-06	4.91E-05	3.04E-05	2335	tags=41%, list=20%, signal=33%	ARHGEF11/SNX27/HES1/MAP4/NDE1/CDK5RAP2/CRKL/MAP7/FEZ1/FBF1/PARD6A/ROCK2/PARVG/PARD6B/NUMA1/ARHGAP35/WEE1/FRMD4B/RIPOR2/ARPC5/RHOBTB1/NDEL1/RHOF/NSFL1C/GSK3B/HSP90AB1/FRMD4A/RPGRIP1L/AMOT/SIPA1L3/SPINT2/WDR1/CDC42BPB/AQP1/BCAS3/ILK/RAC1/JAM3/PTK2B/SLC9A1/SPN/MARK2/STK25/FAM89B/ACTB/CTNNA1/CAP1/PARD3/CLASP1/MYO18A/RHOA/CKAP5/RAC2/LMNA/DOCK2/STK11/DCTN1/GSN/MSN/RHOG/PARVB/PDLIM1/HTT/MYH9/SH3BP1/ANK1
BP	GO:0007186	G protein-coupled receptor signaling pathway	365	-0.40	-1.63	3.75E-06	5.03E-05	3.12E-05	2780	tags=30%, list=24%, signal=24%	GNB1L/CCRL2/FRS2/RGS9/TPRA1/CMKLR1/GABBR1/USP20/OR52K1/GNAQ/PTAFR/SORL1/GNB2/AKT1/PTGIR/GNAO1/POMC/CD3E/BICD1/P2RY14/RGS3/PDE6G/SLC24A4/ARHGEF11/INSL3/CCR1/PCSK1N/CRKL/HRH2/CNR2/PTPN6/GNA11/ROCK2/ADGRE1/NLN/ZDHHC3/PDE2A/ARRB1/LPAR4/RGS10/PDE4B/OR10H1/APLP2/EDN1/PIK3CB/FZD5/DGKZ/GNAZ/GRK2/RGS16/CXCR2/DGKD/ADGRE5/GSK3A/GIT1/CXCR1/PREX1/RPGRIP1L/SPHK1/EZR/GNAS/PLEK/FPR3/FZD2/RAC1/NPFF/PTK2B/GNA15/GNB1/SRC/CA2/YWHAB/GNAI2/VAV1/ADGRL1/PSAP/GPR146/PARD3/RELA/ARRB2/PLCB3/GALR1/ARHGEF12/CALM3/DNM2/FLNA/RAC2/INPP5K/ECE1/SORT1/GNA12/GP1BA/IGF2R/PPP1R9B/HRH4/PDE4A/AKAP13/PIK3CG/RGS6/ATP2B4/SNCA/AHCYL1/ACKR1/PF4/PTGDR2/MGLL/WASF2/TGM2/TBXA2R

BP	GO:0032984	protein-containing complex disassembly	195	-0.47	-1.78	3.78E-06	5.05E-05	3.13E-05	2539	tags=42%, list=22%, signal=33%	UBQLN4/SWAP70/PIK3CA/NSF/TMOD3/DSTN/CLN3/LAMP2/SPTAN1/ATP2A2/ATXN7/TRIOBP/TSG101/LIX1L/ARID2/NCKAP5L/VPS4B/GRWD1/OGFOD1/SMARCD3/SVIL/APEH/TFIP11/MAP1LC3B/SYNJ1/PYM1/CHMP6/SMARCC1/MAP1S/LIMA1/TPX2/PHF23/CHMP4B/TECPR1/GSK3B/SMARCA4/TAOK1/EIF5AL1/DHX8/PLEK/WDR1/KATNB1/IST1/EPG5/SUPT16H/SH3GL1/CHMP1A/EIF5A/RUBCN/FLII/CAMSAP1/PPP1CA/MAP1A/ARHGEF2/UPF1/VCP/CLASP1/CKAP5/MCOLN1/NAPA/GSN/CFL1/GAK/PPP1R9B/SPTBN1/MTPN/SPTA1/NFE2/SPTB/TMOD1/MICAL2/TBC1D25/SCIN/HMGA1/CAPZB/SMARCC2/ARID1A/GSPT1/SH3BP1/DMTN/ADD1
BP	GO:0030855	epithelial cell differentiation	312	-0.42	-1.66	3.91E-06	5.22E-05	3.23E-05	2479	tags=33%, list=21%, signal=26%	JUN/TAGLN2/NOTCH1/EDF1/ZFP36/KRT73/EZH2/TRIOBP/TSG101/PRDM1/HES1/BTG1/MAGI2/SPRED1/MAFG/ICAM1/THRA/IL1B/ROCK2/MYADM/BASP1/PDE2A/HSD17B4/LGALS3/TPP1/NUMA1/SMAD3/MAFF/KEAP1/RBPJ/TGFB1I1/CPT1A/MMP9/KLF4/SOX4/VIM/ACVR2B/GSK3B/GSK3A/CDK6/ST14/EZR/MYO1E/SIPA1L3/SPINT2/MEN1/BCL11B/KDM6B/FZD2/LBH/RARA/ALDOC/KLF2/MGMT/FLNB/BAD/SRC/GREM1/CDKN1C/CEACAM1/NCOA3/FAM20C/PSAP/PIH1D1/NRP1/BCR/VDAC1/TJP2/ABL1/RAPGEF1/BMP6/ACADVL/SIDT2/RHOA/VCL/NOTCH2/ACTA2/TBC1D20/FOXJ2/NF2/FASN/KRT1/PLEC/FZR1/TXNIP/CLDN5/AKT2/IQGAP1/TST/MSN/SHARPIN/ZNF703/TMOD1/GATA1/TUBB/CTSB/GPX1/TOLLIP/MED1/SH3BP1/ADD1/DAB2
BP	GO:0034446	substrate adhesion-dependent cell spreading	66	-0.62	-2.02	4.07E-06	5.42E-05	3.36E-05	1990	tags=41%, list=17%, signal=34%	CRKL/MYADM/ITGB7/ST6GAL1/SRGAP2/PARVG/RCC2/LIMS1/NEDD9/PREX1/CORO1C/ILK/RAC1/SRC/CALR/NRP1/PXN/ABL1/RHOA/DNM2/FLNA/FERMT3/PARVB/ACTN4/ITGB3/DMTN/DAB2
MF	GO:0031267	small GTPase binding	224	-0.45	-1.73	4.38E-06	5.81E-05	3.60E-05	2733	tags=38%, list=23%, signal=30%	DOCK4/TSC2/NCF2/ATP7A/DAAM1/RAB11FIP5/ULK1/EPS8/SORL1/RAB8A/BICD2/LZTR1/BICD1/TRIOBP/RPH3A/PPP6R1/PKN1/PFN1/GCC1/FGD6/FMNL1/BICDL1/EXOC4/PARD6A/ROCK2/IPO4/DIAPH2/RALBP1/NDRG1/FMNL3/SRGAP2/PDE6D/RCC2/ANKFY1/RAP1GAP/IPO8/MAP3K11/YIPF2/XPO6/CYFIP1/RASGRP3/RANGRF/DAPK3/KIF3B/ATP6AP1/CDC42EP4/CORO1C/CDC42BPB/RAB7A/RAB11FIP1/RABGAP1/CDKL5/MICALL1/EVI5L/MYO9B/INF2/DVL3/MYCBP2/RIN3/RAB11FIP4/TBC1D13/RANGAP1/ARHGEF2/RGP1/PAK1/OPTN/FGD4/WAS/XPO7/FLNA/TBC1D20/ARHGAP1/TMEM127/DOCK2/CYFIP2/RASSF5/SYTL4/RILP/AKAP13/IQGAP1/EHD1/GAPVD1/PAK2/YBX3/DIAPH1
BP	GO:0061564	axon development	255	-0.43	-1.69	4.41E-06	5.83E-05	3.61E-05	2622	tags=35%, list=22%, signal=28%	EXT1/ULK1/RAB8A/DIP2B/PLXNC1/JUN/DISC1/PLXNA4/ADNP/PTPN11/NOTCH1/EFNB1/NCAM1/LYPLA2/TSPO/MAP3K13/LMTK2/EPHA4/CDK5/DDR1/FYN/FEZ1/SLITRK5/CSF1R/CERS2/MACF1/PARD6B/ARHGAP35/APLP2/EDN1/KIAA0319/TIAM1/DIXDC1/MAP1S/B4GALT5/NDEL1/KLF4/SEMA4D/GSK3B/BAIAP2/CYFIP1/HSP90AB1/SVBP/BCL11B/IST1/PLXNB2/CDKL5/RAC1/MBP/DPYSL2/PLXND1/TRIO/KIF13B/STK24/TRIM32/SIPA1L1/MARK2/EIF4G2/STK25/MYCBP2/PLXNA1/TAOK2/ACTB/CTNNA1/SEMA4A/NRP1/MAP1A/USP9X/ABL1/PAK1/PARD3/SYNGAP1/XK/VCL/NOTCH2/DNM2/FLNA/FKBP1B/RAC2/BSG/ECE1/NIBAN2/STK11/GRN/VASP/RHOG/PAK2/CTTN/KEL/SCN1B

BP	GO:0043085	positive regulation of catalytic activity	794	-0.34	-1.45	4.61E-06	6.07E-05	3.77E-05	2457	tags=28%, list=21%, signal=23%	TBC1D8/ADNP/MOB3B/BAK1/NCF1/CASP9/DOCK8/EGR1/PPP1R15A/AGAP2/EZH2/FLCN/DLG4/DCP1A/PTPA/PNKP/MAP3K13/DIABLO/LMTK2/SERINC5/IL6R/EPHA4/HLA-DRB1/MAGI2/CDK5/PRKD2/CD81/DDR1/FYN/CASP2/ARAP1/CRKL/IL1B/DOCK10/CSF1R/MAPRE2/MADD/ROCK2/DCUN1D3/VAV2/TLR6/CCNY/RALBP1/PPARGC1B/MOB1B/SRGAP2/PIN1/NR1H2/ITGAM/PRRC1/XRCC6/PKP4/RCC2/RGS10/GCN1/ARHGAP35/SMAD3/SCARB1/PTPRC/EDN1/TIAM1/PIK3CB/CCS/ADAP2/PHACTR4/MAGED1/FZD5/DGKZ/LIMS1/CD74/RAP1GAP/NDEL1/BBC3/TPX2/RGS16/KLF4/SEMA4D/MAP3K11/PSME3/BCAP31/ACVR2B/GSK3B/TBC1D17/NEDD9/NR1H3/GSK3A/PYCARD/DAP/HSP90AB1/TGFB1/MOB3A/TBC1D22A/RASGRP3/ASAP2/LILRA5/TAOK1/PREX1/ASAP1/PRR7/BEX3/SIPA1L3/DAPK1/STX4/SLC27A1/PLEK/CORO1C/TRAF6/MAP3K5/HIP1/VRK3/HIP1R/LYN/BCAS3/SASH1/RABGAP1/CDKL5/PINK1/MBP/PTK2B/GNA15/IGF2/HSPA1A/MAP2K7/EVI5L/BAD/SIPA1L1/MARK2/NCSTN/BCL2L11/SRC/PML/CCND3/GREM1/IQSEC1/YWHAB/SFRP2/GNAI2/DVL3/FIS1/VAV1/LAPTM5/TAOK2/DAXX/PSAP/GMIP/ARHGAP45/TBC1D13/PIH1D1/STAT3/MAP2K2/RANGAP1/VLDLR/BCR/BTRC/PDGFC/CAP1/RGP1/SYK/VCP/ABL1/EGLN3/PAK1/STIM1/LTF/RAPGEF1/DTX3L/DCP1B/PDE5A/RHOA/CALM3/ARHGAP6/TBC1D22B/LGALS9/UBE2S/TBC1D20/RSU1/PSME4/STK11/SDC4/CYFIP2/GNA12/GSN/BCL2L13/FZR1/TERF2/CSK/THBS1/AKT2/UBE2L3/PTPN1/AKAP13/PIK3CG/IQGAP1/RGS6/GRN/ATP2B4/SNCA/MMD/CAMK1/NEURL1/RHOG/TBC1D5/P2RX1/MAPK3/TBC1D25/CDKN1A/TBC1D1/AKIRIN2/CTSD/PAK2/STRADB/TAL1/ITGB3/PDGFA/CLU/SH3BP1/MAP2K3/TGM2/WNK1
BP	GO:0008360	regulation of cell shape	111	-0.54	-1.93	4.68E-06	6.14E-05	3.81E-05	2165	tags=41%, list=18%, signal=34%	FGD6/FYN/FMNL1/ARAP1/PLEKHO1/SH3KBP1/CSF1R/PRAG1/FES/FMNL3/ARHGAP35/RHOBTB1/RHOF/SEMA4D/BAIAP2/CYFIP1/EZR/DAPK3/CDC42EP4/WDR1/ALDOA/TPM1/PLXNB2/RAC1/PTK2B/PLXND1/MARK2/PLXNA1/TAOK2/SEMA4A/FGD4/ARHGEF18/RHOA/RAC2/GNA12/EPB42/EPB41/SPTA1/MSN/PEAK3/RHOG/PARVB/WASF3/MYH9/DMTN/DIAPH1
MF	GO:0005543	phospholipid binding	321	-0.41	-1.65	4.73E-06	6.19E-05	3.84E-05	2827	tags=37%, list=24%, signal=29%	OSBPL1A/OBSCN/PLA2G4C/GAB2/SNX8/SDCBP/ATP13A2/PLD1/CPNE2/KIF16B/PLD2/DAPP1/PTAFR/SBF2/OSBPL5/AKT1/AIDA/GRAMD1B/SYT11/PSD4/NCF1/ATG2B/SNX33/AGAP1/CPNE3/SNX27/RPH3A/PFN1/ARAP1/CPNE1/SNX17/ITPR2/SH3PXD2A/ARHGAP26/ZCCHC2/PCTP/PLA2G15/SVIL/FES/ZCCHC14/TPP1/PACSIN1/NUMA1/SHC1/LPAR4/TOM1/ARHGAP35/SCARB1/HGS/ADAP2/STOML2/ANKFY1/CD300LF/SNX12/KCNJ2/WASHC2A/TECPR1/TEC/PREX1/APOM/ASAP1/DENND1C/ATG2A/MYO1E/SNX10/DENND1A/ESYT1/ALOX15/HIP1/OSBPL10/PITPNC1/HIP1R/BCAS3/GPAA1/SNX15/BSCL2/NME4/MICALL1/DYSF/CGAS/SLC9A1/BAD/PITPNM2/PITPNA/BBS5/CPNE5/RUBCN/SNX3/ARHGAP32/FLII/PLEKHF1/PSAP/MYOF/VDAC1/SNX11/CPTP/ZFYVE1/PARD3/ANXA6/PLEK2/WIPI2/ANXA11/EPN1/LDLRAP1/CAVIN2/SNX29/GSN/SYTL4/THBS1/SPTBN1/EPB41/IQGAP1/SNCA/NRGN/SMPD3/SCIN/WDR45/TLN1
BP	GO:0030833	regulation of actin filament polymerization	106	-0.54	-1.91	4.90E-06	6.38E-05	3.96E-05	1990	tags=41%, list=17%, signal=34%	ADD2/EPS8/CORO1A/TMOD3/SPTAN1/TRIOBP/ARHGAP18/PFN1/SSH1/MYADM/SVIL/SSH2/FMN1/ARPC5/GRB2/BAIAP2/PYCARD/CYFIP1/CDC42EP4/ALOX15/HIP1R/PTK2B/HCLS1/ARPC1B/FLII/SSH3/RHOA/ARPC4/CYFIP2/GSN/SPTBN1/MTPN/SPTA1/COTL1/SPTB/VASP/TMOD1/SCIN/CAPZB/CTTN/ESAM/DMTN/ADD1

BP	GO:0090100	positive regulation of transmembrane receptor protein serine/threonine kinase signaling pathway	64	-0.62	-1.99	4.99E-06	6.46E-05	4.01E-05	1842	tags=41%, list=16%, signal=34%	NUMA1/ACVR1B/KIAA0319/ENG/RBPJ/TGFB1I1/ACVR2B/HSP90AB1/TGFB1/MEN1/TWSG1/PARP1/ILK/C20orf27/CREBBP/CDKN1C/LRG1/BMP6/NOTCH2/NUP93/HIPK2/STK11/THBS1/FKBP8/UBE2O/DAB2
BP	GO:0043087	regulation of GTPase activity	255	-0.43	-1.68	4.99E-06	6.46E-05	4.01E-05	2584	tags=34%, list=22%, signal=27%	RASAL3/SBF2/ARL2/CBLB/PLXNC1/RRP1B/PLXNA4/TBC1D8/DOCK8/EZH2/FLCN/EPHA4/FGD6/ARAP1/CRKL/DOCK10/MAPRE2/VAV2/RALBP1/SRGAP2/PIN1/PKP4/RCC2/RGS10/ARHGAP35/GPSM1/TIAM1/ADAP2/RIPOR2/LIMS1/POLDIP2/RAP1GAP/NDEL1/RGS16/SEMA4D/GSK3B/TBC1D17/NEDD9/PYCARD/TBC1D22A/RASGRP3/ASAP2/PREX1/ASAP1/AMOT/SIPA1L3/CORO1C/BCAS3/PLXNB2/RABGAP1/CDKL5/PTK2B/PLXND1/SBF1/EVI5L/SIPA1L1/RASA3/IQSEC1/DVL3/VAV1/PLXNA1/GMIP/ARHGAP45/TBC1D13/RANGAP1/BCR/RGP1/SYNGAP1/FGD4/RAPGEF1/ARRB2/ARHGAP6/TBC1D22B/TBC1D20/RSU1/AKT2/IQGAP1/RGS6/RHOG/TBC1D5/TBC1D25/TBC1D1/GAPVD1/SH3BP1/TGM2/WNK1
BP	GO:0030219	megakaryocyte differentiation	43	-0.68	-2.05	5.17E-06	6.67E-05	4.13E-05	661	tags=30%, list=6%, signal=29%	PITHD1/MTURN/MEIS1/PIP4K2A/GATA1/PF4/SCIN/TESC/MPIG6B/FAXDC2/MED1/TAL1/WASF2
BP	GO:0051146	striated muscle cell differentiation	147	-0.49	-1.80	5.20E-06	6.67E-05	4.13E-05	1472	tags=44%, list=13%, signal=39%	ATP11A/SLC8A1/MYH3/OBSCN/MSX1/FRS2/TTN/MYOM2/MAPK14/AKT1/TMOD3/NOTCH1/HDAC4/HDAC5/EZH2/G6PD/CD81/PLEKHO1/NLN/BCL9/EDN1/RIPOR2/RBPJ/GSK3A/TGFB1/ACTG1/DYRK1B/CCN3/KDM6B/SKI/SELENON/WDR1/TPM1/YY1/RARA/IGF2/CD9/PLD3/SLC9A1/TRIM32/SOX6/GREM1/CALR/PPARA/FLII/PPP3CA/MYOF/CSRP1/ARRB2/XK/LMNA/RBM38/NIBAN2/SORT1/PLEC/YBX1/AKAP13/MTPN/NFATC2/TMOD1/EHD1/GPX1/MYL9/MYH9/KEL
BP	GO:0006898	receptor-mediated endocytosis	172	-0.48	-1.80	5.19E-06	6.67E-05	4.13E-05	1871	tags=40%, list=16%, signal=34%	SDCBP/DNAJC13/AP2A1/SIGLEC1/SORL1/BMP2K/AP2A2/AP4E1/CLN3/BICD1/DLG4/M6PR/MAGI2/CD81/SNX17/TNK2/ITGAM/PDLIM7/CXCL16/ARRB1/SYNJ1/PI4KB/NEU3/PIK3CB/CD14/GRK2/CANX/AP2B1/CXCR2/DGKD/DTNBP1/GRB2/REPS1/CXCR1/CD63/CLTC/EZR/RAB31/HIP1/AP2S1/ITSN1/HIP1R/RAC1/CD9/MICALL1/ARF1/GREM1/CEACAM1/RIN3/VLDLR/CAP1/SYK/ARRB2/HNRNPK/CLTB/DNM2/LDLRAP1/DNM3/GAK/IGF2R/LRP1/SNCA/TBC1D5/CBL/CTTN/AP2M1/ITGB3/CLU/DAB2
BP	GO:1903844	regulation of cellular response to transforming growth factor beta stimulus	77	-0.59	-1.97	5.26E-06	6.72E-05	4.17E-05	2027	tags=39%, list=17%, signal=32%	HSPA5/ZBTB7A/PIN1/NRROS/SMAD3/ENG/TGFB1I1/BCL9L/HSP90AB1/TGFB1/MEN1/SKI/C20orf27/HSPA1A/CHST11/CREBBP/CDKN1C/PPARA/FAM89B/LRG1/FKBP1A/DNM2/HIPK2/STK11/THBS1/GLG1/ZNF703/PPM1A/LTBP1/DAB2

BP	GO:0048858	cell projection morphogenesis	351	-0.40	-1.62	5.28E-06	6.73E-05	4.17E-05	2622	tags=33%, list=22%, signal=27%	EXT1/CORO1B/CAPRIN1/ULK1/RAB8A/MINK1/DIP2B/TAOK3/MUL1/PLXNC1/DISC1/PLXNA4/ADNP/PTPN11/NOTCH1/EFNB1/DLG4/NCAM1/LYPLA2/MAP3K13/LMTK2/EPHA4/CDK5/MOV10/DDR1/ABI3/FYN/CPNE1/FEZ1/PLEKHO1/SLITRK5/DOCK10/CSF1R/MACF1/SRGAP2/PACSIN1/PARD6B/CUL7/ARHGAP35/WEE1/APLP2/EDN1/KIAA0319/TIAM1/RERE/DIXDC1/MAP1S/B4GALT5/NDEL1/SEMA4D/DTNBP1/GSK3B/BAIAP2/CYFIP1/HSP90AB1/TAOK1/BCL11B/CORO1C/MFSD2A/BCL11A/IST1/NEDD4L/PLXNB2/CDKL5/RAC1/MBP/DPYSL2/PLXND1/TRIO/KIF13B/SIPA1L1/MARK2/FBXO31/SRC/EIF4G2/GAS7/CPNE5/STK25/DVL3/MYCBP2/PLXNA1/PPP3CA/TAOK2/ACTB/SEMA4A/NRP1/MAP1A/VLDLR/YWHAH/USP9X/ABL1/PAK1/PARD3/SYNGAP1/XK/RHOA/VCL/NOTCH2/DNM2/RAC2/BSG/ECE1/NIBAN2/STK11/UBB/CYFIP2/DNM3/CFL1/IQGAP1/CUX1/VASP/RHOG/PAK2/CTTN/WASF2/KEL/SCN1B
CC	GO:0005773	vacuole	658	-0.35	-1.50	5.37E-06	6.82E-05	4.23E-05	2730	tags=32%, list=23%, signal=26%	FUCA2/SDCBP/TSC2/NCF2/SPPL2B/DNAJC13/FAF2/FNBP1/ATP13A2/PLD1/AP2A1/RMC1/TRIM21/AP5S1/ULK1/BPI/GNAQ/SBF2/GNB2/STARD3/ABCA3/UBQLN4/AP2A2/DPP7/ACLY/PCYOX1/ACP2/SYT11/NSF/CPQ/TRPM2/NCF1/CLN3/LAMP2/TMBIM1/CYBRD1/FLCN/CPNE3/M6PR/HGSNAT/AP5B1/ABCB9/HLA-DRB1/PIP4K2C/ABCD1/PI4K2A/IMPDH1/MLC1/WDR24/HYAL2/HLA-DQA2/CMTM6/MARCHF3/CPNE1/IL1B/VPS39/P2RX4/OSBPL7/MFSD12/GNA11/RNASE3/TCN2/NCOA4/ITM2C/PLA2G15/CTSA/TPP1/ARRB1/MAP1LC3B/TOM1/LRBA/SCARB1/ADA2/HGS/RRAGD/NEU3/TFEB/SLC35F6/CHMP6/CD74/ANKFY1/BBC3/VPS41/WDR81/CTSF/CHMP4B/AP2B1/CXCR2/SMPD1/TRAPPC1/HLA-DQA1/HYAL3/TECPR1/STOM/SDC3/TBC1D17/PYCARD/HSP90AB1/CD63/KLHL22/CLTC/SYNGR1/SLC44A2/DNAJC5/STX4/IL4I1/CHID1/GLB1/AP1M1/NAGA/IST1/UNC93B1/UBA52/AP2S1/LNPEP/LYN/GLMP/VTI1B/RAB7A/EPG5/PLEKHM2/IFITM3/PRF1/PLD3/GNB1/SLC22A17/NCSTN/DYNC1H1/SRC/CHMP1A/CALR/RUBCN/YWHAB/ATP6V1A/LRRC8A/HLA-DRB5/VTI1A/PLEKHF1/LAPTM5/PSAP/ARHGAP45/LITAF/IFITM2/UBA1/VLDLR/GABARAP/CAP1/VCP/ATP6V0D1/ZFYVE1/RRAGC/RAB14/OPTN/FNIP2/PYGB/ANXA6/DTX3L/ATP6V0C/LAMTOR1/TUBB4B/CLCN5/GAA/ANXA11/SLC7A5/SIDT2/AP1B1/SLC3A2/GPR137/ARL8A/ZNRF1/RAB3D/ECE1/PA2G4/ATP6V0A1/MCOLN1/CYB5R3/CDIP1/FTH1/SDC4/SORT1/SQSTM1/LRP1/RILP/TRIP10/GNS/MARCHF2/GRN/SNCA/MMD/PRDX6/LAMP1/CAPN1/PIP4K2A/TBC1D5/ELAPOR1/MARCHF8/TBC1D25/CTSD/TUBB/FTL/SLC48A1/CTSB/HPS1/SLC15A3/NPRL3/UBXN6/TOLLIP/AP2M1/HTT/DAB2
BP	GO:0017015	regulation of transforming growth factor beta receptor signaling pathway	76	-0.60	-1.99	5.46E-06	6.87E-05	4.26E-05	2027	tags=39%, list=17%, signal=33%	HSPA5/ZBTB7A/PIN1/NRROS/SMAD3/ENG/TGFB1I1/BCL9L/HSP90AB1/TGFB1/MEN1/SKI/C20orf27/HSPA1A/CHST11/CREBBP/CDKN1C/PPARA/FAM89B/LRG1/FKBP1A/DNM2/HIPK2/STK11/THBS1/GLG1/ZNF703/PPM1A/LTBP1/DAB2
BP	GO:0045766	positive regulation of angiogenesis	97	-0.56	-1.94	5.45E-06	6.87E-05	4.26E-05	1781	tags=30%, list=15%, signal=26%	RUNX1/ENG/KLF4/ETS1/SPHK1/JMJD8/AQP1/SASH1/PTK2B/MTDH/RRAS/GREM1/PIK3CD/SFRP2/PKM/STAT3/NRP1/JAK1/LRG1/ABL1/STIM1/HIPK1/HSPB1/HIPK2/PRKCB/THBS1/SP1/GRN/TBXA2R
BP	GO:1904018	positive regulation of vasculature development	97	-0.56	-1.94	5.45E-06	6.87E-05	4.26E-05	1781	tags=30%, list=15%, signal=26%	RUNX1/ENG/KLF4/ETS1/SPHK1/JMJD8/AQP1/SASH1/PTK2B/MTDH/RRAS/GREM1/PIK3CD/SFRP2/PKM/STAT3/NRP1/JAK1/LRG1/ABL1/STIM1/HIPK1/HSPB1/HIPK2/PRKCB/THBS1/SP1/GRN/TBXA2R

BP	GO:0071345	cellular response to cytokine stimulus	552	-0.37	-1.53	5.50E-06	6.90E-05	4.28E-05	2298	tags=28%, list=20%, signal=23%	USP27X/CCR1/OAS2/SMPD4/OAS3/IKBKE/IL6R/HES1/IRF1/HYAL2/ICAM1/CRKL/CPNE1/IL1B/SPI1/GBP4/RIPK1/CSF1R/OTUD4/PTPN6/SELPLG/PPBP/USP10/HSPA5/IL5RA/SYNCRIP/GSTP1/PDE2A/ZFP36L2/CDK9/NR1H2/SPATA2/CDC37/GBP6/RAB20/KEAP1/PTPRC/EDN1/RIPOR2/LIMS1/CD74/CD300LF/IL2RG/PDE12/KLF4/CXCR2/SIGIRR/VIM/HYAL3/GSK3B/IL2RB/PRKACA/NR1H3/GSK3A/PYCARD/CACTIN/HSP90AB1/ACTG1/CXCR1/LILRA5/TNFRSF1B/SYNGR1/IL1RN/SPHK1/SNX10/DAPK3/DAPK1/STX4/SLC27A1/ALAD/ATIC/CDC42EP4/TRAF6/OXSR1/MAP3K5/ALOX15/ZYX/YY1/MAVS/ILK/IL17RA/ADAR/IFITM3/PTK2B/MT2A/HSPA1A/PIAS4/STIP1/KLF2/IFI27/FLNB/TTLL12/BAD/TRIM32/HCLS1/SRC/PML/ADIPOR2/EIF5A/CEACAM1/PTP4A3/LAPTM5/IFITM2/STAT3/ARHGEF2/UPF1/JAK1/SYK/JAK3/XIAP/AGPAT1/RELA/NFKB1/JARID2/MPL/TLE4/RHOA/LDLRAP1/HIPK1/PTPRJ/LGALS9/WAS/IFIT1B/INPP5K/STAT5A/FASN/CDIP1/PRPF8/GSN/ADIPOR1/PF4V1/PLVAP/THBS1/YBX1/PTPN1/RPS6KA4/SHARPIN/CXCL5/H2BC11/MAPK3/ACKR1/PF4/CBL/SMPD3/ACTN4/KHSRP/YBX3/FOXO3/CDC34/TOLLIP/MED1/HDGF/WNK1
BP	GO:0001952	regulation of cell-matrix adhesion	84	-0.59	-1.99	5.55E-06	6.94E-05	4.30E-05	2019	tags=39%, list=17%, signal=33%	ROCK2/MACF1/RCC2/SMAD3/FMN1/PIK3CB/LIMS1/POLDIP2/GSK3B/ACTG1/CDK6/DAPK3/CORO1C/BCAS3/RAC1/PTK2B/SLC9A1/RRAS/SRC/GREM1/NRP1/ABL1/CLASP1/PLEKHA2/RHOA/VCL/ARHGAP6/PTPRJ/NF2/SDC4/GFUS/THBS1/DMTN
CC	GO:0099513	polymeric cytoskeletal fiber	422	-0.39	-1.60	5.62E-06	7.00E-05	4.34E-05	2296	tags=32%, list=20%, signal=27%	ARHGAP18/SYNM/TUBGCP4/TUBGCP6/HLA-DRB1/MAP4/PCNT/TUBB2A/CDK5/NDE1/ASPM/FYN/NCKAP5L/CDK5RAP2/MAP7/FEZ1/TTLL1/TBCD/FBF1/MTA1/MAPRE2/TUBA1C/NAV1/MACF1/LCP1/TUBG1/TUBG2/DIAPH2/SVIL/EIF6/NDRG1/LZTS2/PDLIM7/RCSD1/NUMA1/RCC2/MAP1LC3B/FMN1/SYNJ1/CDK2AP2/KEAP1/SHROOM1/CHMP6/MAP1S/EPPK1/NDEL1/CCT3/TPX2/EIF3A/CHMP4B/MAP3K11/VIM/SLAIN2/TPM3/KIF1C/BAIAP2/AKNA/ACTG1/FLACC1/KLHL22/PSTPIP2/CLTC/SPECC1L/EML2/RPGRIP1L/EML3/TUBA4A/AMOT/EZR/KIF3B/NEK6/TPM1/KATNB1/BCAS3/RAC1/JAM3/DPYSL2/KIF13B/MARK2/HCLS1/MYO9B/DYNC1H1/SRC/CHMP1A/HOOK3/GAS7/AK1/KIF1B/CAMSAP1/ACTB/NRP1/MAP2K2/SLC1A4/MAP1A/ARHGEF2/GABARAP/TUBA8/XIAP/PAK1/KIFC3/CLASP1/STIM1/TUBB4B/GOLGA2/TPGS2/KLC3/CALM3/DNM2/LDLRAP1/ARHGAP6/CKAP5/WAS/RAB3D/RAC2/LMNA/SPECC1/KRT1/DNM3/RASSF5/DCTN1/PLEC/WIPF1/DYNLL2/IQGAP1/COTL1/DYNLRB1/TMOD1/ESPN/TUBB/ACTN1/PDLIM1/TUBB1/CTTN/GAS2L1/DMTN/DIAPH1
CC	GO:0001725	stress fiber	49	-0.66	-2.05	5.85E-06	7.25E-05	4.50E-05	1992	tags=51%, list=17%, signal=43%	LCP1/PDLIM7/LIMA1/SEPTIN11/TPM3/TRIP6/SIPA1L3/TLNRD1/TPM1/ZYX/DCTN4/ILK/FLNB/FHL3/CNN2/PXN/ABLIM3/ACTA2/MYLK/ACTN4/ACTN1/PDLIM1/MYL9/MYH9/GAS2L1
CC	GO:0097517	contractile actin filament bundle	49	-0.66	-2.05	5.85E-06	7.25E-05	4.50E-05	1992	tags=51%, list=17%, signal=43%	LCP1/PDLIM7/LIMA1/SEPTIN11/TPM3/TRIP6/SIPA1L3/TLNRD1/TPM1/ZYX/DCTN4/ILK/FLNB/FHL3/CNN2/PXN/ABLIM3/ACTA2/MYLK/ACTN4/ACTN1/PDLIM1/MYL9/MYH9/GAS2L1
BP	GO:0034110	regulation of homotypic cell-cell adhesion	27	-0.76	-2.10	6.34E-06	7.83E-05	4.86E-05	1180	tags=37%, list=10%, signal=33%	LYN/CD9/CEACAM1/JAK1/SYK/GP6/ALOX12/UBASH3B/ZNF703/DMTN
BP	GO:0045637	regulation of myeloid cell differentiation	146	-0.50	-1.84	6.64E-06	8.15E-05	5.06E-05	2121	tags=37%, list=18%, signal=31%	MITF/SPI1/RIPK1/ZBTB16/PPARGC1B/EIF6/TRIB1/FES/TFE3/ACVR1B/RUNX1/INPP5D/CD74/FOXP1/TOB2/TGFB1/CTNNBIP1/CDK6/ACIN1/ETS1/CNOT4/TRAF6/LYN/KLF13/PTK2B/RARA/HSPA1A/HCLS1/KAT7/ARNT/CEACAM1/STAT3/MAFB/FOS/PITHD1/LTF/MPL/NOTCH2/GPR137/MTURN/MEIS1/FAM210B/UBASH3B/PAF1/PRKDC/GATA1/PF4/SCIN/FOXO3/TESC/FAXDC2/MED1/TAL1/CUL4A

BP	GO:0098739	import across plasma membrane	96	-0.55	-1.90	7.11E-06	8.70E-05	5.39E-05	2551	tags=39%, list=22%, signal=30%	AKT1/SLC46A1/KCNH2/TRPM2/ATP1A1/SLC24A4/FYN/ATP1B1/SLC9A7/SLC43A2/SLC7A1/SLC19A1/KCNJ2/TSPO2/SLC27A1/ARL6IP1/SLC5A6/SLC9A1/SLC39A4/LRRC8A/SLC7A8/SLC1A5/ATP1B2/SLC1A4/SLC6A20/SLC2A1/SLC7A5/SLC3A2/SLC6A9/THBS1/AKT2/SLC43A1/PER2/ATP2B4/P2RX1/SLC15A3/WNK1
BP	GO:1902905	positive regulation of supramolecular fiber organization	130	-0.52	-1.88	7.53E-06	9.19E-05	5.70E-05	2222	tags=35%, list=19%, signal=28%	PFN1/MECP2/CDK5RAP2/ROCK2/FES/NUMA1/SMAD3/FMN1/EDN1/GRB2/SLAIN2/BAIAP2/SORBS3/COLGALT1/PYCARD/CYFIP1/GIT1/CDC42EP4/PLEK/WDR1/TPM1/KATNB1/ALOX15/RAC1/PTK2B/HSPA1A/NRP1/PXN/ABL1/PAK1/CLASP1/RHOA/CKAP5/WAS/NF2/SDC4/DCTN1/GSN/VASP/WASF3/SCIN/GPX1/CTTN/WASF2/CLU
BP	GO:0018212	peptidyl-tyrosine modification	232	-0.44	-1.72	7.78E-06	9.47E-05	5.87E-05	2915	tags=36%, list=25%, signal=27%	HBEGF/BAZ1B/ITGB2/ABI2/FLT1/TESK1/EPHB1/TTN/EIF2AK2/SH2B3/PECAM1/FGR/CBLB/ADNP/PTPN11/NCF1/CD3E/DLG4/TSG101/IL6R/EPHA4/HES1/SH3BP5L/HYAL2/CD81/DDR1/FYN/CSF1R/PTPN6/TNK2/TESK2/FES/SHC1/WEE1/PTPRC/CHMP6/CD74/SEMA4D/NEDD9/TGFB1/LILRA5/TEC/DYRK1B/MVP/EHD4/LYN/PTK2B/IGF2/MAP2K7/HCLS1/SRC/GREM1/SFRP2/LRRK1/NRP1/MAP2K2/ARHGEF2/BCR/PDGFC/JAK1/SYK/JAK3/ABL1/BMP6/ARRB2/SCYL1/TPST2/HIPK1/PTPRJ/HIPK2/NF2/CSK/PPP2R5B/PTPN1/NEURL1/MAPK3/GATA1/CBL/PAK2/ITGB3/PDGFA/MAP2K3/DMTN
BP	GO:0019221	cytokine-mediated signaling pathway	314	-0.41	-1.63	8.32E-06	0.00010007	6.20E-05	2445	tags=30%, list=21%, signal=24%	PTPN11/NOTCH1/EGR1/YTHDF3/CNOT9/USP27X/CCR1/OAS2/OAS3/IKBKE/IL6R/IRF1/CPNE1/IL1B/SPI1/RIPK1/CSF1R/OTUD4/PTPN6/PPBP/IL5RA/GSTP1/NR1H2/SPATA2/CDC37/PTPRC/EDN1/LIMS1/CD74/CD300LF/IL2RG/CXCR2/SIGIRR/IL2RB/PRKACA/NR1H3/PYCARD/CACTIN/HSP90AB1/CXCR1/LILRA5/TNFRSF1B/IL1RN/SPHK1/SLC27A1/TRAF6/OXSR1/MAVS/ILK/IL17RA/ADAR/IFITM3/PTK2B/HSPA1A/PIAS4/IFI27/TTLL12/BAD/TRIM32/SRC/ADIPOR2/EIF5A/CEACAM1/LAPTM5/IFITM2/STAT3/JAK1/SYK/JAK3/XIAP/AGPAT1/RELA/MPL/HIPK1/PTPRJ/STAT5A/CDIP1/ADIPOR1/PF4V1/PLVAP/PTPN1/RPS6KA4/SHARPIN/CXCL5/H2BC11/MAPK3/ACKR1/PF4/CBL/ACTN4/FOXO3/TOLLIP/MED1/WNK1
BP	GO:0048589	developmental growth	355	-0.40	-1.60	8.36E-06	0.00010011	6.21E-05	2639	tags=32%, list=23%, signal=25%	BNC2/EXT1/ZFY/ULK1/MAPK14/AKT1/DIP2B/MUL1/PIK3CA/CPQ/DISC1/PLXNA4/ADNP/PTPN11/NOTCH1/ACACB/RAI1/EZH2/SGPL1/MAP3K13/G6PD/MAGI2/CDK5/ASPM/CD81/DDR1/ARID2/STK40/CPNE1/MACF1/BASP1/LZTS2/BCL9/SMAD3/FMN1/RUNX1/EDN1/KIAA0319/RBPJ/FTO/EPPK1/NDEL1/SEMA4D/EHMT2/ACVR2B/GSK3B/GSK3A/CYFIP1/HSP90AB1/TEC/CCN3/VGLL4/EZR/GNAS/WDTC1/MFSD2A/BCL11A/HDGFL2/IST1/YY1/NEDD4L/PTPN12/CDKL5/RARA/DPYSL2/IGF2/SP2/CHST11/CD9/KLF2/BCL2L11/TBL1XR1/SRC/CDKN1C/EIF4G2/CPNE5/SFRP2/PPARA/PLXNA1/PPP3CA/TAF8/PSAP/ZMIZ1/TRIM28/STAT3/SEMA4A/NRP1/USP9X/ABL1/ARIH2/ANXA6/JARID2/VCL/NOTCH2/DNM2/STAT5A/STK11/CYFIP2/PLEC/AKAP13/MTPN/IQGAP1/FKBP8/HLX/CDKN1A/NINJ2/SMPD3/GPX1/YBX3/FOXO3/MED1/CTTN/PIM1
BP	GO:1902532	negative regulation of intracellular signal transduction	374	-0.39	-1.60	8.55E-06	0.00010209	6.33E-05	2584	tags=30%, list=22%, signal=24%	RASAL3/MAPK14/SORL1/AKT1/AIDA/TAOK3/MUL1/LZTR1/MAPK7/DUSP8/NR1D1/CD22/KCTD10/CDKN2D/FLCN/FOXO1/TNFAIP8L1/SLC24A4/HYOU1/CHP1/ZNF385A/EPHA4/MAGI2/SPRED1/HYAL2/CPNE1/IL1B/SPI1/RIPK1/PTPN6/USP10/MYADM/GSTP1/NDRG2/PDE2A/CCAR2/PIN1/ARHGAP35/PTPRC/PIK3CB/RIPOR2/CREB3/CD74/MMP9/OTUD7B/EIF3A/SMPD1/DGKD/MARK3/GSK3B/NLRX1/GSK3A/PYCARD/CACTIN/HDAC3/CCN3/VGLL4/EZR/MEN1/PLEK/UBR2/SESN2/VRK3/LYN/PINK1/HSPA1A/TRIM32/SRC/RASA3/SFRP2/PPARA/LITAF/ARHGEF2/PHLPP1/ABL1/SYNGAP1/OPTN/FBP1/RELA/ARRB2/SESN3/RHOA/PTPRJ/INPP5K/TLE1/HSPB1/NF2/TMEM127/BCL2L1/STK11/RIOK3/ADIPOR1/CSK/THBS1/PTPN1/MFN2/SEC14L1/ATP2B4/PPP1R10/ENO1/CASTOR2/GRINA/TNIP1/GPX1/YBX3/NPRL3/CDC34/PPM1A/NPLOC4/CLU/SH3BP1/MAP2K3/DAB2

BP	GO:0032990	cell part morphogenesis	369	-0.39	-1.59	8.58E-06	0.00010213	6.33E-05	2622	tags=33%, list=22%, signal=27%	EXT1/CORO1B/CAPRIN1/SUPV3L1/ULK1/RAB8A/MINK1/DIP2B/TAOK3/MUL1/PLXNC1/DISC1/PLXNA4/ADNP/PTPN11/NOTCH1/EFNB1/DLG4/NCAM1/LYPLA2/MAP3K13/LMTK2/EPHA4/CDK5/MOV10/DDR1/ABI3/FYN/CPNE1/FEZ1/PLEKHO1/SLITRK5/DOCK10/CSF1R/MACF1/SRGAP2/PACSIN1/PARD6B/CUL7/ARHGAP35/WEE1/APLP2/EDN1/KIAA0319/TIAM1/RERE/DIXDC1/MAP1S/B4GALT5/POLDIP2/NDEL1/SEMA4D/DTNBP1/GSK3B/BAIAP2/CYFIP1/HSP90AB1/TAOK1/BCL11B/CORO1C/MFSD2A/BCL11A/IST1/NEDD4L/PLXNB2/CDKL5/RAC1/MBP/DPYSL2/PLXND1/TRIO/KIF13B/SIPA1L1/MARK2/FBXO31/SRC/EIF4G2/GAS7/CPNE5/STK25/DVL3/MYCBP2/FIS1/PLXNA1/PPP3CA/TAOK2/ACTB/SEMA4A/NRP1/MAP1A/VLDLR/YWHAH/USP9X/ABL1/PAK1/PARD3/SYNGAP1/XK/RHOA/VCL/NOTCH2/DNM2/RAC2/BSG/ECE1/NIBAN2/BCL2L1/STK11/UBB/CYFIP2/DNM3/PLEC/CFL1/IQGAP1/CUX1/VASP/RHOG/PAK2/CTTN/WASF2/KEL/SCN1B
BP	GO:0071867	response to monoamine	48	-0.67	-2.05	9.04E-06	0.00010634	6.59E-05	2099	tags=50%, list=18%, signal=41%	HRH2/GNA11/PDE4B/OR10H1/GNAZ/DTNBP1/GSK3B/PRKACA/GSK3A/GNAS/GNA15/SLC9A1/GNB1/NCSTN/ABL1/ARRB2/DNM2/FLNA/HRH4/KLF16/ATP2B4/SNCA/MAPK3/TGM2
BP	GO:0071869	response to catecholamine	48	-0.67	-2.05	9.04E-06	0.00010634	6.59E-05	2099	tags=50%, list=18%, signal=41%	HRH2/GNA11/PDE4B/OR10H1/GNAZ/DTNBP1/GSK3B/PRKACA/GSK3A/GNAS/GNA15/SLC9A1/GNB1/NCSTN/ABL1/ARRB2/DNM2/FLNA/HRH4/KLF16/ATP2B4/SNCA/MAPK3/TGM2
BP	GO:0022414	reproductive process	734	-0.34	-1.45	8.99E-06	0.00010634	6.59E-05	2683	tags=30%, list=23%, signal=25%	PI3/H3-3A/ATP7A/ZNF628/AGFG1/GMCL1/MAGED2/FANCG/NCAPD2/NRIP1/ZFY/PKD1/SMARCA2/MAPK14/SMAD5/PTAFR/RNF38/AKT1/TUBGCP2/NSDHL/AGO4/TIPARP/ADNP/PTPN11/NOTCH1/TRPM2/BAK1/TRPC3/EGR1/YTHDF3/SPIRE1/RAD21/SGPL1/CNOT9/SEPTIN6/KPNA6/CDYL/AAAS/ZSCAN21/INSL3/WASHC5/LHFPL2/ZNF318/TUBGCP4/TOP3A/TUBGCP6/PRDM1/HES1/ATP8B3/BTG1/EME2/SPIN1/ASPM/ACOX1/DDR1/CSDE1/CASP2/CRKL/DEAF1/THRA/SPIRE2/DACH1/IL1B/TTLL1/DHX37/TTC21A/PTPN6/VASH1/HSPA5/GGT1/SPATA5/ZBTB16/PAQR8/TUBG1/TUBG2/DIAPH2/NCOA4/TIMP1/TESK2/BASP1/ENKUR/CHFR/HSD17B4/SPATA2/NUMA1/ALPL/SYNE1/CUL7/BIRC6/MAFF/PTTG1/RHBDD1/ACVR1B/EDN1/TFEB/UBE2Q1/CRTAP/ENO2/FZD5/RBPJ/GTSF1/SLC19A1/TBPL1/MMP9/CCT3/BCAP31/EHMT2/NR6A1/GRB2/TOP2A/PTCHD3/HYAL3/NCOA1/TOB2/SORD/CETN2/PRKACA/SFMBT1/HSP90AB1/ASCL2/SCMH1/ST14/CLIC5/ETV6/SPINT2/MEN1/UCP2/SMC1A/ALDOA/UBR2/SUN2/LNPEP/YY1/EPAS1/PUM1/JAM3/BPTF/PTK2B/RARA/IGF2/DAZAP1/ASF1B/PRSS21/CD9/SBF1/UBAP2L/BAD/DHCR24/DZIP1/BCL2L11/SRC/CDKN1C/ARNT/TAF4/CALR/SFRP2/ZNF296/LRRC8A/SLX4/SEPTIN4/RUVBL1/CDK2/HERC2/PSAP/ZMIZ1/TRIM28/SPOCD1/CTNNA1/USP9X/DPCD/GNPDA1/ALKBH5/FOS/NCAPH/E2F1/PITHD1/SF1/GOLGA3/BMP6/ARRB2/SLC2A1/GOLGA2/PDE5A/KLC3/EPN1/NOTCH2/DNM2/LGALS9/TBC1D20/BSG/PSME4/FOXJ2/KLF1/BCL2L1/STK11/UBB/ABHD2/RPA1/FZR1/IGF2R/PPP1R9B/CCNI/ATP2B4/NEURL1/PRKDC/P2RX1/GATA1/CBL/PTGDR2/CTSB/YBX3/FOXO3/CTDNEP1/MED1/MYH9/PDGFA/OSBP2/NCOR2/ACRBP/MARF1/PBX1/BAG6
BP	GO:0030522	intracellular receptor signaling pathway	190	-0.47	-1.76	9.66E-06	0.00011326	7.02E-05	1955	tags=31%, list=17%, signal=26%	NCOA4/PPARGC1B/ZBTB7A/NR3C1/NR1H2/FAM120B/TRERF1/PDK3/NR6A1/FOXP1/SREBF1/NCOA1/DDX60/ANKRD17/CRY2/NLRX1/NR1H3/SMARCA4/PARP1/TRAF6/ALOX15/PUM1/LBH/RARA/TADA3/HSPA1A/RXRA/KLF2/SRC/PML/ARNT/CALR/PPARA/WBP2/DAXX/ZMIZ1/DHRS3/STAT3/YWHAH/XIAP/PAK1/RELA/RHOA/KANK2/RIOK3/ASXL2/SAFB/SEC14L1/ACTN4/SLC15A3/MED1/ARID1A/NPLOC4/NCOR2/CARM1/PIM1/CNOT1/DAB2

BP	GO:0051056	regulation of small GTPase mediated signal transduction	228	-0.45	-1.72	9.79E-06	0.00011449	7.10E-05	2588	tags=32%, list=22%, signal=25%	EPS8/RASAL3/SWAP70/LZTR1/NOTCH1/PSD4/DOCK8/KCTD10/FLCN/ARHGEF11/ARHGAP18/ARHGAP8/ARAP1/MAPRE2/MADD/VAV2/ARHGAP26/PRAG1/RALBP1/SRGAP2/ARRB1/LPAR4/ARHGAP35/TIAM1/PIK3CB/LRRC59/RIPOR2/RAP1GAP/RHOF/PREX1/AMOT/ARHGAP17/DENND1A/SIPA1L3/ITSN1/RAC1/TRIO/SIPA1L1/MYO9B/SRC/RASA3/IQSEC1/ARHGAP32/VAV1/GMIP/ARHGAP45/NRP1/ARHGEF2/BCR/GABARAP/ABL1/SYNGAP1/FGD4/FBP1/ARHGEF18/CYTH4/ARHGEF12/NOTCH2/DNM2/ARHGAP6/ARHGDIA/ARHGAP31/ARHGAP21/KANK2/ARHGAP1/DOCK2/SQSTM1/AKAP13/PIK3CG/MFN2/CBL/SH3BP1/TGM2
CC	GO:0045202	synapse	715	-0.34	-1.46	9.82E-06	0.00011449	7.10E-05	2253	tags=28%, list=19%, signal=24%	EIF4A3/EPHA4/MAGI2/NETO2/PFN1/MECP2/PI4K2A/CDK5/NDE1/FXR2/ABI3/FYN/ABHD17C/FXYD6/MACO1/CRKL/EXOC4/HRH2/P2RX4/EEF2/SH3KBP1/DOCK10/GNA11/CTNND1/DNAJB1/SNTA1/MPST/PDE2A/SRGAP2/PIN1/MIB1/PKP4/PACSIN1/RGS10/SYNE1/PDE4B/SYNJ1/OR10H1/SLC29A2/PMP22/SNTB1/TIAM1/LRFN1/DIXDC1/DROSHA/NPTXR/ENO2/FZD5/MAP1S/GRK2/NDEL1/CANX/CTBP1/STX6/EIF3A/AP2B1/PPP1R9A/KCNJ2/SEPTIN11/DTNBP1/GSK3B/BAIAP2/PRKACA/GSK3A/RPS6KA1/CYFIP1/ACTG1/GIT1/YWHAG/TPRG1L/SYNGR1/ASAP1/PRR7/DNAJC5/SPHK1/TRAPPC4/DENND1A/STX4/KIF3B/CORO1C/BCL11A/MTHFR/HIP1/UBA52/ITSN1/HIP1R/LYN/VTI1B/RAB7A/PRR12/CDKL5/RAC1/MBP/NPFF/PTK2B/GNA15/ARF1/SH3GL1/EXOC3/SIPA1L1/GNB1/NCSTN/GRIPAP1/SEPTIN1/SRC/DLGAP4/IQSEC1/GNAI2/SLA/KIF1B/SEPTIN4/ARHGAP32/VTI1A/PPP3CA/ATP1B2/ADGRL1/ACTB/RAB6B/PPP1CA/CNIH3/NRP1/SLC1A4/MAP1A/BCR/GABARAP/APBA3/VCP/ATP6V0D1/ABL1/ELAVL1/SYNGAP1/ABLIM3/RELA/RAB27B/C1QA/ARRB2/STAU1/SLC2A1/CLCN5/PLCB3/CLTB/RHOA/CALM3/EIF3B/DNM2/PVALB/SLC3A2/RAB5B/RAB11B/GUCY1A1/ARL8A/ZNRF1/RAB3D/SLC6A9/ELK1/BCL2L1/ROGDI/NAPA/CYFIP2/DNM3/STON2/CLSTN1/GLUL/GAK/DYNLL2/PPP1R9B/PRKCB/SPTBN1/UBE2L3/HRH4/ATP2B4/SHARPIN/SNCA/LRFN3/CAMK1/NEURL1/LAMP1/P2RX1/RNF10/PCBP2/SLC29A1/NRGN/WASF3/CAPN5/PAK2/CAPZB/RNF40/ACTN1/PRKAR2B/CTTN/AP2M1/HTT/MYH9/SHISA7/ITGB3/GUCY1B1/WASF2/CLU/DMTN/ADD1
BP	GO:0018108	peptidyl-tyrosine phosphorylation	229	-0.45	-1.72	9.89E-06	0.00011502	7.13E-05	2915	tags=36%, list=25%, signal=28%	CLK3/HBEGF/BAZ1B/ITGB2/ABI2/FLT1/TESK1/EPHB1/TTN/EIF2AK2/SH2B3/PECAM1/FGR/CBLB/ADNP/PTPN11/NCF1/CD3E/DLG4/TSG101/IL6R/EPHA4/HES1/SH3BP5L/HYAL2/CD81/DDR1/FYN/CSF1R/PTPN6/TNK2/TESK2/FES/SHC1/WEE1/PTPRC/CHMP6/CD74/SEMA4D/NEDD9/TGFB1/LILRA5/TEC/DYRK1B/MVP/EHD4/LYN/PTK2B/IGF2/MAP2K7/HCLS1/SRC/GREM1/SFRP2/LRRK1/NRP1/MAP2K2/ARHGEF2/BCR/PDGFC/JAK1/SYK/JAK3/ABL1/BMP6/ARRB2/SCYL1/HIPK1/PTPRJ/HIPK2/NF2/CSK/PPP2R5B/PTPN1/NEURL1/MAPK3/GATA1/CBL/PAK2/ITGB3/PDGFA/MAP2K3/DMTN
CC	GO:0097447	dendritic tree	336	-0.40	-1.62	1.03E-05	0.0001191	7.38E-05	2567	tags=35%, list=22%, signal=28%	RAB8A/MARK4/MINK1/DIP2B/SYT11/NSF/ADNP/CYBB/NR1D1/NCF1/CD3E/DLG4/RPH3A/ZNF385A/EIF4A3/EPHA4/MAGI2/DMWD/PI4K2A/CDK5/LYNX1/FXR2/ABI3/FYN/ABHD17C/FEZ1/HRH2/YKT6/P2RX4/CNR2/DOCK10/CTNND1/DNAJB1/SRGAP2/TMEM185A/NUMA1/PDE4B/OR10H1/FZD5/GNAZ/TRAK1/MAP1S/RAP1GAP/CANX/PPP1R9A/KCNJ2/MARK3/SEPTIN11/DTNBP1/GSK3B/KIF1C/BAIAP2/PRKACA/GSK3A/CASC3/CYFIP1/HSP90AB1/ASAP1/PRR7/CCN3/TRAPPC4/STX4/KIF3B/HIP1R/CDKL5/RAC1/NPFF/PTK2B/RARA/SIPA1L1/MARK2/GRIPAP1/N4BP3/GNAI2/SLA/KIF1B/SEPTIN4/ARHGAP32/IFT52/PPP3CA/RIN3/TAOK2/AMFR/MAX/PPP1CA/CNIH3/RANGAP1/SLC1A4/MAP1A/BCR/APBA3/MCRS1/TRAK2/ABL1/PAK1/SYNGAP1/ARRB2/STAU1/CKB/RHOA/GLRX5/ELK1/ROGDI/ZDHHC5/DNM3/PLEC/PPP1R9B/HRH4/SHARPIN/LRFN3/NEURL1/NRGN/ACTN1/PRKAR2B/CTTN/HTT/SHISA7/CLU/OSBP2

BP	GO:0006511	ubiquitin-dependent protein catabolic process	552	-0.36	-1.51	1.03E-05	0.0001191	7.38E-05	1917	tags=24%, list=16%, signal=21%	TRIB1/CHFR/CCAR2/MIB1/UBXN7/UBE2J2/ARRB1/CUL7/DERL1/KEAP1/CHMP6/SMARCC1/TAF1/TGFB1I1/CANX/OTUD7B/WDR81/UBE2D4/NSFL1C/VPS37B/USP37/CHMP4B/BCAP31/GSK3B/GSK3A/HSP90AB1/KLHL22/TBX21/CBFA2T3/DET1/EDEM2/KCTD2/CNOT4/UBR2/ZRANB1/UBE2G1/NEDD4L/DDA1/USP31/CDC27/FBXL18/PINK1/PTK2B/HSPA1A/ANKIB1/IFI27/USP12/TRIM32/MARCHF6/TBL1XR1/FBXO31/CHMP1A/UBE2Z/PML/UBE2R2/RNF115/CALR/RBCK1/RMND5A/CDK2/HERC2/LAPTM5/AMFR/UBA1/USP19/TBL1X/PSMD11/MAP1A/BTRC/USP9X/GABARAP/USP34/FBXO6/DDB1/VCP/USP36/ARIH2/KLHL8/UBAP1/N4BP1/FBXW4/PSMC4/RNF213/RAD23A/DTX3L/ARRB2/RNF11/WWP2/GID8/UBE2S/SUFU/MAN1A1/ZNRF1/PSMD2/USP7/HIPK2/VPS28/SIAH2/UBR4/GNA12/USP22/FZR1/HUWE1/PSMF1/SQSTM1/YOD1/UBE2L3/RNF187/ARAF/SHARPIN/ANKRD9/PCBP2/FBXO7/DCAF12/CBL/TRIP12/ASCC2/UBL7/RYBP/ZER1/RNF40/DNAJB2/CDC34/TOLLIP/PTPN23/UBE2H/DCAF11/CUL4A/TRIM58/NPLOC4/CLU/DAB2/BAG6
BP	GO:0051261	protein depolymerization	81	-0.58	-1.96	1.04E-05	0.00011993	7.44E-05	2653	tags=52%, list=23%, signal=40%	ADD2/EPS8/SWAP70/PIK3CA/TMOD3/DSTN/SPTAN1/ATXN7/TRIOBP/NCKAP5L/VPS4B/SVIL/SYNJ1/MAP1S/LIMA1/TPX2/TAOK1/PLEK/WDR1/KATNB1/SH3GL1/FLII/CAMSAP1/MAP1A/ARHGEF2/CLASP1/CKAP5/GSN/CFL1/GAK/PPP1R9B/SPTBN1/MTPN/SPTA1/SPTB/TMOD1/MICAL2/SCIN/CAPZB/SH3BP1/DMTN/ADD1
BP	GO:0051129	negative regulation of cellular component organization	475	-0.37	-1.55	1.04E-05	0.00011993	7.44E-05	2739	tags=34%, list=23%, signal=27%	MYLIP/MCM2/SDCBP/TSC2/PIF1/RPS6KA2/H3-3A/ADD2/RAB3IP/EXOSC10/CORO1B/MAPRE1/ULK1/EPS8/SORL1/CORO1A/AKT1/UBQLN4/DIP2B/AIDA/SWAP70/MUL1/SYT11/PIK3CA/TMOD3/NOTCH1/SPTAN1/ATXN7/RAD21/FLCN/DLG4/SNX33/TRIOBP/TSPO/EPHA4/HES1/MAP4/PFN1/CDK5/ABI3/FYN/XRCC3/CDK5RAP2/SSH1/ARAP1/THRA/IL1B/FEZ1/TBCD/HSPA5/CERS2/ROCK2/DNAJB1/MYADM/PRAG1/ITM2C/PHF8/SVIL/LGALS3/TMC8/SSH2/NR1H2/TFIP11/PACSIN1/RCC2/PTTG1/PMP22/KIAA0319/NEU3/SLC35F6/LIMA1/RAP1GAP/TPX2/CENPF/PHF23/CHMP4B/SEMA4D/DNAJA4/DTNBP1/EHMT2/VIM/TOP2A/GSK3B/NR1H3/TGFB1/CTNNBIP1/KLHL22/TAOK1/TNFRSF1B/EML2/RBM14/APOM/CORO1C/PARP1/BCL11A/KATNB1/HIP1R/BCAS3/RAB7A/PINK1/RAC1/MBP/JAM3/HSPA1A/EVI5L/SRC/RUBCN/SLX4/FLII/PPP3CA/CAMSAP1/RIN3/SEMA4A/NRP1/ARHGEF2/KAT2B/YWHAH/PHF2/CPTP/SYNGAP1/CLASP1/ARHGEF18/SSH3/BMP6/ARRB2/RHOA/DNM2/MAD2L2/ARHGAP6/WAS/FLNA/LMNA/INPP5K/GNL3L/TENT4B/BCL2L1/DNM3/RIOK3/GSN/GAK/HUWE1/TERF2/THBS1/SPTBN1/MTPN/SPTA1/SPTB/SNCA/TMOD1/SCIN/PAK2/CAPZB/GPX1/DNAJB2/PPM1A/ITGB3/WASF2/CLU/DMTN/CARM1/ADD1/DAB2

BP	GO:0045859	regulation of protein kinase activity	452	-0.38	-1.56	1.06E-05	0.00012135	7.52E-05	2856	tags=35%, list=24%, signal=27%	SLC8A1/MAP3K4/FLT1/PRKAR1B/LCP2/SPATC1L/TESK1/CEBPA/TNFRSF10A/TTN/TSC2/PDPK1/CDK12/SH2B3/FGR/TRAF2/PKD1/GNAQ/RALB/SORL1/PKIG/AKT1/AIDA/TAOK3/CBLB/PIK3CA/CCNJL/MLLT1/ADNP/MOB3B/NCF1/EGR1/GADD45B/AGAP2/CDKN2D/EZH2/DLG4/TSG101/CHP1/MAP3K13/IL6R/EPHA4/PKN1/SH3BP5L/CDK5/SPRED1/HYAL2/CDK5RAP2/IL1B/CSF1R/PTPN6/MADD/TLR6/CCNY/GSTP1/MOB1B/TRIB1/PRRC1/XRCC6/CDC37/SHC1/HGS/PTPRC/EDN1/PIK3CB/MAGED1/CHMP6/FZD5/TPX2/MAP3K11/SMPD1/DTNBP1/ACVR2B/NEDD9/PYCARD/HSP90AB1/TGFB1/MOB3A/LILRA5/TAOK1/YWHAG/MEN1/SLC27A1/MVP/SERTAD1/CORO1C/TRAF6/MAP3K5/SESN2/LYN/SASH1/ADAR/PINK1/PTK2B/IGF2/MAP2K7/MARK2/SRC/CCND3/GREM1/CDKN1C/CEACAM1/SFRP2/DVL3/TAOK2/DAXX/ACTB/PIH1D1/MAP2K2/VLDLR/KAT2B/PDGFC/SYK/ABL1/PAK1/LTF/WARS1/PRKAR2A/PDE5A/RHOA/CALM3/PTPRJ/INPP5K/HSPB1/NF2/STK11/SDC4/HEXIM2/PLEC/CSK/THBS1/CCNI/PTPN1/AKAP13/PIK3CG/IQGAP1/UBASH3B/ATP2B4/SNCA/MMD/CAMK1/NEURL1/FBXO7/CDKN1A/CBL/PAK2/STRADB/PRKAR2B/TAL1/HTT/ITGB3/PDGFA/CLU/MAP2K3/WNK1/DAB2/CNPPD1
BP	GO:0040007	growth	565	-0.36	-1.51	1.09E-05	0.00012439	7.71E-05	2622	tags=31%, list=22%, signal=25%	EXT1/ZFY/RFTN1/SUPV3L1/ULK1/SMARCA2/MAPK14/PAK4/AKT1/ATAD3A/DIP2B/MUL1/UBE2E3/PIK3CA/DDX49/CPQ/DISC1/PLXNA4/ZMAT3/EXOSC4/ADNP/PTPN11/NOTCH1/ACACB/RAI1/CDKN2D/EZH2/FLCN/SGPL1/TSG101/ARHGEF11/MAP3K13/G6PD/BTG1/MAGI2/CDK5/HYAL2/ASPM/CD81/DDR1/ARID2/STK40/CRKL/CSNK2A1/CPNE1/ADNP2/DCUN1D3/MACF1/BASP1/CCAR2/TMC8/SERTAD3/SOCS7/EI24/LZTS2/TMEM8B/BCL9/CXCL16/SHC1/SMAD3/FMN1/ACVR1B/RUNX1/EDN1/KIAA0319/RBPJ/FTO/EPPK1/NDEL1/SEMA4D/CDA/EHMT2/ACVR2B/GSK3B/NEDD9/GSK3A/RPS6KA1/CYFIP1/HSP90AB1/TGFB1/SMARCA4/KLHL22/TEC/SPHK1/CCN3/VGLL4/EZR/GNAS/WDTC1/MFSD2A/BCL11A/HDGFL2/SESN2/FHL1/IST1/YY1/NEDD4L/PTPN12/CDKL5/PTK2B/RARA/MT2A/DPYSL2/IGF2/SP2/HSPA1A/CHST11/CD9/KLF2/SLC9A1/TRIM32/BCL2L11/TBL1XR1/SRC/PML/GREM1/KAT7/CDKN1C/DCAF1/CEACAM1/EIF4G2/CPNE5/SFRP2/PPARA/RUVBL1/PLXNA1/PPP3CA/TAF8/TAOK2/PSAP/ZMIZ1/TRIM28/STAT3/SEMA4A/NRP1/USP9X/ABL1/ARIH2/FBP1/ANXA6/LAMTOR1/JARID2/RHOA/VCL/NOTCH2/DNM2/MAD2L2/PTPRJ/STAT5A/BCL2L1/STK11/CYFIP2/PLEC/CLSTN1/ADIPOR1/PPP1R9B/AKAP13/MTPN/IQGAP1/FKBP8/EIF4G1/ENO1/HLX/CDKN1A/NINJ2/SMPD3/PAK2/GPX1/DNAJB2/YBX3/FOXO3/MED1/CTTN/PIM1/DAB2

BP	GO:0051336	regulation of hydrolase activity	644	-0.35	-1.48	1.14E-05	0.0001297	8.04E-05	2590	tags=32%, list=22%, signal=26%	ATP2A3/RASAL3/GNAQ/PTAFR/SORL1/SBF2/AKT1/ARL2/MUL1/CBLB/PLXNC1/RRP1B/SERPINF2/PLXNA4/MAPK7/TBC1D8/KNG1/BAK1/CASP9/DOCK8/BICD1/PPP1R15A/CDKN2D/EZH2/FLCN/PTPA/OAS2/PPP6R1/CHP1/OAS3/DIABLO/EPHA4/MAGI2/PRKD2/FGD6/PCSK1N/FYN/CASP2/ARAP1/CRKL/CSNK2A1/PROS1/DOCK10/MAPRE2/ROCK2/VAV2/RALBP1/TIMP1/PPARGC1B/MGAT5/LGALS3/SRGAP2/PIN1/NR1H2/PKP4/RCC2/RGS10/BIRC6/ARHGAP35/SMAD3/PTTG1/APLP2/GPSM1/PTPRC/CAST/TIAM1/ADAP2/RIPOR2/LIMS1/POLDIP2/RAP1GAP/MMP9/NDEL1/BBC3/RGS16/KLF4/SEMA4D/PSME3/BCAP31/SERPINB6/GSK3B/CRY2/TIMP2/TBC1D17/NEDD9/NR1H3/PYCARD/RPS6KA1/DAP/HSP90AB1/SVBP/TBC1D22A/RASGRP3/ASAP2/PREX1/PCSK6/ASAP1/PRR7/AMOT/BEX3/SIPA1L3/SPINT2/DAPK1/PLEK/CORO1C/ARL6IP1/MAP3K5/HIP1/VRK3/AQP1/HIP1R/LYN/BCAS3/PLXNB2/RABGAP1/SERPING1/CDKL5/PINK1/MBP/PTK2B/GNA15/HSPA1A/PLXND1/SBF1/MGMT/EVI5L/BAD/DHCR24/SIPA1L1/NCSTN/BCL2L11/SRC/UBE2Z/PML/RASA3/IQSEC1/SFRP2/GNAI2/DVL3/FIS1/VAV1/PLXNA1/LAPTM5/PSAP/GMIP/ARHGAP45/TBC1D13/PIH1D1/STAT3/RANGAP1/BCR/RGP1/SYK/VCP/XIAP/ABL1/EGLN3/SYNGAP1/FGD4/FKBP1A/LTF/RAPGEF1/ARRB2/RHOA/CALM3/ARHGAP6/TBC1D22B/LGALS9/TBC1D20/FKBP1B/RSU1/PSME4/SIAH2/PRDX5/CYFIP2/SORT1/GNA12/GSN/BCL2L13/PSMF1/LRP1/TERF2/PPP1R9B/THBS1/AKT2/IQGAP1/RGS6/GPI/GRN/SNCA/RHOG/TBC1D5/P2RX1/TBC1D25/TBC1D1/AKIRIN2/CTSD/GAPVD1/PAK2/UBE2O/CTSB/GPX1/FURIN/HTT/SH3BP1/TGM2/WNK1
BP	GO:0046777	protein autophosphorylation	174	-0.48	-1.79	1.16E-05	0.00013115	8.13E-05	2834	tags=44%, list=24%, signal=34%	FLT1/TESK1/EPHB1/EIF2AK2/ATP13A2/PDPK1/CDK12/CAD/FGR/ULK1/MINK1/AKT1/PIM3/TAOK3/JUN/CHP1/MAP3K13/LMTK2/EPHA4/MAPKAPK3/CDK5/PRKD2/DDR1/FYN/RIPK1/CSF1R/MKNK2/MOB1B/FES/MAP3K3/ACVR1B/PTPRC/ENG/TAF1/MAP3K11/MARK3/GSK3B/PRKACA/TEC/TAOK1/DYRK1B/DAPK3/DAPK1/NEK6/MVP/OXSR1/MAP4K1/LYN/CDKL5/PINK1/PTK2B/STK10/STK24/MARK2/SRC/GREM1/STK25/TAOK2/TRIM28/MAP2K2/PDGFC/SYK/ABL1/PAK1/PIM2/CALM3/MAPKAPK2/STK11/CSK/PPP2R5B/ATG13/MAPK3/PAK2/EIF2AK1/PDGFA/PIM1

BP	GO:0000003	reproduction	738	-0.34	-1.45	1.18E-05	0.0001333	8.26E-05	2683	tags=30%, list=23%, signal=25%	PI3/H3-3A/ATP7A/ZNF628/AGFG1/GMCL1/MAGED2/FANCG/NCAPD2/NRIP1/ZFY/PKD1/SMARCA2/MAPK14/SMAD5/PTAFR/RNF38/AKT1/TUBGCP2/NSDHL/AGO4/TIPARP/ADNP/PTPN11/NOTCH1/TRPM2/BAK1/TRPC3/EGR1/YTHDF3/SPIRE1/RAD21/SGPL1/CNOT9/SEPTIN6/KPNA6/CDYL/AAAS/ZSCAN21/INSL3/WASHC5/LHFPL2/ZNF318/TUBGCP4/TOP3A/TUBGCP6/PRDM1/HES1/ATP8B3/BTG1/EME2/SPIN1/ASPM/ACOX1/DDR1/CSDE1/CASP2/CRKL/DEAF1/THRA/SPIRE2/DACH1/IL1B/TTLL1/DHX37/TTC21A/PTPN6/VASH1/HSPA5/GGT1/SPATA5/ZBTB16/PAQR8/TUBG1/TUBG2/DIAPH2/NCOA4/TIMP1/TESK2/BASP1/ENKUR/CHFR/HSD17B4/SPATA2/NUMA1/ALPL/SYNE1/CUL7/BIRC6/MAFF/PTTG1/RHBDD1/ACVR1B/EDN1/TFEB/UBE2Q1/CRTAP/ENO2/FZD5/RBPJ/GTSF1/SLC19A1/TBPL1/MMP9/CCT3/BCAP31/EHMT2/NR6A1/GRB2/TOP2A/PTCHD3/HYAL3/NCOA1/TOB2/SORD/CETN2/PRKACA/SFMBT1/HSP90AB1/ASCL2/SCMH1/ST14/CLIC5/ETV6/SPINT2/MEN1/UCP2/SMC1A/ALDOA/UBR2/SUN2/LNPEP/YY1/EPAS1/PUM1/JAM3/BPTF/PTK2B/RARA/IGF2/DAZAP1/ASF1B/PRSS21/CD9/SBF1/UBAP2L/BAD/DHCR24/DZIP1/BCL2L11/SRC/CDKN1C/ARNT/TAF4/CALR/SFRP2/ZNF296/LRRC8A/SLX4/SEPTIN4/RUVBL1/CDK2/HERC2/PSAP/ZMIZ1/TRIM28/SPOCD1/STAT3/CTNNA1/USP9X/DPCD/GNPDA1/ALKBH5/FOS/NCAPH/E2F1/PITHD1/SF1/GOLGA3/BMP6/ARRB2/SLC2A1/GOLGA2/PDE5A/KLC3/EPN1/NOTCH2/DNM2/LGALS9/TBC1D20/BSG/PSME4/FOXJ2/KLF1/BCL2L1/STK11/UBB/ABHD2/RPA1/FZR1/IGF2R/PPP1R9B/CCNI/ATP2B4/NEURL1/PRKDC/P2RX1/GATA1/CBL/PTGDR2/CTSB/YBX3/FOXO3/CTDNEP1/MED1/MYH9/PDGFA/OSBP2/NCOR2/ACRBP/MARF1/PBX1/BAG6
BP	GO:0034765	regulation of ion transmembrane transport	233	-0.44	-1.70	1.19E-05	0.00013363	8.29E-05	1587	tags=22%, list=14%, signal=20%	KCNJ2/KCNQ5/EHD3/STOM/PRKACA/HVCN1/CD63/RANGRF/CLIC5/DAPK1/SELENON/ARL6IP1/OXSR1/FHL1/ANO6/LYN/NEDD4L/PINK1/PTK2B/SLC9A1/ATP1B2/CACNG6/CNIH3/YWHAH/VDAC1/ABL1/FKBP1A/STIM1/WWP2/CALM3/DNM2/FLNA/FKBP1B/SLC6A9/GJC2/PIK3CG/SLC43A1/PER2/UBASH3B/ATP2B4/SNCA/AHCYL1/CAPN1/P2RX1/ACTN4/TESC/HTT/SHISA7/KEL/SCN1B/WNK1/DIAPH1
CC	GO:0030055	cell-substrate junction	353	-0.40	-1.61	1.25E-05	0.00013984	8.67E-05	1767	tags=32%, list=15%, signal=28%	SNTB1/PTPRC/DIXDC1/ARPC5/ENG/LIMS1/TGFB1I1/LIMA1/EPPK1/ADGRE5/VIM/EHD3/NEDD9/SORBS3/CYFIP1/ACTG1/TRIP6/ZNF185/GIT1/TNS3/CLTC/YWHAG/EZR/CORO1C/IRF2/PRUNE1/FHL1/ZYX/DCTN4/PTPN12/ILK/FZD2/RAC1/PTK2B/HSPA1A/CD9/ARF1/FLNB/SLC9A1/TSPAN9/NCSTN/RRAS/SRC/ARPC1B/CALR/YWHAB/SLA/FLII/ACTB/FHL3/CTNNA1/NRP1/MAP2K2/CNN2/ARHGEF2/JAK1/CAP1/CSRP1/PXN/PAK1/CLASP1/ANXA6/ATP6V0C/APBB1IP/HNRNPK/PRKAR2A/RHOA/VCL/DNM2/FLNA/ARHGAP31/FERMT3/RAC2/BSG/RSU1/HSPB1/LASP1/SDC4/DCAF6/ITGB5/GNA12/AFAP1/GSN/PLEC/CFL1/GAK/IGF2R/LRP1/CD151/IQGAP1/MSN/MRC2/PEAK3/CAPN1/VASP/RHOG/MAPK3/PARVB/PCBP2/CBL/CAPN5/HMGA1/ACTN4/ACTN1/PDLIM1/CTTN/MYH9/ITGB3/TNS1/ITGA2B/TLN1/TGM2/ADD1/DAB2

BP	GO:0098542	defense response to other organism	690	-0.34	-1.46	1.25E-05	0.00013984	8.67E-05	2262	tags=26%, list=19%, signal=22%	OAS3/CCDC92/IKBKE/PRDM1/SERINC5/IL6R/IRF1/MOV10/HYAL2/BATF2/NUB1/FYN/IL1B/SPI1/GBP4/HLA-A/CSF1R/OTUD4/PTPN6/PPBP/RNASE3/TLR6/CNPY3/SYNCRIP/PGLYRP1/TNK2/FES/LGALS3/NR1H2/ITGAM/XRCC6/CDC37/CXCL16/SHC1/GBP6/RAB20/PTPRC/EDN1/KIR3DL1/DROSHA/RBPJ/CD14/CD74/HLA-C/NCR3/AGBL5/PDE12/SMPD1/VIM/IKBKG/FOXP1/DDX60/ABCF3/ANKRD17/NLRX1/NR1H3/FAM3A/PYCARD/G3BP2/CACTIN/HSP90AB1/ACTG1/MICB/DCD/LILRA5/RBM14/CD180/DAPK3/DAPK1/STX4/CDC42EP4/TRAF6/CHID1/IRF2/TRIM35/MAP3K5/PLD4/APOBEC3D/ZYX/UNC93B1/AQP1/LYN/TRIM26/MAVS/SERPING1/IL17RA/PUM1/ADAR/PLEKHM2/IFITM3/PTK2B/PRF1/IFI27/FLNB/TTLL12/CGAS/CREBBP/SPN/TRIM32/ZC3HAV1/ILRUN/SRC/PML/SRPK2/CEACAM1/RBCK1/PIK3CD/SLA/VAV1/TRIM10/TRIM28/IFITM2/ARHGEF2/JAK1/SYK/JAK3/XIAP/APOBEC3F/PAK1/LCN2/TNFSF4/RAB14/OPTN/N4BP1/RELA/LTF/C1QA/RNF213/DTX3L/ARRB2/ECSIT/TUBB4B/SIGLEC10/WIPI2/SHMT2/MFHAS1/NOTCH2/LGALS9/WAS/HK1/IFIT1B/ATG7/TRIM11/BCL2L1/KRT1/RIOK3/GSN/TRIM14/PTPN1/ZNFX1/PIK3CG/RNF187/SEC14L1/COTL1/MARCHF2/GRN/SHARPIN/ELF4/SELP/SNCA/H2BC11/PRKDC/LAMP1/APOBEC3C/MAPK3/BNIP3L/PCBP2/PF4/AKIRIN2/TUBB/TREML1/PAK2/SLC15A3/TOLLIP/MED1/TRIM58/STMP1/NPLOC4/CLU
BP	GO:0019941	modification-dependent protein catabolic process	562	-0.36	-1.51	1.26E-05	0.00014007	8.68E-05	1712	tags=22%, list=15%, signal=20%	CHMP6/SMARCC1/TAF1/TGFB1I1/CANX/OTUD7B/WDR81/UBE2D4/NSFL1C/VPS37B/USP37/CHMP4B/BCAP31/GSK3B/GSK3A/HSP90AB1/KLHL22/TBX21/CBFA2T3/DET1/EDEM2/KCTD2/CNOT4/UBR2/ZRANB1/UBE2G1/UBA52/NEDD4L/DDA1/USP31/CDC27/FBXL18/PINK1/PTK2B/HSPA1A/ANKIB1/IFI27/USP12/TRIM32/MARCHF6/TBL1XR1/FBXO31/CHMP1A/UBE2Z/PML/UBE2R2/RNF115/CALR/RBCK1/RMND5A/CDK2/HERC2/LAPTM5/AMFR/UBA1/USP19/TBL1X/PSMD11/MAP1A/BTRC/USP9X/GABARAP/USP34/FBXO6/DDB1/VCP/USP36/ARIH2/KLHL8/UBAP1/N4BP1/FBXW4/PSMC4/RNF213/RAD23A/DTX3L/ARRB2/RNF11/WWP2/GID8/UBE2S/SUFU/MAN1A1/ZNRF1/PSMD2/USP7/HIPK2/VPS28/SIAH2/UBB/UBR4/GNA12/USP22/FZR1/HUWE1/PSMF1/SQSTM1/YOD1/UBE2L3/RNF187/ARAF/SHARPIN/ANKRD9/PCBP2/FBXO7/DCAF12/CBL/TRIP12/ASCC2/UBL7/RYBP/ZER1/RNF40/DNAJB2/CDC34/TOLLIP/PTPN23/UBE2H/DCAF11/CUL4A/TRIM58/NPLOC4/CLU/DAB2/BAG6
CC	GO:0030425	dendrite	335	-0.41	-1.63	1.29E-05	0.00014366	8.91E-05	2567	tags=36%, list=22%, signal=29%	RAB8A/MARK4/MINK1/DIP2B/SYT11/NSF/ADNP/CYBB/NR1D1/NCF1/CD3E/DLG4/RPH3A/ZNF385A/EIF4A3/EPHA4/MAGI2/DMWD/PI4K2A/CDK5/LYNX1/FXR2/ABI3/FYN/ABHD17C/FEZ1/HRH2/YKT6/P2RX4/CNR2/DOCK10/CTNND1/DNAJB1/SRGAP2/TMEM185A/NUMA1/PDE4B/OR10H1/FZD5/GNAZ/TRAK1/MAP1S/RAP1GAP/CANX/PPP1R9A/KCNJ2/MARK3/SEPTIN11/DTNBP1/GSK3B/KIF1C/BAIAP2/PRKACA/GSK3A/CASC3/CYFIP1/HSP90AB1/ASAP1/PRR7/CCN3/TRAPPC4/STX4/KIF3B/HIP1R/CDKL5/RAC1/NPFF/PTK2B/RARA/SIPA1L1/MARK2/GRIPAP1/N4BP3/GNAI2/SLA/KIF1B/SEPTIN4/ARHGAP32/IFT52/PPP3CA/RIN3/TAOK2/AMFR/MAX/PPP1CA/CNIH3/RANGAP1/SLC1A4/MAP1A/BCR/APBA3/MCRS1/TRAK2/ABL1/PAK1/SYNGAP1/ARRB2/STAU1/CKB/RHOA/GLRX5/ELK1/ROGDI/ZDHHC5/DNM3/PLEC/PPP1R9B/HRH4/SHARPIN/LRFN3/NEURL1/NRGN/ACTN1/PRKAR2B/CTTN/HTT/SHISA7/CLU/OSBP2

CC	GO:0044297	cell body	306	-0.41	-1.61	1.32E-05	0.00014567	9.03E-05	2705	tags=33%, list=23%, signal=26%	ATP13A2/GABBR1/ATP7A/PTPN13/CAD/SORL1/RAB8A/DIP2B/MUL1/SYT11/DISC1/ADNP/TRPM2/CYBB/NCF1/CD22/CD3E/ZNF385A/EIF4A3/LMTK2/EPHA4/DMWD/PI4K2A/CDK5/FXR2/FYN/TOP1/FEZ1/YKT6/P2RX4/CNR2/SMN1/DNAJB1/NUMA1/DIXDC1/ENO2/FZD5/GNAZ/MAP1S/RAP1GAP/NDEL1/CANX/CCT3/TPX2/KCNJ2/DTNBP1/BAIAP2/GSK3A/PYCARD/CYFIP1/HSP90AB1/TBX21/TNFRSF1B/PDE1A/ENC1/CCN3/KATNB1/HIP1R/VTI1B/PINK1/MBP/NPFF/PTK2B/FBXO31/CPNE5/GNAI2/SEPTIN4/VTI1A/ATP1B2/RIN3/AMFR/PPP1CA/SLC1A4/MAP1A/MCRS1/ABL1/STAU1/MPL/CKB/PDE9A/ACTA2/PVALB/FLNA/SLC3A2/NF2/GLRX5/ELK1/ROGDI/SIAH2/UBB/GNA12/DCTN1/GLUL/PPP1R9B/GJC2/SNCA/NEURL1/NRGN/TUBB/PRKAR2B/SCN1B/HCFC1
MF	GO:0008013	beta-catenin binding	55	-0.64	-2.00	1.34E-05	0.00014748	9.14E-05	1890	tags=45%, list=16%, signal=38%	PIN1/BCL9/SMAD3/MED12L/KLF4/BCL9L/GSK3B/CTNNBIP1/ASH2L/KDM6B/TBL1XR1/DVL3/CTNNA1/TBL1X/BTRC/PXN/FOXO4/CHD8/VCL/PTPRJ/SUFU/SETD1A/TCF7L2/FOXO3/CARM1
CC	GO:0005925	focal adhesion	349	-0.40	-1.59	1.39E-05	0.00015276	9.47E-05	1767	tags=32%, list=15%, signal=28%	SNTB1/PTPRC/DIXDC1/ARPC5/ENG/LIMS1/TGFB1I1/LIMA1/ADGRE5/VIM/EHD3/NEDD9/SORBS3/CYFIP1/ACTG1/TRIP6/ZNF185/GIT1/TNS3/CLTC/YWHAG/EZR/CORO1C/IRF2/PRUNE1/FHL1/ZYX/DCTN4/PTPN12/ILK/FZD2/RAC1/PTK2B/HSPA1A/CD9/ARF1/FLNB/SLC9A1/TSPAN9/NCSTN/RRAS/SRC/ARPC1B/CALR/YWHAB/SLA/FLII/ACTB/FHL3/CTNNA1/NRP1/MAP2K2/CNN2/ARHGEF2/JAK1/CAP1/CSRP1/PXN/PAK1/CLASP1/ANXA6/ATP6V0C/APBB1IP/HNRNPK/PRKAR2A/RHOA/VCL/DNM2/FLNA/ARHGAP31/RAC2/BSG/RSU1/HSPB1/LASP1/SDC4/DCAF6/ITGB5/GNA12/AFAP1/GSN/PLEC/CFL1/GAK/IGF2R/LRP1/CD151/IQGAP1/MSN/MRC2/PEAK3/CAPN1/VASP/RHOG/MAPK3/PARVB/PCBP2/CBL/CAPN5/HMGA1/ACTN4/ACTN1/PDLIM1/CTTN/MYH9/ITGB3/TNS1/ITGA2B/TLN1/TGM2/ADD1/DAB2
BP	GO:0045807	positive regulation of endocytosis	68	-0.59	-1.93	1.43E-05	0.00015649	9.70E-05	1880	tags=44%, list=16%, signal=37%	MIB1/ARRB1/SYNJ1/CD14/ANKFY1/AP2B1/SMPD1/DGKD/DTNBP1/CD63/RAB31/HIP1/ANO6/NEDD4L/GREM1/PPP3CA/SYK/ARRB2/HNRNPK/DNM2/LDLRAP1/VPS28/LRP1/CD151/SNCA/TBC1D5/CBL/AP2M1/CLU/DAB2
CC	GO:0005912	adherens junction	108	-0.53	-1.87	1.48E-05	0.00016141	0.00010008	2276	tags=38%, list=19%, signal=31%	SYNM/EPHA4/CD99L2/TBCD/CTNND1/AJM1/TNK2/NDRG1/PDLIM7/PKP4/FMN1/FRMD4B/SHROOM1/BAIAP2/FRMD4A/EZR/MYO1E/CDC42EP4/ZYX/LYN/DCHS1/CEACAM1/EIF4G2/ACTB/PPP1CA/CTNNA1/TJP2/KIFC3/PARD3/VCL/NIBAN2/NF2/PPP1R9B/TSPAN33/MSN/PDLIM1/MYH9/ESAM/SH3BP1/TLN1/ADD1
BP	GO:0046903	secretion	549	-0.36	-1.49	1.50E-05	0.00016307	0.00010111	1789	tags=20%, list=15%, signal=17%	SYNJ1/SLC29A2/HGS/EDN1/UBE2Q1/ENG/CD74/CPT1A/GRK2/CANX/SOX4/DTNBP1/HYAL3/SREBF1/ACVR2B/GSK3B/CRY2/NR1H3/COPA/TGFB1/ATP9A/FRMD4A/SVBP/GIT1/TNFRSF1B/SYNGR1/PCSK6/IL1RN/DNAJC5/CCN3/EZR/ARHGAP17/SNX10/STX4/UCP2/PLEK/RAB31/AQP1/ITSN1/HIP1R/LYN/VTI1B/RAB7A/RAB11FIP1/PINK1/BAD/PFKL/EXOC3/SEPTIN1/CEACAM1/CA2/PIK3CD/SCFD2/USF2/GNAI2/LRRC8A/SEPTIN4/SLC1A5/PPP3CA/BCR/SYK/ABCC4/PAK1/CLASP1/BLOC1S3/RAB27B/BMP6/PDIA4/RAB3IL1/MYO18A/SIDT2/GALR1/CALM3/LGALS9/RAB11B/FKBP1B/RAB3D/RAC2/STAT5A/NIBAN2/NAPA/SDC4/SYTL4/GLUL/PRKCB/TANGO2/PIK3CG/RAB2B/TCF7L2/PER2/SNCA/NEURL1/LAMP1/P2RX1/GATA1/SLC29A1/CBL/SMPD3/SCIN/FURIN/MED1/PTPN23/MYH9/DMTN/NCOR2/ANK1/WNK1/DAB2
BP	GO:0090257	regulation of muscle system process	122	-0.51	-1.83	1.53E-05	0.00016622	0.00010306	2444	tags=39%, list=21%, signal=31%	NOTCH1/ATP1A1/TRPC3/ATP2A2/HDAC4/FOXO1/G6PD/ATP1B1/P2RX4/ORMDL3/ROCK2/CDK9/SMAD3/PDE4B/EDN1/GRK2/KCNJ2/EHD3/PRKACA/GSK3A/TNFRSF1B/RANGRF/SPHK1/DAPK3/PARP1/TPM1/YY1/SLC9A1/PPARA/PPP3CA/ANXA6/JARID2/PDE5A/RHOA/PDE9A/CALM3/GUCY1A1/FKBP1B/LMNA/MTPN/PIK3CG/ATP2B4/ENO1/P2RX1/FOXO3/MYL9/CTTN/TBXA2R

BP	GO:0043632	modification-dependent macromolecule catabolic process	573	-0.36	-1.50	1.59E-05	0.0001719	0.00010659	1712	tags=22%, list=15%, signal=20%	CHMP6/SMARCC1/TAF1/TGFB1I1/CANX/OTUD7B/WDR81/UBE2D4/NSFL1C/VPS37B/USP37/CHMP4B/BCAP31/GSK3B/GSK3A/HSP90AB1/KLHL22/TBX21/CBFA2T3/DET1/EDEM2/KCTD2/CNOT4/UBR2/ZRANB1/UBE2G1/UBA52/NEDD4L/DDA1/USP31/CDC27/FBXL18/PINK1/PTK2B/HSPA1A/ANKIB1/IFI27/USP12/TRIM32/MARCHF6/TBL1XR1/FBXO31/CHMP1A/UBE2Z/PML/UBE2R2/RNF115/CALR/RBCK1/RMND5A/CDK2/HERC2/LAPTM5/AMFR/UBA1/USP19/TBL1X/PSMD11/MAP1A/BTRC/USP9X/GABARAP/USP34/FBXO6/DDB1/VCP/USP36/ARIH2/KLHL8/UBAP1/N4BP1/FBXW4/PSMC4/RNF213/RAD23A/DTX3L/ARRB2/RNF11/WWP2/GID8/UBE2S/SUFU/MAN1A1/ZNRF1/PSMD2/USP7/HIPK2/TENT4B/VPS28/SIAH2/UBB/UBR4/GNA12/USP22/FZR1/HUWE1/PSMF1/SQSTM1/YOD1/UBE2L3/RNF187/ARAF/SHARPIN/ANKRD9/PCBP2/FBXO7/DCAF12/CBL/TRIP12/ASCC2/UBL7/RYBP/ZER1/RNF40/DNAJB2/CDC34/TOLLIP/PTPN23/UBE2H/DCAF11/CUL4A/TRIM58/NPLOC4/CLU/DAB2/BAG6
BP	GO:0006909	phagocytosis	176	-0.47	-1.74	1.62E-05	0.00017493	0.00010846	1925	tags=31%, list=16%, signal=27%	SIRPG/ITGAM/SCARB1/RAB20/PTPRC/CD14/RHOBTB1/CD300LF/IL2RG/SIRPB1/TM9SF4/IL2RB/NR1H3/PYCARD/MESD/SPHK1/CORO1C/PLD4/ALOX15/RAB31/ANO6/LYN/RAB7A/RAC1/RARA/DYSF/SRC/CALR/RUBCN/SNX3/VAV1/CNN2/BCR/SYK/ABL1/PAK1/RAB14/MYO18A/ITGAL/ANXA11/DNM2/PTPRJ/RAC2/DOCK2/CAMK1D/GSN/LRP1/CSK/THBS1/RHOG/EIF2AK1/MYH9/ITGB3/SH3BP1/TGM2
BP	GO:1990778	protein localization to cell periphery	238	-0.43	-1.68	1.64E-05	0.00017681	0.00010963	2606	tags=31%, list=22%, signal=25%	JUP/BBS1/RAB8A/AKT1/EXOC7/NSF/GOLGA7/CNST/CLN3/TMBIM1/DLG4/CDK5/CD81/ABI3/EXOC4/MAP7/ATP1B1/ROCK2/MACF1/MYADM/ZDHHC3/LGALS3/EPB41L2/PACSIN1/NUMA1/DTNBP1/EHD3/TGFB1/CLTC/RANGRF/EZR/TSPAN17/STX4/ATP6AP1/EHD4/EFR3A/RAB31/VTI1B/RAB7A/DCHS1/GRIPAP1/PLEKHF1/PACS1/ACTB/TTC7B/OPTN/LDLRAP1/FLNA/RAB3D/BSG/INPP5K/BCL2L1/ZDHHC5/TTC7A/CLSTN1/SLC4A1/GAK/SQSTM1/LRP1/PPP1R9B/CSK/SPTBN1/AKT2/EPB41/TSPAN33/ATP2B4/RHOG/EHD1/TSPAN5/TESC/AP2M1/SEC16A/ANK1/DAB2
BP	GO:0071496	cellular response to external stimulus	235	-0.44	-1.69	1.68E-05	0.00018029	0.00011179	2453	tags=33%, list=21%, signal=26%	ADNP/BAK1/LAMP2/FLCN/FOXO1/CBS/PHEX/IRF1/WDR24/CASP2/IL1B/NUAK2/SREBF2/HSPA5/PDE2A/DCPS/RIPOR1/FES/MAP1LC3B/GCN1/ARHGAP35/PTPRC/RRAGD/TFEB/ENG/VPS41/CDA/KCNJ2/EHMT2/SREBF1/NCOA1/DAP/TTC5/CASP5/UCP2/MFSD2A/MAP3K5/SESN2/AQP1/LYN/RXRA/SLC9A1/BAD/STK24/SLC39A4/SFRP2/USF2/PPARA/MAX/PDK2/CNN2/GABARAP/ZFYVE1/RRAGC/FOS/NFKB1/BMP6/SLC2A1/WIPI2/SESN3/KANK2/ATG7/PLEC/GLUL/PPP1R9B/MTPN/EIF4G1/MAPK3/ELAPOR1/CDKN1A/CBL/WDR45/NPRL3/FOXO3/MED1/GAS2L1/PIM1
BP	GO:0009135	purine nucleoside diphosphate metabolic process	80	-0.57	-1.93	1.70E-05	0.00018171	0.00011267	2058	tags=38%, list=18%, signal=31%	AK3/ENTPD5/PGAM1/ZBTB7A/EIF6/FOXK1/ENO2/GALK1/FOXK2/GIT1/CBFA2T3/JMJD8/ALDOA/ALDOC/BAD/PFKL/ARNT/AK1/PPARA/PKM/BPGM/STAT3/TJP2/FBP1/HK1/MPP1/SLC4A1/GPI/ENO1/GUK1
BP	GO:0009179	purine ribonucleoside diphosphate metabolic process	80	-0.57	-1.93	1.70E-05	0.00018171	0.00011267	2058	tags=38%, list=18%, signal=31%	AK3/ENTPD5/PGAM1/ZBTB7A/EIF6/FOXK1/ENO2/GALK1/FOXK2/GIT1/CBFA2T3/JMJD8/ALDOA/ALDOC/BAD/PFKL/ARNT/AK1/PPARA/PKM/BPGM/STAT3/TJP2/FBP1/HK1/MPP1/SLC4A1/GPI/ENO1/GUK1
BP	GO:0046031	ADP metabolic process	70	-0.59	-1.93	1.81E-05	0.00019256	0.00011939	2058	tags=39%, list=18%, signal=32%	AK3/ENTPD5/PGAM1/ZBTB7A/EIF6/FOXK1/ENO2/GALK1/FOXK2/GIT1/CBFA2T3/JMJD8/ALDOA/ALDOC/BAD/PFKL/ARNT/AK1/PPARA/PKM/BPGM/STAT3/FBP1/HK1/SLC4A1/GPI/ENO1

BP	GO:0010595	positive regulation of endothelial cell migration	72	-0.59	-1.97	1.84E-05	0.00019548	0.0001212	2204	tags=42%, list=19%, signal=34%	PRKD2/P2RX4/ROCK2/MAP3K3/SCARB1/EDN1/PIK3CB/FOXP1/TGFB1/ETS1/BCAS3/SASH1/RAC1/PTK2B/RRAS/CALR/PIK3CD/NRP1/ABL1/HSPB1/STAT5A/GFUS/THBS1/PIK3CG/SP1/GPI/GRN/ITGB3/SPARC/MAP2K3
BP	GO:0030879	mammary gland development	70	-0.59	-1.93	1.86E-05	0.00019662	0.00012191	1116	tags=24%, list=10%, signal=22%	LBH/MGMT/BCL2L11/SRC/PML/USF2/BTRC/STAT5A/FASN/AKT2/NEURL1/CAPN1/ZNF703/SLC29A1/GPX1/MED1/NCOR2
MF	GO:0045296	cadherin binding	263	-0.42	-1.66	1.86E-05	0.00019662	0.00012191	2100	tags=35%, list=18%, signal=30%	PLIN3/YKT6/P2RX4/EEF2/EFHD2/HSPA5/CTNND1/MACF1/DNAJB1/NDRG1/PKP4/GCN1/AHSA1/SERBP1/CAST/LRRC59/CCS/LIMA1/CHMP4B/ABCF3/BAIAP2/HSP90AB1/ASAP1/UBAP2/EZR/ATIC/EHD4/ALDOA/IST1/HSPA1A/DCHS1/MICALL1/FLNB/SH3GL1/STK24/EXOC3/MARK2/ZC3HAV1/SRC/EIF4G2/YWHAB/RUVBL1/PKM/PPP1CA/CTNNA1/CTNNAL1/RANGAP1/CNN2/SH3GLB2/EPS15L1/UBFD1/TJP2/GOLGA3/CALD1/STX5/GOLGA2/HNRNPK/PLCB3/SCYL1/VCL/CKAP5/PTPRJ/FLNA/SLC3A2/RAB11B/BSG/ARHGAP1/LASP1/NIBAN2/FASN/SND1/PLEC/SPTBN1/PTPN1/IQGAP1/PRDX6/ENO1/VASP/CBL/EHD1/GAPVD1/PAK2/CAPZB/PDLIM1/CTTN/H3C10/MYH9/WASF2/HDLBP/TLN1/LARP1/ADD1/HCFC1
BP	GO:0031532	actin cytoskeleton reorganization	77	-0.57	-1.91	1.89E-05	0.00019924	0.00012354	2206	tags=45%, list=19%, signal=37%	CDK5/ABI3/ARAP1/CSF1R/FES/PARVG/SHC1/DTNBP1/GRB2/BAIAP2/NEDD9/EZR/DAPK3/PLEK/CDC42BPB/BCAS3/PTK2B/SIPA1L1/HCLS1/NRP1/ABL1/PAK1/PLEK2/RHOA/NOTCH2/ARHGDIA/FLNA/GSN/PLEC/PTPN1/PARVB/CTTN/MYH9/ESAM/DMTN
BP	GO:0032388	positive regulation of intracellular transport	160	-0.47	-1.75	2.04E-05	0.00021355	0.00013241	2267	tags=36%, list=19%, signal=30%	CHP1/PCNT/MLC1/CDK5/HYAL2/CD81/FYN/IL1B/FEZ1/SREBF2/RIPOR1/ITGAM/NUMA1/UBE2J2/SMAD3/FZD5/BBC3/BCAP31/TM9SF4/GSK3B/PRR5L/STOM/PRKACA/GSK3A/HSP90AB1/TGFB1/HDAC3/EDEM2/EZR/BCAS3/MAVS/LEPROT/PINK1/HCLS1/DYNC1H1/FIS1/TRIM28/USP36/ARIH2/ABLIM3/SAE1/LDLRAP1/FLNA/TBC1D20/RAC2/STK11/HUWE1/AKT2/UBE2L3/TCF7L2/MSN/CAMK1/ATG13/EHD1/OAZ1/PTPN23/PPM1A/DAB2
BP	GO:0034332	adherens junction organization	29	-0.74	-2.04	2.11E-05	0.00021994	0.00013637	1108	tags=48%, list=9%, signal=44%	JAM3/DCHS1/SRC/ACTB/KIFC3/BMP6/VCL/HIPK1/PLEC/CSK/ZNF703/PAK2/PTPN23/ADD1
CC	GO:0030424	axon	364	-0.39	-1.59	2.11E-05	0.00021994	0.00013637	2251	tags=30%, list=19%, signal=25%	LMTK2/EPHA4/MAP4/CDK5/FXR2/MACO1/EXOC4/MAP7/FEZ1/P2RX4/SMN1/MADD/NAV1/CTNND1/BASP1/NDRG2/PACSIN1/SYNJ1/DIXDC1/ARPC5/ENO2/FZD5/TRAK1/RAP1GAP/NDEL1/CANX/TPX2/SMIM31/SEPTIN11/DTNBP1/VIM/GSK3B/KIF1C/GSK3A/CYFIP1/COPA/HSP90AB1/ACTG1/GIT1/TNFRSF1B/TPRG1L/CCN3/KIF3B/KATNB1/CDKL5/PINK1/MBP/NPFF/PTK2B/KIF13B/EXOC3/GRIPAP1/DYNC1H1/N4BP3/SH2D3C/EIF4G2/KIF1B/SEPTIN4/MYCBP2/RIN3/ADGRL1/TAOK2/AMFR/ACTB/NRP1/RANGAP1/MAP1A/BCR/USP9X/TRAK2/ATP6V0D1/ABL1/PAK1/PARD3/BLOC1S3/RAB27B/FAM168B/RHOA/CALM3/DNM2/LDLRAP1/PVALB/FLNA/ARL8A/BSG/HSPB1/ELK1/ROGDI/DNM3/DCTN1/PLEC/GLUL/CLDN5/TERF2/PPP1R9B/PRKCB/SPTBN1/GJC2/MTPN/IQGAP1/SPTA1/SNCA/LRFN3/CBL/NRGN/RNF40/CTTN/HTT/SCN1B
BP	GO:0042326	negative regulation of phosphorylation	270	-0.42	-1.63	2.15E-05	0.00022391	0.00013884	2581	tags=33%, list=22%, signal=27%	GNAQ/SORL1/PKIG/AKT1/PIP5KL1/AIDA/CBLB/JUN/MLLT1/BAK1/GADD45B/CDKN2D/HDAC4/TSG101/CHP1/PKN1/SH3BP5L/PIP4K2C/SPRED1/HYAL2/CDK5RAP2/CRKL/IL1B/PARD6A/PTPN6/MYADM/GSTP1/TRIB1/PTPRC/PIK3CB/CHMP6/ENG/SEMA4D/CTDSP1/SMPD1/DTNBP1/PRR5L/PYCARD/CACTIN/TGFB1/GIT1/YWHAG/CBFA2T3/CTDSP2/MEN1/MVP/CORO1C/LYN/ADAR/GREM1/CDKN1C/CEACAM1/RUBCN/SFRP2/PPARA/LRRK1/STAT3/KAT2B/ABL1/PARD3/FBP1/WARS1/ARRB2/NIBAN1/PRKAR2A/CALM3/PPP4C/PTPRJ/INPP5K/HSPB1/NF2/HEXIM2/PLEC/SLC4A1/LRP1/CSK/PTPN1/FKBP8/CTDSPL/UBASH3B/SNCA/EIF4G1/PRKDC/PIP4K2A/FBXO7/CDKN1A/CBL/PAK2/PRKAR2B/DMTN

BP	GO:0010876	lipid localization	262	-0.43	-1.66	2.17E-05	0.00022552	0.00013983	2558	tags=34%, list=22%, signal=27%	OSBPL5/STARD3/AKT1/ABCA3/GRAMD1B/POMC/ABCB11/PTPN11/ACACB/CLN3/ATG2B/TSPO/STARD5/IKBKE/ATP8B3/ABCD1/TEX2/IL1B/PLIN3/P2RX4/OSBPL7/VPS4B/VPS51/SREBF2/PCTP/NR1H2/SCARB1/EDN1/STOML2/FTO/CPT1A/LIMA1/LDAF1/ABCC1/APOBR/SREBF1/CRY2/PLIN2/TSPO2/NR1H3/ATP9A/APOM/FITM1/ATG2A/SLC27A1/MFSD2A/ESYT1/ANO6/OSBPL10/PITPNC1/BSCL2/NME4/GOT2/PITPNM2/PITPNA/CEACAM1/ABCC3/PPARA/FIS1/PSAP/VLDLR/SYK/ABCG2/MEST/CPTP/ABCC4/ZFYVE1/PNPLA2/NFKB1/BMP6/RNF213/SLC2A1/GALR1/LDLRAP1/PLSCR3/SLC4A1/SLC66A2/LRP1/THBS1/AKT2/MFSD2B/PIP4K2A/EHD1/FURIN/ITGB3/CLU/HDLBP/OSBP2/DAB2
BP	GO:0007507	heart development	317	-0.40	-1.61	2.20E-05	0.00022781	0.00014125	2919	tags=34%, list=25%, signal=27%	ATM/PTCH1/IFT122/DVL1/ZFP36L1/SLC8A1/WDR11/MEF2D/MSX1/FRS2/TTN/TSC2/RPS6KA2/DCTN5/CASP7/EXT1/PKD1/MAPK14/MOSPD3/SMAD5/ZFPM1/JUN/TBX19/PLXNA4/PTPN11/NOTCH1/ACACB/G6PD/PRDM1/HES1/SMG9/SPRED1/ARID2/CRKL/SNX17/GNA11/ROCK2/SMARCD3/BASP1/PDE2A/MIB1/PDLIM7/SHC1/SMAD3/RUNX1/EDN1/ADAP2/ENG/RBPJ/GRK2/AP2B1/SOX4/NFATC1/ACVR2B/GSK3A/TGFB1/TNFRSF1B/RPGRIP1L/VGLL4/BCOR/KDM6B/NDST1/NCOA6/TPM1/DAW1/YY1/ILK/FZD2/RARA/PLXND1/DCHS1/SLC9A1/DZIP1/SOX6/GREM1/EIF4G2/BBS5/CALR/SFRP2/PSKH1/PPARA/IFT52/ZMIZ1/DHRS3/NRP1/KAT2B/FKBP1A/ODAD4/ARRB2/JARID2/GAA/NOTCH2/AP1B1/SUFU/FLNA/LMNA/ECE1/PLEC/CLDN5/AKAP13/PRKDC/MAPK3/MICAL2/CDKN1A/PDLIM1/NPRL3/MED1/MAP2K3/PIM1
BP	GO:0051603	proteolysis involved in cellular protein catabolic process	647	-0.34	-1.45	2.22E-05	0.00022894	0.00014195	1917	tags=23%, list=16%, signal=21%	TRIB1/CHFR/CCAR2/MIB1/UBXN7/UBE2J2/ARRB1/CUL7/DERL1/RHBDD1/KEAP1/F8A1/CHMP6/SMARCC1/TAF1/TGFB1I1/CANX/OTUD7B/WDR81/UBE2D4/CTSF/NSFL1C/VPS37B/USP37/CHMP4B/PSME3/BCAP31/PSMB10/GSK3B/PRKACA/GSK3A/HSP90AB1/KLHL22/TBX21/CBFA2T3/DET1/PSMB3/ENC1/EDEM2/KCTD2/CNOT4/ALAD/UBR2/ZRANB1/UBE2G1/UBA52/NEDD4L/DDA1/USP31/CDC27/FBXL18/PINK1/PTK2B/HSPA1A/ANKIB1/IFI27/USP12/TRIM32/MARCHF6/TBL1XR1/FBXO31/CHMP1A/UBE2Z/PML/UBE2R2/RNF115/CALR/RBCK1/RMND5A/CDK2/HERC2/LAPTM5/AMFR/UBA1/USP19/TBL1X/PSMD11/MAP1A/DDI2/BTRC/USP9X/GABARAP/USP34/FBXO6/DDB1/VCP/USP36/ARIH2/KLHL8/UBAP1/N4BP1/PITHD1/FBXW4/PSMC4/RNF213/RAD23A/DTX3L/ARRB2/RNF11/WWP2/GID8/UBE2S/SUFU/MAN1A1/ZNRF1/PSMD2/PSME4/USP7/HIPK2/VPS28/SIAH2/UBB/UBR4/GNA12/USP22/FZR1/HUWE1/PSMF1/SQSTM1/YOD1/UBE2L3/RNF187/ARAF/SHARPIN/ANKRD9/RNF123/PCBP2/FBXO7/DCAF12/CBL/TRIP12/ASCC2/UBL7/RYBP/ZER1/CTSB/GPX1/RNF40/DNAJB2/CDC34/UBXN6/TOLLIP/PTPN23/UBE2H/DCAF11/CUL4A/TRIM58/NPLOC4/CLU/DAB2/BAG6
BP	GO:0016032	viral process	344	-0.40	-1.59	2.25E-05	0.00023154	0.00014356	2484	tags=33%, list=21%, signal=26%	RRP1B/JUN/NOTCH1/KPNA3/BICD1/ZFP36/MVB12B/NCAM1/TSG101/BANF1/KPNA6/OAS2/OAS3/HLA-DRB1/HYAL2/CD81/CSDE1/ICAM1/VPS4B/PARD6A/CSF1R/SELPLG/PABPC1/ROCK2/ITGB7/MGAT5/ST6GAL1/CDK9/MAN2A1/SCARB1/CHMP6/CD74/GRK2/CANX/CTBP1/PDE12/EIF3A/VPS37B/CHMP4B/SMPD1/TOP2A/GSK3B/STOM/GSK3A/HSP90AB1/ST3GAL3/SMARCA4/NFIA/EDEM2/APOBEC3D/IST1/TRIM26/MAVS/RAB7A/ADAR/GYPA/IFITM3/HSPA1A/IFI27/MON1B/TRIM32/ZC3HAV1/CHMP1A/SRPK2/USF2/SNX3/PPARA/SLC1A5/PTBP1/TRIM10/TRIM28/IFITM2/NRP1/DDB1/VCP/APOBEC3F/N4BP1/LTF/RAD23A/STAU1/SETDB1/WWP2/EIF3B/LGALS9/SLC3A2/TBC1D20/BSG/INPP5K/ATG7/TRIM11/ST6GALNAC4/BCL2L1/VPS28/ITGB5/GSN/CFL1/RAB1B/IGF2R/TRIM14/ZNFX1/SP1/MGAT4B/LAMP1/APOBEC3C/PCBP2/TNIP1/ST3GAL1/CTSB/FURIN/ITGB3/LARP1/HCFC1

CC	GO:0009898	cytoplasmic side of plasma membrane	122	-0.51	-1.81	2.30E-05	0.00023631	0.00014652	2642	tags=42%, list=23%, signal=33%	FGR/TRAF2/JUP/GNAQ/GNB2/AKT1/GNAO1/DLG4/DIABLO/G6PD/FYN/CNR2/GNA11/TNK2/FES/PKP4/PTPRC/TIAM1/GNAZ/CHMP4B/SAMD10/EZR/GNAS/MYZAP/TRAF6/ESYT1/ALOX15/HIP1/LYN/RAC1/PTK2B/GNA15/GNB1/SRC/RASA3/GNAI2/SLA/PPP3CA/LITAF/MAP2K2/SYK/RHOA/LDLRAP1/GNA12/SLC4A1/PPP1R9B/IQGAP1/SPTA1/SPTB/MYH9/ANK1
BP	GO:0016358	dendrite development	144	-0.49	-1.78	2.31E-05	0.00023631	0.00014652	2605	tags=38%, list=22%, signal=30%	CAPRIN1/PAK4/MINK1/DISC1/CD3E/EZH2/DLG4/EPHA4/MECP2/CDK5/ABI3/FYN/CRKL/DOCK10/SRGAP2/PACSIN1/CUL7/KIAA0319/RERE/MAP1S/SEMA4D/DTNBP1/GSK3B/BAIAP2/CYFIP1/ASAP1/TRAPPC4/MFSD2A/BCL11A/NEDD4L/CDKL5/SIPA1L1/FBXO31/EIF4G2/IQSEC1/PPP3CA/TAOK2/NRP1/MAP1A/VLDLR/YWHAH/ABL1/SYNGAP1/RHOA/CAMK1D/STK11/DNM3/CFL1/PPP1R9B/CUX1/CAMK1/NEURL1/PAK2/CARM1
BP	GO:0090263	positive regulation of canonical Wnt signaling pathway	68	-0.58	-1.91	2.36E-05	0.00024166	0.00014984	1909	tags=35%, list=16%, signal=30%	CCAR2/PIN1/SMAD3/CSNK1G1/RBPJ/SOX4/TGFB1/DAPK3/ILK/TBL1XR1/SRC/SFRP2/RUVBL1/LRRK1/TBL1X/USP34/VCP/XIAP/NFKB1/GID8/FAM53B/CTDNEP1/PPM1A/WNK1
CC	GO:0043197	dendritic spine	99	-0.54	-1.88	2.38E-05	0.00024267	0.00015046	2567	tags=47%, list=22%, signal=37%	RAB8A/SYT11/NR1D1/CD3E/DLG4/RPH3A/EPHA4/FXR2/ABI3/ABHD17C/P2RX4/DOCK10/CTNND1/DNAJB1/SRGAP2/PDE4B/CANX/PPP1R9A/KCNJ2/SEPTIN11/DTNBP1/BAIAP2/PRKACA/CYFIP1/ASAP1/STX4/KIF3B/HIP1R/RAC1/PTK2B/SIPA1L1/SLA/ARHGAP32/PPP3CA/PPP1CA/BCR/APBA3/ARRB2/RHOA/DNM3/PPP1R9B/NEURL1/NRGN/ACTN1/PRKAR2B/CTTN/SHISA7
BP	GO:0006869	lipid transport	222	-0.43	-1.66	2.39E-05	0.00024289	0.0001506	2558	tags=33%, list=22%, signal=27%	OSBPL5/STARD3/AKT1/ABCA3/GRAMD1B/POMC/ABCB11/PTPN11/CLN3/ATG2B/TSPO/STARD5/ATP8B3/ABCD1/TEX2/IL1B/P2RX4/OSBPL7/VPS4B/VPS51/PCTP/NR1H2/SCARB1/EDN1/CPT1A/LIMA1/ABCC1/APOBR/CRY2/PLIN2/TSPO2/NR1H3/ATP9A/APOM/ATG2A/SLC27A1/MFSD2A/ESYT1/ANO6/OSBPL10/PITPNC1/NME4/GOT2/PITPNM2/PITPNA/CEACAM1/ABCC3/PPARA/FIS1/PSAP/VLDLR/SYK/ABCG2/CPTP/ABCC4/NFKB1/BMP6/SLC2A1/GALR1/LDLRAP1/PLSCR3/SLC4A1/SLC66A2/LRP1/THBS1/AKT2/MFSD2B/PIP4K2A/FURIN/ITGB3/CLU/HDLBP/OSBP2/DAB2
BP	GO:0031668	cellular response to extracellular stimulus	185	-0.45	-1.70	2.49E-05	0.00025219	0.00015636	2057	tags=29%, list=18%, signal=24%	NUAK2/SREBF2/HSPA5/DCPS/RIPOR1/FES/MAP1LC3B/GCN1/ARHGAP35/PTPRC/RRAGD/TFEB/VPS41/EHMT2/SREBF1/NCOA1/DAP/TTC5/UCP2/MFSD2A/MAP3K5/SESN2/LYN/RXRA/STK24/SLC39A4/SFRP2/USF2/PPARA/MAX/PDK2/GABARAP/ZFYVE1/RRAGC/FOS/SLC2A1/WIPI2/SESN3/KANK2/ATG7/PLEC/GLUL/PPP1R9B/EIF4G1/MAPK3/ELAPOR1/CDKN1A/CBL/WDR45/NPRL3/FOXO3/MED1/GAS2L1/PIM1
BP	GO:0010634	positive regulation of epithelial cell migration	100	-0.54	-1.89	2.55E-05	0.00025826	0.00016013	2309	tags=37%, list=20%, signal=30%	INSL3/PFN1/PRKD2/P2RX4/MAPRE2/ROCK2/MAP3K3/SCARB1/EDN1/PIK3CB/MMP9/FOXP1/TGFB1/ETS1/BCAS3/SASH1/RAC1/PTK2B/RRAS/SRC/IQSEC1/CALR/PIK3CD/NRP1/ABL1/CLASP1/HSPB1/STAT5A/GFUS/THBS1/PIK3CG/SP1/GPI/GRN/ITGB3/SPARC/MAP2K3

BP	GO:0048812	neuron projection morphogenesis	338	-0.40	-1.59	2.58E-05	0.00025994	0.00016117	2622	tags=33%, list=22%, signal=26%	EXT1/CAPRIN1/ULK1/RAB8A/MINK1/DIP2B/TAOK3/MUL1/PLXNC1/DISC1/PLXNA4/ADNP/PTPN11/NOTCH1/EFNB1/DLG4/NCAM1/LYPLA2/MAP3K13/LMTK2/EPHA4/CDK5/MOV10/DDR1/ABI3/FYN/CPNE1/FEZ1/SLITRK5/DOCK10/CSF1R/MACF1/SRGAP2/PACSIN1/PARD6B/CUL7/ARHGAP35/WEE1/APLP2/EDN1/KIAA0319/TIAM1/RERE/DIXDC1/MAP1S/B4GALT5/NDEL1/SEMA4D/DTNBP1/GSK3B/BAIAP2/CYFIP1/HSP90AB1/TAOK1/BCL11B/MFSD2A/BCL11A/IST1/NEDD4L/PLXNB2/CDKL5/RAC1/MBP/DPYSL2/PLXND1/TRIO/KIF13B/SIPA1L1/MARK2/FBXO31/EIF4G2/GAS7/CPNE5/STK25/DVL3/MYCBP2/PLXNA1/PPP3CA/TAOK2/ACTB/SEMA4A/NRP1/MAP1A/VLDLR/YWHAH/USP9X/ABL1/PAK1/PARD3/SYNGAP1/XK/RHOA/VCL/NOTCH2/DNM2/RAC2/BSG/ECE1/NIBAN2/STK11/UBB/CYFIP2/DNM3/CFL1/IQGAP1/CUX1/VASP/RHOG/PAK2/CTTN/KEL/SCN1B
BP	GO:0048667	cell morphogenesis involved in neuron differentiation	305	-0.41	-1.61	2.61E-05	0.00026234	0.00016266	2622	tags=32%, list=22%, signal=26%	EXT1/CAPRIN1/ULK1/RAB8A/MINK1/DIP2B/PLXNC1/DISC1/PLXNA4/ADNP/PTPN11/NOTCH1/EFNB1/DLG4/NCAM1/TRIOBP/LYPLA2/MAP3K13/LMTK2/EPHA4/HES1/CDK5/UNK/ABI3/FYN/FEZ1/SLITRK5/TBCD/DOCK10/CSF1R/MACF1/PARD6B/CUL7/ARHGAP35/APLP2/EDN1/KIAA0319/TIAM1/RERE/DIXDC1/MAP1S/B4GALT5/NDEL1/SEMA4D/DTNBP1/GSK3B/BAIAP2/CYFIP1/HSP90AB1/BCL11B/MFSD2A/IST1/NEDD4L/PLXNB2/CDKL5/RAC1/MBP/DPYSL2/PLXND1/TRIO/KIF13B/SIPA1L1/MARK2/FBXO31/EIF4G2/STK25/MYCBP2/PLXNA1/PPP3CA/TAOK2/ACTB/SEMA4A/NRP1/MAP1A/VLDLR/YWHAH/USP9X/ABL1/PAK1/PARD3/SYNGAP1/XK/VCL/NOTCH2/DNM2/RAC2/BSG/ECE1/NIBAN2/STK11/DNM3/CFL1/CUX1/VASP/RHOG/PAK2/CTTN/KEL/SCN1B
BP	GO:0031346	positive regulation of cell projection organization	232	-0.43	-1.67	2.63E-05	0.00026234	0.00016266	2610	tags=42%, list=22%, signal=33%	STYXL1/SRF/EEF2K/STAU2/SF3A2/SEMA7A/SHTN1/PRKCI/ACTR2/WRAP73/KIT/F2RL1/KIDINS220/DVL1/ARSB/FSCN1/ABI2/RETREG3/CROCC/ATP7A/CORO1B/CAPRIN1/EPS8/MARK4/NCKIPSD/PLXNC1/PIK3CA/DISC1/PLXNA4/ADNP/EZH2/DLG4/MAP3K13/EPHA4/MAGI2/PFN1/FYN/ARAP1/FEZ1/HSPA5/MACF1/FES/PACSIN1/CUL7/ARHGAP35/TIAM1/RIPOR2/DIXDC1/NDEL1/SEMA4D/GSK3B/BAIAP2/CYFIP1/ENC1/CDC42EP4/CORO1C/KATNB1/IST1/LYN/BCAS3/PLXNB2/CDKL5/RAC1/PTK2B/PLXND1/STK24/DZIP1/MARK2/FBXO31/SRC/EIF4G2/STK25/SNX3/DVL3/PLXNA1/ATP1B2/NRP1/VLDLR/ABL1/RAPGEF1/PLEK2/DNM2/FLNA/FKBP1B/RAC2/CAMK1D/STK11/DNM3/PPP2R5B/GRN/CUX1/CAMK1/NEURL1/EHD1/HTT/WASF2/SCN1B

CC	GO:0000323	lytic vacuole	589	-0.35	-1.49	2.63E-05	0.00026234	0.00016266	2730	tags=33%, list=23%, signal=26%	FUCA2/SDCBP/TSC2/NCF2/SPPL2B/DNAJC13/FAF2/FNBP1/ATP13A2/PLD1/AP2A1/RMC1/AP5S1/BPI/GNAQ/GNB2/STARD3/ABCA3/AP2A2/DPP7/ACLY/PCYOX1/ACP2/SYT11/NSF/CPQ/TRPM2/NCF1/CLN3/LAMP2/TMBIM1/CYBRD1/FLCN/CPNE3/M6PR/HGSNAT/AP5B1/ABCB9/HLA-DRB1/ABCD1/PI4K2A/IMPDH1/MLC1/WDR24/HYAL2/HLA-DQA2/CMTM6/MARCHF3/CPNE1/IL1B/VPS39/P2RX4/MFSD12/GNA11/RNASE3/TCN2/NCOA4/ITM2C/PLA2G15/CTSA/TPP1/ARRB1/TOM1/LRBA/SCARB1/ADA2/HGS/RRAGD/NEU3/TFEB/SLC35F6/CHMP6/CD74/ANKFY1/BBC3/VPS41/WDR81/CTSF/CHMP4B/AP2B1/CXCR2/SMPD1/TRAPPC1/HLA-DQA1/HYAL3/TECPR1/STOM/SDC3/PYCARD/HSP90AB1/CD63/KLHL22/CLTC/SYNGR1/SLC44A2/DNAJC5/IL4I1/CHID1/GLB1/AP1M1/NAGA/IST1/UNC93B1/UBA52/AP2S1/LNPEP/LYN/GLMP/VTI1B/RAB7A/EPG5/PLEKHM2/IFITM3/PRF1/PLD3/GNB1/NCSTN/DYNC1H1/SRC/CHMP1A/CALR/RUBCN/ATP6V1A/LRRC8A/HLA-DRB5/PLEKHF1/LAPTM5/PSAP/ARHGAP45/LITAF/IFITM2/UBA1/VLDLR/GABARAP/CAP1/VCP/ATP6V0D1/RRAGC/RAB14/FNIP2/PYGB/ANXA6/DTX3L/ATP6V0C/LAMTOR1/TUBB4B/CLCN5/GAA/ANXA11/SLC7A5/SIDT2/AP1B1/SLC3A2/GPR137/ARL8A/ZNRF1/RAB3D/ECE1/PA2G4/ATP6V0A1/MCOLN1/CYB5R3/CDIP1/FTH1/SDC4/SORT1/SQSTM1/LRP1/RILP/TRIP10/GNS/MARCHF2/GRN/SNCA/MMD/PRDX6/LAMP1/CAPN1/PIP4K2A/ELAPOR1/MARCHF8/CTSD/TUBB/FTL/SLC48A1/CTSB/HPS1/SLC15A3/NPRL3/UBXN6/TOLLIP/AP2M1/DAB2
CC	GO:0005764	lysosome	589	-0.35	-1.49	2.63E-05	0.00026234	0.00016266	2730	tags=33%, list=23%, signal=26%	FUCA2/SDCBP/TSC2/NCF2/SPPL2B/DNAJC13/FAF2/FNBP1/ATP13A2/PLD1/AP2A1/RMC1/AP5S1/BPI/GNAQ/GNB2/STARD3/ABCA3/AP2A2/DPP7/ACLY/PCYOX1/ACP2/SYT11/NSF/CPQ/TRPM2/NCF1/CLN3/LAMP2/TMBIM1/CYBRD1/FLCN/CPNE3/M6PR/HGSNAT/AP5B1/ABCB9/HLA-DRB1/ABCD1/PI4K2A/IMPDH1/MLC1/WDR24/HYAL2/HLA-DQA2/CMTM6/MARCHF3/CPNE1/IL1B/VPS39/P2RX4/MFSD12/GNA11/RNASE3/TCN2/NCOA4/ITM2C/PLA2G15/CTSA/TPP1/ARRB1/TOM1/LRBA/SCARB1/ADA2/HGS/RRAGD/NEU3/TFEB/SLC35F6/CHMP6/CD74/ANKFY1/BBC3/VPS41/WDR81/CTSF/CHMP4B/AP2B1/CXCR2/SMPD1/TRAPPC1/HLA-DQA1/HYAL3/TECPR1/STOM/SDC3/PYCARD/HSP90AB1/CD63/KLHL22/CLTC/SYNGR1/SLC44A2/DNAJC5/IL4I1/CHID1/GLB1/AP1M1/NAGA/IST1/UNC93B1/UBA52/AP2S1/LNPEP/LYN/GLMP/VTI1B/RAB7A/EPG5/PLEKHM2/IFITM3/PRF1/PLD3/GNB1/NCSTN/DYNC1H1/SRC/CHMP1A/CALR/RUBCN/ATP6V1A/LRRC8A/HLA-DRB5/PLEKHF1/LAPTM5/PSAP/ARHGAP45/LITAF/IFITM2/UBA1/VLDLR/GABARAP/CAP1/VCP/ATP6V0D1/RRAGC/RAB14/FNIP2/PYGB/ANXA6/DTX3L/ATP6V0C/LAMTOR1/TUBB4B/CLCN5/GAA/ANXA11/SLC7A5/SIDT2/AP1B1/SLC3A2/GPR137/ARL8A/ZNRF1/RAB3D/ECE1/PA2G4/ATP6V0A1/MCOLN1/CYB5R3/CDIP1/FTH1/SDC4/SORT1/SQSTM1/LRP1/RILP/TRIP10/GNS/MARCHF2/GRN/SNCA/MMD/PRDX6/LAMP1/CAPN1/PIP4K2A/ELAPOR1/MARCHF8/CTSD/TUBB/FTL/SLC48A1/CTSB/HPS1/SLC15A3/NPRL3/UBXN6/TOLLIP/AP2M1/DAB2

BP	GO:0032101	regulation of response to external stimulus	625	-0.35	-1.46	2.63E-05	0.00026234	0.00016266	2262	tags=26%, list=19%, signal=22%	OAS3/CCDC92/IKBKE/IL6R/EPHA4/HLA-DRB1/IRF1/PRKD2/HYAL2/CD81/FYN/IL1B/SPI1/P2RX4/CNR2/PROS1/HLA-A/RIPK1/CSF1R/PTPN6/GGT1/CERS2/SCIMP/PGLYRP1/GSTP1/ST6GAL1/PDE2A/TRIB1/NR1H2/SPATA2/XRCC6/CDC37/SMAD3/PTPRC/EDN1/KIAA0319/RIPOR2/DROSHA/CREB3/CD74/MMP9/EPPK1/NDEL1/NCR3/KLF4/SEMA4D/ABCC1/SIGIRR/FOXP1/DDX60/ANKRD17/NLRX1/NAPEPLD/NR1H3/FAM3A/PYCARD/AKNA/CACTIN/HSP90AB1/TGFB1/MICB/GIT1/LILRA5/TNFRSF1B/ETS1/RBM14/SPHK1/CCN3/CD180/STX4/CASP5/TRAF6/OXSR1/ALOX15/ZYX/KARS1/ANO6/LYN/MAVS/SASH1/SERPING1/PIK3AP1/IL17RA/PUM1/ADAR/RAC1/JAM3/PTK2B/RXRA/CD9/TTLL12/CGAS/CREBBP/STK24/ILRUN/SRC/GREM1/CEACAM1/CALR/PPARA/MYCBP2/VAV1/RIN3/SEMA4A/NRP1/BCR/SYK/XIAP/APOBEC3F/PAK1/TNFSF4/OPTN/CLASP1/N4BP1/RELA/LTF/NFKB1/BMP6/DTX3L/ARRB2/SIGLEC10/CHD8/MFHAS1/LGALS9/FLNA/FKBP1B/IFIT1B/RAC2/KANK2/HSPB1/CDK19/CAMK1D/KRT1/MPP1/RIOK3/GP1BA/ALOX12/THBS1/PTPN1/ZNFX1/PIK3CG/SEC14L1/GRN/UBASH3B/SHARPIN/SNCA/PRKDC/LAMP1/MAPK3/PCBP2/BRD4/AKIRIN2/TNIP1/PAK2/GPX1/FURIN/MED1/MGLL/PDGFA/NPLOC4/DMTN/WNK1/TBXA2R
BP	GO:0006096	glycolytic process	61	-0.60	-1.93	2.68E-05	0.00026688	0.00016548	2017	tags=39%, list=17%, signal=33%	ENTPD5/PGAM1/ZBTB7A/EIF6/FOXK1/ENO2/GALK1/FOXK2/GIT1/CBFA2T3/JMJD8/ALDOA/ALDOC/PFKL/ARNT/PPARA/PKM/BPGM/STAT3/FBP1/HK1/SLC4A1/GPI/ENO1
BP	GO:0009132	nucleoside diphosphate metabolic process	98	-0.52	-1.83	2.74E-05	0.00027119	0.00016815	2058	tags=32%, list=18%, signal=26%	AK3/ENTPD5/PGAM1/ZBTB7A/EIF6/FOXK1/ENO2/GALK1/FOXK2/GIT1/CBFA2T3/JMJD8/ALDOA/NME4/ALDOC/BAD/PFKL/ARNT/AK1/PPARA/PKM/BPGM/STAT3/TJP2/FBP1/HK1/MPP1/SLC4A1/GPI/ENO1/GUK1
CC	GO:0043025	neuronal cell body	266	-0.42	-1.63	2.83E-05	0.00028018	0.00017372	2569	tags=32%, list=22%, signal=26%	SORL1/RAB8A/DIP2B/MUL1/SYT11/ADNP/TRPM2/CYBB/NCF1/CD22/ZNF385A/EIF4A3/LMTK2/EPHA4/DMWD/PI4K2A/CDK5/FXR2/TOP1/FEZ1/YKT6/P2RX4/CNR2/SMN1/DNAJB1/NUMA1/DIXDC1/ENO2/FZD5/MAP1S/RAP1GAP/NDEL1/CANX/TPX2/KCNJ2/DTNBP1/BAIAP2/GSK3A/PYCARD/CYFIP1/HSP90AB1/TBX21/TNFRSF1B/PDE1A/ENC1/CCN3/KATNB1/HIP1R/VTI1B/MBP/NPFF/PTK2B/FBXO31/CPNE5/SEPTIN4/VTI1A/RIN3/AMFR/PPP1CA/SLC1A4/MAP1A/MCRS1/ABL1/STAU1/MPL/CKB/PDE9A/PVALB/FLNA/SLC3A2/GLRX5/ELK1/ROGDI/SIAH2/UBB/GNA12/DCTN1/GLUL/PPP1R9B/GJC2/SNCA/NEURL1/NRGN/PRKAR2B/SCN1B/HCFC1
BP	GO:2001236	regulation of extrinsic apoptotic signaling pathway	109	-0.53	-1.86	2.90E-05	0.00028515	0.0001768	2184	tags=36%, list=19%, signal=29%	HYAL2/ICAM1/IL1B/RIPK1/MADD/ITM2C/GSTP1/LGALS3/TMC8/BIRC6/PTPRC/ITPRIP/PSME3/GSK3B/PYCARD/STX4/BCL2L14/HSPA1A/SRC/PML/RBCK1/SFRP2/PPP1CA/CTNNA1/NRP1/ARHGEF2/RELA/LMNA/BCL2L1/SIAH2/THBS1/TCF7L2/GATA1/PF4/PAK2/STRADB/GPX1/CTTN/HTT

BP	GO:0055080	cation homeostasis	435	-0.37	-1.54	2.92E-05	0.00028597	0.00017731	2791	tags=32%, list=24%, signal=25%	TMTC2/SLC40A1/CCRL2/LETM1/CCDC115/COX19/ATP13A2/PDPK1/CMKLR1/ATP7A/EXT1/PKD1/ATP2A3/STEAP3/CORO1A/SELENOK/PTGIR/SWAP70/SLC46A1/KCNH2/DISC1/KNG1/TRPM2/BAK1/ATP1A1/CLN3/CYBRD1/TRPC3/ATP2A2/DLG4/SLC24A4/CCR1/CHP1/CDK5/HYAL2/MAFG/FYN/SLC25A23/ATP1B1/P2RX4/PTPN6/ATP13A3/ITPR2/SLC9A7/NCOA4/TMC8/LPAR4/SMAD3/RAB20/MICU1/PTPRC/EDN1/PIK3CB/STOML2/SLC41A1/FRRS1/KCNJ2/CXCR2/BCAP31/TM9SF4/PRKACA/HVCN1/CXCR1/SNX10/CNNM3/ATP6AP1/SELENON/FPR3/CHERP/LYN/NEDD4L/SPPL3/EPAS1/RAB7A/PTK2B/GNA15/MT2A/ARF1/SLC9A1/SLC22A17/SLC39A4/PML/CA2/RASA3/CALR/GNAI2/ATP6V1A/ATP13A1/SLC7A8/FIS1/ATP1B2/PDK2/JAK3/XIAP/ATP6V0D1/ABL1/LCN2/FKBP1A/STIM1/LTF/ANXA6/BMP6/XK/GALR1/CALM3/FKBP1B/ATP6V0A1/MCOLN1/SLC6A9/SLC24A3/FTH1/TTC7A/SLC4A1/GP1BA/EPB42/PRKCB/GP9/HRH4/PIK3CG/GRN/UBASH3B/ATP2B4/SNCA/ANKRD9/P2RX1/MAPK3/GATA1/GRINA/FTL/ALAS2/SLC25A37/EIF2AK1/HTT/MLLT6/KEL/TGM2/WNK1/DIAPH1/TBXA2R
BP	GO:2001237	negative regulation of extrinsic apoptotic signaling pathway	67	-0.58	-1.88	2.97E-05	0.00029045	0.00018009	2142	tags=40%, list=18%, signal=33%	ICAM1/IL1B/RIPK1/GSTP1/LGALS3/BIRC6/ITPRIP/PSME3/GSK3B/HSPA1A/SRC/SFRP2/CTNNA1/NRP1/ARHGEF2/RELA/LMNA/BCL2L1/SIAH2/THBS1/TCF7L2/GATA1/PF4/STRADB/GPX1/CTTN/HTT
BP	GO:0048010	vascular endothelial growth factor receptor signaling pathway	31	-0.71	-2.00	3.02E-05	0.00029428	0.00018247	2216	tags=58%, list=19%, signal=47%	MAPKAPK3/PRKD2/FYN/IL1B/VAV2/HGS/PTK2B/SRC/ARNT/VAV1/NRP1/MAPKAPK2/HSPB1/NIBAN2/PRKCB/PTPN1/PAK2/ITGB3
BP	GO:0043269	regulation of ion transport	336	-0.40	-1.58	3.09E-05	0.00030073	0.00018646	2571	tags=30%, list=22%, signal=24%	PTAFR/CORO1A/MINK1/AKT1/TMEM109/KCNH2/SYT11/ABCB11/CYBB/BAK1/ATP1A1/TRPC3/DLG4/TSPO/CCR1/CHP1/G6PD/HES1/NETO2/CDK5/FYN/FXYD6/ATP1B1/IL1B/P2RX4/RIPK1/PTPN6/SNTA1/SLC43A2/LGALS3/PDE4B/EDN1/MMP9/KCNJ2/KCNQ5/DTNBP1/EHD3/STOM/CRY2/PRKACA/HVCN1/CD63/LILRA5/RANGRF/CLIC5/DAPK1/SELENON/ARL6IP1/OXSR1/FHL1/ANO6/LYN/NEDD4L/PINK1/PTK2B/SLC9A1/PML/CA2/GNAI2/ATP1B2/CACNG6/CNIH3/YWHAH/VDAC1/SYK/JAK3/ABL1/FKBP1A/STIM1/ARRB2/WWP2/RHOA/CALM3/DNM2/FLNA/RAB11B/FKBP1B/MYLK/INPP5K/SLC6A9/EPB41/GJC2/PIK3CG/SLC43A1/PER2/UBASH3B/ATP2B4/SNCA/AHCYL1/CAPN1/P2RX1/ACTN4/TESC/HTT/MLLT6/SHISA7/ITGB3/KEL/SCN1B/WNK1/DIAPH1
BP	GO:0043547	positive regulation of GTPase activity	189	-0.45	-1.70	3.13E-05	0.00030351	0.00018819	2134	tags=30%, list=18%, signal=25%	ARAP1/CRKL/DOCK10/MAPRE2/RALBP1/SRGAP2/PIN1/PKP4/RCC2/RGS10/ARHGAP35/TIAM1/ADAP2/LIMS1/RAP1GAP/NDEL1/RGS16/SEMA4D/GSK3B/TBC1D17/NEDD9/TBC1D22A/RASGRP3/ASAP2/PREX1/ASAP1/SIPA1L3/CORO1C/BCAS3/RABGAP1/CDKL5/PTK2B/EVI5L/SIPA1L1/IQSEC1/DVL3/VAV1/GMIP/ARHGAP45/TBC1D13/RANGAP1/BCR/RGP1/RAPGEF1/ARHGAP6/TBC1D22B/TBC1D20/RSU1/AKT2/RGS6/RHOG/TBC1D5/TBC1D25/TBC1D1/SH3BP1/TGM2

CC	GO:0010008	endosome membrane	422	-0.38	-1.55	3.17E-05	0.0003065	0.00019004	2756	tags=34%, list=24%, signal=27%	EPHB1/SNX8/SPPL2B/DNAJC13/ATP13A2/PLD1/AP2A1/RMC1/ATP7A/AP5S1/KIF16B/RAB11FIP5/STEAP3/SORL1/RAB8A/SBF2/STARD3/ABCA3/AP2A2/SYT11/LZTR1/ABCB11/CLN3/LAMP2/TMBIM1/MVB12B/TSG101/SNX27/EPHA4/HLA-DRB1/ABHD17C/HLA-DQA2/CMTM6/MARCHF3/VPS39/PLIN3/SNX17/HLA-A/VPS4B/RIPK1/ATP13A3/SLC9A7/NDRG1/RCC2/HGS/KIAA0319/NEU3/CHMP6/FZD5/CD14/ANKFY1/RHOBTB1/HLA-C/VPS41/WDR81/SNX12/VPS37B/CHMP4B/AP2B1/DTNBP1/YIPF2/VPS53/HLA-DQA1/EHD3/NAPEPLD/ATP9A/CD63/CLTC/SPHK1/SNX10/ATP6AP1/EHD4/CORO1C/TRAF6/SUN2/RAB31/UBA52/AP2S1/VTI1B/RAB7A/LEPROT/RAC1/PLEKHM2/IFITM3/TMEM184A/MICALL1/PLD3/SH3GL1/NCSTN/GRIPAP1/SLC39A4/CHMP1A/PML/SNX3/HLA-DRB5/ARHGAP32/VTI1A/PLEKHF1/RAB11FIP4/LITAF/IFITM2/UBA1/CPTP/ATP6V0D1/RAB14/OPTN/UBAP1/RAB27B/ANXA6/DTX3L/ATP6V0C/LAMTOR1/CLCN5/RAB5B/RAB11B/ARL8A/ECE1/ARHGAP1/ATP6V0A1/MCOLN1/CDIP1/VPS28/UBB/MMGT1/SPG21/SORT1/IGF2R/RILP/MARCHF2/MMD/LAMP1/TBC1D5/ELAPOR1/MARCHF8/CTSD/EHD1/SLC48A1/RUFY1/SLC15A3/FURIN/UBXN6/AP2M1/SCAMP2
BP	GO:0010232	vascular transport	59	-0.61	-1.94	3.20E-05	0.0003073	0.00019054	1892	tags=39%, list=16%, signal=33%	SLC7A1/SLC29A2/SLC19A1/ABCC1/SLC27A1/MFSD2A/SLC5A6/ABCC3/SLC7A8/SLC1A5/ATP1B2/SLC1A4/SLC6A20/ABCG2/ABCC4/SLC2A1/SLC7A5/SLC6A9/SLC24A3/LRP1/ATP2B4/SLC29A1/SLC38A5
BP	GO:0150104	transport across blood-brain barrier	59	-0.61	-1.94	3.20E-05	0.0003073	0.00019054	1892	tags=39%, list=16%, signal=33%	SLC7A1/SLC29A2/SLC19A1/ABCC1/SLC27A1/MFSD2A/SLC5A6/ABCC3/SLC7A8/SLC1A5/ATP1B2/SLC1A4/SLC6A20/ABCG2/ABCC4/SLC2A1/SLC7A5/SLC6A9/SLC24A3/LRP1/ATP2B4/SLC29A1/SLC38A5
BP	GO:0097191	extrinsic apoptotic signaling pathway	164	-0.46	-1.71	3.19E-05	0.0003073	0.00019054	2256	tags=37%, list=19%, signal=30%	DIABLO/IL6R/HYAL2/ICAM1/CASP2/IL1B/SPI1/RIPK1/MADD/MKNK2/ITM2C/ZDHHC3/GSTP1/LGALS3/TMC8/BIRC6/SMAD3/ACVR1B/PTPRC/ITPRIP/PSME3/GSK3B/GSK3A/PYCARD/TGFB1/TNFRSF1B/BEX3/DAPK1/STX4/BCL2L14/HSPA1A/IFI27/BAD/BCL2L11/SRC/PML/RBCK1/SFRP2/DAXX/PPP1CA/CTNNA1/NRP1/ARHGEF2/GABARAP/RELA/HIPK1/LMNA/BCL2L1/SIAH2/SORT1/THBS1/TCF7L2/GATA1/PF4/PAK2/STRADB/GPX1/FOXO3/CTTN/HTT
BP	GO:1904062	regulation of cation transmembrane transport	186	-0.46	-1.73	3.28E-05	0.00031413	0.00019477	2267	tags=32%, list=19%, signal=26%	CHP1/G6PD/NETO2/CDK5/FYN/FXYD6/ATP1B1/PTPN6/SNTA1/SLC43A2/PDE4B/EDN1/MMP9/KCNJ2/EHD3/STOM/PRKACA/CD63/RANGRF/DAPK1/SELENON/OXSR1/FHL1/ANO6/LYN/NEDD4L/PINK1/PTK2B/SLC9A1/ATP1B2/CACNG6/CNIH3/YWHAH/VDAC1/ABL1/FKBP1A/STIM1/WWP2/CALM3/DNM2/FLNA/FKBP1B/SLC6A9/GJC2/PIK3CG/SLC43A1/UBASH3B/ATP2B4/SNCA/CAPN1/P2RX1/ACTN4/TESC/HTT/SHISA7/KEL/SCN1B/WNK1/DIAPH1

BP	GO:0050801	ion homeostasis	447	-0.37	-1.53	3.32E-05	0.00031723	0.0001967	2791	tags=32%, list=24%, signal=25%	TMTC2/FGF23/SLC40A1/CCRL2/LETM1/CCDC115/COX19/ATP13A2/PDPK1/CMKLR1/ATP7A/EXT1/PKD1/ATP2A3/STEAP3/CORO1A/SELENOK/PTGIR/SWAP70/SLC46A1/KCNH2/DISC1/KNG1/TRPM2/BAK1/ATP1A1/CLN3/CYBRD1/TRPC3/ATP2A2/DLG4/SLC24A4/CCR1/CHP1/CDK5/HYAL2/MAFG/FYN/SLC25A23/ATP1B1/P2RX4/PTPN6/ATP13A3/ITPR2/SLC9A7/NCOA4/TMC8/LPAR4/SMAD3/RAB20/MICU1/PTPRC/EDN1/PIK3CB/STOML2/SLC41A1/FRRS1/KCNJ2/CXCR2/BCAP31/TM9SF4/PRKACA/HVCN1/CXCR1/SNX10/CNNM3/ATP6AP1/SELENON/FPR3/CHERP/LYN/NEDD4L/SPPL3/EPAS1/RAB7A/PTK2B/GNA15/MT2A/ARF1/SLC9A1/SLC22A17/SLC39A4/PML/CA2/RASA3/CALR/GNAI2/ATP6V1A/ATP13A1/SLC7A8/FIS1/ATP1B2/PDK2/JAK3/XIAP/ATP6V0D1/ABL1/LCN2/FKBP1A/STIM1/LTF/ANXA6/BMP6/XK/CKB/GALR1/CALM3/FKBP1B/ATP6V0A1/MCOLN1/SLC6A9/SLC24A3/FTH1/TTC7A/SLC4A1/GP1BA/EPB42/PRKCB/GP9/HRH4/PIK3CG/GRN/UBASH3B/ATP2B4/SNCA/ANKRD9/P2RX1/MAPK3/GATA1/GRINA/FTL/ALAS2/SLC25A37/EIF2AK1/HTT/MLLT6/KEL/TGM2/WNK1/DIAPH1/TBXA2R
CC	GO:0044309	neuron spine	100	-0.54	-1.88	3.41E-05	0.00032533	0.00020171	2567	tags=47%, list=22%, signal=37%	RAB8A/SYT11/NR1D1/CD3E/DLG4/RPH3A/EPHA4/FXR2/ABI3/ABHD17C/P2RX4/DOCK10/CTNND1/DNAJB1/SRGAP2/PDE4B/CANX/PPP1R9A/KCNJ2/SEPTIN11/DTNBP1/BAIAP2/PRKACA/CYFIP1/ASAP1/STX4/KIF3B/HIP1R/RAC1/PTK2B/SIPA1L1/SLA/ARHGAP32/PPP3CA/PPP1CA/BCR/APBA3/ARRB2/RHOA/DNM3/PPP1R9B/NEURL1/NRGN/ACTN1/PRKAR2B/CTTN/SHISA7
BP	GO:0043244	regulation of protein-containing complex disassembly	98	-0.52	-1.81	3.64E-05	0.00034608	0.00021459	1683	tags=45%, list=14%, signal=39%	ADD2/EPS8/UBQLN4/SWAP70/PIK3CA/TMOD3/DSTN/CLN3/SPTAN1/ATXN7/TRIOBP/OGFOD1/SVIL/MAP1S/LIMA1/TPX2/PHF23/TECPR1/TAOK1/EIF5AL1/PLEK/WDR1/KATNB1/EIF5A/RUBCN/FLII/CAMSAP1/MAP1A/ARHGEF2/UPF1/CLASP1/GSN/SPTBN1/MTPN/SPTA1/SPTB/TMOD1/TBC1D25/SCIN/CAPZB/GSPT1/SH3BP1/DMTN/ADD1
MF	GO:0001221	transcription coregulator binding	91	-0.54	-1.85	3.87E-05	0.00036679	0.00022742	1993	tags=43%, list=17%, signal=36%	ZBTB16/WIZ/SMARCD3/ZBTB7A/CDK9/EHMT1/SMAD3/CTBP1/TRERF1/JUND/EHMT2/NFATC1/SREBF1/BAIAP2/HDAC3/SMARCA4/ETS1/VGLL4/NEK6/EPAS1/RARA/RXRA/CREBBP/PPARA/RUNX3/FAM89B/FOS/RELA/MED25/PER2/SP1/CHD4/ENO1/GATA1/HMGA1/FOXO3/MED1/HDGF/PBX1
BP	GO:0019935	cyclic-nucleotide-mediated signaling	37	-0.68	-1.99	3.93E-05	0.00037021	0.00022954	1186	tags=46%, list=10%, signal=41%	POMC/MAPK7/ADNP/INSL3/PDE2A/AQP1/PTK2B/CGAS/NUDT4/IRAG1/CAP1/PDE9A/GUCY1A1/THBS1/RUNDC3A/CDC34/GUCY1B1
BP	GO:0010506	regulation of autophagy	281	-0.41	-1.62	4.10E-05	0.00038407	0.00023814	1776	tags=26%, list=15%, signal=23%	KEAP1/GPSM1/RRAGD/PIK3CB/TFEB/FZD5/POLDIP2/PHF23/CHMP4B/EHMT2/IKBKG/KDM4A/SREBF1/TECPR1/GSK3B/FOXK2/PRKACA/GSK3A/PYCARD/DAP/KLHL22/ATG2A/DAPK3/DAPK1/VPS13D/SESN2/PINK1/MTDH/BAD/TRIM32/BCL2L11/EIF4G2/RUBCN/ATP6V1A/PLEKHF1/PSAP/STAT3/VDAC1/HERC1/CPTP/USP36/ATP6V0D1/ABL1/RRAGC/OPTN/PIM2/ATP6V0C/CAPNS1/GOLGA2/SESN3/GPR137/ATG7/HSPB1/ATP6V0A1/STK11/TRIM14/SNCA/EIF4G1/CAPN1/PIP4K2A/MAPK3/ELAPOR1/BNIP3L/FBXO7/DCAF12/TBC1D25/WDR45/NPRL3/FOXO3/CTTN/HTT/LARP1/SUPT5H
BP	GO:0019058	viral life cycle	253	-0.42	-1.62	4.15E-05	0.00038814	0.00024066	2359	tags=31%, list=20%, signal=25%	MVB12B/NCAM1/TSG101/BANF1/KPNA6/OAS2/OAS3/HLA-DRB1/HYAL2/CD81/ICAM1/VPS4B/SELPLG/PABPC1/ROCK2/ITGB7/SCARB1/CHMP6/CD74/GRK2/CTBP1/PDE12/VPS37B/CHMP4B/SMPD1/TOP2A/STOM/HSP90AB1/NFIA/APOBEC3D/IST1/TRIM26/MAVS/RAB7A/ADAR/GYPA/IFITM3/HSPA1A/IFI27/ZC3HAV1/CHMP1A/SRPK2/SNX3/PPARA/SLC1A5/TRIM10/TRIM28/IFITM2/NRP1/DDB1/VCP/APOBEC3F/N4BP1/LTF/RAD23A/STAU1/SETDB1/WWP2/LGALS9/SLC3A2/TBC1D20/BSG/INPP5K/TRIM11/VPS28/ITGB5/GSN/RAB1B/TRIM14/ZNFX1/LAMP1/APOBEC3C/PCBP2/TNIP1/CTSB/FURIN/ITGB3/LARP1

MF	GO:0005178	integrin binding	81	-0.56	-1.88	4.16E-05	0.00038872	0.00024102	2169	tags=35%, list=18%, signal=28%	CD81/ICAM1/IL1B/LCP1/ITGB7/ITGAM/LGALS12/CCN3/LYN/JAM3/IGF2/CD9/SRC/CALR/SFRP2/SYK/ITGAL/FERMT3/NF2/ITGB5/THBS1/CD151/ACTN4/ACTN1/MYH9/ITGB3/ITGA2B/TLN1
BP	GO:0030042	actin filament depolymerization	46	-0.65	-2.00	4.21E-05	0.00039211	0.00024312	383	tags=37%, list=3%, signal=36%	PLEK/WDR1/FLII/GSN/CFL1/PPP1R9B/SPTBN1/MTPN/SPTA1/SPTB/TMOD1/MICAL2/SCIN/CAPZB/SH3BP1/DMTN/ADD1
BP	GO:0010811	positive regulation of cell-substrate adhesion	79	-0.55	-1.85	4.23E-05	0.00039309	0.00024373	2606	tags=41%, list=22%, signal=32%	JUP/ARL2/DISC1/CD3E/TRIOBP/CRKL/MYADM/SMAD3/FMN1/LIMS1/POLDIP2/GSK3B/NEDD9/CDK6/PREX1/ALOX15/ILK/RAC1/PTK2B/RRAS/CALR/NRP1/ABL1/PLEKHA2/DNM2/PTPRJ/FLNA/RSU1/SDC4/GFUS/DMTN/DAB2
CC	GO:0031012	extracellular matrix	182	-0.45	-1.68	4.35E-05	0.00040354	0.00025021	1941	tags=24%, list=17%, signal=20%	TIMP1/LGALS3/TFIP11/ALPL/TGFB1I1/MMP9/CTSF/CRISPLD2/SERPINB6/TIMP2/SDC3/TGFB1/PCSK6/CCN3/CD180/F13A1/SERPING1/S100A4/GREM1/CALR/SFRP2/LTBP3/PSAP/PKM/PHOSPHO1/ANXA6/C1QA/ANXA11/LGALS9/HNRNPM/KRT1/GP1BA/THBS1/GLG1/CD151/PF4/CTSD/CTSB/HDGF/SPARC/LTBP1/CLU/TGM2
BP	GO:0006936	muscle contraction	163	-0.46	-1.72	4.40E-05	0.00040566	0.00025152	2735	tags=37%, list=23%, signal=29%	TTN/DOCK4/MYOM2/JUP/SMAD5/PTAFR/KCNH2/GNAO1/PIK3CA/TMOD3/TPM4/ATP1A1/ATP2A2/ARHGEF11/SYNM/ATP1B1/P2RX4/ORMDL3/ROCK2/SNTA1/RCSD1/PDE4B/SNTB1/EDN1/GRK2/KCNJ2/EHD3/TPM3/PRKACA/RANGRF/SPHK1/DAPK3/ALDOA/TPM1/NEDD4L/SLC9A1/MYOF/ANXA6/CALD1/GAA/PDE5A/RHOA/CALM3/ACTA2/FLNA/GUCY1A1/FKBP1B/MYLK/MYL4/GSN/PIK3CG/ATP2B4/ENO1/TMOD1/P2RX1/SLC6A8/MYL9/CTTN/MAP2K3/SCN1B/TBXA2R
BP	GO:0044257	cellular protein catabolic process	694	-0.34	-1.44	4.40E-05	0.00040566	0.00025152	1917	tags=23%, list=16%, signal=21%	TRIB1/CHFR/CCAR2/MIB1/TPP1/UBXN7/UBE2J2/ARRB1/CUL7/DERL1/RHBDD1/KEAP1/F8A1/CHMP6/SMARCC1/TAF1/TGFB1I1/CANX/OTUD7B/WDR81/UBE2D4/CTSF/NSFL1C/VPS37B/USP37/CHMP4B/PSME3/BCAP31/PSMB10/GSK3B/PRKACA/GSK3A/HSP90AB1/KLHL22/TBX21/CBFA2T3/DET1/PSMB3/ENC1/VGLL4/EDEM2/EZR/KCTD2/CNOT4/ALAD/UBR2/ZRANB1/UBE2G1/UBA52/NEDD4L/DDA1/RAB7A/USP31/CDC27/FBXL18/PINK1/PTK2B/HSPA1A/ANKIB1/IFI27/USP12/TRIM32/MARCHF6/TBL1XR1/FBXO31/CHMP1A/UBE2Z/PML/UBE2R2/RNF115/CALR/RBCK1/RMND5A/CDK2/HERC2/LAPTM5/AMFR/UBA1/USP19/TBL1X/PSMD11/MAP1A/DDI2/BTRC/USP9X/GABARAP/USP34/FBXO6/RGP1/DDB1/VCP/USP36/ARIH2/KLHL8/UBAP1/N4BP1/PITHD1/FBXW4/PSMC4/RNF213/RAD23A/DTX3L/ARRB2/RNF11/WWP2/GID8/UBE2S/SUFU/MAN1A1/ZNRF1/PSMD2/PSME4/USP7/HIPK2/VPS28/SIAH2/UBB/UBR4/GNA12/USP22/FZR1/HUWE1/PSMF1/SQSTM1/LRP1/YOD1/UBE2L3/RNF187/MSN/MARCHF2/ARAF/SHARPIN/ANKRD9/RNF123/BNIP3L/PCBP2/FBXO7/DCAF12/CBL/TRIP12/ASCC2/UBL7/RYBP/ZER1/CTSB/GPX1/RNF40/DNAJB2/CDC34/FURIN/UBXN6/TOLLIP/PTPN23/UBE2H/DCAF11/CUL4A/TRIM58/NPLOC4/CLU/DAB2/BAG6
BP	GO:0045165	cell fate commitment	97	-0.53	-1.83	4.78E-05	0.00043964	0.00027259	2622	tags=43%, list=22%, signal=34%	EXT1/SMAD5/LY9/FOXP3/ZFPM1/TBX19/NOTCH1/PRDM1/IL6R/HES1/MITF/SOX13/EDN1/RBPJ/TGFB1I1/KLF4/EHMT2/TBX21/BCL11B/KDM6B/EPAS1/RARA/SPN/SOX6/PML/SFRP2/OLIG2/STAT3/NRP1/ETS2/ARHGEF2/OLIG1/RHOA/NOTCH2/SUFU/TCF7L2/FKBP8/PAF1/PRKDC/SOX12/GATA1/TAL1
BP	GO:1903706	regulation of hemopoiesis	272	-0.41	-1.60	4.83E-05	0.00044355	0.00027502	2121	tags=29%, list=18%, signal=24%	MITF/SPI1/RIPK1/PTPN6/ZBTB16/PGLYRP1/PPARGC1B/ZFP36L2/EIF6/TRIB1/FES/XRCC6/SOX13/TFE3/ACVR1B/RUNX1/PTPRC/DROSHA/INPP5D/CD74/SOX4/FOXP1/TOB2/TGFB1/CTNNBIP1/CDK6/TBX21/TMEM131L/ACIN1/ETS1/CNOT4/IL4I1/TRAF6/LYN/KLF13/PTK2B/RARA/HSPA1A/BAD/HCLS1/KAT7/ARNT/CEACAM1/RUNX3/ZMIZ1/STAT3/SYK/JAK3/ABL1/MAFB/FOS/TNFSF4/PITHD1/LTF/MPL/RHOA/NOTCH2/LGALS9/GPR137/MTURN/MEIS1/FAM210B/UBASH3B/PAF1/PRKDC/SOX12/GATA1/FBXO7/HLX/PF4/SCIN/FOXO3/TESC/FAXDC2/MED1/TAL1/CUL4A/GFI1B

BP	GO:0002684	positive regulation of immune system process	622	-0.34	-1.43	4.85E-05	0.00044398	0.00027528	2248	tags=24%, list=19%, signal=20%	IKBKE/IL6R/HES1/HLA-DRB1/PRKD2/CD81/FYN/CD99L2/HLA-DQA2/ICAM1/CRKL/IL1B/SPI1/P2RX4/HLA-A/SH3KBP1/RIPK1/CSF1R/PTPN6/SCIMP/VAV2/TLR6/ZBTB16/PPARGC1B/SIRPG/TRIB1/FES/LGALS3/SLC7A1/ITGAM/XRCC6/SOX13/PDE4B/RUNX1/PTPRC/SH2B2/EDN1/PIK3CB/RIPOR2/CREB3/KLHL6/FZD5/STOML2/INPP5D/DGKZ/CD74/NCR3/JUND/SIRPB1/SOX4/IKBKG/FOXP1/HLA-DQA1/DDX60/ANKRD17/PYCARD/TGFB1/CTNNBIP1/MICB/TEC/TBX21/ACIN1/RBM14/EZR/STX4/IL4I1/FPR3/TRAF6/OXSR1/BTN3A2/KARS1/ANO6/LYN/SPPL3/MAVS/SERPING1/RAC1/JAM3/PTK2B/RARA/IGF2/CGAS/CREBBP/SPN/BAD/HCLS1/SRC/CEACAM1/CALR/RBCK1/PIK3CD/HLA-DRB5/VAV1/RUNX3/LAPTM5/ZMIZ1/TP53BP1/HMCES/SYK/JAK3/ABL1/PAK1/FOS/TNFSF4/RELA/BLOC1S3/C1QA/MYO18A/MPL/SLC7A5/RHOA/NOTCH2/MAD2L2/PTPRJ/LGALS9/HK1/RAC2/CAMK1D/STK11/KRT1/SPG21/RIOK3/PRKCB/CSK/PLVAP/THBS1/ZNFX1/SPTA1/GPI/NFATC2/SELP/PRKDC/LAMP1/SOX12/MAPK3/GATA1/HLX/CDKN1A/PF4/AKIRIN2/PAK2/FOXO3/TESC/MED1/CLU/WNK1/BAG6
BP	GO:0030162	regulation of proteolysis	491	-0.35	-1.47	4.87E-05	0.00044527	0.00027609	1818	tags=25%, list=16%, signal=22%	BIRC6/SMAD3/PTTG1/RHBDD1/RUNX1/APLP2/KEAP1/F8A1/CAST/SMARCC1/TAF1/MMP9/BBC3/SNX12/KLF4/PSME3/BCAP31/SERPINB6/GSK3B/TIMP2/PRKACA/NR1H3/GSK3A/PYCARD/RPS6KA1/DAP/HSP90AB1/SVBP/TNFRSF1B/CBFA2T3/DET1/PRR7/BEX3/SPINT2/DAPK1/ALAD/ARL6IP1/MAP3K5/IST1/HIP1/AQP1/HIP1R/LYN/DDA1/SERPING1/PINK1/MBP/PTK2B/HSPA1A/ANKIB1/MGMT/BAD/DHCR24/TRIM32/NCSTN/BCL2L11/SRC/UBE2Z/PML/SFRP2/FIS1/IFT52/CDK2/LAPTM5/PIH1D1/STAT3/USP19/MAP1A/BTRC/GABARAP/SYK/VCP/XIAP/EGLN3/ARIH2/N4BP1/PITHD1/LTF/PSMC4/RAD23A/ARRB2/RHOA/LGALS9/SUFU/PSME4/USP7/HIPK2/SIAH2/PRDX5/UBB/CYFIP2/GNA12/GSN/BCL2L13/FZR1/PSMF1/LRP1/THBS1/GLG1/GPI/GRN/ARAF/SNCA/ENO1/P2RX1/AKIRIN2/CTSD/TNIP1/PAK2/UBE2O/RYBP/ZER1/CTSB/GPX1/RNF40/DNAJB2/FURIN/MYH9/CLU/DAB2/BAG6
BP	GO:0051235	maintenance of location	228	-0.43	-1.65	4.94E-05	0.00045055	0.00027936	2206	tags=33%, list=19%, signal=27%	CDK5/ASPM/SLC25A23/IL1B/PLIN3/SREBF2/PTPN6/ITPR2/HSPA5/NR1H2/NRROS/SYNE1/SCARB1/KEAP1/PTPRC/CHCHD10/FTO/CPT1A/LDAF1/SREBF1/CRY2/PRKACA/PLIN2/NR1H3/G3BP2/FITM1/SELENON/VPS13D/SUN2/CHERP/LYN/GPAA1/BSCL2/PINK1/PTK2B/KDELR1/DZIP1/PML/RASA3/CALR/YWHAB/PPARA/TAF8/RANGAP1/MEST/ZFYVE1/ABL1/LCN2/FKBP1A/PNPLA2/NFKB1/CIZ1/ANXA6/RNF213/GAA/CALM3/SUFU/FLNA/HK1/FKBP1B/ARHGAP21/MCOLN1/FTH1/GSN/GP1BA/GP9/UBASH3B/SNCA/EHD1/SCIN/FTL/HTT/ITGB3/LTBP1/DIAPH1
BP	GO:0070482	response to oxygen levels	209	-0.43	-1.64	4.96E-05	0.00045155	0.00027998	1906	tags=25%, list=16%, signal=21%	NDRG1/PIN1/SMAD3/EDN1/PIK3CB/HIF1AN/RBPJ/PDK3/TM9SF4/PLOD1/ASCL2/VHL/CBFA2T3/UCP2/ALAD/ATP6AP1/MTHFR/AQP1/EPAS1/PINK1/PTK2B/CREBBP/SLC9A1/BAD/SRC/PML/ARNT/ATP6V1A/PPARA/USP19/ALKBH5/ATP6V0D1/EGLN3/E2F1/SLC2A1/MPL/RHOA/GUCY1A1/LMNA/ATG7/HIPK2/THBS1/ENO1/BNIP3L/SLC29A1/CBL/ACTN4/ALAS2/PDLIM1/FOXO3/GUCY1B1/PDGFA
CC	GO:0031091	platelet alpha granule	64	-0.59	-1.89	5.47E-05	0.00049474	0.00030676	1261	tags=31%, list=11%, signal=28%	ALDOA/VTI1B/F13A1/SERPING1/IGF2/CD9/FERMT3/SYTL4/THBS1/SELP/SNCA/PF4/TREML1/ACTN4/ACTN1/ITGB3/SPARC/PDGFA/CLU/ITGA2B
BP	GO:0030193	regulation of blood coagulation	37	-0.67	-1.96	5.57E-05	0.00050062	0.0003104	1197	tags=32%, list=10%, signal=29%	ANO6/SERPING1/CD9/CEACAM1/KRT1/GP1BA/ALOX12/THBS1/UBASH3B/PDGFA/DMTN/TBXA2R

BP	GO:1900046	regulation of hemostasis	37	-0.67	-1.96	5.57E-05	0.00050062	0.0003104	1197	tags=32%, list=10%, signal=29%	ANO6/SERPING1/CD9/CEACAM1/KRT1/GP1BA/ALOX12/THBS1/UBASH3B/PDGFA/DMTN/TBXA2R
BP	GO:0000165	MAPK cascade	484	-0.36	-1.48	5.60E-05	0.00050264	0.00031165	2578	tags=30%, list=22%, signal=24%	MAPK14/SORL1/PAK4/MINK1/AIDA/TAOK3/JUN/SERPINF2/MAPK7/DUSP8/PTPN11/NOTCH1/NCF1/YWHAZ/PDE6G/GADD45B/ZFP36/EZH2/FLCN/FOXO1/CCR1/MAP4K5/MAP3K13/EPHA4/HLA-DRB1/ARHGAP8/MAPKAPK3/PRKD2/SPRED1/HYAL2/PELI2/CD81/ICAM1/STK40/CRKL/IL1B/SPI1/RIPK1/CSF1R/PTPN6/MADD/ROCK2/SCIMP/TLR6/GSTP1/NDRG2/ZFP36L2/TRIB1/GPS1/PIN1/MAP3K3/SHC1/ARRB1/SMAD3/TRAF7/PTPRC/EDN1/PIK3CB/DIXDC1/MAGED1/FZD5/CD74/EIF3A/MAP3K11/SMPD1/GRB2/SORBS3/PYCARD/TGFB1/HDAC3/RASGRP3/LILRA5/TAOK1/SPHK1/EZR/MEN1/NDST1/TRAF6/MAP3K5/ALOX15/MAP4K1/KARS1/VRK3/LYN/SASH1/PINK1/LBH/MBP/PPM1L/PTK2B/IGF2/MAP2K7/RRAS/SRC/SH2D3C/CEACAM1/STK25/SFRP2/GNAI2/DVL3/LAPTM5/TAOK2/DAXX/NRP1/MAP2K2/PDGFC/PHLPP1/SYK/XIAP/ABL1/PAK1/SYNGAP1/GADD45G/NFKB1/ARRB2/LAMTOR1/PDE5A/MFHAS1/NOTCH2/ACTA2/PTPRJ/LGALS9/MAPKAPK2/INPP5K/MTURN/HIPK2/NF2/DHX33/CSK/PLVAP/THBS1/HRH4/PTPN1/AKAP13/PIK3CG/IQGAP1/ARAF/MAPK3/TNIP1/PAK2/STRADB/PDGFA/MAP2K3/DAB2
BP	GO:0007188	adenylate cyclase-modulating G protein-coupled receptor signaling pathway	89	-0.54	-1.84	5.63E-05	0.0005029	0.00031182	2088	tags=29%, list=18%, signal=24%	CNR2/GNA11/ADGRE1/PDE2A/PDE4B/EDN1/GNAZ/ADGRE5/GSK3A/GNAS/GNA15/GNB1/GNAI2/ADGRL1/PSAP/GALR1/FLNA/GNA12/HRH4/PDE4A/AKAP13/ATP2B4/PF4/PTGDR2/WASF2/TBXA2R
CC	GO:0005769	early endosome	289	-0.41	-1.61	5.63E-05	0.0005029	0.00031182	2422	tags=32%, list=21%, signal=26%	CD22/CLN3/RUSC1/MVB12B/TSG101/SNX27/WASHC5/LMTK2/EPHA4/MLC1/CMTM6/MARCHF3/SNX17/HLA-A/ATP13A3/USP10/DIAPH2/RCC2/TOM1/DERL1/F8A1/HGS/KIAA0319/NEU3/WDFY4/FZD5/TRAK1/ANKFY1/RAP1GAP/HLA-C/VPS41/STX6/WDR81/SNX12/WASHC2A/TM9SF4/EHD3/HYAL3/NAPEPLD/ATP9A/SPHK1/EHD4/PLD4/RAB31/LNPEP/SNX15/VTI1B/IFITM3/TMEM184A/PLD3/DYSF/SH3GL1/NCSTN/GRIPAP1/CHMP1A/PML/RUBCN/SNX3/PTP4A3/RIN3/LITAF/NRP1/MAP2K2/TRAK2/ATP6V0D1/RAB14/LTF/RAPGEF1/DTX3L/RNF11/CLCN5/LDLRAP1/RAB5B/NF2/TMEM127/VPS28/SIAH2/MMGT1/SORT1/IGF2R/LRP1/AKT2/PTPN1/MAPK3/MARCHF8/TGFBRAP1/ACKR1/EHD1/RUFY1/UBXN6/PTPN23/HTT/WASF2
BP	GO:0001503	ossification	222	-0.42	-1.62	5.66E-05	0.00050425	0.00031265	2974	tags=42%, list=25%, signal=32%	TENT5A/TMEM64/VCAN/CHSY1/LEF1/PTCH1/KREMEN1/SLC8A1/ATP5F1B/CEBPA/FGF23/MEF2D/DNAJC13/GABBR1/H3-3A/SBNO2/FGR/EXT1/MAPK14/SMAD5/AKT1/BMP2K/PTPN11/NOTCH1/TPM4/PHEX/CCR1/IL6R/ALYREF/THRA/TUFT1/ZBTB16/SYNCRIP/XYLT1/PPARGC1B/HSD17B4/PDLIM7/ALPL/SMAD3/RUNX1/ZBTB40/RBPJ/MMP9/JUND/SEMA4D/TOB2/ACVR2B/GSK3B/PRKACA/TGFB1/CTNNBIP1/GIT1/CDK6/CLTC/MESD/SNX10/INPPL1/MEN1/BCOR/SKI/TWSG1/TRAF6/ALOX15/ANO6/ILK/ADAR/PTK2B/IGF2/DCHS1/GREM1/SFRP2/LTBP3/RUNX3/FAM20C/DHRS3/PHOSPHO1/RFLNB/LTF/BMP6/HEMGN/RHOA/SUFU/FASN/SLC24A3/RRBP1/SORT1/SND1/ANKH/SNRNP200/MRC2/MAPK3/GATA1/SMPD3/PBX1

BP	GO:0043534	blood vessel endothelial cell migration	77	-0.55	-1.84	5.80E-05	0.00051618	0.00032005	2217	tags=40%, list=19%, signal=33%	JUP/AKT1/NOTCH1/HDAC5/MECP2/PRKD2/SPRED1/P2RX4/VASH1/MAP3K3/SCARB1/PIK3R3/KLF4/TGFB1/ETS1/AMOT/PTK2B/GREM1/NRP1/ABL1/RHOA/HSPB1/STAT5A/THBS1/SP1/ATP2B4/GPX1/MYH9/SH3BP1/MAP2K3/TBXA2R
CC	GO:0030312	external encapsulating structure	183	-0.45	-1.68	5.83E-05	0.00051744	0.00032083	1941	tags=23%, list=17%, signal=20%	TIMP1/LGALS3/TFIP11/ALPL/TGFB1I1/MMP9/CTSF/CRISPLD2/SERPINB6/TIMP2/SDC3/TGFB1/PCSK6/CCN3/CD180/F13A1/SERPING1/S100A4/GREM1/CALR/SFRP2/LTBP3/PSAP/PKM/PHOSPHO1/ANXA6/C1QA/ANXA11/LGALS9/HNRNPM/KRT1/GP1BA/THBS1/GLG1/CD151/PF4/CTSD/CTSB/HDGF/SPARC/LTBP1/CLU/TGM2
BP	GO:0050818	regulation of coagulation	40	-0.65	-1.94	5.88E-05	0.0005203	0.0003226	1197	tags=30%, list=10%, signal=27%	ANO6/SERPING1/CD9/CEACAM1/KRT1/GP1BA/ALOX12/THBS1/UBASH3B/PDGFA/DMTN/TBXA2R
BP	GO:0045087	innate immune response	541	-0.35	-1.45	5.89E-05	0.0005203	0.0003226	2107	tags=27%, list=18%, signal=23%	USP27X/OAS2/OAS3/CCDC92/IKBKE/PRDM1/SERINC5/IRF1/NUB1/FYN/SPI1/GBP4/HLA-A/CSF1R/OTUD4/PTPN6/RNASE3/CNPY3/SYNCRIP/PGLYRP1/TNK2/FES/LGALS3/NR1H2/ITGAM/XRCC6/CDC37/CXCL16/GBP6/RAB20/EDN1/KIR3DL1/CD14/CD74/HLA-C/NCR3/PDE12/SMPD1/VIM/IKBKG/DDX60/ANKRD17/NLRX1/NR1H3/FAM3A/PYCARD/G3BP2/CACTIN/HSP90AB1/ACTG1/LILRA5/RBM14/CD180/DAPK3/DAPK1/STX4/CDC42EP4/TRAF6/CHID1/TRIM35/MAP3K5/PLD4/APOBEC3D/ZYX/UNC93B1/LYN/TRIM26/MAVS/SERPING1/IL17RA/ADAR/PLEKHM2/IFITM3/PTK2B/IFI27/FLNB/TTLL12/CGAS/CREBBP/TRIM32/ZC3HAV1/ILRUN/SRC/PML/SRPK2/CEACAM1/PIK3CD/SLA/VAV1/TRIM10/TRIM28/IFITM2/ARHGEF2/JAK1/SYK/JAK3/XIAP/APOBEC3F/PAK1/LCN2/OPTN/N4BP1/RELA/LTF/C1QA/DTX3L/ARRB2/ECSIT/TUBB4B/SIGLEC10/SHMT2/MFHAS1/LGALS9/WAS/HK1/IFIT1B/TRIM11/KRT1/RIOK3/GSN/TRIM14/PTPN1/ZNFX1/PIK3CG/RNF187/SEC14L1/MARCHF2/GRN/ELF4/SNCA/H2BC11/PRKDC/LAMP1/APOBEC3C/PCBP2/AKIRIN2/TUBB/TREML1/PAK2/SLC15A3/TOLLIP/MED1/TRIM58/STMP1/CLU
BP	GO:0098771	inorganic ion homeostasis	440	-0.37	-1.51	6.01E-05	0.00052954	0.00032834	2791	tags=32%, list=24%, signal=25%	TMTC2/FGF23/SLC40A1/CCRL2/LETM1/CCDC115/COX19/ATP13A2/PDPK1/CMKLR1/ATP7A/EXT1/PKD1/ATP2A3/STEAP3/CORO1A/SELENOK/PTGIR/SWAP70/SLC46A1/KCNH2/DISC1/KNG1/TRPM2/BAK1/ATP1A1/CLN3/CYBRD1/TRPC3/ATP2A2/DLG4/SLC24A4/CCR1/CHP1/CDK5/HYAL2/MAFG/FYN/SLC25A23/ATP1B1/P2RX4/PTPN6/ATP13A3/ITPR2/SLC9A7/NCOA4/TMC8/LPAR4/SMAD3/RAB20/MICU1/PTPRC/EDN1/PIK3CB/STOML2/SLC41A1/FRRS1/KCNJ2/CXCR2/BCAP31/TM9SF4/PRKACA/HVCN1/CXCR1/SNX10/CNNM3/ATP6AP1/SELENON/FPR3/CHERP/LYN/NEDD4L/SPPL3/EPAS1/RAB7A/PTK2B/GNA15/MT2A/ARF1/SLC9A1/SLC22A17/SLC39A4/PML/CA2/RASA3/CALR/GNAI2/ATP6V1A/ATP13A1/SLC7A8/FIS1/ATP1B2/PDK2/JAK3/XIAP/ATP6V0D1/ABL1/LCN2/FKBP1A/STIM1/LTF/ANXA6/BMP6/XK/CKB/GALR1/CALM3/FKBP1B/ATP6V0A1/MCOLN1/SLC6A9/SLC24A3/FTH1/TTC7A/SLC4A1/GP1BA/EPB42/PRKCB/GP9/HRH4/PIK3CG/GRN/UBASH3B/ATP2B4/SNCA/ANKRD9/P2RX1/MAPK3/GATA1/GRINA/FTL/ALAS2/SLC25A37/EIF2AK1/HTT/MLLT6/KEL/TGM2/DIAPH1/TBXA2R
BP	GO:0045444	fat cell differentiation	142	-0.48	-1.75	6.05E-05	0.00053255	0.0003302	2960	tags=41%, list=25%, signal=31%	TMEM64/SAV1/SYAP1/WDFY2/ZFP36L1/SDF4/CEBPA/RNASEL/CMKLR1/MAPK14/AKT1/ZFPM1/PIAS1/NR1D1/FBXO9/ZFP36/FLCN/FOXO1/ZNF516/ZNF385A/HES1/ALDH6A1/ARL4A/ZBTB16/ZBTB7A/ZFP36L2/SOX13/TFE3/SMAD3/FAM120B/LGALS12/FTO/TGFB1I1/CTBP1/KLF4/SREBF1/NAPEPLD/TGFB1/JDP2/BSCL2/TRIO/TRIM32/TBL1XR1/SFRP2/TAF8/LRG1/MAFB/E2F1/TMEM120A/SORT1/ASXL2/AKT2/TCF7L2/PER2/GPX1/MED1/CARM1/PIM1

BP	GO:0043009	chordate embryonic development	380	-0.37	-1.51	6.08E-05	0.00053321	0.00033061	2253	tags=26%, list=19%, signal=22%	EIF4A3/PRDM1/HES1/SMG9/PFN1/UNK/MAFG/DEAF1/TTLL1/VASH1/SH3PXD2A/PTPN18/XYLT1/MAN2A1/MIB1/BIRC6/ARHGAP35/SMAD3/MAFF/ACVR1B/KEAP1/EDN1/TFEB/PHACTR4/FZD5/RBPJ/NDEL1/OTUD7B/KLF4/SOX4/GRB2/NCOA1/PRKACA/TGFB1/TRIP6/ASCL2/RPGRIP1L/ST14/AMOT/MYO1E/SPINT2/BCOR/NDST1/SKI/WDTC1/TRAF6/SLC39A3/MTHFR/PLXNB2/EPAS1/SUPT6H/FZD2/BPTF/RARA/IGF2/ASF1B/CHST11/KLF2/DCHS1/BCL2L11/TBL1XR1/CDKN1C/ARNT/SFRP2/DVL3/IFT52/TAF8/ZMIZ1/TRIM28/NOLC1/APBA3/ABL1/PSMC4/CMIP/EPN1/CHD8/NOTCH2/SUFU/INPP5K/ITPK1/ECE1/BCL2L1/SDC4/GNA12/TERF2/YBX1/MFN2/GPI/FKBP8/PRKDC/VASP/GATA1/YBX3/FURIN/MED1/CUL4A/MYH9/TENT5C/CNOT1/PBX1
BP	GO:0048871	multicellular organismal homeostasis	292	-0.40	-1.60	6.29E-05	0.0005471	0.00033922	2445	tags=32%, list=21%, signal=26%	PTPN11/NOTCH1/TRPM2/ACACB/NR1D1/EGR1/FLCN/FOXO1/POC1B/ZNF516/HYAL2/THRA/IL1B/P2RX4/CSF1R/PCTP/PPARGC1B/EHMT1/NR1H2/TPP1/TFE3/ALPL/LSR/INPP5D/RBPJ/FTO/NOVA1/CHMP4B/ACVR2B/PRKACA/NAPEPLD/NR1H3/ACTG1/HDAC3/SNX10/GNAS/KDM6B/UCP2/SLC27A1/TRAF6/MFSD2A/AQP1/LNPEP/EPAS1/RAB7A/BSCL2/MTF1/MBP/JAM3/PTK2B/TRIM32/DYNC1H1/SRC/ADIPOR2/LTBP3/LRRK1/ATP1B2/TLE3/ACTB/STAT3/PDGFC/SYK/TJP2/LCN2/GADD45G/LTF/BMP6/SLC2A1/ACADVL/VCL/GPR137/RAB3D/INPP5K/HSPB1/STK11/IP6K1/UBB/KRT1/PLEC/ADIPOR1/ALOX12/CLDN5/SQSTM1/CSK/MFN2/PER2/UBASH3B/EIF4G1/GATA1/GPX1/ITGB3/ESAM
BP	GO:0055082	cellular chemical homeostasis	484	-0.36	-1.47	6.41E-05	0.00055618	0.00034485	2788	tags=31%, list=24%, signal=25%	FGF23/SLC40A1/CCRL2/LETM1/CCDC115/COX19/ATP13A2/PDPK1/CMKLR1/ATP7A/ZBTB20/RAB11FIP5/PKD1/ATP2A3/CORO1A/SELENOK/PIM3/PTGIR/SWAP70/SLC46A1/PIK3CA/DISC1/KNG1/TRPM2/NR1D1/BAK1/ATP1A1/CLN3/CYBRD1/TRPC3/ATP2A2/DLG4/FOXO1/SLC24A4/CCR1/CHP1/HLA-DRB1/CDK5/MAFG/FYN/ICAM1/SLC25A23/ATP1B1/P2RX4/ORMDL3/PTPN6/ATP13A3/ITPR2/SLC9A7/NCOA4/FOXK1/LPAR4/SMAD3/RAB20/MICU1/PTPRC/EDN1/PIK3CB/STOML2/SLC41A1/FRRS1/PDK3/KCNJ2/CXCR2/BCAP31/SOX4/TM9SF4/FOXK2/PRKACA/HVCN1/SMARCA4/CXCR1/MEN1/STX4/UCP2/ATP6AP1/SELENON/WDTC1/FPR3/CHERP/AQP1/LYN/NEDD4L/SPPL3/RAB7A/PTK2B/GNA15/MT2A/ARF1/SLC9A1/BAD/SLC22A17/SLC39A4/PML/CA2/RASA3/CALR/ATP6V1A/LRRC8A/ATP13A1/FIS1/PPP3CA/ATP1B2/PIH1D1/JAK3/ATP6V0D1/ABL1/LCN2/FKBP1A/STIM1/ANXA6/BMP6/XK/CKB/SIDT2/GALR1/CALM3/HK1/RAB11B/FKBP1B/ATP6V0A1/MCOLN1/SLC6A9/SLC24A3/FTH1/TTC7A/SLC4A1/GP1BA/PRKCB/GP9/HRH4/PIK3CG/GRN/UBASH3B/ATP2B4/SNCA/ANKRD9/P2RX1/MAPK3/GATA1/GRINA/SLC29A1/FTL/GPX1/ALAS2/FOXO3/HTT/KEL/TGM2/DIAPH1/TBXA2R
MF	GO:0030695	GTPase regulator activity	377	-0.37	-1.51	6.45E-05	0.00055758	0.00034572	2584	tags=30%, list=22%, signal=24%	RASAL3/GNAQ/SBF2/PTGIR/SWAP70/JUN/TBC1D8/MCF2L2/PSD4/DOCK8/RGS3/AGAP2/FLCN/AGAP1/ARHGEF11/AGAP3/ARHGAP18/ARHGAP8/SH3BP5L/FGD6/ARAP1/TBCD/DOCK10/MADD/VAV2/ARHGAP26/TNK2/RALBP1/SRGAP2/GPS1/PDE6D/RCC2/ARRB1/RGS10/ARHGAP35/GPSM1/TIAM1/SMAP2/ADAP2/RAP1GAP/RGS16/RHOF/TBC1D17/RABIF/TBC1D22A/GIT1/RASGRP3/ASAP2/PREX1/ASAP1/RANGRF/DENND1C/ARHGAP17/DENND1A/SIPA1L3/SESN2/ITSN1/GDPGP1/RABGAP1/SBF1/EVI5L/TRIO/SIPA1L1/MYO9B/SH2D3C/RASA3/IQSEC1/ARHGAP32/MYCBP2/VAV1/RIN3/HERC2/GMIP/ARHGAP45/TBC1D13/ARFGAP2/NRP1/RANGAP1/DENND6B/ARHGEF2/BCR/HERC1/RGP1/SYNGAP1/FGD4/ARHGEF18/CYTH4/RAPGEF1/RAB3IL1/LAMTOR1/ARHGEF12/ARHGAP6/TBC1D22B/ARHGDIA/WAS/TBC1D20/ARHGAP31/ARHGAP21/ARHGAP1/DOCK2/AKAP13/IQGAP1/RGS6/RUNDC3A/TBC1D5/TBC1D25/TBC1D1/GAPVD1/NUCB1/HPS1/NPRL3/SH3BP1/TBXA2R

MF	GO:0060589	nucleoside-triphosphatase regulator activity	377	-0.37	-1.51	6.45E-05	0.00055758	0.00034572	2584	tags=30%, list=22%, signal=24%	RASAL3/GNAQ/SBF2/PTGIR/SWAP70/JUN/TBC1D8/MCF2L2/PSD4/DOCK8/RGS3/AGAP2/FLCN/AGAP1/ARHGEF11/AGAP3/ARHGAP18/ARHGAP8/SH3BP5L/FGD6/ARAP1/TBCD/DOCK10/MADD/VAV2/ARHGAP26/TNK2/RALBP1/SRGAP2/GPS1/PDE6D/RCC2/ARRB1/RGS10/ARHGAP35/GPSM1/TIAM1/SMAP2/ADAP2/RAP1GAP/RGS16/RHOF/TBC1D17/RABIF/TBC1D22A/GIT1/RASGRP3/ASAP2/PREX1/ASAP1/RANGRF/DENND1C/ARHGAP17/DENND1A/SIPA1L3/SESN2/ITSN1/GDPGP1/RABGAP1/SBF1/EVI5L/TRIO/SIPA1L1/MYO9B/SH2D3C/RASA3/IQSEC1/ARHGAP32/MYCBP2/VAV1/RIN3/HERC2/GMIP/ARHGAP45/TBC1D13/ARFGAP2/NRP1/RANGAP1/DENND6B/ARHGEF2/BCR/HERC1/RGP1/SYNGAP1/FGD4/ARHGEF18/CYTH4/RAPGEF1/RAB3IL1/LAMTOR1/ARHGEF12/ARHGAP6/TBC1D22B/ARHGDIA/WAS/TBC1D20/ARHGAP31/ARHGAP21/ARHGAP1/DOCK2/AKAP13/IQGAP1/RGS6/RUNDC3A/TBC1D5/TBC1D25/TBC1D1/GAPVD1/NUCB1/HPS1/NPRL3/SH3BP1/TBXA2R
BP	GO:0010769	regulation of cell morphogenesis involved in differentiation	70	-0.57	-1.86	6.50E-05	0.00056046	0.00034751	2351	tags=39%, list=20%, signal=31%	TRIOBP/EPHA4/CRKL/MYADM/ST6GAL1/RCC2/CUL7/LIMS1/BAIAP2/NEDD9/PREX1/CORO1C/ILK/CDKL5/RAC1/FBXO31/CALR/NRP1/ABL1/MPL/DNM2/FLNA/CUX1/PRKDC/ACTN4/DMTN/DAB2
BP	GO:0061515	myeloid cell development	65	-0.58	-1.89	6.73E-05	0.0005788	0.00035888	1069	tags=32%, list=9%, signal=30%	KLF2/SRC/LRRK1/FAM20C/BPGM/LTF/NOTCH2/PABPC4/SLC4A1/EPB42/MEIS1/FAM210B/PIP4K2A/GATA1/ALAS2/MPIG6B/MED1/TAL1/TRIM58/WASF2/DMTN
MF	GO:0008289	lipid binding	505	-0.36	-1.50	6.74E-05	0.0005788	0.00035888	2804	tags=34%, list=24%, signal=27%	OBSCN/SH3GLB1/PLA2G4C/GAB2/CHPT1/SNX8/SDCBP/FNBP1/ATP13A2/PLD1/CPNE2/KIF16B/TRAF2/PLD2/BPI/DAPP1/PTAFR/SBF2/OSBPL5/STARD3/AKT1/AIDA/GRAMD1B/AP2A2/SYT11/PSD4/NCF1/ATP1A1/CLN3/ATG2B/MVB12B/SNX33/AGAP1/CPNE3/SNX27/TSPO/STARD7/STARD5/RPH3A/PFN1/CDIPT/TEX2/ACOX1/CD81/ARAP1/CPNE1/SNX17/OSBPL7/ITPR2/RNASE3/TLR6/SH3PXD2A/PAQR8/ARHGAP26/ZCCHC2/PCTP/PLA2G15/GSTP1/SVIL/NR3C1/FES/ZCCHC14/TPP1/PACSIN1/NUMA1/SHC1/LPAR4/MAP1LC3B/TOM1/ARHGAP35/SCARB1/HGS/TIAM1/ADAP2/DROSHA/FZD5/STOML2/CD14/ANKFY1/CD300LF/SNX12/NSFL1C/KCNJ2/DGKD/WASHC2A/TECPR1/TSPO2/NR1H3/RASGRP3/TEC/PREX1/APOM/ASAP1/PR7/DENND1C/SPHK1/FITM1/ATG2A/MYO1E/SNX10/DENND1A/ESYT1/ALOX15/HIP1/OSBPL10/PITPNC1/HIP1R/LYN/BCAS3/GPAA1/SNX15/BSCL2/NME4/RARA/RXRA/MICALL1/DYSF/CGAS/SH3GL1/SLC9A1/BAD/PITPNM2/MARK2/PITPNA/BBS5/IQSEC1/CPNE5/RUBCN/SNX3/PPARA/ARHGAP32/FLII/PLEKHF1/PSAP/MYOF/SELL/VDAC1/SNX11/VCP/CPTP/ZFYVE1/PARD3/CYTH4/PLEKHA2/LTF/ANXA6/PLEK2/WIPI2/ANXA11/EPN1/LDLRAP1/CAVIN2/MCOLN1/SNX29/GSN/SYTL4/IGF2R/THBS1/SPTBN1/EPB41/TRIP10/IQGAP1/SELP/SNCA/H2BC11/NRGN/SMPD3/SCIN/RUFY1/WDR45/AP2M1/HDLBP/OSBP2/TLN1
BP	GO:2000146	negative regulation of cell motility	184	-0.44	-1.66	6.79E-05	0.00058217	0.00036097	2610	tags=34%, list=22%, signal=27%	CORO1B/JUP/MACIR/AKT1/PIP5KL1/NOTCH1/HDAC5/FLCN/EPHA4/MAGI2/MECP2/SPRED1/HYAL2/ARID2/MITF/DACH1/VASH1/CERS2/TIMP1/TRIB1/SRGAP2/PIN1/RIPOR2/ENG/CD74/EPPK1/KLF4/DNAJA4/MIIP/TGFB1/SVBP/CCN3/SPINT2/CORO1C/TPM1/RAC1/STK24/RRAS/GREM1/CALR/SFRP2/ATP1B2/RIN3/STAT3/CTNNA1/CNN2/BCR/CLASP1/RHOA/VCL/PTPRJ/WAS/NF2/ABHD2/GNA12/ADIPOR1/CLDN5/LRP1/THBS1/ATP2B4/FOXO3/PTPN23/TBXA2R

BP	GO:0031669	cellular response to nutrient levels	162	-0.46	-1.69	6.87E-05	0.00058732	0.00036416	2057	tags=29%, list=18%, signal=24%	NUAK2/SREBF2/HSPA5/DCPS/RIPOR1/FES/MAP1LC3B/GCN1/RRAGD/TFEB/VPS41/EHMT2/SREBF1/NCOA1/DAP/TTC5/UCP2/MFSD2A/MAP3K5/SESN2/RXRA/STK24/SLC39A4/USF2/PPARA/MAX/PDK2/GABARAP/ZFYVE1/RRAGC/SLC2A1/WIPI2/SESN3/KANK2/ATG7/GLUL/EIF4G1/MAPK3/ELAPOR1/CDKN1A/CBL/WDR45/NPRL3/FOXO3/MED1/GAS2L1/PIM1
BP	GO:0010508	positive regulation of autophagy	116	-0.50	-1.77	6.92E-05	0.0005907	0.00036625	1768	tags=28%, list=15%, signal=24%	GPSM1/PIK3CB/TFEB/IKBKG/GSK3B/GSK3A/ATG2A/DAPK1/VPS13D/SESN2/PINK1/MTDH/BAD/TRIM32/BCL2L11/PLEKHF1/VDAC1/OPTN/PIM2/SESN3/STK11/TRIM14/PIP4K2A/MAPK3/ELAPOR1/BNIP3L/FBXO7/WDR45/NPRL3/FOXO3/HTT/LARP1/SUPT5H
BP	GO:0042176	regulation of protein catabolic process	316	-0.39	-1.55	7.11E-05	0.00060443	0.00037477	1941	tags=27%, list=17%, signal=23%	TIMP1/TRIB1/CHFR/CCAR2/CTSA/PIN1/SMAD3/KEAP1/F8A1/HGS/CHMP6/SMARCC1/TAF1/SNX12/PSME3/BCAP31/GSK3B/TIMP2/PRKACA/GSK3A/HSP90AB1/TNFRSF1B/CBFA2T3/DET1/VGLL4/EZR/ALAD/NEDD4L/DDA1/RAB7A/PINK1/PTK2B/HSPA1A/ANKIB1/TRIM32/PML/SNX3/MYCBP2/CDK2/LAPTM5/USP19/MAP1A/BTRC/GABARAP/RGP1/DDB1/VCP/ARIH2/N4BP1/RELA/PITHD1/PSMC4/STX5/RAD23A/WWP2/ODC1/MAD2L2/SUFU/FLNA/PSMD2/USP7/ATG7/SF3B3/HIPK2/VPS28/UBB/GNA12/FZR1/HUWE1/PSMF1/LRP1/RILP/MSN/MARCHF2/ARAF/SNCA/RYBP/ZER1/GPX1/RNF40/DNAJB2/FURIN/OAZ1/CLU/DAB2/BAG6
MF	GO:0015175	neutral amino acid transmembrane transporter activity	28	-0.71	-1.96	7.18E-05	0.0006085	0.00037729	974	tags=46%, list=8%, signal=43%	SERINC5/MFSD12/SLC43A2/SLC25A38/SLC7A8/SLC1A5/SLC1A4/SLC6A20/SLC7A5/SLC3A2/SLC6A9/SLC43A1/SLC38A5
BP	GO:0045785	positive regulation of cell adhesion	307	-0.39	-1.54	7.27E-05	0.00061465	0.00038111	2606	tags=33%, list=22%, signal=26%	JUP/RASAL3/PTAFR/CORO1A/AKT1/ARL2/SELENOK/FOXP3/PIK3CA/DISC1/SERPINF2/PTPN11/CD3E/EFNB1/DOCK8/FLCN/TRIOBP/EPHA4/HES1/HLA-DRB1/PRKD2/CD81/FYN/HLA-DQA2/CRKL/IL1B/HLA-A/PTPN6/ZBTB16/MYADM/SIRPG/SLC7A1/SOX13/TFE3/SMAD3/FMN1/RUNX1/PTPRC/LIMS1/CD74/POLDIP2/SIRPB1/SOX4/HLA-DQA1/GSK3B/ZFHX3/NEDD9/PYCARD/CDK6/PREX1/ETS1/STX4/IL4I1/TRAF6/TPM1/ALOX15/LYN/ILK/RAC1/PTK2B/RARA/IGF2/BAD/RRAS/SRC/CALR/SFRP2/HLA-DRB5/VAV1/RUNX3/PPP3CA/ZMIZ1/NRP1/JAK1/SYK/JAK3/ABL1/TNFSF4/PLEKHA2/RELA/APBB1IP/GP6/RHOA/DNM2/PTPRJ/LGALS9/FLNA/RSU1/SDC4/GFUS/CSK/SPTA1/SELP/SOX12/HLX/FOXO3/PTPN23/DMTN/TGM2/DAB2

BP	GO:0000122	negative regulation of transcription by RNA polymerase II	625	-0.34	-1.43	7.44E-05	0.00062824	0.00038953	2490	tags=30%, list=21%, signal=25%	ZNF69/ZFPM1/JUN/PIAS1/CHD3/NOTCH1/NR1D1/YWHAZ/EGR1/YEATS2/ZFP36/HDAC4/HDAC5/EZH2/FLCN/FOXO1/TSG101/HOXB3/PRDM1/HES1/ELK4/MECP2/DPF2/KMT5A/ZBTB4/MLX/DEAF1/THRA/MTA2/MITF/DRAP1/DACH1/SPI1/ZNF366/MTA1/SREBF2/DNAJB1/H1-4/ZBTB16/PDE2A/ZBTB7A/NR3C1/EHMT1/NR1H2/FOXK1/SOX13/SMAD3/RUNX1/EDN1/HIF1AN/MAGED1/TAF1/RBPJ/BCORL1/VPS72/CTBP1/ZNF749/OTUD7B/KLF4/JUND/FOXP2/SEMA4D/EHMT2/NFIC/NR6A1/FOXP1/SREBF1/ACVR2B/ZFHX3/RITA1/FOXK2/CRY2/NR1H3/SORBS3/CTNNBIP1/HDAC3/SMARCA4/JDP2/MEPCE/ASCL2/ZNF668/PHF21A/CDK6/TBX21/VHL/CC2D1B/EZR/NCOA2/ETV6/MEN1/BCOR/SKI/TGIF2/WDTC1/HIC1/ZBTB45/PARP1/TRAF6/IRF2/BCL11A/TRPS1/YY1/BPTF/RARA/IGF2/SP2/HSPA1A/RXRA/ATXN1/PIAS4/KLF2/IFI27/CREBBP/MTDH/BRMS1/HCLS1/TBL1XR1/CCND3/CDKN1C/DCAF1/CALR/ZNF296/GATAD2B/OLIG2/PPARA/RUNX3/CDK2/TRIM28/MAX/STAT3/TBL1X/ETS2/USP9X/MAFK/FNIP2/FBP1/RELA/E2F1/NFKB1/BMP6/JARID2/WWP2/TLE4/CHD8/NOTCH2/MAD2L2/SUFU/SAMD1/KANK2/ZBTB42/HIPK2/SAP130/HEXIM2/ZNF282/MED25/TXNIP/SQSTM1/MXD4/YBX1/KLF16/TCF7L2/PER2/NFATC2/CUX1/CHD4/SNCA/PAF1/NFIX/ENO1/GATA1/AKIRIN2/CBX6/RYBP/YBX3/FOXO3/MXI1/MED1/JAZF1/TAL1/HDGF/PPM1A/GFI1B/KLF3/NCOR2/CNOT1/DAB2/HCFC1/SUPT5H
CC	GO:0019898	extrinsic component of membrane	213	-0.43	-1.64	7.50E-05	0.00063182	0.00039175	2642	tags=36%, list=23%, signal=28%	FGR/JUP/ULK1/GNAQ/GNB2/AKT1/MTMR3/GNAO1/PIK3CA/NCF1/ATG2B/DLG4/SNX33/RPH3A/FYN/CRKL/CNR2/GNA11/CTNND1/TNK2/FES/SOCS7/NUMA1/TIAM1/PIK3CB/STOML2/GNAZ/PIK3R3/WDR81/ST14/EZR/ATG2A/SNX10/GNAS/MYZAP/VPS13D/ESYT1/ALOX15/HIP1/LYN/RAB7A/RAC1/PTK2B/GNA15/DCHS1/MICALL1/TRIO/GNB1/BCL2L11/GRIPAP1/SRC/PML/PIK3CD/GNAI2/SLA/PPP3CA/CTNNA1/SYK/ZFYVE1/FKBP1A/GOLGA3/WIPI2/ACADVL/RHOA/UBB/GNA12/SYTL4/PIK3CG/IQGAP1/RGS6/ATG13/RNF10/RNF40/WDR45/UBXN6/MGLL
CC	GO:0030136	clathrin-coated vesicle	129	-0.48	-1.74	7.81E-05	0.00065499	0.00040612	1756	tags=31%, list=15%, signal=27%	EDN1/KIAA0319/FZD5/CD74/VPS41/STX6/AP2B1/BCAP31/HLA-DQA1/SYNRG/CLTC/DENND1C/DNAJC5/MYO1E/DENND1A/AP1M1/HIP1/AP2S1/HIP1R/FZD2/CD9/SNX3/HLA-DRB5/VTI1A/TGOLN2/RAB14/RAB27B/CLTB/EPN1/DNM2/LDLRAP1/ECE1/SPG21/SORT1/STON2/IGF2R/NRGN/FURIN/AP2M1/DAB2
BP	GO:0051271	negative regulation of cellular component movement	190	-0.44	-1.67	7.84E-05	0.00065629	0.00040692	2610	tags=34%, list=22%, signal=27%	CORO1B/JUP/MACIR/AKT1/PIP5KL1/NOTCH1/HDAC5/FLCN/EPHA4/MAGI2/MECP2/SPRED1/HYAL2/ARID2/MITF/DACH1/VASH1/CERS2/TIMP1/TRIB1/SRGAP2/PIN1/RIPOR2/ENG/CD74/EPPK1/KLF4/DNAJA4/MIIP/TGFB1/SVBP/CCN3/SPINT2/CORO1C/TPM1/RAC1/CD9/STK24/RRAS/GREM1/CALR/SFRP2/ATP1B2/RIN3/STAT3/CTNNA1/CNN2/BCR/CLASP1/RHOA/VCL/PTPRJ/WAS/NF2/ABHD2/GNA12/ADIPOR1/CLDN5/LRP1/THBS1/ATP2B4/ACTN1/FOXO3/PTPN23/TBXA2R
MF	GO:0005096	GTPase activator activity	207	-0.43	-1.63	7.92E-05	0.00066181	0.00041035	3148	tags=40%, list=27%, signal=30%	TBC1D24/RANBP1/NF1/NCKAP1L/RAB3GAP1/TBC1D2/TBC1D10A/ARHGEF6/SIPA1/TBC1D2B/HACD3/RIN1/LARS1/RGS9/DOCK4/TSC2/AGFG1/RASAL3/GNAQ/JUN/TBC1D8/RGS3/AGAP2/FLCN/AGAP1/ARHGEF11/AGAP3/ARHGAP18/ARHGAP8/ARAP1/TBCD/ARHGAP26/RALBP1/SRGAP2/ARRB1/RGS10/ARHGAP35/SMAP2/ADAP2/RAP1GAP/RGS16/TBC1D17/TBC1D22A/GIT1/RASGRP3/ASAP2/PREX1/ASAP1/ARHGAP17/SIPA1L3/RABGAP1/EVI5L/SIPA1L1/MYO9B/RASA3/ARHGAP32/RIN3/GMIP/ARHGAP45/TBC1D13/ARFGAP2/NRP1/RANGAP1/BCR/SYNGAP1/ARHGEF12/ARHGAP6/TBC1D22B/ARHGDIA/TBC1D20/ARHGAP31/ARHGAP21/ARHGAP1/DOCK2/IQGAP1/RGS6/TBC1D5/TBC1D25/TBC1D1/GAPVD1/NPRL3/SH3BP1

BP	GO:0007420	brain development	439	-0.37	-1.50	8.03E-05	0.00066954	0.00041514	2476	tags=30%, list=21%, signal=24%	TBX19/DISC1/PLXNA4/PTPN11/NOTCH1/POMK/BAK1/SYNE2/CD3E/EZH2/HOXB3/G6PD/HES1/SMG9/MECP2/CDK5/NDE1/ASPM/FYN/CDK5RAP2/MACO1/CASP2/CRKL/FEZ1/TTLL1/DHX37/CSF1R/VPS51/HSPA5/CTNND1/DNAJB1/SPATA5/TACC1/PHF8/BASP1/NDRG2/SRGAP2/MBOAT7/ITGAM/ARHGAP35/B3GNT5/SYNJ1/RERE/DIXDC1/ARPC5/TAF1/RBPJ/MAP1S/NDEL1/CENPF/FOXP2/DTNBP1/GSK3B/ZFHX3/BAIAP2/AKNA/GIT1/CDK6/RPGRIP1L/SPHK1/MEN1/CASP5/BCL11B/NDST1/SKI/ATIC/NCOA6/TWSG1/SNRPB/MFSD2A/AQP1/PLXNB2/MBP/BPTF/RARA/DPYSL2/ATXN1/TACC3/BAD/NCSTN/BCL2L11/SRC/SOX6/HOOK3/SFRP2/OLIG2/PPP3CA/ATP1B2/ZMIZ1/ACTB/TRAPPC9/NRP1/BCR/YWHAH/DPCD/HERC1/ATP6V0D1/ABL1/MAFB/ODAD4/E2F1/ARRB2/SLC2A1/CKB/RHOA/CHD8/CALM3/AARS1/NF2/ROGDI/NAPA/UBB/ARCN1/PPP1R9B/CSK/UBE2L3/GJC2/SETD1A/MTPN/MEIS1/ATP2B4/SHARPIN/NEURL1/PRKDC/NRGN/FOXO3/MED1/SEC16A/PBX1/BAG6
BP	GO:0043408	regulation of MAPK cascade	406	-0.37	-1.50	8.16E-05	0.0006777	0.0004202	2569	tags=31%, list=22%, signal=25%	SORL1/PAK4/MINK1/AIDA/TAOK3/JUN/SERPINF2/DUSP8/PTPN11/NOTCH1/NCF1/YWHAZ/PDE6G/GADD45B/EZH2/FLCN/FOXO1/CCR1/MAP3K13/EPHA4/HLA-DRB1/ARHGAP8/PRKD2/SPRED1/HYAL2/PELI2/CD81/ICAM1/STK40/CRKL/IL1B/SPI1/RIPK1/CSF1R/PTPN6/MADD/ROCK2/SCIMP/TLR6/GSTP1/NDRG2/PIN1/MAP3K3/SHC1/ARRB1/TRAF7/PTPRC/EDN1/PIK3CB/DIXDC1/MAGED1/FZD5/CD74/EIF3A/MAP3K11/SMPD1/GRB2/SORBS3/PYCARD/TGFB1/HDAC3/LILRA5/TAOK1/SPHK1/EZR/MEN1/NDST1/TRAF6/MAP3K5/ALOX15/MAP4K1/VRK3/LYN/SASH1/PINK1/LBH/PTK2B/IGF2/MAP2K7/RRAS/SRC/CEACAM1/STK25/SFRP2/GNAI2/DVL3/LAPTM5/TAOK2/NRP1/MAP2K2/PDGFC/PHLPP1/SYK/XIAP/ABL1/PAK1/SYNGAP1/GADD45G/ARRB2/LAMTOR1/PDE5A/MFHAS1/NOTCH2/ACTA2/PTPRJ/LGALS9/INPP5K/MTURN/HIPK2/NF2/DHX33/CSK/THBS1/HRH4/PTPN1/AKAP13/PIK3CG/IQGAP1/MAPK3/TNIP1/PAK2/PDGFA/MAP2K3/DAB2
BP	GO:0006325	chromatin organization	454	-0.36	-1.48	8.28E-05	0.0006857	0.00042516	2386	tags=28%, list=20%, signal=23%	BANP/YEATS2/HDAC4/HDAC5/EZH2/WAC/BANF1/CDYL/NAP1L4/ARID1B/PKN1/MECP2/SPIN1/KMT5A/ARID2/RSBN1/MTA2/TOP1/SPI1/MTA1/GRWD1/H1-4/EMSY/EP400/IPO4/SMARCD3/HMG20B/PHF8/ZBTB7A/NR3C1/EHMT1/MCM3AP/RFWD3/RERE/SMARCC1/BCORL1/VPS72/CTBP1/EIF1/WDR5/EHMT2/KDM4A/TRRAP/CHRAC1/UBN1/SFMBT1/BAZ2A/ASH2L/HDAC3/SMARCA4/MKI67/SCMH1/PHF21A/DAPK3/MEN1/BCOR/KDM6B/BABAM2/UBR2/MTHFR/YY1/KMT2B/SETD1B/SUPT6H/BRPF1/BPTF/IGF2/SUPT16H/ASF1B/KLF2/KANSL1/TBL1XR1/KAT7/CDKN1C/DCAF1/GATAD2B/WBP2/RUVBL1/CDK2/DAXX/TRIM28/ACTB/PIH1D1/KAT2B/UPF1/MCRS1/PHF2/PAK1/KMT2A/KDM2A/BABAM1/RELA/DTX3L/KDM4B/JARID2/SETDB1/KMT2C/KDM5C/CHD8/SAMD1/PSME4/PCGF5/USP22/KAT6A/SAFB/PRKCB/CABIN1/ZNFX1/SETD1A/NFE2/CHD4/BRD3/H2BC11/IKZF1/BRD4/HMGA1/CBX6/TET3/GPX1/RNF40/SMARCC2/H3C10/ARID1A/GFI1B/CARM1/HCFC1/BAG6
BP	GO:0009185	ribonucleoside diphosphate metabolic process	89	-0.53	-1.81	8.31E-05	0.00068722	0.0004261	2058	tags=34%, list=18%, signal=28%	AK3/ENTPD5/PGAM1/ZBTB7A/EIF6/FOXK1/ENO2/GALK1/FOXK2/GIT1/CBFA2T3/JMJD8/ALDOA/ALDOC/BAD/PFKL/ARNT/AK1/PPARA/PKM/BPGM/STAT3/TJP2/FBP1/HK1/MPP1/SLC4A1/GPI/ENO1/GUK1

BP	GO:0140352	export from cell	512	-0.36	-1.48	8.54E-05	0.00070429	0.00043668	3038	tags=33%, list=26%, signal=26%	PTGES/SCRN1/NRXN1/KIT/F2RL1/LTBP2/SNPH/VPS18/ANG/VAMP3/STX17/MYO1G/DVL1/ITGB2/PRAM1/SLC8A1/SDF4/NKG7/COPG1/FGF23/SLC40A1/GAB2/TTN/SDCBP/ATP13A2/PDPK1/GABBR1/FGR/SLC16A1/RAB3IP/RAB11FIP5/STEAP3/RALB/PTAFR/RAB8A/RHBDF2/CORO1A/EXOC7/PIM3/FBXL20/KCNH2/POMC/SYT11/NSF/PTPN11/NOTCH1/TRPM2/NR1D1/ATP1A1/ATP2A2/TSG101/M6PR/SLC24A4/CCR1/RPH3A/HLA-DRB1/CDK5/EXOC4/ATP1B1/IL1B/SPI1/YKT6/P2RX4/VPS4B/TXLNA/PARD6A/RALBP1/FES/NR1H2/ITGAM/SYNJ1/HGS/EDN1/UBE2Q1/ENG/CPT1A/GRK2/CANX/ABCC1/SOX4/DTNBP1/HYAL3/SREBF1/ACVR2B/GSK3B/CRY2/NR1H3/TGFB1/ATP9A/FRMD4A/SVBP/GIT1/TNFRSF1B/SYNGR1/PCSK6/IL1RN/DNAJC5/CCN3/EZR/ARHGAP17/STX4/UCP2/SLC27A1/PLEK/RAB31/ANO6/ITSN1/LYN/VTI1B/RAB7A/RAB11FIP1/PINK1/SLC9A1/BAD/PFKL/EXOC3/SEPTIN1/CEACAM1/PIK3CD/SCFD2/GNAI2/LRRC8A/SEPTIN4/SLC1A5/PPP3CA/ATP1B2/BCR/SYK/ABCG2/ABCC4/PAK1/CLASP1/BLOC1S3/RAB27B/BMP6/PDIA4/RAB3IL1/MYO18A/SIDT2/GALR1/CALM3/LGALS9/RAB11B/FKBP1B/RAB3D/RAC2/NIBAN2/NAPA/SDC4/SYTL4/GLUL/PRKCB/TANGO2/PIK3CG/RAB2B/TCF7L2/PER2/SNCA/LAMP1/P2RX1/GATA1/CBL/SMPD3/SCIN/FURIN/PTPN23/MYH9/DMTN/ANK1/DAB2
CC	GO:0005765	lysosomal membrane	352	-0.38	-1.51	8.76E-05	0.00071702	0.00044458	2721	tags=34%, list=23%, signal=27%	SPPL2B/DNAJC13/ATP13A2/PLD1/AP2A1/RMC1/AP5S1/GNAQ/GNB2/STARD3/ABCA3/AP2A2/ACP2/SYT11/NSF/TRPM2/CLN3/LAMP2/TMBIM1/CYBRD1/FLCN/CPNE3/M6PR/HGSNAT/AP5B1/ABCB9/HLA-DRB1/ABCD1/PI4K2A/WDR24/HLA-DQA2/CMTM6/CPNE1/VPS39/P2RX4/MFSD12/GNA11/ITM2C/CTSA/ARRB1/TOM1/SCARB1/NEU3/TFEB/SLC35F6/CHMP6/CD74/ANKFY1/VPS41/WDR81/CHMP4B/AP2B1/HLA-DQA1/TECPR1/STOM/HSP90AB1/CD63/CLTC/SYNGR1/SLC44A2/DNAJC5/AP1M1/UBA52/AP2S1/LNPEP/LYN/GLMP/VTI1B/RAB7A/PLEKHM2/IFITM3/PLD3/GNB1/NCSTN/CHMP1A/ATP6V1A/LRRC8A/HLA-DRB5/PLEKHF1/LAPTM5/PSAP/LITAF/IFITM2/UBA1/VLDLR/ATP6V0D1/RAB14/FNIP2/ANXA6/DTX3L/ATP6V0C/LAMTOR1/CLCN5/GAA/SLC7A5/SIDT2/AP1B1/SLC3A2/GPR137/ARL8A/RAB3D/ECE1/ATP6V0A1/MCOLN1/CDIP1/SORT1/LRP1/RILP/MARCHF2/GRN/MMD/LAMP1/ELAPOR1/MARCHF8/CTSD/SLC48A1/SLC15A3/NPRL3/UBXN6/AP2M1/DAB2
CC	GO:0098852	lytic vacuole membrane	352	-0.38	-1.51	8.76E-05	0.00071702	0.00044458	2721	tags=34%, list=23%, signal=27%	SPPL2B/DNAJC13/ATP13A2/PLD1/AP2A1/RMC1/AP5S1/GNAQ/GNB2/STARD3/ABCA3/AP2A2/ACP2/SYT11/NSF/TRPM2/CLN3/LAMP2/TMBIM1/CYBRD1/FLCN/CPNE3/M6PR/HGSNAT/AP5B1/ABCB9/HLA-DRB1/ABCD1/PI4K2A/WDR24/HLA-DQA2/CMTM6/CPNE1/VPS39/P2RX4/MFSD12/GNA11/ITM2C/CTSA/ARRB1/TOM1/SCARB1/NEU3/TFEB/SLC35F6/CHMP6/CD74/ANKFY1/VPS41/WDR81/CHMP4B/AP2B1/HLA-DQA1/TECPR1/STOM/HSP90AB1/CD63/CLTC/SYNGR1/SLC44A2/DNAJC5/AP1M1/UBA52/AP2S1/LNPEP/LYN/GLMP/VTI1B/RAB7A/PLEKHM2/IFITM3/PLD3/GNB1/NCSTN/CHMP1A/ATP6V1A/LRRC8A/HLA-DRB5/PLEKHF1/LAPTM5/PSAP/LITAF/IFITM2/UBA1/VLDLR/ATP6V0D1/RAB14/FNIP2/ANXA6/DTX3L/ATP6V0C/LAMTOR1/CLCN5/GAA/SLC7A5/SIDT2/AP1B1/SLC3A2/GPR137/ARL8A/RAB3D/ECE1/ATP6V0A1/MCOLN1/CDIP1/SORT1/LRP1/RILP/MARCHF2/GRN/MMD/LAMP1/ELAPOR1/MARCHF8/CTSD/SLC48A1/SLC15A3/NPRL3/UBXN6/AP2M1/DAB2

BP	GO:0010720	positive regulation of cell development	186	-0.44	-1.67	9.09E-05	0.00073798	0.00045758	2509	tags=34%, list=21%, signal=27%	PLXNC1/DISC1/PLXNA4/ADNP/NOTCH1/TRIOBP/TSPO/MAP3K13/EPHA4/HES1/ASPM/CRKL/IL1B/MACF1/MYADM/SMARCD3/TRIB1/MAN2A1/CUL7/SYNJ1/TIAM1/DIXDC1/LIMS1/NDEL1/SEMA4D/BAIAP2/NEDD9/CYFIP1/TGFB1/TNFRSF1B/PREX1/IST1/LYN/PLXNB2/ILK/CDKL5/RAC1/PLXND1/BAD/TRIM32/FBXO31/EIF4G2/STK25/CALR/OLIG2/PLXNA1/NRP1/ABL1/E2F1/MPL/PDE5A/DNM2/FLNA/NIBAN2/STK11/CLDN5/GJC2/CUX1/NEURL1/PRKDC/DMTN/TGM2/ADD1/DAB2
BP	GO:0009792	embryo development ending in birth or egg hatching	390	-0.38	-1.54	9.09E-05	0.00073798	0.00045758	2253	tags=26%, list=19%, signal=22%	EIF4A3/PRDM1/HES1/SMG9/PFN1/UNK/MAFG/DEAF1/TTLL1/VASH1/SH3PXD2A/PTPN18/XYLT1/MAN2A1/MIB1/BIRC6/ARHGAP35/SMAD3/MAFF/ACVR1B/KEAP1/EDN1/TFEB/PHACTR4/FZD5/RBPJ/NDEL1/OTUD7B/KLF4/SOX4/GRB2/NCOA1/PRKACA/TGFB1/TRIP6/ASCL2/RPGRIP1L/ST14/AMOT/MYO1E/SPINT2/BCOR/NDST1/SKI/WDTC1/TRAF6/SLC39A3/MTHFR/PLXNB2/EPAS1/SUPT6H/FZD2/BPTF/RARA/IGF2/ASF1B/CHST11/KLF2/DCHS1/BCL2L11/TBL1XR1/CDKN1C/ARNT/SFRP2/DVL3/IFT52/TAF8/ZMIZ1/TRIM28/NOLC1/APBA3/ABL1/PSMC4/CMIP/EPN1/CHD8/NOTCH2/SUFU/INPP5K/ITPK1/ECE1/BCL2L1/SDC4/GNA12/USP22/TERF2/YBX1/MFN2/GPI/FKBP8/PRKDC/VASP/GATA1/AKIRIN2/YBX3/FURIN/MED1/CUL4A/MYH9/TENT5C/CNOT1/PBX1
BP	GO:0033674	positive regulation of kinase activity	319	-0.39	-1.55	9.12E-05	0.00073846	0.00045787	2642	tags=30%, list=23%, signal=24%	FGR/TRAF2/PKD1/RALB/AKT1/TAOK3/PIK3CA/ADNP/MOB3B/NCF1/EGR1/AGAP2/EZH2/DLG4/MAP3K13/LMTK2/IL6R/EPHA4/HLA-DRB1/CDK5/CD81/DDR1/IL1B/CSF1R/MADD/VAV2/TLR6/CCNY/MOB1B/PRRC1/XRCC6/GCN1/PTPRC/EDN1/MAGED1/FZD5/DGKZ/CD74/TPX2/MAP3K11/ACVR2B/NEDD9/HSP90AB1/TGFB1/MOB3A/LILRA5/TAOK1/SLC27A1/TRAF6/MAP3K5/LYN/SASH1/PINK1/PTK2B/IGF2/MAP2K7/BAD/MARK2/SRC/CCND3/GREM1/DVL3/TAOK2/DAXX/PIH1D1/MAP2K2/VLDLR/PDGFC/SYK/ABL1/PAK1/LTF/PDE5A/RHOA/CALM3/STK11/SDC4/CSK/THBS1/PTPN1/AKAP13/PIK3CG/IQGAP1/ATP2B4/SNCA/MMD/CAMK1/NEURL1/CDKN1A/PAK2/STRADB/TAL1/ITGB3/PDGFA/CLU/MAP2K3/WNK1
BP	GO:0060322	head development	468	-0.36	-1.47	9.49E-05	0.00076654	0.00047528	2476	tags=29%, list=21%, signal=24%	TBX19/TIPARP/DISC1/PLXNA4/PTPN11/NOTCH1/POMK/BAK1/SYNE2/CD3E/EZH2/SGPL1/HOXB3/G6PD/HES1/SMG9/MECP2/CDK5/NDE1/ASPM/FYN/CDK5RAP2/MACO1/CASP2/CRKL/FEZ1/TTLL1/DHX37/CSF1R/VPS51/HSPA5/CTNND1/DNAJB1/SPATA5/TACC1/PHF8/BASP1/NDRG2/SRGAP2/MBOAT7/ITGAM/ARHGAP35/B3GNT5/SYNJ1/RERE/DIXDC1/ARPC5/TAF1/RBPJ/MAP1S/NDEL1/CENPF/FOXP2/CRISPLD2/DTNBP1/GSK3B/ZFHX3/BAIAP2/AKNA/GIT1/CDK6/RPGRIP1L/SPHK1/MEN1/CASP5/BCL11B/NDST1/SKI/ATIC/NCOA6/TWSG1/SNRPB/MFSD2A/AQP1/PLXNB2/MBP/BPTF/RARA/DPYSL2/ATXN1/TACC3/BAD/NCSTN/BCL2L11/RRAS/SRC/SOX6/HOOK3/SFRP2/OLIG2/PPP3CA/ATP1B2/ZMIZ1/ACTB/TRAPPC9/NRP1/BCR/YWHAH/DPCD/HERC1/ATP6V0D1/ABL1/MAFB/ODAD4/E2F1/ARRB2/SLC2A1/CKB/RHOA/CHD8/CALM3/AARS1/NF2/ROGDI/NAPA/UBB/ARCN1/CLDN5/PPP1R9B/CSK/UBE2L3/GJC2/SETD1A/MTPN/MEIS1/ATP2B4/SHARPIN/NEURL1/PRKDC/MAPK3/NRGN/FOXO3/MED1/SEC16A/PBX1/BAG6

BP	GO:0019725	cellular homeostasis	548	-0.35	-1.46	9.74E-05	0.00078397	0.00048609	1845	tags=21%, list=16%, signal=19%	FOXK1/LPAR4/SMAD3/RAB20/MICU1/PTPRC/EDN1/PIK3CB/STOML2/SLC41A1/FRRS1/PDK3/KCNJ2/CXCR2/BCAP31/SOX4/TM9SF4/FOXK2/PRKACA/HVCN1/SMARCA4/GIT1/CXCR1/TAOK1/MEN1/STX4/UCP2/ATP6AP1/SELENON/WDTC1/FPR3/ALDOA/GSR/ANO6/CHERP/AQP1/LYN/NEDD4L/SPPL3/RAB7A/PINK1/PTK2B/GNA15/MT2A/ARF1/SLC9A1/BAD/TRIM32/SLC22A17/SLC39A4/PML/CA2/RASA3/CALR/ATP6V1A/LRRC8A/ATP13A1/FIS1/PPP3CA/ATP1B2/PIH1D1/MAP1A/JAK3/ATP6V0D1/ABL1/LCN2/FKBP1A/STIM1/ANXA6/BMP6/XK/GAA/CKB/SIDT2/GALR1/CALM3/UBE2S/HK1/RAB11B/FKBP1B/ATP6V0A1/MCOLN1/SLC6A9/SLC24A3/FTH1/PRDX5/DCTN1/TTC7A/SLC4A1/GP1BA/PRKCB/GP9/HRH4/PIK3CG/GRN/UBASH3B/ATP2B4/SNCA/ANKRD9/PRDX6/P2RX1/MAPK3/GATA1/GRINA/SLC29A1/FTL/GPX1/DNAJB2/ALAS2/FOXO3/HTT/KEL/TGM2/DIAPH1/TBXA2R
MF	GO:0005200	structural constituent of cytoskeleton	60	-0.59	-1.88	0.00010331	0.00082811	0.00051346	2475	tags=55%, list=21%, signal=44%	CCDC6/SPTAN1/BICD1/SYNM/TUBGCP4/HLA-DRB1/TUBB2A/TUBA1C/TUBG1/TUBG2/ARPC5/VIM/SORBS3/ACTG1/TUBA4A/TPM1/HIP1/ARPC1B/ACTB/TUBA8/TUBB4B/ARPC4/PLEC/EPB42/SPTBN1/EPB41/SPTA1/MSN/SPTB/TUBB/TUBB1/TLN1/ANK1
BP	GO:0032386	regulation of intracellular transport	262	-0.41	-1.60	0.00010545	0.00084363	0.00052308	2233	tags=32%, list=19%, signal=26%	ARHGAP8/PCNT/MLC1/CDK5/HYAL2/CD81/FYN/IL1B/FEZ1/SREBF2/LCP1/RIPOR1/ITGAM/NUMA1/UBE2J2/SMAD3/FZD5/NDEL1/BBC3/SNX12/BCAP31/TM9SF4/SREBF1/GSK3B/PRR5L/STOM/PRKACA/GSK3A/HSP90AB1/TGFB1/ATP9A/HDAC3/EDEM2/EZR/BCAS3/MAVS/LEPROT/SUPT6H/PINK1/IFI27/HCLS1/GRIPAP1/DYNC1H1/SRC/SNX3/FIS1/TRIM28/MAP2K2/RANGAP1/NOLC1/USP36/ARIH2/ABLIM3/SAE1/LDLRAP1/SUFU/FLNA/RAB11B/TBC1D20/RAC2/INPP5K/ARHGAP1/USP7/STK11/HUWE1/YOD1/AKT2/UBE2L3/PTPN1/TCF7L2/MSN/CAMK1/ATG13/LAMP1/MAPK3/BNIP3L/EHD1/OAZ1/PTPN23/PPM1A/TRIM58/DMTN/DAB2
CC	GO:0042470	melanosome	91	-0.52	-1.79	0.00010858	0.00086514	0.00053642	1647	tags=35%, list=14%, signal=30%	CANX/PDIA6/DTNBP1/STOM/HSP90AB1/CD63/CLTC/SYNGR1/CALU/DNAJC5/RAB7A/RAC1/NCSTN/YWHAB/SLC1A5/SLC1A4/RAB27B/ANXA6/PDIA4/SLC2A1/ANXA11/SLC3A2/RAB5B/BSG/FASN/ATP6V0A1/SND1/GANAB/LAMP1/CTSD/CTSB/ITGB3
CC	GO:0048770	pigment granule	91	-0.52	-1.79	0.00010858	0.00086514	0.00053642	1647	tags=35%, list=14%, signal=30%	CANX/PDIA6/DTNBP1/STOM/HSP90AB1/CD63/CLTC/SYNGR1/CALU/DNAJC5/RAB7A/RAC1/NCSTN/YWHAB/SLC1A5/SLC1A4/RAB27B/ANXA6/PDIA4/SLC2A1/ANXA11/SLC3A2/RAB5B/BSG/FASN/ATP6V0A1/SND1/GANAB/LAMP1/CTSD/CTSB/ITGB3
BP	GO:0007409	axonogenesis	229	-0.42	-1.61	0.00011105	0.0008831	0.00054755	2622	tags=34%, list=22%, signal=27%	EXT1/ULK1/RAB8A/DIP2B/PLXNC1/DISC1/PLXNA4/ADNP/PTPN11/NOTCH1/EFNB1/NCAM1/LYPLA2/MAP3K13/LMTK2/EPHA4/CDK5/FYN/FEZ1/SLITRK5/CSF1R/MACF1/PARD6B/ARHGAP35/APLP2/EDN1/KIAA0319/TIAM1/DIXDC1/MAP1S/B4GALT5/NDEL1/SEMA4D/GSK3B/BAIAP2/CYFIP1/HSP90AB1/BCL11B/IST1/PLXNB2/CDKL5/RAC1/MBP/DPYSL2/PLXND1/TRIO/KIF13B/SIPA1L1/MARK2/EIF4G2/STK25/MYCBP2/PLXNA1/TAOK2/ACTB/SEMA4A/NRP1/MAP1A/USP9X/ABL1/PAK1/PARD3/SYNGAP1/XK/VCL/NOTCH2/DNM2/RAC2/BSG/ECE1/NIBAN2/STK11/VASP/RHOG/PAK2/CTTN/KEL/SCN1B
BP	GO:0010959	regulation of metal ion transport	220	-0.43	-1.65	0.00011235	0.00089165	0.00055286	1587	tags=23%, list=14%, signal=20%	KCNJ2/EHD3/STOM/PRKACA/CD63/LILRA5/RANGRF/SELENON/OXSR1/FHL1/ANO6/LYN/NEDD4L/PTK2B/SLC9A1/PML/GNAI2/ATP1B2/CACNG6/YWHAH/VDAC1/JAK3/ABL1/FKBP1A/STIM1/ARRB2/WWP2/RHOA/CALM3/DNM2/FLNA/FKBP1B/MYLK/INPP5K/SLC6A9/EPB41/GJC2/PIK3CG/UBASH3B/ATP2B4/SNCA/AHCYL1/P2RX1/ACTN4/TESC/HTT/MLLT6/KEL/SCN1B/WNK1/DIAPH1

BP	GO:0050730	regulation of peptidyl-tyrosine phosphorylation	159	-0.45	-1.68	0.00011758	0.0009313	0.00057744	2453	tags=34%, list=21%, signal=27%	CBLB/ADNP/PTPN11/NCF1/CD3E/DLG4/TSG101/IL6R/EPHA4/HES1/SH3BP5L/HYAL2/CD81/FYN/CSF1R/PTPN6/TNK2/SHC1/PTPRC/CHMP6/CD74/SEMA4D/NEDD9/TGFB1/LILRA5/TEC/MVP/EHD4/LYN/PTK2B/IGF2/HCLS1/SRC/GREM1/SFRP2/LRRK1/NRP1/ARHGEF2/PDGFC/SYK/ABL1/BMP6/ARRB2/PTPRJ/NF2/PPP2R5B/PTPN1/NEURL1/GATA1/CBL/PAK2/ITGB3/PDGFA/DMTN
BP	GO:0006109	regulation of carbohydrate metabolic process	126	-0.48	-1.74	0.00012205	0.00096474	0.00059817	2017	tags=41%, list=17%, signal=35%	AP2A1/ZBTB20/PTAFR/AKT1/ARL2/POMC/ACACB/HDAC4/FOXO1/ENTPD5/PGAM1/NLN/ZBTB7A/EIF6/FOXK1/PHKG2/PDK3/WDR5/GSK3B/FOXK2/FAM3A/GSK3A/TGFB1/GIT1/CLTC/BCKDK/CBFA2T3/JMJD8/NCOA2/PLEK/SESN2/PTK2B/IGF2/BAD/SRC/ARNT/PPARA/PPP1CA/STAT3/PDK2/KAT2B/DDB1/FBP1/NFKB1/INPP5K/USP7/STK11/ADIPOR1/SLC4A1/AKT2/SNCA/SMPD3
MF	GO:0015171	amino acid transmembrane transporter activity	47	-0.61	-1.89	0.00012373	0.00097612	0.00060523	974	tags=34%, list=8%, signal=31%	SLC7A7/SERINC5/MFSD12/SLC43A2/SLC7A1/SLC25A38/SLC7A8/SLC1A5/SLC1A4/SLC6A20/SLC7A5/SLC3A2/SLC6A9/SLC38A10/SLC43A1/SLC38A5
BP	GO:0051494	negative regulation of cytoskeleton organization	109	-0.51	-1.79	0.00012535	0.00098695	0.00061195	2653	tags=45%, list=23%, signal=35%	ADD2/CORO1B/MAPRE1/EPS8/CORO1A/SWAP70/PIK3CA/TMOD3/SPTAN1/ATXN7/TRIOBP/PFN1/CDK5RAP2/SSH1/ARAP1/TBCD/MYADM/SVIL/SSH2/LIMA1/TPX2/TAOK1/EML2/RBM14/KATNB1/HIP1R/FLII/CAMSAP1/ARHGEF2/KAT2B/CLASP1/ARHGEF18/SSH3/ARHGAP6/WAS/INPP5K/GSN/SPTBN1/MTPN/SPTA1/SPTB/SNCA/TMOD1/SCIN/PAK2/CAPZB/WASF2/DMTN/ADD1
CC	GO:0030139	endocytic vesicle	256	-0.40	-1.55	0.00012663	0.00099502	0.00061695	2429	tags=34%, list=21%, signal=27%	CYBB/NCF1/LAMP2/DLG4/M6PR/HYOU1/HLA-DRB1/HYAL2/FMNL1/HLA-DQA2/HLA-A/SH3KBP1/SCIMP/TLR6/PGLYRP1/SRGAP2/SCARB1/RAB20/HGS/KIAA0319/TAPBP/FZD5/CD74/HLA-C/STX6/AP2B1/VIM/HLA-DQA1/EHD3/HVCN1/ACTG1/CLTC/AMOT/SPHK1/MYO1E/STX4/CDC42EP4/EHD4/PLD4/RAB31/ZYX/UNC93B1/UBA52/AP2S1/LYN/RAB7A/RAB11FIP1/FZD2/CD9/DYSF/FLNB/CALR/SNX3/HLA-DRB5/TGOLN2/RIN3/RAB11FIP4/SYK/ATP6V0D1/RAB14/LTF/ARRB2/ATP6V0C/ANXA11/CLTB/DNM2/LDLRAP1/WAS/RAB5B/RAB11B/RAC2/ATP6V0A1/MCOLN1/ZDHHC5/UBB/ITGB5/GSN/IGF2R/LRP1/RILP/TRIM14/LAMP1/EHD1/GAPVD1/AP2M1/SPARC
BP	GO:1903034	regulation of response to wounding	91	-0.52	-1.78	0.00012926	0.00101367	0.00062851	1816	tags=31%, list=15%, signal=26%	SMAD3/EDN1/KIAA0319/EPPK1/NDEL1/KLF4/ACTG1/GIT1/ANO6/SERPING1/CD9/STK24/CEACAM1/CLASP1/SIGLEC10/ACTA2/FLNA/FKBP1B/MYLK/KRT1/GP1BA/ALOX12/THBS1/GRN/UBASH3B/PDGFA/DMTN/TBXA2R
BP	GO:0044403	biological process involved in symbiotic interaction	211	-0.43	-1.63	0.00012963	0.00101456	0.00062906	2665	tags=34%, list=23%, signal=26%	TRIM21/CR1/SIGLEC1/MID2/EXOC7/RRP1B/JUN/KPNA3/NCAM1/TSG101/ALYREF/HLA-DRB1/HYAL2/CD81/ICAM1/VPS4B/CSF1R/SELPLG/PGLYRP1/ITGB7/CDK9/SCARB1/CHMP6/CD74/GRK2/VPS37B/CHMP4B/SMPD1/STOM/NAPEPLD/HSP90AB1/DCD/SMARCA4/THOC6/IST1/TRIM26/RAB7A/GYPA/IFITM3/HSPA1A/IFI27/SRC/CHMP1A/SNX3/SLC1A5/TRIM10/IFITM2/NRP1/DDB1/LTF/WWP2/LGALS9/SLC3A2/TBC1D20/BSG/INPP5K/ATG7/TRIM11/BCL2L1/ITGB5/GSN/CFL1/IGF2R/TRIM14/SP1/LAMP1/PF4/CBL/CTSB/GPX1/ITGB3

BP	GO:0051345	positive regulation of hydrolase activity	396	-0.37	-1.50	0.00013085	0.00102216	0.00063377	2425	tags=30%, list=21%, signal=25%	BAK1/CASP9/DOCK8/PPP1R15A/EZH2/FLCN/PTPA/DIABLO/EPHA4/MAGI2/PRKD2/FYN/CASP2/ARAP1/CRKL/DOCK10/MAPRE2/ROCK2/RALBP1/PPARGC1B/SRGAP2/PIN1/NR1H2/PKP4/RCC2/RGS10/ARHGAP35/SMAD3/PTPRC/TIAM1/ADAP2/LIMS1/RAP1GAP/NDEL1/BBC3/RGS16/SEMA4D/PSME3/BCAP31/GSK3B/TBC1D17/NEDD9/NR1H3/PYCARD/DAP/HSP90AB1/TBC1D22A/RASGRP3/ASAP2/PREX1/ASAP1/PRR7/BEX3/SIPA1L3/DAPK1/PLEK/CORO1C/MAP3K5/HIP1/VRK3/HIP1R/LYN/BCAS3/RABGAP1/CDKL5/PINK1/MBP/PTK2B/GNA15/HSPA1A/EVI5L/BAD/SIPA1L1/NCSTN/BCL2L11/PML/IQSEC1/SFRP2/DVL3/FIS1/VAV1/LAPTM5/PSAP/GMIP/ARHGAP45/TBC1D13/STAT3/RANGAP1/BCR/RGP1/SYK/VCP/EGLN3/RAPGEF1/RHOA/CALM3/ARHGAP6/TBC1D22B/LGALS9/TBC1D20/RSU1/PSME4/CYFIP2/GNA12/GSN/BCL2L13/AKT2/RGS6/GRN/SNCA/RHOG/TBC1D5/P2RX1/TBC1D25/TBC1D1/AKIRIN2/CTSD/SH3BP1/TGM2
BP	GO:0009991	response to extracellular stimulus	309	-0.39	-1.53	0.00013214	0.00103017	0.00063874	2593	tags=31%, list=22%, signal=25%	ULK1/MAPK14/RALB/SORL1/AKT1/MTMR3/POMC/ADNP/ACACB/CYBB/LAMP2/ZFP36/CDKN2D/FLCN/FOXO1/CBS/TSPO/PHEX/G6PD/WDR24/PCSK1N/NUAK2/SREBF2/HSPA5/DCPS/RIPOR1/FES/NR1H2/FOXK1/ALPL/MAP1LC3B/GCN1/ARHGAP35/PTPRC/RRAGD/TFEB/CPT1A/VPS41/EHMT2/KDM4A/SREBF1/NCOA1/SORD/FOXK2/DAP/TTC5/UCP2/ALAD/MFSD2A/MAP3K5/SESN2/MTHFR/LYN/BCAS3/RARA/RXRA/MGMT/STK24/SLC39A4/TBL1XR1/SRC/SFRP2/USF2/GNAI2/PPARA/MAX/PDK2/GABARAP/ZFYVE1/RRAGC/FOS/FOXO4/SLC2A1/WIPI2/SESN3/FKBP1B/KANK2/ATG7/PLEC/GLUL/PPP1R9B/EIF4G1/TBC1D5/MAPK3/ELAPOR1/CDKN1A/CBL/GPX1/WDR45/NPRL3/FOXO3/MED1/EIF2AK1/GAS2L1/LARP1/PIM1/TBXA2R
BP	GO:0031623	receptor internalization	73	-0.56	-1.84	0.00013267	0.00103224	0.00064003	1665	tags=34%, list=14%, signal=30%	ARRB1/GRK2/CXCR2/DTNBP1/GRB2/CXCR1/CD63/CLTC/EZR/RAB31/CD9/ARF1/GREM1/CEACAM1/RIN3/SYK/ARRB2/DNM2/LDLRAP1/DNM3/LRP1/SNCA/TBC1D5/AP2M1/ITGB3
BP	GO:1903829	positive regulation of protein localization	332	-0.38	-1.54	0.00013408	0.00104122	0.00064559	2439	tags=32%, list=21%, signal=26%	MAPK14/SORL1/MARK4/AKT1/SYT11/TRPM2/CNST/CLN3/BICD1/UBR5/DLG4/SNX33/TSG101/CHP1/HLA-DRB1/PCNT/CDK5/HYAL2/CD81/FYN/ABHD17C/IL1B/ORMDL3/PARD6A/SREBF2/ROCK2/RIPOR1/LGALS3/NR1H2/EPB41L2/ITGAM/NUMA1/UBE2J2/SMAD3/FZD5/BBC3/CCT3/AP2B1/BCAP31/SOX4/TM9SF4/GSK3B/PRR5L/STOM/PRKACA/GSK3A/HSP90AB1/TGFB1/FRMD4A/HDAC3/MEPCE/MESD/RANGRF/EDEM2/EZR/STX4/PARP1/SESN2/BCAS3/MAVS/LEPROT/PINK1/BAD/DZIP1/HCLS1/SRC/KAT7/LRRC8A/FIS1/PTP4A3/TRIM28/MAP1A/MCRS1/USP36/ARIH2/ABLIM3/BMP6/SAE1/DTX3L/MYO18A/FLNA/RAC2/GNL3L/STK11/VPS28/ZDHHC5/SYTL4/GLUL/HUWE1/SQSTM1/LRP1/PPP1R9B/SPTBN1/AKT2/UBE2L3/EPB41/TCF7L2/MSN/ATP2B4/CAMK1/ATG13/RHOG/OAZ1/PTPN23/PPM1A
BP	GO:1901654	response to ketone	110	-0.49	-1.76	0.00013642	0.00105577	0.00065461	2019	tags=34%, list=17%, signal=28%	ROCK2/NCOA4/DCPS/NR3C1/EDN1/TRERF1/CDA/SREBF1/NR1H3/TGFB1/NCOA2/PARP1/GLB1/MAP4K1/AQP1/MBP/KLF2/BAD/GNB1/SRC/CALR/WBP2/FOS/TNFSF4/CALM3/ELK1/BCL2L1/ABHD2/TXNIP/PPP1R9B/THBS1/MSN/GPI/CBL/FOXO3/LARP1/TBXA2R
CC	GO:0062023	collagen-containing extracellular matrix	145	-0.47	-1.71	0.00013649	0.00105577	0.00065461	1679	tags=23%, list=14%, signal=20%	TGFB1I1/MMP9/CTSF/SERPINB6/TIMP2/SDC3/TGFB1/PCSK6/CCN3/F13A1/SERPING1/S100A4/GREM1/CALR/SFRP2/LTBP3/PSAP/PKM/ANXA6/C1QA/ANXA11/LGALS9/HNRNPM/KRT1/THBS1/CD151/PF4/CTSD/CTSB/HDGF/SPARC/LTBP1/CLU/TGM2
MF	GO:0015179	L-amino acid transmembrane transporter activity	35	-0.66	-1.90	0.0001382	0.00106415	0.00065981	974	tags=40%, list=8%, signal=37%	SLC7A7/SERINC5/SLC43A2/SLC7A1/SLC25A38/SLC7A8/SLC1A5/SLC1A4/SLC6A20/SLC7A5/SLC3A2/SLC6A9/SLC43A1/SLC38A5

BP	GO:1902904	negative regulation of supramolecular fiber organization	106	-0.50	-1.77	0.00013837	0.00106415	0.00065981	2653	tags=44%, list=23%, signal=35%	ADD2/CORO1B/MAPRE1/EPS8/CORO1A/SWAP70/PIK3CA/TMOD3/SPTAN1/ATXN7/TRIOBP/PFN1/SSH1/ARAP1/TBCD/MYADM/SVIL/SSH2/LIMA1/TPX2/TAOK1/EML2/KATNB1/HIP1R/FLII/CAMSAP1/ARHGEF2/CLASP1/ARHGEF18/SSH3/ARHGAP6/WAS/INPP5K/GSN/SPTBN1/MTPN/SPTA1/SPTB/SNCA/TMOD1/SCIN/PAK2/CAPZB/WASF2/CLU/DMTN/ADD1
BP	GO:0031667	response to nutrient levels	286	-0.39	-1.55	0.00013836	0.00106415	0.00065981	2593	tags=31%, list=22%, signal=25%	ULK1/MAPK14/RALB/SORL1/AKT1/MTMR3/POMC/ACACB/CYBB/LAMP2/ZFP36/CDKN2D/FLCN/FOXO1/TSPO/PHEX/G6PD/WDR24/PCSK1N/NUAK2/SREBF2/HSPA5/DCPS/RIPOR1/FES/NR1H2/FOXK1/ALPL/MAP1LC3B/GCN1/RRAGD/TFEB/CPT1A/VPS41/EHMT2/KDM4A/SREBF1/NCOA1/SORD/FOXK2/DAP/TTC5/UCP2/ALAD/MFSD2A/MAP3K5/SESN2/MTHFR/BCAS3/RARA/RXRA/MGMT/STK24/SLC39A4/TBL1XR1/SRC/SFRP2/USF2/GNAI2/PPARA/MAX/PDK2/GABARAP/ZFYVE1/RRAGC/FOXO4/SLC2A1/WIPI2/SESN3/FKBP1B/KANK2/ATG7/PLEC/GLUL/EIF4G1/TBC1D5/MAPK3/ELAPOR1/CDKN1A/CBL/GPX1/WDR45/NPRL3/FOXO3/MED1/EIF2AK1/GAS2L1/LARP1/PIM1/TBXA2R
MF	GO:0030674	protein-macromolecule adaptor activity	246	-0.41	-1.59	0.00013951	0.00107084	0.00066396	1857	tags=24%, list=16%, signal=21%	SPATA2/SHC1/SYNE1/SH2B2/ADAP2/STX6/KLF4/AP2B1/GRB2/BAIAP2/G3BP2/FRMD4A/VHL/DET1/MEN1/STX4/AP1M1/SUN2/HIP1/AP2S1/DAW1/HIP1R/MAVS/VTI1B/ZC3H18/ST13/SLC9A1/GNB1/NCSTN/VTI1A/ATP1B2/AMFR/STAT3/MAP2K2/MAP1A/BTRC/NOLC1/DDB1/TJP2/OPTN/STX5/ARPC4/LDLRAP1/DCAF7/NAPA/STON2/SLC4A1/AKAP13/IQGAP1/ANKRD9/FBXO7/DCAF12/AP2M1/CUL4A/MYH9/GAS2L1/ANK1/DAB2/HCFC1
BP	GO:0014896	muscle hypertrophy	50	-0.61	-1.88	0.00014011	0.00107131	0.00066425	2444	tags=56%, list=21%, signal=45%	NOTCH1/TRPC3/ATP2A2/HDAC4/EZH2/FOXO1/G6PD/P2RX4/ROCK2/CDK9/SMAD3/EDN1/KDM4A/GSK3A/TNFRSF1B/PARP1/YY1/SLC9A1/PPARA/PPP3CA/JARID2/PDE5A/PDE9A/LMNA/AKAP13/MTPN/ATP2B4/PAK2
BP	GO:0035725	sodium ion transmembrane transport	80	-0.54	-1.83	0.00014011	0.00107131	0.00066425	2439	tags=36%, list=21%, signal=29%	TRPM2/ATP1A1/SLC24A4/SLC20A1/CHP1/NETO2/FXYD6/ATP1B1/SLC9A7/SNTA1/SLC41A1/STOM/RANGRF/ANO6/NEDD4L/SLC9A1/ATP1B2/SLC6A20/YWHAH/DNM2/SLC3A2/SLC6A9/SLC24A3/ATP2B4/ACTN4/SLC6A8/TESC/SCN1B/WNK1
BP	GO:0042310	vasoconstriction	30	-0.69	-1.93	0.00014167	0.00107911	0.00066909	716	tags=27%, list=6%, signal=25%	ABL1/RHOA/ACTA2/ECE1/PER2/P2RX1/KEL/TBXA2R
CC	GO:0031674	I band	71	-0.56	-1.85	0.00014165	0.00107911	0.00066909	1288	tags=31%, list=11%, signal=28%	MYZAP/ALDOA/FLNB/PPP3CA/FHL3/KAT2B/CSRP1/PAK1/FKBP1A/SLC2A1/FLNA/FKBP1B/HSPB1/STK11/PLEC/SLC4A1/ATP2B4/PARVB/ACTN4/ACTN1/PDLIM1/MYL9
BP	GO:1903900	regulation of viral life cycle	110	-0.49	-1.75	0.00014239	0.00108252	0.0006712	1681	tags=29%, list=14%, signal=25%	CD74/PDE12/VPS37B/TOP2A/APOBEC3D/TRIM26/MAVS/ADAR/IFITM3/ZC3HAV1/SRPK2/SNX3/PPARA/TRIM10/TRIM28/IFITM2/DDB1/APOBEC3F/N4BP1/LTF/RAD23A/STAU1/SETDB1/LGALS9/INPP5K/TRIM11/GSN/TRIM14/ZNFX1/APOBEC3C/TNIP1/LARP1
MF	GO:0005516	calmodulin binding	114	-0.48	-1.73	0.00014785	0.00112183	0.00069558	2970	tags=47%, list=25%, signal=36%	PHKA2/MYO1G/MYO1D/SLC8A1/MYH3/OBSCN/TTN/EWSR1/SNTB2/ADD2/AKT1/EDF1/SPTAN1/SLC24A4/PCNT/MAPKAPK3/MYO1F/ASPM/MYO15A/CDK5RAP2/SNTA1/MKNK2/ENKUR/SNTB1/PHKG2/RGS16/KCNQ5/PDE1A/ITPKC/SPHK1/MYO1E/DAPK1/MBP/SLC9A1/MYO9B/CEACAM1/PPP3CA/CAMSAP1/CNN2/PPP3R1/CALD1/PLCB3/MAPKAPK2/MYLK/CAMK1D/UBR4/SPTBN1/EPB41/IQGAP1/ATP2B4/CAMK1/NRGN/MYH9/ADD1
BP	GO:0003300	cardiac muscle hypertrophy	49	-0.61	-1.89	0.00014841	0.00112185	0.00069559	2444	tags=57%, list=21%, signal=45%	NOTCH1/TRPC3/ATP2A2/HDAC4/EZH2/FOXO1/G6PD/P2RX4/ROCK2/CDK9/SMAD3/EDN1/KDM4A/GSK3A/TNFRSF1B/PARP1/YY1/SLC9A1/PPARA/PPP3CA/JARID2/PDE5A/PDE9A/LMNA/AKAP13/MTPN/ATP2B4/PAK2
BP	GO:0014897	striated muscle hypertrophy	49	-0.61	-1.89	0.00014841	0.00112185	0.00069559	2444	tags=57%, list=21%, signal=45%	NOTCH1/TRPC3/ATP2A2/HDAC4/EZH2/FOXO1/G6PD/P2RX4/ROCK2/CDK9/SMAD3/EDN1/KDM4A/GSK3A/TNFRSF1B/PARP1/YY1/SLC9A1/PPARA/PPP3CA/JARID2/PDE5A/PDE9A/LMNA/AKAP13/MTPN/ATP2B4/PAK2

BP	GO:1902893	regulation of miRNA transcription	40	-0.64	-1.89	0.0001516	0.0011438	0.00070919	2479	tags=55%, list=21%, signal=44%	ZNF512B/JUN/EGR1/SPI1/SREBF2/NR3C1/NR1H2/SMAD3/KLF4/SREBF1/NFATC3/TGFB1/SMARCA4/ETS1/YY1/RARA/MRTFA/PPARA/STAT3/FOS/RELA/FOXO3
BP	GO:0006757	ATP generation from ADP	62	-0.58	-1.85	0.00015417	0.00115873	0.00071845	2017	tags=39%, list=17%, signal=32%	ENTPD5/PGAM1/ZBTB7A/EIF6/FOXK1/ENO2/GALK1/FOXK2/GIT1/CBFA2T3/JMJD8/ALDOA/ALDOC/PFKL/ARNT/PPARA/PKM/BPGM/STAT3/FBP1/HK1/SLC4A1/GPI/ENO1
MF	GO:0044325	transmembrane transporter binding	83	-0.54	-1.82	0.00015389	0.00115873	0.00071845	1470	tags=29%, list=13%, signal=25%	PYCARD/HSP90AB1/RANGRF/FHL1/CHERP/LYN/NEDD4L/SRC/PACS1/YWHAH/VDAC1/FKBP1A/CALM3/FLNA/FKBP1B/PPP1R9B/GRINA/ACTN4/ACTN1/AP2M1/HTT/ANK1/SCN1B/DIAPH1
BP	GO:0045860	positive regulation of protein kinase activity	275	-0.40	-1.56	0.00015475	0.0011609	0.0007198	2573	tags=31%, list=22%, signal=24%	RALB/AKT1/TAOK3/PIK3CA/ADNP/MOB3B/NCF1/EGR1/AGAP2/EZH2/DLG4/MAP3K13/IL6R/EPHA4/CDK5/IL1B/CSF1R/MADD/TLR6/CCNY/MOB1B/PRRC1/XRCC6/PTPRC/EDN1/MAGED1/FZD5/TPX2/MAP3K11/ACVR2B/NEDD9/HSP90AB1/TGFB1/MOB3A/LILRA5/TAOK1/SLC27A1/TRAF6/MAP3K5/SASH1/PINK1/PTK2B/IGF2/MAP2K7/MARK2/SRC/CCND3/GREM1/DVL3/TAOK2/DAXX/PIH1D1/MAP2K2/VLDLR/PDGFC/SYK/ABL1/PAK1/LTF/PDE5A/RHOA/CALM3/STK11/SDC4/CSK/THBS1/PTPN1/AKAP13/PIK3CG/IQGAP1/ATP2B4/SNCA/MMD/CAMK1/NEURL1/CDKN1A/PAK2/STRADB/TAL1/ITGB3/PDGFA/CLU/MAP2K3/WNK1
BP	GO:1900024	regulation of substrate adhesion-dependent cell spreading	40	-0.64	-1.89	0.00015579	0.00116649	0.00072327	2132	tags=48%, list=18%, signal=39%	TRIOBP/CRKL/MYADM/ST6GAL1/RCC2/LIMS1/NEDD9/PREX1/CORO1C/ILK/RAC1/CALR/NRP1/ABL1/DNM2/FLNA/ACTN4/DMTN/DAB2
CC	GO:0098857	membrane microdomain	221	-0.42	-1.61	0.00015736	0.00117387	0.00072784	2758	tags=33%, list=24%, signal=26%	PAG1/EPHB1/TNFRSF10A/SDCBP/GABBR1/ATP7A/PECAM1/ADD2/CR1/TRAF2/RFTN1/CBLB/ATP1A1/CLN3/LAMP2/EFNB1/PI4K2A/MLC1/SPRED1/HYAL2/FYN/ICAM1/ATP1B1/EFHD2/RIPK1/TUBA1C/SELPLG/TLR6/MYADM/ITGAM/TPP1/LIPE/SCARB1/PTPRC/NEU3/ENO2/STOML2/INPP5D/CD14/STOM/PRKACA/TNFRSF1B/RANGRF/EZR/CORO1C/LYN/PTK2B/SLC9A1/SRC/GNAI2/CTNNA1/MYOF/VDAC1/ABCG2/STIM1/LAMTOR1/SLC2A1/PRKAR2A/GP6/HK1/BSG/CAVIN2/SDC4/CSK/PLVAP/ATP2B4/P2RX1/MAPK3/ARID3A/CBL/CTSD/TUBB/PRKAR2B/FURIN
CC	GO:0030133	transport vesicle	264	-0.40	-1.56	0.00015719	0.00117387	0.00072784	2705	tags=35%, list=23%, signal=27%	ATP13A2/AP2A1/GABBR1/ATP7A/SNTB2/COPZ1/RAB3IP/RAB11FIP5/SORL1/RAB8A/SYT11/DISC1/KDELR2/CNST/CLN3/DLG4/M6PR/SEPTIN6/RPH3A/CHP1/HLA-DRB1/HLA-DQA2/PLIN3/YKT6/HLA-A/SREBF2/PDE4B/EDN1/CD74/NDEL1/HLA-C/STX6/SEC23IP/CRISPLD2/DTNBP1/YIPF2/HLA-DQA1/SREBF1/SYNRG/COPA/CLTC/TPRG1L/SYNGR1/DNAJC5/TRAPPC4/STX4/VTI1B/RAB7A/TMEM184A/KDELR1/NCSTN/SEPTIN1/CEACAM1/IQSEC1/KIF1B/ATP6V1A/HLA-DRB5/SEPTIN4/VTI1A/TGOLN2/LAPTM5/COPB2/SEC31A/CNIH3/ATP6V0D1/RAB14/BLOC1S3/RAB27B/STX5/RAB3IL1/CLCN5/CLTB/CALM3/RAB5B/RAB11B/ZNRF1/RAB3D/SLC6A9/BCL2L1/ROGDI/SPG21/SORT1/STON2/SYTL4/RAB1B/ARCN1/IGF2R/SNCA/LAMP1/NRGN/FURIN/SEC16A
BP	GO:0006814	sodium ion transport	113	-0.49	-1.76	0.00015861	0.00118093	0.00073222	2439	tags=31%, list=21%, signal=25%	TRPM2/ATP1A1/SLC24A4/SLC20A1/CHP1/NETO2/FXYD6/ATP1B1/P2RX4/SLC9A7/SNTA1/EDN1/SLC41A1/STOM/RANGRF/SLC5A6/ANO6/NEDD4L/SLC9A1/ATP1B2/SLC6A20/YWHAH/DNM2/SLC3A2/SLC6A9/SLC24A3/SLC38A10/ATP2B4/AHCYL1/ACTN4/SLC6A8/TESC/MLLT6/SCN1B/WNK1
BP	GO:0050773	regulation of dendrite development	67	-0.55	-1.80	0.00016527	0.00122598	0.00076015	1849	tags=37%, list=16%, signal=32%	EZH2/EPHA4/CRKL/PACSIN1/CUL7/KIAA0319/SEMA4D/GSK3B/BAIAP2/CYFIP1/MFSD2A/BCL11A/NEDD4L/CDKL5/SIPA1L1/FBXO31/PPP3CA/VLDLR/YWHAH/ABL1/RHOA/CAMK1D/STK11/CUX1/CARM1

BP	GO:0044092	negative regulation of molecular function	707	-0.32	-1.37	0.00016606	0.0012295	0.00076234	2737	tags=33%, list=23%, signal=27%	PRDX2/MCM2/TSC2/BHLHE40/PIF1/FAF2/PRPSAP2/PDPK1/CMKLR1/SH2B3/GABBR1/PI3/TRIM21/CR1/PPP1R14A/MED13/PKD1/ATP2A3/GNAQ/RALB/SORL1/PKIG/AKT1/ARL2/AIDA/FOXP3/TAOK3/CBLB/RRP1B/ZFPM1/JUN/PEX19/DISC1/SERPINF2/MLLT1/MAPK7/ADNP/KNG1/NOTCH1/ACACB/CLN3/TMBIM1/BICD1/PPP1R15A/ATP2A2/PDE6G/GADD45B/ZFP36/CDKN2D/HDAC4/EZH2/TSG101/PTPA/INKA1/CHP1/LRRC14/EIF4A3/LMTK2/HES1/PKN1/SH3BP5L/PIP4K2C/SPRED1/LYNX1/HYAL2/SUMO3/PCSK1N/CDK5RAP2/CSNK2A1/CPNE1/IL1B/SPI1/CNR2/PROS1/ORMDL3/GTF2F1/PTPN6/ROCK2/TNK2/TIMP1/GSTP1/MGAT5/TRIB1/CCAR2/LGALS3/GPS1/TMC8/PIN1/PDE6D/TFIP11/RCC2/ARRB1/BIRC6/PTTG1/FRY/APLP2/KEAP1/GPSM1/PTPRC/CAST/PIK3CB/RIPOR2/CHMP6/TAF1/RAP1GAP/MMP9/KLF4/SEMA4D/ITPRIP/SIGIRR/SMPD1/DTNBP1/SERPINB6/GSK3B/CRY2/TIMP2/GSK3A/PYCARD/G3BP2/RPS6KA1/CACTIN/DAP/CTNNBIP1/MEPCE/YWHAG/AMOT/SPINT2/MEN1/MVP/WDTC1/CORO1C/ARL6IP1/OXSR1/AQP1/LYN/BCAS3/NEDD4L/SERPING1/ADAR/PIAS4/DHCR24/BRMS1/ILRUN/SRC/UBE2Z/CDKN1C/CEACAM1/RBCK1/RUBCN/YWHAB/SFRP2/GNAI2/PPARA/PPP3CA/GMIP/TFDP1/ACTB/PPP1CA/PIH1D1/KAT2B/BTRC/APBA3/XIAP/FBH1/ABL1/TNFSF4/FKBP1A/E2F1/LTF/NFKB1/WARS1/DTX3L/ARRB2/WWP2/GOLGA2/PRKAR2A/GALR1/CALM3/MAD2L2/PTPRJ/SUFU/FLNA/FKBP1B/INPP5K/MTURN/USP7/HSPB1/NF2/SLC6A9/GNL3L/SIAH2/PRDX5/SORT1/HEXIM2/PLEC/SLC4A1/TXNIP/PSMF1/LRP1/TERF2/PPP1R9B/CSK/THBS1/CABIN1/PTPN1/IQGAP1/TCF7L2/PER2/GPI/UBASH3B/ATP2B4/SNCA/PPP1R10/CAMK1/PIP4K2A/MAPK3/GATA1/FBXO7/CDKN1A/CBL/PAK2/UBE2O/CTSB/GPX1/DNAJB2/PRKAR2B/TESC/FURIN/OAZ1/ITGB3/WNK1/PIM1/DAB2
BP	GO:1901879	regulation of protein depolymerization	63	-0.57	-1.82	0.00016816	0.00124273	0.00077054	2653	tags=52%, list=23%, signal=41%	ADD2/EPS8/SWAP70/PIK3CA/TMOD3/DSTN/SPTAN1/ATXN7/TRIOBP/SVIL/MAP1S/LIMA1/TPX2/TAOK1/PLEK/WDR1/KATNB1/FLII/CAMSAP1/MAP1A/ARHGEF2/CLASP1/GSN/SPTBN1/MTPN/SPTA1/SPTB/TMOD1/SCIN/CAPZB/SH3BP1/DMTN/ADD1
BP	GO:0030336	negative regulation of cell migration	176	-0.44	-1.64	0.00016928	0.00124874	0.00077427	2610	tags=34%, list=22%, signal=26%	CORO1B/JUP/MACIR/AKT1/PIP5KL1/NOTCH1/HDAC5/FLCN/EPHA4/MAGI2/MECP2/SPRED1/HYAL2/ARID2/MITF/DACH1/VASH1/CERS2/TIMP1/TRIB1/SRGAP2/RIPOR2/ENG/CD74/EPPK1/KLF4/DNAJA4/MIIP/TGFB1/SVBP/CCN3/CORO1C/TPM1/RAC1/STK24/RRAS/GREM1/CALR/SFRP2/ATP1B2/RIN3/STAT3/CNN2/BCR/CLASP1/RHOA/VCL/PTPRJ/NF2/ABHD2/GNA12/ADIPOR1/CLDN5/LRP1/THBS1/ATP2B4/FOXO3/PTPN23/TBXA2R
MF	GO:0061630	ubiquitin protein ligase activity	230	-0.41	-1.57	0.00017032	0.00125318	0.00077702	1267	tags=18%, list=11%, signal=17%	TRAF6/TRIM35/UBR2/NEDD4L/TRIM26/ANKIB1/TRIM32/MARCHF6/DTX2/RNF115/RMND5A/MYCBP2/HERC2/TRIM10/AMFR/BTRC/FBXO6/XIAP/ARIH2/RNF213/DTX3L/RNF11/WWP2/ZNRF1/TRIM11/RNF24/SIAH2/UBR4/HUWE1/TRIM14/MKRN1/RNF187/MARCHF2/NEURL1/RNF10/MARCHF8/CBL/TRIP12/UBE2O/RNF40/TRIM58/RNF208
BP	GO:0051693	actin filament capping	32	-0.68	-1.93	0.00017526	0.00128564	0.00079714	383	tags=34%, list=3%, signal=33%	FLII/GSN/SPTBN1/MTPN/SPTA1/SPTB/TMOD1/SCIN/CAPZB/DMTN/ADD1
CC	GO:0098793	presynapse	274	-0.40	-1.56	0.00017667	0.00129363	0.00080209	1792	tags=26%, list=15%, signal=22%	PDE4B/SYNJ1/SLC29A2/DIXDC1/NDEL1/CANX/CTBP1/STX6/DTNBP1/BAIAP2/CYFIP1/ACTG1/GIT1/YWHAG/TPRG1L/SYNGR1/DNAJC5/SPHK1/TRAPPC4/DENND1A/STX4/HIP1/ITSN1/VTI1B/RAB7A/NPFF/SH3GL1/EXOC3/NCSTN/SEPTIN1/IQSEC1/KIF1B/SEPTIN4/VTI1A/ADGRL1/ACTB/RAB6B/PPP1CA/ATP6V0D1/RAB27B/SLC2A1/CLCN5/CLTB/CALM3/DNM2/RAB5B/RAB11B/ZNRF1/RAB3D/SLC6A9/ELK1/BCL2L1/ROGDI/NAPA/DNM3/STON2/GLUL/GAK/PRKCB/UBE2L3/ATP2B4/SNCA/LRFN3/LAMP1/P2RX1/SLC29A1/RNF40/AP2M1/HTT/GUCY1B1

BP	GO:0033144	negative regulation of intracellular steroid hormone receptor signaling pathway	28	-0.69	-1.91	0.00018163	0.00132747	0.00082308	2347	tags=46%, list=20%, signal=37%	CNOT9/ZNF366/ZBTB7A/FOXP1/CRY2/SMARCA4/LBH/CALR/RHOA/KANK2/NCOR2/CNOT1/DAB2
BP	GO:0043242	negative regulation of protein-containing complex disassembly	62	-0.57	-1.83	0.0001848	0.0013452	0.00083407	2653	tags=48%, list=23%, signal=38%	ADD2/EPS8/UBQLN4/SWAP70/PIK3CA/TMOD3/SPTAN1/ATXN7/TRIOBP/SVIL/LIMA1/TPX2/PHF23/TAOK1/KATNB1/RUBCN/FLII/CAMSAP1/ARHGEF2/CLASP1/GSN/SPTBN1/MTPN/SPTA1/SPTB/TMOD1/SCIN/CAPZB/DMTN/ADD1
BP	GO:0018107	peptidyl-threonine phosphorylation	85	-0.51	-1.76	0.00018516	0.0013452	0.00083407	2251	tags=36%, list=19%, signal=30%	LMTK2/PKN1/CDK5/PRKD2/SPRED1/CSNK2A1/ROCK2/ACVR1B/TAF1/GRK2/GSK3B/PRKACA/GSK3A/TGFB1/DYRK1B/SPHK1/OXSR1/CDC42BPB/MARK2/DCAF1/PARD3/CALM3/HIPK1/INPP5K/HIPK2/STK11/PRKCB/EIF4G1/PRKDC/DMTN/WNK1
BP	GO:0060548	negative regulation of cell death	634	-0.33	-1.40	0.00018509	0.0013452	0.00083407	2142	tags=25%, list=18%, signal=22%	ICAM1/STK40/CASP2/CSNK2A1/MITF/IL1B/ORMDL3/VPS4B/RIPK1/CSF1R/NUAK2/ADNP2/HSPA5/TIMP1/GSTP1/ST6GAL1/DCPS/CCAR2/LGALS3/CIAPIN1/KIR3DL2/SHC1/BIRC6/SMAD3/RHBDD1/EDN1/CAST/PIK3CB/CREB3/SLC35F6/CHMP6/ENO2/CD74/MMP9/PDE12/KLF4/CHMP4B/AP2B1/SEMA4D/ITPRIP/PSME3/SOX4/DTNBP1/IKBKG/FOXP1/KDM4A/GSK3B/IL2RB/RPS6KA1/HSP90AB1/HDAC3/TNFRSF1B/VHL/THOC6/DNAJC5/SPHK1/CCN3/DAPK1/BCL11B/UCP2/BABAM2/ARL6IP1/AQP1/HIP1R/ADAR/PINK1/PTK2B/HSPA1A/CHST11/MGMT/SLC9A1/MTDH/DHCR24/TRIM32/PDCD1/HCLS1/SRC/CHMP1A/UBE2Z/GREM1/RBCK1/SFRP2/GNAI2/PPARA/LAPTM5/PIH1D1/CTNNA1/NRP1/ARHGEF2/VDAC1/DDB1/JAK3/XIAP/ABL1/SYNGAP1/RELA/LTF/NFKB1/PIM2/ARRB2/MYO18A/HNRNPK/RHOA/CHD8/NOTCH2/ARHGDIA/FLNA/AARS1/LMNA/KANK2/PA2G4/TLE1/HSPB1/HIPK2/NIBAN2/CAMK1D/BCL2L1/SIAH2/PRDX5/CFL1/ALOX12/THBS1/AKT2/PTPN1/PIK3CG/TCF7L2/GPI/FKBP8/GRN/ARAF/SNCA/PPP1R10/EIF4G1/ENO1/PRKDC/BAG1/GATA1/GRINA/BNIP3L/FBXO7/PF4/CBL/PAK2/UBE2O/STRADB/ST3GAL1/GPX1/YBX3/PTMS/MED1/CTTN/HTT/HDGF/PTCRA/CLU/PIM1/DAB2/BAG6
BP	GO:1903350	response to dopamine	39	-0.65	-1.92	0.00018855	0.0013655	0.00084666	2099	tags=49%, list=18%, signal=40%	HRH2/GNA11/OR10H1/GNAZ/DTNBP1/GSK3B/GSK3A/GNAS/GNA15/GNB1/NCSTN/ABL1/ARRB2/DNM2/FLNA/HRH4/KLF16/MAPK3/TGM2
MF	GO:0060090	molecular adaptor activity	301	-0.39	-1.56	0.00019374	0.00140058	0.00086841	2987	tags=37%, list=25%, signal=28%	ARRDC4/VPS18/SAV1/VAMP3/STX17/WWC3/DEDD2/AIMP2/FSCN1/ABI2/GAB2/PAG1/FRS2/TMEM250/SDCBP/AP2A1/SH2B3/GAS2L3/TRAF2/JUP/EPS8/GNB2/AP2A2/BICD2/DISC1/PTPN11/AP4E1/SYNE2/CD3E/BICD1/SEPTIN6/CDYL/MAGI2/PCNT/ANAPC1/POLD3/CRKL/ATP1B1/YKT6/OTUD4/SCIMP/SPATA2/SHC1/SYNE1/SH2B2/ADAP2/COX15/STX6/KLF4/AP2B1/SEPTIN11/GRB2/BAIAP2/G3BP2/FRMD4A/VHL/DET1/MEN1/STX4/AP1M1/SUN2/HIP1/AP2S1/DAW1/ITSN1/HIP1R/MAVS/VTI1B/SASH1/ZC3H18/ST13/SLC9A1/DZIP1/GNB1/NCSTN/SEPTIN1/DLGAP4/SEPTIN4/VTI1A/ATP1B2/AMFR/STAT3/MAP2K2/MAP1A/BTRC/NOLC1/DDB1/TJP2/OPTN/STX5/LAMTOR1/EIF3B/ARPC4/LDLRAP1/DCAF7/NAPA/STON2/SLC4A1/AKAP13/IQGAP1/ANKRD9/EIF4G1/FBXO7/DCAF12/AP2M1/CUL4A/MYH9/GAS2L1/ANK1/CNOT1/DAB2/HCFC1

BP	GO:0010770	positive regulation of cell morphogenesis involved in differentiation	59	-0.58	-1.83	0.0001975	0.00142261	0.00088207	2351	tags=39%, list=20%, signal=31%	TRIOBP/EPHA4/CRKL/MYADM/CUL7/LIMS1/BAIAP2/NEDD9/PREX1/ILK/CDKL5/RAC1/FBXO31/CALR/NRP1/ABL1/MPL/DNM2/FLNA/CUX1/PRKDC/DMTN/DAB2
BP	GO:0001666	response to hypoxia	182	-0.43	-1.62	0.00019727	0.00142261	0.00088207	2278	tags=30%, list=19%, signal=24%	NOTCH1/CYBB/EGR1/HYOU1/EPHA4/MECP2/ATP1B1/ITPR2/ROCK2/NDRG1/PIN1/SMAD3/EDN1/PIK3CB/HIF1AN/RBPJ/PDK3/TM9SF4/PLOD1/ASCL2/VHL/CBFA2T3/UCP2/ALAD/MTHFR/AQP1/EPAS1/PINK1/PTK2B/CREBBP/SLC9A1/BAD/SRC/PML/ARNT/PPARA/USP19/ALKBH5/EGLN3/E2F1/SLC2A1/MPL/RHOA/LMNA/HIPK2/THBS1/ENO1/BNIP3L/SLC29A1/ACTN4/ALAS2/PDLIM1/FOXO3/PDGFA
BP	GO:0018105	peptidyl-serine phosphorylation	219	-0.41	-1.58	0.00019859	0.00142781	0.00088529	2609	tags=31%, list=22%, signal=24%	PKD1/ULK1/MAPK14/AKT1/PIK3CA/MAPK7/BAK1/CNOT9/MAP3K13/LMTK2/IKBKE/PKN1/MAPKAPK3/CDK5/PRKD2/CSNK2A1/TOP1/RIPK1/ROCK2/MKNK2/CSNK1G1/TAF1/GRK2/PDK3/MARK3/GSK3B/PRKACA/RPS6KA1/HSP90AB1/TGFB1/DYRK1B/NEK6/MAP4K1/VRK3/ILK/PINK1/MARK2/HCLS1/SRC/SH2D3C/SRPK2/SFRP2/MAST3/CDK2/MAP2K2/SYK/PAK1/FNIP2/ARRB2/MAD2L2/HIPK1/MAPKAPK2/INPP5K/HIPK2/CAMK1D/PRKCB/AKT2/RPS6KA4/ARAF/ATP2B4/SNCA/CAMK1/EIF4G1/PRKDC/PAK2/DMTN/WNK1
BP	GO:0006812	cation transport	593	-0.34	-1.43	0.00020139	0.00144534	0.00089616	1587	tags=26%, list=14%, signal=24%	TRPM2/BAK1/ATP1A1/CLN3/CYBRD1/TRPC3/ATP2A2/DLG4/SLC24A4/TSPO/UQCRC1/CCR1/SLC20A1/CHP1/LMTK2/G6PD/SERINC5/HES1/NETO2/CDK5/FYN/FXYD6/SLC25A23/ATP1B1/P2RX4/VPS4B/PTPN6/ATP13A3/ITPR2/MFSD12/SLC9A7/SLC33A1/SNTA1/SLC43A2/TCN2/RALBP1/LGALS3/SLC7A1/SLC25A42/PDE4B/MICU1/PTPRC/EDN1/STOML2/SLC41A1/COX15/MMP9/KCNJ2/KCNQ5/DTNBP1/EHD3/STOM/PRKACA/HVCN1/TSPO2/CD63/LILRA5/CLTC/RANGRF/SLC44A2/FLVCR2/DAPK1/UCP2/SELENON/OXSR1/SLC5A6/SLC39A3/FHL1/ANO6/CHERP/AQP1/LYN/NEDD4L/PINK1/PTK2B/SLC9A1/SLC22A17/SLC39A4/PML/SLC25A38/RASA3/GNAI2/ATP6V1A/ATP13A1/SLC7A8/SLC1A5/CDK2/PPP3CA/ATP1B2/CACNG6/CNIH3/SLC1A4/SLC6A20/YWHAH/VDAC1/SYK/JAK3/ATP6V0D1/ABL1/LCN2/FKBP1A/STIM1/LTF/ANXA6/CYB5A/ARRB2/CLCN5/WWP2/SLC7A5/RHOA/CALM3/DNM2/FLNA/SLC3A2/RAB11B/FKBP1B/MYLK/INPP5K/ARHGAP1/MCOLN1/SLC6A9/SLC24A3/FTH1/MMGT1/SLC38A10/GP1BA/PRKCB/GP9/EPB41/GJC2/PIK3CG/SLC43A1/SEC14L1/UBASH3B/ATP2B4/SNCA/AHCYL1/CAPN1/P2RX1/SLC29A1/FTL/SLC48A1/ACTN4/SLC6A8/SLC15A3/SLC25A37/TESC/HTT/MLLT6/SHISA7/SLC38A5/ITGB3/KEL/TMEM63B/SCN1B/WNK1/DIAPH1
BP	GO:0048598	embryonic morphogenesis	293	-0.39	-1.53	0.00020786	0.00148907	0.00092327	1817	tags=24%, list=15%, signal=21%	ARHGAP35/SMAD3/EDN1/PHACTR4/ENG/FZD5/TGFB1I1/MMP9/KLF4/SOX4/GRB2/NCOA1/ACVR2B/PRKACA/TGFB1/RPGRIP1L/ST14/IL1RN/AMOT/SPINT2/KDM6B/NDST1/SKI/TGIF2/TWSG1/TRAF6/SLC39A3/MTHFR/PLXNB2/FZD2/MBP/RARA/IGF2/CHST11/CREBBP/BCL2L11/GREM1/CDKN1C/BBS5/SFRP2/DVL3/IFT52/TRIM28/ETS2/BCR/ABL1/MAFB/CLASP1/FBXW4/NOTCH2/HIPK1/SUFU/ECE1/HIPK2/NF2/SDC4/ITGB5/GNA12/YBX1/GPI/PAF1/VASP/MAPK3/MICAL2/HLX/MED1/TAL1/ITGB3/WNK1/PBX1
BP	GO:0010761	fibroblast migration	43	-0.62	-1.87	0.00021525	0.00153929	0.00095441	2582	tags=56%, list=22%, signal=44%	TMEM201/MACIR/AKT1/CLN3/SGPL1/HYAL2/MTA2/RCC2/PRR5L/TGFB1/CCN3/CORO1C/ILK/RAC1/PML/ACTA2/SDC4/GNA12/PLEC/THBS1/IQGAP1/PDLIM1/TNS1/DMTN

BP	GO:0002573	myeloid leukocyte differentiation	151	-0.46	-1.69	0.00021717	0.00155023	0.0009612	2294	tags=36%, list=20%, signal=29%	CCR1/HLA-DRB1/BATF2/CD81/MITF/SPI1/RIPK1/CSF1R/SH3PXD2A/OSCAR/PPARGC1B/TRIB1/FES/NRROS/TFE3/RUNX1/INPP5D/RBPJ/CD74/MMP9/FOXP1/TOB2/TGFB1/CTNNBIP1/CDK6/ACIN1/CBFA2T3/SNX10/ATP6AP1/PARP1/TRAF6/LYN/RARA/HCLS1/SRC/CEACAM1/PIK3CD/LRRK1/FAM20C/MAFB/FOS/LTF/NOTCH2/GPR137/FASN/UBASH3B/GATA1/PF4/TESC/MED1/EIF2AK1/TAL1/CUL4A/MYH9
BP	GO:0016052	carbohydrate catabolic process	110	-0.49	-1.73	0.00021815	0.00155163	0.00096207	2017	tags=32%, list=17%, signal=27%	ENTPD5/PGAM1/ZBTB7A/EIF6/FOXK1/NEU3/PHKG2/ENO2/GALK1/SORD/FOXK2/GSK3A/GIT1/CBFA2T3/JMJD8/ALDOA/GLB1/NAGA/ALDOC/BAD/PFKL/ARNT/PPARA/PKM/GALE/BPGM/PPP1CA/STAT3/PYGB/FBP1/GAA/HK1/SLC4A1/GPI/ENO1
MF	GO:0004842	ubiquitin-protein transferase activity	323	-0.38	-1.51	0.0002178	0.00155163	0.00096207	1322	tags=19%, list=11%, signal=17%	TSPAN17/CNOT4/BCOR/TRAF6/TRIM35/UBR2/UBE2G1/NEDD4L/TRIM26/ANKIB1/TRIM32/MARCHF6/DTX2/UBE2Z/PML/UBE2R2/RNF115/RBCK1/RMND5A/MYCBP2/HERC2/TRIM10/TRIM28/AMFR/BTRC/FBXO6/HERC1/XIAP/ARIH2/RNF213/DTX3L/RNF11/WWP2/UBE2M/UBE2S/ZNRF1/TRIM11/RNF24/SIAH2/UBR4/HUWE1/TRIM14/UBE2L3/MKRN1/RNF187/MARCHF2/SHARPIN/NEURL1/RNF123/RNF10/MARCHF8/FBXO7/CBL/TRIP12/UBE2O/RNF40/CDC34/UBE2H/TRIM58/RNF208
BP	GO:0030835	negative regulation of actin filament depolymerization	35	-0.65	-1.87	0.00022179	0.00157469	0.00097637	383	tags=31%, list=3%, signal=30%	FLII/GSN/SPTBN1/MTPN/SPTA1/SPTB/TMOD1/SCIN/CAPZB/DMTN/ADD1
CC	GO:0030018	Z disc	68	-0.55	-1.80	0.00022489	0.00159389	0.00098827	1049	tags=28%, list=9%, signal=26%	FLNB/PPP3CA/FHL3/CSRP1/PAK1/FKBP1A/SLC2A1/FLNA/FKBP1B/HSPB1/STK11/PLEC/SLC4A1/ATP2B4/PARVB/ACTN4/ACTN1/PDLIM1/MYL9
BP	GO:0040013	negative regulation of locomotion	201	-0.43	-1.62	0.00022891	0.00161949	0.00100414	2610	tags=33%, list=22%, signal=26%	CORO1B/JUP/MACIR/AKT1/PIP5KL1/NOTCH1/HDAC5/FLCN/EPHA4/MAGI2/MECP2/SPRED1/HYAL2/ARID2/MITF/DACH1/VASH1/CERS2/TIMP1/ST6GAL1/TRIB1/SRGAP2/PIN1/RIPOR2/ENG/CD74/EPPK1/KLF4/SEMA4D/DNAJA4/MIIP/TGFB1/SVBP/CCN3/SPINT2/CORO1C/TPM1/RAC1/STK24/RRAS/GREM1/CALR/SFRP2/ATP1B2/RIN3/STAT3/CTNNA1/SEMA4A/NRP1/CNN2/BCR/CLASP1/RHOA/VCL/PTPRJ/WAS/NF2/ABHD2/GNA12/ADIPOR1/CLDN5/LRP1/THBS1/ATP2B4/FOXO3/PTPN23/TBXA2R
BP	GO:0022612	gland morphogenesis	55	-0.59	-1.85	0.00023485	0.00165857	0.00102837	1468	tags=31%, list=13%, signal=27%	RPS6KA1/TGFB1/TWSG1/PLXND1/SRC/PML/CEACAM1/PLXNA1/BTBD7/NRP1/BTRC/NOTCH2/MSN/CAPN1/MED1/PDGFA/TGM2
BP	GO:0045600	positive regulation of fat cell differentiation	37	-0.64	-1.88	0.00023766	0.00167246	0.00103698	2960	tags=51%, list=25%, signal=39%	TMEM64/SAV1/SYAP1/WDFY2/ZFP36L1/CEBPA/CMKLR1/MAPK14/AKT1/ZFP36/ZNF385A/ZBTB16/SOX13/TFE3/NAPEPLD/SFRP2/ASXL2/CARM1/PIM1
BP	GO:0006887	exocytosis	242	-0.40	-1.56	0.00023742	0.00167246	0.00103698	3095	tags=40%, list=26%, signal=30%	SCRIB/NCKAP1L/RAB3GAP1/SCRN1/KIT/F2RL1/SNPH/VPS18/VAMP3/STX17/MYO1G/DVL1/ITGB2/PRAM1/SDF4/NKG7/GAB2/SDCBP/ATP13A2/PDPK1/FGR/RAB3IP/RAB11FIP5/RALB/PTAFR/RAB8A/CORO1A/EXOC7/FBXL20/SYT11/NSF/NOTCH1/ATP2A2/TSG101/CCR1/CDK5/EXOC4/SPI1/YKT6/VPS4B/TXLNA/FES/ITGAM/SYNJ1/HGS/DTNBP1/HYAL3/GSK3B/ATP9A/GIT1/SYNGR1/DNAJC5/ARHGAP17/STX4/PLEK/RAB31/ITSN1/LYN/VTI1B/RAB7A/RAB11FIP1/EXOC3/SEPTIN1/CEACAM1/PIK3CD/SCFD2/GNAI2/SEPTIN4/BCR/SYK/ABCC4/PAK1/CLASP1/RAB27B/RAB3IL1/CALM3/LGALS9/RAB11B/RAB3D/RAC2/NAPA/SDC4/SYTL4/PRKCB/PIK3CG/RAB2B/SNCA/LAMP1/P2RX1/GATA1/CBL/SMPD3/SCIN/MYH9/DMTN/ANK1

BP	GO:1901880	negative regulation of protein depolymerization	53	-0.59	-1.82	0.0002424	0.00169979	0.00105393	2653	tags=51%, list=23%, signal=40%	ADD2/EPS8/SWAP70/PIK3CA/TMOD3/SPTAN1/ATXN7/TRIOBP/SVIL/LIMA1/TPX2/TAOK1/KATNB1/FLII/CAMSAP1/ARHGEF2/CLASP1/GSN/SPTBN1/MTPN/SPTA1/SPTB/TMOD1/SCIN/CAPZB/DMTN/ADD1
BP	GO:0009755	hormone-mediated signaling pathway	122	-0.47	-1.68	0.00025375	0.00177625	0.00110134	2445	tags=37%, list=21%, signal=30%	PTPN11/NR1D1/DDX54/UBR5/CNOT9/PKN1/THRA/ZNF366/PAQR8/NCOA4/PPARGC1B/ZBTB7A/NR3C1/TRERF1/FOXP1/NCOA1/CRY2/NR1H3/SMARCA4/SLC27A1/PARP1/LBH/RARA/TADA3/RXRA/SRC/ADIPOR2/CALR/PPARA/WBP2/DAXX/ZMIZ1/YWHAH/PAK1/RHOA/KANK2/ABHD2/ADIPOR1/SAFB/MED1/ARID1A/NCOR2/CARM1/CNOT1/DAB2
BP	GO:0003333	amino acid transmembrane transport	55	-0.59	-1.84	0.00025564	0.00178631	0.00110757	2026	tags=36%, list=17%, signal=30%	CLN3/SLC7A7/MFSD12/SLC43A2/SLC7A1/TSPO2/ARL6IP1/SLC25A38/LRRC8A/SLC7A8/SLC1A5/SLC1A4/SLC6A20/SLC7A5/SLC3A2/SLC6A9/SLC38A10/SLC43A1/PER2/SLC38A5
CC	GO:0030135	coated vesicle	214	-0.41	-1.58	0.00025921	0.00180812	0.0011211	1756	tags=27%, list=15%, signal=23%	EDN1/KIAA0319/FZD5/CD74/HLA-C/VPS41/STX6/SEC23IP/AP2B1/BCAP31/HLA-DQA1/SREBF1/SYNRG/COPA/TEX261/CLTC/DENND1C/DNAJC5/MYO1E/DENND1A/YIF1A/AP1M1/HIP1/AP2S1/HIP1R/VTI1B/FZD2/CD9/KDELR1/SNX3/HLA-DRB5/VTI1A/TGOLN2/PACS1/COPB2/SEC31A/CNIH3/RAB14/RAB27B/STX5/GOLGA2/SCYL1/CLTB/EPN1/DNM2/LDLRAP1/ECE1/SPG21/SORT1/STON2/ARCN1/IGF2R/NRGN/FURIN/AP2M1/SEC16A/DAB2
BP	GO:0030218	erythrocyte differentiation	110	-0.48	-1.72	0.00026218	0.00182561	0.00113194	1481	tags=33%, list=13%, signal=29%	TSPO2/CDK6/ACIN1/ETS1/LYN/KLF13/EPAS1/ADAR/HSPA1A/KLF2/HCLS1/KAT7/SLC25A38/ARNT/TRIM10/BPGM/STAT3/JAK3/MAFB/AHSP/MFHAS1/HIPK2/KLF1/SLC4A1/EPB42/FAM210B/PRKDC/IKZF1/GATA1/ALAS2/FOXO3/MPIG6B/MED1/TAL1/TRIM58/DMTN
BP	GO:0008285	negative regulation of cell population proliferation	439	-0.36	-1.47	0.00026285	0.00182706	0.00113284	2957	tags=36%, list=25%, signal=28%	ANG/SAV1/RC3H1/TAX1BP3/ATM/PTCH1/AIMP2/S100A11/B4GALT1/FLT1/CEBPA/TNFRSF8/CD274/MSX1/EPHB1/EIF2AK2/TSC2/TNFRSF13B/RPS6KA2/SH2B3/ETV3/GABBR1/CR1/PBRM1/ULK1/SMARCA2/TMEM115/FOXP3/PTGIR/E2F3/CBLB/JUN/NOTCH1/BAK1/CDKN2D/FLCN/TSG101/TSPO/INSL3/H2AC8/HES1/PKN1/HLA-DRB1/BTG1/MAGI2/IRF1/DDR1/ARID2/LGALS9C/DACH1/IL1B/CSF1R/PTPN6/VASH1/CERS2/ZBTB16/GSTP1/NDRG2/SIRPG/TRIB1/NDRG1/LZTS2/SMAD3/HGS/PMP22/MAGED1/FZD5/INPP5D/RBPJ/TGFB1I1/RAP1GAP/EPPK1/CTBP1/TP53I11/KLF4/SOX4/TOB2/TGFB1/CTNNBIP1/ASCL2/CDK6/TMEM131L/VHL/ETS1/CBFA2T3/CCN3/VGLL4/SPINT2/INPPL1/MEN1/BCL11B/SKI/IL4I1/TWSG1/TPM1/CHERP/LYN/KLF13/ILK/PTK2B/RARA/HSPA1A/CD9/SPN/DHCR24/TRIM32/PML/GREM1/CDKN1C/CEACAM1/SFRP2/RUNX3/IFT52/UTP20/TFDP1/STAT3/CTNNA1/KAT2B/PAK1/FOXO4/E2F1/SF1/PIM2/WARS1/JARID2/PDE5A/RHOA/PTPRJ/LGALS9/LMNA/KANK2/NIBAN2/NF2/TMEM127/STK11/FTH1/SDC4/RASSF5/GNA12/MED25/WDR13/CSK/THBS1/MFN2/PER2/NEURL1/ATG13/GATA1/RNF10/CDKN1A/SCIN/HMGA1/PTGDR2/DNAJB2/TESC/MED1/EIF2AK1/SPARC/DAB2

BP	GO:0032940	secretion by cell	481	-0.35	-1.45	0.00026881	0.00186199	0.0011545	3038	tags=33%, list=26%, signal=25%	PTGES/SCRN1/NRXN1/KIT/F2RL1/LTBP2/SNPH/VPS18/ANG/VAMP3/STX17/MYO1G/DVL1/ITGB2/PRAM1/SDF4/NKG7/COPG1/FGF23/GAB2/TTN/SDCBP/ATP13A2/PDPK1/GABBR1/FGR/SLC16A1/RAB3IP/RAB11FIP5/STEAP3/RALB/PTAFR/RAB8A/RHBDF2/CORO1A/EXOC7/PIM3/FBXL20/POMC/SYT11/NSF/PTPN11/NOTCH1/TRPM2/NR1D1/ATP2A2/TSG101/M6PR/CCR1/RPH3A/HLA-DRB1/CDK5/EXOC4/IL1B/SPI1/YKT6/P2RX4/VPS4B/TXLNA/PARD6A/FES/NR1H2/ITGAM/SYNJ1/HGS/EDN1/UBE2Q1/ENG/CPT1A/GRK2/CANX/SOX4/DTNBP1/HYAL3/SREBF1/ACVR2B/GSK3B/CRY2/NR1H3/TGFB1/ATP9A/FRMD4A/SVBP/GIT1/TNFRSF1B/SYNGR1/PCSK6/IL1RN/DNAJC5/CCN3/EZR/ARHGAP17/STX4/UCP2/PLEK/RAB31/ITSN1/LYN/VTI1B/RAB7A/RAB11FIP1/PINK1/BAD/PFKL/EXOC3/SEPTIN1/CEACAM1/PIK3CD/SCFD2/GNAI2/LRRC8A/SEPTIN4/SLC1A5/PPP3CA/BCR/SYK/ABCC4/PAK1/CLASP1/BLOC1S3/RAB27B/BMP6/PDIA4/RAB3IL1/MYO18A/SIDT2/GALR1/CALM3/LGALS9/RAB11B/FKBP1B/RAB3D/RAC2/NIBAN2/NAPA/SDC4/SYTL4/GLUL/PRKCB/TANGO2/PIK3CG/RAB2B/TCF7L2/PER2/SNCA/LAMP1/P2RX1/GATA1/CBL/SMPD3/SCIN/FURIN/PTPN23/MYH9/DMTN/ANK1/DAB2
BP	GO:0062012	regulation of small molecule metabolic process	212	-0.41	-1.57	0.00027211	0.0018816	0.00116666	2571	tags=35%, list=22%, signal=28%	PTAFR/AKT1/ARL2/POMC/ABCB11/ACACB/NR1D1/CLN3/EGR1/SLC7A7/HDAC4/FLCN/FOXO1/ABCD1/IL1B/ENTPD5/PGAM1/NLN/ZBTB7A/EIF6/DHCR7/NR1H2/FOXK1/PHKG2/CD74/CPT1A/PDK3/WDR5/CDA/SREBF1/GSK3B/FOXK2/NR1H3/FAM3A/GSK3A/GIT1/BCKDK/CBFA2T3/JMJD8/NCOA2/PLEK/WDTC1/PARP1/MFSD2A/SESN2/PINK1/PTK2B/IGF2/BAD/SRC/ARNT/CEACAM1/PPARA/PPP1CA/STAT3/PDK2/KAT2B/DDB1/VCP/FBP1/NFKB1/BMP6/ACADVL/LDLRAP1/INPP5K/USP7/STK11/ADIPOR1/SLC4A1/AKT2/ATP2B4/SNCA/ENO1/NCOR2/DAB2
MF	GO:0035091	phosphatidylinositol binding	210	-0.42	-1.60	0.00027587	0.00190425	0.0011807	2804	tags=36%, list=24%, signal=28%	OBSCN/GAB2/SNX8/SDCBP/ATP13A2/PLD1/KIF16B/PLD2/DAPP1/SBF2/OSBPL5/AKT1/AIDA/NCF1/ATG2B/SNX33/SNX27/RPH3A/PFN1/ARAP1/SNX17/ITPR2/SH3PXD2A/ZCCHC2/SVIL/FES/ZCCHC14/NUMA1/TOM1/SCARB1/HGS/ADAP2/ANKFY1/SNX12/KCNJ2/WASHC2A/TECPR1/ASAP1/DENND1C/ATG2A/MYO1E/SNX10/DENND1A/ESYT1/ALOX15/HIP1/PITPNC1/HIP1R/BCAS3/GPAA1/SNX15/CGAS/SLC9A1/PITPNM2/PITPNA/BBS5/RUBCN/SNX3/ARHGAP32/FLII/PLEKHF1/SNX11/ZFYVE1/PARD3/PLEK2/WIPI2/LDLRAP1/SNX29/GSN/EPB41/IQGAP1/NRGN/SCIN/WDR45/TLN1

BP	GO:0009628	response to abiotic stimulus	707	-0.32	-1.36	0.00027899	0.00192244	0.00119198	2768	tags=30%, list=24%, signal=24%	LETM1/CCDC115/FOSB/INTS7/EPHB1/TNFRSF10A/EYA3/RGS9/TTN/BHLHE40/PDPK1/ATP7A/FANCG/CASP7/EXT1/RAB11FIP5/PKD1/JUP/GNAQ/MAPK14/PTAFR/AKT1/TMEM109/FOXP3/PIK3CA/JUN/DISC1/NOTCH1/TRPM2/CYBB/BAK1/CLN3/CASP9/TRPC3/EGR1/CDKN2D/FOXO1/SLC24A4/PNKP/ZNF516/TSPO/HYOU1/CHP1/EPHA4/PKN1/MECP2/MLC1/IRF1/CDK5/POLD3/HYAL2/PCSK1N/FYN/CASP2/SLC25A23/THRA/MAP7/ATP1B1/IL1B/MTA1/ITPR2/HSPA5/GNA11/ROCK2/DCUN1D3/DNAJB1/TIMP1/PDE2A/CCAR2/NDRG1/PIN1/XRCC6/RCSD1/SMAD3/RFWD3/RHBDD1/MICU1/PTPRC/EDN1/KIAA0319/PIK3CB/HIF1AN/ENG/TAF1/RBPJ/CD14/MMP9/PDK3/JUND/DNAJA4/KCNJ2/SMPD1/TM9SF4/GRB2/HYAL3/SERPINB6/GSK3B/SORD/CRY2/PRKACA/HVCN1/CYFIP1/HSP90AB1/ACTG1/MICB/PLOD1/ASCL2/VHL/CBFA2T3/MEN1/CASP5/UCP2/SLC27A1/ALAD/ATP6AP1/SMC1A/BABAM2/PARP1/OXSR1/AQP10/MTHFR/KARS1/AQP1/LYN/YY1/EPAS1/PINK1/PTK2B/HSPA1A/MGMT/MAP2K7/CREBBP/SLC9A1/BAD/TRIM32/SRC/PML/ARNT/SFRP2/ATP6V1A/LRRC8A/PPARA/DAXX/TP53BP1/PPP1CA/USP19/CNN2/ARHGEF2/NOLC1/ALKBH5/DDB1/VCP/ATP6V0D1/EGLN3/FOS/SYNGAP1/BABAM1/N4BP1/RELA/E2F1/TMEM120A/NFKB1/BMP6/ARRB2/SLC2A1/MPL/ACADVL/RHOA/DNM2/RAB11B/GUCY1A1/MAPKAPK2/LMNA/MYLK/ATG7/HSPB1/HIPK2/MCOLN1/ELK1/BCL2L1/STK11/GSN/PLEC/TXNIP/THBS1/AKT2/MTPN/PER2/GPI/ATP2B4/ENO1/PRKDC/DYNLRB1/MAPK3/BNIP3L/CDKN1A/SLC29A1/CBL/GMPR/ACTN4/GPX1/ALAS2/YBX3/PDLIM1/FOXO3/FECH/HTT/GUCY1B1/PDGFA
BP	GO:0045055	regulated exocytosis	150	-0.45	-1.66	0.00028026	0.0019279	0.00119537	3095	tags=41%, list=26%, signal=31%	SCRIB/NCKAP1L/RAB3GAP1/KIT/F2RL1/SNPH/VPS18/VAMP3/DVL1/ITGB2/PRAM1/SDF4/NKG7/GAB2/PDPK1/FGR/RAB11FIP5/PTAFR/CORO1A/FBXL20/SYT11/NOTCH1/ATP2A2/CDK5/SPI1/FES/ITGAM/SYNJ1/DTNBP1/HYAL3/GSK3B/GIT1/SYNGR1/DNAJC5/ARHGAP17/STX4/PLEK/RAB31/LYN/RAB11FIP1/CEACAM1/PIK3CD/GNAI2/BCR/SYK/ABCC4/CALM3/LGALS9/RAB11B/RAB3D/RAC2/NAPA/PRKCB/PIK3CG/SNCA/LAMP1/P2RX1/GATA1/CBL/SCIN/MYH9/DMTN
CC	GO:0005905	clathrin-coated pit	57	-0.56	-1.77	0.00028422	0.00195174	0.00121015	1960	tags=39%, list=17%, signal=32%	TNK2/ARRB1/SYNJ1/AP2B1/DGKD/REPS1/CLTC/SPHK1/AP2S1/ITSN1/HIP1R/VLDLR/EPS15L1/ARRB2/CLTB/EPN1/DNM2/SORT1/LRP1/CTTN/AP2M1/DAB2
CC	GO:0045121	membrane raft	220	-0.42	-1.60	0.00028654	0.00196427	0.00121792	2758	tags=33%, list=24%, signal=26%	PAG1/EPHB1/TNFRSF10A/SDCBP/GABBR1/ATP7A/PECAM1/ADD2/CR1/TRAF2/RFTN1/CBLB/ATP1A1/CLN3/EFNB1/PI4K2A/MLC1/SPRED1/HYAL2/FYN/ICAM1/ATP1B1/EFHD2/RIPK1/TUBA1C/SELPLG/TLR6/MYADM/ITGAM/TPP1/LIPE/SCARB1/PTPRC/NEU3/ENO2/STOML2/INPP5D/CD14/STOM/PRKACA/TNFRSF1B/RANGRF/EZR/CORO1C/LYN/PTK2B/SLC9A1/SRC/GNAI2/CTNNA1/MYOF/VDAC1/ABCG2/STIM1/LAMTOR1/SLC2A1/PRKAR2A/GP6/HK1/BSG/CAVIN2/SDC4/CSK/PLVAP/ATP2B4/P2RX1/MAPK3/ARID3A/CBL/CTSD/TUBB/PRKAR2B/FURIN

MF	GO:0003682	chromatin binding	435	-0.35	-1.46	0.00028776	0.00196928	0.00122102	2479	tags=28%, list=21%, signal=23%	JUN/CHD3/ADNP/NOTCH1/EGR1/REPIN1/RAD21/EZH2/FOXO1/WAC/CDYL/NAP1L4/ARID1B/PRDM1/HES1/PKN1/ELK4/MECP2/WDHD1/WDR82/GLYR1/THRA/MTA2/MITF/TOP1/SPI1/MTA1/GRWD1/GTF2F1/H1-4/EP400/PHF8/CDK9/MCM3AP/SMAD3/POLA1/MLLT10/RERE/SMARCC1/RBPJ/CTBP1/CENPF/KLF4/EHMT2/TOP2A/SREBF1/NCOA1/NELFA/ANKRD17/MBD6/SFMBT1/HDAC3/SMARCA4/JDP2/SCMH1/PHF21A/NFIA/TTC5/UBTF/NCOA2/MEN1/KDM6B/SMC1A/NCOA6/YY1/BCAS3/NUP98/SUPT6H/RARA/SUPT16H/CGAS/CREBBP/DHX30/GATAD2B/WBP2/PELP1/TRIM28/ACTB/ZNF609/STAT3/KAT2B/UPF1/DNTTIP1/FOS/RELA/FOXO4/NCAPH/NFKB1/TNRC18/TOX2/JARID2/SETDB1/TLE4/SHMT2/CHD8/SAMD1/ELK1/POLR2A/ASXL2/SAFB/WDR13/PRKCB/CABIN1/YBX1/MEIS1/NFATC2/CHD4/BRD3/PAF1/GATA1/ARID3A/BRD4/HMGA1/ACTN4/FOXO3/SMARCC2/MED1/TAL1/ARID1A/MLLT6/NCOR2/HCFC1/SUPT5H
BP	GO:0043410	positive regulation of MAPK cascade	281	-0.39	-1.53	0.00029121	0.00198942	0.00123351	2479	tags=31%, list=21%, signal=25%	JUN/SERPINF2/PTPN11/NOTCH1/NCF1/PDE6G/GADD45B/EZH2/CCR1/MAP3K13/EPHA4/HLA-DRB1/ARHGAP8/PRKD2/PELI2/CD81/ICAM1/CRKL/IL1B/SPI1/RIPK1/CSF1R/MADD/ROCK2/SCIMP/TLR6/MAP3K3/SHC1/ARRB1/TRAF7/PTPRC/EDN1/DIXDC1/MAGED1/FZD5/CD74/MAP3K11/SORBS3/PYCARD/TGFB1/LILRA5/TAOK1/SPHK1/NDST1/TRAF6/MAP3K5/ALOX15/MAP4K1/SASH1/PTK2B/IGF2/MAP2K7/SRC/STK25/GNAI2/DVL3/LAPTM5/TAOK2/NRP1/MAP2K2/PDGFC/SYK/XIAP/ABL1/PAK1/GADD45G/ARRB2/LAMTOR1/PDE5A/MFHAS1/NOTCH2/ACTA2/PTPRJ/LGALS9/MTURN/HIPK2/DHX33/CSK/THBS1/PTPN1/AKAP13/PIK3CG/IQGAP1/MAPK3/PDGFA/MAP2K3
BP	GO:0030001	metal ion transport	442	-0.36	-1.46	0.0002991	0.00203983	0.00126477	1587	tags=19%, list=14%, signal=17%	KCNJ2/KCNQ5/EHD3/STOM/PRKACA/CD63/LILRA5/CLTC/RANGRF/FLVCR2/SELENON/OXSR1/SLC5A6/SLC39A3/FHL1/ANO6/CHERP/AQP1/LYN/NEDD4L/PTK2B/SLC9A1/SLC22A17/SLC39A4/PML/RASA3/GNAI2/ATP13A1/CDK2/PPP3CA/ATP1B2/CACNG6/SLC6A20/YWHAH/VDAC1/JAK3/ABL1/LCN2/FKBP1A/STIM1/LTF/ANXA6/ARRB2/WWP2/RHOA/CALM3/DNM2/FLNA/SLC3A2/RAB11B/FKBP1B/MYLK/INPP5K/ARHGAP1/MCOLN1/SLC6A9/SLC24A3/FTH1/MMGT1/SLC38A10/GP1BA/PRKCB/GP9/EPB41/GJC2/PIK3CG/UBASH3B/ATP2B4/SNCA/AHCYL1/P2RX1/FTL/SLC48A1/ACTN4/SLC6A8/SLC25A37/TESC/HTT/MLLT6/KEL/SCN1B/WNK1/DIAPH1
BP	GO:0018210	peptidyl-threonine modification	90	-0.50	-1.73	0.00030026	0.00204423	0.00126749	2251	tags=36%, list=19%, signal=29%	LMTK2/PKN1/CDK5/PRKD2/SPRED1/CSNK2A1/ROCK2/ACVR1B/TAF1/GRK2/GSK3B/PRKACA/GSK3A/TGFB1/DYRK1B/SPHK1/OXSR1/CDC42BPB/MARK2/DCAF1/GALNT2/PARD3/CALM3/HIPK1/INPP5K/HIPK2/STK11/PRKCB/EIF4G1/PRKDC/DMTN/WNK1
BP	GO:1905475	regulation of protein localization to membrane	121	-0.48	-1.72	0.00030653	0.00208335	0.00129175	2420	tags=35%, list=21%, signal=28%	CNST/CLN3/TMBIM1/DLG4/CHP1/CDK5/CD81/ABI3/FYN/CRKL/LGALS3/ITGAM/BBC3/AP2B1/STOM/TGFB1/CLTC/MESD/RANGRF/EZR/STX4/VTI1B/GRIPAP1/FIS1/ACTB/LDLRAP1/INPP5K/BCL2L1/ZDHHC5/GSN/SQSTM1/LRP1/PPP1R9B/CSK/SPTBN1/AKT2/ATP2B4/RHOG/AP2M1/SHISA7/DMTN/DAB2

BP	GO:0006811	ion transport	774	-0.32	-1.35	0.00031009	0.00210396	0.00130453	2575	tags=26%, list=22%, signal=22%	STEAP3/PTAFR/CORO1A/MINK1/AKT1/TMEM109/SELENOK/SLC46A1/KCNH2/PCYOX1/SYT11/NSF/ABCB11/TRPM2/CYBB/BAK1/ATP1A1/CLN3/CYBRD1/TRPC3/ATP2A2/SLC7A7/DLG4/SLC24A4/TSPO/UQCRC1/CCR1/SLC20A1/CHP1/LMTK2/G6PD/SERINC5/HES1/NETO2/SLC25A11/MLC1/CDK5/FYN/FXYD6/SLC25A23/ATP1B1/IL1B/P2RX4/VPS4B/RIPK1/PTPN6/SLC25A1/ATP13A3/ITPR2/MFSD12/SLC9A7/SLC33A1/SNTA1/SLC43A2/TCN2/RALBP1/LGALS3/TMC8/SLC7A1/SLC25A42/PDE4B/SLC37A2/MICU1/PTPRC/EDN1/STOML2/SLC41A1/COX15/SLC19A1/MMP9/ABCC1/KCNJ2/KCNQ5/DTNBP1/EHD3/STOM/CRY2/PRKACA/HVCN1/TSPO2/CD63/LILRA5/CLTC/RANGRF/SLC44A2/FLVCR2/CLIC5/DAPK1/UCP2/CNNM3/SLC27A1/SELENON/ARL6IP1/OXSR1/SLC35C2/MFSD2A/SLC5A6/SLC39A3/FHL1/ANO6/CHERP/AQP1/LYN/NEDD4L/PINK1/PTK2B/SLC9A1/SLC22A17/SLC39A4/PML/SLC25A38/CEACAM1/ABCC3/CA2/RASA3/GNAI2/ATP6V1A/LRRC8A/ATP13A1/SLC7A8/SLC1A5/CDK2/PPP3CA/ATP1B2/CACNG6/PSAP/CNIH3/SLC1A4/SLC6A20/YWHAH/VDAC1/SYK/ABCG2/JAK3/CPTP/ABCC4/ATP6V0D1/ABL1/LCN2/FKBP1A/STIM1/LTF/TMEM120A/ANXA6/CYB5A/ARRB2/SLC2A1/CLCN5/WWP2/SLC7A5/RHOA/CALM3/DNM2/FLNA/SLC3A2/RAB11B/FKBP1B/MYLK/INPP5K/ARHGAP1/LASP1/MCOLN1/SLC6A9/SLC24A3/FTH1/MMGT1/SLC38A10/SLC4A1/GP1BA/PRKCB/ANKH/GP9/EPB41/GJC2/PIK3CG/SLC43A1/SEC14L1/PER2/UBASH3B/ATP2B4/SNCA/AHCYL1/CAPN1/P2RX1/TTYH3/SLC29A1/FTL/SLC48A1/ACTN4/SLC6A8/SLC15A3/SLC25A37/TESC/HTT/MLLT6/SHISA7/SLC38A5/ITGB3/KEL/TMEM63B/SCN1B/WNK1/DIAPH1
BP	GO:0006935	chemotaxis	334	-0.37	-1.49	0.00031983	0.00216635	0.00134321	2769	tags=35%, list=24%, signal=28%	LEF1/PTCH1/HBEGF/DVL1/NECTIN1/ITGB2/FLT1/CCRL2/EPHB1/DOCK4/TSC2/PLD1/CMKLR1/EXT1/CORO1B/MAPK14/PTAFR/CORO1A/SWAP70/PLXNC1/PLXNA4/NOTCH1/TRPM2/EFNB1/NCAM1/LYPLA2/CCR1/IL6R/EPHA4/CDK5/PRKD2/FYN/MACO1/CRKL/IL1B/SPI1/FEZ1/P2RX4/CNR2/CSF1R/PPBP/RNASE3/RALBP1/ST6GAL1/FES/LGALS3/CXCL16/HSD3B7/ARHGAP35/SMAD3/PDE4B/EDN1/PIK3CB/RIPOR2/CREB3/CMTM5/CD74/SEMA4D/ABCC1/CXCR2/CYFIP1/TGFB1/CXCR1/PREX1/AMOT/CCN3/STX4/BCL11B/FPR3/OXSR1/ANO6/LYN/PLXNB2/IL17RA/RAC1/JAM3/PTK2B/DPYSL2/PLXND1/TRIO/SPN/GREM1/CALR/PIK3CD/MYCBP2/VAV1/PLXNA1/RIN3/SEMA4A/NRP1/SYK/ARRB2/RHOA/NOTCH2/PTPRJ/LGALS9/RAC2/BSG/ECE1/HSPB1/NIBAN2/DOCK2/CAMK1D/MPP1/PLEC/PF4V1/THBS1/PIK3CG/CXCL5/VASP/RHOG/MAPK3/PF4/PTGDR2/ITGB3/PDGFA/SCN1B/WNK1
BP	GO:0050770	regulation of axonogenesis	90	-0.50	-1.72	0.00032097	0.00217034	0.00134569	2593	tags=41%, list=22%, signal=32%	ULK1/DIP2B/PLXNC1/DISC1/PLXNA4/ADNP/MAP3K13/EPHA4/CDK5/MACF1/ARHGAP35/KIAA0319/TIAM1/DIXDC1/NDEL1/SEMA4D/GSK3B/CYFIP1/IST1/PLXNB2/CDKL5/MBP/PLXND1/KIF13B/SIPA1L1/MARK2/EIF4G2/STK25/PLXNA1/SEMA4A/NRP1/PAK1/SYNGAP1/XK/STK11/PAK2/KEL
BP	GO:0032868	response to insulin	185	-0.42	-1.58	0.00032338	0.00218295	0.00135351	2578	tags=34%, list=22%, signal=27%	MAPK14/SORL1/RAB8A/AKT1/PIK3CA/PTPN11/EGR1/HDAC5/FOXO1/PIP4K2C/IL1B/SRSF4/EIF6/SHC1/SLC29A2/SH2B2/SMARCC1/NDEL1/PIK3R3/GRB2/SREBF1/GSK3B/BAIAP2/CRY2/GSK3A/CYFIP1/YWHAG/ZNF106/INPPL1/UCP2/SLC27A1/WDTC1/PARP1/SESN2/RAB31/LYN/IGF2/SLC9A1/SRC/CCND3/CEACAM1/GNAI2/PPARA/PKM/MAX/PDK2/KAT2B/FBP1/FOXO4/LPIN2/SLC2A1/SESN3/INPP5K/SORT1/ADIPOR1/PRKCB/AKT2/PTPN1/SP1/PRKDC/PIP4K2A/EIF4EBP2

BP	GO:0055065	metal ion homeostasis	382	-0.37	-1.48	0.00033028	0.002222	0.00137772	2791	tags=33%, list=24%, signal=26%	TMTC2/SLC40A1/CCRL2/LETM1/CCDC115/COX19/ATP13A2/PDPK1/CMKLR1/ATP7A/EXT1/PKD1/ATP2A3/STEAP3/CORO1A/SELENOK/PTGIR/SWAP70/SLC46A1/KCNH2/DISC1/KNG1/TRPM2/BAK1/ATP1A1/CLN3/CYBRD1/TRPC3/ATP2A2/DLG4/SLC24A4/CCR1/CDK5/HYAL2/FYN/SLC25A23/ATP1B1/P2RX4/PTPN6/ATP13A3/ITPR2/NCOA4/TMC8/LPAR4/SMAD3/MICU1/PTPRC/EDN1/PIK3CB/STOML2/SLC41A1/FRRS1/KCNJ2/CXCR2/BCAP31/PRKACA/CXCR1/SNX10/CNNM3/ATP6AP1/SELENON/FPR3/CHERP/LYN/NEDD4L/SPPL3/EPAS1/PTK2B/GNA15/MT2A/ARF1/SLC9A1/SLC22A17/SLC39A4/PML/RASA3/CALR/GNAI2/ATP6V1A/ATP13A1/SLC7A8/FIS1/ATP1B2/JAK3/XIAP/ATP6V0D1/ABL1/LCN2/FKBP1A/STIM1/LTF/ANXA6/BMP6/XK/GALR1/CALM3/FKBP1B/MCOLN1/SLC6A9/SLC24A3/FTH1/TTC7A/GP1BA/EPB42/PRKCB/GP9/HRH4/PIK3CG/UBASH3B/ATP2B4/SNCA/ANKRD9/P2RX1/GATA1/GRINA/FTL/ALAS2/SLC25A37/EIF2AK1/HTT/MLLT6/KEL/TGM2/DIAPH1/TBXA2R
BP	GO:0007265	Ras protein signal transduction	261	-0.40	-1.55	0.00033144	0.00222604	0.00138022	2048	tags=25%, list=17%, signal=21%	MAPRE2/MADD/ROCK2/PRAG1/RALBP1/RIPOR1/ARRB1/LPAR4/ARHGAP35/TIAM1/PIK3CB/LRRC59/RIPOR2/PHACTR4/GRB2/G3BP2/CYFIP1/DOK3/RASGRP3/RANGRF/ARHGAP17/DENND1A/CDC42EP4/RAC1/DHCR24/GNB1/MYO9B/RRAS/RASA3/IQSEC1/CDK2/TRIM28/CTNNAL1/NRP1/ARHGEF2/BCR/GABARAP/ABL1/SYNGAP1/FBP1/ARHGEF18/CYTH4/RAPGEF1/SETDB1/RHOA/ARHGEF12/NOTCH2/DNM2/ARHGAP6/ARHGDIA/WAS/RAC2/KANK2/ARHGAP1/GNA12/CFL1/SQSTM1/AKAP13/PIK3CG/MFN2/RHOG/CDKN1A/CBL/WASF2/SH3BP1
BP	GO:0002028	regulation of sodium ion transport	53	-0.58	-1.81	0.00034107	0.00228359	0.00141591	2267	tags=42%, list=19%, signal=34%	AKT1/ATP1A1/CHP1/NETO2/FXYD6/ATP1B1/P2RX4/SNTA1/STOM/RANGRF/NEDD4L/SLC9A1/ATP1B2/YWHAH/DNM2/ATP2B4/AHCYL1/ACTN4/TESC/MLLT6/SCN1B/WNK1
CC	GO:0005774	vacuolar membrane	387	-0.36	-1.48	0.00034116	0.00228359	0.00141591	2977	tags=36%, list=25%, signal=28%	VPS18/ANPEP/BRI3/STX17/KPTN/SLC36A1/ZFYVE26/ATP11A/B4GALT1/CKAP4/WDR11/SH3GLB1/NKG7/CCDC115/SPPL2B/DNAJC13/ATP13A2/PLD1/AP2A1/RMC1/AP5S1/ULK1/GNAQ/SBF2/GNB2/STARD3/ABCA3/AP2A2/ACP2/SYT11/NSF/TRPM2/CLN3/LAMP2/TMBIM1/CYBRD1/FLCN/CPNE3/M6PR/HGSNAT/AP5B1/ABCB9/HLA-DRB1/ABCD1/PI4K2A/WDR24/HLA-DQA2/CMTM6/CPNE1/VPS39/P2RX4/MFSD12/GNA11/ITM2C/CTSA/ARRB1/MAP1LC3B/TOM1/SCARB1/NEU3/TFEB/SLC35F6/CHMP6/CD74/ANKFY1/VPS41/WDR81/CHMP4B/AP2B1/HLA-DQA1/TECPR1/STOM/HSP90AB1/CD63/CLTC/SYNGR1/SLC44A2/DNAJC5/AP1M1/UBA52/AP2S1/LNPEP/LYN/GLMP/VTI1B/RAB7A/PLEKHM2/IFITM3/PLD3/GNB1/SLC22A17/NCSTN/CHMP1A/YWHAB/ATP6V1A/LRRC8A/HLA-DRB5/PLEKHF1/LAPTM5/PSAP/LITAF/IFITM2/UBA1/VLDLR/GABARAP/ATP6V0D1/RAB14/FNIP2/ANXA6/DTX3L/ATP6V0C/LAMTOR1/CLCN5/GAA/SLC7A5/SIDT2/AP1B1/SLC3A2/GPR137/ARL8A/RAB3D/ECE1/ATP6V0A1/MCOLN1/CDIP1/SORT1/LRP1/RILP/MARCHF2/GRN/MMD/LAMP1/ELAPOR1/MARCHF8/CTSD/SLC48A1/SLC15A3/NPRL3/UBXN6/AP2M1/DAB2

CC	GO:0031226	intrinsic component of plasma membrane	761	-0.32	-1.35	0.00034433	0.00230094	0.00142667	2934	tags=28%, list=25%, signal=23%	ICAM4/TMBIM6/HBEGF/XPR1/C17orf99/NECTIN1/PSENEN/ITGB2/ADCY9/ATP11A/SLC8A1/FLT1/GPBAR1/OBSCN/TM7SF2/NKG7/PCDH11X/SLC40A1/TSPAN14/CCRL2/EPHB1/FRS2/SDCBP/NCF2/TNFRSF13B/CMKLR1/ATP8B2/GABBR1/PECAM1/CR1/SLC16A1/TRAF2/PKD1/EPS8/PTAFR/SORL1/PTGIR/PLXNC1/KCNH2/SYT11/PLXNA4/TRPM2/CYBB/NCF1/CD22/ATP1A1/CD3E/TRPC3/EFNB1/ATP2A2/SLC7A7/DLG4/M6PR/SLC24A4/PHEX/CCR1/SLC20A1/DIABLO/LMTK2/IL6R/EPHA4/HLA-DRB1/SLC25A11/PI4K2A/HYAL2/CD81/DDR1/FXYD6/HLA-DQA2/ICAM1/ATP1B1/HRH2/YKT6/P2RX4/CNR2/HLA-A/CSF1R/SELPLG/MFSD12/SLC33A1/TLR6/ADGRE1/ITGB7/TMC8/SLC7A1/FADS2/ITGAM/KIR3DL2/LPAR4/KIR2DS4/SCARB1/ACVR1B/OR10H1/SLC29A2/PTPRC/KIR3DL1/LRFN1/FZD5/CD14/VSIG10/SLC19A1/HLA-C/CANX/NCR3/IL2RG/SEMA4D/SIRPB1/ABCC1/KCNJ2/CXCR2/KCNQ5/BCAP31/ADGRE5/CD37/HLA-DQA1/ACVR2B/IL2RB/STOM/HVCN1/CD63/RASGRP3/ST14/TSPAN17/SLC27A1/TRAF6/MFSD2A/AQP10/SLC5A6/SIT1/AQP1/LNPEP/LYN/PLXNB2/IL17RA/GYPA/JAM3/PTK2B/PLXND1/CD9/SLC9A1/SPN/TSPAN9/SLC22A17/NCSTN/SLC39A4/TMPRSS9/ADIPOR2/CEACAM1/ABCC3/RASA3/LRRC8A/SLC7A8/SLC1A5/SLC14A1/PLXNA1/ATP1B2/ADGRL1/LAPTM5/CNIH3/SEMA4A/NRP1/SLC1A4/SELL/SLC6A20/TNFSF4/STIM1/SLC2A1/CLCN5/ITGAL/PCDH1/GP6/GALR1/CALM3/NOTCH2/PTPRJ/BSG/MCOLN1/SLC6A9/SLC24A3/SDC4/ITGB5/GYPB/ADIPOR1/SLC4A1/GP1BA/IGF2R/LRP1/ANKH/GP9/HRH4/CD151/SPTA1/TSPAN33/SLC43A1/ATP2B4/SPTB/SELP/MFSD2B/MMD/LRFN3/LAMP1/GYPC/P2RX1/ELAPOR1/SLC29A1/NINJ2/PTGDR2/SLC6A8/TSPAN5/SHISA7/SLC38A5/ITGB3/ITGA2B/TGM2/SCN1B/TBXA2R
BP	GO:0048259	regulation of receptor-mediated endocytosis	69	-0.54	-1.76	0.0003509	0.00233304	0.00144657	1960	tags=36%, list=17%, signal=30%	TNK2/ARRB1/SYNJ1/NEU3/PIK3CB/DGKD/DTNBP1/CD63/HIP1/HIP1R/RAC1/ARF1/GREM1/RIN3/SYK/ARRB2/HNRNPK/DNM2/LDLRAP1/TBC1D5/CBL/AP2M1/ITGB3/CLU/DAB2
BP	GO:0006865	amino acid transport	81	-0.52	-1.76	0.0003506	0.00233304	0.00144657	2418	tags=30%, list=21%, signal=24%	CLN3/SLC7A7/SERINC5/MFSD12/SLC43A2/SLC7A1/DTNBP1/TSPO2/ARL6IP1/SLC25A38/LRRC8A/SLC7A8/SLC1A5/SLC1A4/SLC6A20/XK/SLC7A5/SLC3A2/SLC6A9/SLC38A10/SLC43A1/PER2/SNCA/SLC38A5
BP	GO:0018209	peptidyl-serine modification	230	-0.40	-1.55	0.00034989	0.00233304	0.00144657	2703	tags=31%, list=23%, signal=25%	PDPK1/RPS6KA2/POGLUT3/PKD1/ULK1/MAPK14/AKT1/PIK3CA/MAPK7/BAK1/CNOT9/MAP3K13/LMTK2/IKBKE/PKN1/MAPKAPK3/CDK5/PRKD2/CSNK2A1/TOP1/RIPK1/ROCK2/MKNK2/CSNK1G1/TAF1/GRK2/PDK3/MARK3/GSK3B/PRKACA/RPS6KA1/HSP90AB1/TGFB1/DYRK1B/NEK6/PARP1/MAP4K1/VRK3/ILK/PINK1/MARK2/HCLS1/SRC/GALNT2/SH2D3C/SRPK2/SFRP2/MAST3/CDK2/MAP2K2/SYK/PAK1/FNIP2/ARRB2/MAD2L2/HIPK1/MAPKAPK2/INPP5K/HIPK2/CAMK1D/PRKCB/AKT2/RPS6KA4/ARAF/ATP2B4/SNCA/CAMK1/EIF4G1/PRKDC/PAK2/DMTN/WNK1
BP	GO:0030048	actin filament-based movement	66	-0.54	-1.77	0.00035739	0.00236851	0.00146856	2933	tags=47%, list=25%, signal=35%	MYO1G/MYO1D/MYH3/WIPF2/JUP/KCNH2/PIK3CA/SYNE2/ATP1A1/ATP2A2/MYO1F/MYO15A/SNTA1/PDE4B/KCNJ2/RANGRF/MYO1E/TPM1/SUN2/NEDD4L/SLC9A1/MYO9B/ACTA2/WAS/FLNA/GSN/WIPF1/ACTN4/MYH9/WASF2/SCN1B

BP	GO:0055085	transmembrane transport	799	-0.31	-1.33	0.00035783	0.00236851	0.00146856	2451	tags=23%, list=21%, signal=20%	ABCB11/PTPN11/TRPM2/CYBB/BAK1/ATP1A1/CLN3/CYBRD1/TRPC3/ATP2A2/TRAM2/SLC7A7/DLG4/SLC24A4/UQCRC1/ABCB9/SLC20A1/CHP1/G6PD/NETO2/SLC25A11/ABCD1/CDK5/FYN/FXYD6/SLC25A23/ATP1B1/IL1B/P2RX4/TIMM44/RIPK1/PTPN6/SLC25A1/PPBP/ATP13A3/ITPR2/HSPA5/MFSD12/SLC9A7/SLC33A1/SNTA1/SLC43A2/TCN2/RALBP1/TMC8/SLC7A1/SLC25A42/SCARB1/PDE4B/SLC37A2/MICU1/SLC29A2/PTPRC/EDN1/SLC35F6/STOML2/SLC41A1/COX15/CPT1A/SLC19A1/MMP9/ABCC1/KCNJ2/KCNQ5/EHD3/STOM/PRKACA/HVCN1/TSPO2/GSK3A/CD63/RANGRF/SLC44A2/FLVCR2/CLIC5/DAPK1/UCP2/CNNM3/SLC27A1/SELENON/ARL6IP1/OXSR1/SLC35C2/MFSD2A/AQP10/SLC5A6/SLC39A3/SESN2/FHL1/ANO6/CHERP/AQP1/LYN/NEDD4L/PINK1/PTK2B/PRF1/SLC25A51/SLC9A1/SLC22A17/SLC39A4/SLC25A38/SLC35A4/ABCC3/CA2/RASA3/ATP6V1A/LRRC8A/ATP13A1/SLC7A8/SLC1A5/SLC14A1/ATP1B2/CACNG6/ACTB/CNIH3/SLC1A4/BCR/SLC6A20/YWHAH/VDAC1/ABCG2/ABCC4/ATP6V0D1/ABL1/FKBP1A/BLOC1S3/STIM1/TMEM120A/ANXA6/CYB5A/SLC2A1/CLCN5/WWP2/SLC7A5/CALM3/DNM2/FLNA/SLC3A2/FKBP1B/INPP5K/LASP1/MCOLN1/SLC6A9/SLC24A3/MMGT1/SORT1/SLC38A10/SLC4A1/GP1BA/PRKCB/THBS1/AKT2/ANKH/GP9/GJC2/PIK3CG/SLC43A1/TST/PER2/UBASH3B/ATP2B4/MFSD2B/SNCA/AHCYL1/CAPN1/P2RX1/TTYH3/SLC29A1/ACTN4/SLC6A8/SLC15A3/SLC25A37/TESC/OAZ1/HTT/SHISA7/SLC38A5/KEL/TMEM63B/SCN1B/WNK1/PIM1/DIAPH1
BP	GO:0006873	cellular ion homeostasis	395	-0.36	-1.46	0.00035864	0.00236868	0.00146866	2788	tags=31%, list=24%, signal=24%	FGF23/SLC40A1/CCRL2/LETM1/CCDC115/COX19/ATP13A2/PDPK1/CMKLR1/ATP7A/PKD1/ATP2A3/CORO1A/SELENOK/PTGIR/SWAP70/SLC46A1/DISC1/KNG1/TRPM2/BAK1/ATP1A1/CLN3/CYBRD1/TRPC3/ATP2A2/DLG4/SLC24A4/CCR1/CHP1/CDK5/MAFG/FYN/SLC25A23/ATP1B1/P2RX4/PTPN6/ATP13A3/ITPR2/SLC9A7/NCOA4/LPAR4/SMAD3/RAB20/MICU1/PTPRC/EDN1/PIK3CB/STOML2/SLC41A1/FRRS1/KCNJ2/CXCR2/BCAP31/TM9SF4/PRKACA/HVCN1/CXCR1/ATP6AP1/SELENON/FPR3/CHERP/LYN/NEDD4L/SPPL3/RAB7A/PTK2B/GNA15/MT2A/ARF1/SLC9A1/SLC22A17/SLC39A4/PML/CA2/RASA3/CALR/ATP6V1A/ATP13A1/FIS1/ATP1B2/JAK3/ATP6V0D1/ABL1/LCN2/FKBP1A/STIM1/ANXA6/BMP6/XK/CKB/GALR1/CALM3/FKBP1B/ATP6V0A1/MCOLN1/SLC6A9/SLC24A3/FTH1/TTC7A/SLC4A1/GP1BA/PRKCB/GP9/HRH4/PIK3CG/GRN/UBASH3B/ATP2B4/SNCA/ANKRD9/P2RX1/MAPK3/GATA1/GRINA/FTL/ALAS2/HTT/KEL/TGM2/DIAPH1/TBXA2R
MF	GO:0003712	transcription coregulator activity	426	-0.35	-1.45	0.00036788	0.00242571	0.00150403	1955	tags=28%, list=17%, signal=24%	EZH2/TSG101/CNOT9/MAML2/CDYL/ARID1B/PKN1/BTG1/MECP2/DPF2/HYAL2/KMT5A/MTA2/DRAP1/ZNF366/MTA1/DNAJB1/TACC1/NCOA4/SMARCD3/PHF8/BASP1/PPARGC1B/CCAR2/HELZ2/BCL9/ARRB1/MED12L/RERE/MAGED1/SMARCC1/TAF6/TGFB1I1/BCORL1/CTBP1/TRERF1/BCL9L/TRRAP/MED15/NCOA1/TOB2/SFMBT1/HDAC3/SMARCA4/DYRK1B/ETS1/RBM14/CBFA2T3/NCOA2/MAML3/BCOR/NCOA6/HDGFL2/NUP98/TADA3/HSPA1A/PIAS4/CREBBP/MTDH/MED16/TRIM32/TBL1XR1/KAT7/MRTFA/NCOA3/WBP2/RUVBL1/DAXX/ZMIZ1/TRIM28/TP53BP1/TLE3/FHL3/TBL1X/CDYL2/KAT2B/PHF2/PXN/ABL1/MED13L/KDM2A/LPIN2/SF1/TOX2/KMT2C/TLE4/SUFU/PA2G4/TLE1/HIPK2/NIBAN2/ELK1/DPF3/SIAH2/DCAF6/SND1/USP22/KAT6A/PRKCB/TRIM14/UBE2L3/PER2/ENO1/ARID3A/BRD4/AKIRIN2/HMGA1/ACTN4/RYBP/ACTN1/PDLIM1/SMARCC2/MED1/JAZF1/HDGF/ARID1A/NCOR2/CARM1/YY1AP1/HCFC1

BP	GO:0045862	positive regulation of proteolysis	281	-0.39	-1.52	0.00037011	0.00243638	0.00151064	1579	tags=22%, list=13%, signal=20%	PSME3/BCAP31/GSK3B/GSK3A/PYCARD/DAP/TNFRSF1B/CBFA2T3/DET1/PRR7/BEX3/DAPK1/MAP3K5/IST1/HIP1/HIP1R/LYN/DDA1/PINK1/MBP/PTK2B/HSPA1A/ANKIB1/BAD/TRIM32/NCSTN/BCL2L11/SRC/PML/SFRP2/FIS1/LAPTM5/STAT3/BTRC/GABARAP/SYK/VCP/EGLN3/ARIH2/PSMC4/RAD23A/RHOA/LGALS9/PSME4/CYFIP2/GSN/BCL2L13/FZR1/GRN/SNCA/ENO1/P2RX1/AKIRIN2/CTSD/TNIP1/ZER1/RNF40/DNAJB2/FURIN/MYH9/CLU/DAB2/BAG6
CC	GO:0005887	integral component of plasma membrane	725	-0.32	-1.35	0.00037506	0.00246485	0.00152829	2897	tags=28%, list=25%, signal=23%	C17orf99/NECTIN1/PSENEN/ITGB2/ADCY9/ATP11A/SLC8A1/FLT1/GPBAR1/OBSCN/TM7SF2/NKG7/PCDH11X/SLC40A1/TSPAN14/CCRL2/EPHB1/FRS2/SDCBP/NCF2/TNFRSF13B/CMKLR1/ATP8B2/GABBR1/PECAM1/CR1/SLC16A1/TRAF2/PKD1/EPS8/PTAFR/SORL1/PTGIR/PLXNC1/KCNH2/SYT11/PLXNA4/TRPM2/CYBB/NCF1/CD22/ATP1A1/CD3E/TRPC3/EFNB1/ATP2A2/SLC7A7/DLG4/M6PR/SLC24A4/PHEX/CCR1/SLC20A1/DIABLO/LMTK2/IL6R/EPHA4/HLA-DRB1/SLC25A11/PI4K2A/CD81/DDR1/FXYD6/HLA-DQA2/ICAM1/ATP1B1/HRH2/YKT6/P2RX4/CNR2/HLA-A/CSF1R/SELPLG/MFSD12/SLC33A1/TLR6/ADGRE1/ITGB7/TMC8/SLC7A1/FADS2/ITGAM/KIR3DL2/LPAR4/KIR2DS4/SCARB1/ACVR1B/OR10H1/SLC29A2/PTPRC/KIR3DL1/LRFN1/FZD5/VSIG10/SLC19A1/HLA-C/CANX/NCR3/IL2RG/SEMA4D/SIRPB1/ABCC1/KCNJ2/CXCR2/KCNQ5/BCAP31/ADGRE5/CD37/HLA-DQA1/ACVR2B/IL2RB/STOM/HVCN1/CD63/RASGRP3/ST14/TSPAN17/SLC27A1/TRAF6/MFSD2A/AQP10/SLC5A6/SIT1/AQP1/LNPEP/LYN/PLXNB2/IL17RA/GYPA/JAM3/PTK2B/PLXND1/CD9/SLC9A1/SPN/TSPAN9/SLC22A17/NCSTN/SLC39A4/TMPRSS9/CEACAM1/ABCC3/LRRC8A/SLC7A8/SLC1A5/SLC14A1/PLXNA1/ATP1B2/ADGRL1/LAPTM5/CNIH3/SEMA4A/NRP1/SLC1A4/SELL/SLC6A20/TNFSF4/STIM1/SLC2A1/CLCN5/ITGAL/PCDH1/GP6/GALR1/CALM3/NOTCH2/PTPRJ/BSG/MCOLN1/SLC6A9/SLC24A3/SDC4/ITGB5/GYPB/SLC4A1/GP1BA/IGF2R/LRP1/ANKH/GP9/HRH4/CD151/TSPAN33/SLC43A1/ATP2B4/SELP/MFSD2B/MMD/LRFN3/LAMP1/GYPC/P2RX1/ELAPOR1/SLC29A1/NINJ2/PTGDR2/SLC6A8/TSPAN5/SHISA7/SLC38A5/ITGB3/ITGA2B/SCN1B/TBXA2R
BP	GO:0048511	rhythmic process	170	-0.44	-1.64	0.00038017	0.00249429	0.00154655	2453	tags=32%, list=21%, signal=25%	ADNP/TRPM2/NR1D1/EGR1/RAI1/EZH2/SGPL1/HEBP1/CDK5/CASP2/CSNK2A1/TOP1/MTA1/HSPA5/GNA11/ROCK2/CCAR2/GFPT1/MAGED1/JUND/TOP2A/SREBF1/GSK3B/ZFHX3/CRY2/NR1H3/HDAC3/NCOA2/CREBBP/SRC/PML/PPARA/MYCBP2/PPP1CA/KAT2B/BTRC/USP9X/PHLPP1/DDB1/KMT2A/KDM2A/ARRB2/KDM5C/USP7/ATG7/SIAH2/SRRD/HUWE1/PER2/SP1/PRKDC/FOXO3/CUL4A/NCOR2
BP	GO:0045648	positive regulation of erythrocyte differentiation	29	-0.68	-1.89	0.00038103	0.00249585	0.00154752	1786	tags=45%, list=15%, signal=38%	ACVR1B/INPP5D/ETS1/HSPA1A/KAT7/ARNT/STAT3/FAM210B/PRKDC/GATA1/FOXO3/MED1/TAL1
BP	GO:0098751	bone cell development	31	-0.67	-1.88	0.00038404	0.00251143	0.00155718	1550	tags=42%, list=13%, signal=36%	FOXP1/ATP6AP1/SRC/LRRK1/FAM20C/LTF/NOTCH2/MEIS1/PIP4K2A/MPIG6B/MED1/TAL1/WASF2

BP	GO:0003006	developmental process involved in reproduction	504	-0.34	-1.41	0.00038564	0.00251772	0.00156108	2675	tags=30%, list=23%, signal=24%	H3-3A/ZNF628/AGFG1/GMCL1/FANCG/NRIP1/ZFY/PKD1/SMARCA2/MAPK14/SMAD5/RNF38/AKT1/NSDHL/AGO4/TIPARP/PTPN11/NOTCH1/BAK1/SGPL1/CNOT9/SEPTIN6/CDYL/ZSCAN21/INSL3/WASHC5/LHFPL2/PRDM1/HES1/BTG1/ASPM/ACOX1/CSDE1/CASP2/CRKL/DEAF1/DACH1/IL1B/TTLL1/DHX37/TTC21A/PTPN6/VASH1/HSPA5/GGT1/SPATA5/ZBTB16/PAQR8/DIAPH2/NCOA4/TESK2/BASP1/HSD17B4/SPATA2/ALPL/SYNE1/CUL7/BIRC6/PTTG1/RHBDD1/EDN1/TFEB/CRTAP/FZD5/RBPJ/GTSF1/TBPL1/BCAP31/EHMT2/GRB2/PTCHD3/HYAL3/NCOA1/CETN2/PRKACA/SFMBT1/HSP90AB1/ASCL2/SCMH1/ST14/SPINT2/MEN1/UBR2/YY1/EPAS1/PUM1/JAM3/BPTF/PTK2B/RARA/IGF2/DAZAP1/ASF1B/PRSS21/SBF1/BAD/DHCR24/DZIP1/BCL2L11/SRC/CDKN1C/ARNT/TAF4/CALR/SFRP2/ZNF296/LRRC8A/SEPTIN4/RUVBL1/HERC2/PSAP/TRIM28/SPOCD1/CTNNA1/DPCD/ALKBH5/E2F1/PITHD1/SF1/GOLGA3/BMP6/ARRB2/PDE5A/KLC3/NOTCH2/DNM2/TBC1D20/BSG/PSME4/FOXJ2/BCL2L1/STK11/UBB/ABHD2/IGF2R/PPP1R9B/CCNI/ATP2B4/NEURL1/PRKDC/GATA1/CBL/CTSB/YBX3/FOXO3/MED1/PDGFA/OSBP2/ACRBP/MARF1/PBX1/BAG6
CC	GO:0030667	secretory granule membrane	245	-0.39	-1.51	0.00038702	0.00252258	0.00156409	2439	tags=32%, list=21%, signal=26%	TRPM2/NBEAL2/CYBB/LAMP2/TMBIM1/MLEC/CPNE3/HGSNAT/LHFPL2/ATP8B3/CMTM6/CPNE1/ORMDL3/ITPR2/ALDH3B1/LGALS3/ITGAM/TOM1/APLP2/PTPRC/CD14/HLA-C/RHOF/SIRPB1/CXCR2/ADGRE5/HYAL3/SERPINB6/LAIR1/STOM/HVCN1/DOK3/CXCR1/CD63/TNFRSF1B/SYNGR1/SLC44A2/DNAJC5/AP1M1/RAB31/ANO6/RAB7A/RAC1/STK10/TMEM184A/CD9/EXOC3/NCSTN/CEACAM1/PSAP/SELL/ABCC4/RAB14/RAB27B/ATP6V0C/LAMTOR1/ITGAL/GAA/RHOA/PTPRJ/RAB5B/ARL8A/RAB3D/BSG/ATP6V0A1/UBR4/SYTL4/IGF2R/IQGAP1/SELP/SNCA/LAMP1/RHOG/P2RX1/ITGB3/SPARC/ITGA2B/ACRBP/DIAPH1
BP	GO:0022898	regulation of transmembrane transporter activity	143	-0.45	-1.63	0.0003919	0.00255026	0.00158125	1539	tags=25%, list=13%, signal=22%	PDE4B/EDN1/MMP9/EHD3/STOM/PRKACA/RANGRF/DAPK1/SELENON/OXSR1/FHL1/NEDD4L/PINK1/PTK2B/SLC9A1/ATP1B2/ACTB/CNIH3/YWHAH/FKBP1A/STIM1/WWP2/CALM3/DNM2/FKBP1B/SLC6A9/SNCA/AHCYL1/CAPN1/ACTN4/TESC/HTT/SHISA7/SCN1B/WNK1/PIM1
BP	GO:0071902	positive regulation of protein serine/threonine kinase activity	148	-0.44	-1.62	0.00039667	0.00257288	0.00159528	1766	tags=26%, list=15%, signal=22%	PTPRC/EDN1/MAGED1/FZD5/MAP3K11/HSP90AB1/TGFB1/TAOK1/SLC27A1/TRAF6/MAP3K5/SASH1/PTK2B/IGF2/MAP2K7/SRC/CCND3/DVL3/TAOK2/PIH1D1/MAP2K2/PDGFC/SYK/PAK1/LTF/PDE5A/RHOA/CALM3/CSK/THBS1/PTPN1/AKAP13/PIK3CG/IQGAP1/ATP2B4/SNCA/CAMK1/PDGFA
MF	GO:0004713	protein tyrosine kinase activity	89	-0.50	-1.72	0.00039842	0.00257999	0.00159969	3008	tags=40%, list=26%, signal=30%	KIT/CLK3/BAZ1B/FLT1/TESK1/EPHB1/TTN/EIF2AK2/FGR/EPHA4/DDR1/FYN/CSF1R/TNK2/TESK2/FES/WEE1/TEC/DYRK1B/LYN/PTK2B/MAP2K7/SRC/NRP1/MAP2K2/BCR/JAK1/SYK/JAK3/ABL1/SCYL1/HIPK1/HIPK2/IGF2R/CSK/MAP2K3
BP	GO:1903522	regulation of blood circulation	111	-0.48	-1.71	0.00040015	0.00258701	0.00160404	2128	tags=30%, list=18%, signal=25%	THRA/ATP1B1/HRH2/P2RX4/SNTA1/PDE4B/EDN1/GRK2/KCNJ2/EHD3/SREBF1/PRKACA/GSK3A/RANGRF/TPM1/EPAS1/SLC9A1/ATP1B2/ABL1/GAA/PDE5A/RHOA/CALM3/FLNA/FKBP1B/ECE1/MYL4/PIK3CG/PER2/ATP2B4/P2RX1/SCN1B/TBXA2R

BP	GO:0090101	negative regulation of transmembrane receptor protein serine/threonine kinase signaling pathway	65	-0.55	-1.79	0.00040688	0.00262623	0.00162835	2231	tags=40%, list=19%, signal=33%	MAGI2/SPRED1/HSPA5/ZBTB7A/PIN1/NRROS/TGFB1I1/BCL9L/CCN3/SKI/TWSG1/NOMO3/HSPA1A/CHST11/GREM1/SFRP2/PPARA/FAM89B/ABL1/FKBP1A/DNM2/HIPK2/LRP1/GLG1/PPM1A/LTBP1
BP	GO:1902475	L-alpha-amino acid transmembrane transport	38	-0.63	-1.84	0.00040894	0.00263527	0.00163396	1268	tags=42%, list=11%, signal=38%	CLN3/SLC7A7/SLC43A2/SLC7A1/ARL6IP1/SLC25A38/SLC7A8/SLC1A5/SLC1A4/SLC6A20/SLC7A5/SLC3A2/SLC6A9/SLC43A1/PER2/SLC38A5
BP	GO:0043161	proteasome-mediated ubiquitin-dependent protein catabolic process	370	-0.36	-1.46	0.00041435	0.00266579	0.00165288	1917	tags=24%, list=16%, signal=20%	TRIB1/CHFR/CCAR2/UBXN7/UBE2J2/ARRB1/DERL1/KEAP1/SMARCC1/TAF1/CANX/NSFL1C/BCAP31/GSK3B/GSK3A/HSP90AB1/KLHL22/TBX21/CBFA2T3/DET1/EDEM2/KCTD2/UBR2/UBE2G1/NEDD4L/DDA1/CDC27/FBXL18/HSPA1A/ANKIB1/IFI27/MARCHF6/TBL1XR1/FBXO31/PML/CALR/RBCK1/RMND5A/CDK2/HERC2/AMFR/USP19/TBL1X/MAP1A/BTRC/GABARAP/FBXO6/DDB1/VCP/ARIH2/N4BP1/FBXW4/PSMC4/RAD23A/ARRB2/WWP2/GID8/UBE2S/MAN1A1/ZNRF1/PSMD2/USP7/SIAH2/GNA12/FZR1/HUWE1/PSMF1/YOD1/RNF187/ARAF/SHARPIN/ANKRD9/PCBP2/DCAF12/TRIP12/ASCC2/RYBP/ZER1/DNAJB2/CDC34/UBE2H/DCAF11/CUL4A/NPLOC4/CLU/DAB2/BAG6
BP	GO:0051592	response to calcium ion	81	-0.52	-1.74	0.00042113	0.00270502	0.00167721	2763	tags=41%, list=24%, signal=31%	FOSB/TTN/CPNE2/SYT11/JUN/TRPM2/TRPC3/CPNE3/SLC25A23/CPNE1/HSPA5/EDN1/JUND/SMPD1/ACTG1/ALOX15/PTK2B/BAD/NCSTN/CPNE5/PPP3CA/SEC31A/FOS/STIM1/ANXA11/CALM3/MCOLN1/TXNIP/THBS1/IQGAP1/AHCYL1/DMTN/ADD1
BP	GO:0032409	regulation of transporter activity	153	-0.45	-1.65	0.00042424	0.00272059	0.00168686	1539	tags=24%, list=13%, signal=21%	EHD3/STOM/PRKACA/NR1H3/RANGRF/DAPK1/SELENON/OXSR1/FHL1/NEDD4L/PINK1/PTK2B/RXRA/SLC9A1/ATP1B2/ACTB/CNIH3/YWHAH/KMT2A/FKBP1A/STIM1/WWP2/CALM3/DNM2/FKBP1B/SLC6A9/SNCA/AHCYL1/CAPN1/ACTN4/TESC/HTT/SHISA7/SCN1B/WNK1/PIM1
MF	GO:0044389	ubiquitin-like protein ligase binding	278	-0.38	-1.51	0.00042741	0.0027365	0.00169673	1867	tags=27%, list=16%, signal=23%	UBXN7/UBE2J2/ARRB1/MAP1LC3B/CUL7/SMAD3/DERL1/ACVR1B/HGS/FZD5/UBE2D4/IKBKG/KDM4A/GSK3B/PRR5L/PRKACA/CASC3/HSP90AB1/ACTG1/TNFRSF1B/DET1/PRR7/NEK6/SKI/TRAF6/UBE2G1/UBA52/LYN/PINK1/PTK2B/HSPA1A/PIAS4/PML/CALR/HERC2/LAPTM5/DAXX/TRIM28/USP19/RANGAP1/GABARAP/JAK1/VCP/RELA/DTX3L/ARRB2/ATP6V0C/CKB/PRKAR2A/MFHAS1/VCL/PA2G4/USP7/UBB/POLR2A/TXNIP/SQSTM1/YOD1/UBE2L3/MFN2/GPI/UBASH3B/PRDX6/BAG1/PCBP2/FBXO7/CDKN1A/TUBB/RNF40/PRKAR2B/TOLLIP/CUL4A/NPLOC4/CLU/BAG6
BP	GO:0001933	negative regulation of protein phosphorylation	238	-0.40	-1.54	0.00043282	0.00276669	0.00171545	2581	tags=33%, list=22%, signal=26%	GNAQ/SORL1/PKIG/AKT1/PIP5KL1/AIDA/CBLB/JUN/MLLT1/BAK1/GADD45B/CDKN2D/TSG101/CHP1/PKN1/SH3BP5L/SPRED1/HYAL2/CDK5RAP2/CRKL/IL1B/PARD6A/PTPN6/MYADM/GSTP1/TRIB1/PTPRC/PIK3CB/CHMP6/ENG/SEMA4D/CTDSP1/SMPD1/DTNBP1/PRR5L/PYCARD/CACTIN/TGFB1/YWHAG/CTDSP2/MEN1/MVP/CORO1C/LYN/ADAR/GREM1/CDKN1C/CEACAM1/SFRP2/LRRK1/KAT2B/ABL1/PARD3/WARS1/ARRB2/NIBAN1/PRKAR2A/CALM3/PPP4C/PTPRJ/INPP5K/HSPB1/NF2/HEXIM2/PLEC/LRP1/PTPN1/FKBP8/CTDSPL/UBASH3B/SNCA/EIF4G1/PRKDC/FBXO7/CDKN1A/CBL/PAK2/PRKAR2B/DMTN

BP	GO:0048771	tissue remodeling	86	-0.51	-1.76	0.00043971	0.00280627	0.00173999	2121	tags=37%, list=18%, signal=31%	MITF/CSF1R/ROCK2/TIMP1/PPARGC1B/TPP1/RUNX1/INPP5D/RBPJ/ACVR2B/TGFB1/SNX10/TRAF6/EPAS1/RAB7A/PTK2B/SRC/GREM1/CEACAM1/LTBP3/PPP3CA/LRRK1/BCR/SYK/NOTCH2/FLNA/GPR137/RAB3D/CSK/UBASH3B/CAPN1/ITGB3
BP	GO:0030834	regulation of actin filament depolymerization	41	-0.61	-1.83	0.00045193	0.0028796	0.00178545	383	tags=59%, list=3%, signal=57%	ADD2/EPS8/SWAP70/PIK3CA/TMOD3/DSTN/SPTAN1/TRIOBP/SVIL/LIMA1/PLEK/WDR1/FLII/GSN/SPTBN1/MTPN/SPTA1/SPTB/TMOD1/SCIN/CAPZB/SH3BP1/DMTN/ADD1
BP	GO:0045664	regulation of neuron differentiation	95	-0.49	-1.69	0.00046859	0.00296204	0.00183657	2261	tags=39%, list=19%, signal=32%	MAP3K13/HES1/ASPM/CDK5RAP2/CPNE1/FEZ1/CYB5D2/HMG20B/MIB1/TIAM1/DIXDC1/RAP1GAP/CTDSP1/KDM4A/NCOA1/GSK3B/ZFHX3/YWHAG/BCL11B/TGIF2/BCL11A/RARA/DPYSL2/TRIM32/CALR/SFRP2/OLIG2/ARHGEF2/YWHAH/BMP6/RHOA/MEIS1/MMD/EIF4G1/FOXO3/MED1/PBX1
BP	GO:0050808	synapse organization	201	-0.42	-1.59	0.00046795	0.00296204	0.00183657	2468	tags=30%, list=21%, signal=24%	DISC1/ADNP/CLN3/YWHAZ/DLG4/EPHA4/PFN1/MECP2/CDK5/ABI3/FYN/ABHD17C/CRKL/SLITRK5/DOCK10/SNTA1/PIN1/ITGAM/CAST/CHCHD10/FZD5/SEMA4D/DTNBP1/BAIAP2/CYFIP1/ACTG1/NFIA/MESD/PLXNB2/CDKL5/PLXND1/ARF1/SIPA1L1/GRIPAP1/ADGRL1/ACTB/SEMA4A/NRP1/VCP/ABL1/SYNGAP1/C1QA/STAU1/RHOA/FLNA/DNM3/DCTN1/CFL1/CLSTN1/SNCA/LRFN3/CAMK1/EIF4G1/NEURL1/WASF3/TUBB/ACTN1/CTTN/SHISA7/WASF2
MF	GO:0005509	calcium ion binding	309	-0.37	-1.47	0.0004666	0.00296204	0.00183657	1563	tags=22%, list=13%, signal=19%	ADGRE5/EHD3/CETN2/EFCAB2/REPS1/RASGRP3/CALU/MAN1C1/EFCAB14/EDEM2/SELENON/EHD4/CANT1/ESYT1/ITSN1/PRF1/DCHS1/MGMT/DYSF/S100A4/PITPNM2/CALR/LTBP3/PPP3CA/FAM20C/RAB11FIP4/HPCAL1/NHSL2/SELL/VLDLR/EPS15L1/PPP3R1/STIM1/ANXA6/REPS2/CAPNS1/PLCB3/ANXA11/PCDH1/CALM3/NOTCH2/MAN1A1/PVALB/S100Z/MYL4/PLSCR3/GSN/CLSTN1/LRP1/THBS1/IQGAP1/SPTA1/SELP/SNCA/CAPN1/CBL/EHD1/SCIN/ACTN4/NUCB1/ACTN1/MYL9/TESC/SPARC/LTBP1/TGM2/VWCE
BP	GO:0040008	regulation of growth	397	-0.35	-1.44	0.00046737	0.00296204	0.00183657	2597	tags=31%, list=22%, signal=25%	RFTN1/SUPV3L1/ULK1/SMARCA2/MAPK14/PAK4/AKT1/ATAD3A/DIP2B/MUL1/UBE2E3/PIK3CA/DDX49/DISC1/PLXNA4/ZMAT3/EXOSC4/ADNP/NOTCH1/ACACB/RAI1/CDKN2D/FLCN/SGPL1/TSG101/ARHGEF11/MAP3K13/G6PD/BTG1/CDK5/HYAL2/DDR1/CRKL/CSNK2A1/ADNP2/DCUN1D3/MACF1/BASP1/CCAR2/TMC8/SERTAD3/SOCS7/EI24/TMEM8B/CXCL16/SHC1/SMAD3/ACVR1B/RUNX1/EDN1/KIAA0319/RBPJ/FTO/EPPK1/NDEL1/SEMA4D/CDA/GSK3B/NEDD9/GSK3A/RPS6KA1/CYFIP1/TGFB1/SMARCA4/KLHL22/SPHK1/CCN3/VGLL4/EZR/MFSD2A/BCL11A/HDGFL2/SESN2/FHL1/IST1/YY1/NEDD4L/CDKL5/PTK2B/MT2A/DPYSL2/IGF2/HSPA1A/SLC9A1/TRIM32/BCL2L11/PML/GREM1/KAT7/DCAF1/CEACAM1/EIF4G2/CPNE5/SFRP2/PPARA/RUVBL1/TAOK2/STAT3/SEMA4A/NRP1/ABL1/FBP1/LAMTOR1/JARID2/RHOA/DNM2/MAD2L2/PTPRJ/STAT5A/BCL2L1/STK11/CLSTN1/ADIPOR1/PPP1R9B/MTPN/EIF4G1/ENO1/HLX/CDKN1A/PAK2/DNAJB2/YBX3/CTTN/PIM1/DAB2
MF	GO:0061659	ubiquitin-like protein ligase activity	242	-0.39	-1.53	0.00046968	0.00296419	0.0018379	1267	tags=18%, list=11%, signal=16%	TRAF6/TRIM35/UBR2/NEDD4L/TRIM26/ANKIB1/PIAS4/TRIM32/MARCHF6/DTX2/RNF115/RMND5A/MYCBP2/HERC2/TRIM10/AMFR/BTRC/FBXO6/XIAP/ARIH2/RNF213/DTX3L/RNF11/WWP2/ZNRF1/TRIM11/RNF24/SIAH2/UBR4/HUWE1/TRIM14/MKRN1/RNF187/MARCHF2/NEURL1/RNF10/MARCHF8/CBL/TRIP12/UBE2O/RNF40/TRIM58/RNF208
BP	GO:0042752	regulation of circadian rhythm	74	-0.52	-1.72	0.00047203	0.00297428	0.00184416	2065	tags=34%, list=18%, signal=28%	MTA1/GNA11/ROCK2/CCAR2/MAGED1/TOP2A/GSK3B/ZFHX3/CRY2/NR1H3/HDAC3/PML/PPARA/PPP1CA/BTRC/USP9X/PHLPP1/DDB1/KDM2A/USP7/ATG7/SIAH2/SRRD/PER2/PRKDC
BP	GO:0015807	L-amino acid transport	40	-0.62	-1.83	0.00047727	0.00300257	0.0018617	2418	tags=38%, list=21%, signal=30%	CLN3/SLC7A7/SERINC5/SLC43A2/SLC7A1/ARL6IP1/SLC7A8/SLC1A5/SLC1A4/SLC6A20/SLC7A5/SLC3A2/SLC43A1/PER2/SLC38A5

BP	GO:0030003	cellular cation homeostasis	387	-0.36	-1.47	0.00048768	0.00305841	0.00189633	2777	tags=31%, list=24%, signal=24%	SLC40A1/CCRL2/LETM1/CCDC115/COX19/ATP13A2/PDPK1/CMKLR1/ATP7A/PKD1/ATP2A3/CORO1A/SELENOK/PTGIR/SWAP70/SLC46A1/DISC1/KNG1/TRPM2/BAK1/ATP1A1/CLN3/CYBRD1/TRPC3/ATP2A2/DLG4/SLC24A4/CCR1/CHP1/CDK5/MAFG/FYN/SLC25A23/ATP1B1/P2RX4/PTPN6/ATP13A3/ITPR2/SLC9A7/NCOA4/LPAR4/SMAD3/RAB20/MICU1/PTPRC/EDN1/PIK3CB/STOML2/SLC41A1/FRRS1/KCNJ2/CXCR2/BCAP31/TM9SF4/PRKACA/HVCN1/CXCR1/ATP6AP1/SELENON/FPR3/CHERP/LYN/NEDD4L/SPPL3/RAB7A/PTK2B/GNA15/MT2A/ARF1/SLC9A1/SLC22A17/SLC39A4/PML/CA2/RASA3/CALR/ATP6V1A/ATP13A1/FIS1/ATP1B2/JAK3/ATP6V0D1/ABL1/LCN2/FKBP1A/STIM1/ANXA6/BMP6/XK/GALR1/CALM3/FKBP1B/ATP6V0A1/MCOLN1/SLC6A9/SLC24A3/FTH1/TTC7A/SLC4A1/GP1BA/PRKCB/GP9/HRH4/PIK3CG/GRN/UBASH3B/ATP2B4/SNCA/ANKRD9/P2RX1/MAPK3/GATA1/GRINA/FTL/ALAS2/HTT/KEL/TGM2/DIAPH1/TBXA2R
BP	GO:0030865	cortical cytoskeleton organization	47	-0.59	-1.81	0.00049143	0.00307223	0.0019049	2206	tags=40%, list=19%, signal=33%	CDK5/FMNL1/ROCK2/LCP1/FMNL3/EPB41L2/RHOBTB1/RHOF/EZR/PLEK/WDR1/RAC1/CALR/RHOA/RAC2/PLEC/EPB41/RHOG/TLN1
MF	GO:0031625	ubiquitin protein ligase binding	263	-0.39	-1.52	0.00049141	0.00307223	0.0019049	1867	tags=27%, list=16%, signal=24%	UBXN7/UBE2J2/ARRB1/MAP1LC3B/CUL7/SMAD3/DERL1/ACVR1B/FZD5/UBE2D4/IKBKG/KDM4A/GSK3B/PRR5L/PRKACA/CASC3/HSP90AB1/ACTG1/TNFRSF1B/DET1/NEK6/SKI/TRAF6/UBE2G1/UBA52/LYN/PINK1/PTK2B/HSPA1A/PIAS4/PML/CALR/HERC2/LAPTM5/DAXX/TRIM28/USP19/RANGAP1/GABARAP/JAK1/VCP/RELA/ARRB2/ATP6V0C/CKB/PRKAR2A/MFHAS1/VCL/PA2G4/USP7/UBB/POLR2A/TXNIP/SQSTM1/YOD1/UBE2L3/MFN2/GPI/UBASH3B/PRDX6/BAG1/PCBP2/FBXO7/CDKN1A/TUBB/RNF40/PRKAR2B/TOLLIP/CUL4A/NPLOC4/CLU/BAG6
BP	GO:0036293	response to decreased oxygen levels	191	-0.42	-1.58	0.00049599	0.00309096	0.0019165	2030	tags=25%, list=17%, signal=21%	ITPR2/ROCK2/NDRG1/PIN1/SMAD3/EDN1/PIK3CB/HIF1AN/RBPJ/PDK3/TM9SF4/PLOD1/ASCL2/VHL/CBFA2T3/UCP2/ALAD/MTHFR/AQP1/EPAS1/PINK1/PTK2B/CREBBP/SLC9A1/BAD/SRC/PML/ARNT/PPARA/USP19/ALKBH5/EGLN3/E2F1/SLC2A1/MPL/RHOA/LMNA/HIPK2/THBS1/ENO1/BNIP3L/SLC29A1/CBL/ACTN4/ALAS2/PDLIM1/FOXO3/PDGFA
BP	GO:0042330	taxis	335	-0.37	-1.49	0.00049676	0.00309096	0.0019165	2769	tags=35%, list=24%, signal=28%	LEF1/PTCH1/HBEGF/DVL1/NECTIN1/ITGB2/FLT1/CCRL2/EPHB1/DOCK4/TSC2/PLD1/CMKLR1/EXT1/CORO1B/MAPK14/PTAFR/CORO1A/SWAP70/PLXNC1/PLXNA4/NOTCH1/TRPM2/EFNB1/NCAM1/LYPLA2/CCR1/IL6R/EPHA4/CDK5/PRKD2/FYN/MACO1/CRKL/IL1B/SPI1/FEZ1/P2RX4/CNR2/CSF1R/PPBP/RNASE3/RALBP1/ST6GAL1/FES/LGALS3/CXCL16/HSD3B7/ARHGAP35/SMAD3/PDE4B/EDN1/PIK3CB/RIPOR2/CREB3/CMTM5/CD74/SEMA4D/ABCC1/CXCR2/CYFIP1/TGFB1/CXCR1/PREX1/AMOT/CCN3/STX4/BCL11B/FPR3/OXSR1/ANO6/LYN/PLXNB2/IL17RA/RAC1/JAM3/PTK2B/DPYSL2/PLXND1/TRIO/SPN/GREM1/CALR/PIK3CD/MYCBP2/VAV1/PLXNA1/RIN3/SEMA4A/NRP1/SYK/ARRB2/RHOA/NOTCH2/PTPRJ/LGALS9/RAC2/BSG/ECE1/HSPB1/NIBAN2/DOCK2/CAMK1D/MPP1/PLEC/PF4V1/THBS1/PIK3CG/CXCL5/VASP/RHOG/MAPK3/PF4/PTGDR2/ITGB3/PDGFA/SCN1B/WNK1

BP	GO:0010498	proteasomal protein catabolic process	435	-0.35	-1.43	0.00050562	0.00314117	0.00194764	1917	tags=24%, list=16%, signal=21%	TRIB1/CHFR/CCAR2/UBXN7/UBE2J2/ARRB1/DERL1/RHBDD1/KEAP1/F8A1/SMARCC1/TAF1/CANX/NSFL1C/PSME3/BCAP31/PSMB10/GSK3B/PRKACA/GSK3A/HSP90AB1/KLHL22/TBX21/CBFA2T3/DET1/PSMB3/ENC1/EDEM2/KCTD2/ALAD/UBR2/UBE2G1/NEDD4L/DDA1/CDC27/FBXL18/PINK1/HSPA1A/ANKIB1/IFI27/MARCHF6/TBL1XR1/FBXO31/PML/CALR/RBCK1/RMND5A/CDK2/HERC2/AMFR/USP19/TBL1X/MAP1A/DDI2/BTRC/GABARAP/FBXO6/DDB1/VCP/ARIH2/N4BP1/PITHD1/FBXW4/PSMC4/RAD23A/ARRB2/WWP2/GID8/UBE2S/MAN1A1/ZNRF1/PSMD2/PSME4/USP7/SIAH2/UBB/GNA12/FZR1/HUWE1/PSMF1/YOD1/RNF187/ARAF/SHARPIN/ANKRD9/PCBP2/DCAF12/TRIP12/ASCC2/RYBP/ZER1/GPX1/RNF40/DNAJB2/CDC34/UBXN6/UBE2H/DCAF11/CUL4A/NPLOC4/CLU/DAB2/BAG6
BP	GO:0050731	positive regulation of peptidyl-tyrosine phosphorylation	109	-0.48	-1.71	0.00051449	0.00319126	0.00197869	2453	tags=36%, list=21%, signal=29%	ADNP/PTPN11/NCF1/CD3E/DLG4/IL6R/EPHA4/HES1/CD81/FYN/CSF1R/TNK2/PTPRC/CD74/SEMA4D/NEDD9/TGFB1/LILRA5/TEC/EHD4/LYN/PTK2B/IGF2/HCLS1/SRC/GREM1/LRRK1/NRP1/ARHGEF2/SYK/ABL1/BMP6/ARRB2/PTPRJ/PTPN1/NEURL1/GATA1/PAK2/ITGB3
BP	GO:0090630	activation of GTPase activity	80	-0.52	-1.75	0.00051569	0.00319371	0.00198022	1841	tags=31%, list=16%, signal=27%	RCC2/TIAM1/RAP1GAP/NDEL1/TBC1D17/NEDD9/TBC1D22A/SIPA1L3/CORO1C/BCAS3/RABGAP1/PTK2B/EVI5L/SIPA1L1/GMIP/ARHGAP45/TBC1D13/RANGAP1/BCR/TBC1D22B/AKT2/RHOG/TBC1D5/TBC1D25/TBC1D1
BP	GO:0055001	muscle cell development	93	-0.51	-1.75	0.00052215	0.00322873	0.00200193	1756	tags=30%, list=15%, signal=26%	EDN1/RIPOR2/ENG/GSK3A/ACTG1/SKI/SELENON/WDR1/TPM1/YY1/DYSF/PPARA/FLII/PPP3CA/MYOF/CSRP1/XK/LMNA/NIBAN2/PLEC/AKAP13/NFATC2/TMOD1/ACTN4/GPX1/ACTN1/MYL9/KEL
BP	GO:0032869	cellular response to insulin stimulus	150	-0.44	-1.62	0.00053178	0.00328317	0.00203568	1834	tags=27%, list=16%, signal=23%	SHC1/SLC29A2/SH2B2/SMARCC1/NDEL1/PIK3R3/GRB2/SREBF1/GSK3B/BAIAP2/GSK3A/CYFIP1/YWHAG/ZNF106/UCP2/SLC27A1/WDTC1/PARP1/RAB31/IGF2/SLC9A1/SRC/CCND3/CEACAM1/GNAI2/PKM/PDK2/KAT2B/FBP1/FOXO4/LPIN2/SESN3/INPP5K/ADIPOR1/PRKCB/AKT2/PTPN1/SP1/PRKDC/PIP4K2A/EIF4EBP2
BP	GO:0001892	embryonic placenta development	52	-0.58	-1.79	0.00053489	0.00329722	0.00204439	3028	tags=50%, list=26%, signal=37%	CITED2/LEF1/ZFP36L1/CEBPA/PKD1/AKT1/NSDHL/PRDM1/HES1/VASH1/BIRC6/TFEB/FZD5/RBPJ/GRB2/NCOA1/ASCL2/ST14/SPINT2/EPAS1/BPTF/IGF2/CDKN1C/ARNT/TRIM28/MED1
BP	GO:0070988	demethylation	57	-0.55	-1.73	0.00053739	0.00330755	0.0020508	2047	tags=32%, list=17%, signal=26%	OTUD4/PHF8/FTO/KDM4A/KDM6B/APOBEC3D/TRIM28/USP9X/ALKBH5/PHF2/APOBEC3F/KDM2A/KDM4B/JARID2/KDM5C/USP7/APOBEC3C/TET3
BP	GO:1900026	positive regulation of substrate adhesion-dependent cell spreading	30	-0.67	-1.86	0.00053839	0.00330858	0.00205144	2351	tags=50%, list=20%, signal=40%	TRIOBP/CRKL/MYADM/LIMS1/NEDD9/PREX1/ILK/RAC1/CALR/NRP1/ABL1/DNM2/FLNA/DMTN/DAB2
BP	GO:0042445	hormone metabolic process	99	-0.49	-1.70	0.00056814	0.00348064	0.00215813	2595	tags=31%, list=22%, signal=25%	CYP2S1/STARD3/CPQ/TIPARP/PTPN11/ATP1A1/EGR1/SGPL1/TSPO/DHRS13/PCSK1N/HSD17B4/DHCR7/LIPE/PCSK6/IL4I1/BACE2/PAPSS2/ARNT/DHRS3/RDH11/PNPLA2/NFKB1/BMP6/ALDH9A1/ECE1/TCF7L2/CTSB/FURIN/MED1/DAB2

BP	GO:0002521	leukocyte differentiation	420	-0.35	-1.41	0.00056927	0.00348217	0.00215907	2247	tags=28%, list=19%, signal=23%	ZFPM1/JUN/TRPM2/BAK1/CD3E/EGR1/HDAC4/HDAC5/EZH2/CCR1/PRDM1/IL6R/HLA-DRB1/IRF1/BATF2/CD81/MITF/IL1B/SPI1/DOCK10/RIPK1/CSF1R/PTPN6/SH3PXD2A/ZBTB16/PGLYRP1/OSCAR/PPARGC1B/ZBTB7A/ZFP36L2/TRIB1/FES/NRROS/XRCC6/SOX13/TFE3/RUNX1/PTPRC/DROSHA/FZD5/INPP5D/RBPJ/CD74/MMP9/SOX4/FOXP1/TOB2/TGFB1/CTNNBIP1/CDK6/TBX21/TMEM131L/ACIN1/PREX1/CBFA2T3/PRR7/SNX10/BCL11B/ATP6AP1/IL4I1/PARP1/TRAF6/LYN/YY1/PTK2B/RARA/KDELR1/SPN/BAD/HCLS1/RRAS/SRC/KAT7/DCAF1/CEACAM1/PIK3CD/LRRC8A/VAV1/RUNX3/LRRK1/FAM20C/ZMIZ1/STAT3/SEMA4A/SYK/JAK3/ABL1/MAFB/FOS/TNFSF4/LTF/RHOA/NOTCH2/PTPRJ/LGALS9/GPR137/FASN/DOCK2/STK11/UBASH3B/PRKDC/SOX12/IKZF1/GATA1/FBXO7/HLX/LYL1/PF4/ST3GAL1/FOXO3/TESC/MED1/EIF2AK1/TAL1/CUL4A/MYH9/PBX1
BP	GO:0043255	regulation of carbohydrate biosynthetic process	66	-0.53	-1.74	0.00057222	0.00348947	0.00216359	1600	tags=35%, list=14%, signal=30%	WDR5/GSK3B/FAM3A/GSK3A/TGFB1/CLTC/PLEK/SESN2/PTK2B/IGF2/PPARA/PPP1CA/PDK2/KAT2B/DDB1/FBP1/NFKB1/INPP5K/USP7/STK11/AKT2/SNCA/SMPD3
BP	GO:0043069	negative regulation of programmed cell death	571	-0.33	-1.39	0.00057194	0.00348947	0.00216359	1941	tags=23%, list=17%, signal=20%	TIMP1/GSTP1/ST6GAL1/DCPS/CCAR2/LGALS3/CIAPIN1/SHC1/BIRC6/SMAD3/RHBDD1/EDN1/CAST/PIK3CB/CREB3/SLC35F6/CD74/MMP9/KLF4/SEMA4D/ITPRIP/PSME3/FOXP1/GSK3B/IL2RB/RPS6KA1/HSP90AB1/HDAC3/VHL/THOC6/DNAJC5/SPHK1/DAPK1/BCL11B/UCP2/BABAM2/ARL6IP1/AQP1/HIP1R/ADAR/PINK1/PTK2B/HSPA1A/CHST11/MGMT/SLC9A1/MTDH/DHCR24/TRIM32/PDCD1/HCLS1/SRC/UBE2Z/GREM1/RBCK1/SFRP2/GNAI2/PPARA/LAPTM5/PIH1D1/CTNNA1/NRP1/ARHGEF2/VDAC1/DDB1/JAK3/XIAP/ABL1/SYNGAP1/RELA/LTF/NFKB1/PIM2/ARRB2/MYO18A/HNRNPK/RHOA/CHD8/NOTCH2/ARHGDIA/FLNA/AARS1/LMNA/KANK2/PA2G4/TLE1/HSPB1/HIPK2/NIBAN2/CAMK1D/BCL2L1/SIAH2/PRDX5/CFL1/ALOX12/THBS1/AKT2/PTPN1/PIK3CG/TCF7L2/GPI/FKBP8/GRN/ARAF/SNCA/PPP1R10/ENO1/PRKDC/BAG1/GATA1/GRINA/BNIP3L/PF4/CBL/PAK2/UBE2O/STRADB/ST3GAL1/GPX1/YBX3/PTMS/MED1/CTTN/HTT/HDGF/PTCRA/CLU/PIM1/DAB2/BAG6
BP	GO:0016049	cell growth	307	-0.37	-1.46	0.00058991	0.00358638	0.00222368	2622	tags=34%, list=22%, signal=27%	EXT1/SUPV3L1/ULK1/SMARCA2/PAK4/AKT1/ATAD3A/DIP2B/MUL1/DDX49/DISC1/PLXNA4/EXOSC4/ADNP/CDKN2D/FLCN/TSG101/ARHGEF11/MAP3K13/G6PD/BTG1/CDK5/HYAL2/DDR1/CRKL/CSNK2A1/CPNE1/ADNP2/DCUN1D3/MACF1/CCAR2/TMC8/SERTAD3/EI24/CXCL16/SMAD3/ACVR1B/EDN1/KIAA0319/NDEL1/SEMA4D/CDA/GSK3B/GSK3A/RPS6KA1/CYFIP1/HSP90AB1/TGFB1/SMARCA4/KLHL22/SPHK1/CCN3/VGLL4/MFSD2A/BCL11A/HDGFL2/SESN2/FHL1/IST1/YY1/NEDD4L/CDKL5/PTK2B/DPYSL2/HSPA1A/SLC9A1/TRIM32/PML/GREM1/KAT7/CEACAM1/EIF4G2/CPNE5/SFRP2/PPARA/PLXNA1/TAOK2/SEMA4A/NRP1/USP9X/ABL1/ARIH2/FBP1/LAMTOR1/RHOA/VCL/DNM2/MAD2L2/PTPRJ/STK11/CYFIP2/CLSTN1/ADIPOR1/PPP1R9B/AKAP13/MTPN/IQGAP1/EIF4G1/ENO1/CDKN1A/DNAJB2/CTTN/DAB2
BP	GO:1903649	regulation of cytoplasmic transport	27	-0.68	-1.88	0.00059192	0.00359308	0.00222784	1624	tags=41%, list=14%, signal=35%	SNX12/ATP9A/EZR/SRC/SNX3/MAP2K2/USP7/MSN/MAPK3/PTPN23/DAB2
CC	GO:0005903	brush border	59	-0.55	-1.76	0.00059734	0.00362046	0.00224481	1672	tags=31%, list=14%, signal=26%	LIMA1/SLC19A1/HSP90AB1/EZR/MYO1E/SLC5A6/AQP1/FLNB/FLII/ABCG2/FLNA/GNA12/PLEC/ESPN/SCIN/CAPZB/ACTN1/MYH9

BP	GO:0030511	positive regulation of transforming growth factor beta receptor signaling pathway	26	-0.68	-1.86	0.00060238	0.00363993	0.00225689	1679	tags=42%, list=14%, signal=36%	TGFB1I1/HSP90AB1/MEN1/C20orf27/CREBBP/CDKN1C/LRG1/HIPK2/STK11/THBS1/DAB2
BP	GO:1903846	positive regulation of cellular response to transforming growth factor beta stimulus	26	-0.68	-1.86	0.00060238	0.00363993	0.00225689	1679	tags=42%, list=14%, signal=36%	TGFB1I1/HSP90AB1/MEN1/C20orf27/CREBBP/CDKN1C/LRG1/HIPK2/STK11/THBS1/DAB2
BP	GO:0010675	regulation of cellular carbohydrate metabolic process	102	-0.48	-1.67	0.00061389	0.00370384	0.00229651	1989	tags=39%, list=17%, signal=33%	PTAFR/AKT1/POMC/ACACB/HDAC4/FOXO1/PGAM1/NLN/FOXK1/PHKG2/PDK3/WDR5/GSK3B/FOXK2/FAM3A/GSK3A/GIT1/BCKDK/CBFA2T3/NCOA2/PLEK/SESN2/PTK2B/IGF2/BAD/SRC/PPARA/PPP1CA/STAT3/PDK2/KAT2B/DDB1/FBP1/INPP5K/USP7/STK11/ADIPOR1/SLC4A1/AKT2/SNCA
BP	GO:0016525	negative regulation of angiogenesis	51	-0.58	-1.80	0.0006255	0.0037568	0.00232935	2217	tags=43%, list=19%, signal=35%	MECP2/SPRED1/VASH1/ROCK2/AGO1/HGS/PIK3CB/KLF4/AMOT/KLF2/PML/SEMA4A/FOXO4/EPN1/FOXJ2/NIBAN2/CLDN5/THBS1/ATP2B4/E2F2/PF4/SPARC
BP	GO:1901343	negative regulation of vasculature development	51	-0.58	-1.80	0.0006255	0.0037568	0.00232935	2217	tags=43%, list=19%, signal=35%	MECP2/SPRED1/VASH1/ROCK2/AGO1/HGS/PIK3CB/KLF4/AMOT/KLF2/PML/SEMA4A/FOXO4/EPN1/FOXJ2/NIBAN2/CLDN5/THBS1/ATP2B4/E2F2/PF4/SPARC
BP	GO:2000181	negative regulation of blood vessel morphogenesis	51	-0.58	-1.80	0.0006255	0.0037568	0.00232935	2217	tags=43%, list=19%, signal=35%	MECP2/SPRED1/VASH1/ROCK2/AGO1/HGS/PIK3CB/KLF4/AMOT/KLF2/PML/SEMA4A/FOXO4/EPN1/FOXJ2/NIBAN2/CLDN5/THBS1/ATP2B4/E2F2/PF4/SPARC
BP	GO:0010639	negative regulation of organelle organization	264	-0.38	-1.48	0.00062749	0.00376302	0.00233321	2732	tags=34%, list=23%, signal=26%	MCM2/TSC2/PIF1/RPS6KA2/H3-3A/ADD2/EXOSC10/CORO1B/MAPRE1/EPS8/CORO1A/AKT1/SWAP70/MUL1/PIK3CA/TMOD3/SPTAN1/ATXN7/RAD21/FLCN/TRIOBP/TSPO/MAP4/PFN1/XRCC3/CDK5RAP2/SSH1/ARAP1/FEZ1/TBCD/MYADM/SVIL/SSH2/PTTG1/SLC35F6/LIMA1/TPX2/CENPF/PHF23/CHMP4B/EHMT2/TOP2A/KLHL22/TAOK1/EML2/RBM14/PARP1/KATNB1/HIP1R/RAB7A/PINK1/HSPA1A/EVI5L/SRC/SLX4/FLII/CAMSAP1/ARHGEF2/KAT2B/CLASP1/ARHGEF18/SSH3/ARRB2/DNM2/MAD2L2/ARHGAP6/WAS/LMNA/INPP5K/GNL3L/TENT4B/BCL2L1/GSN/HUWE1/TERF2/SPTBN1/MTPN/SPTA1/SPTB/SNCA/TMOD1/SCIN/PAK2/CAPZB/GPX1/WASF2/CLU/DMTN/ADD1
BP	GO:0045732	positive regulation of protein catabolic process	188	-0.41	-1.55	0.00064997	0.00389201	0.00241318	1577	tags=31%, list=13%, signal=28%	SORL1/AKT1/CBLB/NSF/PIAS1/TIPARP/DISC1/RNF144B/FOXO1/SNX33/CD81/NUB1/CSNK2A1/IL1B/OSBPL7/TRIB1/CHFR/KEAP1/TAF1/BCAP31/GSK3B/GSK3A/TNFRSF1B/CBFA2T3/DET1/VGLL4/EZR/NEDD4L/DDA1/RAB7A/PTK2B/HSPA1A/ANKIB1/TRIM32/LAPTM5/GABARAP/DDB1/VCP/ARIH2/PSMC4/STX5/RAD23A/WWP2/ATG7/VPS28/FZR1/HUWE1/LRP1/RILP/MSN/MARCHF2/ZER1/RNF40/DNAJB2/FURIN/OAZ1/CLU/DAB2/BAG6

BP	GO:0050679	positive regulation of epithelial cell proliferation	91	-0.49	-1.68	0.00065582	0.0039211	0.00243123	1094	tags=19%, list=9%, signal=17%	IGF2/BAD/ARNT/PIK3CD/STAT3/NRP1/LRG1/BMP6/NOTCH2/STAT5A/GLUL/TCF7L2/SP1/GRN/ZNF703/MED1/ITGB3
BP	GO:0010976	positive regulation of neuron projection development	99	-0.48	-1.69	0.00065806	0.00392299	0.00243239	3254	tags=45%, list=28%, signal=33%	STYXL1/EEF2K/STAU2/SF3A2/PRKCI/ACTR2/KIDINS220/DVL1/ARSB/RETREG3/CAPRIN1/NCKIPSD/DISC1/ADNP/EZH2/MAGI2/FYN/FEZ1/HSPA5/FES/PACSIN1/ARHGAP35/NDEL1/BAIAP2/CYFIP1/ENC1/KATNB1/LYN/PLXNB2/PTK2B/STK24/MARK2/SNX3/ATP1B2/VLDLR/ABL1/RAPGEF1/FLNA/FKBP1B/CAMK1D/PPP2R5B/GRN/CAMK1/EHD1/SCN1B
BP	GO:0034220	ion transmembrane transport	560	-0.33	-1.37	0.00065811	0.00392299	0.00243239	2439	tags=25%, list=21%, signal=20%	TRPM2/CYBB/BAK1/ATP1A1/CLN3/TRPC3/ATP2A2/DLG4/SLC24A4/UQCRC1/SLC20A1/CHP1/G6PD/NETO2/SLC25A11/CDK5/FYN/FXYD6/SLC25A23/ATP1B1/P2RX4/RIPK1/PTPN6/SLC25A1/ATP13A3/ITPR2/MFSD12/SLC9A7/SLC33A1/SNTA1/SLC43A2/TCN2/TMC8/SLC7A1/SLC25A42/PDE4B/SLC37A2/MICU1/PTPRC/EDN1/STOML2/SLC41A1/COX15/SLC19A1/MMP9/ABCC1/KCNJ2/KCNQ5/EHD3/STOM/PRKACA/HVCN1/TSPO2/CD63/RANGRF/CLIC5/DAPK1/UCP2/SLC27A1/SELENON/ARL6IP1/OXSR1/SLC35C2/SLC5A6/SLC39A3/FHL1/ANO6/CHERP/AQP1/LYN/NEDD4L/PINK1/PTK2B/SLC9A1/SLC39A4/SLC25A38/RASA3/ATP6V1A/LRRC8A/ATP13A1/SLC7A8/SLC1A5/ATP1B2/CACNG6/CNIH3/SLC1A4/SLC6A20/YWHAH/VDAC1/ABCC4/ATP6V0D1/ABL1/FKBP1A/STIM1/TMEM120A/ANXA6/CYB5A/CLCN5/WWP2/SLC7A5/CALM3/DNM2/FLNA/SLC3A2/FKBP1B/LASP1/MCOLN1/SLC6A9/SLC24A3/MMGT1/SLC4A1/GP1BA/ANKH/GP9/GJC2/PIK3CG/SLC43A1/PER2/UBASH3B/ATP2B4/SNCA/AHCYL1/CAPN1/P2RX1/TTYH3/ACTN4/SLC6A8/SLC15A3/SLC25A37/TESC/HTT/SHISA7/SLC38A5/KEL/TMEM63B/SCN1B/WNK1/DIAPH1
BP	GO:0007043	cell-cell junction assembly	76	-0.52	-1.73	0.0006728	0.00400456	0.00248297	2108	tags=34%, list=18%, signal=28%	IL1B/TBCD/FBF1/ROCK2/CTNND1/PKP4/PARD6B/LSR/FZD5/PRKACA/ACTG1/WDR1/JAM3/CD9/DCHS1/ACTB/CTNNA1/PARD3/RHOA/VCL/HIPK1/CLDN5/ZNF703/PAK2/ESAM/TLN1
BP	GO:0001558	regulation of cell growth	266	-0.38	-1.49	0.00068384	0.0040642	0.00251995	2594	tags=35%, list=22%, signal=28%	SUPV3L1/ULK1/SMARCA2/PAK4/AKT1/ATAD3A/DIP2B/MUL1/DDX49/DISC1/PLXNA4/EXOSC4/ADNP/CDKN2D/FLCN/TSG101/ARHGEF11/MAP3K13/G6PD/BTG1/CDK5/HYAL2/DDR1/CRKL/CSNK2A1/ADNP2/DCUN1D3/MACF1/CCAR2/TMC8/SERTAD3/EI24/CXCL16/SMAD3/ACVR1B/EDN1/KIAA0319/NDEL1/SEMA4D/CDA/GSK3B/GSK3A/RPS6KA1/CYFIP1/TGFB1/SMARCA4/KLHL22/SPHK1/CCN3/VGLL4/MFSD2A/BCL11A/HDGFL2/SESN2/FHL1/IST1/YY1/NEDD4L/CDKL5/PTK2B/DPYSL2/HSPA1A/SLC9A1/TRIM32/PML/GREM1/KAT7/CEACAM1/EIF4G2/CPNE5/SFRP2/PPARA/TAOK2/SEMA4A/NRP1/ABL1/FBP1/LAMTOR1/RHOA/DNM2/MAD2L2/PTPRJ/STK11/CLSTN1/ADIPOR1/PPP1R9B/MTPN/EIF4G1/ENO1/CDKN1A/DNAJB2/CTTN/DAB2
MF	GO:1901981	phosphatidylinositol phosphate binding	131	-0.46	-1.65	0.0006886	0.00408638	0.00253371	2804	tags=37%, list=24%, signal=29%	MYO1G/ZFYVE26/OBSCN/GAB2/SDCBP/ATP13A2/KIF16B/DAPP1/OSBPL5/AKT1/ATG2B/SNX27/RPH3A/PFN1/ARAP1/SH3PXD2A/SVIL/ADAP2/ANKFY1/SNX12/KCNJ2/WASHC2A/TECPR1/ASAP1/DENND1C/ATG2A/DENND1A/ALOX15/HIP1/HIP1R/CGAS/SLC9A1/BBS5/RUBCN/SNX3/ARHGAP32/FLII/PLEKHF1/SNX11/ZFYVE1/PARD3/PLEK2/WIPI2/LDLRAP1/GSN/IQGAP1/NRGN/SCIN/WDR45
BP	GO:1904375	regulation of protein localization to cell periphery	85	-0.49	-1.68	0.00069894	0.00413576	0.00256432	1454	tags=25%, list=12%, signal=22%	TGFB1/CLTC/RANGRF/EZR/STX4/VTI1B/GRIPAP1/ACTB/LDLRAP1/BCL2L1/ZDHHC5/SQSTM1/LRP1/PPP1R9B/CSK/SPTBN1/EPB41/ATP2B4/RHOG/AP2M1/DAB2

CC	GO:0005667	transcription regulator complex	353	-0.36	-1.45	0.00070093	0.00414099	0.00256757	2184	tags=28%, list=19%, signal=24%	HYAL2/BATF2/MAFG/CSNK2A1/MTA2/DRAP1/DACH1/SPI1/GTF3C2/MTA1/GTF2F1/ZBTB16/TCEA1/NR1H2/XRCC6/BCL9/SMAD3/MAFF/RUNX1/PRDM10/CREB3/TAF1/TAF6/RBPJ/TBPL1/CTBP1/TRERF1/KLF4/JUND/SOX4/BCL9L/NR6A1/NFATC1/TRRAP/MED15/NCOA1/NFATC3/ZFHX3/NR1H3/CTNNBIP1/HDAC3/ASCL2/RBM14/NCOA2/MEN1/SKI/NCOA6/PARP1/YY1/EPAS1/RARA/TADA3/RXRA/CREBBP/MED16/HCLS1/TBL1XR1/ARNT/TAF4/GATAD2B/RUNX3/CDK2/TAF8/TRIM28/TFDP1/TLE3/MAX/STAT3/TBL1X/MAFB/FOS/RELA/E2F1/TLE4/TLE1/HIPK2/MED25/MEIS1/TCF7L2/SP1/NFE2/NFATC2/CHD4/PRKDC/E2F2/GATA1/AKIRIN2/ASCC2/HMGA1/PDLIM1/FOXO3/MXI1/MED1/JAZF1/TAL1/GTF3C1/HDGF/GFI1B/NCOR2/PBX1
MF	GO:0019209	kinase activator activity	77	-0.44	-1.49	0.01381216	0.04614885	0.02861395	2488	tags=31%, list=21%, signal=25%	PIK3CA/MOB3B/AGAP2/MAP3K13/MADD/CCNY/MOB1B/TPX2/MOB3A/TAOK1/IGF2/MARK2/GREM1/TAOK2/DAXX/MAP2K2/LTF/CALM3/STK11/IQGAP1/CDKN1A/PAK2/TAL1/WNK1
BP	GO:0046486	glycerolipid metabolic process	284	-0.33	-1.31	0.01392566	0.04641108	0.02877654	2569	tags=28%, list=22%, signal=22%	SORL1/OSBPL5/PIP5KL1/ABCA3/MTMR3/PIK3CA/PTDSS1/PTPN11/GDE1/CLN3/LYPLA2/LMF1/SMPD4/SERINC5/PIP4K2C/CDIPT/MECP2/PI4K2A/PLCH2/CSF1R/PLA2G15/MBOAT7/NR1H2/SOCS7/LIPE/SCARB1/SYNJ1/PI4KB/PIK3CB/PNPLA6/STOML2/INPP5D/DGKZ/LPCAT2/CPT1A/PIK3R3/APOBR/DGKD/SREBF1/NAPEPLD/NR1H3/SLC44A2/ITPKC/INPPL1/SLC27A1/PLEK/EFR3A/MFSD2A/ALOX15/OSBPL10/GPAA1/PNPLA1/PITPNM2/TBL1XR1/PIK3CD/PGAP2/PIGT/ABHD4/TTC7B/AGPAT1/PIP5K1B/AGPAT3/PNPLA2/LPIN2/PLCB3/INPP5K/PLSCR3/IP6K1/ABHD2/TTC7A/TMEM86B/PIK3CG/PRDX6/PIP4K2A/DPM2/GPX1/CTDNEP1/MGLL/PDGFA
MF	GO:0019903	protein phosphatase binding	123	-0.40	-1.44	0.01407609	0.04675566	0.02899019	1343	tags=36%, list=11%, signal=32%	TRAF2/JUP/MAPK14/MTMR3/CD22/PPP1R15A/DLG4/FOXO1/PTPA/PPP6R1/IKBKE/CSF1R/GTF2F1/LGALS3/PHACTR4/GRB2/SPHK1/MVP/MAP3K5/VRK3/CDC27/C20orf27/MAP2K7/SLC9A1/BAD/CEACAM1/SNX3/PPP1CA/STAT3/JAK1/VCP/PXN/JAK3/STAU1/MFHAS1/GNA12/PPP1R9B/CSK/PTPN1/IQGAP1/ATP2B4/PPP1R10/SMG7/ANK1
BP	GO:1904705	regulation of vascular associated smooth muscle cell proliferation	33	-0.57	-1.60	0.01456494	0.04821832	0.02989709	1756	tags=33%, list=15%, signal=28%	EDN1/MMP9/MAP3K5/TPM1/GNAI2/RHOA/LDLRAP1/FOXJ2/GNA12/MFN2/CDKN1A
BP	GO:0060419	heart growth	40	-0.53	-1.57	0.01454529	0.04821832	0.02989709	3064	tags=48%, list=26%, signal=35%	PDLIM5/CITED2/SAV1/MAPK14/NOTCH1/ACACB/G6PD/ARID2/BASP1/RUNX1/EDN1/RBPJ/GSK3A/VGLL4/YY1/PPARA/JARID2/AKAP13/PIM1
MF	GO:0016705	oxidoreductase activity, acting on paired donors, with incorporation or reduction of molecular oxygen	96	-0.41	-1.44	0.01455284	0.04821832	0.02989709	2016	tags=21%, list=17%, signal=17%	OGFOD1/PHF8/FADS2/HIF1AN/FTO/KDM4A/DOHH/PLOD1/KDM6B/CYP27A1/ALKBH5/PHF2/EGLN3/KDM2A/KDM4B/KDM5C/MICAL2/TET3/PTGS1/FAXDC2
CC	GO:0005923	bicellular tight junction	72	-0.45	-1.51	0.0147745	0.04871223	0.03020334	2072	tags=32%, list=18%, signal=26%	TBCD/PARD6A/PARD6B/PMP22/FRMD4B/FZD5/EPPK1/UBN1/FRMD4A/RPGRIP1L/AMOT/TJAP1/ARHGAP17/JAM3/MTDH/ARHGEF2/TJP2/PARD3/CLDN5/VASP/YBX3/ESAM/SH3BP1

BP	GO:1903530	regulation of secretion by cell	320	-0.33	-1.31	0.01493652	0.04899968	0.03038157	2788	tags=29%, list=24%, signal=23%	FGF23/GAB2/TTN/SDCBP/ATP13A2/PDPK1/GABBR1/FGR/SLC16A1/RAB11FIP5/PTAFR/RAB8A/RHBDF2/PIM3/FBXL20/POMC/SYT11/NSF/PTPN11/NOTCH1/TRPM2/NR1D1/ATP2A2/TSG101/HLA-DRB1/CDK5/IL1B/SPI1/P2RX4/VPS4B/PARD6A/FES/NR1H2/ITGAM/HGS/EDN1/CPT1A/GRK2/SOX4/DTNBP1/HYAL3/SREBF1/GSK3B/CRY2/NR1H3/TGFB1/ATP9A/FRMD4A/GIT1/TNFRSF1B/CCN3/EZR/STX4/UCP2/LYN/RAB7A/RAB11FIP1/PINK1/BAD/PFKL/SEPTIN1/CEACAM1/GNAI2/LRRC8A/SEPTIN4/PPP3CA/BCR/SYK/CLASP1/RAB27B/BMP6/MYO18A/SIDT2/GALR1/CALM3/LGALS9/FKBP1B/RAB3D/RAC2/SDC4/SYTL4/GLUL/PRKCB/RAB2B/TCF7L2/PER2/SNCA/LAMP1/P2RX1/GATA1/SMPD3/PTPN23/DAB2
BP	GO:1990266	neutrophil migration	80	-0.44	-1.49	0.01526336	0.04990719	0.03094425	2153	tags=32%, list=18%, signal=27%	CD99L2/IL1B/PPBP/LGALS3/PDE4B/EDN1/RIPOR2/CD74/CXCR2/CXCR1/PREX1/WDR1/RAC1/JAM3/PIK3CD/VAV1/SYK/RAC2/BSG/CAMK1D/MPP1/PF4V1/PIK3CG/CXCL5/RHOG/PF4
BP	GO:0051924	regulation of calcium ion transport	143	-0.39	-1.43	0.015295	0.04996952	0.0309829	2425	tags=29%, list=21%, signal=24%	BAK1/TRPC3/TSPO/CCR1/G6PD/HES1/CDK5/FYN/ATP1B1/P2RX4/PTPN6/LGALS3/PDE4B/EHD3/PRKACA/LILRA5/SELENON/LYN/PTK2B/SLC9A1/PML/GNAI2/CACNG6/VDAC1/JAK3/ABL1/FKBP1A/STIM1/ARRB2/RHOA/CALM3/FKBP1B/MYLK/INPP5K/EPB41/GJC2/PIK3CG/UBASH3B/SNCA/P2RX1/HTT/DIAPH1
BP	GO:1903351	cellular response to dopamine	38	-0.66	-1.92	8.96E-05	0.0007298	0.0004525	2099	tags=50%, list=18%, signal=41%	HRH2/GNA11/OR10H1/GNAZ/DTNBP1/GSK3B/GSK3A/GNAS/GNA15/GNB1/NCSTN/ABL1/ARRB2/DNM2/FLNA/HRH4/KLF16/MAPK3/TGM2
BP	GO:0002761	regulation of myeloid leukocyte differentiation	79	-0.50	-1.68	0.00072398	0.0042519	0.00263633	2294	tags=41%, list=20%, signal=33%	CCR1/HLA-DRB1/MITF/RIPK1/PPARGC1B/TRIB1/FES/TFE3/RUNX1/INPP5D/CD74/FOXP1/TOB2/TGFB1/CTNNBIP1/CDK6/ACIN1/TRAF6/LYN/RARA/HCLS1/CEACAM1/MAFB/FOS/LTF/NOTCH2/GPR137/UBASH3B/PF4/TESC/TAL1/CUL4A
BP	GO:0021700	developmental maturation	161	-0.42	-1.56	0.00073337	0.0043007	0.00266659	2468	tags=33%, list=21%, signal=26%	DISC1/YWHAZ/DLG4/SLC24A4/INSL3/WASHC5/MAP3K13/G6PD/HES1/PFN1/MECP2/ZBTB16/XYLT1/ZBTB7A/EDN1/RERE/FZD5/RBPJ/B4GALT5/ANKS1A/SEMA4D/ANKRD17/PRKACA/CD63/NFIA/SNX10/BCL11A/AP1M1/EPAS1/PTK2B/KLF2/DCHS1/GREM1/CDKN1C/FAM20C/ADGRL1/SLFN14/PHOSPHO1/RFLNB/BLOC1S3/LTF/C1QA/RHOA/ABHD2/CLSTN1/EPB42/ARCN1/FAM210B/NEURL1/LYL1/FOXO3/TAL1/TRIM58
BP	GO:0050900	leukocyte migration	246	-0.39	-1.51	0.00074512	0.00436316	0.00270532	2169	tags=28%, list=18%, signal=23%	CD81/FYN/CD99L2/ICAM1/CRKL/IL1B/SPI1/P2RX4/CNR2/CSF1R/SELPLG/PPBP/ITGB7/LGALS3/CXCL16/HSD3B7/PDE4B/EDN1/RIPOR2/CREB3/CD74/MMP9/CXCR2/PYCARD/CXCR1/TBX21/PREX1/CCN3/WDR1/OXSR1/ANO6/LYN/IL17RA/RAC1/JAM3/PTK2B/STK10/CD9/SPN/SRC/GREM1/CALR/PIK3CD/VAV1/RIN3/SELL/BCR/SYK/ITGAL/RHOA/LGALS9/RAC2/BSG/CAMK1D/MPP1/PLEC/PF4V1/PLVAP/THBS1/PIK3CG/MSN/SELP/CXCL5/RHOG/MAPK3/PF4/SMPD3/MYH9/WNK1
BP	GO:0071526	semaphorin-plexin signaling pathway	25	-0.68	-1.85	0.00075475	0.00440657	0.00273223	2509	tags=60%, list=21%, signal=47%	PLXNC1/PLXNA4/NCAM1/EDN1/SEMA4D/PLXNB2/RAC1/PLXND1/PLXNA1/SEMA4A/NRP1/ARHGDIA/FLNA/ECE1/SH3BP1
BP	GO:1903076	regulation of protein localization to plasma membrane	71	-0.53	-1.74	0.00077513	0.00451892	0.00280189	1454	tags=28%, list=12%, signal=25%	TGFB1/CLTC/RANGRF/EZR/STX4/VTI1B/GRIPAP1/ACTB/LDLRAP1/BCL2L1/ZDHHC5/SQSTM1/LRP1/PPP1R9B/CSK/SPTBN1/ATP2B4/RHOG/AP2M1/DAB2

CC	GO:0030140	trans-Golgi network transport vesicle	26	-0.67	-1.84	0.00078517	0.00455744	0.00282578	1477	tags=42%, list=13%, signal=37%	SYNRG/CLTC/TGOLN2/RAB14/RAB27B/CLTB/SPG21/SORT1/IGF2R/NRGN/FURIN
BP	GO:0015804	neutral amino acid transport	30	-0.66	-1.84	0.00078427	0.00455744	0.00282578	974	tags=33%, list=8%, signal=31%	SLC25A38/SLC7A8/SLC1A5/SLC1A4/SLC6A20/SLC7A5/SLC3A2/SLC6A9/SLC43A1/SLC38A5
BP	GO:0015850	organic hydroxy compound transport	116	-0.46	-1.64	0.00081282	0.0046905	0.00290828	2558	tags=37%, list=22%, signal=29%	GABBR1/SLC16A1/OSBPL5/STARD3/GRAMD1B/POMC/SYT11/ABCB11/PTPN11/TSPO/OSBPL7/VPS4B/RALBP1/NR1H2/SCARB1/LIMA1/GRK2/DTNBP1/TSPO2/NR1H3/APOM/AQP10/OSBPL10/AQP1/PINK1/CEACAM1/ABCC3/ACTB/SYK/ABCC4/NFKB1/BMP6/GALR1/LDLRAP1/LRP1/SNCA/PIP4K2A/P2RX1/FURIN/ITGB3/CLU/OSBP2/DAB2
BP	GO:0072001	renal system development	159	-0.43	-1.59	0.00081266	0.0046905	0.00290828	2666	tags=34%, list=23%, signal=27%	PECAM1/EXT1/PKD1/SMAD5/TIPARP/NOTCH1/CASP9/EGR1/FLCN/SGPL1/PRDM1/IL6R/EPHA4/HES1/MAGI2/HYAL2/SNX17/ZBTB16/MPST/BASP1/LZTS2/SMAD3/FMN1/MAGED1/MMP9/CENPF/AP2B1/SOX4/DTNBP1/ACVR2B/CTNNBIP1/NFIA/RPGRIP1L/MYO1E/ILK/RARA/PLXND1/DCHS1/BCL2L11/GREM1/CDKN1C/NRP1/BMP6/RHOA/NOTCH2/ODC1/ACTA2/AP1B1/KANK2/SDC4/IQGAP1/PDGFA/PBX1/BAG6
BP	GO:0007162	negative regulation of cell adhesion	193	-0.41	-1.56	0.00081127	0.0046905	0.00290828	2551	tags=36%, list=22%, signal=28%	AKT1/FOXP3/SWAP70/CBLB/PLXNC1/PLXNA4/MAPK7/KNG1/PTPN11/NOTCH1/EPHA4/HLA-DRB1/IRF1/LGALS9C/SPI1/TBCD/PTPN6/MYADM/LGALS3/RCC2/RUNX1/PTPRC/RIPOR2/CD74/KLF4/SEMA4D/AKNA/TGFB1/TBX21/TMEM131L/IL1RN/SPINT2/MEN1/IL4I1/TWSG1/CORO1C/BCAS3/PLXNB2/MBP/JAM3/PLXND1/CD9/SPN/SRC/CEACAM1/PPARA/RUNX3/PLXNA1/LAPTM5/JAK3/ABL1/TNFSF4/BMP6/PDE5A/RHOA/MAD2L2/ARHGAP6/ARHGDIA/LGALS9/NF2/SDC4/ALOX12/THBS1/UBASH3B/ZNF703/HLX/ACTN4/DMTN/WNK1
BP	GO:0016236	macroautophagy	273	-0.38	-1.48	0.0008216	0.0047343	0.00293544	1875	tags=24%, list=16%, signal=21%	EI24/CDC37/MAP1LC3B/LRBA/HGS/GPSM1/CHMP6/POLDIP2/VPS41/PHF23/WDR81/NSFL1C/VPS37B/CHMP4B/EHMT2/IKBKG/TECPR1/PRKACA/ATG2A/VPS13D/SESN2/VTI1B/RAB7A/EPG5/PINK1/ILRUN/SRC/CHMP1A/RUBCN/ATP6V1A/VTI1A/GABARAP/VDAC1/VCP/USP36/ATP6V0D1/ZFYVE1/OPTN/ATG4D/RNF213/ATP6V0C/CAPNS1/WIPI2/SESN3/ATG7/ATP6V0A1/MCOLN1/VPS28/RAB1B/HUWE1/SQSTM1/YOD1/MFN2/ATG13/CAPN1/PIP4K2A/TBC1D5/MAPK3/ELAPOR1/BNIP3L/TBC1D25/WDR45/UBXN6/HTT/LARP1/SUPT5H
MF	GO:0051213	dioxygenase activity	69	-0.52	-1.70	0.00083723	0.00481046	0.00298266	2133	tags=30%, list=18%, signal=25%	RSBN1/OGFOD1/PHF8/HAAO/HIF1AN/FTO/KDM4A/PLOD1/KDM6B/ALOX15/ASPHD2/ALKBH5/PHF2/EGLN3/KDM2A/KDM4B/KDM5C/HGD/ALOX12/TET3/PTGS1
MF	GO:0019902	phosphatase binding	163	-0.42	-1.57	0.00084976	0.00487541	0.00302293	2637	tags=36%, list=22%, signal=28%	TRAF2/JUP/MAPK14/SBF2/MTMR3/CD22/CNST/ELL/PPP1R15A/DLG4/FOXO1/PTPA/PPP6R1/IKBKE/MAGI2/SPRED1/CSF1R/GTF2F1/LGALS3/SMAD3/PHACTR4/GRB2/CRY2/SPHK1/MVP/MAP3K5/VRK3/CDC27/C20orf27/MAP2K7/SLC9A1/BAD/CEACAM1/SNX3/PPARA/PPP1CA/STAT3/JAK1/PPP3R1/SYK/VCP/PXN/JAK3/STAU1/SIGLEC10/MFHAS1/PPP1R18/GNA12/PPP1R9B/CSK/PTPN1/IQGAP1/NFATC2/ATP2B4/PPP1R10/MAPK3/SMG7/ANK1/WNK1

BP	GO:0010035	response to inorganic substance	332	-0.36	-1.46	0.00086645	0.00496398	0.00307785	2491	tags=30%, list=21%, signal=24%	SYT11/PIK3CA/JUN/MAPK7/ADNP/TRPM2/CYBB/BAK1/NCF1/CASP9/CYBRD1/TRPC3/EZH2/DLG4/FOXO1/CPNE3/TSPO/PHEX/EIF4A3/G6PD/FYN/SLC25A23/CPNE1/P2RX4/RIPK1/HSPA5/D2HGDH/NDRG1/HAAO/EDN1/CCS/SLC41A1/CD14/MMP9/JUND/SMPD1/SORD/HVCN1/ACTG1/SPHK1/KDM6B/UCP2/ALAD/ATIC/PARP1/MAP3K5/ALOX15/AQP1/NEDD4L/MTF1/PINK1/MBP/PTK2B/MT2A/KLF2/BAD/STK24/NCSTN/SRC/CPNE5/STK25/CALR/CDK2/PPP3CA/DAXX/SLFN14/PPP1CA/SEC31A/DDI2/VCP/ABL1/FOS/TNFSF4/FBP1/RELA/STIM1/BMP6/ANXA11/CALM3/DNM2/FKBP1B/BSG/MCOLN1/PLEC/TXNIP/THBS1/IQGAP1/GPI/ATP2B4/SNCA/AHCYL1/MAPK3/SMPD3/GPX1/FOXO3/GUCY1B1/PDGFA/DMTN/ADD1
BP	GO:0006937	regulation of muscle contraction	81	-0.50	-1.70	0.0008967	0.00512988	0.00318071	2110	tags=32%, list=18%, signal=27%	ATP1B1/P2RX4/ORMDL3/PDE4B/EDN1/GRK2/KCNJ2/EHD3/PRKACA/RANGRF/SPHK1/DAPK3/TPM1/SLC9A1/ANXA6/PDE5A/RHOA/CALM3/GUCY1A1/FKBP1B/PIK3CG/ENO1/P2RX1/MYL9/CTTN/TBXA2R
BP	GO:0007009	plasma membrane organization	94	-0.48	-1.66	0.00091846	0.00524686	0.00325324	2551	tags=40%, list=22%, signal=32%	AKT1/SH3TC2/SYT11/NSF/CLN3/ATP2A2/SNX33/ABCD1/NDRG1/PACSIN1/PTPRC/CHMP6/CHMP4B/SMPD1/BAIAP2/ASAP1/STX4/PRX/ANO6/PLEKHM2/CD9/MICALL1/DYSF/CHMP1A/MYOF/ANXA6/DNM2/CAVIN2/PLSCR3/GSN/PLEC/SYTL4/SLC4A1/SPTBN1/AKT2/SPTA1/MYH9/CLU
BP	GO:0010942	positive regulation of cell death	422	-0.35	-1.43	0.00093597	0.00533918	0.00331048	1779	tags=22%, list=15%, signal=19%	TRAF7/PTPRC/PIK3CB/MAGED1/INPP5D/MMP9/BBC3/MAP3K11/BCAP31/SOX4/SMPD1/TOP2A/NCOA1/GSK3B/PYCARD/TEX261/TNFRSF1B/PRR7/DAPK3/DAPK1/UCP2/PARP1/TP53BP2/TRIM35/MAP3K5/KATNB1/ANO6/HIP1R/LYN/PRF1/PIAS4/MAP2K7/SLC9A1/BAD/BRMS1/PDCD1/BCL2L11/SRC/UBE2Z/PML/EIF5A/SRPK2/RBCK1/PIK3CD/SFRP2/SEPTIN4/FIS1/PLEKHF1/LAPTM5/DAXX/PPP1CA/CTNNA1/SYK/FBH1/ABL1/FOS/GADD45G/E2F1/C1QA/ARRB2/RHOA/UBE2M/NOTCH2/DNM2/LGALS9/ATG7/CDK19/ELK1/CAMK1D/UBB/GSN/TXNIP/IGF2R/SQSTM1/LRP1/THBS1/MFN2/GRN/SNCA/EIF4G1/NEURL1/PRKDC/BNIP3L/CDKN1A/CTSD/SCIN/PAK2/RYBP/FOXO3/CDC34/HTT/CLU/TGM2
BP	GO:0009895	negative regulation of catabolic process	247	-0.39	-1.52	0.0009591	0.00546328	0.00338743	2386	tags=35%, list=20%, signal=29%	BANP/AGAP2/ZFP36/HDAC4/WAC/TSPO/IKBKE/EPHA4/FYN/CSDE1/CSNK2A1/IL1B/FEZ1/PABPC1/LARP4B/SYNCRIP/TIMP1/CCAR2/CTSA/PIN1/FOXK1/SMAD3/F8A1/SMARCC1/TAF1/POLDIP2/PHF23/SNX12/CHMP4B/EHMT2/KDM4A/METTL16/FOXK2/TIMP2/GSK3A/DAP/HSP90AB1/GIT1/NBAS/KLHL22/CBFA2T3/ALAD/MFSD2A/BSCL2/PINK1/PML/EIF4G2/RUBCN/SNX3/PPARA/MYCBP2/STAT3/USP19/MAP1A/HERC1/RGP1/CPTP/USP36/ELAVL1/FBP1/N4BP1/RELA/E2F1/GOLGA2/MAD2L2/SUFU/FLNA/MAPKAPK2/USP7/ATG7/SF3B3/HIPK2/TENT4B/SLC4A1/PSMF1/RILP/YBX1/PIK3CG/ATP2B4/SNCA/EIF4G1/RYBP/YBX3/FURIN/TENT5C/LARP1/BAG6
BP	GO:0001822	kidney development	157	-0.43	-1.57	0.00097213	0.00552961	0.00342855	2666	tags=34%, list=23%, signal=26%	PECAM1/EXT1/PKD1/SMAD5/TIPARP/NOTCH1/CASP9/EGR1/FLCN/SGPL1/PRDM1/IL6R/EPHA4/HES1/MAGI2/HYAL2/SNX17/ZBTB16/MPST/BASP1/LZTS2/SMAD3/FMN1/MAGED1/MMP9/CENPF/AP2B1/SOX4/DTNBP1/ACVR2B/CTNNBIP1/RPGRIP1L/MYO1E/ILK/RARA/PLXND1/DCHS1/BCL2L11/GREM1/CDKN1C/NRP1/BMP6/RHOA/NOTCH2/ODC1/ACTA2/AP1B1/KANK2/SDC4/IQGAP1/PDGFA/PBX1/BAG6
BP	GO:0070997	neuron death	238	-0.39	-1.50	0.00097581	0.00554259	0.00343661	2637	tags=32%, list=22%, signal=26%	TRAF2/CASP7/SORL1/CORO1A/AKT1/PIK3CA/JUN/ADNP/TRPM2/CLN3/CASP9/EGR1/AGAP2/HYOU1/CHP1/DIABLO/G6PD/MECP2/CDK5/FYN/CASP2/HSPA5/TLR6/ITGAM/KIR3DL2/ENO2/CHMP4B/AP2B1/MAP3K11/DTNBP1/IKBKG/GSK3B/HSP90AB1/TNFRSF1B/DNAJC5/PARP1/MAP3K5/PINK1/PTK2B/MAP2K7/SLC9A1/BAD/DHCR24/NCSTN/BCL2L11/SRPK2/PPARA/DAXX/MAX/PIGT/NRP1/XIAP/ABL1/EGLN3/FOS/SYNGAP1/C1QA/ARRB2/RHOA/UBE2M/AARS1/HIPK2/ELK1/BCL2L1/UBB/AKT2/GPI/GRN/SNCA/EIF4G1/FBXO7/CBL/GPX1/FOXO3/CDC34/HDGF/CLU

BP	GO:0043066	negative regulation of apoptotic process	557	-0.33	-1.38	0.00098981	0.00561411	0.00348095	1941	tags=23%, list=17%, signal=20%	TIMP1/GSTP1/ST6GAL1/CCAR2/LGALS3/CIAPIN1/SHC1/BIRC6/SMAD3/RHBDD1/EDN1/CAST/PIK3CB/CREB3/SLC35F6/CD74/MMP9/KLF4/SEMA4D/ITPRIP/PSME3/FOXP1/GSK3B/IL2RB/RPS6KA1/HSP90AB1/HDAC3/VHL/THOC6/DNAJC5/SPHK1/DAPK1/BCL11B/UCP2/BABAM2/ARL6IP1/AQP1/HIP1R/ADAR/PINK1/PTK2B/HSPA1A/CHST11/MGMT/SLC9A1/MTDH/DHCR24/TRIM32/PDCD1/HCLS1/SRC/UBE2Z/GREM1/SFRP2/GNAI2/PPARA/PIH1D1/CTNNA1/NRP1/ARHGEF2/VDAC1/DDB1/JAK3/XIAP/ABL1/SYNGAP1/RELA/LTF/NFKB1/PIM2/ARRB2/MYO18A/HNRNPK/RHOA/CHD8/NOTCH2/ARHGDIA/FLNA/AARS1/LMNA/PA2G4/TLE1/HSPB1/HIPK2/NIBAN2/CAMK1D/BCL2L1/SIAH2/PRDX5/CFL1/ALOX12/THBS1/AKT2/PTPN1/PIK3CG/TCF7L2/GPI/FKBP8/GRN/ARAF/SNCA/PPP1R10/ENO1/PRKDC/BAG1/GATA1/GRINA/BNIP3L/PF4/CBL/PAK2/UBE2O/STRADB/ST3GAL1/GPX1/YBX3/PTMS/MED1/CTTN/HTT/HDGF/PTCRA/CLU/PIM1/DAB2/BAG6
BP	GO:0030278	regulation of ossification	60	-0.55	-1.75	0.00102144	0.00578526	0.00358707	2974	tags=52%, list=25%, signal=39%	TENT5A/CHSY1/KREMEN1/SLC8A1/FGF23/MAPK14/BMP2K/PTPN11/NOTCH1/CCR1/ZBTB16/SMAD3/RBPJ/TOB2/ACVR2B/TGFB1/BCOR/ANO6/PTK2B/GREM1/LTBP3/FAM20C/DHRS3/PHOSPHO1/RFLNB/LTF/BMP6/ANKH/MAPK3/GATA1/PBX1
BP	GO:0006970	response to osmotic stress	53	-0.56	-1.73	0.00103119	0.00583217	0.00361616	2951	tags=49%, list=25%, signal=37%	RELB/ZFP36L1/LETM1/PDPK1/CLN3/TSPO/PKN1/MLC1/SLC25A23/MAP7/RCSD1/MICU1/SERPINB6/SORD/HSP90AB1/OXSR1/AQP1/PTK2B/MAP2K7/BAD/LRRC8A/ARHGEF2/NOLC1/SLC2A1/MYLK/YBX3
CC	GO:0043296	apical junction complex	87	-0.49	-1.67	0.00103993	0.00586757	0.00363811	2072	tags=37%, list=18%, signal=31%	TBCD/FBF1/PARD6A/CTNND1/AJM1/PARD6B/PMP22/FRMD4B/SHROOM1/FZD5/EPPK1/UBN1/ACTG1/FRMD4A/RPGRIP1L/AMOT/TJAP1/ARHGAP17/JAM3/MTDH/ACTB/CTNNA1/ARHGEF2/TJP2/KIFC3/PARD3/RHOA/CLDN5/VASP/YBX3/ESAM/SH3BP1
BP	GO:0016482	cytosolic transport	153	-0.43	-1.59	0.0010404	0.00586757	0.00363811	1669	tags=25%, list=14%, signal=22%	ANKFY1/STX6/WDR81/SNX12/WASHC2A/VPS53/EHD3/KIF1C/TBC1D17/ATP9A/CLTC/EZR/VTI1B/RAB7A/SRC/HOOK3/SNX3/VTI1A/LAPTM5/RAB6B/MAP2K2/RGP1/RAB14/STX5/FLNA/USP7/SORT1/DCTN1/SLC66A2/GAK/RILP/MSN/LAMP1/PIP4K2A/TBC1D5/MAPK3/UBE2O/PTPN23/DAB2
CC	GO:0031983	vesicle lumen	264	-0.37	-1.45	0.00106482	0.00598831	0.00371297	2085	tags=27%, list=18%, signal=23%	PROS1/EEF2/PTPN6/PPBP/RNASE3/PGAM1/PGLYRP1/TIMP1/OSCAR/GSTP1/CTSA/XRCC6/ADA2/ARPC5/CDA/CRISPLD2/TRAPPC1/TIMP2/PYCARD/CYFIP1/HSP90AB1/TGFB1/ALAD/MVP/ALDOA/CANT1/GLB1/IST1/VTI1B/F13A1/SERPING1/IGF2/ALDOC/PFKL/DYNC1H1/PKM/ARHGAP45/CNN2/PSMD11/CAP1/VCP/LRG1/LCN2/PYGB/LTF/NFKB1/TUBB4B/VCL/FERMT3/PSMD2/PA2G4/ATG7/DOCK2/CYB5R3/GSN/HUWE1/THBS1/GNS/GPI/COTL1/GRN/PRDX6/PF4/CTSD/TUBB/FTL/ACTN4/ACTN1/TOLLIP/SPARC/PDGFA/CLU
CC	GO:0060205	cytoplasmic vesicle lumen	264	-0.37	-1.45	0.00106482	0.00598831	0.00371297	2085	tags=27%, list=18%, signal=23%	PROS1/EEF2/PTPN6/PPBP/RNASE3/PGAM1/PGLYRP1/TIMP1/OSCAR/GSTP1/CTSA/XRCC6/ADA2/ARPC5/CDA/CRISPLD2/TRAPPC1/TIMP2/PYCARD/CYFIP1/HSP90AB1/TGFB1/ALAD/MVP/ALDOA/CANT1/GLB1/IST1/VTI1B/F13A1/SERPING1/IGF2/ALDOC/PFKL/DYNC1H1/PKM/ARHGAP45/CNN2/PSMD11/CAP1/VCP/LRG1/LCN2/PYGB/LTF/NFKB1/TUBB4B/VCL/FERMT3/PSMD2/PA2G4/ATG7/DOCK2/CYB5R3/GSN/HUWE1/THBS1/GNS/GPI/COTL1/GRN/PRDX6/PF4/CTSD/TUBB/FTL/ACTN4/ACTN1/TOLLIP/SPARC/PDGFA/CLU

BP	GO:0045321	leukocyte activation	687	-0.31	-1.33	0.00111024	0.00623493	0.00386588	2589	tags=28%, list=22%, signal=23%	BPI/RASAL3/PTAFR/CORO1A/AKT1/SELENOK/LY9/FOXP3/TNFSF13/SWAP70/CBLB/SYT11/PIK3CA/ZFPM1/JUN/PTPN11/NR1D1/BAK1/CD22/CD3E/EFNB1/DOCK8/EGR1/HDAC4/HDAC5/EZH2/SNX27/PRDM1/IL6R/HES1/PKN1/HLA-DRB1/IRF1/HYAL2/BATF2/CD81/FYN/HLA-DQA2/ICAM1/LGALS9C/IL1B/SPI1/CNR2/HLA-A/SH3KBP1/DOCK10/TXLNA/PTPN6/SELPLG/TLR6/ZBTB16/LCP1/PGLYRP1/ZBTB7A/SIRPG/ZFP36L2/FES/NDRG1/LGALS3/SLC7A1/ITGAM/XRCC6/SOX13/SMAD3/RUNX1/PTPRC/EDN1/RIPOR2/WDFY4/DROSHA/FZD5/STOML2/INPP5D/RBPJ/CD74/CD300LF/NCR3/JUND/SIRPB1/CXCR2/SOX4/PSMB10/FOXP1/HLA-DQA1/IL2RB/NR1H3/PYCARD/TGFB1/MICB/CDK6/TBX21/TMEM131L/TNFRSF1B/PREX1/PRR7/SPHK1/CD180/STX4/BCL11B/IL4I1/TWSG1/TRAF6/KARS1/SIT1/LYN/YY1/SUPT6H/PTK2B/PRF1/RARA/IGF2/DYSF/KDELR1/CGAS/SPN/BAD/KIF13B/NCSTN/SRC/CCND3/KAT7/DCAF1/CEACAM1/PIK3CD/LRRC8A/HLA-DRB5/VAV1/RUNX3/PPP3CA/LAPTM5/ZMIZ1/TP53BP1/HMCES/STAT3/SEMA4A/BCR/SYK/JAK3/ABL1/MAFB/TNFSF4/FKBP1A/BLOC1S3/C1QA/MYO18A/ITGAL/APBB1IP/MPL/PDE5A/RHOA/MFHAS1/NOTCH2/MAD2L2/PTPRJ/LGALS9/WAS/FKBP1B/RAC2/DOCK2/STK11/SDC4/GSN/PRKCB/CSK/THBS1/PIK3CG/CD151/SPTA1/MSN/NFATC2/GRN/ELF4/SNCA/PRKDC/LAMP1/SOX12/IKZF1/GATA1/FBXO7/HLX/LYL1/CDKN1A/CBL/ST3GAL1/FOXO3/TOLLIP/MYH9/CLU/PBX1/BAG6
BP	GO:0010611	regulation of cardiac muscle hypertrophy	37	-0.61	-1.78	0.00111574	0.00623933	0.00386861	2444	tags=59%, list=21%, signal=47%	NOTCH1/TRPC3/FOXO1/G6PD/P2RX4/ROCK2/CDK9/SMAD3/EDN1/GSK3A/TNFRSF1B/PARP1/YY1/SLC9A1/PPARA/PPP3CA/JARID2/PDE5A/PDE9A/LMNA/MTPN/ATP2B4
BP	GO:0014743	regulation of muscle hypertrophy	37	-0.61	-1.78	0.00111574	0.00623933	0.00386861	2444	tags=59%, list=21%, signal=47%	NOTCH1/TRPC3/FOXO1/G6PD/P2RX4/ROCK2/CDK9/SMAD3/EDN1/GSK3A/TNFRSF1B/PARP1/YY1/SLC9A1/PPARA/PPP3CA/JARID2/PDE5A/PDE9A/LMNA/MTPN/ATP2B4
BP	GO:0051222	positive regulation of protein transport	221	-0.39	-1.51	0.00113826	0.00634744	0.00393564	2439	tags=30%, list=21%, signal=24%	TRPM2/CNST/CLN3/UBR5/TSG101/CHP1/HLA-DRB1/PCNT/CDK5/HYAL2/CD81/FYN/IL1B/PARD6A/SREBF2/RIPOR1/NR1H2/ITGAM/UBE2J2/SMAD3/FZD5/BBC3/BCAP31/SOX4/TM9SF4/GSK3B/PRR5L/STOM/PRKACA/GSK3A/HSP90AB1/TGFB1/FRMD4A/HDAC3/EDEM2/EZR/STX4/BCAS3/MAVS/LEPROT/PINK1/BAD/HCLS1/SRC/LRRC8A/FIS1/TRIM28/USP36/ARIH2/ABLIM3/BMP6/SAE1/MYO18A/FLNA/RAC2/VPS28/SYTL4/GLUL/HUWE1/AKT2/UBE2L3/TCF7L2/CAMK1/ATG13/OAZ1/PTPN23/PPM1A
BP	GO:0043535	regulation of blood vessel endothelial cell migration	62	-0.54	-1.72	0.00118538	0.00659171	0.00408709	2703	tags=40%, list=23%, signal=31%	PDPK1/JUP/AKT1/NOTCH1/HDAC5/MECP2/PRKD2/SPRED1/P2RX4/VASH1/MAP3K3/KLF4/TGFB1/ETS1/NRP1/ABL1/RHOA/HSPB1/STAT5A/THBS1/SP1/ATP2B4/SH3BP1/MAP2K3/TBXA2R
BP	GO:0046849	bone remodeling	49	-0.57	-1.76	0.00118814	0.00659782	0.00409088	2121	tags=43%, list=18%, signal=35%	MITF/CSF1R/PPARGC1B/TPP1/INPP5D/TGFB1/SNX10/TRAF6/RAB7A/PTK2B/SRC/GREM1/LTBP3/LRRK1/SYK/NOTCH2/GPR137/RAB3D/CSK/UBASH3B/ITGB3
MF	GO:0019787	ubiquitin-like protein transferase activity	345	-0.36	-1.43	0.00119867	0.00664703	0.0041214	1322	tags=18%, list=11%, signal=16%	TSPAN17/CNOT4/BCOR/TRAF6/TRIM35/UBR2/UBE2G1/NEDD4L/TRIM26/ANKIB1/PIAS4/TRIM32/MARCHF6/DTX2/UBE2Z/PML/UBE2R2/RNF115/RBCK1/RMND5A/MYCBP2/HERC2/TRIM10/TRIM28/AMFR/BTRC/FBXO6/HERC1/XIAP/ARIH2/RNF213/DTX3L/RNF11/WWP2/UBE2M/UBE2S/ZNRF1/TRIM11/RNF24/SIAH2/UBR4/HUWE1/TRIM14/UBE2L3/MKRN1/RNF187/MARCHF2/SHARPIN/NEURL1/RNF123/RNF10/MARCHF8/FBXO7/CBL/TRIP12/UBE2O/RNF40/CDC34/UBE2H/TRIM58/RNF208

BP	GO:0008284	positive regulation of cell population proliferation	525	-0.33	-1.37	0.00120177	0.00665495	0.00412631	1346	tags=17%, list=11%, signal=15%	RPRD1B/SPHK1/STX4/SERTAD1/TRAF6/MAP3K5/AQP1/LYN/ILK/LBH/PTK2B/RARA/IGF2/BAD/HCLS1/PML/GREM1/KAT7/ARNT/SRPK2/CALR/PIK3CD/SFRP2/GNAI2/LTBP3/NOP2/CDK2/ZMIZ1/STAT3/NRP1/NOLC1/PDGFC/SYK/JAK3/LRG1/ABL1/PAK1/TNFSF4/RELA/E2F1/LTF/BMP6/CAPNS1/MPL/GID8/NOTCH2/ODC1/LDLRAP1/HIPK1/LGALS9/RAC2/FOXJ2/STAT5A/HIPK2/BCL2L1/ROGDI/GLUL/FZR1/CLDN5/THBS1/AKT2/GJC2/SPTA1/RNF187/MFN2/MEIS1/TCF7L2/SP1/NFATC2/GRN/CXCL5/CAPN1/ZNF703/RHOG/MAPK3/GATA1/HLX/CDKN1A/AKIRIN2/SMPD3/MED1/CUL4A/ITGB3/PDGFA/CARM1/PIM1/PBX1
BP	GO:0033627	cell adhesion mediated by integrin	50	-0.56	-1.73	0.00120499	0.00666348	0.0041316	2622	tags=44%, list=22%, signal=34%	EXT1/SWAP70/PTPN11/CD3E/ICAM1/CRKL/PTPN6/ITGB7/ITGAM/CCN3/LYN/JAM3/SFRP2/SYK/ITGAL/FERMT3/ITGB5/PIK3CG/TESC/ITGB3/ITGA2B/WNK1
BP	GO:0048260	positive regulation of receptor-mediated endocytosis	36	-0.62	-1.80	0.00125412	0.0069035	0.00428042	1830	tags=47%, list=16%, signal=40%	ARRB1/SYNJ1/DGKD/DTNBP1/CD63/HIP1/GREM1/SYK/ARRB2/HNRNPK/DNM2/LDLRAP1/TBC1D5/CBL/AP2M1/CLU/DAB2
BP	GO:0090316	positive regulation of intracellular protein transport	128	-0.44	-1.57	0.00125534	0.0069035	0.00428042	2267	tags=36%, list=19%, signal=29%	CHP1/PCNT/CDK5/HYAL2/CD81/FYN/IL1B/SREBF2/RIPOR1/ITGAM/UBE2J2/SMAD3/FZD5/BBC3/BCAP31/TM9SF4/GSK3B/PRR5L/STOM/PRKACA/GSK3A/HSP90AB1/TGFB1/HDAC3/EDEM2/BCAS3/MAVS/LEPROT/PINK1/HCLS1/FIS1/TRIM28/USP36/ARIH2/ABLIM3/SAE1/FLNA/RAC2/HUWE1/AKT2/UBE2L3/TCF7L2/CAMK1/ATG13/OAZ1/PPM1A
CC	GO:0044853	plasma membrane raft	71	-0.52	-1.70	0.00128059	0.00703262	0.00436048	3112	tags=44%, list=27%, signal=32%	FLOT2/HDAC6/PTCH1/ITGB2/LCP2/ADD2/CR1/CLN3/MLC1/SPRED1/ATP1B1/SELPLG/ITGAM/LIPE/SCARB1/NEU3/PRKACA/RANGRF/EZR/CORO1C/SRC/CTNNA1/MYOF/STIM1/SLC2A1/PRKAR2A/CAVIN2/PLVAP/ATP2B4/MAPK3/CBL
BP	GO:0014902	myotube differentiation	64	-0.53	-1.71	0.00129421	0.00707811	0.00438868	2444	tags=41%, list=21%, signal=32%	NOTCH1/HDAC4/HDAC5/CD81/PLEKHO1/NLN/BCL9/RIPOR2/DYRK1B/CCN3/SKI/SELENON/CD9/PLD3/PPP3CA/MYOF/XK/RBM38/NIBAN2/SORT1/PLEC/NFATC2/EHD1/GPX1/MYH9/KEL
BP	GO:0001659	temperature homeostasis	118	-0.45	-1.60	0.00129251	0.00707811	0.00438868	2444	tags=38%, list=21%, signal=30%	NOTCH1/TRPM2/NR1D1/EGR1/FLCN/FOXO1/ZNF516/THRA/IL1B/PCTP/PPARGC1B/EHMT1/NR1H2/TFE3/ALPL/RBPJ/FTO/NOVA1/ACVR2B/NAPEPLD/NR1H3/HDAC3/GNAS/KDM6B/UCP2/SLC27A1/LNPEP/EPAS1/BSCL2/DYNC1H1/ADIPOR2/TLE3/STAT3/PDGFC/SYK/LCN2/GADD45G/ACADVL/STK11/IP6K1/ADIPOR1/SQSTM1/MFN2/PER2/GPX1
BP	GO:0060537	muscle tissue development	198	-0.40	-1.54	0.00129072	0.00707811	0.00438868	1961	tags=27%, list=17%, signal=23%	NLN/HMG20B/SVIL/BCL9/SMAD3/MAFF/RUNX1/EDN1/RIPOR2/ENG/RBPJ/CENPF/GSK3A/TGFB1/VGLL4/KDM6B/SKI/SELENON/TPM1/YY1/RARA/IGF2/FLNB/SLC9A1/SOX6/GREM1/CALR/PPARA/PPP3CA/USP19/CSRP1/FOS/FKBP1A/ARRB2/JARID2/XK/RHOA/NOTCH2/LMNA/MYLK/NIBAN2/PLEC/AKAP13/MTPN/NEURL1/HLX/GPX1/YBX3/NPRL3/MED1/KEL/HIVEP3/PIM1
BP	GO:0016239	positive regulation of macroautophagy	62	-0.53	-1.71	0.00133459	0.00728063	0.00451426	1325	tags=24%, list=11%, signal=22%	ATG2A/VPS13D/SESN2/PINK1/VDAC1/OPTN/SESN3/PIP4K2A/MAPK3/ELAPOR1/BNIP3L/WDR45/HTT/LARP1/SUPT5H
BP	GO:0006638	neutral lipid metabolic process	78	-0.51	-1.71	0.00133491	0.00728063	0.00451426	1951	tags=29%, list=17%, signal=25%	PLA2G15/MBOAT7/NR1H2/LIPE/SCARB1/DGKZ/CPT1A/APOBR/DGKD/SREBF1/NR1H3/SLC27A1/MFSD2A/PNPLA1/TBL1XR1/PNPLA2/LPIN2/ABHD2/PIK3CG/SNCA/GPX1/CTDNEP1/MGLL
BP	GO:0045069	regulation of viral genome replication	77	-0.50	-1.68	0.00136599	0.00742976	0.00460672	1228	tags=27%, list=10%, signal=25%	APOBEC3D/MAVS/ADAR/IFITM3/ZC3HAV1/SRPK2/PPARA/TRIM28/IFITM2/DDB1/APOBEC3F/N4BP1/LTF/RAD23A/STAU1/SETDB1/INPP5K/ZNFX1/APOBEC3C/TNIP1/LARP1

CC	GO:0031234	extrinsic component of cytoplasmic side of plasma membrane	69	-0.51	-1.67	0.00138432	0.00751921	0.00466218	2642	tags=43%, list=23%, signal=34%	FGR/GNAQ/GNB2/AKT1/GNAO1/DLG4/FYN/CNR2/GNA11/TNK2/FES/TIAM1/GNAZ/GNAS/MYZAP/ESYT1/ALOX15/HIP1/LYN/RAC1/PTK2B/GNA15/GNB1/SRC/GNAI2/SLA/SYK/RHOA/GNA12/IQGAP1
BP	GO:0033002	muscle cell proliferation	111	-0.46	-1.63	0.00140737	0.00763398	0.00473334	2957	tags=41%, list=25%, signal=31%	ANG/SAV1/HBEGF/MEF2D/EPHB1/FRS2/MAPK14/PTAFR/AKT1/PTGIR/PIK3CA/JUN/SERPINF2/NOTCH1/IL6R/ARID2/NDRG2/TRIB1/XRCC6/RUNX1/EDN1/RBPJ/POLDIP2/MMP9/FOXP1/TGFB1/CTNNBIP1/CCN3/VGLL4/MAP3K5/TPM1/GNAI2/SF1/JARID2/RHOA/LDLRAP1/FOXJ2/GNA12/THBS1/MFN2/TCF7L2/PRKDC/CDKN1A/SMPD3/PIM1
CC	GO:0070382	exocytic vesicle	129	-0.44	-1.58	0.00141848	0.00767329	0.00475772	1657	tags=26%, list=14%, signal=22%	NDEL1/STX6/DTNBP1/TPRG1L/SYNGR1/DNAJC5/TRAPPC4/STX4/VTI1B/RAB7A/NCSTN/SEPTIN1/IQSEC1/KIF1B/SEPTIN4/VTI1A/ATP6V0D1/RAB27B/RAB3IL1/CLCN5/CLTB/CALM3/RAB5B/RAB11B/ZNRF1/RAB3D/SLC6A9/BCL2L1/ROGDI/STON2/SYTL4/SNCA/LAMP1
BP	GO:1904019	epithelial cell apoptotic process	64	-0.53	-1.70	0.00142781	0.00771326	0.0047825	1251	tags=25%, list=11%, signal=22%	MAP3K5/ANO6/PIAS4/BAD/PPARA/ABL1/E2F1/ARRB2/HIPK1/BCL2L1/GSN/THBS1/PIK3CG/TCF7L2/E2F2/FOXO3
BP	GO:0030308	negative regulation of cell growth	113	-0.46	-1.63	0.00143351	0.00773359	0.0047951	2593	tags=39%, list=22%, signal=31%	ULK1/SMARCA2/DIP2B/MUL1/CDKN2D/FLCN/G6PD/BTG1/CDK5/HYAL2/DCUN1D3/CCAR2/SERTAD3/EI24/SMAD3/ACVR1B/KIAA0319/SEMA4D/CDA/GSK3A/TGFB1/SMARCA4/CCN3/VGLL4/BCL11A/SESN2/FHL1/YY1/HSPA1A/PML/GREM1/SFRP2/PPARA/SEMA4A/NRP1/FBP1/PTPRJ/STK11/ADIPOR1/PPP1R9B/ENO1/CDKN1A/DNAJB2/DAB2
BP	GO:1902895	positive regulation of miRNA transcription	32	-0.64	-1.80	0.00143996	0.00773869	0.00479827	2479	tags=53%, list=21%, signal=42%	JUN/EGR1/SPI1/SREBF2/NR3C1/NR1H2/SMAD3/KLF4/SREBF1/TGFB1/SMARCA4/ETS1/MRTFA/STAT3/FOS/RELA/FOXO3
BP	GO:0006875	cellular metal ion homeostasis	338	-0.36	-1.43	0.00144719	0.00775477	0.00480824	2777	tags=32%, list=24%, signal=25%	SLC40A1/CCRL2/LETM1/CCDC115/COX19/ATP13A2/PDPK1/CMKLR1/ATP7A/PKD1/ATP2A3/CORO1A/SELENOK/PTGIR/SWAP70/SLC46A1/DISC1/KNG1/TRPM2/BAK1/ATP1A1/CLN3/CYBRD1/TRPC3/ATP2A2/DLG4/SLC24A4/CCR1/CDK5/FYN/SLC25A23/ATP1B1/P2RX4/PTPN6/ATP13A3/ITPR2/NCOA4/LPAR4/SMAD3/MICU1/PTPRC/EDN1/PIK3CB/STOML2/SLC41A1/FRRS1/KCNJ2/CXCR2/BCAP31/PRKACA/CXCR1/ATP6AP1/SELENON/FPR3/CHERP/LYN/NEDD4L/SPPL3/PTK2B/GNA15/MT2A/ARF1/SLC9A1/SLC22A17/SLC39A4/PML/RASA3/CALR/ATP6V1A/ATP13A1/FIS1/ATP1B2/JAK3/ATP6V0D1/ABL1/LCN2/FKBP1A/STIM1/ANXA6/BMP6/XK/GALR1/CALM3/FKBP1B/MCOLN1/SLC6A9/SLC24A3/FTH1/TTC7A/GP1BA/PRKCB/GP9/HRH4/PIK3CG/UBASH3B/ATP2B4/SNCA/ANKRD9/P2RX1/GATA1/GRINA/FTL/ALAS2/HTT/KEL/TGM2/DIAPH1/TBXA2R
BP	GO:0051651	maintenance of location in cell	149	-0.43	-1.56	0.00145891	0.00780703	0.00484064	1283	tags=23%, list=11%, signal=21%	SELENON/VPS13D/SUN2/CHERP/LYN/GPAA1/PINK1/PTK2B/KDELR1/PML/RASA3/CALR/TAF8/RANGAP1/ABL1/LCN2/FKBP1A/CIZ1/ANXA6/GAA/CALM3/HK1/FKBP1B/ARHGAP21/MCOLN1/FTH1/GSN/GP1BA/GP9/UBASH3B/SNCA/SCIN/FTL/HTT/DIAPH1
CC	GO:0098562	cytoplasmic side of membrane	142	-0.44	-1.59	0.00146214	0.0078138	0.00484484	2642	tags=39%, list=23%, signal=31%	FGR/TRAF2/JUP/RASAL3/GNAQ/GNB2/AKT1/GNAO1/DLG4/DIABLO/G6PD/FYN/CNR2/GNA11/TNK2/FES/PKP4/PTPRC/TIAM1/GNAZ/CHMP4B/SAMD10/EZR/GNAS/MYZAP/TRAF6/ESYT1/ALOX15/HIP1/LYN/SPPL3/RAC1/PTK2B/GNA15/GNB1/SRC/RASA3/GNAI2/SLA/PPP3CA/LITAF/MAP2K2/SYK/FKBP1A/RHOA/LDLRAP1/CDIP1/GNA12/SLC4A1/PPP1R9B/PTPN1/IQGAP1/SPTA1/SPTB/MYH9/ANK1

BP	GO:0006954	inflammatory response	506	-0.33	-1.38	0.00146895	0.00783066	0.00485529	2592	tags=28%, list=22%, signal=23%	SIGLEC1/MAPK14/PTAFR/RHBDF2/MACIR/AKT1/FOXP3/PTGIR/SYT11/JUN/SERPINF2/MAPK7/KNG1/NOTCH1/CYBB/NR1D1/NCF1/ZFP36/HDAC4/HDAC5/EZH2/KPNA6/CCR1/IL6R/HLA-DRB1/ABCD1/HYAL2/ACER3/CD81/FYN/IL1B/CNR2/RIPK1/CSF1R/PPBP/GGT1/IL5RA/TLR6/PGLYRP1/TIMP1/GSTP1/PDE2A/ITGAM/NRROS/SPATA2/SMAD3/PTPRC/DROSHA/RBPJ/CD14/MMP9/NCR3/KLF4/ABCC1/CXCR2/SIGIRR/ADGRE5/IKBKG/FOXP1/HYAL3/NFATC3/NLRX1/NAPEPLD/NR1H3/PYCARD/AKNA/TGFB1/CTNNBIP1/GIT1/LILRA5/TNFRSF1B/ETS1/IL1RN/SPHK1/CCN3/CD180/CASP5/KDM6B/NDST1/FPR3/CHID1/PLD4/ALOX15/ZYX/KARS1/ANO6/LYN/PIK3AP1/IL17RA/RAC1/JAM3/PLD3/SRC/CERS6/PIK3CD/PPARA/STAT3/BCR/SYK/XIAP/FOS/TNFSF4/RELA/NFKB1/C1QA/BMP6/SIGLEC10/ITGAL/SCYL1/MFHAS1/NOTCH2/LGALS9/HK1/MAPKAPK2/CDK19/FASN/CAMK1D/PRDX5/KRT1/PF4V1/LRP1/THBS1/HRH4/RPS6KA4/PIK3CG/GRN/SHARPIN/SELP/SNCA/CXCL5/P2RX1/BRD4/ACKR1/PF4/TNIP1/PTGS1/GPX1/FURIN/TOLLIP/EIF2AK1/MGLL/CLU/MAP2K3/TBXA2R
BP	GO:1903035	negative regulation of response to wounding	46	-0.58	-1.79	0.00148281	0.00789245	0.0048936	2466	tags=46%, list=21%, signal=36%	SERPINF2/KNG1/EPHA4/PROS1/CERS2/SMAD3/EDN1/KIAA0319/EPPK1/GIT1/SERPING1/CD9/CEACAM1/CLASP1/SIGLEC10/KRT1/GP1BA/ALOX12/THBS1/UBASH3B/PDGFA
BP	GO:0043090	amino acid import	32	-0.64	-1.80	0.00149108	0.00792585	0.00491431	1983	tags=44%, list=17%, signal=36%	SLC43A2/SLC7A1/TSPO2/ARL6IP1/SLC25A38/SLC7A8/SLC1A5/SLC1A4/SLC6A20/SLC7A5/SLC3A2/SLC6A9/SLC43A1/PER2
BP	GO:0032103	positive regulation of response to external stimulus	295	-0.34	-1.36	0.00942776	0.03435819	0.02130331	2298	tags=26%, list=20%, signal=22%	USP27X/CCR1/IKBKE/IL6R/PRKD2/HYAL2/CD81/FYN/IL1B/SPI1/P2RX4/RIPK1/CSF1R/SCIMP/PDE2A/XRCC6/SMAD3/EDN1/RIPOR2/CREB3/CD74/NDEL1/NCR3/ABCC1/DDX60/ANKRD17/NAPEPLD/PYCARD/TGFB1/LILRA5/ETS1/RBM14/CD180/STX4/TRAF6/OXSR1/KARS1/ANO6/LYN/MAVS/SASH1/IL17RA/PUM1/RAC1/PTK2B/CGAS/CREBBP/STK24/SRC/CALR/VAV1/NRP1/SYK/PAK1/TNFSF4/OPTN/BMP6/LGALS9/FLNA/FKBP1B/RAC2/HSPB1/CDK19/CAMK1D/RIOK3/THBS1/ZNFX1/PIK3CG/GRN/SNCA/PRKDC/LAMP1/MAPK3/AKIRIN2/TNIP1/PAK2/MED1/WNK1
BP	GO:0097530	granulocyte migration	94	-0.43	-1.47	0.00947249	0.03445801	0.02136521	2153	tags=32%, list=18%, signal=26%	CD99L2/IL1B/CSF1R/PPBP/LGALS3/PDE4B/EDN1/RIPOR2/CD74/CXCR2/CXCR1/PREX1/WDR1/IL17RA/RAC1/JAM3/PIK3CD/VAV1/SYK/RAC2/BSG/CAMK1D/MPP1/PF4V1/THBS1/PIK3CG/CXCL5/RHOG/MAPK3/PF4
BP	GO:1900542	regulation of purine nucleotide metabolic process	56	-0.51	-1.60	0.00961128	0.03473911	0.0215395	2649	tags=41%, list=23%, signal=32%	ZBTB20/ARL2/HDAC4/FLCN/ENTPD5/PGAM1/ZBTB7A/EIF6/PDK3/GIT1/CBFA2T3/JMJD8/PARP1/PINK1/ARNT/PPARA/STAT3/PDK2/VCP/FBP1/SLC4A1/SNCA/ENO1
CC	GO:0019897	extrinsic component of plasma membrane	107	-0.42	-1.48	0.00961966	0.03473911	0.0215395	2642	tags=37%, list=23%, signal=29%	FGR/JUP/GNAQ/GNB2/AKT1/GNAO1/DLG4/FYN/CRKL/CNR2/GNA11/CTNND1/TNK2/FES/NUMA1/TIAM1/STOML2/GNAZ/ST14/GNAS/MYZAP/ESYT1/ALOX15/HIP1/LYN/RAC1/PTK2B/GNA15/DCHS1/GNB1/SRC/GNAI2/SLA/PPP3CA/CTNNA1/SYK/RHOA/GNA12/IQGAP1/RNF10
BP	GO:1902105	regulation of leukocyte differentiation	210	-0.37	-1.41	0.00968954	0.03495972	0.02167628	2294	tags=29%, list=20%, signal=23%	CCR1/PRDM1/HLA-DRB1/IRF1/MITF/RIPK1/PTPN6/ZBTB16/PGLYRP1/PPARGC1B/ZFP36L2/TRIB1/FES/XRCC6/SOX13/TFE3/RUNX1/PTPRC/DROSHA/INPP5D/CD74/SOX4/FOXP1/TOB2/TGFB1/CTNNBIP1/CDK6/TBX21/TMEM131L/ACIN1/IL4I1/TRAF6/LYN/RARA/BAD/HCLS1/CEACAM1/RUNX3/ZMIZ1/SYK/JAK3/ABL1/MAFB/FOS/TNFSF4/LTF/RHOA/NOTCH2/LGALS9/GPR137/UBASH3B/PRKDC/SOX12/FBXO7/HLX/PF4/FOXO3/TESC/TAL1/CUL4A

BP	GO:0001894	tissue homeostasis	146	-0.40	-1.47	0.00972307	0.03501713	0.02171188	1321	tags=21%, list=11%, signal=19%	SNX10/TRAF6/MFSD2A/EPAS1/RAB7A/MTF1/MBP/JAM3/PTK2B/TRIM32/SRC/LTBP3/LRRK1/ATP1B2/ACTB/SYK/TJP2/LTF/SLC2A1/VCL/GPR137/RAB3D/HSPB1/STK11/KRT1/CLDN5/CSK/UBASH3B/GATA1/ITGB3/ESAM
BP	GO:0034612	response to tumor necrosis factor	163	-0.38	-1.41	0.00993748	0.03563044	0.02209215	2265	tags=30%, list=19%, signal=25%	SMPD4/HES1/HYAL2/NUB1/CPNE1/RIPK1/GSTP1/ZFP36L2/SPATA2/CXCL16/EDN1/LIMS1/CD14/SMPD1/HYAL3/PYCARD/CACTIN/TNFRSF1B/SPHK1/TRAF6/MAP3K5/ILK/MBP/PTK2B/HSPA1A/PIAS4/KLF2/MAP2K7/TRIM32/EIF5A/LAPTM5/ARHGEF2/SYK/XIAP/RELA/NFKB1/HIPK1/INPP5K/CDIP1/PRPF8/PLVAP/THBS1/SHARPIN/H2BC11/MAPK3/SMPD3/ACTN4/YBX3/FOXO3
CC	GO:0009925	basal plasma membrane	146	-0.40	-1.46	0.00998063	0.03575043	0.02216655	1775	tags=24%, list=15%, signal=21%	SLC29A2/SLC41A1/SLC19A1/EPPK1/ABCC1/HSP90AB1/ST14/EZR/STX4/SLC27A1/AQP10/SLC5A6/AQP1/SLC9A1/CEACAM1/ABCC3/SLC7A8/SLC1A5/SLC14A1/ABCC4/ARRB2/SLC2A1/SLC7A5/LDLRAP1/SLC3A2/BSG/SLC6A9/SLC4A1/LRP1/EPB41/MSN/ATP2B4/SLC29A1/WASF2/ANK1
BP	GO:0000422	autophagy of mitochondrion	82	-0.45	-1.54	0.01004619	0.03592056	0.02227204	1840	tags=33%, list=16%, signal=28%	CDC37/MAP1LC3B/LRBA/FZD5/SREBF1/GSK3A/ATG2A/VPS13D/PINK1/MARK2/FIS1/GABARAP/VDAC1/USP36/OPTN/ATG4D/WIPI2/ATG7/HUWE1/SQSTM1/MFN2/ATG13/BNIP3L/FBXO7/WDR45/CTTN/HTT
BP	GO:0061726	mitochondrion disassembly	82	-0.45	-1.54	0.01004619	0.03592056	0.02227204	1840	tags=33%, list=16%, signal=28%	CDC37/MAP1LC3B/LRBA/FZD5/SREBF1/GSK3A/ATG2A/VPS13D/PINK1/MARK2/FIS1/GABARAP/VDAC1/USP36/OPTN/ATG4D/WIPI2/ATG7/HUWE1/SQSTM1/MFN2/ATG13/BNIP3L/FBXO7/WDR45/CTTN/HTT
CC	GO:0097060	synaptic membrane	156	-0.38	-1.41	0.01008122	0.0360134	0.0223296	2291	tags=27%, list=20%, signal=22%	RPH3A/EPHA4/PI4K2A/ABHD17C/FXYD6/CRKL/SNTA1/PDE2A/SRGAP2/SYNE1/SYNJ1/LRFN1/ENO2/CANX/DTNBP1/BAIAP2/PRR7/TRAPPC4/DENND1A/STX4/HIP1/ITSN1/HIP1R/EXOC3/NCSTN/ADGRL1/CNIH3/NRP1/ARRB2/CLTB/DNM2/SLC6A9/DNM3/CLSTN1/PPP1R9B/ATP2B4/LRFN3/P2RX1/RNF10/NRGN/SHISA7/ITGB3
BP	GO:0030851	granulocyte differentiation	29	-0.59	-1.63	0.01017523	0.03625526	0.02247957	2107	tags=45%, list=18%, signal=37%	SPI1/TRIB1/RUNX1/INPP5D/CBFA2T3/RARA/HCLS1/CEACAM1/FASN/GATA1/TESC/TAL1/CUL4A
BP	GO:1903169	regulation of calcium ion transmembrane transport	84	-0.45	-1.54	0.01050784	0.03726955	0.02310846	1539	tags=31%, list=13%, signal=27%	G6PD/CDK5/FYN/ATP1B1/PTPN6/PDE4B/EHD3/PRKACA/SELENON/LYN/PTK2B/SLC9A1/CACNG6/VDAC1/ABL1/FKBP1A/STIM1/CALM3/FKBP1B/GJC2/PIK3CG/UBASH3B/SNCA/P2RX1/HTT/DIAPH1
CC	GO:0005775	vacuolar lumen	128	-0.39	-1.41	0.01071828	0.03784712	0.02346658	2011	tags=27%, list=17%, signal=22%	RNASE3/TCN2/CTSA/TPP1/ADA2/CD74/CTSF/SMPD1/TRAPPC1/SDC3/PYCARD/CHID1/GLB1/IST1/PLD3/DYNC1H1/PSAP/ARHGAP45/CAP1/VCP/PYGB/TUBB4B/GAA/PA2G4/CYB5R3/SDC4/GNS/GRN/PRDX6/CTSD/TUBB/FTL/CTSB/TOLLIP
CC	GO:0014704	intercalated disc	28	-0.58	-1.61	0.01074125	0.03787101	0.02348139	1587	tags=36%, list=14%, signal=31%	KCNJ2/RANGRF/SLC9A1/CTNNA1/YWHAH/PAK1/SLC2A1/VCL/ACTN1/SCN1B
BP	GO:0051209	release of sequestered calcium ion into cytosol	71	-0.45	-1.50	0.01092701	0.03837967	0.02379678	1283	tags=24%, list=11%, signal=21%	PRKACA/SELENON/CHERP/LYN/PTK2B/RASA3/ABL1/FKBP1A/CALM3/FKBP1B/MCOLN1/GP1BA/GP9/UBASH3B/SNCA/HTT/DIAPH1
BP	GO:0031644	regulation of nervous system process	62	-0.48	-1.53	0.01097024	0.03849751	0.02386984	1756	tags=39%, list=15%, signal=33%	BEGAIN/CCL3/DAG1/S1PR2/NRXN1/DVL1/PTAFR/DLG4/EIF4A3/ROCK2/EDN1/NPTXR/BAIAP2/TNFRSF1B/CCN3/PTK2B/SLC9A1/SRC/DLGAP4/PARD3/RNF10/WASF3/SHISA7/MGLL
BP	GO:0000768	syncytium formation by plasma membrane fusion	29	-0.59	-1.63	0.01099469	0.03851534	0.02388089	2658	tags=45%, list=23%, signal=35%	SBNO2/MAPK14/CD81/PLEKHO1/SH3PXD2A/RIPOR2/DYRK1B/CD9/MYOF/NFATC2/CAMK1/EHD1/MYH9

BP	GO:0140253	cell-cell fusion	29	-0.59	-1.63	0.01099469	0.03851534	0.02388089	2658	tags=45%, list=23%, signal=35%	SBNO2/MAPK14/CD81/PLEKHO1/SH3PXD2A/RIPOR2/DYRK1B/CD9/MYOF/NFATC2/CAMK1/EHD1/MYH9
BP	GO:0017157	regulation of exocytosis	131	-0.41	-1.47	0.01110695	0.03881207	0.02406488	3089	tags=42%, list=26%, signal=31%	NCKAP1L/RAB3GAP1/F2RL1/VPS18/DVL1/ITGB2/PRAM1/GAB2/SDCBP/ATP13A2/PDPK1/FGR/PTAFR/RAB8A/FBXL20/SYT11/NSF/NOTCH1/ATP2A2/TSG101/CDK5/SPI1/VPS4B/FES/ITGAM/HGS/DTNBP1/HYAL3/GSK3B/ATP9A/GIT1/STX4/LYN/RAB7A/SEPTIN1/CEACAM1/GNAI2/SEPTIN4/BCR/SYK/CLASP1/RAB27B/CALM3/LGALS9/RAB3D/RAC2/SDC4/SYTL4/PRKCB/RAB2B/SNCA/LAMP1/P2RX1/GATA1/SMPD3
BP	GO:0060078	regulation of postsynaptic membrane potential	46	-0.52	-1.59	0.01121357	0.0391098	0.02424948	3147	tags=59%, list=27%, signal=43%	CHRNA10/LRRK2/P2RX7/MTMR2/APP/SEZ6/S1PR2/RAB3GAP1/NRXN1/DVL1/GABBR1/AKT1/DLG4/EIF4A3/MECP2/CDK5/P2RX4/GSK3B/BAIAP2/GSK3A/NPFF/PTK2B/PPP3CA/ARRB2/SNCA/P2RX1/SLC29A1
BP	GO:0045124	regulation of bone resorption	25	-0.60	-1.62	0.01123945	0.03916568	0.02428413	2059	tags=40%, list=18%, signal=33%	CSF1R/PPARGC1B/INPP5D/SRC/LTBP3/SYK/GPR137/CSK/UBASH3B/ITGB3
CC	GO:0030427	site of polarized growth	121	-0.41	-1.47	0.01165144	0.0404595	0.02508635	2251	tags=31%, list=19%, signal=25%	LMTK2/EPHA4/PI4K2A/CDK5/FXR2/EXOC4/FEZ1/CTNND1/BASP1/NDRG2/FRY/ARPC5/ENO2/NDEL1/DTNBP1/CYFIP1/COPA/HSP90AB1/KATNB1/CDKL5/PINK1/PTK2B/EXOC3/HCLS1/ADGRL1/TAOK2/AMFR/USP9X/ABL1/CALM3/DNM2/FLNA/PPP1R9B/IQGAP1/SNCA/CBL/CTTN
BP	GO:0010591	regulation of lamellipodium assembly	32	-0.57	-1.61	0.01169117	0.04056203	0.02514992	1464	tags=25%, list=12%, signal=22%	CYFIP1/RAC1/DNM2/WAS/RAC2/CAPZB/WASF2/DMTN
BP	GO:1990868	response to chemokine	51	-0.51	-1.57	0.01173479	0.04064246	0.02519979	2769	tags=37%, list=24%, signal=29%	CCRL2/CMKLR1/SH2B3/DOCK8/CCR1/PPBP/EDN1/RIPOR2/CXCR2/CXCR1/OXSR1/PTK2B/MPL/RHOA/PF4V1/CXCL5/ACKR1/PF4/WNK1
BP	GO:1990869	cellular response to chemokine	51	-0.51	-1.57	0.01173479	0.04064246	0.02519979	2769	tags=37%, list=24%, signal=29%	CCRL2/CMKLR1/SH2B3/DOCK8/CCR1/PPBP/EDN1/RIPOR2/CXCR2/CXCR1/OXSR1/PTK2B/MPL/RHOA/PF4V1/CXCL5/ACKR1/PF4/WNK1
BP	GO:0038202	TORC1 signaling	45	-0.50	-1.53	0.01181416	0.04088174	0.02534815	1417	tags=27%, list=12%, signal=24%	KLHL22/SESN2/PIH1D1/RRAGC/OTUD5/SESN3/GPR137/STK11/SIK3/CASTOR2/NPRL3/LARP1
BP	GO:0048821	erythrocyte development	32	-0.57	-1.61	0.01182953	0.0408993	0.02535904	362	tags=34%, list=3%, signal=33%	KLF2/BPGM/SLC4A1/EPB42/FAM210B/GATA1/ALAS2/MED1/TAL1/TRIM58/DMTN
BP	GO:0048017	inositol lipid-mediated signaling	115	-0.41	-1.46	0.01193911	0.04124233	0.02557173	3008	tags=34%, list=26%, signal=25%	KIT/F2RL1/ZFP36L1/FLT1/GAB2/TSC2/PLD1/PTPN13/FGR/PLD2/PTAFR/AKT1/PIK3CA/NCF1/PLCH2/CSF1R/PTPN6/ENTPD5/EDN1/PI4KB/PIK3CB/ADAP2/SEMA4D/PRR5L/EZR/PIK3AP1/PITPNM2/HCLS1/SRC/CEACAM1/PIK3CD/PDGFC/PIP5K1B/PLCB3/PIK3CG/PRR5/SELP/CBL/PDGFA
BP	GO:0060326	cell chemotaxis	188	-0.36	-1.37	0.0122578	0.04212362	0.02611816	2294	tags=28%, list=20%, signal=23%	CCR1/IL6R/PRKD2/CRKL/IL1B/SPI1/P2RX4/CNR2/CSF1R/PPBP/LGALS3/CXCL16/HSD3B7/PDE4B/EDN1/RIPOR2/CREB3/CD74/ABCC1/CXCR2/CXCR1/PREX1/CCN3/OXSR1/ANO6/LYN/IL17RA/RAC1/JAM3/PTK2B/GREM1/CALR/PIK3CD/VAV1/RIN3/NRP1/SYK/ARRB2/LGALS9/RAC2/BSG/HSPB1/CAMK1D/MPP1/PLEC/PF4V1/THBS1/PIK3CG/CXCL5/RHOG/MAPK3/PF4/WNK1
MF	GO:1902936	phosphatidylinositol bisphosphate binding	70	-0.47	-1.53	0.01227042	0.04213056	0.02612247	2291	tags=36%, list=20%, signal=29%	OBSCN/SDCBP/ATP13A2/KIF16B/RPH3A/PFN1/SH3PXD2A/SVIL/ADAP2/KCNJ2/ASAP1/ALOX15/HIP1/HIP1R/CGAS/SLC9A1/SNX3/FLII/PARD3/PLEK2/WIPI2/LDLRAP1/GSN/SCIN/WDR45

BP	GO:0003158	endothelium development	74	-0.45	-1.49	0.01247417	0.04275629	0.02651044	2534	tags=38%, list=22%, signal=30%	FOXP3/NOTCH1/EDF1/BTG1/PRKD2/ICAM1/IL1B/ROCK2/MYADM/PDE2A/RBPJ/FOXP1/ACVR2B/EZR/KDM6B/CEACAM1/NRP1/TJP2/RAPGEF1/BMP6/RHOA/VCL/FOXJ2/FASN/CLDN5/MSN/GPX1/ADD1
BP	GO:1903828	negative regulation of protein localization	145	-0.39	-1.42	0.0124923	0.04278156	0.0265261	2615	tags=35%, list=22%, signal=28%	RAB11FIP5/PKIG/RHBDF2/PIM3/PTPN11/TMBIM1/FLCN/SNX33/CHP1/CDK5/ABI3/ABHD17C/IL1B/SPI1/ROCK2/LZTS2/RIPOR2/MAGED1/OTUD7B/SNX12/NSFL1C/SREBF1/GSK3B/NR1H3/TGFB1/FRMD4A/CLTC/CCN3/UCP2/NEDD4L/RAB11FIP1/LEPROT/PFKL/ILRUN/SNX3/PPP3CA/CTNNA1/RANGAP1/MAP1A/WWP2/MFHAS1/SUFU/FKBP1B/INPP5K/BCL2L1/SYTL4/CSK/YOD1/AP2M1/DMTN/DAB2
BP	GO:0031330	negative regulation of cellular catabolic process	203	-0.36	-1.35	0.01261734	0.04309841	0.02672256	2149	tags=38%, list=18%, signal=32%	SDCBP/TSC2/ATP13A2/FHIT/TRAF2/MAPK14/SORL1/AKT1/UBQLN4/PIK3CA/ACACB/ZFP36/WAC/TSPO/IKBKE/EPHA4/CSDE1/CSNK2A1/FEZ1/PABPC1/LARP4B/SYNCRIP/TIMP1/CCAR2/CTSA/FOXK1/F8A1/SMARCC1/TAF1/POLDIP2/PHF23/CHMP4B/EHMT2/KDM4A/METTL16/FOXK2/TIMP2/GSK3A/DAP/HSP90AB1/NBAS/KLHL22/ALAD/MFSD2A/PINK1/PML/EIF4G2/RUBCN/STAT3/USP19/MAP1A/HERC1/RGP1/CPTP/USP36/ELAVL1/N4BP1/E2F1/GOLGA2/SUFU/MAPKAPK2/USP7/ATG7/HIPK2/TENT4B/PSMF1/YBX1/PIK3CG/ATP2B4/SNCA/EIF4G1/RYBP/YBX3/FURIN/TENT5C/LARP1/BAG6
MF	GO:0030374	nuclear receptor coactivator activity	51	-0.50	-1.57	0.0127069	0.04332986	0.02686607	2350	tags=49%, list=20%, signal=39%	TSG101/CNOT9/PKN1/TACC1/PPARGC1B/CCAR2/HELZ2/TRERF1/NCOA1/ETS1/RBM14/NCOA2/NCOA6/TADA3/NCOA3/ZMIZ1/ELK1/DCAF6/USP22/PRKCB/HMGA1/ACTN4/ACTN1/MED1/CARM1
BP	GO:0044262	cellular carbohydrate metabolic process	197	-0.36	-1.38	0.01300387	0.04422874	0.02742341	2571	tags=30%, list=22%, signal=24%	PTAFR/AKT1/POMC/POMK/ACACB/NR1D1/HDAC4/FOXO1/PGD/GANC/MECP2/STK40/PFKFB4/PGAM1/NLN/FOXK1/SYNJ1/PHKG2/GALK1/B4GALT5/PDK3/WDR5/GSK3B/SORD/FOXK2/FAM3A/GSK3A/GIT1/BCKDK/CBFA2T3/NCOA2/NDST1/PLEK/B3GNT8/GLB1/SESN2/PTK2B/IGF2/BAD/SRC/PPARA/PPP1CA/STAT3/PDK2/KAT2B/DDB1/PYGB/FBP1/IDH2/GAA/HK1/INPP5K/ITPK1/USP7/STK11/ADIPOR1/SLC4A1/AKT2/PER2/SNCA
BP	GO:0060079	excitatory postsynaptic potential	44	-0.52	-1.58	0.01311557	0.04451098	0.02759841	3847	tags=59%, list=33%, signal=40%	CHRNA10/LRRK2/P2RX7/MTMR2/APP/SEZ6/S1PR2/RAB3GAP1/NRXN1/DVL1/AKT1/DLG4/EIF4A3/MECP2/CDK5/P2RX4/GSK3B/BAIAP2/GSK3A/NPFF/PTK2B/PPP3CA/ARRB2/SNCA/P2RX1/SLC29A1
BP	GO:0099601	regulation of neurotransmitter receptor activity	29	-0.58	-1.61	0.01317995	0.04452292	0.02760582	2554	tags=48%, list=22%, signal=38%	MINK1/DLG4/NETO2/LYNX1/NPTXR/DAPK1/PINK1/PTK2B/SRC/DLGAP4/CNIH3/SLC6A9/CAPN1/SHISA7
BP	GO:0060048	cardiac muscle contraction	71	-0.45	-1.48	0.01317861	0.04452292	0.02760582	2606	tags=39%, list=22%, signal=31%	JUP/SMAD5/KCNH2/PIK3CA/ATP1A1/ATP2A2/ATP1B1/P2RX4/SNTA1/PDE4B/GRK2/KCNJ2/EHD3/PRKACA/RANGRF/TPM1/NEDD4L/SLC9A1/GAA/PDE5A/CALM3/FLNA/FKBP1B/MYL4/GSN/PIK3CG/MAP2K3/SCN1B
BP	GO:0006941	striated muscle contraction	87	-0.43	-1.47	0.01330661	0.04487455	0.02782383	2856	tags=40%, list=24%, signal=31%	SLC8A1/MYH3/TTN/JUP/SMAD5/KCNH2/PIK3CA/ATP1A1/ATP2A2/ARHGEF11/SYNM/ATP1B1/P2RX4/SNTA1/RCSD1/PDE4B/GRK2/KCNJ2/EHD3/PRKACA/RANGRF/ALDOA/TPM1/NEDD4L/SLC9A1/GAA/PDE5A/CALM3/FLNA/FKBP1B/MYL4/GSN/PIK3CG/MAP2K3/SCN1B
BP	GO:0046850	regulation of bone remodeling	28	-0.58	-1.59	0.01334101	0.04491435	0.02784851	2059	tags=39%, list=18%, signal=32%	CSF1R/PPARGC1B/INPP5D/SRC/GREM1/LTBP3/SYK/GPR137/CSK/UBASH3B/ITGB3

BP	GO:0050804	modulation of chemical synaptic transmission	219	-0.35	-1.35	0.0133401	0.04491435	0.02784851	1756	tags=28%, list=15%, signal=24%	RAB8A/FBXL20/SYT11/DISC1/ADNP/CLN3/ATP2A2/DLG4/EIF4A3/EPHA4/MECP2/CDK5/FYN/IL1B/CNR2/ZDHHC3/EDN1/NPTXR/DGKZ/PPP1R9A/DTNBP1/GSK3B/BAIAP2/PRKACA/CYFIP1/GIT1/YWHAG/TPRG1L/SYNGR1/STX4/PINK1/PTK2B/RARA/ARF1/SIPA1L1/NCSTN/SRC/DLGAP4/CA2/GNAI2/PPP3CA/MAP1A/BCR/YWHAH/ABL1/SYNGAP1/ARRB2/STAU1/CALM3/CLSTN1/GLUL/SQSTM1/PPP1R9B/PRKCB/SNCA/NEURL1/P2RX1/EIF4EBP2/NRGN/PRKAR2B/SHISA7
BP	GO:0014074	response to purine-containing compound	76	-0.45	-1.51	0.01346476	0.04521613	0.02803563	2143	tags=28%, list=18%, signal=23%	SSH1/IL1B/P2RX4/ITPR2/HSPA5/PPARGC1B/PDE2A/TAF1/SREBF1/EZR/AQP1/PTK2B/FOS/RAPGEF1/BSG/INPP5K/PIK3CG/P2RX1/GATA1/DMTN/CARM1
BP	GO:0030010	establishment of cell polarity	103	-0.42	-1.47	0.0134547	0.04521613	0.02803563	2403	tags=38%, list=20%, signal=30%	DOCK8/ARHGEF11/SNX27/HES1/MAP4/NDE1/CDK5RAP2/CRKL/FEZ1/FBF1/ROCK2/NUMA1/WEE1/FRMD4B/RIPOR2/NDEL1/NSFL1C/GSK3B/HSP90AB1/FRMD4A/AMOT/SIPA1L3/BCAS3/JAM3/PTK2B/MARK2/FAM89B/PARD3/CLASP1/MYO18A/RHOA/DOCK2/STK11/DCTN1/GSN/MSN/HTT/MYH9/SH3BP1
BP	GO:0035265	organ growth	82	-0.45	-1.51	0.01368705	0.04592379	0.0284744	2639	tags=37%, list=23%, signal=29%	BNC2/EXT1/MAPK14/AKT1/PTPN11/NOTCH1/ACACB/G6PD/ARID2/BASP1/RUNX1/EDN1/RBPJ/EHMT2/ACVR2B/GSK3A/VGLL4/YY1/RARA/IGF2/BCL2L11/PPARA/PSAP/ANXA6/JARID2/AKAP13/HLX/SMPD3/YBX3/PIM1
BP	GO:0002062	chondrocyte differentiation	57	-0.47	-1.49	0.01374096	0.04606582	0.02856247	1993	tags=32%, list=17%, signal=26%	ZBTB16/SMAD3/RUNX1/TGFB1/CCN3/TRPS1/CHST11/SOX6/GREM1/SFRP2/LTBP3/RUNX3/RFLNB/ANXA6/BMP6/GLG1/SMPD3/SCIN
CC	GO:0098797	plasma membrane protein complex	283	-0.36	-1.43	0.0015128	0.00803055	0.00497923	2726	tags=31%, list=23%, signal=24%	SDCBP/NCF2/AP2A1/GABBR1/SNTB2/TRAF2/JUP/EPS8/GNAQ/GNB2/AP2A2/KCNH2/GNAO1/CYBB/NCF1/ATP1A1/CD3E/DLG4/DIABLO/IL6R/HLA-DRB1/HLA-DQA2/ATP1B1/HLA-A/RIPK1/PTPN6/GNA11/CTNND1/TLR6/SNTA1/TNK2/ITGB7/ITGAM/SHC1/PDE4B/SYNJ1/ACVR1B/SNTB1/GNAZ/CD74/HLA-C/AP2B1/KCNJ2/KCNQ5/GRB2/HLA-DQA1/ACVR2B/IL2RB/CLTC/GNAS/CORO1C/TRAF6/AP2S1/LYN/PTK2B/GNA15/DCHS1/GNB1/NCSTN/GNAI2/HLA-DRB5/ATP1B2/CACNG6/CTNNA1/CNIH3/EPS15L1/SYK/ATP6V0D1/FKBP1A/ITGAL/APBB1IP/CLTB/SLC7A5/CALM3/SLC3A2/ITGB5/GNA12/GP1BA/LRP1/GP9/GJC2/CBL/AP2M1/SHISA7/ITGB3/ITGA2B/SCN1B
BP	GO:0006641	triglyceride metabolic process	56	-0.56	-1.75	0.00151961	0.00804796	0.00499002	1893	tags=32%, list=16%, signal=27%	MBOAT7/NR1H2/LIPE/SCARB1/CPT1A/APOBR/SREBF1/NR1H3/SLC27A1/MFSD2A/PNPLA1/TBL1XR1/PNPLA2/LPIN2/PIK3CG/GPX1/CTDNEP1/MGLL
BP	GO:0061136	regulation of proteasomal protein catabolic process	164	-0.41	-1.53	0.00152012	0.00804796	0.00499002	1917	tags=27%, list=16%, signal=23%	TRIB1/CHFR/CCAR2/KEAP1/F8A1/SMARCC1/TAF1/PSME3/BCAP31/GSK3B/PRKACA/GSK3A/HSP90AB1/CBFA2T3/DET1/ALAD/DDA1/PINK1/HSPA1A/ANKIB1/CDK2/USP19/MAP1A/BTRC/GABARAP/VCP/ARIH2/N4BP1/PITHD1/PSMC4/RAD23A/USP7/UBB/GNA12/FZR1/PSMF1/ARAF/RYBP/ZER1/GPX1/RNF40/DNAJB2/CLU/DAB2/BAG6
BP	GO:0007266	Rho protein signal transduction	94	-0.47	-1.62	0.00153076	0.00809349	0.00501825	2019	tags=31%, list=17%, signal=26%	ROCK2/PRAG1/RALBP1/RIPOR1/ARRB1/LPAR4/ARHGAP35/RIPOR2/PHACTR4/CDC42EP4/RAC1/MYO9B/CTNNAL1/NRP1/ARHGEF2/BCR/ABL1/ARHGEF18/RHOA/ARHGEF12/ARHGAP6/ARHGDIA/WAS/KANK2/ARHGAP1/GNA12/CFL1/AKAP13/RHOG
CC	GO:0031093	platelet alpha granule lumen	45	-0.57	-1.73	0.00153922	0.00811839	0.00503369	2480	tags=47%, list=21%, signal=37%	APOOL/SERPINF2/KNG1/GTPBP2/PROS1/PPBP/TIMP1/TGFB1/ALDOA/VTI1B/F13A1/SERPING1/IGF2/FERMT3/THBS1/PF4/ACTN4/ACTN1/SPARC/PDGFA/CLU
CC	GO:0005798	Golgi-associated vesicle	64	-0.53	-1.70	0.0015614	0.00821181	0.00509162	1477	tags=44%, list=13%, signal=38%	COPG1/CCDC115/SPPL2B/AP2A1/ATP7A/COPZ1/PKD1/RAB8A/KDELR2/PACSIN1/SYNRG/COPA/CLTC/SPPL3/KDELR1/TGOLN2/PACS1/COPB2/RAB14/RAB27B/SCYL1/CLTB/SPG21/SORT1/ARCN1/IGF2R/NRGN/FURIN

BP	GO:0030510	regulation of BMP signaling pathway	47	-0.56	-1.73	0.00166307	0.00868906	0.00538753	1842	tags=34%, list=16%, signal=29%	NUMA1/ENG/RBPJ/PCSK6/SKI/TWSG1/ILK/GREM1/SFRP2/XIAP/ABL1/NOTCH2/HIPK2/FKBP8/UBE2O/PPM1A
BP	GO:0089718	amino acid import across plasma membrane	25	-0.66	-1.80	0.00166616	0.00869375	0.00539044	1983	tags=52%, list=17%, signal=43%	SLC43A2/SLC7A1/TSPO2/ARL6IP1/SLC7A8/SLC1A5/SLC1A4/SLC6A20/SLC7A5/SLC3A2/SLC6A9/SLC43A1/PER2
BP	GO:1904951	positive regulation of establishment of protein localization	235	-0.38	-1.47	0.00170164	0.00883251	0.00547648	2439	tags=29%, list=21%, signal=24%	TRPM2/CNST/CLN3/UBR5/TSG101/CHP1/HLA-DRB1/PCNT/CDK5/HYAL2/CD81/FYN/IL1B/PARD6A/SREBF2/RIPOR1/NR1H2/ITGAM/UBE2J2/SMAD3/FZD5/BBC3/CCT3/BCAP31/SOX4/TM9SF4/GSK3B/PRR5L/STOM/PRKACA/GSK3A/HSP90AB1/TGFB1/FRMD4A/HDAC3/EDEM2/EZR/STX4/BCAS3/MAVS/LEPROT/PINK1/BAD/HCLS1/SRC/LRRC8A/FIS1/PTP4A3/TRIM28/USP36/ARIH2/ABLIM3/BMP6/SAE1/MYO18A/FLNA/RAC2/VPS28/SYTL4/GLUL/HUWE1/AKT2/UBE2L3/TCF7L2/CAMK1/ATG13/OAZ1/PTPN23/PPM1A
BP	GO:0070371	ERK1 and ERK2 cascade	177	-0.41	-1.54	0.00175271	0.00906213	0.00561884	2479	tags=33%, list=21%, signal=27%	JUN/SERPINF2/PTPN11/NOTCH1/YWHAZ/FLCN/CCR1/EPHA4/HLA-DRB1/ARHGAP8/PRKD2/SPRED1/ICAM1/CRKL/IL1B/CSF1R/PTPN6/SCIMP/GSTP1/NDRG2/ZFP36L2/PIN1/SHC1/ARRB1/TRAF7/PTPRC/EDN1/CD74/EIF3A/PYCARD/TGFB1/EZR/ALOX15/KARS1/VRK3/LYN/PTK2B/MAP2K7/RRAS/SRC/CEACAM1/GNAI2/NRP1/MAP2K2/PDGFC/SYK/ABL1/ARRB2/MFHAS1/NOTCH2/ACTA2/LGALS9/MTURN/CSK/PTPN1/MAPK3/TNIP1/PDGFA/DAB2
BP	GO:0034101	erythrocyte homeostasis	116	-0.45	-1.60	0.0017806	0.00918243	0.00569344	1481	tags=32%, list=13%, signal=28%	TSPO2/CDK6/ACIN1/ETS1/LYN/KLF13/EPAS1/ADAR/HSPA1A/KLF2/HCLS1/KAT7/SLC25A38/ARNT/TRIM10/BPGM/STAT3/JAK3/MAFB/AHSP/MFHAS1/HIPK2/KLF1/SLC4A1/EPB42/FAM210B/GPI/PRKDC/IKZF1/GATA1/ALAS2/FOXO3/MPIG6B/MED1/TAL1/TRIM58/DMTN
CC	GO:0008021	synaptic vesicle	119	-0.43	-1.56	0.0018005	0.00924907	0.00573476	1657	tags=26%, list=14%, signal=23%	NDEL1/STX6/DTNBP1/TPRG1L/SYNGR1/DNAJC5/TRAPPC4/STX4/VTI1B/RAB7A/NCSTN/SEPTIN1/IQSEC1/KIF1B/SEPTIN4/VTI1A/ATP6V0D1/RAB27B/CLCN5/CLTB/CALM3/RAB5B/RAB11B/ZNRF1/RAB3D/SLC6A9/BCL2L1/ROGDI/STON2/SNCA/LAMP1
BP	GO:1904064	positive regulation of cation transmembrane transport	78	-0.50	-1.69	0.00180555	0.00926306	0.00574343	1197	tags=22%, list=10%, signal=20%	ANO6/PINK1/SLC9A1/ATP1B2/ABL1/STIM1/CALM3/DNM2/FLNA/SLC6A9/GJC2/SNCA/P2RX1/ACTN4/TESC/HTT/WNK1
BP	GO:0061448	connective tissue development	129	-0.43	-1.56	0.00185234	0.00949083	0.00588466	2773	tags=36%, list=24%, signal=28%	MEF2D/MSX1/ATP7A/EXT1/PKD1/MAPK14/SMAD5/SORL1/PIK3CA/PTPN11/NOTCH1/EGR1/HOXB3/ZNF516/IL6R/HYAL2/THRA/SPI1/ZBTB16/TIMP1/SMAD3/RUNX1/EDN1/FTO/HYAL3/TGFB1/DYRK1B/CCN3/TRPS1/RARA/CHST11/TBL1XR1/SOX6/GREM1/SFRP2/LTBP3/RUNX3/RFLNB/ANXA6/BMP6/ACTA2/UBB/GLG1/MAPK3/SMPD3/SCIN
BP	GO:0031099	regeneration	99	-0.47	-1.63	0.00193183	0.00988541	0.00612931	2481	tags=37%, list=21%, signal=30%	CPQ/JUN/NOTCH1/BAK1/EZH2/TSPO/EPHA4/CD81/CERS2/LCP1/BCL9/RUNX1/KIAA0319/CPT1A/EPPK1/NDEL1/KLF4/TEC/CCN3/UCP2/ATIC/HDGFL2/PTPN12/CD9/STK24/PPP3CA/CTNNA1/FLNA/FKBP1B/IGF2R/MTPN/GRN/GATA1/CDKN1A/NINJ2/GPX1/MED1
BP	GO:0002262	myeloid cell homeostasis	139	-0.42	-1.53	0.00198119	0.01011196	0.00626978	1481	tags=30%, list=13%, signal=27%	TSPO2/CDK6/ACIN1/ETS1/LYN/KLF13/EPAS1/ADAR/JAM3/HSPA1A/KLF2/HCLS1/NCSTN/BCL2L11/KAT7/SLC25A38/ARNT/PIK3CD/TRIM10/BPGM/STAT3/JAK3/MAFB/AHSP/MPL/MFHAS1/HIPK2/KLF1/SLC4A1/EPB42/FAM210B/GPI/PRKDC/IKZF1/GATA1/ALAS2/FOXO3/MPIG6B/MED1/TAL1/TRIM58/DMTN
BP	GO:0033273	response to vitamin	43	-0.56	-1.71	0.0019852	0.01011942	0.0062744	2372	tags=37%, list=20%, signal=30%	CDKN2D/TSPO/PHEX/DCPS/FES/ALPL/ALAD/MTHFR/RARA/RXRA/MGMT/FKBP1B/KANK2/GPX1/MED1/PIM1

BP	GO:0060840	artery development	48	-0.57	-1.75	0.00199161	0.01012613	0.00627857	2444	tags=58%, list=21%, signal=46%	LDLR/HECTD1/CHD7/STRA6/SRF/NF1/CITED2/DCTN5/NOTCH1/PRDM1/HES1/ARID2/SNX17/PDE2A/EDN1/ENG/RBPJ/AP2B1/SOX4/ACVR2B/NDST1/PLXND1/ZMIZ1/NRP1/SUFU/MYLK/LRP1/NPRL3
BP	GO:0008277	regulation of G protein-coupled receptor signaling pathway	87	-0.47	-1.62	0.00198982	0.01012613	0.00627857	2401	tags=31%, list=20%, signal=25%	BICD1/RGS3/PDE6G/SLC24A4/ROCK2/ZDHHC3/ARRB1/RGS10/PDE4B/EDN1/GRK2/RGS16/GSK3A/GIT1/RPGRIP1L/PLEK/YWHAB/GNAI2/ARRB2/DNM2/PPP1R9B/PDE4A/RGS6/ATP2B4/SNCA/PTGDR2/MGLL
BP	GO:0006639	acylglycerol metabolic process	77	-0.49	-1.65	0.00200437	0.01015709	0.00629776	1951	tags=29%, list=17%, signal=24%	PLA2G15/MBOAT7/NR1H2/LIPE/SCARB1/DGKZ/CPT1A/APOBR/DGKD/SREBF1/NR1H3/SLC27A1/MFSD2A/PNPLA1/TBL1XR1/PNPLA2/LPIN2/ABHD2/PIK3CG/GPX1/CTDNEP1/MGLL
BP	GO:0048525	negative regulation of viral process	74	-0.49	-1.62	0.00200804	0.01015709	0.00629776	1228	tags=30%, list=10%, signal=27%	APOBEC3D/TRIM26/MAVS/IFITM3/TRIM32/ZC3HAV1/SRPK2/SNX3/TRIM10/TRIM28/IFITM2/APOBEC3F/N4BP1/LTF/SETDB1/INPP5K/TRIM11/GSN/TRIM14/ZNFX1/APOBEC3C/TNIP1
BP	GO:0048762	mesenchymal cell differentiation	125	-0.44	-1.58	0.00200929	0.01015709	0.00629776	2444	tags=34%, list=21%, signal=27%	NOTCH1/EFNB1/EZH2/EPHA4/HES1/USF3/SPRED1/IL1B/ROCK2/CUL7/SMAD3/EDN1/TIAM1/PHACTR4/ENG/RBPJ/TGFB1I1/SEMA4D/BCL9L/MCRIP1/GSK3B/AKNA/TGFB1/CORO1C/S100A4/GREM1/SFRP2/TRIM28/SEMA4A/NRP1/NOLC1/RFLNB/CLASP1/OTUD5/ANXA6/MAD2L2/FLNA/ADIPOR1/TCF7L2/ZNF703/MAPK3/DAB2
BP	GO:0002685	regulation of leukocyte migration	144	-0.42	-1.55	0.00201047	0.01015709	0.00629776	2571	tags=33%, list=22%, signal=26%	PTAFR/AKT1/SELENOK/SWAP70/DOCK8/CCR1/IL6R/CD81/CD99L2/ICAM1/CRKL/SPI1/P2RX4/CSF1R/LGALS3/EDN1/RIPOR2/CREB3/CD74/PYCARD/CCN3/OXSR1/ANO6/LYN/RAC1/JAM3/PTK2B/STK10/CD9/SPN/GREM1/CALR/RIN3/BCR/RHOA/LGALS9/RAC2/CAMK1D/MPP1/PLVAP/THBS1/MSN/SELP/RHOG/MAPK3/SMPD3/WNK1
BP	GO:0098655	cation transmembrane transport	450	-0.33	-1.37	0.00200613	0.01015709	0.00629776	2439	tags=25%, list=21%, signal=21%	TRPM2/ATP1A1/CLN3/TRPC3/ATP2A2/DLG4/SLC24A4/UQCRC1/SLC20A1/CHP1/G6PD/NETO2/CDK5/FYN/FXYD6/SLC25A23/ATP1B1/P2RX4/PTPN6/ATP13A3/ITPR2/MFSD12/SLC9A7/SLC33A1/SNTA1/SLC43A2/TCN2/SLC7A1/PDE4B/MICU1/PTPRC/EDN1/STOML2/SLC41A1/COX15/MMP9/KCNJ2/KCNQ5/EHD3/STOM/PRKACA/HVCN1/TSPO2/CD63/RANGRF/DAPK1/UCP2/SELENON/OXSR1/SLC39A3/FHL1/ANO6/CHERP/AQP1/LYN/NEDD4L/PINK1/PTK2B/SLC9A1/SLC39A4/SLC25A38/RASA3/ATP6V1A/ATP13A1/SLC7A8/SLC1A5/ATP1B2/CACNG6/CNIH3/SLC1A4/SLC6A20/YWHAH/VDAC1/ATP6V0D1/ABL1/FKBP1A/STIM1/ANXA6/CYB5A/CLCN5/WWP2/SLC7A5/CALM3/DNM2/FLNA/SLC3A2/FKBP1B/MCOLN1/SLC6A9/SLC24A3/MMGT1/GP1BA/GP9/GJC2/PIK3CG/SLC43A1/UBASH3B/ATP2B4/SNCA/CAPN1/P2RX1/ACTN4/SLC6A8/SLC15A3/SLC25A37/TESC/HTT/SHISA7/SLC38A5/KEL/TMEM63B/SCN1B/WNK1/DIAPH1

BP	GO:0031400	negative regulation of protein modification process	387	-0.35	-1.40	0.00205892	0.01037552	0.0064332	2581	tags=35%, list=22%, signal=28%	GNAQ/SORL1/PKIG/AKT1/PIP5KL1/AIDA/FOXP3/SWAP70/CBLB/JUN/MLLT1/BAK1/PPP1R15A/GADD45B/CDKN2D/UBR5/FLCN/TSG101/PTPA/PNKP/TSPO/CDYL/CHP1/U2AF2/G6PD/PKN1/SH3BP5L/CDK5/SPRED1/HYAL2/FYN/CDK5RAP2/CRKL/IL1B/SPI1/PARD6A/PTPN6/ROCK2/DCUN1D3/MYADM/SNTA1/HMG20B/GSTP1/MGAT5/TRIB1/LGALS3/ARRB1/FRY/PTPRC/PIK3CB/CHMP6/ENG/CRTAP/TAF1/CTBP1/WDR5/SEMA4D/CTDSP1/SOX4/SMPD1/DTNBP1/KDM4A/GSK3B/PRR5L/CRY2/PYCARD/CACTIN/TGFB1/SVBP/YWHAG/CTDSP2/PRR7/MEN1/BCOR/SKI/MVP/CORO1C/LYN/SUPT6H/ADAR/HSPA1A/GREM1/CDKN1C/CEACAM1/YWHAB/SFRP2/GNAI2/LRRK1/PIH1D1/KAT2B/ABL1/PARD3/FKBP1A/N4BP1/RELA/WARS1/DTX3L/ARRB2/JARID2/NIBAN1/PRKAR2A/MFHAS1/CALM3/MAD2L2/PPP4C/PTPRJ/FKBP1B/INPP5K/HSPB1/NF2/GNL3L/VPS28/HEXIM2/PLEC/SQSTM1/LRP1/PPP1R9B/PTPN1/PER2/FKBP8/CTDSPL/UBASH3B/ATP2B4/SNCA/EIF4G1/PRKDC/FBXO7/CDKN1A/CBL/TRIP12/PAK2/PRKAR2B/MLLT6/DMTN
BP	GO:0070372	regulation of ERK1 and ERK2 cascade	162	-0.41	-1.52	0.0020664	0.01039998	0.00644836	2479	tags=34%, list=21%, signal=27%	JUN/SERPINF2/PTPN11/NOTCH1/YWHAZ/FLCN/CCR1/EPHA4/HLA-DRB1/ARHGAP8/PRKD2/SPRED1/ICAM1/CRKL/IL1B/CSF1R/PTPN6/SCIMP/GSTP1/NDRG2/PIN1/SHC1/ARRB1/TRAF7/PTPRC/CD74/EIF3A/PYCARD/TGFB1/EZR/ALOX15/VRK3/LYN/PTK2B/MAP2K7/RRAS/SRC/CEACAM1/GNAI2/NRP1/PDGFC/SYK/ABL1/ARRB2/MFHAS1/NOTCH2/ACTA2/LGALS9/MTURN/CSK/PTPN1/MAPK3/TNIP1/PDGFA/DAB2
BP	GO:0006939	smooth muscle contraction	47	-0.56	-1.71	0.002087	0.01047716	0.00649622	560	tags=23%, list=5%, signal=22%	SPHK1/DAPK3/RHOA/ACTA2/GUCY1A1/FKBP1B/MYLK/ATP2B4/P2RX1/CTTN/TBXA2R
BP	GO:0022407	regulation of cell-cell adhesion	316	-0.36	-1.42	0.00212268	0.01064282	0.00659893	2584	tags=31%, list=22%, signal=25%	RASAL3/MAPK14/PTAFR/CORO1A/MINK1/AKT1/SELENOK/FOXP3/SWAP70/CBLB/PIK3CA/SERPINF2/MAPK7/PTPN11/NOTCH1/CD3E/EFNB1/DOCK8/HES1/HLA-DRB1/IRF1/CD81/FYN/HLA-DQA2/LGALS9C/IL1B/SPI1/HLA-A/PTPN6/ZBTB16/MYADM/SIRPG/LGALS3/SLC7A1/SOX13/RUNX1/PTPRC/RIPOR2/CD74/KLF4/SIRPB1/SOX4/HLA-DQA1/PYCARD/AKNA/TGFB1/TBX21/TMEM131L/ETS1/IL1RN/SPINT2/IL4I1/TWSG1/TRAF6/LYN/MBP/RARA/IGF2/CD9/SPN/BAD/SRC/CEACAM1/HLA-DRB5/PPARA/VAV1/RUNX3/LAPTM5/ZMIZ1/JAK1/SYK/JAK3/ABL1/TNFSF4/RELA/BMP6/GP6/PDE5A/RHOA/MAD2L2/LGALS9/FERMT3/NF2/SDC4/ALOX12/CSK/SPTA1/UBASH3B/SELP/LRFN3/ZNF703/SOX12/HLX/FOXO3/PTPN23/DMTN/WNK1
BP	GO:0051701	biological process involved in interaction with host	146	-0.43	-1.57	0.0021519	0.01074869	0.00666458	2665	tags=36%, list=23%, signal=28%	TRIM21/CR1/SIGLEC1/MID2/EXOC7/KPNA3/NCAM1/TSG101/ALYREF/HLA-DRB1/HYAL2/CD81/ICAM1/VPS4B/SELPLG/PGLYRP1/ITGB7/SCARB1/CHMP6/CD74/GRK2/VPS37B/CHMP4B/SMPD1/THOC6/IST1/TRIM26/RAB7A/GYPA/IFITM3/HSPA1A/SRC/CHMP1A/SNX3/SLC1A5/TRIM10/IFITM2/NRP1/DDB1/WWP2/LGALS9/SLC3A2/BSG/ATG7/TRIM11/BCL2L1/ITGB5/GSN/TRIM14/LAMP1/CBL/CTSB/ITGB3
BP	GO:0007623	circadian rhythm	125	-0.44	-1.58	0.00217265	0.0108387	0.00672038	2428	tags=30%, list=21%, signal=24%	NR1D1/EGR1/RAI1/EZH2/HEBP1/TOP1/MTA1/GNA11/ROCK2/CCAR2/GFPT1/MAGED1/JUND/TOP2A/SREBF1/GSK3B/ZFHX3/CRY2/NR1H3/HDAC3/NCOA2/PML/PPARA/MYCBP2/PPP1CA/BTRC/USP9X/PHLPP1/DDB1/KMT2A/KDM2A/USP7/ATG7/SIAH2/SRRD/HUWE1/PER2/PRKDC
BP	GO:0060485	mesenchyme development	142	-0.43	-1.57	0.00217625	0.01084307	0.00672309	2444	tags=32%, list=21%, signal=26%	NOTCH1/EFNB1/EZH2/EPHA4/HES1/USF3/SPRED1/EXOC4/IL1B/ROCK2/BASP1/CUL7/SMAD3/EDN1/TIAM1/PHACTR4/ENG/RBPJ/TGFB1I1/SEMA4D/BCL9L/MCRIP1/GSK3B/AKNA/TGFB1/CORO1C/DCHS1/S100A4/GREM1/SFRP2/TRIM28/SEMA4A/NRP1/NOLC1/RFLNB/CLASP1/OTUD5/ANXA6/MAD2L2/ACTA2/FLNA/ADIPOR1/TCF7L2/ZNF703/MAPK3/DAB2

MF	GO:0019887	protein kinase regulator activity	154	-0.41	-1.52	0.00221686	0.0109903	0.00681438	2568	tags=34%, list=22%, signal=27%	PKIG/TAOK3/PIK3CA/CCNJL/MOB3B/AGAP2/CDKN2D/INKA1/CHP1/MAP3K13/SH3BP5L/SPRED1/HYAL2/MADD/CCNY/MOB1B/TRIB1/CDC37/GCN1/TPX2/ITPRIP/HSP90AB1/MOB3A/TAOK1/YWHAG/IGF2/MARK2/CCND3/GREM1/CDKN1C/YWHAB/TAOK2/DAXX/MAP2K2/KAT2B/LTF/PRKAR2A/CALM3/RAC2/HSPB1/STK11/HEXIM2/CCNI/IQGAP1/SNCA/CDKN1A/PAK2/PRKAR2B/TESC/TAL1/WNK1/CNPPD1
BP	GO:0050806	positive regulation of synaptic transmission	75	-0.49	-1.64	0.00223608	0.0110443	0.00684786	2360	tags=28%, list=20%, signal=23%	DLG4/EPHA4/MECP2/CDK5/ZDHHC3/DTNBP1/GSK3B/STX4/PINK1/PTK2B/NCSTN/CA2/ABL1/ARRB2/STAU1/CLSTN1/GLUL/SQSTM1/SNCA/NRGN/SHISA7
MF	GO:0008509	anion transmembrane transporter activity	165	-0.40	-1.49	0.00226813	0.01118871	0.0069374	2245	tags=24%, list=19%, signal=20%	SERINC5/SLC25A11/SLC25A23/SLC25A1/MFSD12/SLC9A7/SLC33A1/SLC7A1/SLC25A42/SLC37A2/SLC19A1/ABCC1/CLIC5/SLC27A1/MFSD2A/SLC5A6/ANO6/SLC9A1/SLC25A38/ABCC3/LRRC8A/SLC7A8/SLC1A5/SLC1A4/SLC6A20/VDAC1/ABCG2/ABCC4/SLC2A1/CLCN5/SLC7A5/SLC3A2/SLC6A9/SLC24A3/SLC4A1/ANKH/TTYH3/SLC29A1/SLC6A8/SLC38A5
CC	GO:0005901	caveola	50	-0.55	-1.69	0.00228639	0.01126481	0.00698459	2209	tags=36%, list=19%, signal=29%	CLN3/MLC1/SPRED1/ATP1B1/LIPE/SCARB1/NEU3/RANGRF/CORO1C/SRC/CTNNA1/MYOF/SLC2A1/CAVIN2/PLVAP/ATP2B4/MAPK3/CBL
BP	GO:0060249	anatomical structure homeostasis	174	-0.41	-1.54	0.00234605	0.01154444	0.00715797	1452	tags=22%, list=12%, signal=20%	ACTG1/TAOK1/SNX10/TRAF6/ALDOA/MFSD2A/EPAS1/RAB7A/MTF1/MBP/JAM3/PTK2B/TRIM32/SRC/LTBP3/LRRK1/ATP1B2/ACTB/MAP1A/SYK/TJP2/LTF/SLC2A1/GAA/VCL/GPR137/RAB3D/HSPB1/STK11/KRT1/DCTN1/CLDN5/CSK/UBASH3B/P2RX1/GATA1/DNAJB2/ITGB3/ESAM
BP	GO:0050673	epithelial cell proliferation	200	-0.39	-1.48	0.00239864	0.0117886	0.00730936	1816	tags=22%, list=15%, signal=19%	SMAD3/SCARB1/PIK3CB/MAGED1/RAP1GAP/EPPK1/RPS6KA1/TGFB1/CDK6/CCN3/MEN1/BCL11B/IGF2/BAD/NCSTN/CDKN1C/ARNT/CEACAM1/PIK3CD/SFRP2/RUNX3/IFT52/STAT3/NRP1/BTRC/LRG1/BMP6/SIDT2/NOTCH2/STAT5A/STK11/GLUL/WDR13/THBS1/TCF7L2/SP1/GRN/PRKDC/ZNF703/GPX1/MED1/ITGB3/SPARC/DAB2
BP	GO:1903825	organic acid transmembrane transport	95	-0.46	-1.60	0.00242896	0.01189233	0.00737367	2638	tags=36%, list=22%, signal=28%	SLC16A1/AKT1/SLC46A1/CLN3/SLC7A7/SLC25A11/ABCD1/SLC25A1/MFSD12/SLC43A2/SLC7A1/CPT1A/SLC19A1/ABCC1/TSPO2/SLC27A1/ARL6IP1/SLC5A6/SLC25A38/LRRC8A/SLC7A8/SLC1A5/SLC1A4/SLC6A20/SLC2A1/SLC7A5/SLC3A2/SLC6A9/SLC38A10/THBS1/AKT2/SLC43A1/PER2/SLC38A5
BP	GO:1905039	carboxylic acid transmembrane transport	95	-0.46	-1.60	0.00242896	0.01189233	0.00737367	2638	tags=36%, list=22%, signal=28%	SLC16A1/AKT1/SLC46A1/CLN3/SLC7A7/SLC25A11/ABCD1/SLC25A1/MFSD12/SLC43A2/SLC7A1/CPT1A/SLC19A1/ABCC1/TSPO2/SLC27A1/ARL6IP1/SLC5A6/SLC25A38/LRRC8A/SLC7A8/SLC1A5/SLC1A4/SLC6A20/SLC2A1/SLC7A5/SLC3A2/SLC6A9/SLC38A10/THBS1/AKT2/SLC43A1/PER2/SLC38A5
BP	GO:0045646	regulation of erythrocyte differentiation	41	-0.58	-1.73	0.00247394	0.01203966	0.00746503	1786	tags=41%, list=15%, signal=35%	ACVR1B/INPP5D/CDK6/ETS1/LYN/KLF13/HSPA1A/KAT7/ARNT/STAT3/MAFB/FAM210B/PRKDC/GATA1/FOXO3/MED1/TAL1
BP	GO:0043068	positive regulation of programmed cell death	384	-0.34	-1.37	0.00247367	0.01203966	0.00746503	1779	tags=22%, list=15%, signal=19%	TRAF7/PTPRC/PIK3CB/MAGED1/INPP5D/MMP9/BBC3/MAP3K11/BCAP31/SOX4/SMPD1/TOP2A/NCOA1/PYCARD/TEX261/TNFRSF1B/PRR7/DAPK3/DAPK1/TP53BP2/TRIM35/MAP3K5/ANO6/HIP1R/LYN/PRF1/PIAS4/MAP2K7/SLC9A1/BAD/BRMS1/PDCD1/BCL2L11/SRC/UBE2Z/PML/EIF5A/SRPK2/RBCK1/PIK3CD/SFRP2/SEPTIN4/FIS1/PLEKHF1/LAPTM5/PPP1CA/CTNNA1/SYK/FBH1/ABL1/GADD45G/E2F1/ARRB2/RHOA/UBE2M/NOTCH2/DNM2/LGALS9/ATG7/CDK19/CAMK1D/UBB/GSN/TXNIP/IGF2R/SQSTM1/THBS1/MFN2/GRN/SNCA/NEURL1/PRKDC/BNIP3L/CDKN1A/CTSD/SCIN/PAK2/RYBP/FOXO3/CDC34/HTT/CLU/TGM2
BP	GO:0009743	response to carbohydrate	133	-0.43	-1.56	0.00249225	0.01209932	0.00750201	1609	tags=23%, list=14%, signal=20%	PDK3/SOX4/SREBF1/ACVR2B/PRKACA/SMARCA4/MEN1/STX4/UCP2/SESN2/LYN/PTK2B/MAP2K7/BAD/PFKL/KAT7/PPP3CA/PIH1D1/SIDT2/RHOA/RAB11B/FKBP1B/GLUL/TXNIP/PRKCB/THBS1/TCF7L2/SLC29A1/GPX1/FOXO3/MAP2K3

BP	GO:0032496	response to lipopolysaccharide	223	-0.38	-1.44	0.0024956	0.01209932	0.00750201	2108	tags=26%, list=18%, signal=22%	IL1B/SPI1/CNR2/PPBP/SCIMP/GSTP1/TRIB1/ALPL/SCARB1/PDE4B/EDN1/CD14/JUND/SIGIRR/VIM/FOXP1/NR1H3/PYCARD/CACTIN/TGFB1/GIT1/TNFRSF1B/CD180/ALAD/TRAF6/LYN/SASH1/RARA/MTDH/SRC/LITAF/BCR/UPF1/ABL1/FOS/TNFSF4/RELA/OTUD5/LTF/NFKB1/BMP6/RHOA/LGALS9/MAPKAPK2/CDK19/PLSCR3/PRPF8/PF4V1/SELP/SNCA/PAF1/CXCL5/MAPK3/GATA1/PF4/AKIRIN2/TNIP1/MAP2K3/TBXA2R
MF	GO:0003713	transcription coactivator activity	236	-0.37	-1.43	0.00249878	0.01209932	0.00750201	2665	tags=35%, list=23%, signal=28%	TRIM21/ZMIZ2/NRIP1/MED13/JUP/CCDC124/SMARCA2/MID2/RRP1B/NOTCH1/EDF1/TSG101/CNOT9/MAML2/ARID1B/PKN1/HYAL2/MTA2/DRAP1/MTA1/TACC1/NCOA4/SMARCD3/PPARGC1B/CCAR2/HELZ2/BCL9/ARRB1/MED12L/RERE/MAGED1/SMARCC1/TAF6/TGFB1I1/TRERF1/BCL9L/NCOA1/SMARCA4/DYRK1B/ETS1/RBM14/NCOA2/MAML3/NCOA6/NUP98/TADA3/CREBBP/MTDH/MED16/TRIM32/MRTFA/NCOA3/WBP2/RUVBL1/DAXX/ZMIZ1/TRIM28/KAT2B/PHF2/ABL1/LPIN2/TOX2/KMT2C/HIPK2/NIBAN2/ELK1/DCAF6/USP22/KAT6A/PRKCB/TRIM14/UBE2L3/PER2/BRD4/HMGA1/ACTN4/ACTN1/PDLIM1/SMARCC2/MED1/ARID1A/CARM1/HCFC1
BP	GO:0055074	calcium ion homeostasis	256	-0.36	-1.42	0.00250142	0.01209932	0.00750201	2609	tags=32%, list=22%, signal=25%	TMTC2/CCRL2/LETM1/ATP13A2/PDPK1/CMKLR1/PKD1/ATP2A3/CORO1A/SELENOK/PTGIR/SWAP70/DISC1/KNG1/TRPM2/BAK1/CLN3/TRPC3/ATP2A2/DLG4/SLC24A4/CCR1/CDK5/FYN/SLC25A23/ATP1B1/P2RX4/PTPN6/ATP13A3/ITPR2/LPAR4/SMAD3/MICU1/PTPRC/EDN1/PIK3CB/STOML2/CXCR2/BCAP31/PRKACA/CXCR1/SNX10/SELENON/FPR3/CHERP/LYN/SPPL3/PTK2B/GNA15/PML/RASA3/CALR/ATP13A1/FIS1/JAK3/ABL1/FKBP1A/STIM1/ANXA6/XK/GALR1/CALM3/FKBP1B/MCOLN1/SLC24A3/GP1BA/PRKCB/GP9/HRH4/PIK3CG/UBASH3B/ATP2B4/SNCA/P2RX1/GATA1/GRINA/HTT/KEL/TGM2/DIAPH1/TBXA2R
BP	GO:0045022	early endosome to late endosome transport	41	-0.58	-1.72	0.00250807	0.01211674	0.00751282	1626	tags=34%, list=14%, signal=30%	WDR81/SNX12/EZR/RAB7A/SRC/HOOK3/SNX3/MAP2K2/FLNA/RILP/MSN/MAPK3/PTPN23/DAB2
BP	GO:0007034	vacuolar transport	138	-0.43	-1.55	0.00252001	0.01215968	0.00753944	2418	tags=34%, list=21%, signal=27%	CLN3/LAMP2/TSG101/M6PR/HGSNAT/SNX27/VPS39/VPS4B/VPS51/NCOA4/HGS/CHMP6/TRAK1/ANKFY1/VPS41/VPS37B/CHMP4B/VPS53/EHD3/VPS13D/VTI1B/RAB7A/EPG5/LEPROT/PINK1/HSPA1A/MON1B/CHMP1A/HOOK3/VTI1A/LAPTM5/PSAP/VCP/ATP6V0D1/DTX3L/VPS28/SORT1/GAK/IGF2R/LRP1/RILP/GRN/LAMP1/TGFBRAP1/UBXN6/PTPN23/CLU
BP	GO:0061180	mammary gland epithelium development	35	-0.59	-1.70	0.00253497	0.01220059	0.00756481	1116	tags=54%, list=10%, signal=49%	KDM5B/CEBPB/LATS1/SCRIB/PTCH1/MSX1/AKT1/DDR1/DEAF1/CSF1R/LBH/MGMT/SRC/PML/BTRC/AKT2/ZNF703/GPX1/MED1
BP	GO:0009612	response to mechanical stimulus	120	-0.42	-1.52	0.0025377	0.01220059	0.00756481	3028	tags=40%, list=26%, signal=30%	CITED2/NRXN1/KIT/PTCH1/SLC8A1/TNFRSF8/FOSB/TNFRSF10A/TTN/PKD1/JUP/MAPK14/PIK3CA/JUN/BAK1/IRF1/FYN/CASP2/IL1B/PDE2A/EDN1/KIAA0319/ENG/JUND/KCNJ2/ACTG1/CASP5/AQP1/PTK2B/SLC9A1/BAD/SRC/CNN2/FOS/RELA/TMEM120A/NFKB1/BMP6/SLC2A1/RHOA/GSN/PLEC/TXNIP/THBS1/MTPN/GPI/MAPK3/HTT
BP	GO:0046578	regulation of Ras protein signal transduction	140	-0.43	-1.55	0.00255308	0.01225973	0.00760147	2048	tags=25%, list=17%, signal=21%	MAPRE2/MADD/PRAG1/RALBP1/ARRB1/LPAR4/ARHGAP35/PIK3CB/LRRC59/RIPOR2/ARHGAP17/DENND1A/RAC1/MYO9B/RASA3/IQSEC1/NRP1/ARHGEF2/BCR/GABARAP/ABL1/SYNGAP1/FBP1/ARHGEF18/CYTH4/NOTCH2/DNM2/ARHGDIA/KANK2/SQSTM1/AKAP13/PIK3CG/MFN2/CBL/SH3BP1

BP	GO:1902533	positive regulation of intracellular signal transduction	661	-0.31	-1.31	0.00257409	0.01234571	0.00765479	2271	tags=27%, list=19%, signal=23%	SLC20A1/MAP3K13/ZNF385A/IKBKE/EPHA4/HLA-DRB1/ARHGAP8/PRKD2/SPRED1/WDR24/PELI2/CD81/FYN/XRCC3/ICAM1/CRKL/CPNE1/IL1B/SPI1/P2RX4/RIPK1/CSF1R/MAPRE2/PTPN6/MADD/ROCK2/SCIMP/TLR6/PRAG1/SECTM1/CCAR2/MAP3K3/SHC1/ARRB1/LPAR4/TRAF7/PTPRC/RRAGD/EDN1/PIK3CB/LRRC59/DIXDC1/MAGED1/ENG/FZD5/LIMS1/CD14/CD74/BBC3/SEMA4D/MAP3K11/BCAP31/IKBKG/DDX60/PRR5L/ANKRD17/SORBS3/PYCARD/HSP90AB1/TGFB1/TRIP6/HDAC3/KLHL22/LILRA5/TAOK1/SLC44A2/SPHK1/JMJD8/NDST1/NEK6/HIC1/TRAF6/CANT1/MAP3K5/ALOX15/MAP4K1/HIP1/CHERP/LYN/SPPL3/MAVS/ILK/SASH1/PIK3AP1/PUM1/PINK1/RAC1/PTK2B/IGF2/C20orf27/HSPA1A/PIAS4/MAP2K7/SLC9A1/MTDH/S100A4/BAD/SHISA5/TRIM32/HCLS1/BCL2L11/SRC/PML/GREM1/EIF5A/STK25/CALR/RBCK1/PIK3CD/GNAI2/DVL3/SEPTIN4/FIS1/PTP4A3/PLEKHF1/LRRK1/LAPTM5/TAOK2/PTBP1/LITAF/PIH1D1/NRP1/MAP2K2/PDGFC/SYK/XIAP/FBH1/ABL1/PAK1/RRAGC/FKBP1A/GADD45G/RELA/OTUD5/LTF/PIM2/ARRB2/LAMTOR1/PDE5A/RHOA/MFHAS1/NOTCH2/ACTA2/PTPRJ/LGALS9/FLNA/GUCY1A1/GPR137/MTURN/HIPK2/SIK3/UBB/DHX33/PRKCB/TRIM14/CSK/THBS1/PTPN1/AKAP13/PIK3CG/IQGAP1/PRR5/TCF7L2/SHARPIN/SELP/RUNDC3A/MAPK3/BRD4/CBL/ACTN4/GPX1/SLC15A3/PPM1A/PDGFA/MAP2K3/TGM2
BP	GO:0002064	epithelial cell development	117	-0.45	-1.60	0.00258819	0.01239836	0.00768743	2247	tags=33%, list=19%, signal=27%	PRDM1/MAGI2/ICAM1/IL1B/ROCK2/MYADM/PDE2A/HSD17B4/VIM/GSK3B/GSK3A/CDK6/ST14/EZR/MYO1E/SIPA1L3/SPINT2/BCL11B/RARA/FLNB/BAD/SRC/TJP2/RAPGEF1/BMP6/SIDT2/VCL/NOTCH2/ACTA2/TBC1D20/FASN/PLEC/CLDN5/IQGAP1/MSN/TMOD1/GATA1/GPX1/ADD1
CC	GO:0030658	transport vesicle membrane	125	-0.43	-1.56	0.00263004	0.01256854	0.00779295	1681	tags=26%, list=14%, signal=23%	CD74/HLA-C/STX6/SEC23IP/DTNBP1/HLA-DQA1/SREBF1/SYNRG/CLTC/TPRG1L/SYNGR1/DNAJC5/VTI1B/RAB7A/TMEM184A/CEACAM1/HLA-DRB5/VTI1A/SEC31A/CNIH3/RAB27B/STX5/CLTB/CALM3/RAB5B/RAB11B/ZNRF1/SLC6A9/BCL2L1/SYTL4/SNCA/NRGN/SEC16A
BP	GO:0099536	synaptic signaling	345	-0.35	-1.39	0.0026428	0.01261436	0.00782136	2581	tags=28%, list=22%, signal=22%	GNAQ/RAB8A/MINK1/AKT1/FBXL20/SYT11/DISC1/ADNP/CLN3/ATP2A2/EZH2/DLG4/RPH3A/EIF4A3/EPHA4/MECP2/CDK5/FYN/CRKL/EXOC4/IL1B/HRH2/P2RX4/CNR2/GNA11/ZDHHC3/RGS10/SYNJ1/OR10H1/PMP22/EDN1/NPTXR/DGKZ/GRK2/PPP1R9A/DTNBP1/GSK3B/BAIAP2/PRKACA/GSK3A/RPS6KA1/CYFIP1/GIT1/YWHAG/TPRG1L/SYNGR1/PRR7/DNAJC5/STX4/PINK1/MBP/NPFF/PTK2B/RARA/GNA15/ARF1/SIPA1L1/GNB1/NCSTN/SRC/DLGAP4/CA2/GNAI2/KIF1B/PPP3CA/SLC1A4/MAP1A/BCR/YWHAH/GABARAP/APBA3/ABL1/SYNGAP1/RELA/ARRB2/STAU1/CALM3/PVALB/GUCY1A1/NAPA/CLSTN1/GLUL/SQSTM1/PPP1R9B/PRKCB/HRH4/SNCA/NEURL1/P2RX1/EIF4EBP2/RNF10/SLC29A1/NRGN/PRKAR2B/SHISA7
BP	GO:0019722	calcium-mediated signaling	131	-0.44	-1.57	0.00265304	0.01264808	0.00784227	2545	tags=36%, list=22%, signal=28%	SELENOK/POMC/MAPK7/TRPM2/CD22/CD3E/ATP2A2/SLC24A4/CCR1/CHP1/ATP1B1/P2RX4/PTPRC/EDN1/PPP1R9A/CXCR2/BCAP31/NFATC1/GSK3B/NFATC3/PRKACA/CXCR1/SPHK1/PLEK/CHERP/SPPL3/PTK2B/SLC9A1/NUDT4/FIS1/PPP3CA/ADGRL1/PTBP1/PDK2/PPP3R1/SYK/CALM3/PTPRJ/FKBP1B/MCOLN1/PPP1R9B/NFATC2/ATP2B4/SELP/PTGDR2/HTT/DMTN

BP	GO:0016197	endosomal transport	198	-0.40	-1.50	0.00265708	0.01265214	0.00784478	2377	tags=35%, list=20%, signal=28%	AGAP2/SNX33/SNX27/ARL4C/AP5B1/LMTK2/ARHGAP8/CMTM6/VPS39/YKT6/SNX17/VPS4B/VPS51/RNF126/ANKRD50/TOM1/HGS/CHMP6/ANKFY1/STX6/SNX12/CHMP4B/WASHC2A/VPS53/EHD3/TBC1D17/REPS1/ATP9A/CLTC/DENND1C/DENND1A/ATP6AP1/EHD4/CORO1C/ITSN1/VTI1B/RAB7A/EPG5/LEPROT/MICALL1/GRIPAP1/CHMP1A/TMCC1/SNX3/VTI1A/RAB6B/RAB11FIP4/EPS15L1/RGP1/VCP/RAB14/STX5/REPS2/LAMTOR1/WAS/RAB11B/ARHGAP1/USP7/VPS28/SORT1/DCTN1/SLC66A2/SQSTM1/RILP/TBC1D5/EHD1/UBE2O/UBXN6/PTPN23
MF	GO:0019207	kinase regulator activity	176	-0.40	-1.50	0.00266985	0.01269775	0.00787306	2568	tags=32%, list=22%, signal=26%	PKIG/TAOK3/PIK3CA/CCNJL/MOB3B/AGAP2/CDKN2D/INKA1/CHP1/MAP3K13/SH3BP5L/SPRED1/HYAL2/MADD/CCNY/GSTP1/MOB1B/TRIB1/SOCS7/CDC37/GCN1/TPX2/PIK3R3/WDR81/ITPRIP/HSP90AB1/MOB3A/TAOK1/YWHAG/IGF2/MARK2/CCND3/GREM1/CDKN1C/YWHAB/TAOK2/DAXX/MAP2K2/KAT2B/LTF/WARS1/PRKAR2A/CALM3/RAC2/HSPB1/STK11/HEXIM2/CCNI/IQGAP1/SNCA/CDKN1A/PAK2/PRKAR2B/TESC/TAL1/WNK1/CNPPD1
MF	GO:0030276	clathrin binding	45	-0.55	-1.67	0.00268792	0.01273797	0.007898	1599	tags=36%, list=14%, signal=31%	TOM1/AP2B1/CLTC/AP1M1/HIP1/AP2S1/HIP1R/CLTB/EPN1/LDLRAP1/AP1B1/STON2/GAK/LRP1/AP2M1/DAB2
BP	GO:0006140	regulation of nucleotide metabolic process	57	-0.52	-1.62	0.00268365	0.01273797	0.007898	2649	tags=42%, list=23%, signal=33%	ZBTB20/ARL2/HDAC4/FLCN/ENTPD5/PGAM1/ZBTB7A/EIF6/PDK3/CDA/GIT1/CBFA2T3/JMJD8/PARP1/PINK1/ARNT/PPARA/STAT3/PDK2/VCP/FBP1/SLC4A1/SNCA/ENO1
CC	GO:0150034	distal axon	155	-0.41	-1.53	0.00268786	0.01273797	0.007898	2251	tags=31%, list=19%, signal=25%	LMTK2/EPHA4/CDK5/FXR2/EXOC4/FEZ1/P2RX4/CTNND1/BASP1/NDRG2/PACSIN1/SYNJ1/DIXDC1/ARPC5/ENO2/NDEL1/DTNBP1/CYFIP1/COPA/HSP90AB1/ACTG1/GIT1/TPRG1L/KATNB1/CDKL5/PINK1/NPFF/PTK2B/EXOC3/ADGRL1/TAOK2/AMFR/ACTB/USP9X/ATP6V0D1/ABL1/CALM3/DNM2/FLNA/ELK1/GLUL/PPP1R9B/PRKCB/IQGAP1/SNCA/CBL/RNF40/CTTN
BP	GO:0000209	protein polyubiquitination	212	-0.39	-1.47	0.00271071	0.01283071	0.0079555	1267	tags=19%, list=11%, signal=18%	TRAF6/UBE2G1/DAW1/LNPEP/NEDD4L/DDA1/CDC27/SASH1/ANKIB1/IFI27/TRIM32/MARCHF6/UBE2R2/RNF115/RBCK1/RMND5A/AMFR/BTRC/GABARAP/XIAP/ARIH2/RNF213/DTX3L/WWP2/UBE2S/ZNRF1/FZR1/HUWE1/TRIM14/UBE2L3/MKRN1/RNF187/SHARPIN/MARCHF8/CBL/TRIP12/UBE2O/RNF40/CDC34/UBE2H/TRIM58
BP	GO:0006886	intracellular protein transport	706	-0.31	-1.30	0.00272211	0.01286931	0.00797944	1685	tags=21%, list=14%, signal=19%	TRAK1/CD74/NDEL1/BBC3/IPO8/VPS41/STX6/SEC23IP/ZDHHC19/VPS37B/CHMP4B/AP2B1/BCAP31/TM9SF4/SREBF1/GSK3B/PRR5L/STOM/CRY2/PRKACA/XPO6/SYNRG/NUP188/GSK3A/COPA/HSP90AB1/TGFB1/HDAC3/CLTC/YWHAG/SYNGR1/TECPR2/RANGRF/EDEM2/SNX10/STX4/ATP6AP1/ARL6IP1/VPS13D/AP1M1/KATNB1/RAB31/UNC93B1/AP2S1/BCAS3/NUP98/SNX15/POM121C/MAVS/VTI1B/RAB7A/LEPROT/PINK1/MICALL1/IFI27/ARF1/MON1B/KIF13B/HCLS1/GRIPAP1/PML/CALR/YWHAB/SCFD2/FIS1/VTI1A/PPP3CA/HERC2/LAPTM5/TAOK2/COPB2/TRIM28/RAB6B/TBC1D13/SEC31A/STAT3/RANGAP1/MAP1A/ARHGEF2/BCR/YWHAH/NOLC1/GABARAP/SYK/SNX11/VCP/TRAK2/USP36/ELAVL1/ARIH2/PARD3/RAB14/ABLIM3/RAB27B/SAE1/STX5/CLTB/AP1B1/SUFU/MAN1A1/POM121/XPO7/FLNA/RAB5B/NUP93/RAC2/LMNA/INPP5K/HSPB1/VPS28/NAPA/SORT1/SYTL4/RAB1B/TXNIP/HUWE1/ARCN1/SQSTM1/YOD1/AKT2/UBE2L3/PTPN1/MFN2/TCF7L2/PPP1R10/CAMK1/ATG13/AHCYL1/BNIP3L/FBXO7/TGFBRAP1/CDKN1A/EHD1/FAM53B/STRADB/MED1/OAZ1/PTPN23/CTTN/AP2M1/PPM1A/SEC16A/NPLOC4/CLU/DMTN/BAG6

BP	GO:0002687	positive regulation of leukocyte migration	89	-0.46	-1.58	0.00272673	0.01287586	0.0079835	2571	tags=37%, list=22%, signal=29%	PTAFR/SELENOK/SWAP70/DOCK8/CCR1/IL6R/CD99L2/ICAM1/SPI1/P2RX4/CSF1R/LGALS3/EDN1/RIPOR2/CREB3/CD74/PYCARD/OXSR1/ANO6/RAC1/JAM3/PTK2B/SPN/CALR/RHOA/LGALS9/RAC2/CAMK1D/PLVAP/THBS1/SELP/MAPK3/WNK1
BP	GO:0034764	positive regulation of transmembrane transport	120	-0.42	-1.52	0.00273166	0.01288385	0.00798845	1268	tags=17%, list=11%, signal=15%	ARL6IP1/ANO6/PINK1/SLC9A1/CA2/ATP1B2/ABL1/STIM1/CALM3/DNM2/FLNA/SLC6A9/AKT2/GJC2/SNCA/P2RX1/ACTN4/TESC/HTT/WNK1
BP	GO:0001701	in utero embryonic development	262	-0.37	-1.45	0.00278683	0.01311293	0.00813049	2247	tags=27%, list=19%, signal=22%	PRDM1/HES1/SMG9/UNK/MAFG/TTLL1/VASH1/SH3PXD2A/PTPN18/MAN2A1/MIB1/BIRC6/SMAD3/MAFF/ACVR1B/KEAP1/EDN1/TFEB/FZD5/RBPJ/NDEL1/OTUD7B/KLF4/GRB2/NCOA1/ASCL2/RPGRIP1L/ST14/AMOT/MYO1E/SPINT2/BCOR/WDTC1/TRAF6/SLC39A3/EPAS1/SUPT6H/BPTF/IGF2/ASF1B/CHST11/KLF2/BCL2L11/TBL1XR1/CDKN1C/ARNT/TAF8/ZMIZ1/TRIM28/APBA3/PSMC4/CMIP/EPN1/CHD8/NOTCH2/INPP5K/BCL2L1/GNA12/TERF2/YBX1/MFN2/GPI/GATA1/YBX3/FURIN/MED1/CUL4A/MYH9/TENT5C/CNOT1
BP	GO:0042594	response to starvation	148	-0.41	-1.52	0.00280404	0.01317835	0.00817105	2057	tags=29%, list=18%, signal=24%	NUAK2/SREBF2/HSPA5/RIPOR1/FOXK1/MAP1LC3B/GCN1/RRAGD/TFEB/VPS41/EHMT2/SREBF1/FOXK2/DAP/TTC5/UCP2/MFSD2A/MAP3K5/SESN2/BCAS3/STK24/SLC39A4/PPARA/MAX/GABARAP/ZFYVE1/RRAGC/SLC2A1/WIPI2/SESN3/ATG7/GLUL/TBC1D5/MAPK3/ELAPOR1/CDKN1A/CBL/WDR45/NPRL3/FOXO3/EIF2AK1/GAS2L1/LARP1
BP	GO:0052126	movement in host environment	133	-0.43	-1.55	0.00284599	0.01334393	0.00827372	2665	tags=36%, list=23%, signal=28%	TRIM21/CR1/SIGLEC1/MID2/EXOC7/KPNA3/NCAM1/TSG101/HLA-DRB1/HYAL2/CD81/ICAM1/VPS4B/SELPLG/ITGB7/SCARB1/CHMP6/CD74/GRK2/VPS37B/CHMP4B/SMPD1/IST1/TRIM26/RAB7A/GYPA/IFITM3/HSPA1A/SRC/CHMP1A/SNX3/SLC1A5/TRIM10/IFITM2/NRP1/DDB1/WWP2/LGALS9/SLC3A2/BSG/TRIM11/ITGB5/GSN/TRIM14/LAMP1/CBL/CTSB/ITGB3
BP	GO:0050807	regulation of synapse organization	105	-0.44	-1.57	0.00285367	0.01334848	0.00827654	3236	tags=42%, list=28%, signal=31%	DAG1/EEF2K/STAU2/PGRMC1/ACTR2/PDLIM5/NRXN1/NECTIN1/ABI2/EPHB1/CAPRIN1/MAPK14/DISC1/ADNP/CLN3/YWHAZ/EPHA4/CDK5/ABI3/FYN/ABHD17C/SLITRK5/SEMA4D/DTNBP1/BAIAP2/CYFIP1/CDKL5/SIPA1L1/GRIPAP1/ADGRL1/SEMA4A/VCP/ABL1/RHOA/DNM3/DCTN1/CFL1/CLSTN1/SNCA/LRFN3/CAMK1/EIF4G1/NEURL1/TUBB
BP	GO:0030856	regulation of epithelial cell differentiation	67	-0.49	-1.61	0.0028645	0.01338336	0.00829816	2444	tags=40%, list=21%, signal=32%	MYCL/PTCH1/ZFP36L1/KIAA1109/NOTCH1/ZFP36/EZH2/HES1/BTG1/SPRED1/MAFG/IL1B/ROCK2/NUMA1/MAFF/KEAP1/MMP9/BAD/CDKN1C/CEACAM1/NCOA3/BMP6/VCL/FOXJ2/CLDN5/MED1/ADD1
CC	GO:0034774	secretory granule lumen	261	-0.37	-1.45	0.00287345	0.01340943	0.00831433	2085	tags=28%, list=18%, signal=23%	PROS1/EEF2/PTPN6/PPBP/RNASE3/PGAM1/PGLYRP1/TIMP1/OSCAR/GSTP1/CTSA/XRCC6/ADA2/ARPC5/CDA/CRISPLD2/TRAPPC1/TIMP2/PYCARD/CYFIP1/HSP90AB1/TGFB1/ALAD/MVP/ALDOA/CANT1/GLB1/IST1/VTI1B/F13A1/SERPING1/IGF2/ALDOC/PFKL/DYNC1H1/PKM/ARHGAP45/CNN2/PSMD11/CAP1/VCP/LRG1/LCN2/PYGB/LTF/NFKB1/TUBB4B/VCL/FERMT3/PSMD2/PA2G4/ATG7/DOCK2/CYB5R3/GSN/HUWE1/THBS1/GNS/GPI/COTL1/GRN/PRDX6/PF4/CTSD/TUBB/FTL/ACTN4/ACTN1/TOLLIP/SPARC/PDGFA/CLU
BP	GO:0007568	aging	96	-0.45	-1.57	0.0029315	0.01361641	0.00844267	2425	tags=29%, list=21%, signal=23%	BAK1/CASP9/TSPO/HYAL2/CASP2/GNA11/TIMP1/EDN1/CANX/JUND/GRB2/SREBF1/TNFRSF1B/UCP2/SERPING1/MBP/CDKN1C/PPP3CA/AMFR/CTNNA1/FOS/FOXO4/C1QA/GNA12/PPP1R9B/GPX1/FOXO3/CARM1
BP	GO:0015849	organic acid transport	164	-0.40	-1.48	0.00292862	0.01361641	0.00844267	2245	tags=31%, list=19%, signal=26%	AKT1/SLC46A1/ABCB11/CLN3/SLC7A7/SERINC5/SLC25A11/ABCD1/IL1B/P2RX4/SLC25A1/MFSD12/SLC43A2/SLC7A1/EDN1/CPT1A/SLC19A1/ABCC1/DTNBP1/PLIN2/TSPO2/SLC27A1/ARL6IP1/MFSD2A/SLC5A6/GOT2/SLC25A38/CEACAM1/ABCC3/LRRC8A/SLC7A8/PPARA/FIS1/SLC1A5/SLC1A4/SLC6A20/SYK/ABCG2/ABCC4/SLC2A1/XK/SLC7A5/SLC3A2/SLC6A9/SLC38A10/THBS1/AKT2/SLC43A1/PER2/SNCA/SLC38A5

BP	GO:0050803	regulation of synapse structure or activity	107	-0.45	-1.58	0.00297256	0.013791	0.00855092	3236	tags=42%, list=28%, signal=31%	DAG1/EEF2K/STAU2/PGRMC1/ACTR2/PDLIM5/NRXN1/NECTIN1/ABI2/EPHB1/CAPRIN1/MAPK14/DISC1/ADNP/CLN3/YWHAZ/EPHA4/CDK5/ABI3/FYN/ABHD17C/SLITRK5/SEMA4D/DTNBP1/BAIAP2/CYFIP1/CDKL5/SIPA1L1/GRIPAP1/ADGRL1/SEMA4A/VCP/ABL1/SYNGAP1/RHOA/DNM3/DCTN1/CFL1/CLSTN1/SNCA/LRFN3/CAMK1/EIF4G1/NEURL1/TUBB
BP	GO:0052547	regulation of peptidase activity	263	-0.37	-1.44	0.00301553	0.0139578	0.00865434	1818	tags=27%, list=16%, signal=23%	BIRC6/SMAD3/PTTG1/APLP2/CAST/MMP9/BBC3/KLF4/PSME3/BCAP31/SERPINB6/TIMP2/PYCARD/RPS6KA1/DAP/SVBP/PRR7/BEX3/SPINT2/DAPK1/ARL6IP1/MAP3K5/HIP1/AQP1/HIP1R/LYN/SERPING1/PINK1/MBP/MGMT/BAD/DHCR24/NCSTN/BCL2L11/SRC/UBE2Z/PML/SFRP2/FIS1/LAPTM5/PIH1D1/STAT3/SYK/VCP/XIAP/EGLN3/LTF/ARRB2/RHOA/LGALS9/PSME4/SIAH2/PRDX5/CYFIP2/GSN/BCL2L13/PSMF1/LRP1/THBS1/GPI/GRN/SNCA/P2RX1/AKIRIN2/CTSD/PAK2/UBE2O/CTSB/GPX1/FURIN
BP	GO:0071453	cellular response to oxygen levels	112	-0.44	-1.56	0.00302746	0.0139967	0.00867846	3103	tags=41%, list=26%, signal=30%	IRAK1/ENDOG/CPEB4/CITED2/TMBIM6/ZFP36L1/NPEPPS/SLC8A1/CCDC115/ATP7A/AKT1/NOTCH1/CYBB/EGR1/FOXO1/HYOU1/EPHA4/ROCK2/NDRG1/EDN1/PIK3CB/HIF1AN/RBPJ/PDK3/VHL/ATP6AP1/AQP1/EPAS1/PINK1/SLC9A1/BAD/SRC/ATP6V1A/USP19/ATP6V0D1/EGLN3/E2F1/MPL/LMNA/ATG7/HIPK2/ENO1/BNIP3L/SLC29A1/CBL/FOXO3
BP	GO:0030512	negative regulation of transforming growth factor beta receptor signaling pathway	40	-0.56	-1.68	0.00308816	0.01422771	0.00882169	2200	tags=42%, list=19%, signal=35%	SPRED1/HSPA5/ZBTB7A/PIN1/NRROS/TGFB1I1/BCL9L/SKI/HSPA1A/CHST11/PPARA/FAM89B/FKBP1A/DNM2/GLG1/PPM1A/LTBP1
BP	GO:0034767	positive regulation of ion transmembrane transport	90	-0.46	-1.57	0.00311032	0.01431325	0.00887473	1268	tags=22%, list=11%, signal=20%	KCNJ2/EHD3/ARL6IP1/ANO6/PINK1/SLC9A1/ATP1B2/ABL1/STIM1/CALM3/DNM2/FLNA/SLC6A9/GJC2/SNCA/P2RX1/ACTN4/TESC/HTT/WNK1
BP	GO:0051668	localization within membrane	482	-0.32	-1.34	0.00316517	0.01451526	0.00899998	2606	tags=28%, list=22%, signal=23%	JUP/BBS1/RFTN1/SORL1/RAB8A/OSBPL5/AKT1/EXOC7/NSF/PEX19/GOLGA7/NCF1/CNST/CLN3/TMBIM1/SIL1/TRAM2/DLG4/SNX27/ARL4C/CHP1/LMTK2/SSNA1/ARHGAP8/MAGI2/CDK5/CD81/ABI3/FYN/CRKL/EXOC4/MAP7/CMTM6/ATP1B1/VPS39/SNX17/VPS51/HSPA5/ROCK2/MACF1/MYADM/ANKRD50/ITGB7/ZDHHC3/LGALS3/ITGAM/PACSIN1/HGS/PTPRC/BBC3/STX6/SNX12/ZDHHC19/VPS37B/CHMP4B/AP2B1/TM9SF4/DTNBP1/VPS53/EHD3/STOM/TGFB1/ATP9A/CLTC/MESD/RANGRF/DENND1C/EZR/TSPAN17/DENND1A/STX4/ATP6AP1/EHD4/ARL6IP1/EFR3A/RAB31/VTI1B/RAB7A/EPG5/RAC1/DCHS1/MICALL1/GRIPAP1/SNX3/ATP13A1/FIS1/PLEKHF1/TAOK2/PACS1/PSAP/ACTB/RAB11FIP4/TTC7B/EMC3/PARD3/SYNGAP1/RAB14/OPTN/ITGAL/CALM3/DNM2/LDLRAP1/FLNA/RAB11B/RAB3D/BSG/INPP5K/ARHGAP1/DOCK2/BCL2L1/ZDHHC5/MMGT1/GSN/TTC7A/CLSTN1/SLC4A1/GAK/SQSTM1/LRP1/PPP1R9B/CSK/SPTBN1/AKT2/TSPAN33/ATP2B4/RHOG/EHD1/TSPAN5/TESC/PTPN23/AP2M1/SHISA7/SEC16A/DMTN/ANK1/DAB2/BAG6
BP	GO:0071695	anatomical structure maturation	127	-0.42	-1.51	0.00324889	0.0148649	0.00921677	2340	tags=33%, list=20%, signal=27%	SLC24A4/INSL3/WASHC5/MAP3K13/G6PD/HES1/MECP2/ZBTB16/XYLT1/ZBTB7A/EDN1/RERE/FZD5/RBPJ/B4GALT5/ANKS1A/SEMA4D/ANKRD17/PRKACA/SNX10/BCL11A/EPAS1/PTK2B/KLF2/DCHS1/GREM1/CDKN1C/FAM20C/SLFN14/PHOSPHO1/RFLNB/LTF/C1QA/RHOA/ABHD2/EPB42/ARCN1/FAM210B/LYL1/FOXO3/TAL1/TRIM58

BP	GO:0045453	bone resorption	41	-0.57	-1.70	0.00329289	0.01504891	0.00933086	2059	tags=41%, list=18%, signal=34%	CSF1R/PPARGC1B/TPP1/INPP5D/SNX10/TRAF6/RAB7A/PTK2B/SRC/LTBP3/LRRK1/SYK/GPR137/RAB3D/CSK/UBASH3B/ITGB3
BP	GO:0045921	positive regulation of exocytosis	56	-0.54	-1.68	0.00335795	0.01532861	0.00950429	2883	tags=46%, list=25%, signal=35%	F2RL1/ITGB2/GAB2/SDCBP/ATP13A2/FGR/PTAFR/TSG101/CDK5/VPS4B/ITGAM/HGS/HYAL3/STX4/RAB7A/SYK/CLASP1/RAB27B/RAB3D/SDC4/SYTL4/RAB2B/SNCA/LAMP1/GATA1/SMPD3
BP	GO:0031348	negative regulation of defense response	167	-0.40	-1.48	0.003364	0.01533862	0.0095105	2665	tags=34%, list=23%, signal=27%	TRIM21/CR1/FGR/MAPK14/RHBDF2/MACIR/FOXP3/MUL1/SYT11/MAPK7/NR1D1/YTHDF3/ZFP36/BANF1/OAS3/HLA-DRB1/FYN/CNR2/HLA-A/PGLYRP1/GSTP1/NR1H2/SMAD3/PTPRC/KLF4/NLRX1/NR1H3/FAM3A/CACTIN/MICB/GIT1/TNFRSF1B/CCN3/LYN/SERPING1/ADAR/TTLL12/ILRUN/SRC/CEACAM1/PPARA/BCR/SYK/NFKB1/ARRB2/SIGLEC10/MFHAS1/LGALS9/KRT1/RIOK3/SEC14L1/GRN/SHARPIN/PCBP2/GPX1/FURIN/NPLOC4
BP	GO:0038127	ERBB signaling pathway	82	-0.48	-1.63	0.00338822	0.01543132	0.00956798	2703	tags=48%, list=23%, signal=37%	PDPK1/KIF16B/RHBDF2/AKT1/SH3TC2/CBLB/PIK3CA/PTPN11/NCF1/PDE6G/MVB12B/TSG101/CPNE3/CNOT9/RNF126/PTPN18/SHC1/NEU3/CHMP6/RBPJ/MMP9/DGKD/GRB2/TGFB1/MVP/HIP1/HIP1R/PTPN12/RAB7A/PTK2B/SRC/CEACAM1/RNF115/ABL1/REPS2/PTPRJ/IQGAP1/NEURL1/CBL
BP	GO:0006874	cellular calcium ion homeostasis	253	-0.36	-1.42	0.00340873	0.01548929	0.00960392	2609	tags=29%, list=22%, signal=23%	PKD1/ATP2A3/CORO1A/SELENOK/PTGIR/SWAP70/DISC1/KNG1/TRPM2/BAK1/CLN3/TRPC3/ATP2A2/DLG4/SLC24A4/CCR1/CDK5/FYN/SLC25A23/ATP1B1/P2RX4/PTPN6/ATP13A3/ITPR2/LPAR4/SMAD3/MICU1/PTPRC/EDN1/PIK3CB/STOML2/CXCR2/BCAP31/PRKACA/CXCR1/SELENON/FPR3/CHERP/LYN/SPPL3/PTK2B/GNA15/PML/RASA3/CALR/ATP13A1/FIS1/JAK3/ABL1/FKBP1A/STIM1/ANXA6/XK/GALR1/CALM3/FKBP1B/MCOLN1/SLC24A3/GP1BA/PRKCB/GP9/HRH4/PIK3CG/UBASH3B/ATP2B4/SNCA/P2RX1/GATA1/GRINA/HTT/KEL/TGM2/DIAPH1/TBXA2R
BP	GO:1903532	positive regulation of secretion by cell	167	-0.40	-1.48	0.00342688	0.01553623	0.00963302	2776	tags=30%, list=24%, signal=23%	GAB2/TTN/SDCBP/ATP13A2/GABBR1/FGR/PTAFR/PTPN11/TRPM2/TSG101/HLA-DRB1/CDK5/IL1B/P2RX4/VPS4B/PARD6A/NR1H2/ITGAM/HGS/EDN1/GRK2/SOX4/DTNBP1/HYAL3/TGFB1/FRMD4A/EZR/STX4/RAB7A/PINK1/BAD/LRRC8A/SYK/CLASP1/RAB27B/BMP6/MYO18A/GALR1/RAB3D/SDC4/SYTL4/GLUL/RAB2B/TCF7L2/SNCA/LAMP1/GATA1/SMPD3/PTPN23/DAB2
BP	GO:0008286	insulin receptor signaling pathway	86	-0.47	-1.61	0.00343663	0.01554751	0.00964002	2757	tags=40%, list=24%, signal=30%	SLC27A4/TSC2/SORL1/AKT1/PIK3CA/PTPN11/FOXO1/PIP4K2C/IL1B/SHC1/SH2B2/SMARCC1/NDEL1/PIK3R3/GRB2/SREBF1/GSK3B/BAIAP2/GSK3A/ZNF106/IGF2/SRC/CCND3/GNAI2/PDK2/FOXO4/SESN3/INPP5K/ADIPOR1/PRKCB/AKT2/PTPN1/PIP4K2A/EIF4EBP2
BP	GO:0035904	aorta development	31	-0.62	-1.73	0.00350563	0.01582108	0.00980964	2687	tags=52%, list=23%, signal=40%	DCTN5/NOTCH1/PRDM1/HES1/SNX17/PDE2A/ENG/RBPJ/AP2B1/SOX4/NDST1/PLXND1/SUFU/MYLK/LRP1/NPRL3
BP	GO:0043270	positive regulation of ion transport	151	-0.42	-1.54	0.00354391	0.01597572	0.00990552	2571	tags=30%, list=22%, signal=24%	PTAFR/AKT1/KCNH2/ABCB11/BAK1/TRPC3/TSPO/CCR1/CHP1/G6PD/CDK5/ATP1B1/IL1B/P2RX4/LGALS3/EDN1/KCNJ2/DTNBP1/EHD3/LILRA5/ARL6IP1/FHL1/ANO6/PINK1/SLC9A1/ATP1B2/JAK3/ABL1/STIM1/ARRB2/CALM3/DNM2/FLNA/MYLK/SLC6A9/GJC2/SNCA/AHCYL1/P2RX1/ACTN4/TESC/HTT/MLLT6/SCN1B/WNK1

BP	GO:0061024	membrane organization	571	-0.32	-1.32	0.00356109	0.01603499	0.00994227	1795	tags=20%, list=15%, signal=17%	RAB20/SYNJ1/HGS/PTPRC/CHCHD10/SLC35F6/CHMP6/LPCAT2/ANKFY1/RHOBTB1/NDEL1/BBC3/VPS41/STX6/NSFL1C/CHMP4B/ABCC1/SMPD1/GSK3B/BAIAP2/GSK3A/RABIF/ATP9A/SYNGR1/MESD/ASAP1/SPHK1/ATG2A/STX4/NEK6/PRX/CORO1C/ARL6IP1/MFSD2A/SUN2/ALOX15/RAB31/ANO6/PITPNC1/HIP1R/VTI1B/RAB7A/PINK1/RAC1/PLEKHM2/MBP/HSPA1A/CD9/MICALL1/ARF1/DYSF/SLC9A1/BAD/DHCR24/PITPNM2/PITPNA/BCL2L11/CHMP1A/TMCC1/SNX3/VTI1A/LAPTM5/SEC31A/ARFGAP2/MYOF/SH3GLB2/EMC3/CPTP/ANXA6/STX5/MYO18A/RHOA/CALM3/DNM2/TBC1D20/NUP93/RAC2/CAVIN2/LMNA/DOCK2/BCL2L1/PLSCR3/NAPA/MMGT1/DCTN1/GSN/PLEC/SYTL4/SLC4A1/SLC66A2/GAK/HUWE1/RILP/PRKCB/THBS1/SPTBN1/AKT2/SPTA1/MFN2/SNCA/PIP4K2A/RHOG/BNIP3L/TGFBRAP1/CTDNEP1/AP2M1/MYH9/SHISA7/SEC16A/CLU/SH3BP1/BAG6
BP	GO:0050819	negative regulation of coagulation	26	-0.63	-1.72	0.0036091	0.01621446	0.01005355	1136	tags=54%, list=10%, signal=49%	SH2B3/SERPINF2/KNG1/PROS1/EDN1/SERPING1/CD9/CEACAM1/KRT1/GP1BA/ALOX12/THBS1/UBASH3B/PDGFA
BP	GO:0045071	negative regulation of viral genome replication	49	-0.54	-1.68	0.00364228	0.01634502	0.0101345	1228	tags=31%, list=10%, signal=28%	APOBEC3D/MAVS/IFITM3/ZC3HAV1/SRPK2/TRIM28/IFITM2/APOBEC3F/N4BP1/LTF/SETDB1/INPP5K/ZNFX1/APOBEC3C/TNIP1
BP	GO:0098742	cell-cell adhesion via plasma-membrane adhesion molecules	87	-0.46	-1.59	0.00366322	0.01642046	0.01018128	1356	tags=34%, list=12%, signal=31%	NECTIN1/ITGB2/PCDH11X/PECAM1/SDK2/PKD1/MAPK14/MAPK7/ICAM1/MYADM/ITGAM/PIK3CB/KLF4/IL1RN/PLXNB2/MBP/DCHS1/CEACAM1/ADGRL1/SELL/ITGAL/PCDH1/BSG/CLSTN1/CLDN5/SELP/LRFN3/PTPN23/ESAM/WNK1
BP	GO:0032890	regulation of organic acid transport	38	-0.57	-1.68	0.00373849	0.01666393	0.01033224	2108	tags=37%, list=18%, signal=30%	IL1B/P2RX4/SLC43A2/EDN1/DTNBP1/ARL6IP1/PPARA/FIS1/SYK/THBS1/AKT2/SLC43A1/PER2/SNCA
BP	GO:1901216	positive regulation of neuron death	67	-0.49	-1.58	0.00373721	0.01666393	0.01033224	2439	tags=42%, list=21%, signal=33%	TRPM2/CASP9/EGR1/CDK5/FYN/CASP2/TLR6/ITGAM/MAP3K11/GSK3B/PARP1/MAP3K5/MAP2K7/BAD/BCL2L11/SRPK2/DAXX/ABL1/FOS/C1QA/RHOA/UBE2M/ELK1/GRN/SNCA/FOXO3/CDC34/CLU
BP	GO:0048167	regulation of synaptic plasticity	102	-0.44	-1.55	0.00379596	0.01687775	0.01046481	1521	tags=21%, list=13%, signal=18%	GSK3B/BAIAP2/YWHAG/SYNGR1/STX4/PTK2B/RARA/ARF1/SIPA1L1/NCSTN/MAP1A/YWHAH/ABL1/SYNGAP1/STAU1/SQSTM1/SNCA/NEURL1/EIF4EBP2/NRGN/SHISA7
BP	GO:0044409	entry into host	111	-0.44	-1.56	0.003801	0.01687775	0.01046481	2977	tags=40%, list=25%, signal=30%	VPS18/ANPEP/XPR1/NECTIN1/FUCA2/TRIM21/CR1/SIGLEC1/MID2/EXOC7/KPNA3/NCAM1/HLA-DRB1/HYAL2/CD81/ICAM1/SELPLG/ITGB7/SCARB1/CD74/GRK2/SMPD1/TRIM26/GYPA/IFITM3/HSPA1A/SRC/SNX3/SLC1A5/TRIM10/IFITM2/NRP1/WWP2/LGALS9/SLC3A2/BSG/TRIM11/ITGB5/GSN/TRIM14/LAMP1/CBL/CTSB/ITGB3
CC	GO:0098858	actin-based cell projection	126	-0.43	-1.55	0.00380768	0.01687775	0.01046481	2940	tags=43%, list=25%, signal=32%	KPTN/MYO1G/MYO1D/FSCN1/B4GALT1/UBE2K/ABI2/SLC27A4/EPHB1/DOCK4/CNP/AP2A1/ATP7A/EPS8/SYNE2/TRIOBP/ARL4C/EPHA4/CDK5/FXR2/HYAL2/MYO1F/MYO15A/EXOC4/SCIMP/LCP1/RIPOR2/UBE2Q1/DYNC2I2/PPP1R9A/BAIAP2/CYFIP1/TGFB1/EZR/MYO1E/INPPL1/JAM3/SPN/DYNC1H1/CEACAM1/ATP6V1A/SLC7A8/FGD4/SLC7A5/ACTA2/PVALB/NF2/MPP1/PPP1R9B/MSN/VASP/ESPN/ITGB3/PDGFA

BP	GO:0098876	vesicle-mediated transport to the plasma membrane	115	-0.43	-1.55	0.00381637	0.01689531	0.0104757	2603	tags=40%, list=22%, signal=31%	BBS1/SORL1/RAB8A/OSBPL5/NSF/GOLGA7/CNST/CLN3/SNX27/ARL4C/LMTK2/ARHGAP8/EXOC4/CMTM6/VPS39/SNX17/VPS51/MACF1/ANKRD50/STX6/SNX12/VPS53/EHD3/ATP9A/DENND1C/DENND1A/ATP6AP1/EHD4/RAB31/RAB7A/EPG5/MICALL1/GRIPAP1/SNX3/RAB11FIP4/RAB14/OPTN/DNM2/RAB11B/ARHGAP1/CLSTN1/CSK/SPTBN1/EHD1/PTPN23/SEC16A
BP	GO:0001837	epithelial to mesenchymal transition	91	-0.46	-1.56	0.00383242	0.01691199	0.01048604	2444	tags=35%, list=21%, signal=28%	NOTCH1/EZH2/EPHA4/USF3/SPRED1/IL1B/ROCK2/CUL7/SMAD3/EDN1/TIAM1/ENG/RBPJ/TGFB1I1/BCL9L/MCRIP1/GSK3B/AKNA/TGFB1/S100A4/GREM1/SFRP2/TRIM28/NOLC1/RFLNB/CLASP1/MAD2L2/FLNA/ADIPOR1/TCF7L2/ZNF703/DAB2
BP	GO:0060291	long-term synaptic potentiation	45	-0.54	-1.64	0.00390741	0.01710996	0.01060879	1521	tags=22%, list=13%, signal=19%	GSK3B/STX4/PTK2B/NCSTN/ABL1/STAU1/SQSTM1/SNCA/NRGN/SHISA7
MF	GO:0005342	organic acid transmembrane transporter activity	86	-0.47	-1.60	0.00390373	0.01710996	0.01060879	2901	tags=38%, list=25%, signal=29%	SLC36A1/SLC10A3/SLC27A4/SLC16A1/SLC46A1/ABCB11/SLC7A7/SERINC5/SLC25A11/SLC25A1/MFSD12/SLC43A2/SLC7A1/SLC19A1/ABCC1/SLC27A1/MFSD2A/SLC5A6/SLC25A38/CEACAM1/ABCC3/SLC7A8/SLC1A5/SLC1A4/SLC6A20/ABCG2/ABCC4/SLC7A5/SLC3A2/SLC6A9/SLC38A10/SLC43A1/SLC38A5
MF	GO:0046943	carboxylic acid transmembrane transporter activity	86	-0.47	-1.60	0.00390373	0.01710996	0.01060879	2901	tags=38%, list=25%, signal=29%	SLC36A1/SLC10A3/SLC27A4/SLC16A1/SLC46A1/ABCB11/SLC7A7/SERINC5/SLC25A11/SLC25A1/MFSD12/SLC43A2/SLC7A1/SLC19A1/ABCC1/SLC27A1/MFSD2A/SLC5A6/SLC25A38/CEACAM1/ABCC3/SLC7A8/SLC1A5/SLC1A4/SLC6A20/ABCG2/ABCC4/SLC7A5/SLC3A2/SLC6A9/SLC38A10/SLC43A1/SLC38A5
BP	GO:0048638	regulation of developmental growth	173	-0.39	-1.46	0.0039166	0.01713134	0.01062205	2593	tags=32%, list=22%, signal=25%	ULK1/MAPK14/AKT1/DIP2B/MUL1/PIK3CA/DISC1/PLXNA4/ADNP/NOTCH1/ACACB/RAI1/SGPL1/MAP3K13/G6PD/CDK5/MACF1/BASP1/RUNX1/EDN1/KIAA0319/RBPJ/FTO/EPPK1/NDEL1/SEMA4D/GSK3B/GSK3A/CYFIP1/VGLL4/EZR/MFSD2A/BCL11A/IST1/YY1/NEDD4L/CDKL5/DPYSL2/IGF2/BCL2L11/EIF4G2/CPNE5/PPARA/STAT3/SEMA4A/NRP1/ABL1/JARID2/DNM2/STAT5A/HLX/CDKN1A/YBX3/CTTN/PIM1
BP	GO:0032504	multicellular organism reproduction	444	-0.33	-1.36	0.00395551	0.01726356	0.01070403	2701	tags=29%, list=23%, signal=23%	RPS6KA2/PI3/H3-3A/ZNF628/AGFG1/GMCL1/FANCG/NRIP1/ZFY/SMARCA2/SMAD5/PTAFR/AKT1/AGO4/ADNP/PTPN11/NOTCH1/TRPM2/EGR1/SPIRE1/SGPL1/SEPTIN6/KPNA6/CDYL/ZSCAN21/INSL3/WASHC5/PRDM1/BTG1/SPIN1/ASPM/ACOX1/CASP2/CRKL/DEAF1/THRA/SPIRE2/TTLL1/TTC21A/HSPA5/GGT1/SPATA5/ZBTB16/PAQR8/DIAPH2/TESK2/SPATA2/SYNE1/PTTG1/RHBDD1/EDN1/UBE2Q1/CRTAP/ENO2/GTSF1/TBPL1/BCAP31/EHMT2/NR6A1/TOP2A/PTCHD3/HYAL3/TOB2/CETN2/PRKACA/SFMBT1/SCMH1/ETV6/MEN1/UBR2/YY1/PUM1/JAM3/PTK2B/RARA/DAZAP1/ASF1B/PRSS21/CD9/SBF1/BAD/DZIP1/BCL2L11/SRC/CALR/ZNF296/LRRC8A/SEPTIN4/RUVBL1/HERC2/ZMIZ1/SPOCD1/USP9X/DPCD/ALKBH5/NCAPH/E2F1/PITHD1/GOLGA3/ARRB2/PDE5A/KLC3/DNM2/LGALS9/TBC1D20/BSG/PSME4/FOXJ2/KLF1/BCL2L1/STK11/UBB/ABHD2/IGF2R/PPP1R9B/CCNI/ATP2B4/NEURL1/P2RX1/PTGDR2/CTSB/YBX3/FOXO3/CTDNEP1/MED1/OSBP2/NCOR2/ACRBP/MARF1/BAG6

BP	GO:0050776	regulation of immune response	627	-0.31	-1.30	0.00396603	0.01729048	0.01072072	2262	tags=22%, list=19%, signal=19%	OAS3/LRRC14/IKBKE/PKN1/HLA-DRB1/MAPKAPK3/IRF1/PRKD2/CD81/FYN/CRKL/IL1B/SPI1/HLA-A/OTUD4/PTPN6/SCIMP/VAV2/TLR6/PGLYRP1/FES/LGALS3/NR1H2/ITGAM/XRCC6/CDC37/SMAD3/PDE4B/PTPRC/SH2B2/TAPBP/KLHL6/FZD5/STOML2/INPP5D/DGKZ/CD14/CD74/CD300LF/NCR3/IKBKG/FOXP1/DDX60/ANKRD17/NLRX1/NR1H3/FAM3A/PYCARD/CACTIN/HSP90AB1/TGFB1/MICB/TEC/TBX21/TNFRSF1B/RBM14/EZR/STX4/IL4I1/FPR3/TRAF6/ALOX15/BTN3A2/UNC93B1/KARS1/LYN/SPPL3/MAVS/EPG5/SUPT6H/SERPING1/PIK3AP1/PUM1/ADAR/RARA/HSPA1A/MAP2K7/TTLL12/CGAS/CREBBP/SPN/PDCD1/SRC/CEACAM1/RBCK1/PIK3CD/VAV1/LAPTM5/TP53BP1/HMCES/BCR/SYK/JAK3/XIAP/ABL1/PAK1/TNFSF4/FKBP1A/N4BP1/RELA/LTF/C1QA/ARRB2/MFHAS1/MAD2L2/PTPRJ/LGALS9/WAS/MAPKAPK2/RAC2/STK11/ZDHHC5/KRT1/SPG21/RIOK3/PRKCB/CSK/PTPN1/ZNFX1/SEC14L1/NFATC2/GRN/PRKDC/LAMP1/GATA1/HLX/AKIRIN2/TNIP1/PAK2/GPX1/SLC15A3/FURIN/MED1/NPLOC4/CLU/WNK1/BAG6
CC	GO:0045178	basal part of cell	157	-0.40	-1.48	0.00405473	0.01762676	0.01092922	1775	tags=24%, list=15%, signal=20%	SLC29A2/EDN1/SLC41A1/SLC19A1/EPPK1/ABCC1/HSP90AB1/ST14/EZR/STX4/SLC27A1/AQP10/SLC5A6/AQP1/SLC9A1/CEACAM1/ABCC3/SLC7A8/SLC1A5/SLC14A1/ABCC4/CLASP1/ARRB2/SLC2A1/SLC7A5/LDLRAP1/SLC3A2/BSG/SLC6A9/SLC4A1/LRP1/EPB41/MSN/ATP2B4/SLC29A1/WASF2/ANK1
BP	GO:0050792	regulation of viral process	129	-0.42	-1.52	0.00411144	0.0178267	0.01105319	1681	tags=28%, list=14%, signal=24%	CD74/PDE12/VPS37B/TOP2A/STOM/APOBEC3D/TRIM26/MAVS/RAB7A/ADAR/IFITM3/TRIM32/ZC3HAV1/SRPK2/SNX3/PPARA/TRIM10/TRIM28/IFITM2/DDB1/APOBEC3F/N4BP1/LTF/RAD23A/STAU1/SETDB1/LGALS9/TBC1D20/INPP5K/TRIM11/GSN/TRIM14/ZNFX1/APOBEC3C/TNIP1/LARP1
BP	GO:0110148	biomineralization	82	-0.47	-1.61	0.00419331	0.01808309	0.01121217	2974	tags=46%, list=25%, signal=35%	TENT5A/NECTIN1/SLC8A1/FGF23/SBNO2/FGR/BMP2K/NOTCH1/SLC24A4/PHEX/CCR1/SLC20A1/TUFT1/ROCK2/TFIP11/ALPL/SMAD3/ZBTB40/ACVR2B/TGFB1/SNX10/BCOR/ALOX15/ANO6/PTK2B/GREM1/PPARA/LTBP3/FAM20C/PHOSPHO1/RFLNB/STIM1/LTF/BMP6/SLC24A3/ANKH/GATA1/SMPD3
BP	GO:0045926	negative regulation of growth	142	-0.42	-1.51	0.00418243	0.01808309	0.01121217	2593	tags=33%, list=22%, signal=26%	ULK1/SMARCA2/DIP2B/MUL1/RAI1/CDKN2D/FLCN/G6PD/BTG1/CDK5/HYAL2/DCUN1D3/CCAR2/SERTAD3/EI24/SMAD3/ACVR1B/KIAA0319/SEMA4D/CDA/GSK3A/TGFB1/SMARCA4/CCN3/VGLL4/BCL11A/SESN2/FHL1/YY1/MT2A/HSPA1A/PML/GREM1/SFRP2/PPARA/SEMA4A/NRP1/FBP1/JARID2/PTPRJ/STK11/ADIPOR1/PPP1R9B/ENO1/CDKN1A/DNAJB2/DAB2
BP	GO:0071219	cellular response to molecule of bacterial origin	158	-0.40	-1.48	0.00417524	0.01808309	0.01121217	2108	tags=30%, list=18%, signal=25%	IL1B/SPI1/PPBP/SCIMP/TLR6/GSTP1/TRIB1/SCARB1/PDE4B/FZD5/CD14/SIGIRR/VIM/NR1H3/PYCARD/CACTIN/TGFB1/GIT1/TNFRSF1B/CD180/TRAF6/LYN/SASH1/RARA/MTDH/SRC/LITAF/BCR/UPF1/ABL1/TNFSF4/RELA/LTF/NFKB1/BMP6/RHOA/CDK19/PLSCR3/PRPF8/PF4V1/PAF1/CXCL5/MAPK3/GATA1/PF4/TNIP1/MAP2K3/TBXA2R
BP	GO:0031058	positive regulation of histone modification	86	-0.46	-1.59	0.00423738	0.01825331	0.01131771	2217	tags=30%, list=19%, signal=25%	MECP2/PRKD2/GLYR1/IL1B/CDK9/ARRB1/CTBP1/WDR5/SREBF1/NELFA/JDP2/PINK1/KANSL1/KAT7/WBP2/PIH1D1/MCRS1/KMT2A/JARID2/LMNA/RPS6KA4/PAF1/MAPK3/BRD4/RNF40/HCFC1
CC	GO:0101002	ficolin-1-rich granule	173	-0.39	-1.45	0.00429258	0.01841133	0.01141569	1989	tags=28%, list=17%, signal=24%	PGAM1/GSTP1/LGALS3/APEH/XRCC6/ARPC5/MMP9/CDA/CRISPLD2/SERPINB6/TIMP2/DOK3/HSP90AB1/ALAD/MVP/ALDOA/CANT1/GLB1/RAC1/ALDOC/HSPA1A/PFKL/PKM/PSMD11/VCP/LRG1/ATP6V0C/LAMTOR1/GAA/RHOA/VCL/ARL8A/PSMD2/ATG7/ATP6V0A1/FTH1/KRT1/UBR4/GSN/PLEKHO2/HUWE1/GNS/GPI/COTL1/LAMP1/CAPN1/CTSD/CTSB/DIAPH1

BP	GO:0010927	cellular component assembly involved in morphogenesis	60	-0.51	-1.63	0.00446168	0.01901354	0.01178908	1770	tags=27%, list=15%, signal=23%	PMP22/EDN1/TBPL1/ACTG1/WDR1/TPM1/CD9/FLII/CSRP1/CLASP1/TBC1D20/PLEC/AKAP13/TMOD1/MYL9/ACRBP
BP	GO:0007498	mesoderm development	60	-0.51	-1.63	0.00446168	0.01901354	0.01178908	1929	tags=30%, list=16%, signal=25%	SECTM1/SMAD3/KLF4/ACVR2B/PRKACA/MESD/KDM6B/TWSG1/PUS7/SFRP2/ETS2/MEST/NF2/GPI/IKZF1/CTDNEP1/TAL1/ITGB3
BP	GO:0010717	regulation of epithelial to mesenchymal transition	55	-0.52	-1.64	0.00451071	0.01918137	0.01189314	2444	tags=38%, list=21%, signal=30%	NOTCH1/EZH2/EPHA4/USF3/SPRED1/IL1B/SMAD3/TIAM1/ENG/TGFB1I1/BCL9L/MCRIP1/TGFB1/GREM1/SFRP2/CLASP1/MAD2L2/ADIPOR1/TCF7L2/ZNF703/DAB2
BP	GO:0097581	lamellipodium organization	69	-0.49	-1.59	0.00452058	0.01920281	0.01190643	3008	tags=42%, list=26%, signal=31%	KIT/ARHGEF6/ABLIM1/FSCN1/ARPIN/ABI2/ATP7A/CORO1B/PIK3CA/ABI3/PLEKHO1/VAV2/SRGAP2/ARPC5/CYFIP1/CORO1C/RAC1/SRC/ABLIM3/VCL/DNM2/WAS/RAC2/PARVB/WASF3/CAPZB/CTTN/WASF2/DMTN
BP	GO:0046649	lymphocyte activation	574	-0.31	-1.30	0.00455069	0.01931008	0.01197295	2556	tags=27%, list=22%, signal=22%	CORO1A/AKT1/SELENOK/LY9/FOXP3/TNFSF13/SWAP70/CBLB/PIK3CA/ZFPM1/PTPN11/BAK1/CD22/CD3E/EFNB1/DOCK8/EGR1/HDAC4/HDAC5/EZH2/SNX27/PRDM1/IL6R/HES1/PKN1/HLA-DRB1/IRF1/CD81/FYN/HLA-DQA2/ICAM1/LGALS9C/IL1B/SPI1/HLA-A/SH3KBP1/DOCK10/TXLNA/PTPN6/ZBTB16/LCP1/PGLYRP1/ZBTB7A/SIRPG/ZFP36L2/LGALS3/SLC7A1/XRCC6/SOX13/SMAD3/RUNX1/PTPRC/RIPOR2/WDFY4/DROSHA/FZD5/STOML2/INPP5D/RBPJ/CD74/NCR3/SIRPB1/SOX4/PSMB10/FOXP1/HLA-DQA1/IL2RB/PYCARD/TGFB1/MICB/CDK6/TBX21/TMEM131L/TNFRSF1B/PREX1/PRR7/CD180/BCL11B/IL4I1/TWSG1/TRAF6/SIT1/LYN/YY1/SUPT6H/PTK2B/PRF1/RARA/IGF2/KDELR1/CGAS/SPN/BAD/KIF13B/NCSTN/SRC/CCND3/KAT7/DCAF1/CEACAM1/PIK3CD/LRRC8A/HLA-DRB5/VAV1/RUNX3/PPP3CA/LAPTM5/ZMIZ1/TP53BP1/HMCES/STAT3/SEMA4A/SYK/JAK3/ABL1/MAFB/TNFSF4/FKBP1A/BLOC1S3/ITGAL/APBB1IP/MPL/PDE5A/RHOA/NOTCH2/MAD2L2/PTPRJ/LGALS9/WAS/FKBP1B/RAC2/DOCK2/STK11/SDC4/GSN/PRKCB/CSK/PIK3CG/CD151/SPTA1/MSN/NFATC2/ELF4/PRKDC/LAMP1/SOX12/IKZF1/FBXO7/HLX/LYL1/CDKN1A/ST3GAL1/FOXO3/MYH9/PBX1/BAG6
BP	GO:0072583	clathrin-dependent endocytosis	40	-0.56	-1.66	0.00456083	0.01933246	0.01198682	1960	tags=50%, list=17%, signal=42%	AP2A1/SIGLEC1/BMP2K/AP2A2/TNK2/NEU3/PIK3CB/CANX/AP2B1/DGKD/CLTC/HIP1/AP2S1/ITSN1/HIP1R/CLTB/DNM2/GAK/AP2M1/DAB2
BP	GO:0061458	reproductive system development	228	-0.37	-1.42	0.00462165	0.01950708	0.01209509	2635	tags=33%, list=22%, signal=26%	FANCG/NRIP1/ZFY/PKD1/MAPK14/RNF38/AKT1/NSDHL/AGO4/TIPARP/PTPN11/NOTCH1/BAK1/SGPL1/INSL3/PRDM1/HES1/ASPM/CSDE1/CASP2/CRKL/DHX37/PTPN6/VASH1/HSPA5/NCOA4/BASP1/HSD17B4/SPATA2/CUL7/BIRC6/TFEB/UBE2Q1/FZD5/RBPJ/GRB2/HYAL3/NCOA1/HSP90AB1/ASCL2/ST14/SPINT2/MEN1/EPAS1/BPTF/RARA/IGF2/DAZAP1/DHCR24/BCL2L11/SRC/CDKN1C/ARNT/TAF4/SFRP2/PSAP/TRIM28/CTNNA1/SF1/BMP6/ARRB2/NOTCH2/TBC1D20/BSG/BCL2L1/STK11/UBB/PPP1R9B/GATA1/CBL/CTSB/YBX3/FOXO3/MED1/PDGFA
BP	GO:0034103	regulation of tissue remodeling	42	-0.55	-1.66	0.00464662	0.01956277	0.01212962	2059	tags=40%, list=18%, signal=33%	CSF1R/ROCK2/PPARGC1B/RUNX1/INPP5D/TGFB1/SRC/GREM1/CEACAM1/LTBP3/PPP3CA/BCR/SYK/GPR137/CSK/UBASH3B/ITGB3

BP	GO:1902117	positive regulation of organelle assembly	69	-0.48	-1.58	0.00465452	0.01956277	0.01212962	3084	tags=45%, list=26%, signal=33%	WRAP73/RAB3GAP1/LRSAM1/FSCN1/CNOT6/SH3GLB1/CROCC/SDCBP/ULK1/RALB/MARK4/PIP4K2C/VPS4B/LCP1/NUMA1/ARHGAP35/GSK3B/G3BP2/ASAP1/ATG2A/DZIP1/DYNC1H1/SRC/RHOA/SDC4/MSN/PIP4K2A/ELAPOR1/WDR45/HTT/CNOT1
CC	GO:0009986	cell surface	468	-0.32	-1.32	0.00465162	0.01956277	0.01212962	1871	tags=19%, list=16%, signal=17%	ITGAM/NRROS/TMEM8B/SCARB1/ACVR1B/PTPRC/NEU3/LRFN1/ENG/ENO2/FZD5/CD14/CD74/HLA-C/IL2RG/SIRPB1/CXCR2/FAM234A/IL2RB/SDC3/HSP90AB1/TGFB1/MICB/CXCR1/CD63/LILRA5/PCSK6/AMOT/STX4/MAP3K5/BTN3A2/GSR/ANO6/PLXNB2/MBP/RARA/CD9/SLC9A1/SPN/PDCD1/GREM1/CEACAM1/CALR/LRRC8A/ATP1B2/SLC1A4/PDGFC/ABCG2/ABCC4/TNFSF4/LTF/PDIA4/MYO18A/ITGAL/MPL/GP6/SLC7A5/NOTCH2/PTPRJ/SLC3A2/ECE1/SDC4/ITGB5/SORT1/CLSTN1/GP1BA/IGF2R/PLVAP/THBS1/CD151/TSPAN33/MSN/SELP/LRFN3/ENO1/LAMP1/P2RX1/CAPN5/TREML1/CTSB/SMIM1/FURIN/ITGB3/TNS1/SPARC/PDGFA/CLU/ITGA2B/TLN1
MF	GO:0043130	ubiquitin binding	90	-0.45	-1.54	0.00476457	0.02000416	0.0124033	1867	tags=28%, list=16%, signal=24%	UBXN7/TOM1/SMAD3/HGS/NSFL1C/IKBKG/TOP2A/TRIM32/ILRUN/RBCK1/AMFR/DDI2/UBAP1/N4BP1/RAD23A/JARID2/HSPB1/VPS28/SQSTM1/SHARPIN/FBXO7/ASCC2/DNAJB2/TOLLIP/NPLOC4
BP	GO:0045598	regulation of fat cell differentiation	80	-0.47	-1.59	0.00486768	0.02037254	0.01263171	2960	tags=44%, list=25%, signal=33%	TMEM64/SAV1/SYAP1/WDFY2/ZFP36L1/CEBPA/CMKLR1/MAPK14/AKT1/ZFPM1/NR1D1/ZFP36/FLCN/FOXO1/ZNF385A/HES1/ZBTB16/ZFP36L2/SOX13/TFE3/SMAD3/LGALS12/FTO/TGFB1I1/NAPEPLD/TGFB1/JDP2/TRIO/SFRP2/TAF8/E2F1/SORT1/ASXL2/CARM1/PIM1
BP	GO:0043406	positive regulation of MAP kinase activity	80	-0.47	-1.59	0.00493576	0.02061407	0.01278146	2362	tags=35%, list=20%, signal=28%	EZH2/MAP3K13/EPHA4/IL1B/TLR6/PTPRC/EDN1/MAGED1/FZD5/MAP3K11/TGFB1/TRAF6/MAP3K5/SASH1/PTK2B/MAP2K7/SRC/DVL3/PDGFC/SYK/PAK1/PDE5A/CSK/THBS1/PTPN1/AKAP13/PIK3CG/PDGFA
BP	GO:0031214	biomineral tissue development	81	-0.47	-1.60	0.0049764	0.02076197	0.01287317	2974	tags=47%, list=25%, signal=35%	TENT5A/NECTIN1/SLC8A1/FGF23/SBNO2/FGR/BMP2K/NOTCH1/SLC24A4/PHEX/CCR1/SLC20A1/TUFT1/ROCK2/TFIP11/ALPL/SMAD3/ZBTB40/ACVR2B/TGFB1/SNX10/BCOR/ALOX15/ANO6/PTK2B/GREM1/PPARA/LTBP3/FAM20C/PHOSPHO1/RFLNB/STIM1/LTF/BMP6/SLC24A3/ANKH/GATA1/SMPD3
BP	GO:0051216	cartilage development	89	-0.45	-1.52	0.00500384	0.02084024	0.0129217	2773	tags=38%, list=24%, signal=29%	MEF2D/MSX1/ATP7A/EXT1/PKD1/MAPK14/SMAD5/PTPN11/HOXB3/HYAL2/THRA/ZBTB16/TIMP1/SMAD3/RUNX1/EDN1/HYAL3/TGFB1/CCN3/TRPS1/RARA/CHST11/SOX6/GREM1/SFRP2/LTBP3/RUNX3/RFLNB/ANXA6/BMP6/GLG1/MAPK3/SMPD3/SCIN
BP	GO:0019079	viral genome replication	117	-0.43	-1.55	0.00500564	0.02084024	0.0129217	1665	tags=26%, list=14%, signal=23%	GRK2/CTBP1/PDE12/TOP2A/STOM/NFIA/APOBEC3D/MAVS/ADAR/IFITM3/IFI27/ZC3HAV1/SRPK2/PPARA/TRIM28/IFITM2/DDB1/VCP/APOBEC3F/N4BP1/LTF/RAD23A/STAU1/SETDB1/TBC1D20/INPP5K/ZNFX1/APOBEC3C/PCBP2/TNIP1/LARP1

BP	GO:0048609	multicellular organismal reproductive process	440	-0.33	-1.36	0.00509085	0.02106264	0.0130596	2701	tags=29%, list=23%, signal=23%	RPS6KA2/PI3/H3-3A/ZNF628/AGFG1/GMCL1/FANCG/NRIP1/ZFY/SMARCA2/SMAD5/PTAFR/AKT1/AGO4/ADNP/PTPN11/NOTCH1/TRPM2/EGR1/SPIRE1/SGPL1/SEPTIN6/KPNA6/CDYL/ZSCAN21/INSL3/WASHC5/PRDM1/BTG1/SPIN1/ASPM/ACOX1/CASP2/CRKL/DEAF1/THRA/SPIRE2/TTLL1/TTC21A/HSPA5/GGT1/SPATA5/ZBTB16/PAQR8/DIAPH2/TESK2/SPATA2/SYNE1/PTTG1/RHBDD1/EDN1/UBE2Q1/CRTAP/ENO2/GTSF1/TBPL1/BCAP31/EHMT2/NR6A1/TOP2A/PTCHD3/HYAL3/TOB2/CETN2/PRKACA/SFMBT1/SCMH1/ETV6/MEN1/UBR2/YY1/PUM1/JAM3/PTK2B/RARA/DAZAP1/ASF1B/PRSS21/SBF1/BAD/DZIP1/BCL2L11/SRC/CALR/ZNF296/LRRC8A/SEPTIN4/RUVBL1/HERC2/ZMIZ1/SPOCD1/USP9X/DPCD/ALKBH5/NCAPH/E2F1/PITHD1/GOLGA3/ARRB2/PDE5A/KLC3/DNM2/LGALS9/TBC1D20/BSG/PSME4/FOXJ2/KLF1/BCL2L1/STK11/UBB/ABHD2/IGF2R/PPP1R9B/CCNI/ATP2B4/NEURL1/P2RX1/PTGDR2/CTSB/YBX3/FOXO3/CTDNEP1/MED1/OSBP2/NCOR2/ACRBP/MARF1/BAG6
BP	GO:1902743	regulation of lamellipodium organization	41	-0.56	-1.66	0.00510949	0.02109587	0.0130802	1464	tags=41%, list=12%, signal=36%	FSCN1/ARPIN/ABI2/ATP7A/CORO1B/PIK3CA/ABI3/CYFIP1/CORO1C/RAC1/SRC/DNM2/WAS/RAC2/CAPZB/WASF2/DMTN
BP	GO:0009617	response to bacterium	372	-0.34	-1.36	0.00510727	0.02109587	0.0130802	2108	tags=25%, list=18%, signal=21%	IL1B/SPI1/GBP4/CNR2/HLA-A/PPBP/RNASE3/SCIMP/TLR6/PGLYRP1/GSTP1/TRIB1/SHC1/ALPL/SCARB1/GBP6/PDE4B/EDN1/DROSHA/KLHL6/FZD5/RBPJ/CD14/JUND/SIGIRR/VIM/FOXP1/BAIAP2/ANKRD17/NR1H3/PYCARD/CACTIN/TGFB1/DCD/GIT1/TNFRSF1B/CD180/ALAD/TRAF6/AQP1/LYN/MAVS/SASH1/RARA/ST13/MTDH/SPN/SRC/RBCK1/SNX3/LITAF/BCR/UPF1/SYK/LRG1/ABL1/LCN2/FOS/TNFSF4/RAB14/OPTN/RELA/OTUD5/LTF/NFKB1/BMP6/RNF213/RHOA/NOTCH2/LGALS9/MAPKAPK2/CDK19/PLSCR3/PRPF8/PF4V1/ZNFX1/MARCHF2/GRN/SHARPIN/SELP/SNCA/PAF1/CXCL5/H2BC11/MAPK3/GATA1/PF4/AKIRIN2/TNIP1/GPX1/MAP2K3/TBXA2R
BP	GO:0090288	negative regulation of cellular response to growth factor stimulus	43	-0.54	-1.62	0.00514946	0.02117297	0.01312801	1772	tags=30%, list=15%, signal=26%	HGS/PIK3CB/SKI/TWSG1/GREM1/SFRP2/ABL1/HIPK2/NIBAN2/THBS1/PTPN1/ATP2B4/PPM1A
CC	GO:0045335	phagocytic vesicle	124	-0.43	-1.53	0.00513651	0.02117297	0.01312801	2724	tags=42%, list=23%, signal=33%	NCF2/ATP7A/RAB11FIP5/RAB8A/CORO1A/SYT11/CYBB/NCF1/LAMP2/FMNL1/HLA-A/SCIMP/TLR6/PGLYRP1/SRGAP2/RAB20/HGS/TAPBP/HLA-C/STX6/VIM/HVCN1/ACTG1/STX4/CDC42EP4/PLD4/RAB31/ZYX/UNC93B1/RAB7A/RAB11FIP1/FLNB/CALR/SNX3/SYK/ATP6V0D1/RAB14/LTF/ATP6V0C/ANXA11/DNM2/WAS/RAB11B/RAC2/ATP6V0A1/MCOLN1/ZDHHC5/ITGB5/GSN/RILP/TRIM14/LAMP1
BP	GO:0045744	negative regulation of G protein-coupled receptor signaling pathway	34	-0.58	-1.65	0.00515724	0.02118305	0.01313425	1830	tags=32%, list=16%, signal=27%	ARRB1/PDE4B/GRK2/RPGRIP1L/PLEK/YWHAB/GNAI2/ARRB2/DNM2/ATP2B4/SNCA
BP	GO:0014706	striated muscle tissue development	110	-0.43	-1.53	0.00519276	0.02126306	0.01318386	1816	tags=26%, list=15%, signal=22%	SMAD3/RUNX1/EDN1/ENG/RBPJ/CENPF/GSK3A/TGFB1/VGLL4/KDM6B/TPM1/YY1/RARA/SLC9A1/SOX6/GREM1/CALR/PPARA/FKBP1A/ARRB2/JARID2/NOTCH2/LMNA/PLEC/AKAP13/MTPN/NPRL3/MED1/PIM1

BP	GO:1990874	vascular associated smooth muscle cell proliferation	34	-0.57	-1.64	0.00522646	0.02137904	0.01325577	1756	tags=35%, list=15%, signal=30%	EDN1/POLDIP2/MMP9/MAP3K5/TPM1/GNAI2/RHOA/LDLRAP1/FOXJ2/GNA12/MFN2/CDKN1A
BP	GO:0098927	vesicle-mediated transport between endosomal compartments	44	-0.55	-1.67	0.00529562	0.02161745	0.0134036	1626	tags=32%, list=14%, signal=28%	WDR81/SNX12/EZR/RAB7A/SRC/HOOK3/SNX3/MAP2K2/FLNA/RILP/MSN/MAPK3/PTPN23/DAB2
BP	GO:0043502	regulation of muscle adaptation	49	-0.53	-1.64	0.00534423	0.02172666	0.01347131	2444	tags=51%, list=21%, signal=41%	NOTCH1/TRPC3/ATP2A2/HDAC4/FOXO1/G6PD/P2RX4/ROCK2/CDK9/SMAD3/EDN1/GSK3A/TNFRSF1B/PARP1/YY1/SLC9A1/PPARA/PPP3CA/JARID2/PDE5A/PDE9A/LMNA/MTPN/ATP2B4/FOXO3
BP	GO:0050877	nervous system process	467	-0.32	-1.33	0.00535412	0.02174462	0.01348245	1587	tags=17%, list=14%, signal=15%	KCNJ2/DTNBP1/EHMT2/SERPINB6/GSK3B/BAIAP2/GSK3A/CYFIP1/GIT1/TNFRSF1B/EML2/CCN3/CLIC5/GNAS/WDR1/MFSD2A/DAW1/EPAS1/FZD2/MBP/JAM3/NPFF/PTK2B/ATXN1/SLC9A1/GNB1/PITPNA/NCSTN/SRC/DLGAP4/BBS5/MYCBP2/VTI1A/PPP3CA/DHRS3/AMFR/TBL1X/SLC1A4/MAP1A/VLDLR/BCR/HERC1/ABL1/KIFC3/PARD3/FOS/SYNGAP1/ODAD4/TMEM120A/ARRB2/GAA/CHD8/FKBP1B/AARS1/HIPK2/DCTN1/PLEC/CLDN5/PPP1R9B/PDE4A/GPI/SNCA/P2RX1/MAPK3/EIF4EBP2/RNF10/SLC29A1/NRGN/WASF3/ESPN/GPX1/HPS1/PRKAR2B/HTT/SHISA7/MGLL/SCN1B/DIAPH1
BP	GO:0071216	cellular response to biotic stimulus	182	-0.38	-1.44	0.00538635	0.0218532	0.01354977	2108	tags=32%, list=18%, signal=27%	IL1B/SPI1/PPBP/HSPA5/SCIMP/TLR6/GSTP1/TRIB1/SCARB1/PDE4B/FZD5/CD14/CDA/SIGIRR/VIM/GSK3B/NR1H3/PYCARD/CACTIN/TGFB1/GIT1/TNFRSF1B/CD180/SMC1A/TRAF6/LYN/SASH1/RARA/MTDH/BCL2L11/SRC/SLX4/LITAF/BCR/UPF1/SYK/FBH1/ABL1/TNFSF4/RELA/LTF/NFKB1/BMP6/RHOA/NOTCH2/CDK19/PLSCR3/PRPF8/TXNIP/PF4V1/PAF1/CXCL5/MAPK3/GATA1/PF4/TNIP1/MAP2K3/TBXA2R
BP	GO:0010762	regulation of fibroblast migration	30	-0.60	-1.69	0.00547267	0.02215819	0.01373888	2562	tags=50%, list=22%, signal=39%	MACIR/AKT1/CLN3/HYAL2/MTA2/RCC2/PRR5L/TGFB1/CORO1C/RAC1/ACTA2/SDC4/GNA12/THBS1/DMTN
BP	GO:0048608	reproductive structure development	225	-0.36	-1.39	0.0054925	0.02221585	0.01377463	2635	tags=36%, list=22%, signal=29%	ANG/LEF1/ATM/PTCH1/ZFP36L1/MAP3K4/CEBPA/FRS2/H3-3A/FANCG/NRIP1/ZFY/PKD1/MAPK14/RNF38/AKT1/NSDHL/AGO4/TIPARP/PTPN11/NOTCH1/BAK1/SGPL1/INSL3/PRDM1/HES1/ASPM/CSDE1/CASP2/CRKL/DHX37/PTPN6/VASH1/HSPA5/NCOA4/BASP1/HSD17B4/SPATA2/CUL7/BIRC6/TFEB/FZD5/RBPJ/GRB2/HYAL3/NCOA1/HSP90AB1/ASCL2/ST14/SPINT2/MEN1/EPAS1/BPTF/RARA/IGF2/DAZAP1/DHCR24/BCL2L11/SRC/CDKN1C/ARNT/TAF4/SFRP2/PSAP/TRIM28/CTNNA1/SF1/BMP6/ARRB2/NOTCH2/TBC1D20/BSG/BCL2L1/STK11/UBB/GATA1/CBL/CTSB/YBX3/FOXO3/MED1/PDGFA
BP	GO:0030509	BMP signaling pathway	77	-0.47	-1.56	0.00551804	0.02229066	0.01382101	2622	tags=39%, list=22%, signal=30%	EXT1/SMAD5/SORL1/NOTCH1/EGR1/HES1/SLC33A1/NUMA1/SMAD3/ENG/RBPJ/ACVR2B/TGFB1/PCSK6/SKI/TWSG1/ILK/GREM1/SFRP2/USP9X/XIAP/ABL1/BMP6/NOTCH2/HIPK2/FKBP8/MAPK3/SMPD3/UBE2O/PPM1A
BP	GO:0007041	lysosomal transport	108	-0.44	-1.54	0.00552221	0.02229066	0.01382101	2418	tags=35%, list=21%, signal=28%	CLN3/LAMP2/TSG101/M6PR/HGSNAT/SNX27/VPS39/VPS4B/VPS51/NCOA4/HGS/CHMP6/TRAK1/ANKFY1/VPS41/CHMP4B/VPS53/EHD3/RAB7A/EPG5/PINK1/HSPA1A/CHMP1A/HOOK3/LAPTM5/PSAP/VCP/DTX3L/SORT1/GAK/IGF2R/LRP1/RILP/GRN/LAMP1/TGFBRAP1/UBXN6/CLU

BP	GO:0008610	lipid biosynthetic process	474	-0.32	-1.31	0.00557296	0.02244994	0.01391977	2555	tags=26%, list=22%, signal=21%	STARD3/AKT1/PIP5KL1/ABCA3/MECR/MTMR3/NSDHL/ACLY/PIK3CA/ABCB11/PTDSS1/ACACB/ACSS1/NR1D1/ATP1A1/CLN3/EGR1/SGPL1/TSPO/DECR2/CHP1/SMPD4/G6PD/SERINC5/PIP4K2C/CDIPT/ABCD1/PI4K2A/PRKD2/ACER3/IL1B/OSBPL7/ORMDL3/CERS2/ALDH3B1/GSTM4/PLA2G15/GSTP1/EIF6/DHCR7/MBOAT7/NR1H2/SOCS7/FADS2/HSD3B7/B3GNT5/SCARB1/SYNJ1/EDN1/PI4KB/LSS/PIK3CB/INPP5D/DGKZ/LPCAT2/CD74/B4GALT5/PIK3R3/SMPD1/DGKD/SREBF1/NR1H3/FAM3A/ST3GAL3/SLC44A2/ITPKC/SPHK1/FITM1/INPPL1/SLC27A1/WDTC1/EFR3A/MFSD2A/CYP27A1/ALOX15/GBGT1/GPAA1/PPM1L/PNPLA1/DHCR24/PITPNM2/CEACAM1/CERS6/PIK3CD/PGAP2/PPARA/ACOT7/PIGT/ABHD4/TTC7B/AGPAT1/PIP5K1B/AGPAT3/PNPLA2/NFKB1/LPIN2/SF1/BMP6/ACADVL/INPP5K/FASN/ST6GALNAC4/CYB5R3/PLSCR3/IP6K1/ABHD2/TTC7A/ALOX12/PIK3CG/B4GALT3/PIP4K2A/DPM2/P2RX1/ELOVL7/PTGS1/ST3GAL1/FDFT1/CTDNEP1/FAXDC2/MED1/MGLL/PDGFA/PBX1/DAB2
CC	GO:0070160	tight junction	76	-0.47	-1.57	0.00559994	0.02253578	0.013973	2072	tags=34%, list=18%, signal=28%	TBCD/PARD6A/PARD6B/LSR/PMP22/FRMD4B/FZD5/EPPK1/UBN1/FRMD4A/RPGRIP1L/AMOT/TJAP1/ARHGAP17/SIPA1L3/JAM3/MTDH/ACTB/ARHGEF2/TJP2/PARD3/CLDN5/VASP/YBX3/ESAM/SH3BP1
MF	GO:0005518	collagen binding	36	-0.58	-1.69	0.00562515	0.02261437	0.01402173	709	tags=28%, list=6%, signal=26%	SMAD3/CRTAP/MMP9/PAK1/GP6/THBS1/MRC2/CTSB/SPARC/PDGFA
BP	GO:0043086	negative regulation of catalytic activity	478	-0.32	-1.32	0.00569546	0.02287389	0.01418264	2732	tags=34%, list=23%, signal=27%	MCM2/TSC2/PIF1/FAF2/PRPSAP2/PDPK1/SH2B3/GABBR1/PI3/CR1/PPP1R14A/GNAQ/SORL1/PKIG/AKT1/ARL2/AIDA/TAOK3/CBLB/RRP1B/SERPINF2/MLLT1/MAPK7/KNG1/NOTCH1/ACACB/CLN3/TMBIM1/BICD1/PPP1R15A/PDE6G/GADD45B/ZFP36/CDKN2D/TSG101/PTPA/INKA1/CHP1/LMTK2/PKN1/SH3BP5L/PIP4K2C/SPRED1/HYAL2/PCSK1N/CDK5RAP2/CSNK2A1/IL1B/CNR2/PROS1/ORMDL3/PTPN6/ROCK2/TNK2/TIMP1/GSTP1/MGAT5/TRIB1/CCAR2/LGALS3/GPS1/PDE6D/TFIP11/RCC2/ARRB1/BIRC6/PTTG1/FRY/APLP2/GPSM1/PTPRC/CAST/PIK3CB/CHMP6/MMP9/KLF4/SEMA4D/ITPRIP/SMPD1/DTNBP1/SERPINB6/GSK3B/CRY2/TIMP2/GSK3A/PYCARD/RPS6KA1/YWHAG/AMOT/SPINT2/MEN1/MVP/WDTC1/CORO1C/ARL6IP1/AQP1/LYN/BCAS3/SERPING1/ADAR/DHCR24/SRC/UBE2Z/CDKN1C/CEACAM1/RUBCN/YWHAB/SFRP2/GNAI2/GMIP/PIH1D1/KAT2B/APBA3/XIAP/ABL1/FKBP1A/LTF/NFKB1/WARS1/DTX3L/ARRB2/PRKAR2A/GALR1/MAD2L2/PTPRJ/FKBP1B/INPP5K/HSPB1/NF2/SIAH2/PRDX5/SORT1/HEXIM2/PLEC/SLC4A1/TXNIP/PSMF1/LRP1/TERF2/PPP1R9B/CSK/THBS1/CABIN1/PTPN1/IQGAP1/GPI/UBASH3B/ATP2B4/SNCA/PPP1R10/PIP4K2A/FBXO7/CDKN1A/CBL/PAK2/UBE2O/CTSB/GPX1/PRKAR2B/TESC/FURIN/OAZ1/WNK1
BP	GO:0007204	positive regulation of cytosolic calcium ion concentration	177	-0.39	-1.45	0.00570491	0.0228887	0.01419182	2769	tags=32%, list=24%, signal=25%	CCRL2/LETM1/PDPK1/CMKLR1/PKD1/CORO1A/PTGIR/SWAP70/KNG1/TRPM2/BAK1/TRPC3/DLG4/SLC24A4/CCR1/CDK5/FYN/P2RX4/PTPN6/ITPR2/LPAR4/PTPRC/EDN1/CXCR2/BCAP31/PRKACA/CXCR1/SELENON/FPR3/CHERP/LYN/SPPL3/PTK2B/GNA15/PML/RASA3/FIS1/JAK3/ABL1/FKBP1A/GALR1/CALM3/FKBP1B/MCOLN1/GP1BA/GP9/HRH4/PIK3CG/UBASH3B/ATP2B4/SNCA/P2RX1/GATA1/HTT/DIAPH1/TBXA2R

BP	GO:0051347	positive regulation of transferase activity	389	-0.33	-1.35	0.00571822	0.022919	0.0142106	2453	tags=26%, list=21%, signal=21%	ADNP/MOB3B/NCF1/EGR1/AGAP2/EZH2/DLG4/PNKP/MAP3K13/LMTK2/SERINC5/IL6R/EPHA4/HLA-DRB1/CDK5/CD81/DDR1/IL1B/CSF1R/MADD/DCUN1D3/VAV2/TLR6/CCNY/MOB1B/PIN1/PRRC1/XRCC6/GCN1/PTPRC/EDN1/MAGED1/FZD5/DGKZ/CD74/TPX2/KLF4/MAP3K11/ACVR2B/NEDD9/GSK3A/HSP90AB1/TGFB1/MOB3A/LILRA5/TAOK1/SLC27A1/TRAF6/MAP3K5/LYN/SASH1/PINK1/PTK2B/IGF2/MAP2K7/BAD/MARK2/SRC/CCND3/GREM1/DVL3/TAOK2/DAXX/PIH1D1/MAP2K2/VLDLR/BTRC/PDGFC/SYK/ABL1/PAK1/LTF/DTX3L/PDE5A/RHOA/CALM3/UBE2S/STK11/SDC4/FZR1/CSK/THBS1/UBE2L3/PTPN1/AKAP13/PIK3CG/IQGAP1/ATP2B4/SNCA/MMD/CAMK1/NEURL1/MAPK3/CDKN1A/PAK2/STRADB/TAL1/ITGB3/PDGFA/CLU/MAP2K3/WNK1
BP	GO:0030032	lamellipodium assembly	56	-0.52	-1.64	0.00573163	0.02294961	0.01422959	676	tags=18%, list=6%, signal=17%	ABLIM3/VCL/DNM2/WAS/RAC2/PARVB/WASF3/CAPZB/WASF2/DMTN
BP	GO:0071772	response to BMP	80	-0.47	-1.58	0.0057527	0.02298769	0.01425319	1842	tags=39%, list=16%, signal=33%	EXT1/SMAD5/SORL1/NOTCH1/EGR1/HES1/SLC33A1/NUMA1/SMAD3/ENG/RBPJ/ACVR2B/TGFB1/PCSK6/SPINT2/SKI/TWSG1/ILK/GREM1/SFRP2/USP9X/XIAP/ABL1/BMP6/NOTCH2/HIPK2/FKBP8/MAPK3/SMPD3/UBE2O/PPM1A
BP	GO:0071773	cellular response to BMP stimulus	80	-0.47	-1.58	0.0057527	0.02298769	0.01425319	1842	tags=39%, list=16%, signal=33%	EXT1/SMAD5/SORL1/NOTCH1/EGR1/HES1/SLC33A1/NUMA1/SMAD3/ENG/RBPJ/ACVR2B/TGFB1/PCSK6/SPINT2/SKI/TWSG1/ILK/GREM1/SFRP2/USP9X/XIAP/ABL1/BMP6/NOTCH2/HIPK2/FKBP8/MAPK3/SMPD3/UBE2O/PPM1A
BP	GO:0043405	regulation of MAP kinase activity	124	-0.42	-1.52	0.00579083	0.02311681	0.01433325	2700	tags=38%, list=23%, signal=29%	CDK12/SH2B3/TRAF2/SORL1/AIDA/TAOK3/EZH2/TSG101/MAP3K13/EPHA4/HYAL2/IL1B/PTPN6/TLR6/GSTP1/TRIB1/HGS/PTPRC/EDN1/PIK3CB/MAGED1/FZD5/MAP3K11/SMPD1/DTNBP1/TGFB1/TRAF6/MAP3K5/LYN/SASH1/PTK2B/MAP2K7/SRC/SFRP2/DVL3/PDGFC/SYK/PAK1/PDE5A/PTPRJ/INPP5K/CSK/THBS1/PTPN1/AKAP13/PIK3CG/PDGFA
BP	GO:0030326	embryonic limb morphogenesis	58	-0.50	-1.59	0.00588886	0.02346103	0.01454669	1392	tags=24%, list=12%, signal=21%	RPGRIP1L/SKI/CHST11/CREBBP/BCL2L11/GREM1/SFRP2/IFT52/FBXW4/NOTCH2/ECE1/GNA12/MED1/PBX1
BP	GO:0035113	embryonic appendage morphogenesis	58	-0.50	-1.59	0.00588886	0.02346103	0.01454669	1392	tags=24%, list=12%, signal=21%	RPGRIP1L/SKI/CHST11/CREBBP/BCL2L11/GREM1/SFRP2/IFT52/FBXW4/NOTCH2/ECE1/GNA12/MED1/PBX1
BP	GO:0051279	regulation of release of sequestered calcium ion into cytosol	44	-0.55	-1.66	0.00591059	0.02352403	0.01458575	1491	tags=27%, list=13%, signal=24%	PRKACA/SELENON/LYN/PTK2B/ABL1/FKBP1A/CALM3/FKBP1B/UBASH3B/SNCA/HTT/DIAPH1
BP	GO:0060402	calcium ion transport into cytosol	91	-0.44	-1.53	0.00593512	0.02357446	0.01461701	1283	tags=32%, list=11%, signal=29%	TRPM2/BAK1/TRPC3/SLC24A4/FYN/P2RX4/PTPN6/ITPR2/PTPRC/PRKACA/SELENON/CHERP/LYN/PTK2B/PML/RASA3/ABL1/FKBP1A/CALM3/FKBP1B/MCOLN1/GP1BA/GP9/UBASH3B/ATP2B4/SNCA/P2RX1/HTT/DIAPH1
BP	GO:1904377	positive regulation of protein localization to cell periphery	44	-0.55	-1.66	0.00597892	0.02372474	0.01471019	1903	tags=41%, list=16%, signal=34%	PDPK1/AKT1/CNST/CLN3/LGALS3/EPB41L2/NUMA1/RANGRF/EZR/STX4/ZDHHC5/SQSTM1/LRP1/PPP1R9B/SPTBN1/EPB41/ATP2B4/RHOG
BP	GO:1990089	response to nerve growth factor	30	-0.60	-1.68	0.00602337	0.02382979	0.01477532	2556	tags=40%, list=22%, signal=31%	CORO1A/AKT1/EIF4A3/HES1/MAGI2/HSPA5/E2F1/RAPGEF1/SORT1/CBL/EHD1/FOXO3

BP	GO:1990090	cellular response to nerve growth factor stimulus	30	-0.60	-1.68	0.00602337	0.02382979	0.01477532	2556	tags=40%, list=22%, signal=31%	CORO1A/AKT1/EIF4A3/HES1/MAGI2/HSPA5/E2F1/RAPGEF1/SORT1/CBL/EHD1/FOXO3
BP	GO:0016570	histone modification	414	-0.33	-1.33	0.0060227	0.02382979	0.01477532	2385	tags=27%, list=20%, signal=22%	ATXN7/YEATS2/HDAC4/UBR5/HDAC5/EZH2/FLCN/WAC/PKN1/ELK4/MECP2/CDK5/PRKD2/WDR82/GLYR1/KMT5A/MTA2/IL1B/SPI1/MTA1/H1-4/EP400/PHF8/CDK9/EHMT1/MCM3AP/ARRB1/TAF1/TAF6/VPS72/CTBP1/TRERF1/WDR5/EHMT2/KDM4A/TRRAP/SREBF1/NCOA1/NELFA/BAZ2A/ASH2L/JDP2/CXXC1/RBM14/MEN1/BCOR/KDM6B/SKI/UBR2/MTHFR/VRK3/KMT2B/SETD1B/SUPT6H/PINK1/BRPF1/TADA3/IGF2/CREBBP/KANSL1/BRMS1/TBL1XR1/KAT7/DCAF1/TAF4/NCOA3/GATAD2B/WBP2/RUVBL1/CDK2/ACTB/PIH1D1/TBL1X/KAT2B/MCRS1/DDB1/PHF2/USP36/KMT2A/KDM2A/DTX3L/KDM4B/JARID2/SETDB1/KMT2C/KDM5C/PPP4C/LMNA/USP7/SF3B3/PCGF5/USP22/KAT6A/HUWE1/PRKCB/RPS6KA4/SETD1A/PER2/CHD4/SNCA/PAF1/MAPK3/BRD4/TRIP12/TET3/RYBP/RNF40/MLLT6/CARM1/HCFC1
BP	GO:0002764	immune response-regulating signaling pathway	345	-0.33	-1.35	0.00607527	0.02401121	0.01488782	2262	tags=23%, list=19%, signal=19%	OAS3/LRRC14/HLA-DRB1/MAPKAPK3/IRF1/PRKD2/CD81/FYN/CRKL/HLA-A/OTUD4/PTPN6/SCIMP/VAV2/TLR6/LGALS3/PDE4B/PTPRC/SH2B2/KLHL6/STOML2/INPP5D/DGKZ/CD14/CD300LF/NCR3/IKBKG/FOXP1/DDX60/ANKRD17/NLRX1/NR1H3/CACTIN/MICB/TEC/EZR/FPR3/TRAF6/BTN3A2/UNC93B1/LYN/SPPL3/EPG5/PIK3AP1/PUM1/HSPA1A/MAP2K7/CREBBP/SRC/CEACAM1/RBCK1/PIK3CD/VAV1/LAPTM5/SYK/XIAP/ABL1/PAK1/RELA/LTF/ARRB2/MFHAS1/PTPRJ/MAPKAPK2/STK11/ZDHHC5/SPG21/RIOK3/PRKCB/CSK/SEC14L1/NFATC2/TNIP1/PAK2/SLC15A3/NPLOC4/WNK1/BAG6
CC	GO:0000407	phagophore assembly site	30	-0.60	-1.67	0.00609221	0.02405425	0.0149145	1325	tags=37%, list=11%, signal=33%	ATG2A/BCAS3/RAB7A/ILRUN/ZFYVE1/WIPI2/ATG7/RAB1B/SQSTM1/ATG13/WDR45
BP	GO:2001239	regulation of extrinsic apoptotic signaling pathway in absence of ligand	26	-0.62	-1.68	0.00610506	0.02408108	0.01493114	2751	tags=62%, list=23%, signal=47%	BCL2/PPP2R1A/NF1/EYA3/PRDX2/AKT1/MAPK7/IL1B/RIPK1/HSPA1A/PPP1CA/CTNNA1/BCL2L1/GATA1/PF4/STRADB
BP	GO:0071222	cellular response to lipopolysaccharide	151	-0.41	-1.50	0.00622573	0.02450841	0.0151961	2108	tags=30%, list=18%, signal=25%	IL1B/SPI1/PPBP/SCIMP/GSTP1/TRIB1/SCARB1/PDE4B/CD14/SIGIRR/VIM/NR1H3/PYCARD/CACTIN/TGFB1/GIT1/TNFRSF1B/CD180/TRAF6/LYN/SASH1/RARA/MTDH/SRC/LITAF/BCR/UPF1/ABL1/TNFSF4/RELA/LTF/NFKB1/BMP6/RHOA/CDK19/PLSCR3/PRPF8/PF4V1/PAF1/CXCL5/MAPK3/GATA1/PF4/TNIP1/MAP2K3/TBXA2R
BP	GO:0050886	endocrine process	39	-0.56	-1.65	0.00632057	0.02480808	0.0153819	2701	tags=38%, list=23%, signal=30%	RPS6KA2/RAB11FIP5/POMC/SERPINF2/PTPN11/IL1B/EDN1/CRY2/RAB11FIP1/BMP6/GALR1/RHOA/ECE1/NIBAN2/DAB2
BP	GO:0061045	negative regulation of wound healing	36	-0.58	-1.67	0.0063938	0.02504606	0.01552946	2466	tags=44%, list=21%, signal=35%	SERPINF2/KNG1/PROS1/SMAD3/EDN1/EPPK1/SERPING1/CD9/CEACAM1/CLASP1/KRT1/GP1BA/ALOX12/THBS1/UBASH3B/PDGFA

BP	GO:0007610	behavior	292	-0.35	-1.40	0.00650713	0.02544897	0.01577928	2731	tags=27%, list=23%, signal=21%	UBA6/CNP/PI3/ATP7A/SLC16A1/EXT1/ZFY/EPS8/AKT1/FBXL20/SYT11/ADNP/POMK/NR1D1/CLN3/EGR1/DLG4/SLC24A4/TSPO/AAAS/ZNF385A/EIF4A3/EPHA4/MECP2/CDK5/MAFG/FYN/MYO15A/THRA/DACH1/P2RX4/ORMDL3/MTA1/SYNJ1/UBE2Q1/CPT1A/TRMT1L/DTNBP1/EHMT2/ZFHX3/GIT1/NCOA2/MFSD2A/PUM1/ATXN1/NCSTN/PPARA/ATP1B2/AMFR/STAT3/MAP1A/VLDLR/ABL1/FOS/SYNGAP1/ARRB2/GAA/CHD8/HIPK2/DCTN1/ARCN1/CLDN5/PPP1R9B/THBS1/ANKH/MEIS1/GPI/SNCA/EIF4G1/DYNLRB1/TMOD1/P2RX1/EIF4EBP2/NRGN/PRKAR2B/TAL1/HTT/SHISA7
BP	GO:0009267	cellular response to starvation	126	-0.42	-1.50	0.00654135	0.02554853	0.01584101	2057	tags=29%, list=18%, signal=24%	NUAK2/SREBF2/HSPA5/RIPOR1/MAP1LC3B/GCN1/RRAGD/TFEB/VPS41/EHMT2/SREBF1/DAP/TTC5/UCP2/MFSD2A/MAP3K5/SESN2/STK24/SLC39A4/PPARA/MAX/GABARAP/ZFYVE1/RRAGC/SLC2A1/WIPI2/SESN3/ATG7/GLUL/MAPK3/ELAPOR1/CDKN1A/WDR45/NPRL3/FOXO3/GAS2L1
BP	GO:0005975	carbohydrate metabolic process	414	-0.32	-1.32	0.0065644	0.02561303	0.015881	2578	tags=27%, list=22%, signal=22%	MAPK14/PTAFR/AKT1/ARL2/POMC/PIK3CA/POMK/ACACB/NR1D1/MLEC/HDAC4/FOXO1/PGD/AKR7A2/GANC/G6PD/MECP2/HYAL2/STK40/CHST14/SLC25A1/PFKFB4/ENTPD5/PGAM1/HECTD4/NLN/ZBTB7A/EIF6/MAN2A1/FOXK1/SYNJ1/NEU3/PHKG2/ENO2/GALK1/B4GALT5/CPT1A/PDK3/WDR5/HYAL3/GSK3B/SORD/FOXK2/FAM3A/GSK3A/TGFB1/ST3GAL3/GIT1/CLTC/BCKDK/CBFA2T3/MAN1C1/EDEM2/JMJD8/NCOA2/INPPL1/NDST1/PLEK/WDTC1/CHID1/ALDOA/B3GNT8/GLB1/SESN2/NAGA/GBGT1/GDPGP1/ABO/PTK2B/IGF2/ALDOC/CHST11/BAD/PFKL/MDH2/SRC/TALDO1/ARNT/PPARA/PKM/GALE/BPGM/PPP1CA/STAT3/PDK2/KAT2B/GNPDA1/DDB1/PYGB/FBP1/NFKB1/SLC2A1/IDH2/GAA/MAN1A1/SLC3A2/HK1/INPP5K/ITPK1/USP7/ST6GALNAC4/STK11/ADIPOR1/SLC4A1/AKT2/PER2/GPI/RPIA/SNCA/B4GALT3/ENO1/GANAB/SMPD3
BP	GO:0002763	positive regulation of myeloid leukocyte differentiation	37	-0.56	-1.65	0.00660343	0.02571522	0.01594436	2294	tags=46%, list=20%, signal=37%	CCR1/HLA-DRB1/RIPK1/PPARGC1B/TRIB1/FES/RUNX1/CD74/TGFB1/CTNNBIP1/ACIN1/TRAF6/HCLS1/FOS/NOTCH2/PF4/TESC
BP	GO:1901214	regulation of neuron death	208	-0.38	-1.43	0.00665746	0.02587495	0.0160434	2569	tags=32%, list=22%, signal=25%	SORL1/CORO1A/AKT1/PIK3CA/JUN/ADNP/TRPM2/CLN3/CASP9/EGR1/AGAP2/HYOU1/CHP1/G6PD/MECP2/CDK5/FYN/CASP2/TLR6/ITGAM/KIR3DL2/ENO2/CHMP4B/AP2B1/MAP3K11/DTNBP1/IKBKG/GSK3B/HSP90AB1/TNFRSF1B/DNAJC5/PARP1/MAP3K5/PINK1/PTK2B/MAP2K7/BAD/DHCR24/BCL2L11/SRPK2/PPARA/DAXX/NRP1/ABL1/EGLN3/FOS/SYNGAP1/C1QA/ARRB2/RHOA/UBE2M/AARS1/HIPK2/ELK1/BCL2L1/UBB/GPI/GRN/SNCA/EIF4G1/FBXO7/CBL/FOXO3/CDC34/HDGF/CLU
BP	GO:0007189	adenylate cyclase-activating G protein-coupled receptor signaling pathway	56	-0.52	-1.63	0.00668124	0.02594201	0.01608498	2088	tags=27%, list=18%, signal=22%	CNR2/ADGRE1/PDE4B/ADGRE5/GSK3A/GNAS/GNB1/GNAI2/ADGRL1/GALR1/PDE4A/AKAP13/ATP2B4/PF4/TBXA2R
BP	GO:0010721	negative regulation of cell development	90	-0.44	-1.51	0.00672444	0.02608426	0.01617318	2593	tags=39%, list=22%, signal=31%	ULK1/SORL1/DIP2B/NOTCH1/NR1D1/TSPO/HES1/CDK5/IL1B/CERS2/RCC2/KIAA0319/SEMA4D/CTDSP1/KDM4A/GSK3B/GSK3A/ASCL2/SKI/CORO1C/BCL11A/MBP/HOOK3/PPP3CA/CTNNA1/SEMA4A/NRP1/YWHAH/RFLNB/SYNGAP1/LTF/TRIM11/RNF10/ACTN4/DMTN

BP	GO:0007517	muscle organ development	173	-0.38	-1.42	0.00673835	0.02611275	0.01619085	1961	tags=25%, list=17%, signal=21%	NLN/HMG20B/BASP1/SVIL/FOXK1/BCL9/SMAD3/MAFF/RIPOR2/ENG/RBPJ/CENPF/ZFHX3/SFMBT1/TGFB1/SKI/SELENON/TPM1/FHL1/FZD2/IGF2/FLNB/SOX6/PPP3CA/ZNF609/FHL3/USP19/FOS/FKBP1A/FOXO4/XK/RHOA/MYLK/ZBTB42/NIBAN2/PLEC/MTPN/NEURL1/HLX/GPX1/YBX3/MED1/KEL/HIVEP3
BP	GO:0038066	p38MAPK cascade	42	-0.55	-1.65	0.00678059	0.02625085	0.01627647	2876	tags=48%, list=25%, signal=36%	ZFP36L1/MAP3K4/MAPK14/MINK1/NCF1/GADD45B/ZFP36/IL1B/SPI1/MAP3K3/SPHK1/EZR/MAP3K5/SASH1/PHLPP1/GADD45G/MFHAS1/LGALS9/MAPKAPK2/MAP2K3
BP	GO:0002429	immune response-activating cell surface receptor signaling pathway	197	-0.38	-1.43	0.00685433	0.02648476	0.0164215	2697	tags=29%, list=23%, signal=23%	CMKLR1/CR1/FGR/PLD2/RFTN1/FOXP3/CBLB/PIK3CA/ERMAP/CD22/HLA-DRB1/PRKD2/CD81/FYN/CRKL/HLA-A/PTPN6/VAV2/LGALS3/PDE4B/PTPRC/SH2B2/KLHL6/STOML2/INPP5D/DGKZ/NCR3/IKBKG/FOXP1/MICB/TEC/EZR/FPR3/TRAF6/BTN3A2/LYN/SPPL3/CREBBP/SRC/CEACAM1/RBCK1/PIK3CD/VAV1/LAPTM5/SYK/ABL1/PAK1/RELA/PTPRJ/STK11/SPG21/PRKCB/CSK/NFATC2/PAK2/WNK1/BAG6
BP	GO:0002757	immune response-activating signal transduction	197	-0.38	-1.43	0.00685433	0.02648476	0.0164215	2697	tags=29%, list=23%, signal=23%	CMKLR1/CR1/FGR/PLD2/RFTN1/FOXP3/CBLB/PIK3CA/ERMAP/CD22/HLA-DRB1/PRKD2/CD81/FYN/CRKL/HLA-A/PTPN6/VAV2/LGALS3/PDE4B/PTPRC/SH2B2/KLHL6/STOML2/INPP5D/DGKZ/NCR3/IKBKG/FOXP1/MICB/TEC/EZR/FPR3/TRAF6/BTN3A2/LYN/SPPL3/CREBBP/SRC/CEACAM1/RBCK1/PIK3CD/VAV1/LAPTM5/SYK/ABL1/PAK1/RELA/PTPRJ/STK11/SPG21/PRKCB/CSK/NFATC2/PAK2/WNK1/BAG6
BP	GO:0051480	regulation of cytosolic calcium ion concentration	186	-0.39	-1.45	0.00694658	0.02681513	0.01662635	2769	tags=31%, list=24%, signal=24%	CCRL2/LETM1/PDPK1/CMKLR1/PKD1/CORO1A/PTGIR/SWAP70/KNG1/TRPM2/BAK1/CLN3/TRPC3/DLG4/SLC24A4/CCR1/CDK5/FYN/P2RX4/PTPN6/ITPR2/LPAR4/SMAD3/PTPRC/EDN1/CXCR2/BCAP31/PRKACA/CXCR1/SELENON/FPR3/CHERP/LYN/SPPL3/PTK2B/GNA15/PML/RASA3/FIS1/JAK3/ABL1/FKBP1A/GALR1/CALM3/FKBP1B/MCOLN1/GP1BA/GP9/HRH4/PIK3CG/UBASH3B/ATP2B4/SNCA/P2RX1/GATA1/HTT/DIAPH1/TBXA2R
BP	GO:0061035	regulation of cartilage development	33	-0.59	-1.68	0.00696596	0.02686387	0.01665657	1226	tags=36%, list=10%, signal=33%	ZBTB16/SMAD3/TRPS1/RARA/SOX6/GREM1/LTBP3/RFLNB/BMP6/GLG1/SMPD3/SCIN
CC	GO:0030672	synaptic vesicle membrane	51	-0.52	-1.61	0.00701318	0.02698868	0.01673395	1628	tags=29%, list=14%, signal=25%	STX6/DTNBP1/TPRG1L/SYNGR1/DNAJC5/RAB7A/RAB27B/CLTB/CALM3/RAB5B/RAB11B/ZNRF1/SLC6A9/BCL2L1/SNCA
CC	GO:0099501	exocytic vesicle membrane	51	-0.52	-1.61	0.00701318	0.02698868	0.01673395	1628	tags=29%, list=14%, signal=25%	STX6/DTNBP1/TPRG1L/SYNGR1/DNAJC5/RAB7A/RAB27B/CLTB/CALM3/RAB5B/RAB11B/ZNRF1/SLC6A9/BCL2L1/SNCA
BP	GO:0046718	viral entry into host cell	105	-0.42	-1.48	0.00704843	0.02706578	0.01678176	2665	tags=34%, list=23%, signal=27%	TRIM21/CR1/SIGLEC1/MID2/KPNA3/NCAM1/HLA-DRB1/HYAL2/CD81/ICAM1/SELPLG/ITGB7/SCARB1/CD74/GRK2/SMPD1/TRIM26/GYPA/IFITM3/HSPA1A/SNX3/SLC1A5/TRIM10/IFITM2/NRP1/WWP2/LGALS9/SLC3A2/BSG/TRIM11/ITGB5/GSN/TRIM14/LAMP1/CTSB/ITGB3
BP	GO:0016241	regulation of macroautophagy	138	-0.41	-1.48	0.00719727	0.02756874	0.01709361	1667	tags=22%, list=14%, signal=20%	POLDIP2/PHF23/CHMP4B/EHMT2/IKBKG/TECPR1/PRKACA/ATG2A/VPS13D/SESN2/PINK1/RUBCN/ATP6V1A/VDAC1/USP36/ATP6V0D1/OPTN/ATP6V0C/CAPNS1/SESN3/ATP6V0A1/CAPN1/PIP4K2A/MAPK3/ELAPOR1/BNIP3L/TBC1D25/WDR45/HTT/LARP1/SUPT5H
BP	GO:0048659	smooth muscle cell proliferation	89	-0.44	-1.49	0.00732052	0.02798693	0.0173529	2571	tags=35%, list=22%, signal=27%	PTAFR/AKT1/PTGIR/PIK3CA/JUN/SERPINF2/IL6R/NDRG2/TRIB1/XRCC6/EDN1/POLDIP2/MMP9/FOXP1/TGFB1/CTNNBIP1/CCN3/MAP3K5/TPM1/GNAI2/SF1/RHOA/LDLRAP1/FOXJ2/GNA12/THBS1/MFN2/TCF7L2/PRKDC/CDKN1A/SMPD3

BP	GO:0032456	endocytic recycling	64	-0.49	-1.56	0.00738944	0.02822324	0.01749943	2569	tags=48%, list=22%, signal=38%	SORL1/RAB8A/SNX27/ARL4C/LMTK2/ARHGAP8/CMTM6/VPS39/SNX17/VPS51/ANKRD50/STX6/SNX12/VPS53/EHD3/ATP9A/DENND1C/DENND1A/ATP6AP1/EHD4/RAB7A/EPG5/MICALL1/GRIPAP1/SNX3/RAB11FIP4/RAB14/RAB11B/ARHGAP1/EHD1/PTPN23
BP	GO:0002253	activation of immune response	247	-0.36	-1.41	0.00741912	0.0282823	0.01753604	2236	tags=24%, list=19%, signal=20%	HLA-DRB1/PRKD2/CD81/FYN/CRKL/IL1B/HLA-A/PTPN6/VAV2/LGALS3/XRCC6/PDE4B/PTPRC/SH2B2/KLHL6/STOML2/INPP5D/DGKZ/NCR3/IKBKG/FOXP1/PYCARD/MICB/TEC/RBM14/EZR/FPR3/TRAF6/BTN3A2/LYN/SPPL3/MAVS/SERPING1/CGAS/CREBBP/SRC/CEACAM1/RBCK1/PIK3CD/VAV1/LAPTM5/SYK/ABL1/PAK1/RELA/C1QA/PTPRJ/STK11/KRT1/SPG21/PRKCB/CSK/ZNFX1/NFATC2/PRKDC/PAK2/CLU/WNK1/BAG6
BP	GO:0097553	calcium ion transmembrane import into cytosol	82	-0.46	-1.57	0.00748412	0.02850274	0.01767273	1283	tags=22%, list=11%, signal=20%	SELENON/CHERP/LYN/PTK2B/RASA3/ABL1/FKBP1A/CALM3/FKBP1B/MCOLN1/GP1BA/GP9/UBASH3B/ATP2B4/SNCA/P2RX1/HTT/DIAPH1
BP	GO:0097190	apoptotic signaling pathway	457	-0.32	-1.32	0.00755066	0.02872863	0.01781279	2425	tags=29%, list=21%, signal=24%	BAK1/TMBIM1/CD3E/CASP9/PPP1R15A/CDKN2D/FLCN/SGPL1/HYOU1/ZNF385A/DIABLO/IKBKE/IL6R/DPF2/HYAL2/ICAM1/CASP2/CSNK2A1/IL1B/SPI1/P2RX4/RIPK1/MADD/MKNK2/ITM2C/ZDHHC3/GSTP1/CCAR2/LGALS3/TMC8/ITGAM/BIRC6/SMAD3/ACVR1B/TRAF7/PTPRC/PIK3CB/CHCHD10/LGALS12/MAGED1/CREB3/SLC35F6/CD74/MMP9/BBC3/ITPRIP/PSME3/BCAP31/GSK3B/GSK3A/PYCARD/DAP/TGFB1/TNFRSF1B/BEX3/DAPK3/DAPK1/STX4/CASP5/HIC1/PARP1/TP53BP2/MAP3K5/HIP1/HIP1R/BCL2L14/PINK1/HSPA1A/PIAS4/IFI27/BAD/STK24/SHISA5/TRIM32/BCL2L11/SRC/PML/EIF5A/STK25/RBCK1/SFRP2/GNAI2/SEPTIN4/FIS1/PLEKHF1/DAXX/PPP1CA/PIH1D1/CTNNA1/NRP1/PDK2/ARHGEF2/GABARAP/FBH1/ABL1/FNIP2/RELA/E2F1/ANXA6/ARRB2/HNRNPK/HIPK1/LMNA/HSPB1/HIPK2/CDIP1/BCL2L1/STK11/PLSCR3/SIAH2/UBB/SORT1/THBS1/PTPN1/TCF7L2/ENO1/PRKDC/E2F2/GATA1/GRINA/BNIP3L/CDKN1A/PF4/PAK2/STRADB/GPX1/YBX3/FOXO3/CTTN/HTT/CUL4A/CLU/BAG6
BP	GO:2001234	negative regulation of apoptotic signaling pathway	166	-0.39	-1.45	0.00760711	0.02890591	0.0179227	2142	tags=27%, list=18%, signal=22%	ICAM1/CSNK2A1/IL1B/RIPK1/GSTP1/CCAR2/LGALS3/BIRC6/PIK3CB/CREB3/SLC35F6/CD74/MMP9/ITPRIP/PSME3/GSK3B/PINK1/HSPA1A/TRIM32/SRC/SFRP2/GNAI2/PIH1D1/CTNNA1/NRP1/ARHGEF2/RELA/ARRB2/LMNA/HSPB1/BCL2L1/SIAH2/THBS1/PTPN1/TCF7L2/ENO1/GATA1/GRINA/PF4/STRADB/GPX1/YBX3/CTTN/HTT/CLU
MF	GO:0032266	phosphatidylinositol-3-phosphate binding	37	-0.56	-1.64	0.00765434	0.02903975	0.01800569	2899	tags=51%, list=25%, signal=39%	ZFYVE26/OBSCN/KIF16B/ATG2B/SNX27/SH3PXD2A/SNX12/TECPR1/ATG2A/DENND1A/HIP1/BBS5/SNX3/PLEKHF1/ZFYVE1/PARD3/PLEK2/WIPI2/WDR45
BP	GO:0099565	chemical synaptic transmission, postsynaptic	45	-0.52	-1.57	0.00771682	0.02922104	0.0181181	3147	tags=58%, list=27%, signal=42%	CHRNA10/LRRK2/P2RX7/MTMR2/APP/SEZ6/S1PR2/RAB3GAP1/NRXN1/DVL1/AKT1/DLG4/EIF4A3/MECP2/CDK5/P2RX4/GSK3B/BAIAP2/GSK3A/NPFF/PTK2B/PPP3CA/ARRB2/SNCA/P2RX1/SLC29A1
BP	GO:0030866	cortical actin cytoskeleton organization	32	-0.59	-1.66	0.00781723	0.02957308	0.01833637	2206	tags=38%, list=19%, signal=31%	CDK5/FMNL1/ROCK2/LCP1/FMNL3/EPB41L2/EZR/PLEK/CALR/PLEC/EPB41/TLN1
BP	GO:0051282	regulation of sequestering of calcium ion	74	-0.46	-1.53	0.00783902	0.02962732	0.01837	1283	tags=23%, list=11%, signal=21%	SELENON/CHERP/LYN/PTK2B/RASA3/ABL1/FKBP1A/ANXA6/CALM3/FKBP1B/MCOLN1/GP1BA/GP9/UBASH3B/SNCA/HTT/DIAPH1

BP	GO:0010522	regulation of calcium ion transport into cytosol	55	-0.51	-1.59	0.00797535	0.0301017	0.01866414	1491	tags=25%, list=13%, signal=22%	PRKACA/SELENON/LYN/PTK2B/PML/ABL1/FKBP1A/CALM3/FKBP1B/UBASH3B/SNCA/P2RX1/HTT/DIAPH1
BP	GO:0030282	bone mineralization	63	-0.48	-1.55	0.00799454	0.03012917	0.01868117	2856	tags=46%, list=24%, signal=35%	SLC8A1/FGF23/SBNO2/FGR/BMP2K/PHEX/CCR1/TUFT1/ALPL/SMAD3/ZBTB40/ACVR2B/TGFB1/SNX10/BCOR/ALOX15/ANO6/PTK2B/GREM1/LTBP3/FAM20C/PHOSPHO1/RFLNB/LTF/BMP6/SLC24A3/ANKH/GATA1/SMPD3
BP	GO:0008361	regulation of cell size	113	-0.42	-1.50	0.00808806	0.03045278	0.01888182	2593	tags=35%, list=22%, signal=27%	ATP7A/ULK1/DIP2B/DISC1/PLXNA4/ADNP/CLN3/MAP3K13/CDK5/MACF1/VAV2/EDN1/KIAA0319/NDEL1/SEMA4D/GSK3B/CYFIP1/HSP90AB1/PLEK/WDTC1/IST1/ANO6/AQP1/CDKL5/RAC1/DPYSL2/EIF4G2/LRRC8A/VAV1/SEMA4A/NRP1/ABL1/XK/RHOA/DNM2/MTPN/MSN/CTTN/KEL
BP	GO:0048738	cardiac muscle tissue development	102	-0.43	-1.51	0.00816015	0.03069513	0.01903208	1781	tags=25%, list=15%, signal=22%	RUNX1/EDN1/ENG/RBPJ/GSK3A/TGFB1/VGLL4/KDM6B/TPM1/YY1/RARA/SLC9A1/SOX6/GREM1/CALR/PPARA/FKBP1A/ARRB2/JARID2/NOTCH2/LMNA/PLEC/AKAP13/NPRL3/MED1/PIM1
BP	GO:0019216	regulation of lipid metabolic process	206	-0.37	-1.41	0.00819308	0.03078989	0.01909084	2649	tags=34%, list=23%, signal=27%	ZBTB20/FGR/SORL1/AKT1/ABCA3/MTMR3/ABCB11/ACACB/NR1D1/ATP1A1/EDF1/EGR1/AGAP2/TSPO/CHP1/PIP4K2C/ABCD1/CD81/THRA/IL1B/OPA3/ORMDL3/CERS2/VAV2/EIF6/DHCR7/MBOAT7/NR1H2/SCARB1/LGALS12/STOML2/DGKZ/CD74/CPT1A/PDK3/SREBF1/NR1H3/FAM3A/TGFB1/SPHK1/BCL11B/SLC27A1/WDTC1/MFSD2A/LYN/BSCL2/PTK2B/TBL1XR1/SRC/CEACAM1/RUBCN/PPARA/PSAP/PDK2/PNPLA2/NFKB1/SF1/BMP6/RNF213/LAMTOR1/ACADVL/LDLRAP1/ADIPOR1/AKT2/PIK3CG/SNCA/PIP4K2A/CTDNEP1/PDGFA/DAB2
BP	GO:0006826	iron ion transport	40	-0.54	-1.61	0.0082018	0.03079355	0.01909311	1404	tags=38%, list=12%, signal=33%	CLTC/FLVCR2/SLC22A17/LCN2/LTF/DNM2/RAB11B/ARHGAP1/MCOLN1/SLC6A9/FTH1/MMGT1/FTL/SLC48A1/SLC25A37
BP	GO:0070849	response to epidermal growth factor	33	-0.59	-1.66	0.00823164	0.03087642	0.01914449	485	tags=45%, list=4%, signal=44%	SYAP1/ZFP36L1/PDPK1/AKT1/PTPN11/ZFP36/ZFP36L2/BAIAP2/PTPN12/INPP5K/PPP1R9B/IQGAP1/MAPK3/MED1/DAB2
BP	GO:1905477	positive regulation of protein localization to membrane	74	-0.46	-1.52	0.008248	0.03088089	0.01914726	2420	tags=35%, list=21%, signal=28%	AKT1/CNST/CLN3/DLG4/CHP1/CDK5/CD81/FYN/LGALS3/ITGAM/BBC3/AP2B1/STOM/MESD/RANGRF/EZR/STX4/FIS1/ZDHHC5/SQSTM1/LRP1/PPP1R9B/SPTBN1/AKT2/ATP2B4/RHOG
BP	GO:0032963	collagen metabolic process	44	-0.53	-1.62	0.00830887	0.03104894	0.01925146	1781	tags=23%, list=15%, signal=19%	RUNX1/ENG/MMP9/VIM/TGFB1/ARRB2/MRC2/SMPD3/CTSB/FURIN
BP	GO:0002237	response to molecule of bacterial origin	234	-0.36	-1.39	0.00837409	0.03126326	0.01938435	2108	tags=26%, list=18%, signal=22%	IL1B/SPI1/CNR2/PPBP/SCIMP/TLR6/GSTP1/TRIB1/ALPL/SCARB1/PDE4B/EDN1/FZD5/CD14/JUND/SIGIRR/VIM/FOXP1/NR1H3/PYCARD/CACTIN/TGFB1/GIT1/TNFRSF1B/CD180/ALAD/TRAF6/LYN/SASH1/RARA/MTDH/SRC/LITAF/BCR/UPF1/ABL1/FOS/TNFSF4/RELA/OTUD5/LTF/NFKB1/BMP6/RHOA/LGALS9/MAPKAPK2/CDK19/PLSCR3/PRPF8/PF4V1/SELP/SNCA/PAF1/CXCL5/MAPK3/GATA1/PF4/AKIRIN2/TNIP1/MAP2K3/TBXA2R
BP	GO:0031929	TOR signaling	109	-0.42	-1.49	0.00839558	0.0313141	0.01941587	1516	tags=21%, list=13%, signal=19%	PRR5L/GSK3A/HDAC3/KLHL22/UBR2/SESN2/PINK1/PIH1D1/RRAGC/OTUD5/LAMTOR1/SESN3/GPR137/TMEM127/STK11/SIK3/GNA12/PRR5/ARAF/CASTOR2/EIF4EBP2/NPRL3/LARP1

BP	GO:0050865	regulation of cell activation	457	-0.32	-1.32	0.00850191	0.03159213	0.01958826	2589	tags=28%, list=22%, signal=22%	BPI/RASAL3/PTAFR/CORO1A/AKT1/SELENOK/FOXP3/TNFSF13/CBLB/SYT11/PIK3CA/PTPN11/NR1D1/CD22/CD3E/EFNB1/DOCK8/PRDM1/HES1/PKN1/HLA-DRB1/IRF1/CD81/FYN/HLA-DQA2/LGALS9C/IL1B/SPI1/CNR2/HLA-A/SH3KBP1/PTPN6/TLR6/ZBTB16/PGLYRP1/SIRPG/ZFP36L2/FES/LGALS3/SLC7A1/ITGAM/XRCC6/SOX13/RUNX1/PTPRC/RIPOR2/DROSHA/INPP5D/CD74/CD300LF/JUND/SIRPB1/SOX4/HLA-DQA1/NR1H3/PYCARD/RPS6KA1/TGFB1/LILRA5/TEC/TBX21/TMEM131L/TNFRSF1B/SPHK1/STX4/PLEK/IL4I1/TWSG1/TRAF6/KARS1/SIT1/LYN/SUPT6H/RARA/IGF2/CD9/CGAS/SPN/BAD/SRC/CEACAM1/HLA-DRB5/VAV1/RUNX3/LAPTM5/ZMIZ1/TP53BP1/HMCES/BCR/SYK/JAK3/ABL1/TNFSF4/BLOC1S3/MYO18A/MPL/GP6/PDE5A/RHOA/MFHAS1/MAD2L2/ACTA2/LGALS9/RAC2/SDC4/GP1BA/ALOX12/CSK/THBS1/GP9/SPTA1/NFATC2/GRN/UBASH3B/SELP/SNCA/PRKDC/LAMP1/SOX12/GATA1/FBXO7/HLX/CDKN1A/FOXO3/PDGFA/DMTN
BP	GO:0031670	cellular response to nutrient	27	-0.59	-1.63	0.00857843	0.03184671	0.0197461	1924	tags=37%, list=16%, signal=31%	PHEX/DCPS/FES/NCOA1/RXRA/USF2/PDK2/KANK2/MED1/PIM1
BP	GO:0071470	cellular response to osmotic stress	28	-0.59	-1.63	0.00882564	0.03261221	0.02022075	2951	tags=57%, list=25%, signal=43%	RELB/ZFP36L1/LETM1/CLN3/TSPO/SLC25A23/RCSD1/MICU1/SERPINB6/OXSR1/AQP1/BAD/ARHGEF2/SLC2A1/MYLK/YBX3
CC	GO:0072562	blood microparticle	44	-0.53	-1.62	0.00899554	0.03317835	0.02057177	2466	tags=50%, list=21%, signal=40%	SERPINF2/KNG1/YWHAZ/PFN1/ZBTB38/PROS1/ENG/STOM/TGFB1/ACTG1/F13A1/SERPING1/HSPA1A/GRIPAP1/ACTB/SLC2A1/KRT1/GSN/SLC4A1/MSN/CLU/ITGA2B
BP	GO:0007268	chemical synaptic transmission	321	-0.34	-1.35	0.00901707	0.03319617	0.02058282	2567	tags=26%, list=22%, signal=21%	RAB8A/MINK1/AKT1/FBXL20/SYT11/DISC1/ADNP/CLN3/ATP2A2/EZH2/DLG4/RPH3A/EIF4A3/EPHA4/MECP2/CDK5/FYN/EXOC4/IL1B/HRH2/P2RX4/CNR2/ZDHHC3/SYNJ1/OR10H1/PMP22/EDN1/NPTXR/DGKZ/PPP1R9A/DTNBP1/GSK3B/BAIAP2/PRKACA/GSK3A/RPS6KA1/CYFIP1/GIT1/YWHAG/TPRG1L/SYNGR1/DNAJC5/STX4/PINK1/MBP/NPFF/PTK2B/RARA/ARF1/SIPA1L1/NCSTN/SRC/DLGAP4/CA2/GNAI2/KIF1B/PPP3CA/SLC1A4/MAP1A/BCR/YWHAH/GABARAP/APBA3/ABL1/SYNGAP1/ARRB2/STAU1/CALM3/PVALB/GUCY1A1/NAPA/CLSTN1/GLUL/SQSTM1/PPP1R9B/PRKCB/HRH4/SNCA/NEURL1/P2RX1/EIF4EBP2/SLC29A1/NRGN/PRKAR2B/SHISA7
BP	GO:0098916	anterograde trans-synaptic signaling	321	-0.34	-1.35	0.00901707	0.03319617	0.02058282	2567	tags=26%, list=22%, signal=21%	RAB8A/MINK1/AKT1/FBXL20/SYT11/DISC1/ADNP/CLN3/ATP2A2/EZH2/DLG4/RPH3A/EIF4A3/EPHA4/MECP2/CDK5/FYN/EXOC4/IL1B/HRH2/P2RX4/CNR2/ZDHHC3/SYNJ1/OR10H1/PMP22/EDN1/NPTXR/DGKZ/PPP1R9A/DTNBP1/GSK3B/BAIAP2/PRKACA/GSK3A/RPS6KA1/CYFIP1/GIT1/YWHAG/TPRG1L/SYNGR1/DNAJC5/STX4/PINK1/MBP/NPFF/PTK2B/RARA/ARF1/SIPA1L1/NCSTN/SRC/DLGAP4/CA2/GNAI2/KIF1B/PPP3CA/SLC1A4/MAP1A/BCR/YWHAH/GABARAP/APBA3/ABL1/SYNGAP1/ARRB2/STAU1/CALM3/PVALB/GUCY1A1/NAPA/CLSTN1/GLUL/SQSTM1/PPP1R9B/PRKCB/HRH4/SNCA/NEURL1/P2RX1/EIF4EBP2/SLC29A1/NRGN/PRKAR2B/SHISA7
BP	GO:0051283	negative regulation of sequestering of calcium ion	72	-0.47	-1.55	0.00907275	0.03330865	0.02065256	1283	tags=24%, list=11%, signal=21%	SELENON/CHERP/LYN/PTK2B/RASA3/ABL1/FKBP1A/ANXA6/CALM3/FKBP1B/MCOLN1/GP1BA/GP9/UBASH3B/SNCA/HTT/DIAPH1
BP	GO:0051208	sequestering of calcium ion	78	-0.46	-1.55	0.00906597	0.03330865	0.02065256	1283	tags=23%, list=11%, signal=21%	SELENON/CHERP/LYN/PTK2B/RASA3/CALR/ABL1/FKBP1A/ANXA6/CALM3/FKBP1B/MCOLN1/GP1BA/GP9/UBASH3B/SNCA/HTT/DIAPH1

MF	GO:0030295	protein kinase activator activity	73	-0.46	-1.54	0.00915201	0.0335068	0.02077542	2488	tags=33%, list=21%, signal=26%	PIK3CA/MOB3B/AGAP2/MAP3K13/MADD/CCNY/MOB1B/TPX2/MOB3A/TAOK1/IGF2/MARK2/GREM1/TAOK2/DAXX/MAP2K2/LTF/CALM3/STK11/IQGAP1/CDKN1A/PAK2/TAL1/WNK1
BP	GO:0010565	regulation of cellular ketone metabolic process	77	-0.46	-1.52	0.00918547	0.03359837	0.0208322	2551	tags=38%, list=22%, signal=30%	AKT1/ABCB11/ACACB/NR1D1/CLN3/EGR1/SLC7A7/ABCD1/IL1B/EIF6/NR1H2/CD74/CPT1A/PDK3/SREBF1/NR1H3/BCKDK/WDTC1/MFSD2A/CEACAM1/PPARA/PDK2/BMP6/ACADVL/AKT2/ATP2B4/SNCA/NCOR2/DAB2

Supplementary table 13 Amyloid non-accumulators gene set enrichment analysis gene ontology upreulated

ONTOLOGY	ID	Description	setSize	enrichment Score	NES	pvalue	p.adjust	qvalue	rank	leading_edge	core_enrichment
BP	GO:0034660	ncRNA metabolic process	500	0.44	2.06	1.00E-10	4.19E-09	2.57E-09	2483	tags=35%, list=21%, signal=28%	METTL18/RPS6/EMG1/RPS25/RPL7/RPS16/RPS14/RPL26/RPL27/NSA2/RPS8/RPS7/RPL5/RPS24/NOP58/RPS21/RPL35A/RPL11/RPL35/FBL/RPS27/GTF3A/METTL5/RPS17/UTP14A/WDR4/TRMT10C/RPP30/RPF1/RPL10A/GTF3C3/NIFK/TRMT61B/RTRAF/POP5/LSM6/EXOSC1/RPL14/ZNHIT3/RTCB/MRPL1/TUT1/TSEN54/RARS1/TYW3/TRMT13/TPRKB/KRR1/SSB/ESF1/DDX52/NGDN/BRIX1/SBDS/TRMO/MAK16/PIN4/PRKRA/LARP7/SHQ1/RPL7A/DGCR8/DHX37/RSL1D1/MRM1/THUMPD1/IMP4/UTP20/TRMT12/DDX49/CTU2/POLR1E/REXO4/RPS19/DTWD1/MPHOSPH10/ZBTB8OS/MPHOSPH6/PUS1/WDR75/DARS1/RPS28/NVL/LAGE3/MOCS3/NOP53/HELQ/NHP2/RPUSD3/TARS3/SLFN13/ANKRD16/TRUB1/SNAPC5/TP53RK/WDR3/WDR6/RPS15/EXOSC5/TARBP1/TAF1B/UTP11/MTERF4/RBFA/DDX5/DUS1L/SEPSECS/MARS2/LCMT2/TRMT5/UTP6/DDX47/WDR43/ZCCHC8/DDX1/TRIR/PWP1/DROSHA/GATC/NSUN4/RPP38/FTSJ1/INTS7/PLD6/YTHDF2/TSR2/NSUN6/RRP9/GRSF1/UTP18/GTF3C5/ADAT2/SPOUT1/FARSB/TFB1M/DCAF13/QTRT1/INTS12/USP36/DALRD3/AARS1/DDX17/DDX11/INTS4/ELL2/INTS8/RARS2/MRTO4/PSTK/HSD17B10/CARS2/PDCD11/IARS2/LAS1L/GATB/CDK5RAP1/UTP15/HENMT1/KTI12/TYW1/FDXACB1/PPAN/INTS11/RIOK2/EARS2/CAVIN1/NOL8/KARS1/RBM7/SNU13/TYW5/NOP56/FRG1
CC	GO:0005759	mitochondrial matrix	413	0.46	2.11	1.00E-10	4.19E-09	2.57E-09	2730	tags=41%, list=23%, signal=33%	MRPS22/MRPS21/DNAJC15/MRPS5/MRPL47/MRPL11/DNAJC19/RPS3/MRPL46/SOD1/MRPS17/DNAJA3/HSPE1/MRPL57/TRMT10C/MRPL48/OXA1L/TRMT61B/MRPL30/MRPL4/MRPS33/UQCC2/MRPS18C/ABCE1/PDSS1/MRPS28/ATP5F1A/GCSH/DHFR2/OXCT1/GPT2/REXO2/MRPL39/TBRG4/ATP5PB/MRPL3/MRPL53/DHX30/PIN4/FLAD1/MRPL27/TWNK/SHMT2/PPA2/NAXE/MRPL55/MRPL40/MRPL24/MRPL17/ATP5F1E/COQ5/ACAT1/MTRES1/ALKBH7/MRM1/FASTKD3/MRPL14/MRPL33/MRPL32/NDUFS3/ATP5F1C/TRAP1/SMDT1/PARK7/MRPL22/POLRMT/ACADM/MRPL36/MRPS6/PUS1/MRPS34/MRPL35/NDUFS8/MRPL51/NDUFAB1/MRPL50/BCKDHB/MTRF1L/RPUSD3/FDX1/ETHE1/MRPL58/GRPEL2/MRPL42/GLS/ARL2BP/ACOT2/MRPS16/MPG/HSPA9/MTERF4/MRPL16/ACADSB/MRPL15/MRPL20/PRIMPOL/SSBP1/VDAC2/MRPL18/BCAT2/MARS2/TRMT5/PCCB/MRPS14/MRPL21/DAP3/MRPL9/LIPT1/SDHAF4/MTG1/NSUN4/MRPS30/LRPPRC/TEFM/ISCA2/CHPF/TUFM/HSPD1/MRPL41/MRPS10/GRSF1/NDUFB8/TIMM44/MCEE/FAHD1/MRPL2/ETFBKMT/SDHAF1/SUCLG1/ABHD10/TFB1M/NDUFAF7/NDUFA9/SDHAF3/MTHFD2/NAXD/ACAD8/RARS2/OGG1/MRPS36/HSD17B10/CARS2/IARS2/NUDT1/POLG2/EXD2/FASTKD2/CBR4/SIRT3/EARS2/PDE12/GADD45GIP1/KARS1/PPM1K/NUDT9/MRPS18A/TXN2/ECI1/WARS2/HADHB/DLD/ALDH6A1/ACAA2/MRPS15/LIAS/MRPL19/MMAB/ARL2/MRPS12

CC	GO:0005740	mitochondrial envelope	658	0.47	2.21	1.00E-10	4.19E-09	2.57E-09	1902	tags=34%, list=16%, signal=30%	MRPS22/MRPS21/DNAJC15/MRPS5/UQCRH/MRPL47/LDHB/MRPL11/DNAJC19/UQCRB/COX16/RPS3/AGPAT5/COX7C/MRPL46/SOD1/MRPS17/MCUB/RPS27A/TIMM10/MRPL57/CYCS/MICOS13/MRPL48/C12orf73/MRPS31/COX6C/OXA1L/MRPL30/CYP2U1/NDUFS5/TOMM20/COA6/COX14/ATP5PD/MRPL4/MRPS33/MFF/UQCC2/NDUFAF5/MRPS18C/MRPS23/MRPL1/COX4I1/TIMM9/TRAF3IP3/COX11/TOMM7/ATP5PO/RHOT2/MRPS28/COX7B/ATP5MJ/NDUFB2/UQCC3/ATP5F1A/VAMP1/COQ6/DHFR2/NDUFA4/ATP5MK/REXO2/COX7A2/MRPL39/MICU3/ATP5MF/ATP5PB/TOMM5/MRPL3/AFG3L2/MRPL53/SLC25A28/NDUFB5/TMEM126A/BNIP3/HIGD1A/MRPL27/SHMT2/ATP5MGL/CISD1/MRPL55/PHB2/MRPL40/MRPL24/MRPL17/WDR93/ATP5F1E/COQ5/UQCRQ/COA4/MICOS10/COX5A/NDUFB9/SLC25A19/MPC1/AKAP1/MRPL14/LPIN1/UQCRC2/MRPL33/NDUFA13/PET100/TIMM8B/MRPL32/NDUFS3/CYC1/NDUFS6/NDUFA6/TRIAP1/MIX23/ATP5F1C/TRAP1/COX6A1/PMPCB/NDUFA1/ATP5PF/LETMD1/CHCHD7/SLC25A6/SMDT1/PARK7/IMMP1L/MTX3/RDH13/ATP5MG/MRPL22/ATPSCKMT/TIMM21/MRPS25/SLC25A3/SAMM50/ACADM/MRPL36/MRPS6/NDUFAF4/USP30/MRPS34/SLC25A32/NDUFS4/HAX1/SLC25A25/NDUFAF6/MRPL35/PTCD3/NDUFS8/PMAIP1/PPOX/MRPL51/NDUFAB1/SLC25A1/FAM162A/MRPL50/OGT/SFXN1/CYB5B/IKBKE/BNIP1/UQCR10/SLC25A13/MRPL58/CCDC90B/GRPEL2/YME1L1/TMEM14B/NDUFB3/MRPL42/ARL2BP/ABCB10/UQCRFS1/MRPS16/NDUFA12/HSPA9/TOMM22/SLC25A36/APOO/ATP5MC1/MRPL16/NDUFB1/TIMM17A/CEBPZOS/ATP5ME/MRPL15/HINT2/MRPL20/FLVCR1/ALDH18A1/DMAC1/MPC2/VDAC2/PHB1/MRPL18/VDAC3/COX18/IFI27L2/MRPS14/BCS1L/MRPL21/DAP3/MRPL9/GRAMD4/CYB5A/SFXN4/MTX2/MTG1/MRPS30/NDUFC1/TIMM10B/GNPAT/ANTKMT/SLC25A33/NOA1/NDUFV2/CARD19/PLD6/HSPD1/MICU2/PGAM5/MRPL41/SLC25A14/MRPS10/NDUFB8/TIMM44/MRPL2

CC	GO:0031966	mitochondrial membrane	613	0.48	2.24	1.00E-10	4.19E-09	2.57E-09	1902	tags=35%, list=16%, signal=31%	MRPS22/MRPS21/DNAJC15/MRPS5/UQCRH/MRPL47/LDHB/MRPL11/DNAJC19/UQCRB/COX16/RPS3/AGPAT5/COX7C/MRPL46/MRPS17/MCUB/RPS27A/TIMM10/MRPL57/CYCS/MICOS13/MRPL48/C12orf73/MRPS31/COX6C/OXA1L/MRPL30/CYP2U1/NDUFS5/TOMM20/COX14/ATP5PD/MRPL4/MRPS33/MFF/UQCC2/NDUFAF5/MRPS18C/MRPS23/MRPL1/COX4I1/TIMM9/TRAF3IP3/COX11/TOMM7/ATP5PO/RHOT2/MRPS28/COX7B/ATP5MJ/NDUFB2/UQCC3/ATP5F1A/VAMP1/COQ6/DHFR2/NDUFA4/ATP5MK/COX7A2/MRPL39/MICU3/ATP5MF/ATP5PB/TOMM5/MRPL3/AFG3L2/MRPL53/SLC25A28/NDUFB5/TMEM126A/BNIP3/HIGD1A/MRPL27/SHMT2/ATP5MGL/CISD1/MRPL55/PHB2/MRPL40/MRPL24/MRPL17/WDR93/ATP5F1E/COQ5/UQCRQ/MICOS10/COX5A/NDUFB9/SLC25A19/MPC1/AKAP1/MRPL14/LPIN1/UQCRC2/MRPL33/NDUFA13/PET100/TIMM8B/MRPL32/NDUFS3/CYC1/NDUFS6/NDUFA6/ATP5F1C/TRAP1/COX6A1/PMPCB/NDUFA1/ATP5PF/LETMD1/SLC25A6/SMDT1/IMMP1L/MTX3/RDH13/ATP5MG/MRPL22/ATPSCKMT/TIMM21/MRPS25/SLC25A3/SAMM50/ACADM/MRPL36/MRPS6/NDUFAF4/USP30/MRPS34/SLC25A32/NDUFS4/HAX1/SLC25A25/NDUFAF6/MRPL35/PTCD3/NDUFS8/PMAIP1/PPOX/MRPL51/NDUFAB1/SLC25A1/FAM162A/MRPL50/OGT/SFXN1/CYB5B/IKBKE/BNIP1/UQCR10/SLC25A13/MRPL58/CCDC90B/GRPEL2/YME1L1/TMEM14B/NDUFB3/MRPL42/ABCB10/UQCRFS1/MRPS16/NDUFA12/HSPA9/TOMM22/SLC25A36/APOO/ATP5MC1/MRPL16/NDUFB1/TIMM17A/CEBPZOS/ATP5ME/MRPL15/HINT2/MRPL20/FLVCR1/ALDH18A1/DMAC1/MPC2/VDAC2/PHB1/MRPL18/VDAC3/COX18/IFI27L2/MRPS14/BCS1L/MRPL21/DAP3/MRPL9/GRAMD4/CYB5A/SFXN4/MTX2/MTG1/MRPS30/NDUFC1/TIMM10B/GNPAT/ANTKMT/SLC25A33/NOA1/NDUFV2/CARD19/PLD6/HSPD1/MICU2/PGAM5/MRPL41/SLC25A14/MRPS10/NDUFB8/TIMM44/MRPL2
BP	GO:0006518	peptide metabolic process	697	0.48	2.27	1.00E-10	4.19E-09	2.57E-09	1172	tags=25%, list=10%, signal=24%	METTL18/RPL24/RPL6/RPS6/RPL32/EEF1B2/RPL21/RPL29/RPS13/RPL39/MRPS21/RPL41/RPS18/MRPS5/RPS10/RPS25/RPS20/RPL7/EIF3E/RPS16/MRPL47/RPS14/EEF1G/RPL26/RPL27/RPL34/RPS8/RPL37/MRPL11/AIMP1/RPS5/RPS15A/RPL22/RPL15/RPS7/RPL5/RPL17/RPS24/RPL31/RPL23/RPS3A/RPS3/RPS21/RPL12/RWDD1/RPL30/RPL35A/RPL11/RPL36A/RPL35/RPL9/RPS27/SOD1/MRPS17/RPL13A/RPL37A/EIF4B/DRG1/RPS27A/METTL5/RPL3/RPS17/EIF3F/LSM1/RPL22L1/SARNP/RPS2/MRPL57/TRMT10C/RPL27A/RPLP0/RPS11/RPL10A/RPL38/ZNF540/RPS29/EIF3C/LARP4/SEC11C/DPH5/MRPS33/RPS9/RPL8/NPM1/CTSG/RPL10/UQCC2/EIF3H/RPL14/RPL19/MRPS18C/ABCE1/LARP1B/RSL24D1/RPL36/EIF4A2/RPL26L1/RPL23A/RPLP1/RARS1/EIF3M/UCN/NT5C3B/RPS23/RPS27L/RACK1/EEF1D/MRPS28/EIF1AX/RPS26/EIF4E/EIF2S2/PAIP1/ZC3H15/NGDN/MRPL3/BDH2/RPL4/MRPL27/SHMT2/RPL18A/EEF1A1/MRPL55/MRPL24/RPSA/RPL7A/PASK/EIF2S1/MTRES1/SHMT1/FASTKD3/JMJD4/COPS5/CASP3/EIF2B4/RPL13/USP16/MTIF3/EIF3L/EIF3G/RPL18/MRPL32/CNOT10/TRAP1/RBM3/SLC2A13/EIF5/ERAP2/PARK7/RPS19/METTL14/CLIC5/MRPL36/MRPS6/BIN1/DPP4/MRPS34/SEC11A/MRPL35/DARS1/PTCD3/BZW2/RPS28/MRPL51/UPF3A/TMED2/OGFOD1/MTRF1L/CD28/MCTS1/RPS12/CPA3/RPUSD3/TARS3/TIA1

BP	GO:0034470	ncRNA processing	388	0.48	2.20	1.00E-10	4.19E-09	2.57E-09	2483	tags=38%, list=21%, signal=31%	METTL18/RPS6/EMG1/RPS25/RPL7/RPS16/RPS14/RPL26/RPL27/NSA2/RPS8/RPS7/RPL5/RPS24/NOP58/RPS21/RPL35A/RPL11/RPL35/FBL/RPS27/METTL5/RPS17/UTP14A/WDR4/TRMT10C/RPP30/RPF1/RPL10A/NIFK/TRMT61B/RTRAF/POP5/LSM6/EXOSC1/RPL14/ZNHIT3/RTCB/MRPL1/TUT1/TSEN54/TYW3/TRMT13/TPRKB/KRR1/SSB/ESF1/DDX52/NGDN/BRIX1/SBDS/TRMO/MAK16/PIN4/PRKRA/LARP7/SHQ1/RPL7A/DGCR8/DHX37/RSL1D1/MRM1/THUMPD1/IMP4/UTP20/TRMT12/DDX49/CTU2/REXO4/RPS19/DTWD1/MPHOSPH10/ZBTB8OS/MPHOSPH6/PUS1/WDR75/RPS28/NVL/LAGE3/MOCS3/NOP53/HELQ/NHP2/RPUSD3/ANKRD16/TRUB1/TP53RK/WDR3/WDR6/RPS15/EXOSC5/TARBP1/UTP11/MTERF4/RBFA/DDX5/DUS1L/LCMT2/TRMT5/UTP6/DDX47/WDR43/ZCCHC8/DDX1/PWP1/DROSHA/NSUN4/RPP38/FTSJ1/INTS7/YTHDF2/TSR2/NSUN6/RRP9/GRSF1/UTP18/ADAT2/SPOUT1/TFB1M/DCAF13/QTRT1/INTS12/USP36/DALRD3/AARS1/DDX17/INTS4/INTS8/MRTO4/HSD17B10/PDCD11/LAS1L/CDK5RAP1/UTP15/HENMT1/KTI12/TYW1/FDXACB1/PPAN/INTS11/RIOK2/NOL8/KARS1/SNU13/TYW5/NOP56/FRG1
BP	GO:0043604	amide biosynthetic process	676	0.49	2.31	1.00E-10	4.19E-09	2.57E-09	1172	tags=25%, list=10%, signal=24%	METTL18/RPL24/RPL6/RPS6/RPL32/EEF1B2/RPL21/RPL29/RPS13/RPL39/MRPS21/RPL41/RPS18/MRPS5/RPS10/RPS25/RPS20/RPL7/EIF3E/RPS16/MRPL47/RPS14/EEF1G/RPL26/RPL27/RPL34/RPS8/RPL37/MRPL11/AIMP1/RPS5/RPS15A/RPL22/RPL15/RPS7/RPL5/RPL17/RPS24/RPL31/RPL23/RPS3A/RPS3/RPS21/RPL12/RWDD1/RPL30/RPL35A/RPL11/RPL36A/RPL35/RPL9/RPS27/MRPS17/RPL13A/RPL37A/EIF4B/DRG1/RPS27A/METTL5/RPL3/RPS17/EIF3F/LSM1/RPL22L1/SARNP/RPS2/MRPL57/TRMT10C/RPL27A/RPLP0/RPS11/RPL10A/RPL38/ZNF540/RPS29/EIF3C/LARP4/DPH5/MRPS33/RPS9/RPL8/NPM1/RPL10/UQCC2/EIF3H/RPL14/RPL19/MRPS18C/ABCE1/LARP1B/RSL24D1/RPL36/EIF4A2/RPL26L1/RPL23A/RPLP1/RARS1/EIF3M/UCN/NT5C3B/RPS23/RPS27L/RACK1/EEF1D/MRPS28/EIF1AX/RPS26/EIF4E/EIF2S2/PAIP1/ZC3H15/NGDN/MRPL3/BDH2/RPL4/MRPL27/SHMT2/RPL18A/EEF1A1/MRPL55/MRPL24/RPSA/RPL7A/ACAT1/PASK/EIF2S1/MTRES1/TECR/SHMT1/FASTKD3/JMJD4/COPS5/EIF2B4/RPL13/USP16/MTIF3/EIF3L/EIF3G/RPL18/MRPL32/CNOT10/TRAP1/RBM3/EIF5/RPS19/METTL14/MRPL36/MRPS6/MRPS34/MRPL35/DARS1/PTCD3/BZW2/RPS28/MRPL51/SLC25A1/UPF3A/CERS6/TMED2/OGFOD1/MTRF1L/CD28/MCTS1/RPS12/RPUSD3/TARS3/TIA1

BP	GO:0043043	peptide biosynthetic process	584	0.53	2.48	1.00E-10	4.19E-09	2.57E-09	1172	tags=28%, list=10%, signal=26%	METTL18/RPL24/RPL6/RPS6/RPL32/EEF1B2/RPL21/RPL29/RPS13/RPL39/MRPS21/RPL41/RPS18/MRPS5/RPS10/RPS25/RPS20/RPL7/EIF3E/RPS16/MRPL47/RPS14/EEF1G/RPL26/RPL27/RPL34/RPS8/RPL37/MRPL11/AIMP1/RPS5/RPS15A/RPL22/RPL15/RPS7/RPL5/RPL17/RPS24/RPL31/RPL23/RPS3A/RPS3/RPS21/RPL12/RWDD1/RPL30/RPL35A/RPL11/RPL36A/RPL35/RPL9/RPS27/MRPS17/RPL13A/RPL37A/EIF4B/DRG1/RPS27A/METTL5/RPL3/RPS17/EIF3F/LSM1/RPL22L1/SARNP/RPS2/MRPL57/TRMT10C/RPL27A/RPLP0/RPS11/RPL10A/RPL38/ZNF540/RPS29/EIF3C/LARP4/DPH5/MRPS33/RPS9/RPL8/NPM1/RPL10/UQCC2/EIF3H/RPL14/RPL19/MRPS18C/ABCE1/LARP1B/RSL24D1/RPL36/EIF4A2/RPL26L1/RPL23A/RPLP1/RARS1/EIF3M/UCN/NT5C3B/RPS23/RPS27L/RACK1/EEF1D/MRPS28/EIF1AX/RPS26/EIF4E/EIF2S2/PAIP1/ZC3H15/NGDN/MRPL3/BDH2/RPL4/MRPL27/SHMT2/RPL18A/EEF1A1/MRPL55/MRPL24/RPSA/RPL7A/PASK/EIF2S1/MTRES1/SHMT1/FASTKD3/JMJD4/COPS5/EIF2B4/RPL13/USP16/MTIF3/EIF3L/EIF3G/RPL18/MRPL32/CNOT10/TRAP1/RBM3/EIF5/RPS19/METTL14/MRPL36/MRPS6/MRPS34/MRPL35/DARS1/PTCD3/BZW2/RPS28/MRPL51/UPF3A/TMED2/OGFOD1/MTRF1L/CD28/MCTS1/RPS12/RPUSD3/TARS3/TIA1
BP	GO:0016072	rRNA metabolic process	253	0.54	2.38	1.00E-10	4.19E-09	2.57E-09	1719	tags=32%, list=15%, signal=28%	METTL18/RPS6/EMG1/RPS25/RPL7/RPS16/RPS14/RPL26/RPL27/NSA2/RPS8/RPS7/RPL5/RPS24/NOP58/RPS21/RPL35A/RPL11/RPL35/FBL/RPS27/GTF3A/METTL5/RPS17/UTP14A/RPP30/RPF1/RPL10A/GTF3C3/NIFK/TRMT61B/POP5/LSM6/EXOSC1/RPL14/ZNHIT3/MRPL1/KRR1/ESF1/DDX52/NGDN/BRIX1/SBDS/MAK16/PIN4/SHQ1/RPL7A/DHX37/RSL1D1/MRM1/IMP4/UTP20/DDX49/POLR1E/REXO4/RPS19/MPHOSPH10/MPHOSPH6/WDR75/RPS28/NVL/NOP53/HELQ/NHP2/RPUSD3/SLFN13/WDR3/RPS15/EXOSC5/TAF1B/UTP11/MTERF4/RBFA/UTP6/DDX47/WDR43/TRIR/PWP1/DROSHA/NSUN4/RPP38
BP	GO:0006412	translation	564	0.54	2.54	1.00E-10	4.19E-09	2.57E-09	1172	tags=29%, list=10%, signal=27%	METTL18/RPL24/RPL6/RPS6/RPL32/EEF1B2/RPL21/RPL29/RPS13/RPL39/MRPS21/RPL41/RPS18/MRPS5/RPS10/RPS25/RPS20/RPL7/EIF3E/RPS16/MRPL47/RPS14/EEF1G/RPL26/RPL27/RPL34/RPS8/RPL37/MRPL11/AIMP1/RPS5/RPS15A/RPL22/RPL15/RPS7/RPL5/RPL17/RPS24/RPL31/RPL23/RPS3A/RPS3/RPS21/RPL12/RWDD1/RPL30/RPL35A/RPL11/RPL36A/RPL35/RPL9/RPS27/MRPS17/RPL13A/RPL37A/EIF4B/DRG1/RPS27A/METTL5/RPL3/RPS17/EIF3F/LSM1/RPL22L1/SARNP/RPS2/MRPL57/TRMT10C/RPL27A/RPLP0/RPS11/RPL10A/RPL38/ZNF540/RPS29/EIF3C/LARP4/DPH5/MRPS33/RPS9/RPL8/NPM1/RPL10/UQCC2/EIF3H/RPL14/RPL19/MRPS18C/ABCE1/LARP1B/RSL24D1/RPL36/EIF4A2/RPL26L1/RPL23A/RPLP1/RARS1/EIF3M/UCN/NT5C3B/RPS23/RPS27L/RACK1/EEF1D/MRPS28/EIF1AX/RPS26/EIF4E/EIF2S2/PAIP1/ZC3H15/NGDN/MRPL3/RPL4/MRPL27/SHMT2/RPL18A/EEF1A1/MRPL55/MRPL24/RPSA/RPL7A/PASK/EIF2S1/MTRES1/SHMT1/FASTKD3/JMJD4/COPS5/EIF2B4/RPL13/USP16/MTIF3/EIF3L/EIF3G/RPL18/MRPL32/CNOT10/TRAP1/RBM3/EIF5/RPS19/METTL14/MRPL36/MRPS6/MRPS34/MRPL35/DARS1/PTCD3/BZW2/RPS28/MRPL51/UPF3A/TMED2/OGFOD1/MTRF1L/CD28/MCTS1/RPS12/RPUSD3/TARS3/TIA1

CC	GO:0019866	organelle inner membrane	462	0.54	2.50	1.00E-10	4.19E-09	2.57E-09	1902	tags=40%, list=16%, signal=35%	MRPS22/MRPS21/DNAJC15/MRPS5/UQCRH/MRPL47/LDHB/MRPL11/DNAJC19/UQCRB/COX16/RPS3/COX7C/MRPL46/MRPS17/MCUB/TIMM10/MRPL57/CYCS/MICOS13/MRPL48/C12orf73/MRPS31/COX6C/OXA1L/MRPL30/CYP2U1/NDUFS5/ATP5PD/MRPL4/MRPS33/UQCC2/NDUFAF5/MRPS18C/MRPS23/MRPL1/COX4I1/TIMM9/COX11/ATP5PO/CBX3/MRPS28/COX7B/ATP5MJ/NDUFB2/UQCC3/ATP5F1A/COQ6/DHFR2/NDUFA4/ATP5MK/COX7A2/MRPL39/MICU3/ATP5MF/ATP5PB/MRPL3/AFG3L2/MRPL53/SLC25A28/NDUFB5/TMEM126A/HIGD1A/MRPL27/SHMT2/ATP5MGL/MRPL55/PHB2/MRPL40/MRPL24/MRPL17/WDR93/ATP5F1E/COQ5/UQCRQ/P2RX4/MICOS10/COX5A/NDUFB9/SLC25A19/MPC1/P2RX5/MRPL14/UQCRC2/MRPL33/NDUFA13/PET100/TIMM8B/MRPL32/NDUFS3/CYC1/NDUFS6/NDUFA6/ATP5F1C/TRAP1/COX6A1/PMPCB/NDUFA1/ATP5PF/LETMD1/SLC25A6/SMDT1/IMMP1L/MTX3/RDH13/ATP5MG/MRPL22/ATPSCKMT/TIMM21/MRPS25/SLC25A3/SAMM50/MRPL36/MRPS6/NDUFAF4/MRPS34/SLC25A32/NDUFS4/SLC25A25/NDUFAF6/MRPL35/TRA2B/PTCD3/NDUFS8/PPOX/MRPL51/NDUFAB1/SLC25A1/MRPL50/SFXN1/UQCR10/SLC25A13/MRPL58/GRPEL2/YME1L1/NDUFB3/MRPL42/ABCB10/UQCRFS1/MRPS16/NDUFA12/HSPA9/TMEM120B/SLC25A36/APOO/ATP5MC1/MRPL16/NDUFB1/TIMM17A/ATP5ME/NEMP1/MRPL15/MRPL20/FLVCR1/ALDH18A1/DMAC1/MPC2/PHB1/MRPL18/COX18/MRPS14/BCS1L/MRPL21/DAP3/MRPL9/MATR3/SFXN4/MTX2/MTG1/MRPS30/NDUFC1/LRPPRC/TIMM10B/SLC25A33/NOA1/NDUFV2/DPY19L1/HSPD1/MICU2/MRPL41/SLC25A14/MRPS10/NDUFB8/TIMM44/MRPL2
BP	GO:0022613	ribonucleoprotein complex biogenesis	392	0.55	2.52	1.00E-10	4.19E-09	2.57E-09	1743	tags=34%, list=15%, signal=30%	METTL18/RPL24/RPL6/RPS6/SNRPD2/SNRPE/EMG1/RPS25/RPL7/RPS16/NUP88/RPS14/RPL26/RPL27/NSA2/RPS8/RPS5/RPS7/RPL5/RPS24/NOP58/RPS21/RPL35A/RPL11/RPL35/FBL/RPS27/GTF3A/RPL13A/EIF4B/METTL5/RPL3/RPS17/UTP14A/SNRPG/RPP30/CLNS1A/RPLP0/HSP90AA1/RPF1/RPL10A/RPL38/NOPCHAP1/NIFK/TRMT61B/POP5/NPM1/LSM6/RPL10/EXOSC1/RPL14/ZNHIT3/SF3A3/ABCE1/MRPL1/RSL24D1/RPL26L1/RPL23A/RPS27L/KRR1/ESF1/SNRPD1/EIF2S2/DDX52/PAK1IP1/NGDN/BRIX1/SBDS/MAK16/DHX30/PIN4/RBIS/NMD3/SHQ1/LUC7L3/RPSA/RPL7A/DHX37/RSL1D1/MRM1/IMP4/UTP20/DDX49/TAF9/SRSF10/EIF5/REXO4/SNRPC/RPS19/MRPL22/MPHOSPH10/MRPL36/MPHOSPH6/RUVBL2/WDR75/GEMIN8/RPS28/NVL/SF3B1/SNRPD3/NOP53/HELQ/NHP2/MCTS1/RPUSD3/RAN/PINX1/WDR3/RPS15/ZNF622/EXOSC5/UTP11/PRPF39/MTERF4/RBFA/MRPL20/PSIP1/GPATCH4/UTP6/LTV1/DDX47/WDR43/DDX1/PWP1/DROSHA/RUVBL1/NIP7/NSUN4/LUC7L/RPP38/MALSU1/RBM5

MF	GO:0005198	structural molecule activity	376	0.55	2.50	1.00E-10	4.19E-09	2.57E-09	1607	tags=38%, list=14%, signal=34%	RPL24/MRPS22/RPL6/RPS6/RPL32/RPL21/RPL29/RPS13/RPL39/MRPS21/RPL41/RPS18/MRPS5/RPS10/RPS25/RPS20/RPL7/RPS16/NUP88/MRPL47/RPS14/RPL26/RPL27/RPL34/RPS8/RPL37/MRPL11/RPS5/RPS15A/RPL22/RPL15/RPS7/RPL5/RPL17/RPS24/RPL31/RPL23/RPS3A/RPS3/RPS21/RPL12/RPL30/RPL35A/RPL11/RPL36A/RPL35/RPL9/MRPL46/RPS27/MRPS17/RPL13A/RPL37A/RPS27A/RPL3/RPS17/RPL22L1/RPS2/MRPL57/RPL27A/RPLP0/RPS11/RPL10A/RPL38/RPS29/MRPS31/MRPL30/MRPL4/MRPS33/RPS9/RPL8/RPL10/RPL14/RPL19/MRPS18C/MRPS23/MRPL1/YEATS4/RSL24D1/RPL36/RPL26L1/RPL23A/RPLP1/RPS23/RPS27L/COL19A1/RPS26/MRPL3/RPL4/MRPL27/NUP62/RPL18A/STX2/CMTM8/MRPL55/MRPL24/MRPL17/MYL5/MYL6B/RPSA/RPL7A/NEFL/MRPL14/RPL13/MRPL33/RPL18/MRPL32/LTBP4/NUP54/RPS19/MRPL22/MAL/MRPS25/MRPL36/MRPS6/MRPS34/MRPL35/RPS28/MRPL51/ODF2/UPF3A/RPS12/MYL6/RPS4X/VILL/MRPL42/EPPK1/MRPS16/RPS15/MRPL16/RPLP2/SEC31B/VPS25/MRPL15/MRPL20/CLTA/MRPL18/MRPS14/MRPL21/DAP3/MRPL9/NUP85/MATR3
BP	GO:0022618	ribonucleoprotein complex assembly	153	0.55	2.29	1.00E-10	4.19E-09	2.57E-09	1138	tags=27%, list=10%, signal=25%	RPL24/RPL6/SNRPD2/SNRPE/RPS14/RPS5/RPL5/RPL11/RPS27/RPL13A/EIF4B/RPL3/SNRPG/CLNS1A/RPLP0/HSP90AA1/RPL38/NOPCHAP1/RPL10/ZNHIT3/SF3A3/RPL23A/RPS27L/SNRPD1/EIF2S2/BRIX1/DHX30/SHQ1/LUC7L3/RPSA/TAF9/SRSF10/EIF5/SNRPC/RPS19/RUVBL2/GEMIN8/RPS28/SF3B1/SNRPD3/NOP53/MCTS1
BP	GO:0009060	aerobic respiration	152	0.55	2.30	1.00E-10	4.19E-09	2.57E-09	2627	tags=51%, list=22%, signal=40%	DNAJC15/UQCRH/MYC/UQCRB/COX7C/CYCS/OXA1L/NDUFS5/COA6/ATP5PD/UQCC2/COX4I1/UCN/ATP5PO/NDUFB2/UQCC3/ATP5F1A/NDUFA4/COX7A2/ATP5MF/ATP5PB/NDUFB5/BNIP3/SHMT2/ATP5F1E/UQCRQ/MDH1/COX5A/NDUFB9/UQCRC2/NDUFS3/CYC1/NDUFS6/NDUFA6/ATP5F1C/COX6A1/NDUFA1/ATP5PF/PARK7/ATP5MG/ATPSCKMT/NDUFS4/NDUFS8/NDUFAB1/NOP53/UQCR10/NDUFB3/UQCRFS1/NDUFA12/NDUFB1/ATP5ME/NDUFC1/TEFM/ANTKMT/SLC25A33/NDUFV2/SLC25A14/NDUFB8/SUCLG1/NDUFA5/NDUFA9/NDUFB4/MRPS36/NIPSNAP2/COQ9/COX7A2L/MTFR1L/COX10/SIRT3/PDE12/NDUFB7/ADSL/AFG1L/NDUFB6/TAFAZZIN/DLD/MSH2

CC	GO:0005743	mitochondrial inner membrane	416	0.56	2.59	1.00E-10	4.19E-09	2.57E-09	1902	tags=42%, list=16%, signal=37%	MRPS22/MRPS21/DNAJC15/MRPS5/UQCRH/MRPL47/LDHB/MRPL11/DNAJC19/UQCRB/COX16/RPS3/COX7C/MRPL46/MRPS17/MCUB/TIMM10/MRPL57/CYCS/MICOS13/MRPL48/C12orf73/MRPS31/COX6C/OXA1L/MRPL30/CYP2U1/NDUFS5/ATP5PD/MRPL4/MRPS33/UQCC2/NDUFAF5/MRPS18C/MRPS23/MRPL1/COX4I1/TIMM9/COX11/ATP5PO/MRPS28/COX7B/ATP5MJ/NDUFB2/UQCC3/ATP5F1A/COQ6/DHFR2/NDUFA4/ATP5MK/COX7A2/MRPL39/MICU3/ATP5MF/ATP5PB/MRPL3/AFG3L2/MRPL53/SLC25A28/NDUFB5/TMEM126A/HIGD1A/MRPL27/SHMT2/ATP5MGL/MRPL55/PHB2/MRPL40/MRPL24/MRPL17/WDR93/ATP5F1E/COQ5/UQCRQ/MICOS10/COX5A/NDUFB9/SLC25A19/MPC1/MRPL14/UQCRC2/MRPL33/NDUFA13/PET100/TIMM8B/MRPL32/NDUFS3/CYC1/NDUFS6/NDUFA6/ATP5F1C/TRAP1/COX6A1/PMPCB/NDUFA1/ATP5PF/LETMD1/SLC25A6/SMDT1/IMMP1L/MTX3/RDH13/ATP5MG/MRPL22/ATPSCKMT/TIMM21/MRPS25/SLC25A3/SAMM50/MRPL36/MRPS6/NDUFAF4/MRPS34/SLC25A32/NDUFS4/SLC25A25/NDUFAF6/MRPL35/PTCD3/NDUFS8/PPOX/MRPL51/NDUFAB1/SLC25A1/MRPL50/SFXN1/UQCR10/SLC25A13/MRPL58/GRPEL2/YME1L1/NDUFB3/MRPL42/ABCB10/UQCRFS1/MRPS16/NDUFA12/HSPA9/SLC25A36/APOO/ATP5MC1/MRPL16/NDUFB1/TIMM17A/ATP5ME/MRPL15/MRPL20/FLVCR1/ALDH18A1/DMAC1/MPC2/PHB1/MRPL18/COX18/MRPS14/BCS1L/MRPL21/DAP3/MRPL9/SFXN4/MTX2/MTG1/MRPS30/NDUFC1/TIMM10B/SLC25A33/NOA1/NDUFV2/HSPD1/MICU2/MRPL41/SLC25A14/MRPS10/NDUFB8/TIMM44/MRPL2
BP	GO:0006364	rRNA processing	215	0.57	2.45	1.00E-10	4.19E-09	2.57E-09	1719	tags=34%, list=15%, signal=30%	METTL18/RPS6/EMG1/RPS25/RPL7/RPS16/RPS14/RPL26/RPL27/NSA2/RPS8/RPS7/RPL5/RPS24/NOP58/RPS21/RPL35A/RPL11/RPL35/FBL/RPS27/METTL5/RPS17/UTP14A/RPP30/RPF1/RPL10A/NIFK/TRMT61B/POP5/LSM6/EXOSC1/RPL14/ZNHIT3/MRPL1/KRR1/ESF1/DDX52/NGDN/BRIX1/SBDS/MAK16/PIN4/SHQ1/RPL7A/DHX37/RSL1D1/MRM1/IMP4/UTP20/DDX49/REXO4/RPS19/MPHOSPH10/MPHOSPH6/WDR75/RPS28/NVL/NOP53/HELQ/NHP2/RPUSD3/WDR3/RPS15/EXOSC5/UTP11/MTERF4/RBFA/UTP6/DDX47/WDR43/PWP1/NSUN4/RPP38
BP	GO:0042254	ribosome biogenesis	289	0.57	2.55	1.00E-10	4.19E-09	2.57E-09	1740	tags=36%, list=15%, signal=32%	METTL18/RPL24/RPL6/RPS6/EMG1/RPS25/RPL7/RPS16/NUP88/RPS14/RPL26/RPL27/NSA2/RPS8/RPS5/RPS7/RPL5/RPS24/NOP58/RPS21/RPL35A/RPL11/RPL35/FBL/RPS27/GTF3A/METTL5/RPL3/RPS17/UTP14A/RPP30/RPLP0/RPF1/RPL10A/RPL38/NIFK/TRMT61B/POP5/NPM1/LSM6/RPL10/EXOSC1/RPL14/ZNHIT3/ABCE1/MRPL1/RSL24D1/RPL26L1/RPL23A/RPS27L/KRR1/ESF1/DDX52/PAK1IP1/NGDN/BRIX1/SBDS/MAK16/DHX30/PIN4/RBIS/NMD3/SHQ1/RPSA/RPL7A/DHX37/RSL1D1/MRM1/IMP4/UTP20/DDX49/REXO4/RPS19/MRPL22/MPHOSPH10/MRPL36/MPHOSPH6/WDR75/RPS28/NVL/NOP53/HELQ/NHP2/RPUSD3/RAN/PINX1/WDR3/RPS15/ZNF622/EXOSC5/UTP11/MTERF4/RBFA/MRPL20/GPATCH4/UTP6/LTV1/DDX47/WDR43/PWP1/DROSHA/NIP7/NSUN4/RPP38/MALSU1

CC	GO:1990904	ribonucleoprotein complex	625	0.59	2.80	1.00E-10	4.19E-09	2.57E-09	1743	tags=38%, list=15%, signal=35%	RPL24/MRPS22/RPL6/RPS6/SNRPD2/RPL32/RPL21/RPL29/RPS13/RPL39/MRPS21/SNRPE/EMG1/RPL41/RPS18/MRPS5/RPS10/RPS25/RPS20/RPL7/EIF3E/RPS16/MRPL47/RPS14/RPL26/RPL27/RPL34/NSA2/RPS8/RPL37/MRPL11/RPS5/RPS15A/RPL22/RPL15/RPS7/RPL5/RPL17/RPS24/RPL31/NOP58/RPL23/RPS3A/RPS3/RPS21/RPL12/RWDD1/RPL30/RPL35A/PSMA6/RPL11/RPL36A/RPL35/CWC15/FBL/RPL9/MRPL46/RPS27/MRPS17/RPL13A/RPL37A/PRKRIP1/DRG1/RPS27A/RPL3/RPS17/UTP14A/EIF3F/SNRPG/RPS2/MRPL57/TRMT10C/RPP30/CLNS1A/RPL27A/RPLP0/RPS11/RPF1/MRPL48/RPL10A/RPL38/SF3B6/LSM5/RBMS3/RPS29/EIF3C/MRPL30/HNRNPA1/MRPL4/MRPS33/RPS9/RPL8/POP5/NPM1/LSM6/RPL10/EIF3H/RPL14/ZNHIT3/PPIE/RPL19/SF3A3/SRP14/MRPS18C/LSM3/MRPL1/RPL36/SLIRP/RPL26L1/RPL23A/RPLP1/U2AF1L4/LSM7/TOP2A/EIF3M/SNRNP25/SNRNP70/RPS23/RPS27L/RACK1/MRPS28/KRR1/SSB/ILF2/RPS26/SNRPA1/SNRPD1/EIF4E/NGDN/MRPL39/VBP1/MAK16/MRPL3/MRPL53/HSPA8/RPL4/MRPL27/NUP62/RPL18A/RBMX2/LARP7/PHF5A/MRPL55/MRPL40/MRPL24/LUC7L3/MRPL17/RBMX/RPSA/RPL7A/ACD/EIF2S1/RSL1D1/LSM8/MRPL14/RPL13/MRPL33/DYRK2/IMP4/UTP20/EIF3L/EIF3G/RPL18/MRPL32/TAF9/SMG5/RBM3/SNRPA/SNRPC/RPS19/MRPL22/SRSF2/MPHOSPH10/MRPL36/MRPS6/RUVBL2/MRPS34/BCAS2/MRPL35/TRA2B/RPS28/NVL/SF3B1/MRPL51/MRPL50/RHEB/SNRPD3/ZCCHC17/ZCRB1/NHP2/RPS12/MRPL58/MAGOHB/RPS4X/MRPL42/WDR3/BTF3/DHX15/SNRPN/MRPS16/RPS15/ZNF622/SUGP1/UTP11/PRPF39/MTERF4/MRPL16/RPLP2/PSMA1/MRPL15/DDX5/MRPL20/SRP9/RBM12B/PPWD1/WDR37/HNRNPAB/MRPL18/MRPS14/UTP6/LTV1/MRPL21/DAP3/PABPC4/MRPL9/ZCCHC8/DDX1/PABPC1L/SRP54/AKAP17A/RUVBL1/NIP7/NSUN4/MRPS30/LUC7L/RPP38/JAKMIP1/LRPPRC/TEFM/RBM5
BP	GO:0006119	oxidative phosphorylation	110	0.60	2.37	1.00E-10	4.19E-09	2.57E-09	2627	tags=59%, list=22%, signal=46%	DNAJC15/UQCRH/MYC/UQCRB/COX7C/CYCS/NDUFS5/COA6/ATP5PD/UQCC2/COX4I1/ATP5PO/NDUFB2/UQCC3/ATP5F1A/NDUFA4/COX7A2/ATP5MF/ATP5PB/NDUFB5/SHMT2/ATP5F1E/UQCRQ/COX5A/NDUFB9/UQCRC2/NDUFS3/CYC1/NDUFS6/NDUFA6/ATP5F1C/COX6A1/NDUFA1/ATP5PF/PARK7/ATP5MG/ATPSCKMT/NDUFS4/NDUFS8/NDUFAB1/UQCR10/NDUFB3/UQCRFS1/NDUFA12/NDUFB1/ATP5ME/NDUFC1/TEFM/ANTKMT/SLC25A33/NDUFV2/NDUFB8/NDUFA5/NDUFA9/NDUFB4/NIPSNAP2/COQ9/COX7A2L/PDE12/NDUFB7/AFG1L/NDUFB6/TAFAZZIN/DLD/MSH2
BP	GO:0033108	mitochondrial respiratory chain complex assembly	89	0.66	2.50	1.00E-10	4.19E-09	2.57E-09	2569	tags=61%, list=22%, signal=48%	COX16/C12orf73/OXA1L/NDUFS5/COX14/UQCC2/NDUFAF5/NDUFB2/UQCC3/NDUFB5/TMEM126A/NDUFAF8/COA4/NDUFB9/FASTKD3/NDUFA13/PET100/NDUFS3/NDUFA6/NDUFA1/TIMM21/SAMM50/NDUFAF4/NDUFS4/NDUFAF6/NDUFS8/NDUFAB1/NDUFB3/COA5/UQCRFS1/NDUFA12/NDUFB1/DMAC1/COX18/BCS1L/SDHAF4/DMAC2/NDUFC1/SLC25A33/NDUFB8/SDHAF1/NDUFA5/NDUFAF7/NDUFA9/NDUFB4/SDHAF3/COX17/SCO1/NDUFB7/NDUFA11/TMEM126B/NDUFB6/TAFAZZIN/TACO1

CC	GO:0098798	mitochondrial protein-containing complex	254	0.66	2.93	1.00E-10	4.19E-09	2.57E-09	1965	tags=52%, list=17%, signal=44%	MRPS22/MRPS21/DNAJC15/MRPS5/UQCRH/MRPL47/MRPL11/DNAJC19/UQCRB/COX7C/MRPL46/MRPS17/MCUB/TIMM10/MRPL57/TRMT10C/MICOS13/MRPL48/COX6C/MRPL30/NDUFS5/TOMM20/ATP5PD/MRPL4/MRPS33/MRPS18C/COX4I1/TIMM9/TOMM7/ATP5PO/MRPS28/COX7B/ATP5MJ/NDUFB2/ATP5F1A/NDUFA4/ATP5MK/COX7A2/MRPL39/MICU3/ATP5MF/ATP5PB/TOMM5/MRPL3/AFG3L2/MRPL53/NDUFB5/MRPL27/ATP5MGL/MRPL55/PHB2/MRPL40/MRPL24/MRPL17/WDR93/ATP5F1E/UQCRQ/MICOS10/COX5A/NDUFB9/MRPL14/UQCRC2/MRPL33/NDUFA13/TIMM8B/MRPL32/NDUFS3/CYC1/NDUFS6/NDUFA6/ATP5F1C/COX6A1/NDUFA1/ATP5PF/SLC25A6/SMDT1/IMMP1L/MTX3/ATP5MG/MRPL22/TIMM21/SAMM50/POLRMT/MRPL36/MRPS6/MRPS34/NDUFS4/MRPL35/NDUFS8/MRPL51/NDUFAB1/MRPL50/BCKDHB/UQCR10/MRPL58/GRPEL2/NDUFB3/MRPL42/UQCRFS1/MRPS16/NDUFA12/HSPA9/TOMM22/APOO/ATP5MC1/MTERF4/MRPL16/NDUFB1/TIMM17A/ATP5ME/MRPL15/MRPL20/PHB1/MRPL18/MRPS14/BCS1L/MRPL21/DAP3/MRPL9/MTX2/NSUN4/MRPS30/NDUFC1/TIMM10B/NDUFV2/MICU2/MRPL41/MRPS10/NDUFB8/MRPL2/SUCLG1/NDUFA5
CC	GO:0070469	respirasome	78	0.67	2.47	1.00E-10	4.19E-09	2.57E-09	2046	tags=53%, list=17%, signal=44%	UQCRH/UQCRB/COX7C/CYCS/COX6C/NDUFS5/COA6/COX4I1/COX7B/NDUFB2/NDUFA4/COX7A2/NDUFB5/HIGD1A/WDR93/UQCRQ/COX5A/NDUFB9/UQCRC2/NDUFA13/NDUFS3/CYC1/NDUFS6/NDUFA6/COX6A1/NDUFA1/NDUFS4/NDUFS8/NDUFAB1/UQCR10/NDUFB3/UQCRFS1/NDUFA12/NDUFB1/BCS1L/NDUFC1/NDUFV2/NDUFB8/NDUFA5/NDUFA9/NDUFB4
CC	GO:0098800	inner mitochondrial membrane protein complex	131	0.69	2.79	1.00E-10	4.19E-09	2.57E-09	2046	tags=58%, list=17%, signal=48%	DNAJC15/UQCRH/DNAJC19/UQCRB/COX7C/MCUB/TIMM10/MICOS13/COX6C/NDUFS5/ATP5PD/COX4I1/TIMM9/ATP5PO/COX7B/ATP5MJ/NDUFB2/ATP5F1A/NDUFA4/ATP5MK/COX7A2/MICU3/ATP5MF/ATP5PB/AFG3L2/NDUFB5/ATP5MGL/PHB2/WDR93/ATP5F1E/UQCRQ/MICOS10/COX5A/NDUFB9/UQCRC2/NDUFA13/NDUFS3/CYC1/NDUFS6/NDUFA6/ATP5F1C/COX6A1/NDUFA1/ATP5PF/SLC25A6/SMDT1/IMMP1L/MTX3/ATP5MG/TIMM21/SAMM50/NDUFS4/NDUFS8/NDUFAB1/UQCR10/GRPEL2/NDUFB3/UQCRFS1/NDUFA12/HSPA9/APOO/ATP5MC1/NDUFB1/TIMM17A/ATP5ME/PHB1/BCS1L/MTX2/NDUFC1/TIMM10B/NDUFV2/MICU2/NDUFB8/NDUFA5/NDUFA9/NDUFB4
CC	GO:0098803	respiratory chain complex	68	0.70	2.55	1.00E-10	4.19E-09	2.57E-09	2536	tags=65%, list=22%, signal=51%	UQCRH/UQCRB/COX7C/COX6C/NDUFS5/COA6/COX4I1/COX7B/NDUFB2/NDUFA4/COX7A2/NDUFB5/WDR93/UQCRQ/COX5A/NDUFB9/UQCRC2/NDUFA13/NDUFS3/CYC1/NDUFS6/NDUFA6/COX6A1/NDUFA1/NDUFS4/NDUFS8/NDUFAB1/UQCR10/NDUFB3/UQCRFS1/NDUFA12/NDUFB1/BCS1L/NDUFC1/NDUFV2/NDUFB8/NDUFA5/NDUFA9/NDUFB4/COX7A2L/NDUFB7/NDUFA11/UQCR11/NDUFB6
MF	GO:0019843	rRNA binding	64	0.73	2.62	1.00E-10	4.19E-09	2.57E-09	1539	tags=53%, list=13%, signal=46%	RPS13/EMG1/RPS18/RPS14/RPL37/MRPL11/RPS5/RPL5/RPL23/RPS3/RPL12/RPL11/RPL9/GTF3A/MRPS17/RPL3/RPLP0/RPS11/RPF1/RPS9/RPL8/MRPS18C/RPL23A/BRIX1/SBDS/IMP4/MRPS6/PTCD3/NOP53/RPS4X/MTERF4/MRPL16/MRPL20/MRPL18

CC	GO:0000313	organellar ribosome	80	0.73	2.75	1.00E-10	4.19E-09	2.57E-09	1902	tags=62%, list=16%, signal=53%	MRPS22/MRPS21/MRPS5/MRPL47/MRPL11/MRPL46/MRPS17/MRPL57/MRPL48/MRPL30/MRPL4/MRPS33/MRPS18C/MRPS28/MRPL39/MRPL3/MRPL53/MRPL27/MRPL55/MRPL40/MRPL24/MRPL17/MRPL14/MRPL33/MRPL32/MRPL22/MRPL36/MRPS6/MRPS34/MRPL35/MRPL51/MRPL50/MRPL58/MRPL42/MRPS16/MTERF4/MRPL16/MRPL15/MRPL20/MRPL18/MRPS14/MRPL21/DAP3/MRPL9/MTG1/NSUN4/MRPS30/MRPL41/MRPS10/MRPL2
CC	GO:0005761	mitochondrial ribosome	80	0.73	2.75	1.00E-10	4.19E-09	2.57E-09	1902	tags=62%, list=16%, signal=53%	MRPS22/MRPS21/MRPS5/MRPL47/MRPL11/MRPL46/MRPS17/MRPL57/MRPL48/MRPL30/MRPL4/MRPS33/MRPS18C/MRPS28/MRPL39/MRPL3/MRPL53/MRPL27/MRPL55/MRPL40/MRPL24/MRPL17/MRPL14/MRPL33/MRPL32/MRPL22/MRPL36/MRPS6/MRPS34/MRPL35/MRPL51/MRPL50/MRPL58/MRPL42/MRPS16/MTERF4/MRPL16/MRPL15/MRPL20/MRPL18/MRPS14/MRPL21/DAP3/MRPL9/MTG1/NSUN4/MRPS30/MRPL41/MRPS10/MRPL2
CC	GO:0005844	polysome	61	0.74	2.64	1.00E-10	4.19E-09	2.57E-09	809	tags=48%, list=7%, signal=44%	RPL24/RPL6/RPS6/RPL32/RPL39/RPL41/RPL31/RPS3/RPS21/RWDD1/RPL30/PSMA6/RPL11/RPL36A/DRG1/RPL10A/RPL38/RPS29/RPL8/EIF3H/RPL19/RPL36/RPS23/RPS26/VBP1/RPL18A/RPL7A/EIF2S1/RPL18
BP	GO:0042273	ribosomal large subunit biogenesis	69	0.74	2.70	1.00E-10	4.19E-09	2.57E-09	1134	tags=48%, list=10%, signal=43%	RPL24/RPL6/RPL7/RPL26/NSA2/RPL5/RPL35A/RPL11/RPL35/GTF3A/RPL3/RPLP0/RPF1/RPL10A/RPL38/NIFK/NPM1/RPL10/RPL14/ZNHIT3/MRPL1/RSL24D1/RPL26L1/RPL23A/PAK1IP1/BRIX1/MAK16/DHX30/RPL7A/RSL1D1/NVL/NOP53/NHP2
CC	GO:0000315	organellar large ribosomal subunit	54	0.74	2.60	1.00E-10	4.19E-09	2.57E-09	1902	tags=67%, list=16%, signal=56%	MRPL47/MRPL11/MRPL46/MRPL57/MRPL48/MRPL30/MRPL4/MRPL39/MRPL3/MRPL53/MRPL27/MRPL55/MRPL40/MRPL24/MRPL17/MRPL14/MRPL33/MRPL32/MRPL22/MRPL36/MRPL35/MRPL51/MRPL50/MRPL58/MRPL42/MTERF4/MRPL16/MRPL15/MRPL20/MRPL18/MRPL21/MRPL9/NSUN4/MRPS30/MRPL41/MRPL2
CC	GO:0005762	mitochondrial large ribosomal subunit	54	0.74	2.60	1.00E-10	4.19E-09	2.57E-09	1902	tags=67%, list=16%, signal=56%	MRPL47/MRPL11/MRPL46/MRPL57/MRPL48/MRPL30/MRPL4/MRPL39/MRPL3/MRPL53/MRPL27/MRPL55/MRPL40/MRPL24/MRPL17/MRPL14/MRPL33/MRPL32/MRPL22/MRPL36/MRPL35/MRPL51/MRPL50/MRPL58/MRPL42/MTERF4/MRPL16/MRPL15/MRPL20/MRPL18/MRPL21/MRPL9/NSUN4/MRPS30/MRPL41/MRPL2
CC	GO:0005840	ribosome	195	0.79	3.38	1.00E-10	4.19E-09	2.57E-09	1592	tags=67%, list=14%, signal=59%	RPL24/MRPS22/RPL6/RPS6/RPL32/RPL21/RPL29/RPS13/RPL39/MRPS21/RPL41/RPS18/MRPS5/RPS10/RPS25/RPS20/RPL7/RPS16/MRPL47/RPS14/RPL26/RPL27/RPL34/RPS8/RPL37/MRPL11/RPS5/RPS15A/RPL22/RPL15/RPS7/RPL5/RPL17/RPS24/RPL31/RPL23/RPS3A/RPS3/RPS21/RPL12/RPL30/RPL35A/RPL11/RPL36A/RPL35/RPL9/MRPL46/RPS27/MRPS17/RPL13A/RPL37A/RPS27A/RPL3/RPS17/RPL22L1/RPS2/MRPL57/RPL27A/RPLP0/RPS11/MRPL48/RPL10A/RPL38/RPS29/MRPL30/MRPL4/MRPS33/RPS9/RPL8/RPL10/EIF3H/RPL14/RPL19/MRPS18C/MRPL1/RSL24D1/RPL36/RPL26L1/RPL23A/RPLP1/RPS23/RPS27L/RACK1/MRPS28/RPS26/MRPL39/MRPL3/MRPL53/RPL4/MRPL27/RPL18A/MRPL55/MRPL40/MRPL24/MRPL17/RPSA/RPL7A/MRPL14/RPL13/MRPL33/RPL18/MRPL32/RPS19/MRPL22/MRPL36/MRPS6/MRPS34/MRPL35/RPS28/MRPL51/MRPL50/ZCCHC17/RPS12/MRPL58/RPS4X/MRPL42/BTF3/MRPS16/RPS15/MTERF4/MRPL16/RPLP2/MRPL15/MRPL20/SERP1/APEX1/MRPL18/MRPS14/MRPL21/DAP3/MRPL9

CC	GO:0015934	large ribosomal subunit	108	0.81	3.18	1.00E-10	4.19E-09	2.57E-09	1592	tags=74%, list=14%, signal=65%	RPL24/RPL6/RPL32/RPL21/RPL29/RPL39/RPL41/RPL7/MRPL47/RPL26/RPL27/RPL34/RPL37/MRPL11/RPL22/RPL15/RPL5/RPL17/RPL31/RPL23/RPL12/RPL30/RPL35A/RPL11/RPL36A/RPL35/RPL9/MRPL46/RPL13A/RPL37A/RPL3/MRPL57/RPL27A/RPLP0/MRPL48/RPL10A/RPL38/MRPL30/MRPL4/RPL8/RPL10/RPL14/RPL19/MRPL1/RPL36/RPL26L1/RPL23A/RPLP1/MRPL39/MRPL3/MRPL53/RPL4/MRPL27/RPL18A/MRPL55/MRPL40/MRPL24/MRPL17/RPL7A/MRPL14/RPL13/MRPL33/RPL18/MRPL32/MRPL22/MRPL36/MRPL35/MRPL51/MRPL50/ZCCHC17/MRPL58/MRPL42/MTERF4/MRPL16/RPLP2/MRPL15/MRPL20/MRPL18/MRPL21/MRPL9
BP	GO:0002181	cytoplasmic translation	139	0.81	3.28	1.00E-10	4.19E-09	2.57E-09	918	tags=60%, list=8%, signal=56%	RPL24/RPL6/RPS6/RPL32/RPL21/RPL29/RPS13/RPL39/RPL41/RPS18/RPS10/RPS25/RPS20/RPL7/RPS16/RPS14/RPL26/RPL27/RPL34/RPS8/RPL37/RPS5/RPS15A/RPL22/RPL15/RPS7/RPL5/RPL17/RPS24/RPL31/RPL23/RPS3A/RPS3/RPS21/RPL12/RWDD1/RPL30/RPL35A/RPL11/RPL36A/RPL35/RPL9/RPS27/RPL13A/RPL37A/EIF4B/DRG1/RPS27A/RPL3/RPS17/RPL22L1/RPS2/RPL27A/RPLP0/RPS11/RPL10A/RPL38/RPS29/DPH5/RPS9/RPL8/RPL10/RPL14/RPL19/RPL36/EIF4A2/RPL26L1/RPL23A/RPLP1/EIF3M/RPS23/RPS26/EIF2S2/PAIP1/ZC3H15/RPL4/RPL18A/RPSA/RPL7A/RPL13/RPL18/EIF5/RPS19
CC	GO:0044391	ribosomal subunit	169	0.82	3.44	1.00E-10	4.19E-09	2.57E-09	1592	tags=74%, list=14%, signal=65%	RPL24/MRPS22/RPL6/RPS6/RPL32/RPL21/RPL29/RPS13/RPL39/MRPS21/RPL41/RPS18/MRPS5/RPS10/RPS25/RPS20/RPL7/RPS16/MRPL47/RPS14/RPL26/RPL27/RPL34/RPS8/RPL37/MRPL11/RPS5/RPS15A/RPL22/RPL15/RPS7/RPL5/RPL17/RPS24/RPL31/RPL23/RPS3A/RPS3/RPS21/RPL12/RPL30/RPL35A/RPL11/RPL36A/RPL35/RPL9/MRPL46/RPS27/MRPS17/RPL13A/RPL37A/RPS27A/RPL3/RPS17/RPS2/MRPL57/RPL27A/RPLP0/RPS11/MRPL48/RPL10A/RPL38/RPS29/MRPL30/MRPL4/MRPS33/RPS9/RPL8/RPL10/RPL14/RPL19/MRPS18C/MRPL1/RPL36/RPL26L1/RPL23A/RPLP1/RPS23/RPS27L/RACK1/MRPS28/RPS26/MRPL39/MRPL3/MRPL53/RPL4/MRPL27/RPL18A/MRPL55/MRPL40/MRPL24/MRPL17/RPSA/RPL7A/MRPL14/RPL13/MRPL33/RPL18/MRPL32/RPS19/MRPL22/MRPL36/MRPS6/MRPS34/MRPL35/RPS28/MRPL51/MRPL50/ZCCHC17/RPS12/MRPL58/RPS4X/MRPL42/MRPS16/RPS15/MTERF4/MRPL16/RPLP2/MRPL15/MRPL20/MRPL18/MRPS14/MRPL21/DAP3/MRPL9
MF	GO:0003735	structural constituent of ribosome	154	0.83	3.45	1.00E-10	4.19E-09	2.57E-09	1055	tags=69%, list=9%, signal=63%	RPL24/MRPS22/RPL6/RPS6/RPL32/RPL21/RPL29/RPS13/RPL39/MRPS21/RPL41/RPS18/MRPS5/RPS10/RPS25/RPS20/RPL7/RPS16/MRPL47/RPS14/RPL26/RPL27/RPL34/RPS8/RPL37/MRPL11/RPS5/RPS15A/RPL22/RPL15/RPS7/RPL5/RPL17/RPS24/RPL31/RPL23/RPS3A/RPS3/RPS21/RPL12/RPL30/RPL35A/RPL11/RPL36A/RPL35/RPL9/MRPL46/RPS27/MRPS17/RPL13A/RPL37A/RPS27A/RPL3/RPS17/RPL22L1/RPS2/MRPL57/RPL27A/RPLP0/RPS11/RPL10A/RPL38/RPS29/MRPS31/MRPL30/MRPL4/MRPS33/RPS9/RPL8/RPL10/RPL14/RPL19/MRPS18C/MRPS23/MRPL1/RSL24D1/RPL36/RPL26L1/RPL23A/RPLP1/RPS23/RPS27L/RPS26/MRPL3/RPL4/MRPL27/RPL18A/MRPL55/MRPL24/MRPL17/RPSA/RPL7A/MRPL14/RPL13/MRPL33/RPL18/MRPL32/RPS19/MRPL22/MRPS25/MRPL36/MRPS6/MRPS34/MRPL35/RPS28/MRPL51

CC	GO:0015935	small ribosomal subunit	64	0.83	2.98	1.00E-10	4.19E-09	2.57E-09	1332	tags=69%, list=11%, signal=61%	MRPS22/RPS6/RPS13/MRPS21/RPS18/MRPS5/RPS10/RPS25/RPS20/RPS16/RPS14/RPS8/RPS5/RPS15A/RPS7/RPS24/RPS3A/RPS3/RPS21/RPS27/MRPS17/RPS27A/RPS17/RPS2/RPS11/RPS29/MRPS33/RPS9/MRPS18C/RPS23/RPS27L/RACK1/MRPS28/RPS26/RPSA/RPS19/MRPS6/MRPS34/RPS28/RPS12/RPS4X/MRPL42/MRPS16/RPS15
CC	GO:0022627	cytosolic small ribosomal subunit	39	0.87	2.85	1.00E-10	4.19E-09	2.57E-09	1332	tags=82%, list=11%, signal=73%	RPS6/RPS13/RPS18/RPS10/RPS25/RPS20/RPS16/RPS14/RPS8/RPS5/RPS15A/RPS7/RPS24/RPS3A/RPS3/RPS21/RPS27/RPS27A/RPS17/RPS2/RPS11/RPS29/RPS9/RPS23/RPS27L/RPS26/RPSA/RPS19/RPS28/RPS12/RPS4X/RPS15
CC	GO:0022626	cytosolic ribosome	93	0.88	3.35	1.00E-10	4.19E-09	2.57E-09	809	tags=78%, list=7%, signal=74%	RPL24/RPL6/RPS6/RPL32/RPL21/RPL29/RPS13/RPL39/RPL41/RPS18/RPS10/RPS25/RPS20/RPL7/RPS16/RPS14/RPL26/RPL27/RPL34/RPS8/RPL37/RPS5/RPS15A/RPL22/RPL15/RPS7/RPL5/RPL17/RPS24/RPL31/RPL23/RPS3A/RPS3/RPS21/RPL12/RPL30/RPL35A/RPL11/RPL36A/RPL35/RPL9/RPS27/RPL13A/RPL37A/RPS27A/RPL3/RPS17/RPS2/RPL27A/RPLP0/RPS11/RPL10A/RPL38/RPS29/RPS9/RPL8/RPL10/RPL14/RPL19/MRPL1/RPL36/RPL26L1/RPL23A/RPLP1/RPS23/RPS27L/RPS26/RPL4/RPL18A/RPSA/RPL7A/RPL13/RPL18
CC	GO:0042788	polysomal ribosome	29	0.88	2.70	1.00E-10	4.19E-09	2.57E-09	809	tags=76%, list=7%, signal=71%	RPL24/RPL6/RPL32/RPL39/RPL41/RPL31/RPS21/RPL30/RPL11/RPL36A/RPL10A/RPL38/RPS29/RPL8/EIF3H/RPL19/RPL36/RPS23/RPS26/RPL18A/RPL7A/RPL18
CC	GO:0022625	cytosolic large ribosomal subunit	54	0.89	3.14	1.00E-10	4.19E-09	2.57E-09	809	tags=85%, list=7%, signal=80%	RPL24/RPL6/RPL32/RPL21/RPL29/RPL39/RPL41/RPL7/RPL26/RPL27/RPL34/RPL37/RPL22/RPL15/RPL5/RPL17/RPL31/RPL23/RPL12/RPL30/RPL35A/RPL11/RPL36A/RPL35/RPL9/RPL13A/RPL37A/RPL3/RPL27A/RPLP0/RPL10A/RPL38/RPL8/RPL10/RPL14/RPL19/MRPL1/RPL36/RPL26L1/RPL23A/RPLP1/RPL4/RPL18A/RPL7A/RPL13/RPL18
BP	GO:0071826	ribonucleoprotein complex subunit organization	161	0.53	2.23	1.09E-10	4.47E-09	2.75E-09	1138	tags=27%, list=10%, signal=24%	RPL24/RPL6/SNRPD2/SNRPE/RPS14/RPS5/RPL5/RPL11/RPS27/RPL13A/EIF4B/RPL3/SNRPG/CLNS1A/RPLP0/HSP90AA1/RPL38/NOPCHAP1/RPL10/ZNHIT3/SF3A3/ZFAND1/RPL23A/RPS27L/SNRPD1/EIF2S2/BRIX1/DHX30/SHQ1/LUC7L3/RPSA/TAF9/SRSF10/EIF5/SNRPC/RPS19/RUVBL2/GEMIN8/RPS28/SF3B1/SNRPD3/NOP53/MCTS1
BP	GO:0042255	ribosome assembly	56	0.70	2.46	6.32E-10	2.18E-08	1.34E-08	1472	tags=46%, list=13%, signal=41%	RPL24/RPL6/RPS14/RPS5/RPL5/RPL11/RPS27/RPL3/RPLP0/RPL38/NPM1/RPL10/RPL23A/RPS27L/BRIX1/SBDS/DHX30/RPSA/DHX37/RPS19/MRPL22/RPS28/NOP53/RPS15/MTERF4/MRPL20
CC	GO:0005746	mitochondrial respirasome	72	0.65	2.39	8.97E-10	3.00E-08	1.84E-08	2536	tags=60%, list=22%, signal=47%	UQCRH/UQCRB/COX7C/COX6C/NDUFS5/COX4I1/COX7B/NDUFB2/NDUFA4/COX7A2/NDUFB5/WDR93/UQCRQ/COX5A/NDUFB9/UQCRC2/NDUFA13/NDUFS3/CYC1/NDUFS6/NDUFA6/COX6A1/NDUFA1/NDUFS4/NDUFS8/NDUFAB1/UQCR10/NDUFB3/UQCRFS1/NDUFA12/NDUFB1/BCS1L/NDUFC1/NDUFV2/NDUFB8/NDUFA5/NDUFA9/NDUFB4/COX7A2L/NDUFB7/NDUFA11/UQCR11/NDUFB6
BP	GO:0042274	ribosomal small subunit biogenesis	71	0.65	2.38	1.11E-09	3.63E-08	2.23E-08	1614	tags=39%, list=14%, signal=34%	RPS6/EMG1/RPS25/RPS16/RPS14/RPS8/RPS5/RPS7/RPS24/RPS21/RPS27/RPS17/RPL38/NPM1/LSM6/RPS27L/DDX52/NGDN/RPSA/DHX37/UTP20/RPS19/RPS28/WDR3/RPS15/UTP6/LTV1/WDR43

BP	GO:0045333	cellular respiration	183	0.49	2.08	2.51E-09	7.80E-08	4.80E-08	2627	tags=46%, list=22%, signal=37%	DNAJC15/UQCRH/MYC/UQCRB/COX7C/CYCS/OXA1L/NDUFS5/COA6/ATP5PD/UQCC2/COX4I1/UCN/ATP5PO/NDUFB2/UQCC3/ATP5F1A/NDUFA4/COX7A2/ATP5MF/ATP5PB/NDUFB5/BNIP3/SHMT2/CISD1/ATP5F1E/UQCRQ/MDH1/COX5A/NDUFB9/UQCRC2/NDUFS3/CYC1/NDUFS6/NDUFA6/ATP5F1C/TRAP1/COX6A1/NDUFA1/ATP5PF/PARK7/ATP5MG/ATPSCKMT/NDUFS4/SLC25A25/NDUFS8/NDUFAB1/NOP53/UQCR10/SLC25A13/NDUFB3/UQCRFS1/NDUFA12/NDUFB1/ATP5ME/SDHAF4/NDUFC1/TEFM/ANTKMT/SLC25A33/NOA1/NDUFV2/SLC25A14/NDUFB8/SUCLG1/NDUFA5/NDUFA9/NDUFB4/MRPS36/NIPSNAP2/COQ9/COX7A2L/MTFR1L/POLG2/COX10/SLC25A22/SIRT3/PDE12/NDUFB7/ADSL/AFG1L/NDUFB6/TAFAZZIN/DLD/MSH2
BP	GO:0140053	mitochondrial gene expression	103	0.57	2.23	3.62E-09	1.06E-07	6.55E-08	2938	tags=53%, list=25%, signal=40%	MRPS21/MRPL47/MRPS17/MRPL57/TRMT10C/TRMT61B/UQCC2/MRPS28/TBRG4/TWNK/SHMT2/MTRES1/FASTKD3/MTIF3/POLRMT/MRPS6/PUS1/MRPS34/PTCD3/MRPL51/MTRF1L/RPUSD3/MRPL58/MRPS16/MTERF4/MRPL16/TRMT5/MRPS14/MTG1/GATC/LRPPRC/TEFM/MALSU1/SLC25A33/NOA1/TUFM/MTIF2/MRPL2/TFB1M/RARS2/HSD17B10/IARS2/GATB/CDK5RAP1/FASTKD2/EARS2/MRPS18A/WARS2/TACO1/MRPS15/MRPS12/FASTKD1/GARS1/TRNT1/MRPS18B
BP	GO:0010257	NADH dehydrogenase complex assembly	53	0.69	2.40	4.58E-09	1.30E-07	8.00E-08	2550	tags=66%, list=22%, signal=52%	OXA1L/NDUFS5/NDUFAF5/NDUFB2/NDUFB5/TMEM126A/NDUFAF8/NDUFB9/NDUFA13/NDUFS3/NDUFA6/NDUFA1/TIMM21/NDUFAF4/NDUFS4/NDUFAF6/NDUFS8/NDUFAB1/NDUFB3/NDUFA12/NDUFB1/DMAC1/BCS1L/DMAC2/NDUFC1/NDUFB8/NDUFA5/NDUFAF7/NDUFA9/NDUFB4/NDUFB7/NDUFA11/TMEM126B/NDUFB6/TAFAZZIN
BP	GO:0032981	mitochondrial respiratory chain complex I assembly	53	0.69	2.40	4.58E-09	1.30E-07	8.00E-08	2550	tags=66%, list=22%, signal=52%	OXA1L/NDUFS5/NDUFAF5/NDUFB2/NDUFB5/TMEM126A/NDUFAF8/NDUFB9/NDUFA13/NDUFS3/NDUFA6/NDUFA1/TIMM21/NDUFAF4/NDUFS4/NDUFAF6/NDUFS8/NDUFAB1/NDUFB3/NDUFA12/NDUFB1/DMAC1/BCS1L/DMAC2/NDUFC1/NDUFB8/NDUFA5/NDUFAF7/NDUFA9/NDUFB4/NDUFB7/NDUFA11/TMEM126B/NDUFB6/TAFAZZIN
CC	GO:0031304	intrinsic component of mitochondrial inner membrane	54	0.68	2.38	6.21E-09	1.66E-07	1.02E-07	1636	tags=44%, list=14%, signal=38%	COX16/MCUB/MICOS13/OXA1L/COX11/UQCC3/AFG3L2/MICOS10/SLC25A19/MPC1/PET100/SMDT1/MTX3/SLC25A3/SAMM50/PPOX/SFXN1/HSPA9/APOO/TIMM17A/MPC2/COX18/SFXN4/MTX2
BP	GO:0032543	mitochondrial translation	71	0.64	2.33	6.75E-09	1.77E-07	1.09E-07	2730	tags=55%, list=23%, signal=42%	MRPS21/MRPL47/MRPS17/MRPL57/TRMT10C/UQCC2/MRPS28/SHMT2/FASTKD3/MTIF3/MRPS6/MRPS34/PTCD3/MRPL51/MTRF1L/RPUSD3/MRPL58/MRPS16/MRPL16/MRPS14/MTG1/GATC/LRPPRC/MALSU1/NOA1/TUFM/MTIF2/MRPL2/RARS2/IARS2/GATB/CDK5RAP1/FASTKD2/EARS2/MRPS18A/WARS2/TACO1/MRPS15/MRPS12
MF	GO:0015453	oxidoreduction-driven active transmembrane transporter activity	55	0.67	2.35	2.02E-08	4.94E-07	3.03E-07	2046	tags=55%, list=17%, signal=45%	UQCRH/NDUFS5/COX4I1/COX7B/NDUFB2/NDUFA4/NDUFB5/COX5A/NDUFB9/NDUFA13/NDUFS3/CYC1/NDUFS6/NDUFA6/COX6A1/NDUFA1/NDUFS4/NDUFS8/UQCR10/NDUFB3/UQCRFS1/NDUFA12/NDUFB1/CYB5A/NDUFC1/NDUFV2/NDUFB8/NDUFA5/NDUFA9/NDUFB4
CC	GO:0031305	integral component of mitochondrial inner membrane	53	0.67	2.35	2.02E-08	4.94E-07	3.03E-07	1636	tags=43%, list=14%, signal=38%	COX16/MCUB/MICOS13/OXA1L/COX11/UQCC3/AFG3L2/MICOS10/SLC25A19/MPC1/PET100/SMDT1/MTX3/SLC25A3/SAMM50/SFXN1/HSPA9/APOO/TIMM17A/MPC2/COX18/SFXN4/MTX2

BP	GO:0042773	ATP synthesis coupled electron transport	70	0.63	2.29	2.80E-08	6.50E-07	4.00E-07	2621	tags=61%, list=22%, signal=48%	DNAJC15/UQCRH/UQCRB/COX7C/CYCS/NDUFS5/COA6/COX4I1/NDUFB2/UQCC3/NDUFA4/NDUFB5/UQCRQ/COX5A/NDUFB9/NDUFS3/CYC1/NDUFS6/NDUFA6/COX6A1/NDUFA1/PARK7/NDUFS4/NDUFS8/NDUFAB1/UQCR10/NDUFB3/UQCRFS1/NDUFA12/NDUFB1/NDUFC1/NDUFV2/NDUFB8/NDUFA5/NDUFA9/NDUFB4/COQ9/COX7A2L/NDUFB7/AFG1L/NDUFB6/TAFAZZIN/DLD
BP	GO:0042775	mitochondrial ATP synthesis coupled electron transport	70	0.63	2.29	2.80E-08	6.50E-07	4.00E-07	2621	tags=61%, list=22%, signal=48%	DNAJC15/UQCRH/UQCRB/COX7C/CYCS/NDUFS5/COA6/COX4I1/NDUFB2/UQCC3/NDUFA4/NDUFB5/UQCRQ/COX5A/NDUFB9/NDUFS3/CYC1/NDUFS6/NDUFA6/COX6A1/NDUFA1/PARK7/NDUFS4/NDUFS8/NDUFAB1/UQCR10/NDUFB3/UQCRFS1/NDUFA12/NDUFB1/NDUFC1/NDUFV2/NDUFB8/NDUFA5/NDUFA9/NDUFB4/COQ9/COX7A2L/NDUFB7/AFG1L/NDUFB6/TAFAZZIN/DLD
BP	GO:0019646	aerobic electron transport chain	64	0.64	2.28	2.99E-08	6.83E-07	4.20E-07	2621	tags=62%, list=22%, signal=49%	DNAJC15/UQCRH/UQCRB/COX7C/CYCS/NDUFS5/COX4I1/NDUFB2/UQCC3/NDUFA4/NDUFB5/UQCRQ/COX5A/NDUFB9/NDUFS3/CYC1/NDUFS6/NDUFA6/COX6A1/NDUFA1/PARK7/NDUFS4/NDUFS8/NDUFAB1/UQCR10/NDUFB3/UQCRFS1/NDUFB1/NDUFC1/NDUFV2/NDUFB8/NDUFA5/NDUFA9/NDUFB4/COQ9/COX7A2L/NDUFB7/AFG1L/NDUFB6/DLD
BP	GO:0006399	tRNA metabolic process	178	0.46	1.95	1.02E-07	2.04E-06	1.25E-06	3279	tags=45%, list=28%, signal=33%	WDR4/TRMT10C/RPP30/GTF3C3/TRMT61B/RTRAF/POP5/LSM6/RTCB/TSEN54/RARS1/TYW3/TRMT13/TPRKB/SSB/TRMO/THUMPD1/TRMT12/CTU2/DTWD1/ZBTB8OS/PUS1/DARS1/LAGE3/MOCS3/TARS3/SLFN13/ANKRD16/TRUB1/TP53RK/WDR6/TARBP1/DUS1L/SEPSECS/MARS2/LCMT2/TRMT5/DDX1/GATC/RPP38/FTSJ1/NSUN6/GRSF1/GTF3C5/ADAT2/FARSB/QTRT1/DALRD3/AARS1/RARS2/PSTK/HSD17B10/CARS2/IARS2/GATB/CDK5RAP1/KTI12/TYW1/EARS2/KARS1/TYW5/WARS2/OSGEPL1/DUS4L/EXOSC9/POLR3K/GARS1/TRNT1/RPP25L/URM1/GTF3C2/METTL1/RPP14/TRMT10B/TRMT1/METTL8/ALKBH8/OSGEP/TRMT112/ELAC1
CC	GO:0070069	cytochrome complex	26	0.77	2.31	2.40E-07	4.33E-06	2.66E-06	1579	tags=65%, list=13%, signal=57%	UQCRH/UQCRB/COX7C/COX6C/COA6/COX4I1/COX7B/NDUFA4/COX7A2/UQCRQ/COX5A/UQCRC2/CYC1/COX6A1/UQCR10/UQCRFS1/BCS1L
BP	GO:0022904	respiratory electron transport chain	87	0.56	2.10	5.20E-07	8.65E-06	5.32E-06	2621	tags=53%, list=22%, signal=41%	DNAJC15/UQCRH/UQCRB/COX7C/CYCS/NDUFS5/COA6/COX4I1/NDUFB2/UQCC3/NDUFA4/NDUFB5/UQCRQ/COX5A/NDUFB9/NDUFS3/CYC1/NDUFS6/NDUFA6/COX6A1/NDUFA1/PARK7/NDUFS4/NDUFS8/NDUFAB1/UQCR10/SLC25A13/NDUFB3/UQCRFS1/NDUFA12/NDUFB1/NDUFC1/NDUFV2/NDUFB8/NDUFA5/NDUFA9/NDUFB4/COQ9/COX7A2L/POLG2/SLC25A22/NDUFB7/AFG1L/NDUFB6/TAFAZZIN/DLD
CC	GO:0120114	Sm-like protein family complex	76	0.57	2.11	5.64E-07	9.27E-06	5.70E-06	1378	tags=34%, list=12%, signal=30%	SNRPD2/SNRPE/SNRPG/LSM1/CLNS1A/SF3B6/LSM5/LSM6/SF3A3/LSM3/LSM7/SNRNP70/SNRPA1/SNRPD1/RBMX2/LARP7/PHF5A/LUC7L3/LSM8/SNRPA/SNRPC/GEMIN8/SF3B1/SNRPD3/SNRPN/PRPF39
BP	GO:0000470	maturation of LSU-rRNA	28	0.74	2.27	6.80E-07	1.09E-05	6.73E-06	717	tags=39%, list=6%, signal=37%	RPL7/NSA2/RPL35/RPF1/RPL10A/NIFK/ZNHIT3/MRPL1/MAK16/RPL7A/RSL1D1

BP	GO:0015980	energy derivation by oxidation of organic compounds	235	0.42	1.81	8.94E-07	1.37E-05	8.43E-06	2550	tags=39%, list=22%, signal=31%	DNAJC15/UQCRH/MYC/UQCRB/SELENOS/COX7C/CYCS/OXA1L/NDUFS5/COA6/ATP5PD/UQCC2/COX4I1/UCN/ATP5PO/NDUFB2/UQCC3/ATP5F1A/NDUFA4/COX7A2/ATP5MF/ATP5PB/NDUFB5/BNIP3/SHMT2/CISD1/ATP5F1E/UQCRQ/MDH1/COX5A/NDUFB9/PPP1R3E/PASK/UQCRC2/DYRK2/NDUFS3/CYC1/NDUFS6/NDUFA6/ATP5F1C/TRAP1/COX6A1/NDUFA1/ATP5PF/PARK7/ATP5MG/ATPSCKMT/ACADM/EPM2AIP1/NDUFS4/SLC25A25/NDUFS8/NDUFAB1/NOP53/UQCR10/SLC25A13/NDUFB3/UQCRFS1/NDUFA12/NDUFB1/ATP5ME/SDHAF4/NDUFC1/TEFM/ANTKMT/SLC25A33/NOA1/NDUFV2/SLC25A14/NDUFB8/SUCLG1/NDUFA5/NDUFA9/NDUFB4/MRPS36/NIPSNAP2/COQ9/COX7A2L/MTFR1L/POLG2/COX10/PTGES3/SLC25A22/SIRT3/PDE12/NDUFB7/PPP1CC/ADSL/AFG1L/NDUFB6/TAFAZZIN
MF	GO:0090079	translation regulator activity, nucleic acid binding	96	0.53	2.04	1.10E-06	1.65E-05	1.01E-05	1224	tags=30%, list=10%, signal=27%	EEF1B2/EIF3E/EEF1G/EIF4B/EIF3F/ZNF540/EIF3C/EIF3H/EIF4A2/EIF3M/RPS27L/EEF1D/EIF1AX/EIF4E/EIF2S2/PAIP1/SHMT2/EEF1A1/EIF2S1/SHMT1/COPS5/EIF2B4/MTIF3/EIF3L/EIF3G/EIF5/MTRF1L/MCTS1/MRPL58
CC	GO:0097525	spliceosomal snRNP complex	57	0.61	2.14	2.00E-06	2.84E-05	1.74E-05	1378	tags=39%, list=12%, signal=34%	SNRPD2/SNRPE/SNRPG/SF3B6/LSM5/LSM6/SF3A3/LSM3/LSM7/SNRNP70/SNRPA1/SNRPD1/RBMX2/PHF5A/LUC7L3/LSM8/SNRPA/SNRPC/SF3B1/SNRPD3/SNRPN/PRPF39
BP	GO:0008033	tRNA processing	121	0.48	1.92	3.24E-06	4.42E-05	2.72E-05	3319	tags=50%, list=28%, signal=37%	WDR4/TRMT10C/RPP30/TRMT61B/RTRAF/POP5/LSM6/RTCB/TSEN54/TYW3/TRMT13/TPRKB/SSB/TRMO/THUMPD1/TRMT12/CTU2/DTWD1/ZBTB8OS/PUS1/LAGE3/MOCS3/ANKRD16/TRUB1/TP53RK/WDR6/TARBP1/DUS1L/LCMT2/TRMT5/DDX1/RPP38/FTSJ1/NSUN6/GRSF1/ADAT2/QTRT1/DALRD3/AARS1/HSD17B10/CDK5RAP1/KTI12/TYW1/KARS1/TYW5/OSGEPL1/DUS4L/POLR3K/TRNT1/RPP25L/URM1/METTL1/RPP14/TRMT10B/TRMT1/METTL8/ALKBH8/OSGEP/TRMT112/ELAC1/TRMT11
BP	GO:0008380	RNA splicing	362	0.35	1.59	3.39E-06	4.62E-05	2.84E-05	2129	tags=27%, list=18%, signal=22%	SNRPD2/RPS13/SNRPE/SRSF7/SRSF3/CWC15/PRKRIP1/SNRPG/LSM1/CLNS1A/SF3B6/LSM5/CCNL2/HNRNPA1/RTRAF/LSM6/PPIE/SF3A3/RTCB/LSM3/TSEN54/ARL6IP4/U2AF1L4/LSM7/SNRNP25/SNRNP70/RPS26/SNRPA1/FAM50A/SNRPD1/HSPA8/RBMX2/LARP7/PHF5A/LUC7L3/CLK1/PPIG/RBMX/DDX39A/LSM8/SNRNP27/RBM3/SNRPA/SRSF10/SNRPC/METTL14/SRSF2/MPHOSPH10/ZBTB8OS/GEMIN8/BCAS2/RP9/TRA2B/ZPR1/SF3B1/THOC7/SNRPD3/ZRANB2/ZCRB1/TIA1/MAGOHB/DHX15/SNRPN/SUGP1/PRPF39/SRSF11/DDX5/RBM12B/PPWD1/PSIP1/DDX47/ZCCHC8/DDX1/SRSF8/AKAP17A/PRX/LUC7L/RBM5/PDCD7/PRMT5/SNRPB2/SAP18/THOC6/RNPC3/GRSF1/SFPQ/PNN/PCBP4/LSM4/GEMIN2/ZNF830/DDX17/CIRBP/RBM39/HABP4/SNRPF
CC	GO:0005747	mitochondrial respiratory chain complex I	41	0.67	2.21	3.58E-06	4.76E-05	2.93E-05	2536	tags=63%, list=22%, signal=50%	NDUFS5/NDUFB2/NDUFA4/NDUFB5/WDR93/NDUFB9/NDUFA13/NDUFS3/NDUFS6/NDUFA6/NDUFA1/NDUFS4/NDUFS8/NDUFAB1/NDUFB3/NDUFA12/NDUFB1/NDUFC1/NDUFV2/NDUFB8/NDUFA5/NDUFA9/NDUFB4/NDUFB7/NDUFA11/NDUFB6
CC	GO:0030964	NADH dehydrogenase complex	41	0.67	2.21	3.58E-06	4.76E-05	2.93E-05	2536	tags=63%, list=22%, signal=50%	NDUFS5/NDUFB2/NDUFA4/NDUFB5/WDR93/NDUFB9/NDUFA13/NDUFS3/NDUFS6/NDUFA6/NDUFA1/NDUFS4/NDUFS8/NDUFAB1/NDUFB3/NDUFA12/NDUFB1/NDUFC1/NDUFV2/NDUFB8/NDUFA5/NDUFA9/NDUFB4/NDUFB7/NDUFA11/NDUFB6

CC	GO:0045271	respiratory chain complex I	41	0.67	2.21	3.58E-06	4.76E-05	2.93E-05	2536	tags=63%, list=22%, signal=50%	NDUFS5/NDUFB2/NDUFA4/NDUFB5/WDR93/NDUFB9/NDUFA13/NDUFS3/NDUFS6/NDUFA6/NDUFA1/NDUFS4/NDUFS8/NDUFAB1/NDUFB3/NDUFA12/NDUFB1/NDUFC1/NDUFV2/NDUFB8/NDUFA5/NDUFA9/NDUFB4/NDUFB7/NDUFA11/NDUFB6
CC	GO:0098573	intrinsic component of mitochondrial membrane	83	0.54	2.01	3.61E-06	4.76E-05	2.93E-05	1636	tags=35%, list=14%, signal=30%	COX16/MCUB/MICOS13/OXA1L/TOMM20/MFF/COX11/RHOT2/UQCC3/AFG3L2/BNIP3/MICOS10/SLC25A19/MPC1/PET100/SMDT1/MTX3/SLC25A3/SAMM50/PPOX/SFXN1/ABCB10/HSPA9/APOO/TIMM17A/MPC2/COX18/SFXN4/MTX2
CC	GO:0032592	integral component of mitochondrial membrane	81	0.54	2.01	5.97E-06	7.51E-05	4.62E-05	1636	tags=35%, list=14%, signal=30%	COX16/MCUB/MICOS13/OXA1L/TOMM20/MFF/COX11/RHOT2/UQCC3/AFG3L2/BNIP3/MICOS10/SLC25A19/MPC1/PET100/SMDT1/MTX3/SLC25A3/SAMM50/SFXN1/ABCB10/HSPA9/APOO/TIMM17A/MPC2/COX18/SFXN4/MTX2
MF	GO:0016655	oxidoreductase activity, acting on NAD(P)H, quinone or similar compound as acceptor	45	0.62	2.12	6.41E-06	7.94E-05	4.88E-05	2536	tags=58%, list=22%, signal=45%	CRYZ/NDUFS5/NDUFB2/NDUFA4/NDUFB5/NDUFB9/NDUFA13/NDUFS3/NDUFS6/NDUFA6/NDUFA1/NDUFS4/NDUFS8/NDUFB3/NDUFA12/NDUFB1/AKR1C3/NDUFC1/NDUFV2/NDUFB8/NDUFA5/NDUFA9/NDUFB4/CBR4/NDUFB7/NDUFB6
MF	GO:0003954	NADH dehydrogenase activity	36	0.66	2.12	7.33E-06	8.97E-05	5.51E-05	2046	tags=58%, list=17%, signal=48%	NDUFS5/NDUFB2/NDUFA4/NDUFB5/NDUFB9/NDUFA13/NDUFS3/NDUFS6/NDUFA6/NDUFA1/NDUFS4/NDUFS8/NDUFB3/NDUFA12/NDUFB1/NDUFC1/NDUFV2/NDUFB8/NDUFA5/NDUFA9/NDUFB4
MF	GO:0008137	NADH dehydrogenase (ubiquinone) activity	35	0.68	2.16	8.71E-06	0.00010429	6.41E-05	2046	tags=60%, list=17%, signal=50%	NDUFS5/NDUFB2/NDUFA4/NDUFB5/NDUFB9/NDUFA13/NDUFS3/NDUFS6/NDUFA6/NDUFA1/NDUFS4/NDUFS8/NDUFB3/NDUFA12/NDUFB1/NDUFC1/NDUFV2/NDUFB8/NDUFA5/NDUFA9/NDUFB4
MF	GO:0050136	NADH dehydrogenase (quinone) activity	35	0.68	2.16	8.71E-06	0.00010429	6.41E-05	2046	tags=60%, list=17%, signal=50%	NDUFS5/NDUFB2/NDUFA4/NDUFB5/NDUFB9/NDUFA13/NDUFS3/NDUFS6/NDUFA6/NDUFA1/NDUFS4/NDUFS8/NDUFB3/NDUFA12/NDUFB1/NDUFC1/NDUFV2/NDUFB8/NDUFA5/NDUFA9/NDUFB4
CC	GO:0030532	small nuclear ribonucleoprotein complex	66	0.56	2.03	9.57E-06	0.00011289	6.94E-05	1378	tags=35%, list=12%, signal=31%	SNRPD2/SNRPE/SNRPG/SF3B6/LSM5/LSM6/SF3A3/LSM3/LSM7/SNRNP70/SNRPA1/SNRPD1/RBMX2/LARP7/PHF5A/LUC7L3/LSM8/SNRPA/SNRPC/SF3B1/SNRPD3/SNRPN/PRPF39
MF	GO:0008135	translation factor activity, RNA binding	80	0.53	1.97	9.76E-06	0.00011394	7.00E-05	1224	tags=30%, list=10%, signal=27%	EEF1B2/EIF3E/EEF1G/EIF4B/EIF3F/EIF3C/EIF3H/EIF4A2/EIF3M/EEF1D/EIF1AX/EIF4E/EIF2S2/EEF1A1/EIF2S1/COPS5/EIF2B4/MTIF3/EIF3L/EIF3G/EIF5/MTRF1L/MCTS1/MRPL58
BP	GO:0022900	electron transport chain	125	0.47	1.87	1.02E-05	0.00011865	7.29E-05	2550	tags=44%, list=22%, signal=35%	DNAJC15/UQCRH/UQCRB/COX7C/CYCS/NDUFS5/COA6/COX4I1/COX11/NDUFB2/UQCC3/NDUFA4/NDUFB5/WDR93/UQCRQ/COX5A/AKR1B1/NDUFB9/NDUFA13/NDUFS3/CYC1/NDUFS6/NDUFA6/COX6A1/NDUFA1/PARK7/NDUFS4/NDUFS8/NDUFAB1/PDIA5/QDPR/ADH5/UQCR10/FDX1/SLC25A13/NDUFB3/UQCRFS1/NDUFA12/NDUFB1/CYB5A/NDUFC1/NDUFV2/NDUFB8/NDUFA5/NDUFA9/NDUFB4/COQ9/COX7A2L/POLG2/SLC25A22/PHGDH/NDUFB7/AFG1L/NDUFB6/TAFAZZIN
BP	GO:0017004	cytochrome complex assembly	35	0.67	2.15	1.03E-05	0.00011879	7.30E-05	2669	tags=60%, list=23%, signal=46%	COX16/C12orf73/OXA1L/COA6/COX14/UQCC2/UQCC3/COA4/FASTKD3/PET100/TIMM21/COA5/UQCRFS1/COX18/BCS1L/SLC25A33/COX17/COX10/SCO1/TACO1/UQCC1

CC	GO:0000314	organellar small ribosomal subunit	25	0.73	2.16	1.29E-05	0.00014474	8.90E-05	1585	tags=52%, list=14%, signal=45%	MRPS22/MRPS21/MRPS5/MRPS17/MRPS33/MRPS18C/MRPS28/MRPS6/MRPS34/MRPL42/MRPS16/MRPS14/DAP3
CC	GO:0005763	mitochondrial small ribosomal subunit	25	0.73	2.16	1.29E-05	0.00014474	8.90E-05	1585	tags=52%, list=14%, signal=45%	MRPS22/MRPS21/MRPS5/MRPS17/MRPS33/MRPS18C/MRPS28/MRPS6/MRPS34/MRPL42/MRPS16/MRPS14/DAP3
MF	GO:0048027	mRNA 5'-UTR binding	25	0.72	2.14	1.93E-05	0.00020892	0.00012843	1013	tags=48%, list=9%, signal=44%	RPS13/RPL41/RPS14/RPL26/RPS7/RPL5/RPS3A/SHMT2/GNL3/RSL1D1/SHMT1/CCT5
MF	GO:0016741	transferase activity, transferring one-carbon groups	177	0.41	1.75	2.05E-05	0.00021864	0.00013441	3203	tags=39%, list=27%, signal=29%	METTL18/EMG1/FBL/METTL5/WDR4/TRMT10C/TRMT61B/DPH5/NDUFAF5/HNMT/CSKMT/SMYD2/TYW3/TRMT13/GCSH/TRMO/SHMT2/COQ5/MRM1/SHMT1/PRMT9/TRMT12/CAMKMT/METTL14/ATPSCKMT/ATIC/CMTR2/FAM98A/TARBP1/LCMT2/TRMT5/GART/NSUN4/FTSJ1/SETD6/ANTKMT/CARNMT1/PRMT5/NSUN6/ETFBKMT/SPOUT1/TFB1M/N6AMT1/NDUFAF7/PRMT3/CMTR1/METTL13/HENMT1/FDXACB1/EED/METTL16/VCPKMT/SMYD5/METTL17/WDR5/METTL9/NSUN5/PRDM8/METTL1/AMT/PCMTD2/TRMT10B/SETDB2/TRMT1/METTL8/METTL21A/GAMT/BUD23/ALKBH8
MF	GO:0003955	NAD(P)H dehydrogenase (quinone) activity	37	0.65	2.10	2.08E-05	0.00022161	0.00013623	2536	tags=65%, list=22%, signal=51%	NDUFS5/NDUFB2/NDUFA4/NDUFB5/NDUFB9/NDUFA13/NDUFS3/NDUFS6/NDUFA6/NDUFA1/NDUFS4/NDUFS8/NDUFB3/NDUFA12/NDUFB1/NDUFC1/NDUFV2/NDUFB8/NDUFA5/NDUFA9/NDUFB4/CBR4/NDUFB7/NDUFB6
MF	GO:0045182	translation regulator activity	124	0.46	1.83	2.10E-05	0.00022212	0.00013655	892	tags=24%, list=8%, signal=23%	EEF1B2/EIF3E/RPS14/EEF1G/RPL22/EIF4B/EIF3F/ZNF540/EIF3C/RPS9/RPL10/EIF3H/EIF4A2/EIF3M/RPS27L/EEF1D/EIF1AX/EIF4E/EIF2S2/PAIP1/SHMT2/EEF1A1/EIF2S1/SHMT1/COPS5/EIF2B4/MTIF3/EIF3L/EIF3G/EIF5
BP	GO:0006457	protein folding	167	0.42	1.74	2.28E-05	0.00023729	0.00014587	1827	tags=30%, list=16%, signal=26%	CCT2/TBCA/DNAJC19/TXNDC9/DNAJA3/HSPE1/NUDCD2/HSP90AA1/DNAJA1/PPIE/PDCD5/B2M/DNAJC2/SNRNP70/QSOX2/VBP1/HSPA8/PPIA/PPIG/SACS/DNLZ/MKKS/LTBP4/CCT8/PFDN4/RIC3/TRAP1/CHORDC1/NUDC/RUVBL2/CCT5/PDIA5/DNAJB5/DNAJC10/GRPEL2/CCT3/PRDX4/HSPA9/AIP/HSPA13/DNAJB11/P4HB/CCT4/PFDN1/TCP1/PDRG1/DNAJC21/RP2/ERP29/HSPD1
MF	GO:0009055	electron transfer activity	93	0.50	1.90	2.45E-05	0.00025223	0.00015505	2046	tags=40%, list=17%, signal=33%	UQCRH/CYCS/NDUFS5/COX4I1/COX11/COX7B/NDUFB2/NDUFA4/COX7A2/NDUFB5/COX5A/AKR1B1/NDUFB9/NDUFA13/NDUFS3/CYC1/NDUFS6/NDUFA6/COX6A1/NDUFA1/NDUFS4/NDUFS8/QDPR/ADH5/UQCR10/FDX1/NDUFB3/UQCRFS1/NDUFA12/NDUFB1/CYB5A/NDUFC1/NDUFV2/NDUFB8/NDUFA5/NDUFA9/NDUFB4
MF	GO:0051082	unfolded protein binding	94	0.50	1.90	2.50E-05	0.00025593	0.00015733	1839	tags=41%, list=16%, signal=35%	CCT2/ERLEC1/DNAJA3/HSPE1/NUDCD2/HSP90AA1/TOMM20/DNAJA1/NPM1/VBP1/AFG3L2/HSPA8/SHQ1/PPIA/MKKS/PET100/CCT8/PFDN4/TRAP1/SRSF10/NUDC/RUVBL2/CCT5/DNAJB5/GRPEL2/CCT3/HSPA9/AIP/HSPA13/DNAJB11/CCT4/SERPINH1/PFDN1/TCP1/TIMM10B/PDRG1/RP2/HSPD1/NAP1L4

CC	GO:0005681	spliceosomal complex	185	0.41	1.73	2.54E-05	0.0002589	0.00015916	2129	tags=30%, list=18%, signal=25%	SNRPD2/SNRPE/CWC15/PRKRIP1/SNRPG/CLNS1A/SF3B6/LSM5/HNRNPA1/LSM6/PPIE/SF3A3/LSM3/U2AF1L4/LSM7/SNRNP25/SNRNP70/SNRPA1/SNRPD1/HSPA8/RBMX2/PHF5A/LUC7L3/RBMX/LSM8/RBM3/SNRPA/SNRPC/SRSF2/BCAS2/TRA2B/SF3B1/RHEB/SNRPD3/ZCRB1/MAGOHB/DHX15/SNRPN/SUGP1/PRPF39/DDX5/PPWD1/ZCCHC8/AKAP17A/LUC7L/RBM5/PDCD7/SNRPB2/RNPC3/PNN/LSM4/GEMIN2/CCDC12/ZNF830/CIRBP/SNRPF
BP	GO:0006413	translational initiation	112	0.46	1.84	2.92E-05	0.00028904	0.00017768	1138	tags=24%, list=10%, signal=22%	EIF3E/RPS5/RPS3A/RPS3/RPL13A/EIF4B/RPS17/EIF3F/EIF3C/NPM1/EIF3H/ABCE1/EIF4A2/EIF3M/EIF1AX/EIF2S2/PAIP1/EIF2S1/COPS5/EIF2B4/MTIF3/EIF3L/EIF3G/EIF5/BZW2/TMED2/MCTS1
CC	GO:0030684	preribosome	74	0.53	1.94	3.15E-05	0.00030753	0.00018905	2474	tags=42%, list=21%, signal=33%	EMG1/NSA2/RPS7/NOP58/FBL/UTP14A/RPF1/KRR1/NGDN/MAK16/RSL1D1/IMP4/UTP20/MPHOSPH10/WDR3/ZNF622/UTP11/WDR37/UTP6/LTV1/NIP7/RRP9/UTP18/DCAF13/MRTO4/PDCD11/LAS1L/PPAN/RIOK2/SNU13/NOP56
MF	GO:0003743	translation initiation factor activity	50	0.59	2.05	3.25E-05	0.00031668	0.00019468	1138	tags=36%, list=10%, signal=33%	EIF3E/EIF4B/EIF3F/EIF3C/EIF3H/EIF4A2/EIF3M/EIF1AX/EIF4E/EIF2S2/EIF2S1/COPS5/EIF2B4/MTIF3/EIF3L/EIF3G/EIF5/MCTS1
CC	GO:0071011	precatalytic spliceosome	52	0.58	2.00	3.94E-05	0.00037157	0.00022842	1241	tags=33%, list=11%, signal=29%	SNRPD2/SNRPE/SNRPG/SF3B6/LSM5/LSM6/SF3A3/LSM3/LSM7/SNRPA1/SNRPD1/RBMX2/PHF5A/LSM8/SF3B1/SNRPD3/MAGOHB
CC	GO:0005684	U2-type spliceosomal complex	89	0.50	1.87	4.45E-05	0.00040957	0.00025178	1378	tags=28%, list=12%, signal=25%	SNRPD2/SNRPE/CWC15/SNRPG/SF3B6/LSM5/LSM6/PPIE/SF3A3/LSM3/LSM7/SNRNP70/SNRPA1/SNRPD1/RBMX2/PHF5A/LUC7L3/LSM8/SNRPC/BCAS2/SF3B1/SNRPD3/MAGOHB/SNRPN/PRPF39
BP	GO:0006400	tRNA modification	87	0.50	1.88	5.00E-05	0.00045667	0.00028073	3492	tags=53%, list=30%, signal=37%	WDR4/TRMT10C/TRMT61B/TYW3/TRMT13/TPRKB/SSB/TRMO/THUMPD1/TRMT12/CTU2/DTWD1/PUS1/MOCS3/ANKRD16/TRUB1/WDR6/TARBP1/DUS1L/LCMT2/TRMT5/FTSJ1/NSUN6/ADAT2/QTRT1/DALRD3/AARS1/HSD17B10/CDK5RAP1/KTI12/TYW1/TYW5/OSGEPL1/DUS4L/URM1/METTL1/TRMT10B/TRMT1/METTL8/ALKBH8/OSGEP/TRMT112/TRMT11/THG1L/TRMU/GTPBP3
BP	GO:0009451	RNA modification	153	0.42	1.74	5.57E-05	0.00050418	0.00030994	2364	tags=31%, list=20%, signal=25%	EMG1/FBL/METTL5/WDR4/TRMT10C/TRMT61B/TYW3/TRMT13/TPRKB/SSB/TRMO/LARP7/ADARB1/MRM1/THUMPD1/TRMT12/CTU2/METTL14/DTWD1/PUS1/MOCS3/NHP2/CMTR2/RPUSD3/ANKRD16/TRUB1/WDR6/TARBP1/DUS1L/DNAJB11/HNRNPAB/LCMT2/TRMT5/NSUN4/FTSJ1/NSUN6/ADAT2/TFB1M/QTRT1/DALRD3/AARS1/CMTR1/HSD17B10/CDK5RAP1/HENMT1/KTI12/TYW1/FDXACB1
BP	GO:0008535	respiratory chain complex IV assembly	25	0.70	2.07	6.66E-05	0.00059224	0.00036407	2569	tags=60%, list=22%, signal=47%	COX16/OXA1L/COA6/COX14/COA4/FASTKD3/PET100/TIMM21/COA5/COX18/BCS1L/COX17/COX10/SCO1/TACO1
MF	GO:0008168	methyltransferase activity	171	0.40	1.69	6.82E-05	0.00060366	0.00037109	3319	tags=40%, list=28%, signal=29%	METTL18/EMG1/FBL/METTL5/WDR4/TRMT10C/TRMT61B/DPH5/NDUFAF5/HNMT/CSKMT/SMYD2/TYW3/TRMT13/GCSH/TRMO/COQ5/MRM1/PRMT9/TRMT12/CAMKMT/METTL14/ATPSCKMT/CMTR2/FAM98A/TARBP1/LCMT2/TRMT5/NSUN4/FTSJ1/SETD6/ANTKMT/CARNMT1/PRMT5/NSUN6/ETFBKMT/SPOUT1/TFB1M/N6AMT1/NDUFAF7/PRMT3/CMTR1/METTL13/HENMT1/FDXACB1/EED/METTL16/VCPKMT/SMYD5/METTL17/WDR5/METTL9/NSUN5/PRDM8/METTL1/AMT/PCMTD2/TRMT10B/SETDB2/TRMT1/METTL8/METTL21A/GAMT/BUD23/ALKBH8/TRMT112/COMTD1/TRMT11

BP	GO:0046034	ATP metabolic process	211	0.38	1.66	6.89E-05	0.00060744	0.00037342	1462	tags=27%, list=12%, signal=24%	DNAJC15/UQCRH/MYC/UQCRB/COX7C/CYCS/NDUFS5/COA6/ATP5PD/UQCC2/PRXL2C/COX4I1/ATP5PO/NDUFB2/UQCC3/ATP5F1A/NDUFA4/ATP5IF1/COX7A2/ATP5MF/ATP5PB/NDUFB5/HSPA8/SHMT2/ATP5MGL/OLA1/ATP5F1E/UQCRQ/COX5A/NDUFB9/UQCRC2/NDUFS3/CYC1/NDUFS6/NDUFA6/ATP5F1C/COX6A1/NDUFA1/ATP5PF/PARK7/ATP5MG/ATPSCKMT/NDUFS4/SLC25A25/NDUFS8/NDUFAB1/OGT/UQCR10/SLC25A13/NDUFB3/TPI1/UQCRFS1/NDUFA12/ATP5MC1/NDUFB1/ATP5ME/PPARA
MF	GO:0003729	mRNA binding	248	0.36	1.60	8.10E-05	0.00069668	0.00042828	1172	tags=17%, list=10%, signal=16%	RPL24/CSTF3/RPS13/RPL41/RPL7/RPS14/RPL26/RPS5/RPS7/RPL5/RPS3A/RPS3/RPL35/RPL13A/RPS2/CRYZ/SF3B6/RBMS3/LARP4/PPIE/TUT1/SNRNP70/SSB/RPS26/DHFR2/EIF2S2/SHMT2/LUC7L3/GNL3/RBMX/ADARB1/RSL1D1/SHMT1/NXF1/PARK7/SNRPC/METTL14/CCT5/TRA2B/SF3B1/NHP2/TIA1
BP	GO:0007005	mitochondrion organization	456	0.32	1.46	8.48E-05	0.00072629	0.00044648	2579	tags=33%, list=22%, signal=27%	DNAJC15/RALA/MYC/DNAJC19/COX16/DNAJA3/TIMM10/MICOS13/HSP90AA1/C12orf73/OXA1L/NDUFS5/TOMM20/COX14/MFF/UQCC2/NDUFAF5/PDCD5/SLIRP/TIMM9/TOMM7/RHOT2/NDUFB2/UQCC3/HIP1R/ATP5IF1/COX7A2/TOMM5/AFG3L2/NDUFB5/TMEM126A/BNIP3/HIGD1A/TWNK/PHB2/PSMD10/NDUFAF8/COA4/MICOS10/NDUFB9/DNLZ/ALKBH7/FASTKD3/NDUFA13/PET100/TIMM8B/NDUFS3/NDUFA6/TRIAP1/CAMKMT/PMPCB/NDUFA1/SLC25A6/PARK7/IMMP1L/MTX3/RAB29/TIMM21/SAMM50/NDUFAF4/USP30/NDUFS4/HAX1/NDUFAF6/BLOC1S2/NDUFS8/PMAIP1/NDUFAB1/DDHD2/FAM162A/GRPEL2/YME1L1/NDUFB3/SAE1/COA5/UQCRFS1/NDUFA12/HSPA9/TOMM22/SLC25A36/AIP/APOO/MTERF4/NDUFB1/TIMM17A/TFRC/ATG5/PRIMPOL/DMAC1/SSBP1/VDAC2/PHB1/COX18/RTL10/BCS1L/SDHAF4/PTCD2/MTX2/DMAC2/NDUFC1/SLC35F6/TIMM10B/SLC25A33/STPG1/PLD6/HSPD1/PGAM5/NDUFB8/TIMM44/SDHAF1/NDUFA5/NDUFAF7/NDUFA9/NDUFB4/SDHAF3/USP36/MSTO1/COX17/KAT2A/BCL2/HSD17B10/SLC25A4/BAD/ULK2/NIPSNAP2/TIMM22/UBL5/TMEM242/MFN1/TMEM70/COX7A2L/MTFR1L/POLG2/COX10/ATG12/MAPK8/SCO1/ZDHHC6/NDUFB7/NDUFA11/AFG1L/TMEM126B/ATPAF1/ARMCX3/NDUFB6/TIMM29/GABPB1/TAFAZZIN/TACO1/ATG3
BP	GO:0006091	generation of precursor metabolites and energy	361	0.33	1.49	0.00011312	0.00093122	0.00057246	1326	tags=21%, list=11%, signal=20%	DNAJC15/UQCRH/MYC/UQCRB/SELENOS/COX7C/CYCS/COX6C/OXA1L/NDUFS5/COA6/ATP5PD/UQCC2/PRXL2C/COX4I1/COX11/UCN/ATP5PO/NDUFB2/UQCC3/ATP5F1A/NDUFA4/OXCT1/ATP5IF1/SHPK/COX7A2/ATP5MF/ATP5PB/NDUFB5/BNIP3/SHMT2/CISD1/PHB2/WDR93/ATP5F1E/UQCRQ/MDH1/COX5A/AKR1B1/NDUFB9/ACAT1/PPP1R3E/PASK/UQCRC2/NDUFA13/RPE/DYRK2/NDUFS3/CYC1/NDUFS6/NDUFA6/ATP5F1C/TRAP1/COX6A1/NDUFA1/ATP5PF/PARK7/ATP5MG/ATPSCKMT/ACADM/EPM2AIP1/NDUFS4/SLC25A25/NDUFS8/NDUFAB1/PDIA5/OGT/NOP53/QDPR/ADH5/UQCR10/FDX1/SLC25A13/NDUFB3/TPI1/UQCRFS1/NDUFA12
CC	GO:0032040	small-subunit processome	36	0.61	1.95	0.00012222	0.00099578	0.00061215	2474	tags=53%, list=21%, signal=42%	EMG1/RPS7/NOP58/FBL/UTP14A/KRR1/NGDN/IMP4/UTP20/MPHOSPH10/WDR3/UTP11/UTP6/RRP9/UTP18/DCAF13/PDCD11/SNU13/NOP56
CC	GO:0005689	U12-type spliceosomal complex	26	0.68	2.03	0.00013087	0.0010533	0.00064751	1865	tags=54%, list=16%, signal=45%	SNRPD2/SNRPE/SNRPG/SF3B6/LSM7/SNRNP25/SNRPD1/PHF5A/SF3B1/SNRPD3/ZCRB1/DHX15/PDCD7/RNPC3
CC	GO:0046540	U4/U6 x U5 tri-snRNP complex	31	0.65	2.00	0.00013761	0.00109212	0.00067137	1299	tags=35%, list=11%, signal=32%	SNRPD2/SNRPE/SNRPG/LSM5/LSM6/LSM3/LSM7/SNRPD1/LSM8/SNRPD3/SNRPN

CC	GO:0097526	spliceosomal tri-snRNP complex	31	0.65	2.00	0.00013761	0.00109212	0.00067137	1299	tags=35%, list=11%, signal=32%	SNRPD2/SNRPE/SNRPG/LSM5/LSM6/LSM3/LSM7/SNRPD1/LSM8/SNRPD3/SNRPN
MF	GO:0000049	tRNA binding	70	0.51	1.87	0.00014647	0.00115092	0.00070752	3980	tags=57%, list=34%, signal=38%	AIMP1/RPL35A/TRMT10C/RARS1/XPOT/SSB/EEF1A1/MTRES1/CTU2/PUS1/SLFN13/SEPSECS/NSUN6/DALRD3/AARS1/PSTK/HSD17B10/IARS2/EARS2/KARS1/TYW5/EIF2A/TRNT1/EIF2S3/METTL1/TRMT10B/TRMT1/IGHMBP2/ALKBH8/TRMT11/THG1L/TRMU/EIF2AK4/PTCD1/DARS2/DTD2/ALKBH1/TRMT10A/IARS1/MARS1
CC	GO:1990204	oxidoreductase complex	97	0.45	1.73	0.00016112	0.00124394	0.0007647	2536	tags=44%, list=22%, signal=35%	UQCRH/LDHB/UQCRB/NDUFS5/NDUFB2/GCSH/NDUFA4/NDUFB5/WDR93/UQCRQ/NDUFB9/UQCRC2/NDUFA13/NDUFS3/CYC1/NDUFS6/NDUFA6/NDUFA1/NDUFS4/NDUFS8/NDUFAB1/BCKDHB/CYB5B/RRM2/UQCR10/NDUFB3/UQCRFS1/NDUFA12/NDUFB1/P4HB/BCS1L/NDUFC1/NDUFV2/NDUFB8/NDUFA5/NDUFA9/NDUFB4/MRPS36/CBR4/NDUFB7/NDUFA11/UQCR11/NDUFB6
CC	GO:0071005	U2-type precatalytic spliceosome	50	0.56	1.95	0.00020009	0.00149947	0.00092178	1241	tags=32%, list=11%, signal=29%	SNRPD2/SNRPE/SNRPG/LSM5/LSM6/SF3A3/LSM3/LSM7/SNRPA1/SNRPD1/RBMX2/PHF5A/LSM8/SF3B1/SNRPD3/MAGOHB
BP	GO:0006120	mitochondrial electron transport, NADH to ubiquinone	42	0.58	1.94	0.00020055	0.00149947	0.00092178	2735	tags=64%, list=23%, signal=49%	DNAJC15/NDUFS5/NDUFB2/NDUFA4/NDUFB5/NDUFB9/NDUFS3/NDUFS6/NDUFA6/NDUFA1/PARK7/NDUFS4/NDUFS8/NDUFAB1/NDUFB3/NDUFB1/NDUFC1/NDUFV2/NDUFB8/NDUFA5/NDUFA9/NDUFB4/COQ9/NDUFB7/NDUFB6/DLD/NDUFC2
BP	GO:0000375	RNA splicing, via transesterification reactions	263	0.35	1.54	0.00021814	0.00160023	0.00098373	2587	tags=31%, list=22%, signal=25%	SNRPD2/SNRPE/SRSF7/SRSF3/CWC15/SNRPG/LSM1/CLNS1A/SF3B6/LSM5/HNRNPA1/LSM6/PPIE/SF3A3/LSM3/U2AF1L4/LSM7/SNRNP25/SNRNP70/SNRPA1/SNRPD1/HSPA8/RBMX2/LARP7/PHF5A/LUC7L3/RBMX/DDX39A/LSM8/RBM3/SNRPA/SRSF10/SNRPC/METTL14/SRSF2/MPHOSPH10/GEMIN8/BCAS2/TRA2B/SF3B1/SNRPD3/ZCRB1/TIA1/MAGOHB/SNRPN/PRPF39/DDX5/PPWD1/PSIP1/ZCCHC8/DDX1/SRSF8/LUC7L/RBM5/PRMT5/SNRPB2/SAP18/RNPC3/SFPQ/PNN/PCBP4/LSM4/GEMIN2/DDX17/CIRBP/RBM39/SNRPF/UBL5/HTATSF1/GEMIN6/FXR1/CWC25/WBP4/RNPS1/RBM7/SNU13/FRG1/SRSF6/RBM4/WTAP/PRPF38A/METTL16
MF	GO:0043021	ribonucleoprotein complex binding	145	0.41	1.69	0.00022023	0.00160964	0.00098951	1859	tags=26%, list=16%, signal=22%	EIF4B/SNRPG/NOPCHAP1/EIF3C/OXA1L/NPM1/TMCO1/ABCE1/RPLP1/NME1/DNAJC2/RACK1/GTPBP6/SNRPD1/SBDS/NMD3/OLA1/TMEM147/RPSA/EIF2S1/MTRES1/MTIF3/RBM3/SNRPA/LETMD1/TTC5/PTCD3/NVL/RPN2/DDX5/DERL2/SRP9/SRP54/MALSU1/MTIF2/PRMT5/SNRPB2/PYM1
BP	GO:0000462	maturation of SSU-rRNA from tricistronic rRNA transcript (SSU-rRNA, 5.8S rRNA, LSU-rRNA)	36	0.59	1.90	0.0002585	0.00184859	0.0011364	1996	tags=33%, list=17%, signal=28%	RPS16/RPS14/RPS8/RPS21/NGDN/DHX37/UTP20/RPS19/UTP6/WDR43/TSR2/DCAF13
BP	GO:1990542	mitochondrial transmembrane transport	77	0.48	1.79	0.00030786	0.00214993	0.00132165	1895	tags=38%, list=16%, signal=32%	DNAJC15/DNAJC19/MCUB/TOMM20/TOMM7/MICU3/AFG3L2/SLC25A28/DNLZ/MPC1/PMPCB/SLC25A6/SMDT1/TIMM21/SLC25A3/SLC25A32/SLC25A1/SFXN1/SLC25A13/GRPEL2/SLC25A36/TIMM17A/MPC2/MRPL18/SFXN4/SLC25A33/HSPD1/MICU2/TIMM44

CC	GO:0101031	chaperone complex	28	0.65	1.98	0.0003088	0.00214993	0.00132165	2478	tags=57%, list=21%, signal=45%	CCT2/HSPA8/CCT8/RUVBL2/CCT5/CCT3/DNAJC9/DNAJB11/CCT4/RUVBL1/TCP1/CCT6A/PSMG2/PSMG1/PTGES3/HSP90AB1
BP	GO:0001510	RNA methylation	78	0.49	1.80	0.00032092	0.0022114	0.00135944	3319	tags=50%, list=28%, signal=36%	EMG1/FBL/METTL5/WDR4/TRMT10C/TRMT61B/TYW3/TRMT13/TRMO/LARP7/MRM1/TRMT12/METTL14/CMTR2/WDR6/TARBP1/LCMT2/TRMT5/NSUN4/FTSJ1/NSUN6/TFB1M/DALRD3/CMTR1/HSD17B10/HENMT1/FDXACB1/WTAP/METTL16/NSUN5/RBM15/METTL1/TRMT10B/TRMT1/METTL8/BUD23/ALKBH8/TRMT112/TRMT11
MF	GO:0008173	RNA methyltransferase activity	62	0.51	1.82	0.00032675	0.00224382	0.00137937	2364	tags=37%, list=20%, signal=30%	EMG1/FBL/METTL5/WDR4/TRMT10C/TRMT61B/TYW3/TRMT13/TRMO/MRM1/TRMT12/METTL14/CMTR2/TARBP1/LCMT2/TRMT5/NSUN4/FTSJ1/NSUN6/TFB1M/CMTR1/HENMT1/FDXACB1
BP	GO:0030490	maturation of SSU-rRNA	49	0.55	1.90	0.00035686	0.00241307	0.00148341	1996	tags=33%, list=17%, signal=27%	RPS16/RPS14/RPS8/RPS21/LSM6/DDX52/NGDN/DHX37/UTP20/RPS19/RPS28/WDR3/UTP6/WDR43/TSR2/DCAF13
BP	GO:0000377	RNA splicing, via transesterification reactions with bulged adenosine as nucleophile	259	0.34	1.51	0.00040931	0.00272597	0.00167577	2684	tags=32%, list=23%, signal=25%	SNRPD2/SNRPE/SRSF7/SRSF3/CWC15/SNRPG/CLNS1A/SF3B6/LSM5/HNRNPA1/LSM6/PPIE/SF3A3/LSM3/U2AF1L4/LSM7/SNRNP25/SNRNP70/SNRPA1/SNRPD1/HSPA8/RBMX2/LARP7/PHF5A/LUC7L3/RBMX/DDX39A/LSM8/RBM3/SNRPA/SRSF10/SNRPC/METTL14/SRSF2/GEMIN8/BCAS2/TRA2B/SF3B1/SNRPD3/ZCRB1/TIA1/MAGOHB/SNRPN/PRPF39/DDX5/PPWD1/PSIP1/ZCCHC8/DDX1/SRSF8/LUC7L/RBM5/PRMT5/SNRPB2/SAP18/RNPC3/SFPQ/PNN/PCBP4/LSM4/GEMIN2/DDX17/CIRBP/RBM39/SNRPF/UBL5/HTATSF1/GEMIN6/FXR1/CWC25/WBP4/RNPS1/RBM7/SNU13/FRG1/SRSF6/RBM4/WTAP/PRPF38A/METTL16/MAGOH/ZC3H10/PRPF31
BP	GO:0000398	mRNA splicing, via spliceosome	259	0.34	1.51	0.00040931	0.00272597	0.00167577	2684	tags=32%, list=23%, signal=25%	SNRPD2/SNRPE/SRSF7/SRSF3/CWC15/SNRPG/CLNS1A/SF3B6/LSM5/HNRNPA1/LSM6/PPIE/SF3A3/LSM3/U2AF1L4/LSM7/SNRNP25/SNRNP70/SNRPA1/SNRPD1/HSPA8/RBMX2/LARP7/PHF5A/LUC7L3/RBMX/DDX39A/LSM8/RBM3/SNRPA/SRSF10/SNRPC/METTL14/SRSF2/GEMIN8/BCAS2/TRA2B/SF3B1/SNRPD3/ZCRB1/TIA1/MAGOHB/SNRPN/PRPF39/DDX5/PPWD1/PSIP1/ZCCHC8/DDX1/SRSF8/LUC7L/RBM5/PRMT5/SNRPB2/SAP18/RNPC3/SFPQ/PNN/PCBP4/LSM4/GEMIN2/DDX17/CIRBP/RBM39/SNRPF/UBL5/HTATSF1/GEMIN6/FXR1/CWC25/WBP4/RNPS1/RBM7/SNU13/FRG1/SRSF6/RBM4/WTAP/PRPF38A/METTL16/MAGOH/ZC3H10/PRPF31
MF	GO:0008757	S-adenosylmethionine-dependent methyltransferase activity	129	0.41	1.64	0.00041978	0.00277713	0.00170722	2128	tags=26%, list=18%, signal=22%	METTL18/EMG1/FBL/METTL5/WDR4/TRMT10C/TRMT61B/DPH5/HNMT/CSKMT/SMYD2/TRMO/COQ5/MRM1/PRMT9/CAMKMT/METTL14/ATPSCKMT/CMTR2/TARBP1/TRMT5/NSUN4/FTSJ1/SETD6/ANTKMT/CARNMT1/PRMT5/NSUN6/ETFBKMT/TFB1M/N6AMT1/NDUFAF7/PRMT3/CMTR1
BP	GO:1901796	regulation of signal transduction by p53 class mediator	85	0.46	1.74	0.00045649	0.00298518	0.00183511	2180	tags=34%, list=19%, signal=28%	RPS20/RPL26/RPL37/MYC/RPS7/RPL5/RPL23/RPL11/SMYD2/PAK1IP1/PSMD10/DYRK2/TAF9/TRIAP1/PMAIP1/NOP53/EEF1E1/TP53RK/RPS15/DDX5/TAF9B/SGK1/ING4/ARMC10/PRMT5/TRIM24/PYHIN1/AURKB/BCL2

CC	GO:0031301	integral component of organelle membrane	313	0.32	1.45	0.0005158	0.0033078	0.00203344	2564	tags=29%, list=22%, signal=24%	COX16/SELENOS/MCUB/MICOS13/OXA1L/TOMM20/PIGK/MFF/TMCO1/PEX11B/BFAR/DPM3/SLC35B1/COX11/B2M/HACD3/RHOT2/UQCC3/QSOX2/AFG3L2/BNIP3/P2RX4/MICOS10/KTN1/SLC25A19/DHCR7/MPC1/TECR/P2RX5/PET100/PEX2/SMDT1/DOLK/MTX3/CAMLG/SLC25A3/SAMM50/ATP13A1/SFXN1/TRABD2A/MAN1C1/BNIP1/CASD1/RER1/SLC35A1/ABCB10/LNPK/HSPA9/APOO/BET1/TIMM17A/TBL2/DERL2/PIGG/IER3IP1/MPC2/COX18/TAPT1/ZFYVE27/PEX3/HLA-DRA/SFXN4/MTX2/SPCS1/ANKLE2/ARL6IP1/EMC9/ZDHHC20/HLA-DPB1/TRAM2/UBXN8/SYPL1/DERL3/EMC2/HACD1/LEMD3/SLC25A4/EMC6/TRAM1/MFN1/FICD/TMEM70/PCSK5/PLPP6/SCO1/BET1L/VOPP1/ARMCX3/PEX13/TMEM201/SUN1/EMC10
MF	GO:0140640	catalytic activity, acting on a nucleic acid	510	0.30	1.38	0.00056901	0.00359108	0.00220758	2473	tags=25%, list=21%, signal=21%	EMG1/RPS3/FBL/RNASE2/PCNA/METTL5/WDR4/TRMT10C/RPP30/TRMT61B/POLR2H/POP5/RTCB/TUT1/EIF4A2/RARS1/TOP2A/TYW3/NME1/RAD17/POLR2K/TRMT13/PTRHD1/RTCA/DDX52/REXO2/TRMO/DHX30/TWNK/NTHL1/POLE4/DDX39A/DHX37/RECQL/POLR3D/MRM1/TRMT12/DDX49/POLR1E/METTL14/DTWD1/POLRMT/MCM7/PUS1/RUVBL2/DDX55/POLM/DARS1/HELQ/HLTF/CMTR2/TARS3/SLFN13/MRPL58/FAN1/EXO5/DHX15/DDX50/MPG/EXOSC5/TARBP1/SKIV2L/ANGEL2/MUTYH/POLR1F/DDX5/DUS1L/PRIMPOL/POLA1/APEX1/SEPSECS/TOP3B/MARS2/LCMT2/TRMT5/DDX47/GEN1/POLR2J3/POLN/DDX1/RFC2/DROSHA/CNOT2/RUVBL1/RFC4/GATC/NSUN4/RPP38/FTSJ1/PLD6/NSUN6/SMARCAD1/GTF2F2/FARSB/TFB1M/QTRT1/XRCC2/N6AMT1/POLB/TATDN1/DALRD3/EIF4A1/AARS1/DDX17/DDX11/RARS2/CMTR1/OGG1/PSTK/CARS2/IARS2/GATB/MED21/HENMT1/POLR3C/POLG2/EXD2/PTGES3/TYW1/FDXACB1/INTS11/PPP1R8/EARS2/MCM8/PDE12/KARS1/RAD51C/TYW5/WRNIP1
MF	GO:0140098	catalytic activity, acting on RNA	346	0.32	1.45	0.00058851	0.00366757	0.00225461	2472	tags=26%, list=21%, signal=21%	EMG1/FBL/RNASE2/METTL5/WDR4/TRMT10C/RPP30/TRMT61B/POLR2H/POP5/RTCB/TUT1/EIF4A2/RARS1/TYW3/POLR2K/TRMT13/PTRHD1/RTCA/DDX52/REXO2/TRMO/DHX30/DDX39A/DHX37/POLR3D/MRM1/TRMT12/DDX49/POLR1E/METTL14/DTWD1/POLRMT/PUS1/DDX55/DARS1/CMTR2/TARS3/SLFN13/MRPL58/DHX15/DDX50/EXOSC5/TARBP1/SKIV2L/ANGEL2/POLR1F/DDX5/DUS1L/PRIMPOL/APEX1/SEPSECS/MARS2/LCMT2/TRMT5/DDX47/POLR2J3/DDX1/DROSHA/CNOT2/GATC/NSUN4/RPP38/FTSJ1/PLD6/NSUN6/FARSB/TFB1M/QTRT1/DALRD3/EIF4A1/AARS1/DDX17/RARS2/CMTR1/PSTK/CARS2/IARS2/GATB/MED21/HENMT1/POLR3C/EXD2/TYW1/FDXACB1/INTS11/PPP1R8/EARS2/PDE12/KARS1/TYW5
MF	GO:0140296	general transcription initiation factor binding	47	0.54	1.85	0.00063326	0.00389154	0.00239229	1785	tags=32%, list=15%, signal=27%	NOP58/GTF2B/GTF2A2/FBL/CAND1/TBP/POLR1E/RUVBL2/PSMC5/TAF1B/TAF7/RUVBL1/TAF13/RPTOR/TAF11
BP	GO:0002183	cytoplasmic translational initiation	26	0.64	1.91	0.00078039	0.00458493	0.00281854	2078	tags=42%, list=18%, signal=35%	RPL13A/EIF4B/EIF4A2/EIF3M/EIF2S2/EIF5/MCTS1/YTHDF2/EIF3D/NCK1/EIF4A1
CC	GO:0071007	U2-type catalytic step 2 spliceosome	29	0.63	1.93	0.00079928	0.00467343	0.00287295	1093	tags=31%, list=9%, signal=28%	SNRPD2/SNRPE/CWC15/SNRPG/PPIE/SNRPA1/SNRPD1/BCAS2/SNRPD3

MF	GO:0140101	catalytic activity, acting on a tRNA	120	0.41	1.63	0.00083998	0.0048827	0.0030016	2472	tags=32%, list=21%, signal=26%	WDR4/TRMT10C/RPP30/TRMT61B/POP5/RARS1/TYW3/TRMT13/PTRHD1/TRMO/TRMT12/DTWD1/PUS1/DARS1/TARS3/MRPL58/TARBP1/DUS1L/SEPSECS/MARS2/LCMT2/TRMT5/GATC/RPP38/FTSJ1/NSUN6/FARSB/QTRT1/DALRD3/AARS1/RARS2/PSTK/CARS2/IARS2/GATB/TYW1/EARS2/KARS1/TYW5
CC	GO:0071013	catalytic step 2 spliceosome	86	0.44	1.65	0.00120909	0.00664283	0.00408362	1617	tags=24%, list=14%, signal=21%	SNRPD2/SNRPE/CWC15/SNRPG/SF3B6/HNRNPA1/PPIE/SF3A3/LSM3/LSM7/SNRPA1/SNRPD1/RBMX/BCAS2/SF3B1/SNRPD3/MAGOHB/SNRPN/DDX5/PPWD1/ZCCHC8
BP	GO:0006397	mRNA processing	416	0.31	1.41	0.00122614	0.00670215	0.00412009	2703	tags=29%, list=23%, signal=23%	CSTF3/SNRPD2/SNRPE/SRSF7/SRSF3/CWC15/PRKRIP1/SNRPG/LSM1/CLNS1A/SF3B6/LSM5/HNRNPA1/LSM6/PPIE/SF3A3/LSM3/TUT1/TSEN54/ARL6IP4/U2AF1L4/LSM7/SNRNP25/SNRNP70/SNRPA1/FAM50A/SNRPD1/TBRG4/HSPA8/RBMX2/LARP7/PHF5A/LUC7L3/RBMX/DDX39A/ADARB1/LSM8/PRMT9/SNRNP27/RBM3/SNRPA/SRSF10/SNRPC/METTL14/SRSF2/GEMIN8/BCAS2/NBDY/TRA2B/ZPR1/SF3B1/THOC7/SNRPD3/ZRANB2/ZCRB1/CMTR2/RPUSD3/TIA1/MAGOHB/CDK9/DHX15/SNRPN/SUGP1/PRPF39/SRSF11/DDX5/PPWD1/PSIP1/DDX47/ZCCHC8/DDX1/SRSF8/PTCD2/AKAP17A/BARD1/LUC7L/RBM5/PRMT5/SNRPB2/SAP18/THOC6/RNPC3/GRSF1/SFPQ/PNN/PCBP4/LSM4/RPRD1A/GEMIN2/ZNF830/DDX17/CIRBP/RBM39/HABP4/CMTR1/SNRPF/THOC1/PDCD11/UBL5/HTATSF1/GEMIN6/FXR1/CWC25/PPP1R8/WBP4/RNPS1/PDE12/RBM7/SNU13/FRG1/SRSF6/RBM4/ECD/WTAP/PRPF38A/METTL16/MAGOH/CNOT6L/ZC3H10/PRPF31/CSTF2/THOC3
CC	GO:0098796	membrane protein complex	756	0.27	1.28	0.00130426	0.00709401	0.00436098	1889	tags=22%, list=16%, signal=20%	DNAJC15/CD3D/UQCRH/DNAJC19/UQCRB/SAR1B/SELENOS/COX7C/MCUB/CISH/TIMM10/MICOS13/SOCS2/COX6C/NDUFS5/SEC11C/TOMM20/COA6/ATP5PD/PIGK/KCNA3/IL2RA/VPS29/OSTC/COX4I1/LY96/HLA-DMB/TIMM9/HMGB1/SKAP1/B2M/TOMM7/ATP5PO/ATP6V1G1/ITGAE/UFD1/COX7B/ATP5MJ/NDUFB2/CD79A/SOCS5/CD79B/ATP5F1A/VAMP1/TRAT1/NDUFA4/ATP5MK/CACNA2D4/COX7A2/MICU3/GNB5/ATP5MF/ATP5PB/TOMM5/AFG3L2/SNAP47/NDUFB5/STX8/ATP5MGL/STX2/SSR4/PHB2/WDR93/ATP5F1E/UQCRQ/AP1S2/MICOS10/COX5A/NDUFB9/TMED7/CASP3/STX18/UQCRC2/NDUFA13/NDUFS3/CYC1/NDUFS6/NDUFA6/ATP5F1C/CD3G/TRAF4/CD8B/COX6A1/NDUFA1/ATP5PF/SLC25A6/SMDT1/IMMP1L/MTX3/ATP5MG/CHMP7/CLIC5/TIMM21/SAMM50/SRPRB/NDUFS4/SEC11A/HLA-DOB/PDE4D/RP9/NECAP2/NDUFS8/GNA11/VAMP4/NDUFAB1/KCNC4/SNX2/ZAP70/UBXN1/BNIP1/ITGB7/UQCR10/GRPEL2/NDUFB3/RPN2/DAD1/MLEC/HLA-DOA/UQCRFS1/NDUFA12/TRAF2/HSPA9/USE1/EXT2/TOMM22/PIK3C2B/APOO/BET1/ATP5MC1/TMEM258/NDUFB1/TIMM17A/ATP5ME/SEC31B/TFRC/VPS25/VAMP8/NAPG/DERL2/SRP9/CLTA/VDAC2/PHB1/VDAC3/ITGB1/BCS1L/SEC23A/SPCS3/HLA-DRA/MTX2/LIN7B/NECAP1/SPCS1/NDUFC1/CD8A/TIMM10B/SOCS4/NDUFV2/ARL6IP1/HSPD1/MICU2/EMC9/VAMP7/AP3M2/HLA-DPB1/ORMDL1/NDUFB8
MF	GO:0015078	proton transmembrane transporter activity	79	0.45	1.69	0.00138754	0.00740519	0.00455227	1631	tags=35%, list=14%, signal=31%	UQCRH/ATP5PD/COX4I1/ATP5PO/ATP6V1G1/COX7B/ATP5F1A/ATP5MF/ATP5PB/ATP5MGL/ATP5F1E/COX5A/CYC1/ATP5F1C/COX6A1/SLC2A13/ATP5PF/ATP5MG/SLC25A3/UQCR10/SLC9A9/UQCRFS1/ATP5MC1/ATP5ME/SLC9A3/SLC46A1/CYB5A/SLC15A2

BP	GO:0044743	protein transmembrane import into intracellular organelle	35	0.58	1.84	0.00150722	0.00790593	0.0048601	1895	tags=34%, list=16%, signal=29%	DNAJC15/DNAJC19/TOMM20/TOMM7/DNLZ/PEX2/TIMM21/GRPEL2/TIMM17A/PEX1/HSPD1/TIMM44
BP	GO:0006403	RNA localization	171	0.36	1.50	0.00192124	0.00963285	0.00592171	1731	tags=23%, list=15%, signal=20%	CCT2/NUP88/SRSF7/SRSF3/NOP58/ENY2/FBL/CETN3/SARNP/HNRNPA1/RTRAF/TOMM20/NPM1/ZNHIT3/XPOT/SSB/NUP62/SHQ1/DDX39A/CCT8/SMG5/NUP54/NXF1/RUVBL2/CCT5/UPF3A/THOC7/PCID2/NHP2/RAN/CCT3/CHTOP/HNRNPAB/MRPL18/CCT4/NUP85/RUVBL1/NUP37/TCP1/LRPPRC
BP	GO:0007006	mitochondrial membrane organization	103	0.42	1.62	0.00197382	0.00985918	0.00606084	1782	tags=32%, list=15%, signal=27%	MYC/TIMM10/MICOS13/HSP90AA1/OXA1L/PDCD5/TIMM9/RHOT2/UQCC3/HIP1R/ATP5IF1/AFG3L2/BNIP3/MICOS10/ALKBH7/NDUFA13/TIMM8B/SLC25A6/MTX3/SAMM50/BLOC1S2/PMAIP1/HSPA9/TOMM22/APOO/VDAC2/COX18/RTL10/BCS1L/MTX2/SLC35F6/TIMM10B/STPG1
BP	GO:0072655	establishment of protein localization to mitochondrion	112	0.39	1.57	0.00222243	0.01083374	0.00665995	1579	tags=28%, list=13%, signal=24%	DNAJC15/RALA/DNAJC19/TIMM10/HSP90AA1/OXA1L/TOMM20/DNAJA1/MFF/PDCD5/TIMM9/TOMM7/ATP5IF1/TOMM5/DNLZ/NDUFA13/TIMM8B/PMPCB/IMMP1L/DDIT3/TIMM21/SAMM50/HAX1/GRPEL2/SAE1/TOMM22/AIP/MTERF4/TIMM17A/COX18/BCS1L
CC	GO:1990351	transporter complex	179	0.35	1.48	0.00238123	0.01142073	0.00702079	2046	tags=27%, list=17%, signal=22%	UQCRH/UQCRB/MCUB/TIMM10/NDUFS5/KCNA3/TIMM9/NDUFB2/NDUFA4/CACNA2D4/MICU3/NDUFB5/WDR93/UQCRQ/NDUFB9/UQCRC2/NDUFA13/ATP8B2/TIMM8B/NDUFS3/CYC1/NDUFS6/NDUFA6/NDUFA1/SMDT1/CLIC5/NDUFS4/PDE4D/NDUFS8/NDUFAB1/KCNC4/UQCR10/NDUFB3/UQCRFS1/NDUFA12/NDUFB1/ATP10D/BCS1L/NDUFC1/TIMM10B/NDUFV2/MICU2/NDUFB8/ATP10A/NDUFA5/FXYD1/NDUFA9/NDUFB4
MF	GO:0016887	ATP hydrolysis activity	255	0.33	1.44	0.00240829	0.01148765	0.00706193	2173	tags=26%, list=19%, signal=22%	CCT2/PSMC6/HSPE1/KIF22/HSP90AA1/PMS1/KATNAL1/EIF4A2/ATP6V1G1/ATP5F1A/DDX52/AFG3L2/DHX30/HSPA8/TWNK/NUBP2/OLA1/DDX39A/DHX37/RECQL/MKKS/PSMC2/DDX49/ATP5F1C/CCT8/TRAP1/KIF15/MCM7/RUVBL2/CCT5/ATAD2/DDX55/NVL/PSMC5/HELQ/YME1L1/CCT3/DHX15/ABCB10/DDX50/ABCD3/HSPA9/HSPA13/AK6/DDX5/C10orf88/BCS1L/DDX47/CCT4/DDX1/RFC2/KATNA1/KIF6/PFN2/RUVBL1/RFC4/TCP1/PEX1/HSPD1/SMARCAD1/CCT6A/GTF2F2/EIF4A1/DDX17/DDX11/ATAD1/SWSAP1
BP	GO:2000112	regulation of cellular macromolecule biosynthetic process	403	0.30	1.38	0.00241725	0.01148857	0.0070625	1172	tags=16%, list=10%, signal=15%	METTL18/EIF3E/RPS14/RPL26/SVIP/RPL22/RPL5/RPS3/SELENOS/ITM2A/DBI/RPL13A/EIF4B/METTL5/LSM1/SARNP/TRMT10C/RPL38/ZNF540/CCR7/EIF3C/LARP4/DPH5/RPS9/NPM1/RPL10/UQCC2/EIF3H/ABCE1/LARP1B/EIF4A2/RPLP1/UCN/NT5C3B/RPS27L/RACK1/EIF4E/PAIP1/NGDN/SHMT2/PPP1R3E/PASK/EIF2S1/SHMT1/FASTKD3/JMJD4/EIF2B4/DYRK2/USP16/CNOT10/TRAP1/RBM3/EIF5/METTL14/EPM2AIP1/PTCD3/BZW2/UPF3A/TMED2/OGFOD1/CD28/TMEM59/ITM2C/RPUSD3/TIA1
BP	GO:0070585	protein localization to mitochondrion	116	0.39	1.54	0.00253929	0.01196235	0.00735375	1579	tags=27%, list=13%, signal=23%	DNAJC15/RALA/DNAJC19/TIMM10/HSP90AA1/OXA1L/TOMM20/DNAJA1/MFF/PDCD5/TIMM9/TOMM7/ATP5IF1/TOMM5/DNLZ/NDUFA13/TIMM8B/PMPCB/IMMP1L/DDIT3/TIMM21/SAMM50/HAX1/GRPEL2/SAE1/TOMM22/AIP/MTERF4/TIMM17A/COX18/BCS1L

BP	GO:0006417	regulation of translation	341	0.31	1.38	0.00275921	0.01277137	0.00785109	1172	tags=16%, list=10%, signal=15%	METTL18/EIF3E/RPS14/RPL26/RPL22/RPL5/RPS3/RPL13A/EIF4B/METTL5/LSM1/SARNP/TRMT10C/RPL38/ZNF540/EIF3C/LARP4/DPH5/RPS9/NPM1/RPL10/UQCC2/EIF3H/ABCE1/LARP1B/EIF4A2/RPLP1/UCN/NT5C3B/RPS27L/RACK1/EIF4E/PAIP1/NGDN/SHMT2/PASK/EIF2S1/SHMT1/FASTKD3/JMJD4/EIF2B4/USP16/CNOT10/TRAP1/RBM3/EIF5/METTL14/PTCD3/BZW2/UPF3A/TMED2/OGFOD1/CD28/RPUSD3/TIA1
BP	GO:0009303	rRNA transcription	34	0.54	1.72	0.00297954	0.01349275	0.00829455	3331	tags=44%, list=28%, signal=32%	GTF3A/GTF3C3/NIFK/POLR1E/NOP53/TAF1B/PWP1/GTF3C5/DDX11/CAVIN1/MACROH2A2/SIRT7/GTF3C2/POLR1B/NPM3
MF	GO:0008175	tRNA methyltransferase activity	33	0.55	1.73	0.00316904	0.01415743	0.00870315	1855	tags=36%, list=16%, signal=31%	WDR4/TRMT10C/TRMT61B/TYW3/TRMT13/TRMO/TRMT12/TARBP1/LCMT2/TRMT5/FTSJ1/NSUN6
BP	GO:0000959	mitochondrial RNA metabolic process	49	0.50	1.72	0.00318922	0.01423159	0.00874874	2875	tags=51%, list=25%, signal=39%	TRMT10C/TRMT61B/SLIRP/TBRG4/TWNK/MTRES1/FASTKD3/POLRMT/PUS1/MTERF4/TRMT5/LRPPRC/TEFM/SLC25A33/GRSF1/TFB1M/HSD17B10/CDK5RAP1/FASTKD2/EARS2/PDE12/WARS2/FASTKD1/GARS1/TRNT1
BP	GO:0006839	mitochondrial transport	159	0.37	1.53	0.0034227	0.01493814	0.00918309	1895	tags=30%, list=16%, signal=25%	DNAJC15/DNAJC19/TIMM10/HSP90AA1/OXA1L/TOMM20/MFF/PDCD5/TIMM9/TOMM7/RHOT2/HIP1R/ATP5IF1/TOMM5/BNIP3/DNLZ/ALKBH7/NDUFA13/TIMM8B/PMPCB/SLC25A6/IMMP1L/TIMM21/SAMM50/HAX1/BLOC1S2/PMAIP1/SLC25A13/GRPEL2/SAE1/ABCB10/TOMM22/AIP/MTERF4/TIMM17A/FLVCR1/VDAC2/COX18/RTL10/BCS1L/MTX2/SLC35F6/TIMM10B/STPG1/HSPD1/SLC25A14/TIMM44
MF	GO:0043022	ribosome binding	68	0.44	1.62	0.00393989	0.01674528	0.01029401	972	tags=21%, list=8%, signal=19%	EIF3C/OXA1L/TMCO1/DNAJC2/RACK1/GTPBP6/SBDS/OLA1/TMEM147/RPSA/EIF2S1/MTIF3/LETMD1/TTC5
MF	GO:0015399	primary active transmembrane transporter activity	113	0.39	1.56	0.00438008	0.0183318	0.01126931	2046	tags=32%, list=17%, signal=27%	UQCRH/NDUFS5/TOMM20/ABCC1/COX4I1/ATP6V1G1/COX7B/NDUFB2/NDUFA4/NDUFB5/COX5A/NDUFB9/NDUFA13/NDUFS3/CYC1/NDUFS6/NDUFA6/COX6A1/NDUFA1/NDUFS4/NDUFS8/ATP13A1/UQCR10/NDUFB3/ABCB10/UQCRFS1/NDUFA12/ABCD3/NDUFB1/CYB5A/NDUFC1/NDUFV2/NDUFB8/NDUFA5/NDUFA9/NDUFB4
BP	GO:0007007	inner mitochondrial membrane organization	39	0.54	1.77	0.00453409	0.01870078	0.01149614	1738	tags=38%, list=15%, signal=33%	MYC/TIMM10/MICOS13/TIMM9/UQCC3/AFG3L2/MICOS10/NDUFA13/TIMM8B/MTX3/SAMM50/HSPA9/APOO/MTX2/TIMM10B
BP	GO:0030488	tRNA methylation	41	0.51	1.70	0.00460829	0.01888925	0.011612	2202	tags=37%, list=19%, signal=30%	WDR4/TRMT10C/TRMT61B/TYW3/TRMT13/TRMO/TRMT12/WDR6/TARBP1/LCMT2/TRMT5/FTSJ1/NSUN6/DALRD3/HSD17B10
BP	GO:0000387	spliceosomal snRNP assembly	35	0.54	1.74	0.00499684	0.02016997	0.01239931	2309	tags=40%, list=20%, signal=32%	SNRPD2/SNRPE/SNRPG/CLNS1A/SNRPD1/SRSF10/SNRPC/GEMIN8/SNRPD3/PRMT5/LSM4/GEMIN2/SNRPF/GEMIN6
BP	GO:0006626	protein targeting to mitochondrion	93	0.41	1.55	0.00512192	0.02059124	0.01265828	1409	tags=27%, list=12%, signal=24%	DNAJC15/DNAJC19/TIMM10/HSP90AA1/TOMM20/MFF/PDCD5/TIMM9/TOMM7/ATP5IF1/TOMM5/DNLZ/NDUFA13/TIMM8B/PMPCB/IMMP1L/TIMM21/SAMM50/HAX1/GRPEL2/SAE1/TOMM22/AIP/MTERF4/TIMM17A
BP	GO:0006414	translational elongation	50	0.49	1.69	0.00614467	0.02392871	0.01470996	2232	tags=34%, list=19%, signal=28%	EEF1B2/EEF1G/DPH5/RPLP1/RACK1/EEF1D/EEF1A1/MTRES1/USP16/TRIP4/RPLP2/SRP9/SEPSECS/TUFM/DPH7/AARS1/EFL1

MF	GO:0016651	oxidoreductase activity, acting on NAD(P)H	65	0.44	1.57	0.00728493	0.02766466	0.0170066	2065	tags=38%, list=18%, signal=32%	CRYZ/NDUFS5/NDUFB2/NDUFA4/NDUFB5/WDR93/NDUFB9/NDUFA13/NDUFS3/NDUFS6/NDUFA6/NDUFA1/NDUFS4/NDUFS8/NDUFB3/NDUFA12/NDUFB1/AKR1C3/NDUFC1/NDUFV2/NDUFB8/NDUFA5/NDUFA9/NDUFB4/CYB5RL
BP	GO:0009206	purine ribonucleoside triphosphate biosynthetic process	52	0.46	1.59	0.00815107	0.03045925	0.01872455	1801	tags=42%, list=15%, signal=36%	MYC/NME3/ATP5PD/NME1/ATP5PO/UQCC3/ATP5F1A/ATP5MF/ATP5PB/ATP5MGL/ATP5F1E/ATP5F1C/ATP5PF/ATP5MG/ATPSCKMT/SLC25A13/ATP5MC1/ATP5ME/PPARA/NME7/ANTKMT/AK9
BP	GO:0009201	ribonucleoside triphosphate biosynthetic process	58	0.45	1.58	0.00849778	0.03145919	0.01933926	1801	tags=40%, list=15%, signal=34%	MYC/NME3/ATP5PD/NME1/ATP5PO/UQCC3/ATP5F1A/ATP5MF/ATP5PB/ATP5MGL/ATP5F1E/ATP5F1C/ATP5PF/ATP5MG/ATPSCKMT/SLC25A13/ATP5MC1/ATP5ME/CTPS1/PPARA/NME7/ANTKMT/AK9
BP	GO:0009145	purine nucleoside triphosphate biosynthetic process	53	0.46	1.60	0.00896929	0.03286811	0.02020538	1801	tags=42%, list=15%, signal=35%	MYC/NME3/ATP5PD/NME1/ATP5PO/UQCC3/ATP5F1A/ATP5MF/ATP5PB/ATP5MGL/ATP5F1E/ATP5F1C/ATP5PF/ATP5MG/ATPSCKMT/SLC25A13/ATP5MC1/ATP5ME/PPARA/NME7/ANTKMT/AK9
BP	GO:2000059	negative regulation of ubiquitin-dependent protein catabolic process	43	0.49	1.66	0.00941253	0.03414618	0.02099106	1187	tags=33%, list=10%, signal=29%	SVIP/RPS7/RPL5/RPL23/RPL11/EIF3H/TAF9/PARK7/CAMLG/OGT/NOP53/FHIT/DDRGK1/UBXN1
BP	GO:0071806	protein transmembrane transport	58	0.44	1.56	0.01011181	0.03603457	0.02215193	2837	tags=40%, list=24%, signal=30%	DNAJC15/DNAJC19/TOMM20/TOMM7/DNLZ/PEX2/TIMM21/C2CD5/GRPEL2/TOMM22/TIMM17A/SRP54/PEX1/HSPD1/TIMM44/TRAM2/TIMM22/TRAM1/PEX13/TOMM70/PEX6/SEC61A1/SEC62
BP	GO:0009142	nucleoside triphosphate biosynthetic process	66	0.42	1.53	0.01012797	0.03605086	0.02216195	1801	tags=38%, list=15%, signal=32%	MYC/NME3/ATP5PD/NME1/ATP5PO/UQCC3/ATP5F1A/ATP5MF/ATP5PB/ATP5MGL/ATP5F1E/ATP5F1C/ATP5PF/ATP5MG/ATPSCKMT/SLC25A13/ATP5MC1/ATP5ME/CTPS1/PPARA/CMPK1/DTYMK/NME7/ANTKMT/AK9
BP	GO:0009199	ribonucleoside triphosphate metabolic process	72	0.41	1.50	0.01026422	0.03645356	0.0224095	1801	tags=35%, list=15%, signal=30%	MYC/NME3/GIMAP7/ATP5PD/NME1/ATP5PO/UQCC3/ATP5F1A/ATP5MF/ATP5PB/ATP5MGL/ATP5F1E/ATP5F1C/ATP5PF/ATP5MG/ATPSCKMT/RAN/SLC25A13/ATP5MC1/ATP5ME/CTPS1/PPARA/NME7/ANTKMT/AK9
BP	GO:0070897	transcription preinitiation complex assembly	37	0.50	1.61	0.01030528	0.03645356	0.0224095	1811	tags=35%, list=15%, signal=30%	GTF2B/GTF2A2/CAND1/PSMC6/HMGB1/TBP/TAF9/POLR1E/TAF1B/TAF7/TAF13/TAF11/TAF5
BP	GO:0051204	protein insertion into mitochondrial membrane	26	0.56	1.69	0.01026993	0.03645356	0.0224095	2642	tags=54%, list=23%, signal=42%	TIMM10/HSP90AA1/PDCD5/TIMM9/NDUFA13/TIMM8B/SAMM50/TOMM22/COX18/TIMM10B/TIMM22/MAPK8/TIMM29/TOMM70
BP	GO:0009205	purine ribonucleoside triphosphate metabolic process	65	0.42	1.52	0.01120635	0.03877844	0.0238387	1801	tags=37%, list=15%, signal=31%	MYC/NME3/GIMAP7/ATP5PD/NME1/ATP5PO/UQCC3/ATP5F1A/ATP5MF/ATP5PB/ATP5MGL/ATP5F1E/ATP5F1C/ATP5PF/ATP5MG/ATPSCKMT/RAN/SLC25A13/ATP5MC1/ATP5ME/PPARA/NME7/ANTKMT/AK9

BP	GO:0000154	rRNA modification	32	0.53	1.64	0.01155047	0.03965859	0.02437977	1158	tags=22%, list=10%, signal=20%	EMG1/FBL/METTL5/TRMT61B/MRM1/NHP2/RPUSD3
MF	GO:0051087	chaperone binding	80	0.39	1.47	0.01166168	0.03997142	0.02457208	1895	tags=29%, list=16%, signal=24%	TBCA/SOD1/TIMM10/HSPE1/DNAJA1/TIMM9/GNB5/HSPA8/SACS/DNLZ/DNAJB9/PFDN4/BIN1/DNAJB5/DNAJC10/GRPEL2/DNAJC9/GET4/TFRC/SUGT1/ERP29/HSPD1/TIMM44
BP	GO:0006986	response to unfolded protein	115	0.36	1.45	0.01189908	0.04054048	0.0249219	1479	tags=24%, list=13%, signal=21%	ERLEC1/XBP1/SELENOS/HSPE1/HSP90AA1/DNAJA1/BFAR/RACK1/CREB3/MBTPS1/HSPA8/EIF2S1/COPS5/DNAJB9/DDIT3/PTPN2/MANF/DNAJB5/TMED2/DDRGK1/DNAJC10/KBTBD6/ABCB10/HSPA9/HSPA13/TBL2/DERL2/SERP1
CC	GO:0009295	nucleoid	42	0.48	1.61	0.01237797	0.04172592	0.02565064	2346	tags=43%, list=20%, signal=34%	TRMT10C/UQCC2/DHX30/TWNK/SHMT2/POLRMT/MPG/HSPA9/SSBP1/VDAC2/LRPPRC/TEFM/TUFM/GRSF1/TFB1M/HSD17B10/POLG2/FASTKD2
CC	GO:0042645	mitochondrial nucleoid	42	0.48	1.61	0.01237797	0.04172592	0.02565064	2346	tags=43%, list=20%, signal=34%	TRMT10C/UQCC2/DHX30/TWNK/SHMT2/POLRMT/MPG/HSPA9/SSBP1/VDAC2/LRPPRC/TEFM/TUFM/GRSF1/TFB1M/HSD17B10/POLG2/FASTKD2
BP	GO:0090151	establishment of protein localization to mitochondrial membrane	30	0.54	1.66	0.01237797	0.04172592	0.02565064	2642	tags=53%, list=23%, signal=41%	TIMM10/HSP90AA1/OXA1L/PDCD5/TIMM9/NDUFA13/TIMM8B/SAMM50/TOMM22/COX18/BCS1L/TIMM10B/TIMM22/MAPK8/TIMM29/TOMM70
BP	GO:0051205	protein insertion into membrane	51	0.45	1.56	0.01243385	0.04175422	0.02566803	2642	tags=49%, list=23%, signal=38%	TIMM10/HSP90AA1/OXA1L/PDCD5/TIMM9/NDUFA13/TIMM8B/CAMLG/MAL/SAMM50/TOMM22/GET4/COX18/BCS1L/TIMM10B/EMC9/TRAM2/EMC2/TIMM22/EMC6/TRAM1/MAPK8/TIMM29/EMC10/TOMM70
CC	GO:0016469	proton-transporting two-sector ATPase complex	41	0.48	1.59	0.01249101	0.0418123	0.02570374	1422	tags=37%, list=12%, signal=32%	ATP5PD/ATP5PO/ATP6V1G1/ATP5MJ/ATP5F1A/ATP5MK/ATP5MF/ATP5PB/ATP5MGL/ATP5F1E/ATP5F1C/ATP5PF/ATP5MG/ATP5MC1/ATP5ME
BP	GO:1905897	regulation of response to endoplasmic reticulum stress	71	0.43	1.56	0.01254449	0.04191336	0.02576587	1187	tags=27%, list=10%, signal=24%	SVIP/XBP1/SELENOS/BFAR/TMEM259/RACK1/CREB3/TXNDC12/COPS5/DNAJB9/RNFT1/PARK7/DDIT3/PTPN2/MANF/FCGR2B/PMAIP1/DDRGK1/UBXN1
BP	GO:0065002	intracellular protein transmembrane transport	50	0.47	1.62	0.0128541	0.04258993	0.02618178	2837	tags=42%, list=24%, signal=32%	DNAJC15/DNAJC19/TOMM20/TOMM7/DNLZ/PEX2/TIMM21/C2CD5/GRPEL2/TIMM17A/SRP54/PEX1/HSPD1/TIMM44/TRAM2/TRAM1/PEX13/TOMM70/PEX6/SEC61A1/SEC62
MF	GO:0016874	ligase activity	134	0.34	1.37	0.01314122	0.04336056	0.02665552	2234	tags=28%, list=19%, signal=23%	RIMKLB/ATP5PD/RTCB/RARS1/ATP5PO/ATP5F1A/RTCA/NADSYN1/ATP5MF/ATP5PB/ATP5F1E/ATP5F1C/NAE1/ATP5PF/ATP5MG/UBA3/DARS1/MOCS3/TARS3/PAICS/SAE1/ATP5ME/CTPS1/PPCS/MARS2/PCCB/GART/GATC/SUCLG1/FARSB/SLC27A4/DALRD3/UBA2/AARS1/RARS2/CARS2/IARS2/GATB

BP	GO:0016071	mRNA metabolic process	612	0.26	1.22	0.0132604	0.04352819	0.02675856	2142	tags=22%, list=18%, signal=19%	CSTF3/SNRPD2/SNRPE/EIF3E/SRSF7/SRSF3/CWC15/PRKRIP1/SNRPG/LSM1/TRMT10C/CLNS1A/SF3B6/LSM5/TRMT61B/HNRNPA1/NPM1/LSM6/PPIE/SF3A3/LSM3/TUT1/TSEN54/PNRC2/ARL6IP4/U2AF1L4/LSM7/SNRNP25/NT5C3B/SNRNP70/CEBPG/SSB/SNRPA1/TBP/FAM50A/SNRPD1/PAIP1/TBRG4/HSPA8/RBMX2/LARP7/PHF5A/LUC7L3/RBMX/ZBTB1/DDX39A/ADARB1/LSM8/FASTKD3/PRMT9/TAF9/CNOT10/SMG5/SNRNP27/RBM3/SNRPA/SRSF10/SNRPC/METTL14/SRSF2/PUS1/TTC5/GEMIN8/BCAS2/NBDY/TRA2B/ZPR1/SF3B1/UPF3A/THOC7/SNRPD3/ZRANB2/PCID2/ZCRB1/CMTR2/RPUSD3/IKBKE/TIA1/TRUB1/MAGOHB/CDK9/DHX15/SNRPN/TRAF2/SUGP1/EXOSC5/PRPF39/SKIV2L/ANGEL2/SRSF11/DDX5/PPWD1/DNAJB11/TAF7/APEX1/PSIP1/HNRNPAB/PABPC4/DDX47/ZCCHC8/DDX1/SRSF8/PTCD2/AKAP17A/CNOT2/BARD1/TAF13/LUC7L/RBM5/TAF11/TAF5/PRMT5/YTHDF2/SNRPB2/SAP18/THOC6/PYM1/RNPC3/GRSF1/SFPQ/PNN/PCBP4/LSM4/RPRD1A/GEMIN2/ZNF830/DCP1A/TNRC6A/DDX17/CIRBP/RBM39/HABP4/CMTR1/SNRPF/MRTO4
BP	GO:0009144	purine nucleoside triphosphate metabolic process	70	0.41	1.51	0.0141115	0.04568999	0.02808751	1801	tags=36%, list=15%, signal=30%	MYC/NME3/GIMAP7/ATP5PD/NME1/ATP5PO/UQCC3/ATP5F1A/ATP5MF/ATP5PB/ATP5MGL/ATP5F1E/NUDT15/ATP5F1C/ATP5PF/ATP5MG/ATPSCKMT/RAN/SLC25A13/ATP5MC1/ATP5ME/PPARA/NME7/ANTKMT/AK9
BP	GO:0016556	mRNA modification	25	0.57	1.68	0.01436633	0.04625144	0.02843266	2587	tags=44%, list=22%, signal=34%	TRMT10C/TRMT61B/METTL14/PUS1/CMTR2/TRUB1/DNAJB11/HNRNPAB/CMTR1/WTAP/METTL16
BP	GO:0006405	RNA export from nucleus	73	0.40	1.48	0.01520289	0.04855157	0.02984664	1301	tags=22%, list=11%, signal=20%	NUP88/SRSF3/ENY2/SARNP/HNRNPA1/NPM1/XPOT/SSB/NUP62/DDX39A/SMG5/NXF1/THOC7/PCID2/RAN/CHTOP
MF	GO:0140318	protein transporter activity	27	0.54	1.63	0.01537448	0.04906015	0.03015929	2781	tags=44%, list=24%, signal=34%	TOMM20/TOMM7/TIMM8B/ATP13A1/TOMM22/TIMM17A/TIMM10B/ATAD1/TIMM22/TIMM29/TOMM70/SEC61A1
BP	GO:0043628	ncRNA 3'-end processing	38	0.49	1.59	0.0154506	0.04907487	0.03016834	3307	tags=55%, list=28%, signal=40%	RPS21/FBL/TRMT10C/TUT1/SSB/LARP7/EXOSC5/INTS7/INTS12/INTS8/HSD17B10/INTS1/EXOSC9/POLR3K/TRNT1/PARN/TOE1/INTS2/ELAC1/ERI1/INTS14

Supplementary table 14 Amyloid non-accumulators gene set enrichment analysis gene ontology downregulated

ONTOLOGY	ID	Description	setSize	enrichment Score	NES	pvalue	p.adjust	qvalue	rank	leading_edge	core_enrichment
MF	GO:0051015	actin filament binding	145	-0.61	-2.43	1.00E-10	4.19E-09	2.57E-09	2527	tags=48%, list=22%, signal=38%	ACTR2/TAGLN/SHROOM4/ARPC4/FLNA/CAPZB/ADD1/UTRN/MYH10/CDK5R1/CFL1/SPTA1/COTL1/TWF2/SYNE2/MYH11/FHOD1/ARPC1B/FSCN1/FSCN3/TRIOBP/TPM3/PSTPIP2/GAS7/PSTPIP1/PPP1R9B/CTTN/ESPN/CTNNA1/GAS2L3/ARPC5/ACTN1/MYL4/MYO1D/DBN1/KPTN/ZNF185/GAS2L1/MYO5A/MYO1E/SVIL/TPM1/MYH9/MYO1F/ARPC1A/AFDN/ACTN4/LASP1/TPM4/ERMN/HCLS1/SHROOM1/MAP1S/LCP1/MYOM1/TMOD1/CORO1A/CORO1C/CTNNAL1/ABI3/ABLIM3/TLN1/MARCKS/DMTN/GSN/SPTB/IQGAP1/FMNL1/IQGAP2/HIP1
CC	GO:0030017	sarcomere	110	-0.61	-2.33	1.00E-10	4.19E-09	2.57E-09	1502	tags=37%, list=13%, signal=33%	CACNA1D/MTMR12/MTM1/ALDOA/ARF1/TPM3/PSEN1/FKBP1A/CAPN3/SQSTM1/PDLIM2/SYNPO/PPP2R5A/PALLD/ILK/ACTN1/MYL4/PDLIM5/HSPB1/ABCC9/CALM3/PARVB/TPM1/PPP1R12B/LMOD3/ACTN4/TPM4/PAK1/PPP3CA/RTN2/MYOM1/TMOD1/DNAJB6/CORO1C/SLC4A1/PDLIM7/ANK1/MYL9/SLC8A1/NBR1/MYZAP
CC	GO:0030016	myofibril	124	-0.58	-2.28	1.00E-10	4.19E-09	2.57E-09	1502	tags=38%, list=13%, signal=33%	FHL2/TWF2/SYNE2/PPP1R12A/CACNA1D/MTMR12/MTM1/ALDOA/ARF1/TPM3/PSEN1/FKBP1A/CAPN3/SQSTM1/PDLIM2/SYNPO/PPP2R5A/PALLD/ILK/ACTN1/MYL4/PDLIM5/HSPB1/ABCC9/CALM3/SVIL/PARVB/TPM1/PPP1R12B/LMOD3/ACTN4/TPM4/PAK1/PPP3CA/RTN2/MYOM1/TMOD1/DNAJB6/CORO1C/SLC4A1/PDLIM7/ANK1/MYL9/VCL/SLC8A1/NBR1/MYZAP
BP	GO:0007599	hemostasis	152	-0.57	-2.25	1.00E-10	4.19E-09	2.57E-09	2865	tags=52%, list=24%, signal=40%	ST3GAL4/SRF/GNAS/CD9/GNA13/CLIC1/ANXA5/ANO6/JAK2/HPS6/FCER1G/ACTG1/FLNA/SERPINE2/FERMT3/METAP1/PROS1/PIK3CB/ABAT/GP6/PDGFA/PLSCR1/SYK/F13A1/PIK3CG/PF4V1/PTPN6/SERPING1/JMJD1C/GPI/KNG1/VAV1/MPIG6B/GNA12/GNAQ/F8/PRDX2/RAB27A/ILK/VAV3/SRC/PF4/FLI1/F5/DGKG/HSPB1/ENTPD1/P2RY1/ACTB/PLAUR/SELP/GP1BA/ALOX12/NFE2L2/SERPINE1/P2RX1/SLC7A11/MYH9/TREML1/NFE2/LYN/PLEK/F11R/MAPK14/ITPK1/EPHB2/SLC4A1/WAS/PAFAH2/TLN1/DMTN/ITGB3/MPL/PRKCD/MYL9/VCL/KRT1/TBXA2R/GP9
BP	GO:0050817	coagulation	149	-0.57	-2.24	1.00E-10	4.19E-09	2.57E-09	2865	tags=52%, list=24%, signal=40%	ST3GAL4/SRF/GNAS/CD9/GNA13/CLIC1/ANXA5/ANO6/JAK2/HPS6/FCER1G/ACTG1/FLNA/SERPINE2/FERMT3/METAP1/PROS1/PIK3CB/ABAT/GP6/PDGFA/PLSCR1/SYK/F13A1/PIK3CG/PF4V1/PTPN6/SERPING1/JMJD1C/KNG1/PSEN1/VAV1/MPIG6B/GNA12/GNAQ/F8/PRDX2/RAB27A/ILK/VAV3/SRC/PF4/F5/DGKG/HSPB1/ENTPD1/P2RY1/ACTB/PLAUR/SELP/GP1BA/ALOX12/NFE2L2/SERPINE1/P2RX1/SLC7A11/MYH9/TREML1/LYN/PLEK/F11R/MAPK14/ITPK1/EPHB2/SLC4A1/WAS/PAFAH2/TLN1/DMTN/ITGB3/MPL/PRKCD/MYL9/VCL/KRT1/TBXA2R/GP9
BP	GO:0007596	blood coagulation	148	-0.56	-2.24	1.00E-10	4.19E-09	2.57E-09	2865	tags=51%, list=24%, signal=39%	ST3GAL4/SRF/GNAS/CD9/GNA13/CLIC1/ANXA5/ANO6/JAK2/HPS6/FCER1G/ACTG1/FLNA/SERPINE2/FERMT3/METAP1/PROS1/PIK3CB/ABAT/GP6/PDGFA/PLSCR1/SYK/F13A1/PIK3CG/PF4V1/PTPN6/SERPING1/JMJD1C/KNG1/VAV1/MPIG6B/GNA12/GNAQ/F8/PRDX2/RAB27A/ILK/VAV3/SRC/PF4/F5/DGKG/HSPB1/ENTPD1/P2RY1/ACTB/PLAUR/SELP/GP1BA/ALOX12/NFE2L2/SERPINE1/P2RX1/SLC7A11/MYH9/TREML1/LYN/PLEK/F11R/MAPK14/ITPK1/EPHB2/SLC4A1/WAS/PAFAH2/TLN1/DMTN/ITGB3/MPL/PRKCD/MYL9/VCL/KRT1/TBXA2R/GP9

MF	GO:0003779	actin binding	291	-0.55	-2.34	1.00E-10	4.19E-09	2.57E-09	2332	tags=38%, list=20%, signal=31%	FLNA/CAPZB/MYO9A/LRRK2/ADD1/HNRNPU/MYO9B/UTRN/MYH10/GBP1/CDK5R1/YWHAH/CFL1/SPTA1/COTL1/TWF2/SYNE2/MYH11/FHOD1/ARPC1B/FSCN1/FSCN3/MRTFA/MAP1A/TRIOBP/ALDOA/TPM3/DIAPH2/WASF2/MAPT/PSTPIP2/MSRB2/PDLIM2/GAS7/DIAPH1/PSTPIP1/PPP1R9B/PHACTR4/CTTN/ESPN/CTNNA1/GAS2L3/SYNPO/SNTB1/WIPF2/PHACTR2/ARPC5/PALLD/ACTN1/MYL4/MYO1D/DBN1/KPTN/PDLIM5/ZNF185/GAS2L1/MYO5A/MYO1E/SVIL/PARVB/VPS18/PLEKHG3/TPM1/MYH9/MYO1F/LMOD3/SSH1/ARPC1A/FGD4/EPB41/AFDN/ACTN4/LASP1/TPM4/ERMN/HCLS1/SNCA/SHROOM1/PPP1R18/WIPF1/MSRB1/MAP1S/LCP1/MYOM1/TMOD1/CORO1A/SSH2/CORO1C/WAS/CTNNAL1/ABI3/ABLIM3/VASP/TLN1/MARCKS/DMTN/PDLIM7/MYLK/GSN/MSN/SPTB/CNN2/VCL/MICAL2/IQGAP1/LSP1/FMNL1/TNS1/IQGAP2/HIP1
CC	GO:0015629	actin cytoskeleton	333	-0.53	-2.30	1.00E-10	4.19E-09	2.57E-09	2631	tags=44%, list=22%, signal=35%	ADAM17/PRKCZ/SLC2A1/NEXN/CDC42EP4/DAPK1/ACTR2/SHROOM4/DYNLL2/HAP1/ARPC4/ACTG1/FLNA/CAPZB/MYO9A/FERMT3/ADD1/RHOQ/FBLIM1/PRKCB/DCTN3/MYO9B/UTRN/MYH10/GBP1/BIN2/MYLPF/CFL1/MARK2/MYADM/SPTA1/COTL1/AKAP13/TWF2/MYH11/RAB22A/PPP1R12A/FHOD1/H1-0/ARPC1B/JAM3/RAC2/FSCN1/FSCN3/RFLNB/DUSP22/PVALB/ACTA2/TRIOBP/ALDOA/CDC42BPA/TPM3/DIAPH2/WASF2/RARA/FGR/PSTPIP2/SIPA1L1/PDLIM2/GAS7/DIAPH1/PSTPIP1/PXN/PPP1R9B/CTTN/SPECC1/ESPN/CTNNA1/GAS2L3/SYNPO/HCK/TMEM63B/ARHGAP21/WIPF2/ARPC5/PALLD/ILK/ACTN1/SRC/MYL4/MYO1D/DBN1/ACTR1A/GABARAP/KPTN/PDLIM5/FCHSD1/ZNF185/FLOT1/GAS2L1/ACTB/MYO5A/MYO1E/SVIL/PARVB/PTPN12/VPS18/TPM1/MYH9/SH3GL1/MYO1F/LMOD3/ARPC1A/ACTN4/LASP1/TPM4/FYB1/HCLS1/SNCA/HNRNPK/PAK1/RAC1/SHROOM1/WIPF1/MSRA/MSRB1/LCP1/ASAP1/TMOD1/CORO1A/CORO1C/WAS/ABLIM3/VASP/RAB5A/MARCKS/DMTN/PDLIM7/MTPN/MYLK/GSN/MYL9/SPTB/CNN2/VCL/LPP/FLOT2/ZYX/IQGAP1/LSP1/MYZAP/ARHGAP6/IQGAP2/PEAK3/CLDN5
CC	GO:0005938	cell cortex	212	-0.53	-2.19	1.00E-10	4.19E-09	2.57E-09	2699	tags=42%, list=23%, signal=33%	FRYL/TRAF6/RHOA/PRKCZ/SLC2A1/STIM1/ACTR2/PSEN2/SHROOM4/CSNK1G2/AGTRAP/FLNA/CAPZB/PRKCB/CTSZ/PAFAH1B1/MYO9B/UTRN/MYH10/BIN2/ITPR2/PDZD4/MYADM/SPTA1/COTL1/AKAP13/FNBP1/FRMPD1/EXOC7/GRK4/TRAK1/DLG4/GUCY1B1/RAC2/FSCN1/PVALB/MKLN1/CTBP2/PSEN1/NEDD9/PXN/ASPH/PPP1R9B/CTTN/EXOC6/PTK2B/ACTN1/MYO1D/DBN1/ACTR1A/NRBP1/FCHSD1/EXOC4/FLOT1/ACTB/MYO1E/AKT2/CTNND1/MYH9/EPB41/ACTN4/LASP1/TPM4/ERMN/FRY/HCLS1/SNCA/C2CD2L/RAC1/SHROOM1/RHOG/EPB42/GYPC/TMOD1/CORO1A/CORO1C/SLC4A1/RHOBTB1/MARCKS/DMTN/GSN/MYL9/SPTB/CYTIP/FLOT2/MPP1/IQGAP1/FMNL1/MYZAP/CLDN5
CC	GO:0030667	secretory granule membrane	245	-0.52	-2.18	1.00E-10	4.19E-09	2.57E-09	2884	tags=49%, list=25%, signal=38%	BST2/ORMDL3/ATP6V0C/MMP25/MOSPD2/ADAM8/GAA/MAGT1/CD9/TLR2/CPNE3/FPR2/RHOA/ANO6/CD177/NCKAP1L/SNAP23/CYB5R1/SIGLEC14/FCER1G/CD38/LHFPL2/CYBB/LILRB2/FPR1/BST1/SLCO4C1/KCNAB2/ITPR2/ITGAM/PRCP/ABCC4/AP1M1/STXBP5/ITGA2B/SLC11A1/MANBA/CCDC136/RAB6A/CR1/MGAM/ITGAL/ATAD3B/ATP6AP2/LAMP2/TMBIM1/ADGRE3/STX3/CPNE1/LAMP1/RAB37/FCGR3B/CKAP4/TSPAN14/CD14/SNAP25/ATP6V0A1/FCGR2A/SLC2A3/BRI3/SLC44A2/SNAP29/SYTL4/PSEN1/PTPRJ/IGF2R/ITGB2/CD63/DIAPH1/RAB27B/LGALS3/C5AR1/ARL8A/CXCR2/B4GALT1/CD93/SIRPA/RAB27A/PHACTR2/APLP2/SPARC/SELL/CXCR1/ACP3/RAB7A/ALDH3B1/PSAP/PECAM1/PLAUR/SELP/TNFRSF1B/P2RX1/CA4/RAB5B/TMEM184A/MME/CYSTM1/SIRPB1/SNCA/DNAJC5/TYROBP/RAC1/SIGLEC5/DOK3/PTPRC/RHOG/STK10/PTAFR/RAB5C/ITGB3/CD68/BSG/RAB3D/NFAM1/RAB31/LILRB3/FLOT2/IQGAP1/ACRBP/IQGAP2

BP	GO:0022604	regulation of cell morphogenesis	219	-0.51	-2.10	1.00E-10	4.19E-09	2.57E-09	2756	tags=43%, list=24%, signal=34%	GNA13/SYNE3/ARHGAP18/FES/CAPRIN1/RHOA/CDC42EP4/LPAR1/LST1/KIT/ACTR2/CSF1R/FLNA/CAPZB/PLXNB2/MYO9A/ZRANB1/RHOQ/PLEKHO1/FBLIM1/DNM2/PAFAH1B1/PHIP/ARAP1/ZMYM6/MYH10/GBP1/ZMYM3/CFL1/MARK2/MYADM/SPTA1/SEMA4A/RCC2/RAB21/DLG4/RAC2/TRIOBP/MKLN1/ALDOA/WASHC2C/CDKL5/CCL3/ANKRD27/FGR/ITGB2/DNMBP/SPAG9/SLC23A2/DIAPH1/NEDD9/CUX1/GNA12/PTK2B/HCK/DVL3/DOCK5/CASS4/NTNG2/SPARC/SPRY3/ILK/SRC/DBN1/LIMS1/P2RY1/PARVB/PREX1/TPM1/MYH9/AGO4/FGD4/EPB41/ACTN4/ERMN/UNC13D/RAC1/FGD6/SH3KBP1/F11R/PLXNC1/RHOG/EPB42/CORO1A/EPHB2/CORO1C/RHOBTB1/DMTN/PRKDC/MPL/MSN/BRWD3/DAB2/FMNL1/PEAK3
BP	GO:0050804	modulation of chemical synaptic transmission	219	-0.50	-2.09	1.00E-10	4.19E-09	2.57E-09	2896	tags=45%, list=25%, signal=34%	PRKAR1B/CLN3/SRF/KRAS/BEGAIN/MAPK1/PACSIN2/CRTC1/IL1B/CACNA1A/RAB8A/PLCB1/USP8/NEURL1/PRKCZ/NEUROD2/ADNP/JAK2/KIT/HAP1/CD38/CAMK2A/SERPINE2/CA2/LRRK2/ZDHHC12/GSK3B/LILRB2/PLCL2/FBXL20/SYAP1/PRKCB/ZDHHC2/TNF/KIF5B/MECP2/S1PR2/GRIK2/YWHAH/STXBP5/CPEB3/ABR/APBA1/DLG4/SYN3/GNAI2/STX3/DISC1/MAP1A/SNAP25/ARF1/NPTXR/PTGS2/ZZEF1/MCTP2/PSEN1/RARA/MAPT/RGS14/SQSTM1/SIPA1L1/PPP1R9B/SLC6A6/NLGN3/PTK2B/SYT12/EIF4EBP2/NTNG2/SRC/DBN1/AKAP9/FLOT1/P2RY1/CALM3/PRKACA/VPS18/P2RX1/SLC7A11/MME/SNCA/TYROBP/ARRB2/PPP3CA/PRKAR2B/IQSEC2/NF1/EPHB2/TPRG1L/LRRC4/RAB5A/NRGN/PINK1/ZDHHC3/PTEN/RAPGEF2/STAU1/TSHZ3/GLUL
BP	GO:0030036	actin cytoskeleton organization	484	-0.50	-2.24	1.00E-10	4.19E-09	2.57E-09	2211	tags=35%, list=19%, signal=29%	ADD1/ELMO1/RHOQ/NPHS1/TESK2/PAFAH1B1/TNF/UTRN/ROCK2/BST1/TNXB/MRAS/ARAP1/MYH10/LRP1/CDK5R1/ELMO2/S1PR2/RICTOR/ALOX15/ARHGDIA/CFL1/MYADM/PDGFA/SPTA1/COTL1/AKAP13/TAOK1/TWF2/PIP5K1C/MYH11/FHOD1/ARPC1B/JAM3/RAC2/FSCN1/FSCN3/AGAP2/MRTFA/RFLNB/CUL3/INPP5K/TRIOBP/MKLN1/SHC1/ALDOA/ARF1/CDC42BPA/WASHC2C/TPM3/ROCK1/DIAPH2/WASF2/PSTPIP2/CAPN3/MSRB2/PTPN1/SIPA1L1/PDLIM2/GAS7/DIAPH1/NEDD9/PSTPIP1/PXN/PPP1R9B/PHACTR4/CTTN/SPECC1/ESPN/CTNNA1/GAS2L3/PTK2B/SYNPO/ARHGEF2/HCK/MAD2L2/TF/CASS4/PHACTR2/ARPC5/PALLD/LIMK1/ACTN1/SRC/MYO1D/DBN1/KPTN/PDLIM5/FCHSD1/PAK2/GAB1/ESAM/GAS2L1/ACTB/PLEK2/MYO5A/MYO1E/SVIL/PARVB/PREX1/TPM1/MYH9/MYO1F/ITGB5/ARHGAP25/LMOD3/SSH1/ARPC1A/FGD4/EPB41/ACTN4/TPM4/ERMN/HCLS1/TYROBP/PAK1/SDCBP/RAC1/PYCARD/FGD6/PLEK/SHROOM1/SH3KBP1/WIPF1/CYRIB/F11R/LIMK2/CFLAR/MSRB1/RHOG/LCP1/PPFIA1/TMOD1/DNAJB6/IQSEC2/CORO1A/NF1/SSH2/IQSEC1/CORO1C/WAS/ABI3/ABLIM3/ARHGAP26/VASP/RHOBTB1/TLN1/CYRIA/MARCKS/AQP1/DMTN/PDLIM7/MTPN/PRKAR1A/PRKCD/GSN/MYL9/SPTB/CNN2/NUAK2/ZYX/MICAL2/IQGAP1/FMNL1/ARHGAP6/IQGAP2/HIP1/PEAK3
BP	GO:0007015	actin filament organization	308	-0.50	-2.15	1.00E-10	4.19E-09	2.57E-09	2592	tags=38%, list=22%, signal=31%	CDC42EP4/LPAR1/JAK2/NCKAP1L/ACTR2/PPARGC1B/SLC9A1/SHROOM4/ARPC4/ACTG1/FLNA/CAPZB/GRB2/ADD1/ELMO1/NPHS1/ROCK2/ARAP1/ELMO2/RICTOR/ALOX15/CFL1/MYADM/SPTA1/COTL1/TWF2/FHOD1/ARPC1B/RAC2/FSCN1/FSCN3/RFLNB/CUL3/INPP5K/TRIOBP/ALDOA/ARF1/WASHC2C/TPM3/ROCK1/DIAPH2/WASF2/PSTPIP2/MSRB2/GAS7/DIAPH1/NEDD9/PSTPIP1/PXN/PPP1R9B/CTTN/ESPN/CTNNA1/GAS2L3/PTK2B/SYNPO/ARHGEF2/HCK/MAD2L2/TF/CASS4/ARPC5/LIMK1/ACTN1/SRC/MYO1D/DBN1/KPTN/FCHSD1/PAK2/ESAM/GAS2L1/MYO5A/MYO1E/SVIL/PREX1/TPM1/MYO1F/ITGB5/ARHGAP25/LMOD3/SSH1/ARPC1A/TPM4/ERMN/HCLS1/PAK1/RAC1/PYCARD/PLEK/SHROOM1/SH3KBP1/WIPF1/CYRIB/F11R/MSRB1/RHOG/LCP1/PPFIA1/TMOD1/CORO1A/SSH2/CORO1C/WAS/VASP/RHOBTB1/CYRIA/MARCKS/DMTN/MTPN/PRKCD/GSN/SPTB/ZYX/MICAL2/ARHGAP6/IQGAP2/HIP1

BP	GO:0030029	actin filament-based process	525	-0.49	-2.20	1.00E-10	4.19E-09	2.57E-09	2211	tags=34%, list=19%, signal=29%	ADD1/ELMO1/RHOQ/NPHS1/TESK2/PAFAH1B1/TNF/MYO9B/UTRN/ROCK2/BST1/TNXB/MRAS/ARAP1/MYH10/LRP1/CDK5R1/ELMO2/MYLPF/S1PR2/RICTOR/ALOX15/ARHGDIA/CFL1/MYADM/PDGFA/SPTA1/COTL1/AKAP13/TAOK1/TWF2/PIP5K1C/SYNE2/MYH11/FHOD1/ARPC1B/JAM3/RAC2/FSCN1/FSCN3/AGAP2/MRTFA/RFLNB/CUL3/CACNA1D/INPP5K/ACTA2/TRIOBP/MKLN1/SHC1/ALDOA/ARF1/CDC42BPA/WASHC2C/TPM3/ROCK1/DSC2/DIAPH2/WASF2/PSTPIP2/CAPN3/MSRB2/PTPN1/SIPA1L1/PDLIM2/GAS7/DIAPH1/NEDD9/PSTPIP1/PXN/PPP1R9B/PHACTR4/CTTN/SPECC1/ESPN/CTNNA1/GAS2L3/PTK2B/SYNPO/ARHGEF2/HCK/MAD2L2/TF/WIPF2/KCNQ1/CASS4/PHACTR2/ARPC5/PALLD/LIMK1/ACTN1/SRC/MYO1D/DBN1/KPTN/PDLIM5/FCHSD1/PAK2/GAB1/ESAM/KCNE3/AKAP9/GAS2L1/ACTB/PLEK2/MYO5A/MYO1E/SVIL/PARVB/PREX1/TPM1/MYH9/MYO1F/ITGB5/ARHGAP25/LMOD3/SSH1/ARPC1A/FGD4/EPB41/ACTN4/TPM4/ERMN/HCLS1/TYROBP/PAK1/SDCBP/RAC1/PYCARD/FGD6/PLEK/SHROOM1/SH3KBP1/WIPF1/CYRIB/F11R/LIMK2/CFLAR/KCNJ5/MSRB1/RHOG/LCP1/PPFIA1/TMOD1/DNAJB6/IQSEC2/CORO1A/NF1/SSH2/IQSEC1/CORO1C/WAS/ABI3/ABLIM3/ARHGAP26/VASP/RHOBTB1/TLN1/CYRIA/MARCKS/AQP1/DMTN/PDLIM7/MTPN/PRKAR1A/PRKCD/GSN/MYL9/SPTB/CNN2/NUAK2/ZYX/MICAL2/IQGAP1/FMNL1/ARHGAP6/IQGAP2/HIP1/PEAK3
CC	GO:0031252	cell leading edge	300	-0.48	-2.06	1.00E-10	4.19E-09	2.57E-09	2778	tags=42%, list=24%, signal=33%	SHTN1/PACSIN2/ARHGAP18/MTMR6/CAPRIN1/RHOA/ADAM17/RAPH1/PRKCZ/CD177/ACTR2/SLC9A1/FAM89B/DDX3X/VIM/SNX1/CAPZB/CLIP1/SPATA13/PLEKHO1/DNM2/PAFAH1B1/PLCE1/MYH10/ATP7A/ARHGAP31/CFL1/PDE4A/MYADM/TWF2/PIP5K1C/SYNE2/ITSN1/ITGA5/KNSTRN/RAB22A/ATP6AP2/ATP2B1/RAC2/FSCN1/PSD4/FSCN3/TIRAP/STX3/DUSP22/INPP5K/MTM1/ACTA2/MKLN1/SH2D3C/SH3RF1/ARF1/CDC42BPA/CDKL5/ROCK1/ARAP3/WASF2/MAPT/PTPRJ/FGR/INSR/DIAPH1/NEDD9/PSTPIP1/DPP9/PXN/PPP1R9B/PHACTR4/CTTN/DOCK8/CTNNA1/PTK2B/ARHGEF2/NDEL1/TESC/APBB1IP/SLK/ARPC5/PALLD/LIMK1/ILK/ACTN1/SRC/APC/MYO1D/DBN1/KPTN/NRBP1/FLOT1/ACTB/PLEK2/MYO5A/PARVB/AKT2/CTNND1/TPM1/MYH9/SSH1/FGD4/ATP6V1B2/PAK1/RAC1/FGD6/PLEK/WIPF1/LCP1/BMX/CORO1A/CORO1C/ABI3/ABLIM3/VASP/RAB5A/TLN1/PDLIM7/ITGB3/MYLK/GSN/FRMD4B/RIPOR2/FLOT2/NHS/IQGAP1/S100A11/IQGAP2
BP	GO:0042060	wound healing	273	-0.47	-2.01	1.00E-10	4.19E-09	2.57E-09	2332	tags=35%, list=20%, signal=29%	FLNA/SERPINE2/ERBB2/FERMT3/METAP1/CHMP3/PROS1/C6orf89/PIK3CB/TNF/ABAT/GP6/ALOX15/PRCP/SLC11A1/PDGFA/PLSCR1/SYK/ITGA5/F13A1/PIK3CG/PF4V1/PTPN6/SERPING1/JMJD1C/MRTFA/KNG1/ACTA2/SYTL4/MAP3K5/VAV1/MPIG6B/TIMP1/GNA12/GNAQ/F8/B4GALT1/GPX1/PRDX2/CDKN1A/RAB27A/CHMP1A/ILK/VAV3/SRC/PF4/F5/DGKG/HSPB1/ENTPD1/P2RY1/ACTB/PLAUR/SELP/GP1BA/ALOX12/NFE2L2/SERPINE1/TPM1/P2RX1/SLC7A11/MYH9/TREML1/NOTCH4/LYN/PAK1/PLEK/PPP3CA/F11R/CFLAR/MAPK14/HMOX1/NF1/ITPK1/EPHB2/CHMP2A/SLC4A1/WAS/PAFAH2/TLN1/DMTN/ITGB3/MYLK/MPL/PRKCD/MYL9/CNN2/PTEN/VCL/KRT1/CHMP4B/TBXA2R/DYSF/GP9/TGFA

BP	GO:0006897	endocytosis	389	-0.47	-2.05	1.00E-10	4.19E-09	2.57E-09	2613	tags=38%, list=22%, signal=31%	ANO6/SCARB2/CD177/NCKAP1L/ANGPT1/VPS28/DOCK2/FCER1G/CSNK1G2/EPS15/ACTG1/COLEC12/SNX1/KIAA1109/ABCA2/LRRK2/PPT1/GRB2/TPCN2/DENND1C/ELMO1/RHOQ/SPON2/SORL1/PIK3CB/DNM2/GTF2H2/LRP1/BIN2/ITGAM/ALOX15/LRPAP1/TBC1D5/SYK/PIP5K1C/ANXA2/NECAB2/CXCL16/RAB34/ITSN1/GAK/FNBP1/PIK3CG/RAB22A/RAB21/RIN3/FCN1/GRK4/DLG4/CLEC9A/RAC2/CBL/MICALL1/LRP12/TSC2/RUBCN/AP2M1/GREM1/CD14/EEA1/SNAP25/MKLN1/CLTC/ARF1/NUMB/ZFYVE16/ROCK1/WASF2/VLDLR/CLU/RIN2/IGF2R/ITGB2/CD63/INSR/PTPN1/SMAP1/RAB27B/PSTPIP1/LGALS3/CTTN/ANKRD13A/DNM3/NLGN3/CXCR2/AP2B1/TF/CSNK1G1/SNX10/CXCR1/SRC/DPYSL2/RAB7A/MAPKAPK2/XKR8/FLOT1/ACTB/CALM3/MYO1E/EHD1/MAPKAPK3/RABGAP1L/CUBN/SERPINE1/AP2A1/MARCHF2/MYH9/SH3GL1/LRP10/ARHGAP25/EPS15L1/RAB5B/AP1G1/GPR107/EPN1/GRK3/MRC2/SNCA/HNRNPK/SDCBP/RAC1/BMP2K/ADM/PYCARD/ARRB2/SH3KBP1/SYNJ1/PPP3CA/EHD3/PICALM/RHOG/STON2/RAB5C/RHOBTB1/RAB5A/MAPK3/PDLIM7/SNX18/RALBP1/ITGB3/DENND1A/GSN/BCL2L1/RAB31/APOBR/DAB2/REPS2/HIP1
BP	GO:0048514	blood vessel morphogenesis	342	-0.47	-2.03	1.00E-10	4.19E-09	2.57E-09	2624	tags=37%, list=22%, signal=29%	RHOA/IL1B/VEZF1/BTG1/STIM1/SETD2/SIRT6/QKI/ANGPT1/ALOX5/TGFBI/ACTG1/JAK1/HK2/FLNA/KLF5/ERBB2/CYBB/BMPR1A/HIPK2/BAX/CCBE1/PRKCB/PIK3CB/TNF/ROCK2/LRP1/HIPK1/GPLD1/NRP2/PRKD2/MECP2/SGPL1/PRCP/ITGA2B/PKM/HHEX/PDGFA/FZD5/SEMA4A/SYK/ANXA2/ITGA5/PIK3CG/GADD45A/SP1/JAM3/STK4/HGF/SLC12A6/GREM1/SOX4/MEIS1/YWHAZ/NIBAN2/MAP3K3/JUNB/SHC1/FOXJ2/PTGS2/ROCK1/LRG1/WASF2/RIN2/EPHB4/EGLN1/CHI3L1/AGO2/C5AR1/NR4A1/PTK2B/B4GALT1/HDAC7/GPX1/JMJD8/SPARC/VAV3/GRN/PF4/WARS1/GAB1/HSPB1/CYP1B1/ZFP36L1/MYO1E/PROK2/BRCA1/RBPJ/NFE2L2/SERPINE1/SASH1/MYH9/TAL1/NOTCH4/ZMIZ1/EPN1/CHD7/ADM/PIK3CD/SRPK2/MAPK14/HMOX1/SP100/NF1/EPHB2/AQP1/STAT3/SFRP2/ITGB3/MYLK/BSG/PTEN/KRT1/SPI1/FOXO4/NINJ1/RAPGEF2/NPRL3/TBXA2R/FKBPL/GLUL/AGO1/TGFA/E2F2/CLDN5
BP	GO:0022603	regulation of anatomical structure morphogenesis	546	-0.46	-2.07	1.00E-10	4.19E-09	2.57E-09	2614	tags=37%, list=22%, signal=30%	BTG1/STIM1/CDC42EP4/ADNP/SIRT6/LPAR1/FIS1/LST1/KIT/ACTR2/SEMA7A/ALOX5/CSF1R/HECW2/JAK1/MED12/HK2/FLNA/CAPZB/PLXNB2/MYO9A/ERBB2/MIEF2/LRRK2/CYBB/RTN4R/GSK3B/ZRANB1/BMPR1A/HIPK2/CCBE1/RHOQ/PLEKHO1/FBLIM1/PRKCB/PIK3CB/DNM2/PAFAH1B1/TNF/PHIP/ROCK2/ARAP1/PKN1/ZMYM6/MYH10/GBP1/CST3/TNFSF13B/HIPK1/CDK5R1/PRKD2/BTBD7/MECP2/ZMYM3/YWHAH/CFL1/MARK2/PKM/MYADM/HHEX/PDGFA/SPTA1/SEMA4A/EIF4G2/RCC2/KIAA0319/TWF2/APCDD1/ITGA5/GADD45A/RAB21/SP1/DLG4/RAC2/TMBIM1/DRAXIN/HGF/RTN4/GREM1/DISC1/MSX1/NIBAN2/TRIOBP/PID1/MKLN1/ALDOA/FOXJ2/WASHC2C/CDKL5/ROCK1/LRG1/IST1/MTCH2/CCL3/PSEN1/MAPT/ANKRD27/FGR/ITGB2/EGLN1/ULK1/DNMBP/SPAG9/SIPA1L1/SLC23A2/CHI3L1/AGO2/DIAPH1/NEDD9/C5AR1/CUX1/GNA12/DNM3/PTK2B/NDEL1/HCK/DVL3/DOCK5/JMJD8/CASS4/NIN/NTNG2/SPARC/SPRY3/LIMK1/ILK/SRC/GRN/PF4/DBN1/XK/WARS1/PDLIM5/PAK2/GAB1/LIMS1/HSPB1/CYP1B1/P2RY1/PARVB/PREX1/BRCA1/NFE2L2/SERPINE1/SASH1/TPM1/MYH9/AGO4/FGD4/EPB41/ACTN4/ERMN/EPN1/UNC13D/PAK1/RAC1/ADM/FGD6/SH3KBP1/CYRIB/PIK3CD/PPP3CA/F11R/PLXNC1/RHOG/EPB42/HMOX1/SP100/CORO1A/NF1/EPHB2/CORO1C/ABI3/RHOBTB1/AQP1/STAT3/DMTN/PRKDC/SFRP2/RALBP1/KEL/MPL/PINK1/MSN/PTEN/KRT1/BRWD3/MBP/FOXO4/NINJ1/RAPGEF2/TBXA2R/FKBPL/DAB2/GLUL/AGO1/FMNL1/E2F2/PEAK3/CLDN5

BP	GO:0099536	synaptic signaling	345	-0.46	-2.00	1.00E-10	4.19E-09	2.57E-09	2896	tags=42%, list=25%, signal=32%	PRKAR1B/CLN3/RPH3A/SRF/KRAS/SLC1A2/BEGAIN/PDE7B/MAPK1/PACSIN2/KCMF1/FARP1/CRTC1/IL1B/CACNA1A/RAB8A/PLCB1/USP8/NEURL1/PRKCZ/NEUROD2/ADNP/JAK2/SNAP23/CACNB1/KIT/CEP89/P2RX7/HAP1/CD38/CAMK2A/SERPINE2/CA2/LRRK2/PPT1/ZDHHC12/GSK3B/LILRB2/PLCL2/FBXL20/SYAP1/PRKCB/ZDHHC2/PAFAH1B1/TNF/ABAT/KIF5B/CDK5R1/MECP2/S1PR2/GRIK2/KIF1B/YWHAH/STXBP5/CPEB3/HRH2/PIP5K1C/ABR/HTR3B/APBA1/DLG4/SYN3/MYCBPAP/SLC12A6/GNAI2/STX3/DTNB/DISC1/MAP1A/PVALB/RAB3B/SNAP25/GNB1/ARF1/CTBP2/NPTXR/PTGS2/ZZEF1/SNAP29/MCTP2/GSK3A/PSEN1/RARA/MAPT/RELA/RGS14/PRR7/SQSTM1/SIPA1L1/AGRN/PPP1R9B/NR4A1/SLC6A6/GNAQ/NLGN3/PTK2B/SYT12/RPS6KA1/EIF4EBP2/NTNG2/SRC/STX11/DBN1/GABARAP/FCHSD1/CACNB4/EXOC4/AKAP9/FLOT1/P2RY1/CALM3/STXBP2/PRKACA/VPS18/KPNA1/P2RX1/SLC7A11/MME/SNCA/DNAJC5/TYROBP/SDCBP/ARRB2/SYNJ1/PPP3CA/GUCY1A1/PRKAR2B/IQSEC2/NF1/EPHB2/TPRG1L/LRRC4/RAB5A/NRGN/NAPA/PINK1/ZDHHC3/PTEN/MBP/RNF10/RAPGEF2/STAU1/TSHZ3/GLUL
BP	GO:0001525	angiogenesis	296	-0.46	-1.96	1.00E-10	4.19E-09	2.57E-09	2680	tags=37%, list=23%, signal=29%	RHOA/IL1B/VEZF1/BTG1/STIM1/SETD2/SIRT6/ANGPT1/ALOX5/TGFBI/ACTG1/JAK1/HK2/FLNA/KLF5/ERBB2/CYBB/HIPK2/CCBE1/PRKCB/PIK3CB/TNF/ROCK2/HIPK1/GPLD1/NRP2/PRKD2/MECP2/PRCP/ITGA2B/PKM/HHEX/PDGFA/FZD5/SEMA4A/SYK/ANXA2/ITGA5/PIK3CG/GADD45A/SP1/JAM3/STK4/HGF/SLC12A6/GREM1/MEIS1/YWHAZ/NIBAN2/MAP3K3/SHC1/FOXJ2/PTGS2/ROCK1/LRG1/WASF2/EPHB4/EGLN1/CHI3L1/AGO2/C5AR1/NR4A1/PTK2B/B4GALT1/HDAC7/GPX1/JMJD8/SPARC/VAV3/GRN/PF4/WARS1/GAB1/HSPB1/CYP1B1/PROK2/BRCA1/RBPJ/NFE2L2/SERPINE1/SASH1/MYH9/TAL1/NOTCH4/EPN1/ADM/PIK3CD/SRPK2/MAPK14/HMOX1/SP100/NF1/EPHB2/AQP1/STAT3/SFRP2/ITGB3/BSG/PTEN/KRT1/FOXO4/NINJ1/TBXA2R/FKBPL/GLUL/AGO1/TGFA/E2F2/CLDN5
BP	GO:0001568	blood vessel development	391	-0.46	-2.02	1.00E-10	4.19E-09	2.57E-09	2624	tags=35%, list=22%, signal=28%	VEZF1/BTG1/STIM1/SETD2/SIRT6/QKI/ANGPT1/ALOX5/TGFBI/ACTG1/JAK1/HK2/FLNA/KLF5/ERBB2/CYBB/BMPR1A/HIPK2/BAX/CCBE1/PRKCB/PIK3CB/TNF/ROCK2/LRP1/ATP7A/NKX3-1/HIPK1/GPLD1/NRP2/PRKD2/MECP2/SGPL1/PRCP/ITGA2B/PKM/HHEX/PDGFA/FZD5/SEMA4A/SYK/ANXA2/DYNC2H1/ITGA5/PIK3CG/GADD45A/SP1/JAM3/STK4/HGF/SLC12A6/GREM1/SOX4/MEIS1/YWHAZ/ACTA2/NIBAN2/MAP3K3/JUNB/SHC1/FOXJ2/PTGS2/ROCK1/LRG1/WASF2/SUFU/PSEN1/RIN2/EPHB4/SOCS3/EGLN1/CHI3L1/AGO2/C5AR1/NR4A1/PTK2B/B4GALT1/HDAC7/GPX1/AP2B1/JMJD8/SPARC/VAV3/GRN/PF4/WARS1/GAB1/HSPB1/PECAM1/CYP1B1/IL6R/ZFP36L1/MYO1E/PROK2/BRCA1/RBPJ/NFE2L2/SERPINE1/SASH1/MYH9/NDST1/TAL1/NOTCH4/ZMIZ1/EPN1/CHD7/ADM/PIK3CD/CFLAR/SRPK2/LYL1/MAPK14/HMOX1/SP100/NF1/EPHB2/TCF7L2/AQP1/STAT3/SFRP2/ITGB3/MYLK/BSG/PTEN/KRT1/SPI1/FOXO4/NINJ1/RAPGEF2/NPRL3/TBXA2R/FKBPL/GLUL/AGO1/TGFA/E2F2/CLDN5

BP	GO:0007268	chemical synaptic transmission	321	-0.45	-1.96	1.00E-10	4.19E-09	2.57E-09	2896	tags=41%, list=25%, signal=32%	PRKAR1B/CLN3/RPH3A/SRF/KRAS/SLC1A2/BEGAIN/PDE7B/MAPK1/PACSIN2/CRTC1/IL1B/CACNA1A/RAB8A/PLCB1/USP8/NEURL1/PRKCZ/NEUROD2/ADNP/JAK2/SNAP23/CACNB1/KIT/CEP89/P2RX7/HAP1/CD38/CAMK2A/SERPINE2/CA2/LRRK2/PPT1/ZDHHC12/GSK3B/LILRB2/PLCL2/FBXL20/SYAP1/PRKCB/ZDHHC2/PAFAH1B1/TNF/ABAT/KIF5B/MECP2/S1PR2/GRIK2/KIF1B/YWHAH/STXBP5/CPEB3/HRH2/PIP5K1C/ABR/HTR3B/APBA1/DLG4/SYN3/MYCBPAP/SLC12A6/GNAI2/STX3/DISC1/MAP1A/PVALB/RAB3B/SNAP25/ARF1/CTBP2/NPTXR/PTGS2/ZZEF1/SNAP29/MCTP2/GSK3A/PSEN1/RARA/MAPT/RGS14/SQSTM1/SIPA1L1/PPP1R9B/NR4A1/SLC6A6/NLGN3/PTK2B/SYT12/RPS6KA1/EIF4EBP2/NTNG2/SRC/STX11/DBN1/GABARAP/FCHSD1/CACNB4/EXOC4/AKAP9/FLOT1/P2RY1/CALM3/STXBP2/PRKACA/VPS18/P2RX1/SLC7A11/MME/SNCA/DNAJC5/TYROBP/SDCBP/ARRB2/SYNJ1/PPP3CA/GUCY1A1/PRKAR2B/IQSEC2/NF1/EPHB2/TPRG1L/LRRC4/RAB5A/NRGN/NAPA/PINK1/ZDHHC3/PTEN/MBP/RAPGEF2/STAU1/TSHZ3/GLUL
BP	GO:0098916	anterograde trans-synaptic signaling	321	-0.45	-1.96	1.00E-10	4.19E-09	2.57E-09	2896	tags=41%, list=25%, signal=32%	PRKAR1B/CLN3/RPH3A/SRF/KRAS/SLC1A2/BEGAIN/PDE7B/MAPK1/PACSIN2/CRTC1/IL1B/CACNA1A/RAB8A/PLCB1/USP8/NEURL1/PRKCZ/NEUROD2/ADNP/JAK2/SNAP23/CACNB1/KIT/CEP89/P2RX7/HAP1/CD38/CAMK2A/SERPINE2/CA2/LRRK2/PPT1/ZDHHC12/GSK3B/LILRB2/PLCL2/FBXL20/SYAP1/PRKCB/ZDHHC2/PAFAH1B1/TNF/ABAT/KIF5B/MECP2/S1PR2/GRIK2/KIF1B/YWHAH/STXBP5/CPEB3/HRH2/PIP5K1C/ABR/HTR3B/APBA1/DLG4/SYN3/MYCBPAP/SLC12A6/GNAI2/STX3/DISC1/MAP1A/PVALB/RAB3B/SNAP25/ARF1/CTBP2/NPTXR/PTGS2/ZZEF1/SNAP29/MCTP2/GSK3A/PSEN1/RARA/MAPT/RGS14/SQSTM1/SIPA1L1/PPP1R9B/NR4A1/SLC6A6/NLGN3/PTK2B/SYT12/RPS6KA1/EIF4EBP2/NTNG2/SRC/STX11/DBN1/GABARAP/FCHSD1/CACNB4/EXOC4/AKAP9/FLOT1/P2RY1/CALM3/STXBP2/PRKACA/VPS18/P2RX1/SLC7A11/MME/SNCA/DNAJC5/TYROBP/SDCBP/ARRB2/SYNJ1/PPP3CA/GUCY1A1/PRKAR2B/IQSEC2/NF1/EPHB2/TPRG1L/LRRC4/RAB5A/NRGN/NAPA/PINK1/ZDHHC3/PTEN/MBP/RAPGEF2/STAU1/TSHZ3/GLUL
BP	GO:0001944	vasculature development	413	-0.45	-2.01	1.00E-10	4.19E-09	2.57E-09	2624	tags=34%, list=22%, signal=28%	VEZF1/BTG1/STIM1/SETD2/SIRT6/QKI/KIT/ANGPT1/ALOX5/TGFBI/ACTG1/JAK1/HK2/FLNA/KLF5/ERBB2/CYBB/BMPR1A/HIPK2/BAX/CCBE1/PRKCB/PIK3CB/TNF/ROCK2/LRP1/ATP7A/NKX3-1/HIPK1/GPLD1/NRP2/PRKD2/MECP2/SGPL1/PRCP/ITGA2B/PKM/HHEX/PDGFA/FZD5/SEMA4A/TMEM231/SYK/ANXA2/DYNC2H1/NFATC3/ITGA5/PIK3CG/GADD45A/SP1/JAM3/STK4/HGF/SLC12A6/GREM1/SOX4/MEIS1/YWHAZ/ACTA2/NIBAN2/MAP3K3/JUNB/SHC1/FOXJ2/PTGS2/ROCK1/LRG1/WASF2/SUFU/PSEN1/RIN2/EPHB4/SOCS3/EGLN1/CHI3L1/AGO2/C5AR1/NR4A1/PTK2B/B4GALT1/HDAC7/GPX1/AP2B1/JMJD8/SPARC/VAV3/GRN/PF4/WARS1/GAB1/HSPB1/PECAM1/CYP1B1/IL6R/SOD2/ZFP36L1/MYO1E/PROK2/BRCA1/RBPJ/NFE2L2/SERPINE1/SASH1/MYH9/NDST1/TAL1/NOTCH4/ZMIZ1/EPN1/UNC13D/CHD7/ADM/PIK3CD/CFLAR/SRPK2/LYL1/MAPK14/HMOX1/SP100/NF1/EPHB2/TCF7L2/AQP1/STAT3/SFRP2/ITGB3/MYLK/BSG/PTEN/KRT1/SPI1/FOXO4/NINJ1/RAPGEF2/NPRL3/TBXA2R/FKBPL/GLUL/AGO1/TGFA/E2F2/CLDN5

BP	GO:0097435	supramolecular fiber organization	504	-0.45	-2.02	1.00E-10	4.19E-09	2.57E-09	2212	tags=32%, list=19%, signal=27%	BAX/ADD1/ELMO1/NCKAP5L/NPHS1/NDE1/PAFAH1B1/ROCK2/TNXB/ARAP1/CST3/ATP7A/CDK5R1/ELMO2/CLIP4/MECP2/RICTOR/ALOX15/CFL1/MYADM/PCM1/SPTA1/COTL1/AKAP13/TAOK1/TUBGCP3/TWF2/MYH11/FHOD1/ARPC1B/RAC2/FSCN1/FSCN3/HOOK3/RFLNB/GREM1/CUL3/MAP1A/INPP5K/MTM1/TRIOBP/ALDOA/ARF1/WASHC2C/TPM3/ROCK1/BID/DIAPH2/WASF2/HSPA1B/PSEN1/MAPT/CLU/FKBP1A/PSTPIP2/CAPN3/MSRB2/GAS7/DIAPH1/NEDD9/PSTPIP1/PXN/PPP1R9B/HSPA1A/CTTN/ESPN/CTNNA1/GAS2L3/PTK2B/SYNPO/ARHGEF2/NDEL1/HCK/MAD2L2/GPX1/SLK/TF/CASS4/NIN/ARPC5/LIMK1/ACTN1/SIAH2/SRC/APC/MYO1D/DBN1/KPTN/FCHSD1/PAK2/ESAM/TPPP3/FSD1/AKAP9/CYP1B1/GAS2L1/RIPK1/MYO5A/MYO1E/SVIL/PREX1/TPM1/GOLGA2/MYO1F/ITGB5/ARHGAP25/LMOD3/SSH1/ARPC1A/CCSAP/TPM4/ERMN/CHP1/USP9X/HCLS1/SNCA/PAK1/RAC1/PYCARD/PLEK/SHROOM1/SH3KBP1/WIPF1/CYRIB/F11R/LIMK2/CFLAR/MSRB1/RHOG/MAP1S/LCP1/PPFIA1/TMOD1/DNAJB6/KPNB1/CORO1A/NF1/SSH2/CORO1C/WAS/VASP/RHOBTB1/CYRIA/MARCKS/DMTN/SFRP2/MTPN/PRKAR1A/PRKCD/GSN/MYL9/SPTB/KRT1/LTBP2/ZYX/MICAL2/FURIN/ARHGAP6/IQGAP2/HIP1
BP	GO:0007167	enzyme linked receptor protein signaling pathway	538	-0.45	-2.03	1.00E-10	4.19E-09	2.57E-09	2708	tags=38%, list=23%, signal=30%	FES/CPNE3/FOS/WNT1/PIK3R2/IL1B/PLCB1/ADAM17/NEURL1/PRKCZ/PBLD/JAK2/ZEB2/KIT/ANGPT1/FAM89B/HSPA5/CCND3/HAP1/CSF1R/VIM/JAK1/LCP2/GALNT3/LTBP1/SMARCC1/ERBB2/GRB2/GSK3B/BMPR1A/HIPK2/CCBE1/RHOQ/STAT5A/SORL1/PRKCB/PIK3CB/DNM2/PHIP/EFNB1/PLCE1/LRP1/NKX3-1/GPLD1/CDK5R1/NRP2/PRKD2/GP6/PIP4K2C/SGPL1/RNF165/HHEX/PDGFA/ZC3H3/KIAA0319/SYK/ITGA5/PPM1L/SOGA1/CDKN1C/IL17RD/EP300/DOK4/SULF2/CBL/NCF1/HGF/TSC2/TXNIP/GNAI2/RTN4/GREM1/DUSP22/INPP5K/MSX1/CHST11/GIGYF2/NIBAN2/STAT5B/PID1/SHC1/PDK2/OSBPL8/DSTYK/LRG1/ARNT/RPS6KA5/GSK3A/PSEN1/RGS14/ZEB1/PTPRJ/FGR/ERBIN/FKBP1A/IGF2R/TRIM33/NUP93/EPHB4/FUT7/SOCS3/ZNF703/PTPRE/CD63/INSR/PTPN1/SESN3/SIPA1L1/NEDD9/VAV1/PTP4A3/AKT1S1/PXN/RASSF2/HSPA1A/MBD5/BMP6/PTK2B/NDEL1/HCK/RNF115/DOK1/PPP2R5B/TF/SKI/EIF4EBP2/CASS4/SPRY3/ILK/SRC/APC/SHOC2/RAB7A/MAPKAPK2/ZNF106/PAK2/GAB1/TGFB1I1/HSPB1/PIP4K2A/SETX/UBE2O/PLAUR/MYO1E/ZBTB7A/CSRNP1/AKT2/ZBTB7B/SHKBP1/PTPN12/RBPJ/MAPKAPK3/NDST1/ITGB5/PELO/TAL1/NRROS/ZMIZ1/SLA/LYN/USP9X/SNCA/IGF2/PAK1/SDCBP/RAC1/ARRB2/MMP9/XIAP/ADIPOR1/DOK3/CREBBP/PIK3CD/UBE2D3/STAT6/MAPK14/EPHB2/IRS2/STAT3/MAPK3/SFRP2/RASGRP4/ITGB3/PCSK6/IL13RA1/FLCN/FKBP8/PRKCD/PPM1A/SPI1/LTBP2/FOXO4/ZYX/RAPGEF2/IQGAP1/FURIN/DAB2/REPS2/GAB2/TGFA/HIP1/CLDN5
BP	GO:0099537	trans-synaptic signaling	327	-0.45	-1.94	1.00E-10	4.19E-09	2.57E-09	2896	tags=41%, list=25%, signal=32%	PRKAR1B/CLN3/RPH3A/SRF/KRAS/SLC1A2/BEGAIN/PDE7B/MAPK1/PACSIN2/FARP1/CRTC1/IL1B/CACNA1A/RAB8A/PLCB1/USP8/NEURL1/PRKCZ/NEUROD2/ADNP/JAK2/SNAP23/CACNB1/KIT/CEP89/P2RX7/HAP1/CD38/CAMK2A/SERPINE2/CA2/LRRK2/PPT1/ZDHHC12/GSK3B/LILRB2/PLCL2/FBXL20/SYAP1/PRKCB/ZDHHC2/PAFAH1B1/TNF/ABAT/KIF5B/MECP2/S1PR2/GRIK2/KIF1B/YWHAH/STXBP5/CPEB3/HRH2/PIP5K1C/ABR/HTR3B/APBA1/DLG4/SYN3/MYCBPAP/SLC12A6/GNAI2/STX3/DISC1/MAP1A/PVALB/RAB3B/SNAP25/ARF1/CTBP2/NPTXR/PTGS2/ZZEF1/SNAP29/MCTP2/GSK3A/PSEN1/RARA/MAPT/RGS14/SQSTM1/SIPA1L1/PPP1R9B/NR4A1/SLC6A6/NLGN3/PTK2B/SYT12/RPS6KA1/EIF4EBP2/NTNG2/SRC/STX11/DBN1/GABARAP/FCHSD1/CACNB4/EXOC4/AKAP9/FLOT1/P2RY1/CALM3/STXBP2/PRKACA/VPS18/P2RX1/SLC7A11/MME/SNCA/DNAJC5/TYROBP/SDCBP/ARRB2/SYNJ1/PPP3CA/GUCY1A1/PRKAR2B/IQSEC2/NF1/EPHB2/TPRG1L/LRRC4/RAB5A/NRGN/NAPA/PINK1/ZDHHC3/PTEN/MBP/RAPGEF2/STAU1/TSHZ3/GLUL

BP	GO:0000902	cell morphogenesis	591	-0.45	-2.03	1.00E-10	4.19E-09	2.57E-09	2788	tags=39%, list=24%, signal=31%	ADAM8/SHTN1/PACSIN2/GNA13/SYNE3/ARHGAP18/FARP1/FES/FRYL/CAPRIN1/DICER1/RHOA/RAB8A/RAPH1/PRKCZ/LYPLA2/NEXN/CDC42EP4/TCTN1/ADNP/LPAR1/NCKAP1L/LST1/CASP9/KIT/ACTR2/SCN1B/SEMA7A/SIN3A/P2RX7/CSF1R/HECW2/KLF7/SNX1/FEZ1/ALCAM/FLNA/CAPZB/PLXNB2/MYO9A/ERBB2/LRRK2/FERMT3/RTN4R/GSK3B/ZRANB1/RHOQ/PLEKHO1/FBLIM1/DNM2/PAFAH1B1/PHIP/EFNB1/CFAP418/ARAP1/ZMYM6/MYH10/GBP1/KIF5B/ATP7A/CDK5R1/NRP2/ZMYM3/RNF165/YWHAH/CFL1/MARK2/MYADM/SPTA1/SEMA4A/CERT1/EIF4G2/RCC2/TAOK1/KIAA0319/TWF2/ZFPM1/WEE1/PTPN6/RAB21/RILPL1/DLG4/VPS33A/CREB1/RAC2/EP300/RERE/STK4/NCAM1/DRAXIN/HGF/RTN4/CPNE1/GREM1/CUL3/DISC1/MAP1A/MSX1/SIDT2/NIBAN2/TRIOBP/MKLN1/ALDOA/NUMB/NR4A2/WASHC2C/CDKL5/ROCK1/IST1/RPS6KA5/WASF2/CCL3/PSEN1/MAPT/ANKRD27/FGR/VLDLR/CLU/ITGB2/EPHB4/ULK1/LGR6/DNMBP/B4GALT5/SPAG9/SIPA1L1/SLC23A2/GAS7/DIAPH1/NEDD9/ARL13B/PXN/CUX1/CTTN/VAMP3/MPIG6B/GNA12/DNM3/NLGN3/PTK2B/ARHGEF2/NDEL1/MEGF9/HCK/DVL3/DOCK5/CASS4/NIN/NTNG2/PALLD/APLP2/SPARC/SPRY3/LIMK1/ILK/ACTN1/TAOK3/SRC/DPYSL2/DBN1/UBB/XK/PDLIM5/PAK2/FBXO45/LIMS1/FLOT1/P2RY1/ACTB/VDR/GP1BA/PARVB/FEZ2/PREX1/TPM1/MYH9/SSH1/AGO4/TAL1/FGD4/EPB41/NOTCH4/ACTN4/ERMN/FRY/UNC13D/USP9X/LAMC3/PAK1/RAC1/FGD6/SHROOM1/TBC1D20/SH3KBP1/PPP3CA/F11R/PLXNC1/PICALM/RHOG/EPB42/MAP4K4/MAP1S/MAPK14/CORO1A/EPHB2/CORO1C/RILPL2/ABI3/STRADB/VASP/RHOBTB1/DMTN/PRKDC/ITGB3/KEL/MPL/BSG/MSN/PTEN/VCL/BRWD3/SPI1/MBP/RAPGEF2/IQGAP1/ECE1/DAB2/FMNL1/PEAK3
BP	GO:0007169	transmembrane receptor protein tyrosine kinase signaling pathway	362	-0.44	-1.93	1.00E-10	4.19E-09	2.57E-09	2708	tags=40%, list=23%, signal=32%	FES/CPNE3/WNT1/PIK3R2/IL1B/PLCB1/ADAM17/NEURL1/PRKCZ/JAK2/KIT/ANGPT1/CCND3/HAP1/CSF1R/JAK1/LCP2/GALNT3/SMARCC1/ERBB2/GRB2/GSK3B/CCBE1/RHOQ/STAT5A/SORL1/PRKCB/PIK3CB/PHIP/EFNB1/PLCE1/LRP1/NKX3-1/GPLD1/CDK5R1/NRP2/PRKD2/PIP4K2C/SGPL1/HHEX/PDGFA/SYK/ITGA5/SOGA1/DOK4/SULF2/CBL/NCF1/HGF/TSC2/TXNIP/GNAI2/RTN4/GREM1/INPP5K/GIGYF2/NIBAN2/STAT5B/PID1/SHC1/PDK2/OSBPL8/DSTYK/ARNT/RPS6KA5/GSK3A/PSEN1/RGS14/PTPRJ/FGR/ERBIN/IGF2R/EPHB4/FUT7/SOCS3/PTPRE/CD63/INSR/PTPN1/SESN3/SIPA1L1/NEDD9/VAV1/PTP4A3/AKT1S1/PXN/RASSF2/MBD5/PTK2B/NDEL1/HCK/RNF115/DOK1/PPP2R5B/EIF4EBP2/CASS4/SPRY3/SRC/APC/SHOC2/RAB7A/MAPKAPK2/ZNF106/PAK2/GAB1/HSPB1/PIP4K2A/SETX/PLAUR/MYO1E/CSRNP1/AKT2/ZBTB7B/SHKBP1/PTPN12/RBPJ/MAPKAPK3/NDST1/TAL1/SLA/LYN/SNCA/IGF2/PAK1/RAC1/MMP9/ADIPOR1/DOK3/PIK3CD/STAT6/MAPK14/EPHB2/IRS2/STAT3/RASGRP4/ITGB3/PRKCD/FOXO4/RAPGEF2/IQGAP1/REPS2/GAB2/TGFA/HIP1

BP	GO:0070848	response to growth factor	390	-0.43	-1.91	1.00E-10	4.19E-09	2.57E-09	2394	tags=35%, list=20%, signal=29%	CPNE3/FOS/WNT1/IL1B/USP8/ADAM17/ERN1/PBLD/ZEB2/FAM89B/HSPA5/HAP1/RAB35/GALNT3/FEZ1/LTBP1/ERBB2/KDM5B/BMPR1A/HIPK2/CCBE1/SYAP1/SORL1/PRKCB/PIK3CB/DNM2/ROCK2/CASR/ATP7A/NRP2/PRKD2/RNF165/HHEX/PDGFA/TWF2/ITGA5/KNSTRN/CDKN1C/IL17RD/CREB1/IER2/EP300/GCLM/SULF2/CBL/SOX6/HGF/GREM1/DUSP22/INPP5K/MSX1/CHST11/NIBAN2/STAT5B/SHC1/DSTYK/ROCK1/LRG1/ARNT/MAPT/RELA/ZEB1/FKBP1A/TRIM33/ZNF703/CD63/INSR/PTPN1/NR3C1/RAP1GAP/PTP4A3/AKT1S1/PXN/PPP1R9B/HSPA1A/NR4A1/BMP6/PPP2R5B/ZFHX3/SKI/SPRY3/ILK/SRC/SHOC2/TPR/MAPKAPK2/CDC34/DUSP6/PAK2/GAB1/TGFB1I1/LIMS1/HSPB1/SETX/RIPK1/P2RY1/UBE2O/ZFP36L1/ZBTB7A/EHD1/PTPN12/TNFRSF1B/RBPJ/NDST1/ITGB5/PELO/NRROS/ZMIZ1/APAF1/USP9X/SDCBP/ARRB2/XIAP/CREBBP/PIK3CD/UBE2D3/CFLAR/MAP2K3/MAPK14/CORO1A/FOXO3/STAT3/MAPK3/SFRP2/ITGB3/PCSK6/FLCN/FKBP8/PPM1A/SPI1/LTBP2/ZYX/RAPGEF2/IQGAP1/FURIN/DAB2/CLDN5
BP	GO:0060627	regulation of vesicle-mediated transport	362	-0.43	-1.88	1.00E-10	4.19E-09	2.57E-09	2780	tags=40%, list=24%, signal=31%	USP7/MAPK1/PACSIN2/TLR2/FES/FPR2/IL1B/RAB8A/REST/ANO6/CD177/NCKAP1L/ANGPT1/VPS28/DOCK2/FCER1G/HAP1/CACNA1I/RPH3AL/ABCA2/LRRK2/PPT1/GSK3B/CHMP3/TPCN2/FBXL20/SORL1/PRKCB/PIK3CB/DNM2/ZDHHC2/TNF/PRAM1/LRP1/CDK5R1/ITGAM/ALOX15/LRPAP1/CD300LF/STXBP5/SLC11A1/PLSCR1/TBC1D5/SYK/ANXA2/NECAB2/MAP2K2/RAB21/RIN3/FCN1/APBA1/DLG4/GRIPAP1/RAC2/CBL/CAMK1D/TSC2/RUBCN/GNAI2/AP2M1/LAMP1/GREM1/RAB3B/CD14/MKLN1/ARF1/NUMB/ZFYVE16/ROCK1/C2/SYTL4/ANKRD27/PTPRJ/FGR/CLU/ITGB2/VPS11/CD63/INSR/PTPN1/SMAP1/RAB27B/LGALS3/ANKRD13A/RAB11B/HCK/AP2B1/SYT12/VPS4B/TF/SIRPA/LGALS9/RAB27A/SRC/RAB7A/RAB2B/FLOT1/P2RY1/CALM3/STXBP2/AKT2/EHD1/ARHGAP8/VPS18/SERPINE1/P2RX1/ARHGAP1/RAB5B/AP1G1/LYN/SIRPB1/UNC13D/SNCA/TGM2/DNAJC5/HNRNPK/SDCBP/RAC1/BMP2K/PYCARD/ARRB2/TBC1D20/SYNJ1/PPP3CA/PTPRC/PICALM/HMOX1/CORO1A/PTAFR/CHMP2A/STON2/RAB5C/RAB5A/MAPK3/ITGB3/MSN/RAB3D/CNN2/RAB31/SPI1/DYSF/DAB2/GAB2/HIP1
BP	GO:0072359	circulatory system development	623	-0.43	-1.95	1.00E-10	4.19E-09	2.57E-09	2680	tags=35%, list=23%, signal=28%	RHOA/IL1B/BORCS8/CTDP1/REST/VEZF1/BTG1/STIM1/SETD2/SIRT6/QKI/KIT/ANGPT1/SLC9A1/ALOX5/TGFBI/SMARCD3/MBD2/ACTG1/JAK1/MED12/HK2/FLNA/KLF5/ERBB2/YY1/CYBB/TBX19/SENP2/BMPR1A/HIPK2/BAX/CCBE1/ACACB/PRKCB/TAB2/PIK3CB/HNRNPU/TNF/ROCK2/NSD2/PLCE1/IFT122/LRP1/ATP7A/NKX3-1/RGS2/HIPK1/GPLD1/NRP2/PRKD2/MECP2/MAML1/SGPL1/UTY/PRCP/ITGA2B/PKM/FHL2/HHEX/PDGFA/FZD5/SEMA4A/CERT1/AKAP13/EIF4G2/TMEM231/SYK/ANXA2/ZFPM1/MYH11/DYNC2H1/NFATC3/ITGA5/PIK3CG/TNFRSF1A/GADD45A/SP1/RB1CC1/LMNA/SAV1/CREB1/JAM3/EP300/TMEM65/STK4/PIM1/SOX6/HGF/TSC2/SLC12A6/RTN4/GREM1/SOX4/MEIS1/YWHAZ/MSX1/ACTA2/NIBAN2/MAP3K3/JUNB/SHC1/IFT140/FOXJ2/PTGS2/ROCK1/LRG1/WASF2/TFDP2/SUFU/GSK3A/PSEN1/RARA/FKBP1A/RIN2/EPHB4/SOCS3/EGLN1/INSR/DAW1/CHI3L1/PDLIM2/AGO2/ARL13B/C5AR1/NR4A1/PTK2B/B4GALT1/HDAC7/GPX1/AP2B1/CDKN1A/KCNQ1/JMJD8/SPARC/ILK/VAV3/GRN/NIPBL/PF4/WARS1/DUSP6/PDLIM5/GAB1/HSPB1/PECAM1/CYP1B1/IL6R/PPP1R13L/SOD2/ZFP36L1/MYO1E/TNFRSF1B/PROK2/BRCA1/RBPJ/JARID2/NFE2L2/SERPINE1/SASH1/TPM1/KDM6B/MYH9/NDST1/TAL1/NOTCH4/ZMIZ1/EPN1/UNC13D/CHD7/ADM/ARRB2/PIK3CD/CFLAR/MAP2K3/SRPK2/LYL1/MAPK14/HMOX1/SP100/NF1/EPHB2/AP1B1/G6PD/TCF7L2/AQP1/STAT3/MAPK3/PDLIM7/PRKDC/SFRP2/CCDC39/ITGB3/PRKAR1A/MYLK/BSG/BASP1/PTEN/KRT1/SPI1/SLC8A1/FOXO4/NINJ1/RAPGEF2/NPRL3/MICAL2/TBXA2R/FKBPL/ECE1/GLUL/AGO1/ODAD4/TGFA/E2F2/NCOA6/CLDN5

BP	GO:0030334	regulation of cell migration	568	-0.42	-1.88	1.00E-10	4.19E-09	2.57E-09	3027	tags=39%, list=26%, signal=30%	EDN1/ZNF580/ATOH8/ADAM15/RAP2C/FERMT1/RFFL/CEP43/TIAM1/DAPK2/BCAS3/THBS1/PLAA/SRGAP2B/BST2/CLN3/ST3GAL4/SRF/MOSPD2/WDPCP/HIF1A/ADAM8/SHTN1/MAPK1/CD9/GNA13/TTBK2/CPNE3/FPR2/RHOA/IL1B/PLCB1/ADAM17/ANO6/NEXN/JAK2/NCKAP1L/KIT/ANGPT1/SEMA7A/FAM89B/HSPA5/OXSR1/CSF1R/C5AR2/ACTG1/CAMK2A/ARHGDIB/FADD/MITF/FLNA/SERPINE2/PLXNB2/FERMT3/SPATA13/BMPR1A/CCBE1/STAT5A/PIP5KL1/SORL1/RIPOR1/PIK3CB/TNF/ROCK2/BST1/LRP1/ATP7A/GPLD1/NRP2/PRKD2/MECP2/MIIP/S1PR2/PRCP/MYADM/PDGFA/SEMA4A/MEAK7/RCC2/SYNE2/SRGAP1/CXCL16/ITGA5/PIK3CG/YTHDF3/GADD45A/RIN3/LAMA4/SP1/RNF20/LMNA/JAM3/RAC2/CCR1/CAMK1D/CHST2/STK4/TIRAP/HGF/VSIR/STK24/GNAI2/RTN4/GREM1/GPI/DUSP22/ACTA2/MAP3K3/NUMB/OSBPL8/PTGS2/ROCK1/CCL3/PTPRJ/FGR/RIN2/FUT7/ZNF703/LGR6/INSR/SPAG9/ABHD2/AGO2/DIAPH1/NEDD9/LGALS3/C5AR1/TIMP1/DOCK8/GNA12/PTK2B/ARHGEF2/HDAC7/SLK/GFUS/LGALS9/DOCK5/CASS4/NTNG2/SPARC/SRC/APC/GRN/NIPBL/GAB1/HSPB1/PECAM1/CYP1B1/DUSP1/TNFSF14/IL6R/DLG5/SOD2/SELP/PADI2/AKT2/PLEKHG3/NFE2L2/SERPINE1/SASH1/TPM1/AFDN/ACTN4/LYN/GLIPR2/PAK1/SDCBP/RAC1/WNK1/PYCARD/MMP9/GPSM3/ADIPOR1/CYRIB/PIK3CD/PPP3CA/PLXNC1/MAP2K3/PTPRC/RHOG/MAP4K4/HMOX1/SP100/CORO1A/NF1/FOXO3/STK10/EPHB2/PTAFR/IQSEC1/CORO1C/ABI3/IRS2/DNAJA4/STAT3/MAPK3/DMTN/SFRP2/ITGB3/FLCN/MYLK/MSN/CNN2/PTEN/VCL/SPI1/RIPOR2/SLC8A1/PTP4A1/MPP1/FOXO4/PLVAP/TRIB1/RAPGEF2/TBXA2R/DAB2/GLUL/S100A11/CLDN5
CC	GO:0030141	secretory granule	613	-0.42	-1.89	1.00E-10	4.19E-09	2.57E-09	2884	tags=42%, list=25%, signal=33%	BST2/ORMDL3/RPH3A/ATP6V0C/CFD/MMP25/MOSPD2/GSTP1/ADAM8/GAA/MAGT1/MAPK1/CD9/KCMF1/TLR2/CPNE3/FPR2/RHOA/IL1B/DNAJC3/OSTF1/VTI1B/ANO6/GOLGA1/CD177/CTSA/CANT1/NCKAP1L/SNAP23/IL4I1/KIT/ACTR2/RCBTB2/GCA/ALOX5/DOCK2/ARHGAP9/CYB5R1/SIGLEC14/FCER1G/DDX3X/CD38/LHFPL2/SERPINE2/MAN2B1/CRCP/CYBB/FERMT3/CRISP3/CRISPLD2/LILRB2/PROS1/KNL1/CTSZ/FPR1/BST1/PDAP1/MNDA/SLCO4C1/CST3/ATP7A/KCNAB2/ACLY/BIN2/ITPR2/ITGAM/PRCP/ABCC4/AP1M1/STXBP5/ITGA2B/CDK13/PKM/SLC11A1/PDGFA/MANBA/PGAM1/COTL1/CCDC136/ORM1/RAB6A/ANXA2/PDXK/CR1/MGAM/F13A1/ITGAL/SLC6A9/ATAD3B/NPC2/ATP6AP2/PTPN6/FCN1/LAMP2/PSMD1/TUBB4B/TMBIM1/SERPING1/HGF/ADGRE3/STX3/CPNE1/LAMP1/RAB37/GPI/FCGR3B/KNG1/CKAP4/TSPAN14/CD14/SNAP25/ATP6V0A1/IMPDH1/ALDOA/FCGR2A/SLC2A3/ROCK1/LRG1/IST1/BRI3/SLC44A2/SNAP29/HSPA1B/SYTL4/PSEN1/PTPRJ/FGR/CLU/IGF2R/ITGB2/CD63/SPAG9/ABHD2/CHI3L1/FLG2/DIAPH1/NEU1/RAB27B/LGALS3/ANXA11/C5AR1/HSPA1A/ARL8A/PGM1/VAMP3/TIMP1/RNASET2/CTNNA1/QSOX1/CXCR2/F8/CTSD/B4GALT1/VCP/CD93/ATG7/TF/SIRPA/CYB5R3/RAB27A/PHACTR2/ARPC5/APLP2/SPARC/ACTN1/SELL/SNX10/CXCR1/ACP3/GRN/RAB7A/PF4/F5/TOR4A/ALDH3B1/PSAP/PAK2/PECAM1/STXBP2/PLAUR/SELP/PADI2/MYO5A/OSCAR/PRKACA/PSMD3/TNFRSF1B/SERPINE1/P2RX1/CA4/MYH9/PLEKHO2/NCF2/PYGL/TREML1/RAB5B/TMEM184A/MME/ACTN4/APAF1/TRAPPC1/CYSTM1/DMXL2/SIRPB1/UNC13D/SNCA/IGF2/DNAJC5/TYROBP/SDCBP/RAC1/PYCARD/SIGLEC5/MMP9/CDA/DOK3/CYRIB/GNS/TIMP2/PTPRC/PCSK1N/RHOG/CTSB/MAPK14/KPNB1/STK10/PTAFR/PGLYRP1/PRDX6/RAB5C/TOLLIP/FTL/ITGB3/FTH1/PRKCD/GSN/CD68/BSG/RAB3D/NFAM1/CNN2/CAPN1/VCL/RAB31/KRT1/LILRB3/FLOT2/IQGAP1/TBXA2R/ECE1/ACRBP/CPPED1/S100A11/ATP6V1A/IQGAP2

CC	GO:0099503	secretory vesicle	705	-0.41	-1.90	1.00E-10	4.19E-09	2.57E-09	2884	tags=41%, list=25%, signal=33%	BST2/CLN3/ORMDL3/RPH3A/ATP6V0C/CFD/MMP25/MOSPD2/GSTP1/ADAM8/GAA/BORCS5/MAGT1/MAPK1/CD9/KCMF1/TLR2/CPNE3/FPR2/RHOA/IL1B/RAB8A/DNAJC3/OSTF1/VTI1B/ANO6/GOLGA1/CD177/CTSA/CANT1/NCKAP1L/SNAP23/IL4I1/KIT/ACTR2/RCBTB2/GCA/ALOX5/DOCK2/ARHGAP9/CYB5R1/SIGLEC14/FCER1G/HAP1/DDX3X/RAB35/CD38/LHFPL2/SERPINE2/MAN2B1/LRRK2/CRCP/PPT1/CYBB/FERMT3/CRISP3/CRISPLD2/LILRB2/PROS1/KNL1/CTSZ/FPR1/BST1/PDAP1/MNDA/SLCO4C1/CST3/ATP7A/KCNAB2/ACLY/BIN2/ITPR2/ITGAM/KIF1B/PRCP/ABCC4/AP1M1/STXBP5/ITGA2B/CDK13/PKM/SLC11A1/PDGFA/MANBA/PGAM1/COTL1/CCDC136/ORM1/RAB6A/ANXA2/PDXK/CR1/STX6/MGAM/F13A1/ITGAL/SLC6A9/ATAD3B/NPC2/ATP6AP2/PTPN6/FCN1/APBA1/DLG4/LAMP2/ATP2B1/SYN3/PSMD1/TUBB4B/TMBIM1/SERPING1/HGF/ADGRE3/STX3/CPNE1/LAMP1/RAB37/GPI/DISC1/FCGR3B/KNG1/RAB3B/CKAP4/TSPAN14/CD14/SNAP25/ATP6V0A1/ROGDI/IMPDH1/ALDOA/FCGR2A/SLC2A3/ROCK1/LRG1/IST1/BRI3/SLC44A2/SNAP29/MCTP2/HSPA1B/SYTL4/PSEN1/PTPRJ/FGR/CLU/IGF2R/ITGB2/CD63/SPAG9/ABHD2/CHI3L1/ATP6V0D1/FLG2/DIAPH1/NEU1/RAB27B/LGALS3/ANXA11/C5AR1/HSPA1A/ARL8A/PGM1/VAMP3/TIMP1/RAB11B/RNASET2/CTNNA1/QSOX1/NDEL1/CXCR2/F8/CTSD/B4GALT1/VCP/SLC17A5/CD93/ATG7/SYT12/TF/SIRPA/CYB5R3/RAB27A/PHACTR2/ARPC5/APLP2/SPARC/ACTN1/SELL/SNX10/CXCR1/ACP3/GRN/RAB7A/PF4/STX11/F5/TOR4A/ALDH3B1/PSAP/PAK2/PECAM1/CALM3/STXBP2/PLAUR/SELP/PADI2/MYO5A/OSCAR/PRKACA/PSMD3/TNFRSF1B/SERPINE1/P2RX1/CA4/MYH9/PLEKHO2/NCF2/PYGL/TREML1/RAB5B/TMEM184A/MME/ACTN4/APAF1/TRAPPC1/CYSTM1/DMXL2/SIRPB1/UNC13D/SNCA/IGF2/DNAJC5/TYROBP/SDCBP/RAC1/PYCARD/SIGLEC5/MMP9/CDA/DOK3/CYRIB/GNS/TIMP2/PTPRC/PCSK1N/PICALM/RHOG/CTSB/MAPK14/KPNB1/STK10/PTAFR/STON2/IQSEC1/TPRG1L/PGLYRP1/PRDX6/RAB3IL1/RAB5C/RAB5A/TOLLIP/FTL/ITGB3/FTH1/PRKCD/GSN/CD68/BCL2L1/BSG/RAB3D/NFAM1/CNN2/CAPN1/VCL/RAB31/KRT1/LILRB3/FLOT2/IQGAP1/TBXA2R/ECE1/ACRBP/CPPED1/S100A11/ATP6V1A/IQGAP2
BP	GO:0048646	anatomical structure formation involved in morphogenesis	616	-0.41	-1.88	1.00E-10	4.19E-09	2.57E-09	2803	tags=35%, list=24%, signal=28%	HECTD1/HIF1A/ADAM8/MFN2/MAPK1/CD9/GNA13/PIKFYVE/TLR2/TOPORS/DICER1/TRAF6/WNT1/RHOA/IL1B/WLS/ADAM17/VEZF1/BTG1/STIM1/TCTN1/SETD2/SIRT6/DYRK1B/CNOT1/CASP9/ANGPT1/PPARGC1B/ALOX5/TGFBI/SMARCD3/DUSP5/ACTG1/JAK1/MED12/HK2/FLNA/KLF5/PLXNB2/ERBB2/ABCA2/GRB2/CYBB/NECTIN2/TBX19/BMPR1A/HIPK2/CCBE1/PLEKHO1/NPHS1/KNL1/PRKCB/PIK3CB/PAFAH1B1/TNF/ROCK2/PKN1/IFT122/TNFSF13B/ATP7A/NKX3-1/HIPK1/GPLD1/CDK5R1/NRP2/PRKD2/MECP2/ERCC2/PRCP/ITGA2B/PKM/FHL2/HHEX/PDGFA/FZD5/SEMA4A/CCDC136/AKAP13/SYK/ANXA2/ZFPM1/MYH11/ITGA5/PIK3CG/GADD45A/PTPN6/SP1/VPS33A/NFKB2/JAM3/CTR9/EP300/STK4/PIM1/HGF/TSC2/SLC12A6/SUPT6H/TXNRD1/RTN4/GREM1/GPI/CUL3/SOX4/MEIS1/YWHAZ/MSX1/NIBAN2/MBOAT7/MAP3K3/JUNB/SHC1/FOXJ2/PTGS2/ROCK1/LRG1/WASF2/MTHFR/SUFU/PSEN1/RARA/FKBP1A/ITGB2/EPHB4/EGLN1/CAPN3/RTF1/CHI3L1/AGTPBP1/AGO2/C5AR1/PHACTR4/SBNO2/NR4A1/MPIG6B/PTK2B/B4GALT1/HDAC7/GPX1/DVL3/SKI/JMJD8/SPARC/VAV3/ACTN1/GRN/ETS2/PF4/WARS1/GAB1/EXOC4/HSPB1/RFX2/FLOT1/CYP1B1/DUSP1/TNFSF14/PRKACA/EHD1/PROK2/BRCA1/RBPJ/NFE2L2/SERPINE1/SASH1/TPM1/KDM6B/MYH9/ITGB5/LMOD3/TAL1/NOTCH4/APAF1/EPN1/UNC13D/TGM2/TYROBP/ADM/MMP9/TBC1D20/PIK3CD/CFLAR/SRPK2/MAPK14/TMOD1/HMOX1/SP100/NF1/WDR74/EPHB2/VASP/AQP1/STAT3/MAPK3/PRKDC/SFRP2/MTPN/ITGB3/PRKAR1A/MPL/BSG/MYL9/PTEN/KRT1/BCL3/RIPOR2/FOXO4/NINJ1/TBXA2R/FKBPL/FURIN/GLUL/AGO1/ACRBP/TGFA/E2F2/CLDN5

BP	GO:0035239	tube morphogenesis	471	-0.41	-1.85	1.00E-10	4.19E-09	2.57E-09	2685	tags=34%, list=23%, signal=27%	TRAF6/WNT1/RHOA/IL1B/VEZF1/BTG1/STIM1/TCTN1/SETD2/SIRT6/QKI/ANGPT1/ALOX5/TGFBI/CSF1R/ACTG1/JAK1/MED12/HK2/FLNA/KLF5/PLXNB2/ERBB2/CYBB/KDM5B/BMPR1A/HIPK2/BAX/CCBE1/PRKCB/PIK3CB/CTSZ/TNF/ROCK2/IFT122/CASR/LRP1/NKX3-1/HIPK1/GPLD1/NRP2/PRKD2/MECP2/SGPL1/PRCP/ITGA2B/PKM/HHEX/PDGFA/FZD5/SEMA4A/SYK/ANXA2/ITGA5/PIK3CG/GADD45A/SP1/JAM3/STK4/HGF/TSC2/SLC12A6/GREM1/SOX4/MEIS1/YWHAZ/NIBAN2/MAP3K3/JUNB/SHC1/FOXJ2/PTGS2/ROCK1/LRG1/WASF2/MTHFR/SUFU/PSEN1/RARA/ZEB1/RIN2/EPHB4/EGLN1/CHI3L1/AGO2/ARL13B/C5AR1/PHACTR4/NR4A1/PTK2B/B4GALT1/HDAC7/GPX1/DVL3/SKI/JMJD8/SPARC/ILK/VAV3/SRC/GRN/NIPBL/PF4/WARS1/PBX1/GAB1/HSPB1/CYP1B1/DLG5/ZFP36L1/VDR/MYO1E/PRKACA/PROK2/BRCA1/RBPJ/NFE2L2/SERPINE1/SASH1/MYH9/TAL1/NOTCH4/ZMIZ1/APAF1/CTNNBIP1/EPN1/TGM2/CHD7/PAK1/ADM/PIK3CD/SRPK2/MAPK14/HMOX1/SP100/NF1/EPHB2/VASP/AQP1/STAT3/SFRP2/CCDC39/ITGB3/MYLK/BSG/HLX/PTEN/KRT1/SPI1/FOXO4/NINJ1/RAPGEF2/NPRL3/MICAL2/TBXA2R/FKBPL/GLUL/AGO1/TGFA/E2F2/CLDN5
MF	GO:0008092	cytoskeletal protein binding	686	-0.41	-1.89	1.00E-10	4.19E-09	2.57E-09	2760	tags=35%, list=24%, signal=29%	PACSIN2/CLMN/CCDC69/HDGFL3/SYNE3/FARP1/DNAL1/TTBK2/FES/FARP2/RHOA/RAB8A/PPP5C/STIM1/CCDC88B/NEXN/IFT88/ADNP/SETD2/PLEKHM2/ACTR2/NEK6/TAGLN/SHROOM4/CEP295/DYRK1A/HAP1/DDX3X/ARPC4/FEZ1/RPH3AL/FLNA/CAPZB/MYO9A/LRRK2/IFT81/CLIP1/GSK3B/ADD1/FBLIM1/NPHS1/NDE1/HNRNPU/DNM2/PAFAH1B1/MYO9B/UTRN/MAP1LC3B/ROCK2/MYH10/GBP1/KIF5B/RGS2/GABARAPL2/CDK5R1/CLIP4/KIF1B/YWHAH/STXBP5/CFL1/MARK2/SPTA1/COTL1/GABARAPL1/RCC2/TAOK1/TUBGCP3/TWF2/RAB6A/KIF3C/SYNE2/ANXA2/ATF5/MYH11/ATCAY/KNSTRN/SPATA6/FHOD1/TRAK1/ARPC1B/DLG4/FSCN1/EP300/FSCN3/TTLL5/RAB6B/HOOK3/MRTFA/RFLNB/MX2/DISC1/CACNA1D/MAP1A/RAB3B/CEP350/TRIOBP/MAP1LC3A/ALDOA/TPM3/ROCK1/RAE1/DIAPH2/WASF2/GSK3A/RARA/MAPT/RELA/RGS14/CLU/PSTPIP2/KIF1C/CAPN3/MSRB2/REEP4/SPAG9/PLK1/AGTPBP1/PDLIM2/GAS7/DIAPH1/RAB27B/MAP4/PSTPIP1/PXN/PPP1R9B/PHACTR4/CTTN/ARL8A/ESPN/RAB11B/CTNNA1/GAS2L3/DNM3/MARK4/SYNPO/ARHGEF2/NDEL1/B4GALT1/SNTB1/WIPF2/RAB27A/NIN/PHACTR2/ARPC5/PALLD/ACTN1/APC/DPYSL2/MYL4/MYO1D/RMDN2/TPR/DBN1/GABARAP/KPTN/NEIL2/PDLIM5/ZNF185/TPPP3/KLC3/FSD1/GAS2L1/MARK3/ACTB/DLG5/CALM3/MYO5A/MYO1E/SVIL/PARVB/BRCA1/VPS18/PLEKHG3/TPM1/GOLGA2/MYH9/MYO1F/LMOD3/SSH1/ARPC1A/FGD4/EPB41/AFDN/ACTN4/AP1G1/LASP1/CCSAP/MAST2/TPM4/ERMN/CHP1/LYN/HCLS1/SNCA/PAK1/PYCARD/SHROOM1/PPP1R18/WIPF1/MSRB1/PTPRC/PICALM/MAP4K4/MAP1S/LCP1/MYOM1/TMOD1/MAP1LC3B2/CORO1A/SSH2/CORO1C/SLC4A1/WAS/CTNNAL1/ABI3/ABLIM3/VASP/TLN1/MARCKS/DMTN/PDLIM7/TRAK2/MYLK/GSN/ANK1/MYL9/MSN/RAB3D/SPTB/CNN2/VCL/SLC8A1/MICAL2/IQGAP1/LSP1/FMNL1/TNS1/KIF27/KIF13A/IQGAP2/HIP1

CC	GO:0098590	plasma membrane region	652	-0.41	-1.87	1.00E-10	4.19E-09	2.57E-09	2233	tags=29%, list=19%, signal=25%	BMPR1A/SLC22A4/PLEKHO1/SYAP1/MAGEE1/NDE1/DNM2/DCTN3/UTRN/PI4K2A/PKN1/MYH10/CASR/SLCO4C1/LRP1/ATP7A/NRP2/PRKAR2A/ITGAM/SLC39A4/GRIK2/GASK1A/ABCC4/CFL1/PDE4A/IQCE/TMEM231/PIP5K1C/SYNE2/ANXA2/LPO/CR1/MGAM/ITSN1/HTR3B/ITGA5/FNBP1/SLC6A9/TAS2R4/ATP6AP2/RAB21/APBA1/DLG4/SLC22A1/ATP2B1/FSCN1/PSD4/CBL/FSCN3/ABCB11/TIRAP/LRP12/SLC12A6/STX3/AP2M1/CNIH3/DTNB/GPI/INPP5K/CEACAM5/SNAP25/FFAR4/SH2D3C/CLTC/NUMB/DSTYK/CDKL5/PTGS2/ABCC3/WASF2/SNAP29/PSEN1/MAPT/PTPRJ/FGR/ERBIN/VLDLR/ITGB2/PRR7/AQP9/INSR/SLC23A2/ATP6V0D1/DIAPH1/RAB27B/PSTPIP1/SLC6A20/ARL13B/PPP1R9B/C5AR1/ABHD17C/CTTN/SLC6A6/DOCK8/CTNNA1/GNA12/DNM3/NLGN3/ARHGEF2/B4GALT1/HCK/TESC/AP2B1/TF/RAB27A/KCNQ1/NTNG2/SRC/APC/MYO1D/STX11/DBN1/FBXO45/FSD1/AKAP9/FLOT1/P2RY1/IL6R/PLEK2/RAB11FIP4/SVIL/PRKACA/SLC14A1/AKT2/EHD1/CUBN/AP2A1/TPM1/P2RX1/CA4/SLC7A11/MYH9/LRP10/EPS15L1/SSH1/EPB41/SLC2A9/ATP6V1B2/EPN1/C2CD2L/PAK1/RAC1/PLEK/SHROOM1/ARRB2/SYNJ1/SLC1A5/EHD3/CAVIN2/PICALM/CTSB/LCP1/ASAP1/HMOX1/BMX/ABCG8/ITPK1/EPHB2/CORO1C/SLC4A1/LRRC4/VASP/TLN1/AQP1/MAPK3/DMTN/ITGB3/PRKAR1A/NRGN/MYLK/DENND1A/BSG/ANK1/MSN/PTEN/RIPOR2/SLC8A1/FLOT2/PLVAP/RNF10/NINJ1/RAPGEF2/NHS/DAB2/SLC7A5/LRFN1/TGFA/ATP6V1A/HIP1/AQP10/CLDN5
BP	GO:0051094	positive regulation of developmental process	736	-0.41	-1.88	1.00E-10	4.19E-09	2.57E-09	2708	tags=36%, list=23%, signal=29%	FES/CLIC1/CRTC1/FOS/CAPRIN1/DICER1/TRAF6/WNT1/RHOA/IL1B/REST/PLCB1/ASPM/INPP5D/NEURL1/PRKCZ/VEZF1/BTG1/ANO6/STIM1/NEUROD2/ADNP/EIF4G1/SIRT6/JAK2/NCKAP1L/ZEB2/IL4I1/FIS1/KIT/ACTR2/PPARGC1B/SEMA7A/ALOX5/SIN3A/SMARCD3/P2RX7/EVI2B/SPEN/HAP1/TMEM79/JAK1/FEZ1/CEBPA/TSPO/HK2/UTP25/FADD/FLNA/SERPINE2/KLF5/PLXNB2/MIEF2/CYBB/KDM5B/GSK3B/BMPR1A/WDFY2/TNFSF13/LILRB2/HIPK2/BAX/ADD1/CCBE1/SYAP1/ACACB/SORL1/ZBTB16/PRKCB/HNRNPU/DNM2/PAFAH1B1/TNF/JUND/NSD2/GJC2/TNFSF13B/HIPK1/PRKD2/KCTD11/S1PR2/MAML1/CPEB3/CFL1/PKM/MYADM/METRNL/SEMA4A/EIF4G2/TWF2/SYK/CR1/ITGA5/TNFRSF1A/RAB21/SP1/DLG4/SAV1/CREB1/ATP2B1/CCR1/LRRC8A/STK4/PIM1/SOX6/HGF/CEBPD/VSIR/STK24/RTN4/PDE5A/CPNE1/GREM1/DISC1/SOX4/MTM1/NIBAN2/TRIOBP/STAT5B/MTURN/JUNB/FFAR4/KDM4A/NUMB/CDKL5/PTGS2/LRG1/ARNT/IST1/MTCH2/HSPA1B/PSEN1/RARA/MAPT/RGS14/ANKRD27/ZEB1/VLDLR/RIN2/SOCS3/ZNF703/CAPN3/INSR/B4GALT5/SPAG9/SLC23A2/CHI3L1/MAP3K5/AGO2/NEDD9/AGRN/SMAP1/C5AR1/HSPA1A/CUX1/SLC6A6/BMP6/NLGN3/PTK2B/ARHGEF2/NDEL1/MAD2L2/TESC/RPS6KA1/DVL3/LGALS9/DOCK5/LIN28A/JMJD8/CASS4/NIN/LIMK1/ILK/SRC/GRN/TBX21/NIPBL/PF4/DBN1/XKR8/GAB1/TGFB1I1/LIMS1/HSPB1/FLOT1/CYP1B1/RIPK1/TNFSF14/IL6R/DLG5/PPP1R13L/SOD2/ZFP36L1/VDR/CARM1/EHD1/CLCF1/ZBTB7B/TNFRSF1B/PREX1/BRCA1/RBPJ/NFE2L2/SERPINE1/SASH1/LMOD3/TAL1/POR/MME/ZMIZ1/RCOR1/CTNNBIP1/LYN/MMD/UNC13D/HCLS1/TGM2/IGF2/CHD7/GLIPR2/TYROBP/SDCBP/RAC1/ADM/ARRB2/SYNJ1/PITHD1/PIK3CD/F11R/PLXNC1/CEBPB/PTPRC/STAT6/MAPK14/HMOX1/NCOA1/FAXDC2/FOXO3/EPHB2/CORO1C/TFE3/YBX3/FAM210B/TCF7L2/AQP1/STAT3/DMTN/PDLIM7/PRKDC/SFRP2/RALBP1/MPL/AMBRA1/PINK1/BASP1/HLX/RIPOR2/SLC8A1/TRIB1/NINJ1/RAPGEF2/TBXA2R/DAB2/CLDN5

BP	GO:0045597	positive regulation of cell differentiation	505	-0.41	-1.83	1.00E-10	4.19E-09	2.57E-09	2723	tags=36%, list=23%, signal=29%	TLR2/FES/CLIC1/FOS/CAPRIN1/DICER1/TRAF6/RHOA/IL1B/REST/PLCB1/ASPM/INPP5D/NEURL1/PRKCZ/VEZF1/BTG1/NEUROD2/ADNP/EIF4G1/JAK2/NCKAP1L/ZEB2/IL4I1/KIT/ACTR2/PPARGC1B/SEMA7A/SIN3A/SMARCD3/EVI2B/SPEN/HAP1/FEZ1/CEBPA/TSPO/FADD/FLNA/SERPINE2/KLF5/PLXNB2/GSK3B/BMPR1A/WDFY2/LILRB2/ADD1/SYAP1/ZBTB16/HNRNPU/DNM2/PAFAH1B1/TNF/JUND/GJC2/KCTD11/S1PR2/MAML1/MYADM/METRNL/EIF4G2/TWF2/SYK/CR1/RAB21/SAV1/CREB1/CCR1/LRRC8A/STK4/PIM1/SOX6/HGF/CEBPD/VSIR/PDE5A/CPNE1/GREM1/DISC1/SOX4/NIBAN2/TRIOBP/STAT5B/MTURN/JUNB/FFAR4/KDM4A/NUMB/CDKL5/PTGS2/ARNT/IST1/MTCH2/HSPA1B/RARA/MAPT/RGS14/ANKRD27/ZEB1/RIN2/SOCS3/ZNF703/SPAG9/MAP3K5/NEDD9/SMAP1/HSPA1A/CUX1/SLC6A6/BMP6/ARHGEF2/NDEL1/TESC/RPS6KA1/LGALS9/DOCK5/LIN28A/CASS4/NIN/LIMK1/ILK/PF4/DBN1/XKR8/TGFB1I1/LIMS1/RIPK1/TNFSF14/IL6R/PPP1R13L/SOD2/ZFP36L1/VDR/CARM1/CLCF1/ZBTB7B/TNFRSF1B/PREX1/SERPINE1/LMOD3/TAL1/POR/MME/ZMIZ1/RCOR1/CTNNBIP1/LYN/MMD/UNC13D/HCLS1/TGM2/GLIPR2/TYROBP/SDCBP/RAC1/ADM/ARRB2/SYNJ1/PITHD1/F11R/PLXNC1/CEBPB/PTPRC/MAPK14/NCOA1/FAXDC2/FOXO3/EPHB2/TFE3/FAM210B/TCF7L2/STAT3/DMTN/PDLIM7/PRKDC/SFRP2/MPL/AMBRA1/HLX/RIPOR2/TRIB1/RAPGEF2/DAB2/CLDN5
BP	GO:2000145	regulation of cell motility	596	-0.41	-1.84	1.00E-10	4.19E-09	2.57E-09	3027	tags=38%, list=26%, signal=30%	EDN1/ZNF580/ATOH8/ADAM15/RAP2C/FERMT1/RFFL/CEP43/TIAM1/BBS2/DAPK2/BCAS3/THBS1/PLAA/SRGAP2B/BST2/CLN3/ST3GAL4/SRF/MOSPD2/WDPCP/HIF1A/ADAM8/SHTN1/MAPK1/CD9/GNA13/ARHGAP18/TTBK2/FES/CPNE3/FPR2/RHOA/IL1B/PLCB1/ADAM17/ANO6/NEXN/JAK2/NCKAP1L/KIT/ANGPT1/SEMA7A/FAM89B/HSPA5/OXSR1/CSF1R/C5AR2/ACTG1/CAMK2A/ARHGDIB/FADD/MITF/FLNA/SERPINE2/PLXNB2/FERMT3/SPATA13/BMPR1A/CCBE1/STAT5A/PIP5KL1/SORL1/RIPOR1/PIK3CB/TNF/ROCK2/BST1/PKN1/LRP1/ATP7A/GPLD1/NRP2/PRKD2/MECP2/MIIP/S1PR2/PRCP/MYADM/PDGFA/SEMA4A/MEAK7/RCC2/SYNE2/SRGAP1/CXCL16/ITGA5/PIK3CG/YTHDF3/GADD45A/RIN3/LAMA4/SP1/RNF20/LMNA/JAM3/RAC2/CCR1/CAMK1D/CHST2/STK4/TIRAP/HGF/VSIR/STK24/GNAI2/RTN4/GREM1/GPI/DUSP22/ACTA2/MAP3K3/NUMB/OSBPL8/PTGS2/ROCK1/CCL3/PTPRJ/FGR/RIN2/FUT7/ZNF703/LGR6/INSR/SPAG9/ABHD2/AGO2/DIAPH1/NEDD9/LGALS3/C5AR1/TIMP1/DOCK8/CTNNA1/GNA12/PTK2B/ARHGEF2/HDAC7/SLK/TF/GFUS/LGALS9/DOCK5/CASS4/NTNG2/SPARC/SRC/APC/GRN/NIPBL/GAB1/HSPB1/PECAM1/CYP1B1/DUSP1/TNFSF14/IL6R/DLG5/SOD2/SELP/PADI2/AKT2/PLEKHG3/NFE2L2/SERPINE1/SASH1/TPM1/AFDN/ACTN4/CCSAP/LYN/GLIPR2/PAK1/SDCBP/RAC1/WNK1/PYCARD/MMP9/GPSM3/ADIPOR1/CYRIB/PIK3CD/PPP3CA/PLXNC1/MAP2K3/PTPRC/RHOG/MAP4K4/HMOX1/SP100/CORO1A/NF1/FOXO3/STK10/EPHB2/PTAFR/IQSEC1/CORO1C/WAS/ABI3/IRS2/DNAJA4/STAT3/MAPK3/DMTN/SFRP2/ITGB3/FLCN/MYLK/MSN/CNN2/PTEN/VCL/SPI1/RIPOR2/SLC8A1/PTP4A1/MPP1/FOXO4/PLVAP/TRIB1/RAPGEF2/TBXA2R/DAB2/GLUL/S100A11/CLDN5

BP	GO:0040012	regulation of locomotion	614	-0.41	-1.84	1.00E-10	4.19E-09	2.57E-09	3027	tags=38%, list=26%, signal=30%	EDN1/ZNF580/ATOH8/ADAM15/RAP2C/FERMT1/RFFL/CEP43/TIAM1/BBS2/DAPK2/BCAS3/THBS1/PLAA/ARRDC3/SRGAP2B/BST2/CLN3/ST3GAL4/SRF/MOSPD2/WDPCP/HIF1A/ADAM8/SHTN1/MAPK1/CD9/GNA13/ARHGAP18/TTBK2/FES/CPNE3/FPR2/RHOA/IL1B/PLCB1/ADAM17/ANO6/NEXN/JAK2/NCKAP1L/KIT/ANGPT1/SEMA7A/FAM89B/HSPA5/OXSR1/CSF1R/C5AR2/ACTG1/CAMK2A/ARHGDIB/FADD/MITF/FLNA/SERPINE2/PLXNB2/LRRK2/FERMT3/SPATA13/BMPR1A/CCBE1/STAT5A/PIP5KL1/SORL1/RIPOR1/PIK3CB/TNF/ROCK2/BST1/PKN1/CASR/LRP1/ATP7A/GPLD1/NRP2/PRKD2/MECP2/MIIP/S1PR2/PRCP/MYADM/PDGFA/SEMA4A/MEAK7/RCC2/SYNE2/SRGAP1/CXCL16/ITGA5/PIK3CG/YTHDF3/GADD45A/RIN3/LAMA4/SP1/RNF20/LMNA/JAM3/RAC2/CCR1/CAMK1D/CHST2/STK4/TIRAP/HGF/VSIR/STK24/GNAI2/RTN4/STX3/GREM1/GPI/DUSP22/ACTA2/MAP3K3/NUMB/OSBPL8/PTGS2/ROCK1/CCL3/PTPRJ/FGR/RIN2/FUT7/ZNF703/LGR6/INSR/SPAG9/ABHD2/AGO2/DIAPH1/NEDD9/LGALS3/C5AR1/TIMP1/DOCK8/CTNNA1/GNA12/PTK2B/ARHGEF2/HDAC7/SLK/TF/GFUS/LGALS9/DOCK5/CASS4/NTNG2/SPARC/SRC/APC/GRN/NIPBL/GAB1/HSPB1/PECAM1/CYP1B1/DUSP1/TNFSF14/IL6R/DLG5/SOD2/SELP/PADI2/AKT2/PLEKHG3/NFE2L2/SERPINE1/SASH1/TPM1/AFDN/ACTN4/CCSAP/LYN/SNCA/GLIPR2/PAK1/SDCBP/RAC1/WNK1/PYCARD/MMP9/GPSM3/ADIPOR1/CYRIB/PIK3CD/PPP3CA/PLXNC1/MAP2K3/PTPRC/RHOG/MAP4K4/FBXO7/HMOX1/SP100/CORO1A/NF1/FOXO3/STK10/EPHB2/PTAFR/IQSEC1/CORO1C/WAS/ABI3/IRS2/DNAJA4/STAT3/MAPK3/DMTN/SFRP2/ITGB3/FLCN/MYLK/MSN/CNN2/PTEN/VCL/SPI1/RIPOR2/SLC8A1/PTP4A1/MPP1/FOXO4/PLVAP/TRIB1/RAPGEF2/TBXA2R/DAB2/GLUL/S100A11/CLDN5
BP	GO:0035295	tube development	572	-0.40	-1.83	1.00E-10	4.19E-09	2.57E-09	2685	tags=34%, list=23%, signal=27%	TRAF6/WNT1/RHOA/IL1B/VEZF1/BTG1/STIM1/TCTN1/SETD2/SIRT6/QKI/KIT/ANGPT1/ALOX5/TGFBI/RC3H2/RCN3/CSF1R/ACTG1/JAK1/CEBPA/MED12/HK2/FLNA/SERPINE2/KLF5/PLXNB2/ERBB2/SPATA2/CYBB/NFIA/KDM5B/CRISPLD2/BMPR1A/HIPK2/BAX/CCBE1/PRKCB/YIPF6/PIK3CB/CTSZ/TNF/ROCK2/IFT122/CASR/LRP1/ATP7A/NKX3-1/HIPK1/GPLD1/NRP2/PRKD2/MECP2/SGPL1/PRCP/ITGA2B/PKM/HHEX/PDGFA/FZD5/SEMA4A/SYK/ANXA2/ITGA5/PIK3CG/GADD45A/ATP6AP2/PTPN6/SP1/SAV1/CREB1/JAM3/EP300/STK4/HGF/TSC2/SLC12A6/GREM1/SOX4/MEIS1/YWHAZ/NIBAN2/MAP3K3/JUNB/SHC1/IFT140/FOXJ2/PTGS2/ROCK1/LRG1/NKIRAS2/ATRX/WASF2/MTHFR/SUFU/PSEN1/RARA/ZEB1/RIN2/EPHB4/EGLN1/CHI3L1/AGO2/ARL13B/C5AR1/PHACTR4/NR4A1/PTK2B/B4GALT1/HDAC7/GPX1/DVL3/KCNQ1/SKI/JMJD8/SPARC/ILK/VAV3/SRC/GRN/NIPBL/PF4/UBB/WARS1/PBX1/GAB1/HSPB1/CYP1B1/DLG5/ZFP36L1/VDR/MYO1E/PRKACA/PROK2/BRCA1/RBPJ/NFE2L2/SERPINE1/SASH1/SLC7A11/MYH9/HECA/TAL1/BAG6/MME/NOTCH4/ZMIZ1/APAF1/STK40/CTNNBIP1/EPN1/TGM2/CHD7/PAK1/ADM/TBC1D20/PIK3CD/SRPK2/MAPK14/HMOX1/SP100/NF1/ITPK1/EPHB2/VASP/AQP1/STAT3/MAPK3/SFRP2/CCDC39/ITGB3/FKBP8/MYLK/BSG/BASP1/HLX/PTEN/KRT1/SPI1/FOXO4/NINJ1/RAPGEF2/NPRL3/MICAL2/TBXA2R/FKBPL/ECE1/GLUL/AGO1/ODAD4/TGFA/E2F2/CLDN5

BP	GO:2000026	regulation of multicellular organismal development	775	-0.39	-1.80	1.00E-10	4.19E-09	2.57E-09	2708	tags=34%, list=23%, signal=28%	FES/FOS/CAPRIN1/TRPS1/DICER1/TRAF6/WNT1/RHOA/IL1B/CTDP1/REST/PLCB1/TMEM131L/ASPM/INPP5D/NEURL1/PRKCZ/VEZF1/BTG1/ANO6/STIM1/SOS2/ADNP/SIRT6/NCKAP1L/IL4I1/KIT/ACTR2/PPARGC1B/SEMA7A/ALOX5/GPR137B/SMARCD3/P2RX7/RC3H2/EVI2B/SPEN/HAP1/TRIM11/JAK1/KLF7/TSPO/HK2/UTP25/FADD/MITF/SERPINE2/PLXNB2/ERBB2/KIAA1109/YY1/CYBB/RTN4R/GFI1B/BMPR1A/LILRB2/HIPK2/ADD1/CCBE1/ACACB/SORL1/ZBTB16/PRKCB/PIK3CB/PAFAH1B1/TNF/NFKBIA/ROCK2/GJC2/RGS2/HIPK1/CDK5R1/PRKD2/MECP2/S1PR2/YWHAH/CFL1/PKM/PCM1/HHEX/SEMA4A/CTDSP1/EIF4G2/TMEM176B/KIAA0319/TWF2/SYK/CLPTM1/ZFPM1/CR1/ITGA5/ANKH/TNFRSF1A/GADD45A/PTPN6/RAB21/LAMA4/SP1/CDKN1C/TOB2/SAV1/CREB1/ATP2B1/CTR9/CCR1/PIM1/SOX6/DRAXIN/HGF/VSIR/HOOK3/RTN4/RFLNB/GREM1/DISC1/SOX4/MEIS1/NIBAN2/STAT5B/MTURN/CSF3R/SH3RF1/FOXJ2/KDM4A/NUMB/CDKL5/ROCK1/LRG1/ARNT/IST1/H4C8/HSPA1B/CCL3/GSK3A/PSEN1/RARA/MAPT/RGS14/ANKRD27/ZEB1/EGLN1/ULK1/CAPN3/INSR/B4GALT5/MAFG/CHI3L1/AGO2/AGRN/SMAP1/RASSF2/C5AR1/HSPA1A/CUX1/CTNNA1/BMP6/NLGN3/PTK2B/NDEL1/SLC46A2/NUPR1/TESC/CUL4A/PRDX2/LGALS9/LIN28A/SKI/JMJD8/NIN/SPARC/LIMK1/GRN/TBX21/NIPBL/PF4/DBN1/ASCL2/WARS1/DUSP6/GAB1/HSPB1/CYP1B1/RIPK1/MYCL/DLG5/ZFP36L1/VDR/IRF1/CLCF1/ZBTB7B/TNFRSF1B/BRCA1/RBPJ/JARID2/NFE2L2/SERPINE1/SASH1/PRELID1/TAL1/BAG6/POR/TRIP12/MME/NOTCH4/ZMIZ1/CCSAP/RCOR1/CTNNBIP1/LYN/EPN1/HCLS1/TGM2/CHD7/TYROBP/BMP2K/ADM/MMP9/SYNJ1/PITHD1/PIK3CD/PPP3CA/F11R/PLXNC1/CEBPB/PTPRC/FBXO7/MAPK14/HMOX1/SP100/NF1/FAXDC2/FOXO3/EPHB2/TFE3/PHOSPHO1/PGLYRP1/FAM210B/G6PD/AQP1/STAT3/PRKDC/SFRP2/MPL/AMBRA1/BASP1/HLX/NFAM1/PTEN/VCL/KRT1/SPI1/LILRB3/MBP/SLC8A1/FOXO4/RNF10/TRIB1/NINJ1/RAPGEF2/TBXA2R/FKBPL/NBR1/GLUL/AGO1/E2F2/CLDN5
BP	GO:0051270	regulation of cellular component movement	635	-0.39	-1.78	1.00E-10	4.19E-09	2.57E-09	3027	tags=37%, list=26%, signal=29%	EDN1/BCL6/ZNF580/ATOH8/ADAM15/RAP2C/FERMT1/RFFL/CEP43/TIAM1/BBS2/DAPK2/BCAS3/THBS1/PLAA/SRGAP2B/BST2/CLN3/ST3GAL4/SRF/MOSPD2/WDPCP/HIF1A/ADAM8/SHTN1/BORCS5/MAPK1/CD9/GNA13/ARHGAP18/TTBK2/FES/CPNE3/FPR2/RHOA/IL1B/PLCB1/ADAM17/ANO6/NEXN/JAK2/NCKAP1L/KIT/ANGPT1/SEMA7A/FAM89B/HSPA5/HAP1/OXSR1/CSF1R/C5AR2/ACTG1/CAMK2A/FEZ1/ARHGDIB/FADD/MITF/FLNA/SERPINE2/PLXNB2/FERMT3/SPATA13/BMPR1A/CCBE1/STAT5A/PIP5KL1/SORL1/RIPOR1/PIK3CB/TNF/ROCK2/BST1/ARAP1/PKN1/LRP1/ATP7A/GPLD1/NRP2/PRKD2/MECP2/MIIP/S1PR2/PRCP/MYADM/PDGFA/SEMA4A/MEAK7/RCC2/SYNE2/SRGAP1/CXCL16/ITGA5/PIK3CG/YTHDF3/GADD45A/RIN3/LAMA4/SP1/RNF20/LMNA/JAM3/RAC2/CCR1/CAMK1D/CHST2/STK4/TIRAP/HGF/VSIR/STK24/GNAI2/RTN4/LAMP1/GREM1/GPI/DUSP22/ACTA2/MAP3K3/NUMB/OSBPL8/PTGS2/ROCK1/DSC2/CCL3/PTPRJ/FGR/RIN2/FUT7/ZNF703/LGR6/INSR/SPAG9/ABHD2/AGO2/DIAPH1/NEDD9/LGALS3/C5AR1/TIMP1/DOCK8/CTNNA1/GNA12/PTK2B/ARHGEF2/HDAC7/SLK/TF/GFUS/LGALS9/DOCK5/CASS4/NTNG2/SPARC/ACTN1/SRC/APC/GRN/NIPBL/GAB1/HSPB1/PECAM1/AKAP9/CYP1B1/DUSP1/TNFSF14/IL6R/DLG5/SOD2/SELP/PADI2/AKT2/PLEKHG3/NFE2L2/SERPINE1/SASH1/TPM1/AFDN/ACTN4/CCSAP/LYN/GLIPR2/PAK1/SDCBP/RAC1/WNK1/PYCARD/MMP9/GPSM3/ADIPOR1/CYRIB/PIK3CD/PPP3CA/PLXNC1/MAP2K3/TRIM58/PTPRC/RHOG/MAP4K4/HMOX1/SP100/CORO1A/NF1/FOXO3/STK10/EPHB2/PTAFR/IQSEC1/CORO1C/WAS/ABI3/IRS2/DNAJA4/STAT3/MAPK3/DMTN/SFRP2/CCDC39/ITGB3/FLCN/MYLK/MSN/CNN2/PTEN/VCL/SPI1/RIPOR2/SLC8A1/PTP4A1/MPP1/FOXO4/PLVAP/TRIB1/RAPGEF2/TBXA2R/DAB2/GLUL/S100A11/CLDN5

CC	GO:0099081	supramolecular polymer	539	-0.39	-1.75	1.00E-10	4.19E-09	2.57E-09	2208	tags=30%, list=19%, signal=26%	RHOQ/NCKAP5L/NDE1/HNRNPU/DNM2/PAFAH1B1/MYO9B/MAP1LC3B/MYH10/KIF5B/KCNAB2/CDK5R1/CEP170/CLIP4/MYLPF/KIF1B/MARK2/FHL2/COTL1/GABARAPL1/RCC2/TUBGCP3/TWF2/KIF3C/SYNE2/RASSF1/MYH11/MAP2K2/DYNC2H1/KNSTRN/TUBB6/PPP1R12A/LMNA/JAM3/RAC2/TUBB4B/TTLL5/HOOK3/TUBA1A/MX2/CUL3/DISC1/CACNA1D/MAP1A/DUSP22/MTMR12/MTM1/ACTA2/ZW10/MAP1LC3A/BOD1L1/RASSF3/ALDOA/CLTC/ARF1/ENKD1/TPM3/DIAPH2/PSEN1/MAPT/RGS14/FKBP1A/PSTPIP2/KIF1C/CAPN3/INVS/TPGS2/REEP4/SQSTM1/PLK1/PDLIM2/RASSF5/GAS7/DIAPH1/MAP4/PSTPIP1/DPP9/CTTN/SPECC1/ESPN/GAS2L3/DNM3/SYNPO/ARHGEF2/NDEL1/HCK/TUBB1/WIPF2/PPP2R5A/NIN/CHMP1A/PALLD/ILK/ACTN1/SRC/APC/DPYSL2/MYL4/RMDN2/DBN1/TUBB2A/GABARAP/PDLIM5/TPPP3/HSPB1/KLC3/FSD1/ABCC9/GAS2L1/ACTB/CALM3/MYO5A/SVIL/PARVB/AK1/VPS18/TUBA1C/TPM1/GOLGA2/MYH9/PPP1R12B/NAV1/LMOD3/AKNA/ACTN4/CCSAP/TPM4/HCLS1/SNCA/PAK1/RAC1/SHROOM1/XIAP/SYNJ1/WIPF1/TUBA4A/PPP3CA/RTN2/MAP1S/LCP1/MYOM1/TMOD1/DNAJB6/MAP1LC3B2/CORO1A/CHMP2A/CORO1C/SLC4A1/WAS/DMTN/PDLIM7/ANK1/MYL9/RAB3D/VCL/KRT1/CHMP4B/SLC8A1/IQGAP1/NBR1/MYZAP/KIF27/KIF13A/ARHGAP6
CC	GO:0005768	endosome	749	-0.39	-1.77	1.00E-10	4.19E-09	2.57E-09	2637	tags=32%, list=22%, signal=27%	WLS/USP8/PRKCZ/VTI1B/SCARB2/LPAR1/PLEKHM2/JAK2/YIPF1/HPS6/PHETA1/RAB32/WIPI1/LAMTOR4/VPS28/CLN6/TICAM1/CCDC120/EPS15/HAP1/STEAP4/JAK1/RAB35/SNX1/OPTN/STOML1/SNX14/PRAF2/ERBB2/ABCA2/TMEM150B/LRRK2/GRB2/CLIP1/IFITM3/RFTN1/CHMP3/WDFY2/TPCN2/ADPRH/SORL1/PLEKHM1/TAB2/FCGRT/DNM2/ZDHHC2/TNF/VPS37B/CPTP/PI4K2A/PKN1/SNX8/LRP1/ATP7A/GGA3/TLR8/ITM2B/UBAP1/SLC39A4/LRPAP1/SLC11A1/YIPF2/FZD5/TBC1D5/MCOLN1/MFSD12/RCC2/SLC31A1/KIAA0319/SPG21/PIP5K1C/ANXA2/TMEM127/MAP2K2/HLA-E/STX6/BIRC6/ITSN1/SLC66A2/SLC6A9/RAB22A/ATP6AP2/RAB21/RIN3/SLC15A3/TRAK1/RNF11/VPS33A/GRIPAP1/LAMP2/MICALL1/TMBIM1/ABCB11/RAB11FIP2/AGAP2/RUBCN/NCF4/AP2M1/LAMP1/TUBA1A/RAB37/RAB3B/SNX11/MTM1/GIGYF2/CD14/EEA1/ATP6V0A1/MAP1LC3A/FFAR4/CLTC/TBC1D14/NUMB/WASHC2C/UHRF1BP1L/AOC3/ZFYVE16/IRAK1/DIAPH2/WASF2/SYTL4/PSEN1/ANKRD27/IGF2R/UEVLD/VPS11/MON1B/ULK1/CD63/INSR/SQSTM1/PTPN1/SCAMP4/ATP6V0D1/RAB27B/RAP1GAP/PTP4A3/ABHD17C/ARL8A/VAMP3/CLEC16A/ANKRD13A/RAB11B/IFNAR1/CTSD/AP2B1/VPS4B/TF/ELAPOR1/RILP/RAB27A/KCNQ1/ANTXR2/ARPC5/CHMP1A/TGOLN2/SIAH2/SNX10/PLEKHB2/SRC/MYO1D/GRN/AVL9/RAB7A/UBB/VPS37C/PSAP/FCHSD1/FLOT1/SLC29A3/RIPK1/STAM2/MYO5A/RAB11FIP4/SNX27/AKT2/EHD1/VPS18/RABGAP1L/CUBN/AP2A1/MARCHF2/SH3GL1/ARHGAP1/RAB5B/MMGT1/TMEM184A/MME/SLA/AP1G1/ACKR1/GPR107/UBA1/ABCG1/MMD/USP10/EPN1/UNC13D/RAC1/ARRB2/UBXN6/RAB11FIP1/UBE2D3/EHD3/DTX3L/PICALM/SLC48A1/CTSB/CORO1A/CHMP2A/CORO1C/STS/RAB5C/RHOBTB1/RAB5A/MARCHF8/MAPK3/SBF2/IFITM2/SNX18/PRKAR1A/TRAK2/PRKCD/CD68/BSG/RAB3D/CYTIP/RAB31/CHMP4B/PTP4A1/FLOT2/RAPGEF2/DYSF/ECE1/FURIN/NBR1/LITAF/KIF13A/OSBPL1A/PLIN3

CC	GO:0043005	neuron projection	736	-0.38	-1.77	1.00E-10	4.19E-09	2.57E-09	2415	tags=30%, list=21%, signal=26%	INPP5A/VIM/ACTG1/CACNA1I/CAMK2A/FEZ1/COPA/ALCAM/PDE6A/SNX14/FADD/FLNA/MYO9A/CKB/LRRK2/PPT1/CYBB/RTN4R/TBC1D24/GSK3B/BMPR1A/SYAP1/MAGEE1/PRKCB/HNRNPU/DNM2/PAFAH1B1/GJC2/PI4K2A/IFT122/ABAT/CASR/KIF5B/ATP7A/KCNAB2/RGS2/CDK5R1/NRP2/PTGS1/GRIK2/KIF1B/CPEB3/MARK2/SPTA1/FZD5/EIF4G2/HRH2/TWF2/NECAB2/ATCAY/ABR/ITSN1/HTR3B/ATP6AP2/RAB21/RIN3/SSTR2/CPEB4/EXOC7/GRK4/APBA1/TRAK1/DLG4/GRIPAP1/SAV1/ATP2B1/FSCN1/CBL/FSCN3/ERO1A/NCAM1/NCF1/RNF40/GNAI2/STX3/CNIH3/SLC6A12/DTNB/GPI/DISC1/MAP1A/PVALB/INPP5K/AP3S2/GIGYF2/EEA1/SNAP25/TRIOBP/ROGDI/GNB1/IFT140/SH2D3C/ARF1/CDKL5/PTGS2/VCAN/GSK3A/PSEN1/RARA/MAPT/RGS14/ANKRD27/CLU/EPHB4/PRR7/KIF1C/ULK1/INSR/PRR12/SIPA1L1/MAX/ATP6V0D1/AGTPBP1/AGO2/RAB27B/MAP4/RAP1GAP/PPP1R9B/WDFY3/ABHD17C/CTTN/ARL8A/ESPN/VAMP3/SLC6A6/EXOC6/GNA12/DNM3/MARK4/GNAQ/PTK2B/SYNPO/NDEL1/PTBP2/RAB27A/KCNQ1/NIN/ARPC5/NTNG2/PALLD/LIMK1/ACTN1/SIAH2/SRC/MYO1D/TMEM266/DBN1/UBB/KPTN/EXOC4/HSPB1/KLC3/PIP4K2A/KCNE3/SETX/AKAP9/MARK3/P2RY1/ACTB/CALM3/MYO5A/PRKACA/FXR2/TNFRSF1B/FEZ2/KPNA1/CTNND1/P2RX1/NAV1/RPGR/MME/ACTN4/SLA/CCSAP/GLRX5/SHARPIN/ERMN/USP9X/SNCA/PAK1/RAC1/ARRB2/SH3KBP1/SYNJ1/PPP3CA/OPRL1/FBXO7/MAP1S/ASAP1/PPFIA1/PRKAR2B/CORO1A/NF1/EPHB2/STON2/TPRG1L/LRRC4/OSBP2/ABI3/RAB5A/MTPN/SBF2/TRAK2/NRGN/MPL/PINK1/BSG/BASP1/ANK1/PTEN/MBP/RIPOR2/SLC8A1/FLOT2/MPP1/RAPGEF2/IQGAP1/CASC3/STAU1/TSHZ3/GLUL/CLDN5/GNAZ
CC	GO:0005773	vacuole	658	-0.38	-1.72	1.00E-10	4.19E-09	2.57E-09	2550	tags=32%, list=22%, signal=26%	SNAP23/HPS6/IL4I1/ACTR2/PSEN2/GCA/GNB2/WIPI1/LAMTOR4/GPR137B/TICAM1/HAP1/TMEM79/TECPR1/SNX1/OPTN/ZFYVE26/TRIM21/SNX14/ABCA2/TMEM150B/MAN2B1/LRRK2/PPT1/IFITM3/CHMP3/TPCN2/ADPRH/PLEKHM1/CTSZ/MAP1LC3B/RAB2A/FPR1/PI4K2A/MNDA/SLCO4C1/LRP1/KIF5B/SDC3/ACLY/GGA3/TLR8/GPLD1/GABARAPL2/MYLPF/ATP6V1C1/PIP4K2C/DNASE2/PRCP/CPQ/CALCOCO2/AP1M1/SLC11A1/TBC1D5/MANBA/MCOLN1/MEAK7/MFSD12/GABARAPL1/ANXA2/FNBP1/NPC2/ATP6AP2/SLC15A3/VPS33A/RB1CC1/TP53INP2/LAMP2/PSMD1/TUBB4B/LRRC8A/TMBIM1/SIAE/NCF1/TSC2/RUBCN/NCF4/STX3/AP2M1/CPNE1/LAMP1/ARSG/RAB37/CKAP4/SIDT2/ATP6V0A1/MAP1LC3A/FFAR4/CSF3R/GNB1/IMPDH1/CLTC/TFEB/TBC1D14/VCAN/IST1/BRI3/SLC44A2/SNAP29/YWHAB/PSEN1/ANKRD27/VLDLR/VPS11/ULK1/CD63/INSR/SQSTM1/SPAG9/RNF19B/ATP6V0D1/NEU1/AGRN/ANXA11/WDFY3/ARL8A/CLEC16A/RNASET2/GNAQ/IFNAR1/CXCR2/CTSD/B4GALT1/VCP/HCK/SLC17A5/AP2B1/ELAPOR1/CYB5R3/RILP/RAB27A/KCNQ1/CHMP1A/SRC/ACP3/GRN/RAB7A/GABARAP/KPTN/GLB1L/PGAP6/PSAP/PIP4K2A/FLOT1/SLC29A3/STXBP2/PADI2/MYO5A/SULT1C2/SH3GLB1/SHKBP1/VPS18/CUBN/AP2A1/MARCHF2/NCF2/AP1G1/TRAPPC1/LAPTM5/UBA1/LYN/MMD/ATP6V1B2/UNC13D/SNCA/DNAJC5/SDCBP/PYCARD/UBXN6/BBC3/GNS/NCOA4/DTX3L/RRAGD/SLC48A1/CTSB/MAP1LC3B2/CHMP2A/AP1B1/STS/HPS1/PRDX6/RAB5C/TOLLIP/MARCHF8/FTL/SBF2/IFITM2/GNB4/NRBF2/FLCN/FTH1/AMBRA1/PRKCD/CD68/RAB3D/NFAM1/CAPN1/CHMP4B/NPRL3/AP5B1/ECE1/NBR1/DAB2/LITAF/SLC7A5/CPPED1/ATP6V1A

BP	GO:0071495	cellular response to endogenous stimulus	794	-0.37	-1.70	1.00E-10	4.19E-09	2.57E-09	2697	tags=33%, list=23%, signal=27%	FOS/FPR2/WNT1/RHOA/PIK3R2/ACSL1/IL1B/CACNA1A/LPIN2/RAB8A/REST/PLCB1/USP8/ADAM17/NEURL1/PRKCZ/PBLD/JAK2/ZEB2/CNOT1/CACNB1/CASP9/KIT/ACTR2/PPARGC1B/SLC9A1/SIN3A/LAMTOR4/FAM89B/HSPA5/P2RX7/CCND3/PAQR8/VIM/TAF1/AGTRAP/JAK1/RAB35/LDOC1/GALNT3/CAMK2A/AHCYL1/LTBP1/SMARCC1/FLNA/ERBB2/CA2/LRRK2/CYSLTR2/GRB2/CYBB/KDM5B/GSK3B/RNF14/ARID5A/BMPR1A/HIPK2/RHOQ/STAT5A/NCOR1/SYAP1/SORL1/PRKCB/HNRNPU/DNM2/TNF/PHIP/ROCK2/PKN1/ARID1A/CASR/LRP1/MAS1/ATP7A/NKX3-1/GPLD1/UCP3/PRKD2/UCP2/PIP4K2C/ITPR2/RNF165/PRCP/YWHAH/CPEB3/PKM/PDGFA/HRH2/HTR3B/PIK3CG/KNSTRN/SSTR2/CPEB4/SOGA1/UBR5/SP1/CDKN1C/IL17RD/ATP2B1/EP300/GCLM/SULF2/CBL/SOX6/TSC2/GNAI2/GREM1/DUSP22/TLR6/INPP5K/MSX1/CHST11/STAT5B/PID1/SHC1/PDK2/GNB1/NR4A2/OSBPL8/DSTYK/PTGS2/ROCK1/LRG1/SESN2/HSPA1B/GSK3A/PSEN1/RARA/MAPT/RELA/GOT1/ZEB1/FKBP1A/TRIM33/VPS11/FUT7/SOCS3/ZNF703/AQP9/PTPRE/INSR/PTPN1/SESN3/ABHD2/MAX/NR3C1/DIAPH1/RAP1GAP/PXN/PPP1R9B/HSPA1A/NR4A1/MBD5/CTNNA1/BMP6/ARHGEF2/NDEL1/ZDHHC7/EGLN2/KCNQ1/SKI/EIF4EBP2/SPRY3/ILK/SRC/APC/SHOC2/NCOA2/ZNF106/PAK2/GAB1/TGFB1I1/LIMS1/PIP4K2A/SETX/AKAP9/CYP1B1/GAS2L1/P2RY1/UBE2O/ACTB/ZFP36L1/PADI2/GNAL/MYO5A/ZBTB7A/CARM1/PRKACA/AKT2/RXRA/EHD1/CASTOR2/ZBTB7B/PTPN12/BRCA1/RBPJ/KLF3/VPS18/NFE2L2/UBR2/NDST1/ITGB5/PELO/SSH1/NRROS/POR/ZMIZ1/APAF1/LYN/USP9X/SNCA/TGM2/IGF2/PAK1/SDCBP/ARRB2/XIAP/ADIPOR1/CREBBP/UBE2D3/NCOA4/CFLAR/RRAGD/CEBPB/STAT6/FBP1/CTSB/NCOA1/CORO1A/FOXO3/EPHB2/PTAFR/WBP2/IRS2/AQP1/STAT3/MAPK3/DMTN/PRKDC/SFRP2/PCSK6/FLCN/FKBP8/PRKCD/BCL2L1/PPM1A/WDTC1/RAB31/SPI1/LTBP2/SLC8A1/FOXO4/ZYX/RAPGEF2/IQGAP1/FURIN/DAB2/KLF1/CLDN5/GNAZ
BP	GO:0099177	regulation of trans-synaptic signaling	220	-0.50	-2.08	1.09E-10	4.47E-09	2.75E-09	2896	tags=45%, list=25%, signal=34%	PRKAR1B/CLN3/SRF/KRAS/BEGAIN/MAPK1/PACSIN2/CRTC1/IL1B/CACNA1A/RAB8A/PLCB1/USP8/NEURL1/PRKCZ/NEUROD2/ADNP/JAK2/KIT/HAP1/CD38/CAMK2A/SERPINE2/CA2/LRRK2/ZDHHC12/GSK3B/LILRB2/PLCL2/FBXL20/SYAP1/PRKCB/ZDHHC2/TNF/KIF5B/MECP2/S1PR2/GRIK2/YWHAH/STXBP5/CPEB3/ABR/APBA1/DLG4/SYN3/GNAI2/STX3/DISC1/MAP1A/SNAP25/ARF1/NPTXR/PTGS2/ZZEF1/MCTP2/PSEN1/RARA/MAPT/RGS14/SQSTM1/SIPA1L1/PPP1R9B/SLC6A6/NLGN3/PTK2B/SYT12/EIF4EBP2/NTNG2/SRC/DBN1/AKAP9/FLOT1/P2RY1/CALM3/PRKACA/VPS18/P2RX1/SLC7A11/MME/SNCA/TYROBP/ARRB2/PPP3CA/PRKAR2B/IQSEC2/NF1/EPHB2/TPRG1L/LRRC4/RAB5A/NRGN/PINK1/ZDHHC3/PTEN/RAPGEF2/STAU1/TSHZ3/GLUL
BP	GO:0045055	regulated exocytosis	150	-0.55	-2.18	1.26E-10	5.12E-09	3.15E-09	2708	tags=46%, list=23%, signal=36%	FES/REST/CD177/NCKAP1L/SNAP23/KIT/P2RX7/TMEM79/CACNA1I/RPH3AL/LRRK2/GSK3B/FBXL20/PRKCB/PRAM1/ITGAM/ABCC4/STXBP5/SYK/PIP5K1C/PIK3CG/APBA1/RAC2/CBL/RAB11FIP2/GNAI2/STX3/LAMP1/SNAP25/CTBP2/SNAP29/CCL3/PSEN1/FGR/ITGB2/VAMP3/RAB11B/HCK/SYT12/LGALS9/RAB27A/STX11/LAT2/P2RY1/CALM3/STXBP2/VPS18/P2RX1/MYH9/PTGDS/AP1G1/LYN/UNC13D/SNCA/DNAJC5/PLEK/RAB11FIP1/SYNJ1/PIK3CD/HMOX1/CORO1A/PTAFR/RAB5A/DMTN/NAPA/RAB3D/RAB31/SPI1/GAB2

BP	GO:0001932	regulation of protein phosphorylation	706	-0.37	-1.68	1.66E-10	6.67E-09	4.10E-09	2233	tags=32%, list=19%, signal=28%	FPR2/TRAF6/WNT1/RHOA/ACSL1/IL1B/PRKAG2/HEXIM2/DNAJC3/ADAM17/NEURL1/PRKCZ/ADNP/EIF4G1/ERN1/JAK2/NCKAP1L/KIT/ANGPT1/SEMA7A/P2RX7/CCND3/CSF1R/DDX3X/SH3BP5/LCP2/CEBPA/INCENP/PLXNB2/ERBB2/LRRK2/BMPR1A/WDFY2/MLLT1/CTDSP2/HIPK2/PLCL2/BAX/PIP5KL1/SYAP1/SORL1/TAB2/PIK3CB/TNF/PHIP/ROCK2/BST1/TNXB/GJC2/PLCE1/PKN1/LRP1/MAS1/NKX3-1/RGS2/TLR8/CDK5R1/PRKAR2A/PRKD2/S1PR2/RICTOR/ERN2/OSM/MARK2/SLC11A1/MYADM/HHEX/PDGFA/FZD5/CTDSP1/AKAP13/TAOK1/SYK/SLCO3A1/MAP2K2/UBE2B/ITGA5/PIK3CG/TNFRSF1A/GADD45A/PTPN6/CDKN1C/DLG4/IRAK3/RB1CC1/CDKN2D/CBL/STK4/AGAP2/TIRAP/NCF1/HGF/TSC2/PDE5A/GREM1/DUSP22/TLR6/INPP5K/PID1/SHC1/SH2D3C/OSBPL8/MAVS/PTGS2/PARP14/RPS6KA5/IRAK1/SESN2/CCNY/PSEN1/RGS14/PTPRJ/FGR/VLDLR/CLU/ITGB2/SOCS3/INSR/SQSTM1/SPAG9/PTPN1/ADAR/PLK1/CHI3L1/CCNF/MAP3K5/NEDD9/AKT1S1/RASSF2/PPP1R9B/C5AR1/RALB/HMGCR/BMP6/GNAQ/PTK2B/ARHGEF2/MAD2L2/PPP2R5B/SIRPA/DVL3/CDKN1A/PARP16/MAP2K7/CASS4/HIPK3/SPRY3/ILK/TAOK3/SRC/APC/WARS1/DUSP6/PAK2/PIK3R5/HSPB1/PECAM1/AKAP9/FLOT1/EIF2AK1/DUSP1/RIPK1/P2RY1/IL6R/ACTB/CALM3/DAZAP2/PLAUR/AKT2/FXR2/CLCF1/CCNI/CNPPD1/SASH1/TAL1/SH3BP5L/BRD4/CHP1/LYN/MMD/HCLS1/SNCA/IGF2/STRADA/PAK1/SDCBP/RAC1/WNK1/PYCARD/ARRB2/MMP9/LIMK2/MAP2K3/PTPRC/FBXO7/STK38/PRKAR2B/NF1/FAXDC2/EPHB2/CORO1C/NIBAN1/CACUL1/MOB3A/STRADB/MAPK3/DMTN/PRKDC/SFRP2/RALBP1/ITGB3/PRKAR1A/FLCN/FKBP8/PRKCD/PINK1/ROPN1L/CCNJL/PTEN/MOB3C/PRR5/SLC8A1/PELI2/TRIB1/RAPGEF2/IQGAP1/DAB2/CTDSPL/TGFA
CC	GO:0030863	cortical cytoskeleton	70	-0.66	-2.35	1.71E-10	6.80E-09	4.18E-09	1923	tags=49%, list=16%, signal=41%	MYADM/SPTA1/COTL1/AKAP13/DLG4/PVALB/PPP1R9B/CTTN/ACTN1/DBN1/FCHSD1/FLOT1/ACTB/MYO1E/MYH9/EPB41/ACTN4/LASP1/TPM4/HCLS1/SHROOM1/EPB42/GYPC/TMOD1/CORO1A/SLC4A1/DMTN/GSN/SPTB/FLOT2/MPP1/IQGAP1/MYZAP/CLDN5
CC	GO:0099512	supramolecular fiber	538	-0.39	-1.76	1.81E-10	7.13E-09	4.38E-09	2208	tags=30%, list=19%, signal=26%	RHOQ/NCKAP5L/NDE1/HNRNPU/DNM2/PAFAH1B1/MYO9B/MAP1LC3B/MYH10/KIF5B/KCNAB2/CDK5R1/CEP170/CLIP4/MYLPF/KIF1B/MARK2/FHL2/COTL1/GABARAPL1/RCC2/TUBGCP3/TWF2/KIF3C/SYNE2/RASSF1/MYH11/MAP2K2/DYNC2H1/KNSTRN/TUBB6/PPP1R12A/LMNA/JAM3/RAC2/TUBB4B/TTLL5/HOOK3/TUBA1A/MX2/CUL3/DISC1/CACNA1D/MAP1A/DUSP22/MTMR12/MTM1/ACTA2/ZW10/MAP1LC3A/BOD1L1/RASSF3/ALDOA/CLTC/ARF1/ENKD1/TPM3/DIAPH2/PSEN1/MAPT/RGS14/FKBP1A/PSTPIP2/KIF1C/CAPN3/INVS/TPGS2/REEP4/SQSTM1/PLK1/PDLIM2/RASSF5/GAS7/DIAPH1/MAP4/PSTPIP1/DPP9/CTTN/SPECC1/ESPN/GAS2L3/DNM3/SYNPO/ARHGEF2/NDEL1/HCK/TUBB1/WIPF2/PPP2R5A/NIN/CHMP1A/PALLD/ILK/ACTN1/SRC/APC/DPYSL2/MYL4/RMDN2/DBN1/TUBB2A/GABARAP/PDLIM5/TPPP3/HSPB1/KLC3/FSD1/ABCC9/GAS2L1/ACTB/CALM3/MYO5A/SVIL/PARVB/AK1/VPS18/TUBA1C/TPM1/GOLGA2/MYH9/PPP1R12B/NAV1/LMOD3/AKNA/ACTN4/CCSAP/TPM4/HCLS1/SNCA/PAK1/RAC1/SHROOM1/XIAP/SYNJ1/WIPF1/TUBA4A/PPP3CA/RTN2/MAP1S/LCP1/MYOM1/TMOD1/DNAJB6/MAP1LC3B2/CORO1A/CHMP2A/CORO1C/SLC4A1/WAS/DMTN/PDLIM7/ANK1/MYL9/RAB3D/VCL/KRT1/CHMP4B/SLC8A1/IQGAP1/NBR1/MYZAP/KIF27/KIF13A/ARHGAP6

BP	GO:0071363	cellular response to growth factor stimulus	373	-0.43	-1.87	2.07E-10	8.05E-09	4.95E-09	2707	tags=35%, list=23%, signal=27%	CPNE3/FOS/WNT1/IL1B/USP8/ADAM17/ERN1/PBLD/ZEB2/FAM89B/HSPA5/HAP1/RAB35/GALNT3/FEZ1/LTBP1/ERBB2/KDM5B/BMPR1A/HIPK2/CCBE1/SYAP1/SORL1/PRKCB/PIK3CB/DNM2/CASR/ATP7A/NRP2/PRKD2/RNF165/HHEX/PDGFA/TWF2/ITGA5/KNSTRN/CDKN1C/IL17RD/CREB1/EP300/GCLM/SULF2/CBL/SOX6/HGF/GREM1/DUSP22/INPP5K/MSX1/CHST11/NIBAN2/STAT5B/SHC1/DSTYK/LRG1/ARNT/MAPT/RELA/ZEB1/FKBP1A/TRIM33/ZNF703/CD63/INSR/PTPN1/NR3C1/RAP1GAP/PTP4A3/AKT1S1/PXN/PPP1R9B/HSPA1A/NR4A1/BMP6/PPP2R5B/SKI/SPRY3/ILK/SRC/SHOC2/MAPKAPK2/PAK2/GAB1/TGFB1I1/LIMS1/HSPB1/SETX/RIPK1/UBE2O/ZFP36L1/ZBTB7A/EHD1/PTPN12/TNFRSF1B/RBPJ/NDST1/ITGB5/PELO/NRROS/ZMIZ1/APAF1/USP9X/SDCBP/ARRB2/XIAP/CREBBP/PIK3CD/UBE2D3/CFLAR/MAP2K3/MAPK14/CORO1A/FOXO3/STAT3/MAPK3/SFRP2/ITGB3/PCSK6/FLCN/FKBP8/PPM1A/SPI1/LTBP2/ZYX/RAPGEF2/IQGAP1/FURIN/DAB2/CLDN5
BP	GO:0034330	cell junction organization	354	-0.43	-1.89	2.19E-10	8.45E-09	5.19E-09	2982	tags=37%, list=25%, signal=29%	CSK/TUBB/BCAS3/THBS1/TAOK2/CLN3/SRF/WDPCP/SETD5/CD9/FARP1/TLR2/CAPRIN1/RHOA/IL1B/REST/NEURL1/CD177/NEUROD2/ADNP/EIF4G1/CACNB1/ACTR2/SLC9A1/C1QA/CSF1R/ACTG1/PKP4/FLNA/PLXNB2/MYO9A/ERBB2/CHCHD10/LRRK2/PPFIBP2/ZDHHC12/NFIA/LILRB2/ADD1/TESK2/ZDHHC2/PAFAH1B1/TNF/ROCK2/HIPK1/CDK5R1/NRP2/MECP2/ITGAM/CFL1/FZD5/SEMA4A/RCC2/PIP5K1C/GPBAR1/ITGA5/DLG4/GRIPAP1/JAM3/FSCN1/CAST/GREM1/TUBA1A/DISC1/DUSP22/YWHAZ/ARF1/CTBP2/NUMB/CDKL5/ROCK1/WASF2/PSEN1/MAPT/PTPRJ/ZNF703/INSR/SIPA1L1/AGRN/ADGRF1/C5AR1/ABHD17C/CTTN/CTNNA1/DNM3/BMP6/NLGN3/PTK2B/SYNPO/VCP/HDAC7/SLK/NTNG2/ACTN1/SRC/APC/DBN1/PDLIM5/PAK2/CACNB4/ESAM/FBXO45/LIMS1/PECAM1/FLOT1/ACTB/DLG5/PRKACA/CTNND1/SLC7A11/AFDN/SNCA/SDCBP/RAC1/F11R/MAP4K4/PPFIA1/MAPK14/EPHB2/IQSEC1/CORO1C/LRRC4/ABI3/TLN1/DMTN/FLCN/PTEN/VCL/RAPGEF2/STAU1/ARHGAP6/CLDN5
BP	GO:0032989	cellular component morphogenesis	418	-0.41	-1.83	2.22E-10	8.48E-09	5.21E-09	2778	tags=36%, list=24%, signal=29%	SHTN1/CD9/PACSIN2/PIKFYVE/FARP1/TLR2/CAPRIN1/DICER1/RHOA/RAB8A/RAPH1/PRKCZ/LYPLA2/NEXN/TCTN1/ADNP/NCKAP1L/FIS1/ACTR2/SCN1B/SEMA7A/SIN3A/CSF1R/HECW2/ACTG1/KLF7/SNX1/FEZ1/ALCAM/PLXNB2/MYO9A/ERBB2/ABCA2/LRRK2/NECTIN2/RTN4R/GSK3B/BAX/PLEKHO1/KNL1/DNM2/PAFAH1B1/EFNB1/KIF5B/ATP7A/CDK5R1/NRP2/ERCC2/RNF165/YWHAH/CFL1/MARK2/SEMA4A/CCDC136/CERT1/AKAP13/EIF4G2/TAOK1/KIAA0319/TWF2/MYH11/WEE1/RAB21/DLG4/CREB1/RAC2/RERE/NCAM1/DRAXIN/RTN4/CPNE1/DISC1/MAP1A/MTM1/NIBAN2/PID1/NUMB/NR4A2/CDKL5/ROCK1/IST1/RPS6KA5/WASF2/PSEN1/MAPT/ANKRD27/VLDLR/EPHB4/ULK1/CAPN3/LGR6/B4GALT5/SPAG9/SIPA1L1/SLC23A2/GAS7/CUX1/CTTN/DNM3/NLGN3/NDEL1/DVL3/NIN/NTNG2/PALLD/APLP2/SPRY3/LIMK1/TAOK3/SRC/DPYSL2/DBN1/UBB/XK/PDLIM5/PAK2/FBXO45/RFX2/FLOT1/ACTB/FEZ2/TPM1/LMOD3/USP9X/PAK1/RAC1/TBC1D20/PPP3CA/CFLAR/PLXNC1/PICALM/RHOG/MAP4K4/MAP1S/TMOD1/EPHB2/CORO1C/ABI3/VASP/PRKAR1A/KEL/BCL2L1/BSG/MYL9/PTEN/VCL/MBP/RAPGEF2/IQGAP1/ECE1/ACRBP

MF	GO:0004672	protein kinase activity	407	-0.42	-1.84	2.95E-10	1.12E-08	6.88E-09	3091	tags=40%, list=26%, signal=31%	MKNK1/CLK3/MARK1/MAP3K1/CCL5/STK32C/NEK4/EPHB6/TYK2/CSK/GRK2/DAPK2/STK19/TAOK2/CAMKK2/PIM3/TBCK/STK38L/PDK4/CSNK1A1/RSKR/MAPK1/UHMK1/PIKFYVE/TTBK2/FES/CPNE3/MAP2K4/PRKAG2/PRKCZ/ERN1/CSNK2A1/JAK2/DAPK1/DYRK1B/KIT/NEK6/DYRK1A/CSNK1G2/CCND3/OXSR1/CSF1R/TAF1/JAK1/CAMK2A/LTBP1/ERBB2/LRRK2/GSK3B/BMPR1A/HIPK2/TESK2/PRKCB/ROCK2/DYRK3/PKN1/HIPK1/CDK5R1/NRP2/PRKD2/ERN2/MARK2/CDK13/SNRK/AKAP13/TAOK1/MAST3/SYK/MAP2K2/SIK3/GAK/PIK3CG/WEE1/PPM1D/GRK4/IRAK3/CAMK1D/STK4/PIM1/EIF2AK2/RNASEL/STK24/MKNK2/MAP3K3/GRK6/PDK2/CDC42BPA/DSTYK/CDKL5/ROCK1/RPS6KA5/IRAK1/CCL3/GSK3A/FGR/IGF2R/EPHB4/ULK1/INSR/SQSTM1/VRK3/MAP3K20/PLK1/MAP3K5/RASSF2/PPP1R9B/BCKDK/MARK4/PTK2B/RIOK3/HCK/NEK5/NEK9/SLK/RPS6KA1/CDKN1A/MAP2K7/HIPK3/CSNK1G1/LIMK1/ILK/TAOK3/SRC/ALPK1/MAPKAPK2/PAK2/EIF2AK1/RIPK1/MARK3/PRKACA/AKT2/MAPKAPK3/AATK/SCYL1/BRD4/MAST2/STK40/LYN/MMD/GRK3/PAK1/WNK1/BMP2K/CDK19/MAP3K2/LIMK2/MAP2K3/MAP4K4/SRPK2/STK38/MAPK14/BMX/PDK3/STK10/EPHB2/TOP1/MAPK3/PRKDC/MYLK/PRKCD/PINK1/NUAK2/PEAK3
CC	GO:0005911	cell-cell junction	287	-0.46	-1.95	3.21E-10	1.20E-08	7.40E-09	2631	tags=35%, list=22%, signal=28%	ADAM17/PRKCZ/SLC2A1/NEXN/CDC42EP4/SNAP23/KIT/SCN1B/SLC9A1/SHROOM4/P2RX7/ACTG1/LCP2/PKP4/FLNA/GRB2/NECTIN2/ADD1/NPHS1/GJC2/SYMPK/YWHAH/MYADM/FZD5/EIF4G2/PIP5K1C/ANXA2/MAP2K2/TSPAN33/ITGA5/PTPN6/FHOD1/SAV1/DSG3/JAM3/FSCN1/TMEM65/ABCB11/STX3/NIBAN2/CDC42BPA/DSC2/WASF2/PTPRJ/PCDH1/DNMBP/PDLIM2/VAV1/PXN/PPP1R9B/CTNNA1/SYNPO/ARHGEF2/B4GALT1/ACTN1/APC/DBN1/PDLIM5/PAK2/GAB1/ESAM/LIMS1/PECAM1/FLOT1/ACTB/MYO1E/CTNND1/MYH9/AFDN/UBA1/LYN/PAK1/SDCBP/SHROOM1/PANX2/SH3KBP1/PPP3CA/F11R/CORO1A/WAS/YBX3/VASP/TLN1/PDLIM7/UBN1/ITGB3/FLCN/PRKCD/MSN/FRMD4B/CNN2/VCL/SLC8A1/FLOT2/MPP1/ZYX/RAPGEF2/NHS/IQGAP1/S100A11/CLDN5
BP	GO:0006898	receptor-mediated endocytosis	172	-0.52	-2.11	3.70E-10	1.38E-08	8.46E-09	3275	tags=54%, list=28%, signal=40%	PLCG2/MARCO/DNAJC13/SCYL2/TNK2/FCHO1/ITSN2/AP2S1/ARRB1/TFR2/FCHSD2/GRK2/CLN3/SCARF1/CD9/PIKFYVE/FPR2/SCARB2/ANGPT1/FCER1G/EPS15/SNX1/ABCA2/PPT1/GRB2/TPCN2/SORL1/PIK3CB/DNM2/GTF2H2/LRP1/ITGAM/LRPAP1/TBC1D5/SYK/PIP5K1C/ANXA2/NECAB2/CXCL16/ITSN1/GAK/RAB21/RIN3/GRK4/DLG4/CLEC9A/CBL/MICALL1/AP2M1/GREM1/CD14/SNAP25/MKLN1/CLTC/ARF1/NUMB/VLDLR/CLU/IGF2R/ITGB2/CD63/INSR/SMAP1/CTTN/ANKRD13A/DNM3/NLGN3/CXCR2/AP2B1/TF/CXCR1/FLOT1/CUBN/SERPINE1/AP2A1/AP1G1/GPR107/GRK3/SNCA/HNRNPK/SDCBP/RAC1/BMP2K/ADM/ARRB2/SYNJ1/PICALM/RAB5A/PDLIM7/ITGB3/RAB31/DAB2/HIP1

BP	GO:1901699	cellular response to nitrogen compound	436	-0.41	-1.79	3.88E-10	1.42E-08	8.70E-09	2696	tags=37%, list=23%, signal=30%	FPR2/WNT1/PIK3R2/IL1B/CACNA1A/LPIN2/RAB8A/PLCB1/NEURL1/PRKCZ/JAK2/CACNB1/ACTR2/SLC9A1/SIN3A/LAMTOR4/HSPA5/P2RX7/CCND3/VIM/TAF1/AGTRAP/COLEC12/JAK1/LDOC1/CAMK2A/AHCYL1/SMARCC1/FLNA/CA2/LRRK2/CYSLTR2/GRB2/CYBB/GSK3B/RHOQ/STAT5A/SYAP1/SORL1/PRKCB/DNM2/TNF/PHIP/ROCK2/CASR/LRP1/MAS1/ATP7A/GPLD1/CDK5R1/UCP2/PIP4K2C/ITPR2/CPEB3/PKM/HRH2/HTR3B/PIK3CG/CPEB4/SOGA1/SP1/GUCY1B1/ATP2B1/GCLM/TSC2/GNAI2/TLR6/INPP5K/STAT5B/PID1/SHC1/PDK2/GNB1/NR4A2/OSBPL8/MAVS/PTGS2/ROCK1/SESN2/ATRX/GSK3A/PSEN1/RELA/GOT1/ZEB1/FUT7/SOCS3/AQP9/PTPRE/INSR/PTPN1/SESN3/MAP3K5/MAX/DIAPH1/AGRN/PXN/PPP1R9B/NR4A1/RALB/MBD5/CTNNA1/GNAQ/ARHGEF2/NDEL1/RIOK3/ZDHHC7/KCNQ1/EIF4EBP2/SRC/APC/ZNF106/GAB1/PIP4K2A/AKAP9/FLOT1/CYP1B1/P2RY1/ACTB/ZFP36L1/GNAL/MYO5A/PRKACA/AKT2/CASTOR2/ZBTB7B/BRCA1/KLF3/NFE2L2/UBR2/SSH1/IFIT1B/POR/RTF2/LYN/SNCA/TGM2/IGF2/ARRB2/ADIPOR1/CFLAR/RRAGD/CEBPB/STAT6/FBP1/FOXO3/EPHB2/PTAFR/IRS2/AQP1/STAT3/MAPK3/DMTN/PRKDC/PRKCD/BCL2L1/WDTC1/RAB31/SLC8A1/FOXO4/RAPGEF2/KLF1/GNAZ
BP	GO:0044087	regulation of cellular component biogenesis	679	-0.37	-1.68	3.88E-10	1.42E-08	8.70E-09	2726	tags=35%, list=23%, signal=28%	ARHGAP18/FARP1/TLR2/FES/FARP2/WNT1/RHOA/IL1B/REST/NEURL1/PRKCZ/SLAIN2/CDC42EP4/IFT88/ADNP/EIF4G1/LPAR1/NCKAP1L/CNOT1/HCFC1/KIT/ACTR2/SLC9A1/SIN3A/WIPI1/HSPA5/CEP295/DYRK1A/TICAM1/P2RX7/HAP1/DDX3X/ARPC4/ACTG1/FEZ1/FLNA/CAPZB/KLF5/ABCA2/LRRK2/GRB2/RNF5/CLIP1/GSK3B/CHMP3/BAX/ADD1/RHOQ/FBLIM1/ADPRH/NPHS1/SORL1/PLEKHM1/HNRNPU/DNM2/TNF/CPTP/ROCK2/TBC1D30/PLCE1/ARAP1/ATP7A/CDK5R1/MECP2/PIP4K2C/RICTOR/ALOX15/STXBP5/MYADM/SPTA1/FZD5/COTL1/SEMA4A/RCC2/SETDB1/TWF2/SYK/SYNE2/ZNF445/GPBAR1/FHOD1/ATF7IP/ARPC1B/JAM3/CTR9/RAC2/FSCN1/EP300/MTMR3/TIRAP/EIF2AK2/GREM1/DUSP22/TLR6/INPP5K/MTM1/GNL3L/TRIOBP/CNOT6/IFT140/TBC1D14/WASHC2C/CDKL5/ROCK1/BID/WASF2/NUDT16/HSPA1B/MAPT/ANKRD27/PTPRJ/CLU/RAB1B/VPS11/ULK1/PLK1/AGRN/MAP4/RAP1GAP/LGALS3/PXN/HSPA1A/CTTN/LCAT/RALB/SPICE1/H3-3A/DNM3/MARK4/TFIP11/NLGN3/CEP97/PTK2B/SYNPO/NDEL1/RIOK3/VCP/HCK/NUPR1/SLK/VPS4B/PPP2R5B/ELAPOR1/NIN/ARPC5/CHMP1A/NTNG2/LIMK1/SLX1B/SRC/APC/WDR45/TPR/RAB7A/CDC34/DBN1/AICDA/WARS1/PDLIM5/FCHSD1/PAK2/ESAM/TPPP3/LIMS1/PIP4K2A/AKAP9/FLOT1/DLG5/PLEK2/SELP/SVIL/PRKACA/FXR2/SH3GLB1/FEZ2/BRCA1/TPM1/LMOD3/SSH1/TAL1/STMP1/ARPC1A/CCSAP/CTNNBIP1/HCLS1/SNCA/ATG2A/PAK1/SDCBP/RAC1/PYCARD/PLEK/CYRIB/F11R/LIMK2/RHOG/MAP4K4/H3-3B/LCP1/ASAP1/PPFIA1/TMOD1/DNAJB6/CORO1A/SSH2/EPHB2/CHMP2A/CORO1C/WAS/DNAJB2/ABI3/VASP/DNAJA4/RAB5A/CYRIA/MED25/DMTN/MTPN/SNX18/NAPA/AMBRA1/PRKCD/GSN/PINK1/PPM1A/CCP110/MSN/SPTB/PTEN/VCL/CHMP4B/RIPOR2/RAPGEF2/ARHGAP6/CLDN5

MF	GO:0016773	phosphotransferase activity, alcohol group as acceptor	494	-0.39	-1.75	4.64E-10	1.68E-08	1.03E-08	3091	tags=37%, list=26%, signal=29%	MKNK1/CLK3/MARK1/MAP3K1/CCL5/STK32C/NEK4/EPHB6/TYK2/CSK/IP6K1/GRK2/DAPK2/PIP4K2B/STK19/TAOK2/CAMKK2/PIM3/TBCK/STK38L/PDK4/CSNK1A1/RSKR/MAPK1/UHMK1/PIKFYVE/TKFC/TTBK2/FES/CPNE3/MAP2K4/PRKAG2/PRKCZ/ERN1/CSNK2A1/JAK2/DAPK1/DYRK1B/KIT/NEK6/DYRK1A/CSNK1G2/CCND3/OXSR1/CSF1R/TAF1/JAK1/CAMK2A/HK2/LTBP1/ERBB2/LRRK2/GSK3B/BMPR1A/HIPK2/PIP5KL1/TESK2/PRKCB/PIK3CB/ROCK2/NADK/PI4K2A/DYRK3/PKN1/HIPK1/CDK5R1/NRP2/PRKD2/PIP4K2C/ERN2/MARK2/CDK13/PKM/SNRK/AKAP13/TAOK1/MAST3/SYK/PIP5K1C/PDXK/MAP2K2/SIK3/GAK/PIK3CG/WEE1/PPM1D/GRK4/IRAK3/CAMK1D/STK4/PIM1/EIF2AK2/RNASEL/STK24/MKNK2/MAP3K3/GRK6/PDK2/CDC42BPA/DSTYK/CDKL5/ROCK1/RPS6KA5/IRAK1/CCL3/GSK3A/FGR/IGF2R/EPHB4/ULK1/INSR/SQSTM1/VRK3/MAP3K20/PLK1/MAP3K5/RASSF2/IPMK/PPP1R9B/BCKDK/MARK4/PTK2B/RIOK3/HCK/NEK5/NEK9/SLK/RPS6KA1/CDKN1A/MAP2K7/HIPK3/CSNK1G1/LIMK1/ILK/TAOK3/SRC/ALPK1/MAPKAPK2/DGKG/PAK2/PFKFB3/PIP4K2A/EIF2AK1/RIPK1/MARK3/PRKACA/AKT2/MAPKAPK3/FGGY/AATK/SCYL1/BRD4/MAST2/STK40/LYN/MMD/GRK3/PAK1/WNK1/BMP2K/CDK19/MAP3K2/PIK3CD/LIMK2/MAP2K3/HK1/MAP4K4/SRPK2/STK38/MAPK14/BMX/ITPK1/PDK3/STK10/EPHB2/TOP1/MAPK3/PRKDC/PFKFB4/MYLK/PRKCD/PINK1/NUAK2/PEAK3
BP	GO:0000904	cell morphogenesis involved in differentiation	403	-0.41	-1.83	5.55E-10	1.99E-08	1.22E-08	2690	tags=35%, list=23%, signal=28%	CAPRIN1/RHOA/RAB8A/RAPH1/LYPLA2/NEXN/TCTN1/ADNP/CASP9/ACTR2/SCN1B/SEMA7A/SIN3A/CSF1R/HECW2/KLF7/FEZ1/ALCAM/FLNA/PLXNB2/ERBB2/LRRK2/FERMT3/RTN4R/GSK3B/DNM2/PAFAH1B1/EFNB1/CFAP418/GBP1/KIF5B/CDK5R1/NRP2/RNF165/YWHAH/CFL1/MARK2/MYADM/SEMA4A/EIF4G2/RCC2/KIAA0319/TWF2/ZFPM1/PTPN6/RAB21/RILPL1/DLG4/VPS33A/CREB1/RAC2/EP300/RERE/NCAM1/DRAXIN/RTN4/CUL3/DISC1/MAP1A/NIBAN2/TRIOBP/NUMB/NR4A2/WASHC2C/CDKL5/ROCK1/IST1/RPS6KA5/PSEN1/MAPT/ANKRD27/VLDLR/EPHB4/ULK1/LGR6/B4GALT5/SIPA1L1/NEDD9/ARL13B/PXN/CUX1/CTTN/VAMP3/MPIG6B/DNM3/NLGN3/NDEL1/MEGF9/DOCK5/CASS4/NIN/NTNG2/PALLD/APLP2/LIMK1/ILK/ACTN1/SRC/DPYSL2/DBN1/XK/PDLIM5/PAK2/FBXO45/LIMS1/FLOT1/ACTB/PARVB/FEZ2/PREX1/MYH9/TAL1/NOTCH4/ACTN4/UNC13D/USP9X/LAMC3/PAK1/RAC1/TBC1D20/PPP3CA/PLXNC1/PICALM/RHOG/MAP1S/EPHB2/CORO1C/RILPL2/ABI3/VASP/DMTN/PRKDC/ITGB3/KEL/MPL/BSG/PTEN/VCL/SPI1/MBP/RAPGEF2/ECE1/DAB2
CC	GO:0043292	contractile fiber	132	-0.55	-2.18	5.66E-10	2.01E-08	1.23E-08	2032	tags=39%, list=17%, signal=32%	CDK5R1/MYLPF/FHL2/TWF2/SYNE2/MYH11/PPP1R12A/CACNA1D/MTMR12/MTM1/ACTA2/ALDOA/ARF1/TPM3/PSEN1/FKBP1A/CAPN3/SQSTM1/PDLIM2/SYNPO/PPP2R5A/PALLD/ILK/ACTN1/MYL4/PDLIM5/HSPB1/ABCC9/CALM3/SVIL/PARVB/TPM1/PPP1R12B/LMOD3/ACTN4/TPM4/PAK1/PPP3CA/RTN2/MYOM1/TMOD1/DNAJB6/CORO1C/SLC4A1/PDLIM7/ANK1/MYL9/VCL/SLC8A1/NBR1/MYZAP
BP	GO:0090066	regulation of anatomical structure size	295	-0.45	-1.94	5.72E-10	2.01E-08	1.24E-08	2613	tags=39%, list=22%, signal=31%	ANO6/CDC42EP4/ADNP/NCKAP1L/SEMA7A/SIN3A/LAMTOR4/P2RX7/ARPC4/CD38/CAPZB/LRRK2/GRB2/RTN4R/GSK3B/ADD1/NPHS1/DNM2/PAFAH1B1/TNF/ROCK2/CASR/ATP7A/RGS2/CDK5R1/RICTOR/ALOX15/MYADM/SPTA1/COTL1/SEMA4A/EIF4G2/HRH2/KIAA0319/TWF2/RAB22A/RAB21/RIN3/ARPC1B/RB1CC1/CREB1/ATP2B1/GCLM/LRRC8A/DRAXIN/SLC12A6/RTN4/AP2M1/DISC1/KNG1/RAB3B/ACTA2/TRIOBP/WASHC2C/CDKL5/PTGS2/ROCK1/IST1/MAPT/ULK1/VAV1/AKT1S1/CTTN/PTK2B/NDEL1/HCK/GPX1/RILP/DOCK5/ARPC5/LIMK1/VAV3/DPYSL2/PPP1R15A/DBN1/XK/FCHSD1/ESAM/P2RY1/SOD2/SVIL/TBXAS1/P2RX1/LMOD3/SSH1/ARPC1A/HCLS1/RAC1/ADM/PYCARD/PLEK/CYRIB/GUCY1A1/PICALM/TMOD1/CORO1A/SSH2/WAS/VASP/RAB5A/CYRIA/AQP1/DMTN/MTPN/KEL/PRKCD/GSN/MSN/SPTB/WDTC1/PTEN/SLC8A1/TBXA2R/ECE1

BP	GO:0009611	response to wounding	342	-0.43	-1.86	6.15E-10	2.14E-08	1.32E-08	2865	tags=39%, list=24%, signal=30%	ST3GAL4/SRF/SCARF1/GNAS/SLC1A2/HIF1A/CD9/GNA13/CLIC1/WNT1/RHOA/MAP2K4/ADAM17/ANXA5/ANO6/JAK2/HPS6/ALOX5/FCER1G/ACTG1/TSPO/FLNA/SERPINE2/ERBB2/FERMT3/RTN4R/METAP1/CHMP3/BAX/PROS1/C6orf89/RIPOR1/PIK3CB/TNF/ABAT/ATP7A/GP6/ALOX15/PRCP/SLC11A1/PDGFA/PLSCR1/KIAA0319/SYK/ITGA5/F13A1/PIK3CG/PF4V1/PTPN6/CCR1/SERPING1/JMJD1C/STK24/MRTFA/KNG1/ACTA2/SYTL4/ABHD2/MAP3K5/MAX/VAV1/MPIG6B/TIMP1/CTNNA1/GNA12/GNAQ/NDEL1/F8/B4GALT1/GPX1/PRDX2/CDKN1A/RAB27A/CHMP1A/ILK/VAV3/SRC/GRN/PF4/F5/DGKG/HSPB1/ENTPD1/P2RY1/ACTB/SOD2/PLAUR/ZFP36L1/SELP/GP1BA/ALOX12/NFE2L2/SERPINE1/TPM1/P2RX1/SLC7A11/MYH9/TREML1/NOTCH4/LYN/TYROBP/PAK1/RAC1/ADM/PLEK/PPP3CA/F11R/CFLAR/MAPK14/HMOX1/NF1/ITPK1/EPHB2/CHMP2A/SLC4A1/WAS/PAFAH2/TLN1/DMTN/ITGB3/MYLK/MPL/PRKCD/MYL9/CNN2/PTEN/VCL/KRT1/CHMP4B/TBXA2R/DYSF/GP9/TGFA
CC	GO:0000323	lytic vacuole	589	-0.38	-1.71	7.23E-10	2.46E-08	1.51E-08	2616	tags=32%, list=22%, signal=26%	VTI1B/SCARB2/CTSA/PLEKHM2/SNAP23/HPS6/IL4I1/ACTR2/PSEN2/GCA/GNB2/LAMTOR4/GPR137B/HAP1/TMEM79/TECPR1/SNX1/ZFYVE26/SNX14/ABCA2/MAN2B1/LRRK2/PPT1/IFITM3/CHMP3/TPCN2/PLEKHM1/CTSZ/RAB2A/FPR1/PI4K2A/MNDA/SLCO4C1/LRP1/KIF5B/SDC3/ACLY/GGA3/TLR8/GPLD1/MYLPF/ATP6V1C1/DNASE2/PRCP/CPQ/AP1M1/SLC11A1/MANBA/MCOLN1/MEAK7/MFSD12/ANXA2/FNBP1/NPC2/ATP6AP2/SLC15A3/VPS33A/RB1CC1/LAMP2/PSMD1/TUBB4B/LRRC8A/TMBIM1/SIAE/NCF1/TSC2/RUBCN/NCF4/STX3/AP2M1/CPNE1/LAMP1/ARSG/RAB37/CKAP4/SIDT2/ATP6V0A1/MAP1LC3A/FFAR4/CSF3R/GNB1/IMPDH1/CLTC/TFEB/VCAN/IST1/BRI3/SLC44A2/SNAP29/PSEN1/ANKRD27/VLDLR/VPS11/CD63/INSR/SQSTM1/SPAG9/RNF19B/ATP6V0D1/NEU1/AGRN/ANXA11/ARL8A/CLEC16A/RNASET2/GNAQ/IFNAR1/CXCR2/CTSD/B4GALT1/VCP/HCK/SLC17A5/AP2B1/ELAPOR1/CYB5R3/RILP/RAB27A/KCNQ1/CHMP1A/SRC/ACP3/GRN/RAB7A/GABARAP/KPTN/PGAP6/PSAP/PIP4K2A/FLOT1/SLC29A3/STXBP2/PADI2/MYO5A/SULT1C2/SHKBP1/VPS18/CUBN/AP2A1/MARCHF2/NCF2/AP1G1/TRAPPC1/LAPTM5/UBA1/LYN/MMD/ATP6V1B2/UNC13D/SNCA/DNAJC5/SDCBP/PYCARD/UBXN6/BBC3/GNS/NCOA4/DTX3L/RRAGD/SLC48A1/CTSB/CHMP2A/AP1B1/STS/HPS1/PRDX6/RAB5C/TOLLIP/MARCHF8/FTL/IFITM2/GNB4/FLCN/FTH1/PRKCD/CD68/RAB3D/NFAM1/CAPN1/CHMP4B/NPRL3/AP5B1/ECE1/NBR1/DAB2/LITAF/SLC7A5/CPPED1/ATP6V1A

CC	GO:0005764	lysosome	589	-0.38	-1.71	7.23E-10	2.46E-08	1.51E-08	2616	tags=32%, list=22%, signal=26%	VTI1B/SCARB2/CTSA/PLEKHM2/SNAP23/HPS6/IL4I1/ACTR2/PSEN2/GCA/GNB2/LAMTOR4/GPR137B/HAP1/TMEM79/TECPR1/SNX1/ZFYVE26/SNX14/ABCA2/MAN2B1/LRRK2/PPT1/IFITM3/CHMP3/TPCN2/PLEKHM1/CTSZ/RAB2A/FPR1/PI4K2A/MNDA/SLCO4C1/LRP1/KIF5B/SDC3/ACLY/GGA3/TLR8/GPLD1/MYLPF/ATP6V1C1/DNASE2/PRCP/CPQ/AP1M1/SLC11A1/MANBA/MCOLN1/MEAK7/MFSD12/ANXA2/FNBP1/NPC2/ATP6AP2/SLC15A3/VPS33A/RB1CC1/LAMP2/PSMD1/TUBB4B/LRRC8A/TMBIM1/SIAE/NCF1/TSC2/RUBCN/NCF4/STX3/AP2M1/CPNE1/LAMP1/ARSG/RAB37/CKAP4/SIDT2/ATP6V0A1/MAP1LC3A/FFAR4/CSF3R/GNB1/IMPDH1/CLTC/TFEB/VCAN/IST1/BRI3/SLC44A2/SNAP29/PSEN1/ANKRD27/VLDLR/VPS11/CD63/INSR/SQSTM1/SPAG9/RNF19B/ATP6V0D1/NEU1/AGRN/ANXA11/ARL8A/CLEC16A/RNASET2/GNAQ/IFNAR1/CXCR2/CTSD/B4GALT1/VCP/HCK/SLC17A5/AP2B1/ELAPOR1/CYB5R3/RILP/RAB27A/KCNQ1/CHMP1A/SRC/ACP3/GRN/RAB7A/GABARAP/KPTN/PGAP6/PSAP/PIP4K2A/FLOT1/SLC29A3/STXBP2/PADI2/MYO5A/SULT1C2/SHKBP1/VPS18/CUBN/AP2A1/MARCHF2/NCF2/AP1G1/TRAPPC1/LAPTM5/UBA1/LYN/MMD/ATP6V1B2/UNC13D/SNCA/DNAJC5/SDCBP/PYCARD/UBXN6/BBC3/GNS/NCOA4/DTX3L/RRAGD/SLC48A1/CTSB/CHMP2A/AP1B1/STS/HPS1/PRDX6/RAB5C/TOLLIP/MARCHF8/FTL/IFITM2/GNB4/FLCN/FTH1/PRKCD/CD68/RAB3D/NFAM1/CAPN1/CHMP4B/NPRL3/AP5B1/ECE1/NBR1/DAB2/LITAF/SLC7A5/CPPED1/ATP6V1A
BP	GO:0032940	secretion by cell	481	-0.39	-1.75	8.78E-10	2.96E-08	1.82E-08	2676	tags=32%, list=23%, signal=26%	IL1B/RAB8A/REST/WLS/VTI1B/CD177/JAK2/NCKAP1L/SNAP23/HPS6/KIT/ALOX5/CCDC186/P2RX7/RCN3/ARFGAP3/HAP1/TMEM79/KLF7/PORCN/CD38/CACNA1I/CAMK2A/RPH3AL/SERPINE2/LRRK2/PPT1/KDM5B/GSK3B/TPCN2/FBXL20/PRKCB/PAFAH1B1/TNF/PRAM1/ABAT/PTGES/MYH10/CASR/NKX3-1/GPLD1/UCP2/ITGAM/ABCC4/OSM/STXBP5/P3H1/SYK/PIP5K1C/GPR27/ITSN1/PIK3CG/TNFRSF1A/RAB21/EXOC7/APBA1/CREB1/RAC2/SYN3/CCR1/CBL/LRRC8A/RAB11FIP2/GNAI2/STX3/LAMP1/COPG1/SOX4/RAB3B/SIDT2/SNAP25/NIBAN2/RSAD2/FFAR4/CTBP2/SNAP29/MCTP2/SYTL4/CCL3/PSEN1/FGR/ITGB2/VPS11/RAB27B/NR4A1/VAMP3/RALB/EXOC6/RAB11B/HMGCR/BMP6/HCK/SYT12/VPS4B/LGALS9/RAB27A/KCNQ1/ACSL4/CPT1A/RAB7A/STX11/LAT2/RAB2B/GNPTAB/EXOC4/P2RY1/CALM3/STXBP2/TNFRSF1B/VPS18/IL1RN/P2RX1/MYH9/PTGDS/AP1G1/ABCG1/LYN/UNC13D/SNCA/CHD7/C2CD2L/DNAJC5/PAK1/SDCBP/ADM/PLEK/MIDN/RAB11FIP1/SYNJ1/SLC1A5/TANGO2/PIK3CD/PPP3CA/MYOM1/HMOX1/CORO1A/NF1/PTAFR/CHMP2A/IRS2/RAB3IL1/TCF7L2/RAB5A/DMTN/PCSK6/NAPA/PINK1/ANK1/RAB3D/RAB31/SPI1/LTBP2/FURIN/DAB2/GLUL/GAB2
CC	GO:0098978	glutamatergic synapse	175	-0.51	-2.07	1.06E-09	3.52E-08	2.17E-08	2898	tags=46%, list=25%, signal=35%	PLEKHA5/PRKAR1B/SLC1A2/FXYD6/PACSIN2/FARP1/CRTC1/CAPRIN1/RHOA/RAB8A/PLCB1/USP8/PRKCZ/JAK2/DYNLL2/PORCN/LRRK2/RTN4R/GSK3B/FBXL20/DNM2/NRP2/GRIK2/ABR/APBA1/DLG4/GRIPAP1/GUCY1B1/ATP2B1/SYN3/STX3/AP2M1/YWHAZ/SNAP25/CTBP2/NUMB/CDKL5/NPTXR/RELA/RGS14/ERBIN/PRR7/ABHD17C/DNM3/NLGN3/PTK2B/VCP/AP2B1/NTNG2/ACTN1/SRC/DBN1/PAK2/FBXO45/AKAP9/FLOT1/P2RY1/ACTB/VPS18/KPNA1/CTNND1/P2RX1/SH3GL1/LYN/RAC1/PPP3CA/GUCY1A1/MAPK14/PRKAR2B/CORO1A/EPHB2/LRRC4/ABLIM3/ITGB3/PRKAR1A/NRGN/NAPA/FLOT2/RNF10/STAU1/HIP1

MF	GO:0008289	lipid binding	505	-0.39	-1.73	1.12E-09	3.65E-08	2.25E-08	2723	tags=33%, list=23%, signal=27%	TLR2/PAQR6/FES/CPNE3/WDFY1/PLCB1/PPP5C/SCAP/ARHGAP33/ANXA5/SCARB2/LPAR1/ATG2B/SLC9A1/WIPI1/ARHGAP9/CLN6/STARD8/P2RX7/PAQR8/RAB35/ADAP1/TECPR1/SNX1/TSPO/ZFYVE26/SNX14/SEC14L4/PPT1/RTN4R/CHMP3/TPCN2/DENND1C/BAX/SPON2/MAP1LC3B/CPTP/PRAM1/ARAP1/SNX8/TLR1/GGA3/ACOT11/BIN2/ITPR2/S1PR2/ALOX15/DAPP1/CD300LF/MARK2/FZD5/MCOLN1/CERT1/CPNE2/TWF2/ANXA2/FNBP1/NPC2/PCYT1A/TTPAL/ALOX5AP/PSD4/MICALL1/TIRAP/NCF1/RUBCN/CDIPT/NCF4/STX3/AP2M1/CPNE1/FCGR3B/TLR6/SNX11/MTM1/ERLIN1/CEACAM5/NME4/CD14/PHF12/EEA1/MAP1LC3A/SHC1/FFAR4/OSBPL8/WASHC2C/DBT/ZFYVE16/ARAP3/SYTL4/RARA/MAPT/IGF2R/PRR7/WIPI2/ZCCHC2/NR3C1/TSPO2/ANXA11/WDFY3/HDLBP/VCP/SYT12/KCNQ1/SELL/SNX10/PLEKHB2/WDR45/DGKG/APOL2/PSAP/FCHSD1/PITPNA/STAM2/GLE1/PLEK2/VDR/SELP/MYO1E/SVIL/SNX27/RXRA/HS1BP3/SH3GLB1/PREX1/BSCL2/SH3GL1/PTGDS/EPB41/MME/CYTH4/FYB1/ABCG1/LYN/EPN1/SNCA/ATG2A/C2CD2L/SDCBP/CAVIN2/H2BC11/PICALM/ASAP1/NF1/PTAFR/CHMP2A/IQSEC1/OSBP2/ARHGAP26/PAFAH2/TLN1/ACOX1/SBF2/RASGRP4/SNX18/NRGN/DENND1A/GSN/PTEN/NINJ1/RAPGEF2/IQGAP1/DYSF/GAB2/OSBPL2/OSBPL1A/IQGAP2/HIP1
CC	GO:0010008	endosome membrane	422	-0.40	-1.78	1.20E-09	3.87E-08	2.38E-08	3261	tags=41%, list=28%, signal=31%	DNAJC13/SCYL2/RAB10/SLC6A4/GPNMB/RHOB/ACAP2/MYD88/ARHGAP32/AP2S1/TASL/PSENEN/SLC15A4/AP3D1/LAMTOR1/UBC/CLCN5/RAP2C/RAB11FIP5/RFFL/ATP6V0B/ATP6AP1/PML/CLN3/DOP1B/ATP6V0C/VPS41/ITCH/HLA-DRB1/PACSIN2/PIKFYVE/TRAF6/RAB8A/WLS/USP8/VTI1B/SCARB2/PLEKHM2/YIPF1/HPS6/WIPI1/LAMTOR4/VPS28/TICAM1/EPS15/STEAP4/RAB35/SNX1/OPTN/STOML1/SNX14/PRAF2/ERBB2/ABCA2/TMEM150B/IFITM3/CHMP3/TPCN2/SORL1/PLEKHM1/TAB2/FCGRT/ZDHHC2/VPS37B/CPTP/SNX8/ATP7A/GGA3/TLR8/ITM2B/UBAP1/SLC39A4/SLC11A1/YIPF2/FZD5/TBC1D5/MCOLN1/RCC2/KIAA0319/SPG21/PIP5K1C/ANXA2/HLA-E/RAB22A/ATP6AP2/RAB21/SLC15A3/VPS33A/GRIPAP1/LAMP2/MICALL1/TMBIM1/ABCB11/RAB11FIP2/NCF4/AP2M1/LAMP1/CD14/EEA1/ATP6V0A1/FFAR4/CLTC/NUMB/ZFYVE16/IRAK1/PSEN1/IGF2R/UEVLD/VPS11/CD63/INSR/SCAMP4/ATP6V0D1/RAB27B/ABHD17C/ARL8A/VAMP3/CLEC16A/RAB11B/CTSD/AP2B1/VPS4B/TF/ELAPOR1/RILP/RAB27A/ANTXR2/CHMP1A/SNX10/PLEKHB2/RAB7A/UBB/VPS37C/SLC29A3/RIPK1/STAM2/RAB11FIP4/SNX27/EHD1/VPS18/CUBN/AP2A1/MARCHF2/SH3GL1/ARHGAP1/RAB5B/MMGT1/TMEM184A/UBA1/MMD/RAC1/UBXN6/UBE2D3/EHD3/DTX3L/SLC48A1/CHMP2A/CORO1C/RAB5C/RHOBTB1/RAB5A/MARCHF8/SBF2/IFITM2/SNX18/CD68/RAB31/CHMP4B/ECE1/FURIN/LITAF/KIF13A/PLIN3
BP	GO:0008360	regulation of cell shape	111	-0.57	-2.19	1.83E-09	5.85E-08	3.60E-08	2756	tags=49%, list=24%, signal=38%	TAOK2/WDPCP/GNA13/SYNE3/ARHGAP18/FES/RHOA/CDC42EP4/LPAR1/LST1/KIT/CSF1R/PLXNB2/RHOQ/PLEKHO1/FBLIM1/PHIP/ARAP1/MYH10/MARK2/SPTA1/SEMA4A/RAC2/MKLN1/ALDOA/CCL3/FGR/ITGB2/DNMBP/DIAPH1/GNA12/PTK2B/HCK/P2RY1/PARVB/TPM1/MYH9/FGD4/EPB41/ERMN/RAC1/FGD6/SH3KBP1/F11R/PLXNC1/RHOG/EPB42/CORO1A/RHOBTB1/DMTN/MSN/BRWD3/FMNL1/PEAK3

BP	GO:0009968	negative regulation of signal transduction	796	-0.35	-1.59	1.93E-09	6.14E-08	3.77E-08	2217	tags=28%, list=19%, signal=24%	HIPK2/PLCL2/BAX/NCOR1/MAPKBP1/SORL1/PRKCB/TLE4/PIK3CB/DNM2/ATF3/PAFAH1B1/TNF/PHIP/NFKBIA/DYRK3/IFT122/GTF2H2/WWC3/GBP1/LRP1/RGS3/NKX3-1/RGS2/PPIF/PIP4K2C/S1PR2/LRPAP1/CD300LF/FHL2/MYADM/HHEX/MCL1/WWC2/ZNF653/NMI/NECAB2/TMEM127/KBTBD7/UBE2B/APCDD1/BIRC6/YTHDF3/ELF1/PTPN6/IRAK3/IL17RD/RB1CC1/LMNA/CDKN2D/RGS6/GCLM/SULF2/CBL/TMBIM1/STK4/IVNS1ABP/DRAXIN/HGF/TSC2/GNAI2/CPNE1/GREM1/CUL3/DUSP22/TLR6/INPP5K/MTM1/CHST11/LILRA2/NIBAN2/PID1/FRMD8/SH3RF1/OTUD7B/C16orf72/NR4A2/PSME3/PTGS2/BID/PARP14/SESN2/PELI3/TMEM161A/HSPA1B/YWHAB/GPATCH3/SUFU/GSK3A/PSEN1/RELA/RGS14/PTPRJ/ERBIN/CLU/FKBP1A/TRIM33/VPS11/SOCS3/INVS/PTPRE/PTPN1/SESN3/ADAR/VRK3/ATF6B/LGALS3/AKT1S1/RASSF2/HSPA1A/PHACTR4/CTTN/MPIG6B/WWP2/CTNNA1/HMGCR/ARHGEF2/RIOK3/VCP/HDAC7/MAD2L2/RGS19/GPX1/RNF115/TNIP1/THOC5/SIRPA/PRDX2/SKI/HIPK3/SPRY3/TAOK3/SIAH2/SRC/APC/RAB7A/PF4/PPP1R15A/CDC34/DGKG/KPTN/ITPRIP/DUSP6/TGFB1I1/HSPB1/PPP1R10/PIP4K2A/DUSP1/RIPK1/MARK3/RNF149/DLG5/TMEM88/SOD2/PLAUR/PADI2/ZBTB7A/PRKACA/CASTOR2/PTPN12/BRCA1/VPS18/OTUD5/IL1RN/NFE2L2/SERPINE1/CTNND1/SEC14L1/UBR2/TLE3/PRELID1/ARHGAP25/HIC1/NRROS/LAPTM5/CTNNBIP1/CHP1/LYN/USP10/GRK3/PEA15/SNCA/ADM/PYCARD/PLEK/ARRB2/MMP9/XIAP/ADIPOR1/CYRIB/UBE2D3/GRINA/CFLAR/H2BC11/MAP2K3/PTPRC/OPRL1/FBP1/STK38/MAPK14/HMOX1/NF1/FOXO3/EPHB2/RGS7/YBX3/STRADB/TCF7L2/SFRP2/FLCN/NLRX1/PRKCD/PINK1/BCL2L1/PPM1A/PTEN/SPI1/BCL3/RIPOR2/TRIB1/NPRL3/DAB2/LITAF
BP	GO:0071417	cellular response to organonitrogen compound	396	-0.41	-1.81	2.40E-09	7.59E-08	4.66E-08	2696	tags=38%, list=23%, signal=30%	FPR2/WNT1/PIK3R2/IL1B/CACNA1A/LPIN2/RAB8A/PLCB1/NEURL1/PRKCZ/JAK2/CACNB1/ACTR2/SLC9A1/SIN3A/LAMTOR4/HSPA5/P2RX7/CCND3/VIM/TAF1/AGTRAP/JAK1/LDOC1/CAMK2A/AHCYL1/SMARCC1/FLNA/CA2/LRRK2/CYSLTR2/GRB2/CYBB/GSK3B/RHOQ/STAT5A/SYAP1/SORL1/PRKCB/DNM2/TNF/PHIP/ROCK2/CASR/LRP1/MAS1/ATP7A/GPLD1/UCP2/PIP4K2C/ITPR2/CPEB3/PKM/HRH2/HTR3B/PIK3CG/CPEB4/SOGA1/SP1/ATP2B1/GCLM/TSC2/GNAI2/TLR6/INPP5K/STAT5B/PID1/SHC1/PDK2/GNB1/NR4A2/OSBPL8/PTGS2/ROCK1/SESN2/GSK3A/PSEN1/RELA/GOT1/ZEB1/FUT7/SOCS3/AQP9/PTPRE/INSR/PTPN1/SESN3/MAX/DIAPH1/PXN/PPP1R9B/NR4A1/MBD5/CTNNA1/ARHGEF2/NDEL1/ZDHHC7/KCNQ1/EIF4EBP2/SRC/APC/ZNF106/GAB1/PIP4K2A/AKAP9/CYP1B1/P2RY1/ACTB/ZFP36L1/GNAL/MYO5A/PRKACA/AKT2/CASTOR2/ZBTB7B/BRCA1/KLF3/NFE2L2/UBR2/SSH1/POR/LYN/SNCA/TGM2/IGF2/ARRB2/ADIPOR1/CFLAR/RRAGD/CEBPB/STAT6/FBP1/FOXO3/EPHB2/PTAFR/IRS2/AQP1/STAT3/MAPK3/DMTN/PRKDC/PRKCD/BCL2L1/WDTC1/RAB31/SLC8A1/FOXO4/RAPGEF2/KLF1/GNAZ

BP	GO:1901701	cellular response to oxygen-containing compound	751	-0.35	-1.63	2.51E-09	7.80E-08	4.80E-08	3087	tags=38%, list=26%, signal=30%	BCL10/LAMTOR1/MED1/CDC73/BCL2L11/CCL5/GATA1/EDN1/ZNF580/ABCA1/ADAM15/RAB11FIP5/ADNP2/TYK2/CSK/STAT1/GRK2/PIP4K2B/AIFM1/PLAA/CAMKK2/PIM3/GOLPH3/SRF/PDK4/GSTP1/GNAS/SLC1A2/LCOR/SMARCB1/HLA-DRB1/HIF1A/MAPK1/SLC26A6/TLR2/FES/FOS/FPR2/TRAF6/WNT1/RHOA/PIK3R2/IL1B/CACNA1A/LPIN2/RAB8A/PLCB1/SIGMAR1/USP8/NEURL1/PRKCZ/ERN1/JAK2/DAPK1/CACNB1/CASP9/ACTR2/PPARGC1B/SLC9A1/SIN3A/LAMTOR4/CCDC186/HSPA5/TICAM1/P2RX7/CCND3/VIM/TAF1/AGTRAP/COLEC12/JAK1/LDOC1/KLF7/CAMK2A/TSPO/AHCYL1/SMARCC1/FLNA/CA2/LRRK2/CYSLTR2/GRB2/CYBB/GSK3B/ARID5A/LILRB2/RHOQ/STAT5A/SYAP1/SPON2/SORL1/PRKCB/DNM2/TNF/PHIP/NFKBIA/ROCK2/CASR/LRP1/MAS1/ATP7A/PPIF/GPLD1/CDK5R1/UCP2/PIP4K2C/ITPR2/PRDX5/CPEB3/PKM/CES1/HRH2/TWF2/GPR27/GPBAR1/HTR3B/PIK3CG/PF4V1/SSTR2/CPEB4/SOGA1/SP1/CREB1/GUCY1B1/ATP2B1/CTR9/GCLM/RAB11FIP2/PIM1/TIRAP/NCF1/HGF/TSC2/GNAI2/SOX4/TLR6/INPP5K/SIDT2/CD14/LILRA2/STAT5B/PID1/MAP1LC3A/SHC1/PDK2/GNB1/NR4A2/OSBPL8/PTGS2/ROCK1/IRAK1/SESN2/CCL3/GSK3A/PSEN1/RARA/MAPT/PRPF8/RELA/GOT1/ZEB1/CLU/RDH11/FUT7/SOCS3/ZNF703/AQP9/PTPRE/INSR/PTPN1/SESN3/SLC23A2/MAP3K5/MAX/NR3C1/AGRN/NFKBIB/PXN/PPP1R9B/SBNO2/NR4A1/MBD5/RAB11B/CTNNA1/BMP6/GNAQ/PTK2B/ARHGEF2/NDEL1/VCP/HCK/GPX1/TESC/TNIP1/ZDHHC7/SIRPA/PRDX2/CXCL5/KCNQ1/LIN28A/MAP2K7/EIF4EBP2/SRC/APC/CPT1A/PF4/ZNF106/AICDA/GAB1/FANCC/PIP4K2A/SETX/AKAP9/CYP1B1/RIPK1/P2RY1/ACTB/SOD2/ZFP36L1/VDR/GNAL/MYO5A/PRKACA/AKT2/RXRA/CASTOR2/ZBTB7B/TNFRSF1B/BRCA1/KLF3/DGAT2/KAT5/NFE2L2/SERPINE1/SASH1/TPM1/KDM6B/UBR2/CMPK2/SSH1/POR/LYN/SNCA/TGM2/IGF2/C2CD2L/PYCARD/CDK19/ARRB2/MMP9/ADIPOR1/SIPA1/PPP3CA/NCOA4/CFLAR/RRAGD/MAP2K3/CEBPB/STAT6/FBP1/MAPK14/FOXO3/PDK3/EPHB2/PTAFR/FAM210B/IRS2/AQP1/STAT3/MAPK3/DMTN/PRKDC/PRKCD/CD68/PINK1/BCL2L1/MSN/WDTC1/RAB31/SPI1/SLC8A1/FOXO4/TRIB1/RAPGEF2/TBXA2R/KLF1/LITAF/GNAZ
BP	GO:0140352	export from cell	512	-0.38	-1.72	2.57E-09	7.92E-08	4.87E-08	2676	tags=32%, list=23%, signal=26%	IL1B/RAB8A/REST/WLS/VTI1B/ANO6/CD177/JAK2/NCKAP1L/SNAP23/HPS6/KIT/SLC9A1/ALOX5/CCDC186/P2RX7/RCN3/ARFGAP3/HAP1/TMEM79/KLF7/PORCN/CD38/CACNA1I/CAMK2A/RPH3AL/SERPINE2/LRRK2/PPT1/KDM5B/GSK3B/TPCN2/FBXL20/PRKCB/PAFAH1B1/TNF/PRAM1/ABAT/PTGES/MYH10/CASR/NKX3-1/GPLD1/UCP2/ITGAM/ABCC4/OSM/STXBP5/P3H1/SYK/PIP5K1C/GPR27/ITSN1/PIK3CG/TNFRSF1A/RAB21/EXOC7/APBA1/CREB1/ATP2B1/RAC2/SYN3/CCR1/CBL/LRRC8A/RAB11FIP2/GNAI2/STX3/LAMP1/COPG1/SOX4/RAB3B/SIDT2/SNAP25/NIBAN2/RSAD2/FFAR4/CTBP2/SNAP29/MCTP2/SYTL4/CCL3/PSEN1/FGR/ITGB2/VPS11/RAB27B/NR4A1/VAMP3/RALB/EXOC6/RAB11B/HMGCR/BMP6/HCK/SYT12/VPS4B/LGALS9/RAB27A/KCNQ1/ACSL4/CPT1A/RAB7A/STX11/LAT2/RAB2B/GNPTAB/EXOC4/KCNE3/P2RY1/CALM3/STXBP2/TNFRSF1B/VPS18/IL1RN/P2RX1/MYH9/PTGDS/AP1G1/ABCG1/LYN/UNC13D/SNCA/CHD7/C2CD2L/DNAJC5/PAK1/SDCBP/ADM/PLEK/MIDN/RAB11FIP1/SYNJ1/SLC1A5/TANGO2/PIK3CD/PPP3CA/MYOM1/HMOX1/CORO1A/NF1/PTAFR/CHMP2A/IRS2/RAB3IL1/TCF7L2/RAB5A/DMTN/RALBP1/PCSK6/NAPA/PINK1/ANK1/RAB3D/RAB31/SPI1/LTBP2/SLC8A1/FURIN/DAB2/GLUL/GAB2

BP	GO:0060322	head development	468	-0.39	-1.74	2.69E-09	8.22E-08	5.05E-08	2816	tags=34%, list=24%, signal=27%	SLC1A2/HIF1A/PBX3/CHD8/MAPK1/TTBK2/CASP2/WNT1/RHOA/PLCB1/ASPM/WLS/NEURL1/SLC2A1/NEUROD2/TCTN1/SETD2/SEMA7A/SIN3A/HSPA5/SHROOM4/DPCD/FOXP2/HAP1/CSF1R/SNRPB/TAF1/FEZ1/SERPINE2/PLXNB2/CKB/ATXN1/LRRK2/OTX1/PPT1/RTN4R/TBX19/GSK3B/CRISPLD2/BMPR1A/BAX/ZSWIM6/NDE1/PAFAH1B1/GJC2/ABAT/MAS1/ATP7A/RGS2/CDK5R1/NRP2/MECP2/ITGAM/SGPL1/TACC3/YWHAH/PCM1/ANKRD11/DDX10/SYNE2/ATF5/DYNC2H1/ZNF148/SUDS3/TRAPPC9/ATP6AP2/SSTR2/CREB1/ATP2B1/EP300/RERE/SOX6/DRAXIN/HOOK3/RTN4/TSPAN2/DISC1/MEIS1/MSX1/MBOAT7/ROGDI/NUMB/NR4A2/XRN2/ATRX/XRCC1/PSEN1/RARA/ZEB1/SEC16A/ATP6V0D1/AGTPBP1/ARL13B/ASPH/PPP1R9B/NDEL1/PTBP2/ZFHX3/SKI/NIN/ARPC5/SRC/DPYSL2/NIPBL/UBB/BPTF/PBX1/KDM7A/FANCC/FBXO45/TACC1/PFKFB3/ACTB/DLG5/CALM3/PADI2/CSRNP1/RBPJ/CTNND1/AATK/SLC7A11/NDST1/AKNA/BAG6/ZMIZ1/APAF1/TACC2/SHARPIN/CHD7/TYROBP/ARRB2/PBX2/SYNJ1/PPP3CA/LIMK2/CIC/MAP1S/NF1/FOXO3/EPHB2/RGS7/G6PD/IRS2/AQP1/MAPK3/PRKDC/SFRP2/CCDC39/MTPN/GNB4/NRGN/NAPA/BASP1/PTEN/MBP/SLC8A1/RAPGEF2/ODAD4/KIF27/NCOA6/CLDN5
BP	GO:0030335	positive regulation of cell migration	336	-0.42	-1.84	3.07E-09	9.30E-08	5.72E-08	3027	tags=41%, list=26%, signal=32%	EDN1/ZNF580/ATOH8/FERMT1/CEP43/TIAM1/DAPK2/BCAS3/THBS1/PLAA/MOSPD2/HIF1A/ADAM8/SHTN1/MAPK1/CPNE3/FPR2/RHOA/IL1B/ADAM17/ANO6/JAK2/NCKAP1L/KIT/ANGPT1/SEMA7A/FAM89B/HSPA5/OXSR1/CSF1R/ACTG1/FADD/FERMT3/CCBE1/STAT5A/RIPOR1/PIK3CB/TNF/ROCK2/ATP7A/GPLD1/NRP2/PRKD2/MYADM/PDGFA/SEMA4A/SYNE2/CXCL16/ITGA5/PIK3CG/SP1/JAM3/RAC2/CCR1/CAMK1D/STK4/TIRAP/HGF/VSIR/GNAI2/RTN4/GPI/ACTA2/MAP3K3/NUMB/PTGS2/CCL3/FGR/RIN2/FUT7/ZNF703/LGR6/INSR/SPAG9/AGO2/DIAPH1/NEDD9/LGALS3/C5AR1/DOCK8/PTK2B/ARHGEF2/HDAC7/GFUS/LGALS9/DOCK5/CASS4/SPARC/SRC/APC/GRN/NIPBL/GAB1/HSPB1/PECAM1/CYP1B1/TNFSF14/IL6R/SOD2/SELP/AKT2/NFE2L2/SERPINE1/SASH1/ACTN4/LYN/GLIPR2/PAK1/SDCBP/RAC1/WNK1/PYCARD/MMP9/GPSM3/PIK3CD/PPP3CA/MAP2K3/PTPRC/MAP4K4/HMOX1/CORO1A/EPHB2/PTAFR/IQSEC1/IRS2/STAT3/MAPK3/DMTN/ITGB3/MYLK/SPI1/RIPOR2/SLC8A1/PTP4A1/FOXO4/PLVAP/RAPGEF2/DAB2/S100A11

BP	GO:0048699	generation of neurons	782	-0.35	-1.61	3.09E-09	9.30E-08	5.72E-08	2935	tags=34%, list=25%, signal=27%	CDH23/ENC1/SZT2/PLAA/KATNB1/TAOK2/KIDINS220/SRF/SCLT1/WDPCP/SCARF1/NR2F6/HIF1A/PBX3/SHTN1/UHMK1/CLMN/HDGFL3/FARP1/TOPORS/FES/CRTC1/FRYL/FARP2/CAPRIN1/DICER1/WNT1/RHOA/RAB8A/REST/PIGT/ASPM/RAPH1/NEURL1/PRKCZ/SCARB2/LYPLA2/NEXN/NEUROD2/IFT88/TCTN1/ADNP/EIF4G1/LPAR1/JAK2/NCKAP1L/LST1/ACTR2/SCN1B/SEMA7A/SIN3A/SMARCD3/HSPA5/DDX6/C1QA/CSF1R/HECW2/VIM/RAB35/KLF7/CD38/CAMK2A/FEZ1/TSPO/ALCAM/FLNA/PLXNB2/MYO9A/ERBB2/LRRK2/PPT1/RTN4R/TBC1D24/GSK3B/HIPK2/BAX/ZSWIM6/NDE1/DNM2/PAFAH1B1/EFNB1/CFAP418/KIF5B/ATP7A/RGS2/HIPK1/CDK5R1/NRP2/KCTD11/MECP2/ERCC2/RNF165/YWHAH/CPEB3/CFL1/MARK2/PCM1/FZD5/SEMA4A/CTDSP1/P3H1/EIF4G2/TAOK1/KIAA0319/TWF2/ATF5/ATCAY/DYNC2H1/GAK/WEE1/TRAPPC9/RAB21/CDKN1C/DLG4/CREB1/RAC2/IER2/EP300/RERE/CAMK1D/MICALL1/NCAM1/DRAXIN/HGF/LRP12/STK24/RTN4/STX3/CPNE1/TSPAN2/DTNB/DISC1/MAP1A/SOX4/MEIS1/YWHAZ/GIGYF2/SNAP25/NIBAN2/TRIOBP/POMGNT2/IFT140/SH3RF1/KDM4A/NUMB/NR4A2/XRN2/CDKL5/ROCK1/IST1/DENND5A/RPS6KA5/SUFU/GSK3A/PSEN1/RARA/MAPT/ANKRD27/ZEB1/VLDLR/EPHB4/ULK1/LGR6/B4GALT5/SPAG9/SIPA1L1/SLC23A2/AGTPBP1/GAS7/AGRN/MAP4/RAP1GAP/PPP1R9B/ADGRF1/CUX1/CTTN/CTNNA1/DNM3/BMP6/NLGN3/PTK2B/ARHGEF2/NDEL1/PPP2R5B/DVL3/ZFHX3/KCNQ1/ACSL4/RETREG3/LIN28A/NIN/NTNG2/PALLD/APLP2/SPRY3/LIMK1/TAOK3/DPYSL2/GRN/NIPBL/DBN1/ASCL2/TUBB2A/UBB/DGKG/XK/PDLIM5/PAK2/PBX1/FBXO45/SETX/FLOT1/MYCL/ACTB/DLG5/SOD2/CARM1/EHD1/FEZ2/RBPJ/NFE2L2/NAV1/TAL1/SCYL1/AKNA/ZMIZ1/LYN/MMD/FRY/USP9X/PAK1/RAC1/WNK1/ADM/PBX2/PPP3CA/CFLAR/PLXNC1/CEBPB/PICALM/RHOG/MAP4K4/FBXO7/MAP1S/ASAP1/NCOA1/NF1/FOXO3/EPHB2/IQSEC1/MYEF2/ABI3/VASP/STAT3/SFRP2/MTPN/KEL/NAPA/BSG/PTEN/VCL/MBP/RAPGEF2/IQGAP1/ECE1/DAB2
CC	GO:0005884	actin filament	77	-0.63	-2.28	3.37E-09	1.01E-07	6.19E-08	1933	tags=55%, list=16%, signal=46%	MARK2/COTL1/TWF2/JAM3/RAC2/DUSP22/TPM3/DIAPH2/PSTPIP2/PDLIM2/GAS7/DIAPH1/PSTPIP1/CTTN/SPECC1/ESPN/GAS2L3/HCK/WIPF2/PALLD/ACTN1/SRC/DBN1/PDLIM5/GAS2L1/ACTB/MYO5A/VPS18/TPM1/TPM4/HCLS1/PAK1/RAC1/WIPF1/LCP1/TMOD1/CORO1A/WAS/DMTN/PDLIM7/IQGAP1/ARHGAP6
BP	GO:0048167	regulation of synaptic plasticity	102	-0.58	-2.20	3.53E-09	1.05E-07	6.45E-08	2882	tags=48%, list=25%, signal=37%	CLN3/SRF/KRAS/MAPK1/CRTC1/RAB8A/NEURL1/PRKCZ/NEUROD2/KIT/CD38/CAMK2A/SERPINE2/GSK3B/LILRB2/SYAP1/ZDHHC2/MECP2/GRIK2/YWHAH/CPEB3/DLG4/STX3/MAP1A/SNAP25/ARF1/PTGS2/ZZEF1/PSEN1/RARA/MAPT/RGS14/SQSTM1/SIPA1L1/PTK2B/SYT12/EIF4EBP2/DBN1/MME/SNCA/TYROBP/NF1/EPHB2/RAB5A/NRGN/PTEN/RAPGEF2/STAU1/TSHZ3

BP	GO:0051336	regulation of hydrolase activity	644	-0.36	-1.66	4.13E-09	1.21E-07	7.43E-08	2604	tags=33%, list=22%, signal=27%	SCARB2/PTPA/SOS2/LPAR1/CSNK2A1/JAK2/DAPK1/NCKAP1L/FIS1/CASP9/KIT/PPARGC1B/GPR137B/ARHGAP9/CIDEB/TBC1D22B/RCN3/DDX3X/ADAP1/CAMK2A/PKP4/FADD/BEX3/SERPINE2/PLXNB2/MYO9A/ERBB2/ABCA2/LRRK2/PPT1/RTN4R/GSK3B/CHN2/KDM5A/BAX/TBC1D10B/ADPRH/PROS1/SORL1/C6orf89/PAFAH1B1/TNF/ROCK2/TBC1D30/ARAP1/TBC1D8/LRP1/CST3/NKX3-1/RGS2/GPLD1/PRKD2/RICTOR/PRDX5/RALGDS/TBC1D5/RCC2/SYK/ANXA2/CR1/ABR/BIRC6/PPP1R12A/RALGAPA2/CDKN2D/RGS6/PPP6R1/PI3/RAB11FIP2/SERPING1/CAST/HGF/TSC2/VSIR/GNAI2/GPI/KNG1/ASAP2/BOD1L1/SH3RF1/TBC1D14/PSME3/CDKL5/PTGS2/ROCK1/BID/ARAP3/CCL3/MAPT/RGS14/FKBP1A/IFI16/PRR7/EGLN1/SIPA1L1/VRK3/TBC1D2/EVI5/MAP3K5/NEDD9/AGRN/RAP1GAP/VAV1/LGALS3/ASPH/PPP1R9B/C5AR1/HSPA1A/NR4A1/TIMP1/PSME4/DOCK8/GNA12/GNAQ/PTK2B/NDEL1/CTSD/VCP/EGLN3/GPX1/RPS6KA1/DVL3/LGALS9/APLP2/VAV3/OASL/SIAH2/SRC/GRN/PPP1R15A/PSAP/PAK2/NRDC/LIMS1/TNFSF14/UBE2O/DAP/CALM3/PLAUR/AKT2/PREX1/RABGAP1L/SERPINE1/P2RX1/PRELID1/ARHGAP25/ADRM1/FGD4/POR/AFDN/APAF1/LAPTM5/NLRC4/CHP1/LYN/SNCA/TGM2/WNK1/RSU1/PYCARD/FGD6/PLEK/ARRB2/MMP9/XIAP/TBC1D20/BBC3/SIPA1/F11R/CFLAR/PLXNC1/TIMP2/PTPRC/PCSK1N/PICALM/RHOG/MAP4K4/ABHD5/CTSB/ASAP1/DNAJB6/NF1/PTAFR/IQSEC1/CORO1C/RGS7/DHCR24/TBC1D1/AQP1/STAT3/SFRP2/SBF2/RASGRP4/SNX18/RALBP1/TBC1D3I/PCSK6/FLCN/PRKCD/GSN/PINK1/PSMF1/MBP/SIPA1L2/RIPOR2/RAPGEF2/IQGAP1/FURIN/ARHGAP6/IQGAP2/HIP1
BP	GO:0034329	cell junction assembly	200	-0.49	-2.01	4.22E-09	1.22E-07	7.53E-08	2947	tags=40%, list=25%, signal=30%	BCAS3/THBS1/TAOK2/SRF/WDPCP/SETD5/CD9/FARP1/TLR2/RHOA/IL1B/ADNP/EIF4G1/SLC9A1/ACTG1/PKP4/PLXNB2/MYO9A/ZDHHC12/TESK2/TNF/ROCK2/HIPK1/MECP2/FZD5/SEMA4A/RCC2/PIP5K1C/GPBAR1/ITGA5/JAM3/FSCN1/GREM1/DUSP22/ROCK1/PTPRJ/ZNF703/AGRN/ADGRF1/CTTN/CTNNA1/DNM3/NLGN3/PTK2B/HDAC7/SLK/NTNG2/ACTN1/SRC/APC/PDLIM5/PAK2/ESAM/FBXO45/LIMS1/PECAM1/FLOT1/ACTB/DLG5/PRKACA/CTNND1/AFDN/SNCA/SDCBP/RAC1/F11R/MAP4K4/EPHB2/CORO1C/LRRC4/ABI3/TLN1/DMTN/FLCN/PTEN/VCL/RAPGEF2/ARHGAP6/CLDN5
MF	GO:0004674	protein serine/threonine kinase activity	327	-0.42	-1.84	4.58E-09	1.30E-07	8.00E-08	3308	tags=43%, list=28%, signal=32%	AURKA/MOK/PIM2/MAP4K2/BRAF/PHKG1/TNK2/CAMK1/STK35/SRPK1/MKNK1/CLK3/MARK1/MAP3K1/STK32C/NEK4/GRK2/DAPK2/STK19/TAOK2/CAMKK2/PIM3/STK38L/CSNK1A1/RSKR/MAPK1/UHMK1/PIKFYVE/TTBK2/CPNE3/MAP2K4/PRKAG2/PRKCZ/ERN1/CSNK2A1/DAPK1/DYRK1B/NEK6/DYRK1A/CSNK1G2/CCND3/OXSR1/TAF1/CAMK2A/LTBP1/LRRK2/GSK3B/BMPR1A/HIPK2/TESK2/PRKCB/ROCK2/DYRK3/PKN1/HIPK1/PRKD2/ERN2/MARK2/CDK13/SNRK/AKAP13/TAOK1/MAST3/SYK/MAP2K2/SIK3/GAK/PIK3CG/PPM1D/GRK4/IRAK3/CAMK1D/STK4/PIM1/EIF2AK2/STK24/MKNK2/MAP3K3/GRK6/CDC42BPA/DSTYK/CDKL5/ROCK1/RPS6KA5/IRAK1/CCL3/GSK3A/ULK1/SQSTM1/VRK3/MAP3K20/PLK1/MAP3K5/BCKDK/MARK4/PTK2B/RIOK3/NEK5/NEK9/SLK/RPS6KA1/MAP2K7/HIPK3/CSNK1G1/LIMK1/ILK/TAOK3/ALPK1/MAPKAPK2/PAK2/EIF2AK1/RIPK1/MARK3/PRKACA/AKT2/MAPKAPK3/AATK/BRD4/MAST2/STK40/GRK3/PAK1/WNK1/BMP2K/CDK19/MAP3K2/LIMK2/MAP2K3/MAP4K4/SRPK2/STK38/MAPK14/PDK3/STK10/TOP1/MAPK3/PRKDC/MYLK/PRKCD/PINK1/NUAK2

BP	GO:0051241	negative regulation of multicellular organismal process	575	-0.37	-1.67	4.90E-09	1.38E-07	8.49E-08	3073	tags=38%, list=26%, signal=29%	RAI1/MARK1/IL18R1/GATA1/EDN1/BCL6/FERMT1/RAB11FIP5/CSK/HOMER3/BBS2/IP6K1/STAT1/TNNT1/GRK2/THBS1/OAS3/ARRDC3/BST2/PML/SRF/RELB/ITCH/GSTP1/HLA-DRB1/HIF1A/NLRP12/CD9/TMEM176A/TLR2/LGALS9C/DICER1/RHOA/IL1B/CTDP1/REST/TMEM131L/ADAM17/INPP5D/ANXA5/JAK2/NCKAP1L/ANGPT1/SEMA7A/ALOX5/GPR137B/P2RX7/RC3H2/TRIM11/C5AR2/KLF7/CD38/TSPO/ARHGDIB/SERPINE2/ERBB2/IL1R2/NOVA1/YY1/RTN4R/REL/GSK3B/BMPR1A/PLCL2/PROS1/MAPKBP1/SORL1/PIK3CB/TNF/CPTP/ROCK2/ABAT/WWC3/GBP1/CST3/NKX3-1/RGS2/TLR8/ACOT11/CDK5R1/MECP2/LRPAP1/YWHAH/SLC11A1/PCM1/HHEX/PDGFA/SEMA4A/CTDSP1/WWC2/ORM1/TMEM176B/KIAA0319/NMI/ZFPM1/CR1/PIK3CG/TNFRSF1A/GADD45A/PTPN6/LAMA4/TOB2/IRAK3/IL17RD/LMNA/SAV1/ATP2B1/CHSY1/CCR1/STK4/SERPING1/SOX6/DRAXIN/HGF/VSIR/HOOK3/GNAI2/RTN4/PDE5A/RFLNB/GREM1/KNG1/TLR6/NIBAN2/FFAR4/FOXJ2/KDM4A/OTUD7B/N4BP1/PTGS2/ROCK1/RPS6KA5/GPATCH3/CCL3/GSK3A/PSEN1/RARA/ERBIN/ULK1/RAP1GAP/TIMP1/CTNNA1/HMGCR/PTK2B/HDAC7/MAD2L2/CUL4A/SIRPA/PRDX2/LGALS9/DOCK5/LIN28A/SKI/SPARC/GRN/TBX21/PF4/ASCL2/NRDC/IRF1/GP1BA/ZBTB7B/TNFRSF1B/ALOX12/RBPJ/OTUD5/KAT5/JARID2/USF3/SERPINE1/TLE3/LAPTM5/LYN/EPN1/ILRUN/TYROBP/RAC1/WNK1/ADM/PYCARD/ARRB2/RAB11FIP1/ADIPOR1/PPP3CA/GUCY1A1/PTPRC/FBXO7/HMOX1/SP100/ABCG8/NF1/EPHB2/CORO1C/TFE3/PGLYRP1/G6PD/DNAJA4/SFRP2/FLCN/NLRX1/PRKCD/HLX/PTEN/KRT1/SPI1/LILRB3/BCL3/MBP/FOXO4/RNF10/TRIB1/RAPGEF2/TBXA2R/FURIN/AGO1/E2F2/CLDN5
BP	GO:2000147	positive regulation of cell motility	342	-0.42	-1.81	5.30E-09	1.48E-07	9.12E-08	3027	tags=41%, list=26%, signal=31%	EDN1/ZNF580/ATOH8/FERMT1/CEP43/TIAM1/DAPK2/BCAS3/THBS1/PLAA/MOSPD2/HIF1A/ADAM8/SHTN1/MAPK1/CPNE3/FPR2/RHOA/IL1B/ADAM17/ANO6/JAK2/NCKAP1L/KIT/ANGPT1/SEMA7A/FAM89B/HSPA5/OXSR1/CSF1R/ACTG1/FADD/FERMT3/CCBE1/STAT5A/RIPOR1/PIK3CB/TNF/ROCK2/ATP7A/GPLD1/NRP2/PRKD2/MYADM/PDGFA/SEMA4A/SYNE2/CXCL16/ITGA5/PIK3CG/SP1/JAM3/RAC2/CCR1/CAMK1D/STK4/TIRAP/HGF/VSIR/GNAI2/RTN4/GPI/ACTA2/MAP3K3/NUMB/PTGS2/CCL3/FGR/RIN2/FUT7/ZNF703/LGR6/INSR/SPAG9/AGO2/DIAPH1/NEDD9/LGALS3/C5AR1/DOCK8/PTK2B/ARHGEF2/HDAC7/TF/GFUS/LGALS9/DOCK5/CASS4/SPARC/SRC/APC/GRN/NIPBL/GAB1/HSPB1/PECAM1/CYP1B1/TNFSF14/IL6R/SOD2/SELP/AKT2/NFE2L2/SERPINE1/SASH1/ACTN4/LYN/GLIPR2/PAK1/SDCBP/RAC1/WNK1/PYCARD/MMP9/GPSM3/PIK3CD/PPP3CA/MAP2K3/PTPRC/MAP4K4/HMOX1/CORO1A/EPHB2/PTAFR/IQSEC1/IRS2/STAT3/MAPK3/DMTN/ITGB3/MYLK/SPI1/RIPOR2/SLC8A1/PTP4A1/FOXO4/PLVAP/RAPGEF2/DAB2/S100A11
BP	GO:0045765	regulation of angiogenesis	161	-0.52	-2.07	5.36E-09	1.49E-07	9.17E-08	2614	tags=40%, list=22%, signal=31%	BTG1/STIM1/SIRT6/ALOX5/JAK1/HK2/ERBB2/CYBB/HIPK2/CCBE1/PRKCB/PIK3CB/TNF/ROCK2/HIPK1/PRKD2/MECP2/PKM/HHEX/SEMA4A/ITGA5/GADD45A/SP1/HGF/GREM1/NIBAN2/FOXJ2/ROCK1/LRG1/EGLN1/CHI3L1/AGO2/C5AR1/PTK2B/JMJD8/SPARC/GRN/PF4/WARS1/GAB1/HSPB1/CYP1B1/BRCA1/NFE2L2/SERPINE1/SASH1/EPN1/ADM/PIK3CD/HMOX1/SP100/NF1/AQP1/STAT3/SFRP2/KRT1/FOXO4/NINJ1/TBXA2R/FKBPL/GLUL/AGO1/E2F2/CLDN5
CC	GO:0032432	actin filament bundle	52	-0.68	-2.29	5.64E-09	1.56E-07	9.57E-08	1717	tags=50%, list=15%, signal=43%	FHOD1/FSCN1/RFLNB/ACTA2/TPM3/PDLIM2/PXN/SYNPO/PALLD/ILK/ACTN1/PDLIM5/GAS2L1/TPM1/MYH9/ACTN4/TPM4/LCP1/ABLIM3/MARCKS/PDLIM7/MYLK/MYL9/CNN2/LPP/ZYX

BP	GO:1901342	regulation of vasculature development	162	-0.52	-2.07	5.74E-09	1.57E-07	9.68E-08	2614	tags=40%, list=22%, signal=31%	BTG1/STIM1/SIRT6/ALOX5/JAK1/HK2/ERBB2/CYBB/HIPK2/CCBE1/PRKCB/PIK3CB/TNF/ROCK2/HIPK1/PRKD2/MECP2/PKM/HHEX/SEMA4A/ITGA5/GADD45A/SP1/HGF/GREM1/NIBAN2/FOXJ2/ROCK1/LRG1/EGLN1/CHI3L1/AGO2/C5AR1/PTK2B/JMJD8/SPARC/GRN/PF4/WARS1/GAB1/HSPB1/CYP1B1/BRCA1/NFE2L2/SERPINE1/SASH1/EPN1/ADM/PIK3CD/HMOX1/SP100/NF1/AQP1/STAT3/SFRP2/KRT1/FOXO4/NINJ1/TBXA2R/FKBPL/GLUL/AGO1/E2F2/CLDN5
BP	GO:0007186	G protein-coupled receptor signaling pathway	365	-0.41	-1.78	5.93E-09	1.61E-07	9.92E-08	2696	tags=35%, list=23%, signal=28%	FPR2/PLCB4/ARHGEF11/PLCB1/SIGMAR1/GPR141/GPR160/LPAR1/JAK2/HCAR3/OR7C1/GNB2/C5AR2/AGTRAP/CAMK2A/RPH3AL/AHCYL1/FLNA/CA2/GNG5/CRCP/CYSLTR2/SORL1/PIK3CB/DNM2/ROCK2/FPR1/PLCE1/GTF2H2/CASR/RGS3/MAS1/RGS2/OR2G6/CDK5R1/S1PR2/PDE4A/FZD5/AKAP13/HRH2/NECAB2/GPR27/GPBAR1/P2RY13/PIK3CG/TAS2R4/PTPN6/SSTR2/FRMPD1/FCN1/GRK4/OR6C75/RAC2/RGS6/CCR1/ADGRE3/OR2B11/ARHGEF12/GNAI2/CACNA1D/INPP5K/ADGRA3/GRK6/FFAR4/GNB1/LTB4R/ROCK1/MGLL/WASF2/YWHAB/CCL3/GSK3A/RELA/RGS14/IGF2R/LGR6/INSR/GIT2/GPR85/AGRN/VAV1/PPP1R9B/ADGRF1/C5AR1/GNA12/GNAQ/PTK2B/CXCR2/RGS19/ZDHHC7/KCNQ1/GPR82/APLP2/CXCR1/SRC/ACP3/PF4/DGKG/PSAP/PIK3R5/P2RY1/CALM3/GNAL/GP1BA/PROK2/PREX1/ACKR1/GRK3/SNCA/TGM2/RAC1/ADM/PLEK/ARRB2/OPRL1/PCSK1N/PTAFR/RGS7/RASGRP4/GNB4/ZDHHC3/TAS2R30/CCRL2/RAPGEF2/TBXA2R/ECE1/GPR146/IQGAP2/GNAZ
BP	GO:0010562	positive regulation of phosphorus metabolic process	549	-0.38	-1.69	6.05E-09	1.62E-07	9.98E-08	2696	tags=32%, list=23%, signal=26%	FPR2/TRAF6/WNT1/RHOA/ACSL1/IL1B/PRKAG2/ADAM17/NEURL1/PRKCZ/BTBD10/PTPA/ADNP/EIF4G1/ERN1/JAK2/NCKAP1L/KIT/ANGPT1/GCN1/PPARGC1B/SEMA7A/P2RX7/CCND3/CSF1R/DDX3X/KCTD20/LCP2/INCENP/ERBB2/LRRK2/BMPR1A/WDFY2/LILRB2/HIPK2/SYAP1/TAB2/TNF/RANBP2/ROCK2/ENTPD5/MAS1/NKX3-1/GPLD1/CDK5R1/PRKD2/SYMPK/S1PR2/RICTOR/ERN2/OSM/MARK2/SLC11A1/PDGFA/FZD5/AKAP13/TAOK1/SYK/SLCO3A1/MAP2K2/ITGA5/PIK3CG/TNFRSF1A/PPP1R12A/DLG4/RB1CC1/STK4/AGAP2/TIRAP/NCF1/HGF/PDE5A/GREM1/TLR6/PID1/SH2D3C/OSBPL8/DSTYK/MAVS/PTGS2/ROCK1/ARNT/PARP14/RPS6KA5/IRAK1/CCNY/PSEN1/MAPT/PTPRJ/FGR/VLDLR/CLU/EPHB4/INSR/SQSTM1/PTPN1/VRK3/PLK1/CHI3L1/MAP3K5/NEDD9/RASSF2/RALB/GNA12/BMP6/PTK2B/ARHGEF2/VCP/MAD2L2/TF/DVL3/CDKN1A/PPP2R5A/PARP16/MAP2K7/CASS4/ILK/VAV3/TAOK3/SRC/PPP1R15A/PAK2/PIK3R5/PECAM1/AKAP9/FLOT1/RIPK1/P2RY1/IL6R/CALM3/DAZAP2/PLAUR/AKT2/FXR2/CLCF1/SASH1/TAL1/CHP1/LYN/MMD/HCLS1/SNCA/IGF2/STRADA/PAK1/SDCBP/RAC1/WNK1/PLEK/ARRB2/MMP9/LIMK2/MAP2K3/PTPRC/FAXDC2/EPHB2/PTAFR/NIBAN1/CACUL1/MOB3A/STRADB/MAPK3/SFRP2/RALBP1/ITGB3/FLCN/AMBRA1/PRKCD/PINK1/MOB3C/PRR5/PELI2/RAPGEF2/IQGAP1/DAB2/TGFA

BP	GO:0045937	positive regulation of phosphate metabolic process	549	-0.38	-1.69	6.05E-09	1.62E-07	9.98E-08	2696	tags=32%, list=23%, signal=26%	FPR2/TRAF6/WNT1/RHOA/ACSL1/IL1B/PRKAG2/ADAM17/NEURL1/PRKCZ/BTBD10/PTPA/ADNP/EIF4G1/ERN1/JAK2/NCKAP1L/KIT/ANGPT1/GCN1/PPARGC1B/SEMA7A/P2RX7/CCND3/CSF1R/DDX3X/KCTD20/LCP2/INCENP/ERBB2/LRRK2/BMPR1A/WDFY2/LILRB2/HIPK2/SYAP1/TAB2/TNF/RANBP2/ROCK2/ENTPD5/MAS1/NKX3-1/GPLD1/CDK5R1/PRKD2/SYMPK/S1PR2/RICTOR/ERN2/OSM/MARK2/SLC11A1/PDGFA/FZD5/AKAP13/TAOK1/SYK/SLCO3A1/MAP2K2/ITGA5/PIK3CG/TNFRSF1A/PPP1R12A/DLG4/RB1CC1/STK4/AGAP2/TIRAP/NCF1/HGF/PDE5A/GREM1/TLR6/PID1/SH2D3C/OSBPL8/DSTYK/MAVS/PTGS2/ROCK1/ARNT/PARP14/RPS6KA5/IRAK1/CCNY/PSEN1/MAPT/PTPRJ/FGR/VLDLR/CLU/EPHB4/INSR/SQSTM1/PTPN1/VRK3/PLK1/CHI3L1/MAP3K5/NEDD9/RASSF2/RALB/GNA12/BMP6/PTK2B/ARHGEF2/VCP/MAD2L2/TF/DVL3/CDKN1A/PPP2R5A/PARP16/MAP2K7/CASS4/ILK/VAV3/TAOK3/SRC/PPP1R15A/PAK2/PIK3R5/PECAM1/AKAP9/FLOT1/RIPK1/P2RY1/IL6R/CALM3/DAZAP2/PLAUR/AKT2/FXR2/CLCF1/SASH1/TAL1/CHP1/LYN/MMD/HCLS1/SNCA/IGF2/STRADA/PAK1/SDCBP/RAC1/WNK1/PLEK/ARRB2/MMP9/LIMK2/MAP2K3/PTPRC/FAXDC2/EPHB2/PTAFR/NIBAN1/CACUL1/MOB3A/STRADB/MAPK3/SFRP2/RALBP1/ITGB3/FLCN/AMBRA1/PRKCD/PINK1/MOB3C/PRR5/PELI2/RAPGEF2/IQGAP1/DAB2/TGFA
BP	GO:0050878	regulation of body fluid levels	223	-0.47	-1.95	6.27E-09	1.66E-07	1.02E-07	3126	tags=49%, list=27%, signal=37%	THBD/ELOVL1/MED1/USF2/GATA1/EDN1/SERPINA1/THBS1/MLLT6/ST3GAL4/SRF/GNAS/HIF1A/CD9/GNA13/NCOR2/CLIC1/NEURL1/ANXA5/ANO6/MTX1/JAK2/HPS6/FCER1G/TMEM79/ACTG1/COPA/HK2/FLNA/SERPINE2/FERMT3/METAP1/SLC22A4/STAT5A/PROS1/PIK3CB/ABAT/ATP7A/GP6/PDGFA/PLSCR1/SYK/F13A1/PIK3CG/PF4V1/PTPN6/CREB1/SERPING1/JMJD1C/GNAI2/GPI/KNG1/INPP5K/STAT5B/FLG2/VAV1/MPIG6B/GNA12/GNAQ/F8/PRDX2/RAB27A/ILK/VAV3/SRC/PF4/FLI1/F5/DGKG/HSPB1/ENTPD1/P2RY1/ACTB/PLAUR/VDR/SELP/GP1BA/PRKACA/ZBTB7B/ALOX12/NFE2L2/SERPINE1/P2RX1/SLC7A11/MYH9/TREML1/NFE2/LYN/WNK1/ADM/PLEK/F11R/OPRL1/MAPK14/ITPK1/EPHB2/SLC4A1/WAS/PAFAH2/TLN1/AQP1/DMTN/ITGB3/MPL/PRKCD/MYL9/VCL/KRT1/TBXA2R/GP9
CC	GO:0030027	lamellipodium	156	-0.52	-2.06	6.39E-09	1.68E-07	1.03E-07	2690	tags=45%, list=23%, signal=35%	CAPRIN1/RHOA/RAPH1/CD177/ACTR2/SLC9A1/FAM89B/DDX3X/SNX1/CAPZB/SPATA13/DNM2/PLCE1/MYH10/ARHGAP31/CFL1/TWF2/SYNE2/ITSN1/RAC2/FSCN1/FSCN3/STX3/DUSP22/ACTA2/SH3RF1/CDC42BPA/ROCK1/ARAP3/WASF2/NEDD9/PSTPIP1/PXN/PPP1R9B/PHACTR4/CTTN/DOCK8/CTNNA1/PTK2B/TESC/APBB1IP/ARPC5/PALLD/LIMK1/ILK/APC/DBN1/KPTN/NRBP1/FLOT1/ACTB/PLEK2/PARVB/CTNND1/SSH1/FGD4/PAK1/RAC1/FGD6/CORO1A/CORO1C/ABI3/ABLIM3/VASP/ITGB3/MYLK/GSN/FLOT2/NHS/IQGAP2

BP	GO:0030182	neuron differentiation	745	-0.35	-1.60	7.19E-09	1.87E-07	1.15E-07	2935	tags=34%, list=25%, signal=27%	CDH23/ENC1/SZT2/PLAA/KATNB1/TAOK2/KIDINS220/SRF/SCLT1/WDPCP/SCARF1/NR2F6/HIF1A/PBX3/SHTN1/UHMK1/CLMN/HDGFL3/FARP1/TOPORS/FES/CRTC1/FRYL/FARP2/CAPRIN1/DICER1/WNT1/RHOA/RAB8A/REST/PIGT/ASPM/RAPH1/NEURL1/PRKCZ/SCARB2/LYPLA2/NEXN/NEUROD2/IFT88/TCTN1/ADNP/EIF4G1/LPAR1/JAK2/NCKAP1L/LST1/ACTR2/SCN1B/SEMA7A/SIN3A/HSPA5/DDX6/C1QA/CSF1R/HECW2/VIM/RAB35/KLF7/CD38/CAMK2A/FEZ1/TSPO/ALCAM/FLNA/PLXNB2/MYO9A/ERBB2/LRRK2/PPT1/RTN4R/TBC1D24/GSK3B/HIPK2/ZSWIM6/DNM2/PAFAH1B1/EFNB1/CFAP418/KIF5B/ATP7A/RGS2/HIPK1/CDK5R1/NRP2/KCTD11/MECP2/ERCC2/RNF165/YWHAH/CPEB3/CFL1/MARK2/FZD5/SEMA4A/CTDSP1/P3H1/EIF4G2/TAOK1/KIAA0319/TWF2/ATF5/ATCAY/DYNC2H1/GAK/WEE1/TRAPPC9/RAB21/CDKN1C/DLG4/CREB1/RAC2/IER2/EP300/RERE/CAMK1D/MICALL1/NCAM1/DRAXIN/HGF/LRP12/STK24/RTN4/STX3/CPNE1/TSPAN2/DTNB/DISC1/MAP1A/SOX4/MEIS1/YWHAZ/GIGYF2/SNAP25/NIBAN2/TRIOBP/IFT140/KDM4A/NUMB/NR4A2/XRN2/CDKL5/ROCK1/IST1/DENND5A/RPS6KA5/SUFU/GSK3A/PSEN1/RARA/MAPT/ANKRD27/ZEB1/VLDLR/EPHB4/ULK1/LGR6/B4GALT5/SPAG9/SIPA1L1/SLC23A2/AGTPBP1/GAS7/AGRN/MAP4/RAP1GAP/PPP1R9B/ADGRF1/CUX1/CTTN/CTNNA1/DNM3/BMP6/NLGN3/PTK2B/ARHGEF2/NDEL1/PPP2R5B/DVL3/ZFHX3/KCNQ1/ACSL4/RETREG3/LIN28A/NIN/NTNG2/PALLD/APLP2/SPRY3/LIMK1/TAOK3/DPYSL2/GRN/DBN1/ASCL2/UBB/DGKG/XK/PDLIM5/PAK2/PBX1/FBXO45/SETX/FLOT1/MYCL/ACTB/DLG5/SOD2/CARM1/EHD1/FEZ2/RBPJ/NFE2L2/TAL1/SCYL1/ZMIZ1/LYN/MMD/FRY/USP9X/PAK1/RAC1/WNK1/ADM/PBX2/PPP3CA/CFLAR/PLXNC1/CEBPB/PICALM/RHOG/MAP4K4/FBXO7/MAP1S/ASAP1/NCOA1/FOXO3/EPHB2/IQSEC1/MYEF2/ABI3/VASP/STAT3/SFRP2/MTPN/KEL/NAPA/BSG/PTEN/VCL/MBP/RAPGEF2/IQGAP1/ECE1/DAB2
BP	GO:0007417	central nervous system development	581	-0.37	-1.66	7.59E-09	1.96E-07	1.21E-07	2723	tags=33%, list=23%, signal=26%	TLR2/TTBK2/CASP2/FPR2/WNT1/RHOA/IL1B/PLCB1/ASPM/WLS/NEURL1/SLC2A1/NEUROD2/TCTN1/SETD2/SCN1B/SEMA7A/SIN3A/HSPA5/SHROOM4/DPCD/C1QA/FOXP2/HAP1/CSF1R/SNRPB/VIM/TAF1/FEZ1/MED12/HDAC11/SERPINE2/PLXNB2/ERBB2/ABCA2/CKB/ATXN1/LRRK2/OTX1/PPT1/RTN4R/TBX19/GSK3B/BMPR1A/BAX/ZSWIM6/ZBTB16/NDE1/PAFAH1B1/TNF/GJC2/ABAT/LRP1/MAS1/ATP7A/RGS2/CDK5R1/NRP2/MECP2/ERCC2/ITGAM/TACC3/YWHAH/PCM1/SYNE2/ATF5/DYNC2H1/ZNF148/SUDS3/TRAPPC9/ATP6AP2/SSTR2/CREB1/ATP2B1/RERE/STK4/SOX6/DRAXIN/HOOK3/RTN4/TSPAN2/DISC1/SOX4/MEIS1/MSX1/GIGYF2/MBOAT7/ROGDI/KDM4A/NUMB/NR4A2/XRN2/VCAN/ATRX/XRCC1/SUFU/PSEN1/RARA/MAPT/ATN1/ZEB1/VLDLR/CLU/B4GALT5/INTU/SEC16A/ATP6V0D1/AGTPBP1/ALDH5A1/ARL13B/PPP1R9B/C5AR1/NDEL1/PTBP2/ZFHX3/SKI/NIN/ARPC5/APLP2/SRC/DPYSL2/GRN/NIPBL/UBB/BPTF/PBX1/KDM7A/FANCC/FBXO45/TACC1/PFKFB3/ACTB/DLG5/CALM3/PADI2/CLCF1/TNFRSF1B/RBPJ/JARID2/CTNND1/AATK/SLC7A11/NDST1/SH3GL1/TAL1/SCYL1/AKNA/BAG6/NRROS/ZMIZ1/APAF1/TACC2/SHARPIN/LYN/CHD7/LAMC3/TYROBP/ARRB2/PBX2/SYNJ1/PPP3CA/CIC/MAP1S/NF1/FOXO3/EPHB2/RGS7/G6PD/IRS2/MARCKS/AQP1/STAT3/MAPK3/PRKDC/SFRP2/CCDC39/MTPN/GNB4/NRGN/NAPA/GSN/BASP1/PTEN/MBP/SLC8A1/RAPGEF2/ODAD4/KIF27/NCOA6

BP	GO:0006887	exocytosis	242	-0.46	-1.94	7.89E-09	2.02E-07	1.24E-07	2658	tags=38%, list=23%, signal=30%	RAB8A/REST/VTI1B/CD177/NCKAP1L/SNAP23/KIT/P2RX7/HAP1/TMEM79/CACNA1I/RPH3AL/LRRK2/GSK3B/TPCN2/FBXL20/PRKCB/PRAM1/ITGAM/ABCC4/STXBP5/SYK/PIP5K1C/ITSN1/PIK3CG/RAB21/EXOC7/APBA1/RAC2/CCR1/CBL/RAB11FIP2/GNAI2/STX3/LAMP1/RAB3B/SNAP25/CTBP2/SNAP29/SYTL4/CCL3/PSEN1/FGR/ITGB2/VPS11/RAB27B/VAMP3/RALB/EXOC6/RAB11B/HCK/SYT12/VPS4B/LGALS9/RAB27A/RAB7A/STX11/LAT2/RAB2B/EXOC4/P2RY1/CALM3/STXBP2/VPS18/P2RX1/MYH9/PTGDS/AP1G1/LYN/UNC13D/SNCA/DNAJC5/PAK1/SDCBP/PLEK/RAB11FIP1/SYNJ1/PIK3CD/HMOX1/CORO1A/PTAFR/CHMP2A/RAB3IL1/RAB5A/DMTN/NAPA/ANK1/RAB3D/RAB31/SPI1/GAB2
BP	GO:0043085	positive regulation of catalytic activity	794	-0.34	-1.57	8.62E-09	2.20E-07	1.35E-07	2800	tags=34%, list=24%, signal=27%	HLA-DRB1/HIF1A/AKIRIN2/ADAM8/ATP2A3/NLRP12/MAPK1/TBC1D25/DBF4B/GNA13/TOPORS/CASP2/NAF1/FPR2/TRAF6/RHOA/ACSL1/IL1B/REST/PRKAG2/ADAM17/NEURL1/PRKCZ/STIM1/PTPA/SOS2/ADNP/ERN1/LPAR1/JAK2/DAPK1/NCKAP1L/FIS1/CASP9/KIT/PSEN2/ANGPT1/GCN1/PPARGC1B/CIDEB/TBC1D22B/P2RX7/RCN3/CCND3/CSF1R/DDX3X/LCP2/ADAP1/PKP4/INCENP/FADD/BEX3/MYO9A/ERBB2/LRRK2/RTN4R/GSK3B/BMPR1A/CHN2/BAX/TBC1D10B/ADPRH/SYAP1/SORL1/C6orf89/TAB2/PIK3CB/HNRNPU/TNF/RANBP2/ROCK2/TBC1D30/ARAP1/GTF2H2/TBC1D8/MAS1/ATP7A/NKX3-1/GPLD1/CDK5R1/PRKD2/ITGAM/RICTOR/ERN2/MARK2/SLC11A1/RALGDS/PDGFA/PLSCR1/FZD5/TBC1D5/AKAP13/RCC2/TAOK1/SYK/CR1/UBE2S/MAP2K2/ABR/PIK3CG/PPP1R12A/RALGAPA2/DLG4/MSH6/RGS6/GCLM/STK4/RAB11FIP2/AGAP2/TIRAP/NCF1/TSC2/VSIR/GNAI2/FZR1/PDE5A/GREM1/CACNA1D/TLR6/ASAP2/TBC1D14/NR4A2/OSBPL8/DSTYK/PSME3/CDKL5/ROCK1/BID/IRAK1/ARAP3/CCNY/XRCC1/YWHAB/CCL3/GSK3A/PSEN1/MAPT/RGS14/FGR/VLDLR/CLU/ITGB2/EPHB4/IFI16/PRR7/INSR/PTPN1/SIPA1L1/VRK3/PLK1/TBC1D2/EVI5/CHI3L1/MAP3K5/NEDD9/AGRN/RAP1GAP/VAV1/RASSF2/ASPH/C5AR1/HSPA1A/PHACTR4/RALB/PSME4/DOCK8/GNA12/GNAQ/PTK2B/NDEL1/CTSD/VCP/EGLN3/DVL3/LGALS9/CDKN1A/PARP16/MAP2K7/CASS4/VAV3/TAOK3/SRC/GRN/PPP1R15A/PSAP/PAK2/PIK3R5/LIMS1/DCUN1D3/IL6R/DAP/CALM3/DAZAP2/VDR/AKT2/PREX1/RABGAP1L/SASH1/P2RX1/PRELID1/ARHGAP25/TAL1/ADRM1/POR/AFDN/APAF1/CHTF8/LAPTM5/NLRC4/LYN/MMD/SNCA/TGM2/IGF2/STRADA/PAK1/WNK1/RSU1/PYCARD/PLEK/TBC1D20/BBC3/SIPA1/F11R/DTX3L/CFLAR/MAP2K3/PTPRC/PICALM/RHOG/MAP4K4/ABHD5/ASAP1/MAPK14/NF1/EPHB2/PTAFR/IQSEC1/CORO1C/RGS7/CACUL1/MOB3A/STRADB/TBC1D1/STAT3/MAPK3/SFRP2/RASGRP4/SNX18/RALBP1/TBC1D3I/ITGB3/FLCN/AMBRA1/PRKCD/GSN/PINK1/PTEN/MOB3C/MBP/SIPA1L2/RAPGEF2/IQGAP1/TGFA/ARHGAP6/HIP1
BP	GO:0040017	positive regulation of locomotion	348	-0.42	-1.82	1.04E-08	2.64E-07	1.62E-07	3027	tags=41%, list=26%, signal=31%	EDN1/ZNF580/ATOH8/FERMT1/CEP43/TIAM1/DAPK2/BCAS3/THBS1/PLAA/MOSPD2/HIF1A/ADAM8/SHTN1/MAPK1/CPNE3/FPR2/RHOA/IL1B/ADAM17/ANO6/JAK2/NCKAP1L/KIT/ANGPT1/SEMA7A/FAM89B/HSPA5/OXSR1/CSF1R/ACTG1/FADD/FERMT3/CCBE1/STAT5A/RIPOR1/PIK3CB/TNF/ROCK2/CASR/ATP7A/GPLD1/NRP2/PRKD2/MYADM/PDGFA/SEMA4A/SYNE2/CXCL16/ITGA5/PIK3CG/SP1/JAM3/RAC2/CCR1/CAMK1D/STK4/TIRAP/HGF/VSIR/GNAI2/RTN4/STX3/GPI/ACTA2/MAP3K3/NUMB/PTGS2/CCL3/FGR/RIN2/FUT7/ZNF703/LGR6/INSR/SPAG9/AGO2/DIAPH1/NEDD9/LGALS3/C5AR1/DOCK8/PTK2B/ARHGEF2/HDAC7/TF/GFUS/LGALS9/DOCK5/CASS4/SPARC/SRC/APC/GRN/NIPBL/GAB1/HSPB1/PECAM1/CYP1B1/TNFSF14/IL6R/SOD2/SELP/AKT2/NFE2L2/SERPINE1/SASH1/ACTN4/LYN/GLIPR2/PAK1/SDCBP/RAC1/WNK1/PYCARD/MMP9/GPSM3/PIK3CD/PPP3CA/MAP2K3/PTPRC/MAP4K4/HMOX1/CORO1A/EPHB2/PTAFR/IQSEC1/IRS2/STAT3/MAPK3/DMTN/ITGB3/MYLK/SPI1/RIPOR2/SLC8A1/PTP4A1/FOXO4/PLVAP/RAPGEF2/DAB2/S100A11

BP	GO:0030155	regulation of cell adhesion	495	-0.38	-1.70	1.12E-08	2.82E-07	1.74E-07	3022	tags=38%, list=26%, signal=29%	BCL6/PKD1/ADAM15/FERMT1/TYK2/CSK/PPP2R1A/BCAS3/THBS1/VNN1/PML/ST3GAL4/SRF/ITCH/WDPCP/HLA-DRB1/ADAM8/CD9/LGALS9C/FES/TRAF6/WNT1/RHOA/IL1B/DHPS/TMEM131L/PRKCZ/CCDC88B/JAK2/NCKAP1L/IL4I1/ANGPT1/SLC9A1/ALOX5/TGFBI/RC3H2/ACTG1/JAK1/CD70/PKP4/ARHGDIB/FADD/FLNA/SERPINE2/PLXNB2/ERBB2/FERMT3/GSK3B/LILRB2/ZBTB16/PIK3CB/DNM2/PELI1/ZDHHC2/TNF/UTRN/EFNB1/ROCK2/BST1/ABAT/GBP1/TNFSF13B/PRKD2/NFAT5/GP6/ALOX15/ARHGDIA/MYADM/SPTA1/RCC2/SYK/CR1/HLA-E/ITGA5/PIK3CG/PTPN6/PPP1R12A/LAMA4/JAM3/RAC2/CHST2/STK4/VSIR/STX3/PDE5A/GREM1/DISC1/DUSP22/KNG1/SOX4/TRIOBP/STAT5B/WASHC2C/ROCK1/IRAK1/RARA/RELA/PTPRJ/RIN2/ITGB2/FUT7/ZNF703/NEDD9/VAV1/LGALS3/DOCK8/BMP6/PTK2B/MAD2L2/TESC/APBB1IP/SLK/GFUS/SIRPA/PRDX2/LGALS9/ZFHX3/DOCK5/CASS4/ILK/VAV3/SRC/TBX21/LIMS1/FLOT1/CYP1B1/DUSP1/TNFSF14/DLG5/PLAUR/SELP/IRF1/ZBTB7B/ALOX12/PREX1/IL1RN/SERPINE1/TPM1/AKNA/AFDN/NOTCH4/ZMIZ1/ACTN4/LAPTM5/LYN/SIRPB1/UNC13D/TGM2/IGF2/RAC1/WNK1/RSU1/PYCARD/SIPA1/CYRIB/PPP3CA/F11R/PLXNC1/CEBPB/PTPRC/MAP4K4/MAPK14/CORO1A/NF1/FOXO3/EPHB2/PTAFR/CORO1C/TFE3/DMTN/SFRP2/PRKAR1A/FLCN/AMBRA1/PRKCD/HLX/PTEN/CYTIP/VCL/HSD17B12/SPI1/MBP/RIPOR2/FLOT2/DAB2/ARHGAP6
BP	GO:0030099	myeloid cell differentiation	300	-0.43	-1.83	1.22E-08	3.05E-07	1.88E-07	3106	tags=47%, list=26%, signal=35%	CNOT4/CD4/KLF13/MED1/CDC73/CBFA2T3/GATA1/MFHAS1/BCL6/STAT1/L3MBTL1/IL31RA/ATP6AP1/PML/SRF/RELB/HLA-DRB1/HIF1A/TLR2/FES/FOS/FARP2/TRAF6/INPP5D/IKZF1/JAK2/NCKAP1L/CASP9/KIT/PPARGC1B/GPR137B/EVI2B/CSF1R/IREB2/CEBPA/FADD/MITF/HIPK2/ZBTB16/PAFAH1B1/TNF/NFKBIA/DYRK3/ERCC2/DNASE2/LTBR/SNRK/ANXA2/LBR/ZFPM1/PTPN6/CDKN1C/TOB2/VPS33A/CREB1/CTR9/EP300/CCR1/TIRAP/ARID4A/TSPAN2/MEIS1/TET2/STAT5B/MTURN/JUNB/TRIM10/CSF3R/ARNT/H4C8/WASF2/HSPA1B/CCL3/PSEN1/RARA/IFI16/ADAR/SMAP1/TSPO2/RASSF2/HSPA1A/SBNO2/MPIG6B/KAT6A/PTK2B/TESC/CUL4A/THOC5/TF/KCNQ1/ACTN1/SNX10/SRC/BPGM/KMT2E/PF4/FLI1/PIP4K2A/EIF2AK1/RIPK1/ZFP36L1/ZBTB7A/OSCAR/RBPJ/AHSP/MYH9/TAL1/BATF2/NRROS/RCOR1/CTNNBIP1/LYN/PTBP3/HCLS1/TYROBP/MMP9/PITHD1/PIK3CD/ALAS2/CEBPB/TRIM58/EPB42/MAPK14/NF1/FAXDC2/FOXO3/TFE3/SLC4A1/FAM210B/G6PD/STAT3/DMTN/PRKDC/RASGRP4/MPL/SPI1/LILRB3/TRIB1/KLF1/GAB2/NCOA6
CC	GO:0030424	axon	364	-0.41	-1.78	1.63E-08	4.05E-07	2.49E-07	2680	tags=35%, list=23%, signal=28%	RHOA/SIGMAR1/MAP7/PRKCZ/NEXN/ADNP/PSEN2/SCN1B/DYRK1A/CCDC120/HAP1/VIM/ACTG1/FEZ1/COPA/ALCAM/FLNA/MYO9A/LRRK2/PPT1/RTN4R/TBC1D24/GSK3B/SYAP1/PRKCB/DNM2/PAFAH1B1/GJC2/CASR/KIF5B/ATP7A/KCNAB2/CDK5R1/NRP2/GRIK2/KIF1B/SPTA1/FZD5/EIF4G2/TWF2/NECAB2/ATCAY/ABR/ATP6AP2/RAB21/RIN3/CPEB4/EXOC7/TRAK1/DLG4/GRIPAP1/FSCN1/CBL/FSCN3/RNF40/STX3/DISC1/MAP1A/PVALB/AP3S2/EEA1/SNAP25/ROGDI/SH2D3C/CDKL5/GSK3A/PSEN1/MAPT/KIF1C/ULK1/INSR/ATP6V0D1/AGTPBP1/RAB27B/MAP4/RAP1GAP/PPP1R9B/WDFY3/CTTN/ARL8A/EXOC6/DNM3/PTK2B/NDEL1/PTBP2/NIN/ARPC5/NTNG2/PALLD/MYO1D/DBN1/EXOC4/HSPB1/SETX/ACTB/CALM3/MYO5A/FXR2/TNFRSF1B/FEZ2/CTNND1/NAV1/MME/CCSAP/ERMN/USP9X/SNCA/PAK1/SYNJ1/PPFIA1/CORO1A/NF1/EPHB2/TPRG1L/RAB5A/MTPN/SBF2/TRAK2/NRGN/PINK1/BSG/BASP1/MBP/SLC8A1/IQGAP1/TSHZ3/GLUL/CLDN5

BP	GO:0060284	regulation of cell development	296	-0.43	-1.82	1.64E-08	4.06E-07	2.49E-07	2690	tags=37%, list=23%, signal=29%	CAPRIN1/DICER1/IL1B/REST/PLCB1/ASPM/NEURL1/ADNP/KIT/ACTR2/SEMA7A/SMARCD3/SPEN/HAP1/TRIM11/TSPO/FLNA/SERPINE2/PLXNB2/LRRK2/RTN4R/GSK3B/BMPR1A/ADD1/SORL1/DNM2/PAFAH1B1/TNF/ROCK2/GJC2/GBP1/CDK5R1/S1PR2/YWHAH/MYADM/PCM1/SEMA4A/CTDSP1/EIF4G2/RCC2/KIAA0319/TWF2/TNFRSF1A/RAB21/DRAXIN/HOOK3/RTN4/PDE5A/RFLNB/DISC1/NIBAN2/TRIOBP/KDM4A/NUMB/CDKL5/ROCK1/IST1/GSK3A/PSEN1/MAPT/RGS14/ANKRD27/ULK1/NEDD9/CUX1/CTNNA1/NDEL1/DOCK5/LIN28A/SKI/CASS4/NIN/LIMK1/ILK/GRN/DBN1/ASCL2/LIMS1/CLCF1/TNFRSF1B/PREX1/LMOD3/MME/ACTN4/LYN/UNC13D/TGM2/CHD7/TYROBP/RAC1/SYNJ1/PPP3CA/F11R/PLXNC1/TRIM58/NF1/EPHB2/CORO1C/DMTN/PRKDC/MPL/PTEN/VCL/MBP/RNF10/TRIB1/RAPGEF2/DAB2/CLDN5
CC	GO:0042641	actomyosin	55	-0.67	-2.27	2.24E-08	5.42E-07	3.33E-07	1717	tags=47%, list=15%, signal=41%	FHOD1/FSCN1/ACTA2/CDC42BPA/TPM3/PDLIM2/PXN/SYNPO/PALLD/ILK/ACTN1/DBN1/PDLIM5/GAS2L1/TPM1/MYH9/ACTN4/TPM4/LCP1/ABLIM3/PDLIM7/MYLK/MYL9/CNN2/LPP/ZYX
BP	GO:0006954	inflammatory response	506	-0.37	-1.67	2.39E-08	5.77E-07	3.54E-07	3059	tags=38%, list=26%, signal=29%	IL18R1/CCL5/TFR2/MFHAS1/BCL6/ZNF580/SERPINA1/CST7/IL17RC/THBS1/VNN1/PLAA/IL31RA/BAP1/RELB/NAIP/MMP25/ITCH/NLRP10/GSTP1/HLA-DRB1/HIF1A/ADAM8/NLRP12/TLR2/FOS/FPR2/IL1B/PRKCZ/ANO6/JAK2/SNAP23/KIT/SEMA7A/ALOX5/TICAM1/P2RX7/C1QA/CSF1R/SETD4/C5AR2/CEBPA/PJA2/IL18RAP/SPATA2/IL1R2/THEMIS2/LRRK2/CYBB/REL/TNF/NFKBIA/FPR1/BST1/PTGES/LRP1/TLR1/MAS1/TLR8/PTGS1/ITGAM/RICTOR/HDAC4/ALOX15/PRCP/OSM/PRDX5/SLC11A1/PLSCR1/METRNL/ORM1/SYK/IL10RB/NMI/HLA-E/NFATC3/ITGAL/PIK3CG/TNFRSF1A/PF4V1/JAM3/ALOX5AP/CCR1/CAMK1D/CHST2/TIRAP/NCF1/HGF/TSPAN2/KNG1/TLR6/CD14/STAT5B/FFAR4/LTB4R/AOC3/PTGS2/MGLL/RPS6KA5/NKIRAS2/CCL3/PSEN1/MAPT/RELA/FGR/CLU/ITGB2/FUT7/IFI16/SOCS3/AREL1/CHI3L1/PSTPIP1/NFKBIB/C5AR1/SBNO2/TIMP1/BMP6/CXCR2/F8/B4GALT1/HCK/NUPR1/GPX1/TNIP1/SIRPA/PRDX2/LGALS9/CXCL5/SRC/GRN/PF4/MAPKAPK2/IKBKG/APOL2/EIF2AK1/RIPK1/IL6R/SELP/TNFRSF1B/PROK2/RBPJ/TREM1/IL1RN/NFE2L2/SERPINE1/P2RX1/KDM6B/NDST1/SCYL1/AKNA/NRROS/BRD4/ACKR1/NLRC4/CTNNBIP1/SHARPIN/LYN/UNC13D/SNCA/TYROBP/RAC1/ADM/PYCARD/FCGR3A/CDK19/MMP9/XIAP/GPSM3/PIK3CD/F11R/MAP2K3/CEBPB/PTPRC/HK1/MAPK14/HMOX1/EPHB2/PTAFR/PGLYRP1/IL17RA/TOLLIP/STAT3/NLRX1/PRKCD/CD68/NFAM1/KRT1/CCRL2/ZYX/NINJ1/TBXA2R/FURIN

BP	GO:0045944	positive regulation of transcription by RNA polymerase II	787	-0.34	-1.56	2.48E-08	5.93E-07	3.65E-07	2700	tags=32%, list=23%, signal=26%	CRTC1/FOS/TRAF6/PRDM5/WNT1/PIK3R2/IL1B/MED14/LPIN2/REST/VEZF1/NEUROD2/JAK2/ZEB2/IL4I1/HCFC1/CBX7/ACTR2/GCN1/PPARGC1B/SLC9A1/SIN3A/POU2F2/HSPA5/TAF8/DDX3X/TAF1/MLLT10/KLF7/CEBPA/MED12/SMARCC1/FADD/MITF/NFYA/KLF5/OTX1/YY1/GFI1B/NFIA/TBX19/MOSPD1/REL/SENP2/ARID5A/BMPR1A/BCLAF1/HIPK2/RHOQ/ZBTB16/DR1/KDM1B/HNRNPU/ZNF585B/ATF3/TNF/PHIP/NFKBIA/ZNF107/JUND/RSF1/NKX3-1/ATAD2B/PRKD2/NFAT5/MECP2/PPRC1/MAML1/HDAC4/NAMPT/OSM/CDK13/ZNF784/SLC11A1/HHEX/PLSCR1/FZD5/ZNF746/ZFPM1/ATF5/ZNF445/MED24/ZNF148/NFATC3/KLF6/TNFRSF1A/ELF1/PPP1R12A/FHOD1/SP1/ZGLP1/CREB1/NFKB2/IER2/EP300/PCBP1/THRAP3/HGF/RNASEL/CEBPD/ARID4A/SUPT6H/YBX1/MRTFA/GREM1/KANSL3/ZBED1/TFCP2/SOX4/MEIS1/TET2/MSX1/TET3/STAT5B/PID1/PCBP2/CREBRF/JUNB/FOXJ2/TFEB/CTBP2/NR4A2/MAVS/ARNT/RPS6KA5/SESN2/ATRX/TFDP2/RARA/RELA/ZEB1/IFI16/SQSTM1/ZNF786/HELZ2/MAFG/NFYC/MAX/NR3C1/AGO2/TBL1X/AGRN/SMARCA2/ATF6B/SBNO2/NR4A1/KAT6A/WWP2/IRF2/BMP6/ZNF493/ARHGEF2/PTMS/TNIP1/KMT2C/RPS6KA1/BCL9/PPP2R5B/DVL3/ZFHX3/NPAT/DCAF6/SKI/HDGF/BACH1/NCOA2/TBX21/NIPBL/ETS2/PF4/IKBKG/ASCL2/FLI1/BPTF/PBX1/ZNF48/ETV6/RFX2/SETX/RIPK1/P2RY1/SSBP3/VDR/IRF1/CSRNP1/RXRA/TAF10/ZBTB7B/BRCA1/RBPJ/KAT5/TAF6/NFE2L2/USF3/KDM6B/MEPCE/IRF2BPL/TAL1/AKNA/NOTCH4/ZMIZ1/BRD4/NFIX/PLAGL2/HCLS1/IGF2/CHD7/ARID3A/HNRNPK/ELF4/PBX2/CREBBP/PPP3CA/POU2F1/CEBPB/STAT6/E2F3/ELL/MAPK14/NCOA1/NFIC/FOXO3/TFE3/WAS/PPP3R1/ABLIM3/WBP2/TCF7L2/MED25/STAT3/MAPK3/PRKDC/SFRP2/FLCN/PCGF5/FOSL2/ZNF33A/SPI1/BCL3/FOXO4/RNF10/MICAL2/ARID3B/DAB2/AGO1/LITAF/E2F2/NCOA6
BP	GO:0046903	secretion	549	-0.37	-1.66	2.56E-08	6.06E-07	3.72E-07	2739	tags=33%, list=23%, signal=26%	NCOR2/SLC26A6/TLR2/FES/IL1B/RAB8A/REST/WLS/NEURL1/VTI1B/CD177/MTX1/JAK2/NCKAP1L/SNAP23/HPS6/KIT/ALOX5/CCDC186/P2RX7/RCN3/ARFGAP3/HAP1/TMEM79/KLF7/PORCN/CD38/CACNA1I/CAMK2A/COPA/RPH3AL/HK2/SERPINE2/CA2/LRRK2/PPT1/KDM5B/GSK3B/SLC22A4/TPCN2/STAT5A/FBXL20/NPHS1/PRKCB/PAFAH1B1/TNF/PRAM1/ABAT/PTGES/MYH10/CASR/ATP7A/NKX3-1/GPLD1/UCP2/ITGAM/ABCC4/OSM/STXBP5/CES1/P3H1/HRH2/SYK/PIP5K1C/GPR27/ITSN1/PIK3CG/TNFRSF1A/RAB21/EXOC7/APBA1/CREB1/RAC2/SYN3/CCR1/CBL/LRRC8A/ABCB11/RAB11FIP2/GNAI2/STX3/LAMP1/COPG1/SOX4/RAB3B/SIDT2/SNAP25/NIBAN2/STAT5B/RSAD2/FFAR4/CTBP2/SNAP29/MCTP2/SYTL4/CCL3/PSEN1/FGR/ITGB2/VPS11/AQP9/RAB27B/NR4A1/VAMP3/RALB/EXOC6/RAB11B/HMGCR/BMP6/HCK/SYT12/VPS4B/LGALS9/RAB27A/KCNQ1/ACSL4/SNX10/CPT1A/RAB7A/STX11/LAT2/RAB2B/GNPTAB/EXOC4/P2RY1/CALM3/STXBP2/VDR/ZBTB7B/TNFRSF1B/VPS18/IL1RN/P2RX1/MYH9/PTGDS/AP1G1/ABCG1/LYN/UNC13D/SNCA/CHD7/C2CD2L/DNAJC5/PAK1/SDCBP/WNK1/ADM/PLEK/MIDN/RAB11FIP1/SYNJ1/SLC1A5/TANGO2/PIK3CD/PPP3CA/OPRL1/MYOM1/HMOX1/CORO1A/NF1/PTAFR/CHMP2A/IRS2/RAB3IL1/TCF7L2/RAB5A/AQP1/DMTN/PCSK6/NAPA/PINK1/ANK1/RAB3D/RAB31/SPI1/LTBP2/FURIN/DAB2/GLUL/GAB2

BP	GO:0034097	response to cytokine	619	-0.35	-1.61	2.56E-08	6.06E-07	3.72E-07	2737	tags=38%, list=23%, signal=30%	IL17RC/THBS1/ST3GAL6/OAS3/IL31RA/PARP9/BST2/PML/SRF/RELB/NAIP/GSTP1/HIF1A/NLRP12/MAPK1/LILRA6/SLC26A6/TLR2/FOS/RO60/TRAF6/PRDM5/RHOA/ACSL1/IL1B/PLCB1/SAMHD1/ADAM17/SELPLG/CDC42EP4/SETD2/JAK2/DAPK1/KIT/ACTR2/ANGPT1/HSPA5/FCER1G/OXSR1/CSF1R/VIM/ACTG1/JAK1/CD38/CEBPA/CD70/TRIM21/TUBA1B/HK2/FADD/KLF5/IL18RAP/SPATA2/IL1R2/YY1/REL/KDM5B/IFITM3/GSK3B/LILRB2/BCLAF1/STAT5A/TLE4/HNRNPU/PAFAH1B1/TNF/NFKBIA/MRAS/MNDA/GBP1/KIF5B/TNFSF13B/NKX3-1/HIPK1/NRP2/NFAT5/TRIM25/HDAC4/ALOX15/CD300LF/CALCOCO2/OSM/SLC11A1/PLSCR1/MCL1/SYK/IL10RB/RAB43/LILRA1/NMI/CXCL16/TNFRSF1A/PF4V1/YTHDF3/PTPN6/NUB1/IRAK3/IL17RD/CREB1/CTR9/GCLM/CCR1/AGPAT1/CBL/LSM14A/EIF2AK2/CPNE1/YBX1/MX2/INPP5K/CD14/LILRA2/STAT5B/PID1/CSF3R/GBP2/CTBP2/MAVS/PTGS2/PARP14/RPS6KA5/IRAK1/NKIRAS2/USP27X/PELI3/MTHFR/HSPA1B/CCL3/GSK3A/RARA/PRPF8/RELA/PTPRJ/ERBIN/IFI16/SOCS3/PTPN1/ADAR/CHI3L1/MAP3K5/PTP4A3/MT2A/HSPA1A/SBNO2/VAMP3/TIMP1/DOCK8/PTK2B/IFNAR1/ARHGEF2/CXCR2/HCK/DOK1/SIRPA/LGALS9/CXCL5/PARP16/MAP2K7/ILK/HDGF/OASL/SNX10/CXCR1/SRC/TPR/PF4/MAPKAPK2/CDC34/LIMS1/RFX2/CYP1B1/DUSP1/RIPK1/IL6R/ZFP36L1/SELP/PADI2/IRF1/IFNAR2/PRKACA/GBP4/CLCF1/TNFRSF1B/BRCA1/MAPKAPK3/IL1RN/JARID2/NFE2L2/IFIT1B/MME/ACTN4/ACKR1/LAPTM5/SHARPIN/USP10/HCLS1/SNCA/WNK1/PYCARD/XIAP/RAD23B/ADIPOR1/SYNJ1/TIMP2/H2BC11/CEBPB/PTPRC/STAT6/MAPK14/SP100/CORO1A/WBP1L/FOXO3/WAS/YBX3/IL17RA/TOLLIP/STAT3/MAPK3/IFITM2/IL13RA1/MPL/GSN/BCL2L1/SPI1/CCRL2/LILRB3/MBP/RIPOR2/PLVAP/ZYX/LSP1/NFIL3
BP	GO:0050806	positive regulation of synaptic transmission	75	-0.62	-2.21	2.77E-08	6.50E-07	4.00E-07	2770	tags=49%, list=24%, signal=38%	MAPK1/CRTC1/PRKCZ/HAP1/SERPINE2/CA2/ZDHHC12/GSK3B/LILRB2/ZDHHC2/TNF/KIF5B/MECP2/GRIK2/DLG4/STX3/SNAP25/PTGS2/RGS14/SQSTM1/NLGN3/PTK2B/SYT12/FLOT1/MME/SNCA/TYROBP/ARRB2/NF1/EPHB2/NRGN/PINK1/ZDHHC3/PTEN/STAU1/TSHZ3/GLUL
CC	GO:0001725	stress fiber	49	-0.68	-2.27	3.04E-08	6.83E-07	4.20E-07	1717	tags=49%, list=15%, signal=42%	FHOD1/FSCN1/ACTA2/TPM3/PDLIM2/PXN/SYNPO/PALLD/ILK/ACTN1/PDLIM5/GAS2L1/TPM1/MYH9/ACTN4/TPM4/LCP1/ABLIM3/PDLIM7/MYLK/MYL9/CNN2/LPP/ZYX
CC	GO:0097517	contractile actin filament bundle	49	-0.68	-2.27	3.04E-08	6.83E-07	4.20E-07	1717	tags=49%, list=15%, signal=42%	FHOD1/FSCN1/ACTA2/TPM3/PDLIM2/PXN/SYNPO/PALLD/ILK/ACTN1/PDLIM5/GAS2L1/TPM1/MYH9/ACTN4/TPM4/LCP1/ABLIM3/PDLIM7/MYLK/MYL9/CNN2/LPP/ZYX
MF	GO:0004712	protein serine/threonine/tyrosine kinase activity	325	-0.42	-1.80	2.98E-08	6.83E-07	4.20E-07	3308	tags=42%, list=28%, signal=31%	AURKA/MOK/PIM2/MAP4K2/BRAF/PHKG1/TNK2/CAMK1/STK35/SRPK1/MKNK1/CLK3/MARK1/MAP3K1/STK32C/NEK4/TYK2/CSK/DAPK2/STK19/TAOK2/CAMKK2/PIM3/STK38L/CSNK1A1/RSKR/MAPK1/UHMK1/PIKFYVE/TTBK2/FES/MAP2K4/PRKCZ/ERN1/CSNK2A1/JAK2/DAPK1/DYRK1B/KIT/NEK6/DYRK1A/CSNK1G2/OXSR1/CSF1R/TAF1/JAK1/CAMK2A/ERBB2/LRRK2/GSK3B/HIPK2/TESK2/PRKCB/ROCK2/DYRK3/PKN1/HIPK1/PRKD2/ERN2/MARK2/CDK13/SNRK/TAOK1/MAST3/SYK/MAP2K2/SIK3/GAK/PIK3CG/WEE1/CAMK1D/STK4/PIM1/EIF2AK2/STK24/MKNK2/MAP3K3/CDC42BPA/DSTYK/CDKL5/ROCK1/RPS6KA5/IRAK1/GSK3A/FGR/EPHB4/ULK1/INSR/MAP3K20/PLK1/MAP3K5/MARK4/PTK2B/RIOK3/HCK/NEK5/NEK9/SLK/RPS6KA1/MAP2K7/HIPK3/CSNK1G1/LIMK1/ILK/TAOK3/SRC/ALPK1/MAPKAPK2/PAK2/EIF2AK1/RIPK1/MARK3/PRKACA/AKT2/MAPKAPK3/AATK/MAST2/STK40/LYN/PAK1/WNK1/BMP2K/CDK19/MAP3K2/LIMK2/MAP2K3/MAP4K4/SRPK2/STK38/MAPK14/BMX/STK10/EPHB2/MAPK3/PRKDC/PRKCD/PINK1/NUAK2

CC	GO:0005774	vacuolar membrane	387	-0.40	-1.75	2.99E-08	6.83E-07	4.20E-07	2616	tags=33%, list=22%, signal=27%	VTI1B/SCARB2/CTSA/PLEKHM2/HPS6/PSEN2/GNB2/WIPI1/LAMTOR4/GPR137B/TMEM79/TECPR1/ZFYVE26/SNX14/ABCA2/TMEM150B/IFITM3/CHMP3/TPCN2/PLEKHM1/MAP1LC3B/RAB2A/FPR1/PI4K2A/SLCO4C1/LRP1/KIF5B/TLR8/GPLD1/GABARAPL2/MYLPF/ATP6V1C1/PRCP/CALCOCO2/AP1M1/MANBA/MCOLN1/MEAK7/MFSD12/GABARAPL1/ANXA2/ATP6AP2/SLC15A3/VPS33A/RB1CC1/LAMP2/LRRC8A/TMBIM1/AP2M1/CPNE1/LAMP1/RAB37/CKAP4/SIDT2/ATP6V0A1/MAP1LC3A/FFAR4/CSF3R/GNB1/CLTC/TFEB/BRI3/SLC44A2/SNAP29/YWHAB/PSEN1/VLDLR/VPS11/ULK1/CD63/SPAG9/ATP6V0D1/NEU1/WDFY3/ARL8A/CLEC16A/GNAQ/CTSD/B4GALT1/SLC17A5/AP2B1/ELAPOR1/RILP/CHMP1A/ACP3/GRN/RAB7A/GABARAP/KPTN/PGAP6/PSAP/FLOT1/SLC29A3/SH3GLB1/VPS18/CUBN/AP2A1/MARCHF2/AP1G1/LAPTM5/UBA1/LYN/MMD/ATP6V1B2/DNAJC5/UBXN6/DTX3L/SLC48A1/MAP1LC3B2/CHMP2A/AP1B1/RAB5C/MARCHF8/SBF2/IFITM2/GNB4/FLCN/CD68/RAB3D/NFAM1/CHMP4B/NPRL3/AP5B1/ECE1/DAB2/LITAF/SLC7A5/ATP6V1A
BP	GO:0031401	positive regulation of protein modification process	671	-0.35	-1.59	3.03E-08	6.83E-07	4.20E-07	2916	tags=34%, list=25%, signal=27%	IL31RA/TET1/ARRDC3/TAOK2/CAMKK2/PARP9/PML/CLN3/GLYR1/KRAS/SMARCB1/HLA-DRB1/ADAM8/MAPK1/DBF4B/TOPORS/ALG10B/FPR2/TRAF6/WNT1/RHOA/ACSL1/IL1B/PRKAG2/ADAM17/NEURL1/PRKCZ/PTPA/ADNP/EIF4G1/ERN1/JAK2/NCKAP1L/HCFC1/KIT/ANGPT1/SEMA7A/HSPA5/CEP295/DYRK1A/TICAM1/P2RX7/CCND3/CSF1R/DDX3X/LCP2/INCENP/ERBB2/PTTG1IP/LRRK2/SENP2/ARID5A/BMPR1A/WDFY2/LILRB2/HIPK2/SYAP1/C6orf89/TAB2/PELI1/TNF/PHF20/ROCK2/MAS1/NKX3-1/CDK5R1/PRKD2/SYMPK/NSD3/MECP2/S1PR2/RICTOR/HDAC4/ERN2/OSM/MARK2/SLC11A1/PDGFA/FZD5/AKAP13/TAOK1/SYK/NMI/SLCO3A1/UBE2S/MAP2K2/ITGA5/PIK3CG/TNFRSF1A/PPP1R12A/RNF20/DLG4/RB1CC1/LMNA/CTR9/STK4/FBXO33/AGAP2/TIRAP/NCF1/HGF/RNF40/FZR1/PDE5A/GREM1/KANSL3/CUL3/SOX4/TLR6/PHF19/SH2D3C/OSBPL8/MAVS/PTGS2/ARNT/PARP14/RPS6KA5/IRAK1/CCNY/GSK3A/PSEN1/PTPRJ/VLDLR/CLU/FKBP1A/INSR/SQSTM1/RTF1/PTPN1/VRK3/PLK1/CHI3L1/MAP3K5/RASSF5/NEDD9/RASSF2/RALB/GNA12/BMP6/PTK2B/ARHGEF2/VCP/MAD2L2/NUPR1/TNIP1/ATG7/DVL3/CDKN1A/PPP2R5A/PARP16/MAP2K7/CASS4/ILK/TAOK3/SRC/NIPBL/KMT2E/PPP1R15A/UBB/GABARAP/PAK2/PIK3R5/PECAM1/DCUN1D3/AKAP9/FLOT1/RIPK1/P2RY1/IL6R/CALM3/DAZAP2/PLAUR/PIAS1/AKT2/FXR2/CLCF1/ZBTB7B/BRCA1/KAT5/JARID2/SASH1/GOLGA2/TAL1/BRD4/LAPTM5/CHP1/LYN/MMD/HCLS1/SNCA/IGF2/STRADA/PAK1/SDCBP/RAC1/WNK1/ARRB2/MMP9/XIAP/LIMK2/MAP2K3/PTPRC/WBP1L/FAXDC2/NIBAN1/CACUL1/MOB3A/STRADB/WBP2/TOLLIP/MAPK3/SFRP2/RALBP1/ITGB3/FLCN/AMBRA1/PRKCD/PINK1/PTEN/MOB3C/JDP2/PRR5/PELI2/RAPGEF2/IQGAP1/DAB2/TGFA
MF	GO:0005543	phospholipid binding	321	-0.41	-1.79	3.31E-08	7.39E-07	4.54E-07	2708	tags=34%, list=23%, signal=27%	FES/CPNE3/WDFY1/PLCB1/ARHGAP33/ANXA5/SCARB2/LPAR1/ATG2B/SLC9A1/WIPI1/ARHGAP9/CLN6/RAB35/ADAP1/TECPR1/SNX1/ZFYVE26/SNX14/PPT1/CHMP3/TPCN2/DENND1C/CPTP/ARAP1/SNX8/GGA3/BIN2/ITPR2/ALOX15/DAPP1/CD300LF/CERT1/CPNE2/TWF2/ANXA2/PCYT1A/TTPAL/PSD4/MICALL1/TIRAP/NCF1/RUBCN/NCF4/CPNE1/FCGR3B/SNX11/MTM1/CEACAM5/NME4/PHF12/EEA1/MAP1LC3A/SHC1/OSBPL8/WASHC2C/ZFYVE16/ARAP3/SYTL4/MAPT/WIPI2/ZCCHC2/ANXA11/WDFY3/SYT12/KCNQ1/SNX10/PLEKHB2/WDR45/PSAP/PITPNA/STAM2/GLE1/PLEK2/MYO1E/SVIL/SNX27/HS1BP3/PREX1/BSCL2/EPB41/MME/ABCG1/EPN1/SNCA/ATG2A/C2CD2L/SDCBP/CAVIN2/PICALM/ASAP1/NF1/PTAFR/CHMP2A/ARHGAP26/PAFAH2/TLN1/SBF2/SNX18/NRGN/DENND1A/GSN/RAPGEF2/IQGAP1/DYSF/GAB2/OSBPL2/OSBPL1A/IQGAP2/HIP1

BP	GO:0042327	positive regulation of phosphorylation	504	-0.37	-1.66	3.43E-08	7.62E-07	4.69E-07	2696	tags=32%, list=23%, signal=26%	FPR2/TRAF6/WNT1/RHOA/ACSL1/IL1B/PRKAG2/ADAM17/NEURL1/PRKCZ/BTBD10/ADNP/EIF4G1/ERN1/JAK2/NCKAP1L/KIT/ANGPT1/GCN1/PPARGC1B/SEMA7A/P2RX7/CCND3/CSF1R/DDX3X/KCTD20/LCP2/INCENP/ERBB2/LRRK2/BMPR1A/WDFY2/HIPK2/SYAP1/TAB2/TNF/RANBP2/ROCK2/ENTPD5/MAS1/NKX3-1/CDK5R1/PRKD2/S1PR2/RICTOR/ERN2/OSM/MARK2/SLC11A1/PDGFA/FZD5/AKAP13/TAOK1/SYK/SLCO3A1/MAP2K2/ITGA5/PIK3CG/TNFRSF1A/DLG4/RB1CC1/STK4/AGAP2/TIRAP/NCF1/HGF/PDE5A/GREM1/TLR6/SH2D3C/OSBPL8/DSTYK/MAVS/PTGS2/ARNT/PARP14/RPS6KA5/IRAK1/CCNY/PSEN1/MAPT/PTPRJ/FGR/VLDLR/CLU/EPHB4/INSR/SQSTM1/PTPN1/PLK1/CHI3L1/MAP3K5/NEDD9/RASSF2/RALB/BMP6/PTK2B/ARHGEF2/MAD2L2/TF/DVL3/CDKN1A/PARP16/MAP2K7/CASS4/ILK/VAV3/TAOK3/SRC/PAK2/PIK3R5/PECAM1/AKAP9/FLOT1/RIPK1/P2RY1/IL6R/CALM3/DAZAP2/PLAUR/AKT2/FXR2/CLCF1/SASH1/TAL1/LYN/MMD/HCLS1/SNCA/IGF2/STRADA/PAK1/SDCBP/RAC1/WNK1/ARRB2/MMP9/LIMK2/MAP2K3/PTPRC/FAXDC2/EPHB2/NIBAN1/CACUL1/MOB3A/STRADB/MAPK3/SFRP2/RALBP1/ITGB3/FLCN/AMBRA1/PRKCD/PINK1/MOB3C/PRR5/PELI2/RAPGEF2/IQGAP1/DAB2/TGFA
BP	GO:0051272	positive regulation of cellular component movement	349	-0.41	-1.78	3.76E-08	8.31E-07	5.11E-07	3027	tags=41%, list=26%, signal=31%	EDN1/BCL6/ZNF580/ATOH8/FERMT1/CEP43/TIAM1/DAPK2/BCAS3/THBS1/PLAA/MOSPD2/HIF1A/ADAM8/SHTN1/BORCS5/MAPK1/CPNE3/FPR2/RHOA/IL1B/ADAM17/ANO6/JAK2/NCKAP1L/KIT/ANGPT1/SEMA7A/FAM89B/HSPA5/OXSR1/CSF1R/ACTG1/FEZ1/FADD/FERMT3/CCBE1/STAT5A/RIPOR1/PIK3CB/TNF/ROCK2/ATP7A/GPLD1/NRP2/PRKD2/MYADM/PDGFA/SEMA4A/SYNE2/CXCL16/ITGA5/PIK3CG/SP1/JAM3/RAC2/CCR1/CAMK1D/STK4/TIRAP/HGF/VSIR/GNAI2/RTN4/GPI/ACTA2/MAP3K3/NUMB/PTGS2/CCL3/FGR/RIN2/FUT7/ZNF703/LGR6/INSR/SPAG9/AGO2/DIAPH1/NEDD9/LGALS3/C5AR1/DOCK8/PTK2B/ARHGEF2/HDAC7/TF/GFUS/LGALS9/DOCK5/CASS4/SPARC/SRC/APC/GRN/NIPBL/GAB1/HSPB1/PECAM1/CYP1B1/TNFSF14/IL6R/SOD2/SELP/AKT2/NFE2L2/SERPINE1/SASH1/ACTN4/LYN/GLIPR2/PAK1/SDCBP/RAC1/WNK1/PYCARD/MMP9/GPSM3/PIK3CD/PPP3CA/MAP2K3/PTPRC/MAP4K4/HMOX1/CORO1A/EPHB2/PTAFR/IQSEC1/IRS2/STAT3/MAPK3/DMTN/ITGB3/MYLK/SPI1/RIPOR2/SLC8A1/PTP4A1/FOXO4/PLVAP/RAPGEF2/DAB2/S100A11
BP	GO:0007420	brain development	439	-0.38	-1.68	3.79E-08	8.32E-07	5.11E-07	2718	tags=33%, list=23%, signal=26%	TTBK2/CASP2/WNT1/RHOA/PLCB1/ASPM/WLS/NEURL1/SLC2A1/NEUROD2/TCTN1/SETD2/SEMA7A/SIN3A/HSPA5/SHROOM4/DPCD/FOXP2/HAP1/CSF1R/SNRPB/TAF1/FEZ1/SERPINE2/PLXNB2/CKB/ATXN1/LRRK2/OTX1/PPT1/RTN4R/TBX19/GSK3B/BMPR1A/BAX/ZSWIM6/NDE1/PAFAH1B1/GJC2/ABAT/MAS1/ATP7A/RGS2/CDK5R1/NRP2/MECP2/ITGAM/TACC3/YWHAH/PCM1/SYNE2/ATF5/DYNC2H1/ZNF148/SUDS3/TRAPPC9/SSTR2/CREB1/ATP2B1/RERE/SOX6/DRAXIN/HOOK3/RTN4/TSPAN2/DISC1/MEIS1/MSX1/MBOAT7/ROGDI/NUMB/NR4A2/XRN2/ATRX/XRCC1/PSEN1/RARA/ZEB1/SEC16A/ATP6V0D1/AGTPBP1/ARL13B/PPP1R9B/NDEL1/PTBP2/ZFHX3/SKI/NIN/ARPC5/SRC/DPYSL2/NIPBL/UBB/BPTF/PBX1/KDM7A/FANCC/FBXO45/TACC1/PFKFB3/ACTB/DLG5/CALM3/PADI2/RBPJ/CTNND1/AATK/SLC7A11/NDST1/AKNA/BAG6/ZMIZ1/APAF1/TACC2/SHARPIN/CHD7/TYROBP/ARRB2/PBX2/SYNJ1/PPP3CA/CIC/MAP1S/NF1/FOXO3/EPHB2/RGS7/G6PD/IRS2/AQP1/PRKDC/SFRP2/CCDC39/MTPN/GNB4/NRGN/NAPA/BASP1/PTEN/MBP/SLC8A1/RAPGEF2/ODAD4/KIF27/NCOA6

BP	GO:1901698	response to nitrogen compound	706	-0.34	-1.57	3.92E-08	8.55E-07	5.26E-07	2697	tags=33%, list=23%, signal=27%	FOS/FPR2/WNT1/RHOA/PIK3R2/IL1B/CACNA1A/SRSF4/LPIN2/RAB8A/PLCB1/PPP5C/SCAP/NEURL1/PRKCZ/BTG1/SLC2A1/JAK2/MAN1A1/CACNB1/HADHA/ACTR2/PPARGC1B/SLC9A1/SIN3A/LAMTOR4/HSPA5/FCER1G/TICAM1/P2RX7/RCN3/CCND3/VIM/TAF1/AGTRAP/COLEC12/JAK1/LDOC1/CAMK2A/TSPO/AHCYL1/SMARCC1/FADD/FLNA/UBE4B/CA2/LRRK2/CYSLTR2/GRB2/RNF5/CYBB/GSK3B/RFTN1/RHOQ/STAT5A/SYAP1/SORL1/UQCRC1/PRKCB/DNM2/PELI1/TNF/PHIP/NFKBIA/ROCK2/JUND/UGGT1/ABAT/CASR/TRDMT1/LRP1/MAS1/ATP7A/RGS2/GPLD1/UCP3/CDK5R1/UCP2/TRIM25/PIP4K2C/ITPR2/CPEB3/PKM/CES1/MEAK7/HRH2/SYK/HTR3B/PIK3CG/HM13/CPEB4/SOGA1/SP1/IRAK3/CREB1/GUCY1B1/ATP2B1/GCLM/TSC2/RNF40/OS9/GNAI2/GPI/TLR6/INPP5K/ERLIN1/STAT5B/PID1/MAP1LC3A/SHC1/PDK2/GNB1/TRPC3/NR4A2/OSBPL8/MAVS/PTGS2/ROCK1/SESN2/ATRX/MTHFR/GSK3A/PSEN1/RELA/GOT1/ZEB1/ERBIN/FBXO27/ITGB2/FUT7/SOCS3/EGLN1/AQP9/PTPRE/INSR/PTPN1/SESN3/MAP3K5/MAX/DIAPH1/AGRN/PXN/PPP1R9B/C5AR1/NR4A1/RALB/TIMP1/MBD5/CTNNA1/GNAQ/PTK2B/ARHGEF2/NDEL1/RIOK3/VCP/GPX1/ZDHHC7/KCNQ1/EIF4EBP2/SRC/APC/CPT1A/ZNF106/GAB1/ABCC9/MTARC1/PIP4K2A/AKAP9/FLOT1/CYP1B1/P2RY1/ACTB/CALM3/ZFP36L1/GNAL/MYO5A/CARM1/PRKACA/AKT2/CASTOR2/ZBTB7B/BRCA1/KLF3/NFE2L2/P2RX1/UBR2/SSH1/IFIT1B/BAG6/POR/RTF2/LYN/PEA15/SNCA/TGM2/IGF2/ADM/ARRB2/MMP9/UBXN6/ADIPOR1/CDA/PPP3CA/CFLAR/RRAGD/CEBPB/STAT6/FBP1/MAPK14/PRKAR2B/FOXO3/EPHB2/PTAFR/RGS7/DNAJB2/IRS2/AQP1/STAT3/MAPK3/DMTN/PRKDC/PRKCD/CD68/BCL2L1/BSG/WDTC1/RAB31/SLC8A1/FOXO4/RAPGEF2/KLF1/GNAZ
BP	GO:0009894	regulation of catabolic process	785	-0.34	-1.55	4.30E-08	9.34E-07	5.74E-07	2586	tags=31%, list=22%, signal=26%	EIF4G1/CTSA/SIRT6/ERN1/STX5/CSNK2A1/DAPK1/CNOT1/WIPI1/VPS28/GPR137B/TICAM1/P2RX7/RC3H2/HECW2/VIM/TAF1/TECPR1/SNX1/OPTN/FEZ1/CEBPA/TSPO/TRIM21/DCP2/SMARCC1/FLNA/ZYG11B/SERPINE2/TNRC6B/ABCA2/PTTG1IP/LRRK2/RNF5/CAPNS1/ODC1/GSK3B/RNF14/TPCN2/HIPK2/BAX/FBXL20/NCOR1/ACACB/SORL1/TAB2/PIK3CB/HNRNPU/ZDHHC2/TNF/CPTP/ROCK2/TRDMT1/LRP1/CST3/ENTPD5/GGA3/GPLD1/GABARAPL2/CDK5R1/ATP6V1C1/PIP4K2C/DHRSX/HDAC4/SH3RF3/BNIP3L/CALCOCO2/CPEB3/SLC11A1/FZD5/MCL1/PGAM1/EIF4G2/COP1/ANXA2/PIK3CG/PPP1R3B/YTHDF3/NUB1/SOGA1/EXOC7/IRAK3/SPTLC2/TP53INP2/PATL1/EP300/PSMD1/MTMR3/ABCB11/THRAP3/AGAP2/TIRAP/HGF/RNASEL/TSC2/RNF40/VSIR/RUBCN/FZR1/YBX1/DISC1/MAP1A/MTM1/GIGYF2/ATP6V0A1/CNOT6/SH3RF1/KDM4A/TFEB/TBC1D14/PSME3/N4BP1/ROCK1/ARNT/SESN2/HSPA1B/RYBP/SUFU/GSK3A/PSEN1/MAPT/RELA/CLU/CSDE1/VPS11/IFI16/ULK1/INSR/AREL1/PTPN1/SESN3/PLK1/RNF19B/ATP6V0D1/AGTPBP1/AGO2/HSPA1A/TUT7/CTTN/RALB/TIMP1/CLEC16A/WWP2/QSOX1/GNA12/HMGCR/ZC3HAV1/PTK2B/VCP/MAD2L2/NUPR1/GPX1/ATG7/SAMD4B/ZDHHC7/ELAPOR1/RILP/EGLN2/JMJD8/RAD23A/DDB1/APC/WDR45/CPT1A/RAB7A/MAPKAPK2/IKBKG/UBB/GABARAP/PSAP/ZC3H18/NRDC/GTPBP1/EXOC4/HSPB1/PIP4K2A/WAC/DAP/ZFP36L1/ZBTB7A/PIAS1/PRKACA/AKT2/PSMD3/FXR2/SH3GLB1/TNFRSF1B/FEZ2/KAT5/NFE2L2/BSCL2/GOLGA2/MARCHF2/BAG6/TENT5C/APAF1/LAPTM5/USP10/ATP6V1B2/SNCA/ATG2A/SDCBP/PYCARD/OAZ1/TRIM8/RAD23B/PSMC4/PITHD1/OAZ2/DTX3L/RRAGD/TIMP2/FBP1/ABHD5/FBXO7/MAPK14/HMOX1/FOXO3/SLC4A1/DNAJB2/YBX3/MYEF2/ZER1/PNPLA2/IRS2/STAT3/MAPK3/SNX18/DCAF12/FLCN/AMBRA1/PRKCD/PINK1/MSN/CAPN1/PTEN/PSMF1/CHMP4B/TRIB1/NPRL3/CASC3/FURIN/DAB2/ATP6V1A

BP	GO:0030100	regulation of endocytosis	145	-0.51	-2.01	5.82E-08	1.26E-06	7.73E-07	2613	tags=42%, list=22%, signal=33%	ANO6/CD177/NCKAP1L/ANGPT1/VPS28/ABCA2/LRRK2/PPT1/PIK3CB/DNM2/LRP1/ALOX15/LRPAP1/TBC1D5/SYK/ANXA2/NECAB2/RAB21/RIN3/DLG4/CBL/TSC2/RUBCN/AP2M1/GREM1/CD14/MKLN1/ARF1/NUMB/ZFYVE16/ROCK1/CLU/CD63/INSR/PTPN1/SMAP1/LGALS3/ANKRD13A/AP2B1/TF/SRC/FLOT1/CALM3/SERPINE1/RAB5B/SNCA/HNRNPK/SDCBP/RAC1/BMP2K/ARRB2/SYNJ1/PPP3CA/PICALM/STON2/RAB5C/RAB5A/ITGB3/RAB31/DAB2/HIP1
BP	GO:0006909	phagocytosis	176	-0.48	-1.93	5.86E-08	1.26E-06	7.74E-07	2750	tags=44%, list=23%, signal=34%	PIKFYVE/TLR2/FPR2/IL1B/ANO6/NCKAP1L/DOCK2/FCER1G/P2RX7/COLEC12/KIAA1109/ELMO1/SPON2/DNM2/TNF/LRP1/ELMO2/BIN2/ITGAM/ALOX15/CD300LF/SLC11A1/PLSCR1/SYK/PIP5K1C/RAB34/ITGAL/FCN1/RAC2/CAMK1D/RAB11FIP2/LYST/RUBCN/NCF4/CD14/C2/ICAM3/RARA/PTPRJ/FGR/ITGB2/VAV1/ANXA11/HCK/CD93/SIRPA/RAB27A/LIMK1/VAV3/SRC/RAB7A/XKR8/PECAM1/EIF2AK1/MYH9/ARHGAP25/NCF2/LYN/SIRPB1/UNC13D/TGM2/TYROBP/PAK1/RAC1/PYCARD/PTPRC/RHOG/CORO1A/CORO1C/RHOBTB1/RAB5A/ITGB3/PRKCD/GSN/CNN2/RAB31/DYSF
BP	GO:1990778	protein localization to cell periphery	238	-0.44	-1.85	6.03E-08	1.29E-06	7.93E-07	2153	tags=31%, list=18%, signal=26%	EFR3A/ZDHHC2/TNF/ROCK2/PRAM1/GBP1/LRP1/KIF5B/GGA3/MYADM/RAB34/GAK/TSPAN33/TNFRSF1A/RILPL1/EXOC7/DLG4/GRIPAP1/TMBIM1/STX3/AP2M1/RAB3B/INPP5K/TSPAN14/SNAP25/PID1/CLTC/NUMB/ROCK1/SQSTM1/SEC16A/PLK1/ARL13B/LGALS3/PPP1R9B/VAMP3/TESC/ZDHHC7/PPP2R5A/RAB7A/EXOC4/FLOT1/P2RY1/ACTB/TMEM88/MYO5A/AKT2/EHD1/EPB41/AFDN/FYB1/FAM126B/F11R/EHD3/PICALM/RHOG/TSPAN5/PACS1/PPFIA1/IQSEC2/EPHB2/SLC4A1/RILPL2/ABI3/BCL2L1/BSG/ANK1/RAB3D/ZDHHC3/RAB31/FLOT2/RAPGEF2/DAB2/KIF13A
MF	GO:0016301	kinase activity	536	-0.37	-1.65	6.10E-08	1.30E-06	7.97E-07	3091	tags=36%, list=26%, signal=27%	MKNK1/CLK3/MARK1/MAP3K1/CCL5/STK32C/NEK4/EPHB6/TYK2/CSK/IP6K1/GRK2/DAPK2/PIP4K2B/STK19/TAOK2/CAMKK2/PIM3/TBCK/STK38L/PDK4/CSNK1A1/RSKR/MAPK1/UHMK1/PIKFYVE/TKFC/TTBK2/FES/CPNE3/MAP2K4/PRKAG2/PRKCZ/ERN1/CSNK2A1/JAK2/DAPK1/DYRK1B/KIT/NEK6/DYRK1A/CSNK1G2/CCND3/OXSR1/CSF1R/TAF1/JAK1/CAMK2A/HK2/LTBP1/ERBB2/CKB/LRRK2/GSK3B/BMPR1A/HIPK2/PIP5KL1/TESK2/PRKCB/PIK3CB/ROCK2/NADK/PI4K2A/DYRK3/PKN1/HIPK1/CDK5R1/NRP2/PRKD2/PIP4K2C/ERN2/MARK2/CDK13/PKM/CERT1/SNRK/AKAP13/TAOK1/MAST3/SYK/PIP5K1C/PDXK/MAP2K2/SIK3/GAK/PIK3CG/WEE1/PPM1D/GRK4/IRAK3/CAMK1D/STK4/PIM1/EIF2AK2/RNASEL/STK24/NME4/MKNK2/MAP3K3/GRK6/PDK2/CDC42BPA/DSTYK/CDKL5/ROCK1/RPS6KA5/IRAK1/CCL3/GSK3A/FGR/IGF2R/EPHB4/ULK1/INSR/SQSTM1/VRK3/MAP3K20/PLK1/MAP3K5/RASSF2/IPMK/PPP1R9B/BCKDK/MARK4/PTK2B/RIOK3/HCK/NEK5/NEK9/SLK/RPS6KA1/CDKN1A/MAP2K7/HIPK3/CSNK1G1/LIMK1/ILK/TAOK3/SRC/ALPK1/MAPKAPK2/DGKG/PAK2/PFKFB3/PIP4K2A/EIF2AK1/RIPK1/MARK3/PRKACA/AKT2/AK1/MAPKAPK3/FGGY/AATK/CMPK2/SCYL1/GUK1/BRD4/MAST2/STK40/LYN/MMD/GRK3/PAK1/WNK1/BMP2K/CDK19/MAP3K2/PIK3CD/LIMK2/MAP2K3/HK1/MAP4K4/SRPK2/STK38/MAPK14/BMX/ITPK1/PDK3/STK10/EPHB2/TOP1/MAPK3/PRKDC/PFKFB4/MYLK/PRKCD/PINK1/NUAK2/MPP1/PEAK3

BP	GO:0001934	positive regulation of protein phosphorylation	453	-0.38	-1.69	6.60E-08	1.40E-06	8.58E-07	2696	tags=32%, list=23%, signal=26%	FPR2/TRAF6/WNT1/RHOA/ACSL1/IL1B/PRKAG2/ADAM17/NEURL1/PRKCZ/ADNP/EIF4G1/ERN1/JAK2/NCKAP1L/KIT/ANGPT1/SEMA7A/P2RX7/CCND3/CSF1R/DDX3X/LCP2/INCENP/ERBB2/LRRK2/BMPR1A/WDFY2/HIPK2/SYAP1/TAB2/TNF/ROCK2/MAS1/NKX3-1/CDK5R1/PRKD2/S1PR2/RICTOR/ERN2/OSM/MARK2/SLC11A1/PDGFA/FZD5/AKAP13/TAOK1/SYK/SLCO3A1/MAP2K2/ITGA5/PIK3CG/TNFRSF1A/DLG4/RB1CC1/STK4/AGAP2/TIRAP/NCF1/HGF/PDE5A/GREM1/TLR6/SH2D3C/OSBPL8/MAVS/PTGS2/PARP14/RPS6KA5/IRAK1/CCNY/PSEN1/PTPRJ/VLDLR/CLU/INSR/SQSTM1/PTPN1/PLK1/CHI3L1/MAP3K5/NEDD9/RASSF2/RALB/BMP6/PTK2B/ARHGEF2/MAD2L2/DVL3/CDKN1A/PARP16/MAP2K7/CASS4/ILK/TAOK3/SRC/PAK2/PIK3R5/PECAM1/AKAP9/FLOT1/RIPK1/P2RY1/IL6R/CALM3/DAZAP2/PLAUR/AKT2/FXR2/CLCF1/SASH1/TAL1/LYN/MMD/HCLS1/SNCA/IGF2/STRADA/PAK1/SDCBP/RAC1/WNK1/ARRB2/MMP9/LIMK2/MAP2K3/PTPRC/FAXDC2/NIBAN1/CACUL1/MOB3A/STRADB/MAPK3/SFRP2/RALBP1/ITGB3/FLCN/PRKCD/PINK1/MOB3C/PRR5/PELI2/RAPGEF2/IQGAP1/DAB2/TGFA
BP	GO:0032535	regulation of cellular component size	241	-0.44	-1.85	6.68E-08	1.41E-06	8.64E-07	2613	tags=37%, list=22%, signal=30%	ANO6/CDC42EP4/ADNP/NCKAP1L/SEMA7A/SIN3A/LAMTOR4/P2RX7/ARPC4/CAPZB/GRB2/RTN4R/GSK3B/ADD1/NPHS1/DNM2/PAFAH1B1/ATP7A/CDK5R1/RICTOR/ALOX15/MYADM/SPTA1/COTL1/SEMA4A/EIF4G2/KIAA0319/TWF2/RAB22A/RAB21/RIN3/ARPC1B/RB1CC1/CREB1/LRRC8A/DRAXIN/SLC12A6/RTN4/AP2M1/DISC1/RAB3B/TRIOBP/WASHC2C/CDKL5/IST1/MAPT/ULK1/VAV1/AKT1S1/CTTN/PTK2B/NDEL1/HCK/RILP/ARPC5/LIMK1/VAV3/DPYSL2/PPP1R15A/DBN1/XK/FCHSD1/ESAM/SVIL/LMOD3/SSH1/ARPC1A/HCLS1/RAC1/PYCARD/PLEK/CYRIB/PICALM/TMOD1/CORO1A/SSH2/WAS/VASP/RAB5A/CYRIA/AQP1/DMTN/MTPN/KEL/PRKCD/GSN/MSN/SPTB/WDTC1/PTEN
BP	GO:0048534	hematopoietic or lymphoid organ development	674	-0.34	-1.56	8.59E-08	1.80E-06	1.11E-06	2803	tags=35%, list=24%, signal=28%	HECTD1/HLA-DRB1/HIF1A/ADAM8/MAPK1/RRS1/TMEM176A/WDR7/TLR2/FES/FOS/FARP2/TRAF6/WNT1/RHOA/IL1B/REST/TMEM131L/ADAM17/INPP5D/PRKCZ/IKZF1/SELPLG/SOS2/JAK2/NCKAP1L/IL4I1/C17orf99/CASP9/KIT/ANGPT1/PPARGC1B/CMTM7/SIN3A/DOCK2/GPR137B/ANKLE1/FCER1G/RC3H2/EVI2B/CSF1R/JAK1/IREB2/CEBPA/FADD/DHTKD1/MITF/ERBB2/ZSWIM9/YY1/GFI1B/LILRB2/HIPK2/PLCL2/BAX/ZBTB16/PAFAH1B1/TNF/NFKBIA/DYRK3/PKN1/TNFSF13B/ATP7A/HIPK1/ERCC2/SGPL1/HDAC4/DNASE2/OSM/CDK13/ZNF784/HHEX/SPTA1/FZD5/LTBR/SEMA4A/SNRK/GON4L/TMEM176B/SYK/ANXA2/LBR/CLPTM1/ZFPM1/CR1/KLF6/PTPN6/CDKN1C/TOB2/VPS33A/CREB1/NFKB2/JAM3/CTR9/EP300/CCR1/LRRC8A/STK4/PIM1/TIRAP/EIF2AK2/CEBPD/VSIR/ARID4A/TSPAN2/SOX4/MEIS1/TET2/MKNK2/STAT5B/RSAD2/MTURN/JUNB/TRIM10/ROGDI/CSF3R/SH3RF1/ARNT/H4C8/WASF2/HSPA1B/CCL3/PSEN1/RARA/ZEB1/PTPRJ/FUT7/IFI16/PRR7/ADAR/SMAP1/VAV1/TSPO2/RASSF2/PRRC2C/HSPA1A/SBNO2/MPIG6B/KAT6A/PTK2B/SLC46A2/TESC/CUL4A/THOC5/TF/PRDX2/LGALS9/KCNQ1/ACTN1/SNX10/SRC/TBX21/BPGM/KMT2E/PF4/FLI1/AICDA/PBX1/ETV6/PIP4K2A/EIF2AK1/RIPK1/IL6R/SOD2/ZFP36L1/MYO1E/ZBTB7A/IRF1/OSCAR/TMEM91/CLCF1/ZBTB7B/PREX1/RBPJ/AHSP/JARID2/NFE2L2/MYH9/PRELID1/TAL1/BATF2/NRROS/NOTCH4/ZMIZ1/RCOR1/RBM47/GLRX5/CTNNBIP1/LYN/PTBP3/HCLS1/CHD7/TYROBP/PLEK/MMP9/PITHD1/PIK3CD/ALAS2/CEBPB/TRIM58/PTPRC/PICALM/EPB42/STAT6/FBXO7/LYL1/MAPK14/WBP1L/NF1/FAXDC2/FOXO3/TFE3/SLC4A1/PGLYRP1/FAM210B/G6PD/STAT3/MAPK3/DMTN/PRKDC/SFRP2/RASGRP4/FLCN/MPL/AMBRA1/HLX/NFAM1/CNN2/SPI1/LILRB3/BCL3/TRIB1/KLF1/GAB2/NFIL3/NCOA6

BP	GO:0044057	regulation of system process	263	-0.43	-1.81	8.75E-08	1.82E-06	1.12E-06	2964	tags=38%, list=25%, signal=29%	TNNT1/CST7/GRK2/ORMDL3/SRF/BEGAIN/PBX3/ATP2A3/GAA/DICER1/RHOA/CTDP1/PRKCZ/JAK2/KIT/SCN1B/SLC9A1/CD38/FLNA/LRRK2/YY1/NPHS1/MTG2/TNF/ROCK2/PLCE1/ABAT/CASR/NKX3-1/RGS2/CELF2/MECP2/S1PR2/HDAC4/HRH2/PIK3CG/TNFRSF1A/SSTR2/DLG4/LMNA/ATP2B1/TMEM65/HGF/GNAI2/PDE5A/CACNA1D/INPP5K/TRPC3/NPTXR/PTGS2/ROCK1/DSC2/MGLL/CCL3/GSK3A/NR4A1/CTTN/GTF2IRD1/BMP6/NLGN3/PTK2B/DOCK5/KCNQ1/SRC/MYL4/ACP3/ABCC9/KCNE3/AKAP9/CALM3/PRKACA/TNFRSF1B/PROK2/JARID2/TBXAS1/TPM1/P2RX1/PPP1R12B/EPB41/WNK1/ADM/PPP3CA/EHD3/KCNJ5/GUCY1A1/OPRL1/ABCG8/FOXO3/PTAFR/G6PD/AQP1/MTPN/MYL9/PTEN/SLC8A1/RNF10/TBXA2R/ECE1/TSHZ3/DAB2
BP	GO:0000165	MAPK cascade	484	-0.37	-1.64	9.29E-08	1.92E-06	1.18E-06	2932	tags=35%, list=25%, signal=28%	THBS1/IL31RA/PHLPP1/TAOK2/CAMKK2/KRAS/NAIP/ITCH/GSTP1/HLA-DRB1/ADAM8/NLRP12/MAPK1/FPR2/TRAF6/PIK3R2/IL1B/MAP2K4/PLCB1/PPP5C/PRKCZ/ERN1/LPAR1/JAK2/KIT/ANGPT1/SEMA7A/DUSP5/P2RX7/CSF1R/C5AR2/PJA2/ERBB2/LRRK2/GRB2/HIPK2/NCOR1/NPHS1/MAPKBP1/SORL1/PIK3CB/ATF3/PAFAH1B1/TNF/ROCK2/PLCE1/CASR/GBP1/RGS2/PRKD2/GRIK2/ALOX15/SH3RF3/ERN2/OSM/PDGFA/FZD5/LTBR/AKAP13/TAOK1/SYK/NECAB2/MAP2K2/GPBAR1/PPM1L/PIK3CG/GADD45A/ATP6AP2/PTPN6/IRAK3/RB1CC1/DOK4/CCR1/STK4/TIRAP/EIF2AK2/NCF1/HGF/GNAI2/PDE5A/DUSP22/TLR6/YWHAZ/INPP5K/ACTA2/MTURN/MAP3K3/SHC1/FFAR4/SH2D3C/SH3RF1/DSTYK/ROCK1/IRAK1/CCL3/PSEN1/RGS14/PTPRJ/INSR/SPAG9/PTPN1/VRK3/MAP3K20/CHI3L1/MAP3K5/RASSF2/C5AR1/HMGCR/PTK2B/TNIP1/TF/SIRPA/DVL3/PRDX2/LGALS9/MAP2K7/HIPK3/SPRY3/TAOK3/SRC/MAPKAPK2/DUSP6/PAK2/PIK3R5/SETX/DUSP1/RIPK1/RNF149/P2RY1/ZFP36L1/ARHGAP8/MAPKAPK3/SASH1/NDST1/LAPTM5/STK40/LYN/PEA15/IGF2/GLIPR2/PAK1/SDCBP/PYCARD/ARRB2/XIAP/MAP3K2/CFLAR/MAP2K3/PTPRC/MAP4K4/STK38/MAPK14/NF1/EPHB2/STRADB/MAPK3/SFRP2/FLCN/PRKCD/PINK1/PTEN/SPI1/MBP/PLVAP/PELI2/TRIB1/RAPGEF2/IQGAP1/NBR1/DAB2/TGFA
BP	GO:0032990	cell part morphogenesis	369	-0.39	-1.72	9.61E-08	1.97E-06	1.21E-06	2690	tags=35%, list=23%, signal=28%	CAPRIN1/DICER1/RHOA/RAB8A/RAPH1/PRKCZ/LYPLA2/NEXN/TCTN1/ADNP/NCKAP1L/FIS1/ACTR2/SCN1B/SEMA7A/SIN3A/CSF1R/HECW2/KLF7/SNX1/FEZ1/ALCAM/PLXNB2/MYO9A/ERBB2/LRRK2/NECTIN2/RTN4R/GSK3B/BAX/PLEKHO1/DNM2/PAFAH1B1/EFNB1/KIF5B/ATP7A/CDK5R1/NRP2/RNF165/YWHAH/CFL1/MARK2/SEMA4A/CERT1/EIF4G2/TAOK1/KIAA0319/TWF2/WEE1/RAB21/DLG4/CREB1/RAC2/RERE/NCAM1/DRAXIN/RTN4/CPNE1/DISC1/MAP1A/MTM1/NIBAN2/PID1/NUMB/NR4A2/CDKL5/ROCK1/IST1/RPS6KA5/WASF2/PSEN1/MAPT/ANKRD27/VLDLR/EPHB4/ULK1/LGR6/B4GALT5/SPAG9/SIPA1L1/SLC23A2/GAS7/CUX1/CTTN/DNM3/NLGN3/NDEL1/DVL3/NIN/NTNG2/PALLD/APLP2/SPRY3/LIMK1/TAOK3/SRC/DPYSL2/DBN1/UBB/XK/PDLIM5/PAK2/FBXO45/FLOT1/ACTB/FEZ2/USP9X/PAK1/RAC1/PPP3CA/PLXNC1/PICALM/RHOG/MAP4K4/MAP1S/EPHB2/CORO1C/ABI3/VASP/KEL/BCL2L1/BSG/PTEN/VCL/MBP/RAPGEF2/IQGAP1/ECE1

BP	GO:0048666	neuron development	621	-0.35	-1.58	9.58E-08	1.97E-06	1.21E-06	2935	tags=34%, list=25%, signal=27%	CDH23/ENC1/SZT2/PLAA/KATNB1/TAOK2/KIDINS220/SRF/SCLT1/WDPCP/SCARF1/NR2F6/PBX3/SHTN1/UHMK1/CLMN/HDGFL3/FARP1/TOPORS/FES/CRTC1/FRYL/FARP2/CAPRIN1/DICER1/RHOA/RAB8A/RAPH1/NEURL1/PRKCZ/SCARB2/LYPLA2/NEXN/NEUROD2/IFT88/TCTN1/ADNP/LPAR1/JAK2/NCKAP1L/LST1/ACTR2/SCN1B/SEMA7A/SIN3A/HSPA5/C1QA/CSF1R/HECW2/VIM/RAB35/KLF7/CD38/CAMK2A/FEZ1/TSPO/ALCAM/FLNA/PLXNB2/MYO9A/ERBB2/LRRK2/PPT1/RTN4R/TBC1D24/GSK3B/DNM2/PAFAH1B1/EFNB1/CFAP418/KIF5B/ATP7A/RGS2/CDK5R1/NRP2/MECP2/RNF165/YWHAH/CPEB3/CFL1/MARK2/SEMA4A/P3H1/EIF4G2/TAOK1/KIAA0319/TWF2/ATF5/ATCAY/GAK/WEE1/RAB21/CDKN1C/DLG4/CREB1/RAC2/EP300/RERE/CAMK1D/MICALL1/NCAM1/DRAXIN/HGF/LRP12/STK24/RTN4/STX3/CPNE1/TSPAN2/DISC1/MAP1A/YWHAZ/SNAP25/NIBAN2/TRIOBP/IFT140/NUMB/NR4A2/CDKL5/ROCK1/IST1/DENND5A/RPS6KA5/GSK3A/PSEN1/MAPT/ANKRD27/VLDLR/EPHB4/ULK1/LGR6/B4GALT5/SPAG9/SIPA1L1/SLC23A2/AGTPBP1/GAS7/AGRN/MAP4/PPP1R9B/ADGRF1/CUX1/CTTN/CTNNA1/DNM3/NLGN3/PTK2B/NDEL1/PPP2R5B/DVL3/KCNQ1/RETREG3/NIN/NTNG2/PALLD/APLP2/SPRY3/LIMK1/TAOK3/DPYSL2/GRN/DBN1/UBB/DGKG/XK/PDLIM5/PAK2/PBX1/FBXO45/SETX/FLOT1/ACTB/DLG5/SOD2/CARM1/EHD1/FEZ2/NFE2L2/SCYL1/ZMIZ1/LYN/FRY/USP9X/PAK1/RAC1/WNK1/ADM/PBX2/PPP3CA/CFLAR/PLXNC1/PICALM/RHOG/MAP4K4/FBXO7/MAP1S/ASAP1/EPHB2/IQSEC1/ABI3/VASP/SFRP2/KEL/BSG/PTEN/VCL/MBP/RAPGEF2/IQGAP1/ECE1/DAB2
MF	GO:0031267	small GTPase binding	224	-0.45	-1.88	9.98E-08	2.03E-06	1.25E-06	2592	tags=38%, list=22%, signal=30%	CDC42EP4/YIPF1/HPS6/DOCK2/CCDC186/OPTN/RPH3AL/ARHGDIB/FLNA/LRRK2/SORL1/MYO9B/RANBP2/ROCK2/TBC1D30/PLCE1/XPO7/PKN1/ATP7A/IPO13/GGA3/YIPF2/AKAP13/RCC2/IPO8/TMEM127/RAB34/RIN3/PPP6R1/MICALL1/RAB11FIP2/TSC2/NUTF2/VPS9D1/TNPO1/TRIOBP/CDKL5/ROCK1/DENND5A/DIAPH2/SYTL4/ANKRD27/RIN2/ULK1/EVI5/RASSF5/DIAPH1/RAP1GAP/ARHGEF2/DVL3/RILP/DOCK5/RAB7A/PAK2/EXOC4/MYO5A/RAB11FIP4/EHD1/RABGAP1L/ARHGAP1/NCF2/FGD4/AFDN/GCC1/AP1G1/DMXL2/UNC13D/PAK1/FGD6/TBC1D20/RAB11FIP1/CYRIB/PICALM/KPNB1/CORO1C/WAS/TNPO3/YBX3/CYRIA/RALBP1/XPO6/IQGAP1/FMNL1/IQGAP2
CC	GO:0009898	cytoplasmic side of plasma membrane	122	-0.53	-2.08	1.01E-07	2.04E-06	1.25E-06	2853	tags=47%, list=24%, signal=36%	KRAS/GNAS/GNA13/FARP1/FES/TRAF6/RHOA/KIT/GNB2/SHROOM4/PKP4/GNG5/SYAP1/KCNAB2/RGS2/ALOX15/SPTA1/SYK/MAP2K2/RAB21/DLG4/ATP2B1/TGM3/GNAI2/SNAP25/GNB1/FGR/PPP1R9B/GNA12/GNAQ/PTK2B/HCK/SRC/CACNB4/GNAL/MYH9/SLA/LYN/C2CD2L/RAC1/PPP3CA/PTPRC/SLC4A1/G6PD/SNX18/GNB4/ANK1/SPTB/PTEN/CHMP4B/PTP4A1/IQGAP1/LITAF/MYZAP/OSBPL2/HIP1/GNAZ

BP	GO:0051130	positive regulation of cellular component organization	717	-0.34	-1.55	1.01E-07	2.04E-06	1.25E-06	2708	tags=31%, list=23%, signal=26%	FES/NAF1/CRTC1/CAPRIN1/WNT1/RHOA/IL1B/PLCB1/NEURL1/SLAIN2/ANO6/SCARB2/CDC42EP4/NEUROD2/ADNP/EIF4G1/SIRT6/LPAR1/PLEKHM2/NCKAP1L/CNOT1/FIS1/KIT/ACTR2/ANGPT1/SCN1B/SEMA7A/SIN3A/WIPI1/VPS28/CIDEB/HSPA5/CEP295/P2RX7/HAP1/DDX3X/FEZ1/FLNA/PLXNB2/MIEF2/CHCHD10/LRRK2/PPT1/GRB2/CLIP1/GSK3B/BAX/ADD1/RHOQ/NPHS1/PLEKHM1/DNM2/ZDHHC2/PAFAH1B1/TNF/PHIP/ROCK2/TNXB/PLCE1/ARAP1/GTF2H2/LRP1/ATP7A/RGS2/PPIF/CDK5R1/MECP2/PIP4K2C/RICTOR/ALOX15/CALCOCO2/MARK2/TBC1D5/SEMA4A/P3H1/EIF4G2/SETDB1/TWF2/SYK/ANXA2/UBE2B/RAB21/FHOD1/ATF7IP/DLG4/RAC2/FSCN1/EP300/CBL/CAMK1D/TIRAP/HGF/STK24/AP2M1/GREM1/CUL3/DISC1/TLR6/MSX1/CD14/GNL3L/NABP2/CNOT6/CDC27/WASHC2C/CDKL5/ROCK1/BID/IST1/ATRX/WASF2/HSPA1B/GSK3A/PSEN1/MAPT/ANKRD27/PTPRJ/VLDLR/CLU/ULK1/CD63/INSR/AGRN/LGALS3/PXN/HSPA1A/CUX1/CTTN/RALB/DNM3/MARK4/NLGN3/PTK2B/SYNPO/NDEL1/VCP/HCK/GPX1/AP2B1/VPS4B/PPP2R5B/TF/ELAPOR1/DVL3/RETREG3/MAP2K7/NIN/LIMK1/SLX1B/SRC/APC/GRN/WDR45/TPR/PPP1R15A/CDC34/DBN1/WARS1/FCHSD1/TPPP3/LIMS1/PPP1R10/PIP4K2A/SETX/AKAP9/FLOT1/TNFSF14/DLG5/PLEK2/PLAUR/AKT2/EHD1/SH3GLB1/NFE2L2/SERPINE1/TPM1/TAL1/STMP1/LYN/HCLS1/SNCA/IGF2/ATG2A/HNRNPK/TYROBP/PAK1/SDCBP/RAC1/PYCARD/PLEK/ARRB2/MMP9/BBC3/SYNJ1/PPP3CA/CFLAR/PLXNC1/TRIM58/MAP4K4/FBXO7/LCP1/ASAP1/MAPK14/EPHB2/IQSEC1/CORO1C/WAS/VASP/MED25/MAPK3/SNX18/RALBP1/RAD21/AMBRA1/GSN/PINK1/CCP110/MSN/RAB31/RIPOR2/RAPGEF2/DAB2/TGFA/HIP1/CLDN5
BP	GO:0031589	cell-substrate adhesion	208	-0.46	-1.88	1.02E-07	2.04E-06	1.26E-06	3058	tags=42%, list=26%, signal=31%	BCL2L11/BCL6/PKD1/ADAM15/FERMT1/TIAM1/BCAS3/THBS1/TAOK2/SRF/WDPCP/WNT1/RHOA/PRKCZ/JAK2/ANGPT1/SLC9A1/ACTG1/FLNA/FERMT3/GSK3B/TESK2/PIK3CB/DNM2/UTRN/ROCK2/BST1/TNXB/GBP1/ITGAM/ALOX15/ITGA2B/MYADM/RCC2/ITGA5/ITGAL/JAM3/RAC2/STK4/GREM1/DISC1/DUSP22/TRIOBP/MKLN1/WASHC2C/ROCK1/PTPRJ/RIN2/ITGB2/CD63/NEDD9/PXN/CTTN/VAMP3/PTK2B/MEGF9/SLK/GFUS/DOCK5/CASS4/NTNG2/ILK/ACTN1/SRC/LIMS1/PARVB/PREX1/SERPINE1/ITGB5/ACTN4/UNC13D/LAMC3/RAC1/RSU1/MAP4K4/PPFIA1/CORO1A/NF1/CORO1C/DMTN/ITGB3/PTEN/VCL/HSD17B12/ZYX/DAB2/ARHGAP6
BP	GO:0120039	plasma membrane bounded cell projection morphogenesis	350	-0.40	-1.74	1.13E-07	2.24E-06	1.38E-06	2690	tags=35%, list=23%, signal=27%	CAPRIN1/DICER1/RHOA/RAB8A/RAPH1/PRKCZ/LYPLA2/NEXN/TCTN1/ADNP/NCKAP1L/ACTR2/SCN1B/SEMA7A/SIN3A/CSF1R/HECW2/KLF7/SNX1/FEZ1/ALCAM/PLXNB2/MYO9A/ERBB2/LRRK2/RTN4R/GSK3B/PLEKHO1/DNM2/PAFAH1B1/EFNB1/KIF5B/ATP7A/CDK5R1/NRP2/RNF165/YWHAH/CFL1/MARK2/SEMA4A/EIF4G2/TAOK1/KIAA0319/TWF2/WEE1/RAB21/DLG4/CREB1/RAC2/RERE/NCAM1/DRAXIN/RTN4/CPNE1/DISC1/MAP1A/NIBAN2/NUMB/NR4A2/CDKL5/ROCK1/IST1/RPS6KA5/WASF2/PSEN1/MAPT/ANKRD27/VLDLR/EPHB4/ULK1/LGR6/B4GALT5/SPAG9/SIPA1L1/SLC23A2/GAS7/CUX1/CTTN/DNM3/NLGN3/NDEL1/DVL3/NIN/NTNG2/PALLD/APLP2/SPRY3/LIMK1/TAOK3/SRC/DPYSL2/DBN1/UBB/XK/PDLIM5/PAK2/FBXO45/FLOT1/ACTB/FEZ2/USP9X/PAK1/RAC1/PPP3CA/PLXNC1/PICALM/RHOG/MAP4K4/MAP1S/EPHB2/CORO1C/ABI3/VASP/KEL/BSG/PTEN/VCL/MBP/RAPGEF2/IQGAP1/ECE1

BP	GO:0002520	immune system development	729	-0.34	-1.54	1.15E-07	2.28E-06	1.40E-06	2803	tags=34%, list=24%, signal=27%	HECTD1/HLA-DRB1/HIF1A/ADAM8/MAPK1/RRS1/TMEM176A/WDR7/TLR2/FES/FOS/FARP2/RO60/TRAF6/WNT1/RHOA/IL1B/REST/TMEM131L/SAMHD1/ADAM17/INPP5D/PRKCZ/IKZF1/SELPLG/SOS2/JAK2/NCKAP1L/IL4I1/C17orf99/CASP9/KIT/ANGPT1/PPARGC1B/CMTM7/SIN3A/DOCK2/GPR137B/ANKLE1/FCER1G/RC3H2/EVI2B/CSF1R/JAK1/IREB2/CEBPA/FADD/DHTKD1/MITF/ERBB2/ZSWIM9/YY1/GFI1B/TNFSF13/LILRB2/HIPK2/PLCL2/BAX/ZBTB16/PAFAH1B1/TNF/NFKBIA/NSD2/DYRK3/PKN1/TNFSF13B/ATP7A/HIPK1/ERCC2/SGPL1/HDAC4/DNASE2/OSM/CDK13/ZNF784/HHEX/SPTA1/FZD5/LTBR/SEMA4A/SNRK/GON4L/TMEM176B/SYK/ANXA2/LBR/CLPTM1/ZFPM1/CR1/HLA-E/KLF6/PTPN6/CDKN1C/TOB2/IRAK3/VPS33A/CREB1/NFKB2/JAM3/MSH6/CTR9/EP300/CCR1/LRRC8A/STK4/PIM1/TIRAP/EIF2AK2/CEBPD/VSIR/ARID4A/SUPT6H/TSPAN2/SOX4/MEIS1/TET2/MKNK2/STAT5B/RSAD2/MTURN/JUNB/TRIM10/ROGDI/CSF3R/SH3RF1/POLL/ARNT/H4C8/WASF2/HSPA1B/CCL3/PSEN1/RARA/ZEB1/PTPRJ/FUT7/IFI16/PRR7/ADAR/SMAP1/VAV1/TSPO2/RASSF2/PRRC2C/HSPA1A/SBNO2/MPIG6B/KAT6A/PTK2B/SLC46A2/MAD2L2/TESC/CUL4A/THOC5/TF/PRDX2/LGALS9/KCNQ1/ACTN1/SNX10/SRC/TBX21/BPGM/KMT2E/PF4/FLI1/AICDA/XKR8/PBX1/ETV6/PIP4K2A/EIF2AK1/RIPK1/IL6R/SOD2/ZFP36L1/MYO1E/ZBTB7A/IRF1/OSCAR/TMEM91/CLCF1/ZBTB7B/PREX1/RBPJ/AHSP/JARID2/NFE2L2/MYH9/PRELID1/TAL1/BATF2/NRROS/NOTCH4/ZMIZ1/RCOR1/RBM47/GLRX5/CTNNBIP1/LYN/PTBP3/HCLS1/CHD7/TYROBP/PLEK/MMP9/PITHD1/PIK3CD/ALAS2/CEBPB/TRIM58/PTPRC/PICALM/EPB42/STAT6/FBXO7/LYL1/MAPK14/WBP1L/NF1/FAXDC2/FOXO3/TFE3/SLC4A1/PGLYRP1/FAM210B/G6PD/STAT3/MAPK3/DMTN/PRKDC/SFRP2/RASGRP4/FLCN/MPL/AMBRA1/HLX/NFAM1/CNN2/SPI1/LILRB3/BCL3/TRIB1/KLF1/GAB2/NFIL3/NCOA6
BP	GO:0098609	cell-cell adhesion	521	-0.36	-1.62	1.27E-07	2.50E-06	1.53E-06	2685	tags=32%, list=23%, signal=26%	TRAF6/WNT1/RHOA/IL1B/DHPS/TMEM131L/PRKCZ/SELPLG/CCDC88B/NEXN/CD177/JAK2/NCKAP1L/IL4I1/KIT/ALOX5/RC3H2/ACTG1/JAK1/CD70/PKP4/ALCAM/FADD/FLNA/SERPINE2/PLXNB2/ERBB2/FERMT3/NECTIN2/METAP1/LILRB2/FBLIM1/NPHS1/ZBTB16/PIK3CB/PELI1/ZDHHC2/TNF/EFNB1/TNXB/ABAT/TNFSF13B/CDK5R1/ELMO2/NFAT5/GP6/ITGAM/ITGA2B/MYADM/SPTA1/SYK/PIP5K1C/ANXA2/CR1/HLA-E/ITGA5/ITGAL/PIK3CG/ICAM4/PTPN6/DLG4/DSG3/JAM3/RAC2/CHST2/VSIR/PDE5A/DUSP22/SOX4/CEACAM5/STAT5B/SHC1/ROCK1/DSC2/IRAK1/ICAM3/PSEN1/RARA/RELA/PCDH1/ITGB2/FUT7/ZNF703/VAV1/LGALS3/DOCK8/CTNNA1/BMP6/NLGN3/MAD2L2/CD93/TNIP1/GFUS/SIRPA/PRDX2/LGALS9/NTNG2/PALLD/ILK/SELL/SRC/TBX21/PDLIM5/ESAM/LIMS1/HSPB1/PECAM1/FLOT1/CYP1B1/TNFSF14/PCDHGA8/PCDHB1/ACTB/DLG5/PLAUR/ZFP36L1/SELP/IRF1/GP1BA/ZBTB7B/ALOX12/IL1RN/CTNND1/SLC7A11/MYH9/ITGB5/AKNA/AFDN/NOTCH4/ZMIZ1/NINJ2/LAPTM5/LYN/SIRPB1/IGF2/WNK1/PYCARD/PLEK/CYRIB/F11R/CEBPB/PTPRC/MAPK14/DNAJB6/CORO1A/FOXO3/STK10/PTAFR/LRRC4/TLN1/DMTN/ITGB3/PRKAR1A/MPL/AMBRA1/PRKCD/BSG/MYL9/MSN/HLX/VCL/SPI1/LPP/MBP/RIPOR2/FLOT2/NINJ1/S100A11/CLDN5

BP	GO:0048858	cell projection morphogenesis	351	-0.40	-1.73	1.34E-07	2.62E-06	1.61E-06	2690	tags=34%, list=23%, signal=27%	CAPRIN1/DICER1/RHOA/RAB8A/RAPH1/PRKCZ/LYPLA2/NEXN/TCTN1/ADNP/NCKAP1L/ACTR2/SCN1B/SEMA7A/SIN3A/CSF1R/HECW2/KLF7/SNX1/FEZ1/ALCAM/PLXNB2/MYO9A/ERBB2/LRRK2/RTN4R/GSK3B/PLEKHO1/DNM2/PAFAH1B1/EFNB1/KIF5B/ATP7A/CDK5R1/NRP2/RNF165/YWHAH/CFL1/MARK2/SEMA4A/EIF4G2/TAOK1/KIAA0319/TWF2/WEE1/RAB21/DLG4/CREB1/RAC2/RERE/NCAM1/DRAXIN/RTN4/CPNE1/DISC1/MAP1A/NIBAN2/NUMB/NR4A2/CDKL5/ROCK1/IST1/RPS6KA5/WASF2/PSEN1/MAPT/ANKRD27/VLDLR/EPHB4/ULK1/LGR6/B4GALT5/SPAG9/SIPA1L1/SLC23A2/GAS7/CUX1/CTTN/DNM3/NLGN3/NDEL1/DVL3/NIN/NTNG2/PALLD/APLP2/SPRY3/LIMK1/TAOK3/SRC/DPYSL2/DBN1/UBB/XK/PDLIM5/PAK2/FBXO45/FLOT1/ACTB/FEZ2/USP9X/PAK1/RAC1/PPP3CA/PLXNC1/PICALM/RHOG/MAP4K4/MAP1S/EPHB2/CORO1C/ABI3/VASP/KEL/BSG/PTEN/VCL/MBP/RAPGEF2/IQGAP1/ECE1
BP	GO:0010243	response to organonitrogen compound	655	-0.35	-1.57	1.36E-07	2.65E-06	1.63E-06	2739	tags=33%, list=23%, signal=27%	NCOR2/SLC26A6/TLR2/FOS/FPR2/WNT1/RHOA/PIK3R2/IL1B/CACNA1A/SRSF4/LPIN2/RAB8A/PLCB1/PPP5C/SCAP/NEURL1/PRKCZ/BTG1/SLC2A1/JAK2/MAN1A1/CACNB1/HADHA/ACTR2/PPARGC1B/SLC9A1/SIN3A/LAMTOR4/HSPA5/FCER1G/TICAM1/P2RX7/RCN3/CCND3/VIM/TAF1/AGTRAP/JAK1/LDOC1/CAMK2A/TSPO/AHCYL1/SMARCC1/FADD/FLNA/UBE4B/CA2/LRRK2/CYSLTR2/GRB2/RNF5/CYBB/GSK3B/RHOQ/STAT5A/SYAP1/SORL1/UQCRC1/PRKCB/DNM2/TNF/PHIP/NFKBIA/ROCK2/JUND/UGGT1/ABAT/CASR/TRDMT1/LRP1/MAS1/ATP7A/RGS2/GPLD1/UCP3/UCP2/TRIM25/PIP4K2C/ITPR2/CPEB3/PKM/CES1/MEAK7/HRH2/SYK/HTR3B/PIK3CG/HM13/CPEB4/SOGA1/SP1/IRAK3/CREB1/ATP2B1/GCLM/TSC2/RNF40/OS9/GNAI2/GPI/TLR6/INPP5K/ERLIN1/STAT5B/PID1/MAP1LC3A/SHC1/PDK2/GNB1/TRPC3/NR4A2/OSBPL8/PTGS2/ROCK1/SESN2/MTHFR/GSK3A/PSEN1/RELA/GOT1/ZEB1/ERBIN/FBXO27/ITGB2/FUT7/SOCS3/AQP9/PTPRE/INSR/PTPN1/SESN3/MAX/DIAPH1/PXN/PPP1R9B/C5AR1/NR4A1/TIMP1/MBD5/CTNNA1/PTK2B/ARHGEF2/NDEL1/VCP/GPX1/ZDHHC7/KCNQ1/EIF4EBP2/SRC/APC/CPT1A/ZNF106/GAB1/ABCC9/PIP4K2A/AKAP9/CYP1B1/P2RY1/ACTB/CALM3/ZFP36L1/GNAL/MYO5A/CARM1/PRKACA/AKT2/CASTOR2/ZBTB7B/BRCA1/KLF3/NFE2L2/P2RX1/UBR2/SSH1/BAG6/POR/LYN/PEA15/SNCA/TGM2/IGF2/ADM/ARRB2/MMP9/UBXN6/ADIPOR1/CDA/PPP3CA/CFLAR/RRAGD/CEBPB/STAT6/FBP1/MAPK14/PRKAR2B/FOXO3/EPHB2/PTAFR/RGS7/DNAJB2/IRS2/AQP1/STAT3/MAPK3/DMTN/PRKDC/PRKCD/CD68/BCL2L1/BSG/WDTC1/RAB31/SLC8A1/FOXO4/RAPGEF2/KLF1/GNAZ
MF	GO:0106310	protein serine kinase activity	275	-0.43	-1.81	1.41E-07	2.73E-06	1.68E-06	3308	tags=43%, list=28%, signal=32%	AURKA/MOK/PIM2/MAP4K2/BRAF/PHKG1/TNK2/CAMK1/STK35/SRPK1/MKNK1/CLK3/MARK1/MAP3K1/STK32C/NEK4/DAPK2/STK19/TAOK2/CAMKK2/PIM3/STK38L/CSNK1A1/RSKR/MAPK1/UHMK1/PIKFYVE/TTBK2/MAP2K4/PRKCZ/ERN1/CSNK2A1/DAPK1/DYRK1B/NEK6/DYRK1A/CSNK1G2/OXSR1/TAF1/CAMK2A/LRRK2/GSK3B/HIPK2/TESK2/PRKCB/ROCK2/DYRK3/PKN1/HIPK1/PRKD2/ERN2/MARK2/CDK13/SNRK/TAOK1/MAST3/MAP2K2/SIK3/GAK/PIK3CG/CAMK1D/STK4/PIM1/EIF2AK2/STK24/MKNK2/MAP3K3/CDC42BPA/DSTYK/CDKL5/ROCK1/RPS6KA5/IRAK1/GSK3A/ULK1/MAP3K20/PLK1/MAP3K5/MARK4/RIOK3/NEK5/NEK9/SLK/RPS6KA1/MAP2K7/HIPK3/CSNK1G1/LIMK1/ILK/TAOK3/ALPK1/MAPKAPK2/PAK2/EIF2AK1/RIPK1/MARK3/PRKACA/AKT2/MAPKAPK3/AATK/MAST2/STK40/PAK1/WNK1/BMP2K/CDK19/MAP3K2/LIMK2/MAP2K3/MAP4K4/SRPK2/STK38/MAPK14/STK10/MAPK3/PRKDC/PRKCD/PINK1/NUAK2

BP	GO:0043087	regulation of GTPase activity	255	-0.43	-1.79	1.43E-07	2.75E-06	1.69E-06	2484	tags=33%, list=21%, signal=26%	GPR137B/ARHGAP9/TBC1D22B/ADAP1/PKP4/PLXNB2/MYO9A/ERBB2/LRRK2/RTN4R/GSK3B/CHN2/TBC1D10B/ADPRH/PAFAH1B1/TBC1D30/ARAP1/TBC1D8/RICTOR/RALGDS/TBC1D5/RCC2/ABR/RALGAPA2/RGS6/RAB11FIP2/TSC2/ASAP2/TBC1D14/CDKL5/ARAP3/CCL3/RGS14/SIPA1L1/TBC1D2/EVI5/NEDD9/AGRN/RAP1GAP/VAV1/DOCK8/PTK2B/NDEL1/DVL3/VAV3/LIMS1/AKT2/PREX1/RABGAP1L/ARHGAP25/FGD4/AFDN/TGM2/WNK1/RSU1/PYCARD/FGD6/ARRB2/TBC1D20/SIPA1/F11R/PLXNC1/PICALM/RHOG/MAP4K4/ASAP1/NF1/IQSEC1/CORO1C/RGS7/TBC1D1/SBF2/RASGRP4/SNX18/RALBP1/TBC1D3I/FLCN/SIPA1L2/RIPOR2/RAPGEF2/IQGAP1/ARHGAP6/IQGAP2
BP	GO:0007178	transmembrane receptor protein serine/threonine kinase signaling pathway	196	-0.46	-1.88	1.54E-07	2.96E-06	1.82E-06	2697	tags=34%, list=23%, signal=27%	FOS/WNT1/ADAM17/PBLD/JAK2/ZEB2/FAM89B/HSPA5/VIM/LTBP1/BMPR1A/HIPK2/SORL1/DNM2/LRP1/RNF165/ZC3H3/KIAA0319/PPM1L/CDKN1C/IL17RD/EP300/GREM1/DUSP22/MSX1/CHST11/LRG1/ZEB1/FKBP1A/TRIM33/NUP93/ZNF703/PXN/HSPA1A/BMP6/TF/SKI/ILK/SRC/TGFB1I1/UBE2O/ZBTB7A/RBPJ/ITGB5/PELO/NRROS/ZMIZ1/USP9X/SDCBP/ARRB2/XIAP/CREBBP/UBE2D3/MAPK14/STAT3/MAPK3/SFRP2/PCSK6/FLCN/FKBP8/PPM1A/SPI1/LTBP2/ZYX/FURIN/DAB2/CLDN5
BP	GO:0030097	hemopoiesis	656	-0.34	-1.56	1.56E-07	2.98E-06	1.83E-06	2749	tags=35%, list=23%, signal=28%	RRS1/TMEM176A/WDR7/TLR2/FES/FOS/FARP2/TRAF6/WNT1/RHOA/IL1B/REST/TMEM131L/ADAM17/INPP5D/PRKCZ/IKZF1/SELPLG/SOS2/JAK2/NCKAP1L/IL4I1/C17orf99/CASP9/KIT/ANGPT1/PPARGC1B/CMTM7/SIN3A/DOCK2/GPR137B/ANKLE1/FCER1G/RC3H2/EVI2B/CSF1R/JAK1/IREB2/CEBPA/FADD/DHTKD1/MITF/ERBB2/ZSWIM9/YY1/GFI1B/LILRB2/HIPK2/PLCL2/BAX/ZBTB16/PAFAH1B1/TNF/NFKBIA/DYRK3/TNFSF13B/ATP7A/HIPK1/ERCC2/SGPL1/HDAC4/DNASE2/OSM/CDK13/ZNF784/HHEX/SPTA1/FZD5/LTBR/SEMA4A/SNRK/GON4L/TMEM176B/SYK/ANXA2/LBR/CLPTM1/ZFPM1/CR1/KLF6/PTPN6/CDKN1C/TOB2/VPS33A/CREB1/JAM3/CTR9/EP300/CCR1/LRRC8A/STK4/PIM1/TIRAP/EIF2AK2/CEBPD/VSIR/ARID4A/TSPAN2/SOX4/MEIS1/TET2/MKNK2/STAT5B/RSAD2/MTURN/JUNB/TRIM10/ROGDI/CSF3R/SH3RF1/ARNT/H4C8/WASF2/HSPA1B/CCL3/PSEN1/RARA/ZEB1/PTPRJ/FUT7/IFI16/PRR7/ADAR/SMAP1/VAV1/TSPO2/RASSF2/PRRC2C/HSPA1A/SBNO2/MPIG6B/KAT6A/PTK2B/SLC46A2/TESC/CUL4A/THOC5/TF/PRDX2/LGALS9/KCNQ1/ACTN1/SNX10/SRC/TBX21/BPGM/KMT2E/PF4/FLI1/AICDA/PBX1/ETV6/PIP4K2A/EIF2AK1/RIPK1/IL6R/SOD2/ZFP36L1/MYO1E/ZBTB7A/IRF1/OSCAR/TMEM91/CLCF1/ZBTB7B/PREX1/RBPJ/AHSP/NFE2L2/MYH9/PRELID1/TAL1/BATF2/NRROS/NOTCH4/ZMIZ1/RCOR1/RBM47/GLRX5/CTNNBIP1/LYN/PTBP3/HCLS1/CHD7/TYROBP/PLEK/MMP9/PITHD1/PIK3CD/ALAS2/CEBPB/TRIM58/PTPRC/PICALM/EPB42/STAT6/FBXO7/LYL1/MAPK14/WBP1L/NF1/FAXDC2/FOXO3/TFE3/SLC4A1/PGLYRP1/FAM210B/G6PD/STAT3/DMTN/PRKDC/SFRP2/RASGRP4/FLCN/MPL/AMBRA1/HLX/NFAM1/CNN2/SPI1/LILRB3/BCL3/TRIB1/KLF1/GAB2/NFIL3/NCOA6

BP	GO:0051050	positive regulation of transport	583	-0.35	-1.60	1.57E-07	2.99E-06	1.84E-06	2727	tags=32%, list=23%, signal=26%	SLC26A6/TLR2/ALG10B/FPR2/PIK3R2/ACSL1/IL1B/WLS/PRKCZ/ANO6/STIM1/CD177/JAK2/NCKAP1L/FIS1/ANGPT1/SCN1B/SLC9A1/VPS28/DOCK2/FCER1G/P2RX7/HAP1/CD38/CACNA1I/CAMK2A/FEZ1/TSPO/RPH3AL/AHCYL1/FLNA/ERBB2/MIEF2/CHCHD10/CA2/LRRK2/PPT1/GSK3B/BAX/RHOQ/SORL1/RIPOR1/DNM2/ZDHHC2/TNF/NFKBIA/GJC2/ABAT/PTGES/MYH10/CASR/LRP1/KIF5B/NKX3-1/GPLD1/CDK5R1/ITGAM/CD300LF/STXBP5/SLC11A1/PCM1/FZD5/TBC1D5/CES1/SYK/ANXA2/GPR27/SLC6A9/RAB21/UBR5/FCN1/CREB1/ATP2B1/RAC2/CCR1/CBL/CAMK1D/LRRC8A/ABCB11/RNASEL/GNAI2/AP2M1/LAMP1/GREM1/CACNA1D/SOX4/RAB3B/INPP5K/CD14/LILRA2/FFAR4/TRPC3/OSBPL8/MAVS/PTGS2/C2/SYTL4/CCL3/GSK3A/PSEN1/PTPRJ/FGR/CLU/ITGB2/VPS11/CAPN3/CD63/INSR/RAB27B/LGALS3/DNM3/BMP6/NLGN3/TESC/AP2B1/VPS4B/TF/SIRPA/RAB27A/KCNQ1/ACSL4/SRC/TPR/RAB7A/RAB2B/HSPA1L/AKAP9/FLOT1/P2RY1/ACTB/CALM3/PRKACA/AKT2/RXRA/EHD1/SH3GLB1/NFE2L2/SERPINE1/P2RX1/PRELID1/ACTN4/AP1G1/NPEPPS/ABCG1/CHP1/SIRPB1/UNC13D/HCLS1/SNCA/C2CD2L/HNRNPK/SDCBP/WNK1/PYCARD/OAZ1/ARRB2/TBC1D20/BBC3/SYNJ1/PPP3CA/UBE2D3/EHD3/OAZ2/CEBPB/PTPRC/OPRL1/MAPK14/MYOM1/EPHB2/PTAFR/CHMP2A/RGS7/G6PD/ABLIM3/IRS2/TCF7L2/RAB5A/AQP1/MYLK/PRKCD/PINK1/PPM1A/MSN/RAB3D/RAB31/DAB2/GLUL/GAB2/HIP1
BP	GO:0043547	positive regulation of GTPase activity	189	-0.46	-1.88	1.67E-07	3.17E-06	1.95E-06	2390	tags=34%, list=20%, signal=28%	ADAP1/PKP4/MYO9A/ERBB2/RTN4R/GSK3B/CHN2/TBC1D10B/ADPRH/TBC1D30/ARAP1/TBC1D8/RALGDS/TBC1D5/RCC2/ABR/RALGAPA2/RGS6/RAB11FIP2/TSC2/ASAP2/TBC1D14/CDKL5/ARAP3/CCL3/RGS14/SIPA1L1/TBC1D2/EVI5/NEDD9/AGRN/RAP1GAP/VAV1/DOCK8/PTK2B/NDEL1/DVL3/LIMS1/AKT2/PREX1/RABGAP1L/ARHGAP25/AFDN/TGM2/RSU1/TBC1D20/SIPA1/F11R/PICALM/RHOG/MAP4K4/ASAP1/NF1/IQSEC1/CORO1C/RGS7/TBC1D1/RASGRP4/SNX18/RALBP1/TBC1D3I/FLCN/SIPA1L2/RAPGEF2/ARHGAP6
BP	GO:0048589	developmental growth	355	-0.39	-1.71	1.79E-07	3.37E-06	2.07E-06	2363	tags=31%, list=20%, signal=26%	MED12/ALCAM/KLF5/YY1/RTN4R/KDM5B/GSK3B/BMPR1A/STAT5A/ACACB/DNM2/PAFAH1B1/WWC3/RGS2/CDK5R1/NRP2/ERCC2/SGPL1/CPQ/SEMA4A/WWC2/AKAP13/EIF4G2/KIAA0319/TWF2/ATF5/RAB21/CDKN1C/APBA1/SAV1/CREB1/CTR9/EP300/STK4/PIM1/DRAXIN/RTN4/CPNE1/DISC1/MTM1/CHST11/SP2/GIGYF2/STAT5B/CDKL5/IST1/ATRX/GSK3A/RARA/MAPT/RMI1/ULK1/CAPN3/LGR6/INSR/RTF1/SPAG9/SLC23A2/AGRN/CTTN/MBD5/H3-3A/NLGN3/NDEL1/GPX1/BCL9/CDKN1A/NIN/LIMK1/SRC/DPYSL2/NIPBL/DBN1/DUSP6/PDLIM5/PSAP/PPP1R13L/ZFP36L1/TAF10/PTPN12/RBPJ/KPNA1/JARID2/PELO/POR/ZMIZ1/NINJ2/STK40/USP9X/IGF2/CHD7/ADM/PPP3CA/CFLAR/H3-3B/MAPK14/FOXO3/YBX3/G6PD/STAT3/SFRP2/MTPN/PRKAR1A/FKBP8/BASP1/HLX/WDTC1/PTEN/VCL/NINJ1/IQGAP1
BP	GO:0007179	transforming growth factor beta receptor signaling pathway	117	-0.52	-2.02	1.96E-07	3.67E-06	2.26E-06	2697	tags=37%, list=23%, signal=29%	FOS/WNT1/ADAM17/PBLD/ZEB2/FAM89B/HSPA5/LTBP1/BMPR1A/HIPK2/DNM2/CDKN1C/IL17RD/EP300/DUSP22/CHST11/LRG1/ZEB1/FKBP1A/TRIM33/ZNF703/PXN/HSPA1A/SKI/SRC/TGFB1I1/ZBTB7A/ITGB5/NRROS/ZMIZ1/USP9X/SDCBP/ARRB2/CREBBP/STAT3/FLCN/PPM1A/SPI1/LTBP2/ZYX/FURIN/DAB2/CLDN5
BP	GO:0072659	protein localization to plasma membrane	204	-0.46	-1.89	1.99E-07	3.72E-06	2.29E-06	2208	tags=33%, list=19%, signal=27%	RHOQ/EFR3A/ZDHHC2/TNF/ROCK2/PRAM1/GBP1/LRP1/KIF5B/GGA3/MYADM/RAB34/GAK/TSPAN33/TNFRSF1A/RILPL1/GRIPAP1/TMBIM1/STX3/AP2M1/RAB3B/INPP5K/TSPAN14/PID1/CLTC/NUMB/ROCK1/SQSTM1/SEC16A/LGALS3/PPP1R9B/VAMP3/TESC/ZDHHC7/PPP2R5A/RAB7A/FLOT1/P2RY1/ACTB/TMEM88/MYO5A/AKT2/EHD1/AFDN/FYB1/FAM126B/F11R/EHD3/PICALM/RHOG/TSPAN5/PACS1/PPFIA1/EPHB2/SLC4A1/RILPL2/ABI3/BCL2L1/BSG/ANK1/RAB3D/ZDHHC3/RAB31/FLOT2/RAPGEF2/DAB2/KIF13A

BP	GO:0031623	receptor internalization	73	-0.59	-2.12	2.04E-07	3.79E-06	2.33E-06	2519	tags=53%, list=21%, signal=42%	ANGPT1/FCER1G/SNX1/GRB2/DNM2/GTF2H2/LRP1/LRPAP1/TBC1D5/SYK/ANXA2/NECAB2/RIN3/GRK4/DLG4/AP2M1/GREM1/SNAP25/MKLN1/CLTC/ARF1/NUMB/ITGB2/CD63/INSR/ANKRD13A/DNM3/CXCR2/CXCR1/FLOT1/GRK3/SNCA/SDCBP/ADM/ARRB2/PICALM/RAB5A/ITGB3/RAB31
BP	GO:0048812	neuron projection morphogenesis	338	-0.40	-1.72	2.19E-07	4.06E-06	2.50E-06	2690	tags=34%, list=23%, signal=27%	CAPRIN1/DICER1/RHOA/RAB8A/RAPH1/PRKCZ/LYPLA2/NEXN/TCTN1/ADNP/NCKAP1L/ACTR2/SCN1B/SEMA7A/SIN3A/CSF1R/HECW2/KLF7/FEZ1/ALCAM/PLXNB2/MYO9A/ERBB2/LRRK2/RTN4R/GSK3B/DNM2/PAFAH1B1/EFNB1/KIF5B/ATP7A/CDK5R1/NRP2/RNF165/YWHAH/CFL1/MARK2/SEMA4A/EIF4G2/TAOK1/KIAA0319/TWF2/WEE1/RAB21/DLG4/CREB1/RAC2/RERE/NCAM1/DRAXIN/RTN4/CPNE1/DISC1/MAP1A/NIBAN2/NUMB/NR4A2/CDKL5/ROCK1/IST1/RPS6KA5/PSEN1/MAPT/ANKRD27/VLDLR/EPHB4/ULK1/LGR6/B4GALT5/SPAG9/SIPA1L1/SLC23A2/GAS7/CUX1/CTTN/DNM3/NLGN3/NDEL1/DVL3/NIN/NTNG2/PALLD/APLP2/SPRY3/LIMK1/TAOK3/DPYSL2/DBN1/UBB/XK/PDLIM5/PAK2/FBXO45/FLOT1/ACTB/FEZ2/USP9X/PAK1/RAC1/PPP3CA/PLXNC1/PICALM/RHOG/MAP4K4/MAP1S/EPHB2/ABI3/VASP/KEL/BSG/PTEN/VCL/MBP/RAPGEF2/IQGAP1/ECE1
CC	GO:0030864	cortical actin cytoskeleton	52	-0.64	-2.16	2.40E-07	4.33E-06	2.66E-06	2176	tags=56%, list=19%, signal=46%	SLC2A1/ACTR2/SHROOM4/PRKCB/UTRN/MYADM/SPTA1/COTL1/AKAP13/PVALB/PPP1R9B/CTTN/ACTN1/DBN1/FCHSD1/FLOT1/MYO1E/MYH9/ACTN4/LASP1/HCLS1/SHROOM1/CORO1A/GSN/SPTB/FLOT2/IQGAP1/MYZAP/CLDN5
CC	GO:0031674	I band	71	-0.61	-2.15	2.38E-07	4.33E-06	2.66E-06	1925	tags=39%, list=16%, signal=33%	FHL2/SYNE2/PPP1R12A/CACNA1D/MTM1/ALDOA/PSEN1/FKBP1A/CAPN3/PDLIM2/SYNPO/PPP2R5A/PALLD/ACTN1/PDLIM5/HSPB1/PARVB/PPP1R12B/ACTN4/PAK1/PPP3CA/RTN2/DNAJB6/SLC4A1/PDLIM7/MYL9/SLC8A1/MYZAP
BP	GO:0071375	cellular response to peptide hormone stimulus	194	-0.45	-1.85	2.36E-07	4.33E-06	2.66E-06	2681	tags=44%, list=23%, signal=34%	WNT1/PIK3R2/IL1B/LPIN2/RAB8A/PLCB1/PRKCZ/JAK2/SLC9A1/CCND3/AGTRAP/JAK1/CAMK2A/AHCYL1/SMARCC1/CA2/CYSLTR2/GRB2/GSK3B/RHOQ/STAT5A/SYAP1/SORL1/PRKCB/PHIP/ROCK2/MAS1/GPLD1/UCP2/PIP4K2C/PKM/SOGA1/SP1/ATP2B1/TSC2/GNAI2/INPP5K/STAT5B/PID1/SHC1/PDK2/NR4A2/OSBPL8/ROCK1/GSK3A/RELA/GOT1/FUT7/SOCS3/PTPRE/INSR/PTPN1/SESN3/MAX/PXN/NR4A1/MBD5/NDEL1/ZDHHC7/EIF4EBP2/SRC/APC/ZNF106/GAB1/PIP4K2A/CYP1B1/ZFP36L1/MYO5A/AKT2/ZBTB7B/NFE2L2/POR/LYN/IGF2/ADIPOR1/CFLAR/STAT6/FBP1/IRS2/STAT3/PRKDC/PRKCD/WDTC1/RAB31/FOXO4
BP	GO:0051093	negative regulation of developmental process	469	-0.36	-1.63	2.38E-07	4.33E-06	2.66E-06	2275	tags=29%, list=19%, signal=24%	YY1/RTN4R/GSK3B/BMPR1A/SORL1/ZBTB16/PIK3CB/HNRNPU/TNF/NFKBIA/ROCK2/WWC3/GBP1/CST3/NKX3-1/RGS2/CDK5R1/MECP2/HDAC4/YWHAH/CDK13/PCM1/HHEX/SEMA4A/CTDSP1/WWC2/RCC2/TMEM176B/KIAA0319/ZFPM1/CR1/NFATC3/GADD45A/TOB2/IL17RD/SAV1/CTR9/CCR1/STK4/SOX6/DRAXIN/HOOK3/RTN4/YBX1/RFLNB/GREM1/MEIS1/CEACAM5/MSX1/NIBAN2/STAT5B/FOXJ2/KDM4A/ROCK1/H4C8/CCL3/SUFU/GSK3A/PSEN1/RARA/MAPT/ZEB1/ULK1/SPAG9/CMTM5/RAP1GAP/MBNL3/CTNNA1/DNM3/PTK2B/HDAC7/MAD2L2/CUL4A/PRDX2/CDKN1A/LIN28A/SKI/SPARC/SPRY3/TBX21/PF4/ASCL2/PBX1/TGFB1I1/SOD2/ZFP36L1/IRF1/CARM1/ZBTB7B/RBPJ/JARID2/NFE2L2/USF3/SERPINE1/TENT5C/NOTCH4/ACTN4/ABCG1/LYN/EPN1/IGF2/MMP9/ADIPOR1/PPP3CA/CFLAR/FBXO7/ASAP1/NF1/FOXO3/EPHB2/CORO1C/YBX3/PGLYRP1/G6PD/STAT3/DMTN/SFRP2/ITGB3/FLCN/PINK1/HLX/PTEN/JDP2/SPI1/LILRB3/MBP/FOXO4/RNF10/TRIB1/RAPGEF2/NBR1/AGO1/E2F2/CLDN5

BP	GO:0051345	positive regulation of hydrolase activity	396	-0.39	-1.70	2.41E-07	4.34E-06	2.67E-06	2983	tags=40%, list=25%, signal=31%	BAK1/TIAM1/PLIN5/BCAS3/AIFM1/PLAA/GMIP/TBC1D15/TBCK/PML/RUNDC1/TNFSF10/AKIRIN2/ATP2A3/NLRP12/TBC1D25/GNA13/CASP2/RHOA/REST/PRKCZ/PTPA/SOS2/LPAR1/JAK2/DAPK1/FIS1/CASP9/KIT/PPARGC1B/CIDEB/TBC1D22B/RCN3/DDX3X/ADAP1/PKP4/FADD/BEX3/MYO9A/ERBB2/LRRK2/RTN4R/GSK3B/CHN2/BAX/TBC1D10B/ADPRH/C6orf89/TNF/ROCK2/TBC1D30/ARAP1/TBC1D8/NKX3-1/GPLD1/PRKD2/RALGDS/TBC1D5/RCC2/SYK/CR1/ABR/PPP1R12A/RALGAPA2/RGS6/RAB11FIP2/TSC2/VSIR/ASAP2/TBC1D14/PSME3/CDKL5/ROCK1/BID/ARAP3/CCL3/MAPT/RGS14/IFI16/PRR7/SIPA1L1/VRK3/TBC1D2/EVI5/MAP3K5/NEDD9/AGRN/RAP1GAP/VAV1/ASPH/C5AR1/HSPA1A/PSME4/DOCK8/GNA12/GNAQ/PTK2B/NDEL1/CTSD/VCP/EGLN3/DVL3/LGALS9/GRN/PPP1R15A/PSAP/LIMS1/DAP/CALM3/AKT2/PREX1/RABGAP1L/P2RX1/PRELID1/ARHGAP25/ADRM1/AFDN/APAF1/LAPTM5/NLRC4/LYN/SNCA/TGM2/RSU1/PYCARD/PLEK/TBC1D20/BBC3/SIPA1/F11R/CFLAR/PTPRC/PICALM/RHOG/MAP4K4/ABHD5/ASAP1/NF1/PTAFR/IQSEC1/CORO1C/RGS7/TBC1D1/STAT3/SFRP2/RASGRP4/SNX18/RALBP1/TBC1D3I/FLCN/PRKCD/GSN/PINK1/MBP/SIPA1L2/RAPGEF2/ARHGAP6/HIP1
BP	GO:0007264	small GTPase mediated signal transduction	381	-0.39	-1.71	2.66E-07	4.76E-06	2.93E-06	2782	tags=34%, list=24%, signal=27%	MFN2/SHTN1/GRAP2/GNA13/ARHGAP18/FARP2/RHOA/ARHGEF11/USP8/ARHGAP33/SOS2/CDC42EP4/LPAR1/DOCK2/ARHGAP9/STARD8/RAB35/ARHGDIB/FAM13B/MYO9A/ARHGEF40/GRB2/RTN4R/SPATA13/CHN2/ELMO1/RHOQ/RIPOR1/PIK3CB/DNM2/MYO9B/ROCK2/LRRC59/MRAS/PLCE1/ARAP1/DENND3/ARHGAP31/ARHGEF3/ARHGDIA/CFL1/TAGAP/RALGDS/AKAP13/SETDB1/RASSF1/KBTBD7/ABR/ITSN1/PIK3CG/RAB21/RALGAPA2/RAC2/PSD4/CBL/LRRD1/TSC2/ARHGEF12/CUL3/GNB1/SH2D3C/ROCK1/NKIRAS2/WASF2/ARHGAP30/ERBIN/RIN2/DNMBP/SQSTM1/SIPA1L1/RAP1GAP/VAV1/PHACTR4/RALB/DOCK8/GNA12/ARHGEF2/RGS19/ARHGAP21/DOK1/NUCB1/CDKN1A/DOCK5/SPRY3/LIMK1/VAV3/SIAH2/SRC/SHOC2/GABARAP/FLOT1/ARHGAP8/PREX1/PLEKHG3/ARHGAP1/ARHGAP25/RIT1/FGD4/CYTH4/CHP1/TGM2/SDCBP/RAC1/SIPA1/DOK3/CYRIB/F11R/RHOG/FBP1/MAP4K4/HMOX1/IQSEC2/KPNB1/RUNDC3A/NF1/EPHB2/IQSEC1/WAS/CTNNAL1/DHCR24/ARHGAP26/RHOBTB1/RASGRP4/RALBP1/FLCN/DENND1A/SIPA1L2/RIPOR2/RAPGEF2/ARHGAP6
MF	GO:0051020	GTPase binding	251	-0.42	-1.77	3.03E-07	5.39E-06	3.31E-06	2592	tags=36%, list=22%, signal=29%	CDC42EP4/YIPF1/HPS6/GNB2/DOCK2/CCDC186/OPTN/RPH3AL/ARHGDIB/FLNA/LRRK2/SORL1/MYO9B/RANBP2/ROCK2/TBC1D30/PLCE1/XPO7/PKN1/ATP7A/IPO13/GGA3/YIPF2/AKAP13/RCC2/IPO8/TMEM127/RAB34/RIN3/PPP6R1/MICALL1/RAB11FIP2/TSC2/NUTF2/VPS9D1/YBX1/TNPO1/TRIOBP/GNB1/CDKL5/ROCK1/DENND5A/DIAPH2/SYTL4/ANKRD27/RIN2/ULK1/EVI5/RASSF5/DIAPH1/RAP1GAP/ARHGEF2/DVL3/RILP/DOCK5/RAB7A/PAK2/EXOC4/MYO5A/RAB11FIP4/EHD1/RABGAP1L/ARHGAP1/NCF2/FGD4/AFDN/GCC1/AP1G1/DMXL2/UNC13D/PAK1/FGD6/TBC1D20/RAB11FIP1/CYRIB/RRAGD/PICALM/LCP1/KPNB1/CORO1C/WAS/TNPO3/YBX3/CYRIA/RALBP1/AMBRA1/XPO6/IQGAP1/FMNL1/IQGAP2

BP	GO:0031175	neuron projection development	545	-0.35	-1.60	3.03E-07	5.39E-06	3.31E-06	2778	tags=34%, list=24%, signal=28%	CDH23/ENC1/SZT2/PLAA/KATNB1/TAOK2/KIDINS220/SRF/SCARF1/SHTN1/UHMK1/CLMN/HDGFL3/FARP1/FES/CRTC1/FRYL/CAPRIN1/DICER1/RHOA/RAB8A/RAPH1/NEURL1/PRKCZ/SCARB2/LYPLA2/NEXN/IFT88/TCTN1/ADNP/LPAR1/JAK2/NCKAP1L/LST1/ACTR2/SCN1B/SEMA7A/SIN3A/HSPA5/CSF1R/HECW2/VIM/RAB35/KLF7/CD38/CAMK2A/FEZ1/TSPO/ALCAM/FLNA/PLXNB2/MYO9A/ERBB2/LRRK2/RTN4R/TBC1D24/GSK3B/DNM2/PAFAH1B1/EFNB1/KIF5B/ATP7A/RGS2/CDK5R1/NRP2/MECP2/RNF165/YWHAH/CPEB3/CFL1/MARK2/SEMA4A/P3H1/EIF4G2/TAOK1/KIAA0319/TWF2/ATCAY/GAK/WEE1/RAB21/DLG4/CREB1/RAC2/EP300/RERE/CAMK1D/MICALL1/NCAM1/DRAXIN/HGF/LRP12/STK24/RTN4/STX3/CPNE1/TSPAN2/DISC1/MAP1A/SNAP25/NIBAN2/TRIOBP/NUMB/NR4A2/CDKL5/ROCK1/IST1/DENND5A/RPS6KA5/GSK3A/PSEN1/MAPT/ANKRD27/VLDLR/EPHB4/ULK1/LGR6/B4GALT5/SPAG9/SIPA1L1/SLC23A2/GAS7/MAP4/PPP1R9B/ADGRF1/CUX1/CTTN/CTNNA1/DNM3/NLGN3/PTK2B/NDEL1/PPP2R5B/DVL3/RETREG3/NIN/NTNG2/PALLD/APLP2/SPRY3/LIMK1/TAOK3/DPYSL2/GRN/DBN1/UBB/DGKG/XK/PDLIM5/PAK2/FBXO45/SETX/FLOT1/ACTB/DLG5/CARM1/EHD1/FEZ2/NFE2L2/LYN/FRY/USP9X/PAK1/RAC1/ADM/PPP3CA/CFLAR/PLXNC1/PICALM/RHOG/MAP4K4/FBXO7/MAP1S/ASAP1/EPHB2/IQSEC1/ABI3/VASP/SFRP2/KEL/BSG/PTEN/VCL/MBP/RAPGEF2/IQGAP1/ECE1/DAB2
BP	GO:0032956	regulation of actin cytoskeleton organization	244	-0.43	-1.79	3.14E-07	5.55E-06	3.41E-06	2592	tags=34%, list=22%, signal=27%	CDC42EP4/LPAR1/NCKAP1L/SLC9A1/CSF1R/ARPC4/ACTG1/ARHGDIB/FLNA/CAPZB/GRB2/ADD1/RHOQ/NPHS1/ROCK2/BST1/ARAP1/LRP1/CDK5R1/RICTOR/ALOX15/ARHGDIA/MYADM/PDGFA/SPTA1/COTL1/AKAP13/TAOK1/TWF2/FHOD1/ARPC1B/JAM3/RAC2/FSCN1/INPP5K/TRIOBP/ARF1/WASHC2C/ROCK1/WASF2/PXN/CTTN/PTK2B/SYNPO/HCK/ARPC5/LIMK1/DBN1/FCHSD1/PAK2/ESAM/SVIL/TPM1/LMOD3/SSH1/ARPC1A/HCLS1/PAK1/RAC1/PYCARD/PLEK/CYRIB/F11R/RHOG/PPFIA1/TMOD1/CORO1A/SSH2/WAS/ABI3/VASP/RHOBTB1/CYRIA/DMTN/MTPN/PRKCD/GSN/SPTB/IQGAP1/ARHGAP6/IQGAP2/PEAK3
BP	GO:0048259	regulation of receptor-mediated endocytosis	69	-0.61	-2.15	3.26E-07	5.71E-06	3.51E-06	2309	tags=52%, list=20%, signal=42%	ABCA2/PPT1/PIK3CB/DNM2/LRPAP1/TBC1D5/SYK/ANXA2/NECAB2/RAB21/RIN3/DLG4/CBL/AP2M1/GREM1/MKLN1/ARF1/NUMB/CLU/CD63/INSR/SMAP1/ANKRD13A/TF/FLOT1/SERPINE1/HNRNPK/SDCBP/RAC1/BMP2K/ARRB2/SYNJ1/PICALM/ITGB3/DAB2/HIP1
BP	GO:0019221	cytokine-mediated signaling pathway	314	-0.40	-1.73	3.25E-07	5.71E-06	3.51E-06	3103	tags=40%, list=26%, signal=30%	CD4/EPOR/MED1/IL18R1/CCL5/EDN1/RFFL/TYK2/BBS2/STAT1/IL17RC/OAS3/IL31RA/PARP9/NAIP/GSTP1/HIF1A/LILRA6/TRAF6/ACSL1/IL1B/PLCB1/SAMHD1/ADAM17/JAK2/KIT/ANGPT1/FCER1G/OXSR1/CSF1R/JAK1/CEBPA/CD70/FADD/IL18RAP/SPATA2/IL1R2/IFITM3/LILRB2/STAT5A/PAFAH1B1/TNF/NFKBIA/TNFSF13B/HIPK1/CD300LF/OSM/SYK/IL10RB/LILRA1/NMI/TNFRSF1A/PF4V1/YTHDF3/PTPN6/IRAK3/IL17RD/CTR9/CCR1/AGPAT1/CBL/LSM14A/CPNE1/LILRA2/STAT5B/CSF3R/MAVS/PARP14/RPS6KA5/IRAK1/NKIRAS2/USP27X/PELI3/HSPA1B/CCL3/RELA/PTPRJ/PTPN1/ADAR/HSPA1A/PTK2B/IFNAR1/CXCR2/HCK/DOK1/CXCL5/ILK/OASL/CXCR1/SRC/PF4/LIMS1/RIPK1/IL6R/PADI2/IRF1/IFNAR2/PRKACA/CLCF1/TNFRSF1B/IL1RN/ACTN4/ACKR1/LAPTM5/SHARPIN/WNK1/PYCARD/XIAP/ADIPOR1/H2BC11/PTPRC/STAT6/SP100/WBP1L/FOXO3/IL17RA/TOLLIP/STAT3/MAPK3/IFITM2/IL13RA1/MPL/SPI1/CCRL2/LILRB3/PLVAP

BP	GO:0007507	heart development	317	-0.40	-1.74	3.36E-07	5.85E-06	3.60E-06	2363	tags=30%, list=20%, signal=25%	MED12/FLNA/ERBB2/YY1/TBX19/SENP2/BMPR1A/ACACB/TAB2/HNRNPU/ROCK2/NSD2/PLCE1/IFT122/NKX3-1/RGS2/NRP2/MAML1/UTY/FHL2/CERT1/AKAP13/EIF4G2/ZFPM1/MYH11/DYNC2H1/TNFRSF1A/RB1CC1/LMNA/SAV1/CREB1/EP300/TMEM65/STK4/PIM1/SOX6/TSC2/RTN4/GREM1/SOX4/MSX1/SHC1/IFT140/ROCK1/TFDP2/SUFU/GSK3A/PSEN1/RARA/FKBP1A/EPHB4/INSR/DAW1/PDLIM2/ARL13B/AP2B1/CDKN1A/KCNQ1/ILK/NIPBL/DUSP6/PDLIM5/PPP1R13L/SOD2/ZFP36L1/TNFRSF1B/RBPJ/JARID2/TPM1/KDM6B/NDST1/ZMIZ1/CHD7/ADM/ARRB2/MAP2K3/MAPK14/NF1/AP1B1/G6PD/MAPK3/PDLIM7/PRKDC/SFRP2/CCDC39/PRKAR1A/BASP1/PTEN/SLC8A1/NPRL3/MICAL2/ECE1/ODAD4/NCOA6/CLDN5
BP	GO:0051493	regulation of cytoskeleton organization	381	-0.39	-1.70	3.39E-07	5.89E-06	3.62E-06	2733	tags=33%, list=23%, signal=26%	HDGFL3/ARHGAP18/TTBK2/FES/RHOA/SLAIN2/NEXN/CDC42EP4/LPAR1/NCKAP1L/SLC9A1/CEP295/DYRK1A/P2RX7/CSF1R/ARPC4/ACTG1/ARHGDIB/FLNA/CAPZB/GRB2/CLIP1/GSK3B/CHMP3/ADD1/RHOQ/NPHS1/HNRNPU/PAFAH1B1/ROCK2/BST1/ARAP1/LRP1/CDK5R1/MECP2/RICTOR/ALOX15/ARHGDIA/TACC3/MARK2/MYADM/PDGFA/SPTA1/COTL1/AKAP13/TAOK1/TWF2/RASSF1/FHOD1/ARPC1B/JAM3/RAC2/FSCN1/MAP1A/INPP5K/TRIOBP/CLTC/ARF1/WASHC2C/ROCK1/RAE1/WASF2/HSPA1B/MAPT/PLK1/CCNF/DIAPH1/PXN/HSPA1A/CTTN/CEP97/PTK2B/SYNPO/ARHGEF2/HCK/VPS4B/NIN/ARPC5/CHMP1A/LIMK1/APC/TPR/DBN1/FCHSD1/PAK2/ESAM/FSD1/AKAP9/GAS2L1/SVIL/BRCA1/TPM1/LMOD3/SSH1/ARPC1A/CCSAP/HCLS1/SNCA/PAK1/RAC1/PYCARD/PLEK/CYRIB/F11R/RHOG/MAP1S/PPFIA1/TMOD1/CORO1A/SSH2/CHMP2A/WAS/ABI3/VASP/RHOBTB1/CYRIA/MAPK3/DMTN/MTPN/PRKCD/GSN/SPTB/CHMP4B/IQGAP1/ARHGAP6/IQGAP2/PEAK3
BP	GO:0090287	regulation of cellular response to growth factor stimulus	148	-0.49	-1.95	3.54E-07	6.12E-06	3.76E-06	2681	tags=40%, list=23%, signal=31%	WNT1/IL1B/ADAM17/PBLD/ZEB2/FAM89B/HSPA5/LTBP1/HIPK2/CCBE1/SORL1/PRKCB/PIK3CB/DNM2/PRKD2/RNF165/HHEX/ITGA5/CDKN1C/IL17RD/EP300/SULF2/GREM1/MSX1/CHST11/NIBAN2/DSTYK/LRG1/ARNT/ZEB1/FKBP1A/TRIM33/ZNF703/CD63/PTPN1/PTP4A3/HSPA1A/PPP2R5B/SKI/SPRY3/ILK/TGFB1I1/UBE2O/ZBTB7A/RBPJ/PELO/NRROS/SDCBP/XIAP/CREBBP/UBE2D3/CFLAR/SFRP2/ITGB3/PCSK6/FLCN/FKBP8/PPM1A/DAB2
MF	GO:0005096	GTPase activator activity	207	-0.44	-1.82	3.57E-07	6.12E-06	3.76E-06	3360	tags=48%, list=29%, signal=35%	RINL/TBC1D12/EVI5L/ARHGEF10L/ELMOD1/TBC1D17/SEC23B/ARHGAP45/ACAP2/ARHGAP32/SH3BP1/GDI1/GAPVD1/ARRB1/RANBP3/GMIP/TBC1D15/TBCK/RUNDC1/TBC1D25/ARHGAP18/ARHGEF11/PLCB1/ARHGAP33/NCKAP1L/DOCK2/ARHGAP9/TBC1D22B/STARD8/ARFGAP3/ADAP1/ARHGDIB/FAM13B/MYO9A/LRRK2/TBC1D24/CHN2/TBC1D10B/ADPRH/MYO9B/RANBP2/TBC1D30/ARAP1/TBC1D8/RGS3/RGS2/ARHGAP31/ARHGDIA/STXBP5/TAGAP/TBC1D5/SRGAP1/ABR/RIN3/RALGAPA2/RGS6/AGAP2/TSC2/VPS9D1/ARHGEF12/ASAP2/TBC1D14/ARAP3/ARHGAP30/RGS14/ANKRD27/RIN2/GIT2/SMAP2/SIPA1L1/TBC1D2/EVI5/SMAP1/RAP1GAP/GNAQ/ARHGAP21/DOCK5/VAV3/ARHGAP8/PREX1/RABGAP1L/ARHGAP1/ARHGAP25/TBC1D20/SIPA1/ASAP1/NF1/RGS7/ARHGAP26/TBC1D1/RALBP1/TBC1D3I/FLCN/SIPA1L2/RAPGEF2/NPRL3/IQGAP1/ARHGAP6/IQGAP2
BP	GO:0003012	muscle system process	217	-0.44	-1.83	3.56E-07	6.12E-06	3.76E-06	2148	tags=33%, list=18%, signal=28%	TNF/UTRN/ROCK2/PLCE1/ABAT/RGS2/MYLPF/HDAC4/CERT1/AKAP13/MYH11/PIK3CG/TNFRSF1A/SSTR2/LMNA/ATP2B1/SULF2/PDE5A/CACNA1D/ACTA2/ALDOA/KDM4A/TRPC3/LTB4R/PTGS2/TPM3/ROCK1/DSC2/GSK3A/ASPH/NR4A1/CTTN/GTF2IRD1/SNTB1/DOCK5/KCNQ1/MYL4/PDLIM5/PAK2/KCNE3/AKAP9/P2RY1/CALM3/PPP1R13L/PRKACA/TNFRSF1B/PROK2/JARID2/TPM1/P2RX1/PPP1R12B/LMOD3/TPM4/PPP3CA/EHD3/CFLAR/KCNJ5/GUCY1A1/MAP2K3/MYOM1/TMOD1/HMOX1/FOXO3/PTAFR/G6PD/MTPN/MYLK/GSN/MYL9/SLC8A1/TBXA2R/SLC6A8

CC	GO:0036477	somatodendritic compartment	456	-0.37	-1.65	3.60E-07	6.14E-06	3.77E-06	2700	tags=34%, list=23%, signal=27%	CRTC1/CAPRIN1/RHOA/CACNA1A/RAB8A/USP8/STRN4/NEURL1/PRKCZ/VTI1B/ADNP/HCFC1/PSEN2/SCN1B/DYRK1A/P2RX7/HAP1/INPP5A/CAMK2A/FEZ1/ALCAM/SNX14/FLNA/CKB/LRRK2/PPT1/CYBB/RTN4R/GSK3B/BMPR1A/SYAP1/SORL1/MAGEE1/HNRNPU/PAFAH1B1/TNF/GJC2/PI4K2A/CASR/KIF5B/ATP7A/CDK5R1/GRIK2/KIF1B/CPEB3/MARK2/FZD5/HRH2/NECAB2/ATCAY/ABR/ATP6AP2/RIN3/CPEB4/APBA1/TRAK1/DLG4/GRIPAP1/PDE1A/SAV1/ATP2B1/ERO1A/NCF1/GNAI2/STX3/CNIH3/DTNB/MAP1A/PVALB/GIGYF2/SNAP25/ROGDI/SLC2A3/CDKL5/GSK3A/PSEN1/RARA/MAPT/RGS14/CLU/PRR7/KIF1C/INSR/SIPA1L1/MAX/AGO2/RAP1GAP/PPP1R9B/WDFY3/ABHD17C/CTTN/SLC6A6/GNA12/DNM3/MARK4/PTK2B/SYNPO/NDEL1/PTBP2/RAB27A/KCNQ1/NIN/ACTN1/SIAH2/MYO1D/TBX21/TMEM266/DBN1/UBB/FCHSD1/KCNE3/AKAP9/MARK3/P2RY1/PRKACA/FXR2/TNFRSF1B/KPNA1/CTNND1/MME/SLA/GLRX5/SHARPIN/ERMN/SNCA/PAK1/RAC1/PYCARD/ARRB2/PPP3CA/PICALM/MAP1S/ASAP1/PPFIA1/PRKAR2B/NF1/EPHB2/LRRC4/TOP1/OSBP2/ABI3/RAB5A/TRAK2/NRGN/MPL/MBP/SLC8A1/FLOT2/RAPGEF2/CASC3/STAU1/GLUL/GNAZ
BP	GO:0051129	negative regulation of cellular component organization	475	-0.36	-1.61	3.84E-07	6.53E-06	4.01E-06	2381	tags=30%, list=20%, signal=25%	CD38/FEZ1/TSPO/DCP2/WAPL/FLNA/CAPZB/ABCA2/LRRK2/RTN4R/GSK3B/LILRB2/ADD1/SORL1/HNRNPU/DNM2/PAFAH1B1/TNF/CPTP/ROCK2/TBC1D30/ARAP1/CST3/PPIF/CDK5R1/LRPAP1/YWHAH/MYADM/SPTA1/SEMA4A/RCC2/TAOK1/KIAA0319/TWF2/ANXA2/NECAB2/GAK/TNFRSF1A/RIN3/DLG4/IRAK3/LMNA/JAM3/EP300/DRAXIN/HGF/TSC2/RUBCN/RTN4/DUSP22/INPP5K/MTM1/GNL3L/TRIOBP/ZW10/WASHC2C/ROCK1/DENND5A/ATRX/XRCC1/WASF2/HSPA1B/PSEN1/MAPT/ANKRD27/CLU/ULK1/PLK1/CCNF/MAP4/RAP1GAP/LGALS3/HSPA1A/CLEC16A/ANKRD13A/H3-3A/DNM3/BMP6/TFIP11/CEP97/ARHGEF2/RIOK3/MAD2L2/NUPR1/GPX1/SPRY3/SLX1B/SRC/APC/TPR/RAB7A/AICDA/PAK2/DUSP1/CARM1/SVIL/TNFRSF1B/FEZ2/BRCA1/PRELID1/LMOD3/SSH1/CTNNBIP1/SNCA/SDCBP/RAC1/ARRB2/CYRIB/PPP3CA/F11R/LIMK2/CFLAR/PICALM/H3-3B/PPFIA1/TMOD1/DNAJB6/CORO1A/SSH2/EPHB2/CORO1C/WAS/DNAJB2/ABI3/DNAJA4/DMTN/MTPN/ITGB3/RAD21/FLCN/PRKCD/GSN/PINK1/BCL2L1/PPM1A/CCP110/SPTB/PTEN/CHMP4B/MBP/RAPGEF2/DAB2/ARHGAP6
CC	GO:0030018	Z disc	68	-0.60	-2.11	4.23E-07	7.16E-06	4.40E-06	1925	tags=38%, list=16%, signal=32%	FHL2/SYNE2/PPP1R12A/CACNA1D/PSEN1/FKBP1A/CAPN3/PDLIM2/SYNPO/PPP2R5A/PALLD/ACTN1/PDLIM5/HSPB1/PARVB/PPP1R12B/ACTN4/PAK1/PPP3CA/RTN2/DNAJB6/SLC4A1/PDLIM7/MYL9/SLC8A1/MYZAP

BP	GO:0001775	cell activation	773	-0.33	-1.51	4.55E-07	7.67E-06	4.72E-06	2723	tags=32%, list=23%, signal=27%	TLR2/LGALS9C/FES/CLIC1/FPR2/TRAF6/WNT1/RHOA/IL1B/DHPS/PLCB1/TMEM131L/ADAM17/INPP5D/PRKCZ/IKZF1/SELPLG/CCDC88B/SOS2/CD177/JAK2/NCKAP1L/SNAP23/IL4I1/C17orf99/LST1/KIT/CMTM7/DOCK2/GPR137B/FCER1G/TICAM1/P2RX7/C1QA/RC3H2/CCND3/ACTG1/LCP2/CD38/CEBPA/CD70/PJA2/FADD/FLNA/SERPINE2/KLF5/ERBB2/THEMIS2/LRRK2/YY1/FERMT3/NECTIN2/METAP1/TNFSF13/LILRB2/PLCL2/BAX/ZBTB16/PRKCB/PIK3CB/PELI1/TNF/EFNB1/JUND/PRAM1/BST1/NSD2/PKN1/ABAT/MNDA/LRP1/TNFSF13B/TLR1/ATP7A/GP6/ITGAM/HDAC4/CD300LF/SLC11A1/HHEX/PDGFA/SPTA1/PLSCR1/FZD5/LTBR/SEMA4A/GON4L/SYK/NMI/CLPTM1/ZFPM1/CR1/HLA-E/TREML2/ITGAL/PIK3CG/KLF6/PF4V1/PTPN6/NFKB2/MSH6/RAC2/EP300/GCLM/CBL/LRRC8A/TIRAP/VSIR/SUPT6H/PDE5A/LAMP1/DUSP22/SOX4/TLR6/LILRA2/ACTA2/STAT5B/RSAD2/SH3RF1/HSH2D/CCL3/PSEN1/RARA/MAPT/ZEB1/PTPRJ/FGR/CLU/FKBP1A/ITGB2/FUT7/PRR7/CAPN3/VAV1/LGALS3/C5AR1/SBNO2/TIMP1/DOCK8/PTK2B/CXCR2/SLC46A2/MAD2L2/CD93/APBB1IP/RPS6KA1/SIRPA/PRDX2/LGALS9/CDKN1A/RAB27A/NCR1/ILK/VAV3/SRC/GRN/TBX21/KMT2E/PF4/LAT2/DGKG/AICDA/PBX1/HSPB1/TNFSF14/P2RY1/IL6R/ACTB/DLG5/STXBP2/ZFP36L1/SELP/ZBTB7A/IRF1/GP1BA/SNX27/CLCF1/ZBTB7B/TNFRSF1B/ALOX12/PREX1/RBPJ/KPNA1/P2RX1/SLC7A11/MYH9/PRELID1/TREML1/PTGDS/BATF2/BAG6/ZMIZ1/AP1G1/LAPTM5/LYN/SIRPB1/UNC13D/SNCA/IGF2/CHD7/TYROBP/ELF4/PYCARD/FCGR3A/PLEK/SH3KBP1/CYRIB/PIK3CD/PPP3CA/F11R/CEBPB/PTPRC/STAT6/FBXO7/LYL1/LCP1/MAPK14/HMOX1/CORO1A/FOXO3/EPHB2/PTAFR/WAS/PGLYRP1/IRS2/IL17RA/TOLLIP/TLN1/STAT3/DMTN/PRKDC/ITGB3/PRKAR1A/MPL/AMBRA1/PRKCD/GSN/MYL9/MSN/HLX/NFAM1/VCL/SPI1/BCL3/SKAP2/RIPOR2/FLOT2/DYSF/GP9/GAB2/NFIL3
BP	GO:0060429	epithelium development	565	-0.35	-1.58	4.80E-07	8.05E-06	4.95E-06	2685	tags=30%, list=23%, signal=25%	TRAF6/WNT1/RHOA/IL1B/PLCB1/ADAM17/VEZF1/BTG1/TCTN1/SETD2/SIRT6/JAK2/CEP152/TAGLN/RCN3/CSF1R/TMEM79/VIM/ACTG1/KLF7/MED12/FLNA/SERPINE2/KLF5/PLXNB2/MYO9A/KIAA1109/CA2/GRB2/KDM5B/GSK3B/BMPR1A/BAX/ADD1/NPHS1/YIPF6/CTSZ/PAFAH1B1/TNF/ROCK2/IFT122/CASR/ATP7A/NKX3-1/PRKD2/BTBD7/ERCC2/S1PR2/MYADM/PDGFA/FZD5/CES1/TMEM231/APCDD1/CRYGS/ITGA5/TNFRSF1A/ATP6AP2/RILPL1/CDKN1C/SAV1/CREB1/EP300/TGM3/STK4/HGF/TSC2/ARID4A/TXNIP/FZR1/RTN4/MRTFA/GREM1/SOX4/MSX1/SIDT2/ACTA2/TRIOBP/STAT5B/IFT140/FOXJ2/ROCK1/ATRX/MTHFR/SUFU/GSK3A/PSEN1/RARA/RELA/ZEB1/SOCS3/ZNF703/INTU/MAFG/FLG2/ARL13B/LGALS3/PHACTR4/CNFN/BMP6/B4GALT1/SULT1B1/GPX1/ZDHHC7/DVL3/KCNQ1/SKI/PALLD/ILK/SRC/CPT1A/CEP63/PSAP/PBX1/TGFB1I1/PECAM1/MYCL/ACTB/DLG5/ZFP36L1/VDR/MYO1E/PRKACA/AKT2/TAF10/RBPJ/TAGLN2/SERPINE1/KDM6B/SLC7A11/PELO/AFDN/NOTCH4/APAF1/CTNNBIP1/SHARPIN/TGM2/CHD7/PAK1/RAC1/ADM/MMP9/TST/TBC1D20/PIK3CD/F11R/CEBPB/STAT6/CTSB/TMOD1/NF1/ITPK1/RILPL2/VASP/TOLLIP/PRKDC/SFRP2/CCDC39/FKBP8/BASP1/MSN/PTEN/VCL/FOSL2/KRT1/RAPGEF2/MICAL2/IQGAP1/FKBPL/ECE1/DAB2/CLDN5
BP	GO:0032970	regulation of actin filament-based process	262	-0.42	-1.79	5.13E-07	8.58E-06	5.27E-06	2435	tags=32%, list=21%, signal=26%	CSF1R/ARPC4/ACTG1/ARHGDIB/FLNA/CAPZB/GRB2/ADD1/RHOQ/NPHS1/ROCK2/BST1/ARAP1/LRP1/CDK5R1/RICTOR/ALOX15/ARHGDIA/MYADM/PDGFA/SPTA1/COTL1/AKAP13/TAOK1/TWF2/FHOD1/ARPC1B/JAM3/RAC2/FSCN1/INPP5K/ACTA2/TRIOBP/ARF1/WASHC2C/ROCK1/DSC2/WASF2/PXN/CTTN/PTK2B/SYNPO/HCK/ARPC5/LIMK1/DBN1/FCHSD1/PAK2/ESAM/AKAP9/SVIL/TPM1/MYH9/LMOD3/SSH1/ARPC1A/HCLS1/PAK1/RAC1/PYCARD/PLEK/CYRIB/F11R/RHOG/PPFIA1/TMOD1/CORO1A/SSH2/WAS/ABI3/VASP/RHOBTB1/CYRIA/DMTN/MTPN/PRKCD/GSN/SPTB/CNN2/IQGAP1/ARHGAP6/IQGAP2/PEAK3

MF	GO:0035091	phosphatidylinositol binding	210	-0.44	-1.82	5.32E-07	8.81E-06	5.42E-06	2708	tags=34%, list=23%, signal=27%	FES/WDFY1/PLCB1/ARHGAP33/ATG2B/SLC9A1/WIPI1/ARHGAP9/RAB35/ADAP1/TECPR1/SNX1/ZFYVE26/SNX14/TPCN2/DENND1C/ARAP1/SNX8/GGA3/ITPR2/ALOX15/DAPP1/CERT1/TWF2/ANXA2/TTPAL/TIRAP/NCF1/RUBCN/NCF4/FCGR3B/SNX11/MTM1/CEACAM5/PHF12/EEA1/OSBPL8/WASHC2C/ZFYVE16/ARAP3/MAPT/WIPI2/ZCCHC2/WDFY3/KCNQ1/SNX10/PLEKHB2/WDR45/PITPNA/STAM2/PLEK2/MYO1E/SVIL/SNX27/HS1BP3/EPB41/ATG2A/C2CD2L/SDCBP/PICALM/ASAP1/TLN1/SBF2/SNX18/NRGN/DENND1A/GSN/IQGAP1/GAB2/OSBPL2/IQGAP2/HIP1
CC	GO:0001726	ruffle	141	-0.49	-1.93	5.57E-07	9.20E-06	5.65E-06	1935	tags=36%, list=17%, signal=31%	CFL1/PDE4A/MYADM/PIP5K1C/ITGA5/KNSTRN/RAB22A/FSCN1/PSD4/FSCN3/TIRAP/INPP5K/MTM1/MKLN1/SH2D3C/CDKL5/ROCK1/ARAP3/WASF2/PTPRJ/FGR/DIAPH1/PPP1R9B/CTTN/ARHGEF2/TESC/PALLD/ACTN1/SRC/APC/MYO5A/AKT2/TPM1/MYH9/FGD4/ATP6V1B2/PAK1/RAC1/FGD6/PLEK/WIPF1/LCP1/BMX/CORO1C/RAB5A/TLN1/PDLIM7/ITGB3/FRMD4B/IQGAP1/S100A11
BP	GO:0008154	actin polymerization or depolymerization	152	-0.48	-1.91	5.73E-07	9.37E-06	5.76E-06	1980	tags=32%, list=17%, signal=27%	RICTOR/ALOX15/CFL1/MYADM/SPTA1/COTL1/TWF2/ARPC1B/TRIOBP/WASHC2C/DIAPH2/PSTPIP2/MSRB2/GAS7/DIAPH1/PSTPIP1/PPP1R9B/CTTN/PTK2B/HCK/ARPC5/DBN1/FCHSD1/ESAM/SVIL/PREX1/LMOD3/SSH1/ARPC1A/HCLS1/RAC1/PYCARD/PLEK/WIPF1/CYRIB/MSRB1/TMOD1/CORO1A/SSH2/WAS/VASP/CYRIA/DMTN/MTPN/PRKCD/GSN/SPTB/MICAL2/ARHGAP6
MF	GO:1901981	phosphatidylinositol phosphate binding	131	-0.50	-1.96	6.32E-07	1.03E-05	6.34E-06	3099	tags=43%, list=26%, signal=32%	MAPKAP1/MARK1/PHLDA3/FCHSD2/PHLDA1/PLEKHA5/GOLPH3/RPH3A/PLCB1/ATG2B/SLC9A1/WIPI1/ARHGAP9/RAB35/ADAP1/TECPR1/ZFYVE26/SNX14/TPCN2/DENND1C/ARAP1/ALOX15/DAPP1/CERT1/TWF2/ANXA2/TTPAL/TIRAP/RUBCN/NCF4/SNX11/OSBPL8/WASHC2C/ZFYVE16/ARAP3/MAPT/WIPI2/KCNQ1/PLEKHB2/WDR45/PLEK2/SVIL/SNX27/ATG2A/SDCBP/PICALM/ASAP1/SNX18/NRGN/DENND1A/GSN/IQGAP1/GAB2/OSBPL2/IQGAP2/HIP1
CC	GO:0005765	lysosomal membrane	352	-0.38	-1.68	6.73E-07	1.09E-05	6.68E-06	2616	tags=32%, list=22%, signal=26%	VTI1B/SCARB2/CTSA/PLEKHM2/HPS6/PSEN2/GNB2/LAMTOR4/GPR137B/TMEM79/TECPR1/ZFYVE26/SNX14/ABCA2/IFITM3/CHMP3/TPCN2/PLEKHM1/RAB2A/FPR1/PI4K2A/SLCO4C1/LRP1/KIF5B/TLR8/GPLD1/MYLPF/ATP6V1C1/PRCP/AP1M1/MANBA/MCOLN1/MEAK7/MFSD12/ANXA2/ATP6AP2/SLC15A3/VPS33A/LAMP2/LRRC8A/TMBIM1/AP2M1/CPNE1/LAMP1/RAB37/CKAP4/SIDT2/ATP6V0A1/FFAR4/CSF3R/GNB1/CLTC/TFEB/BRI3/SLC44A2/SNAP29/PSEN1/VLDLR/VPS11/CD63/SPAG9/ATP6V0D1/NEU1/ARL8A/CLEC16A/GNAQ/CTSD/B4GALT1/SLC17A5/AP2B1/ELAPOR1/RILP/CHMP1A/ACP3/GRN/RAB7A/KPTN/PGAP6/PSAP/FLOT1/SLC29A3/VPS18/CUBN/AP2A1/MARCHF2/AP1G1/LAPTM5/UBA1/LYN/MMD/ATP6V1B2/DNAJC5/UBXN6/DTX3L/SLC48A1/CHMP2A/AP1B1/RAB5C/MARCHF8/IFITM2/GNB4/FLCN/CD68/RAB3D/NFAM1/CHMP4B/NPRL3/AP5B1/ECE1/DAB2/LITAF/SLC7A5/ATP6V1A
CC	GO:0098852	lytic vacuole membrane	352	-0.38	-1.68	6.73E-07	1.09E-05	6.68E-06	2616	tags=32%, list=22%, signal=26%	VTI1B/SCARB2/CTSA/PLEKHM2/HPS6/PSEN2/GNB2/LAMTOR4/GPR137B/TMEM79/TECPR1/ZFYVE26/SNX14/ABCA2/IFITM3/CHMP3/TPCN2/PLEKHM1/RAB2A/FPR1/PI4K2A/SLCO4C1/LRP1/KIF5B/TLR8/GPLD1/MYLPF/ATP6V1C1/PRCP/AP1M1/MANBA/MCOLN1/MEAK7/MFSD12/ANXA2/ATP6AP2/SLC15A3/VPS33A/LAMP2/LRRC8A/TMBIM1/AP2M1/CPNE1/LAMP1/RAB37/CKAP4/SIDT2/ATP6V0A1/FFAR4/CSF3R/GNB1/CLTC/TFEB/BRI3/SLC44A2/SNAP29/PSEN1/VLDLR/VPS11/CD63/SPAG9/ATP6V0D1/NEU1/ARL8A/CLEC16A/GNAQ/CTSD/B4GALT1/SLC17A5/AP2B1/ELAPOR1/RILP/CHMP1A/ACP3/GRN/RAB7A/KPTN/PGAP6/PSAP/FLOT1/SLC29A3/VPS18/CUBN/AP2A1/MARCHF2/AP1G1/LAPTM5/UBA1/LYN/MMD/ATP6V1B2/DNAJC5/UBXN6/DTX3L/SLC48A1/CHMP2A/AP1B1/RAB5C/MARCHF8/IFITM2/GNB4/FLCN/CD68/RAB3D/NFAM1/CHMP4B/NPRL3/AP5B1/ECE1/DAB2/LITAF/SLC7A5/ATP6V1A

BP	GO:0071559	response to transforming growth factor beta	153	-0.48	-1.92	7.20E-07	1.15E-05	7.10E-06	1711	tags=27%, list=15%, signal=23%	CDKN1C/IL17RD/EP300/SOX6/DUSP22/CHST11/ROCK1/LRG1/ZEB1/FKBP1A/TRIM33/ZNF703/NR3C1/PXN/HSPA1A/ZFHX3/SKI/SRC/PAK2/TGFB1I1/LIMS1/ZFP36L1/ZBTB7A/ITGB5/NRROS/ZMIZ1/APAF1/USP9X/SDCBP/ARRB2/CREBBP/CFLAR/STAT3/FLCN/PPM1A/SPI1/LTBP2/ZYX/FURIN/DAB2/CLDN5
BP	GO:0071560	cellular response to transforming growth factor beta stimulus	149	-0.48	-1.91	7.26E-07	1.15E-05	7.10E-06	1711	tags=26%, list=15%, signal=23%	CDKN1C/IL17RD/EP300/SOX6/DUSP22/CHST11/LRG1/ZEB1/FKBP1A/TRIM33/ZNF703/NR3C1/PXN/HSPA1A/SKI/SRC/PAK2/TGFB1I1/LIMS1/ZFP36L1/ZBTB7A/ITGB5/NRROS/ZMIZ1/APAF1/USP9X/SDCBP/ARRB2/CREBBP/CFLAR/STAT3/FLCN/PPM1A/SPI1/LTBP2/ZYX/FURIN/DAB2/CLDN5
MF	GO:0019900	kinase binding	583	-0.34	-1.56	7.24E-07	1.15E-05	7.10E-06	2176	tags=26%, list=19%, signal=22%	PRKCB/DNM2/UTRN/PRAM1/PKN1/CASR/WWC3/CDK5R1/ELMO2/PRKAR2A/PRKD2/GP6/MAML1/RICTOR/CDK13/FZD5/PGAM1/WWC2/RCC2/TWF2/SYK/ATF5/GADD45A/PTPN6/PPP1R12A/PPM1D/DLG4/IRAK3/RB1CC1/RAC2/CDKN2D/CBL/RAB11FIP2/LYST/AGAP2/TIRAP/DUSP22/YWHAZ/ACTA2/SHC1/CLTC/CTBP2/TBC1D14/MAVS/IRAK1/BCL2L14/CCNY/SUFU/GSK3A/RARA/MAPT/RELA/RGS14/PTPRJ/FGR/ITGB2/PRR7/SQSTM1/SPAG9/PTPN1/PLK1/MAP3K5/NR3C1/NEDD9/PPP1R9B/PTK2B/HDAC7/MAD2L2/TNIP1/NEK9/SIRPA/CDKN1A/KCNQ1/PPP2R5A/PARP16/SKI/RAD23A/MAP2K7/CASS4/NIN/ILK/SRC/APC/WDR45/TPR/PPP1R15A/MAPKAPK2/WARS1/PDLIM5/PAK2/LIMS1/HSPB1/DUSP1/ACTB/CALM3/DAZAP2/IFNAR2/PRKACA/MAPKAPK3/CIR1/CNPPD1/CTNND1/AP2A1/SASH1/GOLGA2/PPP1R12B/CHP1/HCLS1/STRADA/RAC1/WNK1/PLEK/ARRB2/XIAP/MIDN/ADIPOR1/POLR2A/TUBA4A/MAP3K2/CAVIN2/MAP2K3/CEBPB/PTPRC/RHOG/FBXO7/STK38/MAPK14/MYOM1/PRKAR2B/SP100/FOXO3/PTAFR/WAS/AP1B1/CACUL1/IRS2/TCF7L2/RHOBTB1/TOLLIP/MARCKS/STAT3/PRKAR1A/PRKCD/GSN/PINK1/BCL2L1/MSN/PTEN/TRIB1/IQGAP1/NBR1/GAB2
BP	GO:1901652	response to peptide	318	-0.39	-1.69	7.64E-07	1.21E-05	7.44E-06	2723	tags=39%, list=23%, signal=31%	TLR2/FPR2/WNT1/PIK3R2/IL1B/CACNA1A/SRSF4/LPIN2/RAB8A/PLCB1/SCAP/PRKCZ/BTG1/SLC2A1/JAK2/CACNB1/HADHA/SLC9A1/CCND3/VIM/AGTRAP/JAK1/LDOC1/CAMK2A/AHCYL1/SMARCC1/CA2/CYSLTR2/GRB2/CYBB/GSK3B/RHOQ/STAT5A/SYAP1/SORL1/PRKCB/TNF/PHIP/NFKBIA/ROCK2/JUND/CASR/LRP1/MAS1/GPLD1/UCP3/UCP2/PIP4K2C/PKM/MEAK7/SOGA1/SP1/CREB1/ATP2B1/TSC2/RNF40/GNAI2/TLR6/INPP5K/STAT5B/PID1/SHC1/PDK2/NR4A2/OSBPL8/PTGS2/ROCK1/SESN2/GSK3A/PSEN1/RELA/GOT1/ERBIN/FUT7/SOCS3/PTPRE/INSR/PTPN1/SESN3/MAX/PXN/PPP1R9B/NR4A1/TIMP1/MBD5/ARHGEF2/NDEL1/ZDHHC7/KCNQ1/EIF4EBP2/SRC/APC/ZNF106/GAB1/PIP4K2A/CYP1B1/ZFP36L1/MYO5A/AKT2/ZBTB7B/KLF3/NFE2L2/POR/LYN/IGF2/ADM/MMP9/ADIPOR1/CFLAR/STAT6/FBP1/MAPK14/FOXO3/EPHB2/IRS2/STAT3/PRKDC/PRKCD/BSG/WDTC1/RAB31/FOXO4/KLF1

BP	GO:0098542	defense response to other organism	690	-0.33	-1.52	7.72E-07	1.22E-05	7.49E-06	3231	tags=38%, list=28%, signal=29%	RNF213/MYD88/TNK2/CDC37/AKAP8/ECSIT/FFAR2/PPP1R11/IL1RAP/MICB/SRPK1/TASL/IFIT3/CLEC12B/SLC15A4/PADI4/CD4/BCL10/MED1/DDX21/CCL5/EDN1/MFHAS1/ADAM15/APOBEC3A/TYK2/TRIM38/STAT1/TUBB/IFI44L/H2BC21/VNN1/OAS3/IL31RA/PPP6C/PARP9/BST2/PML/ANKRD17/RELB/AGBL5/CFD/NAIP/ITCH/NLRP10/CLEC4E/AKIRIN2/ADAM8/PUM2/TKFC/HERC5/SLC26A6/TLR2/FES/FPR2/TRAF6/IL1B/SAMHD1/DNAJC3/ADAM17/CCDC88B/CD177/CDC42EP4/SETD2/PLEKHM2/JAK2/DAPK1/ACTR2/SIN3A/FCER1G/EXOSC4/TICAM1/P2RX7/C1QA/APOBEC3C/CSF1R/DDX3X/TRIM11/VIM/ACTG1/COLEC12/JAK1/OPTN/TRIM21/PJA2/FADD/CRCP/CYBB/NECTIN2/REL/IFITM3/CRISP3/CHMP3/ARID5A/MAPKBP1/SPON2/DDX60/TNF/SERINC3/MNDA/GBP1/KIF5B/TLR8/C1RL/TRIM25/ITGAM/BNIP3L/CALCOCO2/SLC11A1/PLSCR1/COTL1/SYK/IL10RB/RAB43/NMI/LPO/CXCL16/CR1/HLA-E/PIK3CG/TNFRSF1A/YTHDF3/PTPN6/NUB1/SLC15A3/FCN1/IRAK3/NCBP3/EP300/TUBB4B/PI3/LYST/SERPING1/LSM14A/TIRAP/EIF2AK2/NCF1/TRIM26/RNASEL/LAMP1/MX2/TLR6/CD14/LILRA2/KLRD1/STAT5B/RSAD2/PCBP2/TRIM10/SHC1/GBP2/N4BP1/MAVS/PARP14/IRAK1/USP27X/C2/GPATCH3/CCL3/RELA/FGR/CLU/IFI16/WIPI2/PTPN1/ADAR/RNF19B/MAP3K5/PSTPIP1/VAV1/LGALS3/C5AR1/VAMP3/RNASET2/IRF2/ZC3HAV1/PTK2B/IFNAR1/ARHGEF2/RIOK3/HCK/ATG7/TF/SIRPA/LGALS9/RAB27A/NCR1/OASL/SRC/GRN/ALPK1/PF4/IKBKG/POLR3H/AICDA/PAK2/ABCC9/IL6R/SELP/IRF1/IFNAR2/GBP4/RBPJ/TREM1/NFE2L2/SERPINE1/SEC14L1/MARCHF2/NCF2/TREML1/IFIT1B/STMP1/DCD/BATF2/SLA/AP1G1/NLRC4/SHARPIN/LYN/UNC13D/ILRUN/RNF187/SNCA/ZNFX1/TYROBP/PAK1/ELF4/FBXO9/PYCARD/FCGR3A/ARRB2/XIAP/TRIM8/CREBBP/PIK3CD/DTX3L/H2BC11/CEBPB/TRIM58/MSRB1/PTPRC/HK1/SRPK2/SP100/CORO1A/WAS/PGLYRP1/IL17RA/TOLLIP/AQP1/MAPK3/PRKDC/IFITM2/DDX60L/NLRX1/PRKCD/GSN/BCL2L1/KRT1/SPI1/BCL3/ZYX/H2BC4
BP	GO:0030168	platelet activation	94	-0.55	-2.05	7.77E-07	1.22E-05	7.50E-06	2861	tags=52%, list=24%, signal=40%	SRF/GNAS/CD9/GNA13/CLIC1/JAK2/FCER1G/ACTG1/FLNA/SERPINE2/FERMT3/METAP1/PIK3CB/ABAT/GP6/PDGFA/PLSCR1/SYK/PIK3CG/PF4V1/PTPN6/VAV1/ILK/VAV3/SRC/PF4/DGKG/HSPB1/P2RY1/ACTB/SELP/GP1BA/ALOX12/P2RX1/SLC7A11/MYH9/TREML1/LYN/PLEK/F11R/MAPK14/TLN1/DMTN/ITGB3/MPL/PRKCD/MYL9/VCL/GP9
MF	GO:0005509	calcium ion binding	309	-0.40	-1.71	7.86E-07	1.23E-05	7.56E-06	2220	tags=28%, list=19%, signal=23%	ALPL/CCBE1/PROS1/USP32/CASR/LRP1/CDK5R1/MYLPF/ITPR2/SPTA1/PLSCR1/ANXA2/NECAB2/ITSN1/DSG3/SULF2/CBL/TGM3/ADGRE3/CRACR2B/CPNE1/PVALB/NHSL2/AOC3/DSC2/VCAN/ZZEF1/MCTP2/VLDLR/PCDH1/CAPN3/SELENON/FLG2/AGRN/ASPH/ANXA11/CD93/TESC/SYT12/NUCB1/NIN/SPARC/EFHD2/ACTN1/VWCE/SELL/MYL4/MICU1/DGKG/GNPTAB/PCDHGA8/PCDHB1/CALM3/TKT/SELP/PADI2/RAB11FIP4/EHD1/CUBN/EPS15L1/NOTCH4/ACTN4/TPM4/CHP1/SNCA/TGM2/PPP3CA/EHD3/PRRG4/LCP1/PPP3R1/HPCAL1/RASGRP4/GSN/MYL9/CAPN1/GPD2/LTBP2/SLC8A1/RAPGEF2/IQGAP1/DYSF/REPS2/CABP5/S100A11

MF	GO:0030695	GTPase regulator activity	377	-0.37	-1.64	8.50E-07	1.32E-05	8.11E-06	2768	tags=34%, list=24%, signal=27%	TBC1D25/GNA13/ARHGAP18/FARP1/FARP2/ARHGEF11/PLCB1/ARHGAP33/SOS2/NCKAP1L/RCBTB2/LAMTOR4/DOCK2/ARHGAP9/TBC1D22B/STARD8/ARFGAP3/SH3BP5/ADAP1/ARHGDIB/FAM13B/MYO9A/ARHGEF40/LRRK2/CYSLTR2/SPATA13/TBC1D24/CHN2/DENND1C/ELMO1/TBC1D10B/ADPRH/MYO9B/RANBP2/TBC1D30/PLCE1/ARAP1/TBC1D8/RGS3/DENND3/RGS2/ARHGAP31/ARHGEF3/ARHGDIA/STXBP5/TAGAP/RALGDS/TBC1D5/AKAP13/RCC2/SRGAP1/ABR/ITSN1/RIN3/RALGAPA2/RGS6/PSD4/AGAP2/TSC2/VPS9D1/ARHGEF12/ASAP2/SH2D3C/TBC1D14/DENND5A/SESN2/ARAP3/ARHGAP30/RGS14/ANKRD27/RIN2/DNMBP/GIT2/SMAP2/SIPA1L1/TBC1D2/EVI5/SMAP1/RAP1GAP/VAV1/DOCK8/GNAQ/ARHGEF2/ARHGAP21/NUCB1/SIRPA/DOCK5/VAV3/ARHGAP8/PREX1/PLEKHG3/RABGAP1L/ARHGAP1/ARHGAP25/RPGR/FGD4/SH3BP5L/CYTH4/FGD6/GPSM3/TBC1D20/SIPA1/ASAP1/IQSEC2/RUNDC3A/NF1/IQSEC1/WAS/RGS7/HPS1/ARHGAP26/RAB3IL1/TBC1D1/SBF2/RASGRP4/RALBP1/TBC1D3I/FLCN/DENND1A/PCP2/SIPA1L2/RAPGEF2/NPRL3/IQGAP1/TBXA2R/ARHGAP6/IQGAP2
MF	GO:0060589	nucleoside-triphosphatase regulator activity	377	-0.37	-1.64	8.50E-07	1.32E-05	8.11E-06	2768	tags=34%, list=24%, signal=27%	TBC1D25/GNA13/ARHGAP18/FARP1/FARP2/ARHGEF11/PLCB1/ARHGAP33/SOS2/NCKAP1L/RCBTB2/LAMTOR4/DOCK2/ARHGAP9/TBC1D22B/STARD8/ARFGAP3/SH3BP5/ADAP1/ARHGDIB/FAM13B/MYO9A/ARHGEF40/LRRK2/CYSLTR2/SPATA13/TBC1D24/CHN2/DENND1C/ELMO1/TBC1D10B/ADPRH/MYO9B/RANBP2/TBC1D30/PLCE1/ARAP1/TBC1D8/RGS3/DENND3/RGS2/ARHGAP31/ARHGEF3/ARHGDIA/STXBP5/TAGAP/RALGDS/TBC1D5/AKAP13/RCC2/SRGAP1/ABR/ITSN1/RIN3/RALGAPA2/RGS6/PSD4/AGAP2/TSC2/VPS9D1/ARHGEF12/ASAP2/SH2D3C/TBC1D14/DENND5A/SESN2/ARAP3/ARHGAP30/RGS14/ANKRD27/RIN2/DNMBP/GIT2/SMAP2/SIPA1L1/TBC1D2/EVI5/SMAP1/RAP1GAP/VAV1/DOCK8/GNAQ/ARHGEF2/ARHGAP21/NUCB1/SIRPA/DOCK5/VAV3/ARHGAP8/PREX1/PLEKHG3/RABGAP1L/ARHGAP1/ARHGAP25/RPGR/FGD4/SH3BP5L/CYTH4/FGD6/GPSM3/TBC1D20/SIPA1/ASAP1/IQSEC2/RUNDC3A/NF1/IQSEC1/WAS/RGS7/HPS1/ARHGAP26/RAB3IL1/TBC1D1/SBF2/RASGRP4/RALBP1/TBC1D3I/FLCN/DENND1A/PCP2/SIPA1L2/RAPGEF2/NPRL3/IQGAP1/TBXA2R/ARHGAP6/IQGAP2
BP	GO:0006914	autophagy	458	-0.36	-1.61	8.74E-07	1.35E-05	8.28E-06	2950	tags=36%, list=25%, signal=28%	DAPK2/BCAS3/PIP4K2B/ATP6V0B/PLAA/CAMKK2/CLN3/ORMDL3/ATP6V0C/VPS41/RIMOC1/HIF1A/VPS13D/MFN2/TBC1D25/EPM2A/PIKFYVE/PIK3R2/RAB8A/VTI1B/IFT88/EIF4G1/CTSA/ERN1/DAPK1/FIS1/ATG2B/WIPI1/VPS28/GPR137B/TICAM1/HAP1/TECPR1/OPTN/FEZ1/TSPO/TRIM21/HK2/SNX14/TMEM150B/LRRK2/RNF5/CAPNS1/GSK3B/CHMP3/TPCN2/ADPRH/PLEKHM1/TAB2/PIK3CB/VPS37B/MAP1LC3B/CPTP/GABARAPL2/CDK5R1/ATP6V1C1/PIP4K2C/DHRSX/BNIP3L/CALCOCO2/MARK2/FZD5/TBC1D5/MCL1/MCOLN1/GABARAPL1/EIF4G2/RAB43/SOGA1/EXOC7/VPS33A/SPTLC2/RB1CC1/TP53INP2/LAMP2/EP300/MTMR3/HGF/TSC2/RUBCN/MTM1/ATP6V0A1/MAP1LC3A/KDM4A/CLTC/TFEB/TBC1D14/ROCK1/SESN2/SNAP29/GSK3A/PSEN1/MAPT/CLU/RAB1B/VPS11/IFI16/ULK1/SQSTM1/WIPI2/SESN3/SEC16A/ATP6V0D1/WDFY3/CTTN/RALB/CLEC16A/QSOX1/VCP/NUPR1/RGS19/ATG7/VPS4B/ELAPOR1/RETREG3/CHMP1A/TECPR2/SRC/WDR45/RAB7A/IKBKG/GABARAP/VPS37C/PSAP/EXOC4/HSPB1/PIP4K2A/WAC/STAM2/DAP/PRKACA/SH3GLB1/FEZ2/VPS18/KAT5/GOLGA2/USP10/ATP6V1B2/ILRUN/SNCA/ATG2A/PYCARD/UBXN6/TRIM8/RRAGD/FBXO7/MAP1S/HMOX1/MAP1LC3B2/FOXO3/CHMP2A/RAB5A/TOLLIP/STAT3/MAPK3/SBF2/SNX18/DCAF12/NRBF2/FLCN/AMBRA1/PINK1/CAPN1/CHMP4B/NPRL3/NBR1/ATP6V1A

BP	GO:0061919	process utilizing autophagic mechanism	458	-0.36	-1.61	8.74E-07	1.35E-05	8.28E-06	2950	tags=36%, list=25%, signal=28%	DAPK2/BCAS3/PIP4K2B/ATP6V0B/PLAA/CAMKK2/CLN3/ORMDL3/ATP6V0C/VPS41/RIMOC1/HIF1A/VPS13D/MFN2/TBC1D25/EPM2A/PIKFYVE/PIK3R2/RAB8A/VTI1B/IFT88/EIF4G1/CTSA/ERN1/DAPK1/FIS1/ATG2B/WIPI1/VPS28/GPR137B/TICAM1/HAP1/TECPR1/OPTN/FEZ1/TSPO/TRIM21/HK2/SNX14/TMEM150B/LRRK2/RNF5/CAPNS1/GSK3B/CHMP3/TPCN2/ADPRH/PLEKHM1/TAB2/PIK3CB/VPS37B/MAP1LC3B/CPTP/GABARAPL2/CDK5R1/ATP6V1C1/PIP4K2C/DHRSX/BNIP3L/CALCOCO2/MARK2/FZD5/TBC1D5/MCL1/MCOLN1/GABARAPL1/EIF4G2/RAB43/SOGA1/EXOC7/VPS33A/SPTLC2/RB1CC1/TP53INP2/LAMP2/EP300/MTMR3/HGF/TSC2/RUBCN/MTM1/ATP6V0A1/MAP1LC3A/KDM4A/CLTC/TFEB/TBC1D14/ROCK1/SESN2/SNAP29/GSK3A/PSEN1/MAPT/CLU/RAB1B/VPS11/IFI16/ULK1/SQSTM1/WIPI2/SESN3/SEC16A/ATP6V0D1/WDFY3/CTTN/RALB/CLEC16A/QSOX1/VCP/NUPR1/RGS19/ATG7/VPS4B/ELAPOR1/RETREG3/CHMP1A/TECPR2/SRC/WDR45/RAB7A/IKBKG/GABARAP/VPS37C/PSAP/EXOC4/HSPB1/PIP4K2A/WAC/STAM2/DAP/PRKACA/SH3GLB1/FEZ2/VPS18/KAT5/GOLGA2/USP10/ATP6V1B2/ILRUN/SNCA/ATG2A/PYCARD/UBXN6/TRIM8/RRAGD/FBXO7/MAP1S/HMOX1/MAP1LC3B2/FOXO3/CHMP2A/RAB5A/TOLLIP/STAT3/MAPK3/SBF2/SNX18/DCAF12/NRBF2/FLCN/AMBRA1/PINK1/CAPN1/CHMP4B/NPRL3/NBR1/ATP6V1A
BP	GO:0001667	ameboidal-type cell migration	254	-0.42	-1.75	9.27E-07	1.42E-05	8.71E-06	2209	tags=28%, list=19%, signal=24%	CCBE1/STAT5A/PIK3CB/PAFAH1B1/TNF/EFNB1/ROCK2/PKN1/GPLD1/NRP2/PRKD2/MECP2/SGPL1/PRCP/SEMA4A/RCC2/PIK3CG/YTHDF3/GADD45A/SP1/RTN4/GREM1/GPI/ACTA2/MAP3K3/PTGS2/ROCK1/WASF2/RIN2/EPHB4/AGO2/PTP4A3/PXN/PHACTR4/NR4A1/TIMP1/GNA12/PTK2B/HDAC7/GPX1/GFUS/DOCK5/SPARC/ILK/SRC/GRN/HSPB1/CYP1B1/NFE2L2/SASH1/MYH9/GLIPR2/RAC1/MMP9/ADIPOR1/PIK3CD/MAP2K3/MAP4K4/HMOX1/SP100/NF1/IQSEC1/CORO1C/DNAJA4/DMTN/ITGB3/PTEN/SLC8A1/IQGAP1/TBXA2R/GLUL/TNS1
BP	GO:0010810	regulation of cell-substrate adhesion	135	-0.49	-1.93	1.01E-06	1.54E-05	9.47E-06	3022	tags=43%, list=26%, signal=32%	BCL6/ADAM15/FERMT1/BCAS3/THBS1/WDPCP/WNT1/RHOA/PRKCZ/JAK2/SLC9A1/ACTG1/FLNA/GSK3B/PIK3CB/DNM2/UTRN/ROCK2/BST1/GBP1/ALOX15/MYADM/RCC2/ITGA5/RAC2/STK4/GREM1/DISC1/DUSP22/TRIOBP/WASHC2C/ROCK1/PTPRJ/RIN2/NEDD9/PTK2B/SLK/GFUS/DOCK5/CASS4/ILK/SRC/LIMS1/PREX1/SERPINE1/ACTN4/UNC13D/RAC1/RSU1/MAP4K4/NF1/CORO1C/DMTN/PTEN/VCL/HSD17B12/DAB2/ARHGAP6
BP	GO:0043408	regulation of MAPK cascade	406	-0.37	-1.64	1.02E-06	1.55E-05	9.50E-06	2848	tags=36%, list=24%, signal=28%	NAIP/ITCH/GSTP1/HLA-DRB1/ADAM8/NLRP12/MAPK1/FPR2/TRAF6/PIK3R2/IL1B/PLCB1/PRKCZ/ERN1/LPAR1/JAK2/KIT/ANGPT1/SEMA7A/DUSP5/P2RX7/CSF1R/C5AR2/PJA2/ERBB2/LRRK2/GRB2/HIPK2/NCOR1/MAPKBP1/SORL1/PIK3CB/ATF3/PAFAH1B1/TNF/ROCK2/PLCE1/CASR/GBP1/RGS2/PRKD2/GRIK2/ALOX15/SH3RF3/ERN2/OSM/PDGFA/FZD5/LTBR/AKAP13/TAOK1/SYK/NECAB2/MAP2K2/GPBAR1/PIK3CG/GADD45A/ATP6AP2/PTPN6/IRAK3/RB1CC1/DOK4/CCR1/STK4/TIRAP/EIF2AK2/NCF1/HGF/GNAI2/PDE5A/DUSP22/TLR6/YWHAZ/INPP5K/ACTA2/MTURN/MAP3K3/SHC1/FFAR4/SH3RF1/DSTYK/ROCK1/IRAK1/CCL3/PSEN1/RGS14/PTPRJ/INSR/SPAG9/PTPN1/VRK3/CHI3L1/MAP3K5/RASSF2/C5AR1/HMGCR/PTK2B/TNIP1/SIRPA/DVL3/PRDX2/LGALS9/MAP2K7/HIPK3/SPRY3/TAOK3/SRC/DUSP6/PAK2/PIK3R5/DUSP1/RIPK1/RNF149/P2RY1/ARHGAP8/SASH1/NDST1/LAPTM5/STK40/LYN/IGF2/GLIPR2/PAK1/SDCBP/PYCARD/ARRB2/XIAP/CFLAR/MAP2K3/PTPRC/MAP4K4/STK38/NF1/EPHB2/MAPK3/SFRP2/FLCN/PRKCD/PINK1/PTEN/SPI1/PELI2/RAPGEF2/IQGAP1/NBR1/DAB2/TGFA

BP	GO:0040007	growth	565	-0.35	-1.56	1.06E-06	1.60E-05	9.81E-06	2680	tags=32%, list=23%, signal=26%	RHOA/ARHGEF11/H3-5/CTDP1/PLCB1/ASPM/ADAM17/RAPH1/PRKCZ/BTG1/ADNP/EIF4G1/CSNK2A1/JADE1/SLC9A1/SEMA7A/SIN3A/EXOSC4/RC3H2/TAF8/DDX3X/CD38/MED12/ALCAM/SERPINE2/KLF5/ERBB2/KIAA1109/PPT1/YY1/RTN4R/KDM5B/GSK3B/RFTN1/BMPR1A/STAT5A/ACACB/DNM2/PAFAH1B1/PLCE1/WWC3/RGS2/CDK5R1/NRP2/KCTD11/ERCC2/SGPL1/CPQ/OSM/SEMA4A/WWC2/AKAP13/EIF4G2/KIAA0319/TWF2/ATF5/CXCL16/RAB21/CDKN1C/APBA1/UBE2E3/SAV1/CREB1/CTR9/EP300/CDKN2D/STK4/PIM1/DRAXIN/LRP12/RTN4/CPNE1/GREM1/DISC1/MTM1/MSX1/CHST11/SP2/GIGYF2/STAT5B/SHC1/NAIF1/CDKL5/IST1/SESN2/ATRX/HSPA1B/SERTAD3/GSK3A/RARA/MAPT/PTPRJ/RMI1/SOCS3/ULK1/CAPN3/LGR6/INSR/RTF1/SPAG9/SLC23A2/NEDD9/AGRN/SMARCA2/NDRG3/MT2A/PPP1R9B/HSPA1A/CTTN/MBD5/H3-3A/NLGN3/PTK2B/NDEL1/MAD2L2/GPX1/RPS6KA1/BCL9/CDKN1A/EGLN2/NIN/LIMK1/SRC/DPYSL2/NIPBL/EXTL3/DBN1/DUSP6/OSGIN2/PDLIM5/PSAP/PAK2/DCUN1D3/TKT/PPP1R13L/ZFP36L1/OGFR/TAF10/PTPN12/RBPJ/KAT5/KPNA1/JARID2/PELO/POR/ZMIZ1/NINJ2/STK40/USP9X/IGF2/CHD7/SDCBP/ADM/ADIPOR1/CDA/SIPA1/PPP3CA/CFLAR/FBP1/H3-3B/MAPK14/FOXO3/DNAJB2/YBX3/G6PD/STAT3/SFRP2/MTPN/PRKAR1A/FLCN/FKBP8/BCL2L1/BASP1/HLX/WDTC1/PTEN/VCL/ING1/NINJ1/IQGAP1/DAB2
BP	GO:0042692	muscle cell differentiation	204	-0.44	-1.83	1.08E-06	1.62E-05	9.96E-06	2568	tags=36%, list=22%, signal=28%	QKI/DYRK1B/KIT/SLC9A1/SMARCD3/ACTG1/KLF5/YY1/BMPR1A/PLEKHO1/NPHS1/HNRNPU/RGS2/MECP2/MAML1/HDAC4/FHL2/AKAP13/MYH11/HIRA/NFATC3/LMNA/SOX6/SUPT6H/YBX1/MRTFA/GREM1/CEACAM5/MSX1/NIBAN2/GSK3A/RARA/ZEB1/CAPN3/SPAG9/SELENON/H3-3A/GPX1/BCL9/SKI/ACTN1/XK/PDLIM5/FLOT1/TNFSF14/SOD2/EHD1/RBPJ/TPM1/KDM6B/MYH9/LMOD3/ACTN4/RBM38/IGF2/ADM/ARRB2/PPP3CA/CFLAR/H3-3B/MAPK14/TMOD1/MYEF2/G6PD/MTPN/PRKAR1A/KEL/MYL9/RIPOR2/SLC8A1/FOXO4/NINJ1/DYSF
BP	GO:0030855	epithelial cell differentiation	312	-0.39	-1.69	1.11E-06	1.66E-05	1.02E-05	2416	tags=32%, list=21%, signal=26%	WNT1/RHOA/IL1B/PLCB1/VEZF1/BTG1/CEP152/TAGLN/TMEM79/VIM/KLF7/KLF5/MYO9A/KIAA1109/KDM5B/GSK3B/ADD1/NPHS1/YIPF6/PAFAH1B1/TNF/ROCK2/ERCC2/S1PR2/MYADM/CES1/TMEM231/TNFRSF1A/RILPL1/CDKN1C/SAV1/CREB1/TGM3/STK4/ARID4A/TXNIP/FZR1/GREM1/SOX4/SIDT2/ACTA2/TRIOBP/STAT5B/FOXJ2/ROCK1/ATRX/GSK3A/RARA/ZEB1/ZNF703/INTU/MAFG/LGALS3/CNFN/BMP6/B4GALT1/SULT1B1/GPX1/ZDHHC7/KCNQ1/PALLD/SRC/CPT1A/CEP63/PSAP/TGFB1I1/PECAM1/MYCL/DLG5/ZFP36L1/VDR/MYO1E/AKT2/TAF10/RBPJ/TAGLN2/SERPINE1/KDM6B/SLC7A11/PELO/AFDN/NOTCH4/SHARPIN/MMP9/TST/TBC1D20/F11R/CEBPB/CTSB/TMOD1/RILPL2/TOLLIP/BASP1/MSN/VCL/FOSL2/KRT1/RAPGEF2/IQGAP1/DAB2/CLDN5

MF	GO:0008047	enzyme activator activity	406	-0.37	-1.64	1.11E-06	1.66E-05	1.02E-05	2919	tags=36%, list=25%, signal=28%	PLAA/RANBP3/TAOK2/GMIP/TBC1D15/TBCK/RUNDC1/GNAS/ATP2A3/NLRP12/TBC1D25/DBF4B/ARHGAP18/ARHGEF11/PLCB1/PRKAG2/ARHGAP33/PTPA/CTSA/NCKAP1L/CASP9/DOCK2/ARHGAP9/TBC1D22B/STARD8/ARFGAP3/DDX3X/ADAP1/ARHGDIB/FAM13B/INCENP/BEX3/MYO9A/LRRK2/TBC1D24/BMPR1A/CHN2/TBC1D10B/ADPRH/MYO9B/RANBP2/TBC1D30/ARAP1/TBC1D8/RGS3/NKX3-1/RGS2/CDK5R1/ARHGAP31/RICTOR/ARHGDIA/STXBP5/MARK2/TAGAP/TBC1D5/TAOK1/SRGAP1/MAP2K2/ABR/RIN3/RALGAPA2/ALOX5AP/RGS6/STK4/AGAP2/NCF1/TSC2/VSIR/VPS9D1/ARHGEF12/NCF4/FZR1/GREM1/ASAP2/TBC1D14/PSME3/ARAP3/CCNY/CCL3/ARHGAP30/RGS14/ANKRD27/RIN2/GIT2/SMAP2/SIPA1L1/TBC1D2/EVI5/SMAP1/RAP1GAP/PHACTR4/PSME4/GNAQ/VCP/NUPR1/ARHGAP21/PPP2R5B/CDKN1A/DOCK5/PPP2R5A/PARP16/VAV3/SRC/PPP1R15A/PSAP/PAK2/BRPF1/DPM2/CALM3/DAZAP2/ARHGAP8/PREX1/RABGAP1L/ARHGAP1/ARHGAP25/NCF2/PPP1R12B/TAL1/ADRM1/APAF1/NLRC4/IGF2/STRADA/WNK1/PYCARD/TBC1D20/SIPA1/DTX3L/CFLAR/ASAP1/NF1/RGS7/MOB3A/ARHGAP26/TBC1D1/SFRP2/RALBP1/TBC1D3I/FLCN/PRKCD/PINK1/MOB3C/SIPA1L2/RAPGEF2/NPRL3/IQGAP1/ARHGAP6/IQGAP2
CC	GO:0099513	polymeric cytoskeletal fiber	422	-0.36	-1.61	1.25E-06	1.85E-05	1.14E-05	2256	tags=30%, list=19%, signal=25%	CLIP1/CHMP3/RHOQ/NCKAP5L/NDE1/HNRNPU/DNM2/PAFAH1B1/MYO9B/MAP1LC3B/KIF5B/KCNAB2/CEP170/CLIP4/KIF1B/MARK2/COTL1/GABARAPL1/RCC2/TUBGCP3/TWF2/KIF3C/RASSF1/MAP2K2/DYNC2H1/KNSTRN/TUBB6/LMNA/JAM3/RAC2/TUBB4B/TTLL5/HOOK3/TUBA1A/MX2/CUL3/DISC1/MAP1A/DUSP22/ZW10/MAP1LC3A/BOD1L1/RASSF3/CLTC/ENKD1/TPM3/DIAPH2/MAPT/RGS14/PSTPIP2/KIF1C/INVS/TPGS2/REEP4/PLK1/PDLIM2/RASSF5/GAS7/DIAPH1/MAP4/PSTPIP1/DPP9/CTTN/SPECC1/ESPN/GAS2L3/DNM3/ARHGEF2/NDEL1/HCK/TUBB1/WIPF2/NIN/CHMP1A/PALLD/ACTN1/SRC/APC/DPYSL2/RMDN2/DBN1/TUBB2A/GABARAP/PDLIM5/TPPP3/KLC3/FSD1/GAS2L1/ACTB/CALM3/MYO5A/SVIL/AK1/VPS18/TUBA1C/TPM1/GOLGA2/NAV1/AKNA/CCSAP/TPM4/HCLS1/PAK1/RAC1/SHROOM1/XIAP/SYNJ1/WIPF1/TUBA4A/RTN2/MAP1S/LCP1/TMOD1/MAP1LC3B2/CORO1A/CHMP2A/WAS/DMTN/PDLIM7/RAB3D/KRT1/CHMP4B/SLC8A1/IQGAP1/KIF27/KIF13A/ARHGAP6
BP	GO:0097191	extrinsic apoptotic signaling pathway	164	-0.46	-1.85	1.29E-06	1.91E-05	1.17E-05	3087	tags=49%, list=26%, signal=36%	BCL10/BCL2L11/GATA1/BAK1/RFFL/PPP2R1A/THBS1/PML/GSTP1/TNFSF10/CASP2/IL1B/JAK2/DAPK1/P2RX7/DDX3X/CD70/FADD/BEX3/GSK3B/BAX/ATF3/TNF/PHIP/HIPK1/G0S2/MCL1/LTBR/BIRC6/TNFRSF1A/TNFRSF10C/RB1CC1/LMNA/GCLM/TMBIM1/STK4/HGF/MKNK2/SH3RF1/PSME3/BID/BCL2L14/PELI3/HSPA1B/GSK3A/RELA/LGALS3/HSPA1A/CTTN/CTNNA1/ARHGEF2/GPX1/PRDX2/SIAH2/SRC/PF4/GABARAP/ITPRIP/PAK2/RIPK1/IL6R/TNFRSF1B/BRCA1/SERPINE1/DEDD2/PEA15/PYCARD/CFLAR/PTPRC/HMOX1/SP100/NF1/FOXO3/STRADB/TCF7L2/SFRP2/BCL2L1/ZDHHC3/PTEN/SPI1

BP	GO:0014070	response to organic cyclic compound	523	-0.35	-1.55	1.31E-06	1.94E-05	1.19E-05	2739	tags=33%, list=23%, signal=26%	NCOR2/SLC26A6/TLR2/FES/FOS/RHOA/ACSL1/IL1B/REST/PLCB1/PPP5C/USP8/JAK2/CNOT1/CASP9/ACTR2/PPARGC1B/SLC9A1/SIN3A/HSPA5/TICAM1/P2RX7/MBD2/PAQR8/TAF1/COLEC12/CD38/CEBPA/TSPO/FADD/FLNA/ABCA2/LRRK2/CYBB/GSK3B/RFTN1/RNF14/ALPL/NCOR1/ACACB/HNRNPU/DNM2/PELI1/TNF/NFKBIA/ROCK2/JUND/PKN1/ARID1A/ABAT/CASR/NKX3-1/GPLD1/UCP3/TRIM25/ITPR2/YWHAH/PDGFA/CES1/HRH2/GPBAR1/P2RY13/HTR3B/PIK3CG/SSTR2/UBR5/ARPC1B/IRAK3/ATP2B1/EP300/CDKN2D/GCLM/CBL/ABCB11/PIM1/TXNIP/GPI/INPP5K/TET2/STAT5B/MAP1LC3A/GNB1/TRPC3/MAVS/PTGS2/RAE1/MTHFR/HSPA1B/CCL3/GSK3A/RARA/GOT1/CLU/VPS11/ZNF703/AQP9/ABHD2/NR3C1/DIAPH1/PPP1R9B/HSPA1A/LCAT/RALB/CTNNA1/BMP6/PTK2B/RIOK3/GPX1/EGLN2/KCNQ1/SRC/NCOA2/CPT1A/DUSP6/PAK2/ABCC9/AKAP9/FLOT1/CYP1B1/P2RY1/ACTB/CALM3/SOD2/ZFP36L1/VDR/PADI2/GNAL/ZBTB7A/CARM1/PRKACA/RXRA/BRCA1/VPS18/KAT5/IL1RN/P2RX1/SLC7A11/SSH1/IFIT1B/POR/ZMIZ1/LYN/PEA15/SNCA/TGM2/PAK1/ADM/ARRB2/CDA/NCOA4/CFLAR/CEBPB/OPRL1/PRKAR2B/FOXO3/PTAFR/FAM210B/G6PD/WBP2/AQP1/STAT3/MAPK3/DMTN/BCL2L1/BSG/MSN/MBP/SLC8A1/RAPGEF2/TBXA2R/DAB2/GNAZ
BP	GO:0030041	actin filament polymerization	130	-0.48	-1.90	1.36E-06	1.99E-05	1.23E-05	1980	tags=40%, list=17%, signal=34%	CDC42EP4/JAK2/NCKAP1L/ARPC4/CAPZB/GRB2/ADD1/NPHS1/RICTOR/ALOX15/MYADM/SPTA1/COTL1/TWF2/ARPC1B/TRIOBP/WASHC2C/DIAPH2/PSTPIP2/MSRB2/GAS7/DIAPH1/PSTPIP1/CTTN/PTK2B/HCK/ARPC5/DBN1/FCHSD1/ESAM/SVIL/PREX1/LMOD3/SSH1/ARPC1A/HCLS1/RAC1/PYCARD/CYRIB/MSRB1/TMOD1/CORO1A/SSH2/WAS/VASP/CYRIA/DMTN/MTPN/PRKCD/GSN/SPTB/ARHGAP6
BP	GO:0071900	regulation of protein serine/threonine kinase activity	256	-0.42	-1.75	1.61E-06	2.36E-05	1.45E-05	2685	tags=39%, list=23%, signal=31%	ADAM8/DBF4B/HERC5/TRAF6/RHOA/ACSL1/IL1B/PRKAG2/HEXIM2/ADAM17/ERN1/KIT/P2RX7/CCND3/CSF1R/DDX3X/CEBPA/INCENP/ERBB2/LRRK2/BMPR1A/SYAP1/SORL1/PIK3CB/TNF/TNXB/RGS2/CDK5R1/PRKAR2A/RICTOR/ERN2/HHEX/PDGFA/FZD5/AKAP13/TAOK1/SYK/MAP2K2/PIK3CG/GADD45A/PTPN6/CDKN1C/IRAK3/CDKN2D/STK4/PDE5A/TLR6/INPP5K/IRAK1/SESN2/CCNY/PSEN1/RGS14/PTPRJ/INSR/PTPN1/PLK1/CCNF/MAP3K5/RALB/HMGCR/PTK2B/DVL3/CDKN1A/PARP16/MAP2K7/HIPK3/SPRY3/TAOK3/SRC/APC/PIK3R5/DUSP1/ACTB/CALM3/DAZAP2/CCNI/CNPPD1/SASH1/LYN/SNCA/IGF2/STRADA/PAK1/PYCARD/PTPRC/FBXO7/STK38/PRKAR2B/NF1/SFRP2/PRKAR1A/PRKCD/CCNJL/PTEN/SLC8A1/TRIB1/RAPGEF2/IQGAP1/DAB2/TGFA
BP	GO:0009896	positive regulation of catabolic process	413	-0.36	-1.61	1.64E-06	2.39E-05	1.47E-05	2580	tags=33%, list=22%, signal=27%	SIRT6/STX5/CSNK2A1/DAPK1/CNOT1/WIPI1/VPS28/TICAM1/P2RX7/RC3H2/HECW2/TAF1/SNX1/OPTN/CEBPA/TRIM21/DCP2/ZYG11B/TNRC6B/ABCA2/PTTG1IP/LRRK2/GSK3B/RNF14/BAX/SORL1/PIK3CB/HNRNPU/TNF/ROCK2/LRP1/ENTPD5/GGA3/GPLD1/PIP4K2C/DHRSX/SH3RF3/BNIP3L/CALCOCO2/CPEB3/COP1/YTHDF3/NUB1/SPTLC2/TP53INP2/PATL1/TSC2/RNF40/VSIR/FZR1/YBX1/DISC1/GIGYF2/CNOT6/SH3RF1/TFEB/ROCK1/ARNT/SESN2/HSPA1B/GSK3A/PSEN1/CLU/CSDE1/VPS11/ULK1/INSR/AREL1/PTPN1/SESN3/PLK1/RNF19B/AGTPBP1/AGO2/HSPA1A/TUT7/RALB/WWP2/ZC3HAV1/PTK2B/VCP/NUPR1/ATG7/SAMD4B/ELAPOR1/RILP/EGLN2/RAD23A/DDB1/APC/WDR45/CPT1A/RAB7A/IKBKG/GABARAP/ZC3H18/NRDC/GTPBP1/PIP4K2A/WAC/ZFP36L1/PIAS1/AKT2/SH3GLB1/TNFRSF1B/KAT5/NFE2L2/MARCHF2/BAG6/LAPTM5/SNCA/ATG2A/OAZ1/TRIM8/PSMC4/OAZ2/DTX3L/ABHD5/FBXO7/HMOX1/FOXO3/DNAJB2/ZER1/PNPLA2/IRS2/MAPK3/SNX18/FLCN/AMBRA1/PRKCD/PINK1/MSN/PTEN/TRIB1/NPRL3/FURIN/DAB2

BP	GO:0061061	muscle structure development	349	-0.38	-1.67	1.69E-06	2.45E-05	1.50E-05	2697	tags=32%, list=23%, signal=26%	FOS/WNT1/RHOA/CTDP1/REST/PLCB1/NEURL1/BTG1/QKI/DYRK1B/KIT/SLC9A1/TAGLN/SMARCD3/ACTG1/KLF5/YY1/COPRS/BMPR1A/PLEKHO1/NPHS1/HNRNPU/ATF3/TNF/UTRN/RGS2/MECP2/MYLPF/MAML1/RNF165/HDAC4/FHL2/AKAP13/ZFPM1/MYH11/HIRA/NFATC3/LMNA/CREB1/EP300/LRRC8A/SOX6/SUPT6H/YBX1/MRTFA/GREM1/MTM1/CEACAM5/MSX1/NIBAN2/GSK3A/RARA/ZEB1/FKBP1A/CAPN3/SPAG9/SELENON/CMTM5/MAP3K5/PDLIM2/MBNL3/NR4A1/H3-3A/NUPR1/GPX1/BCL9/ZFHX3/SKI/ACTN1/XK/PDLIM5/XKR8/FLOT1/TNFSF14/SOD2/ZFP36L1/SVIL/EHD1/RBPJ/TPM1/KDM6B/MYH9/LMOD3/ACTN4/RBM38/IGF2/CHD7/ADM/ARRB2/PPP3CA/CFLAR/H3-3B/MAPK14/TMOD1/NF1/YBX3/MYEF2/G6PD/TCF7L2/PDLIM7/MTPN/PRKAR1A/KEL/MYLK/BASP1/MYL9/HLX/RIPOR2/SLC8A1/FLOT2/FOXO4/NINJ1/DYSF
BP	GO:0060341	regulation of cellular localization	584	-0.34	-1.53	1.81E-06	2.62E-05	1.61E-05	2348	tags=28%, list=20%, signal=24%	WAPL/FLNA/ERBB2/MIEF2/ABCA2/LRRK2/GSK3B/CHMP3/BAX/RHOQ/SORL1/RIPOR1/HNRNPU/ZDHHC2/TNF/ROCK2/GBP1/LRP1/KIF5B/KCNAB2/GPLD1/CDK5R1/UCP2/ITGAM/BNIP3L/PCM1/FZD5/MEAK7/SYK/ANXA2/NECAB2/MAP2K2/GPR27/SLC6A9/PTPN6/RAB21/PPP1R12A/UBR5/DLG4/GRIPAP1/LMNA/RAC2/TMBIM1/SUPT6H/OS9/RTN4/STX3/AP2M1/LAMP1/MX2/MAP1A/INPP5K/SIDT2/GNL3L/PID1/TRPC3/CLTC/OTUD7B/NUMB/MAVS/PTGS2/SESN2/SUFU/GSK3A/PSEN1/MAPT/FGR/FKBP1A/ITGB2/VPS11/CAPN3/SQSTM1/SELENON/PTPN1/PLK1/RASSF5/IWS1/DIAPH1/LGALS3/PPP1R9B/ABHD17C/RALB/ANKRD13A/RAB11B/CTNNA1/MARK4/PTK2B/NDEL1/VCP/ZDHHC7/AP2B1/VPS4B/LGALS9/PPP2R5A/SRC/APC/TPR/RAB7A/FRAT1/HSPA1L/ACTB/CALM3/STXBP2/ZFP36L1/PRKACA/AKT2/EHD1/ARHGAP8/SH3GLB1/P2RX1/SLC7A11/ARHGAP1/MEPCE/EPB41/NPEPPS/CHP1/LYN/UNC13D/ILRUN/HCLS1/SNCA/CHD7/C2CD2L/TYROBP/SDCBP/OAZ1/TRIM8/TBC1D20/BBC3/UBE2D3/LIMK2/OAZ2/DTX3L/TRIM58/PICALM/RHOG/LCP1/PPFIA1/MAPK14/HMOX1/IQSEC2/SP100/CORO1A/NF1/EPHB2/CHMP2A/ABI3/ABLIM3/TCF7L2/MAPK3/DMTN/FLCN/PRKCD/GSN/PINK1/BCL2L1/PPM1A/MSN/PTEN/SPI1/RIPOR2/SLC8A1/DAB2/GLUL/GAB2
BP	GO:1901653	cellular response to peptide	242	-0.43	-1.78	1.84E-06	2.65E-05	1.63E-05	2696	tags=42%, list=23%, signal=33%	FPR2/WNT1/PIK3R2/IL1B/CACNA1A/LPIN2/RAB8A/PLCB1/PRKCZ/JAK2/CACNB1/SLC9A1/CCND3/VIM/AGTRAP/JAK1/LDOC1/CAMK2A/AHCYL1/SMARCC1/CA2/CYSLTR2/GRB2/GSK3B/RHOQ/STAT5A/SYAP1/SORL1/PRKCB/TNF/PHIP/ROCK2/CASR/LRP1/MAS1/GPLD1/UCP2/PIP4K2C/PKM/SOGA1/SP1/ATP2B1/TSC2/GNAI2/TLR6/INPP5K/STAT5B/PID1/SHC1/PDK2/NR4A2/OSBPL8/ROCK1/GSK3A/PSEN1/RELA/GOT1/FUT7/SOCS3/PTPRE/INSR/PTPN1/SESN3/MAX/PXN/PPP1R9B/NR4A1/MBD5/ARHGEF2/NDEL1/ZDHHC7/EIF4EBP2/SRC/APC/ZNF106/GAB1/PIP4K2A/CYP1B1/ZFP36L1/MYO5A/AKT2/ZBTB7B/KLF3/NFE2L2/POR/LYN/IGF2/ADIPOR1/CFLAR/STAT6/FBP1/FOXO3/EPHB2/IRS2/STAT3/PRKDC/PRKCD/WDTC1/RAB31/FOXO4/KLF1
BP	GO:0043434	response to peptide hormone	259	-0.41	-1.71	1.84E-06	2.65E-05	1.63E-05	2681	tags=40%, list=23%, signal=31%	WNT1/PIK3R2/IL1B/SRSF4/LPIN2/RAB8A/PLCB1/SCAP/PRKCZ/BTG1/SLC2A1/JAK2/HADHA/SLC9A1/CCND3/AGTRAP/JAK1/CAMK2A/AHCYL1/SMARCC1/CA2/CYSLTR2/GRB2/CYBB/GSK3B/RHOQ/STAT5A/SYAP1/SORL1/PRKCB/PHIP/ROCK2/JUND/MAS1/GPLD1/UCP3/UCP2/PIP4K2C/PKM/MEAK7/SOGA1/SP1/CREB1/ATP2B1/TSC2/RNF40/GNAI2/INPP5K/STAT5B/PID1/SHC1/PDK2/NR4A2/OSBPL8/PTGS2/ROCK1/SESN2/GSK3A/RELA/GOT1/FUT7/SOCS3/PTPRE/INSR/PTPN1/SESN3/MAX/PXN/NR4A1/TIMP1/MBD5/NDEL1/ZDHHC7/KCNQ1/EIF4EBP2/SRC/APC/ZNF106/GAB1/PIP4K2A/CYP1B1/ZFP36L1/MYO5A/AKT2/ZBTB7B/NFE2L2/POR/LYN/IGF2/ADM/ADIPOR1/CFLAR/STAT6/FBP1/MAPK14/IRS2/STAT3/PRKDC/PRKCD/BSG/WDTC1/RAB31/FOXO4

BP	GO:1901888	regulation of cell junction assembly	101	-0.52	-1.99	1.86E-06	2.65E-05	1.63E-05	2947	tags=45%, list=25%, signal=34%	BCAS3/THBS1/WDPCP/SETD5/FARP1/TLR2/RHOA/IL1B/ADNP/EIF4G1/SLC9A1/ACTG1/TNF/ROCK2/FZD5/SEMA4A/RCC2/GPBAR1/GREM1/DUSP22/ROCK1/PTPRJ/AGRN/NLGN3/SLK/NTNG2/SRC/PDLIM5/LIMS1/FLOT1/DLG5/PRKACA/SNCA/RAC1/F11R/MAP4K4/EPHB2/CORO1C/ABI3/DMTN/PTEN/VCL/RAPGEF2/ARHGAP6/CLDN5
BP	GO:0010563	negative regulation of phosphorus metabolic process	314	-0.39	-1.67	1.92E-06	2.74E-05	1.68E-05	2309	tags=34%, list=20%, signal=28%	ABCA2/LRRK2/GSK3B/MLLT1/CTDSP2/BAX/NCOR1/PIP5KL1/SORL1/PIK3CB/HNRNPU/TNF/ROCK2/PKN1/LRP1/MAS1/RGS2/PRKAR2A/PIP4K2C/HDAC4/MYADM/HHEX/PDGFA/CTDSP1/UBE2B/GADD45A/PTPN6/CDKN1C/IRAK3/CDKN2D/CBL/HGF/TSC2/RUBCN/GNAI2/GREM1/DUSP22/INPP5K/PID1/BOD1L1/ROCK1/PARP14/YWHAB/GSK3A/PSEN1/MAPT/RGS14/PTPRJ/FKBP1A/SOCS3/SPAG9/PTPN1/ADAR/PLK1/LGALS3/AKT1S1/RASSF2/PPP1R9B/HMGCR/GNAQ/NUPR1/SIRPA/CDKN1A/PPP2R5A/HIPK3/SPRY3/APC/PPP1R15A/WARS1/DUSP6/PAK2/HSPB1/PIP4K2A/DUSP1/CALM3/SH3BP5L/CHP1/LYN/SNCA/PYCARD/PLEK/ARRB2/MIDN/CDA/PTPRC/FBP1/FBXO7/STK38/PRKAR2B/NF1/EPHB2/CORO1C/SLC4A1/NIBAN1/IRS2/STAT3/DMTN/PRKDC/SFRP2/PRKAR1A/FLCN/FKBP8/PRKCD/PTEN/SLC8A1/TRIB1/IQGAP1/CTDSPL
BP	GO:0009790	embryo development	604	-0.33	-1.51	2.03E-06	2.87E-05	1.76E-05	2288	tags=27%, list=20%, signal=23%	OTX1/GRB2/TBX19/SENP2/BMPR1A/HIPK2/BAX/SMIM14/ZBTB16/PIK3CB/PAFAH1B1/TNF/EFNB1/ROCK2/IFT122/UBR3/ATP7A/NKX3-1/HIPK1/CDK5R1/NRP2/ERCC2/RICTOR/CFL1/MYADM/PDGFA/CPT2/FZD5/CERT1/TMEM231/ZFPM1/UBE2B/DYNC2H1/BIRC6/HIRA/ITGA5/HM13/ATP6AP2/LAMA4/CDKN1C/APBA1/CTR9/EP300/SULF2/STK4/TSC2/SUPT6H/TXNRD1/RTN4/YBX1/GREM1/GPI/CUL3/SOX4/MEIS1/INPP5K/MSX1/CHST11/TET3/NIBAN2/TRIOBP/JUNB/IFT140/TFEB/OTUD7B/ROCK1/ARNT/MTHFR/SUFU/PSEN1/RARA/ZEB1/ITGB2/SOCS3/INSR/B4GALT5/INTU/RTF1/MAFG/MAP3K20/ARL13B/PHACTR4/GNA12/NDEL1/CUL4A/THOC5/DVL3/NPAT/KCNQ1/ACSL4/SKI/BPNT2/MGAT1/NIPBL/ETS2/DBN1/ASCL2/BPTF/PBX1/TGFB1I1/EXOC4/DUSP1/PPP1R13L/ZFP36L1/MYO1E/USP22/PRKACA/ASF1B/BRCA1/RBPJ/IL1RN/CIR1/NFE2L2/KDM6B/MYH9/NDST1/ITGB5/PELO/TAL1/BAG6/TRIP12/TENT5C/ZMIZ1/CCSAP/APAF1/EPN1/IGF2/CHD7/WNK1/ADM/PBX2/MMP9/PSMC4/CREBBP/CEBPB/ELL/DNAJB6/CMIP/NCOA1/NF1/ITPK1/WDR74/EPHB2/YBX3/TOP1/VASP/MAPK3/PRKDC/SFRP2/CCDC39/ITGB3/PRKAR1A/PCSK6/FLCN/FKBP8/BCL2L1/BASP1/HLX/WDTC1/MBP/MICAL2/ECE1/FURIN
MF	GO:0019904	protein domain specific binding	507	-0.35	-1.58	2.10E-06	2.96E-05	1.82E-05	2689	tags=32%, list=23%, signal=26%	TRPS1/DICER1/WNT1/RHOA/IL1B/USP8/STRN4/ADAM17/INPP5D/OSTF1/IKZF1/QKI/JAK2/CNOT1/CASP9/KIT/PPARGC1B/HSPA5/DDX6/EPS15/TRIM11/SH3BP5/MBD2/INPP5A/VIM/MED12/FADD/GNG5/GRB2/SLC22A4/BAX/ELMO1/RHOQ/ZBTB16/NDE1/DNM2/NKX3-1/ARHGAP31/ELMO2/PRKAR2A/MECP2/GRIK2/USHBP1/YWHAH/PLSCR1/MCL1/ZNF653/RCC2/RAB6A/SYK/LBR/MAP2K2/PTPN6/SSTR2/FHOD1/SP1/DLG4/LAMP2/ATP2B1/CTR9/CBL/NCF1/AP2M1/LAMP1/MRTFA/CUL3/YWHAZ/CLTC/ARF1/MAVS/IST1/H4C8/ATRX/WASF2/TFDP2/HSPA1B/YWHAB/PSEN1/RARA/MAPT/RELA/ATN1/VPS11/INSR/SQSTM1/MAP3K5/TBL1X/RAB27B/HSPA1A/ESPN/VCP/GPX1/LRRFIP2/SNTB1/SIRPA/SH3BGRL2/RAB27A/SKI/DDB1/CHMP1A/ACTN1/SRC/MYO1D/NCOA2/NIPBL/ETS2/IKBKG/SCAF1/LAT2/ZNF106/WARS1/EXOC4/ETV6/RIPK1/DAP/CALM3/DAZAP2/TMEM88/PLAUR/PRKACA/RXRA/ZXDC/PTPN12/NFE2L2/MYH9/ARHGAP1/NFE2/CTNNBIP1/LYN/HCLS1/HNRNPK/PYCARD/ARRB2/SH3KBP1/SYNJ1/WIPF1/F11R/PRRG4/PRKAR2B/SP100/KPNB1/CHMP2A/WAS/PPP3R1/TOP1/ABI3/IRS2/TCF7L2/VASP/TLN1/ACOX1/PRKDC/PRKAR1A/FKBP8/PINK1/BCL2L1/BASP1/PTEN/JDP2/RAPGEF2/IQGAP1/LITAF/ARHGAP6

BP	GO:0009887	animal organ morphogenesis	474	-0.35	-1.57	2.26E-06	3.18E-05	1.95E-05	2363	tags=27%, list=20%, signal=23%	MED12/SMARCC1/FLNA/DGCR2/LRRK2/OTX1/YY1/TBX19/KDM5B/BMPR1A/ALPL/HIPK2/BAX/CTSZ/PAFAH1B1/TNF/NSD2/IFT122/NKX3-1/HIPK1/NRP2/BTBD7/MYLPF/SGPL1/ETV7/FHL2/PDGFA/FZD5/ANKRD11/CERT1/TMEM176B/SYK/ZFPM1/APCDD1/SAV1/CHSY1/EP300/PIM1/SOX6/HGF/RTN4/RFLNB/GREM1/CUL3/SOX4/MEIS1/MSX1/CHST11/ACTA2/TRIOBP/ROGDI/IFT140/SUFU/GSK3A/PSEN1/RARA/RELA/ZEB1/FGR/FKBP1A/EPHB4/INSR/INTU/AGTPBP1/AGRN/ARL13B/PHACTR4/CTNNA1/BMP6/MEGF9/RPS6KA1/DVL3/KCNQ1/SKI/NTNG2/BPNT2/ILK/SNX10/SRC/NIPBL/FLI1/PBX1/FANCC/EXOC4/DLG5/PPP1R13L/VDR/CSRNP1/RBPJ/SERPINE1/TPM1/TLE3/NDST1/POR/FOXN3/ZMIZ1/STK40/CTNNBIP1/TGM2/IGF2/CHD7/LAMC3/RAC1/ADM/PBX2/TBC1D20/CFLAR/CEBPB/STAT6/MAPK14/NF1/FOXO3/EPHB2/PHOSPHO1/AQP1/STAT3/MAPK3/SFRP2/CCDC39/MYLK/BSG/BASP1/MSN/HLX/CAPN1/PTEN/MICAL2/DAB2/TGFA/CLDN5
BP	GO:0009725	response to hormone	524	-0.34	-1.55	2.43E-06	3.40E-05	2.09E-05	2697	tags=35%, list=23%, signal=28%	FOS/WNT1/RHOA/PIK3R2/ACSL1/IL1B/SRSF4/LPIN2/RAB8A/REST/PLCB1/USP8/SCAP/PRKCZ/BTG1/SLC2A1/JAK2/CNOT1/HADHA/CASP9/KIT/PPARGC1B/SLC9A1/CCND3/PAQR8/AGTRAP/JAK1/CD38/CAMK2A/TSPO/AHCYL1/SMARCC1/ABCA2/CA2/CYSLTR2/GRB2/YY1/CYBB/GSK3B/RNF14/ARID5A/ALPL/RHOQ/STAT5A/NCOR1/SYAP1/SORL1/PRKCB/HNRNPU/TNF/PHIP/ROCK2/JUND/PKN1/ARID1A/MAS1/NKX3-1/GPLD1/UCP3/UCP2/TRIM25/PIP4K2C/PRCP/YWHAH/PKM/FHL2/MEAK7/SSTR2/SOGA1/UBR5/SP1/ARPC1B/CREB1/ATP2B1/EP300/GCLM/ABCB11/TSC2/RNF40/TXNIP/GNAI2/GPI/INPP5K/STAT5B/PID1/SHC1/PDK2/GNB1/NR4A2/OSBPL8/PTGS2/ROCK1/SESN2/C2/HSPA1B/GSK3A/RARA/RELA/GOT1/VPS11/FUT7/SOCS3/PTPRE/INSR/PTPN1/SESN3/ABHD2/MAX/NR3C1/PXN/PPP1R9B/HSPA1A/NR4A1/LCAT/TIMP1/MBD5/CTNNA1/BMP6/PTK2B/NDEL1/GPX1/ZDHHC7/EGLN2/KCNQ1/EIF4EBP2/SRC/APC/NCOA2/ZNF106/GAB1/PIP4K2A/CYP1B1/GAS2L1/CALM3/ZFP36L1/PADI2/MYO5A/ZBTB7A/CARM1/PRKACA/AKT2/RXRA/ZBTB7B/BRCA1/VPS18/IL1RN/NFE2L2/POR/ZMIZ1/LYN/HCLS1/IGF2/PAK1/ADM/ADIPOR1/NCOA4/CFLAR/TIMP2/STAT6/FBP1/CTSB/MAPK14/HMOX1/NCOA1/FOXO3/PTAFR/DHCR24/WBP2/IRS2/AQP1/STAT3/PRKDC/PRKCD/BSG/WDTC1/RAB31/CITED4/MBP/FOXO4/DAB2/NCOA6

BP	GO:0071345	cellular response to cytokine stimulus	552	-0.34	-1.56	2.44E-06	3.40E-05	2.09E-05	2737	tags=34%, list=23%, signal=28%	LILRA6/SLC26A6/TLR2/RO60/TRAF6/PRDM5/RHOA/ACSL1/IL1B/PLCB1/SAMHD1/ADAM17/SELPLG/CDC42EP4/JAK2/DAPK1/KIT/ACTR2/ANGPT1/HSPA5/FCER1G/OXSR1/CSF1R/VIM/ACTG1/JAK1/CEBPA/CD70/TUBA1B/HK2/FADD/KLF5/IL18RAP/SPATA2/IL1R2/YY1/KDM5B/IFITM3/GSK3B/LILRB2/BCLAF1/STAT5A/TLE4/HNRNPU/PAFAH1B1/TNF/NFKBIA/MRAS/MNDA/GBP1/KIF5B/TNFSF13B/NKX3-1/HIPK1/NRP2/NFAT5/TRIM25/ALOX15/CD300LF/OSM/SYK/IL10RB/RAB43/LILRA1/NMI/TNFRSF1A/PF4V1/YTHDF3/PTPN6/IRAK3/IL17RD/CREB1/CTR9/GCLM/CCR1/AGPAT1/CBL/LSM14A/CPNE1/YBX1/INPP5K/LILRA2/STAT5B/PID1/CSF3R/GBP2/CTBP2/MAVS/PARP14/RPS6KA5/IRAK1/NKIRAS2/USP27X/PELI3/HSPA1B/CCL3/GSK3A/PRPF8/RELA/PTPRJ/ERBIN/IFI16/SOCS3/PTPN1/ADAR/CHI3L1/MAP3K5/PTP4A3/MT2A/HSPA1A/SBNO2/VAMP3/DOCK8/PTK2B/IFNAR1/ARHGEF2/CXCR2/HCK/DOK1/SIRPA/LGALS9/CXCL5/PARP16/ILK/HDGF/OASL/SNX10/CXCR1/SRC/TPR/PF4/CDC34/LIMS1/RFX2/CYP1B1/DUSP1/RIPK1/IL6R/ZFP36L1/PADI2/IRF1/IFNAR2/PRKACA/GBP4/CLCF1/TNFRSF1B/BRCA1/IL1RN/JARID2/NFE2L2/IFIT1B/MME/ACTN4/ACKR1/LAPTM5/SHARPIN/USP10/HCLS1/WNK1/PYCARD/XIAP/RAD23B/ADIPOR1/H2BC11/CEBPB/PTPRC/STAT6/MAPK14/SP100/CORO1A/WBP1L/FOXO3/WAS/YBX3/IL17RA/TOLLIP/STAT3/MAPK3/IFITM2/IL13RA1/MPL/GSN/SPI1/CCRL2/LILRB3/RIPOR2/PLVAP/ZYX/LSP1/NFIL3
BP	GO:0090092	regulation of transmembrane receptor protein serine/threonine kinase signaling pathway	133	-0.48	-1.90	2.47E-06	3.44E-05	2.11E-05	2681	tags=36%, list=23%, signal=28%	WNT1/ADAM17/PBLD/JAK2/ZEB2/FAM89B/HSPA5/LTBP1/BMPR1A/HIPK2/SORL1/DNM2/LRP1/RNF165/ZC3H3/KIAA0319/CDKN1C/IL17RD/EP300/GREM1/MSX1/CHST11/LRG1/ZEB1/FKBP1A/TRIM33/NUP93/ZNF703/HSPA1A/BMP6/SKI/ILK/TGFB1I1/UBE2O/ZBTB7A/RBPJ/PELO/NRROS/SDCBP/XIAP/CREBBP/UBE2D3/SFRP2/PCSK6/FLCN/FKBP8/PPM1A/DAB2
BP	GO:0003013	circulatory system process	297	-0.39	-1.69	2.57E-06	3.57E-05	2.19E-05	2151	tags=29%, list=18%, signal=24%	CTSZ/TNF/ROCK2/ABAT/TBC1D8/CASR/LRP1/RGS2/CELF2/MECP2/PTGS1/PRCP/ABCC4/SLC44A1/AKAP13/HRH2/SLCO3A1/PIK3CG/SLC6A9/ATP6AP2/SLC22A1/GUCY1B1/ATP2B1/GCLM/TMEM65/SERPING1/GNAI2/PDE5A/CACNA1D/KNG1/ACTA2/SLC2A3/PTGS2/ROCK1/DSC2/ABCC3/GSK3A/PTPRJ/INSR/SLC6A20/PTP4A3/SLC6A6/GNA12/BMP6/GPX1/SLC38A5/CYB5R3/DOCK5/KCNQ1/SRC/MYL4/FLI1/F5/SLC24A3/ABCC9/KCNE3/AKAP9/P2RY1/CALM3/PPP1R13L/SOD2/PRKACA/TBXAS1/TPM1/P2RX1/CTNNBIP1/CHD7/WNK1/ADM/SLC1A5/EHD3/KCNJ5/GUCY1A1/MAP2K3/OPRL1/HMOX1/PTAFR/KEL/GSN/SLC8A1/PLVAP/TBXA2R/ECE1/SLC7A5/CLDN5
BP	GO:0008015	blood circulation	238	-0.41	-1.72	2.93E-06	4.05E-05	2.49E-05	2151	tags=29%, list=18%, signal=24%	CTSZ/TNF/ROCK2/ABAT/TBC1D8/CASR/RGS2/CELF2/MECP2/PTGS1/PRCP/HRH2/PIK3CG/ATP6AP2/GUCY1B1/ATP2B1/GCLM/TMEM65/SERPING1/PDE5A/CACNA1D/KNG1/ACTA2/PTGS2/ROCK1/DSC2/GSK3A/PTPRJ/PTP4A3/GNA12/BMP6/GPX1/CYB5R3/DOCK5/KCNQ1/SRC/MYL4/FLI1/F5/ABCC9/KCNE3/AKAP9/P2RY1/CALM3/PPP1R13L/SOD2/PRKACA/TBXAS1/TPM1/P2RX1/CTNNBIP1/CHD7/WNK1/ADM/EHD3/KCNJ5/GUCY1A1/MAP2K3/OPRL1/HMOX1/PTAFR/KEL/GSN/SLC8A1/PLVAP/TBXA2R/ECE1/CLDN5

BP	GO:0045859	regulation of protein kinase activity	452	-0.35	-1.57	3.06E-06	4.21E-05	2.59E-05	2685	tags=34%, list=23%, signal=27%	TRAF6/RHOA/ACSL1/IL1B/PRKAG2/HEXIM2/ADAM17/NEURL1/PRKCZ/ADNP/ERN1/JAK2/NCKAP1L/KIT/ANGPT1/P2RX7/CCND3/CSF1R/DDX3X/SH3BP5/LCP2/CEBPA/INCENP/ERBB2/LRRK2/BMPR1A/MLLT1/SYAP1/SORL1/TAB2/PIK3CB/TNF/TNXB/PLCE1/PKN1/MAS1/RGS2/CDK5R1/PRKAR2A/RICTOR/ERN2/MARK2/SLC11A1/HHEX/PDGFA/FZD5/AKAP13/TAOK1/SYK/MAP2K2/PIK3CG/GADD45A/PTPN6/CDKN1C/DLG4/IRAK3/CDKN2D/CBL/STK4/AGAP2/TIRAP/NCF1/HGF/TSC2/PDE5A/GREM1/DUSP22/TLR6/INPP5K/SHC1/OSBPL8/IRAK1/SESN2/CCNY/PSEN1/RGS14/PTPRJ/FGR/VLDLR/CLU/INSR/PTPN1/ADAR/PLK1/CHI3L1/CCNF/MAP3K5/NEDD9/AKT1S1/RASSF2/C5AR1/RALB/HMGCR/GNAQ/PTK2B/DVL3/CDKN1A/PARP16/MAP2K7/CASS4/HIPK3/SPRY3/TAOK3/SRC/APC/WARS1/PAK2/PIK3R5/HSPB1/DUSP1/IL6R/ACTB/CALM3/DAZAP2/CCNI/CNPPD1/SASH1/TAL1/SH3BP5L/CHP1/LYN/MMD/SNCA/IGF2/STRADA/PAK1/WNK1/PYCARD/MAP2K3/PTPRC/FBXO7/STK38/PRKAR2B/NF1/EPHB2/CORO1C/CACUL1/MOB3A/STRADB/SFRP2/ITGB3/PRKAR1A/PRKCD/PINK1/CCNJL/PTEN/MOB3C/SLC8A1/TRIB1/RAPGEF2/IQGAP1/DAB2/TGFA
BP	GO:0071496	cellular response to external stimulus	235	-0.41	-1.72	3.12E-06	4.28E-05	2.63E-05	2926	tags=41%, list=25%, signal=31%	AIFM1/SZT2/SRF/VPS41/PDK4/SLC1A2/MAPK1/CASP2/FES/FOS/IL1B/MAP2K4/PRKAG2/SLC2A1/ADNP/EIF4G1/GCN1/SLC9A1/WIPI1/HSPA5/P2RX7/FADD/LRRK2/RIPOR1/ATF3/MAP1LC3B/CASR/TLR8/GABARAPL2/UCP2/SLC39A4/LTBR/GABARAPL1/TNFRSF1A/GADD45A/PPM1D/CPEB4/LAMP2/ATP2B1/CBL/MTMR3/PIM1/EIF2AK2/STK24/MAP1LC3A/PDK2/TFEB/NR4A2/PTGS2/DSC2/SESN2/IFI16/ULK1/WIPI2/SESN3/MAP3K5/MAX/PPP1R9B/RALB/CLEC16A/BMP6/ATG7/ELAPOR1/CDKN1A/WDR45/GABARAP/KPTN/GAS2L1/TNFSF14/DAP/VDR/IRF1/RXRA/SH3GLB1/NFE2L2/LYN/CDA/SIPA1/MAP3K2/F11R/RRAGD/PTPRC/HMOX1/NCOA1/MAP1LC3B2/FOXO3/AQP1/MAPK3/SFRP2/MTPN/FLCN/CD68/CNN2/NUAK2/NPRL3/GLUL
CC	GO:0031253	cell projection membrane	197	-0.43	-1.75	3.43E-06	4.66E-05	2.86E-05	1935	tags=30%, list=17%, signal=25%	CFL1/PDE4A/IQCE/TMEM231/PIP5K1C/SYNE2/ITGA5/TAS2R4/ATP6AP2/ATP2B1/FSCN1/PSD4/FSCN3/TIRAP/GPI/INPP5K/FFAR4/SH2D3C/CDKL5/SNAP29/MAPT/PTPRJ/FGR/INSR/DIAPH1/ARL13B/PPP1R9B/SLC6A6/DOCK8/GNA12/ARHGEF2/B4GALT1/TESC/SRC/APC/MYO1D/PLEK2/AKT2/EHD1/CUBN/TPM1/CA4/SLC7A11/PAK1/RAC1/PLEK/EHD3/LCP1/ASAP1/BMX/CORO1C/VASP/TLN1/AQP1/DMTN/ITGB3/MSN/RIPOR2/SLC7A5
CC	GO:0005769	early endosome	289	-0.40	-1.69	3.46E-06	4.68E-05	2.88E-05	2925	tags=35%, list=25%, signal=27%	MGRN1/ARRDC3/PML/CLN3/DOP1B/VPS41/ITCH/MAPK1/PACSIN2/PIKFYVE/WDFY1/WLS/USP8/VTI1B/HPS6/PHETA1/RAB32/VPS28/CLN6/EPS15/HAP1/STEAP4/SNX1/ERBB2/IFITM3/RFTN1/WDFY2/SORL1/SNX8/LRP1/ATP7A/GGA3/FZD5/RCC2/KIAA0319/TMEM127/MAP2K2/HLA-E/STX6/RAB22A/RAB21/RIN3/TRAK1/RNF11/VPS33A/GRIPAP1/RUBCN/EEA1/NUMB/WASHC2C/UHRF1BP1L/AOC3/ZFYVE16/DIAPH2/WASF2/PSEN1/ANKRD27/IGF2R/VPS11/PTPN1/ATP6V0D1/RAP1GAP/PTP4A3/VAMP3/TF/KCNQ1/CHMP1A/SIAH2/MYO1D/FLOT1/STAM2/MYO5A/SNX27/AKT2/EHD1/VPS18/RABGAP1L/SH3GL1/RAB5B/MMGT1/TMEM184A/MME/ACKR1/GPR107/USP10/UBXN6/EHD3/DTX3L/PICALM/CORO1A/RAB5C/RAB5A/MARCHF8/MAPK3/TRAK2/CYTIP/RAB31/PTP4A1/DYSF/LITAF
CC	GO:0030427	site of polarized growth	121	-0.49	-1.92	3.52E-06	4.73E-05	2.91E-05	2522	tags=42%, list=22%, signal=33%	PSEN2/CCDC120/HAP1/FEZ1/COPA/FLNA/MYO9A/LRRK2/RTN4R/SYAP1/DNM2/PAFAH1B1/PI4K2A/CDK5R1/TWF2/ATCAY/CPEB4/EXOC7/FSCN1/CBL/FSCN3/STX3/DISC1/SNAP25/CDKL5/PSEN1/MAPT/PPP1R9B/CTTN/EXOC6/PTK2B/NDEL1/PTBP2/NIN/ARPC5/PALLD/DBN1/EXOC4/SETX/CALM3/MYO5A/FXR2/CTNND1/FRY/USP9X/HCLS1/SNCA/PINK1/BASP1/IQGAP1/TSHZ3

CC	GO:0070161	anchoring junction	573	-0.34	-1.52	3.52E-06	4.73E-05	2.91E-05	1941	tags=28%, list=17%, signal=24%	YWHAH/CFL1/ITGA2B/FHL2/MYADM/FZD5/EIF4G2/PIP5K1C/SYNE2/ANXA2/MAP2K2/GAK/TSPAN33/ITGA5/PTPN6/RAB21/PPP1R12A/FHOD1/ARPC1B/SAV1/DSG3/JAM3/RAC2/FSCN1/CBL/TMEM65/ABCB11/STX3/YWHAZ/NIBAN2/TRIOBP/PCBP2/CLTC/ARF1/NUMB/CDC42BPA/DSC2/WASF2/HSPA1B/YWHAB/PTPRJ/ERBIN/PCDH1/IGF2R/ITGB2/DNMBP/GIT2/PDLIM2/TSPAN9/NEDD9/VAV1/PXN/PPP1R9B/HSPA1A/CTTN/IRF2/CTNNA1/GNA12/PTK2B/SYNPO/ARHGEF2/B4GALT1/HCK/APBB1IP/SNTB1/DCAF6/CASS4/ARPC5/PALLD/LIMK1/ILK/ACTN1/SRC/APC/DBN1/PDLIM5/PAK2/GAB1/ESAM/ZNF185/TGFB1I1/LIMS1/HSPB1/PECAM1/FLOT1/ACTB/PLAUR/MYO1E/SVIL/PARVB/PTPN12/CTNND1/MYH9/ITGB5/AFDN/MME/ACTN4/SLA/LASP1/TPM4/CAPN5/UBA1/CHP1/LYN/MRC2/TGM2/HNRNPK/PAK1/SDCBP/RAC1/RSU1/SHROOM1/PANX2/SH3KBP1/PPP3CA/F11R/EHD3/PTPRC/RHOG/MAP4K4/LCP1/PPFIA1/CORO1A/CORO1C/WAS/YBX3/ARHGAP26/VASP/TLN1/MARCKS/MAPK3/PDLIM7/UBN1/ITGB3/FLCN/AMBRA1/PRKCD/GSN/BSG/MSN/FRMD4B/CNN2/CAPN1/VCL/LPP/SLC8A1/FLOT2/MPP1/ZYX/RAPGEF2/NHS/IQGAP1/FOCAD/DAB2/TNS1/S100A11/PEAK3/CLDN5
BP	GO:0045936	negative regulation of phosphate metabolic process	313	-0.39	-1.66	3.60E-06	4.76E-05	2.93E-05	2309	tags=34%, list=20%, signal=28%	ABCA2/LRRK2/GSK3B/MLLT1/CTDSP2/BAX/NCOR1/PIP5KL1/SORL1/PIK3CB/HNRNPU/TNF/ROCK2/PKN1/LRP1/MAS1/RGS2/PRKAR2A/PIP4K2C/HDAC4/MYADM/HHEX/PDGFA/CTDSP1/UBE2B/GADD45A/PTPN6/CDKN1C/IRAK3/CDKN2D/CBL/HGF/TSC2/RUBCN/GNAI2/GREM1/DUSP22/INPP5K/PID1/BOD1L1/ROCK1/PARP14/YWHAB/PSEN1/MAPT/RGS14/PTPRJ/FKBP1A/SOCS3/SPAG9/PTPN1/ADAR/PLK1/LGALS3/AKT1S1/RASSF2/PPP1R9B/HMGCR/GNAQ/NUPR1/SIRPA/CDKN1A/PPP2R5A/HIPK3/SPRY3/APC/PPP1R15A/WARS1/DUSP6/PAK2/HSPB1/PIP4K2A/DUSP1/CALM3/SH3BP5L/CHP1/LYN/SNCA/PYCARD/PLEK/ARRB2/MIDN/CDA/PTPRC/FBP1/FBXO7/STK38/PRKAR2B/NF1/EPHB2/CORO1C/SLC4A1/NIBAN1/IRS2/STAT3/DMTN/PRKDC/SFRP2/PRKAR1A/FLCN/FKBP8/PRKCD/PTEN/SLC8A1/TRIB1/IQGAP1/CTDSPL
BP	GO:0051146	striated muscle cell differentiation	147	-0.47	-1.85	3.68E-06	4.85E-05	2.98E-05	2322	tags=35%, list=20%, signal=29%	KLF5/YY1/BMPR1A/PLEKHO1/NPHS1/HNRNPU/RGS2/MAML1/HDAC4/FHL2/AKAP13/MYH11/LMNA/SOX6/YBX1/GREM1/CEACAM5/MSX1/NIBAN2/GSK3A/RARA/CAPN3/SPAG9/SELENON/GPX1/BCL9/SKI/XK/PDLIM5/FLOT1/TNFSF14/EHD1/RBPJ/TPM1/KDM6B/MYH9/LMOD3/RBM38/IGF2/ARRB2/PPP3CA/CFLAR/MAPK14/TMOD1/MYEF2/G6PD/MTPN/PRKAR1A/KEL/MYL9/RIPOR2/SLC8A1

BP	GO:0045087	innate immune response	541	-0.34	-1.53	3.87E-06	5.08E-05	3.12E-05	3225	tags=40%, list=28%, signal=30%	MYD88/TNK2/CDC37/AKAP8/ECSIT/FFAR2/IL1RAP/SRPK1/TASL/IFIT3/CLEC12B/SLC15A4/PADI4/BCL10/MED1/DDX21/CCL5/EDN1/MFHAS1/ADAM15/APOBEC3A/TYK2/TRIM38/STAT1/TUBB/H2BC21/VNN1/OAS3/PPP6C/PARP9/BST2/PML/ANKRD17/RELB/CFD/NAIP/ITCH/NLRP10/CLEC4E/AKIRIN2/ADAM8/TKFC/HERC5/SLC26A6/TLR2/FES/FPR2/TRAF6/SAMHD1/CD177/CDC42EP4/SETD2/PLEKHM2/JAK2/DAPK1/ACTR2/SIN3A/FCER1G/TICAM1/C1QA/APOBEC3C/CSF1R/DDX3X/TRIM11/VIM/ACTG1/COLEC12/JAK1/OPTN/TRIM21/PJA2/FADD/CRCP/CYBB/NECTIN2/REL/IFITM3/CRISP3/ARID5A/SPON2/DDX60/TNF/SERINC3/MNDA/GBP1/KIF5B/TLR8/C1RL/TRIM25/ITGAM/CALCOCO2/SLC11A1/PLSCR1/SYK/RAB43/NMI/CXCL16/CR1/HLA-E/PIK3CG/YTHDF3/PTPN6/NUB1/SLC15A3/FCN1/IRAK3/EP300/TUBB4B/PI3/LYST/SERPING1/LSM14A/TIRAP/EIF2AK2/NCF1/TRIM26/LAMP1/MX2/CD14/LILRA2/KLRD1/STAT5B/RSAD2/PCBP2/TRIM10/GBP2/N4BP1/MAVS/PARP14/IRAK1/USP27X/C2/CCL3/RELA/FGR/CLU/IFI16/PTPN1/ADAR/RNF19B/MAP3K5/PSTPIP1/VAV1/LGALS3/VAMP3/RNASET2/ZC3HAV1/PTK2B/IFNAR1/ARHGEF2/RIOK3/HCK/SIRPA/LGALS9/RAB27A/NCR1/OASL/SRC/GRN/ALPK1/IKBKG/POLR3H/PAK2/IRF1/IFNAR2/GBP4/TREM1/NFE2L2/SEC14L1/MARCHF2/NCF2/TREML1/IFIT1B/STMP1/SLA/AP1G1/NLRC4/LYN/UNC13D/ILRUN/RNF187/SNCA/ZNFX1/TYROBP/PAK1/ELF4/FBXO9/PYCARD/FCGR3A/ARRB2/XIAP/TRIM8/CREBBP/PIK3CD/DTX3L/H2BC11/TRIM58/MSRB1/HK1/SRPK2/SP100/CORO1A/WAS/PGLYRP1/IL17RA/TOLLIP/PRKDC/IFITM2/NLRX1/GSN/KRT1/SPI1/ZYX/H2BC4
CC	GO:0045202	synapse	715	-0.32	-1.47	3.89E-06	5.08E-05	3.12E-05	2816	tags=35%, list=24%, signal=28%	SLC1A2/BEGAIN/PDE7B/FXYD6/BORCS5/MAPK1/PACSIN2/KCMF1/FARP1/SSPN/CRTC1/CAPRIN1/RHOA/CACNA1A/RAB8A/PLCB1/SIGMAR1/USP8/STRN4/NEURL1/PRKCZ/VTI1B/SLC2A1/QKI/JAK2/SNAP23/CACNB1/ACTR2/PSEN2/CEP89/DYNLL2/P2RX7/C1QA/HAP1/ACTG1/RAB35/PORCN/CAMK2A/PKP4/PJA2/CAPZB/SERPINE2/MYO9A/KIAA1109/LRRK2/PPFIBP2/PPT1/RTN4R/TBC1D24/GSK3B/CHN2/ADD1/FBXL20/SYAP1/MAGEE1/PRKCB/NDE1/DNM2/ZDHHC2/PAFAH1B1/UTRN/EFNB1/PI4K2A/CASR/ATP7A/KCNAB2/CDK5R1/NRP2/MECP2/GRIK2/KIF1B/STXBP5/CPEB3/FZD5/HRH2/PIP5K1C/ATCAY/STX6/ABR/ITSN1/GAK/HTR3B/ITGA5/SLC6A9/ATP6AP2/RAB21/CPEB4/APBA1/DLG4/GRIPAP1/SLC22A1/GUCY1B1/ATP2B1/SYN3/MYCBPAP/TSC2/RNF40/RAB6B/SLC12A6/GNAI2/RTN4/STX3/AP2M1/LAMP1/TUBA1A/CNIH3/SLC6A12/DTNB/DISC1/MAP1A/PVALB/RAB3B/YWHAZ/SNAP25/PCBP2/MAP1LC3A/MKLN1/ROGDI/GNB1/ARF1/CTBP2/NUMB/CDKL5/NPTXR/ZZEF1/WASF2/SNAP29/MTHFR/MCTP2/GSK3A/PSEN1/MAPT/RELA/RGS14/ERBIN/CLU/PRR7/DNMBP/PRR12/SIPA1L1/ATP6V0D1/AGRN/RAB27B/PPP1R9B/NR4A1/ABHD17C/CTTN/ARL8A/VAMP3/SLC6A6/RAB11B/DNM3/GNAQ/NLGN3/PTK2B/SYNPO/NDEL1/VCP/SLC17A5/AP2B1/SYT12/RPS6KA1/SNTB1/NTNG2/PALLD/ACTN1/SRC/RAB7A/STX11/DBN1/GABARAP/KPTN/PDLIM5/FCHSD1/PAK2/CACNB4/FBXO45/EXOC4/AKAP9/FLOT1/P2RY1/ACTB/DLG5/CALM3/STXBP2/PRKACA/FXR2/VPS18/KPNA1/CTNND1/P2RX1/MYH9/SH3GL1/RAB5B/MME/SLA/CAPN5/SHARPIN/DMXL2/LYN/RTN3/SNCA/DNAJC5/SDCBP/RAC1/ARRB2/SH3KBP1/SYNJ1/PPP3CA/GUCY1A1/PICALM/MAP1S/ASAP1/PPFIA1/MAPK14/PRKAR2B/IQSEC2/CORO1A/NF1/EPHB2/STON2/IQSEC1/CORO1C/TPRG1L/LRRC4/ABI3/ABLIM3/RAB5A/DMTN/ITGB3/PRKAR1A/NRGN/DENND1A/NAPA/BCL2L1/RAB3D/PTEN/MBP/SLC8A1/FLOT2/RNF10/NINJ1/RAPGEF2/STAU1/GLUL/LRFN1/HIP1

CC	GO:0098858	actin-based cell projection	126	-0.48	-1.88	4.05E-06	5.28E-05	3.25E-05	2289	tags=33%, list=20%, signal=26%	LRRK2/SPATA13/PAFAH1B1/UTRN/ATP7A/PDGFA/TWF2/SYNE2/JAM3/FSCN1/FSCN3/PVALB/MTM1/ACTA2/TRIOBP/AOC3/PPP1R9B/ESPN/SLC6A6/B4GALT1/ACP3/MYO1D/KPTN/EXOC4/MYO5A/MYO1E/FXR2/AP2A1/MYO1F/FGD4/ERMN/ATP6V1B2/LCP1/VASP/ITGB3/MSN/RIPOR2/MPP1/SLC7A5/ATP6V1A/IQGAP2
BP	GO:2001236	regulation of extrinsic apoptotic signaling pathway	109	-0.51	-1.94	4.11E-06	5.34E-05	3.28E-05	2155	tags=44%, list=18%, signal=36%	ATF3/TNF/PHIP/G0S2/MCL1/LTBR/BIRC6/RB1CC1/LMNA/GCLM/TMBIM1/STK4/HGF/SH3RF1/PSME3/BID/BCL2L14/PELI3/HSPA1B/RELA/LGALS3/HSPA1A/CTTN/CTNNA1/ARHGEF2/GPX1/PRDX2/SIAH2/SRC/PF4/ITPRIP/PAK2/RIPK1/BRCA1/SERPINE1/DEDD2/PEA15/PYCARD/CFLAR/PTPRC/HMOX1/SP100/NF1/STRADB/TCF7L2/SFRP2/BCL2L1/PTEN
BP	GO:0007043	cell-cell junction assembly	76	-0.55	-1.98	4.13E-06	5.34E-05	3.28E-05	2861	tags=43%, list=24%, signal=33%	SRF/CD9/RHOA/IL1B/ACTG1/PKP4/TNF/ROCK2/HIPK1/FZD5/PIP5K1C/GPBAR1/JAM3/FSCN1/ROCK1/ZNF703/CTNNA1/HDAC7/APC/PAK2/ESAM/PECAM1/ACTB/DLG5/PRKACA/CTNND1/AFDN/F11R/EPHB2/TLN1/FLCN/VCL/CLDN5
CC	GO:0019897	extrinsic component of plasma membrane	107	-0.52	-1.97	4.95E-06	6.39E-05	3.93E-05	2853	tags=48%, list=24%, signal=36%	TIAM1/CDH23/KRAS/NLRP10/GNAS/GNA13/FARP1/FES/RHOA/USP8/GNB2/SERPINE2/GNG5/SYAP1/KCNAB2/ALOX15/SYK/FCN1/DLG4/TGM3/GNAI2/EEA1/SNAP25/GNB1/NUMB/FGR/CTNNA1/GNA12/GNAQ/PTK2B/HCK/TF/SRC/APC/AKAP9/GNAL/CUBN/CTNND1/SLA/LYN/RAC1/PPP3CA/PICALM/SNX18/GNB4/RNF10/IQGAP1/MYZAP/OSBPL2/HIP1/GNAZ
BP	GO:0045596	negative regulation of cell differentiation	354	-0.37	-1.62	5.04E-06	6.48E-05	3.99E-05	2687	tags=34%, list=23%, signal=27%	DICER1/WNT1/RHOA/IL1B/CTDP1/REST/TMEM131L/ASPM/INPP5D/SEMA7A/GPR137B/DDX6/RC3H2/TRIM11/TSPO/ERBB2/YY1/RTN4R/GSK3B/BMPR1A/SORL1/ZBTB16/HNRNPU/TNF/NFKBIA/GBP1/RGS2/CDK5R1/MECP2/HDAC4/YWHAH/CDK13/PCM1/SEMA4A/CTDSP1/RCC2/TMEM176B/KIAA0319/ZFPM1/CR1/NFATC3/TOB2/IL17RD/CTR9/SOX6/DRAXIN/HOOK3/RTN4/YBX1/RFLNB/GREM1/MEIS1/CEACAM5/MSX1/STAT5B/FOXJ2/KDM4A/H4C8/CCL3/SUFU/GSK3A/PSEN1/RARA/ZEB1/ULK1/SPAG9/CMTM5/RAP1GAP/MBNL3/CTNNA1/PTK2B/HDAC7/MAD2L2/CUL4A/PRDX2/LIN28A/SKI/TBX21/PF4/ASCL2/PBX1/TGFB1I1/SOD2/ZFP36L1/IRF1/ZBTB7B/RBPJ/NFE2L2/USF3/TENT5C/NOTCH4/ACTN4/ABCG1/LYN/IGF2/MMP9/ADIPOR1/PPP3CA/FBXO7/NF1/FOXO3/EPHB2/CORO1C/PGLYRP1/G6PD/STAT3/DMTN/SFRP2/ITGB3/FLCN/HLX/PTEN/JDP2/LILRB3/MBP/FOXO4/RNF10/TRIB1/RAPGEF2/NBR1
BP	GO:0022411	cellular component disassembly	357	-0.37	-1.62	5.52E-06	7.08E-05	4.35E-05	2533	tags=33%, list=22%, signal=27%	FIS1/ATG2B/NEK6/WIPI1/SMARCD3/C1QA/TECPR1/OPTN/TSPO/HK2/SNX14/SMARCC1/CAPZB/DHX8/GSK3B/CHMP3/BAX/ADD1/NCKAP5L/PRKCB/PAFAH1B1/TNF/MAP1LC3B/DYRK3/ARID1A/LRP1/KIF5B/CST3/CTDNEP1/GABARAPL2/ITGAM/DNASE2/ERN2/BNIP3L/CALCOCO2/CFL1/MARK2/SPTA1/FZD5/MCOLN1/GABARAPL1/TAOK1/TWF2/GAK/IRAK3/VPS33A/RB1CC1/LAMP2/FSCN1/TSC2/RUBCN/MAP1A/TRIOBP/MAP1LC3A/WASHC2C/IST1/SNAP29/GSK3A/ULK1/INSR/SQSTM1/WIPI2/PLK1/ASPH/PPP1R9B/CTTN/CLEC16A/TFIP11/ARHGEF2/NDEL1/VCP/GSPT1/ATG7/VPS4B/SMARCD2/RETREG3/CHMP1A/APC/WDR45/GABARAP/FRAT1/SETX/FLOT1/GLE1/SVIL/GOLGA2/SH3GL1/PELO/LMOD3/NFE2/CCSAP/ADAMTS4/APAF1/DEDD2/SHARPIN/ATG2A/PLEK/MMP9/SYNJ1/TRIM58/MAP4K4/FBXO7/MAP1S/LCP1/TMOD1/MAP1LC3B2/CHMP2A/IQSEC1/DMTN/MTPN/NAPA/AMBRA1/GSN/PINK1/SPTB/CHMP4B/MICAL2/FURIN

BP	GO:0051056	regulation of small GTPase mediated signal transduction	228	-0.41	-1.72	5.63E-06	7.20E-05	4.42E-05	3236	tags=42%, list=28%, signal=31%	ARHGAP45/ARHGEF10/DEF6/ARHGAP32/SH3BP1/ARRB1/MAPKAP1/BCL6/ABCA1/KCTD10/ARFGEF1/TIAM1/STK19/GMIP/KRAS/MFN2/GNA13/ARHGAP18/ARHGEF11/SOS2/LPAR1/DOCK2/ARHGAP9/STARD8/ARHGDIB/FAM13B/MYO9A/ARHGEF40/RTN4R/SPATA13/CHN2/PIK3CB/DNM2/MYO9B/LRRC59/PLCE1/ARAP1/DENND3/ARHGAP31/ARHGEF3/ARHGDIA/TAGAP/AKAP13/KBTBD7/ABR/ITSN1/PIK3CG/RALGAPA2/PSD4/CBL/LRRD1/TSC2/ARHGEF12/CUL3/ARHGAP30/ERBIN/DNMBP/SQSTM1/SIPA1L1/RAP1GAP/VAV1/DOCK8/ARHGEF2/ARHGAP21/SPRY3/VAV3/SRC/SHOC2/GABARAP/FLOT1/ARHGAP8/PREX1/PLEKHG3/ARHGAP1/ARHGAP25/FGD4/CYTH4/TGM2/RAC1/SIPA1/CYRIB/F11R/FBP1/MAP4K4/IQSEC2/NF1/EPHB2/IQSEC1/ARHGAP26/RASGRP4/RALBP1/FLCN/DENND1A/SIPA1L2/RIPOR2/ARHGAP6
BP	GO:0045921	positive regulation of exocytosis	56	-0.60	-2.04	5.67E-06	7.22E-05	4.44E-05	1984	tags=38%, list=17%, signal=31%	ITGAM/STXBP5/SYK/LAMP1/SYTL4/FGR/ITGB2/RAB27B/VPS4B/RAB27A/RAB7A/RAB2B/AP1G1/UNC13D/SNCA/SDCBP/PTAFR/CHMP2A/RAB5A/RAB3D/GAB2
CC	GO:0098562	cytoplasmic side of membrane	142	-0.46	-1.81	5.80E-06	7.35E-05	4.52E-05	2853	tags=44%, list=24%, signal=34%	KRAS/GNAS/BORCS5/GNA13/FARP1/FES/TRAF6/RHOA/KIT/GNB2/SHROOM4/PKP4/LRRK2/GNG5/SYAP1/KCNAB2/RGS2/ALOX15/SPTA1/SYK/MAP2K2/HM13/RAB21/DLG4/ATP2B1/TGM3/GNAI2/SNAP25/GNB1/FGR/FKBP1A/PTPN1/PPP1R9B/GNA12/GNAQ/PTK2B/HCK/SRC/CACNB4/GNAL/MYH9/SLA/LYN/C2CD2L/RAC1/PPP3CA/PTPRC/SLC4A1/G6PD/RAB5A/SNX18/GNB4/ANK1/SPTB/PTEN/CHMP4B/PTP4A1/IQGAP1/LITAF/MYZAP/OSBPL2/HIP1/GNAZ
BP	GO:0031329	regulation of cellular catabolic process	686	-0.32	-1.47	5.80E-06	7.35E-05	4.52E-05	2586	tags=30%, list=22%, signal=25%	EIF4G1/CTSA/SIRT6/ERN1/CSNK2A1/DAPK1/CNOT1/WIPI1/GPR137B/TICAM1/P2RX7/RC3H2/VIM/TAF1/TECPR1/OPTN/FEZ1/CEBPA/TSPO/TRIM21/DCP2/SMARCC1/ZYG11B/TNRC6B/ABCA2/PTTG1IP/LRRK2/RNF5/CAPNS1/GSK3B/RNF14/TPCN2/HIPK2/BAX/ACACB/SORL1/TAB2/PIK3CB/HNRNPU/TNF/CPTP/ROCK2/TRDMT1/LRP1/CST3/ENTPD5/GPLD1/GABARAPL2/CDK5R1/ATP6V1C1/PIP4K2C/DHRSX/SH3RF3/BNIP3L/CALCOCO2/CPEB3/SLC11A1/FZD5/MCL1/PGAM1/EIF4G2/COP1/ANXA2/PIK3CG/PPP1R3B/YTHDF3/NUB1/SOGA1/EXOC7/SPTLC2/TP53INP2/PATL1/EP300/MTMR3/ABCB11/THRAP3/TIRAP/HGF/RNASEL/TSC2/RNF40/RUBCN/FZR1/YBX1/DISC1/MAP1A/MTM1/GIGYF2/ATP6V0A1/CNOT6/SH3RF1/KDM4A/TFEB/TBC1D14/PSME3/N4BP1/ROCK1/ARNT/SESN2/HSPA1B/RYBP/SUFU/GSK3A/PSEN1/MAPT/CLU/CSDE1/VPS11/IFI16/ULK1/INSR/PTPN1/SESN3/PLK1/RNF19B/ATP6V0D1/AGTPBP1/AGO2/HSPA1A/TUT7/CTTN/RALB/TIMP1/CLEC16A/QSOX1/GNA12/ZC3HAV1/PTK2B/VCP/NUPR1/GPX1/ATG7/SAMD4B/ELAPOR1/RAD23A/WDR45/CPT1A/MAPKAPK2/IKBKG/UBB/GABARAP/PSAP/ZC3H18/NRDC/GTPBP1/EXOC4/HSPB1/PIP4K2A/WAC/DAP/ZFP36L1/PIAS1/PRKACA/AKT2/FXR2/SH3GLB1/TNFRSF1B/FEZ2/KAT5/NFE2L2/GOLGA2/MARCHF2/BAG6/TENT5C/APAF1/LAPTM5/USP10/ATP6V1B2/SNCA/ATG2A/SDCBP/PYCARD/TRIM8/RAD23B/PSMC4/PITHD1/DTX3L/RRAGD/TIMP2/ABHD5/FBXO7/MAPK14/HMOX1/FOXO3/DNAJB2/YBX3/MYEF2/ZER1/PNPLA2/IRS2/STAT3/MAPK3/SNX18/DCAF12/FLCN/AMBRA1/PRKCD/PINK1/MSN/CAPN1/PTEN/PSMF1/CHMP4B/TRIB1/NPRL3/CASC3/FURIN/DAB2/ATP6V1A

CC	GO:0005887	integral component of plasma membrane	725	-0.32	-1.45	5.84E-06	7.38E-05	4.53E-05	2631	tags=28%, list=22%, signal=23%	ADAM17/KIR2DL1/SELPLG/SLC2A1/STIM1/LPAR1/JAK2/SLC25A11/SIGLEC7/NCKAP1L/HCAR3/C17orf99/SLC28A2/KIT/PSEN2/SCN1B/SLC9A1/SEMA7A/LRRC8B/GPR137B/FCER1G/TNFRSF9/P2RX7/EVI2B/CSF1R/PORCN/CACNA1I/CD70/ALCAM/PLXNB2/ERBB2/IL18RAP/TMEM150B/CYSLTR2/CYBB/RTN4R/SLC22A4/LILRB2/NPHS1/SORL1/ZDHHC2/TNF/EFNB1/PI4K2A/CASR/SLCO4C1/LRP1/IGSF6/TLR1/MAS1/KCNAB2/TMEM130/KCNJ6/NRP2/TMPRSS9/GP6/ITGAM/SLC39A4/GRIK2/STXBP5/ITGA2B/SLC11A1/PLSCR1/FZD5/SEMA4A/MCOLN1/MFSD12/HRH2/SLC31A1/IL10RB/CD37/CLPTM1/SLCO3A1/CR1/GPBAR1/APCDD1/HTR3B/TSPAN33/ITGA5/ITGAL/SLC6A9/CD82/ANKH/ICAM4/TNFRSF1A/SSTR2/DLG4/SLC22A1/JAM3/ATP2B1/CCR1/TMEM11/KIR2DL3/LRRC8A/NCF1/LRP12/ADGRE3/SLC12A6/NCF4/LAMP1/CNIH3/SLC6A12/TSPAN2/FCGR3B/TLR6/CEACAM5/TSPAN14/LILRA2/KLRD1/FFAR4/CSF3R/TRPC3/LTB4R/FCGR2A/SLC2A3/ABCC3/ICAM3/PSEN1/PTPRJ/PCDH1/IGF2R/ITGB2/EPHB4/LGR6/AQP9/CD63/INSR/SLC23A2/TSPAN9/SLC6A20/C5AR1/SLC6A6/NLGN3/PTK2B/IFNAR1/CXCR2/SLC17A5/SIRPA/ELAPOR1/SLC38A5/KCNQ1/NCR1/SELL/TMEM266/SLC24A3/PGAP6/KCNJ15/ABCC9/PECAM1/ENTPD1/AKAP9/PCDHGA8/PCDHB1/P2RY1/IL6R/CALM3/PLAUR/SELP/GP1BA/IFNAR2/SLC14A1/GYPB/P2RX1/ITGB5/NCF2/MME/NOTCH4/SLC2A9/NINJ2/LAPTM5/LYN/SIRPB1/MMD/TYROBP/SDCBP/FCGR3A/SLC1A5/PLXNC1/KCNJ5/PTPRC/OPRL1/GYPC/TSPAN5/ABCG8/EPHB2/PTAFR/SLC4A1/LRRC4/IL17RA/SLC13A4/AQP1/ITGB3/IL13RA1/BSG/PLPPR2/CCRL2/LILRB3/SLC8A1/RAPGEF2/TBXA2R/SLC6A8/GP9/LRFN1/AQP10
BP	GO:1902532	negative regulation of intracellular signal transduction	374	-0.36	-1.58	6.00E-06	7.53E-05	4.63E-05	2198	tags=29%, list=19%, signal=24%	NCOR1/MAPKBP1/SORL1/PIK3CB/ATF3/PAFAH1B1/DYRK3/WWC3/GBP1/RGS2/PPIF/FHL2/MYADM/MCL1/WWC2/NMI/TMEM127/UBE2B/PTPN6/IRAK3/CDKN2D/IVNS1ABP/TSC2/CPNE1/CUL3/INPP5K/MTM1/OTUD7B/C16orf72/PTGS2/BID/SESN2/TMEM161A/GPATCH3/GSK3A/RELA/RGS14/PTPRJ/ERBIN/CLU/PTPN1/SESN3/VRK3/AKT1S1/RASSF2/HSPA1A/HMGCR/ARHGEF2/RIOK3/HDAC7/GPX1/TNIP1/THOC5/SIRPA/HIPK3/SPRY3/TAOK3/SRC/CDC34/DGKG/KPTN/DUSP6/HSPB1/PPP1R10/DUSP1/RIPK1/MARK3/RNF149/DLG5/SOD2/PLAUR/CASTOR2/NFE2L2/SEC14L1/UBR2/ARHGAP25/CHP1/LYN/USP10/PYCARD/PLEK/ARRB2/MMP9/ADIPOR1/CYRIB/GRINA/MAP2K3/PTPRC/OPRL1/FBP1/STK38/MAPK14/NF1/EPHB2/YBX3/SFRP2/FLCN/NLRX1/PRKCD/PINK1/BCL2L1/PPM1A/PTEN/SPI1/RIPOR2/NPRL3/DAB2/LITAF
BP	GO:0002275	myeloid cell activation involved in immune response	76	-0.55	-1.96	6.05E-06	7.53E-05	4.63E-05	2594	tags=46%, list=22%, signal=36%	FES/CD177/SNAP23/KIT/DOCK2/FCER1G/TICAM1/PRAM1/ITGAM/SYK/NMI/PIK3CG/PTPN6/RAC2/CBL/LILRA2/CCL3/FGR/ITGB2/SBNO2/LGALS9/GRN/LAT2/STXBP2/PTGDS/LYN/UNC13D/TYROBP/PYCARD/PIK3CD/HMOX1/PTAFR/SPI1/DYSF/GAB2
BP	GO:0099003	vesicle-mediated transport in synapse	106	-0.51	-1.93	6.06E-06	7.53E-05	4.63E-05	2550	tags=43%, list=22%, signal=34%	SNAP23/P2RX7/ACTG1/KIAA1109/LRRK2/GSK3B/FBXL20/PRKCB/DNM2/CDK5R1/STXBP5/PIP5K1C/ITSN1/GAK/APBA1/GRIPAP1/SYN3/STX3/AP2M1/RAB3B/SNAP25/CTBP2/NUMB/ROCK1/SNAP29/PSEN1/RAB27B/DNM3/NLGN3/SYT12/STX11/P2RY1/ACTB/CALM3/VPS18/P2RX1/SH3GL1/SNCA/DNAJC5/SYNJ1/PICALM/STON2/RAB5A/ITGB3/NAPA/PTEN
BP	GO:0110053	regulation of actin filament organization	192	-0.43	-1.75	6.06E-06	7.53E-05	4.63E-05	2592	tags=33%, list=22%, signal=26%	CDC42EP4/LPAR1/NCKAP1L/SLC9A1/ARPC4/ACTG1/FLNA/CAPZB/GRB2/ADD1/NPHS1/ROCK2/ARAP1/RICTOR/ALOX15/MYADM/SPTA1/COTL1/TWF2/FHOD1/ARPC1B/INPP5K/TRIOBP/ARF1/WASHC2C/ROCK1/WASF2/PXN/CTTN/PTK2B/SYNPO/HCK/ARPC5/LIMK1/DBN1/FCHSD1/PAK2/ESAM/SVIL/TPM1/LMOD3/SSH1/ARPC1A/HCLS1/PAK1/RAC1/PYCARD/PLEK/CYRIB/F11R/PPFIA1/TMOD1/CORO1A/SSH2/WAS/VASP/CYRIA/DMTN/MTPN/PRKCD/GSN/SPTB/ARHGAP6

BP	GO:0043549	regulation of kinase activity	526	-0.34	-1.53	6.56E-06	8.10E-05	4.98E-05	2696	tags=33%, list=23%, signal=26%	FPR2/TRAF6/RHOA/PIK3R2/ACSL1/IL1B/PRKAG2/HEXIM2/ADAM17/NEURL1/PRKCZ/ADNP/ERN1/JAK2/NCKAP1L/KIT/ANGPT1/GCN1/P2RX7/CCND3/CSF1R/DDX3X/SH3BP5/LCP2/CEBPA/INCENP/ERBB2/LRRK2/BMPR1A/MLLT1/SYAP1/SORL1/TAB2/PIK3CB/HNRNPU/TNF/RANBP2/TNXB/PLCE1/PKN1/MAS1/RGS2/CDK5R1/PRKAR2A/PIP4K2C/RICTOR/ERN2/MARK2/SLC11A1/HHEX/PDGFA/FZD5/AKAP13/TAOK1/SYK/MAP2K2/PIK3CG/GADD45A/PTPN6/CDKN1C/DLG4/IRAK3/CDKN2D/CBL/STK4/AGAP2/TIRAP/NCF1/HGF/TSC2/RUBCN/PDE5A/GREM1/DUSP22/TLR6/INPP5K/SHC1/OSBPL8/DSTYK/IRAK1/SESN2/CCNY/PSEN1/MAPT/RGS14/PTPRJ/FGR/VLDLR/CLU/EPHB4/SOCS3/INSR/PTPN1/ADAR/PLK1/CHI3L1/CCNF/MAP3K5/NEDD9/AKT1S1/RASSF2/C5AR1/RALB/HMGCR/GNAQ/PTK2B/DVL3/CDKN1A/PPP2R5A/PARP16/MAP2K7/CASS4/HIPK3/SPRY3/VAV3/TAOK3/SRC/APC/WARS1/PAK2/PIK3R5/HSPB1/PIP4K2A/DUSP1/IL6R/ACTB/CALM3/DAZAP2/CCNI/CNPPD1/SASH1/TAL1/SH3BP5L/CHP1/LYN/MMD/SNCA/IGF2/STRADA/PAK1/WNK1/PYCARD/MIDN/MAP2K3/PTPRC/FBXO7/STK38/PRKAR2B/NF1/EPHB2/CORO1C/CACUL1/MOB3A/STRADB/IRS2/SFRP2/ITGB3/PRKAR1A/NRBF2/AMBRA1/PRKCD/PINK1/CCNJL/PTEN/MOB3C/SLC8A1/TRIB1/RAPGEF2/IQGAP1/DAB2/TGFA
BP	GO:0031344	regulation of cell projection organization	412	-0.36	-1.58	6.64E-06	8.17E-05	5.02E-05	2708	tags=32%, list=23%, signal=25%	FES/CRTC1/CAPRIN1/WNT1/RHOA/NEURL1/SCARB2/CDC42EP4/IFT88/ADNP/LPAR1/KIT/ACTR2/SCN1B/SEMA7A/HSPA5/P2RX7/HAP1/HECW2/VIM/CD38/FEZ1/FLNA/CAPZB/KLF5/PLXNB2/MYO9A/LRRK2/RTN4R/GSK3B/RHOQ/PLEKHM1/DNM2/PAFAH1B1/TBC1D30/PLCE1/ARAP1/ATP7A/RGS2/CDK5R1/YWHAH/CFL1/MARK2/SEMA4A/P3H1/EIF4G2/RCC2/KIAA0319/TWF2/SYNE2/GAK/RAB21/DLG4/RAC2/FSCN1/EP300/CAMK1D/DRAXIN/HGF/STK24/RTN4/DISC1/SNAP25/IFT140/CDKL5/IST1/DENND5A/WASF2/GSK3A/PSEN1/MAPT/ANKRD27/VLDLR/ULK1/SIPA1L1/AGRN/MAP4/RAP1GAP/CUX1/DNM3/MARK4/CEP97/PTK2B/NDEL1/PPP2R5B/DVL3/RETREG3/NIN/NTNG2/SPRY3/LIMK1/SRC/APC/GRN/DBN1/DGKG/XK/PDLIM5/PAK2/SETX/PLEK2/CARM1/FXR2/EHD1/NFE2L2/ERMN/LYN/PAK1/RAC1/PPP3CA/LIMK2/CFLAR/PLXNC1/RHOG/FBXO7/EPHB2/CORO1C/WAS/ABI3/RAB5A/DMTN/SFRP2/KEL/PRKCD/CCP110/PTEN/MBP/RIPOR2/RAPGEF2/DAB2
BP	GO:0051258	protein polymerization	213	-0.41	-1.71	7.23E-06	8.87E-05	5.45E-05	2733	tags=38%, list=23%, signal=29%	HDGFL3/ARHGAP18/FES/RHOA/SLAIN2/CDC42EP4/JAK2/NCKAP1L/DYRK1A/ARPC4/CAPZB/MIEF2/GRB2/CLIP1/CHMP3/ADD1/NPHS1/NDE1/CDK5R1/MECP2/RICTOR/ALOX15/MYADM/SPTA1/COTL1/TUBGCP3/TWF2/UBE2S/ARPC1B/TRIOBP/WASHC2C/DIAPH2/HSPA1B/MAPT/PSTPIP2/MSRB2/GAS7/DIAPH1/PSTPIP1/HSPA1A/CTTN/PTK2B/NDEL1/HCK/NIN/ARPC5/DBN1/FCHSD1/ESAM/TPPP3/AKAP9/SVIL/PREX1/GOLGA2/LMOD3/SSH1/ARPC1A/HCLS1/SNCA/PAK1/RAC1/PYCARD/CYRIB/MSRB1/TMOD1/CORO1A/SSH2/CHMP2A/WAS/VASP/CYRIA/DMTN/MTPN/AMBRA1/PRKCD/GSN/PINK1/SPTB/CHMP4B/ARHGAP6
BP	GO:0034109	homotypic cell-cell adhesion	66	-0.57	-2.00	7.36E-06	8.98E-05	5.52E-05	2819	tags=56%, list=24%, signal=43%	GNAS/CD9/CLIC1/JAK2/ACTG1/JAK1/FLNA/SERPINE2/FERMT3/METAP1/PIK3CB/ABAT/GP6/SYK/PIK3CG/PTPN6/CEACAM5/DSC2/ZNF703/ILK/HSPB1/ACTB/PLAUR/GP1BA/ALOX12/SLC7A11/MYH9/LYN/PLEK/F11R/TLN1/DMTN/ITGB3/MPL/PRKCD/MYL9/VCL
CC	GO:0005776	autophagosome	98	-0.51	-1.92	7.58E-06	9.21E-05	5.66E-05	2493	tags=46%, list=21%, signal=36%	WIPI1/TICAM1/HAP1/TECPR1/OPTN/TRIM21/TMEM150B/LRRK2/CHMP3/ADPRH/PLEKHM1/MAP1LC3B/GABARAPL2/PIP4K2C/CALCOCO2/TBC1D5/GABARAPL1/ATP6AP2/VPS33A/RB1CC1/TP53INP2/LAMP2/LAMP1/MAP1LC3A/TBC1D14/SNAP29/VPS11/ULK1/SQSTM1/WDFY3/CHMP1A/RAB7A/GABARAP/PIP4K2A/SH3GLB1/VPS18/NCOA4/MAP1LC3B2/CHMP2A/FTL/NRBF2/FTH1/AMBRA1/CHMP4B/NBR1

CC	GO:0048471	perinuclear region of cytoplasm	520	-0.34	-1.52	8.01E-06	9.71E-05	5.97E-05	2196	tags=25%, list=19%, signal=21%	SYAP1/MAGEE1/DNM2/DCTN3/PAFAH1B1/MYO9B/SERINC3/PTGES/KIF5B/TNFSF13B/ATP7A/CDK5R1/CALCOCO2/PRDX5/PDE4A/PLSCR1/FZD5/CERT1/AKAP13/TAOK1/TWF2/MAP2K2/RAB34/STX6/GAK/CPEB4/UBR5/APBA1/VPS33A/CTIF/CBL/EIF2AK2/TSC2/LAMP1/DISC1/RAB3B/INPP5K/CKAP4/SNAP25/ATP6V0A1/SH3RF1/ARF1/GBP2/MOGAT3/BRI3/HSPA1B/YWHAB/PSEN1/RARA/ATN1/CLU/RAB1B/IGF2R/PRR7/SPAG9/SEC16A/CHI3L1/CCNF/PSTPIP1/HSPA1A/VAMP3/ANKRD13A/EXOC6/DNM3/PTK2B/VCP/NUPR1/SLK/DOK1/TF/MGAT1/SRC/APC/PAK2/XKR8/TPPP3/LIMS1/DCUN1D3/PRKACA/ZDHHC19/EHD1/GBP4/TAF10/TNFRSF1B/DGAT2/KAT5/AATK/CA4/ARHGAP1/PTGDS/TMEM184A/ACTN4/AP1G1/LAPTM5/ARF3/LYN/SNCA/TGM2/CDK19/SYNJ1/SIPA1/LIMK2/EHD3/PICALM/MAP1S/CTSB/LCP1/PRKAR2B/DNAJB6/HMOX1/YBX3/PRDX6/OSBP2/TOLLIP/DMTN/MTPN/SBF2/AMBRA1/PRKCD/PINK1/MSN/PSMF1/BCL3/FLOT2/PLVAP/RAPGEF2/CASC3/ECE1/DAB2/IRAG1/TGFA
BP	GO:0099504	synaptic vesicle cycle	100	-0.51	-1.92	8.55E-06	0.0001033	6.35E-05	2550	tags=44%, list=22%, signal=35%	SNAP23/P2RX7/ACTG1/KIAA1109/LRRK2/GSK3B/FBXL20/PRKCB/DNM2/CDK5R1/STXBP5/PIP5K1C/ITSN1/GAK/APBA1/GRIPAP1/SYN3/STX3/AP2M1/RAB3B/SNAP25/CTBP2/ROCK1/SNAP29/PSEN1/RAB27B/DNM3/NLGN3/SYT12/STX11/P2RY1/ACTB/CALM3/VPS18/P2RX1/SH3GL1/SNCA/DNAJC5/SYNJ1/PICALM/STON2/RAB5A/NAPA/PTEN
CC	GO:0030426	growth cone	116	-0.48	-1.87	8.59E-06	0.0001035	6.36E-05	2522	tags=41%, list=22%, signal=33%	PSEN2/CCDC120/HAP1/FEZ1/COPA/FLNA/MYO9A/LRRK2/RTN4R/SYAP1/DNM2/PAFAH1B1/CDK5R1/TWF2/ATCAY/CPEB4/EXOC7/FSCN1/CBL/FSCN3/STX3/DISC1/SNAP25/CDKL5/PSEN1/MAPT/PPP1R9B/CTTN/EXOC6/PTK2B/NDEL1/PTBP2/NIN/ARPC5/PALLD/DBN1/EXOC4/SETX/CALM3/MYO5A/FXR2/CTNND1/USP9X/SNCA/PINK1/BASP1/IQGAP1/TSHZ3
CC	GO:0030133	transport vesicle	264	-0.39	-1.66	8.96E-06	0.000107	6.58E-05	3055	tags=38%, list=26%, signal=28%	SEC22B/SLC40A1/EDN1/CLCN5/RAB11FIP5/SEC23IP/GOLGA5/CLN3/RPH3A/SYNRG/HLA-DRB1/BORCS5/TMEM187/SSPN/RAB8A/SCAP/VTI1B/STX5/YIPF1/COPB2/HAP1/RAB35/COPA/RPH3AL/TMEM168/LRRK2/PPT1/CRISPLD2/SORL1/ATP7A/KIF1B/STXBP5/YIPF2/RAB6A/SPG21/HLA-E/STX6/SLC6A9/APBA1/DLG4/ATP2B1/SYN3/STX3/LAMP1/COPG1/CNIH3/DISC1/RAB3B/SNAP25/ROGDI/CLTC/MCTP2/SYTL4/PSEN1/ANKRD27/RAB1B/IGF2R/SEC16A/ATP6V0D1/RAB27B/VAMP3/RAB11B/NDEL1/HCK/SLC17A5/SYT12/RAB27A/KCNQ1/TGOLN2/COPE/RAB7A/STX11/CALM3/AP2A1/CA4/RAB5B/TMEM184A/MME/AP1G1/LAPTM5/CHP1/DMXL2/UNC13D/SNCA/DNAJC5/SIPA1/PICALM/STON2/IQSEC1/TPRG1L/RAB3IL1/RAB5A/NRGN/BCL2L1/RAB3D/FURIN/TGFA/ATP6V1A/PLIN3
MF	GO:0051019	mitogen-activated protein kinase binding	26	-0.72	-2.09	9.14E-06	0.0001088	6.69E-05	1734	tags=42%, list=15%, signal=36%	PPM1D/PTPRJ/TNIP1/TPR/MAPKAPK2/DUSP1/MAPKAPK3/ARRB2/MAPK14/PTAFR/NBR1
CC	GO:0019898	extrinsic component of membrane	213	-0.41	-1.70	9.22E-06	0.0001095	6.73E-05	2883	tags=40%, list=25%, signal=31%	PML/RPH3A/KRAS/NLRP10/GNAS/VPS13D/PACSIN2/GNA13/FARP1/FES/RHOA/PIK3R2/USP8/ATG2B/GNB2/WIPI1/SERPINE2/GNG5/SYAP1/PIK3CB/BST1/KCNAB2/ALOX15/STXBP5/SYK/PIK3CG/FCN1/DLG4/RB1CC1/GRIPAP1/SYN3/RGS6/MTMR3/TGM3/MICALL1/NCF1/RNF40/GNAI2/EEA1/SNAP25/GNB1/NUMB/MGLL/SYTL4/FGR/FKBP1A/SOCS3/ULK1/WIPI2/WDFY3/CTNNA1/GNA12/GNAQ/PTK2B/HCK/TF/SNX10/SRC/APC/WDR45/TPR/RAB7A/UBB/PIK3R5/AKAP9/PLAUR/GNAL/CUBN/CTNND1/SLA/LYN/ATG2A/RAC1/UBXN6/PIK3CD/PPP3CA/PICALM/SNX18/GNB4/RNF10/IQGAP1/MYZAP/OSBPL2/HIP1/GNAZ

BP	GO:0070997	neuron death	238	-0.40	-1.69	9.51E-06	0.0001125	6.92E-05	2930	tags=42%, list=25%, signal=32%	GBE1/AIFM1/CLN3/KRAS/NAIP/HIF1A/ADAM8/CASP2/FOS/WNT1/RHOA/MAP2K4/REST/SIGMAR1/PIGT/BTBD10/ADNP/EIF4G1/JAK2/CASP9/ANGPT1/SLC9A1/HSPA5/C1QA/INPP5A/FADD/LRRK2/PPT1/TBC1D24/GSK3B/HIPK2/BAX/SORL1/CTSZ/TNF/CDK5R1/MECP2/ITGAM/GRIK2/MCL1/ZNF746/MEAK7/CPEB4/RILPL1/GCLM/CBL/AGAP2/DRAXIN/GPI/TLR6/NR4A2/ROCK1/BID/CCL3/PSEN1/MAPT/ATN1/CLU/ITGB2/SLC23A2/MAP3K5/MAX/AKT1S1/PTK2B/EGLN3/NUPR1/GPX1/AP2B1/EGLN2/MAP2K7/HDGF/GRN/CDC34/IKBKG/UBB/SOD2/AKT2/CLCF1/TNFRSF1B/SLC7A11/APAF1/CHP1/SNCA/DNAJC5/TYROBP/ARRB2/XIAP/CEBPB/PICALM/FBXO7/SRPK2/HMOX1/CORO1A/NF1/FOXO3/DHCR24/G6PD/PINK1/BCL2L1/CHMP4B
CC	GO:0000785	chromatin	730	-0.31	-1.44	9.69E-06	0.0001139	7.00E-05	3191	tags=35%, list=27%, signal=27%	ING2/VENTX/PPP4R3B/ZBED3/SRPK1/ARRB1/GMEB2/NR6A1/ZBTB34/MYT1L/KLF13/CHAF1A/USF2/DDX21/GATA1/SIN3B/PBX4/BRD3/ZNF580/ATOH8/ADNP2/STAT1/L3MBTL1/BCAS3/TFDP1/H2BC21/ENC1/BAZ2A/ARGFX/PML/GLYR1/ACTR8/ANKRD17/SRF/RELB/WDR82/NSD1/NR2F6/SMARCB1/HIF1A/SETD5/AKIRIN2/ZBTB18/PBX3/CHD8/NCOR2/POU5F1B/FOS/TRPS1/H2AC19/H3-5/ZBTB8B/PLCB1/PBRM1/IKZF1/STAG1/NEUROD2/ADNP/CSNK2A1/JAK2/ZEB2/CBX7/SIN3A/POU2F2/SMARCD3/EXOSC4/SRCAP/FOXP2/MBD2/MBTD1/TAF1/KLF7/CEBPA/INCENP/WAPL/SMARCC1/BAZ1A/MITF/NFYA/KLF5/OTX1/YY1/NFIA/TBX19/REL/STAT5A/NCOR1/KDM1B/ATF3/JUND/RSF1/NSD2/ARID1A/GATAD2A/NKX3-1/HMGN2/MAFK/NSD3/NFAT5/MECP2/ETV7/HHEX/ZFPM1/ATF5/UBE2B/CBX6/HIRA/NFATC3/KLF6/SUDS3/ELF1/SP1/RNF20/H1-0/CREB1/NFKB2/MSH6/CTR9/RFX1/H3C10/SOX6/JMJD1C/USP3/CEBPD/RNF40/SUPT6H/FAAP24/CHRAC1/ARID1B/ZBED1/TFCP2/PDS5B/SOX4/MEIS1/MSX1/SP2/PHF12/STAT5B/JUNB/FOXJ2/KDM4A/TFEB/NR4A2/CHD4/ARNT/IST1/H4C8/STAG2/ATRX/XRCC1/PDS5A/TFDP2/RARA/RELA/ZEB1/KDM3B/PLK1/NFYC/MAX/NR3C1/SMARCA2/ATF6B/MXI1/CUX1/NR4A1/GATAD2B/KAT6A/IRF2/H3-3A/APTX/DVL3/ZFHX3/SMARCD2/NCR1/AHCTF1/BICRAL/BACH1/NCOA2/NIPBL/ETS2/KMT2E/ASCL2/FLI1/BPTF/PBX1/FANCC/ETV6/PPP1R10/RFX2/TOX4/MYCL/ACTB/VDR/PADI2/IRF1/CSRNP1/ASF1B/RXRA/RBPJ/KLF3/KAT5/NFE2L2/MXD1/H2AC6/UBR2/TAL1/HIC1/BATF2/NFE2/FOXN3/BRD4/NFIX/UBA1/CREB5/TGM2/CHD7/HNRNPK/ELF4/PBX2/CREBBP/POU2F1/H2BC11/CEBPB/CIC/ERF/STAT6/E2F3/SRPK2/LYL1/H3-3B/ELL/NCOA1/NFIC/FOXO3/CHMP2A/TFE3/NACC2/WBP2/TCF7L2/STAT3/RAD21/PINK1/BASP1/HLX/FOSL2/JDP2/SPI1/FOXO4/H2BC4/TSHZ3/KLF1/E2F2/NFIL3
BP	GO:0043269	regulation of ion transport	336	-0.37	-1.61	9.77E-06	0.0001139	7.00E-05	2713	tags=34%, list=23%, signal=27%	ALG10B/CLIC1/RHOA/IL1B/CACNA1A/KCNQ5/ANO6/STIM1/DAPK1/CACNB1/PSEN2/SCN1B/BEST1/SLC9A1/P2RX7/CLIC3/HAP1/OXSR1/HECW2/CACNA1I/CAMK2A/TSPO/AHCYL1/FLNA/SERPINE2/CA2/CYBB/LILRB2/TPCN2/BAX/DNM2/TNF/UTRN/GJC2/ABAT/PTGES/CASR/KIF5B/ATP7A/KCNAB2/RGS2/PPIF/KCNJ6/YWHAH/CES1/SYK/CATSPER2/SLC43A2/PIK3CG/SLC6A9/PTPN6/DLG4/ATP2B1/CCR1/ABCB11/CRACR2B/GNAI2/CNIH3/CACNA1D/RAB3B/INPP5K/LILRA2/TRPC3/PTGS2/CCL3/PSEN1/FKBP1A/CAPN3/CD63/SELENON/DIAPH1/LGALS3/RAB11B/WWP2/NLGN3/PTK2B/TESC/SYT12/TF/KCNQ1/CACNB4/KCNJ15/KCNE3/AKAP9/RIPK1/P2RY1/CALM3/PRKACA/P2RX1/EPB41/ACTN4/CHP1/LYN/SNCA/CHD7/WNK1/ARRB2/MMP9/EHD3/KCNJ5/CEBPB/OPRL1/CORO1A/EPHB2/PTAFR/RGS7/G6PD/ITGB3/KEL/MYLK/PINK1/CAPN1/SLC8A1

BP	GO:0001816	cytokine production	538	-0.33	-1.51	9.77E-06	0.0001139	7.00E-05	3059	tags=36%, list=26%, signal=28%	IL18R1/DDX21/IGF2BP3/BCL6/ZNF580/FERMT1/TYK2/CSK/HOMER3/TRIM38/STAT1/THBS1/OAS3/BST2/PML/BAP1/RELB/ITCH/NLRP10/GSTP1/CLEC4E/HLA-DRB1/HIF1A/AKIRIN2/ADAM8/NLRP12/TLR2/LGALS9C/DICER1/TRAF6/IL1B/PLCB1/ADAM17/INPP5D/PRKCZ/CCDC88B/SETD2/JAK2/NCKAP1L/KIT/ANGPT1/SEMA7A/ALOX5/POU2F2/FCER1G/TICAM1/P2RX7/CSF1R/DDX3X/SETD4/C5AR2/TSPO/TRIM21/LTBP1/FADD/BTNL9/IL1R2/LRRK2/YY1/CYBB/REL/RFTN1/ARID5A/BMPR1A/LILRB2/CCBE1/MAPKBP1/SPON2/SORL1/BTN3A2/PELI1/TNF/CPTP/ROCK2/MNDA/GBP1/TLR1/TLR8/PRKD2/NFAT5/OSM/SLC11A1/FZD5/ORM1/SYK/NMI/ZFPM1/CR1/HLA-E/NFATC3/PIK3CG/ELF1/ATP6AP2/PTPN6/FCN1/IRAK3/CLEC9A/ATP2B1/AGPAT1/SULF2/TIRAP/EIF2AK2/HGF/VSIR/TLR6/CD14/LILRA2/STAT5B/RSAD2/FFAR4/FRMD8/OTUD7B/ERMAP/N4BP1/MAVS/PTGS2/ARNT/RPS6KA5/IRAK1/HSPA1B/GPATCH3/CCL3/PSEN1/RARA/RELA/PTPRJ/FGR/ERBIN/CLU/IFI16/CHI3L1/C5AR1/HSPA1A/ARHGEF2/RIOK3/HDAC7/SIRPA/LGALS9/SRC/TBX21/PF4/MAPKAPK2/HSPB1/FLOT1/CYP1B1/RIPK1/IL6R/IRF1/ZBTB7B/TNFRSF1B/BRCA1/OTUD5/TREM1/KAT5/SERPINE1/STMP1/NRROS/MAST2/ACKR1/LAPTM5/NLRC4/LYN/ILRUN/TYROBP/RAC1/PYCARD/ARRB2/PANX2/GPSM3/CYRIB/PIK3CD/F11R/MAP2K3/CEBPB/PTPRC/HK1/MAPK14/HMOX1/EPHB2/PTAFR/PGLYRP1/IL17RA/STAT3/NLRX1/NFAM1/IGF2BP2/BCL3/MBP/IQGAP1/FURIN/LITAF/SLC7A5
BP	GO:1902903	regulation of supramolecular fiber organization	268	-0.39	-1.65	1.02E-05	0.0001188	7.30E-05	2733	tags=33%, list=23%, signal=26%	HDGFL3/ARHGAP18/TTBK2/FES/RHOA/SLAIN2/CDC42EP4/LPAR1/NCKAP1L/SLC9A1/DYRK1A/ARPC4/ACTG1/FLNA/CAPZB/GRB2/CLIP1/ADD1/NPHS1/ROCK2/TNXB/ARAP1/CDK5R1/MECP2/RICTOR/ALOX15/MYADM/SPTA1/COTL1/AKAP13/TAOK1/TWF2/FHOD1/ARPC1B/MAP1A/INPP5K/TRIOBP/ARF1/WASHC2C/ROCK1/WASF2/HSPA1B/PSEN1/MAPT/CLU/PXN/HSPA1A/CTTN/PTK2B/SYNPO/ARHGEF2/HCK/GPX1/NIN/ARPC5/LIMK1/APC/DBN1/FCHSD1/PAK2/ESAM/AKAP9/SVIL/TPM1/LMOD3/SSH1/ARPC1A/HCLS1/SNCA/PAK1/RAC1/PYCARD/PLEK/CYRIB/F11R/MAP1S/PPFIA1/TMOD1/CORO1A/SSH2/WAS/VASP/CYRIA/DMTN/MTPN/PRKCD/GSN/SPTB/ARHGAP6
CC	GO:0030139	endocytic vesicle	256	-0.40	-1.68	1.07E-05	0.0001239	7.62E-05	2820	tags=37%, list=24%, signal=29%	SCARF1/CLEC4E/HLA-DRB1/ADAM8/CD9/PIKFYVE/TLR2/WNT1/RAB8A/WLS/SCARB2/CDC42EP4/LPAR1/SNAP23/RAB32/VIM/ACTG1/COLEC12/RAB35/CAMK2A/CYBB/DNM2/TAPBP/LRP1/KIF5B/TLR1/ATP7A/SLC11A1/FZD5/MCOLN1/KIAA0319/SYK/RAB43/HLA-E/RAB34/STX6/RAB22A/RIN3/DLG4/LAMP2/RAC2/TIRAP/NCF1/VPS9D1/NCF4/AP2M1/LAMP1/TLR6/ATP6V0A1/FFAR4/CSF3R/CLTC/RIN2/IGF2R/VPS11/ATP6V0D1/ANXA11/VAMP3/RAB11B/NLGN3/AP2B1/TF/RILP/SPARC/TGOLN2/RAB7A/UBB/STAM2/MYO1E/RAB11FIP4/EHD1/CUBN/AP2A1/ITGB5/NCF2/RAB5B/LYN/ARRB2/RAB11FIP1/SH3KBP1/EHD3/PICALM/CORO1A/WAS/PGLYRP1/RAB5C/RAB5A/GSN/RAB31/FLOT2/ZYX/RAPGEF2/DYSF/FMNL1/TGFA
BP	GO:2001237	negative regulation of extrinsic apoptotic signaling pathway	67	-0.56	-1.98	1.16E-05	0.0001328	8.16E-05	2247	tags=54%, list=19%, signal=44%	GSK3B/TNF/PHIP/MCL1/BIRC6/RB1CC1/LMNA/GCLM/TMBIM1/HGF/SH3RF1/PSME3/PELI3/HSPA1B/RELA/LGALS3/HSPA1A/CTTN/CTNNA1/ARHGEF2/GPX1/PRDX2/SIAH2/SRC/PF4/ITPRIP/RIPK1/BRCA1/SERPINE1/PEA15/CFLAR/HMOX1/STRADB/TCF7L2/SFRP2/BCL2L1

MF	GO:0005516	calmodulin binding	114	-0.48	-1.85	1.16E-05	0.0001331	8.18E-05	2660	tags=48%, list=23%, signal=38%	STRN/PHKA2/PDE1B/CAMKK1/MARCKSL1/KCNN1/NOS1/CALD1/PHKG1/CAMK1/MKNK1/FBXL2/DAPK2/CAMKK2/KCNQ5/PLCB1/ASPM/STRN4/DAPK1/SLC9A1/CAMK2A/ADD1/MYO9B/MYH10/RGS2/MYH11/PCYT1A/PDE1A/ATP2B1/CAMK1D/EEA1/MKNK2/INVS/CEP97/SNTB1/KCNQ1/MYO1D/MAPKAPK2/MYO5A/MYO1E/MAPKAPK3/MYH9/MYO1F/RIT1/EPB41/PPP3CA/PPP3R1/MARCKS/NRGN/MYLK/CNN2/MBP/SLC8A1/IQGAP1/IQGAP2
BP	GO:0060537	muscle tissue development	198	-0.42	-1.71	1.17E-05	0.0001342	8.25E-05	2322	tags=31%, list=20%, signal=25%	KLF5/YY1/BMPR1A/NPHS1/HNRNPU/ATF3/RGS2/MYLPF/MAML1/FHL2/AKAP13/ZFPM1/MYH11/LMNA/SAV1/CREB1/EP300/PIM1/SOX6/GREM1/MTM1/MSX1/NIBAN2/GSK3A/RARA/FKBP1A/SELENON/NR4A1/NUPR1/GPX1/BCL9/SKI/XK/PDLIM5/FLOT1/PPP1R13L/SVIL/RBPJ/JARID2/TPM1/KDM6B/LMOD3/IGF2/CHD7/ARRB2/PPP3CA/CFLAR/MAPK14/NF1/YBX3/G6PD/MTPN/PRKAR1A/KEL/FLCN/MYLK/HLX/PTEN/RIPOR2/SLC8A1/NPRL3
BP	GO:0008286	insulin receptor signaling pathway	86	-0.52	-1.91	1.19E-05	0.0001358	8.35E-05	2679	tags=49%, list=23%, signal=38%	PIK3R2/IL1B/PRKCZ/CCND3/SMARCC1/GRB2/GSK3B/RHOQ/SORL1/PRKCB/PHIP/GPLD1/PIP4K2C/SOGA1/TSC2/GNAI2/INPP5K/PID1/SHC1/PDK2/OSBPL8/GSK3A/FUT7/SOCS3/PTPRE/INSR/PTPN1/SESN3/NDEL1/EIF4EBP2/SRC/APC/ZNF106/GAB1/PIP4K2A/AKT2/ZBTB7B/IGF2/ADIPOR1/IRS2/PRKCD/FOXO4
CC	GO:0098793	presynapse	274	-0.39	-1.64	1.21E-05	0.0001376	8.46E-05	2458	tags=34%, list=21%, signal=27%	P2RX7/HAP1/ACTG1/RAB35/KIAA1109/LRRK2/PPFIBP2/PPT1/RTN4R/TBC1D24/FBXL20/SYAP1/PRKCB/DNM2/PI4K2A/CASR/CDK5R1/GRIK2/KIF1B/STXBP5/PIP5K1C/ATCAY/STX6/ITSN1/GAK/SLC6A9/APBA1/DLG4/SLC22A1/GUCY1B1/ATP2B1/SYN3/RNF40/RAB6B/STX3/AP2M1/LAMP1/SLC6A12/DISC1/RAB3B/SNAP25/ROGDI/CTBP2/SNAP29/MCTP2/PSEN1/DNMBP/ATP6V0D1/RAB27B/NR4A1/RAB11B/DNM3/NLGN3/NDEL1/SLC17A5/SYT12/NTNG2/RAB7A/STX11/PDLIM5/FBXO45/FLOT1/P2RY1/ACTB/CALM3/STXBP2/FXR2/VPS18/CTNND1/P2RX1/SH3GL1/RAB5B/MME/DMXL2/SNCA/DNAJC5/SYNJ1/PICALM/PPFIA1/NF1/EPHB2/STON2/IQSEC1/TPRG1L/RAB5A/DENND1A/NAPA/BCL2L1/RAB3D/SLC8A1/GLUL/HIP1
CC	GO:0031226	intrinsic component of plasma membrane	761	-0.31	-1.42	1.22E-05	0.000138	8.48E-05	2631	tags=28%, list=22%, signal=23%	ADAM17/KIR2DL1/SELPLG/SLC2A1/STIM1/CD177/LPAR1/JAK2/SLC25A11/SIGLEC7/NCKAP1L/HCAR3/C17orf99/SLC28A2/KIT/PSEN2/SCN1B/SLC9A1/SEMA7A/LRRC8B/GPR137B/FCER1G/TNFRSF9/P2RX7/EVI2B/CSF1R/PORCN/CACNA1I/CD70/ALCAM/PLXNB2/ERBB2/IL18RAP/TMEM150B/CYSLTR2/CYBB/RTN4R/SLC22A4/LILRB2/NPHS1/SORL1/ZDHHC2/TNF/EFNB1/PI4K2A/CASR/SLCO4C1/LRP1/IGSF6/TLR1/MAS1/KCNAB2/TMEM130/KCNJ6/NRP2/TMPRSS9/GP6/ITGAM/SLC39A4/GRIK2/STXBP5/ITGA2B/SLC11A1/SPTA1/PLSCR1/FZD5/SEMA4A/MCOLN1/MFSD12/HRH2/SLC31A1/IL10RB/CD37/CLPTM1/SLCO3A1/CR1/GPBAR1/APCDD1/HTR3B/TSPAN33/ITGA5/ITGAL/SLC6A9/CD82/ANKH/ICAM4/TNFRSF1A/SSTR2/DLG4/SLC22A1/JAM3/ATP2B1/CCR1/TMEM11/KIR2DL3/LRRC8A/NCF1/LRP12/ADGRE3/SLC12A6/NCF4/LAMP1/CNIH3/SLC6A12/TSPAN2/FCGR3B/TLR6/CEACAM5/TSPAN14/CD14/LILRA2/KLRD1/FFAR4/CSF3R/TRPC3/LTB4R/FCGR2A/SLC2A3/ABCC3/ICAM3/PSEN1/PTPRJ/PCDH1/IGF2R/ITGB2/EPHB4/LGR6/AQP9/CD63/INSR/SLC23A2/TSPAN9/SLC6A20/C5AR1/SLC6A6/NLGN3/PTK2B/IFNAR1/CXCR2/SLC17A5/SIRPA/ELAPOR1/SLC38A5/KCNQ1/NCR1/NTNG2/SELL/TMEM266/SLC24A3/PGAP6/KCNJ15/ABCC9/PECAM1/ENTPD1/AKAP9/PCDHGA8/PCDHB1/P2RY1/IL6R/CALM3/PLAUR/SELP/GP1BA/IFNAR2/SLC14A1/GYPB/P2RX1/CA4/ITGB5/NCF2/MME/NOTCH4/SLC2A9/NINJ2/LAPTM5/LYN/SIRPB1/MMD/TGM2/TYROBP/SDCBP/FCGR3A/ADIPOR1/SLC1A5/PLXNC1/KCNJ5/PTPRC/OPRL1/GYPC/TSPAN5/ABCG8/EPHB2/PTAFR/SLC4A1/LRRC4/IL17RA/SLC13A4/AQP1/ITGB3/IL13RA1/BSG/PLPPR2/SPTB/CCRL2/LILRB3/SLC8A1/RAPGEF2/TBXA2R/SLC6A8/GP9/LRFN1/AQP10

MF	GO:0019901	protein kinase binding	520	-0.34	-1.51	1.25E-05	0.0001414	8.69E-05	2032	tags=31%, list=17%, signal=26%	TRAF6/RHOA/PIK3R2/RAB8A/PRKAG2/DNAJC3/ARHGAP33/PRKCZ/INKA2/JAK2/CASP9/ANGPT1/GCN1/NEK6/CEP152/TICAM1/CCND3/OXSR1/FEZ1/TRIM21/PJA2/FLNA/GRB2/GSK3B/TPCN2/PRKCB/DNM2/UTRN/PRAM1/PKN1/CASR/CDK5R1/ELMO2/PRKAR2A/PRKD2/GP6/MAML1/RICTOR/CDK13/FZD5/PGAM1/RCC2/TWF2/SYK/PTPN6/PPP1R12A/PPM1D/DLG4/IRAK3/RB1CC1/RAC2/CDKN2D/CBL/RAB11FIP2/LYST/AGAP2/TIRAP/DUSP22/YWHAZ/ACTA2/SHC1/CLTC/CTBP2/TBC1D14/MAVS/IRAK1/BCL2L14/CCNY/SUFU/GSK3A/RARA/MAPT/RELA/RGS14/PTPRJ/FGR/ITGB2/PRR7/SQSTM1/SPAG9/PTPN1/PLK1/MAP3K5/NR3C1/NEDD9/PTK2B/HDAC7/MAD2L2/TNIP1/NEK9/SIRPA/CDKN1A/KCNQ1/SKI/MAP2K7/CASS4/ILK/SRC/APC/WDR45/TPR/PPP1R15A/MAPKAPK2/WARS1/PDLIM5/PAK2/LIMS1/HSPB1/DUSP1/ACTB/CALM3/DAZAP2/IFNAR2/PRKACA/MAPKAPK3/CIR1/CNPPD1/CTNND1/AP2A1/SASH1/GOLGA2/PPP1R12B/HCLS1/RAC1/WNK1/PLEK/ARRB2/XIAP/ADIPOR1/TUBA4A/MAP3K2/CAVIN2/MAP2K3/PTPRC/RHOG/FBXO7/STK38/MAPK14/PRKAR2B/FOXO3/PTAFR/WAS/AP1B1/CACUL1/IRS2/TCF7L2/RHOBTB1/MARCKS/STAT3/PRKAR1A/PRKCD/PINK1/BCL2L1/MSN/PTEN/TRIB1/IQGAP1/NBR1/GAB2
BP	GO:0010506	regulation of autophagy	281	-0.38	-1.63	1.34E-05	0.0001503	9.24E-05	2970	tags=38%, list=25%, signal=29%	TRIM38/DAPK2/PIP4K2B/ATP6V0B/CAMKK2/CLN3/ORMDL3/ATP6V0C/HIF1A/VPS13D/TBC1D25/EPM2A/PIK3R2/RAB8A/EIF4G1/CTSA/ERN1/DAPK1/WIPI1/GPR137B/TICAM1/TECPR1/OPTN/FEZ1/TSPO/TRIM21/LRRK2/RNF5/CAPNS1/GSK3B/TPCN2/TAB2/PIK3CB/CPTP/CDK5R1/ATP6V1C1/PIP4K2C/DHRSX/BNIP3L/CALCOCO2/FZD5/MCL1/EIF4G2/SOGA1/EXOC7/SPTLC2/TP53INP2/EP300/MTMR3/HGF/TSC2/RUBCN/MTM1/ATP6V0A1/KDM4A/TFEB/TBC1D14/ROCK1/SESN2/GSK3A/MAPT/IFI16/ULK1/SESN3/ATP6V0D1/CTTN/RALB/CLEC16A/QSOX1/NUPR1/ATG7/ELAPOR1/WDR45/IKBKG/PSAP/EXOC4/HSPB1/PIP4K2A/WAC/DAP/PRKACA/SH3GLB1/FEZ2/KAT5/GOLGA2/USP10/ATP6V1B2/SNCA/ATG2A/PYCARD/TRIM8/RRAGD/FBXO7/HMOX1/FOXO3/STAT3/MAPK3/SNX18/DCAF12/FLCN/AMBRA1/PINK1/CAPN1/CHMP4B/NPRL3/ATP6V1A
BP	GO:0007162	negative regulation of cell adhesion	193	-0.42	-1.72	1.41E-05	0.0001571	9.66E-05	2800	tags=36%, list=24%, signal=28%	ITCH/HLA-DRB1/CD9/LGALS9C/WNT1/RHOA/TMEM131L/JAK2/NCKAP1L/IL4I1/ANGPT1/TGFBI/RC3H2/ARHGDIB/SERPINE2/PLXNB2/ERBB2/LILRB2/PELI1/ABAT/GBP1/ARHGDIA/MYADM/RCC2/CR1/PTPN6/JAM3/VSIR/PDE5A/DUSP22/KNG1/ZNF703/LGALS3/BMP6/MAD2L2/PRDX2/LGALS9/SRC/TBX21/CYP1B1/DUSP1/DLG5/IRF1/ZBTB7B/ALOX12/IL1RN/SERPINE1/AKNA/NOTCH4/ACTN4/LAPTM5/WNK1/SIPA1/PLXNC1/CEBPB/PTPRC/MAP4K4/NF1/EPHB2/CORO1C/DMTN/PRKAR1A/PRKCD/HLX/PTEN/SPI1/MBP/RIPOR2/ARHGAP6
BP	GO:0120035	regulation of plasma membrane bounded cell projection organization	403	-0.35	-1.55	1.41E-05	0.0001571	9.66E-05	2708	tags=31%, list=23%, signal=25%	FES/CRTC1/CAPRIN1/WNT1/RHOA/NEURL1/SCARB2/CDC42EP4/IFT88/ADNP/LPAR1/KIT/ACTR2/SCN1B/SEMA7A/HSPA5/P2RX7/HAP1/HECW2/VIM/CD38/FEZ1/FLNA/CAPZB/KLF5/PLXNB2/LRRK2/RTN4R/GSK3B/RHOQ/PLEKHM1/DNM2/PAFAH1B1/TBC1D30/PLCE1/ARAP1/ATP7A/RGS2/CDK5R1/YWHAH/CFL1/MARK2/SEMA4A/P3H1/EIF4G2/RCC2/KIAA0319/TWF2/SYNE2/GAK/RAB21/RAC2/FSCN1/EP300/CAMK1D/DRAXIN/HGF/STK24/RTN4/DISC1/SNAP25/IFT140/CDKL5/IST1/DENND5A/WASF2/GSK3A/PSEN1/MAPT/ANKRD27/VLDLR/ULK1/SIPA1L1/AGRN/MAP4/RAP1GAP/CUX1/DNM3/MARK4/CEP97/PTK2B/NDEL1/PPP2R5B/RETREG3/NIN/NTNG2/LIMK1/SRC/APC/GRN/DBN1/DGKG/XK/PDLIM5/PAK2/SETX/PLEK2/CARM1/FXR2/EHD1/NFE2L2/LYN/PAK1/RAC1/PPP3CA/LIMK2/CFLAR/PLXNC1/RHOG/FBXO7/EPHB2/CORO1C/WAS/ABI3/RAB5A/DMTN/SFRP2/KEL/PRKCD/CCP110/PTEN/MBP/RIPOR2/RAPGEF2/DAB2

BP	GO:0043410	positive regulation of MAPK cascade	281	-0.38	-1.63	1.44E-05	0.0001594	9.80E-05	2696	tags=36%, list=23%, signal=28%	FPR2/TRAF6/IL1B/PLCB1/PRKCZ/ERN1/LPAR1/JAK2/KIT/ANGPT1/SEMA7A/P2RX7/CSF1R/C5AR2/PJA2/ERBB2/LRRK2/HIPK2/MAPKBP1/TNF/ROCK2/PLCE1/CASR/PRKD2/ALOX15/SH3RF3/ERN2/OSM/PDGFA/FZD5/LTBR/AKAP13/TAOK1/SYK/NECAB2/MAP2K2/GPBAR1/PIK3CG/GADD45A/RB1CC1/DOK4/CCR1/TIRAP/EIF2AK2/NCF1/HGF/GNAI2/PDE5A/DUSP22/TLR6/ACTA2/MTURN/MAP3K3/SHC1/FFAR4/SH3RF1/DSTYK/ROCK1/IRAK1/CCL3/PSEN1/PTPRJ/INSR/SPAG9/PTPN1/CHI3L1/MAP3K5/RASSF2/C5AR1/PTK2B/DVL3/PRDX2/LGALS9/MAP2K7/TAOK3/SRC/PIK3R5/RIPK1/P2RY1/ARHGAP8/SASH1/NDST1/LAPTM5/IGF2/GLIPR2/PAK1/SDCBP/PYCARD/ARRB2/XIAP/CFLAR/MAP2K3/PTPRC/MAPK3/PTEN/SPI1/PELI2/RAPGEF2/IQGAP1/TGFA
BP	GO:0050818	regulation of coagulation	40	-0.64	-2.05	1.46E-05	0.0001616	9.94E-05	3126	tags=60%, list=27%, signal=44%	THBD/EDN1/THBS1/ST3GAL4/CD9/ANXA5/ANO6/SERPINE2/PROS1/ABAT/PDGFA/SERPING1/KNG1/PSEN1/PRDX2/GP1BA/ALOX12/NFE2L2/SERPINE1/EPHB2/DMTN/PRKCD/KRT1/TBXA2R
BP	GO:1903305	regulation of regulated secretory pathway	90	-0.52	-1.92	1.47E-05	0.0001619	9.95E-05	2708	tags=41%, list=23%, signal=32%	FES/REST/CD177/NCKAP1L/CACNA1I/RPH3AL/LRRK2/GSK3B/FBXL20/PRKCB/PRAM1/ITGAM/STXBP5/SYK/APBA1/RAC2/GNAI2/LAMP1/FGR/ITGB2/SYT12/LGALS9/RAB27A/P2RY1/CALM3/STXBP2/VPS18/P2RX1/AP1G1/LYN/UNC13D/HMOX1/PTAFR/RAB5A/RAB3D/SPI1/GAB2
BP	GO:0015850	organic hydroxy compound transport	116	-0.48	-1.85	1.47E-05	0.0001619	9.95E-05	2374	tags=37%, list=20%, signal=30%	TSPO/ABCA2/KDM5B/TPCN2/ABAT/LRP1/ABCC4/CES1/SYK/ANXA2/NPC2/SLC22A1/SERAC1/ABCB11/SLC10A3/RAB3B/OSBPL8/ABCC3/CLU/AQP9/TSPO2/LCAT/BMP6/SYT12/VPS4B/KCNQ1/PIP4K2A/P2RY1/ACTB/P2RX1/ABCG1/SNCA/ABCG8/OSBP2/AQP1/RALBP1/ITGB3/PINK1/FURIN/DAB2/OSBPL2/OSBPL1A/AQP10
BP	GO:0007033	vacuole organization	164	-0.44	-1.74	1.48E-05	0.0001628	0.00010009	2604	tags=38%, list=22%, signal=30%	MFN2/GAA/PIKFYVE/SCARB2/IFT88/ATG2B/WIPI1/CLN6/P2RX7/FEZ1/LRRK2/PPT1/RNF5/CHMP3/TPCN2/ADPRH/MAP1LC3B/GABARAPL2/PIP4K2C/GABARAPL1/RAB43/ANXA2/RAB34/ATP6AP2/VPS33A/RB1CC1/TP53INP2/MTMR3/LYST/HOOK3/MTM1/MAP1LC3A/TFEB/TBC1D14/PSEN1/RAB1B/VPS11/ULK1/WIPI2/RALB/NUPR1/ATG7/ELAPOR1/ACP3/GRN/WDR45/RAB7A/GABARAP/GNPTAB/PIP4K2A/SH3GLB1/FEZ2/VPS18/LAPTM5/ATG2A/MAP1LC3B2/CORO1A/HPS1/RAB5A/SNX18/AMBRA1/PINK1/CHMP4B
CC	GO:0150034	distal axon	155	-0.45	-1.77	1.58E-05	0.000173	0.00010634	2451	tags=39%, list=21%, signal=31%	CCDC120/HAP1/ACTG1/FEZ1/COPA/FLNA/MYO9A/LRRK2/RTN4R/TBC1D24/SYAP1/PRKCB/DNM2/PAFAH1B1/CASR/CDK5R1/GRIK2/TWF2/ATCAY/CPEB4/EXOC7/FSCN1/CBL/FSCN3/RNF40/STX3/DISC1/SNAP25/CDKL5/PSEN1/MAPT/ATP6V0D1/PPP1R9B/CTTN/EXOC6/PTK2B/NDEL1/PTBP2/NIN/ARPC5/PALLD/DBN1/EXOC4/SETX/ACTB/CALM3/MYO5A/FXR2/CTNND1/USP9X/SNCA/SYNJ1/TPRG1L/RAB5A/PINK1/BASP1/SLC8A1/IQGAP1/TSHZ3/GLUL
BP	GO:1901214	regulation of neuron death	208	-0.41	-1.70	1.59E-05	0.0001742	0.00010708	2930	tags=42%, list=25%, signal=32%	GBE1/AIFM1/CLN3/KRAS/NAIP/HIF1A/ADAM8/CASP2/FOS/WNT1/RHOA/MAP2K4/REST/SIGMAR1/BTBD10/ADNP/EIF4G1/JAK2/CASP9/ANGPT1/C1QA/INPP5A/LRRK2/PPT1/GSK3B/HIPK2/BAX/SORL1/CTSZ/TNF/CDK5R1/MECP2/ITGAM/GRIK2/MCL1/ZNF746/MEAK7/CPEB4/RILPL1/GCLM/CBL/AGAP2/DRAXIN/GPI/TLR6/NR4A2/ROCK1/CCL3/PSEN1/MAPT/CLU/ITGB2/SLC23A2/MAP3K5/AKT1S1/PTK2B/EGLN3/NUPR1/AP2B1/EGLN2/MAP2K7/HDGF/GRN/CDC34/IKBKG/UBB/SOD2/CLCF1/TNFRSF1B/SLC7A11/CHP1/SNCA/DNAJC5/TYROBP/ARRB2/CEBPB/PICALM/FBXO7/SRPK2/HMOX1/CORO1A/NF1/FOXO3/DHCR24/G6PD/PINK1/BCL2L1/CHMP4B
BP	GO:0034446	substrate adhesion-dependent cell spreading	66	-0.56	-1.96	1.63E-05	0.0001777	0.00010922	2332	tags=42%, list=20%, signal=34%	FLNA/FERMT3/DNM2/GBP1/MYADM/RCC2/TRIOBP/WASHC2C/NEDD9/PXN/VAMP3/MEGF9/DOCK5/CASS4/NTNG2/ILK/SRC/LIMS1/PARVB/PREX1/ACTN4/UNC13D/LAMC3/RAC1/CORO1C/DMTN/ITGB3/DAB2

CC	GO:0098857	membrane microdomain	221	-0.41	-1.70	1.75E-05	0.0001904	0.00011707	2770	tags=40%, list=24%, signal=31%	MAPK1/PACSIN2/PIKFYVE/TLR2/ADAM17/INPP5D/PRKCZ/SELPLG/SLC2A1/STIM1/CD177/JAK2/PSEN2/ANGPT1/SLC9A1/LAMTOR4/CLN6/LCP2/STOML1/FADD/LRRK2/PPT1/RTN4R/RFTN1/BMPR1A/RHOQ/TNF/EFNB1/PI4K2A/TLR1/ATP7A/PRKAR2A/GP6/ITGAM/GASK1A/MYADM/PLSCR1/ANXA2/CR1/TNFRSF1A/LAMP2/ATP2B1/CBL/GNAI2/TLR6/CD14/PTGS2/PSEN1/MAPT/ITGB2/INSR/CTNNA1/PTK2B/CTSD/HCK/RGS19/KCNQ1/EFHD2/SRC/LAT2/PECAM1/KCNE3/FLOT1/RIPK1/PRKACA/TNFRSF1B/TUBA1C/P2RX1/MME/LYN/ARID3A/SDCBP/CFLAR/CAVIN2/PTPRC/HK1/STAT6/PRKAR2B/HMOX1/CORO1C/RGS7/RAB5A/MAPK3/PRKAR1A/BSG/NFAM1/FLOT2/PLVAP/FURIN
BP	GO:0055001	muscle cell development	93	-0.51	-1.89	1.93E-05	0.0002089	0.00012843	2275	tags=34%, list=19%, signal=28%	YY1/BMPR1A/HNRNPU/RGS2/MAML1/FHL2/AKAP13/MYH11/LMNA/NIBAN2/GSK3A/CAPN3/SELENON/GPX1/SKI/ACTN1/XK/PDLIM5/TPM1/LMOD3/ACTN4/ADM/PPP3CA/CFLAR/TMOD1/G6PD/PRKAR1A/KEL/MYL9/RIPOR2/SLC8A1/DYSF
BP	GO:0001817	regulation of cytokine production	528	-0.33	-1.50	1.99E-05	0.0002142	0.0001317	3059	tags=36%, list=26%, signal=28%	IL18R1/DDX21/IGF2BP3/BCL6/ZNF580/FERMT1/TYK2/CSK/HOMER3/TRIM38/STAT1/THBS1/OAS3/BST2/PML/BAP1/RELB/ITCH/NLRP10/GSTP1/CLEC4E/HLA-DRB1/HIF1A/AKIRIN2/ADAM8/NLRP12/TLR2/LGALS9C/DICER1/TRAF6/IL1B/PLCB1/ADAM17/INPP5D/PRKCZ/CCDC88B/SETD2/JAK2/NCKAP1L/KIT/ANGPT1/SEMA7A/ALOX5/POU2F2/FCER1G/TICAM1/P2RX7/CSF1R/DDX3X/SETD4/C5AR2/TSPO/TRIM21/LTBP1/FADD/BTNL9/IL1R2/LRRK2/YY1/CYBB/REL/RFTN1/ARID5A/BMPR1A/LILRB2/CCBE1/MAPKBP1/SPON2/SORL1/BTN3A2/PELI1/TNF/CPTP/ROCK2/MNDA/GBP1/TLR1/TLR8/PRKD2/OSM/SLC11A1/FZD5/ORM1/SYK/NMI/ZFPM1/CR1/HLA-E/PIK3CG/ELF1/ATP6AP2/PTPN6/FCN1/IRAK3/CLEC9A/ATP2B1/AGPAT1/SULF2/TIRAP/EIF2AK2/HGF/VSIR/TLR6/CD14/LILRA2/STAT5B/RSAD2/FFAR4/FRMD8/OTUD7B/ERMAP/N4BP1/MAVS/PTGS2/ARNT/RPS6KA5/IRAK1/HSPA1B/GPATCH3/CCL3/PSEN1/RARA/RELA/PTPRJ/FGR/ERBIN/CLU/IFI16/CHI3L1/C5AR1/HSPA1A/ARHGEF2/RIOK3/HDAC7/SIRPA/LGALS9/SRC/TBX21/PF4/MAPKAPK2/HSPB1/FLOT1/CYP1B1/RIPK1/IL6R/IRF1/ZBTB7B/TNFRSF1B/BRCA1/OTUD5/KAT5/SERPINE1/STMP1/MAST2/ACKR1/LAPTM5/NLRC4/LYN/ILRUN/TYROBP/RAC1/PYCARD/ARRB2/PANX2/GPSM3/CYRIB/PIK3CD/F11R/MAP2K3/CEBPB/PTPRC/HK1/MAPK14/HMOX1/EPHB2/PTAFR/PGLYRP1/IL17RA/STAT3/NLRX1/NFAM1/IGF2BP2/BCL3/MBP/IQGAP1/FURIN/LITAF/SLC7A5
BP	GO:0034762	regulation of transmembrane transport	293	-0.37	-1.60	2.00E-05	0.0002151	0.00013222	2727	tags=35%, list=23%, signal=28%	SLC26A6/ALG10B/CLIC1/ACSL1/IL1B/CACNA1A/KCNQ5/PRKAG2/ANO6/STIM1/SIRT6/DAPK1/CACNB1/PSEN2/SCN1B/SLC9A1/P2RX7/CLIC3/HAP1/OXSR1/HECW2/CACNA1I/AHCYL1/HK2/FLNA/CA2/CYBB/TPCN2/BAX/RHOQ/PRKCB/DNM2/TNF/UTRN/GJC2/ATP7A/KCNAB2/RGS2/PPIF/KCNJ6/YWHAH/CATSPER2/SLC43A2/PIK3CG/SLC6A9/PTPN6/DLG4/PIM1/RNASEL/CNIH3/CACNA1D/INPP5K/PID1/FFAR4/OSBPL8/GSK3A/PSEN1/FKBP1A/CAPN3/CD63/INSR/SELENON/DIAPH1/WWP2/NLGN3/PTK2B/TESC/KCNQ1/CACNB4/KCNJ15/KCNE3/AKAP9/RIPK1/ACTB/CALM3/PRKACA/AKT2/NFE2L2/P2RX1/ACTN4/CHP1/LYN/PEA15/SNCA/CHD7/WNK1/OAZ1/MMP9/EHD3/OAZ2/KCNJ5/RTN2/OPRL1/MAPK14/CORO1A/EPHB2/PTAFR/RGS7/G6PD/IRS2/KEL/PINK1/CAPN1/SLC8A1

CC	GO:0097447	dendritic tree	336	-0.37	-1.60	2.03E-05	0.0002176	0.00013375	2725	tags=35%, list=23%, signal=28%	FARP1/CRTC1/CAPRIN1/RHOA/RAB8A/USP8/STRN4/NEURL1/ADNP/PSEN2/DYRK1A/HAP1/INPP5A/CAMK2A/FEZ1/ALCAM/SNX14/CKB/LRRK2/PPT1/CYBB/RTN4R/GSK3B/BMPR1A/SYAP1/MAGEE1/HNRNPU/PI4K2A/KIF5B/ATP7A/CDK5R1/GRIK2/KIF1B/CPEB3/MARK2/FZD5/HRH2/NECAB2/ATCAY/ABR/ATP6AP2/RIN3/CPEB4/APBA1/TRAK1/DLG4/GRIPAP1/SAV1/ATP2B1/ERO1A/NCF1/GNAI2/STX3/CNIH3/DTNB/MAP1A/GIGYF2/ROGDI/CDKL5/GSK3A/PSEN1/RARA/MAPT/RGS14/CLU/PRR7/KIF1C/INSR/SIPA1L1/MAX/AGO2/RAP1GAP/PPP1R9B/ABHD17C/CTTN/SLC6A6/DNM3/MARK4/PTK2B/SYNPO/RAB27A/NIN/ACTN1/MYO1D/TMEM266/DBN1/KCNE3/AKAP9/MARK3/P2RY1/PRKACA/FXR2/KPNA1/CTNND1/MME/SLA/GLRX5/SHARPIN/PAK1/RAC1/ARRB2/PPP3CA/MAP1S/ASAP1/PPFIA1/PRKAR2B/NF1/EPHB2/LRRC4/OSBP2/ABI3/RAB5A/TRAK2/NRGN/SLC8A1/FLOT2/CASC3/STAU1/GNAZ
BP	GO:0045639	positive regulation of myeloid cell differentiation	73	-0.54	-1.93	2.05E-05	0.0002186	0.00013441	3103	tags=56%, list=26%, signal=42%	CD4/MED1/GATA1/STAT1/HLA-DRB1/HIF1A/FES/FOS/TRAF6/INPP5D/NCKAP1L/PPARGC1B/EVI2B/FADD/TNF/CREB1/CCR1/STAT5B/MTURN/ARNT/HSPA1B/SMAP1/HSPA1A/TESC/PF4/RIPK1/ZFP36L1/TAL1/RCOR1/CTNNBIP1/HCLS1/TYROBP/PITHD1/MAPK14/FAXDC2/FOXO3/FAM210B/STAT3/PRKDC/MPL/TRIB1
BP	GO:0070527	platelet aggregation	53	-0.60	-2.01	2.11E-05	0.0002221	0.00013655	2819	tags=60%, list=24%, signal=46%	GNAS/CD9/CLIC1/JAK2/ACTG1/FLNA/SERPINE2/FERMT3/METAP1/PIK3CB/ABAT/GP6/SYK/PIK3CG/PTPN6/ILK/HSPB1/ACTB/GP1BA/ALOX12/SLC7A11/MYH9/LYN/PLEK/F11R/TLN1/DMTN/ITGB3/MPL/PRKCD/MYL9/VCL
BP	GO:0061564	axon development	255	-0.39	-1.63	2.10E-05	0.0002221	0.00013655	2658	tags=35%, list=23%, signal=28%	RAB8A/RAPH1/LYPLA2/NEXN/TCTN1/ADNP/JAK2/SCN1B/SEMA7A/SIN3A/CSF1R/KLF7/FEZ1/TSPO/ALCAM/FLNA/PLXNB2/ERBB2/RTN4R/GSK3B/DNM2/PAFAH1B1/EFNB1/KIF5B/CDK5R1/NRP2/RNF165/MARK2/SEMA4A/EIF4G2/KIAA0319/TWF2/RAB21/CREB1/RAC2/NCAM1/DRAXIN/STK24/RTN4/TSPAN2/DISC1/MAP1A/NIBAN2/NUMB/NR4A2/CDKL5/IST1/RPS6KA5/PSEN1/MAPT/EPHB4/ULK1/LGR6/B4GALT5/SIPA1L1/CTTN/CTNNA1/NLGN3/NDEL1/NIN/NTNG2/PALLD/APLP2/SPRY3/LIMK1/DPYSL2/GRN/DBN1/XK/PAK2/FBXO45/FLOT1/ACTB/FEZ2/USP9X/PAK1/RAC1/PLXNC1/PICALM/RHOG/MAP1S/EPHB2/VASP/KEL/BSG/PTEN/VCL/MBP/ECE1
CC	GO:0031234	extrinsic component of cytoplasmic side of plasma membrane	69	-0.55	-1.96	2.12E-05	0.0002233	0.00013728	2853	tags=49%, list=24%, signal=38%	KRAS/GNAS/GNA13/FARP1/FES/RHOA/GNB2/GNG5/SYAP1/KCNAB2/ALOX15/SYK/DLG4/TGM3/GNAI2/SNAP25/GNB1/FGR/GNA12/GNAQ/PTK2B/HCK/SRC/GNAL/SLA/LYN/RAC1/SNX18/GNB4/IQGAP1/MYZAP/OSBPL2/HIP1/GNAZ

BP	GO:0032880	regulation of protein localization	605	-0.32	-1.46	2.15E-05	0.0002251	0.00013836	2679	tags=30%, list=23%, signal=25%	PIK3R2/IL1B/RAB8A/REST/WLS/USP8/PRKCZ/VTI1B/SETD2/PLEKHM2/JAK2/FIS1/ANGPT1/ALOX5/SIN3A/VPS28/GPR137B/CEP295/P2RX7/KLF7/CD38/TRIM21/RPH3AL/WAPL/FLNA/ERBB2/MIEF2/ABCA2/LRRK2/GSK3B/RHOQ/SORL1/RIPOR1/ZDHHC2/TNF/ROCK2/NSD2/ABAT/MYH10/CASR/GBP1/LRP1/KIF5B/NKX3-1/KCNAB2/GPLD1/CDK5R1/UCP2/TRIM25/ITGAM/BNIP3L/ARHGDIA/STXBP5/PCM1/FZD5/MEAK7/P3H1/TMEM231/NECAB2/ATCAY/GPR27/STX6/UBR5/DLG4/GRIPAP1/LMNA/TP53INP2/RAC2/LRRC8A/TMBIM1/OS9/RTN4/STX3/AP2M1/MAP1A/SOX4/INPP5K/SIDT2/GNL3L/SNAP25/PID1/RSAD2/CLTC/OTUD7B/NUMB/MAVS/PTGS2/SESN2/SYTL4/SUFU/GSK3A/PSEN1/MAPT/FKBP1A/ITGB2/VPS11/SQSTM1/PTPN1/PLK1/RASSF5/LGALS3/PTP4A3/PPP1R9B/ABHD17C/ANKRD13A/RAB11B/WWP2/CTNNA1/HMGCR/BMP6/MARK4/NDEL1/CTSD/VCP/ZDHHC7/AP2B1/DVL3/ACSL4/PPP2R5A/SRC/APC/CPT1A/TPR/FRAT1/HSPA1L/PECAM1/ACTB/PRKACA/AKT2/SH3GLB1/RABGAP1L/SLC7A11/MEPCE/EPB41/AFDN/NPEPPS/ABCG1/CHP1/MMD/ILRUN/HCLS1/C2CD2L/TYROBP/OAZ1/TRIM8/MIDN/RAB11FIP1/BBC3/PPP3CA/UBE2D3/LIMK2/OAZ2/DTX3L/PICALM/RHOG/LCP1/PPFIA1/MAPK14/MYOM1/DNAJB6/IQSEC2/SP100/NF1/EPHB2/ABI3/ABLIM3/IRS2/TCF7L2/TBC1D1/DMTN/ITGB3/FLCN/PRKCD/GSN/PINK1/BCL2L1/PPM1A/MSN/VCL/SPI1/RIPOR2/DAB2/GLUL
BP	GO:1903530	regulation of secretion by cell	320	-0.37	-1.58	2.26E-05	0.0002366	0.00014544	2800	tags=32%, list=24%, signal=25%	HLA-DRB1/HIF1A/ADAM8/TLR2/FES/IL1B/RAB8A/REST/WLS/CD177/JAK2/NCKAP1L/ALOX5/P2RX7/HAP1/KLF7/CD38/CACNA1I/CAMK2A/RPH3AL/LRRK2/KDM5B/GSK3B/TPCN2/FBXL20/PRKCB/TNF/PRAM1/ABAT/PTGES/MYH10/CASR/NKX3-1/GPLD1/UCP2/ITGAM/OSM/STXBP5/P3H1/SYK/GPR27/TNFRSF1A/RAB21/APBA1/CREB1/RAC2/LRRC8A/GNAI2/LAMP1/SOX4/RAB3B/SIDT2/SNAP25/RSAD2/FFAR4/MCTP2/SYTL4/FGR/ITGB2/RAB27B/VAMP3/HMGCR/BMP6/SYT12/VPS4B/LGALS9/RAB27A/ACSL4/CPT1A/RAB7A/RAB2B/P2RY1/CALM3/STXBP2/TNFRSF1B/VPS18/P2RX1/AP1G1/ABCG1/LYN/UNC13D/SNCA/CHD7/C2CD2L/SDCBP/MIDN/RAB11FIP1/PPP3CA/MYOM1/HMOX1/NF1/PTAFR/CHMP2A/IRS2/TCF7L2/RAB5A/PINK1/RAB3D/SPI1/DAB2/GLUL/GAB2
BP	GO:0090100	positive regulation of transmembrane receptor protein serine/threonine kinase signaling pathway	64	-0.56	-1.93	2.28E-05	0.0002373	0.00014587	3015	tags=42%, list=26%, signal=32%	NEO1/FERMT1/THBS1/ADAM17/JAK2/ZEB2/BMPR1A/HIPK2/RNF165/ZC3H3/KIAA0319/CDKN1C/EP300/MSX1/LRG1/NUP93/BMP6/ILK/TGFB1I1/UBE2O/RBPJ/PELO/SDCBP/CREBBP/FLCN/FKBP8/DAB2
BP	GO:0010975	regulation of neuron projection development	260	-0.39	-1.63	2.31E-05	0.0002394	0.00014719	2931	tags=35%, list=25%, signal=27%	ENC1/KATNB1/KIDINS220/SRF/SCARF1/SHTN1/FES/CRTC1/CAPRIN1/RHOA/SCARB2/ADNP/LPAR1/ACTR2/SCN1B/SEMA7A/HSPA5/HECW2/VIM/CD38/FEZ1/FLNA/PLXNB2/LRRK2/RTN4R/GSK3B/PAFAH1B1/RGS2/CDK5R1/YWHAH/CFL1/MARK2/SEMA4A/P3H1/EIF4G2/KIAA0319/TWF2/GAK/RAB21/EP300/CAMK1D/DRAXIN/HGF/STK24/RTN4/DISC1/SNAP25/CDKL5/IST1/DENND5A/GSK3A/PSEN1/MAPT/ANKRD27/VLDLR/ULK1/SIPA1L1/CUX1/DNM3/PTK2B/NDEL1/PPP2R5B/RETREG3/NIN/NTNG2/LIMK1/GRN/DBN1/DGKG/XK/PDLIM5/PAK2/SETX/CARM1/EHD1/NFE2L2/LYN/PAK1/PPP3CA/CFLAR/PLXNC1/FBXO7/EPHB2/ABI3/SFRP2/KEL/PTEN/MBP/RAPGEF2/DAB2
BP	GO:0002064	epithelial cell development	117	-0.47	-1.82	2.36E-05	0.0002441	0.00015006	2416	tags=36%, list=21%, signal=29%	IL1B/PLCB1/VEZF1/TMEM79/VIM/KLF5/GSK3B/ADD1/NPHS1/YIPF6/TNF/ROCK2/S1PR2/MYADM/TNFRSF1A/RILPL1/ARID4A/SIDT2/ACTA2/ROCK1/ATRX/GSK3A/RARA/BMP6/B4GALT1/GPX1/PALLD/SRC/PECAM1/MYO1E/AFDN/NOTCH4/TBC1D20/F11R/TMOD1/RILPL2/MSN/VCL/FOSL2/RAPGEF2/IQGAP1/CLDN5

BP	GO:0045216	cell-cell junction organization	113	-0.49	-1.87	2.46E-05	0.0002522	0.00015505	2764	tags=35%, list=24%, signal=27%	CD9/RHOA/IL1B/CD177/CSF1R/ACTG1/PKP4/FLNA/ADD1/TNF/ROCK2/HIPK1/FZD5/PIP5K1C/GPBAR1/JAM3/FSCN1/NUMB/ROCK1/ZNF703/CTNNA1/BMP6/HDAC7/SRC/APC/PAK2/ESAM/LIMS1/PECAM1/ACTB/DLG5/PRKACA/CTNND1/AFDN/F11R/EPHB2/TLN1/FLCN/VCL/CLDN5
BP	GO:0048667	cell morphogenesis involved in neuron differentiation	305	-0.37	-1.59	2.45E-05	0.0002522	0.00015505	3027	tags=36%, list=26%, signal=28%	EDN1/NEO1/EPHB6/TIAM1/CDH23/SZT2/TAOK2/KIDINS220/SRF/WDPCP/SHTN1/FARP1/CAPRIN1/RAB8A/RAPH1/LYPLA2/NEXN/TCTN1/ADNP/ACTR2/SCN1B/SEMA7A/SIN3A/CSF1R/HECW2/KLF7/FEZ1/ALCAM/PLXNB2/ERBB2/LRRK2/RTN4R/GSK3B/DNM2/PAFAH1B1/EFNB1/CFAP418/KIF5B/CDK5R1/NRP2/RNF165/YWHAH/CFL1/MARK2/SEMA4A/EIF4G2/KIAA0319/TWF2/RAB21/DLG4/CREB1/RAC2/RERE/NCAM1/DRAXIN/RTN4/DISC1/MAP1A/NIBAN2/TRIOBP/NUMB/NR4A2/CDKL5/IST1/RPS6KA5/PSEN1/MAPT/ANKRD27/VLDLR/EPHB4/ULK1/LGR6/B4GALT5/SIPA1L1/CUX1/CTTN/DNM3/NLGN3/NDEL1/NIN/NTNG2/PALLD/APLP2/LIMK1/DPYSL2/DBN1/XK/PDLIM5/PAK2/FBXO45/FLOT1/ACTB/FEZ2/USP9X/PAK1/RAC1/PPP3CA/PLXNC1/PICALM/RHOG/MAP1S/EPHB2/ABI3/VASP/KEL/BSG/PTEN/VCL/MBP/RAPGEF2/ECE1
CC	GO:0045335	phagocytic vesicle	124	-0.46	-1.81	2.52E-05	0.0002577	0.00015844	3097	tags=44%, list=26%, signal=32%	HVCN1/SEC22B/ABCA1/RAB11FIP5/ATP6V0B/ATP6V0C/CLEC4E/ADAM8/PIKFYVE/TLR2/RAB8A/CDC42EP4/SNAP23/RAB32/VIM/ACTG1/CYBB/DNM2/TAPBP/KIF5B/TLR1/ATP7A/SLC11A1/MCOLN1/SYK/RAB43/HLA-E/RAB34/STX6/RAB22A/LAMP2/RAC2/NCF1/NCF4/LAMP1/TLR6/ATP6V0A1/ATP6V0D1/ANXA11/VAMP3/RAB11B/RILP/RAB7A/ITGB5/NCF2/RAB11FIP1/CORO1A/WAS/PGLYRP1/RAB5A/GSN/RAB31/ZYX/FMNL1
BP	GO:0045071	negative regulation of viral genome replication	49	-0.59	-1.97	2.57E-05	0.0002611	0.00016054	3156	tags=51%, list=27%, signal=37%	SRPK1/CCL5/APOBEC3A/OAS3/BST2/RESF1/APOBEC3C/IFITM3/TNF/PLSCR1/SETDB1/EIF2AK2/RNASEL/INPP5K/RSAD2/N4BP1/MAVS/IFI16/ZC3HAV1/TNIP1/OASL/AICDA/ZNFX1/SRPK2/IFITM2
BP	GO:0061572	actin filament bundle organization	108	-0.48	-1.82	2.57E-05	0.0002611	0.00016054	1717	tags=29%, list=15%, signal=25%	FHOD1/FSCN1/FSCN3/RFLNB/CUL3/INPP5K/ROCK1/WASF2/NEDD9/PXN/ESPN/PTK2B/SYNPO/LIMK1/ACTN1/SRC/PAK2/TPM1/ITGB5/PAK1/RAC1/PLEK/SHROOM1/F11R/LCP1/PPFIA1/WAS/MARCKS/DMTN/ZYX/ARHGAP6
CC	GO:0045121	membrane raft	220	-0.41	-1.69	2.61E-05	0.0002638	0.00016216	2770	tags=40%, list=24%, signal=31%	MAPK1/PACSIN2/PIKFYVE/TLR2/ADAM17/INPP5D/PRKCZ/SELPLG/SLC2A1/STIM1/CD177/JAK2/PSEN2/ANGPT1/SLC9A1/LAMTOR4/CLN6/LCP2/STOML1/FADD/LRRK2/PPT1/RTN4R/RFTN1/BMPR1A/RHOQ/TNF/EFNB1/PI4K2A/TLR1/ATP7A/PRKAR2A/GP6/ITGAM/GASK1A/MYADM/PLSCR1/ANXA2/CR1/TNFRSF1A/ATP2B1/CBL/GNAI2/TLR6/CD14/PTGS2/PSEN1/MAPT/ITGB2/INSR/CTNNA1/PTK2B/CTSD/HCK/RGS19/KCNQ1/EFHD2/SRC/LAT2/PECAM1/KCNE3/FLOT1/RIPK1/PRKACA/TNFRSF1B/TUBA1C/P2RX1/MME/LYN/ARID3A/SDCBP/CFLAR/CAVIN2/PTPRC/HK1/STAT6/PRKAR2B/HMOX1/CORO1C/RGS7/RAB5A/MAPK3/PRKAR1A/BSG/NFAM1/FLOT2/PLVAP/FURIN
BP	GO:0007409	axonogenesis	229	-0.40	-1.66	2.63E-05	0.0002651	0.00016295	2658	tags=35%, list=23%, signal=28%	RAB8A/RAPH1/LYPLA2/NEXN/TCTN1/ADNP/SCN1B/SEMA7A/SIN3A/CSF1R/KLF7/FEZ1/ALCAM/PLXNB2/ERBB2/RTN4R/GSK3B/DNM2/PAFAH1B1/EFNB1/KIF5B/CDK5R1/NRP2/RNF165/MARK2/SEMA4A/EIF4G2/KIAA0319/TWF2/RAB21/CREB1/RAC2/NCAM1/DRAXIN/RTN4/DISC1/MAP1A/NIBAN2/NUMB/NR4A2/CDKL5/IST1/RPS6KA5/PSEN1/MAPT/EPHB4/ULK1/LGR6/B4GALT5/SIPA1L1/CTTN/NLGN3/NDEL1/NIN/NTNG2/PALLD/APLP2/LIMK1/DPYSL2/DBN1/XK/PAK2/FBXO45/FLOT1/ACTB/FEZ2/USP9X/PAK1/RAC1/PLXNC1/PICALM/RHOG/MAP1S/EPHB2/VASP/KEL/BSG/PTEN/VCL/MBP/ECE1

CC	GO:0031012	extracellular matrix	182	-0.41	-1.67	2.69E-05	0.0002704	0.00016621	2519	tags=36%, list=21%, signal=28%	ANGPT1/SEMA7A/TGFBI/C1QA/DGCR6/COLEC12/LTBP1/SERPINE2/CRISP3/CRISPLD2/ALPL/CCBE1/SPON2/CTSZ/TNXB/SDC3/GPLD1/PKM/PLSCR1/P3H1/ORM1/ANXA2/F13A1/LAMA4/FCN1/SERAC1/PI3/SERPING1/NCAM1/GREM1/KNG1/VCAN/ERBIN/CLU/CHI3L1/AGRN/LGALS3/ANXA11/TIMP1/TFIP11/MEGF9/CTSD/LGALS9/COL9A3/NTNG2/SPARC/HDGF/PF4/PSAP/TGFB1I1/GP1BA/SERPINE1/ADAMTS4/GFOD2/TGM2/LAMC3/MMP9/TIMP2/CTSB/PHOSPHO1/SFRP2/PCSK6/HSD17B12/KRT1/LTBP2
BP	GO:0044089	positive regulation of cellular component biogenesis	349	-0.37	-1.59	2.71E-05	0.0002717	0.00016701	2708	tags=34%, list=23%, signal=27%	FES/WNT1/RHOA/NEURL1/SLAIN2/CDC42EP4/ADNP/EIF4G1/LPAR1/NCKAP1L/CNOT1/KIT/ACTR2/SIN3A/WIPI1/CEP295/P2RX7/HAP1/DDX3X/GRB2/CLIP1/GSK3B/BAX/RHOQ/NPHS1/PLEKHM1/DNM2/TNF/ROCK2/PLCE1/ARAP1/ATP7A/CDK5R1/MECP2/PIP4K2C/RICTOR/ALOX15/SEMA4A/SETDB1/TWF2/SYK/FHOD1/ATF7IP/RAC2/FSCN1/TIRAP/TLR6/GNL3L/CNOT6/WASHC2C/ROCK1/BID/WASF2/HSPA1B/MAPT/PTPRJ/CLU/ULK1/AGRN/LGALS3/PXN/HSPA1A/CTTN/RALB/DNM3/MARK4/NLGN3/PTK2B/SYNPO/NDEL1/VCP/HCK/VPS4B/PPP2R5B/ELAPOR1/NIN/LIMK1/SLX1B/SRC/APC/WDR45/TPR/RAB7A/CDC34/WARS1/FCHSD1/TPPP3/LIMS1/PIP4K2A/AKAP9/FLOT1/DLG5/PLEK2/SH3GLB1/TPM1/TAL1/STMP1/SNCA/ATG2A/PAK1/SDCBP/RAC1/PYCARD/PLEK/MAP4K4/LCP1/ASAP1/EPHB2/CHMP2A/VASP/MED25/SNX18/AMBRA1/PINK1/CCP110/MSN/RIPOR2/CLDN5
BP	GO:0032870	cellular response to hormone stimulus	387	-0.35	-1.54	2.73E-05	0.0002733	0.000168	2697	tags=35%, list=23%, signal=28%	FOS/WNT1/RHOA/PIK3R2/ACSL1/IL1B/LPIN2/RAB8A/REST/PLCB1/USP8/PRKCZ/JAK2/CNOT1/CASP9/KIT/PPARGC1B/SLC9A1/CCND3/PAQR8/AGTRAP/JAK1/CAMK2A/AHCYL1/SMARCC1/CA2/CYSLTR2/GRB2/GSK3B/RNF14/ARID5A/RHOQ/STAT5A/NCOR1/SYAP1/SORL1/PRKCB/HNRNPU/PHIP/ROCK2/PKN1/ARID1A/MAS1/NKX3-1/GPLD1/UCP3/UCP2/PIP4K2C/PRCP/YWHAH/PKM/SSTR2/SOGA1/UBR5/SP1/ATP2B1/EP300/GCLM/TSC2/GNAI2/INPP5K/STAT5B/PID1/SHC1/PDK2/GNB1/NR4A2/OSBPL8/ROCK1/HSPA1B/GSK3A/RARA/RELA/GOT1/VPS11/FUT7/SOCS3/PTPRE/INSR/PTPN1/SESN3/ABHD2/MAX/NR3C1/PXN/HSPA1A/NR4A1/MBD5/NDEL1/ZDHHC7/EGLN2/EIF4EBP2/SRC/APC/NCOA2/ZNF106/GAB1/PIP4K2A/CYP1B1/GAS2L1/ZFP36L1/PADI2/MYO5A/ZBTB7A/CARM1/PRKACA/AKT2/RXRA/ZBTB7B/BRCA1/VPS18/NFE2L2/POR/ZMIZ1/LYN/IGF2/PAK1/ADIPOR1/NCOA4/CFLAR/STAT6/FBP1/CTSB/NCOA1/FOXO3/WBP2/IRS2/AQP1/STAT3/PRKDC/PRKCD/WDTC1/RAB31/FOXO4/DAB2
BP	GO:0017157	regulation of exocytosis	131	-0.46	-1.79	2.77E-05	0.0002763	0.00016983	2708	tags=38%, list=23%, signal=30%	FES/RAB8A/REST/CD177/NCKAP1L/HAP1/CACNA1I/RPH3AL/LRRK2/GSK3B/TPCN2/FBXL20/PRKCB/PRAM1/ITGAM/STXBP5/SYK/RAB21/APBA1/RAC2/GNAI2/LAMP1/RAB3B/SYTL4/FGR/ITGB2/RAB27B/SYT12/VPS4B/LGALS9/RAB27A/RAB7A/RAB2B/P2RY1/CALM3/STXBP2/VPS18/P2RX1/AP1G1/LYN/UNC13D/SNCA/SDCBP/HMOX1/PTAFR/CHMP2A/RAB5A/RAB3D/SPI1/GAB2
BP	GO:1901615	organic hydroxy compound metabolic process	305	-0.37	-1.59	2.77E-05	0.0002763	0.00016983	2671	tags=31%, list=23%, signal=25%	PECR/ACOT8/REST/PLCB1/PRKAG2/SCAP/PCBD2/ZEB2/KIT/CYB5R1/CLN6/INPP5A/CEBPA/ABCA2/ALDH2/CYP27A1/IMPA2/PSAT1/ALPL/TNF/PCBD1/ABAT/MAS1/ATP7A/ACLY/MECP2/ITGAM/ALOX15/CPQ/CES1/MFSD12/LBR/PDXK/NPC2/SPTLC2/ABCB11/CYP3A5/INPP5K/ERLIN1/NR4A2/MOGAT3/ACSS2/GOT1/VLDLR/RDH11/ITGB2/IPMK/HDLBP/LCAT/HMGCR/BMP6/PTK2B/SULT1A1/SULT1B1/AMACR/CYB5R3/BPNT2/ACP3/CYP4F3/ALDH3B1/APOL2/CYP1B1/DPM2/P2RY1/NUDT4/GDE1/RXRA/ALOX12/DGAT2/CUBN/SLC7A11/POR/RETSAT/ABCG1/DHRS13/SNCA/PLEK/SPTLC3/SYNJ1/CTSB/FAXDC2/ITPK1/PTAFR/PNPLA2/DHCR24/G6PD/HAGH/MTPN/PTEN/GPD2/APOBR/RAPGEF2/DAB2/OSBPL2/OSBPL1A

BP	GO:0043299	leukocyte degranulation	65	-0.55	-1.93	2.79E-05	0.0002768	0.00017018	2708	tags=46%, list=23%, signal=36%	FES/CD177/NCKAP1L/SNAP23/KIT/PRAM1/ITGAM/SYK/PIK3CG/RAC2/CBL/LAMP1/CCL3/FGR/ITGB2/HCK/LGALS9/RAB27A/LAT2/STXBP2/PTGDS/AP1G1/LYN/UNC13D/PIK3CD/HMOX1/CORO1A/PTAFR/SPI1/GAB2
BP	GO:0050808	synapse organization	201	-0.41	-1.67	2.93E-05	0.0002894	0.00017793	2725	tags=36%, list=23%, signal=28%	FARP1/TLR2/CAPRIN1/RHOA/REST/NEURL1/NEUROD2/ADNP/EIF4G1/CACNB1/ACTR2/C1QA/ACTG1/FLNA/PLXNB2/ERBB2/CHCHD10/LRRK2/PPFIBP2/ZDHHC12/NFIA/LILRB2/ZDHHC2/PAFAH1B1/TNF/CDK5R1/NRP2/MECP2/ITGAM/CFL1/FZD5/SEMA4A/DLG4/GRIPAP1/CAST/TUBA1A/DISC1/YWHAZ/ARF1/CTBP2/CDKL5/WASF2/PSEN1/MAPT/INSR/SIPA1L1/AGRN/ADGRF1/C5AR1/ABHD17C/CTTN/DNM3/NLGN3/SYNPO/VCP/NTNG2/ACTN1/DBN1/PDLIM5/CACNB4/FBXO45/ACTB/DLG5/SLC7A11/SNCA/SDCBP/PPFIA1/MAPK14/EPHB2/LRRC4/ABI3/PTEN/STAU1
BP	GO:0010639	negative regulation of organelle organization	264	-0.38	-1.61	2.93E-05	0.0002894	0.00017793	2376	tags=30%, list=20%, signal=24%	FEZ1/TSPO/DCP2/WAPL/CAPZB/LRRK2/LILRB2/ADD1/HNRNPU/DNM2/TBC1D30/ARAP1/PPIF/MYADM/SPTA1/TAOK1/TWF2/LMNA/HGF/TSC2/INPP5K/MTM1/GNL3L/TRIOBP/ZW10/WASHC2C/ATRX/XRCC1/WASF2/MAPT/ANKRD27/CLU/PLK1/CCNF/MAP4/HSPA1A/H3-3A/CEP97/ARHGEF2/MAD2L2/NUPR1/GPX1/SLX1B/SRC/APC/TPR/RAB7A/PAK2/DUSP1/SVIL/FEZ2/BRCA1/PRELID1/LMOD3/SSH1/SNCA/ARRB2/CYRIB/F11R/LIMK2/H3-3B/PPFIA1/TMOD1/CORO1A/SSH2/WAS/DMTN/MTPN/RAD21/FLCN/PRKCD/GSN/PINK1/BCL2L1/CCP110/SPTB/CHMP4B/ARHGAP6
BP	GO:0046777	protein autophosphorylation	174	-0.43	-1.72	2.96E-05	0.0002909	0.00017885	3091	tags=41%, list=26%, signal=31%	MKNK1/CLK3/RAP2C/CSK/DAPK2/TAOK2/CAMKK2/PIM3/NLRP12/UHMK1/PIKFYVE/FES/ERN1/JAK2/DAPK1/DYRK1B/KIT/NEK6/DYRK1A/OXSR1/CSF1R/DDX3X/TAF1/CAMK2A/ERBB2/LRRK2/GSK3B/PRKD2/MARK2/PDGFA/TAOK1/SYK/MAP2K2/IRAK3/RB1CC1/STK4/PIM1/EIF2AK2/STK24/GREM1/MKNK2/MAP3K3/CDKL5/IRAK1/FGR/EPHB4/ULK1/INSR/RASSF2/PTK2B/HCK/SLK/PPP2R5B/TAOK3/SRC/MAPKAPK2/PAK2/EIF2AK1/RIPK1/MARK3/CALM3/PRKACA/MAPKAPK3/AATK/CHP1/LYN/PAK1/PTPRC/BMX/STK10/MAPK3/PINK1
BP	GO:0031331	positive regulation of cellular catabolic process	369	-0.35	-1.55	3.08E-05	0.0003028	0.00018612	2461	tags=31%, list=21%, signal=25%	TICAM1/P2RX7/RC3H2/TAF1/OPTN/CEBPA/TRIM21/DCP2/ZYG11B/TNRC6B/ABCA2/PTTG1IP/LRRK2/GSK3B/RNF14/BAX/PIK3CB/HNRNPU/TNF/ROCK2/LRP1/ENTPD5/GPLD1/PIP4K2C/DHRSX/SH3RF3/BNIP3L/CALCOCO2/CPEB3/COP1/YTHDF3/NUB1/SPTLC2/TP53INP2/PATL1/TSC2/RNF40/FZR1/YBX1/DISC1/GIGYF2/CNOT6/SH3RF1/TFEB/ROCK1/ARNT/SESN2/HSPA1B/GSK3A/PSEN1/CLU/CSDE1/VPS11/ULK1/INSR/PTPN1/SESN3/PLK1/RNF19B/AGTPBP1/AGO2/HSPA1A/TUT7/RALB/ZC3HAV1/PTK2B/VCP/NUPR1/SAMD4B/ELAPOR1/RAD23A/WDR45/CPT1A/IKBKG/GABARAP/ZC3H18/NRDC/GTPBP1/PIP4K2A/WAC/ZFP36L1/PIAS1/AKT2/SH3GLB1/TNFRSF1B/KAT5/NFE2L2/MARCHF2/BAG6/LAPTM5/SNCA/ATG2A/TRIM8/PSMC4/DTX3L/ABHD5/FBXO7/HMOX1/FOXO3/DNAJB2/ZER1/PNPLA2/IRS2/MAPK3/SNX18/FLCN/AMBRA1/PRKCD/PINK1/MSN/PTEN/TRIB1/NPRL3/FURIN/DAB2
BP	GO:0010466	negative regulation of peptidase activity	126	-0.46	-1.79	3.15E-05	0.0003075	0.00018905	2199	tags=37%, list=19%, signal=31%	PROS1/SORL1/TNF/LRP1/CST3/PRDX5/ANXA2/CR1/BIRC6/CDKN2D/PI3/SERPING1/CAST/HGF/GPI/KNG1/SH3RF1/PTGS2/ROCK1/IFI16/NR4A1/TIMP1/GPX1/RPS6KA1/APLP2/SIAH2/SRC/PAK2/TNFSF14/UBE2O/PLAUR/SERPINE1/POR/SNCA/ARRB2/MMP9/XIAP/TIMP2/PCSK1N/PICALM/CTSB/DNAJB6/DHCR24/AQP1/SFRP2/PSMF1/FURIN

BP	GO:0007160	cell-matrix adhesion	139	-0.44	-1.76	3.29E-05	0.0003196	0.00019645	3058	tags=43%, list=26%, signal=32%	BCL2L11/BCL6/PKD1/ADAM15/FERMT1/TIAM1/BCAS3/THBS1/TAOK2/SRF/WDPCP/RHOA/PRKCZ/SLC9A1/ACTG1/FERMT3/GSK3B/TESK2/PIK3CB/UTRN/ROCK2/BST1/TNXB/ITGAM/ITGA2B/RCC2/ITGA5/ITGAL/JAM3/GREM1/DISC1/DUSP22/MKLN1/ROCK1/PTPRJ/RIN2/ITGB2/CD63/PXN/CTTN/PTK2B/SLK/GFUS/ILK/ACTN1/SRC/LIMS1/SERPINE1/ITGB5/RAC1/MAP4K4/PPFIA1/NF1/CORO1C/DMTN/ITGB3/PTEN/VCL/ZYX/ARHGAP6
BP	GO:0002090	regulation of receptor internalization	37	-0.64	-2.01	3.34E-05	0.0003239	0.00019909	1954	tags=51%, list=17%, signal=43%	LRPAP1/TBC1D5/SYK/ANXA2/NECAB2/RIN3/DLG4/AP2M1/GREM1/MKLN1/ARF1/NUMB/CD63/INSR/ANKRD13A/FLOT1/SDCBP/ARRB2/ITGB3
BP	GO:0042326	negative regulation of phosphorylation	270	-0.38	-1.63	3.36E-05	0.0003246	0.00019954	2226	tags=33%, list=19%, signal=28%	MLLT1/CTDSP2/BAX/NCOR1/PIP5KL1/SORL1/PIK3CB/HNRNPU/PKN1/LRP1/MAS1/RGS2/PRKAR2A/PIP4K2C/HDAC4/MYADM/HHEX/CTDSP1/UBE2B/GADD45A/PTPN6/CDKN1C/IRAK3/CDKN2D/CBL/HGF/TSC2/RUBCN/GREM1/DUSP22/INPP5K/PID1/ROCK1/PARP14/PSEN1/MAPT/RGS14/PTPRJ/SOCS3/SPAG9/PTPN1/ADAR/PLK1/AKT1S1/RASSF2/HMGCR/GNAQ/NUPR1/SIRPA/CDKN1A/PPP2R5A/HIPK3/SPRY3/APC/WARS1/DUSP6/PAK2/HSPB1/PIP4K2A/DUSP1/CALM3/SH3BP5L/CHP1/LYN/SNCA/PYCARD/ARRB2/MIDN/PTPRC/FBP1/FBXO7/STK38/PRKAR2B/NF1/EPHB2/CORO1C/SLC4A1/NIBAN1/IRS2/STAT3/DMTN/PRKDC/SFRP2/PRKAR1A/FKBP8/PRKCD/PTEN/SLC8A1/TRIB1/CTDSPL
BP	GO:0090130	tissue migration	193	-0.41	-1.69	3.39E-05	0.0003276	0.00020139	2209	tags=30%, list=19%, signal=24%	CCBE1/STAT5A/PIK3CB/TNF/ROCK2/PKN1/GPLD1/NRP2/PRKD2/MECP2/PRCP/SEMA4A/PIK3CG/GADD45A/SP1/RTN4/GREM1/GPI/ACTA2/MAP3K3/PTGS2/ROCK1/RIN2/EPHB4/PTP4A3/PXN/NR4A1/PTK2B/HDAC7/GPX1/GFUS/DOCK5/SPARC/SRC/GRN/HSPB1/CYP1B1/NFE2L2/SASH1/MYH9/GLIPR2/RAC1/MMP9/ADIPOR1/PIK3CD/MAP2K3/MAP4K4/HMOX1/SP100/NF1/IQSEC1/CORO1C/DNAJA4/ITGB3/PTEN/TBXA2R/GLUL
CC	GO:0030425	dendrite	335	-0.37	-1.60	3.49E-05	0.0003358	0.00020646	2725	tags=36%, list=23%, signal=28%	FARP1/CRTC1/CAPRIN1/RHOA/RAB8A/USP8/STRN4/NEURL1/ADNP/PSEN2/DYRK1A/HAP1/INPP5A/CAMK2A/FEZ1/ALCAM/SNX14/CKB/LRRK2/PPT1/CYBB/RTN4R/GSK3B/BMPR1A/SYAP1/MAGEE1/HNRNPU/PI4K2A/KIF5B/ATP7A/CDK5R1/GRIK2/KIF1B/CPEB3/MARK2/FZD5/HRH2/NECAB2/ATCAY/ABR/ATP6AP2/RIN3/CPEB4/APBA1/TRAK1/DLG4/GRIPAP1/SAV1/ATP2B1/ERO1A/NCF1/GNAI2/STX3/CNIH3/DTNB/MAP1A/GIGYF2/ROGDI/CDKL5/GSK3A/PSEN1/RARA/MAPT/RGS14/CLU/PRR7/KIF1C/INSR/SIPA1L1/MAX/AGO2/RAP1GAP/PPP1R9B/ABHD17C/CTTN/SLC6A6/DNM3/MARK4/PTK2B/SYNPO/RAB27A/NIN/ACTN1/MYO1D/TMEM266/DBN1/KCNE3/AKAP9/MARK3/P2RY1/PRKACA/FXR2/KPNA1/CTNND1/MME/SLA/GLRX5/SHARPIN/PAK1/RAC1/ARRB2/PPP3CA/MAP1S/ASAP1/PPFIA1/PRKAR2B/NF1/EPHB2/LRRC4/OSBP2/ABI3/RAB5A/TRAK2/NRGN/SLC8A1/FLOT2/CASC3/STAU1/GNAZ

BP	GO:1902533	positive regulation of intracellular signal transduction	661	-0.32	-1.44	3.51E-05	0.0003371	0.00020722	2696	tags=32%, list=23%, signal=26%	FPR2/TRAF6/RHOA/IL1B/PLCB1/PPP5C/WLS/PRKCZ/SOS2/NEUROD2/ERN1/CANT1/LPAR1/JAK2/NCKAP1L/FIS1/KIT/ANGPT1/NEK6/SLC9A1/SEMA7A/LAMTOR4/GPR137B/TICAM1/P2RX7/RC3H2/CSF1R/DDX3X/C5AR2/TRIM21/PJA2/FADD/FLNA/ERBB2/LRRK2/RTN4R/REL/BCLAF1/HIPK2/BAX/MAPKBP1/PRKCB/TAB2/DDX60/PIK3CB/PELI1/TNF/ROCK2/LRRC59/PLCE1/SERINC3/CASR/NKX3-1/ARHGEF3/PRKD2/NFAT5/TRIM25/RICTOR/ALOX15/SH3RF3/ERN2/OSM/PDGFA/FZD5/MCL1/LTBR/AKAP13/TAOK1/SYK/NMI/NECAB2/MAP2K2/GPBAR1/SIK3/PIK3CG/TNFRSF1A/GADD45A/PTPN6/CC2D1A/SLC15A3/SECTM1/AGO3/RB1CC1/EP300/DOK4/CCR1/CBL/LRRD1/TIRAP/EIF2AK2/NCF1/HGF/GNAI2/RTN4/PDE5A/CPNE1/GREM1/DUSP22/TLR6/MSX1/CD14/ACTA2/MTURN/MAP3K3/SHC1/FFAR4/SH3RF1/OSBPL8/DSTYK/MAVS/ROCK1/BID/IRAK1/SLC44A2/HSPA1B/CCL3/PSEN1/RELA/PTPRJ/FGR/ERBIN/FKBP1A/INSR/SPAG9/PTPN1/MAP3K20/CHI3L1/MAP3K5/PTP4A3/RASSF2/C5AR1/HSPA1A/SHISA5/CLEC16A/PTK2B/SLC46A2/NUPR1/GPX1/DVL3/PRDX2/LGALS9/LIN28A/JMJD8/MAP2K7/CASS4/ILK/OASL/TAOK3/SRC/SHOC2/TPR/ALPK1/IKBKG/UBB/PIK3R5/LIMS1/WAC/RIPK1/TNFSF14/P2RY1/DLG5/SELP/ARHGAP8/OTUD5/SASH1/NDST1/HIC1/ACTN4/BRD4/PLAGL2/LAPTM5/SHARPIN/LYN/HCLS1/TGM2/IGF2/GLIPR2/PAK1/SDCBP/RAC1/PYCARD/ARRB2/XIAP/TRIM8/BBC3/PIK3CD/F11R/CFLAR/RRAGD/GUCY1A1/MAP2K3/PTPRC/MAP4K4/HMOX1/RUNDC3A/NACC2/TCF7L2/MAPK3/RASGRP4/FLCN/PINK1/PPM1A/PTEN/SPI1/PRR5/PELI2/RAPGEF2/IQGAP1/LITAF/TGFA/HIP1
BP	GO:0008285	negative regulation of cell population proliferation	439	-0.34	-1.49	3.54E-05	0.0003395	0.00020871	3084	tags=36%, list=26%, signal=27%	KLF13/MED1/CDC73/CBFA2T3/GATA1/BCL6/ATOH8/FERMT1/BAK1/CSK/STAT1/TFDP1/THBS1/B4GALT7/PML/BAP1/SRF/ITCH/GSTP1/SMARCB1/HLA-DRB1/MFN2/CD9/GNA13/CLMN/TLR2/LGALS9C/DICER1/RHOA/IL1B/REST/TMEM131L/PBRM1/INPP5D/NEURL1/BTG1/SIRT6/JAK2/IL4I1/LST1/ALOX5/TNFRSF9/CSF1R/LDOC1/CEBPA/TSPO/CTCF/SERPINE2/ERBB2/LILRB2/BAX/ZBTB16/PELI1/TNF/PKN1/PTGES/MNDA/NKX3-1/GPLD1/TMEM115/S1PR2/OSM/FZD5/P3H1/NMI/TMEM127/ATF5/CR1/PTPN6/PPM1D/SSTR2/CDKN1C/TOB2/RB1CC1/LMNA/SAV1/CDKN2D/STK4/EIF2AK2/TSC2/VSIR/PDE5A/GREM1/PDS5B/SOX4/MSX1/NIBAN2/CTBP2/PTGS2/HSPA1B/RARA/ZEB1/PTPRJ/ULK1/INTU/AGTPBP1/RASSF5/ZNF503/SMARCA2/RAP1GAP/ASPH/HSPA1A/CTNNA1/GNA12/PTK2B/B4GALT1/NUPR1/TESC/LGALS9/CDKN1A/SKI/SPARC/ILK/APC/ASCL2/WARS1/TGFB1I1/EIF2AK1/CYP1B1/DUSP1/DLG5/SOD2/VDR/IRF1/ZBTB7B/RBPJ/JARID2/TPM1/H2AC6/CTNNBIP1/LYN/TYROBP/PAK1/ADM/CEBPB/E2F3/HMOX1/NF1/DNAJB2/DHCR24/NACC2/MED25/STAT3/SFRP2/PRKAR1A/FLCN/FTH1/TP53I11/PTEN/ING1/SKAP2/FOXO4/RNF10/TRIB1/RAPGEF2/DAB2/S100A11
BP	GO:0002573	myeloid leukocyte differentiation	151	-0.44	-1.74	3.58E-05	0.0003421	0.00021031	2916	tags=46%, list=25%, signal=35%	CD4/MED1/CBFA2T3/GATA1/IL31RA/ATP6AP1/RELB/HLA-DRB1/TLR2/FES/FOS/FARP2/TRAF6/INPP5D/KIT/PPARGC1B/GPR137B/EVI2B/CSF1R/IREB2/CEBPA/FADD/MITF/PAFAH1B1/TNF/LTBR/ANXA2/LBR/ZFPM1/TOB2/CREB1/CCR1/TSPAN2/JUNB/CCL3/PSEN1/RARA/IFI16/RASSF2/SBNO2/TESC/CUL4A/THOC5/TF/SNX10/SRC/PF4/EIF2AK1/RIPK1/ZFP36L1/OSCAR/RBPJ/MYH9/TAL1/BATF2/NRROS/CTNNBIP1/LYN/HCLS1/TYROBP/MMP9/PIK3CD/CEBPB/MAPK14/NF1/TFE3/SPI1/LILRB3/TRIB1/GAB2
BP	GO:0000045	autophagosome assembly	97	-0.49	-1.86	3.60E-05	0.0003435	0.00021118	2589	tags=42%, list=22%, signal=33%	MFN2/PIKFYVE/IFT88/ATG2B/WIPI1/FEZ1/LRRK2/RNF5/ADPRH/MAP1LC3B/GABARAPL2/PIP4K2C/GABARAPL1/RAB43/RB1CC1/TP53INP2/MTMR3/MTM1/MAP1LC3A/TBC1D14/PSEN1/RAB1B/ULK1/WIPI2/RALB/NUPR1/ATG7/ELAPOR1/WDR45/RAB7A/GABARAP/PIP4K2A/SH3GLB1/FEZ2/ATG2A/MAP1LC3B2/RAB5A/SNX18/AMBRA1/PINK1/CHMP4B

BP	GO:0060291	long-term synaptic potentiation	45	-0.62	-2.02	3.63E-05	0.000344	0.00021149	2770	tags=51%, list=24%, signal=39%	MAPK1/CRTC1/PRKCZ/SERPINE2/GSK3B/LILRB2/ZDHHC2/MECP2/STX3/SNAP25/RGS14/SQSTM1/PTK2B/SYT12/MME/SNCA/TYROBP/NF1/EPHB2/NRGN/PTEN/STAU1/TSHZ3
BP	GO:0009991	response to extracellular stimulus	309	-0.37	-1.59	3.63E-05	0.000344	0.00021149	2708	tags=36%, list=23%, signal=29%	FES/FOS/ACSL1/PRKAG2/SLC2A1/ADNP/EIF4G1/GCN1/WIPI1/CIDEB/HSPA5/P2RX7/MBD2/TSPO/LRRK2/CYBB/ALPL/ACACB/SORL1/RIPOR1/ATF3/TNF/MAP1LC3B/CASR/UCP3/GABARAPL2/UCP2/TRIM25/SLC39A4/TBC1D5/MEAK7/GABARAPL1/PPM1D/SSTR2/CPEB4/LAMP2/ATP2B1/CHSY1/CDKN2D/GCLM/CBL/MTMR3/PIM1/EIF2AK2/STK24/GNAI2/MAP1LC3A/PDK2/KDM4A/TFEB/NR4A2/PTGS2/DSC2/SESN2/MTHFR/RARA/ZEB1/RMI1/IFI16/ULK1/WIPI2/SESN3/MAP3K5/MAX/PPP1R9B/RALB/CLEC16A/GPX1/ATG7/ELAPOR1/CDKN1A/SRC/WDR45/CPT1A/F5/GABARAP/KPTN/EIF2AK1/CYP1B1/GAS2L1/DAP/SOD2/VDR/RXRA/SH3GLB1/NFE2L2/POR/APAF1/LYN/ADM/SIPA1/RRAGD/PTPRC/PCSK1N/MAPK14/HMOX1/NCOA1/MAP1LC3B2/ABCG8/FOXO3/G6PD/MAPK3/SFRP2/RASGRP4/FLCN/CD68/NUAK2/SLC8A1/FOXO4/NPRL3/TBXA2R/GLUL
MF	GO:0005102	signaling receptor binding	724	-0.31	-1.43	3.86E-05	0.0003653	0.00022459	2764	tags=32%, list=24%, signal=26%	CD9/GNA13/NCOR2/HDGFL3/TLR2/FES/CPNE3/FPR2/TRAF6/WNT1/PIK3R2/IL1B/ARHGEF11/PECR/MAP7/ADAM17/SELPLG/PTPA/CD177/ERN1/JAK2/SLC39A1/C17orf99/ANGPT1/CMTM7/SEMA7A/TGFBI/WIPI1/DOCK2/SMARCD3/P2RX7/HAP1/JAK1/SNX1/CAMK2A/CD70/COPA/ALCAM/FADD/FLNA/BEX3/SERPINE2/ERBB2/BTNL9/GRB2/FERMT3/TNFSF13/LILRB2/PLCL2/BTN3A2/DNM2/TNF/MYO9B/PHIP/UTRN/EFNB1/FPR1/TNXB/TAPBP/CASR/LRP1/TNFSF13B/TLR1/GABARAPL2/CDK5R1/ELMO2/TRIM25/S1PR2/ITGAM/LRPAP1/CD300LF/NAMPT/OSM/YWHAH/ITGA2B/PDGFA/PLSCR1/METRNL/SEMA4A/GABARAPL1/SYK/SPG21/NECAB2/CXCL16/HLA-E/ITGA5/ITGAL/PIK3CG/ICAM4/PF4V1/LAMA4/SECTM1/FCN1/TRAK1/DLG4/SAV1/JAM3/CBL/TUBB4B/TMBIM1/TIRAP/HGF/PILRA/ARHGEF12/GNAI2/AP2M1/GREM1/GPI/CUL3/KNG1/TLR6/KLRD1/SHC1/CLTC/ERMAP/LRG1/ARNT/BID/DIAPH2/HSPA1B/ICAM3/SYTL4/CCL3/GSK3A/PSEN1/RARA/PTPRJ/FGR/ERBIN/CLU/FKBP1A/ITGB2/LGR6/INSR/SQSTM1/PTPN1/SIPA1L1/CMTM5/DIAPH1/LGALS3/PXN/PPP1R9B/HSPA1A/TIMP1/GNA12/DNM3/BMP6/GNAQ/NLGN3/PTK2B/VCP/HCK/TF/DVL3/CXCL5/VAV3/HDGF/ACTN1/SRC/GRN/NCOA2/PF4/IKBKG/GABARAP/APOL2/OSGIN2/TGFB1I1/TACC1/AKAP9/FLOT1/RIPK1/TNFSF14/P2RY1/IL6R/CALM3/DAZAP2/PLAUR/GNAL/CLCF1/PROK2/TAF6/IL1RN/SERPINE1/CTNND1/AP2A1/SEC14L1/MYH9/ITGB5/BAG6/NOTCH4/ACTN4/SLA/FYB1/LYN/GRK3/IGF2/TYROBP/SDCBP/ADM/PYCARD/ARRB2/CYRIB/F11R/CFLAR/PLXNC1/PTPRC/PCSK1N/PICALM/LCP1/NCOA1/EPHB2/IRS2/IL17RA/TOLLIP/TLN1/MARCHF8/STAT3/DMTN/SFRP2/ITGB3/IL13RA1/TRAK2/MSN/CCRL2/MPP1/RAPGEF2/DAB2/GAB2/TGFA/HIP1/GNAZ

BP	GO:0051046	regulation of secretion	351	-0.35	-1.54	3.91E-05	0.0003696	0.0002272	2800	tags=31%, list=24%, signal=24%	HLA-DRB1/HIF1A/ADAM8/TLR2/FES/IL1B/RAB8A/REST/WLS/CD177/JAK2/NCKAP1L/ALOX5/P2RX7/HAP1/KLF7/CD38/CACNA1I/CAMK2A/RPH3AL/LRRK2/KDM5B/GSK3B/TPCN2/FBXL20/NPHS1/PRKCB/TNF/PRAM1/ABAT/PTGES/MYH10/CASR/NKX3-1/GPLD1/UCP2/ITGAM/OSM/STXBP5/CES1/P3H1/SYK/GPR27/TNFRSF1A/RAB21/APBA1/CREB1/RAC2/LRRC8A/ABCB11/GNAI2/LAMP1/SOX4/RAB3B/SIDT2/SNAP25/RSAD2/FFAR4/MCTP2/SYTL4/FGR/ITGB2/RAB27B/VAMP3/HMGCR/BMP6/SYT12/VPS4B/LGALS9/RAB27A/KCNQ1/ACSL4/CPT1A/RAB7A/RAB2B/P2RY1/CALM3/STXBP2/TNFRSF1B/VPS18/P2RX1/AP1G1/ABCG1/LYN/UNC13D/SNCA/CHD7/C2CD2L/SDCBP/WNK1/MIDN/RAB11FIP1/PPP3CA/OPRL1/MYOM1/HMOX1/NF1/PTAFR/CHMP2A/IRS2/TCF7L2/RAB5A/AQP1/PINK1/RAB3D/SPI1/DAB2/GLUL/GAB2
BP	GO:0007041	lysosomal transport	108	-0.47	-1.81	4.00E-05	0.0003757	0.00023095	2289	tags=40%, list=20%, signal=32%	LRRK2/CHMP3/TPCN2/SORL1/PLEKHM1/LRP1/DENND3/GGA3/GAK/TRAK1/VPS33A/LAMP2/LYST/HOOK3/LAMP1/MTM1/ZFYVE16/CLU/IGF2R/UEVLD/VPS11/HSPA1A/CLEC16A/VCP/VPS4B/RILP/CHMP1A/GRN/RAB7A/PSAP/GNPTAB/SNX27/VPS18/AP1G1/LAPTM5/UBXN6/EHD3/NCOA4/DTX3L/CHMP2A/PINK1/CHMP4B/KIF13A
BP	GO:0030193	regulation of blood coagulation	37	-0.64	-2.00	4.03E-05	0.0003772	0.00023189	3126	tags=59%, list=27%, signal=44%	THBD/EDN1/THBS1/ST3GAL4/CD9/ANO6/SERPINE2/PROS1/ABAT/PDGFA/SERPING1/KNG1/PRDX2/GP1BA/ALOX12/NFE2L2/SERPINE1/EPHB2/DMTN/PRKCD/KRT1/TBXA2R
BP	GO:1900046	regulation of hemostasis	37	-0.64	-2.00	4.03E-05	0.0003772	0.00023189	3126	tags=59%, list=27%, signal=44%	THBD/EDN1/THBS1/ST3GAL4/CD9/ANO6/SERPINE2/PROS1/ABAT/PDGFA/SERPING1/KNG1/PRDX2/GP1BA/ALOX12/NFE2L2/SERPINE1/EPHB2/DMTN/PRKCD/KRT1/TBXA2R
BP	GO:0016236	macroautophagy	273	-0.39	-1.65	4.09E-05	0.0003816	0.00023461	2944	tags=38%, list=25%, signal=29%	PIP4K2B/ATP6V0B/PLAA/CLN3/ATP6V0C/VPS41/RIMOC1/HIF1A/VPS13D/MFN2/TBC1D25/EPM2A/PIKFYVE/VTI1B/IFT88/ERN1/ATG2B/WIPI1/VPS28/TECPR1/OPTN/FEZ1/SNX14/LRRK2/RNF5/CAPNS1/CHMP3/ADPRH/PLEKHM1/VPS37B/MAP1LC3B/GABARAPL2/CDK5R1/ATP6V1C1/PIP4K2C/BNIP3L/CALCOCO2/TBC1D5/MCOLN1/GABARAPL1/RAB43/EXOC7/VPS33A/SPTLC2/RB1CC1/TP53INP2/LAMP2/MTMR3/TSC2/RUBCN/MTM1/ATP6V0A1/MAP1LC3A/TBC1D14/SESN2/SNAP29/PSEN1/RAB1B/ULK1/SQSTM1/WIPI2/SESN3/ATP6V0D1/WDFY3/RALB/CLEC16A/QSOX1/VCP/NUPR1/ATG7/VPS4B/ELAPOR1/RETREG3/CHMP1A/SRC/WDR45/RAB7A/IKBKG/GABARAP/VPS37C/EXOC4/PIP4K2A/WAC/STAM2/PRKACA/SH3GLB1/FEZ2/ATP6V1B2/ILRUN/ATG2A/UBXN6/HMOX1/MAP1LC3B2/CHMP2A/RAB5A/MAPK3/SNX18/AMBRA1/PINK1/CAPN1/CHMP4B/NBR1/ATP6V1A
BP	GO:0010631	epithelial cell migration	192	-0.41	-1.67	4.11E-05	0.000382	0.00023483	2209	tags=29%, list=19%, signal=24%	CCBE1/STAT5A/PIK3CB/TNF/ROCK2/PKN1/GPLD1/NRP2/PRKD2/MECP2/PRCP/SEMA4A/PIK3CG/GADD45A/SP1/RTN4/GREM1/GPI/MAP3K3/PTGS2/ROCK1/RIN2/EPHB4/PTP4A3/PXN/NR4A1/PTK2B/HDAC7/GPX1/GFUS/DOCK5/SPARC/SRC/GRN/HSPB1/CYP1B1/NFE2L2/SASH1/MYH9/GLIPR2/RAC1/MMP9/ADIPOR1/PIK3CD/MAP2K3/MAP4K4/HMOX1/SP100/NF1/IQSEC1/CORO1C/DNAJA4/ITGB3/PTEN/TBXA2R/GLUL
BP	GO:0090132	epithelium migration	192	-0.41	-1.67	4.11E-05	0.000382	0.00023483	2209	tags=29%, list=19%, signal=24%	CCBE1/STAT5A/PIK3CB/TNF/ROCK2/PKN1/GPLD1/NRP2/PRKD2/MECP2/PRCP/SEMA4A/PIK3CG/GADD45A/SP1/RTN4/GREM1/GPI/MAP3K3/PTGS2/ROCK1/RIN2/EPHB4/PTP4A3/PXN/NR4A1/PTK2B/HDAC7/GPX1/GFUS/DOCK5/SPARC/SRC/GRN/HSPB1/CYP1B1/NFE2L2/SASH1/MYH9/GLIPR2/RAC1/MMP9/ADIPOR1/PIK3CD/MAP2K3/MAP4K4/HMOX1/SP100/NF1/IQSEC1/CORO1C/DNAJA4/ITGB3/PTEN/TBXA2R/GLUL

BP	GO:0070482	response to oxygen levels	209	-0.40	-1.64	4.14E-05	0.0003839	0.00023599	2932	tags=37%, list=25%, signal=28%	THBS1/AIFM1/ATP6AP1/PML/PDLIM1/SRF/HIF1A/ADAM8/TLR2/RHOA/REST/SCAP/ADAM17/SLC2A1/SLC9A1/NDUFS2/AGTRAP/CD38/HK2/CYBB/HIPK2/PIK3CB/TNF/ROCK2/ABAT/CASR/ATP7A/NKX3-1/UCP3/UCP2/MECP2/ITPR2/BNIP3L/PDGFA/CPEB4/LMNA/GUCY1B1/EP300/CBL/ERO1A/NR4A2/PTGS2/ARNT/IRAK1/XRCC1/MTHFR/EGLN1/ATP6V0D1/PLOD1/PTK2B/EGLN3/ATG7/EGLN2/SRC/BACH1/ASCL2/SOD2/ZFP36L1/RBPJ/NFE2L2/ACTN4/APAF1/NPEPPS/ADM/CREBBP/CFLAR/ALAS2/GUCY1A1/HMOX1/NF1/FOXO3/PDK3/AQP1/MPL/PINK1/PTEN/SLC8A1/ATP6V1A
MF	GO:0008013	beta-catenin binding	55	-0.57	-1.95	4.24E-05	0.0003918	0.00024087	1655	tags=38%, list=14%, signal=33%	EP300/MED12L/NUMB/NR4A2/SUFU/PSEN1/PTPRJ/TBL1X/PXN/CTNNA1/BCL9/DVL3/APC/DLG5/CARM1/KDM6B/CTNNBIP1/FOXO3/TCF7L2/VCL/FOXO4
CC	GO:0009925	basal plasma membrane	146	-0.44	-1.73	4.29E-05	0.0003959	0.00024336	2513	tags=34%, list=21%, signal=27%	MAP7/SLC2A1/BEST1/SLC9A1/SHROOM4/C5AR2/CD38/ERBB2/CASR/SLCO4C1/LRP1/ATP7A/ABCC4/ANXA2/LPO/SLC6A9/SLC22A1/ATP2B1/SLC12A6/DTNB/CEACAM5/NUMB/DSTYK/ABCC3/WASF2/ERBIN/AQP9/SLC23A2/C5AR1/SLC6A6/B4GALT1/TF/KCNQ1/MYO1D/FLOT1/P2RY1/SLC14A1/EPB41/SLC2A9/ARRB2/SLC1A5/SLC4A1/AQP1/BSG/ANK1/MSN/FLOT2/SLC7A5/TGFA/AQP10
BP	GO:0010721	negative regulation of cell development	90	-0.50	-1.87	4.54E-05	0.0004168	0.00025621	2687	tags=46%, list=23%, signal=35%	DICER1/IL1B/REST/SEMA7A/TRIM11/TSPO/RTN4R/GSK3B/BMPR1A/SORL1/TNF/GBP1/CDK5R1/YWHAH/PCM1/SEMA4A/CTDSP1/RCC2/KIAA0319/DRAXIN/HOOK3/RTN4/RFLNB/KDM4A/GSK3A/PSEN1/ULK1/CTNNA1/LIN28A/SKI/ASCL2/ACTN4/PPP3CA/NF1/EPHB2/CORO1C/DMTN/PTEN/MBP/RNF10/RAPGEF2
BP	GO:0031667	response to nutrient levels	286	-0.37	-1.58	4.88E-05	0.0004466	0.00027457	2289	tags=32%, list=20%, signal=27%	LRRK2/CYBB/ALPL/ACACB/SORL1/RIPOR1/ATF3/TNF/MAP1LC3B/CASR/UCP3/GABARAPL2/UCP2/TRIM25/SLC39A4/TBC1D5/MEAK7/GABARAPL1/PPM1D/SSTR2/CPEB4/LAMP2/ATP2B1/CHSY1/CDKN2D/GCLM/CBL/MTMR3/PIM1/EIF2AK2/STK24/GNAI2/MAP1LC3A/PDK2/KDM4A/TFEB/PTGS2/DSC2/SESN2/MTHFR/RARA/ZEB1/RMI1/IFI16/ULK1/WIPI2/SESN3/MAP3K5/MAX/RALB/CLEC16A/GPX1/ATG7/ELAPOR1/CDKN1A/SRC/WDR45/CPT1A/F5/GABARAP/KPTN/EIF2AK1/CYP1B1/GAS2L1/DAP/SOD2/VDR/RXRA/SH3GLB1/NFE2L2/POR/APAF1/ADM/RRAGD/PCSK1N/MAPK14/HMOX1/NCOA1/MAP1LC3B2/ABCG8/FOXO3/G6PD/MAPK3/SFRP2/FLCN/CD68/NUAK2/SLC8A1/FOXO4/NPRL3/TBXA2R/GLUL
BP	GO:0033993	response to lipid	537	-0.33	-1.46	5.05E-05	0.0004604	0.00028303	2739	tags=33%, list=23%, signal=26%	NCOR2/TLR2/FES/FOS/TRAF6/RHOA/ACSL1/IL1B/REST/PLCB1/PPP5C/USP8/ADAM17/JAK2/CNOT1/CASP9/PPARGC1B/TICAM1/P2RX7/MBD2/PAQR8/VIM/LDOC1/CD38/TSPO/ABCA2/CYBB/GSK3B/RNF14/ARID5A/LILRB2/ALPL/NCOR1/SPON2/TAB2/HNRNPU/PELI1/TNF/NFKBIA/ROCK2/JUND/PKN1/ARID1A/CASR/NKX3-1/GPLD1/UCP3/UCP2/TRIM25/YWHAH/SLC11A1/PDGFA/CES1/TWF2/SYK/GPBAR1/PF4V1/SSTR2/UBR5/ARPC1B/IRAK3/CREB1/ATP2B1/CTR9/EP300/CDKN2D/CBL/ABCB11/PIM1/TIRAP/TXNIP/SLC10A3/GPI/CD14/LILRA2/STAT5B/PID1/PTGS2/IRAK1/C2/TMEM161A/HSPA1B/SCD/CCL3/RARA/PRPF8/RELA/GOT1/ERBIN/CLU/IGF2R/VPS11/ZNF703/ABHD2/NR3C1/NFKBIB/PPP1R9B/HSPA1A/SBNO2/NR4A1/LCAT/BMP6/PTK2B/IFNAR1/HCK/GPX1/TESC/TNIP1/VPS4B/SIRPA/PRDX2/LGALS9/CXCL5/EGLN2/SRC/NCOA2/CPT1A/PF4/MAPKAPK2/AICDA/SETX/CYP1B1/CALM3/SOD2/ZFP36L1/VDR/SELP/PADI2/ZBTB7A/CARM1/RXRA/TNFRSF1B/BRCA1/VPS18/MAPKAPK3/OTUD5/DGAT2/KAT5/IL1RN/SERPINE1/TBXAS1/SASH1/CMPK2/POR/ZMIZ1/ABCG1/LYN/SNCA/PAK1/ADM/PYCARD/CDK19/SYNJ1/NCOA4/CFLAR/MAP2K3/CEBPB/OPRL1/MAPK14/FOXO3/PDK3/EPHB2/PTAFR/FAM210B/WBP2/AQP1/STAT3/MAPK3/CD68/MSN/SPI1/MBP/TRIB1/TBXA2R/DAB2/LITAF

BP	GO:0032101	regulation of response to external stimulus	625	-0.32	-1.44	5.07E-05	0.0004615	0.00028372	3027	tags=33%, list=26%, signal=26%	EDN1/MFHAS1/BCL6/ZNF580/BBS2/TRIM38/STAT1/CST7/DAPK2/THBS1/OAS3/PARP9/BAP1/ST3GAL4/ANKRD17/ITCH/MOSPD2/NLRP10/GSTP1/SCARF1/HLA-DRB1/AKIRIN2/ADAM8/NLRP12/CHD8/MAPK1/CD9/PUM2/TKFC/HERC5/TLR2/FPR2/TRAF6/IL1B/SAMHD1/ADAM17/ANO6/JAK2/NCKAP1L/SEMA7A/ALOX5/SIN3A/OXSR1/CSF1R/SETD4/C5AR2/OPTN/CEBPA/TRIM21/FADD/FLNA/SERPINE2/SPATA2/LRRK2/NECTIN2/RTN4R/PROS1/MAPKBP1/DDX60/TNF/NFKBIA/BST1/ABAT/PTGES/CASR/MNDA/MAS1/TLR8/PRKD2/RICTOR/ALOX15/OSM/PDGFA/PLSCR1/METRNL/SEMA4A/KIAA0319/SYK/NMI/CR1/HLA-E/PIK3CG/TNFRSF1A/YTHDF3/PTPN6/RIN3/FCN1/GRK4/IRAK3/JAM3/RAC2/EP300/ALOX5AP/CCR1/CAMK1D/SERPING1/LSM14A/TIRAP/NCF1/HGF/STK24/STX3/LAMP1/GREM1/KNG1/LILRA2/KLRD1/STAT5B/PCBP2/FFAR4/N4BP1/MAVS/PTGS2/MGLL/PARP14/USP27X/GPATCH3/CCL3/RELA/FGR/FUT7/IFI16/SOCS3/CAPN3/AREL1/PTPN1/ADAR/VAV1/C5AR1/SBNO2/BMP6/PTK2B/NDEL1/RIOK3/HCK/NUPR1/GPX1/TNIP1/SIRPA/PRDX2/LGALS9/NCR1/OASL/SRC/GRN/PAK2/HSPB1/DUSP1/RIPK1/TNFSF14/IL6R/VDR/PADI2/IRF1/GP1BA/RXRA/TNFRSF1B/ALOX12/NFE2L2/SERPINE1/SEC14L1/SASH1/IFIT1B/AKNA/AP1G1/BRD4/NLRC4/SHARPIN/LYN/ILRUN/SNCA/ZNFX1/TYROBP/PAK1/RAC1/WNK1/PYCARD/CDK19/ARRB2/MMP9/XIAP/GPSM3/CREBBP/CYRIB/DTX3L/CEBPB/PTPRC/MAPK14/EPHB2/PGLYRP1/IL17RA/MAPK3/DMTN/PRKDC/NLRX1/PRKCD/PTEN/KRT1/SPI1/RIPOR2/MPP1/TRIB1/ZYX/NINJ1/TBXA2R/FURIN
BP	GO:0000003	reproduction	738	-0.30	-1.40	5.44E-05	0.0004939	0.00030363	2659	tags=28%, list=23%, signal=23%	HERPUD2/PLCB1/ASPM/NEURL1/TTC26/BTG1/SLC2A1/ADNP/SETD2/QKI/OR7C1/KIT/CBX7/ACTR2/HSPA5/DPCD/DDX3X/MBD2/PAQR8/LDOC1/GALNT3/CD38/CACNA1I/CEBPA/LHFPL2/ARHGDIB/INCENP/SERPINE2/SPATA2/LRRK2/IFT81/GRB2/YY1/NECTIN2/KDM5B/SENP2/BMPR1A/ALPL/BAX/KNL1/TESK2/ZBTB16/ARMC2/KDM1B/DNM2/PAFAH1B1/ABAT/SLCO4C1/MAS1/ATP7A/NKX3-1/RGS2/CTDNEP1/CFAP58/UCP2/SGPL1/PDGFA/FZD5/CCDC136/TUBGCP3/UBE2B/BIRC6/ZNF148/CATSPER2/ITGA5/SPATA6/YTHDF3/PTPN6/SSTR2/CDKN1C/ZGLP1/TOB2/NKAPL/JAM3/MSH6/USP42/MYCBPAP/CBL/LRRC8A/FSCN3/PI3/STK4/ARID4A/C3orf62/FZR1/PDE5A/MSX1/TCTE1/STAT5B/ZW10/JUNB/ALDOA/FOXJ2/TRPC3/TFEB/XRN2/PTGS2/ARNT/ATRX/DIAPH2/RARA/RMI1/IGF2R/CSDE1/FUT7/SOCS3/INSR/ABHD2/PLK1/CCNF/AGO2/SMARCA2/NDRG3/TOP3A/PPP1R9B/TUT7/TIMP1/PSME4/CTNNA1/H3-3A/BMP6/PTK2B/B4GALT1/NUPR1/LGALS9/ACSL4/COL9A3/LIN28A/SRC/NIPBL/ASCL2/UBB/APOL2/OSGIN2/BPTF/PSAP/PBX1/TPPP3/FANCC/KLC3/ETV6/HSPA1L/RFX2/SETX/CYP1B1/DUSP1/P2RY1/ZFP36L1/VDR/SPOCD1/PRKACA/ASF1B/PROK2/RBPJ/CCNI/P2RX1/GOLGA2/UBR2/MYH9/IRF2BPL/AGO4/BAG6/MME/ZMIZ1/MAST2/MYCBP/EPN1/USP9X/IGF2/CHD7/ADM/ARRB2/MMP9/RAD23B/TBC1D20/PITHD1/LIMK2/NCOA4/ZMYND15/CEBPB/OPRL1/H3-3B/CTSB/MAPK14/DNAJB6/NCOA1/FOXO3/MARF1/PTAFR/STS/YBX3/DHCR24/OSBP2/ACOX1/STAT3/PRKDC/SFRP2/CCDC39/RAD21/BCL2L1/BSG/BASP1/ROPN1L/PTEN/ACRBP/KLF1
BP	GO:0045444	fat cell differentiation	142	-0.43	-1.71	5.58E-05	0.0005042	0.00030994	2502	tags=37%, list=21%, signal=30%	ALOX5/TAF8/STEAP4/CEBPA/KLF5/SENP2/WDFY2/SYAP1/ZBTB16/HNRNPU/TNF/RGS2/METRNL/ARL4A/ZFPM1/ZNF516/ATF5/SAV1/CREB1/EP300/TMEM120A/STK4/PIM1/ERO1A/RNASEL/CEBPD/FFAR4/CTBP2/NR4A2/OSBPL8/PTGS2/LRG1/FAM120B/NR4A1/GPX1/NIPBL/TGFB1I1/SOD2/ZFP36L1/ZBTB7A/CARM1/PIAS1/AKT2/ZBTB7B/BSCL2/FBXO9/CEBPB/MAPK14/TFE3/TCF7L2/SFRP2/FLCN/JDP2

BP	GO:0022414	reproductive process	734	-0.30	-1.40	5.82E-05	0.0005246	0.00032252	2659	tags=28%, list=23%, signal=23%	HERPUD2/PLCB1/ASPM/NEURL1/TTC26/BTG1/SLC2A1/ADNP/SETD2/QKI/OR7C1/KIT/CBX7/ACTR2/HSPA5/DPCD/DDX3X/MBD2/PAQR8/LDOC1/GALNT3/CD38/CACNA1I/CEBPA/LHFPL2/ARHGDIB/INCENP/SERPINE2/SPATA2/LRRK2/IFT81/GRB2/YY1/NECTIN2/KDM5B/SENP2/BMPR1A/ALPL/BAX/KNL1/TESK2/ZBTB16/ARMC2/KDM1B/DNM2/PAFAH1B1/ABAT/SLCO4C1/MAS1/ATP7A/NKX3-1/RGS2/CTDNEP1/CFAP58/UCP2/SGPL1/PDGFA/FZD5/CCDC136/TUBGCP3/UBE2B/BIRC6/ZNF148/CATSPER2/ITGA5/SPATA6/YTHDF3/PTPN6/SSTR2/CDKN1C/ZGLP1/TOB2/NKAPL/JAM3/MSH6/USP42/MYCBPAP/CBL/LRRC8A/FSCN3/PI3/STK4/ARID4A/C3orf62/FZR1/PDE5A/MSX1/TCTE1/STAT5B/ZW10/JUNB/ALDOA/FOXJ2/TRPC3/TFEB/XRN2/PTGS2/ARNT/ATRX/DIAPH2/RARA/RMI1/IGF2R/CSDE1/FUT7/SOCS3/INSR/ABHD2/PLK1/CCNF/AGO2/SMARCA2/NDRG3/TOP3A/PPP1R9B/TUT7/TIMP1/PSME4/CTNNA1/H3-3A/BMP6/PTK2B/B4GALT1/NUPR1/LGALS9/ACSL4/COL9A3/LIN28A/SRC/NIPBL/ASCL2/UBB/APOL2/OSGIN2/BPTF/PSAP/PBX1/TPPP3/FANCC/KLC3/ETV6/HSPA1L/RFX2/SETX/CYP1B1/DUSP1/P2RY1/ZFP36L1/VDR/SPOCD1/PRKACA/ASF1B/PROK2/RBPJ/CCNI/P2RX1/GOLGA2/UBR2/MYH9/IRF2BPL/AGO4/BAG6/MME/ZMIZ1/MAST2/MYCBP/EPN1/USP9X/IGF2/CHD7/ADM/ARRB2/MMP9/RAD23B/TBC1D20/PITHD1/LIMK2/NCOA4/ZMYND15/CEBPB/OPRL1/H3-3B/CTSB/MAPK14/DNAJB6/NCOA1/FOXO3/MARF1/PTAFR/STS/YBX3/DHCR24/OSBP2/ACOX1/PRKDC/SFRP2/CCDC39/RAD21/BCL2L1/BSG/BASP1/ROPN1L/PTEN/ACRBP/KLF1
BP	GO:0051017	actin filament bundle assembly	106	-0.48	-1.82	5.89E-05	0.0005299	0.00032576	1717	tags=28%, list=15%, signal=24%	FHOD1/FSCN1/FSCN3/CUL3/INPP5K/ROCK1/WASF2/NEDD9/PXN/ESPN/PTK2B/SYNPO/LIMK1/ACTN1/SRC/PAK2/TPM1/ITGB5/PAK1/RAC1/PLEK/SHROOM1/F11R/LCP1/PPFIA1/WAS/MARCKS/DMTN/ZYX/ARHGAP6
BP	GO:0006935	chemotaxis	334	-0.36	-1.55	5.94E-05	0.0005327	0.00032748	2708	tags=36%, list=23%, signal=28%	FES/FPR2/RHOA/IL1B/ADAM17/ANO6/LYPLA2/NEXN/LPAR1/NCKAP1L/KIT/ANGPT1/SCN1B/CMTM7/SEMA7A/ALOX5/DOCK2/FCER1G/OXSR1/CSF1R/C5AR2/KLF7/FEZ1/ALCAM/PLXNB2/ERBB2/PIK3CB/EFNB1/FPR1/BST1/CASR/KIF5B/CDK5R1/ELMO2/NRP2/PRKD2/BIN2/RNF165/PDGFA/SEMA4A/SYK/PIP5K1C/CXCL16/PIK3CG/PF4V1/RIN3/JAM3/RAC2/CCR1/CAMK1D/LYST/TIRAP/NCAM1/DRAXIN/HGF/TSC2/STX3/GREM1/NIBAN2/CSF3R/RPS6KA5/CCL3/PTPRJ/ITGB2/EPHB4/LGR6/CMTM5/VAV1/LGALS3/C5AR1/NR4A1/PTK2B/CXCR2/LGALS9/CXCL5/PALLD/VAV3/CXCR1/DPYSL2/PF4/GAB1/HSPB1/DUSP1/TNFSF14/IL6R/PLAUR/PADI2/FEZ2/PROK2/PREX1/TREM1/SERPINE1/LYN/RAC1/WNK1/ARRB2/GPSM3/CYRIB/PIK3CD/PLXNC1/RHOG/MAPK14/CORO1A/EPHB2/PTAFR/VASP/IL17RA/MAPK3/RALBP1/ITGB3/PRKCD/BSG/SPI1/CCRL2/RIPOR2/MPP1/NINJ1/LSP1/ECE1

BP	GO:0032269	negative regulation of cellular protein metabolic process	699	-0.30	-1.39	6.23E-05	0.0005577	0.00034286	2434	tags=29%, list=21%, signal=25%	DDX3X/SH3BP5/TAF1/IREB2/CEBPA/TSPO/TRIM21/DCP2/SMARCC1/SERPINE2/TNRC6B/IL1R2/LRRK2/GSK3B/SENP2/MLLT1/CTDSP2/HIPK2/KDM5A/BAX/PROS1/PIP5KL1/SORL1/PIK3CB/HNRNPU/CTSZ/TNF/ROCK2/PKN1/LRP1/CST3/MAS1/RGS2/GABARAPL2/PRKAR2A/PRDX5/CNOT11/CPEB3/MYADM/HHEX/CTDSP1/P3H1/ANXA2/CR1/UBE2B/BIRC6/YTHDF3/GADD45A/PTPN6/CPEB4/UBR5/AGO3/CDKN1C/IRAK3/PATL1/CDKN2D/CBL/PI3/EIF4EBP3/SERPING1/LSM14A/CAST/EIF2AK2/IVNS1ABP/HGF/TSC2/SUPT6H/GNAI2/YBX1/GREM1/GPI/MAP1A/DUSP22/KNG1/SOX4/INPP5K/MTM1/GIGYF2/GNL3L/CNOT6/PID1/BOD1L1/SH3RF1/KDM4A/N4BP1/PTGS2/ROCK1/PARP14/SESN2/XRCC1/HSPA1B/YWHAB/RYBP/SUFU/PSEN1/RARA/MAPT/RELA/RGS14/PTPRJ/FKBP1A/CSDE1/IFI16/PRR7/SOCS3/CAPN3/SQSTM1/SPAG9/PTPN1/ADAR/PLK1/AGO2/LGALS3/AKT1S1/RASSF2/PPP1R9B/HSPA1A/TUT7/NR4A1/TIMP1/HMGCR/GNAQ/MAD2L2/GPX1/SAMD4B/RPS6KA1/SIRPA/CDKN1A/LIN28A/SKI/EIF4EBP2/HIPK3/APLP2/SPRY3/LIMK1/SIAH2/SRC/APC/TPR/PPP1R15A/WARS1/DUSP6/PAK2/HSPB1/DCUN1D3/EIF2AK1/DUSP1/WAC/TNFSF14/UBE2O/CALM3/PLAUR/ZFP36L1/FXR2/BRCA1/JARID2/SERPINE1/AGO4/BAG6/POR/TRIP12/SH3BP5L/CHP1/LYN/FRY/SNCA/SDCBP/PYCARD/ARRB2/MMP9/XIAP/DTX3L/TIMP2/PTPRC/PCSK1N/PICALM/FBXO7/STK38/CTSB/PRKAR2B/DNAJB6/NF1/EPHB2/CORO1C/NIBAN1/DHCR24/G6PD/AQP1/DMTN/PRKDC/SFRP2/PRKAR1A/FLCN/FKBP8/PRKCD/PTEN/PSMF1/SPI1/IGF2BP2/SLC8A1/TRIB1/FURIN/AGO1/CTDSPL
CC	GO:0044309	neuron spine	100	-0.48	-1.81	6.36E-05	0.0005679	0.00034909	1802	tags=40%, list=15%, signal=34%	FARP1/RHOA/RAB8A/USP8/STRN4/NEURL1/HAP1/CAMK2A/CDK5R1/ABR/ATP6AP2/CPEB4/APBA1/DLG4/ATP2B1/EEA1/MAPT/RGS14/SIPA1L1/PPP1R9B/ABHD17C/CTTN/DNM3/PTK2B/SYNPO/ACTN1/PRKACA/FXR2/CTNND1/SLA/RAC1/ARRB2/PPP3CA/ASAP1/PRKAR2B/EPHB2/LRRC4/ABI3/NRGN/SLC8A1
MF	GO:0016772	transferase activity, transferring phosphorus-containing groups	668	-0.31	-1.40	6.60E-05	0.0005881	0.00036154	3091	tags=31%, list=26%, signal=25%	MKNK1/CLK3/MARK1/MAP3K1/CCL5/POLR2J/STK32C/NEK4/EPHB6/TYK2/CSK/GDPGP1/IP6K1/GRK2/DAPK2/PIP4K2B/PTDSS2/STK19/OAS3/TAOK2/CAMKK2/PIM3/TBCK/STK38L/PDK4/CSNK1A1/RSKR/MAPK1/UHMK1/PIKFYVE/TKFC/TTBK2/FES/CPNE3/MAP2K4/POLD1/PRKAG2/PRKCZ/ERN1/CSNK2A1/JAK2/DAPK1/DYRK1B/KIT/NEK6/DYRK1A/CSNK1G2/CCND3/OXSR1/CSF1R/TAF1/JAK1/CAMK2A/HK2/LTBP1/ERBB2/CKB/LRRK2/CRCP/GSK3B/BMPR1A/HIPK2/PIP5KL1/TESK2/PRKCB/NMNAT3/PIK3CB/ROCK2/NADK/PI4K2A/DYRK3/PKN1/HIPK1/CDK5R1/NRP2/PRKD2/PIP4K2C/ERN2/MARK2/CDK13/PKM/CERT1/SNRK/AKAP13/TAOK1/MAST3/SYK/PIP5K1C/PDXK/MAP2K2/SIK3/GAK/PIK3CG/WEE1/PCYT1A/PPM1D/GRK4/IRAK3/CAMK1D/STK4/PIM1/POLD3/EIF2AK2/RNASEL/STK24/CDIPT/CHRAC1/REV3L/NME4/MKNK2/MAP3K3/GRK6/PDK2/CDC42BPA/DSTYK/POLL/CDKL5/ROCK1/RPS6KA5/IRAK1/CCL3/GSK3A/FGR/IGF2R/EPHB4/ULK1/INSR/SQSTM1/VRK3/MAP3K20/PLK1/MAP3K5/RASSF2/IPMK/PPP1R9B/TUT7/BCKDK/MARK4/PTK2B/RIOK3/HCK/NEK5/NEK9/SLK/POLR2E/RPS6KA1/CDKN1A/MAP2K7/HIPK3/CSNK1G1/LIMK1/ILK/TAOK3/SRC/ALPK1/MAPKAPK2/POLR3H/DGKG/PAK2/GNPTAB/PFKFB3/PIP4K2A/EIF2AK1/RIPK1/MARK3/PRKACA/AKT2/AK1/MAPKAPK3/FGGY/AATK/CMPK2/SCYL1/TENT5C/GUK1/BRD4/MAST2/STK40/LYN/MMD/GRK3/PAK1/WNK1/BMP2K/CDK19/POLR2A/MAP3K2/PIK3CD/LIMK2/MAP2K3/HK1/MAP4K4/SRPK2/STK38/MAPK14/BMX/ITPK1/PDK3/STK10/EPHB2/TOP1/MAPK3/PRKDC/PFKFB4/MYLK/PRKCD/PINK1/NUAK2/MPP1/PEAK3

CC	GO:0070382	exocytic vesicle	129	-0.45	-1.75	6.70E-05	0.0005944	0.00036541	1967	tags=33%, list=17%, signal=27%	KIF1B/STXBP5/RAB6A/STX6/SLC6A9/APBA1/DLG4/ATP2B1/SYN3/STX3/LAMP1/DISC1/RAB3B/SNAP25/ROGDI/MCTP2/SYTL4/PSEN1/ATP6V0D1/RAB27B/RAB11B/NDEL1/SLC17A5/SYT12/RAB27A/RAB7A/STX11/CALM3/RAB5B/MME/DMXL2/UNC13D/SNCA/DNAJC5/PICALM/STON2/IQSEC1/TPRG1L/RAB3IL1/RAB5A/BCL2L1/RAB3D
BP	GO:0010632	regulation of epithelial cell migration	146	-0.43	-1.71	6.89E-05	0.0006074	0.00037342	2209	tags=32%, list=19%, signal=26%	CCBE1/STAT5A/PIK3CB/TNF/ROCK2/GPLD1/NRP2/PRKD2/MECP2/PRCP/SEMA4A/PIK3CG/GADD45A/SP1/RTN4/GPI/MAP3K3/PTGS2/RIN2/PTK2B/HDAC7/GFUS/DOCK5/SPARC/SRC/GRN/HSPB1/NFE2L2/SASH1/GLIPR2/RAC1/MMP9/ADIPOR1/PIK3CD/MAP2K3/MAP4K4/HMOX1/SP100/NF1/IQSEC1/CORO1C/DNAJA4/ITGB3/PTEN/TBXA2R/GLUL
BP	GO:0007611	learning or memory	135	-0.43	-1.72	6.95E-05	0.0006115	0.00037592	2896	tags=39%, list=25%, signal=30%	PRKAR1B/CLN3/SRF/KRAS/HIF1A/MAPK1/TLR2/CRTC1/FOS/PLCB1/PRKCZ/NEUROD2/ADNP/KIT/ACTR2/PJA2/ATXN1/PPT1/LILRB2/PAFAH1B1/MECP2/CPEB3/ITGA5/DLG4/CREB1/EP300/GPI/MAP1A/SNAP25/PTGS2/PSEN1/MAPT/RGS14/VLDLR/INSR/PPP1R9B/ADGRF1/HMGCR/NLGN3/EIF4EBP2/SLC7A11/MME/SYNJ1/CEBPB/OPRL1/PICALM/CIC/PRKAR2B/NF1/EPHB2/NRGN/PTEN/CLDN5
BP	GO:0044088	regulation of vacuole organization	49	-0.57	-1.91	7.07E-05	0.0006194	0.00038079	2750	tags=57%, list=23%, signal=44%	PIKFYVE/SCARB2/IFT88/WIPI1/FEZ1/LRRK2/RNF5/ADPRH/PIP4K2C/ANXA2/MTMR3/MTM1/TBC1D14/RAB1B/ULK1/RALB/NUPR1/ELAPOR1/GRN/WDR45/PIP4K2A/SH3GLB1/FEZ2/ATG2A/RAB5A/SNX18/PINK1/CHMP4B
CC	GO:0097060	synaptic membrane	156	-0.43	-1.71	7.06E-05	0.0006194	0.00038079	2196	tags=28%, list=19%, signal=23%	SYAP1/MAGEE1/DNM2/UTRN/PI4K2A/NRP2/GRIK2/ITSN1/HTR3B/SLC6A9/ATP6AP2/APBA1/DLG4/ATP2B1/STX3/CNIH3/SNAP25/PSEN1/PRR7/PPP1R9B/ABHD17C/SLC6A6/DNM3/NLGN3/NTNG2/STX11/DBN1/FBXO45/AKAP9/P2RY1/P2RX1/ARRB2/SYNJ1/PICALM/EPHB2/LRRC4/ITGB3/NRGN/DENND1A/SLC8A1/RNF10/NINJ1/LRFN1/HIP1
BP	GO:0051248	negative regulation of protein metabolic process	749	-0.30	-1.39	7.21E-05	0.0006303	0.00038745	2690	tags=32%, list=23%, signal=26%	CAPRIN1/WNT1/IL1B/ACOT8/PRKAG2/HEXIM2/DNAJC3/PRKCZ/PTPA/EIF4G1/CTSA/CSNK2A1/DAPK1/NCKAP1L/CNOT1/ANGPT1/SIN3A/VPS28/DDX6/RC3H2/HAP1/DDX3X/SH3BP5/TAF1/IREB2/CEBPA/TSPO/TRIM21/DCP2/SMARCC1/FLNA/SERPINE2/TNRC6B/IL1R2/LRRK2/RTN1/GSK3B/SENP2/MLLT1/LILRB2/CTDSP2/HIPK2/KDM5A/BAX/PROS1/PIP5KL1/SORL1/PIK3CB/HNRNPU/CTSZ/TNF/ROCK2/PKN1/LRP1/CST3/MAS1/RGS2/GGA3/GABARAPL2/PRKAR2A/ITM2B/PRDX5/CNOT11/CPEB3/MYADM/HHEX/CTDSP1/P3H1/ANXA2/CR1/UBE2B/BIRC6/YTHDF3/GADD45A/PTPN6/CPEB4/UBR5/AGO3/CDKN1C/IRAK3/PATL1/CDKN2D/CBL/PI3/EIF4EBP3/AGAP2/SERPING1/LSM14A/CAST/EIF2AK2/IVNS1ABP/HGF/TSC2/SUPT6H/GNAI2/RTN4/YBX1/GREM1/GPI/MAP1A/DUSP22/KNG1/SOX4/INPP5K/MTM1/GIGYF2/GNL3L/CNOT6/PID1/BOD1L1/SH3RF1/KDM4A/N4BP1/PTGS2/ROCK1/PARP14/SESN2/XRCC1/HSPA1B/YWHAB/RYBP/SUFU/PSEN1/RARA/MAPT/RELA/RGS14/PTPRJ/CLU/FKBP1A/CSDE1/IFI16/PRR7/SOCS3/CAPN3/SQSTM1/SPAG9/PTPN1/ADAR/PLK1/AGO2/LGALS3/AKT1S1/RASSF2/PPP1R9B/HSPA1A/TUT7/NR4A1/TIMP1/HMGCR/GNAQ/MAD2L2/GPX1/SAMD4B/RPS6KA1/SIRPA/RILP/CDKN1A/LIN28A/SKI/EIF4EBP2/HIPK3/APLP2/SPRY3/LIMK1/SIAH2/SRC/APC/TPR/PPP1R15A/WARS1/DUSP6/PAK2/HSPB1/DCUN1D3/EIF2AK1/DUSP1/WAC/TNFSF14/UBE2O/CALM3/PLAUR/ZFP36L1/FXR2/BRCA1/JARID2/SERPINE1/AGO4/BAG6/POR/TRIP12/SH3BP5L/CHP1/LYN/RTN3/FRY/SNCA/SDCBP/PYCARD/ARRB2/MMP9/XIAP/DTX3L/TIMP2/RTN2/PTPRC/PCSK1N/PICALM/FBXO7/STK38/CTSB/PRKAR2B/DNAJB6/NF1/EPHB2/CORO1C/NIBAN1/DHCR24/G6PD/AQP1/DMTN/PRKDC/SFRP2/ITGB3/PRKAR1A/FLCN/FKBP8/PRKCD/PTEN/PSMF1/SPI1/IGF2BP2/SLC8A1/FLOT2/TRIB1/FURIN/AGO1/CTDSPL

CC	GO:0045178	basal part of cell	157	-0.43	-1.73	7.44E-05	0.0006484	0.00039857	2513	tags=32%, list=21%, signal=25%	BEST1/SLC9A1/SHROOM4/C5AR2/CD38/ERBB2/CASR/SLCO4C1/LRP1/ATP7A/PRCP/ABCC4/ANXA2/LPO/SLC6A9/SLC22A1/ATP2B1/SLC12A6/DTNB/CEACAM5/NUMB/DSTYK/ABCC3/WASF2/ERBIN/AQP9/SLC23A2/C5AR1/SLC6A6/B4GALT1/TF/KCNQ1/MYO1D/KCNE3/FLOT1/P2RY1/SLC14A1/EPB41/SLC2A9/ARRB2/SLC1A5/SLC4A1/AQP1/BSG/ANK1/MSN/FLOT2/SLC7A5/TGFA/AQP10
CC	GO:0098797	plasma membrane protein complex	283	-0.37	-1.58	7.56E-05	0.0006574	0.00040412	2819	tags=36%, list=24%, signal=28%	GNAS/HLA-DRB1/ADAM8/GNA13/TLR2/SSPN/TRAF6/CACNA1A/KCNQ5/JAK2/CACNB1/PSEN2/SCN1B/GNB2/FCER1G/EPS15/PORCN/CACNA1I/FADD/ERBB2/IL18RAP/ABCA2/GNG5/GRB2/CYBB/BMPR1A/UTRN/GJC2/LRP1/TLR1/KCNAB2/KCNJ6/ITGAM/GRIK2/STXBP5/ITGA2B/SYK/IL10RB/ANXA2/HLA-E/CATSPER2/HTR3B/ITGA5/ITGAL/PTPN6/DLG4/CBL/NCF1/GNAI2/NCF4/AP2M1/CNIH3/CACNA1D/TLR6/SHC1/GNB1/CLTC/PSEN1/FKBP1A/ITGB2/INSR/ATP6V0D1/SLC6A6/CTNNA1/GNA12/GNAQ/PTK2B/AP2B1/APBB1IP/SNTB1/TF/KCNQ1/APC/CACNB4/ABCC9/AKAP9/FLOT1/RIPK1/IL6R/CALM3/GNAL/GP1BA/CTNND1/AP2A1/ITGB5/EPS15L1/NCF2/LYN/SDCBP/FCGR3A/SYNJ1/CFLAR/KCNJ5/PICALM/ABCG8/CORO1C/GNB4/ITGB3/IL13RA1/FLOT2/GP9/SLC7A5/GNAZ
BP	GO:0045766	positive regulation of angiogenesis	97	-0.48	-1.82	7.82E-05	0.0006767	0.00041602	2676	tags=40%, list=23%, signal=31%	IL1B/BTG1/STIM1/SIRT6/JAK1/HK2/CYBB/HIPK2/CCBE1/PRKCB/HIPK1/PRKD2/PKM/ITGA5/SP1/HGF/GREM1/LRG1/CHI3L1/AGO2/C5AR1/PTK2B/JMJD8/GRN/GAB1/HSPB1/CYP1B1/BRCA1/NFE2L2/SERPINE1/SASH1/ADM/PIK3CD/HMOX1/AQP1/STAT3/SFRP2/NINJ1/TBXA2R
BP	GO:1904018	positive regulation of vasculature development	97	-0.48	-1.82	7.82E-05	0.0006767	0.00041602	2676	tags=40%, list=23%, signal=31%	IL1B/BTG1/STIM1/SIRT6/JAK1/HK2/CYBB/HIPK2/CCBE1/PRKCB/HIPK1/PRKD2/PKM/ITGA5/SP1/HGF/GREM1/LRG1/CHI3L1/AGO2/C5AR1/PTK2B/JMJD8/GRN/GAB1/HSPB1/CYP1B1/BRCA1/NFE2L2/SERPINE1/SASH1/ADM/PIK3CD/HMOX1/AQP1/STAT3/SFRP2/NINJ1/TBXA2R
CC	GO:0030312	external encapsulating structure	183	-0.41	-1.65	7.83E-05	0.0006767	0.00041602	2519	tags=36%, list=21%, signal=28%	ANGPT1/SEMA7A/TGFBI/C1QA/DGCR6/COLEC12/LTBP1/SERPINE2/CRISP3/CRISPLD2/ALPL/CCBE1/SPON2/CTSZ/TNXB/SDC3/GPLD1/PKM/PLSCR1/P3H1/ORM1/ANXA2/F13A1/LAMA4/FCN1/SERAC1/PI3/SERPING1/NCAM1/GREM1/KNG1/VCAN/ERBIN/CLU/CHI3L1/AGRN/LGALS3/ANXA11/TIMP1/TFIP11/MEGF9/CTSD/LGALS9/COL9A3/NTNG2/SPARC/HDGF/PF4/PSAP/TGFB1I1/GP1BA/SERPINE1/ADAMTS4/GFOD2/TGM2/LAMC3/MMP9/TIMP2/CTSB/PHOSPHO1/SFRP2/PCSK6/HSD17B12/KRT1/LTBP2
BP	GO:0032868	response to insulin	185	-0.41	-1.66	7.90E-05	0.0006812	0.00041873	2723	tags=39%, list=23%, signal=30%	TLR2/PIK3R2/IL1B/SRSF4/LPIN2/RAB8A/SCAP/PRKCZ/SLC2A1/HADHA/SLC9A1/CCND3/SMARCC1/GRB2/GSK3B/RHOQ/SYAP1/SORL1/PRKCB/PHIP/GPLD1/UCP3/UCP2/PIP4K2C/PKM/MEAK7/SOGA1/SP1/ATP2B1/TSC2/GNAI2/INPP5K/PID1/SHC1/PDK2/OSBPL8/SESN2/GSK3A/GOT1/FUT7/SOCS3/PTPRE/INSR/PTPN1/SESN3/MAX/NDEL1/ZDHHC7/KCNQ1/EIF4EBP2/SRC/APC/ZNF106/GAB1/PIP4K2A/ZFP36L1/MYO5A/AKT2/ZBTB7B/LYN/IGF2/ADM/ADIPOR1/CFLAR/FBP1/MAPK14/IRS2/PRKDC/PRKCD/WDTC1/RAB31/FOXO4
BP	GO:0010927	cellular component assembly involved in morphogenesis	60	-0.55	-1.88	8.19E-05	0.0007036	0.00043253	3145	tags=45%, list=27%, signal=33%	CLASP1/EDN1/TNNT1/SRF/CD9/PIKFYVE/TLR2/DICER1/ACTG1/ABCA2/NECTIN2/KNL1/PAFAH1B1/ERCC2/CCDC136/AKAP13/MYH11/CAPN3/RFX2/TPM1/LMOD3/TBC1D20/CFLAR/TMOD1/PRKAR1A/MYL9/ACRBP
BP	GO:1905037	autophagosome organization	100	-0.47	-1.80	8.59E-05	0.0007343	0.00045139	2944	tags=43%, list=25%, signal=32%	PIP4K2B/CLN3/MFN2/PIKFYVE/IFT88/ATG2B/WIPI1/FEZ1/LRRK2/RNF5/ADPRH/MAP1LC3B/GABARAPL2/PIP4K2C/GABARAPL1/RAB43/RB1CC1/TP53INP2/MTMR3/MTM1/MAP1LC3A/TBC1D14/PSEN1/RAB1B/ULK1/WIPI2/RALB/NUPR1/ATG7/ELAPOR1/WDR45/RAB7A/GABARAP/PIP4K2A/SH3GLB1/FEZ2/ATG2A/MAP1LC3B2/RAB5A/SNX18/AMBRA1/PINK1/CHMP4B

BP	GO:0051402	neuron apoptotic process	150	-0.43	-1.70	8.63E-05	0.0007365	0.00045276	2930	tags=45%, list=25%, signal=34%	GBE1/AIFM1/CLN3/KRAS/NAIP/HIF1A/ADAM8/CASP2/RHOA/MAP2K4/SIGMAR1/PIGT/ADNP/JAK2/CASP9/ANGPT1/HSPA5/FADD/PPT1/HIPK2/BAX/TNF/CDK5R1/MECP2/ITGAM/GRIK2/MCL1/CPEB4/GCLM/AGAP2/DRAXIN/GPI/NR4A2/ROCK1/BID/CCL3/PSEN1/ATN1/MAX/AKT1S1/PTK2B/EGLN3/NUPR1/GPX1/EGLN2/MAP2K7/HDGF/GRN/CDC34/SOD2/AKT2/CLCF1/APAF1/SNCA/DNAJC5/TYROBP/ARRB2/XIAP/CEBPB/SRPK2/HMOX1/CORO1A/NF1/FOXO3/G6PD/PINK1/BCL2L1
CC	GO:0043197	dendritic spine	99	-0.47	-1.79	8.89E-05	0.0007556	0.0004645	1802	tags=39%, list=15%, signal=34%	FARP1/RHOA/RAB8A/USP8/STRN4/NEURL1/HAP1/CAMK2A/CDK5R1/ABR/ATP6AP2/CPEB4/APBA1/DLG4/ATP2B1/MAPT/RGS14/SIPA1L1/PPP1R9B/ABHD17C/CTTN/DNM3/PTK2B/SYNPO/ACTN1/PRKACA/FXR2/CTNND1/SLA/RAC1/ARRB2/PPP3CA/ASAP1/PRKAR2B/EPHB2/LRRC4/ABI3/NRGN/SLC8A1
BP	GO:0003006	developmental process involved in reproduction	504	-0.32	-1.44	8.88E-05	0.0007556	0.0004645	2676	tags=30%, list=23%, signal=24%	IL1B/HERPUD2/ASPM/NEURL1/TTC26/BTG1/SETD2/QKI/OR7C1/KIT/CBX7/HSPA5/DPCD/PAQR8/LDOC1/GALNT3/CEBPA/LHFPL2/SERPINE2/SPATA2/LRRK2/IFT81/GRB2/YY1/NECTIN2/KDM5B/SENP2/BMPR1A/ALPL/BAX/KNL1/TESK2/ZBTB16/ARMC2/DNM2/PAFAH1B1/SLCO4C1/MAS1/NKX3-1/RGS2/CFAP58/SGPL1/PDGFA/FZD5/CCDC136/UBE2B/BIRC6/CATSPER2/SPATA6/PTPN6/SSTR2/CDKN1C/ZGLP1/NKAPL/JAM3/MSH6/USP42/MYCBPAP/CBL/LRRC8A/FSCN3/STK4/ARID4A/C3orf62/PDE5A/STAT5B/JUNB/FOXJ2/TFEB/XRN2/PTGS2/ARNT/ATRX/DIAPH2/RARA/IGF2R/CSDE1/SOCS3/INSR/ABHD2/CCNF/SMARCA2/NDRG3/PPP1R9B/TUT7/PSME4/CTNNA1/H3-3A/BMP6/PTK2B/B4GALT1/NUPR1/COL9A3/LIN28A/SRC/NIPBL/ASCL2/UBB/BPTF/PSAP/PBX1/TPPP3/FANCC/KLC3/RFX2/SETX/CYP1B1/ZFP36L1/VDR/SPOCD1/PRKACA/ASF1B/PROK2/RBPJ/CCNI/UBR2/IRF2BPL/AGO4/BAG6/MME/MAST2/MYCBP/IGF2/CHD7/ADM/ARRB2/RAD23B/TBC1D20/PITHD1/LIMK2/NCOA4/ZMYND15/CEBPB/H3-3B/CTSB/MAPK14/DNAJB6/NCOA1/FOXO3/MARF1/YBX3/DHCR24/OSBP2/ACOX1/PRKDC/SFRP2/BCL2L1/BSG/BASP1/ROPN1L/PTEN/ACRBP
BP	GO:0003018	vascular process in circulatory system	141	-0.43	-1.70	9.12E-05	0.0007717	0.0004744	2148	tags=33%, list=18%, signal=27%	TNF/ROCK2/CASR/LRP1/RGS2/ABCC4/SLC44A1/HRH2/SLCO3A1/SLC6A9/SLC22A1/ATP2B1/GCLM/KNG1/ACTA2/SLC2A3/PTGS2/ROCK1/ABCC3/PTPRJ/INSR/SLC6A20/PTP4A3/SLC6A6/BMP6/GPX1/SLC38A5/DOCK5/SRC/SLC24A3/ABCC9/P2RY1/SOD2/TBXAS1/P2RX1/CTNNBIP1/ADM/SLC1A5/GUCY1A1/KEL/SLC8A1/PLVAP/TBXA2R/ECE1/SLC7A5/CLDN5
BP	GO:0050877	nervous system process	467	-0.33	-1.46	9.12E-05	0.0007717	0.0004744	2896	tags=33%, list=25%, signal=26%	PRKAR1B/CLN3/DOP1B/ST3GAL4/SRF/KRAS/GNAS/NR2F6/BEGAIN/HIF1A/SETD5/PBX3/GAA/MAGT1/OR4D9/CHD8/MAPK1/TLR2/CRTC1/FOS/DICER1/PLCB1/PRKCZ/SCARB2/NEUROD2/ADNP/OR7C1/KIT/ACTR2/SCN1B/BEST1/SLC9A1/TGFBI/SHROOM4/CLN6/P2RX7/CACNA1I/PDE6A/PJA2/SERPINE2/DGCR2/MYO9A/ATXN1/LRRK2/PPT1/GSK3B/LILRB2/HIPK2/PAFAH1B1/TNF/ROCK2/ABAT/PTGES/UBR3/OPA3/OR2G6/MECP2/S1PR2/GRIK2/CPEB3/PDE4A/KATNIP/LPO/CRYGS/HTR3B/ITGA5/TAS2R4/OR6C75/DLG4/CREB1/JAM3/EP300/CDKN2D/TMEM120A/HGF/OR2B11/GPI/CACNA1D/MAP1A/GIGYF2/SNAP25/TRIOBP/FFAR4/GNB1/NPTXR/PTGS2/MGLL/FBXO11/XRCC1/CCL3/GSK3A/PSEN1/MAPT/RGS14/VLDLR/INSR/DAW1/AGTPBP1/TBL1X/DIAPH1/PPP1R9B/ADGRF1/C5AR1/NR4A1/ESPN/HMGCR/NLGN3/PTK2B/GPX1/KCNQ1/EIF4EBP2/SRC/ACP3/NIPBL/PITPNA/AKAP9/GPR155/P2RY1/SOD2/GNAL/TNFRSF1B/PROK2/P2RX1/SLC7A11/RPGR/MME/SNCA/CHD7/LAMC3/ARRB2/SYNJ1/PPP3CA/CEBPB/OPRL1/PICALM/CIC/PRKAR2B/NF1/EPHB2/PTAFR/HPS1/MAPK3/CCDC39/NRGN/PTEN/TAS2R30/MBP/RIPOR2/RNF10/TSHZ3/ODAD4/CLDN5

BP	GO:0002433	immune response-regulating cell surface receptor signaling pathway involved in phagocytosis	25	-0.69	-1.98	9.20E-05	0.000773	0.00047521	1273	tags=48%, list=11%, signal=43%	SYK/PTPRJ/FGR/VAV1/HCK/LIMK1/VAV3/SRC/LYN/PAK1/PTPRC/PRKCD
BP	GO:0038096	Fc-gamma receptor signaling pathway involved in phagocytosis	25	-0.69	-1.98	9.20E-05	0.000773	0.00047521	1273	tags=48%, list=11%, signal=43%	SYK/PTPRJ/FGR/VAV1/HCK/LIMK1/VAV3/SRC/LYN/PAK1/PTPRC/PRKCD
BP	GO:0042330	taxis	335	-0.36	-1.56	9.18E-05	0.000773	0.00047521	2708	tags=36%, list=23%, signal=28%	FES/FPR2/RHOA/IL1B/ADAM17/ANO6/LYPLA2/NEXN/LPAR1/NCKAP1L/KIT/ANGPT1/SCN1B/CMTM7/SEMA7A/ALOX5/DOCK2/FCER1G/OXSR1/CSF1R/C5AR2/KLF7/FEZ1/ALCAM/PLXNB2/ERBB2/PIK3CB/EFNB1/FPR1/BST1/CASR/KIF5B/CDK5R1/ELMO2/NRP2/PRKD2/BIN2/RNF165/PDGFA/SEMA4A/SYK/PIP5K1C/CXCL16/PIK3CG/PF4V1/RIN3/JAM3/RAC2/EP300/CCR1/CAMK1D/LYST/TIRAP/NCAM1/DRAXIN/HGF/TSC2/STX3/GREM1/NIBAN2/CSF3R/RPS6KA5/CCL3/PTPRJ/ITGB2/EPHB4/LGR6/CMTM5/VAV1/LGALS3/C5AR1/NR4A1/PTK2B/CXCR2/LGALS9/CXCL5/PALLD/VAV3/CXCR1/DPYSL2/PF4/GAB1/HSPB1/DUSP1/TNFSF14/IL6R/PLAUR/PADI2/FEZ2/PROK2/PREX1/TREM1/SERPINE1/LYN/RAC1/WNK1/ARRB2/GPSM3/CYRIB/PIK3CD/PLXNC1/RHOG/MAPK14/CORO1A/EPHB2/PTAFR/VASP/IL17RA/MAPK3/RALBP1/ITGB3/PRKCD/BSG/SPI1/CCRL2/RIPOR2/MPP1/NINJ1/LSP1/ECE1
BP	GO:0044092	negative regulation of molecular function	707	-0.31	-1.41	9.25E-05	0.0007763	0.00047721	2436	tags=29%, list=21%, signal=24%	OXSR1/DDX3X/CABIN1/SH3BP5/HECW2/TAF1/CAMK2A/CEBPA/TRIM21/WAPL/FLNA/SERPINE2/ABCA2/LRRK2/GSK3B/SENP2/MLLT1/KDM5A/BAX/PROS1/ACACB/SORL1/PIK3CB/HNRNPU/PELI1/CTSZ/TNF/NFKBIA/ROCK2/RSF1/PKN1/LRP1/CST3/RGS2/PPIF/PRKAR2A/SYMPK/PIP4K2C/HDAC4/LRPAP1/PRDX5/HHEX/RCC2/ANXA2/ZFPM1/CR1/BIRC6/P2RY13/SLC6A9/GADD45A/PTPN6/PPP1R12A/CDKN1C/IRAK3/CDKN2D/CBL/TMBIM1/PI3/PIM1/SERPING1/CAST/HGF/TSC2/RUBCN/TXNIP/GNAI2/CPNE1/GPI/DISC1/DUSP22/KNG1/INPP5K/MSX1/GNL3L/MTURN/BOD1L1/SH3RF1/AOC3/PTGS2/ROCK1/IRAK1/YWHAB/SUFU/GSK3A/PSEN1/MAPT/RGS14/PTPRJ/ERBIN/FKBP1A/IFI16/SOCS3/EGLN1/PTPN1/ADAR/PLK1/RAP1GAP/NFKBIB/LGALS3/AKT1S1/PPP1R9B/NR4A1/RALB/TIMP1/WWP2/HMGCR/GNAQ/TFIP11/MAD2L2/NUPR1/GPX1/TESC/RPS6KA1/PRDX2/CDKN1A/PPP2R5A/PHACTR2/HIPK3/APLP2/SPRY3/LIMK1/TAOK3/SIAH2/SRC/APC/PPP1R15A/WARS1/ITPRIP/PAK2/HSPB1/PPP1R10/PIP4K2A/KCNE3/AKAP9/CYP1B1/DUSP1/TNFSF14/P2RY1/UBE2O/ACTB/DAP/CALM3/PLAUR/SERPINE1/GOLGA2/PPP1R12B/MEPCE/POR/SH3BP5L/CTNNBIP1/CHP1/LYN/FRY/ILRUN/SNCA/WNK1/PYCARD/OAZ1/ARRB2/MMP9/XIAP/MIDN/PPP3CA/F11R/OAZ2/DTX3L/TIMP2/PTPRC/OPRL1/PCSK1N/PICALM/FBXO7/STK38/CTSB/PRKAR2B/DNAJB6/HMOX1/SP100/NF1/EPHB2/CORO1C/SLC4A1/DNAJB2/DHCR24/IRS2/TCF7L2/AQP1/MAPK3/SFRP2/ITGB3/PRKAR1A/PRKCD/WDTC1/PTEN/PSMF1/SPI1/BCL3/RIPOR2/SLC8A1/TRIB1/IQGAP1/FURIN/DAB2/IQGAP2

BP	GO:0007229	integrin-mediated signaling pathway	73	-0.52	-1.84	9.38E-05	0.0007852	0.0004827	2332	tags=48%, list=20%, signal=39%	FLNA/FERMT3/PRAM1/BST1/ITGAM/ITGA2B/RCC2/SYK/ITGA5/ITGAL/CUL3/FGR/ERBIN/ITGB2/CD63/NEDD9/VAV1/PHACTR4/MPIG6B/TIMP1/CTNNA1/PTK2B/HCK/ILK/VAV3/SRC/LIMS1/MYH9/ITGB5/FYB1/PLEK/TLN1/DMTN/ITGB3/ZYX
BP	GO:0048771	tissue remodeling	86	-0.49	-1.81	9.78E-05	0.0008167	0.00050205	3002	tags=44%, list=26%, signal=33%	BAK1/CSK/PDK4/HIF1A/ADAM8/TRAF6/INPP5D/PPARGC1B/GPR137B/P2RX7/CSF1R/CD38/MITF/FLNA/BAX/PLEKHM1/ROCK2/CST3/ATP7A/SYK/TMBIM1/GREM1/ROCK1/RASSF2/TIMP1/PTK2B/TF/SNX10/SRC/RAB7A/VDR/RBPJ/CHD7/PPP3CA/NF1/ITGB3/RAB3D/CAPN1
BP	GO:0010720	positive regulation of cell development	186	-0.40	-1.65	9.96E-05	0.0008305	0.00051055	2798	tags=37%, list=24%, signal=29%	HIF1A/SHTN1/TLR2/CAPRIN1/DICER1/IL1B/ASPM/NEURL1/ADNP/KIT/ACTR2/SEMA7A/SMARCD3/SPEN/HAP1/TSPO/FLNA/SERPINE2/PLXNB2/ADD1/DNM2/PAFAH1B1/TNF/GJC2/S1PR2/MYADM/EIF4G2/TWF2/RAB21/PDE5A/DISC1/NIBAN2/TRIOBP/NUMB/CDKL5/IST1/MAPT/RGS14/ANKRD27/NEDD9/CUX1/NDEL1/DOCK5/CASS4/NIN/LIMK1/ILK/DBN1/LIMS1/CLCF1/TNFRSF1B/PREX1/LMOD3/MME/LYN/UNC13D/TGM2/TYROBP/RAC1/SYNJ1/F11R/PLXNC1/EPHB2/DMTN/PRKDC/MPL/TRIB1/DAB2/CLDN5
BP	GO:0050770	regulation of axonogenesis	90	-0.49	-1.83	0.00010186	0.0008473	0.00052086	2321	tags=37%, list=20%, signal=30%	ADNP/SEMA7A/PLXNB2/RTN4R/GSK3B/PAFAH1B1/CDK5R1/MARK2/SEMA4A/EIF4G2/KIAA0319/TWF2/DRAXIN/RTN4/DISC1/CDKL5/IST1/PSEN1/MAPT/ULK1/SIPA1L1/NDEL1/NIN/LIMK1/DBN1/XK/PAK2/PAK1/PLXNC1/EPHB2/KEL/PTEN/MBP
CC	GO:0031091	platelet alpha granule	64	-0.53	-1.85	0.00010341	0.0008584	0.00052768	2989	tags=61%, list=25%, signal=46%	SERPINA1/THBS1/CFD/CD9/VTI1B/CYB5R1/LHFPL2/SERPINE2/FERMT3/PROS1/ITGA2B/PDGFA/ORM1/F13A1/SERPING1/HGF/KNG1/ALDOA/SYTL4/CLU/TIMP1/QSOX1/F8/PHACTR2/APLP2/SPARC/ACTN1/PF4/F5/TOR4A/PECAM1/SELP/SERPINE1/TREML1/ACTN4/SNCA/IGF2/CYRIB/ITGB3
BP	GO:0001819	positive regulation of cytokine production	345	-0.35	-1.52	0.00010422	0.0008633	0.0005307	2830	tags=35%, list=24%, signal=27%	NLRP10/CLEC4E/HIF1A/AKIRIN2/ADAM8/NLRP12/TLR2/TRAF6/IL1B/PLCB1/ADAM17/PRKCZ/CCDC88B/SETD2/JAK2/KIT/SEMA7A/POU2F2/FCER1G/TICAM1/P2RX7/CSF1R/DDX3X/SETD4/FADD/LRRK2/CYBB/RFTN1/ARID5A/BMPR1A/LILRB2/CCBE1/SPON2/SORL1/BTN3A2/PELI1/TNF/ROCK2/MNDA/TLR1/TLR8/PRKD2/OSM/SLC11A1/FZD5/ORM1/SYK/ZFPM1/HLA-E/PIK3CG/ATP6AP2/FCN1/IRAK3/CLEC9A/AGPAT1/SULF2/TIRAP/EIF2AK2/HGF/TLR6/CD14/LILRA2/STAT5B/RSAD2/FRMD8/MAVS/PTGS2/ARNT/IRAK1/HSPA1B/CCL3/PSEN1/RARA/RELA/PTPRJ/FGR/CLU/IFI16/CHI3L1/C5AR1/HSPA1A/ARHGEF2/RIOK3/LGALS9/SRC/TBX21/PF4/MAPKAPK2/HSPB1/FLOT1/CYP1B1/RIPK1/IL6R/IRF1/ZBTB7B/BRCA1/SERPINE1/STMP1/LAPTM5/NLRC4/TYROBP/PYCARD/PANX2/GPSM3/CYRIB/PIK3CD/CEBPB/PTPRC/HK1/MAPK14/HMOX1/EPHB2/PTAFR/IL17RA/STAT3/NFAM1/BCL3/MBP/FURIN/SLC7A5
BP	GO:0006936	muscle contraction	163	-0.42	-1.69	0.00010543	0.0008715	0.00053575	2964	tags=42%, list=25%, signal=32%	TNNT1/GRK2/SLMAP/ORMDL3/SRF/GAA/SSPN/RHOA/ARHGEF11/KIT/SCN1B/SLC9A1/CD38/FLNA/TPCN2/TNF/UTRN/ROCK2/PLCE1/ABAT/RGS2/MYLPF/CERT1/MYH11/PIK3CG/SSTR2/ATP2B1/SULF2/PDE5A/CACNA1D/ACTA2/ALDOA/LTB4R/PTGS2/TPM3/ROCK1/DSC2/ASPH/NR4A1/CTTN/SNTB1/DOCK5/KCNQ1/MYL4/KCNE3/AKAP9/CALM3/PPP1R13L/PRKACA/PROK2/TPM1/P2RX1/PPP1R12B/LMOD3/TPM4/EHD3/KCNJ5/GUCY1A1/MAP2K3/MYOM1/TMOD1/PTAFR/MYLK/GSN/MYL9/SLC8A1/TBXA2R/SLC6A8

BP	GO:0018108	peptidyl-tyrosine phosphorylation	229	-0.38	-1.59	0.00011195	0.0009234	0.00056768	3103	tags=40%, list=26%, signal=30%	CD4/CLK3/CCL5/GATA1/RAP2C/EPHB6/TYK2/CEP43/CSK/PPP2R1A/IL31RA/CAMKK2/PARP9/FES/MAP2K4/ADAM17/NEURL1/PRKCZ/ADNP/JAK2/DYRK1B/KIT/ANGPT1/DYRK1A/CSF1R/SH3BP5/JAK1/ERBB2/HIPK2/TESK2/TNF/BST1/DYRK3/HIPK1/RICTOR/OSM/PDGFA/SYK/MAP2K2/ITGA5/WEE1/TNFRSF1A/PTPN6/DLG4/CBL/EIF2AK2/NCF1/HGF/GREM1/DUSP22/SHC1/DSTYK/PARP14/PSEN1/PTPRJ/FGR/ITGB2/EPHB4/SOCS3/INSR/PTPN1/NEDD9/BMP6/PTK2B/ARHGEF2/HCK/PPP2R5B/MAP2K7/CASS4/HIPK3/SRC/PAK2/PECAM1/IL6R/CLCF1/AATK/SCYL1/SH3BP5L/LYN/HCLS1/IGF2/ARRB2/MAP2K3/PTPRC/BMX/EPHB2/MAPK3/DMTN/SFRP2/ITGB3/PRKCD/TGFA
CC	GO:0030670	phagocytic vesicle membrane	65	-0.53	-1.85	0.00011664	0.0009574	0.00058853	3097	tags=54%, list=26%, signal=40%	HVCN1/SEC22B/ATP6V0B/ATP6V0C/CLEC4E/PIKFYVE/TLR2/RAB8A/SNAP23/RAB32/CYBB/DNM2/TAPBP/TLR1/ATP7A/SLC11A1/MCOLN1/RAB43/HLA-E/RAB34/RAB22A/LAMP2/RAC2/LAMP1/TLR6/ATP6V0A1/ATP6V0D1/VAMP3/RAB11B/RILP/RAB7A/RAB11FIP1/CORO1A/RAB5A/RAB31
BP	GO:0043523	regulation of neuron apoptotic process	125	-0.45	-1.75	0.00011678	0.0009574	0.00058853	2930	tags=46%, list=25%, signal=35%	GBE1/AIFM1/CLN3/KRAS/NAIP/HIF1A/ADAM8/CASP2/RHOA/MAP2K4/SIGMAR1/ADNP/JAK2/CASP9/ANGPT1/PPT1/HIPK2/BAX/TNF/CDK5R1/MECP2/ITGAM/GRIK2/MCL1/CPEB4/GCLM/AGAP2/DRAXIN/GPI/NR4A2/ROCK1/CCL3/PSEN1/AKT1S1/PTK2B/EGLN3/NUPR1/EGLN2/MAP2K7/HDGF/GRN/CDC34/SOD2/CLCF1/SNCA/DNAJC5/TYROBP/ARRB2/CEBPB/SRPK2/HMOX1/CORO1A/NF1/FOXO3/G6PD/PINK1/BCL2L1
BP	GO:0010951	negative regulation of endopeptidase activity	122	-0.45	-1.78	0.0001181	0.0009651	0.00059331	2199	tags=37%, list=19%, signal=30%	PROS1/SORL1/TNF/CST3/PRDX5/ANXA2/CR1/BIRC6/CDKN2D/PI3/SERPING1/CAST/HGF/GPI/KNG1/SH3RF1/PTGS2/ROCK1/IFI16/NR4A1/TIMP1/GPX1/RPS6KA1/APLP2/SIAH2/SRC/PAK2/TNFSF14/UBE2O/PLAUR/SERPINE1/POR/SNCA/ARRB2/MMP9/XIAP/TIMP2/PCSK1N/PICALM/DNAJB6/DHCR24/AQP1/SFRP2/PSMF1/FURIN
CC	GO:0016323	basolateral plasma membrane	134	-0.44	-1.72	0.00011822	0.0009651	0.00059331	2084	tags=29%, list=18%, signal=24%	CASR/SLCO4C1/LRP1/ATP7A/ABCC4/ANXA2/LPO/SLC6A9/SLC22A1/ATP2B1/SLC12A6/CEACAM5/NUMB/DSTYK/ABCC3/WASF2/ERBIN/AQP9/SLC23A2/C5AR1/SLC6A6/B4GALT1/KCNQ1/MYO1D/FLOT1/P2RY1/SLC14A1/EPB41/SLC2A9/ARRB2/SLC4A1/AQP1/BSG/ANK1/MSN/FLOT2/SLC7A5/TGFA/AQP10
BP	GO:0051668	localization within membrane	482	-0.32	-1.43	0.00012311	0.001001	0.00061534	2394	tags=27%, list=20%, signal=23%	RAB35/OPTN/YIF1B/SRPRA/FLNA/ERBB2/MIEF2/KIAA1109/ZDHHC12/RFTN1/DENND1C/BAX/RHOQ/SORL1/DNM2/EFR3A/ZDHHC2/TNF/VPS37B/ROCK2/PRAM1/GBP1/LRP1/KIF5B/GGA3/CDK5R1/ITGAM/GRIK2/MYADM/NECAB2/RAB34/STX6/GAK/TSPAN33/ITGAL/TNFRSF1A/RILPL1/FRMPD1/EXOC7/DLG4/GRIPAP1/SAV1/MICALL1/TMBIM1/NCF1/STX3/AP2M1/RAB3B/INPP5K/TSPAN14/SNAP25/PID1/CLTC/NUMB/WASHC2C/ROCK1/BID/ANKRD27/ITGB2/CAPN3/SQSTM1/SEC16A/AGRN/ARL13B/LGALS3/PPP1R9B/VAMP3/EXOC6/RAB11B/EMC3/TESC/ZDHHC7/AP2B1/PPP2R5A/RAB7A/VPS37C/PSAP/EXOC4/FLOT1/P2RY1/ACTB/CALM3/TMEM88/MYO5A/RAB11FIP4/SNX27/ZDHHC19/AKT2/EHD1/ARHGAP8/SH3GLB1/SLC7A11/ARHGAP1/MMGT1/BAG6/AFDN/FYB1/CHP1/FAM126B/SDCBP/RAC1/BBC3/F11R/EHD3/GET3/PTPRC/PICALM/RHOG/TSPAN5/PACS1/PPFIA1/IQSEC2/EPHB2/SLC4A1/RILPL2/LRRC4/ABI3/DMTN/DENND1A/GSN/BCL2L1/BSG/ANK1/RAB3D/ZDHHC3/RAB31/CHMP4B/FLOT2/RAPGEF2/DAB2/KIF13A
MF	GO:0005200	structural constituent of cytoskeleton	60	-0.54	-1.86	0.00012376	0.001003	0.00061661	2527	tags=45%, list=22%, signal=35%	ACTR2/ARPC4/VIM/ACTG1/TUBA1B/SPTA1/TUBGCP3/TUBB6/ARPC1B/TUBB4B/TUBA1A/ERBIN/AGRN/TUBB1/ARPC5/TUBB2A/ACTB/TUBA1C/TPM1/EPB41/TUBA4A/EPB42/TLN1/ANK1/MSN/SPTB/HIP1

BP	GO:0060070	canonical Wnt signaling pathway	182	-0.39	-1.60	0.00012387	0.001003	0.00061661	2681	tags=36%, list=23%, signal=28%	WNT1/TMEM131L/ASPM/WLS/USP8/ZEB2/JADE1/CSNK1G2/DDX3X/PORCN/MED12/MITF/LRRK2/GSK3B/TLE4/CTDNEP1/HHEX/FZD5/UBE2B/USP34/ATP6AP2/UBR5/STK4/DRAXIN/GREM1/DISC1/SOX4/FRMD8/NR4A2/CCNY/GSK3A/PSEN1/ZNF703/INVS/LGR6/TBL1X/GNAQ/VCP/MAD2L2/BCL9/DVL3/CSNK1G1/ILK/SIAH2/SRC/APC/FAM53B/FRAT1/TMEM88/RBPJ/OTUD5/KPNA1/CTNND1/TLE3/CTNNBIP1/WNK1/XIAP/MAPK14/FOXO3/TCF7L2/RAB5A/SFRP2/PPM1A/PTEN/GID8/DAB2
BP	GO:0060996	dendritic spine development	64	-0.53	-1.84	0.00012719	0.0010279	0.00063187	2690	tags=38%, list=23%, signal=29%	CAPRIN1/NEURL1/ACTR2/CAMK2A/LRRK2/PAFAH1B1/CDK5R1/CPEB3/CFL1/EIF4G2/DLG4/DISC1/PSEN1/SIPA1L1/DNM3/DBN1/PDLIM5/PAK2/DLG5/ASAP1/EPHB2/IQSEC1/ABI3/PTEN
BP	GO:0031668	cellular response to extracellular stimulus	185	-0.40	-1.64	0.00012832	0.0010349	0.00063618	2926	tags=42%, list=25%, signal=32%	AIFM1/SZT2/SRF/VPS41/PDK4/SLC1A2/MAPK1/FES/FOS/PRKAG2/SLC2A1/ADNP/EIF4G1/GCN1/WIPI1/HSPA5/P2RX7/LRRK2/RIPOR1/ATF3/MAP1LC3B/CASR/GABARAPL2/UCP2/SLC39A4/GABARAPL1/PPM1D/CPEB4/LAMP2/ATP2B1/CBL/MTMR3/PIM1/EIF2AK2/STK24/MAP1LC3A/PDK2/TFEB/NR4A2/DSC2/SESN2/IFI16/ULK1/WIPI2/SESN3/MAP3K5/MAX/PPP1R9B/RALB/CLEC16A/ATG7/ELAPOR1/CDKN1A/WDR45/GABARAP/KPTN/GAS2L1/DAP/VDR/RXRA/SH3GLB1/NFE2L2/LYN/SIPA1/RRAGD/PTPRC/HMOX1/NCOA1/MAP1LC3B2/FOXO3/MAPK3/SFRP2/FLCN/CD68/NUAK2/NPRL3/GLUL
BP	GO:0008064	regulation of actin polymerization or depolymerization	119	-0.45	-1.75	0.00013279	0.0010666	0.00065569	2726	tags=37%, list=23%, signal=29%	ARHGAP18/RHOA/CDC42EP4/NCKAP1L/ARPC4/CAPZB/GRB2/ADD1/NPHS1/RICTOR/ALOX15/MYADM/SPTA1/COTL1/TWF2/ARPC1B/TRIOBP/WASHC2C/CTTN/PTK2B/HCK/ARPC5/DBN1/FCHSD1/ESAM/SVIL/LMOD3/SSH1/ARPC1A/HCLS1/PYCARD/PLEK/CYRIB/TMOD1/CORO1A/SSH2/WAS/VASP/CYRIA/DMTN/MTPN/PRKCD/GSN/SPTB
BP	GO:0007269	neurotransmitter secretion	78	-0.50	-1.83	0.00013611	0.0010867	0.00066803	2550	tags=41%, list=22%, signal=32%	SNAP23/P2RX7/CAMK2A/LRRK2/PPT1/GSK3B/FBXL20/PRKCB/STXBP5/PIP5K1C/APBA1/SYN3/STX3/SNAP25/CTBP2/SNAP29/MCTP2/PSEN1/NR4A1/SYT12/STX11/P2RY1/CALM3/STXBP2/VPS18/P2RX1/SNCA/DNAJC5/SYNJ1/NF1/RAB5A/NAPA
BP	GO:0099643	signal release from synapse	78	-0.50	-1.83	0.00013611	0.0010867	0.00066803	2550	tags=41%, list=22%, signal=32%	SNAP23/P2RX7/CAMK2A/LRRK2/PPT1/GSK3B/FBXL20/PRKCB/STXBP5/PIP5K1C/APBA1/SYN3/STX3/SNAP25/CTBP2/SNAP29/MCTP2/PSEN1/NR4A1/SYT12/STX11/P2RY1/CALM3/STXBP2/VPS18/P2RX1/SNCA/DNAJC5/SYNJ1/NF1/RAB5A/NAPA
BP	GO:0007163	establishment or maintenance of cell polarity	160	-0.42	-1.67	0.00013594	0.0010867	0.00066803	3597	tags=47%, list=31%, signal=33%	RND1/RPGRIP1L/KRIT1/GBF1/LLGL1/CDC42BPB/PARD3/AMOT/KANK1/SPN/SLC9A3R1/RAB10/RHOB/SH3BP1/CLASP1/PKD1/FERMT1/ARFGEF1/BCAS3/GOLPH3/PDLIM1/WDPCP/SHTN1/RHOA/ARHGEF11/MAP7/PRKCZ/NCKAP1L/ACTR2/SLC9A1/DOCK2/FAM89B/FEZ1/MYO9A/GSK3B/NDE1/PAFAH1B1/ROCK2/RICTOR/MARK2/WEE1/LMNA/JAM3/RAC2/FSCN1/FSCN3/RAB11FIP2/ZW10/ROCK1/ERBIN/PLK1/MAP4/DOCK8/CTNNA1/PTK2B/NDEL1/ARPC5/ILK/ACTB/DLG5/SNX27/PARVB/PLEKHG3/MYH9/RAC1/CYRIB/RHOG/RHOBTB1/AQP1/GSN/ANK1/MSN/FRMD4B/RIPOR2/FLOT2

BP	GO:0033674	positive regulation of kinase activity	319	-0.35	-1.53	0.00013657	0.0010882	0.00066893	2696	tags=33%, list=23%, signal=26%	FPR2/TRAF6/RHOA/ACSL1/IL1B/PRKAG2/ADAM17/NEURL1/PRKCZ/ADNP/ERN1/JAK2/NCKAP1L/KIT/ANGPT1/GCN1/P2RX7/CCND3/CSF1R/DDX3X/LCP2/INCENP/ERBB2/LRRK2/BMPR1A/SYAP1/TAB2/TNF/RANBP2/MAS1/CDK5R1/RICTOR/ERN2/MARK2/SLC11A1/PDGFA/FZD5/AKAP13/TAOK1/SYK/MAP2K2/PIK3CG/DLG4/STK4/AGAP2/TIRAP/NCF1/PDE5A/GREM1/TLR6/OSBPL8/DSTYK/IRAK1/CCNY/PSEN1/MAPT/FGR/VLDLR/CLU/EPHB4/INSR/PTPN1/CHI3L1/MAP3K5/NEDD9/RASSF2/RALB/PTK2B/DVL3/CDKN1A/PARP16/MAP2K7/CASS4/VAV3/TAOK3/SRC/PAK2/PIK3R5/IL6R/CALM3/DAZAP2/SASH1/TAL1/LYN/MMD/SNCA/IGF2/STRADA/PAK1/WNK1/MAP2K3/PTPRC/EPHB2/CACUL1/MOB3A/STRADB/ITGB3/AMBRA1/PRKCD/PINK1/MOB3C/RAPGEF2/IQGAP1/TGFA
BP	GO:0008277	regulation of G protein-coupled receptor signaling pathway	87	-0.48	-1.79	0.00014066	0.0011141	0.00068485	2378	tags=39%, list=20%, signal=31%	CAMK2A/RPH3AL/DNM2/ROCK2/PLCE1/GTF2H2/RGS3/RGS2/PDE4A/NECAB2/GPR27/FRMPD1/GRK4/RGS6/GNAI2/GRK6/ROCK1/MGLL/YWHAB/GSK3A/RGS14/GIT2/PPP1R9B/ZDHHC7/ACP3/GRK3/SNCA/ADM/PLEK/ARRB2/OPRL1/RGS7/RASGRP4/ZDHHC3
BP	GO:0001666	response to hypoxia	182	-0.39	-1.59	0.00014115	0.0011157	0.00068588	3061	tags=39%, list=26%, signal=29%	CBFA2T3/EDN1/ADAM15/THBS1/PML/PDLIM1/SRF/HIF1A/ADAM8/TLR2/RHOA/REST/SCAP/ADAM17/SLC2A1/SLC9A1/AGTRAP/CD38/HK2/CYBB/HIPK2/PIK3CB/TNF/ROCK2/ABAT/CASR/ATP7A/NKX3-1/UCP3/UCP2/MECP2/ITPR2/BNIP3L/PDGFA/LMNA/EP300/ERO1A/NR4A2/PTGS2/ARNT/IRAK1/XRCC1/MTHFR/EGLN1/PLOD1/PTK2B/EGLN3/EGLN2/SRC/BACH1/ASCL2/SOD2/ZFP36L1/RBPJ/NFE2L2/ACTN4/APAF1/NPEPPS/ADM/CREBBP/CFLAR/ALAS2/HMOX1/NF1/FOXO3/PDK3/AQP1/MPL/PINK1/PTEN/SLC8A1
BP	GO:0150115	cell-substrate junction organization	67	-0.52	-1.83	0.00014321	0.0011298	0.00069451	2947	tags=45%, list=25%, signal=34%	BCAS3/THBS1/TAOK2/WDPCP/RHOA/SLC9A1/ACTG1/TESK2/ROCK2/RCC2/ITGA5/GREM1/DUSP22/ROCK1/PTPRJ/CTTN/PTK2B/SLK/ACTN1/SRC/LIMS1/RAC1/MAP4K4/IQSEC1/CORO1C/TLN1/DMTN/PTEN/VCL/ARHGAP6
CC	GO:0005912	adherens junction	108	-0.46	-1.74	0.00014363	0.0011308	0.00069515	1876	tags=27%, list=16%, signal=23%	EIF4G2/PIP5K1C/ANXA2/TSPAN33/NIBAN2/DSC2/PDLIM2/PPP1R9B/CTNNA1/APC/PDLIM5/ESAM/FLOT1/ACTB/MYO1E/CTNND1/MYH9/AFDN/LYN/SDCBP/SHROOM1/TLN1/PDLIM7/MSN/FRMD4B/VCL/FLOT2/ZYX/S100A11
BP	GO:1902414	protein localization to cell junction	61	-0.54	-1.86	0.0001468	0.0011512	0.00070769	2658	tags=41%, list=23%, signal=32%	RAB8A/PRKCZ/ACTG1/JAK1/ZDHHC12/NPHS1/ZDHHC2/KIF5B/DLG4/GRIPAP1/JAM3/STX3/MAP1A/SNAP25/MAPT/RAB27B/ZDHHC7/PAK2/HSPB1/PECAM1/ACTB/DLG5/F11R/IQSEC2/VCL
BP	GO:0017015	regulation of transforming growth factor beta receptor signaling pathway	76	-0.49	-1.77	0.00015158	0.0011864	0.0007293	2681	tags=37%, list=23%, signal=29%	WNT1/ADAM17/PBLD/ZEB2/FAM89B/HSPA5/LTBP1/HIPK2/DNM2/CDKN1C/IL17RD/EP300/CHST11/LRG1/ZEB1/FKBP1A/TRIM33/ZNF703/HSPA1A/SKI/TGFB1I1/ZBTB7A/NRROS/SDCBP/CREBBP/FLCN/PPM1A/DAB2
BP	GO:1903844	regulation of cellular response to transforming growth factor beta stimulus	77	-0.51	-1.82	0.00015333	0.0011978	0.00073631	2681	tags=38%, list=23%, signal=29%	WNT1/ADAM17/PBLD/ZEB2/FAM89B/HSPA5/LTBP1/HIPK2/DNM2/CDKN1C/IL17RD/EP300/CHST11/LRG1/ZEB1/FKBP1A/TRIM33/ZNF703/HSPA1A/SKI/TGFB1I1/ZBTB7A/NRROS/SDCBP/CREBBP/CFLAR/FLCN/PPM1A/DAB2
BP	GO:0097242	amyloid-beta clearance	27	-0.67	-1.97	0.00015655	0.0012205	0.00075029	2148	tags=56%, list=18%, signal=45%	TNF/LRP1/ITGAM/LRPAP1/CLTC/ROCK1/CLU/ITGB2/INSR/C5AR1/RAB11B/HMGCR/MME/PICALM/RAB5A

CC	GO:0005770	late endosome	225	-0.38	-1.57	0.00015758	0.0012261	0.00075376	2896	tags=36%, list=25%, signal=27%	PRKAR1B/KIDINS220/BST2/CLN3/VPS41/HLA-DRB1/MAPK1/PIKFYVE/VTI1B/SCARB2/YIPF1/LAMTOR4/VPS28/STOML1/SNX14/LRRK2/IFITM3/CHMP3/TPCN2/SORL1/PLEKHM1/VPS37B/ATP7A/SLC11A1/YIPF2/MCOLN1/MFSD12/SLC31A1/ANXA2/MAP2K2/RAB22A/VPS33A/LAMP2/MICALL1/RUBCN/LAMP1/MTM1/MAP1LC3A/ANKRD27/IGF2R/VPS11/MON1B/CD63/INSR/SQSTM1/RAB27B/ARL8A/CLEC16A/ANKRD13A/IFNAR1/VPS4B/TF/ELAPOR1/RILP/RAB27A/KCNQ1/CHMP1A/SRC/GRN/RAB7A/VPS37C/PSAP/SLC29A3/MYO5A/VPS18/MMD/UNC13D/UBXN6/CHMP2A/MARCHF8/MAPK3/IFITM2/PRKAR1A/CD68/CHMP4B/RAPGEF2/DYSF/NBR1/LITAF/OSBPL1A
BP	GO:0061515	myeloid cell development	65	-0.52	-1.83	0.00015999	0.0012424	0.00076375	3076	tags=55%, list=26%, signal=41%	MED1/GATA1/BCL6/ATP6AP1/SRF/TLR2/NCKAP1L/KIT/EVI2B/PAFAH1B1/ERCC2/ANXA2/ZFPM1/PTPN6/EP300/ARID4A/TSPAN2/MEIS1/WASF2/MPIG6B/SRC/BPGM/FLI1/PIP4K2A/ZBTB7A/TAL1/NRROS/PTBP3/TYROBP/ALAS2/TRIM58/EPB42/SLC4A1/FAM210B/G6PD/DMTN
BP	GO:0018212	peptidyl-tyrosine modification	232	-0.38	-1.59	0.00016035	0.0012428	0.00076401	3103	tags=40%, list=26%, signal=30%	CD4/CLK3/CCL5/GATA1/RAP2C/EPHB6/TYK2/CEP43/CSK/PPP2R1A/IL31RA/CAMKK2/PARP9/FES/MAP2K4/ADAM17/NEURL1/PRKCZ/ADNP/JAK2/DYRK1B/KIT/ANGPT1/DYRK1A/CSF1R/SH3BP5/JAK1/ERBB2/HIPK2/TESK2/TNF/BST1/DYRK3/HIPK1/RICTOR/OSM/PDGFA/SYK/MAP2K2/ITGA5/WEE1/TNFRSF1A/PTPN6/DLG4/CBL/EIF2AK2/NCF1/HGF/GREM1/DUSP22/SHC1/DSTYK/PARP14/PSEN1/PTPRJ/FGR/ITGB2/EPHB4/SOCS3/INSR/PTPN1/NEDD9/BMP6/PTK2B/ARHGEF2/HCK/PPP2R5B/MAP2K7/CASS4/HIPK3/SRC/PAK2/PECAM1/IL6R/CLCF1/AATK/SCYL1/SH3BP5L/LYN/HCLS1/IGF2/ARRB2/MAP2K3/PTPRC/BMX/EPHB2/MAPK3/DMTN/SFRP2/ITGB3/PRKCD/TGFA
BP	GO:0003158	endothelium development	74	-0.51	-1.83	0.00016088	0.0012439	0.0007647	3320	tags=46%, list=28%, signal=33%	CCM2/RHOB/MYD88/ICAM1/SLC40A1/ATOH8/RAP2C/RHOA/IL1B/PLCB1/VEZF1/BTG1/ADD1/TNF/ROCK2/PRKD2/S1PR2/MYADM/TNFRSF1A/FOXJ2/ROCK1/ZEB1/BMP6/GPX1/PECAM1/RBPJ/KDM6B/AFDN/NOTCH4/F11R/MSN/VCL/RAPGEF2/CLDN5
BP	GO:0038094	Fc-gamma receptor signaling pathway	29	-0.66	-1.95	0.00016408	0.0012633	0.00077659	1273	tags=41%, list=11%, signal=37%	PTPRJ/FGR/VAV1/HCK/LIMK1/VAV3/SRC/LYN/PAK1/FCGR3A/PTPRC/PRKCD
BP	GO:0098742	cell-cell adhesion via plasma-membrane adhesion molecules	87	-0.48	-1.78	0.00016426	0.0012633	0.00077659	2352	tags=34%, list=20%, signal=28%	NEXN/CD177/ALCAM/PLXNB2/NECTIN2/PIK3CB/ITGAM/MYADM/ITGA5/ITGAL/DSG3/CEACAM5/DSC2/PCDH1/ITGB2/NTNG2/PALLD/SELL/ESAM/PECAM1/PCDHGA8/PCDHB1/SELP/IL1RN/WNK1/MAPK14/LRRC4/BSG/MBP/CLDN5
BP	GO:0010959	regulation of metal ion transport	220	-0.39	-1.61	0.00016796	0.0012892	0.00079251	2613	tags=33%, list=22%, signal=26%	ANO6/STIM1/CACNB1/PSEN2/SCN1B/BEST1/SLC9A1/P2RX7/HAP1/OXSR1/HECW2/CAMK2A/TSPO/AHCYL1/FLNA/SERPINE2/LILRB2/BAX/DNM2/UTRN/GJC2/CASR/KIF5B/ATP7A/KCNAB2/YWHAH/PIK3CG/SLC6A9/PTPN6/ATP2B1/CCR1/CRACR2B/GNAI2/CACNA1D/INPP5K/LILRA2/TRPC3/PTGS2/CCL3/FKBP1A/CAPN3/CD63/SELENON/DIAPH1/LGALS3/WWP2/PTK2B/TESC/TF/KCNQ1/CACNB4/KCNE3/AKAP9/CALM3/PRKACA/P2RX1/EPB41/ACTN4/CHP1/LYN/SNCA/CHD7/WNK1/ARRB2/EHD3/OPRL1/CORO1A/RGS7/G6PD/KEL/MYLK/SLC8A1
BP	GO:0016358	dendrite development	144	-0.43	-1.69	0.00017907	0.0013718	0.0008433	2902	tags=36%, list=25%, signal=28%	TAOK2/KIDINS220/SCARF1/FARP1/CRTC1/CAPRIN1/RHOA/NEURL1/LST1/ACTR2/HECW2/KLF7/CAMK2A/LRRK2/GSK3B/PAFAH1B1/CDK5R1/MECP2/YWHAH/CPEB3/CFL1/EIF4G2/KIAA0319/RAB21/DLG4/RERE/CAMK1D/DISC1/MAP1A/CDKL5/PSEN1/ANKRD27/VLDLR/SIPA1L1/PPP1R9B/CUX1/DNM3/DBN1/DGKG/PDLIM5/PAK2/DLG5/CARM1/PPP3CA/PICALM/MAP1S/ASAP1/EPHB2/IQSEC1/ABI3/PTEN/RAPGEF2

BP	GO:0031032	actomyosin structure organization	122	-0.45	-1.75	0.00017956	0.0013729	0.00084399	2132	tags=28%, list=18%, signal=23%	ROCK2/ARAP1/MYH10/AKAP13/MYH11/FHOD1/CUL3/INPP5K/CDC42BPA/ROCK1/WASF2/CAPN3/PXN/PTK2B/LIMK1/SRC/PAK2/TPM1/MYH9/ITGB5/LMOD3/EPB41/PAK1/RAC1/F11R/CFLAR/PPFIA1/TMOD1/WAS/PRKAR1A/MYL9/CNN2/ZYX/ARHGAP6
BP	GO:0030336	negative regulation of cell migration	176	-0.39	-1.59	0.00018465	0.0014092	0.00086628	2414	tags=27%, list=21%, signal=22%	C5AR2/ARHGDIB/MITF/BMPR1A/PIP5KL1/TNF/LRP1/MECP2/MIIP/S1PR2/SRGAP1/GADD45A/RIN3/RNF20/STK24/GREM1/DUSP22/OSBPL8/PTPRJ/ABHD2/TIMP1/GNA12/CYP1B1/DUSP1/DLG5/PADI2/NFE2L2/SERPINE1/TPM1/AFDN/RAC1/ADIPOR1/HMOX1/SP100/NF1/FOXO3/CORO1C/DNAJA4/STAT3/SFRP2/FLCN/CNN2/PTEN/VCL/RIPOR2/TRIB1/TBXA2R/CLDN5
BP	GO:1904705	regulation of vascular associated smooth muscle cell proliferation	33	-0.62	-1.91	0.00018552	0.0014131	0.0008687	2782	tags=58%, list=24%, signal=44%	EDN1/MFN2/GNA13/RHOA/ERN1/JAK2/BMPR1A/TNF/S1PR2/GNAI2/FOXJ2/MAP3K5/GNA12/CDKN1A/SOD2/TPM1/MMP9/HMOX1/PTEN
BP	GO:0045446	endothelial cell differentiation	67	-0.52	-1.81	0.00018648	0.0014177	0.00087151	2676	tags=37%, list=23%, signal=29%	IL1B/PLCB1/VEZF1/BTG1/ADD1/TNF/ROCK2/S1PR2/MYADM/TNFRSF1A/FOXJ2/ROCK1/ZEB1/BMP6/GPX1/PECAM1/RBPJ/KDM6B/AFDN/NOTCH4/F11R/MSN/VCL/RAPGEF2/CLDN5
BP	GO:0030832	regulation of actin filament length	120	-0.45	-1.75	0.00018788	0.0014253	0.00087618	2726	tags=37%, list=23%, signal=28%	ARHGAP18/RHOA/CDC42EP4/NCKAP1L/ARPC4/CAPZB/GRB2/ADD1/NPHS1/RICTOR/ALOX15/MYADM/SPTA1/COTL1/TWF2/ARPC1B/TRIOBP/WASHC2C/CTTN/PTK2B/HCK/ARPC5/DBN1/FCHSD1/ESAM/SVIL/LMOD3/SSH1/ARPC1A/HCLS1/PYCARD/PLEK/CYRIB/TMOD1/CORO1A/SSH2/WAS/VASP/CYRIA/DMTN/MTPN/PRKCD/GSN/SPTB
BP	GO:0050900	leukocyte migration	246	-0.37	-1.57	0.0001882	0.0014253	0.00087618	1855	tags=28%, list=16%, signal=24%	SYK/PIP5K1C/CXCL16/ITGAL/PIK3CG/PF4V1/RIN3/JAM3/RAC2/CCR1/CAMK1D/CHST2/LYST/TIRAP/GREM1/STAT5B/CSF3R/ROCK1/CCL3/ITGB2/FUT7/VAV1/LGALS3/C5AR1/DOCK8/PTK2B/CXCR2/B4GALT1/HCK/SIRPA/LGALS9/CXCL5/VAV3/SELL/CXCR1/SRC/TBX21/PF4/PECAM1/DUSP1/TNFSF14/IL6R/SELP/PADI2/PREX1/TREM1/SERPINE1/MYH9/LYN/RAC1/WNK1/PYCARD/MMP9/GPSM3/PIK3CD/F11R/RHOG/HMOX1/CORO1A/STK10/PTAFR/IL17RA/MAPK3/BSG/MSN/SPI1/RIPOR2/MPP1/PLVAP/NINJ1
BP	GO:0044262	cellular carbohydrate metabolic process	197	-0.38	-1.58	0.00018948	0.0014323	0.00088048	2259	tags=30%, list=19%, signal=25%	IMPA2/GSK3B/NCOR1/ACACB/RANBP2/MAS1/GPLD1/MECP2/HDAC4/PGAM1/MGAM/PPP1R3B/SOGA1/RB1CC1/EP300/INPP5K/PDK2/MOGAT3/SESN2/GSK3A/GOT1/INSR/B4GALT5/BCKDK/PTK2B/B4GALT1/NUPR1/DDB1/BPNT2/PGD/SRC/NCOA2/GNPTAB/PFKFB3/P2RY1/B3GNT8/AKT2/FGGY/DGAT2/NDST1/PYGL/STK40/SNCA/IGF2/PLEK/MIDN/ADIPOR1/SYNJ1/HK1/FBP1/ITPK1/PDK3/PTAFR/SLC4A1/IRS2/STAT3/PFKFB4/PTEN/GPD2
CC	GO:0042581	specific granule	143	-0.43	-1.69	0.00019111	0.0014418	0.00088636	2876	tags=38%, list=25%, signal=29%	ORMDL3/MMP25/MOSPD2/ADAM8/FPR2/ANO6/CD177/CANT1/SNAP23/DOCK2/CYBB/CRISP3/CTSZ/BST1/SLCO4C1/KCNAB2/ITGAM/AP1M1/ORM1/PDXK/ITGAL/PTPN6/TMBIM1/STX3/RAB37/CKAP4/TSPAN14/SNAP25/SLC2A3/LRG1/SLC44A2/PTPRJ/ITGB2/CHI3L1/NEU1/ANXA11/QSOX1/CTSD/CD93/RAB27A/ALDH3B1/STXBP2/PLAUR/OSCAR/TNFRSF1B/P2RX1/DNAJC5/TIMP2/KPNB1/STK10/PGLYRP1/TOLLIP/CNN2/VCL
BP	GO:0002274	myeloid leukocyte activation	179	-0.40	-1.62	0.00019492	0.0014678	0.00090232	2723	tags=41%, list=23%, signal=32%	CCL5/GATA1/MFHAS1/CST7/THBS1/IL31RA/RELB/TLR2/FES/FPR2/TRAF6/CD177/JAK2/SNAP23/KIT/DOCK2/GPR137B/FCER1G/TICAM1/C1QA/LCP2/CEBPA/PJA2/LRRK2/NECTIN2/TNF/JUND/PRAM1/TLR1/ITGAM/CD300LF/SLC11A1/PLSCR1/LTBR/SYK/NMI/PIK3CG/PTPN6/RAC2/CBL/TLR6/LILRA2/CCL3/PSEN1/MAPT/FGR/CLU/ITGB2/C5AR1/SBNO2/CXCR2/CD93/LGALS9/GRN/KMT2E/LAT2/STXBP2/PREX1/RBPJ/PTGDS/BATF2/LYN/UNC13D/SNCA/TYROBP/PYCARD/PIK3CD/PTPRC/HMOX1/PTAFR/PRKCD/SPI1/DYSF/GAB2

BP	GO:0043254	regulation of protein-containing complex assembly	304	-0.35	-1.52	0.00019533	0.0014681	0.00090251	2726	tags=36%, list=23%, signal=28%	ARHGAP18/FES/FARP2/RHOA/PRKCZ/SLAIN2/CDC42EP4/EIF4G1/NCKAP1L/HCFC1/HSPA5/DYRK1A/TICAM1/DDX3X/ARPC4/CAPZB/GRB2/CLIP1/GSK3B/BAX/ADD1/FBLIM1/NPHS1/SORL1/TNF/CPTP/CDK5R1/MECP2/RICTOR/ALOX15/STXBP5/MYADM/SPTA1/COTL1/TWF2/SYK/ATF7IP/ARPC1B/JAM3/FSCN1/EP300/TIRAP/EIF2AK2/TLR6/GNL3L/TRIOBP/WASHC2C/BID/HSPA1B/MAPT/ANKRD27/CLU/ULK1/LGALS3/HSPA1A/CTTN/LCAT/RALB/H3-3A/TFIP11/PTK2B/RIOK3/VCP/HCK/PPP2R5B/NIN/ARPC5/SRC/DBN1/WARS1/FCHSD1/ESAM/TPPP3/AKAP9/SELP/SVIL/SH3GLB1/LMOD3/SSH1/TAL1/STMP1/ARPC1A/CTNNBIP1/HCLS1/SNCA/PAK1/RAC1/PYCARD/PLEK/CYRIB/H3-3B/LCP1/ASAP1/TMOD1/CORO1A/SSH2/VASP/CYRIA/MED25/DMTN/MTPN/NAPA/AMBRA1/PRKCD/GSN/PINK1/PPM1A/MSN/SPTB
CC	GO:0005902	microvillus	49	-0.56	-1.86	0.00020063	0.0014995	0.00092178	2062	tags=37%, list=18%, signal=30%	ATP7A/PDGFA/JAM3/FSCN1/FSCN3/AOC3/ESPN/SLC6A6/MYO1D/EXOC4/MYO1E/MYO1F/ATP6V1B2/ITGB3/MSN/SLC7A5/ATP6V1A/IQGAP2
BP	GO:0008361	regulation of cell size	113	-0.46	-1.75	0.00020327	0.0015164	0.00093216	2882	tags=43%, list=25%, signal=33%	CLN3/SRF/SHTN1/RHOA/ANO6/ADNP/SEMA7A/SIN3A/LAMTOR4/P2RX7/RTN4R/GSK3B/DNM2/PAFAH1B1/ATP7A/CDK5R1/SEMA4A/EIF4G2/KIAA0319/TWF2/RAB21/RB1CC1/CREB1/LRRC8A/DRAXIN/SLC12A6/RTN4/DISC1/CDKL5/IST1/MAPT/ULK1/VAV1/AKT1S1/CTTN/NDEL1/LIMK1/VAV3/DPYSL2/DBN1/XK/RAC1/PLEK/AQP1/MTPN/KEL/MSN/WDTC1/PTEN
BP	GO:0016525	negative regulation of angiogenesis	51	-0.57	-1.90	0.00020614	0.0015291	0.00094001	2162	tags=37%, list=18%, signal=31%	PIK3CB/TNF/ROCK2/MECP2/HHEX/SEMA4A/GADD45A/NIBAN2/FOXJ2/ROCK1/SPARC/PF4/SERPINE1/EPN1/NF1/FOXO4/AGO1/E2F2/CLDN5
BP	GO:1901343	negative regulation of vasculature development	51	-0.57	-1.90	0.00020614	0.0015291	0.00094001	2162	tags=37%, list=18%, signal=31%	PIK3CB/TNF/ROCK2/MECP2/HHEX/SEMA4A/GADD45A/NIBAN2/FOXJ2/ROCK1/SPARC/PF4/SERPINE1/EPN1/NF1/FOXO4/AGO1/E2F2/CLDN5
BP	GO:2000181	negative regulation of blood vessel morphogenesis	51	-0.57	-1.90	0.00020614	0.0015291	0.00094001	2162	tags=37%, list=18%, signal=31%	PIK3CB/TNF/ROCK2/MECP2/HHEX/SEMA4A/GADD45A/NIBAN2/FOXJ2/ROCK1/SPARC/PF4/SERPINE1/EPN1/NF1/FOXO4/AGO1/E2F2/CLDN5
BP	GO:0021700	developmental maturation	161	-0.42	-1.67	0.00020686	0.0015316	0.00094155	2862	tags=39%, list=24%, signal=30%	EDN1/FERMT1/PPP2R1A/ANKRD17/SCLT1/SCARF1/HIF1A/TRPC4AP/SLC26A6/FARP2/WNT1/RHOA/NEURL1/NEUROD2/RAB32/C1QA/TMEM79/CEBPA/LRRK2/NFIA/ZBTB16/ZDHHC2/CDK5R1/MECP2/ERCC2/AP1M1/FZD5/CATSPER2/ATP6AP2/CDKN1C/DLG4/RERE/SLFN14/RFLNB/GREM1/DISC1/YWHAZ/NR4A2/ANKRD27/CD63/B4GALT5/ABHD2/AGRN/TUT7/PTK2B/SNX10/ZBTB7A/PRKACA/RBPJ/TAL1/AP1G1/PTBP3/TRIM58/PICALM/EPB42/LYL1/NF1/FOXO3/PHOSPHO1/FAM210B/G6PD/ROPN1L/PTEN
BP	GO:1990874	vascular associated smooth muscle cell proliferation	34	-0.62	-1.90	0.00021052	0.0015558	0.0009564	2782	tags=56%, list=24%, signal=43%	EDN1/MFN2/GNA13/RHOA/ERN1/JAK2/BMPR1A/TNF/S1PR2/GNAI2/FOXJ2/MAP3K5/GNA12/CDKN1A/SOD2/TPM1/MMP9/HMOX1/PTEN
BP	GO:0072593	reactive oxygen species metabolic process	146	-0.42	-1.65	0.00021216	0.001564	0.00096144	2840	tags=41%, list=24%, signal=32%	PDK4/GSTP1/HIF1A/AATF/PIKFYVE/FPR2/RHOA/CD177/ALOX5/P2RX7/TSPO/HK2/LRRK2/GRB2/CYBB/TNF/BST1/ATP7A/ITGAM/PRCP/PRDX5/SYK/LPO/GADD45A/RAC2/NCF1/GNAI2/NCF4/TLR6/PID1/SESN2/MAPT/ITGB2/VAV1/PTK2B/GPX1/PRDX2/CDKN1A/RAB27A/FANCC/CYP1B1/RIPK1/SOD2/ALOX12/PREX1/BRCA1/NFE2L2/NCF2/NRROS/SNCA/TYROBP/RAC1/CFLAR/MAPK14/FOXO3/PDK3/G6PD/ACOX1/PRKCD/PINK1

BP	GO:0001933	negative regulation of protein phosphorylation	238	-0.37	-1.56	0.00021241	0.001564	0.00096144	2226	tags=32%, list=19%, signal=26%	MLLT1/CTDSP2/BAX/PIP5KL1/SORL1/PIK3CB/PKN1/LRP1/MAS1/RGS2/PRKAR2A/MYADM/HHEX/CTDSP1/UBE2B/GADD45A/PTPN6/CDKN1C/IRAK3/CDKN2D/CBL/HGF/TSC2/GREM1/DUSP22/INPP5K/PID1/PARP14/PSEN1/RGS14/PTPRJ/SOCS3/SPAG9/PTPN1/ADAR/PLK1/AKT1S1/RASSF2/HMGCR/GNAQ/SIRPA/CDKN1A/HIPK3/SPRY3/APC/WARS1/DUSP6/PAK2/HSPB1/DUSP1/CALM3/SH3BP5L/CHP1/LYN/SNCA/PYCARD/ARRB2/PTPRC/FBXO7/STK38/PRKAR2B/NF1/EPHB2/CORO1C/NIBAN1/DMTN/PRKDC/SFRP2/PRKAR1A/FKBP8/PRKCD/PTEN/SLC8A1/TRIB1/CTDSPL
BP	GO:0072657	protein localization to membrane	417	-0.33	-1.46	0.00021775	0.0016002	0.00098373	2383	tags=27%, list=20%, signal=23%	OPTN/YIF1B/SRPRA/FLNA/ERBB2/MIEF2/ZDHHC12/RFTN1/BAX/RHOQ/EFR3A/ZDHHC2/TNF/VPS37B/ROCK2/PRAM1/GBP1/LRP1/KIF5B/GGA3/CDK5R1/ITGAM/GRIK2/MYADM/NECAB2/RAB34/GAK/TSPAN33/ITGAL/TNFRSF1A/RILPL1/FRMPD1/EXOC7/DLG4/GRIPAP1/SAV1/MICALL1/TMBIM1/NCF1/STX3/AP2M1/RAB3B/INPP5K/TSPAN14/SNAP25/PID1/CLTC/NUMB/ROCK1/BID/ITGB2/CAPN3/SQSTM1/SEC16A/AGRN/ARL13B/LGALS3/PPP1R9B/VAMP3/RAB11B/EMC3/TESC/ZDHHC7/AP2B1/PPP2R5A/RAB7A/VPS37C/EXOC4/FLOT1/P2RY1/ACTB/CALM3/TMEM88/MYO5A/ZDHHC19/AKT2/EHD1/SH3GLB1/SLC7A11/MMGT1/BAG6/AFDN/FYB1/CHP1/FAM126B/SDCBP/BBC3/F11R/EHD3/GET3/PICALM/RHOG/TSPAN5/PACS1/PPFIA1/IQSEC2/EPHB2/SLC4A1/RILPL2/LRRC4/ABI3/DMTN/GSN/BCL2L1/BSG/ANK1/RAB3D/ZDHHC3/RAB31/CHMP4B/FLOT2/RAPGEF2/DAB2/KIF13A
BP	GO:1905114	cell surface receptor signaling pathway involved in cell-cell signaling	303	-0.36	-1.54	0.00021957	0.0016078	0.00098837	2681	tags=33%, list=23%, signal=26%	WNT1/RHOA/TMEM131L/ASPM/WLS/USP8/PRKCZ/CSNK2A1/ZEB2/JADE1/P2RX7/CSNK1G2/DDX3X/MBD2/PORCN/MED12/MITF/LRRK2/GSK3B/SENP2/ZRANB1/TLE4/ABAT/LRP1/CTDNEP1/MECP2/S1PR2/ITGAM/GRIK2/MARK2/HHEX/FZD5/UBE2B/APCDD1/USP34/ATP6AP2/UBR5/DLG4/CTR9/SULF2/STK4/DRAXIN/TSC2/GREM1/CUL3/DISC1/SOX4/GRK6/FRMD8/NR4A2/CCNY/GSK3A/PSEN1/ZNF703/INVS/LGR6/RTF1/TBL1X/GNAQ/NLGN3/PTK2B/VCP/MAD2L2/LRRFIP2/BCL9/DVL3/SKI/DDB1/CSNK1G1/ILK/SIAH2/SRC/APC/TGFB1I1/FAM53B/FRAT1/TMEM88/RBPJ/OTUD5/KPNA1/CTNND1/P2RX1/TLE3/HIC1/CTNNBIP1/SNCA/RAC1/WNK1/ARRB2/XIAP/PPP3CA/MAPK14/FOXO3/TCF7L2/RAB5A/SFRP2/PPM1A/PTEN/GID8/DAB2
BP	GO:0006904	vesicle docking involved in exocytosis	26	-0.66	-1.93	0.00022117	0.0016135	0.00099189	1485	tags=38%, list=13%, signal=34%	RAB3B/SNAP25/CTBP2/VPS11/VAMP3/RALB/STXBP2/VPS18/PLEK/RAB3D

BP	GO:0009628	response to abiotic stimulus	707	-0.30	-1.38	0.00023037	0.0016775	0.00103126	2723	tags=29%, list=23%, signal=24%	TLR2/CASP2/CRTC1/FOS/RO60/RHOA/IL1B/MAP2K4/REST/MAP7/POLD1/DNAJC3/SCAP/ADAM17/SLC2A1/NEUROD2/CASP9/KIT/BEST1/SLC9A1/HSPA5/P2RX7/OXSR1/DDX3X/MBD2/TAF1/NDUFS2/ACTG1/AGTRAP/CD38/TSPO/HK2/FADD/SERPINE2/UBE4B/GRB2/YY1/UVSSA/CYBB/GSK3B/HIPK2/BAX/FBXL17/PIK3CB/DNM2/TNF/NFKBIA/ROCK2/JUND/PKN1/ABAT/GTF2H2/CASR/ATP7A/NKX3-1/TLR8/ACOT11/GPLD1/UCP3/NFAT5/UCP2/MECP2/ITPR2/ERCC2/GRIK2/BNIP3L/PDGFA/METRNL/LTBR/MCOLN1/COP1/KIAA0319/FECH/ZNF516/UBE2B/BABAM1/TNFRSF1A/GADD45A/PPM1D/CPEB4/GRK4/LMNA/GUCY1B1/MSH6/ATP2B1/EP300/CDKN2D/TMEM120A/CBL/LRRC8A/POLD3/ERO1A/SLC12A6/TXNIP/GPI/DISC1/CD14/NABP2/TRPC3/NR4A2/N4BP1/PTGS2/ARNT/IRAK1/XRCC1/MTHFR/TMEM161A/HSPA1B/MAPT/RELA/RGS14/IFI16/EGLN1/CAPN3/AQP9/MAP3K20/CHI3L1/ATP6V0D1/HSPA1A/NR4A1/TIMP1/PLOD1/RAB11B/HMGCR/BMP6/GNAQ/PTK2B/ARHGEF2/TSC22D4/VCP/EGLN3/GPX1/ATG7/CDKN1A/EGLN2/KCNQ1/MAP2K7/DDB1/SRC/BACH1/MICU1/NIPBL/TPR/MAPKAPK2/ASCL2/HSPB1/DCUN1D3/AKAP9/TNFSF14/P2RY1/SOD2/ZFP36L1/IRF1/PRKACA/AKT2/BRCA1/RBPJ/KAT5/NFE2L2/TSC22D3/SLC7A11/MME/ACTN4/APAF1/NPEPPS/CHP1/LYN/ADM/ARRB2/MMP9/SIPA1/CREBBP/MAP3K2/F11R/CFLAR/ALAS2/GMPR/GUCY1A1/PTPRC/PCSK1N/MAPK14/DNAJB6/HMOX1/NF1/FOXO3/PDK3/PTAFR/RGS7/YBX3/DNAJA4/AQP1/MAPK3/PRKDC/SFRP2/MTPN/MYLK/MPL/PRKCD/GSN/PINK1/BCL2L1/CNN2/PTEN/BCL3/SLC8A1/FKBPL/ATP6V1A/AQP10
BP	GO:0150116	regulation of cell-substrate junction organization	49	-0.55	-1.84	0.00023303	0.0016939	0.00104129	2947	tags=47%, list=25%, signal=35%	BCAS3/THBS1/WDPCP/RHOA/SLC9A1/ACTG1/ROCK2/RCC2/GREM1/DUSP22/ROCK1/PTPRJ/SLK/SRC/LIMS1/RAC1/MAP4K4/IQSEC1/CORO1C/DMTN/PTEN/VCL/ARHGAP6
BP	GO:2000785	regulation of autophagosome assembly	44	-0.58	-1.89	0.00023375	0.0016959	0.00104257	2750	tags=64%, list=23%, signal=49%	PHF23/SEC22B/PIP4K2B/PIKFYVE/IFT88/WIPI1/FEZ1/LRRK2/RNF5/ADPRH/PIP4K2C/MTMR3/MTM1/TBC1D14/RAB1B/ULK1/RALB/NUPR1/ELAPOR1/WDR45/PIP4K2A/SH3GLB1/FEZ2/ATG2A/RAB5A/SNX18/PINK1/CHMP4B
BP	GO:2000146	negative regulation of cell motility	184	-0.40	-1.61	0.00023657	0.0017133	0.00105324	2414	tags=27%, list=21%, signal=22%	C5AR2/ARHGDIB/MITF/BMPR1A/PIP5KL1/TNF/LRP1/MECP2/MIIP/S1PR2/SRGAP1/GADD45A/RIN3/RNF20/STK24/GREM1/DUSP22/OSBPL8/PTPRJ/ABHD2/TIMP1/CTNNA1/GNA12/CYP1B1/DUSP1/DLG5/PADI2/NFE2L2/SERPINE1/TPM1/AFDN/RAC1/ADIPOR1/HMOX1/SP100/NF1/FOXO3/CORO1C/WAS/DNAJA4/STAT3/SFRP2/FLCN/CNN2/PTEN/VCL/RIPOR2/TRIB1/TBXA2R/CLDN5
CC	GO:0030175	filopodium	68	-0.51	-1.79	0.00023901	0.0017278	0.00106216	1860	tags=29%, list=16%, signal=25%	TWF2/SYNE2/FSCN1/FSCN3/MTM1/ACTA2/PPP1R9B/B4GALT1/ACP3/MYO5A/FXR2/AP2A1/FGD4/ERMN/LCP1/VASP/ITGB3/MSN/RIPOR2/IQGAP2
BP	GO:1902115	regulation of organelle assembly	169	-0.41	-1.66	0.00024165	0.0017435	0.00107183	2589	tags=37%, list=22%, signal=29%	IFT88/CNOT1/WIPI1/CEP295/HAP1/FEZ1/LRRK2/RNF5/GSK3B/CHMP3/ADPRH/HNRNPU/DNM2/TNF/TBC1D30/PIP4K2C/SYNE2/FSCN1/MTMR3/MTM1/CNOT6/IFT140/TBC1D14/CDKL5/HSPA1B/RAB1B/VPS11/ULK1/PLK1/MAP4/HSPA1A/RALB/SPICE1/MARK4/CEP97/HCK/NUPR1/VPS4B/ELAPOR1/CHMP1A/SRC/WDR45/TPR/PIP4K2A/SH3GLB1/FEZ2/BRCA1/CCSAP/ATG2A/SDCBP/LIMK2/LCP1/ASAP1/CHMP2A/ABI3/RAB5A/SNX18/GSN/PINK1/CCP110/MSN/CHMP4B

BP	GO:0048878	chemical homeostasis	684	-0.30	-1.37	0.00024206	0.0017435	0.00107183	3125	tags=34%, list=27%, signal=27%	IMMT/AP3D1/ELOVL1/HVCN1/LRRC8D/LAMTOR1/DMXL1/USF2/CCL5/GATA1/SLC40A1/TFR2/EDN1/PKD1/NEO1/ABCA1/BAK1/RAB11FIP5/STAT1/CDH23/MLLT6/PIM3/ATP6AP1/PML/CLN3/ORMDL3/SRF/PDK4/GSTP1/SMARCB1/HLA-DRB1/HIF1A/ATP2A3/MAPK1/GNA13/SLC26A6/SLC9A8/FPR2/PIK3R2/DHPS/CACNA1A/POLD1/STIM1/ADNP/SIRT6/ERN1/LPAR1/JAK2/HPS6/FIS1/SLC9A1/ALOX5/SIN3A/HECTD4/CCDC186/CLN6/P2RX7/RCN3/HAP1/DDX3X/TMEM79/C5AR2/STEAP4/KLF7/IREB2/CD38/CEBPA/RPH3AL/HK2/ABCA2/CKB/CA2/LRRK2/PPT1/TPCN2/BAX/PRKCB/PIK3CB/FPR1/PLCE1/CASR/ATP7A/GPLD1/UCP2/ITPR2/SLC39A4/GRIK2/PRCP/SLC11A1/CES1/MCOLN1/CERT1/SLC31A1/GPR27/PIK3CG/SLC6A9/NPC2/ATP6AP2/PTPN6/SLC39A7/DLG4/VPS33A/ATP2B1/GCLM/CCR1/LRRC8A/ABCB11/RAB11FIP2/ERO1A/SLC12A6/TMTC2/GNAI2/RTN4/ANKRD9/GPI/DISC1/CACNA1D/KNG1/SOX4/TLCD2/INPP5K/SIDT2/ATP6V0A1/TLCD4/FFAR4/PDK2/TRPC3/ARF1/SESN2/NKIRAS2/MTCH2/CCL3/PSEN1/GOT1/FKBP1A/RMI1/EGLN1/CAPN3/AQP9/INSR/SELENON/SESN3/MAFG/ATP6V0D1/FLG2/DIAPH1/MT2A/C5AR1/LCAT/MBD5/RAB11B/BMP6/PTK2B/CXCR2/GPX1/TF/KCNQ1/LIN28A/DDB1/SNX10/CXCR1/MICU1/GRN/RAB7A/XK/SLC24A3/KCNE3/EIF2AK1/P2RY1/CALM3/SOD2/VDR/GP1BA/PRKACA/EHD1/ALOX12/PROK2/DGAT2/TBXAS1/ABHD4/P2RX1/SLC7A11/PYGL/ABCG1/CHP1/DMXL2/LYN/SNCA/TGM2/CHD7/C2CD2L/SLC25A37/WNK1/ADM/XIAP/TBC1D20/ADIPOR1/PPP3CA/GRINA/NCOA4/ALAS2/PTPRC/OPRL1/PICALM/HK1/EPB42/ABHD5/HMOX1/ABCG8/CORO1A/ACO1/FOXO3/PDK3/SLC4A1/PNPLA2/IRS2/TCF7L2/ACOX1/AQP1/STAT3/MAPK3/FTL/KEL/FTH1/WDTC1/RALY/KRT1/CCRL2/SLC8A1/TBXA2R/STAU1/GP9/ATP6V1A
BP	GO:0007034	vacuolar transport	138	-0.43	-1.69	0.00025153	0.0018085	0.00111175	2289	tags=35%, list=20%, signal=28%	LRRK2/CHMP3/TPCN2/SORL1/PLEKHM1/VPS37B/LRP1/DENND3/GGA3/GAK/TRAK1/VPS33A/LAMP2/LYST/HOOK3/LAMP1/MTM1/AP3S2/ZFYVE16/CLU/IGF2R/UEVLD/VPS11/MON1B/ATP6V0D1/HSPA1A/CLEC16A/VCP/VPS4B/RILP/CHMP1A/GRN/RAB7A/VPS37C/PSAP/GNPTAB/SNX27/VPS18/AP1G1/LAPTM5/UBXN6/EHD3/NCOA4/DTX3L/CHMP2A/PINK1/CHMP4B/KIF13A
BP	GO:0051961	negative regulation of nervous system development	73	-0.50	-1.79	0.00025604	0.0018376	0.00112963	2723	tags=47%, list=23%, signal=36%	TLR2/DICER1/IL1B/REST/SEMA7A/TRIM11/TSPO/RTN4R/BMPR1A/SORL1/TNF/CDK5R1/YWHAH/PCM1/SEMA4A/CTDSP1/KIAA0319/DRAXIN/HOOK3/RTN4/KDM4A/PSEN1/ULK1/CTNNA1/LIN28A/SKI/ASCL2/PPP3CA/NF1/EPHB2/PTEN/MBP/RNF10/RAPGEF2
BP	GO:0052547	regulation of peptidase activity	263	-0.36	-1.54	0.00025654	0.0018378	0.0011298	3002	tags=41%, list=26%, signal=32%	BAK1/RFFL/SERPINA1/CST7/THBS1/AIFM1/BST2/PML/NAIP/TNFSF10/AKIRIN2/ATP2A3/NLRP12/CASP2/RHOA/REST/CSNK2A1/JAK2/DAPK1/FIS1/CASP9/CIDEB/RCN3/DDX3X/FADD/BEX3/SERPINE2/ABCA2/BAX/PROS1/SORL1/TNF/ROCK2/LRP1/CST3/NKX3-1/PRDX5/SYK/ANXA2/CR1/BIRC6/CDKN2D/PI3/SERPING1/CAST/HGF/VSIR/GPI/KNG1/SH3RF1/PSME3/PTGS2/ROCK1/BID/MAPT/IFI16/PRR7/MAP3K5/ASPH/NR4A1/TIMP1/PSME4/CTSD/VCP/EGLN3/GPX1/RPS6KA1/LGALS9/APLP2/SIAH2/SRC/GRN/PAK2/NRDC/TNFSF14/UBE2O/DAP/PLAUR/SERPINE1/P2RX1/PRELID1/ADRM1/POR/APAF1/LAPTM5/NLRC4/LYN/SNCA/PYCARD/ARRB2/MMP9/XIAP/BBC3/CFLAR/TIMP2/PCSK1N/PICALM/CTSB/DNAJB6/DHCR24/AQP1/STAT3/SFRP2/GSN/PINK1/PSMF1/MBP/FURIN/HIP1

BP	GO:0043086	negative regulation of catalytic activity	478	-0.32	-1.43	0.00026306	0.0018778	0.00115435	2247	tags=29%, list=19%, signal=25%	GSK3B/MLLT1/KDM5A/PROS1/ACACB/SORL1/PIK3CB/HNRNPU/TNF/ROCK2/PKN1/LRP1/CST3/RGS2/PRKAR2A/PIP4K2C/PRDX5/HHEX/RCC2/ANXA2/CR1/BIRC6/P2RY13/GADD45A/PTPN6/PPP1R12A/CDKN1C/IRAK3/CDKN2D/CBL/TMBIM1/PI3/SERPING1/CAST/HGF/TSC2/RUBCN/TXNIP/GNAI2/GPI/DUSP22/KNG1/INPP5K/BOD1L1/SH3RF1/AOC3/PTGS2/ROCK1/YWHAB/GSK3A/PSEN1/MAPT/RGS14/PTPRJ/FKBP1A/IFI16/SOCS3/EGLN1/PTPN1/ADAR/PLK1/LGALS3/AKT1S1/PPP1R9B/NR4A1/TIMP1/HMGCR/GNAQ/TFIP11/MAD2L2/GPX1/TESC/RPS6KA1/CDKN1A/PPP2R5A/PHACTR2/HIPK3/APLP2/SPRY3/LIMK1/TAOK3/SIAH2/SRC/APC/PPP1R15A/WARS1/ITPRIP/PAK2/HSPB1/PPP1R10/PIP4K2A/AKAP9/DUSP1/TNFSF14/UBE2O/PLAUR/SERPINE1/PPP1R12B/POR/SH3BP5L/CHP1/LYN/FRY/SNCA/WNK1/PYCARD/OAZ1/ARRB2/MMP9/XIAP/MIDN/F11R/OAZ2/DTX3L/TIMP2/PTPRC/PCSK1N/PICALM/FBXO7/STK38/CTSB/PRKAR2B/DNAJB6/NF1/EPHB2/CORO1C/SLC4A1/DHCR24/IRS2/AQP1/SFRP2/PRKAR1A/PRKCD/WDTC1/PTEN/PSMF1/SLC8A1/TRIB1/IQGAP1/FURIN/IQGAP2
BP	GO:0030833	regulation of actin filament polymerization	106	-0.46	-1.73	0.0002655	0.0018884	0.0011609	2726	tags=40%, list=23%, signal=31%	ARHGAP18/RHOA/CDC42EP4/NCKAP1L/ARPC4/CAPZB/GRB2/ADD1/NPHS1/RICTOR/ALOX15/MYADM/SPTA1/COTL1/TWF2/ARPC1B/TRIOBP/WASHC2C/CTTN/PTK2B/HCK/ARPC5/DBN1/FCHSD1/ESAM/SVIL/LMOD3/SSH1/ARPC1A/HCLS1/PYCARD/CYRIB/TMOD1/CORO1A/SSH2/VASP/CYRIA/DMTN/MTPN/PRKCD/GSN/SPTB
BP	GO:0001503	ossification	222	-0.37	-1.56	0.0002651	0.0018884	0.0011609	2804	tags=36%, list=24%, signal=28%	ALYREF/HIF1A/MAPK1/CLIC1/TRAF6/RHOA/REST/OSTF1/ANO6/PPARGC1B/SEMA7A/ALOX5/P2RX7/CEBPA/GSK3B/BMPR1A/ALPL/ZBTB16/HNRNPU/TNF/JUND/CASR/GPLD1/ERCC2/ALOX15/FHL2/HIRA/ANKH/TOB2/TP53INP2/ATP2B1/CHSY1/RRBP1/CCR1/HEMGN/HGF/CEBPD/RFLNB/GREM1/JUNB/FFAR4/CLTC/PTGS2/VCAN/CCL3/SUFU/FGR/INTU/ADAR/RASSF2/SBNO2/H3-3A/BMP6/PTK2B/HDAC7/SKI/BPNT2/ILK/SNX10/NIPBL/SLC24A3/PBX1/IL6R/PRKACA/RBPJ/TPM4/CTNNBIP1/MRC2/IGF2/BMP2K/MMP9/CEBPB/H3-3B/MAPK14/NF1/PHOSPHO1/MAPK3/PDLIM7/SFRP2/SLC8A1/NBR1
BP	GO:0016241	regulation of macroautophagy	138	-0.43	-1.69	0.00026689	0.0018916	0.00116282	2944	tags=38%, list=25%, signal=29%	PIP4K2B/ATP6V0B/CLN3/ATP6V0C/HIF1A/VPS13D/TBC1D25/EPM2A/ERN1/WIPI1/TECPR1/OPTN/FEZ1/LRRK2/CAPNS1/CDK5R1/ATP6V1C1/PIP4K2C/BNIP3L/CALCOCO2/EXOC7/SPTLC2/TSC2/RUBCN/MTM1/ATP6V0A1/SESN2/ULK1/SESN3/ATP6V0D1/RALB/CLEC16A/QSOX1/NUPR1/ELAPOR1/WDR45/IKBKG/EXOC4/PIP4K2A/WAC/PRKACA/SH3GLB1/FEZ2/ATP6V1B2/ATG2A/HMOX1/MAPK3/SNX18/AMBRA1/PINK1/CAPN1/CHMP4B/ATP6V1A
BP	GO:0045637	regulation of myeloid cell differentiation	146	-0.41	-1.64	0.00026666	0.0018916	0.00116282	1700	tags=45%, list=14%, signal=39%	CNOT4/CD4/KLF13/MED1/CDC73/GATA1/STAT1/L3MBTL1/HLA-DRB1/HIF1A/FES/FOS/TRAF6/INPP5D/NCKAP1L/PPARGC1B/GPR137B/EVI2B/FADD/MITF/ZBTB16/TNF/NFKBIA/ZFPM1/TOB2/CREB1/CTR9/CCR1/MEIS1/STAT5B/MTURN/CSF3R/ARNT/H4C8/HSPA1B/CCL3/RARA/SMAP1/RASSF2/HSPA1A/PTK2B/TESC/CUL4A/PF4/RIPK1/ZFP36L1/TAL1/RCOR1/CTNNBIP1/LYN/HCLS1/TYROBP/PITHD1/CEBPB/MAPK14/NF1/FAXDC2/FOXO3/TFE3/FAM210B/STAT3/PRKDC/MPL/SPI1/LILRB3/TRIB1
BP	GO:1900024	regulation of substrate adhesion-dependent cell spreading	40	-0.58	-1.87	0.00027819	0.0019681	0.00120988	2332	tags=48%, list=20%, signal=38%	FLNA/DNM2/GBP1/MYADM/RCC2/TRIOBP/WASHC2C/NEDD9/DOCK5/CASS4/ILK/LIMS1/PREX1/ACTN4/UNC13D/RAC1/CORO1C/DMTN/DAB2

BP	GO:0032271	regulation of protein polymerization	152	-0.41	-1.64	0.00027875	0.0019686	0.00121017	2726	tags=39%, list=23%, signal=30%	ARHGAP18/FES/RHOA/SLAIN2/CDC42EP4/NCKAP1L/DYRK1A/ARPC4/CAPZB/GRB2/CLIP1/ADD1/NPHS1/CDK5R1/MECP2/RICTOR/ALOX15/MYADM/SPTA1/COTL1/TWF2/ARPC1B/TRIOBP/WASHC2C/HSPA1B/MAPT/HSPA1A/CTTN/PTK2B/HCK/NIN/ARPC5/DBN1/FCHSD1/ESAM/TPPP3/AKAP9/SVIL/LMOD3/SSH1/ARPC1A/HCLS1/SNCA/PAK1/RAC1/PYCARD/CYRIB/TMOD1/CORO1A/SSH2/VASP/CYRIA/DMTN/MTPN/AMBRA1/PRKCD/GSN/PINK1/SPTB
BP	GO:0032984	protein-containing complex disassembly	195	-0.38	-1.58	0.00028895	0.002037	0.00125225	2387	tags=33%, list=20%, signal=27%	TECPR1/SNX14/SMARCC1/CAPZB/DHX8/GSK3B/CHMP3/ADD1/NCKAP5L/TNF/MAP1LC3B/DYRK3/ARID1A/KIF5B/CALCOCO2/CFL1/SPTA1/MCOLN1/TAOK1/TWF2/GAK/IRAK3/VPS33A/LAMP2/RUBCN/MAP1A/TRIOBP/MAP1LC3A/WASHC2C/IST1/SNAP29/INSR/ASPH/PPP1R9B/CLEC16A/TFIP11/ARHGEF2/VCP/GSPT1/VPS4B/SMARCD2/CHMP1A/APC/FRAT1/SETX/GLE1/SVIL/SH3GL1/LMOD3/NFE2/CCSAP/PLEK/SYNJ1/MAP1S/TMOD1/MAP1LC3B2/CHMP2A/DMTN/MTPN/NAPA/GSN/SPTB/CHMP4B/MICAL2
BP	GO:2000377	regulation of reactive oxygen species metabolic process	105	-0.46	-1.74	0.00029594	0.0020826	0.00128025	2967	tags=42%, list=25%, signal=32%	PLIN5/THBS1/GSTP1/HIF1A/AATF/PIKFYVE/FPR2/RHOA/CD177/ALOX5/TSPO/HK2/GRB2/TNF/BST1/ITGAM/PRCP/SYK/GADD45A/RAC2/GNAI2/TLR6/PID1/MAPT/ITGB2/PTK2B/PRDX2/CDKN1A/RAB27A/CYP1B1/RIPK1/SOD2/BRCA1/NFE2L2/SNCA/TYROBP/RAC1/CFLAR/MAPK14/FOXO3/PDK3/G6PD/PRKCD/PINK1
BP	GO:0034765	regulation of ion transmembrane transport	233	-0.37	-1.56	0.00029862	0.0020978	0.00128957	2713	tags=33%, list=23%, signal=26%	ALG10B/CLIC1/CACNA1A/KCNQ5/ANO6/STIM1/DAPK1/CACNB1/PSEN2/SCN1B/SLC9A1/P2RX7/CLIC3/HAP1/OXSR1/HECW2/CACNA1I/AHCYL1/FLNA/CYBB/TPCN2/BAX/DNM2/TNF/UTRN/GJC2/ATP7A/KCNAB2/RGS2/PPIF/KCNJ6/YWHAH/CATSPER2/SLC43A2/PIK3CG/SLC6A9/PTPN6/DLG4/CNIH3/CACNA1D/PSEN1/FKBP1A/CAPN3/CD63/SELENON/DIAPH1/WWP2/NLGN3/PTK2B/TESC/KCNQ1/CACNB4/KCNJ15/KCNE3/AKAP9/RIPK1/CALM3/PRKACA/P2RX1/ACTN4/CHP1/LYN/SNCA/CHD7/WNK1/MMP9/EHD3/KCNJ5/OPRL1/CORO1A/EPHB2/PTAFR/RGS7/G6PD/KEL/PINK1/CAPN1/SLC8A1
BP	GO:0014902	myotube differentiation	64	-0.51	-1.79	0.00030136	0.0021132	0.00129909	2322	tags=42%, list=20%, signal=34%	CD9/WNT1/DYRK1B/KLF5/PLEKHO1/NPHS1/MAML1/HDAC4/CEACAM5/NIBAN2/SELENON/GPX1/BCL9/SKI/XK/FLOT1/TNFSF14/EHD1/MYH9/LMOD3/RBM38/PPP3CA/CFLAR/MAPK14/MYEF2/KEL/RIPOR2
BP	GO:0050768	negative regulation of neurogenesis	71	-0.51	-1.80	0.0003093	0.0021499	0.00132165	2687	tags=46%, list=23%, signal=36%	DICER1/IL1B/REST/SEMA7A/TRIM11/TSPO/RTN4R/BMPR1A/SORL1/TNF/CDK5R1/YWHAH/PCM1/SEMA4A/CTDSP1/KIAA0319/DRAXIN/HOOK3/RTN4/KDM4A/PSEN1/ULK1/CTNNA1/LIN28A/SKI/ASCL2/PPP3CA/NF1/EPHB2/PTEN/MBP/RNF10/RAPGEF2
BP	GO:0006836	neurotransmitter transport	103	-0.46	-1.74	0.00030877	0.0021499	0.00132165	2550	tags=40%, list=22%, signal=31%	SNAP23/SLC28A2/P2RX7/CAMK2A/LRRK2/PPT1/GSK3B/FBXL20/PRKCB/STXBP5/PIP5K1C/SLC6A9/APBA1/SLC22A1/SYN3/STX3/SLC6A12/RAB3B/SNAP25/CTBP2/SNAP29/MCTP2/PSEN1/NR4A1/SLC6A6/SYT12/STX11/FLOT1/P2RY1/CALM3/STXBP2/VPS18/P2RX1/SNCA/DNAJC5/SYNJ1/NF1/RAB5A/ITGB3/NAPA/SLC6A8
CC	GO:0045211	postsynaptic membrane	106	-0.45	-1.73	0.00030877	0.0021499	0.00132165	2196	tags=29%, list=19%, signal=24%	SYAP1/MAGEE1/DNM2/UTRN/NRP2/GRIK2/HTR3B/SLC6A9/ATP6AP2/DLG4/CNIH3/PRR7/ABHD17C/SLC6A6/DNM3/NLGN3/DBN1/FBXO45/AKAP9/P2RY1/P2RX1/ARRB2/PICALM/EPHB2/LRRC4/ITGB3/NRGN/SLC8A1/RNF10/LRFN1/HIP1

BP	GO:0040008	regulation of growth	397	-0.33	-1.46	0.00030998	0.0021509	0.00132225	3082	tags=37%, list=26%, signal=28%	LAMTOR1/RAI1/CDC73/BCL2L11/EDN1/BCL6/ADAM15/ADNP2/CEP43/BBS2/PPP2R1A/PLAA/TAOK2/BST2/PML/BAP1/SRF/ITCH/HIF1A/SHTN1/KCTD21/RHOA/ARHGEF11/H3-5/CTDP1/PLCB1/ADAM17/BTG1/ADNP/EIF4G1/CSNK2A1/JADE1/SLC9A1/SEMA7A/SIN3A/EXOSC4/DDX3X/CD38/SERPINE2/ERBB2/KIAA1109/PPT1/YY1/RTN4R/GSK3B/RFTN1/BMPR1A/STAT5A/ACACB/DNM2/PAFAH1B1/PLCE1/WWC3/RGS2/CDK5R1/KCTD11/SGPL1/OSM/SEMA4A/WWC2/EIF4G2/KIAA0319/TWF2/CXCL16/RAB21/UBE2E3/SAV1/CREB1/CDKN2D/STK4/PIM1/DRAXIN/LRP12/RTN4/GREM1/DISC1/MTM1/MSX1/STAT5B/SHC1/NAIF1/CDKL5/IST1/SESN2/HSPA1B/SERTAD3/GSK3A/MAPT/PTPRJ/SOCS3/ULK1/CAPN3/INSR/SPAG9/SLC23A2/NEDD9/AGRN/SMARCA2/NDRG3/MT2A/PPP1R9B/HSPA1A/CTTN/MBD5/H3-3A/PTK2B/NDEL1/MAD2L2/RPS6KA1/CDKN1A/EGLN2/LIMK1/DPYSL2/NIPBL/EXTL3/DBN1/DUSP6/OSGIN2/PAK2/DCUN1D3/TKT/OGFR/RBPJ/KAT5/JARID2/IGF2/CHD7/SDCBP/ADIPOR1/CDA/SIPA1/FBP1/H3-3B/MAPK14/DNAJB2/YBX3/G6PD/STAT3/SFRP2/MTPN/FLCN/BCL2L1/BASP1/HLX/PTEN/ING1/DAB2
BP	GO:0036293	response to decreased oxygen levels	191	-0.39	-1.60	0.00031244	0.0021643	0.00133045	3061	tags=39%, list=26%, signal=29%	CBFA2T3/EDN1/ADAM15/THBS1/AIFM1/PML/PDLIM1/SRF/HIF1A/ADAM8/TLR2/RHOA/REST/SCAP/ADAM17/SLC2A1/SLC9A1/AGTRAP/CD38/HK2/CYBB/HIPK2/PIK3CB/TNF/ROCK2/ABAT/CASR/ATP7A/NKX3-1/UCP3/UCP2/MECP2/ITPR2/BNIP3L/PDGFA/CPEB4/LMNA/EP300/CBL/ERO1A/NR4A2/PTGS2/ARNT/IRAK1/XRCC1/MTHFR/EGLN1/PLOD1/PTK2B/EGLN3/EGLN2/SRC/BACH1/ASCL2/SOD2/ZFP36L1/RBPJ/NFE2L2/ACTN4/APAF1/NPEPPS/ADM/CREBBP/CFLAR/ALAS2/HMOX1/NF1/FOXO3/PDK3/AQP1/MPL/PINK1/PTEN/SLC8A1
CC	GO:0070820	tertiary granule	142	-0.41	-1.63	0.00031554	0.0021819	0.0013413	2788	tags=41%, list=24%, signal=32%	ADAM8/GAA/FPR2/RHOA/ANO6/CD177/CANT1/NCKAP1L/SNAP23/SIGLEC14/FCER1G/CYBB/CRISP3/LILRB2/FPR1/CST3/KCNAB2/ITGAM/PRCP/SLC11A1/ORM1/CR1/MGAM/ATAD3B/ATP6AP2/PTPN6/LAMP2/ADGRE3/LAMP1/TSPAN14/SNAP25/ATP6V0A1/ALDOA/SLC2A3/LRG1/ITGB2/FLG2/DIAPH1/LGALS3/ARL8A/PGM1/QSOX1/CTSD/CD93/SIRPA/STXBP2/OSCAR/CYSTM1/RAC1/SIGLEC5/MMP9/CDA/DOK3/TIMP2/PTAFR/PGLYRP1/FTH1/CNN2
BP	GO:0032731	positive regulation of interleukin-1 beta production	48	-0.57	-1.86	0.00031625	0.002183	0.00134197	2779	tags=54%, list=24%, signal=42%	TLR4/TMEM106A/NLRP3/EGR1/NLRP1/MYD88/NLRP12/JAK2/P2RX7/TNF/MNDA/TLR8/FZD5/ORM1/TLR6/LILRA2/CCL3/IFI16/LGALS9/HSPB1/STMP1/NLRC4/TYROBP/PYCARD/HK1/STAT3
BP	GO:0045834	positive regulation of lipid metabolic process	87	-0.47	-1.74	0.0003262	0.0022438	0.00137937	2696	tags=36%, list=23%, signal=28%	FPR2/IL1B/SCAP/LPGAT1/KIT/TNF/CTDNEP1/GPLD1/CES1/CREB1/AGAP2/PTGS2/FGR/BMP6/PTK2B/VAV3/SRC/CPT1A/AKT2/DGAT2/POR/ABCG1/CHP1/LYN/ADM/ABHD5/PNPLA2/IRS2/AMBRA1/PRKCD/DAB2
BP	GO:0010976	positive regulation of neuron projection development	99	-0.45	-1.71	0.00032787	0.0022476	0.00138168	2931	tags=40%, list=25%, signal=31%	ENC1/KATNB1/KIDINS220/SCARF1/FES/CRTC1/CAPRIN1/SCARB2/ADNP/ACTR2/SCN1B/HSPA5/FEZ1/FLNA/PLXNB2/PAFAH1B1/RGS2/MARK2/P3H1/TWF2/EP300/CAMK1D/HGF/STK24/DISC1/ANKRD27/VLDLR/PTK2B/NDEL1/PPP2R5B/RETREG3/GRN/DBN1/SETX/EHD1/NFE2L2/LYN/CFLAR/EPHB2/RAPGEF2
BP	GO:0051261	protein depolymerization	81	-0.49	-1.76	0.00032919	0.0022527	0.00138485	2331	tags=36%, list=20%, signal=29%	CAPZB/ADD1/NCKAP5L/CFL1/SPTA1/TAOK1/TWF2/GAK/MAP1A/TRIOBP/WASHC2C/ASPH/PPP1R9B/ARHGEF2/VPS4B/APC/SVIL/SH3GL1/LMOD3/CCSAP/PLEK/SYNJ1/MAP1S/TMOD1/DMTN/MTPN/GSN/SPTB/MICAL2

BP	GO:0050890	cognition	159	-0.41	-1.63	0.00033806	0.0023095	0.00141973	2896	tags=40%, list=25%, signal=31%	PRKAR1B/CLN3/DOP1B/ST3GAL4/SRF/KRAS/HIF1A/SETD5/MAGT1/MAPK1/TLR2/CRTC1/FOS/PLCB1/PRKCZ/NEUROD2/ADNP/KIT/ACTR2/SHROOM4/PJA2/DGCR2/ATXN1/PPT1/LILRB2/PAFAH1B1/TNF/MECP2/CPEB3/ITGA5/DLG4/CREB1/EP300/GPI/MAP1A/SNAP25/PTGS2/PSEN1/MAPT/RGS14/VLDLR/INSR/PPP1R9B/ADGRF1/C5AR1/HMGCR/NLGN3/EIF4EBP2/NIPBL/GPR155/SLC7A11/MME/CHD7/SYNJ1/CEBPB/OPRL1/PICALM/CIC/PRKAR2B/NF1/EPHB2/NRGN/PTEN/CLDN5
BP	GO:0045807	positive regulation of endocytosis	68	-0.50	-1.77	0.00033877	0.0023104	0.00142027	2613	tags=46%, list=22%, signal=36%	ANO6/NCKAP1L/ANGPT1/VPS28/LRRK2/PPT1/DNM2/LRP1/TBC1D5/SYK/ANXA2/RAB21/CBL/AP2M1/GREM1/CD14/CLU/CD63/INSR/AP2B1/TF/FLOT1/SERPINE1/SNCA/HNRNPK/ARRB2/SYNJ1/PPP3CA/RAB31/DAB2/HIP1
BP	GO:0031346	positive regulation of cell projection organization	232	-0.37	-1.55	0.00034159	0.0023256	0.00142965	2983	tags=35%, list=25%, signal=27%	TIAM1/BCAS3/ENC1/KATNB1/KIDINS220/SRF/SCARF1/SHTN1/FES/CRTC1/CAPRIN1/WNT1/NEURL1/SCARB2/CDC42EP4/ADNP/KIT/ACTR2/SCN1B/SEMA7A/HSPA5/P2RX7/HAP1/FEZ1/FLNA/PLXNB2/GSK3B/RHOQ/PLEKHM1/DNM2/PAFAH1B1/PLCE1/ARAP1/ATP7A/RGS2/MARK2/P3H1/EIF4G2/TWF2/RAB21/DLG4/RAC2/FSCN1/EP300/CAMK1D/HGF/STK24/DISC1/CDKL5/IST1/WASF2/MAPT/ANKRD27/VLDLR/AGRN/CUX1/DNM3/MARK4/PTK2B/NDEL1/PPP2R5B/DVL3/RETREG3/NIN/LIMK1/SRC/APC/GRN/DBN1/SETX/PLEK2/EHD1/NFE2L2/LYN/RAC1/CFLAR/PLXNC1/EPHB2/CORO1C/CCP110/RIPOR2/RAPGEF2
BP	GO:0009617	response to bacterium	372	-0.33	-1.43	0.00034414	0.0023389	0.00143785	2862	tags=36%, list=24%, signal=28%	ANKRD17/CFD/NAIP/NLRP10/GSTP1/CLEC4E/HLA-DRB1/AKIRIN2/MAPK1/TLR2/FOS/FPR2/TRAF6/RHOA/IL1B/ADAM17/JAK2/FKBP5/CASP9/CCDC186/FCER1G/TICAM1/P2RX7/VIM/COLEC12/LDOC1/OPTN/TSPO/RNF5/ARID5A/LILRB2/ALPL/MAPKBP1/SPON2/TAB2/PELI1/TNF/NFKBIA/JUND/TLR1/ST13/SLC11A1/FZD5/SYK/LPO/HLA-E/TNFRSF1A/PF4V1/PPM1D/IRAK3/CTR9/PI3/LYST/TIRAP/TLR6/CD14/LILRA2/SHC1/GBP2/MAVS/PTGS2/LRG1/IRAK1/C2/CCL3/RARA/PRPF8/RELA/FGR/ERBIN/NFKBIB/C5AR1/SBNO2/NR4A1/BMP6/IFNAR1/HCK/SLC17A5/GPX1/TNIP1/TF/SIRPA/PRDX2/LGALS9/CXCL5/SRC/GRN/PF4/MAPKAPK2/AICDA/IL6R/SOD2/SELP/GBP4/TNFRSF1B/RBPJ/MAPKAPK3/OTUD5/TREM1/CUBN/SERPINE1/SASH1/MARCHF2/CMPK2/PYGL/DCD/NLRC4/SHARPIN/LYN/SNCA/CHD7/ZNFX1/ADM/PYCARD/CDK19/CYRIB/H2BC11/MAP2K3/CEBPB/MAPK14/EPHB2/PTAFR/PGLYRP1/AQP1/MAPK3/PRKCD/CD68/SPI1/BCL3/TRIB1/H2BC4/TBXA2R/LITAF
BP	GO:0045229	external encapsulating structure organization	123	-0.43	-1.70	0.0003511	0.0023822	0.00146444	2287	tags=30%, list=20%, signal=24%	COLGALT2/CRISPLD2/NPHS1/TNF/TNXB/LRP1/CST3/ATP7A/ERCC2/MYH11/TNFRSF1A/NFKB2/FSCN1/SULF2/TGM3/SERAC1/ERO1A/GREM1/QSOX1/B4GALT1/COL9A3/NTNG2/FLOT1/CYP1B1/MYO1E/TNFRSF1B/ADAMTS4/GFOD2/MMP9/CFLAR/LCP1/DNAJB6/NF1/SFRP2/HSD17B12/BCL3/FURIN
BP	GO:1903532	positive regulation of secretion by cell	167	-0.40	-1.62	0.00035582	0.0024101	0.00148159	3049	tags=36%, list=26%, signal=27%	GATA1/TFR2/EDN1/GRK2/GOLPH3/HLA-DRB1/HIF1A/ADAM8/TLR2/IL1B/WLS/CD177/JAK2/P2RX7/CD38/CACNA1I/RPH3AL/ABAT/PTGES/MYH10/CASR/NKX3-1/GPLD1/ITGAM/STXBP5/SYK/GPR27/CREB1/LRRC8A/LAMP1/SOX4/FFAR4/SYTL4/FGR/ITGB2/RAB27B/BMP6/VPS4B/RAB27A/ACSL4/RAB7A/RAB2B/P2RY1/AP1G1/ABCG1/UNC13D/SNCA/C2CD2L/SDCBP/MYOM1/PTAFR/CHMP2A/IRS2/TCF7L2/RAB5A/PINK1/RAB3D/DAB2/GLUL/GAB2
CC	GO:0070160	tight junction	76	-0.48	-1.70	0.0003619	0.002443	0.00150179	1065	tags=18%, list=9%, signal=17%	SYNPO/ARHGEF2/APC/ESAM/ACTB/AFDN/F11R/YBX3/VASP/UBN1/FRMD4B/RAPGEF2/NHS/CLDN5
BP	GO:0030278	regulation of ossification	60	-0.53	-1.81	0.0003762	0.0025309	0.00155586	2798	tags=45%, list=24%, signal=34%	HIF1A/MAPK1/ANO6/ALOX5/P2RX7/BMPR1A/ZBTB16/ANKH/TOB2/ATP2B1/CHSY1/CCR1/RFLNB/GREM1/CCL3/INTU/BMP6/PTK2B/NIPBL/PBX1/RBPJ/BMP2K/MAPK14/PHOSPHO1/MAPK3/SLC8A1/NBR1

BP	GO:1904062	regulation of cation transmembrane transport	186	-0.39	-1.59	0.00037571	0.0025309	0.00155586	2613	tags=32%, list=22%, signal=25%	ANO6/STIM1/DAPK1/CACNB1/PSEN2/SCN1B/SLC9A1/HAP1/OXSR1/HECW2/FLNA/BAX/DNM2/UTRN/GJC2/ATP7A/KCNAB2/RGS2/PPIF/YWHAH/SLC43A2/PIK3CG/SLC6A9/PTPN6/DLG4/CNIH3/CACNA1D/FKBP1A/CAPN3/CD63/SELENON/DIAPH1/WWP2/NLGN3/PTK2B/TESC/KCNQ1/CACNB4/KCNE3/AKAP9/CALM3/PRKACA/P2RX1/ACTN4/CHP1/LYN/SNCA/CHD7/WNK1/MMP9/EHD3/OPRL1/CORO1A/EPHB2/RGS7/G6PD/KEL/PINK1/CAPN1/SLC8A1
BP	GO:0043542	endothelial cell migration	140	-0.42	-1.65	0.00039817	0.0026742	0.00164393	2209	tags=32%, list=19%, signal=26%	CCBE1/STAT5A/PIK3CB/TNF/ROCK2/GPLD1/NRP2/PRKD2/MECP2/PRCP/SEMA4A/PIK3CG/GADD45A/SP1/GREM1/GPI/MAP3K3/PTGS2/RIN2/EPHB4/PTP4A3/PXN/NR4A1/PTK2B/HDAC7/GPX1/GFUS/SPARC/GRN/HSPB1/CYP1B1/NFE2L2/SASH1/MYH9/RAC1/PIK3CD/MAP2K3/HMOX1/SP100/NF1/DNAJA4/ITGB3/PTEN/TBXA2R/GLUL
MF	GO:0017124	SH3 domain binding	87	-0.47	-1.72	0.00040132	0.0026908	0.00165415	2636	tags=40%, list=22%, signal=31%	USP8/ADAM17/INPP5D/OSTF1/QKI/CASP9/EPS15/SH3BP5/GRB2/ELMO1/DNM2/ARHGAP31/ELMO2/PLSCR1/PTPN6/CBL/NCF1/WASF2/MAPT/ESPN/GPX1/SIRPA/SH3BGRL2/ZNF106/PTPN12/ARHGAP1/LYN/HCLS1/SH3KBP1/SYNJ1/WIPF1/WAS/ABI3/VASP/ARHGAP6
BP	GO:0016079	synaptic vesicle exocytosis	55	-0.53	-1.79	0.00040441	0.002707	0.00166408	2550	tags=45%, list=22%, signal=36%	SNAP23/P2RX7/LRRK2/GSK3B/FBXL20/PRKCB/STXBP5/PIP5K1C/APBA1/STX3/SNAP25/CTBP2/SNAP29/PSEN1/SYT12/STX11/P2RY1/CALM3/VPS18/P2RX1/SNCA/DNAJC5/SYNJ1/RAB5A/NAPA
CC	GO:0030666	endocytic vesicle membrane	134	-0.42	-1.65	0.00040878	0.002726	0.00167577	3270	tags=48%, list=28%, signal=35%	MARCO/RAB10/AP2S1/CD4/HVCN1/SEC22B/UBC/ATP6V0B/ATP6V0C/SCARF1/CLEC4E/HLA-DRB1/CD9/PIKFYVE/TLR2/WNT1/RAB8A/WLS/SCARB2/SNAP23/RAB32/COLEC12/RAB35/CAMK2A/CYBB/DNM2/TAPBP/LRP1/TLR1/ATP7A/SLC11A1/FZD5/MCOLN1/KIAA0319/RAB43/HLA-E/RAB34/RAB22A/DLG4/LAMP2/RAC2/AP2M1/LAMP1/TLR6/ATP6V0A1/CSF3R/CLTC/IGF2R/ATP6V0D1/VAMP3/RAB11B/AP2B1/TF/RILP/TGOLN2/RAB7A/UBB/AP2A1/LYN/RAB11FIP1/CORO1A/RAB5A/RAB31/TGFA
BP	GO:0051051	negative regulation of transport	271	-0.36	-1.51	0.00041024	0.0027276	0.00167676	2436	tags=32%, list=21%, signal=26%	SIRT6/NCKAP1L/FIS1/ANGPT1/OXSR1/HECW2/KLF7/SERPINE2/ABCA2/LRRK2/LILRB2/PRKCB/TNF/ABAT/ATP7A/RGS2/PPIF/UCP2/LRPAP1/CD300LF/OSM/PLSCR1/ANXA2/NECAB2/SLC43A2/SLC6A9/TNFRSF1A/RIN3/DLG4/RUBCN/OS9/GNAI2/INPP5K/PID1/RSAD2/FFAR4/PTGS2/SYTL4/SUFU/GSK3A/ANKRD27/LGALS3/VAMP3/ANKRD13A/WWP2/HMGCR/PTK2B/VPS4B/SIRPA/LGALS9/TPR/RAB7A/KCNE3/P2RY1/CALM3/AKT2/ARHGAP8/TNFRSF1B/ARHGAP1/CHP1/PEA15/SNCA/SDCBP/RAC1/WNK1/OAZ1/MMP9/MIDN/RAB11FIP1/PPP3CA/OAZ2/OPRL1/PICALM/HMOX1/SP100/ABCG8/CORO1A/NF1/EPHB2/IRS2/DMTN/ITGB3/KEL/CNN2/PTEN/SPI1/DYSF
BP	GO:0007044	cell-substrate junction assembly	61	-0.52	-1.79	0.00041471	0.0027527	0.0016922	2947	tags=48%, list=25%, signal=36%	BCAS3/THBS1/TAOK2/WDPCP/RHOA/SLC9A1/ACTG1/TESK2/ROCK2/RCC2/ITGA5/GREM1/DUSP22/ROCK1/PTPRJ/CTTN/PTK2B/SLK/ACTN1/SRC/LIMS1/RAC1/MAP4K4/CORO1C/TLN1/DMTN/PTEN/VCL/ARHGAP6
BP	GO:0050819	negative regulation of coagulation	26	-0.65	-1.89	0.00041546	0.0027531	0.00169246	3126	tags=62%, list=27%, signal=45%	THBD/EDN1/THBS1/CD9/ANXA5/SERPINE2/PROS1/ABAT/PDGFA/SERPING1/KNG1/GP1BA/ALOX12/SERPINE1/PRKCD/KRT1
CC	GO:0035577	azurophil granule membrane	53	-0.55	-1.84	0.000421	0.0027806	0.00170933	2927	tags=53%, list=25%, signal=40%	VNN1/BST2/ATP6V0C/GAA/MAGT1/CPNE3/FPR1/SLCO4C1/PRCP/MANBA/LAMP2/CPNE1/LAMP1/RAB37/CKAP4/BRI3/SNAP29/PSEN1/CD63/ARL8A/B4GALT1/ACP3/PSAP/DNAJC5/RAB5C/CD68/RAB3D/NFAM1

BP	GO:0045860	positive regulation of protein kinase activity	275	-0.35	-1.50	0.00042194	0.0027821	0.0017103	2685	tags=34%, list=23%, signal=27%	TRAF6/RHOA/ACSL1/IL1B/PRKAG2/ADAM17/NEURL1/PRKCZ/ADNP/ERN1/JAK2/NCKAP1L/KIT/ANGPT1/P2RX7/CCND3/CSF1R/DDX3X/LCP2/INCENP/ERBB2/LRRK2/BMPR1A/SYAP1/TAB2/TNF/MAS1/CDK5R1/RICTOR/ERN2/MARK2/SLC11A1/PDGFA/FZD5/AKAP13/TAOK1/SYK/MAP2K2/PIK3CG/DLG4/STK4/AGAP2/TIRAP/NCF1/PDE5A/GREM1/TLR6/OSBPL8/IRAK1/CCNY/PSEN1/VLDLR/CLU/INSR/PTPN1/CHI3L1/MAP3K5/NEDD9/RASSF2/RALB/PTK2B/DVL3/CDKN1A/PARP16/MAP2K7/CASS4/TAOK3/SRC/PAK2/PIK3R5/IL6R/CALM3/DAZAP2/SASH1/TAL1/MMD/SNCA/IGF2/STRADA/PAK1/WNK1/MAP2K3/PTPRC/CACUL1/MOB3A/STRADB/ITGB3/PRKCD/PINK1/MOB3C/RAPGEF2/IQGAP1/TGFA
CC	GO:0030136	clathrin-coated vesicle	129	-0.42	-1.64	0.00042642	0.002807	0.0017256	1943	tags=29%, list=17%, signal=24%	AP1M1/FZD5/KIAA0319/SPG21/STX6/VPS33A/AP2M1/CLTC/NUMB/IGF2R/VPS11/RAB27B/VAMP3/RGS19/AP2B1/TF/RAB27A/TGOLN2/MYO1E/VPS18/AP2A1/AP1G1/GPR107/EPN1/UNC13D/DNAJC5/PICALM/STON2/RAB5A/SNX18/NRGN/DENND1A/ECE1/FURIN/DAB2/TGFA/HIP1
BP	GO:0016049	cell growth	307	-0.34	-1.48	0.00043532	0.0028609	0.00175868	3085	tags=37%, list=26%, signal=28%	KMT2D/LAMTOR1/CDC73/EDN1/BCL6/ADAM15/ADNP2/CEP43/PPP2R1A/PLAA/TAOK2/BST2/PML/BAP1/SRF/ITCH/SHTN1/RHOA/ARHGEF11/H3-5/CTDP1/ADAM17/RAPH1/PRKCZ/BTG1/ADNP/EIF4G1/CSNK2A1/JADE1/SLC9A1/SEMA7A/SIN3A/EXOSC4/DDX3X/CD38/ALCAM/SERPINE2/ERBB2/KIAA1109/PPT1/YY1/RTN4R/GSK3B/DNM2/PAFAH1B1/PLCE1/RGS2/CDK5R1/NRP2/SEMA4A/AKAP13/EIF4G2/KIAA0319/TWF2/CXCL16/RAB21/CDKN2D/DRAXIN/RTN4/CPNE1/GREM1/DISC1/MSX1/NAIF1/CDKL5/IST1/SESN2/HSPA1B/SERTAD3/GSK3A/MAPT/PTPRJ/ULK1/SPAG9/SLC23A2/SMARCA2/NDRG3/PPP1R9B/HSPA1A/CTTN/H3-3A/NLGN3/PTK2B/NDEL1/MAD2L2/RPS6KA1/CDKN1A/EGLN2/NIN/LIMK1/DPYSL2/EXTL3/DBN1/OSGIN2/PDLIM5/DCUN1D3/OGFR/USP9X/SDCBP/ADIPOR1/CDA/SIPA1/FBP1/H3-3B/DNAJB2/G6PD/SFRP2/MTPN/FLCN/VCL/ING1/IQGAP1/DAB2
BP	GO:0032869	cellular response to insulin stimulus	150	-0.41	-1.63	0.00043952	0.0028837	0.00177274	2679	tags=39%, list=23%, signal=31%	PIK3R2/IL1B/LPIN2/RAB8A/PRKCZ/SLC9A1/CCND3/SMARCC1/GRB2/GSK3B/RHOQ/SYAP1/SORL1/PRKCB/PHIP/GPLD1/UCP2/PIP4K2C/PKM/SOGA1/SP1/ATP2B1/TSC2/GNAI2/INPP5K/PID1/SHC1/PDK2/OSBPL8/GSK3A/GOT1/FUT7/SOCS3/PTPRE/INSR/PTPN1/SESN3/NDEL1/ZDHHC7/EIF4EBP2/SRC/APC/ZNF106/GAB1/PIP4K2A/ZFP36L1/MYO5A/AKT2/ZBTB7B/IGF2/ADIPOR1/CFLAR/FBP1/IRS2/PRKDC/PRKCD/WDTC1/RAB31/FOXO4
CC	GO:0045177	apical part of cell	213	-0.37	-1.52	0.00044531	0.0029169	0.00179313	2639	tags=30%, list=23%, signal=23%	ASPM/ADAM17/PRKCZ/SLC2A1/PSEN2/SLC9A1/SHROOM4/C5AR2/ACTG1/AHCYL1/ERBB2/CA2/SLC22A4/CASR/ATP7A/ATP6V1C1/SLC39A4/ABCC4/PCM1/DYNC2H1/MGAM/SLC6A9/ATP6AP2/SLC22A1/ATP2B1/ABCB11/STX3/CEACAM5/NUMB/DSTYK/PSEN1/SLC23A2/ATP6V0D1/RAB27B/SLC6A20/C5AR1/SLC6A6/TF/RAB27A/KCNQ1/NIN/SNX10/P2RY1/IL6R/CUBN/CA4/SLC7A11/SLC2A9/ATP6V1B2/C2CD2L/SHROOM1/CTSB/ABCG8/ITPK1/AQP1/MSN/PTEN/RIPOR2/RAPGEF2/NHS/SLC7A5/ATP6V1A/AQP10
BP	GO:0048598	embryonic morphogenesis	293	-0.35	-1.48	0.0004614	0.0030124	0.00185183	2321	tags=28%, list=20%, signal=23%	PLXNB2/OTX1/GRB2/TBX19/BMPR1A/HIPK2/BAX/ZBTB16/ROCK2/IFT122/HIPK1/MYADM/FZD5/TMEM231/DYNC2H1/HIRA/ITGA5/CDKN1C/CTR9/STK4/TSC2/TXNRD1/YBX1/GREM1/GPI/CUL3/SOX4/MSX1/CHST11/TRIOBP/IFT140/ROCK1/MTHFR/SUFU/PSEN1/RARA/ZEB1/ITGB2/SOCS3/INTU/RTF1/MAP3K20/ARL13B/PHACTR4/GNA12/DVL3/KCNQ1/SKI/BPNT2/NIPBL/ETS2/PBX1/TGFB1I1/EXOC4/DUSP1/ZFP36L1/PRKACA/IL1RN/KDM6B/NDST1/ITGB5/TAL1/APAF1/IGF2/CHD7/WNK1/ADM/PBX2/MMP9/CREBBP/DNAJB6/NCOA1/EPHB2/VASP/MAPK3/SFRP2/CCDC39/ITGB3/PRKAR1A/HLX/MBP/MICAL2/ECE1

BP	GO:0051271	negative regulation of cellular component movement	190	-0.39	-1.59	0.00046538	0.0030334	0.00186474	2233	tags=27%, list=19%, signal=22%	C5AR2/ARHGDIB/MITF/BMPR1A/PIP5KL1/TNF/LRP1/MECP2/MIIP/S1PR2/SRGAP1/GADD45A/RIN3/RNF20/STK24/GREM1/DUSP22/OSBPL8/PTPRJ/ABHD2/TIMP1/CTNNA1/GNA12/ACTN1/CYP1B1/DUSP1/DLG5/PADI2/NFE2L2/SERPINE1/TPM1/AFDN/RAC1/ADIPOR1/HMOX1/SP100/NF1/FOXO3/CORO1C/WAS/DNAJA4/STAT3/SFRP2/FLCN/CNN2/PTEN/VCL/RIPOR2/TRIB1/TBXA2R/CLDN5
BP	GO:0030593	neutrophil chemotaxis	64	-0.51	-1.76	0.00047203	0.0030716	0.00188826	3053	tags=58%, list=26%, signal=43%	CCL5/EDN1/JAML/DAPK2/MOSPD2/PIKFYVE/IL1B/NCKAP1L/FCER1G/C5AR2/BST1/SYK/PIP5K1C/PIK3CG/PF4V1/JAM3/RAC2/CAMK1D/TIRAP/CSF3R/CCL3/ITGB2/VAV1/LGALS3/C5AR1/CXCR2/CXCL5/VAV3/CXCR1/PF4/PREX1/TREM1/RAC1/PIK3CD/BSG/RIPOR2/MPP1
BP	GO:0048638	regulation of developmental growth	173	-0.39	-1.57	0.00048158	0.0031287	0.00192333	2275	tags=33%, list=19%, signal=27%	YY1/RTN4R/GSK3B/BMPR1A/STAT5A/ACACB/DNM2/PAFAH1B1/WWC3/RGS2/CDK5R1/SGPL1/SEMA4A/WWC2/EIF4G2/KIAA0319/TWF2/RAB21/SAV1/CREB1/STK4/PIM1/DRAXIN/RTN4/DISC1/MTM1/STAT5B/CDKL5/IST1/GSK3A/MAPT/ULK1/CAPN3/INSR/SPAG9/SLC23A2/AGRN/CTTN/MBD5/NDEL1/CDKN1A/LIMK1/DPYSL2/NIPBL/DBN1/DUSP6/RBPJ/JARID2/IGF2/CHD7/MAPK14/YBX3/G6PD/STAT3/BASP1/HLX/PTEN
BP	GO:0007265	Ras protein signal transduction	261	-0.36	-1.53	0.00050056	0.0032467	0.00199588	3255	tags=38%, list=28%, signal=28%	RRAS/RHOB/ARHGEF10/SH3BP1/GDI1/ARRB1/MAPKAP1/RAB42/BCL6/ABCA1/RAP2C/KCTD10/ARFGEF1/TIAM1/STK19/KRAS/MFN2/SHTN1/GRAP2/GNA13/FARP2/RHOA/ARHGEF11/USP8/SOS2/CDC42EP4/LPAR1/STARD8/RAB35/ARHGDIB/GRB2/RTN4R/ELMO1/RIPOR1/PIK3CB/DNM2/MYO9B/ROCK2/LRRC59/MRAS/PLCE1/DENND3/ARHGEF3/ARHGDIA/CFL1/RALGDS/AKAP13/SETDB1/RASSF1/KBTBD7/PIK3CG/RAB21/RAC2/PSD4/CBL/LRRD1/ARHGEF12/CUL3/GNB1/ROCK1/NKIRAS2/WASF2/ERBIN/SQSTM1/PHACTR4/RALB/GNA12/ARHGEF2/DOK1/CDKN1A/SPRY3/LIMK1/SHOC2/GABARAP/FLOT1/ARHGAP1/RIT1/CYTH4/SDCBP/RAC1/DOK3/F11R/RHOG/FBP1/MAP4K4/IQSEC2/KPNB1/NF1/EPHB2/IQSEC1/WAS/CTNNAL1/DHCR24/RASGRP4/RALBP1/FLCN/DENND1A/RIPOR2/RAPGEF2/ARHGAP6
BP	GO:0140029	exocytic process	51	-0.55	-1.84	0.00050456	0.0032629	0.00200582	1940	tags=43%, list=17%, signal=36%	STXBP5/SYK/STX3/RAB3B/SNAP25/CTBP2/SNAP29/PSEN1/VPS11/VAMP3/RALB/STX11/STXBP2/VPS18/P2RX1/LYN/SNCA/PLEK/SYNJ1/DMTN/NAPA/RAB3D
BP	GO:0009612	response to mechanical stimulus	120	-0.43	-1.68	0.00050469	0.0032629	0.00200582	3267	tags=44%, list=28%, signal=32%	CASP5/ABHD12/MYD88/BCL10/MAP3K1/EDN1/PKD1/BAK1/STAT1/THBS1/CASP2/FOS/RHOA/IL1B/MAP2K4/SLC2A1/KIT/SLC9A1/P2RX7/MBD2/ACTG1/FADD/SERPINE2/TNF/NFKBIA/JUND/TLR8/LTBR/KIAA0319/TNFRSF1A/GADD45A/TMEM120A/TXNIP/GPI/PTGS2/RELA/CHI3L1/BMP6/PTK2B/KCNQ1/SRC/TNFSF14/P2RY1/IRF1/MAP3K2/F11R/MAPK14/AQP1/MAPK3/MTPN/GSN/CNN2/SLC8A1
BP	GO:0048260	positive regulation of receptor-mediated endocytosis	36	-0.60	-1.86	0.00050638	0.0032685	0.00200927	2280	tags=56%, list=19%, signal=45%	ANGPT1/PPT1/DNM2/TBC1D5/SYK/ANXA2/RAB21/CBL/AP2M1/GREM1/CLU/CD63/INSR/TF/SERPINE1/HNRNPK/ARRB2/SYNJ1/DAB2/HIP1

BP	GO:0048609	multicellular organismal reproductive process	440	-0.32	-1.40	0.00050723	0.0032686	0.00200937	2659	tags=28%, list=23%, signal=23%	HERPUD2/ASPM/NEURL1/TTC26/BTG1/ADNP/QKI/OR7C1/KIT/HSPA5/DPCD/DDX3X/MBD2/PAQR8/LDOC1/GALNT3/SERPINE2/SPATA2/LRRK2/IFT81/YY1/NECTIN2/BAX/KNL1/TESK2/ZBTB16/ARMC2/KDM1B/DNM2/PAFAH1B1/ABAT/SLCO4C1/MAS1/RGS2/CTDNEP1/CFAP58/SGPL1/CCDC136/UBE2B/ZNF148/CATSPER2/SPATA6/SSTR2/ZGLP1/TOB2/NKAPL/JAM3/MSH6/USP42/MYCBPAP/LRRC8A/FSCN3/PI3/ARID4A/C3orf62/PDE5A/STAT5B/JUNB/FOXJ2/XRN2/PTGS2/ATRX/DIAPH2/RARA/IGF2R/ABHD2/PLK1/SMARCA2/NDRG3/PPP1R9B/TUT7/PSME4/H3-3A/PTK2B/B4GALT1/LGALS9/ACSL4/LIN28A/SRC/UBB/APOL2/TPPP3/FANCC/KLC3/ETV6/RFX2/SETX/CYP1B1/P2RY1/VDR/SPOCD1/PRKACA/ASF1B/PROK2/CCNI/P2RX1/UBR2/AGO4/BAG6/ZMIZ1/MAST2/MYCBP/USP9X/ARRB2/RAD23B/TBC1D20/PITHD1/LIMK2/ZMYND15/OPRL1/H3-3B/CTSB/FOXO3/MARF1/PTAFR/YBX3/OSBP2/ACOX1/BCL2L1/BSG/ROPN1L/PTEN/ACRBP/KLF1
BP	GO:0032760	positive regulation of tumor necrosis factor production	78	-0.48	-1.75	0.0005091	0.0032754	0.00201349	2564	tags=44%, list=22%, signal=34%	JAK2/TICAM1/SETD4/FADD/LRRK2/CYBB/ARID5A/SPON2/TLR1/FZD5/ORM1/SYK/HLA-E/TIRAP/CD14/LILRA2/FRMD8/MAVS/CCL3/PSEN1/PTPRJ/CLU/ARHGEF2/LGALS9/PF4/MAPKAPK2/HSPB1/RIPK1/TYROBP/PYCARD/PTPRC/EPHB2/PTAFR/STAT3
BP	GO:0030162	regulation of proteolysis	491	-0.31	-1.40	0.00051264	0.0032929	0.00202425	2884	tags=35%, list=25%, signal=27%	BST2/PML/CLN3/NAIP/ITCH/TNFSF10/CSNK1A1/HECTD1/AKIRIN2/ADAM8/ATP2A3/USP7/NLRP12/CASP2/WNT1/RHOA/IL1B/REST/SIRT6/CSNK2A1/JAK2/DAPK1/FIS1/CASP9/CIDEB/CLN6/RCN3/DDX3X/TAF1/CEBPA/TRIM21/SMARCC1/FADD/BEX3/ZYG11B/SERPINE2/ABCA2/IL1R2/LRRK2/GSK3B/RNF14/HIPK2/BAX/CCBE1/PROS1/SORL1/CTSZ/TNF/ROCK2/LRP1/CST3/NKX3-1/GPLD1/GABARAPL2/SH3RF3/PRDX5/COP1/SYK/ANXA2/CR1/BIRC6/NUB1/CDKN2D/PI3/SERPING1/CAST/HGF/RNF40/VSIR/FZR1/GPI/DISC1/MAP1A/KNG1/MTM1/SH3RF1/PSME3/N4BP1/PTGS2/ROCK1/BID/IST1/HSPA1B/RYBP/SUFU/GSK3A/PSEN1/MAPT/CLU/IFI16/PRR7/CAPN3/PLK1/RNF19B/MAP3K5/AGTPBP1/ASPH/HSPA1A/NR4A1/TIMP1/PSME4/GNA12/PTK2B/CTSD/VCP/EGLN3/NUPR1/GPX1/TNIP1/RPS6KA1/LGALS9/RAD23A/APLP2/SIAH2/SRC/GRN/UBB/GABARAP/PAK2/NRDC/WAC/TNFSF14/UBE2O/DAP/PLAUR/PIAS1/PRKACA/TNFRSF1B/NFE2L2/SERPINE1/P2RX1/MYH9/PRELID1/ADRM1/BAG6/POR/APAF1/LAPTM5/NLRC4/LYN/SNCA/SDCBP/PYCARD/ARRB2/MMP9/XIAP/RAD23B/PSMC4/BBC3/PITHD1/CFLAR/TIMP2/PCSK1N/PICALM/CTSB/DNAJB6/DNAJB2/ZER1/DHCR24/AQP1/STAT3/SFRP2/GSN/PINK1/PTEN/PSMF1/MBP/TRIB1/FURIN/DAB2/HIP1
BP	GO:0030308	negative regulation of cell growth	113	-0.44	-1.70	0.00051961	0.0033269	0.00204517	3022	tags=42%, list=26%, signal=32%	BCL6/ADAM15/PPP2R1A/BST2/PML/CTDP1/BTG1/JADE1/SEMA7A/DDX3X/SERPINE2/PPT1/YY1/RTN4R/RGS2/CDK5R1/SEMA4A/KIAA0319/CDKN2D/DRAXIN/RTN4/GREM1/MSX1/NAIF1/SESN2/HSPA1B/SERTAD3/GSK3A/PTPRJ/ULK1/SPAG9/SMARCA2/NDRG3/PPP1R9B/HSPA1A/CDKN1A/OSGIN2/DCUN1D3/ADIPOR1/CDA/SIPA1/FBP1/DNAJB2/G6PD/SFRP2/FLCN/ING1/DAB2
CC	GO:0005901	caveola	50	-0.55	-1.83	0.00052633	0.0033644	0.00206826	2882	tags=42%, list=25%, signal=32%	CLN3/MAPK1/PACSIN2/SLC2A1/JAK2/LRRK2/BMPR1A/GASK1A/CBL/PTGS2/INSR/CTNNA1/HCK/SRC/FLOT1/CAVIN2/HMOX1/CORO1C/MAPK3/FLOT2/PLVAP

BP	GO:0045321	leukocyte activation	687	-0.29	-1.33	0.00053194	0.0033949	0.00208696	2723	tags=30%, list=23%, signal=25%	TLR2/LGALS9C/FES/FPR2/TRAF6/WNT1/RHOA/IL1B/DHPS/TMEM131L/ADAM17/INPP5D/PRKCZ/IKZF1/SELPLG/CCDC88B/SOS2/CD177/JAK2/NCKAP1L/SNAP23/IL4I1/C17orf99/LST1/KIT/CMTM7/DOCK2/GPR137B/FCER1G/TICAM1/P2RX7/C1QA/RC3H2/CCND3/LCP2/CD38/CEBPA/CD70/PJA2/FADD/ERBB2/THEMIS2/LRRK2/YY1/NECTIN2/TNFSF13/LILRB2/PLCL2/BAX/ZBTB16/PRKCB/PELI1/TNF/EFNB1/JUND/PRAM1/BST1/NSD2/PKN1/MNDA/TNFSF13B/TLR1/ATP7A/ITGAM/HDAC4/CD300LF/SLC11A1/HHEX/SPTA1/PLSCR1/FZD5/LTBR/SEMA4A/GON4L/SYK/NMI/CLPTM1/ZFPM1/CR1/HLA-E/TREML2/ITGAL/PIK3CG/KLF6/PTPN6/MSH6/RAC2/EP300/CBL/LRRC8A/TIRAP/VSIR/SUPT6H/PDE5A/LAMP1/DUSP22/SOX4/TLR6/LILRA2/STAT5B/RSAD2/SH3RF1/HSH2D/CCL3/PSEN1/RARA/MAPT/ZEB1/PTPRJ/FGR/CLU/FKBP1A/ITGB2/FUT7/PRR7/VAV1/LGALS3/C5AR1/SBNO2/DOCK8/PTK2B/CXCR2/SLC46A2/MAD2L2/CD93/APBB1IP/SIRPA/PRDX2/LGALS9/CDKN1A/RAB27A/NCR1/VAV3/SRC/GRN/TBX21/KMT2E/LAT2/AICDA/PBX1/TNFSF14/IL6R/DLG5/STXBP2/ZFP36L1/ZBTB7A/IRF1/SNX27/CLCF1/ZBTB7B/TNFRSF1B/PREX1/RBPJ/MYH9/PRELID1/PTGDS/BATF2/BAG6/ZMIZ1/AP1G1/LAPTM5/LYN/SIRPB1/UNC13D/SNCA/IGF2/CHD7/TYROBP/ELF4/PYCARD/FCGR3A/SH3KBP1/CYRIB/PIK3CD/PPP3CA/CEBPB/PTPRC/STAT6/FBXO7/LYL1/LCP1/HMOX1/CORO1A/FOXO3/EPHB2/PTAFR/WAS/PGLYRP1/IRS2/TOLLIP/STAT3/PRKDC/PRKAR1A/MPL/AMBRA1/PRKCD/GSN/MSN/HLX/NFAM1/SPI1/BCL3/SKAP2/RIPOR2/FLOT2/DYSF/GAB2/NFIL3
BP	GO:0050807	regulation of synapse organization	105	-0.44	-1.69	0.00053966	0.0034331	0.00211047	2959	tags=39%, list=25%, signal=29%	TUBB/CLN3/SETD5/FARP1/TLR2/CAPRIN1/RHOA/NEURL1/NEUROD2/ADNP/EIF4G1/ACTR2/LRRK2/LILRB2/PAFAH1B1/TNF/CDK5R1/NRP2/CFL1/SEMA4A/GRIPAP1/TUBA1A/DISC1/YWHAZ/CDKL5/SIPA1L1/AGRN/ABHD17C/DNM3/NLGN3/VCP/NTNG2/DBN1/PDLIM5/DLG5/SLC7A11/SNCA/MAPK14/EPHB2/ABI3/PTEN
BP	GO:0007610	behavior	292	-0.35	-1.49	0.00053949	0.0034331	0.00211047	2935	tags=36%, list=25%, signal=27%	CDH23/THBS1/ARRDC3/PRKAR1B/CLN3/ORMDL3/SRF/KRAS/SLC1A2/HIF1A/PBX3/GAA/CHD8/MAPK1/TLR2/CRTC1/FOS/PLCB1/PRKCZ/NEUROD2/ADNP/EIF4G1/KIT/ACTR2/CLN6/MBD2/TSPO/PJA2/FADD/SERPINE2/ABCA2/ATXN1/LRRK2/PPT1/LILRB2/HIPK2/FBXL20/NCOR1/PAFAH1B1/ABAT/CASR/UBR3/ATP7A/MECP2/GRIK2/TRMT1L/CPEB3/PCM1/ITGA5/ANKH/APBA1/DLG4/CREB1/EP300/PI3/GPI/MAP1A/MEIS1/GIGYF2/SNAP25/NR4A2/PTGS2/CCL3/PSEN1/MAPT/RGS14/VLDLR/RMI1/INSR/MAFG/AGTPBP1/PPP1R9B/ADGRF1/MBD5/HMGCR/NLGN3/ZFHX3/KCNQ1/EIF4EBP2/NCOA2/CPT1A/P2RY1/SOD2/P2RX1/SLC7A11/PTGDS/TAL1/MME/SNCA/CHD7/ARRB2/SYNJ1/CEBPB/OPRL1/PICALM/CIC/PRKAR2B/TMOD1/NF1/EPHB2/STAT3/NRGN/PTEN/CLDN5
BP	GO:0010769	regulation of cell morphogenesis involved in differentiation	70	-0.49	-1.74	0.00054225	0.0034441	0.0021172	2158	tags=39%, list=18%, signal=32%	DNM2/PAFAH1B1/GBP1/MYADM/RCC2/RAB21/TRIOBP/WASHC2C/CDKL5/ANKRD27/NEDD9/CUX1/DOCK5/CASS4/ILK/DBN1/LIMS1/PREX1/ACTN4/UNC13D/RAC1/EPHB2/CORO1C/DMTN/PRKDC/MPL/DAB2

BP	GO:0002252	immune effector process	441	-0.32	-1.42	0.00054609	0.0034629	0.00212882	2631	tags=30%, list=22%, signal=24%	ADAM17/INPP5D/PRKCZ/CD177/PLEKHM2/NCKAP1L/SNAP23/IL4I1/C17orf99/KIT/ANGPT1/SEMA7A/DOCK2/FCER1G/TICAM1/P2RX7/C1QA/RC3H2/CD70/FADD/NECTIN2/RFTN1/ARID5A/TNFSF13/PLCL2/SPON2/BTN3A2/DDX60/TNF/PRAM1/NSD2/PKN1/LRP1/KIF5B/ATP7A/TLR8/C1RL/ITGAM/SLC11A1/FZD5/SEMA4A/SYK/NMI/ZFPM1/CR1/HLA-E/ITGAL/PIK3CG/PTPN6/FCN1/IRAK3/MSH6/RAC2/CBL/TUBB4B/LYST/SERPING1/NCF1/CR1L/SUPT6H/LAMP1/GPI/DUSP22/LILRA2/KLRD1/STAT5B/RSAD2/MAVS/C2/CCL3/PSEN1/RARA/PTPRJ/FGR/CLU/ITGB2/FUT7/RNF19B/VAV1/LGALS3/SBNO2/PTK2B/HCK/MAD2L2/APBB1IP/PRDX2/LGALS9/RAB27A/NCR1/LIMK1/VAV3/SRC/GRN/TBX21/KMT2E/LAT2/AICDA/IL6R/STXBP2/CLCF1/ZBTB7B/TNFRSF1B/TREM1/PTGDS/AP1G1/LAPTM5/LYN/UNC13D/TYROBP/PAK1/PYCARD/FCGR3A/ARRB2/CYRIB/PIK3CD/PTPRC/HK1/STAT6/LCP1/HMOX1/CORO1A/EPHB2/PTAFR/WAS/PGLYRP1/STAT3/PRKCD/HLX/KRT1/SPI1/BCL3/DYSF/SLC7A5/GAB2
BP	GO:0071695	anatomical structure maturation	127	-0.41	-1.62	0.00054754	0.0034666	0.00213105	3027	tags=39%, list=26%, signal=29%	EDN1/FERMT1/PPP2R1A/ANKRD17/SCLT1/SCARF1/HIF1A/TRPC4AP/SLC26A6/FARP2/WNT1/RHOA/C1QA/TMEM79/CEBPA/LRRK2/ZBTB16/CDK5R1/MECP2/ERCC2/FZD5/CATSPER2/ATP6AP2/CDKN1C/RERE/SLFN14/RFLNB/GREM1/NR4A2/B4GALT5/ABHD2/AGRN/TUT7/PTK2B/SNX10/ZBTB7A/PRKACA/RBPJ/TAL1/PTBP3/TRIM58/EPB42/LYL1/NF1/FOXO3/PHOSPHO1/FAM210B/G6PD/ROPN1L
BP	GO:0098927	vesicle-mediated transport between endosomal compartments	44	-0.55	-1.81	0.00056617	0.0035789	0.00220007	2332	tags=41%, list=20%, signal=33%	FLNA/CHMP3/SORL1/MAP2K2/RAB21/HOOK3/EEA1/ANKRD27/VPS11/RILP/SRC/MYO1D/RAB7A/CORO1A/RAB5A/MAPK3/MSN/DAB2
BP	GO:0048041	focal adhesion assembly	55	-0.52	-1.77	0.00057019	0.0035928	0.00220865	2947	tags=49%, list=25%, signal=37%	BCAS3/THBS1/TAOK2/WDPCP/RHOA/SLC9A1/ACTG1/TESK2/ROCK2/RCC2/GREM1/DUSP22/ROCK1/PTPRJ/CTTN/PTK2B/SLK/ACTN1/SRC/LIMS1/RAC1/MAP4K4/CORO1C/DMTN/PTEN/VCL/ARHGAP6
CC	GO:0031256	leading edge membrane	108	-0.43	-1.66	0.00057219	0.0035997	0.00221289	1935	tags=34%, list=17%, signal=29%	CFL1/PDE4A/PIP5K1C/SYNE2/ITGA5/ATP6AP2/ATP2B1/PSD4/TIRAP/INPP5K/SH2D3C/CDKL5/MAPT/PTPRJ/FGR/INSR/DIAPH1/PPP1R9B/DOCK8/ARHGEF2/TESC/SRC/APC/MYO1D/PLEK2/AKT2/TPM1/PAK1/RAC1/PLEK/LCP1/BMX/CORO1C/VASP/TLN1/ITGB3/RIPOR2
BP	GO:0052548	regulation of endopeptidase activity	250	-0.36	-1.51	0.00057311	0.0035998	0.00221296	3002	tags=41%, list=26%, signal=31%	BAK1/RFFL/SERPINA1/CST7/THBS1/AIFM1/BST2/PML/NAIP/TNFSF10/AKIRIN2/ATP2A3/NLRP12/CASP2/RHOA/REST/CSNK2A1/JAK2/DAPK1/FIS1/CASP9/CIDEB/DDX3X/FADD/BEX3/SERPINE2/ABCA2/BAX/PROS1/SORL1/TNF/ROCK2/CST3/NKX3-1/PRDX5/SYK/ANXA2/CR1/BIRC6/CDKN2D/PI3/SERPING1/CAST/HGF/VSIR/GPI/KNG1/SH3RF1/PSME3/PTGS2/ROCK1/BID/MAPT/IFI16/PRR7/MAP3K5/ASPH/NR4A1/TIMP1/CTSD/VCP/EGLN3/GPX1/RPS6KA1/LGALS9/APLP2/SIAH2/SRC/PAK2/NRDC/TNFSF14/UBE2O/DAP/PLAUR/SERPINE1/P2RX1/PRELID1/ADRM1/POR/APAF1/LAPTM5/NLRC4/LYN/SNCA/PYCARD/ARRB2/MMP9/XIAP/BBC3/CFLAR/TIMP2/PCSK1N/PICALM/DNAJB6/DHCR24/AQP1/STAT3/SFRP2/GSN/PSMF1/MBP/FURIN/HIP1
BP	GO:0038066	p38MAPK cascade	42	-0.57	-1.86	0.00057426	0.0036014	0.0022139	1749	tags=33%, list=15%, signal=28%	GADD45A/NCF1/HGF/MAP3K3/MAP3K20/MAP3K5/LGALS9/MAPKAPK2/DUSP1/ZFP36L1/SASH1/MAP2K3/MAPK14/SPI1

BP	GO:0050803	regulation of synapse structure or activity	107	-0.45	-1.71	0.00057706	0.0036132	0.00222118	2959	tags=39%, list=25%, signal=30%	TUBB/CLN3/SETD5/FARP1/TLR2/CAPRIN1/RHOA/NEURL1/NEUROD2/ADNP/EIF4G1/ACTR2/LRRK2/PPT1/LILRB2/PAFAH1B1/TNF/CDK5R1/NRP2/CFL1/SEMA4A/GRIPAP1/TUBA1A/DISC1/YWHAZ/CDKL5/SIPA1L1/AGRN/ABHD17C/DNM3/NLGN3/VCP/NTNG2/DBN1/PDLIM5/DLG5/SLC7A11/SNCA/MAPK14/EPHB2/ABI3/PTEN
BP	GO:0002684	positive regulation of immune system process	622	-0.30	-1.36	0.00057797	0.0036132	0.00222121	2708	tags=31%, list=23%, signal=25%	FES/FOS/FPR2/TRAF6/RHOA/IL1B/DHPS/ADAM17/INPP5D/PRKCZ/ANO6/CCDC88B/CD177/JAK2/NCKAP1L/IL4I1/C17orf99/KIT/PPARGC1B/SEMA7A/SIN3A/FCER1G/TICAM1/P2RX7/C1QA/RC3H2/EVI2B/OXSR1/CSF1R/C5AR2/LCP2/CD38/CEBPA/CD70/FADD/BTNL9/THEMIS2/LRRK2/NECTIN2/RFTN1/ARID5A/TNFSF13/LILRB2/PLCL2/BAX/SPON2/ZBTB16/BTN3A2/PRKCB/DDX60/PIK3CB/PELI1/TNF/EFNB1/JUND/PRAM1/FPR1/BST1/NSD2/MNDA/GBP1/TNFSF13B/TLR8/C1RL/GPLD1/PRKD2/ITGAM/SLC11A1/SPTA1/PLSCR1/FZD5/SYK/SPG21/NMI/CR1/HLA-E/ELF1/PTPN6/FCN1/IRAK3/CREB1/JAM3/RAC2/EP300/CCR1/CAMK1D/SERPING1/LSM14A/TIRAP/VSIR/CR1L/LAMP1/GPI/DUSP22/SOX4/TLR6/LILRA2/KLRD1/STAT5B/RSAD2/ERMAP/MAVS/USP27X/C2/CCL3/PSEN1/RARA/RELA/PTPRJ/FGR/CLU/ITGB2/FUT7/IFI16/VAV1/LGALS3/C5AR1/DOCK8/PTK2B/RIOK3/HCK/MAD2L2/TESC/SIRPA/LGALS9/CDKN1A/LIMK1/VAV3/SRC/TBX21/PF4/IKBKG/LAT2/PAK2/RIPK1/TNFSF14/IL6R/STXBP2/ZFP36L1/SELP/CLCF1/ZBTB7B/SERPINE1/BAG6/ZMIZ1/AP1G1/LAPTM5/NLRC4/FYB1/CTNNBIP1/LYN/SIRPB1/UNC13D/HCLS1/IGF2/ZNFX1/TYROBP/PAK1/RAC1/WNK1/PYCARD/FCGR3A/GPSM3/SH3KBP1/CREBBP/CYRIB/PIK3CD/PTPRC/HK1/STAT6/HMOX1/BMX/CORO1A/FOXO3/EPHB2/PTAFR/IRS2/MAPK3/PRKDC/MPL/AMBRA1/PRKCD/HLX/NFAM1/KRT1/SPI1/RIPOR2/FLOT2/PLVAP/TRIB1/SLC7A5/GAB2
BP	GO:0045862	positive regulation of proteolysis	281	-0.35	-1.47	0.00058549	0.0036545	0.00224658	2826	tags=38%, list=24%, signal=29%	TNFSF10/CSNK1A1/HECTD1/AKIRIN2/ADAM8/ATP2A3/NLRP12/CASP2/RHOA/IL1B/REST/SIRT6/JAK2/DAPK1/FIS1/CASP9/CIDEB/CLN6/RCN3/DDX3X/TAF1/CEBPA/FADD/BEX3/ZYG11B/LRRK2/GSK3B/RNF14/BAX/CCBE1/TNF/ROCK2/NKX3-1/GPLD1/SH3RF3/COP1/SYK/ANXA2/CR1/NUB1/RNF40/VSIR/FZR1/DISC1/SH3RF1/PSME3/BID/IST1/HSPA1B/GSK3A/PSEN1/MAPT/CLU/IFI16/PRR7/CAPN3/PLK1/RNF19B/MAP3K5/AGTPBP1/ASPH/HSPA1A/PSME4/PTK2B/CTSD/VCP/EGLN3/NUPR1/TNIP1/LGALS9/RAD23A/SRC/GRN/GABARAP/NRDC/DAP/PIAS1/TNFRSF1B/NFE2L2/P2RX1/MYH9/PRELID1/ADRM1/BAG6/APAF1/LAPTM5/NLRC4/LYN/SNCA/PYCARD/PSMC4/BBC3/CFLAR/PICALM/DNAJB2/ZER1/STAT3/SFRP2/GSN/PINK1/PTEN/MBP/TRIB1/FURIN/DAB2/HIP1
BP	GO:0008625	extrinsic apoptotic signaling pathway via death domain receptors	70	-0.49	-1.73	0.0006002	0.0037346	0.00229579	2434	tags=44%, list=21%, signal=35%	DDX3X/FADD/BEX3/GSK3B/BAX/ATF3/TNF/TNFRSF1A/TNFRSF10C/TMBIM1/STK4/HGF/BID/LGALS3/ARHGEF2/GPX1/GABARAP/ITPRIP/BRCA1/SERPINE1/DEDD2/PEA15/CFLAR/HMOX1/SP100/NF1/SFRP2/BCL2L1/ZDHHC3/PTEN/SPI1
BP	GO:0071621	granulocyte chemotaxis	76	-0.47	-1.67	0.00061231	0.003804	0.00233845	3053	tags=55%, list=26%, signal=41%	CCL5/EDN1/JAML/DAPK2/THBS1/MOSPD2/MAPK1/PIKFYVE/IL1B/NCKAP1L/FCER1G/CSF1R/C5AR2/BST1/SYK/PIP5K1C/PIK3CG/PF4V1/JAM3/RAC2/CAMK1D/TIRAP/CSF3R/CCL3/ITGB2/VAV1/LGALS3/C5AR1/CXCR2/CXCL5/VAV3/CXCR1/PF4/PREX1/TREM1/RAC1/PIK3CD/IL17RA/MAPK3/BSG/RIPOR2/MPP1
MF	GO:1902936	phosphatidylinositol bisphosphate binding	70	-0.49	-1.73	0.00061675	0.0038256	0.00235176	3576	tags=53%, list=30%, signal=37%	KRIT1/GBF1/ADAP2/SNX3/PARD3/CAPG/MAPKAP1/MARK1/PHLDA3/PLEKHA5/RPH3A/PLCB1/SLC9A1/WIPI1/RAB35/ADAP1/SNX14/TPCN2/ALOX15/TWF2/ANXA2/TTPAL/TIRAP/WASHC2C/MAPT/WIPI2/KCNQ1/WDR45/PLEK2/SVIL/SDCBP/PICALM/ASAP1/SNX18/GSN/OSBPL2/HIP1

MF	GO:0003924	GTPase activity	243	-0.36	-1.49	0.00061818	0.0038285	0.00235353	2511	tags=31%, list=21%, signal=25%	GNB2/RAB32/DDX3X/RAB35/TUBA1B/ARHGDIB/SRPRA/LRRK2/GNG5/RHOQ/MTG2/DNM2/RAB2A/MRAS/GBP1/RGS3/RGS2/ARL4A/RAB6A/RAB43/RAB34/RAB22A/TUBB6/RAB21/RAC2/RGS6/TUBB4B/AGAP2/RAB6B/GNAI2/TUBA1A/MX2/RAB37/RAB3B/GNB1/ARF1/GBP2/NKIRAS2/RGS14/RAB1B/RAB27B/ARL17B/ARL13B/RAP1GAP/ARL8A/RALB/RAB11B/GNA12/DNM3/GNAQ/TUBB1/RGS19/GSPT1/RAB27A/RAB7A/TUBB2A/RAB36/RAB2B/GTPBP1/GNAL/GBP4/TUBA1C/RAB5B/RIT1/ARF3/RAC1/TUBA4A/RRAGD/RHOG/RGS7/RAB5C/RHOBTB1/RAB5A/RAB3D/RAB31/GNAZ
BP	GO:0043297	apical junction assembly	42	-0.57	-1.85	0.00062313	0.0038496	0.00236649	2148	tags=38%, list=18%, signal=31%	TNF/ROCK2/FZD5/GPBAR1/ROCK1/CTNNA1/APC/PAK2/ESAM/PECAM1/DLG5/PRKACA/AFDN/F11R/VCL/CLDN5
BP	GO:0030198	extracellular matrix organization	122	-0.43	-1.68	0.00062449	0.0038496	0.00236649	2287	tags=30%, list=20%, signal=24%	COLGALT2/CRISPLD2/NPHS1/TNF/TNXB/LRP1/CST3/ATP7A/ERCC2/MYH11/TNFRSF1A/NFKB2/FSCN1/SULF2/SERAC1/ERO1A/GREM1/QSOX1/B4GALT1/COL9A3/NTNG2/FLOT1/CYP1B1/MYO1E/TNFRSF1B/ADAMTS4/GFOD2/MMP9/CFLAR/LCP1/DNAJB6/NF1/SFRP2/HSD17B12/BCL3/FURIN
BP	GO:0043062	extracellular structure organization	122	-0.43	-1.68	0.00062449	0.0038496	0.00236649	2287	tags=30%, list=20%, signal=24%	COLGALT2/CRISPLD2/NPHS1/TNF/TNXB/LRP1/CST3/ATP7A/ERCC2/MYH11/TNFRSF1A/NFKB2/FSCN1/SULF2/SERAC1/ERO1A/GREM1/QSOX1/B4GALT1/COL9A3/NTNG2/FLOT1/CYP1B1/MYO1E/TNFRSF1B/ADAMTS4/GFOD2/MMP9/CFLAR/LCP1/DNAJB6/NF1/SFRP2/HSD17B12/BCL3/FURIN
MF	GO:0001228	DNA-binding transcription activator activity, RNA polymerase II-specific	250	-0.36	-1.50	0.00062769	0.0038633	0.00237494	2392	tags=31%, list=20%, signal=25%	KLF7/CEBPA/MITF/KLF5/OTX1/YY1/GFI1B/NFIA/TBX19/REL/ZBTB16/ZNF585B/ATF3/ZNF107/JUND/NFAT5/ZNF784/HHEX/PLSCR1/ATF5/ZNF445/NFATC3/KLF6/ELF1/SP1/CREB1/NFKB2/CEBPD/ZBED1/TFCP2/SOX4/MEIS1/MSX1/STAT5B/CREBRF/JUNB/FOXJ2/NR4A2/TFDP2/RELA/ZNF786/MAFG/NFYC/NR3C1/ATF6B/NR4A1/IRF2/ZNF493/BACH1/TBX21/FLI1/PBX1/ZNF48/ETV6/RFX2/IRF1/CSRNP1/ZBTB7B/RBPJ/NFE2L2/USF3/PLAGL2/ELF4/PBX2/CEBPB/STAT6/E2F3/NFIC/FOXO3/TFE3/STAT3/FOSL2/ZNF33A/SPI1/FOXO4/LITAF/E2F2
BP	GO:0061045	negative regulation of wound healing	36	-0.59	-1.84	0.00063602	0.0039025	0.00239901	3145	tags=53%, list=27%, signal=39%	CLASP1/THBD/EDN1/THBS1/CD9/ALOX5/SERPINE2/PROS1/TNF/ABAT/PDGFA/SERPING1/KNG1/GP1BA/ALOX12/SERPINE1/PRKCD/PTEN/KRT1
BP	GO:0034332	adherens junction organization	29	-0.63	-1.86	0.00065458	0.0040102	0.00246523	2211	tags=41%, list=19%, signal=34%	ADD1/HIPK1/PIP5K1C/JAM3/NUMB/ZNF703/BMP6/SRC/PAK2/ACTB/DLG5/VCL
BP	GO:0051494	negative regulation of cytoskeleton organization	109	-0.44	-1.67	0.00065561	0.0040103	0.00246532	1923	tags=28%, list=16%, signal=23%	MYADM/SPTA1/TAOK1/TWF2/INPP5K/TRIOBP/WASHC2C/WASF2/CCNF/ARHGEF2/APC/PAK2/SVIL/BRCA1/LMOD3/SSH1/SNCA/CYRIB/F11R/PPFIA1/TMOD1/CORO1A/SSH2/WAS/DMTN/MTPN/PRKCD/GSN/SPTB/ARHGAP6
BP	GO:0051893	regulation of focal adhesion assembly	44	-0.55	-1.81	0.00066254	0.0040403	0.00248372	2947	tags=50%, list=25%, signal=38%	BCAS3/THBS1/WDPCP/RHOA/SLC9A1/ACTG1/ROCK2/RCC2/GREM1/DUSP22/ROCK1/PTPRJ/SLK/SRC/LIMS1/RAC1/MAP4K4/CORO1C/DMTN/PTEN/VCL/ARHGAP6
BP	GO:0090109	regulation of cell-substrate junction assembly	44	-0.55	-1.81	0.00066254	0.0040403	0.00248372	2947	tags=50%, list=25%, signal=38%	BCAS3/THBS1/WDPCP/RHOA/SLC9A1/ACTG1/ROCK2/RCC2/GREM1/DUSP22/ROCK1/PTPRJ/SLK/SRC/LIMS1/RAC1/MAP4K4/CORO1C/DMTN/PTEN/VCL/ARHGAP6

MF	GO:0038023	signaling receptor activity	532	-0.30	-1.35	0.00066694	0.0040547	0.00249258	2849	tags=31%, list=24%, signal=24%	PAQR7/INTS6/SCARF1/CLEC4E/NR2F6/HLA-DRB1/OR4D9/LILRA6/TLR2/PAQR6/FPR2/SIGMAR1/GPR141/KIR2DL1/SCARB2/GPR160/LPAR1/SIGLEC7/HCAR3/OR7C1/C17orf99/KIT/FCER1G/TNFRSF9/P2RX7/CSF1R/PAQR8/C5AR2/AGTRAP/COLEC12/TSPO/LTBP1/PLXNB2/ERBB2/IL18RAP/IL1R2/CRCP/CYSLTR2/NECTIN2/RTN4R/BMPR1A/LILRB2/SORL1/FPR1/CASR/LRP1/IGSF6/TLR1/MAS1/NKX3-1/OR2G6/TLR8/NRP2/PAQR4/GP6/S1PR2/GRIK2/CD300LF/FZD5/HRH2/IL10RB/LILRA1/CR1/GPR27/GPBAR1/TREML2/P2RY13/HTR3B/ITGAL/TAS2R4/TNFRSF1A/TNFRSF10C/ATP6AP2/PTPN6/SSTR2/FCN1/OR6C75/IL17RD/GUCY1B1/RRBP1/CCR1/KIR2DL3/ADGRE3/OR2B11/FCGR3B/TLR6/INPP5K/CD14/LILRA2/KLRD1/ADGRA3/FFAR4/CSF3R/LTB4R/NR4A2/FCGR2A/ARNT/RARA/VLDLR/IGF2R/ITGB2/EPHB4/LGR6/PTPRE/INSR/ABHD2/GPR85/NR3C1/ADGRF1/C5AR1/NR4A1/NLGN3/PTK2B/IFNAR1/CXCR2/TMEM63B/CD93/ANTXR2/GPR82/CXCR1/ABCC9/PECAM1/P2RY1/IL6R/PLAUR/VDR/OGFR/GP1BA/IFNAR2/OSCAR/RXRA/TNFRSF1B/TREM1/CUBN/P2RX1/ITGB5/TREML1/NOTCH4/ACKR1/MMD/MRC2/FCGR3A/ADIPOR1/SLC1A5/PLXNC1/OGFRL1/GUCY1A1/PTPRC/OPRL1/EPHB2/PTAFR/PGLYRP1/IL17RA/STAT3/ITGB3/IL13RA1/MPL/NFAM1/TAS2R30/KRT1/CCRL2/LILRB3/TBXA2R/GPR146
MF	GO:0060089	molecular transducer activity	532	-0.30	-1.35	0.00066694	0.0040547	0.00249258	2849	tags=31%, list=24%, signal=24%	PAQR7/INTS6/SCARF1/CLEC4E/NR2F6/HLA-DRB1/OR4D9/LILRA6/TLR2/PAQR6/FPR2/SIGMAR1/GPR141/KIR2DL1/SCARB2/GPR160/LPAR1/SIGLEC7/HCAR3/OR7C1/C17orf99/KIT/FCER1G/TNFRSF9/P2RX7/CSF1R/PAQR8/C5AR2/AGTRAP/COLEC12/TSPO/LTBP1/PLXNB2/ERBB2/IL18RAP/IL1R2/CRCP/CYSLTR2/NECTIN2/RTN4R/BMPR1A/LILRB2/SORL1/FPR1/CASR/LRP1/IGSF6/TLR1/MAS1/NKX3-1/OR2G6/TLR8/NRP2/PAQR4/GP6/S1PR2/GRIK2/CD300LF/FZD5/HRH2/IL10RB/LILRA1/CR1/GPR27/GPBAR1/TREML2/P2RY13/HTR3B/ITGAL/TAS2R4/TNFRSF1A/TNFRSF10C/ATP6AP2/PTPN6/SSTR2/FCN1/OR6C75/IL17RD/GUCY1B1/RRBP1/CCR1/KIR2DL3/ADGRE3/OR2B11/FCGR3B/TLR6/INPP5K/CD14/LILRA2/KLRD1/ADGRA3/FFAR4/CSF3R/LTB4R/NR4A2/FCGR2A/ARNT/RARA/VLDLR/IGF2R/ITGB2/EPHB4/LGR6/PTPRE/INSR/ABHD2/GPR85/NR3C1/ADGRF1/C5AR1/NR4A1/NLGN3/PTK2B/IFNAR1/CXCR2/TMEM63B/CD93/ANTXR2/GPR82/CXCR1/ABCC9/PECAM1/P2RY1/IL6R/PLAUR/VDR/OGFR/GP1BA/IFNAR2/OSCAR/RXRA/TNFRSF1B/TREM1/CUBN/P2RX1/ITGB5/TREML1/NOTCH4/ACKR1/MMD/MRC2/FCGR3A/ADIPOR1/SLC1A5/PLXNC1/OGFRL1/GUCY1A1/PTPRC/OPRL1/EPHB2/PTAFR/PGLYRP1/IL17RA/STAT3/ITGB3/IL13RA1/MPL/NFAM1/TAS2R30/KRT1/CCRL2/LILRB3/TBXA2R/GPR146
BP	GO:0032732	positive regulation of interleukin-1 production	56	-0.51	-1.75	0.00066864	0.0040588	0.00249509	3675	tags=52%, list=31%, signal=36%	TLR4/TMEM106A/NLRP3/EGR1/NLRP1/HAVCR2/MYD88/NLRP10/NLRP12/JAK2/P2RX7/TNF/MNDA/TLR8/FZD5/ORM1/TLR6/LILRA2/CCL3/IFI16/LGALS9/HSPB1/STMP1/NLRC4/TYROBP/PYCARD/PANX2/HK1/STAT3

MF	GO:0050839	cell adhesion molecule binding	372	-0.32	-1.41	0.00067039	0.0040632	0.00249782	2788	tags=36%, list=24%, signal=28%	ADAM8/SHTN1/CD9/PACSIN2/ARHGAP18/CLIC1/IL1B/USP8/ADAM17/LYPLA2/NEXN/CD177/STX5/HCFC1/GCN1/SEMA7A/TGFBI/HSPA5/DDX6/EPS15/DDX3X/SNX1/PKP4/FLNA/CAPZB/FERMT3/NECTIN2/SH3GLB2/LILRB2/ADD1/NPHS1/UTRN/TNXB/LRRC59/CASR/KIF5B/CDK5R1/TRIM25/S1PR2/ITGAM/ITGA2B/MARK2/PKM/EIF4G2/TWF2/SYK/ANXA2/ITGA5/ITGAL/ICAM4/PTPN6/CC2D1A/JAM3/FSCN1/CBL/MICALL1/PCBP1/H3C10/CAST/STK24/RTN4/YWHAZ/GIGYF2/NIBAN2/ALDOA/NUMB/DSC2/IST1/WASF2/YWHAB/ICAM3/PSEN1/PTPRJ/ITGB2/PTPN1/TBC1D2/HSPA1A/HDLBP/CTTN/RAB11B/CTNNA1/NLGN3/ZC3HAV1/SLK/SIRPA/PALLD/EFHD2/ACTN1/SRC/DBN1/PDLIM5/PAK2/ESAM/PPP1R13L/EHD1/SH3GLB1/TAGLN2/CTNND1/GOLGA2/MYH9/SH3GL1/ARHGAP1/ITGB5/EPS15L1/SCYL1/AFDN/ACTN4/LASP1/LYN/IGF2/HNRNPK/SDCBP/LRRFIP1/F11R/PICALM/STK38/LCP1/ASAP1/CTNNAL1/PRDX6/VASP/TLN1/SFRP2/ITGB3/BSG/MSN/CNN2/VCL/CHMP4B/NINJ1/IQGAP1/S100A11/PLIN3
BP	GO:0010657	muscle cell apoptotic process	42	-0.57	-1.84	0.000672	0.0040668	0.00250003	1188	tags=31%, list=10%, signal=28%	MAP3K5/PTK2B/NUPR1/PPP1R10/SOD2/ALOX12/NFE2L2/APAF1/ARRB2/CFLAR/E2F3/HMOX1/SFRP2
BP	GO:0090257	regulation of muscle system process	122	-0.43	-1.68	0.00067879	0.0041016	0.00252145	2132	tags=33%, list=18%, signal=27%	ROCK2/PLCE1/ABAT/RGS2/HDAC4/PIK3CG/TNFRSF1A/SSTR2/LMNA/ATP2B1/PDE5A/TRPC3/PTGS2/ROCK1/DSC2/GSK3A/NR4A1/CTTN/GTF2IRD1/DOCK5/KCNQ1/AKAP9/CALM3/PRKACA/TNFRSF1B/PROK2/JARID2/TPM1/P2RX1/PPP1R12B/PPP3CA/EHD3/GUCY1A1/FOXO3/PTAFR/G6PD/MTPN/MYL9/SLC8A1/TBXA2R
CC	GO:0062023	collagen-containing extracellular matrix	145	-0.40	-1.59	0.00068147	0.0041115	0.00252753	2519	tags=34%, list=21%, signal=27%	ANGPT1/SEMA7A/TGFBI/C1QA/LTBP1/SERPINE2/CTSZ/TNXB/SDC3/PKM/PLSCR1/P3H1/ORM1/ANXA2/F13A1/LAMA4/FCN1/SERPING1/NCAM1/GREM1/KNG1/VCAN/ERBIN/CLU/AGRN/LGALS3/ANXA11/TIMP1/MEGF9/CTSD/LGALS9/COL9A3/NTNG2/SPARC/HDGF/PF4/PSAP/TGFB1I1/SERPINE1/ADAMTS4/TGM2/LAMC3/MMP9/TIMP2/CTSB/SFRP2/PCSK6/KRT1/LTBP2
BP	GO:0060548	negative regulation of cell death	634	-0.29	-1.34	0.00068251	0.0041116	0.00252756	2681	tags=32%, list=23%, signal=26%	WNT1/RHOA/IL1B/MAP2K4/REST/DNAJC3/PRKCZ/ANXA5/BTBD10/CBX4/ADNP/EIF4G1/CSNK2A1/JAK2/DAPK1/NCKAP1L/KIT/PSEN2/ANGPT1/SLC9A1/SIN3A/HSPA5/EVI2B/CSF1R/DDX3X/INPP5A/CD38/FADD/MITF/FLNA/PTTG1IP/LRRK2/PPT1/GSK3B/CHMP3/HIPK2/BAX/SORL1/PIK3CB/PELI1/TNF/PHIP/DYRK3/PPIF/UCP2/MECP2/GRIK2/BNIP3L/ARHGDIA/LRPAP1/PRDX5/CFL1/FHL2/MCL1/HYPK/MEAK7/DDAH2/ATF5/UBE2B/BIRC6/ITGA5/PIK3CG/CPEB4/RB1CC1/LMNA/CDKN2D/GCLM/CBL/CAMK1D/TMBIM1/AGAP2/PIM1/CAST/EIF2AK2/IVNS1ABP/DRAXIN/HGF/GNAI2/GREM1/GPI/SOX4/YWHAZ/CEACAM5/MSX1/CHST11/NIBAN2/STAT5B/SHC1/FFAR4/SH3RF1/KDM4A/NR4A2/DSTYK/PSME3/PTGS2/ROCK1/BID/PELI3/HSH2D/TMEM161A/HSPA1B/PSEN1/RELA/CLU/SOCS3/IER3/CAPN3/AREL1/PTPN1/ADAR/SLC23A2/PLK1/LGALS3/HSPA1A/NR4A1/CTTN/TIMP1/DOCK8/CTNNA1/PTK2B/ARHGEF2/HCK/SLC46A2/NUPR1/PTMS/GPX1/SON/AP2B1/RPS6KA1/VPS4B/PRDX2/PARP16/DDB1/HIPK3/CHMP1A/HDGF/SIAH2/SRC/GRN/PF4/IKBKG/ITPRIP/PAK2/HSPB1/PPP1R10/SETX/RIPK1/TNFSF14/UBE2O/SOD2/PLAUR/AKT2/CLCF1/TNFRSF1B/ALOX12/PROK2/BRCA1/NFE2L2/SERPINE1/TSC22D3/SLC7A11/PRELID1/BAG1/AGO4/BAG6/POR/LAPTM5/STK40/HCLS1/PEA15/SNCA/DNAJC5/HNRNPK/ARRB2/MMP9/XIAP/GRINA/CFLAR/CEBPB/MAP4K4/FBXO7/DNAJB6/HMOX1/CORO1A/CHMP2A/YBX3/DHCR24/STRADB/IRS2/TCF7L2/AQP1/PRKDC/SFRP2/PTCRA/FKBP8/PRKCD/PINK1/BCL2L1/PTEN/NUAK2/CHMP4B/BCL3/DAB2/TGFA

BP	GO:0051047	positive regulation of secretion	181	-0.39	-1.57	0.00068655	0.0041297	0.00253869	2092	tags=33%, list=18%, signal=27%	HLA-DRB1/HIF1A/ADAM8/TLR2/IL1B/WLS/CD177/JAK2/P2RX7/CD38/CACNA1I/RPH3AL/ABAT/PTGES/MYH10/CASR/NKX3-1/GPLD1/ITGAM/STXBP5/SYK/GPR27/CREB1/LRRC8A/ABCB11/GNAI2/LAMP1/SOX4/FFAR4/SYTL4/FGR/ITGB2/RAB27B/BMP6/VPS4B/RAB27A/ACSL4/RAB7A/RAB2B/P2RY1/AP1G1/ABCG1/UNC13D/SNCA/C2CD2L/SDCBP/OPRL1/MYOM1/PTAFR/CHMP2A/IRS2/TCF7L2/RAB5A/AQP1/PINK1/RAB3D/DAB2/GLUL/GAB2
BP	GO:0051235	maintenance of location	228	-0.36	-1.51	0.00069054	0.0041474	0.00254957	2475	tags=29%, list=21%, signal=23%	HSPA5/P2RX7/HAP1/TAF8/TSPO/HK2/LTBP1/FLNA/CHCHD10/TPCN2/BAX/ACACB/SORL1/HNRNPU/PAFAH1B1/TNF/NFKBIA/ITPR2/CES1/MCOLN1/TWF2/PTPN6/ERO1A/OS9/STAT5B/OSBPL8/YWHAB/CCL3/SUFU/PSEN1/FKBP1A/CAPN3/SELENON/DIAPH1/NFKBIB/PTK2B/ARHGAP21/CPT1A/DBN1/AKAP9/CALM3/GP1BA/PRKACA/EHD1/DGAT2/BSCL2/NRROS/ABCG1/LYN/SNCA/CHD7/PTPRC/HK1/ABHD5/SP100/CORO1A/PNPLA2/FTL/ITGB3/FTH1/GSN/PINK1/SLC8A1/GP9/PLIN3
BP	GO:0005975	carbohydrate metabolic process	414	-0.32	-1.41	0.00069383	0.0041609	0.00255786	2419	tags=26%, list=21%, signal=22%	RPIA/INPP5A/GALNT3/HK2/DHTKD1/GALM/MAN2B1/ALDH2/IMPA2/GSK3B/NCOR1/ACACB/ATF3/TNF/RANBP2/ENTPD5/MAS1/GPLD1/MECP2/HDAC4/PKM/MANBA/PGAM1/MGAM/PPP1R3B/SOGA1/RB1CC1/EP300/CHST2/SIAE/GPI/INPP5K/CHST11/CHST13/PDK2/ALDOA/CLTC/MOGAT3/SLC2A3/TPD52L2/ST6GALNAC4/ARNT/SESN2/PARG/GSK3A/PSEN1/GOT1/UEVLD/FUT7/INSR/B4GALT5/SLC23A2/CHI3L1/CHST7/NEU1/BCKDK/PGM1/ST3GAL2/PTK2B/B4GALT1/MAN2A2/NUPR1/KCNQ1/JMJD8/DDB1/BPNT2/PGD/SRC/NCOA2/CPT1A/BPGM/GLB1L/ABO/GNPTAB/PFKFB3/P2RY1/ZBTB7A/B3GNT8/AKT2/FGGY/DGAT2/GBGT1/AP2A1/NDST1/PYGL/STK40/SNCA/IGF2/TALDO1/PLEK/NPL/MIDN/ADIPOR1/SYNJ1/HK1/FBP1/MAPK14/B3GAT1/ITPK1/PDK3/PTAFR/SLC4A1/G6PD/IRS2/STAT3/PFKFB4/WDTC1/PTEN/GPD2
BP	GO:0048762	mesenchymal cell differentiation	125	-0.42	-1.65	0.00069787	0.0041788	0.00256888	3158	tags=39%, list=27%, signal=29%	SPRED1/CLASP1/S100A4/MARK1/EDN1/ADAM15/TIAM1/STAT1/ZFP64/HIF1A/MAPK1/IL1B/SEMA7A/MCRIP1/FLNA/GSK3B/BMPR1A/FBXL17/EFNB1/ROCK2/NRP2/SEMA4A/IL17RD/HGF/RTN4/RFLNB/GREM1/MSX1/ROCK1/ZNF703/PHACTR4/MAD2L2/FAM172A/TGFB1I1/DLG5/RBPJ/OTUD5/USF3/AKNA/NOTCH4/GLIPR2/SDCBP/ADIPOR1/CORO1C/TCF7L2/MAPK3/SFRP2/PTEN/DAB2
MF	GO:0001216	DNA-binding transcription activator activity	253	-0.36	-1.49	0.00069906	0.0041797	0.00256941	2392	tags=30%, list=20%, signal=25%	KLF7/CEBPA/MITF/KLF5/OTX1/YY1/GFI1B/NFIA/TBX19/REL/ZBTB16/ZNF585B/ATF3/ZNF107/JUND/NFAT5/ZNF784/HHEX/PLSCR1/ATF5/ZNF445/NFATC3/KLF6/ELF1/SP1/CREB1/NFKB2/CEBPD/ZBED1/TFCP2/SOX4/MEIS1/MSX1/STAT5B/CREBRF/JUNB/FOXJ2/NR4A2/TFDP2/RELA/ZNF786/MAFG/NFYC/NR3C1/ATF6B/NR4A1/IRF2/ZNF493/BACH1/TBX21/FLI1/PBX1/ZNF48/ETV6/RFX2/IRF1/CSRNP1/ZBTB7B/RBPJ/NFE2L2/USF3/PLAGL2/ELF4/PBX2/CEBPB/STAT6/E2F3/NFIC/FOXO3/TFE3/STAT3/FOSL2/ZNF33A/SPI1/FOXO4/LITAF/E2F2
BP	GO:0016055	Wnt signaling pathway	263	-0.35	-1.50	0.00070379	0.0041952	0.00257895	2681	tags=33%, list=23%, signal=26%	WNT1/RHOA/TMEM131L/ASPM/WLS/USP8/CSNK2A1/ZEB2/JADE1/CSNK1G2/DDX3X/MBD2/PORCN/MED12/MITF/LRRK2/GSK3B/SENP2/ZRANB1/TLE4/LRP1/CTDNEP1/MARK2/HHEX/FZD5/UBE2B/APCDD1/USP34/ATP6AP2/UBR5/CTR9/SULF2/STK4/DRAXIN/TSC2/GREM1/CUL3/DISC1/SOX4/GRK6/FRMD8/NR4A2/CCNY/GSK3A/PSEN1/ZNF703/INVS/LGR6/RTF1/TBL1X/GNAQ/VCP/MAD2L2/LRRFIP2/BCL9/DVL3/SKI/DDB1/CSNK1G1/ILK/SIAH2/SRC/APC/TGFB1I1/FAM53B/FRAT1/TMEM88/RBPJ/OTUD5/KPNA1/CTNND1/TLE3/HIC1/CTNNBIP1/RAC1/WNK1/XIAP/MAPK14/FOXO3/TCF7L2/RAB5A/SFRP2/PPM1A/PTEN/GID8/DAB2

BP	GO:0048871	multicellular organismal homeostasis	292	-0.35	-1.48	0.0007032	0.0041952	0.00257895	3059	tags=37%, list=26%, signal=28%	IL18R1/GATA1/SLC40A1/DYNC1H1/CSK/BBS2/IP6K1/CDH23/ARRDC3/SRF/KRAS/PDK4/GNAS/ADAM8/MFN2/CRTC1/TRAF6/ACSL1/IL1B/ADAM17/INPP5D/POC1B/SLC2A1/EIF4G1/SIRT6/PBLD/JAK2/SLC28A2/ANGPT1/PPARGC1B/GPR137B/P2RX7/RCN3/CSF1R/TMEM79/ACTG1/CD38/NOVA1/ALPL/PLCL2/BAX/ACACB/PLEKHM1/TNF/AMPD2/ABAT/PTGES/ACOT11/UCP2/G0S2/PRCP/SLC11A1/CPT2/METRNL/SYK/ZNF516/LAMA4/TP53INP2/JAM3/INPP5K/GIGYF2/FFAR4/PTGS2/NKIRAS2/SCD/SQSTM1/FLG2/BMP6/PTK2B/GPX1/TF/SLC25A44/SNX10/SRC/APC/RAB7A/UBB/ESAM/NRDC/HSPB1/PECAM1/ACTB/PPP1R13L/PRKACA/ZBTB7B/ALOX12/RBPJ/CUBN/BSCL2/KDM6B/TLE3/ADIPOR1/F11R/CEBPB/STAT6/CORO1A/NF1/TFE3/AQP1/STAT3/ITGB3/FLCN/RAB3D/VCL/KRT1/CHMP4B/MBP/CLDN5
BP	GO:0120031	plasma membrane bounded cell projection assembly	384	-0.32	-1.40	0.00070482	0.0041952	0.00257895	2722	tags=29%, list=23%, signal=23%	DNAL1/TTBK2/RO60/WNT1/RAB8A/NEURL1/TTC26/POC1B/ANO6/CDC42EP4/IFT88/TCTN1/KIT/ACTR2/CEP89/DYNLL2/P2RX7/HAP1/FLNA/CAPZB/KLF5/IFT81/SPATA13/GSK3B/RHOQ/ARMC2/PLEKHM1/DNM2/TBC1D30/PLCE1/ARAP1/IFT122/ATP7A/CFAP58/S1PR2/ABCC4/PCM1/RCC2/TWF2/TMEM231/SYNE2/UBE2B/DYNC2H1/SPATA6/CEP19/RILPL1/ARMC9/RAC2/FSCN1/DISC1/INPP5K/CEP350/BBOF1/IFT140/CDKL5/ROCK1/WASF2/SNAP29/DAW1/INTU/ATP6V0D1/AGRN/MAP4/ARL13B/RAP1GAP/PPP1R9B/CTTN/ESPN/DNM3/MARK4/CEP97/NDEL1/RILP/KCNQ1/VAV3/SNX10/APC/ALPK1/EXOC4/KLC3/RFX2/PLEK2/PARVB/FXR2/EHD1/RPGR/FGD4/SDCBP/RAC1/FGD6/LIMK2/EHD3/RHOG/ASAP1/EPHB2/CORO1C/WAS/RILPL2/ABI3/ABLIM3/RAB5A/DMTN/CCDC39/MYLK/PRKCD/GSN/CCP110/VCL/RIPOR2/RAPGEF2/ODAD4/KIF27
BP	GO:1900026	positive regulation of substrate adhesion-dependent cell spreading	30	-0.62	-1.86	0.00071273	0.0042359	0.00260399	2332	tags=47%, list=20%, signal=37%	FLNA/DNM2/MYADM/TRIOBP/NEDD9/DOCK5/CASS4/ILK/LIMS1/PREX1/UNC13D/RAC1/DMTN/DAB2
BP	GO:1903557	positive regulation of tumor necrosis factor superfamily cytokine production	81	-0.48	-1.73	0.00071915	0.0042677	0.00262352	3675	tags=59%, list=31%, signal=41%	TLR4/TMEM106A/SASH3/ZBTB20/HAVCR2/DDX58/SPN/PLCG2/MYD88/BCL10/THBS1/OAS3/ADAM8/TLR2/JAK2/TICAM1/SETD4/FADD/LRRK2/CYBB/ARID5A/SPON2/TLR1/FZD5/ORM1/SYK/HLA-E/TIRAP/CD14/LILRA2/FRMD8/MAVS/CCL3/PSEN1/PTPRJ/CLU/ARHGEF2/LGALS9/PF4/MAPKAPK2/HSPB1/RIPK1/TYROBP/PYCARD/PTPRC/EPHB2/PTAFR/STAT3
BP	GO:0032504	multicellular organism reproduction	444	-0.31	-1.40	0.00072117	0.0042733	0.00262695	2659	tags=28%, list=23%, signal=22%	HERPUD2/ASPM/NEURL1/TTC26/BTG1/ADNP/QKI/OR7C1/KIT/HSPA5/DPCD/DDX3X/MBD2/PAQR8/LDOC1/GALNT3/SERPINE2/SPATA2/LRRK2/IFT81/YY1/NECTIN2/BAX/KNL1/TESK2/ZBTB16/ARMC2/KDM1B/DNM2/PAFAH1B1/ABAT/SLCO4C1/MAS1/RGS2/CTDNEP1/CFAP58/SGPL1/CCDC136/UBE2B/ZNF148/CATSPER2/SPATA6/SSTR2/ZGLP1/TOB2/NKAPL/JAM3/MSH6/USP42/MYCBPAP/LRRC8A/FSCN3/PI3/ARID4A/C3orf62/PDE5A/STAT5B/JUNB/FOXJ2/XRN2/PTGS2/ATRX/DIAPH2/RARA/IGF2R/ABHD2/PLK1/SMARCA2/NDRG3/PPP1R9B/TUT7/PSME4/H3-3A/PTK2B/B4GALT1/LGALS9/ACSL4/LIN28A/SRC/UBB/APOL2/TPPP3/FANCC/KLC3/ETV6/RFX2/SETX/CYP1B1/P2RY1/VDR/SPOCD1/PRKACA/ASF1B/PROK2/CCNI/P2RX1/UBR2/AGO4/BAG6/ZMIZ1/MAST2/MYCBP/USP9X/ARRB2/RAD23B/TBC1D20/PITHD1/LIMK2/ZMYND15/OPRL1/H3-3B/CTSB/FOXO3/MARF1/PTAFR/YBX3/OSBP2/ACOX1/BCL2L1/BSG/ROPN1L/PTEN/ACRBP/KLF1

BP	GO:0070373	negative regulation of ERK1 and ERK2 cascade	41	-0.55	-1.79	0.00074003	0.0043785	0.00269164	1278	tags=32%, list=11%, signal=28%	RGS14/PTPN1/VRK3/TNIP1/SIRPA/SPRY3/DUSP6/LYN/PTPRC/EPHB2/FLCN/PTEN/DAB2
BP	GO:0001952	regulation of cell-matrix adhesion	84	-0.46	-1.70	0.00074247	0.0043864	0.00269652	3022	tags=43%, list=26%, signal=32%	BCL6/ADAM15/FERMT1/BCAS3/THBS1/WDPCP/RHOA/PRKCZ/SLC9A1/ACTG1/GSK3B/PIK3CB/UTRN/ROCK2/BST1/RCC2/GREM1/DISC1/DUSP22/ROCK1/PTPRJ/RIN2/PTK2B/SLK/GFUS/SRC/LIMS1/SERPINE1/RAC1/MAP4K4/NF1/CORO1C/DMTN/PTEN/VCL/ARHGAP6
BP	GO:0030177	positive regulation of Wnt signaling pathway	89	-0.46	-1.71	0.00076156	0.0044925	0.00276175	2723	tags=40%, list=23%, signal=31%	TLR2/ASPM/WLS/USP8/CSNK2A1/ZEB2/CSNK1G2/DDX3X/MBD2/LRRK2/ZRANB1/CTDNEP1/HHEX/UBE2B/USP34/ATP6AP2/UBR5/SULF2/DISC1/SOX4/LGR6/TBL1X/VCP/SKI/CSNK1G1/ILK/SRC/FAM53B/FRAT1/RBPJ/WNK1/XIAP/SFRP2/PPM1A/GID8/DAB2
BP	GO:0045601	regulation of endothelial cell differentiation	27	-0.63	-1.87	0.00076297	0.0044942	0.00276274	2676	tags=63%, list=23%, signal=49%	IL1B/PLCB1/VEZF1/BTG1/ADD1/TNF/ROCK2/S1PR2/TNFRSF1A/FOXJ2/ROCK1/ZEB1/BMP6/NOTCH4/F11R/VCL/CLDN5
CC	GO:0101003	ficolin-1-rich granule membrane	53	-0.53	-1.79	0.00078068	0.0045849	0.00281854	2860	tags=64%, list=24%, signal=49%	TMEM179B/CD300A/CD55/LAMTOR1/ATP6V0C/ADAM8/GAA/FPR2/RHOA/NCKAP1L/SIGLEC14/FCER1G/LILRB2/FPR1/PRCP/SLC11A1/CR1/MGAM/ATAD3B/ATP6AP2/LAMP2/ADGRE3/LAMP1/ATP6V0A1/SLC2A3/ITGB2/DIAPH1/LGALS3/ARL8A/CD93/SIRPA/RAC1/SIGLEC5/DOK3
BP	GO:0060348	bone development	130	-0.41	-1.59	0.00079071	0.0046301	0.00284633	2220	tags=28%, list=19%, signal=23%	ALPL/ZBTB16/PAFAH1B1/NSD2/DYM/AKAP13/P3H1/ANXA2/ZFPM1/PTPN6/CHSY1/EP300/SULF2/RFLNB/GREM1/MEIS1/MSX1/WASF2/RARA/MPIG6B/BMP6/SKI/BPNT2/SNX10/SRC/FLI1/PIP4K2A/TAL1/POR/FOXN3/TGM2/TYROBP/PTPRC/MAPK14/PHOSPHO1/SFRP2
BP	GO:0016197	endosomal transport	198	-0.37	-1.52	0.00079009	0.0046301	0.00284633	2658	tags=32%, list=23%, signal=25%	RAB8A/VTI1B/STX5/PHETA1/VPS28/EPS15/RAB35/SNX1/KIAA1109/LRRK2/CHMP3/DENND1C/TBC1D10B/SORL1/ZDHHC2/SNX8/GGA3/TBC1D5/RAB6A/STX6/ITSN1/SLC66A2/GRIPAP1/MICALL1/LYST/AGAP2/RAB6B/EEA1/CLTC/TBC1D14/WASHC2C/ZFYVE16/DENND5A/ANKRD27/SQSTM1/SPAG9/EVI5/VAMP3/CLEC16A/RAB11B/TMCC1/VCP/VPS4B/RILP/CHMP1A/RAB7A/UBE2O/RAB11FIP4/SNX27/EHD1/ARHGAP8/ARHGAP1/EPS15L1/UBXN6/EHD3/PICALM/CHMP2A/CORO1C/WAS/SNX18/DENND1A/CHMP4B/AP5B1/REPS2
BP	GO:0002431	Fc receptor mediated stimulatory signaling pathway	31	-0.61	-1.85	0.00082236	0.0048013	0.00295158	1915	tags=42%, list=16%, signal=35%	PLSCR1/SYK/PTPRJ/FGR/VAV1/HCK/LIMK1/VAV3/SRC/LYN/PAK1/PTPRC/PRKCD
BP	GO:0098739	import across plasma membrane	96	-0.44	-1.67	0.00082545	0.0048053	0.00295399	2678	tags=33%, list=23%, signal=26%	ACSL1/CACNA1A/SLC2A1/SLC9A1/SLC22A4/TNF/RGS2/KCNJ6/SLC39A4/SLC43A2/SLC6A9/SLC15A3/LRRC8A/SLC12A6/CACNA1D/SLC2A3/PSEN1/SLC6A20/TSPO2/SLC6A6/KCNQ1/KCNJ15/ABCC9/AKT2/P2RX1/SLC7A11/WNK1/SLC1A5/KCNJ5/IRS2/SLC8A1/SLC7A5

BP	GO:0051640	organelle localization	409	-0.32	-1.41	0.00082484	0.0048053	0.00295399	2813	tags=32%, list=24%, signal=25%	EML3/HIF1A/MFN2/BORCS5/CLMN/RRS1/SYNE3/FES/RAB8A/ASPM/PRKCZ/VTI1B/STX5/PLEKHM2/SNAP23/HPS6/KIT/ACTR2/PSEN2/CDCA8/WIPI1/CCDC186/ARFGAP3/HAP1/KXD1/FEZ1/AHCYL1/INCENP/LRRK2/NECTIN2/CHMP3/PLEKHM1/RIPOR1/NDE1/HNRNPU/DNM2/PAFAH1B1/KIF5B/CFL1/MARK2/PCM1/NOP9/RAB6A/SYK/KIF3C/SYNE2/ATCAY/UBE2B/RAB34/STX6/PIK3CG/CEP19/FHOD1/TRAK1/VPS33A/LMNA/RAC2/SYN3/CBL/HOOK3/STX3/LAMP1/COPG1/CUL3/RAB3B/YWHAZ/MTM1/AP3S2/SNAP25/ZW10/TFEB/CTBP2/SNAP29/SYTL4/PSEN1/MAPT/FGR/VPS11/KIF1C/SPAG9/SEC16A/PLK1/AGTPBP1/RAB27B/MAP4/VAMP3/RALB/SPICE1/RAB11B/NDEL1/ARHGAP21/VPS4B/LGALS9/RAB27A/CHMP1A/MYO1D/RAB7A/STX11/IKBKG/LAT2/UBB/AKAP9/STXBP2/MYO5A/MYO1E/VPS18/MYH9/MYO1F/PTGDS/ACTN4/LYN/UNC13D/SNCA/SDCBP/PLEK/SYNJ1/PIK3CD/LIMK2/TRIM58/PICALM/MAP1S/HMOX1/KPNB1/CHMP2A/FLCN/TRAK2/PINK1/RAB3D/PTEN/CHMP4B/GAB2/KIF13A
CC	GO:0044291	cell-cell contact zone	45	-0.56	-1.82	0.00084465	0.0049027	0.00301386	2607	tags=42%, list=22%, signal=33%	SLC2A1/SCN1B/SLC9A1/PKP4/NECTIN2/YWHAH/FHOD1/JAM3/TMEM65/DSC2/CTNNA1/ACTN1/FLOT1/AFDN/PAK1/FLCN/VCL/SLC8A1/FLOT2
BP	GO:1901879	regulation of protein depolymerization	63	-0.51	-1.76	0.00086003	0.0049847	0.00306429	1918	tags=48%, list=16%, signal=40%	CAMSAP1/TMOD3/SPAST/CAPG/HDAC6/SH3BP1/CLASP1/KATNB1/HDGFL3/TTBK2/CAPZB/ADD1/SPTA1/TAOK1/TWF2/MAP1A/TRIOBP/WASHC2C/ASPH/ARHGEF2/APC/SVIL/LMOD3/PLEK/MAP1S/TMOD1/DMTN/MTPN/GSN/SPTB
MF	GO:0044325	transmembrane transporter binding	83	-0.46	-1.68	0.00086259	0.0049922	0.00306893	2515	tags=35%, list=21%, signal=28%	SCN1B/HAP1/TSPO/FLNA/LRRK2/CASR/KCNAB2/ITPR2/YWHAH/AP2M1/YWHAZ/FKBP1A/DIAPH1/PPP1R9B/KCNQ1/ACTN1/SRC/ABCC9/KCNE3/AKAP9/CALM3/ACTN4/LYN/USP10/PYCARD/GRINA/PACS1/ANK1/SLC8A1
BP	GO:1900076	regulation of cellular response to insulin stimulus	45	-0.55	-1.81	0.00087745	0.0050708	0.00311725	2189	tags=44%, list=19%, signal=36%	SORL1/PRKCB/GPLD1/PIP4K2C/ATP2B1/TSC2/GNAI2/INPP5K/PID1/OSBPL8/GSK3A/SOCS3/PTPRE/PTPN1/SRC/PIP4K2A/ZBTB7B/IGF2/ADIPOR1/PRKCD
BP	GO:0048588	developmental cell growth	127	-0.41	-1.59	0.00087881	0.0050713	0.00311754	3408	tags=44%, list=29%, signal=32%	NEDD4L/TTL/AURKA/HDAC6/DIP2B/GDI1/ITSN2/KMT2D/EDN1/PLAA/SRF/SHTN1/CTDP1/RAPH1/PRKCZ/ADNP/SEMA7A/SIN3A/ALCAM/YY1/RTN4R/GSK3B/DNM2/PAFAH1B1/RGS2/CDK5R1/NRP2/SEMA4A/AKAP13/EIF4G2/KIAA0319/TWF2/RAB21/DRAXIN/RTN4/CPNE1/DISC1/CDKL5/IST1/GSK3A/MAPT/ULK1/SPAG9/SLC23A2/CTTN/NLGN3/NDEL1/NIN/LIMK1/DPYSL2/DBN1/PDLIM5/USP9X/G6PD/VCL/IQGAP1

BP	GO:0006325	chromatin organization	454	-0.31	-1.38	0.00089017	0.0051294	0.00315328	2972	tags=33%, list=25%, signal=26%	MPND/L3MBTL1/ALKBH4/H2BC21/BAZ2A/TET1/MKI67/ACTR8/BAP1/RESF1/NSD1/RBM15B/SMARCB1/SETD5/CHD8/SETD1B/PRDM5/PBRM1/IKZF1/CBX4/SETD2/SIRT6/JAK2/HCFC1/CBX7/SIN3A/SMARCD3/SRCAP/CABIN1/MBD2/MBTD1/CTCF/L3MBTL4/SMARCC1/HDAC11/BAZ1A/YY1/GFI1B/KDM5B/COPRS/MLLT1/METTL4/KDM5A/NCOR1/DR1/PRKCB/KDM1B/HNRNPU/PHF20/RSF1/NSD2/PKN1/ARID1A/GATAD2A/HMGN2/ATXN7L3/NSD3/MECP2/HDAC4/EIF1/UTY/BCORL1/SETDB1/ZNF445/CBX6/HIRA/BABAM1/SUDS3/USP49/PPM1D/CDKN1C/RNF20/H1-0/ATF7IP/CTR9/EP300/RERE/H3C10/JMJD1C/USP3/MSL1/RNF40/ARID4A/SUPT6H/CHRAC1/KANSL3/ARID1B/TET2/PHF19/TET3/KDM4A/SMCHD1/CHD4/H4C8/ATRX/SETD7/MTHFR/RELA/IFI16/KDM3B/CENPP/NR3C1/SMARCA2/GATAD2B/PSME4/KAT6A/H3-3A/HDAC7/GPX1/KMT2C/SMARCD2/UBN2/KCNQ1/TPR/KMT2E/AICDA/FAM172A/BPTF/KDM7A/BRPF1/PHF21A/WAC/ACTB/PADI2/ZBTB7A/USP22/CARM1/ASF1B/BRCA1/KAT5/JARID2/KDM6B/UBR2/BAG6/NFE2/BRD4/RCOR1/KDM4B/IGF2/CHD7/ZNFX1/PAK1/DTX3L/H2BC11/H3-3B/TOP1/BAZ2B/WBP2/UBN1/PCGF5/SPI1/H2BC4
BP	GO:0032418	lysosome localization	67	-0.49	-1.71	0.00089506	0.0051427	0.00316144	2775	tags=46%, list=24%, signal=36%	BORCS5/FES/PLEKHM2/SNAP23/HPS6/KIT/KXD1/PLEKHM1/KIF5B/SYK/RAB34/PIK3CG/VPS33A/RAC2/CBL/TFEB/SYTL4/FGR/SPAG9/NDEL1/LGALS9/LAT2/STXBP2/MYH9/PTGDS/LYN/UNC13D/PIK3CD/HMOX1/FLCN/GAB2
BP	GO:1990849	vacuolar localization	67	-0.49	-1.71	0.00089506	0.0051427	0.00316144	2775	tags=46%, list=24%, signal=36%	BORCS5/FES/PLEKHM2/SNAP23/HPS6/KIT/KXD1/PLEKHM1/KIF5B/SYK/RAB34/PIK3CG/VPS33A/RAC2/CBL/TFEB/SYTL4/FGR/SPAG9/NDEL1/LGALS9/LAT2/STXBP2/MYH9/PTGDS/LYN/UNC13D/PIK3CD/HMOX1/FLCN/GAB2
BP	GO:0071902	positive regulation of protein serine/threonine kinase activity	148	-0.40	-1.60	0.00089807	0.0051525	0.00316748	3027	tags=42%, list=26%, signal=31%	EDN1/PKD1/CSK/THBS1/TAOK2/ADAM8/DBF4B/TRAF6/RHOA/ACSL1/IL1B/ADAM17/ERN1/KIT/P2RX7/CCND3/CSF1R/DDX3X/INCENP/ERBB2/LRRK2/BMPR1A/SYAP1/TNF/CDK5R1/RICTOR/ERN2/PDGFA/FZD5/AKAP13/TAOK1/SYK/MAP2K2/PIK3CG/STK4/PDE5A/TLR6/IRAK1/CCNY/PSEN1/INSR/PTPN1/MAP3K5/RALB/PTK2B/DVL3/PARP16/MAP2K7/TAOK3/SRC/PIK3R5/CALM3/DAZAP2/SASH1/SNCA/IGF2/STRADA/PAK1/PTPRC/RAPGEF2/IQGAP1/TGFA
BP	GO:0071407	cellular response to organic cyclic compound	363	-0.33	-1.42	0.00090095	0.0051616	0.00317307	2819	tags=32%, list=24%, signal=25%	GNAS/SLC1A2/LCOR/MAPK1/NCOR2/SLC26A6/FES/RHOA/IL1B/REST/PLCB1/USP8/JAK2/CNOT1/CASP9/ACTR2/PPARGC1B/SLC9A1/SIN3A/HSPA5/P2RX7/MBD2/PAQR8/TAF1/COLEC12/CEBPA/FLNA/LRRK2/GSK3B/RNF14/ALPL/NCOR1/HNRNPU/DNM2/TNF/ROCK2/PKN1/ARID1A/CASR/NKX3-1/GPLD1/ITPR2/YWHAH/CES1/HRH2/GPBAR1/P2RY13/HTR3B/PIK3CG/SSTR2/UBR5/ATP2B1/EP300/GCLM/PIM1/INPP5K/GNB1/MAVS/PTGS2/RAE1/HSPA1B/CCL3/GSK3A/CLU/VPS11/ZNF703/AQP9/ABHD2/NR3C1/DIAPH1/PPP1R9B/HSPA1A/RALB/CTNNA1/RIOK3/EGLN2/KCNQ1/SRC/PAK2/AKAP9/FLOT1/CYP1B1/P2RY1/ACTB/ZFP36L1/VDR/PADI2/GNAL/ZBTB7A/CARM1/PRKACA/RXRA/BRCA1/VPS18/KAT5/SSH1/IFIT1B/ZMIZ1/SNCA/TGM2/PAK1/ARRB2/NCOA4/CFLAR/CEBPB/FOXO3/PTAFR/FAM210B/WBP2/AQP1/MAPK3/DMTN/MSN/SLC8A1/RAPGEF2/DAB2/GNAZ
CC	GO:0043296	apical junction complex	87	-0.45	-1.68	0.00090243	0.0051627	0.00317372	1087	tags=18%, list=9%, signal=17%	CTNNA1/SYNPO/ARHGEF2/APC/ESAM/ACTB/CTNND1/SHROOM1/F11R/YBX3/VASP/UBN1/FRMD4B/RAPGEF2/NHS/CLDN5

BP	GO:0010876	lipid localization	262	-0.35	-1.48	0.00091379	0.0052201	0.00320903	2678	tags=30%, list=23%, signal=24%	ACSL1/IL1B/PRELID2/SIGMAR1/SLC27A3/ANO6/SLC2A1/SCARB2/FIS1/ATG2B/P2RX7/TSPO/STOML1/ABCA2/KDM5B/TPCN2/ACACB/TNF/NFKBIA/CPTP/PTGES/LRP1/NKX3-1/ABCC4/PLSCR1/CPT2/CES1/CERT1/SYK/ANXA2/SLCO3A1/SLC66A2/NPC2/SERAC1/ABCB11/SLC10A3/NME4/STAT5B/OSBPL8/ANO7/ABCC3/VLDLR/CLU/AQP9/TSPO2/HDLBP/LCAT/BMP6/VPS4B/KCNQ1/CPT1A/APOL2/PSAP/XKR8/PIP4K2A/PITPNA/AKT2/EHD1/DGAT2/BSCL2/PRELID1/LRP10/ABCG1/ATG2A/C2CD2L/ABHD5/ABCG8/SLC4A1/PNPLA2/OSBP2/IRS2/ITGB3/PRKCD/APOBR/FURIN/DAB2/OSBPL2/OSBPL1A/PLIN3
CC	GO:0005811	lipid droplet	79	-0.46	-1.68	0.00094814	0.0054086	0.00332489	2051	tags=32%, list=17%, signal=26%	CTDNEP1/G0S2/ALOX15/CES1/ANXA2/CKAP4/ZW10/RSAD2/IRAK1/VCP/CYB5R3/ACSL4/RAB7A/EHD1/FAAH2/DGAT2/ABHD4/BSCL2/ATG2A/ABHD5/PNPLA2/RAB5C/OSBPL2/PLIN3/AQP10
BP	GO:0198738	cell-cell signaling by wnt	265	-0.35	-1.49	0.00095752	0.0054543	0.00335298	2681	tags=32%, list=23%, signal=26%	WNT1/RHOA/TMEM131L/ASPM/WLS/USP8/CSNK2A1/ZEB2/JADE1/CSNK1G2/DDX3X/MBD2/PORCN/MED12/MITF/LRRK2/GSK3B/SENP2/ZRANB1/TLE4/LRP1/CTDNEP1/MARK2/HHEX/FZD5/UBE2B/APCDD1/USP34/ATP6AP2/UBR5/CTR9/SULF2/STK4/DRAXIN/TSC2/GREM1/CUL3/DISC1/SOX4/GRK6/FRMD8/NR4A2/CCNY/GSK3A/PSEN1/ZNF703/INVS/LGR6/RTF1/TBL1X/GNAQ/VCP/MAD2L2/LRRFIP2/BCL9/DVL3/SKI/DDB1/CSNK1G1/ILK/SIAH2/SRC/APC/TGFB1I1/FAM53B/FRAT1/TMEM88/RBPJ/OTUD5/KPNA1/CTNND1/TLE3/HIC1/CTNNBIP1/RAC1/WNK1/XIAP/MAPK14/FOXO3/TCF7L2/RAB5A/SFRP2/PPM1A/PTEN/GID8/DAB2
BP	GO:0040013	negative regulation of locomotion	201	-0.37	-1.52	0.00097782	0.005562	0.00341918	2233	tags=24%, list=19%, signal=20%	BMPR1A/PIP5KL1/TNF/LRP1/MECP2/MIIP/S1PR2/SEMA4A/SRGAP1/GADD45A/RIN3/RNF20/STK24/GREM1/DUSP22/OSBPL8/PTPRJ/ABHD2/TIMP1/CTNNA1/GNA12/CYP1B1/DUSP1/DLG5/PADI2/NFE2L2/SERPINE1/TPM1/AFDN/RAC1/ADIPOR1/HMOX1/SP100/NF1/FOXO3/CORO1C/WAS/DNAJA4/STAT3/SFRP2/FLCN/CNN2/PTEN/VCL/RIPOR2/TRIB1/TBXA2R/CLDN5
BP	GO:0040029	regulation of gene expression, epigenetic	113	-0.43	-1.66	0.00098058	0.0055697	0.00342394	3104	tags=48%, list=26%, signal=35%	DNMT3A/RB1/YTHDC1/PHF2/UPF1/L3MBTL3/CTCFL/HDAC6/LRIF1/KMT2D/L3MBTL1/BAZ2A/TET1/RESF1/RBM15B/SIRT6/SIN3A/MBD2/CTCF/KDM1B/HNRNPU/ARID1A/MECP2/HDAC4/EIF1/SETDB1/ZNF445/PPM1D/CDKN1C/H1-0/ATF7IP/CTR9/EP300/H3C10/ARID4A/ARID1B/SMCHD1/IFI16/H3-3A/GPX1/KCNQ1/TPR/AICDA/FAM172A/PADI2/BRCA1/JARID2/UBR2/IGF2/ZNFX1/H3-3B/WBP2/PCGF5/SPI1
MF	GO:0048029	monosaccharide binding	50	-0.53	-1.78	0.00102505	0.005814	0.0035741	2419	tags=44%, list=21%, signal=35%	RPIA/COLEC12/HK2/MANBA/P3H1/GPI/ALDOA/SLC2A3/IGF2R/EGLN1/PLOD1/EGLN3/LGALS9/EGLN2/ALPK1/SELP/PYGL/TALDO1/HK1/FBP1/G6PD/BSG
BP	GO:0030865	cortical cytoskeleton organization	47	-0.54	-1.78	0.00104145	0.0058986	0.00362611	2208	tags=36%, list=19%, signal=29%	RHOA/NCKAP1L/RHOQ/PAFAH1B1/TNF/ROCK2/FHOD1/RAC2/ROCK1/EPB41/RAC1/PLEK/RHOG/LCP1/RHOBTB1/TLN1/FMNL1
CC	GO:0008021	synaptic vesicle	119	-0.42	-1.63	0.00106262	0.0060053	0.00369168	1967	tags=31%, list=17%, signal=26%	KIF1B/STXBP5/STX6/SLC6A9/APBA1/DLG4/ATP2B1/SYN3/STX3/LAMP1/DISC1/RAB3B/SNAP25/ROGDI/MCTP2/PSEN1/ATP6V0D1/RAB27B/RAB11B/NDEL1/SLC17A5/SYT12/RAB7A/STX11/CALM3/RAB5B/MME/DMXL2/SNCA/DNAJC5/PICALM/STON2/IQSEC1/TPRG1L/RAB5A/BCL2L1/RAB3D
BP	GO:0043405	regulation of MAP kinase activity	124	-0.41	-1.60	0.0010633	0.0060053	0.00369168	2685	tags=40%, list=23%, signal=31%	TRAF6/IL1B/ERN1/KIT/P2RX7/ERBB2/LRRK2/SORL1/PIK3CB/TNF/TNXB/RGS2/ERN2/PDGFA/FZD5/AKAP13/SYK/PIK3CG/PTPN6/IRAK3/PDE5A/TLR6/INPP5K/IRAK1/PSEN1/RGS14/PTPRJ/INSR/PTPN1/MAP3K5/HMGCR/PTK2B/DVL3/MAP2K7/HIPK3/SPRY3/TAOK3/SRC/PIK3R5/DUSP1/SASH1/LYN/PAK1/PTPRC/STK38/NF1/SFRP2/PRKCD/TRIB1/TGFA

BP	GO:0090263	positive regulation of canonical Wnt signaling pathway	68	-0.48	-1.70	0.00106625	0.0060089	0.00369391	2639	tags=41%, list=23%, signal=32%	ASPM/WLS/USP8/ZEB2/CSNK1G2/DDX3X/LRRK2/CTDNEP1/HHEX/UBE2B/USP34/ATP6AP2/UBR5/SOX4/LGR6/TBL1X/VCP/CSNK1G1/ILK/SRC/FAM53B/FRAT1/RBPJ/WNK1/XIAP/SFRP2/PPM1A/GID8
BP	GO:0033002	muscle cell proliferation	111	-0.43	-1.67	0.00106697	0.0060089	0.00369391	1682	tags=23%, list=14%, signal=20%	SAV1/PIM1/GNAI2/FOXJ2/PTGS2/IRAK1/MAP3K5/GNA12/CDKN1A/IL6R/SOD2/RBPJ/KPNA1/JARID2/TPM1/CTNNBIP1/MMP9/CFLAR/MAPK14/HMOX1/PTAFR/TCF7L2/PRKDC/PRKAR1A/PTEN/TRIB1
BP	GO:0031669	cellular response to nutrient levels	162	-0.40	-1.58	0.00108841	0.0061167	0.0037602	2926	tags=41%, list=25%, signal=31%	AIFM1/SZT2/SRF/VPS41/PDK4/MAPK1/FES/PRKAG2/SLC2A1/EIF4G1/GCN1/WIPI1/HSPA5/LRRK2/RIPOR1/ATF3/MAP1LC3B/CASR/GABARAPL2/UCP2/SLC39A4/GABARAPL1/PPM1D/CPEB4/LAMP2/ATP2B1/CBL/MTMR3/PIM1/EIF2AK2/STK24/MAP1LC3A/PDK2/TFEB/DSC2/SESN2/IFI16/ULK1/WIPI2/SESN3/MAP3K5/MAX/RALB/CLEC16A/ATG7/ELAPOR1/CDKN1A/WDR45/GABARAP/KPTN/GAS2L1/DAP/VDR/RXRA/SH3GLB1/NFE2L2/RRAGD/HMOX1/NCOA1/MAP1LC3B2/FOXO3/MAPK3/FLCN/CD68/NUAK2/NPRL3/GLUL
BP	GO:0019932	second-messenger-mediated signaling	175	-0.38	-1.54	0.0010892	0.0061167	0.0037602	2590	tags=33%, list=22%, signal=26%	NEUROD2/ADNP/FIS1/SLC9A1/P2RX7/INPP5A/LRRK2/GSK3B/TPCN2/TNF/FPR1/BST1/PLCE1/GBP1/NFAT5/ITPR2/FHL2/MCOLN1/SYK/DDAH2/UBE2B/NFATC3/GUCY1B1/CCR1/PDE5A/FFAR4/PDK2/MCTP2/CCL3/MAPT/PTPRJ/PPP1R9B/PTK2B/CXCR2/CXCR1/CDC34/LAT2/CALM3/SELP/NUDT4/PRKACA/CHP1/ADM/PLEK/PPP3CA/GUCY1A1/PTPRC/OPRL1/RUNDC3A/PPP3R1/AQP1/DMTN/CCRL2/SLC8A1/RAPGEF2/TBXA2R/IRAG1
BP	GO:0038034	signal transduction in absence of ligand	42	-0.56	-1.81	0.00110508	0.0061797	0.00379893	3058	tags=55%, list=26%, signal=41%	BCL2L11/GATA1/BAK1/PPP2R1A/CASP2/IL1B/FADD/GSK3B/BAX/TNF/MCL1/MKNK2/HSPA1B/GSK3A/HSPA1A/CTNNA1/PRDX2/PF4/RIPK1/NF1/FOXO3/STRADB/BCL2L1
BP	GO:0097192	extrinsic apoptotic signaling pathway in absence of ligand	42	-0.56	-1.81	0.00110508	0.0061797	0.00379893	3058	tags=55%, list=26%, signal=41%	BCL2L11/GATA1/BAK1/PPP2R1A/CASP2/IL1B/FADD/GSK3B/BAX/TNF/MCL1/MKNK2/HSPA1B/GSK3A/HSPA1A/CTNNA1/PRDX2/PF4/RIPK1/NF1/FOXO3/STRADB/BCL2L1
BP	GO:0018105	peptidyl-serine phosphorylation	219	-0.37	-1.53	0.00110356	0.0061797	0.00379893	3099	tags=41%, list=26%, signal=30%	MAPKAP1/MKNK1/MARK1/STK32C/PKD1/BAK1/GRK2/STK38L/NSD1/CSNK1A1/MAPK1/UHMK1/PIKFYVE/TTBK2/PRKCZ/EIF4G1/ERN1/CSNK2A1/DYRK1B/ANGPT1/NEK6/DYRK1A/CSNK1G2/TAF1/CAMK2A/LRRK2/GSK3B/HIPK2/PLCL2/BAX/PRKCB/TNF/ROCK2/DYRK3/PKN1/HIPK1/CDK5R1/PRKD2/RICTOR/OSM/MARK2/MAST3/SYK/MAP2K2/GADD45A/CAMK1D/STK4/HGF/INPP5K/MKNK2/SH2D3C/PTGS2/ROCK1/RPS6KA5/ULK1/VRK3/PLK1/RASSF2/MAD2L2/RPS6KA1/HIPK3/CSNK1G1/ILK/SRC/MAPKAPK2/PAK2/AKAP9/RIPK1/MARK3/PRKACA/AKT2/MAPKAPK3/MAST2/HCLS1/SNCA/PAK1/WNK1/ARRB2/SRPK2/STK38/MAPK14/PDK3/TOP1/DMTN/PRKDC/SFRP2/PRKCD/PINK1/PTEN
BP	GO:0071453	cellular response to oxygen levels	112	-0.42	-1.61	0.00110814	0.0061881	0.0038041	2270	tags=31%, list=19%, signal=25%	CYBB/HIPK2/PIK3CB/ROCK2/CASR/ATP7A/NKX3-1/BNIP3L/CPEB4/LMNA/CBL/ERO1A/PTGS2/IRAK1/EGLN1/ATP6V0D1/EGLN3/ATG7/EGLN2/SRC/BACH1/ZFP36L1/RBPJ/NFE2L2/NPEPPS/CFLAR/HMOX1/FOXO3/PDK3/AQP1/MPL/PINK1/PTEN/SLC8A1/ATP6V1A
BP	GO:0030218	erythrocyte differentiation	110	-0.42	-1.62	0.00112631	0.0062808	0.00386108	1545	tags=31%, list=13%, signal=27%	ARID4A/STAT5B/TRIM10/ARNT/HSPA1B/ADAR/SMAP1/TSPO2/HSPA1A/MPIG6B/KCNQ1/BPGM/KMT2E/ZFP36L1/ZBTB7A/AHSP/TAL1/RCOR1/LYN/PTBP3/HCLS1/ALAS2/TRIM58/EPB42/MAPK14/FOXO3/SLC4A1/FAM210B/G6PD/STAT3/DMTN/PRKDC/SPI1/KLF1

BP	GO:0043242	negative regulation of protein-containing complex disassembly	62	-0.49	-1.70	0.00113359	0.0063037	0.00387517	2331	tags=44%, list=20%, signal=35%	CAPG/HDAC6/PHF23/CLASP1/KATNB1/HDGFL3/TTBK2/CAPZB/ADD1/TNF/SPTA1/TAOK1/TWF2/IRAK3/RUBCN/TRIOBP/WASHC2C/CLEC16A/ARHGEF2/APC/SVIL/LMOD3/TMOD1/DMTN/MTPN/GSN/SPTB
BP	GO:0016239	positive regulation of macroautophagy	62	-0.49	-1.70	0.00113359	0.0063037	0.00387517	1998	tags=42%, list=17%, signal=35%	HIF1A/VPS13D/EPM2A/WIPI1/OPTN/PIP4K2C/BNIP3L/CALCOCO2/SPTLC2/TSC2/SESN2/ULK1/SESN3/RALB/ELAPOR1/WDR45/IKBKG/PIP4K2A/WAC/SH3GLB1/ATG2A/HMOX1/MAPK3/SNX18/AMBRA1/PINK1
CC	GO:0005766	primary lysosome	138	-0.41	-1.61	0.00114019	0.0063139	0.00388142	2126	tags=45%, list=18%, signal=37%	TUBB/VNN1/BST2/ATP6V0C/GAA/MAGT1/MAPK1/CPNE3/DNAJC3/CTSA/SNAP23/ACTR2/GCA/MAN2B1/FPR1/MNDA/SLCO4C1/ACLY/PRCP/MANBA/ANXA2/NPC2/LAMP2/PSMD1/TUBB4B/STX3/CPNE1/LAMP1/RAB37/CKAP4/IMPDH1/IST1/BRI3/SNAP29/PSEN1/CD63/ANXA11/ARL8A/RNASET2/B4GALT1/VCP/CYB5R3/ACP3/GRN/PSAP/STXBP2/PADI2/TRAPPC1/UNC13D/DNAJC5/SDCBP/PYCARD/GNS/PRDX6/RAB5C/TOLLIP/FTL/PRKCD/CD68/RAB3D/NFAM1/CPPED1
CC	GO:0042582	azurophil granule	138	-0.41	-1.61	0.00114019	0.0063139	0.00388142	2126	tags=45%, list=18%, signal=37%	TUBB/VNN1/BST2/ATP6V0C/GAA/MAGT1/MAPK1/CPNE3/DNAJC3/CTSA/SNAP23/ACTR2/GCA/MAN2B1/FPR1/MNDA/SLCO4C1/ACLY/PRCP/MANBA/ANXA2/NPC2/LAMP2/PSMD1/TUBB4B/STX3/CPNE1/LAMP1/RAB37/CKAP4/IMPDH1/IST1/BRI3/SNAP29/PSEN1/CD63/ANXA11/ARL8A/RNASET2/B4GALT1/VCP/CYB5R3/ACP3/GRN/PSAP/STXBP2/PADI2/TRAPPC1/UNC13D/DNAJC5/SDCBP/PYCARD/GNS/PRDX6/RAB5C/TOLLIP/FTL/PRKCD/CD68/RAB3D/NFAM1/CPPED1
BP	GO:0030031	cell projection assembly	392	-0.32	-1.41	0.00113875	0.0063139	0.00388142	2722	tags=29%, list=23%, signal=23%	DNAL1/TTBK2/RO60/WNT1/RAB8A/NEURL1/TTC26/POC1B/ANO6/CDC42EP4/IFT88/TCTN1/NCKAP1L/KIT/ACTR2/CEP89/DYNLL2/P2RX7/HAP1/FLNA/CAPZB/KLF5/IFT81/SPATA13/GSK3B/RHOQ/ARMC2/PLEKHM1/DNM2/TBC1D30/PLCE1/ARAP1/IFT122/ATP7A/CFAP58/S1PR2/ABCC4/PCM1/PDGFA/RCC2/TWF2/TMEM231/SYNE2/UBE2B/DYNC2H1/SPATA6/CEP19/RILPL1/ARMC9/RAC2/FSCN1/DISC1/INPP5K/CEP350/BBOF1/IFT140/CDKL5/ROCK1/WASF2/SNAP29/DAW1/INTU/ATP6V0D1/AGRN/MAP4/ARL13B/RAP1GAP/PPP1R9B/CTTN/ESPN/DNM3/MARK4/CEP97/NDEL1/RILP/KCNQ1/VAV3/SNX10/SRC/APC/ALPK1/EXOC4/KLC3/RFX2/PLEK2/PARVB/FXR2/EHD1/RPGR/FGD4/SDCBP/RAC1/FGD6/LIMK2/EHD3/RHOG/ASAP1/EPHB2/CORO1C/WAS/RILPL2/ABI3/ABLIM3/RAB5A/DMTN/CCDC39/MYLK/PRKCD/GSN/CCP110/VCL/RIPOR2/RAPGEF2/ODAD4/KIF27
BP	GO:0030101	natural killer cell activation	62	-0.49	-1.70	0.00116669	0.0064517	0.00396612	1523	tags=29%, list=13%, signal=25%	LAMP1/STAT5B/FGR/RAB27A/NCR1/PBX1/SNX27/BAG6/AP1G1/UNC13D/TYROBP/ELF4/FCGR3A/PIK3CD/PTPRC/CORO1A/PGLYRP1/NFIL3
BP	GO:0098751	bone cell development	31	-0.60	-1.83	0.00118061	0.0065015	0.00399675	3076	tags=55%, list=26%, signal=41%	MED1/ATP6AP1/SRF/KIT/PAFAH1B1/ANXA2/ZFPM1/PTPN6/EP300/MEIS1/WASF2/MPIG6B/SRC/FLI1/PIP4K2A/TAL1/TYROBP
BP	GO:0010812	negative regulation of cell-substrate adhesion	36	-0.58	-1.79	0.00118061	0.0065015	0.00399675	3022	tags=50%, list=26%, signal=37%	BCL6/ADAM15/BCAS3/THBS1/WNT1/RHOA/GBP1/RCC2/DUSP22/SRC/SERPINE1/ACTN4/MAP4K4/NF1/CORO1C/DMTN/PTEN/ARHGAP6
BP	GO:0051648	vesicle localization	142	-0.40	-1.56	0.00117965	0.0065015	0.00399675	2550	tags=31%, list=22%, signal=25%	PRKCZ/SNAP23/WIPI1/CCDC186/ARFGAP3/HAP1/LRRK2/NDE1/DNM2/PAFAH1B1/KIF5B/PIK3CG/CEP19/TRAK1/VPS33A/SYN3/CUL3/AP3S2/SNAP29/PSEN1/KIF1C/SEC16A/RAB27B/RAB11B/NDEL1/VPS4B/RAB27A/MYO1D/RAB7A/IKBKG/MYO5A/MYO1E/MYO1F/ACTN4/SNCA/SDCBP/SYNJ1/LIMK2/PICALM/CHMP2A/TRAK2/PINK1/PTEN/KIF13A

BP	GO:0007568	aging	96	-0.44	-1.64	0.00121012	0.0066428	0.00408362	2816	tags=44%, list=24%, signal=34%	SLC1A2/GNA13/CASP2/FOS/SCAP/CASP9/SIN3A/C1QA/MBD2/TSPO/GRB2/JUND/ABAT/UCP3/UCP2/ERCC2/RNF165/CDKN1C/ATP2B1/GCLM/SERPING1/PTGS2/IRAK1/DNMBP/PPP1R9B/TIMP1/CTNNA1/GNA12/GPX1/P2RY1/SOD2/CARM1/TNFRSF1B/NFE2L2/MME/APAF1/ADM/PPP3CA/FOXO3/CD68/MBP/FOXO4
BP	GO:0010256	endomembrane system organization	406	-0.32	-1.40	0.00121128	0.0066428	0.00408362	2636	tags=28%, list=22%, signal=22%	USP8/VTI1B/ANO6/SCARB2/STX5/PLEKHM2/GET1/SNAP23/PHETA1/NEK6/VPS28/P2RX7/OPTN/SERPINE2/LRRK2/NECTIN2/CHMP3/KNL1/PRKCB/DNM2/PAFAH1B1/VPS37B/RAB2A/PI4K2A/DYM/CTDNEP1/BIN2/SPTA1/PLSCR1/CCDC136/CERT1/RAB43/TMEM127/CR1/MAP2K2/DYNC2H1/STX6/GAK/RAB22A/YIPF4/LMNA/CREB1/MICALL1/LYST/HOOK3/RTN4/STX3/TLCD2/YWHAZ/EEA1/SNAP25/ZW10/ANO7/IST1/SNAP29/SYTL4/CLU/VPS11/NUP93/REEP4/SQSTM1/SEC16A/PLK1/EMC3/TOR1AIP2/NDEL1/TMCC1/ARHGAP21/VPS4B/RILP/RAB27A/CHMP1A/SNX10/RAB7A/STX11/VPS37C/XKR8/RFX2/AKAP9/FLOT1/STAM2/AKT2/SH3GLB1/VPS18/GOLGA2/MYH9/RAB5B/MMGT1/BAG6/RTN3/UNC13D/GOLGB1/TBC1D20/SYNJ1/TANGO2/EHD3/CAVIN2/GET3/PTPRC/ASAP1/CHMP2A/CORO1C/SLC4A1/MAPK3/SNX18/PRKCD/GSN/PTEN/CHMP4B/DYSF/ACRBP/OSBPL2
BP	GO:0048608	reproductive structure development	225	-0.35	-1.48	0.00121665	0.0066631	0.00409607	2393	tags=30%, list=20%, signal=25%	LDOC1/CEBPA/SERPINE2/SPATA2/GRB2/KDM5B/SENP2/BAX/MAS1/NKX3-1/SGPL1/PDGFA/FZD5/BIRC6/PTPN6/CDKN1C/CBL/STK4/ARID4A/STAT5B/JUNB/TFEB/PTGS2/ARNT/ATRX/RARA/CSDE1/SOCS3/INSR/CCNF/CTNNA1/H3-3A/BMP6/NUPR1/COL9A3/SRC/NIPBL/ASCL2/UBB/BPTF/PSAP/TPPP3/CYP1B1/ZFP36L1/VDR/RBPJ/AGO4/MME/IGF2/CHD7/ADM/ARRB2/TBC1D20/NCOA4/CEBPB/H3-3B/CTSB/MAPK14/DNAJB6/NCOA1/FOXO3/YBX3/DHCR24/SFRP2/BCL2L1/BSG/BASP1/PTEN
BP	GO:0060485	mesenchyme development	142	-0.40	-1.56	0.00122715	0.0067022	0.00412009	3158	tags=37%, list=27%, signal=28%	SPRED1/CLASP1/S100A4/MARK1/EDN1/ADAM15/TIAM1/STAT1/ZFP64/HIF1A/MAPK1/IL1B/SEMA7A/MCRIP1/FLNA/GSK3B/BMPR1A/FBXL17/EFNB1/ROCK2/NRP2/SEMA4A/IL17RD/HGF/RTN4/RFLNB/GREM1/MSX1/ACTA2/ROCK1/ZNF703/PHACTR4/MAD2L2/FAM172A/TGFB1I1/EXOC4/DLG5/ZFP36L1/RBPJ/OTUD5/USF3/AKNA/NOTCH4/GLIPR2/SDCBP/ADIPOR1/CORO1C/TCF7L2/MAPK3/SFRP2/BASP1/PTEN/DAB2
BP	GO:0051693	actin filament capping	32	-0.58	-1.75	0.00123696	0.0067464	0.0041473	2331	tags=41%, list=20%, signal=33%	CAPZB/ADD1/SPTA1/TWF2/TRIOBP/WASHC2C/SVIL/LMOD3/TMOD1/DMTN/MTPN/GSN/SPTB
BP	GO:0048729	tissue morphogenesis	305	-0.33	-1.43	0.00128503	0.006999	0.00430257	2685	tags=31%, list=23%, signal=24%	TRAF6/WNT1/RHOA/WLS/ADAM17/TCTN1/SETD2/SIRT6/CSF1R/TMEM79/ACTG1/MED12/FLNA/PLXNB2/MYO9A/CA2/GRB2/TBX19/KDM5B/BMPR1A/CTSZ/PAFAH1B1/TNF/IFT122/CASR/ATP7A/NKX3-1/NRP2/PRKD2/BTBD7/MYLPF/PDGFA/FZD5/ZFPM1/CRYGS/ITGA5/TGM3/STK4/HGF/TSC2/TXNRD1/MRTFA/GREM1/GPI/SOX4/MSX1/ACTA2/MTHFR/SUFU/PSEN1/RARA/FKBP1A/SOCS3/INTU/FLG2/ARL13B/PHACTR4/ZDHHC7/DVL3/SKI/ILK/SRC/PBX1/TGFB1I1/EXOC4/ACTB/DLG5/VDR/PRKACA/RBPJ/TPM1/KDM6B/TAL1/NOTCH4/APAF1/CTNNBIP1/TGM2/CHD7/PAK1/RAC1/ADM/PIK3CD/VASP/SFRP2/CCDC39/ITGB3/PRKAR1A/MYLK/MSN/PTEN/VCL/MICAL2/FKBPL/DAB2
BP	GO:0098657	import into cell	125	-0.41	-1.61	0.0013086	0.0071079	0.00436952	2678	tags=34%, list=23%, signal=26%	ACSL1/CACNA1A/SLC2A1/SLC9A1/VPS28/STEAP4/SLC22A4/TNF/RGS2/KCNJ6/SLC39A4/SLC43A2/SLC6A9/SLC15A3/SLC22A1/LRRC8A/SLC12A6/SLC6A12/CACNA1D/RAB3B/SNAP25/SLC2A3/PSEN1/SLC6A20/TSPO2/SLC6A6/KCNQ1/KCNJ15/ABCC9/FLOT1/ACTB/AKT2/P2RX1/SLC7A11/SNCA/WNK1/SLC1A5/KCNJ5/IRS2/ITGB3/SLC8A1/SLC7A5

BP	GO:0031347	regulation of defense response	441	-0.31	-1.38	0.00131279	0.0071209	0.00437754	2148	tags=24%, list=18%, signal=20%	TNF/NFKBIA/BST1/PTGES/MNDA/MAS1/TLR8/RICTOR/ALOX15/OSM/PLSCR1/METRNL/SYK/NMI/CR1/HLA-E/PIK3CG/TNFRSF1A/YTHDF3/PTPN6/FCN1/IRAK3/EP300/ALOX5AP/SERPING1/LSM14A/TIRAP/NCF1/HGF/LAMP1/LILRA2/KLRD1/STAT5B/PCBP2/FFAR4/N4BP1/MAVS/PTGS2/MGLL/PARP14/USP27X/GPATCH3/CCL3/RELA/FGR/FUT7/IFI16/SOCS3/AREL1/PTPN1/ADAR/VAV1/SBNO2/RIOK3/HCK/NUPR1/GPX1/TNIP1/SIRPA/LGALS9/NCR1/SRC/GRN/PAK2/RIPK1/IRF1/TNFRSF1B/NFE2L2/SERPINE1/SEC14L1/IFIT1B/AKNA/AP1G1/BRD4/NLRC4/SHARPIN/LYN/ILRUN/SNCA/ZNFX1/TYROBP/PAK1/PYCARD/CDK19/ARRB2/MMP9/XIAP/GPSM3/CREBBP/DTX3L/CEBPB/PTPRC/MAPK14/EPHB2/PGLYRP1/IL17RA/MAPK3/PRKDC/NLRX1/PRKCD/KRT1/SPI1/ZYX/NINJ1/FURIN
BP	GO:0007173	epidermal growth factor receptor signaling pathway	73	-0.47	-1.67	0.00131563	0.0071266	0.00438103	1632	tags=29%, list=14%, signal=25%	CBL/NCF1/SHC1/RPS6KA5/PSEN1/PTPRJ/ERBIN/RASSF2/PTK2B/RNF115/SRC/RAB7A/GAB1/PLAUR/SHKBP1/PTPN12/MMP9/IQGAP1/REPS2/TGFA/HIP1
BP	GO:1990266	neutrophil migration	80	-0.47	-1.68	0.0013204	0.0071425	0.00439081	3053	tags=52%, list=26%, signal=39%	CCL5/EDN1/JAML/DAPK2/MOSPD2/ADAM8/PIKFYVE/IL1B/CD177/NCKAP1L/FCER1G/C5AR2/BST1/SYK/PIP5K1C/PIK3CG/PF4V1/JAM3/RAC2/CAMK1D/TIRAP/CSF3R/CCL3/ITGB2/FUT7/VAV1/LGALS3/C5AR1/CXCR2/CXCL5/VAV3/CXCR1/PF4/PECAM1/PREX1/TREM1/RAC1/PIK3CD/RHOG/BSG/RIPOR2/MPP1
BP	GO:0045926	negative regulation of growth	142	-0.39	-1.55	0.00132216	0.0071425	0.00439081	3073	tags=41%, list=26%, signal=31%	RAI1/BCL6/ADAM15/BBS2/PPP2R1A/BST2/PML/HIF1A/CTDP1/BTG1/JADE1/SEMA7A/DDX3X/SERPINE2/PPT1/YY1/RTN4R/WWC3/RGS2/CDK5R1/SEMA4A/WWC2/KIAA0319/SAV1/CDKN2D/STK4/DRAXIN/RTN4/GREM1/MSX1/NAIF1/SESN2/HSPA1B/SERTAD3/GSK3A/PTPRJ/ULK1/SPAG9/SMARCA2/NDRG3/MT2A/PPP1R9B/HSPA1A/CDKN1A/OSGIN2/DCUN1D3/JARID2/ADIPOR1/CDA/SIPA1/FBP1/DNAJB2/G6PD/SFRP2/FLCN/PTEN/ING1/DAB2
BP	GO:0099072	regulation of postsynaptic membrane neurotransmitter receptor levels	32	-0.57	-1.75	0.00133397	0.0071966	0.00442403	2885	tags=47%, list=25%, signal=35%	FRRS1L/RAB8A/PRKCZ/PORCN/DLG4/GRIPAP1/STX3/SNAP25/NUMB/ERBIN/DNM3/CTNND1/IQSEC2/ITGB3/FLOT2
BP	GO:0030048	actin filament-based movement	66	-0.49	-1.70	0.00134187	0.0072293	0.00444418	2515	tags=41%, list=21%, signal=32%	SCN1B/SLC9A1/FLNA/MYO9B/MYH10/MYLPF/SYNE2/CACNA1D/ACTA2/ROCK1/DSC2/WASF2/WIPF2/KCNQ1/MYO1D/KCNE3/AKAP9/MYO5A/MYO1E/TPM1/MYH9/MYO1F/ACTN4/WIPF1/KCNJ5/WAS/GSN
BP	GO:0006970	response to osmotic stress	53	-0.52	-1.75	0.00135549	0.007283	0.00447716	2882	tags=49%, list=25%, signal=37%	CLN3/RELB/MAP7/SLC2A1/OXSR1/DDX3X/TSPO/BAX/TNF/PKN1/NFAT5/LRRC8A/SLC12A6/PTGS2/CAPN3/AQP9/PTK2B/ARHGEF2/TSC22D4/MAP2K7/MICU1/ZFP36L1/TSC22D3/YBX3/AQP1/MYLK
BP	GO:0006066	alcohol metabolic process	221	-0.36	-1.51	0.00135454	0.007283	0.00447716	1553	tags=21%, list=13%, signal=19%	CYP3A5/INPP5K/ERLIN1/MOGAT3/ACSS2/GOT1/VLDLR/RDH11/IPMK/HDLBP/LCAT/HMGCR/BMP6/PTK2B/SULT1A1/SULT1B1/CYB5R3/BPNT2/ACP3/ALDH3B1/APOL2/CYP1B1/DPM2/P2RY1/NUDT4/GDE1/RXRA/DGAT2/CUBN/POR/RETSAT/ABCG1/DHRS13/SNCA/PLEK/SPTLC3/SYNJ1/ITPK1/PTAFR/PNPLA2/DHCR24/G6PD/PTEN/GPD2/APOBR/DAB2/OSBPL1A
BP	GO:0035107	appendage morphogenesis	72	-0.47	-1.68	0.00136036	0.0072895	0.00448115	2233	tags=38%, list=19%, signal=31%	BMPR1A/BAX/ZBTB16/RNF165/IQCE/TMEM231/DYNC2H1/GREM1/SOX4/MSX1/CHST11/IFT140/ATRX/PSEN1/INTU/MAP3K20/ASPH/GNA12/SKI/BPNT2/NIPBL/PBX1/CHD7/PBX2/CREBBP/SFRP2/ECE1
BP	GO:0035108	limb morphogenesis	72	-0.47	-1.68	0.00136036	0.0072895	0.00448115	2233	tags=38%, list=19%, signal=31%	BMPR1A/BAX/ZBTB16/RNF165/IQCE/TMEM231/DYNC2H1/GREM1/SOX4/MSX1/CHST11/IFT140/ATRX/PSEN1/INTU/MAP3K20/ASPH/GNA12/SKI/BPNT2/NIPBL/PBX1/CHD7/PBX2/CREBBP/SFRP2/ECE1

CC	GO:0000421	autophagosome membrane	44	-0.54	-1.76	0.0013639	0.0072986	0.00448677	2493	tags=52%, list=21%, signal=41%	WIPI1/TECPR1/TMEM150B/CHMP3/MAP1LC3B/GABARAPL2/CALCOCO2/GABARAPL1/ATP6AP2/RB1CC1/LAMP2/LAMP1/MAP1LC3A/SNAP29/ULK1/WDFY3/CHMP1A/RAB7A/GABARAP/SH3GLB1/MAP1LC3B2/CHMP2A/CHMP4B
BP	GO:0045785	positive regulation of cell adhesion	307	-0.33	-1.43	0.00138343	0.0073932	0.00454488	2685	tags=33%, list=23%, signal=26%	TRAF6/RHOA/IL1B/DHPS/PRKCZ/CCDC88B/JAK2/NCKAP1L/IL4I1/ANGPT1/ALOX5/JAK1/CD70/FADD/FLNA/ERBB2/GSK3B/LILRB2/ZBTB16/DNM2/TNF/UTRN/EFNB1/TNFSF13B/PRKD2/NFAT5/GP6/ALOX15/MYADM/SPTA1/SYK/CR1/HLA-E/ITGA5/PTPN6/CHST2/STK4/VSIR/STX3/DISC1/SOX4/TRIOBP/STAT5B/ROCK1/IRAK1/RARA/RELA/PTPRJ/RIN2/ITGB2/FUT7/NEDD9/VAV1/DOCK8/PTK2B/APBB1IP/GFUS/SIRPA/LGALS9/ZFHX3/DOCK5/CASS4/ILK/VAV3/SRC/LIMS1/FLOT1/TNFSF14/PLAUR/SELP/ZBTB7B/PREX1/TPM1/AFDN/ZMIZ1/LYN/SIRPB1/UNC13D/TGM2/IGF2/RAC1/RSU1/PYCARD/CYRIB/PPP3CA/F11R/PTPRC/MAP4K4/CORO1A/FOXO3/PTAFR/TFE3/DMTN/SFRP2/FLCN/AMBRA1/HLX/HSD17B12/FLOT2/DAB2
BP	GO:0070372	regulation of ERK1 and ERK2 cascade	162	-0.39	-1.57	0.0013903	0.0074099	0.0045552	3289	tags=44%, list=28%, signal=32%	SLC9A3R1/MARCO/RRAS/GPNMB/BRAF/ICAM1/SPRED1/ARRB1/CD4/CCL5/MFHAS1/CSK/GSTP1/HLA-DRB1/NLRP12/FPR2/IL1B/PRKCZ/ANGPT1/SEMA7A/CSF1R/C5AR2/ERBB2/ATF3/TNF/CASR/GBP1/PRKD2/ALOX15/PDGFA/SYK/NECAB2/GPBAR1/PTPN6/CCR1/TIRAP/GNAI2/YWHAZ/ACTA2/MTURN/SHC1/FFAR4/DSTYK/CCL3/RGS14/PTPN1/VRK3/CHI3L1/C5AR1/PTK2B/TNIP1/SIRPA/LGALS9/MAP2K7/SPRY3/SRC/DUSP6/P2RY1/ARHGAP8/LYN/GLIPR2/PYCARD/ARRB2/CFLAR/PTPRC/EPHB2/MAPK3/FLCN/PTEN/RAPGEF2/DAB2
BP	GO:0030900	forebrain development	212	-0.36	-1.50	0.00140263	0.0074657	0.00458945	2681	tags=37%, list=23%, signal=29%	WNT1/RHOA/PLCB1/ASPM/SLC2A1/TCTN1/SETD2/SEMA7A/SIN3A/DPCD/FOXP2/HAP1/CSF1R/FEZ1/LRRK2/OTX1/RTN4R/TBX19/GSK3B/BMPR1A/BAX/ZSWIM6/NDE1/PAFAH1B1/MAS1/ATP7A/CDK5R1/NRP2/ITGAM/TACC3/PCM1/SYNE2/ATF5/DYNC2H1/TRAPPC9/SSTR2/CREB1/DRAXIN/HOOK3/RTN4/DISC1/MSX1/MBOAT7/NUMB/NR4A2/XRN2/ATRX/XRCC1/PSEN1/RARA/ZEB1/AGTPBP1/ARL13B/PPP1R9B/NDEL1/SKI/NIN/ARPC5/SRC/UBB/FBXO45/TACC1/RBPJ/SLC7A11/NDST1/AKNA/ZMIZ1/APAF1/TACC2/CHD7/TYROBP/NF1/EPHB2/AQP1/NRGN/PTEN/SLC8A1/RAPGEF2
BP	GO:0010811	positive regulation of cell-substrate adhesion	79	-0.46	-1.65	0.00140983	0.0074842	0.00460087	2332	tags=35%, list=20%, signal=29%	FLNA/GSK3B/DNM2/UTRN/ALOX15/MYADM/ITGA5/STK4/DISC1/TRIOBP/ROCK1/PTPRJ/RIN2/NEDD9/PTK2B/GFUS/DOCK5/CASS4/ILK/LIMS1/PREX1/UNC13D/RAC1/RSU1/MAP4K4/DMTN/HSD17B12/DAB2
CC	GO:0043025	neuronal cell body	266	-0.35	-1.47	0.00140988	0.0074842	0.00460087	2700	tags=31%, list=23%, signal=24%	CRTC1/CACNA1A/RAB8A/NEURL1/PRKCZ/VTI1B/ADNP/HCFC1/PSEN2/SCN1B/P2RX7/FEZ1/ALCAM/FLNA/CKB/LRRK2/PPT1/CYBB/RTN4R/BMPR1A/SYAP1/SORL1/PAFAH1B1/TNF/GJC2/PI4K2A/CASR/ATP7A/CDK5R1/GRIK2/FZD5/RIN3/PDE1A/ATP2B1/NCF1/MAP1A/PVALB/GIGYF2/ROGDI/SLC2A3/GSK3A/PSEN1/MAPT/INSR/RAP1GAP/PPP1R9B/WDFY3/SLC6A6/GNA12/PTK2B/SYNPO/NDEL1/PTBP2/KCNQ1/SIAH2/MYO1D/TBX21/TMEM266/UBB/FCHSD1/KCNE3/AKAP9/FXR2/TNFRSF1B/MME/GLRX5/ERMN/SNCA/PYCARD/PICALM/MAP1S/PRKAR2B/EPHB2/TOP1/RAB5A/NRGN/MPL/MBP/SLC8A1/RAPGEF2/STAU1/GLUL
BP	GO:0045600	positive regulation of fat cell differentiation	37	-0.56	-1.77	0.00141983	0.007507	0.00461483	2375	tags=54%, list=20%, signal=43%	CEBPA/KLF5/WDFY2/SYAP1/ZBTB16/HNRNPU/METRNL/SAV1/CREB1/STK4/PIM1/FFAR4/PTGS2/ZFP36L1/CARM1/ZBTB7B/CEBPB/MAPK14/TFE3/SFRP2
BP	GO:0051588	regulation of neurotransmitter transport	50	-0.53	-1.75	0.00141866	0.007507	0.00461483	2378	tags=38%, list=20%, signal=30%	CAMK2A/LRRK2/GSK3B/FBXL20/PRKCB/STXBP5/APBA1/RAB3B/MCTP2/SYT12/FLOT1/P2RY1/CALM3/VPS18/P2RX1/SNCA/NF1/RAB5A/ITGB3

CC	GO:0101002	ficolin-1-rich granule	173	-0.37	-1.52	0.00141702	0.007507	0.00461483	2860	tags=45%, list=24%, signal=35%	ATP6V0C/CFD/GSTP1/ADAM8/GAA/MAPK1/KCMF1/FPR2/RHOA/OSTF1/CANT1/NCKAP1L/ACTR2/ALOX5/SIGLEC14/FCER1G/DDX3X/CRISPLD2/LILRB2/CTSZ/FPR1/PDAP1/MNDA/CST3/ACLY/BIN2/PRCP/CDK13/PKM/SLC11A1/PGAM1/COTL1/CR1/MGAM/ATAD3B/ATP6AP2/FCN1/LAMP2/ADGRE3/LAMP1/GPI/ATP6V0A1/IMPDH1/ALDOA/SLC2A3/LRG1/HSPA1B/ITGB2/DIAPH1/LGALS3/HSPA1A/ARL8A/PGM1/CTSD/VCP/CD93/ATG7/SIRPA/ARPC5/PSMD3/PLEKHO2/PYGL/APAF1/RAC1/SIGLEC5/MMP9/CDA/DOK3/GNS/TIMP2/CTSB/MAPK14/KPNB1/FTH1/GSN/CAPN1/VCL/KRT1
BP	GO:0048168	regulation of neuronal synaptic plasticity	30	-0.60	-1.81	0.00144887	0.0076504	0.00470299	3514	tags=60%, list=30%, signal=42%	NPTN/RAB3GAP1/MCTP1/CLN3/KRAS/RAB8A/NEURL1/KIT/CAMK2A/SYAP1/ZDHHC2/GRIK2/DLG4/DBN1/SNCA/NF1/EPHB2/RAB5A
BP	GO:1902991	regulation of amyloid precursor protein catabolic process	33	-0.58	-1.79	0.00145744	0.0076854	0.00472451	2439	tags=61%, list=21%, signal=48%	HAP1/ABCA2/RTN1/SORL1/TNF/ROCK2/GGA3/SP1/RTN4/ROCK1/GSK3A/RELA/CLU/FKBP1A/ABCG1/LYN/RTN3/RTN2/PICALM/FLOT2
BP	GO:0018209	peptidyl-serine modification	230	-0.35	-1.45	0.00148099	0.0077992	0.00479449	3099	tags=39%, list=26%, signal=29%	MAPKAP1/MKNK1/MARK1/STK32C/PKD1/BAK1/GRK2/STK38L/NSD1/CSNK1A1/MAPK1/UHMK1/PIKFYVE/TTBK2/PRKCZ/EIF4G1/ERN1/CSNK2A1/DYRK1B/ANGPT1/NEK6/DYRK1A/CSNK1G2/TAF1/GALNT3/CAMK2A/LRRK2/GSK3B/HIPK2/PLCL2/BAX/PRKCB/TNF/ROCK2/DYRK3/PKN1/HIPK1/CDK5R1/PRKD2/RICTOR/OSM/MARK2/MAST3/SYK/MAP2K2/GADD45A/CAMK1D/STK4/HGF/INPP5K/MKNK2/SH2D3C/PTGS2/ROCK1/RPS6KA5/ULK1/VRK3/PLK1/RASSF2/MAD2L2/RPS6KA1/HIPK3/CSNK1G1/ILK/SRC/MAPKAPK2/PAK2/AKAP9/RIPK1/MARK3/PRKACA/AKT2/MAPKAPK3/MAST2/HCLS1/SNCA/PAK1/WNK1/ARRB2/SRPK2/STK38/MAPK14/PDK3/TOP1/DMTN/PRKDC/SFRP2/PRKCD/PINK1/PTEN
CC	GO:0042383	sarcolemma	71	-0.48	-1.70	0.00149766	0.0078728	0.00483974	1523	tags=23%, list=13%, signal=20%	LAMP1/CACNA1D/PSEN1/CAPN3/SNTB1/FLOT1/ALOX12/PPP3CA/RTN2/CORO1C/PPP3R1/AQP1/BSG/VCL/SLC8A1/DYSF
BP	GO:0061458	reproductive system development	228	-0.36	-1.48	0.00149892	0.0078728	0.00483974	2393	tags=30%, list=20%, signal=25%	LDOC1/CEBPA/SERPINE2/SPATA2/GRB2/KDM5B/SENP2/BAX/MAS1/NKX3-1/SGPL1/PDGFA/FZD5/BIRC6/PTPN6/CDKN1C/CBL/STK4/ARID4A/STAT5B/JUNB/TFEB/PTGS2/ARNT/ATRX/RARA/CSDE1/SOCS3/INSR/CCNF/PPP1R9B/CTNNA1/H3-3A/BMP6/NUPR1/COL9A3/SRC/NIPBL/ASCL2/UBB/BPTF/PSAP/TPPP3/CYP1B1/ZFP36L1/VDR/RBPJ/AGO4/MME/IGF2/CHD7/ADM/ARRB2/TBC1D20/NCOA4/CEBPB/H3-3B/CTSB/MAPK14/DNAJB6/NCOA1/FOXO3/YBX3/DHCR24/SFRP2/BCL2L1/BSG/BASP1/PTEN
BP	GO:0010508	positive regulation of autophagy	116	-0.41	-1.58	0.00150993	0.0079097	0.00486244	2970	tags=41%, list=25%, signal=31%	TRIM38/PIP4K2B/CAMKK2/ORMDL3/HIF1A/VPS13D/EPM2A/DAPK1/WIPI1/TICAM1/OPTN/TRIM21/LRRK2/GSK3B/PIK3CB/PIP4K2C/DHRSX/BNIP3L/CALCOCO2/SPTLC2/TP53INP2/TSC2/TFEB/ROCK1/SESN2/GSK3A/ULK1/SESN3/RALB/ELAPOR1/WDR45/IKBKG/PIP4K2A/WAC/SH3GLB1/KAT5/ATG2A/TRIM8/FBXO7/HMOX1/FOXO3/MAPK3/SNX18/FLCN/AMBRA1/PINK1/NPRL3

BP	GO:0019725	cellular homeostasis	548	-0.30	-1.34	0.00153556	0.0080334	0.00493847	2894	tags=31%, list=25%, signal=24%	PIM3/ATP6AP1/PML/CLN3/ORMDL3/SRF/SMARCB1/HLA-DRB1/HIF1A/ATP2A3/GAA/MAPK1/GNA13/SLC26A6/SLC9A8/FPR2/PIK3R2/CACNA1A/ANO6/STIM1/ERN1/LPAR1/JAK2/FIS1/SLC9A1/SIN3A/CCDC186/CLN6/P2RX7/HAP1/C5AR2/STEAP4/KLF7/IREB2/CD38/HK2/ABCA2/CKB/CA2/LRRK2/PPT1/TPCN2/BAX/PRKCB/PIK3CB/FPR1/PLCE1/CASR/ATP7A/GPLD1/UCP2/ITPR2/SLC39A4/GRIK2/PRDX5/SLC11A1/MCL1/MCOLN1/GSR/TAOK1/SLC31A1/UBE2S/GPR27/PIK3CG/SLC6A9/ATP6AP2/PTPN6/SLC39A7/DLG4/VPS33A/LAMP2/ATP2B1/GCLM/CCR1/LRRC8A/RAB11FIP2/ERO1A/SLC12A6/TXNRD1/RTN4/ANKRD9/DISC1/CACNA1D/MAP1A/KNG1/SOX4/MTM1/SIDT2/ATP6V0A1/FFAR4/ALDOA/TRPC3/ARF1/CCL3/PSEN1/FKBP1A/CAPN3/SELENON/MAFG/ATP6V0D1/DIAPH1/MT2A/C5AR1/RAB11B/BMP6/PTK2B/CXCR2/GPX1/TF/PRDX2/EGLN2/KCNQ1/LIN28A/CXCR1/MICU1/GRN/RAB7A/XK/SLC24A3/KCNE3/P2RY1/CALM3/VDR/GP1BA/PRKACA/PROK2/FGGY/DGAT2/NFE2L2/TBXAS1/P2RX1/DEDD2/CHP1/DMXL2/LYN/SNCA/TGM2/CHD7/C2CD2L/ADM/PPP3CA/GRINA/NCOA4/ALAS2/PTPRC/OPRL1/HK1/HMOX1/CORO1A/ACO1/FOXO3/PDK3/SLC4A1/DNAJB2/PRDX6/IRS2/AQP1/MAPK3/FTL/KEL/FTH1/AMBRA1/PINK1/WDTC1/CCRL2/SLC8A1/TBXA2R/GP9/ATP6V1A
BP	GO:0033673	negative regulation of kinase activity	169	-0.39	-1.56	0.00155403	0.0081194	0.0049913	2226	tags=35%, list=19%, signal=29%	MLLT1/SORL1/PIK3CB/HNRNPU/PKN1/RGS2/PRKAR2A/PIP4K2C/HHEX/GADD45A/PTPN6/CDKN1C/IRAK3/CDKN2D/CBL/TSC2/RUBCN/DUSP22/INPP5K/PSEN1/MAPT/RGS14/PTPRJ/PTPN1/ADAR/PLK1/AKT1S1/HMGCR/GNAQ/CDKN1A/PPP2R5A/HIPK3/SPRY3/APC/WARS1/PAK2/HSPB1/PIP4K2A/DUSP1/SH3BP5L/CHP1/LYN/SNCA/PYCARD/MIDN/PTPRC/FBXO7/STK38/PRKAR2B/NF1/EPHB2/CORO1C/IRS2/SFRP2/PRKAR1A/PRKCD/PTEN/SLC8A1/TRIB1
BP	GO:0010591	regulation of lamellipodium assembly	32	-0.57	-1.73	0.00156034	0.0081416	0.00500499	2681	tags=44%, list=23%, signal=34%	WNT1/ACTR2/CAPZB/DNM2/PLCE1/ATP7A/TWF2/RAC2/FSCN1/WASF2/RAC1/WAS/ABI3/DMTN
[truncated: 433,947 more chars]
